# RNA-seq *de novo* Assembly Reveals Differential Gene Expression in *Glossina palpalis gambiensis* Infected with *Trypanosoma brucei gambiense* vs. Non-Infected and Self-Cured Flies

**DOI:** 10.3389/fmicb.2015.01259

**Published:** 2015-11-13

**Authors:** Illiassou Hamidou Soumana, Christophe Klopp, Sophie Ravel, Ibouniyamine Nabihoudine, Bernadette Tchicaya, Hugues Parrinello, Luc Abate, Stéphanie Rialle, Anne Geiger

**Affiliations:** ^1^UMR 177, Institut de Recherche Pour le Développement-CIRAD, CIRAD TA A-17/GMontpellier, France; ^2^Institut National de la Recherche Agronomique, GenoToul, UR875Castanet-Tolosan, France; ^3^Centre National de la Recherche Scientifique, Unité Mixte de Recherche 5203, Institut de Génomique FonctionnelleMontpellier, France; ^4^Institut National de la Santé et de la Recherche Médicale U661Montpellier, France; ^5^Universités de Montpellier 1 and 2, UMR 5203Montpellier, France; ^6^Montpellier GenomiX, Institut de Génomique FonctionnelleMontpellier, France; ^7^UMR MIVEGEC (Institut de Recherche pour le Développement 224-Centre National de la Recherche Scientifique 5290-UM1-UM2), Institut de Recherche pour le DéveloppementMontpellier, France

**Keywords:** *de novo* assembly, *Glossina palpalis gambiensis*, human African trypanosomiasis, *in vivo* metatranscriptomics, RNA-seq, *Trypanosoma brucei gambiense*

## Abstract

*Trypanosoma brucei gambiense* (Tbg), causing the sleeping sickness chronic form, completes its developmental cycle within the tsetse fly vector *Glossina palpalis gambiensis* (Gpg) before its transmission to humans. Within the framework of an anti-vector disease control strategy, a global gene expression profiling of trypanosome infected (susceptible), non-infected, and self-cured (refractory) tsetse flies was performed, on their midguts, to determine differential genes expression resulting from *in vivo* trypanosomes, tsetse flies (and their microbiome) interactions. An RNAseq *de novo* assembly was achieved. The assembled transcripts were mapped to reference sequences for functional annotation. Twenty-four percent of the 16,936 contigs could not be annotated, possibly representing untranslated mRNA regions, or Gpg- or Tbg-specific ORFs. The remaining contigs were classified into 65 functional groups. Only a few transposable elements were present in the Gpg midgut transcriptome, which may represent active transpositions and play regulatory roles. One thousand three hundred and seventy three genes differentially expressed (DEGs) between stimulated and non-stimulated flies were identified at day-3 post-feeding; 52 and 1025 between infected and self-cured flies at 10 and 20 days post-feeding, respectively. The possible roles of several DEGs regarding fly susceptibility and refractoriness are discussed. The results provide new means to decipher fly infection mechanisms, crucial to develop anti-vector control strategies.

## Introduction

Human African Trypanosomiasis (HAT), one of the most neglected tropical diseases in the world (Brun et al., 2010), is endemic to 36 countries of sub-Saharan Africa, where it results in a loss of 1.5 million disability-adjusted life years every year (Hotez et al., 2009). This devasting disease has been targeted for elimination by the WHO and PATTEC (Pan-African Tsetse and Trypanosomiasis Eradication Campaign), and subsequently by the London declaration on neglected tropical diseases. In terms of mortality, the disease is ranked ninth out of 25 human infectious and parasitic diseases in Africa (Welburn et al., 2009). Sleeping sickness remains responsible to this day for major hindrances to social, agricultural, and economic development in Africa.

HAT is caused by two subspecies of African trypanosomes transmitted by tsetse flies: *Trypanosoma brucei gambiense* (Tbg) is responsible for the chronic form of HAT in Western and Central Africa, while *Trypanosoma brucei rhodesiense* is responsible for the acute form of the disease in East Africa (Kennedy, 2008; Franco et al., 2014). In recent years, the number of new cases has begun to decrease, mirroring a situation previously observed in the 1960s and which preceded the last heavy outbreak in the 1990s.

To date, no vaccine is available to prevent sleeping sickness. Moreover, several currently used drugs cause harmful side effects, in addition to inducing trypanosome-resistant strains (Baker et al., 2013). Furthermore, some diagnostic tools are inefficient for proper HAT detection (Simarro et al., 2008; Geiger et al., 2011). The search for novel strategies, including alternative vector-based strategies (Rio et al., 2004; Aksoy et al., 2008; Medlock et al., 2013), must therefore be pursued further. One such approach, the release of sterile *Glossina* males to drastically decrease the targeted population size, was successfully tested in Zanzibar (Vreysen et al., 2000; Abd-Alla et al., 2013). However, even though these sterile males are not trypanosome-infected, they are still able to acquire trypanosomes from an infected host and transmit them to non-infected humans. Therefore, releasing flies that are both sterile and resistant to trypanosome infection (refractory flies) could be more effective and a lesser risk for humans. This type of approach requires deciphering the physiological mechanisms that govern fly refractoriness to trypanosome infection, in order to develop methodologies for enhancing tsetse fly resistance.

Refractoriness is the status of most tsetse flies, as shown by the typical low prevalence of trypanosome infections in natural fly populations in HAT foci, as well as in flies submitted to an experimental infection. In the latter case, flies are fed on trypanosome-infected mice displaying high levels of parasitemia. Even though all the flies ingested trypanosomes, typically only around 15% become infected; the others were either self-cured from the ingested parasites, or they did not produce mature parasites and therefore never became infective (Moloo et al., 1986; Dukes et al., 1989; Frézil and Cuisance, 1994; Maudlin and Welburn, 1994; Jamonneau et al., 2004). Understanding the biological processes leading to the elimination of ingested trypanosomes or parasite maturation failure, identifying the key steps and the key factors involved, and investigating different means to stimulate refractoriness will all help to effectively combat sleeping sickness.

The existence of two distinct pathways, one in which ingested trypanosomes are eliminated by refractory flies and the other in which trypanosomes are established in the gut and achieve their developmental cycle in susceptible flies, clearly demonstrates the occurrence of complex molecular interactions. These interactions are not restricted to cross-talk between the invading trypanosomes and the tsetse fly. For example, tsetse can harbor the primary obligate symbiont *Wigglesworthia glossinidia* and the secondary symbiont *Sodalis glossinidius*, which are known to favor fly infection by trypanosomes (Geiger et al., 2007; Farikou et al., 2010). Significant modulation of *Wigglesworthia* and *Sodalis* gene expression was previously recorded following fly trypanosome invasion (Hamidou Soumana et al., 2014a,b). Finally, field flies have been shown to harbor a large diversity of bacterial species (Geiger et al., 2013; Hamidou Soumana et al., 2013), suggesting that the whole microbiome may be involved in modulating the fly global response to trypanosome invasion, and consequently the fly vector competence.

The physiological mechanisms involved in this vector competence (i.e., its ability to acquire the parasite, to favor its maturation, and to transmit it to a mammalian host) are not well understood, and the genes that control it remain largely unknown. Nevertheless, some responses have been identified (reviewed in Geiger et al., 2015). For example, several studies have reported that an RNAi approach used to silence genes controlling the Imd pathway (Hao et al., 2001) and the tsetse fly immune-responsive glutamine/proline-rich (EP) protein (Haines et al., 2010) increase midgut colonization efficiency. The importance of reactive oxygen species as determinants of resistance have been similarly demonstrated (MacLeod et al., 2007a; Macleod et al., 2007b; Nayduch and Aksoy, 2007). Recently, Weiss et al. (2013, 2014) demonstrated the importance of microbiome-regulated host immune barriers in establishing the trypanosome infection. In addition, as trypanosomes migrate from the gut toward the salivary glands they reach the proventriculus, an immune-active tissue expressing the nitric oxide synthase gene and containing increased levels of nitric oxide, reactive oxygen intermediates, and hydrogen peroxide (Hao et al., 2003). Only a few trypanosomes will survive and complete their migration to the salivary glands, where they multiply and evolve into their infectious metacyclic form.

We previously investigated 12 immune genes selected from those formerly reported by Lehane et al. (2003) to be highly over-expressed in *Glossina morsitans morsitans* challenged with *T. b. brucei* (Hamidou Soumana et al., 2014c). Nevertheless, deciphering the mechanisms that allow trypanosomes to adapt to the different tsetse fly microenvironments and thereby escape insect immune responses requires a more global approach. We have therefore performed a large comparative transcriptome analysis of trypanosome-infected, non-infected and self-cured (refractory) *Glossina palpalis gambiensis* (Gpg) flies, the vector of *T. b. gambiense*, the trypanosome causing the chronic form of HAT in West Africa. The present work follows our previous investigations of differential gene expression in *Sodalis* and *Wigglesworthia* (Hamidou Soumana et al., 2014a,b) by focusing on the differentially expressed genes (DEGs) from both flies and trypanosomes, since some genes could be used as targets to enhance tsetse fly refractoriness to trypanosome infection. Since the establishment step is fundamental to the trypanosome life cycle within its vector, our investigation has once again focused on the tsetse fly midgut, where the ingested trypanosome is established (or not). To investigate global infection dynamics at key early time points, samples were collected at 3, 10, and 20 days post-feeding on either trypanosome-infected or non-infected bloodmeals.

The analyses were performed using an RNA-seq *de novo* assembly approach—“*a revolutionary tool for transcriptomics*” (Wang et al., 2009). Our report presents results of transcriptome read assembly. *G. p. gambiensis* and *G. m. morsitans* being two different species, functional annotation was performed with reference to a broad panel of insect data bases including *G. m. morsitans*. Here, we identified DEGs in susceptible and refractory tsetse. In addition, we have identified single nucleotide polymorphisms (SNPs) and their variants (insertions and deletions) and have evaluated their relationships within the levels of gene expression in the different samples. Finally, this study highlights molecular interactions on the basis of biosynthesis pathways controlled by genes shown to be differentially expressed.

## Materials and methods

### Ethical statement

All reported experiments on animals were conducted according to internationally recognized guidelines. The experimental protocols (numbers 12TRYP03, 12TRYP04, and 12TRYP06) were approved by the Ethics Committee on Animal Experiments and the Veterinary Department of the Centre International de Recherche Agronomique pour le Développement (CIRAD), Montpellier, France.

### Glossina species and trypanosome strains used for experimental infections

*G. p. gambiensis* flies and the *T. b. gambiense* isolate T.b.g. S7/2/2 used in this study have been previously described (Hamidou Soumana et al., 2014a,b).

### Experimental design and sampling procedures

Preliminary note: the samples analyzed in the present study were previously used to identify DEGs in *Sodalis* and *Wigglesworthia* (Hamidou Soumana et al., 2014a,b). The experimental steps described in this Section (“Experimental Design and Sampling Procedures”) are similar to those described in the latter studies. Additional experimental steps (described in “RNA-seq: Sample Preparation and Sequencing” and the following Sections) are specific to the present study.

Briefly, a set of 100 randomly chosen *G. p. gambiensis* teneral (< 32 h old) female flies were fed on non-infected mice. Three days after feeding, two biological replicates of seven flies each were dissected and the seven midguts from each replicate were pooled (= sample NS3 in two replicates). A second set of 900 *G. p. gambiensis* teneral (< 32 h old) female flies were fed on *T. b. gambiense*-infected mice (averaging 20 flies per mouse), which displayed parasitemia levels ranging between 16 and 64 × 10^6^ parasites/ml of blood. Flies were then randomly separated into three groups.

The first group of flies was recovered 3 days after the infective feeding (= “stimulated flies” or S3 samples) and randomly separated into two biological replicates of seven flies each. The flies from each replicate were dissected separately and the corresponding midguts were pooled in RNAlater (Ambion) and stored at −80°C until RNA extraction.

The second group of flies was recovered 10 days after the infective feeding. DNA was extracted from anal drops by the chelex method (Ravel et al., 2003), and the presence of *T. b. gambiense* in their anal drops was confirmed by PCR using specific primers (Moser et al., 1989). Based on the PCR results, flies were separated into one of two subgroups: (a) those with trypanosomes in their anal drops (= infected flies or I10 samples); and (b) those not displaying trypanosomes in their anal drops (= self-cured flies or NI10 samples). Each subgroup was further divided randomly into two biological replicates of three flies each (at this sampling time the prevalence of infected flies was < 5%). The flies from each replicate were then processed as above.

The third group of flies was recovered 20 days after feeding on trypanosome-infected mice and was processed similarly to the second group. Infection prevalence was high enough at this sampling time to establish two replicates of seven flies each (infected flies = I20; self-cured flies = NI20 samples).

Finally, transcriptome analyses were performed on a total of 12 samples, representing six “categories” of differently treated flies (S3 and NS3; I10 and NI10; I20 and NI20). Each category was further subdivided into two biological replicates.

### RNA-seq: sample preparation and sequencing

RNA was extracted from the pooled midguts of each biological replicate using TRIzol reagent (Gibco-BRL, Life Technologies), according to the manufacturer's protocols. RNA pellets were resuspended in nuclease-free water and the concentration was quantified using a NanoDrop spectrophotometer. RNA quality and the absence of DNA contamination were confirmed on a 2100 Bioanalyzer chip (Agilent Technologies, Santa Clara, CA, USA) prior to cDNA library synthesis.

cDNA libraries were prepared (using 4 μg of total RNA from each sample) for subsequent Illumina sequencing with the mRNA-seq Sample Prep kit (Illumina, San Diego, CA, USA). Specifically, RNA was fragmented and used as a template for a randomly primed PCR. After amplification, ends were repaired and ligated to Illumina adapters. The cDNA library was then verified for appropriate fragment size (200–300 bp) on a BioAnalyzer chip. Libraries were amplified onto flow cells using an Illumina cBot and the fragments were sequenced, using a paired-ends strategy, on an Illumina HiSeq2000 (Illumina, San Diego, CA, USA) for 2 × 101 cycles, according to the manufacturer's protocols. The barcoded libraries were multiplexed by 4 on a single lane. Paired-end raw reads were automatically trimmed and validated by screening for low quality (e.g., short sequences or ambiguous nucleotides), low complexity, and contaminants. These false reads were removed from the study and the remaining reads were assembled *de novo*.

The 2.91 × 10^8^ raw sequencing reads were filtered to remove bad quality bases and reads, resulting in 2.76 × 10^8^ remaining reads (95.14%). All reads were then used for *de novo* assembly of the transcriptome. Datasets for the reads are available from the NCBI Short Read Archive (SRA), accession number SRP046074.

### *De novo* assembly and transcriptome analysis

To construct the *G. p. gambiensis* assembled whole transcriptome, all short reads obtained from infected, stimulated and non-infected, non-stimulated tsetse flies at 3, 10, and 20 days post-feeding were first assembled into contigs with no gap, using the *de novo* transcriptome assembly software programs Velvet and Oases (Zerbino and Birney, 2008). Each read was then mapped back to the contigs using the Bwa short-read aligner (Li and Durbin, 2009), to generate the gross count per contig for each biological replicate representing the different conditions. The assembled contigs were annotated by BLASTX alignment (*E* < 0.00001) to protein databases such as the NCBI NR (http://www.ncbi.nlm.nih.gov), Swiss-Prot (http://www.expasy.ch/sprot), ensembl-pep, refseq-rna, refseq-protein, and FlyBase databases. Contig sequences were deposited at the NCBI Transcriptome Shotgun Assembly (TSA) Database under BioProject PRJNA260242. Gene Ontology (GO) annotation assignment (Ashburner et al., 2000) was used to perform functional gene annotation by mapping GO terms using the NCBI NR, GO (http://www.geneontology.org/), and UniProts (http://www.ebi.ac.uk/UniProt/) databases; *E*-value cutoff of 10^−5^ (Conesa et al., 2005).

### Technical description of the assembly process

#### Per condition assembly

Read pairs were first cleaned from remaining sequencing adapter sequences using the trim_galore script (http://www.bioinformatics.babraham.ac.uk/projects/trim_galore/; Smallwood et al., 2014). Over-represented reads were then filtered out using the normalize_by_kmer_coverage.pl script from the Trinity software package (Haas et al., 2013). In the next step, invalid base calls were discarded by extracting the longest sub-sequence without Ns from each read. Specifically, if the length of the longest sub-sequence did not exceed half of the sequence length, the read, and its pair were removed. The final step was performed using an in-house script.

Nine assemblies using nine different k-mers (25, 31, 37, 43, 49, 55, 61, 65, and 69) were performed on pre-processed input data. Each assembly produces a transcripts.fa file and each raw transcripts.fa file is organized into loci. Rather than referring to genetic loci, each locus is actually a collection of similar sequences including (but not limited to) splice variations and partial assemblies of the longer transcripts in the locus. We chose to keep only the best transcript for each locus, using the script OasesV0.2.04OutputToCsvDataBase.py (http://code.google.com/p/oases-to-csv/; Schulz et al., 2012). Subsequently, all files were merged and anti-sense chimeras (accidentally produced by the assembly step) were cut with a homemade script.

Identical contigs produced by different k-mers were removed using the cd-hit-est program (Li and Godzik, 2006). Because different k-mers sometimes construct different transcript parts, we used TGICL (Pertea et al., 2003), an OLC (overlap layout consensus) assembler, to assemble contigs displaying significant overlaps. The contigs were also filtered to a minimum length of 200 bp.

Input reads were then mapped back to the contigs using the bwa aln function (Li and Durbin, 2009). The resulting alignment files were used to correct contig sequences from spurious insertions and deletions resulting from an in-house script, and to filter out contigs with very low coverage. The filter excludes contigs with less than two mapped reads per million.

#### Meta-assembly

All single condition contig fasta files were concatenated, and each contig was renamed by adding the condition name to the beginning of its name. The longest open reading frame (ORF) of each contig was then searched using the getorf program from EMBOSS (Rice et al., 2000). A cd-hit clustering was performed on ORFs with a sequence identity ≥0.9, in order to extract from each cluster the contig with the longest ORF, or the longest contig (if the ORF sizes were identical). A clustering using cd-hit-est was then done on selected ORFs with a sequence identity ≥0.95. Input reads from all conditions were then mapped back to the contigs using bwa (Li and Durbin, 2009). Contigs with very low coverage (< 1 mapped read per million) were filtered out.

### Analysis of DEGs

DEG's were identified using the DESeq software, version 1.16.0 (Anders and Huber, 2010). This method represents widely accepted and complementary analytical approaches for RNASeq data. The raw read counts were produced by realigning the read on the contigs. These counts were used as inputs for DESeq to calculate the normalized expression for each contig in the different samples (e.g., trypanosome-stimulated and non-stimulated tsetse flies at 3 days and trypanosome-infected and non-infected tsetse flies at 10 and 20 days). Differential expression was then reported as fold change, with associated *p*-values. DESeq calculates *p*-values using a negative binomial distribution that accounts for technical as well as biological variability. The resulting raw *p*-values were corrected for multiple tests using the False Discovery Rate (Benjamini and Hochberg, 1995). Contig pairs whose read numbers displayed a greater than two-fold difference between the selected conditions (with *p* < 0.05) were identified as DEGs. The DESeq approach is well suited for count data (i.e., read counts), as is the case for RNA-Seq experiments, and the method estimates variance in a local fashion for varying signal strength (Trapnell et al., 2010).

For functional analysis, all DEGs were mapped to terms in the GO database, which requires *E*-values adjusted for multiple testing to be < 0.001. The annotation of all significant genes was further supplemented by BLASTX, conserved domains, and literature searches. Using these combined approaches resulted in a functionally driven classification.

### SNP identification

Many SNP panels have been build using an RNA-Seq assembly reference for species without reference genome (Salem et al., 2012; Swaminathan et al., 2012; see also GATK Program which is the classical tool to evidence SNPs).

In order to call putative variants (SNPs, insertions and deletions), the alignment files were cleaned from reads with low mapping quality and PCR duplicates using the samtools software, v 0.1.19-44428cd. The remaining reads were recalibrated and realigned with the GATK Program 2.4-9-g532efad (DePristo et al., 2011), which was also used for variant calling (Unifiedgenotyperalgorithm). Variants were filtered using a Phred quality score of 20.

Variants were deposited at the NCBI single nucleotide polymorphism database (dbSNP) with the accession number SUB833398.

## Results

### Infection time course

At day 10 after fly feeding, the anal drops from 13 out of 262 tsetse flies (4.96%) were PCR-positive for trypanosomes. At day 20 after fly feeding, the anal drops of 43 out of 349 flies (12.32%) were PCR-positive for trypanosomes.

### Transcriptome sequencing by RNA-seq and *de novo* assembly

Twelve RNA-seq libraries were prepared from total RNA extracted from pooled midguts of non-stimulated and non-infected (refractory) tsetse flies [representing 3 (NS3), 10 (NI10), and 20 days (NI20) post-bloodmeal uptake] and from pooled midguts of stimulated or infected tsetse flies [representing 3 (S3), 10 (I10), and 20 days (I20) post-bloodmeal uptake].

A total of approximately 520 million raw reads (50 million paired-end reads, 2 × 100 bp) representing approximately 165 GB of sequence data were generated from 12 independent 200 bp insert libraries. Prior to *de novo* assembly, the quality of the reads was assessed using the base-calling quality scores (Cock et al., 2010) from Illumina's base-caller RTA software. Most reads displayed Phred-like quality scores at the Q20 level, indicating a sequencing error probability of 0.01%. After trimming and cleaning, between 58,370,072 and 87,387,674 read pairs were kept, depending on the sample library.

*De novo* assembly was then performed using the Velvet software. Oases is a *de novo* transcriptome assembler designed to produce extended contigs from short read sequencing technologies in the absence of any genomic reference. It clusters the contigs from a preliminary assembly by Velvet into small groups and uses a de Bruijn graph-based algorithm to construct transcript isoforms (Schulz and Zerbino, 2010). The contigs, produced by Velvet, were post-processed using Oases.

**Figure 1:**
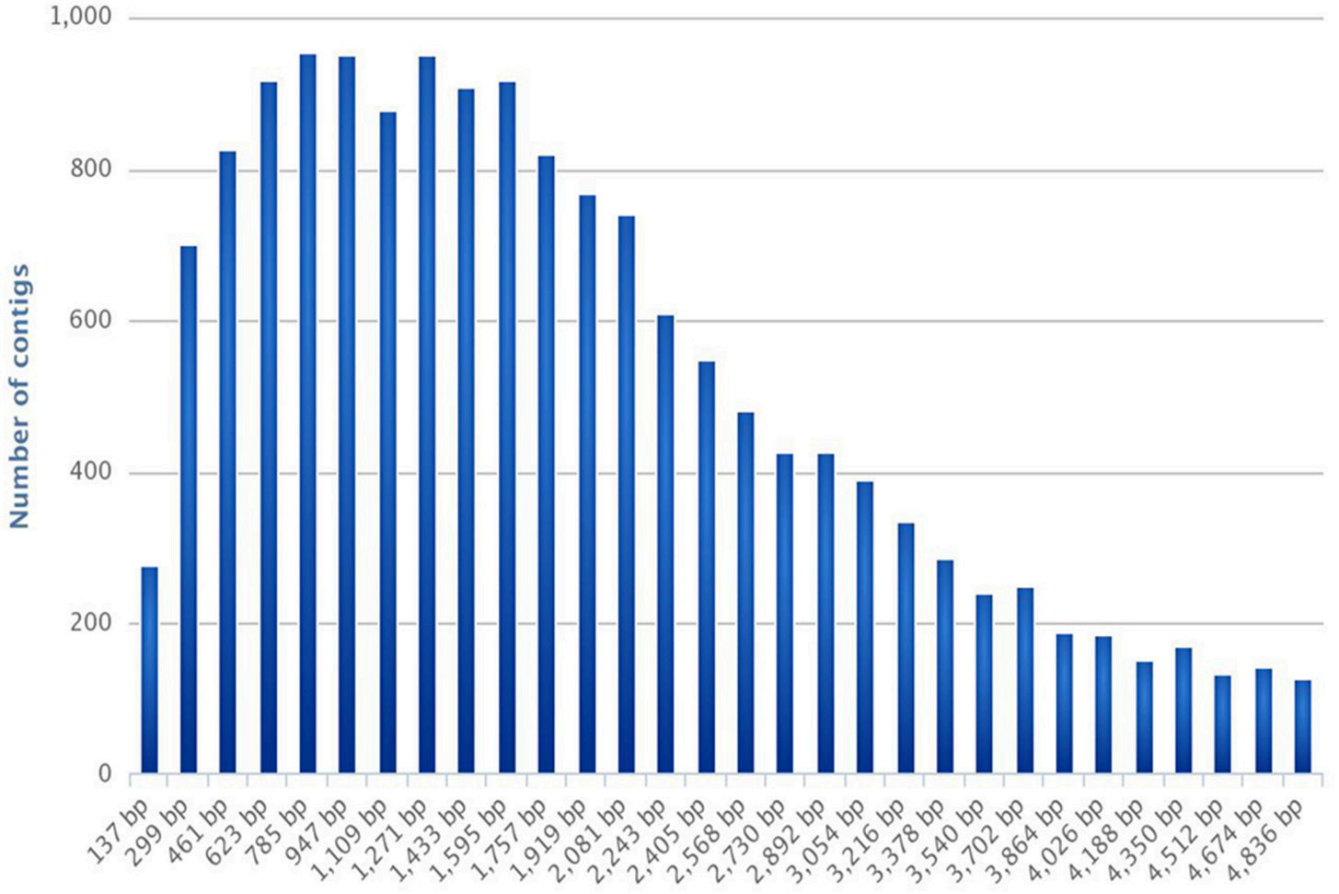
**Frequencies of contig length distribution**. The histogram represents the number of contigs per contig length (expressed in bp).

This yielded a total of 16,936 contigs ranging from 137 to 4836 bp (average length: 2302 bp), with an N50 at 3036 and a N90 at 1215 (Table 1, Figure 1). The assembled transcriptome size is 38,986,687 bp.

**Table 1:** **Measurement of contigs from *de novo* assembly**.

**Sample ID**	**N75**	**N50**	**N25**	**Mini**	**Maxi**	**Ave**	**Count**	**Total (bp)**
Assembly from 12 combined libraries	2910	1796.5	1042	137	4836	2302	16,936	38,986,687

### Functional annotation and classification of assembled contigs

**Figure 2:**
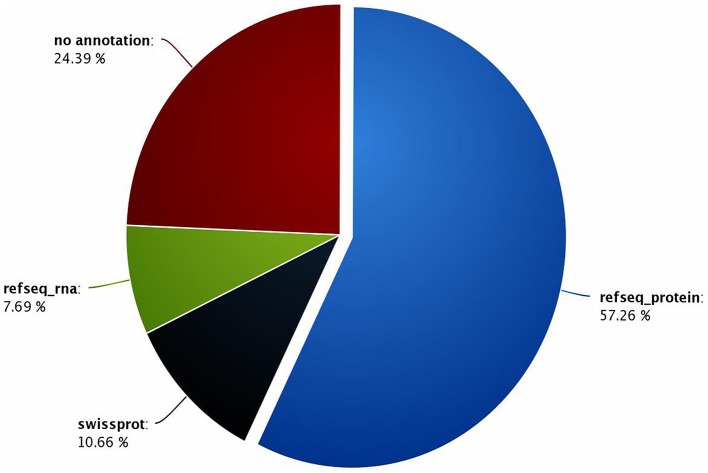
**Percentage of contigs annotated with reference to the Refseq-proteins, Refseq-rna, and Swiss-Prot databases**.

A total of 12,806 contigs could be annotated (out of 16,936) from which 9698 were annotated with reference to the sequences recorded in the Refseq-proteins database. Subsequently, 1303 and 1805 contigs were annotated with reference to the sequences recorded in the Refseq-rna and Swiss-Prot databases, respectively. In the end, 24.39% of the contigs could not be annotated. Nevertheless, these orphan sequences may be of great interest, as they could refer to putative *G. p. gambiensis* and *T. b. gambiense* specific biological functions (Figure 2), and therefore specific genes.

**Figure 3:**
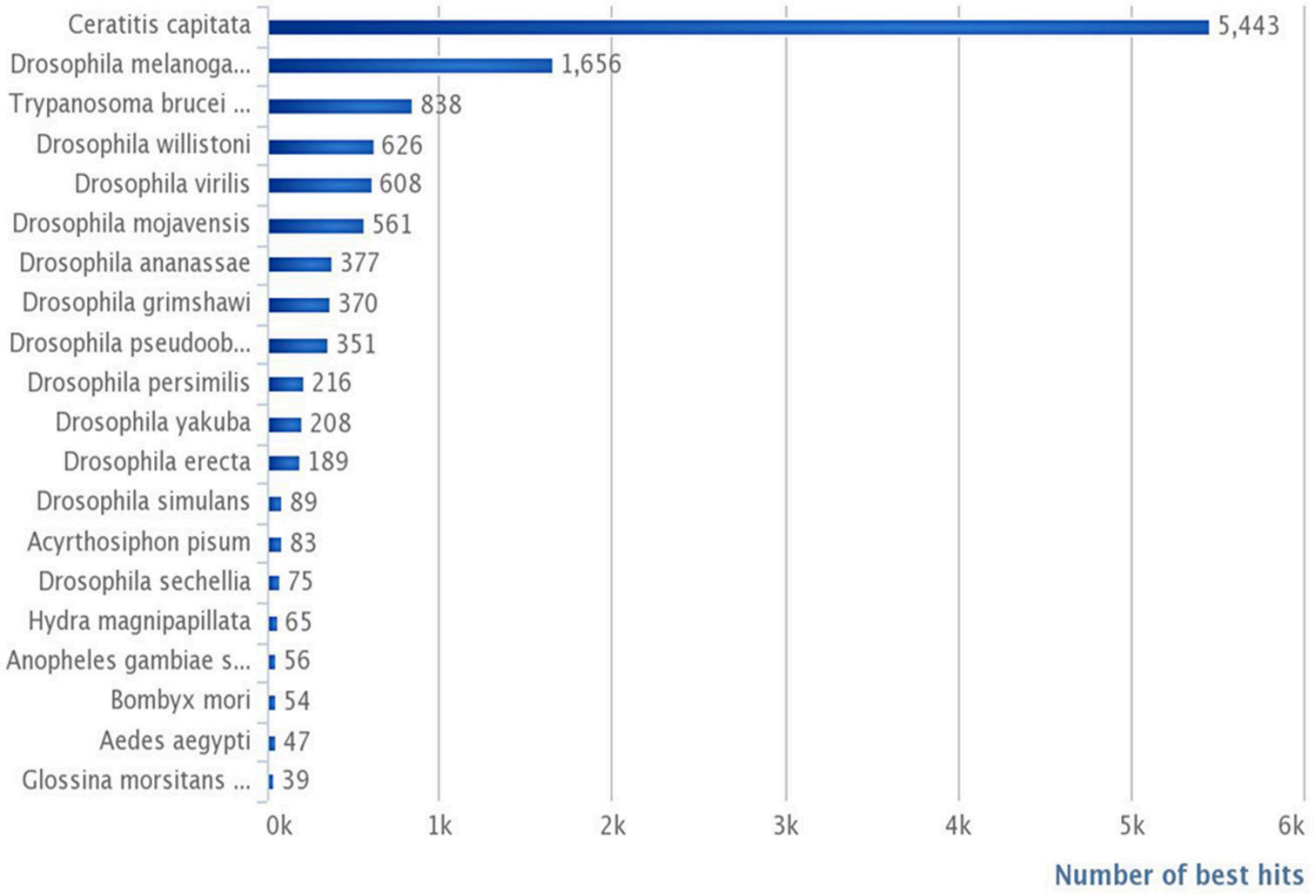
**Number of best hits per species, in reference to the total number of contigs**.

Our annotated dataset including 16,936 contigs (Supplementary Table S1) is most likely representative of the *G. p. gambiensis* gene catalog. In terms of the total number of hits, BLASTX hits and top hits were mostly identified with *Ceratitis capitata* (5443 hits), *Drosophila melanogaster* (1656 hits), *Trypanosoma brucei* (838 hits), *Drosophila willistoni* (626 hits), *Drosophila virilis* (608 hits), and *Drosophila mojavensis* (561 hits). Less than 24% of the *G. p. gambiensis* annotated consensus transcriptome had orthologous hits in 14 other species, including several *Drosophila* species*, Acyrthosiphon pisum, Hydra magnipapillata, Anopheles* sp., *Bombyx* sp., *Aedes* sp., and *Glossina morsitans* (Figure 3).

**Figure 4:**
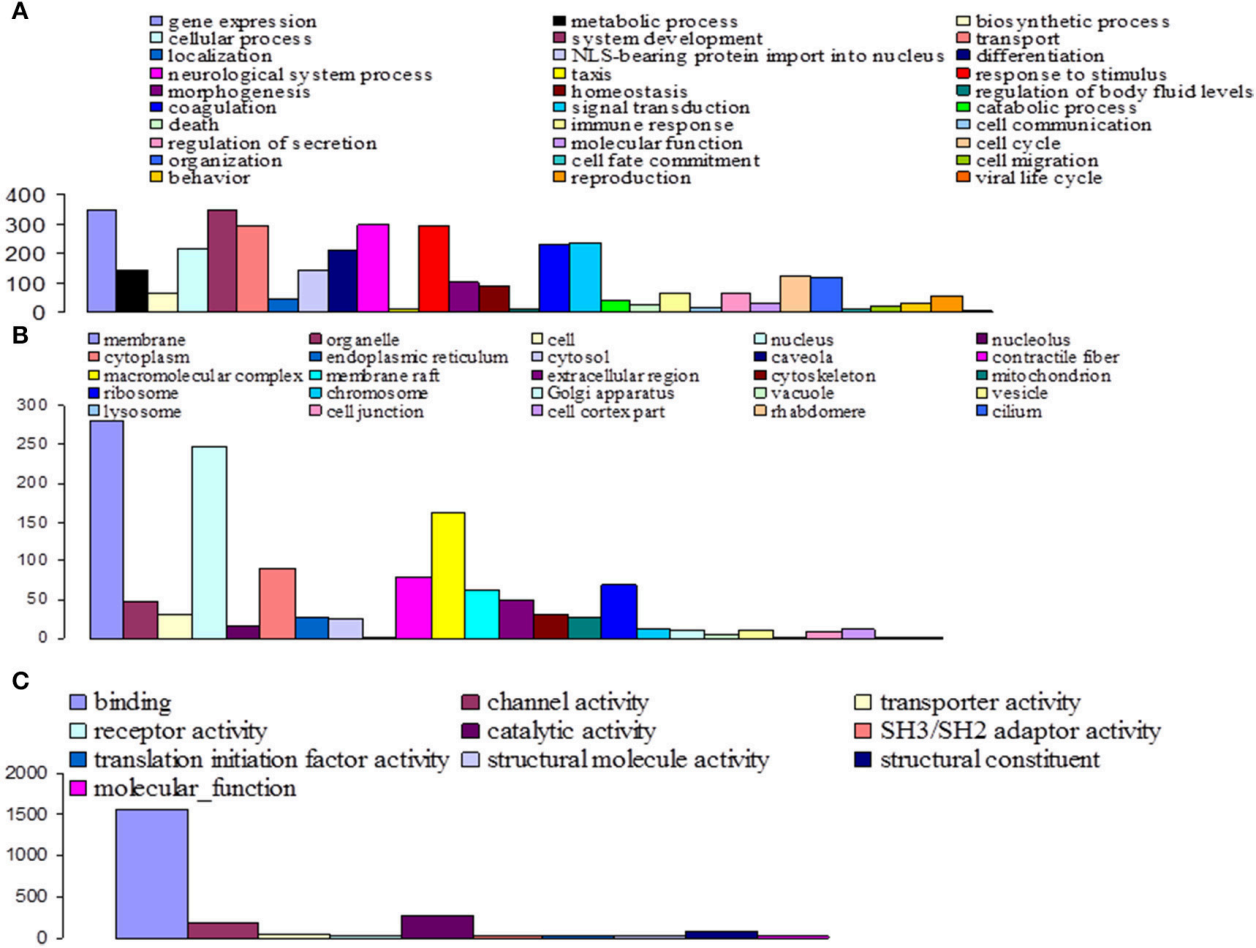
**Functional classification of *G. p. gambiensis* and *T. b. gambiense* assembled contigs based on Gene Ontology (GO) categorization**. The results are presented in three main GO categories: **(A)** Biological process, **(B)** cellular component, and **(C)** molecular function. The x-axis indicates the subcategories and the y-axis indicates the contig numbers assigned to a given GO terms.

Among the 16,936 contigs, 7207 could be assigned to three main GO: “biological process” (3702 contigs) was the predominant domain, followed by the “molecular function” (2191 contigs) and “cellular component” (1314 contigs) domains (Figure 4). GO annotation assignments classified contigs into 30 subcategories within the biological process domain, 10 within the molecular function domain, and 25 within the cellular component domain (Figure 4). The “biological process” domain subcategories that displayed the most highly abundant transcripts include: gene expression (348 transcripts—9.4% of the “biological process” domain transcripts), system development (345 transcripts—9.3%), neurological system process (300 transcripts—8.1%), responses to stimuli (293 transcripts—7.9%), transport (292 transcripts—7.9%), signal transduction (234 transcripts—6.3%), coagulation (230 transcripts—6.2%), cellular process (216 transcripts—5.8%), and differentiation (207 transcripts—5.6%). The “molecular function” domain subcategories that displayed the most highly abundant transcripts include: binding (1559 transcripts—71.2% of the “molecular function” domain transcripts), catalytic activity (260 transcripts—11.9%), and channel activity (191 transcripts—8.7%). Finally, the “cellular component” domain subcategories that displayed the most common groups of proteins include: membrane (281 transcripts—21.4% of the “cellular component” domain transcripts), nucleus (246 transcripts—18.7%), and macromolecular complex (161 transcripts—12.3%) (Figure 4).

### Detection and identification of DEGs in response to tsetse infection by trypanosomes

We compared 12 tsetse fly (*G. p. gambiensis*) transcriptome profiles to better understand their pathosystem at the transcriptome level (S3 vs. NS3; I10 vs. NI10; I20 vs. NI 20 tsetse flies).

**Figure 5:**
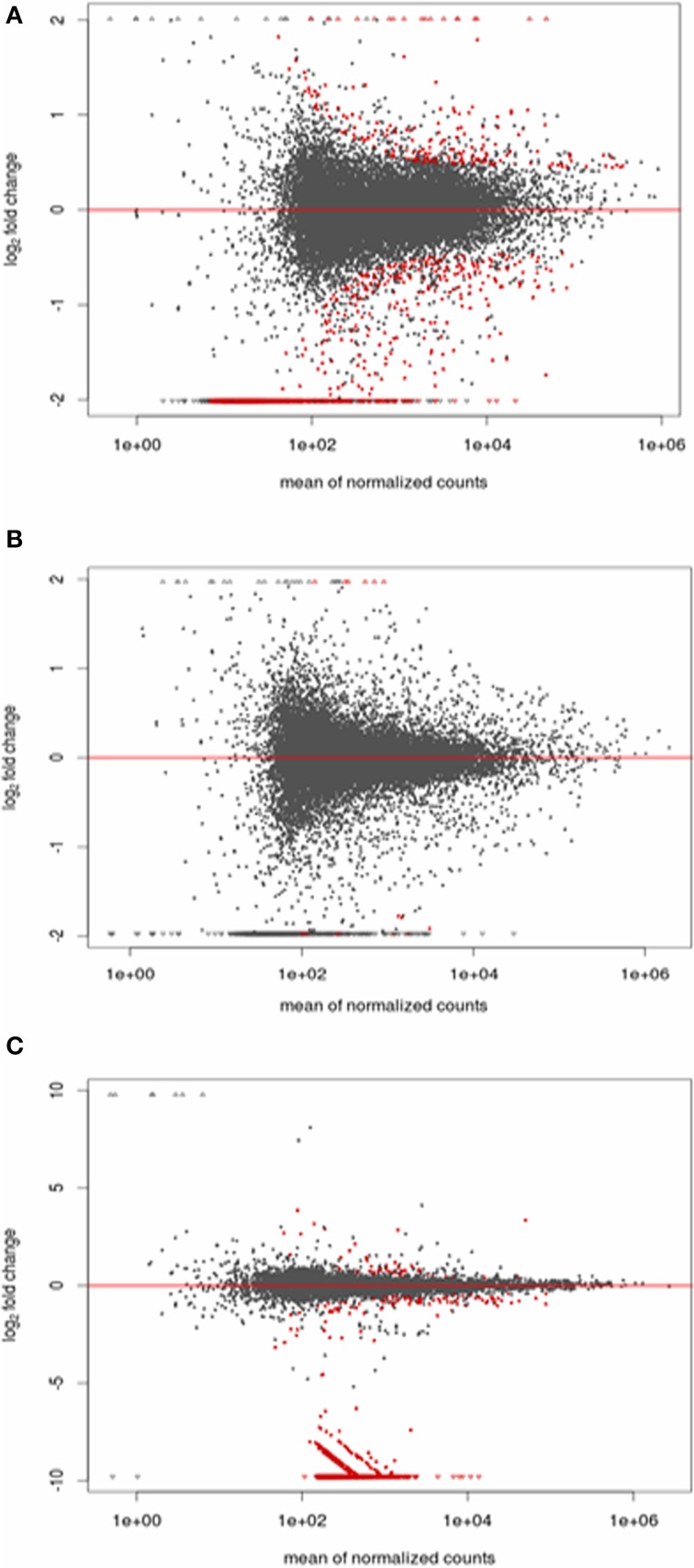
**Plot of the normalized mean vs. the log_2_-fold change for the infected vs. non-infected conditions**. Genes detected as differentially expressed are indicated in red. **(A)** Stimulated vs. non-stimulated 3 days post-bloodmeal; **(B)** infected vs. non-infected 10 days post-infected bloodmeal; **(C)** infected vs. non-infected 20 days post-infected bloodmeal.

**Figure 6:**
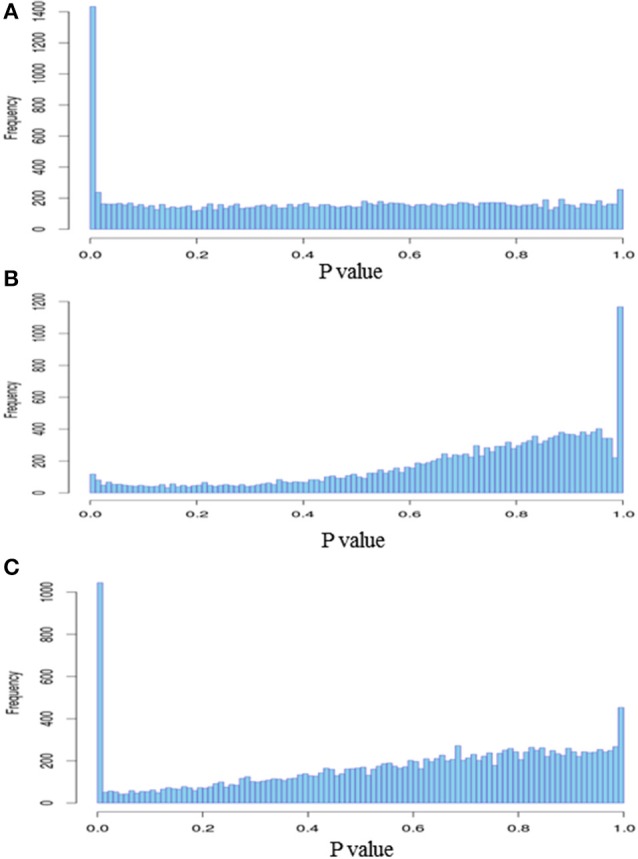
**Histogram of DEGs *p*-values**. **(A)** Stimulated vs. non-stimulated 3 days post-bloodmeal; **(B)** infected vs. non-infected 10 days post-infected bloodmeal; **(C)** infected vs. non-infected 20 days post-infected bloodmeal.

We observed significantly differentially expressed genes (Figures 5, 6; *p* < 0.05) between S3 and NS3 flies (1373 genes), I10 and NI10 flies (52 genes), and I20 and NI20 flies (1025 genes) (Supplementary Tables S2–S4). Among the DEGs identified for 3-day samples, names could be assigned to 797 contigs with reference to the *T. brucei* database, and to 435 contigs with reference to the insect database; 141 contigs remained “hypothetical.” Among the DEGs identified for 10-day samples, names could be assigned to 39 contigs with reference to the insect database, and 13 remained “hypothetical.” Finally, among the DEGs identified for the 20-day samples, names could be assigned to 866 contigs with reference to the *T. brucei* database, and 112 contigs with reference to the insect database; 47 remained “hypothetical.”

When comparing day three sampled flies that ingested a non-infected bloodmeal (NS3 flies) with flies that ingested an infected bloodmeal (S3 flies), 208 transcripts showed an up-regulated expression (Fold Change > 1) in non-stimulated flies, whereas 1165 transcripts were down-regulated (Fold change < 1) (Supplementary Table S2). In self-cured flies sampled 10 days after ingesting an infected bloodmeal (NI10 flies), 19 transcripts were up-regulated and 33 were down-regulated when compared to the corresponding genes of infected flies (I10 flies) (Supplementary Table S3). Finally, in self-cured flies sampled 20 days after ingesting an infected bloodmeal (NI20 flies), 49 contig-derived transcripts were up-regulated and 976 were down-regulated when compared to the corresponding genes of infected (I20) flies (Supplementary Table S4).

**Figure 7:**
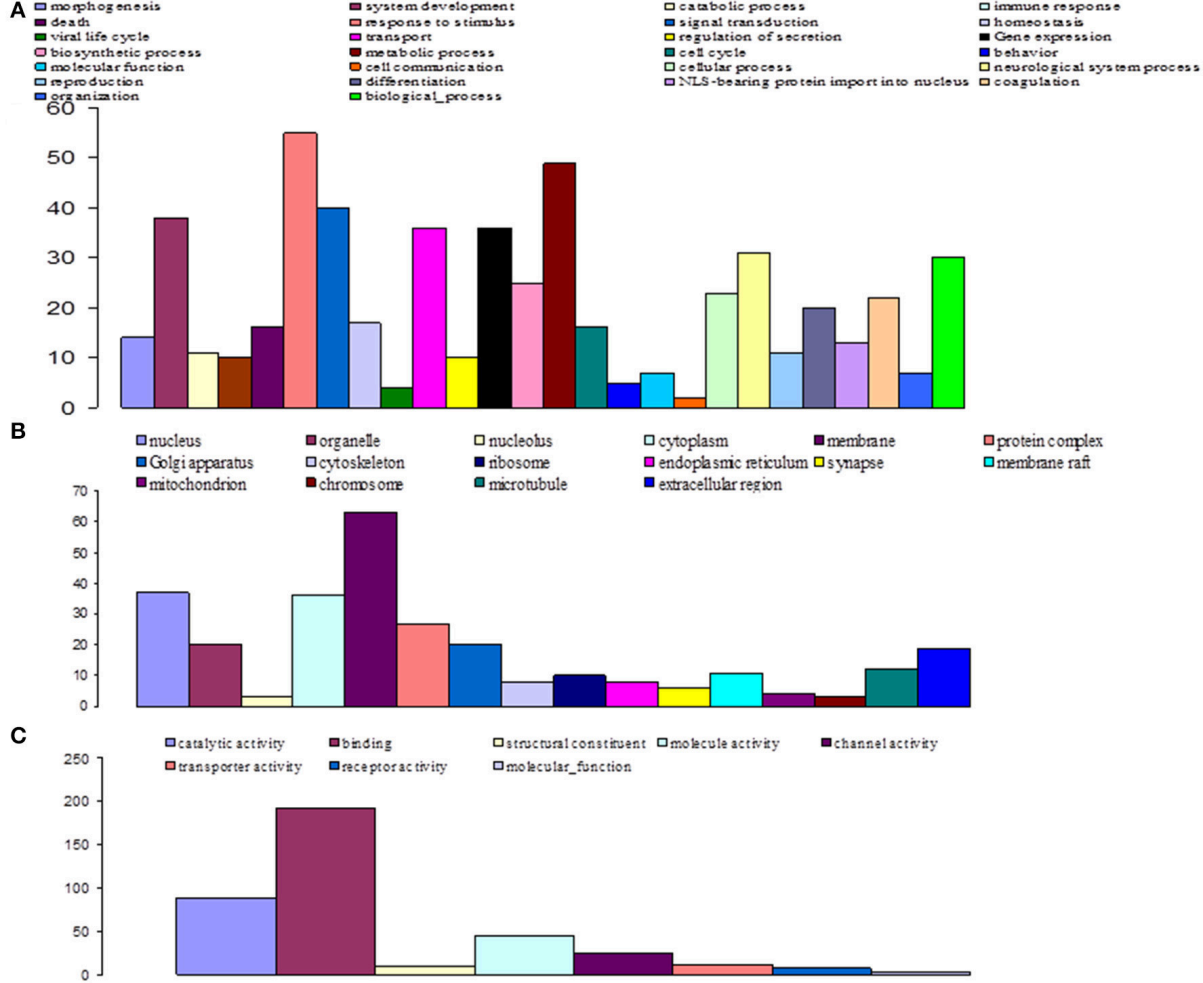
**Distribution of statistically significant contigs for *Glossina palpalis gambiensis*, 3 days post-bloodmeal, based on Gene Ontology (GO) categorization**. The results are presented in three main GO categories: **(A)** biological process, **(B)** cellular component, and **(C)** molecular function. The x-axis indicates the subcategories and the y-axis indicates the number of contigs assigned to a given GO term.

**Figure 8:**
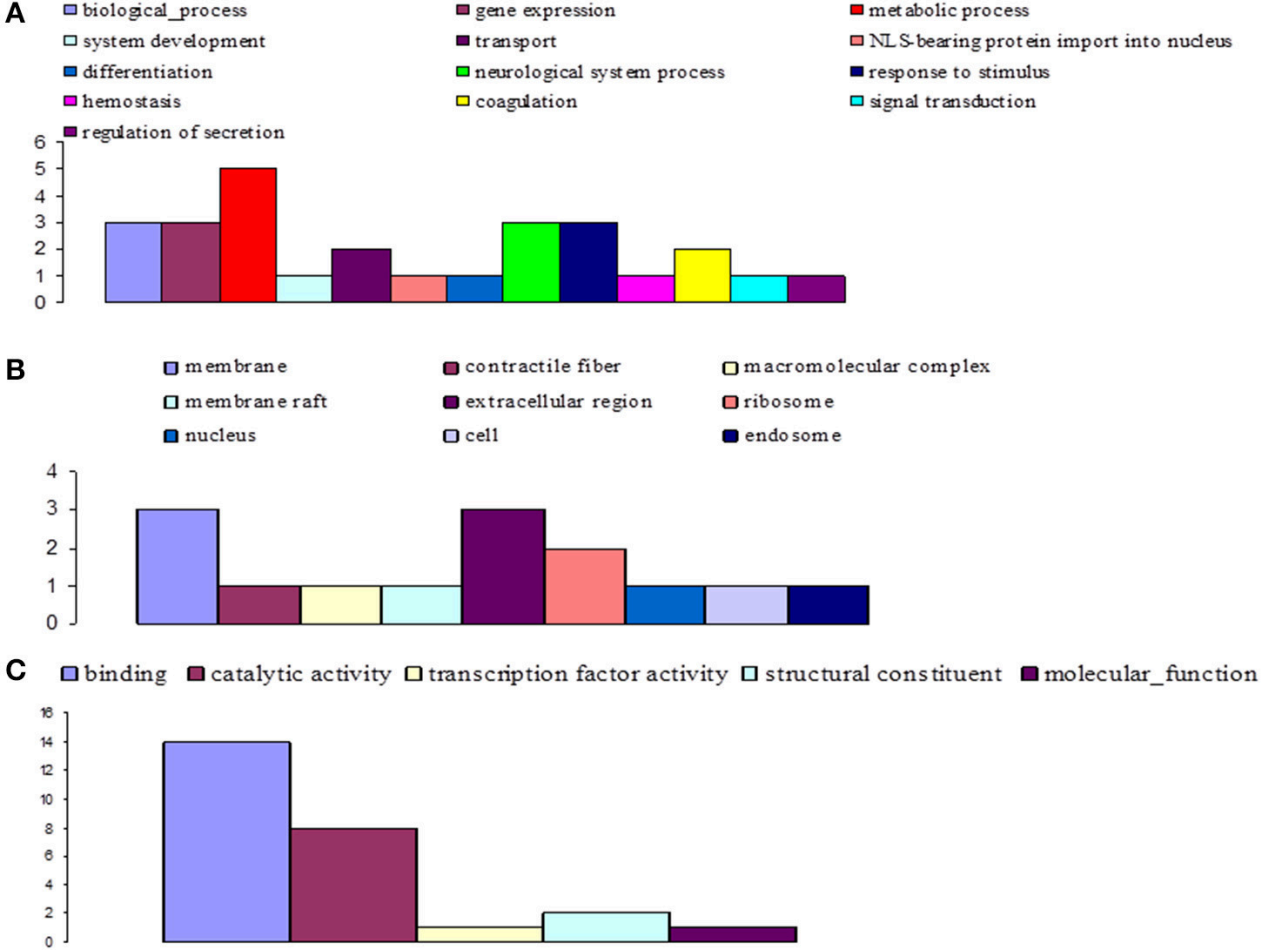
**Distribution of statistically significant contigs for *Glossina palpalis gambiensis*, 10 days post-bloodmeal, based on Gene Ontology (GO) categorization**. The results are presented in three main GO categories: **(A)** biological process, **(B)** cellular component, and **(C)** molecular function. The x-axis indicates the subcategories and the y-axis indicates the number of contigs assigned to a given GO term.

**Figure 9:**
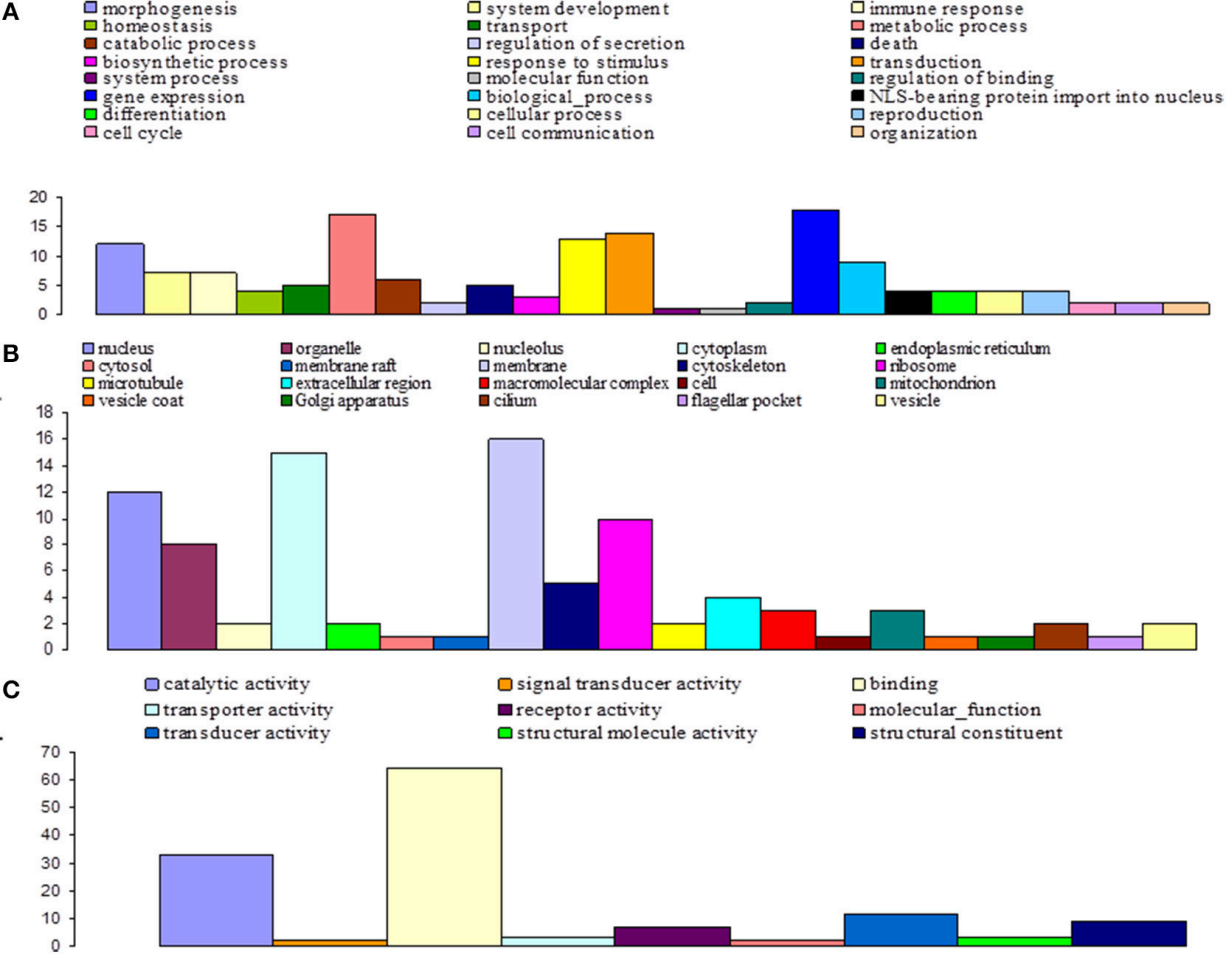
**Distribution of statistically significant contigs for *Glossina palpalis gambiensis*, 20 days post-bloodmeal, based on Gene Ontology (GO) categorization**. The results are presented in three main GO categories: **(A)** biological process, **(B)** cellular component, and **(C)** molecular function. The x-axis indicates the subcategories and the y-axis indicates the number of contigs assigned to a given GO term.

GO-based classification was performed on the characterized DEGs and categories, in order to identify which ones were significantly altered during invasion and infection of tsetse flies by trypanosomes (Figures 7–9). At day 3 sampling, GO analysis classified 151 of the annotated DEGs into 26, 8, and 16 subgroups within the biological process, molecular function, and cellular component categories, respectively (Figure 7). In the biological process category, the subcategories that were most affected by trypanosome stimulation were: metabolic process (8.9%), system development (6.9%), response to stimulus (10%), signal transduction (7.3%), transport (6.6%), and gene expression (6.6%); in addition, 18 identified metabolic pathways were significantly affected by trypanosome stimulation.

At day 10 sampling, GO analysis classified 17 of the annotated DEGs into 13, 5, and 9 subgroups within the biological process, molecular function, and cellular component categories, respectively (Figure 8). In the biological process category, the subcategories most affected by trypanosome infection were: metabolic process (e.g., carbohydrate metabolic process; 18.5%), biological process, gene expression, neurological system process, and response to stimuli (11.1% each). The binding subcategory was the most affected by trypanosome infection (53.8%) within the molecular function category.

Finally, at day 20 post infected blood meal uptake, GO analysis classified 57 of the annotated DEGs into 24, 9, and 20 subgroups within the biological process, molecular function, and cellular component categories, respectively (Figure 9). In the biological process category, the subcategories that were most affected by trypanosome infection were: gene expression (12.1%), metabolic process (11.5%), transduction (9.5%), response to stimulus (8.8%), and morphogenesis (8.1%). In the molecular function category, the most affected subcategories were: binding (47.4%) and catalytic activity (24.4%). Finally, in the cellular component category, the most affected subcategories were: membrane (17.4%), cytoplasm (16.3%), nucleus (13%), and ribosome (10.9%).

### Refined list of DEGs of interest

Some DEGs appear *a priori* to be of greater interest than others, owing either to their level of over-expression or down-expression in S3, I10 and I20 samples vs. NS3, NI10, and NI20 samples, or the protein function that they encode. These particular DEGs selected for days 3, 10, and 20 are presented together in Table 2. As expected, trypanosome genes (noted in the identification column as “GLOS_TB …”) were expressed only in samples from flies that had ingested a trypanosome-infected bloodmeal; similar results are presented for the overall DEGs in Supplementary Table S2 (day 3 samples), Supplementary Table S3 (day 10 samples), and Supplementary Table S4 (day 20 samples). However, we could not detect any evidence for trypanosome gene expression in the I10 samples (see the Discussion section). Some DEGs were observed to be expressed in both S3 and I20 flies; their mean expression levels are compared in Table 3. The set of genes that were previously identified as being mostly trypanosome genes were expressed much higher in I20 vs. S3 samples (with the exception of two genes). Finally, the set of genes reported in Table 2 was sorted on the basis of the function (mostly catalytic activity) of the proteins they encode. Interestingly, genes encoding proteases were predominant, whether they belong to the tsetse or trypanosome genome.

**Table 2:** **DEGs distribution in stimulated vs. non-stimulated (S3 vs. NS3) flies, and in infected vs. non-infected (refractory) flies either 10 days (I10 vs. NI10) or 20 days (I20 vs. NI20) post-bloodmeal**.

**Identification**	**Base mean A**	**Base mean B**	**Fold change**	**Best hit description**
	**(S) or (I)**	**(NS) or (NI)**	**NS/S or NI/I**	
**PROTEASES AND PROTEASE INHIBITORS**				
GLOS_TB10.61.1870.1.1	552.2672	0.0000	0.000	Aminopeptidase—partial mRNA—Tbb strain 927/4
GLOS_TB10.61.1870.1.1	28.0979	0.0000	0.000	Aminopeptidase—partial mRNA—Tbb strain 927/4
GLOS_TB11.02.0070.1.1	341.6449	0.0000	0.000	Aminopeptidase—partial mRNA—Tbb strain 927/4
GLOS_TB11.02.1070.1.1	64.4924	0.0000	0.000	Aminopeptidase—partial Tbb strain 927/4
GLOS_TB927.3.4750.1.1	789.7420	0.0000	0.000	Aminopeptidase—Tbb strain 927/4
GLOS_TB927.6.1520.1.1	1555.5389	2.2036	0.001	Aquaporin 3 partial mRNA—Tbb strain 927/4
GLOS_TB927.6.1520.1.1	67.2871	0.0000	0.000	Aquaporin 3 partial mRNA—Tbb strain 927/4
GLOS_TB927.3.3410.1.1	337.3614	0.0000	0.000	Aspartyl aminopeptidase—Tbb strain 927/4
GLOS_TB927.3.3410.1.1	29.1621	0.0000	0.000	Aspartyl aminopeptidase—Tbb strain 927/4
GLOS_TB11.47.0036.1.1	440.7008	0.0000	0.000	Calpain, partial mRNA—Tbb strain 927/4
GLOS_TB11.47.0036.1.1	14.0309	0.0000	0.000	Calpain, partial mRNA—Tbb strain 927/4
GLOS_TB927.7.4070.1.1	951.2004	0.0000	0.000	Calpain-like cysteine peptidase—Tbb strain 927/4
GLOS_TB927.7.4070.1.1	50.4615	0.0000	0.000	Calpain-like cysteine peptidase—Tbb strain 927/4
GLOS_TB927.1.2100.1.1	976.7119	1.1018	0.001	Calpain-like cysteine peptidase. cysteine peptidase – Tbb
GLOS_TB927.1.2230.1.1	582.3075	0.0000	0.000	Calpain-like protein fragment—partial mRNA—Tbb
GLOS_LOC101462601.1.5	8394.7979	4561.9074	0.543	Chymotrypsin-1-like [*Ceratitis capitata*]
GLOS_TB927.6.1020.1.1	261.7561	0.0000	0.000	Cysteine peptidase precursor partial mRNA—Tbb strain 927/4
GLOS_TB927.8.3060.1.1	467.3909	0.0000	0.000	Cytosolic leucyl aminopeptidase—partial mRNA—Tbb
GLOS_TB10.389.1480.1.1	801.1016	0.0000	0.000	Cytosolic nonspecific dipeptidase—partial mRNA—Tbb
GLOS_GPL.11.22	28498.3972	40606.4756	1.425	Lectizyme Glossina austeni (lectin and protease activity)
GLOS_GPL.16.22	88429.6303	127653.4331	1.444	Lectizyme Glossina fuscipes fuscipes (lectin and protease activity)
GLOS_LOC101461571.2.2	2911.7794	1267.1640	0.435	Lysosomal aspartic protease-like [*Ceratitis capitata*]
GLOS_TB927.8.1620.1.1	1486.2183	0.0000	0.000	Major surface protease gp63—Tbb strain 927/4
GLOS_TB09.211.4760.1.1	42.3819	0.0000	0.000	Metacaspase 5 partial mRNA (protease arg-lys specific)—Tbb
GLOS_TB11.01.6360.1.1	327.1803	0.0000	0.000	Metalloprotease—partial mRNA—Tbb strain 927/4
GLOS_TB11.01.6360.1.1	34.1576	0.0000	0.000	Metalloprotease—partial mRNA—Tbb strain 927/4
GLOS_TB11.52.0003.1.1	587.8647	0.0000	0.000	Oligopeptidase b—Tbb strain 927/4
GLOS_TB11.52.0003.1.1	14.1393	0.0000	0.000	Oligopeptidase b—Tbb strain 927/4
GLOS_MTRNR21.10.1.3	875.3765	451.0249	0.515	Pan troglodytes MTRNR2-like 10, mRNA
GLOS_LOC101456159.4.4	10357.1870	14556.9484	1.405	Retinoid-inducible serine carboxypeptidase-like [*C. capitata*]
GLOS_TB10.70.7100.1.1	1243.2538	0.9682	0.001	Serine carboxypeptidase III precursor—partial mRNA—Tbb
GLOS_TB10.70.7100.1.1	29.2706	0.0000	0.000	Serine carboxypeptidase III precursor—partial mRNA—Tbb
GLOS_LOC101457953.1.5	467.5594	28.6144	0.061	Serine protease easter-like [*Ceratitis capitata*]
GLOS_LOC101457953.2.5	1048.7914	149.3232	0.142	Serine protease easter-like [*Ceratitis capitata*]
GLOS_LOC101457953.3.5	257.1584	104.7158	0.407	Serine protease easter-like [*Ceratitis capitata*]
GLOS_LOC101457953.5.5	535.7352	31.6210	0.059	Serine protease easter-like [*Ceratitis capitata*]
GLOS_LOC101455604.4.10	4147.7876	8446.3133	2.036	Serine protease SP24D-like [*Ceratitis capitata*]
GLOS_LOC101455604.7.10	7256.5753	4107.4021	0.566	Serine protease SP24D-like [*Ceratitis capitata*]
GLOS_TB10.6K15.3800.1.1	675.8737	0.0000	0.000	Dipeptidyl-peptidase 8-like serine peptidase—Tbb strain 927/4
GLOS_TB927.7.190.1.1	471.9197	0.0000	0.000	Thimet oligopeptidase A—Tbb strain 927/4
GLOS_TB927.7.190.1.1	20.0182	0.0000	0.000	Thimet oligopeptidase A—Tbb strain 927/4
GLOS_LOC101450759.15.17	101392.5605	76275.2604	0.752	Transmembrane protease serine 9-like [*Ceratitis capitata*]
GLOS_TRYDG.4.5	205.3854	100.1940	0.488	Trypsin delta/gamma [*Drosophila melanogaster*]
GLOS_TRYDG.5.5	25628.5380	15491.6912	0.604	Trypsin delta/gamma [*Drosophila erecta*]
GLOS_LOC101463325.1.1	70961.0232	106614.8609	1.502	Trypsin-like [*Ceratitis capitata*]
GLOS_LOC101460475.2.2	622.8445	282.1185	0.453	Venom carboxylesterase-6-like [*Ceratitis capitata*]
GLOS_CBPA1.4.11	81.9328	203.9497	2.489	Zinc carboxypeptidase A 1 [*Drosophila p. pseudoobscura*]
GLOS_CBPA1.11.11	12571.5017	25447.6241	2.024	Zinc carboxypeptidase A [*Drosophila p. pseudoobscura*]
GLOS_LOC101459622.1.22	1301.8232	2242.4272	1.723	zinc metalloproteinase nas-4-like [*Ceratitis capitata*]
GLOS_TB927.8.6450.1.1	439.0594	0.0000	0.000	Inhibitor of cysteine peptidase—Tbb strain 927/4
GLOS_LOC101459846.1.2	2433.4913	1664.4399	0.684	Alaserpin-like (Serin protease inhibitor) [*Ceratitis capitata*]
GLOS_LOC101459846.2.2	1420.6193	838.5583	0.590	Alaserpin-like (Serin protease inhibitor) [*Ceratitis capitata*]
**OXYDASES AND DEHYDROGENASES**				
GLOS_LOC101457181.1.5	73109.9421	21931.6983	0.300	Laccase-2-like [*Ceratitis capitata*]
GLOS_LOC101457181.2.5	17482.0138	5829.7342	0.333	Laccase-2-like [*Ceratitis capitata*]
GLOS_LOC101457181.3.5	1802.2092	1078.3896	0.598	Laccase-2-like [*Ceratitis capitata*]
GLOS_LOC101457181.3.5	4844.5886	1509.7455	0.312	Laccase-2-like [*Ceratitis capitata*]
GLOS_LOC101457181.4.5	25283.1929	8470.9491	0.335	Laccase-2-like [*Ceratitis capitata*]
GLOS_LOC101457181.5.5	11790.1166	7254.7118	0.615	Laccase-2-like [*Ceratitis capitata*]
GLOS_LOC101457181.5.5	34357.2827	8198.3799	0.239	Laccase-2-like [*Ceratitis capitata*]
GLOS_TB927.7.210.1.1	411.4912	0.0000	0.000	Proline oxidase—Tbb strain 927/4
GLOS_TB10.70.4280.1.1	841.4256	0.0000	0.000	Delta-1-pyrroline-5-carboxylate dehydrogenase [*T. brucei*]
GLOS_TB10.70.4280.1.1	25.2307	0.0000	0.000	Delta-1-pyrroline-5-carboxylate dehydrogenase [*T. brucei*]
GLOS_TB11.02.1990.1.1	319.6615	0.0000	0.000	Ferric reductase—Tbb strain 927/4
GLOS_ND1.2.4	9129.9740	5081.8960	0.557	NADH dehydrogenase subunit 1 (mit.) [*C. megacephala*]
GLOS_ND3.1.1	1793.2711	869.6530	0.485	NADH dehydrogenase subunit 3 (mitochondrion) [*P. utilis*]
GLOS_ND4.5.6	12286.9040	7978.0201	0.649	NADH dehydrogenase subunit 4 (mitochondrion) [*C. bezziana*]
**LECTINS**				
GLOS_TB11.02.1680.1.1	456.9371	0.0000	0.000	Lectin—partial mRNA—Tbb strain 927/4
GLOS_TB11.02.1680.1.1	29.1621	0.0000	0.000	Lectin—partial mRNA—Tbb strain 927/4
GLOS_LECA.10.13	3842.8084	1551.5134	0.404	Lectin subunit alpha [*Sarcophaga peregrina*]
GLOS_LECA.3.13	8003.5218	3988.4579	0.498	Lectin subunit alpha [*Sarcophaga peregrina*]
GLOS_LECA.4.13	1427.7877	656.6802	0.460	Lectin subunit alpha [*Sarcophaga peregrina*]
GLOS_LECA.5.13	2385.9247	1113.9812	0.467	Lectin subunit alpha [*Sarcophaga peregrina*]
GLOS_LECA.6.13	1794.0384	740.5790	0.413	Lectin subunit alpha [*Sarcophaga peregrina*]
GLOS_LECA.9.13	3360.6422	1983.1933	0.590	Lectin subunit alpha [*Sarcophaga peregrina*]
GLOS_GPL.11.22	28498.3972	40606.4756	1.425	Lectizyme [*Glossina austeni*] (lectin and protease activity)
GLOS_GPL.16.22	88429.6303	127653.4331	1.444	Lectizyme [*G. fuscipes fuscipes*] (lectin and protease activity)
**HYDROLASES**				
GLOS_LOC101452734.1.1	299.0185	126.9519	0.425	Alpha-N-acetylgalactosaminidase-like [*Ceratitis capitata*]
GLOS_LOC101462140.1.1	5318.9872	2975.0751	0.559	Chitinase 3-like [*Ceratitis capitata*]
GLOS_FDL.1.2	34480.1169	48644.5776	1.411	Probable beta-hexosaminidase [*D. melanogaster*]
GLOS_TB927.7.6850.1.1	1626.9116	0.0000	0.000	Trans-sialidase partial mRNA—Tbb strain 927/4
GLOS_TB927.7.6850.1.1	102.4366	0.0000	0.000	Trans-sialidase partial mRNA—Tbb strain 927/4
**INHIBITORS**				
GLOS_LOC101459846.1.2	2433.4913	1664.4399	0.684	Alaserpin-like (Serine protease inhibitor) [*C. capitata*]
GLOS_LOC101459846.2.2	1420.6193	838.5583	0.590	Alaserpin-like (Serine protease inhibitor) [*C. capitata*]
GLOS_TTI.10.16	988.9930	513.9447	0.520	Thrombin inhibitor (tsetse) [*G. morsitans morsitans*]
GLOS_TTI.3.16	55.7102	131.7672	2.365	Thrombin inhibitor (tsetse) [*G. morsitans morsitans*]
GLOS_TTI.4.16	9152.8285	3778.1122	0.413	Thrombin inhibitor (tsetse) [*G. morsitans morsitans*]
GLOS_TTI.1.16	1304.6401	185.2590	0.142	Thrombin inhibitor (tsetse) [*G. morsitans morsitans*]
**OTHER FUNCTIONS**				
GLOS_LOC101454505.3.12	8857.1384	5131.7479	0.579	Acyl-CoA-binding protein homolog isoform X1 [*Ceratitis capitata*]
GLOS_LOC101461142.7.9	6823.2923	10238.5963	1.501	Acyl-CoA Delta(11) desaturase-like isoform X3 [*Ceratitis capitata*]
GLOS_TB927.8.7410.1.1	49.3250	0.0000	0.000	Calreticulin, putative (signaling)—partial mRNA—Tbb strain 927/4
GLOS_TB927.8.7410.1.1	1024.9149	1.1018	0.001	Calreticulin, putative (signaling)—partial mRNA—Tbb strain 927/4
GLOS_CEC.2.2	761.3134	160.3734	0.211	Cecropin G.m.m. (antimicrobial peptide)
GLOS_CECC.1.1	679.9491	104.6283	0.154	Cecropin-C D. yakuba (antimicrobial peptide)
GLOS_CC2H.1.1	321.8132	0.0000	0.000	Cell division control protein 2 homolog—Tbb
GLOS_TB10.26.0510.1.1	860.7592	0.0000	0.000	CYC2-like cyclin—partial mRNA—Tbb strain 927/4
GLOS_HYPB.1.2	4304.3151	8966.5053	2.083	Hypodermin—[*Hypoderma lineatum*]
GLOS_GST.1.1	1624.8670	583.4978	0.359	Glutathione S-transferase [*Musca domestica*]
GLOS_TB927.7.3980.1.1	380.9405	1.1018	0.003	Immunodominant antigen—partial mRNA—Tbb
GLOS_TB09.211.4513.1.1	49.1803	0.0000	0.000	Kinetoplastid membrane protein KMP-11—Tbb strain 927/4
GLOS_TB927.3.3580.1.1	1112.2710	1.1018	0.001	Lipophosphoglycan biosynthetic protein—partial mRNA—Tbb
GLOS_TB927.3.3580.1.1	43.0844	0.0000	0.000	Lipophosphoglycan biosynthetic protein—partial mRNA—Tbb
GLOS_LOC101449088.1.1	3335.7392	6030.1811	1.808	LysM and peptidoglycan-binding domain-containing protein 1-like isoform X1
GLOS_LOC101460827.1.2	2004.2841	643.2004	0.321	Mucin-5AC-like (glycosylated protein)[*Ceratitis capitata*]
GLOS_TB927.8.2160.1.1	24.3474	0.0000	0.000	Multidrug resistance protein A—partial mRNA—Tbb strain 927/4
GLOS_TB11.03.0140.1.1	448.9753	0.0000	0.000	Nucleoporin—partial mRNA—Tbb strain 927/4
GLOS_PMP3.2.2	20992.2730	13567.5959	0.646	Peritrophic matrix protein 3 precursor [*Tribolium castaneum*]
GLOS_LOC101453268.1.1	733.2798	1317.6387	1.797	Period circadian protein-like [*Ceratitis capitata*]
GLOS_LOC101456033.1.1	1049.0807	701.7895	0.669	Platelet binding protein GspB-like [*Ceratitis capitata*]
GLOS_LOC101456285.1.1	32755.6635	60800.4454	1.856	Protein FAM188A homolog [*Ceratitis capitata*]
GLOS_LTV1.1.1	1777.9362	2547.2329	1.433	Protein LTV1 homolog—[*Drosophila melanogaster*]
GLOS_PMAR_PMAR029216.1.2	1356.3842	2415.7386	1.781	Protein conserved hypothetical[*Perkinsus marinus* ATCC 50983]
GLOS_LOC101461327.1.1	631.4029	1439.7920	2.280	Protein Uncharacterized LOC101461327 [*C. capitata*]
GLOS_LOC101450586.2.9	7835.2507	4885.1378	0.623	Protein uncharacterized—LOC101450586 [*Ceratitis capitata*]
GLOS_LOC101462034.1.2	1260.9956	595.6506	0.472	Protein uncharacterized—LOC101462034 [*Ceratitis capitata*]
GLOS_TB11.01.5310.1.1	638.3697	0.9682	0.002	Receptor-type adenylate cyclase—partial mRNA—Tbb
GLOS_RS25.5.10	44.5773	300.9004	6.750	Ribosomal protein (40S) [*Drosophila melanogaster*]
GLOS_RL31.2.3	5894.0883	3872.9890	0.657	Ribosomal protein (60S) [*Drosophila melanogaster*]
GLOS_LOC101450592.2.2	381.2922	201.2523	0.528	Sialin-like (transporter) [*Ceratitis capitata*]
GLOS_LOC101461145.1.1	623.4847	291.2207	0.467	Stress response protein NST1-like [*Ceratitis capitata*]
GLOS_TB927.5.2940.1.1	455.0980	0.0000	0.000	Stress induced protein sti1 partial mRNA—Tbb strain 927/4
GLOS_TRF.1.1	5009.4602	116.1075	0.023	Transferrin—[*Sarcophaga peregrina*]
GLOS_TB927.8.6750.1.1	483.9673	0.0000	0.000	Translationally controlled tumor protein (TCTP)—Tbb strain 927/4
GLOS_TB927.8.6750.1.1	35.2218	0.0000	0.000	Translationally controlled tumor protein (TCTP)—Tbb strain 927/4
GLOS_MLC_9020.1.1	318.8201	1124.6933	3.528	Transmembrane protein [*Mycoplasma mycoides* subsp. Capri]
GLOS_TVAG_198570.1.2	1670.6333	838.7765	0.502	Viral A-type inclusion protein [*Trichomonas vaginalis* G3]

*DEGs are sorted within each section with reference to the proteins that they encode (listed alphabetically). The gene sets are a part of those presented in Supplementary Tables S2 (day 3 samples), S3 (day 10 samples), and S4 (day 20 samples)*.

*Fonts colors indicate genes differentially expressed between tsetse flies sampled at day 3, day 10, and day 20 post-fly feeding*.

*The text “GLOS_TB …  ” refers to Trypanosoma genes; all others are Glossina genes*.

*For day three samples, S, refers to flies that ingested a trypanosome-infected bloodmeal; NS, refers to flies that ingested a non-infected blood meal*.

“*I10” and “NI10” are 10 day samples; “I20” and “NI20” are 20 day samples. I, susceptible flies, which received an infected bloodmeal and became infected. NS, refractory flies that were self-cured following an infected bloodmeal*.

**Table 3:** **Comparison of gene expression levels differentially expressed in both stimulated (S3) and infected (I20) flies**.

**Identification**	**Base mean S3**	**Base mean I20**	**I20/S3**	**Best hit description**
GLOS_TB10.61.1870.1.1	28.0979	552.2672	19.655	Aminopeptidase—partial mRNA—Tbb strain 927/4
GLOS_TB927.6.1520.1.1	67.2871	1555.5389	23.118	Aquaporin three partial mRNA—Tbb strain 927/4
GLOS_TB927.3.3410.1.1	29.1621	337.3614	11.568	Aspartyl aminopeptidase—Tbb strain 927/4
GLOS_TB11.47.0036.1.1	14.0309	440.7008	31.409	Calpain, partial mRNA—Tbb strain 927/4
GLOS_TB927.7.4070.1.1	50.4615	951.2004	18.850	Calpain-like cysteine peptidase—Tbb strain 927/4
GLOS_TB11.01.6360.1.1	34.1576	327.1803	9.579	Metalloprotease—partial mRNA—Tbb strain 927/4
GLOS_TB11.52.0003.1.1	14.1393	587.8647	41.577	Oligopeptidase b—Tbb strain 927/4
GLOS_TB10.70.7100.1.1	29.2706	1243.2538	42.475	Serine carboxypeptidase III precursor—partial mRNA –Tbb
GLOS_TB927.7.190.1.1	20.0182	471.9197	23.574	Thimet oligopeptidase A—Tbb strain 927/4
GLOS_LOC101457181.3.5	4844.5886	1802.2092	0.372	Laccase-2-like [*Ceratitis capitata*]
GLOS_LOC101457181.5.5	34357.2827	11790.1166	0.343	Laccase-2-like [*Ceratitis capitata*]
GLOS_TB10.70.4280.1.1	25.2307	841.4256	33.349	Delta-1-pyrroline-5-carboxylate dehydrogenase [*T. brucei*]
GLOS_TB11.02.1680.1.1	29.1621	456.9371	15.669	Lectin—partial mRNA—Tbb strain 927/4
GLOS_TB927.7.6850.1.1	102.4366	1626.9119	15.882	Trans-sialidase partial mRNA—Tbb strain 927/4
GLOS_TB927.3.3580.1.1	43.0844	1112.2710	25.816	Lipophosphoglycan biosynthetic protein, putative—mRNA—Tbb
GLOS_TB927.8.6750.1.1	35.2218	483.9673	13.741	Translationally controlled tumor protein—Tbb strain 927/4
GLOS_TB927.8.7410.1.1	49.3250	1024.9149	20.779	Tbb strain 927/4 calreticulin, putative—partial mRNA

*This list of genes is extracted from the genes presented in Table 2*.

*I20/S3 indicates the fold change in gene expression levels in I20 vs. S3 samples*.

### SNP detection

In our study, SNPs were identified after realigning the reads on the 16,936 contigs. After applying filters, the analysis performed on all 16,936 contigs resulted in the identification of 195,464 high confidence SNPs from 14,929 contigs (average = 11 SNPs per contig). Detected polymorphisms were more due to transition (178,328) than to insertion (11,269) and deletion (5867) processes.

SNPs were also revealed in the DEGs from 3-, 10-, and 20-day tsetse fly samples (Supplementary Tables S5–S7, respectively). SNPs could be assigned to 625, 17, and 480 annotated contigs from the 3-, 10-, and 20-day tsetse samples, respectively. Three hundred and ninety nine annotated contigs showing SNPs were identified both in 3 and 20-days tsetse samples.

DEGs from 3-day samples in which SNPs were identified encode such proteins as proteases, antimicrobial peptides, glucose metabolism enzymes, nucleotide metabolism enzymes, proteins involved in transcription process, chitinases, and aquaporins (Supplementary Table S5). DEGs from 10-day samples displaying SNPs were found to encode glucose metabolism enzymes, lectizyme, glutathione S-transferase, and thrombin inhibitor (Supplementary Table S6). Finally, DEGs from 20-day samples displaying SNPs were found to encode such proteins as proteases, glucose metabolism enzymes, nucleotide metabolism enzymes, and proteins involved in transcription process, as well as the trypanosome reactive oxygen species detoxification system (Supplementary Table S7). Sequences encoding stress-related proteins, such as heat-shock proteins, were also identified among the DEGs that carried SNPs; these were referenced as belonging to the *G. p. gambiensis* and *T. b. gambiense* transcriptomes.

## Discussion

### General aspects

Deciphering the mechanisms involved in the facilitation of (or refractoriness to) tsetse fly infection by trypanosomes is crucial for developing anti-vector strategies to fight sleeping sickness. In this frame, *G. p. gambiensis* (Gpg) and *T. b. gambiense* (Tbg) transcripts were identified using the RNAseq *de novo* assembly approach. The transcripts were mapped not only on the *G. morsitans morsitans* (Gmm) genome but on a panel of other reference sequences allowing the identification of Gpg genes that may not be represented into the Gmm genome.

The sampling times were chosen according to a previously determined time course of susceptible fly infection by trypanosomes (Van den Abbeele et al., 1999; Ravel et al., 2003): 3 days post-feeding to target DEGs involved in early events associated with trypanosome entry into the midgut; 10 days post-feeding to target DEGs involved in the establishment of infection; and 20 days post-infected bloodmeal feeding, in order to target genes involved in events occurring relatively late during trypanosome infection.

A limited number of transposable element sequences (such as protein LTV1 homologs and the viral A-type inclusion protein) were present in the *G. p. gambiensis* midgut transcriptome. This is in contrast to data reported for the *G. m. morsitans* sialotranscriptome (Alves-Silva et al., 2010), and which were obtained following the sequencing of the *G. morsitans* genome (International Glossina Genome Initiative, 2014). As suggested by Alves-Silva et al. (2010), these sequences may represent active transposition, as well as expression of regulatory sequences (Silva et al., 2004).

Numerous DEGs were identified that may be specific to the post-infected bloodmeal time. Nevertheless, some DEGs appear to be of greater interest than others regarding the objectives of the study, and are presented in Table 2.

Although S3, I10, and I20 flies were all fed on trypanosome-infected bloodmeal, we preliminarily observed that trypanosome gene expression was only recorded in S3 and I20 samples, even though the anal drops from I10 flies were positive. This apparent discrepancy is likely due to the attrition phenomenon (Gibson and Bailey, 2003) that occurs several days after trypanosome ingestion, which leads to the elimination of most of the ingested trypanosomes (even in susceptible flies). In contrast, the number of trypanosomes surviving in I10 flies is probably too low to be detected by an indirect and less sensitive detection method, i.e., recording the transcripts resulting from the expression of some of their genes.

In our experiment, the evolvement of trypanosome populations between the day 3 and day 20 sampling points could not be measured (i.e., by trypanosome counting or use of specific DNA probes), since the total midgut extracts were dedicated to total mRNA extraction. Nevertheless, data shown in Table 3 for genes expressed in both S3 and I20 flies support the hypothesis of an increased trypanosome population in I20 vs. S3 (and I10) flies. In fact, the trypanosome expression levels of these genes are 10- to 40-fold higher (depending on the gene) in I20 flies than in S3 flies. However, these differences in gene expression between the different genes also support the idea that gene expression could be differentially stimulated, thus leading to the noted differences in expression levels. Thus, both the increase in the midgut trypanosome population and the modulation in trypanosome gene expression could contribute to the differences in trypanosome gene expression recorded in I20 flies, as compared to S3 flies. Finally, as expected, no trypanosome expression could be recorded in NI20 flies. This is in agreement with the absence of trypanosome detection in the anal drops of these flies, and confirms their refractoriness to trypanosome infection. Non-infected control flies (NS3) also did not display any trypanosome gene expression (Table 2).

In Table 2, the set of DEGs were classified according to a major function of the proteins they encode. We observed a very high percentage of both tsetse and trypanosome genes encoding a wide array of proteases. Trypanosome genes were only expressed in S3 and I20, whereas tsetse genes were also expressed in the day 10 samples. The high number of trypanosome protease genes identified is in agreement with previous studies of the trypanosome secretome, which characterized a number of proteases suspected to be involved in the trypanosome infective process (Atyame Nten et al., 2010; Geiger et al., 2010). The number of tsetse (but not trypanosome) laccase encoding genes was also surprising. One trypanosome lectin gene was identified and is expressed in S3 and I20 samples, whereas several tsetse lectin genes are only expressed in I10.

As illustrated by our results, several representatives can be listed for a given protein (e.g., laccase, lectin, or serine protease). Each of these representatives (i.e., an isoprotein/isoenzyme) appears to be encoded by a specific gene, suggesting that the isoproteins do not result from posttranscriptional events, and that their expression could be differentially regulated.

### Specific aspects

Thrombin inhibitor was under-expressed in stimulated flies as compared to flies fed on a non-infected bloodmeal. By contrast, thrombin was over-expressed in infected flies as compared to self-cured flies at 10 and 20 days post-infected bloodmeal. Adult tsetse flies require several molecules that are essential for efficient blood feeding, which counteract the coagulation and blood platelet aggregation responses of the host (Alves-Silva et al., 2010). Thrombin inhibitor may be associated with such anti-clotting activities (Parker and Mant, 1979; Cappello et al., 1998; Alves-Silva et al., 2010). Furthermore, its under-expression early in the midgut invasion process could represent a defense mechanism to immobilize parasites and avoid their dissemination into other tissues.

The peritrophic matrix protein three precursor and mucin genes were over-expressed in stimulated flies. *Glossina* possess a peritrophic membrane, continuously built by the proventriculus, which separates the lumen of the midgut from the epithelial cells (Lehane, 1997). It is generally composed of chitin, peritrophin proteins, glycosaminoglycans, and mucin-like molecules (International Glossina Genome Initiative, 2014). Importantly, the peritrophic membrane is involved in regulating the host immune induction timing, following the parasite challenge (Weiss et al., 2013). Thus, over-expression of these genes in stimulated *G. p. gambiensis* flies could delay the activation of immune gene expression, which would further favor *T. b. gambiense* establishment.

Serine proteases could be involved in such diverse functions as digestion, clotting activity, control of proteolytic cascades in the immunity process, and control of pro-phenoloxidase activation, which causes pathogen melanization (Stark and James, 1998; Kanost, 1999; Alves-Silva et al., 2010). Serine proteases and serpin were previously reported in the *G. m. morsitans* transcriptome (Lehane et al., 2003) and sialotranscriptome (Alves-Silva et al., 2010). Several serine proteases as well as serpin were over-expressed in *G. p. gambiensis*, although they were under-expressed in stimulated or infected flies (depending on the sampling time post-infected bloodmeal ingestion).

Innate immune response products have previously been considered as contributors to fly refractoriness (Hao et al., 2001), and we observed that the antimicrobial peptide cecropin was over-expressed in stimulated flies. There is evidence that innate immune responses, particularly the antimicrobial peptides regulated *via* the Imd pathway, are among the factors contributing to the tsetse refractoriness to trypanosomes (Hu and Aksoy, 2006).

Lectins display carbohydrate recognition domains associated with innate immunity (Kanost et al., 2004). *S. glossinidius* was previously shown to favor parasite establishment in the insect midgut through a complex biochemical mechanism involving N-acetyl glucosamine, which would result from pupal chitin hydrolysis by a *S. glossinidius*-produced endochitinase; this product could then inhibit the tsetse midgut lectin otherwise lethal to procyclic forms of the trypanosome (Welburn and Maudlin, 1999). In our experiments, the insect chitinase gene was over-expressed in day 3 stimulated tsetse flies, which is in line with previous hypotheses. However, the lectin gene was over-expressed in infected flies 10 days post-infected bloodmeal ingestion contradicting previous hypotheses. Lectins have also been suggested to display anti-clotting activities (Alves-Silva et al., 2010); thus, the possibility of differential catalytic specificities between the insect and the *Sodalis* chitinase and lectins.

In contrast, the lectizyme gene was over-expressed in flies that cleared the infection 10 days post-infected bloodmeal. This enzyme was previously reported to display lectin (trypanosome agglutination capability) and protease activity involved in the establishment of trypanosomes in tsetse flies (Abubakar et al., 2006).

In our study we identified the presence of both LysM and the putative peptidoglycan-binding domain-containing protein 1-like isoform X1. This protein, homologous to the *C. capitata* protein, was under-expressed in day 3 stimulated *G. p. gambiensis* flies. Such proteins, closely related to *Drosophila*, have been found in the sialotranscriptome of *G. m. morsitans* (Alves-Silva et al., 2010). A pathogen recognition protein implicated in the initiation of innate defense mechanisms was also previously identified in *G. m. morsitans* fat body (Attardo et al., 2006).

Laccase genes were over-expressed in both stimulated and day 20 infected flies. In *Anopheles gambiae*, laccases have been suggested to oxidize toxic molecules in the bloodmeal, resulting in detoxification or cross-linking of the molecules to the peritrophic matrix, and thus targeting them for excretion (Lang et al., 2012).

For a successful *Glossina* infection, trypanosomes must be transferred from a mammal to an insect host, and therefore they must express specialized proteins to escape multispecies host immune responses (Atyame Nten et al., 2010). Tsetse flies use a proline-alanine shuttle system for energy distribution instead of carbohydrate metabolism (International Glossina Genome Initiative, 2014). Proline is used as a major carbon source during tsetse flight, as well as by trypanosomes (Bursell, 1963). Delta-1-pyrroline-5-carboxylate dehydrogenase is involved in proline catabolism. In all flies, whether stimulated (3-day samples) or infected (10 and 20 days post-infected bloodmeal), the gene encoding this dehydrogenase was over-expressed. This gene was found to be homologous with that of *T. brucei*.

Several different peptidase families were shown in the present study to be expressed by trypanosomes, including members of the serine and cysteine proteinases, and metallopeptidases. These peptidases could: (a) act as virulence factors thus favoring parasite invasion and growth in the host environment; (b) allow trypanosomes to evade the host immune defenses; (c) produce nutrients by hydrolyzing host proteins (Atyame Nten et al., 2010); and (d) be involved in the blood clotting, thus in immobilizing invading parasites (Lehane et al., 2003; Alves-Silva et al., 2010; International Glossina Genome Initiative, 2014).

Proteins involved in signaling were also identified in the secretome of procyclic trypanosomes (Atyame Nten et al., 2010). Several of these proteins, such as calreticulin, could play physiopathological roles. In the present study, transcripts corresponding to these proteins were found expressed by trypanosomes.

Transferrin has also been demonstrated as an important part of the immune system in insects and vertebrates (Nichol et al., 2002; Guz et al., 2007). Up-regulation of its transcription following an immune challenge is reported for a number of insects including *Aedes aegypti, Bombyx mori*, and *Drosophila* (Yoshiga et al., 1997, 1999; Yun et al., 1999). In the present study, tsetse flies stimulated by trypanosomes (S-3days) were observed to over-express the transferrin gene. These results are in agreement with Guz et al. (2007), who reported an increase in transferrin expression levels upon microbial challenge in tsetse flies.

As previously reported (Hamidou Soumana et al., 2014b), genes encoding ribosomal proteins can also be differentially expressed (either up-regulated or down-regulated). For instance, in NI10 samples (i.e., self-cured flies) a tsetse fly 40S ribosomal protein gene was 6.75-fold over-expressed. In contrast, a 60S ribosomal protein gene was under-expressed as compared to the expression levels of the same genes recorded in I10 samples (NI10/I10 = 0.657). Wang et al. (2013) reported similar results for phosphate- or iron-deficient *Arabidopsis* roots vs. control roots, in response to a changing environment. These findings raise the question of whether modulations of ribosomal protein gene expression could also be involved in tsetse fly adaptation to the stress of trypanosome invasion.

We identified over 195,464 high confidence SNPs from 14,929 contigs across the whole transcriptome assemblies of *G. p. gambiensis* and *T. b. gambiense*. These SNPs overlap genes that exhibit both up- and down-regulation of homologous transcripts from different insect and parasite species. The SNP genetic sites identified in our dataset will provide useful marker resources for fine mapping experiments and marker-assisted *G. p. gambiensis* control programs.

These results will have immediate applications for exploring *G. p. gambiensis* genome diversity and co-expression networks involved in tsetse infection by trypanosomes, as well as the development of stochastic and metabolic networks. In addition, these resources can be used to identify novel genes, transcript models and eQTLs, and to study trypanosome adaptation to diverse fly tissue environments. These findings will also be useful when undertaking comparative studies with the *G. morsitans* transcriptome (Attardo et al., 2006; Alves-Silva et al., 2010) and genome (International Glossina Genome Initiative, 2014).

To conclude: our study is the first to investigate key steps of tsetse fly infection by trypanosomes through characterization of the *G. p. gambiensis* transcriptome and the complete set of tsetse fly and trypanosome DEGs. This approach revealed genes that interact in well-defined patterns and the various characterized DEGs provide insights into the complexity of the host-parasite interactions. Future investigations should aim to characterize the involvement of identified genes in tsetse refractoriness to trypanosome infection.

### Conflict of interest statement

The authors declare that the research was conducted in the absence of any commercial or financial relationships that could be construed as a potential conflict of interest.
