# Supplementary material for: RNA-seq de novo Assembly Reveals Differential Gene Expression in Glossina palpalis gambiensis Infected with Trypanosoma brucei gambiense vs. Non-Infected and Self-Cured Flies
Source: Front Microbiol. 2015 Nov 13;6:1259. doi: 10.3389/fmicb.2015.01259 (PMC4643127; doi:10.3389/fmicb.2015.01259)
Supplement: Supplementary file 1 [file Table1.PDF]

Supplementary Table S1: annotated dataset with16936 contigs

Best hit description	Best hit accession	Best hit evalue	Name	Code	Onto-logy	Source	Go name
NM_001273490.1 D. m. bunched (bun), transcript variant O, mRNA	NM_001273490.1	5,00E-06	GLOS_BUN.1.2	GO:0006357	B	.	regulation of transcription from RNA polymerase II promoter
NM_001273490.1 D. m. bunched (bun), transcript variant O, mRNA	NM_001273490.1	5,00E-06	GLOS_BUN.1.2	GO:0006355	B	.	regulation of transcription, DNA-dependent
NM_001273490.1 D. m. bunched (bun), transcript variant O, mRNA	NM_001273490.1	5,00E-06	GLOS_BUN.1.2	GO:0010468	B	.	regulation of gene expression
NM_001273490.1 D. m. bunched (bun), transcript variant O, mRNA	NM_001273490.1	5,00E-06	GLOS_BUN.1.2	GO:0060255	B	.	regulation of macromolecule metabolic process
NM_001273490.1 D. m. bunched (bun), transcript variant O, mRNA	NM_001273490.1	5,00E-06	GLOS_BUN.1.2	GO:0019222	B	.	regulation of metabolic process
NM_001273490.1 D. m. bunched (bun), transcript variant O, mRNA	NM_001273490.1	5,00E-06	GLOS_BUN.1.2	GO:0050789	B	.	regulation of biological process
NM_001273490.1 D. m. bunched (bun), transcript variant O, mRNA	NM_001273490.1	5,00E-06	GLOS_BUN.1.2	GO:0065007	B	.	biological regulation
NM_001273490.1 D. m. bunched (bun), transcript variant O, mRNA	NM_001273490.1	5,00E-06	GLOS_BUN.1.2	GO:0008150	B	.	biological_process
NM_001273490.1 D. m. bunched (bun), transcript variant O, mRNA	NM_001273490.1	5,00E-06	GLOS_BUN.1.2	GO:2000112	B	.	regulation of cellular macromolecule biosynthetic process
NM_001273490.1 D. m. bunched (bun), transcript variant O, mRNA	NM_001273490.1	5,00E-06	GLOS_BUN.1.2	GO:0010556	B	.	regulation of macromolecule biosynthetic process
NM_001273490.1 D. m. bunched (bun), transcript variant O, mRNA	NM_001273490.1	5,00E-06	GLOS_BUN.1.2	GO:0009889	B	.	regulation of biosynthetic process
NM_001273490.1 D. m. bunched (bun), transcript variant O, mRNA	NM_001273490.1	5,00E-06	GLOS_BUN.1.2	GO:0031326	B	.	regulation of cellular biosynthetic process
NM_001273490.1 D. m. bunched (bun), transcript variant O, mRNA	NM_001273490.1	5,00E-06	GLOS_BUN.1.2	GO:0031323	B	.	regulation of cellular metabolic process
NM_001273490.1 D. m. bunched (bun), transcript variant O, mRNA	NM_001273490.1	5,00E-06	GLOS_BUN.1.2	GO:0050794	B	.	regulation of cellular process
NM_001273490.1 D. m. bunched (bun), transcript variant O, mRNA	NM_001273490.1	5,00E-06	GLOS_BUN.1.2	GO:2001141	B	.	regulation of RNA biosynthetic process
NM_001273490.1 D. m. bunched (bun), transcript variant O, mRNA	NM_001273490.1	5,00E-06	GLOS_BUN.1.2	GO:0051252	B	.	regulation of RNA metabolic process
NM_001273490.1 D. m. bunched (bun), transcript variant O, mRNA	NM_001273490.1	5,00E-06	GLOS_BUN.1.2	GO:0019219	B	.	regulation of nucleobase-containing compound metabolic process
NM_001273490.1 D. m. bunched (bun), transcript variant O, mRNA	NM_001273490.1	5,00E-06	GLOS_BUN.1.2	GO:0051171	B	.	regulation of nitrogen compound metabolic process
NM_001273490.1 D. m. bunched (bun), transcript variant O, mRNA	NM_001273490.1	5,00E-06	GLOS_BUN.1.2	GO:0080090	B	.	regulation of primary metabolic process
NM_001273490.1 D. m. bunched (bun), transcript variant O, mRNA	NM_001273490.1	5,00E-06	GLOS_BUN.1.2	GO:0045892	B	.	negative regulation of transcription, DNA-dependent
NM_001273490.1 D. m. bunched (bun), transcript variant O, mRNA	NM_001273490.1	5,00E-06	GLOS_BUN.1.2	GO:0010629	B	.	negative regulation of gene expression
NM_001273490.1 D. m. bunched (bun), transcript variant O, mRNA	NM_001273490.1	5,00E-06	GLOS_BUN.1.2	GO:0010605	B	.	negative regulation of macromolecule metabolic process
NM_001273490.1 D. m. bunched (bun), transcript variant O, mRNA	NM_001273490.1	5,00E-06	GLOS_BUN.1.2	GO:0009892	B	.	negative regulation of metabolic process
NM_001273490.1 D. m. bunched (bun), transcript variant O, mRNA	NM_001273490.1	5,00E-06	GLOS_BUN.1.2	GO:0048519	B	.	negative regulation of biological process
NM_001273490.1 D. m. bunched (bun), transcript variant O, mRNA	NM_001273490.1	5,00E-06	GLOS_BUN.1.2	GO:0051253	B	.	negative regulation of RNA metabolic process
NM_001273490.1 D. m. bunched (bun), transcript variant O, mRNA	NM_001273490.1	5,00E-06	GLOS_BUN.1.2	GO:0045934	B	.	negative regulation of nucleobase-containing compound metabolic process
NM_001273490.1 D. m. bunched (bun), transcript variant O, mRNA	NM_001273490.1	5,00E-06	GLOS_BUN.1.2	GO:0031324	B	.	negative regulation of cellular metabolic process
NM_001273490.1 D. m. bunched (bun), transcript variant O, mRNA	NM_001273490.1	5,00E-06	GLOS_BUN.1.2	GO:0048523	B	.	negative regulation of cellular process
NM_001273490.1 D. m. bunched (bun), transcript variant O, mRNA	NM_001273490.1	5,00E-06	GLOS_BUN.1.2	GO:0051172	B	.	negative regulation of nitrogen compound metabolic process
NM_001273490.1 D. m. bunched (bun), transcript variant O, mRNA	NM_001273490.1	5,00E-06	GLOS_BUN.1.2	GO:2000113	B	.	negative regulation of cellular macromolecule biosynthetic process
NM_001273490.1 D. m. bunched (bun), transcript variant O, mRNA	NM_001273490.1	5,00E-06	GLOS_BUN.1.2	GO:0010558	B	.	negative regulation of macromolecule biosynthetic process
NM_001273490.1 D. m. bunched (bun), transcript variant O, mRNA	NM_001273490.1	5,00E-06	GLOS_BUN.1.2	GO:0009890	B	.	negative regulation of biosynthetic process
NM_001273490.1 D. m. bunched (bun), transcript variant O, mRNA	NM_001273490.1	5,00E-06	GLOS_BUN.1.2	GO:0031327	B	.	negative regulation of cellular biosynthetic process
NM_001273490.1 D. m. bunched (bun), transcript variant O, mRNA	NM_001273490.1	5,00E-06	GLOS_BUN.1.2	GO:0000122	B	Refseq	negative regulation of transcription from RNA polymerase II promoter
NM_001273490.1 D. m. bunched (bun), transcript variant O, mRNA	NM_001273490.1	5,00E-06	GLOS_BUN.1.2	GO:0048731	B	.	system development
NM_001273490.1 D. m. bunched (bun), transcript variant O, mRNA	NM_001273490.1	5,00E-06	GLOS_BUN.1.2	GO:0048856	B	.	anatomical structure development
NM_001273490.1 D. m. bunched (bun), transcript variant O, mRNA	NM_001273490.1	5,00E-06	GLOS_BUN.1.2	GO:0032502	B	.	developmental process
NM_001273490.1 D. m. bunched (bun), transcript variant O, mRNA	NM_001273490.1	5,00E-06	GLOS_BUN.1.2	GO:0007275	B	.	multicellular organismal development
NM_001273490.1 D. m. bunched (bun), transcript variant O, mRNA	NM_001273490.1	5,00E-06	GLOS_BUN.1.2	GO:0044707	B	.	single-multicellular organism process
NM_001273490.1 D. m. bunched (bun), transcript variant O, mRNA	NM_001273490.1	5,00E-06	GLOS_BUN.1.2	GO:0032501	B	.	multicellular organismal process
NM_001273490.1 D. m. bunched (bun), transcript variant O, mRNA	NM_001273490.1	5,00E-06	GLOS_BUN.1.2	GO:0044699	B	.	single-organism process
NM_001273490.1 D. m. bunched (bun), transcript variant O, mRNA	NM_001273490.1	5,00E-06	GLOS_BUN.1.2	GO:0001501	B	Refseq	skeletal system development
NM_001273490.1 D. m. bunched (bun), transcript variant O, mRNA	NM_001273490.1	5,00E-06	GLOS_BUN.1.2	GO:0001071	M	.	nucleic acid binding transcription factor activity
NM_001273490.1 D. m. bunched (bun), transcript variant O, mRNA	NM_001273490.1	5,00E-06	GLOS_BUN.1.2	GO:0003674	M	.	molecular_function
NM_001273490.1 D. m. bunched (bun), transcript variant O, mRNA	NM_001273490.1	5,00E-06	GLOS_BUN.1.2	GO:0003700	M	Refseq	sequence-specific DNA binding transcription factor activity
NM_001273490.1 D. m. bunched (bun), transcript variant O, mRNA	NM_001273490.1	5,00E-06	GLOS_BUN.1.2	GO:0005488	M	.	binding
NM_001273490.1 D. m. bunched (bun), transcript variant O, mRNA	NM_001273490.1	5,00E-06	GLOS_BUN.1.2	GO:0005515	M	Refseq	protein binding
NM_001273490.1 D. m. bunched (bun), transcript variant O, mRNA	NM_001273490.1	5,00E-06	GLOS_BUN.1.2	GO:0043231	C	.	intracellular membrane-bounded organelle
NM_001273490.1 D. m. bunched (bun), transcript variant O, mRNA	NM_001273490.1	5,00E-06	GLOS_BUN.1.2	GO:0043227	C	.	membrane-bounded organelle
NM_001273490.1 D. m. bunched (bun), transcript variant O, mRNA	NM_001273490.1	5,00E-06	GLOS_BUN.1.2	GO:0043226	C	.	organelle
NM_001273490.1 D. m. bunched (bun), transcript variant O, mRNA	NM_001273490.1	5,00E-06	GLOS_BUN.1.2	GO:0005575	C	.	cellular_component

NM_001273490.1 D. m. bunched (bun), transcript variant O, mRNA	NM_001273490.1	5,00E-06	GLOS_BUN.1.2	GO:0043229	C	.	intracellular organelle
NM_001273490.1 D. m. bunched (bun), transcript variant O, mRNA	NM_001273490.1	5,00E-06	GLOS_BUN.1.2	GO:0044424	C	.	intracellular part
NM_001273490.1 D. m. bunched (bun), transcript variant O, mRNA	NM_001273490.1	5,00E-06	GLOS_BUN.1.2	GO:0044464	C	.	cell part
NM_001273490.1 D. m. bunched (bun), transcript variant O, mRNA	NM_001273490.1	5,00E-06	GLOS_BUN.1.2	GO:0005623	C	.	cell
NM_001273490.1 D. m. bunched (bun), transcript variant O, mRNA	NM_001273490.1	5,00E-06	GLOS_BUN.1.2	GO:0005622	C	.	intracellular
NM_001273490.1 D. m. bunched (bun), transcript variant O, mRNA	NM_001273490.1	5,00E-06	GLOS_BUN.1.2	GO:0005634	C	Refseq	nucleus
NM_001273490.1 D. m. bunched (bun), transcript variant O, mRNA	NM_001273490.1	5,00E-06	GLOS_BUN.1.2	GO:0006351	B	.	transcription, DNA-dependent
NM_001273490.1 D. m. bunched (bun), transcript variant O, mRNA	NM_001273490.1	5,00E-06	GLOS_BUN.1.2	GO:0032774	B	.	RNA biosynthetic process
NM_001273490.1 D. m. bunched (bun), transcript variant O, mRNA	NM_001273490.1	5,00E-06	GLOS_BUN.1.2	GO:0009059	B	.	macromolecule biosynthetic process
NM_001273490.1 D. m. bunched (bun), transcript variant O, mRNA	NM_001273490.1	5,00E-06	GLOS_BUN.1.2	GO:0043170	B	.	macromolecule metabolic process
NM_001273490.1 D. m. bunched (bun), transcript variant O, mRNA	NM_001273490.1	5,00E-06	GLOS_BUN.1.2	GO:0071704	B	.	organic substance metabolic process
NM_001273490.1 D. m. bunched (bun), transcript variant O, mRNA	NM_001273490.1	5,00E-06	GLOS_BUN.1.2	GO:0008152	B	.	metabolic process
NM_001273490.1 D. m. bunched (bun), transcript variant O, mRNA	NM_001273490.1	5,00E-06	GLOS_BUN.1.2	GO:1901576	B	.	organic substance biosynthetic process
NM_001273490.1 D. m. bunched (bun), transcript variant O, mRNA	NM_001273490.1	5,00E-06	GLOS_BUN.1.2	GO:0009058	B	.	biosynthetic process
NM_001273490.1 D. m. bunched (bun), transcript variant O, mRNA	NM_001273490.1	5,00E-06	GLOS_BUN.1.2	GO:0016070	B	.	RNA metabolic process
NM_001273490.1 D. m. bunched (bun), transcript variant O, mRNA	NM_001273490.1	5,00E-06	GLOS_BUN.1.2	GO:0044260	B	.	cellular macromolecule metabolic process
NM_001273490.1 D. m. bunched (bun), transcript variant O, mRNA	NM_001273490.1	5,00E-06	GLOS_BUN.1.2	GO:0044237	B	.	cellular metabolic process
NM_001273490.1 D. m. bunched (bun), transcript variant O, mRNA	NM_001273490.1	5,00E-06	GLOS_BUN.1.2	GO:0009987	B	.	cellular process
NM_001273490.1 D. m. bunched (bun), transcript variant O, mRNA	NM_001273490.1	5,00E-06	GLOS_BUN.1.2	GO:0090304	B	.	nucleic acid metabolic process
NM_001273490.1 D. m. bunched (bun), transcript variant O, mRNA	NM_001273490.1	5,00E-06	GLOS_BUN.1.2	GO:0006139	B	.	nucleobase-containing compound metabolic process
NM_001273490.1 D. m. bunched (bun), transcript variant O, mRNA	NM_001273490.1	5,00E-06	GLOS_BUN.1.2	GO:0006725	B	.	cellular aromatic compound metabolic process
NM_001273490.1 D. m. bunched (bun), transcript variant O, mRNA	NM_001273490.1	5,00E-06	GLOS_BUN.1.2	GO:0034641	B	.	cellular nitrogen compound metabolic process
NM_001273490.1 D. m. bunched (bun), transcript variant O, mRNA	NM_001273490.1	5,00E-06	GLOS_BUN.1.2	GO:0006807	B	.	nitrogen compound metabolic process
NM_001273490.1 D. m. bunched (bun), transcript variant O, mRNA	NM_001273490.1	5,00E-06	GLOS_BUN.1.2	GO:0044238	B	.	primary metabolic process
NM_001273490.1 D. m. bunched (bun), transcript variant O, mRNA	NM_001273490.1	5,00E-06	GLOS_BUN.1.2	GO:0046483	B	.	heterocycle metabolic process
NM_001273490.1 D. m. bunched (bun), transcript variant O, mRNA	NM_001273490.1	5,00E-06	GLOS_BUN.1.2	GO:1901360	B	.	organic cyclic compound metabolic process
NM_001273490.1 D. m. bunched (bun), transcript variant O, mRNA	NM_001273490.1	5,00E-06	GLOS_BUN.1.2	GO:0034654	B	.	nucleobase-containing compound biosynthetic process
NM_001273490.1 D. m. bunched (bun), transcript variant O, mRNA	NM_001273490.1	5,00E-06	GLOS_BUN.1.2	GO:0018130	B	.	heterocycle biosynthetic process
NM_001273490.1 D. m. bunched (bun), transcript variant O, mRNA	NM_001273490.1	5,00E-06	GLOS_BUN.1.2	GO:0044249	B	.	cellular biosynthetic process
NM_001273490.1 D. m. bunched (bun), transcript variant O, mRNA	NM_001273490.1	5,00E-06	GLOS_BUN.1.2	GO:0019438	B	.	aromatic compound biosynthetic process
NM_001273490.1 D. m. bunched (bun), transcript variant O, mRNA	NM_001273490.1	5,00E-06	GLOS_BUN.1.2	GO:0044271	B	.	cellular nitrogen compound biosynthetic process
NM_001273490.1 D. m. bunched (bun), transcript variant O, mRNA	NM_001273490.1	5,00E-06	GLOS_BUN.1.2	GO:1901362	B	.	organic cyclic compound biosynthetic process
NM_001273490.1 D. m. bunched (bun), transcript variant O, mRNA	NM_001273490.1	5,00E-06	GLOS_BUN.1.2	GO:0034645	B	.	cellular macromolecule biosynthetic process
NM_001273490.1 D. m. bunched (bun), transcript variant O, mRNA	NM_001273490.1	5,00E-06	GLOS_BUN.1.2	GO:0010467	B	.	gene expression
NM_001273490.1 D. m. bunched (bun), transcript variant O, mRNA	NM_001273490.1	5,00E-06	GLOS_BUN.1.2	GO:0006366	B	Refseq	transcription from RNA polymerase II promoter
NM_001273490.1 D. m. bunched (bun), transcript variant O, mRNA	NM_001273490.1	5,00E-06	GLOS_BUN.1.2	GO:0006606	B	.	protein import into nucleus
NM_001273490.1 D. m. bunched (bun), transcript variant O, mRNA	NM_001273490.1	5,00E-06	GLOS_BUN.1.2	GO:0006886	B	.	intracellular protein transport
NM_001273490.1 D. m. bunched (bun), transcript variant O, mRNA	NM_001273490.1	5,00E-06	GLOS_BUN.1.2	GO:0015031	B	.	protein transport
NM_001273490.1 D. m. bunched (bun), transcript variant O, mRNA	NM_001273490.1	5,00E-06	GLOS_BUN.1.2	GO:0045184	B	.	establishment of protein localization
NM_001273490.1 D. m. bunched (bun), transcript variant O, mRNA	NM_001273490.1	5,00E-06	GLOS_BUN.1.2	GO:0051234	B	.	establishment of localization
NM_001273490.1 D. m. bunched (bun), transcript variant O, mRNA	NM_001273490.1	5,00E-06	GLOS_BUN.1.2	GO:0051179	B	.	localization
NM_001273490.1 D. m. bunched (bun), transcript variant O, mRNA	NM_001273490.1	5,00E-06	GLOS_BUN.1.2	GO:0008104	B	.	protein localization
NM_001273490.1 D. m. bunched (bun), transcript variant O, mRNA	NM_001273490.1	5,00E-06	GLOS_BUN.1.2	GO:0033036	B	.	macromolecule localization
NM_001273490.1 D. m. bunched (bun), transcript variant O, mRNA	NM_001273490.1	5,00E-06	GLOS_BUN.1.2	GO:0071702	B	.	organic substance transport
NM_001273490.1 D. m. bunched (bun), transcript variant O, mRNA	NM_001273490.1	5,00E-06	GLOS_BUN.1.2	GO:0006810	B	.	transport
NM_001273490.1 D. m. bunched (bun), transcript variant O, mRNA	NM_001273490.1	5,00E-06	GLOS_BUN.1.2	GO:0046907	B	.	intracellular transport
NM_001273490.1 D. m. bunched (bun), transcript variant O, mRNA	NM_001273490.1	5,00E-06	GLOS_BUN.1.2	GO:0051649	B	.	establishment of localization in cell
NM_001273490.1 D. m. bunched (bun), transcript variant O, mRNA	NM_001273490.1	5,00E-06	GLOS_BUN.1.2	GO:0051641	B	.	cellular localization
NM_001273490.1 D. m. bunched (bun), transcript variant O, mRNA	NM_001273490.1	5,00E-06	GLOS_BUN.1.2	GO:0044763	B	.	single-organism cellular process
NM_001273490.1 D. m. bunched (bun), transcript variant O, mRNA	NM_001273490.1	5,00E-06	GLOS_BUN.1.2	GO:0034613	B	.	cellular protein localization
NM_001273490.1 D. m. bunched (bun), transcript variant O, mRNA	NM_001273490.1	5,00E-06	GLOS_BUN.1.2	GO:0070727	B	.	cellular macromolecule localization
NM_001273490.1 D. m. bunched (bun), transcript variant O, mRNA	NM_001273490.1	5,00E-06	GLOS_BUN.1.2	GO:0017038	B	.	protein import
NM_001273490.1 D. m. bunched (bun), transcript variant O, mRNA	NM_001273490.1	5,00E-06	GLOS_BUN.1.2	GO:0044765	B	.	single-organism transport
NM_001273490.1 D. m. bunched (bun), transcript variant O, mRNA	NM_001273490.1	5,00E-06	GLOS_BUN.1.2	GO:0051170	B	.	nuclear import

NM_001273490.1 D. m. bunched (bun), transcript variant O, mRNA	NM_001273490.1	5,00E-06	GLOS_BUN.1.2	GO:0006913	B	.	nucleocytoplasmic transport
NM_001273490.1 D. m. bunched (bun), transcript variant O, mRNA	NM_001273490.1	5,00E-06	GLOS_BUN.1.2	GO:0016482	B	.	cytoplasmic transport
NM_001273490.1 D. m. bunched (bun), transcript variant O, mRNA	NM_001273490.1	5,00E-06	GLOS_BUN.1.2	GO:0051169	B	.	nuclear transport
NM_001273490.1 D. m. bunched (bun), transcript variant O, mRNA	NM_001273490.1	5,00E-06	GLOS_BUN.1.2	GO:0072594	B	.	establishment of protein localization to organelle
NM_001273490.1 D. m. bunched (bun), transcript variant O, mRNA	NM_001273490.1	5,00E-06	GLOS_BUN.1.2	GO:0033365	B	.	protein localization to organelle
NM_001273490.1 D. m. bunched (bun), transcript variant O, mRNA	NM_001273490.1	5,00E-06	GLOS_BUN.1.2	GO:0034504	B	.	protein localization to nucleus
NM_001273490.1 D. m. bunched (bun), transcript variant O, mRNA	NM_001273490.1	5,00E-06	GLOS_BUN.1.2	GO:0044744	B	.	protein targeting to nucleus
NM_001273490.1 D. m. bunched (bun), transcript variant O, mRNA	NM_001273490.1	5,00E-06	GLOS_BUN.1.2	GO:0006605	B	.	protein targeting
NM_001273490.1 D. m. bunched (bun), transcript variant O, mRNA	NM_001273490.1	5,00E-06	GLOS_BUN.1.2	GO:0006607	B	Refseq	NLS-bearing protein import into nucleus
NM_001273490.1 D. m. bunched (bun), transcript variant O, mRNA	NM_001273490.1	5,00E-06	GLOS_BUN.1.2	GO:0046914	M	.	transition metal ion binding
NM_001273490.1 D. m. bunched (bun), transcript variant O, mRNA	NM_001273490.1	5,00E-06	GLOS_BUN.1.2	GO:0046872	M	.	metal ion binding
NM_001273490.1 D. m. bunched (bun), transcript variant O, mRNA	NM_001273490.1	5,00E-06	GLOS_BUN.1.2	GO:0043169	M	.	cation binding
NM_001273490.1 D. m. bunched (bun), transcript variant O, mRNA	NM_001273490.1	5,00E-06	GLOS_BUN.1.2	GO:0043167	M	.	ion binding
NM_001273490.1 D. m. bunched (bun), transcript variant O, mRNA	NM_001273490.1	5,00E-06	GLOS_BUN.1.2	GO:0008270	M	Refseq	zinc ion binding
NM_001273490.1 D. m. bunched (bun), transcript variant O, mRNA	NM_001273490.1	5,00E-06	GLOS_BUN.1.2	GO:0045595	B	.	regulation of cell differentiation
NM_001273490.1 D. m. bunched (bun), transcript variant O, mRNA	NM_001273490.1	5,00E-06	GLOS_BUN.1.2	GO:0050793	B	.	regulation of developmental process
NM_001273490.1 D. m. bunched (bun), transcript variant O, mRNA	NM_001273490.1	5,00E-06	GLOS_BUN.1.2	GO:0061035	B	.	regulation of cartilage development
NM_001273490.1 D. m. bunched (bun), transcript variant O, mRNA	NM_001273490.1	5,00E-06	GLOS_BUN.1.2	GO:2000026	B	.	regulation of multicellular organismal development
NM_001273490.1 D. m. bunched (bun), transcript variant O, mRNA	NM_001273490.1	5,00E-06	GLOS_BUN.1.2	GO:0051239	B	.	regulation of multicellular organismal process
NM_001273490.1 D. m. bunched (bun), transcript variant O, mRNA	NM_001273490.1	5,00E-06	GLOS_BUN.1.2	GO:0032330	B	Refseq	regulation of chondrocyte differentiation
NM_001273490.1 D. m. bunched (bun), transcript variant O, mRNA	NM_001273490.1	5,00E-06	GLOS_BUN.1.2	GO:0003677	M	.	DNA binding
NM_001273490.1 D. m. bunched (bun), transcript variant O, mRNA	NM_001273490.1	5,00E-06	GLOS_BUN.1.2	GO:0003676	M	.	nucleic acid binding
NM_001273490.1 D. m. bunched (bun), transcript variant O, mRNA	NM_001273490.1	5,00E-06	GLOS_BUN.1.2	GO:0097159	M	.	organic cyclic compound binding
NM_001273490.1 D. m. bunched (bun), transcript variant O, mRNA	NM_001273490.1	5,00E-06	GLOS_BUN.1.2	GO:1901363	M	.	heterocyclic compound binding
NM_001273490.1 D. m. bunched (bun), transcript variant O, mRNA	NM_001273490.1	5,00E-06	GLOS_BUN.1.2	GO:0043565	M	Refseq	sequence-specific DNA binding
NP_733313.2 CG31033, isoform C [Drosophila melanogaster]	NP_733313.2	0	GLOS_CG31033.1	GO:0000122	B	Refseq	negative regulation of transcription from RNA polymerase II promoter
NP_733313.2 CG31033, isoform C [Drosophila melanogaster]	NP_733313.2	0	GLOS_CG31033.1	GO:0001501	B	Refseq	skeletal system development
NP_733313.2 CG31033, isoform C [Drosophila melanogaster]	NP_733313.2	0	GLOS_CG31033.1	GO:0003700	M	Refseq	sequence-specific DNA binding transcription factor activity
NP_733313.2 CG31033, isoform C [Drosophila melanogaster]	NP_733313.2	0	GLOS_CG31033.1	GO:0005515	M	Refseq	protein binding
NP_733313.2 CG31033, isoform C [Drosophila melanogaster]	NP_733313.2	0	GLOS_CG31033.1	GO:0005634	C	Refseq	nucleus
NP_733313.2 CG31033, isoform C [Drosophila melanogaster]	NP_733313.2	0	GLOS_CG31033.1	GO:0006366	B	Refseq	transcription from RNA polymerase II promoter
NP_733313.2 CG31033, isoform C [Drosophila melanogaster]	NP_733313.2	0	GLOS_CG31033.1	GO:0006607	B	Refseq	NLS-bearing protein import into nucleus
NP_733313.2 CG31033, isoform C [Drosophila melanogaster]	NP_733313.2	0	GLOS_CG31033.1	GO:0008270	M	Refseq	zinc ion binding
NP_733313.2 CG31033, isoform C [Drosophila melanogaster]	NP_733313.2	0	GLOS_CG31033.1	GO:0032330	B	Refseq	regulation of chondrocyte differentiation
NP_733313.2 CG31033, isoform C [Drosophila melanogaster]	NP_733313.2	0	GLOS_CG31033.1	GO:0043565	M	Refseq	sequence-specific DNA binding
NP_001260615.1 CG43861, isoform C [Drosophila melanogaster]	NP_001260615.1	0	GLOS_CG43861.1	GO:0000122	B	Refseq	negative regulation of transcription from RNA polymerase II promoter
NP_001260615.1 CG43861, isoform C [Drosophila melanogaster]	NP_001260615.1	0	GLOS_CG43861.1	GO:0001501	B	Refseq	skeletal system development
NP_001260615.1 CG43861, isoform C [Drosophila melanogaster]	NP_001260615.1	0	GLOS_CG43861.1	GO:0003700	M	Refseq	sequence-specific DNA binding transcription factor activity
NP_001260615.1 CG43861, isoform C [Drosophila melanogaster]	NP_001260615.1	0	GLOS_CG43861.1	GO:0005515	M	Refseq	protein binding
NP_001260615.1 CG43861, isoform C [Drosophila melanogaster]	NP_001260615.1	0	GLOS_CG43861.1	GO:0005634	C	Refseq	nucleus
NP_001260615.1 CG43861, isoform C [Drosophila melanogaster]	NP_001260615.1	0	GLOS_CG43861.1	GO:0006366	B	Refseq	transcription from RNA polymerase II promoter
NP_001260615.1 CG43861, isoform C [Drosophila melanogaster]	NP_001260615.1	0	GLOS_CG43861.1	GO:0006607	B	Refseq	NLS-bearing protein import into nucleus
NP_001260615.1 CG43861, isoform C [Drosophila melanogaster]	NP_001260615.1	0	GLOS_CG43861.1	GO:0008270	M	Refseq	zinc ion binding
NP_001260615.1 CG43861, isoform C [Drosophila melanogaster]	NP_001260615.1	0	GLOS_CG43861.1	GO:0032330	B	Refseq	regulation of chondrocyte differentiation
NP_001260615.1 CG43861, isoform C [Drosophila melanogaster]	NP_001260615.1	0	GLOS_CG43861.1	GO:0043565	M	Refseq	sequence-specific DNA binding
NP_648639.1 CG10171, isoform A [Drosophila melanogaster]	NP_648639.1	1,00E-140	GLOS_CG10171.1	GO:0000122	B	Refseq	negative regulation of transcription from RNA polymerase II promoter
NP_648639.1 CG10171, isoform A [Drosophila melanogaster]	NP_648639.1	1,00E-140	GLOS_CG10171.1	GO:0001501	B	Refseq	skeletal system development
NP_648639.1 CG10171, isoform A [Drosophila melanogaster]	NP_648639.1	1,00E-140	GLOS_CG10171.1	GO:0003700	M	Refseq	sequence-specific DNA binding transcription factor activity
NP_648639.1 CG10171, isoform A [Drosophila melanogaster]	NP_648639.1	1,00E-140	GLOS_CG10171.1	GO:0005515	M	Refseq	protein binding
NP_648639.1 CG10171, isoform A [Drosophila melanogaster]	NP_648639.1	1,00E-140	GLOS_CG10171.1	GO:0005634	C	Refseq	nucleus
NP_648639.1 CG10171, isoform A [Drosophila melanogaster]	NP_648639.1	1,00E-140	GLOS_CG10171.1	GO:0006366	B	Refseq	transcription from RNA polymerase II promoter
NP_648639.1 CG10171, isoform A [Drosophila melanogaster]	NP_648639.1	1,00E-140	GLOS_CG10171.1	GO:0006607	B	Refseq	NLS-bearing protein import into nucleus
NP_648639.1 CG10171, isoform A [Drosophila melanogaster]	NP_648639.1	1,00E-140	GLOS_CG10171.1	GO:0008270	M	Refseq	zinc ion binding
NP_648639.1 CG10171, isoform A [Drosophila melanogaster]	NP_648639.1	1,00E-140	GLOS_CG10171.1	GO:0032330	B	Refseq	regulation of chondrocyte differentiation

NP_648639.1 CG10171, isoform A [Drosophila melanogaster]	NP_648639.1	1,00E-140	GLOS_CG10171.1	GO:0043565	M	Refseq	sequence-specific DNA binding
XP_001986263.1 GH20619 [Drosophila grimshawi]	XP_001986263.1	8,00E-34	GLOS_DGRI_GH2	GO:0005261	M	.	cation channel activity
XP_001986263.1 GH20619 [Drosophila grimshawi]	XP_001986263.1	8,00E-34	GLOS_DGRI_GH2	GO:0005216	M	.	ion channel activity
XP_001986263.1 GH20619 [Drosophila grimshawi]	XP_001986263.1	8,00E-34	GLOS_DGRI_GH2	GO:0015075	M	.	ion transmembrane transporter activity
XP_001986263.1 GH20619 [Drosophila grimshawi]	XP_001986263.1	8,00E-34	GLOS_DGRI_GH2	GO:0022891	M	.	substrate-specific transmembrane transporter activity
XP_001986263.1 GH20619 [Drosophila grimshawi]	XP_001986263.1	8,00E-34	GLOS_DGRI_GH2	GO:0022857	M	.	transmembrane transporter activity
XP_001986263.1 GH20619 [Drosophila grimshawi]	XP_001986263.1	8,00E-34	GLOS_DGRI_GH2	GO:0005215	M	.	transporter activity
XP_001986263.1 GH20619 [Drosophila grimshawi]	XP_001986263.1	8,00E-34	GLOS_DGRI_GH2	GO:0022892	M	.	substrate-specific transporter activity
XP_001986263.1 GH20619 [Drosophila grimshawi]	XP_001986263.1	8,00E-34	GLOS_DGRI_GH2	GO:0022838	M	.	substrate-specific channel activity
XP_001986263.1 GH20619 [Drosophila grimshawi]	XP_001986263.1	8,00E-34	GLOS_DGRI_GH2	GO:0015267	M	.	channel activity
XP_001986263.1 GH20619 [Drosophila grimshawi]	XP_001986263.1	8,00E-34	GLOS_DGRI_GH2	GO:0022803	M	.	passive transmembrane transporter activity
XP_001986263.1 GH20619 [Drosophila grimshawi]	XP_001986263.1	8,00E-34	GLOS_DGRI_GH2	GO:0008324	M	.	cation transmembrane transporter activity
XP_001986263.1 GH20619 [Drosophila grimshawi]	XP_001986263.1	8,00E-34	GLOS_DGRI_GH2	GO:0015085	M	.	calcium ion transmembrane transporter activity
XP_001986263.1 GH20619 [Drosophila grimshawi]	XP_001986263.1	8,00E-34	GLOS_DGRI_GH2	GO:0046873	M	.	metal ion transmembrane transporter activity
XP_001986263.1 GH20619 [Drosophila grimshawi]	XP_001986263.1	8,00E-34	GLOS_DGRI_GH2	GO:0022890	M	.	inorganic cation transmembrane transporter activity
XP_001986263.1 GH20619 [Drosophila grimshawi]	XP_001986263.1	8,00E-34	GLOS_DGRI_GH2	GO:0072509	M	.	divalent inorganic cation transmembrane transporter activity
XP_001986263.1 GH20619 [Drosophila grimshawi]	XP_001986263.1	8,00E-34	GLOS_DGRI_GH2	GO:0005262	M	Refseq	calcium channel activity
XP_001986263.1 GH20619 [Drosophila grimshawi]	XP_001986263.1	8,00E-34	GLOS_DGRI_GH2	GO:0005515	M	Refseq	protein binding
XP_001986263.1 GH20619 [Drosophila grimshawi]	XP_001986263.1	8,00E-34	GLOS_DGRI_GH2	GO:0016020	C	.	membrane
XP_001986263.1 GH20619 [Drosophila grimshawi]	XP_001986263.1	8,00E-34	GLOS_DGRI_GH2	GO:0071944	C	.	cell periphery
XP_001986263.1 GH20619 [Drosophila grimshawi]	XP_001986263.1	8,00E-34	GLOS_DGRI_GH2	GO:0005886	C	Refseq	plasma membrane
XP_001986263.1 GH20619 [Drosophila grimshawi]	XP_001986263.1	8,00E-34	GLOS_DGRI_GH2	GO:0016021	C	.	integral to membrane
XP_001986263.1 GH20619 [Drosophila grimshawi]	XP_001986263.1	8,00E-34	GLOS_DGRI_GH2	GO:0031224	C	.	intrinsic to membrane
XP_001986263.1 GH20619 [Drosophila grimshawi]	XP_001986263.1	8,00E-34	GLOS_DGRI_GH2	GO:0044425	C	.	membrane part
XP_001986263.1 GH20619 [Drosophila grimshawi]	XP_001986263.1	8,00E-34	GLOS_DGRI_GH2	GO:0031226	C	.	intrinsic to plasma membrane
XP_001986263.1 GH20619 [Drosophila grimshawi]	XP_001986263.1	8,00E-34	GLOS_DGRI_GH2	GO:0044459	C	.	plasma membrane part
XP_001986263.1 GH20619 [Drosophila grimshawi]	XP_001986263.1	8,00E-34	GLOS_DGRI_GH2	GO:0005886	C	.	plasma membrane
XP_001986263.1 GH20619 [Drosophila grimshawi]	XP_001986263.1	8,00E-34	GLOS_DGRI_GH2	GO:0005887	C	Refseq	integral to plasma membrane
XP_001986263.1 GH20619 [Drosophila grimshawi]	XP_001986263.1	8,00E-34	GLOS_DGRI_GH2	GO:0070838	B	.	divalent metal ion transport
XP_001986263.1 GH20619 [Drosophila grimshawi]	XP_001986263.1	8,00E-34	GLOS_DGRI_GH2	GO:0030001	B	.	metal ion transport
XP_001986263.1 GH20619 [Drosophila grimshawi]	XP_001986263.1	8,00E-34	GLOS_DGRI_GH2	GO:0006812	B	.	cation transport
XP_001986263.1 GH20619 [Drosophila grimshawi]	XP_001986263.1	8,00E-34	GLOS_DGRI_GH2	GO:0006811	B	.	ion transport
XP_001986263.1 GH20619 [Drosophila grimshawi]	XP_001986263.1	8,00E-34	GLOS_DGRI_GH2	GO:0072511	B	.	divalent inorganic cation transport
XP_001986263.1 GH20619 [Drosophila grimshawi]	XP_001986263.1	8,00E-34	GLOS_DGRI_GH2	GO:0006816	B	Refseq	calcium ion transport
XP_001986263.1 GH20619 [Drosophila grimshawi]	XP_001986263.1	8,00E-34	GLOS_DGRI_GH2	GO:0097485	B	.	neuron projection guidance
XP_001986263.1 GH20619 [Drosophila grimshawi]	XP_001986263.1	8,00E-34	GLOS_DGRI_GH2	GO:0006928	B	.	cellular component movement
XP_001986263.1 GH20619 [Drosophila grimshawi]	XP_001986263.1	8,00E-34	GLOS_DGRI_GH2	GO:0006935	B	.	chemotaxis
XP_001986263.1 GH20619 [Drosophila grimshawi]	XP_001986263.1	8,00E-34	GLOS_DGRI_GH2	GO:0042221	B	.	response to chemical stimulus
XP_001986263.1 GH20619 [Drosophila grimshawi]	XP_001986263.1	8,00E-34	GLOS_DGRI_GH2	GO:0050896	B	.	response to stimulus
XP_001986263.1 GH20619 [Drosophila grimshawi]	XP_001986263.1	8,00E-34	GLOS_DGRI_GH2	GO:0042330	B	.	taxis
XP_001986263.1 GH20619 [Drosophila grimshawi]	XP_001986263.1	8,00E-34	GLOS_DGRI_GH2	GO:0009605	B	.	response to external stimulus
XP_001986263.1 GH20619 [Drosophila grimshawi]	XP_001986263.1	8,00E-34	GLOS_DGRI_GH2	GO:0040011	B	.	locomotion
XP_001986263.1 GH20619 [Drosophila grimshawi]	XP_001986263.1	8,00E-34	GLOS_DGRI_GH2	GO:0048812	B	.	neuron projection morphogenesis
XP_001986263.1 GH20619 [Drosophila grimshawi]	XP_001986263.1	8,00E-34	GLOS_DGRI_GH2	GO:0048858	B	.	cell projection morphogenesis
XP_001986263.1 GH20619 [Drosophila grimshawi]	XP_001986263.1	8,00E-34	GLOS_DGRI_GH2	GO:0030030	B	.	cell projection organization
XP_001986263.1 GH20619 [Drosophila grimshawi]	XP_001986263.1	8,00E-34	GLOS_DGRI_GH2	GO:0016043	B	.	cellular component organization
XP_001986263.1 GH20619 [Drosophila grimshawi]	XP_001986263.1	8,00E-34	GLOS_DGRI_GH2	GO:0071840	B	.	cellular component organization or biogenesis
XP_001986263.1 GH20619 [Drosophila grimshawi]	XP_001986263.1	8,00E-34	GLOS_DGRI_GH2	GO:0032990	B	.	cell part morphogenesis
XP_001986263.1 GH20619 [Drosophila grimshawi]	XP_001986263.1	8,00E-34	GLOS_DGRI_GH2	GO:0032989	B	.	cellular component morphogenesis
XP_001986263.1 GH20619 [Drosophila grimshawi]	XP_001986263.1	8,00E-34	GLOS_DGRI_GH2	GO:0009653	B	.	anatomical structure morphogenesis
XP_001986263.1 GH20619 [Drosophila grimshawi]	XP_001986263.1	8,00E-34	GLOS_DGRI_GH2	GO:0044767	B	.	single-organism developmental process
XP_001986263.1 GH20619 [Drosophila grimshawi]	XP_001986263.1	8,00E-34	GLOS_DGRI_GH2	GO:0048869	B	.	cellular developmental process
XP_001986263.1 GH20619 [Drosophila grimshawi]	XP_001986263.1	8,00E-34	GLOS_DGRI_GH2	GO:0000902	B	.	cell morphogenesis
XP_001986263.1 GH20619 [Drosophila grimshawi]	XP_001986263.1	8,00E-34	GLOS_DGRI_GH2	GO:0031175	B	.	neuron projection development

XP_001986263.1 GH20619 [Drosophila grimshawi]	XP_001986263.1	8,00E-34	GLOS_DGRI_GH2	GO:0048666	B	.	neuron development
XP_001986263.1 GH20619 [Drosophila grimshawi]	XP_001986263.1	8,00E-34	GLOS_DGRI_GH2	GO:0048468	B	.	cell development
XP_001986263.1 GH20619 [Drosophila grimshawi]	XP_001986263.1	8,00E-34	GLOS_DGRI_GH2	GO:0030154	B	.	cell differentiation
XP_001986263.1 GH20619 [Drosophila grimshawi]	XP_001986263.1	8,00E-34	GLOS_DGRI_GH2	GO:0030182	B	.	neuron differentiation
XP_001986263.1 GH20619 [Drosophila grimshawi]	XP_001986263.1	8,00E-34	GLOS_DGRI_GH2	GO:0048699	B	.	generation of neurons
XP_001986263.1 GH20619 [Drosophila grimshawi]	XP_001986263.1	8,00E-34	GLOS_DGRI_GH2	GO:0022008	B	.	neurogenesis
XP_001986263.1 GH20619 [Drosophila grimshawi]	XP_001986263.1	8,00E-34	GLOS_DGRI_GH2	GO:0007399	B	.	nervous system development
XP_001986263.1 GH20619 [Drosophila grimshawi]	XP_001986263.1	8,00E-34	GLOS_DGRI_GH2	GO:0007409	B	.	axonogenesis
XP_001986263.1 GH20619 [Drosophila grimshawi]	XP_001986263.1	8,00E-34	GLOS_DGRI_GH2	GO:0048667	B	.	cell morphogenesis involved in neuron differentiation
XP_001986263.1 GH20619 [Drosophila grimshawi]	XP_001986263.1	8,00E-34	GLOS_DGRI_GH2	GO:0000904	B	.	cell morphogenesis involved in differentiation
XP_001986263.1 GH20619 [Drosophila grimshawi]	XP_001986263.1	8,00E-34	GLOS_DGRI_GH2	GO:0061564	B	.	axon development
XP_001986263.1 GH20619 [Drosophila grimshawi]	XP_001986263.1	8,00E-34	GLOS_DGRI_GH2	GO:0007411	B	Refseq	axon guidance
XP_001986263.1 GH20619 [Drosophila grimshawi]	XP_001986263.1	8,00E-34	GLOS_DGRI_GH2	GO:0007599	B	.	hemostasis
XP_001986263.1 GH20619 [Drosophila grimshawi]	XP_001986263.1	8,00E-34	GLOS_DGRI_GH2	GO:0050878	B	.	regulation of body fluid levels
XP_001986263.1 GH20619 [Drosophila grimshawi]	XP_001986263.1	8,00E-34	GLOS_DGRI_GH2	GO:0065008	B	.	regulation of biological quality
XP_001986263.1 GH20619 [Drosophila grimshawi]	XP_001986263.1	8,00E-34	GLOS_DGRI_GH2	GO:0050817	B	.	coagulation
XP_001986263.1 GH20619 [Drosophila grimshawi]	XP_001986263.1	8,00E-34	GLOS_DGRI_GH2	GO:0042060	B	.	wound healing
XP_001986263.1 GH20619 [Drosophila grimshawi]	XP_001986263.1	8,00E-34	GLOS_DGRI_GH2	GO:0009611	B	.	response to wounding
XP_001986263.1 GH20619 [Drosophila grimshawi]	XP_001986263.1	8,00E-34	GLOS_DGRI_GH2	GO:0006950	B	.	response to stress
XP_001986263.1 GH20619 [Drosophila grimshawi]	XP_001986263.1	8,00E-34	GLOS_DGRI_GH2	GO:0007596	B	Refseq	blood coagulation
XP_001986263.1 GH20619 [Drosophila grimshawi]	XP_001986263.1	8,00E-34	GLOS_DGRI_GH2	GO:0007165	B	.	signal transduction
XP_001986263.1 GH20619 [Drosophila grimshawi]	XP_001986263.1	8,00E-34	GLOS_DGRI_GH2	GO:0051716	B	.	cellular response to stimulus
XP_001986263.1 GH20619 [Drosophila grimshawi]	XP_001986263.1	8,00E-34	GLOS_DGRI_GH2	GO:0007154	B	.	cell communication
XP_001986263.1 GH20619 [Drosophila grimshawi]	XP_001986263.1	8,00E-34	GLOS_DGRI_GH2	GO:0044700	B	.	single organism signaling
XP_001986263.1 GH20619 [Drosophila grimshawi]	XP_001986263.1	8,00E-34	GLOS_DGRI_GH2	GO:0023052	B	.	signaling
XP_001986263.1 GH20619 [Drosophila grimshawi]	XP_001986263.1	8,00E-34	GLOS_DGRI_GH2	GO:0009583	B	.	detection of light stimulus
XP_001986263.1 GH20619 [Drosophila grimshawi]	XP_001986263.1	8,00E-34	GLOS_DGRI_GH2	GO:0009416	B	.	response to light stimulus
XP_001986263.1 GH20619 [Drosophila grimshawi]	XP_001986263.1	8,00E-34	GLOS_DGRI_GH2	GO:0009314	B	.	response to radiation
XP_001986263.1 GH20619 [Drosophila grimshawi]	XP_001986263.1	8,00E-34	GLOS_DGRI_GH2	GO:0009628	B	.	response to abiotic stimulus
XP_001986263.1 GH20619 [Drosophila grimshawi]	XP_001986263.1	8,00E-34	GLOS_DGRI_GH2	GO:0009581	B	.	detection of external stimulus
XP_001986263.1 GH20619 [Drosophila grimshawi]	XP_001986263.1	8,00E-34	GLOS_DGRI_GH2	GO:0051606	B	.	detection of stimulus
XP_001986263.1 GH20619 [Drosophila grimshawi]	XP_001986263.1	8,00E-34	GLOS_DGRI_GH2	GO:0009582	B	.	detection of abiotic stimulus
XP_001986263.1 GH20619 [Drosophila grimshawi]	XP_001986263.1	8,00E-34	GLOS_DGRI_GH2	GO:0007602	B	Refseq	phototransduction
XP_001986263.1 GH20619 [Drosophila grimshawi]	XP_001986263.1	8,00E-34	GLOS_DGRI_GH2	GO:0010522	B	.	regulation of calcium ion transport into cytosol
XP_001986263.1 GH20619 [Drosophila grimshawi]	XP_001986263.1	8,00E-34	GLOS_DGRI_GH2	GO:0032386	B	.	regulation of intracellular transport
XP_001986263.1 GH20619 [Drosophila grimshawi]	XP_001986263.1	8,00E-34	GLOS_DGRI_GH2	GO:0051049	B	.	regulation of transport
XP_001986263.1 GH20619 [Drosophila grimshawi]	XP_001986263.1	8,00E-34	GLOS_DGRI_GH2	GO:0032879	B	.	regulation of localization
XP_001986263.1 GH20619 [Drosophila grimshawi]	XP_001986263.1	8,00E-34	GLOS_DGRI_GH2	GO:0060341	B	.	regulation of cellular localization
XP_001986263.1 GH20619 [Drosophila grimshawi]	XP_001986263.1	8,00E-34	GLOS_DGRI_GH2	GO:0051924	B	.	regulation of calcium ion transport
XP_001986263.1 GH20619 [Drosophila grimshawi]	XP_001986263.1	8,00E-34	GLOS_DGRI_GH2	GO:0010959	B	.	regulation of metal ion transport
XP_001986263.1 GH20619 [Drosophila grimshawi]	XP_001986263.1	8,00E-34	GLOS_DGRI_GH2	GO:0043269	B	.	regulation of ion transport
XP_001986263.1 GH20619 [Drosophila grimshawi]	XP_001986263.1	8,00E-34	GLOS_DGRI_GH2	GO:2000021	B	.	regulation of ion homeostasis
XP_001986263.1 GH20619 [Drosophila grimshawi]	XP_001986263.1	8,00E-34	GLOS_DGRI_GH2	GO:0032844	B	.	regulation of homeostatic process
XP_001986263.1 GH20619 [Drosophila grimshawi]	XP_001986263.1	8,00E-34	GLOS_DGRI_GH2	GO:0032388	B	.	positive regulation of intracellular transport
XP_001986263.1 GH20619 [Drosophila grimshawi]	XP_001986263.1	8,00E-34	GLOS_DGRI_GH2	GO:0051050	B	.	positive regulation of transport
XP_001986263.1 GH20619 [Drosophila grimshawi]	XP_001986263.1	8,00E-34	GLOS_DGRI_GH2	GO:0048518	B	.	positive regulation of biological process
XP_001986263.1 GH20619 [Drosophila grimshawi]	XP_001986263.1	8,00E-34	GLOS_DGRI_GH2	GO:0032846	B	.	positive regulation of homeostatic process
XP_001986263.1 GH20619 [Drosophila grimshawi]	XP_001986263.1	8,00E-34	GLOS_DGRI_GH2	GO:0048522	B	.	positive regulation of cellular process
XP_001986263.1 GH20619 [Drosophila grimshawi]	XP_001986263.1	8,00E-34	GLOS_DGRI_GH2	GO:0051928	B	.	positive regulation of calcium ion transport
XP_001986263.1 GH20619 [Drosophila grimshawi]	XP_001986263.1	8,00E-34	GLOS_DGRI_GH2	GO:0043270	B	.	positive regulation of ion transport
XP_001986263.1 GH20619 [Drosophila grimshawi]	XP_001986263.1	8,00E-34	GLOS_DGRI_GH2	GO:0010524	B	Refseq	positive regulation of calcium ion transport into cytosol
XP_001986263.1 GH20619 [Drosophila grimshawi]	XP_001986263.1	8,00E-34	GLOS_DGRI_GH2	GO:0005262	M	.	calcium channel activity
XP_001986263.1 GH20619 [Drosophila grimshawi]	XP_001986263.1	8,00E-34	GLOS_DGRI_GH2	GO:0015276	M	.	ligand-gated ion channel activity
XP_001986263.1 GH20619 [Drosophila grimshawi]	XP_001986263.1	8,00E-34	GLOS_DGRI_GH2	GO:0022834	M	.	ligand-gated channel activity

XP_001986263.1 GH20619 [Drosophila grimshawi]	XP_001986263.1	8,00E-34	GLOS_DGRI_GH2	GO:0022836	M	.	gated channel activity
XP_001986263.1 GH20619 [Drosophila grimshawi]	XP_001986263.1	8,00E-34	GLOS_DGRI_GH2	GO:0022839	M	.	ion gated channel activity
XP_001986263.1 GH20619 [Drosophila grimshawi]	XP_001986263.1	8,00E-34	GLOS_DGRI_GH2	GO:0015279	M	Refseq	store-operated calcium channel activity
XP_001986263.1 GH20619 [Drosophila grimshawi]	XP_001986263.1	8,00E-34	GLOS_DGRI_GH2	GO:0001775	B	.	cell activation
XP_001986263.1 GH20619 [Drosophila grimshawi]	XP_001986263.1	8,00E-34	GLOS_DGRI_GH2	GO:0007596	B	.	blood coagulation
XP_001986263.1 GH20619 [Drosophila grimshawi]	XP_001986263.1	8,00E-34	GLOS_DGRI_GH2	GO:0030168	B	Refseq	platelet activation
XP_001986263.1 GH20619 [Drosophila grimshawi]	XP_001986263.1	8,00E-34	GLOS_DGRI_GH2	GO:0014074	B	.	response to purine-containing compound
XP_001986263.1 GH20619 [Drosophila grimshawi]	XP_001986263.1	8,00E-34	GLOS_DGRI_GH2	GO:0010243	B	.	response to organonitrogen compound
XP_001986263.1 GH20619 [Drosophila grimshawi]	XP_001986263.1	8,00E-34	GLOS_DGRI_GH2	GO:0009719	B	.	response to endogenous stimulus
XP_001986263.1 GH20619 [Drosophila grimshawi]	XP_001986263.1	8,00E-34	GLOS_DGRI_GH2	GO:0010033	B	.	response to organic substance
XP_001986263.1 GH20619 [Drosophila grimshawi]	XP_001986263.1	8,00E-34	GLOS_DGRI_GH2	GO:1901698	B	.	response to nitrogen compound
XP_001986263.1 GH20619 [Drosophila grimshawi]	XP_001986263.1	8,00E-34	GLOS_DGRI_GH2	GO:0014070	B	.	response to organic cyclic compound
XP_001986263.1 GH20619 [Drosophila grimshawi]	XP_001986263.1	8,00E-34	GLOS_DGRI_GH2	GO:0046683	B	.	response to organophosphorus
XP_001986263.1 GH20619 [Drosophila grimshawi]	XP_001986263.1	8,00E-34	GLOS_DGRI_GH2	GO:1901700	B	.	response to oxygen-containing compound
XP_001986263.1 GH20619 [Drosophila grimshawi]	XP_001986263.1	8,00E-34	GLOS_DGRI_GH2	GO:0033198	B	Refseq	response to ATP
XP_001986263.1 GH20619 [Drosophila grimshawi]	XP_001986263.1	8,00E-34	GLOS_DGRI_GH2	GO:0010038	B	.	response to metal ion
XP_001986263.1 GH20619 [Drosophila grimshawi]	XP_001986263.1	8,00E-34	GLOS_DGRI_GH2	GO:0010035	B	.	response to inorganic substance
XP_001986263.1 GH20619 [Drosophila grimshawi]	XP_001986263.1	8,00E-34	GLOS_DGRI_GH2	GO:0051592	B	Refseq	response to calcium ion
XP_001986263.1 GH20619 [Drosophila grimshawi]	XP_001986263.1	8,00E-34	GLOS_DGRI_GH2	GO:0006816	B	.	calcium ion transport
XP_001986263.1 GH20619 [Drosophila grimshawi]	XP_001986263.1	8,00E-34	GLOS_DGRI_GH2	GO:0034220	B	.	ion transmembrane transport
XP_001986263.1 GH20619 [Drosophila grimshawi]	XP_001986263.1	8,00E-34	GLOS_DGRI_GH2	GO:0055085	B	.	transmembrane transport
XP_001986263.1 GH20619 [Drosophila grimshawi]	XP_001986263.1	8,00E-34	GLOS_DGRI_GH2	GO:0070588	B	Refseq	calcium ion transmembrane transport
XP_001986263.1 GH20619 [Drosophila grimshawi]	XP_001986263.1	8,00E-34	GLOS_DGRI_GH2	GO:0043168	M	.	anion binding
XP_001986263.1 GH20619 [Drosophila grimshawi]	XP_001986263.1	8,00E-34	GLOS_DGRI_GH2	GO:0043178	M	.	alcohol binding
XP_001986263.1 GH20619 [Drosophila grimshawi]	XP_001986263.1	8,00E-34	GLOS_DGRI_GH2	GO:0036094	M	.	small molecule binding
XP_001986263.1 GH20619 [Drosophila grimshawi]	XP_001986263.1	8,00E-34	GLOS_DGRI_GH2	GO:0070679	M	Refseq	inositol 1,4,5 trisphosphate binding
XM_003581200.1 PREDIC: B. distachyon uncharact. (LOC100843429), mRNA	XM_003581200.1	6,00E-19	GLOS_LOC10084	GO:0048646	B	.	anatomical structure formation involved in morphogenesis
XM_003581200.1 PREDIC: B. distachyon uncharact. (LOC100843429), mRNA	XM_003581200.1	6,00E-19	GLOS_LOC10084	GO:0048514	B	.	blood vessel morphogenesis
XM_003581200.1 PREDIC: B. distachyon uncharact. (LOC100843429), mRNA	XM_003581200.1	6,00E-19	GLOS_LOC10084	GO:0001568	B	.	blood vessel development
XM_003581200.1 PREDIC: B. distachyon uncharact. (LOC100843429), mRNA	XM_003581200.1	6,00E-19	GLOS_LOC10084	GO:0001944	B	.	vasculature development
XM_003581200.1 PREDIC: B. distachyon uncharact. (LOC100843429), mRNA	XM_003581200.1	6,00E-19	GLOS_LOC10084	GO:0072358	B	.	cardiovascular system development
XM_003581200.1 PREDIC: B. distachyon uncharact. (LOC100843429), mRNA	XM_003581200.1	6,00E-19	GLOS_LOC10084	GO:0072359	B	.	circulatory system development
XM_003581200.1 PREDIC: B. distachyon uncharact. (LOC100843429), mRNA	XM_003581200.1	6,00E-19	GLOS_LOC10084	GO:0001525	B	Refseq	angiogenesis
XM_003581200.1 PREDIC: B. distachyon uncharact. (LOC100843429), mRNA	XM_003581200.1	6,00E-19	GLOS_LOC10084	GO:0006954	B	.	inflammatory response
XM_003581200.1 PREDIC: B. distachyon uncharact. (LOC100843429), mRNA	XM_003581200.1	6,00E-19	GLOS_LOC10084	GO:0006952	B	.	defense response
XM_003581200.1 PREDIC: B. distachyon uncharact. (LOC100843429), mRNA	XM_003581200.1	6,00E-19	GLOS_LOC10084	GO:0002246	B	Refseq	wound healing involved in inflammatory response
XM_003581200.1 PREDIC: B. distachyon uncharact. (LOC100843429), mRNA	XM_003581200.1	6,00E-19	GLOS_LOC10084	GO:0016705	M	.	oxidored. activ, on paired donors, with incorporation or reduct of O2
XM_003581200.1 PREDIC: B. distachyon uncharact. (LOC100843429), mRNA	XM_003581200.1	6,00E-19	GLOS_LOC10084	GO:0016491	M	.	oxidoreductase activity
XM_003581200.1 PREDIC: B. distachyon uncharact. (LOC100843429), mRNA	XM_003581200.1	6,00E-19	GLOS_LOC10084	GO:0003824	M	.	catalytic activity
XM_003581200.1 PREDIC: B. distachyon uncharact. (LOC100843429), mRNA	XM_003581200.1	6,00E-19	GLOS_LOC10084	GO:0004392	M	Refseq	heme oxygenase (decyclizing) activity
XM_003581200.1 PREDIC: B. distachyon uncharact. (LOC100843429), mRNA	XM_003581200.1	6,00E-19	GLOS_LOC10084	GO:0004620	M	.	phospholipase activity
XM_003581200.1 PREDIC: B. distachyon uncharact. (LOC100843429), mRNA	XM_003581200.1	6,00E-19	GLOS_LOC10084	GO:0016298	M	.	lipase activity
XM_003581200.1 PREDIC: B. distachyon uncharact. (LOC100843429), mRNA	XM_003581200.1	6,00E-19	GLOS_LOC10084	GO:0016788	M	.	hydrolase activity, acting on ester bonds
XM_003581200.1 PREDIC: B. distachyon uncharact. (LOC100843429), mRNA	XM_003581200.1	6,00E-19	GLOS_LOC10084	GO:0016787	M	.	hydrolase activity
XM_003581200.1 PREDIC: B. distachyon uncharact. (LOC100843429), mRNA	XM_003581200.1	6,00E-19	GLOS_LOC10084	GO:0008081	M	.	phosphoric diester hydrolase activity
XM_003581200.1 PREDIC: B. distachyon uncharact. (LOC100843429), mRNA	XM_003581200.1	6,00E-19	GLOS_LOC10084	GO:0042578	M	.	phosphoric ester hydrolase activity
XM_003581200.1 PREDIC: B. distachyon uncharact. (LOC100843429), mRNA	XM_003581200.1	6,00E-19	GLOS_LOC10084	GO:0004630	M	Refseq	phospholipase D activity
XM_003581200.1 PREDIC: B. distachyon uncharact. (LOC100843429), mRNA	XM_003581200.1	6,00E-19	GLOS_LOC10084	GO:0060089	M	.	molecular transducer activity
XM_003581200.1 PREDIC: B. distachyon uncharact. (LOC100843429), mRNA	XM_003581200.1	6,00E-19	GLOS_LOC10084	GO:0004871	M	Refseq	signal transducer activity
XM_003581200.1 PREDIC: B. distachyon uncharact. (LOC100843429), mRNA	XM_003581200.1	6,00E-19	GLOS_LOC10084	GO:0005634	C	Refseq	nucleus
XM_003581200.1 PREDIC: B. distachyon uncharact. (LOC100843429), mRNA	XM_003581200.1	6,00E-19	GLOS_LOC10084	GO:0043232	C	.	intracellular non-membrane-bounded organelle
XM_003581200.1 PREDIC: B. distachyon uncharact. (LOC100843429), mRNA	XM_003581200.1	6,00E-19	GLOS_LOC10084	GO:0043228	C	.	non-membrane-bounded organelle
XM_003581200.1 PREDIC: B. distachyon uncharact. (LOC100843429), mRNA	XM_003581200.1	6,00E-19	GLOS_LOC10084	GO:0044428	C	.	nuclear part
XM_003581200.1 PREDIC: B. distachyon uncharact. (LOC100843429), mRNA	XM_003581200.1	6,00E-19	GLOS_LOC10084	GO:0044446	C	.	intracellular organelle part

XM_003581200.1 PREDIC: B. distachyon uncharact. (LOC100843429), mRNA	XM_003581200.1	6,00E-19	GLOS_LOC10084:	GO:0044422	C	.	organelle part
XM_003581200.1 PREDIC: B. distachyon uncharact. (LOC100843429), mRNA	XM_003581200.1	6,00E-19	GLOS_LOC10084:	GO:0005634	C	.	nucleus
XM_003581200.1 PREDIC: B. distachyon uncharact. (LOC100843429), mRNA	XM_003581200.1	6,00E-19	GLOS_LOC10084:	GO:0031981	C	.	nuclear lumen
XM_003581200.1 PREDIC: B. distachyon uncharact. (LOC100843429), mRNA	XM_003581200.1	6,00E-19	GLOS_LOC10084:	GO:0070013	C	.	intracellular organelle lumen
XM_003581200.1 PREDIC: B. distachyon uncharact. (LOC100843429), mRNA	XM_003581200.1	6,00E-19	GLOS_LOC10084:	GO:0043233	C	.	organelle lumen
XM_003581200.1 PREDIC: B. distachyon uncharact. (LOC100843429), mRNA	XM_003581200.1	6,00E-19	GLOS_LOC10084:	GO:0031974	C	.	membrane-enclosed lumen
XM_003581200.1 PREDIC: B. distachyon uncharact. (LOC100843429), mRNA	XM_003581200.1	6,00E-19	GLOS_LOC10084:	GO:0005730	C	Refseq	nucleolus
XM_003581200.1 PREDIC: B. distachyon uncharact. (LOC100843429), mRNA	XM_003581200.1	6,00E-19	GLOS_LOC10084:	GO:0044444	C	.	cytoplasmic part
XM_003581200.1 PREDIC: B. distachyon uncharact. (LOC100843429), mRNA	XM_003581200.1	6,00E-19	GLOS_LOC10084:	GO:0005737	C	.	cytoplasm
XM_003581200.1 PREDIC: B. distachyon uncharact. (LOC100843429), mRNA	XM_003581200.1	6,00E-19	GLOS_LOC10084:	GO:0005783	C	Refseq	endoplasmic reticulum
XM_003581200.1 PREDIC: B. distachyon uncharact. (LOC100843429), mRNA	XM_003581200.1	6,00E-19	GLOS_LOC10084:	GO:0005829	C	Refseq	cytosol
XM_003581200.1 PREDIC: B. distachyon uncharact. (LOC100843429), mRNA	XM_003581200.1	6,00E-19	GLOS_LOC10084:	GO:0045121	C	.	membrane raft
XM_003581200.1 PREDIC: B. distachyon uncharact. (LOC100843429), mRNA	XM_003581200.1	6,00E-19	GLOS_LOC10084:	GO:0005901	C	Refseq	caveola
XM_003581200.1 PREDIC: B. distachyon uncharact. (LOC100843429), mRNA	XM_003581200.1	6,00E-19	GLOS_LOC10084:	GO:0042168	B	.	heme metabolic process
XM_003581200.1 PREDIC: B. distachyon uncharact. (LOC100843429), mRNA	XM_003581200.1	6,00E-19	GLOS_LOC10084:	GO:0006778	B	.	porphyrin-containing compound metabolic process
XM_003581200.1 PREDIC: B. distachyon uncharact. (LOC100843429), mRNA	XM_003581200.1	6,00E-19	GLOS_LOC10084:	GO:0033013	B	.	tetrapyrrole metabolic process
XM_003581200.1 PREDIC: B. distachyon uncharact. (LOC100843429), mRNA	XM_003581200.1	6,00E-19	GLOS_LOC10084:	GO:1901564	B	.	organonitrogen compound metabolic process
XM_003581200.1 PREDIC: B. distachyon uncharact. (LOC100843429), mRNA	XM_003581200.1	6,00E-19	GLOS_LOC10084:	GO:0051186	B	.	cofactor metabolic process
XM_003581200.1 PREDIC: B. distachyon uncharact. (LOC100843429), mRNA	XM_003581200.1	6,00E-19	GLOS_LOC10084:	GO:0042440	B	.	pigment metabolic process
XM_003581200.1 PREDIC: B. distachyon uncharact. (LOC100843429), mRNA	XM_003581200.1	6,00E-19	GLOS_LOC10084:	GO:0044710	B	.	single-organism metabolic process
XM_003581200.1 PREDIC: B. distachyon uncharact. (LOC100843429), mRNA	XM_003581200.1	6,00E-19	GLOS_LOC10084:	GO:0055114	B	.	oxidation-reduction process
XM_003581200.1 PREDIC: B. distachyon uncharact. (LOC100843429), mRNA	XM_003581200.1	6,00E-19	GLOS_LOC10084:	GO:0006787	B	.	porphyrin-containing compound catabolic process
XM_003581200.1 PREDIC: B. distachyon uncharact. (LOC100843429), mRNA	XM_003581200.1	6,00E-19	GLOS_LOC10084:	GO:0033015	B	.	tetrapyrrole catabolic process
XM_003581200.1 PREDIC: B. distachyon uncharact. (LOC100843429), mRNA	XM_003581200.1	6,00E-19	GLOS_LOC10084:	GO:0019439	B	.	aromatic compound catabolic process
XM_003581200.1 PREDIC: B. distachyon uncharact. (LOC100843429), mRNA	XM_003581200.1	6,00E-19	GLOS_LOC10084:	GO:0044248	B	.	cellular catabolic process
XM_003581200.1 PREDIC: B. distachyon uncharact. (LOC100843429), mRNA	XM_003581200.1	6,00E-19	GLOS_LOC10084:	GO:0009056	B	.	catabolic process
XM_003581200.1 PREDIC: B. distachyon uncharact. (LOC100843429), mRNA	XM_003581200.1	6,00E-19	GLOS_LOC10084:	GO:0044270	B	.	cellular nitrogen compound catabolic process
XM_003581200.1 PREDIC: B. distachyon uncharact. (LOC100843429), mRNA	XM_003581200.1	6,00E-19	GLOS_LOC10084:	GO:0046700	B	.	heterocycle catabolic process
XM_003581200.1 PREDIC: B. distachyon uncharact. (LOC100843429), mRNA	XM_003581200.1	6,00E-19	GLOS_LOC10084:	GO:1901361	B	.	organic cyclic compound catabolic process
XM_003581200.1 PREDIC: B. distachyon uncharact. (LOC100843429), mRNA	XM_003581200.1	6,00E-19	GLOS_LOC10084:	GO:1901575	B	.	organic substance catabolic process
XM_003581200.1 PREDIC: B. distachyon uncharact. (LOC100843429), mRNA	XM_003581200.1	6,00E-19	GLOS_LOC10084:	GO:1901565	B	.	organonitrogen compound catabolic process
XM_003581200.1 PREDIC: B. distachyon uncharact. (LOC100843429), mRNA	XM_003581200.1	6,00E-19	GLOS_LOC10084:	GO:0051187	B	.	cofactor catabolic process
XM_003581200.1 PREDIC: B. distachyon uncharact. (LOC100843429), mRNA	XM_003581200.1	6,00E-19	GLOS_LOC10084:	GO:0006788	B	Refseq	heme oxidation
XM_003581200.1 PREDIC: B. distachyon uncharact. (LOC100843429), mRNA	XM_003581200.1	6,00E-19	GLOS_LOC10084:	GO:0012501	B	.	programmed cell death
XM_003581200.1 PREDIC: B. distachyon uncharact. (LOC100843429), mRNA	XM_003581200.1	6,00E-19	GLOS_LOC10084:	GO:0008219	B	.	cell death
XM_003581200.1 PREDIC: B. distachyon uncharact. (LOC100843429), mRNA	XM_003581200.1	6,00E-19	GLOS_LOC10084:	GO:0016265	B	.	death
XM_003581200.1 PREDIC: B. distachyon uncharact. (LOC100843429), mRNA	XM_003581200.1	6,00E-19	GLOS_LOC10084:	GO:0006915	B	Refseq	apoptotic process
XM_003581200.1 PREDIC: B. distachyon uncharact. (LOC100843429), mRNA	XM_003581200.1	6,00E-19	GLOS_LOC10084:	GO:0006979	B	Refseq	response to oxidative stress
XM_003581200.1 PREDIC: B. distachyon uncharact. (LOC100843429), mRNA	XM_003581200.1	6,00E-19	GLOS_LOC10084:	GO:0035556	B	.	intracellular signal transduction
XM_003581200.1 PREDIC: B. distachyon uncharact. (LOC100843429), mRNA	XM_003581200.1	6,00E-19	GLOS_LOC10084:	GO:0007264	B	Refseq	small GTPase mediated signal transduction
XM_003581200.1 PREDIC: B. distachyon uncharact. (LOC100843429), mRNA	XM_003581200.1	6,00E-19	GLOS_LOC10084:	GO:0008015	B	.	blood circulation
XM_003581200.1 PREDIC: B. distachyon uncharact. (LOC100843429), mRNA	XM_003581200.1	6,00E-19	GLOS_LOC10084:	GO:0003013	B	.	circulatory system process
XM_003581200.1 PREDIC: B. distachyon uncharact. (LOC100843429), mRNA	XM_003581200.1	6,00E-19	GLOS_LOC10084:	GO:0003008	B	.	system process
XM_003581200.1 PREDIC: B. distachyon uncharact. (LOC100843429), mRNA	XM_003581200.1	6,00E-19	GLOS_LOC10084:	GO:0008217	B	Refseq	regulation of blood pressure
XM_003581200.1 PREDIC: B. distachyon uncharact. (LOC100843429), mRNA	XM_003581200.1	6,00E-19	GLOS_LOC10084:	GO:0008219	B	Refseq	cell death
XM_003581200.1 PREDIC: B. distachyon uncharact. (LOC100843429), mRNA	XM_003581200.1	6,00E-19	GLOS_LOC10084:	GO:0042127	B	.	regulation of cell proliferation
XM_003581200.1 PREDIC: B. distachyon uncharact. (LOC100843429), mRNA	XM_003581200.1	6,00E-19	GLOS_LOC10084:	GO:0008285	B	Refseq	negative regulation of cell proliferation
XM_003581200.1 PREDIC: B. distachyon uncharact. (LOC100843429), mRNA	XM_003581200.1	6,00E-19	GLOS_LOC10084:	GO:0006974	B	.	cellular response to DNA damage stimulus
XM_003581200.1 PREDIC: B. distachyon uncharact. (LOC100843429), mRNA	XM_003581200.1	6,00E-19	GLOS_LOC10084:	GO:0033554	B	.	cellular response to stress
XM_003581200.1 PREDIC: B. distachyon uncharact. (LOC100843429), mRNA	XM_003581200.1	6,00E-19	GLOS_LOC10084:	GO:0097193	B	.	intrinsic apoptotic signaling pathway
XM_003581200.1 PREDIC: B. distachyon uncharact. (LOC100843429), mRNA	XM_003581200.1	6,00E-19	GLOS_LOC10084:	GO:0097190	B	.	apoptotic signaling pathway
XM_003581200.1 PREDIC: B. distachyon uncharact. (LOC100843429), mRNA	XM_003581200.1	6,00E-19	GLOS_LOC10084:	GO:0006915	B	.	apoptotic process
XM_003581200.1 PREDIC: B. distachyon uncharact. (LOC100843429), mRNA	XM_003581200.1	6,00E-19	GLOS_LOC10084:	GO:0008630	B	Refseq	intrinsic apoptotic signaling pathway in response to DNA damage
XM_003581200.1 PREDIC: B. distachyon uncharact. (LOC100843429), mRNA	XM_003581200.1	6,00E-19	GLOS_LOC10084:	GO:0010660	B	.	regulation of muscle cell apoptotic process

XM_003581200.1 PREDIC: B. distachyon uncharact. (LOC100843429), mRNA	XM_003581200.1	6,00E-19	GLOS_LOC10084:	GO:0042981	B	.	regulation of apoptotic process
XM_003581200.1 PREDIC: B. distachyon uncharact. (LOC100843429), mRNA	XM_003581200.1	6,00E-19	GLOS_LOC10084:	GO:0043067	B	.	regulation of programmed cell death
XM_003581200.1 PREDIC: B. distachyon uncharact. (LOC100843429), mRNA	XM_003581200.1	6,00E-19	GLOS_LOC10084:	GO:0010941	B	.	regulation of cell death
XM_003581200.1 PREDIC: B. distachyon uncharact. (LOC100843429), mRNA	XM_003581200.1	6,00E-19	GLOS_LOC10084:	GO:0043066	B	.	negative regulation of apoptotic process
XM_003581200.1 PREDIC: B. distachyon uncharact. (LOC100843429), mRNA	XM_003581200.1	6,00E-19	GLOS_LOC10084:	GO:0043069	B	.	negative regulation of programmed cell death
XM_003581200.1 PREDIC: B. distachyon uncharact. (LOC100843429), mRNA	XM_003581200.1	6,00E-19	GLOS_LOC10084:	GO:0060548	B	.	negative regulation of cell death
XM_003581200.1 PREDIC: B. distachyon uncharact. (LOC100843429), mRNA	XM_003581200.1	6,00E-19	GLOS_LOC10084:	GO:0010656	B	Refseq	negative regulation of muscle cell apoptotic process
XM_003581200.1 PREDIC: B. distachyon uncharact. (LOC100843429), mRNA	XM_003581200.1	6,00E-19	GLOS_LOC10084:	GO:0016020	C	Refseq	membrane
XM_003581200.1 PREDIC: B. distachyon uncharact. (LOC100843429), mRNA	XM_003581200.1	6,00E-19	GLOS_LOC10084:	GO:0016491	M	Refseq	oxidoreductase activity
XM_003581200.1 PREDIC: B. distachyon uncharact. (LOC100843429), mRNA	XM_003581200.1	6,00E-19	GLOS_LOC10084:	GO:0005515	M	.	protein binding
XM_003581200.1 PREDIC: B. distachyon uncharact. (LOC100843429), mRNA	XM_003581200.1	6,00E-19	GLOS_LOC10084:	GO:0019899	M	Refseq	enzyme binding
XM_003581200.1 PREDIC: B. distachyon uncharact. (LOC100843429), mRNA	XM_003581200.1	6,00E-19	GLOS_LOC10084:	GO:0046906	M	.	tetrapyrrole binding
XM_003581200.1 PREDIC: B. distachyon uncharact. (LOC100843429), mRNA	XM_003581200.1	6,00E-19	GLOS_LOC10084:	GO:0020037	M	Refseq	heme binding
XM_003581200.1 PREDIC: B. distachyon uncharact. (LOC100843429), mRNA	XM_003581200.1	6,00E-19	GLOS_LOC10084:	GO:0002719	B	.	negative regul. of cytokine product. involved in immune resp.
XM_003581200.1 PREDIC: B. distachyon uncharact. (LOC100843429), mRNA	XM_003581200.1	6,00E-19	GLOS_LOC10084:	GO:0001818	B	.	negative regulation of cytokine production
XM_003581200.1 PREDIC: B. distachyon uncharact. (LOC100843429), mRNA	XM_003581200.1	6,00E-19	GLOS_LOC10084:	GO:0001817	B	.	regulation of cytokine production
XM_003581200.1 PREDIC: B. distachyon uncharact. (LOC100843429), mRNA	XM_003581200.1	6,00E-19	GLOS_LOC10084:	GO:0051241	B	.	negative regulation of multicellular organismal process
XM_003581200.1 PREDIC: B. distachyon uncharact. (LOC100843429), mRNA	XM_003581200.1	6,00E-19	GLOS_LOC10084:	GO:0002701	B	.	negative regul. of prod. of molecular mediator of immune resp.
XM_003581200.1 PREDIC: B. distachyon uncharact. (LOC100843429), mRNA	XM_003581200.1	6,00E-19	GLOS_LOC10084:	GO:0002698	B	.	negative regulation of immune effector process
XM_003581200.1 PREDIC: B. distachyon uncharact. (LOC100843429), mRNA	XM_003581200.1	6,00E-19	GLOS_LOC10084:	GO:0002683	B	.	negative regulation of immune system process
XM_003581200.1 PREDIC: B. distachyon uncharact. (LOC100843429), mRNA	XM_003581200.1	6,00E-19	GLOS_LOC10084:	GO:0002682	B	.	regulation of immune system process
XM_003581200.1 PREDIC: B. distachyon uncharact. (LOC100843429), mRNA	XM_003581200.1	6,00E-19	GLOS_LOC10084:	GO:0002697	B	.	regulation of immune effector process
XM_003581200.1 PREDIC: B. distachyon uncharact. (LOC100843429), mRNA	XM_003581200.1	6,00E-19	GLOS_LOC10084:	GO:0002700	B	.	regul. of prod. of molecular mediator of immune response
XM_003581200.1 PREDIC: B. distachyon uncharact. (LOC100843429), mRNA	XM_003581200.1	6,00E-19	GLOS_LOC10084:	GO:0002718	B	.	regul. of cytokine production involved in immune response
XM_003581200.1 PREDIC: B. distachyon uncharact. (LOC100843429), mRNA	XM_003581200.1	6,00E-19	GLOS_LOC10084:	GO:0050776	B	.	regulation of immune response
XM_003581200.1 PREDIC: B. distachyon uncharact. (LOC100843429), mRNA	XM_003581200.1	6,00E-19	GLOS_LOC10084:	GO:0048583	B	.	regulation of response to stimulus
XM_003581200.1 PREDIC: B. distachyon uncharact. (LOC100843429), mRNA	XM_003581200.1	6,00E-19	GLOS_LOC10084:	GO:0032763	B	.	regulation of mast cell cytokine production
XM_003581200.1 PREDIC: B. distachyon uncharact. (LOC100843429), mRNA	XM_003581200.1	6,00E-19	GLOS_LOC10084:	GO:0032764	B	Refseq	negative regulation of mast cell cytokine production
XM_003581200.1 PREDIC: B. distachyon uncharact. (LOC100843429), mRNA	XM_003581200.1	6,00E-19	GLOS_LOC10084:	GO:0002262	B	.	myeloid cell homeostasis
XM_003581200.1 PREDIC: B. distachyon uncharact. (LOC100843429), mRNA	XM_003581200.1	6,00E-19	GLOS_LOC10084:	GO:0002376	B	.	immune system process
XM_003581200.1 PREDIC: B. distachyon uncharact. (LOC100843429), mRNA	XM_003581200.1	6,00E-19	GLOS_LOC10084:	GO:0048872	B	.	homeostasis of number of cells
XM_003581200.1 PREDIC: B. distachyon uncharact. (LOC100843429), mRNA	XM_003581200.1	6,00E-19	GLOS_LOC10084:	GO:0042592	B	.	homeostatic process
XM_003581200.1 PREDIC: B. distachyon uncharact. (LOC100843429), mRNA	XM_003581200.1	6,00E-19	GLOS_LOC10084:	GO:0034101	B	Refseq	erythrocyte homeostasis
XM_003581200.1 PREDIC: B. distachyon uncharact. (LOC100843429), mRNA	XM_003581200.1	6,00E-19	GLOS_LOC10084:	GO:0043279	B	.	response to alkaloid
XM_003581200.1 PREDIC: B. distachyon uncharact. (LOC100843429), mRNA	XM_003581200.1	6,00E-19	GLOS_LOC10084:	GO:0035094	B	Refseq	response to nicotine
XM_003581200.1 PREDIC: B. distachyon uncharact. (LOC100843429), mRNA	XM_003581200.1	6,00E-19	GLOS_LOC10084:	GO:0035556	B	Refseq	intracellular signal transduction
XM_003581200.1 PREDIC: B. distachyon uncharact. (LOC100843429), mRNA	XM_003581200.1	6,00E-19	GLOS_LOC10084:	GO:0046149	B	.	pigment catabolic process
XM_003581200.1 PREDIC: B. distachyon uncharact. (LOC100843429), mRNA	XM_003581200.1	6,00E-19	GLOS_LOC10084:	GO:0042167	B	Refseq	heme catabolic process
XM_003581200.1 PREDIC: B. distachyon uncharact. (LOC100843429), mRNA	XM_003581200.1	6,00E-19	GLOS_LOC10084:	GO:0042168	B	Refseq	heme metabolic process
XM_003581200.1 PREDIC: B. distachyon uncharact. (LOC100843429), mRNA	XM_003581200.1	6,00E-19	GLOS_LOC10084:	GO:0000302	B	.	response to reactive oxygen species
XM_003581200.1 PREDIC: B. distachyon uncharact. (LOC100843429), mRNA	XM_003581200.1	6,00E-19	GLOS_LOC10084:	GO:0006979	B	.	response to oxidative stress
XM_003581200.1 PREDIC: B. distachyon uncharact. (LOC100843429), mRNA	XM_003581200.1	6,00E-19	GLOS_LOC10084:	GO:0042542	B	Refseq	response to hydrogen peroxide
XM_003581200.1 PREDIC: B. distachyon uncharact. (LOC100843429), mRNA	XM_003581200.1	6,00E-19	GLOS_LOC10084:	GO:0042802	M	.	identical protein binding
XM_003581200.1 PREDIC: B. distachyon uncharact. (LOC100843429), mRNA	XM_003581200.1	6,00E-19	GLOS_LOC10084:	GO:0046983	M	.	protein dimerization activity
XM_003581200.1 PREDIC: B. distachyon uncharact. (LOC100843429), mRNA	XM_003581200.1	6,00E-19	GLOS_LOC10084:	GO:0042803	M	Refseq	protein homodimerization activity
XM_003581200.1 PREDIC: B. distachyon uncharact. (LOC100843429), mRNA	XM_003581200.1	6,00E-19	GLOS_LOC10084:	GO:0010740	B	.	positive regulation of intracellular protein kinase cascade
XM_003581200.1 PREDIC: B. distachyon uncharact. (LOC100843429), mRNA	XM_003581200.1	6,00E-19	GLOS_LOC10084:	GO:0009967	B	.	positive regulation of signal transduction
XM_003581200.1 PREDIC: B. distachyon uncharact. (LOC100843429), mRNA	XM_003581200.1	6,00E-19	GLOS_LOC10084:	GO:0009966	B	.	regulation of signal transduction
XM_003581200.1 PREDIC: B. distachyon uncharact. (LOC100843429), mRNA	XM_003581200.1	6,00E-19	GLOS_LOC10084:	GO:0010646	B	.	regulation of cell communication
XM_003581200.1 PREDIC: B. distachyon uncharact. (LOC100843429), mRNA	XM_003581200.1	6,00E-19	GLOS_LOC10084:	GO:0023051	B	.	regulation of signaling
XM_003581200.1 PREDIC: B. distachyon uncharact. (LOC100843429), mRNA	XM_003581200.1	6,00E-19	GLOS_LOC10084:	GO:0010647	B	.	positive regulation of cell communication
XM_003581200.1 PREDIC: B. distachyon uncharact. (LOC100843429), mRNA	XM_003581200.1	6,00E-19	GLOS_LOC10084:	GO:0023056	B	.	positive regulation of signaling
XM_003581200.1 PREDIC: B. distachyon uncharact. (LOC100843429), mRNA	XM_003581200.1	6,00E-19	GLOS_LOC10084:	GO:0048584	B	.	positive regulation of response to stimulus
XM_003581200.1 PREDIC: B. distachyon uncharact. (LOC100843429), mRNA	XM_003581200.1	6,00E-19	GLOS_LOC10084:	GO:0010627	B	.	regulation of intracellular protein kinase cascade

XM_003581200.1 PREDIC: B. distachyon uncharact. (LOC100843429), mRNA	XM_003581200.1	6,00E-19	GLOS_LOC10084:	GO:0043122	B	.	regulation of I-kappaB kinase/NF-kappaB cascade
XM_003581200.1 PREDIC: B. distachyon uncharact. (LOC100843429), mRNA	XM_003581200.1	6,00E-19	GLOS_LOC10084:	GO:0043123	B	Refseq	positive regulation of I-kappaB kinase/NF-kappaB cascade
XM_003581200.1 PREDIC: B. distachyon uncharact. (LOC100843429), mRNA	XM_003581200.1	6,00E-19	GLOS_LOC10084:	GO:0043231	C	Refseq	intracellular membrane-bounded organelle
XM_003581200.1 PREDIC: B. distachyon uncharact. (LOC100843429), mRNA	XM_003581200.1	6,00E-19	GLOS_LOC10084:	GO:0032387	B	.	negative regulation of intracellular transport
XM_003581200.1 PREDIC: B. distachyon uncharact. (LOC100843429), mRNA	XM_003581200.1	6,00E-19	GLOS_LOC10084:	GO:0051051	B	.	negative regulation of transport
XM_003581200.1 PREDIC: B. distachyon uncharact. (LOC100843429), mRNA	XM_003581200.1	6,00E-19	GLOS_LOC10084:	GO:0033007	B	.	negative regul. of mast cell activation involved in immune resp.
XM_003581200.1 PREDIC: B. distachyon uncharact. (LOC100843429), mRNA	XM_003581200.1	6,00E-19	GLOS_LOC10084:	GO:0033004	B	.	negative regulation of mast cell activation
XM_003581200.1 PREDIC: B. distachyon uncharact. (LOC100843429), mRNA	XM_003581200.1	6,00E-19	GLOS_LOC10084:	GO:0002695	B	.	negative regulation of leukocyte activation
XM_003581200.1 PREDIC: B. distachyon uncharact. (LOC100843429), mRNA	XM_003581200.1	6,00E-19	GLOS_LOC10084:	GO:0002694	B	.	regulation of leukocyte activation
XM_003581200.1 PREDIC: B. distachyon uncharact. (LOC100843429), mRNA	XM_003581200.1	6,00E-19	GLOS_LOC10084:	GO:0050865	B	.	regulation of cell activation
XM_003581200.1 PREDIC: B. distachyon uncharact. (LOC100843429), mRNA	XM_003581200.1	6,00E-19	GLOS_LOC10084:	GO:0050866	B	.	negative regulation of cell activation
XM_003581200.1 PREDIC: B. distachyon uncharact. (LOC100843429), mRNA	XM_003581200.1	6,00E-19	GLOS_LOC10084:	GO:0033003	B	.	regulation of mast cell activation
XM_003581200.1 PREDIC: B. distachyon uncharact. (LOC100843429), mRNA	XM_003581200.1	6,00E-19	GLOS_LOC10084:	GO:0033006	B	.	regulation of mast cell activation involved in immune response
XM_003581200.1 PREDIC: B. distachyon uncharact. (LOC100843429), mRNA	XM_003581200.1	6,00E-19	GLOS_LOC10084:	GO:0002699	B	.	positive regulation of immune effector process
XM_003581200.1 PREDIC: B. distachyon uncharact. (LOC100843429), mRNA	XM_003581200.1	6,00E-19	GLOS_LOC10084:	GO:0002684	B	.	positive regulation of immune system process
XM_003581200.1 PREDIC: B. distachyon uncharact. (LOC100843429), mRNA	XM_003581200.1	6,00E-19	GLOS_LOC10084:	GO:0043301	B	.	negative regulation of leukocyte degranulation
XM_003581200.1 PREDIC: B. distachyon uncharact. (LOC100843429), mRNA	XM_003581200.1	6,00E-19	GLOS_LOC10084:	GO:0043300	B	.	regulation of leukocyte degranulation
XM_003581200.1 PREDIC: B. distachyon uncharact. (LOC100843429), mRNA	XM_003581200.1	6,00E-19	GLOS_LOC10084:	GO:0017157	B	.	regulation of exocytosis
XM_003581200.1 PREDIC: B. distachyon uncharact. (LOC100843429), mRNA	XM_003581200.1	6,00E-19	GLOS_LOC10084:	GO:0051046	B	.	regulation of secretion
XM_003581200.1 PREDIC: B. distachyon uncharact. (LOC100843429), mRNA	XM_003581200.1	6,00E-19	GLOS_LOC10084:	GO:0060627	B	.	regulation of vesicle-mediated transport
XM_003581200.1 PREDIC: B. distachyon uncharact. (LOC100843429), mRNA	XM_003581200.1	6,00E-19	GLOS_LOC10084:	GO:0045920	B	.	negative regulation of exocytosis
XM_003581200.1 PREDIC: B. distachyon uncharact. (LOC100843429), mRNA	XM_003581200.1	6,00E-19	GLOS_LOC10084:	GO:0051048	B	.	negative regulation of secretion
XM_003581200.1 PREDIC: B. distachyon uncharact. (LOC100843429), mRNA	XM_003581200.1	6,00E-19	GLOS_LOC10084:	GO:0043304	B	.	regulation of mast cell degranulation
XM_003581200.1 PREDIC: B. distachyon uncharact. (LOC100843429), mRNA	XM_003581200.1	6,00E-19	GLOS_LOC10084:	GO:0002886	B	.	regulation of myeloid leukocyte mediated immunity
XM_003581200.1 PREDIC: B. distachyon uncharact. (LOC100843429), mRNA	XM_003581200.1	6,00E-19	GLOS_LOC10084:	GO:0002703	B	.	regulation of leukocyte mediated immunity
XM_003581200.1 PREDIC: B. distachyon uncharact. (LOC100843429), mRNA	XM_003581200.1	6,00E-19	GLOS_LOC10084:	GO:0043305	B	Refseq	negative regulation of mast cell degranulation
XM_003581200.1 PREDIC: B. distachyon uncharact. (LOC100843429), mRNA	XM_003581200.1	6,00E-19	GLOS_LOC10084:	GO:0051100	B	.	negative regulation of binding
XM_003581200.1 PREDIC: B. distachyon uncharact. (LOC100843429), mRNA	XM_003581200.1	6,00E-19	GLOS_LOC10084:	GO:0044092	B	.	negative regulation of molecular function
XM_003581200.1 PREDIC: B. distachyon uncharact. (LOC100843429), mRNA	XM_003581200.1	6,00E-19	GLOS_LOC10084:	GO:0065009	B	.	regulation of molecular function
XM_003581200.1 PREDIC: B. distachyon uncharact. (LOC100843429), mRNA	XM_003581200.1	6,00E-19	GLOS_LOC10084:	GO:0051098	B	.	regulation of binding
XM_003581200.1 PREDIC: B. distachyon uncharact. (LOC100843429), mRNA	XM_003581200.1	6,00E-19	GLOS_LOC10084:	GO:0051101	B	.	regulation of DNA binding
XM_003581200.1 PREDIC: B. distachyon uncharact. (LOC100843429), mRNA	XM_003581200.1	6,00E-19	GLOS_LOC10084:	GO:0043392	B	Refseq	negative regulation of DNA binding
XM_003581200.1 PREDIC: B. distachyon uncharact. (LOC100843429), mRNA	XM_003581200.1	6,00E-19	GLOS_LOC10084:	GO:0051090	B	.	regul. of sequence-specific DNA binding transcription fact. activity
XM_003581200.1 PREDIC: B. distachyon uncharact. (LOC100843429), mRNA	XM_003581200.1	6,00E-19	GLOS_LOC10084:	GO:0043433	B	Refseq	negative regul. of sequence-specific DNA binding transcription fact. Act.
XM_003581200.1 PREDIC: B. distachyon uncharact. (LOC100843429), mRNA	XM_003581200.1	6,00E-19	GLOS_LOC10084:	GO:0043523	B	.	regulation of neuron apoptotic process
XM_003581200.1 PREDIC: B. distachyon uncharact. (LOC100843429), mRNA	XM_003581200.1	6,00E-19	GLOS_LOC10084:	GO:1901214	B	.	regulation of neuron death
XM_003581200.1 PREDIC: B. distachyon uncharact. (LOC100843429), mRNA	XM_003581200.1	6,00E-19	GLOS_LOC10084:	GO:1901215	B	.	negative regulation of neuron death
XM_003581200.1 PREDIC: B. distachyon uncharact. (LOC100843429), mRNA	XM_003581200.1	6,00E-19	GLOS_LOC10084:	GO:0043524	B	Refseq	negative regulation of neuron apoptotic process
XM_003581200.1 PREDIC: B. distachyon uncharact. (LOC100843429), mRNA	XM_003581200.1	6,00E-19	GLOS_LOC10084:	GO:0034599	B	.	cellular response to oxidative stress
XM_003581200.1 PREDIC: B. distachyon uncharact. (LOC100843429), mRNA	XM_003581200.1	6,00E-19	GLOS_LOC10084:	GO:0070887	B	.	cellular response to chemical stimulus
XM_003581200.1 PREDIC: B. distachyon uncharact. (LOC100843429), mRNA	XM_003581200.1	6,00E-19	GLOS_LOC10084:	GO:0043618	B	.	regul. of transcrip from RNA polymerase II promoter in resp. to stress
XM_003581200.1 PREDIC: B. distachyon uncharact. (LOC100843429), mRNA	XM_003581200.1	6,00E-19	GLOS_LOC10084:	GO:0043620	B	.	regulation of DNA-dependent transcription in response to stress
XM_003581200.1 PREDIC: B. distachyon uncharact. (LOC100843429), mRNA	XM_003581200.1	6,00E-19	GLOS_LOC10084:	GO:0043619	B	Refseq	regul of transcrip from RNA polymerase II promoter in resp to oxid stress
XM_003581200.1 PREDIC: B. distachyon uncharact. (LOC100843429), mRNA	XM_003581200.1	6,00E-19	GLOS_LOC10084:	GO:0048545	B	.	response to steroid hormone stimulus
XM_003581200.1 PREDIC: B. distachyon uncharact. (LOC100843429), mRNA	XM_003581200.1	6,00E-19	GLOS_LOC10084:	GO:0009725	B	.	response to hormone stimulus
XM_003581200.1 PREDIC: B. distachyon uncharact. (LOC100843429), mRNA	XM_003581200.1	6,00E-19	GLOS_LOC10084:	GO:0033993	B	.	response to lipid
XM_003581200.1 PREDIC: B. distachyon uncharact. (LOC100843429), mRNA	XM_003581200.1	6,00E-19	GLOS_LOC10084:	GO:0043627	B	Refseq	response to estrogen stimulus
XM_003581200.1 PREDIC: B. distachyon uncharact. (LOC100843429), mRNA	XM_003581200.1	6,00E-19	GLOS_LOC10084:	GO:0045765	B	.	regulation of angiogenesis
XM_003581200.1 PREDIC: B. distachyon uncharact. (LOC100843429), mRNA	XM_003581200.1	6,00E-19	GLOS_LOC10084:	GO:0022603	B	.	regulation of anatomical structure morphogenesis
XM_003581200.1 PREDIC: B. distachyon uncharact. (LOC100843429), mRNA	XM_003581200.1	6,00E-19	GLOS_LOC10084:	GO:1901342	B	.	regulation of vasculature development
XM_003581200.1 PREDIC: B. distachyon uncharact. (LOC100843429), mRNA	XM_003581200.1	6,00E-19	GLOS_LOC10084:	GO:0051094	B	.	positive regulation of developmental process
XM_003581200.1 PREDIC: B. distachyon uncharact. (LOC100843429), mRNA	XM_003581200.1	6,00E-19	GLOS_LOC10084:	GO:0045766	B	Refseq	positive regulation of angiogenesis
XM_003581200.1 PREDIC: B. distachyon uncharact. (LOC100843429), mRNA	XM_003581200.1	6,00E-19	GLOS_LOC10084:	GO:0046872	M	Refseq	metal ion binding
XM_003581200.1 PREDIC: B. distachyon uncharact. (LOC100843429), mRNA	XM_003581200.1	6,00E-19	GLOS_LOC10084:	GO:0008284	B	.	positive regulation of cell proliferation

XM_003581200.1 PREDIC: B. distachyon uncharact. (LOC100843429), mRNA	XM_003581200.1	6,00E-19	GLOS_LOC10084	GO:0048660	B	.	regulation of smooth muscle cell proliferation
XM_003581200.1 PREDIC: B. distachyon uncharact. (LOC100843429), mRNA	XM_003581200.1	6,00E-19	GLOS_LOC10084	GO:0048661	B	Refseq	positive regulation of smooth muscle cell proliferation
XM_003581200.1 PREDIC: B. distachyon uncharact. (LOC100843429), mRNA	XM_003581200.1	6,00E-19	GLOS_LOC10084	GO:0008285	B	.	negative regulation of cell proliferation
XM_003581200.1 PREDIC: B. distachyon uncharact. (LOC100843429), mRNA	XM_003581200.1	6,00E-19	GLOS_LOC10084	GO:0048662	B	Refseq	negative regulation of smooth muscle cell proliferation
XM_003581200.1 PREDIC: B. distachyon uncharact. (LOC100843429), mRNA	XM_003581200.1	6,00E-19	GLOS_LOC10084	GO:0051090	B	Refseq	regulation of sequence-specific DNA binding transcription factor activity
XM_003581200.1 PREDIC: B. distachyon uncharact. (LOC100843429), mRNA	XM_003581200.1	6,00E-19	GLOS_LOC10084	GO:0051259	B	.	protein oligomerization
XM_003581200.1 PREDIC: B. distachyon uncharact. (LOC100843429), mRNA	XM_003581200.1	6,00E-19	GLOS_LOC10084	GO:0006461	B	.	protein complex assembly
XM_003581200.1 PREDIC: B. distachyon uncharact. (LOC100843429), mRNA	XM_003581200.1	6,00E-19	GLOS_LOC10084	GO:0065003	B	.	macromolecular complex assembly
XM_003581200.1 PREDIC: B. distachyon uncharact. (LOC100843429), mRNA	XM_003581200.1	6,00E-19	GLOS_LOC10084	GO:0022607	B	.	cellular component assembly
XM_003581200.1 PREDIC: B. distachyon uncharact. (LOC100843429), mRNA	XM_003581200.1	6,00E-19	GLOS_LOC10084	GO:0044085	B	.	cellular component biogenesis
XM_003581200.1 PREDIC: B. distachyon uncharact. (LOC100843429), mRNA	XM_003581200.1	6,00E-19	GLOS_LOC10084	GO:0043933	B	.	macromolecular complex subunit organization
XM_003581200.1 PREDIC: B. distachyon uncharact. (LOC100843429), mRNA	XM_003581200.1	6,00E-19	GLOS_LOC10084	GO:0071822	B	.	protein complex subunit organization
XM_003581200.1 PREDIC: B. distachyon uncharact. (LOC100843429), mRNA	XM_003581200.1	6,00E-19	GLOS_LOC10084	GO:0070271	B	.	protein complex biogenesis
XM_003581200.1 PREDIC: B. distachyon uncharact. (LOC100843429), mRNA	XM_003581200.1	6,00E-19	GLOS_LOC10084	GO:0051260	B	Refseq	protein homooligomerization
XM_003581200.1 PREDIC: B. distachyon uncharact. (LOC100843429), mRNA	XM_003581200.1	6,00E-19	GLOS_LOC10084	GO:0055076	B	.	transition metal ion homeostasis
XM_003581200.1 PREDIC: B. distachyon uncharact. (LOC100843429), mRNA	XM_003581200.1	6,00E-19	GLOS_LOC10084	GO:0055065	B	.	metal ion homeostasis
XM_003581200.1 PREDIC: B. distachyon uncharact. (LOC100843429), mRNA	XM_003581200.1	6,00E-19	GLOS_LOC10084	GO:0055080	B	.	cation homeostasis
XM_003581200.1 PREDIC: B. distachyon uncharact. (LOC100843429), mRNA	XM_003581200.1	6,00E-19	GLOS_LOC10084	GO:0050801	B	.	ion homeostasis
XM_003581200.1 PREDIC: B. distachyon uncharact. (LOC100843429), mRNA	XM_003581200.1	6,00E-19	GLOS_LOC10084	GO:0048878	B	.	chemical homeostasis
XM_003581200.1 PREDIC: B. distachyon uncharact. (LOC100843429), mRNA	XM_003581200.1	6,00E-19	GLOS_LOC10084	GO:0055072	B	Refseq	iron ion homeostasis
XM_003581200.1 PREDIC: B. distachyon uncharact. (LOC100843429), mRNA	XM_003581200.1	6,00E-19	GLOS_LOC10084	GO:0055114	B	Refseq	oxidation-reduction process
XM_003581200.1 PREDIC: B. distachyon uncharact. (LOC100843429), mRNA	XM_003581200.1	6,00E-19	GLOS_LOC10084	GO:0046685	B	.	response to arsenic-containing substance
XM_003581200.1 PREDIC: B. distachyon uncharact. (LOC100843429), mRNA	XM_003581200.1	6,00E-19	GLOS_LOC10084	GO:0071243	B	Refseq	cellular response to arsenic-containing substance
XM_003581200.1 PREDIC: B. distachyon uncharact. (LOC100843429), mRNA	XM_003581200.1	6,00E-19	GLOS_LOC10084	GO:0046686	B	.	response to cadmium ion
XM_003581200.1 PREDIC: B. distachyon uncharact. (LOC100843429), mRNA	XM_003581200.1	6,00E-19	GLOS_LOC10084	GO:0071248	B	.	cellular response to metal ion
XM_003581200.1 PREDIC: B. distachyon uncharact. (LOC100843429), mRNA	XM_003581200.1	6,00E-19	GLOS_LOC10084	GO:0071241	B	.	cellular response to inorganic substance
XM_003581200.1 PREDIC: B. distachyon uncharact. (LOC100843429), mRNA	XM_003581200.1	6,00E-19	GLOS_LOC10084	GO:0071276	B	Refseq	cellular response to cadmium ion
XM_003581200.1 PREDIC: B. distachyon uncharact. (LOC100843429), mRNA	XM_003581200.1	6,00E-19	GLOS_LOC10084	GO:1902041	B	.	regulation of extrinsic apoptotic signaling pathway via death domain receptors
XM_003581200.1 PREDIC: B. distachyon uncharact. (LOC100843429), mRNA	XM_003581200.1	6,00E-19	GLOS_LOC10084	GO:2001236	B	.	regulation of extrinsic apoptotic signaling pathway
XM_003581200.1 PREDIC: B. distachyon uncharact. (LOC100843429), mRNA	XM_003581200.1	6,00E-19	GLOS_LOC10084	GO:2001233	B	.	regulation of apoptotic signaling pathway
XM_003581200.1 PREDIC: B. distachyon uncharact. (LOC100843429), mRNA	XM_003581200.1	6,00E-19	GLOS_LOC10084	GO:2001237	B	.	negative regulation of extrinsic apoptotic signaling pathway
XM_003581200.1 PREDIC: B. distachyon uncharact. (LOC100843429), mRNA	XM_003581200.1	6,00E-19	GLOS_LOC10084	GO:2001234	B	.	negative regulation of apoptotic signaling pathway
XM_003581200.1 PREDIC: B. distachyon uncharact. (LOC100843429), mRNA	XM_003581200.1	6,00E-19	GLOS_LOC10084	GO:0009968	B	.	negative regulation of signal transduction
XM_003581200.1 PREDIC: B. distachyon uncharact. (LOC100843429), mRNA	XM_003581200.1	6,00E-19	GLOS_LOC10084	GO:0010648	B	.	negative regulation of cell communication
XM_003581200.1 PREDIC: B. distachyon uncharact. (LOC100843429), mRNA	XM_003581200.1	6,00E-19	GLOS_LOC10084	GO:0023057	B	.	negative regulation of signaling
XM_003581200.1 PREDIC: B. distachyon uncharact. (LOC100843429), mRNA	XM_003581200.1	6,00E-19	GLOS_LOC10084	GO:0048585	B	.	negative regulation of response to stimulus
XM_003581200.1 PREDIC: B. distachyon uncharact. (LOC100843429), mRNA	XM_003581200.1	6,00E-19	GLOS_LOC10084	GO:1902042	B	Refseq	negat. regulat. of extrinsic apoptotic signaling pathway via death domain recept.
NP_612045.1 CG3402 [D. melanogaster] ref|XP_001352805.1| GA17428 [D. p. pseudoobscura] ref|XP_001956682.1| GF10058 [D. ananassae] ref|XP_001971037.1| GG14653 [D. erecta] ref|XP_001983721.1| GH16044 [D. grimshawi] ref|XP_002007574.1| GI12308 [D. mojavensis] ref|XP_002034808.1 | GM14267 [D. sechellia] ref|XP_002047106.1| GJ13244 [D. virilis] ref|XP_002062386.1| GK16689 [D. willistoni] ref|XP_002093025.1| GE21011 [D. yakuba]	NP_612045.1	9,00E-82	GLOS_CG3402.1.	GO:0000122	B	Refseq	negative regulation of transcription from RNA polymerase II promoter
NP_612045.1 CG3402 [D. m.] ref|XP_001352805.1| GA17428 … same as above	NP_612045.1	9,00E-82	GLOS_CG3402.1.	GO:0001501	B	Refseq	skeletal system development
NP_612045.1 CG3402 [D. m.] ref|XP_001352805.1| GA17428 … same as above	NP_612045.1	9,00E-82	GLOS_CG3402.1.	GO:0003700	M	Refseq	sequence-specific DNA binding transcription factor activity
NP_612045.1 CG3402 [D. m.] ref|XP_001352805.1| GA17428 … same as above	NP_612045.1	9,00E-82	GLOS_CG3402.1.	GO:0005515	M	Refseq	protein binding
NP_612045.1 CG3402 [D. m.] ref|XP_001352805.1| GA17428 … same as above	NP_612045.1	9,00E-82	GLOS_CG3402.1.	GO:0005634	C	Refseq	nucleus
NP_612045.1 CG3402 [D. m.] ref|XP_001352805.1| GA17428 … same as above	NP_612045.1	9,00E-82	GLOS_CG3402.1.	GO:0006366	B	Refseq	transcription from RNA polymerase II promoter
NP_612045.1 CG3402 [D. m.] ref|XP_001352805.1| GA17428 … same as above	NP_612045.1	9,00E-82	GLOS_CG3402.1.	GO:0006607	B	Refseq	NLS-bearing protein import into nucleus
NP_612045.1 CG3402 [D. m.] ref|XP_001352805.1| GA17428 … same as above	NP_612045.1	9,00E-82	GLOS_CG3402.1.	GO:0008270	M	Refseq	zinc ion binding
NP_612045.1 CG3402 [D. m.] ref|XP_001352805.1| GA17428 … same as above	NP_612045.1	9,00E-82	GLOS_CG3402.1.	GO:0032330	B	Refseq	regulation of chondrocyte differentiation
NP_612045.1 CG3402 [D. m.] ref|XP_001352805.1| GA17428 … same as above	NP_612045.1	9,00E-82	GLOS_CG3402.1.	GO:0043565	M	Refseq	sequence-specific DNA binding
NP_651629.1 huntingtin, isoform A, ref|NP_001263041.1| huntingtin, isoform B	NP_651629.1	1,00E-109	GLOS_HTT.1.1	GO:0000122	B	Refseq	negative regulation of transcription from RNA polymerase II promoter

NP_651629.1 huntingtin, isoform A, ref|NP_001263041.1| huntingtin, isoform B	NP_651629.1	1,00E-109	GLOS_HTT.1.1	GO:0001501	B	Refseq	skeletal system development
NP_651629.1 huntingtin, isoform A, ref|NP_001263041.1| huntingtin, isoform B	NP_651629.1	1,00E-109	GLOS_HTT.1.1	GO:0003700	M	Refseq	sequence-specific DNA binding transcription factor activity
NP_651629.1 huntingtin, isoform A, ref|NP_001263041.1| huntingtin, isoform B	NP_651629.1	1,00E-109	GLOS_HTT.1.1	GO:0005515	M	Refseq	protein binding
NP_651629.1 huntingtin, isoform A, ref|NP_001263041.1| huntingtin, isoform B	NP_651629.1	1,00E-109	GLOS_HTT.1.1	GO:0005634	C	Refseq	nucleus
NP_651629.1 huntingtin, isoform A, ref|NP_001263041.1| huntingtin, isoform B	NP_651629.1	1,00E-109	GLOS_HTT.1.1	GO:0006366	B	Refseq	transcription from RNA polymerase II promoter
NP_651629.1 huntingtin, isoform A, ref|NP_001263041.1| huntingtin, isoform B	NP_651629.1	1,00E-109	GLOS_HTT.1.1	GO:0006607	B	Refseq	NLS-bearing protein import into nucleus
NP_651629.1 huntingtin, isoform A, ref|NP_001263041.1| huntingtin, isoform B	NP_651629.1	1,00E-109	GLOS_HTT.1.1	GO:0008270	M	Refseq	zinc ion binding
NP_651629.1 huntingtin, isoform A, ref|NP_001263041.1| huntingtin, isoform B	NP_651629.1	1,00E-109	GLOS_HTT.1.1	GO:0032330	B	Refseq	regulation of chondrocyte differentiation
NP_651629.1 huntingtin, isoform A, ref|NP_001263041.1| huntingtin, isoform B	NP_651629.1	1,00E-109	GLOS_HTT.1.1	GO:0043565	M	Refseq	sequence-specific DNA binding
XP_001978049.1 GG19380 [Drosophila erecta]	XP_001978049.1	1,00E-35	GLOS_DERE_GG	GO:0005261	M	Refseq	cation channel activity
XP_001978049.1 GG19380 [Drosophila erecta]	XP_001978049.1	1,00E-35	GLOS_DERE_GG	GO:0005262	M	Refseq	calcium channel activity
XP_001978049.1 GG19380 [Drosophila erecta]	XP_001978049.1	1,00E-35	GLOS_DERE_GG	GO:0005515	M	Refseq	protein binding
XP_001978049.1 GG19380 [Drosophila erecta]	XP_001978049.1	1,00E-35	GLOS_DERE_GG	GO:0005886	C	Refseq	plasma membrane
XP_001978049.1 GG19380 [Drosophila erecta]	XP_001978049.1	1,00E-35	GLOS_DERE_GG	GO:0005887	C	Refseq	integral to plasma membrane
XP_001978049.1 GG19380 [Drosophila erecta]	XP_001978049.1	1,00E-35	GLOS_DERE_GG	GO:0006816	B	Refseq	calcium ion transport
XP_001978049.1 GG19380 [Drosophila erecta]	XP_001978049.1	1,00E-35	GLOS_DERE_GG	GO:0007411	B	Refseq	axon guidance
XP_001978049.1 GG19380 [Drosophila erecta]	XP_001978049.1	1,00E-35	GLOS_DERE_GG	GO:0015279	M	Refseq	store-operated calcium channel activity
XP_001978049.1 GG19380 [Drosophila erecta]	XP_001978049.1	1,00E-35	GLOS_DERE_GG	GO:0016323	C	Refseq	basolateral plasma membrane
XP_001978049.1 GG19380 [Drosophila erecta]	XP_001978049.1	1,00E-35	GLOS_DERE_GG	GO:0044449	C	.	contractile fiber part
XP_001978049.1 GG19380 [Drosophila erecta]	XP_001978049.1	1,00E-35	GLOS_DERE_GG	GO:0043292	C	.	contractile fiber
XP_001978049.1 GG19380 [Drosophila erecta]	XP_001978049.1	1,00E-35	GLOS_DERE_GG	GO:0030016	C	.	myofibril
XP_001978049.1 GG19380 [Drosophila erecta]	XP_001978049.1	1,00E-35	GLOS_DERE_GG	GO:0030017	C	Refseq	sarcomere
XP_001978049.1 GG19380 [Drosophila erecta]	XP_001978049.1	1,00E-35	GLOS_DERE_GG	GO:0043034	C	Refseq	costamere
XP_001978049.1 GG19380 [Drosophila erecta]	XP_001978049.1	1,00E-35	GLOS_DERE_GG	GO:0032991	C	.	macromolecular complex
XP_001978049.1 GG19380 [Drosophila erecta]	XP_001978049.1	1,00E-35	GLOS_DERE_GG	GO:0043234	C	Refseq	protein complex
XP_001978049.1 GG19380 [Drosophila erecta]	XP_001978049.1	1,00E-35	GLOS_DERE_GG	GO:0044325	M	Refseq	ion channel binding
XP_001978049.1 GG19380 [Drosophila erecta]	XP_001978049.1	1,00E-35	GLOS_DERE_GG	GO:0045121	C	Refseq	membrane raft
XP_001978049.1 GG19380 [Drosophila erecta]	XP_001978049.1	1,00E-35	GLOS_DERE_GG	GO:0007589	B	.	body fluid secretion
XP_001978049.1 GG19380 [Drosophila erecta]	XP_001978049.1	1,00E-35	GLOS_DERE_GG	GO:0046903	B	.	secretion
XP_001978049.1 GG19380 [Drosophila erecta]	XP_001978049.1	1,00E-35	GLOS_DERE_GG	GO:0022600	B	.	digestive system process
XP_001978049.1 GG19380 [Drosophila erecta]	XP_001978049.1	1,00E-35	GLOS_DERE_GG	GO:0007586	B	.	digestion
XP_001978049.1 GG19380 [Drosophila erecta]	XP_001978049.1	1,00E-35	GLOS_DERE_GG	GO:0032941	B	.	secretion by tissue
XP_001978049.1 GG19380 [Drosophila erecta]	XP_001978049.1	1,00E-35	GLOS_DERE_GG	GO:0046541	B	Refseq	saliva secretion
XP_001978049.1 GG19380 [Drosophila erecta]	XP_001978049.1	1,00E-35	GLOS_DERE_GG	GO:0010524	B	.	positive regulation of calcium ion transport into cytosol
XP_001978049.1 GG19380 [Drosophila erecta]	XP_001978049.1	1,00E-35	GLOS_DERE_GG	GO:0051279	B	.	regulation of release of sequestered calcium ion into cytosol
XP_001978049.1 GG19380 [Drosophila erecta]	XP_001978049.1	1,00E-35	GLOS_DERE_GG	GO:0051281	B	Refseq	positive regulation of release of sequestered calcium ion into cytosol
XP_001978049.1 GG19380 [Drosophila erecta]	XP_001978049.1	1,00E-35	GLOS_DERE_GG	GO:0006874	B	.	cellular calcium ion homeostasis
XP_001978049.1 GG19380 [Drosophila erecta]	XP_001978049.1	1,00E-35	GLOS_DERE_GG	GO:0006875	B	.	cellular metal ion homeostasis
XP_001978049.1 GG19380 [Drosophila erecta]	XP_001978049.1	1,00E-35	GLOS_DERE_GG	GO:0030003	B	.	cellular cation homeostasis
XP_001978049.1 GG19380 [Drosophila erecta]	XP_001978049.1	1,00E-35	GLOS_DERE_GG	GO:0006873	B	.	cellular ion homeostasis
XP_001978049.1 GG19380 [Drosophila erecta]	XP_001978049.1	1,00E-35	GLOS_DERE_GG	GO:0055082	B	.	cellular chemical homeostasis
XP_001978049.1 GG19380 [Drosophila erecta]	XP_001978049.1	1,00E-35	GLOS_DERE_GG	GO:0019725	B	.	cellular homeostasis
XP_001978049.1 GG19380 [Drosophila erecta]	XP_001978049.1	1,00E-35	GLOS_DERE_GG	GO:0055074	B	.	calcium ion homeostasis
XP_001978049.1 GG19380 [Drosophila erecta]	XP_001978049.1	1,00E-35	GLOS_DERE_GG	GO:0072507	B	.	divalent inorganic cation homeostasis
XP_001978049.1 GG19380 [Drosophila erecta]	XP_001978049.1	1,00E-35	GLOS_DERE_GG	GO:0072503	B	.	cellular divalent inorganic cation homeostasis
XP_001978049.1 GG19380 [Drosophila erecta]	XP_001978049.1	1,00E-35	GLOS_DERE_GG	GO:0051480	B	Refseq	cytosolic calcium ion homeostasis
XP_001978049.1 GG19380 [Drosophila erecta]	XP_001978049.1	1,00E-35	GLOS_DERE_GG	GO:0051592	B	Refseq	response to calcium ion
XP_001978049.1 GG19380 [Drosophila erecta]	XP_001978049.1	1,00E-35	GLOS_DERE_GG	GO:0070679	M	Refseq	inositol 1,4,5 trisphosphate binding
XP_001979691.1 GG22681 [Drosophila erecta]	XP_001979691.1	0	GLOS_DERE_GG	GO:0005261	M	Refseq	cation channel activity
XP_001979691.1 GG22681 [Drosophila erecta]	XP_001979691.1	0	GLOS_DERE_GG	GO:0005262	M	Refseq	calcium channel activity
XP_001979691.1 GG22681 [Drosophila erecta]	XP_001979691.1	0	GLOS_DERE_GG	GO:0005515	M	Refseq	protein binding
XP_001979691.1 GG22681 [Drosophila erecta]	XP_001979691.1	0	GLOS_DERE_GG	GO:0005886	C	Refseq	plasma membrane
XP_001979691.1 GG22681 [Drosophila erecta]	XP_001979691.1	0	GLOS_DERE_GG	GO:0005887	C	Refseq	integral to plasma membrane
XP_001979691.1 GG22681 [Drosophila erecta]	XP_001979691.1	0	GLOS_DERE_GG	GO:0006816	B	Refseq	calcium ion transport

XP_001979691.1 GG22681 [Drosophila erecta]	XP_001979691.1	0	GLOS_DERE_GG	GO:0007411	B	Refseq	axon guidance
XP_001979691.1 GG22681 [Drosophila erecta]	XP_001979691.1	0	GLOS_DERE_GG	GO:0015279	M	Refseq	store-operated calcium channel activity
XP_001979691.1 GG22681 [Drosophila erecta]	XP_001979691.1	0	GLOS_DERE_GG	GO:0016323	C	Refseq	basolateral plasma membrane
XP_001979691.1 GG22681 [Drosophila erecta]	XP_001979691.1	0	GLOS_DERE_GG	GO:0030017	C	Refseq	sarcomere
XP_001979691.1 GG22681 [Drosophila erecta]	XP_001979691.1	0	GLOS_DERE_GG	GO:0043034	C	Refseq	costamere
XP_001979691.1 GG22681 [Drosophila erecta]	XP_001979691.1	0	GLOS_DERE_GG	GO:0043234	C	Refseq	protein complex
XP_001979691.1 GG22681 [Drosophila erecta]	XP_001979691.1	0	GLOS_DERE_GG	GO:0044325	M	Refseq	ion channel binding
XP_001979691.1 GG22681 [Drosophila erecta]	XP_001979691.1	0	GLOS_DERE_GG	GO:0045121	C	Refseq	membrane raft
XP_001979691.1 GG22681 [Drosophila erecta]	XP_001979691.1	0	GLOS_DERE_GG	GO:0046541	B	Refseq	saliva secretion
XP_001979691.1 GG22681 [Drosophila erecta]	XP_001979691.1	0	GLOS_DERE_GG	GO:0051281	B	Refseq	positive regulation of release of sequestered calcium ion into cytosol
XP_001979691.1 GG22681 [Drosophila erecta]	XP_001979691.1	0	GLOS_DERE_GG	GO:0051480	B	Refseq	cytosolic calcium ion homeostasis
XP_001979691.1 GG22681 [Drosophila erecta]	XP_001979691.1	0	GLOS_DERE_GG	GO:0051592	B	Refseq	response to calcium ion
XP_001979691.1 GG22681 [Drosophila erecta]	XP_001979691.1	0	GLOS_DERE_GG	GO:0070679	M	Refseq	inositol 1,4,5 trisphosphate binding
XP_001995236.1 GH23040 [Drosophila grimshawi]	XP_001995236.1	3,00E-16	GLOS_DGRI_GH2	GO:0005262	M	Refseq	calcium channel activity
XP_001995236.1 GH23040 [Drosophila grimshawi]	XP_001995236.1	3,00E-16	GLOS_DGRI_GH2	GO:0005515	M	Refseq	protein binding
XP_001995236.1 GH23040 [Drosophila grimshawi]	XP_001995236.1	3,00E-16	GLOS_DGRI_GH2	GO:0005886	C	Refseq	plasma membrane
XP_001995236.1 GH23040 [Drosophila grimshawi]	XP_001995236.1	3,00E-16	GLOS_DGRI_GH2	GO:0005887	C	Refseq	integral to plasma membrane
XP_001995236.1 GH23040 [Drosophila grimshawi]	XP_001995236.1	3,00E-16	GLOS_DGRI_GH2	GO:0006816	B	Refseq	calcium ion transport
XP_001995236.1 GH23040 [Drosophila grimshawi]	XP_001995236.1	3,00E-16	GLOS_DGRI_GH2	GO:0007411	B	Refseq	axon guidance
XP_001995236.1 GH23040 [Drosophila grimshawi]	XP_001995236.1	3,00E-16	GLOS_DGRI_GH2	GO:0007596	B	Refseq	blood coagulation
XP_001995236.1 GH23040 [Drosophila grimshawi]	XP_001995236.1	3,00E-16	GLOS_DGRI_GH2	GO:0007602	B	Refseq	phototransduction
XP_001995236.1 GH23040 [Drosophila grimshawi]	XP_001995236.1	3,00E-16	GLOS_DGRI_GH2	GO:0010524	B	Refseq	positive regulation of calcium ion transport into cytosol
XP_001995236.1 GH23040 [Drosophila grimshawi]	XP_001995236.1	3,00E-16	GLOS_DGRI_GH2	GO:0015279	M	Refseq	store-operated calcium channel activity
XP_001995236.1 GH23040 [Drosophila grimshawi]	XP_001995236.1	3,00E-16	GLOS_DGRI_GH2	GO:0030168	B	Refseq	platelet activation
XP_001995236.1 GH23040 [Drosophila grimshawi]	XP_001995236.1	3,00E-16	GLOS_DGRI_GH2	GO:0033198	B	Refseq	response to ATP
XP_001995236.1 GH23040 [Drosophila grimshawi]	XP_001995236.1	3,00E-16	GLOS_DGRI_GH2	GO:0051592	B	Refseq	response to calcium ion
XP_001995236.1 GH23040 [Drosophila grimshawi]	XP_001995236.1	3,00E-16	GLOS_DGRI_GH2	GO:0070588	B	Refseq	calcium ion transmembrane transport
XP_001995236.1 GH23040 [Drosophila grimshawi]	XP_001995236.1	3,00E-16	GLOS_DGRI_GH2	GO:0070679	M	Refseq	inositol 1,4,5 trisphosphate binding
XP_001993733.1 GH19571 [Drosophila grimshawi]	XP_001993733.1	0	GLOS_DGRI_GH1	GO:0005262	M	Refseq	calcium channel activity
XP_001993733.1 GH19571 [Drosophila grimshawi]	XP_001993733.1	0	GLOS_DGRI_GH1	GO:0005515	M	Refseq	protein binding
XP_001993733.1 GH19571 [Drosophila grimshawi]	XP_001993733.1	0	GLOS_DGRI_GH1	GO:0005886	C	Refseq	plasma membrane
XP_001993733.1 GH19571 [Drosophila grimshawi]	XP_001993733.1	0	GLOS_DGRI_GH1	GO:0005887	C	Refseq	integral to plasma membrane
XP_001993733.1 GH19571 [Drosophila grimshawi]	XP_001993733.1	0	GLOS_DGRI_GH1	GO:0006816	B	Refseq	calcium ion transport
XP_001993733.1 GH19571 [Drosophila grimshawi]	XP_001993733.1	0	GLOS_DGRI_GH1	GO:0007411	B	Refseq	axon guidance
XP_001993733.1 GH19571 [Drosophila grimshawi]	XP_001993733.1	0	GLOS_DGRI_GH1	GO:0007596	B	Refseq	blood coagulation
XP_001993733.1 GH19571 [Drosophila grimshawi]	XP_001993733.1	0	GLOS_DGRI_GH1	GO:0007602	B	Refseq	phototransduction
XP_001993733.1 GH19571 [Drosophila grimshawi]	XP_001993733.1	0	GLOS_DGRI_GH1	GO:0010524	B	Refseq	positive regulation of calcium ion transport into cytosol
XP_001993733.1 GH19571 [Drosophila grimshawi]	XP_001993733.1	0	GLOS_DGRI_GH1	GO:0015279	M	Refseq	store-operated calcium channel activity
XP_001993733.1 GH19571 [Drosophila grimshawi]	XP_001993733.1	0	GLOS_DGRI_GH1	GO:0030168	B	Refseq	platelet activation
XP_001993733.1 GH19571 [Drosophila grimshawi]	XP_001993733.1	0	GLOS_DGRI_GH1	GO:0033198	B	Refseq	response to ATP
XP_001993733.1 GH19571 [Drosophila grimshawi]	XP_001993733.1	0	GLOS_DGRI_GH1	GO:0051592	B	Refseq	response to calcium ion
XP_001993733.1 GH19571 [Drosophila grimshawi]	XP_001993733.1	0	GLOS_DGRI_GH1	GO:0070588	B	Refseq	calcium ion transmembrane transport
XP_001993733.1 GH19571 [Drosophila grimshawi]	XP_001993733.1	0	GLOS_DGRI_GH1	GO:0070679	M	Refseq	inositol 1,4,5 trisphosphate binding
XM_003395475.1PRED.:B.t.40S ribosomal prot. S29-like(LOC100646020),mRNA	XM_003395475.1	5,00E-12	GLOS_LOC10064(	GO:0005576	C	Refseq	extracellular region
XM_003395475.1PRED.:B.t.40S ribosomal prot. S29-like(LOC100646020),mRNA	XM_003395475.1	5,00E-12	GLOS_LOC10064(	GO:0005102	M	.	receptor binding
XM_003395475.1PRED.:B.t.40S ribosomal prot. S29-like(LOC100646020),mRNA	XM_003395475.1	5,00E-12	GLOS_LOC10064(	GO:0008083	M	Refseq	growth factor activity
XM_003395475.1PRED.:B.t.40S ribosomal prot. S29-like(LOC100646020),mRNA	XM_003395475.1	5,00E-12	GLOS_LOC10064(	GO:0040007	B	Refseq	growth
NP_001097872.1 CG34376, isoform B [Drosophila melanogaster]	NP_001097872.1	1,00E-154	GLOS_CG34376.1	GO:0000122	B	Refseq	negative regulation of transcription from RNA polymerase II promoter
NP_001097872.1 CG34376, isoform B [Drosophila melanogaster]	NP_001097872.1	1,00E-154	GLOS_CG34376.1	GO:0001501	B	Refseq	skeletal system development
NP_001097872.1 CG34376, isoform B [Drosophila melanogaster]	NP_001097872.1	1,00E-154	GLOS_CG34376.1	GO:0003700	M	Refseq	sequence-specific DNA binding transcription factor activity
NP_001097872.1 CG34376, isoform B [Drosophila melanogaster]	NP_001097872.1	1,00E-154	GLOS_CG34376.1	GO:0005515	M	Refseq	protein binding
NP_001097872.1 CG34376, isoform B [Drosophila melanogaster]	NP_001097872.1	1,00E-154	GLOS_CG34376.1	GO:0005634	C	Refseq	nucleus
NP_001097872.1 CG34376, isoform B [Drosophila melanogaster]	NP_001097872.1	1,00E-154	GLOS_CG34376.1	GO:0006366	B	Refseq	transcription from RNA polymerase II promoter
NP_001097872.1 CG34376, isoform B [Drosophila melanogaster]	NP_001097872.1	1,00E-154	GLOS_CG34376.1	GO:0006607	B	Refseq	NLS-bearing protein import into nucleus

NP_001097872.1 CG34376, isoform B [Drosophila melanogaster]	NP_001097872.1	1,00E-154	GLOS_CG34376.1	GO:0008270	M	Refseq	zinc ion binding
NP_001097872.1 CG34376, isoform B [Drosophila melanogaster]	NP_001097872.1	1,00E-154	GLOS_CG34376.1	GO:0032330	B	Refseq	regulation of chondrocyte differentiation
NP_001097872.1 CG34376, isoform B [Drosophila melanogaster]	NP_001097872.1	1,00E-154	GLOS_CG34376.1	GO:0043565	M	Refseq	sequence-specific DNA binding
XP_001987559.1 GH21984 [Drosophila grimshawi]	XP_001987559.1	7,00E-31	GLOS_DGRI_GH2	GO:0005262	M	Refseq	calcium channel activity
XP_001987559.1 GH21984 [Drosophila grimshawi]	XP_001987559.1	7,00E-31	GLOS_DGRI_GH2	GO:0005515	M	Refseq	protein binding
XP_001987559.1 GH21984 [Drosophila grimshawi]	XP_001987559.1	7,00E-31	GLOS_DGRI_GH2	GO:0005886	C	Refseq	plasma membrane
XP_001987559.1 GH21984 [Drosophila grimshawi]	XP_001987559.1	7,00E-31	GLOS_DGRI_GH2	GO:0005887	C	Refseq	integral to plasma membrane
XP_001987559.1 GH21984 [Drosophila grimshawi]	XP_001987559.1	7,00E-31	GLOS_DGRI_GH2	GO:0006816	B	Refseq	calcium ion transport
XP_001987559.1 GH21984 [Drosophila grimshawi]	XP_001987559.1	7,00E-31	GLOS_DGRI_GH2	GO:0007411	B	Refseq	axon guidance
XP_001987559.1 GH21984 [Drosophila grimshawi]	XP_001987559.1	7,00E-31	GLOS_DGRI_GH2	GO:0007596	B	Refseq	blood coagulation
XP_001987559.1 GH21984 [Drosophila grimshawi]	XP_001987559.1	7,00E-31	GLOS_DGRI_GH2	GO:0007602	B	Refseq	phototransduction
XP_001987559.1 GH21984 [Drosophila grimshawi]	XP_001987559.1	7,00E-31	GLOS_DGRI_GH2	GO:0010524	B	Refseq	positive regulation of calcium ion transport into cytosol
XP_001987559.1 GH21984 [Drosophila grimshawi]	XP_001987559.1	7,00E-31	GLOS_DGRI_GH2	GO:0015279	M	Refseq	store-operated calcium channel activity
XP_001987559.1 GH21984 [Drosophila grimshawi]	XP_001987559.1	7,00E-31	GLOS_DGRI_GH2	GO:0030168	B	Refseq	platelet activation
XP_001987559.1 GH21984 [Drosophila grimshawi]	XP_001987559.1	7,00E-31	GLOS_DGRI_GH2	GO:0033198	B	Refseq	response to ATP
XP_001987559.1 GH21984 [Drosophila grimshawi]	XP_001987559.1	7,00E-31	GLOS_DGRI_GH2	GO:0051592	B	Refseq	response to calcium ion
XP_001987559.1 GH21984 [Drosophila grimshawi]	XP_001987559.1	7,00E-31	GLOS_DGRI_GH2	GO:0070588	B	Refseq	calcium ion transmembrane transport
XP_001987559.1 GH21984 [Drosophila grimshawi]	XP_001987559.1	7,00E-31	GLOS_DGRI_GH2	GO:0070679	M	Refseq	inositol 1,4,5 trisphosphate binding
XP_001985733.1 GH20961 [Drosophila grimshawi]	XP_001985733.1	1,00E-68	GLOS_DGRI_GH2	GO:0005262	M	Refseq	calcium channel activity
XP_001985733.1 GH20961 [Drosophila grimshawi]	XP_001985733.1	1,00E-68	GLOS_DGRI_GH2	GO:0005515	M	Refseq	protein binding
XP_001985733.1 GH20961 [Drosophila grimshawi]	XP_001985733.1	1,00E-68	GLOS_DGRI_GH2	GO:0005886	C	Refseq	plasma membrane
XP_001985733.1 GH20961 [Drosophila grimshawi]	XP_001985733.1	1,00E-68	GLOS_DGRI_GH2	GO:0005887	C	Refseq	integral to plasma membrane
XP_001985733.1 GH20961 [Drosophila grimshawi]	XP_001985733.1	1,00E-68	GLOS_DGRI_GH2	GO:0006816	B	Refseq	calcium ion transport
XP_001985733.1 GH20961 [Drosophila grimshawi]	XP_001985733.1	1,00E-68	GLOS_DGRI_GH2	GO:0007411	B	Refseq	axon guidance
XP_001985733.1 GH20961 [Drosophila grimshawi]	XP_001985733.1	1,00E-68	GLOS_DGRI_GH2	GO:0007596	B	Refseq	blood coagulation
XP_001985733.1 GH20961 [Drosophila grimshawi]	XP_001985733.1	1,00E-68	GLOS_DGRI_GH2	GO:0007602	B	Refseq	phototransduction
XP_001985733.1 GH20961 [Drosophila grimshawi]	XP_001985733.1	1,00E-68	GLOS_DGRI_GH2	GO:0010524	B	Refseq	positive regulation of calcium ion transport into cytosol
XP_001985733.1 GH20961 [Drosophila grimshawi]	XP_001985733.1	1,00E-68	GLOS_DGRI_GH2	GO:0015279	M	Refseq	store-operated calcium channel activity
XP_001985733.1 GH20961 [Drosophila grimshawi]	XP_001985733.1	1,00E-68	GLOS_DGRI_GH2	GO:0030168	B	Refseq	platelet activation
XP_001985733.1 GH20961 [Drosophila grimshawi]	XP_001985733.1	1,00E-68	GLOS_DGRI_GH2	GO:0033198	B	Refseq	response to ATP
XP_001985733.1 GH20961 [Drosophila grimshawi]	XP_001985733.1	1,00E-68	GLOS_DGRI_GH2	GO:0051592	B	Refseq	response to calcium ion
XP_001985733.1 GH20961 [Drosophila grimshawi]	XP_001985733.1	1,00E-68	GLOS_DGRI_GH2	GO:0070588	B	Refseq	calcium ion transmembrane transport
XP_001985733.1 GH20961 [Drosophila grimshawi]	XP_001985733.1	1,00E-68	GLOS_DGRI_GH2	GO:0070679	M	Refseq	inositol 1,4,5 trisphosphate binding
XP_001976101.1 GG22677 [Drosophila erecta]	XP_001976101.1	0	GLOS_DERE_GG	GO:0005261	M	Refseq	cation channel activity
XP_001976101.1 GG22677 [Drosophila erecta]	XP_001976101.1	0	GLOS_DERE_GG	GO:0005262	M	Refseq	calcium channel activity
XP_001976101.1 GG22677 [Drosophila erecta]	XP_001976101.1	0	GLOS_DERE_GG	GO:0005515	M	Refseq	protein binding
XP_001976101.1 GG22677 [Drosophila erecta]	XP_001976101.1	0	GLOS_DERE_GG	GO:0005886	C	Refseq	plasma membrane
XP_001976101.1 GG22677 [Drosophila erecta]	XP_001976101.1	0	GLOS_DERE_GG	GO:0005887	C	Refseq	integral to plasma membrane
XP_001976101.1 GG22677 [Drosophila erecta]	XP_001976101.1	0	GLOS_DERE_GG	GO:0006816	B	Refseq	calcium ion transport
XP_001976101.1 GG22677 [Drosophila erecta]	XP_001976101.1	0	GLOS_DERE_GG	GO:0007411	B	Refseq	axon guidance
XP_001976101.1 GG22677 [Drosophila erecta]	XP_001976101.1	0	GLOS_DERE_GG	GO:0015279	M	Refseq	store-operated calcium channel activity
XP_001976101.1 GG22677 [Drosophila erecta]	XP_001976101.1	0	GLOS_DERE_GG	GO:0016323	C	Refseq	basolateral plasma membrane
XP_001976101.1 GG22677 [Drosophila erecta]	XP_001976101.1	0	GLOS_DERE_GG	GO:0030017	C	Refseq	sarcomere
XP_001976101.1 GG22677 [Drosophila erecta]	XP_001976101.1	0	GLOS_DERE_GG	GO:0043034	C	Refseq	costamere
XP_001976101.1 GG22677 [Drosophila erecta]	XP_001976101.1	0	GLOS_DERE_GG	GO:0043234	C	Refseq	protein complex
XP_001976101.1 GG22677 [Drosophila erecta]	XP_001976101.1	0	GLOS_DERE_GG	GO:0044325	M	Refseq	ion channel binding
XP_001976101.1 GG22677 [Drosophila erecta]	XP_001976101.1	0	GLOS_DERE_GG	GO:0045121	C	Refseq	membrane raft
XP_001976101.1 GG22677 [Drosophila erecta]	XP_001976101.1	0	GLOS_DERE_GG	GO:0046541	B	Refseq	saliva secretion
XP_001976101.1 GG22677 [Drosophila erecta]	XP_001976101.1	0	GLOS_DERE_GG	GO:0051281	B	Refseq	positive regulation of release of sequestered calcium ion into cytosol
XP_001976101.1 GG22677 [Drosophila erecta]	XP_001976101.1	0	GLOS_DERE_GG	GO:0051480	B	Refseq	cytosolic calcium ion homeostasis
XP_001976101.1 GG22677 [Drosophila erecta]	XP_001976101.1	0	GLOS_DERE_GG	GO:0051592	B	Refseq	response to calcium ion
XP_001976101.1 GG22677 [Drosophila erecta]	XP_001976101.1	0	GLOS_DERE_GG	GO:0070679	M	Refseq	inositol 1,4,5 trisphosphate binding
XP_001658531.1 cation efflux protein/ zinc transporter [Aedes aegypti]	XP_001658531.1	1,00E-121	GLOS_AAEL_AAE	GO:0030674	M	.	protein binding, bridging
XP_001658531.1 cation efflux protein/ zinc transporter [Aedes aegypti]	XP_001658531.1	1,00E-121	GLOS_AAEL_AAE	GO:0060090	M	.	binding, bridging

XP_001658531.1 cation efflux protein/ zinc transporter [Aedes aegypti]	XP_001658531.1	1,00E-121	GLOS_AAEL_AAE	GO:0035591	M	.	signaling adaptor activity
XP_001658531.1 cation efflux protein/ zinc transporter [Aedes aegypti]	XP_001658531.1	1,00E-121	GLOS_AAEL_AAE	GO:0005070	M	Refseq	SH3/SH2 adaptor activity
XP_001658531.1 cation efflux protein/ zinc transporter [Aedes aegypti]	XP_001658531.1	1,00E-121	GLOS_AAEL_AAE	GO:0005515	M	Refseq	protein binding
XP_001658531.1 cation efflux protein/ zinc transporter [Aedes aegypti]	XP_001658531.1	1,00E-121	GLOS_AAEL_AAE	GO:0005634	C	Refseq	nucleus
XP_001658531.1 cation efflux protein/ zinc transporter [Aedes aegypti]	XP_001658531.1	1,00E-121	GLOS_AAEL_AAE	GO:0005737	C	Refseq	cytoplasm
XP_001658531.1 cation efflux protein/ zinc transporter [Aedes aegypti]	XP_001658531.1	1,00E-121	GLOS_AAEL_AAE	GO:0007049	B	Refseq	cell cycle
XP_001658531.1 cation efflux protein/ zinc transporter [Aedes aegypti]	XP_001658531.1	1,00E-121	GLOS_AAEL_AAE	GO:0007165	B	Refseq	signal transduction
XP_001658531.1 cation efflux protein/ zinc transporter [Aedes aegypti]	XP_001658531.1	1,00E-121	GLOS_AAEL_AAE	GO:0007417	B	Refseq	central nervous system development
XP_001658531.1 cation efflux protein/ zinc transporter [Aedes aegypti]	XP_001658531.1	1,00E-121	GLOS_AAEL_AAE	GO:0048513	B	.	organ development
XP_001658531.1 cation efflux protein/ zinc transporter [Aedes aegypti]	XP_001658531.1	1,00E-121	GLOS_AAEL_AAE	GO:0007507	B	Refseq	heart development
XP_001658531.1 cation efflux protein/ zinc transporter [Aedes aegypti]	XP_001658531.1	1,00E-121	GLOS_AAEL_AAE	GO:0009790	B	.	embryo development
XP_001658531.1 cation efflux protein/ zinc transporter [Aedes aegypti]	XP_001658531.1	1,00E-121	GLOS_AAEL_AAE	GO:0009792	B	Refseq	embryo development ending in birth or egg hatching
XP_001658531.1 cation efflux protein/ zinc transporter [Aedes aegypti]	XP_001658531.1	1,00E-121	GLOS_AAEL_AAE	GO:0010212	B	Refseq	response to ionizing radiation
XP_001658531.1 cation efflux protein/ zinc transporter [Aedes aegypti]	XP_001658531.1	1,00E-121	GLOS_AAEL_AAE	GO:0019904	M	.	protein domain specific binding
XP_001658531.1 cation efflux protein/ zinc transporter [Aedes aegypti]	XP_001658531.1	1,00E-121	GLOS_AAEL_AAE	GO:0017124	M	Refseq	SH3 domain binding
XP_001658531.1 cation efflux protein/ zinc transporter [Aedes aegypti]	XP_001658531.1	1,00E-121	GLOS_AAEL_AAE	GO:0042802	M	Refseq	identical protein binding
XP_001658531.1 cation efflux protein/ zinc transporter [Aedes aegypti]	XP_001658531.1	1,00E-121	GLOS_AAEL_AAE	GO:0051726	B	.	regulation of cell cycle
XP_001658531.1 cation efflux protein/ zinc transporter [Aedes aegypti]	XP_001658531.1	1,00E-121	GLOS_AAEL_AAE	GO:0045786	B	Refseq	negative regulation of cell cycle
XP_001658531.1 cation efflux protein/ zinc transporter [Aedes aegypti]	XP_001658531.1	1,00E-121	GLOS_AAEL_AAE	GO:0048471	C	Refseq	perinuclear region of cytoplasm
XP_001658531.1 cation efflux protein/ zinc transporter [Aedes aegypti]	XP_001658531.1	1,00E-121	GLOS_AAEL_AAE	GO:0008134	M	.	transcription factor binding
XP_001658531.1 cation efflux protein/ zinc transporter [Aedes aegypti]	XP_001658531.1	1,00E-121	GLOS_AAEL_AAE	GO:0051059	M	Refseq	NF-kappaB binding
XP_001658531.1 cation efflux protein/ zinc transporter [Aedes aegypti]	XP_001658531.1	1,00E-121	GLOS_AAEL_AAE	GO:0072331	B	.	signal transduction by p53 class mediator
XP_001658531.1 cation efflux protein/ zinc transporter [Aedes aegypti]	XP_001658531.1	1,00E-121	GLOS_AAEL_AAE	GO:0072332	B	Refseq	intrinsic apoptotic signaling pathway by p53 class mediator
XP_001664151.1 RP58 protein, putative [Aedes aegypti]	XP_001664151.1	2,00E-19	GLOS_AAEL_AAE	GO:0005070	M	Refseq	SH3/SH2 adaptor activity
XP_001664151.1 RP58 protein, putative [Aedes aegypti]	XP_001664151.1	2,00E-19	GLOS_AAEL_AAE	GO:0005515	M	Refseq	protein binding
XP_001664151.1 RP58 protein, putative [Aedes aegypti]	XP_001664151.1	2,00E-19	GLOS_AAEL_AAE	GO:0005634	C	Refseq	nucleus
XP_001664151.1 RP58 protein, putative [Aedes aegypti]	XP_001664151.1	2,00E-19	GLOS_AAEL_AAE	GO:0005737	C	Refseq	cytoplasm
XP_001664151.1 RP58 protein, putative [Aedes aegypti]	XP_001664151.1	2,00E-19	GLOS_AAEL_AAE	GO:0007049	B	Refseq	cell cycle
XP_001664151.1 RP58 protein, putative [Aedes aegypti]	XP_001664151.1	2,00E-19	GLOS_AAEL_AAE	GO:0007165	B	Refseq	signal transduction
XP_001664151.1 RP58 protein, putative [Aedes aegypti]	XP_001664151.1	2,00E-19	GLOS_AAEL_AAE	GO:0007417	B	Refseq	central nervous system development
XP_001664151.1 RP58 protein, putative [Aedes aegypti]	XP_001664151.1	2,00E-19	GLOS_AAEL_AAE	GO:0007507	B	Refseq	heart development
XP_001664151.1 RP58 protein, putative [Aedes aegypti]	XP_001664151.1	2,00E-19	GLOS_AAEL_AAE	GO:0009792	B	Refseq	embryo development ending in birth or egg hatching
XP_001664151.1 RP58 protein, putative [Aedes aegypti]	XP_001664151.1	2,00E-19	GLOS_AAEL_AAE	GO:0010212	B	Refseq	response to ionizing radiation
XP_001664151.1 RP58 protein, putative [Aedes aegypti]	XP_001664151.1	2,00E-19	GLOS_AAEL_AAE	GO:0017124	M	Refseq	SH3 domain binding
XP_001664151.1 RP58 protein, putative [Aedes aegypti]	XP_001664151.1	2,00E-19	GLOS_AAEL_AAE	GO:0042802	M	Refseq	identical protein binding
XP_001664151.1 RP58 protein, putative [Aedes aegypti]	XP_001664151.1	2,00E-19	GLOS_AAEL_AAE	GO:0045786	B	Refseq	negative regulation of cell cycle
XP_001664151.1 RP58 protein, putative [Aedes aegypti]	XP_001664151.1	2,00E-19	GLOS_AAEL_AAE	GO:0048471	C	Refseq	perinuclear region of cytoplasm
XP_001664151.1 RP58 protein, putative [Aedes aegypti]	XP_001664151.1	2,00E-19	GLOS_AAEL_AAE	GO:0051059	M	Refseq	NF-kappaB binding
XP_001664151.1 RP58 protein, putative [Aedes aegypti]	XP_001664151.1	2,00E-19	GLOS_AAEL_AAE	GO:0072332	B	Refseq	intrinsic apoptotic signaling pathway by p53 class mediator
NP_001262107.1 RhoBTB, isoform B [Drosophila melanogaster]	NP_001262107.1	0	GLOS_RHOBTB.1	GO:0000122	B	Refseq	negative regulation of transcription from RNA polymerase II promoter
NP_001262107.1 RhoBTB, isoform B [Drosophila melanogaster]	NP_001262107.1	0	GLOS_RHOBTB.1	GO:0001501	B	Refseq	skeletal system development
NP_001262107.1 RhoBTB, isoform B [Drosophila melanogaster]	NP_001262107.1	0	GLOS_RHOBTB.1	GO:0003700	M	Refseq	sequence-specific DNA binding transcription factor activity
NP_001262107.1 RhoBTB, isoform B [Drosophila melanogaster]	NP_001262107.1	0	GLOS_RHOBTB.1	GO:0005515	M	Refseq	protein binding
NP_001262107.1 RhoBTB, isoform B [Drosophila melanogaster]	NP_001262107.1	0	GLOS_RHOBTB.1	GO:0005634	C	Refseq	nucleus
NP_001262107.1 RhoBTB, isoform B [Drosophila melanogaster]	NP_001262107.1	0	GLOS_RHOBTB.1	GO:0006366	B	Refseq	transcription from RNA polymerase II promoter
NP_001262107.1 RhoBTB, isoform B [Drosophila melanogaster]	NP_001262107.1	0	GLOS_RHOBTB.1	GO:0006607	B	Refseq	NLS-bearing protein import into nucleus
NP_001262107.1 RhoBTB, isoform B [Drosophila melanogaster]	NP_001262107.1	0	GLOS_RHOBTB.1	GO:0008270	M	Refseq	zinc ion binding
NP_001262107.1 RhoBTB, isoform B [Drosophila melanogaster]	NP_001262107.1	0	GLOS_RHOBTB.1	GO:0032330	B	Refseq	regulation of chondrocyte differentiation
NP_001262107.1 RhoBTB, isoform B [Drosophila melanogaster]	NP_001262107.1	0	GLOS_RHOBTB.1	GO:0043565	M	Refseq	sequence-specific DNA binding
NP_001137942.2 sosondowah, isoform J [Drosophila melanogaster]	NP_001137942.2	1,00E-164	GLOS_contig_004	GO:0000122	B	Refseq	negative regulation of transcription from RNA polymerase II promoter
NP_001137942.2 sosondowah, isoform J [Drosophila melanogaster]	NP_001137942.2	1,00E-164	GLOS_contig_004	GO:0001501	B	Refseq	skeletal system development
NP_001137942.2 sosondowah, isoform J [Drosophila melanogaster]	NP_001137942.2	1,00E-164	GLOS_contig_004	GO:0003700	M	Refseq	sequence-specific DNA binding transcription factor activity
NP_001137942.2 sosondowah, isoform J [Drosophila melanogaster]	NP_001137942.2	1,00E-164	GLOS_contig_004	GO:0005515	M	Refseq	protein binding
NP_001137942.2 sosondowah, isoform J [Drosophila melanogaster]	NP_001137942.2	1,00E-164	GLOS_contig_004	GO:0005634	C	Refseq	nucleus

NP_001137942.2 sosondowah, isoform J [Drosophila melanogaster]	NP_001137942.2	1,00E-164	GLOS_contig_004	GO:0006366	B	Refseq	transcription from RNA polymerase II promoter
NP_001137942.2 sosondowah, isoform J [Drosophila melanogaster]	NP_001137942.2	1,00E-164	GLOS_contig_004	GO:0006607	B	Refseq	NLS-bearing protein import into nucleus
NP_001137942.2 sosondowah, isoform J [Drosophila melanogaster]	NP_001137942.2	1,00E-164	GLOS_contig_004	GO:0008270	M	Refseq	zinc ion binding
NP_001137942.2 sosondowah, isoform J [Drosophila melanogaster]	NP_001137942.2	1,00E-164	GLOS_contig_004	GO:0032330	B	Refseq	regulation of chondrocyte differentiation
NP_001137942.2 sosondowah, isoform J [Drosophila melanogaster]	NP_001137942.2	1,00E-164	GLOS_contig_004	GO:0043565	M	Refseq	sequence-specific DNA binding
NP_647813.1 CG12012, isoform A [D. m.] ref|XP_002083495.1| GD13762 [D. s.]	NP_647813.1	5,00E-35	GLOS_CG12012.1	GO:0000122	B	Refseq	negative regulation of transcription from RNA polymerase II promoter
NP_647813.1 CG12012, isoform A [D. m.] ref|XP_002083495.1| GD13762 [D. s.]	NP_647813.1	5,00E-35	GLOS_CG12012.1	GO:0001501	B	Refseq	skeletal system development
NP_647813.1 CG12012, isoform A [D. m.] ref|XP_002083495.1| GD13762 [D. s.]	NP_647813.1	5,00E-35	GLOS_CG12012.1	GO:0003700	M	Refseq	sequence-specific DNA binding transcription factor activity
NP_647813.1 CG12012, isoform A [D. m.] ref|XP_002083495.1| GD13762 [D. s.]	NP_647813.1	5,00E-35	GLOS_CG12012.1	GO:0005515	M	Refseq	protein binding
NP_647813.1 CG12012, isoform A [D. m.] ref|XP_002083495.1| GD13762 [D. s.]	NP_647813.1	5,00E-35	GLOS_CG12012.1	GO:0005634	C	Refseq	nucleus
NP_647813.1 CG12012, isoform A [D. m.] ref|XP_002083495.1| GD13762 [D. s.]	NP_647813.1	5,00E-35	GLOS_CG12012.1	GO:0006366	B	Refseq	transcription from RNA polymerase II promoter
NP_647813.1 CG12012, isoform A [D. m.] ref|XP_002083495.1| GD13762 [D. s.]	NP_647813.1	5,00E-35	GLOS_CG12012.1	GO:0006607	B	Refseq	NLS-bearing protein import into nucleus
NP_647813.1 CG12012, isoform A [D. m.] ref|XP_002083495.1| GD13762 [D. s.]	NP_647813.1	5,00E-35	GLOS_CG12012.1	GO:0008270	M	Refseq	zinc ion binding
NP_647813.1 CG12012, isoform A [D. m.] ref|XP_002083495.1| GD13762 [D. s.]	NP_647813.1	5,00E-35	GLOS_CG12012.1	GO:0032330	B	Refseq	regulation of chondrocyte differentiation
NP_647813.1 CG12012, isoform A [D. m.] ref|XP_002083495.1| GD13762 [D. s.]	NP_647813.1	5,00E-35	GLOS_CG12012.1	GO:0043565	M	Refseq	sequence-specific DNA binding
[BBH] ACT1_TRYBB (sp|P12432) Actin A OS=T. b. brucei PE=3 SV=1	ACT1_TRYBB	0	GLOS_ACT1.1.1	GO:0005737	C	UniProtKB-K	cytoplasm
[BBH] ACT1_TRYBB (sp|P12432) Actin A OS=T. b. brucei PE=3 SV=1	ACT1_TRYBB	0	GLOS_ACT1.1.1	GO:0005856	C	UniProtKB-S	cytoskeleton
[BBH] ACT1_TRYBB (sp|P12432) Actin A OS=T. b. brucei PE=3 SV=1	ACT1_TRYBB	0	GLOS_ACT1.1.1	GO:0032550	M	.	purine ribonucleoside binding
[BBH] ACT1_TRYBB (sp|P12432) Actin A OS=T. b. brucei PE=3 SV=1	ACT1_TRYBB	0	GLOS_ACT1.1.1	GO:0001883	M	.	purine nucleoside binding
[BBH] ACT1_TRYBB (sp|P12432) Actin A OS=T. b. brucei PE=3 SV=1	ACT1_TRYBB	0	GLOS_ACT1.1.1	GO:0001882	M	.	nucleoside binding
[BBH] ACT1_TRYBB (sp|P12432) Actin A OS=T. b. brucei PE=3 SV=1	ACT1_TRYBB	0	GLOS_ACT1.1.1	GO:0097367	M	.	carbohydrate derivative binding
[BBH] ACT1_TRYBB (sp|P12432) Actin A OS=T. b. brucei PE=3 SV=1	ACT1_TRYBB	0	GLOS_ACT1.1.1	GO:0032549	M	.	ribonucleoside binding
[BBH] ACT1_TRYBB (sp|P12432) Actin A OS=T. b. brucei PE=3 SV=1	ACT1_TRYBB	0	GLOS_ACT1.1.1	GO:0032559	M	.	adenyl ribonucleotide binding
[BBH] ACT1_TRYBB (sp|P12432) Actin A OS=T. b. brucei PE=3 SV=1	ACT1_TRYBB	0	GLOS_ACT1.1.1	GO:0030554	M	.	adenyl nucleotide binding
[BBH] ACT1_TRYBB (sp|P12432) Actin A OS=T. b. brucei PE=3 SV=1	ACT1_TRYBB	0	GLOS_ACT1.1.1	GO:0017076	M	.	purine nucleotide binding
[BBH] ACT1_TRYBB (sp|P12432) Actin A OS=T. b. brucei PE=3 SV=1	ACT1_TRYBB	0	GLOS_ACT1.1.1	GO:0000166	M	.	nucleotide binding
[BBH] ACT1_TRYBB (sp|P12432) Actin A OS=T. b. brucei PE=3 SV=1	ACT1_TRYBB	0	GLOS_ACT1.1.1	GO:1901265	M	.	nucleoside phosphate binding
[BBH] ACT1_TRYBB (sp|P12432) Actin A OS=T. b. brucei PE=3 SV=1	ACT1_TRYBB	0	GLOS_ACT1.1.1	GO:0032555	M	.	purine ribonucleotide binding
[BBH] ACT1_TRYBB (sp|P12432) Actin A OS=T. b. brucei PE=3 SV=1	ACT1_TRYBB	0	GLOS_ACT1.1.1	GO:0032553	M	.	ribonucleotide binding
[BBH] ACT1_TRYBB (sp|P12432) Actin A OS=T. b. brucei PE=3 SV=1	ACT1_TRYBB	0	GLOS_ACT1.1.1	GO:0035639	M	.	purine ribonucleoside triphosphate binding
[BBH] ACT1_TRYBB (sp|P12432) Actin A OS=T. b. brucei PE=3 SV=1	ACT1_TRYBB	0	GLOS_ACT1.1.1	GO:0005524	M	UniProtKB-K	ATP binding
[BBH] CALM_TRYBG (sp|P69098) Calmodulin OS=T. b. gambiense	CALM_TRYBG	6,00E-99	GLOS_CALM.1.1	GO:0005509	M	InterPro	calcium ion binding
DGK2_DROME (sp|Q09103) Eye-spec. diacylglycerol kinase OS=D. m.	DGK2_DROME	0	GLOS_DGK2.1.2	GO:0016020	C	UniProtKB-S	membrane
DGK2_DROME (sp|Q09103) Eye-spec. diacylglycerol kinase OS=D. m.	DGK2_DROME	0	GLOS_DGK2.1.2	GO:0043234	C	.	protein complex
DGK2_DROME (sp|Q09103) Eye-spec. diacylglycerol kinase OS=D. m.	DGK2_DROME	0	GLOS_DGK2.1.2	GO:0044430	C	.	cytoskeletal part
DGK2_DROME (sp|Q09103) Eye-spec. diacylglycerol kinase OS=D. m.	DGK2_DROME	0	GLOS_DGK2.1.2	GO:0005856	C	.	cytoskeleton
DGK2_DROME (sp|Q09103) Eye-spec. diacylglycerol kinase OS=D. m.	DGK2_DROME	0	GLOS_DGK2.1.2	GO:0015630	C	.	microtubule cytoskeleton
DGK2_DROME (sp|Q09103) Eye-spec. diacylglycerol kinase OS=D. m.	DGK2_DROME	0	GLOS_DGK2.1.2	GO:0005875	C	FlyBase	microtubule associated complex
DGK2_DROME (sp|Q09103) Eye-spec. diacylglycerol kinase OS=D. m.	DGK2_DROME	0	GLOS_DGK2.1.2	GO:0005524	M	UniProtKB-K	ATP binding
DGK2_DROME (sp|Q09103) Eye-spec. diacylglycerol kinase OS=D. m.	DGK2_DROME	0	GLOS_DGK2.1.2	GO:0016301	M	.	kinase activity
DGK2_DROME (sp|Q09103) Eye-spec. diacylglycerol kinase OS=D. m.	DGK2_DROME	0	GLOS_DGK2.1.2	GO:0016772	M	.	transferase activity, transferring phosphorus-containing groups
DGK2_DROME (sp|Q09103) Eye-spec. diacylglycerol kinase OS=D. m.	DGK2_DROME	0	GLOS_DGK2.1.2	GO:0016740	M	.	transferase activity
DGK2_DROME (sp|Q09103) Eye-spec. diacylglycerol kinase OS=D. m.	DGK2_DROME	0	GLOS_DGK2.1.2	GO:0016773	M	.	phosphotransferase activity, alcohol group as acceptor
DGK2_DROME (sp|Q09103) Eye-spec. diacylglycerol kinase OS=D. m.	DGK2_DROME	0	GLOS_DGK2.1.2	GO:0004143	M	FlyBase	diacylglycerol kinase activity
DGK2_DROME (sp|Q09103) Eye-spec. diacylglycerol kinase OS=D. m.	DGK2_DROME	0	GLOS_DGK2.1.2	GO:0046872	M	UniProtKB-K	metal ion binding
DGK2_DROME (sp|Q09103) Eye-spec. diacylglycerol kinase OS=D. m.	DGK2_DROME	0	GLOS_DGK2.1.2	GO:0030036	B	.	actin cytoskeleton organization
DGK2_DROME (sp|Q09103) Eye-spec. diacylglycerol kinase OS=D. m.	DGK2_DROME	0	GLOS_DGK2.1.2	GO:0007010	B	.	cytoskeleton organization
DGK2_DROME (sp|Q09103) Eye-spec. diacylglycerol kinase OS=D. m.	DGK2_DROME	0	GLOS_DGK2.1.2	GO:0006996	B	.	organelle organization
DGK2_DROME (sp|Q09103) Eye-spec. diacylglycerol kinase OS=D. m.	DGK2_DROME	0	GLOS_DGK2.1.2	GO:0030029	B	.	actin filament-based process
DGK2_DROME (sp|Q09103) Eye-spec. diacylglycerol kinase OS=D. m.	DGK2_DROME	0	GLOS_DGK2.1.2	GO:0007015	B	FlyBase	actin filament organization
DGK2_DROME (sp|Q09103) Eye-spec. diacylglycerol kinase OS=D. m.	DGK2_DROME	0	GLOS_DGK2.1.2	GO:0022400	B	.	regulation of rhodopsin mediated signaling pathway
DGK2_DROME (sp|Q09103) Eye-spec. diacylglycerol kinase OS=D. m.	DGK2_DROME	0	GLOS_DGK2.1.2	GO:0008277	B	.	regulation of G-protein coupled receptor protein signaling pathway
DGK2_DROME (sp|Q09103) Eye-spec. diacylglycerol kinase OS=D. m.	DGK2_DROME	0	GLOS_DGK2.1.2	GO:0032101	B	.	regulation of response to external stimulus
DGK2_DROME (sp|Q09103) Eye-spec. diacylglycerol kinase OS=D. m.	DGK2_DROME	0	GLOS_DGK2.1.2	GO:0016059	B	FlyBase	deactivation of rhodopsin mediated signaling

DGK2_DROME (sp|Q09103) Eye-spec. diacylglycerol kinase OS=D. m.	DGK2_DROME	0	GLOS_DGK2.1.2	GO:0035556	B	InterPro	intracellular signal transduction
DGK2_DROME (sp|Q09103) Eye-spec. diacylglycerol kinase OS=D. m.	DGK2_DROME	0	GLOS_DGK2.1.2	GO:0016310	B	.	phosphorylation
DGK2_DROME (sp|Q09103) Eye-spec. diacylglycerol kinase OS=D. m.	DGK2_DROME	0	GLOS_DGK2.1.2	GO:0006796	B	.	phosphate-containing compound metabolic process
DGK2_DROME (sp|Q09103) Eye-spec. diacylglycerol kinase OS=D. m.	DGK2_DROME	0	GLOS_DGK2.1.2	GO:0006793	B	.	phosphorus metabolic process
DGK2_DROME (sp|Q09103) Eye-spec. diacylglycerol kinase OS=D. m.	DGK2_DROME	0	GLOS_DGK2.1.2	GO:0030258	B	.	lipid modification
DGK2_DROME (sp|Q09103) Eye-spec. diacylglycerol kinase OS=D. m.	DGK2_DROME	0	GLOS_DGK2.1.2	GO:0044255	B	.	cellular lipid metabolic process
DGK2_DROME (sp|Q09103) Eye-spec. diacylglycerol kinase OS=D. m.	DGK2_DROME	0	GLOS_DGK2.1.2	GO:0006629	B	.	lipid metabolic process
DGK2_DROME (sp|Q09103) Eye-spec. diacylglycerol kinase OS=D. m.	DGK2_DROME	0	GLOS_DGK2.1.2	GO:0046834	B	FlyBase	lipid phosphorylation
DGK2_DROME (sp|Q09103) Eye-spec. diacylglycerol kinase OS=D. m.	DGK2_DROME	0	GLOS_DGK2.1.2	GO:0046473	B	.	phosphatidic acid metabolic process
DGK2_DROME (sp|Q09103) Eye-spec. diacylglycerol kinase OS=D. m.	DGK2_DROME	0	GLOS_DGK2.1.2	GO:0006650	B	.	glycerophospholipid metabolic process
DGK2_DROME (sp|Q09103) Eye-spec. diacylglycerol kinase OS=D. m.	DGK2_DROME	0	GLOS_DGK2.1.2	GO:0006644	B	.	phospholipid metabolic process
DGK2_DROME (sp|Q09103) Eye-spec. diacylglycerol kinase OS=D. m.	DGK2_DROME	0	GLOS_DGK2.1.2	GO:0019637	B	.	organophosphate metabolic process
DGK2_DROME (sp|Q09103) Eye-spec. diacylglycerol kinase OS=D. m.	DGK2_DROME	0	GLOS_DGK2.1.2	GO:0046486	B	.	glycerolipid metabolic process
DGK2_DROME (sp|Q09103) Eye-spec. diacylglycerol kinase OS=D. m.	DGK2_DROME	0	GLOS_DGK2.1.2	GO:0046474	B	.	glycerophospholipid biosynthetic process
DGK2_DROME (sp|Q09103) Eye-spec. diacylglycerol kinase OS=D. m.	DGK2_DROME	0	GLOS_DGK2.1.2	GO:0008654	B	.	phospholipid biosynthetic process
DGK2_DROME (sp|Q09103) Eye-spec. diacylglycerol kinase OS=D. m.	DGK2_DROME	0	GLOS_DGK2.1.2	GO:0008610	B	.	lipid biosynthetic process
DGK2_DROME (sp|Q09103) Eye-spec. diacylglycerol kinase OS=D. m.	DGK2_DROME	0	GLOS_DGK2.1.2	GO:0090407	B	.	organophosphate biosynthetic process
DGK2_DROME (sp|Q09103) Eye-spec. diacylglycerol kinase OS=D. m.	DGK2_DROME	0	GLOS_DGK2.1.2	GO:0045017	B	.	glycerolipid biosynthetic process
DGK2_DROME (sp|Q09103) Eye-spec. diacylglycerol kinase OS=D. m.	DGK2_DROME	0	GLOS_DGK2.1.2	GO:0006654	B	FlyBase	phosphatidic acid biosynthetic process
DGK2_DROME (sp|Q09103) Eye-spec. diacylglycerol kinase OS=D. m.	DGK2_DROME	0	GLOS_DGK2.1.2	GO:0046488	B	.	phosphatidylinositol metabolic process
DGK2_DROME (sp|Q09103) Eye-spec. diacylglycerol kinase OS=D. m.	DGK2_DROME	0	GLOS_DGK2.1.2	GO:0006661	B	FlyBase	phosphatidylinositol biosynthetic process
DGK2_DROME (sp|Q09103) Eye-spec. diacylglycerol kinase OS=D. m.	DGK2_DROME	0	GLOS_DGK2.1.2	GO:0001895	B	.	retina homeostasis
DGK2_DROME (sp|Q09103) Eye-spec. diacylglycerol kinase OS=D. m.	DGK2_DROME	0	GLOS_DGK2.1.2	GO:0001894	B	.	tissue homeostasis
DGK2_DROME (sp|Q09103) Eye-spec. diacylglycerol kinase OS=D. m.	DGK2_DROME	0	GLOS_DGK2.1.2	GO:0060249	B	.	anatomical structure homeostasis
DGK2_DROME (sp|Q09103) Eye-spec. diacylglycerol kinase OS=D. m.	DGK2_DROME	0	GLOS_DGK2.1.2	GO:0048871	B	.	multicellular organismal homeostasis
DGK2_DROME (sp|Q09103) Eye-spec. diacylglycerol kinase OS=D. m.	DGK2_DROME	0	GLOS_DGK2.1.2	GO:0045494	B	FlyBase	photoreceptor cell maintenance
DGK2_DROME (sp|Q09103) Eye-spec. diacylglycerol kinase OS=D. m.	DGK2_DROME	0	GLOS_DGK2.1.2	GO:0007186	B	.	G-protein coupled receptor signaling pathway
DGK2_DROME (sp|Q09103) Eye-spec. diacylglycerol kinase OS=D. m.	DGK2_DROME	0	GLOS_DGK2.1.2	GO:0007166	B	.	cell surface receptor signaling pathway
DGK2_DROME (sp|Q09103) Eye-spec. diacylglycerol kinase OS=D. m.	DGK2_DROME	0	GLOS_DGK2.1.2	GO:0007205	B	InterPro	protein kinase C-activating G-protein coupled receptor signaling pathway
DGK2_DROME (sp|Q09103) Eye-spec. diacylglycerol kinase OS=D. m.	DGK2_DROME	0	GLOS_DGK2.1.2	GO:0071482	B	.	cellular response to light stimulus
DGK2_DROME (sp|Q09103) Eye-spec. diacylglycerol kinase OS=D. m.	DGK2_DROME	0	GLOS_DGK2.1.2	GO:0071478	B	.	cellular response to radiation
DGK2_DROME (sp|Q09103) Eye-spec. diacylglycerol kinase OS=D. m.	DGK2_DROME	0	GLOS_DGK2.1.2	GO:0071214	B	.	cellular response to abiotic stimulus
DGK2_DROME (sp|Q09103) Eye-spec. diacylglycerol kinase OS=D. m.	DGK2_DROME	0	GLOS_DGK2.1.2	GO:0007603	B	.	phototransduction, visible light
DGK2_DROME (sp|Q09103) Eye-spec. diacylglycerol kinase OS=D. m.	DGK2_DROME	0	GLOS_DGK2.1.2	GO:0007602	B	.	phototransduction
DGK2_DROME (sp|Q09103) Eye-spec. diacylglycerol kinase OS=D. m.	DGK2_DROME	0	GLOS_DGK2.1.2	GO:0009584	B	.	detection of visible light
DGK2_DROME (sp|Q09103) Eye-spec. diacylglycerol kinase OS=D. m.	DGK2_DROME	0	GLOS_DGK2.1.2	GO:0016056	B	FlyBase	rhodopsin mediated signaling pathway
DGK2_DROME (sp|Q09103) Eye-spec. diacylglycerol kinase OS=D. m.	DGK2_DROME	0	GLOS_DGK2.1.2	GO:0007606	B	.	sensory perception of chemical stimulus
DGK2_DROME (sp|Q09103) Eye-spec. diacylglycerol kinase OS=D. m.	DGK2_DROME	0	GLOS_DGK2.1.2	GO:0007600	B	.	sensory perception
DGK2_DROME (sp|Q09103) Eye-spec. diacylglycerol kinase OS=D. m.	DGK2_DROME	0	GLOS_DGK2.1.2	GO:0050877	B	.	neurological system process
DGK2_DROME (sp|Q09103) Eye-spec. diacylglycerol kinase OS=D. m.	DGK2_DROME	0	GLOS_DGK2.1.2	GO:0007608	B	FlyBase	sensory perception of smell
DGK2_DROME (sp|Q09103) Eye-spec. diacylglycerol kinase OS=D. m.	DGK2_DROME	0	GLOS_DGK2.1.2	GO:0050954	B	.	sensory perception of mechanical stimulus
DGK2_DROME (sp|Q09103) Eye-spec. diacylglycerol kinase OS=D. m.	DGK2_DROME	0	GLOS_DGK2.1.2	GO:0007605	B	FlyBase	sensory perception of sound
DGK2_DROME (sp|Q09103) Eye-spec. diacylglycerol kinase OS=D. m.	DGK2_DROME	0	GLOS_DGK2.1.2	GO:0009266	B	.	response to temperature stimulus
DGK2_DROME (sp|Q09103) Eye-spec. diacylglycerol kinase OS=D. m.	DGK2_DROME	0	GLOS_DGK2.1.2	GO:0043052	B	FlyBase	thermotaxis
DGK2_DROME (sp|Q09103) Eye-spec. diacylglycerol kinase OS=D. m.	DGK2_DROME	0	GLOS_DGK2.1.2	GO:0050953	B	.	sensory perception of light stimulus
DGK2_DROME (sp|Q09103) Eye-spec. diacylglycerol kinase OS=D. m.	DGK2_DROME	0	GLOS_DGK2.1.2	GO:0007601	B	UniProtKB-k	visual perception
[BBH] DOS_DROME (sp|Q9VZZ9) Prot daughter of sevenless D. m.	DOS_DROME	0	GLOS_DOS.1.1	GO:0005737	C	UniProtKB	cytoplasm
[BBH] DOS_DROME (sp|Q9VZZ9) Prot daughter of sevenless D. m.	DOS_DROME	0	GLOS_DOS.1.1	GO:0005886	C	FlyBase	plasma membrane
[BBH] DOS_DROME (sp|Q9VZZ9) Prot daughter of sevenless D. m.	DOS_DROME	0	GLOS_DOS.1.1	GO:0008289	M	.	lipid binding
[BBH] DOS_DROME (sp|Q9VZZ9) Prot daughter of sevenless D. m.	DOS_DROME	0	GLOS_DOS.1.1	GO:0005543	M	InterPro	phospholipid binding
[BBH] DOS_DROME (sp|Q9VZZ9) Prot daughter of sevenless D. m.	DOS_DROME	0	GLOS_DOS.1.1	GO:0005070	M	FlyBase	SH3/SH2 adaptor activity
[BBH] DOS_DROME (sp|Q9VZZ9) Prot daughter of sevenless D. m.	DOS_DROME	0	GLOS_DOS.1.1	GO:0000578	B	.	embryonic axis specification
[BBH] DOS_DROME (sp|Q9VZZ9) Prot daughter of sevenless D. m.	DOS_DROME	0	GLOS_DOS.1.1	GO:0009798	B	.	axis specification
[BBH] DOS_DROME (sp|Q9VZZ9) Prot daughter of sevenless D. m.	DOS_DROME	0	GLOS_DOS.1.1	GO:0007389	B	.	pattern specification process

[BBH] DOS_DROME (sp|Q9VZZ9) Prot daughter of sevenless D. m.	DOS_DROME	0	GLOS_DOS.1.1	GO:0009880	B	.	embryonic pattern specification
[BBH] DOS_DROME (sp|Q9VZZ9) Prot daughter of sevenless D. m.	DOS_DROME	0	GLOS_DOS.1.1	GO:0009948	B	.	anterior/posterior axis specification
[BBH] DOS_DROME (sp|Q9VZZ9) Prot daughter of sevenless D. m.	DOS_DROME	0	GLOS_DOS.1.1	GO:0009952	B	.	anterior/posterior pattern specification
[BBH] DOS_DROME (sp|Q9VZZ9) Prot daughter of sevenless D. m.	DOS_DROME	0	GLOS_DOS.1.1	GO:0003002	B	.	regionalization
[BBH] DOS_DROME (sp|Q9VZZ9) Prot daughter of sevenless D. m.	DOS_DROME	0	GLOS_DOS.1.1	GO:0007351	B	.	tripartite regional subdivision
[BBH] DOS_DROME (sp|Q9VZZ9) Prot daughter of sevenless D. m.	DOS_DROME	0	GLOS_DOS.1.1	GO:0007350	B	.	blastoderm segmentation
[BBH] DOS_DROME (sp|Q9VZZ9) Prot daughter of sevenless D. m.	DOS_DROME	0	GLOS_DOS.1.1	GO:0035282	B	.	segmentation
[BBH] DOS_DROME (sp|Q9VZZ9) Prot daughter of sevenless D. m.	DOS_DROME	0	GLOS_DOS.1.1	GO:0008595	B	FlyBase	anterior/posterior axis specification, embryo
[BBH] DOS_DROME (sp|Q9VZZ9) Prot daughter of sevenless D. m.	DOS_DROME	0	GLOS_DOS.1.1	GO:0006959	B	.	humoral immune response
[BBH] DOS_DROME (sp|Q9VZZ9) Prot daughter of sevenless D. m.	DOS_DROME	0	GLOS_DOS.1.1	GO:0006955	B	.	immune response
[BBH] DOS_DROME (sp|Q9VZZ9) Prot daughter of sevenless D. m.	DOS_DROME	0	GLOS_DOS.1.1	GO:0051707	B	.	response to other organism
[BBH] DOS_DROME (sp|Q9VZZ9) Prot daughter of sevenless D. m.	DOS_DROME	0	GLOS_DOS.1.1	GO:0009607	B	.	response to biotic stimulus
[BBH] DOS_DROME (sp|Q9VZZ9) Prot daughter of sevenless D. m.	DOS_DROME	0	GLOS_DOS.1.1	GO:0051704	B	.	multi-organism process
[BBH] DOS_DROME (sp|Q9VZZ9) Prot daughter of sevenless D. m.	DOS_DROME	0	GLOS_DOS.1.1	GO:0019730	B	FlyBase	antimicrobial humoral response
[BBH] DOS_DROME (sp|Q9VZZ9) Prot daughter of sevenless D. m.	DOS_DROME	0	GLOS_DOS.1.1	GO:0046530	B	.	photoreceptor cell differentiation
[BBH] DOS_DROME (sp|Q9VZZ9) Prot daughter of sevenless D. m.	DOS_DROME	0	GLOS_DOS.1.1	GO:0042461	B	UniProtKB	photoreceptor cell development
[BBH] DOS_DROME (sp|Q9VZZ9) Prot daughter of sevenless D. m.	DOS_DROME	0	GLOS_DOS.1.1	GO:0051056	B	.	regulation of small GTPase mediated signal transduction
[BBH] DOS_DROME (sp|Q9VZZ9) Prot daughter of sevenless D. m.	DOS_DROME	0	GLOS_DOS.1.1	GO:0046578	B	UniProtKB	regulation of Ras protein signal transduction
[BBH] DOS_DROME (sp|Q9VZZ9) Prot daughter of sevenless D. m.	DOS_DROME	0	GLOS_DOS.1.1	GO:0007169	B	.	transmembrane receptor protein tyrosine kinase signaling pathway
[BBH] DOS_DROME (sp|Q9VZZ9) Prot daughter of sevenless D. m.	DOS_DROME	0	GLOS_DOS.1.1	GO:0007167	B	.	enzyme linked receptor protein signaling pathway
[BBH] DOS_DROME (sp|Q9VZZ9) Prot daughter of sevenless D. m.	DOS_DROME	0	GLOS_DOS.1.1	GO:0007465	B	.	R7 cell fate commitment
[BBH] DOS_DROME (sp|Q9VZZ9) Prot daughter of sevenless D. m.	DOS_DROME	0	GLOS_DOS.1.1	GO:0001752	B	.	compound eye photoreceptor fate commitment
[BBH] DOS_DROME (sp|Q9VZZ9) Prot daughter of sevenless D. m.	DOS_DROME	0	GLOS_DOS.1.1	GO:0042706	B	.	eye photoreceptor cell fate commitment
[BBH] DOS_DROME (sp|Q9VZZ9) Prot daughter of sevenless D. m.	DOS_DROME	0	GLOS_DOS.1.1	GO:0046552	B	.	photoreceptor cell fate commitment
[BBH] DOS_DROME (sp|Q9VZZ9) Prot daughter of sevenless D. m.	DOS_DROME	0	GLOS_DOS.1.1	GO:0048663	B	.	neuron fate commitment
[BBH] DOS_DROME (sp|Q9VZZ9) Prot daughter of sevenless D. m.	DOS_DROME	0	GLOS_DOS.1.1	GO:0045165	B	.	cell fate commitment
[BBH] DOS_DROME (sp|Q9VZZ9) Prot daughter of sevenless D. m.	DOS_DROME	0	GLOS_DOS.1.1	GO:0001754	B	.	eye photoreceptor cell differentiation
[BBH] DOS_DROME (sp|Q9VZZ9) Prot daughter of sevenless D. m.	DOS_DROME	0	GLOS_DOS.1.1	GO:0048592	B	.	eye morphogenesis
[BBH] DOS_DROME (sp|Q9VZZ9) Prot daughter of sevenless D. m.	DOS_DROME	0	GLOS_DOS.1.1	GO:0009887	B	.	organ morphogenesis
[BBH] DOS_DROME (sp|Q9VZZ9) Prot daughter of sevenless D. m.	DOS_DROME	0	GLOS_DOS.1.1	GO:0001654	B	.	eye development
[BBH] DOS_DROME (sp|Q9VZZ9) Prot daughter of sevenless D. m.	DOS_DROME	0	GLOS_DOS.1.1	GO:0007423	B	.	sensory organ development
[BBH] DOS_DROME (sp|Q9VZZ9) Prot daughter of sevenless D. m.	DOS_DROME	0	GLOS_DOS.1.1	GO:0001751	B	.	compound eye photoreceptor cell differentiation
[BBH] DOS_DROME (sp|Q9VZZ9) Prot daughter of sevenless D. m.	DOS_DROME	0	GLOS_DOS.1.1	GO:0001745	B	.	compound eye morphogenesis
[BBH] DOS_DROME (sp|Q9VZZ9) Prot daughter of sevenless D. m.	DOS_DROME	0	GLOS_DOS.1.1	GO:0048749	B	.	compound eye development
[BBH] DOS_DROME (sp|Q9VZZ9) Prot daughter of sevenless D. m.	DOS_DROME	0	GLOS_DOS.1.1	GO:0045466	B	.	R7 cell differentiation
[BBH] DOS_DROME (sp|Q9VZZ9) Prot daughter of sevenless D. m.	DOS_DROME	0	GLOS_DOS.1.1	GO:0045500	B	FlyBase	sevenless signaling pathway
[BBH] DOS_DROME (sp|Q9VZZ9) Prot daughter of sevenless D. m.	DOS_DROME	0	GLOS_DOS.1.1	GO:0008293	B	FlyBase	torso signaling pathway
[BBH] DOS_DROME (sp|Q9VZZ9) Prot daughter of sevenless D. m.	DOS_DROME	0	GLOS_DOS.1.1	GO:0007444	B	.	imaginal disc development
[BBH] DOS_DROME (sp|Q9VZZ9) Prot daughter of sevenless D. m.	DOS_DROME	0	GLOS_DOS.1.1	GO:0035220	B	UniProtKB	wing disc development
[BBH] EGH_DROME (sp|O01346) B-1,4-mannosyltransf. egh D. m.	EGH_DROME	0	GLOS_EGH.1.1	GO:0016021	C	UniProtKB	integral to membrane
[BBH] EGH_DROME (sp|O01346) B-1,4-mannosyltransf. egh D. m.	EGH_DROME	0	GLOS_EGH.1.1	GO:0000030	M	.	mannosyltransferase activity
[BBH] EGH_DROME (sp|O01346) B-1,4-mannosyltransf. egh D. m.	EGH_DROME	0	GLOS_EGH.1.1	GO:0016758	M	.	transferase activity, transferring hexosyl groups
[BBH] EGH_DROME (sp|O01346) B-1,4-mannosyltransf. egh D. m.	EGH_DROME	0	GLOS_EGH.1.1	GO:0016757	M	.	transferase activity, transferring glycosyl groups
[BBH] EGH_DROME (sp|O01346) B-1,4-mannosyltransf. egh D. m.	EGH_DROME	0	GLOS_EGH.1.1	GO:0019187	M	UniProtKB	beta-1,4-mannosyltransferase activity
[BBH] EGH_DROME (sp|O01346) B-1,4-mannosyltransf. egh D. m.	EGH_DROME	0	GLOS_EGH.1.1	GO:0007411	B	FlyBase	axon guidance
[BBH] EGH_DROME (sp|O01346) B-1,4-mannosyltransf. egh D. m.	EGH_DROME	0	GLOS_EGH.1.1	GO:0007297	B	.	ovarian follicle cell migration
[BBH] EGH_DROME (sp|O01346) B-1,4-mannosyltransf. egh D. m.	EGH_DROME	0	GLOS_EGH.1.1	GO:0010631	B	.	epithelial cell migration
[BBH] EGH_DROME (sp|O01346) B-1,4-mannosyltransf. egh D. m.	EGH_DROME	0	GLOS_EGH.1.1	GO:0001667	B	.	ameboidal cell migration
[BBH] EGH_DROME (sp|O01346) B-1,4-mannosyltransf. egh D. m.	EGH_DROME	0	GLOS_EGH.1.1	GO:0016477	B	.	cell migration
[BBH] EGH_DROME (sp|O01346) B-1,4-mannosyltransf. egh D. m.	EGH_DROME	0	GLOS_EGH.1.1	GO:0048870	B	.	cell motility
[BBH] EGH_DROME (sp|O01346) B-1,4-mannosyltransf. egh D. m.	EGH_DROME	0	GLOS_EGH.1.1	GO:0051674	B	.	localization of cell
[BBH] EGH_DROME (sp|O01346) B-1,4-mannosyltransf. egh D. m.	EGH_DROME	0	GLOS_EGH.1.1	GO:0090132	B	.	epithelium migration
[BBH] EGH_DROME (sp|O01346) B-1,4-mannosyltransf. egh D. m.	EGH_DROME	0	GLOS_EGH.1.1	GO:0090130	B	.	tissue migration
[BBH] EGH_DROME (sp|O01346) B-1,4-mannosyltransf. egh D. m.	EGH_DROME	0	GLOS_EGH.1.1	GO:0022412	B	.	cellular process involved in reproduction in multicellular organism

[BBH] EGH_DROME (sp|O01346) B-1,4-mannosyltransf. egh D. m.	EGH_DROME	0	GLOS_EGH.1.1	GO:0048610	B	.	cellular process involved in reproduction
[BBH] EGH_DROME (sp|O01346) B-1,4-mannosyltransf. egh D. m.	EGH_DROME	0	GLOS_EGH.1.1	GO:0000003	B	.	reproduction
[BBH] EGH_DROME (sp|O01346) B-1,4-mannosyltransf. egh D. m.	EGH_DROME	0	GLOS_EGH.1.1	GO:0032504	B	.	multicellular organism reproduction
[BBH] EGH_DROME (sp|O01346) B-1,4-mannosyltransf. egh D. m.	EGH_DROME	0	GLOS_EGH.1.1	GO:0030707	B	.	ovarian follicle cell development
[BBH] EGH_DROME (sp|O01346) B-1,4-mannosyltransf. egh D. m.	EGH_DROME	0	GLOS_EGH.1.1	GO:0003006	B	.	developmental process involved in reproduction
[BBH] EGH_DROME (sp|O01346) B-1,4-mannosyltransf. egh D. m.	EGH_DROME	0	GLOS_EGH.1.1	GO:0022414	B	.	reproductive process
[BBH] EGH_DROME (sp|O01346) B-1,4-mannosyltransf. egh D. m.	EGH_DROME	0	GLOS_EGH.1.1	GO:0048477	B	.	oogenesis
[BBH] EGH_DROME (sp|O01346) B-1,4-mannosyltransf. egh D. m.	EGH_DROME	0	GLOS_EGH.1.1	GO:0007292	B	.	female gamete generation
[BBH] EGH_DROME (sp|O01346) B-1,4-mannosyltransf. egh D. m.	EGH_DROME	0	GLOS_EGH.1.1	GO:0007276	B	.	gamete generation
[BBH] EGH_DROME (sp|O01346) B-1,4-mannosyltransf. egh D. m.	EGH_DROME	0	GLOS_EGH.1.1	GO:0044702	B	.	single organism reproductive process
[BBH] EGH_DROME (sp|O01346) B-1,4-mannosyltransf. egh D. m.	EGH_DROME	0	GLOS_EGH.1.1	GO:0048609	B	.	multicellular organismal reproductive process
[BBH] EGH_DROME (sp|O01346) B-1,4-mannosyltransf. egh D. m.	EGH_DROME	0	GLOS_EGH.1.1	GO:0019953	B	.	sexual reproduction
[BBH] EGH_DROME (sp|O01346) B-1,4-mannosyltransf. egh D. m.	EGH_DROME	0	GLOS_EGH.1.1	GO:0007298	B	FlyBase	border follicle cell migration
[BBH] EGH_DROME (sp|O01346) B-1,4-mannosyltransf. egh D. m.	EGH_DROME	0	GLOS_EGH.1.1	GO:0045165	B	UniProtKB	cell fate commitment
[BBH] EGH_DROME (sp|O01346) B-1,4-mannosyltransf. egh D. m.	EGH_DROME	0	GLOS_EGH.1.1	GO:0050658	B	.	RNA transport
[BBH] EGH_DROME (sp|O01346) B-1,4-mannosyltransf. egh D. m.	EGH_DROME	0	GLOS_EGH.1.1	GO:0050657	B	.	nucleic acid transport
[BBH] EGH_DROME (sp|O01346) B-1,4-mannosyltransf. egh D. m.	EGH_DROME	0	GLOS_EGH.1.1	GO:0015931	B	.	nucleobase-containing compound transport
[BBH] EGH_DROME (sp|O01346) B-1,4-mannosyltransf. egh D. m.	EGH_DROME	0	GLOS_EGH.1.1	GO:0071705	B	.	nitrogen compound transport
[BBH] EGH_DROME (sp|O01346) B-1,4-mannosyltransf. egh D. m.	EGH_DROME	0	GLOS_EGH.1.1	GO:0051236	B	.	establishment of RNA localization
[BBH] EGH_DROME (sp|O01346) B-1,4-mannosyltransf. egh D. m.	EGH_DROME	0	GLOS_EGH.1.1	GO:0006403	B	.	RNA localization
[BBH] EGH_DROME (sp|O01346) B-1,4-mannosyltransf. egh D. m.	EGH_DROME	0	GLOS_EGH.1.1	GO:0033227	B	FlyBase	dsRNA transport
[BBH] EGH_DROME (sp|O01346) B-1,4-mannosyltransf. egh D. m.	EGH_DROME	0	GLOS_EGH.1.1	GO:0006687	B	.	glycosphingolipid metabolic process
[BBH] EGH_DROME (sp|O01346) B-1,4-mannosyltransf. egh D. m.	EGH_DROME	0	GLOS_EGH.1.1	GO:0006664	B	.	glycolipid metabolic process
[BBH] EGH_DROME (sp|O01346) B-1,4-mannosyltransf. egh D. m.	EGH_DROME	0	GLOS_EGH.1.1	GO:0006643	B	.	membrane lipid metabolic process
[BBH] EGH_DROME (sp|O01346) B-1,4-mannosyltransf. egh D. m.	EGH_DROME	0	GLOS_EGH.1.1	GO:1901135	B	.	carbohydrate derivative metabolic process
[BBH] EGH_DROME (sp|O01346) B-1,4-mannosyltransf. egh D. m.	EGH_DROME	0	GLOS_EGH.1.1	GO:0006665	B	.	sphingolipid metabolic process
[BBH] EGH_DROME (sp|O01346) B-1,4-mannosyltransf. egh D. m.	EGH_DROME	0	GLOS_EGH.1.1	GO:0009247	B	.	glycolipid biosynthetic process
[BBH] EGH_DROME (sp|O01346) B-1,4-mannosyltransf. egh D. m.	EGH_DROME	0	GLOS_EGH.1.1	GO:0046467	B	.	membrane lipid biosynthetic process
[BBH] EGH_DROME (sp|O01346) B-1,4-mannosyltransf. egh D. m.	EGH_DROME	0	GLOS_EGH.1.1	GO:1901137	B	.	carbohydrate derivative biosynthetic process
[BBH] EGH_DROME (sp|O01346) B-1,4-mannosyltransf. egh D. m.	EGH_DROME	0	GLOS_EGH.1.1	GO:0030148	B	.	sphingolipid biosynthetic process
[BBH] EGH_DROME (sp|O01346) B-1,4-mannosyltransf. egh D. m.	EGH_DROME	0	GLOS_EGH.1.1	GO:1901566	B	.	organonitrogen compound biosynthetic process
[BBH] EGH_DROME (sp|O01346) B-1,4-mannosyltransf. egh D. m.	EGH_DROME	0	GLOS_EGH.1.1	GO:0006688	B	UniProtKB	glycosphingolipid biosynthetic process
[BBH] EGH_DROME (sp|O01346) B-1,4-mannosyltransf. egh D. m.	EGH_DROME	0	GLOS_EGH.1.1	GO:0016334	B	.	establishment or maintenance of polarity of follicular epithelium
[BBH] EGH_DROME (sp|O01346) B-1,4-mannosyltransf. egh D. m.	EGH_DROME	0	GLOS_EGH.1.1	GO:0007163	B	.	establishment or maintenance of cell polarity
[BBH] EGH_DROME (sp|O01346) B-1,4-mannosyltransf. egh D. m.	EGH_DROME	0	GLOS_EGH.1.1	GO:0016333	B	.	morphogenesis of follicular epithelium
[BBH] EGH_DROME (sp|O01346) B-1,4-mannosyltransf. egh D. m.	EGH_DROME	0	GLOS_EGH.1.1	GO:0002009	B	.	morphogenesis of an epithelium
[BBH] EGH_DROME (sp|O01346) B-1,4-mannosyltransf. egh D. m.	EGH_DROME	0	GLOS_EGH.1.1	GO:0048729	B	.	tissue morphogenesis
[BBH] EGH_DROME (sp|O01346) B-1,4-mannosyltransf. egh D. m.	EGH_DROME	0	GLOS_EGH.1.1	GO:0009888	B	.	tissue development
[BBH] EGH_DROME (sp|O01346) B-1,4-mannosyltransf. egh D. m.	EGH_DROME	0	GLOS_EGH.1.1	GO:0060429	B	.	epithelium development
[BBH] EGH_DROME (sp|O01346) B-1,4-mannosyltransf. egh D. m.	EGH_DROME	0	GLOS_EGH.1.1	GO:0042248	B	FlyBase	maintenance of polarity of follicular epithelium
[BBH] EGH_DROME (sp|O01346) B-1,4-mannosyltransf. egh D. m.	EGH_DROME	0	GLOS_EGH.1.1	GO:0060179	B	.	male mating behavior
[BBH] EGH_DROME (sp|O01346) B-1,4-mannosyltransf. egh D. m.	EGH_DROME	0	GLOS_EGH.1.1	GO:0033057	B	.	multicellular organismal reproductive behavior
[BBH] EGH_DROME (sp|O01346) B-1,4-mannosyltransf. egh D. m.	EGH_DROME	0	GLOS_EGH.1.1	GO:0019098	B	.	reproductive behavior
[BBH] EGH_DROME (sp|O01346) B-1,4-mannosyltransf. egh D. m.	EGH_DROME	0	GLOS_EGH.1.1	GO:0007610	B	.	behavior
[BBH] EGH_DROME (sp|O01346) B-1,4-mannosyltransf. egh D. m.	EGH_DROME	0	GLOS_EGH.1.1	GO:0044706	B	.	multi-multicellular organism process
[BBH] EGH_DROME (sp|O01346) B-1,4-mannosyltransf. egh D. m.	EGH_DROME	0	GLOS_EGH.1.1	GO:0007617	B	.	mating behavior
[BBH] EGH_DROME (sp|O01346) B-1,4-mannosyltransf. egh D. m.	EGH_DROME	0	GLOS_EGH.1.1	GO:0051705	B	.	multi-organism behavior
[BBH] EGH_DROME (sp|O01346) B-1,4-mannosyltransf. egh D. m.	EGH_DROME	0	GLOS_EGH.1.1	GO:0007618	B	.	mating
[BBH] EGH_DROME (sp|O01346) B-1,4-mannosyltransf. egh D. m.	EGH_DROME	0	GLOS_EGH.1.1	GO:0044703	B	.	multi-organism reproductive process
[BBH] EGH_DROME (sp|O01346) B-1,4-mannosyltransf. egh D. m.	EGH_DROME	0	GLOS_EGH.1.1	GO:0007619	B	.	courtship behavior
[BBH] EGH_DROME (sp|O01346) B-1,4-mannosyltransf. egh D. m.	EGH_DROME	0	GLOS_EGH.1.1	GO:0008049	B	FlyBase	male courtship behavior
[BBH] EGH_DROME (sp|O01346) B-1,4-mannosyltransf. egh D. m.	EGH_DROME	0	GLOS_EGH.1.1	GO:0007621	B	.	negative regulation of female receptivity
[BBH] EGH_DROME (sp|O01346) B-1,4-mannosyltransf. egh D. m.	EGH_DROME	0	GLOS_EGH.1.1	GO:0045924	B	.	regulation of female receptivity
[BBH] EGH_DROME (sp|O01346) B-1,4-mannosyltransf. egh D. m.	EGH_DROME	0	GLOS_EGH.1.1	GO:0060180	B	.	female mating behavior

[BBH] EGH_DROME (sp|O01346) B-1,4-mannosyltransf. egh D. m.	EGH_DROME	0	GLOS_EGH.1.1	GO:0046008	B	.	regulation of female receptivity, post-mating
[BBH] EGH_DROME (sp|O01346) B-1,4-mannosyltransf. egh D. m.	EGH_DROME	0	GLOS_EGH.1.1	GO:0045297	B	.	post-mating behavior
[BBH] EGH_DROME (sp|O01346) B-1,4-mannosyltransf. egh D. m.	EGH_DROME	0	GLOS_EGH.1.1	GO:0045434	B	FlyBase	negative regulation of female receptivity, post-mating
[BBH] EGH_DROME (sp|O01346) B-1,4-mannosyltransf. egh D. m.	EGH_DROME	0	GLOS_EGH.1.1	GO:0007293	B	.	germarium-derived egg chamber formation
[BBH] EGH_DROME (sp|O01346) B-1,4-mannosyltransf. egh D. m.	EGH_DROME	0	GLOS_EGH.1.1	GO:0030720	B	FlyBase	oocyte localization involved in germarium-derived egg chamber formation
[BBH] EGH_DROME (sp|O01346) B-1,4-mannosyltransf. egh D. m.	EGH_DROME	0	GLOS_EGH.1.1	GO:0030951	B	.	establishment or maintenance of microtubule cytoskeleton polarity
[BBH] EGH_DROME (sp|O01346) B-1,4-mannosyltransf. egh D. m.	EGH_DROME	0	GLOS_EGH.1.1	GO:0000226	B	.	microtubule cytoskeleton organization
[BBH] EGH_DROME (sp|O01346) B-1,4-mannosyltransf. egh D. m.	EGH_DROME	0	GLOS_EGH.1.1	GO:0007017	B	.	microtubule-based process
[BBH] EGH_DROME (sp|O01346) B-1,4-mannosyltransf. egh D. m.	EGH_DROME	0	GLOS_EGH.1.1	GO:0030952	B	.	establishment or maintenance of cytoskeleton polarity
[BBH] EGH_DROME (sp|O01346) B-1,4-mannosyltransf. egh D. m.	EGH_DROME	0	GLOS_EGH.1.1	GO:0007308	B	.	oocyte construction
[BBH] EGH_DROME (sp|O01346) B-1,4-mannosyltransf. egh D. m.	EGH_DROME	0	GLOS_EGH.1.1	GO:0048469	B	.	cell maturation
[BBH] EGH_DROME (sp|O01346) B-1,4-mannosyltransf. egh D. m.	EGH_DROME	0	GLOS_EGH.1.1	GO:0021700	B	.	developmental maturation
[BBH] EGH_DROME (sp|O01346) B-1,4-mannosyltransf. egh D. m.	EGH_DROME	0	GLOS_EGH.1.1	GO:0048599	B	.	oocyte development
[BBH] EGH_DROME (sp|O01346) B-1,4-mannosyltransf. egh D. m.	EGH_DROME	0	GLOS_EGH.1.1	GO:0007281	B	.	germ cell development
[BBH] EGH_DROME (sp|O01346) B-1,4-mannosyltransf. egh D. m.	EGH_DROME	0	GLOS_EGH.1.1	GO:0009994	B	.	oocyte differentiation
[BBH] EGH_DROME (sp|O01346) B-1,4-mannosyltransf. egh D. m.	EGH_DROME	0	GLOS_EGH.1.1	GO:0016325	B	FlyBase	oocyte microtubule cytoskeleton organization
[BBH] EGH_DROME (sp|O01346) B-1,4-mannosyltransf. egh D. m.	EGH_DROME	0	GLOS_EGH.1.1	GO:0001743	B	.	optic placode formation
[BBH] EGH_DROME (sp|O01346) B-1,4-mannosyltransf. egh D. m.	EGH_DROME	0	GLOS_EGH.1.1	GO:0060788	B	.	ectodermal placode formation
[BBH] EGH_DROME (sp|O01346) B-1,4-mannosyltransf. egh D. m.	EGH_DROME	0	GLOS_EGH.1.1	GO:0071697	B	.	ectodermal placode morphogenesis
[BBH] EGH_DROME (sp|O01346) B-1,4-mannosyltransf. egh D. m.	EGH_DROME	0	GLOS_EGH.1.1	GO:0071696	B	.	ectodermal placode development
[BBH] EGH_DROME (sp|O01346) B-1,4-mannosyltransf. egh D. m.	EGH_DROME	0	GLOS_EGH.1.1	GO:0048598	B	.	embryonic morphogenesis
[BBH] EGH_DROME (sp|O01346) B-1,4-mannosyltransf. egh D. m.	EGH_DROME	0	GLOS_EGH.1.1	GO:0001748	B	.	optic lobe placode development
[BBH] EGH_DROME (sp|O01346) B-1,4-mannosyltransf. egh D. m.	EGH_DROME	0	GLOS_EGH.1.1	GO:0001744	B	FlyBase	optic lobe placode formation
[BBH] EGH_DROME (sp|O01346) B-1,4-mannosyltransf. egh D. m.	EGH_DROME	0	GLOS_EGH.1.1	GO:0090136	B	.	epithelial cell-cell adhesion
[BBH] EGH_DROME (sp|O01346) B-1,4-mannosyltransf. egh D. m.	EGH_DROME	0	GLOS_EGH.1.1	GO:0016337	B	.	cell-cell adhesion
[BBH] EGH_DROME (sp|O01346) B-1,4-mannosyltransf. egh D. m.	EGH_DROME	0	GLOS_EGH.1.1	GO:0007155	B	.	cell adhesion
[BBH] EGH_DROME (sp|O01346) B-1,4-mannosyltransf. egh D. m.	EGH_DROME	0	GLOS_EGH.1.1	GO:0022610	B	.	biological adhesion
[BBH] EGH_DROME (sp|O01346) B-1,4-mannosyltransf. egh D. m.	EGH_DROME	0	GLOS_EGH.1.1	GO:0007299	B	UniProtKB	ovarian follicle cell-cell adhesion
[BBH] EGH_DROME (sp|O01346) B-1,4-mannosyltransf. egh D. m.	EGH_DROME	0	GLOS_EGH.1.1	GO:0043900	B	.	regulation of multi-organism process
[BBH] EGH_DROME (sp|O01346) B-1,4-mannosyltransf. egh D. m.	EGH_DROME	0	GLOS_EGH.1.1	GO:0050795	B	.	regulation of behavior
[BBH] EGH_DROME (sp|O01346) B-1,4-mannosyltransf. egh D. m.	EGH_DROME	0	GLOS_EGH.1.1	GO:2000241	B	.	regulation of reproductive process
[BBH] EGH_DROME (sp|O01346) B-1,4-mannosyltransf. egh D. m.	EGH_DROME	0	GLOS_EGH.1.1	GO:0046662	B	FlyBase	regulation of oviposition
[BBH] EIF3J_DROMO (sp|B4KTH6) Eukaryotic transl. Init. fact. 3 subunit J	EIF3J_DROMO	9,00E-62	GLOS_EIF3J.1.1	GO:0070993	C	.	translation preinitiation complex
[BBH] EIF3J_DROMO (sp|B4KTH6) Eukaryotic transl. Init. fact. 3 subunit J	EIF3J_DROMO	9,00E-62	GLOS_EIF3J.1.1	GO:0030529	C	.	ribonucleoprotein complex
[BBH] EIF3J_DROMO (sp|B4KTH6) Eukaryotic transl. Init. fact. 3 subunit J	EIF3J_DROMO	9,00E-62	GLOS_EIF3J.1.1	GO:0016282	C	UniProtKB-I	eukaryotic 43S preinitiation complex
[BBH] EIF3J_DROMO (sp|B4KTH6) Eukaryotic transl. Init. fact. 3 subunit J	EIF3J_DROMO	9,00E-62	GLOS_EIF3J.1.1	GO:0033290	C	UniProtKB-I	eukaryotic 48S preinitiation complex
[BBH] EIF3J_DROMO (sp|B4KTH6) Eukaryotic transl. Init. fact. 3 subunit J	EIF3J_DROMO	9,00E-62	GLOS_EIF3J.1.1	GO:0005852	C	UniProtKB-I	eukaryotic translation initiation factor 3 complex
[BBH] EIF3J_DROMO (sp|B4KTH6) Eukaryotic transl. Init. fact. 3 subunit J	EIF3J_DROMO	9,00E-62	GLOS_EIF3J.1.1	GO:0008135	M	.	translation factor activity, nucleic acid binding
[BBH] EIF3J_DROMO (sp|B4KTH6) Eukaryotic transl. Init. fact. 3 subunit J	EIF3J_DROMO	9,00E-62	GLOS_EIF3J.1.1	GO:0003723	M	.	RNA binding
[BBH] EIF3J_DROMO (sp|B4KTH6) Eukaryotic transl. Init. fact. 3 subunit J	EIF3J_DROMO	9,00E-62	GLOS_EIF3J.1.1	GO:0003743	M	UniProtKB-I	translation initiation factor activity
[BBH] EIF3J_DROMO (sp|B4KTH6) Eukaryotic transl. Init. fact. 3 subunit J	EIF3J_DROMO	9,00E-62	GLOS_EIF3J.1.1	GO:0022618	B	.	ribonucleoprotein complex assembly
[BBH] EIF3J_DROMO (sp|B4KTH6) Eukaryotic transl. Init. fact. 3 subunit J	EIF3J_DROMO	9,00E-62	GLOS_EIF3J.1.1	GO:0034622	B	.	cellular macromolecular complex assembly
[BBH] EIF3J_DROMO (sp|B4KTH6) Eukaryotic transl. Init. fact. 3 subunit J	EIF3J_DROMO	9,00E-62	GLOS_EIF3J.1.1	GO:0071826	B	.	ribonucleoprotein complex subunit organization
[BBH] EIF3J_DROMO (sp|B4KTH6) Eukaryotic transl. Init. fact. 3 subunit J	EIF3J_DROMO	9,00E-62	GLOS_EIF3J.1.1	GO:0022613	B	.	ribonucleoprotein complex biogenesis
[BBH] EIF3J_DROMO (sp|B4KTH6) Eukaryotic transl. Init. fact. 3 subunit J	EIF3J_DROMO	9,00E-62	GLOS_EIF3J.1.1	GO:0006413	B	.	translational initiation
[BBH] EIF3J_DROMO (sp|B4KTH6) Eukaryotic transl. Init. fact. 3 subunit J	EIF3J_DROMO	9,00E-62	GLOS_EIF3J.1.1	GO:0006412	B	.	translation
[BBH] EIF3J_DROMO (sp|B4KTH6) Eukaryotic transl. Init. fact. 3 subunit J	EIF3J_DROMO	9,00E-62	GLOS_EIF3J.1.1	GO:0044267	B	.	cellular protein metabolic process
[BBH] EIF3J_DROMO (sp|B4KTH6) Eukaryotic transl. Init. fact. 3 subunit J	EIF3J_DROMO	9,00E-62	GLOS_EIF3J.1.1	GO:0019538	B	.	protein metabolic process
[BBH] EIF3J_DROMO (sp|B4KTH6) Eukaryotic transl. Init. fact. 3 subunit J	EIF3J_DROMO	9,00E-62	GLOS_EIF3J.1.1	GO:0001731	B	UniProtKB-I	formation of translation preinitiation complex
[BBH] EIF3J_DROMO (sp|B4KTH6) Eukaryotic transl. Init. fact. 3 subunit J	EIF3J_DROMO	9,00E-62	GLOS_EIF3J.1.1	GO:0006417	B	.	regulation of translation
[BBH] EIF3J_DROMO (sp|B4KTH6) Eukaryotic transl. Init. fact. 3 subunit J	EIF3J_DROMO	9,00E-62	GLOS_EIF3J.1.1	GO:0010608	B	.	posttranscriptional regulation of gene expression
[BBH] EIF3J_DROMO (sp|B4KTH6) Eukaryotic transl. Init. fact. 3 subunit J	EIF3J_DROMO	9,00E-62	GLOS_EIF3J.1.1	GO:0032268	B	.	regulation of cellular protein metabolic process
[BBH] EIF3J_DROMO (sp|B4KTH6) Eukaryotic transl. Init. fact. 3 subunit J	EIF3J_DROMO	9,00E-62	GLOS_EIF3J.1.1	GO:0051246	B	.	regulation of protein metabolic process
[BBH] EIF3J_DROMO (sp|B4KTH6) Eukaryotic transl. Init. fact. 3 subunit J	EIF3J_DROMO	9,00E-62	GLOS_EIF3J.1.1	GO:0006446	B	UniProtKB-I	regulation of translational initiation

[BBH] ELP2_DROME (sp|Q7K4B3) Prob elongator complex prot 2; D. m.	ELP2_DROME	0	GLOS_ELP2.1.1	GO:0005737	C	UniProtKB-S	cytoplasm
[BBH] ELP2_DROME (sp|Q7K4B3) Prob elongator complex prot 2; D. m.	ELP2_DROME	0	GLOS_ELP2.1.1	GO:0005634	C	UniProtKB-S	nucleus
[BBH] ELP2_DROME (sp|Q7K4B3) Prob elongator complex prot 2; D. m.	ELP2_DROME	0	GLOS_ELP2.1.1	GO:0000502	C	UniProtKB	proteasome complex
[BBH] ELP2_DROME (sp|Q7K4B3) Prob elongator complex prot 2; D. m.	ELP2_DROME	0	GLOS_ELP2.1.1	GO:0016504	M	.	peptidase activator activity
[BBH] ELP2_DROME (sp|Q7K4B3) Prob elongator complex prot 2; D. m.	ELP2_DROME	0	GLOS_ELP2.1.1	GO:0008047	M	.	enzyme activator activity
[BBH] ELP2_DROME (sp|Q7K4B3) Prob elongator complex prot 2; D. m.	ELP2_DROME	0	GLOS_ELP2.1.1	GO:0030234	M	.	enzyme regulator activity
[BBH] ELP2_DROME (sp|Q7K4B3) Prob elongator complex prot 2; D. m.	ELP2_DROME	0	GLOS_ELP2.1.1	GO:0061134	M	.	peptidase regulator activity
[BBH] ELP2_DROME (sp|Q7K4B3) Prob elongator complex prot 2; D. m.	ELP2_DROME	0	GLOS_ELP2.1.1	GO:0061135	M	.	endopeptidase regulator activity
[BBH] ELP2_DROME (sp|Q7K4B3) Prob elongator complex prot 2; D. m.	ELP2_DROME	0	GLOS_ELP2.1.1	GO:0061133	M	UniProtKB	endopeptidase activator activity
[BBH] ELP2_DROME (sp|Q7K4B3) Prob elongator complex prot 2; D. m.	ELP2_DROME	0	GLOS_ELP2.1.1	GO:0043623	B	.	cellular protein complex assembly
[BBH] ELP2_DROME (sp|Q7K4B3) Prob elongator complex prot 2; D. m.	ELP2_DROME	0	GLOS_ELP2.1.1	GO:0043248	B	UniProtKB	proteasome assembly
[BBH] ELP2_DROME (sp|Q7K4B3) Prob elongator complex prot 2; D. m.	ELP2_DROME	0	GLOS_ELP2.1.1	GO:0006355	B	UniProtKB-k	regulation of transcription, DNA-dependent
[BBH] ELP2_DROME (sp|Q7K4B3) Prob elongator complex prot 2; D. m.	ELP2_DROME	0	GLOS_ELP2.1.1	GO:0006354	B	.	DNA-dependent transcription, elongation
[BBH] ELP2_DROME (sp|Q7K4B3) Prob elongator complex prot 2; D. m.	ELP2_DROME	0	GLOS_ELP2.1.1	GO:0006366	B	.	transcription from RNA polymerase II promoter
[BBH] ELP2_DROME (sp|Q7K4B3) Prob elongator complex prot 2; D. m.	ELP2_DROME	0	GLOS_ELP2.1.1	GO:0006368	B	UniProtKB	transcription elongation from RNA polymerase II promoter
[BBH] EMC89_DROME (sp|Q9W1Y1) ER memb prot complex subunit 8/9 homolog	EMC89_DROME	1,00E-89	GLOS_EMC89.1.1	GO:0005875	C	FlyBase	microtubule associated complex
[BBH] GALE_DROME (sp|Q9W0P5) Probable UDP-glucose 4-epimerase	GALE_DROME	1,00E-177	GLOS_GALE.1.1	GO:0048037	M	.	cofactor binding
[BBH] GALE_DROME (sp|Q9W0P5) Probable UDP-glucose 4-epimerase	GALE_DROME	1,00E-177	GLOS_GALE.1.1	GO:0050662	M	InterPro	coenzyme binding
[BBH] GALE_DROME (sp|Q9W0P5) Probable UDP-glucose 4-epimerase	GALE_DROME	1,00E-177	GLOS_GALE.1.1	GO:0016857	M	.	racemase and epimerase activity, acting on carbohydrates and derivatives
[BBH] GALE_DROME (sp|Q9W0P5) Probable UDP-glucose 4-epimerase	GALE_DROME	1,00E-177	GLOS_GALE.1.1	GO:0016854	M	.	racemase and epimerase activity
[BBH] GALE_DROME (sp|Q9W0P5) Probable UDP-glucose 4-epimerase	GALE_DROME	1,00E-177	GLOS_GALE.1.1	GO:0016853	M	.	isomerase activity
[BBH] GALE_DROME (sp|Q9W0P5) Probable UDP-glucose 4-epimerase	GALE_DROME	1,00E-177	GLOS_GALE.1.1	GO:0003978	M	FlyBase	UDP-glucose 4-epimerase activity
[BBH] GALE_DROME (sp|Q9W0P5) Probable UDP-glucose 4-epimerase	GALE_DROME	1,00E-177	GLOS_GALE.1.1	GO:0044237	B	InterPro	cellular metabolic process
[BBH] GALE_DROME (sp|Q9W0P5) Probable UDP-glucose 4-epimerase	GALE_DROME	1,00E-177	GLOS_GALE.1.1	GO:0019318	B	.	hexose metabolic process
[BBH] GALE_DROME (sp|Q9W0P5) Probable UDP-glucose 4-epimerase	GALE_DROME	1,00E-177	GLOS_GALE.1.1	GO:0005996	B	.	monosaccharide metabolic process
[BBH] GALE_DROME (sp|Q9W0P5) Probable UDP-glucose 4-epimerase	GALE_DROME	1,00E-177	GLOS_GALE.1.1	GO:0044723	B	.	single-organism carbohydrate metabolic process
[BBH] GALE_DROME (sp|Q9W0P5) Probable UDP-glucose 4-epimerase	GALE_DROME	1,00E-177	GLOS_GALE.1.1	GO:0005975	B	.	carbohydrate metabolic process
[BBH] GALE_DROME (sp|Q9W0P5) Probable UDP-glucose 4-epimerase	GALE_DROME	1,00E-177	GLOS_GALE.1.1	GO:0006012	B	FlyBase	galactose metabolic process
[BBH] GBG1_DROME (sp|P38040) Guanine nucleotide-binding prot. subunit gamma-1 OS=D. melanogaster	GBG1_DROME	7,00E-36	GLOS_GBG1.1.3	GO:0031234	C	.	extrinsic to internal side of plasma membrane
[BBH] GBG1_DROME (sp|P38040) Guanine nucleotide-bind … Same as above	GBG1_DROME	7,00E-36	GLOS_GBG1.1.3	GO:0019897	C	.	extrinsic to plasma membrane
[BBH] GBG1_DROME (sp|P38040) Guanine nucleotide-bind … Same as above	GBG1_DROME	7,00E-36	GLOS_GBG1.1.3	GO:0019898	C	.	extrinsic to membrane
[BBH] GBG1_DROME (sp|P38040) Guanine nucleotide-bind … Same as above	GBG1_DROME	7,00E-36	GLOS_GBG1.1.3	GO:0009898	C	.	internal side of plasma membrane
[BBH] GBG1_DROME (sp|P38040) Guanine nucleotide-bind … Same as above	GBG1_DROME	7,00E-36	GLOS_GBG1.1.3	GO:0005834	C	FlyBase	heterotrimeric G-protein complex
[BBH] GBG1_DROME (sp|P38040) Guanine nucleotide-bind … Same as above	GBG1_DROME	7,00E-36	GLOS_GBG1.1.3	GO:0017111	M	.	nucleoside-triphosphatase activity
[BBH] GBG1_DROME (sp|P38040) Guanine nucleotide-bind … Same as above	GBG1_DROME	7,00E-36	GLOS_GBG1.1.3	GO:0016462	M	.	pyrophosphatase activity
[BBH] GBG1_DROME (sp|P38040) Guanine nucleotide-bind … Same as above	GBG1_DROME	7,00E-36	GLOS_GBG1.1.3	GO:0016818	M	.	hydrolase act., acting on acid anhydrides, in phosphorus-containing anhydrides
[BBH] GBG1_DROME (sp|P38040) Guanine nucleotide-bind … Same as above	GBG1_DROME	7,00E-36	GLOS_GBG1.1.3	GO:0016817	M	.	hydrolase activity, acting on acid anhydrides
[BBH] GBG1_DROME (sp|P38040) Guanine nucleotide-bind … Same as above	GBG1_DROME	7,00E-36	GLOS_GBG1.1.3	GO:0003924	M	FlyBase	GTPase activity
[BBH] GBG1_DROME (sp|P38040) Guanine nucleotide-bind … Same as above	GBG1_DROME	7,00E-36	GLOS_GBG1.1.3	GO:0004871	M	UniProtKB-k	signal transducer activity
[BBH] GBG1_DROME (sp|P38040) Guanine nucleotide-bind … Same as above	GBG1_DROME	7,00E-36	GLOS_GBG1.1.3	GO:0007015	B	FlyBase	actin filament organization
[BBH] GBG1_DROME (sp|P38040) Guanine nucleotide-bind … Same as above	GBG1_DROME	7,00E-36	GLOS_GBG1.1.3	GO:0008105	B	.	asymmetric protein localization
[BBH] GBG1_DROME (sp|P38040) Guanine nucleotide-bind … Same as above	GBG1_DROME	7,00E-36	GLOS_GBG1.1.3	GO:0045176	B	FlyBase	apical protein localization
[BBH] GBG1_DROME (sp|P38040) Guanine nucleotide-bind … Same as above	GBG1_DROME	7,00E-36	GLOS_GBG1.1.3	GO:0055057	B	.	neuroblast division
[BBH] GBG1_DROME (sp|P38040) Guanine nucleotide-bind … Same as above	GBG1_DROME	7,00E-36	GLOS_GBG1.1.3	GO:0048103	B	.	somatic stem cell division
[BBH] GBG1_DROME (sp|P38040) Guanine nucleotide-bind … Same as above	GBG1_DROME	7,00E-36	GLOS_GBG1.1.3	GO:0017145	B	.	stem cell division
[BBH] GBG1_DROME (sp|P38040) Guanine nucleotide-bind … Same as above	GBG1_DROME	7,00E-36	GLOS_GBG1.1.3	GO:0051301	B	.	cell division
[BBH] GBG1_DROME (sp|P38040) Guanine nucleotide-bind … Same as above	GBG1_DROME	7,00E-36	GLOS_GBG1.1.3	GO:0007405	B	.	neuroblast proliferation
[BBH] GBG1_DROME (sp|P38040) Guanine nucleotide-bind … Same as above	GBG1_DROME	7,00E-36	GLOS_GBG1.1.3	GO:0061351	B	.	neural precursor cell proliferation
[BBH] GBG1_DROME (sp|P38040) Guanine nucleotide-bind … Same as above	GBG1_DROME	7,00E-36	GLOS_GBG1.1.3	GO:0008283	B	.	cell proliferation
[BBH] GBG1_DROME (sp|P38040) Guanine nucleotide-bind … Same as above	GBG1_DROME	7,00E-36	GLOS_GBG1.1.3	GO:0072089	B	.	stem cell proliferation
[BBH] GBG1_DROME (sp|P38040) Guanine nucleotide-bind … Same as above	GBG1_DROME	7,00E-36	GLOS_GBG1.1.3	GO:0055059	B	FlyBase	asymmetric neuroblast division
[BBH] GBG1_DROME (sp|P38040) Guanine nucleotide-bind … Same as above	GBG1_DROME	7,00E-36	GLOS_GBG1.1.3	GO:0003007	B	.	heart morphogenesis
[BBH] GBG1_DROME (sp|P38040) Guanine nucleotide-bind … Same as above	GBG1_DROME	7,00E-36	GLOS_GBG1.1.3	GO:0007507	B	.	heart development

[BBH] GBG1_DROME (sp|P38040) Guanine nucleotide-bind … Same as above	GBG1_DROME	7,00E-36	GLOS_GBG1.1.3	GO:0061343	B	FlyBase	cell adhesion involved in heart morphogenesis
[BBH] GBG1_DROME (sp|P38040) Guanine nucleotide-bind … Same as above	GBG1_DROME	7,00E-36	GLOS_GBG1.1.3	GO:0016331	B	.	morphogenesis of embryonic epithelium
[BBH] GBG1_DROME (sp|P38040) Guanine nucleotide-bind … Same as above	GBG1_DROME	7,00E-36	GLOS_GBG1.1.3	GO:0001700	B	.	embryonic development via the syncytial blastoderm
[BBH] GBG1_DROME (sp|P38040) Guanine nucleotide-bind … Same as above	GBG1_DROME	7,00E-36	GLOS_GBG1.1.3	GO:0009792	B	.	embryo development ending in birth or egg hatching
[BBH] GBG1_DROME (sp|P38040) Guanine nucleotide-bind … Same as above	GBG1_DROME	7,00E-36	GLOS_GBG1.1.3	GO:0007391	B	FlyBase	dorsal closure
[BBH] GBG1_DROME (sp|P38040) Guanine nucleotide-bind … Same as above	GBG1_DROME	7,00E-36	GLOS_GBG1.1.3	GO:0035295	B	.	tube development
[BBH] GBG1_DROME (sp|P38040) Guanine nucleotide-bind … Same as above	GBG1_DROME	7,00E-36	GLOS_GBG1.1.3	GO:0048568	B	.	embryonic organ development
[BBH] GBG1_DROME (sp|P38040) Guanine nucleotide-bind … Same as above	GBG1_DROME	7,00E-36	GLOS_GBG1.1.3	GO:0035050	B	FlyBase	embryonic heart tube development
[BBH] GBG1_DROME (sp|P38040) Guanine nucleotide-bind … Same as above	GBG1_DROME	7,00E-36	GLOS_GBG1.1.3	GO:0007186	B	FlyBase	G-protein coupled receptor signaling pathway
[BBH] GBG1_DROME (sp|P38040) Guanine nucleotide-bind … Same as above	GBG1_DROME	7,00E-36	GLOS_GBG1.1.3	GO:0003015	B	FlyBase	heart process
[BBH] GBG1_DROME (sp|P38040) Guanine nucleotide-bind … Same as above	GBG1_DROME	7,00E-36	GLOS_GBG1.1.3	GO:0007635	B	.	chemosensory behavior
[BBH] GBG1_DROME (sp|P38040) Guanine nucleotide-bind … Same as above	GBG1_DROME	7,00E-36	GLOS_GBG1.1.3	GO:0044708	B	.	single-organism behavior
[BBH] GBG1_DROME (sp|P38040) Guanine nucleotide-bind … Same as above	GBG1_DROME	7,00E-36	GLOS_GBG1.1.3	GO:0051780	B	.	behavioral response to nutrient
[BBH] GBG1_DROME (sp|P38040) Guanine nucleotide-bind … Same as above	GBG1_DROME	7,00E-36	GLOS_GBG1.1.3	GO:0007584	B	.	response to nutrient
[BBH] GBG1_DROME (sp|P38040) Guanine nucleotide-bind … Same as above	GBG1_DROME	7,00E-36	GLOS_GBG1.1.3	GO:0031667	B	.	response to nutrient levels
[BBH] GBG1_DROME (sp|P38040) Guanine nucleotide-bind … Same as above	GBG1_DROME	7,00E-36	GLOS_GBG1.1.3	GO:0009991	B	.	response to extracellular stimulus
[BBH] GBG1_DROME (sp|P38040) Guanine nucleotide-bind … Same as above	GBG1_DROME	7,00E-36	GLOS_GBG1.1.3	GO:0060004	B	.	reflex
[BBH] GBG1_DROME (sp|P38040) Guanine nucleotide-bind … Same as above	GBG1_DROME	7,00E-36	GLOS_GBG1.1.3	GO:0007637	B	FlyBase	proboscis extension reflex
[BBH] GBG1_DROME (sp|P38040) Guanine nucleotide-bind … Same as above	GBG1_DROME	7,00E-36	GLOS_GBG1.1.3	GO:0022604	B	.	regulation of cell morphogenesis
[BBH] GBG1_DROME (sp|P38040) Guanine nucleotide-bind … Same as above	GBG1_DROME	7,00E-36	GLOS_GBG1.1.3	GO:0051128	B	.	regulation of cellular component organization
[BBH] GBG1_DROME (sp|P38040) Guanine nucleotide-bind … Same as above	GBG1_DROME	7,00E-36	GLOS_GBG1.1.3	GO:0008360	B	FlyBase	regulation of cell shape
[BBH] GEK_DROME (sp|Q9W1B0) Serine/threonine-prot. kinase Genghis Khan OS=D. melanogaster	GEK_DROME	0	GLOS_GEK.1.1	GO:0043005	C	.	neuron projection
[BBH] GEK_DROME (sp|Q9W1B0) Serine/threonine-prot. … Same as above	GEK_DROME	0	GLOS_GEK.1.1	GO:0042995	C	.	cell projection
[BBH] GEK_DROME (sp|Q9W1B0) Serine/threonine-prot. … Same as above	GEK_DROME	0	GLOS_GEK.1.1	GO:0097458	C	.	neuron part
[BBH] GEK_DROME (sp|Q9W1B0) Serine/threonine-prot. … Same as above	GEK_DROME	0	GLOS_GEK.1.1	GO:0030424	C	FlyBase	axon
[BBH] GEK_DROME (sp|Q9W1B0) Serine/threonine-prot. … Same as above	GEK_DROME	0	GLOS_GEK.1.1	GO:0030427	C	.	site of polarized growth
[BBH] GEK_DROME (sp|Q9W1B0) Serine/threonine-prot. … Same as above	GEK_DROME	0	GLOS_GEK.1.1	GO:0044463	C	.	cell projection part
[BBH] GEK_DROME (sp|Q9W1B0) Serine/threonine-prot. … Same as above	GEK_DROME	0	GLOS_GEK.1.1	GO:0030426	C	FlyBase	growth cone
[BBH] GEK_DROME (sp|Q9W1B0) Serine/threonine-prot. … Same as above	GEK_DROME	0	GLOS_GEK.1.1	GO:0005524	M	UniProtKB-k	ATP binding
[BBH] GEK_DROME (sp|Q9W1B0) Serine/threonine-prot. … Same as above	GEK_DROME	0	GLOS_GEK.1.1	GO:0046872	M	UniProtKB-k	metal ion binding
[BBH] GEK_DROME (sp|Q9W1B0) Serine/threonine-prot. … Same as above	GEK_DROME	0	GLOS_GEK.1.1	GO:0005543	M	InterPro	phospholipid binding
[BBH] GEK_DROME (sp|Q9W1B0) Serine/threonine-prot. … Same as above	GEK_DROME	0	GLOS_GEK.1.1	GO:0004672	M	.	protein kinase activity
[BBH] GEK_DROME (sp|Q9W1B0) Serine/threonine-prot. … Same as above	GEK_DROME	0	GLOS_GEK.1.1	GO:0004674	M	UniProtKB	protein serine/threonine kinase activity
[BBH] GEK_DROME (sp|Q9W1B0) Serine/threonine-prot. … Same as above	GEK_DROME	0	GLOS_GEK.1.1	GO:0030695	M	.	GTPase regulator activity
[BBH] GEK_DROME (sp|Q9W1B0) Serine/threonine-prot. … Same as above	GEK_DROME	0	GLOS_GEK.1.1	GO:0060589	M	.	nucleoside-triphosphatase regulator activity
[BBH] GEK_DROME (sp|Q9W1B0) Serine/threonine-prot. … Same as above	GEK_DROME	0	GLOS_GEK.1.1	GO:0005083	M	InterPro	small GTPase regulator activity
[BBH] GEK_DROME (sp|Q9W1B0) Serine/threonine-prot. … Same as above	GEK_DROME	0	GLOS_GEK.1.1	GO:0031532	B	InterPro	actin cytoskeleton reorganization
[BBH] GEK_DROME (sp|Q9W1B0) Serine/threonine-prot. … Same as above	GEK_DROME	0	GLOS_GEK.1.1	GO:0007015	B	.	actin filament organization
[BBH] GEK_DROME (sp|Q9W1B0) Serine/threonine-prot. … Same as above	GEK_DROME	0	GLOS_GEK.1.1	GO:0008154	B	FlyBase	actin polymerization or depolymerization
[BBH] GEK_DROME (sp|Q9W1B0) Serine/threonine-prot. … Same as above	GEK_DROME	0	GLOS_GEK.1.1	GO:0035556	B	InterPro	intracellular signal transduction
[BBH] GEK_DROME (sp|Q9W1B0) Serine/threonine-prot. … Same as above	GEK_DROME	0	GLOS_GEK.1.1	GO:0008064	B	.	regulation of actin polymerization or depolymerization
[BBH] GEK_DROME (sp|Q9W1B0) Serine/threonine-prot. … Same as above	GEK_DROME	0	GLOS_GEK.1.1	GO:0030832	B	.	regulation of actin filament length
[BBH] GEK_DROME (sp|Q9W1B0) Serine/threonine-prot. … Same as above	GEK_DROME	0	GLOS_GEK.1.1	GO:0032535	B	.	regulation of cellular component size
[BBH] GEK_DROME (sp|Q9W1B0) Serine/threonine-prot. … Same as above	GEK_DROME	0	GLOS_GEK.1.1	GO:0090066	B	.	regulation of anatomical structure size
[BBH] GEK_DROME (sp|Q9W1B0) Serine/threonine-prot. … Same as above	GEK_DROME	0	GLOS_GEK.1.1	GO:0032956	B	.	regulation of actin cytoskeleton organization
[BBH] GEK_DROME (sp|Q9W1B0) Serine/threonine-prot. … Same as above	GEK_DROME	0	GLOS_GEK.1.1	GO:0032970	B	.	regulation of actin filament-based process
[BBH] GEK_DROME (sp|Q9W1B0) Serine/threonine-prot. … Same as above	GEK_DROME	0	GLOS_GEK.1.1	GO:0051493	B	.	regulation of cytoskeleton organization
[BBH] GEK_DROME (sp|Q9W1B0) Serine/threonine-prot. … Same as above	GEK_DROME	0	GLOS_GEK.1.1	GO:0033043	B	.	regulation of organelle organization
[BBH] GEK_DROME (sp|Q9W1B0) Serine/threonine-prot. … Same as above	GEK_DROME	0	GLOS_GEK.1.1	GO:0032271	B	.	regulation of protein polymerization
[BBH] GEK_DROME (sp|Q9W1B0) Serine/threonine-prot. … Same as above	GEK_DROME	0	GLOS_GEK.1.1	GO:0043254	B	.	regulation of protein complex assembly
[BBH] GEK_DROME (sp|Q9W1B0) Serine/threonine-prot. … Same as above	GEK_DROME	0	GLOS_GEK.1.1	GO:0044087	B	.	regulation of cellular component biogenesis
[BBH] GEK_DROME (sp|Q9W1B0) Serine/threonine-prot. … Same as above	GEK_DROME	0	GLOS_GEK.1.1	GO:0030833	B	UniProtKB	regulation of actin filament polymerization
[BBH] GEK_DROME (sp|Q9W1B0) Serine/threonine-prot. … Same as above	GEK_DROME	0	GLOS_GEK.1.1	GO:0008038	B	.	neuron recognition

[BBH] GEK_DROME (sp|Q9W1B0) Serine/threonine-prot. … Same as above	GEK_DROME	0	GLOS_GEK.1.1	GO:0008037	B	.	cell recognition
[BBH] GEK_DROME (sp|Q9W1B0) Serine/threonine-prot. … Same as above	GEK_DROME	0	GLOS_GEK.1.1	GO:0008039	B	FlyBase	synaptic target recognition
[BBH] GSK3B_TRYB2 (sp|Q388M1) Glycogen synthase kinase 3; T.b.b.	GSK3B_TRYB2	0	GLOS_GSK3B.1.1	GO:0005524	M	UniProtKB-k	ATP binding
[BBH] GSK3B_TRYB2 (sp|Q388M1) Glycogen synthase kinase 3;T.b.b.	GSK3B_TRYB2	0	GLOS_GSK3B.1.1	GO:0004674	M	UniProtKB-k	protein serine/threonine kinase activity
[BBH] GSK3B_TRYB2 (sp|Q388M1) Glycogen synthase kinase 3; T.b.b.	GSK3B_TRYB2	0	GLOS_GSK3B.1.1	GO:0050321	M	UniProtKB	tau-protein kinase activity
[BBH] HAIR_DROME (sp|P14003) Protein hairy OS=D. m.	HAIR_DROME	5,00E-96	GLOS_HAIR.1.1	GO:0005634	C	UniProtKB	nucleus
[BBH] HAIR_DROME (sp|P14003) Protein hairy OS=D. m.	HAIR_DROME	5,00E-96	GLOS_HAIR.1.1	GO:0044212	M	.	transcription regulatory region DNA binding
[BBH] HAIR_DROME (sp|P14003) Protein hairy OS=D. m.	HAIR_DROME	5,00E-96	GLOS_HAIR.1.1	GO:0000975	M	.	regulatory region DNA binding
[BBH] HAIR_DROME (sp|P14003) Protein hairy OS=D. m.	HAIR_DROME	5,00E-96	GLOS_HAIR.1.1	GO:0001067	M	.	regulatory region nucleic acid binding
[BBH] HAIR_DROME (sp|P14003) Protein hairy OS=D. m.	HAIR_DROME	5,00E-96	GLOS_HAIR.1.1	GO:0070888	M	FlyBase	E-box binding
[BBH] HAIR_DROME (sp|P14003) Protein hairy OS=D. m.	HAIR_DROME	5,00E-96	GLOS_HAIR.1.1	GO:0000977	M	.	RNA polymerase II regulatory region sequence-specific DNA binding
[BBH] HAIR_DROME (sp|P14003) Protein hairy OS=D. m.	HAIR_DROME	5,00E-96	GLOS_HAIR.1.1	GO:0000976	M	.	transcription regulatory region sequence-specific DNA binding
[BBH] HAIR_DROME (sp|P14003) Protein hairy OS=D. m.	HAIR_DROME	5,00E-96	GLOS_HAIR.1.1	GO:0043565	M	.	sequence-specific DNA binding
[BBH] HAIR_DROME (sp|P14003) Protein hairy OS=D. m.	HAIR_DROME	5,00E-96	GLOS_HAIR.1.1	GO:0001012	M	.	RNA polymerase II regulatory region DNA binding
[BBH] HAIR_DROME (sp|P14003) Protein hairy OS=D. m.	HAIR_DROME	5,00E-96	GLOS_HAIR.1.1	GO:0000987	M	.	core promoter proximal region sequence-specific DNA binding
[BBH] HAIR_DROME (sp|P14003) Protein hairy OS=D. m.	HAIR_DROME	5,00E-96	GLOS_HAIR.1.1	GO:0001159	M	.	core promoter proximal region DNA binding
[BBH] HAIR_DROME (sp|P14003) Protein hairy OS=D. m.	HAIR_DROME	5,00E-96	GLOS_HAIR.1.1	GO:0000978	M	FlyBase	RNA polymerase II core promoter proximal region sequence-specific DNA binding
[BBH] HAIR_DROME (sp|P14003) Protein hairy OS=D. m.	HAIR_DROME	5,00E-96	GLOS_HAIR.1.1	GO:0000982	M	.	RNA polymerase II core promoter proximal region sequence-specific DNA binding transcription factor activity
[BBH] HAIR_DROME (sp|P14003) Protein hairy OS=D. m.	HAIR_DROME	5,00E-96	GLOS_HAIR.1.1	GO:0000981	M	.	sequence-specific DNA binding RNA polymerase II transcription factor activity
[BBH] HAIR_DROME (sp|P14003) Protein hairy OS=D. m.	HAIR_DROME	5,00E-96	GLOS_HAIR.1.1	GO:0003700	M	.	sequence-specific DNA binding transcription factor activity
[BBH] HAIR_DROME (sp|P14003) Protein hairy OS=D. m.	HAIR_DROME	5,00E-96	GLOS_HAIR.1.1	GO:0001227	M	.	RNA polymerase II transcription regulatory region sequence-specific DNA binding transcription factor activity involved in negative regulation of transcription
[BBH] HAIR_DROME (sp|P14003) Protein hairy OS=D. m.	HAIR_DROME	5,00E-96	GLOS_HAIR.1.1	GO:0001078	M	FlyBase	RNA polymerase II core promoter proximal region sequence-specific DNA binding transcription factor activity involved in negative regulation of transcription
[BBH] HAIR_DROME (sp|P14003) Protein hairy OS=D. m.	HAIR_DROME	5,00E-96	GLOS_HAIR.1.1	GO:0000902	B	FlyBase	cell morphogenesis
[BBH] HAIR_DROME (sp|P14003) Protein hairy OS=D. m.	HAIR_DROME	5,00E-96	GLOS_HAIR.1.1	GO:0016044	B	FlyBase	.
[BBH] HAIR_DROME (sp|P14003) Protein hairy OS=D. m.	HAIR_DROME	5,00E-96	GLOS_HAIR.1.1	GO:0060541	B	.	respiratory system development
[BBH] HAIR_DROME (sp|P14003) Protein hairy OS=D. m.	HAIR_DROME	5,00E-96	GLOS_HAIR.1.1	GO:0007424	B	FlyBase	open tracheal system development
[BBH] HAIR_DROME (sp|P14003) Protein hairy OS=D. m.	HAIR_DROME	5,00E-96	GLOS_HAIR.1.1	GO:0007365	B	.	periodic partitioning
[BBH] HAIR_DROME (sp|P14003) Protein hairy OS=D. m.	HAIR_DROME	5,00E-96	GLOS_HAIR.1.1	GO:0007366	B	FlyBase	periodic partitioning by pair rule gene
[BBH] HAIR_DROME (sp|P14003) Protein hairy OS=D. m.	HAIR_DROME	5,00E-96	GLOS_HAIR.1.1	GO:0035287	B	.	head segmentation
[BBH] HAIR_DROME (sp|P14003) Protein hairy OS=D. m.	HAIR_DROME	5,00E-96	GLOS_HAIR.1.1	GO:0060322	B	.	head development
[BBH] HAIR_DROME (sp|P14003) Protein hairy OS=D. m.	HAIR_DROME	5,00E-96	GLOS_HAIR.1.1	GO:0035289	B	FlyBase	posterior head segmentation
[BBH] HAIR_DROME (sp|P14003) Protein hairy OS=D. m.	HAIR_DROME	5,00E-96	GLOS_HAIR.1.1	GO:0045465	B	.	R8 cell differentiation
[BBH] HAIR_DROME (sp|P14003) Protein hairy OS=D. m.	HAIR_DROME	5,00E-96	GLOS_HAIR.1.1	GO:0007460	B	FlyBase	R8 cell fate commitment
[BBH] HAIR_DROME (sp|P14003) Protein hairy OS=D. m.	HAIR_DROME	5,00E-96	GLOS_HAIR.1.1	GO:0036293	B	.	response to decreased oxygen levels
[BBH] HAIR_DROME (sp|P14003) Protein hairy OS=D. m.	HAIR_DROME	5,00E-96	GLOS_HAIR.1.1	GO:0070482	B	.	response to oxygen levels
[BBH] HAIR_DROME (sp|P14003) Protein hairy OS=D. m.	HAIR_DROME	5,00E-96	GLOS_HAIR.1.1	GO:0001666	B	FlyBase	response to hypoxia
[BBH] HAIR_DROME (sp|P14003) Protein hairy OS=D. m.	HAIR_DROME	5,00E-96	GLOS_HAIR.1.1	GO:0022612	B	.	gland morphogenesis
[BBH] HAIR_DROME (sp|P14003) Protein hairy OS=D. m.	HAIR_DROME	5,00E-96	GLOS_HAIR.1.1	GO:0048732	B	.	gland development
[BBH] HAIR_DROME (sp|P14003) Protein hairy OS=D. m.	HAIR_DROME	5,00E-96	GLOS_HAIR.1.1	GO:0007431	B	.	salivary gland development
[BBH] HAIR_DROME (sp|P14003) Protein hairy OS=D. m.	HAIR_DROME	5,00E-96	GLOS_HAIR.1.1	GO:0035272	B	.	exocrine system development
[BBH] HAIR_DROME (sp|P14003) Protein hairy OS=D. m.	HAIR_DROME	5,00E-96	GLOS_HAIR.1.1	GO:0007435	B	FlyBase	salivary gland morphogenesis
[BBH] HAIR_DROME (sp|P14003) Protein hairy OS=D. m.	HAIR_DROME	5,00E-96	GLOS_HAIR.1.1	GO:0035290	B	FlyBase	trunk segmentation
[BBH] HAIR_DROME (sp|P14003) Protein hairy OS=D. m.	HAIR_DROME	5,00E-96	GLOS_HAIR.1.1	GO:0035239	B	FlyBase	tube morphogenesis
[BBH] HPRT_TRYBB (sp|Q07010) Hypoxanthine-guanine phosphoribosyltransferase OS=Trypanosoma b.brucei	HPRT_TRYBB	1,00E-145	GLOS_HPRT.1.1	GO:0005737	C	UniProtKB-S	cytoplasm
[BBH] HPRT_TRYBB (sp|Q07010) Hypoxanthine-guanine … Same as above	HPRT_TRYBB	1,00E-145	GLOS_HPRT.1.1	GO:0016763	M	.	transferase activity, transferring pentosyl groups
[BBH] HPRT_TRYBB (sp|Q07010) Hypoxanthine-guanine … Same as above	HPRT_TRYBB	1,00E-145	GLOS_HPRT.1.1	GO:0052657	M	UniProtKB-E	guanine phosphoribosyltransferase activity
[BBH] HPRT_TRYBB (sp|Q07010) Hypoxanthine-guanine … Same as above	HPRT_TRYBB	1,00E-145	GLOS_HPRT.1.1	GO:0004422	M	UniProtKB-E	hypoxanthine phosphoribosyltransferase activity
[BBH] HPRT_TRYBB (sp|Q07010) Hypoxanthine-guanine … Same as above	HPRT_TRYBB	1,00E-145	GLOS_HPRT.1.1	GO:0046872	M	UniProtKB-k	metal ion binding
[BBH] HPRT_TRYBB (sp|Q07010) Hypoxanthine-guanine … Same as above	HPRT_TRYBB	1,00E-145	GLOS_HPRT.1.1	GO:0000166	M	UniProtKB-k	nucleotide binding
[BBH] HPRT_TRYBB (sp|Q07010) Hypoxanthine-guanine … Same as above	HPRT_TRYBB	1,00E-145	GLOS_HPRT.1.1	GO:0006188	B	.	IMP biosynthetic process

[BBH] HPRT_TRYBB (sp|Q07010) Hypoxanthine-guanine …	Same as above	HPRT_TRYBB	1,00E-145	GLOS_HPRT.1.1	GO:0009152	B	.	purine ribonucleotide biosynthetic process
[BBH] HPRT_TRYBB (sp|Q07010) Hypoxanthine-guanine …	Same as above	HPRT_TRYBB	1,00E-145	GLOS_HPRT.1.1	GO:0006164	B	.	purine nucleotide biosynthetic process
[BBH] HPRT_TRYBB (sp|Q07010) Hypoxanthine-guanine …	Same as above	HPRT_TRYBB	1,00E-145	GLOS_HPRT.1.1	GO:0006163	B	.	purine nucleotide metabolic process
[BBH] HPRT_TRYBB (sp|Q07010) Hypoxanthine-guanine …	Same as above	HPRT_TRYBB	1,00E-145	GLOS_HPRT.1.1	GO:0009117	B	.	nucleotide metabolic process
[BBH] HPRT_TRYBB (sp|Q07010) Hypoxanthine-guanine …	Same as above	HPRT_TRYBB	1,00E-145	GLOS_HPRT.1.1	GO:0006753	B	.	nucleoside phosphate metabolic process
[BBH] HPRT_TRYBB (sp|Q07010) Hypoxanthine-guanine …	Same as above	HPRT_TRYBB	1,00E-145	GLOS_HPRT.1.1	GO:0055086	B	.	nucleobase-containing small molecule metabolic process
[BBH] HPRT_TRYBB (sp|Q07010) Hypoxanthine-guanine …	Same as above	HPRT_TRYBB	1,00E-145	GLOS_HPRT.1.1	GO:0044281	B	.	small molecule metabolic process
[BBH] HPRT_TRYBB (sp|Q07010) Hypoxanthine-guanine …	Same as above	HPRT_TRYBB	1,00E-145	GLOS_HPRT.1.1	GO:0072521	B	.	purine-containing compound metabolic process
[BBH] HPRT_TRYBB (sp|Q07010) Hypoxanthine-guanine …	Same as above	HPRT_TRYBB	1,00E-145	GLOS_HPRT.1.1	GO:0009165	B	.	nucleotide biosynthetic process
[BBH] HPRT_TRYBB (sp|Q07010) Hypoxanthine-guanine …	Same as above	HPRT_TRYBB	1,00E-145	GLOS_HPRT.1.1	GO:1901293	B	.	nucleoside phosphate biosynthetic process
[BBH] HPRT_TRYBB (sp|Q07010) Hypoxanthine-guanine …	Same as above	HPRT_TRYBB	1,00E-145	GLOS_HPRT.1.1	GO:0072522	B	.	purine-containing compound biosynthetic process
[BBH] HPRT_TRYBB (sp|Q07010) Hypoxanthine-guanine …	Same as above	HPRT_TRYBB	1,00E-145	GLOS_HPRT.1.1	GO:0009150	B	.	purine ribonucleotide metabolic process
[BBH] HPRT_TRYBB (sp|Q07010) Hypoxanthine-guanine …	Same as above	HPRT_TRYBB	1,00E-145	GLOS_HPRT.1.1	GO:0009259	B	.	ribonucleotide metabolic process
[BBH] HPRT_TRYBB (sp|Q07010) Hypoxanthine-guanine …	Same as above	HPRT_TRYBB	1,00E-145	GLOS_HPRT.1.1	GO:0019693	B	.	ribose phosphate metabolic process
[BBH] HPRT_TRYBB (sp|Q07010) Hypoxanthine-guanine …	Same as above	HPRT_TRYBB	1,00E-145	GLOS_HPRT.1.1	GO:0009260	B	.	ribonucleotide biosynthetic process
[BBH] HPRT_TRYBB (sp|Q07010) Hypoxanthine-guanine …	Same as above	HPRT_TRYBB	1,00E-145	GLOS_HPRT.1.1	GO:0046390	B	.	ribose phosphate biosynthetic process
[BBH] HPRT_TRYBB (sp|Q07010) Hypoxanthine-guanine …	Same as above	HPRT_TRYBB	1,00E-145	GLOS_HPRT.1.1	GO:0009168	B	.	purine ribonucleoside monophosphate biosynthetic process
[BBH] HPRT_TRYBB (sp|Q07010) Hypoxanthine-guanine …	Same as above	HPRT_TRYBB	1,00E-145	GLOS_HPRT.1.1	GO:0009127	B	.	purine nucleoside monophosphate biosynthetic process
[BBH] HPRT_TRYBB (sp|Q07010) Hypoxanthine-guanine …	Same as above	HPRT_TRYBB	1,00E-145	GLOS_HPRT.1.1	GO:0009124	B	.	nucleoside monophosphate biosynthetic process
[BBH] HPRT_TRYBB (sp|Q07010) Hypoxanthine-guanine …	Same as above	HPRT_TRYBB	1,00E-145	GLOS_HPRT.1.1	GO:0009123	B	.	nucleoside monophosphate metabolic process
[BBH] HPRT_TRYBB (sp|Q07010) Hypoxanthine-guanine …	Same as above	HPRT_TRYBB	1,00E-145	GLOS_HPRT.1.1	GO:0009126	B	.	purine nucleoside monophosphate metabolic process
[BBH] HPRT_TRYBB (sp|Q07010) Hypoxanthine-guanine …	Same as above	HPRT_TRYBB	1,00E-145	GLOS_HPRT.1.1	GO:0009156	B	.	ribonucleoside monophosphate biosynthetic process
[BBH] HPRT_TRYBB (sp|Q07010) Hypoxanthine-guanine …	Same as above	HPRT_TRYBB	1,00E-145	GLOS_HPRT.1.1	GO:0009161	B	.	ribonucleoside monophosphate metabolic process
[BBH] HPRT_TRYBB (sp|Q07010) Hypoxanthine-guanine …	Same as above	HPRT_TRYBB	1,00E-145	GLOS_HPRT.1.1	GO:0009167	B	.	purine ribonucleoside monophosphate metabolic process
[BBH] HPRT_TRYBB (sp|Q07010) Hypoxanthine-guanine …	Same as above	HPRT_TRYBB	1,00E-145	GLOS_HPRT.1.1	GO:0046040	B	.	IMP metabolic process
[BBH] HPRT_TRYBB (sp|Q07010) Hypoxanthine-guanine …	Same as above	HPRT_TRYBB	1,00E-145	GLOS_HPRT.1.1	GO:0032261	B	.	purine nucleotide salvage
[BBH] HPRT_TRYBB (sp|Q07010) Hypoxanthine-guanine …	Same as above	HPRT_TRYBB	1,00E-145	GLOS_HPRT.1.1	GO:0043101	B	.	purine-containing compound salvage
[BBH] HPRT_TRYBB (sp|Q07010) Hypoxanthine-guanine …	Same as above	HPRT_TRYBB	1,00E-145	GLOS_HPRT.1.1	GO:0043094	B	.	cellular metabolic compound salvage
[BBH] HPRT_TRYBB (sp|Q07010) Hypoxanthine-guanine …	Same as above	HPRT_TRYBB	1,00E-145	GLOS_HPRT.1.1	GO:0043173	B	.	nucleotide salvage
[BBH] HPRT_TRYBB (sp|Q07010) Hypoxanthine-guanine …	Same as above	HPRT_TRYBB	1,00E-145	GLOS_HPRT.1.1	GO:0032264	B	UniProtKB-U	IMP salvage
[BBH] HPRT_TRYBB (sp|Q07010) Hypoxanthine-guanine …	Same as above	HPRT_TRYBB	1,00E-145	GLOS_HPRT.1.1	GO:0043174	B	.	nucleoside salvage
[BBH] HPRT_TRYBB (sp|Q07010) Hypoxanthine-guanine …	Same as above	HPRT_TRYBB	1,00E-145	GLOS_HPRT.1.1	GO:0009163	B	.	nucleoside biosynthetic process
[BBH] HPRT_TRYBB (sp|Q07010) Hypoxanthine-guanine …	Same as above	HPRT_TRYBB	1,00E-145	GLOS_HPRT.1.1	GO:0009116	B	.	nucleoside metabolic process
[BBH] HPRT_TRYBB (sp|Q07010) Hypoxanthine-guanine …	Same as above	HPRT_TRYBB	1,00E-145	GLOS_HPRT.1.1	GO:1901657	B	.	glycosyl compound metabolic process
[BBH] HPRT_TRYBB (sp|Q07010) Hypoxanthine-guanine …	Same as above	HPRT_TRYBB	1,00E-145	GLOS_HPRT.1.1	GO:1901659	B	.	glycosyl compound biosynthetic process
[BBH] HPRT_TRYBB (sp|Q07010) Hypoxanthine-guanine …	Same as above	HPRT_TRYBB	1,00E-145	GLOS_HPRT.1.1	GO:0046129	B	.	purine ribonucleoside biosynthetic process
[BBH] HPRT_TRYBB (sp|Q07010) Hypoxanthine-guanine …	Same as above	HPRT_TRYBB	1,00E-145	GLOS_HPRT.1.1	GO:0042451	B	.	purine nucleoside biosynthetic process
[BBH] HPRT_TRYBB (sp|Q07010) Hypoxanthine-guanine …	Same as above	HPRT_TRYBB	1,00E-145	GLOS_HPRT.1.1	GO:0042278	B	.	purine nucleoside metabolic process
[BBH] HPRT_TRYBB (sp|Q07010) Hypoxanthine-guanine …	Same as above	HPRT_TRYBB	1,00E-145	GLOS_HPRT.1.1	GO:0042455	B	.	ribonucleoside biosynthetic process
[BBH] HPRT_TRYBB (sp|Q07010) Hypoxanthine-guanine …	Same as above	HPRT_TRYBB	1,00E-145	GLOS_HPRT.1.1	GO:0009119	B	.	ribonucleoside metabolic process
[BBH] HPRT_TRYBB (sp|Q07010) Hypoxanthine-guanine …	Same as above	HPRT_TRYBB	1,00E-145	GLOS_HPRT.1.1	GO:0046128	B	.	purine ribonucleoside metabolic process
[BBH] HPRT_TRYBB (sp|Q07010) Hypoxanthine-guanine …	Same as above	HPRT_TRYBB	1,00E-145	GLOS_HPRT.1.1	GO:0006166	B	UniProtKB-k	purine ribonucleoside salvage
[BBH] KEN_DROPS (sp|Q292R5) Transcription factor Ken OS=D. p. p.		KEN_DROPS	0	GLOS_KEN.1.1	GO:0005634	C	UniProtKB	nucleus
[BBH] KEN_DROPS (sp|Q292R5) Transcription factor Ken OS=D. p. p.		KEN_DROPS	0	GLOS_KEN.1.1	GO:0003677	M	UniProtKB	DNA binding
[BBH] KEN_DROPS (sp|Q292R5) Transcription factor Ken OS=D. p. p.		KEN_DROPS	0	GLOS_KEN.1.1	GO:0046872	M	UniProtKB-k	metal ion binding
[BBH] KEN_DROPS (sp|Q292R5) Transcription factor Ken OS=D. p. p.		KEN_DROPS	0	GLOS_KEN.1.1	GO:0003700	M	UniProtKB	sequence-specific DNA binding transcription factor activity
[BBH] KEN_DROPS (sp|Q292R5) Transcription factor Ken OS=D. p. p.		KEN_DROPS	0	GLOS_KEN.1.1	GO:0007487	B	.	analia development
[BBH] KEN_DROPS (sp|Q292R5) Transcription factor Ken OS=D. p. p.		KEN_DROPS	0	GLOS_KEN.1.1	GO:0035215	B	.	genital disc development
[BBH] KEN_DROPS (sp|Q292R5) Transcription factor Ken OS=D. p. p.		KEN_DROPS	0	GLOS_KEN.1.1	GO:0045497	B	UniProtKB	female analia development
[BBH] KEN_DROPS (sp|Q292R5) Transcription factor Ken OS=D. p. p.		KEN_DROPS	0	GLOS_KEN.1.1	GO:0048806	B	.	genitalia development
[BBH] KEN_DROPS (sp|Q292R5) Transcription factor Ken OS=D. p. p.		KEN_DROPS	0	GLOS_KEN.1.1	GO:0048608	B	.	reproductive structure development
[BBH] KEN_DROPS (sp|Q292R5) Transcription factor Ken OS=D. p. p.		KEN_DROPS	0	GLOS_KEN.1.1	GO:0061458	B	.	reproductive system development
[BBH] KEN_DROPS (sp|Q292R5) Transcription factor Ken OS=D. p. p.		KEN_DROPS	0	GLOS_KEN.1.1	GO:0007548	B	.	sex differentiation
[BBH] KEN_DROPS (sp|Q292R5) Transcription factor Ken OS=D. p. p.		KEN_DROPS	0	GLOS_KEN.1.1	GO:0046545	B	.	development of primary female sexual characteristics

[BBH] KEN_DROPS (sp|Q292R5) Transcription factor Ken OS=D. p. p.	KEN_DROPS	0	GLOS_KEN.1.1	GO:0045137	B	.	development of primary sexual characteristics
[BBH] KEN_DROPS (sp|Q292R5) Transcription factor Ken OS=D. p. p.	KEN_DROPS	0	GLOS_KEN.1.1	GO:0046660	B	.	female sex differentiation
[BBH] KEN_DROPS (sp|Q292R5) Transcription factor Ken OS=D. p. p.	KEN_DROPS	0	GLOS_KEN.1.1	GO:0030540	B	UniProtKB	female genitalia development
[BBH] KEN_DROPS (sp|Q292R5) Transcription factor Ken OS=D. p. p.	KEN_DROPS	0	GLOS_KEN.1.1	GO:0045496	B	UniProtKB	male analia development
[BBH] KEN_DROPS (sp|Q292R5) Transcription factor Ken OS=D. p. p.	KEN_DROPS	0	GLOS_KEN.1.1	GO:0046546	B	.	development of primary male sexual characteristics
[BBH] KEN_DROPS (sp|Q292R5) Transcription factor Ken OS=D. p. p.	KEN_DROPS	0	GLOS_KEN.1.1	GO:0046661	B	.	male sex differentiation
[BBH] KEN_DROPS (sp|Q292R5) Transcription factor Ken OS=D. p. p.	KEN_DROPS	0	GLOS_KEN.1.1	GO:0030539	B	UniProtKB	male genitalia development
[BBH] KEN_DROPS (sp|Q292R5) Transcription factor Ken OS=D. p. p.	KEN_DROPS	0	GLOS_KEN.1.1	GO:0010741	B	.	negative regulation of intracellular protein kinase cascade
[BBH] KEN_DROPS (sp|Q292R5) Transcription factor Ken OS=D. p. p.	KEN_DROPS	0	GLOS_KEN.1.1	GO:0046425	B	.	regulation of JAK-STAT cascade
[BBH] KEN_DROPS (sp|Q292R5) Transcription factor Ken OS=D. p. p.	KEN_DROPS	0	GLOS_KEN.1.1	GO:0046426	B	UniProtKB	negative regulation of JAK-STAT cascade
[BBH] KEN_DROPS (sp|Q292R5) Transcription factor Ken OS=D. p. p.	KEN_DROPS	0	GLOS_KEN.1.1	GO:0006351	B	UniProtKB-K	transcription, DNA-dependent
[BBH] MECR_DROME (sp|Q9V6U9) Prob trans-2-enoyl-CoA reductase, mito.	MECR_DROME	1,00E-173	GLOS_MECR.1.1	GO:0005875	C	FlyBase	microtubule associated complex
[BBH] MECR_DROME (sp|Q9V6U9) Prob trans-2-enoyl-CoA reductase, mito.	MECR_DROME	1,00E-173	GLOS_MECR.1.1	GO:0005739	C	UniProtKB	mitochondrion
[BBH] MECR_DROME (sp|Q9V6U9) Prob trans-2-enoyl-CoA reductase, mito.	MECR_DROME	1,00E-173	GLOS_MECR.1.1	GO:0016628	M	.	oxidoreductase activity, acting on the CH-CH group of donors, NAD or NADP as acceptor
[BBH] MECR_DROME (sp|Q9V6U9) Prob trans-2-enoyl-CoA reductase, mito.	MECR_DROME	1,00E-173	GLOS_MECR.1.1	GO:0016627	M	.	oxidoreductase activity, acting on the CH-CH group of donors
[BBH] MECR_DROME (sp|Q9V6U9) Prob trans-2-enoyl-CoA reductase, mito.	MECR_DROME	1,00E-173	GLOS_MECR.1.1	GO:0019166	M	UniProtKB	trans-2-enoyl-CoA reductase (NADPH) activity
[BBH] MECR_DROME (sp|Q9V6U9) Prob trans-2-enoyl-CoA reductase, mito.	MECR_DROME	1,00E-173	GLOS_MECR.1.1	GO:0008270	M	InterPro	zinc ion binding
[BBH] MECR_DROME (sp|Q9V6U9) Prob trans-2-enoyl-CoA reductase, mito.	MECR_DROME	1,00E-173	GLOS_MECR.1.1	GO:0006631	B	.	fatty acid metabolic process
[BBH] MECR_DROME (sp|Q9V6U9) Prob trans-2-enoyl-CoA reductase, mito.	MECR_DROME	1,00E-173	GLOS_MECR.1.1	GO:0032787	B	.	monocarboxylic acid metabolic process
[BBH] MECR_DROME (sp|Q9V6U9) Prob trans-2-enoyl-CoA reductase, mito.	MECR_DROME	1,00E-173	GLOS_MECR.1.1	GO:0019752	B	.	carboxylic acid metabolic process
[BBH] MECR_DROME (sp|Q9V6U9) Prob trans-2-enoyl-CoA reductase, mito.	MECR_DROME	1,00E-173	GLOS_MECR.1.1	GO:0043436	B	.	oxoacid metabolic process
[BBH] MECR_DROME (sp|Q9V6U9) Prob trans-2-enoyl-CoA reductase, mito.	MECR_DROME	1,00E-173	GLOS_MECR.1.1	GO:0006082	B	.	organic acid metabolic process
[BBH] MECR_DROME (sp|Q9V6U9) Prob trans-2-enoyl-CoA reductase, mito.	MECR_DROME	1,00E-173	GLOS_MECR.1.1	GO:0072330	B	.	monocarboxylic acid biosynthetic process
[BBH] MECR_DROME (sp|Q9V6U9) Prob trans-2-enoyl-CoA reductase, mito.	MECR_DROME	1,00E-173	GLOS_MECR.1.1	GO:0046394	B	.	carboxylic acid biosynthetic process
[BBH] MECR_DROME (sp|Q9V6U9) Prob trans-2-enoyl-CoA reductase, mito.	MECR_DROME	1,00E-173	GLOS_MECR.1.1	GO:0016053	B	.	organic acid biosynthetic process
[BBH] MECR_DROME (sp|Q9V6U9) Prob trans-2-enoyl-CoA reductase, mito.	MECR_DROME	1,00E-173	GLOS_MECR.1.1	GO:0044283	B	.	small molecule biosynthetic process
[BBH] MECR_DROME (sp|Q9V6U9) Prob trans-2-enoyl-CoA reductase, mito.	MECR_DROME	1,00E-173	GLOS_MECR.1.1	GO:0044711	B	.	single-organism biosynthetic process
[BBH] MECR_DROME (sp|Q9V6U9) Prob trans-2-enoyl-CoA reductase, mito.	MECR_DROME	1,00E-173	GLOS_MECR.1.1	GO:0006633	B	UniProtKB-K	fatty acid biosynthetic process
[BBH] MECR_DROME (sp|Q9V6U9) Prob trans-2-enoyl-CoA reductase, mito.	MECR_DROME	1,00E-173	GLOS_MECR.1.1	GO:0006631	B	UniProtKB	fatty acid metabolic process
MOS1T_DROMA (sp|Q7JQ07) Mariner Mos1 transposase OS=D mauritiana	MOS1T_DROMA	3,00E-23	GLOS_MOS1T.18.	GO:0005634	C	UniProtKB-S	nucleus
MOS1T_DROMA (sp|Q7JQ07) Mariner Mos1 transposase OS=D mauritiana	MOS1T_DROMA	3,00E-23	GLOS_MOS1T.18.	GO:0003677	M	UniProtKB-K	DNA binding
MOS1T_DROMA (sp|Q7JQ07) Mariner Mos1 transposase OS=D mauritiana	MOS1T_DROMA	3,00E-23	GLOS_MOS1T.18.	GO:0004518	M	.	nuclease activity
MOS1T_DROMA (sp|Q7JQ07) Mariner Mos1 transposase OS=D mauritiana	MOS1T_DROMA	3,00E-23	GLOS_MOS1T.18.	GO:0004519	M	UniProtKB-K	endonuclease activity
MOS1T_DROMA (sp|Q7JQ07) Mariner Mos1 transposase OS=D mauritiana	MOS1T_DROMA	3,00E-23	GLOS_MOS1T.18.	GO:0046872	M	UniProtKB-K	metal ion binding
MOS1T_DROMA (sp|Q7JQ07) Mariner Mos1 transposase OS=D mauritiana	MOS1T_DROMA	3,00E-23	GLOS_MOS1T.18.	GO:0006259	B	.	DNA metabolic process
MOS1T_DROMA (sp|Q7JQ07) Mariner Mos1 transposase OS=D mauritiana	MOS1T_DROMA	3,00E-23	GLOS_MOS1T.18.	GO:0015074	B	UniProtKB-K	DNA integration
MOS1T_DROMA (sp|Q7JQ07) Mariner Mos1 transposase OS=D mauritiana	MOS1T_DROMA	3,00E-23	GLOS_MOS1T.18.	GO:0006310	B	UniProtKB-K	DNA recombination
MOS1T_DROMA (sp|Q7JQ07) Mariner Mos1 transposase OS=D mauritiana	MOS1T_DROMA	3,00E-23	GLOS_MOS1T.18.	GO:0090305	B	GOC	nucleic acid phosphodiester bond hydrolysis
NESSY_DROME (sp|Q9VVX5) Transmembrane protein nessy OS=D. m.	NESSY_DROME	0	GLOS_NESSY.1.1	GO:0016021	C	UniProtKB	integral to membrane
NESSY_DROME (sp|Q9VVX5) Transmembrane protein nessy OS=D. m.	NESSY_DROME	0	GLOS_NESSY.1.1	GO:0016747	M	.	transferase activity, transferring acyl groups other than amino-acyl groups
NESSY_DROME (sp|Q9VVX5) Transmembrane protein nessy OS=D. m.	NESSY_DROME	0	GLOS_NESSY.1.1	GO:0016746	M	.	transferase activity, transferring acyl groups
NESSY_DROME (sp|Q9VVX5) Transmembrane protein nessy OS=D. m.	NESSY_DROME	0	GLOS_NESSY.1.1	GO:0071617	M	FlyBase	lysophospholipid acyltransferase activity
NESSY_DROME (sp|Q9VVX5) Transmembrane protein nessy OS=D. m.	NESSY_DROME	0	GLOS_NESSY.1.1	GO:0008354	B	FlyBase	germ cell migration
NESSY_DROME (sp|Q9VVX5) Transmembrane protein nessy OS=D. m.	NESSY_DROME	0	GLOS_NESSY.1.1	GO:0030258	B	FlyBase	lipid modification
NESSY_DROME (sp|Q9VVX5) Transmembrane protein nessy OS=D. m.	NESSY_DROME	0	GLOS_NESSY.1.1	GO:0044802	B	.	single-organism membrane organization
NESSY_DROME (sp|Q9VVX5) Transmembrane protein nessy OS=D. m.	NESSY_DROME	0	GLOS_NESSY.1.1	GO:0061024	B	.	membrane organization
NESSY_DROME (sp|Q9VVX5) Transmembrane protein nessy OS=D. m.	NESSY_DROME	0	GLOS_NESSY.1.1	GO:0007009	B	FlyBase	plasma membrane organization
NESSY_DROME (sp|Q9VVX5) Transmembrane protein nessy OS=D. m.	NESSY_DROME	0	GLOS_NESSY.1.1	GO:0007349	B	.	cellularization
NESSY_DROME (sp|Q9VVX5) Transmembrane protein nessy OS=D. m.	NESSY_DROME	0	GLOS_NESSY.1.1	GO:0007286	B	.	spermatid development
NESSY_DROME (sp|Q9VVX5) Transmembrane protein nessy OS=D. m.	NESSY_DROME	0	GLOS_NESSY.1.1	GO:0002064	B	.	epithelial cell development
NESSY_DROME (sp|Q9VVX5) Transmembrane protein nessy OS=D. m.	NESSY_DROME	0	GLOS_NESSY.1.1	GO:0030855	B	.	epithelial cell differentiation
NESSY_DROME (sp|Q9VVX5) Transmembrane protein nessy OS=D. m.	NESSY_DROME	0	GLOS_NESSY.1.1	GO:0048515	B	.	spermatid differentiation
NESSY_DROME (sp|Q9VVX5) Transmembrane protein nessy OS=D. m.	NESSY_DROME	0	GLOS_NESSY.1.1	GO:0007283	B	.	spermatogenesis

NESSY_DROME (sp|Q9VVX5) Transmembrane protein nessy OS=D. m.	NESSY_DROME	0	GLOS_NESSY.1.1	GO:0048232	B	.	male gamete generation
NESSY_DROME (sp|Q9VVX5) Transmembrane protein nessy OS=D. m.	NESSY_DROME	0	GLOS_NESSY.1.1	GO:0007291	B	FlyBase	sperm individualization
[BBH] NNRD_DROPS (sp|B5DHB2) ATP-depend. (S)-NAD(P)H-hydrate dehydratase OS=D.p.pseudoobscura	NNRD_DROPS	1,00E-116	GLOS_NNRD.1.1	GO:0005524	M	UniProtKB-K	ATP binding
[BBH] NNRD_DROPS (sp|B5DHB2) ATP-depend. … Same as abobe	NNRD_DROPS	1,00E-116	GLOS_NNRD.1.1	GO:0016836	M	.	hydro-lyase activity
[BBH] NNRD_DROPS (sp|B5DHB2) ATP-depend. … Same as abobe	NNRD_DROPS	1,00E-116	GLOS_NNRD.1.1	GO:0016835	M	.	carbon-oxygen lyase activity
[BBH] NNRD_DROPS (sp|B5DHB2) ATP-depend. … Same as abobe	NNRD_DROPS	1,00E-116	GLOS_NNRD.1.1	GO:0016829	M	.	lyase activity
[BBH] NNRD_DROPS (sp|B5DHB2) ATP-depend. … Same as abobe	NNRD_DROPS	1,00E-116	GLOS_NNRD.1.1	GO:0047453	M	UniProtKB-H	ATP-dependent NAD(P)H-hydrate dehydratase activity
[BBH] NNRD_DROPS (sp|B5DHB2) ATP-depend. … Same as abobe	NNRD_DROPS	1,00E-116	GLOS_NNRD.1.1	GO:0019362	B	.	pyridine nucleotide metabolic process
[BBH] NNRD_DROPS (sp|B5DHB2) ATP-depend. … Same as abobe	NNRD_DROPS	1,00E-116	GLOS_NNRD.1.1	GO:0006733	B	.	oxidoreduction coenzyme metabolic process
[BBH] NNRD_DROPS (sp|B5DHB2) ATP-depend. … Same as abobe	NNRD_DROPS	1,00E-116	GLOS_NNRD.1.1	GO:0006732	B	.	coenzyme metabolic process
[BBH] NNRD_DROPS (sp|B5DHB2) ATP-depend. … Same as abobe	NNRD_DROPS	1,00E-116	GLOS_NNRD.1.1	GO:0072524	B	.	pyridine-containing compound metabolic process
[BBH] NNRD_DROPS (sp|B5DHB2) ATP-depend. … Same as abobe	NNRD_DROPS	1,00E-116	GLOS_NNRD.1.1	GO:0046496	B	UniProtKB-H	nicotinamide nucleotide metabolic process
[BBH] NXF1_DROME (sp|Q9U1H9) Nuclear RNA export fact 1; D.m.	NXF1_DROME	0	GLOS_NXF1.1.1	GO:0005737	C	UniProtKB-S	cytoplasm
[BBH] NXF1_DROME (sp|Q9U1H9) Nuclear RNA export fact 1; D.m.	NXF1_DROME	0	GLOS_NXF1.1.1	GO:0031090	C	.	organelle membrane
[BBH] NXF1_DROME (sp|Q9U1H9) Nuclear RNA export fact 1; D.m.	NXF1_DROME	0	GLOS_NXF1.1.1	GO:0005635	C	.	nuclear envelope
[BBH] NXF1_DROME (sp|Q9U1H9) Nuclear RNA export fact 1; D.m.	NXF1_DROME	0	GLOS_NXF1.1.1	GO:0031967	C	.	organelle envelope
[BBH] NXF1_DROME (sp|Q9U1H9) Nuclear RNA export fact 1; D.m.	NXF1_DROME	0	GLOS_NXF1.1.1	GO:0031975	C	.	envelope
[BBH] NXF1_DROME (sp|Q9U1H9) Nuclear RNA export fact 1; D.m.	NXF1_DROME	0	GLOS_NXF1.1.1	GO:0012505	C	.	endomembrane system
[BBH] NXF1_DROME (sp|Q9U1H9) Nuclear RNA export fact 1; D.m.	NXF1_DROME	0	GLOS_NXF1.1.1	GO:0031965	C	FlyBase	nuclear membrane
[BBH] NXF1_DROME (sp|Q9U1H9) Nuclear RNA export fact 1; D.m.	NXF1_DROME	0	GLOS_NXF1.1.1	GO:0005654	C	FlyBase	nucleoplasm
[BBH] NXF1_DROME (sp|Q9U1H9) Nuclear RNA export fact 1; D.m.	NXF1_DROME	0	GLOS_NXF1.1.1	GO:0000166	M	InterPro	nucleotide binding
[BBH] NXF1_DROME (sp|Q9U1H9) Nuclear RNA export fact 1; D.m.	NXF1_DROME	0	GLOS_NXF1.1.1	GO:0003723	M	UniProtKB-K	RNA binding
[BBH] NXF1_DROME (sp|Q9U1H9) Nuclear RNA export fact 1; D.m.	NXF1_DROME	0	GLOS_NXF1.1.1	GO:0031124	B	.	mRNA 3'-end processing
[BBH] NXF1_DROME (sp|Q9U1H9) Nuclear RNA export fact 1; D.m.	NXF1_DROME	0	GLOS_NXF1.1.1	GO:0006397	B	.	mRNA processing
[BBH] NXF1_DROME (sp|Q9U1H9) Nuclear RNA export fact 1; D.m.	NXF1_DROME	0	GLOS_NXF1.1.1	GO:0006396	B	.	RNA processing
[BBH] NXF1_DROME (sp|Q9U1H9) Nuclear RNA export fact 1; D.m.	NXF1_DROME	0	GLOS_NXF1.1.1	GO:0016071	B	.	mRNA metabolic process
[BBH] NXF1_DROME (sp|Q9U1H9) Nuclear RNA export fact 1; D.m.	NXF1_DROME	0	GLOS_NXF1.1.1	GO:0031123	B	.	RNA 3'-end processing
[BBH] NXF1_DROME (sp|Q9U1H9) Nuclear RNA export fact 1; D.m.	NXF1_DROME	0	GLOS_NXF1.1.1	GO:0043631	B	.	RNA polyadenylation
[BBH] NXF1_DROME (sp|Q9U1H9) Nuclear RNA export fact 1; D.m.	NXF1_DROME	0	GLOS_NXF1.1.1	GO:0006378	B	FlyBase	mRNA polyadenylation
[BBH] NXF1_DROME (sp|Q9U1H9) Nuclear RNA export fact 1; D.m.	NXF1_DROME	0	GLOS_NXF1.1.1	GO:0006406	B	.	mRNA export from nucleus
[BBH] NXF1_DROME (sp|Q9U1H9) Nuclear RNA export fact 1; D.m.	NXF1_DROME	0	GLOS_NXF1.1.1	GO:0006405	B	.	RNA export from nucleus
[BBH] NXF1_DROME (sp|Q9U1H9) Nuclear RNA export fact 1; D.m.	NXF1_DROME	0	GLOS_NXF1.1.1	GO:0051168	B	.	nuclear export
[BBH] NXF1_DROME (sp|Q9U1H9) Nuclear RNA export fact 1; D.m.	NXF1_DROME	0	GLOS_NXF1.1.1	GO:0051028	B	.	mRNA transport
[BBH] NXF1_DROME (sp|Q9U1H9) Nuclear RNA export fact 1; D.m.	NXF1_DROME	0	GLOS_NXF1.1.1	GO:0016973	B	FlyBase	poly(A)+ mRNA export from nucleus
[BBH] RCL1_DROME (sp|P56175)ProbRNA 3'-terminal phosph. cyclase-like prot.	RCL1_DROME	0	GLOS_RCL1.1.1	GO:0005730	C	UniProtKB-S	nucleolus
[BBH] RCL1_DROME (sp|P56175)ProbRNA 3'-terminal phosph. cyclase-like prot.	RCL1_DROME	0	GLOS_RCL1.1.1	GO:0003824	M	InterPro	catalytic activity
[BBH] RCL1_DROME (sp|P56175)ProbRNA 3'-terminal phosph. cyclase-like prot.	RCL1_DROME	0	GLOS_RCL1.1.1	GO:0042254	B	UniProtKB-K	ribosome biogenesis
[BBH] RCL1_DROME (sp|P56175)ProbRNA 3'-terminal phosph. cyclase-like prot.	RCL1_DROME	0	GLOS_RCL1.1.1	GO:0006396	B	InterPro	RNA processing
[BBH] RIR2_TRYBB (sp|O15910)Ribonucleoside-diphosphate reduct. small chain	RIR2_TRYBB	0	GLOS_RIR2.1.2	GO:0016728	M	.	oxidoreductase activity, acting on CH or CH2 groups, disulfide as acceptor
[BBH] RIR2_TRYBB (sp|O15910)Ribonucleoside-diphosphate reduct. small chain	RIR2_TRYBB	0	GLOS_RIR2.1.2	GO:0016725	M	.	oxidoreductase activity, acting on CH or CH2 groups
[BBH] RIR2_TRYBB (sp|O15910)Ribonucleoside-diphosphate reduct. small chain	RIR2_TRYBB	0	GLOS_RIR2.1.2	GO:0004748	M	UniProtKB-E	ribonucleoside-diphosphate reductase activity, thioredoxin disulfide as acceptor
[BBH] RIR2_TRYBB (sp|O15910)Ribonucleoside-diphosphate reduct. small chain	RIR2_TRYBB	0	GLOS_RIR2.1.2	GO:0046914	M	InterPro	transition metal ion binding
[BBH] RIR2_TRYBB (sp|O15910)Ribonucleoside-diphosphate reduct. small chain	RIR2_TRYBB	0	GLOS_RIR2.1.2	GO:0009132	B	.	nucleoside diphosphate metabolic process
[BBH] RIR2_TRYBB (sp|O15910)Ribonucleoside-diphosphate reduct. small chain	RIR2_TRYBB	0	GLOS_RIR2.1.2	GO:0009186	B	InterPro	deoxyribonucleoside diphosphate metabolic process
[BBH] RIR2_TRYBB (sp|O15910)Ribonucleoside-diphosphate reduct. small chain	RIR2_TRYBB	0	GLOS_RIR2.1.2	GO:0006260	B	UniProtKB-U	DNA replication
RL23_DROME (sp|P48159) 60S ribosomal protein L23 OS=D.m.	RL23_DROME	3,00E-77	GLOS_RL23.5.16	GO:0005840	C	UniProtKB-K	ribosome
RL23_DROME (sp|P48159) 60S ribosomal protein L23 OS=D.m.	RL23_DROME	3,00E-77	GLOS_RL23.5.16	GO:0005198	M	.	structural molecule activity
RL23_DROME (sp|P48159) 60S ribosomal protein L23 OS=D.m.	RL23_DROME	3,00E-77	GLOS_RL23.5.16	GO:0003735	M	InterPro	structural constituent of ribosome
RL23_DROME (sp|P48159) 60S ribosomal protein L23 OS=D.m.	RL23_DROME	3,00E-77	GLOS_RL23.5.16	GO:0051231	B	.	spindle elongation
RL23_DROME (sp|P48159) 60S ribosomal protein L23 OS=D.m.	RL23_DROME	3,00E-77	GLOS_RL23.5.16	GO:0022402	B	.	cell cycle process
RL23_DROME (sp|P48159) 60S ribosomal protein L23 OS=D.m.	RL23_DROME	3,00E-77	GLOS_RL23.5.16	GO:0007049	B	.	cell cycle
RL23_DROME (sp|P48159) 60S ribosomal protein L23 OS=D.m.	RL23_DROME	3,00E-77	GLOS_RL23.5.16	GO:0007051	B	.	spindle organization
RL23_DROME (sp|P48159) 60S ribosomal protein L23 OS=D.m.	RL23_DROME	3,00E-77	GLOS_RL23.5.16	GO:0007052	B	.	mitotic spindle organization

RL23_DROME (sp|P48159) 60S ribosomal protein L23 OS=D.m.	RL23_DROME	3,00E-77	GLOS_RL23.5.16	GO:0000278	B	.	mitotic cell cycle
RL23_DROME (sp|P48159) 60S ribosomal protein L23 OS=D.m.	RL23_DROME	3,00E-77	GLOS_RL23.5.16	GO:0000022	B	FlyBase	mitotic spindle elongation
RL23_DROME (sp|P48159) 60S ribosomal protein L23 OS=D.m.	RL23_DROME	3,00E-77	GLOS_RL23.5.16	GO:0022008	B	FlyBase	neurogenesis
RL23_DROME (sp|P48159) 60S ribosomal protein L23 OS=D.m.	RL23_DROME	3,00E-77	GLOS_RL23.5.16	GO:0006412	B	InterPro	translation
RS20_DROME (sp|P55828) 40S ribosomal protein S20 OS=D.m.	RS20_DROME	8,00E-73	GLOS_RS20.3.14	GO:0044391	C	.	ribosomal subunit
RS20_DROME (sp|P55828) 40S ribosomal protein S20 OS=D.m.	RS20_DROME	8,00E-73	GLOS_RS20.3.14	GO:0005840	C	.	ribosome
RS20_DROME (sp|P55828) 40S ribosomal protein S20 OS=D.m.	RS20_DROME	8,00E-73	GLOS_RS20.3.14	GO:0015935	C	UniProtKB	small ribosomal subunit
RS20_DROME (sp|P55828) 40S ribosomal protein S20 OS=D.m.	RS20_DROME	8,00E-73	GLOS_RS20.3.14	GO:0003723	M	InterPro	RNA binding
RS20_DROME (sp|P55828) 40S ribosomal protein S20 OS=D.m.	RS20_DROME	8,00E-73	GLOS_RS20.3.14	GO:0003735	M	UniProtKB	structural constituent of ribosome
RS20_DROME (sp|P55828) 40S ribosomal protein S20 OS=D.m.	RS20_DROME	8,00E-73	GLOS_RS20.3.14	GO:0006412	B	UniProtKB	translation
RS7_DROYA (sp|P62085) 40S ribosomal prot. S7 OS=D. yakuba	RS7_DROYA	3,00E-90	GLOS_RS7.3.9	GO:0005840	C	UniProtKB-K	ribosome
RS7_DROYA (sp|P62085) 40S ribosomal prot. S7 OS=D. yakuba	RS7_DROYA	3,00E-90	GLOS_RS7.3.9	GO:0003735	M	InterPro	structural constituent of ribosome
RS7_DROYA (sp|P62085) 40S ribosomal prot. S7 OS=D. yakuba	RS7_DROYA	3,00E-90	GLOS_RS7.3.9	GO:0006412	B	InterPro	translation
RSSA_DROWI (sp|B4NPT0) 40S ribosomal prot. SA OS=D. willistoni	RSSA_DROWI	5,00E-93	GLOS_RSSA.2.8	GO:0015935	C	.	small ribosomal subunit
RSSA_DROWI (sp|B4NPT0) 40S ribosomal prot. SA OS=D. willistoni	RSSA_DROWI	5,00E-93	GLOS_RSSA.2.8	GO:0044445	C	.	cytosolic part
RSSA_DROWI (sp|B4NPT0) 40S ribosomal prot. SA OS=D. willistoni	RSSA_DROWI	5,00E-93	GLOS_RSSA.2.8	GO:0005829	C	.	cytosol
RSSA_DROWI (sp|B4NPT0) 40S ribosomal prot. SA OS=D. willistoni	RSSA_DROWI	5,00E-93	GLOS_RSSA.2.8	GO:0022626	C	.	cytosolic ribosome
RSSA_DROWI (sp|B4NPT0) 40S ribosomal prot. SA OS=D. willistoni	RSSA_DROWI	5,00E-93	GLOS_RSSA.2.8	GO:0022627	C	UniProtKB-H	cytosolic small ribosomal subunit
RSSA_DROWI (sp|B4NPT0) 40S ribosomal prot. SA OS=D. willistoni	RSSA_DROWI	5,00E-93	GLOS_RSSA.2.8	GO:0005634	C	UniProtKB-S	nucleus
RSSA_DROWI (sp|B4NPT0) 40S ribosomal prot. SA OS=D. willistoni	RSSA_DROWI	5,00E-93	GLOS_RSSA.2.8	GO:0003735	M	UniProtKB-H	structural constituent of ribosome
RSSA_DROWI (sp|B4NPT0) 40S ribosomal prot. SA OS=D. willistoni	RSSA_DROWI	5,00E-93	GLOS_RSSA.2.8	GO:0007275	B	UniProtKB-K	multicellular organismal development
RSSA_DROWI (sp|B4NPT0) 40S ribosomal prot. SA OS=D. willistoni	RSSA_DROWI	5,00E-93	GLOS_RSSA.2.8	GO:0042255	B	.	ribosome assembly
RSSA_DROWI (sp|B4NPT0) 40S ribosomal prot. SA OS=D. willistoni	RSSA_DROWI	5,00E-93	GLOS_RSSA.2.8	GO:0070925	B	.	organelle assembly
RSSA_DROWI (sp|B4NPT0) 40S ribosomal prot. SA OS=D. willistoni	RSSA_DROWI	5,00E-93	GLOS_RSSA.2.8	GO:0042254	B	.	ribosome biogenesis
RSSA_DROWI (sp|B4NPT0) 40S ribosomal prot. SA OS=D. willistoni	RSSA_DROWI	5,00E-93	GLOS_RSSA.2.8	GO:0042274	B	.	ribosomal small subunit biogenesis
RSSA_DROWI (sp|B4NPT0) 40S ribosomal prot. SA OS=D. willistoni	RSSA_DROWI	5,00E-93	GLOS_RSSA.2.8	GO:0000028	B	UniProtKB-H	ribosomal small subunit assembly
RSSA_DROWI (sp|B4NPT0) 40S ribosomal prot. SA OS=D. willistoni	RSSA_DROWI	5,00E-93	GLOS_RSSA.2.8	GO:0006412	B	UniProtKB-H	translation
SAHH2_DROME (sp|P50245) Putative adenosylhomocysteinase 2 OS=D.m.	SAHH2_DROME	0	GLOS_SAHH2.1.1	GO:0016802	M	.	trialkylsulfonium hydrolase activity
SAHH2_DROME (sp|P50245) Putative adenosylhomocysteinase 2 OS=D.m.	SAHH2_DROME	0	GLOS_SAHH2.1.1	GO:0016801	M	.	hydrolase activity, acting on ether bonds
SAHH2_DROME (sp|P50245) Putative adenosylhomocysteinase 2 OS=D.m.	SAHH2_DROME	0	GLOS_SAHH2.1.1	GO:0004013	M	UniProtKB-E	adenosylhomocysteinase activity
SAHH2_DROME (sp|P50245) Putative adenosylhomocysteinase 2 OS=D.m.	SAHH2_DROME	0	GLOS_SAHH2.1.1	GO:0006730	B	UniProtKB-K	one-carbon metabolic process
[BBH] SCC4_DROVI (sp|B4M4L4) MAU2 chromatid cohesion factor homolog	SCC4_DROVI	0	GLOS_SCC4.2.2	GO:0044427	C	.	chromosomal part
[BBH] SCC4_DROVI (sp|B4M4L4) MAU2 chromatid cohesion factor homolog	SCC4_DROVI	0	GLOS_SCC4.2.2	GO:0005694	C	.	chromosome
[BBH] SCC4_DROVI (sp|B4M4L4) MAU2 chromatid cohesion factor homolog	SCC4_DROVI	0	GLOS_SCC4.2.2	GO:0000785	C	UniProtKB	chromatin
[BBH] SCC4_DROVI (sp|B4M4L4) MAU2 chromatid cohesion factor homolog	SCC4_DROVI	0	GLOS_SCC4.2.2	GO:0005654	C	UniProtKB	nucleoplasm
[BBH] SCC4_DROVI (sp|B4M4L4) MAU2 chromatid cohesion factor homolog	SCC4_DROVI	0	GLOS_SCC4.2.2	GO:0032116	C	UniProtKB	SMC loading complex
[BBH] SCC4_DROVI (sp|B4M4L4) MAU2 chromatid cohesion factor homolog	SCC4_DROVI	0	GLOS_SCC4.2.2	GO:0051301	B	UniProtKB-K	cell division
[BBH] SCC4_DROVI (sp|B4M4L4) MAU2 chromatid cohesion factor homolog	SCC4_DROVI	0	GLOS_SCC4.2.2	GO:0034086	B	.	maintenance of sister chromatid cohesion
[BBH] SCC4_DROVI (sp|B4M4L4) MAU2 chromatid cohesion factor homolog	SCC4_DROVI	0	GLOS_SCC4.2.2	GO:0007062	B	.	sister chromatid cohesion
[BBH] SCC4_DROVI (sp|B4M4L4) MAU2 chromatid cohesion factor homolog	SCC4_DROVI	0	GLOS_SCC4.2.2	GO:0051276	B	.	chromosome organization
[BBH] SCC4_DROVI (sp|B4M4L4) MAU2 chromatid cohesion factor homolog	SCC4_DROVI	0	GLOS_SCC4.2.2	GO:0007059	B	.	chromosome segregation
[BBH] SCC4_DROVI (sp|B4M4L4) MAU2 chromatid cohesion factor homolog	SCC4_DROVI	0	GLOS_SCC4.2.2	GO:0007064	B	.	mitotic sister chromatid cohesion
[BBH] SCC4_DROVI (sp|B4M4L4) MAU2 chromatid cohesion factor homolog	SCC4_DROVI	0	GLOS_SCC4.2.2	GO:0000070	B	.	mitotic sister chromatid segregation
[BBH] SCC4_DROVI (sp|B4M4L4) MAU2 chromatid cohesion factor homolog	SCC4_DROVI	0	GLOS_SCC4.2.2	GO:0000819	B	.	sister chromatid segregation
[BBH] SCC4_DROVI (sp|B4M4L4) MAU2 chromatid cohesion factor homolog	SCC4_DROVI	0	GLOS_SCC4.2.2	GO:0007067	B	.	mitosis
[BBH] SCC4_DROVI (sp|B4M4L4) MAU2 chromatid cohesion factor homolog	SCC4_DROVI	0	GLOS_SCC4.2.2	GO:0000280	B	.	nuclear division
[BBH] SCC4_DROVI (sp|B4M4L4) MAU2 chromatid cohesion factor homolog	SCC4_DROVI	0	GLOS_SCC4.2.2	GO:0048285	B	.	organelle fission
[BBH] SCC4_DROVI (sp|B4M4L4) MAU2 chromatid cohesion factor homolog	SCC4_DROVI	0	GLOS_SCC4.2.2	GO:0034088	B	UniProtKB	maintenance of mitotic sister chromatid cohesion
[BBH] SDA1_DROME (sp|Q7KKH3) Protein SDA1 homolog OS=D.m.	SDA1_DROME	0	GLOS_SDA1.1.1	GO:0005730	C	UniProtKB	nucleolus
[BBH] SDA1_DROME (sp|Q7KKH3) Protein SDA1 homolog OS=D.m.	SDA1_DROME	0	GLOS_SDA1.1.1	GO:0030036	B	InterPro	actin cytoskeleton organization
[BBH] SDA1_DROME (sp|Q7KKH3) Protein SDA1 homolog OS=D.m.	SDA1_DROME	0	GLOS_SDA1.1.1	GO:0022008	B	FlyBase	neurogenesis
[BBH] SDA1_DROME (sp|Q7KKH3) Protein SDA1 homolog OS=D.m.	SDA1_DROME	0	GLOS_SDA1.1.1	GO:0015031	B	UniProtKB-K	protein transport
[BBH] SDA1_DROME (sp|Q7KKH3) Protein SDA1 homolog OS=D.m.	SDA1_DROME	0	GLOS_SDA1.1.1	GO:0042273	B	InterPro	ribosomal large subunit biogenesis
[BBH] SDA1_DROME (sp|Q7KKH3) Protein SDA1 homolog OS=D.m.	SDA1_DROME	0	GLOS_SDA1.1.1	GO:0000054	B	.	ribosomal subunit export from nucleus

[BBH] SDA1_DROME (sp|Q7KKH3) Protein SDA1 homolog OS=D.m.	SDA1_DROME	0	GLOS_SDA1.1.1	GO:0033753	B	.	establishment of ribosome localization
[BBH] SDA1_DROME (sp|Q7KKH3) Protein SDA1 homolog OS=D.m.	SDA1_DROME	0	GLOS_SDA1.1.1	GO:0051656	B	.	establishment of organelle localization
[BBH] SDA1_DROME (sp|Q7KKH3) Protein SDA1 homolog OS=D.m.	SDA1_DROME	0	GLOS_SDA1.1.1	GO:0051640	B	.	organelle localization
[BBH] SDA1_DROME (sp|Q7KKH3) Protein SDA1 homolog OS=D.m.	SDA1_DROME	0	GLOS_SDA1.1.1	GO:0033750	B	.	ribosome localization
[BBH] SDA1_DROME (sp|Q7KKH3) Protein SDA1 homolog OS=D.m.	SDA1_DROME	0	GLOS_SDA1.1.1	GO:0071428	B	.	rRNA-containing ribonucleoprotein complex export from nucleus
[BBH] SDA1_DROME (sp|Q7KKH3) Protein SDA1 homolog OS=D.m.	SDA1_DROME	0	GLOS_SDA1.1.1	GO:0071426	B	.	ribonucleoprotein complex export from nucleus
[BBH] SDA1_DROME (sp|Q7KKH3) Protein SDA1 homolog OS=D.m.	SDA1_DROME	0	GLOS_SDA1.1.1	GO:0071166	B	.	ribonucleoprotein complex localization
[BBH] SDA1_DROME (sp|Q7KKH3) Protein SDA1 homolog OS=D.m.	SDA1_DROME	0	GLOS_SDA1.1.1	GO:0000055	B	InterPro	ribosomal large subunit export from nucleus
[BBH] SIMA_DROME (sp|Q24167) Protein similar OS=D.m.	SIMA_DROME	1,00E-130	GLOS_SIMA.1.1	GO:0005737	C	FlyBase	cytoplasm
[BBH] SIMA_DROME (sp|Q24167) Protein similar OS=D.m.	SIMA_DROME	1,00E-130	GLOS_SIMA.1.1	GO:0005634	C	FlyBase	nucleus
[BBH] SIMA_DROME (sp|Q24167) Protein similar OS=D.m.	SIMA_DROME	1,00E-130	GLOS_SIMA.1.1	GO:0003677	M	UniProtKB-K	DNA binding
[BBH] SIMA_DROME (sp|Q24167) Protein similar OS=D.m.	SIMA_DROME	1,00E-130	GLOS_SIMA.1.1	GO:0004871	M	InterPro	signal transducer activity
[BBH] SIMA_DROME (sp|Q24167) Protein similar OS=D.m.	SIMA_DROME	1,00E-130	GLOS_SIMA.1.1	GO:0001666	B	.	response to hypoxia
[BBH] SIMA_DROME (sp|Q24167) Protein similar OS=D.m.	SIMA_DROME	1,00E-130	GLOS_SIMA.1.1	GO:0036294	B	.	cellular response to decreased oxygen levels
[BBH] SIMA_DROME (sp|Q24167) Protein similar OS=D.m.	SIMA_DROME	1,00E-130	GLOS_SIMA.1.1	GO:0071453	B	.	cellular response to oxygen levels
[BBH] SIMA_DROME (sp|Q24167) Protein similar OS=D.m.	SIMA_DROME	1,00E-130	GLOS_SIMA.1.1	GO:0071456	B	FlyBase	cellular response to hypoxia
[BBH] SIMA_DROME (sp|Q24167) Protein similar OS=D.m.	SIMA_DROME	1,00E-130	GLOS_SIMA.1.1	GO:0032868	B	.	response to insulin stimulus
[BBH] SIMA_DROME (sp|Q24167) Protein similar OS=D.m.	SIMA_DROME	1,00E-130	GLOS_SIMA.1.1	GO:0043434	B	.	response to peptide hormone stimulus
[BBH] SIMA_DROME (sp|Q24167) Protein similar OS=D.m.	SIMA_DROME	1,00E-130	GLOS_SIMA.1.1	GO:1901652	B	.	response to peptide
[BBH] SIMA_DROME (sp|Q24167) Protein similar OS=D.m.	SIMA_DROME	1,00E-130	GLOS_SIMA.1.1	GO:0071375	B	.	cellular response to peptide hormone stimulus
[BBH] SIMA_DROME (sp|Q24167) Protein similar OS=D.m.	SIMA_DROME	1,00E-130	GLOS_SIMA.1.1	GO:0032870	B	.	cellular response to hormone stimulus
[BBH] SIMA_DROME (sp|Q24167) Protein similar OS=D.m.	SIMA_DROME	1,00E-130	GLOS_SIMA.1.1	GO:0071310	B	.	cellular response to organic substance
[BBH] SIMA_DROME (sp|Q24167) Protein similar OS=D.m.	SIMA_DROME	1,00E-130	GLOS_SIMA.1.1	GO:0071495	B	.	cellular response to endogenous stimulus
[BBH] SIMA_DROME (sp|Q24167) Protein similar OS=D.m.	SIMA_DROME	1,00E-130	GLOS_SIMA.1.1	GO:1901653	B	.	cellular response to peptide
[BBH] SIMA_DROME (sp|Q24167) Protein similar OS=D.m.	SIMA_DROME	1,00E-130	GLOS_SIMA.1.1	GO:0071417	B	.	cellular response to organonitrogen compound
[BBH] SIMA_DROME (sp|Q24167) Protein similar OS=D.m.	SIMA_DROME	1,00E-130	GLOS_SIMA.1.1	GO:1901699	B	.	cellular response to nitrogen compound
[BBH] SIMA_DROME (sp|Q24167) Protein similar OS=D.m.	SIMA_DROME	1,00E-130	GLOS_SIMA.1.1	GO:1901701	B	.	cellular response to oxygen-containing compound
[BBH] SIMA_DROME (sp|Q24167) Protein similar OS=D.m.	SIMA_DROME	1,00E-130	GLOS_SIMA.1.1	GO:0032869	B	FlyBase	cellular response to insulin stimulus
[BBH] SIMA_DROME (sp|Q24167) Protein similar OS=D.m.	SIMA_DROME	1,00E-130	GLOS_SIMA.1.1	GO:0001558	B	.	regulation of cell growth
[BBH] SIMA_DROME (sp|Q24167) Protein similar OS=D.m.	SIMA_DROME	1,00E-130	GLOS_SIMA.1.1	GO:0040008	B	.	regulation of growth
[BBH] SIMA_DROME (sp|Q24167) Protein similar OS=D.m.	SIMA_DROME	1,00E-130	GLOS_SIMA.1.1	GO:0045926	B	.	negative regulation of growth
[BBH] SIMA_DROME (sp|Q24167) Protein similar OS=D.m.	SIMA_DROME	1,00E-130	GLOS_SIMA.1.1	GO:0030308	B	FlyBase	negative regulation of cell growth
[BBH] SIMA_DROME (sp|Q24167) Protein similar OS=D.m.	SIMA_DROME	1,00E-130	GLOS_SIMA.1.1	GO:0048477	B	FlyBase	oogenesis
[BBH] SIMA_DROME (sp|Q24167) Protein similar OS=D.m.	SIMA_DROME	1,00E-130	GLOS_SIMA.1.1	GO:0045893	B	.	positive regulation of transcription, DNA-dependent
[BBH] SIMA_DROME (sp|Q24167) Protein similar OS=D.m.	SIMA_DROME	1,00E-130	GLOS_SIMA.1.1	GO:0010557	B	.	positive regulation of macromolecule biosynthetic process
[BBH] SIMA_DROME (sp|Q24167) Protein similar OS=D.m.	SIMA_DROME	1,00E-130	GLOS_SIMA.1.1	GO:0009891	B	.	positive regulation of biosynthetic process
[BBH] SIMA_DROME (sp|Q24167) Protein similar OS=D.m.	SIMA_DROME	1,00E-130	GLOS_SIMA.1.1	GO:0009893	B	.	positive regulation of metabolic process
[BBH] SIMA_DROME (sp|Q24167) Protein similar OS=D.m.	SIMA_DROME	1,00E-130	GLOS_SIMA.1.1	GO:0010604	B	.	positive regulation of macromolecule metabolic process
[BBH] SIMA_DROME (sp|Q24167) Protein similar OS=D.m.	SIMA_DROME	1,00E-130	GLOS_SIMA.1.1	GO:0010628	B	.	positive regulation of gene expression
[BBH] SIMA_DROME (sp|Q24167) Protein similar OS=D.m.	SIMA_DROME	1,00E-130	GLOS_SIMA.1.1	GO:0031328	B	.	positive regulation of cellular biosynthetic process
[BBH] SIMA_DROME (sp|Q24167) Protein similar OS=D.m.	SIMA_DROME	1,00E-130	GLOS_SIMA.1.1	GO:0031325	B	.	positive regulation of cellular metabolic process
[BBH] SIMA_DROME (sp|Q24167) Protein similar OS=D.m.	SIMA_DROME	1,00E-130	GLOS_SIMA.1.1	GO:0051254	B	.	positive regulation of RNA metabolic process
[BBH] SIMA_DROME (sp|Q24167) Protein similar OS=D.m.	SIMA_DROME	1,00E-130	GLOS_SIMA.1.1	GO:0045935	B	.	positive regulation of nucleobase-containing compound metabolic process
[BBH] SIMA_DROME (sp|Q24167) Protein similar OS=D.m.	SIMA_DROME	1,00E-130	GLOS_SIMA.1.1	GO:0051173	B	.	positive regulation of nitrogen compound metabolic process
[BBH] SIMA_DROME (sp|Q24167) Protein similar OS=D.m.	SIMA_DROME	1,00E-130	GLOS_SIMA.1.1	GO:0045944	B	FlyBase	positive regulation of transcription from RNA polymerase II promoter
[BBH] SIMA_DROME (sp|Q24167) Protein similar OS=D.m.	SIMA_DROME	1,00E-130	GLOS_SIMA.1.1	GO:0051726	B	FlyBase	regulation of cell cycle
[BBH] SIMA_DROME (sp|Q24167) Protein similar OS=D.m.	SIMA_DROME	1,00E-130	GLOS_SIMA.1.1	GO:0006974	B	FlyBase	cellular response to DNA damage stimulus
[BBH] SIMA_DROME (sp|Q24167) Protein similar OS=D.m.	SIMA_DROME	1,00E-130	GLOS_SIMA.1.1	GO:0060438	B	FlyBase	trachea development
[BBH] SIMA_DROME (sp|Q24167) Protein similar OS=D.m.	SIMA_DROME	1,00E-130	GLOS_SIMA.1.1	GO:0006351	B	UniProtKB-K	transcription, DNA-dependent
SWS_DROME (sp|Q9U969) Neuropathy target esterase sws OS=D.m.	SWS_DROME	0	GLOS_SWS.2.2	GO:0044432	C	.	endoplasmic reticulum part
SWS_DROME (sp|Q9U969) Neuropathy target esterase sws OS=D.m.	SWS_DROME	0	GLOS_SWS.2.2	GO:0005783	C	.	endoplasmic reticulum
SWS_DROME (sp|Q9U969) Neuropathy target esterase sws OS=D.m.	SWS_DROME	0	GLOS_SWS.2.2	GO:0042175	C	.	nuclear outer membrane-endoplasmic reticulum membrane network
SWS_DROME (sp|Q9U969) Neuropathy target esterase sws OS=D.m.	SWS_DROME	0	GLOS_SWS.2.2	GO:0005789	C	UniProtKB	endoplasmic reticulum membrane
SWS_DROME (sp|Q9U969) Neuropathy target esterase sws OS=D.m.	SWS_DROME	0	GLOS_SWS.2.2	GO:0016021	C	UniProtKB-K	integral to membrane

SWS_DROME (sp|Q9U969) Neuropathy target esterase sws OS=D.m.	SWS_DROME	0	GLOS_SWS.2.2	GO:0052689	M	.	carboxylic ester hydrolase activity
SWS_DROME (sp|Q9U969) Neuropathy target esterase sws OS=D.m.	SWS_DROME	0	GLOS_SWS.2.2	GO:0004622	M	UniProtKB	lysophospholipase activity
SWS_DROME (sp|Q9U969) Neuropathy target esterase sws OS=D.m.	SWS_DROME	0	GLOS_SWS.2.2	GO:0016044	B	FlyBase	.
SWS_DROME (sp|Q9U969) Neuropathy target esterase sws OS=D.m.	SWS_DROME	0	GLOS_SWS.2.2	GO:0097285	B	.	cell-type specific apoptotic process
SWS_DROME (sp|Q9U969) Neuropathy target esterase sws OS=D.m.	SWS_DROME	0	GLOS_SWS.2.2	GO:0034349	B	UniProtKB	glial cell apoptotic process
SWS_DROME (sp|Q9U969) Neuropathy target esterase sws OS=D.m.	SWS_DROME	0	GLOS_SWS.2.2	GO:0006643	B	FlyBase	membrane lipid metabolic process
SWS_DROME (sp|Q9U969) Neuropathy target esterase sws OS=D.m.	SWS_DROME	0	GLOS_SWS.2.2	GO:0007399	B	UniProtKB-K	nervous system development
SWS_DROME (sp|Q9U969) Neuropathy target esterase sws OS=D.m.	SWS_DROME	0	GLOS_SWS.2.2	GO:0070997	B	.	neuron death
SWS_DROME (sp|Q9U969) Neuropathy target esterase sws OS=D.m.	SWS_DROME	0	GLOS_SWS.2.2	GO:0051402	B	FlyBase	neuron apoptotic process
SWS_DROME (sp|Q9U969) Neuropathy target esterase sws OS=D.m.	SWS_DROME	0	GLOS_SWS.2.2	GO:0042439	B	.	ethanolamine-containing compound metabolic process
SWS_DROME (sp|Q9U969) Neuropathy target esterase sws OS=D.m.	SWS_DROME	0	GLOS_SWS.2.2	GO:0006066	B	.	alcohol metabolic process
SWS_DROME (sp|Q9U969) Neuropathy target esterase sws OS=D.m.	SWS_DROME	0	GLOS_SWS.2.2	GO:1901615	B	.	organic hydroxy compound metabolic process
SWS_DROME (sp|Q9U969) Neuropathy target esterase sws OS=D.m.	SWS_DROME	0	GLOS_SWS.2.2	GO:0006576	B	.	cellular biogenic amine metabolic process
SWS_DROME (sp|Q9U969) Neuropathy target esterase sws OS=D.m.	SWS_DROME	0	GLOS_SWS.2.2	GO:0044106	B	.	cellular amine metabolic process
SWS_DROME (sp|Q9U969) Neuropathy target esterase sws OS=D.m.	SWS_DROME	0	GLOS_SWS.2.2	GO:0009308	B	.	amine metabolic process
SWS_DROME (sp|Q9U969) Neuropathy target esterase sws OS=D.m.	SWS_DROME	0	GLOS_SWS.2.2	GO:0046470	B	FlyBase	phosphatidylcholine metabolic process
SWS_DROME (sp|Q9U969) Neuropathy target esterase sws OS=D.m.	SWS_DROME	0	GLOS_SWS.2.2	GO:0007608	B	FlyBase	sensory perception of smell
[BBH] TAMO_DROME (sp|Q9W1A4) Protein tamozhennic	TAMO_DROME	1,00E-114	GLOS_TAMO.1.1	GO:0005737	C	UniProtKB	cytoplasm
[BBH] TAMO_DROME (sp|Q9W1A4) Protein tamozhennic	TAMO_DROME	1,00E-114	GLOS_TAMO.1.1	GO:0008270	M	InterPro	zinc ion binding
[BBH] TAMO_DROME (sp|Q9W1A4) Protein tamozhennic	TAMO_DROME	1,00E-114	GLOS_TAMO.1.1	GO:0007275	B	UniProtKB	multicellular organismal development
[BBH] TAMO_DROME (sp|Q9W1A4) Protein tamozhennic	TAMO_DROME	1,00E-114	GLOS_TAMO.1.1	GO:0015031	B	UniProtKB	protein transport
[BBH] TM120_DROME (sp|Q9U1M2) Transmembrane protein 120 homolog .	TM120_DROME	0	GLOS_TM120.1.1	GO:0016021	C	UniProtKB-K	integral to membrane
TRYA4_LUCCU (sp|P35044) Trypsin alpha-4 OS=L. cuprina PE=3 SV=1	TRYA4_LUCCU	2,00E-06	GLOS_TRYA4.1.6	GO:0044421	C	.	extracellular region part
TRYA4_LUCCU (sp|P35044) Trypsin alpha-4 OS=L. cuprina PE=3 SV=1	TRYA4_LUCCU	2,00E-06	GLOS_TRYA4.1.6	GO:0005576	C	.	extracellular region
TRYA4_LUCCU (sp|P35044) Trypsin alpha-4 OS=L. cuprina PE=3 SV=1	TRYA4_LUCCU	2,00E-06	GLOS_TRYA4.1.6	GO:0005615	C	UniProtKB-S	extracellular space
TRYA4_LUCCU (sp|P35044) Trypsin alpha-4 OS=L. cuprina PE=3 SV=1	TRYA4_LUCCU	2,00E-06	GLOS_TRYA4.1.6	GO:0004175	M	.	endopeptidase activity
TRYA4_LUCCU (sp|P35044) Trypsin alpha-4 OS=L. cuprina PE=3 SV=1	TRYA4_LUCCU	2,00E-06	GLOS_TRYA4.1.6	GO:0070011	M	.	peptidase activity, acting on L-amino acid peptides
TRYA4_LUCCU (sp|P35044) Trypsin alpha-4 OS=L. cuprina PE=3 SV=1	TRYA4_LUCCU	2,00E-06	GLOS_TRYA4.1.6	GO:0008233	M	.	peptidase activity
TRYA4_LUCCU (sp|P35044) Trypsin alpha-4 OS=L. cuprina PE=3 SV=1	TRYA4_LUCCU	2,00E-06	GLOS_TRYA4.1.6	GO:0008236	M	.	serine-type peptidase activity
TRYA4_LUCCU (sp|P35044) Trypsin alpha-4 OS=L. cuprina PE=3 SV=1	TRYA4_LUCCU	2,00E-06	GLOS_TRYA4.1.6	GO:0017171	M	.	serine hydrolase activity
TRYA4_LUCCU (sp|P35044) Trypsin alpha-4 OS=L. cuprina PE=3 SV=1	TRYA4_LUCCU	2,00E-06	GLOS_TRYA4.1.6	GO:0004252	M	InterPro	serine-type endopeptidase activity
TRYA4_LUCCU (sp|P35044) Trypsin alpha-4 OS=L. cuprina PE=3 SV=1	TRYA4_LUCCU	2,00E-06	GLOS_TRYA4.1.6	GO:0006508	B	UniProtKB-K	proteolysis
[BBH] TYTR_TRYBB (sp|P39051) Trypanothione reductase OS=T.b.b.	TYTR_TRYBB	0	GLOS_TYTR.1.1	GO:0005737	C	UniProtKB-S	cytoplasm
[BBH] TYTR_TRYBB (sp|P39051) Trypanothione reductase OS=T.b.b.	TYTR_TRYBB	0	GLOS_TYTR.1.1	GO:0016667	M	.	oxidoreductase activity, acting on a sulfur group of donors
[BBH] TYTR_TRYBB (sp|P39051) Trypanothione reductase OS=T.b.b.	TYTR_TRYBB	0	GLOS_TYTR.1.1	GO:0015036	M	InterPro	disulfide oxidoreductase activity
[BBH] TYTR_TRYBB (sp|P39051) Trypanothione reductase OS=T.b.b.	TYTR_TRYBB	0	GLOS_TYTR.1.1	GO:0050662	M	.	coenzyme binding
[BBH] TYTR_TRYBB (sp|P39051) Trypanothione reductase OS=T.b.b.	TYTR_TRYBB	0	GLOS_TYTR.1.1	GO:0050660	M	InterPro	flavin adenine dinucleotide binding
[BBH] TYTR_TRYBB (sp|P39051) Trypanothione reductase OS=T.b.b.	TYTR_TRYBB	0	GLOS_TYTR.1.1	GO:0016668	M	.	oxidoreductase activity, acting on a sulfur group of donors, NAD(P) as acceptor
[BBH] TYTR_TRYBB (sp|P39051) Trypanothione reductase OS=T.b.b.	TYTR_TRYBB	0	GLOS_TYTR.1.1	GO:0016651	M	.	oxidoreductase activity, acting on NAD(P)H
[BBH] TYTR_TRYBB (sp|P39051) Trypanothione reductase OS=T.b.b.	TYTR_TRYBB	0	GLOS_TYTR.1.1	GO:0015042	M	UniProtKB-E	trypanothione-disulfide reductase activity
[BBH] TYTR_TRYBB (sp|P39051) Trypanothione reductase OS=T.b.b.	TYTR_TRYBB	0	GLOS_TYTR.1.1	GO:0045454	B	InterPro	cell redox homeostasis
[BBH] Y9705_DROME (sp|Q9VVA0) Cold shock domain-containing protein	Y9705_DROME	4,00E-55	GLOS_Y9705.1.1	GO:0003677	M	InterPro	DNA binding
[BBH] Y9705_DROME (sp|Q9VVA0) Cold shock domain-containing protein	Y9705_DROME	4,00E-55	GLOS_Y9705.1.1	GO:0006355	B	InterPro	regulation of transcription, DNA-dependent
ZNF41_HUMAN (sp|P51814) Zinc finger protein 41 OS=H. sapiens	ZNF41_HUMAN	2,00E-39	GLOS_ZNF41.1.1	GO:0005634	C	UniProtKB	nucleus
ZNF41_HUMAN (sp|P51814) Zinc finger protein 41 OS=H. sapiens	ZNF41_HUMAN	2,00E-39	GLOS_ZNF41.1.1	GO:0003677	M	UniProtKB	DNA binding
ZNF41_HUMAN (sp|P51814) Zinc finger protein 41 OS=H. sapiens	ZNF41_HUMAN	2,00E-39	GLOS_ZNF41.1.1	GO:0046872	M	UniProtKB-K	metal ion binding
ZNF41_HUMAN (sp|P51814) Zinc finger protein 41 OS=H. sapiens	ZNF41_HUMAN	2,00E-39	GLOS_ZNF41.1.1	GO:0003700	M	UniProtKB	sequence-specific DNA binding transcription factor activity
ZNF41_HUMAN (sp|P51814) Zinc finger protein 41 OS=H. sapiens	ZNF41_HUMAN	2,00E-39	GLOS_ZNF41.1.1	GO:0006351	B	UniProtKB-K	transcription, DNA-dependent
XP_001986435.1 GH20533 [Drosophila grimshawi]	XP_001986435.1	0	GLOS_DGRI_GH2	GO:0005261	M	.	cation channel activity
XP_001986435.1 GH20533 [Drosophila grimshawi]	XP_001986435.1	0	GLOS_DGRI_GH2	GO:0005216	M	.	ion channel activity
XP_001986435.1 GH20533 [Drosophila grimshawi]	XP_001986435.1	0	GLOS_DGRI_GH2	GO:0015075	M	.	ion transmembrane transporter activity
XP_001986435.1 GH20533 [Drosophila grimshawi]	XP_001986435.1	0	GLOS_DGRI_GH2	GO:0022891	M	.	substrate-specific transmembrane transporter activity
XP_001986435.1 GH20533 [Drosophila grimshawi]	XP_001986435.1	0	GLOS_DGRI_GH2	GO:0022857	M	.	transmembrane transporter activity
XP_001986435.1 GH20533 [Drosophila grimshawi]	XP_001986435.1	0	GLOS_DGRI_GH2	GO:0005215	M	.	transporter activity

XP_001986435.1 GH20533 [Drosophila grimshawi]	XP_001986435.1	0	GLOS_DGRI_GH2	GO:0003674	M	.	molecular_function
XP_001986435.1 GH20533 [Drosophila grimshawi]	XP_001986435.1	0	GLOS_DGRI_GH2	GO:0022892	M	.	substrate-specific transporter activity
XP_001986435.1 GH20533 [Drosophila grimshawi]	XP_001986435.1	0	GLOS_DGRI_GH2	GO:0022838	M	.	substrate-specific channel activity
XP_001986435.1 GH20533 [Drosophila grimshawi]	XP_001986435.1	0	GLOS_DGRI_GH2	GO:0015267	M	.	channel activity
XP_001986435.1 GH20533 [Drosophila grimshawi]	XP_001986435.1	0	GLOS_DGRI_GH2	GO:0022803	M	.	passive transmembrane transporter activity
XP_001986435.1 GH20533 [Drosophila grimshawi]	XP_001986435.1	0	GLOS_DGRI_GH2	GO:0008324	M	.	cation transmembrane transporter activity
XP_001986435.1 GH20533 [Drosophila grimshawi]	XP_001986435.1	0	GLOS_DGRI_GH2	GO:0015085	M	.	calcium ion transmembrane transporter activity
XP_001986435.1 GH20533 [Drosophila grimshawi]	XP_001986435.1	0	GLOS_DGRI_GH2	GO:0046873	M	.	metal ion transmembrane transporter activity
XP_001986435.1 GH20533 [Drosophila grimshawi]	XP_001986435.1	0	GLOS_DGRI_GH2	GO:0022890	M	.	inorganic cation transmembrane transporter activity
XP_001986435.1 GH20533 [Drosophila grimshawi]	XP_001986435.1	0	GLOS_DGRI_GH2	GO:0072509	M	.	divalent inorganic cation transmembrane transporter activity
XP_001986435.1 GH20533 [Drosophila grimshawi]	XP_001986435.1	0	GLOS_DGRI_GH2	GO:0005262	M	Refseq	calcium channel activity
XP_001986435.1 GH20533 [Drosophila grimshawi]	XP_001986435.1	0	GLOS_DGRI_GH2	GO:0005488	M	.	binding
XP_001986435.1 GH20533 [Drosophila grimshawi]	XP_001986435.1	0	GLOS_DGRI_GH2	GO:0005515	M	Refseq	protein binding
XP_001986435.1 GH20533 [Drosophila grimshawi]	XP_001986435.1	0	GLOS_DGRI_GH2	GO:0016020	C	.	membrane
XP_001986435.1 GH20533 [Drosophila grimshawi]	XP_001986435.1	0	GLOS_DGRI_GH2	GO:0005575	C	.	cellular_component
XP_001986435.1 GH20533 [Drosophila grimshawi]	XP_001986435.1	0	GLOS_DGRI_GH2	GO:0044464	C	.	cell part
XP_001986435.1 GH20533 [Drosophila grimshawi]	XP_001986435.1	0	GLOS_DGRI_GH2	GO:0005623	C	.	cell
XP_001986435.1 GH20533 [Drosophila grimshawi]	XP_001986435.1	0	GLOS_DGRI_GH2	GO:0071944	C	.	cell periphery
XP_001986435.1 GH20533 [Drosophila grimshawi]	XP_001986435.1	0	GLOS_DGRI_GH2	GO:0005886	C	Refseq	plasma membrane
XP_001986435.1 GH20533 [Drosophila grimshawi]	XP_001986435.1	0	GLOS_DGRI_GH2	GO:0016021	C	.	integral to membrane
XP_001986435.1 GH20533 [Drosophila grimshawi]	XP_001986435.1	0	GLOS_DGRI_GH2	GO:0031224	C	.	intrinsic to membrane
XP_001986435.1 GH20533 [Drosophila grimshawi]	XP_001986435.1	0	GLOS_DGRI_GH2	GO:0044425	C	.	membrane part
XP_001986435.1 GH20533 [Drosophila grimshawi]	XP_001986435.1	0	GLOS_DGRI_GH2	GO:0031226	C	.	intrinsic to plasma membrane
XP_001986435.1 GH20533 [Drosophila grimshawi]	XP_001986435.1	0	GLOS_DGRI_GH2	GO:0044459	C	.	plasma membrane part
XP_001986435.1 GH20533 [Drosophila grimshawi]	XP_001986435.1	0	GLOS_DGRI_GH2	GO:0005886	C	.	plasma membrane
XP_001986435.1 GH20533 [Drosophila grimshawi]	XP_001986435.1	0	GLOS_DGRI_GH2	GO:0005887	C	Refseq	integral to plasma membrane
XP_001986435.1 GH20533 [Drosophila grimshawi]	XP_001986435.1	0	GLOS_DGRI_GH2	GO:0070838	B	.	divalent metal ion transport
XP_001986435.1 GH20533 [Drosophila grimshawi]	XP_001986435.1	0	GLOS_DGRI_GH2	GO:0030001	B	.	metal ion transport
XP_001986435.1 GH20533 [Drosophila grimshawi]	XP_001986435.1	0	GLOS_DGRI_GH2	GO:0006812	B	.	cation transport
XP_001986435.1 GH20533 [Drosophila grimshawi]	XP_001986435.1	0	GLOS_DGRI_GH2	GO:0006811	B	.	ion transport
XP_001986435.1 GH20533 [Drosophila grimshawi]	XP_001986435.1	0	GLOS_DGRI_GH2	GO:0044765	B	.	single-organism transport
XP_001986435.1 GH20533 [Drosophila grimshawi]	XP_001986435.1	0	GLOS_DGRI_GH2	GO:0006810	B	.	transport
XP_001986435.1 GH20533 [Drosophila grimshawi]	XP_001986435.1	0	GLOS_DGRI_GH2	GO:0051234	B	.	establishment of localization
XP_001986435.1 GH20533 [Drosophila grimshawi]	XP_001986435.1	0	GLOS_DGRI_GH2	GO:0008150	B	.	biological_process
XP_001986435.1 GH20533 [Drosophila grimshawi]	XP_001986435.1	0	GLOS_DGRI_GH2	GO:0051179	B	.	localization
XP_001986435.1 GH20533 [Drosophila grimshawi]	XP_001986435.1	0	GLOS_DGRI_GH2	GO:0044699	B	.	single-organism process
XP_001986435.1 GH20533 [Drosophila grimshawi]	XP_001986435.1	0	GLOS_DGRI_GH2	GO:0072511	B	.	divalent inorganic cation transport
XP_001986435.1 GH20533 [Drosophila grimshawi]	XP_001986435.1	0	GLOS_DGRI_GH2	GO:0006816	B	Refseq	calcium ion transport
XP_001986435.1 GH20533 [Drosophila grimshawi]	XP_001986435.1	0	GLOS_DGRI_GH2	GO:0097485	B	.	neuron projection guidance
XP_001986435.1 GH20533 [Drosophila grimshawi]	XP_001986435.1	0	GLOS_DGRI_GH2	GO:0006928	B	.	cellular component movement
XP_001986435.1 GH20533 [Drosophila grimshawi]	XP_001986435.1	0	GLOS_DGRI_GH2	GO:0044763	B	.	single-organism cellular process
XP_001986435.1 GH20533 [Drosophila grimshawi]	XP_001986435.1	0	GLOS_DGRI_GH2	GO:0009987	B	.	cellular process
XP_001986435.1 GH20533 [Drosophila grimshawi]	XP_001986435.1	0	GLOS_DGRI_GH2	GO:0006935	B	.	chemotaxis
XP_001986435.1 GH20533 [Drosophila grimshawi]	XP_001986435.1	0	GLOS_DGRI_GH2	GO:0042221	B	.	response to chemical stimulus
XP_001986435.1 GH20533 [Drosophila grimshawi]	XP_001986435.1	0	GLOS_DGRI_GH2	GO:0050896	B	.	response to stimulus
XP_001986435.1 GH20533 [Drosophila grimshawi]	XP_001986435.1	0	GLOS_DGRI_GH2	GO:0042330	B	.	taxis
XP_001986435.1 GH20533 [Drosophila grimshawi]	XP_001986435.1	0	GLOS_DGRI_GH2	GO:0009605	B	.	response to external stimulus
XP_001986435.1 GH20533 [Drosophila grimshawi]	XP_001986435.1	0	GLOS_DGRI_GH2	GO:0040011	B	.	locomotion
XP_001986435.1 GH20533 [Drosophila grimshawi]	XP_001986435.1	0	GLOS_DGRI_GH2	GO:0048812	B	.	neuron projection morphogenesis
XP_001986435.1 GH20533 [Drosophila grimshawi]	XP_001986435.1	0	GLOS_DGRI_GH2	GO:0048858	B	.	cell projection morphogenesis
XP_001986435.1 GH20533 [Drosophila grimshawi]	XP_001986435.1	0	GLOS_DGRI_GH2	GO:0030030	B	.	cell projection organization
XP_001986435.1 GH20533 [Drosophila grimshawi]	XP_001986435.1	0	GLOS_DGRI_GH2	GO:0016043	B	.	cellular component organization
XP_001986435.1 GH20533 [Drosophila grimshawi]	XP_001986435.1	0	GLOS_DGRI_GH2	GO:0071840	B	.	cellular component organization or biogenesis
XP_001986435.1 GH20533 [Drosophila grimshawi]	XP_001986435.1	0	GLOS_DGRI_GH2	GO:0032990	B	.	cell part morphogenesis

XP_001986435.1 GH20533 [Drosophila grimshawi]	XP_001986435.1	0	GLOS_DGRI_GH2	GO:0032989	B	.	cellular component morphogenesis
XP_001986435.1 GH20533 [Drosophila grimshawi]	XP_001986435.1	0	GLOS_DGRI_GH2	GO:0009653	B	.	anatomical structure morphogenesis
XP_001986435.1 GH20533 [Drosophila grimshawi]	XP_001986435.1	0	GLOS_DGRI_GH2	GO:0032502	B	.	developmental process
XP_001986435.1 GH20533 [Drosophila grimshawi]	XP_001986435.1	0	GLOS_DGRI_GH2	GO:0048856	B	.	anatomical structure development
XP_001986435.1 GH20533 [Drosophila grimshawi]	XP_001986435.1	0	GLOS_DGRI_GH2	GO:0044767	B	.	single-organism developmental process
XP_001986435.1 GH20533 [Drosophila grimshawi]	XP_001986435.1	0	GLOS_DGRI_GH2	GO:0048869	B	.	cellular developmental process
XP_001986435.1 GH20533 [Drosophila grimshawi]	XP_001986435.1	0	GLOS_DGRI_GH2	GO:0000902	B	.	cell morphogenesis
XP_001986435.1 GH20533 [Drosophila grimshawi]	XP_001986435.1	0	GLOS_DGRI_GH2	GO:0031175	B	.	neuron projection development
XP_001986435.1 GH20533 [Drosophila grimshawi]	XP_001986435.1	0	GLOS_DGRI_GH2	GO:0048666	B	.	neuron development
XP_001986435.1 GH20533 [Drosophila grimshawi]	XP_001986435.1	0	GLOS_DGRI_GH2	GO:0048468	B	.	cell development
XP_001986435.1 GH20533 [Drosophila grimshawi]	XP_001986435.1	0	GLOS_DGRI_GH2	GO:0030154	B	.	cell differentiation
XP_001986435.1 GH20533 [Drosophila grimshawi]	XP_001986435.1	0	GLOS_DGRI_GH2	GO:0030182	B	.	neuron differentiation
XP_001986435.1 GH20533 [Drosophila grimshawi]	XP_001986435.1	0	GLOS_DGRI_GH2	GO:0048699	B	.	generation of neurons
XP_001986435.1 GH20533 [Drosophila grimshawi]	XP_001986435.1	0	GLOS_DGRI_GH2	GO:0022008	B	.	neurogenesis
XP_001986435.1 GH20533 [Drosophila grimshawi]	XP_001986435.1	0	GLOS_DGRI_GH2	GO:0007399	B	.	nervous system development
XP_001986435.1 GH20533 [Drosophila grimshawi]	XP_001986435.1	0	GLOS_DGRI_GH2	GO:0048731	B	.	system development
XP_001986435.1 GH20533 [Drosophila grimshawi]	XP_001986435.1	0	GLOS_DGRI_GH2	GO:0007275	B	.	multicellular organismal development
XP_001986435.1 GH20533 [Drosophila grimshawi]	XP_001986435.1	0	GLOS_DGRI_GH2	GO:0044707	B	.	single-multicellular organism process
XP_001986435.1 GH20533 [Drosophila grimshawi]	XP_001986435.1	0	GLOS_DGRI_GH2	GO:0032501	B	.	multicellular organismal process
XP_001986435.1 GH20533 [Drosophila grimshawi]	XP_001986435.1	0	GLOS_DGRI_GH2	GO:0007409	B	.	axonogenesis
XP_001986435.1 GH20533 [Drosophila grimshawi]	XP_001986435.1	0	GLOS_DGRI_GH2	GO:0048667	B	.	cell morphogenesis involved in neuron differentiation
XP_001986435.1 GH20533 [Drosophila grimshawi]	XP_001986435.1	0	GLOS_DGRI_GH2	GO:0000904	B	.	cell morphogenesis involved in differentiation
XP_001986435.1 GH20533 [Drosophila grimshawi]	XP_001986435.1	0	GLOS_DGRI_GH2	GO:0061564	B	.	axon development
XP_001986435.1 GH20533 [Drosophila grimshawi]	XP_001986435.1	0	GLOS_DGRI_GH2	GO:0007411	B	Refseq	axon guidance
XP_001986435.1 GH20533 [Drosophila grimshawi]	XP_001986435.1	0	GLOS_DGRI_GH2	GO:0007599	B	.	hemostasis
XP_001986435.1 GH20533 [Drosophila grimshawi]	XP_001986435.1	0	GLOS_DGRI_GH2	GO:0050878	B	.	regulation of body fluid levels
XP_001986435.1 GH20533 [Drosophila grimshawi]	XP_001986435.1	0	GLOS_DGRI_GH2	GO:0065008	B	.	regulation of biological quality
XP_001986435.1 GH20533 [Drosophila grimshawi]	XP_001986435.1	0	GLOS_DGRI_GH2	GO:0065007	B	.	biological regulation
XP_001986435.1 GH20533 [Drosophila grimshawi]	XP_001986435.1	0	GLOS_DGRI_GH2	GO:0050817	B	.	coagulation
XP_001986435.1 GH20533 [Drosophila grimshawi]	XP_001986435.1	0	GLOS_DGRI_GH2	GO:0042060	B	.	wound healing
XP_001986435.1 GH20533 [Drosophila grimshawi]	XP_001986435.1	0	GLOS_DGRI_GH2	GO:0009611	B	.	response to wounding
XP_001986435.1 GH20533 [Drosophila grimshawi]	XP_001986435.1	0	GLOS_DGRI_GH2	GO:0006950	B	.	response to stress
XP_001986435.1 GH20533 [Drosophila grimshawi]	XP_001986435.1	0	GLOS_DGRI_GH2	GO:0007596	B	Refseq	blood coagulation
XP_001986435.1 GH20533 [Drosophila grimshawi]	XP_001986435.1	0	GLOS_DGRI_GH2	GO:0007165	B	.	signal transduction
XP_001986435.1 GH20533 [Drosophila grimshawi]	XP_001986435.1	0	GLOS_DGRI_GH2	GO:0050794	B	.	regulation of cellular process
XP_001986435.1 GH20533 [Drosophila grimshawi]	XP_001986435.1	0	GLOS_DGRI_GH2	GO:0050789	B	.	regulation of biological process
XP_001986435.1 GH20533 [Drosophila grimshawi]	XP_001986435.1	0	GLOS_DGRI_GH2	GO:0051716	B	.	cellular response to stimulus
XP_001986435.1 GH20533 [Drosophila grimshawi]	XP_001986435.1	0	GLOS_DGRI_GH2	GO:0007154	B	.	cell communication
XP_001986435.1 GH20533 [Drosophila grimshawi]	XP_001986435.1	0	GLOS_DGRI_GH2	GO:0044700	B	.	single organism signaling
XP_001986435.1 GH20533 [Drosophila grimshawi]	XP_001986435.1	0	GLOS_DGRI_GH2	GO:0023052	B	.	signaling
XP_001986435.1 GH20533 [Drosophila grimshawi]	XP_001986435.1	0	GLOS_DGRI_GH2	GO:0009583	B	.	detection of light stimulus
XP_001986435.1 GH20533 [Drosophila grimshawi]	XP_001986435.1	0	GLOS_DGRI_GH2	GO:0009416	B	.	response to light stimulus
XP_001986435.1 GH20533 [Drosophila grimshawi]	XP_001986435.1	0	GLOS_DGRI_GH2	GO:0009314	B	.	response to radiation
XP_001986435.1 GH20533 [Drosophila grimshawi]	XP_001986435.1	0	GLOS_DGRI_GH2	GO:0009628	B	.	response to abiotic stimulus
XP_001986435.1 GH20533 [Drosophila grimshawi]	XP_001986435.1	0	GLOS_DGRI_GH2	GO:0009581	B	.	detection of external stimulus
XP_001986435.1 GH20533 [Drosophila grimshawi]	XP_001986435.1	0	GLOS_DGRI_GH2	GO:0051606	B	.	detection of stimulus
XP_001986435.1 GH20533 [Drosophila grimshawi]	XP_001986435.1	0	GLOS_DGRI_GH2	GO:0009582	B	.	detection of abiotic stimulus
XP_001986435.1 GH20533 [Drosophila grimshawi]	XP_001986435.1	0	GLOS_DGRI_GH2	GO:0007602	B	Refseq	phototransduction
XP_001986435.1 GH20533 [Drosophila grimshawi]	XP_001986435.1	0	GLOS_DGRI_GH2	GO:0010522	B	.	regulation of calcium ion transport into cytosol
XP_001986435.1 GH20533 [Drosophila grimshawi]	XP_001986435.1	0	GLOS_DGRI_GH2	GO:0032386	B	.	regulation of intracellular transport
XP_001986435.1 GH20533 [Drosophila grimshawi]	XP_001986435.1	0	GLOS_DGRI_GH2	GO:0051049	B	.	regulation of transport
XP_001986435.1 GH20533 [Drosophila grimshawi]	XP_001986435.1	0	GLOS_DGRI_GH2	GO:0032879	B	.	regulation of localization
XP_001986435.1 GH20533 [Drosophila grimshawi]	XP_001986435.1	0	GLOS_DGRI_GH2	GO:0060341	B	.	regulation of cellular localization
XP_001986435.1 GH20533 [Drosophila grimshawi]	XP_001986435.1	0	GLOS_DGRI_GH2	GO:0051924	B	.	regulation of calcium ion transport

XP_001986435.1 GH20533 [Drosophila grimshawi]	XP_001986435.1	0	GLOS_DGRI_GH2	GO:0010959	B	.	regulation of metal ion transport
XP_001986435.1 GH20533 [Drosophila grimshawi]	XP_001986435.1	0	GLOS_DGRI_GH2	GO:0043269	B	.	regulation of ion transport
XP_001986435.1 GH20533 [Drosophila grimshawi]	XP_001986435.1	0	GLOS_DGRI_GH2	GO:2000021	B	.	regulation of ion homeostasis
XP_001986435.1 GH20533 [Drosophila grimshawi]	XP_001986435.1	0	GLOS_DGRI_GH2	GO:0032844	B	.	regulation of homeostatic process
XP_001986435.1 GH20533 [Drosophila grimshawi]	XP_001986435.1	0	GLOS_DGRI_GH2	GO:0032388	B	.	positive regulation of intracellular transport
XP_001986435.1 GH20533 [Drosophila grimshawi]	XP_001986435.1	0	GLOS_DGRI_GH2	GO:0051050	B	.	positive regulation of transport
XP_001986435.1 GH20533 [Drosophila grimshawi]	XP_001986435.1	0	GLOS_DGRI_GH2	GO:0048518	B	.	positive regulation of biological process
XP_001986435.1 GH20533 [Drosophila grimshawi]	XP_001986435.1	0	GLOS_DGRI_GH2	GO:0032846	B	.	positive regulation of homeostatic process
XP_001986435.1 GH20533 [Drosophila grimshawi]	XP_001986435.1	0	GLOS_DGRI_GH2	GO:0048522	B	.	positive regulation of cellular process
XP_001986435.1 GH20533 [Drosophila grimshawi]	XP_001986435.1	0	GLOS_DGRI_GH2	GO:0051928	B	.	positive regulation of calcium ion transport
XP_001986435.1 GH20533 [Drosophila grimshawi]	XP_001986435.1	0	GLOS_DGRI_GH2	GO:0043270	B	.	positive regulation of ion transport
XP_001986435.1 GH20533 [Drosophila grimshawi]	XP_001986435.1	0	GLOS_DGRI_GH2	GO:0010524	B	Refseq	positive regulation of calcium ion transport into cytosol
XP_001986435.1 GH20533 [Drosophila grimshawi]	XP_001986435.1	0	GLOS_DGRI_GH2	GO:0005262	M	.	calcium channel activity
XP_001986435.1 GH20533 [Drosophila grimshawi]	XP_001986435.1	0	GLOS_DGRI_GH2	GO:0015276	M	.	ligand-gated ion channel activity
XP_001986435.1 GH20533 [Drosophila grimshawi]	XP_001986435.1	0	GLOS_DGRI_GH2	GO:0022834	M	.	ligand-gated channel activity
XP_001986435.1 GH20533 [Drosophila grimshawi]	XP_001986435.1	0	GLOS_DGRI_GH2	GO:0022836	M	.	gated channel activity
XP_001986435.1 GH20533 [Drosophila grimshawi]	XP_001986435.1	0	GLOS_DGRI_GH2	GO:0022839	M	.	ion gated channel activity
XP_001986435.1 GH20533 [Drosophila grimshawi]	XP_001986435.1	0	GLOS_DGRI_GH2	GO:0015279	M	Refseq	store-operated calcium channel activity
XP_001986435.1 GH20533 [Drosophila grimshawi]	XP_001986435.1	0	GLOS_DGRI_GH2	GO:0001775	B	.	cell activation
XP_001986435.1 GH20533 [Drosophila grimshawi]	XP_001986435.1	0	GLOS_DGRI_GH2	GO:0007596	B	.	blood coagulation
XP_001986435.1 GH20533 [Drosophila grimshawi]	XP_001986435.1	0	GLOS_DGRI_GH2	GO:0030168	B	Refseq	platelet activation
XP_001986435.1 GH20533 [Drosophila grimshawi]	XP_001986435.1	0	GLOS_DGRI_GH2	GO:0014074	B	.	response to purine-containing compound
XP_001986435.1 GH20533 [Drosophila grimshawi]	XP_001986435.1	0	GLOS_DGRI_GH2	GO:0010243	B	.	response to organonitrogen compound
XP_001986435.1 GH20533 [Drosophila grimshawi]	XP_001986435.1	0	GLOS_DGRI_GH2	GO:0009719	B	.	response to endogenous stimulus
XP_001986435.1 GH20533 [Drosophila grimshawi]	XP_001986435.1	0	GLOS_DGRI_GH2	GO:0010033	B	.	response to organic substance
XP_001986435.1 GH20533 [Drosophila grimshawi]	XP_001986435.1	0	GLOS_DGRI_GH2	GO:1901698	B	.	response to nitrogen compound
XP_001986435.1 GH20533 [Drosophila grimshawi]	XP_001986435.1	0	GLOS_DGRI_GH2	GO:0014070	B	.	response to organic cyclic compound
XP_001986435.1 GH20533 [Drosophila grimshawi]	XP_001986435.1	0	GLOS_DGRI_GH2	GO:0046683	B	.	response to organophosphorus
XP_001986435.1 GH20533 [Drosophila grimshawi]	XP_001986435.1	0	GLOS_DGRI_GH2	GO:1901700	B	.	response to oxygen-containing compound
XP_001986435.1 GH20533 [Drosophila grimshawi]	XP_001986435.1	0	GLOS_DGRI_GH2	GO:0033198	B	Refseq	response to ATP
XP_001986435.1 GH20533 [Drosophila grimshawi]	XP_001986435.1	0	GLOS_DGRI_GH2	GO:0010038	B	.	response to metal ion
XP_001986435.1 GH20533 [Drosophila grimshawi]	XP_001986435.1	0	GLOS_DGRI_GH2	GO:0010035	B	.	response to inorganic substance
XP_001986435.1 GH20533 [Drosophila grimshawi]	XP_001986435.1	0	GLOS_DGRI_GH2	GO:0051592	B	Refseq	response to calcium ion
XP_001986435.1 GH20533 [Drosophila grimshawi]	XP_001986435.1	0	GLOS_DGRI_GH2	GO:0006816	B	.	calcium ion transport
XP_001986435.1 GH20533 [Drosophila grimshawi]	XP_001986435.1	0	GLOS_DGRI_GH2	GO:0034220	B	.	ion transmembrane transport
XP_001986435.1 GH20533 [Drosophila grimshawi]	XP_001986435.1	0	GLOS_DGRI_GH2	GO:0055085	B	.	transmembrane transport
XP_001986435.1 GH20533 [Drosophila grimshawi]	XP_001986435.1	0	GLOS_DGRI_GH2	GO:0070588	B	Refseq	calcium ion transmembrane transport
XP_001986435.1 GH20533 [Drosophila grimshawi]	XP_001986435.1	0	GLOS_DGRI_GH2	GO:0043168	M	.	anion binding
XP_001986435.1 GH20533 [Drosophila grimshawi]	XP_001986435.1	0	GLOS_DGRI_GH2	GO:0043167	M	.	ion binding
XP_001986435.1 GH20533 [Drosophila grimshawi]	XP_001986435.1	0	GLOS_DGRI_GH2	GO:0043178	M	.	alcohol binding
XP_001986435.1 GH20533 [Drosophila grimshawi]	XP_001986435.1	0	GLOS_DGRI_GH2	GO:0036094	M	.	small molecule binding
XP_001986435.1 GH20533 [Drosophila grimshawi]	XP_001986435.1	0	GLOS_DGRI_GH2	GO:0070679	M	Refseq	inositol 1,4,5 trisphosphate binding
XP_001985481.1 GH17085 [Drosophila grimshawi]	XP_001985481.1	2,00E-48	GLOS_DGRI_GH1	GO:0005262	M	Refseq	calcium channel activity
XP_001985481.1 GH17085 [Drosophila grimshawi]	XP_001985481.1	2,00E-48	GLOS_DGRI_GH1	GO:0005515	M	Refseq	protein binding
XP_001985481.1 GH17085 [Drosophila grimshawi]	XP_001985481.1	2,00E-48	GLOS_DGRI_GH1	GO:0005886	C	Refseq	plasma membrane
XP_001985481.1 GH17085 [Drosophila grimshawi]	XP_001985481.1	2,00E-48	GLOS_DGRI_GH1	GO:0005887	C	Refseq	integral to plasma membrane
XP_001985481.1 GH17085 [Drosophila grimshawi]	XP_001985481.1	2,00E-48	GLOS_DGRI_GH1	GO:0006816	B	Refseq	calcium ion transport
XP_001985481.1 GH17085 [Drosophila grimshawi]	XP_001985481.1	2,00E-48	GLOS_DGRI_GH1	GO:0007411	B	Refseq	axon guidance
XP_001985481.1 GH17085 [Drosophila grimshawi]	XP_001985481.1	2,00E-48	GLOS_DGRI_GH1	GO:0007596	B	Refseq	blood coagulation
XP_001985481.1 GH17085 [Drosophila grimshawi]	XP_001985481.1	2,00E-48	GLOS_DGRI_GH1	GO:0007602	B	Refseq	phototransduction
XP_001985481.1 GH17085 [Drosophila grimshawi]	XP_001985481.1	2,00E-48	GLOS_DGRI_GH1	GO:0010524	B	Refseq	positive regulation of calcium ion transport into cytosol
XP_001985481.1 GH17085 [Drosophila grimshawi]	XP_001985481.1	2,00E-48	GLOS_DGRI_GH1	GO:0015279	M	Refseq	store-operated calcium channel activity
XP_001985481.1 GH17085 [Drosophila grimshawi]	XP_001985481.1	2,00E-48	GLOS_DGRI_GH1	GO:0030168	B	Refseq	platelet activation
XP_001985481.1 GH17085 [Drosophila grimshawi]	XP_001985481.1	2,00E-48	GLOS_DGRI_GH1	GO:0033198	B	Refseq	response to ATP

XP_001985481.1 GH17085 [Drosophila grimshawi]	XP_001985481.1	2,00E-48	GLOS_DGRI_GH1	GO:0051592	B	Refseq	response to calcium ion
XP_001985481.1 GH17085 [Drosophila grimshawi]	XP_001985481.1	2,00E-48	GLOS_DGRI_GH1	GO:0070588	B	Refseq	calcium ion transmembrane transport
XP_001985481.1 GH17085 [Drosophila grimshawi]	XP_001985481.1	2,00E-48	GLOS_DGRI_GH1	GO:0070679	M	Refseq	inositol 1,4,5 trisphosphate binding
XP_001986067.1 GH21159 [Drosophila grimshawi]	XP_001986067.1	1,00E-130	GLOS_DGRI_GH2	GO:0005262	M	Refseq	calcium channel activity
XP_001986067.1 GH21159 [Drosophila grimshawi]	XP_001986067.1	1,00E-130	GLOS_DGRI_GH2	GO:0005515	M	Refseq	protein binding
XP_001986067.1 GH21159 [Drosophila grimshawi]	XP_001986067.1	1,00E-130	GLOS_DGRI_GH2	GO:0005886	C	Refseq	plasma membrane
XP_001986067.1 GH21159 [Drosophila grimshawi]	XP_001986067.1	1,00E-130	GLOS_DGRI_GH2	GO:0005887	C	Refseq	integral to plasma membrane
XP_001986067.1 GH21159 [Drosophila grimshawi]	XP_001986067.1	1,00E-130	GLOS_DGRI_GH2	GO:0006816	B	Refseq	calcium ion transport
XP_001986067.1 GH21159 [Drosophila grimshawi]	XP_001986067.1	1,00E-130	GLOS_DGRI_GH2	GO:0007411	B	Refseq	axon guidance
XP_001986067.1 GH21159 [Drosophila grimshawi]	XP_001986067.1	1,00E-130	GLOS_DGRI_GH2	GO:0007596	B	Refseq	blood coagulation
XP_001986067.1 GH21159 [Drosophila grimshawi]	XP_001986067.1	1,00E-130	GLOS_DGRI_GH2	GO:0007602	B	Refseq	phototransduction
XP_001986067.1 GH21159 [Drosophila grimshawi]	XP_001986067.1	1,00E-130	GLOS_DGRI_GH2	GO:0010524	B	Refseq	positive regulation of calcium ion transport into cytosol
XP_001986067.1 GH21159 [Drosophila grimshawi]	XP_001986067.1	1,00E-130	GLOS_DGRI_GH2	GO:0015279	M	Refseq	store-operated calcium channel activity
XP_001986067.1 GH21159 [Drosophila grimshawi]	XP_001986067.1	1,00E-130	GLOS_DGRI_GH2	GO:0030168	B	Refseq	platelet activation
XP_001986067.1 GH21159 [Drosophila grimshawi]	XP_001986067.1	1,00E-130	GLOS_DGRI_GH2	GO:0033198	B	Refseq	response to ATP
XP_001986067.1 GH21159 [Drosophila grimshawi]	XP_001986067.1	1,00E-130	GLOS_DGRI_GH2	GO:0051592	B	Refseq	response to calcium ion
XP_001986067.1 GH21159 [Drosophila grimshawi]	XP_001986067.1	1,00E-130	GLOS_DGRI_GH2	GO:0070588	B	Refseq	calcium ion transmembrane transport
XP_001986067.1 GH21159 [Drosophila grimshawi]	XP_001986067.1	1,00E-130	GLOS_DGRI_GH2	GO:0070679	M	Refseq	inositol 1,4,5 trisphosphate binding
XP_004929373.1 PREDICT.: uncharact. protein LOC101742112 [Bombyx mori]	XP_004929373.1	2,00E-08	GLOS_LOC10174:	GO:0006357	B	.	regulation of transcription from RNA polymerase II promoter
XP_004929373.1 PREDICT.: uncharact. protein LOC101742112 [Bombyx mori]	XP_004929373.1	2,00E-08	GLOS_LOC10174:	GO:0006355	B	.	regulation of transcription, DNA-dependent
XP_004929373.1 PREDICT.: uncharact. protein LOC101742112 [Bombyx mori]	XP_004929373.1	2,00E-08	GLOS_LOC10174:	GO:0010468	B	.	regulation of gene expression
XP_004929373.1 PREDICT.: uncharact. protein LOC101742112 [Bombyx mori]	XP_004929373.1	2,00E-08	GLOS_LOC10174:	GO:0060255	B	.	regulation of macromolecule metabolic process
XP_004929373.1 PREDICT.: uncharact. protein LOC101742112 [Bombyx mori]	XP_004929373.1	2,00E-08	GLOS_LOC10174:	GO:0019222	B	.	regulation of metabolic process
XP_004929373.1 PREDICT.: uncharact. protein LOC101742112 [Bombyx mori]	XP_004929373.1	2,00E-08	GLOS_LOC10174:	GO:2000112	B	.	regulation of cellular macromolecule biosynthetic process
XP_004929373.1 PREDICT.: uncharact. protein LOC101742112 [Bombyx mori]	XP_004929373.1	2,00E-08	GLOS_LOC10174:	GO:0010556	B	.	regulation of macromolecule biosynthetic process
XP_004929373.1 PREDICT.: uncharact. protein LOC101742112 [Bombyx mori]	XP_004929373.1	2,00E-08	GLOS_LOC10174:	GO:0009889	B	.	regulation of biosynthetic process
XP_004929373.1 PREDICT.: uncharact. protein LOC101742112 [Bombyx mori]	XP_004929373.1	2,00E-08	GLOS_LOC10174:	GO:0031326	B	.	regulation of cellular biosynthetic process
XP_004929373.1 PREDICT.: uncharact. protein LOC101742112 [Bombyx mori]	XP_004929373.1	2,00E-08	GLOS_LOC10174:	GO:0031323	B	.	regulation of cellular metabolic process
XP_004929373.1 PREDICT.: uncharact. protein LOC101742112 [Bombyx mori]	XP_004929373.1	2,00E-08	GLOS_LOC10174:	GO:2001141	B	.	regulation of RNA biosynthetic process
XP_004929373.1 PREDICT.: uncharact. protein LOC101742112 [Bombyx mori]	XP_004929373.1	2,00E-08	GLOS_LOC10174:	GO:0051252	B	.	regulation of RNA metabolic process
XP_004929373.1 PREDICT.: uncharact. protein LOC101742112 [Bombyx mori]	XP_004929373.1	2,00E-08	GLOS_LOC10174:	GO:0019219	B	.	regulation of nucleobase-containing compound metabolic process
XP_004929373.1 PREDICT.: uncharact. protein LOC101742112 [Bombyx mori]	XP_004929373.1	2,00E-08	GLOS_LOC10174:	GO:0051171	B	.	regulation of nitrogen compound metabolic process
XP_004929373.1 PREDICT.: uncharact. protein LOC101742112 [Bombyx mori]	XP_004929373.1	2,00E-08	GLOS_LOC10174:	GO:0080090	B	.	regulation of primary metabolic process
XP_004929373.1 PREDICT.: uncharact. protein LOC101742112 [Bombyx mori]	XP_004929373.1	2,00E-08	GLOS_LOC10174:	GO:0045892	B	.	negative regulation of transcription, DNA-dependent
XP_004929373.1 PREDICT.: uncharact. protein LOC101742112 [Bombyx mori]	XP_004929373.1	2,00E-08	GLOS_LOC10174:	GO:0010629	B	.	negative regulation of gene expression
XP_004929373.1 PREDICT.: uncharact. protein LOC101742112 [Bombyx mori]	XP_004929373.1	2,00E-08	GLOS_LOC10174:	GO:0010605	B	.	negative regulation of macromolecule metabolic process
XP_004929373.1 PREDICT.: uncharact. protein LOC101742112 [Bombyx mori]	XP_004929373.1	2,00E-08	GLOS_LOC10174:	GO:0009892	B	.	negative regulation of metabolic process
XP_004929373.1 PREDICT.: uncharact. protein LOC101742112 [Bombyx mori]	XP_004929373.1	2,00E-08	GLOS_LOC10174:	GO:0048519	B	.	negative regulation of biological process
XP_004929373.1 PREDICT.: uncharact. protein LOC101742112 [Bombyx mori]	XP_004929373.1	2,00E-08	GLOS_LOC10174:	GO:0051253	B	.	negative regulation of RNA metabolic process
XP_004929373.1 PREDICT.: uncharact. protein LOC101742112 [Bombyx mori]	XP_004929373.1	2,00E-08	GLOS_LOC10174:	GO:0045934	B	.	negative regulation of nucleobase-containing compound metabolic process
XP_004929373.1 PREDICT.: uncharact. protein LOC101742112 [Bombyx mori]	XP_004929373.1	2,00E-08	GLOS_LOC10174:	GO:0031324	B	.	negative regulation of cellular metabolic process
XP_004929373.1 PREDICT.: uncharact. protein LOC101742112 [Bombyx mori]	XP_004929373.1	2,00E-08	GLOS_LOC10174:	GO:0048523	B	.	negative regulation of cellular process
XP_004929373.1 PREDICT.: uncharact. protein LOC101742112 [Bombyx mori]	XP_004929373.1	2,00E-08	GLOS_LOC10174:	GO:0051172	B	.	negative regulation of nitrogen compound metabolic process
XP_004929373.1 PREDICT.: uncharact. protein LOC101742112 [Bombyx mori]	XP_004929373.1	2,00E-08	GLOS_LOC10174:	GO:2000113	B	.	negative regulation of cellular macromolecule biosynthetic process
XP_004929373.1 PREDICT.: uncharact. protein LOC101742112 [Bombyx mori]	XP_004929373.1	2,00E-08	GLOS_LOC10174:	GO:0010558	B	.	negative regulation of macromolecule biosynthetic process
XP_004929373.1 PREDICT.: uncharact. protein LOC101742112 [Bombyx mori]	XP_004929373.1	2,00E-08	GLOS_LOC10174:	GO:0009890	B	.	negative regulation of biosynthetic process
XP_004929373.1 PREDICT.: uncharact. protein LOC101742112 [Bombyx mori]	XP_004929373.1	2,00E-08	GLOS_LOC10174:	GO:0031327	B	.	negative regulation of cellular biosynthetic process
XP_004929373.1 PREDICT.: uncharact. protein LOC101742112 [Bombyx mori]	XP_004929373.1	2,00E-08	GLOS_LOC10174:	GO:0000122	B	Refseq	negative regulation of transcription from RNA polymerase II promoter
XP_004929373.1 PREDICT.: uncharact. protein LOC101742112 [Bombyx mori]	XP_004929373.1	2,00E-08	GLOS_LOC10174:	GO:0003674	M	Refseq	molecular_function
XP_004929373.1 PREDICT.: uncharact. protein LOC101742112 [Bombyx mori]	XP_004929373.1	2,00E-08	GLOS_LOC10174:	GO:0003682	M	Refseq	chromatin binding
XP_004929373.1 PREDICT.: uncharact. protein LOC101742112 [Bombyx mori]	XP_004929373.1	2,00E-08	GLOS_LOC10174:	GO:0000981	M	.	sequence-specific DNA binding RNA polymerase II transcription factor activity
XP_004929373.1 PREDICT.: uncharact. protein LOC101742112 [Bombyx mori]	XP_004929373.1	2,00E-08	GLOS_LOC10174:	GO:0003700	M	.	sequence-specific DNA binding transcription factor activity
XP_004929373.1 PREDICT.: uncharact. protein LOC101742112 [Bombyx mori]	XP_004929373.1	2,00E-08	GLOS_LOC10174:	GO:0001071	M	.	nucleic acid binding transcription factor activity
XP_004929373.1 PREDICT.: uncharact. protein LOC101742112 [Bombyx mori]	XP_004929373.1	2,00E-08	GLOS_LOC10174:	GO:0003705	M	Refseq	RNA polymerase II distal enhancer sequence-specific DNA binding

							transcription factor activity
XP_004929373.1 PREDICT.: uncharact. protein LOC101742112 [Bombyx mori]	XP_004929373.1	2,00E-08	GLOS_LOC10174	GO:0003712	M	.	transcription cofactor activity
XP_004929373.1 PREDICT.: uncharact. protein LOC101742112 [Bombyx mori]	XP_004929373.1	2,00E-08	GLOS_LOC10174	GO:0000989	M	.	transcription factor binding transcription factor activity
XP_004929373.1 PREDICT.: uncharact. protein LOC101742112 [Bombyx mori]	XP_004929373.1	2,00E-08	GLOS_LOC10174	GO:0000988	M	.	protein binding transcription factor activity
XP_004929373.1 PREDICT.: uncharact. protein LOC101742112 [Bombyx mori]	XP_004929373.1	2,00E-08	GLOS_LOC10174	GO:0003714	M	Refseq	transcription corepressor activity
XP_004929373.1 PREDICT.: uncharact. protein LOC101742112 [Bombyx mori]	XP_004929373.1	2,00E-08	GLOS_LOC10174	GO:0043231	C	.	intracellular membrane-bounded organelle
XP_004929373.1 PREDICT.: uncharact. protein LOC101742112 [Bombyx mori]	XP_004929373.1	2,00E-08	GLOS_LOC10174	GO:0043227	C	.	membrane-bounded organelle
XP_004929373.1 PREDICT.: uncharact. protein LOC101742112 [Bombyx mori]	XP_004929373.1	2,00E-08	GLOS_LOC10174	GO:0043226	C	.	organelle
XP_004929373.1 PREDICT.: uncharact. protein LOC101742112 [Bombyx mori]	XP_004929373.1	2,00E-08	GLOS_LOC10174	GO:0043229	C	.	intracellular organelle
XP_004929373.1 PREDICT.: uncharact. protein LOC101742112 [Bombyx mori]	XP_004929373.1	2,00E-08	GLOS_LOC10174	GO:0044424	C	.	intracellular part
XP_004929373.1 PREDICT.: uncharact. protein LOC101742112 [Bombyx mori]	XP_004929373.1	2,00E-08	GLOS_LOC10174	GO:0005622	C	.	intracellular
XP_004929373.1 PREDICT.: uncharact. protein LOC101742112 [Bombyx mori]	XP_004929373.1	2,00E-08	GLOS_LOC10174	GO:0005634	C	Refseq	nucleus
XP_004929373.1 PREDICT.: uncharact. protein LOC101742112 [Bombyx mori]	XP_004929373.1	2,00E-08	GLOS_LOC10174	GO:0044428	C	.	nuclear part
XP_004929373.1 PREDICT.: uncharact. protein LOC101742112 [Bombyx mori]	XP_004929373.1	2,00E-08	GLOS_LOC10174	GO:0044446	C	.	intracellular organelle part
XP_004929373.1 PREDICT.: uncharact. protein LOC101742112 [Bombyx mori]	XP_004929373.1	2,00E-08	GLOS_LOC10174	GO:0044422	C	.	organelle part
XP_004929373.1 PREDICT.: uncharact. protein LOC101742112 [Bombyx mori]	XP_004929373.1	2,00E-08	GLOS_LOC10174	GO:0005634	C	.	nucleus
XP_004929373.1 PREDICT.: uncharact. protein LOC101742112 [Bombyx mori]	XP_004929373.1	2,00E-08	GLOS_LOC10174	GO:0031981	C	.	nuclear lumen
XP_004929373.1 PREDICT.: uncharact. protein LOC101742112 [Bombyx mori]	XP_004929373.1	2,00E-08	GLOS_LOC10174	GO:0070013	C	.	intracellular organelle lumen
XP_004929373.1 PREDICT.: uncharact. protein LOC101742112 [Bombyx mori]	XP_004929373.1	2,00E-08	GLOS_LOC10174	GO:0043233	C	.	organelle lumen
XP_004929373.1 PREDICT.: uncharact. protein LOC101742112 [Bombyx mori]	XP_004929373.1	2,00E-08	GLOS_LOC10174	GO:0031974	C	.	membrane-enclosed lumen
XP_004929373.1 PREDICT.: uncharact. protein LOC101742112 [Bombyx mori]	XP_004929373.1	2,00E-08	GLOS_LOC10174	GO:0005654	C	Refseq	nucleoplasm
XP_004929373.1 PREDICT.: uncharact. protein LOC101742112 [Bombyx mori]	XP_004929373.1	2,00E-08	GLOS_LOC10174	GO:0007166	B	.	cell surface receptor signaling pathway
XP_004929373.1 PREDICT.: uncharact. protein LOC101742112 [Bombyx mori]	XP_004929373.1	2,00E-08	GLOS_LOC10174	GO:0007219	B	Refseq	Notch signaling pathway
XP_004929373.1 PREDICT.: uncharact. protein LOC101742112 [Bombyx mori]	XP_004929373.1	2,00E-08	GLOS_LOC10174	GO:0008150	B	Refseq	biological_process
XP_004929373.1 PREDICT.: uncharact. protein LOC101742112 [Bombyx mori]	XP_004929373.1	2,00E-08	GLOS_LOC10174	GO:0016055	B	Refseq	Wnt receptor signaling pathway
XP_004929373.1 PREDICT.: uncharact. protein LOC101742112 [Bombyx mori]	XP_004929373.1	2,00E-08	GLOS_LOC10174	GO:0008134	M	.	transcription factor binding
XP_004929373.1 PREDICT.: uncharact. protein LOC101742112 [Bombyx mori]	XP_004929373.1	2,00E-08	GLOS_LOC10174	GO:0005515	M	.	protein binding
XP_004929373.1 PREDICT.: uncharact. protein LOC101742112 [Bombyx mori]	XP_004929373.1	2,00E-08	GLOS_LOC10174	GO:0070491	M	Refseq	repressing transcription factor binding
NP_001097982.1 tropomodulin, isoform G [Drosophila melanogaster]	NP_001097982.1	0	GLOS_TMOD.1.1	GO:0000122	B	Refseq	negative regulation of transcription from RNA polymerase II promoter
NP_001097982.1 tropomodulin, isoform G [Drosophila melanogaster]	NP_001097982.1	0	GLOS_TMOD.1.1	GO:0001501	B	Refseq	skeletal system development
NP_001097982.1 tropomodulin, isoform G [Drosophila melanogaster]	NP_001097982.1	0	GLOS_TMOD.1.1	GO:0003700	M	Refseq	sequence-specific DNA binding transcription factor activity
NP_001097982.1 tropomodulin, isoform G [Drosophila melanogaster]	NP_001097982.1	0	GLOS_TMOD.1.1	GO:0005515	M	Refseq	protein binding
NP_001097982.1 tropomodulin, isoform G [Drosophila melanogaster]	NP_001097982.1	0	GLOS_TMOD.1.1	GO:0005634	C	Refseq	nucleus
NP_001097982.1 tropomodulin, isoform G [Drosophila melanogaster]	NP_001097982.1	0	GLOS_TMOD.1.1	GO:0006351	B	.	transcription, DNA-dependent
NP_001097982.1 tropomodulin, isoform G [Drosophila melanogaster]	NP_001097982.1	0	GLOS_TMOD.1.1	GO:0032774	B	.	RNA biosynthetic process
NP_001097982.1 tropomodulin, isoform G [Drosophila melanogaster]	NP_001097982.1	0	GLOS_TMOD.1.1	GO:0009059	B	.	macromolecule biosynthetic process
NP_001097982.1 tropomodulin, isoform G [Drosophila melanogaster]	NP_001097982.1	0	GLOS_TMOD.1.1	GO:0043170	B	.	macromolecule metabolic process
NP_001097982.1 tropomodulin, isoform G [Drosophila melanogaster]	NP_001097982.1	0	GLOS_TMOD.1.1	GO:0071704	B	.	organic substance metabolic process
NP_001097982.1 tropomodulin, isoform G [Drosophila melanogaster]	NP_001097982.1	0	GLOS_TMOD.1.1	GO:0008152	B	.	metabolic process
NP_001097982.1 tropomodulin, isoform G [Drosophila melanogaster]	NP_001097982.1	0	GLOS_TMOD.1.1	GO:1901576	B	.	organic substance biosynthetic process
NP_001097982.1 tropomodulin, isoform G [Drosophila melanogaster]	NP_001097982.1	0	GLOS_TMOD.1.1	GO:0009058	B	.	biosynthetic process
NP_001097982.1 tropomodulin, isoform G [Drosophila melanogaster]	NP_001097982.1	0	GLOS_TMOD.1.1	GO:0016070	B	.	RNA metabolic process
NP_001097982.1 tropomodulin, isoform G [Drosophila melanogaster]	NP_001097982.1	0	GLOS_TMOD.1.1	GO:0044260	B	.	cellular macromolecule metabolic process
NP_001097982.1 tropomodulin, isoform G [Drosophila melanogaster]	NP_001097982.1	0	GLOS_TMOD.1.1	GO:0044237	B	.	cellular metabolic process
NP_001097982.1 tropomodulin, isoform G [Drosophila melanogaster]	NP_001097982.1	0	GLOS_TMOD.1.1	GO:0090304	B	.	nucleic acid metabolic process
NP_001097982.1 tropomodulin, isoform G [Drosophila melanogaster]	NP_001097982.1	0	GLOS_TMOD.1.1	GO:0006139	B	.	nucleobase-containing compound metabolic process
NP_001097982.1 tropomodulin, isoform G [Drosophila melanogaster]	NP_001097982.1	0	GLOS_TMOD.1.1	GO:0006725	B	.	cellular aromatic compound metabolic process
NP_001097982.1 tropomodulin, isoform G [Drosophila melanogaster]	NP_001097982.1	0	GLOS_TMOD.1.1	GO:0034641	B	.	cellular nitrogen compound metabolic process
NP_001097982.1 tropomodulin, isoform G [Drosophila melanogaster]	NP_001097982.1	0	GLOS_TMOD.1.1	GO:0006807	B	.	nitrogen compound metabolic process
NP_001097982.1 tropomodulin, isoform G [Drosophila melanogaster]	NP_001097982.1	0	GLOS_TMOD.1.1	GO:0044238	B	.	primary metabolic process
NP_001097982.1 tropomodulin, isoform G [Drosophila melanogaster]	NP_001097982.1	0	GLOS_TMOD.1.1	GO:0046483	B	.	heterocycle metabolic process
NP_001097982.1 tropomodulin, isoform G [Drosophila melanogaster]	NP_001097982.1	0	GLOS_TMOD.1.1	GO:1901360	B	.	organic cyclic compound metabolic process
NP_001097982.1 tropomodulin, isoform G [Drosophila melanogaster]	NP_001097982.1	0	GLOS_TMOD.1.1	GO:0034654	B	.	nucleobase-containing compound biosynthetic process
NP_001097982.1 tropomodulin, isoform G [Drosophila melanogaster]	NP_001097982.1	0	GLOS_TMOD.1.1	GO:0018130	B	.	heterocycle biosynthetic process

NP_001097982.1 tropomodulin, isoform G [Drosophila melanogaster]	NP_001097982.1	0	GLOS_TMOD.1.1	GO:0044249	B	.	cellular biosynthetic process
NP_001097982.1 tropomodulin, isoform G [Drosophila melanogaster]	NP_001097982.1	0	GLOS_TMOD.1.1	GO:0019438	B	.	aromatic compound biosynthetic process
NP_001097982.1 tropomodulin, isoform G [Drosophila melanogaster]	NP_001097982.1	0	GLOS_TMOD.1.1	GO:0044271	B	.	cellular nitrogen compound biosynthetic process
NP_001097982.1 tropomodulin, isoform G [Drosophila melanogaster]	NP_001097982.1	0	GLOS_TMOD.1.1	GO:1901362	B	.	organic cyclic compound biosynthetic process
NP_001097982.1 tropomodulin, isoform G [Drosophila melanogaster]	NP_001097982.1	0	GLOS_TMOD.1.1	GO:0034645	B	.	cellular macromolecule biosynthetic process
NP_001097982.1 tropomodulin, isoform G [Drosophila melanogaster]	NP_001097982.1	0	GLOS_TMOD.1.1	GO:0010467	B	.	gene expression
NP_001097982.1 tropomodulin, isoform G [Drosophila melanogaster]	NP_001097982.1	0	GLOS_TMOD.1.1	GO:0006366	B	Refseq	transcription from RNA polymerase II promoter
NP_001097982.1 tropomodulin, isoform G [Drosophila melanogaster]	NP_001097982.1	0	GLOS_TMOD.1.1	GO:0006606	B	.	protein import into nucleus
NP_001097982.1 tropomodulin, isoform G [Drosophila melanogaster]	NP_001097982.1	0	GLOS_TMOD.1.1	GO:0006886	B	.	intracellular protein transport
NP_001097982.1 tropomodulin, isoform G [Drosophila melanogaster]	NP_001097982.1	0	GLOS_TMOD.1.1	GO:0015031	B	.	protein transport
NP_001097982.1 tropomodulin, isoform G [Drosophila melanogaster]	NP_001097982.1	0	GLOS_TMOD.1.1	GO:0045184	B	.	establishment of protein localization
NP_001097982.1 tropomodulin, isoform G [Drosophila melanogaster]	NP_001097982.1	0	GLOS_TMOD.1.1	GO:0008104	B	.	protein localization
NP_001097982.1 tropomodulin, isoform G [Drosophila melanogaster]	NP_001097982.1	0	GLOS_TMOD.1.1	GO:0033036	B	.	macromolecule localization
NP_001097982.1 tropomodulin, isoform G [Drosophila melanogaster]	NP_001097982.1	0	GLOS_TMOD.1.1	GO:0071702	B	.	organic substance transport
NP_001097982.1 tropomodulin, isoform G [Drosophila melanogaster]	NP_001097982.1	0	GLOS_TMOD.1.1	GO:0046907	B	.	intracellular transport
NP_001097982.1 tropomodulin, isoform G [Drosophila melanogaster]	NP_001097982.1	0	GLOS_TMOD.1.1	GO:0051649	B	.	establishment of localization in cell
NP_001097982.1 tropomodulin, isoform G [Drosophila melanogaster]	NP_001097982.1	0	GLOS_TMOD.1.1	GO:0051641	B	.	cellular localization
NP_001097982.1 tropomodulin, isoform G [Drosophila melanogaster]	NP_001097982.1	0	GLOS_TMOD.1.1	GO:0034613	B	.	cellular protein localization
NP_001097982.1 tropomodulin, isoform G [Drosophila melanogaster]	NP_001097982.1	0	GLOS_TMOD.1.1	GO:0070727	B	.	cellular macromolecule localization
NP_001097982.1 tropomodulin, isoform G [Drosophila melanogaster]	NP_001097982.1	0	GLOS_TMOD.1.1	GO:0017038	B	.	protein import
NP_001097982.1 tropomodulin, isoform G [Drosophila melanogaster]	NP_001097982.1	0	GLOS_TMOD.1.1	GO:0051170	B	.	nuclear import
NP_001097982.1 tropomodulin, isoform G [Drosophila melanogaster]	NP_001097982.1	0	GLOS_TMOD.1.1	GO:0006913	B	.	nucleocytoplasmic transport
NP_001097982.1 tropomodulin, isoform G [Drosophila melanogaster]	NP_001097982.1	0	GLOS_TMOD.1.1	GO:0016482	B	.	cytoplasmic transport
NP_001097982.1 tropomodulin, isoform G [Drosophila melanogaster]	NP_001097982.1	0	GLOS_TMOD.1.1	GO:0051169	B	.	nuclear transport
NP_001097982.1 tropomodulin, isoform G [Drosophila melanogaster]	NP_001097982.1	0	GLOS_TMOD.1.1	GO:0072594	B	.	establishment of protein localization to organelle
NP_001097982.1 tropomodulin, isoform G [Drosophila melanogaster]	NP_001097982.1	0	GLOS_TMOD.1.1	GO:0033365	B	.	protein localization to organelle
NP_001097982.1 tropomodulin, isoform G [Drosophila melanogaster]	NP_001097982.1	0	GLOS_TMOD.1.1	GO:0034504	B	.	protein localization to nucleus
NP_001097982.1 tropomodulin, isoform G [Drosophila melanogaster]	NP_001097982.1	0	GLOS_TMOD.1.1	GO:0044744	B	.	protein targeting to nucleus
NP_001097982.1 tropomodulin, isoform G [Drosophila melanogaster]	NP_001097982.1	0	GLOS_TMOD.1.1	GO:0006605	B	.	protein targeting
NP_001097982.1 tropomodulin, isoform G [Drosophila melanogaster]	NP_001097982.1	0	GLOS_TMOD.1.1	GO:0006607	B	Refseq	NLS-bearing protein import into nucleus
NP_001097982.1 tropomodulin, isoform G [Drosophila melanogaster]	NP_001097982.1	0	GLOS_TMOD.1.1	GO:0046914	M	.	transition metal ion binding
NP_001097982.1 tropomodulin, isoform G [Drosophila melanogaster]	NP_001097982.1	0	GLOS_TMOD.1.1	GO:0046872	M	.	metal ion binding
NP_001097982.1 tropomodulin, isoform G [Drosophila melanogaster]	NP_001097982.1	0	GLOS_TMOD.1.1	GO:0043169	M	.	cation binding
NP_001097982.1 tropomodulin, isoform G [Drosophila melanogaster]	NP_001097982.1	0	GLOS_TMOD.1.1	GO:0008270	M	Refseq	zinc ion binding
NP_001097982.1 tropomodulin, isoform G [Drosophila melanogaster]	NP_001097982.1	0	GLOS_TMOD.1.1	GO:0045595	B	.	regulation of cell differentiation
NP_001097982.1 tropomodulin, isoform G [Drosophila melanogaster]	NP_001097982.1	0	GLOS_TMOD.1.1	GO:0050793	B	.	regulation of developmental process
NP_001097982.1 tropomodulin, isoform G [Drosophila melanogaster]	NP_001097982.1	0	GLOS_TMOD.1.1	GO:0061035	B	.	regulation of cartilage development
NP_001097982.1 tropomodulin, isoform G [Drosophila melanogaster]	NP_001097982.1	0	GLOS_TMOD.1.1	GO:2000026	B	.	regulation of multicellular organismal development
NP_001097982.1 tropomodulin, isoform G [Drosophila melanogaster]	NP_001097982.1	0	GLOS_TMOD.1.1	GO:0051239	B	.	regulation of multicellular organismal process
NP_001097982.1 tropomodulin, isoform G [Drosophila melanogaster]	NP_001097982.1	0	GLOS_TMOD.1.1	GO:0032330	B	Refseq	regulation of chondrocyte differentiation
NP_001097982.1 tropomodulin, isoform G [Drosophila melanogaster]	NP_001097982.1	0	GLOS_TMOD.1.1	GO:0003677	M	.	DNA binding
NP_001097982.1 tropomodulin, isoform G [Drosophila melanogaster]	NP_001097982.1	0	GLOS_TMOD.1.1	GO:0003676	M	.	nucleic acid binding
NP_001097982.1 tropomodulin, isoform G [Drosophila melanogaster]	NP_001097982.1	0	GLOS_TMOD.1.1	GO:0097159	M	.	organic cyclic compound binding
NP_001097982.1 tropomodulin, isoform G [Drosophila melanogaster]	NP_001097982.1	0	GLOS_TMOD.1.1	GO:1901363	M	.	heterocyclic compound binding
NP_001097982.1 tropomodulin, isoform G [Drosophila melanogaster]	NP_001097982.1	0	GLOS_TMOD.1.1	GO:0043565	M	Refseq	sequence-specific DNA binding
NP_001247216.1 CG43446, isoform A [Drosophila melanogaster]	NP_001247216.1	4,00E-06	GLOS_CG43446.1	GO:0000122	B	Refseq	negative regulation of transcription from RNA polymerase II promoter
NP_001247216.1 CG43446, isoform A [Drosophila melanogaster]	NP_001247216.1	4,00E-06	GLOS_CG43446.1	GO:0001501	B	Refseq	skeletal system development
NP_001247216.1 CG43446, isoform A [Drosophila melanogaster]	NP_001247216.1	4,00E-06	GLOS_CG43446.1	GO:0003700	M	Refseq	sequence-specific DNA binding transcription factor activity
NP_001247216.1 CG43446, isoform A [Drosophila melanogaster]	NP_001247216.1	4,00E-06	GLOS_CG43446.1	GO:0005515	M	Refseq	protein binding
NP_001247216.1 CG43446, isoform A [Drosophila melanogaster]	NP_001247216.1	4,00E-06	GLOS_CG43446.1	GO:0005634	C	Refseq	nucleus
NP_001247216.1 CG43446, isoform A [Drosophila melanogaster]	NP_001247216.1	4,00E-06	GLOS_CG43446.1	GO:0006366	B	Refseq	transcription from RNA polymerase II promoter
NP_001247216.1 CG43446, isoform A [Drosophila melanogaster]	NP_001247216.1	4,00E-06	GLOS_CG43446.1	GO:0006607	B	Refseq	NLS-bearing protein import into nucleus
NP_001247216.1 CG43446, isoform A [Drosophila melanogaster]	NP_001247216.1	4,00E-06	GLOS_CG43446.1	GO:0008270	M	Refseq	zinc ion binding
NP_001247216.1 CG43446, isoform A [Drosophila melanogaster]	NP_001247216.1	4,00E-06	GLOS_CG43446.1	GO:0032330	B	Refseq	regulation of chondrocyte differentiation

NP_001247216.1 CG43446, isoform A [Drosophila melanogaster]	NP_001247216.1	4,00E-06	GLOS_CG43446.1	GO:0043565	M	Refseq	sequence-specific DNA binding
XP_001979606.1 GG16338 [Drosophila erecta]	XP_001979606.1	0	GLOS_DERE_GG	GO:0005261	M	Refseq	cation channel activity
XP_001979606.1 GG16338 [Drosophila erecta]	XP_001979606.1	0	GLOS_DERE_GG	GO:0005262	M	Refseq	calcium channel activity
XP_001979606.1 GG16338 [Drosophila erecta]	XP_001979606.1	0	GLOS_DERE_GG	GO:0005515	M	Refseq	protein binding
XP_001979606.1 GG16338 [Drosophila erecta]	XP_001979606.1	0	GLOS_DERE_GG	GO:0005886	C	Refseq	plasma membrane
XP_001979606.1 GG16338 [Drosophila erecta]	XP_001979606.1	0	GLOS_DERE_GG	GO:0005887	C	Refseq	integral to plasma membrane
XP_001979606.1 GG16338 [Drosophila erecta]	XP_001979606.1	0	GLOS_DERE_GG	GO:0006816	B	Refseq	calcium ion transport
XP_001979606.1 GG16338 [Drosophila erecta]	XP_001979606.1	0	GLOS_DERE_GG	GO:0007411	B	Refseq	axon guidance
XP_001979606.1 GG16338 [Drosophila erecta]	XP_001979606.1	0	GLOS_DERE_GG	GO:0015279	M	Refseq	store-operated calcium channel activity
XP_001979606.1 GG16338 [Drosophila erecta]	XP_001979606.1	0	GLOS_DERE_GG	GO:0016323	C	Refseq	basolateral plasma membrane
XP_001979606.1 GG16338 [Drosophila erecta]	XP_001979606.1	0	GLOS_DERE_GG	GO:0044449	C	.	contractile fiber part
XP_001979606.1 GG16338 [Drosophila erecta]	XP_001979606.1	0	GLOS_DERE_GG	GO:0044444	C	.	cytoplasmic part
XP_001979606.1 GG16338 [Drosophila erecta]	XP_001979606.1	0	GLOS_DERE_GG	GO:0005737	C	.	cytoplasm
XP_001979606.1 GG16338 [Drosophila erecta]	XP_001979606.1	0	GLOS_DERE_GG	GO:0043292	C	.	contractile fiber
XP_001979606.1 GG16338 [Drosophila erecta]	XP_001979606.1	0	GLOS_DERE_GG	GO:0043232	C	.	intracellular non-membrane-bounded organelle
XP_001979606.1 GG16338 [Drosophila erecta]	XP_001979606.1	0	GLOS_DERE_GG	GO:0043228	C	.	non-membrane-bounded organelle
XP_001979606.1 GG16338 [Drosophila erecta]	XP_001979606.1	0	GLOS_DERE_GG	GO:0030016	C	.	myofibril
XP_001979606.1 GG16338 [Drosophila erecta]	XP_001979606.1	0	GLOS_DERE_GG	GO:0030017	C	Refseq	sarcomere
XP_001979606.1 GG16338 [Drosophila erecta]	XP_001979606.1	0	GLOS_DERE_GG	GO:0043034	C	Refseq	costamere
XP_001979606.1 GG16338 [Drosophila erecta]	XP_001979606.1	0	GLOS_DERE_GG	GO:0032991	C	.	macromolecular complex
XP_001979606.1 GG16338 [Drosophila erecta]	XP_001979606.1	0	GLOS_DERE_GG	GO:0043234	C	Refseq	protein complex
XP_001979606.1 GG16338 [Drosophila erecta]	XP_001979606.1	0	GLOS_DERE_GG	GO:0044325	M	Refseq	ion channel binding
XP_001979606.1 GG16338 [Drosophila erecta]	XP_001979606.1	0	GLOS_DERE_GG	GO:0045121	C	Refseq	membrane raft
XP_001979606.1 GG16338 [Drosophila erecta]	XP_001979606.1	0	GLOS_DERE_GG	GO:0007589	B	.	body fluid secretion
XP_001979606.1 GG16338 [Drosophila erecta]	XP_001979606.1	0	GLOS_DERE_GG	GO:0046903	B	.	secretion
XP_001979606.1 GG16338 [Drosophila erecta]	XP_001979606.1	0	GLOS_DERE_GG	GO:0022600	B	.	digestive system process
XP_001979606.1 GG16338 [Drosophila erecta]	XP_001979606.1	0	GLOS_DERE_GG	GO:0003008	B	.	system process
XP_001979606.1 GG16338 [Drosophila erecta]	XP_001979606.1	0	GLOS_DERE_GG	GO:0007586	B	.	digestion
XP_001979606.1 GG16338 [Drosophila erecta]	XP_001979606.1	0	GLOS_DERE_GG	GO:0032941	B	.	secretion by tissue
XP_001979606.1 GG16338 [Drosophila erecta]	XP_001979606.1	0	GLOS_DERE_GG	GO:0046541	B	Refseq	saliva secretion
XP_001979606.1 GG16338 [Drosophila erecta]	XP_001979606.1	0	GLOS_DERE_GG	GO:0010524	B	.	positive regulation of calcium ion transport into cytosol
XP_001979606.1 GG16338 [Drosophila erecta]	XP_001979606.1	0	GLOS_DERE_GG	GO:0051279	B	.	regulation of release of sequestered calcium ion into cytosol
XP_001979606.1 GG16338 [Drosophila erecta]	XP_001979606.1	0	GLOS_DERE_GG	GO:0051281	B	Refseq	positive regulation of release of sequestered calcium ion into cytosol
XP_001979606.1 GG16338 [Drosophila erecta]	XP_001979606.1	0	GLOS_DERE_GG	GO:0006874	B	.	cellular calcium ion homeostasis
XP_001979606.1 GG16338 [Drosophila erecta]	XP_001979606.1	0	GLOS_DERE_GG	GO:0006875	B	.	cellular metal ion homeostasis
XP_001979606.1 GG16338 [Drosophila erecta]	XP_001979606.1	0	GLOS_DERE_GG	GO:0030003	B	.	cellular cation homeostasis
XP_001979606.1 GG16338 [Drosophila erecta]	XP_001979606.1	0	GLOS_DERE_GG	GO:0006873	B	.	cellular ion homeostasis
XP_001979606.1 GG16338 [Drosophila erecta]	XP_001979606.1	0	GLOS_DERE_GG	GO:0050801	B	.	ion homeostasis
XP_001979606.1 GG16338 [Drosophila erecta]	XP_001979606.1	0	GLOS_DERE_GG	GO:0048878	B	.	chemical homeostasis
XP_001979606.1 GG16338 [Drosophila erecta]	XP_001979606.1	0	GLOS_DERE_GG	GO:0042592	B	.	homeostatic process
XP_001979606.1 GG16338 [Drosophila erecta]	XP_001979606.1	0	GLOS_DERE_GG	GO:0055082	B	.	cellular chemical homeostasis
XP_001979606.1 GG16338 [Drosophila erecta]	XP_001979606.1	0	GLOS_DERE_GG	GO:0019725	B	.	cellular homeostasis
XP_001979606.1 GG16338 [Drosophila erecta]	XP_001979606.1	0	GLOS_DERE_GG	GO:0055080	B	.	cation homeostasis
XP_001979606.1 GG16338 [Drosophila erecta]	XP_001979606.1	0	GLOS_DERE_GG	GO:0055065	B	.	metal ion homeostasis
XP_001979606.1 GG16338 [Drosophila erecta]	XP_001979606.1	0	GLOS_DERE_GG	GO:0055074	B	.	calcium ion homeostasis
XP_001979606.1 GG16338 [Drosophila erecta]	XP_001979606.1	0	GLOS_DERE_GG	GO:0072507	B	.	divalent inorganic cation homeostasis
XP_001979606.1 GG16338 [Drosophila erecta]	XP_001979606.1	0	GLOS_DERE_GG	GO:0072503	B	.	cellular divalent inorganic cation homeostasis
XP_001979606.1 GG16338 [Drosophila erecta]	XP_001979606.1	0	GLOS_DERE_GG	GO:0051480	B	Refseq	cytosolic calcium ion homeostasis
XP_001979606.1 GG16338 [Drosophila erecta]	XP_001979606.1	0	GLOS_DERE_GG	GO:0051592	B	Refseq	response to calcium ion
XP_001979606.1 GG16338 [Drosophila erecta]	XP_001979606.1	0	GLOS_DERE_GG	GO:0070679	M	Refseq	inositol 1,4,5 trisphosphate binding
NP_731796.1 CG9813, isoform C [D. m.] ref|NP_731797.1| CG9813, isoform F	NP_731796.1	1,00E-75	GLOS_CG9813.1.:	GO:0000122	B	Refseq	negative regulation of transcription from RNA polymerase II promoter
NP_731796.1 CG9813, isoform C [D. m.] ref|NP_731797.1| CG9813, isoform F	NP_731796.1	1,00E-75	GLOS_CG9813.1.:	GO:0001501	B	Refseq	skeletal system development
NP_731796.1 CG9813, isoform C [D. m.] ref|NP_731797.1| CG9813, isoform F	NP_731796.1	1,00E-75	GLOS_CG9813.1.:	GO:0003700	M	Refseq	sequence-specific DNA binding transcription factor activity
NP_731796.1 CG9813, isoform C [D. m.] ref|NP_731797.1| CG9813, isoform F	NP_731796.1	1,00E-75	GLOS_CG9813.1.:	GO:0005515	M	Refseq	protein binding

NP_731796.1 CG9813, isoform C [D. m.] ref|NP_731797.1| CG9813, isoform F	NP_731796.1	1,00E-75	GLOS_CG9813.1.:	GO:0005634	C	Refseq	nucleus
NP_731796.1 CG9813, isoform C [D. m.] ref|NP_731797.1| CG9813, isoform F	NP_731796.1	1,00E-75	GLOS_CG9813.1.:	GO:0006366	B	Refseq	transcription from RNA polymerase II promoter
NP_731796.1 CG9813, isoform C [D. m.] ref|NP_731797.1| CG9813, isoform F	NP_731796.1	1,00E-75	GLOS_CG9813.1.:	GO:0006607	B	Refseq	NLS-bearing protein import into nucleus
NP_731796.1 CG9813, isoform C [D. m.] ref|NP_731797.1| CG9813, isoform F	NP_731796.1	1,00E-75	GLOS_CG9813.1.:	GO:0008270	M	Refseq	zinc ion binding
NP_731796.1 CG9813, isoform C [D. m.] ref|NP_731797.1| CG9813, isoform F	NP_731796.1	1,00E-75	GLOS_CG9813.1.:	GO:0032330	B	Refseq	regulation of chondrocyte differentiation
NP_731796.1 CG9813, isoform C [D. m.] ref|NP_731797.1| CG9813, isoform F	NP_731796.1	1,00E-75	GLOS_CG9813.1.:	GO:0043565	M	Refseq	sequence-specific DNA binding
XP_001979918.1 GG16851 [Drosophila erecta]	XP_001979918.1	2,00E-31	GLOS_DERE_GG	GO:0005261	M	Refseq	cation channel activity
XP_001979918.1 GG16851 [Drosophila erecta]	XP_001979918.1	2,00E-31	GLOS_DERE_GG	GO:0005262	M	Refseq	calcium channel activity
XP_001979918.1 GG16851 [Drosophila erecta]	XP_001979918.1	2,00E-31	GLOS_DERE_GG	GO:0005515	M	Refseq	protein binding
XP_001979918.1 GG16851 [Drosophila erecta]	XP_001979918.1	2,00E-31	GLOS_DERE_GG	GO:0005886	C	Refseq	plasma membrane
XP_001979918.1 GG16851 [Drosophila erecta]	XP_001979918.1	2,00E-31	GLOS_DERE_GG	GO:0005887	C	Refseq	integral to plasma membrane
XP_001979918.1 GG16851 [Drosophila erecta]	XP_001979918.1	2,00E-31	GLOS_DERE_GG	GO:0006816	B	Refseq	calcium ion transport
XP_001979918.1 GG16851 [Drosophila erecta]	XP_001979918.1	2,00E-31	GLOS_DERE_GG	GO:0007411	B	Refseq	axon guidance
XP_001979918.1 GG16851 [Drosophila erecta]	XP_001979918.1	2,00E-31	GLOS_DERE_GG	GO:0015279	M	Refseq	store-operated calcium channel activity
XP_001979918.1 GG16851 [Drosophila erecta]	XP_001979918.1	2,00E-31	GLOS_DERE_GG	GO:0016323	C	Refseq	basolateral plasma membrane
XP_001979918.1 GG16851 [Drosophila erecta]	XP_001979918.1	2,00E-31	GLOS_DERE_GG	GO:0030017	C	Refseq	sarcomere
XP_001979918.1 GG16851 [Drosophila erecta]	XP_001979918.1	2,00E-31	GLOS_DERE_GG	GO:0043034	C	Refseq	costamere
XP_001979918.1 GG16851 [Drosophila erecta]	XP_001979918.1	2,00E-31	GLOS_DERE_GG	GO:0043234	C	Refseq	protein complex
XP_001979918.1 GG16851 [Drosophila erecta]	XP_001979918.1	2,00E-31	GLOS_DERE_GG	GO:0044325	M	Refseq	ion channel binding
XP_001979918.1 GG16851 [Drosophila erecta]	XP_001979918.1	2,00E-31	GLOS_DERE_GG	GO:0045121	C	Refseq	membrane raft
XP_001979918.1 GG16851 [Drosophila erecta]	XP_001979918.1	2,00E-31	GLOS_DERE_GG	GO:0046541	B	Refseq	saliva secretion
XP_001979918.1 GG16851 [Drosophila erecta]	XP_001979918.1	2,00E-31	GLOS_DERE_GG	GO:0051281	B	Refseq	positive regulation of release of sequestered calcium ion into cytosol
XP_001979918.1 GG16851 [Drosophila erecta]	XP_001979918.1	2,00E-31	GLOS_DERE_GG	GO:0051480	B	Refseq	cytosolic calcium ion homeostasis
XP_001979918.1 GG16851 [Drosophila erecta]	XP_001979918.1	2,00E-31	GLOS_DERE_GG	GO:0051592	B	Refseq	response to calcium ion
XP_001979918.1 GG16851 [Drosophila erecta]	XP_001979918.1	2,00E-31	GLOS_DERE_GG	GO:0070679	M	Refseq	inositol 1,4,5 trisphosphate binding
NP_001093282.1 mariner transposase [Bombyx mori]	NP_001093282.1	5,00E-12	GLOS_LOC10010:	GO:0000122	B	Refseq	negative regulation of transcription from RNA polymerase II promoter
NP_001093282.1 mariner transposase [Bombyx mori]	NP_001093282.1	5,00E-12	GLOS_LOC10010:	GO:0003674	M	Refseq	molecular_function
NP_001093282.1 mariner transposase [Bombyx mori]	NP_001093282.1	5,00E-12	GLOS_LOC10010:	GO:0003682	M	Refseq	chromatin binding
NP_001093282.1 mariner transposase [Bombyx mori]	NP_001093282.1	5,00E-12	GLOS_LOC10010:	GO:0003705	M	Refseq	RNA polymerase II distal enhancer sequence-specific DNA binding transcription factor activity
NP_001093282.1 mariner transposase [Bombyx mori]	NP_001093282.1	5,00E-12	GLOS_LOC10010:	GO:0003714	M	Refseq	transcription corepressor activity
NP_001093282.1 mariner transposase [Bombyx mori]	NP_001093282.1	5,00E-12	GLOS_LOC10010:	GO:0005634	C	Refseq	nucleus
NP_001093282.1 mariner transposase [Bombyx mori]	NP_001093282.1	5,00E-12	GLOS_LOC10010:	GO:0005654	C	Refseq	nucleoplasm
NP_001093282.1 mariner transposase [Bombyx mori]	NP_001093282.1	5,00E-12	GLOS_LOC10010:	GO:0007219	B	Refseq	Notch signaling pathway
NP_001093282.1 mariner transposase [Bombyx mori]	NP_001093282.1	5,00E-12	GLOS_LOC10010:	GO:0008150	B	Refseq	biological_process
NP_001093282.1 mariner transposase [Bombyx mori]	NP_001093282.1	5,00E-12	GLOS_LOC10010:	GO:0016055	B	Refseq	Wnt receptor signaling pathway
NP_001093282.1 mariner transposase [Bombyx mori]	NP_001093282.1	5,00E-12	GLOS_LOC10010:	GO:0070491	M	Refseq	repressing transcription factor binding
XP_001992923.1 GH13545 [Drosophila grimshawi]	XP_001992923.1	8,00E-21	GLOS_DGRI_GH1	GO:0005262	M	Refseq	calcium channel activity
XP_001992923.1 GH13545 [Drosophila grimshawi]	XP_001992923.1	8,00E-21	GLOS_DGRI_GH1	GO:0005515	M	Refseq	protein binding
XP_001992923.1 GH13545 [Drosophila grimshawi]	XP_001992923.1	8,00E-21	GLOS_DGRI_GH1	GO:0005886	C	Refseq	plasma membrane
XP_001992923.1 GH13545 [Drosophila grimshawi]	XP_001992923.1	8,00E-21	GLOS_DGRI_GH1	GO:0005887	C	Refseq	integral to plasma membrane
XP_001992923.1 GH13545 [Drosophila grimshawi]	XP_001992923.1	8,00E-21	GLOS_DGRI_GH1	GO:0006816	B	Refseq	calcium ion transport
XP_001992923.1 GH13545 [Drosophila grimshawi]	XP_001992923.1	8,00E-21	GLOS_DGRI_GH1	GO:0007411	B	Refseq	axon guidance
XP_001992923.1 GH13545 [Drosophila grimshawi]	XP_001992923.1	8,00E-21	GLOS_DGRI_GH1	GO:0007596	B	Refseq	blood coagulation
XP_001992923.1 GH13545 [Drosophila grimshawi]	XP_001992923.1	8,00E-21	GLOS_DGRI_GH1	GO:0007602	B	Refseq	phototransduction
XP_001992923.1 GH13545 [Drosophila grimshawi]	XP_001992923.1	8,00E-21	GLOS_DGRI_GH1	GO:0010524	B	Refseq	positive regulation of calcium ion transport into cytosol
XP_001992923.1 GH13545 [Drosophila grimshawi]	XP_001992923.1	8,00E-21	GLOS_DGRI_GH1	GO:0015279	M	Refseq	store-operated calcium channel activity
XP_001992923.1 GH13545 [Drosophila grimshawi]	XP_001992923.1	8,00E-21	GLOS_DGRI_GH1	GO:0030168	B	Refseq	platelet activation
XP_001992923.1 GH13545 [Drosophila grimshawi]	XP_001992923.1	8,00E-21	GLOS_DGRI_GH1	GO:0033198	B	Refseq	response to ATP
XP_001992923.1 GH13545 [Drosophila grimshawi]	XP_001992923.1	8,00E-21	GLOS_DGRI_GH1	GO:0051592	B	Refseq	response to calcium ion
XP_001992923.1 GH13545 [Drosophila grimshawi]	XP_001992923.1	8,00E-21	GLOS_DGRI_GH1	GO:0070588	B	Refseq	calcium ion transmembrane transport
XP_001992923.1 GH13545 [Drosophila grimshawi]	XP_001992923.1	8,00E-21	GLOS_DGRI_GH1	GO:0070679	M	Refseq	inositol 1,4,5 trisphosphate binding
NP_731611.2 C-terminal Src kinase, isoform G [D.m.] ref|NP_001262476.1|; isoform L [Drosophila melanogaster]	NP_731611.2	2,00E-49	GLOS_CSK.1.1	GO:0000122	B	Refseq	negative regulation of transcription from RNA polymerase II promoter

NP_731611.2 C-terminal Src kinase, isoform G .. Same as above	NP_731611.2	2,00E-49	GLOS_CSK.1.1	GO:0001501	B	Refseq	skeletal system development
NP_731611.2 C-terminal Src kinase, isoform G .. Same as above	NP_731611.2	2,00E-49	GLOS_CSK.1.1	GO:0003700	M	Refseq	sequence-specific DNA binding transcription factor activity
NP_731611.2 C-terminal Src kinase, isoform G .. Same as above	NP_731611.2	2,00E-49	GLOS_CSK.1.1	GO:0005515	M	Refseq	protein binding
NP_731611.2 C-terminal Src kinase, isoform G .. Same as above	NP_731611.2	2,00E-49	GLOS_CSK.1.1	GO:0005634	C	Refseq	nucleus
NP_731611.2 C-terminal Src kinase, isoform G .. Same as above	NP_731611.2	2,00E-49	GLOS_CSK.1.1	GO:0006366	B	Refseq	transcription from RNA polymerase II promoter
NP_731611.2 C-terminal Src kinase, isoform G .. Same as above	NP_731611.2	2,00E-49	GLOS_CSK.1.1	GO:0006607	B	Refseq	NLS-bearing protein import into nucleus
NP_731611.2 C-terminal Src kinase, isoform G .. Same as above	NP_731611.2	2,00E-49	GLOS_CSK.1.1	GO:0008270	M	Refseq	zinc ion binding
NP_731611.2 C-terminal Src kinase, isoform G .. Same as above	NP_731611.2	2,00E-49	GLOS_CSK.1.1	GO:0032330	B	Refseq	regulation of chondrocyte differentiation
NP_731611.2 C-terminal Src kinase, isoform G .. Same as above	NP_731611.2	2,00E-49	GLOS_CSK.1.1	GO:0043565	M	Refseq	sequence-specific DNA binding
XP_001987246.1 GH21812 [Drosophila grimshawi]	XP_001987246.1	2,00E-28	GLOS_DGRI_GH2	GO:0005262	M	Refseq	calcium channel activity
XP_001987246.1 GH21812 [Drosophila grimshawi]	XP_001987246.1	2,00E-28	GLOS_DGRI_GH2	GO:0005515	M	Refseq	protein binding
XP_001987246.1 GH21812 [Drosophila grimshawi]	XP_001987246.1	2,00E-28	GLOS_DGRI_GH2	GO:0005886	C	Refseq	plasma membrane
XP_001987246.1 GH21812 [Drosophila grimshawi]	XP_001987246.1	2,00E-28	GLOS_DGRI_GH2	GO:0005887	C	Refseq	integral to plasma membrane
XP_001987246.1 GH21812 [Drosophila grimshawi]	XP_001987246.1	2,00E-28	GLOS_DGRI_GH2	GO:0006816	B	Refseq	calcium ion transport
XP_001987246.1 GH21812 [Drosophila grimshawi]	XP_001987246.1	2,00E-28	GLOS_DGRI_GH2	GO:0007411	B	Refseq	axon guidance
XP_001987246.1 GH21812 [Drosophila grimshawi]	XP_001987246.1	2,00E-28	GLOS_DGRI_GH2	GO:0007596	B	Refseq	blood coagulation
XP_001987246.1 GH21812 [Drosophila grimshawi]	XP_001987246.1	2,00E-28	GLOS_DGRI_GH2	GO:0007602	B	Refseq	phototransduction
XP_001987246.1 GH21812 [Drosophila grimshawi]	XP_001987246.1	2,00E-28	GLOS_DGRI_GH2	GO:0010524	B	Refseq	positive regulation of calcium ion transport into cytosol
XP_001987246.1 GH21812 [Drosophila grimshawi]	XP_001987246.1	2,00E-28	GLOS_DGRI_GH2	GO:0015279	M	Refseq	store-operated calcium channel activity
XP_001987246.1 GH21812 [Drosophila grimshawi]	XP_001987246.1	2,00E-28	GLOS_DGRI_GH2	GO:0030168	B	Refseq	platelet activation
XP_001987246.1 GH21812 [Drosophila grimshawi]	XP_001987246.1	2,00E-28	GLOS_DGRI_GH2	GO:0033198	B	Refseq	response to ATP
XP_001987246.1 GH21812 [Drosophila grimshawi]	XP_001987246.1	2,00E-28	GLOS_DGRI_GH2	GO:0051592	B	Refseq	response to calcium ion
XP_001987246.1 GH21812 [Drosophila grimshawi]	XP_001987246.1	2,00E-28	GLOS_DGRI_GH2	GO:0070588	B	Refseq	calcium ion transmembrane transport
XP_001987246.1 GH21812 [Drosophila grimshawi]	XP_001987246.1	2,00E-28	GLOS_DGRI_GH2	GO:0070679	M	Refseq	inositol 1,4,5 trisphosphate binding
XP_001658535.1 ribosomal pseudouridine synthase [Aedes aegypti]	XP_001658535.1	0	GLOS_AAEL_AAE	GO:0030674	M	.	protein binding, bridging
XP_001658535.1 ribosomal pseudouridine synthase [Aedes aegypti]	XP_001658535.1	0	GLOS_AAEL_AAE	GO:0060090	M	.	binding, bridging
XP_001658535.1 ribosomal pseudouridine synthase [Aedes aegypti]	XP_001658535.1	0	GLOS_AAEL_AAE	GO:0035591	M	.	signaling adaptor activity
XP_001658535.1 ribosomal pseudouridine synthase [Aedes aegypti]	XP_001658535.1	0	GLOS_AAEL_AAE	GO:0005070	M	Refseq	SH3/SH2 adaptor activity
XP_001658535.1 ribosomal pseudouridine synthase [Aedes aegypti]	XP_001658535.1	0	GLOS_AAEL_AAE	GO:0005515	M	Refseq	protein binding
XP_001658535.1 ribosomal pseudouridine synthase [Aedes aegypti]	XP_001658535.1	0	GLOS_AAEL_AAE	GO:0005634	C	Refseq	nucleus
XP_001658535.1 ribosomal pseudouridine synthase [Aedes aegypti]	XP_001658535.1	0	GLOS_AAEL_AAE	GO:0005737	C	Refseq	cytoplasm
XP_001658535.1 ribosomal pseudouridine synthase [Aedes aegypti]	XP_001658535.1	0	GLOS_AAEL_AAE	GO:0007049	B	Refseq	cell cycle
XP_001658535.1 ribosomal pseudouridine synthase [Aedes aegypti]	XP_001658535.1	0	GLOS_AAEL_AAE	GO:0007165	B	Refseq	signal transduction
XP_001658535.1 ribosomal pseudouridine synthase [Aedes aegypti]	XP_001658535.1	0	GLOS_AAEL_AAE	GO:0007417	B	Refseq	central nervous system development
XP_001658535.1 ribosomal pseudouridine synthase [Aedes aegypti]	XP_001658535.1	0	GLOS_AAEL_AAE	GO:0048513	B	.	organ development
XP_001658535.1 ribosomal pseudouridine synthase [Aedes aegypti]	XP_001658535.1	0	GLOS_AAEL_AAE	GO:0072358	B	.	cardiovascular system development
XP_001658535.1 ribosomal pseudouridine synthase [Aedes aegypti]	XP_001658535.1	0	GLOS_AAEL_AAE	GO:0072359	B	.	circulatory system development
XP_001658535.1 ribosomal pseudouridine synthase [Aedes aegypti]	XP_001658535.1	0	GLOS_AAEL_AAE	GO:0007507	B	Refseq	heart development
XP_001658535.1 ribosomal pseudouridine synthase [Aedes aegypti]	XP_001658535.1	0	GLOS_AAEL_AAE	GO:0009790	B	.	embryo development
XP_001658535.1 ribosomal pseudouridine synthase [Aedes aegypti]	XP_001658535.1	0	GLOS_AAEL_AAE	GO:0009792	B	Refseq	embryo development ending in birth or egg hatching
XP_001658535.1 ribosomal pseudouridine synthase [Aedes aegypti]	XP_001658535.1	0	GLOS_AAEL_AAE	GO:0010212	B	Refseq	response to ionizing radiation
XP_001658535.1 ribosomal pseudouridine synthase [Aedes aegypti]	XP_001658535.1	0	GLOS_AAEL_AAE	GO:0019904	M	.	protein domain specific binding
XP_001658535.1 ribosomal pseudouridine synthase [Aedes aegypti]	XP_001658535.1	0	GLOS_AAEL_AAE	GO:0017124	M	Refseq	SH3 domain binding
XP_001658535.1 ribosomal pseudouridine synthase [Aedes aegypti]	XP_001658535.1	0	GLOS_AAEL_AAE	GO:0042802	M	Refseq	identical protein binding
XP_001658535.1 ribosomal pseudouridine synthase [Aedes aegypti]	XP_001658535.1	0	GLOS_AAEL_AAE	GO:0051726	B	.	regulation of cell cycle
XP_001658535.1 ribosomal pseudouridine synthase [Aedes aegypti]	XP_001658535.1	0	GLOS_AAEL_AAE	GO:0045786	B	Refseq	negative regulation of cell cycle
XP_001658535.1 ribosomal pseudouridine synthase [Aedes aegypti]	XP_001658535.1	0	GLOS_AAEL_AAE	GO:0048471	C	Refseq	perinuclear region of cytoplasm
XP_001658535.1 ribosomal pseudouridine synthase [Aedes aegypti]	XP_001658535.1	0	GLOS_AAEL_AAE	GO:0051059	M	Refseq	NF-kappaB binding
XP_001658535.1 ribosomal pseudouridine synthase [Aedes aegypti]	XP_001658535.1	0	GLOS_AAEL_AAE	GO:0072331	B	.	signal transduction by p53 class mediator
XP_001658535.1 ribosomal pseudouridine synthase [Aedes aegypti]	XP_001658535.1	0	GLOS_AAEL_AAE	GO:0035556	B	.	intracellular signal transduction
XP_001658535.1 ribosomal pseudouridine synthase [Aedes aegypti]	XP_001658535.1	0	GLOS_AAEL_AAE	GO:0097193	B	.	intrinsic apoptotic signaling pathway
XP_001658535.1 ribosomal pseudouridine synthase [Aedes aegypti]	XP_001658535.1	0	GLOS_AAEL_AAE	GO:0097190	B	.	apoptotic signaling pathway
XP_001658535.1 ribosomal pseudouridine synthase [Aedes aegypti]	XP_001658535.1	0	GLOS_AAEL_AAE	GO:0006915	B	.	apoptotic process
XP_001658535.1 ribosomal pseudouridine synthase [Aedes aegypti]	XP_001658535.1	0	GLOS_AAEL_AAE	GO:0012501	B	.	programmed cell death

XP_001658535.1 ribosomal pseudouridine synthase [Aedes aegypti]	XP_001658535.1	0	GLOS_AAEL_AAE	GO:0008219	B	.	cell death
XP_001658535.1 ribosomal pseudouridine synthase [Aedes aegypti]	XP_001658535.1	0	GLOS_AAEL_AAE	GO:0016265	B	.	death
XP_001658535.1 ribosomal pseudouridine synthase [Aedes aegypti]	XP_001658535.1	0	GLOS_AAEL_AAE	GO:0072332	B	Refseq	intrinsic apoptotic signaling pathway by p53 class mediator
NP_001137631.1 KCNQ potassium channel, isoform C [D.m.]	NP_001137631.1	2,00E-20	GLOS_contig_005	GO:0000122	B	Refseq	negative regulation of transcription from RNA polymerase II promoter
NP_001137631.1 KCNQ potassium channel, isoform C [D.m.]	NP_001137631.1	2,00E-20	GLOS_contig_005	GO:0001501	B	Refseq	skeletal system development
NP_001137631.1 KCNQ potassium channel, isoform C [D.m.]	NP_001137631.1	2,00E-20	GLOS_contig_005	GO:0003700	M	Refseq	sequence-specific DNA binding transcription factor activity
NP_001137631.1 KCNQ potassium channel, isoform C [D.m.]	NP_001137631.1	2,00E-20	GLOS_contig_005	GO:0005515	M	Refseq	protein binding
NP_001137631.1 KCNQ potassium channel, isoform C [D.m.]	NP_001137631.1	2,00E-20	GLOS_contig_005	GO:0005634	C	Refseq	nucleus
NP_001137631.1 KCNQ potassium channel, isoform C [D.m.]	NP_001137631.1	2,00E-20	GLOS_contig_005	GO:0006366	B	Refseq	transcription from RNA polymerase II promoter
NP_001137631.1 KCNQ potassium channel, isoform C [D.m.]	NP_001137631.1	2,00E-20	GLOS_contig_005	GO:0006607	B	Refseq	NLS-bearing protein import into nucleus
NP_001137631.1 KCNQ potassium channel, isoform C [D.m.]	NP_001137631.1	2,00E-20	GLOS_contig_005	GO:0008270	M	Refseq	zinc ion binding
NP_001137631.1 KCNQ potassium channel, isoform C [D.m.]	NP_001137631.1	2,00E-20	GLOS_contig_005	GO:0032330	B	Refseq	regulation of chondrocyte differentiation
NP_001137631.1 KCNQ potassium channel, isoform C [D.m.]	NP_001137631.1	2,00E-20	GLOS_contig_005	GO:0043565	M	Refseq	sequence-specific DNA binding
XP_004925906.1 PREDICT.: piggyBac transpos element-derived prot 3-like	XP_004925906.1	1,00E-115	GLOS_LOC10174	GO:0000122	B	Refseq	negative regulation of transcription from RNA polymerase II promoter
XP_004925906.1 PREDICT.: piggyBac transpos element-derived prot 3-like	XP_004925906.1	1,00E-115	GLOS_LOC10174	GO:0003674	M	Refseq	molecular_function
XP_004925906.1 PREDICT.: piggyBac transpos element-derived prot 3-like	XP_004925906.1	1,00E-115	GLOS_LOC10174	GO:0003682	M	Refseq	chromatin binding
XP_004925906.1 PREDICT.: piggyBac transpos element-derived prot 3-like	XP_004925906.1	1,00E-115	GLOS_LOC10174	GO:0003705	M	Refseq	RNA polymerase II distal enhancer sequence-specific DNA binding transcription factor activity
XP_004925906.1 PREDICT.: piggyBac transpos element-derived prot 3-like	XP_004925906.1	1,00E-115	GLOS_LOC10174	GO:0003714	M	Refseq	transcription corepressor activity
XP_004925906.1 PREDICT.: piggyBac transpos element-derived prot 3-like	XP_004925906.1	1,00E-115	GLOS_LOC10174	GO:0005634	C	Refseq	nucleus
XP_004925906.1 PREDICT.: piggyBac transpos element-derived prot 3-like	XP_004925906.1	1,00E-115	GLOS_LOC10174	GO:0005654	C	Refseq	nucleoplasm
XP_004925906.1 PREDICT.: piggyBac transpos element-derived prot 3-like	XP_004925906.1	1,00E-115	GLOS_LOC10174	GO:0007219	B	Refseq	Notch signaling pathway
XP_004925906.1 PREDICT.: piggyBac transpos element-derived prot 3-like	XP_004925906.1	1,00E-115	GLOS_LOC10174	GO:0008150	B	Refseq	biological_process
XP_004925906.1 PREDICT.: piggyBac transpos element-derived prot 3-like	XP_004925906.1	1,00E-115	GLOS_LOC10174	GO:0016055	B	Refseq	Wnt receptor signaling pathway
XP_004925906.1 PREDICT.: piggyBac transpos element-derived prot 3-like	XP_004925906.1	1,00E-115	GLOS_LOC10174	GO:0070491	M	Refseq	repressing transcription factor binding
XP_001984442.1 GH16460 [Drosophila grimshawi]	XP_001984442.1	3,00E-06	GLOS_DGRI_GH1	GO:0005262	M	Refseq	calcium channel activity
XP_001984442.1 GH16460 [Drosophila grimshawi]	XP_001984442.1	3,00E-06	GLOS_DGRI_GH1	GO:0005515	M	Refseq	protein binding
XP_001984442.1 GH16460 [Drosophila grimshawi]	XP_001984442.1	3,00E-06	GLOS_DGRI_GH1	GO:0005886	C	Refseq	plasma membrane
XP_001984442.1 GH16460 [Drosophila grimshawi]	XP_001984442.1	3,00E-06	GLOS_DGRI_GH1	GO:0005887	C	Refseq	integral to plasma membrane
XP_001984442.1 GH16460 [Drosophila grimshawi]	XP_001984442.1	3,00E-06	GLOS_DGRI_GH1	GO:0006816	B	Refseq	calcium ion transport
XP_001984442.1 GH16460 [Drosophila grimshawi]	XP_001984442.1	3,00E-06	GLOS_DGRI_GH1	GO:0007411	B	Refseq	axon guidance
XP_001984442.1 GH16460 [Drosophila grimshawi]	XP_001984442.1	3,00E-06	GLOS_DGRI_GH1	GO:0007596	B	Refseq	blood coagulation
XP_001984442.1 GH16460 [Drosophila grimshawi]	XP_001984442.1	3,00E-06	GLOS_DGRI_GH1	GO:0007602	B	Refseq	phototransduction
XP_001984442.1 GH16460 [Drosophila grimshawi]	XP_001984442.1	3,00E-06	GLOS_DGRI_GH1	GO:0010524	B	Refseq	positive regulation of calcium ion transport into cytosol
XP_001984442.1 GH16460 [Drosophila grimshawi]	XP_001984442.1	3,00E-06	GLOS_DGRI_GH1	GO:0015279	M	Refseq	store-operated calcium channel activity
XP_001984442.1 GH16460 [Drosophila grimshawi]	XP_001984442.1	3,00E-06	GLOS_DGRI_GH1	GO:0030168	B	Refseq	platelet activation
XP_001984442.1 GH16460 [Drosophila grimshawi]	XP_001984442.1	3,00E-06	GLOS_DGRI_GH1	GO:0033198	B	Refseq	response to ATP
XP_001984442.1 GH16460 [Drosophila grimshawi]	XP_001984442.1	3,00E-06	GLOS_DGRI_GH1	GO:0051592	B	Refseq	response to calcium ion
XP_001984442.1 GH16460 [Drosophila grimshawi]	XP_001984442.1	3,00E-06	GLOS_DGRI_GH1	GO:0070588	B	Refseq	calcium ion transmembrane transport
XP_001984442.1 GH16460 [Drosophila grimshawi]	XP_001984442.1	3,00E-06	GLOS_DGRI_GH1	GO:0070679	M	Refseq	inositol 1,4,5 trisphosphate binding
NP_572801.2 CG42258, isoform C [Drosophila melanogaster]	NP_572801.2	9,00E-76	GLOS_CG42258.1	GO:0000122	B	Refseq	negative regulation of transcription from RNA polymerase II promoter
NP_572801.2 CG42258, isoform C [Drosophila melanogaster]	NP_572801.2	9,00E-76	GLOS_CG42258.1	GO:0001501	B	Refseq	skeletal system development
NP_572801.2 CG42258, isoform C [Drosophila melanogaster]	NP_572801.2	9,00E-76	GLOS_CG42258.1	GO:0003700	M	Refseq	sequence-specific DNA binding transcription factor activity
NP_572801.2 CG42258, isoform C [Drosophila melanogaster]	NP_572801.2	9,00E-76	GLOS_CG42258.1	GO:0005515	M	Refseq	protein binding
NP_572801.2 CG42258, isoform C [Drosophila melanogaster]	NP_572801.2	9,00E-76	GLOS_CG42258.1	GO:0005634	C	Refseq	nucleus
NP_572801.2 CG42258, isoform C [Drosophila melanogaster]	NP_572801.2	9,00E-76	GLOS_CG42258.1	GO:0006366	B	Refseq	transcription from RNA polymerase II promoter
NP_572801.2 CG42258, isoform C [Drosophila melanogaster]	NP_572801.2	9,00E-76	GLOS_CG42258.1	GO:0006607	B	Refseq	NLS-bearing protein import into nucleus
NP_572801.2 CG42258, isoform C [Drosophila melanogaster]	NP_572801.2	9,00E-76	GLOS_CG42258.1	GO:0008270	M	Refseq	zinc ion binding
NP_572801.2 CG42258, isoform C [Drosophila melanogaster]	NP_572801.2	9,00E-76	GLOS_CG42258.1	GO:0032330	B	Refseq	regulation of chondrocyte differentiation
NP_572801.2 CG42258, isoform C [Drosophila melanogaster]	NP_572801.2	9,00E-76	GLOS_CG42258.1	GO:0043565	M	Refseq	sequence-specific DNA binding
NP_650306.1 flyers-cup, isoform B [Drosophila melanogaster]	NP_650306.1	0	GLOS_F-CUP.1.1	GO:0000122	B	Refseq	negative regulation of transcription from RNA polymerase II promoter
NP_650306.1 flyers-cup, isoform B [Drosophila melanogaster]	NP_650306.1	0	GLOS_F-CUP.1.1	GO:0001501	B	Refseq	skeletal system development
NP_650306.1 flyers-cup, isoform B [Drosophila melanogaster]	NP_650306.1	0	GLOS_F-CUP.1.1	GO:0003700	M	Refseq	sequence-specific DNA binding transcription factor activity
NP_650306.1 flyers-cup, isoform B [Drosophila melanogaster]	NP_650306.1	0	GLOS_F-CUP.1.1	GO:0005515	M	Refseq	protein binding

NP_650306.1 flyers-cup, isoform B [Drosophila melanogaster]	NP_650306.1	0	GLOS_F-CUP.1.1	GO:0005634	C	Refseq	nucleus
NP_650306.1 flyers-cup, isoform B [Drosophila melanogaster]	NP_650306.1	0	GLOS_F-CUP.1.1	GO:0006366	B	Refseq	transcription from RNA polymerase II promoter
NP_650306.1 flyers-cup, isoform B [Drosophila melanogaster]	NP_650306.1	0	GLOS_F-CUP.1.1	GO:0006607	B	Refseq	NLS-bearing protein import into nucleus
NP_650306.1 flyers-cup, isoform B [Drosophila melanogaster]	NP_650306.1	0	GLOS_F-CUP.1.1	GO:0008270	M	Refseq	zinc ion binding
NP_650306.1 flyers-cup, isoform B [Drosophila melanogaster]	NP_650306.1	0	GLOS_F-CUP.1.1	GO:0032330	B	Refseq	regulation of chondrocyte differentiation
NP_650306.1 flyers-cup, isoform B [Drosophila melanogaster]	NP_650306.1	0	GLOS_F-CUP.1.1	GO:0043565	M	Refseq	sequence-specific DNA binding
XP_001979909.1 GG21380 [Drosophila erecta]	XP_001979909.1	0	GLOS_DERE_GG	GO:0005261	M	Refseq	cation channel activity
XP_001979909.1 GG21380 [Drosophila erecta]	XP_001979909.1	0	GLOS_DERE_GG	GO:0005262	M	Refseq	calcium channel activity
XP_001979909.1 GG21380 [Drosophila erecta]	XP_001979909.1	0	GLOS_DERE_GG	GO:0005515	M	Refseq	protein binding
XP_001979909.1 GG21380 [Drosophila erecta]	XP_001979909.1	0	GLOS_DERE_GG	GO:0005886	C	Refseq	plasma membrane
XP_001979909.1 GG21380 [Drosophila erecta]	XP_001979909.1	0	GLOS_DERE_GG	GO:0005887	C	Refseq	integral to plasma membrane
XP_001979909.1 GG21380 [Drosophila erecta]	XP_001979909.1	0	GLOS_DERE_GG	GO:0006816	B	Refseq	calcium ion transport
XP_001979909.1 GG21380 [Drosophila erecta]	XP_001979909.1	0	GLOS_DERE_GG	GO:0007411	B	Refseq	axon guidance
XP_001979909.1 GG21380 [Drosophila erecta]	XP_001979909.1	0	GLOS_DERE_GG	GO:0015279	M	Refseq	store-operated calcium channel activity
XP_001979909.1 GG21380 [Drosophila erecta]	XP_001979909.1	0	GLOS_DERE_GG	GO:0016323	C	Refseq	basolateral plasma membrane
XP_001979909.1 GG21380 [Drosophila erecta]	XP_001979909.1	0	GLOS_DERE_GG	GO:0030017	C	Refseq	sarcomere
XP_001979909.1 GG21380 [Drosophila erecta]	XP_001979909.1	0	GLOS_DERE_GG	GO:0043034	C	Refseq	costamere
XP_001979909.1 GG21380 [Drosophila erecta]	XP_001979909.1	0	GLOS_DERE_GG	GO:0043234	C	Refseq	protein complex
XP_001979909.1 GG21380 [Drosophila erecta]	XP_001979909.1	0	GLOS_DERE_GG	GO:0044325	M	Refseq	ion channel binding
XP_001979909.1 GG21380 [Drosophila erecta]	XP_001979909.1	0	GLOS_DERE_GG	GO:0045121	C	Refseq	membrane raft
XP_001979909.1 GG21380 [Drosophila erecta]	XP_001979909.1	0	GLOS_DERE_GG	GO:0046541	B	Refseq	saliva secretion
XP_001979909.1 GG21380 [Drosophila erecta]	XP_001979909.1	0	GLOS_DERE_GG	GO:0051281	B	Refseq	positive regulation of release of sequestered calcium ion into cytosol
XP_001979909.1 GG21380 [Drosophila erecta]	XP_001979909.1	0	GLOS_DERE_GG	GO:0051480	B	Refseq	cytosolic calcium ion homeostasis
XP_001979909.1 GG21380 [Drosophila erecta]	XP_001979909.1	0	GLOS_DERE_GG	GO:0051592	B	Refseq	response to calcium ion
XP_001979909.1 GG21380 [Drosophila erecta]	XP_001979909.1	0	GLOS_DERE_GG	GO:0070679	M	Refseq	inositol 1,4,5 trisphosphate binding
NP_728540.1 CG32483 [Drosophila melanogaster]	NP_728540.1	5,00E-99	GLOS_CG32483.1	GO:0000122	B	Refseq	negative regulation of transcription from RNA polymerase II promoter
NP_728540.1 CG32483 [Drosophila melanogaster]	NP_728540.1	5,00E-99	GLOS_CG32483.1	GO:0001501	B	Refseq	skeletal system development
NP_728540.1 CG32483 [Drosophila melanogaster]	NP_728540.1	5,00E-99	GLOS_CG32483.1	GO:0003700	M	Refseq	sequence-specific DNA binding transcription factor activity
NP_728540.1 CG32483 [Drosophila melanogaster]	NP_728540.1	5,00E-99	GLOS_CG32483.1	GO:0005515	M	Refseq	protein binding
NP_728540.1 CG32483 [Drosophila melanogaster]	NP_728540.1	5,00E-99	GLOS_CG32483.1	GO:0005634	C	Refseq	nucleus
NP_728540.1 CG32483 [Drosophila melanogaster]	NP_728540.1	5,00E-99	GLOS_CG32483.1	GO:0006366	B	Refseq	transcription from RNA polymerase II promoter
NP_728540.1 CG32483 [Drosophila melanogaster]	NP_728540.1	5,00E-99	GLOS_CG32483.1	GO:0006607	B	Refseq	NLS-bearing protein import into nucleus
NP_728540.1 CG32483 [Drosophila melanogaster]	NP_728540.1	5,00E-99	GLOS_CG32483.1	GO:0008270	M	Refseq	zinc ion binding
NP_728540.1 CG32483 [Drosophila melanogaster]	NP_728540.1	5,00E-99	GLOS_CG32483.1	GO:0032330	B	Refseq	regulation of chondrocyte differentiation
NP_728540.1 CG32483 [Drosophila melanogaster]	NP_728540.1	5,00E-99	GLOS_CG32483.1	GO:0043565	M	Refseq	sequence-specific DNA binding
XP_004921797.1 PREDICT: bifunctional glutamate/proline--tRNA ligase-like	XP_004921797.1	0	GLOS_LOC10173	GO:0000122	B	Refseq	negative regulation of transcription from RNA polymerase II promoter
XP_004921797.1 PREDICT: bifunctional glutamate/proline--tRNA ligase-like	XP_004921797.1	0	GLOS_LOC10173	GO:0003674	M	Refseq	molecular_function
XP_004921797.1 PREDICT: bifunctional glutamate/proline--tRNA ligase-like	XP_004921797.1	0	GLOS_LOC10173	GO:0003682	M	Refseq	chromatin binding
XP_004921797.1 PREDICT: bifunctional glutamate/proline--tRNA ligase-like	XP_004921797.1	0	GLOS_LOC10173	GO:0003705	M	Refseq	RNA polymerase II distal enhancer sequence-specific DNA binding transcription factor activity
XP_004921797.1 PREDICT: bifunctional glutamate/proline--tRNA ligase-like	XP_004921797.1	0	GLOS_LOC10173	GO:0003714	M	Refseq	transcription corepressor activity
XP_004921797.1 PREDICT: bifunctional glutamate/proline--tRNA ligase-like	XP_004921797.1	0	GLOS_LOC10173	GO:0005634	C	Refseq	nucleus
XP_004921797.1 PREDICT: bifunctional glutamate/proline--tRNA ligase-like	XP_004921797.1	0	GLOS_LOC10173	GO:0005654	C	Refseq	nucleoplasm
XP_004921797.1 PREDICT: bifunctional glutamate/proline--tRNA ligase-like	XP_004921797.1	0	GLOS_LOC10173	GO:0007219	B	Refseq	Notch signaling pathway
XP_004921797.1 PREDICT: bifunctional glutamate/proline--tRNA ligase-like	XP_004921797.1	0	GLOS_LOC10173	GO:0008150	B	Refseq	biological_process
XP_004921797.1 PREDICT: bifunctional glutamate/proline--tRNA ligase-like	XP_004921797.1	0	GLOS_LOC10173	GO:0016055	B	Refseq	Wnt receptor signaling pathway
XP_004921797.1 PREDICT: bifunctional glutamate/proline--tRNA ligase-like	XP_004921797.1	0	GLOS_LOC10173	GO:0070491	M	Refseq	repressing transcription factor binding
XP_809984.1 hypothetical protein [Trypanosoma cruzi strain CL Brener]	XP_809984.1	1,00E-142	GLOS_TC00.1047	GO:0060089	M	.	molecular transducer activity
XP_809984.1 hypothetical protein [Trypanosoma cruzi strain CL Brener]	XP_809984.1	1,00E-142	GLOS_TC00.1047	GO:0004871	M	Refseq	signal transducer activity
XP_809984.1 hypothetical protein [Trypanosoma cruzi strain CL Brener]	XP_809984.1	1,00E-142	GLOS_TC00.1047	GO:0004888	M	.	transmembrane signaling receptor activity
XP_809984.1 hypothetical protein [Trypanosoma cruzi strain CL Brener]	XP_809984.1	1,00E-142	GLOS_TC00.1047	GO:0038023	M	.	signaling receptor activity
XP_809984.1 hypothetical protein [Trypanosoma cruzi strain CL Brener]	XP_809984.1	1,00E-142	GLOS_TC00.1047	GO:0004871	M	.	signal transducer activity
XP_809984.1 hypothetical protein [Trypanosoma cruzi strain CL Brener]	XP_809984.1	1,00E-142	GLOS_TC00.1047	GO:0004872	M	.	receptor activity
XP_809984.1 hypothetical protein [Trypanosoma cruzi strain CL Brener]	XP_809984.1	1,00E-142	GLOS_TC00.1047	GO:0004930	M	Refseq	G-protein coupled receptor activity

XP_809984.1 hypothetical protein [Trypanosoma cruzi strain CL Brener]	XP_809984.1	1,00E-142	GLOS_TC00.1047	GO:0008528	M	.	G-protein coupled peptide receptor activity
XP_809984.1 hypothetical protein [Trypanosoma cruzi strain CL Brener]	XP_809984.1	1,00E-142	GLOS_TC00.1047	GO:0001653	M	.	peptide receptor activity
XP_809984.1 hypothetical protein [Trypanosoma cruzi strain CL Brener]	XP_809984.1	1,00E-142	GLOS_TC00.1047	GO:0004930	M	.	G-protein coupled receptor activity
XP_809984.1 hypothetical protein [Trypanosoma cruzi strain CL Brener]	XP_809984.1	1,00E-142	GLOS_TC00.1047	GO:0004977	M	Refseq	melanocortin receptor activity
XP_809984.1 hypothetical protein [Trypanosoma cruzi strain CL Brener]	XP_809984.1	1,00E-142	GLOS_TC00.1047	GO:0005886	C	Refseq	plasma membrane
XP_809984.1 hypothetical protein [Trypanosoma cruzi strain CL Brener]	XP_809984.1	1,00E-142	GLOS_TC00.1047	GO:0007165	B	Refseq	signal transduction
XP_809984.1 hypothetical protein [Trypanosoma cruzi strain CL Brener]	XP_809984.1	1,00E-142	GLOS_TC00.1047	GO:0007186	B	Refseq	G-protein coupled receptor signaling pathway
XP_809984.1 hypothetical protein [Trypanosoma cruzi strain CL Brener]	XP_809984.1	1,00E-142	GLOS_TC00.1047	GO:0016020	C	Refseq	membrane
XP_809984.1 hypothetical protein [Trypanosoma cruzi strain CL Brener]	XP_809984.1	1,00E-142	GLOS_TC00.1047	GO:0016021	C	Refseq	integral to membrane
NP_611058.4 Z band alternatively spliced PDZ-motif prot 52, isoform I	NP_611058.4	0	GLOS_contig_005	GO:0000122	B	Refseq	negative regulation of transcription from RNA polymerase II promoter
NP_611058.4 Z band alternatively spliced PDZ-motif prot 52, isoform I	NP_611058.4	0	GLOS_contig_005	GO:0001501	B	Refseq	skeletal system development
NP_611058.4 Z band alternatively spliced PDZ-motif prot 52, isoform I	NP_611058.4	0	GLOS_contig_005	GO:0003700	M	Refseq	sequence-specific DNA binding transcription factor activity
NP_611058.4 Z band alternatively spliced PDZ-motif prot 52, isoform I	NP_611058.4	0	GLOS_contig_005	GO:0005515	M	Refseq	protein binding
NP_611058.4 Z band alternatively spliced PDZ-motif prot 52, isoform I	NP_611058.4	0	GLOS_contig_005	GO:0005634	C	Refseq	nucleus
NP_611058.4 Z band alternatively spliced PDZ-motif prot 52, isoform I	NP_611058.4	0	GLOS_contig_005	GO:0006366	B	Refseq	transcription from RNA polymerase II promoter
NP_611058.4 Z band alternatively spliced PDZ-motif prot 52, isoform I	NP_611058.4	0	GLOS_contig_005	GO:0006607	B	Refseq	NLS-bearing protein import into nucleus
NP_611058.4 Z band alternatively spliced PDZ-motif prot 52, isoform I	NP_611058.4	0	GLOS_contig_005	GO:0008270	M	Refseq	zinc ion binding
NP_611058.4 Z band alternatively spliced PDZ-motif prot 52, isoform I	NP_611058.4	0	GLOS_contig_005	GO:0032330	B	Refseq	regulation of chondrocyte differentiation
NP_611058.4 Z band alternatively spliced PDZ-motif prot 52, isoform I	NP_611058.4	0	GLOS_contig_005	GO:0043565	M	Refseq	sequence-specific DNA binding
XP_001988283.1 GH11081 [Drosophila grimshawi]	XP_001988283.1	1,00E-70	GLOS_DGRI_GH1	GO:0005262	M	Refseq	calcium channel activity
XP_001988283.1 GH11081 [Drosophila grimshawi]	XP_001988283.1	1,00E-70	GLOS_DGRI_GH1	GO:0005515	M	Refseq	protein binding
XP_001988283.1 GH11081 [Drosophila grimshawi]	XP_001988283.1	1,00E-70	GLOS_DGRI_GH1	GO:0005886	C	Refseq	plasma membrane
XP_001988283.1 GH11081 [Drosophila grimshawi]	XP_001988283.1	1,00E-70	GLOS_DGRI_GH1	GO:0005887	C	Refseq	integral to plasma membrane
XP_001988283.1 GH11081 [Drosophila grimshawi]	XP_001988283.1	1,00E-70	GLOS_DGRI_GH1	GO:0006816	B	Refseq	calcium ion transport
XP_001988283.1 GH11081 [Drosophila grimshawi]	XP_001988283.1	1,00E-70	GLOS_DGRI_GH1	GO:0007411	B	Refseq	axon guidance
XP_001988283.1 GH11081 [Drosophila grimshawi]	XP_001988283.1	1,00E-70	GLOS_DGRI_GH1	GO:0007596	B	Refseq	blood coagulation
XP_001988283.1 GH11081 [Drosophila grimshawi]	XP_001988283.1	1,00E-70	GLOS_DGRI_GH1	GO:0007602	B	Refseq	phototransduction
XP_001988283.1 GH11081 [Drosophila grimshawi]	XP_001988283.1	1,00E-70	GLOS_DGRI_GH1	GO:0010524	B	Refseq	positive regulation of calcium ion transport into cytosol
XP_001988283.1 GH11081 [Drosophila grimshawi]	XP_001988283.1	1,00E-70	GLOS_DGRI_GH1	GO:0015279	M	Refseq	store-operated calcium channel activity
XP_001988283.1 GH11081 [Drosophila grimshawi]	XP_001988283.1	1,00E-70	GLOS_DGRI_GH1	GO:0030168	B	Refseq	platelet activation
XP_001988283.1 GH11081 [Drosophila grimshawi]	XP_001988283.1	1,00E-70	GLOS_DGRI_GH1	GO:0033198	B	Refseq	response to ATP
XP_001988283.1 GH11081 [Drosophila grimshawi]	XP_001988283.1	1,00E-70	GLOS_DGRI_GH1	GO:0051592	B	Refseq	response to calcium ion
XP_001988283.1 GH11081 [Drosophila grimshawi]	XP_001988283.1	1,00E-70	GLOS_DGRI_GH1	GO:0070588	B	Refseq	calcium ion transmembrane transport
XP_001988283.1 GH11081 [Drosophila grimshawi]	XP_001988283.1	1,00E-70	GLOS_DGRI_GH1	GO:0070679	M	Refseq	inositol 1,4,5 trisphosphate binding
XP_003248683.1 PREDICT: hypoth.l prot LOC100573436 [A. pisum]	XP_003248683.1	4,00E-08	GLOS_LOC10057:	GO:0007049	B	.	cell cycle
XP_003248683.1 PREDICT: hypoth.l prot LOC100573436 [A. pisum]	XP_003248683.1	4,00E-08	GLOS_LOC10057:	GO:0000278	B	Refseq	mitotic cell cycle
XP_003248683.1 PREDICT: hypoth.l prot LOC100573436 [A. pisum]	XP_003248683.1	4,00E-08	GLOS_LOC10057:	GO:0003677	M	Refseq	DNA binding
XP_003248683.1 PREDICT: hypoth.l prot LOC100573436 [A. pisum]	XP_003248683.1	4,00E-08	GLOS_LOC10057:	GO:0003700	M	Refseq	sequence-specific DNA binding transcription factor activity
XP_003248683.1 PREDICT: hypoth.l prot LOC100573436 [A. pisum]	XP_003248683.1	4,00E-08	GLOS_LOC10057:	GO:0003712	M	Refseq	transcription cofactor activity
XP_003248683.1 PREDICT: hypoth.l prot LOC100573436 [A. pisum]	XP_003248683.1	4,00E-08	GLOS_LOC10057:	GO:0005654	C	Refseq	nucleoplasm
XP_003248683.1 PREDICT: hypoth.l prot LOC100573436 [A. pisum]	XP_003248683.1	4,00E-08	GLOS_LOC10057:	GO:0043234	C	.	protein complex
XP_003248683.1 PREDICT: hypoth.l prot LOC100573436 [A. pisum]	XP_003248683.1	4,00E-08	GLOS_LOC10057:	GO:0005667	C	Refseq	transcription factor complex
XP_003248683.1 PREDICT: hypoth.l prot LOC100573436 [A. pisum]	XP_003248683.1	4,00E-08	GLOS_LOC10057:	GO:0006351	B	Refseq	transcription, DNA-dependent
XP_003248683.1 PREDICT: hypoth.l prot LOC100573436 [A. pisum]	XP_003248683.1	4,00E-08	GLOS_LOC10057:	GO:0006352	B	.	DNA-dependent transcription, initiation
XP_003248683.1 PREDICT: hypoth.l prot LOC100573436 [A. pisum]	XP_003248683.1	4,00E-08	GLOS_LOC10057:	GO:0006366	B	.	transcription from RNA polymerase II promoter
XP_003248683.1 PREDICT: hypoth.l prot LOC100573436 [A. pisum]	XP_003248683.1	4,00E-08	GLOS_LOC10057:	GO:0006367	B	Refseq	transcription initiation from RNA polymerase II promoter
XP_003248683.1 PREDICT: hypoth.l prot LOC100573436 [A. pisum]	XP_003248683.1	4,00E-08	GLOS_LOC10057:	GO:0007178	B	.	transmembrane receptor protein serine/threonine kinase signaling pathway
XP_003248683.1 PREDICT: hypoth.l prot LOC100573436 [A. pisum]	XP_003248683.1	4,00E-08	GLOS_LOC10057:	GO:0007167	B	.	enzyme linked receptor protein signaling pathway
XP_003248683.1 PREDICT: hypoth.l prot LOC100573436 [A. pisum]	XP_003248683.1	4,00E-08	GLOS_LOC10057:	GO:0071560	B	.	cellular response to transforming growth factor beta stimulus
XP_003248683.1 PREDICT: hypoth.l prot LOC100573436 [A. pisum]	XP_003248683.1	4,00E-08	GLOS_LOC10057:	GO:0071363	B	.	cellular response to growth factor stimulus
XP_003248683.1 PREDICT: hypoth.l prot LOC100573436 [A. pisum]	XP_003248683.1	4,00E-08	GLOS_LOC10057:	GO:0070848	B	.	response to growth factor stimulus
XP_003248683.1 PREDICT: hypoth.l prot LOC100573436 [A. pisum]	XP_003248683.1	4,00E-08	GLOS_LOC10057:	GO:0071310	B	.	cellular response to organic substance
XP_003248683.1 PREDICT: hypoth.l prot LOC100573436 [A. pisum]	XP_003248683.1	4,00E-08	GLOS_LOC10057:	GO:0070887	B	.	cellular response to chemical stimulus
XP_003248683.1 PREDICT: hypoth.l prot LOC100573436 [A. pisum]	XP_003248683.1	4,00E-08	GLOS_LOC10057:	GO:0071495	B	.	cellular response to endogenous stimulus

XP_003248683.1 PREDICT: hypoth.l prot LOC100573436 [A. pisum]	XP_003248683.1	4,00E-08	GLOS_LOC10057:	GO:0071559	B	.	response to transforming growth factor beta stimulus
XP_003248683.1 PREDICT: hypoth.l prot LOC100573436 [A. pisum]	XP_003248683.1	4,00E-08	GLOS_LOC10057:	GO:0007179	B	Refseq	transforming growth factor beta receptor signaling pathway
XP_003248683.1 PREDICT: hypoth.l prot LOC100573436 [A. pisum]	XP_003248683.1	4,00E-08	GLOS_LOC10057:	GO:0007507	B	Refseq	heart development
XP_003248683.1 PREDICT: hypoth.l prot LOC100573436 [A. pisum]	XP_003248683.1	4,00E-08	GLOS_LOC10057:	GO:0008134	M	Refseq	transcription factor binding
XP_003248683.1 PREDICT: hypoth.l prot LOC100573436 [A. pisum]	XP_003248683.1	4,00E-08	GLOS_LOC10057:	GO:0010467	B	Refseq	gene expression
XP_003248683.1 PREDICT: hypoth.l prot LOC100573436 [A. pisum]	XP_003248683.1	4,00E-08	GLOS_LOC10057:	GO:0019904	M	Refseq	protein domain specific binding
XP_003248683.1 PREDICT: hypoth.l prot LOC100573436 [A. pisum]	XP_003248683.1	4,00E-08	GLOS_LOC10057:	GO:0045893	B	.	positive regulation of transcription, DNA-dependent
XP_003248683.1 PREDICT: hypoth.l prot LOC100573436 [A. pisum]	XP_003248683.1	4,00E-08	GLOS_LOC10057:	GO:0010557	B	.	positive regulation of macromolecule biosynthetic process
XP_003248683.1 PREDICT: hypoth.l prot LOC100573436 [A. pisum]	XP_003248683.1	4,00E-08	GLOS_LOC10057:	GO:0009891	B	.	positive regulation of biosynthetic process
XP_003248683.1 PREDICT: hypoth.l prot LOC100573436 [A. pisum]	XP_003248683.1	4,00E-08	GLOS_LOC10057:	GO:0009893	B	.	positive regulation of metabolic process
XP_003248683.1 PREDICT: hypoth.l prot LOC100573436 [A. pisum]	XP_003248683.1	4,00E-08	GLOS_LOC10057:	GO:0010604	B	.	positive regulation of macromolecule metabolic process
XP_003248683.1 PREDICT: hypoth.l prot LOC100573436 [A. pisum]	XP_003248683.1	4,00E-08	GLOS_LOC10057:	GO:0010628	B	.	positive regulation of gene expression
XP_003248683.1 PREDICT: hypoth.l prot LOC100573436 [A. pisum]	XP_003248683.1	4,00E-08	GLOS_LOC10057:	GO:0031328	B	.	positive regulation of cellular biosynthetic process
XP_003248683.1 PREDICT: hypoth.l prot LOC100573436 [A. pisum]	XP_003248683.1	4,00E-08	GLOS_LOC10057:	GO:0031325	B	.	positive regulation of cellular metabolic process
XP_003248683.1 PREDICT: hypoth.l prot LOC100573436 [A. pisum]	XP_003248683.1	4,00E-08	GLOS_LOC10057:	GO:0051254	B	.	positive regulation of RNA metabolic process
XP_003248683.1 PREDICT: hypoth.l prot LOC100573436 [A. pisum]	XP_003248683.1	4,00E-08	GLOS_LOC10057:	GO:0045935	B	.	positive regulation of nucleobase-containing compound metabolic process
XP_003248683.1 PREDICT: hypoth.l prot LOC100573436 [A. pisum]	XP_003248683.1	4,00E-08	GLOS_LOC10057:	GO:0051173	B	.	positive regulation of nitrogen compound metabolic process
XP_003248683.1 PREDICT: hypoth.l prot LOC100573436 [A. pisum]	XP_003248683.1	4,00E-08	GLOS_LOC10057:	GO:0045944	B	Refseq	positive regulation of transcription from RNA polymerase II promoter
NP_001262624.1 gilgamesh, isoform L [Drosophila melanogaster]	NP_001262624.1	0	GLOS_GISH.1.4	GO:0000122	B	Refseq	negative regulation of transcription from RNA polymerase II promoter
NP_001262624.1 gilgamesh, isoform L [Drosophila melanogaster]	NP_001262624.1	0	GLOS_GISH.1.4	GO:0001501	B	Refseq	skeletal system development
NP_001262624.1 gilgamesh, isoform L [Drosophila melanogaster]	NP_001262624.1	0	GLOS_GISH.1.4	GO:0003700	M	Refseq	sequence-specific DNA binding transcription factor activity
NP_001262624.1 gilgamesh, isoform L [Drosophila melanogaster]	NP_001262624.1	0	GLOS_GISH.1.4	GO:0005515	M	Refseq	protein binding
NP_001262624.1 gilgamesh, isoform L [Drosophila melanogaster]	NP_001262624.1	0	GLOS_GISH.1.4	GO:0005634	C	Refseq	nucleus
NP_001262624.1 gilgamesh, isoform L [Drosophila melanogaster]	NP_001262624.1	0	GLOS_GISH.1.4	GO:0006366	B	Refseq	transcription from RNA polymerase II promoter
NP_001262624.1 gilgamesh, isoform L [Drosophila melanogaster]	NP_001262624.1	0	GLOS_GISH.1.4	GO:0006607	B	Refseq	NLS-bearing protein import into nucleus
NP_001262624.1 gilgamesh, isoform L [Drosophila melanogaster]	NP_001262624.1	0	GLOS_GISH.1.4	GO:0008270	M	Refseq	zinc ion binding
NP_001262624.1 gilgamesh, isoform L [Drosophila melanogaster]	NP_001262624.1	0	GLOS_GISH.1.4	GO:0032330	B	Refseq	regulation of chondrocyte differentiation
NP_001262624.1 gilgamesh, isoform L [Drosophila melanogaster]	NP_001262624.1	0	GLOS_GISH.1.4	GO:0043565	M	Refseq	sequence-specific DNA binding
NP_001163175.1 patronin, isoform F [Drosophila melanogaster]	NP_001163175.1	1,00E-104	GLOS_PATRONIN	GO:0000122	B	Refseq	negative regulation of transcription from RNA polymerase II promoter
NP_001163175.1 patronin, isoform F [Drosophila melanogaster]	NP_001163175.1	1,00E-104	GLOS_PATRONIN	GO:0001501	B	Refseq	skeletal system development
NP_001163175.1 patronin, isoform F [Drosophila melanogaster]	NP_001163175.1	1,00E-104	GLOS_PATRONIN	GO:0003700	M	Refseq	sequence-specific DNA binding transcription factor activity
NP_001163175.1 patronin, isoform F [Drosophila melanogaster]	NP_001163175.1	1,00E-104	GLOS_PATRONIN	GO:0005515	M	Refseq	protein binding
NP_001163175.1 patronin, isoform F [Drosophila melanogaster]	NP_001163175.1	1,00E-104	GLOS_PATRONIN	GO:0005634	C	Refseq	nucleus
NP_001163175.1 patronin, isoform F [Drosophila melanogaster]	NP_001163175.1	1,00E-104	GLOS_PATRONIN	GO:0006366	B	Refseq	transcription from RNA polymerase II promoter
NP_001163175.1 patronin, isoform F [Drosophila melanogaster]	NP_001163175.1	1,00E-104	GLOS_PATRONIN	GO:0006607	B	Refseq	NLS-bearing protein import into nucleus
NP_001163175.1 patronin, isoform F [Drosophila melanogaster]	NP_001163175.1	1,00E-104	GLOS_PATRONIN	GO:0008270	M	Refseq	zinc ion binding
NP_001163175.1 patronin, isoform F [Drosophila melanogaster]	NP_001163175.1	1,00E-104	GLOS_PATRONIN	GO:0032330	B	Refseq	regulation of chondrocyte differentiation
NP_001163175.1 patronin, isoform F [Drosophila melanogaster]	NP_001163175.1	1,00E-104	GLOS_PATRONIN	GO:0043565	M	Refseq	sequence-specific DNA binding
XP_001994210.1 GH13487 [Drosophila grimshawi]	XP_001994210.1	2,00E-70	GLOS_DGRI_GH1	GO:0005262	M	Refseq	calcium channel activity
XP_001994210.1 GH13487 [Drosophila grimshawi]	XP_001994210.1	2,00E-70	GLOS_DGRI_GH1	GO:0005515	M	Refseq	protein binding
XP_001994210.1 GH13487 [Drosophila grimshawi]	XP_001994210.1	2,00E-70	GLOS_DGRI_GH1	GO:0005886	C	Refseq	plasma membrane
XP_001994210.1 GH13487 [Drosophila grimshawi]	XP_001994210.1	2,00E-70	GLOS_DGRI_GH1	GO:0005887	C	Refseq	integral to plasma membrane
XP_001994210.1 GH13487 [Drosophila grimshawi]	XP_001994210.1	2,00E-70	GLOS_DGRI_GH1	GO:0006816	B	Refseq	calcium ion transport
XP_001994210.1 GH13487 [Drosophila grimshawi]	XP_001994210.1	2,00E-70	GLOS_DGRI_GH1	GO:0007411	B	Refseq	axon guidance
XP_001994210.1 GH13487 [Drosophila grimshawi]	XP_001994210.1	2,00E-70	GLOS_DGRI_GH1	GO:0007596	B	Refseq	blood coagulation
XP_001994210.1 GH13487 [Drosophila grimshawi]	XP_001994210.1	2,00E-70	GLOS_DGRI_GH1	GO:0007602	B	Refseq	phototransduction
XP_001994210.1 GH13487 [Drosophila grimshawi]	XP_001994210.1	2,00E-70	GLOS_DGRI_GH1	GO:0010524	B	Refseq	positive regulation of calcium ion transport into cytosol
XP_001994210.1 GH13487 [Drosophila grimshawi]	XP_001994210.1	2,00E-70	GLOS_DGRI_GH1	GO:0015279	M	Refseq	store-operated calcium channel activity
XP_001994210.1 GH13487 [Drosophila grimshawi]	XP_001994210.1	2,00E-70	GLOS_DGRI_GH1	GO:0030168	B	Refseq	platelet activation
XP_001994210.1 GH13487 [Drosophila grimshawi]	XP_001994210.1	2,00E-70	GLOS_DGRI_GH1	GO:0033198	B	Refseq	response to ATP
XP_001994210.1 GH13487 [Drosophila grimshawi]	XP_001994210.1	2,00E-70	GLOS_DGRI_GH1	GO:0051592	B	Refseq	response to calcium ion
XP_001994210.1 GH13487 [Drosophila grimshawi]	XP_001994210.1	2,00E-70	GLOS_DGRI_GH1	GO:0070588	B	Refseq	calcium ion transmembrane transport
XP_001994210.1 GH13487 [Drosophila grimshawi]	XP_001994210.1	2,00E-70	GLOS_DGRI_GH1	GO:0070679	M	Refseq	inositol 1,4,5 trisphosphate binding
NP_001188868.1 missing-in-metastasis, isoform E [D.m.]	NP_001188868.1	3,00E-19	GLOS_contig_005	GO:0000122	B	Refseq	negative regulation of transcription from RNA polymerase II promoter

NP_001188868.1 missing-in-metastasis, isoform E [D.m.]	NP_001188868.1	3,00E-19	GLOS_contig_005	GO:0001501	B	Refseq	skeletal system development
NP_001188868.1 missing-in-metastasis, isoform E [D.m.]	NP_001188868.1	3,00E-19	GLOS_contig_005	GO:0003700	M	Refseq	sequence-specific DNA binding transcription factor activity
NP_001188868.1 missing-in-metastasis, isoform E [D.m.]	NP_001188868.1	3,00E-19	GLOS_contig_005	GO:0005515	M	Refseq	protein binding
NP_001188868.1 missing-in-metastasis, isoform E [D.m.]	NP_001188868.1	3,00E-19	GLOS_contig_005	GO:0005634	C	Refseq	nucleus
NP_001188868.1 missing-in-metastasis, isoform E [D.m.]	NP_001188868.1	3,00E-19	GLOS_contig_005	GO:0006366	B	Refseq	transcription from RNA polymerase II promoter
NP_001188868.1 missing-in-metastasis, isoform E [D.m.]	NP_001188868.1	3,00E-19	GLOS_contig_005	GO:0006607	B	Refseq	NLS-bearing protein import into nucleus
NP_001188868.1 missing-in-metastasis, isoform E [D.m.]	NP_001188868.1	3,00E-19	GLOS_contig_005	GO:0008270	M	Refseq	zinc ion binding
NP_001188868.1 missing-in-metastasis, isoform E [D.m.]	NP_001188868.1	3,00E-19	GLOS_contig_005	GO:0032330	B	Refseq	regulation of chondrocyte differentiation
NP_001188868.1 missing-in-metastasis, isoform E [D.m.]	NP_001188868.1	3,00E-19	GLOS_contig_005	GO:0043565	M	Refseq	sequence-specific DNA binding
NP_611188.3 CG30456, isoform B [Drosophila melanogaster]	NP_611188.3	0	GLOS_CG30456.2	GO:0000122	B	Refseq	negative regulation of transcription from RNA polymerase II promoter
NP_611188.3 CG30456, isoform B [Drosophila melanogaster]	NP_611188.3	0	GLOS_CG30456.2	GO:0001501	B	Refseq	skeletal system development
NP_611188.3 CG30456, isoform B [Drosophila melanogaster]	NP_611188.3	0	GLOS_CG30456.2	GO:0003700	M	Refseq	sequence-specific DNA binding transcription factor activity
NP_611188.3 CG30456, isoform B [Drosophila melanogaster]	NP_611188.3	0	GLOS_CG30456.2	GO:0005515	M	Refseq	protein binding
NP_611188.3 CG30456, isoform B [Drosophila melanogaster]	NP_611188.3	0	GLOS_CG30456.2	GO:0005634	C	Refseq	nucleus
NP_611188.3 CG30456, isoform B [Drosophila melanogaster]	NP_611188.3	0	GLOS_CG30456.2	GO:0006366	B	Refseq	transcription from RNA polymerase II promoter
NP_611188.3 CG30456, isoform B [Drosophila melanogaster]	NP_611188.3	0	GLOS_CG30456.2	GO:0006607	B	Refseq	NLS-bearing protein import into nucleus
NP_611188.3 CG30456, isoform B [Drosophila melanogaster]	NP_611188.3	0	GLOS_CG30456.2	GO:0008270	M	Refseq	zinc ion binding
NP_611188.3 CG30456, isoform B [Drosophila melanogaster]	NP_611188.3	0	GLOS_CG30456.2	GO:0032330	B	Refseq	regulation of chondrocyte differentiation
NP_611188.3 CG30456, isoform B [Drosophila melanogaster]	NP_611188.3	0	GLOS_CG30456.2	GO:0043565	M	Refseq	sequence-specific DNA binding
XP_001988235.1 GH10676 [Drosophila grimshawi]	XP_001988235.1	4,00E-18	GLOS_DGRI_GH1	GO:0005262	M	Refseq	calcium channel activity
XP_001988235.1 GH10676 [Drosophila grimshawi]	XP_001988235.1	4,00E-18	GLOS_DGRI_GH1	GO:0005515	M	Refseq	protein binding
XP_001988235.1 GH10676 [Drosophila grimshawi]	XP_001988235.1	4,00E-18	GLOS_DGRI_GH1	GO:0005886	C	Refseq	plasma membrane
XP_001988235.1 GH10676 [Drosophila grimshawi]	XP_001988235.1	4,00E-18	GLOS_DGRI_GH1	GO:0005887	C	Refseq	integral to plasma membrane
XP_001988235.1 GH10676 [Drosophila grimshawi]	XP_001988235.1	4,00E-18	GLOS_DGRI_GH1	GO:0006816	B	Refseq	calcium ion transport
XP_001988235.1 GH10676 [Drosophila grimshawi]	XP_001988235.1	4,00E-18	GLOS_DGRI_GH1	GO:0007411	B	Refseq	axon guidance
XP_001988235.1 GH10676 [Drosophila grimshawi]	XP_001988235.1	4,00E-18	GLOS_DGRI_GH1	GO:0007596	B	Refseq	blood coagulation
XP_001988235.1 GH10676 [Drosophila grimshawi]	XP_001988235.1	4,00E-18	GLOS_DGRI_GH1	GO:0007602	B	Refseq	phototransduction
XP_001988235.1 GH10676 [Drosophila grimshawi]	XP_001988235.1	4,00E-18	GLOS_DGRI_GH1	GO:0010524	B	Refseq	positive regulation of calcium ion transport into cytosol
XP_001988235.1 GH10676 [Drosophila grimshawi]	XP_001988235.1	4,00E-18	GLOS_DGRI_GH1	GO:0015279	M	Refseq	store-operated calcium channel activity
XP_001988235.1 GH10676 [Drosophila grimshawi]	XP_001988235.1	4,00E-18	GLOS_DGRI_GH1	GO:0030168	B	Refseq	platelet activation
XP_001988235.1 GH10676 [Drosophila grimshawi]	XP_001988235.1	4,00E-18	GLOS_DGRI_GH1	GO:0033198	B	Refseq	response to ATP
XP_001988235.1 GH10676 [Drosophila grimshawi]	XP_001988235.1	4,00E-18	GLOS_DGRI_GH1	GO:0051592	B	Refseq	response to calcium ion
XP_001988235.1 GH10676 [Drosophila grimshawi]	XP_001988235.1	4,00E-18	GLOS_DGRI_GH1	GO:0070588	B	Refseq	calcium ion transmembrane transport
XP_001988235.1 GH10676 [Drosophila grimshawi]	XP_001988235.1	4,00E-18	GLOS_DGRI_GH1	GO:0070679	M	Refseq	inositol 1,4,5 trisphosphate binding
XP_001970032.1 GG10419 [Drosophila erecta]	XP_001970032.1	5,00E-77	GLOS_DERE_GG	GO:0005261	M	Refseq	cation channel activity
XP_001970032.1 GG10419 [Drosophila erecta]	XP_001970032.1	5,00E-77	GLOS_DERE_GG	GO:0005262	M	Refseq	calcium channel activity
XP_001970032.1 GG10419 [Drosophila erecta]	XP_001970032.1	5,00E-77	GLOS_DERE_GG	GO:0005515	M	Refseq	protein binding
XP_001970032.1 GG10419 [Drosophila erecta]	XP_001970032.1	5,00E-77	GLOS_DERE_GG	GO:0005886	C	Refseq	plasma membrane
XP_001970032.1 GG10419 [Drosophila erecta]	XP_001970032.1	5,00E-77	GLOS_DERE_GG	GO:0005887	C	Refseq	integral to plasma membrane
XP_001970032.1 GG10419 [Drosophila erecta]	XP_001970032.1	5,00E-77	GLOS_DERE_GG	GO:0006816	B	Refseq	calcium ion transport
XP_001970032.1 GG10419 [Drosophila erecta]	XP_001970032.1	5,00E-77	GLOS_DERE_GG	GO:0007411	B	Refseq	axon guidance
XP_001970032.1 GG10419 [Drosophila erecta]	XP_001970032.1	5,00E-77	GLOS_DERE_GG	GO:0015279	M	Refseq	store-operated calcium channel activity
XP_001970032.1 GG10419 [Drosophila erecta]	XP_001970032.1	5,00E-77	GLOS_DERE_GG	GO:0016323	C	Refseq	basolateral plasma membrane
XP_001970032.1 GG10419 [Drosophila erecta]	XP_001970032.1	5,00E-77	GLOS_DERE_GG	GO:0030017	C	Refseq	sarcomere
XP_001970032.1 GG10419 [Drosophila erecta]	XP_001970032.1	5,00E-77	GLOS_DERE_GG	GO:0043034	C	Refseq	costamere
XP_001970032.1 GG10419 [Drosophila erecta]	XP_001970032.1	5,00E-77	GLOS_DERE_GG	GO:0043234	C	Refseq	protein complex
XP_001970032.1 GG10419 [Drosophila erecta]	XP_001970032.1	5,00E-77	GLOS_DERE_GG	GO:0044325	M	Refseq	ion channel binding
XP_001970032.1 GG10419 [Drosophila erecta]	XP_001970032.1	5,00E-77	GLOS_DERE_GG	GO:0045121	C	Refseq	membrane raft
XP_001970032.1 GG10419 [Drosophila erecta]	XP_001970032.1	5,00E-77	GLOS_DERE_GG	GO:0046541	B	Refseq	saliva secretion
XP_001970032.1 GG10419 [Drosophila erecta]	XP_001970032.1	5,00E-77	GLOS_DERE_GG	GO:0051281	B	Refseq	positive regulation of release of sequestered calcium ion into cytosol
XP_001970032.1 GG10419 [Drosophila erecta]	XP_001970032.1	5,00E-77	GLOS_DERE_GG	GO:0051480	B	Refseq	cytosolic calcium ion homeostasis
XP_001970032.1 GG10419 [Drosophila erecta]	XP_001970032.1	5,00E-77	GLOS_DERE_GG	GO:0051592	B	Refseq	response to calcium ion
XP_001970032.1 GG10419 [Drosophila erecta]	XP_001970032.1	5,00E-77	GLOS_DERE_GG	GO:0070679	M	Refseq	inositol 1,4,5 trisphosphate binding
XP_001984700.1 GH16615 [Drosophila grimshawi]	XP_001984700.1	6,00E-11	GLOS_DGRI_GH1	GO:0005262	M	Refseq	calcium channel activity

XP_001984700.1 GH16615 [Drosophila grimshawi]	XP_001984700.1	6,00E-11	GLOS_DGRI_GH1	GO:0005515	M	Refseq	protein binding
XP_001984700.1 GH16615 [Drosophila grimshawi]	XP_001984700.1	6,00E-11	GLOS_DGRI_GH1	GO:0005886	C	Refseq	plasma membrane
XP_001984700.1 GH16615 [Drosophila grimshawi]	XP_001984700.1	6,00E-11	GLOS_DGRI_GH1	GO:0005887	C	Refseq	integral to plasma membrane
XP_001984700.1 GH16615 [Drosophila grimshawi]	XP_001984700.1	6,00E-11	GLOS_DGRI_GH1	GO:0006816	B	Refseq	calcium ion transport
XP_001984700.1 GH16615 [Drosophila grimshawi]	XP_001984700.1	6,00E-11	GLOS_DGRI_GH1	GO:0007411	B	Refseq	axon guidance
XP_001984700.1 GH16615 [Drosophila grimshawi]	XP_001984700.1	6,00E-11	GLOS_DGRI_GH1	GO:0007596	B	Refseq	blood coagulation
XP_001984700.1 GH16615 [Drosophila grimshawi]	XP_001984700.1	6,00E-11	GLOS_DGRI_GH1	GO:0007602	B	Refseq	phototransduction
XP_001984700.1 GH16615 [Drosophila grimshawi]	XP_001984700.1	6,00E-11	GLOS_DGRI_GH1	GO:0010524	B	Refseq	positive regulation of calcium ion transport into cytosol
XP_001984700.1 GH16615 [Drosophila grimshawi]	XP_001984700.1	6,00E-11	GLOS_DGRI_GH1	GO:0015279	M	Refseq	store-operated calcium channel activity
XP_001984700.1 GH16615 [Drosophila grimshawi]	XP_001984700.1	6,00E-11	GLOS_DGRI_GH1	GO:0030168	B	Refseq	platelet activation
XP_001984700.1 GH16615 [Drosophila grimshawi]	XP_001984700.1	6,00E-11	GLOS_DGRI_GH1	GO:0033198	B	Refseq	response to ATP
XP_001984700.1 GH16615 [Drosophila grimshawi]	XP_001984700.1	6,00E-11	GLOS_DGRI_GH1	GO:0051592	B	Refseq	response to calcium ion
XP_001984700.1 GH16615 [Drosophila grimshawi]	XP_001984700.1	6,00E-11	GLOS_DGRI_GH1	GO:0070588	B	Refseq	calcium ion transmembrane transport
XP_001984700.1 GH16615 [Drosophila grimshawi]	XP_001984700.1	6,00E-11	GLOS_DGRI_GH1	GO:0070679	M	Refseq	inositol 1,4,5 trisphosphate binding
[BBH] BRU_DROME (sp|Q9VIL0) Prot brunelleschi OS=D.m.	BRU_DROME	0	GLOS_BRU.1.1	GO:0044431	C	.	Golgi apparatus part
[BBH] BRU_DROME (sp|Q9VIL0) Prot brunelleschi OS=D.m.	BRU_DROME	0	GLOS_BRU.1.1	GO:0005794	C	.	Golgi apparatus
[BBH] BRU_DROME (sp|Q9VIL0) Prot brunelleschi OS=D.m.	BRU_DROME	0	GLOS_BRU.1.1	GO:0036063	C	FlyBase	acroblast
[BBH] BRU_DROME (sp|Q9VIL0) Prot brunelleschi OS=D.m.	BRU_DROME	0	GLOS_BRU.1.1	GO:0036213	B	.	contractile ring contraction
[BBH] BRU_DROME (sp|Q9VIL0) Prot brunelleschi OS=D.m.	BRU_DROME	0	GLOS_BRU.1.1	GO:0032506	B	.	cytokinetic process
[BBH] BRU_DROME (sp|Q9VIL0) Prot brunelleschi OS=D.m.	BRU_DROME	0	GLOS_BRU.1.1	GO:0022402	B	.	cell cycle process
[BBH] BRU_DROME (sp|Q9VIL0) Prot brunelleschi OS=D.m.	BRU_DROME	0	GLOS_BRU.1.1	GO:0000910	B	.	cytokinesis
[BBH] BRU_DROME (sp|Q9VIL0) Prot brunelleschi OS=D.m.	BRU_DROME	0	GLOS_BRU.1.1	GO:0051301	B	.	cell division
[BBH] BRU_DROME (sp|Q9VIL0) Prot brunelleschi OS=D.m.	BRU_DROME	0	GLOS_BRU.1.1	GO:0000916	B	FlyBase	actomyosin contractile ring contraction
[BBH] BRU_DROME (sp|Q9VIL0) Prot brunelleschi OS=D.m.	BRU_DROME	0	GLOS_BRU.1.1	GO:0033206	B	.	meiotic cytokinesis
[BBH] BRU_DROME (sp|Q9VIL0) Prot brunelleschi OS=D.m.	BRU_DROME	0	GLOS_BRU.1.1	GO:0048610	B	.	cellular process involved in reproduction
[BBH] BRU_DROME (sp|Q9VIL0) Prot brunelleschi OS=D.m.	BRU_DROME	0	GLOS_BRU.1.1	GO:0000003	B	.	reproduction
[BBH] BRU_DROME (sp|Q9VIL0) Prot brunelleschi OS=D.m.	BRU_DROME	0	GLOS_BRU.1.1	GO:0051321	B	.	meiotic cell cycle
[BBH] BRU_DROME (sp|Q9VIL0) Prot brunelleschi OS=D.m.	BRU_DROME	0	GLOS_BRU.1.1	GO:0007112	B	FlyBase	male meiosis cytokinesis
[BBH] BRU_DROME (sp|Q9VIL0) Prot brunelleschi OS=D.m.	BRU_DROME	0	GLOS_BRU.1.1	GO:0007127	B	.	meiosis I
[BBH] BRU_DROME (sp|Q9VIL0) Prot brunelleschi OS=D.m.	BRU_DROME	0	GLOS_BRU.1.1	GO:0007126	B	.	meiosis
[BBH] BRU_DROME (sp|Q9VIL0) Prot brunelleschi OS=D.m.	BRU_DROME	0	GLOS_BRU.1.1	GO:0007110	B	FlyBase	meiosis I cytokinesis
[BBH] BRU_DROME (sp|Q9VIL0) Prot brunelleschi OS=D.m.	BRU_DROME	0	GLOS_BRU.1.1	GO:0007135	B	.	meiosis II
[BBH] BRU_DROME (sp|Q9VIL0) Prot brunelleschi OS=D.m.	BRU_DROME	0	GLOS_BRU.1.1	GO:0007111	B	FlyBase	meiosis II cytokinesis
[BBH] BRU_DROME (sp|Q9VIL0) Prot brunelleschi OS=D.m.	BRU_DROME	0	GLOS_BRU.1.1	GO:0043146	B	.	spindle stabilization
[BBH] BRU_DROME (sp|Q9VIL0) Prot brunelleschi OS=D.m.	BRU_DROME	0	GLOS_BRU.1.1	GO:0007026	B	.	negative regulation of microtubule depolymerization
[BBH] BRU_DROME (sp|Q9VIL0) Prot brunelleschi OS=D.m.	BRU_DROME	0	GLOS_BRU.1.1	GO:0031111	B	.	negative regulation of microtubule polymerization or depolymerization
[BBH] BRU_DROME (sp|Q9VIL0) Prot brunelleschi OS=D.m.	BRU_DROME	0	GLOS_BRU.1.1	GO:0031110	B	.	regulation of microtubule polymerization or depolymerization
[BBH] BRU_DROME (sp|Q9VIL0) Prot brunelleschi OS=D.m.	BRU_DROME	0	GLOS_BRU.1.1	GO:0070507	B	.	regulation of microtubule cytoskeleton organization
[BBH] BRU_DROME (sp|Q9VIL0) Prot brunelleschi OS=D.m.	BRU_DROME	0	GLOS_BRU.1.1	GO:0032886	B	.	regulation of microtubule-based process
[BBH] BRU_DROME (sp|Q9VIL0) Prot brunelleschi OS=D.m.	BRU_DROME	0	GLOS_BRU.1.1	GO:0051493	B	.	regulation of cytoskeleton organization
[BBH] BRU_DROME (sp|Q9VIL0) Prot brunelleschi OS=D.m.	BRU_DROME	0	GLOS_BRU.1.1	GO:0033043	B	.	regulation of organelle organization
[BBH] BRU_DROME (sp|Q9VIL0) Prot brunelleschi OS=D.m.	BRU_DROME	0	GLOS_BRU.1.1	GO:0051128	B	.	regulation of cellular component organization
[BBH] BRU_DROME (sp|Q9VIL0) Prot brunelleschi OS=D.m.	BRU_DROME	0	GLOS_BRU.1.1	GO:0051494	B	.	negative regulation of cytoskeleton organization
[BBH] BRU_DROME (sp|Q9VIL0) Prot brunelleschi OS=D.m.	BRU_DROME	0	GLOS_BRU.1.1	GO:0010639	B	.	negative regulation of organelle organization
[BBH] BRU_DROME (sp|Q9VIL0) Prot brunelleschi OS=D.m.	BRU_DROME	0	GLOS_BRU.1.1	GO:0051129	B	.	negative regulation of cellular component organization
[BBH] BRU_DROME (sp|Q9VIL0) Prot brunelleschi OS=D.m.	BRU_DROME	0	GLOS_BRU.1.1	GO:0031114	B	.	regulation of microtubule depolymerization
[BBH] BRU_DROME (sp|Q9VIL0) Prot brunelleschi OS=D.m.	BRU_DROME	0	GLOS_BRU.1.1	GO:1901879	B	.	regulation of protein depolymerization
[BBH] BRU_DROME (sp|Q9VIL0) Prot brunelleschi OS=D.m.	BRU_DROME	0	GLOS_BRU.1.1	GO:0043244	B	.	regulation of protein complex disassembly
[BBH] BRU_DROME (sp|Q9VIL0) Prot brunelleschi OS=D.m.	BRU_DROME	0	GLOS_BRU.1.1	GO:1901880	B	.	negative regulation of protein depolymerization
[BBH] BRU_DROME (sp|Q9VIL0) Prot brunelleschi OS=D.m.	BRU_DROME	0	GLOS_BRU.1.1	GO:0043242	B	.	negative regulation of protein complex disassembly
[BBH] BRU_DROME (sp|Q9VIL0) Prot brunelleschi OS=D.m.	BRU_DROME	0	GLOS_BRU.1.1	GO:0090224	B	.	regulation of spindle organization
[BBH] BRU_DROME (sp|Q9VIL0) Prot brunelleschi OS=D.m.	BRU_DROME	0	GLOS_BRU.1.1	GO:0010564	B	.	regulation of cell cycle process
[BBH] BRU_DROME (sp|Q9VIL0) Prot brunelleschi OS=D.m.	BRU_DROME	0	GLOS_BRU.1.1	GO:0000212	B	.	meiotic spindle organization
[BBH] BRU_DROME (sp|Q9VIL0) Prot brunelleschi OS=D.m.	BRU_DROME	0	GLOS_BRU.1.1	GO:0007051	B	.	spindle organization

[BBH] BRU_DROME (sp|Q9VIL0) Prot brunelleschi OS=D.m.	BRU_DROME	0	GLOS_BRU.1.1	GO:0000226	B	.	microtubule cytoskeleton organization
[BBH] BRU_DROME (sp|Q9VIL0) Prot brunelleschi OS=D.m.	BRU_DROME	0	GLOS_BRU.1.1	GO:0007010	B	.	cytoskeleton organization
[BBH] BRU_DROME (sp|Q9VIL0) Prot brunelleschi OS=D.m.	BRU_DROME	0	GLOS_BRU.1.1	GO:0006996	B	.	organelle organization
[BBH] BRU_DROME (sp|Q9VIL0) Prot brunelleschi OS=D.m.	BRU_DROME	0	GLOS_BRU.1.1	GO:0007017	B	.	microtubule-based process
[BBH] BRU_DROME (sp|Q9VIL0) Prot brunelleschi OS=D.m.	BRU_DROME	0	GLOS_BRU.1.1	GO:0071822	B	.	protein complex subunit organization
[BBH] BRU_DROME (sp|Q9VIL0) Prot brunelleschi OS=D.m.	BRU_DROME	0	GLOS_BRU.1.1	GO:0043933	B	.	macromolecular complex subunit organization
[BBH] BRU_DROME (sp|Q9VIL0) Prot brunelleschi OS=D.m.	BRU_DROME	0	GLOS_BRU.1.1	GO:0043147	B	FlyBase	meiotic spindle stabilization
[BBH] BRU_DROME (sp|Q9VIL0) Prot brunelleschi OS=D.m.	BRU_DROME	0	GLOS_BRU.1.1	GO:0022412	B	.	cellular process involved in reproduction in multicellular organism
[BBH] BRU_DROME (sp|Q9VIL0) Prot brunelleschi OS=D.m.	BRU_DROME	0	GLOS_BRU.1.1	GO:0032504	B	.	multicellular organism reproduction
[BBH] BRU_DROME (sp|Q9VIL0) Prot brunelleschi OS=D.m.	BRU_DROME	0	GLOS_BRU.1.1	GO:0007283	B	.	spermatogenesis
[BBH] BRU_DROME (sp|Q9VIL0) Prot brunelleschi OS=D.m.	BRU_DROME	0	GLOS_BRU.1.1	GO:0048232	B	.	male gamete generation
[BBH] BRU_DROME (sp|Q9VIL0) Prot brunelleschi OS=D.m.	BRU_DROME	0	GLOS_BRU.1.1	GO:0007276	B	.	gamete generation
[BBH] BRU_DROME (sp|Q9VIL0) Prot brunelleschi OS=D.m.	BRU_DROME	0	GLOS_BRU.1.1	GO:0044702	B	.	single organism reproductive process
[BBH] BRU_DROME (sp|Q9VIL0) Prot brunelleschi OS=D.m.	BRU_DROME	0	GLOS_BRU.1.1	GO:0022414	B	.	reproductive process
[BBH] BRU_DROME (sp|Q9VIL0) Prot brunelleschi OS=D.m.	BRU_DROME	0	GLOS_BRU.1.1	GO:0048609	B	.	multicellular organismal reproductive process
[BBH] BRU_DROME (sp|Q9VIL0) Prot brunelleschi OS=D.m.	BRU_DROME	0	GLOS_BRU.1.1	GO:0019953	B	.	sexual reproduction
[BBH] BRU_DROME (sp|Q9VIL0) Prot brunelleschi OS=D.m.	BRU_DROME	0	GLOS_BRU.1.1	GO:0048137	B	FlyBase	spermatocyte division
[BBH] CSN4_DROME (sp|Q9V345) COP9 signalosome complex subunit 4	CSN4_DROME	0	GLOS_CSN4.1.4	GO:0008180	C	UniProtKB	COP9 signalosome
[BBH] CSN4_DROME (sp|Q9V345) COP9 signalosome complex subunit 4	CSN4_DROME	0	GLOS_CSN4.1.4	GO:0005737	C	UniProtKB-S	cytoplasm
[BBH] CSN4_DROME (sp|Q9V345) COP9 signalosome complex subunit 4	CSN4_DROME	0	GLOS_CSN4.1.4	GO:0008641	M	.	small protein activating enzyme activity
[BBH] CSN4_DROME (sp|Q9V345) COP9 signalosome complex subunit 4	CSN4_DROME	0	GLOS_CSN4.1.4	GO:0003824	M	.	catalytic activity
[BBH] CSN4_DROME (sp|Q9V345) COP9 signalosome complex subunit 4	CSN4_DROME	0	GLOS_CSN4.1.4	GO:0019781	M	UniProtKB	NEDD8 activating enzyme activity
[BBH] CSN4_DROME (sp|Q9V345) COP9 signalosome complex subunit 4	CSN4_DROME	0	GLOS_CSN4.1.4	GO:0000975	M	.	regulatory region DNA binding
[BBH] CSN4_DROME (sp|Q9V345) COP9 signalosome complex subunit 4	CSN4_DROME	0	GLOS_CSN4.1.4	GO:0001067	M	.	regulatory region nucleic acid binding
[BBH] CSN4_DROME (sp|Q9V345) COP9 signalosome complex subunit 4	CSN4_DROME	0	GLOS_CSN4.1.4	GO:0044212	M	FlyBase	transcription regulatory region DNA binding
[BBH] CSN4_DROME (sp|Q9V345) COP9 signalosome complex subunit 4	CSN4_DROME	0	GLOS_CSN4.1.4	GO:0001754	B	.	eye photoreceptor cell differentiation
[BBH] CSN4_DROME (sp|Q9V345) COP9 signalosome complex subunit 4	CSN4_DROME	0	GLOS_CSN4.1.4	GO:0046530	B	.	photoreceptor cell differentiation
[BBH] CSN4_DROME (sp|Q9V345) COP9 signalosome complex subunit 4	CSN4_DROME	0	GLOS_CSN4.1.4	GO:0048592	B	.	eye morphogenesis
[BBH] CSN4_DROME (sp|Q9V345) COP9 signalosome complex subunit 4	CSN4_DROME	0	GLOS_CSN4.1.4	GO:0009887	B	.	organ morphogenesis
[BBH] CSN4_DROME (sp|Q9V345) COP9 signalosome complex subunit 4	CSN4_DROME	0	GLOS_CSN4.1.4	GO:0001654	B	.	eye development
[BBH] CSN4_DROME (sp|Q9V345) COP9 signalosome complex subunit 4	CSN4_DROME	0	GLOS_CSN4.1.4	GO:0007423	B	.	sensory organ development
[BBH] CSN4_DROME (sp|Q9V345) COP9 signalosome complex subunit 4	CSN4_DROME	0	GLOS_CSN4.1.4	GO:0001745	B	.	compound eye morphogenesis
[BBH] CSN4_DROME (sp|Q9V345) COP9 signalosome complex subunit 4	CSN4_DROME	0	GLOS_CSN4.1.4	GO:0048749	B	.	compound eye development
[BBH] CSN4_DROME (sp|Q9V345) COP9 signalosome complex subunit 4	CSN4_DROME	0	GLOS_CSN4.1.4	GO:0001751	B	FlyBase	compound eye photoreceptor cell differentiation
[BBH] CSN4_DROME (sp|Q9V345) COP9 signalosome complex subunit 4	CSN4_DROME	0	GLOS_CSN4.1.4	GO:0045168	B	.	cell-cell signaling involved in cell fate commitment
[BBH] CSN4_DROME (sp|Q9V345) COP9 signalosome complex subunit 4	CSN4_DROME	0	GLOS_CSN4.1.4	GO:0007267	B	.	cell-cell signaling
[BBH] CSN4_DROME (sp|Q9V345) COP9 signalosome complex subunit 4	CSN4_DROME	0	GLOS_CSN4.1.4	GO:0045165	B	.	cell fate commitment
[BBH] CSN4_DROME (sp|Q9V345) COP9 signalosome complex subunit 4	CSN4_DROME	0	GLOS_CSN4.1.4	GO:0046331	B	FlyBase	lateral inhibition
[BBH] CSN4_DROME (sp|Q9V345) COP9 signalosome complex subunit 4	CSN4_DROME	0	GLOS_CSN4.1.4	GO:0031572	B	.	G2 DNA damage checkpoint
[BBH] CSN4_DROME (sp|Q9V345) COP9 signalosome complex subunit 4	CSN4_DROME	0	GLOS_CSN4.1.4	GO:0000077	B	.	DNA damage checkpoint
[BBH] CSN4_DROME (sp|Q9V345) COP9 signalosome complex subunit 4	CSN4_DROME	0	GLOS_CSN4.1.4	GO:0006974	B	.	cellular response to DNA damage stimulus
[BBH] CSN4_DROME (sp|Q9V345) COP9 signalosome complex subunit 4	CSN4_DROME	0	GLOS_CSN4.1.4	GO:0033554	B	.	cellular response to stress
[BBH] CSN4_DROME (sp|Q9V345) COP9 signalosome complex subunit 4	CSN4_DROME	0	GLOS_CSN4.1.4	GO:0031570	B	.	DNA integrity checkpoint
[BBH] CSN4_DROME (sp|Q9V345) COP9 signalosome complex subunit 4	CSN4_DROME	0	GLOS_CSN4.1.4	GO:0000075	B	.	cell cycle checkpoint
[BBH] CSN4_DROME (sp|Q9V345) COP9 signalosome complex subunit 4	CSN4_DROME	0	GLOS_CSN4.1.4	GO:0044773	B	.	mitotic DNA damage checkpoint
[BBH] CSN4_DROME (sp|Q9V345) COP9 signalosome complex subunit 4	CSN4_DROME	0	GLOS_CSN4.1.4	GO:0044774	B	.	mitotic DNA integrity checkpoint
[BBH] CSN4_DROME (sp|Q9V345) COP9 signalosome complex subunit 4	CSN4_DROME	0	GLOS_CSN4.1.4	GO:0007093	B	.	mitotic cell cycle checkpoint
[BBH] CSN4_DROME (sp|Q9V345) COP9 signalosome complex subunit 4	CSN4_DROME	0	GLOS_CSN4.1.4	GO:1901991	B	.	negative regulation of mitotic cell cycle phase transition
[BBH] CSN4_DROME (sp|Q9V345) COP9 signalosome complex subunit 4	CSN4_DROME	0	GLOS_CSN4.1.4	GO:1901988	B	.	negative regulation of cell cycle phase transition
[BBH] CSN4_DROME (sp|Q9V345) COP9 signalosome complex subunit 4	CSN4_DROME	0	GLOS_CSN4.1.4	GO:0010948	B	.	negative regulation of cell cycle process
[BBH] CSN4_DROME (sp|Q9V345) COP9 signalosome complex subunit 4	CSN4_DROME	0	GLOS_CSN4.1.4	GO:1901987	B	.	regulation of cell cycle phase transition
[BBH] CSN4_DROME (sp|Q9V345) COP9 signalosome complex subunit 4	CSN4_DROME	0	GLOS_CSN4.1.4	GO:1901990	B	.	regulation of mitotic cell cycle phase transition
[BBH] CSN4_DROME (sp|Q9V345) COP9 signalosome complex subunit 4	CSN4_DROME	0	GLOS_CSN4.1.4	GO:0007346	B	.	regulation of mitotic cell cycle
[BBH] CSN4_DROME (sp|Q9V345) COP9 signalosome complex subunit 4	CSN4_DROME	0	GLOS_CSN4.1.4	GO:0007095	B	FlyBase	mitotic G2 DNA damage checkpoint

[BBH] CSN4_DROME (sp|Q9V345) COP9 signalosome complex subunit 4	CSN4_DROME	0	GLOS_CSN4.1.4	GO:0031330	B	.	negative regulation of cellular catabolic process
[BBH] CSN4_DROME (sp|Q9V345) COP9 signalosome complex subunit 4	CSN4_DROME	0	GLOS_CSN4.1.4	GO:0009895	B	.	negative regulation of catabolic process
[BBH] CSN4_DROME (sp|Q9V345) COP9 signalosome complex subunit 4	CSN4_DROME	0	GLOS_CSN4.1.4	GO:0009894	B	.	regulation of catabolic process
[BBH] CSN4_DROME (sp|Q9V345) COP9 signalosome complex subunit 4	CSN4_DROME	0	GLOS_CSN4.1.4	GO:0031329	B	.	regulation of cellular catabolic process
[BBH] CSN4_DROME (sp|Q9V345) COP9 signalosome complex subunit 4	CSN4_DROME	0	GLOS_CSN4.1.4	GO:0032434	B	.	regulation of proteasomal ubiquitin-dependent protein catabolic process
[BBH] CSN4_DROME (sp|Q9V345) COP9 signalosome complex subunit 4	CSN4_DROME	0	GLOS_CSN4.1.4	GO:0061136	B	.	regulation of proteasomal protein catabolic process
[BBH] CSN4_DROME (sp|Q9V345) COP9 signalosome complex subunit 4	CSN4_DROME	0	GLOS_CSN4.1.4	GO:0030162	B	.	regulation of proteolysis
[BBH] CSN4_DROME (sp|Q9V345) COP9 signalosome complex subunit 4	CSN4_DROME	0	GLOS_CSN4.1.4	GO:0051246	B	.	regulation of protein metabolic process
[BBH] CSN4_DROME (sp|Q9V345) COP9 signalosome complex subunit 4	CSN4_DROME	0	GLOS_CSN4.1.4	GO:0032268	B	.	regulation of cellular protein metabolic process
[BBH] CSN4_DROME (sp|Q9V345) COP9 signalosome complex subunit 4	CSN4_DROME	0	GLOS_CSN4.1.4	GO:0042176	B	.	regulation of protein catabolic process
[BBH] CSN4_DROME (sp|Q9V345) COP9 signalosome complex subunit 4	CSN4_DROME	0	GLOS_CSN4.1.4	GO:1901799	B	.	negative regulation of proteasomal protein catabolic process
[BBH] CSN4_DROME (sp|Q9V345) COP9 signalosome complex subunit 4	CSN4_DROME	0	GLOS_CSN4.1.4	GO:0042177	B	.	negative regulation of protein catabolic process
[BBH] CSN4_DROME (sp|Q9V345) COP9 signalosome complex subunit 4	CSN4_DROME	0	GLOS_CSN4.1.4	GO:0051248	B	.	negative regulation of protein metabolic process
[BBH] CSN4_DROME (sp|Q9V345) COP9 signalosome complex subunit 4	CSN4_DROME	0	GLOS_CSN4.1.4	GO:0045861	B	.	negative regulation of proteolysis
[BBH] CSN4_DROME (sp|Q9V345) COP9 signalosome complex subunit 4	CSN4_DROME	0	GLOS_CSN4.1.4	GO:0032269	B	.	negative regulation of cellular protein metabolic process
[BBH] CSN4_DROME (sp|Q9V345) COP9 signalosome complex subunit 4	CSN4_DROME	0	GLOS_CSN4.1.4	GO:0032435	B	FlyBase	negat. Regul. of proteasomal ubiquitin-dependent protein catabolic process
[BBH] CSN4_DROME (sp|Q9V345) COP9 signalosome complex subunit 4	CSN4_DROME	0	GLOS_CSN4.1.4	GO:0007292	B	.	female gamete generation
[BBH] CSN4_DROME (sp|Q9V345) COP9 signalosome complex subunit 4	CSN4_DROME	0	GLOS_CSN4.1.4	GO:0048477	B	UniProtKB-k	oogenesis
[BBH] CSN4_DROME (sp|Q9V345) COP9 signalosome complex subunit 4	CSN4_DROME	0	GLOS_CSN4.1.4	GO:0070646	B	.	protein modification by small protein removal
[BBH] CSN4_DROME (sp|Q9V345) COP9 signalosome complex subunit 4	CSN4_DROME	0	GLOS_CSN4.1.4	GO:0070647	B	.	protein modification by small protein conjugation or removal
[BBH] CSN4_DROME (sp|Q9V345) COP9 signalosome complex subunit 4	CSN4_DROME	0	GLOS_CSN4.1.4	GO:0006464	B	.	cellular protein modification process
[BBH] CSN4_DROME (sp|Q9V345) COP9 signalosome complex subunit 4	CSN4_DROME	0	GLOS_CSN4.1.4	GO:0036211	B	.	protein modification process
[BBH] CSN4_DROME (sp|Q9V345) COP9 signalosome complex subunit 4	CSN4_DROME	0	GLOS_CSN4.1.4	GO:0019538	B	.	protein metabolic process
[BBH] CSN4_DROME (sp|Q9V345) COP9 signalosome complex subunit 4	CSN4_DROME	0	GLOS_CSN4.1.4	GO:0043412	B	.	macromolecule modification
[BBH] CSN4_DROME (sp|Q9V345) COP9 signalosome complex subunit 4	CSN4_DROME	0	GLOS_CSN4.1.4	GO:0044267	B	.	cellular protein metabolic process
[BBH] CSN4_DROME (sp|Q9V345) COP9 signalosome complex subunit 4	CSN4_DROME	0	GLOS_CSN4.1.4	GO:0000338	B	UniProtKB	protein deneddylation
[BBH] CSN4_DROME (sp|Q9V345) COP9 signalosome complex subunit 4	CSN4_DROME	0	GLOS_CSN4.1.4	GO:0031647	B	.	regulation of protein stability
[BBH] CSN4_DROME (sp|Q9V345) COP9 signalosome complex subunit 4	CSN4_DROME	0	GLOS_CSN4.1.4	GO:0010608	B	.	posttranscriptional regulation of gene expression
[BBH] CSN4_DROME (sp|Q9V345) COP9 signalosome complex subunit 4	CSN4_DROME	0	GLOS_CSN4.1.4	GO:0050821	B	FlyBase	protein stabilization
[BBH] CSW_DROME (sp|P29349) Tyrosine-protein phosphatase corkscrew	CSW_DROME	0	GLOS_CSW.1.1	GO:0005737	C	UniProtKB	cytoplasm
[BBH] CSW_DROME (sp|P29349) Tyrosine-protein phosphatase corkscrew	CSW_DROME	0	GLOS_CSW.1.1	GO:0004725	M	.	protein tyrosine phosphatase activity
[BBH] CSW_DROME (sp|P29349) Tyrosine-protein phosphatase corkscrew	CSW_DROME	0	GLOS_CSW.1.1	GO:0004721	M	.	phosphoprotein phosphatase activity
[BBH] CSW_DROME (sp|P29349) Tyrosine-protein phosphatase corkscrew	CSW_DROME	0	GLOS_CSW.1.1	GO:0016791	M	.	phosphatase activity
[BBH] CSW_DROME (sp|P29349) Tyrosine-protein phosphatase corkscrew	CSW_DROME	0	GLOS_CSW.1.1	GO:0042578	M	.	phosphoric ester hydrolase activity
[BBH] CSW_DROME (sp|P29349) Tyrosine-protein phosphatase corkscrew	CSW_DROME	0	GLOS_CSW.1.1	GO:0016788	M	.	hydrolase activity, acting on ester bonds
[BBH] CSW_DROME (sp|P29349) Tyrosine-protein phosphatase corkscrew	CSW_DROME	0	GLOS_CSW.1.1	GO:0016787	M	.	hydrolase activity
[BBH] CSW_DROME (sp|P29349) Tyrosine-protein phosphatase corkscrew	CSW_DROME	0	GLOS_CSW.1.1	GO:0004726	M	UniProtKB	non-membrane spanning protein tyrosine phosphatase activity
[BBH] CSW_DROME (sp|P29349) Tyrosine-protein phosphatase corkscrew	CSW_DROME	0	GLOS_CSW.1.1	GO:0005057	M	.	receptor signaling protein activity
[BBH] CSW_DROME (sp|P29349) Tyrosine-protein phosphatase corkscrew	CSW_DROME	0	GLOS_CSW.1.1	GO:0004728	M	FlyBase	receptor signaling protein tyrosine phosphatase activity
[BBH] CSW_DROME (sp|P29349) Tyrosine-protein phosphatase corkscrew	CSW_DROME	0	GLOS_CSW.1.1	GO:0003006	B	.	developmental process involved in reproduction
[BBH] CSW_DROME (sp|P29349) Tyrosine-protein phosphatase corkscrew	CSW_DROME	0	GLOS_CSW.1.1	GO:0009950	B	.	dorsal/ventral axis specification
[BBH] CSW_DROME (sp|P29349) Tyrosine-protein phosphatase corkscrew	CSW_DROME	0	GLOS_CSW.1.1	GO:0009798	B	.	axis specification
[BBH] CSW_DROME (sp|P29349) Tyrosine-protein phosphatase corkscrew	CSW_DROME	0	GLOS_CSW.1.1	GO:0007389	B	.	pattern specification process
[BBH] CSW_DROME (sp|P29349) Tyrosine-protein phosphatase corkscrew	CSW_DROME	0	GLOS_CSW.1.1	GO:0009953	B	.	dorsal/ventral pattern formation
[BBH] CSW_DROME (sp|P29349) Tyrosine-protein phosphatase corkscrew	CSW_DROME	0	GLOS_CSW.1.1	GO:0003002	B	.	regionalization
[BBH] CSW_DROME (sp|P29349) Tyrosine-protein phosphatase corkscrew	CSW_DROME	0	GLOS_CSW.1.1	GO:0016334	B	.	establishment or maintenance of polarity of follicular epithelium
[BBH] CSW_DROME (sp|P29349) Tyrosine-protein phosphatase corkscrew	CSW_DROME	0	GLOS_CSW.1.1	GO:0007163	B	.	establishment or maintenance of cell polarity
[BBH] CSW_DROME (sp|P29349) Tyrosine-protein phosphatase corkscrew	CSW_DROME	0	GLOS_CSW.1.1	GO:0016333	B	.	morphogenesis of follicular epithelium
[BBH] CSW_DROME (sp|P29349) Tyrosine-protein phosphatase corkscrew	CSW_DROME	0	GLOS_CSW.1.1	GO:0002009	B	.	morphogenesis of an epithelium
[BBH] CSW_DROME (sp|P29349) Tyrosine-protein phosphatase corkscrew	CSW_DROME	0	GLOS_CSW.1.1	GO:0048729	B	.	tissue morphogenesis
[BBH] CSW_DROME (sp|P29349) Tyrosine-protein phosphatase corkscrew	CSW_DROME	0	GLOS_CSW.1.1	GO:0009888	B	.	tissue development
[BBH] CSW_DROME (sp|P29349) Tyrosine-protein phosphatase corkscrew	CSW_DROME	0	GLOS_CSW.1.1	GO:0060429	B	.	epithelium development
[BBH] CSW_DROME (sp|P29349) Tyrosine-protein phosphatase corkscrew	CSW_DROME	0	GLOS_CSW.1.1	GO:0030707	B	.	ovarian follicle cell development
[BBH] CSW_DROME (sp|P29349) Tyrosine-protein phosphatase corkscrew	CSW_DROME	0	GLOS_CSW.1.1	GO:0048477	B	.	oogenesis

[BBH] CSW_DROME (sp|P29349) Tyrosine-protein phosphatase corkscrew	CSW_DROME	0	GLOS_CSW.1.1	GO:0008069	B	FlyBase	dorsal/ventral axis specification, ovarian follicular epithelium
[BBH] CSW_DROME (sp|P29349) Tyrosine-protein phosphatase corkscrew	CSW_DROME	0	GLOS_CSW.1.1	GO:0038127	B	.	ERBB signaling pathway
[BBH] CSW_DROME (sp|P29349) Tyrosine-protein phosphatase corkscrew	CSW_DROME	0	GLOS_CSW.1.1	GO:0007169	B	.	transmembrane receptor protein tyrosine kinase signaling pathway
[BBH] CSW_DROME (sp|P29349) Tyrosine-protein phosphatase corkscrew	CSW_DROME	0	GLOS_CSW.1.1	GO:0007173	B	FlyBase	epidermal growth factor receptor signaling pathway
[BBH] CSW_DROME (sp|P29349) Tyrosine-protein phosphatase corkscrew	CSW_DROME	0	GLOS_CSW.1.1	GO:0010631	B	.	epithelial cell migration
[BBH] CSW_DROME (sp|P29349) Tyrosine-protein phosphatase corkscrew	CSW_DROME	0	GLOS_CSW.1.1	GO:0001667	B	.	ameboidal cell migration
[BBH] CSW_DROME (sp|P29349) Tyrosine-protein phosphatase corkscrew	CSW_DROME	0	GLOS_CSW.1.1	GO:0016477	B	.	cell migration
[BBH] CSW_DROME (sp|P29349) Tyrosine-protein phosphatase corkscrew	CSW_DROME	0	GLOS_CSW.1.1	GO:0048870	B	.	cell motility
[BBH] CSW_DROME (sp|P29349) Tyrosine-protein phosphatase corkscrew	CSW_DROME	0	GLOS_CSW.1.1	GO:0051674	B	.	localization of cell
[BBH] CSW_DROME (sp|P29349) Tyrosine-protein phosphatase corkscrew	CSW_DROME	0	GLOS_CSW.1.1	GO:0090132	B	.	epithelium migration
[BBH] CSW_DROME (sp|P29349) Tyrosine-protein phosphatase corkscrew	CSW_DROME	0	GLOS_CSW.1.1	GO:0090130	B	.	tissue migration
[BBH] CSW_DROME (sp|P29349) Tyrosine-protein phosphatase corkscrew	CSW_DROME	0	GLOS_CSW.1.1	GO:0007424	B	.	open tracheal system development
[BBH] CSW_DROME (sp|P29349) Tyrosine-protein phosphatase corkscrew	CSW_DROME	0	GLOS_CSW.1.1	GO:0060541	B	.	respiratory system development
[BBH] CSW_DROME (sp|P29349) Tyrosine-protein phosphatase corkscrew	CSW_DROME	0	GLOS_CSW.1.1	GO:0007427	B	FlyBase	epithelial cell migration, open tracheal system
[BBH] CSW_DROME (sp|P29349) Tyrosine-protein phosphatase corkscrew	CSW_DROME	0	GLOS_CSW.1.1	GO:0044344	B	.	cellular response to fibroblast growth factor stimulus
[BBH] CSW_DROME (sp|P29349) Tyrosine-protein phosphatase corkscrew	CSW_DROME	0	GLOS_CSW.1.1	GO:0071774	B	.	response to fibroblast growth factor stimulus
[BBH] CSW_DROME (sp|P29349) Tyrosine-protein phosphatase corkscrew	CSW_DROME	0	GLOS_CSW.1.1	GO:0008543	B	FlyBase	fibroblast growth factor receptor signaling pathway
[BBH] CSW_DROME (sp|P29349) Tyrosine-protein phosphatase corkscrew	CSW_DROME	0	GLOS_CSW.1.1	GO:0007444	B	FlyBase	imaginal disc development
[BBH] CSW_DROME (sp|P29349) Tyrosine-protein phosphatase corkscrew	CSW_DROME	0	GLOS_CSW.1.1	GO:0007498	B	FlyBase	mesoderm development
[BBH] CSW_DROME (sp|P29349) Tyrosine-protein phosphatase corkscrew	CSW_DROME	0	GLOS_CSW.1.1	GO:0000278	B	FlyBase	mitotic cell cycle
[BBH] CSW_DROME (sp|P29349) Tyrosine-protein phosphatase corkscrew	CSW_DROME	0	GLOS_CSW.1.1	GO:0060446	B	.	branching involved in open tracheal system development
[BBH] CSW_DROME (sp|P29349) Tyrosine-protein phosphatase corkscrew	CSW_DROME	0	GLOS_CSW.1.1	GO:0048754	B	.	branching morphogenesis of an epithelial tube
[BBH] CSW_DROME (sp|P29349) Tyrosine-protein phosphatase corkscrew	CSW_DROME	0	GLOS_CSW.1.1	GO:0061138	B	.	morphogenesis of a branching epithelium
[BBH] CSW_DROME (sp|P29349) Tyrosine-protein phosphatase corkscrew	CSW_DROME	0	GLOS_CSW.1.1	GO:0001763	B	.	morphogenesis of a branching structure
[BBH] CSW_DROME (sp|P29349) Tyrosine-protein phosphatase corkscrew	CSW_DROME	0	GLOS_CSW.1.1	GO:0060562	B	.	epithelial tube morphogenesis
[BBH] CSW_DROME (sp|P29349) Tyrosine-protein phosphatase corkscrew	CSW_DROME	0	GLOS_CSW.1.1	GO:0035239	B	.	tube morphogenesis
[BBH] CSW_DROME (sp|P29349) Tyrosine-protein phosphatase corkscrew	CSW_DROME	0	GLOS_CSW.1.1	GO:0035295	B	.	tube development
[BBH] CSW_DROME (sp|P29349) Tyrosine-protein phosphatase corkscrew	CSW_DROME	0	GLOS_CSW.1.1	GO:0007428	B	FlyBase	primary branching, open tracheal system
[BBH] CSW_DROME (sp|P29349) Tyrosine-protein phosphatase corkscrew	CSW_DROME	0	GLOS_CSW.1.1	GO:0042478	B	.	regulation of eye photoreceptor cell development
[BBH] CSW_DROME (sp|P29349) Tyrosine-protein phosphatase corkscrew	CSW_DROME	0	GLOS_CSW.1.1	GO:0046532	B	.	regulation of photoreceptor cell differentiation
[BBH] CSW_DROME (sp|P29349) Tyrosine-protein phosphatase corkscrew	CSW_DROME	0	GLOS_CSW.1.1	GO:0045664	B	.	regulation of neuron differentiation
[BBH] CSW_DROME (sp|P29349) Tyrosine-protein phosphatase corkscrew	CSW_DROME	0	GLOS_CSW.1.1	GO:0050767	B	.	regulation of neurogenesis
[BBH] CSW_DROME (sp|P29349) Tyrosine-protein phosphatase corkscrew	CSW_DROME	0	GLOS_CSW.1.1	GO:0051960	B	.	regulation of nervous system development
[BBH] CSW_DROME (sp|P29349) Tyrosine-protein phosphatase corkscrew	CSW_DROME	0	GLOS_CSW.1.1	GO:0060284	B	.	regulation of cell development
[BBH] CSW_DROME (sp|P29349) Tyrosine-protein phosphatase corkscrew	CSW_DROME	0	GLOS_CSW.1.1	GO:2000027	B	.	regulation of organ morphogenesis
[BBH] CSW_DROME (sp|P29349) Tyrosine-protein phosphatase corkscrew	CSW_DROME	0	GLOS_CSW.1.1	GO:0022603	B	.	regulation of anatomical structure morphogenesis
[BBH] CSW_DROME (sp|P29349) Tyrosine-protein phosphatase corkscrew	CSW_DROME	0	GLOS_CSW.1.1	GO:0045314	B	FlyBase	regulation of compound eye photoreceptor development
[BBH] CSW_DROME (sp|P29349) Tyrosine-protein phosphatase corkscrew	CSW_DROME	0	GLOS_CSW.1.1	GO:0007465	B	.	R7 cell fate commitment
[BBH] CSW_DROME (sp|P29349) Tyrosine-protein phosphatase corkscrew	CSW_DROME	0	GLOS_CSW.1.1	GO:0001752	B	.	compound eye photoreceptor fate commitment
[BBH] CSW_DROME (sp|P29349) Tyrosine-protein phosphatase corkscrew	CSW_DROME	0	GLOS_CSW.1.1	GO:0042706	B	.	eye photoreceptor cell fate commitment
[BBH] CSW_DROME (sp|P29349) Tyrosine-protein phosphatase corkscrew	CSW_DROME	0	GLOS_CSW.1.1	GO:0046552	B	.	photoreceptor cell fate commitment
[BBH] CSW_DROME (sp|P29349) Tyrosine-protein phosphatase corkscrew	CSW_DROME	0	GLOS_CSW.1.1	GO:0048663	B	.	neuron fate commitment
[BBH] CSW_DROME (sp|P29349) Tyrosine-protein phosphatase corkscrew	CSW_DROME	0	GLOS_CSW.1.1	GO:0001751	B	.	compound eye photoreceptor cell differentiation
[BBH] CSW_DROME (sp|P29349) Tyrosine-protein phosphatase corkscrew	CSW_DROME	0	GLOS_CSW.1.1	GO:0045466	B	.	R7 cell differentiation
[BBH] CSW_DROME (sp|P29349) Tyrosine-protein phosphatase corkscrew	CSW_DROME	0	GLOS_CSW.1.1	GO:0045500	B	FlyBase	sevenless signaling pathway
[BBH] CSW_DROME (sp|P29349) Tyrosine-protein phosphatase corkscrew	CSW_DROME	0	GLOS_CSW.1.1	GO:0009880	B	.	embryonic pattern specification
[BBH] CSW_DROME (sp|P29349) Tyrosine-protein phosphatase corkscrew	CSW_DROME	0	GLOS_CSW.1.1	GO:0007354	B	.	zygotic determination of anterior/posterior axis, embryo
[BBH] CSW_DROME (sp|P29349) Tyrosine-protein phosphatase corkscrew	CSW_DROME	0	GLOS_CSW.1.1	GO:0008595	B	.	anterior/posterior axis specification, embryo
[BBH] CSW_DROME (sp|P29349) Tyrosine-protein phosphatase corkscrew	CSW_DROME	0	GLOS_CSW.1.1	GO:0000578	B	.	embryonic axis specification
[BBH] CSW_DROME (sp|P29349) Tyrosine-protein phosphatase corkscrew	CSW_DROME	0	GLOS_CSW.1.1	GO:0009948	B	.	anterior/posterior axis specification
[BBH] CSW_DROME (sp|P29349) Tyrosine-protein phosphatase corkscrew	CSW_DROME	0	GLOS_CSW.1.1	GO:0009952	B	.	anterior/posterior pattern specification
[BBH] CSW_DROME (sp|P29349) Tyrosine-protein phosphatase corkscrew	CSW_DROME	0	GLOS_CSW.1.1	GO:0007351	B	.	tripartite regional subdivision
[BBH] CSW_DROME (sp|P29349) Tyrosine-protein phosphatase corkscrew	CSW_DROME	0	GLOS_CSW.1.1	GO:0007350	B	.	blastoderm segmentation
[BBH] CSW_DROME (sp|P29349) Tyrosine-protein phosphatase corkscrew	CSW_DROME	0	GLOS_CSW.1.1	GO:0035282	B	.	segmentation

[BBH] CSW_DROME (sp|P29349) Tyrosine-protein phosphatase corkscrew	CSW_DROME	0	GLOS_CSW.1.1	GO:0007362	B	UniProtKB	terminal region determination
[BBH] CSW_DROME (sp|P29349) Tyrosine-protein phosphatase corkscrew	CSW_DROME	0	GLOS_CSW.1.1	GO:0008293	B	UniProtKB	torso signaling pathway
[BBH] CSW_DROME (sp|P29349) Tyrosine-protein phosphatase corkscrew	CSW_DROME	0	GLOS_CSW.1.1	GO:0007417	B	.	central nervous system development
[BBH] CSW_DROME (sp|P29349) Tyrosine-protein phosphatase corkscrew	CSW_DROME	0	GLOS_CSW.1.1	GO:0007418	B	FlyBase	ventral midline development
[BBH] CUE_DROPS (sp|Q29FE9) Protein cueball OS=D.p.p.	CUE_DROPS	0	GLOS_CUE.1.1	GO:0016021	C	UniProtKB-K	integral to membrane
[BBH] CUE_DROPS (sp|Q29FE9) Protein cueball OS=D.p.p.	CUE_DROPS	0	GLOS_CUE.1.1	GO:0005886	C	UniProtKB-S	plasma membrane
[BBH] CUE_DROPS (sp|Q29FE9) Protein cueball OS=D.p.p.	CUE_DROPS	0	GLOS_CUE.1.1	GO:0030154	B	UniProtKB-K	cell differentiation
[BBH] CUE_DROPS (sp|Q29FE9) Protein cueball OS=D.p.p.	CUE_DROPS	0	GLOS_CUE.1.1	GO:0048477	B	UniProtKB	oogenesis
[BBH] CUE_DROPS (sp|Q29FE9) Protein cueball OS=D.p.p.	CUE_DROPS	0	GLOS_CUE.1.1	GO:0007283	B	UniProtKB	spermatogenesis
[BBH] DMDA_DROME (sp|Q9VDW6) Dystrophin, isoforms A/C/F/G/H	DMDA_DROME	0	GLOS_DMDA.1.3	GO:0005737	C	FlyBase	cytoplasm
[BBH] DMDA_DROME (sp|Q9VDW6) Dystrophin, isoforms A/C/F/G/H	DMDA_DROME	0	GLOS_DMDA.1.3	GO:0005856	C	UniProtKB	cytoskeleton
[BBH] DMDA_DROME (sp|Q9VDW6) Dystrophin, isoforms A/C/F/G/H	DMDA_DROME	0	GLOS_DMDA.1.3	GO:0016010	C	FlyBase	dystrophin-associated glycoprotein complex
[BBH] DMDA_DROME (sp|Q9VDW6) Dystrophin, isoforms A/C/F/G/H	DMDA_DROME	0	GLOS_DMDA.1.3	GO:0042383	C	UniProtKB-S	sarcolemma
[BBH] DMDA_DROME (sp|Q9VDW6) Dystrophin, isoforms A/C/F/G/H	DMDA_DROME	0	GLOS_DMDA.1.3	GO:0045202	C	UniProtKB	synapse
[BBH] DMDA_DROME (sp|Q9VDW6) Dystrophin, isoforms A/C/F/G/H	DMDA_DROME	0	GLOS_DMDA.1.3	GO:0008092	M	.	cytoskeletal protein binding
[BBH] DMDA_DROME (sp|Q9VDW6) Dystrophin, isoforms A/C/F/G/H	DMDA_DROME	0	GLOS_DMDA.1.3	GO:0003779	M	FlyBase	actin binding
[BBH] DMDA_DROME (sp|Q9VDW6) Dystrophin, isoforms A/C/F/G/H	DMDA_DROME	0	GLOS_DMDA.1.3	GO:0005509	M	InterPro	calcium ion binding
[BBH] DMDA_DROME (sp|Q9VDW6) Dystrophin, isoforms A/C/F/G/H	DMDA_DROME	0	GLOS_DMDA.1.3	GO:0005198	M	FlyBase	structural molecule activity
[BBH] DMDA_DROME (sp|Q9VDW6) Dystrophin, isoforms A/C/F/G/H	DMDA_DROME	0	GLOS_DMDA.1.3	GO:0008270	M	InterPro	zinc ion binding
[BBH] DMDA_DROME (sp|Q9VDW6) Dystrophin, isoforms A/C/F/G/H	DMDA_DROME	0	GLOS_DMDA.1.3	GO:0030010	B	FlyBase	establishment of cell polarity
[BBH] DMDA_DROME (sp|Q9VDW6) Dystrophin, isoforms A/C/F/G/H	DMDA_DROME	0	GLOS_DMDA.1.3	GO:0009886	B	.	post-embryonic morphogenesis
[BBH] DMDA_DROME (sp|Q9VDW6) Dystrophin, isoforms A/C/F/G/H	DMDA_DROME	0	GLOS_DMDA.1.3	GO:0009791	B	.	post-embryonic development
[BBH] DMDA_DROME (sp|Q9VDW6) Dystrophin, isoforms A/C/F/G/H	DMDA_DROME	0	GLOS_DMDA.1.3	GO:0007476	B	.	imaginal disc-derived wing morphogenesis
[BBH] DMDA_DROME (sp|Q9VDW6) Dystrophin, isoforms A/C/F/G/H	DMDA_DROME	0	GLOS_DMDA.1.3	GO:0035114	B	.	imaginal disc-derived appendage morphogenesis
[BBH] DMDA_DROME (sp|Q9VDW6) Dystrophin, isoforms A/C/F/G/H	DMDA_DROME	0	GLOS_DMDA.1.3	GO:0035107	B	.	appendage morphogenesis
[BBH] DMDA_DROME (sp|Q9VDW6) Dystrophin, isoforms A/C/F/G/H	DMDA_DROME	0	GLOS_DMDA.1.3	GO:0048736	B	.	appendage development
[BBH] DMDA_DROME (sp|Q9VDW6) Dystrophin, isoforms A/C/F/G/H	DMDA_DROME	0	GLOS_DMDA.1.3	GO:0048737	B	.	imaginal disc-derived appendage development
[BBH] DMDA_DROME (sp|Q9VDW6) Dystrophin, isoforms A/C/F/G/H	DMDA_DROME	0	GLOS_DMDA.1.3	GO:0035120	B	.	post-embryonic appendage morphogenesis
[BBH] DMDA_DROME (sp|Q9VDW6) Dystrophin, isoforms A/C/F/G/H	DMDA_DROME	0	GLOS_DMDA.1.3	GO:0007472	B	.	wing disc morphogenesis
[BBH] DMDA_DROME (sp|Q9VDW6) Dystrophin, isoforms A/C/F/G/H	DMDA_DROME	0	GLOS_DMDA.1.3	GO:0007560	B	.	imaginal disc morphogenesis
[BBH] DMDA_DROME (sp|Q9VDW6) Dystrophin, isoforms A/C/F/G/H	DMDA_DROME	0	GLOS_DMDA.1.3	GO:0048563	B	.	post-embryonic organ morphogenesis
[BBH] DMDA_DROME (sp|Q9VDW6) Dystrophin, isoforms A/C/F/G/H	DMDA_DROME	0	GLOS_DMDA.1.3	GO:0048569	B	.	post-embryonic organ development
[BBH] DMDA_DROME (sp|Q9VDW6) Dystrophin, isoforms A/C/F/G/H	DMDA_DROME	0	GLOS_DMDA.1.3	GO:0007444	B	.	imaginal disc development
[BBH] DMDA_DROME (sp|Q9VDW6) Dystrophin, isoforms A/C/F/G/H	DMDA_DROME	0	GLOS_DMDA.1.3	GO:0007552	B	.	metamorphosis
[BBH] DMDA_DROME (sp|Q9VDW6) Dystrophin, isoforms A/C/F/G/H	DMDA_DROME	0	GLOS_DMDA.1.3	GO:0048707	B	.	instar larval or pupal morphogenesis
[BBH] DMDA_DROME (sp|Q9VDW6) Dystrophin, isoforms A/C/F/G/H	DMDA_DROME	0	GLOS_DMDA.1.3	GO:0002165	B	.	instar larval or pupal development
[BBH] DMDA_DROME (sp|Q9VDW6) Dystrophin, isoforms A/C/F/G/H	DMDA_DROME	0	GLOS_DMDA.1.3	GO:0035220	B	.	wing disc development
[BBH] DMDA_DROME (sp|Q9VDW6) Dystrophin, isoforms A/C/F/G/H	DMDA_DROME	0	GLOS_DMDA.1.3	GO:0008586	B	FlyBase	imaginal disc-derived wing vein morphogenesis
[BBH] DMDA_DROME (sp|Q9VDW6) Dystrophin, isoforms A/C/F/G/H	DMDA_DROME	0	GLOS_DMDA.1.3	GO:0007474	B	UniProtKB	imaginal disc-derived wing vein specification
[BBH] DMDA_DROME (sp|Q9VDW6) Dystrophin, isoforms A/C/F/G/H	DMDA_DROME	0	GLOS_DMDA.1.3	GO:0060249	B	.	anatomical structure homeostasis
[BBH] DMDA_DROME (sp|Q9VDW6) Dystrophin, isoforms A/C/F/G/H	DMDA_DROME	0	GLOS_DMDA.1.3	GO:0046716	B	FlyBase	muscle cell cellular homeostasis
[BBH] DMDA_DROME (sp|Q9VDW6) Dystrophin, isoforms A/C/F/G/H	DMDA_DROME	0	GLOS_DMDA.1.3	GO:0007268	B	.	synaptic transmission
[BBH] DMDA_DROME (sp|Q9VDW6) Dystrophin, isoforms A/C/F/G/H	DMDA_DROME	0	GLOS_DMDA.1.3	GO:0019226	B	.	transmission of nerve impulse
[BBH] DMDA_DROME (sp|Q9VDW6) Dystrophin, isoforms A/C/F/G/H	DMDA_DROME	0	GLOS_DMDA.1.3	GO:0035637	B	.	multicellular organismal signaling
[BBH] DMDA_DROME (sp|Q9VDW6) Dystrophin, isoforms A/C/F/G/H	DMDA_DROME	0	GLOS_DMDA.1.3	GO:0050877	B	.	neurological system process
[BBH] DMDA_DROME (sp|Q9VDW6) Dystrophin, isoforms A/C/F/G/H	DMDA_DROME	0	GLOS_DMDA.1.3	GO:0007274	B	FlyBase	neuromuscular synaptic transmission
[BBH] DMDA_DROME (sp|Q9VDW6) Dystrophin, isoforms A/C/F/G/H	DMDA_DROME	0	GLOS_DMDA.1.3	GO:0050804	B	.	regulation of synaptic transmission
[BBH] DMDA_DROME (sp|Q9VDW6) Dystrophin, isoforms A/C/F/G/H	DMDA_DROME	0	GLOS_DMDA.1.3	GO:0051969	B	.	regulation of transmission of nerve impulse
[BBH] DMDA_DROME (sp|Q9VDW6) Dystrophin, isoforms A/C/F/G/H	DMDA_DROME	0	GLOS_DMDA.1.3	GO:0010646	B	.	regulation of cell communication
[BBH] DMDA_DROME (sp|Q9VDW6) Dystrophin, isoforms A/C/F/G/H	DMDA_DROME	0	GLOS_DMDA.1.3	GO:0023051	B	.	regulation of signaling
[BBH] DMDA_DROME (sp|Q9VDW6) Dystrophin, isoforms A/C/F/G/H	DMDA_DROME	0	GLOS_DMDA.1.3	GO:0031644	B	.	regulation of neurological system process
[BBH] DMDA_DROME (sp|Q9VDW6) Dystrophin, isoforms A/C/F/G/H	DMDA_DROME	0	GLOS_DMDA.1.3	GO:0044057	B	.	regulation of system process
[BBH] DMDA_DROME (sp|Q9VDW6) Dystrophin, isoforms A/C/F/G/H	DMDA_DROME	0	GLOS_DMDA.1.3	GO:0051046	B	.	regulation of secretion
[BBH] DMDA_DROME (sp|Q9VDW6) Dystrophin, isoforms A/C/F/G/H	DMDA_DROME	0	GLOS_DMDA.1.3	GO:0051588	B	.	regulation of neurotransmitter transport

[BBH] DMDA_DROME (sp|Q9VDW6) Dystrophin, isoforms A/C/F/G/H	DMDA_DROME	0	GLOS_DMDA.1.3	GO:0046928	B	UniProtKB	regulation of neurotransmitter secretion
[BBH] DMDA_DROME (sp|Q9VDW6) Dystrophin, isoforms A/C/F/G/H	DMDA_DROME	0	GLOS_DMDA.1.3	GO:0048168	B	.	regulation of neuronal synaptic plasticity
[BBH] DMDA_DROME (sp|Q9VDW6) Dystrophin, isoforms A/C/F/G/H	DMDA_DROME	0	GLOS_DMDA.1.3	GO:0048167	B	.	regulation of synaptic plasticity
[BBH] DMDA_DROME (sp|Q9VDW6) Dystrophin, isoforms A/C/F/G/H	DMDA_DROME	0	GLOS_DMDA.1.3	GO:0048172	B	FlyBase	regulation of short-term neuronal synaptic plasticity
DRM_DROME (sp|Q9VQS6) Protein drumstick OS=D.m.	DRM_DROME	5,00E-48	GLOS_DRM.2.2	GO:0005634	C	UniProtKB-S	nucleus
DRM_DROME (sp|Q9VQS6) Protein drumstick OS=D.m.	DRM_DROME	5,00E-48	GLOS_DRM.2.2	GO:0003677	M	UniProtKB-K	DNA binding
DRM_DROME (sp|Q9VQS6) Protein drumstick OS=D.m.	DRM_DROME	5,00E-48	GLOS_DRM.2.2	GO:0046872	M	UniProtKB-K	metal ion binding
DRM_DROME (sp|Q9VQS6) Protein drumstick OS=D.m.	DRM_DROME	5,00E-48	GLOS_DRM.2.2	GO:0048598	B	.	embryonic morphogenesis
DRM_DROME (sp|Q9VQS6) Protein drumstick OS=D.m.	DRM_DROME	5,00E-48	GLOS_DRM.2.2	GO:0007440	B	.	foregut morphogenesis
DRM_DROME (sp|Q9VQS6) Protein drumstick OS=D.m.	DRM_DROME	5,00E-48	GLOS_DRM.2.2	GO:0048546	B	.	digestive tract morphogenesis
DRM_DROME (sp|Q9VQS6) Protein drumstick OS=D.m.	DRM_DROME	5,00E-48	GLOS_DRM.2.2	GO:0048565	B	.	digestive tract development
DRM_DROME (sp|Q9VQS6) Protein drumstick OS=D.m.	DRM_DROME	5,00E-48	GLOS_DRM.2.2	GO:0055123	B	.	digestive system development
DRM_DROME (sp|Q9VQS6) Protein drumstick OS=D.m.	DRM_DROME	5,00E-48	GLOS_DRM.2.2	GO:0048617	B	UniProtKB	embryonic foregut morphogenesis
DRM_DROME (sp|Q9VQS6) Protein drumstick OS=D.m.	DRM_DROME	5,00E-48	GLOS_DRM.2.2	GO:0007442	B	.	hindgut morphogenesis
DRM_DROME (sp|Q9VQS6) Protein drumstick OS=D.m.	DRM_DROME	5,00E-48	GLOS_DRM.2.2	GO:0061525	B	.	hindgut development
DRM_DROME (sp|Q9VQS6) Protein drumstick OS=D.m.	DRM_DROME	5,00E-48	GLOS_DRM.2.2	GO:0048619	B	UniProtKB	embryonic hindgut morphogenesis
DRM_DROME (sp|Q9VQS6) Protein drumstick OS=D.m.	DRM_DROME	5,00E-48	GLOS_DRM.2.2	GO:0009880	B	UniProtKB	embryonic pattern specification
DRM_DROME (sp|Q9VQS6) Protein drumstick OS=D.m.	DRM_DROME	5,00E-48	GLOS_DRM.2.2	GO:0036011	B	.	imaginal disc-derived leg segmentation
DRM_DROME (sp|Q9VQS6) Protein drumstick OS=D.m.	DRM_DROME	5,00E-48	GLOS_DRM.2.2	GO:0035285	B	.	appendage segmentation
DRM_DROME (sp|Q9VQS6) Protein drumstick OS=D.m.	DRM_DROME	5,00E-48	GLOS_DRM.2.2	GO:0007480	B	.	imaginal disc-derived leg morphogenesis
DRM_DROME (sp|Q9VQS6) Protein drumstick OS=D.m.	DRM_DROME	5,00E-48	GLOS_DRM.2.2	GO:0007478	B	.	leg disc morphogenesis
DRM_DROME (sp|Q9VQS6) Protein drumstick OS=D.m.	DRM_DROME	5,00E-48	GLOS_DRM.2.2	GO:0035218	B	.	leg disc development
DRM_DROME (sp|Q9VQS6) Protein drumstick OS=D.m.	DRM_DROME	5,00E-48	GLOS_DRM.2.2	GO:0016348	B	FlyBase	imaginal disc-derived leg joint morphogenesis
DRM_DROME (sp|Q9VQS6) Protein drumstick OS=D.m.	DRM_DROME	5,00E-48	GLOS_DRM.2.2	GO:0000122	B	UniProtKB	negative regulation of transcription from RNA polymerase II promoter
DRM_DROME (sp|Q9VQS6) Protein drumstick OS=D.m.	DRM_DROME	5,00E-48	GLOS_DRM.2.2	GO:0007365	B	.	periodic partitioning
DRM_DROME (sp|Q9VQS6) Protein drumstick OS=D.m.	DRM_DROME	5,00E-48	GLOS_DRM.2.2	GO:0007366	B	UniProtKB-K	periodic partitioning by pair rule gene
DRM_DROME (sp|Q9VQS6) Protein drumstick OS=D.m.	DRM_DROME	5,00E-48	GLOS_DRM.2.2	GO:0045944	B	UniProtKB	positive regulation of transcription from RNA polymerase II promoter
DRM_DROME (sp|Q9VQS6) Protein drumstick OS=D.m.	DRM_DROME	5,00E-48	GLOS_DRM.2.2	GO:0006351	B	UniProtKB-K	transcription, DNA-dependent
[BBH] ERF1_TRYBB (sp|Q9NAX8) Eukary. pept chain release fact subunit 1;	ERF1_TRYBB	0	GLOS_ERF1.1.2	GO:0005737	C	UniProtKB-S	cytoplasm
[BBH] ERF1_TRYBB (sp|Q9NAX8) Eukary. pept chain release fact subunit 1;	ERF1_TRYBB	0	GLOS_ERF1.1.2	GO:0003747	M	.	translation release factor activity
[BBH] ERF1_TRYBB (sp|Q9NAX8) Eukary. pept chain release fact subunit 1;	ERF1_TRYBB	0	GLOS_ERF1.1.2	GO:0008079	M	.	translation termination factor activity
[BBH] ERF1_TRYBB (sp|Q9NAX8) Eukary. pept chain release fact subunit 1;	ERF1_TRYBB	0	GLOS_ERF1.1.2	GO:0008135	M	.	translation factor activity, nucleic acid binding
[BBH] ERF1_TRYBB (sp|Q9NAX8) Eukary. pept chain release fact subunit 1;	ERF1_TRYBB	0	GLOS_ERF1.1.2	GO:0003723	M	.	RNA binding
[BBH] ERF1_TRYBB (sp|Q9NAX8) Eukary. pept chain release fact subunit 1;	ERF1_TRYBB	0	GLOS_ERF1.1.2	GO:0016149	M	InterPro	translation release factor activity, codon specific
[BBH] FBSP1_DROSE (sp|B4HQ29) F-box/SPRY domain-contain. prot 1	FBSP1_DROSE	1,00E-164	GLOS_FBSP1.1.1	GO:0030054	C	UniProtKB-K	cell junction
[BBH] FBSP1_DROSE (sp|B4HQ29) F-box/SPRY domain-contain. prot 1	FBSP1_DROSE	1,00E-164	GLOS_FBSP1.1.1	GO:0045202	C	.	synapse
[BBH] FBSP1_DROSE (sp|B4HQ29) F-box/SPRY domain-contain. prot 1	FBSP1_DROSE	1,00E-164	GLOS_FBSP1.1.1	GO:0031594	C	UniProtKB	neuromuscular junction
[BBH] FBSP1_DROSE (sp|B4HQ29) F-box/SPRY domain-contain. prot 1	FBSP1_DROSE	1,00E-164	GLOS_FBSP1.1.1	GO:0008582	B	.	regulation of synaptic growth at neuromuscular junction
[BBH] FBSP1_DROSE (sp|B4HQ29) F-box/SPRY domain-contain. prot 1	FBSP1_DROSE	1,00E-164	GLOS_FBSP1.1.1	GO:0048638	B	.	regulation of developmental growth
[BBH] FBSP1_DROSE (sp|B4HQ29) F-box/SPRY domain-contain. prot 1	FBSP1_DROSE	1,00E-164	GLOS_FBSP1.1.1	GO:0040008	B	.	regulation of growth
[BBH] FBSP1_DROSE (sp|B4HQ29) F-box/SPRY domain-contain. prot 1	FBSP1_DROSE	1,00E-164	GLOS_FBSP1.1.1	GO:0048742	B	.	regulation of skeletal muscle fiber development
[BBH] FBSP1_DROSE (sp|B4HQ29) F-box/SPRY domain-contain. prot 1	FBSP1_DROSE	1,00E-164	GLOS_FBSP1.1.1	GO:0048641	B	.	regulation of skeletal muscle tissue development
[BBH] FBSP1_DROSE (sp|B4HQ29) F-box/SPRY domain-contain. prot 1	FBSP1_DROSE	1,00E-164	GLOS_FBSP1.1.1	GO:0016202	B	.	regulation of striated muscle tissue development
[BBH] FBSP1_DROSE (sp|B4HQ29) F-box/SPRY domain-contain. prot 1	FBSP1_DROSE	1,00E-164	GLOS_FBSP1.1.1	GO:0048634	B	.	regulation of muscle organ development
[BBH] FBSP1_DROSE (sp|B4HQ29) F-box/SPRY domain-contain. prot 1	FBSP1_DROSE	1,00E-164	GLOS_FBSP1.1.1	GO:1901861	B	.	regulation of muscle tissue development
[BBH] FBSP1_DROSE (sp|B4HQ29) F-box/SPRY domain-contain. prot 1	FBSP1_DROSE	1,00E-164	GLOS_FBSP1.1.1	GO:0051153	B	.	regulation of striated muscle cell differentiation
[BBH] FBSP1_DROSE (sp|B4HQ29) F-box/SPRY domain-contain. prot 1	FBSP1_DROSE	1,00E-164	GLOS_FBSP1.1.1	GO:0051147	B	.	regulation of muscle cell differentiation
[BBH] FBSP1_DROSE (sp|B4HQ29) F-box/SPRY domain-contain. prot 1	FBSP1_DROSE	1,00E-164	GLOS_FBSP1.1.1	GO:0051963	B	.	regulation of synapse assembly
[BBH] FBSP1_DROSE (sp|B4HQ29) F-box/SPRY domain-contain. prot 1	FBSP1_DROSE	1,00E-164	GLOS_FBSP1.1.1	GO:0044087	B	.	regulation of cellular component biogenesis
[BBH] FBSP1_DROSE (sp|B4HQ29) F-box/SPRY domain-contain. prot 1	FBSP1_DROSE	1,00E-164	GLOS_FBSP1.1.1	GO:0050807	B	.	regulation of synapse organization
[BBH] FBSP1_DROSE (sp|B4HQ29) F-box/SPRY domain-contain. prot 1	FBSP1_DROSE	1,00E-164	GLOS_FBSP1.1.1	GO:0050803	B	.	regulation of synapse structure and activity
[BBH] FBSP1_DROSE (sp|B4HQ29) F-box/SPRY domain-contain. prot 1	FBSP1_DROSE	1,00E-164	GLOS_FBSP1.1.1	GO:0048640	B	.	negative regulation of developmental growth
[BBH] FBSP1_DROSE (sp|B4HQ29) F-box/SPRY domain-contain. prot 1	FBSP1_DROSE	1,00E-164	GLOS_FBSP1.1.1	GO:0045926	B	.	negative regulation of growth
[BBH] FBSP1_DROSE (sp|B4HQ29) F-box/SPRY domain-contain. prot 1	FBSP1_DROSE	1,00E-164	GLOS_FBSP1.1.1	GO:0051093	B	.	negative regulation of developmental process

[BBH] FBSP1_DROSE (sp|B4HQ29) F-box/SPRY domain-contain. prot 1	FBSP1_DROSE	1,00E-164	GLOS_FBSP1.1.1	GO:0051964	B	.	negative regulation of synapse assembly
[BBH] FBSP1_DROSE (sp|B4HQ29) F-box/SPRY domain-contain. prot 1	FBSP1_DROSE	1,00E-164	GLOS_FBSP1.1.1	GO:0051961	B	.	negative regulation of nervous system development
[BBH] FBSP1_DROSE (sp|B4HQ29) F-box/SPRY domain-contain. prot 1	FBSP1_DROSE	1,00E-164	GLOS_FBSP1.1.1	GO:0045886	B	UniProtKB	negative regulation of synaptic growth at neuromuscular junction
[BBH] FBSP1_DROSE (sp|B4HQ29) F-box/SPRY domain-contain. prot 1	FBSP1_DROSE	1,00E-164	GLOS_FBSP1.1.1	GO:0007399	B	UniProtKB-k	nervous system development
[BBH] FBSP1_DROSE (sp|B4HQ29) F-box/SPRY domain-contain. prot 1	FBSP1_DROSE	1,00E-164	GLOS_FBSP1.1.1	GO:0007274	B	UniProtKB	neuromuscular synaptic transmission
[BBH] FBSP1_DROSE (sp|B4HQ29) F-box/SPRY domain-contain. prot 1	FBSP1_DROSE	1,00E-164	GLOS_FBSP1.1.1	GO:0032446	B	.	protein modification by small protein conjugation
[BBH] FBSP1_DROSE (sp|B4HQ29) F-box/SPRY domain-contain. prot 1	FBSP1_DROSE	1,00E-164	GLOS_FBSP1.1.1	GO:0016567	B	UniProtKB-U	protein ubiquitination
[BBH] FLOT2_DROME (sp|O61492) Flotillin-2 OS=D.m.	FLOT2_DROME	0	GLOS_FLOT2.1.1	GO:0005901	C	.	caveola
[BBH] FLOT2_DROME (sp|O61492) Flotillin-2 OS=D.m.	FLOT2_DROME	0	GLOS_FLOT2.1.1	GO:0045121	C	.	membrane raft
[BBH] FLOT2_DROME (sp|O61492) Flotillin-2 OS=D.m.	FLOT2_DROME	0	GLOS_FLOT2.1.1	GO:0016600	C	UniProtKB	flotillin complex
[BBH] FLOT2_DROME (sp|O61492) Flotillin-2 OS=D.m.	FLOT2_DROME	0	GLOS_FLOT2.1.1	GO:0005198	M	UniProtKB	structural molecule activity
[BBH] FLOT2_DROME (sp|O61492) Flotillin-2 OS=D.m.	FLOT2_DROME	0	GLOS_FLOT2.1.1	GO:0022610	B	.	biological adhesion
[BBH] FLOT2_DROME (sp|O61492) Flotillin-2 OS=D.m.	FLOT2_DROME	0	GLOS_FLOT2.1.1	GO:0007155	B	UniProtKB	cell adhesion
[BBH] FLOT2_DROME (sp|O61492) Flotillin-2 OS=D.m.	FLOT2_DROME	0	GLOS_FLOT2.1.1	GO:0035006	B	.	melanization defense response
[BBH] FLOT2_DROME (sp|O61492) Flotillin-2 OS=D.m.	FLOT2_DROME	0	GLOS_FLOT2.1.1	GO:0006582	B	.	melanin metabolic process
[BBH] FLOT2_DROME (sp|O61492) Flotillin-2 OS=D.m.	FLOT2_DROME	0	GLOS_FLOT2.1.1	GO:0018958	B	.	phenol-containing compound metabolic process
[BBH] FLOT2_DROME (sp|O61492) Flotillin-2 OS=D.m.	FLOT2_DROME	0	GLOS_FLOT2.1.1	GO:1901615	B	.	organic hydroxy compound metabolic process
[BBH] FLOT2_DROME (sp|O61492) Flotillin-2 OS=D.m.	FLOT2_DROME	0	GLOS_FLOT2.1.1	GO:0019748	B	.	secondary metabolic process
[BBH] FLOT2_DROME (sp|O61492) Flotillin-2 OS=D.m.	FLOT2_DROME	0	GLOS_FLOT2.1.1	GO:0044710	B	.	single-organism metabolic process
[BBH] FLOT2_DROME (sp|O61492) Flotillin-2 OS=D.m.	FLOT2_DROME	0	GLOS_FLOT2.1.1	GO:0042440	B	.	pigment metabolic process
[BBH] FLOT2_DROME (sp|O61492) Flotillin-2 OS=D.m.	FLOT2_DROME	0	GLOS_FLOT2.1.1	GO:0045087	B	.	innate immune response
[BBH] FLOT2_DROME (sp|O61492) Flotillin-2 OS=D.m.	FLOT2_DROME	0	GLOS_FLOT2.1.1	GO:0006952	B	.	defense response
[BBH] FLOT2_DROME (sp|O61492) Flotillin-2 OS=D.m.	FLOT2_DROME	0	GLOS_FLOT2.1.1	GO:0006955	B	.	immune response
[BBH] FLOT2_DROME (sp|O61492) Flotillin-2 OS=D.m.	FLOT2_DROME	0	GLOS_FLOT2.1.1	GO:0002376	B	.	immune system process
[BBH] FLOT2_DROME (sp|O61492) Flotillin-2 OS=D.m.	FLOT2_DROME	0	GLOS_FLOT2.1.1	GO:0035010	B	.	encapsulation of foreign target
[BBH] FLOT2_DROME (sp|O61492) Flotillin-2 OS=D.m.	FLOT2_DROME	0	GLOS_FLOT2.1.1	GO:0002252	B	.	immune effector process
[BBH] FLOT2_DROME (sp|O61492) Flotillin-2 OS=D.m.	FLOT2_DROME	0	GLOS_FLOT2.1.1	GO:0035011	B	FlyBase	melanotic encapsulation of foreign target
GBG1_DROME (sp|P38040) Guanine nucleotide-binding prot subunit gamma-1	GBG1_DROME	6,00E-33	GLOS_GBG1.2.3	GO:0031234	C	.	extrinsic to internal side of plasma membrane
GBG1_DROME (sp|P38040) Guanine nucleotide-binding prot subunit gamma-1	GBG1_DROME	6,00E-33	GLOS_GBG1.2.3	GO:0019897	C	.	extrinsic to plasma membrane
GBG1_DROME (sp|P38040) Guanine nucleotide-binding prot subunit gamma-1	GBG1_DROME	6,00E-33	GLOS_GBG1.2.3	GO:0019898	C	.	extrinsic to membrane
GBG1_DROME (sp|P38040) Guanine nucleotide-binding prot subunit gamma-1	GBG1_DROME	6,00E-33	GLOS_GBG1.2.3	GO:0009898	C	.	internal side of plasma membrane
GBG1_DROME (sp|P38040) Guanine nucleotide-binding prot subunit gamma-1	GBG1_DROME	6,00E-33	GLOS_GBG1.2.3	GO:0005834	C	FlyBase	heterotrimeric G-protein complex
GBG1_DROME (sp|P38040) Guanine nucleotide-binding prot subunit gamma-1	GBG1_DROME	6,00E-33	GLOS_GBG1.2.3	GO:0017111	M	.	nucleoside-triphosphatase activity
GBG1_DROME (sp|P38040) Guanine nucleotide-binding prot subunit gamma-1	GBG1_DROME	6,00E-33	GLOS_GBG1.2.3	GO:0016462	M	.	pyrophosphatase activity
GBG1_DROME (sp|P38040) Guanine nucleotide-binding prot subunit gamma-1	GBG1_DROME	6,00E-33	GLOS_GBG1.2.3	GO:0016818	M	.	hydrolase act., acting on acid anhydrides, in phosphorus-containing anhydrides
GBG1_DROME (sp|P38040) Guanine nucleotide-binding prot subunit gamma-1	GBG1_DROME	6,00E-33	GLOS_GBG1.2.3	GO:0016817	M	.	hydrolase activity, acting on acid anhydrides
GBG1_DROME (sp|P38040) Guanine nucleotide-binding prot subunit gamma-1	GBG1_DROME	6,00E-33	GLOS_GBG1.2.3	GO:0003924	M	FlyBase	GTPase activity
GBG1_DROME (sp|P38040) Guanine nucleotide-binding prot subunit gamma-1	GBG1_DROME	6,00E-33	GLOS_GBG1.2.3	GO:0004871	M	UniProtKB-k	signal transducer activity
GBG1_DROME (sp|P38040) Guanine nucleotide-binding prot subunit gamma-1	GBG1_DROME	6,00E-33	GLOS_GBG1.2.3	GO:0030036	B	.	actin cytoskeleton organization
GBG1_DROME (sp|P38040) Guanine nucleotide-binding prot subunit gamma-1	GBG1_DROME	6,00E-33	GLOS_GBG1.2.3	GO:0030029	B	.	actin filament-based process
GBG1_DROME (sp|P38040) Guanine nucleotide-binding prot subunit gamma-1	GBG1_DROME	6,00E-33	GLOS_GBG1.2.3	GO:0007015	B	FlyBase	actin filament organization
GBG1_DROME (sp|P38040) Guanine nucleotide-binding prot subunit gamma-1	GBG1_DROME	6,00E-33	GLOS_GBG1.2.3	GO:0008105	B	.	asymmetric protein localization
GBG1_DROME (sp|P38040) Guanine nucleotide-binding prot subunit gamma-1	GBG1_DROME	6,00E-33	GLOS_GBG1.2.3	GO:0045176	B	FlyBase	apical protein localization
GBG1_DROME (sp|P38040) Guanine nucleotide-binding prot subunit gamma-1	GBG1_DROME	6,00E-33	GLOS_GBG1.2.3	GO:0055057	B	.	neuroblast division
GBG1_DROME (sp|P38040) Guanine nucleotide-binding prot subunit gamma-1	GBG1_DROME	6,00E-33	GLOS_GBG1.2.3	GO:0048103	B	.	somatic stem cell division
GBG1_DROME (sp|P38040) Guanine nucleotide-binding prot subunit gamma-1	GBG1_DROME	6,00E-33	GLOS_GBG1.2.3	GO:0017145	B	.	stem cell division
GBG1_DROME (sp|P38040) Guanine nucleotide-binding prot subunit gamma-1	GBG1_DROME	6,00E-33	GLOS_GBG1.2.3	GO:0007405	B	.	neuroblast proliferation
GBG1_DROME (sp|P38040) Guanine nucleotide-binding prot subunit gamma-1	GBG1_DROME	6,00E-33	GLOS_GBG1.2.3	GO:0061351	B	.	neural precursor cell proliferation
GBG1_DROME (sp|P38040) Guanine nucleotide-binding prot subunit gamma-1	GBG1_DROME	6,00E-33	GLOS_GBG1.2.3	GO:0008283	B	.	cell proliferation
GBG1_DROME (sp|P38040) Guanine nucleotide-binding prot subunit gamma-1	GBG1_DROME	6,00E-33	GLOS_GBG1.2.3	GO:0072089	B	.	stem cell proliferation
GBG1_DROME (sp|P38040) Guanine nucleotide-binding prot subunit gamma-1	GBG1_DROME	6,00E-33	GLOS_GBG1.2.3	GO:0055059	B	FlyBase	asymmetric neuroblast division
GBG1_DROME (sp|P38040) Guanine nucleotide-binding prot subunit gamma-1	GBG1_DROME	6,00E-33	GLOS_GBG1.2.3	GO:0007155	B	.	cell adhesion
GBG1_DROME (sp|P38040) Guanine nucleotide-binding prot subunit gamma-1	GBG1_DROME	6,00E-33	GLOS_GBG1.2.3	GO:0003007	B	.	heart morphogenesis
GBG1_DROME (sp|P38040) Guanine nucleotide-binding prot subunit gamma-1	GBG1_DROME	6,00E-33	GLOS_GBG1.2.3	GO:0007507	B	.	heart development

GBG1_DROME (sp|P38040) Guanine nucleotide-binding prot subunit gamma-1	GBG1_DROME	6,00E-33	GLOS_GBG1.2.3	GO:0061343	B	FlyBase	cell adhesion involved in heart morphogenesis
GBG1_DROME (sp|P38040) Guanine nucleotide-binding prot subunit gamma-1	GBG1_DROME	6,00E-33	GLOS_GBG1.2.3	GO:0016331	B	.	morphogenesis of embryonic epithelium
GBG1_DROME (sp|P38040) Guanine nucleotide-binding prot subunit gamma-1	GBG1_DROME	6,00E-33	GLOS_GBG1.2.3	GO:0001700	B	.	embryonic development via the syncytial blastoderm
GBG1_DROME (sp|P38040) Guanine nucleotide-binding prot subunit gamma-1	GBG1_DROME	6,00E-33	GLOS_GBG1.2.3	GO:0009792	B	.	embryo development ending in birth or egg hatching
GBG1_DROME (sp|P38040) Guanine nucleotide-binding prot subunit gamma-1	GBG1_DROME	6,00E-33	GLOS_GBG1.2.3	GO:0007391	B	FlyBase	dorsal closure
GBG1_DROME (sp|P38040) Guanine nucleotide-binding prot subunit gamma-1	GBG1_DROME	6,00E-33	GLOS_GBG1.2.3	GO:0048568	B	.	embryonic organ development
GBG1_DROME (sp|P38040) Guanine nucleotide-binding prot subunit gamma-1	GBG1_DROME	6,00E-33	GLOS_GBG1.2.3	GO:0035050	B	FlyBase	embryonic heart tube development
GBG1_DROME (sp|P38040) Guanine nucleotide-binding prot subunit gamma-1	GBG1_DROME	6,00E-33	GLOS_GBG1.2.3	GO:0007186	B	FlyBase	G-protein coupled receptor signaling pathway
GBG1_DROME (sp|P38040) Guanine nucleotide-binding prot subunit gamma-1	GBG1_DROME	6,00E-33	GLOS_GBG1.2.3	GO:0003013	B	.	circulatory system process
GBG1_DROME (sp|P38040) Guanine nucleotide-binding prot subunit gamma-1	GBG1_DROME	6,00E-33	GLOS_GBG1.2.3	GO:0003015	B	FlyBase	heart process
GBG1_DROME (sp|P38040) Guanine nucleotide-binding prot subunit gamma-1	GBG1_DROME	6,00E-33	GLOS_GBG1.2.3	GO:0007635	B	.	chemosensory behavior
GBG1_DROME (sp|P38040) Guanine nucleotide-binding prot subunit gamma-1	GBG1_DROME	6,00E-33	GLOS_GBG1.2.3	GO:0044708	B	.	single-organism behavior
GBG1_DROME (sp|P38040) Guanine nucleotide-binding prot subunit gamma-1	GBG1_DROME	6,00E-33	GLOS_GBG1.2.3	GO:0007610	B	.	behavior
GBG1_DROME (sp|P38040) Guanine nucleotide-binding prot subunit gamma-1	GBG1_DROME	6,00E-33	GLOS_GBG1.2.3	GO:0051780	B	.	behavioral response to nutrient
GBG1_DROME (sp|P38040) Guanine nucleotide-binding prot subunit gamma-1	GBG1_DROME	6,00E-33	GLOS_GBG1.2.3	GO:0007584	B	.	response to nutrient
GBG1_DROME (sp|P38040) Guanine nucleotide-binding prot subunit gamma-1	GBG1_DROME	6,00E-33	GLOS_GBG1.2.3	GO:0031667	B	.	response to nutrient levels
GBG1_DROME (sp|P38040) Guanine nucleotide-binding prot subunit gamma-1	GBG1_DROME	6,00E-33	GLOS_GBG1.2.3	GO:0009991	B	.	response to extracellular stimulus
GBG1_DROME (sp|P38040) Guanine nucleotide-binding prot subunit gamma-1	GBG1_DROME	6,00E-33	GLOS_GBG1.2.3	GO:0060004	B	.	reflex
GBG1_DROME (sp|P38040) Guanine nucleotide-binding prot subunit gamma-1	GBG1_DROME	6,00E-33	GLOS_GBG1.2.3	GO:0007637	B	FlyBase	proboscis extension reflex
GBG1_DROME (sp|P38040) Guanine nucleotide-binding prot subunit gamma-1	GBG1_DROME	6,00E-33	GLOS_GBG1.2.3	GO:0022604	B	.	regulation of cell morphogenesis
GBG1_DROME (sp|P38040) Guanine nucleotide-binding prot subunit gamma-1	GBG1_DROME	6,00E-33	GLOS_GBG1.2.3	GO:0008360	B	FlyBase	regulation of cell shape
[BBH] HSP83_DROAV (sp|O02192) Heat shock prot. 83 OS=D. auraria	HSP83_DROAV	0	GLOS_HSP83.1.1	GO:0005737	C	UniProtKB-S	cytoplasm
[BBH] HSP83_DROAV (sp|O02192) Heat shock prot. 83 OS=D. auraria	HSP83_DROAV	0	GLOS_HSP83.1.1	GO:0032550	M	.	purine ribonucleoside binding
[BBH] HSP83_DROAV (sp|O02192) Heat shock prot. 83 OS=D. auraria	HSP83_DROAV	0	GLOS_HSP83.1.1	GO:0001883	M	.	purine nucleoside binding
[BBH] HSP83_DROAV (sp|O02192) Heat shock prot. 83 OS=D. auraria	HSP83_DROAV	0	GLOS_HSP83.1.1	GO:0001882	M	.	nucleoside binding
[BBH] HSP83_DROAV (sp|O02192) Heat shock prot. 83 OS=D. auraria	HSP83_DROAV	0	GLOS_HSP83.1.1	GO:0097367	M	.	carbohydrate derivative binding
[BBH] HSP83_DROAV (sp|O02192) Heat shock prot. 83 OS=D. auraria	HSP83_DROAV	0	GLOS_HSP83.1.1	GO:0032549	M	.	ribonucleoside binding
[BBH] HSP83_DROAV (sp|O02192) Heat shock prot. 83 OS=D. auraria	HSP83_DROAV	0	GLOS_HSP83.1.1	GO:0032559	M	.	adenyl ribonucleotide binding
[BBH] HSP83_DROAV (sp|O02192) Heat shock prot. 83 OS=D. auraria	HSP83_DROAV	0	GLOS_HSP83.1.1	GO:0030554	M	.	adenyl nucleotide binding
[BBH] HSP83_DROAV (sp|O02192) Heat shock prot. 83 OS=D. auraria	HSP83_DROAV	0	GLOS_HSP83.1.1	GO:0017076	M	.	purine nucleotide binding
[BBH] HSP83_DROAV (sp|O02192) Heat shock prot. 83 OS=D. auraria	HSP83_DROAV	0	GLOS_HSP83.1.1	GO:0000166	M	.	nucleotide binding
[BBH] HSP83_DROAV (sp|O02192) Heat shock prot. 83 OS=D. auraria	HSP83_DROAV	0	GLOS_HSP83.1.1	GO:1901265	M	.	nucleoside phosphate binding
[BBH] HSP83_DROAV (sp|O02192) Heat shock prot. 83 OS=D. auraria	HSP83_DROAV	0	GLOS_HSP83.1.1	GO:0032555	M	.	purine ribonucleotide binding
[BBH] HSP83_DROAV (sp|O02192) Heat shock prot. 83 OS=D. auraria	HSP83_DROAV	0	GLOS_HSP83.1.1	GO:0032553	M	.	ribonucleotide binding
[BBH] HSP83_DROAV (sp|O02192) Heat shock prot. 83 OS=D. auraria	HSP83_DROAV	0	GLOS_HSP83.1.1	GO:0035639	M	.	purine ribonucleoside triphosphate binding
[BBH] HSP83_DROAV (sp|O02192) Heat shock prot. 83 OS=D. auraria	HSP83_DROAV	0	GLOS_HSP83.1.1	GO:0005524	M	UniProtKB-K	ATP binding
[BBH] HSP83_DROAV (sp|O02192) Heat shock prot. 83 OS=D. auraria	HSP83_DROAV	0	GLOS_HSP83.1.1	GO:0006457	B	InterPro	protein folding
[BBH] HSP83_DROAV (sp|O02192) Heat shock prot. 83 OS=D. auraria	HSP83_DROAV	0	GLOS_HSP83.1.1	GO:0006950	B	UniProtKB-K	response to stress
[BBH] IF4A_TRYB2 (sp|Q38F76) Prob. eukaryotic initiation factor 4A	IF4A_TRYB2	0	GLOS_IF4A.1.2	GO:0005524	M	UniProtKB-K	ATP binding
[BBH] IF4A_TRYB2 (sp|Q38F76) Prob. eukaryotic initiation factor 4A	IF4A_TRYB2	0	GLOS_IF4A.1.2	GO:0042623	M	.	ATPase activity, coupled
[BBH] IF4A_TRYB2 (sp|Q38F76) Prob. eukaryotic initiation factor 4A	IF4A_TRYB2	0	GLOS_IF4A.1.2	GO:0016887	M	.	ATPase activity
[BBH] IF4A_TRYB2 (sp|Q38F76) Prob. eukaryotic initiation factor 4A	IF4A_TRYB2	0	GLOS_IF4A.1.2	GO:0070035	M	.	purine NTP-dependent helicase activity
[BBH] IF4A_TRYB2 (sp|Q38F76) Prob. eukaryotic initiation factor 4A	IF4A_TRYB2	0	GLOS_IF4A.1.2	GO:0004386	M	.	helicase activity
[BBH] IF4A_TRYB2 (sp|Q38F76) Prob. eukaryotic initiation factor 4A	IF4A_TRYB2	0	GLOS_IF4A.1.2	GO:0008026	M	InterPro	ATP-dependent helicase activity
[BBH] IF4A_TRYB2 (sp|Q38F76) Prob. eukaryotic initiation factor 4A	IF4A_TRYB2	0	GLOS_IF4A.1.2	GO:0003743	M	UniProtKB-K	translation initiation factor activity
[BBH] IMDH_DROME (sp|Q07152) Inosine-5'-monophosphate dehydrogenase	IMDH_DROME	0	GLOS_IMDH.1.2	GO:0005737	C	UniProtKB-S	cytoplasm
[BBH] IMDH_DROME (sp|Q07152) Inosine-5'-monophosphate dehydrogenase	IMDH_DROME	0	GLOS_IMDH.1.2	GO:0030554	M	InterPro	adenyl nucleotide binding
[BBH] IMDH_DROME (sp|Q07152) Inosine-5'-monophosphate dehydrogenase	IMDH_DROME	0	GLOS_IMDH.1.2	GO:0016616	M	.	oxidoreductase activity, acting on the CH-OH group of donors, NAD or NADP as acceptor
[BBH] IMDH_DROME (sp|Q07152) Inosine-5'-monophosphate dehydrogenase	IMDH_DROME	0	GLOS_IMDH.1.2	GO:0016614	M	.	oxidoreductase activity, acting on CH-OH group of donors
[BBH] IMDH_DROME (sp|Q07152) Inosine-5'-monophosphate dehydrogenase	IMDH_DROME	0	GLOS_IMDH.1.2	GO:0016491	M	.	oxidoreductase activity
[BBH] IMDH_DROME (sp|Q07152) Inosine-5'-monophosphate dehydrogenase	IMDH_DROME	0	GLOS_IMDH.1.2	GO:0003938	M	UniProtKB-K	IMP dehydrogenase activity
[BBH] IMDH_DROME (sp|Q07152) Inosine-5'-monophosphate dehydrogenase	IMDH_DROME	0	GLOS_IMDH.1.2	GO:0046872	M	UniProtKB-K	metal ion binding
[BBH] IMDH_DROME (sp|Q07152) Inosine-5'-monophosphate dehydrogenase	IMDH_DROME	0	GLOS_IMDH.1.2	GO:0007411	B	FlyBase	axon guidance

[BBH] IMDH_DROME (sp|Q07152) Inosine-5'-monophosphate dehydrogenase	IMDH_DROME	0	GLOS_IMDH.1.2	GO:0009152	B	.	purine ribonucleotide biosynthetic process
[BBH] IMDH_DROME (sp|Q07152) Inosine-5'-monophosphate dehydrogenase	IMDH_DROME	0	GLOS_IMDH.1.2	GO:0006164	B	.	purine nucleotide biosynthetic process
[BBH] IMDH_DROME (sp|Q07152) Inosine-5'-monophosphate dehydrogenase	IMDH_DROME	0	GLOS_IMDH.1.2	GO:0006163	B	.	purine nucleotide metabolic process
[BBH] IMDH_DROME (sp|Q07152) Inosine-5'-monophosphate dehydrogenase	IMDH_DROME	0	GLOS_IMDH.1.2	GO:0009117	B	.	nucleotide metabolic process
[BBH] IMDH_DROME (sp|Q07152) Inosine-5'-monophosphate dehydrogenase	IMDH_DROME	0	GLOS_IMDH.1.2	GO:0006753	B	.	nucleoside phosphate metabolic process
[BBH] IMDH_DROME (sp|Q07152) Inosine-5'-monophosphate dehydrogenase	IMDH_DROME	0	GLOS_IMDH.1.2	GO:0006796	B	.	phosphate-containing compound metabolic process
[BBH] IMDH_DROME (sp|Q07152) Inosine-5'-monophosphate dehydrogenase	IMDH_DROME	0	GLOS_IMDH.1.2	GO:0006793	B	.	phosphorus metabolic process
[BBH] IMDH_DROME (sp|Q07152) Inosine-5'-monophosphate dehydrogenase	IMDH_DROME	0	GLOS_IMDH.1.2	GO:0019637	B	.	organophosphate metabolic process
[BBH] IMDH_DROME (sp|Q07152) Inosine-5'-monophosphate dehydrogenase	IMDH_DROME	0	GLOS_IMDH.1.2	GO:0055086	B	.	nucleobase-containing small molecule metabolic process
[BBH] IMDH_DROME (sp|Q07152) Inosine-5'-monophosphate dehydrogenase	IMDH_DROME	0	GLOS_IMDH.1.2	GO:0044281	B	.	small molecule metabolic process
[BBH] IMDH_DROME (sp|Q07152) Inosine-5'-monophosphate dehydrogenase	IMDH_DROME	0	GLOS_IMDH.1.2	GO:0072521	B	.	purine-containing compound metabolic process
[BBH] IMDH_DROME (sp|Q07152) Inosine-5'-monophosphate dehydrogenase	IMDH_DROME	0	GLOS_IMDH.1.2	GO:1901564	B	.	organonitrogen compound metabolic process
[BBH] IMDH_DROME (sp|Q07152) Inosine-5'-monophosphate dehydrogenase	IMDH_DROME	0	GLOS_IMDH.1.2	GO:0009165	B	.	nucleotide biosynthetic process
[BBH] IMDH_DROME (sp|Q07152) Inosine-5'-monophosphate dehydrogenase	IMDH_DROME	0	GLOS_IMDH.1.2	GO:1901293	B	.	nucleoside phosphate biosynthetic process
[BBH] IMDH_DROME (sp|Q07152) Inosine-5'-monophosphate dehydrogenase	IMDH_DROME	0	GLOS_IMDH.1.2	GO:0090407	B	.	organophosphate biosynthetic process
[BBH] IMDH_DROME (sp|Q07152) Inosine-5'-monophosphate dehydrogenase	IMDH_DROME	0	GLOS_IMDH.1.2	GO:0072522	B	.	purine-containing compound biosynthetic process
[BBH] IMDH_DROME (sp|Q07152) Inosine-5'-monophosphate dehydrogenase	IMDH_DROME	0	GLOS_IMDH.1.2	GO:1901566	B	.	organonitrogen compound biosynthetic process
[BBH] IMDH_DROME (sp|Q07152) Inosine-5'-monophosphate dehydrogenase	IMDH_DROME	0	GLOS_IMDH.1.2	GO:0009150	B	.	purine ribonucleotide metabolic process
[BBH] IMDH_DROME (sp|Q07152) Inosine-5'-monophosphate dehydrogenase	IMDH_DROME	0	GLOS_IMDH.1.2	GO:0009259	B	.	ribonucleotide metabolic process
[BBH] IMDH_DROME (sp|Q07152) Inosine-5'-monophosphate dehydrogenase	IMDH_DROME	0	GLOS_IMDH.1.2	GO:0019693	B	.	ribose phosphate metabolic process
[BBH] IMDH_DROME (sp|Q07152) Inosine-5'-monophosphate dehydrogenase	IMDH_DROME	0	GLOS_IMDH.1.2	GO:1901135	B	.	carbohydrate derivative metabolic process
[BBH] IMDH_DROME (sp|Q07152) Inosine-5'-monophosphate dehydrogenase	IMDH_DROME	0	GLOS_IMDH.1.2	GO:0009260	B	.	ribonucleotide biosynthetic process
[BBH] IMDH_DROME (sp|Q07152) Inosine-5'-monophosphate dehydrogenase	IMDH_DROME	0	GLOS_IMDH.1.2	GO:0046390	B	.	ribose phosphate biosynthetic process
[BBH] IMDH_DROME (sp|Q07152) Inosine-5'-monophosphate dehydrogenase	IMDH_DROME	0	GLOS_IMDH.1.2	GO:1901137	B	.	carbohydrate derivative biosynthetic process
[BBH] IMDH_DROME (sp|Q07152) Inosine-5'-monophosphate dehydrogenase	IMDH_DROME	0	GLOS_IMDH.1.2	GO:0009168	B	.	purine ribonucleoside monophosphate biosynthetic process
[BBH] IMDH_DROME (sp|Q07152) Inosine-5'-monophosphate dehydrogenase	IMDH_DROME	0	GLOS_IMDH.1.2	GO:0009127	B	.	purine nucleoside monophosphate biosynthetic process
[BBH] IMDH_DROME (sp|Q07152) Inosine-5'-monophosphate dehydrogenase	IMDH_DROME	0	GLOS_IMDH.1.2	GO:0009124	B	.	nucleoside monophosphate biosynthetic process
[BBH] IMDH_DROME (sp|Q07152) Inosine-5'-monophosphate dehydrogenase	IMDH_DROME	0	GLOS_IMDH.1.2	GO:0009123	B	.	nucleoside monophosphate metabolic process
[BBH] IMDH_DROME (sp|Q07152) Inosine-5'-monophosphate dehydrogenase	IMDH_DROME	0	GLOS_IMDH.1.2	GO:0009126	B	.	purine nucleoside monophosphate metabolic process
[BBH] IMDH_DROME (sp|Q07152) Inosine-5'-monophosphate dehydrogenase	IMDH_DROME	0	GLOS_IMDH.1.2	GO:0009156	B	.	ribonucleoside monophosphate biosynthetic process
[BBH] IMDH_DROME (sp|Q07152) Inosine-5'-monophosphate dehydrogenase	IMDH_DROME	0	GLOS_IMDH.1.2	GO:0009161	B	.	ribonucleoside monophosphate metabolic process
[BBH] IMDH_DROME (sp|Q07152) Inosine-5'-monophosphate dehydrogenase	IMDH_DROME	0	GLOS_IMDH.1.2	GO:0009167	B	.	purine ribonucleoside monophosphate metabolic process
[BBH] IMDH_DROME (sp|Q07152) Inosine-5'-monophosphate dehydrogenase	IMDH_DROME	0	GLOS_IMDH.1.2	GO:0046037	B	.	GMP metabolic process
[BBH] IMDH_DROME (sp|Q07152) Inosine-5'-monophosphate dehydrogenase	IMDH_DROME	0	GLOS_IMDH.1.2	GO:1901068	B	.	guanosine-containing compound metabolic process
[BBH] IMDH_DROME (sp|Q07152) Inosine-5'-monophosphate dehydrogenase	IMDH_DROME	0	GLOS_IMDH.1.2	GO:0046128	B	.	purine ribonucleoside metabolic process
[BBH] IMDH_DROME (sp|Q07152) Inosine-5'-monophosphate dehydrogenase	IMDH_DROME	0	GLOS_IMDH.1.2	GO:0009119	B	.	ribonucleoside metabolic process
[BBH] IMDH_DROME (sp|Q07152) Inosine-5'-monophosphate dehydrogenase	IMDH_DROME	0	GLOS_IMDH.1.2	GO:0009116	B	.	nucleoside metabolic process
[BBH] IMDH_DROME (sp|Q07152) Inosine-5'-monophosphate dehydrogenase	IMDH_DROME	0	GLOS_IMDH.1.2	GO:1901657	B	.	glycosyl compound metabolic process
[BBH] IMDH_DROME (sp|Q07152) Inosine-5'-monophosphate dehydrogenase	IMDH_DROME	0	GLOS_IMDH.1.2	GO:0042278	B	.	purine nucleoside metabolic process
[BBH] IMDH_DROME (sp|Q07152) Inosine-5'-monophosphate dehydrogenase	IMDH_DROME	0	GLOS_IMDH.1.2	GO:1901070	B	.	guanosine-containing compound biosynthetic process
[BBH] IMDH_DROME (sp|Q07152) Inosine-5'-monophosphate dehydrogenase	IMDH_DROME	0	GLOS_IMDH.1.2	GO:0046129	B	.	purine ribonucleoside biosynthetic process
[BBH] IMDH_DROME (sp|Q07152) Inosine-5'-monophosphate dehydrogenase	IMDH_DROME	0	GLOS_IMDH.1.2	GO:0042451	B	.	purine nucleoside biosynthetic process
[BBH] IMDH_DROME (sp|Q07152) Inosine-5'-monophosphate dehydrogenase	IMDH_DROME	0	GLOS_IMDH.1.2	GO:0009163	B	.	nucleoside biosynthetic process
[BBH] IMDH_DROME (sp|Q07152) Inosine-5'-monophosphate dehydrogenase	IMDH_DROME	0	GLOS_IMDH.1.2	GO:1901659	B	.	glycosyl compound biosynthetic process
[BBH] IMDH_DROME (sp|Q07152) Inosine-5'-monophosphate dehydrogenase	IMDH_DROME	0	GLOS_IMDH.1.2	GO:0042455	B	.	ribonucleoside biosynthetic process
[BBH] IMDH_DROME (sp|Q07152) Inosine-5'-monophosphate dehydrogenase	IMDH_DROME	0	GLOS_IMDH.1.2	GO:0006177	B	UniProtKB-I	GMP biosynthetic process
[BBH] IMDH_DROME (sp|Q07152) Inosine-5'-monophosphate dehydrogenase	IMDH_DROME	0	GLOS_IMDH.1.2	GO:0048477	B	FlyBase	oogenesis
[BBH] IMDH_DROME (sp|Q07152) Inosine-5'-monophosphate dehydrogenase	IMDH_DROME	0	GLOS_IMDH.1.2	GO:0006897	B	.	endocytosis
[BBH] IMDH_DROME (sp|Q07152) Inosine-5'-monophosphate dehydrogenase	IMDH_DROME	0	GLOS_IMDH.1.2	GO:0016192	B	.	vesicle-mediated transport
[BBH] IMDH_DROME (sp|Q07152) Inosine-5'-monophosphate dehydrogenase	IMDH_DROME	0	GLOS_IMDH.1.2	GO:0006909	B	FlyBase	phagocytosis
KCC2A_DROME (sp|Q00168) Ca/calmod.-depend prot kinase type II alpha chain	KCC2A_DROME	0	GLOS_KCC2A.1.2	GO:0043005	C	.	neuron projection
KCC2A_DROME (sp|Q00168) Ca/calmod.-depend prot kinase type II alpha chain	KCC2A_DROME	0	GLOS_KCC2A.1.2	GO:0042995	C	.	cell projection
KCC2A_DROME (sp|Q00168) Ca/calmod.-depend prot kinase type II alpha chain	KCC2A_DROME	0	GLOS_KCC2A.1.2	GO:0097458	C	.	neuron part
KCC2A_DROME (sp|Q00168) Ca/calmod.-depend prot kinase type II alpha chain	KCC2A_DROME	0	GLOS_KCC2A.1.2	GO:0030424	C	FlyBase	axon

KCC2A_DROME (sp|Q00168) Ca/calmod.-depend prot kinase type II alpha chain	KCC2A_DROME	0	GLOS_KCC2A.1.2	GO:0005954	C	UniProtKB	calcium- and calmodulin-dependent protein kinase complex
KCC2A_DROME (sp|Q00168) Ca/calmod.-depend prot kinase type II alpha chain	KCC2A_DROME	0	GLOS_KCC2A.1.2	GO:0030425	C	FlyBase	dendrite
KCC2A_DROME (sp|Q00168) Ca/calmod.-depend prot kinase type II alpha chain	KCC2A_DROME	0	GLOS_KCC2A.1.2	GO:0005886	C	FlyBase	plasma membrane
KCC2A_DROME (sp|Q00168) Ca/calmod.-depend prot kinase type II alpha chain	KCC2A_DROME	0	GLOS_KCC2A.1.2	GO:0097060	C	.	synaptic membrane
KCC2A_DROME (sp|Q00168) Ca/calmod.-depend prot kinase type II alpha chain	KCC2A_DROME	0	GLOS_KCC2A.1.2	GO:0044456	C	.	synapse part
KCC2A_DROME (sp|Q00168) Ca/calmod.-depend prot kinase type II alpha chain	KCC2A_DROME	0	GLOS_KCC2A.1.2	GO:0045211	C	FlyBase	postsynaptic membrane
KCC2A_DROME (sp|Q00168) Ca/calmod.-depend prot kinase type II alpha chain	KCC2A_DROME	0	GLOS_KCC2A.1.2	GO:0048786	C	FlyBase	presynaptic active zone
KCC2A_DROME (sp|Q00168) Ca/calmod.-depend prot kinase type II alpha chain	KCC2A_DROME	0	GLOS_KCC2A.1.2	GO:0005524	M	UniProtKB-k	ATP binding
KCC2A_DROME (sp|Q00168) Ca/calmod.-depend prot kinase type II alpha chain	KCC2A_DROME	0	GLOS_KCC2A.1.2	GO:0005516	M	FlyBase	calmodulin binding
KCC2A_DROME (sp|Q00168) Ca/calmod.-depend prot kinase type II alpha chain	KCC2A_DROME	0	GLOS_KCC2A.1.2	GO:0004674	M	.	protein serine/threonine kinase activity
KCC2A_DROME (sp|Q00168) Ca/calmod.-depend prot kinase type II alpha chain	KCC2A_DROME	0	GLOS_KCC2A.1.2	GO:0004672	M	.	protein kinase activity
KCC2A_DROME (sp|Q00168) Ca/calmod.-depend prot kinase type II alpha chain	KCC2A_DROME	0	GLOS_KCC2A.1.2	GO:0016301	M	.	kinase activity
KCC2A_DROME (sp|Q00168) Ca/calmod.-depend prot kinase type II alpha chain	KCC2A_DROME	0	GLOS_KCC2A.1.2	GO:0016772	M	.	transferase activity, transferring phosphorus-containing groups
KCC2A_DROME (sp|Q00168) Ca/calmod.-depend prot kinase type II alpha chain	KCC2A_DROME	0	GLOS_KCC2A.1.2	GO:0016740	M	.	transferase activity
KCC2A_DROME (sp|Q00168) Ca/calmod.-depend prot kinase type II alpha chain	KCC2A_DROME	0	GLOS_KCC2A.1.2	GO:0016773	M	.	phosphotransferase activity, alcohol group as acceptor
KCC2A_DROME (sp|Q00168) Ca/calmod.-depend prot kinase type II alpha chain	KCC2A_DROME	0	GLOS_KCC2A.1.2	GO:0004683	M	FlyBase	calmodulin-dependent protein kinase activity
KCC2A_DROME (sp|Q00168) Ca/calmod.-depend prot kinase type II alpha chain	KCC2A_DROME	0	GLOS_KCC2A.1.2	GO:0007613	B	.	memory
KCC2A_DROME (sp|Q00168) Ca/calmod.-depend prot kinase type II alpha chain	KCC2A_DROME	0	GLOS_KCC2A.1.2	GO:0007611	B	.	learning or memory
KCC2A_DROME (sp|Q00168) Ca/calmod.-depend prot kinase type II alpha chain	KCC2A_DROME	0	GLOS_KCC2A.1.2	GO:0050890	B	.	cognition
KCC2A_DROME (sp|Q00168) Ca/calmod.-depend prot kinase type II alpha chain	KCC2A_DROME	0	GLOS_KCC2A.1.2	GO:0007616	B	FlyBase	long-term memory
KCC2A_DROME (sp|Q00168) Ca/calmod.-depend prot kinase type II alpha chain	KCC2A_DROME	0	GLOS_KCC2A.1.2	GO:0060179	B	.	male mating behavior
KCC2A_DROME (sp|Q00168) Ca/calmod.-depend prot kinase type II alpha chain	KCC2A_DROME	0	GLOS_KCC2A.1.2	GO:0033057	B	.	multicellular organismal reproductive behavior
KCC2A_DROME (sp|Q00168) Ca/calmod.-depend prot kinase type II alpha chain	KCC2A_DROME	0	GLOS_KCC2A.1.2	GO:0019098	B	.	reproductive behavior
KCC2A_DROME (sp|Q00168) Ca/calmod.-depend prot kinase type II alpha chain	KCC2A_DROME	0	GLOS_KCC2A.1.2	GO:0044706	B	.	multi-multicellular organism process
KCC2A_DROME (sp|Q00168) Ca/calmod.-depend prot kinase type II alpha chain	KCC2A_DROME	0	GLOS_KCC2A.1.2	GO:0051704	B	.	multi-organism process
KCC2A_DROME (sp|Q00168) Ca/calmod.-depend prot kinase type II alpha chain	KCC2A_DROME	0	GLOS_KCC2A.1.2	GO:0007617	B	.	mating behavior
KCC2A_DROME (sp|Q00168) Ca/calmod.-depend prot kinase type II alpha chain	KCC2A_DROME	0	GLOS_KCC2A.1.2	GO:0051705	B	.	multi-organism behavior
KCC2A_DROME (sp|Q00168) Ca/calmod.-depend prot kinase type II alpha chain	KCC2A_DROME	0	GLOS_KCC2A.1.2	GO:0007618	B	.	mating
KCC2A_DROME (sp|Q00168) Ca/calmod.-depend prot kinase type II alpha chain	KCC2A_DROME	0	GLOS_KCC2A.1.2	GO:0044703	B	.	multi-organism reproductive process
KCC2A_DROME (sp|Q00168) Ca/calmod.-depend prot kinase type II alpha chain	KCC2A_DROME	0	GLOS_KCC2A.1.2	GO:0007619	B	.	courtship behavior
KCC2A_DROME (sp|Q00168) Ca/calmod.-depend prot kinase type II alpha chain	KCC2A_DROME	0	GLOS_KCC2A.1.2	GO:0008049	B	FlyBase	male courtship behavior
KCC2A_DROME (sp|Q00168) Ca/calmod.-depend prot kinase type II alpha chain	KCC2A_DROME	0	GLOS_KCC2A.1.2	GO:0050808	B	.	synapse organization
KCC2A_DROME (sp|Q00168) Ca/calmod.-depend prot kinase type II alpha chain	KCC2A_DROME	0	GLOS_KCC2A.1.2	GO:0007528	B	FlyBase	neuromuscular junction development
KCC2A_DROME (sp|Q00168) Ca/calmod.-depend prot kinase type II alpha chain	KCC2A_DROME	0	GLOS_KCC2A.1.2	GO:0060491	B	.	regulation of cell projection assembly
KCC2A_DROME (sp|Q00168) Ca/calmod.-depend prot kinase type II alpha chain	KCC2A_DROME	0	GLOS_KCC2A.1.2	GO:0031344	B	.	regulation of cell projection organization
KCC2A_DROME (sp|Q00168) Ca/calmod.-depend prot kinase type II alpha chain	KCC2A_DROME	0	GLOS_KCC2A.1.2	GO:0051489	B	FlyBase	regulation of filopodium assembly
KCC2A_DROME (sp|Q00168) Ca/calmod.-depend prot kinase type II alpha chain	KCC2A_DROME	0	GLOS_KCC2A.1.2	GO:2000241	B	.	regulation of reproductive process
KCC2A_DROME (sp|Q00168) Ca/calmod.-depend prot kinase type II alpha chain	KCC2A_DROME	0	GLOS_KCC2A.1.2	GO:0060278	B	FlyBase	regulation of ovulation
KCC2A_DROME (sp|Q00168) Ca/calmod.-depend prot kinase type II alpha chain	KCC2A_DROME	0	GLOS_KCC2A.1.2	GO:0007268	B	FlyBase	synaptic transmission
[BBH] KDM5_DROME (sp|Q9VMJ7) Lysine-specific demethylase lid OS=D.m.	KDM5_DROME	0	GLOS_KDM5.1.1	GO:0000118	C	.	histone deacetylase complex
[BBH] KDM5_DROME (sp|Q9VMJ7) Lysine-specific demethylase lid OS=D.m.	KDM5_DROME	0	GLOS_KDM5.1.1	GO:0044451	C	.	nucleoplasm part
[BBH] KDM5_DROME (sp|Q9VMJ7) Lysine-specific demethylase lid OS=D.m.	KDM5_DROME	0	GLOS_KDM5.1.1	GO:0005654	C	.	nucleoplasm
[BBH] KDM5_DROME (sp|Q9VMJ7) Lysine-specific demethylase lid OS=D.m.	KDM5_DROME	0	GLOS_KDM5.1.1	GO:0044454	C	.	nuclear chromosome part
[BBH] KDM5_DROME (sp|Q9VMJ7) Lysine-specific demethylase lid OS=D.m.	KDM5_DROME	0	GLOS_KDM5.1.1	GO:0044427	C	.	chromosomal part
[BBH] KDM5_DROME (sp|Q9VMJ7) Lysine-specific demethylase lid OS=D.m.	KDM5_DROME	0	GLOS_KDM5.1.1	GO:0005694	C	.	chromosome
[BBH] KDM5_DROME (sp|Q9VMJ7) Lysine-specific demethylase lid OS=D.m.	KDM5_DROME	0	GLOS_KDM5.1.1	GO:0000228	C	.	nuclear chromosome
[BBH] KDM5_DROME (sp|Q9VMJ7) Lysine-specific demethylase lid OS=D.m.	KDM5_DROME	0	GLOS_KDM5.1.1	GO:0000790	C	.	nuclear chromatin
[BBH] KDM5_DROME (sp|Q9VMJ7) Lysine-specific demethylase lid OS=D.m.	KDM5_DROME	0	GLOS_KDM5.1.1	GO:0000785	C	.	chromatin
[BBH] KDM5_DROME (sp|Q9VMJ7) Lysine-specific demethylase lid OS=D.m.	KDM5_DROME	0	GLOS_KDM5.1.1	GO:0070822	C	FlyBase	Sin3-type complex
[BBH] KDM5_DROME (sp|Q9VMJ7) Lysine-specific demethylase lid OS=D.m.	KDM5_DROME	0	GLOS_KDM5.1.1	GO:0003677	M	InterPro	DNA binding
[BBH] KDM5_DROME (sp|Q9VMJ7) Lysine-specific demethylase lid OS=D.m.	KDM5_DROME	0	GLOS_KDM5.1.1	GO:0010484	M	.	H3 histone acetyltransferase activity
[BBH] KDM5_DROME (sp|Q9VMJ7) Lysine-specific demethylase lid OS=D.m.	KDM5_DROME	0	GLOS_KDM5.1.1	GO:0004402	M	.	histone acetyltransferase activity
[BBH] KDM5_DROME (sp|Q9VMJ7) Lysine-specific demethylase lid OS=D.m.	KDM5_DROME	0	GLOS_KDM5.1.1	GO:0008080	M	.	N-acetyltransferase activity
[BBH] KDM5_DROME (sp|Q9VMJ7) Lysine-specific demethylase lid OS=D.m.	KDM5_DROME	0	GLOS_KDM5.1.1	GO:0016407	M	.	acetyltransferase activity

[BBH] KDM5_DROME (sp|Q9VMJ7) Lysine-specific demethylase lid OS=D.m.	KDM5_DROME	0	GLOS_KDM5.1.1	GO:0016747	M	.	transferase activity, transferring acyl groups other than amino-acyl groups
[BBH] KDM5_DROME (sp|Q9VMJ7) Lysine-specific demethylase lid OS=D.m.	KDM5_DROME	0	GLOS_KDM5.1.1	GO:0016746	M	.	transferase activity, transferring acyl groups
[BBH] KDM5_DROME (sp|Q9VMJ7) Lysine-specific demethylase lid OS=D.m.	KDM5_DROME	0	GLOS_KDM5.1.1	GO:0016410	M	.	N-acyltransferase activity
[BBH] KDM5_DROME (sp|Q9VMJ7) Lysine-specific demethylase lid OS=D.m.	KDM5_DROME	0	GLOS_KDM5.1.1	GO:0043992	M	FlyBase	histone acetyltransferase activity (H3-K9 specific)
[BBH] KDM5_DROME (sp|Q9VMJ7) Lysine-specific demethylase lid OS=D.m.	KDM5_DROME	0	GLOS_KDM5.1.1	GO:0032453	M	.	histone demethylase activity (H3-K4 specific)
[BBH] KDM5_DROME (sp|Q9VMJ7) Lysine-specific demethylase lid OS=D.m.	KDM5_DROME	0	GLOS_KDM5.1.1	GO:0032452	M	.	histone demethylase activity
[BBH] KDM5_DROME (sp|Q9VMJ7) Lysine-specific demethylase lid OS=D.m.	KDM5_DROME	0	GLOS_KDM5.1.1	GO:0032451	M	.	demethylase activity
[BBH] KDM5_DROME (sp|Q9VMJ7) Lysine-specific demethylase lid OS=D.m.	KDM5_DROME	0	GLOS_KDM5.1.1	GO:0034647	M	FlyBase	histone demethylase activity (H3-trimethyl-K4 specific)
[BBH] KDM5_DROME (sp|Q9VMJ7) Lysine-specific demethylase lid OS=D.m.	KDM5_DROME	0	GLOS_KDM5.1.1	GO:0016705	M	.	oxidoreductase activity, acting on paired donors, with incorporation or reduction of molecular oxygen
[BBH] KDM5_DROME (sp|Q9VMJ7) Lysine-specific demethylase lid OS=D.m.	KDM5_DROME	0	GLOS_KDM5.1.1	GO:0051213	M	.	dioxygenase activity
[BBH] KDM5_DROME (sp|Q9VMJ7) Lysine-specific demethylase lid OS=D.m.	KDM5_DROME	0	GLOS_KDM5.1.1	GO:0016706	M	InterPro	oxidoreductase activity, acting on paired donors, with incorporation or reduction of molecular oxygen, 2-oxoglutarate as one donor, and incorporation of one atom atom each of oxygen into both donors
[BBH] KDM5_DROME (sp|Q9VMJ7) Lysine-specific demethylase lid OS=D.m.	KDM5_DROME	0	GLOS_KDM5.1.1	GO:0008270	M	InterPro	zinc ion binding
[BBH] KDM5_DROME (sp|Q9VMJ7) Lysine-specific demethylase lid OS=D.m.	KDM5_DROME	0	GLOS_KDM5.1.1	GO:0007626	B	.	locomotory behavior
[BBH] KDM5_DROME (sp|Q9VMJ7) Lysine-specific demethylase lid OS=D.m.	KDM5_DROME	0	GLOS_KDM5.1.1	GO:0048512	B	.	circadian behavior
[BBH] KDM5_DROME (sp|Q9VMJ7) Lysine-specific demethylase lid OS=D.m.	KDM5_DROME	0	GLOS_KDM5.1.1	GO:0007622	B	.	rhythmic behavior
[BBH] KDM5_DROME (sp|Q9VMJ7) Lysine-specific demethylase lid OS=D.m.	KDM5_DROME	0	GLOS_KDM5.1.1	GO:0048511	B	.	rhythmic process
[BBH] KDM5_DROME (sp|Q9VMJ7) Lysine-specific demethylase lid OS=D.m.	KDM5_DROME	0	GLOS_KDM5.1.1	GO:0007623	B	.	circadian rhythm
[BBH] KDM5_DROME (sp|Q9VMJ7) Lysine-specific demethylase lid OS=D.m.	KDM5_DROME	0	GLOS_KDM5.1.1	GO:0045475	B	FlyBase	locomotor rhythm
[BBH] KDM5_DROME (sp|Q9VMJ7) Lysine-specific demethylase lid OS=D.m.	KDM5_DROME	0	GLOS_KDM5.1.1	GO:0007275	B	UniProtKB-K	multicellular organismal development
[BBH] KDM5_DROME (sp|Q9VMJ7) Lysine-specific demethylase lid OS=D.m.	KDM5_DROME	0	GLOS_KDM5.1.1	GO:0045893	B	UniProtKB	positive regulation of transcription, DNA-dependent
[BBH] KDM5_DROME (sp|Q9VMJ7) Lysine-specific demethylase lid OS=D.m.	KDM5_DROME	0	GLOS_KDM5.1.1	GO:0006351	B	UniProtKB-K	transcription, DNA-dependent
[BBH] KRR1_DROMO(sp|B4KF66)KRR1 small subunit processome comp. homol.	KRR1_DROMO	1,00E-171	GLOS_KRR1.1.1	GO:0005730	C	UniProtKB-S	nucleolus
[BBH] KRR1_DROMO(sp|B4KF66)KRR1 small subunit processome comp. homol.	KRR1_DROMO	1,00E-171	GLOS_KRR1.1.1	GO:0030529	C	UniProtKB-K	ribonucleoprotein complex
[BBH] KRR1_DROMO(sp|B4KF66)KRR1 small subunit processome comp. homol.	KRR1_DROMO	1,00E-171	GLOS_KRR1.1.1	GO:0003723	M	UniProtKB-K	RNA binding
[BBH] KRR1_DROMO(sp|B4KF66)KRR1 small subunit processome comp. homol.	KRR1_DROMO	1,00E-171	GLOS_KRR1.1.1	GO:0007275	B	UniProtKB-K	multicellular organismal development
[BBH] KRR1_DROMO(sp|B4KF66)KRR1 small subunit processome comp. homol.	KRR1_DROMO	1,00E-171	GLOS_KRR1.1.1	GO:0016072	B	.	rRNA metabolic process
[BBH] KRR1_DROMO(sp|B4KF66)KRR1 small subunit processome comp. homol.	KRR1_DROMO	1,00E-171	GLOS_KRR1.1.1	GO:0034660	B	.	ncRNA metabolic process
[BBH] KRR1_DROMO(sp|B4KF66)KRR1 small subunit processome comp. homol.	KRR1_DROMO	1,00E-171	GLOS_KRR1.1.1	GO:0034470	B	.	ncRNA processing
[BBH] KRR1_DROMO(sp|B4KF66)KRR1 small subunit processome comp. homol.	KRR1_DROMO	1,00E-171	GLOS_KRR1.1.1	GO:0006396	B	.	RNA processing
[BBH] KRR1_DROMO(sp|B4KF66)KRR1 small subunit processome comp. homol.	KRR1_DROMO	1,00E-171	GLOS_KRR1.1.1	GO:0042254	B	.	ribosome biogenesis
[BBH] KRR1_DROMO(sp|B4KF66)KRR1 small subunit processome comp. homol.	KRR1_DROMO	1,00E-171	GLOS_KRR1.1.1	GO:0022613	B	.	ribonucleoprotein complex biogenesis
[BBH] KRR1_DROMO(sp|B4KF66)KRR1 small subunit processome comp. homol.	KRR1_DROMO	1,00E-171	GLOS_KRR1.1.1	GO:0044085	B	.	cellular component biogenesis
[BBH] KRR1_DROMO(sp|B4KF66)KRR1 small subunit processome comp. homol.	KRR1_DROMO	1,00E-171	GLOS_KRR1.1.1	GO:0006364	B	UniProtKB-K	rRNA processing
[BBH] MED28_DROPS (sp|Q294G7)Mediator of RNA polym.II transcrip. subunit28	MED28_DROPS	4,00E-62	GLOS_MED28.1.1	GO:0016592	C	UniProtKB	mediator complex
[BBH] MED28_DROPS (sp|Q294G7)Mediator of RNA polym.II transcrip. subunit28	MED28_DROPS	4,00E-62	GLOS_MED28.1.1	GO:0001076	M	.	RNA polymerase II transcription factor binding transcription factor activity
[BBH] MED28_DROPS (sp|Q294G7)Mediator of RNA polym.II transcrip. subunit28	MED28_DROPS	4,00E-62	GLOS_MED28.1.1	GO:0001104	M	UniProtKB	RNA polymerase II transcription cofactor activity
NFU1_DROER (sp|B3NYF7) NFU1 iron-sulfur cluster scaffold homolog, mitoch.	NFU1_DROER	1,00E-121	GLOS_NFU1.1.1	GO:0005739	C	UniProtKB-S	mitochondrion
NFU1_DROER (sp|B3NYF7) NFU1 iron-sulfur cluster scaffold homolog, mitoch.	NFU1_DROER	1,00E-121	GLOS_NFU1.1.1	GO:0005524	M	InterPro	ATP binding
NFU1_DROER (sp|B3NYF7) NFU1 iron-sulfur cluster scaffold homolog, mitoch.	NFU1_DROER	1,00E-121	GLOS_NFU1.1.1	GO:0008026	M	InterPro	ATP-dependent helicase activity
NFU1_DROER (sp|B3NYF7) NFU1 iron-sulfur cluster scaffold homolog, mitoch.	NFU1_DROER	1,00E-121	GLOS_NFU1.1.1	GO:0005506	M	InterPro	iron ion binding
NFU1_DROER (sp|B3NYF7) NFU1 iron-sulfur cluster scaffold homolog, mitoch.	NFU1_DROER	1,00E-121	GLOS_NFU1.1.1	GO:0051540	M	.	metal cluster binding
NFU1_DROER (sp|B3NYF7) NFU1 iron-sulfur cluster scaffold homolog, mitoch.	NFU1_DROER	1,00E-121	GLOS_NFU1.1.1	GO:0051536	M	InterPro	iron-sulfur cluster binding
NFU1_DROER (sp|B3NYF7) NFU1 iron-sulfur cluster scaffold homolog, mitoch.	NFU1_DROER	1,00E-121	GLOS_NFU1.1.1	GO:0003676	M	InterPro	nucleic acid binding
NFU1_DROER (sp|B3NYF7) NFU1 iron-sulfur cluster scaffold homolog, mitoch.	NFU1_DROER	1,00E-121	GLOS_NFU1.1.1	GO:0031163	B	.	metallo-sulfur cluster assembly
NFU1_DROER (sp|B3NYF7) NFU1 iron-sulfur cluster scaffold homolog, mitoch.	NFU1_DROER	1,00E-121	GLOS_NFU1.1.1	GO:0022607	B	.	cellular component assembly
NFU1_DROER (sp|B3NYF7) NFU1 iron-sulfur cluster scaffold homolog, mitoch.	NFU1_DROER	1,00E-121	GLOS_NFU1.1.1	GO:0016226	B	InterPro	iron-sulfur cluster assembly
[BBH] PCNA_SARCR (sp|O16852)Proliferating cell nuclear antigen; S.crassipalpis	PCNA_SARCR	1,00E-169	GLOS_PCNA.1.1	GO:0005634	C	UniProtKB-S	nucleus
[BBH] PCNA_SARCR (sp|O16852)Proliferating cell nuclear antigen; S.crassipalpis	PCNA_SARCR	1,00E-169	GLOS_PCNA.1.1	GO:0044796	C	.	DNA polymerase processivity factor complex
[BBH] PCNA_SARCR (sp|O16852)Proliferating cell nuclear antigen; S.crassipalpis	PCNA_SARCR	1,00E-169	GLOS_PCNA.1.1	GO:0043626	C	InterPro	PCNA complex
[BBH] PCNA_SARCR (sp|O16852)Proliferating cell nuclear antigen; S.crassipalpis	PCNA_SARCR	1,00E-169	GLOS_PCNA.1.1	GO:0003677	M	UniProtKB-K	DNA binding
[BBH] PCNA_SARCR (sp|O16852)Proliferating cell nuclear antigen; S.crassipalpis	PCNA_SARCR	1,00E-169	GLOS_PCNA.1.1	GO:0030234	M	.	enzyme regulator activity

[BBH] PCNA_SARCR (sp|O16852)Proliferating cell nuclear antigen; S.crassipalpis	PCNA_SARCR	1,00E-169	GLOS_PCNA.1.1	GO:0030337	M	InterPro	DNA polymerase processivity factor activity
[BBH] PCNA_SARCR (sp|O16852)Proliferating cell nuclear antigen; S.crassipalpis	PCNA_SARCR	1,00E-169	GLOS_PCNA.1.1	GO:0006259	B	.	DNA metabolic process
[BBH] PCNA_SARCR (sp|O16852)Proliferating cell nuclear antigen; S.crassipalpis	PCNA_SARCR	1,00E-169	GLOS_PCNA.1.1	GO:0006260	B	UniProtKB-K	DNA replication
[BBH] PCNA_SARCR (sp|O16852)Proliferating cell nuclear antigen; S.crassipalpis	PCNA_SARCR	1,00E-169	GLOS_PCNA.1.1	GO:0051052	B	.	regulation of DNA metabolic process
[BBH] PCNA_SARCR (sp|O16852)Proliferating cell nuclear antigen; S.crassipalpis	PCNA_SARCR	1,00E-169	GLOS_PCNA.1.1	GO:0006275	B	InterPro	regulation of DNA replication
[BBH] PGKE_TRYBB (sp|P08893) Phosphoglycerate kinase, cytosolic OS=T.b.b.	PGKE_TRYBB	0	GLOS_PGKE.1.1	GO:0005737	C	UniProtKB-S	cytoplasm
[BBH] PGKE_TRYBB (sp|P08893) Phosphoglycerate kinase, cytosolic OS=T.b.b.	PGKE_TRYBB	0	GLOS_PGKE.1.1	GO:0005524	M	UniProtKB-K	ATP binding
[BBH] PGKE_TRYBB (sp|P08893) Phosphoglycerate kinase, cytosolic OS=T.b.b.	PGKE_TRYBB	0	GLOS_PGKE.1.1	GO:0016774	M	.	phosphotransferase activity, carboxyl group as acceptor
[BBH] PGKE_TRYBB (sp|P08893) Phosphoglycerate kinase, cytosolic OS=T.b.b.	PGKE_TRYBB	0	GLOS_PGKE.1.1	GO:0004618	M	UniProtKB-E	phosphoglycerate kinase activity
[BBH] PGKE_TRYBB (sp|P08893) Phosphoglycerate kinase, cytosolic OS=T.b.b.	PGKE_TRYBB	0	GLOS_PGKE.1.1	GO:0006007	B	.	glucose catabolic process
[BBH] PGKE_TRYBB (sp|P08893) Phosphoglycerate kinase, cytosolic OS=T.b.b.	PGKE_TRYBB	0	GLOS_PGKE.1.1	GO:0006006	B	.	glucose metabolic process
[BBH] PGKE_TRYBB (sp|P08893) Phosphoglycerate kinase, cytosolic OS=T.b.b.	PGKE_TRYBB	0	GLOS_PGKE.1.1	GO:0019318	B	.	hexose metabolic process
[BBH] PGKE_TRYBB (sp|P08893) Phosphoglycerate kinase, cytosolic OS=T.b.b.	PGKE_TRYBB	0	GLOS_PGKE.1.1	GO:0005996	B	.	monosaccharide metabolic process
[BBH] PGKE_TRYBB (sp|P08893) Phosphoglycerate kinase, cytosolic OS=T.b.b.	PGKE_TRYBB	0	GLOS_PGKE.1.1	GO:0044723	B	.	single-organism carbohydrate metabolic process
[BBH] PGKE_TRYBB (sp|P08893) Phosphoglycerate kinase, cytosolic OS=T.b.b.	PGKE_TRYBB	0	GLOS_PGKE.1.1	GO:0005975	B	.	carbohydrate metabolic process
[BBH] PGKE_TRYBB (sp|P08893) Phosphoglycerate kinase, cytosolic OS=T.b.b.	PGKE_TRYBB	0	GLOS_PGKE.1.1	GO:0019320	B	.	hexose catabolic process
[BBH] PGKE_TRYBB (sp|P08893) Phosphoglycerate kinase, cytosolic OS=T.b.b.	PGKE_TRYBB	0	GLOS_PGKE.1.1	GO:0046365	B	.	monosaccharide catabolic process
[BBH] PGKE_TRYBB (sp|P08893) Phosphoglycerate kinase, cytosolic OS=T.b.b.	PGKE_TRYBB	0	GLOS_PGKE.1.1	GO:0044724	B	.	single-organism carbohydrate catabolic process
[BBH] PGKE_TRYBB (sp|P08893) Phosphoglycerate kinase, cytosolic OS=T.b.b.	PGKE_TRYBB	0	GLOS_PGKE.1.1	GO:0016052	B	.	carbohydrate catabolic process
[BBH] PGKE_TRYBB (sp|P08893) Phosphoglycerate kinase, cytosolic OS=T.b.b.	PGKE_TRYBB	0	GLOS_PGKE.1.1	GO:1901575	B	.	organic substance catabolic process
[BBH] PGKE_TRYBB (sp|P08893) Phosphoglycerate kinase, cytosolic OS=T.b.b.	PGKE_TRYBB	0	GLOS_PGKE.1.1	GO:0009056	B	.	catabolic process
[BBH] PGKE_TRYBB (sp|P08893) Phosphoglycerate kinase, cytosolic OS=T.b.b.	PGKE_TRYBB	0	GLOS_PGKE.1.1	GO:0006091	B	.	generation of precursor metabolites and energy
[BBH] PGKE_TRYBB (sp|P08893) Phosphoglycerate kinase, cytosolic OS=T.b.b.	PGKE_TRYBB	0	GLOS_PGKE.1.1	GO:0006096	B	UniProtKB-U	glycolysis
POL5_DROME (sp|Q8I7P9)Retrovirus-related Pol polyprot. from transposon opus	POL5_DROME	5,00E-06	GLOS_POL5.2.3	GO:0005634	C	UniProtKB	nucleus
POL5_DROME (sp|Q8I7P9)Retrovirus-related Pol polyprot. from transposon opus	POL5_DROME	5,00E-06	GLOS_POL5.2.3	GO:0004518	M	.	nuclease activity
POL5_DROME (sp|Q8I7P9)Retrovirus-related Pol polyprot. from transposon opus	POL5_DROME	5,00E-06	GLOS_POL5.2.3	GO:0004519	M	UniProtKB-K	endonuclease activity
POL5_DROME (sp|Q8I7P9)Retrovirus-related Pol polyprot. from transposon opus	POL5_DROME	5,00E-06	GLOS_POL5.2.3	GO:0008233	M	UniProtKB-K	peptidase activity
POL5_DROME (sp|Q8I7P9)Retrovirus-related Pol polyprot. from transposon opus	POL5_DROME	5,00E-06	GLOS_POL5.2.3	GO:0003723	M	InterPro	RNA binding
POL5_DROME (sp|Q8I7P9)Retrovirus-related Pol polyprot. from transposon opus	POL5_DROME	5,00E-06	GLOS_POL5.2.3	GO:0034061	M	.	DNA polymerase activity
POL5_DROME (sp|Q8I7P9)Retrovirus-related Pol polyprot. from transposon opus	POL5_DROME	5,00E-06	GLOS_POL5.2.3	GO:0016779	M	.	nucleotidyltransferase activity
POL5_DROME (sp|Q8I7P9)Retrovirus-related Pol polyprot. from transposon opus	POL5_DROME	5,00E-06	GLOS_POL5.2.3	GO:0003964	M	UniProtKB	RNA-directed DNA polymerase activity
POL5_DROME (sp|Q8I7P9)Retrovirus-related Pol polyprot. from transposon opus	POL5_DROME	5,00E-06	GLOS_POL5.2.3	GO:0015074	B	InterPro	DNA integration
POL5_DROME (sp|Q8I7P9)Retrovirus-related Pol polyprot. from transposon opus	POL5_DROME	5,00E-06	GLOS_POL5.2.3	GO:0090305	B	GOC	nucleic acid phosphodiester bond hydrolysis
POL5_DROME (sp|Q8I7P9)Retrovirus-related Pol polyprot. from transposon opus	POL5_DROME	5,00E-06	GLOS_POL5.2.3	GO:0006508	B	UniProtKB-K	proteolysis
POL5_DROME (sp|Q8I7P9)Retrovirus-related Pol polyprot. from transposon opus	POL5_DROME	5,00E-06	GLOS_POL5.2.3	GO:0006260	B	.	DNA replication
POL5_DROME (sp|Q8I7P9)Retrovirus-related Pol polyprot. from transposon opus	POL5_DROME	5,00E-06	GLOS_POL5.2.3	GO:0006278	B	InterPro	RNA-dependent DNA replication
POL5_DROME (sp|Q8I7P9)Retrovirus-related Pol polyprot. from transposon opus	POL5_DROME	5,00E-06	GLOS_POL5.2.3	GO:0006310	B	.	DNA recombination
POL5_DROME (sp|Q8I7P9)Retrovirus-related Pol polyprot. from transposon opus	POL5_DROME	5,00E-06	GLOS_POL5.2.3	GO:0032196	B	.	transposition
POL5_DROME (sp|Q8I7P9)Retrovirus-related Pol polyprot. from transposon opus	POL5_DROME	5,00E-06	GLOS_POL5.2.3	GO:0006313	B	UniProtKB	transposition, DNA-mediated
[BBH] PSA5_DROME (sp|Q95083) Proteasome subunit alpha type-5 OS=D.m.	PSA5_DROME	1,00E-145	GLOS_PSA5.1.1	GO:0005737	C	UniProtKB-S	cytoplasm
[BBH] PSA5_DROME (sp|Q95083) Proteasome subunit alpha type-5 OS=D.m.	PSA5_DROME	1,00E-145	GLOS_PSA5.1.1	GO:0005634	C	UniProtKB-S	nucleus
[BBH] PSA5_DROME (sp|Q95083) Proteasome subunit alpha type-5 OS=D.m.	PSA5_DROME	1,00E-145	GLOS_PSA5.1.1	GO:0000502	C	.	proteasome complex
[BBH] PSA5_DROME (sp|Q95083) Proteasome subunit alpha type-5 OS=D.m.	PSA5_DROME	1,00E-145	GLOS_PSA5.1.1	GO:0005839	C	FlyBase	proteasome core complex
[BBH] PSA5_DROME (sp|Q95083) Proteasome subunit alpha type-5 OS=D.m.	PSA5_DROME	1,00E-145	GLOS_PSA5.1.1	GO:0005839	C	.	proteasome core complex
[BBH] PSA5_DROME (sp|Q95083) Proteasome subunit alpha type-5 OS=D.m.	PSA5_DROME	1,00E-145	GLOS_PSA5.1.1	GO:0019773	C	InterPro	proteasome core complex, alpha-subunit complex
[BBH] PSA5_DROME (sp|Q95083) Proteasome subunit alpha type-5 OS=D.m.	PSA5_DROME	1,00E-145	GLOS_PSA5.1.1	GO:0004175	M	.	endopeptidase activity
[BBH] PSA5_DROME (sp|Q95083) Proteasome subunit alpha type-5 OS=D.m.	PSA5_DROME	1,00E-145	GLOS_PSA5.1.1	GO:0070011	M	.	peptidase activity, acting on L-amino acid peptides
[BBH] PSA5_DROME (sp|Q95083) Proteasome subunit alpha type-5 OS=D.m.	PSA5_DROME	1,00E-145	GLOS_PSA5.1.1	GO:0008233	M	.	peptidase activity
[BBH] PSA5_DROME (sp|Q95083) Proteasome subunit alpha type-5 OS=D.m.	PSA5_DROME	1,00E-145	GLOS_PSA5.1.1	GO:0070003	M	.	threonine-type peptidase activity
[BBH] PSA5_DROME (sp|Q95083) Proteasome subunit alpha type-5 OS=D.m.	PSA5_DROME	1,00E-145	GLOS_PSA5.1.1	GO:0004298	M	UniProtKB-K	threonine-type endopeptidase activity
[BBH] PSA5_DROME (sp|Q95083) Proteasome subunit alpha type-5 OS=D.m.	PSA5_DROME	1,00E-145	GLOS_PSA5.1.1	GO:0006511	B	.	ubiquitin-dependent protein catabolic process
[BBH] PSA5_DROME (sp|Q95083) Proteasome subunit alpha type-5 OS=D.m.	PSA5_DROME	1,00E-145	GLOS_PSA5.1.1	GO:0019941	B	.	modification-dependent protein catabolic process
[BBH] PSA5_DROME (sp|Q95083) Proteasome subunit alpha type-5 OS=D.m.	PSA5_DROME	1,00E-145	GLOS_PSA5.1.1	GO:0043632	B	.	modification-dependent macromolecule catabolic process
[BBH] PSA5_DROME (sp|Q95083) Proteasome subunit alpha type-5 OS=D.m.	PSA5_DROME	1,00E-145	GLOS_PSA5.1.1	GO:0044265	B	.	cellular macromolecule catabolic process

[BBH] PSA5_DROME (sp|Q95083) Proteasome subunit alpha type-5 OS=D.m.	PSA5_DROME	1,00E-145	GLOS_PSA5.1.1	GO:0009057	B	.	macromolecule catabolic process
[BBH] PSA5_DROME (sp|Q95083) Proteasome subunit alpha type-5 OS=D.m.	PSA5_DROME	1,00E-145	GLOS_PSA5.1.1	GO:0044248	B	.	cellular catabolic process
[BBH] PSA5_DROME (sp|Q95083) Proteasome subunit alpha type-5 OS=D.m.	PSA5_DROME	1,00E-145	GLOS_PSA5.1.1	GO:0051603	B	.	proteolysis involved in cellular protein catabolic process
[BBH] PSA5_DROME (sp|Q95083) Proteasome subunit alpha type-5 OS=D.m.	PSA5_DROME	1,00E-145	GLOS_PSA5.1.1	GO:0006508	B	.	proteolysis
[BBH] PSA5_DROME (sp|Q95083) Proteasome subunit alpha type-5 OS=D.m.	PSA5_DROME	1,00E-145	GLOS_PSA5.1.1	GO:0044257	B	.	cellular protein catabolic process
[BBH] PSA5_DROME (sp|Q95083) Proteasome subunit alpha type-5 OS=D.m.	PSA5_DROME	1,00E-145	GLOS_PSA5.1.1	GO:0030163	B	.	protein catabolic process
[BBH] PSA5_DROME (sp|Q95083) Proteasome subunit alpha type-5 OS=D.m.	PSA5_DROME	1,00E-145	GLOS_PSA5.1.1	GO:0010498	B	.	proteasomal protein catabolic process
[BBH] PSA5_DROME (sp|Q95083) Proteasome subunit alpha type-5 OS=D.m.	PSA5_DROME	1,00E-145	GLOS_PSA5.1.1	GO:0043161	B	FlyBase	proteasomal ubiquitin-dependent protein catabolic process
RHO1_DROME (sp|P48148) Ras-like GTP-binding protein Rho1 OS=D.m.	RHO1_DROME	1,00E-129	GLOS_RHO1.1.3	GO:0005938	C	FlyBase	cell cortex
RHO1_DROME (sp|P48148) Ras-like GTP-binding protein Rho1 OS=D.m.	RHO1_DROME	1,00E-129	GLOS_RHO1.1.3	GO:0070451	C	FlyBase	cell hair
RHO1_DROME (sp|P48148) Ras-like GTP-binding protein Rho1 OS=D.m.	RHO1_DROME	1,00E-129	GLOS_RHO1.1.3	GO:0005856	C	UniProtKB-S	cytoskeleton
RHO1_DROME (sp|P48148) Ras-like GTP-binding protein Rho1 OS=D.m.	RHO1_DROME	1,00E-129	GLOS_RHO1.1.3	GO:0005886	C	UniProtKB-S	plasma membrane
RHO1_DROME (sp|P48148) Ras-like GTP-binding protein Rho1 OS=D.m.	RHO1_DROME	1,00E-129	GLOS_RHO1.1.3	GO:0032561	M	.	guanyl ribonucleotide binding
RHO1_DROME (sp|P48148) Ras-like GTP-binding protein Rho1 OS=D.m.	RHO1_DROME	1,00E-129	GLOS_RHO1.1.3	GO:0019001	M	.	guanyl nucleotide binding
RHO1_DROME (sp|P48148) Ras-like GTP-binding protein Rho1 OS=D.m.	RHO1_DROME	1,00E-129	GLOS_RHO1.1.3	GO:0005525	M	UniProtKB-K	GTP binding
RHO1_DROME (sp|P48148) Ras-like GTP-binding protein Rho1 OS=D.m.	RHO1_DROME	1,00E-129	GLOS_RHO1.1.3	GO:0003924	M	FlyBase	GTPase activity
RHO1_DROME (sp|P48148) Ras-like GTP-binding protein Rho1 OS=D.m.	RHO1_DROME	1,00E-129	GLOS_RHO1.1.3	GO:0061572	B	.	actin filament bundle organization
RHO1_DROME (sp|P48148) Ras-like GTP-binding protein Rho1 OS=D.m.	RHO1_DROME	1,00E-129	GLOS_RHO1.1.3	GO:0007015	B	.	actin filament organization
RHO1_DROME (sp|P48148) Ras-like GTP-binding protein Rho1 OS=D.m.	RHO1_DROME	1,00E-129	GLOS_RHO1.1.3	GO:0051017	B	FlyBase	actin filament bundle assembly
RHO1_DROME (sp|P48148) Ras-like GTP-binding protein Rho1 OS=D.m.	RHO1_DROME	1,00E-129	GLOS_RHO1.1.3	GO:0007297	B	.	ovarian follicle cell migration
RHO1_DROME (sp|P48148) Ras-like GTP-binding protein Rho1 OS=D.m.	RHO1_DROME	1,00E-129	GLOS_RHO1.1.3	GO:0007298	B	FlyBase	border follicle cell migration
RHO1_DROME (sp|P48148) Ras-like GTP-binding protein Rho1 OS=D.m.	RHO1_DROME	1,00E-129	GLOS_RHO1.1.3	GO:0035146	B	.	tube fusion
RHO1_DROME (sp|P48148) Ras-like GTP-binding protein Rho1 OS=D.m.	RHO1_DROME	1,00E-129	GLOS_RHO1.1.3	GO:0035147	B	FlyBase	branch fusion, open tracheal system
RHO1_DROME (sp|P48148) Ras-like GTP-binding protein Rho1 OS=D.m.	RHO1_DROME	1,00E-129	GLOS_RHO1.1.3	GO:0009826	B	.	unidimensional cell growth
RHO1_DROME (sp|P48148) Ras-like GTP-binding protein Rho1 OS=D.m.	RHO1_DROME	1,00E-129	GLOS_RHO1.1.3	GO:0016049	B	.	cell growth
RHO1_DROME (sp|P48148) Ras-like GTP-binding protein Rho1 OS=D.m.	RHO1_DROME	1,00E-129	GLOS_RHO1.1.3	GO:0040007	B	.	growth
RHO1_DROME (sp|P48148) Ras-like GTP-binding protein Rho1 OS=D.m.	RHO1_DROME	1,00E-129	GLOS_RHO1.1.3	GO:0060560	B	.	developmental growth involved in morphogenesis
RHO1_DROME (sp|P48148) Ras-like GTP-binding protein Rho1 OS=D.m.	RHO1_DROME	1,00E-129	GLOS_RHO1.1.3	GO:0048589	B	.	developmental growth
RHO1_DROME (sp|P48148) Ras-like GTP-binding protein Rho1 OS=D.m.	RHO1_DROME	1,00E-129	GLOS_RHO1.1.3	GO:0090254	B	FlyBase	cell elongation involved in imaginal disc-derived wing morphogenesis
RHO1_DROME (sp|P48148) Ras-like GTP-binding protein Rho1 OS=D.m.	RHO1_DROME	1,00E-129	GLOS_RHO1.1.3	GO:0048646	B	.	anatomical structure formation involved in morphogenesis
RHO1_DROME (sp|P48148) Ras-like GTP-binding protein Rho1 OS=D.m.	RHO1_DROME	1,00E-129	GLOS_RHO1.1.3	GO:0007349	B	FlyBase	cellularization
RHO1_DROME (sp|P48148) Ras-like GTP-binding protein Rho1 OS=D.m.	RHO1_DROME	1,00E-129	GLOS_RHO1.1.3	GO:0030865	B	.	cortical cytoskeleton organization
RHO1_DROME (sp|P48148) Ras-like GTP-binding protein Rho1 OS=D.m.	RHO1_DROME	1,00E-129	GLOS_RHO1.1.3	GO:0030866	B	FlyBase	cortical actin cytoskeleton organization
RHO1_DROME (sp|P48148) Ras-like GTP-binding protein Rho1 OS=D.m.	RHO1_DROME	1,00E-129	GLOS_RHO1.1.3	GO:0000910	B	FlyBase	cytokinesis
RHO1_DROME (sp|P48148) Ras-like GTP-binding protein Rho1 OS=D.m.	RHO1_DROME	1,00E-129	GLOS_RHO1.1.3	GO:0016358	B	.	dendrite development
RHO1_DROME (sp|P48148) Ras-like GTP-binding protein Rho1 OS=D.m.	RHO1_DROME	1,00E-129	GLOS_RHO1.1.3	GO:0048813	B	FlyBase	dendrite morphogenesis
RHO1_DROME (sp|P48148) Ras-like GTP-binding protein Rho1 OS=D.m.	RHO1_DROME	1,00E-129	GLOS_RHO1.1.3	GO:0008038	B	.	neuron recognition
RHO1_DROME (sp|P48148) Ras-like GTP-binding protein Rho1 OS=D.m.	RHO1_DROME	1,00E-129	GLOS_RHO1.1.3	GO:0008037	B	.	cell recognition
RHO1_DROME (sp|P48148) Ras-like GTP-binding protein Rho1 OS=D.m.	RHO1_DROME	1,00E-129	GLOS_RHO1.1.3	GO:0070593	B	FlyBase	dendrite self-avoidance
RHO1_DROME (sp|P48148) Ras-like GTP-binding protein Rho1 OS=D.m.	RHO1_DROME	1,00E-129	GLOS_RHO1.1.3	GO:0009855	B	.	determination of bilateral symmetry
RHO1_DROME (sp|P48148) Ras-like GTP-binding protein Rho1 OS=D.m.	RHO1_DROME	1,00E-129	GLOS_RHO1.1.3	GO:0009799	B	.	specification of symmetry
RHO1_DROME (sp|P48148) Ras-like GTP-binding protein Rho1 OS=D.m.	RHO1_DROME	1,00E-129	GLOS_RHO1.1.3	GO:0007368	B	FlyBase	determination of left/right symmetry
RHO1_DROME (sp|P48148) Ras-like GTP-binding protein Rho1 OS=D.m.	RHO1_DROME	1,00E-129	GLOS_RHO1.1.3	GO:0035026	B	.	leading edge cell differentiation
RHO1_DROME (sp|P48148) Ras-like GTP-binding protein Rho1 OS=D.m.	RHO1_DROME	1,00E-129	GLOS_RHO1.1.3	GO:0030855	B	.	epithelial cell differentiation
RHO1_DROME (sp|P48148) Ras-like GTP-binding protein Rho1 OS=D.m.	RHO1_DROME	1,00E-129	GLOS_RHO1.1.3	GO:0007392	B	.	initiation of dorsal closure
RHO1_DROME (sp|P48148) Ras-like GTP-binding protein Rho1 OS=D.m.	RHO1_DROME	1,00E-129	GLOS_RHO1.1.3	GO:0007391	B	.	dorsal closure
RHO1_DROME (sp|P48148) Ras-like GTP-binding protein Rho1 OS=D.m.	RHO1_DROME	1,00E-129	GLOS_RHO1.1.3	GO:0046663	B	FlyBase	dorsal closure, leading edge cell differentiation
RHO1_DROME (sp|P48148) Ras-like GTP-binding protein Rho1 OS=D.m.	RHO1_DROME	1,00E-129	GLOS_RHO1.1.3	GO:0007395	B	FlyBase	dorsal closure, spreading of leading edge cells
RHO1_DROME (sp|P48148) Ras-like GTP-binding protein Rho1 OS=D.m.	RHO1_DROME	1,00E-129	GLOS_RHO1.1.3	GO:0006897	B	FlyBase	endocytosis
RHO1_DROME (sp|P48148) Ras-like GTP-binding protein Rho1 OS=D.m.	RHO1_DROME	1,00E-129	GLOS_RHO1.1.3	GO:0007173	B	FlyBase	epidermal growth factor receptor signaling pathway
RHO1_DROME (sp|P48148) Ras-like GTP-binding protein Rho1 OS=D.m.	RHO1_DROME	1,00E-129	GLOS_RHO1.1.3	GO:0001736	B	.	establishment of planar polarity
RHO1_DROME (sp|P48148) Ras-like GTP-binding protein Rho1 OS=D.m.	RHO1_DROME	1,00E-129	GLOS_RHO1.1.3	GO:0007164	B	.	establishment of tissue polarity
RHO1_DROME (sp|P48148) Ras-like GTP-binding protein Rho1 OS=D.m.	RHO1_DROME	1,00E-129	GLOS_RHO1.1.3	GO:0001738	B	.	morphogenesis of a polarized epithelium
RHO1_DROME (sp|P48148) Ras-like GTP-binding protein Rho1 OS=D.m.	RHO1_DROME	1,00E-129	GLOS_RHO1.1.3	GO:0035317	B	.	imaginal disc-derived wing hair organization

RHO1_DROME (sp|P48148) Ras-like GTP-binding protein Rho1 OS=D.m.	RHO1_DROME	1,00E-129	GLOS_RHO1.1.3	GO:0035316	B	.	non-sensory hair organization
RHO1_DROME (sp|P48148) Ras-like GTP-binding protein Rho1 OS=D.m.	RHO1_DROME	1,00E-129	GLOS_RHO1.1.3	GO:0035315	B	.	hair cell differentiation
RHO1_DROME (sp|P48148) Ras-like GTP-binding protein Rho1 OS=D.m.	RHO1_DROME	1,00E-129	GLOS_RHO1.1.3	GO:0009913	B	.	epidermal cell differentiation
RHO1_DROME (sp|P48148) Ras-like GTP-binding protein Rho1 OS=D.m.	RHO1_DROME	1,00E-129	GLOS_RHO1.1.3	GO:0008544	B	.	epidermis development
RHO1_DROME (sp|P48148) Ras-like GTP-binding protein Rho1 OS=D.m.	RHO1_DROME	1,00E-129	GLOS_RHO1.1.3	GO:0043588	B	.	skin development
RHO1_DROME (sp|P48148) Ras-like GTP-binding protein Rho1 OS=D.m.	RHO1_DROME	1,00E-129	GLOS_RHO1.1.3	GO:0001737	B	FlyBase	establishment of imaginal disc-derived wing hair orientation
RHO1_DROME (sp|P48148) Ras-like GTP-binding protein Rho1 OS=D.m.	RHO1_DROME	1,00E-129	GLOS_RHO1.1.3	GO:0045184	B	FlyBase	establishment of protein localization
RHO1_DROME (sp|P48148) Ras-like GTP-binding protein Rho1 OS=D.m.	RHO1_DROME	1,00E-129	GLOS_RHO1.1.3	GO:0008354	B	FlyBase	germ cell migration
RHO1_DROME (sp|P48148) Ras-like GTP-binding protein Rho1 OS=D.m.	RHO1_DROME	1,00E-129	GLOS_RHO1.1.3	GO:0010004	B	.	gastrulation involving germ band extension
RHO1_DROME (sp|P48148) Ras-like GTP-binding protein Rho1 OS=D.m.	RHO1_DROME	1,00E-129	GLOS_RHO1.1.3	GO:0001703	B	.	gastrulation with mouth forming first
RHO1_DROME (sp|P48148) Ras-like GTP-binding protein Rho1 OS=D.m.	RHO1_DROME	1,00E-129	GLOS_RHO1.1.3	GO:0007369	B	.	gastrulation
RHO1_DROME (sp|P48148) Ras-like GTP-binding protein Rho1 OS=D.m.	RHO1_DROME	1,00E-129	GLOS_RHO1.1.3	GO:0007377	B	FlyBase	germ-band extension
RHO1_DROME (sp|P48148) Ras-like GTP-binding protein Rho1 OS=D.m.	RHO1_DROME	1,00E-129	GLOS_RHO1.1.3	GO:0042063	B	.	gliogenesis
RHO1_DROME (sp|P48148) Ras-like GTP-binding protein Rho1 OS=D.m.	RHO1_DROME	1,00E-129	GLOS_RHO1.1.3	GO:0008347	B	FlyBase	glial cell migration
RHO1_DROME (sp|P48148) Ras-like GTP-binding protein Rho1 OS=D.m.	RHO1_DROME	1,00E-129	GLOS_RHO1.1.3	GO:0035162	B	.	embryonic hemopoiesis
RHO1_DROME (sp|P48148) Ras-like GTP-binding protein Rho1 OS=D.m.	RHO1_DROME	1,00E-129	GLOS_RHO1.1.3	GO:0030097	B	.	hemopoiesis
RHO1_DROME (sp|P48148) Ras-like GTP-binding protein Rho1 OS=D.m.	RHO1_DROME	1,00E-129	GLOS_RHO1.1.3	GO:0048534	B	.	hematopoietic or lymphoid organ development
RHO1_DROME (sp|P48148) Ras-like GTP-binding protein Rho1 OS=D.m.	RHO1_DROME	1,00E-129	GLOS_RHO1.1.3	GO:0002520	B	.	immune system development
RHO1_DROME (sp|P48148) Ras-like GTP-binding protein Rho1 OS=D.m.	RHO1_DROME	1,00E-129	GLOS_RHO1.1.3	GO:0035099	B	FlyBase	hemocyte migration
RHO1_DROME (sp|P48148) Ras-like GTP-binding protein Rho1 OS=D.m.	RHO1_DROME	1,00E-129	GLOS_RHO1.1.3	GO:0051403	B	.	stress-activated MAPK cascade
RHO1_DROME (sp|P48148) Ras-like GTP-binding protein Rho1 OS=D.m.	RHO1_DROME	1,00E-129	GLOS_RHO1.1.3	GO:0000165	B	.	MAPK cascade
RHO1_DROME (sp|P48148) Ras-like GTP-binding protein Rho1 OS=D.m.	RHO1_DROME	1,00E-129	GLOS_RHO1.1.3	GO:0007243	B	.	intracellular protein kinase cascade
RHO1_DROME (sp|P48148) Ras-like GTP-binding protein Rho1 OS=D.m.	RHO1_DROME	1,00E-129	GLOS_RHO1.1.3	GO:0031098	B	.	stress-activated protein kinase signaling cascade
RHO1_DROME (sp|P48148) Ras-like GTP-binding protein Rho1 OS=D.m.	RHO1_DROME	1,00E-129	GLOS_RHO1.1.3	GO:0007254	B	FlyBase	JNK cascade
RHO1_DROME (sp|P48148) Ras-like GTP-binding protein Rho1 OS=D.m.	RHO1_DROME	1,00E-129	GLOS_RHO1.1.3	GO:0035148	B	.	tube formation
RHO1_DROME (sp|P48148) Ras-like GTP-binding protein Rho1 OS=D.m.	RHO1_DROME	1,00E-129	GLOS_RHO1.1.3	GO:0035152	B	.	regulation of tube architecture, open tracheal system
RHO1_DROME (sp|P48148) Ras-like GTP-binding protein Rho1 OS=D.m.	RHO1_DROME	1,00E-129	GLOS_RHO1.1.3	GO:0035149	B	FlyBase	lumen formation, open tracheal system
RHO1_DROME (sp|P48148) Ras-like GTP-binding protein Rho1 OS=D.m.	RHO1_DROME	1,00E-129	GLOS_RHO1.1.3	GO:0035090	B	.	maintenance of apical/basal cell polarity
RHO1_DROME (sp|P48148) Ras-like GTP-binding protein Rho1 OS=D.m.	RHO1_DROME	1,00E-129	GLOS_RHO1.1.3	GO:0030011	B	.	maintenance of cell polarity
RHO1_DROME (sp|P48148) Ras-like GTP-binding protein Rho1 OS=D.m.	RHO1_DROME	1,00E-129	GLOS_RHO1.1.3	GO:0035088	B	.	establishment or maintenance of apical/basal cell polarity
RHO1_DROME (sp|P48148) Ras-like GTP-binding protein Rho1 OS=D.m.	RHO1_DROME	1,00E-129	GLOS_RHO1.1.3	GO:0061245	B	.	establishment or maintenance of bipolar cell polarity
RHO1_DROME (sp|P48148) Ras-like GTP-binding protein Rho1 OS=D.m.	RHO1_DROME	1,00E-129	GLOS_RHO1.1.3	GO:0045197	B	.	establishment or maintenance of epithelial cell apical/basal polarity
RHO1_DROME (sp|P48148) Ras-like GTP-binding protein Rho1 OS=D.m.	RHO1_DROME	1,00E-129	GLOS_RHO1.1.3	GO:0045199	B	FlyBase	maintenance of epithelial cell apical/basal polarity
RHO1_DROME (sp|P48148) Ras-like GTP-binding protein Rho1 OS=D.m.	RHO1_DROME	1,00E-129	GLOS_RHO1.1.3	GO:0035006	B	FlyBase	melanization defense response
RHO1_DROME (sp|P48148) Ras-like GTP-binding protein Rho1 OS=D.m.	RHO1_DROME	1,00E-129	GLOS_RHO1.1.3	GO:0007411	B	.	axon guidance
RHO1_DROME (sp|P48148) Ras-like GTP-binding protein Rho1 OS=D.m.	RHO1_DROME	1,00E-129	GLOS_RHO1.1.3	GO:0008045	B	FlyBase	motor neuron axon guidance
RHO1_DROME (sp|P48148) Ras-like GTP-binding protein Rho1 OS=D.m.	RHO1_DROME	1,00E-129	GLOS_RHO1.1.3	GO:0007405	B	FlyBase	neuroblast proliferation
RHO1_DROME (sp|P48148) Ras-like GTP-binding protein Rho1 OS=D.m.	RHO1_DROME	1,00E-129	GLOS_RHO1.1.3	GO:0042067	B	.	establishment of ommatidial planar polarity
RHO1_DROME (sp|P48148) Ras-like GTP-binding protein Rho1 OS=D.m.	RHO1_DROME	1,00E-129	GLOS_RHO1.1.3	GO:0016318	B	FlyBase	ommatidial rotation
RHO1_DROME (sp|P48148) Ras-like GTP-binding protein Rho1 OS=D.m.	RHO1_DROME	1,00E-129	GLOS_RHO1.1.3	GO:0007422	B	FlyBase	peripheral nervous system development
RHO1_DROME (sp|P48148) Ras-like GTP-binding protein Rho1 OS=D.m.	RHO1_DROME	1,00E-129	GLOS_RHO1.1.3	GO:0045860	B	.	positive regulation of protein kinase activity
RHO1_DROME (sp|P48148) Ras-like GTP-binding protein Rho1 OS=D.m.	RHO1_DROME	1,00E-129	GLOS_RHO1.1.3	GO:0001934	B	.	positive regulation of protein phosphorylation
RHO1_DROME (sp|P48148) Ras-like GTP-binding protein Rho1 OS=D.m.	RHO1_DROME	1,00E-129	GLOS_RHO1.1.3	GO:0001932	B	.	regulation of protein phosphorylation
RHO1_DROME (sp|P48148) Ras-like GTP-binding protein Rho1 OS=D.m.	RHO1_DROME	1,00E-129	GLOS_RHO1.1.3	GO:0031399	B	.	regulation of protein modification process
RHO1_DROME (sp|P48148) Ras-like GTP-binding protein Rho1 OS=D.m.	RHO1_DROME	1,00E-129	GLOS_RHO1.1.3	GO:0042325	B	.	regulation of phosphorylation
RHO1_DROME (sp|P48148) Ras-like GTP-binding protein Rho1 OS=D.m.	RHO1_DROME	1,00E-129	GLOS_RHO1.1.3	GO:0019220	B	.	regulation of phosphate metabolic process
RHO1_DROME (sp|P48148) Ras-like GTP-binding protein Rho1 OS=D.m.	RHO1_DROME	1,00E-129	GLOS_RHO1.1.3	GO:0051174	B	.	regulation of phosphorus metabolic process
RHO1_DROME (sp|P48148) Ras-like GTP-binding protein Rho1 OS=D.m.	RHO1_DROME	1,00E-129	GLOS_RHO1.1.3	GO:0031401	B	.	positive regulation of protein modification process
RHO1_DROME (sp|P48148) Ras-like GTP-binding protein Rho1 OS=D.m.	RHO1_DROME	1,00E-129	GLOS_RHO1.1.3	GO:0032270	B	.	positive regulation of cellular protein metabolic process
RHO1_DROME (sp|P48148) Ras-like GTP-binding protein Rho1 OS=D.m.	RHO1_DROME	1,00E-129	GLOS_RHO1.1.3	GO:0051247	B	.	positive regulation of protein metabolic process
RHO1_DROME (sp|P48148) Ras-like GTP-binding protein Rho1 OS=D.m.	RHO1_DROME	1,00E-129	GLOS_RHO1.1.3	GO:0042327	B	.	positive regulation of phosphorylation
RHO1_DROME (sp|P48148) Ras-like GTP-binding protein Rho1 OS=D.m.	RHO1_DROME	1,00E-129	GLOS_RHO1.1.3	GO:0045937	B	.	positive regulation of phosphate metabolic process
RHO1_DROME (sp|P48148) Ras-like GTP-binding protein Rho1 OS=D.m.	RHO1_DROME	1,00E-129	GLOS_RHO1.1.3	GO:0010562	B	.	positive regulation of phosphorus metabolic process
RHO1_DROME (sp|P48148) Ras-like GTP-binding protein Rho1 OS=D.m.	RHO1_DROME	1,00E-129	GLOS_RHO1.1.3	GO:0033674	B	.	positive regulation of kinase activity

RHO1_DROME (sp|P48148) Ras-like GTP-binding protein Rho1 OS=D.m.	RHO1_DROME	1,00E-129	GLOS_RHO1.1.3	GO:0043549	B	.	regulation of kinase activity
RHO1_DROME (sp|P48148) Ras-like GTP-binding protein Rho1 OS=D.m.	RHO1_DROME	1,00E-129	GLOS_RHO1.1.3	GO:0051338	B	.	regulation of transferase activity
RHO1_DROME (sp|P48148) Ras-like GTP-binding protein Rho1 OS=D.m.	RHO1_DROME	1,00E-129	GLOS_RHO1.1.3	GO:0050790	B	.	regulation of catalytic activity
RHO1_DROME (sp|P48148) Ras-like GTP-binding protein Rho1 OS=D.m.	RHO1_DROME	1,00E-129	GLOS_RHO1.1.3	GO:0065009	B	.	regulation of molecular function
RHO1_DROME (sp|P48148) Ras-like GTP-binding protein Rho1 OS=D.m.	RHO1_DROME	1,00E-129	GLOS_RHO1.1.3	GO:0051347	B	.	positive regulation of transferase activity
RHO1_DROME (sp|P48148) Ras-like GTP-binding protein Rho1 OS=D.m.	RHO1_DROME	1,00E-129	GLOS_RHO1.1.3	GO:0043085	B	.	positive regulation of catalytic activity
RHO1_DROME (sp|P48148) Ras-like GTP-binding protein Rho1 OS=D.m.	RHO1_DROME	1,00E-129	GLOS_RHO1.1.3	GO:0044093	B	.	positive regulation of molecular function
RHO1_DROME (sp|P48148) Ras-like GTP-binding protein Rho1 OS=D.m.	RHO1_DROME	1,00E-129	GLOS_RHO1.1.3	GO:0045859	B	.	regulation of protein kinase activity
RHO1_DROME (sp|P48148) Ras-like GTP-binding protein Rho1 OS=D.m.	RHO1_DROME	1,00E-129	GLOS_RHO1.1.3	GO:0071900	B	.	regulation of protein serine/threonine kinase activity
RHO1_DROME (sp|P48148) Ras-like GTP-binding protein Rho1 OS=D.m.	RHO1_DROME	1,00E-129	GLOS_RHO1.1.3	GO:0071902	B	UniProtKB	positive regulation of protein serine/threonine kinase activity
RHO1_DROME (sp|P48148) Ras-like GTP-binding protein Rho1 OS=D.m.	RHO1_DROME	1,00E-129	GLOS_RHO1.1.3	GO:0060571	B	.	morphogenesis of an epithelial fold
RHO1_DROME (sp|P48148) Ras-like GTP-binding protein Rho1 OS=D.m.	RHO1_DROME	1,00E-129	GLOS_RHO1.1.3	GO:0007374	B	FlyBase	posterior midgut invagination
RHO1_DROME (sp|P48148) Ras-like GTP-binding protein Rho1 OS=D.m.	RHO1_DROME	1,00E-129	GLOS_RHO1.1.3	GO:0030588	B	.	pseudocleavage
RHO1_DROME (sp|P48148) Ras-like GTP-binding protein Rho1 OS=D.m.	RHO1_DROME	1,00E-129	GLOS_RHO1.1.3	GO:0030589	B	FlyBase	pseudocleavage involved in syncytial blastoderm formation
RHO1_DROME (sp|P48148) Ras-like GTP-binding protein Rho1 OS=D.m.	RHO1_DROME	1,00E-129	GLOS_RHO1.1.3	GO:0010769	B	.	regulation of cell morphogenesis involved in differentiation
RHO1_DROME (sp|P48148) Ras-like GTP-binding protein Rho1 OS=D.m.	RHO1_DROME	1,00E-129	GLOS_RHO1.1.3	GO:0010975	B	.	regulation of neuron projection development
RHO1_DROME (sp|P48148) Ras-like GTP-binding protein Rho1 OS=D.m.	RHO1_DROME	1,00E-129	GLOS_RHO1.1.3	GO:0050770	B	FlyBase	regulation of axonogenesis
RHO1_DROME (sp|P48148) Ras-like GTP-binding protein Rho1 OS=D.m.	RHO1_DROME	1,00E-129	GLOS_RHO1.1.3	GO:0051493	B	FlyBase	regulation of cytoskeleton organization
RHO1_DROME (sp|P48148) Ras-like GTP-binding protein Rho1 OS=D.m.	RHO1_DROME	1,00E-129	GLOS_RHO1.1.3	GO:0008360	B	.	regulation of cell shape
RHO1_DROME (sp|P48148) Ras-like GTP-binding protein Rho1 OS=D.m.	RHO1_DROME	1,00E-129	GLOS_RHO1.1.3	GO:0045995	B	.	regulation of embryonic development
RHO1_DROME (sp|P48148) Ras-like GTP-binding protein Rho1 OS=D.m.	RHO1_DROME	1,00E-129	GLOS_RHO1.1.3	GO:0016476	B	FlyBase	regulation of embryonic cell shape
RHO1_DROME (sp|P48148) Ras-like GTP-binding protein Rho1 OS=D.m.	RHO1_DROME	1,00E-129	GLOS_RHO1.1.3	GO:0035150	B	.	regulation of tube size
RHO1_DROME (sp|P48148) Ras-like GTP-binding protein Rho1 OS=D.m.	RHO1_DROME	1,00E-129	GLOS_RHO1.1.3	GO:0090066	B	.	regulation of anatomical structure size
RHO1_DROME (sp|P48148) Ras-like GTP-binding protein Rho1 OS=D.m.	RHO1_DROME	1,00E-129	GLOS_RHO1.1.3	GO:0007443	B	.	Malpighian tubule morphogenesis
RHO1_DROME (sp|P48148) Ras-like GTP-binding protein Rho1 OS=D.m.	RHO1_DROME	1,00E-129	GLOS_RHO1.1.3	GO:0061333	B	.	renal tubule morphogenesis
RHO1_DROME (sp|P48148) Ras-like GTP-binding protein Rho1 OS=D.m.	RHO1_DROME	1,00E-129	GLOS_RHO1.1.3	GO:0061326	B	.	renal tubule development
RHO1_DROME (sp|P48148) Ras-like GTP-binding protein Rho1 OS=D.m.	RHO1_DROME	1,00E-129	GLOS_RHO1.1.3	GO:0072001	B	.	renal system development
RHO1_DROME (sp|P48148) Ras-like GTP-binding protein Rho1 OS=D.m.	RHO1_DROME	1,00E-129	GLOS_RHO1.1.3	GO:0001655	B	.	urogenital system development
RHO1_DROME (sp|P48148) Ras-like GTP-binding protein Rho1 OS=D.m.	RHO1_DROME	1,00E-129	GLOS_RHO1.1.3	GO:0048619	B	.	embryonic hindgut morphogenesis
RHO1_DROME (sp|P48148) Ras-like GTP-binding protein Rho1 OS=D.m.	RHO1_DROME	1,00E-129	GLOS_RHO1.1.3	GO:0072002	B	.	Malpighian tubule development
RHO1_DROME (sp|P48148) Ras-like GTP-binding protein Rho1 OS=D.m.	RHO1_DROME	1,00E-129	GLOS_RHO1.1.3	GO:0035298	B	FlyBase	regulation of Malpighian tubule size
RHO1_DROME (sp|P48148) Ras-like GTP-binding protein Rho1 OS=D.m.	RHO1_DROME	1,00E-129	GLOS_RHO1.1.3	GO:0035151	B	.	regulation of tube size, open tracheal system
RHO1_DROME (sp|P48148) Ras-like GTP-binding protein Rho1 OS=D.m.	RHO1_DROME	1,00E-129	GLOS_RHO1.1.3	GO:0035159	B	FlyBase	regulation of tube length, open tracheal system
RHO1_DROME (sp|P48148) Ras-like GTP-binding protein Rho1 OS=D.m.	RHO1_DROME	1,00E-129	GLOS_RHO1.1.3	GO:0006974	B	FlyBase	cellular response to DNA damage stimulus
RHO1_DROME (sp|P48148) Ras-like GTP-binding protein Rho1 OS=D.m.	RHO1_DROME	1,00E-129	GLOS_RHO1.1.3	GO:0022612	B	.	gland morphogenesis
RHO1_DROME (sp|P48148) Ras-like GTP-binding protein Rho1 OS=D.m.	RHO1_DROME	1,00E-129	GLOS_RHO1.1.3	GO:0048732	B	.	gland development
RHO1_DROME (sp|P48148) Ras-like GTP-binding protein Rho1 OS=D.m.	RHO1_DROME	1,00E-129	GLOS_RHO1.1.3	GO:0007431	B	.	salivary gland development
RHO1_DROME (sp|P48148) Ras-like GTP-binding protein Rho1 OS=D.m.	RHO1_DROME	1,00E-129	GLOS_RHO1.1.3	GO:0035272	B	.	exocrine system development
RHO1_DROME (sp|P48148) Ras-like GTP-binding protein Rho1 OS=D.m.	RHO1_DROME	1,00E-129	GLOS_RHO1.1.3	GO:0007435	B	FlyBase	salivary gland morphogenesis
RHO1_DROME (sp|P48148) Ras-like GTP-binding protein Rho1 OS=D.m.	RHO1_DROME	1,00E-129	GLOS_RHO1.1.3	GO:0007264	B	InterPro	small GTPase mediated signal transduction
RHO1_DROME (sp|P48148) Ras-like GTP-binding protein Rho1 OS=D.m.	RHO1_DROME	1,00E-129	GLOS_RHO1.1.3	GO:0035277	B	FlyBase	spiracle morphogenesis, open tracheal system
RHO1_DROME (sp|P48148) Ras-like GTP-binding protein Rho1 OS=D.m.	RHO1_DROME	1,00E-129	GLOS_RHO1.1.3	GO:0007370	B	FlyBase	ventral furrow formation
RHO1_DROME (sp|P48148) Ras-like GTP-binding protein Rho1 OS=D.m.	RHO1_DROME	1,00E-129	GLOS_RHO1.1.3	GO:0050953	B	.	sensory perception of light stimulus
RHO1_DROME (sp|P48148) Ras-like GTP-binding protein Rho1 OS=D.m.	RHO1_DROME	1,00E-129	GLOS_RHO1.1.3	GO:0007600	B	.	sensory perception
RHO1_DROME (sp|P48148) Ras-like GTP-binding protein Rho1 OS=D.m.	RHO1_DROME	1,00E-129	GLOS_RHO1.1.3	GO:0007601	B	UniProtKB-K	visual perception
RHO1_DROME (sp|P48148) Ras-like GTP-binding protein Rho1 OS=D.m.	RHO1_DROME	1,00E-129	GLOS_RHO1.1.3	GO:0016055	B	FlyBase	Wnt receptor signaling pathway
RHO1_DROME (sp|P48148) Ras-like GTP-binding protein Rho1 OS=D.m.	RHO1_DROME	1,00E-129	GLOS_RHO1.1.3	GO:0042060	B	FlyBase	wound healing
RL30_TRYBB (sp|P49153) 60S ribosomal protein L30 OS=T.b.b.	RL30_TRYBB	3,00E-70	GLOS_RL30.1.1	GO:0030529	C	.	ribonucleoprotein complex
RL30_TRYBB (sp|P49153) 60S ribosomal protein L30 OS=T.b.b.	RL30_TRYBB	3,00E-70	GLOS_RL30.1.1	GO:0005840	C	UniProtKB-K	ribosome
RL30_TRYBB (sp|P49153) 60S ribosomal protein L30 OS=T.b.b.	RL30_TRYBB	3,00E-70	GLOS_RL30.1.1	GO:0005198	M	.	structural molecule activity
RL30_TRYBB (sp|P49153) 60S ribosomal protein L30 OS=T.b.b.	RL30_TRYBB	3,00E-70	GLOS_RL30.1.1	GO:0003735	M	InterPro	structural constituent of ribosome
RL30_TRYBB (sp|P49153) 60S ribosomal protein L30 OS=T.b.b.	RL30_TRYBB	3,00E-70	GLOS_RL30.1.1	GO:0006412	B	InterPro	translation
RL39_DROME (sp|O16130) 60S ribosomal protein L39 OS=D.m.	RL39_DROME	6,00E-30	GLOS_RL39.3.7	GO:0005840	C	UniProtKB-K	ribosome
RL39_DROME (sp|O16130) 60S ribosomal protein L39 OS=D.m.	RL39_DROME	6,00E-30	GLOS_RL39.3.7	GO:0003735	M	InterPro	structural constituent of ribosome

RL39_DROME (sp|O16130) 60S ribosomal protein L39 OS=D.m.	RL39_DROME	6,00E-30	GLOS_RL39.3.7	GO:0051231	B	.	spindle elongation
RL39_DROME (sp|O16130) 60S ribosomal protein L39 OS=D.m.	RL39_DROME	6,00E-30	GLOS_RL39.3.7	GO:0007052	B	.	mitotic spindle organization
RL39_DROME (sp|O16130) 60S ribosomal protein L39 OS=D.m.	RL39_DROME	6,00E-30	GLOS_RL39.3.7	GO:0000278	B	.	mitotic cell cycle
RL39_DROME (sp|O16130) 60S ribosomal protein L39 OS=D.m.	RL39_DROME	6,00E-30	GLOS_RL39.3.7	GO:0000022	B	FlyBase	mitotic spindle elongation
RL39_DROME (sp|O16130) 60S ribosomal protein L39 OS=D.m.	RL39_DROME	6,00E-30	GLOS_RL39.3.7	GO:0006412	B	InterPro	translation
RL5_BOMMO (sp|O76190) 60S ribosomal protein L5 OS=B. mori	RL5_BOMMO	1,00E-10	GLOS_RL5.1.5	GO:0005840	C	UniProtKB-k	ribosome
RL5_BOMMO (sp|O76190) 60S ribosomal protein L5 OS=B. mori	RL5_BOMMO	1,00E-10	GLOS_RL5.1.5	GO:0019843	M	.	rRNA binding
RL5_BOMMO (sp|O76190) 60S ribosomal protein L5 OS=B. mori	RL5_BOMMO	1,00E-10	GLOS_RL5.1.5	GO:0008097	M	InterPro	5S rRNA binding
RL5_BOMMO (sp|O76190) 60S ribosomal protein L5 OS=B. mori	RL5_BOMMO	1,00E-10	GLOS_RL5.1.5	GO:0003735	M	InterPro	structural constituent of ribosome
RL5_BOMMO (sp|O76190) 60S ribosomal protein L5 OS=B. mori	RL5_BOMMO	1,00E-10	GLOS_RL5.1.5	GO:0006412	B	InterPro	translation
RL7_DROME (sp|P32100) 60S ribosomal protein L7 OS=D. m.	RL7_DROME	1,00E-106	GLOS_RL7.1.5	GO:0005840	C	FlyBase	ribosome
RL7_DROME (sp|P32100) 60S ribosomal protein L7 OS=D. m.	RL7_DROME	1,00E-106	GLOS_RL7.1.5	GO:0003723	M	UniProtKB-k	RNA binding
RL7_DROME (sp|P32100) 60S ribosomal protein L7 OS=D. m.	RL7_DROME	1,00E-106	GLOS_RL7.1.5	GO:0003735	M	FlyBase	structural constituent of ribosome
RL7_DROME (sp|P32100) 60S ribosomal protein L7 OS=D. m.	RL7_DROME	1,00E-106	GLOS_RL7.1.5	GO:0051297	B	.	centrosome organization
RL7_DROME (sp|P32100) 60S ribosomal protein L7 OS=D. m.	RL7_DROME	1,00E-106	GLOS_RL7.1.5	GO:0031023	B	.	microtubule organizing center organization
RL7_DROME (sp|P32100) 60S ribosomal protein L7 OS=D. m.	RL7_DROME	1,00E-106	GLOS_RL7.1.5	GO:0007098	B	.	centrosome cycle
RL7_DROME (sp|P32100) 60S ribosomal protein L7 OS=D. m.	RL7_DROME	1,00E-106	GLOS_RL7.1.5	GO:0051298	B	FlyBase	centrosome duplication
RL7_DROME (sp|P32100) 60S ribosomal protein L7 OS=D. m.	RL7_DROME	1,00E-106	GLOS_RL7.1.5	GO:0000022	B	FlyBase	mitotic spindle elongation
RL7_DROME (sp|P32100) 60S ribosomal protein L7 OS=D. m.	RL7_DROME	1,00E-106	GLOS_RL7.1.5	GO:0035210	B	.	prepupal development
RL7_DROME (sp|P32100) 60S ribosomal protein L7 OS=D. m.	RL7_DROME	1,00E-106	GLOS_RL7.1.5	GO:0035073	B	FlyBase	pupariation
RS20_DROME (sp|P55828) 40S ribosomal prot S20 OS=D.m.	RS20_DROME	5,00E-48	GLOS_RS20.4.14	GO:0044391	C	.	ribosomal subunit
RS20_DROME (sp|P55828) 40S ribosomal prot S20 OS=D.m.	RS20_DROME	5,00E-48	GLOS_RS20.4.14	GO:0005840	C	.	ribosome
RS20_DROME (sp|P55828) 40S ribosomal prot S20 OS=D.m.	RS20_DROME	5,00E-48	GLOS_RS20.4.14	GO:0015935	C	UniProtKB	small ribosomal subunit
RS20_DROME (sp|P55828) 40S ribosomal prot S20 OS=D.m.	RS20_DROME	5,00E-48	GLOS_RS20.4.14	GO:0003723	M	InterPro	RNA binding
RS20_DROME (sp|P55828) 40S ribosomal prot S20 OS=D.m.	RS20_DROME	5,00E-48	GLOS_RS20.4.14	GO:0003735	M	UniProtKB	structural constituent of ribosome
RS20_DROME (sp|P55828) 40S ribosomal prot S20 OS=D.m.	RS20_DROME	5,00E-48	GLOS_RS20.4.14	GO:0006412	B	UniProtKB	translation
RS23_DROME (sp|Q8T3U2) 40S ribosomal prot S23 OS=D. m.	RS23_DROME	1,00E-74	GLOS_RS23.2.4	GO:0005840	C	FlyBase	ribosome
RS23_DROME (sp|Q8T3U2) 40S ribosomal prot S23 OS=D. m.	RS23_DROME	1,00E-74	GLOS_RS23.2.4	GO:0015935	C	InterPro	small ribosomal subunit
RS23_DROME (sp|Q8T3U2) 40S ribosomal prot S23 OS=D. m.	RS23_DROME	1,00E-74	GLOS_RS23.2.4	GO:0003735	M	FlyBase	structural constituent of ribosome
RS23_DROME (sp|Q8T3U2) 40S ribosomal prot S23 OS=D. m.	RS23_DROME	1,00E-74	GLOS_RS23.2.4	GO:0051298	B	FlyBase	centrosome duplication
RS23_DROME (sp|Q8T3U2) 40S ribosomal prot S23 OS=D. m.	RS23_DROME	1,00E-74	GLOS_RS23.2.4	GO:0006412	B	InterPro	translation
RS25_DROME (sp|P48588) 40S ribosomal prot S25 OS=D. m.	RS25_DROME	2,00E-38	GLOS_RS25.3.10	GO:0005840	C	FlyBase	ribosome
RS25_DROME (sp|P48588) 40S ribosomal prot S25 OS=D. m.	RS25_DROME	2,00E-38	GLOS_RS25.3.10	GO:0003735	M	FlyBase	structural constituent of ribosome
RS29_DROME (sp|Q9VH69) 40S ribosomal prot S29 OS=D. m.	RS29_DROME	6,00E-37	GLOS_RS29.4.12	GO:0005840	C	UniProtKB-k	ribosome
RS29_DROME (sp|Q9VH69) 40S ribosomal prot S29 OS=D. m.	RS29_DROME	6,00E-37	GLOS_RS29.4.12	GO:0046872	M	UniProtKB-k	metal ion binding
RS29_DROME (sp|Q9VH69) 40S ribosomal prot S29 OS=D. m.	RS29_DROME	6,00E-37	GLOS_RS29.4.12	GO:0003735	M	InterPro	structural constituent of ribosome
RS29_DROME (sp|Q9VH69) 40S ribosomal prot S29 OS=D. m.	RS29_DROME	6,00E-37	GLOS_RS29.4.12	GO:0048666	B	FlyBase	neuron development
RS29_DROME (sp|Q9VH69) 40S ribosomal prot S29 OS=D. m.	RS29_DROME	6,00E-37	GLOS_RS29.4.12	GO:0006412	B	InterPro	translation
[BBH] S35B1_DROME (sp|Q9VDD7) Solute carrier family 35 member B1 homolog	S35B1_DROME	1,00E-163	GLOS_S35B1.1.1	GO:0031090	C	.	organelle membrane
[BBH] S35B1_DROME (sp|Q9VDD7) Solute carrier family 35 member B1 homolog	S35B1_DROME	1,00E-163	GLOS_S35B1.1.1	GO:0044432	C	.	endoplasmic reticulum part
[BBH] S35B1_DROME (sp|Q9VDD7) Solute carrier family 35 member B1 homolog	S35B1_DROME	1,00E-163	GLOS_S35B1.1.1	GO:0005783	C	.	endoplasmic reticulum
[BBH] S35B1_DROME (sp|Q9VDD7) Solute carrier family 35 member B1 homolog	S35B1_DROME	1,00E-163	GLOS_S35B1.1.1	GO:0042175	C	.	nuclear outer membrane-endoplasmic reticulum membrane network
[BBH] S35B1_DROME (sp|Q9VDD7) Solute carrier family 35 member B1 homolog	S35B1_DROME	1,00E-163	GLOS_S35B1.1.1	GO:0012505	C	.	endomembrane system
[BBH] S35B1_DROME (sp|Q9VDD7) Solute carrier family 35 member B1 homolog	S35B1_DROME	1,00E-163	GLOS_S35B1.1.1	GO:0005789	C	FlyBase	endoplasmic reticulum membrane
[BBH] S35B1_DROME (sp|Q9VDD7) Solute carrier family 35 member B1 homolog	S35B1_DROME	1,00E-163	GLOS_S35B1.1.1	GO:0016021	C	UniProtKB-k	integral to membrane
[BBH] S35B1_DROME (sp|Q9VDD7) Solute carrier family 35 member B1 homolog	S35B1_DROME	1,00E-163	GLOS_S35B1.1.1	GO:0015215	M	.	nucleotide transmembrane transporter activity
[BBH] S35B1_DROME (sp|Q9VDD7) Solute carrier family 35 member B1 homolog	S35B1_DROME	1,00E-163	GLOS_S35B1.1.1	GO:0015605	M	.	organophosphate ester transmembrane transporter activity
[BBH] S35B1_DROME (sp|Q9VDD7) Solute carrier family 35 member B1 homolog	S35B1_DROME	1,00E-163	GLOS_S35B1.1.1	GO:0008514	M	.	organic anion transmembrane transporter activity
[BBH] S35B1_DROME (sp|Q9VDD7) Solute carrier family 35 member B1 homolog	S35B1_DROME	1,00E-163	GLOS_S35B1.1.1	GO:0008509	M	.	anion transmembrane transporter activity
[BBH] S35B1_DROME (sp|Q9VDD7) Solute carrier family 35 member B1 homolog	S35B1_DROME	1,00E-163	GLOS_S35B1.1.1	GO:1901677	M	.	phosphate transmembrane transporter activity
[BBH] S35B1_DROME (sp|Q9VDD7) Solute carrier family 35 member B1 homolog	S35B1_DROME	1,00E-163	GLOS_S35B1.1.1	GO:0015932	M	.	nucleobase-containing compound transmembrane transporter activity
[BBH] S35B1_DROME (sp|Q9VDD7) Solute carrier family 35 member B1 homolog	S35B1_DROME	1,00E-163	GLOS_S35B1.1.1	GO:1901505	M	.	carbohydrate derivative transporter activity
[BBH] S35B1_DROME (sp|Q9VDD7) Solute carrier family 35 member B1 homolog	S35B1_DROME	1,00E-163	GLOS_S35B1.1.1	GO:0005338	M	UniProtKB	nucleotide-sugar transmembrane transporter activity
[BBH] S35B1_DROME (sp|Q9VDD7) Solute carrier family 35 member B1 homolog	S35B1_DROME	1,00E-163	GLOS_S35B1.1.1	GO:0007411	B	FlyBase	axon guidance

[BBH] S35B1_DROME (sp|Q9VDD7) Solute carrier family 35 member B1 homolog	S35B1_DROME	1,00E-163	GLOS_S35B1.1.1	GO:0008643	B	UniProtKB-K	carbohydrate transport
[BBH] S35B1_DROME (sp|Q9VDD7) Solute carrier family 35 member B1 homolog	S35B1_DROME	1,00E-163	GLOS_S35B1.1.1	GO:0048813	B	.	dendrite morphogenesis
[BBH] S35B1_DROME (sp|Q9VDD7) Solute carrier family 35 member B1 homolog	S35B1_DROME	1,00E-163	GLOS_S35B1.1.1	GO:0070983	B	FlyBase	dendrite guidance
[BBH] S35B1_DROME (sp|Q9VDD7) Solute carrier family 35 member B1 homolog	S35B1_DROME	1,00E-163	GLOS_S35B1.1.1	GO:0006862	B	.	nucleotide transport
[BBH] S35B1_DROME (sp|Q9VDD7) Solute carrier family 35 member B1 homolog	S35B1_DROME	1,00E-163	GLOS_S35B1.1.1	GO:0015748	B	.	organophosphate ester transport
[BBH] S35B1_DROME (sp|Q9VDD7) Solute carrier family 35 member B1 homolog	S35B1_DROME	1,00E-163	GLOS_S35B1.1.1	GO:0015931	B	.	nucleobase-containing compound transport
[BBH] S35B1_DROME (sp|Q9VDD7) Solute carrier family 35 member B1 homolog	S35B1_DROME	1,00E-163	GLOS_S35B1.1.1	GO:0071705	B	.	nitrogen compound transport
[BBH] S35B1_DROME (sp|Q9VDD7) Solute carrier family 35 member B1 homolog	S35B1_DROME	1,00E-163	GLOS_S35B1.1.1	GO:1901264	B	.	carbohydrate derivative transport
[BBH] S35B1_DROME (sp|Q9VDD7) Solute carrier family 35 member B1 homolog	S35B1_DROME	1,00E-163	GLOS_S35B1.1.1	GO:0015780	B	UniProtKB	nucleotide-sugar transport
[BBH] S35B1_DROME (sp|Q9VDD7) Solute carrier family 35 member B1 homolog	S35B1_DROME	1,00E-163	GLOS_S35B1.1.1	GO:0006486	B	.	protein glycosylation
[BBH] S35B1_DROME (sp|Q9VDD7) Solute carrier family 35 member B1 homolog	S35B1_DROME	1,00E-163	GLOS_S35B1.1.1	GO:0043413	B	.	macromolecule glycosylation
[BBH] S35B1_DROME (sp|Q9VDD7) Solute carrier family 35 member B1 homolog	S35B1_DROME	1,00E-163	GLOS_S35B1.1.1	GO:0070085	B	.	glycosylation
[BBH] S35B1_DROME (sp|Q9VDD7) Solute carrier family 35 member B1 homolog	S35B1_DROME	1,00E-163	GLOS_S35B1.1.1	GO:0009101	B	.	glycoprotein biosynthetic process
[BBH] S35B1_DROME (sp|Q9VDD7) Solute carrier family 35 member B1 homolog	S35B1_DROME	1,00E-163	GLOS_S35B1.1.1	GO:0009100	B	.	glycoprotein metabolic process
[BBH] S35B1_DROME (sp|Q9VDD7) Solute carrier family 35 member B1 homolog	S35B1_DROME	1,00E-163	GLOS_S35B1.1.1	GO:0006487	B	FlyBase	protein N-linked glycosylation
[BBH] S35B1_DROME (sp|Q9VDD7) Solute carrier family 35 member B1 homolog	S35B1_DROME	1,00E-163	GLOS_S35B1.1.1	GO:0034976	B	FlyBase	response to endoplasmic reticulum stress
SLU7_DROME (sp|Q9VAQ7) Pre-mRNA-splicing factor Slu7	SLU7_DROME	1,00E-06	GLOS_SLU7.3.13	GO:0016604	C	.	nuclear body
SLU7_DROME (sp|Q9VAQ7) Pre-mRNA-splicing factor Slu7	SLU7_DROME	1,00E-06	GLOS_SLU7.3.13	GO:0016607	C	UniProtKB	nuclear speck
SLU7_DROME (sp|Q9VAQ7) Pre-mRNA-splicing factor Slu7	SLU7_DROME	1,00E-06	GLOS_SLU7.3.13	GO:0005681	C	FlyBase	spliceosomal complex
SLU7_DROME (sp|Q9VAQ7) Pre-mRNA-splicing factor Slu7	SLU7_DROME	1,00E-06	GLOS_SLU7.3.13	GO:0036002	M	.	pre-mRNA binding
SLU7_DROME (sp|Q9VAQ7) Pre-mRNA-splicing factor Slu7	SLU7_DROME	1,00E-06	GLOS_SLU7.3.13	GO:0030628	M	UniProtKB	pre-mRNA 3'-splice site binding
SLU7_DROME (sp|Q9VAQ7) Pre-mRNA-splicing factor Slu7	SLU7_DROME	1,00E-06	GLOS_SLU7.3.13	GO:0008270	M	InterPro	zinc ion binding
SLU7_DROME (sp|Q9VAQ7) Pre-mRNA-splicing factor Slu7	SLU7_DROME	1,00E-06	GLOS_SLU7.3.13	GO:0007052	B	FlyBase	mitotic spindle organization
SLU7_DROME (sp|Q9VAQ7) Pre-mRNA-splicing factor Slu7	SLU7_DROME	1,00E-06	GLOS_SLU7.3.13	GO:0006376	B	.	mRNA splice site selection
SLU7_DROME (sp|Q9VAQ7) Pre-mRNA-splicing factor Slu7	SLU7_DROME	1,00E-06	GLOS_SLU7.3.13	GO:0022618	B	.	ribonucleoprotein complex assembly
SLU7_DROME (sp|Q9VAQ7) Pre-mRNA-splicing factor Slu7	SLU7_DROME	1,00E-06	GLOS_SLU7.3.13	GO:0034622	B	.	cellular macromolecular complex assembly
SLU7_DROME (sp|Q9VAQ7) Pre-mRNA-splicing factor Slu7	SLU7_DROME	1,00E-06	GLOS_SLU7.3.13	GO:0065003	B	.	macromolecular complex assembly
SLU7_DROME (sp|Q9VAQ7) Pre-mRNA-splicing factor Slu7	SLU7_DROME	1,00E-06	GLOS_SLU7.3.13	GO:0071826	B	.	ribonucleoprotein complex subunit organization
SLU7_DROME (sp|Q9VAQ7) Pre-mRNA-splicing factor Slu7	SLU7_DROME	1,00E-06	GLOS_SLU7.3.13	GO:0000245	B	.	spliceosomal complex assembly
SLU7_DROME (sp|Q9VAQ7) Pre-mRNA-splicing factor Slu7	SLU7_DROME	1,00E-06	GLOS_SLU7.3.13	GO:0000398	B	.	mRNA splicing, via spliceosome
SLU7_DROME (sp|Q9VAQ7) Pre-mRNA-splicing factor Slu7	SLU7_DROME	1,00E-06	GLOS_SLU7.3.13	GO:0000377	B	.	RNA splicing, via transesterification react. with bulged adenosine as nucleophile
SLU7_DROME (sp|Q9VAQ7) Pre-mRNA-splicing factor Slu7	SLU7_DROME	1,00E-06	GLOS_SLU7.3.13	GO:0000375	B	.	RNA splicing, via transesterification reactions
SLU7_DROME (sp|Q9VAQ7) Pre-mRNA-splicing factor Slu7	SLU7_DROME	1,00E-06	GLOS_SLU7.3.13	GO:0008380	B	.	RNA splicing
SLU7_DROME (sp|Q9VAQ7) Pre-mRNA-splicing factor Slu7	SLU7_DROME	1,00E-06	GLOS_SLU7.3.13	GO:0006397	B	.	mRNA processing
SLU7_DROME (sp|Q9VAQ7) Pre-mRNA-splicing factor Slu7	SLU7_DROME	1,00E-06	GLOS_SLU7.3.13	GO:0016071	B	.	mRNA metabolic process
SLU7_DROME (sp|Q9VAQ7) Pre-mRNA-splicing factor Slu7	SLU7_DROME	1,00E-06	GLOS_SLU7.3.13	GO:0000389	B	UniProtKB	mRNA 3'-splice site recognition
[BBH] TBA_TRYBR (sp|P04106) Tubulin alpha chain OS=T.b.b. PE=3 SV=1	TBA_TRYBR	0	GLOS_TBA.1.1	GO:0005737	C	UniProtKB-K	cytoplasm
[BBH] TBA_TRYBR (sp|P04106) Tubulin alpha chain OS=T.b.b. PE=3 SV=1	TBA_TRYBR	0	GLOS_TBA.1.1	GO:0044430	C	.	cytoskeletal part
[BBH] TBA_TRYBR (sp|P04106) Tubulin alpha chain OS=T.b.b. PE=3 SV=1	TBA_TRYBR	0	GLOS_TBA.1.1	GO:0005856	C	.	cytoskeleton
[BBH] TBA_TRYBR (sp|P04106) Tubulin alpha chain OS=T.b.b. PE=3 SV=1	TBA_TRYBR	0	GLOS_TBA.1.1	GO:0015630	C	.	microtubule cytoskeleton
[BBH] TBA_TRYBR (sp|P04106) Tubulin alpha chain OS=T.b.b. PE=3 SV=1	TBA_TRYBR	0	GLOS_TBA.1.1	GO:0005874	C	UniProtKB-K	microtubule
[BBH] TBA_TRYBR (sp|P04106) Tubulin alpha chain OS=T.b.b. PE=3 SV=1	TBA_TRYBR	0	GLOS_TBA.1.1	GO:0005525	M	UniProtKB-K	GTP binding
[BBH] TBA_TRYBR (sp|P04106) Tubulin alpha chain OS=T.b.b. PE=3 SV=1	TBA_TRYBR	0	GLOS_TBA.1.1	GO:0003924	M	InterPro	GTPase activity
[BBH] TBA_TRYBR (sp|P04106) Tubulin alpha chain OS=T.b.b. PE=3 SV=1	TBA_TRYBR	0	GLOS_TBA.1.1	GO:0005200	M	InterPro	structural constituent of cytoskeleton
[BBH] TBA_TRYBR (sp|P04106) Tubulin alpha chain OS=T.b.b. PE=3 SV=1	TBA_TRYBR	0	GLOS_TBA.1.1	GO:0007017	B	InterPro	microtubule-based process
[BBH] TBA_TRYBR (sp|P04106) Tubulin alpha chain OS=T.b.b. PE=3 SV=1	TBA_TRYBR	0	GLOS_TBA.1.1	GO:0043623	B	.	cellular protein complex assembly
[BBH] TBA_TRYBR (sp|P04106) Tubulin alpha chain OS=T.b.b. PE=3 SV=1	TBA_TRYBR	0	GLOS_TBA.1.1	GO:0006461	B	.	protein complex assembly
[BBH] TBA_TRYBR (sp|P04106) Tubulin alpha chain OS=T.b.b. PE=3 SV=1	TBA_TRYBR	0	GLOS_TBA.1.1	GO:0070271	B	.	protein complex biogenesis
[BBH] TBA_TRYBR (sp|P04106) Tubulin alpha chain OS=T.b.b. PE=3 SV=1	TBA_TRYBR	0	GLOS_TBA.1.1	GO:0051258	B	InterPro	protein polymerization
TMEDA_DROVI (sp|B4MGF8) Transmembrane emp24 domain-containing prot bai	TMEDA_DROVI	5,00E-23	GLOS_TMEDA.1.3	GO:0016021	C	UniProtKB-K	integral to membrane
TMEDA_DROVI (sp|B4MGF8) Transmembrane emp24 domain-containing prot bai	TMEDA_DROVI	5,00E-23	GLOS_TMEDA.1.3	GO:0009953	B	UniProtKB	dorsal/ventral pattern formation
TMEDA_DROVI (sp|B4MGF8) Transmembrane emp24 domain-containing prot bai	TMEDA_DROVI	5,00E-23	GLOS_TMEDA.1.3	GO:0006810	B	InterPro	transport
[BBH] TNNC3_DROME (sp|P47949) Troponin C, isoform 3 OS=D.m.	TNNC3_DROME	3,00E-88	GLOS_TNNC3.1.4	GO:0005509	M	InterPro	calcium ion binding
TSEP_GLOPP (sp|Q8T4N5) Protein TsetseEP OS=Gl. P. palpalis	TSEP_GLOPP	4,00E-32	GLOS_TSEP.5.20	GO:0005576	C	UniProtKB-S	extracellular region

TTI_GLOMM (sp|O97373) Tsetse thrombin inhibitor OS=G. m. morsitans	TTI_GLOMM	9,00E-21	GLOS_TTI.3.16	GO:0005576	C	UniProtKB-S	extracellular region
TTI_GLOMM (sp|O97373) Tsetse thrombin inhibitor OS=G. m. morsitans	TTI_GLOMM	9,00E-21	GLOS_TTI.3.16	GO:0004866	M	.	endopeptidase inhibitor activity
TTI_GLOMM (sp|O97373) Tsetse thrombin inhibitor OS=G. m. morsitans	TTI_GLOMM	9,00E-21	GLOS_TTI.3.16	GO:0030414	M	.	peptidase inhibitor activity
TTI_GLOMM (sp|O97373) Tsetse thrombin inhibitor OS=G. m. morsitans	TTI_GLOMM	9,00E-21	GLOS_TTI.3.16	GO:0004857	M	.	enzyme inhibitor activity
TTI_GLOMM (sp|O97373) Tsetse thrombin inhibitor OS=G. m. morsitans	TTI_GLOMM	9,00E-21	GLOS_TTI.3.16	GO:0061134	M	.	peptidase regulator activity
TTI_GLOMM (sp|O97373) Tsetse thrombin inhibitor OS=G. m. morsitans	TTI_GLOMM	9,00E-21	GLOS_TTI.3.16	GO:0061135	M	.	endopeptidase regulator activity
TTI_GLOMM (sp|O97373) Tsetse thrombin inhibitor OS=G. m. morsitans	TTI_GLOMM	9,00E-21	GLOS_TTI.3.16	GO:0004867	M	UniProtKB-k	serine-type endopeptidase inhibitor activity
UBIQ_LUMTE (sp|P84589) Ubiquitin (Fragment) OS=L. terrestris PE=1 SV=2	UBIQ_LUMTE	3,00E-25	GLOS_UBIQ.1.2	GO:0005737	C	UniProtKB-S	cytoplasm
UBIQ_LUMTE (sp|P84589) Ubiquitin (Fragment) OS=L. terrestris PE=1 SV=2	UBIQ_LUMTE	3,00E-25	GLOS_UBIQ.1.2	GO:0005634	C	UniProtKB-S	nucleus
[BBH] VATD1_DROME (sp|Q9V7D2) V-type proton ATPase subunit D 1 OS=D.m.	VATD1_DROME	1,00E-157	GLOS_VATD1.1.1	GO:0033176	C	.	proton-transporting V-type ATPase complex
[BBH] VATD1_DROME (sp|Q9V7D2) V-type proton ATPase subunit D 1 OS=D.m.	VATD1_DROME	1,00E-157	GLOS_VATD1.1.1	GO:0016469	C	.	proton-transporting two-sector ATPase complex
[BBH] VATD1_DROME (sp|Q9V7D2) V-type proton ATPase subunit D 1 OS=D.m.	VATD1_DROME	1,00E-157	GLOS_VATD1.1.1	GO:0033181	C	FlyBase	plasma membrane proton-transporting V-type ATPase complex
[BBH] VATD1_DROME (sp|Q9V7D2) V-type proton ATPase subunit D 1 OS=D.m.	VATD1_DROME	1,00E-157	GLOS_VATD1.1.1	GO:0033180	C	.	proton-transporting V-type ATPase, V1 domain
[BBH] VATD1_DROME (sp|Q9V7D2) V-type proton ATPase subunit D 1 OS=D.m.	VATD1_DROME	1,00E-157	GLOS_VATD1.1.1	GO:0033178	C	.	proton-transporting two-sector ATPase complex, catalytic domain
[BBH] VATD1_DROME (sp|Q9V7D2) V-type proton ATPase subunit D 1 OS=D.m.	VATD1_DROME	1,00E-157	GLOS_VATD1.1.1	GO:0044437	C	.	vacuolar part
[BBH] VATD1_DROME (sp|Q9V7D2) V-type proton ATPase subunit D 1 OS=D.m.	VATD1_DROME	1,00E-157	GLOS_VATD1.1.1	GO:0005773	C	.	vacuole
[BBH] VATD1_DROME (sp|Q9V7D2) V-type proton ATPase subunit D 1 OS=D.m.	VATD1_DROME	1,00E-157	GLOS_VATD1.1.1	GO:0016471	C	.	vacuolar proton-transporting V-type ATPase complex
[BBH] VATD1_DROME (sp|Q9V7D2) V-type proton ATPase subunit D 1 OS=D.m.	VATD1_DROME	1,00E-157	GLOS_VATD1.1.1	GO:0005774	C	.	vacuolar membrane
[BBH] VATD1_DROME (sp|Q9V7D2) V-type proton ATPase subunit D 1 OS=D.m.	VATD1_DROME	1,00E-157	GLOS_VATD1.1.1	GO:0000221	C	FlyBase	vacuolar proton-transporting V-type ATPase, V1 domain
[BBH] VATD1_DROME (sp|Q9V7D2) V-type proton ATPase subunit D 1 OS=D.m.	VATD1_DROME	1,00E-157	GLOS_VATD1.1.1	GO:0015405	M	.	P-P-bond-hydrolysis-driven transmembrane transporter activity
[BBH] VATD1_DROME (sp|Q9V7D2) V-type proton ATPase subunit D 1 OS=D.m.	VATD1_DROME	1,00E-157	GLOS_VATD1.1.1	GO:0015399	M	.	primary active transmembrane transporter activity
[BBH] VATD1_DROME (sp|Q9V7D2) V-type proton ATPase subunit D 1 OS=D.m.	VATD1_DROME	1,00E-157	GLOS_VATD1.1.1	GO:0022804	M	.	active transmembrane transporter activity
[BBH] VATD1_DROME (sp|Q9V7D2) V-type proton ATPase subunit D 1 OS=D.m.	VATD1_DROME	1,00E-157	GLOS_VATD1.1.1	GO:0016820	M	.	hydrolase activity, acting on acid anhydrides, catalyzing transmembrane movement of substances
[BBH] VATD1_DROME (sp|Q9V7D2) V-type proton ATPase subunit D 1 OS=D.m.	VATD1_DROME	1,00E-157	GLOS_VATD1.1.1	GO:0043492	M	.	ATPase activity, coupled to movement of substances
[BBH] VATD1_DROME (sp|Q9V7D2) V-type proton ATPase subunit D 1 OS=D.m.	VATD1_DROME	1,00E-157	GLOS_VATD1.1.1	GO:0042626	M	InterPro	ATPase activity, coupled to transmembrane movement of substances
[BBH] VATD1_DROME (sp|Q9V7D2) V-type proton ATPase subunit D 1 OS=D.m.	VATD1_DROME	1,00E-157	GLOS_VATD1.1.1	GO:0015988	B	.	energy coupled proton transmemb. Transp., against electrochemical gradient
[BBH] VATD1_DROME (sp|Q9V7D2) V-type proton ATPase subunit D 1 OS=D.m.	VATD1_DROME	1,00E-157	GLOS_VATD1.1.1	GO:0015992	B	.	proton transport
[BBH] VATD1_DROME (sp|Q9V7D2) V-type proton ATPase subunit D 1 OS=D.m.	VATD1_DROME	1,00E-157	GLOS_VATD1.1.1	GO:0006818	B	.	hydrogen transport
[BBH] VATD1_DROME (sp|Q9V7D2) V-type proton ATPase subunit D 1 OS=D.m.	VATD1_DROME	1,00E-157	GLOS_VATD1.1.1	GO:0015672	B	.	monovalent inorganic cation transport
[BBH] VATD1_DROME (sp|Q9V7D2) V-type proton ATPase subunit D 1 OS=D.m.	VATD1_DROME	1,00E-157	GLOS_VATD1.1.1	GO:0015991	B	FlyBase	ATP hydrolysis coupled proton transport
[BBH] XRN2_DROME (sp|Q9VM71) 5'-3' exoribonuclease 2 homolog OS=D.m.	XRN2_DROME	0	GLOS_XRN2.1.1	GO:0005634	C	UniProtKB	nucleus
[BBH] XRN2_DROME (sp|Q9VM71) 5'-3' exoribonuclease 2 homolog OS=D.m.	XRN2_DROME	0	GLOS_XRN2.1.1	GO:0004527	M	.	exonuclease activity
[BBH] XRN2_DROME (sp|Q9VM71) 5'-3' exoribonuclease 2 homolog OS=D.m.	XRN2_DROME	0	GLOS_XRN2.1.1	GO:0008409	M	UniProtKB	5'-3' exonuclease activity
[BBH] XRN2_DROME (sp|Q9VM71) 5'-3' exoribonuclease 2 homolog OS=D.m.	XRN2_DROME	0	GLOS_XRN2.1.1	GO:0008409	M	.	5'-3' exonuclease activity
[BBH] XRN2_DROME (sp|Q9VM71) 5'-3' exoribonuclease 2 homolog OS=D.m.	XRN2_DROME	0	GLOS_XRN2.1.1	GO:0016896	M	.	exoribonuclease activity, producing 5'-phosphomonoesters
[BBH] XRN2_DROME (sp|Q9VM71) 5'-3' exoribonuclease 2 homolog OS=D.m.	XRN2_DROME	0	GLOS_XRN2.1.1	GO:0004532	M	.	exoribonuclease activity
[BBH] XRN2_DROME (sp|Q9VM71) 5'-3' exoribonuclease 2 homolog OS=D.m.	XRN2_DROME	0	GLOS_XRN2.1.1	GO:0004540	M	.	ribonuclease activity
[BBH] XRN2_DROME (sp|Q9VM71) 5'-3' exoribonuclease 2 homolog OS=D.m.	XRN2_DROME	0	GLOS_XRN2.1.1	GO:0016796	M	.	exonuclease activity, active with either ribo- or deoxyribonucleic acids and producing 5'-phosphomonoesters
[BBH] XRN2_DROME (sp|Q9VM71) 5'-3' exoribonuclease 2 homolog OS=D.m.	XRN2_DROME	0	GLOS_XRN2.1.1	GO:0004534	M	InterPro	5'-3' exoribonuclease activity
[BBH] XRN2_DROME (sp|Q9VM71) 5'-3' exoribonuclease 2 homolog OS=D.m.	XRN2_DROME	0	GLOS_XRN2.1.1	GO:0046872	M	UniProtKB-k	metal ion binding
[BBH] XRN2_DROME (sp|Q9VM71) 5'-3' exoribonuclease 2 homolog OS=D.m.	XRN2_DROME	0	GLOS_XRN2.1.1	GO:0003676	M	InterPro	nucleic acid binding
[BBH] XRN2_DROME (sp|Q9VM71) 5'-3' exoribonuclease 2 homolog OS=D.m.	XRN2_DROME	0	GLOS_XRN2.1.1	GO:0006308	B	.	DNA catabolic process
[BBH] XRN2_DROME (sp|Q9VM71) 5'-3' exoribonuclease 2 homolog OS=D.m.	XRN2_DROME	0	GLOS_XRN2.1.1	GO:0034655	B	.	nucleobase-containing compound catabolic process
[BBH] XRN2_DROME (sp|Q9VM71) 5'-3' exoribonuclease 2 homolog OS=D.m.	XRN2_DROME	0	GLOS_XRN2.1.1	GO:0019439	B	.	aromatic compound catabolic process
[BBH] XRN2_DROME (sp|Q9VM71) 5'-3' exoribonuclease 2 homolog OS=D.m.	XRN2_DROME	0	GLOS_XRN2.1.1	GO:0044270	B	.	cellular nitrogen compound catabolic process
[BBH] XRN2_DROME (sp|Q9VM71) 5'-3' exoribonuclease 2 homolog OS=D.m.	XRN2_DROME	0	GLOS_XRN2.1.1	GO:0046700	B	.	heterocycle catabolic process
[BBH] XRN2_DROME (sp|Q9VM71) 5'-3' exoribonuclease 2 homolog OS=D.m.	XRN2_DROME	0	GLOS_XRN2.1.1	GO:1901361	B	.	organic cyclic compound catabolic process
[BBH] XRN2_DROME (sp|Q9VM71) 5'-3' exoribonuclease 2 homolog OS=D.m.	XRN2_DROME	0	GLOS_XRN2.1.1	GO:0090305	B	.	nucleic acid phosphodiester bond hydrolysis
[BBH] XRN2_DROME (sp|Q9VM71) 5'-3' exoribonuclease 2 homolog OS=D.m.	XRN2_DROME	0	GLOS_XRN2.1.1	GO:0000738	B	UniProtKB	DNA catabolic process, exonucleolytic
[BBH] XRN2_DROME (sp|Q9VM71) 5'-3' exoribonuclease 2 homolog OS=D.m.	XRN2_DROME	0	GLOS_XRN2.1.1	GO:0006353	B	UniProtKB-k	DNA-dependent transcription, termination
[BBH] XRN2_DROME (sp|Q9VM71) 5'-3' exoribonuclease 2 homolog OS=D.m.	XRN2_DROME	0	GLOS_XRN2.1.1	GO:0006397	B	UniProtKB-k	mRNA processing
[BBH] XRN2_DROME (sp|Q9VM71) 5'-3' exoribonuclease 2 homolog OS=D.m.	XRN2_DROME	0	GLOS_XRN2.1.1	GO:0022008	B	FlyBase	neurogenesis

[BBH] XRN2_DROME (sp|Q9VM71) 5'-3' exoribonuclease 2 homolog OS=D.m.	XRN2_DROME	0	GLOS_XRN2.1.1	GO:0006355	B	UniProtKB-k	regulation of transcription, DNA-dependent
ZN761_HUMAN (sp|Q86XN6) Zinc finger protein 761 OS=Homo sapiens	ZN761_HUMAN	5,00E-44	GLOS_ZN761.1.1	GO:0005634	C	UniProtKB-S	nucleus
ZN761_HUMAN (sp|Q86XN6) Zinc finger protein 761 OS=Homo sapiens	ZN761_HUMAN	5,00E-44	GLOS_ZN761.1.1	GO:0003677	M	UniProtKB-k	DNA binding
ZN761_HUMAN (sp|Q86XN6) Zinc finger protein 761 OS=Homo sapiens	ZN761_HUMAN	5,00E-44	GLOS_ZN761.1.1	GO:0046872	M	UniProtKB-k	metal ion binding
ZN761_HUMAN (sp|Q86XN6) Zinc finger protein 761 OS=Homo sapiens	ZN761_HUMAN	5,00E-44	GLOS_ZN761.1.1	GO:0006355	B	UniProtKB-k	regulation of transcription, DNA-dependent
ZN761_HUMAN (sp|Q86XN6) Zinc finger protein 761 OS=Homo sapiens	ZN761_HUMAN	5,00E-44	GLOS_ZN761.1.1	GO:0006351	B	UniProtKB-k	transcription, DNA-dependent
XP_001983838.1 GH16119 [Drosophila grimshawi]	XP_001983838.1	6,00E-47	GLOS_DGRI_GH1	GO:0005261	M	.	cation channel activity
XP_001983838.1 GH16119 [Drosophila grimshawi]	XP_001983838.1	6,00E-47	GLOS_DGRI_GH1	GO:0005216	M	.	ion channel activity
XP_001983838.1 GH16119 [Drosophila grimshawi]	XP_001983838.1	6,00E-47	GLOS_DGRI_GH1	GO:0015075	M	.	ion transmembrane transporter activity
XP_001983838.1 GH16119 [Drosophila grimshawi]	XP_001983838.1	6,00E-47	GLOS_DGRI_GH1	GO:0022891	M	.	substrate-specific transmembrane transporter activity
XP_001983838.1 GH16119 [Drosophila grimshawi]	XP_001983838.1	6,00E-47	GLOS_DGRI_GH1	GO:0022857	M	.	transmembrane transporter activity
XP_001983838.1 GH16119 [Drosophila grimshawi]	XP_001983838.1	6,00E-47	GLOS_DGRI_GH1	GO:0005215	M	.	transporter activity
XP_001983838.1 GH16119 [Drosophila grimshawi]	XP_001983838.1	6,00E-47	GLOS_DGRI_GH1	GO:0003674	M	.	molecular_function
XP_001983838.1 GH16119 [Drosophila grimshawi]	XP_001983838.1	6,00E-47	GLOS_DGRI_GH1	GO:0022892	M	.	substrate-specific transporter activity
XP_001983838.1 GH16119 [Drosophila grimshawi]	XP_001983838.1	6,00E-47	GLOS_DGRI_GH1	GO:0022838	M	.	substrate-specific channel activity
XP_001983838.1 GH16119 [Drosophila grimshawi]	XP_001983838.1	6,00E-47	GLOS_DGRI_GH1	GO:0015267	M	.	channel activity
XP_001983838.1 GH16119 [Drosophila grimshawi]	XP_001983838.1	6,00E-47	GLOS_DGRI_GH1	GO:0022803	M	.	passive transmembrane transporter activity
XP_001983838.1 GH16119 [Drosophila grimshawi]	XP_001983838.1	6,00E-47	GLOS_DGRI_GH1	GO:0008324	M	.	cation transmembrane transporter activity
XP_001983838.1 GH16119 [Drosophila grimshawi]	XP_001983838.1	6,00E-47	GLOS_DGRI_GH1	GO:0015085	M	.	calcium ion transmembrane transporter activity
XP_001983838.1 GH16119 [Drosophila grimshawi]	XP_001983838.1	6,00E-47	GLOS_DGRI_GH1	GO:0046873	M	.	metal ion transmembrane transporter activity
XP_001983838.1 GH16119 [Drosophila grimshawi]	XP_001983838.1	6,00E-47	GLOS_DGRI_GH1	GO:0022890	M	.	inorganic cation transmembrane transporter activity
XP_001983838.1 GH16119 [Drosophila grimshawi]	XP_001983838.1	6,00E-47	GLOS_DGRI_GH1	GO:0072509	M	.	divalent inorganic cation transmembrane transporter activity
XP_001983838.1 GH16119 [Drosophila grimshawi]	XP_001983838.1	6,00E-47	GLOS_DGRI_GH1	GO:0005262	M	Refseq	calcium channel activity
XP_001983838.1 GH16119 [Drosophila grimshawi]	XP_001983838.1	6,00E-47	GLOS_DGRI_GH1	GO:0005488	M	.	binding
XP_001983838.1 GH16119 [Drosophila grimshawi]	XP_001983838.1	6,00E-47	GLOS_DGRI_GH1	GO:0005515	M	Refseq	protein binding
XP_001983838.1 GH16119 [Drosophila grimshawi]	XP_001983838.1	6,00E-47	GLOS_DGRI_GH1	GO:0016020	C	.	membrane
XP_001983838.1 GH16119 [Drosophila grimshawi]	XP_001983838.1	6,00E-47	GLOS_DGRI_GH1	GO:0005575	C	.	cellular_component
XP_001983838.1 GH16119 [Drosophila grimshawi]	XP_001983838.1	6,00E-47	GLOS_DGRI_GH1	GO:0044464	C	.	cell part
XP_001983838.1 GH16119 [Drosophila grimshawi]	XP_001983838.1	6,00E-47	GLOS_DGRI_GH1	GO:0005623	C	.	cell
XP_001983838.1 GH16119 [Drosophila grimshawi]	XP_001983838.1	6,00E-47	GLOS_DGRI_GH1	GO:0071944	C	.	cell periphery
XP_001983838.1 GH16119 [Drosophila grimshawi]	XP_001983838.1	6,00E-47	GLOS_DGRI_GH1	GO:0005886	C	Refseq	plasma membrane
XP_001983838.1 GH16119 [Drosophila grimshawi]	XP_001983838.1	6,00E-47	GLOS_DGRI_GH1	GO:0016021	C	.	integral to membrane
XP_001983838.1 GH16119 [Drosophila grimshawi]	XP_001983838.1	6,00E-47	GLOS_DGRI_GH1	GO:0031224	C	.	intrinsic to membrane
XP_001983838.1 GH16119 [Drosophila grimshawi]	XP_001983838.1	6,00E-47	GLOS_DGRI_GH1	GO:0044425	C	.	membrane part
XP_001983838.1 GH16119 [Drosophila grimshawi]	XP_001983838.1	6,00E-47	GLOS_DGRI_GH1	GO:0031226	C	.	intrinsic to plasma membrane
XP_001983838.1 GH16119 [Drosophila grimshawi]	XP_001983838.1	6,00E-47	GLOS_DGRI_GH1	GO:0044459	C	.	plasma membrane part
XP_001983838.1 GH16119 [Drosophila grimshawi]	XP_001983838.1	6,00E-47	GLOS_DGRI_GH1	GO:0005886	C	.	plasma membrane
XP_001983838.1 GH16119 [Drosophila grimshawi]	XP_001983838.1	6,00E-47	GLOS_DGRI_GH1	GO:0005887	C	Refseq	integral to plasma membrane
XP_001983838.1 GH16119 [Drosophila grimshawi]	XP_001983838.1	6,00E-47	GLOS_DGRI_GH1	GO:0070838	B	.	divalent metal ion transport
XP_001983838.1 GH16119 [Drosophila grimshawi]	XP_001983838.1	6,00E-47	GLOS_DGRI_GH1	GO:0030001	B	.	metal ion transport
XP_001983838.1 GH16119 [Drosophila grimshawi]	XP_001983838.1	6,00E-47	GLOS_DGRI_GH1	GO:0006812	B	.	cation transport
XP_001983838.1 GH16119 [Drosophila grimshawi]	XP_001983838.1	6,00E-47	GLOS_DGRI_GH1	GO:0006811	B	.	ion transport
XP_001983838.1 GH16119 [Drosophila grimshawi]	XP_001983838.1	6,00E-47	GLOS_DGRI_GH1	GO:0044765	B	.	single-organism transport
XP_001983838.1 GH16119 [Drosophila grimshawi]	XP_001983838.1	6,00E-47	GLOS_DGRI_GH1	GO:0006810	B	.	transport
XP_001983838.1 GH16119 [Drosophila grimshawi]	XP_001983838.1	6,00E-47	GLOS_DGRI_GH1	GO:0051234	B	.	establishment of localization
XP_001983838.1 GH16119 [Drosophila grimshawi]	XP_001983838.1	6,00E-47	GLOS_DGRI_GH1	GO:0008150	B	.	biological_process
XP_001983838.1 GH16119 [Drosophila grimshawi]	XP_001983838.1	6,00E-47	GLOS_DGRI_GH1	GO:0051179	B	.	localization
XP_001983838.1 GH16119 [Drosophila grimshawi]	XP_001983838.1	6,00E-47	GLOS_DGRI_GH1	GO:0044699	B	.	single-organism process
XP_001983838.1 GH16119 [Drosophila grimshawi]	XP_001983838.1	6,00E-47	GLOS_DGRI_GH1	GO:0072511	B	.	divalent inorganic cation transport
XP_001983838.1 GH16119 [Drosophila grimshawi]	XP_001983838.1	6,00E-47	GLOS_DGRI_GH1	GO:0006816	B	Refseq	calcium ion transport
XP_001983838.1 GH16119 [Drosophila grimshawi]	XP_001983838.1	6,00E-47	GLOS_DGRI_GH1	GO:0097485	B	.	neuron projection guidance
XP_001983838.1 GH16119 [Drosophila grimshawi]	XP_001983838.1	6,00E-47	GLOS_DGRI_GH1	GO:0006928	B	.	cellular component movement
XP_001983838.1 GH16119 [Drosophila grimshawi]	XP_001983838.1	6,00E-47	GLOS_DGRI_GH1	GO:0044763	B	.	single-organism cellular process
XP_001983838.1 GH16119 [Drosophila grimshawi]	XP_001983838.1	6,00E-47	GLOS_DGRI_GH1	GO:0009987	B	.	cellular process

XP_001983838.1 GH16119 [Drosophila grimshawi]	XP_001983838.1	6,00E-47	GLOS_DGRI_GH1	GO:0006935	B	.	chemotaxis
XP_001983838.1 GH16119 [Drosophila grimshawi]	XP_001983838.1	6,00E-47	GLOS_DGRI_GH1	GO:0042221	B	.	response to chemical stimulus
XP_001983838.1 GH16119 [Drosophila grimshawi]	XP_001983838.1	6,00E-47	GLOS_DGRI_GH1	GO:0050896	B	.	response to stimulus
XP_001983838.1 GH16119 [Drosophila grimshawi]	XP_001983838.1	6,00E-47	GLOS_DGRI_GH1	GO:0042330	B	.	taxis
XP_001983838.1 GH16119 [Drosophila grimshawi]	XP_001983838.1	6,00E-47	GLOS_DGRI_GH1	GO:0009605	B	.	response to external stimulus
XP_001983838.1 GH16119 [Drosophila grimshawi]	XP_001983838.1	6,00E-47	GLOS_DGRI_GH1	GO:0040011	B	.	locomotion
XP_001983838.1 GH16119 [Drosophila grimshawi]	XP_001983838.1	6,00E-47	GLOS_DGRI_GH1	GO:0048812	B	.	neuron projection morphogenesis
XP_001983838.1 GH16119 [Drosophila grimshawi]	XP_001983838.1	6,00E-47	GLOS_DGRI_GH1	GO:0048858	B	.	cell projection morphogenesis
XP_001983838.1 GH16119 [Drosophila grimshawi]	XP_001983838.1	6,00E-47	GLOS_DGRI_GH1	GO:0030030	B	.	cell projection organization
XP_001983838.1 GH16119 [Drosophila grimshawi]	XP_001983838.1	6,00E-47	GLOS_DGRI_GH1	GO:0016043	B	.	cellular component organization
XP_001983838.1 GH16119 [Drosophila grimshawi]	XP_001983838.1	6,00E-47	GLOS_DGRI_GH1	GO:0071840	B	.	cellular component organization or biogenesis
XP_001983838.1 GH16119 [Drosophila grimshawi]	XP_001983838.1	6,00E-47	GLOS_DGRI_GH1	GO:0032990	B	.	cell part morphogenesis
XP_001983838.1 GH16119 [Drosophila grimshawi]	XP_001983838.1	6,00E-47	GLOS_DGRI_GH1	GO:0032989	B	.	cellular component morphogenesis
XP_001983838.1 GH16119 [Drosophila grimshawi]	XP_001983838.1	6,00E-47	GLOS_DGRI_GH1	GO:0009653	B	.	anatomical structure morphogenesis
XP_001983838.1 GH16119 [Drosophila grimshawi]	XP_001983838.1	6,00E-47	GLOS_DGRI_GH1	GO:0032502	B	.	developmental process
XP_001983838.1 GH16119 [Drosophila grimshawi]	XP_001983838.1	6,00E-47	GLOS_DGRI_GH1	GO:0048856	B	.	anatomical structure development
XP_001983838.1 GH16119 [Drosophila grimshawi]	XP_001983838.1	6,00E-47	GLOS_DGRI_GH1	GO:0044767	B	.	single-organism developmental process
XP_001983838.1 GH16119 [Drosophila grimshawi]	XP_001983838.1	6,00E-47	GLOS_DGRI_GH1	GO:0048869	B	.	cellular developmental process
XP_001983838.1 GH16119 [Drosophila grimshawi]	XP_001983838.1	6,00E-47	GLOS_DGRI_GH1	GO:0000902	B	.	cell morphogenesis
XP_001983838.1 GH16119 [Drosophila grimshawi]	XP_001983838.1	6,00E-47	GLOS_DGRI_GH1	GO:0031175	B	.	neuron projection development
XP_001983838.1 GH16119 [Drosophila grimshawi]	XP_001983838.1	6,00E-47	GLOS_DGRI_GH1	GO:0048666	B	.	neuron development
XP_001983838.1 GH16119 [Drosophila grimshawi]	XP_001983838.1	6,00E-47	GLOS_DGRI_GH1	GO:0048468	B	.	cell development
XP_001983838.1 GH16119 [Drosophila grimshawi]	XP_001983838.1	6,00E-47	GLOS_DGRI_GH1	GO:0030154	B	.	cell differentiation
XP_001983838.1 GH16119 [Drosophila grimshawi]	XP_001983838.1	6,00E-47	GLOS_DGRI_GH1	GO:0030182	B	.	neuron differentiation
XP_001983838.1 GH16119 [Drosophila grimshawi]	XP_001983838.1	6,00E-47	GLOS_DGRI_GH1	GO:0048699	B	.	generation of neurons
XP_001983838.1 GH16119 [Drosophila grimshawi]	XP_001983838.1	6,00E-47	GLOS_DGRI_GH1	GO:0022008	B	.	neurogenesis
XP_001983838.1 GH16119 [Drosophila grimshawi]	XP_001983838.1	6,00E-47	GLOS_DGRI_GH1	GO:0007399	B	.	nervous system development
XP_001983838.1 GH16119 [Drosophila grimshawi]	XP_001983838.1	6,00E-47	GLOS_DGRI_GH1	GO:0048731	B	.	system development
XP_001983838.1 GH16119 [Drosophila grimshawi]	XP_001983838.1	6,00E-47	GLOS_DGRI_GH1	GO:0007275	B	.	multicellular organismal development
XP_001983838.1 GH16119 [Drosophila grimshawi]	XP_001983838.1	6,00E-47	GLOS_DGRI_GH1	GO:0044707	B	.	single-multicellular organism process
XP_001983838.1 GH16119 [Drosophila grimshawi]	XP_001983838.1	6,00E-47	GLOS_DGRI_GH1	GO:0032501	B	.	multicellular organismal process
XP_001983838.1 GH16119 [Drosophila grimshawi]	XP_001983838.1	6,00E-47	GLOS_DGRI_GH1	GO:0007409	B	.	axonogenesis
XP_001983838.1 GH16119 [Drosophila grimshawi]	XP_001983838.1	6,00E-47	GLOS_DGRI_GH1	GO:0048667	B	.	cell morphogenesis involved in neuron differentiation
XP_001983838.1 GH16119 [Drosophila grimshawi]	XP_001983838.1	6,00E-47	GLOS_DGRI_GH1	GO:0000904	B	.	cell morphogenesis involved in differentiation
XP_001983838.1 GH16119 [Drosophila grimshawi]	XP_001983838.1	6,00E-47	GLOS_DGRI_GH1	GO:0061564	B	.	axon development
XP_001983838.1 GH16119 [Drosophila grimshawi]	XP_001983838.1	6,00E-47	GLOS_DGRI_GH1	GO:0007411	B	Refseq	axon guidance
XP_001983838.1 GH16119 [Drosophila grimshawi]	XP_001983838.1	6,00E-47	GLOS_DGRI_GH1	GO:0007599	B	.	hemostasis
XP_001983838.1 GH16119 [Drosophila grimshawi]	XP_001983838.1	6,00E-47	GLOS_DGRI_GH1	GO:0050878	B	.	regulation of body fluid levels
XP_001983838.1 GH16119 [Drosophila grimshawi]	XP_001983838.1	6,00E-47	GLOS_DGRI_GH1	GO:0065008	B	.	regulation of biological quality
XP_001983838.1 GH16119 [Drosophila grimshawi]	XP_001983838.1	6,00E-47	GLOS_DGRI_GH1	GO:0065007	B	.	biological regulation
XP_001983838.1 GH16119 [Drosophila grimshawi]	XP_001983838.1	6,00E-47	GLOS_DGRI_GH1	GO:0050817	B	.	coagulation
XP_001983838.1 GH16119 [Drosophila grimshawi]	XP_001983838.1	6,00E-47	GLOS_DGRI_GH1	GO:0042060	B	.	wound healing
XP_001983838.1 GH16119 [Drosophila grimshawi]	XP_001983838.1	6,00E-47	GLOS_DGRI_GH1	GO:0009611	B	.	response to wounding
XP_001983838.1 GH16119 [Drosophila grimshawi]	XP_001983838.1	6,00E-47	GLOS_DGRI_GH1	GO:0006950	B	.	response to stress
XP_001983838.1 GH16119 [Drosophila grimshawi]	XP_001983838.1	6,00E-47	GLOS_DGRI_GH1	GO:0007596	B	Refseq	blood coagulation
XP_001983838.1 GH16119 [Drosophila grimshawi]	XP_001983838.1	6,00E-47	GLOS_DGRI_GH1	GO:0007165	B	.	signal transduction
XP_001983838.1 GH16119 [Drosophila grimshawi]	XP_001983838.1	6,00E-47	GLOS_DGRI_GH1	GO:0050794	B	.	regulation of cellular process
XP_001983838.1 GH16119 [Drosophila grimshawi]	XP_001983838.1	6,00E-47	GLOS_DGRI_GH1	GO:0050789	B	.	regulation of biological process
XP_001983838.1 GH16119 [Drosophila grimshawi]	XP_001983838.1	6,00E-47	GLOS_DGRI_GH1	GO:0051716	B	.	cellular response to stimulus
XP_001983838.1 GH16119 [Drosophila grimshawi]	XP_001983838.1	6,00E-47	GLOS_DGRI_GH1	GO:0007154	B	.	cell communication
XP_001983838.1 GH16119 [Drosophila grimshawi]	XP_001983838.1	6,00E-47	GLOS_DGRI_GH1	GO:0044700	B	.	single organism signaling
XP_001983838.1 GH16119 [Drosophila grimshawi]	XP_001983838.1	6,00E-47	GLOS_DGRI_GH1	GO:0023052	B	.	signaling
XP_001983838.1 GH16119 [Drosophila grimshawi]	XP_001983838.1	6,00E-47	GLOS_DGRI_GH1	GO:0009583	B	.	detection of light stimulus
XP_001983838.1 GH16119 [Drosophila grimshawi]	XP_001983838.1	6,00E-47	GLOS_DGRI_GH1	GO:0009416	B	.	response to light stimulus

XP_001983838.1 GH16119 [Drosophila grimshawi]	XP_001983838.1	6,00E-47	GLOS_DGRI_GH1	GO:0009314	B	.	response to radiation
XP_001983838.1 GH16119 [Drosophila grimshawi]	XP_001983838.1	6,00E-47	GLOS_DGRI_GH1	GO:0009628	B	.	response to abiotic stimulus
XP_001983838.1 GH16119 [Drosophila grimshawi]	XP_001983838.1	6,00E-47	GLOS_DGRI_GH1	GO:0009581	B	.	detection of external stimulus
XP_001983838.1 GH16119 [Drosophila grimshawi]	XP_001983838.1	6,00E-47	GLOS_DGRI_GH1	GO:0051606	B	.	detection of stimulus
XP_001983838.1 GH16119 [Drosophila grimshawi]	XP_001983838.1	6,00E-47	GLOS_DGRI_GH1	GO:0009582	B	.	detection of abiotic stimulus
XP_001983838.1 GH16119 [Drosophila grimshawi]	XP_001983838.1	6,00E-47	GLOS_DGRI_GH1	GO:0007602	B	Refseq	phototransduction
XP_001983838.1 GH16119 [Drosophila grimshawi]	XP_001983838.1	6,00E-47	GLOS_DGRI_GH1	GO:0010522	B	.	regulation of calcium ion transport into cytosol
XP_001983838.1 GH16119 [Drosophila grimshawi]	XP_001983838.1	6,00E-47	GLOS_DGRI_GH1	GO:0032386	B	.	regulation of intracellular transport
XP_001983838.1 GH16119 [Drosophila grimshawi]	XP_001983838.1	6,00E-47	GLOS_DGRI_GH1	GO:0051049	B	.	regulation of transport
XP_001983838.1 GH16119 [Drosophila grimshawi]	XP_001983838.1	6,00E-47	GLOS_DGRI_GH1	GO:0032879	B	.	regulation of localization
XP_001983838.1 GH16119 [Drosophila grimshawi]	XP_001983838.1	6,00E-47	GLOS_DGRI_GH1	GO:0060341	B	.	regulation of cellular localization
XP_001983838.1 GH16119 [Drosophila grimshawi]	XP_001983838.1	6,00E-47	GLOS_DGRI_GH1	GO:0051924	B	.	regulation of calcium ion transport
XP_001983838.1 GH16119 [Drosophila grimshawi]	XP_001983838.1	6,00E-47	GLOS_DGRI_GH1	GO:0010959	B	.	regulation of metal ion transport
XP_001983838.1 GH16119 [Drosophila grimshawi]	XP_001983838.1	6,00E-47	GLOS_DGRI_GH1	GO:0043269	B	.	regulation of ion transport
XP_001983838.1 GH16119 [Drosophila grimshawi]	XP_001983838.1	6,00E-47	GLOS_DGRI_GH1	GO:2000021	B	.	regulation of ion homeostasis
XP_001983838.1 GH16119 [Drosophila grimshawi]	XP_001983838.1	6,00E-47	GLOS_DGRI_GH1	GO:0032844	B	.	regulation of homeostatic process
XP_001983838.1 GH16119 [Drosophila grimshawi]	XP_001983838.1	6,00E-47	GLOS_DGRI_GH1	GO:0032388	B	.	positive regulation of intracellular transport
XP_001983838.1 GH16119 [Drosophila grimshawi]	XP_001983838.1	6,00E-47	GLOS_DGRI_GH1	GO:0051050	B	.	positive regulation of transport
XP_001983838.1 GH16119 [Drosophila grimshawi]	XP_001983838.1	6,00E-47	GLOS_DGRI_GH1	GO:0048518	B	.	positive regulation of biological process
XP_001983838.1 GH16119 [Drosophila grimshawi]	XP_001983838.1	6,00E-47	GLOS_DGRI_GH1	GO:0032846	B	.	positive regulation of homeostatic process
XP_001983838.1 GH16119 [Drosophila grimshawi]	XP_001983838.1	6,00E-47	GLOS_DGRI_GH1	GO:0048522	B	.	positive regulation of cellular process
XP_001983838.1 GH16119 [Drosophila grimshawi]	XP_001983838.1	6,00E-47	GLOS_DGRI_GH1	GO:0051928	B	.	positive regulation of calcium ion transport
XP_001983838.1 GH16119 [Drosophila grimshawi]	XP_001983838.1	6,00E-47	GLOS_DGRI_GH1	GO:0043270	B	.	positive regulation of ion transport
XP_001983838.1 GH16119 [Drosophila grimshawi]	XP_001983838.1	6,00E-47	GLOS_DGRI_GH1	GO:0010524	B	Refseq	positive regulation of calcium ion transport into cytosol
XP_001983838.1 GH16119 [Drosophila grimshawi]	XP_001983838.1	6,00E-47	GLOS_DGRI_GH1	GO:0005262	M	.	calcium channel activity
XP_001983838.1 GH16119 [Drosophila grimshawi]	XP_001983838.1	6,00E-47	GLOS_DGRI_GH1	GO:0015276	M	.	ligand-gated ion channel activity
XP_001983838.1 GH16119 [Drosophila grimshawi]	XP_001983838.1	6,00E-47	GLOS_DGRI_GH1	GO:0022834	M	.	ligand-gated channel activity
XP_001983838.1 GH16119 [Drosophila grimshawi]	XP_001983838.1	6,00E-47	GLOS_DGRI_GH1	GO:0022836	M	.	gated channel activity
XP_001983838.1 GH16119 [Drosophila grimshawi]	XP_001983838.1	6,00E-47	GLOS_DGRI_GH1	GO:0022839	M	.	ion gated channel activity
XP_001983838.1 GH16119 [Drosophila grimshawi]	XP_001983838.1	6,00E-47	GLOS_DGRI_GH1	GO:0015279	M	Refseq	store-operated calcium channel activity
XP_001983838.1 GH16119 [Drosophila grimshawi]	XP_001983838.1	6,00E-47	GLOS_DGRI_GH1	GO:0001775	B	.	cell activation
XP_001983838.1 GH16119 [Drosophila grimshawi]	XP_001983838.1	6,00E-47	GLOS_DGRI_GH1	GO:0007596	B	.	blood coagulation
XP_001983838.1 GH16119 [Drosophila grimshawi]	XP_001983838.1	6,00E-47	GLOS_DGRI_GH1	GO:0030168	B	Refseq	platelet activation
XP_001983838.1 GH16119 [Drosophila grimshawi]	XP_001983838.1	6,00E-47	GLOS_DGRI_GH1	GO:0014074	B	.	response to purine-containing compound
XP_001983838.1 GH16119 [Drosophila grimshawi]	XP_001983838.1	6,00E-47	GLOS_DGRI_GH1	GO:0010243	B	.	response to organonitrogen compound
XP_001983838.1 GH16119 [Drosophila grimshawi]	XP_001983838.1	6,00E-47	GLOS_DGRI_GH1	GO:0009719	B	.	response to endogenous stimulus
XP_001983838.1 GH16119 [Drosophila grimshawi]	XP_001983838.1	6,00E-47	GLOS_DGRI_GH1	GO:0010033	B	.	response to organic substance
XP_001983838.1 GH16119 [Drosophila grimshawi]	XP_001983838.1	6,00E-47	GLOS_DGRI_GH1	GO:1901698	B	.	response to nitrogen compound
XP_001983838.1 GH16119 [Drosophila grimshawi]	XP_001983838.1	6,00E-47	GLOS_DGRI_GH1	GO:0014070	B	.	response to organic cyclic compound
XP_001983838.1 GH16119 [Drosophila grimshawi]	XP_001983838.1	6,00E-47	GLOS_DGRI_GH1	GO:0046683	B	.	response to organophosphorus
XP_001983838.1 GH16119 [Drosophila grimshawi]	XP_001983838.1	6,00E-47	GLOS_DGRI_GH1	GO:1901700	B	.	response to oxygen-containing compound
XP_001983838.1 GH16119 [Drosophila grimshawi]	XP_001983838.1	6,00E-47	GLOS_DGRI_GH1	GO:0033198	B	Refseq	response to ATP
XP_001983838.1 GH16119 [Drosophila grimshawi]	XP_001983838.1	6,00E-47	GLOS_DGRI_GH1	GO:0010038	B	.	response to metal ion
XP_001983838.1 GH16119 [Drosophila grimshawi]	XP_001983838.1	6,00E-47	GLOS_DGRI_GH1	GO:0010035	B	.	response to inorganic substance
XP_001983838.1 GH16119 [Drosophila grimshawi]	XP_001983838.1	6,00E-47	GLOS_DGRI_GH1	GO:0051592	B	Refseq	response to calcium ion
XP_001983838.1 GH16119 [Drosophila grimshawi]	XP_001983838.1	6,00E-47	GLOS_DGRI_GH1	GO:0006816	B	.	calcium ion transport
XP_001983838.1 GH16119 [Drosophila grimshawi]	XP_001983838.1	6,00E-47	GLOS_DGRI_GH1	GO:0034220	B	.	ion transmembrane transport
XP_001983838.1 GH16119 [Drosophila grimshawi]	XP_001983838.1	6,00E-47	GLOS_DGRI_GH1	GO:0055085	B	.	transmembrane transport
XP_001983838.1 GH16119 [Drosophila grimshawi]	XP_001983838.1	6,00E-47	GLOS_DGRI_GH1	GO:0070588	B	Refseq	calcium ion transmembrane transport
XP_001983838.1 GH16119 [Drosophila grimshawi]	XP_001983838.1	6,00E-47	GLOS_DGRI_GH1	GO:0043168	M	.	anion binding
XP_001983838.1 GH16119 [Drosophila grimshawi]	XP_001983838.1	6,00E-47	GLOS_DGRI_GH1	GO:0043167	M	.	ion binding
XP_001983838.1 GH16119 [Drosophila grimshawi]	XP_001983838.1	6,00E-47	GLOS_DGRI_GH1	GO:0043178	M	.	alcohol binding
XP_001983838.1 GH16119 [Drosophila grimshawi]	XP_001983838.1	6,00E-47	GLOS_DGRI_GH1	GO:0036094	M	.	small molecule binding
XP_001983838.1 GH16119 [Drosophila grimshawi]	XP_001983838.1	6,00E-47	GLOS_DGRI_GH1	GO:0070679	M	Refseq	inositol 1,4,5 trisphosphate binding

XP_001984700.1 GH16615 [Drosophila grimshawi]	XP_001984700.1	1,00E-156	GLOS_DGRI_GH1	GO:0005262	M	Refseq	calcium channel activity
XP_001984700.1 GH16615 [Drosophila grimshawi]	XP_001984700.1	1,00E-156	GLOS_DGRI_GH1	GO:0005515	M	Refseq	protein binding
XP_001984700.1 GH16615 [Drosophila grimshawi]	XP_001984700.1	1,00E-156	GLOS_DGRI_GH1	GO:0005886	C	Refseq	plasma membrane
XP_001984700.1 GH16615 [Drosophila grimshawi]	XP_001984700.1	1,00E-156	GLOS_DGRI_GH1	GO:0005887	C	Refseq	integral to plasma membrane
XP_001984700.1 GH16615 [Drosophila grimshawi]	XP_001984700.1	1,00E-156	GLOS_DGRI_GH1	GO:0006816	B	Refseq	calcium ion transport
XP_001984700.1 GH16615 [Drosophila grimshawi]	XP_001984700.1	1,00E-156	GLOS_DGRI_GH1	GO:0007411	B	Refseq	axon guidance
XP_001984700.1 GH16615 [Drosophila grimshawi]	XP_001984700.1	1,00E-156	GLOS_DGRI_GH1	GO:0007596	B	Refseq	blood coagulation
XP_001984700.1 GH16615 [Drosophila grimshawi]	XP_001984700.1	1,00E-156	GLOS_DGRI_GH1	GO:0007602	B	Refseq	phototransduction
XP_001984700.1 GH16615 [Drosophila grimshawi]	XP_001984700.1	1,00E-156	GLOS_DGRI_GH1	GO:0010524	B	Refseq	positive regulation of calcium ion transport into cytosol
XP_001984700.1 GH16615 [Drosophila grimshawi]	XP_001984700.1	1,00E-156	GLOS_DGRI_GH1	GO:0015279	M	Refseq	store-operated calcium channel activity
XP_001984700.1 GH16615 [Drosophila grimshawi]	XP_001984700.1	1,00E-156	GLOS_DGRI_GH1	GO:0030168	B	Refseq	platelet activation
XP_001984700.1 GH16615 [Drosophila grimshawi]	XP_001984700.1	1,00E-156	GLOS_DGRI_GH1	GO:0033198	B	Refseq	response to ATP
XP_001984700.1 GH16615 [Drosophila grimshawi]	XP_001984700.1	1,00E-156	GLOS_DGRI_GH1	GO:0051592	B	Refseq	response to calcium ion
XP_001984700.1 GH16615 [Drosophila grimshawi]	XP_001984700.1	1,00E-156	GLOS_DGRI_GH1	GO:0070588	B	Refseq	calcium ion transmembrane transport
XP_001984700.1 GH16615 [Drosophila grimshawi]	XP_001984700.1	1,00E-156	GLOS_DGRI_GH1	GO:0070679	M	Refseq	inositol 1,4,5 trisphosphate binding
NP_731505.1 fau, isoform C [Drosophila melanogaster]	NP_731505.1	2,00E-20	GLOS_FAU.1.1	GO:0006357	B	.	regulation of transcription from RNA polymerase II promoter
NP_731505.1 fau, isoform C [Drosophila melanogaster]	NP_731505.1	2,00E-20	GLOS_FAU.1.1	GO:0006355	B	.	regulation of transcription, DNA-dependent
NP_731505.1 fau, isoform C [Drosophila melanogaster]	NP_731505.1	2,00E-20	GLOS_FAU.1.1	GO:0010468	B	.	regulation of gene expression
NP_731505.1 fau, isoform C [Drosophila melanogaster]	NP_731505.1	2,00E-20	GLOS_FAU.1.1	GO:0060255	B	.	regulation of macromolecule metabolic process
NP_731505.1 fau, isoform C [Drosophila melanogaster]	NP_731505.1	2,00E-20	GLOS_FAU.1.1	GO:0019222	B	.	regulation of metabolic process
NP_731505.1 fau, isoform C [Drosophila melanogaster]	NP_731505.1	2,00E-20	GLOS_FAU.1.1	GO:2000112	B	.	regulation of cellular macromolecule biosynthetic process
NP_731505.1 fau, isoform C [Drosophila melanogaster]	NP_731505.1	2,00E-20	GLOS_FAU.1.1	GO:0010556	B	.	regulation of macromolecule biosynthetic process
NP_731505.1 fau, isoform C [Drosophila melanogaster]	NP_731505.1	2,00E-20	GLOS_FAU.1.1	GO:0009889	B	.	regulation of biosynthetic process
NP_731505.1 fau, isoform C [Drosophila melanogaster]	NP_731505.1	2,00E-20	GLOS_FAU.1.1	GO:0031326	B	.	regulation of cellular biosynthetic process
NP_731505.1 fau, isoform C [Drosophila melanogaster]	NP_731505.1	2,00E-20	GLOS_FAU.1.1	GO:0031323	B	.	regulation of cellular metabolic process
NP_731505.1 fau, isoform C [Drosophila melanogaster]	NP_731505.1	2,00E-20	GLOS_FAU.1.1	GO:2001141	B	.	regulation of RNA biosynthetic process
NP_731505.1 fau, isoform C [Drosophila melanogaster]	NP_731505.1	2,00E-20	GLOS_FAU.1.1	GO:0051252	B	.	regulation of RNA metabolic process
NP_731505.1 fau, isoform C [Drosophila melanogaster]	NP_731505.1	2,00E-20	GLOS_FAU.1.1	GO:0019219	B	.	regulation of nucleobase-containing compound metabolic process
NP_731505.1 fau, isoform C [Drosophila melanogaster]	NP_731505.1	2,00E-20	GLOS_FAU.1.1	GO:0051171	B	.	regulation of nitrogen compound metabolic process
NP_731505.1 fau, isoform C [Drosophila melanogaster]	NP_731505.1	2,00E-20	GLOS_FAU.1.1	GO:0080090	B	.	regulation of primary metabolic process
NP_731505.1 fau, isoform C [Drosophila melanogaster]	NP_731505.1	2,00E-20	GLOS_FAU.1.1	GO:0045892	B	.	negative regulation of transcription, DNA-dependent
NP_731505.1 fau, isoform C [Drosophila melanogaster]	NP_731505.1	2,00E-20	GLOS_FAU.1.1	GO:0010629	B	.	negative regulation of gene expression
NP_731505.1 fau, isoform C [Drosophila melanogaster]	NP_731505.1	2,00E-20	GLOS_FAU.1.1	GO:0010605	B	.	negative regulation of macromolecule metabolic process
NP_731505.1 fau, isoform C [Drosophila melanogaster]	NP_731505.1	2,00E-20	GLOS_FAU.1.1	GO:0009892	B	.	negative regulation of metabolic process
NP_731505.1 fau, isoform C [Drosophila melanogaster]	NP_731505.1	2,00E-20	GLOS_FAU.1.1	GO:0048519	B	.	negative regulation of biological process
NP_731505.1 fau, isoform C [Drosophila melanogaster]	NP_731505.1	2,00E-20	GLOS_FAU.1.1	GO:0051253	B	.	negative regulation of RNA metabolic process
NP_731505.1 fau, isoform C [Drosophila melanogaster]	NP_731505.1	2,00E-20	GLOS_FAU.1.1	GO:0045934	B	.	negative regulation of nucleobase-containing compound metabolic process
NP_731505.1 fau, isoform C [Drosophila melanogaster]	NP_731505.1	2,00E-20	GLOS_FAU.1.1	GO:0031324	B	.	negative regulation of cellular metabolic process
NP_731505.1 fau, isoform C [Drosophila melanogaster]	NP_731505.1	2,00E-20	GLOS_FAU.1.1	GO:0048523	B	.	negative regulation of cellular process
NP_731505.1 fau, isoform C [Drosophila melanogaster]	NP_731505.1	2,00E-20	GLOS_FAU.1.1	GO:0051172	B	.	negative regulation of nitrogen compound metabolic process
NP_731505.1 fau, isoform C [Drosophila melanogaster]	NP_731505.1	2,00E-20	GLOS_FAU.1.1	GO:2000113	B	.	negative regulation of cellular macromolecule biosynthetic process
NP_731505.1 fau, isoform C [Drosophila melanogaster]	NP_731505.1	2,00E-20	GLOS_FAU.1.1	GO:0010558	B	.	negative regulation of macromolecule biosynthetic process
NP_731505.1 fau, isoform C [Drosophila melanogaster]	NP_731505.1	2,00E-20	GLOS_FAU.1.1	GO:0009890	B	.	negative regulation of biosynthetic process
NP_731505.1 fau, isoform C [Drosophila melanogaster]	NP_731505.1	2,00E-20	GLOS_FAU.1.1	GO:0031327	B	.	negative regulation of cellular biosynthetic process
NP_731505.1 fau, isoform C [Drosophila melanogaster]	NP_731505.1	2,00E-20	GLOS_FAU.1.1	GO:0000122	B	Refseq	negative regulation of transcription from RNA polymerase II promoter
NP_731505.1 fau, isoform C [Drosophila melanogaster]	NP_731505.1	2,00E-20	GLOS_FAU.1.1	GO:0001501	B	Refseq	skeletal system development
NP_731505.1 fau, isoform C [Drosophila melanogaster]	NP_731505.1	2,00E-20	GLOS_FAU.1.1	GO:0001071	M	.	nucleic acid binding transcription factor activity
NP_731505.1 fau, isoform C [Drosophila melanogaster]	NP_731505.1	2,00E-20	GLOS_FAU.1.1	GO:0003700	M	Refseq	sequence-specific DNA binding transcription factor activity
NP_731505.1 fau, isoform C [Drosophila melanogaster]	NP_731505.1	2,00E-20	GLOS_FAU.1.1	GO:0005515	M	Refseq	protein binding
NP_731505.1 fau, isoform C [Drosophila melanogaster]	NP_731505.1	2,00E-20	GLOS_FAU.1.1	GO:0043231	C	.	intracellular membrane-bounded organelle
NP_731505.1 fau, isoform C [Drosophila melanogaster]	NP_731505.1	2,00E-20	GLOS_FAU.1.1	GO:0043227	C	.	membrane-bounded organelle
NP_731505.1 fau, isoform C [Drosophila melanogaster]	NP_731505.1	2,00E-20	GLOS_FAU.1.1	GO:0043226	C	.	organelle
NP_731505.1 fau, isoform C [Drosophila melanogaster]	NP_731505.1	2,00E-20	GLOS_FAU.1.1	GO:0043229	C	.	intracellular organelle
NP_731505.1 fau, isoform C [Drosophila melanogaster]	NP_731505.1	2,00E-20	GLOS_FAU.1.1	GO:0044424	C	.	intracellular part

NP_731505.1 fau, isoform C [Drosophila melanogaster]	NP_731505.1	2,00E-20	GLOS_FAU.1.1	GO:0005622	C	.	intracellular
NP_731505.1 fau, isoform C [Drosophila melanogaster]	NP_731505.1	2,00E-20	GLOS_FAU.1.1	GO:0005634	C	Refseq	nucleus
NP_731505.1 fau, isoform C [Drosophila melanogaster]	NP_731505.1	2,00E-20	GLOS_FAU.1.1	GO:0006351	B	.	transcription, DNA-dependent
NP_731505.1 fau, isoform C [Drosophila melanogaster]	NP_731505.1	2,00E-20	GLOS_FAU.1.1	GO:0032774	B	.	RNA biosynthetic process
NP_731505.1 fau, isoform C [Drosophila melanogaster]	NP_731505.1	2,00E-20	GLOS_FAU.1.1	GO:0009059	B	.	macromolecule biosynthetic process
NP_731505.1 fau, isoform C [Drosophila melanogaster]	NP_731505.1	2,00E-20	GLOS_FAU.1.1	GO:0043170	B	.	macromolecule metabolic process
NP_731505.1 fau, isoform C [Drosophila melanogaster]	NP_731505.1	2,00E-20	GLOS_FAU.1.1	GO:0071704	B	.	organic substance metabolic process
NP_731505.1 fau, isoform C [Drosophila melanogaster]	NP_731505.1	2,00E-20	GLOS_FAU.1.1	GO:0008152	B	.	metabolic process
NP_731505.1 fau, isoform C [Drosophila melanogaster]	NP_731505.1	2,00E-20	GLOS_FAU.1.1	GO:1901576	B	.	organic substance biosynthetic process
NP_731505.1 fau, isoform C [Drosophila melanogaster]	NP_731505.1	2,00E-20	GLOS_FAU.1.1	GO:0009058	B	.	biosynthetic process
NP_731505.1 fau, isoform C [Drosophila melanogaster]	NP_731505.1	2,00E-20	GLOS_FAU.1.1	GO:0016070	B	.	RNA metabolic process
NP_731505.1 fau, isoform C [Drosophila melanogaster]	NP_731505.1	2,00E-20	GLOS_FAU.1.1	GO:0044260	B	.	cellular macromolecule metabolic process
NP_731505.1 fau, isoform C [Drosophila melanogaster]	NP_731505.1	2,00E-20	GLOS_FAU.1.1	GO:0044237	B	.	cellular metabolic process
NP_731505.1 fau, isoform C [Drosophila melanogaster]	NP_731505.1	2,00E-20	GLOS_FAU.1.1	GO:0090304	B	.	nucleic acid metabolic process
NP_731505.1 fau, isoform C [Drosophila melanogaster]	NP_731505.1	2,00E-20	GLOS_FAU.1.1	GO:0006139	B	.	nucleobase-containing compound metabolic process
NP_731505.1 fau, isoform C [Drosophila melanogaster]	NP_731505.1	2,00E-20	GLOS_FAU.1.1	GO:0006725	B	.	cellular aromatic compound metabolic process
NP_731505.1 fau, isoform C [Drosophila melanogaster]	NP_731505.1	2,00E-20	GLOS_FAU.1.1	GO:0034641	B	.	cellular nitrogen compound metabolic process
NP_731505.1 fau, isoform C [Drosophila melanogaster]	NP_731505.1	2,00E-20	GLOS_FAU.1.1	GO:0006807	B	.	nitrogen compound metabolic process
NP_731505.1 fau, isoform C [Drosophila melanogaster]	NP_731505.1	2,00E-20	GLOS_FAU.1.1	GO:0044238	B	.	primary metabolic process
NP_731505.1 fau, isoform C [Drosophila melanogaster]	NP_731505.1	2,00E-20	GLOS_FAU.1.1	GO:0046483	B	.	heterocycle metabolic process
NP_731505.1 fau, isoform C [Drosophila melanogaster]	NP_731505.1	2,00E-20	GLOS_FAU.1.1	GO:1901360	B	.	organic cyclic compound metabolic process
NP_731505.1 fau, isoform C [Drosophila melanogaster]	NP_731505.1	2,00E-20	GLOS_FAU.1.1	GO:0034654	B	.	nucleobase-containing compound biosynthetic process
NP_731505.1 fau, isoform C [Drosophila melanogaster]	NP_731505.1	2,00E-20	GLOS_FAU.1.1	GO:0018130	B	.	heterocycle biosynthetic process
NP_731505.1 fau, isoform C [Drosophila melanogaster]	NP_731505.1	2,00E-20	GLOS_FAU.1.1	GO:0044249	B	.	cellular biosynthetic process
NP_731505.1 fau, isoform C [Drosophila melanogaster]	NP_731505.1	2,00E-20	GLOS_FAU.1.1	GO:0019438	B	.	aromatic compound biosynthetic process
NP_731505.1 fau, isoform C [Drosophila melanogaster]	NP_731505.1	2,00E-20	GLOS_FAU.1.1	GO:0044271	B	.	cellular nitrogen compound biosynthetic process
NP_731505.1 fau, isoform C [Drosophila melanogaster]	NP_731505.1	2,00E-20	GLOS_FAU.1.1	GO:1901362	B	.	organic cyclic compound biosynthetic process
NP_731505.1 fau, isoform C [Drosophila melanogaster]	NP_731505.1	2,00E-20	GLOS_FAU.1.1	GO:0034645	B	.	cellular macromolecule biosynthetic process
NP_731505.1 fau, isoform C [Drosophila melanogaster]	NP_731505.1	2,00E-20	GLOS_FAU.1.1	GO:0010467	B	.	gene expression
NP_731505.1 fau, isoform C [Drosophila melanogaster]	NP_731505.1	2,00E-20	GLOS_FAU.1.1	GO:0006366	B	Refseq	transcription from RNA polymerase II promoter
NP_731505.1 fau, isoform C [Drosophila melanogaster]	NP_731505.1	2,00E-20	GLOS_FAU.1.1	GO:0006606	B	.	protein import into nucleus
NP_731505.1 fau, isoform C [Drosophila melanogaster]	NP_731505.1	2,00E-20	GLOS_FAU.1.1	GO:0006886	B	.	intracellular protein transport
NP_731505.1 fau, isoform C [Drosophila melanogaster]	NP_731505.1	2,00E-20	GLOS_FAU.1.1	GO:0015031	B	.	protein transport
NP_731505.1 fau, isoform C [Drosophila melanogaster]	NP_731505.1	2,00E-20	GLOS_FAU.1.1	GO:0045184	B	.	establishment of protein localization
NP_731505.1 fau, isoform C [Drosophila melanogaster]	NP_731505.1	2,00E-20	GLOS_FAU.1.1	GO:0008104	B	.	protein localization
NP_731505.1 fau, isoform C [Drosophila melanogaster]	NP_731505.1	2,00E-20	GLOS_FAU.1.1	GO:0033036	B	.	macromolecule localization
NP_731505.1 fau, isoform C [Drosophila melanogaster]	NP_731505.1	2,00E-20	GLOS_FAU.1.1	GO:0071702	B	.	organic substance transport
NP_731505.1 fau, isoform C [Drosophila melanogaster]	NP_731505.1	2,00E-20	GLOS_FAU.1.1	GO:0046907	B	.	intracellular transport
NP_731505.1 fau, isoform C [Drosophila melanogaster]	NP_731505.1	2,00E-20	GLOS_FAU.1.1	GO:0051649	B	.	establishment of localization in cell
NP_731505.1 fau, isoform C [Drosophila melanogaster]	NP_731505.1	2,00E-20	GLOS_FAU.1.1	GO:0051641	B	.	cellular localization
NP_731505.1 fau, isoform C [Drosophila melanogaster]	NP_731505.1	2,00E-20	GLOS_FAU.1.1	GO:0034613	B	.	cellular protein localization
NP_731505.1 fau, isoform C [Drosophila melanogaster]	NP_731505.1	2,00E-20	GLOS_FAU.1.1	GO:0070727	B	.	cellular macromolecule localization
NP_731505.1 fau, isoform C [Drosophila melanogaster]	NP_731505.1	2,00E-20	GLOS_FAU.1.1	GO:0017038	B	.	protein import
NP_731505.1 fau, isoform C [Drosophila melanogaster]	NP_731505.1	2,00E-20	GLOS_FAU.1.1	GO:0051170	B	.	nuclear import
NP_731505.1 fau, isoform C [Drosophila melanogaster]	NP_731505.1	2,00E-20	GLOS_FAU.1.1	GO:0006913	B	.	nucleocytoplasmic transport
NP_731505.1 fau, isoform C [Drosophila melanogaster]	NP_731505.1	2,00E-20	GLOS_FAU.1.1	GO:0016482	B	.	cytoplasmic transport
NP_731505.1 fau, isoform C [Drosophila melanogaster]	NP_731505.1	2,00E-20	GLOS_FAU.1.1	GO:0051169	B	.	nuclear transport
NP_731505.1 fau, isoform C [Drosophila melanogaster]	NP_731505.1	2,00E-20	GLOS_FAU.1.1	GO:0072594	B	.	establishment of protein localization to organelle
NP_731505.1 fau, isoform C [Drosophila melanogaster]	NP_731505.1	2,00E-20	GLOS_FAU.1.1	GO:0033365	B	.	protein localization to organelle
NP_731505.1 fau, isoform C [Drosophila melanogaster]	NP_731505.1	2,00E-20	GLOS_FAU.1.1	GO:0034504	B	.	protein localization to nucleus
NP_731505.1 fau, isoform C [Drosophila melanogaster]	NP_731505.1	2,00E-20	GLOS_FAU.1.1	GO:0044744	B	.	protein targeting to nucleus
NP_731505.1 fau, isoform C [Drosophila melanogaster]	NP_731505.1	2,00E-20	GLOS_FAU.1.1	GO:0006605	B	.	protein targeting
NP_731505.1 fau, isoform C [Drosophila melanogaster]	NP_731505.1	2,00E-20	GLOS_FAU.1.1	GO:0006607	B	Refseq	NLS-bearing protein import into nucleus
NP_731505.1 fau, isoform C [Drosophila melanogaster]	NP_731505.1	2,00E-20	GLOS_FAU.1.1	GO:0046914	M	.	transition metal ion binding

NP_731505.1 fau, isoform C [Drosophila melanogaster]	NP_731505.1	2,00E-20	GLOS_FAU.1.1	GO:0046872	M	.	metal ion binding
NP_731505.1 fau, isoform C [Drosophila melanogaster]	NP_731505.1	2,00E-20	GLOS_FAU.1.1	GO:0043169	M	.	cation binding
NP_731505.1 fau, isoform C [Drosophila melanogaster]	NP_731505.1	2,00E-20	GLOS_FAU.1.1	GO:0008270	M	Refseq	zinc ion binding
NP_731505.1 fau, isoform C [Drosophila melanogaster]	NP_731505.1	2,00E-20	GLOS_FAU.1.1	GO:0045595	B	.	regulation of cell differentiation
NP_731505.1 fau, isoform C [Drosophila melanogaster]	NP_731505.1	2,00E-20	GLOS_FAU.1.1	GO:0050793	B	.	regulation of developmental process
NP_731505.1 fau, isoform C [Drosophila melanogaster]	NP_731505.1	2,00E-20	GLOS_FAU.1.1	GO:0061035	B	.	regulation of cartilage development
NP_731505.1 fau, isoform C [Drosophila melanogaster]	NP_731505.1	2,00E-20	GLOS_FAU.1.1	GO:2000026	B	.	regulation of multicellular organismal development
NP_731505.1 fau, isoform C [Drosophila melanogaster]	NP_731505.1	2,00E-20	GLOS_FAU.1.1	GO:0051239	B	.	regulation of multicellular organismal process
NP_731505.1 fau, isoform C [Drosophila melanogaster]	NP_731505.1	2,00E-20	GLOS_FAU.1.1	GO:0032330	B	Refseq	regulation of chondrocyte differentiation
NP_731505.1 fau, isoform C [Drosophila melanogaster]	NP_731505.1	2,00E-20	GLOS_FAU.1.1	GO:0003677	M	.	DNA binding
NP_731505.1 fau, isoform C [Drosophila melanogaster]	NP_731505.1	2,00E-20	GLOS_FAU.1.1	GO:0003676	M	.	nucleic acid binding
NP_731505.1 fau, isoform C [Drosophila melanogaster]	NP_731505.1	2,00E-20	GLOS_FAU.1.1	GO:0097159	M	.	organic cyclic compound binding
NP_731505.1 fau, isoform C [Drosophila melanogaster]	NP_731505.1	2,00E-20	GLOS_FAU.1.1	GO:1901363	M	.	heterocyclic compound binding
NP_731505.1 fau, isoform C [Drosophila melanogaster]	NP_731505.1	2,00E-20	GLOS_FAU.1.1	GO:0043565	M	Refseq	sequence-specific DNA binding
NP_001246157.1 missing-in-metastasis, isoform H [D. melanogaster]	NP_001246157.1	1,00E-87	GLOS_contig_005	GO:0000122	B	Refseq	negative regulation of transcription from RNA polymerase II promoter
NP_001246157.1 missing-in-metastasis, isoform H [D. melanogaster]	NP_001246157.1	1,00E-87	GLOS_contig_005	GO:0001501	B	Refseq	skeletal system development
NP_001246157.1 missing-in-metastasis, isoform H [D. melanogaster]	NP_001246157.1	1,00E-87	GLOS_contig_005	GO:0003700	M	Refseq	sequence-specific DNA binding transcription factor activity
NP_001246157.1 missing-in-metastasis, isoform H [D. melanogaster]	NP_001246157.1	1,00E-87	GLOS_contig_005	GO:0005515	M	Refseq	protein binding
NP_001246157.1 missing-in-metastasis, isoform H [D. melanogaster]	NP_001246157.1	1,00E-87	GLOS_contig_005	GO:0005634	C	Refseq	nucleus
NP_001246157.1 missing-in-metastasis, isoform H [D. melanogaster]	NP_001246157.1	1,00E-87	GLOS_contig_005	GO:0006366	B	Refseq	transcription from RNA polymerase II promoter
NP_001246157.1 missing-in-metastasis, isoform H [D. melanogaster]	NP_001246157.1	1,00E-87	GLOS_contig_005	GO:0006607	B	Refseq	NLS-bearing protein import into nucleus
NP_001246157.1 missing-in-metastasis, isoform H [D. melanogaster]	NP_001246157.1	1,00E-87	GLOS_contig_005	GO:0008270	M	Refseq	zinc ion binding
NP_001246157.1 missing-in-metastasis, isoform H [D. melanogaster]	NP_001246157.1	1,00E-87	GLOS_contig_005	GO:0032330	B	Refseq	regulation of chondrocyte differentiation
NP_001246157.1 missing-in-metastasis, isoform H [D. melanogaster]	NP_001246157.1	1,00E-87	GLOS_contig_005	GO:0043565	M	Refseq	sequence-specific DNA binding
XP_001992234.1 GH24314 [Drosophila grimshawi]	XP_001992234.1	2,00E-34	GLOS_DGRI_GH2	GO:0005262	M	Refseq	calcium channel activity
XP_001992234.1 GH24314 [Drosophila grimshawi]	XP_001992234.1	2,00E-34	GLOS_DGRI_GH2	GO:0005515	M	Refseq	protein binding
XP_001992234.1 GH24314 [Drosophila grimshawi]	XP_001992234.1	2,00E-34	GLOS_DGRI_GH2	GO:0005886	C	Refseq	plasma membrane
XP_001992234.1 GH24314 [Drosophila grimshawi]	XP_001992234.1	2,00E-34	GLOS_DGRI_GH2	GO:0005887	C	Refseq	integral to plasma membrane
XP_001992234.1 GH24314 [Drosophila grimshawi]	XP_001992234.1	2,00E-34	GLOS_DGRI_GH2	GO:0006816	B	Refseq	calcium ion transport
XP_001992234.1 GH24314 [Drosophila grimshawi]	XP_001992234.1	2,00E-34	GLOS_DGRI_GH2	GO:0007411	B	Refseq	axon guidance
XP_001992234.1 GH24314 [Drosophila grimshawi]	XP_001992234.1	2,00E-34	GLOS_DGRI_GH2	GO:0007596	B	Refseq	blood coagulation
XP_001992234.1 GH24314 [Drosophila grimshawi]	XP_001992234.1	2,00E-34	GLOS_DGRI_GH2	GO:0007602	B	Refseq	phototransduction
XP_001992234.1 GH24314 [Drosophila grimshawi]	XP_001992234.1	2,00E-34	GLOS_DGRI_GH2	GO:0010524	B	Refseq	positive regulation of calcium ion transport into cytosol
XP_001992234.1 GH24314 [Drosophila grimshawi]	XP_001992234.1	2,00E-34	GLOS_DGRI_GH2	GO:0015279	M	Refseq	store-operated calcium channel activity
XP_001992234.1 GH24314 [Drosophila grimshawi]	XP_001992234.1	2,00E-34	GLOS_DGRI_GH2	GO:0030168	B	Refseq	platelet activation
XP_001992234.1 GH24314 [Drosophila grimshawi]	XP_001992234.1	2,00E-34	GLOS_DGRI_GH2	GO:0033198	B	Refseq	response to ATP
XP_001992234.1 GH24314 [Drosophila grimshawi]	XP_001992234.1	2,00E-34	GLOS_DGRI_GH2	GO:0051592	B	Refseq	response to calcium ion
XP_001992234.1 GH24314 [Drosophila grimshawi]	XP_001992234.1	2,00E-34	GLOS_DGRI_GH2	GO:0070588	B	Refseq	calcium ion transmembrane transport
XP_001992234.1 GH24314 [Drosophila grimshawi]	XP_001992234.1	2,00E-34	GLOS_DGRI_GH2	GO:0070679	M	Refseq	inositol 1,4,5 trisphosphate binding
NP_001097854.1 SNF4/AMP-activated protein kinase gamma subunit, isoform M [D. melanogaster] ref|NP_001097855.1| SNF4/AMP-activated protein kinase gamma subunit, isoform N [D. mel.] ref|NP_001036736.2| SNF4/AMP-activated protein kinase gamma subunit, isoform O [D. melanogaster]	NP_001097854.1	9,00E-25	GLOS_SNF4AGAI	GO:0000122	B	Refseq	negative regulation of transcription from RNA polymerase II promoter
NP_001097854.1 SNF4/AMP-activated protein kinase … Same as above	NP_001097854.1	9,00E-25	GLOS_SNF4AGAI	GO:0001501	B	Refseq	skeletal system development
NP_001097854.1 SNF4/AMP-activated protein kinase … Same as above	NP_001097854.1	9,00E-25	GLOS_SNF4AGAI	GO:0003700	M	Refseq	sequence-specific DNA binding transcription factor activity
NP_001097854.1 SNF4/AMP-activated protein kinase … Same as above	NP_001097854.1	9,00E-25	GLOS_SNF4AGAI	GO:0005515	M	Refseq	protein binding
NP_001097854.1 SNF4/AMP-activated protein kinase … Same as above	NP_001097854.1	9,00E-25	GLOS_SNF4AGAI	GO:0005634	C	Refseq	nucleus
NP_001097854.1 SNF4/AMP-activated protein kinase … Same as above	NP_001097854.1	9,00E-25	GLOS_SNF4AGAI	GO:0006366	B	Refseq	transcription from RNA polymerase II promoter
NP_001097854.1 SNF4/AMP-activated protein kinase … Same as above	NP_001097854.1	9,00E-25	GLOS_SNF4AGAI	GO:0006607	B	Refseq	NLS-bearing protein import into nucleus
NP_001097854.1 SNF4/AMP-activated protein kinase … Same as above	NP_001097854.1	9,00E-25	GLOS_SNF4AGAI	GO:0008270	M	Refseq	zinc ion binding
NP_001097854.1 SNF4/AMP-activated protein kinase … Same as above	NP_001097854.1	9,00E-25	GLOS_SNF4AGAI	GO:0032330	B	Refseq	regulation of chondrocyte differentiation
NP_001097854.1 SNF4/AMP-activated protein kinase … Same as above	NP_001097854.1	9,00E-25	GLOS_SNF4AGAI	GO:0043565	M	Refseq	sequence-specific DNA binding
XR_046822.1 Drosophila erecta GG25432 (DereGG25432), ncRNA	XR_046822.1	7,00E-60	GLOS_DERE_GG	GO:0005261	M	Refseq	cation channel activity
XR_046822.1 Drosophila erecta GG25432 (DereGG25432), ncRNA	XR_046822.1	7,00E-60	GLOS_DERE_GG	GO:0005262	M	Refseq	calcium channel activity

XR_046822.1 Drosophila erecta GG25432 (DereGG25432), ncRNA	XR_046822.1	7,00E-60	GLOS_DERE_GG	GO:0005515	M	Refseq	protein binding
XR_046822.1 Drosophila erecta GG25432 (DereGG25432), ncRNA	XR_046822.1	7,00E-60	GLOS_DERE_GG	GO:0005886	C	Refseq	plasma membrane
XR_046822.1 Drosophila erecta GG25432 (DereGG25432), ncRNA	XR_046822.1	7,00E-60	GLOS_DERE_GG	GO:0005887	C	Refseq	integral to plasma membrane
XR_046822.1 Drosophila erecta GG25432 (DereGG25432), ncRNA	XR_046822.1	7,00E-60	GLOS_DERE_GG	GO:0006816	B	Refseq	calcium ion transport
XR_046822.1 Drosophila erecta GG25432 (DereGG25432), ncRNA	XR_046822.1	7,00E-60	GLOS_DERE_GG	GO:0007411	B	Refseq	axon guidance
XR_046822.1 Drosophila erecta GG25432 (DereGG25432), ncRNA	XR_046822.1	7,00E-60	GLOS_DERE_GG	GO:0015279	M	Refseq	store-operated calcium channel activity
XR_046822.1 Drosophila erecta GG25432 (DereGG25432), ncRNA	XR_046822.1	7,00E-60	GLOS_DERE_GG	GO:0016323	C	Refseq	basolateral plasma membrane
XR_046822.1 Drosophila erecta GG25432 (DereGG25432), ncRNA	XR_046822.1	7,00E-60	GLOS_DERE_GG	GO:0044449	C	.	contractile fiber part
XR_046822.1 Drosophila erecta GG25432 (DereGG25432), ncRNA	XR_046822.1	7,00E-60	GLOS_DERE_GG	GO:0044422	C	.	organelle part
XR_046822.1 Drosophila erecta GG25432 (DereGG25432), ncRNA	XR_046822.1	7,00E-60	GLOS_DERE_GG	GO:0044444	C	.	cytoplasmic part
XR_046822.1 Drosophila erecta GG25432 (DereGG25432), ncRNA	XR_046822.1	7,00E-60	GLOS_DERE_GG	GO:0005737	C	.	cytoplasm
XR_046822.1 Drosophila erecta GG25432 (DereGG25432), ncRNA	XR_046822.1	7,00E-60	GLOS_DERE_GG	GO:0043292	C	.	contractile fiber
XR_046822.1 Drosophila erecta GG25432 (DereGG25432), ncRNA	XR_046822.1	7,00E-60	GLOS_DERE_GG	GO:0043232	C	.	intracellular non-membrane-bounded organelle
XR_046822.1 Drosophila erecta GG25432 (DereGG25432), ncRNA	XR_046822.1	7,00E-60	GLOS_DERE_GG	GO:0043228	C	.	non-membrane-bounded organelle
XR_046822.1 Drosophila erecta GG25432 (DereGG25432), ncRNA	XR_046822.1	7,00E-60	GLOS_DERE_GG	GO:0030016	C	.	myofibril
XR_046822.1 Drosophila erecta GG25432 (DereGG25432), ncRNA	XR_046822.1	7,00E-60	GLOS_DERE_GG	GO:0030017	C	Refseq	sarcomere
XR_046822.1 Drosophila erecta GG25432 (DereGG25432), ncRNA	XR_046822.1	7,00E-60	GLOS_DERE_GG	GO:0043034	C	Refseq	costamere
XR_046822.1 Drosophila erecta GG25432 (DereGG25432), ncRNA	XR_046822.1	7,00E-60	GLOS_DERE_GG	GO:0032991	C	.	macromolecular complex
XR_046822.1 Drosophila erecta GG25432 (DereGG25432), ncRNA	XR_046822.1	7,00E-60	GLOS_DERE_GG	GO:0043234	C	Refseq	protein complex
XR_046822.1 Drosophila erecta GG25432 (DereGG25432), ncRNA	XR_046822.1	7,00E-60	GLOS_DERE_GG	GO:0005515	M	.	protein binding
XR_046822.1 Drosophila erecta GG25432 (DereGG25432), ncRNA	XR_046822.1	7,00E-60	GLOS_DERE_GG	GO:0044325	M	Refseq	ion channel binding
XR_046822.1 Drosophila erecta GG25432 (DereGG25432), ncRNA	XR_046822.1	7,00E-60	GLOS_DERE_GG	GO:0045121	C	Refseq	membrane raft
XR_046822.1 Drosophila erecta GG25432 (DereGG25432), ncRNA	XR_046822.1	7,00E-60	GLOS_DERE_GG	GO:0007589	B	.	body fluid secretion
XR_046822.1 Drosophila erecta GG25432 (DereGG25432), ncRNA	XR_046822.1	7,00E-60	GLOS_DERE_GG	GO:0046903	B	.	secretion
XR_046822.1 Drosophila erecta GG25432 (DereGG25432), ncRNA	XR_046822.1	7,00E-60	GLOS_DERE_GG	GO:0022600	B	.	digestive system process
XR_046822.1 Drosophila erecta GG25432 (DereGG25432), ncRNA	XR_046822.1	7,00E-60	GLOS_DERE_GG	GO:0003008	B	.	system process
XR_046822.1 Drosophila erecta GG25432 (DereGG25432), ncRNA	XR_046822.1	7,00E-60	GLOS_DERE_GG	GO:0007586	B	.	digestion
XR_046822.1 Drosophila erecta GG25432 (DereGG25432), ncRNA	XR_046822.1	7,00E-60	GLOS_DERE_GG	GO:0032941	B	.	secretion by tissue
XR_046822.1 Drosophila erecta GG25432 (DereGG25432), ncRNA	XR_046822.1	7,00E-60	GLOS_DERE_GG	GO:0046541	B	Refseq	saliva secretion
XR_046822.1 Drosophila erecta GG25432 (DereGG25432), ncRNA	XR_046822.1	7,00E-60	GLOS_DERE_GG	GO:0010524	B	.	positive regulation of calcium ion transport into cytosol
XR_046822.1 Drosophila erecta GG25432 (DereGG25432), ncRNA	XR_046822.1	7,00E-60	GLOS_DERE_GG	GO:0051279	B	.	regulation of release of sequestered calcium ion into cytosol
XR_046822.1 Drosophila erecta GG25432 (DereGG25432), ncRNA	XR_046822.1	7,00E-60	GLOS_DERE_GG	GO:0051281	B	Refseq	positive regulation of release of sequestered calcium ion into cytosol
XR_046822.1 Drosophila erecta GG25432 (DereGG25432), ncRNA	XR_046822.1	7,00E-60	GLOS_DERE_GG	GO:0006874	B	.	cellular calcium ion homeostasis
XR_046822.1 Drosophila erecta GG25432 (DereGG25432), ncRNA	XR_046822.1	7,00E-60	GLOS_DERE_GG	GO:0006875	B	.	cellular metal ion homeostasis
XR_046822.1 Drosophila erecta GG25432 (DereGG25432), ncRNA	XR_046822.1	7,00E-60	GLOS_DERE_GG	GO:0030003	B	.	cellular cation homeostasis
XR_046822.1 Drosophila erecta GG25432 (DereGG25432), ncRNA	XR_046822.1	7,00E-60	GLOS_DERE_GG	GO:0006873	B	.	cellular ion homeostasis
XR_046822.1 Drosophila erecta GG25432 (DereGG25432), ncRNA	XR_046822.1	7,00E-60	GLOS_DERE_GG	GO:0050801	B	.	ion homeostasis
XR_046822.1 Drosophila erecta GG25432 (DereGG25432), ncRNA	XR_046822.1	7,00E-60	GLOS_DERE_GG	GO:0048878	B	.	chemical homeostasis
XR_046822.1 Drosophila erecta GG25432 (DereGG25432), ncRNA	XR_046822.1	7,00E-60	GLOS_DERE_GG	GO:0042592	B	.	homeostatic process
XR_046822.1 Drosophila erecta GG25432 (DereGG25432), ncRNA	XR_046822.1	7,00E-60	GLOS_DERE_GG	GO:0055082	B	.	cellular chemical homeostasis
XR_046822.1 Drosophila erecta GG25432 (DereGG25432), ncRNA	XR_046822.1	7,00E-60	GLOS_DERE_GG	GO:0019725	B	.	cellular homeostasis
XR_046822.1 Drosophila erecta GG25432 (DereGG25432), ncRNA	XR_046822.1	7,00E-60	GLOS_DERE_GG	GO:0055080	B	.	cation homeostasis
XR_046822.1 Drosophila erecta GG25432 (DereGG25432), ncRNA	XR_046822.1	7,00E-60	GLOS_DERE_GG	GO:0055065	B	.	metal ion homeostasis
XR_046822.1 Drosophila erecta GG25432 (DereGG25432), ncRNA	XR_046822.1	7,00E-60	GLOS_DERE_GG	GO:0055074	B	.	calcium ion homeostasis
XR_046822.1 Drosophila erecta GG25432 (DereGG25432), ncRNA	XR_046822.1	7,00E-60	GLOS_DERE_GG	GO:0072507	B	.	divalent inorganic cation homeostasis
XR_046822.1 Drosophila erecta GG25432 (DereGG25432), ncRNA	XR_046822.1	7,00E-60	GLOS_DERE_GG	GO:0072503	B	.	cellular divalent inorganic cation homeostasis
XR_046822.1 Drosophila erecta GG25432 (DereGG25432), ncRNA	XR_046822.1	7,00E-60	GLOS_DERE_GG	GO:0051480	B	Refseq	cytosolic calcium ion homeostasis
XR_046822.1 Drosophila erecta GG25432 (DereGG25432), ncRNA	XR_046822.1	7,00E-60	GLOS_DERE_GG	GO:0051592	B	Refseq	response to calcium ion
XR_046822.1 Drosophila erecta GG25432 (DereGG25432), ncRNA	XR_046822.1	7,00E-60	GLOS_DERE_GG	GO:0070679	M	Refseq	inositol 1,4,5 trisphosphate binding
NP_650034.1 thiamine pyrophosphate carrier protein 1, isoform A [D. melanogaster] ref|NP_731527.1| thiamine pyrophosphate carrier protein 1, isoform B [D. melanogaster]	NP_650034.1	1,00E-156	GLOS_TPC1.1.1	GO:0000122	B	Refseq	negative regulation of transcription from RNA polymerase II promoter
NP_650034.1 thiamine pyrophosphate carrier prot. 1, isoform A , .. Same as above	NP_650034.1	1,00E-156	GLOS_TPC1.1.1	GO:0001501	B	Refseq	skeletal system development
NP_650034.1 thiamine pyrophosphate carrier prot. 1, isoform A , .. Same as above	NP_650034.1	1,00E-156	GLOS_TPC1.1.1	GO:0003700	M	Refseq	sequence-specific DNA binding transcription factor activity

NP_650034.1 thiamine pyrophosphate carrier prot. 1, isoform A , .. Same as above	NP_650034.1	1,00E-156	GLOS_TPC1.1.1	GO:0005515	M	Refseq	protein binding
NP_650034.1 thiamine pyrophosphate carrier prot. 1, isoform A , .. Same as above	NP_650034.1	1,00E-156	GLOS_TPC1.1.1	GO:0005634	C	Refseq	nucleus
NP_650034.1 thiamine pyrophosphate carrier prot. 1, isoform A , .. Same as above	NP_650034.1	1,00E-156	GLOS_TPC1.1.1	GO:0006366	B	Refseq	transcription from RNA polymerase II promoter
NP_650034.1 thiamine pyrophosphate carrier prot. 1, isoform A , .. Same as above	NP_650034.1	1,00E-156	GLOS_TPC1.1.1	GO:0006607	B	Refseq	NLS-bearing protein import into nucleus
NP_650034.1 thiamine pyrophosphate carrier prot. 1, isoform A , .. Same as above	NP_650034.1	1,00E-156	GLOS_TPC1.1.1	GO:0008270	M	Refseq	zinc ion binding
NP_650034.1 thiamine pyrophosphate carrier prot. 1, isoform A , .. Same as above	NP_650034.1	1,00E-156	GLOS_TPC1.1.1	GO:0032330	B	Refseq	regulation of chondrocyte differentiation
NP_650034.1 thiamine pyrophosphate carrier prot. 1, isoform A , .. Same as above	NP_650034.1	1,00E-156	GLOS_TPC1.1.1	GO:0043565	M	Refseq	sequence-specific DNA binding
NP_001260550.1 ribosomal protein L30, isoform D [Drosophila melanogaster]	NP_001260550.1	4,00E-27	GLOS_RPL30.1.3	GO:0000122	B	Refseq	negative regulation of transcription from RNA polymerase II promoter
NP_001260550.1 ribosomal protein L30, isoform D [Drosophila melanogaster]	NP_001260550.1	4,00E-27	GLOS_RPL30.1.3	GO:0001501	B	Refseq	skeletal system development
NP_001260550.1 ribosomal protein L30, isoform D [Drosophila melanogaster]	NP_001260550.1	4,00E-27	GLOS_RPL30.1.3	GO:0003700	M	Refseq	sequence-specific DNA binding transcription factor activity
NP_001260550.1 ribosomal protein L30, isoform D [Drosophila melanogaster]	NP_001260550.1	4,00E-27	GLOS_RPL30.1.3	GO:0005515	M	Refseq	protein binding
NP_001260550.1 ribosomal protein L30, isoform D [Drosophila melanogaster]	NP_001260550.1	4,00E-27	GLOS_RPL30.1.3	GO:0005634	C	Refseq	nucleus
NP_001260550.1 ribosomal protein L30, isoform D [Drosophila melanogaster]	NP_001260550.1	4,00E-27	GLOS_RPL30.1.3	GO:0006366	B	Refseq	transcription from RNA polymerase II promoter
NP_001260550.1 ribosomal protein L30, isoform D [Drosophila melanogaster]	NP_001260550.1	4,00E-27	GLOS_RPL30.1.3	GO:0006607	B	Refseq	NLS-bearing protein import into nucleus
NP_001260550.1 ribosomal protein L30, isoform D [Drosophila melanogaster]	NP_001260550.1	4,00E-27	GLOS_RPL30.1.3	GO:0008270	M	Refseq	zinc ion binding
NP_001260550.1 ribosomal protein L30, isoform D [Drosophila melanogaster]	NP_001260550.1	4,00E-27	GLOS_RPL30.1.3	GO:0032330	B	Refseq	regulation of chondrocyte differentiation
NP_001260550.1 ribosomal protein L30, isoform D [Drosophila melanogaster]	NP_001260550.1	4,00E-27	GLOS_RPL30.1.3	GO:0043565	M	Refseq	sequence-specific DNA binding
NP_001188589.1 maternal gene required for meiosis, isoform E [D. melanogaster] ref|NP_001259539.1] isoform D [D. melanogaster]	NP_001188589.1	9,00E-32	GLOS_MAMO.1.1	GO:0000122	B	Refseq	negative regulation of transcription from RNA polymerase II promoter
NP_001188589.1 maternal gene required for meiosis, isoform E...Same as above	NP_001188589.1	9,00E-32	GLOS_MAMO.1.1	GO:0001501	B	Refseq	skeletal system development
NP_001188589.1 maternal gene required for meiosis, isoform E...Same as above	NP_001188589.1	9,00E-32	GLOS_MAMO.1.1	GO:0003700	M	Refseq	sequence-specific DNA binding transcription factor activity
NP_001188589.1 maternal gene required for meiosis, isoform E...Same as above	NP_001188589.1	9,00E-32	GLOS_MAMO.1.1	GO:0005515	M	Refseq	protein binding
NP_001188589.1 maternal gene required for meiosis, isoform E...Same as above	NP_001188589.1	9,00E-32	GLOS_MAMO.1.1	GO:0005634	C	Refseq	nucleus
NP_001188589.1 maternal gene required for meiosis, isoform E...Same as above	NP_001188589.1	9,00E-32	GLOS_MAMO.1.1	GO:0006366	B	Refseq	transcription from RNA polymerase II promoter
NP_001188589.1 maternal gene required for meiosis, isoform E...Same as above	NP_001188589.1	9,00E-32	GLOS_MAMO.1.1	GO:0006607	B	Refseq	NLS-bearing protein import into nucleus
NP_001188589.1 maternal gene required for meiosis, isoform E...Same as above	NP_001188589.1	9,00E-32	GLOS_MAMO.1.1	GO:0008270	M	Refseq	zinc ion binding
NP_001188589.1 maternal gene required for meiosis, isoform E...Same as above	NP_001188589.1	9,00E-32	GLOS_MAMO.1.1	GO:0032330	B	Refseq	regulation of chondrocyte differentiation
NP_001188589.1 maternal gene required for meiosis, isoform E...Same as above	NP_001188589.1	9,00E-32	GLOS_MAMO.1.1	GO:0043565	M	Refseq	sequence-specific DNA binding
XP_001983524.1 GH15943 [Drosophila grimshawi]	XP_001983524.1	0	GLOS_DGRI_GH1	GO:0005262	M	Refseq	calcium channel activity
XP_001983524.1 GH15943 [Drosophila grimshawi]	XP_001983524.1	0	GLOS_DGRI_GH1	GO:0005515	M	Refseq	protein binding
XP_001983524.1 GH15943 [Drosophila grimshawi]	XP_001983524.1	0	GLOS_DGRI_GH1	GO:0005886	C	Refseq	plasma membrane
XP_001983524.1 GH15943 [Drosophila grimshawi]	XP_001983524.1	0	GLOS_DGRI_GH1	GO:0005887	C	Refseq	integral to plasma membrane
XP_001983524.1 GH15943 [Drosophila grimshawi]	XP_001983524.1	0	GLOS_DGRI_GH1	GO:0006816	B	Refseq	calcium ion transport
XP_001983524.1 GH15943 [Drosophila grimshawi]	XP_001983524.1	0	GLOS_DGRI_GH1	GO:0007411	B	Refseq	axon guidance
XP_001983524.1 GH15943 [Drosophila grimshawi]	XP_001983524.1	0	GLOS_DGRI_GH1	GO:0007596	B	Refseq	blood coagulation
XP_001983524.1 GH15943 [Drosophila grimshawi]	XP_001983524.1	0	GLOS_DGRI_GH1	GO:0007602	B	Refseq	phototransduction
XP_001983524.1 GH15943 [Drosophila grimshawi]	XP_001983524.1	0	GLOS_DGRI_GH1	GO:0010524	B	Refseq	positive regulation of calcium ion transport into cytosol
XP_001983524.1 GH15943 [Drosophila grimshawi]	XP_001983524.1	0	GLOS_DGRI_GH1	GO:0015279	M	Refseq	store-operated calcium channel activity
XP_001983524.1 GH15943 [Drosophila grimshawi]	XP_001983524.1	0	GLOS_DGRI_GH1	GO:0030168	B	Refseq	platelet activation
XP_001983524.1 GH15943 [Drosophila grimshawi]	XP_001983524.1	0	GLOS_DGRI_GH1	GO:0033198	B	Refseq	response to ATP
XP_001983524.1 GH15943 [Drosophila grimshawi]	XP_001983524.1	0	GLOS_DGRI_GH1	GO:0051592	B	Refseq	response to calcium ion
XP_001983524.1 GH15943 [Drosophila grimshawi]	XP_001983524.1	0	GLOS_DGRI_GH1	GO:0070588	B	Refseq	calcium ion transmembrane transport
XP_001983524.1 GH15943 [Drosophila grimshawi]	XP_001983524.1	0	GLOS_DGRI_GH1	GO:0070679	M	Refseq	inositol 1,4,5 trisphosphate binding
NP_608746.1 CG17221, isoform A; ref|NP_001259984.1| CG17221, isoform C	NP_608746.1	1,00E-149	GLOS_CG17221.1	GO:0000122	B	Refseq	negative regulation of transcription from RNA polymerase II promoter
NP_608746.1 CG17221, isoform A; ref|NP_001259984.1| CG17221, isoform C	NP_608746.1	1,00E-149	GLOS_CG17221.1	GO:0001501	B	Refseq	skeletal system development
NP_608746.1 CG17221, isoform A; ref|NP_001259984.1| CG17221, isoform C	NP_608746.1	1,00E-149	GLOS_CG17221.1	GO:0003700	M	Refseq	sequence-specific DNA binding transcription factor activity
NP_608746.1 CG17221, isoform A; ref|NP_001259984.1| CG17221, isoform C	NP_608746.1	1,00E-149	GLOS_CG17221.1	GO:0005515	M	Refseq	protein binding
NP_608746.1 CG17221, isoform A; ref|NP_001259984.1| CG17221, isoform C	NP_608746.1	1,00E-149	GLOS_CG17221.1	GO:0005634	C	Refseq	nucleus
NP_608746.1 CG17221, isoform A; ref|NP_001259984.1| CG17221, isoform C	NP_608746.1	1,00E-149	GLOS_CG17221.1	GO:0006366	B	Refseq	transcription from RNA polymerase II promoter
NP_608746.1 CG17221, isoform A; ref|NP_001259984.1| CG17221, isoform C	NP_608746.1	1,00E-149	GLOS_CG17221.1	GO:0006607	B	Refseq	NLS-bearing protein import into nucleus
NP_608746.1 CG17221, isoform A; ref|NP_001259984.1| CG17221, isoform C	NP_608746.1	1,00E-149	GLOS_CG17221.1	GO:0008270	M	Refseq	zinc ion binding
NP_608746.1 CG17221, isoform A; ref|NP_001259984.1| CG17221, isoform C	NP_608746.1	1,00E-149	GLOS_CG17221.1	GO:0032330	B	Refseq	regulation of chondrocyte differentiation
NP_608746.1 CG17221, isoform A; ref|NP_001259984.1| CG17221, isoform C	NP_608746.1	1,00E-149	GLOS_CG17221.1	GO:0043565	M	Refseq	sequence-specific DNA binding
XP_003246116.1 PREDICTED: hypothetical protein LOC100572450 [A. pisum]	XP_003246116.1	4,00E-98	GLOS_LOC10057	GO:0007049	B	.	cell cycle

XP_003246116.1 PREDICTED: hypothetical protein LOC100572450 [A. pisum]	XP_003246116.1	4,00E-98	GLOS_LOC10057	GO:0000278	B	Refseq	mitotic cell cycle
XP_003246116.1 PREDICTED: hypothetical protein LOC100572450 [A. pisum]	XP_003246116.1	4,00E-98	GLOS_LOC10057	GO:0003677	M	Refseq	DNA binding
XP_003246116.1 PREDICTED: hypothetical protein LOC100572450 [A. pisum]	XP_003246116.1	4,00E-98	GLOS_LOC10057	GO:0003700	M	Refseq	sequence-specific DNA binding transcription factor activity
XP_003246116.1 PREDICTED: hypothetical protein LOC100572450 [A. pisum]	XP_003246116.1	4,00E-98	GLOS_LOC10057	GO:0000989	M	.	transcription factor binding transcription factor activity
XP_003246116.1 PREDICTED: hypothetical protein LOC100572450 [A. pisum]	XP_003246116.1	4,00E-98	GLOS_LOC10057	GO:0000988	M	.	protein binding transcription factor activity
XP_003246116.1 PREDICTED: hypothetical protein LOC100572450 [A. pisum]	XP_003246116.1	4,00E-98	GLOS_LOC10057	GO:0003712	M	Refseq	transcription cofactor activity
XP_003246116.1 PREDICTED: hypothetical protein LOC100572450 [A. pisum]	XP_003246116.1	4,00E-98	GLOS_LOC10057	GO:0044428	C	.	nuclear part
XP_003246116.1 PREDICTED: hypothetical protein LOC100572450 [A. pisum]	XP_003246116.1	4,00E-98	GLOS_LOC10057	GO:0044446	C	.	intracellular organelle part
XP_003246116.1 PREDICTED: hypothetical protein LOC100572450 [A. pisum]	XP_003246116.1	4,00E-98	GLOS_LOC10057	GO:0005634	C	.	nucleus
XP_003246116.1 PREDICTED: hypothetical protein LOC100572450 [A. pisum]	XP_003246116.1	4,00E-98	GLOS_LOC10057	GO:0031981	C	.	nuclear lumen
XP_003246116.1 PREDICTED: hypothetical protein LOC100572450 [A. pisum]	XP_003246116.1	4,00E-98	GLOS_LOC10057	GO:0070013	C	.	intracellular organelle lumen
XP_003246116.1 PREDICTED: hypothetical protein LOC100572450 [A. pisum]	XP_003246116.1	4,00E-98	GLOS_LOC10057	GO:0043233	C	.	organelle lumen
XP_003246116.1 PREDICTED: hypothetical protein LOC100572450 [A. pisum]	XP_003246116.1	4,00E-98	GLOS_LOC10057	GO:0031974	C	.	membrane-enclosed lumen
XP_003246116.1 PREDICTED: hypothetical protein LOC100572450 [A. pisum]	XP_003246116.1	4,00E-98	GLOS_LOC10057	GO:0005654	C	Refseq	nucleoplasm
XP_003246116.1 PREDICTED: hypothetical protein LOC100572450 [A. pisum]	XP_003246116.1	4,00E-98	GLOS_LOC10057	GO:0043234	C	.	protein complex
XP_003246116.1 PREDICTED: hypothetical protein LOC100572450 [A. pisum]	XP_003246116.1	4,00E-98	GLOS_LOC10057	GO:0005667	C	Refseq	transcription factor complex
XP_003246116.1 PREDICTED: hypothetical protein LOC100572450 [A. pisum]	XP_003246116.1	4,00E-98	GLOS_LOC10057	GO:0006351	B	Refseq	transcription, DNA-dependent
XP_003246116.1 PREDICTED: hypothetical protein LOC100572450 [A. pisum]	XP_003246116.1	4,00E-98	GLOS_LOC10057	GO:0006352	B	.	DNA-dependent transcription, initiation
XP_003246116.1 PREDICTED: hypothetical protein LOC100572450 [A. pisum]	XP_003246116.1	4,00E-98	GLOS_LOC10057	GO:0006366	B	.	transcription from RNA polymerase II promoter
XP_003246116.1 PREDICTED: hypothetical protein LOC100572450 [A. pisum]	XP_003246116.1	4,00E-98	GLOS_LOC10057	GO:0006367	B	Refseq	transcription initiation from RNA polymerase II promoter
XP_003246116.1 PREDICTED: hypothetical protein LOC100572450 [A. pisum]	XP_003246116.1	4,00E-98	GLOS_LOC10057	GO:0007178	B	.	transmembrane receptor protein serine/threonine kinase signaling pathway
XP_003246116.1 PREDICTED: hypothetical protein LOC100572450 [A. pisum]	XP_003246116.1	4,00E-98	GLOS_LOC10057	GO:0007167	B	.	enzyme linked receptor protein signaling pathway
XP_003246116.1 PREDICTED: hypothetical protein LOC100572450 [A. pisum]	XP_003246116.1	4,00E-98	GLOS_LOC10057	GO:0007166	B	.	cell surface receptor signaling pathway
XP_003246116.1 PREDICTED: hypothetical protein LOC100572450 [A. pisum]	XP_003246116.1	4,00E-98	GLOS_LOC10057	GO:0071560	B	.	cellular response to transforming growth factor beta stimulus
XP_003246116.1 PREDICTED: hypothetical protein LOC100572450 [A. pisum]	XP_003246116.1	4,00E-98	GLOS_LOC10057	GO:0071363	B	.	cellular response to growth factor stimulus
XP_003246116.1 PREDICTED: hypothetical protein LOC100572450 [A. pisum]	XP_003246116.1	4,00E-98	GLOS_LOC10057	GO:0070848	B	.	response to growth factor stimulus
XP_003246116.1 PREDICTED: hypothetical protein LOC100572450 [A. pisum]	XP_003246116.1	4,00E-98	GLOS_LOC10057	GO:0071310	B	.	cellular response to organic substance
XP_003246116.1 PREDICTED: hypothetical protein LOC100572450 [A. pisum]	XP_003246116.1	4,00E-98	GLOS_LOC10057	GO:0070887	B	.	cellular response to chemical stimulus
XP_003246116.1 PREDICTED: hypothetical protein LOC100572450 [A. pisum]	XP_003246116.1	4,00E-98	GLOS_LOC10057	GO:0071495	B	.	cellular response to endogenous stimulus
XP_003246116.1 PREDICTED: hypothetical protein LOC100572450 [A. pisum]	XP_003246116.1	4,00E-98	GLOS_LOC10057	GO:0071559	B	.	response to transforming growth factor beta stimulus
XP_003246116.1 PREDICTED: hypothetical protein LOC100572450 [A. pisum]	XP_003246116.1	4,00E-98	GLOS_LOC10057	GO:0007179	B	Refseq	transforming growth factor beta receptor signaling pathway
XP_003246116.1 PREDICTED: hypothetical protein LOC100572450 [A. pisum]	XP_003246116.1	4,00E-98	GLOS_LOC10057	GO:0048513	B	.	organ development
XP_003246116.1 PREDICTED: hypothetical protein LOC100572450 [A. pisum]	XP_003246116.1	4,00E-98	GLOS_LOC10057	GO:0072358	B	.	cardiovascular system development
XP_003246116.1 PREDICTED: hypothetical protein LOC100572450 [A. pisum]	XP_003246116.1	4,00E-98	GLOS_LOC10057	GO:0072359	B	.	circulatory system development
XP_003246116.1 PREDICTED: hypothetical protein LOC100572450 [A. pisum]	XP_003246116.1	4,00E-98	GLOS_LOC10057	GO:0007507	B	Refseq	heart development
XP_003246116.1 PREDICTED: hypothetical protein LOC100572450 [A. pisum]	XP_003246116.1	4,00E-98	GLOS_LOC10057	GO:0008134	M	Refseq	transcription factor binding
XP_003246116.1 PREDICTED: hypothetical protein LOC100572450 [A. pisum]	XP_003246116.1	4,00E-98	GLOS_LOC10057	GO:0010467	B	Refseq	gene expression
XP_003246116.1 PREDICTED: hypothetical protein LOC100572450 [A. pisum]	XP_003246116.1	4,00E-98	GLOS_LOC10057	GO:0019904	M	Refseq	protein domain specific binding
XP_003246116.1 PREDICTED: hypothetical protein LOC100572450 [A. pisum]	XP_003246116.1	4,00E-98	GLOS_LOC10057	GO:0045893	B	.	positive regulation of transcription, DNA-dependent
XP_003246116.1 PREDICTED: hypothetical protein LOC100572450 [A. pisum]	XP_003246116.1	4,00E-98	GLOS_LOC10057	GO:0010557	B	.	positive regulation of macromolecule biosynthetic process
XP_003246116.1 PREDICTED: hypothetical protein LOC100572450 [A. pisum]	XP_003246116.1	4,00E-98	GLOS_LOC10057	GO:0009891	B	.	positive regulation of biosynthetic process
XP_003246116.1 PREDICTED: hypothetical protein LOC100572450 [A. pisum]	XP_003246116.1	4,00E-98	GLOS_LOC10057	GO:0009893	B	.	positive regulation of metabolic process
XP_003246116.1 PREDICTED: hypothetical protein LOC100572450 [A. pisum]	XP_003246116.1	4,00E-98	GLOS_LOC10057	GO:0010604	B	.	positive regulation of macromolecule metabolic process
XP_003246116.1 PREDICTED: hypothetical protein LOC100572450 [A. pisum]	XP_003246116.1	4,00E-98	GLOS_LOC10057	GO:0010628	B	.	positive regulation of gene expression
XP_003246116.1 PREDICTED: hypothetical protein LOC100572450 [A. pisum]	XP_003246116.1	4,00E-98	GLOS_LOC10057	GO:0031328	B	.	positive regulation of cellular biosynthetic process
XP_003246116.1 PREDICTED: hypothetical protein LOC100572450 [A. pisum]	XP_003246116.1	4,00E-98	GLOS_LOC10057	GO:0031325	B	.	positive regulation of cellular metabolic process
XP_003246116.1 PREDICTED: hypothetical protein LOC100572450 [A. pisum]	XP_003246116.1	4,00E-98	GLOS_LOC10057	GO:0051254	B	.	positive regulation of RNA metabolic process
XP_003246116.1 PREDICTED: hypothetical protein LOC100572450 [A. pisum]	XP_003246116.1	4,00E-98	GLOS_LOC10057	GO:0045935	B	.	positive regulation of nucleobase-containing compound metabolic process
XP_003246116.1 PREDICTED: hypothetical protein LOC100572450 [A. pisum]	XP_003246116.1	4,00E-98	GLOS_LOC10057	GO:0051173	B	.	positive regulation of nitrogen compound metabolic process
XP_003246116.1 PREDICTED: hypothetical protein LOC100572450 [A. pisum]	XP_003246116.1	4,00E-98	GLOS_LOC10057	GO:0045944	B	Refseq	positive regulation of transcription from RNA polymerase II promoter
NP_001262624.1 gilgamesh, isoform L [Drosophila melanogaster]	NP_001262624.1	0	GLOS_GISH.2.4	GO:0000122	B	Refseq	negative regulation of transcription from RNA polymerase II promoter
NP_001262624.1 gilgamesh, isoform L [Drosophila melanogaster]	NP_001262624.1	0	GLOS_GISH.2.4	GO:0001501	B	Refseq	skeletal system development
NP_001262624.1 gilgamesh, isoform L [Drosophila melanogaster]	NP_001262624.1	0	GLOS_GISH.2.4	GO:0003700	M	Refseq	sequence-specific DNA binding transcription factor activity
NP_001262624.1 gilgamesh, isoform L [Drosophila melanogaster]	NP_001262624.1	0	GLOS_GISH.2.4	GO:0005515	M	Refseq	protein binding

NP_001262624.1 gilgamesh, isoform L [Drosophila melanogaster]	NP_001262624.1	0	GLOS_GISH.2.4	GO:0005634	C	Refseq	nucleus
NP_001262624.1 gilgamesh, isoform L [Drosophila melanogaster]	NP_001262624.1	0	GLOS_GISH.2.4	GO:0006366	B	Refseq	transcription from RNA polymerase II promoter
NP_001262624.1 gilgamesh, isoform L [Drosophila melanogaster]	NP_001262624.1	0	GLOS_GISH.2.4	GO:0006607	B	Refseq	NLS-bearing protein import into nucleus
NP_001262624.1 gilgamesh, isoform L [Drosophila melanogaster]	NP_001262624.1	0	GLOS_GISH.2.4	GO:0008270	M	Refseq	zinc ion binding
NP_001262624.1 gilgamesh, isoform L [Drosophila melanogaster]	NP_001262624.1	0	GLOS_GISH.2.4	GO:0032330	B	Refseq	regulation of chondrocyte differentiation
NP_001262624.1 gilgamesh, isoform L [Drosophila melanogaster]	NP_001262624.1	0	GLOS_GISH.2.4	GO:0043565	M	Refseq	sequence-specific DNA binding
XP_001969675.1 GG23818 [Drosophila erecta]	XP_001969675.1	1,00E-173	GLOS_DERE_GG	GO:0005261	M	Refseq	cation channel activity
XP_001969675.1 GG23818 [Drosophila erecta]	XP_001969675.1	1,00E-173	GLOS_DERE_GG	GO:0005262	M	Refseq	calcium channel activity
XP_001969675.1 GG23818 [Drosophila erecta]	XP_001969675.1	1,00E-173	GLOS_DERE_GG	GO:0005515	M	Refseq	protein binding
XP_001969675.1 GG23818 [Drosophila erecta]	XP_001969675.1	1,00E-173	GLOS_DERE_GG	GO:0005886	C	Refseq	plasma membrane
XP_001969675.1 GG23818 [Drosophila erecta]	XP_001969675.1	1,00E-173	GLOS_DERE_GG	GO:0005887	C	Refseq	integral to plasma membrane
XP_001969675.1 GG23818 [Drosophila erecta]	XP_001969675.1	1,00E-173	GLOS_DERE_GG	GO:0006816	B	Refseq	calcium ion transport
XP_001969675.1 GG23818 [Drosophila erecta]	XP_001969675.1	1,00E-173	GLOS_DERE_GG	GO:0007411	B	Refseq	axon guidance
XP_001969675.1 GG23818 [Drosophila erecta]	XP_001969675.1	1,00E-173	GLOS_DERE_GG	GO:0015279	M	Refseq	store-operated calcium channel activity
XP_001969675.1 GG23818 [Drosophila erecta]	XP_001969675.1	1,00E-173	GLOS_DERE_GG	GO:0016323	C	Refseq	basolateral plasma membrane
XP_001969675.1 GG23818 [Drosophila erecta]	XP_001969675.1	1,00E-173	GLOS_DERE_GG	GO:0030017	C	Refseq	sarcomere
XP_001969675.1 GG23818 [Drosophila erecta]	XP_001969675.1	1,00E-173	GLOS_DERE_GG	GO:0043034	C	Refseq	costamere
XP_001969675.1 GG23818 [Drosophila erecta]	XP_001969675.1	1,00E-173	GLOS_DERE_GG	GO:0043234	C	Refseq	protein complex
XP_001969675.1 GG23818 [Drosophila erecta]	XP_001969675.1	1,00E-173	GLOS_DERE_GG	GO:0044325	M	Refseq	ion channel binding
XP_001969675.1 GG23818 [Drosophila erecta]	XP_001969675.1	1,00E-173	GLOS_DERE_GG	GO:0045121	C	Refseq	membrane raft
XP_001969675.1 GG23818 [Drosophila erecta]	XP_001969675.1	1,00E-173	GLOS_DERE_GG	GO:0046541	B	Refseq	saliva secretion
XP_001969675.1 GG23818 [Drosophila erecta]	XP_001969675.1	1,00E-173	GLOS_DERE_GG	GO:0051281	B	Refseq	positive regulation of release of sequestered calcium ion into cytosol
XP_001969675.1 GG23818 [Drosophila erecta]	XP_001969675.1	1,00E-173	GLOS_DERE_GG	GO:0051480	B	Refseq	cytosolic calcium ion homeostasis
XP_001969675.1 GG23818 [Drosophila erecta]	XP_001969675.1	1,00E-173	GLOS_DERE_GG	GO:0051592	B	Refseq	response to calcium ion
XP_001969675.1 GG23818 [Drosophila erecta]	XP_001969675.1	1,00E-173	GLOS_DERE_GG	GO:0070679	M	Refseq	inositol 1,4,5 trisphosphate binding
XP_001655838.1 secreted modular calcium-binding protein [Aedes aegypti]	XP_001655838.1	1,00E-179	GLOS_AAEL_AAE	GO:0030674	M	.	protein binding, bridging
XP_001655838.1 secreted modular calcium-binding protein [Aedes aegypti]	XP_001655838.1	1,00E-179	GLOS_AAEL_AAE	GO:0060090	M	.	binding, bridging
XP_001655838.1 secreted modular calcium-binding protein [Aedes aegypti]	XP_001655838.1	1,00E-179	GLOS_AAEL_AAE	GO:0035591	M	.	signaling adaptor activity
XP_001655838.1 secreted modular calcium-binding protein [Aedes aegypti]	XP_001655838.1	1,00E-179	GLOS_AAEL_AAE	GO:0005070	M	Refseq	SH3/SH2 adaptor activity
XP_001655838.1 secreted modular calcium-binding protein [Aedes aegypti]	XP_001655838.1	1,00E-179	GLOS_AAEL_AAE	GO:0005515	M	Refseq	protein binding
XP_001655838.1 secreted modular calcium-binding protein [Aedes aegypti]	XP_001655838.1	1,00E-179	GLOS_AAEL_AAE	GO:0005634	C	Refseq	nucleus
XP_001655838.1 secreted modular calcium-binding protein [Aedes aegypti]	XP_001655838.1	1,00E-179	GLOS_AAEL_AAE	GO:0005737	C	Refseq	cytoplasm
XP_001655838.1 secreted modular calcium-binding protein [Aedes aegypti]	XP_001655838.1	1,00E-179	GLOS_AAEL_AAE	GO:0007049	B	Refseq	cell cycle
XP_001655838.1 secreted modular calcium-binding protein [Aedes aegypti]	XP_001655838.1	1,00E-179	GLOS_AAEL_AAE	GO:0007165	B	Refseq	signal transduction
XP_001655838.1 secreted modular calcium-binding protein [Aedes aegypti]	XP_001655838.1	1,00E-179	GLOS_AAEL_AAE	GO:0007417	B	Refseq	central nervous system development
XP_001655838.1 secreted modular calcium-binding protein [Aedes aegypti]	XP_001655838.1	1,00E-179	GLOS_AAEL_AAE	GO:0007507	B	Refseq	heart development
XP_001655838.1 secreted modular calcium-binding protein [Aedes aegypti]	XP_001655838.1	1,00E-179	GLOS_AAEL_AAE	GO:0009790	B	.	embryo development
XP_001655838.1 secreted modular calcium-binding protein [Aedes aegypti]	XP_001655838.1	1,00E-179	GLOS_AAEL_AAE	GO:0009792	B	Refseq	embryo development ending in birth or egg hatching
XP_001655838.1 secreted modular calcium-binding protein [Aedes aegypti]	XP_001655838.1	1,00E-179	GLOS_AAEL_AAE	GO:0010212	B	Refseq	response to ionizing radiation
XP_001655838.1 secreted modular calcium-binding protein [Aedes aegypti]	XP_001655838.1	1,00E-179	GLOS_AAEL_AAE	GO:0019904	M	.	protein domain specific binding
XP_001655838.1 secreted modular calcium-binding protein [Aedes aegypti]	XP_001655838.1	1,00E-179	GLOS_AAEL_AAE	GO:0017124	M	Refseq	SH3 domain binding
XP_001655838.1 secreted modular calcium-binding protein [Aedes aegypti]	XP_001655838.1	1,00E-179	GLOS_AAEL_AAE	GO:0042802	M	Refseq	identical protein binding
XP_001655838.1 secreted modular calcium-binding protein [Aedes aegypti]	XP_001655838.1	1,00E-179	GLOS_AAEL_AAE	GO:0051726	B	.	regulation of cell cycle
XP_001655838.1 secreted modular calcium-binding protein [Aedes aegypti]	XP_001655838.1	1,00E-179	GLOS_AAEL_AAE	GO:0045786	B	Refseq	negative regulation of cell cycle
XP_001655838.1 secreted modular calcium-binding protein [Aedes aegypti]	XP_001655838.1	1,00E-179	GLOS_AAEL_AAE	GO:0048471	C	Refseq	perinuclear region of cytoplasm
XP_001655838.1 secreted modular calcium-binding protein [Aedes aegypti]	XP_001655838.1	1,00E-179	GLOS_AAEL_AAE	GO:0008134	M	.	transcription factor binding
XP_001655838.1 secreted modular calcium-binding protein [Aedes aegypti]	XP_001655838.1	1,00E-179	GLOS_AAEL_AAE	GO:0051059	M	Refseq	NF-kappaB binding
XP_001655838.1 secreted modular calcium-binding protein [Aedes aegypti]	XP_001655838.1	1,00E-179	GLOS_AAEL_AAE	GO:0072331	B	.	signal transduction by p53 class mediator
XP_001655838.1 secreted modular calcium-binding protein [Aedes aegypti]	XP_001655838.1	1,00E-179	GLOS_AAEL_AAE	GO:0035556	B	.	intracellular signal transduction
XP_001655838.1 secreted modular calcium-binding protein [Aedes aegypti]	XP_001655838.1	1,00E-179	GLOS_AAEL_AAE	GO:0097193	B	.	intrinsic apoptotic signaling pathway
XP_001655838.1 secreted modular calcium-binding protein [Aedes aegypti]	XP_001655838.1	1,00E-179	GLOS_AAEL_AAE	GO:0097190	B	.	apoptotic signaling pathway
XP_001655838.1 secreted modular calcium-binding protein [Aedes aegypti]	XP_001655838.1	1,00E-179	GLOS_AAEL_AAE	GO:0006915	B	.	apoptotic process
XP_001655838.1 secreted modular calcium-binding protein [Aedes aegypti]	XP_001655838.1	1,00E-179	GLOS_AAEL_AAE	GO:0012501	B	.	programmed cell death
XP_001655838.1 secreted modular calcium-binding protein [Aedes aegypti]	XP_001655838.1	1,00E-179	GLOS_AAEL_AAE	GO:0008219	B	.	cell death

XP_001655838.1 secreted modular calcium-binding protein [Aedes aegypti]	XP_001655838.1	1,00E-179	GLOS_AAEL_AAE	GO:0016265	B	.	death
XP_001655838.1 secreted modular calcium-binding protein [Aedes aegypti]	XP_001655838.1	1,00E-179	GLOS_AAEL_AAE	GO:0072332	B	Refseq	intrinsic apoptotic signaling pathway by p53 class mediator
XP_003243964.1 PREDICTED: hypothetical protein LOC100568954 [A. pisum]	XP_003243964.1	2,00E-15	GLOS_LOC10056	GO:0000278	B	Refseq	mitotic cell cycle
XP_003243964.1 PREDICTED: hypothetical protein LOC100568954 [A. pisum]	XP_003243964.1	2,00E-15	GLOS_LOC10056	GO:0003677	M	Refseq	DNA binding
XP_003243964.1 PREDICTED: hypothetical protein LOC100568954 [A. pisum]	XP_003243964.1	2,00E-15	GLOS_LOC10056	GO:0003700	M	Refseq	sequence-specific DNA binding transcription factor activity
XP_003243964.1 PREDICTED: hypothetical protein LOC100568954 [A. pisum]	XP_003243964.1	2,00E-15	GLOS_LOC10056	GO:0003712	M	Refseq	transcription cofactor activity
XP_003243964.1 PREDICTED: hypothetical protein LOC100568954 [A. pisum]	XP_003243964.1	2,00E-15	GLOS_LOC10056	GO:0005654	C	Refseq	nucleoplasm
XP_003243964.1 PREDICTED: hypothetical protein LOC100568954 [A. pisum]	XP_003243964.1	2,00E-15	GLOS_LOC10056	GO:0005667	C	Refseq	transcription factor complex
XP_003243964.1 PREDICTED: hypothetical protein LOC100568954 [A. pisum]	XP_003243964.1	2,00E-15	GLOS_LOC10056	GO:0006351	B	Refseq	transcription, DNA-dependent
XP_003243964.1 PREDICTED: hypothetical protein LOC100568954 [A. pisum]	XP_003243964.1	2,00E-15	GLOS_LOC10056	GO:0006367	B	Refseq	transcription initiation from RNA polymerase II promoter
XP_003243964.1 PREDICTED: hypothetical protein LOC100568954 [A. pisum]	XP_003243964.1	2,00E-15	GLOS_LOC10056	GO:0007179	B	Refseq	transforming growth factor beta receptor signaling pathway
XP_003243964.1 PREDICTED: hypothetical protein LOC100568954 [A. pisum]	XP_003243964.1	2,00E-15	GLOS_LOC10056	GO:0007507	B	Refseq	heart development
XP_003243964.1 PREDICTED: hypothetical protein LOC100568954 [A. pisum]	XP_003243964.1	2,00E-15	GLOS_LOC10056	GO:0008134	M	Refseq	transcription factor binding
XP_003243964.1 PREDICTED: hypothetical protein LOC100568954 [A. pisum]	XP_003243964.1	2,00E-15	GLOS_LOC10056	GO:0010467	B	Refseq	gene expression
XP_003243964.1 PREDICTED: hypothetical protein LOC100568954 [A. pisum]	XP_003243964.1	2,00E-15	GLOS_LOC10056	GO:0019904	M	Refseq	protein domain specific binding
XP_003243964.1 PREDICTED: hypothetical protein LOC100568954 [A. pisum]	XP_003243964.1	2,00E-15	GLOS_LOC10056	GO:0045944	B	Refseq	positive regulation of transcription from RNA polymerase II promoter
XP_004926945.1 PREDICTED: uncharact prot. LOC101740280 [B. mori]	XP_004926945.1	8,00E-09	GLOS_LOC10174	GO:0000122	B	Refseq	negative regulation of transcription from RNA polymerase II promoter
XP_004926945.1 PREDICTED: uncharact prot. LOC101740280 [B. mori]	XP_004926945.1	8,00E-09	GLOS_LOC10174	GO:0003674	M	Refseq	molecular_function
XP_004926945.1 PREDICTED: uncharact prot. LOC101740280 [B. mori]	XP_004926945.1	8,00E-09	GLOS_LOC10174	GO:0003682	M	Refseq	chromatin binding
XP_004926945.1 PREDICTED: uncharact prot. LOC101740280 [B. mori]	XP_004926945.1	8,00E-09	GLOS_LOC10174	GO:0000981	M	.	sequence-specific DNA binding RNA polymerase II transcription factor activity
XP_004926945.1 PREDICTED: uncharact prot. LOC101740280 [B. mori]	XP_004926945.1	8,00E-09	GLOS_LOC10174	GO:0003700	M	.	sequence-specific DNA binding transcription factor activity
XP_004926945.1 PREDICTED: uncharact prot. LOC101740280 [B. mori]	XP_004926945.1	8,00E-09	GLOS_LOC10174	GO:0003705	M	Refseq	RNA polymerase II distal enhancer sequence-specific DNA binding transcription factor activity
XP_004926945.1 PREDICTED: uncharact prot. LOC101740280 [B. mori]	XP_004926945.1	8,00E-09	GLOS_LOC10174	GO:0003712	M	.	transcription cofactor activity
XP_004926945.1 PREDICTED: uncharact prot. LOC101740280 [B. mori]	XP_004926945.1	8,00E-09	GLOS_LOC10174	GO:0003714	M	Refseq	transcription corepressor activity
XP_004926945.1 PREDICTED: uncharact prot. LOC101740280 [B. mori]	XP_004926945.1	8,00E-09	GLOS_LOC10174	GO:0005634	C	Refseq	nucleus
XP_004926945.1 PREDICTED: uncharact prot. LOC101740280 [B. mori]	XP_004926945.1	8,00E-09	GLOS_LOC10174	GO:0005654	C	Refseq	nucleoplasm
XP_004926945.1 PREDICTED: uncharact prot. LOC101740280 [B. mori]	XP_004926945.1	8,00E-09	GLOS_LOC10174	GO:0007219	B	Refseq	Notch signaling pathway
XP_004926945.1 PREDICTED: uncharact prot. LOC101740280 [B. mori]	XP_004926945.1	8,00E-09	GLOS_LOC10174	GO:0008150	B	Refseq	biological_process
XP_004926945.1 PREDICTED: uncharact prot. LOC101740280 [B. mori]	XP_004926945.1	8,00E-09	GLOS_LOC10174	GO:0016055	B	Refseq	Wnt receptor signaling pathway
XP_004926945.1 PREDICTED: uncharact prot. LOC101740280 [B. mori]	XP_004926945.1	8,00E-09	GLOS_LOC10174	GO:0070491	M	Refseq	repressing transcription factor binding
XP_003245892.1 PREDICTED: hypoth. Prot. LOC100575767 [A. pisum]	XP_003245892.1	9,00E-31	GLOS_LOC10057	GO:0000278	B	Refseq	mitotic cell cycle
XP_003245892.1 PREDICTED: hypoth. Prot. LOC100575767 [A. pisum]	XP_003245892.1	9,00E-31	GLOS_LOC10057	GO:0003677	M	Refseq	DNA binding
XP_003245892.1 PREDICTED: hypoth. Prot. LOC100575767 [A. pisum]	XP_003245892.1	9,00E-31	GLOS_LOC10057	GO:0003700	M	Refseq	sequence-specific DNA binding transcription factor activity
XP_003245892.1 PREDICTED: hypoth. Prot. LOC100575767 [A. pisum]	XP_003245892.1	9,00E-31	GLOS_LOC10057	GO:0003712	M	Refseq	transcription cofactor activity
XP_003245892.1 PREDICTED: hypoth. Prot. LOC100575767 [A. pisum]	XP_003245892.1	9,00E-31	GLOS_LOC10057	GO:0005654	C	Refseq	nucleoplasm
XP_003245892.1 PREDICTED: hypoth. Prot. LOC100575767 [A. pisum]	XP_003245892.1	9,00E-31	GLOS_LOC10057	GO:0005667	C	Refseq	transcription factor complex
XP_003245892.1 PREDICTED: hypoth. Prot. LOC100575767 [A. pisum]	XP_003245892.1	9,00E-31	GLOS_LOC10057	GO:0006351	B	Refseq	transcription, DNA-dependent
XP_003245892.1 PREDICTED: hypoth. Prot. LOC100575767 [A. pisum]	XP_003245892.1	9,00E-31	GLOS_LOC10057	GO:0006367	B	Refseq	transcription initiation from RNA polymerase II promoter
XP_003245892.1 PREDICTED: hypoth. Prot. LOC100575767 [A. pisum]	XP_003245892.1	9,00E-31	GLOS_LOC10057	GO:0007179	B	Refseq	transforming growth factor beta receptor signaling pathway
XP_003245892.1 PREDICTED: hypoth. Prot. LOC100575767 [A. pisum]	XP_003245892.1	9,00E-31	GLOS_LOC10057	GO:0007507	B	Refseq	heart development
XP_003245892.1 PREDICTED: hypoth. Prot. LOC100575767 [A. pisum]	XP_003245892.1	9,00E-31	GLOS_LOC10057	GO:0008134	M	Refseq	transcription factor binding
XP_003245892.1 PREDICTED: hypoth. Prot. LOC100575767 [A. pisum]	XP_003245892.1	9,00E-31	GLOS_LOC10057	GO:0010467	B	Refseq	gene expression
XP_003245892.1 PREDICTED: hypoth. Prot. LOC100575767 [A. pisum]	XP_003245892.1	9,00E-31	GLOS_LOC10057	GO:0019904	M	Refseq	protein domain specific binding
XP_003245892.1 PREDICTED: hypoth. Prot. LOC100575767 [A. pisum]	XP_003245892.1	9,00E-31	GLOS_LOC10057	GO:0045944	B	Refseq	positive regulation of transcription from RNA polymerase II promoter
XP_001807351.1 PREDICTED: similar to pol polyprotein [Tribolium castaneum]	XP_001807351.1	3,00E-16	GLOS_LOC10014	GO:0048646	B	.	anatomical structure formation involved in morphogenesis
XP_001807351.1 PREDICTED: similar to pol polyprotein [Tribolium castaneum]	XP_001807351.1	3,00E-16	GLOS_LOC10014	GO:0048514	B	.	blood vessel morphogenesis
XP_001807351.1 PREDICTED: similar to pol polyprotein [Tribolium castaneum]	XP_001807351.1	3,00E-16	GLOS_LOC10014	GO:0001568	B	.	blood vessel development
XP_001807351.1 PREDICTED: similar to pol polyprotein [Tribolium castaneum]	XP_001807351.1	3,00E-16	GLOS_LOC10014	GO:0001944	B	.	vasculature development
XP_001807351.1 PREDICTED: similar to pol polyprotein [Tribolium castaneum]	XP_001807351.1	3,00E-16	GLOS_LOC10014	GO:0001525	B	Refseq	angiogenesis
XP_001807351.1 PREDICTED: similar to pol polyprotein [Tribolium castaneum]	XP_001807351.1	3,00E-16	GLOS_LOC10014	GO:0005099	M	.	Ras GTPase activator activity
XP_001807351.1 PREDICTED: similar to pol polyprotein [Tribolium castaneum]	XP_001807351.1	3,00E-16	GLOS_LOC10014	GO:0005083	M	.	small GTPase regulator activity
XP_001807351.1 PREDICTED: similar to pol polyprotein [Tribolium castaneum]	XP_001807351.1	3,00E-16	GLOS_LOC10014	GO:0030695	M	.	GTPase regulator activity
XP_001807351.1 PREDICTED: similar to pol polyprotein [Tribolium castaneum]	XP_001807351.1	3,00E-16	GLOS_LOC10014	GO:0060589	M	.	nucleoside-triphosphatase regulator activity

XP_001807351.1 PREDICTED: similar to pol polyprotein [Tribolium castaneum]	XP_001807351.1	3,00E-16	GLOS_LOC10014:	GO:0030234	M	.	enzyme regulator activity
XP_001807351.1 PREDICTED: similar to pol polyprotein [Tribolium castaneum]	XP_001807351.1	3,00E-16	GLOS_LOC10014:	GO:0005096	M	.	GTPase activator activity
XP_001807351.1 PREDICTED: similar to pol polyprotein [Tribolium castaneum]	XP_001807351.1	3,00E-16	GLOS_LOC10014:	GO:0008047	M	.	enzyme activator activity
XP_001807351.1 PREDICTED: similar to pol polyprotein [Tribolium castaneum]	XP_001807351.1	3,00E-16	GLOS_LOC10014:	GO:0005100	M	Refseq	Rho GTPase activator activity
XP_001807351.1 PREDICTED: similar to pol polyprotein [Tribolium castaneum]	XP_001807351.1	3,00E-16	GLOS_LOC10014:	GO:0005102	M	.	receptor binding
XP_001807351.1 PREDICTED: similar to pol polyprotein [Tribolium castaneum]	XP_001807351.1	3,00E-16	GLOS_LOC10014:	GO:0032403	M	.	protein complex binding
XP_001807351.1 PREDICTED: similar to pol polyprotein [Tribolium castaneum]	XP_001807351.1	3,00E-16	GLOS_LOC10014:	GO:0005178	M	Refseq	integrin binding
XP_001807351.1 PREDICTED: similar to pol polyprotein [Tribolium castaneum]	XP_001807351.1	3,00E-16	GLOS_LOC10014:	GO:0005515	M	Refseq	protein binding
XP_001807351.1 PREDICTED: similar to pol polyprotein [Tribolium castaneum]	XP_001807351.1	3,00E-16	GLOS_LOC10014:	GO:0005783	C	Refseq	endoplasmic reticulum
XP_001807351.1 PREDICTED: similar to pol polyprotein [Tribolium castaneum]	XP_001807351.1	3,00E-16	GLOS_LOC10014:	GO:0005829	C	Refseq	cytosol
XP_001807351.1 PREDICTED: similar to pol polyprotein [Tribolium castaneum]	XP_001807351.1	3,00E-16	GLOS_LOC10014:	GO:0005886	C	Refseq	plasma membrane
XP_001807351.1 PREDICTED: similar to pol polyprotein [Tribolium castaneum]	XP_001807351.1	3,00E-16	GLOS_LOC10014:	GO:0005887	C	Refseq	integral to plasma membrane
XP_001807351.1 PREDICTED: similar to pol polyprotein [Tribolium castaneum]	XP_001807351.1	3,00E-16	GLOS_LOC10014:	GO:0001933	B	.	negative regulation of protein phosphorylation
XP_001807351.1 PREDICTED: similar to pol polyprotein [Tribolium castaneum]	XP_001807351.1	3,00E-16	GLOS_LOC10014:	GO:0001932	B	.	regulation of protein phosphorylation
XP_001807351.1 PREDICTED: similar to pol polyprotein [Tribolium castaneum]	XP_001807351.1	3,00E-16	GLOS_LOC10014:	GO:0031399	B	.	regulation of protein modification process
XP_001807351.1 PREDICTED: similar to pol polyprotein [Tribolium castaneum]	XP_001807351.1	3,00E-16	GLOS_LOC10014:	GO:0032268	B	.	regulation of cellular protein metabolic process
XP_001807351.1 PREDICTED: similar to pol polyprotein [Tribolium castaneum]	XP_001807351.1	3,00E-16	GLOS_LOC10014:	GO:0051246	B	.	regulation of protein metabolic process
XP_001807351.1 PREDICTED: similar to pol polyprotein [Tribolium castaneum]	XP_001807351.1	3,00E-16	GLOS_LOC10014:	GO:0042325	B	.	regulation of phosphorylation
XP_001807351.1 PREDICTED: similar to pol polyprotein [Tribolium castaneum]	XP_001807351.1	3,00E-16	GLOS_LOC10014:	GO:0019220	B	.	regulation of phosphate metabolic process
XP_001807351.1 PREDICTED: similar to pol polyprotein [Tribolium castaneum]	XP_001807351.1	3,00E-16	GLOS_LOC10014:	GO:0051174	B	.	regulation of phosphorus metabolic process
XP_001807351.1 PREDICTED: similar to pol polyprotein [Tribolium castaneum]	XP_001807351.1	3,00E-16	GLOS_LOC10014:	GO:0031400	B	.	negative regulation of protein modification process
XP_001807351.1 PREDICTED: similar to pol polyprotein [Tribolium castaneum]	XP_001807351.1	3,00E-16	GLOS_LOC10014:	GO:0032269	B	.	negative regulation of cellular protein metabolic process
XP_001807351.1 PREDICTED: similar to pol polyprotein [Tribolium castaneum]	XP_001807351.1	3,00E-16	GLOS_LOC10014:	GO:0051248	B	.	negative regulation of protein metabolic process
XP_001807351.1 PREDICTED: similar to pol polyprotein [Tribolium castaneum]	XP_001807351.1	3,00E-16	GLOS_LOC10014:	GO:0042326	B	.	negative regulation of phosphorylation
XP_001807351.1 PREDICTED: similar to pol polyprotein [Tribolium castaneum]	XP_001807351.1	3,00E-16	GLOS_LOC10014:	GO:0045936	B	.	negative regulation of phosphate metabolic process
XP_001807351.1 PREDICTED: similar to pol polyprotein [Tribolium castaneum]	XP_001807351.1	3,00E-16	GLOS_LOC10014:	GO:0010563	B	.	negative regulation of phosphorus metabolic process
XP_001807351.1 PREDICTED: similar to pol polyprotein [Tribolium castaneum]	XP_001807351.1	3,00E-16	GLOS_LOC10014:	GO:0033673	B	.	negative regulation of kinase activity
XP_001807351.1 PREDICTED: similar to pol polyprotein [Tribolium castaneum]	XP_001807351.1	3,00E-16	GLOS_LOC10014:	GO:0043549	B	.	regulation of kinase activity
XP_001807351.1 PREDICTED: similar to pol polyprotein [Tribolium castaneum]	XP_001807351.1	3,00E-16	GLOS_LOC10014:	GO:0051338	B	.	regulation of transferase activity
XP_001807351.1 PREDICTED: similar to pol polyprotein [Tribolium castaneum]	XP_001807351.1	3,00E-16	GLOS_LOC10014:	GO:0050790	B	.	regulation of catalytic activity
XP_001807351.1 PREDICTED: similar to pol polyprotein [Tribolium castaneum]	XP_001807351.1	3,00E-16	GLOS_LOC10014:	GO:0065009	B	.	regulation of molecular function
XP_001807351.1 PREDICTED: similar to pol polyprotein [Tribolium castaneum]	XP_001807351.1	3,00E-16	GLOS_LOC10014:	GO:0051348	B	.	negative regulation of transferase activity
XP_001807351.1 PREDICTED: similar to pol polyprotein [Tribolium castaneum]	XP_001807351.1	3,00E-16	GLOS_LOC10014:	GO:0043086	B	.	negative regulation of catalytic activity
XP_001807351.1 PREDICTED: similar to pol polyprotein [Tribolium castaneum]	XP_001807351.1	3,00E-16	GLOS_LOC10014:	GO:0044092	B	.	negative regulation of molecular function
XP_001807351.1 PREDICTED: similar to pol polyprotein [Tribolium castaneum]	XP_001807351.1	3,00E-16	GLOS_LOC10014:	GO:0045859	B	.	regulation of protein kinase activity
XP_001807351.1 PREDICTED: similar to pol polyprotein [Tribolium castaneum]	XP_001807351.1	3,00E-16	GLOS_LOC10014:	GO:0006469	B	Refseq	negative regulation of protein kinase activity
XP_001807351.1 PREDICTED: similar to pol polyprotein [Tribolium castaneum]	XP_001807351.1	3,00E-16	GLOS_LOC10014:	GO:0006996	B	.	organelle organization
XP_001807351.1 PREDICTED: similar to pol polyprotein [Tribolium castaneum]	XP_001807351.1	3,00E-16	GLOS_LOC10014:	GO:0007010	B	Refseq	cytoskeleton organization
XP_001807351.1 PREDICTED: similar to pol polyprotein [Tribolium castaneum]	XP_001807351.1	3,00E-16	GLOS_LOC10014:	GO:0009986	C	.	cell surface
XP_001807351.1 PREDICTED: similar to pol polyprotein [Tribolium castaneum]	XP_001807351.1	3,00E-16	GLOS_LOC10014:	GO:0009897	C	Refseq	external side of plasma membrane
XP_001807351.1 PREDICTED: similar to pol polyprotein [Tribolium castaneum]	XP_001807351.1	3,00E-16	GLOS_LOC10014:	GO:0045177	C	.	apical part of cell
XP_001807351.1 PREDICTED: similar to pol polyprotein [Tribolium castaneum]	XP_001807351.1	3,00E-16	GLOS_LOC10014:	GO:0016324	C	Refseq	apical plasma membrane
XP_001807351.1 PREDICTED: similar to pol polyprotein [Tribolium castaneum]	XP_001807351.1	3,00E-16	GLOS_LOC10014:	GO:0007155	B	.	cell adhesion
XP_001807351.1 PREDICTED: similar to pol polyprotein [Tribolium castaneum]	XP_001807351.1	3,00E-16	GLOS_LOC10014:	GO:0022610	B	.	biological adhesion
XP_001807351.1 PREDICTED: similar to pol polyprotein [Tribolium castaneum]	XP_001807351.1	3,00E-16	GLOS_LOC10014:	GO:0016337	B	Refseq	cell-cell adhesion
XP_001807351.1 PREDICTED: similar to pol polyprotein [Tribolium castaneum]	XP_001807351.1	3,00E-16	GLOS_LOC10014:	GO:0019900	M	.	kinase binding
XP_001807351.1 PREDICTED: similar to pol polyprotein [Tribolium castaneum]	XP_001807351.1	3,00E-16	GLOS_LOC10014:	GO:0019899	M	.	enzyme binding
XP_001807351.1 PREDICTED: similar to pol polyprotein [Tribolium castaneum]	XP_001807351.1	3,00E-16	GLOS_LOC10014:	GO:0019901	M	Refseq	protein kinase binding
XP_001807351.1 PREDICTED: similar to pol polyprotein [Tribolium castaneum]	XP_001807351.1	3,00E-16	GLOS_LOC10014:	GO:0030334	B	.	regulation of cell migration
XP_001807351.1 PREDICTED: similar to pol polyprotein [Tribolium castaneum]	XP_001807351.1	3,00E-16	GLOS_LOC10014:	GO:2000145	B	.	regulation of cell motility
XP_001807351.1 PREDICTED: similar to pol polyprotein [Tribolium castaneum]	XP_001807351.1	3,00E-16	GLOS_LOC10014:	GO:0040012	B	.	regulation of locomotion
XP_001807351.1 PREDICTED: similar to pol polyprotein [Tribolium castaneum]	XP_001807351.1	3,00E-16	GLOS_LOC10014:	GO:0051270	B	.	regulation of cellular component movement
XP_001807351.1 PREDICTED: similar to pol polyprotein [Tribolium castaneum]	XP_001807351.1	3,00E-16	GLOS_LOC10014:	GO:2000146	B	.	negative regulation of cell motility
XP_001807351.1 PREDICTED: similar to pol polyprotein [Tribolium castaneum]	XP_001807351.1	3,00E-16	GLOS_LOC10014:	GO:0040013	B	.	negative regulation of locomotion

XP_001807351.1 PREDICTED: similar to pol polyprotein [Tribolium castaneum]	XP_001807351.1	3,00E-16	GLOS_LOC10014:	GO:0051271	B	.	negative regulation of cellular component movement
XP_001807351.1 PREDICTED: similar to pol polyprotein [Tribolium castaneum]	XP_001807351.1	3,00E-16	GLOS_LOC10014:	GO:0030336	B	Refseq	negative regulation of cell migration
XP_001807351.1 PREDICTED: similar to pol polyprotein [Tribolium castaneum]	XP_001807351.1	3,00E-16	GLOS_LOC10014:	GO:0043005	C	.	neuron projection
XP_001807351.1 PREDICTED: similar to pol polyprotein [Tribolium castaneum]	XP_001807351.1	3,00E-16	GLOS_LOC10014:	GO:0042995	C	.	cell projection
XP_001807351.1 PREDICTED: similar to pol polyprotein [Tribolium castaneum]	XP_001807351.1	3,00E-16	GLOS_LOC10014:	GO:0097458	C	.	neuron part
XP_001807351.1 PREDICTED: similar to pol polyprotein [Tribolium castaneum]	XP_001807351.1	3,00E-16	GLOS_LOC10014:	GO:0030425	C	Refseq	dendrite
XP_001807351.1 PREDICTED: similar to pol polyprotein [Tribolium castaneum]	XP_001807351.1	3,00E-16	GLOS_LOC10014:	GO:0030427	C	.	site of polarized growth
XP_001807351.1 PREDICTED: similar to pol polyprotein [Tribolium castaneum]	XP_001807351.1	3,00E-16	GLOS_LOC10014:	GO:0044463	C	.	cell projection part
XP_001807351.1 PREDICTED: similar to pol polyprotein [Tribolium castaneum]	XP_001807351.1	3,00E-16	GLOS_LOC10014:	GO:0030426	C	Refseq	growth cone
XP_001807351.1 PREDICTED: similar to pol polyprotein [Tribolium castaneum]	XP_001807351.1	3,00E-16	GLOS_LOC10014:	GO:0031233	C	.	intrinsic to external side of plasma membrane
XP_001807351.1 PREDICTED: similar to pol polyprotein [Tribolium castaneum]	XP_001807351.1	3,00E-16	GLOS_LOC10014:	GO:0009897	C	.	external side of plasma membrane
XP_001807351.1 PREDICTED: similar to pol polyprotein [Tribolium castaneum]	XP_001807351.1	3,00E-16	GLOS_LOC10014:	GO:0046658	C	.	anchored to plasma membrane
XP_001807351.1 PREDICTED: similar to pol polyprotein [Tribolium castaneum]	XP_001807351.1	3,00E-16	GLOS_LOC10014:	GO:0031225	C	.	anchored to membrane
XP_001807351.1 PREDICTED: similar to pol polyprotein [Tribolium castaneum]	XP_001807351.1	3,00E-16	GLOS_LOC10014:	GO:0031362	C	Refseq	anchored to external side of plasma membrane
XP_001807351.1 PREDICTED: similar to pol polyprotein [Tribolium castaneum]	XP_001807351.1	3,00E-16	GLOS_LOC10014:	GO:0035091	M	.	phosphatidylinositol binding
XP_001807351.1 PREDICTED: similar to pol polyprotein [Tribolium castaneum]	XP_001807351.1	3,00E-16	GLOS_LOC10014:	GO:0005543	M	.	phospholipid binding
XP_001807351.1 PREDICTED: similar to pol polyprotein [Tribolium castaneum]	XP_001807351.1	3,00E-16	GLOS_LOC10014:	GO:0008289	M	.	lipid binding
XP_001807351.1 PREDICTED: similar to pol polyprotein [Tribolium castaneum]	XP_001807351.1	3,00E-16	GLOS_LOC10014:	GO:0051861	M	.	glycolipid binding
XP_001807351.1 PREDICTED: similar to pol polyprotein [Tribolium castaneum]	XP_001807351.1	3,00E-16	GLOS_LOC10014:	GO:0097367	M	.	carbohydrate derivative binding
XP_001807351.1 PREDICTED: similar to pol polyprotein [Tribolium castaneum]	XP_001807351.1	3,00E-16	GLOS_LOC10014:	GO:0034235	M	Refseq	GPI anchor binding
XP_001807351.1 PREDICTED: similar to pol polyprotein [Tribolium castaneum]	XP_001807351.1	3,00E-16	GLOS_LOC10014:	GO:0043087	B	.	regulation of GTPase activity
XP_001807351.1 PREDICTED: similar to pol polyprotein [Tribolium castaneum]	XP_001807351.1	3,00E-16	GLOS_LOC10014:	GO:0033124	B	.	regulation of GTP catabolic process
XP_001807351.1 PREDICTED: similar to pol polyprotein [Tribolium castaneum]	XP_001807351.1	3,00E-16	GLOS_LOC10014:	GO:0009118	B	.	regulation of nucleoside metabolic process
XP_001807351.1 PREDICTED: similar to pol polyprotein [Tribolium castaneum]	XP_001807351.1	3,00E-16	GLOS_LOC10014:	GO:0033121	B	.	regulation of purine nucleotide catabolic process
XP_001807351.1 PREDICTED: similar to pol polyprotein [Tribolium castaneum]	XP_001807351.1	3,00E-16	GLOS_LOC10014:	GO:0030811	B	.	regulation of nucleotide catabolic process
XP_001807351.1 PREDICTED: similar to pol polyprotein [Tribolium castaneum]	XP_001807351.1	3,00E-16	GLOS_LOC10014:	GO:0006140	B	.	regulation of nucleotide metabolic process
XP_001807351.1 PREDICTED: similar to pol polyprotein [Tribolium castaneum]	XP_001807351.1	3,00E-16	GLOS_LOC10014:	GO:0031329	B	.	regulation of cellular catabolic process
XP_001807351.1 PREDICTED: similar to pol polyprotein [Tribolium castaneum]	XP_001807351.1	3,00E-16	GLOS_LOC10014:	GO:0009894	B	.	regulation of catabolic process
XP_001807351.1 PREDICTED: similar to pol polyprotein [Tribolium castaneum]	XP_001807351.1	3,00E-16	GLOS_LOC10014:	GO:1900542	B	.	regulation of purine nucleotide metabolic process
XP_001807351.1 PREDICTED: similar to pol polyprotein [Tribolium castaneum]	XP_001807351.1	3,00E-16	GLOS_LOC10014:	GO:0051336	B	.	regulation of hydrolase activity
XP_001807351.1 PREDICTED: similar to pol polyprotein [Tribolium castaneum]	XP_001807351.1	3,00E-16	GLOS_LOC10014:	GO:0051345	B	.	positive regulation of hydrolase activity
XP_001807351.1 PREDICTED: similar to pol polyprotein [Tribolium castaneum]	XP_001807351.1	3,00E-16	GLOS_LOC10014:	GO:0043085	B	.	positive regulation of catalytic activity
XP_001807351.1 PREDICTED: similar to pol polyprotein [Tribolium castaneum]	XP_001807351.1	3,00E-16	GLOS_LOC10014:	GO:0044093	B	.	positive regulation of molecular function
XP_001807351.1 PREDICTED: similar to pol polyprotein [Tribolium castaneum]	XP_001807351.1	3,00E-16	GLOS_LOC10014:	GO:0043547	B	Refseq	positive regulation of GTPase activity
XP_001807351.1 PREDICTED: similar to pol polyprotein [Tribolium castaneum]	XP_001807351.1	3,00E-16	GLOS_LOC10014:	GO:0045121	C	Refseq	membrane raft
XP_001807351.1 PREDICTED: similar to pol polyprotein [Tribolium castaneum]	XP_001807351.1	3,00E-16	GLOS_LOC10014:	GO:0042462	B	.	eye photoreceptor cell development
XP_001807351.1 PREDICTED: similar to pol polyprotein [Tribolium castaneum]	XP_001807351.1	3,00E-16	GLOS_LOC10014:	GO:0042461	B	.	photoreceptor cell development
XP_001807351.1 PREDICTED: similar to pol polyprotein [Tribolium castaneum]	XP_001807351.1	3,00E-16	GLOS_LOC10014:	GO:0046530	B	.	photoreceptor cell differentiation
XP_001807351.1 PREDICTED: similar to pol polyprotein [Tribolium castaneum]	XP_001807351.1	3,00E-16	GLOS_LOC10014:	GO:0001754	B	.	eye photoreceptor cell differentiation
XP_001807351.1 PREDICTED: similar to pol polyprotein [Tribolium castaneum]	XP_001807351.1	3,00E-16	GLOS_LOC10014:	GO:0048592	B	.	eye morphogenesis
XP_001807351.1 PREDICTED: similar to pol polyprotein [Tribolium castaneum]	XP_001807351.1	3,00E-16	GLOS_LOC10014:	GO:0009887	B	.	organ morphogenesis
XP_001807351.1 PREDICTED: similar to pol polyprotein [Tribolium castaneum]	XP_001807351.1	3,00E-16	GLOS_LOC10014:	GO:0001654	B	.	eye development
XP_001807351.1 PREDICTED: similar to pol polyprotein [Tribolium castaneum]	XP_001807351.1	3,00E-16	GLOS_LOC10014:	GO:0007423	B	.	sensory organ development
XP_001807351.1 PREDICTED: similar to pol polyprotein [Tribolium castaneum]	XP_001807351.1	3,00E-16	GLOS_LOC10014:	GO:0042670	B	.	retinal cone cell differentiation
XP_001807351.1 PREDICTED: similar to pol polyprotein [Tribolium castaneum]	XP_001807351.1	3,00E-16	GLOS_LOC10014:	GO:0060219	B	.	camera-type eye photoreceptor cell differentiation
XP_001807351.1 PREDICTED: similar to pol polyprotein [Tribolium castaneum]	XP_001807351.1	3,00E-16	GLOS_LOC10014:	GO:0003407	B	.	neural retina development
XP_001807351.1 PREDICTED: similar to pol polyprotein [Tribolium castaneum]	XP_001807351.1	3,00E-16	GLOS_LOC10014:	GO:0060041	B	.	retina development in camera-type eye
XP_001807351.1 PREDICTED: similar to pol polyprotein [Tribolium castaneum]	XP_001807351.1	3,00E-16	GLOS_LOC10014:	GO:0043010	B	.	camera-type eye development
XP_001807351.1 PREDICTED: similar to pol polyprotein [Tribolium castaneum]	XP_001807351.1	3,00E-16	GLOS_LOC10014:	GO:0060042	B	.	retina morphogenesis in camera-type eye
XP_001807351.1 PREDICTED: similar to pol polyprotein [Tribolium castaneum]	XP_001807351.1	3,00E-16	GLOS_LOC10014:	GO:0048593	B	.	camera-type eye morphogenesis
XP_001807351.1 PREDICTED: similar to pol polyprotein [Tribolium castaneum]	XP_001807351.1	3,00E-16	GLOS_LOC10014:	GO:0046549	B	Refseq	retinal cone cell development
XP_001807351.1 PREDICTED: similar to pol polyprotein [Tribolium castaneum]	XP_001807351.1	3,00E-16	GLOS_LOC10014:	GO:0007045	B	.	cell-substrate adherens junction assembly
XP_001807351.1 PREDICTED: similar to pol polyprotein [Tribolium castaneum]	XP_001807351.1	3,00E-16	GLOS_LOC10014:	GO:0007044	B	.	cell-substrate junction assembly
XP_001807351.1 PREDICTED: similar to pol polyprotein [Tribolium castaneum]	XP_001807351.1	3,00E-16	GLOS_LOC10014:	GO:0034329	B	.	cell junction assembly

XP_001807351.1 PREDICTED: similar to pol polyprotein [Tribolium castaneum]	XP_001807351.1	3,00E-16	GLOS_LOC10014:	GO:0022607	B	.	cellular component assembly
XP_001807351.1 PREDICTED: similar to pol polyprotein [Tribolium castaneum]	XP_001807351.1	3,00E-16	GLOS_LOC10014:	GO:0044085	B	.	cellular component biogenesis
XP_001807351.1 PREDICTED: similar to pol polyprotein [Tribolium castaneum]	XP_001807351.1	3,00E-16	GLOS_LOC10014:	GO:0034330	B	.	cell junction organization
XP_001807351.1 PREDICTED: similar to pol polyprotein [Tribolium castaneum]	XP_001807351.1	3,00E-16	GLOS_LOC10014:	GO:0034333	B	.	adherens junction assembly
XP_001807351.1 PREDICTED: similar to pol polyprotein [Tribolium castaneum]	XP_001807351.1	3,00E-16	GLOS_LOC10014:	GO:0034332	B	.	adherens junction organization
XP_001807351.1 PREDICTED: similar to pol polyprotein [Tribolium castaneum]	XP_001807351.1	3,00E-16	GLOS_LOC10014:	GO:0045216	B	.	cell-cell junction organization
XP_001807351.1 PREDICTED: similar to pol polyprotein [Tribolium castaneum]	XP_001807351.1	3,00E-16	GLOS_LOC10014:	GO:0007160	B	.	cell-matrix adhesion
XP_001807351.1 PREDICTED: similar to pol polyprotein [Tribolium castaneum]	XP_001807351.1	3,00E-16	GLOS_LOC10014:	GO:0031589	B	.	cell-substrate adhesion
XP_001807351.1 PREDICTED: similar to pol polyprotein [Tribolium castaneum]	XP_001807351.1	3,00E-16	GLOS_LOC10014:	GO:0048041	B	Refseq	focal adhesion assembly
XP_001807351.1 PREDICTED: similar to pol polyprotein [Tribolium castaneum]	XP_001807351.1	3,00E-16	GLOS_LOC10014:	GO:0031345	B	.	negative regulation of cell projection organization
XP_001807351.1 PREDICTED: similar to pol polyprotein [Tribolium castaneum]	XP_001807351.1	3,00E-16	GLOS_LOC10014:	GO:0031344	B	.	regulation of cell projection organization
XP_001807351.1 PREDICTED: similar to pol polyprotein [Tribolium castaneum]	XP_001807351.1	3,00E-16	GLOS_LOC10014:	GO:0051128	B	.	regulation of cellular component organization
XP_001807351.1 PREDICTED: similar to pol polyprotein [Tribolium castaneum]	XP_001807351.1	3,00E-16	GLOS_LOC10014:	GO:0051129	B	.	negative regulation of cellular component organization
XP_001807351.1 PREDICTED: similar to pol polyprotein [Tribolium castaneum]	XP_001807351.1	3,00E-16	GLOS_LOC10014:	GO:0050768	B	.	negative regulation of neurogenesis
XP_001807351.1 PREDICTED: similar to pol polyprotein [Tribolium castaneum]	XP_001807351.1	3,00E-16	GLOS_LOC10014:	GO:0010721	B	.	negative regulation of cell development
XP_001807351.1 PREDICTED: similar to pol polyprotein [Tribolium castaneum]	XP_001807351.1	3,00E-16	GLOS_LOC10014:	GO:0045596	B	.	negative regulation of cell differentiation
XP_001807351.1 PREDICTED: similar to pol polyprotein [Tribolium castaneum]	XP_001807351.1	3,00E-16	GLOS_LOC10014:	GO:0051093	B	.	negative regulation of developmental process
XP_001807351.1 PREDICTED: similar to pol polyprotein [Tribolium castaneum]	XP_001807351.1	3,00E-16	GLOS_LOC10014:	GO:0060284	B	.	regulation of cell development
XP_001807351.1 PREDICTED: similar to pol polyprotein [Tribolium castaneum]	XP_001807351.1	3,00E-16	GLOS_LOC10014:	GO:0050767	B	.	regulation of neurogenesis
XP_001807351.1 PREDICTED: similar to pol polyprotein [Tribolium castaneum]	XP_001807351.1	3,00E-16	GLOS_LOC10014:	GO:0051960	B	.	regulation of nervous system development
XP_001807351.1 PREDICTED: similar to pol polyprotein [Tribolium castaneum]	XP_001807351.1	3,00E-16	GLOS_LOC10014:	GO:0050770	B	.	regulation of axonogenesis
XP_001807351.1 PREDICTED: similar to pol polyprotein [Tribolium castaneum]	XP_001807351.1	3,00E-16	GLOS_LOC10014:	GO:0010769	B	.	regulation of cell morphogenesis involved in differentiation
XP_001807351.1 PREDICTED: similar to pol polyprotein [Tribolium castaneum]	XP_001807351.1	3,00E-16	GLOS_LOC10014:	GO:0022604	B	.	regulation of cell morphogenesis
XP_001807351.1 PREDICTED: similar to pol polyprotein [Tribolium castaneum]	XP_001807351.1	3,00E-16	GLOS_LOC10014:	GO:0022603	B	.	regulation of anatomical structure morphogenesis
XP_001807351.1 PREDICTED: similar to pol polyprotein [Tribolium castaneum]	XP_001807351.1	3,00E-16	GLOS_LOC10014:	GO:0010975	B	.	regulation of neuron projection development
XP_001807351.1 PREDICTED: similar to pol polyprotein [Tribolium castaneum]	XP_001807351.1	3,00E-16	GLOS_LOC10014:	GO:0045664	B	.	regulation of neuron differentiation
XP_001807351.1 PREDICTED: similar to pol polyprotein [Tribolium castaneum]	XP_001807351.1	3,00E-16	GLOS_LOC10014:	GO:0050771	B	Refseq	negative regulation of axonogenesis
XP_001807351.1 PREDICTED: similar to pol polyprotein [Tribolium castaneum]	XP_001807351.1	3,00E-16	GLOS_LOC10014:	GO:0050851	B	.	antigen receptor-mediated signaling pathway
XP_001807351.1 PREDICTED: similar to pol polyprotein [Tribolium castaneum]	XP_001807351.1	3,00E-16	GLOS_LOC10014:	GO:0002429	B	.	immune response-activating cell surface receptor signaling pathway
XP_001807351.1 PREDICTED: similar to pol polyprotein [Tribolium castaneum]	XP_001807351.1	3,00E-16	GLOS_LOC10014:	GO:0002757	B	.	immune response-activating signal transduction
XP_001807351.1 PREDICTED: similar to pol polyprotein [Tribolium castaneum]	XP_001807351.1	3,00E-16	GLOS_LOC10014:	GO:0002253	B	.	activation of immune response
XP_001807351.1 PREDICTED: similar to pol polyprotein [Tribolium castaneum]	XP_001807351.1	3,00E-16	GLOS_LOC10014:	GO:0002376	B	.	immune system process
XP_001807351.1 PREDICTED: similar to pol polyprotein [Tribolium castaneum]	XP_001807351.1	3,00E-16	GLOS_LOC10014:	GO:0050778	B	.	positive regulation of immune response
XP_001807351.1 PREDICTED: similar to pol polyprotein [Tribolium castaneum]	XP_001807351.1	3,00E-16	GLOS_LOC10014:	GO:0002684	B	.	positive regulation of immune system process
XP_001807351.1 PREDICTED: similar to pol polyprotein [Tribolium castaneum]	XP_001807351.1	3,00E-16	GLOS_LOC10014:	GO:0002682	B	.	regulation of immune system process
XP_001807351.1 PREDICTED: similar to pol polyprotein [Tribolium castaneum]	XP_001807351.1	3,00E-16	GLOS_LOC10014:	GO:0048584	B	.	positive regulation of response to stimulus
XP_001807351.1 PREDICTED: similar to pol polyprotein [Tribolium castaneum]	XP_001807351.1	3,00E-16	GLOS_LOC10014:	GO:0048583	B	.	regulation of response to stimulus
XP_001807351.1 PREDICTED: similar to pol polyprotein [Tribolium castaneum]	XP_001807351.1	3,00E-16	GLOS_LOC10014:	GO:0050776	B	.	regulation of immune response
XP_001807351.1 PREDICTED: similar to pol polyprotein [Tribolium castaneum]	XP_001807351.1	3,00E-16	GLOS_LOC10014:	GO:0002764	B	.	immune response-regulating signaling pathway
XP_001807351.1 PREDICTED: similar to pol polyprotein [Tribolium castaneum]	XP_001807351.1	3,00E-16	GLOS_LOC10014:	GO:0002768	B	.	immune response-regulating cell surface receptor signaling pathway
XP_001807351.1 PREDICTED: similar to pol polyprotein [Tribolium castaneum]	XP_001807351.1	3,00E-16	GLOS_LOC10014:	GO:0050852	B	Refseq	T cell receptor signaling pathway
XP_001807351.1 PREDICTED: similar to pol polyprotein [Tribolium castaneum]	XP_001807351.1	3,00E-16	GLOS_LOC10014:	GO:0050856	B	.	regulation of T cell receptor signaling pathway
XP_001807351.1 PREDICTED: similar to pol polyprotein [Tribolium castaneum]	XP_001807351.1	3,00E-16	GLOS_LOC10014:	GO:0050854	B	.	regulation of antigen receptor-mediated signaling pathway
XP_001807351.1 PREDICTED: similar to pol polyprotein [Tribolium castaneum]	XP_001807351.1	3,00E-16	GLOS_LOC10014:	GO:0009966	B	.	regulation of signal transduction
XP_001807351.1 PREDICTED: similar to pol polyprotein [Tribolium castaneum]	XP_001807351.1	3,00E-16	GLOS_LOC10014:	GO:0010646	B	.	regulation of cell communication
XP_001807351.1 PREDICTED: similar to pol polyprotein [Tribolium castaneum]	XP_001807351.1	3,00E-16	GLOS_LOC10014:	GO:0023051	B	.	regulation of signaling
XP_001807351.1 PREDICTED: similar to pol polyprotein [Tribolium castaneum]	XP_001807351.1	3,00E-16	GLOS_LOC10014:	GO:0050858	B	.	negative regulation of antigen receptor-mediated signaling pathway
XP_001807351.1 PREDICTED: similar to pol polyprotein [Tribolium castaneum]	XP_001807351.1	3,00E-16	GLOS_LOC10014:	GO:0002683	B	.	negative regulation of immune system process
XP_001807351.1 PREDICTED: similar to pol polyprotein [Tribolium castaneum]	XP_001807351.1	3,00E-16	GLOS_LOC10014:	GO:0009968	B	.	negative regulation of signal transduction
XP_001807351.1 PREDICTED: similar to pol polyprotein [Tribolium castaneum]	XP_001807351.1	3,00E-16	GLOS_LOC10014:	GO:0010648	B	.	negative regulation of cell communication
XP_001807351.1 PREDICTED: similar to pol polyprotein [Tribolium castaneum]	XP_001807351.1	3,00E-16	GLOS_LOC10014:	GO:0023057	B	.	negative regulation of signaling
XP_001807351.1 PREDICTED: similar to pol polyprotein [Tribolium castaneum]	XP_001807351.1	3,00E-16	GLOS_LOC10014:	GO:0048585	B	.	negative regulation of response to stimulus
XP_001807351.1 PREDICTED: similar to pol polyprotein [Tribolium castaneum]	XP_001807351.1	3,00E-16	GLOS_LOC10014:	GO:0050860	B	Refseq	negative regulation of T cell receptor signaling pathway
XP_001807351.1 PREDICTED: similar to pol polyprotein [Tribolium castaneum]	XP_001807351.1	3,00E-16	GLOS_LOC10014:	GO:0050863	B	.	regulation of T cell activation

XP_001807351.1 PREDICTED: similar to pol polyprotein [Tribolium castaneum]	XP_001807351.1	3,00E-16	GLOS_LOC10014:	GO:0051249	B	.	regulation of lymphocyte activation
XP_001807351.1 PREDICTED: similar to pol polyprotein [Tribolium castaneum]	XP_001807351.1	3,00E-16	GLOS_LOC10014:	GO:0002694	B	.	regulation of leukocyte activation
XP_001807351.1 PREDICTED: similar to pol polyprotein [Tribolium castaneum]	XP_001807351.1	3,00E-16	GLOS_LOC10014:	GO:0050865	B	.	regulation of cell activation
XP_001807351.1 PREDICTED: similar to pol polyprotein [Tribolium castaneum]	XP_001807351.1	3,00E-16	GLOS_LOC10014:	GO:0051251	B	.	positive regulation of lymphocyte activation
XP_001807351.1 PREDICTED: similar to pol polyprotein [Tribolium castaneum]	XP_001807351.1	3,00E-16	GLOS_LOC10014:	GO:0002696	B	.	positive regulation of leukocyte activation
XP_001807351.1 PREDICTED: similar to pol polyprotein [Tribolium castaneum]	XP_001807351.1	3,00E-16	GLOS_LOC10014:	GO:0050867	B	.	positive regulation of cell activation
XP_001807351.1 PREDICTED: similar to pol polyprotein [Tribolium castaneum]	XP_001807351.1	3,00E-16	GLOS_LOC10014:	GO:0050870	B	Refseq	positive regulation of T cell activation
XP_001807351.1 PREDICTED: similar to pol polyprotein [Tribolium castaneum]	XP_001807351.1	3,00E-16	GLOS_LOC10014:	GO:0051281	B	Refseq	positive regulation of release of sequestered calcium ion into cytosol
XP_970252.2 PREDICTED: similar to formin 3 CG33556-PB [Tribolium castaneum]	XP_970252.2	4,00E-06	GLOS_LOC65880	GO:0001525	B	Refseq	angiogenesis
XP_970252.2 PREDICTED: similar to formin 3 CG33556-PB [Tribolium castaneum]	XP_970252.2	4,00E-06	GLOS_LOC65880	GO:0005100	M	Refseq	Rho GTPase activator activity
XP_970252.2 PREDICTED: similar to formin 3 CG33556-PB [Tribolium castaneum]	XP_970252.2	4,00E-06	GLOS_LOC65880	GO:0005178	M	Refseq	integrin binding
XP_970252.2 PREDICTED: similar to formin 3 CG33556-PB [Tribolium castaneum]	XP_970252.2	4,00E-06	GLOS_LOC65880	GO:0005515	M	Refseq	protein binding
XP_970252.2 PREDICTED: similar to formin 3 CG33556-PB [Tribolium castaneum]	XP_970252.2	4,00E-06	GLOS_LOC65880	GO:0005783	C	Refseq	endoplasmic reticulum
XP_970252.2 PREDICTED: similar to formin 3 CG33556-PB [Tribolium castaneum]	XP_970252.2	4,00E-06	GLOS_LOC65880	GO:0005829	C	Refseq	cytosol
XP_970252.2 PREDICTED: similar to formin 3 CG33556-PB [Tribolium castaneum]	XP_970252.2	4,00E-06	GLOS_LOC65880	GO:0005886	C	Refseq	plasma membrane
XP_970252.2 PREDICTED: similar to formin 3 CG33556-PB [Tribolium castaneum]	XP_970252.2	4,00E-06	GLOS_LOC65880	GO:0005887	C	Refseq	integral to plasma membrane
XP_970252.2 PREDICTED: similar to formin 3 CG33556-PB [Tribolium castaneum]	XP_970252.2	4,00E-06	GLOS_LOC65880	GO:0006469	B	Refseq	negative regulation of protein kinase activity
XP_970252.2 PREDICTED: similar to formin 3 CG33556-PB [Tribolium castaneum]	XP_970252.2	4,00E-06	GLOS_LOC65880	GO:0007010	B	Refseq	cytoskeleton organization
XP_970252.2 PREDICTED: similar to formin 3 CG33556-PB [Tribolium castaneum]	XP_970252.2	4,00E-06	GLOS_LOC65880	GO:0009897	C	Refseq	external side of plasma membrane
XP_970252.2 PREDICTED: similar to formin 3 CG33556-PB [Tribolium castaneum]	XP_970252.2	4,00E-06	GLOS_LOC65880	GO:0016324	C	Refseq	apical plasma membrane
XP_970252.2 PREDICTED: similar to formin 3 CG33556-PB [Tribolium castaneum]	XP_970252.2	4,00E-06	GLOS_LOC65880	GO:0016337	B	Refseq	cell-cell adhesion
XP_970252.2 PREDICTED: similar to formin 3 CG33556-PB [Tribolium castaneum]	XP_970252.2	4,00E-06	GLOS_LOC65880	GO:0019901	M	Refseq	protein kinase binding
XP_970252.2 PREDICTED: similar to formin 3 CG33556-PB [Tribolium castaneum]	XP_970252.2	4,00E-06	GLOS_LOC65880	GO:0030336	B	Refseq	negative regulation of cell migration
XP_970252.2 PREDICTED: similar to formin 3 CG33556-PB [Tribolium castaneum]	XP_970252.2	4,00E-06	GLOS_LOC65880	GO:0030425	C	Refseq	dendrite
XP_970252.2 PREDICTED: similar to formin 3 CG33556-PB [Tribolium castaneum]	XP_970252.2	4,00E-06	GLOS_LOC65880	GO:0030426	C	Refseq	growth cone
XP_970252.2 PREDICTED: similar to formin 3 CG33556-PB [Tribolium castaneum]	XP_970252.2	4,00E-06	GLOS_LOC65880	GO:0031362	C	Refseq	anchored to external side of plasma membrane
XP_970252.2 PREDICTED: similar to formin 3 CG33556-PB [Tribolium castaneum]	XP_970252.2	4,00E-06	GLOS_LOC65880	GO:0034235	M	Refseq	GPI anchor binding
XP_970252.2 PREDICTED: similar to formin 3 CG33556-PB [Tribolium castaneum]	XP_970252.2	4,00E-06	GLOS_LOC65880	GO:0043547	B	Refseq	positive regulation of GTPase activity
XP_970252.2 PREDICTED: similar to formin 3 CG33556-PB [Tribolium castaneum]	XP_970252.2	4,00E-06	GLOS_LOC65880	GO:0045121	C	Refseq	membrane raft
XP_970252.2 PREDICTED: similar to formin 3 CG33556-PB [Tribolium castaneum]	XP_970252.2	4,00E-06	GLOS_LOC65880	GO:0046549	B	Refseq	retinal cone cell development
XP_970252.2 PREDICTED: similar to formin 3 CG33556-PB [Tribolium castaneum]	XP_970252.2	4,00E-06	GLOS_LOC65880	GO:0048041	B	Refseq	focal adhesion assembly
XP_970252.2 PREDICTED: similar to formin 3 CG33556-PB [Tribolium castaneum]	XP_970252.2	4,00E-06	GLOS_LOC65880	GO:0050771	B	Refseq	negative regulation of axonogenesis
XP_970252.2 PREDICTED: similar to formin 3 CG33556-PB [Tribolium castaneum]	XP_970252.2	4,00E-06	GLOS_LOC65880	GO:0050852	B	Refseq	T cell receptor signaling pathway
XP_970252.2 PREDICTED: similar to formin 3 CG33556-PB [Tribolium castaneum]	XP_970252.2	4,00E-06	GLOS_LOC65880	GO:0050860	B	Refseq	negative regulation of T cell receptor signaling pathway
XP_970252.2 PREDICTED: similar to formin 3 CG33556-PB [Tribolium castaneum]	XP_970252.2	4,00E-06	GLOS_LOC65880	GO:0050870	B	Refseq	positive regulation of T cell activation
XP_970252.2 PREDICTED: similar to formin 3 CG33556-PB [Tribolium castaneum]	XP_970252.2	4,00E-06	GLOS_LOC65880	GO:0051281	B	Refseq	positive regulation of release of sequestered calcium ion into cytosol
XM_003395475.1 PRED: B. terrestris 40S ribos. prot S29-like (LOC100646020), mRNA	XM_003395475.1	4,00E-13	GLOS_LOC10064	GO:0005576	C	Refseq	extracellular region
XM_003395475.1 PRED: B. terrestris 40S ribos. prot S29-like (LOC100646020), mRNA	XM_003395475.1	4,00E-13	GLOS_LOC10064	GO:0008083	M	Refseq	growth factor activity
XM_003395475.1 PRED: B. terrestris 40S ribos. prot S29-like (LOC100646020), mRNA	XM_003395475.1	4,00E-13	GLOS_LOC10064	GO:0040007	B	Refseq	growth
NP_651109.3 CG34375, isoform A [Drosophila melanogaster]	NP_651109.3	9,00E-87	GLOS_CG34375.1	GO:0000122	B	Refseq	negative regulation of transcription from RNA polymerase II promoter
NP_651109.3 CG34375, isoform A [Drosophila melanogaster]	NP_651109.3	9,00E-87	GLOS_CG34375.1	GO:0001501	B	Refseq	skeletal system development
NP_651109.3 CG34375, isoform A [Drosophila melanogaster]	NP_651109.3	9,00E-87	GLOS_CG34375.1	GO:0003700	M	Refseq	sequence-specific DNA binding transcription factor activity
NP_651109.3 CG34375, isoform A [Drosophila melanogaster]	NP_651109.3	9,00E-87	GLOS_CG34375.1	GO:0005515	M	Refseq	protein binding
NP_651109.3 CG34375, isoform A [Drosophila melanogaster]	NP_651109.3	9,00E-87	GLOS_CG34375.1	GO:0005634	C	Refseq	nucleus
NP_651109.3 CG34375, isoform A [Drosophila melanogaster]	NP_651109.3	9,00E-87	GLOS_CG34375.1	GO:0006366	B	Refseq	transcription from RNA polymerase II promoter
NP_651109.3 CG34375, isoform A [Drosophila melanogaster]	NP_651109.3	9,00E-87	GLOS_CG34375.1	GO:0006607	B	Refseq	NLS-bearing protein import into nucleus
NP_651109.3 CG34375, isoform A [Drosophila melanogaster]	NP_651109.3	9,00E-87	GLOS_CG34375.1	GO:0008270	M	Refseq	zinc ion binding
NP_651109.3 CG34375, isoform A [Drosophila melanogaster]	NP_651109.3	9,00E-87	GLOS_CG34375.1	GO:0032330	B	Refseq	regulation of chondrocyte differentiation
NP_651109.3 CG34375, isoform A [Drosophila melanogaster]	NP_651109.3	9,00E-87	GLOS_CG34375.1	GO:0043565	M	Refseq	sequence-specific DNA binding
NP_001036706.2 CG42327 [Drosophila melanogaster]	NP_001036706.2	2,00E-11	GLOS_CG42327.1	GO:0000122	B	Refseq	negative regulation of transcription from RNA polymerase II promoter
NP_001036706.2 CG42327 [Drosophila melanogaster]	NP_001036706.2	2,00E-11	GLOS_CG42327.1	GO:0001501	B	Refseq	skeletal system development
NP_001036706.2 CG42327 [Drosophila melanogaster]	NP_001036706.2	2,00E-11	GLOS_CG42327.1	GO:0003700	M	Refseq	sequence-specific DNA binding transcription factor activity
NP_001036706.2 CG42327 [Drosophila melanogaster]	NP_001036706.2	2,00E-11	GLOS_CG42327.1	GO:0005515	M	Refseq	protein binding
NP_001036706.2 CG42327 [Drosophila melanogaster]	NP_001036706.2	2,00E-11	GLOS_CG42327.1	GO:0005634	C	Refseq	nucleus

NP_001036706.2 CG42327 [Drosophila melanogaster]	NP_001036706.2	2,00E-11	GLOS_CG42327.1	GO:0006366	B	Refseq	transcription from RNA polymerase II promoter
NP_001036706.2 CG42327 [Drosophila melanogaster]	NP_001036706.2	2,00E-11	GLOS_CG42327.1	GO:0006607	B	Refseq	NLS-bearing protein import into nucleus
NP_001036706.2 CG42327 [Drosophila melanogaster]	NP_001036706.2	2,00E-11	GLOS_CG42327.1	GO:0008270	M	Refseq	zinc ion binding
NP_001036706.2 CG42327 [Drosophila melanogaster]	NP_001036706.2	2,00E-11	GLOS_CG42327.1	GO:0032330	B	Refseq	regulation of chondrocyte differentiation
NP_001036706.2 CG42327 [Drosophila melanogaster]	NP_001036706.2	2,00E-11	GLOS_CG42327.1	GO:0043565	M	Refseq	sequence-specific DNA binding
XP_001986508.1 GH21400 [Drosophila grimshawi]	XP_001986508.1	1,00E-117	GLOS_DGRI_GH2	GO:0005262	M	Refseq	calcium channel activity
XP_001986508.1 GH21400 [Drosophila grimshawi]	XP_001986508.1	1,00E-117	GLOS_DGRI_GH2	GO:0005515	M	Refseq	protein binding
XP_001986508.1 GH21400 [Drosophila grimshawi]	XP_001986508.1	1,00E-117	GLOS_DGRI_GH2	GO:0005886	C	Refseq	plasma membrane
XP_001986508.1 GH21400 [Drosophila grimshawi]	XP_001986508.1	1,00E-117	GLOS_DGRI_GH2	GO:0005887	C	Refseq	integral to plasma membrane
XP_001986508.1 GH21400 [Drosophila grimshawi]	XP_001986508.1	1,00E-117	GLOS_DGRI_GH2	GO:0006816	B	Refseq	calcium ion transport
XP_001986508.1 GH21400 [Drosophila grimshawi]	XP_001986508.1	1,00E-117	GLOS_DGRI_GH2	GO:0007411	B	Refseq	axon guidance
XP_001986508.1 GH21400 [Drosophila grimshawi]	XP_001986508.1	1,00E-117	GLOS_DGRI_GH2	GO:0007596	B	Refseq	blood coagulation
XP_001986508.1 GH21400 [Drosophila grimshawi]	XP_001986508.1	1,00E-117	GLOS_DGRI_GH2	GO:0007602	B	Refseq	phototransduction
XP_001986508.1 GH21400 [Drosophila grimshawi]	XP_001986508.1	1,00E-117	GLOS_DGRI_GH2	GO:0010524	B	Refseq	positive regulation of calcium ion transport into cytosol
XP_001986508.1 GH21400 [Drosophila grimshawi]	XP_001986508.1	1,00E-117	GLOS_DGRI_GH2	GO:0015279	M	Refseq	store-operated calcium channel activity
XP_001986508.1 GH21400 [Drosophila grimshawi]	XP_001986508.1	1,00E-117	GLOS_DGRI_GH2	GO:0030168	B	Refseq	platelet activation
XP_001986508.1 GH21400 [Drosophila grimshawi]	XP_001986508.1	1,00E-117	GLOS_DGRI_GH2	GO:0033198	B	Refseq	response to ATP
XP_001986508.1 GH21400 [Drosophila grimshawi]	XP_001986508.1	1,00E-117	GLOS_DGRI_GH2	GO:0051592	B	Refseq	response to calcium ion
XP_001986508.1 GH21400 [Drosophila grimshawi]	XP_001986508.1	1,00E-117	GLOS_DGRI_GH2	GO:0070588	B	Refseq	calcium ion transmembrane transport
XP_001986508.1 GH21400 [Drosophila grimshawi]	XP_001986508.1	1,00E-117	GLOS_DGRI_GH2	GO:0070679	M	Refseq	inositol 1,4,5 trisphosphate binding
NP_572805.1 CG2556 [Drosophila melanogaster]	NP_572805.1	2,00E-64	GLOS_CG2556.1.:	GO:0000122	B	Refseq	negative regulation of transcription from RNA polymerase II promoter
NP_572805.1 CG2556 [Drosophila melanogaster]	NP_572805.1	2,00E-64	GLOS_CG2556.1.:	GO:0001501	B	Refseq	skeletal system development
NP_572805.1 CG2556 [Drosophila melanogaster]	NP_572805.1	2,00E-64	GLOS_CG2556.1.:	GO:0003700	M	Refseq	sequence-specific DNA binding transcription factor activity
NP_572805.1 CG2556 [Drosophila melanogaster]	NP_572805.1	2,00E-64	GLOS_CG2556.1.:	GO:0005515	M	Refseq	protein binding
NP_572805.1 CG2556 [Drosophila melanogaster]	NP_572805.1	2,00E-64	GLOS_CG2556.1.:	GO:0005634	C	Refseq	nucleus
NP_572805.1 CG2556 [Drosophila melanogaster]	NP_572805.1	2,00E-64	GLOS_CG2556.1.:	GO:0006366	B	Refseq	transcription from RNA polymerase II promoter
NP_572805.1 CG2556 [Drosophila melanogaster]	NP_572805.1	2,00E-64	GLOS_CG2556.1.:	GO:0006607	B	Refseq	NLS-bearing protein import into nucleus
NP_572805.1 CG2556 [Drosophila melanogaster]	NP_572805.1	2,00E-64	GLOS_CG2556.1.:	GO:0008270	M	Refseq	zinc ion binding
NP_572805.1 CG2556 [Drosophila melanogaster]	NP_572805.1	2,00E-64	GLOS_CG2556.1.:	GO:0032330	B	Refseq	regulation of chondrocyte differentiation
NP_572805.1 CG2556 [Drosophila melanogaster]	NP_572805.1	2,00E-64	GLOS_CG2556.1.:	GO:0043565	M	Refseq	sequence-specific DNA binding
XP_001989945.1 GH19073 /001999168.1| GI23217 [D.mo]/002054301.1| GJ22877	XP_001989945.1	1,00E-126	GLOS_DGRI_GH1	GO:0005262	M	Refseq	calcium channel activity
XP_001989945.1 GH19073 /001999168.1| GI23217 [D.mo]/002054301.1| GJ22877	XP_001989945.1	1,00E-126	GLOS_DGRI_GH1	GO:0005515	M	Refseq	protein binding
XP_001989945.1 GH19073 /001999168.1| GI23217 [D.mo]/002054301.1| GJ22877	XP_001989945.1	1,00E-126	GLOS_DGRI_GH1	GO:0005886	C	Refseq	plasma membrane
XP_001989945.1 GH19073 /001999168.1| GI23217 [D.mo]/002054301.1| GJ22877	XP_001989945.1	1,00E-126	GLOS_DGRI_GH1	GO:0005887	C	Refseq	integral to plasma membrane
XP_001989945.1 GH19073 /001999168.1| GI23217 [D.mo]/002054301.1| GJ22877	XP_001989945.1	1,00E-126	GLOS_DGRI_GH1	GO:0006816	B	Refseq	calcium ion transport
XP_001989945.1 GH19073 /001999168.1| GI23217 [D.mo]/002054301.1| GJ22877	XP_001989945.1	1,00E-126	GLOS_DGRI_GH1	GO:0007411	B	Refseq	axon guidance
XP_001989945.1 GH19073 /001999168.1| GI23217 [D.mo]/002054301.1| GJ22877	XP_001989945.1	1,00E-126	GLOS_DGRI_GH1	GO:0007596	B	Refseq	blood coagulation
XP_001989945.1 GH19073 /001999168.1| GI23217 [D.mo]/002054301.1| GJ22877	XP_001989945.1	1,00E-126	GLOS_DGRI_GH1	GO:0007602	B	Refseq	phototransduction
XP_001989945.1 GH19073 /001999168.1| GI23217 [D.mo]/002054301.1| GJ22877	XP_001989945.1	1,00E-126	GLOS_DGRI_GH1	GO:0010524	B	Refseq	positive regulation of calcium ion transport into cytosol
XP_001989945.1 GH19073 /001999168.1| GI23217 [D.mo]/002054301.1| GJ22877	XP_001989945.1	1,00E-126	GLOS_DGRI_GH1	GO:0015279	M	Refseq	store-operated calcium channel activity
XP_001989945.1 GH19073 /001999168.1| GI23217 [D.mo]/002054301.1| GJ22877	XP_001989945.1	1,00E-126	GLOS_DGRI_GH1	GO:0030168	B	Refseq	platelet activation
XP_001989945.1 GH19073 /001999168.1| GI23217 [D.mo]/002054301.1| GJ22877	XP_001989945.1	1,00E-126	GLOS_DGRI_GH1	GO:0033198	B	Refseq	response to ATP
XP_001989945.1 GH19073 /001999168.1| GI23217 [D.mo]/002054301.1| GJ22877	XP_001989945.1	1,00E-126	GLOS_DGRI_GH1	GO:0051592	B	Refseq	response to calcium ion
XP_001989945.1 GH19073 /001999168.1| GI23217 [D.mo]/002054301.1| GJ22877	XP_001989945.1	1,00E-126	GLOS_DGRI_GH1	GO:0070588	B	Refseq	calcium ion transmembrane transport
XP_001989945.1 GH19073 /001999168.1| GI23217 [D.mo]/002054301.1| GJ22877	XP_001989945.1	1,00E-126	GLOS_DGRI_GH1	GO:0070679	M	Refseq	inositol 1,4,5 trisphosphate binding
NP_001260273.1 CG9541, isoform D [Drosophila melanogaster]	NP_001260273.1	1,00E-151	GLOS_CG9541.1.:	GO:0000122	B	Refseq	negative regulation of transcription from RNA polymerase II promoter
NP_001260273.1 CG9541, isoform D [Drosophila melanogaster]	NP_001260273.1	1,00E-151	GLOS_CG9541.1.:	GO:0001501	B	Refseq	skeletal system development
NP_001260273.1 CG9541, isoform D [Drosophila melanogaster]	NP_001260273.1	1,00E-151	GLOS_CG9541.1.:	GO:0003700	M	Refseq	sequence-specific DNA binding transcription factor activity
NP_001260273.1 CG9541, isoform D [Drosophila melanogaster]	NP_001260273.1	1,00E-151	GLOS_CG9541.1.:	GO:0005515	M	Refseq	protein binding
NP_001260273.1 CG9541, isoform D [Drosophila melanogaster]	NP_001260273.1	1,00E-151	GLOS_CG9541.1.:	GO:0005634	C	Refseq	nucleus
NP_001260273.1 CG9541, isoform D [Drosophila melanogaster]	NP_001260273.1	1,00E-151	GLOS_CG9541.1.:	GO:0006366	B	Refseq	transcription from RNA polymerase II promoter
NP_001260273.1 CG9541, isoform D [Drosophila melanogaster]	NP_001260273.1	1,00E-151	GLOS_CG9541.1.:	GO:0006607	B	Refseq	NLS-bearing protein import into nucleus
NP_001260273.1 CG9541, isoform D [Drosophila melanogaster]	NP_001260273.1	1,00E-151	GLOS_CG9541.1.:	GO:0008270	M	Refseq	zinc ion binding
NP_001260273.1 CG9541, isoform D [Drosophila melanogaster]	NP_001260273.1	1,00E-151	GLOS_CG9541.1.:	GO:0032330	B	Refseq	regulation of chondrocyte differentiation

NP_001260273.1 CG9541, isoform D [Drosophila melanogaster]	NP_001260273.1	1,00E-151	GLOS_CG9541.1.:	GO:0043565	M	Refseq	sequence-specific DNA binding
XP_001986777.1 GH21555 [Drosophila grimshawi]	XP_001986777.1	1,00E-107	GLOS_DGRI_GH2	GO:0005262	M	Refseq	calcium channel activity
XP_001986777.1 GH21555 [Drosophila grimshawi]	XP_001986777.1	1,00E-107	GLOS_DGRI_GH2	GO:0005515	M	Refseq	protein binding
XP_001986777.1 GH21555 [Drosophila grimshawi]	XP_001986777.1	1,00E-107	GLOS_DGRI_GH2	GO:0005886	C	Refseq	plasma membrane
XP_001986777.1 GH21555 [Drosophila grimshawi]	XP_001986777.1	1,00E-107	GLOS_DGRI_GH2	GO:0005887	C	Refseq	integral to plasma membrane
XP_001986777.1 GH21555 [Drosophila grimshawi]	XP_001986777.1	1,00E-107	GLOS_DGRI_GH2	GO:0006816	B	Refseq	calcium ion transport
XP_001986777.1 GH21555 [Drosophila grimshawi]	XP_001986777.1	1,00E-107	GLOS_DGRI_GH2	GO:0007411	B	Refseq	axon guidance
XP_001986777.1 GH21555 [Drosophila grimshawi]	XP_001986777.1	1,00E-107	GLOS_DGRI_GH2	GO:0007596	B	Refseq	blood coagulation
XP_001986777.1 GH21555 [Drosophila grimshawi]	XP_001986777.1	1,00E-107	GLOS_DGRI_GH2	GO:0007602	B	Refseq	phototransduction
XP_001986777.1 GH21555 [Drosophila grimshawi]	XP_001986777.1	1,00E-107	GLOS_DGRI_GH2	GO:0010524	B	Refseq	positive regulation of calcium ion transport into cytosol
XP_001986777.1 GH21555 [Drosophila grimshawi]	XP_001986777.1	1,00E-107	GLOS_DGRI_GH2	GO:0015279	M	Refseq	store-operated calcium channel activity
XP_001986777.1 GH21555 [Drosophila grimshawi]	XP_001986777.1	1,00E-107	GLOS_DGRI_GH2	GO:0030168	B	Refseq	platelet activation
XP_001986777.1 GH21555 [Drosophila grimshawi]	XP_001986777.1	1,00E-107	GLOS_DGRI_GH2	GO:0033198	B	Refseq	response to ATP
XP_001986777.1 GH21555 [Drosophila grimshawi]	XP_001986777.1	1,00E-107	GLOS_DGRI_GH2	GO:0051592	B	Refseq	response to calcium ion
XP_001986777.1 GH21555 [Drosophila grimshawi]	XP_001986777.1	1,00E-107	GLOS_DGRI_GH2	GO:0070588	B	Refseq	calcium ion transmembrane transport
XP_001986777.1 GH21555 [Drosophila grimshawi]	XP_001986777.1	1,00E-107	GLOS_DGRI_GH2	GO:0070679	M	Refseq	inositol 1,4,5 trisphosphate binding
NP_787956.1 CG33127 [Drosophila melanogaster]	NP_787956.1	1,00E-81	GLOS_CG33127.1	GO:0000122	B	Refseq	negative regulation of transcription from RNA polymerase II promoter
NP_787956.1 CG33127 [Drosophila melanogaster]	NP_787956.1	1,00E-81	GLOS_CG33127.1	GO:0001501	B	Refseq	skeletal system development
NP_787956.1 CG33127 [Drosophila melanogaster]	NP_787956.1	1,00E-81	GLOS_CG33127.1	GO:0003700	M	Refseq	sequence-specific DNA binding transcription factor activity
NP_787956.1 CG33127 [Drosophila melanogaster]	NP_787956.1	1,00E-81	GLOS_CG33127.1	GO:0005515	M	Refseq	protein binding
NP_787956.1 CG33127 [Drosophila melanogaster]	NP_787956.1	1,00E-81	GLOS_CG33127.1	GO:0005634	C	Refseq	nucleus
NP_787956.1 CG33127 [Drosophila melanogaster]	NP_787956.1	1,00E-81	GLOS_CG33127.1	GO:0006366	B	Refseq	transcription from RNA polymerase II promoter
NP_787956.1 CG33127 [Drosophila melanogaster]	NP_787956.1	1,00E-81	GLOS_CG33127.1	GO:0006607	B	Refseq	NLS-bearing protein import into nucleus
NP_787956.1 CG33127 [Drosophila melanogaster]	NP_787956.1	1,00E-81	GLOS_CG33127.1	GO:0008270	M	Refseq	zinc ion binding
NP_787956.1 CG33127 [Drosophila melanogaster]	NP_787956.1	1,00E-81	GLOS_CG33127.1	GO:0032330	B	Refseq	regulation of chondrocyte differentiation
NP_787956.1 CG33127 [Drosophila melanogaster]	NP_787956.1	1,00E-81	GLOS_CG33127.1	GO:0043565	M	Refseq	sequence-specific DNA binding
XP_001657469.1 hypothetical protein AaeL_AAEL000958 [Aedes aegypti]	XP_001657469.1	1,00E-11	GLOS_AAEL_AAE	GO:0005070	M	Refseq	SH3/SH2 adaptor activity
XP_001657469.1 hypothetical protein AaeL_AAEL000958 [Aedes aegypti]	XP_001657469.1	1,00E-11	GLOS_AAEL_AAE	GO:0005515	M	Refseq	protein binding
XP_001657469.1 hypothetical protein AaeL_AAEL000958 [Aedes aegypti]	XP_001657469.1	1,00E-11	GLOS_AAEL_AAE	GO:0005634	C	Refseq	nucleus
XP_001657469.1 hypothetical protein AaeL_AAEL000958 [Aedes aegypti]	XP_001657469.1	1,00E-11	GLOS_AAEL_AAE	GO:0005737	C	Refseq	cytoplasm
XP_001657469.1 hypothetical protein AaeL_AAEL000958 [Aedes aegypti]	XP_001657469.1	1,00E-11	GLOS_AAEL_AAE	GO:0007049	B	Refseq	cell cycle
XP_001657469.1 hypothetical protein AaeL_AAEL000958 [Aedes aegypti]	XP_001657469.1	1,00E-11	GLOS_AAEL_AAE	GO:0007165	B	Refseq	signal transduction
XP_001657469.1 hypothetical protein AaeL_AAEL000958 [Aedes aegypti]	XP_001657469.1	1,00E-11	GLOS_AAEL_AAE	GO:0007417	B	Refseq	central nervous system development
XP_001657469.1 hypothetical protein AaeL_AAEL000958 [Aedes aegypti]	XP_001657469.1	1,00E-11	GLOS_AAEL_AAE	GO:0007507	B	Refseq	heart development
XP_001657469.1 hypothetical protein AaeL_AAEL000958 [Aedes aegypti]	XP_001657469.1	1,00E-11	GLOS_AAEL_AAE	GO:0009792	B	Refseq	embryo development ending in birth or egg hatching
XP_001657469.1 hypothetical protein AaeL_AAEL000958 [Aedes aegypti]	XP_001657469.1	1,00E-11	GLOS_AAEL_AAE	GO:0010212	B	Refseq	response to ionizing radiation
XP_001657469.1 hypothetical protein AaeL_AAEL000958 [Aedes aegypti]	XP_001657469.1	1,00E-11	GLOS_AAEL_AAE	GO:0017124	M	Refseq	SH3 domain binding
XP_001657469.1 hypothetical protein AaeL_AAEL000958 [Aedes aegypti]	XP_001657469.1	1,00E-11	GLOS_AAEL_AAE	GO:0042802	M	Refseq	identical protein binding
XP_001657469.1 hypothetical protein AaeL_AAEL000958 [Aedes aegypti]	XP_001657469.1	1,00E-11	GLOS_AAEL_AAE	GO:0045786	B	Refseq	negative regulation of cell cycle
XP_001657469.1 hypothetical protein AaeL_AAEL000958 [Aedes aegypti]	XP_001657469.1	1,00E-11	GLOS_AAEL_AAE	GO:0048471	C	Refseq	perinuclear region of cytoplasm
XP_001657469.1 hypothetical protein AaeL_AAEL000958 [Aedes aegypti]	XP_001657469.1	1,00E-11	GLOS_AAEL_AAE	GO:0051059	M	Refseq	NF-kappaB binding
XP_001657469.1 hypothetical protein AaeL_AAEL000958 [Aedes aegypti]	XP_001657469.1	1,00E-11	GLOS_AAEL_AAE	GO:0072332	B	Refseq	intrinsic apoptotic signaling pathway by p53 class mediator
XP_001991088.1 GH12482 [Drosophila grimshawi]	XP_001991088.1	1,00E-108	GLOS_DGRI_GH1	GO:0005262	M	Refseq	calcium channel activity
XP_001991088.1 GH12482 [Drosophila grimshawi]	XP_001991088.1	1,00E-108	GLOS_DGRI_GH1	GO:0005515	M	Refseq	protein binding
XP_001991088.1 GH12482 [Drosophila grimshawi]	XP_001991088.1	1,00E-108	GLOS_DGRI_GH1	GO:0005886	C	Refseq	plasma membrane
XP_001991088.1 GH12482 [Drosophila grimshawi]	XP_001991088.1	1,00E-108	GLOS_DGRI_GH1	GO:0005887	C	Refseq	integral to plasma membrane
XP_001991088.1 GH12482 [Drosophila grimshawi]	XP_001991088.1	1,00E-108	GLOS_DGRI_GH1	GO:0006816	B	Refseq	calcium ion transport
XP_001991088.1 GH12482 [Drosophila grimshawi]	XP_001991088.1	1,00E-108	GLOS_DGRI_GH1	GO:0007411	B	Refseq	axon guidance
XP_001991088.1 GH12482 [Drosophila grimshawi]	XP_001991088.1	1,00E-108	GLOS_DGRI_GH1	GO:0007596	B	Refseq	blood coagulation
XP_001991088.1 GH12482 [Drosophila grimshawi]	XP_001991088.1	1,00E-108	GLOS_DGRI_GH1	GO:0007602	B	Refseq	phototransduction
XP_001991088.1 GH12482 [Drosophila grimshawi]	XP_001991088.1	1,00E-108	GLOS_DGRI_GH1	GO:0010524	B	Refseq	positive regulation of calcium ion transport into cytosol
XP_001991088.1 GH12482 [Drosophila grimshawi]	XP_001991088.1	1,00E-108	GLOS_DGRI_GH1	GO:0015279	M	Refseq	store-operated calcium channel activity
XP_001991088.1 GH12482 [Drosophila grimshawi]	XP_001991088.1	1,00E-108	GLOS_DGRI_GH1	GO:0030168	B	Refseq	platelet activation
XP_001991088.1 GH12482 [Drosophila grimshawi]	XP_001991088.1	1,00E-108	GLOS_DGRI_GH1	GO:0033198	B	Refseq	response to ATP

XP_001991088.1 GH12482 [Drosophila grimshawi]	XP_001991088.1	1,00E-108	GLOS_DGRI_GH1	GO:0051592	B	Refseq	response to calcium ion
XP_001991088.1 GH12482 [Drosophila grimshawi]	XP_001991088.1	1,00E-108	GLOS_DGRI_GH1	GO:0070588	B	Refseq	calcium ion transmembrane transport
XP_001991088.1 GH12482 [Drosophila grimshawi]	XP_001991088.1	1,00E-108	GLOS_DGRI_GH1	GO:0070679	M	Refseq	inositol 1,4,5 trisphosphate binding
XP_003426074.1 PREDICTED: hypoth. Prot. LOC100678720 [N. vitripennis]	XP_003426074.1	1,00E-174	GLOS_LOC10067	GO:0022602	B	.	ovulation cycle process
XP_003426074.1 PREDICTED: hypoth. Prot. LOC100678720 [N. vitripennis]	XP_003426074.1	1,00E-174	GLOS_LOC10067	GO:0048511	B	.	rhythmic process
XP_003426074.1 PREDICTED: hypoth. Prot. LOC100678720 [N. vitripennis]	XP_003426074.1	1,00E-174	GLOS_LOC10067	GO:0042698	B	.	ovulation cycle
XP_003426074.1 PREDICTED: hypoth. Prot. LOC100678720 [N. vitripennis]	XP_003426074.1	1,00E-174	GLOS_LOC10067	GO:0044702	B	.	single organism reproductive process
XP_003426074.1 PREDICTED: hypoth. Prot. LOC100678720 [N. vitripennis]	XP_003426074.1	1,00E-174	GLOS_LOC10067	GO:0022414	B	.	reproductive process
XP_003426074.1 PREDICTED: hypoth. Prot. LOC100678720 [N. vitripennis]	XP_003426074.1	1,00E-174	GLOS_LOC10067	GO:0000003	B	.	reproduction
XP_003426074.1 PREDICTED: hypoth. Prot. LOC100678720 [N. vitripennis]	XP_003426074.1	1,00E-174	GLOS_LOC10067	GO:0048609	B	.	multicellular organismal reproductive process
XP_003426074.1 PREDICTED: hypoth. Prot. LOC100678720 [N. vitripennis]	XP_003426074.1	1,00E-174	GLOS_LOC10067	GO:0032504	B	.	multicellular organism reproduction
XP_003426074.1 PREDICTED: hypoth. Prot. LOC100678720 [N. vitripennis]	XP_003426074.1	1,00E-174	GLOS_LOC10067	GO:0008585	B	.	female gonad development
XP_003426074.1 PREDICTED: hypoth. Prot. LOC100678720 [N. vitripennis]	XP_003426074.1	1,00E-174	GLOS_LOC10067	GO:0008406	B	.	gonad development
XP_003426074.1 PREDICTED: hypoth. Prot. LOC100678720 [N. vitripennis]	XP_003426074.1	1,00E-174	GLOS_LOC10067	GO:0048608	B	.	reproductive structure development
XP_003426074.1 PREDICTED: hypoth. Prot. LOC100678720 [N. vitripennis]	XP_003426074.1	1,00E-174	GLOS_LOC10067	GO:0003006	B	.	developmental process involved in reproduction
XP_003426074.1 PREDICTED: hypoth. Prot. LOC100678720 [N. vitripennis]	XP_003426074.1	1,00E-174	GLOS_LOC10067	GO:0061458	B	.	reproductive system development
XP_003426074.1 PREDICTED: hypoth. Prot. LOC100678720 [N. vitripennis]	XP_003426074.1	1,00E-174	GLOS_LOC10067	GO:0045137	B	.	development of primary sexual characteristics
XP_003426074.1 PREDICTED: hypoth. Prot. LOC100678720 [N. vitripennis]	XP_003426074.1	1,00E-174	GLOS_LOC10067	GO:0007548	B	.	sex differentiation
XP_003426074.1 PREDICTED: hypoth. Prot. LOC100678720 [N. vitripennis]	XP_003426074.1	1,00E-174	GLOS_LOC10067	GO:0046545	B	.	development of primary female sexual characteristics
XP_003426074.1 PREDICTED: hypoth. Prot. LOC100678720 [N. vitripennis]	XP_003426074.1	1,00E-174	GLOS_LOC10067	GO:0046660	B	.	female sex differentiation
XP_003426074.1 PREDICTED: hypoth. Prot. LOC100678720 [N. vitripennis]	XP_003426074.1	1,00E-174	GLOS_LOC10067	GO:0001541	B	Refseq	ovarian follicle development
XP_003426074.1 PREDICTED: hypoth. Prot. LOC100678720 [N. vitripennis]	XP_003426074.1	1,00E-174	GLOS_LOC10067	GO:0040014	B	.	regulation of multicellular organism growth
XP_003426074.1 PREDICTED: hypoth. Prot. LOC100678720 [N. vitripennis]	XP_003426074.1	1,00E-174	GLOS_LOC10067	GO:0040008	B	.	regulation of growth
XP_003426074.1 PREDICTED: hypoth. Prot. LOC100678720 [N. vitripennis]	XP_003426074.1	1,00E-174	GLOS_LOC10067	GO:0002021	B	Refseq	response to dietary excess
XP_003426074.1 PREDICTED: hypoth. Prot. LOC100678720 [N. vitripennis]	XP_003426074.1	1,00E-174	GLOS_LOC10067	GO:0003674	M	Refseq	molecular_function
XP_003426074.1 PREDICTED: hypoth. Prot. LOC100678720 [N. vitripennis]	XP_003426074.1	1,00E-174	GLOS_LOC10067	GO:0005179	M	.	hormone activity
XP_003426074.1 PREDICTED: hypoth. Prot. LOC100678720 [N. vitripennis]	XP_003426074.1	1,00E-174	GLOS_LOC10067	GO:0005184	M	Refseq	neuropeptide hormone activity
XP_003426074.1 PREDICTED: hypoth. Prot. LOC100678720 [N. vitripennis]	XP_003426074.1	1,00E-174	GLOS_LOC10067	GO:0044421	C	.	extracellular region part
XP_003426074.1 PREDICTED: hypoth. Prot. LOC100678720 [N. vitripennis]	XP_003426074.1	1,00E-174	GLOS_LOC10067	GO:0005576	C	.	extracellular region
XP_003426074.1 PREDICTED: hypoth. Prot. LOC100678720 [N. vitripennis]	XP_003426074.1	1,00E-174	GLOS_LOC10067	GO:0005615	C	Refseq	extracellular space
XP_003426074.1 PREDICTED: hypoth. Prot. LOC100678720 [N. vitripennis]	XP_003426074.1	1,00E-174	GLOS_LOC10067	GO:0006091	B	Refseq	generation of precursor metabolites and energy
XP_003426074.1 PREDICTED: hypoth. Prot. LOC100678720 [N. vitripennis]	XP_003426074.1	1,00E-174	GLOS_LOC10067	GO:0008083	M	Refseq	growth factor activity
XP_003426074.1 PREDICTED: hypoth. Prot. LOC100678720 [N. vitripennis]	XP_003426074.1	1,00E-174	GLOS_LOC10067	GO:0009266	B	.	response to temperature stimulus
XP_003426074.1 PREDICTED: hypoth. Prot. LOC100678720 [N. vitripennis]	XP_003426074.1	1,00E-174	GLOS_LOC10067	GO:0009409	B	Refseq	response to cold
XP_003426074.1 PREDICTED: hypoth. Prot. LOC100678720 [N. vitripennis]	XP_003426074.1	1,00E-174	GLOS_LOC10067	GO:0019953	B	Refseq	sexual reproduction
XP_003426074.1 PREDICTED: hypoth. Prot. LOC100678720 [N. vitripennis]	XP_003426074.1	1,00E-174	GLOS_LOC10067	GO:0030072	B	.	peptide hormone secretion
XP_003426074.1 PREDICTED: hypoth. Prot. LOC100678720 [N. vitripennis]	XP_003426074.1	1,00E-174	GLOS_LOC10067	GO:0002790	B	.	peptide secretion
XP_003426074.1 PREDICTED: hypoth. Prot. LOC100678720 [N. vitripennis]	XP_003426074.1	1,00E-174	GLOS_LOC10067	GO:0015833	B	.	peptide transport
XP_003426074.1 PREDICTED: hypoth. Prot. LOC100678720 [N. vitripennis]	XP_003426074.1	1,00E-174	GLOS_LOC10067	GO:0042886	B	.	amide transport
XP_003426074.1 PREDICTED: hypoth. Prot. LOC100678720 [N. vitripennis]	XP_003426074.1	1,00E-174	GLOS_LOC10067	GO:0071705	B	.	nitrogen compound transport
XP_003426074.1 PREDICTED: hypoth. Prot. LOC100678720 [N. vitripennis]	XP_003426074.1	1,00E-174	GLOS_LOC10067	GO:0046879	B	.	hormone secretion
XP_003426074.1 PREDICTED: hypoth. Prot. LOC100678720 [N. vitripennis]	XP_003426074.1	1,00E-174	GLOS_LOC10067	GO:0009914	B	.	hormone transport
XP_003426074.1 PREDICTED: hypoth. Prot. LOC100678720 [N. vitripennis]	XP_003426074.1	1,00E-174	GLOS_LOC10067	GO:0010817	B	.	regulation of hormone levels
XP_003426074.1 PREDICTED: hypoth. Prot. LOC100678720 [N. vitripennis]	XP_003426074.1	1,00E-174	GLOS_LOC10067	GO:0023061	B	.	signal release
XP_003426074.1 PREDICTED: hypoth. Prot. LOC100678720 [N. vitripennis]	XP_003426074.1	1,00E-174	GLOS_LOC10067	GO:0032940	B	.	secretion by cell
XP_003426074.1 PREDICTED: hypoth. Prot. LOC100678720 [N. vitripennis]	XP_003426074.1	1,00E-174	GLOS_LOC10067	GO:0003001	B	.	generation of a signal involved in cell-cell signaling
XP_003426074.1 PREDICTED: hypoth. Prot. LOC100678720 [N. vitripennis]	XP_003426074.1	1,00E-174	GLOS_LOC10067	GO:0007267	B	.	cell-cell signaling
XP_003426074.1 PREDICTED: hypoth. Prot. LOC100678720 [N. vitripennis]	XP_003426074.1	1,00E-174	GLOS_LOC10067	GO:0030073	B	Refseq	insulin secretion
XP_003426074.1 PREDICTED: hypoth. Prot. LOC100678720 [N. vitripennis]	XP_003426074.1	1,00E-174	GLOS_LOC10067	GO:0016023	C	.	cytoplasmic membrane-bounded vesicle
XP_003426074.1 PREDICTED: hypoth. Prot. LOC100678720 [N. vitripennis]	XP_003426074.1	1,00E-174	GLOS_LOC10067	GO:0031410	C	.	cytoplasmic vesicle
XP_003426074.1 PREDICTED: hypoth. Prot. LOC100678720 [N. vitripennis]	XP_003426074.1	1,00E-174	GLOS_LOC10067	GO:0031982	C	.	vesicle
XP_003426074.1 PREDICTED: hypoth. Prot. LOC100678720 [N. vitripennis]	XP_003426074.1	1,00E-174	GLOS_LOC10067	GO:0031988	C	.	membrane-bounded vesicle
XP_003426074.1 PREDICTED: hypoth. Prot. LOC100678720 [N. vitripennis]	XP_003426074.1	1,00E-174	GLOS_LOC10067	GO:0030133	C	Refseq	transport vesicle
XP_003426074.1 PREDICTED: hypoth. Prot. LOC100678720 [N. vitripennis]	XP_003426074.1	1,00E-174	GLOS_LOC10067	GO:0031410	C	Refseq	cytoplasmic vesicle

XP_003426074.1 PREDICTED: hypoth. Prot. LOC100678720 [N. vitripennis]	XP_003426074.1	1,00E-174	GLOS_LOC10067	GO:0043434	B	.	response to peptide hormone stimulus
XP_003426074.1 PREDICTED: hypoth. Prot. LOC100678720 [N. vitripennis]	XP_003426074.1	1,00E-174	GLOS_LOC10067	GO:0009725	B	.	response to hormone stimulus
XP_003426074.1 PREDICTED: hypoth. Prot. LOC100678720 [N. vitripennis]	XP_003426074.1	1,00E-174	GLOS_LOC10067	GO:1901652	B	.	response to peptide
XP_003426074.1 PREDICTED: hypoth. Prot. LOC100678720 [N. vitripennis]	XP_003426074.1	1,00E-174	GLOS_LOC10067	GO:0032868	B	Refseq	response to insulin stimulus
XP_003426074.1 PREDICTED: hypoth. Prot. LOC100678720 [N. vitripennis]	XP_003426074.1	1,00E-174	GLOS_LOC10067	GO:0033500	B	.	carbohydrate homeostasis
XP_003426074.1 PREDICTED: hypoth. Prot. LOC100678720 [N. vitripennis]	XP_003426074.1	1,00E-174	GLOS_LOC10067	GO:0042593	B	Refseq	glucose homeostasis
XP_003426074.1 PREDICTED: hypoth. Prot. LOC100678720 [N. vitripennis]	XP_003426074.1	1,00E-174	GLOS_LOC10067	GO:0006952	B	.	defense response
XP_003426074.1 PREDICTED: hypoth. Prot. LOC100678720 [N. vitripennis]	XP_003426074.1	1,00E-174	GLOS_LOC10067	GO:0009617	B	.	response to bacterium
XP_003426074.1 PREDICTED: hypoth. Prot. LOC100678720 [N. vitripennis]	XP_003426074.1	1,00E-174	GLOS_LOC10067	GO:0051707	B	.	response to other organism
XP_003426074.1 PREDICTED: hypoth. Prot. LOC100678720 [N. vitripennis]	XP_003426074.1	1,00E-174	GLOS_LOC10067	GO:0009607	B	.	response to biotic stimulus
XP_003426074.1 PREDICTED: hypoth. Prot. LOC100678720 [N. vitripennis]	XP_003426074.1	1,00E-174	GLOS_LOC10067	GO:0051704	B	.	multi-organism process
XP_003426074.1 PREDICTED: hypoth. Prot. LOC100678720 [N. vitripennis]	XP_003426074.1	1,00E-174	GLOS_LOC10067	GO:0042742	B	Refseq	defense response to bacterium
XP_003426074.1 PREDICTED: hypoth. Prot. LOC100678720 [N. vitripennis]	XP_003426074.1	1,00E-174	GLOS_LOC10067	GO:0051591	B	Refseq	response to cAMP
NP_648894.2 CG4573 [Drosophila melanogaster]	NP_648894.2	0	GLOS_CG4573.1.	GO:0000122	B	Refseq	negative regulation of transcription from RNA polymerase II promoter
NP_648894.2 CG4573 [Drosophila melanogaster]	NP_648894.2	0	GLOS_CG4573.1.	GO:0001501	B	Refseq	skeletal system development
NP_648894.2 CG4573 [Drosophila melanogaster]	NP_648894.2	0	GLOS_CG4573.1.	GO:0003700	M	Refseq	sequence-specific DNA binding transcription factor activity
NP_648894.2 CG4573 [Drosophila melanogaster]	NP_648894.2	0	GLOS_CG4573.1.	GO:0005515	M	Refseq	protein binding
NP_648894.2 CG4573 [Drosophila melanogaster]	NP_648894.2	0	GLOS_CG4573.1.	GO:0005634	C	Refseq	nucleus
NP_648894.2 CG4573 [Drosophila melanogaster]	NP_648894.2	0	GLOS_CG4573.1.	GO:0006366	B	Refseq	transcription from RNA polymerase II promoter
NP_648894.2 CG4573 [Drosophila melanogaster]	NP_648894.2	0	GLOS_CG4573.1.	GO:0006607	B	Refseq	NLS-bearing protein import into nucleus
NP_648894.2 CG4573 [Drosophila melanogaster]	NP_648894.2	0	GLOS_CG4573.1.	GO:0008270	M	Refseq	zinc ion binding
NP_648894.2 CG4573 [Drosophila melanogaster]	NP_648894.2	0	GLOS_CG4573.1.	GO:0032330	B	Refseq	regulation of chondrocyte differentiation
NP_648894.2 CG4573 [Drosophila melanogaster]	NP_648894.2	0	GLOS_CG4573.1.	GO:0043565	M	Refseq	sequence-specific DNA binding
XP_001992786.1 GH13451 [Drosophila grimshawi]	XP_001992786.1	1,00E-121	GLOS_DGRI_GH1	GO:0005262	M	Refseq	calcium channel activity
XP_001992786.1 GH13451 [Drosophila grimshawi]	XP_001992786.1	1,00E-121	GLOS_DGRI_GH1	GO:0005515	M	Refseq	protein binding
XP_001992786.1 GH13451 [Drosophila grimshawi]	XP_001992786.1	1,00E-121	GLOS_DGRI_GH1	GO:0005886	C	Refseq	plasma membrane
XP_001992786.1 GH13451 [Drosophila grimshawi]	XP_001992786.1	1,00E-121	GLOS_DGRI_GH1	GO:0005887	C	Refseq	integral to plasma membrane
XP_001992786.1 GH13451 [Drosophila grimshawi]	XP_001992786.1	1,00E-121	GLOS_DGRI_GH1	GO:0006816	B	Refseq	calcium ion transport
XP_001992786.1 GH13451 [Drosophila grimshawi]	XP_001992786.1	1,00E-121	GLOS_DGRI_GH1	GO:0007411	B	Refseq	axon guidance
XP_001992786.1 GH13451 [Drosophila grimshawi]	XP_001992786.1	1,00E-121	GLOS_DGRI_GH1	GO:0007596	B	Refseq	blood coagulation
XP_001992786.1 GH13451 [Drosophila grimshawi]	XP_001992786.1	1,00E-121	GLOS_DGRI_GH1	GO:0007602	B	Refseq	phototransduction
XP_001992786.1 GH13451 [Drosophila grimshawi]	XP_001992786.1	1,00E-121	GLOS_DGRI_GH1	GO:0010524	B	Refseq	positive regulation of calcium ion transport into cytosol
XP_001992786.1 GH13451 [Drosophila grimshawi]	XP_001992786.1	1,00E-121	GLOS_DGRI_GH1	GO:0015279	M	Refseq	store-operated calcium channel activity
XP_001992786.1 GH13451 [Drosophila grimshawi]	XP_001992786.1	1,00E-121	GLOS_DGRI_GH1	GO:0030168	B	Refseq	platelet activation
XP_001992786.1 GH13451 [Drosophila grimshawi]	XP_001992786.1	1,00E-121	GLOS_DGRI_GH1	GO:0033198	B	Refseq	response to ATP
XP_001992786.1 GH13451 [Drosophila grimshawi]	XP_001992786.1	1,00E-121	GLOS_DGRI_GH1	GO:0051592	B	Refseq	response to calcium ion
XP_001992786.1 GH13451 [Drosophila grimshawi]	XP_001992786.1	1,00E-121	GLOS_DGRI_GH1	GO:0070588	B	Refseq	calcium ion transmembrane transport
XP_001992786.1 GH13451 [Drosophila grimshawi]	XP_001992786.1	1,00E-121	GLOS_DGRI_GH1	GO:0070679	M	Refseq	inositol 1,4,5 trisphosphate binding
[BBH] ACADM_DROME (sp|Q9VSA3) Prob. medium-chain spec. acyl-CoA DHase, mito.	ACADM_DROME	0	GLOS_ACADM.1.	GO:0005811	C	FlyBase	lipid particle
[BBH] ACADM_DROME (sp|Q9VSA3) Prob. medium-chain spec. acyl-CoA DHase, mito.	ACADM_DROME	0	GLOS_ACADM.1.	GO:0044430	C	.	cytoskeletal part
[BBH] ACADM_DROME (sp|Q9VSA3) Prob. medium-chain spec. acyl-CoA DHase, mito.	ACADM_DROME	0	GLOS_ACADM.1.	GO:0005856	C	.	cytoskeleton
[BBH] ACADM_DROME (sp|Q9VSA3) Prob. medium-chain spec. acyl-CoA DHase, mito.	ACADM_DROME	0	GLOS_ACADM.1.	GO:0015630	C	.	microtubule cytoskeleton
[BBH] ACADM_DROME (sp|Q9VSA3) Prob. medium-chain spec. acyl-CoA DHase, mito.	ACADM_DROME	0	GLOS_ACADM.1.	GO:0005875	C	FlyBase	microtubule associated complex
[BBH] ACADM_DROME (sp|Q9VSA3) Prob. medium-chain spec. acyl-CoA DHase, mito.	ACADM_DROME	0	GLOS_ACADM.1.	GO:0044429	C	.	mitochondrial part
[BBH] ACADM_DROME (sp|Q9VSA3) Prob. medium-chain spec. acyl-CoA DHase, mito.	ACADM_DROME	0	GLOS_ACADM.1.	GO:0005739	C	.	mitochondrion
[BBH] ACADM_DROME (sp|Q9VSA3) Prob. medium-chain spec. acyl-CoA DHase, mito.	ACADM_DROME	0	GLOS_ACADM.1.	GO:0005759	C	UniProtKB-S	mitochondrial matrix
[BBH] ACADM_DROME (sp|Q9VSA3) Prob. medium-chain spec. acyl-CoA DHase, mito.	ACADM_DROME	0	GLOS_ACADM.1.	GO:0005739	C	FlyBase	mitochondrion
[BBH] ACADM_DROME (sp|Q9VSA3) Prob. medium-chain spec. acyl-CoA DHase, mito.	ACADM_DROME	0	GLOS_ACADM.1.	GO:0016627	M	.	oxidoreductase activity, acting on the CH-CH group of donors
[BBH] ACADM_DROME (sp|Q9VSA3) Prob. medium-chain spec. acyl-CoA DHase, mito.	ACADM_DROME	0	GLOS_ACADM.1.	GO:0016491	M	.	oxidoreductase activity
[BBH] ACADM_DROME (sp|Q9VSA3) Prob. medium-chain spec. acyl-CoA DHase, mito.	ACADM_DROME	0	GLOS_ACADM.1.	GO:0003824	M	.	catalytic activity
[BBH] ACADM_DROME (sp|Q9VSA3) Prob. medium-chain spec. acyl-CoA DHase, mito.	ACADM_DROME	0	GLOS_ACADM.1.	GO:0003995	M	UniProtKB	acyl-CoA dehydrogenase activity
[BBH] ACADM_DROME (sp|Q9VSA3) Prob. medium-chain spec. acyl-CoA DHase, mito.	ACADM_DROME	0	GLOS_ACADM.1.	GO:0000166	M	.	nucleotide binding
[BBH] ACADM_DROME (sp|Q9VSA3) Prob. medium-chain spec. acyl-CoA DHase, mito.	ACADM_DROME	0	GLOS_ACADM.1.	GO:1901265	M	.	nucleoside phosphate binding
[BBH] ACADM_DROME (sp|Q9VSA3) Prob. medium-chain spec. acyl-CoA DHase, mito.	ACADM_DROME	0	GLOS_ACADM.1.	GO:0050662	M	.	coenzyme binding

[BBH] ACADM_DROME (sp|Q9VSA3) Prob. medium-chain spec. acyl-CoA DHase, mito.	ACADM_DROME	0	GLOS_ACADM.1.1	GO:0048037	M	.	cofactor binding
[BBH] ACADM_DROME (sp|Q9VSA3) Prob. medium-chain spec. acyl-CoA DHase, mito.	ACADM_DROME	0	GLOS_ACADM.1.1	GO:0050660	M	InterPro	flavin adenine dinucleotide binding
[BBH] ACADM_DROME (sp|Q9VSA3) Prob. medium-chain spec. acyl-CoA DHase, mito.	ACADM_DROME	0	GLOS_ACADM.1.1	GO:0009062	B	.	fatty acid catabolic process
[BBH] ACADM_DROME (sp|Q9VSA3) Prob. medium-chain spec. acyl-CoA DHase, mito.	ACADM_DROME	0	GLOS_ACADM.1.1	GO:0006631	B	.	fatty acid metabolic process
[BBH] ACADM_DROME (sp|Q9VSA3) Prob. medium-chain spec. acyl-CoA DHase, mito.	ACADM_DROME	0	GLOS_ACADM.1.1	GO:0032787	B	.	monocarboxylic acid metabolic process
[BBH] ACADM_DROME (sp|Q9VSA3) Prob. medium-chain spec. acyl-CoA DHase, mito.	ACADM_DROME	0	GLOS_ACADM.1.1	GO:0019752	B	.	carboxylic acid metabolic process
[BBH] ACADM_DROME (sp|Q9VSA3) Prob. medium-chain spec. acyl-CoA DHase, mito.	ACADM_DROME	0	GLOS_ACADM.1.1	GO:0043436	B	.	oxoacid metabolic process
[BBH] ACADM_DROME (sp|Q9VSA3) Prob. medium-chain spec. acyl-CoA DHase, mito.	ACADM_DROME	0	GLOS_ACADM.1.1	GO:0006082	B	.	organic acid metabolic process
[BBH] ACADM_DROME (sp|Q9VSA3) Prob. medium-chain spec. acyl-CoA DHase, mito.	ACADM_DROME	0	GLOS_ACADM.1.1	GO:0044281	B	.	small molecule metabolic process
[BBH] ACADM_DROME (sp|Q9VSA3) Prob. medium-chain spec. acyl-CoA DHase, mito.	ACADM_DROME	0	GLOS_ACADM.1.1	GO:0044710	B	.	single-organism metabolic process
[BBH] ACADM_DROME (sp|Q9VSA3) Prob. medium-chain spec. acyl-CoA DHase, mito.	ACADM_DROME	0	GLOS_ACADM.1.1	GO:0044255	B	.	cellular lipid metabolic process
[BBH] ACADM_DROME (sp|Q9VSA3) Prob. medium-chain spec. acyl-CoA DHase, mito.	ACADM_DROME	0	GLOS_ACADM.1.1	GO:0006629	B	.	lipid metabolic process
[BBH] ACADM_DROME (sp|Q9VSA3) Prob. medium-chain spec. acyl-CoA DHase, mito.	ACADM_DROME	0	GLOS_ACADM.1.1	GO:0044242	B	.	cellular lipid catabolic process
[BBH] ACADM_DROME (sp|Q9VSA3) Prob. medium-chain spec. acyl-CoA DHase, mito.	ACADM_DROME	0	GLOS_ACADM.1.1	GO:0016042	B	.	lipid catabolic process
[BBH] ACADM_DROME (sp|Q9VSA3) Prob. medium-chain spec. acyl-CoA DHase, mito.	ACADM_DROME	0	GLOS_ACADM.1.1	GO:1901575	B	.	organic substance catabolic process
[BBH] ACADM_DROME (sp|Q9VSA3) Prob. medium-chain spec. acyl-CoA DHase, mito.	ACADM_DROME	0	GLOS_ACADM.1.1	GO:0009056	B	.	catabolic process
[BBH] ACADM_DROME (sp|Q9VSA3) Prob. medium-chain spec. acyl-CoA DHase, mito.	ACADM_DROME	0	GLOS_ACADM.1.1	GO:0044248	B	.	cellular catabolic process
[BBH] ACADM_DROME (sp|Q9VSA3) Prob. medium-chain spec. acyl-CoA DHase, mito.	ACADM_DROME	0	GLOS_ACADM.1.1	GO:0072329	B	.	monocarboxylic acid catabolic process
[BBH] ACADM_DROME (sp|Q9VSA3) Prob. medium-chain spec. acyl-CoA DHase, mito.	ACADM_DROME	0	GLOS_ACADM.1.1	GO:0046395	B	.	carboxylic acid catabolic process
[BBH] ACADM_DROME (sp|Q9VSA3) Prob. medium-chain spec. acyl-CoA DHase, mito.	ACADM_DROME	0	GLOS_ACADM.1.1	GO:0016054	B	.	organic acid catabolic process
[BBH] ACADM_DROME (sp|Q9VSA3) Prob. medium-chain spec. acyl-CoA DHase, mito.	ACADM_DROME	0	GLOS_ACADM.1.1	GO:0044282	B	.	small molecule catabolic process
[BBH] ACADM_DROME (sp|Q9VSA3) Prob. medium-chain spec. acyl-CoA DHase, mito.	ACADM_DROME	0	GLOS_ACADM.1.1	GO:0044712	B	.	single-organism catabolic process
[BBH] ACADM_DROME (sp|Q9VSA3) Prob. medium-chain spec. acyl-CoA DHase, mito.	ACADM_DROME	0	GLOS_ACADM.1.1	GO:0019395	B	.	fatty acid oxidation
[BBH] ACADM_DROME (sp|Q9VSA3) Prob. medium-chain spec. acyl-CoA DHase, mito.	ACADM_DROME	0	GLOS_ACADM.1.1	GO:0034440	B	.	lipid oxidation
[BBH] ACADM_DROME (sp|Q9VSA3) Prob. medium-chain spec. acyl-CoA DHase, mito.	ACADM_DROME	0	GLOS_ACADM.1.1	GO:0030258	B	.	lipid modification
[BBH] ACADM_DROME (sp|Q9VSA3) Prob. medium-chain spec. acyl-CoA DHase, mito.	ACADM_DROME	0	GLOS_ACADM.1.1	GO:0055114	B	.	oxidation-reduction process
[BBH] ACADM_DROME (sp|Q9VSA3) Prob. medium-chain spec. acyl-CoA DHase, mito.	ACADM_DROME	0	GLOS_ACADM.1.1	GO:0006635	B	UniProtKB	fatty acid beta-oxidation
[BBH] ATLAS_DROME (sp|Q9VC57) Atlastin OS=D. m.	ATLAS_DROME	0	GLOS_ATLAS.1.1	GO:0005794	C	UniProtKB	Golgi apparatus
[BBH] ATLAS_DROME (sp|Q9VC57) Atlastin OS=D. m.	ATLAS_DROME	0	GLOS_ATLAS.1.1	GO:0031090	C	.	organelle membrane
[BBH] ATLAS_DROME (sp|Q9VC57) Atlastin OS=D. m.	ATLAS_DROME	0	GLOS_ATLAS.1.1	GO:0044431	C	.	Golgi apparatus part
[BBH] ATLAS_DROME (sp|Q9VC57) Atlastin OS=D. m.	ATLAS_DROME	0	GLOS_ATLAS.1.1	GO:0005794	C	.	Golgi apparatus
[BBH] ATLAS_DROME (sp|Q9VC57) Atlastin OS=D. m.	ATLAS_DROME	0	GLOS_ATLAS.1.1	GO:0012505	C	.	endomembrane system
[BBH] ATLAS_DROME (sp|Q9VC57) Atlastin OS=D. m.	ATLAS_DROME	0	GLOS_ATLAS.1.1	GO:0000139	C	UniProtKB-S	Golgi membrane
[BBH] ATLAS_DROME (sp|Q9VC57) Atlastin OS=D. m.	ATLAS_DROME	0	GLOS_ATLAS.1.1	GO:0016021	C	UniProtKB-k	integral to membrane
[BBH] ATLAS_DROME (sp|Q9VC57) Atlastin OS=D. m.	ATLAS_DROME	0	GLOS_ATLAS.1.1	GO:0031300	C	.	intrinsic to organelle membrane
[BBH] ATLAS_DROME (sp|Q9VC57) Atlastin OS=D. m.	ATLAS_DROME	0	GLOS_ATLAS.1.1	GO:0044432	C	.	endoplasmic reticulum part
[BBH] ATLAS_DROME (sp|Q9VC57) Atlastin OS=D. m.	ATLAS_DROME	0	GLOS_ATLAS.1.1	GO:0005783	C	.	endoplasmic reticulum
[BBH] ATLAS_DROME (sp|Q9VC57) Atlastin OS=D. m.	ATLAS_DROME	0	GLOS_ATLAS.1.1	GO:0005789	C	.	endoplasmic reticulum membrane
[BBH] ATLAS_DROME (sp|Q9VC57) Atlastin OS=D. m.	ATLAS_DROME	0	GLOS_ATLAS.1.1	GO:0042175	C	.	nuclear outer membrane-endoplasmic reticulum membrane network
[BBH] ATLAS_DROME (sp|Q9VC57) Atlastin OS=D. m.	ATLAS_DROME	0	GLOS_ATLAS.1.1	GO:0031227	C	FlyBase	intrinsic to endoplasmic reticulum membrane
[BBH] ATLAS_DROME (sp|Q9VC57) Atlastin OS=D. m.	ATLAS_DROME	0	GLOS_ATLAS.1.1	GO:0032550	M	.	purine ribonucleoside binding
[BBH] ATLAS_DROME (sp|Q9VC57) Atlastin OS=D. m.	ATLAS_DROME	0	GLOS_ATLAS.1.1	GO:0001883	M	.	purine nucleoside binding
[BBH] ATLAS_DROME (sp|Q9VC57) Atlastin OS=D. m.	ATLAS_DROME	0	GLOS_ATLAS.1.1	GO:0001882	M	.	nucleoside binding
[BBH] ATLAS_DROME (sp|Q9VC57) Atlastin OS=D. m.	ATLAS_DROME	0	GLOS_ATLAS.1.1	GO:0032549	M	.	ribonucleoside binding
[BBH] ATLAS_DROME (sp|Q9VC57) Atlastin OS=D. m.	ATLAS_DROME	0	GLOS_ATLAS.1.1	GO:0032561	M	.	guanyl ribonucleotide binding
[BBH] ATLAS_DROME (sp|Q9VC57) Atlastin OS=D. m.	ATLAS_DROME	0	GLOS_ATLAS.1.1	GO:0019001	M	.	guanyl nucleotide binding
[BBH] ATLAS_DROME (sp|Q9VC57) Atlastin OS=D. m.	ATLAS_DROME	0	GLOS_ATLAS.1.1	GO:0017076	M	.	purine nucleotide binding
[BBH] ATLAS_DROME (sp|Q9VC57) Atlastin OS=D. m.	ATLAS_DROME	0	GLOS_ATLAS.1.1	GO:0032555	M	.	purine ribonucleotide binding
[BBH] ATLAS_DROME (sp|Q9VC57) Atlastin OS=D. m.	ATLAS_DROME	0	GLOS_ATLAS.1.1	GO:0032553	M	.	ribonucleotide binding
[BBH] ATLAS_DROME (sp|Q9VC57) Atlastin OS=D. m.	ATLAS_DROME	0	GLOS_ATLAS.1.1	GO:0035639	M	.	purine ribonucleoside triphosphate binding
[BBH] ATLAS_DROME (sp|Q9VC57) Atlastin OS=D. m.	ATLAS_DROME	0	GLOS_ATLAS.1.1	GO:0005525	M	UniProtKB	GTP binding
[BBH] ATLAS_DROME (sp|Q9VC57) Atlastin OS=D. m.	ATLAS_DROME	0	GLOS_ATLAS.1.1	GO:0017111	M	.	nucleoside-triphosphatase activity
[BBH] ATLAS_DROME (sp|Q9VC57) Atlastin OS=D. m.	ATLAS_DROME	0	GLOS_ATLAS.1.1	GO:0016462	M	.	pyrophosphatase activity
[BBH] ATLAS_DROME (sp|Q9VC57) Atlastin OS=D. m.	ATLAS_DROME	0	GLOS_ATLAS.1.1	GO:0016818	M	.	hydrolase act., acting on acid anhydrides, in phosphorus-containing anhydrides

[BBH] ATLAS_DROME (sp|Q9VC57) Atlastin OS=D. m.	ATLAS_DROME	0	GLOS_ATLAS.1.1	GO:0016817	M	.	hydrolase activity, acting on acid anhydrides
[BBH] ATLAS_DROME (sp|Q9VC57) Atlastin OS=D. m.	ATLAS_DROME	0	GLOS_ATLAS.1.1	GO:0016787	M	.	hydrolase activity
[BBH] ATLAS_DROME (sp|Q9VC57) Atlastin OS=D. m.	ATLAS_DROME	0	GLOS_ATLAS.1.1	GO:0003924	M	UniProtKB	GTPase activity
[BBH] ATLAS_DROME (sp|Q9VC57) Atlastin OS=D. m.	ATLAS_DROME	0	GLOS_ATLAS.1.1	GO:0042802	M	.	identical protein binding
[BBH] ATLAS_DROME (sp|Q9VC57) Atlastin OS=D. m.	ATLAS_DROME	0	GLOS_ATLAS.1.1	GO:0046983	M	.	protein dimerization activity
[BBH] ATLAS_DROME (sp|Q9VC57) Atlastin OS=D. m.	ATLAS_DROME	0	GLOS_ATLAS.1.1	GO:0042803	M	FlyBase	protein homodimerization activity
[BBH] ATLAS_DROME (sp|Q9VC57) Atlastin OS=D. m.	ATLAS_DROME	0	GLOS_ATLAS.1.1	GO:0048284	B	.	organelle fusion
[BBH] ATLAS_DROME (sp|Q9VC57) Atlastin OS=D. m.	ATLAS_DROME	0	GLOS_ATLAS.1.1	GO:0090174	B	.	organelle membrane fusion
[BBH] ATLAS_DROME (sp|Q9VC57) Atlastin OS=D. m.	ATLAS_DROME	0	GLOS_ATLAS.1.1	GO:0044801	B	.	single-organism membrane fusion
[BBH] ATLAS_DROME (sp|Q9VC57) Atlastin OS=D. m.	ATLAS_DROME	0	GLOS_ATLAS.1.1	GO:0044802	B	.	single-organism membrane organization
[BBH] ATLAS_DROME (sp|Q9VC57) Atlastin OS=D. m.	ATLAS_DROME	0	GLOS_ATLAS.1.1	GO:0061024	B	.	membrane organization
[BBH] ATLAS_DROME (sp|Q9VC57) Atlastin OS=D. m.	ATLAS_DROME	0	GLOS_ATLAS.1.1	GO:0061025	B	.	membrane fusion
[BBH] ATLAS_DROME (sp|Q9VC57) Atlastin OS=D. m.	ATLAS_DROME	0	GLOS_ATLAS.1.1	GO:0016320	B	UniProtKB	endoplasmic reticulum membrane fusion
[BBH] ATLAS_DROME (sp|Q9VC57) Atlastin OS=D. m.	ATLAS_DROME	0	GLOS_ATLAS.1.1	GO:0007029	B	UniProtKB	endoplasmic reticulum organization
[BBH] ATLAS_DROME (sp|Q9VC57) Atlastin OS=D. m.	ATLAS_DROME	0	GLOS_ATLAS.1.1	GO:0007030	B	UniProtKB	Golgi organization
[BBH] ATLAS_DROME (sp|Q9VC57) Atlastin OS=D. m.	ATLAS_DROME	0	GLOS_ATLAS.1.1	GO:0031109	B	.	microtubule polymerization or depolymerization
[BBH] ATLAS_DROME (sp|Q9VC57) Atlastin OS=D. m.	ATLAS_DROME	0	GLOS_ATLAS.1.1	GO:0000226	B	.	microtubule cytoskeleton organization
[BBH] ATLAS_DROME (sp|Q9VC57) Atlastin OS=D. m.	ATLAS_DROME	0	GLOS_ATLAS.1.1	GO:0007010	B	.	cytoskeleton organization
[BBH] ATLAS_DROME (sp|Q9VC57) Atlastin OS=D. m.	ATLAS_DROME	0	GLOS_ATLAS.1.1	GO:0007017	B	.	microtubule-based process
[BBH] ATLAS_DROME (sp|Q9VC57) Atlastin OS=D. m.	ATLAS_DROME	0	GLOS_ATLAS.1.1	GO:0051261	B	.	protein depolymerization
[BBH] ATLAS_DROME (sp|Q9VC57) Atlastin OS=D. m.	ATLAS_DROME	0	GLOS_ATLAS.1.1	GO:0043624	B	.	cellular protein complex disassembly
[BBH] ATLAS_DROME (sp|Q9VC57) Atlastin OS=D. m.	ATLAS_DROME	0	GLOS_ATLAS.1.1	GO:0043241	B	.	protein complex disassembly
[BBH] ATLAS_DROME (sp|Q9VC57) Atlastin OS=D. m.	ATLAS_DROME	0	GLOS_ATLAS.1.1	GO:0032984	B	.	macromolecular complex disassembly
[BBH] ATLAS_DROME (sp|Q9VC57) Atlastin OS=D. m.	ATLAS_DROME	0	GLOS_ATLAS.1.1	GO:0022411	B	.	cellular component disassembly
[BBH] ATLAS_DROME (sp|Q9VC57) Atlastin OS=D. m.	ATLAS_DROME	0	GLOS_ATLAS.1.1	GO:0043933	B	.	macromolecular complex subunit organization
[BBH] ATLAS_DROME (sp|Q9VC57) Atlastin OS=D. m.	ATLAS_DROME	0	GLOS_ATLAS.1.1	GO:0071822	B	.	protein complex subunit organization
[BBH] ATLAS_DROME (sp|Q9VC57) Atlastin OS=D. m.	ATLAS_DROME	0	GLOS_ATLAS.1.1	GO:0007019	B	UniProtKB	microtubule depolymerization
[BBH] ATLAS_DROME (sp|Q9VC57) Atlastin OS=D. m.	ATLAS_DROME	0	GLOS_ATLAS.1.1	GO:0051259	B	.	protein oligomerization
[BBH] ATLAS_DROME (sp|Q9VC57) Atlastin OS=D. m.	ATLAS_DROME	0	GLOS_ATLAS.1.1	GO:0006461	B	.	protein complex assembly
[BBH] ATLAS_DROME (sp|Q9VC57) Atlastin OS=D. m.	ATLAS_DROME	0	GLOS_ATLAS.1.1	GO:0065003	B	.	macromolecular complex assembly
[BBH] ATLAS_DROME (sp|Q9VC57) Atlastin OS=D. m.	ATLAS_DROME	0	GLOS_ATLAS.1.1	GO:0070271	B	.	protein complex biogenesis
[BBH] ATLAS_DROME (sp|Q9VC57) Atlastin OS=D. m.	ATLAS_DROME	0	GLOS_ATLAS.1.1	GO:0051260	B	UniProtKB	protein homooligomerization
[BBH] ATLAS_DROME (sp|Q9VC57) Atlastin OS=D. m.	ATLAS_DROME	0	GLOS_ATLAS.1.1	GO:0031110	B	.	regulation of microtubule polymerization or depolymerization
[BBH] ATLAS_DROME (sp|Q9VC57) Atlastin OS=D. m.	ATLAS_DROME	0	GLOS_ATLAS.1.1	GO:0070507	B	.	regulation of microtubule cytoskeleton organization
[BBH] ATLAS_DROME (sp|Q9VC57) Atlastin OS=D. m.	ATLAS_DROME	0	GLOS_ATLAS.1.1	GO:0032886	B	.	regulation of microtubule-based process
[BBH] ATLAS_DROME (sp|Q9VC57) Atlastin OS=D. m.	ATLAS_DROME	0	GLOS_ATLAS.1.1	GO:0051493	B	.	regulation of cytoskeleton organization
[BBH] ATLAS_DROME (sp|Q9VC57) Atlastin OS=D. m.	ATLAS_DROME	0	GLOS_ATLAS.1.1	GO:0033043	B	.	regulation of organelle organization
[BBH] ATLAS_DROME (sp|Q9VC57) Atlastin OS=D. m.	ATLAS_DROME	0	GLOS_ATLAS.1.1	GO:1901879	B	.	regulation of protein depolymerization
[BBH] ATLAS_DROME (sp|Q9VC57) Atlastin OS=D. m.	ATLAS_DROME	0	GLOS_ATLAS.1.1	GO:0043244	B	.	regulation of protein complex disassembly
[BBH] ATLAS_DROME (sp|Q9VC57) Atlastin OS=D. m.	ATLAS_DROME	0	GLOS_ATLAS.1.1	GO:0031114	B	FlyBase	regulation of microtubule depolymerization
[BBH] ATLAS_DROME (sp|Q9VC57) Atlastin OS=D. m.	ATLAS_DROME	0	GLOS_ATLAS.1.1	GO:0048638	B	.	regulation of developmental growth
[BBH] ATLAS_DROME (sp|Q9VC57) Atlastin OS=D. m.	ATLAS_DROME	0	GLOS_ATLAS.1.1	GO:0048742	B	.	regulation of skeletal muscle fiber development
[BBH] ATLAS_DROME (sp|Q9VC57) Atlastin OS=D. m.	ATLAS_DROME	0	GLOS_ATLAS.1.1	GO:0048641	B	.	regulation of skeletal muscle tissue development
[BBH] ATLAS_DROME (sp|Q9VC57) Atlastin OS=D. m.	ATLAS_DROME	0	GLOS_ATLAS.1.1	GO:0016202	B	.	regulation of striated muscle tissue development
[BBH] ATLAS_DROME (sp|Q9VC57) Atlastin OS=D. m.	ATLAS_DROME	0	GLOS_ATLAS.1.1	GO:0048634	B	.	regulation of muscle organ development
[BBH] ATLAS_DROME (sp|Q9VC57) Atlastin OS=D. m.	ATLAS_DROME	0	GLOS_ATLAS.1.1	GO:1901861	B	.	regulation of muscle tissue development
[BBH] ATLAS_DROME (sp|Q9VC57) Atlastin OS=D. m.	ATLAS_DROME	0	GLOS_ATLAS.1.1	GO:0051153	B	.	regulation of striated muscle cell differentiation
[BBH] ATLAS_DROME (sp|Q9VC57) Atlastin OS=D. m.	ATLAS_DROME	0	GLOS_ATLAS.1.1	GO:0051147	B	.	regulation of muscle cell differentiation
[BBH] ATLAS_DROME (sp|Q9VC57) Atlastin OS=D. m.	ATLAS_DROME	0	GLOS_ATLAS.1.1	GO:0051963	B	.	regulation of synapse assembly
[BBH] ATLAS_DROME (sp|Q9VC57) Atlastin OS=D. m.	ATLAS_DROME	0	GLOS_ATLAS.1.1	GO:0044087	B	.	regulation of cellular component biogenesis
[BBH] ATLAS_DROME (sp|Q9VC57) Atlastin OS=D. m.	ATLAS_DROME	0	GLOS_ATLAS.1.1	GO:0050807	B	.	regulation of synapse organization
[BBH] ATLAS_DROME (sp|Q9VC57) Atlastin OS=D. m.	ATLAS_DROME	0	GLOS_ATLAS.1.1	GO:0050803	B	.	regulation of synapse structure and activity
[BBH] ATLAS_DROME (sp|Q9VC57) Atlastin OS=D. m.	ATLAS_DROME	0	GLOS_ATLAS.1.1	GO:0007268	B	.	synaptic transmission
[BBH] ATLAS_DROME (sp|Q9VC57) Atlastin OS=D. m.	ATLAS_DROME	0	GLOS_ATLAS.1.1	GO:0019226	B	.	transmission of nerve impulse

[BBH] ATLAS_DROME (sp|Q9VC57) Atlastin OS=D. m.	ATLAS_DROME	0	GLOS_ATLAS.1.1	GO:0035637	B	.	multicellular organismal signaling
[BBH] ATLAS_DROME (sp|Q9VC57) Atlastin OS=D. m.	ATLAS_DROME	0	GLOS_ATLAS.1.1	GO:0050877	B	.	neurological system process
[BBH] ATLAS_DROME (sp|Q9VC57) Atlastin OS=D. m.	ATLAS_DROME	0	GLOS_ATLAS.1.1	GO:0008582	B	FlyBase	regulation of synaptic growth at neuromuscular junction
[BBH] ATLAS_DROME (sp|Q9VC57) Atlastin OS=D. m.	ATLAS_DROME	0	GLOS_ATLAS.1.1	GO:0007416	B	.	synapse assembly
[BBH] ATLAS_DROME (sp|Q9VC57) Atlastin OS=D. m.	ATLAS_DROME	0	GLOS_ATLAS.1.1	GO:0050808	B	.	synapse organization
[BBH] ATLAS_DROME (sp|Q9VC57) Atlastin OS=D. m.	ATLAS_DROME	0	GLOS_ATLAS.1.1	GO:0007528	B	.	neuromuscular junction development
[BBH] ATLAS_DROME (sp|Q9VC57) Atlastin OS=D. m.	ATLAS_DROME	0	GLOS_ATLAS.1.1	GO:0048589	B	.	developmental growth
[BBH] ATLAS_DROME (sp|Q9VC57) Atlastin OS=D. m.	ATLAS_DROME	0	GLOS_ATLAS.1.1	GO:0040007	B	.	growth
[BBH] ATLAS_DROME (sp|Q9VC57) Atlastin OS=D. m.	ATLAS_DROME	0	GLOS_ATLAS.1.1	GO:0051124	B	UniProtKB	synaptic growth at neuromuscular junction
CADF_DROME (sp|P45594) Cofilin/actin-depolymerizing factor homolog OS=D.m.	CADF_DROME	1,00E-25	GLOS_CADF.1.7	GO:0015629	C	InterPro	actin cytoskeleton
CADF_DROME (sp|P45594) Cofilin/actin-depolymerizing factor homolog OS=D.m.	CADF_DROME	1,00E-25	GLOS_CADF.1.7	GO:0005737	C	UniProtKB-K	cytoplasm
CADF_DROME (sp|P45594) Cofilin/actin-depolymerizing factor homolog OS=D.m.	CADF_DROME	1,00E-25	GLOS_CADF.1.7	GO:0005875	C	FlyBase	microtubule associated complex
CADF_DROME (sp|P45594) Cofilin/actin-depolymerizing factor homolog OS=D.m.	CADF_DROME	1,00E-25	GLOS_CADF.1.7	GO:0034399	C	.	nuclear periphery
CADF_DROME (sp|P45594) Cofilin/actin-depolymerizing factor homolog OS=D.m.	CADF_DROME	1,00E-25	GLOS_CADF.1.7	GO:0016363	C	UniProtKB-S	nuclear matrix
CADF_DROME (sp|P45594) Cofilin/actin-depolymerizing factor homolog OS=D.m.	CADF_DROME	1,00E-25	GLOS_CADF.1.7	GO:0008092	M	.	cytoskeletal protein binding
CADF_DROME (sp|P45594) Cofilin/actin-depolymerizing factor homolog OS=D.m.	CADF_DROME	1,00E-25	GLOS_CADF.1.7	GO:0003779	M	FlyBase	actin binding
CADF_DROME (sp|P45594) Cofilin/actin-depolymerizing factor homolog OS=D.m.	CADF_DROME	1,00E-25	GLOS_CADF.1.7	GO:0008154	B	.	actin polymerization or depolymerization
CADF_DROME (sp|P45594) Cofilin/actin-depolymerizing factor homolog OS=D.m.	CADF_DROME	1,00E-25	GLOS_CADF.1.7	GO:0007015	B	.	actin filament organization
CADF_DROME (sp|P45594) Cofilin/actin-depolymerizing factor homolog OS=D.m.	CADF_DROME	1,00E-25	GLOS_CADF.1.7	GO:0030036	B	.	actin cytoskeleton organization
CADF_DROME (sp|P45594) Cofilin/actin-depolymerizing factor homolog OS=D.m.	CADF_DROME	1,00E-25	GLOS_CADF.1.7	GO:0030029	B	.	actin filament-based process
CADF_DROME (sp|P45594) Cofilin/actin-depolymerizing factor homolog OS=D.m.	CADF_DROME	1,00E-25	GLOS_CADF.1.7	GO:0030042	B	InterPro	actin filament depolymerization
CADF_DROME (sp|P45594) Cofilin/actin-depolymerizing factor homolog OS=D.m.	CADF_DROME	1,00E-25	GLOS_CADF.1.7	GO:0051258	B	.	protein polymerization
CADF_DROME (sp|P45594) Cofilin/actin-depolymerizing factor homolog OS=D.m.	CADF_DROME	1,00E-25	GLOS_CADF.1.7	GO:0043623	B	.	cellular protein complex assembly
CADF_DROME (sp|P45594) Cofilin/actin-depolymerizing factor homolog OS=D.m.	CADF_DROME	1,00E-25	GLOS_CADF.1.7	GO:0034622	B	.	cellular macromolecular complex assembly
CADF_DROME (sp|P45594) Cofilin/actin-depolymerizing factor homolog OS=D.m.	CADF_DROME	1,00E-25	GLOS_CADF.1.7	GO:0030041	B	FlyBase	actin filament polymerization
CADF_DROME (sp|P45594) Cofilin/actin-depolymerizing factor homolog OS=D.m.	CADF_DROME	1,00E-25	GLOS_CADF.1.7	GO:0007409	B	FlyBase	axonogenesis
CADF_DROME (sp|P45594) Cofilin/actin-depolymerizing factor homolog OS=D.m.	CADF_DROME	1,00E-25	GLOS_CADF.1.7	GO:0007297	B	.	ovarian follicle cell migration
CADF_DROME (sp|P45594) Cofilin/actin-depolymerizing factor homolog OS=D.m.	CADF_DROME	1,00E-25	GLOS_CADF.1.7	GO:0010631	B	.	epithelial cell migration
CADF_DROME (sp|P45594) Cofilin/actin-depolymerizing factor homolog OS=D.m.	CADF_DROME	1,00E-25	GLOS_CADF.1.7	GO:0001667	B	.	ameboidal cell migration
CADF_DROME (sp|P45594) Cofilin/actin-depolymerizing factor homolog OS=D.m.	CADF_DROME	1,00E-25	GLOS_CADF.1.7	GO:0016477	B	.	cell migration
CADF_DROME (sp|P45594) Cofilin/actin-depolymerizing factor homolog OS=D.m.	CADF_DROME	1,00E-25	GLOS_CADF.1.7	GO:0048870	B	.	cell motility
CADF_DROME (sp|P45594) Cofilin/actin-depolymerizing factor homolog OS=D.m.	CADF_DROME	1,00E-25	GLOS_CADF.1.7	GO:0051674	B	.	localization of cell
CADF_DROME (sp|P45594) Cofilin/actin-depolymerizing factor homolog OS=D.m.	CADF_DROME	1,00E-25	GLOS_CADF.1.7	GO:0090132	B	.	epithelium migration
CADF_DROME (sp|P45594) Cofilin/actin-depolymerizing factor homolog OS=D.m.	CADF_DROME	1,00E-25	GLOS_CADF.1.7	GO:0090130	B	.	tissue migration
CADF_DROME (sp|P45594) Cofilin/actin-depolymerizing factor homolog OS=D.m.	CADF_DROME	1,00E-25	GLOS_CADF.1.7	GO:0022412	B	.	cellular process involved in reproduction in multicellular organism
CADF_DROME (sp|P45594) Cofilin/actin-depolymerizing factor homolog OS=D.m.	CADF_DROME	1,00E-25	GLOS_CADF.1.7	GO:0048610	B	.	cellular process involved in reproduction
CADF_DROME (sp|P45594) Cofilin/actin-depolymerizing factor homolog OS=D.m.	CADF_DROME	1,00E-25	GLOS_CADF.1.7	GO:0030707	B	.	ovarian follicle cell development
CADF_DROME (sp|P45594) Cofilin/actin-depolymerizing factor homolog OS=D.m.	CADF_DROME	1,00E-25	GLOS_CADF.1.7	GO:0048477	B	.	oogenesis
CADF_DROME (sp|P45594) Cofilin/actin-depolymerizing factor homolog OS=D.m.	CADF_DROME	1,00E-25	GLOS_CADF.1.7	GO:0007292	B	.	female gamete generation
CADF_DROME (sp|P45594) Cofilin/actin-depolymerizing factor homolog OS=D.m.	CADF_DROME	1,00E-25	GLOS_CADF.1.7	GO:0007276	B	.	gamete generation
CADF_DROME (sp|P45594) Cofilin/actin-depolymerizing factor homolog OS=D.m.	CADF_DROME	1,00E-25	GLOS_CADF.1.7	GO:0019953	B	.	sexual reproduction
CADF_DROME (sp|P45594) Cofilin/actin-depolymerizing factor homolog OS=D.m.	CADF_DROME	1,00E-25	GLOS_CADF.1.7	GO:0007298	B	FlyBase	border follicle cell migration
CADF_DROME (sp|P45594) Cofilin/actin-depolymerizing factor homolog OS=D.m.	CADF_DROME	1,00E-25	GLOS_CADF.1.7	GO:0000912	B	.	assembly of actomyosin apparatus involved in cytokinesis
CADF_DROME (sp|P45594) Cofilin/actin-depolymerizing factor homolog OS=D.m.	CADF_DROME	1,00E-25	GLOS_CADF.1.7	GO:0031032	B	.	actomyosin structure organization
CADF_DROME (sp|P45594) Cofilin/actin-depolymerizing factor homolog OS=D.m.	CADF_DROME	1,00E-25	GLOS_CADF.1.7	GO:0032506	B	.	cytokinetic process
CADF_DROME (sp|P45594) Cofilin/actin-depolymerizing factor homolog OS=D.m.	CADF_DROME	1,00E-25	GLOS_CADF.1.7	GO:0022402	B	.	cell cycle process
CADF_DROME (sp|P45594) Cofilin/actin-depolymerizing factor homolog OS=D.m.	CADF_DROME	1,00E-25	GLOS_CADF.1.7	GO:0000910	B	.	cytokinesis
CADF_DROME (sp|P45594) Cofilin/actin-depolymerizing factor homolog OS=D.m.	CADF_DROME	1,00E-25	GLOS_CADF.1.7	GO:0051301	B	.	cell division
CADF_DROME (sp|P45594) Cofilin/actin-depolymerizing factor homolog OS=D.m.	CADF_DROME	1,00E-25	GLOS_CADF.1.7	GO:0000915	B	FlyBase	cytokinesis, actomyosin contractile ring assembly
CADF_DROME (sp|P45594) Cofilin/actin-depolymerizing factor homolog OS=D.m.	CADF_DROME	1,00E-25	GLOS_CADF.1.7	GO:0001736	B	.	establishment of planar polarity
CADF_DROME (sp|P45594) Cofilin/actin-depolymerizing factor homolog OS=D.m.	CADF_DROME	1,00E-25	GLOS_CADF.1.7	GO:0007164	B	.	establishment of tissue polarity
CADF_DROME (sp|P45594) Cofilin/actin-depolymerizing factor homolog OS=D.m.	CADF_DROME	1,00E-25	GLOS_CADF.1.7	GO:0001738	B	.	morphogenesis of a polarized epithelium
CADF_DROME (sp|P45594) Cofilin/actin-depolymerizing factor homolog OS=D.m.	CADF_DROME	1,00E-25	GLOS_CADF.1.7	GO:0002009	B	.	morphogenesis of an epithelium
CADF_DROME (sp|P45594) Cofilin/actin-depolymerizing factor homolog OS=D.m.	CADF_DROME	1,00E-25	GLOS_CADF.1.7	GO:0048729	B	.	tissue morphogenesis

CADF_DROME (sp|P45594) Cofilin/actin-depolymerizing factor homolog OS=D.m.	CADF_DROME	1,00E-25	GLOS_CADF.1.7	GO:0009888	B	.	tissue development
CADF_DROME (sp|P45594) Cofilin/actin-depolymerizing factor homolog OS=D.m.	CADF_DROME	1,00E-25	GLOS_CADF.1.7	GO:0060429	B	.	epithelium development
CADF_DROME (sp|P45594) Cofilin/actin-depolymerizing factor homolog OS=D.m.	CADF_DROME	1,00E-25	GLOS_CADF.1.7	GO:0009886	B	.	post-embryonic morphogenesis
CADF_DROME (sp|P45594) Cofilin/actin-depolymerizing factor homolog OS=D.m.	CADF_DROME	1,00E-25	GLOS_CADF.1.7	GO:0009791	B	.	post-embryonic development
CADF_DROME (sp|P45594) Cofilin/actin-depolymerizing factor homolog OS=D.m.	CADF_DROME	1,00E-25	GLOS_CADF.1.7	GO:0035317	B	.	imaginal disc-derived wing hair organization
CADF_DROME (sp|P45594) Cofilin/actin-depolymerizing factor homolog OS=D.m.	CADF_DROME	1,00E-25	GLOS_CADF.1.7	GO:0035316	B	.	non-sensory hair organization
CADF_DROME (sp|P45594) Cofilin/actin-depolymerizing factor homolog OS=D.m.	CADF_DROME	1,00E-25	GLOS_CADF.1.7	GO:0035315	B	.	hair cell differentiation
CADF_DROME (sp|P45594) Cofilin/actin-depolymerizing factor homolog OS=D.m.	CADF_DROME	1,00E-25	GLOS_CADF.1.7	GO:0009913	B	.	epidermal cell differentiation
CADF_DROME (sp|P45594) Cofilin/actin-depolymerizing factor homolog OS=D.m.	CADF_DROME	1,00E-25	GLOS_CADF.1.7	GO:0030855	B	.	epithelial cell differentiation
CADF_DROME (sp|P45594) Cofilin/actin-depolymerizing factor homolog OS=D.m.	CADF_DROME	1,00E-25	GLOS_CADF.1.7	GO:0008544	B	.	epidermis development
CADF_DROME (sp|P45594) Cofilin/actin-depolymerizing factor homolog OS=D.m.	CADF_DROME	1,00E-25	GLOS_CADF.1.7	GO:0043588	B	.	skin development
CADF_DROME (sp|P45594) Cofilin/actin-depolymerizing factor homolog OS=D.m.	CADF_DROME	1,00E-25	GLOS_CADF.1.7	GO:0007476	B	.	imaginal disc-derived wing morphogenesis
CADF_DROME (sp|P45594) Cofilin/actin-depolymerizing factor homolog OS=D.m.	CADF_DROME	1,00E-25	GLOS_CADF.1.7	GO:0035114	B	.	imaginal disc-derived appendage morphogenesis
CADF_DROME (sp|P45594) Cofilin/actin-depolymerizing factor homolog OS=D.m.	CADF_DROME	1,00E-25	GLOS_CADF.1.7	GO:0035107	B	.	appendage morphogenesis
CADF_DROME (sp|P45594) Cofilin/actin-depolymerizing factor homolog OS=D.m.	CADF_DROME	1,00E-25	GLOS_CADF.1.7	GO:0048736	B	.	appendage development
CADF_DROME (sp|P45594) Cofilin/actin-depolymerizing factor homolog OS=D.m.	CADF_DROME	1,00E-25	GLOS_CADF.1.7	GO:0048737	B	.	imaginal disc-derived appendage development
CADF_DROME (sp|P45594) Cofilin/actin-depolymerizing factor homolog OS=D.m.	CADF_DROME	1,00E-25	GLOS_CADF.1.7	GO:0035120	B	.	post-embryonic appendage morphogenesis
CADF_DROME (sp|P45594) Cofilin/actin-depolymerizing factor homolog OS=D.m.	CADF_DROME	1,00E-25	GLOS_CADF.1.7	GO:0007472	B	.	wing disc morphogenesis
CADF_DROME (sp|P45594) Cofilin/actin-depolymerizing factor homolog OS=D.m.	CADF_DROME	1,00E-25	GLOS_CADF.1.7	GO:0007560	B	.	imaginal disc morphogenesis
CADF_DROME (sp|P45594) Cofilin/actin-depolymerizing factor homolog OS=D.m.	CADF_DROME	1,00E-25	GLOS_CADF.1.7	GO:0048563	B	.	post-embryonic organ morphogenesis
CADF_DROME (sp|P45594) Cofilin/actin-depolymerizing factor homolog OS=D.m.	CADF_DROME	1,00E-25	GLOS_CADF.1.7	GO:0048569	B	.	post-embryonic organ development
CADF_DROME (sp|P45594) Cofilin/actin-depolymerizing factor homolog OS=D.m.	CADF_DROME	1,00E-25	GLOS_CADF.1.7	GO:0007444	B	.	imaginal disc development
CADF_DROME (sp|P45594) Cofilin/actin-depolymerizing factor homolog OS=D.m.	CADF_DROME	1,00E-25	GLOS_CADF.1.7	GO:0007552	B	.	metamorphosis
CADF_DROME (sp|P45594) Cofilin/actin-depolymerizing factor homolog OS=D.m.	CADF_DROME	1,00E-25	GLOS_CADF.1.7	GO:0048707	B	.	instar larval or pupal morphogenesis
CADF_DROME (sp|P45594) Cofilin/actin-depolymerizing factor homolog OS=D.m.	CADF_DROME	1,00E-25	GLOS_CADF.1.7	GO:0002165	B	.	instar larval or pupal development
CADF_DROME (sp|P45594) Cofilin/actin-depolymerizing factor homolog OS=D.m.	CADF_DROME	1,00E-25	GLOS_CADF.1.7	GO:0035220	B	.	wing disc development
CADF_DROME (sp|P45594) Cofilin/actin-depolymerizing factor homolog OS=D.m.	CADF_DROME	1,00E-25	GLOS_CADF.1.7	GO:0001737	B	FlyBase	establishment of imaginal disc-derived wing hair orientation
CADF_DROME (sp|P45594) Cofilin/actin-depolymerizing factor homolog OS=D.m.	CADF_DROME	1,00E-25	GLOS_CADF.1.7	GO:0007163	B	.	establishment or maintenance of cell polarity
CADF_DROME (sp|P45594) Cofilin/actin-depolymerizing factor homolog OS=D.m.	CADF_DROME	1,00E-25	GLOS_CADF.1.7	GO:0001745	B	.	compound eye morphogenesis
CADF_DROME (sp|P45594) Cofilin/actin-depolymerizing factor homolog OS=D.m.	CADF_DROME	1,00E-25	GLOS_CADF.1.7	GO:0048749	B	.	compound eye development
CADF_DROME (sp|P45594) Cofilin/actin-depolymerizing factor homolog OS=D.m.	CADF_DROME	1,00E-25	GLOS_CADF.1.7	GO:0042067	B	FlyBase	establishment of ommatidial planar polarity
CADF_DROME (sp|P45594) Cofilin/actin-depolymerizing factor homolog OS=D.m.	CADF_DROME	1,00E-25	GLOS_CADF.1.7	GO:0008585	B	FlyBase	female gonad development
CADF_DROME (sp|P45594) Cofilin/actin-depolymerizing factor homolog OS=D.m.	CADF_DROME	1,00E-25	GLOS_CADF.1.7	GO:0035285	B	.	appendage segmentation
CADF_DROME (sp|P45594) Cofilin/actin-depolymerizing factor homolog OS=D.m.	CADF_DROME	1,00E-25	GLOS_CADF.1.7	GO:0035282	B	.	segmentation
CADF_DROME (sp|P45594) Cofilin/actin-depolymerizing factor homolog OS=D.m.	CADF_DROME	1,00E-25	GLOS_CADF.1.7	GO:0003002	B	.	regionalization
CADF_DROME (sp|P45594) Cofilin/actin-depolymerizing factor homolog OS=D.m.	CADF_DROME	1,00E-25	GLOS_CADF.1.7	GO:0007389	B	.	pattern specification process
CADF_DROME (sp|P45594) Cofilin/actin-depolymerizing factor homolog OS=D.m.	CADF_DROME	1,00E-25	GLOS_CADF.1.7	GO:0007480	B	.	imaginal disc-derived leg morphogenesis
CADF_DROME (sp|P45594) Cofilin/actin-depolymerizing factor homolog OS=D.m.	CADF_DROME	1,00E-25	GLOS_CADF.1.7	GO:0007478	B	.	leg disc morphogenesis
CADF_DROME (sp|P45594) Cofilin/actin-depolymerizing factor homolog OS=D.m.	CADF_DROME	1,00E-25	GLOS_CADF.1.7	GO:0035218	B	.	leg disc development
CADF_DROME (sp|P45594) Cofilin/actin-depolymerizing factor homolog OS=D.m.	CADF_DROME	1,00E-25	GLOS_CADF.1.7	GO:0036011	B	FlyBase	imaginal disc-derived leg segmentation
CADF_DROME (sp|P45594) Cofilin/actin-depolymerizing factor homolog OS=D.m.	CADF_DROME	1,00E-25	GLOS_CADF.1.7	GO:0007420	B	.	brain development
CADF_DROME (sp|P45594) Cofilin/actin-depolymerizing factor homolog OS=D.m.	CADF_DROME	1,00E-25	GLOS_CADF.1.7	GO:0007417	B	.	central nervous system development
CADF_DROME (sp|P45594) Cofilin/actin-depolymerizing factor homolog OS=D.m.	CADF_DROME	1,00E-25	GLOS_CADF.1.7	GO:0016319	B	FlyBase	mushroom body development
CADF_DROME (sp|P45594) Cofilin/actin-depolymerizing factor homolog OS=D.m.	CADF_DROME	1,00E-25	GLOS_CADF.1.7	GO:0060491	B	.	regulation of cell projection assembly
CADF_DROME (sp|P45594) Cofilin/actin-depolymerizing factor homolog OS=D.m.	CADF_DROME	1,00E-25	GLOS_CADF.1.7	GO:0010591	B	FlyBase	regulation of lamellipodium assembly
CADF_DROME (sp|P45594) Cofilin/actin-depolymerizing factor homolog OS=D.m.	CADF_DROME	1,00E-25	GLOS_CADF.1.7	GO:0042051	B	.	compound eye photoreceptor development
CADF_DROME (sp|P45594) Cofilin/actin-depolymerizing factor homolog OS=D.m.	CADF_DROME	1,00E-25	GLOS_CADF.1.7	GO:0001751	B	.	compound eye photoreceptor cell differentiation
CADF_DROME (sp|P45594) Cofilin/actin-depolymerizing factor homolog OS=D.m.	CADF_DROME	1,00E-25	GLOS_CADF.1.7	GO:0042052	B	FlyBase	rhabdomere development
CP9F2_DROME (sp|Q9VG82) Probable cytochrome P450 9f2 OS=D.m.	CP9F2_DROME	1,00E-177	GLOS_CP9F2.3.9	GO:0005789	C	UniProtKB-S	endoplasmic reticulum membrane
CP9F2_DROME (sp|Q9VG82) Probable cytochrome P450 9f2 OS=D.m.	CP9F2_DROME	1,00E-177	GLOS_CP9F2.3.9	GO:0009055	M	InterPro	electron carrier activity
CP9F2_DROME (sp|Q9VG82) Probable cytochrome P450 9f2 OS=D.m.	CP9F2_DROME	1,00E-177	GLOS_CP9F2.3.9	GO:0046906	M	.	tetrapyrrole binding
CP9F2_DROME (sp|Q9VG82) Probable cytochrome P450 9f2 OS=D.m.	CP9F2_DROME	1,00E-177	GLOS_CP9F2.3.9	GO:0020037	M	InterPro	heme binding
CP9F2_DROME (sp|Q9VG82) Probable cytochrome P450 9f2 OS=D.m.	CP9F2_DROME	1,00E-177	GLOS_CP9F2.3.9	GO:0005506	M	InterPro	iron ion binding
CP9F2_DROME (sp|Q9VG82) Probable cytochrome P450 9f2 OS=D.m.	CP9F2_DROME	1,00E-177	GLOS_CP9F2.3.9	GO:0004497	M	UniProtKB-K	monooxygenase activity

CP9F2_DROME (sp|Q9VG82) Probable cytochrome P450 9f2 OS=D.m.	CP9F2_DROME	1,00E-177	GLOS_CP9F2.3.9	GO:0016705	M	InterPro	oxidoreductase activity, acting on paired donors, with incorporation or reduction of molecular oxygen
CP9F2_DROME (sp|Q9VG82) Probable cytochrome P450 9f2 OS=D.m.	CP9F2_DROME	1,00E-177	GLOS_CP9F2.3.9	GO:0035220	B	FlyBase	wing disc development
[BBH] CRY1_DROME (sp|O77059) Cryptochrome-1 OS=D.m.	CRY1_DROME	0	GLOS_CRY1.1.1	GO:0005737	C	FlyBase	cytoplasm
[BBH] CRY1_DROME (sp|O77059) Cryptochrome-1 OS=D.m.	CRY1_DROME	0	GLOS_CRY1.1.1	GO:0031970	C	.	organelle envelope lumen
[BBH] CRY1_DROME (sp|O77059) Cryptochrome-1 OS=D.m.	CRY1_DROME	0	GLOS_CRY1.1.1	GO:0031967	C	.	organelle envelope
[BBH] CRY1_DROME (sp|O77059) Cryptochrome-1 OS=D.m.	CRY1_DROME	0	GLOS_CRY1.1.1	GO:0031975	C	.	envelope
[BBH] CRY1_DROME (sp|O77059) Cryptochrome-1 OS=D.m.	CRY1_DROME	0	GLOS_CRY1.1.1	GO:0005635	C	.	nuclear envelope
[BBH] CRY1_DROME (sp|O77059) Cryptochrome-1 OS=D.m.	CRY1_DROME	0	GLOS_CRY1.1.1	GO:0005641	C	FlyBase	nuclear envelope lumen
[BBH] CRY1_DROME (sp|O77059) Cryptochrome-1 OS=D.m.	CRY1_DROME	0	GLOS_CRY1.1.1	GO:0048471	C	UniProtKB-S	perinuclear region of cytoplasm
[BBH] CRY1_DROME (sp|O77059) Cryptochrome-1 OS=D.m.	CRY1_DROME	0	GLOS_CRY1.1.1	GO:0043234	C	FlyBase	protein complex
[BBH] CRY1_DROME (sp|O77059) Cryptochrome-1 OS=D.m.	CRY1_DROME	0	GLOS_CRY1.1.1	GO:0009881	M	.	photoreceptor activity
[BBH] CRY1_DROME (sp|O77059) Cryptochrome-1 OS=D.m.	CRY1_DROME	0	GLOS_CRY1.1.1	GO:0038023	M	.	signaling receptor activity
[BBH] CRY1_DROME (sp|O77059) Cryptochrome-1 OS=D.m.	CRY1_DROME	0	GLOS_CRY1.1.1	GO:0004871	M	.	signal transducer activity
[BBH] CRY1_DROME (sp|O77059) Cryptochrome-1 OS=D.m.	CRY1_DROME	0	GLOS_CRY1.1.1	GO:0060089	M	.	molecular transducer activity
[BBH] CRY1_DROME (sp|O77059) Cryptochrome-1 OS=D.m.	CRY1_DROME	0	GLOS_CRY1.1.1	GO:0004872	M	.	receptor activity
[BBH] CRY1_DROME (sp|O77059) Cryptochrome-1 OS=D.m.	CRY1_DROME	0	GLOS_CRY1.1.1	GO:0009882	M	UniProtKB	blue light photoreceptor activity
[BBH] CRY1_DROME (sp|O77059) Cryptochrome-1 OS=D.m.	CRY1_DROME	0	GLOS_CRY1.1.1	GO:0050660	M	UniProtKB	flavin adenine dinucleotide binding
[BBH] CRY1_DROME (sp|O77059) Cryptochrome-1 OS=D.m.	CRY1_DROME	0	GLOS_CRY1.1.1	GO:0050962	B	.	detection of light stimulus involved in sensory perception
[BBH] CRY1_DROME (sp|O77059) Cryptochrome-1 OS=D.m.	CRY1_DROME	0	GLOS_CRY1.1.1	GO:0050906	B	.	detection of stimulus involved in sensory perception
[BBH] CRY1_DROME (sp|O77059) Cryptochrome-1 OS=D.m.	CRY1_DROME	0	GLOS_CRY1.1.1	GO:0007600	B	.	sensory perception
[BBH] CRY1_DROME (sp|O77059) Cryptochrome-1 OS=D.m.	CRY1_DROME	0	GLOS_CRY1.1.1	GO:0050953	B	.	sensory perception of light stimulus
[BBH] CRY1_DROME (sp|O77059) Cryptochrome-1 OS=D.m.	CRY1_DROME	0	GLOS_CRY1.1.1	GO:0050958	B	.	magnetoreception
[BBH] CRY1_DROME (sp|O77059) Cryptochrome-1 OS=D.m.	CRY1_DROME	0	GLOS_CRY1.1.1	GO:0050980	B	UniProtKB	detection of light stimulus involved in magnetoreception
[BBH] CRY1_DROME (sp|O77059) Cryptochrome-1 OS=D.m.	CRY1_DROME	0	GLOS_CRY1.1.1	GO:0006259	B	.	DNA metabolic process
[BBH] CRY1_DROME (sp|O77059) Cryptochrome-1 OS=D.m.	CRY1_DROME	0	GLOS_CRY1.1.1	GO:0006974	B	.	cellular response to DNA damage stimulus
[BBH] CRY1_DROME (sp|O77059) Cryptochrome-1 OS=D.m.	CRY1_DROME	0	GLOS_CRY1.1.1	GO:0033554	B	.	cellular response to stress
[BBH] CRY1_DROME (sp|O77059) Cryptochrome-1 OS=D.m.	CRY1_DROME	0	GLOS_CRY1.1.1	GO:0006281	B	InterPro	DNA repair
[BBH] CRY1_DROME (sp|O77059) Cryptochrome-1 OS=D.m.	CRY1_DROME	0	GLOS_CRY1.1.1	GO:0009648	B	.	photoperiodism
[BBH] CRY1_DROME (sp|O77059) Cryptochrome-1 OS=D.m.	CRY1_DROME	0	GLOS_CRY1.1.1	GO:0009649	B	.	entrainment of circadian clock
[BBH] CRY1_DROME (sp|O77059) Cryptochrome-1 OS=D.m.	CRY1_DROME	0	GLOS_CRY1.1.1	GO:0042752	B	.	regulation of circadian rhythm
[BBH] CRY1_DROME (sp|O77059) Cryptochrome-1 OS=D.m.	CRY1_DROME	0	GLOS_CRY1.1.1	GO:0043153	B	FlyBase	entrainment of circadian clock by photoperiod
[BBH] CRY1_DROME (sp|O77059) Cryptochrome-1 OS=D.m.	CRY1_DROME	0	GLOS_CRY1.1.1	GO:0009629	B	.	response to gravity
[BBH] CRY1_DROME (sp|O77059) Cryptochrome-1 OS=D.m.	CRY1_DROME	0	GLOS_CRY1.1.1	GO:0042332	B	FlyBase	gravitaxis
[BBH] CRY1_DROME (sp|O77059) Cryptochrome-1 OS=D.m.	CRY1_DROME	0	GLOS_CRY1.1.1	GO:0007626	B	.	locomotory behavior
[BBH] CRY1_DROME (sp|O77059) Cryptochrome-1 OS=D.m.	CRY1_DROME	0	GLOS_CRY1.1.1	GO:0044708	B	.	single-organism behavior
[BBH] CRY1_DROME (sp|O77059) Cryptochrome-1 OS=D.m.	CRY1_DROME	0	GLOS_CRY1.1.1	GO:0007610	B	.	behavior
[BBH] CRY1_DROME (sp|O77059) Cryptochrome-1 OS=D.m.	CRY1_DROME	0	GLOS_CRY1.1.1	GO:0048512	B	.	circadian behavior
[BBH] CRY1_DROME (sp|O77059) Cryptochrome-1 OS=D.m.	CRY1_DROME	0	GLOS_CRY1.1.1	GO:0007622	B	.	rhythmic behavior
[BBH] CRY1_DROME (sp|O77059) Cryptochrome-1 OS=D.m.	CRY1_DROME	0	GLOS_CRY1.1.1	GO:0007623	B	.	circadian rhythm
[BBH] CRY1_DROME (sp|O77059) Cryptochrome-1 OS=D.m.	CRY1_DROME	0	GLOS_CRY1.1.1	GO:0045475	B	FlyBase	locomotor rhythm
[BBH] CRY1_DROME (sp|O77059) Cryptochrome-1 OS=D.m.	CRY1_DROME	0	GLOS_CRY1.1.1	GO:0045892	B	UniProtKB	negative regulation of transcription, DNA-dependent
[BBH] CRY1_DROME (sp|O77059) Cryptochrome-1 OS=D.m.	CRY1_DROME	0	GLOS_CRY1.1.1	GO:0007602	B	FlyBase	phototransduction
[BBH] CRY1_DROME (sp|O77059) Cryptochrome-1 OS=D.m.	CRY1_DROME	0	GLOS_CRY1.1.1	GO:0000060	B	UniProtKB	protein import into nucleus, translocation
[BBH] CRY1_DROME (sp|O77059) Cryptochrome-1 OS=D.m.	CRY1_DROME	0	GLOS_CRY1.1.1	GO:0006464	B	.	cellular protein modification process
[BBH] CRY1_DROME (sp|O77059) Cryptochrome-1 OS=D.m.	CRY1_DROME	0	GLOS_CRY1.1.1	GO:0036211	B	.	protein modification process
[BBH] CRY1_DROME (sp|O77059) Cryptochrome-1 OS=D.m.	CRY1_DROME	0	GLOS_CRY1.1.1	GO:0019538	B	.	protein metabolic process
[BBH] CRY1_DROME (sp|O77059) Cryptochrome-1 OS=D.m.	CRY1_DROME	0	GLOS_CRY1.1.1	GO:0043412	B	.	macromolecule modification
[BBH] CRY1_DROME (sp|O77059) Cryptochrome-1 OS=D.m.	CRY1_DROME	0	GLOS_CRY1.1.1	GO:0044267	B	.	cellular protein metabolic process
[BBH] CRY1_DROME (sp|O77059) Cryptochrome-1 OS=D.m.	CRY1_DROME	0	GLOS_CRY1.1.1	GO:0018298	B	UniProtKB-k	protein-chromophore linkage
[BBH] CRY1_DROME (sp|O77059) Cryptochrome-1 OS=D.m.	CRY1_DROME	0	GLOS_CRY1.1.1	GO:0071000	B	FlyBase	response to magnetism
[BBH] CRY1_DROME (sp|O77059) Cryptochrome-1 OS=D.m.	CRY1_DROME	0	GLOS_CRY1.1.1	GO:0006351	B	UniProtKB-k	transcription, DNA-dependent
DYHC_ONCMY (sp|P15305) Dynein heavy chain (Fragment) OS=O. mykiss	DYHC_ONCMY	1,00E-09	GLOS_DYHC.2.2	GO:0005930	C	.	axoneme
DYHC_ONCMY (sp|P15305) Dynein heavy chain (Fragment) OS=O. mykiss	DYHC_ONCMY	1,00E-09	GLOS_DYHC.2.2	GO:0044441	C	.	cilium part

DYHC_ONCMY (sp|P15305) Dynein heavy chain (Fragment) OS=O. mykiss	DYHC_ONCMY	1,00E-09	GLOS_DYHC.2.2	GO:0005929	C	.	cilium
DYHC_ONCMY (sp|P15305) Dynein heavy chain (Fragment) OS=O. mykiss	DYHC_ONCMY	1,00E-09	GLOS_DYHC.2.2	GO:0031514	C	.	motile cilium
DYHC_ONCMY (sp|P15305) Dynein heavy chain (Fragment) OS=O. mykiss	DYHC_ONCMY	1,00E-09	GLOS_DYHC.2.2	GO:0097014	C	.	cilium cytoplasm
DYHC_ONCMY (sp|P15305) Dynein heavy chain (Fragment) OS=O. mykiss	DYHC_ONCMY	1,00E-09	GLOS_DYHC.2.2	GO:0032838	C	.	cell projection cytoplasm
DYHC_ONCMY (sp|P15305) Dynein heavy chain (Fragment) OS=O. mykiss	DYHC_ONCMY	1,00E-09	GLOS_DYHC.2.2	GO:0035085	C	UniProtKB-S	cilium axoneme
DYHC_ONCMY (sp|P15305) Dynein heavy chain (Fragment) OS=O. mykiss	DYHC_ONCMY	1,00E-09	GLOS_DYHC.2.2	GO:0005875	C	.	microtubule associated complex
DYHC_ONCMY (sp|P15305) Dynein heavy chain (Fragment) OS=O. mykiss	DYHC_ONCMY	1,00E-09	GLOS_DYHC.2.2	GO:0030286	C	UniProtKB-K	dynein complex
DYHC_ONCMY (sp|P15305) Dynein heavy chain (Fragment) OS=O. mykiss	DYHC_ONCMY	1,00E-09	GLOS_DYHC.2.2	GO:0005874	C	UniProtKB-K	microtubule
DYHC_ONCMY (sp|P15305) Dynein heavy chain (Fragment) OS=O. mykiss	DYHC_ONCMY	1,00E-09	GLOS_DYHC.2.2	GO:0003774	M	UniProtKB-K	motor activity
[BBH] EDC3_DROME (sp|Q9VVI2) Enhancer of mRNA-decapping prot 3 OS=D.m.	EDC3_DROME	1,00E-174	GLOS_EDC3.1.1	GO:0035770	C	.	ribonucleoprotein granule
[BBH] EDC3_DROME (sp|Q9VVI2) Enhancer of mRNA-decapping prot 3 OS=D.m.	EDC3_DROME	1,00E-174	GLOS_EDC3.1.1	GO:0030529	C	.	ribonucleoprotein complex
[BBH] EDC3_DROME (sp|Q9VVI2) Enhancer of mRNA-decapping prot 3 OS=D.m.	EDC3_DROME	1,00E-174	GLOS_EDC3.1.1	GO:0000932	C	UniProtKB	cytoplasmic mRNA processing body
[BBH] EDC3_DROME (sp|Q9VVI2) Enhancer of mRNA-decapping prot 3 OS=D.m.	EDC3_DROME	1,00E-174	GLOS_EDC3.1.1	GO:0005875	C	FlyBase	microtubule associated complex
[BBH] EDC3_DROME (sp|Q9VVI2) Enhancer of mRNA-decapping prot 3 OS=D.m.	EDC3_DROME	1,00E-174	GLOS_EDC3.1.1	GO:0006401	B	.	RNA catabolic process
[BBH] EDC3_DROME (sp|Q9VVI2) Enhancer of mRNA-decapping prot 3 OS=D.m.	EDC3_DROME	1,00E-174	GLOS_EDC3.1.1	GO:0034655	B	.	nucleobase-containing compound catabolic process
[BBH] EDC3_DROME (sp|Q9VVI2) Enhancer of mRNA-decapping prot 3 OS=D.m.	EDC3_DROME	1,00E-174	GLOS_EDC3.1.1	GO:0019439	B	.	aromatic compound catabolic process
[BBH] EDC3_DROME (sp|Q9VVI2) Enhancer of mRNA-decapping prot 3 OS=D.m.	EDC3_DROME	1,00E-174	GLOS_EDC3.1.1	GO:0044270	B	.	cellular nitrogen compound catabolic process
[BBH] EDC3_DROME (sp|Q9VVI2) Enhancer of mRNA-decapping prot 3 OS=D.m.	EDC3_DROME	1,00E-174	GLOS_EDC3.1.1	GO:0046700	B	.	heterocycle catabolic process
[BBH] EDC3_DROME (sp|Q9VVI2) Enhancer of mRNA-decapping prot 3 OS=D.m.	EDC3_DROME	1,00E-174	GLOS_EDC3.1.1	GO:1901361	B	.	organic cyclic compound catabolic process
[BBH] EDC3_DROME (sp|Q9VVI2) Enhancer of mRNA-decapping prot 3 OS=D.m.	EDC3_DROME	1,00E-174	GLOS_EDC3.1.1	GO:0044265	B	.	cellular macromolecule catabolic process
[BBH] EDC3_DROME (sp|Q9VVI2) Enhancer of mRNA-decapping prot 3 OS=D.m.	EDC3_DROME	1,00E-174	GLOS_EDC3.1.1	GO:0009057	B	.	macromolecule catabolic process
[BBH] EDC3_DROME (sp|Q9VVI2) Enhancer of mRNA-decapping prot 3 OS=D.m.	EDC3_DROME	1,00E-174	GLOS_EDC3.1.1	GO:0016071	B	.	mRNA metabolic process
[BBH] EDC3_DROME (sp|Q9VVI2) Enhancer of mRNA-decapping prot 3 OS=D.m.	EDC3_DROME	1,00E-174	GLOS_EDC3.1.1	GO:0006402	B	UniProtKB	mRNA catabolic process
[BBH] EF2_DROME (sp|P13060) Elongation factor 2 OS=D.m.	EF2_DROME	0	GLOS_EF2.1.1	GO:0005829	C	FlyBase	cytosol
[BBH] EF2_DROME (sp|P13060) Elongation factor 2 OS=D.m.	EF2_DROME	0	GLOS_EF2.1.1	GO:0005811	C	FlyBase	lipid particle
[BBH] EF2_DROME (sp|P13060) Elongation factor 2 OS=D.m.	EF2_DROME	0	GLOS_EF2.1.1	GO:0005875	C	FlyBase	microtubule associated complex
[BBH] EF2_DROME (sp|P13060) Elongation factor 2 OS=D.m.	EF2_DROME	0	GLOS_EF2.1.1	GO:0005525	M	UniProtKB-K	GTP binding
[BBH] EF2_DROME (sp|P13060) Elongation factor 2 OS=D.m.	EF2_DROME	0	GLOS_EF2.1.1	GO:0003924	M	InterPro	GTPase activity
[BBH] EF2_DROME (sp|P13060) Elongation factor 2 OS=D.m.	EF2_DROME	0	GLOS_EF2.1.1	GO:0008135	M	.	translation factor activity, nucleic acid binding
[BBH] EF2_DROME (sp|P13060) Elongation factor 2 OS=D.m.	EF2_DROME	0	GLOS_EF2.1.1	GO:0003723	M	.	RNA binding
[BBH] EF2_DROME (sp|P13060) Elongation factor 2 OS=D.m.	EF2_DROME	0	GLOS_EF2.1.1	GO:0003746	M	FlyBase	translation elongation factor activity
[BBH] EF2_DROME (sp|P13060) Elongation factor 2 OS=D.m.	EF2_DROME	0	GLOS_EF2.1.1	GO:0009154	B	.	purine ribonucleotide catabolic process
[BBH] EF2_DROME (sp|P13060) Elongation factor 2 OS=D.m.	EF2_DROME	0	GLOS_EF2.1.1	GO:0006195	B	.	purine nucleotide catabolic process
[BBH] EF2_DROME (sp|P13060) Elongation factor 2 OS=D.m.	EF2_DROME	0	GLOS_EF2.1.1	GO:0006163	B	.	purine nucleotide metabolic process
[BBH] EF2_DROME (sp|P13060) Elongation factor 2 OS=D.m.	EF2_DROME	0	GLOS_EF2.1.1	GO:0009117	B	.	nucleotide metabolic process
[BBH] EF2_DROME (sp|P13060) Elongation factor 2 OS=D.m.	EF2_DROME	0	GLOS_EF2.1.1	GO:0006753	B	.	nucleoside phosphate metabolic process
[BBH] EF2_DROME (sp|P13060) Elongation factor 2 OS=D.m.	EF2_DROME	0	GLOS_EF2.1.1	GO:0006796	B	.	phosphate-containing compound metabolic process
[BBH] EF2_DROME (sp|P13060) Elongation factor 2 OS=D.m.	EF2_DROME	0	GLOS_EF2.1.1	GO:0006793	B	.	phosphorus metabolic process
[BBH] EF2_DROME (sp|P13060) Elongation factor 2 OS=D.m.	EF2_DROME	0	GLOS_EF2.1.1	GO:0019637	B	.	organophosphate metabolic process
[BBH] EF2_DROME (sp|P13060) Elongation factor 2 OS=D.m.	EF2_DROME	0	GLOS_EF2.1.1	GO:0055086	B	.	nucleobase-containing small molecule metabolic process
[BBH] EF2_DROME (sp|P13060) Elongation factor 2 OS=D.m.	EF2_DROME	0	GLOS_EF2.1.1	GO:0072521	B	.	purine-containing compound metabolic process
[BBH] EF2_DROME (sp|P13060) Elongation factor 2 OS=D.m.	EF2_DROME	0	GLOS_EF2.1.1	GO:1901564	B	.	organonitrogen compound metabolic process
[BBH] EF2_DROME (sp|P13060) Elongation factor 2 OS=D.m.	EF2_DROME	0	GLOS_EF2.1.1	GO:0009166	B	.	nucleotide catabolic process
[BBH] EF2_DROME (sp|P13060) Elongation factor 2 OS=D.m.	EF2_DROME	0	GLOS_EF2.1.1	GO:1901292	B	.	nucleoside phosphate catabolic process
[BBH] EF2_DROME (sp|P13060) Elongation factor 2 OS=D.m.	EF2_DROME	0	GLOS_EF2.1.1	GO:0046434	B	.	organophosphate catabolic process
[BBH] EF2_DROME (sp|P13060) Elongation factor 2 OS=D.m.	EF2_DROME	0	GLOS_EF2.1.1	GO:0072523	B	.	purine-containing compound catabolic process
[BBH] EF2_DROME (sp|P13060) Elongation factor 2 OS=D.m.	EF2_DROME	0	GLOS_EF2.1.1	GO:1901565	B	.	organonitrogen compound catabolic process
[BBH] EF2_DROME (sp|P13060) Elongation factor 2 OS=D.m.	EF2_DROME	0	GLOS_EF2.1.1	GO:0009150	B	.	purine ribonucleotide metabolic process
[BBH] EF2_DROME (sp|P13060) Elongation factor 2 OS=D.m.	EF2_DROME	0	GLOS_EF2.1.1	GO:0009259	B	.	ribonucleotide metabolic process
[BBH] EF2_DROME (sp|P13060) Elongation factor 2 OS=D.m.	EF2_DROME	0	GLOS_EF2.1.1	GO:0019693	B	.	ribose phosphate metabolic process
[BBH] EF2_DROME (sp|P13060) Elongation factor 2 OS=D.m.	EF2_DROME	0	GLOS_EF2.1.1	GO:1901135	B	.	carbohydrate derivative metabolic process
[BBH] EF2_DROME (sp|P13060) Elongation factor 2 OS=D.m.	EF2_DROME	0	GLOS_EF2.1.1	GO:0009261	B	.	ribonucleotide catabolic process
[BBH] EF2_DROME (sp|P13060) Elongation factor 2 OS=D.m.	EF2_DROME	0	GLOS_EF2.1.1	GO:1901136	B	.	carbohydrate derivative catabolic process
[BBH] EF2_DROME (sp|P13060) Elongation factor 2 OS=D.m.	EF2_DROME	0	GLOS_EF2.1.1	GO:0009207	B	.	purine ribonucleoside triphosphate catabolic process

[BBH] EF2_DROME (sp|P13060) Elongation factor 2 OS=D.m.	EF2_DROME	0	GLOS_EF2.1.1	GO:0009146	B	.	purine nucleoside triphosphate catabolic process
[BBH] EF2_DROME (sp|P13060) Elongation factor 2 OS=D.m.	EF2_DROME	0	GLOS_EF2.1.1	GO:0009143	B	.	nucleoside triphosphate catabolic process
[BBH] EF2_DROME (sp|P13060) Elongation factor 2 OS=D.m.	EF2_DROME	0	GLOS_EF2.1.1	GO:0009141	B	.	nucleoside triphosphate metabolic process
[BBH] EF2_DROME (sp|P13060) Elongation factor 2 OS=D.m.	EF2_DROME	0	GLOS_EF2.1.1	GO:0009144	B	.	purine nucleoside triphosphate metabolic process
[BBH] EF2_DROME (sp|P13060) Elongation factor 2 OS=D.m.	EF2_DROME	0	GLOS_EF2.1.1	GO:0009203	B	.	ribonucleoside triphosphate catabolic process
[BBH] EF2_DROME (sp|P13060) Elongation factor 2 OS=D.m.	EF2_DROME	0	GLOS_EF2.1.1	GO:0009199	B	.	ribonucleoside triphosphate metabolic process
[BBH] EF2_DROME (sp|P13060) Elongation factor 2 OS=D.m.	EF2_DROME	0	GLOS_EF2.1.1	GO:0009205	B	.	purine ribonucleoside triphosphate metabolic process
[BBH] EF2_DROME (sp|P13060) Elongation factor 2 OS=D.m.	EF2_DROME	0	GLOS_EF2.1.1	GO:0046039	B	.	GTP metabolic process
[BBH] EF2_DROME (sp|P13060) Elongation factor 2 OS=D.m.	EF2_DROME	0	GLOS_EF2.1.1	GO:1901068	B	.	guanosine-containing compound metabolic process
[BBH] EF2_DROME (sp|P13060) Elongation factor 2 OS=D.m.	EF2_DROME	0	GLOS_EF2.1.1	GO:0046128	B	.	purine ribonucleoside metabolic process
[BBH] EF2_DROME (sp|P13060) Elongation factor 2 OS=D.m.	EF2_DROME	0	GLOS_EF2.1.1	GO:0009119	B	.	ribonucleoside metabolic process
[BBH] EF2_DROME (sp|P13060) Elongation factor 2 OS=D.m.	EF2_DROME	0	GLOS_EF2.1.1	GO:0009116	B	.	nucleoside metabolic process
[BBH] EF2_DROME (sp|P13060) Elongation factor 2 OS=D.m.	EF2_DROME	0	GLOS_EF2.1.1	GO:1901657	B	.	glycosyl compound metabolic process
[BBH] EF2_DROME (sp|P13060) Elongation factor 2 OS=D.m.	EF2_DROME	0	GLOS_EF2.1.1	GO:0042278	B	.	purine nucleoside metabolic process
[BBH] EF2_DROME (sp|P13060) Elongation factor 2 OS=D.m.	EF2_DROME	0	GLOS_EF2.1.1	GO:1901069	B	.	guanosine-containing compound catabolic process
[BBH] EF2_DROME (sp|P13060) Elongation factor 2 OS=D.m.	EF2_DROME	0	GLOS_EF2.1.1	GO:0046130	B	.	purine ribonucleoside catabolic process
[BBH] EF2_DROME (sp|P13060) Elongation factor 2 OS=D.m.	EF2_DROME	0	GLOS_EF2.1.1	GO:0006152	B	.	purine nucleoside catabolic process
[BBH] EF2_DROME (sp|P13060) Elongation factor 2 OS=D.m.	EF2_DROME	0	GLOS_EF2.1.1	GO:0009164	B	.	nucleoside catabolic process
[BBH] EF2_DROME (sp|P13060) Elongation factor 2 OS=D.m.	EF2_DROME	0	GLOS_EF2.1.1	GO:1901658	B	.	glycosyl compound catabolic process
[BBH] EF2_DROME (sp|P13060) Elongation factor 2 OS=D.m.	EF2_DROME	0	GLOS_EF2.1.1	GO:0042454	B	.	ribonucleoside catabolic process
[BBH] EF2_DROME (sp|P13060) Elongation factor 2 OS=D.m.	EF2_DROME	0	GLOS_EF2.1.1	GO:0006184	B	GOC	GTP catabolic process
[BBH] EF2_DROME (sp|P13060) Elongation factor 2 OS=D.m.	EF2_DROME	0	GLOS_EF2.1.1	GO:0051231	B	.	spindle elongation
[BBH] EF2_DROME (sp|P13060) Elongation factor 2 OS=D.m.	EF2_DROME	0	GLOS_EF2.1.1	GO:0007051	B	.	spindle organization
[BBH] EF2_DROME (sp|P13060) Elongation factor 2 OS=D.m.	EF2_DROME	0	GLOS_EF2.1.1	GO:0007052	B	.	mitotic spindle organization
[BBH] EF2_DROME (sp|P13060) Elongation factor 2 OS=D.m.	EF2_DROME	0	GLOS_EF2.1.1	GO:0000278	B	.	mitotic cell cycle
[BBH] EF2_DROME (sp|P13060) Elongation factor 2 OS=D.m.	EF2_DROME	0	GLOS_EF2.1.1	GO:0000022	B	FlyBase	mitotic spindle elongation
GBLP_DROME (sp|O18640) Guanine nucleot.-binding prot subunit beta-like prot	GBLP_DROME	1,00E-148	GLOS_GBLP.2.4	GO:0005773	C	.	vacuole
GBLP_DROME (sp|O18640) Guanine nucleot.-binding prot subunit beta-like prot	GBLP_DROME	1,00E-148	GLOS_GBLP.2.4	GO:0005776	C	FlyBase	autophagic vacuole
GBLP_DROME (sp|O18640) Guanine nucleot.-binding prot subunit beta-like prot	GBLP_DROME	1,00E-148	GLOS_GBLP.2.4	GO:0005681	C	.	spliceosomal complex
GBLP_DROME (sp|O18640) Guanine nucleot.-binding prot subunit beta-like prot	GBLP_DROME	1,00E-148	GLOS_GBLP.2.4	GO:0071013	C	FlyBase	catalytic step 2 spliceosome
GBLP_DROME (sp|O18640) Guanine nucleot.-binding prot subunit beta-like prot	GBLP_DROME	1,00E-148	GLOS_GBLP.2.4	GO:0005875	C	FlyBase	microtubule associated complex
GBLP_DROME (sp|O18640) Guanine nucleot.-binding prot subunit beta-like prot	GBLP_DROME	1,00E-148	GLOS_GBLP.2.4	GO:0071011	C	FlyBase	precatalytic spliceosome
GBLP_DROME (sp|O18640) Guanine nucleot.-binding prot subunit beta-like prot	GBLP_DROME	1,00E-148	GLOS_GBLP.2.4	GO:0042335	B	FlyBase	cuticle development
GBLP_DROME (sp|O18640) Guanine nucleot.-binding prot subunit beta-like prot	GBLP_DROME	1,00E-148	GLOS_GBLP.2.4	GO:0007626	B	FlyBase	locomotory behavior
GBLP_DROME (sp|O18640) Guanine nucleot.-binding prot subunit beta-like prot	GBLP_DROME	1,00E-148	GLOS_GBLP.2.4	GO:0000377	B	.	RNA splicing, via transesterificat. reactions with bulged adenosine as nucleophile
GBLP_DROME (sp|O18640) Guanine nucleot.-binding prot subunit beta-like prot	GBLP_DROME	1,00E-148	GLOS_GBLP.2.4	GO:0000375	B	.	RNA splicing, via transesterification reactions
GBLP_DROME (sp|O18640) Guanine nucleot.-binding prot subunit beta-like prot	GBLP_DROME	1,00E-148	GLOS_GBLP.2.4	GO:0008380	B	.	RNA splicing
GBLP_DROME (sp|O18640) Guanine nucleot.-binding prot subunit beta-like prot	GBLP_DROME	1,00E-148	GLOS_GBLP.2.4	GO:0006396	B	.	RNA processing
GBLP_DROME (sp|O18640) Guanine nucleot.-binding prot subunit beta-like prot	GBLP_DROME	1,00E-148	GLOS_GBLP.2.4	GO:0006397	B	.	mRNA processing
GBLP_DROME (sp|O18640) Guanine nucleot.-binding prot subunit beta-like prot	GBLP_DROME	1,00E-148	GLOS_GBLP.2.4	GO:0000398	B	FlyBase	mRNA splicing, via spliceosome
GBLP_DROME (sp|O18640) Guanine nucleot.-binding prot subunit beta-like prot	GBLP_DROME	1,00E-148	GLOS_GBLP.2.4	GO:0060249	B	.	anatomical structure homeostasis
GBLP_DROME (sp|O18640) Guanine nucleot.-binding prot subunit beta-like prot	GBLP_DROME	1,00E-148	GLOS_GBLP.2.4	GO:0046716	B	FlyBase	muscle cell cellular homeostasis
GBLP_DROME (sp|O18640) Guanine nucleot.-binding prot subunit beta-like prot	GBLP_DROME	1,00E-148	GLOS_GBLP.2.4	GO:0048477	B	FlyBase	oogenesis
GBLP_DROME (sp|O18640) Guanine nucleot.-binding prot subunit beta-like prot	GBLP_DROME	1,00E-148	GLOS_GBLP.2.4	GO:0033057	B	.	multicellular organismal reproductive behavior
GBLP_DROME (sp|O18640) Guanine nucleot.-binding prot subunit beta-like prot	GBLP_DROME	1,00E-148	GLOS_GBLP.2.4	GO:0019098	B	.	reproductive behavior
GBLP_DROME (sp|O18640) Guanine nucleot.-binding prot subunit beta-like prot	GBLP_DROME	1,00E-148	GLOS_GBLP.2.4	GO:0044706	B	.	multi-multicellular organism process
GBLP_DROME (sp|O18640) Guanine nucleot.-binding prot subunit beta-like prot	GBLP_DROME	1,00E-148	GLOS_GBLP.2.4	GO:0018991	B	FlyBase	oviposition
GBLP_DROME (sp|O18640) Guanine nucleot.-binding prot subunit beta-like prot	GBLP_DROME	1,00E-148	GLOS_GBLP.2.4	GO:0005979	B	.	regulation of glycogen biosynthetic process
GBLP_DROME (sp|O18640) Guanine nucleot.-binding prot subunit beta-like prot	GBLP_DROME	1,00E-148	GLOS_GBLP.2.4	GO:0010962	B	.	regulation of glucan biosynthetic process
GBLP_DROME (sp|O18640) Guanine nucleot.-binding prot subunit beta-like prot	GBLP_DROME	1,00E-148	GLOS_GBLP.2.4	GO:0010675	B	.	regulation of cellular carbohydrate metabolic process
GBLP_DROME (sp|O18640) Guanine nucleot.-binding prot subunit beta-like prot	GBLP_DROME	1,00E-148	GLOS_GBLP.2.4	GO:0006109	B	.	regulation of carbohydrate metabolic process
GBLP_DROME (sp|O18640) Guanine nucleot.-binding prot subunit beta-like prot	GBLP_DROME	1,00E-148	GLOS_GBLP.2.4	GO:0032885	B	.	regulation of polysaccharide biosynthetic process
GBLP_DROME (sp|O18640) Guanine nucleot.-binding prot subunit beta-like prot	GBLP_DROME	1,00E-148	GLOS_GBLP.2.4	GO:0032881	B	.	regulation of polysaccharide metabolic process
GBLP_DROME (sp|O18640) Guanine nucleot.-binding prot subunit beta-like prot	GBLP_DROME	1,00E-148	GLOS_GBLP.2.4	GO:0043255	B	.	regulation of carbohydrate biosynthetic process

GBLP_DROME (sp|O18640) Guanine nucleot.-binding prot subunit beta-like prot	GBLP_DROME	1,00E-148	GLOS_GBLP.2.4	GO:0070873	B	.	regulation of glycogen metabolic process
GBLP_DROME (sp|O18640) Guanine nucleot.-binding prot subunit beta-like prot	GBLP_DROME	1,00E-148	GLOS_GBLP.2.4	GO:0010906	B	.	regulation of glucose metabolic process
GBLP_DROME (sp|O18640) Guanine nucleot.-binding prot subunit beta-like prot	GBLP_DROME	1,00E-148	GLOS_GBLP.2.4	GO:0043467	B	.	regulation of generation of precursor metabolites and energy
GBLP_DROME (sp|O18640) Guanine nucleot.-binding prot subunit beta-like prot	GBLP_DROME	1,00E-148	GLOS_GBLP.2.4	GO:0070875	B	.	positive regulation of glycogen metabolic process
GBLP_DROME (sp|O18640) Guanine nucleot.-binding prot subunit beta-like prot	GBLP_DROME	1,00E-148	GLOS_GBLP.2.4	GO:0010907	B	.	positive regulation of glucose metabolic process
GBLP_DROME (sp|O18640) Guanine nucleot.-binding prot subunit beta-like prot	GBLP_DROME	1,00E-148	GLOS_GBLP.2.4	GO:0010676	B	.	positive regulation of cellular carbohydrate metabolic process
GBLP_DROME (sp|O18640) Guanine nucleot.-binding prot subunit beta-like prot	GBLP_DROME	1,00E-148	GLOS_GBLP.2.4	GO:0045913	B	.	positive regulation of carbohydrate metabolic process
GBLP_DROME (sp|O18640) Guanine nucleot.-binding prot subunit beta-like prot	GBLP_DROME	1,00E-148	GLOS_GBLP.2.4	GO:0045725	B	FlyBase	positive regulation of glycogen biosynthetic process
GBLP_DROME (sp|O18640) Guanine nucleot.-binding prot subunit beta-like prot	GBLP_DROME	1,00E-148	GLOS_GBLP.2.4	GO:0032535	B	.	regulation of cellular component size
GBLP_DROME (sp|O18640) Guanine nucleot.-binding prot subunit beta-like prot	GBLP_DROME	1,00E-148	GLOS_GBLP.2.4	GO:0090066	B	.	regulation of anatomical structure size
GBLP_DROME (sp|O18640) Guanine nucleot.-binding prot subunit beta-like prot	GBLP_DROME	1,00E-148	GLOS_GBLP.2.4	GO:0016236	B	.	macroautophagy
GBLP_DROME (sp|O18640) Guanine nucleot.-binding prot subunit beta-like prot	GBLP_DROME	1,00E-148	GLOS_GBLP.2.4	GO:0006914	B	.	autophagy
GBLP_DROME (sp|O18640) Guanine nucleot.-binding prot subunit beta-like prot	GBLP_DROME	1,00E-148	GLOS_GBLP.2.4	GO:0009267	B	.	cellular response to starvation
GBLP_DROME (sp|O18640) Guanine nucleot.-binding prot subunit beta-like prot	GBLP_DROME	1,00E-148	GLOS_GBLP.2.4	GO:0031669	B	.	cellular response to nutrient levels
GBLP_DROME (sp|O18640) Guanine nucleot.-binding prot subunit beta-like prot	GBLP_DROME	1,00E-148	GLOS_GBLP.2.4	GO:0031667	B	.	response to nutrient levels
GBLP_DROME (sp|O18640) Guanine nucleot.-binding prot subunit beta-like prot	GBLP_DROME	1,00E-148	GLOS_GBLP.2.4	GO:0009991	B	.	response to extracellular stimulus
GBLP_DROME (sp|O18640) Guanine nucleot.-binding prot subunit beta-like prot	GBLP_DROME	1,00E-148	GLOS_GBLP.2.4	GO:0031668	B	.	cellular response to extracellular stimulus
GBLP_DROME (sp|O18640) Guanine nucleot.-binding prot subunit beta-like prot	GBLP_DROME	1,00E-148	GLOS_GBLP.2.4	GO:0071496	B	.	cellular response to external stimulus
GBLP_DROME (sp|O18640) Guanine nucleot.-binding prot subunit beta-like prot	GBLP_DROME	1,00E-148	GLOS_GBLP.2.4	GO:0042594	B	.	response to starvation
GBLP_DROME (sp|O18640) Guanine nucleot.-binding prot subunit beta-like prot	GBLP_DROME	1,00E-148	GLOS_GBLP.2.4	GO:0016243	B	FlyBase	regulation of autophagic vacuole size
GBLP_DROME (sp|O18640) Guanine nucleot.-binding prot subunit beta-like prot	GBLP_DROME	1,00E-148	GLOS_GBLP.2.4	GO:0035220	B	FlyBase	wing disc development
GPL_GLOFF (sp|Q8MUG0) Lectizyme OS=G. f. fuscipes GN=Gpl PE=2 SV=1	GPL_GLOFF	4,00E-14	GLOS_GPL.6.22	GO:0005576	C	UniProtKB-S	extracellular region
GPL_GLOFF (sp|Q8MUG0) Lectizyme OS=G. f. fuscipes GN=Gpl PE=2 SV=1	GPL_GLOFF	4,00E-14	GLOS_GPL.6.22	GO:0004175	M	.	endopeptidase activity
GPL_GLOFF (sp|Q8MUG0) Lectizyme OS=G. f. fuscipes GN=Gpl PE=2 SV=1	GPL_GLOFF	4,00E-14	GLOS_GPL.6.22	GO:0070011	M	.	peptidase activity, acting on L-amino acid peptides
GPL_GLOFF (sp|Q8MUG0) Lectizyme OS=G. f. fuscipes GN=Gpl PE=2 SV=1	GPL_GLOFF	4,00E-14	GLOS_GPL.6.22	GO:0008233	M	.	peptidase activity
GPL_GLOFF (sp|Q8MUG0) Lectizyme OS=G. f. fuscipes GN=Gpl PE=2 SV=1	GPL_GLOFF	4,00E-14	GLOS_GPL.6.22	GO:0008236	M	.	serine-type peptidase activity
GPL_GLOFF (sp|Q8MUG0) Lectizyme OS=G. f. fuscipes GN=Gpl PE=2 SV=1	GPL_GLOFF	4,00E-14	GLOS_GPL.6.22	GO:0017171	M	.	serine hydrolase activity
GPL_GLOFF (sp|Q8MUG0) Lectizyme OS=G. f. fuscipes GN=Gpl PE=2 SV=1	GPL_GLOFF	4,00E-14	GLOS_GPL.6.22	GO:0004252	M	InterPro	serine-type endopeptidase activity
GPL_GLOFF (sp|Q8MUG0) Lectizyme OS=G. f. fuscipes GN=Gpl PE=2 SV=1	GPL_GLOFF	4,00E-14	GLOS_GPL.6.22	GO:0006508	B	UniProtKB-k	proteolysis
[BBH] K10_DROME (sp|P13468) DNA-binding prot K10 OS=D.m.	K10_DROME	2,00E-25	GLOS_K10.2.2	GO:0005634	C	FlyBase	nucleus
[BBH] K10_DROME (sp|P13468) DNA-binding prot K10 OS=D.m.	K10_DROME	2,00E-25	GLOS_K10.2.2	GO:0003677	M	FlyBase	DNA binding
[BBH] K10_DROME (sp|P13468) DNA-binding prot K10 OS=D.m.	K10_DROME	2,00E-25	GLOS_K10.2.2	GO:0006417	B	.	regulation of translation
[BBH] K10_DROME (sp|P13468) DNA-binding prot K10 OS=D.m.	K10_DROME	2,00E-25	GLOS_K10.2.2	GO:0010608	B	.	posttranscriptional regulation of gene expression
[BBH] K10_DROME (sp|P13468) DNA-binding prot K10 OS=D.m.	K10_DROME	2,00E-25	GLOS_K10.2.2	GO:0017148	B	FlyBase	negative regulation of translation
[BBH] K10_DROME (sp|P13468) DNA-binding prot K10 OS=D.m.	K10_DROME	2,00E-25	GLOS_K10.2.2	GO:0007309	B	.	oocyte axis specification
[BBH] K10_DROME (sp|P13468) DNA-binding prot K10 OS=D.m.	K10_DROME	2,00E-25	GLOS_K10.2.2	GO:0009798	B	.	axis specification
[BBH] K10_DROME (sp|P13468) DNA-binding prot K10 OS=D.m.	K10_DROME	2,00E-25	GLOS_K10.2.2	GO:0007308	B	.	oocyte construction
[BBH] K10_DROME (sp|P13468) DNA-binding prot K10 OS=D.m.	K10_DROME	2,00E-25	GLOS_K10.2.2	GO:0048469	B	.	cell maturation
[BBH] K10_DROME (sp|P13468) DNA-binding prot K10 OS=D.m.	K10_DROME	2,00E-25	GLOS_K10.2.2	GO:0021700	B	.	developmental maturation
[BBH] K10_DROME (sp|P13468) DNA-binding prot K10 OS=D.m.	K10_DROME	2,00E-25	GLOS_K10.2.2	GO:0048599	B	.	oocyte development
[BBH] K10_DROME (sp|P13468) DNA-binding prot K10 OS=D.m.	K10_DROME	2,00E-25	GLOS_K10.2.2	GO:0007281	B	.	germ cell development
[BBH] K10_DROME (sp|P13468) DNA-binding prot K10 OS=D.m.	K10_DROME	2,00E-25	GLOS_K10.2.2	GO:0009994	B	.	oocyte differentiation
[BBH] K10_DROME (sp|P13468) DNA-binding prot K10 OS=D.m.	K10_DROME	2,00E-25	GLOS_K10.2.2	GO:0009950	B	.	dorsal/ventral axis specification
[BBH] K10_DROME (sp|P13468) DNA-binding prot K10 OS=D.m.	K10_DROME	2,00E-25	GLOS_K10.2.2	GO:0009953	B	.	dorsal/ventral pattern formation
[BBH] K10_DROME (sp|P13468) DNA-binding prot K10 OS=D.m.	K10_DROME	2,00E-25	GLOS_K10.2.2	GO:0007310	B	FlyBase	oocyte dorsal/ventral axis specification
[BBH] K10_DROME (sp|P13468) DNA-binding prot K10 OS=D.m.	K10_DROME	2,00E-25	GLOS_K10.2.2	GO:0030707	B	FlyBase	ovarian follicle cell development
[BBH] K10_DROME (sp|P13468) DNA-binding prot K10 OS=D.m.	K10_DROME	2,00E-25	GLOS_K10.2.2	GO:0007316	B	.	pole plasm RNA localization
[BBH] K10_DROME (sp|P13468) DNA-binding prot K10 OS=D.m.	K10_DROME	2,00E-25	GLOS_K10.2.2	GO:0006403	B	.	RNA localization
[BBH] K10_DROME (sp|P13468) DNA-binding prot K10 OS=D.m.	K10_DROME	2,00E-25	GLOS_K10.2.2	GO:0007315	B	.	pole plasm assembly
[BBH] K10_DROME (sp|P13468) DNA-binding prot K10 OS=D.m.	K10_DROME	2,00E-25	GLOS_K10.2.2	GO:0007028	B	.	cytoplasm organization
[BBH] K10_DROME (sp|P13468) DNA-binding prot K10 OS=D.m.	K10_DROME	2,00E-25	GLOS_K10.2.2	GO:0007314	B	.	oocyte anterior/posterior axis specification
[BBH] K10_DROME (sp|P13468) DNA-binding prot K10 OS=D.m.	K10_DROME	2,00E-25	GLOS_K10.2.2	GO:0009948	B	.	anterior/posterior axis specification
[BBH] K10_DROME (sp|P13468) DNA-binding prot K10 OS=D.m.	K10_DROME	2,00E-25	GLOS_K10.2.2	GO:0009952	B	.	anterior/posterior pattern specification
[BBH] K10_DROME (sp|P13468) DNA-binding prot K10 OS=D.m.	K10_DROME	2,00E-25	GLOS_K10.2.2	GO:0008358	B	.	maternal determination of anterior/posterior axis, embryo

[BBH] K10_DROME (sp|P13468) DNA-binding prot K10 OS=D.m.	K10_DROME	2,00E-25	GLOS_K10.2.2	GO:0008595	B	.	anterior/posterior axis specification, embryo
[BBH] K10_DROME (sp|P13468) DNA-binding prot K10 OS=D.m.	K10_DROME	2,00E-25	GLOS_K10.2.2	GO:0000578	B	.	embryonic axis specification
[BBH] K10_DROME (sp|P13468) DNA-binding prot K10 OS=D.m.	K10_DROME	2,00E-25	GLOS_K10.2.2	GO:0009880	B	.	embryonic pattern specification
[BBH] K10_DROME (sp|P13468) DNA-binding prot K10 OS=D.m.	K10_DROME	2,00E-25	GLOS_K10.2.2	GO:0007351	B	.	tripartite regional subdivision
[BBH] K10_DROME (sp|P13468) DNA-binding prot K10 OS=D.m.	K10_DROME	2,00E-25	GLOS_K10.2.2	GO:0007350	B	.	blastoderm segmentation
[BBH] K10_DROME (sp|P13468) DNA-binding prot K10 OS=D.m.	K10_DROME	2,00E-25	GLOS_K10.2.2	GO:0060811	B	.	intracellular mRNA localization involved in anterior/posterior axis specification
[BBH] K10_DROME (sp|P13468) DNA-binding prot K10 OS=D.m.	K10_DROME	2,00E-25	GLOS_K10.2.2	GO:0060810	B	.	intracellular mRNA localization involved in pattern specification process
[BBH] K10_DROME (sp|P13468) DNA-binding prot K10 OS=D.m.	K10_DROME	2,00E-25	GLOS_K10.2.2	GO:0008298	B	.	intracellular mRNA localization
[BBH] K10_DROME (sp|P13468) DNA-binding prot K10 OS=D.m.	K10_DROME	2,00E-25	GLOS_K10.2.2	GO:0019094	B	FlyBase	pole plasm mRNA localization
[BBH] K10_DROME (sp|P13468) DNA-binding prot K10 OS=D.m.	K10_DROME	2,00E-25	GLOS_K10.2.2	GO:0008104	B	FlyBase	protein localization
[BBH] KCC2A_DROME (sp|Q00168) Ca/calmodulin-dep prot kinase type II a-chain	KCC2A_DROME	0	GLOS_KCC2A.2.2	GO:0030424	C	FlyBase	axon
[BBH] KCC2A_DROME (sp|Q00168) Ca/calmodulin-dep prot kinase type II a-chain	KCC2A_DROME	0	GLOS_KCC2A.2.2	GO:0005954	C	UniProtKB	calcium- and calmodulin-dependent protein kinase complex
[BBH] KCC2A_DROME (sp|Q00168) Ca/calmodulin-dep prot kinase type II a-chain	KCC2A_DROME	0	GLOS_KCC2A.2.2	GO:0030425	C	FlyBase	dendrite
[BBH] KCC2A_DROME (sp|Q00168) Ca/calmodulin-dep prot kinase type II a-chain	KCC2A_DROME	0	GLOS_KCC2A.2.2	GO:0005886	C	FlyBase	plasma membrane
[BBH] KCC2A_DROME (sp|Q00168) Ca/calmodulin-dep prot kinase type II a-chain	KCC2A_DROME	0	GLOS_KCC2A.2.2	GO:0097060	C	.	synaptic membrane
[BBH] KCC2A_DROME (sp|Q00168) Ca/calmodulin-dep prot kinase type II a-chain	KCC2A_DROME	0	GLOS_KCC2A.2.2	GO:0044456	C	.	synapse part
[BBH] KCC2A_DROME (sp|Q00168) Ca/calmodulin-dep prot kinase type II a-chain	KCC2A_DROME	0	GLOS_KCC2A.2.2	GO:0045202	C	.	synapse
[BBH] KCC2A_DROME (sp|Q00168) Ca/calmodulin-dep prot kinase type II a-chain	KCC2A_DROME	0	GLOS_KCC2A.2.2	GO:0045211	C	FlyBase	postsynaptic membrane
[BBH] KCC2A_DROME (sp|Q00168) Ca/calmodulin-dep prot kinase type II a-chain	KCC2A_DROME	0	GLOS_KCC2A.2.2	GO:0048786	C	FlyBase	presynaptic active zone
[BBH] KCC2A_DROME (sp|Q00168) Ca/calmodulin-dep prot kinase type II a-chain	KCC2A_DROME	0	GLOS_KCC2A.2.2	GO:0032559	M	.	adenyl ribonucleotide binding
[BBH] KCC2A_DROME (sp|Q00168) Ca/calmodulin-dep prot kinase type II a-chain	KCC2A_DROME	0	GLOS_KCC2A.2.2	GO:0030554	M	.	adenyl nucleotide binding
[BBH] KCC2A_DROME (sp|Q00168) Ca/calmodulin-dep prot kinase type II a-chain	KCC2A_DROME	0	GLOS_KCC2A.2.2	GO:0005524	M	UniProtKB-k	ATP binding
[BBH] KCC2A_DROME (sp|Q00168) Ca/calmodulin-dep prot kinase type II a-chain	KCC2A_DROME	0	GLOS_KCC2A.2.2	GO:0005516	M	FlyBase	calmodulin binding
[BBH] KCC2A_DROME (sp|Q00168) Ca/calmodulin-dep prot kinase type II a-chain	KCC2A_DROME	0	GLOS_KCC2A.2.2	GO:0004674	M	.	protein serine/threonine kinase activity
[BBH] KCC2A_DROME (sp|Q00168) Ca/calmodulin-dep prot kinase type II a-chain	KCC2A_DROME	0	GLOS_KCC2A.2.2	GO:0004672	M	.	protein kinase activity
[BBH] KCC2A_DROME (sp|Q00168) Ca/calmodulin-dep prot kinase type II a-chain	KCC2A_DROME	0	GLOS_KCC2A.2.2	GO:0016301	M	.	kinase activity
[BBH] KCC2A_DROME (sp|Q00168) Ca/calmodulin-dep prot kinase type II a-chain	KCC2A_DROME	0	GLOS_KCC2A.2.2	GO:0016772	M	.	transferase activity, transferring phosphorus-containing groups
[BBH] KCC2A_DROME (sp|Q00168) Ca/calmodulin-dep prot kinase type II a-chain	KCC2A_DROME	0	GLOS_KCC2A.2.2	GO:0016740	M	.	transferase activity
[BBH] KCC2A_DROME (sp|Q00168) Ca/calmodulin-dep prot kinase type II a-chain	KCC2A_DROME	0	GLOS_KCC2A.2.2	GO:0016773	M	.	phosphotransferase activity, alcohol group as acceptor
[BBH] KCC2A_DROME (sp|Q00168) Ca/calmodulin-dep prot kinase type II a-chain	KCC2A_DROME	0	GLOS_KCC2A.2.2	GO:0004683	M	FlyBase	calmodulin-dependent protein kinase activity
[BBH] KCC2A_DROME (sp|Q00168) Ca/calmodulin-dep prot kinase type II a-chain	KCC2A_DROME	0	GLOS_KCC2A.2.2	GO:0007613	B	.	memory
[BBH] KCC2A_DROME (sp|Q00168) Ca/calmodulin-dep prot kinase type II a-chain	KCC2A_DROME	0	GLOS_KCC2A.2.2	GO:0007611	B	.	learning or memory
[BBH] KCC2A_DROME (sp|Q00168) Ca/calmodulin-dep prot kinase type II a-chain	KCC2A_DROME	0	GLOS_KCC2A.2.2	GO:0050890	B	.	cognition
[BBH] KCC2A_DROME (sp|Q00168) Ca/calmodulin-dep prot kinase type II a-chain	KCC2A_DROME	0	GLOS_KCC2A.2.2	GO:0007616	B	FlyBase	long-term memory
[BBH] KCC2A_DROME (sp|Q00168) Ca/calmodulin-dep prot kinase type II a-chain	KCC2A_DROME	0	GLOS_KCC2A.2.2	GO:0060179	B	.	male mating behavior
[BBH] KCC2A_DROME (sp|Q00168) Ca/calmodulin-dep prot kinase type II a-chain	KCC2A_DROME	0	GLOS_KCC2A.2.2	GO:0007617	B	.	mating behavior
[BBH] KCC2A_DROME (sp|Q00168) Ca/calmodulin-dep prot kinase type II a-chain	KCC2A_DROME	0	GLOS_KCC2A.2.2	GO:0051705	B	.	multi-organism behavior
[BBH] KCC2A_DROME (sp|Q00168) Ca/calmodulin-dep prot kinase type II a-chain	KCC2A_DROME	0	GLOS_KCC2A.2.2	GO:0007618	B	.	mating
[BBH] KCC2A_DROME (sp|Q00168) Ca/calmodulin-dep prot kinase type II a-chain	KCC2A_DROME	0	GLOS_KCC2A.2.2	GO:0044703	B	.	multi-organism reproductive process
[BBH] KCC2A_DROME (sp|Q00168) Ca/calmodulin-dep prot kinase type II a-chain	KCC2A_DROME	0	GLOS_KCC2A.2.2	GO:0007619	B	.	courtship behavior
[BBH] KCC2A_DROME (sp|Q00168) Ca/calmodulin-dep prot kinase type II a-chain	KCC2A_DROME	0	GLOS_KCC2A.2.2	GO:0008049	B	FlyBase	male courtship behavior
[BBH] KCC2A_DROME (sp|Q00168) Ca/calmodulin-dep prot kinase type II a-chain	KCC2A_DROME	0	GLOS_KCC2A.2.2	GO:0007528	B	FlyBase	neuromuscular junction development
[BBH] KCC2A_DROME (sp|Q00168) Ca/calmodulin-dep prot kinase type II a-chain	KCC2A_DROME	0	GLOS_KCC2A.2.2	GO:0051489	B	FlyBase	regulation of filopodium assembly
[BBH] KCC2A_DROME (sp|Q00168) Ca/calmodulin-dep prot kinase type II a-chain	KCC2A_DROME	0	GLOS_KCC2A.2.2	GO:2000241	B	.	regulation of reproductive process
[BBH] KCC2A_DROME (sp|Q00168) Ca/calmodulin-dep prot kinase type II a-chain	KCC2A_DROME	0	GLOS_KCC2A.2.2	GO:0060278	B	FlyBase	regulation of ovulation
[BBH] KCC2A_DROME (sp|Q00168) Ca/calmodulin-dep prot kinase type II a-chain	KCC2A_DROME	0	GLOS_KCC2A.2.2	GO:0007268	B	FlyBase	synaptic transmission
[BBH] KU70_DROME (sp|Q23976) ATP-dependent DNA helicase 2 subunit 1	KU70_DROME	0	GLOS_KU70.1.1	GO:0005694	C	UniProtKB-S	chromosome
[BBH] KU70_DROME (sp|Q23976) ATP-dependent DNA helicase 2 subunit 1	KU70_DROME	0	GLOS_KU70.1.1	GO:0043564	C	FlyBase	Ku70:Ku80 complex
[BBH] KU70_DROME (sp|Q23976) ATP-dependent DNA helicase 2 subunit 1	KU70_DROME	0	GLOS_KU70.1.1	GO:0005524	M	UniProtKB-k	ATP binding
[BBH] KU70_DROME (sp|Q23976) ATP-dependent DNA helicase 2 subunit 1	KU70_DROME	0	GLOS_KU70.1.1	GO:0003678	M	.	DNA helicase activity
[BBH] KU70_DROME (sp|Q23976) ATP-dependent DNA helicase 2 subunit 1	KU70_DROME	0	GLOS_KU70.1.1	GO:0004386	M	.	helicase activity
[BBH] KU70_DROME (sp|Q23976) ATP-dependent DNA helicase 2 subunit 1	KU70_DROME	0	GLOS_KU70.1.1	GO:0008026	M	.	ATP-dependent helicase activity
[BBH] KU70_DROME (sp|Q23976) ATP-dependent DNA helicase 2 subunit 1	KU70_DROME	0	GLOS_KU70.1.1	GO:0042623	M	.	ATPase activity, coupled
[BBH] KU70_DROME (sp|Q23976) ATP-dependent DNA helicase 2 subunit 1	KU70_DROME	0	GLOS_KU70.1.1	GO:0016887	M	.	ATPase activity

[BBH] KU70_DROME (sp|Q23976) ATP-dependent DNA helicase 2 subunit 1	KU70_DROME	0	GLOS_KU70.1.1	GO:0070035	M	.	purine NTP-dependent helicase activity
[BBH] KU70_DROME (sp|Q23976) ATP-dependent DNA helicase 2 subunit 1	KU70_DROME	0	GLOS_KU70.1.1	GO:0008094	M	.	DNA-dependent ATPase activity
[BBH] KU70_DROME (sp|Q23976) ATP-dependent DNA helicase 2 subunit 1	KU70_DROME	0	GLOS_KU70.1.1	GO:0004003	M	InterPro	ATP-dependent DNA helicase activity
[BBH] KU70_DROME (sp|Q23976) ATP-dependent DNA helicase 2 subunit 1	KU70_DROME	0	GLOS_KU70.1.1	GO:0003684	M	InterPro	damaged DNA binding
[BBH] KU70_DROME (sp|Q23976) ATP-dependent DNA helicase 2 subunit 1	KU70_DROME	0	GLOS_KU70.1.1	GO:0003677	M	FlyBase	DNA binding
[BBH] KU70_DROME (sp|Q23976) ATP-dependent DNA helicase 2 subunit 1	KU70_DROME	0	GLOS_KU70.1.1	GO:0046982	M	FlyBase	protein heterodimerization activity
[BBH] KU70_DROME (sp|Q23976) ATP-dependent DNA helicase 2 subunit 1	KU70_DROME	0	GLOS_KU70.1.1	GO:0043565	M	.	sequence-specific DNA binding
[BBH] KU70_DROME (sp|Q23976) ATP-dependent DNA helicase 2 subunit 1	KU70_DROME	0	GLOS_KU70.1.1	GO:0042162	M	InterPro	telomeric DNA binding
[BBH] KU70_DROME (sp|Q23976) ATP-dependent DNA helicase 2 subunit 1	KU70_DROME	0	GLOS_KU70.1.1	GO:0006310	B	UniProtKB-K	DNA recombination
[BBH] KU70_DROME (sp|Q23976) ATP-dependent DNA helicase 2 subunit 1	KU70_DROME	0	GLOS_KU70.1.1	GO:0000726	B	.	non-recombinational repair
[BBH] KU70_DROME (sp|Q23976) ATP-dependent DNA helicase 2 subunit 1	KU70_DROME	0	GLOS_KU70.1.1	GO:0006281	B	.	DNA repair
[BBH] KU70_DROME (sp|Q23976) ATP-dependent DNA helicase 2 subunit 1	KU70_DROME	0	GLOS_KU70.1.1	GO:0006302	B	.	double-strand break repair
[BBH] KU70_DROME (sp|Q23976) ATP-dependent DNA helicase 2 subunit 1	KU70_DROME	0	GLOS_KU70.1.1	GO:0006303	B	InterPro	double-strand break repair via nonhomologous end joining
[BBH] KU70_DROME (sp|Q23976) ATP-dependent DNA helicase 2 subunit 1	KU70_DROME	0	GLOS_KU70.1.1	GO:0032200	B	.	telomere organization
[BBH] KU70_DROME (sp|Q23976) ATP-dependent DNA helicase 2 subunit 1	KU70_DROME	0	GLOS_KU70.1.1	GO:0051276	B	.	chromosome organization
[BBH] KU70_DROME (sp|Q23976) ATP-dependent DNA helicase 2 subunit 1	KU70_DROME	0	GLOS_KU70.1.1	GO:0000723	B	FlyBase	telomere maintenance
[BBH] LAB_DROME (sp|P10105) Homeotic protein labial OS=D.m.	LAB_DROME	4,00E-92	GLOS_LAB.1.1	GO:0005634	C	FlyBase	nucleus
[BBH] LAB_DROME (sp|P10105) Homeotic protein labial OS=D.m.	LAB_DROME	4,00E-92	GLOS_LAB.1.1	GO:0043565	M	InterPro	sequence-specific DNA binding
[BBH] LAB_DROME (sp|P10105) Homeotic protein labial OS=D.m.	LAB_DROME	4,00E-92	GLOS_LAB.1.1	GO:0003700	M	FlyBase	sequence-specific DNA binding transcription factor activity
[BBH] LAB_DROME (sp|P10105) Homeotic protein labial OS=D.m.	LAB_DROME	4,00E-92	GLOS_LAB.1.1	GO:0035283	B	.	central nervous system segmentation
[BBH] LAB_DROME (sp|P10105) Homeotic protein labial OS=D.m.	LAB_DROME	4,00E-92	GLOS_LAB.1.1	GO:0035284	B	FlyBase	brain segmentation
[BBH] LAB_DROME (sp|P10105) Homeotic protein labial OS=D.m.	LAB_DROME	4,00E-92	GLOS_LAB.1.1	GO:0045165	B	.	cell fate commitment
[BBH] LAB_DROME (sp|P10105) Homeotic protein labial OS=D.m.	LAB_DROME	4,00E-92	GLOS_LAB.1.1	GO:0001709	B	FlyBase	cell fate determination
[BBH] LAB_DROME (sp|P10105) Homeotic protein labial OS=D.m.	LAB_DROME	4,00E-92	GLOS_LAB.1.1	GO:0009790	B	FlyBase	embryo development
[BBH] LAB_DROME (sp|P10105) Homeotic protein labial OS=D.m.	LAB_DROME	4,00E-92	GLOS_LAB.1.1	GO:0048565	B	.	digestive tract development
[BBH] LAB_DROME (sp|P10105) Homeotic protein labial OS=D.m.	LAB_DROME	4,00E-92	GLOS_LAB.1.1	GO:0055123	B	.	digestive system development
[BBH] LAB_DROME (sp|P10105) Homeotic protein labial OS=D.m.	LAB_DROME	4,00E-92	GLOS_LAB.1.1	GO:0007494	B	FlyBase	midgut development
[BBH] LAB_DROME (sp|P10105) Homeotic protein labial OS=D.m.	LAB_DROME	4,00E-92	GLOS_LAB.1.1	GO:0045944	B	FlyBase	positive regulation of transcription from RNA polymerase II promoter
[BBH] LAB_DROME (sp|P10105) Homeotic protein labial OS=D.m.	LAB_DROME	4,00E-92	GLOS_LAB.1.1	GO:0006351	B	UniProtKB-K	transcription, DNA-dependent
LIN1_NYCCO (sp|P08548) LINE-1 reverse transcriptase homolog OS=N. coucang	LIN1_NYCCO	5,00E-10	GLOS_LIN1.1.1	GO:0046872	M	UniProtKB-K	metal ion binding
LIN1_NYCCO (sp|P08548) LINE-1 reverse transcriptase homolog OS=N. coucang	LIN1_NYCCO	5,00E-10	GLOS_LIN1.1.1	GO:0003723	M	InterPro	RNA binding
LIN1_NYCCO (sp|P08548) LINE-1 reverse transcriptase homolog OS=N. coucang	LIN1_NYCCO	5,00E-10	GLOS_LIN1.1.1	GO:0034061	M	.	DNA polymerase activity
LIN1_NYCCO (sp|P08548) LINE-1 reverse transcriptase homolog OS=N. coucang	LIN1_NYCCO	5,00E-10	GLOS_LIN1.1.1	GO:0016779	M	.	nucleotidyltransferase activity
LIN1_NYCCO (sp|P08548) LINE-1 reverse transcriptase homolog OS=N. coucang	LIN1_NYCCO	5,00E-10	GLOS_LIN1.1.1	GO:0003964	M	UniProtKB-K	RNA-directed DNA polymerase activity
LIN1_NYCCO (sp|P08548) LINE-1 reverse transcriptase homolog OS=N. coucang	LIN1_NYCCO	5,00E-10	GLOS_LIN1.1.1	GO:0006260	B	.	DNA replication
LIN1_NYCCO (sp|P08548) LINE-1 reverse transcriptase homolog OS=N. coucang	LIN1_NYCCO	5,00E-10	GLOS_LIN1.1.1	GO:0006278	B	InterPro	RNA-dependent DNA replication
LOLA5_DROME (sp|Q9V5M6) Longitudinals lacking prot, isoforms J/P/Q/S/Z	LOLA5_DROME	3,00E-29	GLOS_LOLA5.1.1	GO:0005634	C	UniProtKB	nucleus
LOLA5_DROME (sp|Q9V5M6) Longitudinals lacking prot, isoforms J/P/Q/S/Z	LOLA5_DROME	3,00E-29	GLOS_LOLA5.1.1	GO:0003677	M	UniProtKB-K	DNA binding
LOLA5_DROME (sp|Q9V5M6) Longitudinals lacking prot, isoforms J/P/Q/S/Z	LOLA5_DROME	3,00E-29	GLOS_LOLA5.1.1	GO:0046872	M	UniProtKB-K	metal ion binding
LOLA5_DROME (sp|Q9V5M6) Longitudinals lacking prot, isoforms J/P/Q/S/Z	LOLA5_DROME	3,00E-29	GLOS_LOLA5.1.1	GO:0003700	M	FlyBase	sequence-specific DNA binding transcription factor activity
LOLA5_DROME (sp|Q9V5M6) Longitudinals lacking prot, isoforms J/P/Q/S/Z	LOLA5_DROME	3,00E-29	GLOS_LOLA5.1.1	GO:0006959	B	.	humoral immune response
LOLA5_DROME (sp|Q9V5M6) Longitudinals lacking prot, isoforms J/P/Q/S/Z	LOLA5_DROME	3,00E-29	GLOS_LOLA5.1.1	GO:0006955	B	.	immune response
LOLA5_DROME (sp|Q9V5M6) Longitudinals lacking prot, isoforms J/P/Q/S/Z	LOLA5_DROME	3,00E-29	GLOS_LOLA5.1.1	GO:0019730	B	FlyBase	antimicrobial humoral response
LOLA5_DROME (sp|Q9V5M6) Longitudinals lacking prot, isoforms J/P/Q/S/Z	LOLA5_DROME	3,00E-29	GLOS_LOLA5.1.1	GO:0016198	B	.	axon choice point recognition
LOLA5_DROME (sp|Q9V5M6) Longitudinals lacking prot, isoforms J/P/Q/S/Z	LOLA5_DROME	3,00E-29	GLOS_LOLA5.1.1	GO:0008038	B	.	neuron recognition
LOLA5_DROME (sp|Q9V5M6) Longitudinals lacking prot, isoforms J/P/Q/S/Z	LOLA5_DROME	3,00E-29	GLOS_LOLA5.1.1	GO:0008037	B	.	cell recognition
LOLA5_DROME (sp|Q9V5M6) Longitudinals lacking prot, isoforms J/P/Q/S/Z	LOLA5_DROME	3,00E-29	GLOS_LOLA5.1.1	GO:0007411	B	.	axon guidance
LOLA5_DROME (sp|Q9V5M6) Longitudinals lacking prot, isoforms J/P/Q/S/Z	LOLA5_DROME	3,00E-29	GLOS_LOLA5.1.1	GO:0016199	B	UniProtKB	axon midline choice point recognition
LOLA5_DROME (sp|Q9V5M6) Longitudinals lacking prot, isoforms J/P/Q/S/Z	LOLA5_DROME	3,00E-29	GLOS_LOLA5.1.1	GO:0048854	B	FlyBase	brain morphogenesis
LOLA5_DROME (sp|Q9V5M6) Longitudinals lacking prot, isoforms J/P/Q/S/Z	LOLA5_DROME	3,00E-29	GLOS_LOLA5.1.1	GO:0008406	B	FlyBase	gonad development
LOLA5_DROME (sp|Q9V5M6) Longitudinals lacking prot, isoforms J/P/Q/S/Z	LOLA5_DROME	3,00E-29	GLOS_LOLA5.1.1	GO:0002118	B	.	aggressive behavior
LOLA5_DROME (sp|Q9V5M6) Longitudinals lacking prot, isoforms J/P/Q/S/Z	LOLA5_DROME	3,00E-29	GLOS_LOLA5.1.1	GO:0002121	B	FlyBase	inter-male aggressive behavior
LOLA5_DROME (sp|Q9V5M6) Longitudinals lacking prot, isoforms J/P/Q/S/Z	LOLA5_DROME	3,00E-29	GLOS_LOLA5.1.1	GO:0031987	B	FlyBase	locomotion involved in locomotory behavior
LOLA5_DROME (sp|Q9V5M6) Longitudinals lacking prot, isoforms J/P/Q/S/Z	LOLA5_DROME	3,00E-29	GLOS_LOLA5.1.1	GO:0097285	B	.	cell-type specific apoptotic process

LOLA5_DROME (sp|Q9V5M6) Longitudinals lacking prot, isoforms J/P/Q/S/Z	LOLA5_DROME	3,00E-29	GLOS_LOLA5.1.1	GO:0045476	B	FlyBase	nurse cell apoptotic process
LOLA5_DROME (sp|Q9V5M6) Longitudinals lacking prot, isoforms J/P/Q/S/Z	LOLA5_DROME	3,00E-29	GLOS_LOLA5.1.1	GO:0045893	B	UniProtKB	positive regulation of transcription, DNA-dependent
LOLA5_DROME (sp|Q9V5M6) Longitudinals lacking prot, isoforms J/P/Q/S/Z	LOLA5_DROME	3,00E-29	GLOS_LOLA5.1.1	GO:0001752	B	.	compound eye photoreceptor fate commitment
LOLA5_DROME (sp|Q9V5M6) Longitudinals lacking prot, isoforms J/P/Q/S/Z	LOLA5_DROME	3,00E-29	GLOS_LOLA5.1.1	GO:0042706	B	.	eye photoreceptor cell fate commitment
LOLA5_DROME (sp|Q9V5M6) Longitudinals lacking prot, isoforms J/P/Q/S/Z	LOLA5_DROME	3,00E-29	GLOS_LOLA5.1.1	GO:0046552	B	.	photoreceptor cell fate commitment
LOLA5_DROME (sp|Q9V5M6) Longitudinals lacking prot, isoforms J/P/Q/S/Z	LOLA5_DROME	3,00E-29	GLOS_LOLA5.1.1	GO:0048663	B	.	neuron fate commitment
LOLA5_DROME (sp|Q9V5M6) Longitudinals lacking prot, isoforms J/P/Q/S/Z	LOLA5_DROME	3,00E-29	GLOS_LOLA5.1.1	GO:0042067	B	.	establishment of ommatidial planar polarity
LOLA5_DROME (sp|Q9V5M6) Longitudinals lacking prot, isoforms J/P/Q/S/Z	LOLA5_DROME	3,00E-29	GLOS_LOLA5.1.1	GO:0048056	B	.	R3/R4 cell differentiation
LOLA5_DROME (sp|Q9V5M6) Longitudinals lacking prot, isoforms J/P/Q/S/Z	LOLA5_DROME	3,00E-29	GLOS_LOLA5.1.1	GO:0007464	B	FlyBase	R3/R4 cell fate commitment
LOLA5_DROME (sp|Q9V5M6) Longitudinals lacking prot, isoforms J/P/Q/S/Z	LOLA5_DROME	3,00E-29	GLOS_LOLA5.1.1	GO:0045466	B	.	R7 cell differentiation
LOLA5_DROME (sp|Q9V5M6) Longitudinals lacking prot, isoforms J/P/Q/S/Z	LOLA5_DROME	3,00E-29	GLOS_LOLA5.1.1	GO:0045467	B	FlyBase	R7 cell development
LOLA5_DROME (sp|Q9V5M6) Longitudinals lacking prot, isoforms J/P/Q/S/Z	LOLA5_DROME	3,00E-29	GLOS_LOLA5.1.1	GO:0050905	B	.	neuromuscular process
LOLA5_DROME (sp|Q9V5M6) Longitudinals lacking prot, isoforms J/P/Q/S/Z	LOLA5_DROME	3,00E-29	GLOS_LOLA5.1.1	GO:0001964	B	FlyBase	startle response
LOLA5_DROME (sp|Q9V5M6) Longitudinals lacking prot, isoforms J/P/Q/S/Z	LOLA5_DROME	3,00E-29	GLOS_LOLA5.1.1	GO:0006351	B	UniProtKB-k	transcription, DNA-dependent
[BBH] LTN1_DROME (sp|Q9VW09) E3 ubiquitin-protein ligase listerin OS=D.m.	LTN1_DROME	0	GLOS_LTN1.1.1	GO:0016874	M	UniProtKB-k	ligase activity
[BBH] LTN1_DROME (sp|Q9VW09) E3 ubiquitin-protein ligase listerin OS=D.m.	LTN1_DROME	0	GLOS_LTN1.1.1	GO:0008270	M	InterPro	zinc ion binding
[BBH] LTN1_DROME (sp|Q9VW09) E3 ubiquitin-protein ligase listerin OS=D.m.	LTN1_DROME	0	GLOS_LTN1.1.1	GO:0032446	B	.	protein modification by small protein conjugation
[BBH] LTN1_DROME (sp|Q9VW09) E3 ubiquitin-protein ligase listerin OS=D.m.	LTN1_DROME	0	GLOS_LTN1.1.1	GO:0070647	B	.	protein modification by small protein conjugation or removal
[BBH] LTN1_DROME (sp|Q9VW09) E3 ubiquitin-protein ligase listerin OS=D.m.	LTN1_DROME	0	GLOS_LTN1.1.1	GO:0016567	B	UniProtKB-U	protein ubiquitination
MCM5_DROME (sp|Q9VGW6) DNA replication licensing factor Mcm5 OS=D.m.	MCM5_DROME	2,00E-60	GLOS_MCM5.2.5	GO:0042555	C	InterPro	MCM complex
MCM5_DROME (sp|Q9VGW6) DNA replication licensing factor Mcm5 OS=D.m.	MCM5_DROME	2,00E-60	GLOS_MCM5.2.5	GO:0005634	C	UniProtKB-S	nucleus
MCM5_DROME (sp|Q9VGW6) DNA replication licensing factor Mcm5 OS=D.m.	MCM5_DROME	2,00E-60	GLOS_MCM5.2.5	GO:0005524	M	UniProtKB-k	ATP binding
MCM5_DROME (sp|Q9VGW6) DNA replication licensing factor Mcm5 OS=D.m.	MCM5_DROME	2,00E-60	GLOS_MCM5.2.5	GO:0003677	M	UniProtKB-k	DNA binding
MCM5_DROME (sp|Q9VGW6) DNA replication licensing factor Mcm5 OS=D.m.	MCM5_DROME	2,00E-60	GLOS_MCM5.2.5	GO:0003678	M	InterPro	DNA helicase activity
MCM5_DROME (sp|Q9VGW6) DNA replication licensing factor Mcm5 OS=D.m.	MCM5_DROME	2,00E-60	GLOS_MCM5.2.5	GO:0006323	B	.	DNA packaging
MCM5_DROME (sp|Q9VGW6) DNA replication licensing factor Mcm5 OS=D.m.	MCM5_DROME	2,00E-60	GLOS_MCM5.2.5	GO:0071103	B	.	DNA conformation change
MCM5_DROME (sp|Q9VGW6) DNA replication licensing factor Mcm5 OS=D.m.	MCM5_DROME	2,00E-60	GLOS_MCM5.2.5	GO:0030261	B	FlyBase	chromosome condensation
MCM5_DROME (sp|Q9VGW6) DNA replication licensing factor Mcm5 OS=D.m.	MCM5_DROME	2,00E-60	GLOS_MCM5.2.5	GO:0032392	B	.	DNA geometric change
MCM5_DROME (sp|Q9VGW6) DNA replication licensing factor Mcm5 OS=D.m.	MCM5_DROME	2,00E-60	GLOS_MCM5.2.5	GO:0032508	B	GOC	DNA duplex unwinding
MCM5_DROME (sp|Q9VGW6) DNA replication licensing factor Mcm5 OS=D.m.	MCM5_DROME	2,00E-60	GLOS_MCM5.2.5	GO:0044786	B	.	cell cycle DNA replication
MCM5_DROME (sp|Q9VGW6) DNA replication licensing factor Mcm5 OS=D.m.	MCM5_DROME	2,00E-60	GLOS_MCM5.2.5	GO:0006261	B	.	DNA-dependent DNA replication
MCM5_DROME (sp|Q9VGW6) DNA replication licensing factor Mcm5 OS=D.m.	MCM5_DROME	2,00E-60	GLOS_MCM5.2.5	GO:0042023	B	FlyBase	DNA endoreduplication
MCM5_DROME (sp|Q9VGW6) DNA replication licensing factor Mcm5 OS=D.m.	MCM5_DROME	2,00E-60	GLOS_MCM5.2.5	GO:0006270	B	InterPro	DNA replication initiation
MCM5_DROME (sp|Q9VGW6) DNA replication licensing factor Mcm5 OS=D.m.	MCM5_DROME	2,00E-60	GLOS_MCM5.2.5	GO:0051307	B	.	meiotic chromosome separation
MCM5_DROME (sp|Q9VGW6) DNA replication licensing factor Mcm5 OS=D.m.	MCM5_DROME	2,00E-60	GLOS_MCM5.2.5	GO:0051304	B	.	chromosome separation
MCM5_DROME (sp|Q9VGW6) DNA replication licensing factor Mcm5 OS=D.m.	MCM5_DROME	2,00E-60	GLOS_MCM5.2.5	GO:0007059	B	.	chromosome segregation
MCM5_DROME (sp|Q9VGW6) DNA replication licensing factor Mcm5 OS=D.m.	MCM5_DROME	2,00E-60	GLOS_MCM5.2.5	GO:0045132	B	.	meiotic chromosome segregation
MCM5_DROME (sp|Q9VGW6) DNA replication licensing factor Mcm5 OS=D.m.	MCM5_DROME	2,00E-60	GLOS_MCM5.2.5	GO:0007126	B	.	meiosis
MCM5_DROME (sp|Q9VGW6) DNA replication licensing factor Mcm5 OS=D.m.	MCM5_DROME	2,00E-60	GLOS_MCM5.2.5	GO:0051321	B	.	meiotic cell cycle
MCM5_DROME (sp|Q9VGW6) DNA replication licensing factor Mcm5 OS=D.m.	MCM5_DROME	2,00E-60	GLOS_MCM5.2.5	GO:0007131	B	.	reciprocal meiotic recombination
MCM5_DROME (sp|Q9VGW6) DNA replication licensing factor Mcm5 OS=D.m.	MCM5_DROME	2,00E-60	GLOS_MCM5.2.5	GO:0035825	B	.	reciprocal DNA recombination
MCM5_DROME (sp|Q9VGW6) DNA replication licensing factor Mcm5 OS=D.m.	MCM5_DROME	2,00E-60	GLOS_MCM5.2.5	GO:0006310	B	.	DNA recombination
MCM5_DROME (sp|Q9VGW6) DNA replication licensing factor Mcm5 OS=D.m.	MCM5_DROME	2,00E-60	GLOS_MCM5.2.5	GO:0007127	B	.	meiosis I
MCM5_DROME (sp|Q9VGW6) DNA replication licensing factor Mcm5 OS=D.m.	MCM5_DROME	2,00E-60	GLOS_MCM5.2.5	GO:0000712	B	FlyBase	resolution of meiotic recombination intermediates
MOS1T_DROMA (sp|Q7JQ07) Mariner Mos1 transposase OS=D. mauritiana	MOS1T_DROMA	1,00E-08	GLOS_MOS1T.19.	GO:0005634	C	UniProtKB-S	nucleus
MOS1T_DROMA (sp|Q7JQ07) Mariner Mos1 transposase OS=D. mauritiana	MOS1T_DROMA	1,00E-08	GLOS_MOS1T.19.	GO:0003677	M	UniProtKB-k	DNA binding
MOS1T_DROMA (sp|Q7JQ07) Mariner Mos1 transposase OS=D. mauritiana	MOS1T_DROMA	1,00E-08	GLOS_MOS1T.19.	GO:0004518	M	.	nuclease activity
MOS1T_DROMA (sp|Q7JQ07) Mariner Mos1 transposase OS=D. mauritiana	MOS1T_DROMA	1,00E-08	GLOS_MOS1T.19.	GO:0016788	M	.	hydrolase activity, acting on ester bonds
MOS1T_DROMA (sp|Q7JQ07) Mariner Mos1 transposase OS=D. mauritiana	MOS1T_DROMA	1,00E-08	GLOS_MOS1T.19.	GO:0004519	M	UniProtKB-k	endonuclease activity
MOS1T_DROMA (sp|Q7JQ07) Mariner Mos1 transposase OS=D. mauritiana	MOS1T_DROMA	1,00E-08	GLOS_MOS1T.19.	GO:0046872	M	UniProtKB-k	metal ion binding
MOS1T_DROMA (sp|Q7JQ07) Mariner Mos1 transposase OS=D. mauritiana	MOS1T_DROMA	1,00E-08	GLOS_MOS1T.19.	GO:0015074	B	UniProtKB-k	DNA integration
MOS1T_DROMA (sp|Q7JQ07) Mariner Mos1 transposase OS=D. mauritiana	MOS1T_DROMA	1,00E-08	GLOS_MOS1T.19.	GO:0006310	B	UniProtKB-k	DNA recombination
MOS1T_DROMA (sp|Q7JQ07) Mariner Mos1 transposase OS=D. mauritiana	MOS1T_DROMA	1,00E-08	GLOS_MOS1T.19.	GO:0090305	B	GOC	nucleic acid phosphodiester bond hydrolysis
NETB_DROME (sp|Q24568) Netrin-B OS=D.m. GN=NetB PE=1 SV=1	NETB_DROME	0	GLOS_NETB.1.1	GO:0031012	C	.	extracellular matrix

NETB_DROME (sp|Q24568) Netrin-B OS=D.m. GN=NetB PE=1 SV=1	NETB_DROME	0	GLOS_NETB.1.1	GO:0005578	C	FlyBase	proteinaceous extracellular matrix
NETB_DROME (sp|Q24568) Netrin-B OS=D.m. GN=NetB PE=1 SV=1	NETB_DROME	0	GLOS_NETB.1.1	GO:0048813	B	.	dendrite morphogenesis
NETB_DROME (sp|Q24568) Netrin-B OS=D.m. GN=NetB PE=1 SV=1	NETB_DROME	0	GLOS_NETB.1.1	GO:0016358	B	.	dendrite development
NETB_DROME (sp|Q24568) Netrin-B OS=D.m. GN=NetB PE=1 SV=1	NETB_DROME	0	GLOS_NETB.1.1	GO:0070983	B	FlyBase	dendrite guidance
NETB_DROME (sp|Q24568) Netrin-B OS=D.m. GN=NetB PE=1 SV=1	NETB_DROME	0	GLOS_NETB.1.1	GO:0042063	B	.	gliogenesis
NETB_DROME (sp|Q24568) Netrin-B OS=D.m. GN=NetB PE=1 SV=1	NETB_DROME	0	GLOS_NETB.1.1	GO:0008347	B	FlyBase	glial cell migration
NETB_DROME (sp|Q24568) Netrin-B OS=D.m. GN=NetB PE=1 SV=1	NETB_DROME	0	GLOS_NETB.1.1	GO:0008045	B	FlyBase	motor neuron axon guidance
NETB_DROME (sp|Q24568) Netrin-B OS=D.m. GN=NetB PE=1 SV=1	NETB_DROME	0	GLOS_NETB.1.1	GO:0050920	B	.	regulation of chemotaxis
NETB_DROME (sp|Q24568) Netrin-B OS=D.m. GN=NetB PE=1 SV=1	NETB_DROME	0	GLOS_NETB.1.1	GO:0032101	B	.	regulation of response to external stimulus
NETB_DROME (sp|Q24568) Netrin-B OS=D.m. GN=NetB PE=1 SV=1	NETB_DROME	0	GLOS_NETB.1.1	GO:0050795	B	.	regulation of behavior
NETB_DROME (sp|Q24568) Netrin-B OS=D.m. GN=NetB PE=1 SV=1	NETB_DROME	0	GLOS_NETB.1.1	GO:2000289	B	FlyBase	regulation of photoreceptor cell axon guidance
NETB_DROME (sp|Q24568) Netrin-B OS=D.m. GN=NetB PE=1 SV=1	NETB_DROME	0	GLOS_NETB.1.1	GO:0010160	B	.	formation of organ boundary
NETB_DROME (sp|Q24568) Netrin-B OS=D.m. GN=NetB PE=1 SV=1	NETB_DROME	0	GLOS_NETB.1.1	GO:0048859	B	.	formation of anatomical boundary
NETB_DROME (sp|Q24568) Netrin-B OS=D.m. GN=NetB PE=1 SV=1	NETB_DROME	0	GLOS_NETB.1.1	GO:0048645	B	.	organ formation
NETB_DROME (sp|Q24568) Netrin-B OS=D.m. GN=NetB PE=1 SV=1	NETB_DROME	0	GLOS_NETB.1.1	GO:0007431	B	.	salivary gland development
NETB_DROME (sp|Q24568) Netrin-B OS=D.m. GN=NetB PE=1 SV=1	NETB_DROME	0	GLOS_NETB.1.1	GO:0048732	B	.	gland development
NETB_DROME (sp|Q24568) Netrin-B OS=D.m. GN=NetB PE=1 SV=1	NETB_DROME	0	GLOS_NETB.1.1	GO:0035272	B	.	exocrine system development
NETB_DROME (sp|Q24568) Netrin-B OS=D.m. GN=NetB PE=1 SV=1	NETB_DROME	0	GLOS_NETB.1.1	GO:0007432	B	FlyBase	salivary gland boundary specification
NETB_DROME (sp|Q24568) Netrin-B OS=D.m. GN=NetB PE=1 SV=1	NETB_DROME	0	GLOS_NETB.1.1	GO:0050918	B	.	positive chemotaxis
NETB_DROME (sp|Q24568) Netrin-B OS=D.m. GN=NetB PE=1 SV=1	NETB_DROME	0	GLOS_NETB.1.1	GO:0008039	B	.	synaptic target recognition
NETB_DROME (sp|Q24568) Netrin-B OS=D.m. GN=NetB PE=1 SV=1	NETB_DROME	0	GLOS_NETB.1.1	GO:0016200	B	FlyBase	synaptic target attraction
NU3M_DROYA (sp|P07705) NADH-ubiquinone oxidored. chain 3 OS=D. y.	NU3M_DROYA	6,00E-15	GLOS_NU3M.1.2	GO:0016021	C	UniProtKB-k	integral to membrane
NU3M_DROYA (sp|P07705) NADH-ubiquinone oxidored. chain 3 OS=D. y.	NU3M_DROYA	6,00E-15	GLOS_NU3M.1.2	GO:0005740	C	.	mitochondrial envelope
NU3M_DROYA (sp|P07705) NADH-ubiquinone oxidored. chain 3 OS=D. y.	NU3M_DROYA	6,00E-15	GLOS_NU3M.1.2	GO:0031966	C	UniProtKB-S	mitochondrial membrane
NU3M_DROYA (sp|P07705) NADH-ubiquinone oxidored. chain 3 OS=D. y.	NU3M_DROYA	6,00E-15	GLOS_NU3M.1.2	GO:0070469	C	UniProtKB-k	respiratory chain
NU3M_DROYA (sp|P07705) NADH-ubiquinone oxidored. chain 3 OS=D. y.	NU3M_DROYA	6,00E-15	GLOS_NU3M.1.2	GO:0050136	M	.	NADH dehydrogenase (quinone) activity
NU3M_DROYA (sp|P07705) NADH-ubiquinone oxidored. chain 3 OS=D. y.	NU3M_DROYA	6,00E-15	GLOS_NU3M.1.2	GO:0003954	M	.	NADH dehydrogenase activity
NU3M_DROYA (sp|P07705) NADH-ubiquinone oxidored. chain 3 OS=D. y.	NU3M_DROYA	6,00E-15	GLOS_NU3M.1.2	GO:0016651	M	.	oxidoreductase activity, acting on NAD(P)H
NU3M_DROYA (sp|P07705) NADH-ubiquinone oxidored. chain 3 OS=D. y.	NU3M_DROYA	6,00E-15	GLOS_NU3M.1.2	GO:0016655	M	.	oxidoreductase act., acting on NAD(P)H, quinone or similar compound as acceptor
NU3M_DROYA (sp|P07705) NADH-ubiquinone oxidored. chain 3 OS=D. y.	NU3M_DROYA	6,00E-15	GLOS_NU3M.1.2	GO:0008137	M	UniProtKB-E	NADH dehydrogenase (ubiquinone) activity
[BBH] PSMD7_DROME (sp|P26270) 26S proteas non-ATPase regulatory subunit7	PSMD7_DROME	1,00E-179	GLOS_PSMD7.1.1	GO:0030425	C	FlyBase	dendrite
[BBH] PSMD7_DROME (sp|P26270) 26S proteas non-ATPase regulatory subunit7	PSMD7_DROME	1,00E-179	GLOS_PSMD7.1.1	GO:0005838	C	.	proteasome regulatory particle
[BBH] PSMD7_DROME (sp|P26270) 26S proteas non-ATPase regulatory subunit7	PSMD7_DROME	1,00E-179	GLOS_PSMD7.1.1	GO:0022624	C	.	proteasome accessory complex
[BBH] PSMD7_DROME (sp|P26270) 26S proteas non-ATPase regulatory subunit7	PSMD7_DROME	1,00E-179	GLOS_PSMD7.1.1	GO:0000502	C	.	proteasome complex
[BBH] PSMD7_DROME (sp|P26270) 26S proteas non-ATPase regulatory subunit7	PSMD7_DROME	1,00E-179	GLOS_PSMD7.1.1	GO:0008541	C	FlyBase	proteasome regulatory particle, lid subcomplex
[BBH] PSMD7_DROME (sp|P26270) 26S proteas non-ATPase regulatory subunit7	PSMD7_DROME	1,00E-179	GLOS_PSMD7.1.1	GO:0004175	M	FlyBase	endopeptidase activity
[BBH] PSMD7_DROME (sp|P26270) 26S proteas non-ATPase regulatory subunit7	PSMD7_DROME	1,00E-179	GLOS_PSMD7.1.1	GO:0008283	B	FlyBase	cell proliferation
[BBH] PSMD7_DROME (sp|P26270) 26S proteas non-ATPase regulatory subunit7	PSMD7_DROME	1,00E-179	GLOS_PSMD7.1.1	GO:0000022	B	FlyBase	mitotic spindle elongation
[BBH] PSMD7_DROME (sp|P26270) 26S proteas non-ATPase regulatory subunit7	PSMD7_DROME	1,00E-179	GLOS_PSMD7.1.1	GO:0022008	B	FlyBase	neurogenesis
[BBH] PSMD7_DROME (sp|P26270) 26S proteas non-ATPase regulatory subunit7	PSMD7_DROME	1,00E-179	GLOS_PSMD7.1.1	GO:0006511	B	.	ubiquitin-dependent protein catabolic process
[BBH] PSMD7_DROME (sp|P26270) 26S proteas non-ATPase regulatory subunit7	PSMD7_DROME	1,00E-179	GLOS_PSMD7.1.1	GO:0019941	B	.	modification-dependent protein catabolic process
[BBH] PSMD7_DROME (sp|P26270) 26S proteas non-ATPase regulatory subunit7	PSMD7_DROME	1,00E-179	GLOS_PSMD7.1.1	GO:0043632	B	.	modification-dependent macromolecule catabolic process
[BBH] PSMD7_DROME (sp|P26270) 26S proteas non-ATPase regulatory subunit7	PSMD7_DROME	1,00E-179	GLOS_PSMD7.1.1	GO:0051603	B	.	proteolysis involved in cellular protein catabolic process
[BBH] PSMD7_DROME (sp|P26270) 26S proteas non-ATPase regulatory subunit7	PSMD7_DROME	1,00E-179	GLOS_PSMD7.1.1	GO:0006508	B	.	proteolysis
[BBH] PSMD7_DROME (sp|P26270) 26S proteas non-ATPase regulatory subunit7	PSMD7_DROME	1,00E-179	GLOS_PSMD7.1.1	GO:0044257	B	.	cellular protein catabolic process
[BBH] PSMD7_DROME (sp|P26270) 26S proteas non-ATPase regulatory subunit7	PSMD7_DROME	1,00E-179	GLOS_PSMD7.1.1	GO:0030163	B	.	protein catabolic process
[BBH] PSMD7_DROME (sp|P26270) 26S proteas non-ATPase regulatory subunit7	PSMD7_DROME	1,00E-179	GLOS_PSMD7.1.1	GO:0010498	B	.	proteasomal protein catabolic process
[BBH] PSMD7_DROME (sp|P26270) 26S proteas non-ATPase regulatory subunit7	PSMD7_DROME	1,00E-179	GLOS_PSMD7.1.1	GO:0043161	B	FlyBase	proteasomal ubiquitin-dependent protein catabolic process
RL19_DROME (sp|P36241) 60S ribosomal prot. L19 OS=D.m.	RL19_DROME	2,00E-76	GLOS_RL19.1.9	GO:0005840	C	FlyBase	ribosome
RL19_DROME (sp|P36241) 60S ribosomal prot. L19 OS=D.m.	RL19_DROME	2,00E-76	GLOS_RL19.1.9	GO:0005198	M	.	structural molecule activity
RL19_DROME (sp|P36241) 60S ribosomal prot. L19 OS=D.m.	RL19_DROME	2,00E-76	GLOS_RL19.1.9	GO:0003735	M	FlyBase	structural constituent of ribosome
RL19_DROME (sp|P36241) 60S ribosomal prot. L19 OS=D.m.	RL19_DROME	2,00E-76	GLOS_RL19.1.9	GO:0051297	B	.	centrosome organization
RL19_DROME (sp|P36241) 60S ribosomal prot. L19 OS=D.m.	RL19_DROME	2,00E-76	GLOS_RL19.1.9	GO:0031023	B	.	microtubule organizing center organization
RL19_DROME (sp|P36241) 60S ribosomal prot. L19 OS=D.m.	RL19_DROME	2,00E-76	GLOS_RL19.1.9	GO:0007098	B	.	centrosome cycle

RL19_DROME (sp|P36241) 60S ribosomal prot. L19 OS=D.m.	RL19_DROME	2,00E-76	GLOS_RL19.1.9	GO:0051298	B	FlyBase	centrosome duplication
RL19_DROME (sp|P36241) 60S ribosomal prot. L19 OS=D.m.	RL19_DROME	2,00E-76	GLOS_RL19.1.9	GO:0000022	B	FlyBase	mitotic spindle elongation
RL19_DROME (sp|P36241) 60S ribosomal prot. L19 OS=D.m.	RL19_DROME	2,00E-76	GLOS_RL19.1.9	GO:0006412	B	InterPro	translation
RL371_DROME (sp|Q9VXX8) Prob. 60S ribos. Prot. L37-A OS=D.m.	RL371_DROME	4,00E-43	GLOS_RL371.2.8	GO:0005840	C	UniProtKB-K	ribosome
RL371_DROME (sp|Q9VXX8) Prob. 60S ribos. Prot. L37-A OS=D.m.	RL371_DROME	4,00E-43	GLOS_RL371.2.8	GO:0046872	M	UniProtKB-K	metal ion binding
RL371_DROME (sp|Q9VXX8) Prob. 60S ribos. Prot. L37-A OS=D.m.	RL371_DROME	4,00E-43	GLOS_RL371.2.8	GO:0019843	M	UniProtKB-K	rRNA binding
RL371_DROME (sp|Q9VXX8) Prob. 60S ribos. Prot. L37-A OS=D.m.	RL371_DROME	4,00E-43	GLOS_RL371.2.8	GO:0003735	M	InterPro	structural constituent of ribosome
RL371_DROME (sp|Q9VXX8) Prob. 60S ribos. Prot. L37-A OS=D.m.	RL371_DROME	4,00E-43	GLOS_RL371.2.8	GO:0006412	B	InterPro	translation
[BBH] RPF2_DROME (sp|Q9VEB3) Ribosome product. Fact. 2 homolog OS=D.m.	RPF2_DROME	1,00E-161	GLOS_RPF2.1.1	GO:0005730	C	UniProtKB-S	nucleolus
[BBH] RPF2_DROME (sp|Q9VEB3) Ribosome product. Fact. 2 homolog OS=D.m.	RPF2_DROME	1,00E-161	GLOS_RPF2.1.1	GO:0022008	B	FlyBase	neurogenesis
RS16_DROME (sp|Q9W237) 40S ribosomal prot. S16 OS=D.m.	RS16_DROME	6,00E-45	GLOS_RS16.2.5	GO:0005840	C	FlyBase	ribosome
RS16_DROME (sp|Q9W237) 40S ribosomal prot. S16 OS=D.m.	RS16_DROME	6,00E-45	GLOS_RS16.2.5	GO:0003735	M	FlyBase	structural constituent of ribosome
RS16_DROME (sp|Q9W237) 40S ribosomal prot. S16 OS=D.m.	RS16_DROME	6,00E-45	GLOS_RS16.2.5	GO:0000022	B	FlyBase	mitotic spindle elongation
RS16_DROME (sp|Q9W237) 40S ribosomal prot. S16 OS=D.m.	RS16_DROME	6,00E-45	GLOS_RS16.2.5	GO:0006412	B	InterPro	translation
[BBH] RS17_DROME (sp|P17704) 40S ribos. prot. S17 OS=D.m.	RS17_DROME	3,00E-67	GLOS_RS17.2.3	GO:0015935	C	.	small ribosomal subunit
[BBH] RS17_DROME (sp|P17704) 40S ribos. prot. S17 OS=D.m.	RS17_DROME	3,00E-67	GLOS_RS17.2.3	GO:0044391	C	.	ribosomal subunit
[BBH] RS17_DROME (sp|P17704) 40S ribos. prot. S17 OS=D.m.	RS17_DROME	3,00E-67	GLOS_RS17.2.3	GO:0005840	C	.	ribosome
[BBH] RS17_DROME (sp|P17704) 40S ribos. prot. S17 OS=D.m.	RS17_DROME	3,00E-67	GLOS_RS17.2.3	GO:0044445	C	.	cytosolic part
[BBH] RS17_DROME (sp|P17704) 40S ribos. prot. S17 OS=D.m.	RS17_DROME	3,00E-67	GLOS_RS17.2.3	GO:0005829	C	.	cytosol
[BBH] RS17_DROME (sp|P17704) 40S ribos. prot. S17 OS=D.m.	RS17_DROME	3,00E-67	GLOS_RS17.2.3	GO:0022626	C	.	cytosolic ribosome
[BBH] RS17_DROME (sp|P17704) 40S ribos. prot. S17 OS=D.m.	RS17_DROME	3,00E-67	GLOS_RS17.2.3	GO:0022627	C	RefGenome	cytosolic small ribosomal subunit
[BBH] RS17_DROME (sp|P17704) 40S ribos. prot. S17 OS=D.m.	RS17_DROME	3,00E-67	GLOS_RS17.2.3	GO:0003735	M	FlyBase	structural constituent of ribosome
[BBH] RS17_DROME (sp|P17704) 40S ribos. prot. S17 OS=D.m.	RS17_DROME	3,00E-67	GLOS_RS17.2.3	GO:0022618	B	.	ribonucleoprotein complex assembly
[BBH] RS17_DROME (sp|P17704) 40S ribos. prot. S17 OS=D.m.	RS17_DROME	3,00E-67	GLOS_RS17.2.3	GO:0071826	B	.	ribonucleoprotein complex subunit organization
[BBH] RS17_DROME (sp|P17704) 40S ribos. prot. S17 OS=D.m.	RS17_DROME	3,00E-67	GLOS_RS17.2.3	GO:0022613	B	.	ribonucleoprotein complex biogenesis
[BBH] RS17_DROME (sp|P17704) 40S ribos. prot. S17 OS=D.m.	RS17_DROME	3,00E-67	GLOS_RS17.2.3	GO:0042255	B	.	ribosome assembly
[BBH] RS17_DROME (sp|P17704) 40S ribos. prot. S17 OS=D.m.	RS17_DROME	3,00E-67	GLOS_RS17.2.3	GO:0070925	B	.	organelle assembly
[BBH] RS17_DROME (sp|P17704) 40S ribos. prot. S17 OS=D.m.	RS17_DROME	3,00E-67	GLOS_RS17.2.3	GO:0042254	B	.	ribosome biogenesis
[BBH] RS17_DROME (sp|P17704) 40S ribos. prot. S17 OS=D.m.	RS17_DROME	3,00E-67	GLOS_RS17.2.3	GO:0042274	B	.	ribosomal small subunit biogenesis
[BBH] RS17_DROME (sp|P17704) 40S ribos. prot. S17 OS=D.m.	RS17_DROME	3,00E-67	GLOS_RS17.2.3	GO:0000028	B	RefGenome	ribosomal small subunit assembly
[BBH] RS17_DROME (sp|P17704) 40S ribos. prot. S17 OS=D.m.	RS17_DROME	3,00E-67	GLOS_RS17.2.3	GO:0006412	B	.	translation
[BBH] RS17_DROME (sp|P17704) 40S ribos. prot. S17 OS=D.m.	RS17_DROME	3,00E-67	GLOS_RS17.2.3	GO:0006414	B	RefGenome	translational elongation
RS5A_DROME (sp|Q24186) 40S ribosomal prot. S5a OS=D.m.	RS5A_DROME	1,00E-104	GLOS_RS5A.1.8	GO:0005694	C	.	chromosome
RS5A_DROME (sp|Q24186) 40S ribosomal prot. S5a OS=D.m.	RS5A_DROME	1,00E-104	GLOS_RS5A.1.8	GO:0000228	C	FlyBase	nuclear chromosome
RS5A_DROME (sp|Q24186) 40S ribosomal prot. S5a OS=D.m.	RS5A_DROME	1,00E-104	GLOS_RS5A.1.8	GO:0005730	C	FlyBase	nucleolus
RS5A_DROME (sp|Q24186) 40S ribosomal prot. S5a OS=D.m.	RS5A_DROME	1,00E-104	GLOS_RS5A.1.8	GO:0005840	C	FlyBase	ribosome
RS5A_DROME (sp|Q24186) 40S ribosomal prot. S5a OS=D.m.	RS5A_DROME	1,00E-104	GLOS_RS5A.1.8	GO:0015935	C	InterPro	small ribosomal subunit
RS5A_DROME (sp|Q24186) 40S ribosomal prot. S5a OS=D.m.	RS5A_DROME	1,00E-104	GLOS_RS5A.1.8	GO:0003723	M	InterPro	RNA binding
RS5A_DROME (sp|Q24186) 40S ribosomal prot. S5a OS=D.m.	RS5A_DROME	1,00E-104	GLOS_RS5A.1.8	GO:0003735	M	FlyBase	structural constituent of ribosome
RS5A_DROME (sp|Q24186) 40S ribosomal prot. S5a OS=D.m.	RS5A_DROME	1,00E-104	GLOS_RS5A.1.8	GO:0000022	B	FlyBase	mitotic spindle elongation
RS5A_DROME (sp|Q24186) 40S ribosomal prot. S5a OS=D.m.	RS5A_DROME	1,00E-104	GLOS_RS5A.1.8	GO:0006412	B	InterPro	translation
TBB1_GLOMM (sp|Q27U48) Tubulin beta-1 chain OS=G. morsitans PE=2 SV=1	TBB1_GLOMM	0	GLOS_TBB1.1.1	GO:0005737	C	UniProtKB-K	cytoplasm
TBB1_GLOMM (sp|Q27U48) Tubulin beta-1 chain OS=G. morsitans PE=2 SV=1	TBB1_GLOMM	0	GLOS_TBB1.1.1	GO:0005874	C	UniProtKB-K	microtubule
TBB1_GLOMM (sp|Q27U48) Tubulin beta-1 chain OS=G. morsitans PE=2 SV=1	TBB1_GLOMM	0	GLOS_TBB1.1.1	GO:0005525	M	UniProtKB-K	GTP binding
TBB1_GLOMM (sp|Q27U48) Tubulin beta-1 chain OS=G. morsitans PE=2 SV=1	TBB1_GLOMM	0	GLOS_TBB1.1.1	GO:0003924	M	InterPro	GTPase activity
TBB1_GLOMM (sp|Q27U48) Tubulin beta-1 chain OS=G. morsitans PE=2 SV=1	TBB1_GLOMM	0	GLOS_TBB1.1.1	GO:0005200	M	InterPro	structural constituent of cytoskeleton
TBB1_GLOMM (sp|Q27U48) Tubulin beta-1 chain OS=G. morsitans PE=2 SV=1	TBB1_GLOMM	0	GLOS_TBB1.1.1	GO:0007017	B	InterPro	microtubule-based process
TBB1_GLOMM (sp|Q27U48) Tubulin beta-1 chain OS=G. morsitans PE=2 SV=1	TBB1_GLOMM	0	GLOS_TBB1.1.1	GO:0051258	B	InterPro	protein polymerization
TRYP_SARBU (sp|P51588) Trypsin OS=Sarcophaga bullata PE=1 SV=1	TRYP_SARBU	4,00E-85	GLOS_TRYP.6.26	GO:0005615	C	UniProtKB-S	extracellular space
TRYP_SARBU (sp|P51588) Trypsin OS=Sarcophaga bullata PE=1 SV=1	TRYP_SARBU	4,00E-85	GLOS_TRYP.6.26	GO:0004252	M	InterPro	serine-type endopeptidase activity
TRYP_SARBU (sp|P51588) Trypsin OS=Sarcophaga bullata PE=1 SV=1	TRYP_SARBU	4,00E-85	GLOS_TRYP.6.26	GO:0007586	B	UniProtKB-K	digestion
TRYP_SARBU (sp|P51588) Trypsin OS=Sarcophaga bullata PE=1 SV=1	TRYP_SARBU	4,00E-85	GLOS_TRYP.6.26	GO:0006508	B	UniProtKB-K	proteolysis
TSEP_GLOPP (sp|Q8T4N5) Protein TsetseEP OS=G.p. palpalis PE=4 SV=1	TSEP_GLOPP	3,00E-45	GLOS_TSEP.6.20	GO:0005576	C	UniProtKB-S	extracellular region
UBCD1_DROME (sp|P25867) Ubiquitin-conjugating enzyme E2-17 kDa OS=D.m.	UBCD1_DROME	1,00E-100	GLOS_UBCD1.1.2	GO:0005875	C	FlyBase	microtubule associated complex

UBCD1_DROME (sp|P25867) Ubiquitin-conjugating enzyme E2-17 kDa OS=D.m.	UBCD1_DROME	1,00E-100	GLOS_UBCD1.1.2	GO:0005524	M	UniProtKB-k	ATP binding
UBCD1_DROME (sp|P25867) Ubiquitin-conjugating enzyme E2-17 kDa OS=D.m.	UBCD1_DROME	1,00E-100	GLOS_UBCD1.1.2	GO:0019787	M	.	small conjugating protein ligase activity
UBCD1_DROME (sp|P25867) Ubiquitin-conjugating enzyme E2-17 kDa OS=D.m.	UBCD1_DROME	1,00E-100	GLOS_UBCD1.1.2	GO:0016881	M	.	acid-amino acid ligase activity
UBCD1_DROME (sp|P25867) Ubiquitin-conjugating enzyme E2-17 kDa OS=D.m.	UBCD1_DROME	1,00E-100	GLOS_UBCD1.1.2	GO:0016879	M	.	ligase activity, forming carbon-nitrogen bonds
UBCD1_DROME (sp|P25867) Ubiquitin-conjugating enzyme E2-17 kDa OS=D.m.	UBCD1_DROME	1,00E-100	GLOS_UBCD1.1.2	GO:0016874	M	.	ligase activity
UBCD1_DROME (sp|P25867) Ubiquitin-conjugating enzyme E2-17 kDa OS=D.m.	UBCD1_DROME	1,00E-100	GLOS_UBCD1.1.2	GO:0004842	M	FlyBase	ubiquitin-protein ligase activity
UBCD1_DROME (sp|P25867) Ubiquitin-conjugating enzyme E2-17 kDa OS=D.m.	UBCD1_DROME	1,00E-100	GLOS_UBCD1.1.2	GO:0051276	B	FlyBase	chromosome organization
UBCD1_DROME (sp|P25867) Ubiquitin-conjugating enzyme E2-17 kDa OS=D.m.	UBCD1_DROME	1,00E-100	GLOS_UBCD1.1.2	GO:0001751	B	FlyBase	compound eye photoreceptor cell differentiation
UBCD1_DROME (sp|P25867) Ubiquitin-conjugating enzyme E2-17 kDa OS=D.m.	UBCD1_DROME	1,00E-100	GLOS_UBCD1.1.2	GO:0043161	B	.	proteasomal ubiquitin-dependent protein catabolic process
UBCD1_DROME (sp|P25867) Ubiquitin-conjugating enzyme E2-17 kDa OS=D.m.	UBCD1_DROME	1,00E-100	GLOS_UBCD1.1.2	GO:0008054	B	FlyBase	cyclin catabolic process
UBCD1_DROME (sp|P25867) Ubiquitin-conjugating enzyme E2-17 kDa OS=D.m.	UBCD1_DROME	1,00E-100	GLOS_UBCD1.1.2	GO:0042078	B	.	germ-line stem cell division
UBCD1_DROME (sp|P25867) Ubiquitin-conjugating enzyme E2-17 kDa OS=D.m.	UBCD1_DROME	1,00E-100	GLOS_UBCD1.1.2	GO:0008356	B	.	asymmetric cell division
UBCD1_DROME (sp|P25867) Ubiquitin-conjugating enzyme E2-17 kDa OS=D.m.	UBCD1_DROME	1,00E-100	GLOS_UBCD1.1.2	GO:0017145	B	.	stem cell division
UBCD1_DROME (sp|P25867) Ubiquitin-conjugating enzyme E2-17 kDa OS=D.m.	UBCD1_DROME	1,00E-100	GLOS_UBCD1.1.2	GO:0048132	B	FlyBase	female germ-line stem cell division
UBCD1_DROME (sp|P25867) Ubiquitin-conjugating enzyme E2-17 kDa OS=D.m.	UBCD1_DROME	1,00E-100	GLOS_UBCD1.1.2	GO:0019827	B	.	stem cell maintenance
UBCD1_DROME (sp|P25867) Ubiquitin-conjugating enzyme E2-17 kDa OS=D.m.	UBCD1_DROME	1,00E-100	GLOS_UBCD1.1.2	GO:0048864	B	.	stem cell development
UBCD1_DROME (sp|P25867) Ubiquitin-conjugating enzyme E2-17 kDa OS=D.m.	UBCD1_DROME	1,00E-100	GLOS_UBCD1.1.2	GO:0048863	B	.	stem cell differentiation
UBCD1_DROME (sp|P25867) Ubiquitin-conjugating enzyme E2-17 kDa OS=D.m.	UBCD1_DROME	1,00E-100	GLOS_UBCD1.1.2	GO:0030718	B	FlyBase	germ-line stem cell maintenance
UBCD1_DROME (sp|P25867) Ubiquitin-conjugating enzyme E2-17 kDa OS=D.m.	UBCD1_DROME	1,00E-100	GLOS_UBCD1.1.2	GO:0007140	B	FlyBase	male meiosis
UBCD1_DROME (sp|P25867) Ubiquitin-conjugating enzyme E2-17 kDa OS=D.m.	UBCD1_DROME	1,00E-100	GLOS_UBCD1.1.2	GO:0000280	B	.	nuclear division
UBCD1_DROME (sp|P25867) Ubiquitin-conjugating enzyme E2-17 kDa OS=D.m.	UBCD1_DROME	1,00E-100	GLOS_UBCD1.1.2	GO:0048285	B	.	organelle fission
UBCD1_DROME (sp|P25867) Ubiquitin-conjugating enzyme E2-17 kDa OS=D.m.	UBCD1_DROME	1,00E-100	GLOS_UBCD1.1.2	GO:0007067	B	FlyBase	mitosis
UBCD1_DROME (sp|P25867) Ubiquitin-conjugating enzyme E2-17 kDa OS=D.m.	UBCD1_DROME	1,00E-100	GLOS_UBCD1.1.2	GO:0042551	B	.	neuron maturation
UBCD1_DROME (sp|P25867) Ubiquitin-conjugating enzyme E2-17 kDa OS=D.m.	UBCD1_DROME	1,00E-100	GLOS_UBCD1.1.2	GO:0016322	B	FlyBase	neuron remodeling
UBCD1_DROME (sp|P25867) Ubiquitin-conjugating enzyme E2-17 kDa OS=D.m.	UBCD1_DROME	1,00E-100	GLOS_UBCD1.1.2	GO:0031647	B	FlyBase	regulation of protein stability
UBCD1_DROME (sp|P25867) Ubiquitin-conjugating enzyme E2-17 kDa OS=D.m.	UBCD1_DROME	1,00E-100	GLOS_UBCD1.1.2	GO:0046532	B	.	regulation of photoreceptor cell differentiation
UBCD1_DROME (sp|P25867) Ubiquitin-conjugating enzyme E2-17 kDa OS=D.m.	UBCD1_DROME	1,00E-100	GLOS_UBCD1.1.2	GO:2000027	B	.	regulation of organ morphogenesis
UBCD1_DROME (sp|P25867) Ubiquitin-conjugating enzyme E2-17 kDa OS=D.m.	UBCD1_DROME	1,00E-100	GLOS_UBCD1.1.2	GO:0045676	B	FlyBase	regulation of R7 cell differentiation
UBCD1_DROME (sp|P25867) Ubiquitin-conjugating enzyme E2-17 kDa OS=D.m.	UBCD1_DROME	1,00E-100	GLOS_UBCD1.1.2	GO:0002064	B	.	epithelial cell development
UBCD1_DROME (sp|P25867) Ubiquitin-conjugating enzyme E2-17 kDa OS=D.m.	UBCD1_DROME	1,00E-100	GLOS_UBCD1.1.2	GO:0048515	B	.	spermatid differentiation
UBCD1_DROME (sp|P25867) Ubiquitin-conjugating enzyme E2-17 kDa OS=D.m.	UBCD1_DROME	1,00E-100	GLOS_UBCD1.1.2	GO:0007283	B	.	spermatogenesis
UBCD1_DROME (sp|P25867) Ubiquitin-conjugating enzyme E2-17 kDa OS=D.m.	UBCD1_DROME	1,00E-100	GLOS_UBCD1.1.2	GO:0048232	B	.	male gamete generation
UBCD1_DROME (sp|P25867) Ubiquitin-conjugating enzyme E2-17 kDa OS=D.m.	UBCD1_DROME	1,00E-100	GLOS_UBCD1.1.2	GO:0007286	B	FlyBase	spermatid development
[BBH] UN112_DROME (sp|Q9VZI3) Unc-112-related prot. OS=D.m.	UN112_DROME	0	GLOS_UN112.1.1	GO:0005737	C	UniProtKB	cytoplasm
[BBH] UN112_DROME (sp|Q9VZI3) Unc-112-related prot. OS=D.m.	UN112_DROME	0	GLOS_UN112.1.1	GO:0050839	M	UniProtKB	cell adhesion molecule binding
[BBH] UN112_DROME (sp|Q9VZI3) Unc-112-related prot. OS=D.m.	UN112_DROME	0	GLOS_UN112.1.1	GO:0005543	M	InterPro	phospholipid binding
[BBH] UN112_DROME (sp|Q9VZI3) Unc-112-related prot. OS=D.m.	UN112_DROME	0	GLOS_UN112.1.1	GO:0014706	B	.	striated muscle tissue development
[BBH] UN112_DROME (sp|Q9VZI3) Unc-112-related prot. OS=D.m.	UN112_DROME	0	GLOS_UN112.1.1	GO:0060537	B	.	muscle tissue development
[BBH] UN112_DROME (sp|Q9VZI3) Unc-112-related prot. OS=D.m.	UN112_DROME	0	GLOS_UN112.1.1	GO:0007507	B	.	heart development
[BBH] UN112_DROME (sp|Q9VZI3) Unc-112-related prot. OS=D.m.	UN112_DROME	0	GLOS_UN112.1.1	GO:0048738	B	FlyBase	cardiac muscle tissue development
[BBH] UN112_DROME (sp|Q9VZI3) Unc-112-related prot. OS=D.m.	UN112_DROME	0	GLOS_UN112.1.1	GO:0007160	B	UniProtKB	cell-matrix adhesion
[BBH] UN112_DROME (sp|Q9VZI3) Unc-112-related prot. OS=D.m.	UN112_DROME	0	GLOS_UN112.1.1	GO:0042742	B	.	defense response to bacterium
[BBH] UN112_DROME (sp|Q9VZI3) Unc-112-related prot. OS=D.m.	UN112_DROME	0	GLOS_UN112.1.1	GO:0050829	B	FlyBase	defense response to Gram-negative bacterium
[BBH] VIAF1_DROME (sp|Q8MR62) Viral IAP-associated factor homolog OS=D.m.	VIAF1_DROME	1,00E-108	GLOS_VIAF1.1.1	GO:0005737	C	UniProtKB	cytoplasm
[BBH] VIAF1_DROME (sp|Q8MR62) Viral IAP-associated factor homolog OS=D.m.	VIAF1_DROME	1,00E-108	GLOS_VIAF1.1.1	GO:0042981	B	.	regulation of apoptotic process
[BBH] VIAF1_DROME (sp|Q8MR62) Viral IAP-associated factor homolog OS=D.m.	VIAF1_DROME	1,00E-108	GLOS_VIAF1.1.1	GO:0043067	B	.	regulation of programmed cell death
[BBH] VIAF1_DROME (sp|Q8MR62) Viral IAP-associated factor homolog OS=D.m.	VIAF1_DROME	1,00E-108	GLOS_VIAF1.1.1	GO:0010941	B	.	regulation of cell death
[BBH] VIAF1_DROME (sp|Q8MR62) Viral IAP-associated factor homolog OS=D.m.	VIAF1_DROME	1,00E-108	GLOS_VIAF1.1.1	GO:2000116	B	.	regulation of cysteine-type endopeptidase activity
[BBH] VIAF1_DROME (sp|Q8MR62) Viral IAP-associated factor homolog OS=D.m.	VIAF1_DROME	1,00E-108	GLOS_VIAF1.1.1	GO:0052548	B	.	regulation of endopeptidase activity
[BBH] VIAF1_DROME (sp|Q8MR62) Viral IAP-associated factor homolog OS=D.m.	VIAF1_DROME	1,00E-108	GLOS_VIAF1.1.1	GO:0052547	B	.	regulation of peptidase activity
[BBH] VIAF1_DROME (sp|Q8MR62) Viral IAP-associated factor homolog OS=D.m.	VIAF1_DROME	1,00E-108	GLOS_VIAF1.1.1	GO:0043281	B	FlyBase	regulation of cysteine-type endopeptidase activity involved in apoptotic process
NP_001097174.1 fasciclin3, isoformD / ref|NP_001163005.1| fasciclin3, isoformE	NP_001097174.1	0	GLOS_FAS3.1.1	GO:0006357	B	.	regulation of transcription from RNA polymerase II promoter
NP_001097174.1 fasciclin3, isoformD / ref|NP_001163005.1| fasciclin3, isoformE	NP_001097174.1	0	GLOS_FAS3.1.1	GO:0006355	B	.	regulation of transcription, DNA-dependent
NP_001097174.1 fasciclin3, isoformD / ref|NP_001163005.1| fasciclin3, isoformE	NP_001097174.1	0	GLOS_FAS3.1.1	GO:0010468	B	.	regulation of gene expression

NP_001097174.1 fasciclin3, isoformD / ref|NP_001163005.1| fasciclin3, isoformE	NP_001097174.1	0	GLOS_FAS3.1.1	GO:0060255	B	.	regulation of macromolecule metabolic process
NP_001097174.1 fasciclin3, isoformD / ref|NP_001163005.1| fasciclin3, isoformE	NP_001097174.1	0	GLOS_FAS3.1.1	GO:0019222	B	.	regulation of metabolic process
NP_001097174.1 fasciclin3, isoformD / ref|NP_001163005.1| fasciclin3, isoformE	NP_001097174.1	0	GLOS_FAS3.1.1	GO:0050789	B	.	regulation of biological process
NP_001097174.1 fasciclin3, isoformD / ref|NP_001163005.1| fasciclin3, isoformE	NP_001097174.1	0	GLOS_FAS3.1.1	GO:0065007	B	.	biological regulation
NP_001097174.1 fasciclin3, isoformD / ref|NP_001163005.1| fasciclin3, isoformE	NP_001097174.1	0	GLOS_FAS3.1.1	GO:0008150	B	.	biological_process
NP_001097174.1 fasciclin3, isoformD / ref|NP_001163005.1| fasciclin3, isoformE	NP_001097174.1	0	GLOS_FAS3.1.1	GO:2000112	B	.	regulation of cellular macromolecule biosynthetic process
NP_001097174.1 fasciclin3, isoformD / ref|NP_001163005.1| fasciclin3, isoformE	NP_001097174.1	0	GLOS_FAS3.1.1	GO:0010556	B	.	regulation of macromolecule biosynthetic process
NP_001097174.1 fasciclin3, isoformD / ref|NP_001163005.1| fasciclin3, isoformE	NP_001097174.1	0	GLOS_FAS3.1.1	GO:0009889	B	.	regulation of biosynthetic process
NP_001097174.1 fasciclin3, isoformD / ref|NP_001163005.1| fasciclin3, isoformE	NP_001097174.1	0	GLOS_FAS3.1.1	GO:0031326	B	.	regulation of cellular biosynthetic process
NP_001097174.1 fasciclin3, isoformD / ref|NP_001163005.1| fasciclin3, isoformE	NP_001097174.1	0	GLOS_FAS3.1.1	GO:0031323	B	.	regulation of cellular metabolic process
NP_001097174.1 fasciclin3, isoformD / ref|NP_001163005.1| fasciclin3, isoformE	NP_001097174.1	0	GLOS_FAS3.1.1	GO:0050794	B	.	regulation of cellular process
NP_001097174.1 fasciclin3, isoformD / ref|NP_001163005.1| fasciclin3, isoformE	NP_001097174.1	0	GLOS_FAS3.1.1	GO:2001141	B	.	regulation of RNA biosynthetic process
NP_001097174.1 fasciclin3, isoformD / ref|NP_001163005.1| fasciclin3, isoformE	NP_001097174.1	0	GLOS_FAS3.1.1	GO:0051252	B	.	regulation of RNA metabolic process
NP_001097174.1 fasciclin3, isoformD / ref|NP_001163005.1| fasciclin3, isoformE	NP_001097174.1	0	GLOS_FAS3.1.1	GO:0019219	B	.	regulation of nucleobase-containing compound metabolic process
NP_001097174.1 fasciclin3, isoformD / ref|NP_001163005.1| fasciclin3, isoformE	NP_001097174.1	0	GLOS_FAS3.1.1	GO:0051171	B	.	regulation of nitrogen compound metabolic process
NP_001097174.1 fasciclin3, isoformD / ref|NP_001163005.1| fasciclin3, isoformE	NP_001097174.1	0	GLOS_FAS3.1.1	GO:0080090	B	.	regulation of primary metabolic process
NP_001097174.1 fasciclin3, isoformD / ref|NP_001163005.1| fasciclin3, isoformE	NP_001097174.1	0	GLOS_FAS3.1.1	GO:0045892	B	.	negative regulation of transcription, DNA-dependent
NP_001097174.1 fasciclin3, isoformD / ref|NP_001163005.1| fasciclin3, isoformE	NP_001097174.1	0	GLOS_FAS3.1.1	GO:0010629	B	.	negative regulation of gene expression
NP_001097174.1 fasciclin3, isoformD / ref|NP_001163005.1| fasciclin3, isoformE	NP_001097174.1	0	GLOS_FAS3.1.1	GO:0010605	B	.	negative regulation of macromolecule metabolic process
NP_001097174.1 fasciclin3, isoformD / ref|NP_001163005.1| fasciclin3, isoformE	NP_001097174.1	0	GLOS_FAS3.1.1	GO:0009892	B	.	negative regulation of metabolic process
NP_001097174.1 fasciclin3, isoformD / ref|NP_001163005.1| fasciclin3, isoformE	NP_001097174.1	0	GLOS_FAS3.1.1	GO:0048519	B	.	negative regulation of biological process
NP_001097174.1 fasciclin3, isoformD / ref|NP_001163005.1| fasciclin3, isoformE	NP_001097174.1	0	GLOS_FAS3.1.1	GO:0051253	B	.	negative regulation of RNA metabolic process
NP_001097174.1 fasciclin3, isoformD / ref|NP_001163005.1| fasciclin3, isoformE	NP_001097174.1	0	GLOS_FAS3.1.1	GO:0045934	B	.	negative regulation of nucleobase-containing compound metabolic process
NP_001097174.1 fasciclin3, isoformD / ref|NP_001163005.1| fasciclin3, isoformE	NP_001097174.1	0	GLOS_FAS3.1.1	GO:0031324	B	.	negative regulation of cellular metabolic process
NP_001097174.1 fasciclin3, isoformD / ref|NP_001163005.1| fasciclin3, isoformE	NP_001097174.1	0	GLOS_FAS3.1.1	GO:0048523	B	.	negative regulation of cellular process
NP_001097174.1 fasciclin3, isoformD / ref|NP_001163005.1| fasciclin3, isoformE	NP_001097174.1	0	GLOS_FAS3.1.1	GO:0051172	B	.	negative regulation of nitrogen compound metabolic process
NP_001097174.1 fasciclin3, isoformD / ref|NP_001163005.1| fasciclin3, isoformE	NP_001097174.1	0	GLOS_FAS3.1.1	GO:2000113	B	.	negative regulation of cellular macromolecule biosynthetic process
NP_001097174.1 fasciclin3, isoformD / ref|NP_001163005.1| fasciclin3, isoformE	NP_001097174.1	0	GLOS_FAS3.1.1	GO:0010558	B	.	negative regulation of macromolecule biosynthetic process
NP_001097174.1 fasciclin3, isoformD / ref|NP_001163005.1| fasciclin3, isoformE	NP_001097174.1	0	GLOS_FAS3.1.1	GO:0009890	B	.	negative regulation of biosynthetic process
NP_001097174.1 fasciclin3, isoformD / ref|NP_001163005.1| fasciclin3, isoformE	NP_001097174.1	0	GLOS_FAS3.1.1	GO:0031327	B	.	negative regulation of cellular biosynthetic process
NP_001097174.1 fasciclin3, isoformD / ref|NP_001163005.1| fasciclin3, isoformE	NP_001097174.1	0	GLOS_FAS3.1.1	GO:0000122	B	Refseq	negative regulation of transcription from RNA polymerase II promoter
NP_001097174.1 fasciclin3, isoformD / ref|NP_001163005.1| fasciclin3, isoformE	NP_001097174.1	0	GLOS_FAS3.1.1	GO:0048731	B	.	system development
NP_001097174.1 fasciclin3, isoformD / ref|NP_001163005.1| fasciclin3, isoformE	NP_001097174.1	0	GLOS_FAS3.1.1	GO:0048856	B	.	anatomical structure development
NP_001097174.1 fasciclin3, isoformD / ref|NP_001163005.1| fasciclin3, isoformE	NP_001097174.1	0	GLOS_FAS3.1.1	GO:0032502	B	.	developmental process
NP_001097174.1 fasciclin3, isoformD / ref|NP_001163005.1| fasciclin3, isoformE	NP_001097174.1	0	GLOS_FAS3.1.1	GO:0007275	B	.	multicellular organismal development
NP_001097174.1 fasciclin3, isoformD / ref|NP_001163005.1| fasciclin3, isoformE	NP_001097174.1	0	GLOS_FAS3.1.1	GO:0044707	B	.	single-multicellular organism process
NP_001097174.1 fasciclin3, isoformD / ref|NP_001163005.1| fasciclin3, isoformE	NP_001097174.1	0	GLOS_FAS3.1.1	GO:0032501	B	.	multicellular organismal process
NP_001097174.1 fasciclin3, isoformD / ref|NP_001163005.1| fasciclin3, isoformE	NP_001097174.1	0	GLOS_FAS3.1.1	GO:0044699	B	.	single-organism process
NP_001097174.1 fasciclin3, isoformD / ref|NP_001163005.1| fasciclin3, isoformE	NP_001097174.1	0	GLOS_FAS3.1.1	GO:0001501	B	Refseq	skeletal system development
NP_001097174.1 fasciclin3, isoformD / ref|NP_001163005.1| fasciclin3, isoformE	NP_001097174.1	0	GLOS_FAS3.1.1	GO:0001071	M	.	nucleic acid binding transcription factor activity
NP_001097174.1 fasciclin3, isoformD / ref|NP_001163005.1| fasciclin3, isoformE	NP_001097174.1	0	GLOS_FAS3.1.1	GO:0003674	M	.	molecular_function
NP_001097174.1 fasciclin3, isoformD / ref|NP_001163005.1| fasciclin3, isoformE	NP_001097174.1	0	GLOS_FAS3.1.1	GO:0003700	M	Refseq	sequence-specific DNA binding transcription factor activity
NP_001097174.1 fasciclin3, isoformD / ref|NP_001163005.1| fasciclin3, isoformE	NP_001097174.1	0	GLOS_FAS3.1.1	GO:0005488	M	.	binding
NP_001097174.1 fasciclin3, isoformD / ref|NP_001163005.1| fasciclin3, isoformE	NP_001097174.1	0	GLOS_FAS3.1.1	GO:0005515	M	Refseq	protein binding
NP_001097174.1 fasciclin3, isoformD / ref|NP_001163005.1| fasciclin3, isoformE	NP_001097174.1	0	GLOS_FAS3.1.1	GO:0043231	C	.	intracellular membrane-bounded organelle
NP_001097174.1 fasciclin3, isoformD / ref|NP_001163005.1| fasciclin3, isoformE	NP_001097174.1	0	GLOS_FAS3.1.1	GO:0043227	C	.	membrane-bounded organelle
NP_001097174.1 fasciclin3, isoformD / ref|NP_001163005.1| fasciclin3, isoformE	NP_001097174.1	0	GLOS_FAS3.1.1	GO:0043226	C	.	organelle
NP_001097174.1 fasciclin3, isoformD / ref|NP_001163005.1| fasciclin3, isoformE	NP_001097174.1	0	GLOS_FAS3.1.1	GO:0005575	C	.	cellular_component
NP_001097174.1 fasciclin3, isoformD / ref|NP_001163005.1| fasciclin3, isoformE	NP_001097174.1	0	GLOS_FAS3.1.1	GO:0043229	C	.	intracellular organelle
NP_001097174.1 fasciclin3, isoformD / ref|NP_001163005.1| fasciclin3, isoformE	NP_001097174.1	0	GLOS_FAS3.1.1	GO:0044424	C	.	intracellular part
NP_001097174.1 fasciclin3, isoformD / ref|NP_001163005.1| fasciclin3, isoformE	NP_001097174.1	0	GLOS_FAS3.1.1	GO:0044464	C	.	cell part
NP_001097174.1 fasciclin3, isoformD / ref|NP_001163005.1| fasciclin3, isoformE	NP_001097174.1	0	GLOS_FAS3.1.1	GO:0005623	C	.	cell
NP_001097174.1 fasciclin3, isoformD / ref|NP_001163005.1| fasciclin3, isoformE	NP_001097174.1	0	GLOS_FAS3.1.1	GO:0005622	C	.	intracellular
NP_001097174.1 fasciclin3, isoformD / ref|NP_001163005.1| fasciclin3, isoformE	NP_001097174.1	0	GLOS_FAS3.1.1	GO:0005634	C	Refseq	nucleus

NP_001097174.1 fasciclin3, isoformD / ref|NP_001163005.1| fasciclin3, isoformE	NP_001097174.1	0	GLOS_FAS3.1.1	GO:0006351	B	.	transcription, DNA-dependent
NP_001097174.1 fasciclin3, isoformD / ref|NP_001163005.1| fasciclin3, isoformE	NP_001097174.1	0	GLOS_FAS3.1.1	GO:0032774	B	.	RNA biosynthetic process
NP_001097174.1 fasciclin3, isoformD / ref|NP_001163005.1| fasciclin3, isoformE	NP_001097174.1	0	GLOS_FAS3.1.1	GO:0009059	B	.	macromolecule biosynthetic process
NP_001097174.1 fasciclin3, isoformD / ref|NP_001163005.1| fasciclin3, isoformE	NP_001097174.1	0	GLOS_FAS3.1.1	GO:0043170	B	.	macromolecule metabolic process
NP_001097174.1 fasciclin3, isoformD / ref|NP_001163005.1| fasciclin3, isoformE	NP_001097174.1	0	GLOS_FAS3.1.1	GO:0071704	B	.	organic substance metabolic process
NP_001097174.1 fasciclin3, isoformD / ref|NP_001163005.1| fasciclin3, isoformE	NP_001097174.1	0	GLOS_FAS3.1.1	GO:0008152	B	.	metabolic process
NP_001097174.1 fasciclin3, isoformD / ref|NP_001163005.1| fasciclin3, isoformE	NP_001097174.1	0	GLOS_FAS3.1.1	GO:1901576	B	.	organic substance biosynthetic process
NP_001097174.1 fasciclin3, isoformD / ref|NP_001163005.1| fasciclin3, isoformE	NP_001097174.1	0	GLOS_FAS3.1.1	GO:0009058	B	.	biosynthetic process
NP_001097174.1 fasciclin3, isoformD / ref|NP_001163005.1| fasciclin3, isoformE	NP_001097174.1	0	GLOS_FAS3.1.1	GO:0016070	B	.	RNA metabolic process
NP_001097174.1 fasciclin3, isoformD / ref|NP_001163005.1| fasciclin3, isoformE	NP_001097174.1	0	GLOS_FAS3.1.1	GO:0044260	B	.	cellular macromolecule metabolic process
NP_001097174.1 fasciclin3, isoformD / ref|NP_001163005.1| fasciclin3, isoformE	NP_001097174.1	0	GLOS_FAS3.1.1	GO:0044237	B	.	cellular metabolic process
NP_001097174.1 fasciclin3, isoformD / ref|NP_001163005.1| fasciclin3, isoformE	NP_001097174.1	0	GLOS_FAS3.1.1	GO:0009987	B	.	cellular process
NP_001097174.1 fasciclin3, isoformD / ref|NP_001163005.1| fasciclin3, isoformE	NP_001097174.1	0	GLOS_FAS3.1.1	GO:0090304	B	.	nucleic acid metabolic process
NP_001097174.1 fasciclin3, isoformD / ref|NP_001163005.1| fasciclin3, isoformE	NP_001097174.1	0	GLOS_FAS3.1.1	GO:0006139	B	.	nucleobase-containing compound metabolic process
NP_001097174.1 fasciclin3, isoformD / ref|NP_001163005.1| fasciclin3, isoformE	NP_001097174.1	0	GLOS_FAS3.1.1	GO:0006725	B	.	cellular aromatic compound metabolic process
NP_001097174.1 fasciclin3, isoformD / ref|NP_001163005.1| fasciclin3, isoformE	NP_001097174.1	0	GLOS_FAS3.1.1	GO:0034641	B	.	cellular nitrogen compound metabolic process
NP_001097174.1 fasciclin3, isoformD / ref|NP_001163005.1| fasciclin3, isoformE	NP_001097174.1	0	GLOS_FAS3.1.1	GO:0006807	B	.	nitrogen compound metabolic process
NP_001097174.1 fasciclin3, isoformD / ref|NP_001163005.1| fasciclin3, isoformE	NP_001097174.1	0	GLOS_FAS3.1.1	GO:0044238	B	.	primary metabolic process
NP_001097174.1 fasciclin3, isoformD / ref|NP_001163005.1| fasciclin3, isoformE	NP_001097174.1	0	GLOS_FAS3.1.1	GO:0046483	B	.	heterocycle metabolic process
NP_001097174.1 fasciclin3, isoformD / ref|NP_001163005.1| fasciclin3, isoformE	NP_001097174.1	0	GLOS_FAS3.1.1	GO:1901360	B	.	organic cyclic compound metabolic process
NP_001097174.1 fasciclin3, isoformD / ref|NP_001163005.1| fasciclin3, isoformE	NP_001097174.1	0	GLOS_FAS3.1.1	GO:0034654	B	.	nucleobase-containing compound biosynthetic process
NP_001097174.1 fasciclin3, isoformD / ref|NP_001163005.1| fasciclin3, isoformE	NP_001097174.1	0	GLOS_FAS3.1.1	GO:0018130	B	.	heterocycle biosynthetic process
NP_001097174.1 fasciclin3, isoformD / ref|NP_001163005.1| fasciclin3, isoformE	NP_001097174.1	0	GLOS_FAS3.1.1	GO:0044249	B	.	cellular biosynthetic process
NP_001097174.1 fasciclin3, isoformD / ref|NP_001163005.1| fasciclin3, isoformE	NP_001097174.1	0	GLOS_FAS3.1.1	GO:0019438	B	.	aromatic compound biosynthetic process
NP_001097174.1 fasciclin3, isoformD / ref|NP_001163005.1| fasciclin3, isoformE	NP_001097174.1	0	GLOS_FAS3.1.1	GO:0044271	B	.	cellular nitrogen compound biosynthetic process
NP_001097174.1 fasciclin3, isoformD / ref|NP_001163005.1| fasciclin3, isoformE	NP_001097174.1	0	GLOS_FAS3.1.1	GO:1901362	B	.	organic cyclic compound biosynthetic process
NP_001097174.1 fasciclin3, isoformD / ref|NP_001163005.1| fasciclin3, isoformE	NP_001097174.1	0	GLOS_FAS3.1.1	GO:0034645	B	.	cellular macromolecule biosynthetic process
NP_001097174.1 fasciclin3, isoformD / ref|NP_001163005.1| fasciclin3, isoformE	NP_001097174.1	0	GLOS_FAS3.1.1	GO:0010467	B	.	gene expression
NP_001097174.1 fasciclin3, isoformD / ref|NP_001163005.1| fasciclin3, isoformE	NP_001097174.1	0	GLOS_FAS3.1.1	GO:0006366	B	Refseq	transcription from RNA polymerase II promoter
NP_001097174.1 fasciclin3, isoformD / ref|NP_001163005.1| fasciclin3, isoformE	NP_001097174.1	0	GLOS_FAS3.1.1	GO:0006606	B	.	protein import into nucleus
NP_001097174.1 fasciclin3, isoformD / ref|NP_001163005.1| fasciclin3, isoformE	NP_001097174.1	0	GLOS_FAS3.1.1	GO:0006886	B	.	intracellular protein transport
NP_001097174.1 fasciclin3, isoformD / ref|NP_001163005.1| fasciclin3, isoformE	NP_001097174.1	0	GLOS_FAS3.1.1	GO:0015031	B	.	protein transport
NP_001097174.1 fasciclin3, isoformD / ref|NP_001163005.1| fasciclin3, isoformE	NP_001097174.1	0	GLOS_FAS3.1.1	GO:0045184	B	.	establishment of protein localization
NP_001097174.1 fasciclin3, isoformD / ref|NP_001163005.1| fasciclin3, isoformE	NP_001097174.1	0	GLOS_FAS3.1.1	GO:0051234	B	.	establishment of localization
NP_001097174.1 fasciclin3, isoformD / ref|NP_001163005.1| fasciclin3, isoformE	NP_001097174.1	0	GLOS_FAS3.1.1	GO:0051179	B	.	localization
NP_001097174.1 fasciclin3, isoformD / ref|NP_001163005.1| fasciclin3, isoformE	NP_001097174.1	0	GLOS_FAS3.1.1	GO:0008104	B	.	protein localization
NP_001097174.1 fasciclin3, isoformD / ref|NP_001163005.1| fasciclin3, isoformE	NP_001097174.1	0	GLOS_FAS3.1.1	GO:0033036	B	.	macromolecule localization
NP_001097174.1 fasciclin3, isoformD / ref|NP_001163005.1| fasciclin3, isoformE	NP_001097174.1	0	GLOS_FAS3.1.1	GO:0071702	B	.	organic substance transport
NP_001097174.1 fasciclin3, isoformD / ref|NP_001163005.1| fasciclin3, isoformE	NP_001097174.1	0	GLOS_FAS3.1.1	GO:0006810	B	.	transport
NP_001097174.1 fasciclin3, isoformD / ref|NP_001163005.1| fasciclin3, isoformE	NP_001097174.1	0	GLOS_FAS3.1.1	GO:0046907	B	.	intracellular transport
NP_001097174.1 fasciclin3, isoformD / ref|NP_001163005.1| fasciclin3, isoformE	NP_001097174.1	0	GLOS_FAS3.1.1	GO:0051649	B	.	establishment of localization in cell
NP_001097174.1 fasciclin3, isoformD / ref|NP_001163005.1| fasciclin3, isoformE	NP_001097174.1	0	GLOS_FAS3.1.1	GO:0051641	B	.	cellular localization
NP_001097174.1 fasciclin3, isoformD / ref|NP_001163005.1| fasciclin3, isoformE	NP_001097174.1	0	GLOS_FAS3.1.1	GO:0044763	B	.	single-organism cellular process
NP_001097174.1 fasciclin3, isoformD / ref|NP_001163005.1| fasciclin3, isoformE	NP_001097174.1	0	GLOS_FAS3.1.1	GO:0034613	B	.	cellular protein localization
NP_001097174.1 fasciclin3, isoformD / ref|NP_001163005.1| fasciclin3, isoformE	NP_001097174.1	0	GLOS_FAS3.1.1	GO:0070727	B	.	cellular macromolecule localization
NP_001097174.1 fasciclin3, isoformD / ref|NP_001163005.1| fasciclin3, isoformE	NP_001097174.1	0	GLOS_FAS3.1.1	GO:0017038	B	.	protein import
NP_001097174.1 fasciclin3, isoformD / ref|NP_001163005.1| fasciclin3, isoformE	NP_001097174.1	0	GLOS_FAS3.1.1	GO:0044765	B	.	single-organism transport
NP_001097174.1 fasciclin3, isoformD / ref|NP_001163005.1| fasciclin3, isoformE	NP_001097174.1	0	GLOS_FAS3.1.1	GO:0051170	B	.	nuclear import
NP_001097174.1 fasciclin3, isoformD / ref|NP_001163005.1| fasciclin3, isoformE	NP_001097174.1	0	GLOS_FAS3.1.1	GO:0006913	B	.	nucleocytoplasmic transport
NP_001097174.1 fasciclin3, isoformD / ref|NP_001163005.1| fasciclin3, isoformE	NP_001097174.1	0	GLOS_FAS3.1.1	GO:0016482	B	.	cytoplasmic transport
NP_001097174.1 fasciclin3, isoformD / ref|NP_001163005.1| fasciclin3, isoformE	NP_001097174.1	0	GLOS_FAS3.1.1	GO:0051169	B	.	nuclear transport
NP_001097174.1 fasciclin3, isoformD / ref|NP_001163005.1| fasciclin3, isoformE	NP_001097174.1	0	GLOS_FAS3.1.1	GO:0072594	B	.	establishment of protein localization to organelle
NP_001097174.1 fasciclin3, isoformD / ref|NP_001163005.1| fasciclin3, isoformE	NP_001097174.1	0	GLOS_FAS3.1.1	GO:0033365	B	.	protein localization to organelle
NP_001097174.1 fasciclin3, isoformD / ref|NP_001163005.1| fasciclin3, isoformE	NP_001097174.1	0	GLOS_FAS3.1.1	GO:0034504	B	.	protein localization to nucleus

NP_001097174.1 fasciclin3, isoformD / ref|NP_001163005.1| fasciclin3, isoformE	NP_001097174.1	0	GLOS_FAS3.1.1	GO:0044744	B	.	protein targeting to nucleus
NP_001097174.1 fasciclin3, isoformD / ref|NP_001163005.1| fasciclin3, isoformE	NP_001097174.1	0	GLOS_FAS3.1.1	GO:0006605	B	.	protein targeting
NP_001097174.1 fasciclin3, isoformD / ref|NP_001163005.1| fasciclin3, isoformE	NP_001097174.1	0	GLOS_FAS3.1.1	GO:0006607	B	Refseq	NLS-bearing protein import into nucleus
NP_001097174.1 fasciclin3, isoformD / ref|NP_001163005.1| fasciclin3, isoformE	NP_001097174.1	0	GLOS_FAS3.1.1	GO:0046914	M	.	transition metal ion binding
NP_001097174.1 fasciclin3, isoformD / ref|NP_001163005.1| fasciclin3, isoformE	NP_001097174.1	0	GLOS_FAS3.1.1	GO:0046872	M	.	metal ion binding
NP_001097174.1 fasciclin3, isoformD / ref|NP_001163005.1| fasciclin3, isoformE	NP_001097174.1	0	GLOS_FAS3.1.1	GO:0043169	M	.	cation binding
NP_001097174.1 fasciclin3, isoformD / ref|NP_001163005.1| fasciclin3, isoformE	NP_001097174.1	0	GLOS_FAS3.1.1	GO:0043167	M	.	ion binding
NP_001097174.1 fasciclin3, isoformD / ref|NP_001163005.1| fasciclin3, isoformE	NP_001097174.1	0	GLOS_FAS3.1.1	GO:0008270	M	Refseq	zinc ion binding
NP_001097174.1 fasciclin3, isoformD / ref|NP_001163005.1| fasciclin3, isoformE	NP_001097174.1	0	GLOS_FAS3.1.1	GO:0045595	B	.	regulation of cell differentiation
NP_001097174.1 fasciclin3, isoformD / ref|NP_001163005.1| fasciclin3, isoformE	NP_001097174.1	0	GLOS_FAS3.1.1	GO:0050793	B	.	regulation of developmental process
NP_001097174.1 fasciclin3, isoformD / ref|NP_001163005.1| fasciclin3, isoformE	NP_001097174.1	0	GLOS_FAS3.1.1	GO:0061035	B	.	regulation of cartilage development
NP_001097174.1 fasciclin3, isoformD / ref|NP_001163005.1| fasciclin3, isoformE	NP_001097174.1	0	GLOS_FAS3.1.1	GO:2000026	B	.	regulation of multicellular organismal development
NP_001097174.1 fasciclin3, isoformD / ref|NP_001163005.1| fasciclin3, isoformE	NP_001097174.1	0	GLOS_FAS3.1.1	GO:0051239	B	.	regulation of multicellular organismal process
NP_001097174.1 fasciclin3, isoformD / ref|NP_001163005.1| fasciclin3, isoformE	NP_001097174.1	0	GLOS_FAS3.1.1	GO:0032330	B	Refseq	regulation of chondrocyte differentiation
NP_001097174.1 fasciclin3, isoformD / ref|NP_001163005.1| fasciclin3, isoformE	NP_001097174.1	0	GLOS_FAS3.1.1	GO:0003677	M	.	DNA binding
NP_001097174.1 fasciclin3, isoformD / ref|NP_001163005.1| fasciclin3, isoformE	NP_001097174.1	0	GLOS_FAS3.1.1	GO:0003676	M	.	nucleic acid binding
NP_001097174.1 fasciclin3, isoformD / ref|NP_001163005.1| fasciclin3, isoformE	NP_001097174.1	0	GLOS_FAS3.1.1	GO:0097159	M	.	organic cyclic compound binding
NP_001097174.1 fasciclin3, isoformD / ref|NP_001163005.1| fasciclin3, isoformE	NP_001097174.1	0	GLOS_FAS3.1.1	GO:1901363	M	.	heterocyclic compound binding
NP_001097174.1 fasciclin3, isoformD / ref|NP_001163005.1| fasciclin3, isoformE	NP_001097174.1	0	GLOS_FAS3.1.1	GO:0043565	M	Refseq	sequence-specific DNA binding
NP_001246951.1 CG15186, isoform G [Drosophila melanogaster]	NP_001246951.1	0	GLOS_CG15186.1	GO:0000122	B	Refseq	negative regulation of transcription from RNA polymerase II promoter
NP_001246951.1 CG15186, isoform G [Drosophila melanogaster]	NP_001246951.1	0	GLOS_CG15186.1	GO:0001501	B	Refseq	skeletal system development
NP_001246951.1 CG15186, isoform G [Drosophila melanogaster]	NP_001246951.1	0	GLOS_CG15186.1	GO:0003700	M	Refseq	sequence-specific DNA binding transcription factor activity
NP_001246951.1 CG15186, isoform G [Drosophila melanogaster]	NP_001246951.1	0	GLOS_CG15186.1	GO:0005515	M	Refseq	protein binding
NP_001246951.1 CG15186, isoform G [Drosophila melanogaster]	NP_001246951.1	0	GLOS_CG15186.1	GO:0005634	C	Refseq	nucleus
NP_001246951.1 CG15186, isoform G [Drosophila melanogaster]	NP_001246951.1	0	GLOS_CG15186.1	GO:0006366	B	Refseq	transcription from RNA polymerase II promoter
NP_001246951.1 CG15186, isoform G [Drosophila melanogaster]	NP_001246951.1	0	GLOS_CG15186.1	GO:0006607	B	Refseq	NLS-bearing protein import into nucleus
NP_001246951.1 CG15186, isoform G [Drosophila melanogaster]	NP_001246951.1	0	GLOS_CG15186.1	GO:0008270	M	Refseq	zinc ion binding
NP_001246951.1 CG15186, isoform G [Drosophila melanogaster]	NP_001246951.1	0	GLOS_CG15186.1	GO:0032330	B	Refseq	regulation of chondrocyte differentiation
NP_001246951.1 CG15186, isoform G [Drosophila melanogaster]	NP_001246951.1	0	GLOS_CG15186.1	GO:0043565	M	Refseq	sequence-specific DNA binding
XP_001976430.1 GG22867 [D. erecta] ref|XP_002092642.1| GE14305 [D. yakuba]	XP_001976430.1	0	GLOS_DERE_GG	GO:0005216	M	.	ion channel activity
XP_001976430.1 GG22867 [D. erecta] ref|XP_002092642.1| GE14305 [D. yakuba]	XP_001976430.1	0	GLOS_DERE_GG	GO:0015075	M	.	ion transmembrane transporter activity
XP_001976430.1 GG22867 [D. erecta] ref|XP_002092642.1| GE14305 [D. yakuba]	XP_001976430.1	0	GLOS_DERE_GG	GO:0022891	M	.	substrate-specific transmembrane transporter activity
XP_001976430.1 GG22867 [D. erecta] ref|XP_002092642.1| GE14305 [D. yakuba]	XP_001976430.1	0	GLOS_DERE_GG	GO:0022857	M	.	transmembrane transporter activity
XP_001976430.1 GG22867 [D. erecta] ref|XP_002092642.1| GE14305 [D. yakuba]	XP_001976430.1	0	GLOS_DERE_GG	GO:0005215	M	.	transporter activity
XP_001976430.1 GG22867 [D. erecta] ref|XP_002092642.1| GE14305 [D. yakuba]	XP_001976430.1	0	GLOS_DERE_GG	GO:0022892	M	.	substrate-specific transporter activity
XP_001976430.1 GG22867 [D. erecta] ref|XP_002092642.1| GE14305 [D. yakuba]	XP_001976430.1	0	GLOS_DERE_GG	GO:0022838	M	.	substrate-specific channel activity
XP_001976430.1 GG22867 [D. erecta] ref|XP_002092642.1| GE14305 [D. yakuba]	XP_001976430.1	0	GLOS_DERE_GG	GO:0015267	M	.	channel activity
XP_001976430.1 GG22867 [D. erecta] ref|XP_002092642.1| GE14305 [D. yakuba]	XP_001976430.1	0	GLOS_DERE_GG	GO:0022803	M	.	passive transmembrane transporter activity
XP_001976430.1 GG22867 [D. erecta] ref|XP_002092642.1| GE14305 [D. yakuba]	XP_001976430.1	0	GLOS_DERE_GG	GO:0008324	M	.	cation transmembrane transporter activity
XP_001976430.1 GG22867 [D. erecta] ref|XP_002092642.1| GE14305 [D. yakuba]	XP_001976430.1	0	GLOS_DERE_GG	GO:0005261	M	Refseq	cation channel activity
XP_001976430.1 GG22867 [D. erecta] ref|XP_002092642.1| GE14305 [D. yakuba]	XP_001976430.1	0	GLOS_DERE_GG	GO:0005261	M	.	cation channel activity
XP_001976430.1 GG22867 [D. erecta] ref|XP_002092642.1| GE14305 [D. yakuba]	XP_001976430.1	0	GLOS_DERE_GG	GO:0015085	M	.	calcium ion transmembrane transporter activity
XP_001976430.1 GG22867 [D. erecta] ref|XP_002092642.1| GE14305 [D. yakuba]	XP_001976430.1	0	GLOS_DERE_GG	GO:0046873	M	.	metal ion transmembrane transporter activity
XP_001976430.1 GG22867 [D. erecta] ref|XP_002092642.1| GE14305 [D. yakuba]	XP_001976430.1	0	GLOS_DERE_GG	GO:0022890	M	.	inorganic cation transmembrane transporter activity
XP_001976430.1 GG22867 [D. erecta] ref|XP_002092642.1| GE14305 [D. yakuba]	XP_001976430.1	0	GLOS_DERE_GG	GO:0072509	M	.	divalent inorganic cation transmembrane transporter activity
XP_001976430.1 GG22867 [D. erecta] ref|XP_002092642.1| GE14305 [D. yakuba]	XP_001976430.1	0	GLOS_DERE_GG	GO:0005262	M	Refseq	calcium channel activity
XP_001976430.1 GG22867 [D. erecta] ref|XP_002092642.1| GE14305 [D. yakuba]	XP_001976430.1	0	GLOS_DERE_GG	GO:0005515	M	Refseq	protein binding
XP_001976430.1 GG22867 [D. erecta] ref|XP_002092642.1| GE14305 [D. yakuba]	XP_001976430.1	0	GLOS_DERE_GG	GO:0016020	C	.	membrane
XP_001976430.1 GG22867 [D. erecta] ref|XP_002092642.1| GE14305 [D. yakuba]	XP_001976430.1	0	GLOS_DERE_GG	GO:0071944	C	.	cell periphery
XP_001976430.1 GG22867 [D. erecta] ref|XP_002092642.1| GE14305 [D. yakuba]	XP_001976430.1	0	GLOS_DERE_GG	GO:0005886	C	Refseq	plasma membrane
XP_001976430.1 GG22867 [D. erecta] ref|XP_002092642.1| GE14305 [D. yakuba]	XP_001976430.1	0	GLOS_DERE_GG	GO:0016021	C	.	integral to membrane
XP_001976430.1 GG22867 [D. erecta] ref|XP_002092642.1| GE14305 [D. yakuba]	XP_001976430.1	0	GLOS_DERE_GG	GO:0031224	C	.	intrinsic to membrane
XP_001976430.1 GG22867 [D. erecta] ref|XP_002092642.1| GE14305 [D. yakuba]	XP_001976430.1	0	GLOS_DERE_GG	GO:0044425	C	.	membrane part
XP_001976430.1 GG22867 [D. erecta] ref|XP_002092642.1| GE14305 [D. yakuba]	XP_001976430.1	0	GLOS_DERE_GG	GO:0031226	C	.	intrinsic to plasma membrane

XP_001976430.1 GG22867 [D. erecta] ref|XP_002092642.1| GE14305 [D. yakuba]	XP_001976430.1	0	GLOS_DERE_GG GO:0044459	C	.	plasma membrane part
XP_001976430.1 GG22867 [D. erecta] ref|XP_002092642.1| GE14305 [D. yakuba]	XP_001976430.1	0	GLOS_DERE_GG GO:0005886	C	.	plasma membrane
XP_001976430.1 GG22867 [D. erecta] ref|XP_002092642.1| GE14305 [D. yakuba]	XP_001976430.1	0	GLOS_DERE_GG GO:0005887	C	Refseq	integral to plasma membrane
XP_001976430.1 GG22867 [D. erecta] ref|XP_002092642.1| GE14305 [D. yakuba]	XP_001976430.1	0	GLOS_DERE_GG GO:0070838	B	.	divalent metal ion transport
XP_001976430.1 GG22867 [D. erecta] ref|XP_002092642.1| GE14305 [D. yakuba]	XP_001976430.1	0	GLOS_DERE_GG GO:0030001	B	.	metal ion transport
XP_001976430.1 GG22867 [D. erecta] ref|XP_002092642.1| GE14305 [D. yakuba]	XP_001976430.1	0	GLOS_DERE_GG GO:0006812	B	.	cation transport
XP_001976430.1 GG22867 [D. erecta] ref|XP_002092642.1| GE14305 [D. yakuba]	XP_001976430.1	0	GLOS_DERE_GG GO:0006811	B	.	ion transport
XP_001976430.1 GG22867 [D. erecta] ref|XP_002092642.1| GE14305 [D. yakuba]	XP_001976430.1	0	GLOS_DERE_GG GO:0072511	B	.	divalent inorganic cation transport
XP_001976430.1 GG22867 [D. erecta] ref|XP_002092642.1| GE14305 [D. yakuba]	XP_001976430.1	0	GLOS_DERE_GG GO:0006816	B	Refseq	calcium ion transport
XP_001976430.1 GG22867 [D. erecta] ref|XP_002092642.1| GE14305 [D. yakuba]	XP_001976430.1	0	GLOS_DERE_GG GO:0097485	B	.	neuron projection guidance
XP_001976430.1 GG22867 [D. erecta] ref|XP_002092642.1| GE14305 [D. yakuba]	XP_001976430.1	0	GLOS_DERE_GG GO:0006928	B	.	cellular component movement
XP_001976430.1 GG22867 [D. erecta] ref|XP_002092642.1| GE14305 [D. yakuba]	XP_001976430.1	0	GLOS_DERE_GG GO:0006935	B	.	chemotaxis
XP_001976430.1 GG22867 [D. erecta] ref|XP_002092642.1| GE14305 [D. yakuba]	XP_001976430.1	0	GLOS_DERE_GG GO:0042221	B	.	response to chemical stimulus
XP_001976430.1 GG22867 [D. erecta] ref|XP_002092642.1| GE14305 [D. yakuba]	XP_001976430.1	0	GLOS_DERE_GG GO:0050896	B	.	response to stimulus
XP_001976430.1 GG22867 [D. erecta] ref|XP_002092642.1| GE14305 [D. yakuba]	XP_001976430.1	0	GLOS_DERE_GG GO:0042330	B	.	taxis
XP_001976430.1 GG22867 [D. erecta] ref|XP_002092642.1| GE14305 [D. yakuba]	XP_001976430.1	0	GLOS_DERE_GG GO:0009605	B	.	response to external stimulus
XP_001976430.1 GG22867 [D. erecta] ref|XP_002092642.1| GE14305 [D. yakuba]	XP_001976430.1	0	GLOS_DERE_GG GO:0040011	B	.	locomotion
XP_001976430.1 GG22867 [D. erecta] ref|XP_002092642.1| GE14305 [D. yakuba]	XP_001976430.1	0	GLOS_DERE_GG GO:0048812	B	.	neuron projection morphogenesis
XP_001976430.1 GG22867 [D. erecta] ref|XP_002092642.1| GE14305 [D. yakuba]	XP_001976430.1	0	GLOS_DERE_GG GO:0048858	B	.	cell projection morphogenesis
XP_001976430.1 GG22867 [D. erecta] ref|XP_002092642.1| GE14305 [D. yakuba]	XP_001976430.1	0	GLOS_DERE_GG GO:0030030	B	.	cell projection organization
XP_001976430.1 GG22867 [D. erecta] ref|XP_002092642.1| GE14305 [D. yakuba]	XP_001976430.1	0	GLOS_DERE_GG GO:0016043	B	.	cellular component organization
XP_001976430.1 GG22867 [D. erecta] ref|XP_002092642.1| GE14305 [D. yakuba]	XP_001976430.1	0	GLOS_DERE_GG GO:0071840	B	.	cellular component organization or biogenesis
XP_001976430.1 GG22867 [D. erecta] ref|XP_002092642.1| GE14305 [D. yakuba]	XP_001976430.1	0	GLOS_DERE_GG GO:0032990	B	.	cell part morphogenesis
XP_001976430.1 GG22867 [D. erecta] ref|XP_002092642.1| GE14305 [D. yakuba]	XP_001976430.1	0	GLOS_DERE_GG GO:0032989	B	.	cellular component morphogenesis
XP_001976430.1 GG22867 [D. erecta] ref|XP_002092642.1| GE14305 [D. yakuba]	XP_001976430.1	0	GLOS_DERE_GG GO:0009653	B	.	anatomical structure morphogenesis
XP_001976430.1 GG22867 [D. erecta] ref|XP_002092642.1| GE14305 [D. yakuba]	XP_001976430.1	0	GLOS_DERE_GG GO:0044767	B	.	single-organism developmental process
XP_001976430.1 GG22867 [D. erecta] ref|XP_002092642.1| GE14305 [D. yakuba]	XP_001976430.1	0	GLOS_DERE_GG GO:0048869	B	.	cellular developmental process
XP_001976430.1 GG22867 [D. erecta] ref|XP_002092642.1| GE14305 [D. yakuba]	XP_001976430.1	0	GLOS_DERE_GG GO:0000902	B	.	cell morphogenesis
XP_001976430.1 GG22867 [D. erecta] ref|XP_002092642.1| GE14305 [D. yakuba]	XP_001976430.1	0	GLOS_DERE_GG GO:0031175	B	.	neuron projection development
XP_001976430.1 GG22867 [D. erecta] ref|XP_002092642.1| GE14305 [D. yakuba]	XP_001976430.1	0	GLOS_DERE_GG GO:0048666	B	.	neuron development
XP_001976430.1 GG22867 [D. erecta] ref|XP_002092642.1| GE14305 [D. yakuba]	XP_001976430.1	0	GLOS_DERE_GG GO:0048468	B	.	cell development
XP_001976430.1 GG22867 [D. erecta] ref|XP_002092642.1| GE14305 [D. yakuba]	XP_001976430.1	0	GLOS_DERE_GG GO:0030154	B	.	cell differentiation
XP_001976430.1 GG22867 [D. erecta] ref|XP_002092642.1| GE14305 [D. yakuba]	XP_001976430.1	0	GLOS_DERE_GG GO:0030182	B	.	neuron differentiation
XP_001976430.1 GG22867 [D. erecta] ref|XP_002092642.1| GE14305 [D. yakuba]	XP_001976430.1	0	GLOS_DERE_GG GO:0048699	B	.	generation of neurons
XP_001976430.1 GG22867 [D. erecta] ref|XP_002092642.1| GE14305 [D. yakuba]	XP_001976430.1	0	GLOS_DERE_GG GO:0022008	B	.	neurogenesis
XP_001976430.1 GG22867 [D. erecta] ref|XP_002092642.1| GE14305 [D. yakuba]	XP_001976430.1	0	GLOS_DERE_GG GO:0007399	B	.	nervous system development
XP_001976430.1 GG22867 [D. erecta] ref|XP_002092642.1| GE14305 [D. yakuba]	XP_001976430.1	0	GLOS_DERE_GG GO:0007409	B	.	axonogenesis
XP_001976430.1 GG22867 [D. erecta] ref|XP_002092642.1| GE14305 [D. yakuba]	XP_001976430.1	0	GLOS_DERE_GG GO:0048667	B	.	cell morphogenesis involved in neuron differentiation
XP_001976430.1 GG22867 [D. erecta] ref|XP_002092642.1| GE14305 [D. yakuba]	XP_001976430.1	0	GLOS_DERE_GG GO:0000904	B	.	cell morphogenesis involved in differentiation
XP_001976430.1 GG22867 [D. erecta] ref|XP_002092642.1| GE14305 [D. yakuba]	XP_001976430.1	0	GLOS_DERE_GG GO:0061564	B	.	axon development
XP_001976430.1 GG22867 [D. erecta] ref|XP_002092642.1| GE14305 [D. yakuba]	XP_001976430.1	0	GLOS_DERE_GG GO:0007411	B	Refseq	axon guidance
XP_001976430.1 GG22867 [D. erecta] ref|XP_002092642.1| GE14305 [D. yakuba]	XP_001976430.1	0	GLOS_DERE_GG GO:0005262	M	.	calcium channel activity
XP_001976430.1 GG22867 [D. erecta] ref|XP_002092642.1| GE14305 [D. yakuba]	XP_001976430.1	0	GLOS_DERE_GG GO:0015276	M	.	ligand-gated ion channel activity
XP_001976430.1 GG22867 [D. erecta] ref|XP_002092642.1| GE14305 [D. yakuba]	XP_001976430.1	0	GLOS_DERE_GG GO:0022834	M	.	ligand-gated channel activity
XP_001976430.1 GG22867 [D. erecta] ref|XP_002092642.1| GE14305 [D. yakuba]	XP_001976430.1	0	GLOS_DERE_GG GO:0022836	M	.	gated channel activity
XP_001976430.1 GG22867 [D. erecta] ref|XP_002092642.1| GE14305 [D. yakuba]	XP_001976430.1	0	GLOS_DERE_GG GO:0022839	M	.	ion gated channel activity
XP_001976430.1 GG22867 [D. erecta] ref|XP_002092642.1| GE14305 [D. yakuba]	XP_001976430.1	0	GLOS_DERE_GG GO:0015279	M	Refseq	store-operated calcium channel activity
XP_001976430.1 GG22867 [D. erecta] ref|XP_002092642.1| GE14305 [D. yakuba]	XP_001976430.1	0	GLOS_DERE_GG GO:0016323	C	Refseq	basolateral plasma membrane
XP_001976430.1 GG22867 [D. erecta] ref|XP_002092642.1| GE14305 [D. yakuba]	XP_001976430.1	0	GLOS_DERE_GG GO:0044449	C	.	contractile fiber part
XP_001976430.1 GG22867 [D. erecta] ref|XP_002092642.1| GE14305 [D. yakuba]	XP_001976430.1	0	GLOS_DERE_GG GO:0044422	C	.	organelle part
XP_001976430.1 GG22867 [D. erecta] ref|XP_002092642.1| GE14305 [D. yakuba]	XP_001976430.1	0	GLOS_DERE_GG GO:0044444	C	.	cytoplasmic part
XP_001976430.1 GG22867 [D. erecta] ref|XP_002092642.1| GE14305 [D. yakuba]	XP_001976430.1	0	GLOS_DERE_GG GO:0005737	C	.	cytoplasm
XP_001976430.1 GG22867 [D. erecta] ref|XP_002092642.1| GE14305 [D. yakuba]	XP_001976430.1	0	GLOS_DERE_GG GO:0043292	C	.	contractile fiber
XP_001976430.1 GG22867 [D. erecta] ref|XP_002092642.1| GE14305 [D. yakuba]	XP_001976430.1	0	GLOS_DERE_GG GO:0043232	C	.	intracellular non-membrane-bounded organelle

XP_001976430.1 GG22867 [D. erecta] ref|XP_002092642.1| GE14305 [D. yakuba]	XP_001976430.1	0	GLOS_DERE_GG	GO:0043228	C	.	non-membrane-bounded organelle
XP_001976430.1 GG22867 [D. erecta] ref|XP_002092642.1| GE14305 [D. yakuba]	XP_001976430.1	0	GLOS_DERE_GG	GO:0030016	C	.	myofibril
XP_001976430.1 GG22867 [D. erecta] ref|XP_002092642.1| GE14305 [D. yakuba]	XP_001976430.1	0	GLOS_DERE_GG	GO:0030017	C	Refseq	sarcomere
XP_001976430.1 GG22867 [D. erecta] ref|XP_002092642.1| GE14305 [D. yakuba]	XP_001976430.1	0	GLOS_DERE_GG	GO:0043034	C	Refseq	costamere
XP_001976430.1 GG22867 [D. erecta] ref|XP_002092642.1| GE14305 [D. yakuba]	XP_001976430.1	0	GLOS_DERE_GG	GO:0032991	C	.	macromolecular complex
XP_001976430.1 GG22867 [D. erecta] ref|XP_002092642.1| GE14305 [D. yakuba]	XP_001976430.1	0	GLOS_DERE_GG	GO:0043234	C	Refseq	protein complex
XP_001976430.1 GG22867 [D. erecta] ref|XP_002092642.1| GE14305 [D. yakuba]	XP_001976430.1	0	GLOS_DERE_GG	GO:0005515	M	.	protein binding
XP_001976430.1 GG22867 [D. erecta] ref|XP_002092642.1| GE14305 [D. yakuba]	XP_001976430.1	0	GLOS_DERE_GG	GO:0044325	M	Refseq	ion channel binding
XP_001976430.1 GG22867 [D. erecta] ref|XP_002092642.1| GE14305 [D. yakuba]	XP_001976430.1	0	GLOS_DERE_GG	GO:0045121	C	Refseq	membrane raft
XP_001976430.1 GG22867 [D. erecta] ref|XP_002092642.1| GE14305 [D. yakuba]	XP_001976430.1	0	GLOS_DERE_GG	GO:0007589	B	.	body fluid secretion
XP_001976430.1 GG22867 [D. erecta] ref|XP_002092642.1| GE14305 [D. yakuba]	XP_001976430.1	0	GLOS_DERE_GG	GO:0046903	B	.	secretion
XP_001976430.1 GG22867 [D. erecta] ref|XP_002092642.1| GE14305 [D. yakuba]	XP_001976430.1	0	GLOS_DERE_GG	GO:0050878	B	.	regulation of body fluid levels
XP_001976430.1 GG22867 [D. erecta] ref|XP_002092642.1| GE14305 [D. yakuba]	XP_001976430.1	0	GLOS_DERE_GG	GO:0065008	B	.	regulation of biological quality
XP_001976430.1 GG22867 [D. erecta] ref|XP_002092642.1| GE14305 [D. yakuba]	XP_001976430.1	0	GLOS_DERE_GG	GO:0022600	B	.	digestive system process
XP_001976430.1 GG22867 [D. erecta] ref|XP_002092642.1| GE14305 [D. yakuba]	XP_001976430.1	0	GLOS_DERE_GG	GO:0003008	B	.	system process
XP_001976430.1 GG22867 [D. erecta] ref|XP_002092642.1| GE14305 [D. yakuba]	XP_001976430.1	0	GLOS_DERE_GG	GO:0007586	B	.	digestion
XP_001976430.1 GG22867 [D. erecta] ref|XP_002092642.1| GE14305 [D. yakuba]	XP_001976430.1	0	GLOS_DERE_GG	GO:0032941	B	.	secretion by tissue
XP_001976430.1 GG22867 [D. erecta] ref|XP_002092642.1| GE14305 [D. yakuba]	XP_001976430.1	0	GLOS_DERE_GG	GO:0046541	B	Refseq	saliva secretion
XP_001976430.1 GG22867 [D. erecta] ref|XP_002092642.1| GE14305 [D. yakuba]	XP_001976430.1	0	GLOS_DERE_GG	GO:0010524	B	.	positive regulation of calcium ion transport into cytosol
XP_001976430.1 GG22867 [D. erecta] ref|XP_002092642.1| GE14305 [D. yakuba]	XP_001976430.1	0	GLOS_DERE_GG	GO:0010522	B	.	regulation of calcium ion transport into cytosol
XP_001976430.1 GG22867 [D. erecta] ref|XP_002092642.1| GE14305 [D. yakuba]	XP_001976430.1	0	GLOS_DERE_GG	GO:0032386	B	.	regulation of intracellular transport
XP_001976430.1 GG22867 [D. erecta] ref|XP_002092642.1| GE14305 [D. yakuba]	XP_001976430.1	0	GLOS_DERE_GG	GO:0051049	B	.	regulation of transport
XP_001976430.1 GG22867 [D. erecta] ref|XP_002092642.1| GE14305 [D. yakuba]	XP_001976430.1	0	GLOS_DERE_GG	GO:0032879	B	.	regulation of localization
XP_001976430.1 GG22867 [D. erecta] ref|XP_002092642.1| GE14305 [D. yakuba]	XP_001976430.1	0	GLOS_DERE_GG	GO:0060341	B	.	regulation of cellular localization
XP_001976430.1 GG22867 [D. erecta] ref|XP_002092642.1| GE14305 [D. yakuba]	XP_001976430.1	0	GLOS_DERE_GG	GO:0051924	B	.	regulation of calcium ion transport
XP_001976430.1 GG22867 [D. erecta] ref|XP_002092642.1| GE14305 [D. yakuba]	XP_001976430.1	0	GLOS_DERE_GG	GO:0010959	B	.	regulation of metal ion transport
XP_001976430.1 GG22867 [D. erecta] ref|XP_002092642.1| GE14305 [D. yakuba]	XP_001976430.1	0	GLOS_DERE_GG	GO:0043269	B	.	regulation of ion transport
XP_001976430.1 GG22867 [D. erecta] ref|XP_002092642.1| GE14305 [D. yakuba]	XP_001976430.1	0	GLOS_DERE_GG	GO:2000021	B	.	regulation of ion homeostasis
XP_001976430.1 GG22867 [D. erecta] ref|XP_002092642.1| GE14305 [D. yakuba]	XP_001976430.1	0	GLOS_DERE_GG	GO:0032844	B	.	regulation of homeostatic process
XP_001976430.1 GG22867 [D. erecta] ref|XP_002092642.1| GE14305 [D. yakuba]	XP_001976430.1	0	GLOS_DERE_GG	GO:0032388	B	.	positive regulation of intracellular transport
XP_001976430.1 GG22867 [D. erecta] ref|XP_002092642.1| GE14305 [D. yakuba]	XP_001976430.1	0	GLOS_DERE_GG	GO:0051050	B	.	positive regulation of transport
XP_001976430.1 GG22867 [D. erecta] ref|XP_002092642.1| GE14305 [D. yakuba]	XP_001976430.1	0	GLOS_DERE_GG	GO:0048518	B	.	positive regulation of biological process
XP_001976430.1 GG22867 [D. erecta] ref|XP_002092642.1| GE14305 [D. yakuba]	XP_001976430.1	0	GLOS_DERE_GG	GO:0032846	B	.	positive regulation of homeostatic process
XP_001976430.1 GG22867 [D. erecta] ref|XP_002092642.1| GE14305 [D. yakuba]	XP_001976430.1	0	GLOS_DERE_GG	GO:0048522	B	.	positive regulation of cellular process
XP_001976430.1 GG22867 [D. erecta] ref|XP_002092642.1| GE14305 [D. yakuba]	XP_001976430.1	0	GLOS_DERE_GG	GO:0051928	B	.	positive regulation of calcium ion transport
XP_001976430.1 GG22867 [D. erecta] ref|XP_002092642.1| GE14305 [D. yakuba]	XP_001976430.1	0	GLOS_DERE_GG	GO:0043270	B	.	positive regulation of ion transport
XP_001976430.1 GG22867 [D. erecta] ref|XP_002092642.1| GE14305 [D. yakuba]	XP_001976430.1	0	GLOS_DERE_GG	GO:0051279	B	.	regulation of release of sequestered calcium ion into cytosol
XP_001976430.1 GG22867 [D. erecta] ref|XP_002092642.1| GE14305 [D. yakuba]	XP_001976430.1	0	GLOS_DERE_GG	GO:0051281	B	Refseq	positive regulation of release of sequestered calcium ion into cytosol
XP_001976430.1 GG22867 [D. erecta] ref|XP_002092642.1| GE14305 [D. yakuba]	XP_001976430.1	0	GLOS_DERE_GG	GO:0006874	B	.	cellular calcium ion homeostasis
XP_001976430.1 GG22867 [D. erecta] ref|XP_002092642.1| GE14305 [D. yakuba]	XP_001976430.1	0	GLOS_DERE_GG	GO:0006875	B	.	cellular metal ion homeostasis
XP_001976430.1 GG22867 [D. erecta] ref|XP_002092642.1| GE14305 [D. yakuba]	XP_001976430.1	0	GLOS_DERE_GG	GO:0030003	B	.	cellular cation homeostasis
XP_001976430.1 GG22867 [D. erecta] ref|XP_002092642.1| GE14305 [D. yakuba]	XP_001976430.1	0	GLOS_DERE_GG	GO:0006873	B	.	cellular ion homeostasis
XP_001976430.1 GG22867 [D. erecta] ref|XP_002092642.1| GE14305 [D. yakuba]	XP_001976430.1	0	GLOS_DERE_GG	GO:0050801	B	.	ion homeostasis
XP_001976430.1 GG22867 [D. erecta] ref|XP_002092642.1| GE14305 [D. yakuba]	XP_001976430.1	0	GLOS_DERE_GG	GO:0048878	B	.	chemical homeostasis
XP_001976430.1 GG22867 [D. erecta] ref|XP_002092642.1| GE14305 [D. yakuba]	XP_001976430.1	0	GLOS_DERE_GG	GO:0042592	B	.	homeostatic process
XP_001976430.1 GG22867 [D. erecta] ref|XP_002092642.1| GE14305 [D. yakuba]	XP_001976430.1	0	GLOS_DERE_GG	GO:0055082	B	.	cellular chemical homeostasis
XP_001976430.1 GG22867 [D. erecta] ref|XP_002092642.1| GE14305 [D. yakuba]	XP_001976430.1	0	GLOS_DERE_GG	GO:0019725	B	.	cellular homeostasis
XP_001976430.1 GG22867 [D. erecta] ref|XP_002092642.1| GE14305 [D. yakuba]	XP_001976430.1	0	GLOS_DERE_GG	GO:0055080	B	.	cation homeostasis
XP_001976430.1 GG22867 [D. erecta] ref|XP_002092642.1| GE14305 [D. yakuba]	XP_001976430.1	0	GLOS_DERE_GG	GO:0055065	B	.	metal ion homeostasis
XP_001976430.1 GG22867 [D. erecta] ref|XP_002092642.1| GE14305 [D. yakuba]	XP_001976430.1	0	GLOS_DERE_GG	GO:0055074	B	.	calcium ion homeostasis
XP_001976430.1 GG22867 [D. erecta] ref|XP_002092642.1| GE14305 [D. yakuba]	XP_001976430.1	0	GLOS_DERE_GG	GO:0072507	B	.	divalent inorganic cation homeostasis
XP_001976430.1 GG22867 [D. erecta] ref|XP_002092642.1| GE14305 [D. yakuba]	XP_001976430.1	0	GLOS_DERE_GG	GO:0072503	B	.	cellular divalent inorganic cation homeostasis
XP_001976430.1 GG22867 [D. erecta] ref|XP_002092642.1| GE14305 [D. yakuba]	XP_001976430.1	0	GLOS_DERE_GG	GO:0051480	B	Refseq	cytosolic calcium ion homeostasis
XP_001976430.1 GG22867 [D. erecta] ref|XP_002092642.1| GE14305 [D. yakuba]	XP_001976430.1	0	GLOS_DERE_GG	GO:0010038	B	.	response to metal ion

XP_001976430.1 GG22867 [D. erecta] ref|XP_002092642.1| GE14305 [D. yakuba]	XP_001976430.1	0	GLOS_DERE_GG	GO:0010035	B	.	response to inorganic substance
XP_001976430.1 GG22867 [D. erecta] ref|XP_002092642.1| GE14305 [D. yakuba]	XP_001976430.1	0	GLOS_DERE_GG	GO:0051592	B	Refseq	response to calcium ion
XP_001976430.1 GG22867 [D. erecta] ref|XP_002092642.1| GE14305 [D. yakuba]	XP_001976430.1	0	GLOS_DERE_GG	GO:0043168	M	.	anion binding
XP_001976430.1 GG22867 [D. erecta] ref|XP_002092642.1| GE14305 [D. yakuba]	XP_001976430.1	0	GLOS_DERE_GG	GO:0043178	M	.	alcohol binding
XP_001976430.1 GG22867 [D. erecta] ref|XP_002092642.1| GE14305 [D. yakuba]	XP_001976430.1	0	GLOS_DERE_GG	GO:0036094	M	.	small molecule binding
XP_001976430.1 GG22867 [D. erecta] ref|XP_002092642.1| GE14305 [D. yakuba]	XP_001976430.1	0	GLOS_DERE_GG	GO:0070679	M	Refseq	inositol 1,4,5 trisphosphate binding
XP_003243964.1 PREDICTED: hypothetical prot. LOC100568954 [A. pisum]	XP_003243964.1	1,00E-15	GLOS_LOC10056	GO:0007049	B	.	cell cycle
XP_003243964.1 PREDICTED: hypothetical prot. LOC100568954 [A. pisum]	XP_003243964.1	1,00E-15	GLOS_LOC10056	GO:0000278	B	Refseq	mitotic cell cycle
XP_003243964.1 PREDICTED: hypothetical prot. LOC100568954 [A. pisum]	XP_003243964.1	1,00E-15	GLOS_LOC10056	GO:0003677	M	Refseq	DNA binding
XP_003243964.1 PREDICTED: hypothetical prot. LOC100568954 [A. pisum]	XP_003243964.1	1,00E-15	GLOS_LOC10056	GO:0003700	M	Refseq	sequence-specific DNA binding transcription factor activity
XP_003243964.1 PREDICTED: hypothetical prot. LOC100568954 [A. pisum]	XP_003243964.1	1,00E-15	GLOS_LOC10056	GO:0000989	M	.	transcription factor binding transcription factor activity
XP_003243964.1 PREDICTED: hypothetical prot. LOC100568954 [A. pisum]	XP_003243964.1	1,00E-15	GLOS_LOC10056	GO:0000988	M	.	protein binding transcription factor activity
XP_003243964.1 PREDICTED: hypothetical prot. LOC100568954 [A. pisum]	XP_003243964.1	1,00E-15	GLOS_LOC10056	GO:0003712	M	Refseq	transcription cofactor activity
XP_003243964.1 PREDICTED: hypothetical prot. LOC100568954 [A. pisum]	XP_003243964.1	1,00E-15	GLOS_LOC10056	GO:0044428	C	.	nuclear part
XP_003243964.1 PREDICTED: hypothetical prot. LOC100568954 [A. pisum]	XP_003243964.1	1,00E-15	GLOS_LOC10056	GO:0044446	C	.	intracellular organelle part
XP_003243964.1 PREDICTED: hypothetical prot. LOC100568954 [A. pisum]	XP_003243964.1	1,00E-15	GLOS_LOC10056	GO:0005634	C	.	nucleus
XP_003243964.1 PREDICTED: hypothetical prot. LOC100568954 [A. pisum]	XP_003243964.1	1,00E-15	GLOS_LOC10056	GO:0031981	C	.	nuclear lumen
XP_003243964.1 PREDICTED: hypothetical prot. LOC100568954 [A. pisum]	XP_003243964.1	1,00E-15	GLOS_LOC10056	GO:0070013	C	.	intracellular organelle lumen
XP_003243964.1 PREDICTED: hypothetical prot. LOC100568954 [A. pisum]	XP_003243964.1	1,00E-15	GLOS_LOC10056	GO:0043233	C	.	organelle lumen
XP_003243964.1 PREDICTED: hypothetical prot. LOC100568954 [A. pisum]	XP_003243964.1	1,00E-15	GLOS_LOC10056	GO:0031974	C	.	membrane-enclosed lumen
XP_003243964.1 PREDICTED: hypothetical prot. LOC100568954 [A. pisum]	XP_003243964.1	1,00E-15	GLOS_LOC10056	GO:0005654	C	Refseq	nucleoplasm
XP_003243964.1 PREDICTED: hypothetical prot. LOC100568954 [A. pisum]	XP_003243964.1	1,00E-15	GLOS_LOC10056	GO:0043234	C	.	protein complex
XP_003243964.1 PREDICTED: hypothetical prot. LOC100568954 [A. pisum]	XP_003243964.1	1,00E-15	GLOS_LOC10056	GO:0005667	C	Refseq	transcription factor complex
XP_003243964.1 PREDICTED: hypothetical prot. LOC100568954 [A. pisum]	XP_003243964.1	1,00E-15	GLOS_LOC10056	GO:0006351	B	Refseq	transcription, DNA-dependent
XP_003243964.1 PREDICTED: hypothetical prot. LOC100568954 [A. pisum]	XP_003243964.1	1,00E-15	GLOS_LOC10056	GO:0006352	B	.	DNA-dependent transcription, initiation
XP_003243964.1 PREDICTED: hypothetical prot. LOC100568954 [A. pisum]	XP_003243964.1	1,00E-15	GLOS_LOC10056	GO:0006366	B	.	transcription from RNA polymerase II promoter
XP_003243964.1 PREDICTED: hypothetical prot. LOC100568954 [A. pisum]	XP_003243964.1	1,00E-15	GLOS_LOC10056	GO:0006367	B	Refseq	transcription initiation from RNA polymerase II promoter
XP_003243964.1 PREDICTED: hypothetical prot. LOC100568954 [A. pisum]	XP_003243964.1	1,00E-15	GLOS_LOC10056	GO:0007178	B	.	transmembrane receptor protein serine/threonine kinase signaling pathway
XP_003243964.1 PREDICTED: hypothetical prot. LOC100568954 [A. pisum]	XP_003243964.1	1,00E-15	GLOS_LOC10056	GO:0007167	B	.	enzyme linked receptor protein signaling pathway
XP_003243964.1 PREDICTED: hypothetical prot. LOC100568954 [A. pisum]	XP_003243964.1	1,00E-15	GLOS_LOC10056	GO:0007166	B	.	cell surface receptor signaling pathway
XP_003243964.1 PREDICTED: hypothetical prot. LOC100568954 [A. pisum]	XP_003243964.1	1,00E-15	GLOS_LOC10056	GO:0007165	B	.	signal transduction
XP_003243964.1 PREDICTED: hypothetical prot. LOC100568954 [A. pisum]	XP_003243964.1	1,00E-15	GLOS_LOC10056	GO:0051716	B	.	cellular response to stimulus
XP_003243964.1 PREDICTED: hypothetical prot. LOC100568954 [A. pisum]	XP_003243964.1	1,00E-15	GLOS_LOC10056	GO:0007154	B	.	cell communication
XP_003243964.1 PREDICTED: hypothetical prot. LOC100568954 [A. pisum]	XP_003243964.1	1,00E-15	GLOS_LOC10056	GO:0044700	B	.	single organism signaling
XP_003243964.1 PREDICTED: hypothetical prot. LOC100568954 [A. pisum]	XP_003243964.1	1,00E-15	GLOS_LOC10056	GO:0023052	B	.	signaling
XP_003243964.1 PREDICTED: hypothetical prot. LOC100568954 [A. pisum]	XP_003243964.1	1,00E-15	GLOS_LOC10056	GO:0071560	B	.	cellular response to transforming growth factor beta stimulus
XP_003243964.1 PREDICTED: hypothetical prot. LOC100568954 [A. pisum]	XP_003243964.1	1,00E-15	GLOS_LOC10056	GO:0071363	B	.	cellular response to growth factor stimulus
XP_003243964.1 PREDICTED: hypothetical prot. LOC100568954 [A. pisum]	XP_003243964.1	1,00E-15	GLOS_LOC10056	GO:0070848	B	.	response to growth factor stimulus
XP_003243964.1 PREDICTED: hypothetical prot. LOC100568954 [A. pisum]	XP_003243964.1	1,00E-15	GLOS_LOC10056	GO:0010033	B	.	response to organic substance
XP_003243964.1 PREDICTED: hypothetical prot. LOC100568954 [A. pisum]	XP_003243964.1	1,00E-15	GLOS_LOC10056	GO:0071310	B	.	cellular response to organic substance
XP_003243964.1 PREDICTED: hypothetical prot. LOC100568954 [A. pisum]	XP_003243964.1	1,00E-15	GLOS_LOC10056	GO:0070887	B	.	cellular response to chemical stimulus
XP_003243964.1 PREDICTED: hypothetical prot. LOC100568954 [A. pisum]	XP_003243964.1	1,00E-15	GLOS_LOC10056	GO:0071495	B	.	cellular response to endogenous stimulus
XP_003243964.1 PREDICTED: hypothetical prot. LOC100568954 [A. pisum]	XP_003243964.1	1,00E-15	GLOS_LOC10056	GO:0009719	B	.	response to endogenous stimulus
XP_003243964.1 PREDICTED: hypothetical prot. LOC100568954 [A. pisum]	XP_003243964.1	1,00E-15	GLOS_LOC10056	GO:0071559	B	.	response to transforming growth factor beta stimulus
XP_003243964.1 PREDICTED: hypothetical prot. LOC100568954 [A. pisum]	XP_003243964.1	1,00E-15	GLOS_LOC10056	GO:0007179	B	Refseq	transforming growth factor beta receptor signaling pathway
XP_003243964.1 PREDICTED: hypothetical prot. LOC100568954 [A. pisum]	XP_003243964.1	1,00E-15	GLOS_LOC10056	GO:0048513	B	.	organ development
XP_003243964.1 PREDICTED: hypothetical prot. LOC100568954 [A. pisum]	XP_003243964.1	1,00E-15	GLOS_LOC10056	GO:0072358	B	.	cardiovascular system development
XP_003243964.1 PREDICTED: hypothetical prot. LOC100568954 [A. pisum]	XP_003243964.1	1,00E-15	GLOS_LOC10056	GO:0072359	B	.	circulatory system development
XP_003243964.1 PREDICTED: hypothetical prot. LOC100568954 [A. pisum]	XP_003243964.1	1,00E-15	GLOS_LOC10056	GO:0007507	B	Refseq	heart development
XP_003243964.1 PREDICTED: hypothetical prot. LOC100568954 [A. pisum]	XP_003243964.1	1,00E-15	GLOS_LOC10056	GO:0008134	M	Refseq	transcription factor binding
XP_003243964.1 PREDICTED: hypothetical prot. LOC100568954 [A. pisum]	XP_003243964.1	1,00E-15	GLOS_LOC10056	GO:0010467	B	Refseq	gene expression
XP_003243964.1 PREDICTED: hypothetical prot. LOC100568954 [A. pisum]	XP_003243964.1	1,00E-15	GLOS_LOC10056	GO:0019904	M	Refseq	protein domain specific binding
XP_003243964.1 PREDICTED: hypothetical prot. LOC100568954 [A. pisum]	XP_003243964.1	1,00E-15	GLOS_LOC10056	GO:0045893	B	.	positive regulation of transcription, DNA-dependent
XP_003243964.1 PREDICTED: hypothetical prot. LOC100568954 [A. pisum]	XP_003243964.1	1,00E-15	GLOS_LOC10056	GO:0010557	B	.	positive regulation of macromolecule biosynthetic process

XP_003243964.1 PREDICTED: hypothetical prot. LOC100568954 [A. pisum]	XP_003243964.1	1,00E-15	GLOS_LOC10056{	GO:0009891	B	.	positive regulation of biosynthetic process
XP_003243964.1 PREDICTED: hypothetical prot. LOC100568954 [A. pisum]	XP_003243964.1	1,00E-15	GLOS_LOC10056{	GO:0009893	B	.	positive regulation of metabolic process
XP_003243964.1 PREDICTED: hypothetical prot. LOC100568954 [A. pisum]	XP_003243964.1	1,00E-15	GLOS_LOC10056{	GO:0010604	B	.	positive regulation of macromolecule metabolic process
XP_003243964.1 PREDICTED: hypothetical prot. LOC100568954 [A. pisum]	XP_003243964.1	1,00E-15	GLOS_LOC10056{	GO:0010628	B	.	positive regulation of gene expression
XP_003243964.1 PREDICTED: hypothetical prot. LOC100568954 [A. pisum]	XP_003243964.1	1,00E-15	GLOS_LOC10056{	GO:0031328	B	.	positive regulation of cellular biosynthetic process
XP_003243964.1 PREDICTED: hypothetical prot. LOC100568954 [A. pisum]	XP_003243964.1	1,00E-15	GLOS_LOC10056{	GO:0031325	B	.	positive regulation of cellular metabolic process
XP_003243964.1 PREDICTED: hypothetical prot. LOC100568954 [A. pisum]	XP_003243964.1	1,00E-15	GLOS_LOC10056{	GO:0051254	B	.	positive regulation of RNA metabolic process
XP_003243964.1 PREDICTED: hypothetical prot. LOC100568954 [A. pisum]	XP_003243964.1	1,00E-15	GLOS_LOC10056{	GO:0045935	B	.	positive regulation of nucleobase-containing compound metabolic process
XP_003243964.1 PREDICTED: hypothetical prot. LOC100568954 [A. pisum]	XP_003243964.1	1,00E-15	GLOS_LOC10056{	GO:0051173	B	.	positive regulation of nitrogen compound metabolic process
XP_003243964.1 PREDICTED: hypothetical prot. LOC100568954 [A. pisum]	XP_003243964.1	1,00E-15	GLOS_LOC10056{	GO:0045944	B	Refseq	positive regulation of transcription from RNA polymerase II promoter
XP_001996461.1 GH25201 [Drosophila grimshawi]	XP_001996461.1	1,00E-127	GLOS_DGRI_GH2	GO:0005262	M	Refseq	calcium channel activity
XP_001996461.1 GH25201 [Drosophila grimshawi]	XP_001996461.1	1,00E-127	GLOS_DGRI_GH2	GO:0005515	M	Refseq	protein binding
XP_001996461.1 GH25201 [Drosophila grimshawi]	XP_001996461.1	1,00E-127	GLOS_DGRI_GH2	GO:0005886	C	Refseq	plasma membrane
XP_001996461.1 GH25201 [Drosophila grimshawi]	XP_001996461.1	1,00E-127	GLOS_DGRI_GH2	GO:0005887	C	Refseq	integral to plasma membrane
XP_001996461.1 GH25201 [Drosophila grimshawi]	XP_001996461.1	1,00E-127	GLOS_DGRI_GH2	GO:0006816	B	Refseq	calcium ion transport
XP_001996461.1 GH25201 [Drosophila grimshawi]	XP_001996461.1	1,00E-127	GLOS_DGRI_GH2	GO:0007411	B	Refseq	axon guidance
XP_001996461.1 GH25201 [Drosophila grimshawi]	XP_001996461.1	1,00E-127	GLOS_DGRI_GH2	GO:0007599	B	.	hemostasis
XP_001996461.1 GH25201 [Drosophila grimshawi]	XP_001996461.1	1,00E-127	GLOS_DGRI_GH2	GO:0050817	B	.	coagulation
XP_001996461.1 GH25201 [Drosophila grimshawi]	XP_001996461.1	1,00E-127	GLOS_DGRI_GH2	GO:0042060	B	.	wound healing
XP_001996461.1 GH25201 [Drosophila grimshawi]	XP_001996461.1	1,00E-127	GLOS_DGRI_GH2	GO:0009611	B	.	response to wounding
XP_001996461.1 GH25201 [Drosophila grimshawi]	XP_001996461.1	1,00E-127	GLOS_DGRI_GH2	GO:0006950	B	.	response to stress
XP_001996461.1 GH25201 [Drosophila grimshawi]	XP_001996461.1	1,00E-127	GLOS_DGRI_GH2	GO:0007596	B	Refseq	blood coagulation
XP_001996461.1 GH25201 [Drosophila grimshawi]	XP_001996461.1	1,00E-127	GLOS_DGRI_GH2	GO:0009583	B	.	detection of light stimulus
XP_001996461.1 GH25201 [Drosophila grimshawi]	XP_001996461.1	1,00E-127	GLOS_DGRI_GH2	GO:0009416	B	.	response to light stimulus
XP_001996461.1 GH25201 [Drosophila grimshawi]	XP_001996461.1	1,00E-127	GLOS_DGRI_GH2	GO:0009314	B	.	response to radiation
XP_001996461.1 GH25201 [Drosophila grimshawi]	XP_001996461.1	1,00E-127	GLOS_DGRI_GH2	GO:0009628	B	.	response to abiotic stimulus
XP_001996461.1 GH25201 [Drosophila grimshawi]	XP_001996461.1	1,00E-127	GLOS_DGRI_GH2	GO:0009581	B	.	detection of external stimulus
XP_001996461.1 GH25201 [Drosophila grimshawi]	XP_001996461.1	1,00E-127	GLOS_DGRI_GH2	GO:0051606	B	.	detection of stimulus
XP_001996461.1 GH25201 [Drosophila grimshawi]	XP_001996461.1	1,00E-127	GLOS_DGRI_GH2	GO:0009582	B	.	detection of abiotic stimulus
XP_001996461.1 GH25201 [Drosophila grimshawi]	XP_001996461.1	1,00E-127	GLOS_DGRI_GH2	GO:0007602	B	Refseq	phototransduction
XP_001996461.1 GH25201 [Drosophila grimshawi]	XP_001996461.1	1,00E-127	GLOS_DGRI_GH2	GO:0010524	B	Refseq	positive regulation of calcium ion transport into cytosol
XP_001996461.1 GH25201 [Drosophila grimshawi]	XP_001996461.1	1,00E-127	GLOS_DGRI_GH2	GO:0015279	M	Refseq	store-operated calcium channel activity
XP_001996461.1 GH25201 [Drosophila grimshawi]	XP_001996461.1	1,00E-127	GLOS_DGRI_GH2	GO:0001775	B	.	cell activation
XP_001996461.1 GH25201 [Drosophila grimshawi]	XP_001996461.1	1,00E-127	GLOS_DGRI_GH2	GO:0007596	B	.	blood coagulation
XP_001996461.1 GH25201 [Drosophila grimshawi]	XP_001996461.1	1,00E-127	GLOS_DGRI_GH2	GO:0030168	B	Refseq	platelet activation
XP_001996461.1 GH25201 [Drosophila grimshawi]	XP_001996461.1	1,00E-127	GLOS_DGRI_GH2	GO:0014074	B	.	response to purine-containing compound
XP_001996461.1 GH25201 [Drosophila grimshawi]	XP_001996461.1	1,00E-127	GLOS_DGRI_GH2	GO:0010243	B	.	response to organonitrogen compound
XP_001996461.1 GH25201 [Drosophila grimshawi]	XP_001996461.1	1,00E-127	GLOS_DGRI_GH2	GO:1901698	B	.	response to nitrogen compound
XP_001996461.1 GH25201 [Drosophila grimshawi]	XP_001996461.1	1,00E-127	GLOS_DGRI_GH2	GO:0014070	B	.	response to organic cyclic compound
XP_001996461.1 GH25201 [Drosophila grimshawi]	XP_001996461.1	1,00E-127	GLOS_DGRI_GH2	GO:0046683	B	.	response to organophosphorus
XP_001996461.1 GH25201 [Drosophila grimshawi]	XP_001996461.1	1,00E-127	GLOS_DGRI_GH2	GO:1901700	B	.	response to oxygen-containing compound
XP_001996461.1 GH25201 [Drosophila grimshawi]	XP_001996461.1	1,00E-127	GLOS_DGRI_GH2	GO:0033198	B	Refseq	response to ATP
XP_001996461.1 GH25201 [Drosophila grimshawi]	XP_001996461.1	1,00E-127	GLOS_DGRI_GH2	GO:0051592	B	Refseq	response to calcium ion
XP_001996461.1 GH25201 [Drosophila grimshawi]	XP_001996461.1	1,00E-127	GLOS_DGRI_GH2	GO:0006816	B	.	calcium ion transport
XP_001996461.1 GH25201 [Drosophila grimshawi]	XP_001996461.1	1,00E-127	GLOS_DGRI_GH2	GO:0034220	B	.	ion transmembrane transport
XP_001996461.1 GH25201 [Drosophila grimshawi]	XP_001996461.1	1,00E-127	GLOS_DGRI_GH2	GO:0055085	B	.	transmembrane transport
XP_001996461.1 GH25201 [Drosophila grimshawi]	XP_001996461.1	1,00E-127	GLOS_DGRI_GH2	GO:0070588	B	Refseq	calcium ion transmembrane transport
XP_001996461.1 GH25201 [Drosophila grimshawi]	XP_001996461.1	1,00E-127	GLOS_DGRI_GH2	GO:0070679	M	Refseq	inositol 1,4,5 trisphosphate binding
XP_001970283.1 GG10538 [Drosophila erecta]	XP_001970283.1	2,00E-17	GLOS_DERE_GG	GO:0005261	M	Refseq	cation channel activity
XP_001970283.1 GG10538 [Drosophila erecta]	XP_001970283.1	2,00E-17	GLOS_DERE_GG	GO:0005262	M	Refseq	calcium channel activity
XP_001970283.1 GG10538 [Drosophila erecta]	XP_001970283.1	2,00E-17	GLOS_DERE_GG	GO:0005515	M	Refseq	protein binding
XP_001970283.1 GG10538 [Drosophila erecta]	XP_001970283.1	2,00E-17	GLOS_DERE_GG	GO:0005886	C	Refseq	plasma membrane
XP_001970283.1 GG10538 [Drosophila erecta]	XP_001970283.1	2,00E-17	GLOS_DERE_GG	GO:0005887	C	Refseq	integral to plasma membrane
XP_001970283.1 GG10538 [Drosophila erecta]	XP_001970283.1	2,00E-17	GLOS_DERE_GG	GO:0006816	B	Refseq	calcium ion transport

XP_001970283.1 GG10538 [Drosophila erecta]	XP_001970283.1	2,00E-17	GLOS_DERE_GG	GO:0007411	B	Refseq	axon guidance
XP_001970283.1 GG10538 [Drosophila erecta]	XP_001970283.1	2,00E-17	GLOS_DERE_GG	GO:0015279	M	Refseq	store-operated calcium channel activity
XP_001970283.1 GG10538 [Drosophila erecta]	XP_001970283.1	2,00E-17	GLOS_DERE_GG	GO:0016323	C	Refseq	basolateral plasma membrane
XP_001970283.1 GG10538 [Drosophila erecta]	XP_001970283.1	2,00E-17	GLOS_DERE_GG	GO:0030017	C	Refseq	sarcomere
XP_001970283.1 GG10538 [Drosophila erecta]	XP_001970283.1	2,00E-17	GLOS_DERE_GG	GO:0043034	C	Refseq	costamere
XP_001970283.1 GG10538 [Drosophila erecta]	XP_001970283.1	2,00E-17	GLOS_DERE_GG	GO:0043234	C	Refseq	protein complex
XP_001970283.1 GG10538 [Drosophila erecta]	XP_001970283.1	2,00E-17	GLOS_DERE_GG	GO:0044325	M	Refseq	ion channel binding
XP_001970283.1 GG10538 [Drosophila erecta]	XP_001970283.1	2,00E-17	GLOS_DERE_GG	GO:0045121	C	Refseq	membrane raft
XP_001970283.1 GG10538 [Drosophila erecta]	XP_001970283.1	2,00E-17	GLOS_DERE_GG	GO:0046541	B	Refseq	saliva secretion
XP_001970283.1 GG10538 [Drosophila erecta]	XP_001970283.1	2,00E-17	GLOS_DERE_GG	GO:0051281	B	Refseq	positive regulation of release of sequestered calcium ion into cytosol
XP_001970283.1 GG10538 [Drosophila erecta]	XP_001970283.1	2,00E-17	GLOS_DERE_GG	GO:0051480	B	Refseq	cytosolic calcium ion homeostasis
XP_001970283.1 GG10538 [Drosophila erecta]	XP_001970283.1	2,00E-17	GLOS_DERE_GG	GO:0051592	B	Refseq	response to calcium ion
XP_001970283.1 GG10538 [Drosophila erecta]	XP_001970283.1	2,00E-17	GLOS_DERE_GG	GO:0070679	M	Refseq	inositol 1,4,5 trisphosphate binding
NP_728868.1 PHGPx, isoform C [Drosophila melanogaster]	NP_728868.1	9,00E-93	GLOS_PHGPX.1.1	GO:0000122	B	Refseq	negative regulation of transcription from RNA polymerase II promoter
NP_728868.1 PHGPx, isoform C [Drosophila melanogaster]	NP_728868.1	9,00E-93	GLOS_PHGPX.1.1	GO:0001501	B	Refseq	skeletal system development
NP_728868.1 PHGPx, isoform C [Drosophila melanogaster]	NP_728868.1	9,00E-93	GLOS_PHGPX.1.1	GO:0003700	M	Refseq	sequence-specific DNA binding transcription factor activity
NP_728868.1 PHGPx, isoform C [Drosophila melanogaster]	NP_728868.1	9,00E-93	GLOS_PHGPX.1.1	GO:0005515	M	Refseq	protein binding
NP_728868.1 PHGPx, isoform C [Drosophila melanogaster]	NP_728868.1	9,00E-93	GLOS_PHGPX.1.1	GO:0005634	C	Refseq	nucleus
NP_728868.1 PHGPx, isoform C [Drosophila melanogaster]	NP_728868.1	9,00E-93	GLOS_PHGPX.1.1	GO:0006366	B	Refseq	transcription from RNA polymerase II promoter
NP_728868.1 PHGPx, isoform C [Drosophila melanogaster]	NP_728868.1	9,00E-93	GLOS_PHGPX.1.1	GO:0006607	B	Refseq	NLS-bearing protein import into nucleus
NP_728868.1 PHGPx, isoform C [Drosophila melanogaster]	NP_728868.1	9,00E-93	GLOS_PHGPX.1.1	GO:0008270	M	Refseq	zinc ion binding
NP_728868.1 PHGPx, isoform C [Drosophila melanogaster]	NP_728868.1	9,00E-93	GLOS_PHGPX.1.1	GO:0032330	B	Refseq	regulation of chondrocyte differentiation
NP_728868.1 PHGPx, isoform C [Drosophila melanogaster]	NP_728868.1	9,00E-93	GLOS_PHGPX.1.1	GO:0043565	M	Refseq	sequence-specific DNA binding
XP_001972515.1 GG13845 [Drosophila erecta]	XP_001972515.1	2,00E-66	GLOS_DERE_GG	GO:0005261	M	Refseq	cation channel activity
XP_001972515.1 GG13845 [Drosophila erecta]	XP_001972515.1	2,00E-66	GLOS_DERE_GG	GO:0005262	M	Refseq	calcium channel activity
XP_001972515.1 GG13845 [Drosophila erecta]	XP_001972515.1	2,00E-66	GLOS_DERE_GG	GO:0005515	M	Refseq	protein binding
XP_001972515.1 GG13845 [Drosophila erecta]	XP_001972515.1	2,00E-66	GLOS_DERE_GG	GO:0005886	C	Refseq	plasma membrane
XP_001972515.1 GG13845 [Drosophila erecta]	XP_001972515.1	2,00E-66	GLOS_DERE_GG	GO:0005887	C	Refseq	integral to plasma membrane
XP_001972515.1 GG13845 [Drosophila erecta]	XP_001972515.1	2,00E-66	GLOS_DERE_GG	GO:0006816	B	Refseq	calcium ion transport
XP_001972515.1 GG13845 [Drosophila erecta]	XP_001972515.1	2,00E-66	GLOS_DERE_GG	GO:0007411	B	Refseq	axon guidance
XP_001972515.1 GG13845 [Drosophila erecta]	XP_001972515.1	2,00E-66	GLOS_DERE_GG	GO:0015279	M	Refseq	store-operated calcium channel activity
XP_001972515.1 GG13845 [Drosophila erecta]	XP_001972515.1	2,00E-66	GLOS_DERE_GG	GO:0016323	C	Refseq	basolateral plasma membrane
XP_001972515.1 GG13845 [Drosophila erecta]	XP_001972515.1	2,00E-66	GLOS_DERE_GG	GO:0030017	C	Refseq	sarcomere
XP_001972515.1 GG13845 [Drosophila erecta]	XP_001972515.1	2,00E-66	GLOS_DERE_GG	GO:0043034	C	Refseq	costamere
XP_001972515.1 GG13845 [Drosophila erecta]	XP_001972515.1	2,00E-66	GLOS_DERE_GG	GO:0043234	C	Refseq	protein complex
XP_001972515.1 GG13845 [Drosophila erecta]	XP_001972515.1	2,00E-66	GLOS_DERE_GG	GO:0044325	M	Refseq	ion channel binding
XP_001972515.1 GG13845 [Drosophila erecta]	XP_001972515.1	2,00E-66	GLOS_DERE_GG	GO:0045121	C	Refseq	membrane raft
XP_001972515.1 GG13845 [Drosophila erecta]	XP_001972515.1	2,00E-66	GLOS_DERE_GG	GO:0046541	B	Refseq	saliva secretion
XP_001972515.1 GG13845 [Drosophila erecta]	XP_001972515.1	2,00E-66	GLOS_DERE_GG	GO:0051281	B	Refseq	positive regulation of release of sequestered calcium ion into cytosol
XP_001972515.1 GG13845 [Drosophila erecta]	XP_001972515.1	2,00E-66	GLOS_DERE_GG	GO:0051480	B	Refseq	cytosolic calcium ion homeostasis
XP_001972515.1 GG13845 [Drosophila erecta]	XP_001972515.1	2,00E-66	GLOS_DERE_GG	GO:0051592	B	Refseq	response to calcium ion
XP_001972515.1 GG13845 [Drosophila erecta]	XP_001972515.1	2,00E-66	GLOS_DERE_GG	GO:0070679	M	Refseq	inositol 1,4,5 trisphosphate binding
NP_732112.1 CG32855 [Drosophila melanogaster]	NP_732112.1	3,00E-66	GLOS_CG32855.1	GO:0000122	B	Refseq	negative regulation of transcription from RNA polymerase II promoter
NP_732112.1 CG32855 [Drosophila melanogaster]	NP_732112.1	3,00E-66	GLOS_CG32855.1	GO:0001501	B	Refseq	skeletal system development
NP_732112.1 CG32855 [Drosophila melanogaster]	NP_732112.1	3,00E-66	GLOS_CG32855.1	GO:0003700	M	Refseq	sequence-specific DNA binding transcription factor activity
NP_732112.1 CG32855 [Drosophila melanogaster]	NP_732112.1	3,00E-66	GLOS_CG32855.1	GO:0005515	M	Refseq	protein binding
NP_732112.1 CG32855 [Drosophila melanogaster]	NP_732112.1	3,00E-66	GLOS_CG32855.1	GO:0005634	C	Refseq	nucleus
NP_732112.1 CG32855 [Drosophila melanogaster]	NP_732112.1	3,00E-66	GLOS_CG32855.1	GO:0006366	B	Refseq	transcription from RNA polymerase II promoter
NP_732112.1 CG32855 [Drosophila melanogaster]	NP_732112.1	3,00E-66	GLOS_CG32855.1	GO:0006607	B	Refseq	NLS-bearing protein import into nucleus
NP_732112.1 CG32855 [Drosophila melanogaster]	NP_732112.1	3,00E-66	GLOS_CG32855.1	GO:0008270	M	Refseq	zinc ion binding
NP_732112.1 CG32855 [Drosophila melanogaster]	NP_732112.1	3,00E-66	GLOS_CG32855.1	GO:0032330	B	Refseq	regulation of chondrocyte differentiation
NP_732112.1 CG32855 [Drosophila melanogaster]	NP_732112.1	3,00E-66	GLOS_CG32855.1	GO:0043565	M	Refseq	sequence-specific DNA binding
XP_001987332.1 GH21864 [Drosophila grimshawi]	XP_001987332.1	0	GLOS_DGRI_GH2	GO:0005262	M	Refseq	calcium channel activity
XP_001987332.1 GH21864 [Drosophila grimshawi]	XP_001987332.1	0	GLOS_DGRI_GH2	GO:0005515	M	Refseq	protein binding

XP_001987332.1 GH21864 [Drosophila grimshawi]	XP_001987332.1	0	GLOS_DGRI_GH2	GO:0005886	C	Refseq	plasma membrane
XP_001987332.1 GH21864 [Drosophila grimshawi]	XP_001987332.1	0	GLOS_DGRI_GH2	GO:0005887	C	Refseq	integral to plasma membrane
XP_001987332.1 GH21864 [Drosophila grimshawi]	XP_001987332.1	0	GLOS_DGRI_GH2	GO:0006816	B	Refseq	calcium ion transport
XP_001987332.1 GH21864 [Drosophila grimshawi]	XP_001987332.1	0	GLOS_DGRI_GH2	GO:0007411	B	Refseq	axon guidance
XP_001987332.1 GH21864 [Drosophila grimshawi]	XP_001987332.1	0	GLOS_DGRI_GH2	GO:0007596	B	Refseq	blood coagulation
XP_001987332.1 GH21864 [Drosophila grimshawi]	XP_001987332.1	0	GLOS_DGRI_GH2	GO:0007602	B	Refseq	phototransduction
XP_001987332.1 GH21864 [Drosophila grimshawi]	XP_001987332.1	0	GLOS_DGRI_GH2	GO:0010524	B	Refseq	positive regulation of calcium ion transport into cytosol
XP_001987332.1 GH21864 [Drosophila grimshawi]	XP_001987332.1	0	GLOS_DGRI_GH2	GO:0015279	M	Refseq	store-operated calcium channel activity
XP_001987332.1 GH21864 [Drosophila grimshawi]	XP_001987332.1	0	GLOS_DGRI_GH2	GO:0030168	B	Refseq	platelet activation
XP_001987332.1 GH21864 [Drosophila grimshawi]	XP_001987332.1	0	GLOS_DGRI_GH2	GO:0033198	B	Refseq	response to ATP
XP_001987332.1 GH21864 [Drosophila grimshawi]	XP_001987332.1	0	GLOS_DGRI_GH2	GO:0051592	B	Refseq	response to calcium ion
XP_001987332.1 GH21864 [Drosophila grimshawi]	XP_001987332.1	0	GLOS_DGRI_GH2	GO:0070588	B	Refseq	calcium ion transmembrane transport
XP_001987332.1 GH21864 [Drosophila grimshawi]	XP_001987332.1	0	GLOS_DGRI_GH2	GO:0070679	M	Refseq	inositol 1,4,5 trisphosphate binding
XP_001991349.1 GH12103 [Drosophila grimshawi]	XP_001991349.1	1,00E-92	GLOS_DGRI_GH1	GO:0005262	M	Refseq	calcium channel activity
XP_001991349.1 GH12103 [Drosophila grimshawi]	XP_001991349.1	1,00E-92	GLOS_DGRI_GH1	GO:0005515	M	Refseq	protein binding
XP_001991349.1 GH12103 [Drosophila grimshawi]	XP_001991349.1	1,00E-92	GLOS_DGRI_GH1	GO:0005886	C	Refseq	plasma membrane
XP_001991349.1 GH12103 [Drosophila grimshawi]	XP_001991349.1	1,00E-92	GLOS_DGRI_GH1	GO:0005887	C	Refseq	integral to plasma membrane
XP_001991349.1 GH12103 [Drosophila grimshawi]	XP_001991349.1	1,00E-92	GLOS_DGRI_GH1	GO:0006816	B	Refseq	calcium ion transport
XP_001991349.1 GH12103 [Drosophila grimshawi]	XP_001991349.1	1,00E-92	GLOS_DGRI_GH1	GO:0007411	B	Refseq	axon guidance
XP_001991349.1 GH12103 [Drosophila grimshawi]	XP_001991349.1	1,00E-92	GLOS_DGRI_GH1	GO:0007596	B	Refseq	blood coagulation
XP_001991349.1 GH12103 [Drosophila grimshawi]	XP_001991349.1	1,00E-92	GLOS_DGRI_GH1	GO:0007602	B	Refseq	phototransduction
XP_001991349.1 GH12103 [Drosophila grimshawi]	XP_001991349.1	1,00E-92	GLOS_DGRI_GH1	GO:0010524	B	Refseq	positive regulation of calcium ion transport into cytosol
XP_001991349.1 GH12103 [Drosophila grimshawi]	XP_001991349.1	1,00E-92	GLOS_DGRI_GH1	GO:0015279	M	Refseq	store-operated calcium channel activity
XP_001991349.1 GH12103 [Drosophila grimshawi]	XP_001991349.1	1,00E-92	GLOS_DGRI_GH1	GO:0030168	B	Refseq	platelet activation
XP_001991349.1 GH12103 [Drosophila grimshawi]	XP_001991349.1	1,00E-92	GLOS_DGRI_GH1	GO:0033198	B	Refseq	response to ATP
XP_001991349.1 GH12103 [Drosophila grimshawi]	XP_001991349.1	1,00E-92	GLOS_DGRI_GH1	GO:0051592	B	Refseq	response to calcium ion
XP_001991349.1 GH12103 [Drosophila grimshawi]	XP_001991349.1	1,00E-92	GLOS_DGRI_GH1	GO:0070588	B	Refseq	calcium ion transmembrane transport
XP_001991349.1 GH12103 [Drosophila grimshawi]	XP_001991349.1	1,00E-92	GLOS_DGRI_GH1	GO:0070679	M	Refseq	inositol 1,4,5 trisphosphate binding
XP_001978711.1 GG17524 [Drosophila erecta]	XP_001978711.1	3,00E-33	GLOS_DERE_GG	GO:0005261	M	Refseq	cation channel activity
XP_001978711.1 GG17524 [Drosophila erecta]	XP_001978711.1	3,00E-33	GLOS_DERE_GG	GO:0005262	M	Refseq	calcium channel activity
XP_001978711.1 GG17524 [Drosophila erecta]	XP_001978711.1	3,00E-33	GLOS_DERE_GG	GO:0005515	M	Refseq	protein binding
XP_001978711.1 GG17524 [Drosophila erecta]	XP_001978711.1	3,00E-33	GLOS_DERE_GG	GO:0005886	C	Refseq	plasma membrane
XP_001978711.1 GG17524 [Drosophila erecta]	XP_001978711.1	3,00E-33	GLOS_DERE_GG	GO:0005887	C	Refseq	integral to plasma membrane
XP_001978711.1 GG17524 [Drosophila erecta]	XP_001978711.1	3,00E-33	GLOS_DERE_GG	GO:0006816	B	Refseq	calcium ion transport
XP_001978711.1 GG17524 [Drosophila erecta]	XP_001978711.1	3,00E-33	GLOS_DERE_GG	GO:0007411	B	Refseq	axon guidance
XP_001978711.1 GG17524 [Drosophila erecta]	XP_001978711.1	3,00E-33	GLOS_DERE_GG	GO:0015279	M	Refseq	store-operated calcium channel activity
XP_001978711.1 GG17524 [Drosophila erecta]	XP_001978711.1	3,00E-33	GLOS_DERE_GG	GO:0016323	C	Refseq	basolateral plasma membrane
XP_001978711.1 GG17524 [Drosophila erecta]	XP_001978711.1	3,00E-33	GLOS_DERE_GG	GO:0030017	C	Refseq	sarcomere
XP_001978711.1 GG17524 [Drosophila erecta]	XP_001978711.1	3,00E-33	GLOS_DERE_GG	GO:0043034	C	Refseq	costamere
XP_001978711.1 GG17524 [Drosophila erecta]	XP_001978711.1	3,00E-33	GLOS_DERE_GG	GO:0043234	C	Refseq	protein complex
XP_001978711.1 GG17524 [Drosophila erecta]	XP_001978711.1	3,00E-33	GLOS_DERE_GG	GO:0044325	M	Refseq	ion channel binding
XP_001978711.1 GG17524 [Drosophila erecta]	XP_001978711.1	3,00E-33	GLOS_DERE_GG	GO:0045121	C	Refseq	membrane raft
XP_001978711.1 GG17524 [Drosophila erecta]	XP_001978711.1	3,00E-33	GLOS_DERE_GG	GO:0046541	B	Refseq	saliva secretion
XP_001978711.1 GG17524 [Drosophila erecta]	XP_001978711.1	3,00E-33	GLOS_DERE_GG	GO:0051281	B	Refseq	positive regulation of release of sequestered calcium ion into cytosol
XP_001978711.1 GG17524 [Drosophila erecta]	XP_001978711.1	3,00E-33	GLOS_DERE_GG	GO:0051480	B	Refseq	cytosolic calcium ion homeostasis
XP_001978711.1 GG17524 [Drosophila erecta]	XP_001978711.1	3,00E-33	GLOS_DERE_GG	GO:0051592	B	Refseq	response to calcium ion
XP_001978711.1 GG17524 [Drosophila erecta]	XP_001978711.1	3,00E-33	GLOS_DERE_GG	GO:0070679	M	Refseq	inositol 1,4,5 trisphosphate binding
XP_001994318.1 GH23882 [Drosophila grimshawi]	XP_001994318.1	0	GLOS_DGRI_GH2	GO:0005262	M	Refseq	calcium channel activity
XP_001994318.1 GH23882 [Drosophila grimshawi]	XP_001994318.1	0	GLOS_DGRI_GH2	GO:0005515	M	Refseq	protein binding
XP_001994318.1 GH23882 [Drosophila grimshawi]	XP_001994318.1	0	GLOS_DGRI_GH2	GO:0005886	C	Refseq	plasma membrane
XP_001994318.1 GH23882 [Drosophila grimshawi]	XP_001994318.1	0	GLOS_DGRI_GH2	GO:0005887	C	Refseq	integral to plasma membrane
XP_001994318.1 GH23882 [Drosophila grimshawi]	XP_001994318.1	0	GLOS_DGRI_GH2	GO:0006816	B	Refseq	calcium ion transport
XP_001994318.1 GH23882 [Drosophila grimshawi]	XP_001994318.1	0	GLOS_DGRI_GH2	GO:0007411	B	Refseq	axon guidance
XP_001994318.1 GH23882 [Drosophila grimshawi]	XP_001994318.1	0	GLOS_DGRI_GH2	GO:0007596	B	Refseq	blood coagulation

XP_001994318.1 GH23882 [Drosophila grimshawi]	XP_001994318.1	0	GLOS_DGRI_GH2	GO:0007602	B	Refseq	phototransduction
XP_001994318.1 GH23882 [Drosophila grimshawi]	XP_001994318.1	0	GLOS_DGRI_GH2	GO:0010524	B	Refseq	positive regulation of calcium ion transport into cytosol
XP_001994318.1 GH23882 [Drosophila grimshawi]	XP_001994318.1	0	GLOS_DGRI_GH2	GO:0015279	M	Refseq	store-operated calcium channel activity
XP_001994318.1 GH23882 [Drosophila grimshawi]	XP_001994318.1	0	GLOS_DGRI_GH2	GO:0030168	B	Refseq	platelet activation
XP_001994318.1 GH23882 [Drosophila grimshawi]	XP_001994318.1	0	GLOS_DGRI_GH2	GO:0033198	B	Refseq	response to ATP
XP_001994318.1 GH23882 [Drosophila grimshawi]	XP_001994318.1	0	GLOS_DGRI_GH2	GO:0051592	B	Refseq	response to calcium ion
XP_001994318.1 GH23882 [Drosophila grimshawi]	XP_001994318.1	0	GLOS_DGRI_GH2	GO:0070588	B	Refseq	calcium ion transmembrane transport
XP_001994318.1 GH23882 [Drosophila grimshawi]	XP_001994318.1	0	GLOS_DGRI_GH2	GO:0070679	M	Refseq	inositol 1,4,5 trisphosphate binding
XP_003246116.1 PREDICTED: hypothetical prot. LOC100572450 [A. pisum]	XP_003246116.1	4,00E-07	GLOS_LOC10057	GO:0000278	B	Refseq	mitotic cell cycle
XP_003246116.1 PREDICTED: hypothetical prot. LOC100572450 [A. pisum]	XP_003246116.1	4,00E-07	GLOS_LOC10057	GO:0003677	M	Refseq	DNA binding
XP_003246116.1 PREDICTED: hypothetical prot. LOC100572450 [A. pisum]	XP_003246116.1	4,00E-07	GLOS_LOC10057	GO:0003700	M	Refseq	sequence-specific DNA binding transcription factor activity
XP_003246116.1 PREDICTED: hypothetical prot. LOC100572450 [A. pisum]	XP_003246116.1	4,00E-07	GLOS_LOC10057	GO:0003712	M	Refseq	transcription cofactor activity
XP_003246116.1 PREDICTED: hypothetical prot. LOC100572450 [A. pisum]	XP_003246116.1	4,00E-07	GLOS_LOC10057	GO:0005654	C	Refseq	nucleoplasm
XP_003246116.1 PREDICTED: hypothetical prot. LOC100572450 [A. pisum]	XP_003246116.1	4,00E-07	GLOS_LOC10057	GO:0005667	C	Refseq	transcription factor complex
XP_003246116.1 PREDICTED: hypothetical prot. LOC100572450 [A. pisum]	XP_003246116.1	4,00E-07	GLOS_LOC10057	GO:0006351	B	Refseq	transcription, DNA-dependent
XP_003246116.1 PREDICTED: hypothetical prot. LOC100572450 [A. pisum]	XP_003246116.1	4,00E-07	GLOS_LOC10057	GO:0006367	B	Refseq	transcription initiation from RNA polymerase II promoter
XP_003246116.1 PREDICTED: hypothetical prot. LOC100572450 [A. pisum]	XP_003246116.1	4,00E-07	GLOS_LOC10057	GO:0007179	B	Refseq	transforming growth factor beta receptor signaling pathway
XP_003246116.1 PREDICTED: hypothetical prot. LOC100572450 [A. pisum]	XP_003246116.1	4,00E-07	GLOS_LOC10057	GO:0007507	B	Refseq	heart development
XP_003246116.1 PREDICTED: hypothetical prot. LOC100572450 [A. pisum]	XP_003246116.1	4,00E-07	GLOS_LOC10057	GO:0008134	M	Refseq	transcription factor binding
XP_003246116.1 PREDICTED: hypothetical prot. LOC100572450 [A. pisum]	XP_003246116.1	4,00E-07	GLOS_LOC10057	GO:0010467	B	Refseq	gene expression
XP_003246116.1 PREDICTED: hypothetical prot. LOC100572450 [A. pisum]	XP_003246116.1	4,00E-07	GLOS_LOC10057	GO:0019904	M	Refseq	protein domain specific binding
XP_003246116.1 PREDICTED: hypothetical prot. LOC100572450 [A. pisum]	XP_003246116.1	4,00E-07	GLOS_LOC10057	GO:0045944	B	Refseq	positive regulation of transcription from RNA polymerase II promoter
XP_001657418.1 U3 small nucleolar ribonucleoprot. prot. imp4 [A. aegypti]	XP_001657418.1	1,00E-154	GLOS_AAEL_AAE	GO:0030674	M	.	protein binding, bridging
XP_001657418.1 U3 small nucleolar ribonucleoprot. prot. imp4 [A. aegypti]	XP_001657418.1	1,00E-154	GLOS_AAEL_AAE	GO:0060090	M	.	binding, bridging
XP_001657418.1 U3 small nucleolar ribonucleoprot. prot. imp4 [A. aegypti]	XP_001657418.1	1,00E-154	GLOS_AAEL_AAE	GO:0035591	M	.	signaling adaptor activity
XP_001657418.1 U3 small nucleolar ribonucleoprot. prot. imp4 [A. aegypti]	XP_001657418.1	1,00E-154	GLOS_AAEL_AAE	GO:0005070	M	Refseq	SH3/SH2 adaptor activity
XP_001657418.1 U3 small nucleolar ribonucleoprot. prot. imp4 [A. aegypti]	XP_001657418.1	1,00E-154	GLOS_AAEL_AAE	GO:0005515	M	Refseq	protein binding
XP_001657418.1 U3 small nucleolar ribonucleoprot. prot. imp4 [A. aegypti]	XP_001657418.1	1,00E-154	GLOS_AAEL_AAE	GO:0005634	C	Refseq	nucleus
XP_001657418.1 U3 small nucleolar ribonucleoprot. prot. imp4 [A. aegypti]	XP_001657418.1	1,00E-154	GLOS_AAEL_AAE	GO:0005737	C	Refseq	cytoplasm
XP_001657418.1 U3 small nucleolar ribonucleoprot. prot. imp4 [A. aegypti]	XP_001657418.1	1,00E-154	GLOS_AAEL_AAE	GO:0007049	B	Refseq	cell cycle
XP_001657418.1 U3 small nucleolar ribonucleoprot. prot. imp4 [A. aegypti]	XP_001657418.1	1,00E-154	GLOS_AAEL_AAE	GO:0007165	B	Refseq	signal transduction
XP_001657418.1 U3 small nucleolar ribonucleoprot. prot. imp4 [A. aegypti]	XP_001657418.1	1,00E-154	GLOS_AAEL_AAE	GO:0007417	B	Refseq	central nervous system development
XP_001657418.1 U3 small nucleolar ribonucleoprot. prot. imp4 [A. aegypti]	XP_001657418.1	1,00E-154	GLOS_AAEL_AAE	GO:0007507	B	Refseq	heart development
XP_001657418.1 U3 small nucleolar ribonucleoprot. prot. imp4 [A. aegypti]	XP_001657418.1	1,00E-154	GLOS_AAEL_AAE	GO:0009790	B	.	embryo development
XP_001657418.1 U3 small nucleolar ribonucleoprot. prot. imp4 [A. aegypti]	XP_001657418.1	1,00E-154	GLOS_AAEL_AAE	GO:0009792	B	Refseq	embryo development ending in birth or egg hatching
XP_001657418.1 U3 small nucleolar ribonucleoprot. prot. imp4 [A. aegypti]	XP_001657418.1	1,00E-154	GLOS_AAEL_AAE	GO:0010212	B	Refseq	response to ionizing radiation
XP_001657418.1 U3 small nucleolar ribonucleoprot. prot. imp4 [A. aegypti]	XP_001657418.1	1,00E-154	GLOS_AAEL_AAE	GO:0019904	M	.	protein domain specific binding
XP_001657418.1 U3 small nucleolar ribonucleoprot. prot. imp4 [A. aegypti]	XP_001657418.1	1,00E-154	GLOS_AAEL_AAE	GO:0017124	M	Refseq	SH3 domain binding
XP_001657418.1 U3 small nucleolar ribonucleoprot. prot. imp4 [A. aegypti]	XP_001657418.1	1,00E-154	GLOS_AAEL_AAE	GO:0042802	M	Refseq	identical protein binding
XP_001657418.1 U3 small nucleolar ribonucleoprot. prot. imp4 [A. aegypti]	XP_001657418.1	1,00E-154	GLOS_AAEL_AAE	GO:0051726	B	.	regulation of cell cycle
XP_001657418.1 U3 small nucleolar ribonucleoprot. prot. imp4 [A. aegypti]	XP_001657418.1	1,00E-154	GLOS_AAEL_AAE	GO:0045786	B	Refseq	negative regulation of cell cycle
XP_001657418.1 U3 small nucleolar ribonucleoprot. prot. imp4 [A. aegypti]	XP_001657418.1	1,00E-154	GLOS_AAEL_AAE	GO:0048471	C	Refseq	perinuclear region of cytoplasm
XP_001657418.1 U3 small nucleolar ribonucleoprot. prot. imp4 [A. aegypti]	XP_001657418.1	1,00E-154	GLOS_AAEL_AAE	GO:0008134	M	.	transcription factor binding
XP_001657418.1 U3 small nucleolar ribonucleoprot. prot. imp4 [A. aegypti]	XP_001657418.1	1,00E-154	GLOS_AAEL_AAE	GO:0051059	M	Refseq	NF-kappaB binding
XP_001657418.1 U3 small nucleolar ribonucleoprot. prot. imp4 [A. aegypti]	XP_001657418.1	1,00E-154	GLOS_AAEL_AAE	GO:0072331	B	.	signal transduction by p53 class mediator
XP_001657418.1 U3 small nucleolar ribonucleoprot. prot. imp4 [A. aegypti]	XP_001657418.1	1,00E-154	GLOS_AAEL_AAE	GO:0035556	B	.	intracellular signal transduction
XP_001657418.1 U3 small nucleolar ribonucleoprot. prot. imp4 [A. aegypti]	XP_001657418.1	1,00E-154	GLOS_AAEL_AAE	GO:0097193	B	.	intrinsic apoptotic signaling pathway
XP_001657418.1 U3 small nucleolar ribonucleoprot. prot. imp4 [A. aegypti]	XP_001657418.1	1,00E-154	GLOS_AAEL_AAE	GO:0097190	B	.	apoptotic signaling pathway
XP_001657418.1 U3 small nucleolar ribonucleoprot. prot. imp4 [A. aegypti]	XP_001657418.1	1,00E-154	GLOS_AAEL_AAE	GO:0006915	B	.	apoptotic process
XP_001657418.1 U3 small nucleolar ribonucleoprot. prot. imp4 [A. aegypti]	XP_001657418.1	1,00E-154	GLOS_AAEL_AAE	GO:0012501	B	.	programmed cell death
XP_001657418.1 U3 small nucleolar ribonucleoprot. prot. imp4 [A. aegypti]	XP_001657418.1	1,00E-154	GLOS_AAEL_AAE	GO:0008219	B	.	cell death
XP_001657418.1 U3 small nucleolar ribonucleoprot. prot. imp4 [A. aegypti]	XP_001657418.1	1,00E-154	GLOS_AAEL_AAE	GO:0016265	B	.	death
XP_001657418.1 U3 small nucleolar ribonucleoprot. prot. imp4 [A. aegypti]	XP_001657418.1	1,00E-154	GLOS_AAEL_AAE	GO:0072332	B	Refseq	intrinsic apoptotic signaling pathway by p53 class mediator
XP_001973917.1 GG21449 [Drosophila erecta]	XP_001973917.1	2,00E-10	GLOS_DERE_GG	GO:0005261	M	Refseq	cation channel activity

XP_001973917.1 GG21449 [Drosophila erecta]	XP_001973917.1	2,00E-10	GLOS_DERE_GG	GO:0005262	M	Refseq	calcium channel activity
XP_001973917.1 GG21449 [Drosophila erecta]	XP_001973917.1	2,00E-10	GLOS_DERE_GG	GO:0005515	M	Refseq	protein binding
XP_001973917.1 GG21449 [Drosophila erecta]	XP_001973917.1	2,00E-10	GLOS_DERE_GG	GO:0005886	C	Refseq	plasma membrane
XP_001973917.1 GG21449 [Drosophila erecta]	XP_001973917.1	2,00E-10	GLOS_DERE_GG	GO:0005887	C	Refseq	integral to plasma membrane
XP_001973917.1 GG21449 [Drosophila erecta]	XP_001973917.1	2,00E-10	GLOS_DERE_GG	GO:0006816	B	Refseq	calcium ion transport
XP_001973917.1 GG21449 [Drosophila erecta]	XP_001973917.1	2,00E-10	GLOS_DERE_GG	GO:0007411	B	Refseq	axon guidance
XP_001973917.1 GG21449 [Drosophila erecta]	XP_001973917.1	2,00E-10	GLOS_DERE_GG	GO:0015279	M	Refseq	store-operated calcium channel activity
XP_001973917.1 GG21449 [Drosophila erecta]	XP_001973917.1	2,00E-10	GLOS_DERE_GG	GO:0016323	C	Refseq	basolateral plasma membrane
XP_001973917.1 GG21449 [Drosophila erecta]	XP_001973917.1	2,00E-10	GLOS_DERE_GG	GO:0030017	C	Refseq	sarcomere
XP_001973917.1 GG21449 [Drosophila erecta]	XP_001973917.1	2,00E-10	GLOS_DERE_GG	GO:0043034	C	Refseq	costamere
XP_001973917.1 GG21449 [Drosophila erecta]	XP_001973917.1	2,00E-10	GLOS_DERE_GG	GO:0043234	C	Refseq	protein complex
XP_001973917.1 GG21449 [Drosophila erecta]	XP_001973917.1	2,00E-10	GLOS_DERE_GG	GO:0044325	M	Refseq	ion channel binding
XP_001973917.1 GG21449 [Drosophila erecta]	XP_001973917.1	2,00E-10	GLOS_DERE_GG	GO:0045121	C	Refseq	membrane raft
XP_001973917.1 GG21449 [Drosophila erecta]	XP_001973917.1	2,00E-10	GLOS_DERE_GG	GO:0046541	B	Refseq	saliva secretion
XP_001973917.1 GG21449 [Drosophila erecta]	XP_001973917.1	2,00E-10	GLOS_DERE_GG	GO:0051281	B	Refseq	positive regulation of release of sequestered calcium ion into cytosol
XP_001973917.1 GG21449 [Drosophila erecta]	XP_001973917.1	2,00E-10	GLOS_DERE_GG	GO:0051480	B	Refseq	cytosolic calcium ion homeostasis
XP_001973917.1 GG21449 [Drosophila erecta]	XP_001973917.1	2,00E-10	GLOS_DERE_GG	GO:0051592	B	Refseq	response to calcium ion
XP_001973917.1 GG21449 [Drosophila erecta]	XP_001973917.1	2,00E-10	GLOS_DERE_GG	GO:0070679	M	Refseq	inositol 1,4,5 trisphosphate binding
XP_001985572.1 GH17139 [Drosophila grimshawi]	XP_001985572.1	3,00E-73	GLOS_DGRI_GH1	GO:0005262	M	Refseq	calcium channel activity
XP_001985572.1 GH17139 [Drosophila grimshawi]	XP_001985572.1	3,00E-73	GLOS_DGRI_GH1	GO:0005515	M	Refseq	protein binding
XP_001985572.1 GH17139 [Drosophila grimshawi]	XP_001985572.1	3,00E-73	GLOS_DGRI_GH1	GO:0005886	C	Refseq	plasma membrane
XP_001985572.1 GH17139 [Drosophila grimshawi]	XP_001985572.1	3,00E-73	GLOS_DGRI_GH1	GO:0005887	C	Refseq	integral to plasma membrane
XP_001985572.1 GH17139 [Drosophila grimshawi]	XP_001985572.1	3,00E-73	GLOS_DGRI_GH1	GO:0006816	B	Refseq	calcium ion transport
XP_001985572.1 GH17139 [Drosophila grimshawi]	XP_001985572.1	3,00E-73	GLOS_DGRI_GH1	GO:0007411	B	Refseq	axon guidance
XP_001985572.1 GH17139 [Drosophila grimshawi]	XP_001985572.1	3,00E-73	GLOS_DGRI_GH1	GO:0007596	B	Refseq	blood coagulation
XP_001985572.1 GH17139 [Drosophila grimshawi]	XP_001985572.1	3,00E-73	GLOS_DGRI_GH1	GO:0007602	B	Refseq	phototransduction
XP_001985572.1 GH17139 [Drosophila grimshawi]	XP_001985572.1	3,00E-73	GLOS_DGRI_GH1	GO:0010524	B	Refseq	positive regulation of calcium ion transport into cytosol
XP_001985572.1 GH17139 [Drosophila grimshawi]	XP_001985572.1	3,00E-73	GLOS_DGRI_GH1	GO:0015279	M	Refseq	store-operated calcium channel activity
XP_001985572.1 GH17139 [Drosophila grimshawi]	XP_001985572.1	3,00E-73	GLOS_DGRI_GH1	GO:0030168	B	Refseq	platelet activation
XP_001985572.1 GH17139 [Drosophila grimshawi]	XP_001985572.1	3,00E-73	GLOS_DGRI_GH1	GO:0033198	B	Refseq	response to ATP
XP_001985572.1 GH17139 [Drosophila grimshawi]	XP_001985572.1	3,00E-73	GLOS_DGRI_GH1	GO:0051592	B	Refseq	response to calcium ion
XP_001985572.1 GH17139 [Drosophila grimshawi]	XP_001985572.1	3,00E-73	GLOS_DGRI_GH1	GO:0070588	B	Refseq	calcium ion transmembrane transport
XP_001985572.1 GH17139 [Drosophila grimshawi]	XP_001985572.1	3,00E-73	GLOS_DGRI_GH1	GO:0070679	M	Refseq	inositol 1,4,5 trisphosphate binding
NM_143699.4 D. melanogaster jim (jim), transcript variant C, mRNA	NM_143699.4	5,00E-06	GLOS_JIM.2.2	GO:0000122	B	Refseq	negative regulation of transcription from RNA polymerase II promoter
NM_143699.4 D. melanogaster jim (jim), transcript variant C, mRNA	NM_143699.4	5,00E-06	GLOS_JIM.2.2	GO:0001501	B	Refseq	skeletal system development
NM_143699.4 D. melanogaster jim (jim), transcript variant C, mRNA	NM_143699.4	5,00E-06	GLOS_JIM.2.2	GO:0003700	M	Refseq	sequence-specific DNA binding transcription factor activity
NM_143699.4 D. melanogaster jim (jim), transcript variant C, mRNA	NM_143699.4	5,00E-06	GLOS_JIM.2.2	GO:0005515	M	Refseq	protein binding
NM_143699.4 D. melanogaster jim (jim), transcript variant C, mRNA	NM_143699.4	5,00E-06	GLOS_JIM.2.2	GO:0005634	C	Refseq	nucleus
NM_143699.4 D. melanogaster jim (jim), transcript variant C, mRNA	NM_143699.4	5,00E-06	GLOS_JIM.2.2	GO:0006366	B	Refseq	transcription from RNA polymerase II promoter
NM_143699.4 D. melanogaster jim (jim), transcript variant C, mRNA	NM_143699.4	5,00E-06	GLOS_JIM.2.2	GO:0006607	B	Refseq	NLS-bearing protein import into nucleus
NM_143699.4 D. melanogaster jim (jim), transcript variant C, mRNA	NM_143699.4	5,00E-06	GLOS_JIM.2.2	GO:0008270	M	Refseq	zinc ion binding
NM_143699.4 D. melanogaster jim (jim), transcript variant C, mRNA	NM_143699.4	5,00E-06	GLOS_JIM.2.2	GO:0032330	B	Refseq	regulation of chondrocyte differentiation
NM_143699.4 D. melanogaster jim (jim), transcript variant C, mRNA	NM_143699.4	5,00E-06	GLOS_JIM.2.2	GO:0043565	M	Refseq	sequence-specific DNA binding
XP_001989108.1 GH10228 [Drosophila grimshawi]	XP_001989108.1	1,00E-155	GLOS_DGRI_GH1	GO:0005262	M	Refseq	calcium channel activity
XP_001989108.1 GH10228 [Drosophila grimshawi]	XP_001989108.1	1,00E-155	GLOS_DGRI_GH1	GO:0005515	M	Refseq	protein binding
XP_001989108.1 GH10228 [Drosophila grimshawi]	XP_001989108.1	1,00E-155	GLOS_DGRI_GH1	GO:0005886	C	Refseq	plasma membrane
XP_001989108.1 GH10228 [Drosophila grimshawi]	XP_001989108.1	1,00E-155	GLOS_DGRI_GH1	GO:0005887	C	Refseq	integral to plasma membrane
XP_001989108.1 GH10228 [Drosophila grimshawi]	XP_001989108.1	1,00E-155	GLOS_DGRI_GH1	GO:0006816	B	Refseq	calcium ion transport
XP_001989108.1 GH10228 [Drosophila grimshawi]	XP_001989108.1	1,00E-155	GLOS_DGRI_GH1	GO:0007411	B	Refseq	axon guidance
XP_001989108.1 GH10228 [Drosophila grimshawi]	XP_001989108.1	1,00E-155	GLOS_DGRI_GH1	GO:0007596	B	Refseq	blood coagulation
XP_001989108.1 GH10228 [Drosophila grimshawi]	XP_001989108.1	1,00E-155	GLOS_DGRI_GH1	GO:0007602	B	Refseq	phototransduction
XP_001989108.1 GH10228 [Drosophila grimshawi]	XP_001989108.1	1,00E-155	GLOS_DGRI_GH1	GO:0010524	B	Refseq	positive regulation of calcium ion transport into cytosol
XP_001989108.1 GH10228 [Drosophila grimshawi]	XP_001989108.1	1,00E-155	GLOS_DGRI_GH1	GO:0015279	M	Refseq	store-operated calcium channel activity
XP_001989108.1 GH10228 [Drosophila grimshawi]	XP_001989108.1	1,00E-155	GLOS_DGRI_GH1	GO:0030168	B	Refseq	platelet activation

XP_001989108.1 GH10228 [Drosophila grimshawi]	XP_001989108.1	1,00E-155	GLOS_DGRI_GH1	GO:0033198	B	Refseq	response to ATP
XP_001989108.1 GH10228 [Drosophila grimshawi]	XP_001989108.1	1,00E-155	GLOS_DGRI_GH1	GO:0051592	B	Refseq	response to calcium ion
XP_001989108.1 GH10228 [Drosophila grimshawi]	XP_001989108.1	1,00E-155	GLOS_DGRI_GH1	GO:0070588	B	Refseq	calcium ion transmembrane transport
XP_001989108.1 GH10228 [Drosophila grimshawi]	XP_001989108.1	1,00E-155	GLOS_DGRI_GH1	GO:0070679	M	Refseq	inositol 1,4,5 trisphosphate binding
NP_001097568.1 nudE, isoform B [Drosophila melanogaster]	NP_001097568.1	1,00E-111	GLOS_NUDE.1.1	GO:0000122	B	Refseq	negative regulation of transcription from RNA polymerase II promoter
NP_001097568.1 nudE, isoform B [Drosophila melanogaster]	NP_001097568.1	1,00E-111	GLOS_NUDE.1.1	GO:0001501	B	Refseq	skeletal system development
NP_001097568.1 nudE, isoform B [Drosophila melanogaster]	NP_001097568.1	1,00E-111	GLOS_NUDE.1.1	GO:0003700	M	Refseq	sequence-specific DNA binding transcription factor activity
NP_001097568.1 nudE, isoform B [Drosophila melanogaster]	NP_001097568.1	1,00E-111	GLOS_NUDE.1.1	GO:0005515	M	Refseq	protein binding
NP_001097568.1 nudE, isoform B [Drosophila melanogaster]	NP_001097568.1	1,00E-111	GLOS_NUDE.1.1	GO:0005634	C	Refseq	nucleus
NP_001097568.1 nudE, isoform B [Drosophila melanogaster]	NP_001097568.1	1,00E-111	GLOS_NUDE.1.1	GO:0006366	B	Refseq	transcription from RNA polymerase II promoter
NP_001097568.1 nudE, isoform B [Drosophila melanogaster]	NP_001097568.1	1,00E-111	GLOS_NUDE.1.1	GO:0006607	B	Refseq	NLS-bearing protein import into nucleus
NP_001097568.1 nudE, isoform B [Drosophila melanogaster]	NP_001097568.1	1,00E-111	GLOS_NUDE.1.1	GO:0008270	M	Refseq	zinc ion binding
NP_001097568.1 nudE, isoform B [Drosophila melanogaster]	NP_001097568.1	1,00E-111	GLOS_NUDE.1.1	GO:0032330	B	Refseq	regulation of chondrocyte differentiation
NP_001097568.1 nudE, isoform B [Drosophila melanogaster]	NP_001097568.1	1,00E-111	GLOS_NUDE.1.1	GO:0043565	M	Refseq	sequence-specific DNA binding
NP_001261414.1 syntaxin 17, isoform B [D. melanogaster]	NP_001261414.1	1,00E-123	GLOS_SYX17.1.1	GO:0000122	B	Refseq	negative regulation of transcription from RNA polymerase II promoter
NP_001261414.1 syntaxin 17, isoform B [D. melanogaster]	NP_001261414.1	1,00E-123	GLOS_SYX17.1.1	GO:0001501	B	Refseq	skeletal system development
NP_001261414.1 syntaxin 17, isoform B [D. melanogaster]	NP_001261414.1	1,00E-123	GLOS_SYX17.1.1	GO:0003700	M	Refseq	sequence-specific DNA binding transcription factor activity
NP_001261414.1 syntaxin 17, isoform B [D. melanogaster]	NP_001261414.1	1,00E-123	GLOS_SYX17.1.1	GO:0005515	M	Refseq	protein binding
NP_001261414.1 syntaxin 17, isoform B [D. melanogaster]	NP_001261414.1	1,00E-123	GLOS_SYX17.1.1	GO:0005634	C	Refseq	nucleus
NP_001261414.1 syntaxin 17, isoform B [D. melanogaster]	NP_001261414.1	1,00E-123	GLOS_SYX17.1.1	GO:0006366	B	Refseq	transcription from RNA polymerase II promoter
NP_001261414.1 syntaxin 17, isoform B [D. melanogaster]	NP_001261414.1	1,00E-123	GLOS_SYX17.1.1	GO:0006607	B	Refseq	NLS-bearing protein import into nucleus
NP_001261414.1 syntaxin 17, isoform B [D. melanogaster]	NP_001261414.1	1,00E-123	GLOS_SYX17.1.1	GO:0008270	M	Refseq	zinc ion binding
NP_001261414.1 syntaxin 17, isoform B [D. melanogaster]	NP_001261414.1	1,00E-123	GLOS_SYX17.1.1	GO:0032330	B	Refseq	regulation of chondrocyte differentiation
NP_001261414.1 syntaxin 17, isoform B [D. melanogaster]	NP_001261414.1	1,00E-123	GLOS_SYX17.1.1	GO:0043565	M	Refseq	sequence-specific DNA binding
XP_001981250.1 GG11725 [Drosophila erecta]	XP_001981250.1	1,00E-155	GLOS_DERE_GG	GO:0005261	M	Refseq	cation channel activity
XP_001981250.1 GG11725 [Drosophila erecta]	XP_001981250.1	1,00E-155	GLOS_DERE_GG	GO:0005262	M	Refseq	calcium channel activity
XP_001981250.1 GG11725 [Drosophila erecta]	XP_001981250.1	1,00E-155	GLOS_DERE_GG	GO:0005515	M	Refseq	protein binding
XP_001981250.1 GG11725 [Drosophila erecta]	XP_001981250.1	1,00E-155	GLOS_DERE_GG	GO:0005886	C	Refseq	plasma membrane
XP_001981250.1 GG11725 [Drosophila erecta]	XP_001981250.1	1,00E-155	GLOS_DERE_GG	GO:0005887	C	Refseq	integral to plasma membrane
XP_001981250.1 GG11725 [Drosophila erecta]	XP_001981250.1	1,00E-155	GLOS_DERE_GG	GO:0006816	B	Refseq	calcium ion transport
XP_001981250.1 GG11725 [Drosophila erecta]	XP_001981250.1	1,00E-155	GLOS_DERE_GG	GO:0007411	B	Refseq	axon guidance
XP_001981250.1 GG11725 [Drosophila erecta]	XP_001981250.1	1,00E-155	GLOS_DERE_GG	GO:0015279	M	Refseq	store-operated calcium channel activity
XP_001981250.1 GG11725 [Drosophila erecta]	XP_001981250.1	1,00E-155	GLOS_DERE_GG	GO:0016323	C	Refseq	basolateral plasma membrane
XP_001981250.1 GG11725 [Drosophila erecta]	XP_001981250.1	1,00E-155	GLOS_DERE_GG	GO:0030017	C	Refseq	sarcomere
XP_001981250.1 GG11725 [Drosophila erecta]	XP_001981250.1	1,00E-155	GLOS_DERE_GG	GO:0043034	C	Refseq	costamere
XP_001981250.1 GG11725 [Drosophila erecta]	XP_001981250.1	1,00E-155	GLOS_DERE_GG	GO:0043234	C	Refseq	protein complex
XP_001981250.1 GG11725 [Drosophila erecta]	XP_001981250.1	1,00E-155	GLOS_DERE_GG	GO:0044325	M	Refseq	ion channel binding
XP_001981250.1 GG11725 [Drosophila erecta]	XP_001981250.1	1,00E-155	GLOS_DERE_GG	GO:0045121	C	Refseq	membrane raft
XP_001981250.1 GG11725 [Drosophila erecta]	XP_001981250.1	1,00E-155	GLOS_DERE_GG	GO:0046541	B	Refseq	saliva secretion
XP_001981250.1 GG11725 [Drosophila erecta]	XP_001981250.1	1,00E-155	GLOS_DERE_GG	GO:0051281	B	Refseq	positive regulation of release of sequestered calcium ion into cytosol
XP_001981250.1 GG11725 [Drosophila erecta]	XP_001981250.1	1,00E-155	GLOS_DERE_GG	GO:0051480	B	Refseq	cytosolic calcium ion homeostasis
XP_001981250.1 GG11725 [Drosophila erecta]	XP_001981250.1	1,00E-155	GLOS_DERE_GG	GO:0051592	B	Refseq	response to calcium ion
XP_001981250.1 GG11725 [Drosophila erecta]	XP_001981250.1	1,00E-155	GLOS_DERE_GG	GO:0070679	M	Refseq	inositol 1,4,5 trisphosphate binding
NP_001260550.1 ribosomal protein L30, isoform D [D. melanogaster]	NP_001260550.1	3,00E-27	GLOS_RPL30.2.3	GO:0000122	B	Refseq	negative regulation of transcription from RNA polymerase II promoter
NP_001260550.1 ribosomal protein L30, isoform D [D. melanogaster]	NP_001260550.1	3,00E-27	GLOS_RPL30.2.3	GO:0001501	B	Refseq	skeletal system development
NP_001260550.1 ribosomal protein L30, isoform D [D. melanogaster]	NP_001260550.1	3,00E-27	GLOS_RPL30.2.3	GO:0003700	M	Refseq	sequence-specific DNA binding transcription factor activity
NP_001260550.1 ribosomal protein L30, isoform D [D. melanogaster]	NP_001260550.1	3,00E-27	GLOS_RPL30.2.3	GO:0005515	M	Refseq	protein binding
NP_001260550.1 ribosomal protein L30, isoform D [D. melanogaster]	NP_001260550.1	3,00E-27	GLOS_RPL30.2.3	GO:0005634	C	Refseq	nucleus
NP_001260550.1 ribosomal protein L30, isoform D [D. melanogaster]	NP_001260550.1	3,00E-27	GLOS_RPL30.2.3	GO:0006366	B	Refseq	transcription from RNA polymerase II promoter
NP_001260550.1 ribosomal protein L30, isoform D [D. melanogaster]	NP_001260550.1	3,00E-27	GLOS_RPL30.2.3	GO:0006607	B	Refseq	NLS-bearing protein import into nucleus
NP_001260550.1 ribosomal protein L30, isoform D [D. melanogaster]	NP_001260550.1	3,00E-27	GLOS_RPL30.2.3	GO:0008270	M	Refseq	zinc ion binding
NP_001260550.1 ribosomal protein L30, isoform D [D. melanogaster]	NP_001260550.1	3,00E-27	GLOS_RPL30.2.3	GO:0032330	B	Refseq	regulation of chondrocyte differentiation
NP_001260550.1 ribosomal protein L30, isoform D [D. melanogaster]	NP_001260550.1	3,00E-27	GLOS_RPL30.2.3	GO:0043565	M	Refseq	sequence-specific DNA binding
NP_610551.1 CG1371 [Drosophila melanogaster]	NP_610551.1	0	GLOS_CG1371.1.:	GO:0000122	B	Refseq	negative regulation of transcription from RNA polymerase II promoter

NP_610551.1 CG1371 [Drosophila melanogaster]	NP_610551.1	0	GLOS_CG1371.1.:	GO:0001501	B	Refseq	skeletal system development
NP_610551.1 CG1371 [Drosophila melanogaster]	NP_610551.1	0	GLOS_CG1371.1.:	GO:0003700	M	Refseq	sequence-specific DNA binding transcription factor activity
NP_610551.1 CG1371 [Drosophila melanogaster]	NP_610551.1	0	GLOS_CG1371.1.:	GO:0005515	M	Refseq	protein binding
NP_610551.1 CG1371 [Drosophila melanogaster]	NP_610551.1	0	GLOS_CG1371.1.:	GO:0005634	C	Refseq	nucleus
NP_610551.1 CG1371 [Drosophila melanogaster]	NP_610551.1	0	GLOS_CG1371.1.:	GO:0006366	B	Refseq	transcription from RNA polymerase II promoter
NP_610551.1 CG1371 [Drosophila melanogaster]	NP_610551.1	0	GLOS_CG1371.1.:	GO:0006607	B	Refseq	NLS-bearing protein import into nucleus
NP_610551.1 CG1371 [Drosophila melanogaster]	NP_610551.1	0	GLOS_CG1371.1.:	GO:0008270	M	Refseq	zinc ion binding
NP_610551.1 CG1371 [Drosophila melanogaster]	NP_610551.1	0	GLOS_CG1371.1.:	GO:0032330	B	Refseq	regulation of chondrocyte differentiation
NP_610551.1 CG1371 [Drosophila melanogaster]	NP_610551.1	0	GLOS_CG1371.1.:	GO:0043565	M	Refseq	sequence-specific DNA binding
XP_001991059.1 GH12467 [Drosophila grimshawi]	XP_001991059.1	5,00E-09	GLOS_DGRI_GH1	GO:0005262	M	Refseq	calcium channel activity
XP_001991059.1 GH12467 [Drosophila grimshawi]	XP_001991059.1	5,00E-09	GLOS_DGRI_GH1	GO:0005515	M	Refseq	protein binding
XP_001991059.1 GH12467 [Drosophila grimshawi]	XP_001991059.1	5,00E-09	GLOS_DGRI_GH1	GO:0005886	C	Refseq	plasma membrane
XP_001991059.1 GH12467 [Drosophila grimshawi]	XP_001991059.1	5,00E-09	GLOS_DGRI_GH1	GO:0005887	C	Refseq	integral to plasma membrane
XP_001991059.1 GH12467 [Drosophila grimshawi]	XP_001991059.1	5,00E-09	GLOS_DGRI_GH1	GO:0006816	B	Refseq	calcium ion transport
XP_001991059.1 GH12467 [Drosophila grimshawi]	XP_001991059.1	5,00E-09	GLOS_DGRI_GH1	GO:0007411	B	Refseq	axon guidance
XP_001991059.1 GH12467 [Drosophila grimshawi]	XP_001991059.1	5,00E-09	GLOS_DGRI_GH1	GO:0007596	B	Refseq	blood coagulation
XP_001991059.1 GH12467 [Drosophila grimshawi]	XP_001991059.1	5,00E-09	GLOS_DGRI_GH1	GO:0007602	B	Refseq	phototransduction
XP_001991059.1 GH12467 [Drosophila grimshawi]	XP_001991059.1	5,00E-09	GLOS_DGRI_GH1	GO:0010524	B	Refseq	positive regulation of calcium ion transport into cytosol
XP_001991059.1 GH12467 [Drosophila grimshawi]	XP_001991059.1	5,00E-09	GLOS_DGRI_GH1	GO:0015279	M	Refseq	store-operated calcium channel activity
XP_001991059.1 GH12467 [Drosophila grimshawi]	XP_001991059.1	5,00E-09	GLOS_DGRI_GH1	GO:0030168	B	Refseq	platelet activation
XP_001991059.1 GH12467 [Drosophila grimshawi]	XP_001991059.1	5,00E-09	GLOS_DGRI_GH1	GO:0033198	B	Refseq	response to ATP
XP_001991059.1 GH12467 [Drosophila grimshawi]	XP_001991059.1	5,00E-09	GLOS_DGRI_GH1	GO:0051592	B	Refseq	response to calcium ion
XP_001991059.1 GH12467 [Drosophila grimshawi]	XP_001991059.1	5,00E-09	GLOS_DGRI_GH1	GO:0070588	B	Refseq	calcium ion transmembrane transport
XP_001991059.1 GH12467 [Drosophila grimshawi]	XP_001991059.1	5,00E-09	GLOS_DGRI_GH1	GO:0070679	M	Refseq	inositol 1,4,5 trisphosphate binding
NP_001262988.1 scribbled, isoform T [Drosophila melanogaster]	NP_001262988.1	0	GLOS_contig_006	GO:0000122	B	Refseq	negative regulation of transcription from RNA polymerase II promoter
NP_001262988.1 scribbled, isoform T [Drosophila melanogaster]	NP_001262988.1	0	GLOS_contig_006	GO:0001501	B	Refseq	skeletal system development
NP_001262988.1 scribbled, isoform T [Drosophila melanogaster]	NP_001262988.1	0	GLOS_contig_006	GO:0003700	M	Refseq	sequence-specific DNA binding transcription factor activity
NP_001262988.1 scribbled, isoform T [Drosophila melanogaster]	NP_001262988.1	0	GLOS_contig_006	GO:0005515	M	Refseq	protein binding
NP_001262988.1 scribbled, isoform T [Drosophila melanogaster]	NP_001262988.1	0	GLOS_contig_006	GO:0005634	C	Refseq	nucleus
NP_001262988.1 scribbled, isoform T [Drosophila melanogaster]	NP_001262988.1	0	GLOS_contig_006	GO:0006366	B	Refseq	transcription from RNA polymerase II promoter
NP_001262988.1 scribbled, isoform T [Drosophila melanogaster]	NP_001262988.1	0	GLOS_contig_006	GO:0006607	B	Refseq	NLS-bearing protein import into nucleus
NP_001262988.1 scribbled, isoform T [Drosophila melanogaster]	NP_001262988.1	0	GLOS_contig_006	GO:0008270	M	Refseq	zinc ion binding
NP_001262988.1 scribbled, isoform T [Drosophila melanogaster]	NP_001262988.1	0	GLOS_contig_006	GO:0032330	B	Refseq	regulation of chondrocyte differentiation
NP_001262988.1 scribbled, isoform T [Drosophila melanogaster]	NP_001262988.1	0	GLOS_contig_006	GO:0043565	M	Refseq	sequence-specific DNA binding
XP_001982123.1 GG12419 [Drosophila erecta]	XP_001982123.1	0	GLOS_DERE_GG	GO:0005261	M	Refseq	cation channel activity
XP_001982123.1 GG12419 [Drosophila erecta]	XP_001982123.1	0	GLOS_DERE_GG	GO:0005262	M	Refseq	calcium channel activity
XP_001982123.1 GG12419 [Drosophila erecta]	XP_001982123.1	0	GLOS_DERE_GG	GO:0005515	M	Refseq	protein binding
XP_001982123.1 GG12419 [Drosophila erecta]	XP_001982123.1	0	GLOS_DERE_GG	GO:0005886	C	Refseq	plasma membrane
XP_001982123.1 GG12419 [Drosophila erecta]	XP_001982123.1	0	GLOS_DERE_GG	GO:0005887	C	Refseq	integral to plasma membrane
XP_001982123.1 GG12419 [Drosophila erecta]	XP_001982123.1	0	GLOS_DERE_GG	GO:0006816	B	Refseq	calcium ion transport
XP_001982123.1 GG12419 [Drosophila erecta]	XP_001982123.1	0	GLOS_DERE_GG	GO:0007411	B	Refseq	axon guidance
XP_001982123.1 GG12419 [Drosophila erecta]	XP_001982123.1	0	GLOS_DERE_GG	GO:0015279	M	Refseq	store-operated calcium channel activity
XP_001982123.1 GG12419 [Drosophila erecta]	XP_001982123.1	0	GLOS_DERE_GG	GO:0016323	C	Refseq	basolateral plasma membrane
XP_001982123.1 GG12419 [Drosophila erecta]	XP_001982123.1	0	GLOS_DERE_GG	GO:0030017	C	Refseq	sarcomere
XP_001982123.1 GG12419 [Drosophila erecta]	XP_001982123.1	0	GLOS_DERE_GG	GO:0043034	C	Refseq	costamere
XP_001982123.1 GG12419 [Drosophila erecta]	XP_001982123.1	0	GLOS_DERE_GG	GO:0043234	C	Refseq	protein complex
XP_001982123.1 GG12419 [Drosophila erecta]	XP_001982123.1	0	GLOS_DERE_GG	GO:0044325	M	Refseq	ion channel binding
XP_001982123.1 GG12419 [Drosophila erecta]	XP_001982123.1	0	GLOS_DERE_GG	GO:0045121	C	Refseq	membrane raft
XP_001982123.1 GG12419 [Drosophila erecta]	XP_001982123.1	0	GLOS_DERE_GG	GO:0046541	B	Refseq	saliva secretion
XP_001982123.1 GG12419 [Drosophila erecta]	XP_001982123.1	0	GLOS_DERE_GG	GO:0051281	B	Refseq	positive regulation of release of sequestered calcium ion into cytosol
XP_001982123.1 GG12419 [Drosophila erecta]	XP_001982123.1	0	GLOS_DERE_GG	GO:0051480	B	Refseq	cytosolic calcium ion homeostasis
XP_001982123.1 GG12419 [Drosophila erecta]	XP_001982123.1	0	GLOS_DERE_GG	GO:0051592	B	Refseq	response to calcium ion
XP_001982123.1 GG12419 [Drosophila erecta]	XP_001982123.1	0	GLOS_DERE_GG	GO:0070679	M	Refseq	inositol 1,4,5 trisphosphate binding
XP_001650367.1 hypothetical prot. AaeL_AAEL015047 [A. aegypti]	XP_001650367.1	6,00E-06	GLOS_AAEL_AAE	GO:0005070	M	Refseq	SH3/SH2 adaptor activity

XP_001650367.1 hypothetical prot. AaeL_AAEL015047 [A. aegypti]	XP_001650367.1	6,00E-06	GLOS_AAEL_AAE	GO:0005515	M	Refseq	protein binding
XP_001650367.1 hypothetical prot. AaeL_AAEL015047 [A. aegypti]	XP_001650367.1	6,00E-06	GLOS_AAEL_AAE	GO:0005634	C	Refseq	nucleus
XP_001650367.1 hypothetical prot. AaeL_AAEL015047 [A. aegypti]	XP_001650367.1	6,00E-06	GLOS_AAEL_AAE	GO:0005737	C	Refseq	cytoplasm
XP_001650367.1 hypothetical prot. AaeL_AAEL015047 [A. aegypti]	XP_001650367.1	6,00E-06	GLOS_AAEL_AAE	GO:0007049	B	Refseq	cell cycle
XP_001650367.1 hypothetical prot. AaeL_AAEL015047 [A. aegypti]	XP_001650367.1	6,00E-06	GLOS_AAEL_AAE	GO:0007165	B	Refseq	signal transduction
XP_001650367.1 hypothetical prot. AaeL_AAEL015047 [A. aegypti]	XP_001650367.1	6,00E-06	GLOS_AAEL_AAE	GO:0007417	B	Refseq	central nervous system development
XP_001650367.1 hypothetical prot. AaeL_AAEL015047 [A. aegypti]	XP_001650367.1	6,00E-06	GLOS_AAEL_AAE	GO:0007507	B	Refseq	heart development
XP_001650367.1 hypothetical prot. AaeL_AAEL015047 [A. aegypti]	XP_001650367.1	6,00E-06	GLOS_AAEL_AAE	GO:0009792	B	Refseq	embryo development ending in birth or egg hatching
XP_001650367.1 hypothetical prot. AaeL_AAEL015047 [A. aegypti]	XP_001650367.1	6,00E-06	GLOS_AAEL_AAE	GO:0010212	B	Refseq	response to ionizing radiation
XP_001650367.1 hypothetical prot. AaeL_AAEL015047 [A. aegypti]	XP_001650367.1	6,00E-06	GLOS_AAEL_AAE	GO:0017124	M	Refseq	SH3 domain binding
XP_001650367.1 hypothetical prot. AaeL_AAEL015047 [A. aegypti]	XP_001650367.1	6,00E-06	GLOS_AAEL_AAE	GO:0042802	M	Refseq	identical protein binding
XP_001650367.1 hypothetical prot. AaeL_AAEL015047 [A. aegypti]	XP_001650367.1	6,00E-06	GLOS_AAEL_AAE	GO:0045786	B	Refseq	negative regulation of cell cycle
XP_001650367.1 hypothetical prot. AaeL_AAEL015047 [A. aegypti]	XP_001650367.1	6,00E-06	GLOS_AAEL_AAE	GO:0048471	C	Refseq	perinuclear region of cytoplasm
XP_001650367.1 hypothetical prot. AaeL_AAEL015047 [A. aegypti]	XP_001650367.1	6,00E-06	GLOS_AAEL_AAE	GO:0051059	M	Refseq	NF-kappaB binding
XP_001650367.1 hypothetical prot. AaeL_AAEL015047 [A. aegypti]	XP_001650367.1	6,00E-06	GLOS_AAEL_AAE	GO:0072332	B	Refseq	intrinsic apoptotic signaling pathway by p53 class mediator
NP_001260176.1 CG13784, isoform F [Drosophila melanogaster]	NP_001260176.1	2,00E-23	GLOS_CG13784.1	GO:0000122	B	Refseq	negative regulation of transcription from RNA polymerase II promoter
NP_001260176.1 CG13784, isoform F [Drosophila melanogaster]	NP_001260176.1	2,00E-23	GLOS_CG13784.1	GO:0001501	B	Refseq	skeletal system development
NP_001260176.1 CG13784, isoform F [Drosophila melanogaster]	NP_001260176.1	2,00E-23	GLOS_CG13784.1	GO:0003700	M	Refseq	sequence-specific DNA binding transcription factor activity
NP_001260176.1 CG13784, isoform F [Drosophila melanogaster]	NP_001260176.1	2,00E-23	GLOS_CG13784.1	GO:0005515	M	Refseq	protein binding
NP_001260176.1 CG13784, isoform F [Drosophila melanogaster]	NP_001260176.1	2,00E-23	GLOS_CG13784.1	GO:0005634	C	Refseq	nucleus
NP_001260176.1 CG13784, isoform F [Drosophila melanogaster]	NP_001260176.1	2,00E-23	GLOS_CG13784.1	GO:0006366	B	Refseq	transcription from RNA polymerase II promoter
NP_001260176.1 CG13784, isoform F [Drosophila melanogaster]	NP_001260176.1	2,00E-23	GLOS_CG13784.1	GO:0006607	B	Refseq	NLS-bearing protein import into nucleus
NP_001260176.1 CG13784, isoform F [Drosophila melanogaster]	NP_001260176.1	2,00E-23	GLOS_CG13784.1	GO:0008270	M	Refseq	zinc ion binding
NP_001260176.1 CG13784, isoform F [Drosophila melanogaster]	NP_001260176.1	2,00E-23	GLOS_CG13784.1	GO:0032330	B	Refseq	regulation of chondrocyte differentiation
NP_001260176.1 CG13784, isoform F [Drosophila melanogaster]	NP_001260176.1	2,00E-23	GLOS_CG13784.1	GO:0043565	M	Refseq	sequence-specific DNA binding
XP_001990087.1 GH18428 [Drosophila grimshawi]	XP_001990087.1	9,00E-57	GLOS_DGRI_GH1	GO:0005262	M	Refseq	calcium channel activity
XP_001990087.1 GH18428 [Drosophila grimshawi]	XP_001990087.1	9,00E-57	GLOS_DGRI_GH1	GO:0005515	M	Refseq	protein binding
XP_001990087.1 GH18428 [Drosophila grimshawi]	XP_001990087.1	9,00E-57	GLOS_DGRI_GH1	GO:0005886	C	Refseq	plasma membrane
XP_001990087.1 GH18428 [Drosophila grimshawi]	XP_001990087.1	9,00E-57	GLOS_DGRI_GH1	GO:0005887	C	Refseq	integral to plasma membrane
XP_001990087.1 GH18428 [Drosophila grimshawi]	XP_001990087.1	9,00E-57	GLOS_DGRI_GH1	GO:0006816	B	Refseq	calcium ion transport
XP_001990087.1 GH18428 [Drosophila grimshawi]	XP_001990087.1	9,00E-57	GLOS_DGRI_GH1	GO:0007411	B	Refseq	axon guidance
XP_001990087.1 GH18428 [Drosophila grimshawi]	XP_001990087.1	9,00E-57	GLOS_DGRI_GH1	GO:0007596	B	Refseq	blood coagulation
XP_001990087.1 GH18428 [Drosophila grimshawi]	XP_001990087.1	9,00E-57	GLOS_DGRI_GH1	GO:0007602	B	Refseq	phototransduction
XP_001990087.1 GH18428 [Drosophila grimshawi]	XP_001990087.1	9,00E-57	GLOS_DGRI_GH1	GO:0010524	B	Refseq	positive regulation of calcium ion transport into cytosol
XP_001990087.1 GH18428 [Drosophila grimshawi]	XP_001990087.1	9,00E-57	GLOS_DGRI_GH1	GO:0015279	M	Refseq	store-operated calcium channel activity
XP_001990087.1 GH18428 [Drosophila grimshawi]	XP_001990087.1	9,00E-57	GLOS_DGRI_GH1	GO:0030168	B	Refseq	platelet activation
XP_001990087.1 GH18428 [Drosophila grimshawi]	XP_001990087.1	9,00E-57	GLOS_DGRI_GH1	GO:0033198	B	Refseq	response to ATP
XP_001990087.1 GH18428 [Drosophila grimshawi]	XP_001990087.1	9,00E-57	GLOS_DGRI_GH1	GO:0051592	B	Refseq	response to calcium ion
XP_001990087.1 GH18428 [Drosophila grimshawi]	XP_001990087.1	9,00E-57	GLOS_DGRI_GH1	GO:0070588	B	Refseq	calcium ion transmembrane transport
XP_001990087.1 GH18428 [Drosophila grimshawi]	XP_001990087.1	9,00E-57	GLOS_DGRI_GH1	GO:0070679	M	Refseq	inositol 1,4,5 trisphosphate binding
XP_001977597.1 GG18161 [Drosophila erecta]	XP_001977597.1	0	GLOS_DERE_GG	GO:0005261	M	Refseq	cation channel activity
XP_001977597.1 GG18161 [Drosophila erecta]	XP_001977597.1	0	GLOS_DERE_GG	GO:0005262	M	Refseq	calcium channel activity
XP_001977597.1 GG18161 [Drosophila erecta]	XP_001977597.1	0	GLOS_DERE_GG	GO:0005515	M	Refseq	protein binding
XP_001977597.1 GG18161 [Drosophila erecta]	XP_001977597.1	0	GLOS_DERE_GG	GO:0005886	C	Refseq	plasma membrane
XP_001977597.1 GG18161 [Drosophila erecta]	XP_001977597.1	0	GLOS_DERE_GG	GO:0005887	C	Refseq	integral to plasma membrane
XP_001977597.1 GG18161 [Drosophila erecta]	XP_001977597.1	0	GLOS_DERE_GG	GO:0006816	B	Refseq	calcium ion transport
XP_001977597.1 GG18161 [Drosophila erecta]	XP_001977597.1	0	GLOS_DERE_GG	GO:0007411	B	Refseq	axon guidance
XP_001977597.1 GG18161 [Drosophila erecta]	XP_001977597.1	0	GLOS_DERE_GG	GO:0015279	M	Refseq	store-operated calcium channel activity
XP_001977597.1 GG18161 [Drosophila erecta]	XP_001977597.1	0	GLOS_DERE_GG	GO:0016323	C	Refseq	basolateral plasma membrane
XP_001977597.1 GG18161 [Drosophila erecta]	XP_001977597.1	0	GLOS_DERE_GG	GO:0030017	C	Refseq	sarcomere
XP_001977597.1 GG18161 [Drosophila erecta]	XP_001977597.1	0	GLOS_DERE_GG	GO:0043034	C	Refseq	costamere
XP_001977597.1 GG18161 [Drosophila erecta]	XP_001977597.1	0	GLOS_DERE_GG	GO:0043234	C	Refseq	protein complex
XP_001977597.1 GG18161 [Drosophila erecta]	XP_001977597.1	0	GLOS_DERE_GG	GO:0044325	M	Refseq	ion channel binding
XP_001977597.1 GG18161 [Drosophila erecta]	XP_001977597.1	0	GLOS_DERE_GG	GO:0045121	C	Refseq	membrane raft

XP_001977597.1 GG18161 [Drosophila erecta]	XP_001977597.1	0	GLOS_DERE_GG	GO:0046541	B	Refseq	saliva secretion
XP_001977597.1 GG18161 [Drosophila erecta]	XP_001977597.1	0	GLOS_DERE_GG	GO:0051281	B	Refseq	positive regulation of release of sequestered calcium ion into cytosol
XP_001977597.1 GG18161 [Drosophila erecta]	XP_001977597.1	0	GLOS_DERE_GG	GO:0051480	B	Refseq	cytosolic calcium ion homeostasis
XP_001977597.1 GG18161 [Drosophila erecta]	XP_001977597.1	0	GLOS_DERE_GG	GO:0051592	B	Refseq	response to calcium ion
XP_001977597.1 GG18161 [Drosophila erecta]	XP_001977597.1	0	GLOS_DERE_GG	GO:0070679	M	Refseq	inositol 1,4,5 trisphosphate binding
XP_001970177.1 GG23519 [Drosophila erecta]	XP_001970177.1	1,00E-130	GLOS_DERE_GG	GO:0005261	M	Refseq	cation channel activity
XP_001970177.1 GG23519 [Drosophila erecta]	XP_001970177.1	1,00E-130	GLOS_DERE_GG	GO:0005262	M	Refseq	calcium channel activity
XP_001970177.1 GG23519 [Drosophila erecta]	XP_001970177.1	1,00E-130	GLOS_DERE_GG	GO:0005515	M	Refseq	protein binding
XP_001970177.1 GG23519 [Drosophila erecta]	XP_001970177.1	1,00E-130	GLOS_DERE_GG	GO:0005886	C	Refseq	plasma membrane
XP_001970177.1 GG23519 [Drosophila erecta]	XP_001970177.1	1,00E-130	GLOS_DERE_GG	GO:0005887	C	Refseq	integral to plasma membrane
XP_001970177.1 GG23519 [Drosophila erecta]	XP_001970177.1	1,00E-130	GLOS_DERE_GG	GO:0006816	B	Refseq	calcium ion transport
XP_001970177.1 GG23519 [Drosophila erecta]	XP_001970177.1	1,00E-130	GLOS_DERE_GG	GO:0007411	B	Refseq	axon guidance
XP_001970177.1 GG23519 [Drosophila erecta]	XP_001970177.1	1,00E-130	GLOS_DERE_GG	GO:0015279	M	Refseq	store-operated calcium channel activity
XP_001970177.1 GG23519 [Drosophila erecta]	XP_001970177.1	1,00E-130	GLOS_DERE_GG	GO:0016323	C	Refseq	basolateral plasma membrane
XP_001970177.1 GG23519 [Drosophila erecta]	XP_001970177.1	1,00E-130	GLOS_DERE_GG	GO:0030017	C	Refseq	sarcomere
XP_001970177.1 GG23519 [Drosophila erecta]	XP_001970177.1	1,00E-130	GLOS_DERE_GG	GO:0043034	C	Refseq	costamere
XP_001970177.1 GG23519 [Drosophila erecta]	XP_001970177.1	1,00E-130	GLOS_DERE_GG	GO:0043234	C	Refseq	protein complex
XP_001970177.1 GG23519 [Drosophila erecta]	XP_001970177.1	1,00E-130	GLOS_DERE_GG	GO:0044325	M	Refseq	ion channel binding
XP_001970177.1 GG23519 [Drosophila erecta]	XP_001970177.1	1,00E-130	GLOS_DERE_GG	GO:0045121	C	Refseq	membrane raft
XP_001970177.1 GG23519 [Drosophila erecta]	XP_001970177.1	1,00E-130	GLOS_DERE_GG	GO:0046541	B	Refseq	saliva secretion
XP_001970177.1 GG23519 [Drosophila erecta]	XP_001970177.1	1,00E-130	GLOS_DERE_GG	GO:0051281	B	Refseq	positive regulation of release of sequestered calcium ion into cytosol
XP_001970177.1 GG23519 [Drosophila erecta]	XP_001970177.1	1,00E-130	GLOS_DERE_GG	GO:0051480	B	Refseq	cytosolic calcium ion homeostasis
XP_001970177.1 GG23519 [Drosophila erecta]	XP_001970177.1	1,00E-130	GLOS_DERE_GG	GO:0051592	B	Refseq	response to calcium ion
XP_001970177.1 GG23519 [Drosophila erecta]	XP_001970177.1	1,00E-130	GLOS_DERE_GG	GO:0070679	M	Refseq	inositol 1,4,5 trisphosphate binding
XP_001989735.1 GH18956 [Drosophila grimshawi]	XP_001989735.1	1,00E-107	GLOS_DGRI_GH1	GO:0005262	M	Refseq	calcium channel activity
XP_001989735.1 GH18956 [Drosophila grimshawi]	XP_001989735.1	1,00E-107	GLOS_DGRI_GH1	GO:0005515	M	Refseq	protein binding
XP_001989735.1 GH18956 [Drosophila grimshawi]	XP_001989735.1	1,00E-107	GLOS_DGRI_GH1	GO:0005886	C	Refseq	plasma membrane
XP_001989735.1 GH18956 [Drosophila grimshawi]	XP_001989735.1	1,00E-107	GLOS_DGRI_GH1	GO:0005887	C	Refseq	integral to plasma membrane
XP_001989735.1 GH18956 [Drosophila grimshawi]	XP_001989735.1	1,00E-107	GLOS_DGRI_GH1	GO:0006816	B	Refseq	calcium ion transport
XP_001989735.1 GH18956 [Drosophila grimshawi]	XP_001989735.1	1,00E-107	GLOS_DGRI_GH1	GO:0007411	B	Refseq	axon guidance
XP_001989735.1 GH18956 [Drosophila grimshawi]	XP_001989735.1	1,00E-107	GLOS_DGRI_GH1	GO:0007596	B	Refseq	blood coagulation
XP_001989735.1 GH18956 [Drosophila grimshawi]	XP_001989735.1	1,00E-107	GLOS_DGRI_GH1	GO:0007602	B	Refseq	phototransduction
XP_001989735.1 GH18956 [Drosophila grimshawi]	XP_001989735.1	1,00E-107	GLOS_DGRI_GH1	GO:0010524	B	Refseq	positive regulation of calcium ion transport into cytosol
XP_001989735.1 GH18956 [Drosophila grimshawi]	XP_001989735.1	1,00E-107	GLOS_DGRI_GH1	GO:0015279	M	Refseq	store-operated calcium channel activity
XP_001989735.1 GH18956 [Drosophila grimshawi]	XP_001989735.1	1,00E-107	GLOS_DGRI_GH1	GO:0030168	B	Refseq	platelet activation
XP_001989735.1 GH18956 [Drosophila grimshawi]	XP_001989735.1	1,00E-107	GLOS_DGRI_GH1	GO:0033198	B	Refseq	response to ATP
XP_001989735.1 GH18956 [Drosophila grimshawi]	XP_001989735.1	1,00E-107	GLOS_DGRI_GH1	GO:0051592	B	Refseq	response to calcium ion
XP_001989735.1 GH18956 [Drosophila grimshawi]	XP_001989735.1	1,00E-107	GLOS_DGRI_GH1	GO:0070588	B	Refseq	calcium ion transmembrane transport
XP_001989735.1 GH18956 [Drosophila grimshawi]	XP_001989735.1	1,00E-107	GLOS_DGRI_GH1	GO:0070679	M	Refseq	inositol 1,4,5 trisphosphate binding
NP_572699.2 CG42683, isoform A [Drosophila melanogaster]	NP_572699.2	1,00E-116	GLOS_CG42683.1	GO:0000122	B	Refseq	negative regulation of transcription from RNA polymerase II promoter
NP_572699.2 CG42683, isoform A [Drosophila melanogaster]	NP_572699.2	1,00E-116	GLOS_CG42683.1	GO:0001501	B	Refseq	skeletal system development
NP_572699.2 CG42683, isoform A [Drosophila melanogaster]	NP_572699.2	1,00E-116	GLOS_CG42683.1	GO:0003700	M	Refseq	sequence-specific DNA binding transcription factor activity
NP_572699.2 CG42683, isoform A [Drosophila melanogaster]	NP_572699.2	1,00E-116	GLOS_CG42683.1	GO:0005515	M	Refseq	protein binding
NP_572699.2 CG42683, isoform A [Drosophila melanogaster]	NP_572699.2	1,00E-116	GLOS_CG42683.1	GO:0005634	C	Refseq	nucleus
NP_572699.2 CG42683, isoform A [Drosophila melanogaster]	NP_572699.2	1,00E-116	GLOS_CG42683.1	GO:0006366	B	Refseq	transcription from RNA polymerase II promoter
NP_572699.2 CG42683, isoform A [Drosophila melanogaster]	NP_572699.2	1,00E-116	GLOS_CG42683.1	GO:0006607	B	Refseq	NLS-bearing protein import into nucleus
NP_572699.2 CG42683, isoform A [Drosophila melanogaster]	NP_572699.2	1,00E-116	GLOS_CG42683.1	GO:0008270	M	Refseq	zinc ion binding
NP_572699.2 CG42683, isoform A [Drosophila melanogaster]	NP_572699.2	1,00E-116	GLOS_CG42683.1	GO:0032330	B	Refseq	regulation of chondrocyte differentiation
NP_572699.2 CG42683, isoform A [Drosophila melanogaster]	NP_572699.2	1,00E-116	GLOS_CG42683.1	GO:0043565	M	Refseq	sequence-specific DNA binding
NP_665700.2 Z band alternatively spliced PDZ-motif prot. 52, isoform C [D.m.]	NP_665700.2	0	GLOS_ZASP52.1.	GO:0000122	B	Refseq	negative regulation of transcription from RNA polymerase II promoter
NP_665700.2 Z band alternatively spliced PDZ-motif prot. 52, isoform C [D.m.]	NP_665700.2	0	GLOS_ZASP52.1.	GO:0001501	B	Refseq	skeletal system development
NP_665700.2 Z band alternatively spliced PDZ-motif prot. 52, isoform C [D.m.]	NP_665700.2	0	GLOS_ZASP52.1.	GO:0003700	M	Refseq	sequence-specific DNA binding transcription factor activity
NP_665700.2 Z band alternatively spliced PDZ-motif prot. 52, isoform C [D.m.]	NP_665700.2	0	GLOS_ZASP52.1.	GO:0005515	M	Refseq	protein binding
NP_665700.2 Z band alternatively spliced PDZ-motif prot. 52, isoform C [D.m.]	NP_665700.2	0	GLOS_ZASP52.1.	GO:0005634	C	Refseq	nucleus

NP_665700.2 Z band alternatively spliced PDZ-motif prot. 52, isoform C [D.m.]	NP_665700.2	0	GLOS_ZASP52.1.	GO:0006366	B	Refseq	transcription from RNA polymerase II promoter
NP_665700.2 Z band alternatively spliced PDZ-motif prot. 52, isoform C [D.m.]	NP_665700.2	0	GLOS_ZASP52.1.	GO:0006607	B	Refseq	NLS-bearing protein import into nucleus
NP_665700.2 Z band alternatively spliced PDZ-motif prot. 52, isoform C [D.m.]	NP_665700.2	0	GLOS_ZASP52.1.	GO:0008270	M	Refseq	zinc ion binding
NP_665700.2 Z band alternatively spliced PDZ-motif prot. 52, isoform C [D.m.]	NP_665700.2	0	GLOS_ZASP52.1.	GO:0032330	B	Refseq	regulation of chondrocyte differentiation
NP_665700.2 Z band alternatively spliced PDZ-motif prot. 52, isoform C [D.m.]	NP_665700.2	0	GLOS_ZASP52.1.	GO:0043565	M	Refseq	sequence-specific DNA binding
ADF1_DROME (sp|P05552) Transcrip. Fact. Adf-1 OS=D.m. GN=Adf1 PE=2 SV=2	ADF1_DROME	1,00E-116	GLOS_ADF1.1.3	GO:0005634	C	FlyBase	nucleus
ADF1_DROME (sp|P05552) Transcrip. Fact. Adf-1 OS=D.m. GN=Adf1 PE=2 SV=2	ADF1_DROME	1,00E-116	GLOS_ADF1.1.3	GO:0003677	M	UniProtKB-K	DNA binding
ADF1_DROME (sp|P05552) Transcrip. Fact. Adf-1 OS=D.m. GN=Adf1 PE=2 SV=2	ADF1_DROME	1,00E-116	GLOS_ADF1.1.3	GO:0000982	M	.	RNA polymerase II core promoter proximal region sequence-specific DNA binding transcription factor activity
ADF1_DROME (sp|P05552) Transcrip. Fact. Adf-1 OS=D.m. GN=Adf1 PE=2 SV=2	ADF1_DROME	1,00E-116	GLOS_ADF1.1.3	GO:0000981	M	.	sequence-specific DNA binding RNA polymerase II transcription factor activity
ADF1_DROME (sp|P05552) Transcrip. Fact. Adf-1 OS=D.m. GN=Adf1 PE=2 SV=2	ADF1_DROME	1,00E-116	GLOS_ADF1.1.3	GO:0003700	M	.	sequence-specific DNA binding transcription factor activity
ADF1_DROME (sp|P05552) Transcrip. Fact. Adf-1 OS=D.m. GN=Adf1 PE=2 SV=2	ADF1_DROME	1,00E-116	GLOS_ADF1.1.3	GO:0001228	M	.	RNA polymerase II transcript. regulatory region sequence-specific DNA binding transcription factor activity involved in positive regulation of transcription
ADF1_DROME (sp|P05552) Transcrip. Fact. Adf-1 OS=D.m. GN=Adf1 PE=2 SV=2	ADF1_DROME	1,00E-116	GLOS_ADF1.1.3	GO:0001077	M	FlyBase	RNA polymerase II core promoter proximal region sequence-specific DNA binding transcription factor activity involved in positive regulation of transcription
ADF1_DROME (sp|P05552) Transcrip. Fact. Adf-1 OS=D.m. GN=Adf1 PE=2 SV=2	ADF1_DROME	1,00E-116	GLOS_ADF1.1.3	GO:0016358	B	.	dendrite development
ADF1_DROME (sp|P05552) Transcrip. Fact. Adf-1 OS=D.m. GN=Adf1 PE=2 SV=2	ADF1_DROME	1,00E-116	GLOS_ADF1.1.3	GO:0048813	B	FlyBase	dendrite morphogenesis
ADF1_DROME (sp|P05552) Transcrip. Fact. Adf-1 OS=D.m. GN=Adf1 PE=2 SV=2	ADF1_DROME	1,00E-116	GLOS_ADF1.1.3	GO:0007626	B	.	locomotory behavior
ADF1_DROME (sp|P05552) Transcrip. Fact. Adf-1 OS=D.m. GN=Adf1 PE=2 SV=2	ADF1_DROME	1,00E-116	GLOS_ADF1.1.3	GO:0044708	B	.	single-organism behavior
ADF1_DROME (sp|P05552) Transcrip. Fact. Adf-1 OS=D.m. GN=Adf1 PE=2 SV=2	ADF1_DROME	1,00E-116	GLOS_ADF1.1.3	GO:0007610	B	.	behavior
ADF1_DROME (sp|P05552) Transcrip. Fact. Adf-1 OS=D.m. GN=Adf1 PE=2 SV=2	ADF1_DROME	1,00E-116	GLOS_ADF1.1.3	GO:0030537	B	.	larval behavior
ADF1_DROME (sp|P05552) Transcrip. Fact. Adf-1 OS=D.m. GN=Adf1 PE=2 SV=2	ADF1_DROME	1,00E-116	GLOS_ADF1.1.3	GO:0008345	B	FlyBase	larval locomotory behavior
ADF1_DROME (sp|P05552) Transcrip. Fact. Adf-1 OS=D.m. GN=Adf1 PE=2 SV=2	ADF1_DROME	1,00E-116	GLOS_ADF1.1.3	GO:0040011	B	FlyBase	locomotion
ADF1_DROME (sp|P05552) Transcrip. Fact. Adf-1 OS=D.m. GN=Adf1 PE=2 SV=2	ADF1_DROME	1,00E-116	GLOS_ADF1.1.3	GO:0007613	B	.	memory
ADF1_DROME (sp|P05552) Transcrip. Fact. Adf-1 OS=D.m. GN=Adf1 PE=2 SV=2	ADF1_DROME	1,00E-116	GLOS_ADF1.1.3	GO:0007611	B	.	learning or memory
ADF1_DROME (sp|P05552) Transcrip. Fact. Adf-1 OS=D.m. GN=Adf1 PE=2 SV=2	ADF1_DROME	1,00E-116	GLOS_ADF1.1.3	GO:0050890	B	.	cognition
ADF1_DROME (sp|P05552) Transcrip. Fact. Adf-1 OS=D.m. GN=Adf1 PE=2 SV=2	ADF1_DROME	1,00E-116	GLOS_ADF1.1.3	GO:0050877	B	.	neurological system process
ADF1_DROME (sp|P05552) Transcrip. Fact. Adf-1 OS=D.m. GN=Adf1 PE=2 SV=2	ADF1_DROME	1,00E-116	GLOS_ADF1.1.3	GO:0007616	B	FlyBase	long-term memory
ADF1_DROME (sp|P05552) Transcrip. Fact. Adf-1 OS=D.m. GN=Adf1 PE=2 SV=2	ADF1_DROME	1,00E-116	GLOS_ADF1.1.3	GO:0008306	B	.	associative learning
ADF1_DROME (sp|P05552) Transcrip. Fact. Adf-1 OS=D.m. GN=Adf1 PE=2 SV=2	ADF1_DROME	1,00E-116	GLOS_ADF1.1.3	GO:0007612	B	.	learning
ADF1_DROME (sp|P05552) Transcrip. Fact. Adf-1 OS=D.m. GN=Adf1 PE=2 SV=2	ADF1_DROME	1,00E-116	GLOS_ADF1.1.3	GO:0042048	B	.	olfactory behavior
ADF1_DROME (sp|P05552) Transcrip. Fact. Adf-1 OS=D.m. GN=Adf1 PE=2 SV=2	ADF1_DROME	1,00E-116	GLOS_ADF1.1.3	GO:0007635	B	.	chemosensory behavior
ADF1_DROME (sp|P05552) Transcrip. Fact. Adf-1 OS=D.m. GN=Adf1 PE=2 SV=2	ADF1_DROME	1,00E-116	GLOS_ADF1.1.3	GO:0008355	B	FlyBase	olfactory learning
ADF1_DROME (sp|P05552) Transcrip. Fact. Adf-1 OS=D.m. GN=Adf1 PE=2 SV=2	ADF1_DROME	1,00E-116	GLOS_ADF1.1.3	GO:0022607	B	.	cellular component assembly
ADF1_DROME (sp|P05552) Transcrip. Fact. Adf-1 OS=D.m. GN=Adf1 PE=2 SV=2	ADF1_DROME	1,00E-116	GLOS_ADF1.1.3	GO:0044085	B	.	cellular component biogenesis
ADF1_DROME (sp|P05552) Transcrip. Fact. Adf-1 OS=D.m. GN=Adf1 PE=2 SV=2	ADF1_DROME	1,00E-116	GLOS_ADF1.1.3	GO:0050808	B	.	synapse organization
ADF1_DROME (sp|P05552) Transcrip. Fact. Adf-1 OS=D.m. GN=Adf1 PE=2 SV=2	ADF1_DROME	1,00E-116	GLOS_ADF1.1.3	GO:0007416	B	FlyBase	synapse assembly
[BBH] ARR1_CALVI (sp|P51486) Phosrestin-2 OS=C. vicina GN=ARR1 PE=2 SV=1	ARR1_CALVI	0	GLOS_ARR1.1.1	GO:0007165	B	InterPro	signal transduction
[BBH] ARR1_CALVI (sp|P51486) Phosrestin-2 OS=C. vicina GN=ARR1 PE=2 SV=1	ARR1_CALVI	0	GLOS_ARR1.1.1	GO:0050953	B	.	sensory perception of light stimulus
[BBH] ARR1_CALVI (sp|P51486) Phosrestin-2 OS=C. vicina GN=ARR1 PE=2 SV=1	ARR1_CALVI	0	GLOS_ARR1.1.1	GO:0007600	B	.	sensory perception
[BBH] ARR1_CALVI (sp|P51486) Phosrestin-2 OS=C. vicina GN=ARR1 PE=2 SV=1	ARR1_CALVI	0	GLOS_ARR1.1.1	GO:0007601	B	UniProtKB-K	visual perception
[BBH] ATG5_DROME (sp|Q9W3R7) Autophagy prot. 5 OS=D.m.	ATG5_DROME	1,00E-144	GLOS_ATG5.1.1	GO:0005737	C	UniProtKB	cytoplasm
[BBH] ATG5_DROME (sp|Q9W3R7) Autophagy prot. 5 OS=D.m.	ATG5_DROME	1,00E-144	GLOS_ATG5.1.1	GO:0000407	C	.	pre-autophagosomal structure
[BBH] ATG5_DROME (sp|Q9W3R7) Autophagy prot. 5 OS=D.m.	ATG5_DROME	1,00E-144	GLOS_ATG5.1.1	GO:0034045	C	UniProtKB-S	pre-autophagosomal structure membrane
[BBH] ATG5_DROME (sp|Q9W3R7) Autophagy prot. 5 OS=D.m.	ATG5_DROME	1,00E-144	GLOS_ATG5.1.1	GO:0006914	B	.	autophagy
[BBH] ATG5_DROME (sp|Q9W3R7) Autophagy prot. 5 OS=D.m.	ATG5_DROME	1,00E-144	GLOS_ATG5.1.1	GO:0044248	B	.	cellular catabolic process
[BBH] ATG5_DROME (sp|Q9W3R7) Autophagy prot. 5 OS=D.m.	ATG5_DROME	1,00E-144	GLOS_ATG5.1.1	GO:0009056	B	.	catabolic process
[BBH] ATG5_DROME (sp|Q9W3R7) Autophagy prot. 5 OS=D.m.	ATG5_DROME	1,00E-144	GLOS_ATG5.1.1	GO:0009267	B	.	cellular response to starvation
[BBH] ATG5_DROME (sp|Q9W3R7) Autophagy prot. 5 OS=D.m.	ATG5_DROME	1,00E-144	GLOS_ATG5.1.1	GO:0031669	B	.	cellular response to nutrient levels
[BBH] ATG5_DROME (sp|Q9W3R7) Autophagy prot. 5 OS=D.m.	ATG5_DROME	1,00E-144	GLOS_ATG5.1.1	GO:0031667	B	.	response to nutrient levels
[BBH] ATG5_DROME (sp|Q9W3R7) Autophagy prot. 5 OS=D.m.	ATG5_DROME	1,00E-144	GLOS_ATG5.1.1	GO:0009991	B	.	response to extracellular stimulus
[BBH] ATG5_DROME (sp|Q9W3R7) Autophagy prot. 5 OS=D.m.	ATG5_DROME	1,00E-144	GLOS_ATG5.1.1	GO:0031668	B	.	cellular response to extracellular stimulus
[BBH] ATG5_DROME (sp|Q9W3R7) Autophagy prot. 5 OS=D.m.	ATG5_DROME	1,00E-144	GLOS_ATG5.1.1	GO:0071496	B	.	cellular response to external stimulus
[BBH] ATG5_DROME (sp|Q9W3R7) Autophagy prot. 5 OS=D.m.	ATG5_DROME	1,00E-144	GLOS_ATG5.1.1	GO:0033554	B	.	cellular response to stress

[BBH] ATG5_DROME (sp|Q9W3R7) Autophagy prot. 5 OS=D.m.	ATG5_DROME	1,00E-144	GLOS_ATG5.1.1	GO:0042594	B	.	response to starvation
[BBH] ATG5_DROME (sp|Q9W3R7) Autophagy prot. 5 OS=D.m.	ATG5_DROME	1,00E-144	GLOS_ATG5.1.1	GO:0016236	B	FlyBase	macroautophagy
[BBH] ATG5_DROME (sp|Q9W3R7) Autophagy prot. 5 OS=D.m.	ATG5_DROME	1,00E-144	GLOS_ATG5.1.1	GO:0015031	B	UniProtKB-K	protein transport
[BBH] ATG5_DROME (sp|Q9W3R7) Autophagy prot. 5 OS=D.m.	ATG5_DROME	1,00E-144	GLOS_ATG5.1.1	GO:0048102	B	.	autophagic cell death
[BBH] ATG5_DROME (sp|Q9W3R7) Autophagy prot. 5 OS=D.m.	ATG5_DROME	1,00E-144	GLOS_ATG5.1.1	GO:0035070	B	.	salivary gland histolysis
[BBH] ATG5_DROME (sp|Q9W3R7) Autophagy prot. 5 OS=D.m.	ATG5_DROME	1,00E-144	GLOS_ATG5.1.1	GO:0007435	B	.	salivary gland morphogenesis
[BBH] ATG5_DROME (sp|Q9W3R7) Autophagy prot. 5 OS=D.m.	ATG5_DROME	1,00E-144	GLOS_ATG5.1.1	GO:0022612	B	.	gland morphogenesis
[BBH] ATG5_DROME (sp|Q9W3R7) Autophagy prot. 5 OS=D.m.	ATG5_DROME	1,00E-144	GLOS_ATG5.1.1	GO:0048732	B	.	gland development
[BBH] ATG5_DROME (sp|Q9W3R7) Autophagy prot. 5 OS=D.m.	ATG5_DROME	1,00E-144	GLOS_ATG5.1.1	GO:0007431	B	.	salivary gland development
[BBH] ATG5_DROME (sp|Q9W3R7) Autophagy prot. 5 OS=D.m.	ATG5_DROME	1,00E-144	GLOS_ATG5.1.1	GO:0035272	B	.	exocrine system development
[BBH] ATG5_DROME (sp|Q9W3R7) Autophagy prot. 5 OS=D.m.	ATG5_DROME	1,00E-144	GLOS_ATG5.1.1	GO:0007559	B	.	histolysis
[BBH] ATG5_DROME (sp|Q9W3R7) Autophagy prot. 5 OS=D.m.	ATG5_DROME	1,00E-144	GLOS_ATG5.1.1	GO:0016271	B	.	tissue death
[BBH] ATG5_DROME (sp|Q9W3R7) Autophagy prot. 5 OS=D.m.	ATG5_DROME	1,00E-144	GLOS_ATG5.1.1	GO:0009888	B	.	tissue development
[BBH] ATG5_DROME (sp|Q9W3R7) Autophagy prot. 5 OS=D.m.	ATG5_DROME	1,00E-144	GLOS_ATG5.1.1	GO:0009886	B	.	post-embryonic morphogenesis
[BBH] ATG5_DROME (sp|Q9W3R7) Autophagy prot. 5 OS=D.m.	ATG5_DROME	1,00E-144	GLOS_ATG5.1.1	GO:0009791	B	.	post-embryonic development
[BBH] ATG5_DROME (sp|Q9W3R7) Autophagy prot. 5 OS=D.m.	ATG5_DROME	1,00E-144	GLOS_ATG5.1.1	GO:0007552	B	.	metamorphosis
[BBH] ATG5_DROME (sp|Q9W3R7) Autophagy prot. 5 OS=D.m.	ATG5_DROME	1,00E-144	GLOS_ATG5.1.1	GO:0048707	B	.	instar larval or pupal morphogenesis
[BBH] ATG5_DROME (sp|Q9W3R7) Autophagy prot. 5 OS=D.m.	ATG5_DROME	1,00E-144	GLOS_ATG5.1.1	GO:0002165	B	.	instar larval or pupal development
[BBH] ATG5_DROME (sp|Q9W3R7) Autophagy prot. 5 OS=D.m.	ATG5_DROME	1,00E-144	GLOS_ATG5.1.1	GO:0035071	B	FlyBase	salivary gland cell autophagic cell death
ATTA_GLOMM (sp|Q8WTD3) Attacin-A OS=G. m. morsitans PE=1 SV=1	ATTA_GLOMM	1,00E-131	GLOS_ATTA.3.7	GO:0005576	C	UniProtKB	extracellular region
ATTA_GLOMM (sp|Q8WTD3) Attacin-A OS=G. m. morsitans PE=1 SV=1	ATTA_GLOMM	1,00E-131	GLOS_ATTA.3.7	GO:0019730	B	.	antimicrobial humoral response
ATTA_GLOMM (sp|Q8WTD3) Attacin-A OS=G. m. morsitans PE=1 SV=1	ATTA_GLOMM	1,00E-131	GLOS_ATTA.3.7	GO:0006959	B	.	humoral immune response
ATTA_GLOMM (sp|Q8WTD3) Attacin-A OS=G. m. morsitans PE=1 SV=1	ATTA_GLOMM	1,00E-131	GLOS_ATTA.3.7	GO:0006955	B	.	immune response
ATTA_GLOMM (sp|Q8WTD3) Attacin-A OS=G. m. morsitans PE=1 SV=1	ATTA_GLOMM	1,00E-131	GLOS_ATTA.3.7	GO:0002376	B	.	immune system process
ATTA_GLOMM (sp|Q8WTD3) Attacin-A OS=G. m. morsitans PE=1 SV=1	ATTA_GLOMM	1,00E-131	GLOS_ATTA.3.7	GO:0051707	B	.	response to other organism
ATTA_GLOMM (sp|Q8WTD3) Attacin-A OS=G. m. morsitans PE=1 SV=1	ATTA_GLOMM	1,00E-131	GLOS_ATTA.3.7	GO:0009607	B	.	response to biotic stimulus
ATTA_GLOMM (sp|Q8WTD3) Attacin-A OS=G. m. morsitans PE=1 SV=1	ATTA_GLOMM	1,00E-131	GLOS_ATTA.3.7	GO:0051704	B	.	multi-organism process
ATTA_GLOMM (sp|Q8WTD3) Attacin-A OS=G. m. morsitans PE=1 SV=1	ATTA_GLOMM	1,00E-131	GLOS_ATTA.3.7	GO:0042742	B	.	defense response to bacterium
ATTA_GLOMM (sp|Q8WTD3) Attacin-A OS=G. m. morsitans PE=1 SV=1	ATTA_GLOMM	1,00E-131	GLOS_ATTA.3.7	GO:0006952	B	.	defense response
ATTA_GLOMM (sp|Q8WTD3) Attacin-A OS=G. m. morsitans PE=1 SV=1	ATTA_GLOMM	1,00E-131	GLOS_ATTA.3.7	GO:0009617	B	.	response to bacterium
ATTA_GLOMM (sp|Q8WTD3) Attacin-A OS=G. m. morsitans PE=1 SV=1	ATTA_GLOMM	1,00E-131	GLOS_ATTA.3.7	GO:0019731	B	UniProtKB	antibacterial humoral response
ATTA_GLOMM (sp|Q8WTD3) Attacin-A OS=G. m. morsitans PE=1 SV=1	ATTA_GLOMM	1,00E-131	GLOS_ATTA.3.7	GO:0050829	B	UniProtKB	defense response to Gram-negative bacterium
ATTA_GLOMM (sp|Q8WTD3) Attacin-A OS=G. m. morsitans PE=1 SV=1	ATTA_GLOMM	1,00E-131	GLOS_ATTA.3.7	GO:0045087	B	UniProtKB-K	innate immune response
[BBH] BGB_DROME (sp|Q24040) Prot. big brother OS=D.m. GN=Bgb PE=2 SV=3	BGB_DROME	1,00E-146	GLOS_BGB.1.1	GO:0005634	C	FlyBase	nucleus
[BBH] BGB_DROME (sp|Q24040) Prot. big brother OS=D.m. GN=Bgb PE=2 SV=3	BGB_DROME	1,00E-146	GLOS_BGB.1.1	GO:0046983	M	.	protein dimerization activity
[BBH] BGB_DROME (sp|Q24040) Prot. big brother OS=D.m. GN=Bgb PE=2 SV=3	BGB_DROME	1,00E-146	GLOS_BGB.1.1	GO:0046982	M	FlyBase	protein heterodimerization activity
[BBH] BGB_DROME (sp|Q24040) Prot. big brother OS=D.m. GN=Bgb PE=2 SV=3	BGB_DROME	1,00E-146	GLOS_BGB.1.1	GO:0003712	M	.	transcription cofactor activity
[BBH] BGB_DROME (sp|Q24040) Prot. big brother OS=D.m. GN=Bgb PE=2 SV=3	BGB_DROME	1,00E-146	GLOS_BGB.1.1	GO:0003713	M	FlyBase	transcription coactivator activity
[BBH] BGB_DROME (sp|Q24040) Prot. big brother OS=D.m. GN=Bgb PE=2 SV=3	BGB_DROME	1,00E-146	GLOS_BGB.1.1	GO:0008134	M	FlyBase	transcription factor binding
[BBH] BGB_DROME (sp|Q24040) Prot. big brother OS=D.m. GN=Bgb PE=2 SV=3	BGB_DROME	1,00E-146	GLOS_BGB.1.1	GO:0002682	B	.	regulation of immune system process
[BBH] BGB_DROME (sp|Q24040) Prot. big brother OS=D.m. GN=Bgb PE=2 SV=3	BGB_DROME	1,00E-146	GLOS_BGB.1.1	GO:0042127	B	.	regulation of cell proliferation
[BBH] BGB_DROME (sp|Q24040) Prot. big brother OS=D.m. GN=Bgb PE=2 SV=3	BGB_DROME	1,00E-146	GLOS_BGB.1.1	GO:0035206	B	FlyBase	regulation of hemocyte proliferation
[BBH] BGB_DROME (sp|Q24040) Prot. big brother OS=D.m. GN=Bgb PE=2 SV=3	BGB_DROME	1,00E-146	GLOS_BGB.1.1	GO:0006357	B	FlyBase	regulation of transcription from RNA polymerase II promoter
[BBH] CAND1_DROME (sp|Q9VKY2) Cullin-associated NEDD8-dissociated prot 1	CAND1_DROME	0	GLOS_CAND1.1.1	GO:0031400	B	.	negative regulation of protein modification process
[BBH] CAND1_DROME (sp|Q9VKY2) Cullin-associated NEDD8-dissociated prot 1	CAND1_DROME	0	GLOS_CAND1.1.1	GO:0031399	B	.	regulation of protein modification process
[BBH] CAND1_DROME (sp|Q9VKY2) Cullin-associated NEDD8-dissociated prot 1	CAND1_DROME	0	GLOS_CAND1.1.1	GO:0032268	B	.	regulation of cellular protein metabolic process
[BBH] CAND1_DROME (sp|Q9VKY2) Cullin-associated NEDD8-dissociated prot 1	CAND1_DROME	0	GLOS_CAND1.1.1	GO:0051246	B	.	regulation of protein metabolic process
[BBH] CAND1_DROME (sp|Q9VKY2) Cullin-associated NEDD8-dissociated prot 1	CAND1_DROME	0	GLOS_CAND1.1.1	GO:0032269	B	.	negative regulation of cellular protein metabolic process
[BBH] CAND1_DROME (sp|Q9VKY2) Cullin-associated NEDD8-dissociated prot 1	CAND1_DROME	0	GLOS_CAND1.1.1	GO:0051248	B	.	negative regulation of protein metabolic process
[BBH] CAND1_DROME (sp|Q9VKY2) Cullin-associated NEDD8-dissociated prot 1	CAND1_DROME	0	GLOS_CAND1.1.1	GO:2000434	B	.	regulation of protein neddylation
[BBH] CAND1_DROME (sp|Q9VKY2) Cullin-associated NEDD8-dissociated prot 1	CAND1_DROME	0	GLOS_CAND1.1.1	GO:2000435	B	FlyBase	negative regulation of protein neddylation
[BBH] CAND1_DROME (sp|Q9VKY2) Cullin-associated NEDD8-dissociated prot 1	CAND1_DROME	0	GLOS_CAND1.1.1	GO:0010608	B	.	posttranscriptional regulation of gene expression
[BBH] CAND1_DROME (sp|Q9VKY2) Cullin-associated NEDD8-dissociated prot 1	CAND1_DROME	0	GLOS_CAND1.1.1	GO:0031647	B	FlyBase	regulation of protein stability
[BBH] CP301_DROME (sp|Q9V6D6) Probable cyt P450 301a1, mito; OS=D.m.	CP301_DROME	0	GLOS_CP301.1.1	GO:0031090	C	.	organelle membrane

[BBH] CP301_DROME (sp|Q9V6D6) Probable cyt P450 301a1, mito; OS=D.m.	CP301_DROME	0	GLOS_CP301.1.1	GO:0044429	C	.	mitochondrial part
[BBH] CP301_DROME (sp|Q9V6D6) Probable cyt P450 301a1, mito; OS=D.m.	CP301_DROME	0	GLOS_CP301.1.1	GO:0005739	C	.	mitochondrion
[BBH] CP301_DROME (sp|Q9V6D6) Probable cyt P450 301a1, mito; OS=D.m.	CP301_DROME	0	GLOS_CP301.1.1	GO:0005740	C	.	mitochondrial envelope
[BBH] CP301_DROME (sp|Q9V6D6) Probable cyt P450 301a1, mito; OS=D.m.	CP301_DROME	0	GLOS_CP301.1.1	GO:0031967	C	.	organelle envelope
[BBH] CP301_DROME (sp|Q9V6D6) Probable cyt P450 301a1, mito; OS=D.m.	CP301_DROME	0	GLOS_CP301.1.1	GO:0031975	C	.	envelope
[BBH] CP301_DROME (sp|Q9V6D6) Probable cyt P450 301a1, mito; OS=D.m.	CP301_DROME	0	GLOS_CP301.1.1	GO:0031966	C	UniProtKB-S	mitochondrial membrane
[BBH] CP301_DROME (sp|Q9V6D6) Probable cyt P450 301a1, mito; OS=D.m.	CP301_DROME	0	GLOS_CP301.1.1	GO:0009055	M	InterPro	electron carrier activity
[BBH] CP301_DROME (sp|Q9V6D6) Probable cyt P450 301a1, mito; OS=D.m.	CP301_DROME	0	GLOS_CP301.1.1	GO:0046906	M	.	tetrapyrrole binding
[BBH] CP301_DROME (sp|Q9V6D6) Probable cyt P450 301a1, mito; OS=D.m.	CP301_DROME	0	GLOS_CP301.1.1	GO:0020037	M	InterPro	heme binding
[BBH] CP301_DROME (sp|Q9V6D6) Probable cyt P450 301a1, mito; OS=D.m.	CP301_DROME	0	GLOS_CP301.1.1	GO:0005506	M	InterPro	iron ion binding
[BBH] CP301_DROME (sp|Q9V6D6) Probable cyt P450 301a1, mito; OS=D.m.	CP301_DROME	0	GLOS_CP301.1.1	GO:0016491	M	.	oxidoreductase activity
[BBH] CP301_DROME (sp|Q9V6D6) Probable cyt P450 301a1, mito; OS=D.m.	CP301_DROME	0	GLOS_CP301.1.1	GO:0003824	M	.	catalytic activity
[BBH] CP301_DROME (sp|Q9V6D6) Probable cyt P450 301a1, mito; OS=D.m.	CP301_DROME	0	GLOS_CP301.1.1	GO:0004497	M	UniProtKB-K	monooxygenase activity
[BBH] CP301_DROME (sp|Q9V6D6) Probable cyt P450 301a1, mito; OS=D.m.	CP301_DROME	0	GLOS_CP301.1.1	GO:0016705	M	InterPro	oxidoreductase activity, acting on paired donors, with incorporation or reduction of molecular oxygen
[BBH] CP301_DROME (sp|Q9V6D6) Probable cyt P450 301a1, mito; OS=D.m.	CP301_DROME	0	GLOS_CP301.1.1	GO:0007488	B	.	histoblast morphogenesis
[BBH] CP301_DROME (sp|Q9V6D6) Probable cyt P450 301a1, mito; OS=D.m.	CP301_DROME	0	GLOS_CP301.1.1	GO:0007560	B	.	imaginal disc morphogenesis
[BBH] CP301_DROME (sp|Q9V6D6) Probable cyt P450 301a1, mito; OS=D.m.	CP301_DROME	0	GLOS_CP301.1.1	GO:0048563	B	.	post-embryonic organ morphogenesis
[BBH] CP301_DROME (sp|Q9V6D6) Probable cyt P450 301a1, mito; OS=D.m.	CP301_DROME	0	GLOS_CP301.1.1	GO:0009887	B	.	organ morphogenesis
[BBH] CP301_DROME (sp|Q9V6D6) Probable cyt P450 301a1, mito; OS=D.m.	CP301_DROME	0	GLOS_CP301.1.1	GO:0048569	B	.	post-embryonic organ development
[BBH] CP301_DROME (sp|Q9V6D6) Probable cyt P450 301a1, mito; OS=D.m.	CP301_DROME	0	GLOS_CP301.1.1	GO:0007444	B	.	imaginal disc development
[BBH] CP301_DROME (sp|Q9V6D6) Probable cyt P450 301a1, mito; OS=D.m.	CP301_DROME	0	GLOS_CP301.1.1	GO:0007490	B	FlyBase	tergite morphogenesis
[BBH] CSK2A_DROME (sp|P08181) Casein kinase II subunit alpha OS=D.m.	CSK2A_DROME	0	GLOS_CSK2A.1.1	GO:0005829	C	FlyBase	cytosol
[BBH] CSK2A_DROME (sp|P08181) Casein kinase II subunit alpha OS=D.m.	CSK2A_DROME	0	GLOS_CSK2A.1.1	GO:0005634	C	FlyBase	nucleus
[BBH] CSK2A_DROME (sp|P08181) Casein kinase II subunit alpha OS=D.m.	CSK2A_DROME	0	GLOS_CSK2A.1.1	GO:0005956	C	FlyBase	protein kinase CK2 complex
[BBH] CSK2A_DROME (sp|P08181) Casein kinase II subunit alpha OS=D.m.	CSK2A_DROME	0	GLOS_CSK2A.1.1	GO:0032550	M	.	purine ribonucleoside binding
[BBH] CSK2A_DROME (sp|P08181) Casein kinase II subunit alpha OS=D.m.	CSK2A_DROME	0	GLOS_CSK2A.1.1	GO:0001883	M	.	purine nucleoside binding
[BBH] CSK2A_DROME (sp|P08181) Casein kinase II subunit alpha OS=D.m.	CSK2A_DROME	0	GLOS_CSK2A.1.1	GO:0001882	M	.	nucleoside binding
[BBH] CSK2A_DROME (sp|P08181) Casein kinase II subunit alpha OS=D.m.	CSK2A_DROME	0	GLOS_CSK2A.1.1	GO:0097367	M	.	carbohydrate derivative binding
[BBH] CSK2A_DROME (sp|P08181) Casein kinase II subunit alpha OS=D.m.	CSK2A_DROME	0	GLOS_CSK2A.1.1	GO:0032549	M	.	ribonucleoside binding
[BBH] CSK2A_DROME (sp|P08181) Casein kinase II subunit alpha OS=D.m.	CSK2A_DROME	0	GLOS_CSK2A.1.1	GO:0032559	M	.	adenyl ribonucleotide binding
[BBH] CSK2A_DROME (sp|P08181) Casein kinase II subunit alpha OS=D.m.	CSK2A_DROME	0	GLOS_CSK2A.1.1	GO:0030554	M	.	adenyl nucleotide binding
[BBH] CSK2A_DROME (sp|P08181) Casein kinase II subunit alpha OS=D.m.	CSK2A_DROME	0	GLOS_CSK2A.1.1	GO:0017076	M	.	purine nucleotide binding
[BBH] CSK2A_DROME (sp|P08181) Casein kinase II subunit alpha OS=D.m.	CSK2A_DROME	0	GLOS_CSK2A.1.1	GO:0000166	M	.	nucleotide binding
[BBH] CSK2A_DROME (sp|P08181) Casein kinase II subunit alpha OS=D.m.	CSK2A_DROME	0	GLOS_CSK2A.1.1	GO:1901265	M	.	nucleoside phosphate binding
[BBH] CSK2A_DROME (sp|P08181) Casein kinase II subunit alpha OS=D.m.	CSK2A_DROME	0	GLOS_CSK2A.1.1	GO:0032555	M	.	purine ribonucleotide binding
[BBH] CSK2A_DROME (sp|P08181) Casein kinase II subunit alpha OS=D.m.	CSK2A_DROME	0	GLOS_CSK2A.1.1	GO:0032553	M	.	ribonucleotide binding
[BBH] CSK2A_DROME (sp|P08181) Casein kinase II subunit alpha OS=D.m.	CSK2A_DROME	0	GLOS_CSK2A.1.1	GO:0035639	M	.	purine ribonucleoside triphosphate binding
[BBH] CSK2A_DROME (sp|P08181) Casein kinase II subunit alpha OS=D.m.	CSK2A_DROME	0	GLOS_CSK2A.1.1	GO:0005524	M	UniProtKB-K	ATP binding
[BBH] CSK2A_DROME (sp|P08181) Casein kinase II subunit alpha OS=D.m.	CSK2A_DROME	0	GLOS_CSK2A.1.1	GO:0004672	M	.	protein kinase activity
[BBH] CSK2A_DROME (sp|P08181) Casein kinase II subunit alpha OS=D.m.	CSK2A_DROME	0	GLOS_CSK2A.1.1	GO:0016301	M	.	kinase activity
[BBH] CSK2A_DROME (sp|P08181) Casein kinase II subunit alpha OS=D.m.	CSK2A_DROME	0	GLOS_CSK2A.1.1	GO:0016772	M	.	transferase activity, transferring phosphorus-containing groups
[BBH] CSK2A_DROME (sp|P08181) Casein kinase II subunit alpha OS=D.m.	CSK2A_DROME	0	GLOS_CSK2A.1.1	GO:0016740	M	.	transferase activity
[BBH] CSK2A_DROME (sp|P08181) Casein kinase II subunit alpha OS=D.m.	CSK2A_DROME	0	GLOS_CSK2A.1.1	GO:0016773	M	.	phosphotransferase activity, alcohol group as acceptor
[BBH] CSK2A_DROME (sp|P08181) Casein kinase II subunit alpha OS=D.m.	CSK2A_DROME	0	GLOS_CSK2A.1.1	GO:0004674	M	FlyBase	protein serine/threonine kinase activity
[BBH] CSK2A_DROME (sp|P08181) Casein kinase II subunit alpha OS=D.m.	CSK2A_DROME	0	GLOS_CSK2A.1.1	GO:0007411	B	FlyBase	axon guidance
[BBH] CSK2A_DROME (sp|P08181) Casein kinase II subunit alpha OS=D.m.	CSK2A_DROME	0	GLOS_CSK2A.1.1	GO:0007423	B	.	sensory organ development
[BBH] CSK2A_DROME (sp|P08181) Casein kinase II subunit alpha OS=D.m.	CSK2A_DROME	0	GLOS_CSK2A.1.1	GO:0022416	B	FlyBase	chaeta development
[BBH] CSK2A_DROME (sp|P08181) Casein kinase II subunit alpha OS=D.m.	CSK2A_DROME	0	GLOS_CSK2A.1.1	GO:0001654	B	.	eye development
[BBH] CSK2A_DROME (sp|P08181) Casein kinase II subunit alpha OS=D.m.	CSK2A_DROME	0	GLOS_CSK2A.1.1	GO:0048749	B	FlyBase	compound eye development
[BBH] CSK2A_DROME (sp|P08181) Casein kinase II subunit alpha OS=D.m.	CSK2A_DROME	0	GLOS_CSK2A.1.1	GO:0045168	B	.	cell-cell signaling involved in cell fate commitment
[BBH] CSK2A_DROME (sp|P08181) Casein kinase II subunit alpha OS=D.m.	CSK2A_DROME	0	GLOS_CSK2A.1.1	GO:0007267	B	.	cell-cell signaling
[BBH] CSK2A_DROME (sp|P08181) Casein kinase II subunit alpha OS=D.m.	CSK2A_DROME	0	GLOS_CSK2A.1.1	GO:0045165	B	.	cell fate commitment
[BBH] CSK2A_DROME (sp|P08181) Casein kinase II subunit alpha OS=D.m.	CSK2A_DROME	0	GLOS_CSK2A.1.1	GO:0046331	B	FlyBase	lateral inhibition

[BBH] CSK2A_DROME (sp|P08181) Casein kinase II subunit alpha OS=D.m.	CSK2A_DROME	0	GLOS_CSK2A.1.1	GO:0048512	B	.	circadian behavior
[BBH] CSK2A_DROME (sp|P08181) Casein kinase II subunit alpha OS=D.m.	CSK2A_DROME	0	GLOS_CSK2A.1.1	GO:0007622	B	.	rhythmic behavior
[BBH] CSK2A_DROME (sp|P08181) Casein kinase II subunit alpha OS=D.m.	CSK2A_DROME	0	GLOS_CSK2A.1.1	GO:0048511	B	.	rhythmic process
[BBH] CSK2A_DROME (sp|P08181) Casein kinase II subunit alpha OS=D.m.	CSK2A_DROME	0	GLOS_CSK2A.1.1	GO:0007623	B	.	circadian rhythm
[BBH] CSK2A_DROME (sp|P08181) Casein kinase II subunit alpha OS=D.m.	CSK2A_DROME	0	GLOS_CSK2A.1.1	GO:0045475	B	FlyBase	locomotor rhythm
[BBH] CSK2A_DROME (sp|P08181) Casein kinase II subunit alpha OS=D.m.	CSK2A_DROME	0	GLOS_CSK2A.1.1	GO:0000280	B	.	nuclear division
[BBH] CSK2A_DROME (sp|P08181) Casein kinase II subunit alpha OS=D.m.	CSK2A_DROME	0	GLOS_CSK2A.1.1	GO:0048285	B	.	organelle fission
[BBH] CSK2A_DROME (sp|P08181) Casein kinase II subunit alpha OS=D.m.	CSK2A_DROME	0	GLOS_CSK2A.1.1	GO:0006996	B	.	organelle organization
[BBH] CSK2A_DROME (sp|P08181) Casein kinase II subunit alpha OS=D.m.	CSK2A_DROME	0	GLOS_CSK2A.1.1	GO:0022402	B	.	cell cycle process
[BBH] CSK2A_DROME (sp|P08181) Casein kinase II subunit alpha OS=D.m.	CSK2A_DROME	0	GLOS_CSK2A.1.1	GO:0000278	B	.	mitotic cell cycle
[BBH] CSK2A_DROME (sp|P08181) Casein kinase II subunit alpha OS=D.m.	CSK2A_DROME	0	GLOS_CSK2A.1.1	GO:0007067	B	FlyBase	mitosis
[BBH] CSK2A_DROME (sp|P08181) Casein kinase II subunit alpha OS=D.m.	CSK2A_DROME	0	GLOS_CSK2A.1.1	GO:0031396	B	.	regulation of protein ubiquitination
[BBH] CSK2A_DROME (sp|P08181) Casein kinase II subunit alpha OS=D.m.	CSK2A_DROME	0	GLOS_CSK2A.1.1	GO:0031397	B	FlyBase	negative regulation of protein ubiquitination
[BBH] CSK2A_DROME (sp|P08181) Casein kinase II subunit alpha OS=D.m.	CSK2A_DROME	0	GLOS_CSK2A.1.1	GO:0051091	B	.	positive regulation of sequence-specific DNA binding transcription factor activity
[BBH] CSK2A_DROME (sp|P08181) Casein kinase II subunit alpha OS=D.m.	CSK2A_DROME	0	GLOS_CSK2A.1.1	GO:0044093	B	.	positive regulation of molecular function
[BBH] CSK2A_DROME (sp|P08181) Casein kinase II subunit alpha OS=D.m.	CSK2A_DROME	0	GLOS_CSK2A.1.1	GO:0065009	B	.	regulation of molecular function
[BBH] CSK2A_DROME (sp|P08181) Casein kinase II subunit alpha OS=D.m.	CSK2A_DROME	0	GLOS_CSK2A.1.1	GO:0051090	B	.	regulation of sequence-specific DNA binding transcription factor activity
[BBH] CSK2A_DROME (sp|P08181) Casein kinase II subunit alpha OS=D.m.	CSK2A_DROME	0	GLOS_CSK2A.1.1	GO:0007227	B	.	signal transduction downstream of smoothened
[BBH] CSK2A_DROME (sp|P08181) Casein kinase II subunit alpha OS=D.m.	CSK2A_DROME	0	GLOS_CSK2A.1.1	GO:0007224	B	.	smoothened signaling pathway
[BBH] CSK2A_DROME (sp|P08181) Casein kinase II subunit alpha OS=D.m.	CSK2A_DROME	0	GLOS_CSK2A.1.1	GO:0007228	B	FlyBase	positive regulation of hh target transcription factor activity
[BBH] CSK2A_DROME (sp|P08181) Casein kinase II subunit alpha OS=D.m.	CSK2A_DROME	0	GLOS_CSK2A.1.1	GO:0008589	B	.	regulation of smoothened signaling pathway
[BBH] CSK2A_DROME (sp|P08181) Casein kinase II subunit alpha OS=D.m.	CSK2A_DROME	0	GLOS_CSK2A.1.1	GO:0009966	B	.	regulation of signal transduction
[BBH] CSK2A_DROME (sp|P08181) Casein kinase II subunit alpha OS=D.m.	CSK2A_DROME	0	GLOS_CSK2A.1.1	GO:0010646	B	.	regulation of cell communication
[BBH] CSK2A_DROME (sp|P08181) Casein kinase II subunit alpha OS=D.m.	CSK2A_DROME	0	GLOS_CSK2A.1.1	GO:0023051	B	.	regulation of signaling
[BBH] CSK2A_DROME (sp|P08181) Casein kinase II subunit alpha OS=D.m.	CSK2A_DROME	0	GLOS_CSK2A.1.1	GO:0048583	B	.	regulation of response to stimulus
[BBH] CSK2A_DROME (sp|P08181) Casein kinase II subunit alpha OS=D.m.	CSK2A_DROME	0	GLOS_CSK2A.1.1	GO:0009967	B	.	positive regulation of signal transduction
[BBH] CSK2A_DROME (sp|P08181) Casein kinase II subunit alpha OS=D.m.	CSK2A_DROME	0	GLOS_CSK2A.1.1	GO:0010647	B	.	positive regulation of cell communication
[BBH] CSK2A_DROME (sp|P08181) Casein kinase II subunit alpha OS=D.m.	CSK2A_DROME	0	GLOS_CSK2A.1.1	GO:0023056	B	.	positive regulation of signaling
[BBH] CSK2A_DROME (sp|P08181) Casein kinase II subunit alpha OS=D.m.	CSK2A_DROME	0	GLOS_CSK2A.1.1	GO:0048584	B	.	positive regulation of response to stimulus
[BBH] CSK2A_DROME (sp|P08181) Casein kinase II subunit alpha OS=D.m.	CSK2A_DROME	0	GLOS_CSK2A.1.1	GO:0045880	B	FlyBase	positive regulation of smoothened signaling pathway
[BBH] CSK2A_DROME (sp|P08181) Casein kinase II subunit alpha OS=D.m.	CSK2A_DROME	0	GLOS_CSK2A.1.1	GO:0031647	B	FlyBase	regulation of protein stability
[BBH] CSK2A_DROME (sp|P08181) Casein kinase II subunit alpha OS=D.m.	CSK2A_DROME	0	GLOS_CSK2A.1.1	GO:0016055	B	FlyBase	Wnt receptor signaling pathway
[BBH] DDX49_DROME (sp|Q07886) Prob. ATP-dependent RNA helicase Dbp45A	DDX49_DROME	0	GLOS_DDX49.1.1	GO:0005634	C	FlyBase	nucleus
[BBH] DDX49_DROME (sp|Q07886) Prob. ATP-dependent RNA helicase Dbp45A	DDX49_DROME	0	GLOS_DDX49.1.1	GO:0005524	M	UniProtKB-k	ATP binding
[BBH] DDX49_DROME (sp|Q07886) Prob. ATP-dependent RNA helicase Dbp45A	DDX49_DROME	0	GLOS_DDX49.1.1	GO:0003724	M	.	RNA helicase activity
[BBH] DDX49_DROME (sp|Q07886) Prob. ATP-dependent RNA helicase Dbp45A	DDX49_DROME	0	GLOS_DDX49.1.1	GO:0004386	M	.	helicase activity
[BBH] DDX49_DROME (sp|Q07886) Prob. ATP-dependent RNA helicase Dbp45A	DDX49_DROME	0	GLOS_DDX49.1.1	GO:0017111	M	.	nucleoside-triphosphatase activity
[BBH] DDX49_DROME (sp|Q07886) Prob. ATP-dependent RNA helicase Dbp45A	DDX49_DROME	0	GLOS_DDX49.1.1	GO:0016462	M	.	pyrophosphatase activity
[BBH] DDX49_DROME (sp|Q07886) Prob. ATP-dependent RNA helicase Dbp45A	DDX49_DROME	0	GLOS_DDX49.1.1	GO:0016818	M	.	hydrolase act., acting on acid anhydrides, in phosphorus-containing anhydrides
[BBH] DDX49_DROME (sp|Q07886) Prob. ATP-dependent RNA helicase Dbp45A	DDX49_DROME	0	GLOS_DDX49.1.1	GO:0016817	M	.	hydrolase activity, acting on acid anhydrides
[BBH] DDX49_DROME (sp|Q07886) Prob. ATP-dependent RNA helicase Dbp45A	DDX49_DROME	0	GLOS_DDX49.1.1	GO:0016787	M	.	hydrolase activity
[BBH] DDX49_DROME (sp|Q07886) Prob. ATP-dependent RNA helicase Dbp45A	DDX49_DROME	0	GLOS_DDX49.1.1	GO:0008026	M	.	ATP-dependent helicase activity
[BBH] DDX49_DROME (sp|Q07886) Prob. ATP-dependent RNA helicase Dbp45A	DDX49_DROME	0	GLOS_DDX49.1.1	GO:0042623	M	.	ATPase activity, coupled
[BBH] DDX49_DROME (sp|Q07886) Prob. ATP-dependent RNA helicase Dbp45A	DDX49_DROME	0	GLOS_DDX49.1.1	GO:0016887	M	.	ATPase activity
[BBH] DDX49_DROME (sp|Q07886) Prob. ATP-dependent RNA helicase Dbp45A	DDX49_DROME	0	GLOS_DDX49.1.1	GO:0070035	M	.	purine NTP-dependent helicase activity
[BBH] DDX49_DROME (sp|Q07886) Prob. ATP-dependent RNA helicase Dbp45A	DDX49_DROME	0	GLOS_DDX49.1.1	GO:0008186	M	.	RNA-dependent ATPase activity
[BBH] DDX49_DROME (sp|Q07886) Prob. ATP-dependent RNA helicase Dbp45A	DDX49_DROME	0	GLOS_DDX49.1.1	GO:0004004	M	FlyBase	ATP-dependent RNA helicase activity
[BBH] DDX49_DROME (sp|Q07886) Prob. ATP-dependent RNA helicase Dbp45A	DDX49_DROME	0	GLOS_DDX49.1.1	GO:0003723	M	UniProtKB-k	RNA binding
[BBH] DDX49_DROME (sp|Q07886) Prob. ATP-dependent RNA helicase Dbp45A	DDX49_DROME	0	GLOS_DDX49.1.1	GO:0048024	B	.	regulation of mRNA splicing, via spliceosome
[BBH] DDX49_DROME (sp|Q07886) Prob. ATP-dependent RNA helicase Dbp45A	DDX49_DROME	0	GLOS_DDX49.1.1	GO:0043484	B	.	regulation of RNA splicing
[BBH] DDX49_DROME (sp|Q07886) Prob. ATP-dependent RNA helicase Dbp45A	DDX49_DROME	0	GLOS_DDX49.1.1	GO:0050684	B	.	regulation of mRNA processing
[BBH] DDX49_DROME (sp|Q07886) Prob. ATP-dependent RNA helicase Dbp45A	DDX49_DROME	0	GLOS_DDX49.1.1	GO:0000381	B	FlyBase	regulation of alternative mRNA splicing, via spliceosome
[BBH] DUOX_DROME (sp|Q9VQH2) Dual oxidase OS=D.m. GN=Duox PE=1 SV=2	DUOX_DROME	0	GLOS_DUOX.1.1	GO:0016021	C	UniProtKB-k	integral to membrane
[BBH] DUOX_DROME (sp|Q9VQH2) Dual oxidase OS=D.m. GN=Duox PE=1 SV=2	DUOX_DROME	0	GLOS_DUOX.1.1	GO:0005509	M	InterPro	calcium ion binding

[BBH] DUOX_DROME (sp|Q9VQH2) Dual oxidase OS=D.m. GN=Duox PE=1 SV=2	DUOX_DROME	0	GLOS_DUOX.1.1	GO:0020037	M	InterPro	heme binding
[BBH] DUOX_DROME (sp|Q9VQH2) Dual oxidase OS=D.m. GN=Duox PE=1 SV=2	DUOX_DROME	0	GLOS_DUOX.1.1	GO:0050664	M	.	oxidoreductase activity, acting on NAD(P)H, oxygen as acceptor
[BBH] DUOX_DROME (sp|Q9VQH2) Dual oxidase OS=D.m. GN=Duox PE=1 SV=2	DUOX_DROME	0	GLOS_DUOX.1.1	GO:0016651	M	.	oxidoreductase activity, acting on NAD(P)H
[BBH] DUOX_DROME (sp|Q9VQH2) Dual oxidase OS=D.m. GN=Duox PE=1 SV=2	DUOX_DROME	0	GLOS_DUOX.1.1	GO:0016174	M	UniProtKB-E	NAD(P)H oxidase activity
[BBH] DUOX_DROME (sp|Q9VQH2) Dual oxidase OS=D.m. GN=Duox PE=1 SV=2	DUOX_DROME	0	GLOS_DUOX.1.1	GO:0016209	M	.	antioxidant activity
[BBH] DUOX_DROME (sp|Q9VQH2) Dual oxidase OS=D.m. GN=Duox PE=1 SV=2	DUOX_DROME	0	GLOS_DUOX.1.1	GO:0016684	M	.	oxidoreductase activity, acting on peroxide as acceptor
[BBH] DUOX_DROME (sp|Q9VQH2) Dual oxidase OS=D.m. GN=Duox PE=1 SV=2	DUOX_DROME	0	GLOS_DUOX.1.1	GO:0004601	M	FlyBase	peroxidase activity
[BBH] DUOX_DROME (sp|Q9VQH2) Dual oxidase OS=D.m. GN=Duox PE=1 SV=2	DUOX_DROME	0	GLOS_DUOX.1.1	GO:0040003	B	.	chitin-based cuticle development
[BBH] DUOX_DROME (sp|Q9VQH2) Dual oxidase OS=D.m. GN=Duox PE=1 SV=2	DUOX_DROME	0	GLOS_DUOX.1.1	GO:0042335	B	.	cuticle development
[BBH] DUOX_DROME (sp|Q9VQH2) Dual oxidase OS=D.m. GN=Duox PE=1 SV=2	DUOX_DROME	0	GLOS_DUOX.1.1	GO:0008365	B	FlyBase	adult chitin-based cuticle development
[BBH] DUOX_DROME (sp|Q9VQH2) Dual oxidase OS=D.m. GN=Duox PE=1 SV=2	DUOX_DROME	0	GLOS_DUOX.1.1	GO:0048067	B	.	cuticle pigmentation
[BBH] DUOX_DROME (sp|Q9VQH2) Dual oxidase OS=D.m. GN=Duox PE=1 SV=2	DUOX_DROME	0	GLOS_DUOX.1.1	GO:0048066	B	.	developmental pigmentation
[BBH] DUOX_DROME (sp|Q9VQH2) Dual oxidase OS=D.m. GN=Duox PE=1 SV=2	DUOX_DROME	0	GLOS_DUOX.1.1	GO:0043473	B	.	pigmentation
[BBH] DUOX_DROME (sp|Q9VQH2) Dual oxidase OS=D.m. GN=Duox PE=1 SV=2	DUOX_DROME	0	GLOS_DUOX.1.1	GO:0007593	B	.	chitin-based cuticle sclerotization
[BBH] DUOX_DROME (sp|Q9VQH2) Dual oxidase OS=D.m. GN=Duox PE=1 SV=2	DUOX_DROME	0	GLOS_DUOX.1.1	GO:0007591	B	.	molting cycle, chitin-based cuticle
[BBH] DUOX_DROME (sp|Q9VQH2) Dual oxidase OS=D.m. GN=Duox PE=1 SV=2	DUOX_DROME	0	GLOS_DUOX.1.1	GO:0042303	B	.	molting cycle
[BBH] DUOX_DROME (sp|Q9VQH2) Dual oxidase OS=D.m. GN=Duox PE=1 SV=2	DUOX_DROME	0	GLOS_DUOX.1.1	GO:0021700	B	.	developmental maturation
[BBH] DUOX_DROME (sp|Q9VQH2) Dual oxidase OS=D.m. GN=Duox PE=1 SV=2	DUOX_DROME	0	GLOS_DUOX.1.1	GO:0022404	B	.	molting cycle process
[BBH] DUOX_DROME (sp|Q9VQH2) Dual oxidase OS=D.m. GN=Duox PE=1 SV=2	DUOX_DROME	0	GLOS_DUOX.1.1	GO:0048085	B	FlyBase	adult chitin-containing cuticle pigmentation
[BBH] DUOX_DROME (sp|Q9VQH2) Dual oxidase OS=D.m. GN=Duox PE=1 SV=2	DUOX_DROME	0	GLOS_DUOX.1.1	GO:0071345	B	.	cellular response to cytokine stimulus
[BBH] DUOX_DROME (sp|Q9VQH2) Dual oxidase OS=D.m. GN=Duox PE=1 SV=2	DUOX_DROME	0	GLOS_DUOX.1.1	GO:0034097	B	.	response to cytokine stimulus
[BBH] DUOX_DROME (sp|Q9VQH2) Dual oxidase OS=D.m. GN=Duox PE=1 SV=2	DUOX_DROME	0	GLOS_DUOX.1.1	GO:0019221	B	UniProtKB	cytokine-mediated signaling pathway
[BBH] DUOX_DROME (sp|Q9VQH2) Dual oxidase OS=D.m. GN=Duox PE=1 SV=2	DUOX_DROME	0	GLOS_DUOX.1.1	GO:0042742	B	FlyBase	defense response to bacterium
[BBH] DUOX_DROME (sp|Q9VQH2) Dual oxidase OS=D.m. GN=Duox PE=1 SV=2	DUOX_DROME	0	GLOS_DUOX.1.1	GO:0042743	B	.	hydrogen peroxide metabolic process
[BBH] DUOX_DROME (sp|Q9VQH2) Dual oxidase OS=D.m. GN=Duox PE=1 SV=2	DUOX_DROME	0	GLOS_DUOX.1.1	GO:0072593	B	.	reactive oxygen species metabolic process
[BBH] DUOX_DROME (sp|Q9VQH2) Dual oxidase OS=D.m. GN=Duox PE=1 SV=2	DUOX_DROME	0	GLOS_DUOX.1.1	GO:0070301	B	.	cellular response to hydrogen peroxide
[BBH] DUOX_DROME (sp|Q9VQH2) Dual oxidase OS=D.m. GN=Duox PE=1 SV=2	DUOX_DROME	0	GLOS_DUOX.1.1	GO:0034614	B	.	cellular response to reactive oxygen species
[BBH] DUOX_DROME (sp|Q9VQH2) Dual oxidase OS=D.m. GN=Duox PE=1 SV=2	DUOX_DROME	0	GLOS_DUOX.1.1	GO:0000302	B	.	response to reactive oxygen species
[BBH] DUOX_DROME (sp|Q9VQH2) Dual oxidase OS=D.m. GN=Duox PE=1 SV=2	DUOX_DROME	0	GLOS_DUOX.1.1	GO:0006979	B	.	response to oxidative stress
[BBH] DUOX_DROME (sp|Q9VQH2) Dual oxidase OS=D.m. GN=Duox PE=1 SV=2	DUOX_DROME	0	GLOS_DUOX.1.1	GO:0034599	B	.	cellular response to oxidative stress
[BBH] DUOX_DROME (sp|Q9VQH2) Dual oxidase OS=D.m. GN=Duox PE=1 SV=2	DUOX_DROME	0	GLOS_DUOX.1.1	GO:1901701	B	.	cellular response to oxygen-containing compound
[BBH] DUOX_DROME (sp|Q9VQH2) Dual oxidase OS=D.m. GN=Duox PE=1 SV=2	DUOX_DROME	0	GLOS_DUOX.1.1	GO:0042542	B	.	response to hydrogen peroxide
[BBH] DUOX_DROME (sp|Q9VQH2) Dual oxidase OS=D.m. GN=Duox PE=1 SV=2	DUOX_DROME	0	GLOS_DUOX.1.1	GO:0042744	B	UniProtKB-k	hydrogen peroxide catabolic process
[BBH] DUOX_DROME (sp|Q9VQH2) Dual oxidase OS=D.m. GN=Duox PE=1 SV=2	DUOX_DROME	0	GLOS_DUOX.1.1	GO:0002251	B	.	organ or tissue specific immune response
[BBH] DUOX_DROME (sp|Q9VQH2) Dual oxidase OS=D.m. GN=Duox PE=1 SV=2	DUOX_DROME	0	GLOS_DUOX.1.1	GO:0002385	B	FlyBase	mucosal immune response
[BBH] DUOX_DROME (sp|Q9VQH2) Dual oxidase OS=D.m. GN=Duox PE=1 SV=2	DUOX_DROME	0	GLOS_DUOX.1.1	GO:0042981	B	.	regulation of apoptotic process
[BBH] DUOX_DROME (sp|Q9VQH2) Dual oxidase OS=D.m. GN=Duox PE=1 SV=2	DUOX_DROME	0	GLOS_DUOX.1.1	GO:0043067	B	.	regulation of programmed cell death
[BBH] DUOX_DROME (sp|Q9VQH2) Dual oxidase OS=D.m. GN=Duox PE=1 SV=2	DUOX_DROME	0	GLOS_DUOX.1.1	GO:0010941	B	.	regulation of cell death
[BBH] DUOX_DROME (sp|Q9VQH2) Dual oxidase OS=D.m. GN=Duox PE=1 SV=2	DUOX_DROME	0	GLOS_DUOX.1.1	GO:0043069	B	.	negative regulation of programmed cell death
[BBH] DUOX_DROME (sp|Q9VQH2) Dual oxidase OS=D.m. GN=Duox PE=1 SV=2	DUOX_DROME	0	GLOS_DUOX.1.1	GO:0060548	B	.	negative regulation of cell death
[BBH] DUOX_DROME (sp|Q9VQH2) Dual oxidase OS=D.m. GN=Duox PE=1 SV=2	DUOX_DROME	0	GLOS_DUOX.1.1	GO:0043066	B	FlyBase	negative regulation of apoptotic process
[BBH] DUOX_DROME (sp|Q9VQH2) Dual oxidase OS=D.m. GN=Duox PE=1 SV=2	DUOX_DROME	0	GLOS_DUOX.1.1	GO:0072593	B	FlyBase	reactive oxygen species metabolic process
[BBH] DUOX_DROME (sp|Q9VQH2) Dual oxidase OS=D.m. GN=Duox PE=1 SV=2	DUOX_DROME	0	GLOS_DUOX.1.1	GO:0051591	B	UniProtKB	response to cAMP
[BBH] DUOX_DROME (sp|Q9VQH2) Dual oxidase OS=D.m. GN=Duox PE=1 SV=2	DUOX_DROME	0	GLOS_DUOX.1.1	GO:0035220	B	FlyBase	wing disc development
DYL2_DROME (sp|O96860) Dynein light chain 2, cytopl. OS=D.m.	DYL2_DROME	2,00E-55	GLOS_DYL2.2.3	GO:0005737	C	UniProtKB-k	cytoplasm
DYL2_DROME (sp|O96860) Dynein light chain 2, cytopl. OS=D.m.	DYL2_DROME	2,00E-55	GLOS_DYL2.2.3	GO:0005875	C	.	microtubule associated complex
DYL2_DROME (sp|O96860) Dynein light chain 2, cytopl. OS=D.m.	DYL2_DROME	2,00E-55	GLOS_DYL2.2.3	GO:0044430	C	.	cytoskeletal part
DYL2_DROME (sp|O96860) Dynein light chain 2, cytopl. OS=D.m.	DYL2_DROME	2,00E-55	GLOS_DYL2.2.3	GO:0005856	C	.	cytoskeleton
DYL2_DROME (sp|O96860) Dynein light chain 2, cytopl. OS=D.m.	DYL2_DROME	2,00E-55	GLOS_DYL2.2.3	GO:0015630	C	.	microtubule cytoskeleton
DYL2_DROME (sp|O96860) Dynein light chain 2, cytopl. OS=D.m.	DYL2_DROME	2,00E-55	GLOS_DYL2.2.3	GO:0030286	C	FlyBase	dynein complex
DYL2_DROME (sp|O96860) Dynein light chain 2, cytopl. OS=D.m.	DYL2_DROME	2,00E-55	GLOS_DYL2.2.3	GO:0005874	C	UniProtKB-k	microtubule
DYL2_DROME (sp|O96860) Dynein light chain 2, cytopl. OS=D.m.	DYL2_DROME	2,00E-55	GLOS_DYL2.2.3	GO:0042623	M	FlyBase	ATPase activity, coupled
DYL2_DROME (sp|O96860) Dynein light chain 2, cytopl. OS=D.m.	DYL2_DROME	2,00E-55	GLOS_DYL2.2.3	GO:0003774	M	UniProtKB-k	motor activity
DYL2_DROME (sp|O96860) Dynein light chain 2, cytopl. OS=D.m.	DYL2_DROME	2,00E-55	GLOS_DYL2.2.3	GO:0007017	B	.	microtubule-based process

DYL2_DROME (sp|O96860) Dynein light chain 2, cytopl. OS=D.m.	DYL2_DROME	2,00E-55	GLOS_DYL2.2.3	GO:0007018	B	FlyBase	microtubule-based movement
[BBH] DYN_DROME (sp|P27619) Dynamin OS=D.m. GN=shi PE=1 SV=2	DYN_DROME	0	GLOS_DYN.1.1	GO:0005737	C	UniProtKB-S	cytoplasm
[BBH] DYN_DROME (sp|P27619) Dynamin OS=D.m. GN=shi PE=1 SV=2	DYN_DROME	0	GLOS_DYN.1.1	GO:0005874	C	UniProtKB-K	microtubule
[BBH] DYN_DROME (sp|P27619) Dynamin OS=D.m. GN=shi PE=1 SV=2	DYN_DROME	0	GLOS_DYN.1.1	GO:0005886	C	FlyBase	plasma membrane
[BBH] DYN_DROME (sp|P27619) Dynamin OS=D.m. GN=shi PE=1 SV=2	DYN_DROME	0	GLOS_DYN.1.1	GO:0070864	C	FlyBase	sperm individualization complex
[BBH] DYN_DROME (sp|P27619) Dynamin OS=D.m. GN=shi PE=1 SV=2	DYN_DROME	0	GLOS_DYN.1.1	GO:0045202	C	FlyBase	synapse
[BBH] DYN_DROME (sp|P27619) Dynamin OS=D.m. GN=shi PE=1 SV=2	DYN_DROME	0	GLOS_DYN.1.1	GO:0008092	M	.	cytoskeletal protein binding
[BBH] DYN_DROME (sp|P27619) Dynamin OS=D.m. GN=shi PE=1 SV=2	DYN_DROME	0	GLOS_DYN.1.1	GO:0003779	M	FlyBase	actin binding
[BBH] DYN_DROME (sp|P27619) Dynamin OS=D.m. GN=shi PE=1 SV=2	DYN_DROME	0	GLOS_DYN.1.1	GO:0032561	M	.	guanyl ribonucleotide binding
[BBH] DYN_DROME (sp|P27619) Dynamin OS=D.m. GN=shi PE=1 SV=2	DYN_DROME	0	GLOS_DYN.1.1	GO:0019001	M	.	guanyl nucleotide binding
[BBH] DYN_DROME (sp|P27619) Dynamin OS=D.m. GN=shi PE=1 SV=2	DYN_DROME	0	GLOS_DYN.1.1	GO:0005525	M	UniProtKB-K	GTP binding
[BBH] DYN_DROME (sp|P27619) Dynamin OS=D.m. GN=shi PE=1 SV=2	DYN_DROME	0	GLOS_DYN.1.1	GO:0003924	M	FlyBase	GTPase activity
[BBH] DYN_DROME (sp|P27619) Dynamin OS=D.m. GN=shi PE=1 SV=2	DYN_DROME	0	GLOS_DYN.1.1	GO:0015631	M	.	tubulin binding
[BBH] DYN_DROME (sp|P27619) Dynamin OS=D.m. GN=shi PE=1 SV=2	DYN_DROME	0	GLOS_DYN.1.1	GO:0032403	M	.	protein complex binding
[BBH] DYN_DROME (sp|P27619) Dynamin OS=D.m. GN=shi PE=1 SV=2	DYN_DROME	0	GLOS_DYN.1.1	GO:0008017	M	FlyBase	microtubule binding
[BBH] DYN_DROME (sp|P27619) Dynamin OS=D.m. GN=shi PE=1 SV=2	DYN_DROME	0	GLOS_DYN.1.1	GO:0008289	M	.	lipid binding
[BBH] DYN_DROME (sp|P27619) Dynamin OS=D.m. GN=shi PE=1 SV=2	DYN_DROME	0	GLOS_DYN.1.1	GO:0005543	M	InterPro	phospholipid binding
[BBH] DYN_DROME (sp|P27619) Dynamin OS=D.m. GN=shi PE=1 SV=2	DYN_DROME	0	GLOS_DYN.1.1	GO:0034331	B	.	cell junction maintenance
[BBH] DYN_DROME (sp|P27619) Dynamin OS=D.m. GN=shi PE=1 SV=2	DYN_DROME	0	GLOS_DYN.1.1	GO:0034330	B	.	cell junction organization
[BBH] DYN_DROME (sp|P27619) Dynamin OS=D.m. GN=shi PE=1 SV=2	DYN_DROME	0	GLOS_DYN.1.1	GO:0043954	B	.	cellular component maintenance
[BBH] DYN_DROME (sp|P27619) Dynamin OS=D.m. GN=shi PE=1 SV=2	DYN_DROME	0	GLOS_DYN.1.1	GO:0034332	B	.	adherens junction organization
[BBH] DYN_DROME (sp|P27619) Dynamin OS=D.m. GN=shi PE=1 SV=2	DYN_DROME	0	GLOS_DYN.1.1	GO:0045216	B	.	cell-cell junction organization
[BBH] DYN_DROME (sp|P27619) Dynamin OS=D.m. GN=shi PE=1 SV=2	DYN_DROME	0	GLOS_DYN.1.1	GO:0034334	B	FlyBase	adherens junction maintenance
[BBH] DYN_DROME (sp|P27619) Dynamin OS=D.m. GN=shi PE=1 SV=2	DYN_DROME	0	GLOS_DYN.1.1	GO:0007297	B	.	ovarian follicle cell migration
[BBH] DYN_DROME (sp|P27619) Dynamin OS=D.m. GN=shi PE=1 SV=2	DYN_DROME	0	GLOS_DYN.1.1	GO:0010631	B	.	epithelial cell migration
[BBH] DYN_DROME (sp|P27619) Dynamin OS=D.m. GN=shi PE=1 SV=2	DYN_DROME	0	GLOS_DYN.1.1	GO:0001667	B	.	ameboidal cell migration
[BBH] DYN_DROME (sp|P27619) Dynamin OS=D.m. GN=shi PE=1 SV=2	DYN_DROME	0	GLOS_DYN.1.1	GO:0016477	B	.	cell migration
[BBH] DYN_DROME (sp|P27619) Dynamin OS=D.m. GN=shi PE=1 SV=2	DYN_DROME	0	GLOS_DYN.1.1	GO:0048870	B	.	cell motility
[BBH] DYN_DROME (sp|P27619) Dynamin OS=D.m. GN=shi PE=1 SV=2	DYN_DROME	0	GLOS_DYN.1.1	GO:0051674	B	.	localization of cell
[BBH] DYN_DROME (sp|P27619) Dynamin OS=D.m. GN=shi PE=1 SV=2	DYN_DROME	0	GLOS_DYN.1.1	GO:0090132	B	.	epithelium migration
[BBH] DYN_DROME (sp|P27619) Dynamin OS=D.m. GN=shi PE=1 SV=2	DYN_DROME	0	GLOS_DYN.1.1	GO:0090130	B	.	tissue migration
[BBH] DYN_DROME (sp|P27619) Dynamin OS=D.m. GN=shi PE=1 SV=2	DYN_DROME	0	GLOS_DYN.1.1	GO:0022412	B	.	cellular process involved in reproduction in multicellular organism
[BBH] DYN_DROME (sp|P27619) Dynamin OS=D.m. GN=shi PE=1 SV=2	DYN_DROME	0	GLOS_DYN.1.1	GO:0048610	B	.	cellular process involved in reproduction
[BBH] DYN_DROME (sp|P27619) Dynamin OS=D.m. GN=shi PE=1 SV=2	DYN_DROME	0	GLOS_DYN.1.1	GO:0000003	B	.	reproduction
[BBH] DYN_DROME (sp|P27619) Dynamin OS=D.m. GN=shi PE=1 SV=2	DYN_DROME	0	GLOS_DYN.1.1	GO:0032504	B	.	multicellular organism reproduction
[BBH] DYN_DROME (sp|P27619) Dynamin OS=D.m. GN=shi PE=1 SV=2	DYN_DROME	0	GLOS_DYN.1.1	GO:0030707	B	.	ovarian follicle cell development
[BBH] DYN_DROME (sp|P27619) Dynamin OS=D.m. GN=shi PE=1 SV=2	DYN_DROME	0	GLOS_DYN.1.1	GO:0003006	B	.	developmental process involved in reproduction
[BBH] DYN_DROME (sp|P27619) Dynamin OS=D.m. GN=shi PE=1 SV=2	DYN_DROME	0	GLOS_DYN.1.1	GO:0022414	B	.	reproductive process
[BBH] DYN_DROME (sp|P27619) Dynamin OS=D.m. GN=shi PE=1 SV=2	DYN_DROME	0	GLOS_DYN.1.1	GO:0048477	B	.	oogenesis
[BBH] DYN_DROME (sp|P27619) Dynamin OS=D.m. GN=shi PE=1 SV=2	DYN_DROME	0	GLOS_DYN.1.1	GO:0007292	B	.	female gamete generation
[BBH] DYN_DROME (sp|P27619) Dynamin OS=D.m. GN=shi PE=1 SV=2	DYN_DROME	0	GLOS_DYN.1.1	GO:0007276	B	.	gamete generation
[BBH] DYN_DROME (sp|P27619) Dynamin OS=D.m. GN=shi PE=1 SV=2	DYN_DROME	0	GLOS_DYN.1.1	GO:0044702	B	.	single organism reproductive process
[BBH] DYN_DROME (sp|P27619) Dynamin OS=D.m. GN=shi PE=1 SV=2	DYN_DROME	0	GLOS_DYN.1.1	GO:0048609	B	.	multicellular organismal reproductive process
[BBH] DYN_DROME (sp|P27619) Dynamin OS=D.m. GN=shi PE=1 SV=2	DYN_DROME	0	GLOS_DYN.1.1	GO:0019953	B	.	sexual reproduction
[BBH] DYN_DROME (sp|P27619) Dynamin OS=D.m. GN=shi PE=1 SV=2	DYN_DROME	0	GLOS_DYN.1.1	GO:0007298	B	FlyBase	border follicle cell migration
[BBH] DYN_DROME (sp|P27619) Dynamin OS=D.m. GN=shi PE=1 SV=2	DYN_DROME	0	GLOS_DYN.1.1	GO:0046666	B	.	retinal cell programmed cell death
[BBH] DYN_DROME (sp|P27619) Dynamin OS=D.m. GN=shi PE=1 SV=2	DYN_DROME	0	GLOS_DYN.1.1	GO:0010623	B	.	developmental programmed cell death
[BBH] DYN_DROME (sp|P27619) Dynamin OS=D.m. GN=shi PE=1 SV=2	DYN_DROME	0	GLOS_DYN.1.1	GO:0048592	B	.	eye morphogenesis
[BBH] DYN_DROME (sp|P27619) Dynamin OS=D.m. GN=shi PE=1 SV=2	DYN_DROME	0	GLOS_DYN.1.1	GO:0001745	B	.	compound eye morphogenesis
[BBH] DYN_DROME (sp|P27619) Dynamin OS=D.m. GN=shi PE=1 SV=2	DYN_DROME	0	GLOS_DYN.1.1	GO:0048749	B	.	compound eye development
[BBH] DYN_DROME (sp|P27619) Dynamin OS=D.m. GN=shi PE=1 SV=2	DYN_DROME	0	GLOS_DYN.1.1	GO:0046667	B	FlyBase	compound eye retinal cell programmed cell death
[BBH] DYN_DROME (sp|P27619) Dynamin OS=D.m. GN=shi PE=1 SV=2	DYN_DROME	0	GLOS_DYN.1.1	GO:0007631	B	.	feeding behavior
[BBH] DYN_DROME (sp|P27619) Dynamin OS=D.m. GN=shi PE=1 SV=2	DYN_DROME	0	GLOS_DYN.1.1	GO:0001661	B	FlyBase	conditioned taste aversion
[BBH] DYN_DROME (sp|P27619) Dynamin OS=D.m. GN=shi PE=1 SV=2	DYN_DROME	0	GLOS_DYN.1.1	GO:0030036	B	.	actin cytoskeleton organization

[BBH] DYN_DROME (sp|P27619) Dynamin OS=D.m. GN=shi PE=1 SV=2	DYN_DROME	0	GLOS_DYN.1.1	GO:0007010	B	.	cytoskeleton organization
[BBH] DYN_DROME (sp|P27619) Dynamin OS=D.m. GN=shi PE=1 SV=2	DYN_DROME	0	GLOS_DYN.1.1	GO:0030029	B	.	actin filament-based process
[BBH] DYN_DROME (sp|P27619) Dynamin OS=D.m. GN=shi PE=1 SV=2	DYN_DROME	0	GLOS_DYN.1.1	GO:0030865	B	.	cortical cytoskeleton organization
[BBH] DYN_DROME (sp|P27619) Dynamin OS=D.m. GN=shi PE=1 SV=2	DYN_DROME	0	GLOS_DYN.1.1	GO:0030866	B	FlyBase	cortical actin cytoskeleton organization
[BBH] DYN_DROME (sp|P27619) Dynamin OS=D.m. GN=shi PE=1 SV=2	DYN_DROME	0	GLOS_DYN.1.1	GO:0051301	B	.	cell division
[BBH] DYN_DROME (sp|P27619) Dynamin OS=D.m. GN=shi PE=1 SV=2	DYN_DROME	0	GLOS_DYN.1.1	GO:0000910	B	FlyBase	cytokinesis
[BBH] DYN_DROME (sp|P27619) Dynamin OS=D.m. GN=shi PE=1 SV=2	DYN_DROME	0	GLOS_DYN.1.1	GO:0007424	B	.	open tracheal system development
[BBH] DYN_DROME (sp|P27619) Dynamin OS=D.m. GN=shi PE=1 SV=2	DYN_DROME	0	GLOS_DYN.1.1	GO:0060541	B	.	respiratory system development
[BBH] DYN_DROME (sp|P27619) Dynamin OS=D.m. GN=shi PE=1 SV=2	DYN_DROME	0	GLOS_DYN.1.1	GO:0007427	B	FlyBase	epithelial cell migration, open tracheal system
[BBH] DYN_DROME (sp|P27619) Dynamin OS=D.m. GN=shi PE=1 SV=2	DYN_DROME	0	GLOS_DYN.1.1	GO:0043062	B	.	extracellular structure organization
[BBH] DYN_DROME (sp|P27619) Dynamin OS=D.m. GN=shi PE=1 SV=2	DYN_DROME	0	GLOS_DYN.1.1	GO:0030198	B	FlyBase	extracellular matrix organization
[BBH] DYN_DROME (sp|P27619) Dynamin OS=D.m. GN=shi PE=1 SV=2	DYN_DROME	0	GLOS_DYN.1.1	GO:0030536	B	FlyBase	larval feeding behavior
[BBH] DYN_DROME (sp|P27619) Dynamin OS=D.m. GN=shi PE=1 SV=2	DYN_DROME	0	GLOS_DYN.1.1	GO:0002009	B	.	morphogenesis of an epithelium
[BBH] DYN_DROME (sp|P27619) Dynamin OS=D.m. GN=shi PE=1 SV=2	DYN_DROME	0	GLOS_DYN.1.1	GO:0048729	B	.	tissue morphogenesis
[BBH] DYN_DROME (sp|P27619) Dynamin OS=D.m. GN=shi PE=1 SV=2	DYN_DROME	0	GLOS_DYN.1.1	GO:0060429	B	.	epithelium development
[BBH] DYN_DROME (sp|P27619) Dynamin OS=D.m. GN=shi PE=1 SV=2	DYN_DROME	0	GLOS_DYN.1.1	GO:0001738	B	FlyBase	morphogenesis of a polarized epithelium
[BBH] DYN_DROME (sp|P27619) Dynamin OS=D.m. GN=shi PE=1 SV=2	DYN_DROME	0	GLOS_DYN.1.1	GO:0008355	B	FlyBase	olfactory learning
[BBH] DYN_DROME (sp|P27619) Dynamin OS=D.m. GN=shi PE=1 SV=2	DYN_DROME	0	GLOS_DYN.1.1	GO:0007349	B	.	cellularization
[BBH] DYN_DROME (sp|P27619) Dynamin OS=D.m. GN=shi PE=1 SV=2	DYN_DROME	0	GLOS_DYN.1.1	GO:0048646	B	.	anatomical structure formation involved in morphogenesis
[BBH] DYN_DROME (sp|P27619) Dynamin OS=D.m. GN=shi PE=1 SV=2	DYN_DROME	0	GLOS_DYN.1.1	GO:0007277	B	.	pole cell development
[BBH] DYN_DROME (sp|P27619) Dynamin OS=D.m. GN=shi PE=1 SV=2	DYN_DROME	0	GLOS_DYN.1.1	GO:0007279	B	FlyBase	pole cell formation
[BBH] DYN_DROME (sp|P27619) Dynamin OS=D.m. GN=shi PE=1 SV=2	DYN_DROME	0	GLOS_DYN.1.1	GO:0008593	B	.	regulation of Notch signaling pathway
[BBH] DYN_DROME (sp|P27619) Dynamin OS=D.m. GN=shi PE=1 SV=2	DYN_DROME	0	GLOS_DYN.1.1	GO:0045747	B	FlyBase	positive regulation of Notch signaling pathway
[BBH] DYN_DROME (sp|P27619) Dynamin OS=D.m. GN=shi PE=1 SV=2	DYN_DROME	0	GLOS_DYN.1.1	GO:0051780	B	.	behavioral response to nutrient
[BBH] DYN_DROME (sp|P27619) Dynamin OS=D.m. GN=shi PE=1 SV=2	DYN_DROME	0	GLOS_DYN.1.1	GO:0007584	B	.	response to nutrient
[BBH] DYN_DROME (sp|P27619) Dynamin OS=D.m. GN=shi PE=1 SV=2	DYN_DROME	0	GLOS_DYN.1.1	GO:0060004	B	.	reflex
[BBH] DYN_DROME (sp|P27619) Dynamin OS=D.m. GN=shi PE=1 SV=2	DYN_DROME	0	GLOS_DYN.1.1	GO:0007637	B	FlyBase	proboscis extension reflex
[BBH] DYN_DROME (sp|P27619) Dynamin OS=D.m. GN=shi PE=1 SV=2	DYN_DROME	0	GLOS_DYN.1.1	GO:0006897	B	.	endocytosis
[BBH] DYN_DROME (sp|P27619) Dynamin OS=D.m. GN=shi PE=1 SV=2	DYN_DROME	0	GLOS_DYN.1.1	GO:0016192	B	.	vesicle-mediated transport
[BBH] DYN_DROME (sp|P27619) Dynamin OS=D.m. GN=shi PE=1 SV=2	DYN_DROME	0	GLOS_DYN.1.1	GO:0006898	B	FlyBase	receptor-mediated endocytosis
[BBH] DYN_DROME (sp|P27619) Dynamin OS=D.m. GN=shi PE=1 SV=2	DYN_DROME	0	GLOS_DYN.1.1	GO:0032970	B	.	regulation of actin filament-based process
[BBH] DYN_DROME (sp|P27619) Dynamin OS=D.m. GN=shi PE=1 SV=2	DYN_DROME	0	GLOS_DYN.1.1	GO:0051493	B	.	regulation of cytoskeleton organization
[BBH] DYN_DROME (sp|P27619) Dynamin OS=D.m. GN=shi PE=1 SV=2	DYN_DROME	0	GLOS_DYN.1.1	GO:0033043	B	.	regulation of organelle organization
[BBH] DYN_DROME (sp|P27619) Dynamin OS=D.m. GN=shi PE=1 SV=2	DYN_DROME	0	GLOS_DYN.1.1	GO:0051128	B	.	regulation of cellular component organization
[BBH] DYN_DROME (sp|P27619) Dynamin OS=D.m. GN=shi PE=1 SV=2	DYN_DROME	0	GLOS_DYN.1.1	GO:0032956	B	FlyBase	regulation of actin cytoskeleton organization
[BBH] DYN_DROME (sp|P27619) Dynamin OS=D.m. GN=shi PE=1 SV=2	DYN_DROME	0	GLOS_DYN.1.1	GO:0040008	B	FlyBase	regulation of growth
[BBH] DYN_DROME (sp|P27619) Dynamin OS=D.m. GN=shi PE=1 SV=2	DYN_DROME	0	GLOS_DYN.1.1	GO:0048168	B	.	regulation of neuronal synaptic plasticity
[BBH] DYN_DROME (sp|P27619) Dynamin OS=D.m. GN=shi PE=1 SV=2	DYN_DROME	0	GLOS_DYN.1.1	GO:0048167	B	.	regulation of synaptic plasticity
[BBH] DYN_DROME (sp|P27619) Dynamin OS=D.m. GN=shi PE=1 SV=2	DYN_DROME	0	GLOS_DYN.1.1	GO:0050804	B	.	regulation of synaptic transmission
[BBH] DYN_DROME (sp|P27619) Dynamin OS=D.m. GN=shi PE=1 SV=2	DYN_DROME	0	GLOS_DYN.1.1	GO:0051969	B	.	regulation of transmission of nerve impulse
[BBH] DYN_DROME (sp|P27619) Dynamin OS=D.m. GN=shi PE=1 SV=2	DYN_DROME	0	GLOS_DYN.1.1	GO:0031644	B	.	regulation of neurological system process
[BBH] DYN_DROME (sp|P27619) Dynamin OS=D.m. GN=shi PE=1 SV=2	DYN_DROME	0	GLOS_DYN.1.1	GO:0044057	B	.	regulation of system process
[BBH] DYN_DROME (sp|P27619) Dynamin OS=D.m. GN=shi PE=1 SV=2	DYN_DROME	0	GLOS_DYN.1.1	GO:0048172	B	FlyBase	regulation of short-term neuronal synaptic plasticity
[BBH] DYN_DROME (sp|P27619) Dynamin OS=D.m. GN=shi PE=1 SV=2	DYN_DROME	0	GLOS_DYN.1.1	GO:0007268	B	.	synaptic transmission
[BBH] DYN_DROME (sp|P27619) Dynamin OS=D.m. GN=shi PE=1 SV=2	DYN_DROME	0	GLOS_DYN.1.1	GO:0019226	B	.	transmission of nerve impulse
[BBH] DYN_DROME (sp|P27619) Dynamin OS=D.m. GN=shi PE=1 SV=2	DYN_DROME	0	GLOS_DYN.1.1	GO:0035637	B	.	multicellular organismal signaling
[BBH] DYN_DROME (sp|P27619) Dynamin OS=D.m. GN=shi PE=1 SV=2	DYN_DROME	0	GLOS_DYN.1.1	GO:0050803	B	FlyBase	regulation of synapse structure and activity
[BBH] DYN_DROME (sp|P27619) Dynamin OS=D.m. GN=shi PE=1 SV=2	DYN_DROME	0	GLOS_DYN.1.1	GO:0035152	B	FlyBase	regulation of tube architecture, open tracheal system
[BBH] DYN_DROME (sp|P27619) Dynamin OS=D.m. GN=shi PE=1 SV=2	DYN_DROME	0	GLOS_DYN.1.1	GO:0009266	B	.	response to temperature stimulus
[BBH] DYN_DROME (sp|P27619) Dynamin OS=D.m. GN=shi PE=1 SV=2	DYN_DROME	0	GLOS_DYN.1.1	GO:0009408	B	FlyBase	response to heat
[BBH] DYN_DROME (sp|P27619) Dynamin OS=D.m. GN=shi PE=1 SV=2	DYN_DROME	0	GLOS_DYN.1.1	GO:0007435	B	FlyBase	salivary gland morphogenesis
[BBH] DYN_DROME (sp|P27619) Dynamin OS=D.m. GN=shi PE=1 SV=2	DYN_DROME	0	GLOS_DYN.1.1	GO:0007614	B	FlyBase	short-term memory
[BBH] DYN_DROME (sp|P27619) Dynamin OS=D.m. GN=shi PE=1 SV=2	DYN_DROME	0	GLOS_DYN.1.1	GO:0007286	B	.	spermatid development
[BBH] DYN_DROME (sp|P27619) Dynamin OS=D.m. GN=shi PE=1 SV=2	DYN_DROME	0	GLOS_DYN.1.1	GO:0002064	B	.	epithelial cell development

[BBH] DYN_DROME (sp|P27619) Dynamin OS=D.m. GN=shi PE=1 SV=2	DYN_DROME	0	GLOS_DYN.1.1	GO:0030855	B	.	epithelial cell differentiation
[BBH] DYN_DROME (sp|P27619) Dynamin OS=D.m. GN=shi PE=1 SV=2	DYN_DROME	0	GLOS_DYN.1.1	GO:0007281	B	.	germ cell development
[BBH] DYN_DROME (sp|P27619) Dynamin OS=D.m. GN=shi PE=1 SV=2	DYN_DROME	0	GLOS_DYN.1.1	GO:0048515	B	.	spermatid differentiation
[BBH] DYN_DROME (sp|P27619) Dynamin OS=D.m. GN=shi PE=1 SV=2	DYN_DROME	0	GLOS_DYN.1.1	GO:0007283	B	.	spermatogenesis
[BBH] DYN_DROME (sp|P27619) Dynamin OS=D.m. GN=shi PE=1 SV=2	DYN_DROME	0	GLOS_DYN.1.1	GO:0048232	B	.	male gamete generation
[BBH] DYN_DROME (sp|P27619) Dynamin OS=D.m. GN=shi PE=1 SV=2	DYN_DROME	0	GLOS_DYN.1.1	GO:0007291	B	FlyBase	sperm individualization
[BBH] DYN_DROME (sp|P27619) Dynamin OS=D.m. GN=shi PE=1 SV=2	DYN_DROME	0	GLOS_DYN.1.1	GO:0070142	B	.	synaptic vesicle budding
[BBH] DYN_DROME (sp|P27619) Dynamin OS=D.m. GN=shi PE=1 SV=2	DYN_DROME	0	GLOS_DYN.1.1	GO:0006900	B	.	membrane budding
[BBH] DYN_DROME (sp|P27619) Dynamin OS=D.m. GN=shi PE=1 SV=2	DYN_DROME	0	GLOS_DYN.1.1	GO:0016050	B	.	vesicle organization
[BBH] DYN_DROME (sp|P27619) Dynamin OS=D.m. GN=shi PE=1 SV=2	DYN_DROME	0	GLOS_DYN.1.1	GO:0044802	B	.	single-organism membrane organization
[BBH] DYN_DROME (sp|P27619) Dynamin OS=D.m. GN=shi PE=1 SV=2	DYN_DROME	0	GLOS_DYN.1.1	GO:0061024	B	.	membrane organization
[BBH] DYN_DROME (sp|P27619) Dynamin OS=D.m. GN=shi PE=1 SV=2	DYN_DROME	0	GLOS_DYN.1.1	GO:0048489	B	.	synaptic vesicle transport
[BBH] DYN_DROME (sp|P27619) Dynamin OS=D.m. GN=shi PE=1 SV=2	DYN_DROME	0	GLOS_DYN.1.1	GO:0097480	B	.	establishment of synaptic vesicle localization
[BBH] DYN_DROME (sp|P27619) Dynamin OS=D.m. GN=shi PE=1 SV=2	DYN_DROME	0	GLOS_DYN.1.1	GO:0051650	B	.	establishment of vesicle localization
[BBH] DYN_DROME (sp|P27619) Dynamin OS=D.m. GN=shi PE=1 SV=2	DYN_DROME	0	GLOS_DYN.1.1	GO:0051656	B	.	establishment of organelle localization
[BBH] DYN_DROME (sp|P27619) Dynamin OS=D.m. GN=shi PE=1 SV=2	DYN_DROME	0	GLOS_DYN.1.1	GO:0051640	B	.	organelle localization
[BBH] DYN_DROME (sp|P27619) Dynamin OS=D.m. GN=shi PE=1 SV=2	DYN_DROME	0	GLOS_DYN.1.1	GO:0051648	B	.	vesicle localization
[BBH] DYN_DROME (sp|P27619) Dynamin OS=D.m. GN=shi PE=1 SV=2	DYN_DROME	0	GLOS_DYN.1.1	GO:0097479	B	.	synaptic vesicle localization
[BBH] DYN_DROME (sp|P27619) Dynamin OS=D.m. GN=shi PE=1 SV=2	DYN_DROME	0	GLOS_DYN.1.1	GO:0048488	B	.	synaptic vesicle endocytosis
[BBH] DYN_DROME (sp|P27619) Dynamin OS=D.m. GN=shi PE=1 SV=2	DYN_DROME	0	GLOS_DYN.1.1	GO:0016185	B	FlyBase	synaptic vesicle budding from presynaptic membrane
[BBH] DYN_DROME (sp|P27619) Dynamin OS=D.m. GN=shi PE=1 SV=2	DYN_DROME	0	GLOS_DYN.1.1	GO:0010256	B	.	endomembrane system organization
[BBH] DYN_DROME (sp|P27619) Dynamin OS=D.m. GN=shi PE=1 SV=2	DYN_DROME	0	GLOS_DYN.1.1	GO:0048499	B	FlyBase	synaptic vesicle membrane organization
[BBH] ECM29_DROME (sp|Q9V677) Proteasome-assoc. prot ECM29 homolog	ECM29_DROME	0	GLOS_ECM29.1.1	GO:0005737	C	UniProtKB	cytoplasm
[BBH] ECM29_DROME (sp|Q9V677) Proteasome-assoc. prot ECM29 homolog	ECM29_DROME	0	GLOS_ECM29.1.1	GO:0005634	C	UniProtKB	nucleus
[BBH] ECM29_DROME (sp|Q9V677) Proteasome-assoc. prot ECM29 homolog	ECM29_DROME	0	GLOS_ECM29.1.1	GO:0000502	C	UniProtKB	proteasome complex
[BBH] EIF3B_DROMO (sp|B4KNN9) Eukar. Transl. initiation fact. 3 subunit B	EIF3B_DROMO	0	GLOS_EIF3B.1.1	GO:0070993	C	.	translation preinitiation complex
[BBH] EIF3B_DROMO (sp|B4KNN9) Eukar. Transl. initiation fact. 3 subunit B	EIF3B_DROMO	0	GLOS_EIF3B.1.1	GO:0030529	C	.	ribonucleoprotein complex
[BBH] EIF3B_DROMO (sp|B4KNN9) Eukar. Transl. initiation fact. 3 subunit B	EIF3B_DROMO	0	GLOS_EIF3B.1.1	GO:0016282	C	UniProtKB-H	eukaryotic 43S preinitiation complex
[BBH] EIF3B_DROMO (sp|B4KNN9) Eukar. Transl. initiation fact. 3 subunit B	EIF3B_DROMO	0	GLOS_EIF3B.1.1	GO:0033290	C	UniProtKB-H	eukaryotic 48S preinitiation complex
[BBH] EIF3B_DROMO (sp|B4KNN9) Eukar. Transl. initiation fact. 3 subunit B	EIF3B_DROMO	0	GLOS_EIF3B.1.1	GO:0005852	C	UniProtKB	eukaryotic translation initiation factor 3 complex
[BBH] EIF3B_DROMO (sp|B4KNN9) Eukar. Transl. initiation fact. 3 subunit B	EIF3B_DROMO	0	GLOS_EIF3B.1.1	GO:0000166	M	InterPro	nucleotide binding
[BBH] EIF3B_DROMO (sp|B4KNN9) Eukar. Transl. initiation fact. 3 subunit B	EIF3B_DROMO	0	GLOS_EIF3B.1.1	GO:0008135	M	.	translation factor activity, nucleic acid binding
[BBH] EIF3B_DROMO (sp|B4KNN9) Eukar. Transl. initiation fact. 3 subunit B	EIF3B_DROMO	0	GLOS_EIF3B.1.1	GO:0003723	M	.	RNA binding
[BBH] EIF3B_DROMO (sp|B4KNN9) Eukar. Transl. initiation fact. 3 subunit B	EIF3B_DROMO	0	GLOS_EIF3B.1.1	GO:0003743	M	UniProtKB	translation initiation factor activity
[BBH] EIF3B_DROMO (sp|B4KNN9) Eukar. Transl. initiation fact. 3 subunit B	EIF3B_DROMO	0	GLOS_EIF3B.1.1	GO:0022618	B	.	ribonucleoprotein complex assembly
[BBH] EIF3B_DROMO (sp|B4KNN9) Eukar. Transl. initiation fact. 3 subunit B	EIF3B_DROMO	0	GLOS_EIF3B.1.1	GO:0034622	B	.	cellular macromolecular complex assembly

Best hit description	Best hit accession	Best hit evalu	Name	Code	Ontolo	Source	Go name
NP_730457.1 PAPS synthetase, isoform A [D. melanogaster] ref|NP_730458.1| isoform B [D. melanogaster] ref|NP_730459.1| isoform C [D. melanogaster] ref|NP_001262072.1| isoform G [D. melanogaster]	NP_730457.1	1,00E-151	GLOS_PAPSS.1.1	GO:0000122	B	Refseq	negative regulation of transcription from RNA polymerase II promoter
NP_730457.1 PAPS synthetase, isoform A [D. melanogaster] … Same as above	NP_730457.1	1,00E-151	GLOS_PAPSS.1.1	GO:0001501	B	Refseq	skeletal system development
NP_730457.1 PAPS synthetase, isoform A [D. melanogaster] … Same as above	NP_730457.1	1,00E-151	GLOS_PAPSS.1.1	GO:0003700	M	Refseq	sequence-specific DNA binding transcription factor activity
NP_730457.1 PAPS synthetase, isoform A [D. melanogaster] … Same as above	NP_730457.1	1,00E-151	GLOS_PAPSS.1.1	GO:0005515	M	Refseq	protein binding
NP_730457.1 PAPS synthetase, isoform A [D. melanogaster] … Same as above	NP_730457.1	1,00E-151	GLOS_PAPSS.1.1	GO:0005634	C	Refseq	nucleus
NP_730457.1 PAPS synthetase, isoform A [D. melanogaster] … Same as above	NP_730457.1	1,00E-151	GLOS_PAPSS.1.1	GO:0006366	B	Refseq	transcription from RNA polymerase II promoter
NP_730457.1 PAPS synthetase, isoform A [D. melanogaster] … Same as above	NP_730457.1	1,00E-151	GLOS_PAPSS.1.1	GO:0006607	B	Refseq	NLS-bearing protein import into nucleus
NP_730457.1 PAPS synthetase, isoform A [D. melanogaster] … Same as above	NP_730457.1	1,00E-151	GLOS_PAPSS.1.1	GO:0008270	M	Refseq	zinc ion binding
NP_730457.1 PAPS synthetase, isoform A [D. melanogaster] … Same as above	NP_730457.1	1,00E-151	GLOS_PAPSS.1.1	GO:0032330	B	Refseq	regulation of chondrocyte differentiation
NP_730457.1 PAPS synthetase, isoform A [D. melanogaster] … Same as above	NP_730457.1	1,00E-151	GLOS_PAPSS.1.1	GO:0043565	M	Refseq	sequence-specific DNA binding
NP_724931.1 peroxiredoxin 2540-1 [Drosophila melanogaster]	NP_724931.1	2,00E-53	GLOS_PRX2540-1	GO:0000122	B	Refseq	negative regulation of transcription from RNA polymerase II promoter
NP_724931.1 peroxiredoxin 2540-1 [Drosophila melanogaster]	NP_724931.1	2,00E-53	GLOS_PRX2540-1	GO:0001501	B	Refseq	skeletal system development
NP_724931.1 peroxiredoxin 2540-1 [Drosophila melanogaster]	NP_724931.1	2,00E-53	GLOS_PRX2540-1	GO:0003700	M	Refseq	sequence-specific DNA binding transcription factor activity

NP_724931.1 peroxiredoxin 2540-1 [Drosophila melanogaster]	NP_724931.1	2,00E-53	GLOS_PRX2540-1	GO:0005515	M	Refseq	protein binding
NP_724931.1 peroxiredoxin 2540-1 [Drosophila melanogaster]	NP_724931.1	2,00E-53	GLOS_PRX2540-1	GO:0005634	C	Refseq	nucleus
NP_724931.1 peroxiredoxin 2540-1 [Drosophila melanogaster]	NP_724931.1	2,00E-53	GLOS_PRX2540-1	GO:0006366	B	Refseq	transcription from RNA polymerase II promoter
NP_724931.1 peroxiredoxin 2540-1 [Drosophila melanogaster]	NP_724931.1	2,00E-53	GLOS_PRX2540-1	GO:0006607	B	Refseq	NLS-bearing protein import into nucleus
NP_724931.1 peroxiredoxin 2540-1 [Drosophila melanogaster]	NP_724931.1	2,00E-53	GLOS_PRX2540-1	GO:0008270	M	Refseq	zinc ion binding
NP_724931.1 peroxiredoxin 2540-1 [Drosophila melanogaster]	NP_724931.1	2,00E-53	GLOS_PRX2540-1	GO:0032330	B	Refseq	regulation of chondrocyte differentiation
NP_724931.1 peroxiredoxin 2540-1 [Drosophila melanogaster]	NP_724931.1	2,00E-53	GLOS_PRX2540-1	GO:0043565	M	Refseq	sequence-specific DNA binding
XM_001996457.1 Drosophila grimshawi GH23965 (DgriGH23965), mRNA	XM_001996457.1	0	GLOS_DGRI_GH2	GO:0005262	M	Refseq	calcium channel activity
XM_001996457.1 Drosophila grimshawi GH23965 (DgriGH23965), mRNA	XM_001996457.1	0	GLOS_DGRI_GH2	GO:0005515	M	Refseq	protein binding
XM_001996457.1 Drosophila grimshawi GH23965 (DgriGH23965), mRNA	XM_001996457.1	0	GLOS_DGRI_GH2	GO:0005886	C	Refseq	plasma membrane
XM_001996457.1 Drosophila grimshawi GH23965 (DgriGH23965), mRNA	XM_001996457.1	0	GLOS_DGRI_GH2	GO:0005887	C	Refseq	integral to plasma membrane
XM_001996457.1 Drosophila grimshawi GH23965 (DgriGH23965), mRNA	XM_001996457.1	0	GLOS_DGRI_GH2	GO:0006816	B	Refseq	calcium ion transport
XM_001996457.1 Drosophila grimshawi GH23965 (DgriGH23965), mRNA	XM_001996457.1	0	GLOS_DGRI_GH2	GO:0007411	B	Refseq	axon guidance
XM_001996457.1 Drosophila grimshawi GH23965 (DgriGH23965), mRNA	XM_001996457.1	0	GLOS_DGRI_GH2	GO:0007596	B	Refseq	blood coagulation
XM_001996457.1 Drosophila grimshawi GH23965 (DgriGH23965), mRNA	XM_001996457.1	0	GLOS_DGRI_GH2	GO:0007602	B	Refseq	phototransduction
XM_001996457.1 Drosophila grimshawi GH23965 (DgriGH23965), mRNA	XM_001996457.1	0	GLOS_DGRI_GH2	GO:0010524	B	Refseq	positive regulation of calcium ion transport into cytosol
XM_001996457.1 Drosophila grimshawi GH23965 (DgriGH23965), mRNA	XM_001996457.1	0	GLOS_DGRI_GH2	GO:0015279	M	Refseq	store-operated calcium channel activity
XM_001996457.1 Drosophila grimshawi GH23965 (DgriGH23965), mRNA	XM_001996457.1	0	GLOS_DGRI_GH2	GO:0030168	B	Refseq	platelet activation
XM_001996457.1 Drosophila grimshawi GH23965 (DgriGH23965), mRNA	XM_001996457.1	0	GLOS_DGRI_GH2	GO:0033198	B	Refseq	response to ATP
XM_001996457.1 Drosophila grimshawi GH23965 (DgriGH23965), mRNA	XM_001996457.1	0	GLOS_DGRI_GH2	GO:0051592	B	Refseq	response to calcium ion
XM_001996457.1 Drosophila grimshawi GH23965 (DgriGH23965), mRNA	XM_001996457.1	0	GLOS_DGRI_GH2	GO:0070588	B	Refseq	calcium ion transmembrane transport
XM_001996457.1 Drosophila grimshawi GH23965 (DgriGH23965), mRNA	XM_001996457.1	0	GLOS_DGRI_GH2	GO:0070679	M	Refseq	inositol 1,4,5 trisphosphate binding
XP_001988758.1 GH10404 [Drosophila grimshawi]	XP_001988758.1	0	GLOS_DGRI_GH1	GO:0005262	M	Refseq	calcium channel activity
XP_001988758.1 GH10404 [Drosophila grimshawi]	XP_001988758.1	0	GLOS_DGRI_GH1	GO:0005515	M	Refseq	protein binding
XP_001988758.1 GH10404 [Drosophila grimshawi]	XP_001988758.1	0	GLOS_DGRI_GH1	GO:0005886	C	Refseq	plasma membrane
XP_001988758.1 GH10404 [Drosophila grimshawi]	XP_001988758.1	0	GLOS_DGRI_GH1	GO:0005887	C	Refseq	integral to plasma membrane
XP_001988758.1 GH10404 [Drosophila grimshawi]	XP_001988758.1	0	GLOS_DGRI_GH1	GO:0006816	B	Refseq	calcium ion transport
XP_001988758.1 GH10404 [Drosophila grimshawi]	XP_001988758.1	0	GLOS_DGRI_GH1	GO:0007411	B	Refseq	axon guidance
XP_001988758.1 GH10404 [Drosophila grimshawi]	XP_001988758.1	0	GLOS_DGRI_GH1	GO:0007596	B	Refseq	blood coagulation
XP_001988758.1 GH10404 [Drosophila grimshawi]	XP_001988758.1	0	GLOS_DGRI_GH1	GO:0007602	B	Refseq	phototransduction
XP_001988758.1 GH10404 [Drosophila grimshawi]	XP_001988758.1	0	GLOS_DGRI_GH1	GO:0010524	B	Refseq	positive regulation of calcium ion transport into cytosol
XP_001988758.1 GH10404 [Drosophila grimshawi]	XP_001988758.1	0	GLOS_DGRI_GH1	GO:0015279	M	Refseq	store-operated calcium channel activity
XP_001988758.1 GH10404 [Drosophila grimshawi]	XP_001988758.1	0	GLOS_DGRI_GH1	GO:0030168	B	Refseq	platelet activation
XP_001988758.1 GH10404 [Drosophila grimshawi]	XP_001988758.1	0	GLOS_DGRI_GH1	GO:0033198	B	Refseq	response to ATP
XP_001988758.1 GH10404 [Drosophila grimshawi]	XP_001988758.1	0	GLOS_DGRI_GH1	GO:0051592	B	Refseq	response to calcium ion
XP_001988758.1 GH10404 [Drosophila grimshawi]	XP_001988758.1	0	GLOS_DGRI_GH1	GO:0070588	B	Refseq	calcium ion transmembrane transport
XP_001988758.1 GH10404 [Drosophila grimshawi]	XP_001988758.1	0	GLOS_DGRI_GH1	GO:0070679	M	Refseq	inositol 1,4,5 trisphosphate binding
XP_001994847.1 GH13879 [Drosophila grimshawi]	XP_001994847.1	2,00E-10	GLOS_DGRI_GH1	GO:0005262	M	Refseq	calcium channel activity
XP_001994847.1 GH13879 [Drosophila grimshawi]	XP_001994847.1	2,00E-10	GLOS_DGRI_GH1	GO:0005515	M	Refseq	protein binding
XP_001994847.1 GH13879 [Drosophila grimshawi]	XP_001994847.1	2,00E-10	GLOS_DGRI_GH1	GO:0005886	C	Refseq	plasma membrane
XP_001994847.1 GH13879 [Drosophila grimshawi]	XP_001994847.1	2,00E-10	GLOS_DGRI_GH1	GO:0005887	C	Refseq	integral to plasma membrane
XP_001994847.1 GH13879 [Drosophila grimshawi]	XP_001994847.1	2,00E-10	GLOS_DGRI_GH1	GO:0006816	B	Refseq	calcium ion transport
XP_001994847.1 GH13879 [Drosophila grimshawi]	XP_001994847.1	2,00E-10	GLOS_DGRI_GH1	GO:0007411	B	Refseq	axon guidance
XP_001994847.1 GH13879 [Drosophila grimshawi]	XP_001994847.1	2,00E-10	GLOS_DGRI_GH1	GO:0007596	B	Refseq	blood coagulation
XP_001994847.1 GH13879 [Drosophila grimshawi]	XP_001994847.1	2,00E-10	GLOS_DGRI_GH1	GO:0007602	B	Refseq	phototransduction
XP_001994847.1 GH13879 [Drosophila grimshawi]	XP_001994847.1	2,00E-10	GLOS_DGRI_GH1	GO:0010524	B	Refseq	positive regulation of calcium ion transport into cytosol
XP_001994847.1 GH13879 [Drosophila grimshawi]	XP_001994847.1	2,00E-10	GLOS_DGRI_GH1	GO:0015279	M	Refseq	store-operated calcium channel activity
XP_001994847.1 GH13879 [Drosophila grimshawi]	XP_001994847.1	2,00E-10	GLOS_DGRI_GH1	GO:0030168	B	Refseq	platelet activation
XP_001994847.1 GH13879 [Drosophila grimshawi]	XP_001994847.1	2,00E-10	GLOS_DGRI_GH1	GO:0033198	B	Refseq	response to ATP
XP_001994847.1 GH13879 [Drosophila grimshawi]	XP_001994847.1	2,00E-10	GLOS_DGRI_GH1	GO:0051592	B	Refseq	response to calcium ion
XP_001994847.1 GH13879 [Drosophila grimshawi]	XP_001994847.1	2,00E-10	GLOS_DGRI_GH1	GO:0070588	B	Refseq	calcium ion transmembrane transport
XP_001994847.1 GH13879 [Drosophila grimshawi]	XP_001994847.1	2,00E-10	GLOS_DGRI_GH1	GO:0070679	M	Refseq	inositol 1,4,5 trisphosphate binding
NP_001260954.1 bb in a boxcar, isoform D [Drosophila melanogaster]	NP_001260954.1	6,00E-25	GLOS_BBC.3.5	GO:0000122	B	Refseq	negative regulation of transcription from RNA polymerase II promoter
NP_001260954.1 bb in a boxcar, isoform D [Drosophila melanogaster]	NP_001260954.1	6,00E-25	GLOS_BBC.3.5	GO:0001501	B	Refseq	skeletal system development

NP_001260954.1 bb in a boxcar, isoform D [Drosophila melanogaster]	NP_001260954.1	6,00E-25	GLOS_BBC.3.5	GO:0003700	M	Refseq	sequence-specific DNA binding transcription factor activity
NP_001260954.1 bb in a boxcar, isoform D [Drosophila melanogaster]	NP_001260954.1	6,00E-25	GLOS_BBC.3.5	GO:0005515	M	Refseq	protein binding
NP_001260954.1 bb in a boxcar, isoform D [Drosophila melanogaster]	NP_001260954.1	6,00E-25	GLOS_BBC.3.5	GO:0005634	C	Refseq	nucleus
NP_001260954.1 bb in a boxcar, isoform D [Drosophila melanogaster]	NP_001260954.1	6,00E-25	GLOS_BBC.3.5	GO:0006366	B	Refseq	transcription from RNA polymerase II promoter
NP_001260954.1 bb in a boxcar, isoform D [Drosophila melanogaster]	NP_001260954.1	6,00E-25	GLOS_BBC.3.5	GO:0006607	B	Refseq	NLS-bearing protein import into nucleus
NP_001260954.1 bb in a boxcar, isoform D [Drosophila melanogaster]	NP_001260954.1	6,00E-25	GLOS_BBC.3.5	GO:0008270	M	Refseq	zinc ion binding
NP_001260954.1 bb in a boxcar, isoform D [Drosophila melanogaster]	NP_001260954.1	6,00E-25	GLOS_BBC.3.5	GO:0032330	B	Refseq	regulation of chondrocyte differentiation
NP_001260954.1 bb in a boxcar, isoform D [Drosophila melanogaster]	NP_001260954.1	6,00E-25	GLOS_BBC.3.5	GO:0043565	M	Refseq	sequence-specific DNA binding
NP_647774.2 BTB-protein-VII, isoform F [Drosophila melanogaster]	NP_647774.2	1,00E-105	GLOS_BTBVII.1.1	GO:0000122	B	Refseq	negative regulation of transcription from RNA polymerase II promoter
NP_647774.2 BTB-protein-VII, isoform F [Drosophila melanogaster]	NP_647774.2	1,00E-105	GLOS_BTBVII.1.1	GO:0001501	B	Refseq	skeletal system development
NP_647774.2 BTB-protein-VII, isoform F [Drosophila melanogaster]	NP_647774.2	1,00E-105	GLOS_BTBVII.1.1	GO:0003700	M	Refseq	sequence-specific DNA binding transcription factor activity
NP_647774.2 BTB-protein-VII, isoform F [Drosophila melanogaster]	NP_647774.2	1,00E-105	GLOS_BTBVII.1.1	GO:0005515	M	Refseq	protein binding
NP_647774.2 BTB-protein-VII, isoform F [Drosophila melanogaster]	NP_647774.2	1,00E-105	GLOS_BTBVII.1.1	GO:0005634	C	Refseq	nucleus
NP_647774.2 BTB-protein-VII, isoform F [Drosophila melanogaster]	NP_647774.2	1,00E-105	GLOS_BTBVII.1.1	GO:0006366	B	Refseq	transcription from RNA polymerase II promoter
NP_647774.2 BTB-protein-VII, isoform F [Drosophila melanogaster]	NP_647774.2	1,00E-105	GLOS_BTBVII.1.1	GO:0006607	B	Refseq	NLS-bearing protein import into nucleus
NP_647774.2 BTB-protein-VII, isoform F [Drosophila melanogaster]	NP_647774.2	1,00E-105	GLOS_BTBVII.1.1	GO:0008270	M	Refseq	zinc ion binding
NP_647774.2 BTB-protein-VII, isoform F [Drosophila melanogaster]	NP_647774.2	1,00E-105	GLOS_BTBVII.1.1	GO:0032330	B	Refseq	regulation of chondrocyte differentiation
NP_647774.2 BTB-protein-VII, isoform F [Drosophila melanogaster]	NP_647774.2	1,00E-105	GLOS_BTBVII.1.1	GO:0043565	M	Refseq	sequence-specific DNA binding
XP_001988793.1 GH11353 [Drosophila grimshawi]	XP_001988793.1	8,00E-07	GLOS_DGRI_GH1	GO:0005262	M	Refseq	calcium channel activity
XP_001988793.1 GH11353 [Drosophila grimshawi]	XP_001988793.1	8,00E-07	GLOS_DGRI_GH1	GO:0005515	M	Refseq	protein binding
XP_001988793.1 GH11353 [Drosophila grimshawi]	XP_001988793.1	8,00E-07	GLOS_DGRI_GH1	GO:0005886	C	Refseq	plasma membrane
XP_001988793.1 GH11353 [Drosophila grimshawi]	XP_001988793.1	8,00E-07	GLOS_DGRI_GH1	GO:0005887	C	Refseq	integral to plasma membrane
XP_001988793.1 GH11353 [Drosophila grimshawi]	XP_001988793.1	8,00E-07	GLOS_DGRI_GH1	GO:0006816	B	Refseq	calcium ion transport
XP_001988793.1 GH11353 [Drosophila grimshawi]	XP_001988793.1	8,00E-07	GLOS_DGRI_GH1	GO:0007411	B	Refseq	axon guidance
XP_001988793.1 GH11353 [Drosophila grimshawi]	XP_001988793.1	8,00E-07	GLOS_DGRI_GH1	GO:0007596	B	Refseq	blood coagulation
XP_001988793.1 GH11353 [Drosophila grimshawi]	XP_001988793.1	8,00E-07	GLOS_DGRI_GH1	GO:0007602	B	Refseq	phototransduction
XP_001988793.1 GH11353 [Drosophila grimshawi]	XP_001988793.1	8,00E-07	GLOS_DGRI_GH1	GO:0010524	B	Refseq	positive regulation of calcium ion transport into cytosol
XP_001988793.1 GH11353 [Drosophila grimshawi]	XP_001988793.1	8,00E-07	GLOS_DGRI_GH1	GO:0015279	M	Refseq	store-operated calcium channel activity
XP_001988793.1 GH11353 [Drosophila grimshawi]	XP_001988793.1	8,00E-07	GLOS_DGRI_GH1	GO:0030168	B	Refseq	platelet activation
XP_001988793.1 GH11353 [Drosophila grimshawi]	XP_001988793.1	8,00E-07	GLOS_DGRI_GH1	GO:0033198	B	Refseq	response to ATP
XP_001988793.1 GH11353 [Drosophila grimshawi]	XP_001988793.1	8,00E-07	GLOS_DGRI_GH1	GO:0051592	B	Refseq	response to calcium ion
XP_001988793.1 GH11353 [Drosophila grimshawi]	XP_001988793.1	8,00E-07	GLOS_DGRI_GH1	GO:0070588	B	Refseq	calcium ion transmembrane transport
XP_001988793.1 GH11353 [Drosophila grimshawi]	XP_001988793.1	8,00E-07	GLOS_DGRI_GH1	GO:0070679	M	Refseq	inositol 1,4,5 trisphosphate binding
NP_001033997.1 CG43897, isoform H [Drosophila melanogaster]	NP_001033997.1	2,00E-95	GLOS_contig_008	GO:0000122	B	Refseq	negative regulation of transcription from RNA polymerase II promoter
NP_001033997.1 CG43897, isoform H [Drosophila melanogaster]	NP_001033997.1	2,00E-95	GLOS_contig_008	GO:0001501	B	Refseq	skeletal system development
NP_001033997.1 CG43897, isoform H [Drosophila melanogaster]	NP_001033997.1	2,00E-95	GLOS_contig_008	GO:0003700	M	Refseq	sequence-specific DNA binding transcription factor activity
NP_001033997.1 CG43897, isoform H [Drosophila melanogaster]	NP_001033997.1	2,00E-95	GLOS_contig_008	GO:0005515	M	Refseq	protein binding
NP_001033997.1 CG43897, isoform H [Drosophila melanogaster]	NP_001033997.1	2,00E-95	GLOS_contig_008	GO:0005634	C	Refseq	nucleus
NP_001033997.1 CG43897, isoform H [Drosophila melanogaster]	NP_001033997.1	2,00E-95	GLOS_contig_008	GO:0006366	B	Refseq	transcription from RNA polymerase II promoter
NP_001033997.1 CG43897, isoform H [Drosophila melanogaster]	NP_001033997.1	2,00E-95	GLOS_contig_008	GO:0006607	B	Refseq	NLS-bearing protein import into nucleus
NP_001033997.1 CG43897, isoform H [Drosophila melanogaster]	NP_001033997.1	2,00E-95	GLOS_contig_008	GO:0008270	M	Refseq	zinc ion binding
NP_001033997.1 CG43897, isoform H [Drosophila melanogaster]	NP_001033997.1	2,00E-95	GLOS_contig_008	GO:0032330	B	Refseq	regulation of chondrocyte differentiation
NP_001033997.1 CG43897, isoform H [Drosophila melanogaster]	NP_001033997.1	2,00E-95	GLOS_contig_008	GO:0043565	M	Refseq	sequence-specific DNA binding
XP_001987640.1GH19845/ref|XP_002005363.1|GI20435/ref|XP_002050608.1|GJ20107	XP_001987640.1	4,00E-96	GLOS_DGRI_GH1	GO:0005262	M	Refseq	calcium channel activity
XP_001987640.1GH19845/ref|XP_002005363.1|GI20435/ref|XP_002050608.1|GJ20107	XP_001987640.1	4,00E-96	GLOS_DGRI_GH1	GO:0005515	M	Refseq	protein binding
XP_001987640.1GH19845/ref|XP_002005363.1|GI20435/ref|XP_002050608.1|GJ20107	XP_001987640.1	4,00E-96	GLOS_DGRI_GH1	GO:0005886	C	Refseq	plasma membrane
XP_001987640.1GH19845/ref|XP_002005363.1|GI20435/ref|XP_002050608.1|GJ20107	XP_001987640.1	4,00E-96	GLOS_DGRI_GH1	GO:0005887	C	Refseq	integral to plasma membrane
XP_001987640.1GH19845/ref|XP_002005363.1|GI20435/ref|XP_002050608.1|GJ20107	XP_001987640.1	4,00E-96	GLOS_DGRI_GH1	GO:0006816	B	Refseq	calcium ion transport
XP_001987640.1GH19845/ref|XP_002005363.1|GI20435/ref|XP_002050608.1|GJ20107	XP_001987640.1	4,00E-96	GLOS_DGRI_GH1	GO:0007411	B	Refseq	axon guidance
XP_001987640.1GH19845/ref|XP_002005363.1|GI20435/ref|XP_002050608.1|GJ20107	XP_001987640.1	4,00E-96	GLOS_DGRI_GH1	GO:0007596	B	Refseq	blood coagulation
XP_001987640.1GH19845/ref|XP_002005363.1|GI20435/ref|XP_002050608.1|GJ20107	XP_001987640.1	4,00E-96	GLOS_DGRI_GH1	GO:0007602	B	Refseq	phototransduction
XP_001987640.1GH19845/ref|XP_002005363.1|GI20435/ref|XP_002050608.1|GJ20107	XP_001987640.1	4,00E-96	GLOS_DGRI_GH1	GO:0010524	B	Refseq	positive regulation of calcium ion transport into cytosol
XP_001987640.1GH19845/ref|XP_002005363.1|GI20435/ref|XP_002050608.1|GJ20107	XP_001987640.1	4,00E-96	GLOS_DGRI_GH1	GO:0015279	M	Refseq	store-operated calcium channel activity
XP_001987640.1GH19845/ref|XP_002005363.1|GI20435/ref|XP_002050608.1|GJ20107	XP_001987640.1	4,00E-96	GLOS_DGRI_GH1	GO:0030168	B	Refseq	platelet activation

XP_001987640.1GH19845/ref|XP_002005363.1|GI20435/ref|XP_002050608.1|GJ20107	XP_001987640.1	4,00E-96	GLOS_DGRI_GH1	GO:0033198	B	Refseq	response to ATP
XP_001987640.1GH19845/ref|XP_002005363.1|GI20435/ref|XP_002050608.1|GJ20107	XP_001987640.1	4,00E-96	GLOS_DGRI_GH1	GO:0051592	B	Refseq	response to calcium ion
XP_001987640.1GH19845/ref|XP_002005363.1|GI20435/ref|XP_002050608.1|GJ20107	XP_001987640.1	4,00E-96	GLOS_DGRI_GH1	GO:0070588	B	Refseq	calcium ion transmembrane transport
XP_001987640.1GH19845/ref|XP_002005363.1|GI20435/ref|XP_002050608.1|GJ20107	XP_001987640.1	4,00E-96	GLOS_DGRI_GH1	GO:0070679	M	Refseq	inositol 1,4,5 trisphosphate binding
NP_001245994.1 Pde1c, isoform H [Drosophila melanogaster]	NP_001245994.1	0	GLOS_PDE1C.1.1	GO:0000122	B	Refseq	negative regulation of transcription from RNA polymerase II promoter
NP_001245994.1 Pde1c, isoform H [Drosophila melanogaster]	NP_001245994.1	0	GLOS_PDE1C.1.1	GO:0001501	B	Refseq	skeletal system development
NP_001245994.1 Pde1c, isoform H [Drosophila melanogaster]	NP_001245994.1	0	GLOS_PDE1C.1.1	GO:0003700	M	Refseq	sequence-specific DNA binding transcription factor activity
NP_001245994.1 Pde1c, isoform H [Drosophila melanogaster]	NP_001245994.1	0	GLOS_PDE1C.1.1	GO:0005515	M	Refseq	protein binding
NP_001245994.1 Pde1c, isoform H [Drosophila melanogaster]	NP_001245994.1	0	GLOS_PDE1C.1.1	GO:0005634	C	Refseq	nucleus
NP_001245994.1 Pde1c, isoform H [Drosophila melanogaster]	NP_001245994.1	0	GLOS_PDE1C.1.1	GO:0006366	B	Refseq	transcription from RNA polymerase II promoter
NP_001245994.1 Pde1c, isoform H [Drosophila melanogaster]	NP_001245994.1	0	GLOS_PDE1C.1.1	GO:0006607	B	Refseq	NLS-bearing protein import into nucleus
NP_001245994.1 Pde1c, isoform H [Drosophila melanogaster]	NP_001245994.1	0	GLOS_PDE1C.1.1	GO:0008270	M	Refseq	zinc ion binding
NP_001245994.1 Pde1c, isoform H [Drosophila melanogaster]	NP_001245994.1	0	GLOS_PDE1C.1.1	GO:0032330	B	Refseq	regulation of chondrocyte differentiation
NP_001245994.1 Pde1c, isoform H [Drosophila melanogaster]	NP_001245994.1	0	GLOS_PDE1C.1.1	GO:0043565	M	Refseq	sequence-specific DNA binding
XP_001980898.1 GG13434 [Drosophila erecta]	XP_001980898.1	0	GLOS_DERE_GG	GO:0005261	M	Refseq	cation channel activity
XP_001980898.1 GG13434 [Drosophila erecta]	XP_001980898.1	0	GLOS_DERE_GG	GO:0005262	M	Refseq	calcium channel activity
XP_001980898.1 GG13434 [Drosophila erecta]	XP_001980898.1	0	GLOS_DERE_GG	GO:0005515	M	Refseq	protein binding
XP_001980898.1 GG13434 [Drosophila erecta]	XP_001980898.1	0	GLOS_DERE_GG	GO:0005886	C	Refseq	plasma membrane
XP_001980898.1 GG13434 [Drosophila erecta]	XP_001980898.1	0	GLOS_DERE_GG	GO:0005887	C	Refseq	integral to plasma membrane
XP_001980898.1 GG13434 [Drosophila erecta]	XP_001980898.1	0	GLOS_DERE_GG	GO:0006816	B	Refseq	calcium ion transport
XP_001980898.1 GG13434 [Drosophila erecta]	XP_001980898.1	0	GLOS_DERE_GG	GO:0007411	B	Refseq	axon guidance
XP_001980898.1 GG13434 [Drosophila erecta]	XP_001980898.1	0	GLOS_DERE_GG	GO:0015279	M	Refseq	store-operated calcium channel activity
XP_001980898.1 GG13434 [Drosophila erecta]	XP_001980898.1	0	GLOS_DERE_GG	GO:0016323	C	Refseq	basolateral plasma membrane
XP_001980898.1 GG13434 [Drosophila erecta]	XP_001980898.1	0	GLOS_DERE_GG	GO:0044449	C	.	contractile fiber part
XP_001980898.1 GG13434 [Drosophila erecta]	XP_001980898.1	0	GLOS_DERE_GG	GO:0044422	C	.	organelle part
XP_001980898.1 GG13434 [Drosophila erecta]	XP_001980898.1	0	GLOS_DERE_GG	GO:0044444	C	.	cytoplasmic part
XP_001980898.1 GG13434 [Drosophila erecta]	XP_001980898.1	0	GLOS_DERE_GG	GO:0005737	C	.	cytoplasm
XP_001980898.1 GG13434 [Drosophila erecta]	XP_001980898.1	0	GLOS_DERE_GG	GO:0043292	C	.	contractile fiber
XP_001980898.1 GG13434 [Drosophila erecta]	XP_001980898.1	0	GLOS_DERE_GG	GO:0043232	C	.	intracellular non-membrane-bounded organelle
XP_001980898.1 GG13434 [Drosophila erecta]	XP_001980898.1	0	GLOS_DERE_GG	GO:0043228	C	.	non-membrane-bounded organelle
XP_001980898.1 GG13434 [Drosophila erecta]	XP_001980898.1	0	GLOS_DERE_GG	GO:0030016	C	.	myofibril
XP_001980898.1 GG13434 [Drosophila erecta]	XP_001980898.1	0	GLOS_DERE_GG	GO:0030017	C	Refseq	sarcomere
XP_001980898.1 GG13434 [Drosophila erecta]	XP_001980898.1	0	GLOS_DERE_GG	GO:0043034	C	Refseq	costamere
XP_001980898.1 GG13434 [Drosophila erecta]	XP_001980898.1	0	GLOS_DERE_GG	GO:0032991	C	.	macromolecular complex
XP_001980898.1 GG13434 [Drosophila erecta]	XP_001980898.1	0	GLOS_DERE_GG	GO:0043234	C	Refseq	protein complex
XP_001980898.1 GG13434 [Drosophila erecta]	XP_001980898.1	0	GLOS_DERE_GG	GO:0005515	M	.	protein binding
XP_001980898.1 GG13434 [Drosophila erecta]	XP_001980898.1	0	GLOS_DERE_GG	GO:0044325	M	Refseq	ion channel binding
XP_001980898.1 GG13434 [Drosophila erecta]	XP_001980898.1	0	GLOS_DERE_GG	GO:0045121	C	Refseq	membrane raft
XP_001980898.1 GG13434 [Drosophila erecta]	XP_001980898.1	0	GLOS_DERE_GG	GO:0007589	B	.	body fluid secretion
XP_001980898.1 GG13434 [Drosophila erecta]	XP_001980898.1	0	GLOS_DERE_GG	GO:0046903	B	.	secretion
XP_001980898.1 GG13434 [Drosophila erecta]	XP_001980898.1	0	GLOS_DERE_GG	GO:0022600	B	.	digestive system process
XP_001980898.1 GG13434 [Drosophila erecta]	XP_001980898.1	0	GLOS_DERE_GG	GO:0003008	B	.	system process
XP_001980898.1 GG13434 [Drosophila erecta]	XP_001980898.1	0	GLOS_DERE_GG	GO:0007586	B	.	digestion
XP_001980898.1 GG13434 [Drosophila erecta]	XP_001980898.1	0	GLOS_DERE_GG	GO:0032941	B	.	secretion by tissue
XP_001980898.1 GG13434 [Drosophila erecta]	XP_001980898.1	0	GLOS_DERE_GG	GO:0046541	B	Refseq	saliva secretion
XP_001980898.1 GG13434 [Drosophila erecta]	XP_001980898.1	0	GLOS_DERE_GG	GO:0010524	B	.	positive regulation of calcium ion transport into cytosol
XP_001980898.1 GG13434 [Drosophila erecta]	XP_001980898.1	0	GLOS_DERE_GG	GO:0051279	B	.	regulation of release of sequestered calcium ion into cytosol
XP_001980898.1 GG13434 [Drosophila erecta]	XP_001980898.1	0	GLOS_DERE_GG	GO:0051281	B	Refseq	positive regulation of release of sequestered calcium ion into cytosol
XP_001980898.1 GG13434 [Drosophila erecta]	XP_001980898.1	0	GLOS_DERE_GG	GO:0006874	B	.	cellular calcium ion homeostasis
XP_001980898.1 GG13434 [Drosophila erecta]	XP_001980898.1	0	GLOS_DERE_GG	GO:0006875	B	.	cellular metal ion homeostasis
XP_001980898.1 GG13434 [Drosophila erecta]	XP_001980898.1	0	GLOS_DERE_GG	GO:0030003	B	.	cellular cation homeostasis
XP_001980898.1 GG13434 [Drosophila erecta]	XP_001980898.1	0	GLOS_DERE_GG	GO:0006873	B	.	cellular ion homeostasis
XP_001980898.1 GG13434 [Drosophila erecta]	XP_001980898.1	0	GLOS_DERE_GG	GO:0050801	B	.	ion homeostasis
XP_001980898.1 GG13434 [Drosophila erecta]	XP_001980898.1	0	GLOS_DERE_GG	GO:0048878	B	.	chemical homeostasis

XP_001980898.1 GG13434 [Drosophila erecta]	XP_001980898.1	0	GLOS_DERE_GG	GO:0042592	B	.	homeostatic process
XP_001980898.1 GG13434 [Drosophila erecta]	XP_001980898.1	0	GLOS_DERE_GG	GO:0055082	B	.	cellular chemical homeostasis
XP_001980898.1 GG13434 [Drosophila erecta]	XP_001980898.1	0	GLOS_DERE_GG	GO:0019725	B	.	cellular homeostasis
XP_001980898.1 GG13434 [Drosophila erecta]	XP_001980898.1	0	GLOS_DERE_GG	GO:0055080	B	.	cation homeostasis
XP_001980898.1 GG13434 [Drosophila erecta]	XP_001980898.1	0	GLOS_DERE_GG	GO:0055065	B	.	metal ion homeostasis
XP_001980898.1 GG13434 [Drosophila erecta]	XP_001980898.1	0	GLOS_DERE_GG	GO:0055074	B	.	calcium ion homeostasis
XP_001980898.1 GG13434 [Drosophila erecta]	XP_001980898.1	0	GLOS_DERE_GG	GO:0072507	B	.	divalent inorganic cation homeostasis
XP_001980898.1 GG13434 [Drosophila erecta]	XP_001980898.1	0	GLOS_DERE_GG	GO:0072503	B	.	cellular divalent inorganic cation homeostasis
XP_001980898.1 GG13434 [Drosophila erecta]	XP_001980898.1	0	GLOS_DERE_GG	GO:0051480	B	Refseq	cytosolic calcium ion homeostasis
XP_001980898.1 GG13434 [Drosophila erecta]	XP_001980898.1	0	GLOS_DERE_GG	GO:0051592	B	Refseq	response to calcium ion
XP_001980898.1 GG13434 [Drosophila erecta]	XP_001980898.1	0	GLOS_DERE_GG	GO:0070679	M	Refseq	inositol 1,4,5 trisphosphate binding
NP_001097474.1 CG12004, isoform C [Drosophila melanogaster]	NP_001097474.1	0	GLOS_CG12004.1	GO:0000122	B	Refseq	negative regulation of transcription from RNA polymerase II promoter
NP_001097474.1 CG12004, isoform C [Drosophila melanogaster]	NP_001097474.1	0	GLOS_CG12004.1	GO:0001501	B	Refseq	skeletal system development
NP_001097474.1 CG12004, isoform C [Drosophila melanogaster]	NP_001097474.1	0	GLOS_CG12004.1	GO:0003700	M	Refseq	sequence-specific DNA binding transcription factor activity
NP_001097474.1 CG12004, isoform C [Drosophila melanogaster]	NP_001097474.1	0	GLOS_CG12004.1	GO:0005515	M	Refseq	protein binding
NP_001097474.1 CG12004, isoform C [Drosophila melanogaster]	NP_001097474.1	0	GLOS_CG12004.1	GO:0005634	C	Refseq	nucleus
NP_001097474.1 CG12004, isoform C [Drosophila melanogaster]	NP_001097474.1	0	GLOS_CG12004.1	GO:0006366	B	Refseq	transcription from RNA polymerase II promoter
NP_001097474.1 CG12004, isoform C [Drosophila melanogaster]	NP_001097474.1	0	GLOS_CG12004.1	GO:0006607	B	Refseq	NLS-bearing protein import into nucleus
NP_001097474.1 CG12004, isoform C [Drosophila melanogaster]	NP_001097474.1	0	GLOS_CG12004.1	GO:0008270	M	Refseq	zinc ion binding
NP_001097474.1 CG12004, isoform C [Drosophila melanogaster]	NP_001097474.1	0	GLOS_CG12004.1	GO:0032330	B	Refseq	regulation of chondrocyte differentiation
NP_001097474.1 CG12004, isoform C [Drosophila melanogaster]	NP_001097474.1	0	GLOS_CG12004.1	GO:0043565	M	Refseq	sequence-specific DNA binding
XP_003424941.1 PREDICTED: hypoth. prot. LOC100678979 [N. vitripennis]	XP_003424941.1	6,00E-17	GLOS_LOC10067	GO:0022602	B	.	ovulation cycle process
XP_003424941.1 PREDICTED: hypoth. prot. LOC100678979 [N. vitripennis]	XP_003424941.1	6,00E-17	GLOS_LOC10067	GO:0048511	B	.	rhythmic process
XP_003424941.1 PREDICTED: hypoth. prot. LOC100678979 [N. vitripennis]	XP_003424941.1	6,00E-17	GLOS_LOC10067	GO:0042698	B	.	ovulation cycle
XP_003424941.1 PREDICTED: hypoth. prot. LOC100678979 [N. vitripennis]	XP_003424941.1	6,00E-17	GLOS_LOC10067	GO:0044702	B	.	single organism reproductive process
XP_003424941.1 PREDICTED: hypoth. prot. LOC100678979 [N. vitripennis]	XP_003424941.1	6,00E-17	GLOS_LOC10067	GO:0022414	B	.	reproductive process
XP_003424941.1 PREDICTED: hypoth. prot. LOC100678979 [N. vitripennis]	XP_003424941.1	6,00E-17	GLOS_LOC10067	GO:0000003	B	.	reproduction
XP_003424941.1 PREDICTED: hypoth. prot. LOC100678979 [N. vitripennis]	XP_003424941.1	6,00E-17	GLOS_LOC10067	GO:0048609	B	.	multicellular organismal reproductive process
XP_003424941.1 PREDICTED: hypoth. prot. LOC100678979 [N. vitripennis]	XP_003424941.1	6,00E-17	GLOS_LOC10067	GO:0032504	B	.	multicellular organism reproduction
XP_003424941.1 PREDICTED: hypoth. prot. LOC100678979 [N. vitripennis]	XP_003424941.1	6,00E-17	GLOS_LOC10067	GO:0008585	B	.	female gonad development
XP_003424941.1 PREDICTED: hypoth. prot. LOC100678979 [N. vitripennis]	XP_003424941.1	6,00E-17	GLOS_LOC10067	GO:0008406	B	.	gonad development
XP_003424941.1 PREDICTED: hypoth. prot. LOC100678979 [N. vitripennis]	XP_003424941.1	6,00E-17	GLOS_LOC10067	GO:0048513	B	.	organ development
XP_003424941.1 PREDICTED: hypoth. prot. LOC100678979 [N. vitripennis]	XP_003424941.1	6,00E-17	GLOS_LOC10067	GO:0048608	B	.	reproductive structure development
XP_003424941.1 PREDICTED: hypoth. prot. LOC100678979 [N. vitripennis]	XP_003424941.1	6,00E-17	GLOS_LOC10067	GO:0003006	B	.	developmental process involved in reproduction
XP_003424941.1 PREDICTED: hypoth. prot. LOC100678979 [N. vitripennis]	XP_003424941.1	6,00E-17	GLOS_LOC10067	GO:0061458	B	.	reproductive system development
XP_003424941.1 PREDICTED: hypoth. prot. LOC100678979 [N. vitripennis]	XP_003424941.1	6,00E-17	GLOS_LOC10067	GO:0045137	B	.	development of primary sexual characteristics
XP_003424941.1 PREDICTED: hypoth. prot. LOC100678979 [N. vitripennis]	XP_003424941.1	6,00E-17	GLOS_LOC10067	GO:0007548	B	.	sex differentiation
XP_003424941.1 PREDICTED: hypoth. prot. LOC100678979 [N. vitripennis]	XP_003424941.1	6,00E-17	GLOS_LOC10067	GO:0046545	B	.	development of primary female sexual characteristics
XP_003424941.1 PREDICTED: hypoth. prot. LOC100678979 [N. vitripennis]	XP_003424941.1	6,00E-17	GLOS_LOC10067	GO:0046660	B	.	female sex differentiation
XP_003424941.1 PREDICTED: hypoth. prot. LOC100678979 [N. vitripennis]	XP_003424941.1	6,00E-17	GLOS_LOC10067	GO:0001541	B	Refseq	ovarian follicle development
XP_003424941.1 PREDICTED: hypoth. prot. LOC100678979 [N. vitripennis]	XP_003424941.1	6,00E-17	GLOS_LOC10067	GO:0040014	B	.	regulation of multicellular organism growth
XP_003424941.1 PREDICTED: hypoth. prot. LOC100678979 [N. vitripennis]	XP_003424941.1	6,00E-17	GLOS_LOC10067	GO:0040008	B	.	regulation of growth
XP_003424941.1 PREDICTED: hypoth. prot. LOC100678979 [N. vitripennis]	XP_003424941.1	6,00E-17	GLOS_LOC10067	GO:0002021	B	Refseq	response to dietary excess
XP_003424941.1 PREDICTED: hypoth. prot. LOC100678979 [N. vitripennis]	XP_003424941.1	6,00E-17	GLOS_LOC10067	GO:0003674	M	Refseq	molecular_function
XP_003424941.1 PREDICTED: hypoth. prot. LOC100678979 [N. vitripennis]	XP_003424941.1	6,00E-17	GLOS_LOC10067	GO:0005179	M	.	hormone activity
XP_003424941.1 PREDICTED: hypoth. prot. LOC100678979 [N. vitripennis]	XP_003424941.1	6,00E-17	GLOS_LOC10067	GO:0005102	M	.	receptor binding
XP_003424941.1 PREDICTED: hypoth. prot. LOC100678979 [N. vitripennis]	XP_003424941.1	6,00E-17	GLOS_LOC10067	GO:0005184	M	Refseq	neuropeptide hormone activity
XP_003424941.1 PREDICTED: hypoth. prot. LOC100678979 [N. vitripennis]	XP_003424941.1	6,00E-17	GLOS_LOC10067	GO:0044421	C	.	extracellular region part
XP_003424941.1 PREDICTED: hypoth. prot. LOC100678979 [N. vitripennis]	XP_003424941.1	6,00E-17	GLOS_LOC10067	GO:0005576	C	.	extracellular region
XP_003424941.1 PREDICTED: hypoth. prot. LOC100678979 [N. vitripennis]	XP_003424941.1	6,00E-17	GLOS_LOC10067	GO:0005615	C	Refseq	extracellular space
XP_003424941.1 PREDICTED: hypoth. prot. LOC100678979 [N. vitripennis]	XP_003424941.1	6,00E-17	GLOS_LOC10067	GO:0006091	B	Refseq	generation of precursor metabolites and energy
XP_003424941.1 PREDICTED: hypoth. prot. LOC100678979 [N. vitripennis]	XP_003424941.1	6,00E-17	GLOS_LOC10067	GO:0008083	M	Refseq	growth factor activity
XP_003424941.1 PREDICTED: hypoth. prot. LOC100678979 [N. vitripennis]	XP_003424941.1	6,00E-17	GLOS_LOC10067	GO:0009266	B	.	response to temperature stimulus
XP_003424941.1 PREDICTED: hypoth. prot. LOC100678979 [N. vitripennis]	XP_003424941.1	6,00E-17	GLOS_LOC10067	GO:0009409	B	Refseq	response to cold

XP_003424941.1 PREDICTED: hypoth. prot. LOC100678979 [N. vitripennis]	XP_003424941.1	6,00E-17	GLOS_LOC10067[illegible]	GO:0019953	B	Refseq	sexual reproduction
XP_003424941.1 PREDICTED: hypoth. prot. LOC100678979 [N. vitripennis]	XP_003424941.1	6,00E-17	GLOS_LOC10067[illegible]	GO:0030072	B	.	peptide hormone secretion
XP_003424941.1 PREDICTED: hypoth. prot. LOC100678979 [N. vitripennis]	XP_003424941.1	6,00E-17	GLOS_LOC10067[illegible]	GO:0002790	B	.	peptide secretion
XP_003424941.1 PREDICTED: hypoth. prot. LOC100678979 [N. vitripennis]	XP_003424941.1	6,00E-17	GLOS_LOC10067[illegible]	GO:0015833	B	.	peptide transport
XP_003424941.1 PREDICTED: hypoth. prot. LOC100678979 [N. vitripennis]	XP_003424941.1	6,00E-17	GLOS_LOC10067[illegible]	GO:0042886	B	.	amide transport
XP_003424941.1 PREDICTED: hypoth. prot. LOC100678979 [N. vitripennis]	XP_003424941.1	6,00E-17	GLOS_LOC10067[illegible]	GO:0071705	B	.	nitrogen compound transport
XP_003424941.1 PREDICTED: hypoth. prot. LOC100678979 [N. vitripennis]	XP_003424941.1	6,00E-17	GLOS_LOC10067[illegible]	GO:0046879	B	.	hormone secretion
XP_003424941.1 PREDICTED: hypoth. prot. LOC100678979 [N. vitripennis]	XP_003424941.1	6,00E-17	GLOS_LOC10067[illegible]	GO:0009914	B	.	hormone transport
XP_003424941.1 PREDICTED: hypoth. prot. LOC100678979 [N. vitripennis]	XP_003424941.1	6,00E-17	GLOS_LOC10067[illegible]	GO:0010817	B	.	regulation of hormone levels
XP_003424941.1 PREDICTED: hypoth. prot. LOC100678979 [N. vitripennis]	XP_003424941.1	6,00E-17	GLOS_LOC10067[illegible]	GO:0023061	B	.	signal release
XP_003424941.1 PREDICTED: hypoth. prot. LOC100678979 [N. vitripennis]	XP_003424941.1	6,00E-17	GLOS_LOC10067[illegible]	GO:0032940	B	.	secretion by cell
XP_003424941.1 PREDICTED: hypoth. prot. LOC100678979 [N. vitripennis]	XP_003424941.1	6,00E-17	GLOS_LOC10067[illegible]	GO:0003001	B	.	generation of a signal involved in cell-cell signaling
XP_003424941.1 PREDICTED: hypoth. prot. LOC100678979 [N. vitripennis]	XP_003424941.1	6,00E-17	GLOS_LOC10067[illegible]	GO:0007267	B	.	cell-cell signaling
XP_003424941.1 PREDICTED: hypoth. prot. LOC100678979 [N. vitripennis]	XP_003424941.1	6,00E-17	GLOS_LOC10067[illegible]	GO:0030073	B	Refseq	insulin secretion
XP_003424941.1 PREDICTED: hypoth. prot. LOC100678979 [N. vitripennis]	XP_003424941.1	6,00E-17	GLOS_LOC10067[illegible]	GO:0016023	C	.	cytoplasmic membrane-bounded vesicle
XP_003424941.1 PREDICTED: hypoth. prot. LOC100678979 [N. vitripennis]	XP_003424941.1	6,00E-17	GLOS_LOC10067[illegible]	GO:0031410	C	.	cytoplasmic vesicle
XP_003424941.1 PREDICTED: hypoth. prot. LOC100678979 [N. vitripennis]	XP_003424941.1	6,00E-17	GLOS_LOC10067[illegible]	GO:0031982	C	.	vesicle
XP_003424941.1 PREDICTED: hypoth. prot. LOC100678979 [N. vitripennis]	XP_003424941.1	6,00E-17	GLOS_LOC10067[illegible]	GO:0031988	C	.	membrane-bounded vesicle
XP_003424941.1 PREDICTED: hypoth. prot. LOC100678979 [N. vitripennis]	XP_003424941.1	6,00E-17	GLOS_LOC10067[illegible]	GO:0030133	C	Refseq	transport vesicle
XP_003424941.1 PREDICTED: hypoth. prot. LOC100678979 [N. vitripennis]	XP_003424941.1	6,00E-17	GLOS_LOC10067[illegible]	GO:0031410	C	Refseq	cytoplasmic vesicle
XP_003424941.1 PREDICTED: hypoth. prot. LOC100678979 [N. vitripennis]	XP_003424941.1	6,00E-17	GLOS_LOC10067[illegible]	GO:0043434	B	.	response to peptide hormone stimulus
XP_003424941.1 PREDICTED: hypoth. prot. LOC100678979 [N. vitripennis]	XP_003424941.1	6,00E-17	GLOS_LOC10067[illegible]	GO:0009725	B	.	response to hormone stimulus
XP_003424941.1 PREDICTED: hypoth. prot. LOC100678979 [N. vitripennis]	XP_003424941.1	6,00E-17	GLOS_LOC10067[illegible]	GO:1901652	B	.	response to peptide
XP_003424941.1 PREDICTED: hypoth. prot. LOC100678979 [N. vitripennis]	XP_003424941.1	6,00E-17	GLOS_LOC10067[illegible]	GO:0032868	B	Refseq	response to insulin stimulus
XP_003424941.1 PREDICTED: hypoth. prot. LOC100678979 [N. vitripennis]	XP_003424941.1	6,00E-17	GLOS_LOC10067[illegible]	GO:0033500	B	.	carbohydrate homeostasis
XP_003424941.1 PREDICTED: hypoth. prot. LOC100678979 [N. vitripennis]	XP_003424941.1	6,00E-17	GLOS_LOC10067[illegible]	GO:0042593	B	Refseq	glucose homeostasis
XP_003424941.1 PREDICTED: hypoth. prot. LOC100678979 [N. vitripennis]	XP_003424941.1	6,00E-17	GLOS_LOC10067[illegible]	GO:0006952	B	.	defense response
XP_003424941.1 PREDICTED: hypoth. prot. LOC100678979 [N. vitripennis]	XP_003424941.1	6,00E-17	GLOS_LOC10067[illegible]	GO:0009617	B	.	response to bacterium
XP_003424941.1 PREDICTED: hypoth. prot. LOC100678979 [N. vitripennis]	XP_003424941.1	6,00E-17	GLOS_LOC10067[illegible]	GO:0051707	B	.	response to other organism
XP_003424941.1 PREDICTED: hypoth. prot. LOC100678979 [N. vitripennis]	XP_003424941.1	6,00E-17	GLOS_LOC10067[illegible]	GO:0009607	B	.	response to biotic stimulus
XP_003424941.1 PREDICTED: hypoth. prot. LOC100678979 [N. vitripennis]	XP_003424941.1	6,00E-17	GLOS_LOC10067[illegible]	GO:0051704	B	.	multi-organism process
XP_003424941.1 PREDICTED: hypoth. prot. LOC100678979 [N. vitripennis]	XP_003424941.1	6,00E-17	GLOS_LOC10067[illegible]	GO:0042742	B	Refseq	defense response to bacterium
XP_003424941.1 PREDICTED: hypoth. prot. LOC100678979 [N. vitripennis]	XP_003424941.1	6,00E-17	GLOS_LOC10067[illegible]	GO:0051591	B	Refseq	response to cAMP
XP_001988357.1 GH11121 [Drosophila grimshawi]	XP_001988357.1	3,00E-49	GLOS_DGRI_GH1	GO:0005262	M	Refseq	calcium channel activity
XP_001988357.1 GH11121 [Drosophila grimshawi]	XP_001988357.1	3,00E-49	GLOS_DGRI_GH1	GO:0005515	M	Refseq	protein binding
XP_001988357.1 GH11121 [Drosophila grimshawi]	XP_001988357.1	3,00E-49	GLOS_DGRI_GH1	GO:0005886	C	Refseq	plasma membrane
XP_001988357.1 GH11121 [Drosophila grimshawi]	XP_001988357.1	3,00E-49	GLOS_DGRI_GH1	GO:0005887	C	Refseq	integral to plasma membrane
XP_001988357.1 GH11121 [Drosophila grimshawi]	XP_001988357.1	3,00E-49	GLOS_DGRI_GH1	GO:0006816	B	Refseq	calcium ion transport
XP_001988357.1 GH11121 [Drosophila grimshawi]	XP_001988357.1	3,00E-49	GLOS_DGRI_GH1	GO:0007411	B	Refseq	axon guidance
XP_001988357.1 GH11121 [Drosophila grimshawi]	XP_001988357.1	3,00E-49	GLOS_DGRI_GH1	GO:0007596	B	Refseq	blood coagulation
XP_001988357.1 GH11121 [Drosophila grimshawi]	XP_001988357.1	3,00E-49	GLOS_DGRI_GH1	GO:0007602	B	Refseq	phototransduction
XP_001988357.1 GH11121 [Drosophila grimshawi]	XP_001988357.1	3,00E-49	GLOS_DGRI_GH1	GO:0010524	B	Refseq	positive regulation of calcium ion transport into cytosol
XP_001988357.1 GH11121 [Drosophila grimshawi]	XP_001988357.1	3,00E-49	GLOS_DGRI_GH1	GO:0015279	M	Refseq	store-operated calcium channel activity
XP_001988357.1 GH11121 [Drosophila grimshawi]	XP_001988357.1	3,00E-49	GLOS_DGRI_GH1	GO:0030168	B	Refseq	platelet activation
XP_001988357.1 GH11121 [Drosophila grimshawi]	XP_001988357.1	3,00E-49	GLOS_DGRI_GH1	GO:0033198	B	Refseq	response to ATP
XP_001988357.1 GH11121 [Drosophila grimshawi]	XP_001988357.1	3,00E-49	GLOS_DGRI_GH1	GO:0051592	B	Refseq	response to calcium ion
XP_001988357.1 GH11121 [Drosophila grimshawi]	XP_001988357.1	3,00E-49	GLOS_DGRI_GH1	GO:0070588	B	Refseq	calcium ion transmembrane transport
XP_001988357.1 GH11121 [Drosophila grimshawi]	XP_001988357.1	3,00E-49	GLOS_DGRI_GH1	GO:0070679	M	Refseq	inositol 1,4,5 trisphosphate binding
XP_001980073.1 GG20486 [Drosophila erecta]	XP_001980073.1	3,00E-14	GLOS_DERE_GG	GO:0005261	M	Refseq	cation channel activity
XP_001980073.1 GG20486 [Drosophila erecta]	XP_001980073.1	3,00E-14	GLOS_DERE_GG	GO:0005262	M	Refseq	calcium channel activity
XP_001980073.1 GG20486 [Drosophila erecta]	XP_001980073.1	3,00E-14	GLOS_DERE_GG	GO:0005515	M	Refseq	protein binding
XP_001980073.1 GG20486 [Drosophila erecta]	XP_001980073.1	3,00E-14	GLOS_DERE_GG	GO:0005886	C	Refseq	plasma membrane
XP_001980073.1 GG20486 [Drosophila erecta]	XP_001980073.1	3,00E-14	GLOS_DERE_GG	GO:0005887	C	Refseq	integral to plasma membrane
XP_001980073.1 GG20486 [Drosophila erecta]	XP_001980073.1	3,00E-14	GLOS_DERE_GG	GO:0006816	B	Refseq	calcium ion transport

XP_001980073.1 GG20486 [Drosophila erecta]	XP_001980073.1	3,00E-14	GLOS_DERE_GG	GO:0007411	B	Refseq	axon guidance
XP_001980073.1 GG20486 [Drosophila erecta]	XP_001980073.1	3,00E-14	GLOS_DERE_GG	GO:0015279	M	Refseq	store-operated calcium channel activity
XP_001980073.1 GG20486 [Drosophila erecta]	XP_001980073.1	3,00E-14	GLOS_DERE_GG	GO:0016323	C	Refseq	basolateral plasma membrane
XP_001980073.1 GG20486 [Drosophila erecta]	XP_001980073.1	3,00E-14	GLOS_DERE_GG	GO:0030017	C	Refseq	sarcomere
XP_001980073.1 GG20486 [Drosophila erecta]	XP_001980073.1	3,00E-14	GLOS_DERE_GG	GO:0043034	C	Refseq	costamere
XP_001980073.1 GG20486 [Drosophila erecta]	XP_001980073.1	3,00E-14	GLOS_DERE_GG	GO:0043234	C	Refseq	protein complex
XP_001980073.1 GG20486 [Drosophila erecta]	XP_001980073.1	3,00E-14	GLOS_DERE_GG	GO:0044325	M	Refseq	ion channel binding
XP_001980073.1 GG20486 [Drosophila erecta]	XP_001980073.1	3,00E-14	GLOS_DERE_GG	GO:0045121	C	Refseq	membrane raft
XP_001980073.1 GG20486 [Drosophila erecta]	XP_001980073.1	3,00E-14	GLOS_DERE_GG	GO:0046541	B	Refseq	saliva secretion
XP_001980073.1 GG20486 [Drosophila erecta]	XP_001980073.1	3,00E-14	GLOS_DERE_GG	GO:0051281	B	Refseq	positive regulation of release of sequestered calcium ion into cytosol
XP_001980073.1 GG20486 [Drosophila erecta]	XP_001980073.1	3,00E-14	GLOS_DERE_GG	GO:0051480	B	Refseq	cytosolic calcium ion homeostasis
XP_001980073.1 GG20486 [Drosophila erecta]	XP_001980073.1	3,00E-14	GLOS_DERE_GG	GO:0051592	B	Refseq	response to calcium ion
XP_001980073.1 GG20486 [Drosophila erecta]	XP_001980073.1	3,00E-14	GLOS_DERE_GG	GO:0070679	M	Refseq	inositol 1,4,5 trisphosphate binding
XP_001943543.2 PRED.: collagen alpha-1(XXII) chain-like [A. pisum]	XP_001943543.2	5,00E-55	GLOS_LOC10015	GO:0007049	B	.	cell cycle
XP_001943543.2 PRED.: collagen alpha-1(XXII) chain-like [A. pisum]	XP_001943543.2	5,00E-55	GLOS_LOC10015	GO:0000278	B	Refseq	mitotic cell cycle
XP_001943543.2 PRED.: collagen alpha-1(XXII) chain-like [A. pisum]	XP_001943543.2	5,00E-55	GLOS_LOC10015	GO:0003677	M	Refseq	DNA binding
XP_001943543.2 PRED.: collagen alpha-1(XXII) chain-like [A. pisum]	XP_001943543.2	5,00E-55	GLOS_LOC10015	GO:0003700	M	Refseq	sequence-specific DNA binding transcription factor activity
XP_001943543.2 PRED.: collagen alpha-1(XXII) chain-like [A. pisum]	XP_001943543.2	5,00E-55	GLOS_LOC10015	GO:0000989	M	.	transcription factor binding transcription factor activity
XP_001943543.2 PRED.: collagen alpha-1(XXII) chain-like [A. pisum]	XP_001943543.2	5,00E-55	GLOS_LOC10015	GO:0000988	M	.	protein binding transcription factor activity
XP_001943543.2 PRED.: collagen alpha-1(XXII) chain-like [A. pisum]	XP_001943543.2	5,00E-55	GLOS_LOC10015	GO:0003712	M	Refseq	transcription cofactor activity
XP_001943543.2 PRED.: collagen alpha-1(XXII) chain-like [A. pisum]	XP_001943543.2	5,00E-55	GLOS_LOC10015	GO:0044428	C	.	nuclear part
XP_001943543.2 PRED.: collagen alpha-1(XXII) chain-like [A. pisum]	XP_001943543.2	5,00E-55	GLOS_LOC10015	GO:0044446	C	.	intracellular organelle part
XP_001943543.2 PRED.: collagen alpha-1(XXII) chain-like [A. pisum]	XP_001943543.2	5,00E-55	GLOS_LOC10015	GO:0005634	C	.	nucleus
XP_001943543.2 PRED.: collagen alpha-1(XXII) chain-like [A. pisum]	XP_001943543.2	5,00E-55	GLOS_LOC10015	GO:0031981	C	.	nuclear lumen
XP_001943543.2 PRED.: collagen alpha-1(XXII) chain-like [A. pisum]	XP_001943543.2	5,00E-55	GLOS_LOC10015	GO:0070013	C	.	intracellular organelle lumen
XP_001943543.2 PRED.: collagen alpha-1(XXII) chain-like [A. pisum]	XP_001943543.2	5,00E-55	GLOS_LOC10015	GO:0043233	C	.	organelle lumen
XP_001943543.2 PRED.: collagen alpha-1(XXII) chain-like [A. pisum]	XP_001943543.2	5,00E-55	GLOS_LOC10015	GO:0031974	C	.	membrane-enclosed lumen
XP_001943543.2 PRED.: collagen alpha-1(XXII) chain-like [A. pisum]	XP_001943543.2	5,00E-55	GLOS_LOC10015	GO:0005654	C	Refseq	nucleoplasm
XP_001943543.2 PRED.: collagen alpha-1(XXII) chain-like [A. pisum]	XP_001943543.2	5,00E-55	GLOS_LOC10015	GO:0043234	C	.	protein complex
XP_001943543.2 PRED.: collagen alpha-1(XXII) chain-like [A. pisum]	XP_001943543.2	5,00E-55	GLOS_LOC10015	GO:0005667	C	Refseq	transcription factor complex
XP_001943543.2 PRED.: collagen alpha-1(XXII) chain-like [A. pisum]	XP_001943543.2	5,00E-55	GLOS_LOC10015	GO:0006351	B	Refseq	transcription, DNA-dependent
XP_001943543.2 PRED.: collagen alpha-1(XXII) chain-like [A. pisum]	XP_001943543.2	5,00E-55	GLOS_LOC10015	GO:0006352	B	.	DNA-dependent transcription, initiation
XP_001943543.2 PRED.: collagen alpha-1(XXII) chain-like [A. pisum]	XP_001943543.2	5,00E-55	GLOS_LOC10015	GO:0006366	B	.	transcription from RNA polymerase II promoter
XP_001943543.2 PRED.: collagen alpha-1(XXII) chain-like [A. pisum]	XP_001943543.2	5,00E-55	GLOS_LOC10015	GO:0006367	B	Refseq	transcription initiation from RNA polymerase II promoter
XP_001943543.2 PRED.: collagen alpha-1(XXII) chain-like [A. pisum]	XP_001943543.2	5,00E-55	GLOS_LOC10015	GO:0007178	B	.	transmembrane receptor protein serine/threonine kinase signaling pathway
XP_001943543.2 PRED.: collagen alpha-1(XXII) chain-like [A. pisum]	XP_001943543.2	5,00E-55	GLOS_LOC10015	GO:0007167	B	.	enzyme linked receptor protein signaling pathway
XP_001943543.2 PRED.: collagen alpha-1(XXII) chain-like [A. pisum]	XP_001943543.2	5,00E-55	GLOS_LOC10015	GO:0007166	B	.	cell surface receptor signaling pathway
XP_001943543.2 PRED.: collagen alpha-1(XXII) chain-like [A. pisum]	XP_001943543.2	5,00E-55	GLOS_LOC10015	GO:0071560	B	.	cellular response to transforming growth factor beta stimulus
XP_001943543.2 PRED.: collagen alpha-1(XXII) chain-like [A. pisum]	XP_001943543.2	5,00E-55	GLOS_LOC10015	GO:0071363	B	.	cellular response to growth factor stimulus
XP_001943543.2 PRED.: collagen alpha-1(XXII) chain-like [A. pisum]	XP_001943543.2	5,00E-55	GLOS_LOC10015	GO:0070848	B	.	response to growth factor stimulus
XP_001943543.2 PRED.: collagen alpha-1(XXII) chain-like [A. pisum]	XP_001943543.2	5,00E-55	GLOS_LOC10015	GO:0071310	B	.	cellular response to organic substance
XP_001943543.2 PRED.: collagen alpha-1(XXII) chain-like [A. pisum]	XP_001943543.2	5,00E-55	GLOS_LOC10015	GO:0070887	B	.	cellular response to chemical stimulus
XP_001943543.2 PRED.: collagen alpha-1(XXII) chain-like [A. pisum]	XP_001943543.2	5,00E-55	GLOS_LOC10015	GO:0071495	B	.	cellular response to endogenous stimulus
XP_001943543.2 PRED.: collagen alpha-1(XXII) chain-like [A. pisum]	XP_001943543.2	5,00E-55	GLOS_LOC10015	GO:0071559	B	.	response to transforming growth factor beta stimulus
XP_001943543.2 PRED.: collagen alpha-1(XXII) chain-like [A. pisum]	XP_001943543.2	5,00E-55	GLOS_LOC10015	GO:0007179	B	Refseq	transforming growth factor beta receptor signaling pathway
XP_001943543.2 PRED.: collagen alpha-1(XXII) chain-like [A. pisum]	XP_001943543.2	5,00E-55	GLOS_LOC10015	GO:0072358	B	.	cardiovascular system development
XP_001943543.2 PRED.: collagen alpha-1(XXII) chain-like [A. pisum]	XP_001943543.2	5,00E-55	GLOS_LOC10015	GO:0072359	B	.	circulatory system development
XP_001943543.2 PRED.: collagen alpha-1(XXII) chain-like [A. pisum]	XP_001943543.2	5,00E-55	GLOS_LOC10015	GO:0007507	B	Refseq	heart development
XP_001943543.2 PRED.: collagen alpha-1(XXII) chain-like [A. pisum]	XP_001943543.2	5,00E-55	GLOS_LOC10015	GO:0008134	M	Refseq	transcription factor binding
XP_001943543.2 PRED.: collagen alpha-1(XXII) chain-like [A. pisum]	XP_001943543.2	5,00E-55	GLOS_LOC10015	GO:0010467	B	Refseq	gene expression
XP_001943543.2 PRED.: collagen alpha-1(XXII) chain-like [A. pisum]	XP_001943543.2	5,00E-55	GLOS_LOC10015	GO:0019904	M	Refseq	protein domain specific binding
XP_001943543.2 PRED.: collagen alpha-1(XXII) chain-like [A. pisum]	XP_001943543.2	5,00E-55	GLOS_LOC10015	GO:0045893	B	.	positive regulation of transcription, DNA-dependent
XP_001943543.2 PRED.: collagen alpha-1(XXII) chain-like [A. pisum]	XP_001943543.2	5,00E-55	GLOS_LOC10015	GO:0010557	B	.	positive regulation of macromolecule biosynthetic process
XP_001943543.2 PRED.: collagen alpha-1(XXII) chain-like [A. pisum]	XP_001943543.2	5,00E-55	GLOS_LOC10015	GO:0009891	B	.	positive regulation of biosynthetic process

XP_001943543.2 PRED.: collagen alpha-1(XXII) chain-like [A. pisum]	XP_001943543.2	5,00E-55	GLOS_LOC10015!	GO:0009893	B	.	positive regulation of metabolic process
XP_001943543.2 PRED.: collagen alpha-1(XXII) chain-like [A. pisum]	XP_001943543.2	5,00E-55	GLOS_LOC10015!	GO:0010604	B	.	positive regulation of macromolecule metabolic process
XP_001943543.2 PRED.: collagen alpha-1(XXII) chain-like [A. pisum]	XP_001943543.2	5,00E-55	GLOS_LOC10015!	GO:0010628	B	.	positive regulation of gene expression
XP_001943543.2 PRED.: collagen alpha-1(XXII) chain-like [A. pisum]	XP_001943543.2	5,00E-55	GLOS_LOC10015!	GO:0031328	B	.	positive regulation of cellular biosynthetic process
XP_001943543.2 PRED.: collagen alpha-1(XXII) chain-like [A. pisum]	XP_001943543.2	5,00E-55	GLOS_LOC10015!	GO:0031325	B	.	positive regulation of cellular metabolic process
XP_001943543.2 PRED.: collagen alpha-1(XXII) chain-like [A. pisum]	XP_001943543.2	5,00E-55	GLOS_LOC10015!	GO:0051254	B	.	positive regulation of RNA metabolic process
XP_001943543.2 PRED.: collagen alpha-1(XXII) chain-like [A. pisum]	XP_001943543.2	5,00E-55	GLOS_LOC10015!	GO:0045935	B	.	positive regulation of nucleobase-containing compound metabolic process
XP_001943543.2 PRED.: collagen alpha-1(XXII) chain-like [A. pisum]	XP_001943543.2	5,00E-55	GLOS_LOC10015!	GO:0051173	B	.	positive regulation of nitrogen compound metabolic process
XP_001943543.2 PRED.: collagen alpha-1(XXII) chain-like [A. pisum]	XP_001943543.2	5,00E-55	GLOS_LOC10015!	GO:0045944	B	Refseq	positive regulation of transcription from RNA polymerase II promoter
NM_206469.3 D.m. CG43143 (CG43143), transcript variant D, mRNA	NM_206469.3	1,00E-09	GLOS_CG43143.1	GO:0000122	B	Refseq	negative regulation of transcription from RNA polymerase II promoter
NM_206469.3 D.m. CG43143 (CG43143), transcript variant D, mRNA	NM_206469.3	1,00E-09	GLOS_CG43143.1	GO:0001501	B	Refseq	skeletal system development
NM_206469.3 D.m. CG43143 (CG43143), transcript variant D, mRNA	NM_206469.3	1,00E-09	GLOS_CG43143.1	GO:0003700	M	Refseq	sequence-specific DNA binding transcription factor activity
NM_206469.3 D.m. CG43143 (CG43143), transcript variant D, mRNA	NM_206469.3	1,00E-09	GLOS_CG43143.1	GO:0005515	M	Refseq	protein binding
NM_206469.3 D.m. CG43143 (CG43143), transcript variant D, mRNA	NM_206469.3	1,00E-09	GLOS_CG43143.1	GO:0005634	C	Refseq	nucleus
NM_206469.3 D.m. CG43143 (CG43143), transcript variant D, mRNA	NM_206469.3	1,00E-09	GLOS_CG43143.1	GO:0006366	B	Refseq	transcription from RNA polymerase II promoter
NM_206469.3 D.m. CG43143 (CG43143), transcript variant D, mRNA	NM_206469.3	1,00E-09	GLOS_CG43143.1	GO:0006607	B	Refseq	NLS-bearing protein import into nucleus
NM_206469.3 D.m. CG43143 (CG43143), transcript variant D, mRNA	NM_206469.3	1,00E-09	GLOS_CG43143.1	GO:0008270	M	Refseq	zinc ion binding
NM_206469.3 D.m. CG43143 (CG43143), transcript variant D, mRNA	NM_206469.3	1,00E-09	GLOS_CG43143.1	GO:0032330	B	Refseq	regulation of chondrocyte differentiation
NM_206469.3 D.m. CG43143 (CG43143), transcript variant D, mRNA	NM_206469.3	1,00E-09	GLOS_CG43143.1	GO:0043565	M	Refseq	sequence-specific DNA binding
XP_001991144.1 GH12228 [Drosophila grimshawi]	XP_001991144.1	4,00E-87	GLOS_DGRI_GH1	GO:0005262	M	Refseq	calcium channel activity
XP_001991144.1 GH12228 [Drosophila grimshawi]	XP_001991144.1	4,00E-87	GLOS_DGRI_GH1	GO:0005515	M	Refseq	protein binding
XP_001991144.1 GH12228 [Drosophila grimshawi]	XP_001991144.1	4,00E-87	GLOS_DGRI_GH1	GO:0005886	C	Refseq	plasma membrane
XP_001991144.1 GH12228 [Drosophila grimshawi]	XP_001991144.1	4,00E-87	GLOS_DGRI_GH1	GO:0005887	C	Refseq	integral to plasma membrane
XP_001991144.1 GH12228 [Drosophila grimshawi]	XP_001991144.1	4,00E-87	GLOS_DGRI_GH1	GO:0006816	B	Refseq	calcium ion transport
XP_001991144.1 GH12228 [Drosophila grimshawi]	XP_001991144.1	4,00E-87	GLOS_DGRI_GH1	GO:0007411	B	Refseq	axon guidance
XP_001991144.1 GH12228 [Drosophila grimshawi]	XP_001991144.1	4,00E-87	GLOS_DGRI_GH1	GO:0007596	B	Refseq	blood coagulation
XP_001991144.1 GH12228 [Drosophila grimshawi]	XP_001991144.1	4,00E-87	GLOS_DGRI_GH1	GO:0007602	B	Refseq	phototransduction
XP_001991144.1 GH12228 [Drosophila grimshawi]	XP_001991144.1	4,00E-87	GLOS_DGRI_GH1	GO:0010524	B	Refseq	positive regulation of calcium ion transport into cytosol
XP_001991144.1 GH12228 [Drosophila grimshawi]	XP_001991144.1	4,00E-87	GLOS_DGRI_GH1	GO:0015279	M	Refseq	store-operated calcium channel activity
XP_001991144.1 GH12228 [Drosophila grimshawi]	XP_001991144.1	4,00E-87	GLOS_DGRI_GH1	GO:0030168	B	Refseq	platelet activation
XP_001991144.1 GH12228 [Drosophila grimshawi]	XP_001991144.1	4,00E-87	GLOS_DGRI_GH1	GO:0033198	B	Refseq	response to ATP
XP_001991144.1 GH12228 [Drosophila grimshawi]	XP_001991144.1	4,00E-87	GLOS_DGRI_GH1	GO:0051592	B	Refseq	response to calcium ion
XP_001991144.1 GH12228 [Drosophila grimshawi]	XP_001991144.1	4,00E-87	GLOS_DGRI_GH1	GO:0070588	B	Refseq	calcium ion transmembrane transport
XP_001991144.1 GH12228 [Drosophila grimshawi]	XP_001991144.1	4,00E-87	GLOS_DGRI_GH1	GO:0070679	M	Refseq	inositol 1,4,5 trisphosphate binding
NP_612572.3 metallophosphoesterase,isoform A /ref|XP_002081111.1|GD25866	NP_612572.3	1,00E-161	GLOS_MPPE.1.2	GO:0000122	B	Refseq	negative regulation of transcription from RNA polymerase II promoter
NP_612572.3 metallophosphoesterase,isoform A /ref|XP_002081111.1|GD25866	NP_612572.3	1,00E-161	GLOS_MPPE.1.2	GO:0001501	B	Refseq	skeletal system development
NP_612572.3 metallophosphoesterase,isoform A /ref|XP_002081111.1|GD25866	NP_612572.3	1,00E-161	GLOS_MPPE.1.2	GO:0003700	M	Refseq	sequence-specific DNA binding transcription factor activity
NP_612572.3 metallophosphoesterase,isoform A /ref|XP_002081111.1|GD25866	NP_612572.3	1,00E-161	GLOS_MPPE.1.2	GO:0005515	M	Refseq	protein binding
NP_612572.3 metallophosphoesterase,isoform A /ref|XP_002081111.1|GD25866	NP_612572.3	1,00E-161	GLOS_MPPE.1.2	GO:0005634	C	Refseq	nucleus
NP_612572.3 metallophosphoesterase,isoform A /ref|XP_002081111.1|GD25866	NP_612572.3	1,00E-161	GLOS_MPPE.1.2	GO:0006366	B	Refseq	transcription from RNA polymerase II promoter
NP_612572.3 metallophosphoesterase,isoform A /ref|XP_002081111.1|GD25866	NP_612572.3	1,00E-161	GLOS_MPPE.1.2	GO:0006607	B	Refseq	NLS-bearing protein import into nucleus
NP_612572.3 metallophosphoesterase,isoform A /ref|XP_002081111.1|GD25866	NP_612572.3	1,00E-161	GLOS_MPPE.1.2	GO:0008270	M	Refseq	zinc ion binding
NP_612572.3 metallophosphoesterase,isoform A /ref|XP_002081111.1|GD25866	NP_612572.3	1,00E-161	GLOS_MPPE.1.2	GO:0032330	B	Refseq	regulation of chondrocyte differentiation
NP_612572.3 metallophosphoesterase,isoform A /ref|XP_002081111.1|GD25866	NP_612572.3	1,00E-161	GLOS_MPPE.1.2	GO:0043565	M	Refseq	sequence-specific DNA binding
XP_001979880.1 GG21523 [Drosophila erecta]	XP_001979880.1	2,00E-09	GLOS_DERE_GG	GO:0005261	M	Refseq	cation channel activity
XP_001979880.1 GG21523 [Drosophila erecta]	XP_001979880.1	2,00E-09	GLOS_DERE_GG	GO:0005262	M	Refseq	calcium channel activity
XP_001979880.1 GG21523 [Drosophila erecta]	XP_001979880.1	2,00E-09	GLOS_DERE_GG	GO:0005515	M	Refseq	protein binding
XP_001979880.1 GG21523 [Drosophila erecta]	XP_001979880.1	2,00E-09	GLOS_DERE_GG	GO:0005886	C	Refseq	plasma membrane
XP_001979880.1 GG21523 [Drosophila erecta]	XP_001979880.1	2,00E-09	GLOS_DERE_GG	GO:0005887	C	Refseq	integral to plasma membrane
XP_001979880.1 GG21523 [Drosophila erecta]	XP_001979880.1	2,00E-09	GLOS_DERE_GG	GO:0006816	B	Refseq	calcium ion transport
XP_001979880.1 GG21523 [Drosophila erecta]	XP_001979880.1	2,00E-09	GLOS_DERE_GG	GO:0007411	B	Refseq	axon guidance
XP_001979880.1 GG21523 [Drosophila erecta]	XP_001979880.1	2,00E-09	GLOS_DERE_GG	GO:0015279	M	Refseq	store-operated calcium channel activity
XP_001979880.1 GG21523 [Drosophila erecta]	XP_001979880.1	2,00E-09	GLOS_DERE_GG	GO:0016323	C	Refseq	basolateral plasma membrane
XP_001979880.1 GG21523 [Drosophila erecta]	XP_001979880.1	2,00E-09	GLOS_DERE_GG	GO:0030017	C	Refseq	sarcomere

XP_001979880.1 GG21523 [Drosophila erecta]	XP_001979880.1	2,00E-09	GLOS_DERE_GG	GO:0043034	C	Refseq	costamere
XP_001979880.1 GG21523 [Drosophila erecta]	XP_001979880.1	2,00E-09	GLOS_DERE_GG	GO:0043234	C	Refseq	protein complex
XP_001979880.1 GG21523 [Drosophila erecta]	XP_001979880.1	2,00E-09	GLOS_DERE_GG	GO:0044325	M	Refseq	ion channel binding
XP_001979880.1 GG21523 [Drosophila erecta]	XP_001979880.1	2,00E-09	GLOS_DERE_GG	GO:0045121	C	Refseq	membrane raft
XP_001979880.1 GG21523 [Drosophila erecta]	XP_001979880.1	2,00E-09	GLOS_DERE_GG	GO:0046541	B	Refseq	saliva secretion
XP_001979880.1 GG21523 [Drosophila erecta]	XP_001979880.1	2,00E-09	GLOS_DERE_GG	GO:0051281	B	Refseq	positive regulation of release of sequestered calcium ion into cytosol
XP_001979880.1 GG21523 [Drosophila erecta]	XP_001979880.1	2,00E-09	GLOS_DERE_GG	GO:0051480	B	Refseq	cytosolic calcium ion homeostasis
XP_001979880.1 GG21523 [Drosophila erecta]	XP_001979880.1	2,00E-09	GLOS_DERE_GG	GO:0051592	B	Refseq	response to calcium ion
XP_001979880.1 GG21523 [Drosophila erecta]	XP_001979880.1	2,00E-09	GLOS_DERE_GG	GO:0070679	M	Refseq	inositol 1,4,5 trisphosphate binding
NP_001188973.1 CG15097, isoform C [Drosophila melanogaster]	NP_001188973.1	1,00E-106	GLOS_CG15097.3	GO:0000122	B	Refseq	negative regulation of transcription from RNA polymerase II promoter
NP_001188973.1 CG15097, isoform C [Drosophila melanogaster]	NP_001188973.1	1,00E-106	GLOS_CG15097.3	GO:0001501	B	Refseq	skeletal system development
NP_001188973.1 CG15097, isoform C [Drosophila melanogaster]	NP_001188973.1	1,00E-106	GLOS_CG15097.3	GO:0003700	M	Refseq	sequence-specific DNA binding transcription factor activity
NP_001188973.1 CG15097, isoform C [Drosophila melanogaster]	NP_001188973.1	1,00E-106	GLOS_CG15097.3	GO:0005515	M	Refseq	protein binding
NP_001188973.1 CG15097, isoform C [Drosophila melanogaster]	NP_001188973.1	1,00E-106	GLOS_CG15097.3	GO:0005634	C	Refseq	nucleus
NP_001188973.1 CG15097, isoform C [Drosophila melanogaster]	NP_001188973.1	1,00E-106	GLOS_CG15097.3	GO:0006366	B	Refseq	transcription from RNA polymerase II promoter
NP_001188973.1 CG15097, isoform C [Drosophila melanogaster]	NP_001188973.1	1,00E-106	GLOS_CG15097.3	GO:0006607	B	Refseq	NLS-bearing protein import into nucleus
NP_001188973.1 CG15097, isoform C [Drosophila melanogaster]	NP_001188973.1	1,00E-106	GLOS_CG15097.3	GO:0008270	M	Refseq	zinc ion binding
NP_001188973.1 CG15097, isoform C [Drosophila melanogaster]	NP_001188973.1	1,00E-106	GLOS_CG15097.3	GO:0032330	B	Refseq	regulation of chondrocyte differentiation
NP_001188973.1 CG15097, isoform C [Drosophila melanogaster]	NP_001188973.1	1,00E-106	GLOS_CG15097.3	GO:0043565	M	Refseq	sequence-specific DNA binding
XP_001973574.1 GG16158 [Drosophila erecta]	XP_001973574.1	0	GLOS_DERE_GG	GO:0005261	M	Refseq	cation channel activity
XP_001973574.1 GG16158 [Drosophila erecta]	XP_001973574.1	0	GLOS_DERE_GG	GO:0005262	M	Refseq	calcium channel activity
XP_001973574.1 GG16158 [Drosophila erecta]	XP_001973574.1	0	GLOS_DERE_GG	GO:0005515	M	Refseq	protein binding
XP_001973574.1 GG16158 [Drosophila erecta]	XP_001973574.1	0	GLOS_DERE_GG	GO:0005886	C	Refseq	plasma membrane
XP_001973574.1 GG16158 [Drosophila erecta]	XP_001973574.1	0	GLOS_DERE_GG	GO:0005887	C	Refseq	integral to plasma membrane
XP_001973574.1 GG16158 [Drosophila erecta]	XP_001973574.1	0	GLOS_DERE_GG	GO:0006816	B	Refseq	calcium ion transport
XP_001973574.1 GG16158 [Drosophila erecta]	XP_001973574.1	0	GLOS_DERE_GG	GO:0007411	B	Refseq	axon guidance
XP_001973574.1 GG16158 [Drosophila erecta]	XP_001973574.1	0	GLOS_DERE_GG	GO:0015279	M	Refseq	store-operated calcium channel activity
XP_001973574.1 GG16158 [Drosophila erecta]	XP_001973574.1	0	GLOS_DERE_GG	GO:0016323	C	Refseq	basolateral plasma membrane
XP_001973574.1 GG16158 [Drosophila erecta]	XP_001973574.1	0	GLOS_DERE_GG	GO:0030017	C	Refseq	sarcomere
XP_001973574.1 GG16158 [Drosophila erecta]	XP_001973574.1	0	GLOS_DERE_GG	GO:0043034	C	Refseq	costamere
XP_001973574.1 GG16158 [Drosophila erecta]	XP_001973574.1	0	GLOS_DERE_GG	GO:0043234	C	Refseq	protein complex
XP_001973574.1 GG16158 [Drosophila erecta]	XP_001973574.1	0	GLOS_DERE_GG	GO:0044325	M	Refseq	ion channel binding
XP_001973574.1 GG16158 [Drosophila erecta]	XP_001973574.1	0	GLOS_DERE_GG	GO:0045121	C	Refseq	membrane raft
XP_001973574.1 GG16158 [Drosophila erecta]	XP_001973574.1	0	GLOS_DERE_GG	GO:0046541	B	Refseq	saliva secretion
XP_001973574.1 GG16158 [Drosophila erecta]	XP_001973574.1	0	GLOS_DERE_GG	GO:0051281	B	Refseq	positive regulation of release of sequestered calcium ion into cytosol
XP_001973574.1 GG16158 [Drosophila erecta]	XP_001973574.1	0	GLOS_DERE_GG	GO:0051480	B	Refseq	cytosolic calcium ion homeostasis
XP_001973574.1 GG16158 [Drosophila erecta]	XP_001973574.1	0	GLOS_DERE_GG	GO:0051592	B	Refseq	response to calcium ion
XP_001973574.1 GG16158 [Drosophila erecta]	XP_001973574.1	0	GLOS_DERE_GG	GO:0070679	M	Refseq	inositol 1,4,5 trisphosphate binding
NP_524327.1 sarcoglycan beta [Drosophila melanogaster]	NP_524327.1	2,00E-93	GLOS_SCGBETA.	GO:0000122	B	Refseq	negative regulation of transcription from RNA polymerase II promoter
NP_524327.1 sarcoglycan beta [Drosophila melanogaster]	NP_524327.1	2,00E-93	GLOS_SCGBETA.	GO:0001501	B	Refseq	skeletal system development
NP_524327.1 sarcoglycan beta [Drosophila melanogaster]	NP_524327.1	2,00E-93	GLOS_SCGBETA.	GO:0003700	M	Refseq	sequence-specific DNA binding transcription factor activity
NP_524327.1 sarcoglycan beta [Drosophila melanogaster]	NP_524327.1	2,00E-93	GLOS_SCGBETA.	GO:0005515	M	Refseq	protein binding
NP_524327.1 sarcoglycan beta [Drosophila melanogaster]	NP_524327.1	2,00E-93	GLOS_SCGBETA.	GO:0005634	C	Refseq	nucleus
NP_524327.1 sarcoglycan beta [Drosophila melanogaster]	NP_524327.1	2,00E-93	GLOS_SCGBETA.	GO:0006366	B	Refseq	transcription from RNA polymerase II promoter
NP_524327.1 sarcoglycan beta [Drosophila melanogaster]	NP_524327.1	2,00E-93	GLOS_SCGBETA.	GO:0006607	B	Refseq	NLS-bearing protein import into nucleus
NP_524327.1 sarcoglycan beta [Drosophila melanogaster]	NP_524327.1	2,00E-93	GLOS_SCGBETA.	GO:0008270	M	Refseq	zinc ion binding
NP_524327.1 sarcoglycan beta [Drosophila melanogaster]	NP_524327.1	2,00E-93	GLOS_SCGBETA.	GO:0032330	B	Refseq	regulation of chondrocyte differentiation
NP_524327.1 sarcoglycan beta [Drosophila melanogaster]	NP_524327.1	2,00E-93	GLOS_SCGBETA.	GO:0043565	M	Refseq	sequence-specific DNA binding
NP_650802.1 CG3734 [Drosophila melanogaster]	NP_650802.1	8,00E-44	GLOS_CG3734.1.:	GO:0000122	B	Refseq	negative regulation of transcription from RNA polymerase II promoter
NP_650802.1 CG3734 [Drosophila melanogaster]	NP_650802.1	8,00E-44	GLOS_CG3734.1.:	GO:0001501	B	Refseq	skeletal system development
NP_650802.1 CG3734 [Drosophila melanogaster]	NP_650802.1	8,00E-44	GLOS_CG3734.1.:	GO:0003700	M	Refseq	sequence-specific DNA binding transcription factor activity
NP_650802.1 CG3734 [Drosophila melanogaster]	NP_650802.1	8,00E-44	GLOS_CG3734.1.:	GO:0005515	M	Refseq	protein binding
NP_650802.1 CG3734 [Drosophila melanogaster]	NP_650802.1	8,00E-44	GLOS_CG3734.1.:	GO:0005634	C	Refseq	nucleus
NP_650802.1 CG3734 [Drosophila melanogaster]	NP_650802.1	8,00E-44	GLOS_CG3734.1.:	GO:0006366	B	Refseq	transcription from RNA polymerase II promoter

NP_650802.1 CG3734 [Drosophila melanogaster]	NP_650802.1	8,00E-44	GLOS_CG3734.1.:	GO:0006607	B	Refseq	NLS-bearing protein import into nucleus
NP_650802.1 CG3734 [Drosophila melanogaster]	NP_650802.1	8,00E-44	GLOS_CG3734.1.:	GO:0008270	M	Refseq	zinc ion binding
NP_650802.1 CG3734 [Drosophila melanogaster]	NP_650802.1	8,00E-44	GLOS_CG3734.1.:	GO:0032330	B	Refseq	regulation of chondrocyte differentiation
NP_650802.1 CG3734 [Drosophila melanogaster]	NP_650802.1	8,00E-44	GLOS_CG3734.1.:	GO:0043565	M	Refseq	sequence-specific DNA binding
XP_004929373.1 PREDICTED: uncharact. prot. LOC101742112 [B. mori]	XP_004929373.1	8,00E-26	GLOS_LOC10174:	GO:0000122	B	Refseq	negative regulation of transcription from RNA polymerase II promoter
XP_004929373.1 PREDICTED: uncharact. prot. LOC101742112 [B. mori]	XP_004929373.1	8,00E-26	GLOS_LOC10174:	GO:0003674	M	Refseq	molecular_function
XP_004929373.1 PREDICTED: uncharact. prot. LOC101742112 [B. mori]	XP_004929373.1	8,00E-26	GLOS_LOC10174:	GO:0003682	M	Refseq	chromatin binding
XP_004929373.1 PREDICTED: uncharact. prot. LOC101742112 [B. mori]	XP_004929373.1	8,00E-26	GLOS_LOC10174:	GO:0000981	M	.	sequence-specific DNA binding RNA polymerase II transcription factor activity
XP_004929373.1 PREDICTED: uncharact. prot. LOC101742112 [B. mori]	XP_004929373.1	8,00E-26	GLOS_LOC10174:	GO:0003700	M	.	sequence-specific DNA binding transcription factor activity
XP_004929373.1 PREDICTED: uncharact. prot. LOC101742112 [B. mori]	XP_004929373.1	8,00E-26	GLOS_LOC10174:	GO:0003705	M	Refseq	RNA polymerase II distal enhancer sequence-specific DNA binding transcription factor activity
XP_004929373.1 PREDICTED: uncharact. prot. LOC101742112 [B. mori]	XP_004929373.1	8,00E-26	GLOS_LOC10174:	GO:0003712	M	.	transcription cofactor activity
XP_004929373.1 PREDICTED: uncharact. prot. LOC101742112 [B. mori]	XP_004929373.1	8,00E-26	GLOS_LOC10174:	GO:0003714	M	Refseq	transcription corepressor activity
XP_004929373.1 PREDICTED: uncharact. prot. LOC101742112 [B. mori]	XP_004929373.1	8,00E-26	GLOS_LOC10174:	GO:0005634	C	Refseq	nucleus
XP_004929373.1 PREDICTED: uncharact. prot. LOC101742112 [B. mori]	XP_004929373.1	8,00E-26	GLOS_LOC10174:	GO:0005654	C	Refseq	nucleoplasm
XP_004929373.1 PREDICTED: uncharact. prot. LOC101742112 [B. mori]	XP_004929373.1	8,00E-26	GLOS_LOC10174:	GO:0007219	B	Refseq	Notch signaling pathway
XP_004929373.1 PREDICTED: uncharact. prot. LOC101742112 [B. mori]	XP_004929373.1	8,00E-26	GLOS_LOC10174:	GO:0008150	B	Refseq	biological_process
XP_004929373.1 PREDICTED: uncharact. prot. LOC101742112 [B. mori]	XP_004929373.1	8,00E-26	GLOS_LOC10174:	GO:0016055	B	Refseq	Wnt receptor signaling pathway
XP_004929373.1 PREDICTED: uncharact. prot. LOC101742112 [B. mori]	XP_004929373.1	8,00E-26	GLOS_LOC10174:	GO:0008134	M	.	transcription factor binding
XP_004929373.1 PREDICTED: uncharact. prot. LOC101742112 [B. mori]	XP_004929373.1	8,00E-26	GLOS_LOC10174:	GO:0070491	M	Refseq	repressing transcription factor binding
NP_477338.3 deformed wings [Drosophila melanogaster]	NP_477338.3	2,00E-77	GLOS_DWG.1.1	GO:0000122	B	Refseq	negative regulation of transcription from RNA polymerase II promoter
NP_477338.3 deformed wings [Drosophila melanogaster]	NP_477338.3	2,00E-77	GLOS_DWG.1.1	GO:0001501	B	Refseq	skeletal system development
NP_477338.3 deformed wings [Drosophila melanogaster]	NP_477338.3	2,00E-77	GLOS_DWG.1.1	GO:0003700	M	Refseq	sequence-specific DNA binding transcription factor activity
NP_477338.3 deformed wings [Drosophila melanogaster]	NP_477338.3	2,00E-77	GLOS_DWG.1.1	GO:0005515	M	Refseq	protein binding
NP_477338.3 deformed wings [Drosophila melanogaster]	NP_477338.3	2,00E-77	GLOS_DWG.1.1	GO:0005634	C	Refseq	nucleus
NP_477338.3 deformed wings [Drosophila melanogaster]	NP_477338.3	2,00E-77	GLOS_DWG.1.1	GO:0006366	B	Refseq	transcription from RNA polymerase II promoter
NP_477338.3 deformed wings [Drosophila melanogaster]	NP_477338.3	2,00E-77	GLOS_DWG.1.1	GO:0006607	B	Refseq	NLS-bearing protein import into nucleus
NP_477338.3 deformed wings [Drosophila melanogaster]	NP_477338.3	2,00E-77	GLOS_DWG.1.1	GO:0008270	M	Refseq	zinc ion binding
NP_477338.3 deformed wings [Drosophila melanogaster]	NP_477338.3	2,00E-77	GLOS_DWG.1.1	GO:0032330	B	Refseq	regulation of chondrocyte differentiation
NP_477338.3 deformed wings [Drosophila melanogaster]	NP_477338.3	2,00E-77	GLOS_DWG.1.1	GO:0043565	M	Refseq	sequence-specific DNA binding
XP_003977328.1 PRED.: histone H3.2-like, partial [T. rubripes]	XP_003977328.1	1,00E-88	GLOS_LOC10106(	GO:0004175	M	.	endopeptidase activity
XP_003977328.1 PRED.: histone H3.2-like, partial [T. rubripes]	XP_003977328.1	1,00E-88	GLOS_LOC10106(	GO:0070011	M	.	peptidase activity, acting on L-amino acid peptides
XP_003977328.1 PRED.: histone H3.2-like, partial [T. rubripes]	XP_003977328.1	1,00E-88	GLOS_LOC10106(	GO:0008233	M	.	peptidase activity
XP_003977328.1 PRED.: histone H3.2-like, partial [T. rubripes]	XP_003977328.1	1,00E-88	GLOS_LOC10106(	GO:0016787	M	.	hydrolase activity
XP_003977328.1 PRED.: histone H3.2-like, partial [T. rubripes]	XP_003977328.1	1,00E-88	GLOS_LOC10106(	GO:0003824	M	.	catalytic activity
XP_003977328.1 PRED.: histone H3.2-like, partial [T. rubripes]	XP_003977328.1	1,00E-88	GLOS_LOC10106(	GO:0008236	M	.	serine-type peptidase activity
XP_003977328.1 PRED.: histone H3.2-like, partial [T. rubripes]	XP_003977328.1	1,00E-88	GLOS_LOC10106(	GO:0017171	M	.	serine hydrolase activity
XP_003977328.1 PRED.: histone H3.2-like, partial [T. rubripes]	XP_003977328.1	1,00E-88	GLOS_LOC10106(	GO:0004252	M	Refseq	serine-type endopeptidase activity
XP_003977328.1 PRED.: histone H3.2-like, partial [T. rubripes]	XP_003977328.1	1,00E-88	GLOS_LOC10106(	GO:0005615	C	Refseq	extracellular space
XP_003977328.1 PRED.: histone H3.2-like, partial [T. rubripes]	XP_003977328.1	1,00E-88	GLOS_LOC10106(	GO:0019538	B	.	protein metabolic process
XP_003977328.1 PRED.: histone H3.2-like, partial [T. rubripes]	XP_003977328.1	1,00E-88	GLOS_LOC10106(	GO:0006508	B	Refseq	proteolysis
XP_003977328.1 PRED.: histone H3.2-like, partial [T. rubripes]	XP_003977328.1	1,00E-88	GLOS_LOC10106(	GO:0032504	B	Refseq	multicellular organism reproduction
NP_524742.1 mitoch. Ribos.lprot. L12 [D.m.] ref|XP_002084197.1|GD14139 [D.s.]	NP_524742.1	3,00E-30	GLOS_MRPL12.1.	GO:0000122	B	Refseq	negative regulation of transcription from RNA polymerase II promoter
NP_524742.1 mitoch. Ribos.lprot. L12 [D.m.] ref|XP_002084197.1|GD14139 [D.s.]	NP_524742.1	3,00E-30	GLOS_MRPL12.1.	GO:0001501	B	Refseq	skeletal system development
NP_524742.1 mitoch. Ribos.lprot. L12 [D.m.] ref|XP_002084197.1|GD14139 [D.s.]	NP_524742.1	3,00E-30	GLOS_MRPL12.1.	GO:0003700	M	Refseq	sequence-specific DNA binding transcription factor activity
NP_524742.1 mitoch. Ribos.lprot. L12 [D.m.] ref|XP_002084197.1|GD14139 [D.s.]	NP_524742.1	3,00E-30	GLOS_MRPL12.1.	GO:0005515	M	Refseq	protein binding
NP_524742.1 mitoch. Ribos.lprot. L12 [D.m.] ref|XP_002084197.1|GD14139 [D.s.]	NP_524742.1	3,00E-30	GLOS_MRPL12.1.	GO:0005634	C	Refseq	nucleus
NP_524742.1 mitoch. Ribos.lprot. L12 [D.m.] ref|XP_002084197.1|GD14139 [D.s.]	NP_524742.1	3,00E-30	GLOS_MRPL12.1.	GO:0006366	B	Refseq	transcription from RNA polymerase II promoter
NP_524742.1 mitoch. Ribos.lprot. L12 [D.m.] ref|XP_002084197.1|GD14139 [D.s.]	NP_524742.1	3,00E-30	GLOS_MRPL12.1.	GO:0006607	B	Refseq	NLS-bearing protein import into nucleus
NP_524742.1 mitoch. Ribos.lprot. L12 [D.m.] ref|XP_002084197.1|GD14139 [D.s.]	NP_524742.1	3,00E-30	GLOS_MRPL12.1.	GO:0008270	M	Refseq	zinc ion binding
NP_524742.1 mitoch. Ribos.lprot. L12 [D.m.] ref|XP_002084197.1|GD14139 [D.s.]	NP_524742.1	3,00E-30	GLOS_MRPL12.1.	GO:0032330	B	Refseq	regulation of chondrocyte differentiation
NP_524742.1 mitoch. Ribos.lprot. L12 [D.m.] ref|XP_002084197.1|GD14139 [D.s.]	NP_524742.1	3,00E-30	GLOS_MRPL12.1.	GO:0043565	M	Refseq	sequence-specific DNA binding
XP_001990083.1 GH19143 [Drosophila grimshawi]	XP_001990083.1	1,00E-173	GLOS_DGRI_GH1	GO:0005262	M	Refseq	calcium channel activity
XP_001990083.1 GH19143 [Drosophila grimshawi]	XP_001990083.1	1,00E-173	GLOS_DGRI_GH1	GO:0005515	M	Refseq	protein binding

XP_001990083.1 GH19143 [Drosophila grimshawi]	XP_001990083.1	1,00E-173	GLOS_DGRI_GH1	GO:0005886	C	Refseq	plasma membrane
XP_001990083.1 GH19143 [Drosophila grimshawi]	XP_001990083.1	1,00E-173	GLOS_DGRI_GH1	GO:0005887	C	Refseq	integral to plasma membrane
XP_001990083.1 GH19143 [Drosophila grimshawi]	XP_001990083.1	1,00E-173	GLOS_DGRI_GH1	GO:0006816	B	Refseq	calcium ion transport
XP_001990083.1 GH19143 [Drosophila grimshawi]	XP_001990083.1	1,00E-173	GLOS_DGRI_GH1	GO:0007411	B	Refseq	axon guidance
XP_001990083.1 GH19143 [Drosophila grimshawi]	XP_001990083.1	1,00E-173	GLOS_DGRI_GH1	GO:0007596	B	Refseq	blood coagulation
XP_001990083.1 GH19143 [Drosophila grimshawi]	XP_001990083.1	1,00E-173	GLOS_DGRI_GH1	GO:0007602	B	Refseq	phototransduction
XP_001990083.1 GH19143 [Drosophila grimshawi]	XP_001990083.1	1,00E-173	GLOS_DGRI_GH1	GO:0010524	B	Refseq	positive regulation of calcium ion transport into cytosol
XP_001990083.1 GH19143 [Drosophila grimshawi]	XP_001990083.1	1,00E-173	GLOS_DGRI_GH1	GO:0015279	M	Refseq	store-operated calcium channel activity
XP_001990083.1 GH19143 [Drosophila grimshawi]	XP_001990083.1	1,00E-173	GLOS_DGRI_GH1	GO:0030168	B	Refseq	platelet activation
XP_001990083.1 GH19143 [Drosophila grimshawi]	XP_001990083.1	1,00E-173	GLOS_DGRI_GH1	GO:0033198	B	Refseq	response to ATP
XP_001990083.1 GH19143 [Drosophila grimshawi]	XP_001990083.1	1,00E-173	GLOS_DGRI_GH1	GO:0051592	B	Refseq	response to calcium ion
XP_001990083.1 GH19143 [Drosophila grimshawi]	XP_001990083.1	1,00E-173	GLOS_DGRI_GH1	GO:0070588	B	Refseq	calcium ion transmembrane transport
XP_001990083.1 GH19143 [Drosophila grimshawi]	XP_001990083.1	1,00E-173	GLOS_DGRI_GH1	GO:0070679	M	Refseq	inositol 1,4,5 trisphosphate binding
XP_001988170.1 GH10713 [Drosophila grimshawi]	XP_001988170.1	2,00E-97	GLOS_DGRI_GH1	GO:0005262	M	Refseq	calcium channel activity
XP_001988170.1 GH10713 [Drosophila grimshawi]	XP_001988170.1	2,00E-97	GLOS_DGRI_GH1	GO:0005515	M	Refseq	protein binding
XP_001988170.1 GH10713 [Drosophila grimshawi]	XP_001988170.1	2,00E-97	GLOS_DGRI_GH1	GO:0005886	C	Refseq	plasma membrane
XP_001988170.1 GH10713 [Drosophila grimshawi]	XP_001988170.1	2,00E-97	GLOS_DGRI_GH1	GO:0005887	C	Refseq	integral to plasma membrane
XP_001988170.1 GH10713 [Drosophila grimshawi]	XP_001988170.1	2,00E-97	GLOS_DGRI_GH1	GO:0006816	B	Refseq	calcium ion transport
XP_001988170.1 GH10713 [Drosophila grimshawi]	XP_001988170.1	2,00E-97	GLOS_DGRI_GH1	GO:0007411	B	Refseq	axon guidance
XP_001988170.1 GH10713 [Drosophila grimshawi]	XP_001988170.1	2,00E-97	GLOS_DGRI_GH1	GO:0007596	B	Refseq	blood coagulation
XP_001988170.1 GH10713 [Drosophila grimshawi]	XP_001988170.1	2,00E-97	GLOS_DGRI_GH1	GO:0007602	B	Refseq	phototransduction
XP_001988170.1 GH10713 [Drosophila grimshawi]	XP_001988170.1	2,00E-97	GLOS_DGRI_GH1	GO:0010524	B	Refseq	positive regulation of calcium ion transport into cytosol
XP_001988170.1 GH10713 [Drosophila grimshawi]	XP_001988170.1	2,00E-97	GLOS_DGRI_GH1	GO:0015279	M	Refseq	store-operated calcium channel activity
XP_001988170.1 GH10713 [Drosophila grimshawi]	XP_001988170.1	2,00E-97	GLOS_DGRI_GH1	GO:0030168	B	Refseq	platelet activation
XP_001988170.1 GH10713 [Drosophila grimshawi]	XP_001988170.1	2,00E-97	GLOS_DGRI_GH1	GO:0033198	B	Refseq	response to ATP
XP_001988170.1 GH10713 [Drosophila grimshawi]	XP_001988170.1	2,00E-97	GLOS_DGRI_GH1	GO:0051592	B	Refseq	response to calcium ion
XP_001988170.1 GH10713 [Drosophila grimshawi]	XP_001988170.1	2,00E-97	GLOS_DGRI_GH1	GO:0070588	B	Refseq	calcium ion transmembrane transport
XP_001988170.1 GH10713 [Drosophila grimshawi]	XP_001988170.1	2,00E-97	GLOS_DGRI_GH1	GO:0070679	M	Refseq	inositol 1,4,5 trisphosphate binding
XP_003241296.1 PRED.: putative nuclease HARBI1-like [A. pisum]	XP_003241296.1	6,00E-06	GLOS_LOC10056!	GO:0000278	B	Refseq	mitotic cell cycle
XP_003241296.1 PRED.: putative nuclease HARBI1-like [A. pisum]	XP_003241296.1	6,00E-06	GLOS_LOC10056!	GO:0003677	M	Refseq	DNA binding
XP_003241296.1 PRED.: putative nuclease HARBI1-like [A. pisum]	XP_003241296.1	6,00E-06	GLOS_LOC10056!	GO:0003700	M	Refseq	sequence-specific DNA binding transcription factor activity
XP_003241296.1 PRED.: putative nuclease HARBI1-like [A. pisum]	XP_003241296.1	6,00E-06	GLOS_LOC10056!	GO:0003712	M	Refseq	transcription cofactor activity
XP_003241296.1 PRED.: putative nuclease HARBI1-like [A. pisum]	XP_003241296.1	6,00E-06	GLOS_LOC10056!	GO:0005654	C	Refseq	nucleoplasm
XP_003241296.1 PRED.: putative nuclease HARBI1-like [A. pisum]	XP_003241296.1	6,00E-06	GLOS_LOC10056!	GO:0005667	C	Refseq	transcription factor complex
XP_003241296.1 PRED.: putative nuclease HARBI1-like [A. pisum]	XP_003241296.1	6,00E-06	GLOS_LOC10056!	GO:0006351	B	Refseq	transcription, DNA-dependent
XP_003241296.1 PRED.: putative nuclease HARBI1-like [A. pisum]	XP_003241296.1	6,00E-06	GLOS_LOC10056!	GO:0006367	B	Refseq	transcription initiation from RNA polymerase II promoter
XP_003241296.1 PRED.: putative nuclease HARBI1-like [A. pisum]	XP_003241296.1	6,00E-06	GLOS_LOC10056!	GO:0007179	B	Refseq	transforming growth factor beta receptor signaling pathway
XP_003241296.1 PRED.: putative nuclease HARBI1-like [A. pisum]	XP_003241296.1	6,00E-06	GLOS_LOC10056!	GO:0007507	B	Refseq	heart development
XP_003241296.1 PRED.: putative nuclease HARBI1-like [A. pisum]	XP_003241296.1	6,00E-06	GLOS_LOC10056!	GO:0008134	M	Refseq	transcription factor binding
XP_003241296.1 PRED.: putative nuclease HARBI1-like [A. pisum]	XP_003241296.1	6,00E-06	GLOS_LOC10056!	GO:0010467	B	Refseq	gene expression
XP_003241296.1 PRED.: putative nuclease HARBI1-like [A. pisum]	XP_003241296.1	6,00E-06	GLOS_LOC10056!	GO:0019904	M	Refseq	protein domain specific binding
XP_003241296.1 PRED.: putative nuclease HARBI1-like [A. pisum]	XP_003241296.1	6,00E-06	GLOS_LOC10056!	GO:0045944	B	Refseq	positive regulation of transcription from RNA polymerase II promoter
NP_001162964.1 bunched, isoform D / ref|NP_723755.2| bunched, isoform H	NP_001162964.1	1,00E-35	GLOS_contig_008	GO:0000122	B	Refseq	negative regulation of transcription from RNA polymerase II promoter
NP_001162964.1 bunched, isoform D / ref|NP_723755.2| bunched, isoform H	NP_001162964.1	1,00E-35	GLOS_contig_008	GO:0001501	B	Refseq	skeletal system development
NP_001162964.1 bunched, isoform D / ref|NP_723755.2| bunched, isoform H	NP_001162964.1	1,00E-35	GLOS_contig_008	GO:0003700	M	Refseq	sequence-specific DNA binding transcription factor activity
NP_001162964.1 bunched, isoform D / ref|NP_723755.2| bunched, isoform H	NP_001162964.1	1,00E-35	GLOS_contig_008	GO:0005515	M	Refseq	protein binding
NP_001162964.1 bunched, isoform D / ref|NP_723755.2| bunched, isoform H	NP_001162964.1	1,00E-35	GLOS_contig_008	GO:0005634	C	Refseq	nucleus
NP_001162964.1 bunched, isoform D / ref|NP_723755.2| bunched, isoform H	NP_001162964.1	1,00E-35	GLOS_contig_008	GO:0006366	B	Refseq	transcription from RNA polymerase II promoter
NP_001162964.1 bunched, isoform D / ref|NP_723755.2| bunched, isoform H	NP_001162964.1	1,00E-35	GLOS_contig_008	GO:0006607	B	Refseq	NLS-bearing protein import into nucleus
NP_001162964.1 bunched, isoform D / ref|NP_723755.2| bunched, isoform H	NP_001162964.1	1,00E-35	GLOS_contig_008	GO:0008270	M	Refseq	zinc ion binding
NP_001162964.1 bunched, isoform D / ref|NP_723755.2| bunched, isoform H	NP_001162964.1	1,00E-35	GLOS_contig_008	GO:0032330	B	Refseq	regulation of chondrocyte differentiation
NP_001162964.1 bunched, isoform D / ref|NP_723755.2| bunched, isoform H	NP_001162964.1	1,00E-35	GLOS_contig_008	GO:0043565	M	Refseq	sequence-specific DNA binding
NP_649099.1 CG9629 [Drosophila melanogaster]	NP_649099.1	0	GLOS_CG9629.1.:	GO:0000122	B	Refseq	negative regulation of transcription from RNA polymerase II promoter
NP_649099.1 CG9629 [Drosophila melanogaster]	NP_649099.1	0	GLOS_CG9629.1.:	GO:0001501	B	Refseq	skeletal system development

NP_649099.1 CG9629 [Drosophila melanogaster]	NP_649099.1	0	GLOS_CG9629.1.:	GO:0003700	M	Refseq	sequence-specific DNA binding transcription factor activity
NP_649099.1 CG9629 [Drosophila melanogaster]	NP_649099.1	0	GLOS_CG9629.1.:	GO:0005515	M	Refseq	protein binding
NP_649099.1 CG9629 [Drosophila melanogaster]	NP_649099.1	0	GLOS_CG9629.1.:	GO:0005634	C	Refseq	nucleus
NP_649099.1 CG9629 [Drosophila melanogaster]	NP_649099.1	0	GLOS_CG9629.1.:	GO:0006366	B	Refseq	transcription from RNA polymerase II promoter
NP_649099.1 CG9629 [Drosophila melanogaster]	NP_649099.1	0	GLOS_CG9629.1.:	GO:0006607	B	Refseq	NLS-bearing protein import into nucleus
NP_649099.1 CG9629 [Drosophila melanogaster]	NP_649099.1	0	GLOS_CG9629.1.:	GO:0008270	M	Refseq	zinc ion binding
NP_649099.1 CG9629 [Drosophila melanogaster]	NP_649099.1	0	GLOS_CG9629.1.:	GO:0032330	B	Refseq	regulation of chondrocyte differentiation
NP_649099.1 CG9629 [Drosophila melanogaster]	NP_649099.1	0	GLOS_CG9629.1.:	GO:0043565	M	Refseq	sequence-specific DNA binding
XP_001984984.1 GH14753 [Drosophila grimshawi]	XP_001984984.1	0	GLOS_DGRI_GH1	GO:0005262	M	Refseq	calcium channel activity
XP_001984984.1 GH14753 [Drosophila grimshawi]	XP_001984984.1	0	GLOS_DGRI_GH1	GO:0005515	M	Refseq	protein binding
XP_001984984.1 GH14753 [Drosophila grimshawi]	XP_001984984.1	0	GLOS_DGRI_GH1	GO:0005886	C	Refseq	plasma membrane
XP_001984984.1 GH14753 [Drosophila grimshawi]	XP_001984984.1	0	GLOS_DGRI_GH1	GO:0005887	C	Refseq	integral to plasma membrane
XP_001984984.1 GH14753 [Drosophila grimshawi]	XP_001984984.1	0	GLOS_DGRI_GH1	GO:0006816	B	Refseq	calcium ion transport
XP_001984984.1 GH14753 [Drosophila grimshawi]	XP_001984984.1	0	GLOS_DGRI_GH1	GO:0007411	B	Refseq	axon guidance
XP_001984984.1 GH14753 [Drosophila grimshawi]	XP_001984984.1	0	GLOS_DGRI_GH1	GO:0007596	B	Refseq	blood coagulation
XP_001984984.1 GH14753 [Drosophila grimshawi]	XP_001984984.1	0	GLOS_DGRI_GH1	GO:0007602	B	Refseq	phototransduction
XP_001984984.1 GH14753 [Drosophila grimshawi]	XP_001984984.1	0	GLOS_DGRI_GH1	GO:0010524	B	Refseq	positive regulation of calcium ion transport into cytosol
XP_001984984.1 GH14753 [Drosophila grimshawi]	XP_001984984.1	0	GLOS_DGRI_GH1	GO:0015279	M	Refseq	store-operated calcium channel activity
XP_001984984.1 GH14753 [Drosophila grimshawi]	XP_001984984.1	0	GLOS_DGRI_GH1	GO:0030168	B	Refseq	platelet activation
XP_001984984.1 GH14753 [Drosophila grimshawi]	XP_001984984.1	0	GLOS_DGRI_GH1	GO:0033198	B	Refseq	response to ATP
XP_001984984.1 GH14753 [Drosophila grimshawi]	XP_001984984.1	0	GLOS_DGRI_GH1	GO:0051592	B	Refseq	response to calcium ion
XP_001984984.1 GH14753 [Drosophila grimshawi]	XP_001984984.1	0	GLOS_DGRI_GH1	GO:0070588	B	Refseq	calcium ion transmembrane transport
XP_001984984.1 GH14753 [Drosophila grimshawi]	XP_001984984.1	0	GLOS_DGRI_GH1	GO:0070679	M	Refseq	inositol 1,4,5 trisphosphate binding
XP_001989100.1 GH11536 [D.g.] ref|XP_001997710.1| GH25231 [D.g.]	XP_001989100.1	6,00E-29	GLOS_DGRI_GH1	GO:0005262	M	Refseq	calcium channel activity
XP_001989100.1 GH11536 [D.g.] ref|XP_001997710.1| GH25231 [D.g.]	XP_001989100.1	6,00E-29	GLOS_DGRI_GH1	GO:0005515	M	Refseq	protein binding
XP_001989100.1 GH11536 [D.g.] ref|XP_001997710.1| GH25231 [D.g.]	XP_001989100.1	6,00E-29	GLOS_DGRI_GH1	GO:0005886	C	Refseq	plasma membrane
XP_001989100.1 GH11536 [D.g.] ref|XP_001997710.1| GH25231 [D.g.]	XP_001989100.1	6,00E-29	GLOS_DGRI_GH1	GO:0005887	C	Refseq	integral to plasma membrane
XP_001989100.1 GH11536 [D.g.] ref|XP_001997710.1| GH25231 [D.g.]	XP_001989100.1	6,00E-29	GLOS_DGRI_GH1	GO:0006816	B	Refseq	calcium ion transport
XP_001989100.1 GH11536 [D.g.] ref|XP_001997710.1| GH25231 [D.g.]	XP_001989100.1	6,00E-29	GLOS_DGRI_GH1	GO:0007411	B	Refseq	axon guidance
XP_001989100.1 GH11536 [D.g.] ref|XP_001997710.1| GH25231 [D.g.]	XP_001989100.1	6,00E-29	GLOS_DGRI_GH1	GO:0007596	B	Refseq	blood coagulation
XP_001989100.1 GH11536 [D.g.] ref|XP_001997710.1| GH25231 [D.g.]	XP_001989100.1	6,00E-29	GLOS_DGRI_GH1	GO:0007602	B	Refseq	phototransduction
XP_001989100.1 GH11536 [D.g.] ref|XP_001997710.1| GH25231 [D.g.]	XP_001989100.1	6,00E-29	GLOS_DGRI_GH1	GO:0010524	B	Refseq	positive regulation of calcium ion transport into cytosol
XP_001989100.1 GH11536 [D.g.] ref|XP_001997710.1| GH25231 [D.g.]	XP_001989100.1	6,00E-29	GLOS_DGRI_GH1	GO:0015279	M	Refseq	store-operated calcium channel activity
XP_001989100.1 GH11536 [D.g.] ref|XP_001997710.1| GH25231 [D.g.]	XP_001989100.1	6,00E-29	GLOS_DGRI_GH1	GO:0030168	B	Refseq	platelet activation
XP_001989100.1 GH11536 [D.g.] ref|XP_001997710.1| GH25231 [D.g.]	XP_001989100.1	6,00E-29	GLOS_DGRI_GH1	GO:0033198	B	Refseq	response to ATP
XP_001989100.1 GH11536 [D.g.] ref|XP_001997710.1| GH25231 [D.g.]	XP_001989100.1	6,00E-29	GLOS_DGRI_GH1	GO:0051592	B	Refseq	response to calcium ion
XP_001989100.1 GH11536 [D.g.] ref|XP_001997710.1| GH25231 [D.g.]	XP_001989100.1	6,00E-29	GLOS_DGRI_GH1	GO:0070588	B	Refseq	calcium ion transmembrane transport
XP_001989100.1 GH11536 [D.g.] ref|XP_001997710.1| GH25231 [D.g.]	XP_001989100.1	6,00E-29	GLOS_DGRI_GH1	GO:0070679	M	Refseq	inositol 1,4,5 trisphosphate binding
NP_001262684.1 CG43102, isoform F [Drosophila melanogaster]	NP_001262684.1	0	GLOS_CG43102.1	GO:0000122	B	Refseq	negative regulation of transcription from RNA polymerase II promoter
NP_001262684.1 CG43102, isoform F [Drosophila melanogaster]	NP_001262684.1	0	GLOS_CG43102.1	GO:0001501	B	Refseq	skeletal system development
NP_001262684.1 CG43102, isoform F [Drosophila melanogaster]	NP_001262684.1	0	GLOS_CG43102.1	GO:0003700	M	Refseq	sequence-specific DNA binding transcription factor activity
NP_001262684.1 CG43102, isoform F [Drosophila melanogaster]	NP_001262684.1	0	GLOS_CG43102.1	GO:0005515	M	Refseq	protein binding
NP_001262684.1 CG43102, isoform F [Drosophila melanogaster]	NP_001262684.1	0	GLOS_CG43102.1	GO:0005634	C	Refseq	nucleus
NP_001262684.1 CG43102, isoform F [Drosophila melanogaster]	NP_001262684.1	0	GLOS_CG43102.1	GO:0006366	B	Refseq	transcription from RNA polymerase II promoter
NP_001262684.1 CG43102, isoform F [Drosophila melanogaster]	NP_001262684.1	0	GLOS_CG43102.1	GO:0006607	B	Refseq	NLS-bearing protein import into nucleus
NP_001262684.1 CG43102, isoform F [Drosophila melanogaster]	NP_001262684.1	0	GLOS_CG43102.1	GO:0008270	M	Refseq	zinc ion binding
NP_001262684.1 CG43102, isoform F [Drosophila melanogaster]	NP_001262684.1	0	GLOS_CG43102.1	GO:0032330	B	Refseq	regulation of chondrocyte differentiation
NP_001262684.1 CG43102, isoform F [Drosophila melanogaster]	NP_001262684.1	0	GLOS_CG43102.1	GO:0043565	M	Refseq	sequence-specific DNA binding
[BBH] 2AAA_DROME (sp|P36179) Serine/threonine-protein phosphatase PP2A 65 kDa regulatory subunit OS=D. melanogaster	2AAA_DROME	0	GLOS_2AAA.1.1	GO:0044450	C	.	microtubule organizing center part
[BBH] 2AAA_DROME (sp|P36179) Serine/threonine-protein ph.. … Same as above	2AAA_DROME	0	GLOS_2AAA.1.1	GO:0044430	C	.	cytoskeletal part
[BBH] 2AAA_DROME (sp|P36179) Serine/threonine-protein ph.. … Same as above	2AAA_DROME	0	GLOS_2AAA.1.1	GO:0005856	C	.	cytoskeleton
[BBH] 2AAA_DROME (sp|P36179) Serine/threonine-protein ph.. … Same as above	2AAA_DROME	0	GLOS_2AAA.1.1	GO:0005815	C	.	microtubule organizing center
[BBH] 2AAA_DROME (sp|P36179) Serine/threonine-protein ph.. … Same as above	2AAA_DROME	0	GLOS_2AAA.1.1	GO:0015630	C	.	microtubule cytoskeleton

[BBH] 2AAA_DROME (sp|P36179) Serine/threonine-protein ph.. …	Same as above	2AAA_DROME	0	GLOS_2AAA.1.1	GO:0005813	C	.	centrosome
[BBH] 2AAA_DROME (sp|P36179) Serine/threonine-protein ph.. …	Same as above	2AAA_DROME	0	GLOS_2AAA.1.1	GO:0005814	C	FlyBase	centriole
[BBH] 2AAA_DROME (sp|P36179) Serine/threonine-protein ph.. …	Same as above	2AAA_DROME	0	GLOS_2AAA.1.1	GO:0008287	C	.	protein serine/threonine phosphatase complex
[BBH] 2AAA_DROME (sp|P36179) Serine/threonine-protein ph.. …	Same as above	2AAA_DROME	0	GLOS_2AAA.1.1	GO:0000159	C	FlyBase	protein phosphatase type 2A complex
[BBH] 2AAA_DROME (sp|P36179) Serine/threonine-protein ph.. …	Same as above	2AAA_DROME	0	GLOS_2AAA.1.1	GO:0019888	M	.	protein phosphatase regulator activity
[BBH] 2AAA_DROME (sp|P36179) Serine/threonine-protein ph.. …	Same as above	2AAA_DROME	0	GLOS_2AAA.1.1	GO:0019208	M	.	phosphatase regulator activity
[BBH] 2AAA_DROME (sp|P36179) Serine/threonine-protein ph.. …	Same as above	2AAA_DROME	0	GLOS_2AAA.1.1	GO:0030234	M	.	enzyme regulator activity
[BBH] 2AAA_DROME (sp|P36179) Serine/threonine-protein ph.. …	Same as above	2AAA_DROME	0	GLOS_2AAA.1.1	GO:0008601	M	FlyBase	protein phosphatase type 2A regulator activity
[BBH] 2AAA_DROME (sp|P36179) Serine/threonine-protein ph.. …	Same as above	2AAA_DROME	0	GLOS_2AAA.1.1	GO:0022402	B	.	cell cycle process
[BBH] 2AAA_DROME (sp|P36179) Serine/threonine-protein ph.. …	Same as above	2AAA_DROME	0	GLOS_2AAA.1.1	GO:0051297	B	.	centrosome organization
[BBH] 2AAA_DROME (sp|P36179) Serine/threonine-protein ph.. …	Same as above	2AAA_DROME	0	GLOS_2AAA.1.1	GO:0031023	B	.	microtubule organizing center organization
[BBH] 2AAA_DROME (sp|P36179) Serine/threonine-protein ph.. …	Same as above	2AAA_DROME	0	GLOS_2AAA.1.1	GO:0006996	B	.	organelle organization
[BBH] 2AAA_DROME (sp|P36179) Serine/threonine-protein ph.. …	Same as above	2AAA_DROME	0	GLOS_2AAA.1.1	GO:0000226	B	.	microtubule cytoskeleton organization
[BBH] 2AAA_DROME (sp|P36179) Serine/threonine-protein ph.. …	Same as above	2AAA_DROME	0	GLOS_2AAA.1.1	GO:0007010	B	.	cytoskeleton organization
[BBH] 2AAA_DROME (sp|P36179) Serine/threonine-protein ph.. …	Same as above	2AAA_DROME	0	GLOS_2AAA.1.1	GO:0007017	B	.	microtubule-based process
[BBH] 2AAA_DROME (sp|P36179) Serine/threonine-protein ph.. …	Same as above	2AAA_DROME	0	GLOS_2AAA.1.1	GO:0007098	B	.	centrosome cycle
[BBH] 2AAA_DROME (sp|P36179) Serine/threonine-protein ph.. …	Same as above	2AAA_DROME	0	GLOS_2AAA.1.1	GO:0051298	B	FlyBase	centrosome duplication
[BBH] 2AAA_DROME (sp|P36179) Serine/threonine-protein ph.. …	Same as above	2AAA_DROME	0	GLOS_2AAA.1.1	GO:0007059	B	FlyBase	chromosome segregation
[BBH] 2AAA_DROME (sp|P36179) Serine/threonine-protein ph.. …	Same as above	2AAA_DROME	0	GLOS_2AAA.1.1	GO:0007051	B	.	spindle organization
[BBH] 2AAA_DROME (sp|P36179) Serine/threonine-protein ph.. …	Same as above	2AAA_DROME	0	GLOS_2AAA.1.1	GO:0071822	B	.	protein complex subunit organization
[BBH] 2AAA_DROME (sp|P36179) Serine/threonine-protein ph.. …	Same as above	2AAA_DROME	0	GLOS_2AAA.1.1	GO:0043933	B	.	macromolecular complex subunit organization
[BBH] 2AAA_DROME (sp|P36179) Serine/threonine-protein ph.. …	Same as above	2AAA_DROME	0	GLOS_2AAA.1.1	GO:0000278	B	.	mitotic cell cycle
[BBH] 2AAA_DROME (sp|P36179) Serine/threonine-protein ph.. …	Same as above	2AAA_DROME	0	GLOS_2AAA.1.1	GO:0007052	B	FlyBase	mitotic spindle organization
[BBH] 2AAA_DROME (sp|P36179) Serine/threonine-protein ph.. …	Same as above	2AAA_DROME	0	GLOS_2AAA.1.1	GO:0022008	B	FlyBase	neurogenesis
[BBH] 2AAA_DROME (sp|P36179) Serine/threonine-protein ph.. …	Same as above	2AAA_DROME	0	GLOS_2AAA.1.1	GO:0006897	B	.	endocytosis
[BBH] 2AAA_DROME (sp|P36179) Serine/threonine-protein ph.. …	Same as above	2AAA_DROME	0	GLOS_2AAA.1.1	GO:0016192	B	.	vesicle-mediated transport
[BBH] 2AAA_DROME (sp|P36179) Serine/threonine-protein ph.. …	Same as above	2AAA_DROME	0	GLOS_2AAA.1.1	GO:0006909	B	FlyBase	phagocytosis
[BBH] 2AAA_DROME (sp|P36179) Serine/threonine-protein ph.. …	Same as above	2AAA_DROME	0	GLOS_2AAA.1.1	GO:0006464	B	.	cellular protein modification process
[BBH] 2AAA_DROME (sp|P36179) Serine/threonine-protein ph.. …	Same as above	2AAA_DROME	0	GLOS_2AAA.1.1	GO:0036211	B	.	protein modification process
[BBH] 2AAA_DROME (sp|P36179) Serine/threonine-protein ph.. …	Same as above	2AAA_DROME	0	GLOS_2AAA.1.1	GO:0043412	B	.	macromolecule modification
[BBH] 2AAA_DROME (sp|P36179) Serine/threonine-protein ph.. …	Same as above	2AAA_DROME	0	GLOS_2AAA.1.1	GO:0044267	B	.	cellular protein metabolic process
[BBH] 2AAA_DROME (sp|P36179) Serine/threonine-protein ph.. …	Same as above	2AAA_DROME	0	GLOS_2AAA.1.1	GO:0016311	B	.	dephosphorylation
[BBH] 2AAA_DROME (sp|P36179) Serine/threonine-protein ph.. …	Same as above	2AAA_DROME	0	GLOS_2AAA.1.1	GO:0006796	B	.	phosphate-containing compound metabolic process
[BBH] 2AAA_DROME (sp|P36179) Serine/threonine-protein ph.. …	Same as above	2AAA_DROME	0	GLOS_2AAA.1.1	GO:0006793	B	.	phosphorus metabolic process
[BBH] 2AAA_DROME (sp|P36179) Serine/threonine-protein ph.. …	Same as above	2AAA_DROME	0	GLOS_2AAA.1.1	GO:0006470	B	FlyBase	protein dephosphorylation
[BBH] 2AAA_DROME (sp|P36179) Serine/threonine-protein ph.. …	Same as above	2AAA_DROME	0	GLOS_2AAA.1.1	GO:0006461	B	.	protein complex assembly
[BBH] 2AAA_DROME (sp|P36179) Serine/threonine-protein ph.. …	Same as above	2AAA_DROME	0	GLOS_2AAA.1.1	GO:0065003	B	.	macromolecular complex assembly
[BBH] 2AAA_DROME (sp|P36179) Serine/threonine-protein ph.. …	Same as above	2AAA_DROME	0	GLOS_2AAA.1.1	GO:0022607	B	.	cellular component assembly
[BBH] 2AAA_DROME (sp|P36179) Serine/threonine-protein ph.. …	Same as above	2AAA_DROME	0	GLOS_2AAA.1.1	GO:0044085	B	.	cellular component biogenesis
[BBH] 2AAA_DROME (sp|P36179) Serine/threonine-protein ph.. …	Same as above	2AAA_DROME	0	GLOS_2AAA.1.1	GO:0070271	B	.	protein complex biogenesis
[BBH] 2AAA_DROME (sp|P36179) Serine/threonine-protein ph.. …	Same as above	2AAA_DROME	0	GLOS_2AAA.1.1	GO:0070925	B	.	organelle assembly
[BBH] 2AAA_DROME (sp|P36179) Serine/threonine-protein ph.. …	Same as above	2AAA_DROME	0	GLOS_2AAA.1.1	GO:0051225	B	FlyBase	spindle assembly
ACE_DROME (sp|Q10714) Angiotensin-converting enzyme OS=D.m.		ACE_DROME	1,00E-104	GLOS_ACE.5.16	GO:0005615	C	FlyBase	extracellular space
ACE_DROME (sp|Q10714) Angiotensin-converting enzyme OS=D.m.		ACE_DROME	1,00E-104	GLOS_ACE.5.16	GO:0016020	C	InterPro	membrane
ACE_DROME (sp|Q10714) Angiotensin-converting enzyme OS=D.m.		ACE_DROME	1,00E-104	GLOS_ACE.5.16	GO:0008238	M	.	exopeptidase activity
ACE_DROME (sp|Q10714) Angiotensin-converting enzyme OS=D.m.		ACE_DROME	1,00E-104	GLOS_ACE.5.16	GO:0004180	M	UniProtKB-k	carboxypeptidase activity
ACE_DROME (sp|Q10714) Angiotensin-converting enzyme OS=D.m.		ACE_DROME	1,00E-104	GLOS_ACE.5.16	GO:0046872	M	UniProtKB-k	metal ion binding
ACE_DROME (sp|Q10714) Angiotensin-converting enzyme OS=D.m.		ACE_DROME	1,00E-104	GLOS_ACE.5.16	GO:0008237	M	UniProtKB-k	metallopeptidase activity
ACE_DROME (sp|Q10714) Angiotensin-converting enzyme OS=D.m.		ACE_DROME	1,00E-104	GLOS_ACE.5.16	GO:0008241	M	FlyBase	peptidyl-dipeptidase activity
ACE_DROME (sp|Q10714) Angiotensin-converting enzyme OS=D.m.		ACE_DROME	1,00E-104	GLOS_ACE.5.16	GO:0007552	B	FlyBase	metamorphosis
ACE_DROME (sp|Q10714) Angiotensin-converting enzyme OS=D.m.		ACE_DROME	1,00E-104	GLOS_ACE.5.16	GO:0006508	B	FlyBase	proteolysis
ACE_DROME (sp|Q10714) Angiotensin-converting enzyme OS=D.m.		ACE_DROME	1,00E-104	GLOS_ACE.5.16	GO:0009608	B	.	response to symbiont
ACE_DROME (sp|Q10714) Angiotensin-converting enzyme OS=D.m.		ACE_DROME	1,00E-104	GLOS_ACE.5.16	GO:0009609	B	FlyBase	response to symbiotic bacterium
ACE_DROME (sp|Q10714) Angiotensin-converting enzyme OS=D.m.		ACE_DROME	1,00E-104	GLOS_ACE.5.16	GO:0007349	B	.	cellularization

ACE_DROME (sp|Q10714) Angiotensin-converting enzyme OS=D.m.	ACE_DROME	1,00E-104	GLOS_ACE.5.16	GO:0048646	B	.	anatomical structure formation involved in morphogenesis
ACE_DROME (sp|Q10714) Angiotensin-converting enzyme OS=D.m.	ACE_DROME	1,00E-104	GLOS_ACE.5.16	GO:0007286	B	.	spermatid development
ACE_DROME (sp|Q10714) Angiotensin-converting enzyme OS=D.m.	ACE_DROME	1,00E-104	GLOS_ACE.5.16	GO:0002064	B	.	epithelial cell development
ACE_DROME (sp|Q10714) Angiotensin-converting enzyme OS=D.m.	ACE_DROME	1,00E-104	GLOS_ACE.5.16	GO:0030855	B	.	epithelial cell differentiation
ACE_DROME (sp|Q10714) Angiotensin-converting enzyme OS=D.m.	ACE_DROME	1,00E-104	GLOS_ACE.5.16	GO:0060429	B	.	epithelium development
ACE_DROME (sp|Q10714) Angiotensin-converting enzyme OS=D.m.	ACE_DROME	1,00E-104	GLOS_ACE.5.16	GO:0009888	B	.	tissue development
ACE_DROME (sp|Q10714) Angiotensin-converting enzyme OS=D.m.	ACE_DROME	1,00E-104	GLOS_ACE.5.16	GO:0007281	B	.	germ cell development
ACE_DROME (sp|Q10714) Angiotensin-converting enzyme OS=D.m.	ACE_DROME	1,00E-104	GLOS_ACE.5.16	GO:0022412	B	.	cellular process involved in reproduction in multicellular organism
ACE_DROME (sp|Q10714) Angiotensin-converting enzyme OS=D.m.	ACE_DROME	1,00E-104	GLOS_ACE.5.16	GO:0048610	B	.	cellular process involved in reproduction
ACE_DROME (sp|Q10714) Angiotensin-converting enzyme OS=D.m.	ACE_DROME	1,00E-104	GLOS_ACE.5.16	GO:0007276	B	.	gamete generation
ACE_DROME (sp|Q10714) Angiotensin-converting enzyme OS=D.m.	ACE_DROME	1,00E-104	GLOS_ACE.5.16	GO:0019953	B	.	sexual reproduction
ACE_DROME (sp|Q10714) Angiotensin-converting enzyme OS=D.m.	ACE_DROME	1,00E-104	GLOS_ACE.5.16	GO:0048515	B	.	spermatid differentiation
ACE_DROME (sp|Q10714) Angiotensin-converting enzyme OS=D.m.	ACE_DROME	1,00E-104	GLOS_ACE.5.16	GO:0007283	B	.	spermatogenesis
ACE_DROME (sp|Q10714) Angiotensin-converting enzyme OS=D.m.	ACE_DROME	1,00E-104	GLOS_ACE.5.16	GO:0048232	B	.	male gamete generation
ACE_DROME (sp|Q10714) Angiotensin-converting enzyme OS=D.m.	ACE_DROME	1,00E-104	GLOS_ACE.5.16	GO:0007291	B	FlyBase	sperm individualization
ACE_DROME (sp|Q10714) Angiotensin-converting enzyme OS=D.m.	ACE_DROME	1,00E-104	GLOS_ACE.5.16	GO:0006997	B	.	nucleus organization
ACE_DROME (sp|Q10714) Angiotensin-converting enzyme OS=D.m.	ACE_DROME	1,00E-104	GLOS_ACE.5.16	GO:0007289	B	FlyBase	spermatid nucleus differentiation
ACOD_SHEEP (sp|O62849) Acyl-CoA desaturase OS=Ovis aries	ACOD_SHEEP	3,00E-33	GLOS_ACOD.2.2	GO:0031090	C	.	organelle membrane
ACOD_SHEEP (sp|O62849) Acyl-CoA desaturase OS=Ovis aries	ACOD_SHEEP	3,00E-33	GLOS_ACOD.2.2	GO:0044432	C	.	endoplasmic reticulum part
ACOD_SHEEP (sp|O62849) Acyl-CoA desaturase OS=Ovis aries	ACOD_SHEEP	3,00E-33	GLOS_ACOD.2.2	GO:0005783	C	.	endoplasmic reticulum
ACOD_SHEEP (sp|O62849) Acyl-CoA desaturase OS=Ovis aries	ACOD_SHEEP	3,00E-33	GLOS_ACOD.2.2	GO:0042175	C	.	nuclear outer membrane-endoplasmic reticulum membrane network
ACOD_SHEEP (sp|O62849) Acyl-CoA desaturase OS=Ovis aries	ACOD_SHEEP	3,00E-33	GLOS_ACOD.2.2	GO:0012505	C	.	endomembrane system
ACOD_SHEEP (sp|O62849) Acyl-CoA desaturase OS=Ovis aries	ACOD_SHEEP	3,00E-33	GLOS_ACOD.2.2	GO:0005789	C	UniProtKB-S	endoplasmic reticulum membrane
ACOD_SHEEP (sp|O62849) Acyl-CoA desaturase OS=Ovis aries	ACOD_SHEEP	3,00E-33	GLOS_ACOD.2.2	GO:0016021	C	UniProtKB-K	integral to membrane
ACOD_SHEEP (sp|O62849) Acyl-CoA desaturase OS=Ovis aries	ACOD_SHEEP	3,00E-33	GLOS_ACOD.2.2	GO:0005506	M	InterPro	iron ion binding
ACOD_SHEEP (sp|O62849) Acyl-CoA desaturase OS=Ovis aries	ACOD_SHEEP	3,00E-33	GLOS_ACOD.2.2	GO:0016215	M	.	acyl-CoA desaturase activity
ACOD_SHEEP (sp|O62849) Acyl-CoA desaturase OS=Ovis aries	ACOD_SHEEP	3,00E-33	GLOS_ACOD.2.2	GO:0016717	M	.	oxidoreductase activity, acting on paired donors, with oxidation of a pair of donors resulting in the reduction of molecular oxygen to two molecules of water
ACOD_SHEEP (sp|O62849) Acyl-CoA desaturase OS=Ovis aries	ACOD_SHEEP	3,00E-33	GLOS_ACOD.2.2	GO:0016705	M	.	oxidoreductase activity, acting on paired donors, with incorporation or reduction of molecular oxygen
ACOD_SHEEP (sp|O62849) Acyl-CoA desaturase OS=Ovis aries	ACOD_SHEEP	3,00E-33	GLOS_ACOD.2.2	GO:0016491	M	.	oxidoreductase activity
ACOD_SHEEP (sp|O62849) Acyl-CoA desaturase OS=Ovis aries	ACOD_SHEEP	3,00E-33	GLOS_ACOD.2.2	GO:0004768	M	UniProtKB-E	stearoyl-CoA 9-desaturase activity
ACOD_SHEEP (sp|O62849) Acyl-CoA desaturase OS=Ovis aries	ACOD_SHEEP	3,00E-33	GLOS_ACOD.2.2	GO:0006631	B	.	fatty acid metabolic process
ACOD_SHEEP (sp|O62849) Acyl-CoA desaturase OS=Ovis aries	ACOD_SHEEP	3,00E-33	GLOS_ACOD.2.2	GO:0032787	B	.	monocarboxylic acid metabolic process
ACOD_SHEEP (sp|O62849) Acyl-CoA desaturase OS=Ovis aries	ACOD_SHEEP	3,00E-33	GLOS_ACOD.2.2	GO:0019752	B	.	carboxylic acid metabolic process
ACOD_SHEEP (sp|O62849) Acyl-CoA desaturase OS=Ovis aries	ACOD_SHEEP	3,00E-33	GLOS_ACOD.2.2	GO:0043436	B	.	oxoacid metabolic process
ACOD_SHEEP (sp|O62849) Acyl-CoA desaturase OS=Ovis aries	ACOD_SHEEP	3,00E-33	GLOS_ACOD.2.2	GO:0006082	B	.	organic acid metabolic process
ACOD_SHEEP (sp|O62849) Acyl-CoA desaturase OS=Ovis aries	ACOD_SHEEP	3,00E-33	GLOS_ACOD.2.2	GO:0044281	B	.	small molecule metabolic process
ACOD_SHEEP (sp|O62849) Acyl-CoA desaturase OS=Ovis aries	ACOD_SHEEP	3,00E-33	GLOS_ACOD.2.2	GO:0044710	B	.	single-organism metabolic process
ACOD_SHEEP (sp|O62849) Acyl-CoA desaturase OS=Ovis aries	ACOD_SHEEP	3,00E-33	GLOS_ACOD.2.2	GO:0044255	B	.	cellular lipid metabolic process
ACOD_SHEEP (sp|O62849) Acyl-CoA desaturase OS=Ovis aries	ACOD_SHEEP	3,00E-33	GLOS_ACOD.2.2	GO:0006629	B	.	lipid metabolic process
ACOD_SHEEP (sp|O62849) Acyl-CoA desaturase OS=Ovis aries	ACOD_SHEEP	3,00E-33	GLOS_ACOD.2.2	GO:0008610	B	.	lipid biosynthetic process
ACOD_SHEEP (sp|O62849) Acyl-CoA desaturase OS=Ovis aries	ACOD_SHEEP	3,00E-33	GLOS_ACOD.2.2	GO:0072330	B	.	monocarboxylic acid biosynthetic process
ACOD_SHEEP (sp|O62849) Acyl-CoA desaturase OS=Ovis aries	ACOD_SHEEP	3,00E-33	GLOS_ACOD.2.2	GO:0046394	B	.	carboxylic acid biosynthetic process
ACOD_SHEEP (sp|O62849) Acyl-CoA desaturase OS=Ovis aries	ACOD_SHEEP	3,00E-33	GLOS_ACOD.2.2	GO:0016053	B	.	organic acid biosynthetic process
ACOD_SHEEP (sp|O62849) Acyl-CoA desaturase OS=Ovis aries	ACOD_SHEEP	3,00E-33	GLOS_ACOD.2.2	GO:0044283	B	.	small molecule biosynthetic process
ACOD_SHEEP (sp|O62849) Acyl-CoA desaturase OS=Ovis aries	ACOD_SHEEP	3,00E-33	GLOS_ACOD.2.2	GO:0044711	B	.	single-organism biosynthetic process
ACOD_SHEEP (sp|O62849) Acyl-CoA desaturase OS=Ovis aries	ACOD_SHEEP	3,00E-33	GLOS_ACOD.2.2	GO:0006633	B	UniProtKB-K	fatty acid biosynthetic process
[BBH] ACT6_DROSI (sp|P83968) Actin, indirect flight muscle OS=D. sim.	ACT6_DROSI	0	GLOS_ACT6.1.1	GO:0005737	C	UniProtKB-K	cytoplasm
[BBH] ACT6_DROSI (sp|P83968) Actin, indirect flight muscle OS=D. sim.	ACT6_DROSI	0	GLOS_ACT6.1.1	GO:0005856	C	UniProtKB-S	cytoskeleton
[BBH] ACT6_DROSI (sp|P83968) Actin, indirect flight muscle OS=D. sim.	ACT6_DROSI	0	GLOS_ACT6.1.1	GO:0032550	M	.	purine ribonucleoside binding
[BBH] ACT6_DROSI (sp|P83968) Actin, indirect flight muscle OS=D. sim.	ACT6_DROSI	0	GLOS_ACT6.1.1	GO:0001883	M	.	purine nucleoside binding
[BBH] ACT6_DROSI (sp|P83968) Actin, indirect flight muscle OS=D. sim.	ACT6_DROSI	0	GLOS_ACT6.1.1	GO:0001882	M	.	nucleoside binding
[BBH] ACT6_DROSI (sp|P83968) Actin, indirect flight muscle OS=D. sim.	ACT6_DROSI	0	GLOS_ACT6.1.1	GO:0097367	M	.	carbohydrate derivative binding

[BBH] ACT6_DROSI (sp|P83968) Actin, indirect flight muscle OS=D. sim.	ACT6_DROSI	0	GLOS_ACT6.1.1	GO:0032549	M	.	ribonucleoside binding
[BBH] ACT6_DROSI (sp|P83968) Actin, indirect flight muscle OS=D. sim.	ACT6_DROSI	0	GLOS_ACT6.1.1	GO:0032559	M	.	adenyl ribonucleotide binding
[BBH] ACT6_DROSI (sp|P83968) Actin, indirect flight muscle OS=D. sim.	ACT6_DROSI	0	GLOS_ACT6.1.1	GO:0030554	M	.	adenyl nucleotide binding
[BBH] ACT6_DROSI (sp|P83968) Actin, indirect flight muscle OS=D. sim.	ACT6_DROSI	0	GLOS_ACT6.1.1	GO:0017076	M	.	purine nucleotide binding
[BBH] ACT6_DROSI (sp|P83968) Actin, indirect flight muscle OS=D. sim.	ACT6_DROSI	0	GLOS_ACT6.1.1	GO:0000166	M	.	nucleotide binding
[BBH] ACT6_DROSI (sp|P83968) Actin, indirect flight muscle OS=D. sim.	ACT6_DROSI	0	GLOS_ACT6.1.1	GO:1901265	M	.	nucleoside phosphate binding
[BBH] ACT6_DROSI (sp|P83968) Actin, indirect flight muscle OS=D. sim.	ACT6_DROSI	0	GLOS_ACT6.1.1	GO:0032555	M	.	purine ribonucleotide binding
[BBH] ACT6_DROSI (sp|P83968) Actin, indirect flight muscle OS=D. sim.	ACT6_DROSI	0	GLOS_ACT6.1.1	GO:0032553	M	.	ribonucleotide binding
[BBH] ACT6_DROSI (sp|P83968) Actin, indirect flight muscle OS=D. sim.	ACT6_DROSI	0	GLOS_ACT6.1.1	GO:0035639	M	.	purine ribonucleoside triphosphate binding
[BBH] ACT6_DROSI (sp|P83968) Actin, indirect flight muscle OS=D. sim.	ACT6_DROSI	0	GLOS_ACT6.1.1	GO:0005524	M	UniProtKB-k	ATP binding
[BBH] ADF1_DROME (sp|P05552) Transcription factor Adf-1 OS=D.m.	ADF1_DROME	1,00E-119	GLOS_ADF1.2.3	GO:0005634	C	FlyBase	nucleus
[BBH] ADF1_DROME (sp|P05552) Transcription factor Adf-1 OS=D.m.	ADF1_DROME	1,00E-119	GLOS_ADF1.2.3	GO:0003677	M	UniProtKB-k	DNA binding
[BBH] ADF1_DROME (sp|P05552) Transcription factor Adf-1 OS=D.m.	ADF1_DROME	1,00E-119	GLOS_ADF1.2.3	GO:0000982	M	.	RNA polymerase II core promoter proximal region sequence-specific DNA binding transcription factor activity
[BBH] ADF1_DROME (sp|P05552) Transcription factor Adf-1 OS=D.m.	ADF1_DROME	1,00E-119	GLOS_ADF1.2.3	GO:0001228	M	.	RNA polymerase II transcription regulatory region sequence-specific DNA binding transcription factor activity involved in positive regulation of transcription
[BBH] ADF1_DROME (sp|P05552) Transcription factor Adf-1 OS=D.m.	ADF1_DROME	1,00E-119	GLOS_ADF1.2.3	GO:0001077	M	FlyBase	RNA polymerase II core promoter proximal region sequence-specific DNA binding binding transcription factor activity involved in positive regulation of transcription
[BBH] ADF1_DROME (sp|P05552) Transcription factor Adf-1 OS=D.m.	ADF1_DROME	1,00E-119	GLOS_ADF1.2.3	GO:0016358	B	.	dendrite development
[BBH] ADF1_DROME (sp|P05552) Transcription factor Adf-1 OS=D.m.	ADF1_DROME	1,00E-119	GLOS_ADF1.2.3	GO:0048813	B	FlyBase	dendrite morphogenesis
[BBH] ADF1_DROME (sp|P05552) Transcription factor Adf-1 OS=D.m.	ADF1_DROME	1,00E-119	GLOS_ADF1.2.3	GO:0007626	B	.	locomotory behavior
[BBH] ADF1_DROME (sp|P05552) Transcription factor Adf-1 OS=D.m.	ADF1_DROME	1,00E-119	GLOS_ADF1.2.3	GO:0044708	B	.	single-organism behavior
[BBH] ADF1_DROME (sp|P05552) Transcription factor Adf-1 OS=D.m.	ADF1_DROME	1,00E-119	GLOS_ADF1.2.3	GO:0007610	B	.	behavior
[BBH] ADF1_DROME (sp|P05552) Transcription factor Adf-1 OS=D.m.	ADF1_DROME	1,00E-119	GLOS_ADF1.2.3	GO:0030537	B	.	larval behavior
[BBH] ADF1_DROME (sp|P05552) Transcription factor Adf-1 OS=D.m.	ADF1_DROME	1,00E-119	GLOS_ADF1.2.3	GO:0008345	B	FlyBase	larval locomotory behavior
[BBH] ADF1_DROME (sp|P05552) Transcription factor Adf-1 OS=D.m.	ADF1_DROME	1,00E-119	GLOS_ADF1.2.3	GO:0040011	B	FlyBase	locomotion
[BBH] ADF1_DROME (sp|P05552) Transcription factor Adf-1 OS=D.m.	ADF1_DROME	1,00E-119	GLOS_ADF1.2.3	GO:0007613	B	.	memory
[BBH] ADF1_DROME (sp|P05552) Transcription factor Adf-1 OS=D.m.	ADF1_DROME	1,00E-119	GLOS_ADF1.2.3	GO:0007611	B	.	learning or memory
[BBH] ADF1_DROME (sp|P05552) Transcription factor Adf-1 OS=D.m.	ADF1_DROME	1,00E-119	GLOS_ADF1.2.3	GO:0050890	B	.	cognition
[BBH] ADF1_DROME (sp|P05552) Transcription factor Adf-1 OS=D.m.	ADF1_DROME	1,00E-119	GLOS_ADF1.2.3	GO:0050877	B	.	neurological system process
[BBH] ADF1_DROME (sp|P05552) Transcription factor Adf-1 OS=D.m.	ADF1_DROME	1,00E-119	GLOS_ADF1.2.3	GO:0007616	B	FlyBase	long-term memory
[BBH] ADF1_DROME (sp|P05552) Transcription factor Adf-1 OS=D.m.	ADF1_DROME	1,00E-119	GLOS_ADF1.2.3	GO:0008306	B	.	associative learning
[BBH] ADF1_DROME (sp|P05552) Transcription factor Adf-1 OS=D.m.	ADF1_DROME	1,00E-119	GLOS_ADF1.2.3	GO:0007612	B	.	learning
[BBH] ADF1_DROME (sp|P05552) Transcription factor Adf-1 OS=D.m.	ADF1_DROME	1,00E-119	GLOS_ADF1.2.3	GO:0042048	B	.	olfactory behavior
[BBH] ADF1_DROME (sp|P05552) Transcription factor Adf-1 OS=D.m.	ADF1_DROME	1,00E-119	GLOS_ADF1.2.3	GO:0007635	B	.	chemosensory behavior
[BBH] ADF1_DROME (sp|P05552) Transcription factor Adf-1 OS=D.m.	ADF1_DROME	1,00E-119	GLOS_ADF1.2.3	GO:0008355	B	FlyBase	olfactory learning
[BBH] ADF1_DROME (sp|P05552) Transcription factor Adf-1 OS=D.m.	ADF1_DROME	1,00E-119	GLOS_ADF1.2.3	GO:0050808	B	.	synapse organization
[BBH] ADF1_DROME (sp|P05552) Transcription factor Adf-1 OS=D.m.	ADF1_DROME	1,00E-119	GLOS_ADF1.2.3	GO:0007416	B	FlyBase	synapse assembly
ATP5J_DROME (sp|Q24407) ATP synthase-coupling fact 6, mitoch OS=D.m.	ATP5J_DROME	5,00E-20	GLOS_ATP5J.1.1	GO:0044455	C	.	mitochondrial membrane part
ATP5J_DROME (sp|Q24407) ATP synthase-coupling fact 6, mitoch OS=D.m.	ATP5J_DROME	5,00E-20	GLOS_ATP5J.1.1	GO:0044429	C	.	mitochondrial part
ATP5J_DROME (sp|Q24407) ATP synthase-coupling fact 6, mitoch OS=D.m.	ATP5J_DROME	5,00E-20	GLOS_ATP5J.1.1	GO:0005739	C	.	mitochondrion
ATP5J_DROME (sp|Q24407) ATP synthase-coupling fact 6, mitoch OS=D.m.	ATP5J_DROME	5,00E-20	GLOS_ATP5J.1.1	GO:0031966	C	.	mitochondrial membrane
ATP5J_DROME (sp|Q24407) ATP synthase-coupling fact 6, mitoch OS=D.m.	ATP5J_DROME	5,00E-20	GLOS_ATP5J.1.1	GO:0005740	C	.	mitochondrial envelope
ATP5J_DROME (sp|Q24407) ATP synthase-coupling fact 6, mitoch OS=D.m.	ATP5J_DROME	5,00E-20	GLOS_ATP5J.1.1	GO:0031967	C	.	organelle envelope
ATP5J_DROME (sp|Q24407) ATP synthase-coupling fact 6, mitoch OS=D.m.	ATP5J_DROME	5,00E-20	GLOS_ATP5J.1.1	GO:0031975	C	.	envelope
ATP5J_DROME (sp|Q24407) ATP synthase-coupling fact 6, mitoch OS=D.m.	ATP5J_DROME	5,00E-20	GLOS_ATP5J.1.1	GO:0045263	C	.	proton-transporting ATP synthase complex, coupling factor F(o)
ATP5J_DROME (sp|Q24407) ATP synthase-coupling fact 6, mitoch OS=D.m.	ATP5J_DROME	5,00E-20	GLOS_ATP5J.1.1	GO:0033177	C	.	proton-transporting two-sector ATPase complex, proton-transporting domain
ATP5J_DROME (sp|Q24407) ATP synthase-coupling fact 6, mitoch OS=D.m.	ATP5J_DROME	5,00E-20	GLOS_ATP5J.1.1	GO:0016469	C	.	proton-transporting two-sector ATPase complex
ATP5J_DROME (sp|Q24407) ATP synthase-coupling fact 6, mitoch OS=D.m.	ATP5J_DROME	5,00E-20	GLOS_ATP5J.1.1	GO:0045259	C	.	proton-transporting ATP synthase complex
ATP5J_DROME (sp|Q24407) ATP synthase-coupling fact 6, mitoch OS=D.m.	ATP5J_DROME	5,00E-20	GLOS_ATP5J.1.1	GO:0005753	C	.	mitochondrial proton-transporting ATP synthase complex
ATP5J_DROME (sp|Q24407) ATP synthase-coupling fact 6, mitoch OS=D.m.	ATP5J_DROME	5,00E-20	GLOS_ATP5J.1.1	GO:0005743	C	.	mitochondrial inner membrane
ATP5J_DROME (sp|Q24407) ATP synthase-coupling fact 6, mitoch OS=D.m.	ATP5J_DROME	5,00E-20	GLOS_ATP5J.1.1	GO:0019866	C	.	organelle inner membrane
ATP5J_DROME (sp|Q24407) ATP synthase-coupling fact 6, mitoch OS=D.m.	ATP5J_DROME	5,00E-20	GLOS_ATP5J.1.1	GO:0000276	C	InterPro	mitochondrial proton-transporting ATP synthase complex, coupling factor F(o)
ATP5J_DROME (sp|Q24407) ATP synthase-coupling fact 6, mitoch OS=D.m.	ATP5J_DROME	5,00E-20	GLOS_ATP5J.1.1	GO:0015077	M	.	monovalent inorganic cation transmembrane transporter activity

ATP5J_DROME (sp|Q24407) ATP synthase-coupling fact 6, mitoch OS=D.m.	ATP5J_DROME	5,00E-20	GLOS_ATP5J.1.1	GO:0015078	M	InterPro	hydrogen ion transmembrane transporter activity
ATP5J_DROME (sp|Q24407) ATP synthase-coupling fact 6, mitoch OS=D.m.	ATP5J_DROME	5,00E-20	GLOS_ATP5J.1.1	GO:0006754	B	.	ATP biosynthetic process
ATP5J_DROME (sp|Q24407) ATP synthase-coupling fact 6, mitoch OS=D.m.	ATP5J_DROME	5,00E-20	GLOS_ATP5J.1.1	GO:0009152	B	.	purine ribonucleotide biosynthetic process
ATP5J_DROME (sp|Q24407) ATP synthase-coupling fact 6, mitoch OS=D.m.	ATP5J_DROME	5,00E-20	GLOS_ATP5J.1.1	GO:0006164	B	.	purine nucleotide biosynthetic process
ATP5J_DROME (sp|Q24407) ATP synthase-coupling fact 6, mitoch OS=D.m.	ATP5J_DROME	5,00E-20	GLOS_ATP5J.1.1	GO:0006163	B	.	purine nucleotide metabolic process
ATP5J_DROME (sp|Q24407) ATP synthase-coupling fact 6, mitoch OS=D.m.	ATP5J_DROME	5,00E-20	GLOS_ATP5J.1.1	GO:0009117	B	.	nucleotide metabolic process
ATP5J_DROME (sp|Q24407) ATP synthase-coupling fact 6, mitoch OS=D.m.	ATP5J_DROME	5,00E-20	GLOS_ATP5J.1.1	GO:0006753	B	.	nucleoside phosphate metabolic process
ATP5J_DROME (sp|Q24407) ATP synthase-coupling fact 6, mitoch OS=D.m.	ATP5J_DROME	5,00E-20	GLOS_ATP5J.1.1	GO:0019637	B	.	organophosphate metabolic process
ATP5J_DROME (sp|Q24407) ATP synthase-coupling fact 6, mitoch OS=D.m.	ATP5J_DROME	5,00E-20	GLOS_ATP5J.1.1	GO:0055086	B	.	nucleobase-containing small molecule metabolic process
ATP5J_DROME (sp|Q24407) ATP synthase-coupling fact 6, mitoch OS=D.m.	ATP5J_DROME	5,00E-20	GLOS_ATP5J.1.1	GO:0072521	B	.	purine-containing compound metabolic process
ATP5J_DROME (sp|Q24407) ATP synthase-coupling fact 6, mitoch OS=D.m.	ATP5J_DROME	5,00E-20	GLOS_ATP5J.1.1	GO:0009165	B	.	nucleotide biosynthetic process
ATP5J_DROME (sp|Q24407) ATP synthase-coupling fact 6, mitoch OS=D.m.	ATP5J_DROME	5,00E-20	GLOS_ATP5J.1.1	GO:1901293	B	.	nucleoside phosphate biosynthetic process
ATP5J_DROME (sp|Q24407) ATP synthase-coupling fact 6, mitoch OS=D.m.	ATP5J_DROME	5,00E-20	GLOS_ATP5J.1.1	GO:0090407	B	.	organophosphate biosynthetic process
ATP5J_DROME (sp|Q24407) ATP synthase-coupling fact 6, mitoch OS=D.m.	ATP5J_DROME	5,00E-20	GLOS_ATP5J.1.1	GO:0072522	B	.	purine-containing compound biosynthetic process
ATP5J_DROME (sp|Q24407) ATP synthase-coupling fact 6, mitoch OS=D.m.	ATP5J_DROME	5,00E-20	GLOS_ATP5J.1.1	GO:1901566	B	.	organonitrogen compound biosynthetic process
ATP5J_DROME (sp|Q24407) ATP synthase-coupling fact 6, mitoch OS=D.m.	ATP5J_DROME	5,00E-20	GLOS_ATP5J.1.1	GO:0009150	B	.	purine ribonucleotide metabolic process
ATP5J_DROME (sp|Q24407) ATP synthase-coupling fact 6, mitoch OS=D.m.	ATP5J_DROME	5,00E-20	GLOS_ATP5J.1.1	GO:0009259	B	.	ribonucleotide metabolic process
ATP5J_DROME (sp|Q24407) ATP synthase-coupling fact 6, mitoch OS=D.m.	ATP5J_DROME	5,00E-20	GLOS_ATP5J.1.1	GO:0019693	B	.	ribose phosphate metabolic process
ATP5J_DROME (sp|Q24407) ATP synthase-coupling fact 6, mitoch OS=D.m.	ATP5J_DROME	5,00E-20	GLOS_ATP5J.1.1	GO:1901135	B	.	carbohydrate derivative metabolic process
ATP5J_DROME (sp|Q24407) ATP synthase-coupling fact 6, mitoch OS=D.m.	ATP5J_DROME	5,00E-20	GLOS_ATP5J.1.1	GO:0009260	B	.	ribonucleotide biosynthetic process
ATP5J_DROME (sp|Q24407) ATP synthase-coupling fact 6, mitoch OS=D.m.	ATP5J_DROME	5,00E-20	GLOS_ATP5J.1.1	GO:0046390	B	.	ribose phosphate biosynthetic process
ATP5J_DROME (sp|Q24407) ATP synthase-coupling fact 6, mitoch OS=D.m.	ATP5J_DROME	5,00E-20	GLOS_ATP5J.1.1	GO:1901137	B	.	carbohydrate derivative biosynthetic process
ATP5J_DROME (sp|Q24407) ATP synthase-coupling fact 6, mitoch OS=D.m.	ATP5J_DROME	5,00E-20	GLOS_ATP5J.1.1	GO:0009168	B	.	purine ribonucleoside monophosphate biosynthetic process
ATP5J_DROME (sp|Q24407) ATP synthase-coupling fact 6, mitoch OS=D.m.	ATP5J_DROME	5,00E-20	GLOS_ATP5J.1.1	GO:0009127	B	.	purine nucleoside monophosphate biosynthetic process
ATP5J_DROME (sp|Q24407) ATP synthase-coupling fact 6, mitoch OS=D.m.	ATP5J_DROME	5,00E-20	GLOS_ATP5J.1.1	GO:0009124	B	.	nucleoside monophosphate biosynthetic process
ATP5J_DROME (sp|Q24407) ATP synthase-coupling fact 6, mitoch OS=D.m.	ATP5J_DROME	5,00E-20	GLOS_ATP5J.1.1	GO:0009123	B	.	nucleoside monophosphate metabolic process
ATP5J_DROME (sp|Q24407) ATP synthase-coupling fact 6, mitoch OS=D.m.	ATP5J_DROME	5,00E-20	GLOS_ATP5J.1.1	GO:0009126	B	.	purine nucleoside monophosphate metabolic process
ATP5J_DROME (sp|Q24407) ATP synthase-coupling fact 6, mitoch OS=D.m.	ATP5J_DROME	5,00E-20	GLOS_ATP5J.1.1	GO:0009156	B	.	ribonucleoside monophosphate biosynthetic process
ATP5J_DROME (sp|Q24407) ATP synthase-coupling fact 6, mitoch OS=D.m.	ATP5J_DROME	5,00E-20	GLOS_ATP5J.1.1	GO:0009161	B	.	ribonucleoside monophosphate metabolic process
ATP5J_DROME (sp|Q24407) ATP synthase-coupling fact 6, mitoch OS=D.m.	ATP5J_DROME	5,00E-20	GLOS_ATP5J.1.1	GO:0009167	B	.	purine ribonucleoside monophosphate metabolic process
ATP5J_DROME (sp|Q24407) ATP synthase-coupling fact 6, mitoch OS=D.m.	ATP5J_DROME	5,00E-20	GLOS_ATP5J.1.1	GO:0009206	B	.	purine ribonucleoside triphosphate biosynthetic process
ATP5J_DROME (sp|Q24407) ATP synthase-coupling fact 6, mitoch OS=D.m.	ATP5J_DROME	5,00E-20	GLOS_ATP5J.1.1	GO:0009145	B	.	purine nucleoside triphosphate biosynthetic process
ATP5J_DROME (sp|Q24407) ATP synthase-coupling fact 6, mitoch OS=D.m.	ATP5J_DROME	5,00E-20	GLOS_ATP5J.1.1	GO:0009142	B	.	nucleoside triphosphate biosynthetic process
ATP5J_DROME (sp|Q24407) ATP synthase-coupling fact 6, mitoch OS=D.m.	ATP5J_DROME	5,00E-20	GLOS_ATP5J.1.1	GO:0009141	B	.	nucleoside triphosphate metabolic process
ATP5J_DROME (sp|Q24407) ATP synthase-coupling fact 6, mitoch OS=D.m.	ATP5J_DROME	5,00E-20	GLOS_ATP5J.1.1	GO:0009144	B	.	purine nucleoside triphosphate metabolic process
ATP5J_DROME (sp|Q24407) ATP synthase-coupling fact 6, mitoch OS=D.m.	ATP5J_DROME	5,00E-20	GLOS_ATP5J.1.1	GO:0009201	B	.	ribonucleoside triphosphate biosynthetic process
ATP5J_DROME (sp|Q24407) ATP synthase-coupling fact 6, mitoch OS=D.m.	ATP5J_DROME	5,00E-20	GLOS_ATP5J.1.1	GO:0009199	B	.	ribonucleoside triphosphate metabolic process
ATP5J_DROME (sp|Q24407) ATP synthase-coupling fact 6, mitoch OS=D.m.	ATP5J_DROME	5,00E-20	GLOS_ATP5J.1.1	GO:0009205	B	.	purine ribonucleoside triphosphate metabolic process
ATP5J_DROME (sp|Q24407) ATP synthase-coupling fact 6, mitoch OS=D.m.	ATP5J_DROME	5,00E-20	GLOS_ATP5J.1.1	GO:0046034	B	.	ATP metabolic process
ATP5J_DROME (sp|Q24407) ATP synthase-coupling fact 6, mitoch OS=D.m.	ATP5J_DROME	5,00E-20	GLOS_ATP5J.1.1	GO:0046128	B	.	purine ribonucleoside metabolic process
ATP5J_DROME (sp|Q24407) ATP synthase-coupling fact 6, mitoch OS=D.m.	ATP5J_DROME	5,00E-20	GLOS_ATP5J.1.1	GO:0009119	B	.	ribonucleoside metabolic process
ATP5J_DROME (sp|Q24407) ATP synthase-coupling fact 6, mitoch OS=D.m.	ATP5J_DROME	5,00E-20	GLOS_ATP5J.1.1	GO:0009116	B	.	nucleoside metabolic process
ATP5J_DROME (sp|Q24407) ATP synthase-coupling fact 6, mitoch OS=D.m.	ATP5J_DROME	5,00E-20	GLOS_ATP5J.1.1	GO:1901657	B	.	glycosyl compound metabolic process
ATP5J_DROME (sp|Q24407) ATP synthase-coupling fact 6, mitoch OS=D.m.	ATP5J_DROME	5,00E-20	GLOS_ATP5J.1.1	GO:0042278	B	.	purine nucleoside metabolic process
ATP5J_DROME (sp|Q24407) ATP synthase-coupling fact 6, mitoch OS=D.m.	ATP5J_DROME	5,00E-20	GLOS_ATP5J.1.1	GO:0046129	B	.	purine ribonucleoside biosynthetic process
ATP5J_DROME (sp|Q24407) ATP synthase-coupling fact 6, mitoch OS=D.m.	ATP5J_DROME	5,00E-20	GLOS_ATP5J.1.1	GO:0042451	B	.	purine nucleoside biosynthetic process
ATP5J_DROME (sp|Q24407) ATP synthase-coupling fact 6, mitoch OS=D.m.	ATP5J_DROME	5,00E-20	GLOS_ATP5J.1.1	GO:0009163	B	.	nucleoside biosynthetic process
ATP5J_DROME (sp|Q24407) ATP synthase-coupling fact 6, mitoch OS=D.m.	ATP5J_DROME	5,00E-20	GLOS_ATP5J.1.1	GO:1901659	B	.	glycosyl compound biosynthetic process
ATP5J_DROME (sp|Q24407) ATP synthase-coupling fact 6, mitoch OS=D.m.	ATP5J_DROME	5,00E-20	GLOS_ATP5J.1.1	GO:0042455	B	.	ribonucleoside biosynthetic process
ATP5J_DROME (sp|Q24407) ATP synthase-coupling fact 6, mitoch OS=D.m.	ATP5J_DROME	5,00E-20	GLOS_ATP5J.1.1	GO:0015985	B	.	energy coupled proton transport, down electrochemical gradient
ATP5J_DROME (sp|Q24407) ATP synthase-coupling fact 6, mitoch OS=D.m.	ATP5J_DROME	5,00E-20	GLOS_ATP5J.1.1	GO:0015992	B	.	proton transport
ATP5J_DROME (sp|Q24407) ATP synthase-coupling fact 6, mitoch OS=D.m.	ATP5J_DROME	5,00E-20	GLOS_ATP5J.1.1	GO:0006818	B	.	hydrogen transport
ATP5J_DROME (sp|Q24407) ATP synthase-coupling fact 6, mitoch OS=D.m.	ATP5J_DROME	5,00E-20	GLOS_ATP5J.1.1	GO:0015672	B	.	monovalent inorganic cation transport
ATP5J_DROME (sp|Q24407) ATP synthase-coupling fact 6, mitoch OS=D.m.	ATP5J_DROME	5,00E-20	GLOS_ATP5J.1.1	GO:0015986	B	InterPro	ATP synthesis coupled proton transport

ATP5J_DROME (sp|Q24407) ATP synthase-coupling fact 6, mitoch OS=D.m.	ATP5J_DROME	5,00E-20	GLOS_ATP5J.1.1	GO:0045168	B	.	cell-cell signaling involved in cell fate commitment
ATP5J_DROME (sp|Q24407) ATP synthase-coupling fact 6, mitoch OS=D.m.	ATP5J_DROME	5,00E-20	GLOS_ATP5J.1.1	GO:0045165	B	.	cell fate commitment
ATP5J_DROME (sp|Q24407) ATP synthase-coupling fact 6, mitoch OS=D.m.	ATP5J_DROME	5,00E-20	GLOS_ATP5J.1.1	GO:0046331	B	FlyBase	lateral inhibition
[BBH] ATTA_GLOMM (sp|Q8WTD3) Attacin-A OS=G.m. morsitans PE=1 SV=1	ATTA_GLOMM	1,00E-142	GLOS_ATTA.4.7	GO:0005576	C	UniProtKB	extracellular region
[BBH] ATTA_GLOMM (sp|Q8WTD3) Attacin-A OS=G.m. morsitans PE=1 SV=1	ATTA_GLOMM	1,00E-142	GLOS_ATTA.4.7	GO:0019730	B	.	antimicrobial humoral response
[BBH] ATTA_GLOMM (sp|Q8WTD3) Attacin-A OS=G.m. morsitans PE=1 SV=1	ATTA_GLOMM	1,00E-142	GLOS_ATTA.4.7	GO:0006959	B	.	humoral immune response
[BBH] ATTA_GLOMM (sp|Q8WTD3) Attacin-A OS=G.m. morsitans PE=1 SV=1	ATTA_GLOMM	1,00E-142	GLOS_ATTA.4.7	GO:0006955	B	.	immune response
[BBH] ATTA_GLOMM (sp|Q8WTD3) Attacin-A OS=G.m. morsitans PE=1 SV=1	ATTA_GLOMM	1,00E-142	GLOS_ATTA.4.7	GO:0002376	B	.	immune system process
[BBH] ATTA_GLOMM (sp|Q8WTD3) Attacin-A OS=G.m. morsitans PE=1 SV=1	ATTA_GLOMM	1,00E-142	GLOS_ATTA.4.7	GO:0042742	B	.	defense response to bacterium
[BBH] ATTA_GLOMM (sp|Q8WTD3) Attacin-A OS=G.m. morsitans PE=1 SV=1	ATTA_GLOMM	1,00E-142	GLOS_ATTA.4.7	GO:0019731	B	UniProtKB	antibacterial humoral response
[BBH] ATTA_GLOMM (sp|Q8WTD3) Attacin-A OS=G.m. morsitans PE=1 SV=1	ATTA_GLOMM	1,00E-142	GLOS_ATTA.4.7	GO:0050829	B	UniProtKB	defense response to Gram-negative bacterium
[BBH] ATTA_GLOMM (sp|Q8WTD3) Attacin-A OS=G.m. morsitans PE=1 SV=1	ATTA_GLOMM	1,00E-142	GLOS_ATTA.4.7	GO:0045087	B	UniProtKB-k	innate immune response
[BBH] CF2_DROME (sp|P20385) Chorion transcrip. Fact. Cf2 OS=D.m.	CF2_DROME	1,00E-45	GLOS_CF2.1.1	GO:0005634	C	FlyBase	nucleus
[BBH] CF2_DROME (sp|P20385) Chorion transcrip. Fact. Cf2 OS=D.m.	CF2_DROME	1,00E-45	GLOS_CF2.1.1	GO:0046872	M	UniProtKB-k	metal ion binding
[BBH] CF2_DROME (sp|P20385) Chorion transcrip. Fact. Cf2 OS=D.m.	CF2_DROME	1,00E-45	GLOS_CF2.1.1	GO:0001077	M	FlyBase	RNA polymerase II core promoter proximal region sequence-specific DNA binding transcription factor activity involved in positive regulation of transcription
[BBH] CF2_DROME (sp|P20385) Chorion transcrip. Fact. Cf2 OS=D.m.	CF2_DROME	1,00E-45	GLOS_CF2.1.1	GO:0043565	M	FlyBase	sequence-specific DNA binding
[BBH] CF2_DROME (sp|P20385) Chorion transcrip. Fact. Cf2 OS=D.m.	CF2_DROME	1,00E-45	GLOS_CF2.1.1	GO:0007525	B	.	somatic muscle development
[BBH] CF2_DROME (sp|P20385) Chorion transcrip. Fact. Cf2 OS=D.m.	CF2_DROME	1,00E-45	GLOS_CF2.1.1	GO:0061061	B	.	muscle structure development
[BBH] CF2_DROME (sp|P20385) Chorion transcrip. Fact. Cf2 OS=D.m.	CF2_DROME	1,00E-45	GLOS_CF2.1.1	GO:0007527	B	FlyBase	adult somatic muscle development
[BBH] CF2_DROME (sp|P20385) Chorion transcrip. Fact. Cf2 OS=D.m.	CF2_DROME	1,00E-45	GLOS_CF2.1.1	GO:0032786	B	.	positive regulation of DNA-dependent transcription, elongation
[BBH] CF2_DROME (sp|P20385) Chorion transcrip. Fact. Cf2 OS=D.m.	CF2_DROME	1,00E-45	GLOS_CF2.1.1	GO:0032784	B	.	regulation of DNA-dependent transcription, elongation
[BBH] CF2_DROME (sp|P20385) Chorion transcrip. Fact. Cf2 OS=D.m.	CF2_DROME	1,00E-45	GLOS_CF2.1.1	GO:0034243	B	.	regulation of transcription elongation from RNA polymerase II promoter
[BBH] CF2_DROME (sp|P20385) Chorion transcrip. Fact. Cf2 OS=D.m.	CF2_DROME	1,00E-45	GLOS_CF2.1.1	GO:0032968	B	FlyBase	positive regulation of transcription elongation from RNA polymerase II promoter
[BBH] CISY_GLOMM (sp|Q0QHL3) Prob. citrate synthase, mitoch. OS=G.m.m.	CISY_GLOMM	0	GLOS_CISY.1.1	GO:0005759	C	UniProtKB	mitochondrial matrix
[BBH] CISY_GLOMM (sp|Q0QHL3) Prob. citrate synthase, mitoch. OS=G.m.m.	CISY_GLOMM	0	GLOS_CISY.1.1	GO:0036440	M	.	citrate synthase activity
[BBH] CISY_GLOMM (sp|Q0QHL3) Prob. citrate synthase, mitoch. OS=G.m.m.	CISY_GLOMM	0	GLOS_CISY.1.1	GO:0046912	M	.	transferase act, transferring acyl groups, acyl groups converted into alkyl on transfer
[BBH] CISY_GLOMM (sp|Q0QHL3) Prob. citrate synthase, mitoch. OS=G.m.m.	CISY_GLOMM	0	GLOS_CISY.1.1	GO:0016746	M	.	transferase activity, transferring acyl groups
[BBH] CISY_GLOMM (sp|Q0QHL3) Prob. citrate synthase, mitoch. OS=G.m.m.	CISY_GLOMM	0	GLOS_CISY.1.1	GO:0016740	M	.	transferase activity
[BBH] CISY_GLOMM (sp|Q0QHL3) Prob. citrate synthase, mitoch. OS=G.m.m.	CISY_GLOMM	0	GLOS_CISY.1.1	GO:0004108	M	UniProtKB	citrate (Si)-synthase activity
[BBH] CISY_GLOMM (sp|Q0QHL3) Prob. citrate synthase, mitoch. OS=G.m.m.	CISY_GLOMM	0	GLOS_CISY.1.1	GO:0005975	B	UniProtKB	carbohydrate metabolic process
[BBH] CISY_GLOMM (sp|Q0QHL3) Prob. citrate synthase, mitoch. OS=G.m.m.	CISY_GLOMM	0	GLOS_CISY.1.1	GO:0005975	B	.	carbohydrate metabolic process
[BBH] CISY_GLOMM (sp|Q0QHL3) Prob. citrate synthase, mitoch. OS=G.m.m.	CISY_GLOMM	0	GLOS_CISY.1.1	GO:0044262	B	InterPro	cellular carbohydrate metabolic process
[BBH] CISY_GLOMM (sp|Q0QHL3) Prob. citrate synthase, mitoch. OS=G.m.m.	CISY_GLOMM	0	GLOS_CISY.1.1	GO:0009060	B	.	aerobic respiration
[BBH] CISY_GLOMM (sp|Q0QHL3) Prob. citrate synthase, mitoch. OS=G.m.m.	CISY_GLOMM	0	GLOS_CISY.1.1	GO:0045333	B	.	cellular respiration
[BBH] CISY_GLOMM (sp|Q0QHL3) Prob. citrate synthase, mitoch. OS=G.m.m.	CISY_GLOMM	0	GLOS_CISY.1.1	GO:0015980	B	.	energy derivation by oxidation of organic compounds
[BBH] CISY_GLOMM (sp|Q0QHL3) Prob. citrate synthase, mitoch. OS=G.m.m.	CISY_GLOMM	0	GLOS_CISY.1.1	GO:0006091	B	.	generation of precursor metabolites and energy
[BBH] CISY_GLOMM (sp|Q0QHL3) Prob. citrate synthase, mitoch. OS=G.m.m.	CISY_GLOMM	0	GLOS_CISY.1.1	GO:0055114	B	.	oxidation-reduction process
[BBH] CISY_GLOMM (sp|Q0QHL3) Prob. citrate synthase, mitoch. OS=G.m.m.	CISY_GLOMM	0	GLOS_CISY.1.1	GO:0006099	B	UniProtKB-U	tricarboxylic acid cycle
[BBH] CNI_DROME (sp|P49858) Protein cornichon OS=D.m. GN=cni PE=1 SV=1	CNI_DROME	1,00E-97	GLOS_CNI.1.1	GO:0005789	C	UniProtKB	endoplasmic reticulum membrane
[BBH] CNI_DROME (sp|P49858) Protein cornichon OS=D.m. GN=cni PE=1 SV=1	CNI_DROME	1,00E-97	GLOS_CNI.1.1	GO:0016021	C	UniProtKB-k	integral to membrane
[BBH] CNI_DROME (sp|P49858) Protein cornichon OS=D.m. GN=cni PE=1 SV=1	CNI_DROME	1,00E-97	GLOS_CNI.1.1	GO:0060811	B	.	intracellular mRNA localization involved in anterior/posterior axis specification
[BBH] CNI_DROME (sp|P49858) Protein cornichon OS=D.m. GN=cni PE=1 SV=1	CNI_DROME	1,00E-97	GLOS_CNI.1.1	GO:0060810	B	.	intracellular mRNA localization involved in pattern specification process
[BBH] CNI_DROME (sp|P49858) Protein cornichon OS=D.m. GN=cni PE=1 SV=1	CNI_DROME	1,00E-97	GLOS_CNI.1.1	GO:0008298	B	.	intracellular mRNA localization
[BBH] CNI_DROME (sp|P49858) Protein cornichon OS=D.m. GN=cni PE=1 SV=1	CNI_DROME	1,00E-97	GLOS_CNI.1.1	GO:0006403	B	.	RNA localization
[BBH] CNI_DROME (sp|P49858) Protein cornichon OS=D.m. GN=cni PE=1 SV=1	CNI_DROME	1,00E-97	GLOS_CNI.1.1	GO:0007389	B	.	pattern specification process
[BBH] CNI_DROME (sp|P49858) Protein cornichon OS=D.m. GN=cni PE=1 SV=1	CNI_DROME	1,00E-97	GLOS_CNI.1.1	GO:0009948	B	.	anterior/posterior axis specification
[BBH] CNI_DROME (sp|P49858) Protein cornichon OS=D.m. GN=cni PE=1 SV=1	CNI_DROME	1,00E-97	GLOS_CNI.1.1	GO:0009798	B	.	axis specification
[BBH] CNI_DROME (sp|P49858) Protein cornichon OS=D.m. GN=cni PE=1 SV=1	CNI_DROME	1,00E-97	GLOS_CNI.1.1	GO:0009952	B	.	anterior/posterior pattern specification
[BBH] CNI_DROME (sp|P49858) Protein cornichon OS=D.m. GN=cni PE=1 SV=1	CNI_DROME	1,00E-97	GLOS_CNI.1.1	GO:0003002	B	.	regionalization
[BBH] CNI_DROME (sp|P49858) Protein cornichon OS=D.m. GN=cni PE=1 SV=1	CNI_DROME	1,00E-97	GLOS_CNI.1.1	GO:0007314	B	.	oocyte anterior/posterior axis specification
[BBH] CNI_DROME (sp|P49858) Protein cornichon OS=D.m. GN=cni PE=1 SV=1	CNI_DROME	1,00E-97	GLOS_CNI.1.1	GO:0007309	B	.	oocyte axis specification
[BBH] CNI_DROME (sp|P49858) Protein cornichon OS=D.m. GN=cni PE=1 SV=1	CNI_DROME	1,00E-97	GLOS_CNI.1.1	GO:0007308	B	.	oocyte construction
[BBH] CNI_DROME (sp|P49858) Protein cornichon OS=D.m. GN=cni PE=1 SV=1	CNI_DROME	1,00E-97	GLOS_CNI.1.1	GO:0048469	B	.	cell maturation

[BBH] CNI_DROME (sp|P49858) Protein cornichon OS=D.m. GN=cni PE=1 SV=1	CNI_DROME	1,00E-97	GLOS_CNI.1.1	GO:0021700	B	.	developmental maturation
[BBH] CNI_DROME (sp|P49858) Protein cornichon OS=D.m. GN=cni PE=1 SV=1	CNI_DROME	1,00E-97	GLOS_CNI.1.1	GO:0048599	B	.	oocyte development
[BBH] CNI_DROME (sp|P49858) Protein cornichon OS=D.m. GN=cni PE=1 SV=1	CNI_DROME	1,00E-97	GLOS_CNI.1.1	GO:0009994	B	.	oocyte differentiation
[BBH] CNI_DROME (sp|P49858) Protein cornichon OS=D.m. GN=cni PE=1 SV=1	CNI_DROME	1,00E-97	GLOS_CNI.1.1	GO:0048477	B	.	oogenesis
[BBH] CNI_DROME (sp|P49858) Protein cornichon OS=D.m. GN=cni PE=1 SV=1	CNI_DROME	1,00E-97	GLOS_CNI.1.1	GO:0007292	B	.	female gamete generation
[BBH] CNI_DROME (sp|P49858) Protein cornichon OS=D.m. GN=cni PE=1 SV=1	CNI_DROME	1,00E-97	GLOS_CNI.1.1	GO:0008358	B	.	maternal determination of anterior/posterior axis, embryo
[BBH] CNI_DROME (sp|P49858) Protein cornichon OS=D.m. GN=cni PE=1 SV=1	CNI_DROME	1,00E-97	GLOS_CNI.1.1	GO:0008595	B	.	anterior/posterior axis specification, embryo
[BBH] CNI_DROME (sp|P49858) Protein cornichon OS=D.m. GN=cni PE=1 SV=1	CNI_DROME	1,00E-97	GLOS_CNI.1.1	GO:0000578	B	.	embryonic axis specification
[BBH] CNI_DROME (sp|P49858) Protein cornichon OS=D.m. GN=cni PE=1 SV=1	CNI_DROME	1,00E-97	GLOS_CNI.1.1	GO:0009880	B	.	embryonic pattern specification
[BBH] CNI_DROME (sp|P49858) Protein cornichon OS=D.m. GN=cni PE=1 SV=1	CNI_DROME	1,00E-97	GLOS_CNI.1.1	GO:0009790	B	.	embryo development
[BBH] CNI_DROME (sp|P49858) Protein cornichon OS=D.m. GN=cni PE=1 SV=1	CNI_DROME	1,00E-97	GLOS_CNI.1.1	GO:0007351	B	.	tripartite regional subdivision
[BBH] CNI_DROME (sp|P49858) Protein cornichon OS=D.m. GN=cni PE=1 SV=1	CNI_DROME	1,00E-97	GLOS_CNI.1.1	GO:0007350	B	.	blastoderm segmentation
[BBH] CNI_DROME (sp|P49858) Protein cornichon OS=D.m. GN=cni PE=1 SV=1	CNI_DROME	1,00E-97	GLOS_CNI.1.1	GO:0035282	B	.	segmentation
[BBH] CNI_DROME (sp|P49858) Protein cornichon OS=D.m. GN=cni PE=1 SV=1	CNI_DROME	1,00E-97	GLOS_CNI.1.1	GO:0045450	B	FlyBase	bicoid mRNA localization
[BBH] CNI_DROME (sp|P49858) Protein cornichon OS=D.m. GN=cni PE=1 SV=1	CNI_DROME	1,00E-97	GLOS_CNI.1.1	GO:0007306	B	.	eggshell chorion assembly
[BBH] CNI_DROME (sp|P49858) Protein cornichon OS=D.m. GN=cni PE=1 SV=1	CNI_DROME	1,00E-97	GLOS_CNI.1.1	GO:0010927	B	.	cellular component assembly involved in morphogenesis
[BBH] CNI_DROME (sp|P49858) Protein cornichon OS=D.m. GN=cni PE=1 SV=1	CNI_DROME	1,00E-97	GLOS_CNI.1.1	GO:0007304	B	.	chorion-containing eggshell formation
[BBH] CNI_DROME (sp|P49858) Protein cornichon OS=D.m. GN=cni PE=1 SV=1	CNI_DROME	1,00E-97	GLOS_CNI.1.1	GO:0030703	B	.	eggshell formation
[BBH] CNI_DROME (sp|P49858) Protein cornichon OS=D.m. GN=cni PE=1 SV=1	CNI_DROME	1,00E-97	GLOS_CNI.1.1	GO:0030707	B	.	ovarian follicle cell development
[BBH] CNI_DROME (sp|P49858) Protein cornichon OS=D.m. GN=cni PE=1 SV=1	CNI_DROME	1,00E-97	GLOS_CNI.1.1	GO:0046843	B	FlyBase	dorsal appendage formation
[BBH] CNI_DROME (sp|P49858) Protein cornichon OS=D.m. GN=cni PE=1 SV=1	CNI_DROME	1,00E-97	GLOS_CNI.1.1	GO:0048193	B	.	Golgi vesicle transport
[BBH] CNI_DROME (sp|P49858) Protein cornichon OS=D.m. GN=cni PE=1 SV=1	CNI_DROME	1,00E-97	GLOS_CNI.1.1	GO:0006888	B	UniProtKB	ER to Golgi vesicle-mediated transport
[BBH] CNI_DROME (sp|P49858) Protein cornichon OS=D.m. GN=cni PE=1 SV=1	CNI_DROME	1,00E-97	GLOS_CNI.1.1	GO:0040023	B	.	establishment of nucleus localization
[BBH] CNI_DROME (sp|P49858) Protein cornichon OS=D.m. GN=cni PE=1 SV=1	CNI_DROME	1,00E-97	GLOS_CNI.1.1	GO:0051656	B	.	establishment of organelle localization
[BBH] CNI_DROME (sp|P49858) Protein cornichon OS=D.m. GN=cni PE=1 SV=1	CNI_DROME	1,00E-97	GLOS_CNI.1.1	GO:0051640	B	.	organelle localization
[BBH] CNI_DROME (sp|P49858) Protein cornichon OS=D.m. GN=cni PE=1 SV=1	CNI_DROME	1,00E-97	GLOS_CNI.1.1	GO:0051647	B	.	nucleus localization
[BBH] CNI_DROME (sp|P49858) Protein cornichon OS=D.m. GN=cni PE=1 SV=1	CNI_DROME	1,00E-97	GLOS_CNI.1.1	GO:0051663	B	.	oocyte nucleus localization involved in oocyte dorsal/ventral axis specification
[BBH] CNI_DROME (sp|P49858) Protein cornichon OS=D.m. GN=cni PE=1 SV=1	CNI_DROME	1,00E-97	GLOS_CNI.1.1	GO:0007310	B	.	oocyte dorsal/ventral axis specification
[BBH] CNI_DROME (sp|P49858) Protein cornichon OS=D.m. GN=cni PE=1 SV=1	CNI_DROME	1,00E-97	GLOS_CNI.1.1	GO:0009950	B	.	dorsal/ventral axis specification
[BBH] CNI_DROME (sp|P49858) Protein cornichon OS=D.m. GN=cni PE=1 SV=1	CNI_DROME	1,00E-97	GLOS_CNI.1.1	GO:0009953	B	.	dorsal/ventral pattern formation
[BBH] CNI_DROME (sp|P49858) Protein cornichon OS=D.m. GN=cni PE=1 SV=1	CNI_DROME	1,00E-97	GLOS_CNI.1.1	GO:0030722	B	FlyBase	establishment of oocyte nucleus localization involved in oocyte dorsal/ventral axis specification
[BBH] CNI_DROME (sp|P49858) Protein cornichon OS=D.m. GN=cni PE=1 SV=1	CNI_DROME	1,00E-97	GLOS_CNI.1.1	GO:0007173	B	.	epidermal growth factor receptor signaling pathway
[BBH] CNI_DROME (sp|P49858) Protein cornichon OS=D.m. GN=cni PE=1 SV=1	CNI_DROME	1,00E-97	GLOS_CNI.1.1	GO:0038127	B	.	ERBB signaling pathway
[BBH] CNI_DROME (sp|P49858) Protein cornichon OS=D.m. GN=cni PE=1 SV=1	CNI_DROME	1,00E-97	GLOS_CNI.1.1	GO:0007169	B	.	transmembrane receptor protein tyrosine kinase signaling pathway
[BBH] CNI_DROME (sp|P49858) Protein cornichon OS=D.m. GN=cni PE=1 SV=1	CNI_DROME	1,00E-97	GLOS_CNI.1.1	GO:0008314	B	FlyBase	gurken receptor signaling pathway
[BBH] CNI_DROME (sp|P49858) Protein cornichon OS=D.m. GN=cni PE=1 SV=1	CNI_DROME	1,00E-97	GLOS_CNI.1.1	GO:0035556	B	InterPro	intracellular signal transduction
[BBH] CNI_DROME (sp|P49858) Protein cornichon OS=D.m. GN=cni PE=1 SV=1	CNI_DROME	1,00E-97	GLOS_CNI.1.1	GO:0019094	B	.	pole plasm mRNA localization
[BBH] CNI_DROME (sp|P49858) Protein cornichon OS=D.m. GN=cni PE=1 SV=1	CNI_DROME	1,00E-97	GLOS_CNI.1.1	GO:0007316	B	.	pole plasm RNA localization
[BBH] CNI_DROME (sp|P49858) Protein cornichon OS=D.m. GN=cni PE=1 SV=1	CNI_DROME	1,00E-97	GLOS_CNI.1.1	GO:0007315	B	.	pole plasm assembly
[BBH] CNI_DROME (sp|P49858) Protein cornichon OS=D.m. GN=cni PE=1 SV=1	CNI_DROME	1,00E-97	GLOS_CNI.1.1	GO:0007028	B	.	cytoplasm organization
[BBH] CNI_DROME (sp|P49858) Protein cornichon OS=D.m. GN=cni PE=1 SV=1	CNI_DROME	1,00E-97	GLOS_CNI.1.1	GO:0045451	B	FlyBase	pole plasm oskar mRNA localization
[BBH] CNI_DROME (sp|P49858) Protein cornichon OS=D.m. GN=cni PE=1 SV=1	CNI_DROME	1,00E-97	GLOS_CNI.1.1	GO:0015031	B	UniProtKB-k	protein transport
[BBH] CP4D2_DROME (sp|Q27589) Cyt P450 4d2 OS=D.m.	CP4D2_DROME	0	GLOS_CP4D2.1.1	GO:0005789	C	UniProtKB-S	endoplasmic reticulum membrane
[BBH] CP4D2_DROME (sp|Q27589) Cyt P450 4d2 OS=D.m.	CP4D2_DROME	0	GLOS_CP4D2.1.1	GO:0009055	M	InterPro	electron carrier activity
[BBH] CP4D2_DROME (sp|Q27589) Cyt P450 4d2 OS=D.m.	CP4D2_DROME	0	GLOS_CP4D2.1.1	GO:0020037	M	InterPro	heme binding
[BBH] CP4D2_DROME (sp|Q27589) Cyt P450 4d2 OS=D.m.	CP4D2_DROME	0	GLOS_CP4D2.1.1	GO:0005506	M	InterPro	iron ion binding
[BBH] CP4D2_DROME (sp|Q27589) Cyt P450 4d2 OS=D.m.	CP4D2_DROME	0	GLOS_CP4D2.1.1	GO:0004497	M	UniProtKB-k	monooxygenase activity
[BBH] CP4D2_DROME (sp|Q27589) Cyt P450 4d2 OS=D.m.	CP4D2_DROME	0	GLOS_CP4D2.1.1	GO:0016705	M	InterPro	oxidoreductase activity, acting on paired donors, with incorporation or reduction of molecular oxygen
[BBH] DDX3_DROME (sp|Q9VHP0) ATP-dependent RNA helicase bel OS=D.m.	DDX3_DROME	1,00E-08	GLOS_DDX3.1.2	GO:0005811	C	FlyBase	lipid particle
[BBH] DDX3_DROME (sp|Q9VHP0) ATP-dependent RNA helicase bel OS=D.m.	DDX3_DROME	1,00E-08	GLOS_DDX3.1.2	GO:0005875	C	FlyBase	microtubule associated complex
[BBH] DDX3_DROME (sp|Q9VHP0) ATP-dependent RNA helicase bel OS=D.m.	DDX3_DROME	1,00E-08	GLOS_DDX3.1.2	GO:0044297	C	.	cell body
[BBH] DDX3_DROME (sp|Q9VHP0) ATP-dependent RNA helicase bel OS=D.m.	DDX3_DROME	1,00E-08	GLOS_DDX3.1.2	GO:0097458	C	.	neuron part

[BBH] DDX3_DROME (sp|Q9VHP0) ATP-dependent RNA helicase bel OS=D.m.	DDX3_DROME	1,00E-08	GLOS_DDX3.1.2	GO:0043025	C	FlyBase	neuronal cell body
[BBH] DDX3_DROME (sp|Q9VHP0) ATP-dependent RNA helicase bel OS=D.m.	DDX3_DROME	1,00E-08	GLOS_DDX3.1.2	GO:0035770	C	.	ribonucleoprotein granule
[BBH] DDX3_DROME (sp|Q9VHP0) ATP-dependent RNA helicase bel OS=D.m.	DDX3_DROME	1,00E-08	GLOS_DDX3.1.2	GO:0030529	C	.	ribonucleoprotein complex
[BBH] DDX3_DROME (sp|Q9VHP0) ATP-dependent RNA helicase bel OS=D.m.	DDX3_DROME	1,00E-08	GLOS_DDX3.1.2	GO:0060293	C	.	germ plasm
[BBH] DDX3_DROME (sp|Q9VHP0) ATP-dependent RNA helicase bel OS=D.m.	DDX3_DROME	1,00E-08	GLOS_DDX3.1.2	GO:0045495	C	.	pole plasm
[BBH] DDX3_DROME (sp|Q9VHP0) ATP-dependent RNA helicase bel OS=D.m.	DDX3_DROME	1,00E-08	GLOS_DDX3.1.2	GO:0043186	C	UniProtKB	P granule
[BBH] DDX3_DROME (sp|Q9VHP0) ATP-dependent RNA helicase bel OS=D.m.	DDX3_DROME	1,00E-08	GLOS_DDX3.1.2	GO:0031332	C	.	RNAi effector complex
[BBH] DDX3_DROME (sp|Q9VHP0) ATP-dependent RNA helicase bel OS=D.m.	DDX3_DROME	1,00E-08	GLOS_DDX3.1.2	GO:0016442	C	UniProtKB	RISC complex
[BBH] DDX3_DROME (sp|Q9VHP0) ATP-dependent RNA helicase bel OS=D.m.	DDX3_DROME	1,00E-08	GLOS_DDX3.1.2	GO:0005524	M	UniProtKB-k	ATP binding
[BBH] DDX3_DROME (sp|Q9VHP0) ATP-dependent RNA helicase bel OS=D.m.	DDX3_DROME	1,00E-08	GLOS_DDX3.1.2	GO:0003724	M	.	RNA helicase activity
[BBH] DDX3_DROME (sp|Q9VHP0) ATP-dependent RNA helicase bel OS=D.m.	DDX3_DROME	1,00E-08	GLOS_DDX3.1.2	GO:0004386	M	.	helicase activity
[BBH] DDX3_DROME (sp|Q9VHP0) ATP-dependent RNA helicase bel OS=D.m.	DDX3_DROME	1,00E-08	GLOS_DDX3.1.2	GO:0017111	M	.	nucleoside-triphosphatase activity
[BBH] DDX3_DROME (sp|Q9VHP0) ATP-dependent RNA helicase bel OS=D.m.	DDX3_DROME	1,00E-08	GLOS_DDX3.1.2	GO:0016462	M	.	pyrophosphatase activity
[BBH] DDX3_DROME (sp|Q9VHP0) ATP-dependent RNA helicase bel OS=D.m.	DDX3_DROME	1,00E-08	GLOS_DDX3.1.2	GO:0016818	M	.	hydrolase activity, acting on acid anhydrides, in phosphorus-containing anhydrides
[BBH] DDX3_DROME (sp|Q9VHP0) ATP-dependent RNA helicase bel OS=D.m.	DDX3_DROME	1,00E-08	GLOS_DDX3.1.2	GO:0016817	M	.	hydrolase activity, acting on acid anhydrides
[BBH] DDX3_DROME (sp|Q9VHP0) ATP-dependent RNA helicase bel OS=D.m.	DDX3_DROME	1,00E-08	GLOS_DDX3.1.2	GO:0008026	M	.	ATP-dependent helicase activity
[BBH] DDX3_DROME (sp|Q9VHP0) ATP-dependent RNA helicase bel OS=D.m.	DDX3_DROME	1,00E-08	GLOS_DDX3.1.2	GO:0042623	M	.	ATPase activity, coupled
[BBH] DDX3_DROME (sp|Q9VHP0) ATP-dependent RNA helicase bel OS=D.m.	DDX3_DROME	1,00E-08	GLOS_DDX3.1.2	GO:0016887	M	.	ATPase activity
[BBH] DDX3_DROME (sp|Q9VHP0) ATP-dependent RNA helicase bel OS=D.m.	DDX3_DROME	1,00E-08	GLOS_DDX3.1.2	GO:0070035	M	.	purine NTP-dependent helicase activity
[BBH] DDX3_DROME (sp|Q9VHP0) ATP-dependent RNA helicase bel OS=D.m.	DDX3_DROME	1,00E-08	GLOS_DDX3.1.2	GO:0008186	M	.	RNA-dependent ATPase activity
[BBH] DDX3_DROME (sp|Q9VHP0) ATP-dependent RNA helicase bel OS=D.m.	DDX3_DROME	1,00E-08	GLOS_DDX3.1.2	GO:0004004	M	UniProtKB	ATP-dependent RNA helicase activity
[BBH] DDX3_DROME (sp|Q9VHP0) ATP-dependent RNA helicase bel OS=D.m.	DDX3_DROME	1,00E-08	GLOS_DDX3.1.2	GO:0003723	M	UniProtKB-k	RNA binding
[BBH] DDX3_DROME (sp|Q9VHP0) ATP-dependent RNA helicase bel OS=D.m.	DDX3_DROME	1,00E-08	GLOS_DDX3.1.2	GO:0002164	B	.	larval development
[BBH] DDX3_DROME (sp|Q9VHP0) ATP-dependent RNA helicase bel OS=D.m.	DDX3_DROME	1,00E-08	GLOS_DDX3.1.2	GO:0002168	B	FlyBase	instar larval development
[BBH] DDX3_DROME (sp|Q9VHP0) ATP-dependent RNA helicase bel OS=D.m.	DDX3_DROME	1,00E-08	GLOS_DDX3.1.2	GO:0000819	B	.	sister chromatid segregation
[BBH] DDX3_DROME (sp|Q9VHP0) ATP-dependent RNA helicase bel OS=D.m.	DDX3_DROME	1,00E-08	GLOS_DDX3.1.2	GO:0007059	B	.	chromosome segregation
[BBH] DDX3_DROME (sp|Q9VHP0) ATP-dependent RNA helicase bel OS=D.m.	DDX3_DROME	1,00E-08	GLOS_DDX3.1.2	GO:0051276	B	.	chromosome organization
[BBH] DDX3_DROME (sp|Q9VHP0) ATP-dependent RNA helicase bel OS=D.m.	DDX3_DROME	1,00E-08	GLOS_DDX3.1.2	GO:0007067	B	.	mitosis
[BBH] DDX3_DROME (sp|Q9VHP0) ATP-dependent RNA helicase bel OS=D.m.	DDX3_DROME	1,00E-08	GLOS_DDX3.1.2	GO:0000280	B	.	nuclear division
[BBH] DDX3_DROME (sp|Q9VHP0) ATP-dependent RNA helicase bel OS=D.m.	DDX3_DROME	1,00E-08	GLOS_DDX3.1.2	GO:0048285	B	.	organelle fission
[BBH] DDX3_DROME (sp|Q9VHP0) ATP-dependent RNA helicase bel OS=D.m.	DDX3_DROME	1,00E-08	GLOS_DDX3.1.2	GO:0000070	B	FlyBase	mitotic sister chromatid segregation
[BBH] DDX3_DROME (sp|Q9VHP0) ATP-dependent RNA helicase bel OS=D.m.	DDX3_DROME	1,00E-08	GLOS_DDX3.1.2	GO:0048477	B	FlyBase	oogenesis
[BBH] DDX3_DROME (sp|Q9VHP0) ATP-dependent RNA helicase bel OS=D.m.	DDX3_DROME	1,00E-08	GLOS_DDX3.1.2	GO:0035194	B	.	posttranscriptional gene silencing by RNA
[BBH] DDX3_DROME (sp|Q9VHP0) ATP-dependent RNA helicase bel OS=D.m.	DDX3_DROME	1,00E-08	GLOS_DDX3.1.2	GO:0016441	B	.	posttranscriptional gene silencing
[BBH] DDX3_DROME (sp|Q9VHP0) ATP-dependent RNA helicase bel OS=D.m.	DDX3_DROME	1,00E-08	GLOS_DDX3.1.2	GO:0010608	B	.	posttranscriptional regulation of gene expression
[BBH] DDX3_DROME (sp|Q9VHP0) ATP-dependent RNA helicase bel OS=D.m.	DDX3_DROME	1,00E-08	GLOS_DDX3.1.2	GO:0016458	B	.	gene silencing
[BBH] DDX3_DROME (sp|Q9VHP0) ATP-dependent RNA helicase bel OS=D.m.	DDX3_DROME	1,00E-08	GLOS_DDX3.1.2	GO:0040029	B	.	regulation of gene expression, epigenetic
[BBH] DDX3_DROME (sp|Q9VHP0) ATP-dependent RNA helicase bel OS=D.m.	DDX3_DROME	1,00E-08	GLOS_DDX3.1.2	GO:0031047	B	.	gene silencing by RNA
[BBH] DDX3_DROME (sp|Q9VHP0) ATP-dependent RNA helicase bel OS=D.m.	DDX3_DROME	1,00E-08	GLOS_DDX3.1.2	GO:0016246	B	UniProtKB	RNA interference
[BBH] DDX3_DROME (sp|Q9VHP0) ATP-dependent RNA helicase bel OS=D.m.	DDX3_DROME	1,00E-08	GLOS_DDX3.1.2	GO:0007286	B	FlyBase	spermatid development
[BBH] DKC1_DROME (sp|O44081) H/ACA ribonucleoprot complex subunit 4 D.m.	DKC1_DROME	0	GLOS_DKC1.1.1	GO:0005730	C	FlyBase	nucleolus
[BBH] DKC1_DROME (sp|O44081) H/ACA ribonucleoprot complex subunit 4 D.m.	DKC1_DROME	0	GLOS_DKC1.1.1	GO:0030529	C	UniProtKB-k	ribonucleoprotein complex
[BBH] DKC1_DROME (sp|O44081) H/ACA ribonucleoprot complex subunit 4 D.m.	DKC1_DROME	0	GLOS_DKC1.1.1	GO:0016866	M	.	intramolecular transferase activity
[BBH] DKC1_DROME (sp|O44081) H/ACA ribonucleoprot complex subunit 4 D.m.	DKC1_DROME	0	GLOS_DKC1.1.1	GO:0016853	M	.	isomerase activity
[BBH] DKC1_DROME (sp|O44081) H/ACA ribonucleoprot complex subunit 4 D.m.	DKC1_DROME	0	GLOS_DKC1.1.1	GO:0009982	M	InterPro	pseudouridine synthase activity
[BBH] DKC1_DROME (sp|O44081) H/ACA ribonucleoprot complex subunit 4 D.m.	DKC1_DROME	0	GLOS_DKC1.1.1	GO:0003723	M	UniProtKB-k	RNA binding
[BBH] DKC1_DROME (sp|O44081) H/ACA ribonucleoprot complex subunit 4 D.m.	DKC1_DROME	0	GLOS_DKC1.1.1	GO:0007281	B	FlyBase	germ cell development
[BBH] DKC1_DROME (sp|O44081) H/ACA ribonucleoprot complex subunit 4 D.m.	DKC1_DROME	0	GLOS_DKC1.1.1	GO:0022008	B	FlyBase	neurogenesis
[BBH] DKC1_DROME (sp|O44081) H/ACA ribonucleoprot complex subunit 4 D.m.	DKC1_DROME	0	GLOS_DKC1.1.1	GO:0009451	B	.	RNA modification
[BBH] DKC1_DROME (sp|O44081) H/ACA ribonucleoprot complex subunit 4 D.m.	DKC1_DROME	0	GLOS_DKC1.1.1	GO:0001522	B	FlyBase	pseudouridine synthesis
[BBH] DKC1_DROME (sp|O44081) H/ACA ribonucleoprot complex subunit 4 D.m.	DKC1_DROME	0	GLOS_DKC1.1.1	GO:0016072	B	.	rRNA metabolic process
[BBH] DKC1_DROME (sp|O44081) H/ACA ribonucleoprot complex subunit 4 D.m.	DKC1_DROME	0	GLOS_DKC1.1.1	GO:0034660	B	.	ncRNA metabolic process
[BBH] DKC1_DROME (sp|O44081) H/ACA ribonucleoprot complex subunit 4 D.m.	DKC1_DROME	0	GLOS_DKC1.1.1	GO:0034470	B	.	ncRNA processing
[BBH] DKC1_DROME (sp|O44081) H/ACA ribonucleoprot complex subunit 4 D.m.	DKC1_DROME	0	GLOS_DKC1.1.1	GO:0006396	B	.	RNA processing

[BBH] DKC1_DROME (sp|O44081) H/ACA ribonucleoprot complex subunit 4 D.m.	DKC1_DROME	0	GLOS_DKC1.1.1	GO:0042254	B	.	ribosome biogenesis
[BBH] DKC1_DROME (sp|O44081) H/ACA ribonucleoprot complex subunit 4 D.m.	DKC1_DROME	0	GLOS_DKC1.1.1	GO:0022613	B	.	ribonucleoprotein complex biogenesis
[BBH] DKC1_DROME (sp|O44081) H/ACA ribonucleoprot complex subunit 4 D.m.	DKC1_DROME	0	GLOS_DKC1.1.1	GO:0006364	B	FlyBase	rRNA processing
[BBH] DKC1_DROME (sp|O44081) H/ACA ribonucleoprot complex subunit 4 D.m.	DKC1_DROME	0	GLOS_DKC1.1.1	GO:0007444	B	.	imaginal disc development
[BBH] DKC1_DROME (sp|O44081) H/ACA ribonucleoprot complex subunit 4 D.m.	DKC1_DROME	0	GLOS_DKC1.1.1	GO:0035220	B	FlyBase	wing disc development
[BBH] DYLT_DROME (sp|Q94524) Dynein light chain Tctex-type OS=D.m.	DYLT_DROME	1,00E-73	GLOS_DYLT.1.1	GO:0005737	C	UniProtKB-K	cytoplasm
[BBH] DYLT_DROME (sp|Q94524) Dynein light chain Tctex-type OS=D.m.	DYLT_DROME	1,00E-73	GLOS_DYLT.1.1	GO:0005875	C	.	microtubule associated complex
[BBH] DYLT_DROME (sp|Q94524) Dynein light chain Tctex-type OS=D.m.	DYLT_DROME	1,00E-73	GLOS_DYLT.1.1	GO:0030286	C	FlyBase	dynein complex
[BBH] DYLT_DROME (sp|Q94524) Dynein light chain Tctex-type OS=D.m.	DYLT_DROME	1,00E-73	GLOS_DYLT.1.1	GO:0005874	C	UniProtKB-K	microtubule
[BBH] DYLT_DROME (sp|Q94524) Dynein light chain Tctex-type OS=D.m.	DYLT_DROME	1,00E-73	GLOS_DYLT.1.1	GO:0042623	M	FlyBase	ATPase activity, coupled
[BBH] DYLT_DROME (sp|Q94524) Dynein light chain Tctex-type OS=D.m.	DYLT_DROME	1,00E-73	GLOS_DYLT.1.1	GO:0003774	M	UniProtKB-K	motor activity
[BBH] DYLT_DROME (sp|Q94524) Dynein light chain Tctex-type OS=D.m.	DYLT_DROME	1,00E-73	GLOS_DYLT.1.1	GO:0042802	M	.	identical protein binding
[BBH] DYLT_DROME (sp|Q94524) Dynein light chain Tctex-type OS=D.m.	DYLT_DROME	1,00E-73	GLOS_DYLT.1.1	GO:0046983	M	.	protein dimerization activity
[BBH] DYLT_DROME (sp|Q94524) Dynein light chain Tctex-type OS=D.m.	DYLT_DROME	1,00E-73	GLOS_DYLT.1.1	GO:0042803	M	FlyBase	protein homodimerization activity
[BBH] DYLT_DROME (sp|Q94524) Dynein light chain Tctex-type OS=D.m.	DYLT_DROME	1,00E-73	GLOS_DYLT.1.1	GO:0010259	B	.	multicellular organismal aging
[BBH] DYLT_DROME (sp|Q94524) Dynein light chain Tctex-type OS=D.m.	DYLT_DROME	1,00E-73	GLOS_DYLT.1.1	GO:0007568	B	.	aging
[BBH] DYLT_DROME (sp|Q94524) Dynein light chain Tctex-type OS=D.m.	DYLT_DROME	1,00E-73	GLOS_DYLT.1.1	GO:0008340	B	FlyBase	determination of adult lifespan
[BBH] DYLT_DROME (sp|Q94524) Dynein light chain Tctex-type OS=D.m.	DYLT_DROME	1,00E-73	GLOS_DYLT.1.1	GO:0007018	B	FlyBase	microtubule-based movement
[BBH] DYLT_DROME (sp|Q94524) Dynein light chain Tctex-type OS=D.m.	DYLT_DROME	1,00E-73	GLOS_DYLT.1.1	GO:0007067	B	FlyBase	mitosis
[BBH] DYLT_DROME (sp|Q94524) Dynein light chain Tctex-type OS=D.m.	DYLT_DROME	1,00E-73	GLOS_DYLT.1.1	GO:0051272	B	.	positive regulation of cellular component movement
[BBH] DYLT_DROME (sp|Q94524) Dynein light chain Tctex-type OS=D.m.	DYLT_DROME	1,00E-73	GLOS_DYLT.1.1	GO:0051270	B	.	regulation of cellular component movement
[BBH] DYLT_DROME (sp|Q94524) Dynein light chain Tctex-type OS=D.m.	DYLT_DROME	1,00E-73	GLOS_DYLT.1.1	GO:2001017	B	.	regulation of retrograde axon cargo transport
[BBH] DYLT_DROME (sp|Q94524) Dynein light chain Tctex-type OS=D.m.	DYLT_DROME	1,00E-73	GLOS_DYLT.1.1	GO:0060632	B	.	regulation of microtubule-based movement
[BBH] DYLT_DROME (sp|Q94524) Dynein light chain Tctex-type OS=D.m.	DYLT_DROME	1,00E-73	GLOS_DYLT.1.1	GO:0032886	B	.	regulation of microtubule-based process
[BBH] DYLT_DROME (sp|Q94524) Dynein light chain Tctex-type OS=D.m.	DYLT_DROME	1,00E-73	GLOS_DYLT.1.1	GO:2001019	B	FlyBase	positive regulation of retrograde axon cargo transport
[BBH] DYLT_DROME (sp|Q94524) Dynein light chain Tctex-type OS=D.m.	DYLT_DROME	1,00E-73	GLOS_DYLT.1.1	GO:0007286	B	FlyBase	spermatid development
[BBH] EF1G_DROME (sp|Q9NJH0) Elong facr 1-gamma D.m. GN=Ef1gamma	EF1G_DROME	1,00E-170	GLOS_EF1G.2.2	GO:0005853	C	FlyBase	eukaryotic translation elongation factor 1 complex
[BBH] EF1G_DROME (sp|Q9NJH0) Elong facr 1-gamma D.m. GN=Ef1gamma	EF1G_DROME	1,00E-170	GLOS_EF1G.2.2	GO:0005811	C	FlyBase	lipid particle
[BBH] EF1G_DROME (sp|Q9NJH0) Elong facr 1-gamma D.m. GN=Ef1gamma	EF1G_DROME	1,00E-170	GLOS_EF1G.2.2	GO:0005875	C	FlyBase	microtubule associated complex
[BBH] EF1G_DROME (sp|Q9NJH0) Elong facr 1-gamma D.m. GN=Ef1gamma	EF1G_DROME	1,00E-170	GLOS_EF1G.2.2	GO:0005634	C	FlyBase	nucleus
[BBH] EF1G_DROME (sp|Q9NJH0) Elong facr 1-gamma D.m. GN=Ef1gamma	EF1G_DROME	1,00E-170	GLOS_EF1G.2.2	GO:0008135	M	.	translation factor activity, nucleic acid binding
[BBH] EF1G_DROME (sp|Q9NJH0) Elong facr 1-gamma D.m. GN=Ef1gamma	EF1G_DROME	1,00E-170	GLOS_EF1G.2.2	GO:0003723	M	.	RNA binding
[BBH] EF1G_DROME (sp|Q9NJH0) Elong facr 1-gamma D.m. GN=Ef1gamma	EF1G_DROME	1,00E-170	GLOS_EF1G.2.2	GO:0003746	M	FlyBase	translation elongation factor activity
[BBH] EF1G_DROME (sp|Q9NJH0) Elong facr 1-gamma D.m. GN=Ef1gamma	EF1G_DROME	1,00E-170	GLOS_EF1G.2.2	GO:0035071	B	FlyBase	salivary gland cell autophagic cell death
[BBH] EIF3E_DROMO (sp|B4KY00) Eukaryotic translation initiation factor 3 subunit E OS=D. mojavensis	EIF3E_DROMO	0	GLOS_EIF3E.1.1	GO:0070993	C	.	translation preinitiation complex
[BBH] EIF3E_DROMO (sp|B4KY00) Eukar. translation initia. … Same as above	EIF3E_DROMO	0	GLOS_EIF3E.1.1	GO:0016282	C	UniProtKB-H	eukaryotic 43S preinitiation complex
[BBH] EIF3E_DROMO (sp|B4KY00) Eukar. translation initia. … Same as above	EIF3E_DROMO	0	GLOS_EIF3E.1.1	GO:0033290	C	UniProtKB-H	eukaryotic 48S preinitiation complex
[BBH] EIF3E_DROMO (sp|B4KY00) Eukar. translation initia. … Same as above	EIF3E_DROMO	0	GLOS_EIF3E.1.1	GO:0005852	C	UniProtKB-H	eukaryotic translation initiation factor 3 complex
[BBH] EIF3E_DROMO (sp|B4KY00) Eukar. translation initia. … Same as above	EIF3E_DROMO	0	GLOS_EIF3E.1.1	GO:0003743	M	UniProtKB-H	translation initiation factor activity
[BBH] EIF3E_DROMO (sp|B4KY00) Eukar. translation initia. … Same as above	EIF3E_DROMO	0	GLOS_EIF3E.1.1	GO:0022618	B	.	ribonucleoprotein complex assembly
[BBH] EIF3E_DROMO (sp|B4KY00) Eukar. translation initia. … Same as above	EIF3E_DROMO	0	GLOS_EIF3E.1.1	GO:0034622	B	.	cellular macromolecular complex assembly
[BBH] EIF3E_DROMO (sp|B4KY00) Eukar. translation initia. … Same as above	EIF3E_DROMO	0	GLOS_EIF3E.1.1	GO:0071826	B	.	ribonucleoprotein complex subunit organization
[BBH] EIF3E_DROMO (sp|B4KY00) Eukar. translation initia. … Same as above	EIF3E_DROMO	0	GLOS_EIF3E.1.1	GO:0006413	B	.	translational initiation
[BBH] EIF3E_DROMO (sp|B4KY00) Eukar. translation initia. … Same as above	EIF3E_DROMO	0	GLOS_EIF3E.1.1	GO:0006412	B	.	translation
[BBH] EIF3E_DROMO (sp|B4KY00) Eukar. translation initia. … Same as above	EIF3E_DROMO	0	GLOS_EIF3E.1.1	GO:0001731	B	UniProtKB-H	formation of translation preinitiation complex
[BBH] EIF3E_DROMO (sp|B4KY00) Eukar. translation initia. … Same as above	EIF3E_DROMO	0	GLOS_EIF3E.1.1	GO:0006417	B	.	regulation of translation
[BBH] EIF3E_DROMO (sp|B4KY00) Eukar. translation initia. … Same as above	EIF3E_DROMO	0	GLOS_EIF3E.1.1	GO:0006446	B	UniProtKB-H	regulation of translational initiation
[BBH] ERD2_DROME (sp|O76767) ER lumen prot retaining recept D.m.	ERD2_DROME	1,00E-125	GLOS_ERD2.1.1	GO:0005789	C	UniProtKB-S	endoplasmic reticulum membrane
[BBH] ERD2_DROME (sp|O76767) ER lumen prot retaining recept D.m.	ERD2_DROME	1,00E-125	GLOS_ERD2.1.1	GO:0016021	C	UniProtKB-K	integral to membrane
[BBH] ERD2_DROME (sp|O76767) ER lumen prot retaining recept D.m.	ERD2_DROME	1,00E-125	GLOS_ERD2.1.1	GO:0005798	C	.	Golgi-associated vesicle
[BBH] ERD2_DROME (sp|O76767) ER lumen prot retaining recept D.m.	ERD2_DROME	1,00E-125	GLOS_ERD2.1.1	GO:0044431	C	.	Golgi apparatus part
[BBH] ERD2_DROME (sp|O76767) ER lumen prot retaining recept D.m.	ERD2_DROME	1,00E-125	GLOS_ERD2.1.1	GO:0005794	C	.	Golgi apparatus
[BBH] ERD2_DROME (sp|O76767) ER lumen prot retaining recept D.m.	ERD2_DROME	1,00E-125	GLOS_ERD2.1.1	GO:0030133	C	.	transport vesicle

[BBH] ERD2_DROME (sp|O76767) ER lumen prot retaining recept D.m.	ERD2_DROME	1,00E-125	GLOS_ERD2.1.1	GO:0030136	C	.	clathrin-coated vesicle
[BBH] ERD2_DROME (sp|O76767) ER lumen prot retaining recept D.m.	ERD2_DROME	1,00E-125	GLOS_ERD2.1.1	GO:0030135	C	.	coated vesicle
[BBH] ERD2_DROME (sp|O76767) ER lumen prot retaining recept D.m.	ERD2_DROME	1,00E-125	GLOS_ERD2.1.1	GO:0030140	C	FlyBase	trans-Golgi network transport vesicle
[BBH] ERD2_DROME (sp|O76767) ER lumen prot retaining recept D.m.	ERD2_DROME	1,00E-125	GLOS_ERD2.1.1	GO:0046923	M	.	ER retention sequence binding
[BBH] ERD2_DROME (sp|O76767) ER lumen prot retaining recept D.m.	ERD2_DROME	1,00E-125	GLOS_ERD2.1.1	GO:0005048	M	.	signal sequence binding
[BBH] ERD2_DROME (sp|O76767) ER lumen prot retaining recept D.m.	ERD2_DROME	1,00E-125	GLOS_ERD2.1.1	GO:0042277	M	.	peptide binding
[BBH] ERD2_DROME (sp|O76767) ER lumen prot retaining recept D.m.	ERD2_DROME	1,00E-125	GLOS_ERD2.1.1	GO:0033218	M	.	amide binding
[BBH] ERD2_DROME (sp|O76767) ER lumen prot retaining recept D.m.	ERD2_DROME	1,00E-125	GLOS_ERD2.1.1	GO:0005046	M	FlyBase	KDEL sequence binding
[BBH] ERD2_DROME (sp|O76767) ER lumen prot retaining recept D.m.	ERD2_DROME	1,00E-125	GLOS_ERD2.1.1	GO:0035437	B	.	maintenance of protein localization in endoplasmic reticulum
[BBH] ERD2_DROME (sp|O76767) ER lumen prot retaining recept D.m.	ERD2_DROME	1,00E-125	GLOS_ERD2.1.1	GO:0072595	B	.	maintenance of protein localization in organelle
[BBH] ERD2_DROME (sp|O76767) ER lumen prot retaining recept D.m.	ERD2_DROME	1,00E-125	GLOS_ERD2.1.1	GO:0032507	B	.	maintenance of protein location in cell
[BBH] ERD2_DROME (sp|O76767) ER lumen prot retaining recept D.m.	ERD2_DROME	1,00E-125	GLOS_ERD2.1.1	GO:0045185	B	.	maintenance of protein location
[BBH] ERD2_DROME (sp|O76767) ER lumen prot retaining recept D.m.	ERD2_DROME	1,00E-125	GLOS_ERD2.1.1	GO:0051235	B	.	maintenance of location
[BBH] ERD2_DROME (sp|O76767) ER lumen prot retaining recept D.m.	ERD2_DROME	1,00E-125	GLOS_ERD2.1.1	GO:0051651	B	.	maintenance of location in cell
[BBH] ERD2_DROME (sp|O76767) ER lumen prot retaining recept D.m.	ERD2_DROME	1,00E-125	GLOS_ERD2.1.1	GO:0070972	B	.	protein localization to endoplasmic reticulum
[BBH] ERD2_DROME (sp|O76767) ER lumen prot retaining recept D.m.	ERD2_DROME	1,00E-125	GLOS_ERD2.1.1	GO:0006621	B	InterPro	protein retention in ER lumen
[BBH] ERD2_DROME (sp|O76767) ER lumen prot retaining recept D.m.	ERD2_DROME	1,00E-125	GLOS_ERD2.1.1	GO:0015031	B	UniProtKB-k	protein transport
[BBH] ERD2_DROME (sp|O76767) ER lumen prot retaining recept D.m.	ERD2_DROME	1,00E-125	GLOS_ERD2.1.1	GO:0006890	B	FlyBase	retrograde vesicle-mediated transport, Golgi to ER
GRAU_DROME (sp|Q9U405) Transcription factor grauzone OS=D.m.	GRAU_DROME	5,00E-52	GLOS_GRAU.3.4	GO:0005634	C	UniProtKB	nucleus
GRAU_DROME (sp|Q9U405) Transcription factor grauzone OS=D.m.	GRAU_DROME	5,00E-52	GLOS_GRAU.3.4	GO:0000976	M	.	transcription regulatory region sequence-specific DNA binding
GRAU_DROME (sp|Q9U405) Transcription factor grauzone OS=D.m.	GRAU_DROME	5,00E-52	GLOS_GRAU.3.4	GO:0043565	M	.	sequence-specific DNA binding
GRAU_DROME (sp|Q9U405) Transcription factor grauzone OS=D.m.	GRAU_DROME	5,00E-52	GLOS_GRAU.3.4	GO:0044212	M	.	transcription regulatory region DNA binding
GRAU_DROME (sp|Q9U405) Transcription factor grauzone OS=D.m.	GRAU_DROME	5,00E-52	GLOS_GRAU.3.4	GO:0000975	M	.	regulatory region DNA binding
GRAU_DROME (sp|Q9U405) Transcription factor grauzone OS=D.m.	GRAU_DROME	5,00E-52	GLOS_GRAU.3.4	GO:0001067	M	.	regulatory region nucleic acid binding
GRAU_DROME (sp|Q9U405) Transcription factor grauzone OS=D.m.	GRAU_DROME	5,00E-52	GLOS_GRAU.3.4	GO:0001159	M	.	core promoter proximal region DNA binding
GRAU_DROME (sp|Q9U405) Transcription factor grauzone OS=D.m.	GRAU_DROME	5,00E-52	GLOS_GRAU.3.4	GO:0000987	M	FlyBase	core promoter proximal region sequence-specific DNA binding
GRAU_DROME (sp|Q9U405) Transcription factor grauzone OS=D.m.	GRAU_DROME	5,00E-52	GLOS_GRAU.3.4	GO:0008270	M	InterPro	zinc ion binding
GRAU_DROME (sp|Q9U405) Transcription factor grauzone OS=D.m.	GRAU_DROME	5,00E-52	GLOS_GRAU.3.4	GO:0007338	B	.	single fertilization
GRAU_DROME (sp|Q9U405) Transcription factor grauzone OS=D.m.	GRAU_DROME	5,00E-52	GLOS_GRAU.3.4	GO:0009566	B	.	fertilization
GRAU_DROME (sp|Q9U405) Transcription factor grauzone OS=D.m.	GRAU_DROME	5,00E-52	GLOS_GRAU.3.4	GO:0007343	B	FlyBase	egg activation
GRAU_DROME (sp|Q9U405) Transcription factor grauzone OS=D.m.	GRAU_DROME	5,00E-52	GLOS_GRAU.3.4	GO:0007126	B	.	meiosis
GRAU_DROME (sp|Q9U405) Transcription factor grauzone OS=D.m.	GRAU_DROME	5,00E-52	GLOS_GRAU.3.4	GO:0051321	B	.	meiotic cell cycle
GRAU_DROME (sp|Q9U405) Transcription factor grauzone OS=D.m.	GRAU_DROME	5,00E-52	GLOS_GRAU.3.4	GO:0007143	B	UniProtKB	female meiosis
GRAU_DROME (sp|Q9U405) Transcription factor grauzone OS=D.m.	GRAU_DROME	5,00E-52	GLOS_GRAU.3.4	GO:0045132	B	.	meiotic chromosome segregation
GRAU_DROME (sp|Q9U405) Transcription factor grauzone OS=D.m.	GRAU_DROME	5,00E-52	GLOS_GRAU.3.4	GO:0007127	B	.	meiosis I
GRAU_DROME (sp|Q9U405) Transcription factor grauzone OS=D.m.	GRAU_DROME	5,00E-52	GLOS_GRAU.3.4	GO:0045143	B	FlyBase	homologous chromosome segregation
GRAU_DROME (sp|Q9U405) Transcription factor grauzone OS=D.m.	GRAU_DROME	5,00E-52	GLOS_GRAU.3.4	GO:0007277	B	.	pole cell development
GRAU_DROME (sp|Q9U405) Transcription factor grauzone OS=D.m.	GRAU_DROME	5,00E-52	GLOS_GRAU.3.4	GO:0007279	B	FlyBase	pole cell formation
GRAU_DROME (sp|Q9U405) Transcription factor grauzone OS=D.m.	GRAU_DROME	5,00E-52	GLOS_GRAU.3.4	GO:0045944	B	FlyBase	positive regulation of transcription from RNA polymerase II promoter
GRAU_DROME (sp|Q9U405) Transcription factor grauzone OS=D.m.	GRAU_DROME	5,00E-52	GLOS_GRAU.3.4	GO:0006366	B	UniProtKB	transcription from RNA polymerase II promoter
GRAU_DROME (sp|Q9U405) Transcription factor grauzone OS=D.m.	GST_MUSDO	1,00E-134	GLOS_GST.1.1	GO:0016765	M	.	transferase activity, transferring alkyl or aryl (other than methyl) groups
GRAU_DROME (sp|Q9U405) Transcription factor grauzone OS=D.m.	GST_MUSDO	1,00E-134	GLOS_GST.1.1	GO:0004364	M	UniProtKB-E	glutathione transferase activity
[BBH] GUS_DROME (sp|A1Z6E0) Protein gustavus OS=D.m. GN=gus PE=1 SV=1	GUS_DROME	0	GLOS_GUS.1.1	GO:0005938	C	UniProtKB	cell cortex
[BBH] GUS_DROME (sp|A1Z6E0) Protein gustavus OS=D.m. GN=gus PE=1 SV=1	GUS_DROME	0	GLOS_GUS.1.1	GO:0008023	C	.	transcription elongation factor complex
[BBH] GUS_DROME (sp|A1Z6E0) Protein gustavus OS=D.m. GN=gus PE=1 SV=1	GUS_DROME	0	GLOS_GUS.1.1	GO:0070449	C	UniProtKB	elongin complex
[BBH] GUS_DROME (sp|A1Z6E0) Protein gustavus OS=D.m. GN=gus PE=1 SV=1	GUS_DROME	0	GLOS_GUS.1.1	GO:0048471	C	UniProtKB	perinuclear region of cytoplasm
[BBH] GUS_DROME (sp|A1Z6E0) Protein gustavus OS=D.m. GN=gus PE=1 SV=1	GUS_DROME	0	GLOS_GUS.1.1	GO:0045495	C	UniProtKB	pole plasm
[BBH] GUS_DROME (sp|A1Z6E0) Protein gustavus OS=D.m. GN=gus PE=1 SV=1	GUS_DROME	0	GLOS_GUS.1.1	GO:0042335	B	.	cuticle development
[BBH] GUS_DROME (sp|A1Z6E0) Protein gustavus OS=D.m. GN=gus PE=1 SV=1	GUS_DROME	0	GLOS_GUS.1.1	GO:0035017	B	UniProtKB	cuticle pattern formation
[BBH] GUS_DROME (sp|A1Z6E0) Protein gustavus OS=D.m. GN=gus PE=1 SV=1	GUS_DROME	0	GLOS_GUS.1.1	GO:0046843	B	FlyBase	dorsal appendage formation
[BBH] GUS_DROME (sp|A1Z6E0) Protein gustavus OS=D.m. GN=gus PE=1 SV=1	GUS_DROME	0	GLOS_GUS.1.1	GO:0035556	B	InterPro	intracellular signal transduction
[BBH] GUS_DROME (sp|A1Z6E0) Protein gustavus OS=D.m. GN=gus PE=1 SV=1	GUS_DROME	0	GLOS_GUS.1.1	GO:0008354	B	.	germ cell migration
[BBH] GUS_DROME (sp|A1Z6E0) Protein gustavus OS=D.m. GN=gus PE=1 SV=1	GUS_DROME	0	GLOS_GUS.1.1	GO:0016477	B	.	cell migration
[BBH] GUS_DROME (sp|A1Z6E0) Protein gustavus OS=D.m. GN=gus PE=1 SV=1	GUS_DROME	0	GLOS_GUS.1.1	GO:0048870	B	.	cell motility

[BBH] GUS_DROME (sp|A1Z6E0) Protein gustavus OS=D.m. GN=gus PE=1 SV=1	GUS_DROME	0	GLOS_GUS.1.1	GO:0051674	B	.	localization of cell
[BBH] GUS_DROME (sp|A1Z6E0) Protein gustavus OS=D.m. GN=gus PE=1 SV=1	GUS_DROME	0	GLOS_GUS.1.1	GO:0007280	B	UniProtKB	pole cell migration
[BBH] GUS_DROME (sp|A1Z6E0) Protein gustavus OS=D.m. GN=gus PE=1 SV=1	GUS_DROME	0	GLOS_GUS.1.1	GO:0007315	B	UniProtKB	pole plasm assembly
[BBH] GUS_DROME (sp|A1Z6E0) Protein gustavus OS=D.m. GN=gus PE=1 SV=1	GUS_DROME	0	GLOS_GUS.1.1	GO:0008104	B	UniProtKB	protein localization
[BBH] GUS_DROME (sp|A1Z6E0) Protein gustavus OS=D.m. GN=gus PE=1 SV=1	GUS_DROME	0	GLOS_GUS.1.1	GO:0007560	B	.	imaginal disc morphogenesis
[BBH] GUS_DROME (sp|A1Z6E0) Protein gustavus OS=D.m. GN=gus PE=1 SV=1	GUS_DROME	0	GLOS_GUS.1.1	GO:0048563	B	.	post-embryonic organ morphogenesis
[BBH] GUS_DROME (sp|A1Z6E0) Protein gustavus OS=D.m. GN=gus PE=1 SV=1	GUS_DROME	0	GLOS_GUS.1.1	GO:0009887	B	.	organ morphogenesis
[BBH] GUS_DROME (sp|A1Z6E0) Protein gustavus OS=D.m. GN=gus PE=1 SV=1	GUS_DROME	0	GLOS_GUS.1.1	GO:0048569	B	.	post-embryonic organ development
[BBH] GUS_DROME (sp|A1Z6E0) Protein gustavus OS=D.m. GN=gus PE=1 SV=1	GUS_DROME	0	GLOS_GUS.1.1	GO:0035220	B	.	wing disc development
[BBH] GUS_DROME (sp|A1Z6E0) Protein gustavus OS=D.m. GN=gus PE=1 SV=1	GUS_DROME	0	GLOS_GUS.1.1	GO:0007472	B	UniProtKB	wing disc morphogenesis
LECA_SARPE (sp|P05047) Lectin subunit alpha OS=S. peregrina PE=1 SV=1	LECA_SARPE	1,00E-22	GLOS_LECA.6.13	GO:0030246	M	InterPro	carbohydrate binding
[BBH] MED23_DROME (sp|Q9W1X7)Mediator of RNA polymII transcrip subunit 23	MED23_DROME	0	GLOS_MED23.1.1	GO:0016592	C	UniProtKB	mediator complex
[BBH] MED23_DROME (sp|Q9W1X7)Mediator of RNA polymII transcrip subunit 23	MED23_DROME	0	GLOS_MED23.1.1	GO:0001104	M	UniProtKB	RNA polymerase II transcription cofactor activity
[BBH] MED23_DROME (sp|Q9W1X7)Mediator of RNA polymII transcrip subunit 23	MED23_DROME	0	GLOS_MED23.1.1	GO:0006367	B	FlyBase	transcription initiation from RNA polymerase II promoter
MOS1T_DROMA (sp|Q7JQ07) Mariner Mos1 transposase OS=D. maur.	MOS1T_DROMA	1,00E-52	GLOS_MOS1T.25.	GO:0005634	C	UniProtKB-S	nucleus
MOS1T_DROMA (sp|Q7JQ07) Mariner Mos1 transposase OS=D. maur.	MOS1T_DROMA	1,00E-52	GLOS_MOS1T.25.	GO:0003677	M	UniProtKB-K	DNA binding
MOS1T_DROMA (sp|Q7JQ07) Mariner Mos1 transposase OS=D. maur.	MOS1T_DROMA	1,00E-52	GLOS_MOS1T.25.	GO:0004518	M	.	nuclease activity
MOS1T_DROMA (sp|Q7JQ07) Mariner Mos1 transposase OS=D. maur.	MOS1T_DROMA	1,00E-52	GLOS_MOS1T.25.	GO:0016788	M	.	hydrolase activity, acting on ester bonds
MOS1T_DROMA (sp|Q7JQ07) Mariner Mos1 transposase OS=D. maur.	MOS1T_DROMA	1,00E-52	GLOS_MOS1T.25.	GO:0004519	M	UniProtKB-K	endonuclease activity
MOS1T_DROMA (sp|Q7JQ07) Mariner Mos1 transposase OS=D. maur.	MOS1T_DROMA	1,00E-52	GLOS_MOS1T.25.	GO:0046872	M	UniProtKB-K	metal ion binding
MOS1T_DROMA (sp|Q7JQ07) Mariner Mos1 transposase OS=D. maur.	MOS1T_DROMA	1,00E-52	GLOS_MOS1T.25.	GO:0006259	B	.	DNA metabolic process
MOS1T_DROMA (sp|Q7JQ07) Mariner Mos1 transposase OS=D. maur.	MOS1T_DROMA	1,00E-52	GLOS_MOS1T.25.	GO:0015074	B	UniProtKB-K	DNA integration
MOS1T_DROMA (sp|Q7JQ07) Mariner Mos1 transposase OS=D. maur.	MOS1T_DROMA	1,00E-52	GLOS_MOS1T.25.	GO:0006310	B	UniProtKB-K	DNA recombination
MOS1T_DROMA (sp|Q7JQ07) Mariner Mos1 transposase OS=D. maur.	MOS1T_DROMA	1,00E-52	GLOS_MOS1T.25.	GO:0090305	B	GOC	nucleic acid phosphodiester bond hydrolysis
MP20_DROME (sp|P14318) Muscle-specific protein 20 OS=D.m.	MP20_DROME	4,00E-71	GLOS_MP20.1.4	GO:0008092	M	.	cytoskeletal protein binding
MP20_DROME (sp|P14318) Muscle-specific protein 20 OS=D.m.	MP20_DROME	4,00E-71	GLOS_MP20.1.4	GO:0003779	M	FlyBase	actin binding
MP20_DROME (sp|P14318) Muscle-specific protein 20 OS=D.m.	MP20_DROME	4,00E-71	GLOS_MP20.1.4	GO:0007155	B	FlyBase	cell adhesion
MP20_DROME (sp|P14318) Muscle-specific protein 20 OS=D.m.	MP20_DROME	4,00E-71	GLOS_MP20.1.4	GO:0000768	B	.	syncytium formation by plasma membrane fusion
MP20_DROME (sp|P14318) Muscle-specific protein 20 OS=D.m.	MP20_DROME	4,00E-71	GLOS_MP20.1.4	GO:0006949	B	.	syncytium formation
MP20_DROME (sp|P14318) Muscle-specific protein 20 OS=D.m.	MP20_DROME	4,00E-71	GLOS_MP20.1.4	GO:0014902	B	.	myotube differentiation
MP20_DROME (sp|P14318) Muscle-specific protein 20 OS=D.m.	MP20_DROME	4,00E-71	GLOS_MP20.1.4	GO:0051146	B	.	striated muscle cell differentiation
MP20_DROME (sp|P14318) Muscle-specific protein 20 OS=D.m.	MP20_DROME	4,00E-71	GLOS_MP20.1.4	GO:0042692	B	.	muscle cell differentiation
MP20_DROME (sp|P14318) Muscle-specific protein 20 OS=D.m.	MP20_DROME	4,00E-71	GLOS_MP20.1.4	GO:0007520	B	FlyBase	myoblast fusion
MP20_DROME (sp|P14318) Muscle-specific protein 20 OS=D.m.	MP20_DROME	4,00E-71	GLOS_MP20.1.4	GO:0022604	B	.	regulation of cell morphogenesis
MP20_DROME (sp|P14318) Muscle-specific protein 20 OS=D.m.	MP20_DROME	4,00E-71	GLOS_MP20.1.4	GO:0022603	B	.	regulation of anatomical structure morphogenesis
MP20_DROME (sp|P14318) Muscle-specific protein 20 OS=D.m.	MP20_DROME	4,00E-71	GLOS_MP20.1.4	GO:0051128	B	.	regulation of cellular component organization
MP20_DROME (sp|P14318) Muscle-specific protein 20 OS=D.m.	MP20_DROME	4,00E-71	GLOS_MP20.1.4	GO:0008360	B	FlyBase	regulation of cell shape
[BBH] ORD_DROME (sp|Q24434) Protein ORD OS=D.m. GN=ord PE=1 SV=2	ORD_DROME	2,00E-17	GLOS_ORD.1.1	GO:0044427	C	.	chromosomal part
[BBH] ORD_DROME (sp|Q24434) Protein ORD OS=D.m. GN=ord PE=1 SV=2	ORD_DROME	2,00E-17	GLOS_ORD.1.1	GO:0005694	C	.	chromosome
[BBH] ORD_DROME (sp|Q24434) Protein ORD OS=D.m. GN=ord PE=1 SV=2	ORD_DROME	2,00E-17	GLOS_ORD.1.1	GO:0000775	C	UniProtKB-S	chromosome, centromeric region
[BBH] ORD_DROME (sp|Q24434) Protein ORD OS=D.m. GN=ord PE=1 SV=2	ORD_DROME	2,00E-17	GLOS_ORD.1.1	GO:0000793	C	.	condensed chromosome
[BBH] ORD_DROME (sp|Q24434) Protein ORD OS=D.m. GN=ord PE=1 SV=2	ORD_DROME	2,00E-17	GLOS_ORD.1.1	GO:0008278	C	UniProtKB	cohesin complex
[BBH] ORD_DROME (sp|Q24434) Protein ORD OS=D.m. GN=ord PE=1 SV=2	ORD_DROME	2,00E-17	GLOS_ORD.1.1	GO:0043073	C	.	germ cell nucleus
[BBH] ORD_DROME (sp|Q24434) Protein ORD OS=D.m. GN=ord PE=1 SV=2	ORD_DROME	2,00E-17	GLOS_ORD.1.1	GO:0001674	C	UniProtKB	female germ cell nucleus
[BBH] ORD_DROME (sp|Q24434) Protein ORD OS=D.m. GN=ord PE=1 SV=2	ORD_DROME	2,00E-17	GLOS_ORD.1.1	GO:0001673	C	UniProtKB	male germ cell nucleus
[BBH] ORD_DROME (sp|Q24434) Protein ORD OS=D.m. GN=ord PE=1 SV=2	ORD_DROME	2,00E-17	GLOS_ORD.1.1	GO:0051177	B	.	meiotic sister chromatid cohesion
[BBH] ORD_DROME (sp|Q24434) Protein ORD OS=D.m. GN=ord PE=1 SV=2	ORD_DROME	2,00E-17	GLOS_ORD.1.1	GO:0007062	B	.	sister chromatid cohesion
[BBH] ORD_DROME (sp|Q24434) Protein ORD OS=D.m. GN=ord PE=1 SV=2	ORD_DROME	2,00E-17	GLOS_ORD.1.1	GO:0070192	B	.	chromosome organization involved in meiosis
[BBH] ORD_DROME (sp|Q24434) Protein ORD OS=D.m. GN=ord PE=1 SV=2	ORD_DROME	2,00E-17	GLOS_ORD.1.1	GO:0007143	B	.	female meiosis
[BBH] ORD_DROME (sp|Q24434) Protein ORD OS=D.m. GN=ord PE=1 SV=2	ORD_DROME	2,00E-17	GLOS_ORD.1.1	GO:0007066	B	UniProtKB	female meiosis sister chromatid cohesion
[BBH] ORD_DROME (sp|Q24434) Protein ORD OS=D.m. GN=ord PE=1 SV=2	ORD_DROME	2,00E-17	GLOS_ORD.1.1	GO:0007276	B	FlyBase	gamete generation
[BBH] ORD_DROME (sp|Q24434) Protein ORD OS=D.m. GN=ord PE=1 SV=2	ORD_DROME	2,00E-17	GLOS_ORD.1.1	GO:0007140	B	.	male meiosis
[BBH] ORD_DROME (sp|Q24434) Protein ORD OS=D.m. GN=ord PE=1 SV=2	ORD_DROME	2,00E-17	GLOS_ORD.1.1	GO:0007060	B	FlyBase	male meiosis chromosome segregation
[BBH] ORD_DROME (sp|Q24434) Protein ORD OS=D.m. GN=ord PE=1 SV=2	ORD_DROME	2,00E-17	GLOS_ORD.1.1	GO:0007065	B	UniProtKB	male meiosis sister chromatid cohesion

[BBH] ORD_DROME (sp|Q24434) Protein ORD OS=D.m. GN=ord PE=1 SV=2	ORD_DROME	2,00E-17	GLOS_ORD.1.1	GO:0007067	B	FlyBase	mitosis
PGK_DROME (sp|Q01604) Phosphoglycerate kinase; D.m. GN=Pgk PE=2 SV=2	PGK_DROME	0	GLOS_PGK.2.3	GO:0031672	C	.	A band
PGK_DROME (sp|Q01604) Phosphoglycerate kinase; D.m. GN=Pgk PE=2 SV=2	PGK_DROME	0	GLOS_PGK.2.3	GO:0030017	C	.	sarcomere
PGK_DROME (sp|Q01604) Phosphoglycerate kinase; D.m. GN=Pgk PE=2 SV=2	PGK_DROME	0	GLOS_PGK.2.3	GO:0031430	C	FlyBase	M band
PGK_DROME (sp|Q01604) Phosphoglycerate kinase; D.m. GN=Pgk PE=2 SV=2	PGK_DROME	0	GLOS_PGK.2.3	GO:0031674	C	.	I band
PGK_DROME (sp|Q01604) Phosphoglycerate kinase; D.m. GN=Pgk PE=2 SV=2	PGK_DROME	0	GLOS_PGK.2.3	GO:0030018	C	FlyBase	Z disc
PGK_DROME (sp|Q01604) Phosphoglycerate kinase; D.m. GN=Pgk PE=2 SV=2	PGK_DROME	0	GLOS_PGK.2.3	GO:0005524	M	UniProtKB	ATP binding
PGK_DROME (sp|Q01604) Phosphoglycerate kinase; D.m. GN=Pgk PE=2 SV=2	PGK_DROME	0	GLOS_PGK.2.3	GO:0016301	M	.	kinase activity
PGK_DROME (sp|Q01604) Phosphoglycerate kinase; D.m. GN=Pgk PE=2 SV=2	PGK_DROME	0	GLOS_PGK.2.3	GO:0016772	M	.	transferase activity, transferring phosphorus-containing groups
PGK_DROME (sp|Q01604) Phosphoglycerate kinase; D.m. GN=Pgk PE=2 SV=2	PGK_DROME	0	GLOS_PGK.2.3	GO:0016774	M	.	phosphotransferase activity, carboxyl group as acceptor
PGK_DROME (sp|Q01604) Phosphoglycerate kinase; D.m. GN=Pgk PE=2 SV=2	PGK_DROME	0	GLOS_PGK.2.3	GO:0004618	M	UniProtKB	phosphoglycerate kinase activity
PGK_DROME (sp|Q01604) Phosphoglycerate kinase; D.m. GN=Pgk PE=2 SV=2	PGK_DROME	0	GLOS_PGK.2.3	GO:0006007	B	.	glucose catabolic process
PGK_DROME (sp|Q01604) Phosphoglycerate kinase; D.m. GN=Pgk PE=2 SV=2	PGK_DROME	0	GLOS_PGK.2.3	GO:0006006	B	.	glucose metabolic process
PGK_DROME (sp|Q01604) Phosphoglycerate kinase; D.m. GN=Pgk PE=2 SV=2	PGK_DROME	0	GLOS_PGK.2.3	GO:0019318	B	.	hexose metabolic process
PGK_DROME (sp|Q01604) Phosphoglycerate kinase; D.m. GN=Pgk PE=2 SV=2	PGK_DROME	0	GLOS_PGK.2.3	GO:0005996	B	.	monosaccharide metabolic process
PGK_DROME (sp|Q01604) Phosphoglycerate kinase; D.m. GN=Pgk PE=2 SV=2	PGK_DROME	0	GLOS_PGK.2.3	GO:0044723	B	.	single-organism carbohydrate metabolic process
PGK_DROME (sp|Q01604) Phosphoglycerate kinase; D.m. GN=Pgk PE=2 SV=2	PGK_DROME	0	GLOS_PGK.2.3	GO:0019320	B	.	hexose catabolic process
PGK_DROME (sp|Q01604) Phosphoglycerate kinase; D.m. GN=Pgk PE=2 SV=2	PGK_DROME	0	GLOS_PGK.2.3	GO:0046365	B	.	monosaccharide catabolic process
PGK_DROME (sp|Q01604) Phosphoglycerate kinase; D.m. GN=Pgk PE=2 SV=2	PGK_DROME	0	GLOS_PGK.2.3	GO:0044724	B	.	single-organism carbohydrate catabolic process
PGK_DROME (sp|Q01604) Phosphoglycerate kinase; D.m. GN=Pgk PE=2 SV=2	PGK_DROME	0	GLOS_PGK.2.3	GO:0016052	B	.	carbohydrate catabolic process
PGK_DROME (sp|Q01604) Phosphoglycerate kinase; D.m. GN=Pgk PE=2 SV=2	PGK_DROME	0	GLOS_PGK.2.3	GO:0006096	B	UniProtKB-U	glycolysis
PGK_DROME (sp|Q01604) Phosphoglycerate kinase; D.m. GN=Pgk PE=2 SV=2	PGK_DROME	0	GLOS_PGK.2.3	GO:0046716	B	FlyBase	muscle cell cellular homeostasis
PGK_DROME (sp|Q01604) Phosphoglycerate kinase; D.m. GN=Pgk PE=2 SV=2	PGK_DROME	0	GLOS_PGK.2.3	GO:0019226	B	.	transmission of nerve impulse
PGK_DROME (sp|Q01604) Phosphoglycerate kinase; D.m. GN=Pgk PE=2 SV=2	PGK_DROME	0	GLOS_PGK.2.3	GO:0035637	B	.	multicellular organismal signaling
PGK_DROME (sp|Q01604) Phosphoglycerate kinase; D.m. GN=Pgk PE=2 SV=2	PGK_DROME	0	GLOS_PGK.2.3	GO:0007268	B	FlyBase	synaptic transmission
PH4H_DROME (sp|P17276) Protein henna OS=D.m. GN=Hn PE=2 SV=3	PH4H_DROME	0	GLOS_PH4H.1.2	GO:0005811	C	FlyBase	lipid particle
PH4H_DROME (sp|P17276) Protein henna OS=D.m. GN=Hn PE=2 SV=3	PH4H_DROME	0	GLOS_PH4H.1.2	GO:0005506	M	InterPro	iron ion binding
PH4H_DROME (sp|P17276) Protein henna OS=D.m. GN=Hn PE=2 SV=3	PH4H_DROME	0	GLOS_PH4H.1.2	GO:0016714	M	.	oxidoreductase activity, acting on paired donors, with incorporation or reduction<br>of mol. oxygen, reduced pteridine as one donor, and incorpor. of 1 atom of oxygen
PH4H_DROME (sp|P17276) Protein henna OS=D.m. GN=Hn PE=2 SV=3	PH4H_DROME	0	GLOS_PH4H.1.2	GO:0004497	M	.	monooxygenase activity
PH4H_DROME (sp|P17276) Protein henna OS=D.m. GN=Hn PE=2 SV=3	PH4H_DROME	0	GLOS_PH4H.1.2	GO:0004505	M	FlyBase	phenylalanine 4-monooxygenase activity
PH4H_DROME (sp|P17276) Protein henna OS=D.m. GN=Hn PE=2 SV=3	PH4H_DROME	0	GLOS_PH4H.1.2	GO:0004510	M	UniProtKB-E	tryptophan 5-monooxygenase activity
PH4H_DROME (sp|P17276) Protein henna OS=D.m. GN=Hn PE=2 SV=3	PH4H_DROME	0	GLOS_PH4H.1.2	GO:0006558	B	.	L-phenylalanine metabolic process
PH4H_DROME (sp|P17276) Protein henna OS=D.m. GN=Hn PE=2 SV=3	PH4H_DROME	0	GLOS_PH4H.1.2	GO:0009072	B	.	aromatic amino acid family metabolic process
PH4H_DROME (sp|P17276) Protein henna OS=D.m. GN=Hn PE=2 SV=3	PH4H_DROME	0	GLOS_PH4H.1.2	GO:1902221	B	.	erythrose 4-phosphate/phosphoenolpyruvate family amino acid metabolic process
PH4H_DROME (sp|P17276) Protein henna OS=D.m. GN=Hn PE=2 SV=3	PH4H_DROME	0	GLOS_PH4H.1.2	GO:0009074	B	.	aromatic amino acid family catabolic process
PH4H_DROME (sp|P17276) Protein henna OS=D.m. GN=Hn PE=2 SV=3	PH4H_DROME	0	GLOS_PH4H.1.2	GO:0019439	B	.	aromatic compound catabolic process
PH4H_DROME (sp|P17276) Protein henna OS=D.m. GN=Hn PE=2 SV=3	PH4H_DROME	0	GLOS_PH4H.1.2	GO:1901361	B	.	organic cyclic compound catabolic process
PH4H_DROME (sp|P17276) Protein henna OS=D.m. GN=Hn PE=2 SV=3	PH4H_DROME	0	GLOS_PH4H.1.2	GO:1902222	B	.	erythrose 4-phosphate/phosphoenolpyruvate family amino acid catabolic process
PH4H_DROME (sp|P17276) Protein henna OS=D.m. GN=Hn PE=2 SV=3	PH4H_DROME	0	GLOS_PH4H.1.2	GO:0006559	B	FlyBase	L-phenylalanine catabolic process
PH4H_DROME (sp|P17276) Protein henna OS=D.m. GN=Hn PE=2 SV=3	PH4H_DROME	0	GLOS_PH4H.1.2	GO:0007616	B	FlyBase	long-term memory
PH4H_DROME (sp|P17276) Protein henna OS=D.m. GN=Hn PE=2 SV=3	PH4H_DROME	0	GLOS_PH4H.1.2	GO:0006909	B	FlyBase	phagocytosis
PH4H_DROME (sp|P17276) Protein henna OS=D.m. GN=Hn PE=2 SV=3	PH4H_DROME	0	GLOS_PH4H.1.2	GO:0042428	B	.	serotonin metabolic process
PH4H_DROME (sp|P17276) Protein henna OS=D.m. GN=Hn PE=2 SV=3	PH4H_DROME	0	GLOS_PH4H.1.2	GO:0042430	B	.	indole-containing compound metabolic process
PH4H_DROME (sp|P17276) Protein henna OS=D.m. GN=Hn PE=2 SV=3	PH4H_DROME	0	GLOS_PH4H.1.2	GO:1901160	B	.	primary amino compound metabolic process
PH4H_DROME (sp|P17276) Protein henna OS=D.m. GN=Hn PE=2 SV=3	PH4H_DROME	0	GLOS_PH4H.1.2	GO:0042435	B	.	indole-containing compound biosynthetic process
PH4H_DROME (sp|P17276) Protein henna OS=D.m. GN=Hn PE=2 SV=3	PH4H_DROME	0	GLOS_PH4H.1.2	GO:1901162	B	.	primary amino compound biosynthetic process
PH4H_DROME (sp|P17276) Protein henna OS=D.m. GN=Hn PE=2 SV=3	PH4H_DROME	0	GLOS_PH4H.1.2	GO:0042427	B	UniProtKB-k	serotonin biosynthetic process
POLG_CILVC (sp|Q1KZ59) RNA replication protein OS=C. leprosis virus C<br>(isolate Citrus sinesis/Brazil/Cordeiropolis/2003) PE=4 SV=1	POLG_CILVC	1,00E-21	GLOS_POLG.3.4	GO:0005524	M	UniProtKB-k	ATP binding
POLG_CILVC (sp|Q1KZ59) RNA replication protein …Same as above	POLG_CILVC	1,00E-21	GLOS_POLG.3.4	GO:0004386	M	UniProtKB-k	helicase activity
POLG_CILVC (sp|Q1KZ59) RNA replication protein …Same as above	POLG_CILVC	1,00E-21	GLOS_POLG.3.4	GO:0008173	M	.	RNA methyltransferase activity
POLG_CILVC (sp|Q1KZ59) RNA replication protein …Same as above	POLG_CILVC	1,00E-21	GLOS_POLG.3.4	GO:0008168	M	.	methyltransferase activity
POLG_CILVC (sp|Q1KZ59) RNA replication protein …Same as above	POLG_CILVC	1,00E-21	GLOS_POLG.3.4	GO:0016741	M	.	transferase activity, transferring one-carbon groups

POLG_CILVC (sp|Q1KZ59) RNA replication protein …Same as above	POLG_CILVC	1,00E-21	GLOS_POLG.3.4	GO:0008757	M	.	S-adenosylmethionine-dependent methyltransferase activity
POLG_CILVC (sp|Q1KZ59) RNA replication protein …Same as above	POLG_CILVC	1,00E-21	GLOS_POLG.3.4	GO:0008174	M	InterPro	mRNA methyltransferase activity
POLG_CILVC (sp|Q1KZ59) RNA replication protein …Same as above	POLG_CILVC	1,00E-21	GLOS_POLG.3.4	GO:0003723	M	InterPro	RNA binding
POLG_CILVC (sp|Q1KZ59) RNA replication protein …Same as above	POLG_CILVC	1,00E-21	GLOS_POLG.3.4	GO:0034062	M	.	RNA polymerase activity
POLG_CILVC (sp|Q1KZ59) RNA replication protein …Same as above	POLG_CILVC	1,00E-21	GLOS_POLG.3.4	GO:0016779	M	.	nucleotidyltransferase activity
POLG_CILVC (sp|Q1KZ59) RNA replication protein …Same as above	POLG_CILVC	1,00E-21	GLOS_POLG.3.4	GO:0003968	M	UniProtKB-k	RNA-directed RNA polymerase activity
POLG_CILVC (sp|Q1KZ59) RNA replication protein …Same as above	POLG_CILVC	1,00E-21	GLOS_POLG.3.4	GO:0006396	B	InterPro	RNA processing
POLG_CILVC (sp|Q1KZ59) RNA replication protein …Same as above	POLG_CILVC	1,00E-21	GLOS_POLG.3.4	GO:0006351	B	InterPro	transcription, DNA-dependent
POLG_CILVC (sp|Q1KZ59) RNA replication protein …Same as above	POLG_CILVC	1,00E-21	GLOS_POLG.3.4	GO:0016032	B	.	viral process
POLG_CILVC (sp|Q1KZ59) RNA replication protein …Same as above	POLG_CILVC	1,00E-21	GLOS_POLG.3.4	GO:0044403	B	.	symbiosis, encompassing mutualism through parasitism
POLG_CILVC (sp|Q1KZ59) RNA replication protein …Same as above	POLG_CILVC	1,00E-21	GLOS_POLG.3.4	GO:0044419	B	.	interspecies interaction between organisms
POLG_CILVC (sp|Q1KZ59) RNA replication protein …Same as above	POLG_CILVC	1,00E-21	GLOS_POLG.3.4	GO:0044764	B	.	multi-organism cellular process
POLG_CILVC (sp|Q1KZ59) RNA replication protein …Same as above	POLG_CILVC	1,00E-21	GLOS_POLG.3.4	GO:0019058	B	.	viral life cycle
POLG_CILVC (sp|Q1KZ59) RNA replication protein …Same as above	POLG_CILVC	1,00E-21	GLOS_POLG.3.4	GO:0019079	B	InterPro	viral genome replication
PPIB_CHICK (sp|P24367) Peptidyl-prolyl cis-trans isomerase B OS=G. gallus	PPIB_CHICK	5,00E-69	GLOS_PPIB.1.1	GO:0005788	C	UniProtKB-S	endoplasmic reticulum lumen
PPIB_CHICK (sp|P24367) Peptidyl-prolyl cis-trans isomerase B OS=G. gallus	PPIB_CHICK	5,00E-69	GLOS_PPIB.1.1	GO:0042277	M	UniProtKB-k	peptide binding
PPIB_CHICK (sp|P24367) Peptidyl-prolyl cis-trans isomerase B OS=G. gallus	PPIB_CHICK	5,00E-69	GLOS_PPIB.1.1	GO:0016859	M	.	cis-trans isomerase activity
PPIB_CHICK (sp|P24367) Peptidyl-prolyl cis-trans isomerase B OS=G. gallus	PPIB_CHICK	5,00E-69	GLOS_PPIB.1.1	GO:0003755	M	UniProtKB-k	peptidyl-prolyl cis-trans isomerase activity
PPIB_CHICK (sp|P24367) Peptidyl-prolyl cis-trans isomerase B OS=G. gallus	PPIB_CHICK	5,00E-69	GLOS_PPIB.1.1	GO:0006457	B	UniProtKB-k	protein folding
PPIB_CHICK (sp|P24367) Peptidyl-prolyl cis-trans isomerase B OS=G. gallus	PPIB_CHICK	5,00E-69	GLOS_PPIB.1.1	GO:0018208	B	.	peptidyl-proline modification
PPIB_CHICK (sp|P24367) Peptidyl-prolyl cis-trans isomerase B OS=G. gallus	PPIB_CHICK	5,00E-69	GLOS_PPIB.1.1	GO:0018193	B	.	peptidyl-amino acid modification
PPIB_CHICK (sp|P24367) Peptidyl-prolyl cis-trans isomerase B OS=G. gallus	PPIB_CHICK	5,00E-69	GLOS_PPIB.1.1	GO:0000413	B	GOC	protein peptidyl-prolyl isomerization
[BBH] PR2_DROME (sp|Q9I7F7) Tyrosine-prot kinase PR2 OS=D.m.	PR2_DROME	0	GLOS_PR2.1.1	GO:0005524	M	UniProtKB-k	ATP binding
[BBH] PR2_DROME (sp|Q9I7F7) Tyrosine-prot kinase PR2 OS=D.m.	PR2_DROME	0	GLOS_PR2.1.1	GO:0004713	M	.	protein tyrosine kinase activity
[BBH] PR2_DROME (sp|Q9I7F7) Tyrosine-prot kinase PR2 OS=D.m.	PR2_DROME	0	GLOS_PR2.1.1	GO:0004672	M	.	protein kinase activity
[BBH] PR2_DROME (sp|Q9I7F7) Tyrosine-prot kinase PR2 OS=D.m.	PR2_DROME	0	GLOS_PR2.1.1	GO:0016773	M	.	phosphotransferase activity, alcohol group as acceptor
[BBH] PR2_DROME (sp|Q9I7F7) Tyrosine-prot kinase PR2 OS=D.m.	PR2_DROME	0	GLOS_PR2.1.1	GO:0004715	M	UniProtKB-E	non-membrane spanning protein tyrosine kinase activity
[BBH] PR2_DROME (sp|Q9I7F7) Tyrosine-prot kinase PR2 OS=D.m.	PR2_DROME	0	GLOS_PR2.1.1	GO:0004713	M	UniProtKB	protein tyrosine kinase activity
[BBH] PR2_DROME (sp|Q9I7F7) Tyrosine-prot kinase PR2 OS=D.m.	PR2_DROME	0	GLOS_PR2.1.1	GO:0035071	B	FlyBase	salivary gland cell autophagic cell death
[BBH] PYG_DROME (sp|Q9XTL9) Glycogen phosphorylase OS=D.m.	PYG_DROME	0	GLOS_PYG.1.1	GO:0004645	M	.	phosphorylase activity
[BBH] PYG_DROME (sp|Q9XTL9) Glycogen phosphorylase OS=D.m.	PYG_DROME	0	GLOS_PYG.1.1	GO:0016758	M	.	transferase activity, transferring hexosyl groups
[BBH] PYG_DROME (sp|Q9XTL9) Glycogen phosphorylase OS=D.m.	PYG_DROME	0	GLOS_PYG.1.1	GO:0016757	M	.	transferase activity, transferring glycosyl groups
[BBH] PYG_DROME (sp|Q9XTL9) Glycogen phosphorylase OS=D.m.	PYG_DROME	0	GLOS_PYG.1.1	GO:0008184	M	FlyBase	glycogen phosphorylase activity
[BBH] PYG_DROME (sp|Q9XTL9) Glycogen phosphorylase OS=D.m.	PYG_DROME	0	GLOS_PYG.1.1	GO:0048037	M	.	cofactor binding
[BBH] PYG_DROME (sp|Q9XTL9) Glycogen phosphorylase OS=D.m.	PYG_DROME	0	GLOS_PYG.1.1	GO:0030170	M	FlyBase	pyridoxal phosphate binding
[BBH] PYG_DROME (sp|Q9XTL9) Glycogen phosphorylase OS=D.m.	PYG_DROME	0	GLOS_PYG.1.1	GO:0060361	B	FlyBase	flight
[BBH] PYG_DROME (sp|Q9XTL9) Glycogen phosphorylase OS=D.m.	PYG_DROME	0	GLOS_PYG.1.1	GO:0005977	B	.	glycogen metabolic process
[BBH] PYG_DROME (sp|Q9XTL9) Glycogen phosphorylase OS=D.m.	PYG_DROME	0	GLOS_PYG.1.1	GO:0006073	B	.	cellular glucan metabolic process
[BBH] PYG_DROME (sp|Q9XTL9) Glycogen phosphorylase OS=D.m.	PYG_DROME	0	GLOS_PYG.1.1	GO:0044042	B	.	glucan metabolic process
[BBH] PYG_DROME (sp|Q9XTL9) Glycogen phosphorylase OS=D.m.	PYG_DROME	0	GLOS_PYG.1.1	GO:0005976	B	.	polysaccharide metabolic process
[BBH] PYG_DROME (sp|Q9XTL9) Glycogen phosphorylase OS=D.m.	PYG_DROME	0	GLOS_PYG.1.1	GO:0044264	B	.	cellular polysaccharide metabolic process
[BBH] PYG_DROME (sp|Q9XTL9) Glycogen phosphorylase OS=D.m.	PYG_DROME	0	GLOS_PYG.1.1	GO:0044262	B	.	cellular carbohydrate metabolic process
[BBH] PYG_DROME (sp|Q9XTL9) Glycogen phosphorylase OS=D.m.	PYG_DROME	0	GLOS_PYG.1.1	GO:0006112	B	.	energy reserve metabolic process
[BBH] PYG_DROME (sp|Q9XTL9) Glycogen phosphorylase OS=D.m.	PYG_DROME	0	GLOS_PYG.1.1	GO:0009251	B	.	glucan catabolic process
[BBH] PYG_DROME (sp|Q9XTL9) Glycogen phosphorylase OS=D.m.	PYG_DROME	0	GLOS_PYG.1.1	GO:0000272	B	.	polysaccharide catabolic process
[BBH] PYG_DROME (sp|Q9XTL9) Glycogen phosphorylase OS=D.m.	PYG_DROME	0	GLOS_PYG.1.1	GO:0009057	B	.	macromolecule catabolic process
[BBH] PYG_DROME (sp|Q9XTL9) Glycogen phosphorylase OS=D.m.	PYG_DROME	0	GLOS_PYG.1.1	GO:0044247	B	.	cellular polysaccharide catabolic process
[BBH] PYG_DROME (sp|Q9XTL9) Glycogen phosphorylase OS=D.m.	PYG_DROME	0	GLOS_PYG.1.1	GO:0044275	B	.	cellular carbohydrate catabolic process
[BBH] PYG_DROME (sp|Q9XTL9) Glycogen phosphorylase OS=D.m.	PYG_DROME	0	GLOS_PYG.1.1	GO:0005980	B	FlyBase	glycogen catabolic process
RL13A_DROME (sp|Q9VNE9) 60S ribosomal prot L13a OS=D.m.	RL13A_DROME	1,00E-97	GLOS_RL13A.3.1	GO:0044391	C	.	ribosomal subunit
RL13A_DROME (sp|Q9VNE9) 60S ribosomal prot L13a OS=D.m.	RL13A_DROME	1,00E-97	GLOS_RL13A.3.1	GO:0005840	C	.	ribosome
RL13A_DROME (sp|Q9VNE9) 60S ribosomal prot L13a OS=D.m.	RL13A_DROME	1,00E-97	GLOS_RL13A.3.1	GO:0015934	C	InterPro	large ribosomal subunit
RL13A_DROME (sp|Q9VNE9) 60S ribosomal prot L13a OS=D.m.	RL13A_DROME	1,00E-97	GLOS_RL13A.3.1	GO:0005840	C	FlyBase	ribosome
RL13A_DROME (sp|Q9VNE9) 60S ribosomal prot L13a OS=D.m.	RL13A_DROME	1,00E-97	GLOS_RL13A.3.1	GO:0005198	M	.	structural molecule activity

RL13A_DROME (sp|Q9VNE9) 60S ribosomal prot L13a OS=D.m.	RL13A_DROME	1,00E-97	GLOS_RL13A.3.1: GO:0003735	M	FlyBase	structural constituent of ribosome
RL13A_DROME (sp|Q9VNE9) 60S ribosomal prot L13a OS=D.m.	RL13A_DROME	1,00E-97	GLOS_RL13A.3.1: GO:0051298	B	FlyBase	centrosome duplication
RL13A_DROME (sp|Q9VNE9) 60S ribosomal prot L13a OS=D.m.	RL13A_DROME	1,00E-97	GLOS_RL13A.3.1: GO:0022416	B	.	chaeta development
RL13A_DROME (sp|Q9VNE9) 60S ribosomal prot L13a OS=D.m.	RL13A_DROME	1,00E-97	GLOS_RL13A.3.1: GO:0007423	B	.	sensory organ development
RL13A_DROME (sp|Q9VNE9) 60S ribosomal prot L13a OS=D.m.	RL13A_DROME	1,00E-97	GLOS_RL13A.3.1: GO:0008407	B	FlyBase	chaeta morphogenesis
RL13A_DROME (sp|Q9VNE9) 60S ribosomal prot L13a OS=D.m.	RL13A_DROME	1,00E-97	GLOS_RL13A.3.1: GO:0007219	B	FlyBase	Notch signaling pathway
RL13A_DROME (sp|Q9VNE9) 60S ribosomal prot L13a OS=D.m.	RL13A_DROME	1,00E-97	GLOS_RL13A.3.1: GO:0006412	B	InterPro	translation
RL13_DROME (sp|P41126) 60S ribosomal prot L13 D.m. GN=RpL13 PE=1 SV=1	RL13_DROME	1,00E-114	GLOS_RL13.2.12 GO:0005840	C	FlyBase	ribosome
RL13_DROME (sp|P41126) 60S ribosomal prot L13 D.m. GN=RpL13 PE=1 SV=1	RL13_DROME	1,00E-114	GLOS_RL13.2.12 GO:0003735	M	FlyBase	structural constituent of ribosome
RL13_DROME (sp|P41126) 60S ribosomal prot L13 D.m. GN=RpL13 PE=1 SV=1	RL13_DROME	1,00E-114	GLOS_RL13.2.12 GO:0051298	B	FlyBase	centrosome duplication
RL13_DROME (sp|P41126) 60S ribosomal prot L13 D.m. GN=RpL13 PE=1 SV=1	RL13_DROME	1,00E-114	GLOS_RL13.2.12 GO:0051231	B	.	spindle elongation
RL13_DROME (sp|P41126) 60S ribosomal prot L13 D.m. GN=RpL13 PE=1 SV=1	RL13_DROME	1,00E-114	GLOS_RL13.2.12 GO:0007052	B	.	mitotic spindle organization
RL13_DROME (sp|P41126) 60S ribosomal prot L13 D.m. GN=RpL13 PE=1 SV=1	RL13_DROME	1,00E-114	GLOS_RL13.2.12 GO:0000022	B	FlyBase	mitotic spindle elongation
RL13_DROME (sp|P41126) 60S ribosomal prot L13 D.m. GN=RpL13 PE=1 SV=1	RL13_DROME	1,00E-114	GLOS_RL13.2.12 GO:0006412	B	InterPro	translation
RS21_DROWI (sp|B4N002) 40S ribosomal prot S21 OS=D. willistoni	RS21_DROWI	1,00E-47	GLOS_RS21.2.3 GO:0005840	C	UniProtKB	ribosome
RS21_DROWI (sp|B4N002) 40S ribosomal prot S21 OS=D. willistoni	RS21_DROWI	1,00E-47	GLOS_RS21.2.3 GO:0043021	M	.	ribonucleoprotein complex binding
RS21_DROWI (sp|B4N002) 40S ribosomal prot S21 OS=D. willistoni	RS21_DROWI	1,00E-47	GLOS_RS21.2.3 GO:0043022	M	UniProtKB	ribosome binding
RS21_DROWI (sp|B4N002) 40S ribosomal prot S21 OS=D. willistoni	RS21_DROWI	1,00E-47	GLOS_RS21.2.3 GO:0003735	M	InterPro	structural constituent of ribosome
RS21_DROWI (sp|B4N002) 40S ribosomal prot S21 OS=D. willistoni	RS21_DROWI	1,00E-47	GLOS_RS21.2.3 GO:0007275	B	UniProtKB-k	multicellular organismal development
RS21_DROWI (sp|B4N002) 40S ribosomal prot S21 OS=D. willistoni	RS21_DROWI	1,00E-47	GLOS_RS21.2.3 GO:0042127	B	UniProtKB	regulation of cell proliferation
RS21_DROWI (sp|B4N002) 40S ribosomal prot S21 OS=D. willistoni	RS21_DROWI	1,00E-47	GLOS_RS21.2.3 GO:0006417	B	UniProtKB-k	regulation of translation
RS21_DROWI (sp|B4N002) 40S ribosomal prot S21 OS=D. willistoni	RS21_DROWI	1,00E-47	GLOS_RS21.2.3 GO:0006364	B	UniProtKB-k	rRNA processing
RS21_DROWI (sp|B4N002) 40S ribosomal prot S21 OS=D. willistoni	RS21_DROWI	1,00E-47	GLOS_RS21.2.3 GO:0006412	B	InterPro	translation
[BBH] RS29_DROME (sp|Q9VH69) 40S ribosomal prot S29 OS=D.m.	RS29_DROME	1,00E-37	GLOS_RS29.7.12 GO:0005840	C	UniProtKB-k	ribosome
[BBH] RS29_DROME (sp|Q9VH69) 40S ribosomal prot S29 OS=D.m.	RS29_DROME	1,00E-37	GLOS_RS29.7.12 GO:0046872	M	UniProtKB-k	metal ion binding
[BBH] RS29_DROME (sp|Q9VH69) 40S ribosomal prot S29 OS=D.m.	RS29_DROME	1,00E-37	GLOS_RS29.7.12 GO:0003735	M	InterPro	structural constituent of ribosome
[BBH] RS29_DROME (sp|Q9VH69) 40S ribosomal prot S29 OS=D.m.	RS29_DROME	1,00E-37	GLOS_RS29.7.12 GO:0048666	B	FlyBase	neuron development
[BBH] RS29_DROME (sp|Q9VH69) 40S ribosomal prot S29 OS=D.m.	RS29_DROME	1,00E-37	GLOS_RS29.7.12 GO:0006412	B	InterPro	translation
SF3B5_DROME (sp|Q9VHI4) Prob splicing fact 3B subunit 5 OS=D.m.	SF3B5_DROME	6,00E-32	GLOS_SF3B5.2.3 GO:0005681	C	.	spliceosomal complex
SF3B5_DROME (sp|Q9VHI4) Prob splicing fact 3B subunit 5 OS=D.m.	SF3B5_DROME	6,00E-32	GLOS_SF3B5.2.3 GO:0071011	C	FlyBase	precatalytic spliceosome
SF3B5_DROME (sp|Q9VHI4) Prob splicing fact 3B subunit 5 OS=D.m.	SF3B5_DROME	6,00E-32	GLOS_SF3B5.2.3 GO:0007052	B	FlyBase	mitotic spindle organization
SF3B5_DROME (sp|Q9VHI4) Prob splicing fact 3B subunit 5 OS=D.m.	SF3B5_DROME	6,00E-32	GLOS_SF3B5.2.3 GO:0000377	B	.	RNA splicing, via transesterification react. with bulged adenosine as nucleophile
SF3B5_DROME (sp|Q9VHI4) Prob splicing fact 3B subunit 5 OS=D.m.	SF3B5_DROME	6,00E-32	GLOS_SF3B5.2.3 GO:0000375	B	.	RNA splicing, via transesterification reactions
SF3B5_DROME (sp|Q9VHI4) Prob splicing fact 3B subunit 5 OS=D.m.	SF3B5_DROME	6,00E-32	GLOS_SF3B5.2.3 GO:0008380	B	.	RNA splicing
SF3B5_DROME (sp|Q9VHI4) Prob splicing fact 3B subunit 5 OS=D.m.	SF3B5_DROME	6,00E-32	GLOS_SF3B5.2.3 GO:0006397	B	.	mRNA processing
SF3B5_DROME (sp|Q9VHI4) Prob splicing fact 3B subunit 5 OS=D.m.	SF3B5_DROME	6,00E-32	GLOS_SF3B5.2.3 GO:0016071	B	.	mRNA metabolic process
SF3B5_DROME (sp|Q9VHI4) Prob splicing fact 3B subunit 5 OS=D.m.	SF3B5_DROME	6,00E-32	GLOS_SF3B5.2.3 GO:0000398	B	FlyBase	mRNA splicing, via spliceosome
SHEP_DROMO (sp|B4KX02) Protein alan shepard OS=D. mojavensis	SHEP_DROMO	1,00E-107	GLOS_SHEP.2.2 GO:0000166	M	InterPro	nucleotide binding
SHEP_DROMO (sp|B4KX02) Protein alan shepard OS=D. mojavensis	SHEP_DROMO	1,00E-107	GLOS_SHEP.2.2 GO:0003723	M	UniProtKB-k	RNA binding
SHEP_DROMO (sp|B4KX02) Protein alan shepard OS=D. mojavensis	SHEP_DROMO	1,00E-107	GLOS_SHEP.2.2 GO:0009629	B	UniProtKB	response to gravity
SPCS2_DROME (sp|Q9VYY2) Signal peptidase complex subunit 2 D.m.	SPCS2_DROME	2,00E-39	GLOS_SPCS2.3.3 GO:0016021	C	UniProtKB-k	integral to membrane
SPCS2_DROME (sp|Q9VYY2) Signal peptidase complex subunit 2 D.m.	SPCS2_DROME	2,00E-39	GLOS_SPCS2.3.3 GO:0005789	C	.	endoplasmic reticulum membrane
SPCS2_DROME (sp|Q9VYY2) Signal peptidase complex subunit 2 D.m.	SPCS2_DROME	2,00E-39	GLOS_SPCS2.3.3 GO:0005787	C	InterPro	signal peptidase complex
SPCS2_DROME (sp|Q9VYY2) Signal peptidase complex subunit 2 D.m.	SPCS2_DROME	2,00E-39	GLOS_SPCS2.3.3 GO:0008233	M	UniProtKB-k	peptidase activity
SPCS2_DROME (sp|Q9VYY2) Signal peptidase complex subunit 2 D.m.	SPCS2_DROME	2,00E-39	GLOS_SPCS2.3.3 GO:0006508	B	UniProtKB-k	proteolysis
SPCS2_DROME (sp|Q9VYY2) Signal peptidase complex subunit 2 D.m.	SPCS2_DROME	2,00E-39	GLOS_SPCS2.3.3 GO:0006518	B	.	peptide metabolic process
SPCS2_DROME (sp|Q9VYY2) Signal peptidase complex subunit 2 D.m.	SPCS2_DROME	2,00E-39	GLOS_SPCS2.3.3 GO:0006465	B	InterPro	signal peptide processing
TNNC3_DROME (sp|P47949) Troponin C, isoform 3 OS=D.m. GN=TpnC73F	TNNC3_DROME	4,00E-81	GLOS_TNNC3.2.4 GO:0005509	M	InterPro	calcium ion binding
TNNC3_DROME (sp|P47949) Troponin C, isoform 3 OS=D.m. GN=TpnC73F	U195A_DROME	5,00E-81	GLOS_U195A.3.4 GO:0007059	B	UniProtKB-k	chromosome segregation
VATF1_DROME (sp|Q24583) V-type proton ATPase subunit F 1 D.m.	VATF1_DROME	1,00E-80	GLOS_VATF1.1.2 GO:0033176	C	.	proton-transporting V-type ATPase complex
VATF1_DROME (sp|Q24583) V-type proton ATPase subunit F 1 D.m.	VATF1_DROME	1,00E-80	GLOS_VATF1.1.2 GO:0033181	C	FlyBase	plasma membrane proton-transporting V-type ATPase complex
VATF1_DROME (sp|Q24583) V-type proton ATPase subunit F 1 D.m.	VATF1_DROME	1,00E-80	GLOS_VATF1.1.2 GO:0033180	C	.	proton-transporting V-type ATPase, V1 domain
VATF1_DROME (sp|Q24583) V-type proton ATPase subunit F 1 D.m.	VATF1_DROME	1,00E-80	GLOS_VATF1.1.2 GO:0033178	C	.	proton-transporting two-sector ATPase complex, catalytic domain
VATF1_DROME (sp|Q24583) V-type proton ATPase subunit F 1 D.m.	VATF1_DROME	1,00E-80	GLOS_VATF1.1.2 GO:0044437	C	.	vacuolar part

VATF1_DROME (sp|Q24583) V-type proton ATPase subunit F 1 D.m.	VATF1_DROME	1,00E-80	GLOS_VATF1.1.2	GO:0005773	C	.	vacuole
VATF1_DROME (sp|Q24583) V-type proton ATPase subunit F 1 D.m.	VATF1_DROME	1,00E-80	GLOS_VATF1.1.2	GO:0016471	C	.	vacuolar proton-transporting V-type ATPase complex
VATF1_DROME (sp|Q24583) V-type proton ATPase subunit F 1 D.m.	VATF1_DROME	1,00E-80	GLOS_VATF1.1.2	GO:0005774	C	.	vacuolar membrane
VATF1_DROME (sp|Q24583) V-type proton ATPase subunit F 1 D.m.	VATF1_DROME	1,00E-80	GLOS_VATF1.1.2	GO:0000221	C	FlyBase	vacuolar proton-transporting V-type ATPase, V1 domain
VATF1_DROME (sp|Q24583) V-type proton ATPase subunit F 1 D.m.	VATF1_DROME	1,00E-80	GLOS_VATF1.1.2	GO:0015078	M	.	hydrogen ion transmembrane transporter activity
VATF1_DROME (sp|Q24583) V-type proton ATPase subunit F 1 D.m.	VATF1_DROME	1,00E-80	GLOS_VATF1.1.2	GO:0036442	M	.	hydrogen-exporting ATPase activity
VATF1_DROME (sp|Q24583) V-type proton ATPase subunit F 1 D.m.	VATF1_DROME	1,00E-80	GLOS_VATF1.1.2	GO:0019829	M	.	cation-transporting ATPase activity
VATF1_DROME (sp|Q24583) V-type proton ATPase subunit F 1 D.m.	VATF1_DROME	1,00E-80	GLOS_VATF1.1.2	GO:0042625	M	.	ATPase activity, coupled to transmembrane movement of ions
VATF1_DROME (sp|Q24583) V-type proton ATPase subunit F 1 D.m.	VATF1_DROME	1,00E-80	GLOS_VATF1.1.2	GO:0042626	M	.	ATPase activity, coupled to transmembrane movement of substances
VATF1_DROME (sp|Q24583) V-type proton ATPase subunit F 1 D.m.	VATF1_DROME	1,00E-80	GLOS_VATF1.1.2	GO:0015405	M	.	P-P-bond-hydrolysis-driven transmembrane transporter activity
VATF1_DROME (sp|Q24583) V-type proton ATPase subunit F 1 D.m.	VATF1_DROME	1,00E-80	GLOS_VATF1.1.2	GO:0015399	M	.	primary active transmembrane transporter activity
VATF1_DROME (sp|Q24583) V-type proton ATPase subunit F 1 D.m.	VATF1_DROME	1,00E-80	GLOS_VATF1.1.2	GO:0022804	M	.	active transmembrane transporter activity
VATF1_DROME (sp|Q24583) V-type proton ATPase subunit F 1 D.m.	VATF1_DROME	1,00E-80	GLOS_VATF1.1.2	GO:0016820	M	.	hydrolase activity, acting on acid anhydrides, catalyzing transmembrane movement of substances
VATF1_DROME (sp|Q24583) V-type proton ATPase subunit F 1 D.m.	VATF1_DROME	1,00E-80	GLOS_VATF1.1.2	GO:0043492	M	.	ATPase activity, coupled to movement of substances
VATF1_DROME (sp|Q24583) V-type proton ATPase subunit F 1 D.m.	VATF1_DROME	1,00E-80	GLOS_VATF1.1.2	GO:0044769	M	.	ATPase act., coupled to transmembrane movement of ions, rotational mechanism
VATF1_DROME (sp|Q24583) V-type proton ATPase subunit F 1 D.m.	VATF1_DROME	1,00E-80	GLOS_VATF1.1.2	GO:0046961	M	InterPro	proton-transporting ATPase activity, rotational mechanism
VATF1_DROME (sp|Q24583) V-type proton ATPase subunit F 1 D.m.	VATF1_DROME	1,00E-80	GLOS_VATF1.1.2	GO:0015988	B	.	energy coupled proton transmembrane transport, against electrochemical gradient
VATF1_DROME (sp|Q24583) V-type proton ATPase subunit F 1 D.m.	VATF1_DROME	1,00E-80	GLOS_VATF1.1.2	GO:0015991	B	FlyBase	ATP hydrolysis coupled proton transport
XP_001971990.1 GG15272 [Drosophila erecta]	XP_001971990.1	9,00E-10	GLOS_DERE_GG	GO:0005216	M	.	ion channel activity
XP_001971990.1 GG15272 [Drosophila erecta]	XP_001971990.1	9,00E-10	GLOS_DERE_GG	GO:0015075	M	.	ion transmembrane transporter activity
XP_001971990.1 GG15272 [Drosophila erecta]	XP_001971990.1	9,00E-10	GLOS_DERE_GG	GO:0022891	M	.	substrate-specific transmembrane transporter activity
XP_001971990.1 GG15272 [Drosophila erecta]	XP_001971990.1	9,00E-10	GLOS_DERE_GG	GO:0022857	M	.	transmembrane transporter activity
XP_001971990.1 GG15272 [Drosophila erecta]	XP_001971990.1	9,00E-10	GLOS_DERE_GG	GO:0005215	M	.	transporter activity
XP_001971990.1 GG15272 [Drosophila erecta]	XP_001971990.1	9,00E-10	GLOS_DERE_GG	GO:0003674	M	.	molecular_function
XP_001971990.1 GG15272 [Drosophila erecta]	XP_001971990.1	9,00E-10	GLOS_DERE_GG	GO:0022892	M	.	substrate-specific transporter activity
XP_001971990.1 GG15272 [Drosophila erecta]	XP_001971990.1	9,00E-10	GLOS_DERE_GG	GO:0022838	M	.	substrate-specific channel activity
XP_001971990.1 GG15272 [Drosophila erecta]	XP_001971990.1	9,00E-10	GLOS_DERE_GG	GO:0015267	M	.	channel activity
XP_001971990.1 GG15272 [Drosophila erecta]	XP_001971990.1	9,00E-10	GLOS_DERE_GG	GO:0022803	M	.	passive transmembrane transporter activity
XP_001971990.1 GG15272 [Drosophila erecta]	XP_001971990.1	9,00E-10	GLOS_DERE_GG	GO:0008324	M	.	cation transmembrane transporter activity
XP_001971990.1 GG15272 [Drosophila erecta]	XP_001971990.1	9,00E-10	GLOS_DERE_GG	GO:0005261	M	Refseq	cation channel activity
XP_001971990.1 GG15272 [Drosophila erecta]	XP_001971990.1	9,00E-10	GLOS_DERE_GG	GO:0005261	M	.	cation channel activity
XP_001971990.1 GG15272 [Drosophila erecta]	XP_001971990.1	9,00E-10	GLOS_DERE_GG	GO:0015085	M	.	calcium ion transmembrane transporter activity
XP_001971990.1 GG15272 [Drosophila erecta]	XP_001971990.1	9,00E-10	GLOS_DERE_GG	GO:0046873	M	.	metal ion transmembrane transporter activity
XP_001971990.1 GG15272 [Drosophila erecta]	XP_001971990.1	9,00E-10	GLOS_DERE_GG	GO:0022890	M	.	inorganic cation transmembrane transporter activity
XP_001971990.1 GG15272 [Drosophila erecta]	XP_001971990.1	9,00E-10	GLOS_DERE_GG	GO:0072509	M	.	divalent inorganic cation transmembrane transporter activity
XP_001971990.1 GG15272 [Drosophila erecta]	XP_001971990.1	9,00E-10	GLOS_DERE_GG	GO:0005262	M	Refseq	calcium channel activity
XP_001971990.1 GG15272 [Drosophila erecta]	XP_001971990.1	9,00E-10	GLOS_DERE_GG	GO:0005488	M	.	binding
XP_001971990.1 GG15272 [Drosophila erecta]	XP_001971990.1	9,00E-10	GLOS_DERE_GG	GO:0005515	M	Refseq	protein binding
XP_001971990.1 GG15272 [Drosophila erecta]	XP_001971990.1	9,00E-10	GLOS_DERE_GG	GO:0016020	C	.	membrane
XP_001971990.1 GG15272 [Drosophila erecta]	XP_001971990.1	9,00E-10	GLOS_DERE_GG	GO:0005575	C	.	cellular_component
XP_001971990.1 GG15272 [Drosophila erecta]	XP_001971990.1	9,00E-10	GLOS_DERE_GG	GO:0044464	C	.	cell part
XP_001971990.1 GG15272 [Drosophila erecta]	XP_001971990.1	9,00E-10	GLOS_DERE_GG	GO:0005623	C	.	cell
XP_001971990.1 GG15272 [Drosophila erecta]	XP_001971990.1	9,00E-10	GLOS_DERE_GG	GO:0071944	C	.	cell periphery
XP_001971990.1 GG15272 [Drosophila erecta]	XP_001971990.1	9,00E-10	GLOS_DERE_GG	GO:0005886	C	Refseq	plasma membrane
XP_001971990.1 GG15272 [Drosophila erecta]	XP_001971990.1	9,00E-10	GLOS_DERE_GG	GO:0016021	C	.	integral to membrane
XP_001971990.1 GG15272 [Drosophila erecta]	XP_001971990.1	9,00E-10	GLOS_DERE_GG	GO:0031224	C	.	intrinsic to membrane
XP_001971990.1 GG15272 [Drosophila erecta]	XP_001971990.1	9,00E-10	GLOS_DERE_GG	GO:0044425	C	.	membrane part
XP_001971990.1 GG15272 [Drosophila erecta]	XP_001971990.1	9,00E-10	GLOS_DERE_GG	GO:0031226	C	.	intrinsic to plasma membrane
XP_001971990.1 GG15272 [Drosophila erecta]	XP_001971990.1	9,00E-10	GLOS_DERE_GG	GO:0044459	C	.	plasma membrane part
XP_001971990.1 GG15272 [Drosophila erecta]	XP_001971990.1	9,00E-10	GLOS_DERE_GG	GO:0005886	C	.	plasma membrane
XP_001971990.1 GG15272 [Drosophila erecta]	XP_001971990.1	9,00E-10	GLOS_DERE_GG	GO:0005887	C	Refseq	integral to plasma membrane
XP_001971990.1 GG15272 [Drosophila erecta]	XP_001971990.1	9,00E-10	GLOS_DERE_GG	GO:0070838	B	.	divalent metal ion transport
XP_001971990.1 GG15272 [Drosophila erecta]	XP_001971990.1	9,00E-10	GLOS_DERE_GG	GO:0030001	B	.	metal ion transport

XP_001971990.1 GG15272 [Drosophila erecta]	XP_001971990.1	9,00E-10	GLOS_DERE_GG	GO:0006812	B	.	cation transport
XP_001971990.1 GG15272 [Drosophila erecta]	XP_001971990.1	9,00E-10	GLOS_DERE_GG	GO:0006811	B	.	ion transport
XP_001971990.1 GG15272 [Drosophila erecta]	XP_001971990.1	9,00E-10	GLOS_DERE_GG	GO:0044765	B	.	single-organism transport
XP_001971990.1 GG15272 [Drosophila erecta]	XP_001971990.1	9,00E-10	GLOS_DERE_GG	GO:0006810	B	.	transport
XP_001971990.1 GG15272 [Drosophila erecta]	XP_001971990.1	9,00E-10	GLOS_DERE_GG	GO:0051234	B	.	establishment of localization
XP_001971990.1 GG15272 [Drosophila erecta]	XP_001971990.1	9,00E-10	GLOS_DERE_GG	GO:0008150	B	.	biological_process
XP_001971990.1 GG15272 [Drosophila erecta]	XP_001971990.1	9,00E-10	GLOS_DERE_GG	GO:0051179	B	.	localization
XP_001971990.1 GG15272 [Drosophila erecta]	XP_001971990.1	9,00E-10	GLOS_DERE_GG	GO:0044699	B	.	single-organism process
XP_001971990.1 GG15272 [Drosophila erecta]	XP_001971990.1	9,00E-10	GLOS_DERE_GG	GO:0072511	B	.	divalent inorganic cation transport
XP_001971990.1 GG15272 [Drosophila erecta]	XP_001971990.1	9,00E-10	GLOS_DERE_GG	GO:0006816	B	Refseq	calcium ion transport
XP_001971990.1 GG15272 [Drosophila erecta]	XP_001971990.1	9,00E-10	GLOS_DERE_GG	GO:0097485	B	.	neuron projection guidance
XP_001971990.1 GG15272 [Drosophila erecta]	XP_001971990.1	9,00E-10	GLOS_DERE_GG	GO:0006928	B	.	cellular component movement
XP_001971990.1 GG15272 [Drosophila erecta]	XP_001971990.1	9,00E-10	GLOS_DERE_GG	GO:0044763	B	.	single-organism cellular process
XP_001971990.1 GG15272 [Drosophila erecta]	XP_001971990.1	9,00E-10	GLOS_DERE_GG	GO:0009987	B	.	cellular process
XP_001971990.1 GG15272 [Drosophila erecta]	XP_001971990.1	9,00E-10	GLOS_DERE_GG	GO:0006935	B	.	chemotaxis
XP_001971990.1 GG15272 [Drosophila erecta]	XP_001971990.1	9,00E-10	GLOS_DERE_GG	GO:0042221	B	.	response to chemical stimulus
XP_001971990.1 GG15272 [Drosophila erecta]	XP_001971990.1	9,00E-10	GLOS_DERE_GG	GO:0050896	B	.	response to stimulus
XP_001971990.1 GG15272 [Drosophila erecta]	XP_001971990.1	9,00E-10	GLOS_DERE_GG	GO:0042330	B	.	taxis
XP_001971990.1 GG15272 [Drosophila erecta]	XP_001971990.1	9,00E-10	GLOS_DERE_GG	GO:0009605	B	.	response to external stimulus
XP_001971990.1 GG15272 [Drosophila erecta]	XP_001971990.1	9,00E-10	GLOS_DERE_GG	GO:0040011	B	.	locomotion
XP_001971990.1 GG15272 [Drosophila erecta]	XP_001971990.1	9,00E-10	GLOS_DERE_GG	GO:0048812	B	.	neuron projection morphogenesis
XP_001971990.1 GG15272 [Drosophila erecta]	XP_001971990.1	9,00E-10	GLOS_DERE_GG	GO:0048858	B	.	cell projection morphogenesis
XP_001971990.1 GG15272 [Drosophila erecta]	XP_001971990.1	9,00E-10	GLOS_DERE_GG	GO:0030030	B	.	cell projection organization
XP_001971990.1 GG15272 [Drosophila erecta]	XP_001971990.1	9,00E-10	GLOS_DERE_GG	GO:0016043	B	.	cellular component organization
XP_001971990.1 GG15272 [Drosophila erecta]	XP_001971990.1	9,00E-10	GLOS_DERE_GG	GO:0071840	B	.	cellular component organization or biogenesis
XP_001971990.1 GG15272 [Drosophila erecta]	XP_001971990.1	9,00E-10	GLOS_DERE_GG	GO:0032990	B	.	cell part morphogenesis
XP_001971990.1 GG15272 [Drosophila erecta]	XP_001971990.1	9,00E-10	GLOS_DERE_GG	GO:0032989	B	.	cellular component morphogenesis
XP_001971990.1 GG15272 [Drosophila erecta]	XP_001971990.1	9,00E-10	GLOS_DERE_GG	GO:0009653	B	.	anatomical structure morphogenesis
XP_001971990.1 GG15272 [Drosophila erecta]	XP_001971990.1	9,00E-10	GLOS_DERE_GG	GO:0032502	B	.	developmental process
XP_001971990.1 GG15272 [Drosophila erecta]	XP_001971990.1	9,00E-10	GLOS_DERE_GG	GO:0048856	B	.	anatomical structure development
XP_001971990.1 GG15272 [Drosophila erecta]	XP_001971990.1	9,00E-10	GLOS_DERE_GG	GO:0044767	B	.	single-organism developmental process
XP_001971990.1 GG15272 [Drosophila erecta]	XP_001971990.1	9,00E-10	GLOS_DERE_GG	GO:0048869	B	.	cellular developmental process
XP_001971990.1 GG15272 [Drosophila erecta]	XP_001971990.1	9,00E-10	GLOS_DERE_GG	GO:0000902	B	.	cell morphogenesis
XP_001971990.1 GG15272 [Drosophila erecta]	XP_001971990.1	9,00E-10	GLOS_DERE_GG	GO:0031175	B	.	neuron projection development
XP_001971990.1 GG15272 [Drosophila erecta]	XP_001971990.1	9,00E-10	GLOS_DERE_GG	GO:0048666	B	.	neuron development
XP_001971990.1 GG15272 [Drosophila erecta]	XP_001971990.1	9,00E-10	GLOS_DERE_GG	GO:0048468	B	.	cell development
XP_001971990.1 GG15272 [Drosophila erecta]	XP_001971990.1	9,00E-10	GLOS_DERE_GG	GO:0030154	B	.	cell differentiation
XP_001971990.1 GG15272 [Drosophila erecta]	XP_001971990.1	9,00E-10	GLOS_DERE_GG	GO:0030182	B	.	neuron differentiation
XP_001971990.1 GG15272 [Drosophila erecta]	XP_001971990.1	9,00E-10	GLOS_DERE_GG	GO:0048699	B	.	generation of neurons
XP_001971990.1 GG15272 [Drosophila erecta]	XP_001971990.1	9,00E-10	GLOS_DERE_GG	GO:0022008	B	.	neurogenesis
XP_001971990.1 GG15272 [Drosophila erecta]	XP_001971990.1	9,00E-10	GLOS_DERE_GG	GO:0007399	B	.	nervous system development
XP_001971990.1 GG15272 [Drosophila erecta]	XP_001971990.1	9,00E-10	GLOS_DERE_GG	GO:0048731	B	.	system development
XP_001971990.1 GG15272 [Drosophila erecta]	XP_001971990.1	9,00E-10	GLOS_DERE_GG	GO:0007275	B	.	multicellular organismal development
XP_001971990.1 GG15272 [Drosophila erecta]	XP_001971990.1	9,00E-10	GLOS_DERE_GG	GO:0044707	B	.	single-multicellular organism process
XP_001971990.1 GG15272 [Drosophila erecta]	XP_001971990.1	9,00E-10	GLOS_DERE_GG	GO:0032501	B	.	multicellular organismal process
XP_001971990.1 GG15272 [Drosophila erecta]	XP_001971990.1	9,00E-10	GLOS_DERE_GG	GO:0007409	B	.	axonogenesis
XP_001971990.1 GG15272 [Drosophila erecta]	XP_001971990.1	9,00E-10	GLOS_DERE_GG	GO:0048667	B	.	cell morphogenesis involved in neuron differentiation
XP_001971990.1 GG15272 [Drosophila erecta]	XP_001971990.1	9,00E-10	GLOS_DERE_GG	GO:0000904	B	.	cell morphogenesis involved in differentiation
XP_001971990.1 GG15272 [Drosophila erecta]	XP_001971990.1	9,00E-10	GLOS_DERE_GG	GO:0061564	B	.	axon development
XP_001971990.1 GG15272 [Drosophila erecta]	XP_001971990.1	9,00E-10	GLOS_DERE_GG	GO:0007411	B	Refseq	axon guidance
XP_001971990.1 GG15272 [Drosophila erecta]	XP_001971990.1	9,00E-10	GLOS_DERE_GG	GO:0005262	M	.	calcium channel activity
XP_001971990.1 GG15272 [Drosophila erecta]	XP_001971990.1	9,00E-10	GLOS_DERE_GG	GO:0015276	M	.	ligand-gated ion channel activity
XP_001971990.1 GG15272 [Drosophila erecta]	XP_001971990.1	9,00E-10	GLOS_DERE_GG	GO:0022834	M	.	ligand-gated channel activity
XP_001971990.1 GG15272 [Drosophila erecta]	XP_001971990.1	9,00E-10	GLOS_DERE_GG	GO:0022836	M	.	gated channel activity

XP_001971990.1 GG15272 [Drosophila erecta]	XP_001971990.1	9,00E-10	GLOS_DERE_GG	GO:0022839	M	.	ion gated channel activity
XP_001971990.1 GG15272 [Drosophila erecta]	XP_001971990.1	9,00E-10	GLOS_DERE_GG	GO:0015279	M	Refseq	store-operated calcium channel activity
XP_001971990.1 GG15272 [Drosophila erecta]	XP_001971990.1	9,00E-10	GLOS_DERE_GG	GO:0016323	C	Refseq	basolateral plasma membrane
XP_001971990.1 GG15272 [Drosophila erecta]	XP_001971990.1	9,00E-10	GLOS_DERE_GG	GO:0044449	C	.	contractile fiber part
XP_001971990.1 GG15272 [Drosophila erecta]	XP_001971990.1	9,00E-10	GLOS_DERE_GG	GO:0044422	C	.	organelle part
XP_001971990.1 GG15272 [Drosophila erecta]	XP_001971990.1	9,00E-10	GLOS_DERE_GG	GO:0043226	C	.	organelle
XP_001971990.1 GG15272 [Drosophila erecta]	XP_001971990.1	9,00E-10	GLOS_DERE_GG	GO:0044444	C	.	cytoplasmic part
XP_001971990.1 GG15272 [Drosophila erecta]	XP_001971990.1	9,00E-10	GLOS_DERE_GG	GO:0044424	C	.	intracellular part
XP_001971990.1 GG15272 [Drosophila erecta]	XP_001971990.1	9,00E-10	GLOS_DERE_GG	GO:0005622	C	.	intracellular
XP_001971990.1 GG15272 [Drosophila erecta]	XP_001971990.1	9,00E-10	GLOS_DERE_GG	GO:0005737	C	.	cytoplasm
XP_001971990.1 GG15272 [Drosophila erecta]	XP_001971990.1	9,00E-10	GLOS_DERE_GG	GO:0043292	C	.	contractile fiber
XP_001971990.1 GG15272 [Drosophila erecta]	XP_001971990.1	9,00E-10	GLOS_DERE_GG	GO:0043232	C	.	intracellular non-membrane-bounded organelle
XP_001971990.1 GG15272 [Drosophila erecta]	XP_001971990.1	9,00E-10	GLOS_DERE_GG	GO:0043228	C	.	non-membrane-bounded organelle
XP_001971990.1 GG15272 [Drosophila erecta]	XP_001971990.1	9,00E-10	GLOS_DERE_GG	GO:0043229	C	.	intracellular organelle
XP_001971990.1 GG15272 [Drosophila erecta]	XP_001971990.1	9,00E-10	GLOS_DERE_GG	GO:0030016	C	.	myofibril
XP_001971990.1 GG15272 [Drosophila erecta]	XP_001971990.1	9,00E-10	GLOS_DERE_GG	GO:0030017	C	Refseq	sarcomere
XP_001971990.1 GG15272 [Drosophila erecta]	XP_001971990.1	9,00E-10	GLOS_DERE_GG	GO:0043034	C	Refseq	costamere
XP_001971990.1 GG15272 [Drosophila erecta]	XP_001971990.1	9,00E-10	GLOS_DERE_GG	GO:0032991	C	.	macromolecular complex
XP_001971990.1 GG15272 [Drosophila erecta]	XP_001971990.1	9,00E-10	GLOS_DERE_GG	GO:0043234	C	Refseq	protein complex
XP_001971990.1 GG15272 [Drosophila erecta]	XP_001971990.1	9,00E-10	GLOS_DERE_GG	GO:0005515	M	.	protein binding
XP_001971990.1 GG15272 [Drosophila erecta]	XP_001971990.1	9,00E-10	GLOS_DERE_GG	GO:0044325	M	Refseq	ion channel binding
XP_001971990.1 GG15272 [Drosophila erecta]	XP_001971990.1	9,00E-10	GLOS_DERE_GG	GO:0045121	C	Refseq	membrane raft
XP_001971990.1 GG15272 [Drosophila erecta]	XP_001971990.1	9,00E-10	GLOS_DERE_GG	GO:0007589	B	.	body fluid secretion
XP_001971990.1 GG15272 [Drosophila erecta]	XP_001971990.1	9,00E-10	GLOS_DERE_GG	GO:0046903	B	.	secretion
XP_001971990.1 GG15272 [Drosophila erecta]	XP_001971990.1	9,00E-10	GLOS_DERE_GG	GO:0050878	B	.	regulation of body fluid levels
XP_001971990.1 GG15272 [Drosophila erecta]	XP_001971990.1	9,00E-10	GLOS_DERE_GG	GO:0065008	B	.	regulation of biological quality
XP_001971990.1 GG15272 [Drosophila erecta]	XP_001971990.1	9,00E-10	GLOS_DERE_GG	GO:0065007	B	.	biological regulation
XP_001971990.1 GG15272 [Drosophila erecta]	XP_001971990.1	9,00E-10	GLOS_DERE_GG	GO:0022600	B	.	digestive system process
XP_001971990.1 GG15272 [Drosophila erecta]	XP_001971990.1	9,00E-10	GLOS_DERE_GG	GO:0003008	B	.	system process
XP_001971990.1 GG15272 [Drosophila erecta]	XP_001971990.1	9,00E-10	GLOS_DERE_GG	GO:0007586	B	.	digestion
XP_001971990.1 GG15272 [Drosophila erecta]	XP_001971990.1	9,00E-10	GLOS_DERE_GG	GO:0032941	B	.	secretion by tissue
XP_001971990.1 GG15272 [Drosophila erecta]	XP_001971990.1	9,00E-10	GLOS_DERE_GG	GO:0046541	B	Refseq	saliva secretion
XP_001971990.1 GG15272 [Drosophila erecta]	XP_001971990.1	9,00E-10	GLOS_DERE_GG	GO:0010524	B	.	positive regulation of calcium ion transport into cytosol
XP_001971990.1 GG15272 [Drosophila erecta]	XP_001971990.1	9,00E-10	GLOS_DERE_GG	GO:0010522	B	.	regulation of calcium ion transport into cytosol
XP_001971990.1 GG15272 [Drosophila erecta]	XP_001971990.1	9,00E-10	GLOS_DERE_GG	GO:0032386	B	.	regulation of intracellular transport
XP_001971990.1 GG15272 [Drosophila erecta]	XP_001971990.1	9,00E-10	GLOS_DERE_GG	GO:0051049	B	.	regulation of transport
XP_001971990.1 GG15272 [Drosophila erecta]	XP_001971990.1	9,00E-10	GLOS_DERE_GG	GO:0032879	B	.	regulation of localization
XP_001971990.1 GG15272 [Drosophila erecta]	XP_001971990.1	9,00E-10	GLOS_DERE_GG	GO:0050789	B	.	regulation of biological process
XP_001971990.1 GG15272 [Drosophila erecta]	XP_001971990.1	9,00E-10	GLOS_DERE_GG	GO:0060341	B	.	regulation of cellular localization
XP_001971990.1 GG15272 [Drosophila erecta]	XP_001971990.1	9,00E-10	GLOS_DERE_GG	GO:0050794	B	.	regulation of cellular process
XP_001971990.1 GG15272 [Drosophila erecta]	XP_001971990.1	9,00E-10	GLOS_DERE_GG	GO:0051924	B	.	regulation of calcium ion transport
XP_001971990.1 GG15272 [Drosophila erecta]	XP_001971990.1	9,00E-10	GLOS_DERE_GG	GO:0010959	B	.	regulation of metal ion transport
XP_001971990.1 GG15272 [Drosophila erecta]	XP_001971990.1	9,00E-10	GLOS_DERE_GG	GO:0043269	B	.	regulation of ion transport
XP_001971990.1 GG15272 [Drosophila erecta]	XP_001971990.1	9,00E-10	GLOS_DERE_GG	GO:2000021	B	.	regulation of ion homeostasis
XP_001971990.1 GG15272 [Drosophila erecta]	XP_001971990.1	9,00E-10	GLOS_DERE_GG	GO:0032844	B	.	regulation of homeostatic process
XP_001971990.1 GG15272 [Drosophila erecta]	XP_001971990.1	9,00E-10	GLOS_DERE_GG	GO:0032388	B	.	positive regulation of intracellular transport
XP_001971990.1 GG15272 [Drosophila erecta]	XP_001971990.1	9,00E-10	GLOS_DERE_GG	GO:0051050	B	.	positive regulation of transport
XP_001971990.1 GG15272 [Drosophila erecta]	XP_001971990.1	9,00E-10	GLOS_DERE_GG	GO:0048518	B	.	positive regulation of biological process
XP_001971990.1 GG15272 [Drosophila erecta]	XP_001971990.1	9,00E-10	GLOS_DERE_GG	GO:0032846	B	.	positive regulation of homeostatic process
XP_001971990.1 GG15272 [Drosophila erecta]	XP_001971990.1	9,00E-10	GLOS_DERE_GG	GO:0048522	B	.	positive regulation of cellular process
XP_001971990.1 GG15272 [Drosophila erecta]	XP_001971990.1	9,00E-10	GLOS_DERE_GG	GO:0051928	B	.	positive regulation of calcium ion transport
XP_001971990.1 GG15272 [Drosophila erecta]	XP_001971990.1	9,00E-10	GLOS_DERE_GG	GO:0043270	B	.	positive regulation of ion transport
XP_001971990.1 GG15272 [Drosophila erecta]	XP_001971990.1	9,00E-10	GLOS_DERE_GG	GO:0051279	B	.	regulation of release of sequestered calcium ion into cytosol
XP_001971990.1 GG15272 [Drosophila erecta]	XP_001971990.1	9,00E-10	GLOS_DERE_GG	GO:0051281	B	Refseq	positive regulation of release of sequestered calcium ion into cytosol

XP_001971990.1 GG15272 [Drosophila erecta]	XP_001971990.1	9,00E-10	GLOS_DERE_GG	GO:0006874	B	.	cellular calcium ion homeostasis
XP_001971990.1 GG15272 [Drosophila erecta]	XP_001971990.1	9,00E-10	GLOS_DERE_GG	GO:0006875	B	.	cellular metal ion homeostasis
XP_001971990.1 GG15272 [Drosophila erecta]	XP_001971990.1	9,00E-10	GLOS_DERE_GG	GO:0030003	B	.	cellular cation homeostasis
XP_001971990.1 GG15272 [Drosophila erecta]	XP_001971990.1	9,00E-10	GLOS_DERE_GG	GO:0006873	B	.	cellular ion homeostasis
XP_001971990.1 GG15272 [Drosophila erecta]	XP_001971990.1	9,00E-10	GLOS_DERE_GG	GO:0050801	B	.	ion homeostasis
XP_001971990.1 GG15272 [Drosophila erecta]	XP_001971990.1	9,00E-10	GLOS_DERE_GG	GO:0048878	B	.	chemical homeostasis
XP_001971990.1 GG15272 [Drosophila erecta]	XP_001971990.1	9,00E-10	GLOS_DERE_GG	GO:0042592	B	.	homeostatic process
XP_001971990.1 GG15272 [Drosophila erecta]	XP_001971990.1	9,00E-10	GLOS_DERE_GG	GO:0055082	B	.	cellular chemical homeostasis
XP_001971990.1 GG15272 [Drosophila erecta]	XP_001971990.1	9,00E-10	GLOS_DERE_GG	GO:0019725	B	.	cellular homeostasis
XP_001971990.1 GG15272 [Drosophila erecta]	XP_001971990.1	9,00E-10	GLOS_DERE_GG	GO:0055080	B	.	cation homeostasis
XP_001971990.1 GG15272 [Drosophila erecta]	XP_001971990.1	9,00E-10	GLOS_DERE_GG	GO:0055065	B	.	metal ion homeostasis
XP_001971990.1 GG15272 [Drosophila erecta]	XP_001971990.1	9,00E-10	GLOS_DERE_GG	GO:0055074	B	.	calcium ion homeostasis
XP_001971990.1 GG15272 [Drosophila erecta]	XP_001971990.1	9,00E-10	GLOS_DERE_GG	GO:0072507	B	.	divalent inorganic cation homeostasis
XP_001971990.1 GG15272 [Drosophila erecta]	XP_001971990.1	9,00E-10	GLOS_DERE_GG	GO:0072503	B	.	cellular divalent inorganic cation homeostasis
XP_001971990.1 GG15272 [Drosophila erecta]	XP_001971990.1	9,00E-10	GLOS_DERE_GG	GO:0051480	B	Refseq	cytosolic calcium ion homeostasis
XP_001971990.1 GG15272 [Drosophila erecta]	XP_001971990.1	9,00E-10	GLOS_DERE_GG	GO:0010038	B	.	response to metal ion
XP_001971990.1 GG15272 [Drosophila erecta]	XP_001971990.1	9,00E-10	GLOS_DERE_GG	GO:0010035	B	.	response to inorganic substance
XP_001971990.1 GG15272 [Drosophila erecta]	XP_001971990.1	9,00E-10	GLOS_DERE_GG	GO:0051592	B	Refseq	response to calcium ion
XP_001971990.1 GG15272 [Drosophila erecta]	XP_001971990.1	9,00E-10	GLOS_DERE_GG	GO:0043168	M	.	anion binding
XP_001971990.1 GG15272 [Drosophila erecta]	XP_001971990.1	9,00E-10	GLOS_DERE_GG	GO:0043167	M	.	ion binding
XP_001971990.1 GG15272 [Drosophila erecta]	XP_001971990.1	9,00E-10	GLOS_DERE_GG	GO:0043178	M	.	alcohol binding
XP_001971990.1 GG15272 [Drosophila erecta]	XP_001971990.1	9,00E-10	GLOS_DERE_GG	GO:0036094	M	.	small molecule binding
XP_001971990.1 GG15272 [Drosophila erecta]	XP_001971990.1	9,00E-10	GLOS_DERE_GG	GO:0070679	M	Refseq	inositol 1,4,5 trisphosphate binding
XP_001989232.1 GH11610 [Drosophila grimshawi]	XP_001989232.1	2,00E-23	GLOS_DGRI_GH1	GO:0005262	M	Refseq	calcium channel activity
XP_001989232.1 GH11610 [Drosophila grimshawi]	XP_001989232.1	2,00E-23	GLOS_DGRI_GH1	GO:0005515	M	Refseq	protein binding
XP_001989232.1 GH11610 [Drosophila grimshawi]	XP_001989232.1	2,00E-23	GLOS_DGRI_GH1	GO:0005886	C	Refseq	plasma membrane
XP_001989232.1 GH11610 [Drosophila grimshawi]	XP_001989232.1	2,00E-23	GLOS_DGRI_GH1	GO:0005887	C	Refseq	integral to plasma membrane
XP_001989232.1 GH11610 [Drosophila grimshawi]	XP_001989232.1	2,00E-23	GLOS_DGRI_GH1	GO:0006816	B	Refseq	calcium ion transport
XP_001989232.1 GH11610 [Drosophila grimshawi]	XP_001989232.1	2,00E-23	GLOS_DGRI_GH1	GO:0007411	B	Refseq	axon guidance
XP_001989232.1 GH11610 [Drosophila grimshawi]	XP_001989232.1	2,00E-23	GLOS_DGRI_GH1	GO:0007599	B	.	hemostasis
XP_001989232.1 GH11610 [Drosophila grimshawi]	XP_001989232.1	2,00E-23	GLOS_DGRI_GH1	GO:0050817	B	.	coagulation
XP_001989232.1 GH11610 [Drosophila grimshawi]	XP_001989232.1	2,00E-23	GLOS_DGRI_GH1	GO:0042060	B	.	wound healing
XP_001989232.1 GH11610 [Drosophila grimshawi]	XP_001989232.1	2,00E-23	GLOS_DGRI_GH1	GO:0009611	B	.	response to wounding
XP_001989232.1 GH11610 [Drosophila grimshawi]	XP_001989232.1	2,00E-23	GLOS_DGRI_GH1	GO:0006950	B	.	response to stress
XP_001989232.1 GH11610 [Drosophila grimshawi]	XP_001989232.1	2,00E-23	GLOS_DGRI_GH1	GO:0007596	B	Refseq	blood coagulation
XP_001989232.1 GH11610 [Drosophila grimshawi]	XP_001989232.1	2,00E-23	GLOS_DGRI_GH1	GO:0007165	B	.	signal transduction
XP_001989232.1 GH11610 [Drosophila grimshawi]	XP_001989232.1	2,00E-23	GLOS_DGRI_GH1	GO:0051716	B	.	cellular response to stimulus
XP_001989232.1 GH11610 [Drosophila grimshawi]	XP_001989232.1	2,00E-23	GLOS_DGRI_GH1	GO:0007154	B	.	cell communication
XP_001989232.1 GH11610 [Drosophila grimshawi]	XP_001989232.1	2,00E-23	GLOS_DGRI_GH1	GO:0044700	B	.	single organism signaling
XP_001989232.1 GH11610 [Drosophila grimshawi]	XP_001989232.1	2,00E-23	GLOS_DGRI_GH1	GO:0023052	B	.	signaling
XP_001989232.1 GH11610 [Drosophila grimshawi]	XP_001989232.1	2,00E-23	GLOS_DGRI_GH1	GO:0009583	B	.	detection of light stimulus
XP_001989232.1 GH11610 [Drosophila grimshawi]	XP_001989232.1	2,00E-23	GLOS_DGRI_GH1	GO:0009416	B	.	response to light stimulus
XP_001989232.1 GH11610 [Drosophila grimshawi]	XP_001989232.1	2,00E-23	GLOS_DGRI_GH1	GO:0009314	B	.	response to radiation
XP_001989232.1 GH11610 [Drosophila grimshawi]	XP_001989232.1	2,00E-23	GLOS_DGRI_GH1	GO:0009628	B	.	response to abiotic stimulus
XP_001989232.1 GH11610 [Drosophila grimshawi]	XP_001989232.1	2,00E-23	GLOS_DGRI_GH1	GO:0009581	B	.	detection of external stimulus
XP_001989232.1 GH11610 [Drosophila grimshawi]	XP_001989232.1	2,00E-23	GLOS_DGRI_GH1	GO:0051606	B	.	detection of stimulus
XP_001989232.1 GH11610 [Drosophila grimshawi]	XP_001989232.1	2,00E-23	GLOS_DGRI_GH1	GO:0009582	B	.	detection of abiotic stimulus
XP_001989232.1 GH11610 [Drosophila grimshawi]	XP_001989232.1	2,00E-23	GLOS_DGRI_GH1	GO:0007602	B	Refseq	phototransduction
XP_001989232.1 GH11610 [Drosophila grimshawi]	XP_001989232.1	2,00E-23	GLOS_DGRI_GH1	GO:0010524	B	Refseq	positive regulation of calcium ion transport into cytosol
XP_001989232.1 GH11610 [Drosophila grimshawi]	XP_001989232.1	2,00E-23	GLOS_DGRI_GH1	GO:0015279	M	Refseq	store-operated calcium channel activity
XP_001989232.1 GH11610 [Drosophila grimshawi]	XP_001989232.1	2,00E-23	GLOS_DGRI_GH1	GO:0001775	B	.	cell activation
XP_001989232.1 GH11610 [Drosophila grimshawi]	XP_001989232.1	2,00E-23	GLOS_DGRI_GH1	GO:0007596	B	.	blood coagulation
XP_001989232.1 GH11610 [Drosophila grimshawi]	XP_001989232.1	2,00E-23	GLOS_DGRI_GH1	GO:0030168	B	Refseq	platelet activation
XP_001989232.1 GH11610 [Drosophila grimshawi]	XP_001989232.1	2,00E-23	GLOS_DGRI_GH1	GO:0014074	B	.	response to purine-containing compound

XP_001989232.1 GH11610 [Drosophila grimshawi]	XP_001989232.1	2,00E-23	GLOS_DGRI_GH1	GO:0010243	B	.	response to organonitrogen compound
XP_001989232.1 GH11610 [Drosophila grimshawi]	XP_001989232.1	2,00E-23	GLOS_DGRI_GH1	GO:0009719	B	.	response to endogenous stimulus
XP_001989232.1 GH11610 [Drosophila grimshawi]	XP_001989232.1	2,00E-23	GLOS_DGRI_GH1	GO:0010033	B	.	response to organic substance
XP_001989232.1 GH11610 [Drosophila grimshawi]	XP_001989232.1	2,00E-23	GLOS_DGRI_GH1	GO:1901698	B	.	response to nitrogen compound
XP_001989232.1 GH11610 [Drosophila grimshawi]	XP_001989232.1	2,00E-23	GLOS_DGRI_GH1	GO:0014070	B	.	response to organic cyclic compound
XP_001989232.1 GH11610 [Drosophila grimshawi]	XP_001989232.1	2,00E-23	GLOS_DGRI_GH1	GO:0046683	B	.	response to organophosphorus
XP_001989232.1 GH11610 [Drosophila grimshawi]	XP_001989232.1	2,00E-23	GLOS_DGRI_GH1	GO:1901700	B	.	response to oxygen-containing compound
XP_001989232.1 GH11610 [Drosophila grimshawi]	XP_001989232.1	2,00E-23	GLOS_DGRI_GH1	GO:0033198	B	Refseq	response to ATP
XP_001989232.1 GH11610 [Drosophila grimshawi]	XP_001989232.1	2,00E-23	GLOS_DGRI_GH1	GO:0051592	B	Refseq	response to calcium ion
XP_001989232.1 GH11610 [Drosophila grimshawi]	XP_001989232.1	2,00E-23	GLOS_DGRI_GH1	GO:0006816	B	.	calcium ion transport
XP_001989232.1 GH11610 [Drosophila grimshawi]	XP_001989232.1	2,00E-23	GLOS_DGRI_GH1	GO:0034220	B	.	ion transmembrane transport
XP_001989232.1 GH11610 [Drosophila grimshawi]	XP_001989232.1	2,00E-23	GLOS_DGRI_GH1	GO:0055085	B	.	transmembrane transport
XP_001989232.1 GH11610 [Drosophila grimshawi]	XP_001989232.1	2,00E-23	GLOS_DGRI_GH1	GO:0070588	B	Refseq	calcium ion transmembrane transport
XP_001989232.1 GH11610 [Drosophila grimshawi]	XP_001989232.1	2,00E-23	GLOS_DGRI_GH1	GO:0070679	M	Refseq	inositol 1,4,5 trisphosphate binding
XP_001990213.1 GH18354 [Drosophila grimshawi]	XP_001990213.1	9,00E-66	GLOS_DGRI_GH1	GO:0005262	M	Refseq	calcium channel activity
XP_001990213.1 GH18354 [Drosophila grimshawi]	XP_001990213.1	9,00E-66	GLOS_DGRI_GH1	GO:0005515	M	Refseq	protein binding
XP_001990213.1 GH18354 [Drosophila grimshawi]	XP_001990213.1	9,00E-66	GLOS_DGRI_GH1	GO:0005886	C	Refseq	plasma membrane
XP_001990213.1 GH18354 [Drosophila grimshawi]	XP_001990213.1	9,00E-66	GLOS_DGRI_GH1	GO:0005887	C	Refseq	integral to plasma membrane
XP_001990213.1 GH18354 [Drosophila grimshawi]	XP_001990213.1	9,00E-66	GLOS_DGRI_GH1	GO:0006816	B	Refseq	calcium ion transport
XP_001990213.1 GH18354 [Drosophila grimshawi]	XP_001990213.1	9,00E-66	GLOS_DGRI_GH1	GO:0007411	B	Refseq	axon guidance
XP_001990213.1 GH18354 [Drosophila grimshawi]	XP_001990213.1	9,00E-66	GLOS_DGRI_GH1	GO:0007596	B	Refseq	blood coagulation
XP_001990213.1 GH18354 [Drosophila grimshawi]	XP_001990213.1	9,00E-66	GLOS_DGRI_GH1	GO:0007602	B	Refseq	phototransduction
XP_001990213.1 GH18354 [Drosophila grimshawi]	XP_001990213.1	9,00E-66	GLOS_DGRI_GH1	GO:0010524	B	Refseq	positive regulation of calcium ion transport into cytosol
XP_001990213.1 GH18354 [Drosophila grimshawi]	XP_001990213.1	9,00E-66	GLOS_DGRI_GH1	GO:0015279	M	Refseq	store-operated calcium channel activity
XP_001990213.1 GH18354 [Drosophila grimshawi]	XP_001990213.1	9,00E-66	GLOS_DGRI_GH1	GO:0030168	B	Refseq	platelet activation
XP_001990213.1 GH18354 [Drosophila grimshawi]	XP_001990213.1	9,00E-66	GLOS_DGRI_GH1	GO:0033198	B	Refseq	response to ATP
XP_001990213.1 GH18354 [Drosophila grimshawi]	XP_001990213.1	9,00E-66	GLOS_DGRI_GH1	GO:0051592	B	Refseq	response to calcium ion
XP_001990213.1 GH18354 [Drosophila grimshawi]	XP_001990213.1	9,00E-66	GLOS_DGRI_GH1	GO:0070588	B	Refseq	calcium ion transmembrane transport
XP_001990213.1 GH18354 [Drosophila grimshawi]	XP_001990213.1	9,00E-66	GLOS_DGRI_GH1	GO:0070679	M	Refseq	inositol 1,4,5 trisphosphate binding
NP_001262659.1 cheerio, isoform N [Drosophila melanogaster]	NP_001262659.1	0	GLOS_CHER.1.1	GO:0006357	B	.	regulation of transcription from RNA polymerase II promoter
NP_001262659.1 cheerio, isoform N [Drosophila melanogaster]	NP_001262659.1	0	GLOS_CHER.1.1	GO:0006355	B	.	regulation of transcription, DNA-dependent
NP_001262659.1 cheerio, isoform N [Drosophila melanogaster]	NP_001262659.1	0	GLOS_CHER.1.1	GO:0010468	B	.	regulation of gene expression
NP_001262659.1 cheerio, isoform N [Drosophila melanogaster]	NP_001262659.1	0	GLOS_CHER.1.1	GO:0060255	B	.	regulation of macromolecule metabolic process
NP_001262659.1 cheerio, isoform N [Drosophila melanogaster]	NP_001262659.1	0	GLOS_CHER.1.1	GO:0019222	B	.	regulation of metabolic process
NP_001262659.1 cheerio, isoform N [Drosophila melanogaster]	NP_001262659.1	0	GLOS_CHER.1.1	GO:2000112	B	.	regulation of cellular macromolecule biosynthetic process
NP_001262659.1 cheerio, isoform N [Drosophila melanogaster]	NP_001262659.1	0	GLOS_CHER.1.1	GO:0010556	B	.	regulation of macromolecule biosynthetic process
NP_001262659.1 cheerio, isoform N [Drosophila melanogaster]	NP_001262659.1	0	GLOS_CHER.1.1	GO:0009889	B	.	regulation of biosynthetic process
NP_001262659.1 cheerio, isoform N [Drosophila melanogaster]	NP_001262659.1	0	GLOS_CHER.1.1	GO:0031326	B	.	regulation of cellular biosynthetic process
NP_001262659.1 cheerio, isoform N [Drosophila melanogaster]	NP_001262659.1	0	GLOS_CHER.1.1	GO:0031323	B	.	regulation of cellular metabolic process
NP_001262659.1 cheerio, isoform N [Drosophila melanogaster]	NP_001262659.1	0	GLOS_CHER.1.1	GO:2001141	B	.	regulation of RNA biosynthetic process
NP_001262659.1 cheerio, isoform N [Drosophila melanogaster]	NP_001262659.1	0	GLOS_CHER.1.1	GO:0051252	B	.	regulation of RNA metabolic process
NP_001262659.1 cheerio, isoform N [Drosophila melanogaster]	NP_001262659.1	0	GLOS_CHER.1.1	GO:0019219	B	.	regulation of nucleobase-containing compound metabolic process
NP_001262659.1 cheerio, isoform N [Drosophila melanogaster]	NP_001262659.1	0	GLOS_CHER.1.1	GO:0051171	B	.	regulation of nitrogen compound metabolic process
NP_001262659.1 cheerio, isoform N [Drosophila melanogaster]	NP_001262659.1	0	GLOS_CHER.1.1	GO:0080090	B	.	regulation of primary metabolic process
NP_001262659.1 cheerio, isoform N [Drosophila melanogaster]	NP_001262659.1	0	GLOS_CHER.1.1	GO:0045892	B	.	negative regulation of transcription, DNA-dependent
NP_001262659.1 cheerio, isoform N [Drosophila melanogaster]	NP_001262659.1	0	GLOS_CHER.1.1	GO:0010629	B	.	negative regulation of gene expression
NP_001262659.1 cheerio, isoform N [Drosophila melanogaster]	NP_001262659.1	0	GLOS_CHER.1.1	GO:0010605	B	.	negative regulation of macromolecule metabolic process
NP_001262659.1 cheerio, isoform N [Drosophila melanogaster]	NP_001262659.1	0	GLOS_CHER.1.1	GO:0009892	B	.	negative regulation of metabolic process
NP_001262659.1 cheerio, isoform N [Drosophila melanogaster]	NP_001262659.1	0	GLOS_CHER.1.1	GO:0048519	B	.	negative regulation of biological process
NP_001262659.1 cheerio, isoform N [Drosophila melanogaster]	NP_001262659.1	0	GLOS_CHER.1.1	GO:0051253	B	.	negative regulation of RNA metabolic process
NP_001262659.1 cheerio, isoform N [Drosophila melanogaster]	NP_001262659.1	0	GLOS_CHER.1.1	GO:0045934	B	.	negative regulation of nucleobase-containing compound metabolic process
NP_001262659.1 cheerio, isoform N [Drosophila melanogaster]	NP_001262659.1	0	GLOS_CHER.1.1	GO:0031324	B	.	negative regulation of cellular metabolic process
NP_001262659.1 cheerio, isoform N [Drosophila melanogaster]	NP_001262659.1	0	GLOS_CHER.1.1	GO:0048523	B	.	negative regulation of cellular process
NP_001262659.1 cheerio, isoform N [Drosophila melanogaster]	NP_001262659.1	0	GLOS_CHER.1.1	GO:0051172	B	.	negative regulation of nitrogen compound metabolic process

NP_001262659.1 cheerio, isoform N [Drosophila melanogaster]	NP_001262659.1	0	GLOS_CHER.1.1	GO:2000113	B	.	negative regulation of cellular macromolecule biosynthetic process
NP_001262659.1 cheerio, isoform N [Drosophila melanogaster]	NP_001262659.1	0	GLOS_CHER.1.1	GO:0010558	B	.	negative regulation of macromolecule biosynthetic process
NP_001262659.1 cheerio, isoform N [Drosophila melanogaster]	NP_001262659.1	0	GLOS_CHER.1.1	GO:0009890	B	.	negative regulation of biosynthetic process
NP_001262659.1 cheerio, isoform N [Drosophila melanogaster]	NP_001262659.1	0	GLOS_CHER.1.1	GO:0031327	B	.	negative regulation of cellular biosynthetic process
NP_001262659.1 cheerio, isoform N [Drosophila melanogaster]	NP_001262659.1	0	GLOS_CHER.1.1	GO:0000122	B	Refseq	negative regulation of transcription from RNA polymerase II promoter
NP_001262659.1 cheerio, isoform N [Drosophila melanogaster]	NP_001262659.1	0	GLOS_CHER.1.1	GO:0001501	B	Refseq	skeletal system development
NP_001262659.1 cheerio, isoform N [Drosophila melanogaster]	NP_001262659.1	0	GLOS_CHER.1.1	GO:0001071	M	.	nucleic acid binding transcription factor activity
NP_001262659.1 cheerio, isoform N [Drosophila melanogaster]	NP_001262659.1	0	GLOS_CHER.1.1	GO:0003700	M	Refseq	sequence-specific DNA binding transcription factor activity
NP_001262659.1 cheerio, isoform N [Drosophila melanogaster]	NP_001262659.1	0	GLOS_CHER.1.1	GO:0005515	M	Refseq	protein binding
NP_001262659.1 cheerio, isoform N [Drosophila melanogaster]	NP_001262659.1	0	GLOS_CHER.1.1	GO:0043231	C	.	intracellular membrane-bounded organelle
NP_001262659.1 cheerio, isoform N [Drosophila melanogaster]	NP_001262659.1	0	GLOS_CHER.1.1	GO:0043227	C	.	membrane-bounded organelle
NP_001262659.1 cheerio, isoform N [Drosophila melanogaster]	NP_001262659.1	0	GLOS_CHER.1.1	GO:0005634	C	Refseq	nucleus
NP_001262659.1 cheerio, isoform N [Drosophila melanogaster]	NP_001262659.1	0	GLOS_CHER.1.1	GO:0006351	B	.	transcription, DNA-dependent
NP_001262659.1 cheerio, isoform N [Drosophila melanogaster]	NP_001262659.1	0	GLOS_CHER.1.1	GO:0032774	B	.	RNA biosynthetic process
NP_001262659.1 cheerio, isoform N [Drosophila melanogaster]	NP_001262659.1	0	GLOS_CHER.1.1	GO:0009059	B	.	macromolecule biosynthetic process
NP_001262659.1 cheerio, isoform N [Drosophila melanogaster]	NP_001262659.1	0	GLOS_CHER.1.1	GO:0043170	B	.	macromolecule metabolic process
NP_001262659.1 cheerio, isoform N [Drosophila melanogaster]	NP_001262659.1	0	GLOS_CHER.1.1	GO:0071704	B	.	organic substance metabolic process
NP_001262659.1 cheerio, isoform N [Drosophila melanogaster]	NP_001262659.1	0	GLOS_CHER.1.1	GO:0008152	B	.	metabolic process
NP_001262659.1 cheerio, isoform N [Drosophila melanogaster]	NP_001262659.1	0	GLOS_CHER.1.1	GO:1901576	B	.	organic substance biosynthetic process
NP_001262659.1 cheerio, isoform N [Drosophila melanogaster]	NP_001262659.1	0	GLOS_CHER.1.1	GO:0009058	B	.	biosynthetic process
NP_001262659.1 cheerio, isoform N [Drosophila melanogaster]	NP_001262659.1	0	GLOS_CHER.1.1	GO:0016070	B	.	RNA metabolic process
NP_001262659.1 cheerio, isoform N [Drosophila melanogaster]	NP_001262659.1	0	GLOS_CHER.1.1	GO:0044260	B	.	cellular macromolecule metabolic process
NP_001262659.1 cheerio, isoform N [Drosophila melanogaster]	NP_001262659.1	0	GLOS_CHER.1.1	GO:0044237	B	.	cellular metabolic process
NP_001262659.1 cheerio, isoform N [Drosophila melanogaster]	NP_001262659.1	0	GLOS_CHER.1.1	GO:0090304	B	.	nucleic acid metabolic process
NP_001262659.1 cheerio, isoform N [Drosophila melanogaster]	NP_001262659.1	0	GLOS_CHER.1.1	GO:0006139	B	.	nucleobase-containing compound metabolic process
NP_001262659.1 cheerio, isoform N [Drosophila melanogaster]	NP_001262659.1	0	GLOS_CHER.1.1	GO:0006725	B	.	cellular aromatic compound metabolic process
NP_001262659.1 cheerio, isoform N [Drosophila melanogaster]	NP_001262659.1	0	GLOS_CHER.1.1	GO:0034641	B	.	cellular nitrogen compound metabolic process
NP_001262659.1 cheerio, isoform N [Drosophila melanogaster]	NP_001262659.1	0	GLOS_CHER.1.1	GO:0006807	B	.	nitrogen compound metabolic process
NP_001262659.1 cheerio, isoform N [Drosophila melanogaster]	NP_001262659.1	0	GLOS_CHER.1.1	GO:0044238	B	.	primary metabolic process
NP_001262659.1 cheerio, isoform N [Drosophila melanogaster]	NP_001262659.1	0	GLOS_CHER.1.1	GO:0046483	B	.	heterocycle metabolic process
NP_001262659.1 cheerio, isoform N [Drosophila melanogaster]	NP_001262659.1	0	GLOS_CHER.1.1	GO:1901360	B	.	organic cyclic compound metabolic process
NP_001262659.1 cheerio, isoform N [Drosophila melanogaster]	NP_001262659.1	0	GLOS_CHER.1.1	GO:0034654	B	.	nucleobase-containing compound biosynthetic process
NP_001262659.1 cheerio, isoform N [Drosophila melanogaster]	NP_001262659.1	0	GLOS_CHER.1.1	GO:0018130	B	.	heterocycle biosynthetic process
NP_001262659.1 cheerio, isoform N [Drosophila melanogaster]	NP_001262659.1	0	GLOS_CHER.1.1	GO:0044249	B	.	cellular biosynthetic process
NP_001262659.1 cheerio, isoform N [Drosophila melanogaster]	NP_001262659.1	0	GLOS_CHER.1.1	GO:0019438	B	.	aromatic compound biosynthetic process
NP_001262659.1 cheerio, isoform N [Drosophila melanogaster]	NP_001262659.1	0	GLOS_CHER.1.1	GO:0044271	B	.	cellular nitrogen compound biosynthetic process
NP_001262659.1 cheerio, isoform N [Drosophila melanogaster]	NP_001262659.1	0	GLOS_CHER.1.1	GO:1901362	B	.	organic cyclic compound biosynthetic process
NP_001262659.1 cheerio, isoform N [Drosophila melanogaster]	NP_001262659.1	0	GLOS_CHER.1.1	GO:0034645	B	.	cellular macromolecule biosynthetic process
NP_001262659.1 cheerio, isoform N [Drosophila melanogaster]	NP_001262659.1	0	GLOS_CHER.1.1	GO:0010467	B	.	gene expression
NP_001262659.1 cheerio, isoform N [Drosophila melanogaster]	NP_001262659.1	0	GLOS_CHER.1.1	GO:0006366	B	Refseq	transcription from RNA polymerase II promoter
NP_001262659.1 cheerio, isoform N [Drosophila melanogaster]	NP_001262659.1	0	GLOS_CHER.1.1	GO:0006606	B	.	protein import into nucleus
NP_001262659.1 cheerio, isoform N [Drosophila melanogaster]	NP_001262659.1	0	GLOS_CHER.1.1	GO:0006886	B	.	intracellular protein transport
NP_001262659.1 cheerio, isoform N [Drosophila melanogaster]	NP_001262659.1	0	GLOS_CHER.1.1	GO:0015031	B	.	protein transport
NP_001262659.1 cheerio, isoform N [Drosophila melanogaster]	NP_001262659.1	0	GLOS_CHER.1.1	GO:0045184	B	.	establishment of protein localization
NP_001262659.1 cheerio, isoform N [Drosophila melanogaster]	NP_001262659.1	0	GLOS_CHER.1.1	GO:0008104	B	.	protein localization
NP_001262659.1 cheerio, isoform N [Drosophila melanogaster]	NP_001262659.1	0	GLOS_CHER.1.1	GO:0033036	B	.	macromolecule localization
NP_001262659.1 cheerio, isoform N [Drosophila melanogaster]	NP_001262659.1	0	GLOS_CHER.1.1	GO:0071702	B	.	organic substance transport
NP_001262659.1 cheerio, isoform N [Drosophila melanogaster]	NP_001262659.1	0	GLOS_CHER.1.1	GO:0046907	B	.	intracellular transport
NP_001262659.1 cheerio, isoform N [Drosophila melanogaster]	NP_001262659.1	0	GLOS_CHER.1.1	GO:0051649	B	.	establishment of localization in cell
NP_001262659.1 cheerio, isoform N [Drosophila melanogaster]	NP_001262659.1	0	GLOS_CHER.1.1	GO:0051641	B	.	cellular localization
NP_001262659.1 cheerio, isoform N [Drosophila melanogaster]	NP_001262659.1	0	GLOS_CHER.1.1	GO:0034613	B	.	cellular protein localization
NP_001262659.1 cheerio, isoform N [Drosophila melanogaster]	NP_001262659.1	0	GLOS_CHER.1.1	GO:0070727	B	.	cellular macromolecule localization
NP_001262659.1 cheerio, isoform N [Drosophila melanogaster]	NP_001262659.1	0	GLOS_CHER.1.1	GO:0017038	B	.	protein import
NP_001262659.1 cheerio, isoform N [Drosophila melanogaster]	NP_001262659.1	0	GLOS_CHER.1.1	GO:0051170	B	.	nuclear import

NP_001262659.1 cheerio, isoform N [Drosophila melanogaster]	NP_001262659.1	0	GLOS_CHER.1.1	GO:0006913	B	.	nucleocytoplasmic transport
NP_001262659.1 cheerio, isoform N [Drosophila melanogaster]	NP_001262659.1	0	GLOS_CHER.1.1	GO:0016482	B	.	cytoplasmic transport
NP_001262659.1 cheerio, isoform N [Drosophila melanogaster]	NP_001262659.1	0	GLOS_CHER.1.1	GO:0051169	B	.	nuclear transport
NP_001262659.1 cheerio, isoform N [Drosophila melanogaster]	NP_001262659.1	0	GLOS_CHER.1.1	GO:0072594	B	.	establishment of protein localization to organelle
NP_001262659.1 cheerio, isoform N [Drosophila melanogaster]	NP_001262659.1	0	GLOS_CHER.1.1	GO:0033365	B	.	protein localization to organelle
NP_001262659.1 cheerio, isoform N [Drosophila melanogaster]	NP_001262659.1	0	GLOS_CHER.1.1	GO:0034504	B	.	protein localization to nucleus
NP_001262659.1 cheerio, isoform N [Drosophila melanogaster]	NP_001262659.1	0	GLOS_CHER.1.1	GO:0044744	B	.	protein targeting to nucleus
NP_001262659.1 cheerio, isoform N [Drosophila melanogaster]	NP_001262659.1	0	GLOS_CHER.1.1	GO:0006605	B	.	protein targeting
NP_001262659.1 cheerio, isoform N [Drosophila melanogaster]	NP_001262659.1	0	GLOS_CHER.1.1	GO:0006607	B	Refseq	NLS-bearing protein import into nucleus
NP_001262659.1 cheerio, isoform N [Drosophila melanogaster]	NP_001262659.1	0	GLOS_CHER.1.1	GO:0046914	M	.	transition metal ion binding
NP_001262659.1 cheerio, isoform N [Drosophila melanogaster]	NP_001262659.1	0	GLOS_CHER.1.1	GO:0046872	M	.	metal ion binding
NP_001262659.1 cheerio, isoform N [Drosophila melanogaster]	NP_001262659.1	0	GLOS_CHER.1.1	GO:0043169	M	.	cation binding
NP_001262659.1 cheerio, isoform N [Drosophila melanogaster]	NP_001262659.1	0	GLOS_CHER.1.1	GO:0008270	M	Refseq	zinc ion binding
NP_001262659.1 cheerio, isoform N [Drosophila melanogaster]	NP_001262659.1	0	GLOS_CHER.1.1	GO:0045595	B	.	regulation of cell differentiation
NP_001262659.1 cheerio, isoform N [Drosophila melanogaster]	NP_001262659.1	0	GLOS_CHER.1.1	GO:0050793	B	.	regulation of developmental process
NP_001262659.1 cheerio, isoform N [Drosophila melanogaster]	NP_001262659.1	0	GLOS_CHER.1.1	GO:0061035	B	.	regulation of cartilage development
NP_001262659.1 cheerio, isoform N [Drosophila melanogaster]	NP_001262659.1	0	GLOS_CHER.1.1	GO:2000026	B	.	regulation of multicellular organismal development
NP_001262659.1 cheerio, isoform N [Drosophila melanogaster]	NP_001262659.1	0	GLOS_CHER.1.1	GO:0051239	B	.	regulation of multicellular organismal process
NP_001262659.1 cheerio, isoform N [Drosophila melanogaster]	NP_001262659.1	0	GLOS_CHER.1.1	GO:0032330	B	Refseq	regulation of chondrocyte differentiation
NP_001262659.1 cheerio, isoform N [Drosophila melanogaster]	NP_001262659.1	0	GLOS_CHER.1.1	GO:0003677	M	.	DNA binding
NP_001262659.1 cheerio, isoform N [Drosophila melanogaster]	NP_001262659.1	0	GLOS_CHER.1.1	GO:0003676	M	.	nucleic acid binding
NP_001262659.1 cheerio, isoform N [Drosophila melanogaster]	NP_001262659.1	0	GLOS_CHER.1.1	GO:0097159	M	.	organic cyclic compound binding
NP_001262659.1 cheerio, isoform N [Drosophila melanogaster]	NP_001262659.1	0	GLOS_CHER.1.1	GO:1901363	M	.	heterocyclic compound binding
NP_001262659.1 cheerio, isoform N [Drosophila melanogaster]	NP_001262659.1	0	GLOS_CHER.1.1	GO:0043565	M	Refseq	sequence-specific DNA binding
XP_003243964.1 PREDICTED: hypothetical prot. LOC100568954 [A. pisum]	XP_003243964.1	6,00E-19	GLOS_LOC10056	GO:0007049	B	.	cell cycle
XP_003243964.1 PREDICTED: hypothetical prot. LOC100568954 [A. pisum]	XP_003243964.1	6,00E-19	GLOS_LOC10056	GO:0000278	B	Refseq	mitotic cell cycle
XP_003243964.1 PREDICTED: hypothetical prot. LOC100568954 [A. pisum]	XP_003243964.1	6,00E-19	GLOS_LOC10056	GO:0003677	M	Refseq	DNA binding
XP_003243964.1 PREDICTED: hypothetical prot. LOC100568954 [A. pisum]	XP_003243964.1	6,00E-19	GLOS_LOC10056	GO:0003700	M	Refseq	sequence-specific DNA binding transcription factor activity
XP_003243964.1 PREDICTED: hypothetical prot. LOC100568954 [A. pisum]	XP_003243964.1	6,00E-19	GLOS_LOC10056	GO:0000989	M	.	transcription factor binding transcription factor activity
XP_003243964.1 PREDICTED: hypothetical prot. LOC100568954 [A. pisum]	XP_003243964.1	6,00E-19	GLOS_LOC10056	GO:0000988	M	.	protein binding transcription factor activity
XP_003243964.1 PREDICTED: hypothetical prot. LOC100568954 [A. pisum]	XP_003243964.1	6,00E-19	GLOS_LOC10056	GO:0003712	M	Refseq	transcription cofactor activity
XP_003243964.1 PREDICTED: hypothetical prot. LOC100568954 [A. pisum]	XP_003243964.1	6,00E-19	GLOS_LOC10056	GO:0044428	C	.	nuclear part
XP_003243964.1 PREDICTED: hypothetical prot. LOC100568954 [A. pisum]	XP_003243964.1	6,00E-19	GLOS_LOC10056	GO:0044446	C	.	intracellular organelle part
XP_003243964.1 PREDICTED: hypothetical prot. LOC100568954 [A. pisum]	XP_003243964.1	6,00E-19	GLOS_LOC10056	GO:0005634	C	.	nucleus
XP_003243964.1 PREDICTED: hypothetical prot. LOC100568954 [A. pisum]	XP_003243964.1	6,00E-19	GLOS_LOC10056	GO:0031981	C	.	nuclear lumen
XP_003243964.1 PREDICTED: hypothetical prot. LOC100568954 [A. pisum]	XP_003243964.1	6,00E-19	GLOS_LOC10056	GO:0070013	C	.	intracellular organelle lumen
XP_003243964.1 PREDICTED: hypothetical prot. LOC100568954 [A. pisum]	XP_003243964.1	6,00E-19	GLOS_LOC10056	GO:0043233	C	.	organelle lumen
XP_003243964.1 PREDICTED: hypothetical prot. LOC100568954 [A. pisum]	XP_003243964.1	6,00E-19	GLOS_LOC10056	GO:0031974	C	.	membrane-enclosed lumen
XP_003243964.1 PREDICTED: hypothetical prot. LOC100568954 [A. pisum]	XP_003243964.1	6,00E-19	GLOS_LOC10056	GO:0005654	C	Refseq	nucleoplasm
XP_003243964.1 PREDICTED: hypothetical prot. LOC100568954 [A. pisum]	XP_003243964.1	6,00E-19	GLOS_LOC10056	GO:0043234	C	.	protein complex
XP_003243964.1 PREDICTED: hypothetical prot. LOC100568954 [A. pisum]	XP_003243964.1	6,00E-19	GLOS_LOC10056	GO:0005667	C	Refseq	transcription factor complex
XP_003243964.1 PREDICTED: hypothetical prot. LOC100568954 [A. pisum]	XP_003243964.1	6,00E-19	GLOS_LOC10056	GO:0006351	B	Refseq	transcription, DNA-dependent
XP_003243964.1 PREDICTED: hypothetical prot. LOC100568954 [A. pisum]	XP_003243964.1	6,00E-19	GLOS_LOC10056	GO:0006352	B	.	DNA-dependent transcription, initiation
XP_003243964.1 PREDICTED: hypothetical prot. LOC100568954 [A. pisum]	XP_003243964.1	6,00E-19	GLOS_LOC10056	GO:0006366	B	.	transcription from RNA polymerase II promoter
XP_003243964.1 PREDICTED: hypothetical prot. LOC100568954 [A. pisum]	XP_003243964.1	6,00E-19	GLOS_LOC10056	GO:0006367	B	Refseq	transcription initiation from RNA polymerase II promoter
XP_003243964.1 PREDICTED: hypothetical prot. LOC100568954 [A. pisum]	XP_003243964.1	6,00E-19	GLOS_LOC10056	GO:0007178	B	.	transmembrane receptor protein serine/threonine kinase signaling pathway
XP_003243964.1 PREDICTED: hypothetical prot. LOC100568954 [A. pisum]	XP_003243964.1	6,00E-19	GLOS_LOC10056	GO:0007167	B	.	enzyme linked receptor protein signaling pathway
XP_003243964.1 PREDICTED: hypothetical prot. LOC100568954 [A. pisum]	XP_003243964.1	6,00E-19	GLOS_LOC10056	GO:0007166	B	.	cell surface receptor signaling pathway
XP_003243964.1 PREDICTED: hypothetical prot. LOC100568954 [A. pisum]	XP_003243964.1	6,00E-19	GLOS_LOC10056	GO:0071560	B	.	cellular response to transforming growth factor beta stimulus
XP_003243964.1 PREDICTED: hypothetical prot. LOC100568954 [A. pisum]	XP_003243964.1	6,00E-19	GLOS_LOC10056	GO:0071363	B	.	cellular response to growth factor stimulus
XP_003243964.1 PREDICTED: hypothetical prot. LOC100568954 [A. pisum]	XP_003243964.1	6,00E-19	GLOS_LOC10056	GO:0070848	B	.	response to growth factor stimulus
XP_003243964.1 PREDICTED: hypothetical prot. LOC100568954 [A. pisum]	XP_003243964.1	6,00E-19	GLOS_LOC10056	GO:0071310	B	.	cellular response to organic substance
XP_003243964.1 PREDICTED: hypothetical prot. LOC100568954 [A. pisum]	XP_003243964.1	6,00E-19	GLOS_LOC10056	GO:0070887	B	.	cellular response to chemical stimulus
XP_003243964.1 PREDICTED: hypothetical prot. LOC100568954 [A. pisum]	XP_003243964.1	6,00E-19	GLOS_LOC10056	GO:0071495	B	.	cellular response to endogenous stimulus

XP_003243964.1 PREDICTED: hypothetical prot. LOC100568954 [A. pisum]	XP_003243964.1	6,00E-19	GLOS_LOC10056	GO:0071559	B	.	response to transforming growth factor beta stimulus
XP_003243964.1 PREDICTED: hypothetical prot. LOC100568954 [A. pisum]	XP_003243964.1	6,00E-19	GLOS_LOC10056	GO:0007179	B	Refseq	transforming growth factor beta receptor signaling pathway
XP_003243964.1 PREDICTED: hypothetical prot. LOC100568954 [A. pisum]	XP_003243964.1	6,00E-19	GLOS_LOC10056	GO:0048513	B	.	organ development
XP_003243964.1 PREDICTED: hypothetical prot. LOC100568954 [A. pisum]	XP_003243964.1	6,00E-19	GLOS_LOC10056	GO:0072358	B	.	cardiovascular system development
XP_003243964.1 PREDICTED: hypothetical prot. LOC100568954 [A. pisum]	XP_003243964.1	6,00E-19	GLOS_LOC10056	GO:0072359	B	.	circulatory system development
XP_003243964.1 PREDICTED: hypothetical prot. LOC100568954 [A. pisum]	XP_003243964.1	6,00E-19	GLOS_LOC10056	GO:0007507	B	Refseq	heart development
XP_003243964.1 PREDICTED: hypothetical prot. LOC100568954 [A. pisum]	XP_003243964.1	6,00E-19	GLOS_LOC10056	GO:0008134	M	Refseq	transcription factor binding
XP_003243964.1 PREDICTED: hypothetical prot. LOC100568954 [A. pisum]	XP_003243964.1	6,00E-19	GLOS_LOC10056	GO:0010467	B	Refseq	gene expression
XP_003243964.1 PREDICTED: hypothetical prot. LOC100568954 [A. pisum]	XP_003243964.1	6,00E-19	GLOS_LOC10056	GO:0019904	M	Refseq	protein domain specific binding
XP_003243964.1 PREDICTED: hypothetical prot. LOC100568954 [A. pisum]	XP_003243964.1	6,00E-19	GLOS_LOC10056	GO:0045893	B	.	positive regulation of transcription, DNA-dependent
XP_003243964.1 PREDICTED: hypothetical prot. LOC100568954 [A. pisum]	XP_003243964.1	6,00E-19	GLOS_LOC10056	GO:0010557	B	.	positive regulation of macromolecule biosynthetic process
XP_003243964.1 PREDICTED: hypothetical prot. LOC100568954 [A. pisum]	XP_003243964.1	6,00E-19	GLOS_LOC10056	GO:0009891	B	.	positive regulation of biosynthetic process
XP_003243964.1 PREDICTED: hypothetical prot. LOC100568954 [A. pisum]	XP_003243964.1	6,00E-19	GLOS_LOC10056	GO:0009893	B	.	positive regulation of metabolic process
XP_003243964.1 PREDICTED: hypothetical prot. LOC100568954 [A. pisum]	XP_003243964.1	6,00E-19	GLOS_LOC10056	GO:0010604	B	.	positive regulation of macromolecule metabolic process
XP_003243964.1 PREDICTED: hypothetical prot. LOC100568954 [A. pisum]	XP_003243964.1	6,00E-19	GLOS_LOC10056	GO:0010628	B	.	positive regulation of gene expression
XP_003243964.1 PREDICTED: hypothetical prot. LOC100568954 [A. pisum]	XP_003243964.1	6,00E-19	GLOS_LOC10056	GO:0031328	B	.	positive regulation of cellular biosynthetic process
XP_003243964.1 PREDICTED: hypothetical prot. LOC100568954 [A. pisum]	XP_003243964.1	6,00E-19	GLOS_LOC10056	GO:0031325	B	.	positive regulation of cellular metabolic process
XP_003243964.1 PREDICTED: hypothetical prot. LOC100568954 [A. pisum]	XP_003243964.1	6,00E-19	GLOS_LOC10056	GO:0051254	B	.	positive regulation of RNA metabolic process
XP_003243964.1 PREDICTED: hypothetical prot. LOC100568954 [A. pisum]	XP_003243964.1	6,00E-19	GLOS_LOC10056	GO:0045935	B	.	positive regulation of nucleobase-containing compound metabolic process
XP_003243964.1 PREDICTED: hypothetical prot. LOC100568954 [A. pisum]	XP_003243964.1	6,00E-19	GLOS_LOC10056	GO:0051173	B	.	positive regulation of nitrogen compound metabolic process
XP_003243964.1 PREDICTED: hypothetical prot. LOC100568954 [A. pisum]	XP_003243964.1	6,00E-19	GLOS_LOC10056	GO:0045944	B	Refseq	positive regulation of transcription from RNA polymerase II promoter
XP_001657526.1 hypothetical protein AaeL_AAEL006153 [Aedes aegypti]	XP_001657526.1	1,00E-16	GLOS_AAEL_AAE	GO:0030674	M	.	protein binding, bridging
XP_001657526.1 hypothetical protein AaeL_AAEL006153 [Aedes aegypti]	XP_001657526.1	1,00E-16	GLOS_AAEL_AAE	GO:0060090	M	.	binding, bridging
XP_001657526.1 hypothetical protein AaeL_AAEL006153 [Aedes aegypti]	XP_001657526.1	1,00E-16	GLOS_AAEL_AAE	GO:0035591	M	.	signaling adaptor activity
XP_001657526.1 hypothetical protein AaeL_AAEL006153 [Aedes aegypti]	XP_001657526.1	1,00E-16	GLOS_AAEL_AAE	GO:0005070	M	Refseq	SH3/SH2 adaptor activity
XP_001657526.1 hypothetical protein AaeL_AAEL006153 [Aedes aegypti]	XP_001657526.1	1,00E-16	GLOS_AAEL_AAE	GO:0005515	M	Refseq	protein binding
XP_001657526.1 hypothetical protein AaeL_AAEL006153 [Aedes aegypti]	XP_001657526.1	1,00E-16	GLOS_AAEL_AAE	GO:0005634	C	Refseq	nucleus
XP_001657526.1 hypothetical protein AaeL_AAEL006153 [Aedes aegypti]	XP_001657526.1	1,00E-16	GLOS_AAEL_AAE	GO:0005737	C	Refseq	cytoplasm
XP_001657526.1 hypothetical protein AaeL_AAEL006153 [Aedes aegypti]	XP_001657526.1	1,00E-16	GLOS_AAEL_AAE	GO:0007049	B	Refseq	cell cycle
XP_001657526.1 hypothetical protein AaeL_AAEL006153 [Aedes aegypti]	XP_001657526.1	1,00E-16	GLOS_AAEL_AAE	GO:0007165	B	Refseq	signal transduction
XP_001657526.1 hypothetical protein AaeL_AAEL006153 [Aedes aegypti]	XP_001657526.1	1,00E-16	GLOS_AAEL_AAE	GO:0007417	B	Refseq	central nervous system development
XP_001657526.1 hypothetical protein AaeL_AAEL006153 [Aedes aegypti]	XP_001657526.1	1,00E-16	GLOS_AAEL_AAE	GO:0007507	B	Refseq	heart development
XP_001657526.1 hypothetical protein AaeL_AAEL006153 [Aedes aegypti]	XP_001657526.1	1,00E-16	GLOS_AAEL_AAE	GO:0009790	B	.	embryo development
XP_001657526.1 hypothetical protein AaeL_AAEL006153 [Aedes aegypti]	XP_001657526.1	1,00E-16	GLOS_AAEL_AAE	GO:0009792	B	Refseq	embryo development ending in birth or egg hatching
XP_001657526.1 hypothetical protein AaeL_AAEL006153 [Aedes aegypti]	XP_001657526.1	1,00E-16	GLOS_AAEL_AAE	GO:0010212	B	Refseq	response to ionizing radiation
XP_001657526.1 hypothetical protein AaeL_AAEL006153 [Aedes aegypti]	XP_001657526.1	1,00E-16	GLOS_AAEL_AAE	GO:0019904	M	.	protein domain specific binding
XP_001657526.1 hypothetical protein AaeL_AAEL006153 [Aedes aegypti]	XP_001657526.1	1,00E-16	GLOS_AAEL_AAE	GO:0017124	M	Refseq	SH3 domain binding
XP_001657526.1 hypothetical protein AaeL_AAEL006153 [Aedes aegypti]	XP_001657526.1	1,00E-16	GLOS_AAEL_AAE	GO:0042802	M	Refseq	identical protein binding
XP_001657526.1 hypothetical protein AaeL_AAEL006153 [Aedes aegypti]	XP_001657526.1	1,00E-16	GLOS_AAEL_AAE	GO:0051726	B	.	regulation of cell cycle
XP_001657526.1 hypothetical protein AaeL_AAEL006153 [Aedes aegypti]	XP_001657526.1	1,00E-16	GLOS_AAEL_AAE	GO:0045786	B	Refseq	negative regulation of cell cycle
XP_001657526.1 hypothetical protein AaeL_AAEL006153 [Aedes aegypti]	XP_001657526.1	1,00E-16	GLOS_AAEL_AAE	GO:0048471	C	Refseq	perinuclear region of cytoplasm
XP_001657526.1 hypothetical protein AaeL_AAEL006153 [Aedes aegypti]	XP_001657526.1	1,00E-16	GLOS_AAEL_AAE	GO:0008134	M	.	transcription factor binding
XP_001657526.1 hypothetical protein AaeL_AAEL006153 [Aedes aegypti]	XP_001657526.1	1,00E-16	GLOS_AAEL_AAE	GO:0051059	M	Refseq	NF-kappaB binding
XP_001657526.1 hypothetical protein AaeL_AAEL006153 [Aedes aegypti]	XP_001657526.1	1,00E-16	GLOS_AAEL_AAE	GO:0072331	B	.	signal transduction by p53 class mediator
XP_001657526.1 hypothetical protein AaeL_AAEL006153 [Aedes aegypti]	XP_001657526.1	1,00E-16	GLOS_AAEL_AAE	GO:0035556	B	.	intracellular signal transduction
XP_001657526.1 hypothetical protein AaeL_AAEL006153 [Aedes aegypti]	XP_001657526.1	1,00E-16	GLOS_AAEL_AAE	GO:0097193	B	.	intrinsic apoptotic signaling pathway
XP_001657526.1 hypothetical protein AaeL_AAEL006153 [Aedes aegypti]	XP_001657526.1	1,00E-16	GLOS_AAEL_AAE	GO:0097190	B	.	apoptotic signaling pathway
XP_001657526.1 hypothetical protein AaeL_AAEL006153 [Aedes aegypti]	XP_001657526.1	1,00E-16	GLOS_AAEL_AAE	GO:0006915	B	.	apoptotic process
XP_001657526.1 hypothetical protein AaeL_AAEL006153 [Aedes aegypti]	XP_001657526.1	1,00E-16	GLOS_AAEL_AAE	GO:0012501	B	.	programmed cell death
XP_001657526.1 hypothetical protein AaeL_AAEL006153 [Aedes aegypti]	XP_001657526.1	1,00E-16	GLOS_AAEL_AAE	GO:0008219	B	.	cell death
XP_001657526.1 hypothetical protein AaeL_AAEL006153 [Aedes aegypti]	XP_001657526.1	1,00E-16	GLOS_AAEL_AAE	GO:0016265	B	.	death
XP_001657526.1 hypothetical protein AaeL_AAEL006153 [Aedes aegypti]	XP_001657526.1	1,00E-16	GLOS_AAEL_AAE	GO:0072332	B	Refseq	intrinsic apoptotic signaling pathway by p53 class mediator
NP_609865.1 CG15152 [Drosophila melanogaster]	NP_609865.1	7,00E-19	GLOS_CG15152.1	GO:0000122	B	Refseq	negative regulation of transcription from RNA polymerase II promoter
NP_609865.1 CG15152 [Drosophila melanogaster]	NP_609865.1	7,00E-19	GLOS_CG15152.1	GO:0001501	B	Refseq	skeletal system development

NP_609865.1 CG15152 [Drosophila melanogaster]	NP_609865.1	7,00E-19	GLOS_CG15152.1	GO:0003700	M	Refseq	sequence-specific DNA binding transcription factor activity
NP_609865.1 CG15152 [Drosophila melanogaster]	NP_609865.1	7,00E-19	GLOS_CG15152.1	GO:0005515	M	Refseq	protein binding
NP_609865.1 CG15152 [Drosophila melanogaster]	NP_609865.1	7,00E-19	GLOS_CG15152.1	GO:0005634	C	Refseq	nucleus
NP_609865.1 CG15152 [Drosophila melanogaster]	NP_609865.1	7,00E-19	GLOS_CG15152.1	GO:0006366	B	Refseq	transcription from RNA polymerase II promoter
NP_609865.1 CG15152 [Drosophila melanogaster]	NP_609865.1	7,00E-19	GLOS_CG15152.1	GO:0006607	B	Refseq	NLS-bearing protein import into nucleus
NP_609865.1 CG15152 [Drosophila melanogaster]	NP_609865.1	7,00E-19	GLOS_CG15152.1	GO:0008270	M	Refseq	zinc ion binding
NP_609865.1 CG15152 [Drosophila melanogaster]	NP_609865.1	7,00E-19	GLOS_CG15152.1	GO:0032330	B	Refseq	regulation of chondrocyte differentiation
NP_609865.1 CG15152 [Drosophila melanogaster]	NP_609865.1	7,00E-19	GLOS_CG15152.1	GO:0043565	M	Refseq	sequence-specific DNA binding
XP_001982933.1 GG13013 [Drosophila erecta]	XP_001982933.1	9,00E-47	GLOS_DERE_GG	GO:0005261	M	Refseq	cation channel activity
XP_001982933.1 GG13013 [Drosophila erecta]	XP_001982933.1	9,00E-47	GLOS_DERE_GG	GO:0005262	M	Refseq	calcium channel activity
XP_001982933.1 GG13013 [Drosophila erecta]	XP_001982933.1	9,00E-47	GLOS_DERE_GG	GO:0005515	M	Refseq	protein binding
XP_001982933.1 GG13013 [Drosophila erecta]	XP_001982933.1	9,00E-47	GLOS_DERE_GG	GO:0005886	C	Refseq	plasma membrane
XP_001982933.1 GG13013 [Drosophila erecta]	XP_001982933.1	9,00E-47	GLOS_DERE_GG	GO:0005887	C	Refseq	integral to plasma membrane
XP_001982933.1 GG13013 [Drosophila erecta]	XP_001982933.1	9,00E-47	GLOS_DERE_GG	GO:0006816	B	Refseq	calcium ion transport
XP_001982933.1 GG13013 [Drosophila erecta]	XP_001982933.1	9,00E-47	GLOS_DERE_GG	GO:0007411	B	Refseq	axon guidance
XP_001982933.1 GG13013 [Drosophila erecta]	XP_001982933.1	9,00E-47	GLOS_DERE_GG	GO:0015279	M	Refseq	store-operated calcium channel activity
XP_001982933.1 GG13013 [Drosophila erecta]	XP_001982933.1	9,00E-47	GLOS_DERE_GG	GO:0016323	C	Refseq	basolateral plasma membrane
XP_001982933.1 GG13013 [Drosophila erecta]	XP_001982933.1	9,00E-47	GLOS_DERE_GG	GO:0030017	C	Refseq	sarcomere
XP_001982933.1 GG13013 [Drosophila erecta]	XP_001982933.1	9,00E-47	GLOS_DERE_GG	GO:0043034	C	Refseq	costamere
XP_001982933.1 GG13013 [Drosophila erecta]	XP_001982933.1	9,00E-47	GLOS_DERE_GG	GO:0043234	C	Refseq	protein complex
XP_001982933.1 GG13013 [Drosophila erecta]	XP_001982933.1	9,00E-47	GLOS_DERE_GG	GO:0044325	M	Refseq	ion channel binding
XP_001982933.1 GG13013 [Drosophila erecta]	XP_001982933.1	9,00E-47	GLOS_DERE_GG	GO:0045121	C	Refseq	membrane raft
XP_001982933.1 GG13013 [Drosophila erecta]	XP_001982933.1	9,00E-47	GLOS_DERE_GG	GO:0046541	B	Refseq	saliva secretion
XP_001982933.1 GG13013 [Drosophila erecta]	XP_001982933.1	9,00E-47	GLOS_DERE_GG	GO:0051281	B	Refseq	positive regulation of release of sequestered calcium ion into cytosol
XP_001982933.1 GG13013 [Drosophila erecta]	XP_001982933.1	9,00E-47	GLOS_DERE_GG	GO:0051480	B	Refseq	cytosolic calcium ion homeostasis
XP_001982933.1 GG13013 [Drosophila erecta]	XP_001982933.1	9,00E-47	GLOS_DERE_GG	GO:0051592	B	Refseq	response to calcium ion
XP_001982933.1 GG13013 [Drosophila erecta]	XP_001982933.1	9,00E-47	GLOS_DERE_GG	GO:0070679	M	Refseq	inositol 1,4,5 trisphosphate binding
NP_001097103.1 CG34310, isoform A /ref|NP_001097104.2|CG34310, isoformC	NP_001097103.1	4,00E-10	GLOS_CG34310.1	GO:0000122	B	Refseq	negative regulation of transcription from RNA polymerase II promoter
NP_001097103.1 CG34310, isoform A /ref|NP_001097104.2|CG34310, isoformC	NP_001097103.1	4,00E-10	GLOS_CG34310.1	GO:0001501	B	Refseq	skeletal system development
NP_001097103.1 CG34310, isoform A /ref|NP_001097104.2|CG34310, isoformC	NP_001097103.1	4,00E-10	GLOS_CG34310.1	GO:0003700	M	Refseq	sequence-specific DNA binding transcription factor activity
NP_001097103.1 CG34310, isoform A /ref|NP_001097104.2|CG34310, isoformC	NP_001097103.1	4,00E-10	GLOS_CG34310.1	GO:0005515	M	Refseq	protein binding
NP_001097103.1 CG34310, isoform A /ref|NP_001097104.2|CG34310, isoformC	NP_001097103.1	4,00E-10	GLOS_CG34310.1	GO:0005634	C	Refseq	nucleus
NP_001097103.1 CG34310, isoform A /ref|NP_001097104.2|CG34310, isoformC	NP_001097103.1	4,00E-10	GLOS_CG34310.1	GO:0006366	B	Refseq	transcription from RNA polymerase II promoter
NP_001097103.1 CG34310, isoform A /ref|NP_001097104.2|CG34310, isoformC	NP_001097103.1	4,00E-10	GLOS_CG34310.1	GO:0006607	B	Refseq	NLS-bearing protein import into nucleus
NP_001097103.1 CG34310, isoform A /ref|NP_001097104.2|CG34310, isoformC	NP_001097103.1	4,00E-10	GLOS_CG34310.1	GO:0008270	M	Refseq	zinc ion binding
NP_001097103.1 CG34310, isoform A /ref|NP_001097104.2|CG34310, isoformC	NP_001097103.1	4,00E-10	GLOS_CG34310.1	GO:0032330	B	Refseq	regulation of chondrocyte differentiation
NP_001097103.1 CG34310, isoform A /ref|NP_001097104.2|CG34310, isoformC	NP_001097103.1	4,00E-10	GLOS_CG34310.1	GO:0043565	M	Refseq	sequence-specific DNA binding
XP_974127.2 PREDICTED: similar to AGAP009641-PA [Tribolium castaneum]	XP_974127.2	2,00E-79	GLOS_LOC66296	GO:0048646	B	.	anatomical structure formation involved in morphogenesis
XP_974127.2 PREDICTED: similar to AGAP009641-PA [Tribolium castaneum]	XP_974127.2	2,00E-79	GLOS_LOC66296	GO:0048514	B	.	blood vessel morphogenesis
XP_974127.2 PREDICTED: similar to AGAP009641-PA [Tribolium castaneum]	XP_974127.2	2,00E-79	GLOS_LOC66296	GO:0001568	B	.	blood vessel development
XP_974127.2 PREDICTED: similar to AGAP009641-PA [Tribolium castaneum]	XP_974127.2	2,00E-79	GLOS_LOC66296	GO:0001944	B	.	vasculature development
XP_974127.2 PREDICTED: similar to AGAP009641-PA [Tribolium castaneum]	XP_974127.2	2,00E-79	GLOS_LOC66296	GO:0001525	B	Refseq	angiogenesis
XP_974127.2 PREDICTED: similar to AGAP009641-PA [Tribolium castaneum]	XP_974127.2	2,00E-79	GLOS_LOC66296	GO:0005099	M	.	Ras GTPase activator activity
XP_974127.2 PREDICTED: similar to AGAP009641-PA [Tribolium castaneum]	XP_974127.2	2,00E-79	GLOS_LOC66296	GO:0005083	M	.	small GTPase regulator activity
XP_974127.2 PREDICTED: similar to AGAP009641-PA [Tribolium castaneum]	XP_974127.2	2,00E-79	GLOS_LOC66296	GO:0030695	M	.	GTPase regulator activity
XP_974127.2 PREDICTED: similar to AGAP009641-PA [Tribolium castaneum]	XP_974127.2	2,00E-79	GLOS_LOC66296	GO:0060589	M	.	nucleoside-triphosphatase regulator activity
XP_974127.2 PREDICTED: similar to AGAP009641-PA [Tribolium castaneum]	XP_974127.2	2,00E-79	GLOS_LOC66296	GO:0030234	M	.	enzyme regulator activity
XP_974127.2 PREDICTED: similar to AGAP009641-PA [Tribolium castaneum]	XP_974127.2	2,00E-79	GLOS_LOC66296	GO:0005096	M	.	GTPase activator activity
XP_974127.2 PREDICTED: similar to AGAP009641-PA [Tribolium castaneum]	XP_974127.2	2,00E-79	GLOS_LOC66296	GO:0008047	M	.	enzyme activator activity
XP_974127.2 PREDICTED: similar to AGAP009641-PA [Tribolium castaneum]	XP_974127.2	2,00E-79	GLOS_LOC66296	GO:0005100	M	Refseq	Rho GTPase activator activity
XP_974127.2 PREDICTED: similar to AGAP009641-PA [Tribolium castaneum]	XP_974127.2	2,00E-79	GLOS_LOC66296	GO:0005102	M	.	receptor binding
XP_974127.2 PREDICTED: similar to AGAP009641-PA [Tribolium castaneum]	XP_974127.2	2,00E-79	GLOS_LOC66296	GO:0032403	M	.	protein complex binding
XP_974127.2 PREDICTED: similar to AGAP009641-PA [Tribolium castaneum]	XP_974127.2	2,00E-79	GLOS_LOC66296	GO:0005178	M	Refseq	integrin binding
XP_974127.2 PREDICTED: similar to AGAP009641-PA [Tribolium castaneum]	XP_974127.2	2,00E-79	GLOS_LOC66296	GO:0005515	M	Refseq	protein binding

XP_974127.2 PREDICTED: similar to AGAP009641-PA [Tribolium castaneum]	XP_974127.2	2,00E-79	GLOS_LOC66296	GO:0005783	C	Refseq	endoplasmic reticulum
XP_974127.2 PREDICTED: similar to AGAP009641-PA [Tribolium castaneum]	XP_974127.2	2,00E-79	GLOS_LOC66296	GO:0005829	C	Refseq	cytosol
XP_974127.2 PREDICTED: similar to AGAP009641-PA [Tribolium castaneum]	XP_974127.2	2,00E-79	GLOS_LOC66296	GO:0005886	C	Refseq	plasma membrane
XP_974127.2 PREDICTED: similar to AGAP009641-PA [Tribolium castaneum]	XP_974127.2	2,00E-79	GLOS_LOC66296	GO:0005887	C	Refseq	integral to plasma membrane
XP_974127.2 PREDICTED: similar to AGAP009641-PA [Tribolium castaneum]	XP_974127.2	2,00E-79	GLOS_LOC66296	GO:0001933	B	.	negative regulation of protein phosphorylation
XP_974127.2 PREDICTED: similar to AGAP009641-PA [Tribolium castaneum]	XP_974127.2	2,00E-79	GLOS_LOC66296	GO:0001932	B	.	regulation of protein phosphorylation
XP_974127.2 PREDICTED: similar to AGAP009641-PA [Tribolium castaneum]	XP_974127.2	2,00E-79	GLOS_LOC66296	GO:0031399	B	.	regulation of protein modification process
XP_974127.2 PREDICTED: similar to AGAP009641-PA [Tribolium castaneum]	XP_974127.2	2,00E-79	GLOS_LOC66296	GO:0032268	B	.	regulation of cellular protein metabolic process
XP_974127.2 PREDICTED: similar to AGAP009641-PA [Tribolium castaneum]	XP_974127.2	2,00E-79	GLOS_LOC66296	GO:0051246	B	.	regulation of protein metabolic process
XP_974127.2 PREDICTED: similar to AGAP009641-PA [Tribolium castaneum]	XP_974127.2	2,00E-79	GLOS_LOC66296	GO:0042325	B	.	regulation of phosphorylation
XP_974127.2 PREDICTED: similar to AGAP009641-PA [Tribolium castaneum]	XP_974127.2	2,00E-79	GLOS_LOC66296	GO:0019220	B	.	regulation of phosphate metabolic process
XP_974127.2 PREDICTED: similar to AGAP009641-PA [Tribolium castaneum]	XP_974127.2	2,00E-79	GLOS_LOC66296	GO:0051174	B	.	regulation of phosphorus metabolic process
XP_974127.2 PREDICTED: similar to AGAP009641-PA [Tribolium castaneum]	XP_974127.2	2,00E-79	GLOS_LOC66296	GO:0031400	B	.	negative regulation of protein modification process
XP_974127.2 PREDICTED: similar to AGAP009641-PA [Tribolium castaneum]	XP_974127.2	2,00E-79	GLOS_LOC66296	GO:0032269	B	.	negative regulation of cellular protein metabolic process
XP_974127.2 PREDICTED: similar to AGAP009641-PA [Tribolium castaneum]	XP_974127.2	2,00E-79	GLOS_LOC66296	GO:0051248	B	.	negative regulation of protein metabolic process
XP_974127.2 PREDICTED: similar to AGAP009641-PA [Tribolium castaneum]	XP_974127.2	2,00E-79	GLOS_LOC66296	GO:0042326	B	.	negative regulation of phosphorylation
XP_974127.2 PREDICTED: similar to AGAP009641-PA [Tribolium castaneum]	XP_974127.2	2,00E-79	GLOS_LOC66296	GO:0045936	B	.	negative regulation of phosphate metabolic process
XP_974127.2 PREDICTED: similar to AGAP009641-PA [Tribolium castaneum]	XP_974127.2	2,00E-79	GLOS_LOC66296	GO:0010563	B	.	negative regulation of phosphorus metabolic process
XP_974127.2 PREDICTED: similar to AGAP009641-PA [Tribolium castaneum]	XP_974127.2	2,00E-79	GLOS_LOC66296	GO:0033673	B	.	negative regulation of kinase activity
XP_974127.2 PREDICTED: similar to AGAP009641-PA [Tribolium castaneum]	XP_974127.2	2,00E-79	GLOS_LOC66296	GO:0043549	B	.	regulation of kinase activity
XP_974127.2 PREDICTED: similar to AGAP009641-PA [Tribolium castaneum]	XP_974127.2	2,00E-79	GLOS_LOC66296	GO:0051338	B	.	regulation of transferase activity
XP_974127.2 PREDICTED: similar to AGAP009641-PA [Tribolium castaneum]	XP_974127.2	2,00E-79	GLOS_LOC66296	GO:0050790	B	.	regulation of catalytic activity
XP_974127.2 PREDICTED: similar to AGAP009641-PA [Tribolium castaneum]	XP_974127.2	2,00E-79	GLOS_LOC66296	GO:0065009	B	.	regulation of molecular function
XP_974127.2 PREDICTED: similar to AGAP009641-PA [Tribolium castaneum]	XP_974127.2	2,00E-79	GLOS_LOC66296	GO:0051348	B	.	negative regulation of transferase activity
XP_974127.2 PREDICTED: similar to AGAP009641-PA [Tribolium castaneum]	XP_974127.2	2,00E-79	GLOS_LOC66296	GO:0043086	B	.	negative regulation of catalytic activity
XP_974127.2 PREDICTED: similar to AGAP009641-PA [Tribolium castaneum]	XP_974127.2	2,00E-79	GLOS_LOC66296	GO:0044092	B	.	negative regulation of molecular function
XP_974127.2 PREDICTED: similar to AGAP009641-PA [Tribolium castaneum]	XP_974127.2	2,00E-79	GLOS_LOC66296	GO:0045859	B	.	regulation of protein kinase activity
XP_974127.2 PREDICTED: similar to AGAP009641-PA [Tribolium castaneum]	XP_974127.2	2,00E-79	GLOS_LOC66296	GO:0006469	B	Refseq	negative regulation of protein kinase activity
XP_974127.2 PREDICTED: similar to AGAP009641-PA [Tribolium castaneum]	XP_974127.2	2,00E-79	GLOS_LOC66296	GO:0006996	B	.	organelle organization
XP_974127.2 PREDICTED: similar to AGAP009641-PA [Tribolium castaneum]	XP_974127.2	2,00E-79	GLOS_LOC66296	GO:0007010	B	Refseq	cytoskeleton organization
XP_974127.2 PREDICTED: similar to AGAP009641-PA [Tribolium castaneum]	XP_974127.2	2,00E-79	GLOS_LOC66296	GO:0009986	C	.	cell surface
XP_974127.2 PREDICTED: similar to AGAP009641-PA [Tribolium castaneum]	XP_974127.2	2,00E-79	GLOS_LOC66296	GO:0009897	C	Refseq	external side of plasma membrane
XP_974127.2 PREDICTED: similar to AGAP009641-PA [Tribolium castaneum]	XP_974127.2	2,00E-79	GLOS_LOC66296	GO:0045177	C	.	apical part of cell
XP_974127.2 PREDICTED: similar to AGAP009641-PA [Tribolium castaneum]	XP_974127.2	2,00E-79	GLOS_LOC66296	GO:0016324	C	Refseq	apical plasma membrane
XP_974127.2 PREDICTED: similar to AGAP009641-PA [Tribolium castaneum]	XP_974127.2	2,00E-79	GLOS_LOC66296	GO:0007155	B	.	cell adhesion
XP_974127.2 PREDICTED: similar to AGAP009641-PA [Tribolium castaneum]	XP_974127.2	2,00E-79	GLOS_LOC66296	GO:0022610	B	.	biological adhesion
XP_974127.2 PREDICTED: similar to AGAP009641-PA [Tribolium castaneum]	XP_974127.2	2,00E-79	GLOS_LOC66296	GO:0016337	B	Refseq	cell-cell adhesion
XP_974127.2 PREDICTED: similar to AGAP009641-PA [Tribolium castaneum]	XP_974127.2	2,00E-79	GLOS_LOC66296	GO:0019900	M	.	kinase binding
XP_974127.2 PREDICTED: similar to AGAP009641-PA [Tribolium castaneum]	XP_974127.2	2,00E-79	GLOS_LOC66296	GO:0019899	M	.	enzyme binding
XP_974127.2 PREDICTED: similar to AGAP009641-PA [Tribolium castaneum]	XP_974127.2	2,00E-79	GLOS_LOC66296	GO:0019901	M	Refseq	protein kinase binding
XP_974127.2 PREDICTED: similar to AGAP009641-PA [Tribolium castaneum]	XP_974127.2	2,00E-79	GLOS_LOC66296	GO:0030334	B	.	regulation of cell migration
XP_974127.2 PREDICTED: similar to AGAP009641-PA [Tribolium castaneum]	XP_974127.2	2,00E-79	GLOS_LOC66296	GO:2000145	B	.	regulation of cell motility
XP_974127.2 PREDICTED: similar to AGAP009641-PA [Tribolium castaneum]	XP_974127.2	2,00E-79	GLOS_LOC66296	GO:0040012	B	.	regulation of locomotion
XP_974127.2 PREDICTED: similar to AGAP009641-PA [Tribolium castaneum]	XP_974127.2	2,00E-79	GLOS_LOC66296	GO:0051270	B	.	regulation of cellular component movement
XP_974127.2 PREDICTED: similar to AGAP009641-PA [Tribolium castaneum]	XP_974127.2	2,00E-79	GLOS_LOC66296	GO:2000146	B	.	negative regulation of cell motility
XP_974127.2 PREDICTED: similar to AGAP009641-PA [Tribolium castaneum]	XP_974127.2	2,00E-79	GLOS_LOC66296	GO:0040013	B	.	negative regulation of locomotion
XP_974127.2 PREDICTED: similar to AGAP009641-PA [Tribolium castaneum]	XP_974127.2	2,00E-79	GLOS_LOC66296	GO:0051271	B	.	negative regulation of cellular component movement
XP_974127.2 PREDICTED: similar to AGAP009641-PA [Tribolium castaneum]	XP_974127.2	2,00E-79	GLOS_LOC66296	GO:0030336	B	Refseq	negative regulation of cell migration
XP_974127.2 PREDICTED: similar to AGAP009641-PA [Tribolium castaneum]	XP_974127.2	2,00E-79	GLOS_LOC66296	GO:0043005	C	.	neuron projection
XP_974127.2 PREDICTED: similar to AGAP009641-PA [Tribolium castaneum]	XP_974127.2	2,00E-79	GLOS_LOC66296	GO:0042995	C	.	cell projection
XP_974127.2 PREDICTED: similar to AGAP009641-PA [Tribolium castaneum]	XP_974127.2	2,00E-79	GLOS_LOC66296	GO:0097458	C	.	neuron part
XP_974127.2 PREDICTED: similar to AGAP009641-PA [Tribolium castaneum]	XP_974127.2	2,00E-79	GLOS_LOC66296	GO:0030425	C	Refseq	dendrite
XP_974127.2 PREDICTED: similar to AGAP009641-PA [Tribolium castaneum]	XP_974127.2	2,00E-79	GLOS_LOC66296	GO:0030427	C	.	site of polarized growth
XP_974127.2 PREDICTED: similar to AGAP009641-PA [Tribolium castaneum]	XP_974127.2	2,00E-79	GLOS_LOC66296	GO:0044463	C	.	cell projection part

XP_974127.2 PREDICTED: similar to AGAP009641-PA [Tribolium castaneum]	XP_974127.2	2,00E-79	GLOS_LOC66296	GO:0030426	C	Refseq	growth cone
XP_974127.2 PREDICTED: similar to AGAP009641-PA [Tribolium castaneum]	XP_974127.2	2,00E-79	GLOS_LOC66296	GO:0031233	C	.	intrinsic to external side of plasma membrane
XP_974127.2 PREDICTED: similar to AGAP009641-PA [Tribolium castaneum]	XP_974127.2	2,00E-79	GLOS_LOC66296	GO:0009897	C	.	external side of plasma membrane
XP_974127.2 PREDICTED: similar to AGAP009641-PA [Tribolium castaneum]	XP_974127.2	2,00E-79	GLOS_LOC66296	GO:0046658	C	.	anchored to plasma membrane
XP_974127.2 PREDICTED: similar to AGAP009641-PA [Tribolium castaneum]	XP_974127.2	2,00E-79	GLOS_LOC66296	GO:0031225	C	.	anchored to membrane
XP_974127.2 PREDICTED: similar to AGAP009641-PA [Tribolium castaneum]	XP_974127.2	2,00E-79	GLOS_LOC66296	GO:0031362	C	Refseq	anchored to external side of plasma membrane
XP_974127.2 PREDICTED: similar to AGAP009641-PA [Tribolium castaneum]	XP_974127.2	2,00E-79	GLOS_LOC66296	GO:0035091	M	.	phosphatidylinositol binding
XP_974127.2 PREDICTED: similar to AGAP009641-PA [Tribolium castaneum]	XP_974127.2	2,00E-79	GLOS_LOC66296	GO:0005543	M	.	phospholipid binding
XP_974127.2 PREDICTED: similar to AGAP009641-PA [Tribolium castaneum]	XP_974127.2	2,00E-79	GLOS_LOC66296	GO:0008289	M	.	lipid binding
XP_974127.2 PREDICTED: similar to AGAP009641-PA [Tribolium castaneum]	XP_974127.2	2,00E-79	GLOS_LOC66296	GO:0051861	M	.	glycolipid binding
XP_974127.2 PREDICTED: similar to AGAP009641-PA [Tribolium castaneum]	XP_974127.2	2,00E-79	GLOS_LOC66296	GO:0097367	M	.	carbohydrate derivative binding
XP_974127.2 PREDICTED: similar to AGAP009641-PA [Tribolium castaneum]	XP_974127.2	2,00E-79	GLOS_LOC66296	GO:0034235	M	Refseq	GPI anchor binding
XP_974127.2 PREDICTED: similar to AGAP009641-PA [Tribolium castaneum]	XP_974127.2	2,00E-79	GLOS_LOC66296	GO:0043087	B	.	regulation of GTPase activity
XP_974127.2 PREDICTED: similar to AGAP009641-PA [Tribolium castaneum]	XP_974127.2	2,00E-79	GLOS_LOC66296	GO:0033124	B	.	regulation of GTP catabolic process
XP_974127.2 PREDICTED: similar to AGAP009641-PA [Tribolium castaneum]	XP_974127.2	2,00E-79	GLOS_LOC66296	GO:0009118	B	.	regulation of nucleoside metabolic process
XP_974127.2 PREDICTED: similar to AGAP009641-PA [Tribolium castaneum]	XP_974127.2	2,00E-79	GLOS_LOC66296	GO:0033121	B	.	regulation of purine nucleotide catabolic process
XP_974127.2 PREDICTED: similar to AGAP009641-PA [Tribolium castaneum]	XP_974127.2	2,00E-79	GLOS_LOC66296	GO:0030811	B	.	regulation of nucleotide catabolic process
XP_974127.2 PREDICTED: similar to AGAP009641-PA [Tribolium castaneum]	XP_974127.2	2,00E-79	GLOS_LOC66296	GO:0006140	B	.	regulation of nucleotide metabolic process
XP_974127.2 PREDICTED: similar to AGAP009641-PA [Tribolium castaneum]	XP_974127.2	2,00E-79	GLOS_LOC66296	GO:0031329	B	.	regulation of cellular catabolic process
XP_974127.2 PREDICTED: similar to AGAP009641-PA [Tribolium castaneum]	XP_974127.2	2,00E-79	GLOS_LOC66296	GO:0009894	B	.	regulation of catabolic process
XP_974127.2 PREDICTED: similar to AGAP009641-PA [Tribolium castaneum]	XP_974127.2	2,00E-79	GLOS_LOC66296	GO:1900542	B	.	regulation of purine nucleotide metabolic process
XP_974127.2 PREDICTED: similar to AGAP009641-PA [Tribolium castaneum]	XP_974127.2	2,00E-79	GLOS_LOC66296	GO:0051336	B	.	regulation of hydrolase activity
XP_974127.2 PREDICTED: similar to AGAP009641-PA [Tribolium castaneum]	XP_974127.2	2,00E-79	GLOS_LOC66296	GO:0051345	B	.	positive regulation of hydrolase activity
XP_974127.2 PREDICTED: similar to AGAP009641-PA [Tribolium castaneum]	XP_974127.2	2,00E-79	GLOS_LOC66296	GO:0043085	B	.	positive regulation of catalytic activity
XP_974127.2 PREDICTED: similar to AGAP009641-PA [Tribolium castaneum]	XP_974127.2	2,00E-79	GLOS_LOC66296	GO:0044093	B	.	positive regulation of molecular function
XP_974127.2 PREDICTED: similar to AGAP009641-PA [Tribolium castaneum]	XP_974127.2	2,00E-79	GLOS_LOC66296	GO:0043547	B	Refseq	positive regulation of GTPase activity
XP_974127.2 PREDICTED: similar to AGAP009641-PA [Tribolium castaneum]	XP_974127.2	2,00E-79	GLOS_LOC66296	GO:0045121	C	Refseq	membrane raft
XP_974127.2 PREDICTED: similar to AGAP009641-PA [Tribolium castaneum]	XP_974127.2	2,00E-79	GLOS_LOC66296	GO:0042462	B	.	eye photoreceptor cell development
XP_974127.2 PREDICTED: similar to AGAP009641-PA [Tribolium castaneum]	XP_974127.2	2,00E-79	GLOS_LOC66296	GO:0042461	B	.	photoreceptor cell development
XP_974127.2 PREDICTED: similar to AGAP009641-PA [Tribolium castaneum]	XP_974127.2	2,00E-79	GLOS_LOC66296	GO:0046530	B	.	photoreceptor cell differentiation
XP_974127.2 PREDICTED: similar to AGAP009641-PA [Tribolium castaneum]	XP_974127.2	2,00E-79	GLOS_LOC66296	GO:0001754	B	.	eye photoreceptor cell differentiation
XP_974127.2 PREDICTED: similar to AGAP009641-PA [Tribolium castaneum]	XP_974127.2	2,00E-79	GLOS_LOC66296	GO:0048592	B	.	eye morphogenesis
XP_974127.2 PREDICTED: similar to AGAP009641-PA [Tribolium castaneum]	XP_974127.2	2,00E-79	GLOS_LOC66296	GO:0009887	B	.	organ morphogenesis
XP_974127.2 PREDICTED: similar to AGAP009641-PA [Tribolium castaneum]	XP_974127.2	2,00E-79	GLOS_LOC66296	GO:0001654	B	.	eye development
XP_974127.2 PREDICTED: similar to AGAP009641-PA [Tribolium castaneum]	XP_974127.2	2,00E-79	GLOS_LOC66296	GO:0007423	B	.	sensory organ development
XP_974127.2 PREDICTED: similar to AGAP009641-PA [Tribolium castaneum]	XP_974127.2	2,00E-79	GLOS_LOC66296	GO:0042670	B	.	retinal cone cell differentiation
XP_974127.2 PREDICTED: similar to AGAP009641-PA [Tribolium castaneum]	XP_974127.2	2,00E-79	GLOS_LOC66296	GO:0060219	B	.	camera-type eye photoreceptor cell differentiation
XP_974127.2 PREDICTED: similar to AGAP009641-PA [Tribolium castaneum]	XP_974127.2	2,00E-79	GLOS_LOC66296	GO:0003407	B	.	neural retina development
XP_974127.2 PREDICTED: similar to AGAP009641-PA [Tribolium castaneum]	XP_974127.2	2,00E-79	GLOS_LOC66296	GO:0060041	B	.	retina development in camera-type eye
XP_974127.2 PREDICTED: similar to AGAP009641-PA [Tribolium castaneum]	XP_974127.2	2,00E-79	GLOS_LOC66296	GO:0043010	B	.	camera-type eye development
XP_974127.2 PREDICTED: similar to AGAP009641-PA [Tribolium castaneum]	XP_974127.2	2,00E-79	GLOS_LOC66296	GO:0060042	B	.	retina morphogenesis in camera-type eye
XP_974127.2 PREDICTED: similar to AGAP009641-PA [Tribolium castaneum]	XP_974127.2	2,00E-79	GLOS_LOC66296	GO:0048593	B	.	camera-type eye morphogenesis
XP_974127.2 PREDICTED: similar to AGAP009641-PA [Tribolium castaneum]	XP_974127.2	2,00E-79	GLOS_LOC66296	GO:0046549	B	Refseq	retinal cone cell development
XP_974127.2 PREDICTED: similar to AGAP009641-PA [Tribolium castaneum]	XP_974127.2	2,00E-79	GLOS_LOC66296	GO:0007045	B	.	cell-substrate adherens junction assembly
XP_974127.2 PREDICTED: similar to AGAP009641-PA [Tribolium castaneum]	XP_974127.2	2,00E-79	GLOS_LOC66296	GO:0007044	B	.	cell-substrate junction assembly
XP_974127.2 PREDICTED: similar to AGAP009641-PA [Tribolium castaneum]	XP_974127.2	2,00E-79	GLOS_LOC66296	GO:0034329	B	.	cell junction assembly
XP_974127.2 PREDICTED: similar to AGAP009641-PA [Tribolium castaneum]	XP_974127.2	2,00E-79	GLOS_LOC66296	GO:0022607	B	.	cellular component assembly
XP_974127.2 PREDICTED: similar to AGAP009641-PA [Tribolium castaneum]	XP_974127.2	2,00E-79	GLOS_LOC66296	GO:0044085	B	.	cellular component biogenesis
XP_974127.2 PREDICTED: similar to AGAP009641-PA [Tribolium castaneum]	XP_974127.2	2,00E-79	GLOS_LOC66296	GO:0034330	B	.	cell junction organization
XP_974127.2 PREDICTED: similar to AGAP009641-PA [Tribolium castaneum]	XP_974127.2	2,00E-79	GLOS_LOC66296	GO:0034333	B	.	adherens junction assembly
XP_974127.2 PREDICTED: similar to AGAP009641-PA [Tribolium castaneum]	XP_974127.2	2,00E-79	GLOS_LOC66296	GO:0034332	B	.	adherens junction organization
XP_974127.2 PREDICTED: similar to AGAP009641-PA [Tribolium castaneum]	XP_974127.2	2,00E-79	GLOS_LOC66296	GO:0045216	B	.	cell-cell junction organization
XP_974127.2 PREDICTED: similar to AGAP009641-PA [Tribolium castaneum]	XP_974127.2	2,00E-79	GLOS_LOC66296	GO:0007160	B	.	cell-matrix adhesion
XP_974127.2 PREDICTED: similar to AGAP009641-PA [Tribolium castaneum]	XP_974127.2	2,00E-79	GLOS_LOC66296	GO:0031589	B	.	cell-substrate adhesion

XP_974127.2 PREDICTED: similar to AGAP009641-PA [Tribolium castaneum]	XP_974127.2	2,00E-79	GLOS_LOC66296	GO:0048041	B	Refseq	focal adhesion assembly
XP_974127.2 PREDICTED: similar to AGAP009641-PA [Tribolium castaneum]	XP_974127.2	2,00E-79	GLOS_LOC66296	GO:0031345	B	.	negative regulation of cell projection organization
XP_974127.2 PREDICTED: similar to AGAP009641-PA [Tribolium castaneum]	XP_974127.2	2,00E-79	GLOS_LOC66296	GO:0031344	B	.	regulation of cell projection organization
XP_974127.2 PREDICTED: similar to AGAP009641-PA [Tribolium castaneum]	XP_974127.2	2,00E-79	GLOS_LOC66296	GO:0051128	B	.	regulation of cellular component organization
XP_974127.2 PREDICTED: similar to AGAP009641-PA [Tribolium castaneum]	XP_974127.2	2,00E-79	GLOS_LOC66296	GO:0051129	B	.	negative regulation of cellular component organization
XP_974127.2 PREDICTED: similar to AGAP009641-PA [Tribolium castaneum]	XP_974127.2	2,00E-79	GLOS_LOC66296	GO:0050768	B	.	negative regulation of neurogenesis
XP_974127.2 PREDICTED: similar to AGAP009641-PA [Tribolium castaneum]	XP_974127.2	2,00E-79	GLOS_LOC66296	GO:0010721	B	.	negative regulation of cell development
XP_974127.2 PREDICTED: similar to AGAP009641-PA [Tribolium castaneum]	XP_974127.2	2,00E-79	GLOS_LOC66296	GO:0045596	B	.	negative regulation of cell differentiation
XP_974127.2 PREDICTED: similar to AGAP009641-PA [Tribolium castaneum]	XP_974127.2	2,00E-79	GLOS_LOC66296	GO:0051093	B	.	negative regulation of developmental process
XP_974127.2 PREDICTED: similar to AGAP009641-PA [Tribolium castaneum]	XP_974127.2	2,00E-79	GLOS_LOC66296	GO:0060284	B	.	regulation of cell development
XP_974127.2 PREDICTED: similar to AGAP009641-PA [Tribolium castaneum]	XP_974127.2	2,00E-79	GLOS_LOC66296	GO:0050767	B	.	regulation of neurogenesis
XP_974127.2 PREDICTED: similar to AGAP009641-PA [Tribolium castaneum]	XP_974127.2	2,00E-79	GLOS_LOC66296	GO:0051960	B	.	regulation of nervous system development
XP_974127.2 PREDICTED: similar to AGAP009641-PA [Tribolium castaneum]	XP_974127.2	2,00E-79	GLOS_LOC66296	GO:0050770	B	.	regulation of axonogenesis
XP_974127.2 PREDICTED: similar to AGAP009641-PA [Tribolium castaneum]	XP_974127.2	2,00E-79	GLOS_LOC66296	GO:0010769	B	.	regulation of cell morphogenesis involved in differentiation
XP_974127.2 PREDICTED: similar to AGAP009641-PA [Tribolium castaneum]	XP_974127.2	2,00E-79	GLOS_LOC66296	GO:0022604	B	.	regulation of cell morphogenesis
XP_974127.2 PREDICTED: similar to AGAP009641-PA [Tribolium castaneum]	XP_974127.2	2,00E-79	GLOS_LOC66296	GO:0022603	B	.	regulation of anatomical structure morphogenesis
XP_974127.2 PREDICTED: similar to AGAP009641-PA [Tribolium castaneum]	XP_974127.2	2,00E-79	GLOS_LOC66296	GO:0010975	B	.	regulation of neuron projection development
XP_974127.2 PREDICTED: similar to AGAP009641-PA [Tribolium castaneum]	XP_974127.2	2,00E-79	GLOS_LOC66296	GO:0045664	B	.	regulation of neuron differentiation
XP_974127.2 PREDICTED: similar to AGAP009641-PA [Tribolium castaneum]	XP_974127.2	2,00E-79	GLOS_LOC66296	GO:0050771	B	Refseq	negative regulation of axonogenesis
XP_974127.2 PREDICTED: similar to AGAP009641-PA [Tribolium castaneum]	XP_974127.2	2,00E-79	GLOS_LOC66296	GO:0050851	B	.	antigen receptor-mediated signaling pathway
XP_974127.2 PREDICTED: similar to AGAP009641-PA [Tribolium castaneum]	XP_974127.2	2,00E-79	GLOS_LOC66296	GO:0002429	B	.	immune response-activating cell surface receptor signaling pathway
XP_974127.2 PREDICTED: similar to AGAP009641-PA [Tribolium castaneum]	XP_974127.2	2,00E-79	GLOS_LOC66296	GO:0002757	B	.	immune response-activating signal transduction
XP_974127.2 PREDICTED: similar to AGAP009641-PA [Tribolium castaneum]	XP_974127.2	2,00E-79	GLOS_LOC66296	GO:0002253	B	.	activation of immune response
XP_974127.2 PREDICTED: similar to AGAP009641-PA [Tribolium castaneum]	XP_974127.2	2,00E-79	GLOS_LOC66296	GO:0002376	B	.	immune system process
XP_974127.2 PREDICTED: similar to AGAP009641-PA [Tribolium castaneum]	XP_974127.2	2,00E-79	GLOS_LOC66296	GO:0050778	B	.	positive regulation of immune response
XP_974127.2 PREDICTED: similar to AGAP009641-PA [Tribolium castaneum]	XP_974127.2	2,00E-79	GLOS_LOC66296	GO:0002684	B	.	positive regulation of immune system process
XP_974127.2 PREDICTED: similar to AGAP009641-PA [Tribolium castaneum]	XP_974127.2	2,00E-79	GLOS_LOC66296	GO:0002682	B	.	regulation of immune system process
XP_974127.2 PREDICTED: similar to AGAP009641-PA [Tribolium castaneum]	XP_974127.2	2,00E-79	GLOS_LOC66296	GO:0048584	B	.	positive regulation of response to stimulus
XP_974127.2 PREDICTED: similar to AGAP009641-PA [Tribolium castaneum]	XP_974127.2	2,00E-79	GLOS_LOC66296	GO:0048583	B	.	regulation of response to stimulus
XP_974127.2 PREDICTED: similar to AGAP009641-PA [Tribolium castaneum]	XP_974127.2	2,00E-79	GLOS_LOC66296	GO:0050776	B	.	regulation of immune response
XP_974127.2 PREDICTED: similar to AGAP009641-PA [Tribolium castaneum]	XP_974127.2	2,00E-79	GLOS_LOC66296	GO:0002764	B	.	immune response-regulating signaling pathway
XP_974127.2 PREDICTED: similar to AGAP009641-PA [Tribolium castaneum]	XP_974127.2	2,00E-79	GLOS_LOC66296	GO:0002768	B	.	immune response-regulating cell surface receptor signaling pathway
XP_974127.2 PREDICTED: similar to AGAP009641-PA [Tribolium castaneum]	XP_974127.2	2,00E-79	GLOS_LOC66296	GO:0050852	B	Refseq	T cell receptor signaling pathway
XP_974127.2 PREDICTED: similar to AGAP009641-PA [Tribolium castaneum]	XP_974127.2	2,00E-79	GLOS_LOC66296	GO:0050856	B	.	regulation of T cell receptor signaling pathway
XP_974127.2 PREDICTED: similar to AGAP009641-PA [Tribolium castaneum]	XP_974127.2	2,00E-79	GLOS_LOC66296	GO:0050854	B	.	regulation of antigen receptor-mediated signaling pathway
XP_974127.2 PREDICTED: similar to AGAP009641-PA [Tribolium castaneum]	XP_974127.2	2,00E-79	GLOS_LOC66296	GO:0009966	B	.	regulation of signal transduction
XP_974127.2 PREDICTED: similar to AGAP009641-PA [Tribolium castaneum]	XP_974127.2	2,00E-79	GLOS_LOC66296	GO:0010646	B	.	regulation of cell communication
XP_974127.2 PREDICTED: similar to AGAP009641-PA [Tribolium castaneum]	XP_974127.2	2,00E-79	GLOS_LOC66296	GO:0023051	B	.	regulation of signaling
XP_974127.2 PREDICTED: similar to AGAP009641-PA [Tribolium castaneum]	XP_974127.2	2,00E-79	GLOS_LOC66296	GO:0050858	B	.	negative regulation of antigen receptor-mediated signaling pathway
XP_974127.2 PREDICTED: similar to AGAP009641-PA [Tribolium castaneum]	XP_974127.2	2,00E-79	GLOS_LOC66296	GO:0002683	B	.	negative regulation of immune system process
XP_974127.2 PREDICTED: similar to AGAP009641-PA [Tribolium castaneum]	XP_974127.2	2,00E-79	GLOS_LOC66296	GO:0009968	B	.	negative regulation of signal transduction
XP_974127.2 PREDICTED: similar to AGAP009641-PA [Tribolium castaneum]	XP_974127.2	2,00E-79	GLOS_LOC66296	GO:0010648	B	.	negative regulation of cell communication
XP_974127.2 PREDICTED: similar to AGAP009641-PA [Tribolium castaneum]	XP_974127.2	2,00E-79	GLOS_LOC66296	GO:0023057	B	.	negative regulation of signaling
XP_974127.2 PREDICTED: similar to AGAP009641-PA [Tribolium castaneum]	XP_974127.2	2,00E-79	GLOS_LOC66296	GO:0048585	B	.	negative regulation of response to stimulus
XP_974127.2 PREDICTED: similar to AGAP009641-PA [Tribolium castaneum]	XP_974127.2	2,00E-79	GLOS_LOC66296	GO:0050860	B	Refseq	negative regulation of T cell receptor signaling pathway
XP_974127.2 PREDICTED: similar to AGAP009641-PA [Tribolium castaneum]	XP_974127.2	2,00E-79	GLOS_LOC66296	GO:0050863	B	.	regulation of T cell activation
XP_974127.2 PREDICTED: similar to AGAP009641-PA [Tribolium castaneum]	XP_974127.2	2,00E-79	GLOS_LOC66296	GO:0051249	B	.	regulation of lymphocyte activation
XP_974127.2 PREDICTED: similar to AGAP009641-PA [Tribolium castaneum]	XP_974127.2	2,00E-79	GLOS_LOC66296	GO:0002694	B	.	regulation of leukocyte activation
XP_974127.2 PREDICTED: similar to AGAP009641-PA [Tribolium castaneum]	XP_974127.2	2,00E-79	GLOS_LOC66296	GO:0050865	B	.	regulation of cell activation
XP_974127.2 PREDICTED: similar to AGAP009641-PA [Tribolium castaneum]	XP_974127.2	2,00E-79	GLOS_LOC66296	GO:0051251	B	.	positive regulation of lymphocyte activation
XP_974127.2 PREDICTED: similar to AGAP009641-PA [Tribolium castaneum]	XP_974127.2	2,00E-79	GLOS_LOC66296	GO:0002696	B	.	positive regulation of leukocyte activation
XP_974127.2 PREDICTED: similar to AGAP009641-PA [Tribolium castaneum]	XP_974127.2	2,00E-79	GLOS_LOC66296	GO:0050867	B	.	positive regulation of cell activation
XP_974127.2 PREDICTED: similar to AGAP009641-PA [Tribolium castaneum]	XP_974127.2	2,00E-79	GLOS_LOC66296	GO:0050870	B	Refseq	positive regulation of T cell activation
XP_974127.2 PREDICTED: similar to AGAP009641-PA [Tribolium castaneum]	XP_974127.2	2,00E-79	GLOS_LOC66296	GO:0051281	B	Refseq	positive regulation of release of sequestered calcium ion into cytosol

XR_134092.1 PREDICT: cyt c oxidase subunit 1-like (LOC100113910), miscRNA	XR_134092.1	1,00E-15	GLOS_LOC10011:	GO:0022602	B	.	ovulation cycle process
XR_134092.1 PREDICT: cyt c oxidase subunit 1-like (LOC100113910), miscRNA	XR_134092.1	1,00E-15	GLOS_LOC10011:	GO:0048511	B	.	rhythmic process
XR_134092.1 PREDICT: cyt c oxidase subunit 1-like (LOC100113910), miscRNA	XR_134092.1	1,00E-15	GLOS_LOC10011:	GO:0042698	B	.	ovulation cycle
XR_134092.1 PREDICT: cyt c oxidase subunit 1-like (LOC100113910), miscRNA	XR_134092.1	1,00E-15	GLOS_LOC10011:	GO:0044702	B	.	single organism reproductive process
XR_134092.1 PREDICT: cyt c oxidase subunit 1-like (LOC100113910), miscRNA	XR_134092.1	1,00E-15	GLOS_LOC10011:	GO:0022414	B	.	reproductive process
XR_134092.1 PREDICT: cyt c oxidase subunit 1-like (LOC100113910), miscRNA	XR_134092.1	1,00E-15	GLOS_LOC10011:	GO:0000003	B	.	reproduction
XR_134092.1 PREDICT: cyt c oxidase subunit 1-like (LOC100113910), miscRNA	XR_134092.1	1,00E-15	GLOS_LOC10011:	GO:0048609	B	.	multicellular organismal reproductive process
XR_134092.1 PREDICT: cyt c oxidase subunit 1-like (LOC100113910), miscRNA	XR_134092.1	1,00E-15	GLOS_LOC10011:	GO:0032504	B	.	multicellular organism reproduction
XR_134092.1 PREDICT: cyt c oxidase subunit 1-like (LOC100113910), miscRNA	XR_134092.1	1,00E-15	GLOS_LOC10011:	GO:0008585	B	.	female gonad development
XR_134092.1 PREDICT: cyt c oxidase subunit 1-like (LOC100113910), miscRNA	XR_134092.1	1,00E-15	GLOS_LOC10011:	GO:0008406	B	.	gonad development
XR_134092.1 PREDICT: cyt c oxidase subunit 1-like (LOC100113910), miscRNA	XR_134092.1	1,00E-15	GLOS_LOC10011:	GO:0048608	B	.	reproductive structure development
XR_134092.1 PREDICT: cyt c oxidase subunit 1-like (LOC100113910), miscRNA	XR_134092.1	1,00E-15	GLOS_LOC10011:	GO:0003006	B	.	developmental process involved in reproduction
XR_134092.1 PREDICT: cyt c oxidase subunit 1-like (LOC100113910), miscRNA	XR_134092.1	1,00E-15	GLOS_LOC10011:	GO:0061458	B	.	reproductive system development
XR_134092.1 PREDICT: cyt c oxidase subunit 1-like (LOC100113910), miscRNA	XR_134092.1	1,00E-15	GLOS_LOC10011:	GO:0045137	B	.	development of primary sexual characteristics
XR_134092.1 PREDICT: cyt c oxidase subunit 1-like (LOC100113910), miscRNA	XR_134092.1	1,00E-15	GLOS_LOC10011:	GO:0007548	B	.	sex differentiation
XR_134092.1 PREDICT: cyt c oxidase subunit 1-like (LOC100113910), miscRNA	XR_134092.1	1,00E-15	GLOS_LOC10011:	GO:0046545	B	.	development of primary female sexual characteristics
XR_134092.1 PREDICT: cyt c oxidase subunit 1-like (LOC100113910), miscRNA	XR_134092.1	1,00E-15	GLOS_LOC10011:	GO:0046660	B	.	female sex differentiation
XR_134092.1 PREDICT: cyt c oxidase subunit 1-like (LOC100113910), miscRNA	XR_134092.1	1,00E-15	GLOS_LOC10011:	GO:0001541	B	Refseq	ovarian follicle development
XR_134092.1 PREDICT: cyt c oxidase subunit 1-like (LOC100113910), miscRNA	XR_134092.1	1,00E-15	GLOS_LOC10011:	GO:0040014	B	.	regulation of multicellular organism growth
XR_134092.1 PREDICT: cyt c oxidase subunit 1-like (LOC100113910), miscRNA	XR_134092.1	1,00E-15	GLOS_LOC10011:	GO:0040008	B	.	regulation of growth
XR_134092.1 PREDICT: cyt c oxidase subunit 1-like (LOC100113910), miscRNA	XR_134092.1	1,00E-15	GLOS_LOC10011:	GO:0002021	B	Refseq	response to dietary excess
XR_134092.1 PREDICT: cyt c oxidase subunit 1-like (LOC100113910), miscRNA	XR_134092.1	1,00E-15	GLOS_LOC10011:	GO:0003674	M	Refseq	molecular_function
XR_134092.1 PREDICT: cyt c oxidase subunit 1-like (LOC100113910), miscRNA	XR_134092.1	1,00E-15	GLOS_LOC10011:	GO:0005179	M	.	hormone activity
XR_134092.1 PREDICT: cyt c oxidase subunit 1-like (LOC100113910), miscRNA	XR_134092.1	1,00E-15	GLOS_LOC10011:	GO:0005184	M	Refseq	neuropeptide hormone activity
XR_134092.1 PREDICT: cyt c oxidase subunit 1-like (LOC100113910), miscRNA	XR_134092.1	1,00E-15	GLOS_LOC10011:	GO:0044421	C	.	extracellular region part
XR_134092.1 PREDICT: cyt c oxidase subunit 1-like (LOC100113910), miscRNA	XR_134092.1	1,00E-15	GLOS_LOC10011:	GO:0005576	C	.	extracellular region
XR_134092.1 PREDICT: cyt c oxidase subunit 1-like (LOC100113910), miscRNA	XR_134092.1	1,00E-15	GLOS_LOC10011:	GO:0005615	C	Refseq	extracellular space
XR_134092.1 PREDICT: cyt c oxidase subunit 1-like (LOC100113910), miscRNA	XR_134092.1	1,00E-15	GLOS_LOC10011:	GO:0006091	B	Refseq	generation of precursor metabolites and energy
XR_134092.1 PREDICT: cyt c oxidase subunit 1-like (LOC100113910), miscRNA	XR_134092.1	1,00E-15	GLOS_LOC10011:	GO:0008083	M	Refseq	growth factor activity
XR_134092.1 PREDICT: cyt c oxidase subunit 1-like (LOC100113910), miscRNA	XR_134092.1	1,00E-15	GLOS_LOC10011:	GO:0009266	B	.	response to temperature stimulus
XR_134092.1 PREDICT: cyt c oxidase subunit 1-like (LOC100113910), miscRNA	XR_134092.1	1,00E-15	GLOS_LOC10011:	GO:0009409	B	Refseq	response to cold
XR_134092.1 PREDICT: cyt c oxidase subunit 1-like (LOC100113910), miscRNA	XR_134092.1	1,00E-15	GLOS_LOC10011:	GO:0019953	B	Refseq	sexual reproduction
XR_134092.1 PREDICT: cyt c oxidase subunit 1-like (LOC100113910), miscRNA	XR_134092.1	1,00E-15	GLOS_LOC10011:	GO:0030072	B	.	peptide hormone secretion
XR_134092.1 PREDICT: cyt c oxidase subunit 1-like (LOC100113910), miscRNA	XR_134092.1	1,00E-15	GLOS_LOC10011:	GO:0002790	B	.	peptide secretion
XR_134092.1 PREDICT: cyt c oxidase subunit 1-like (LOC100113910), miscRNA	XR_134092.1	1,00E-15	GLOS_LOC10011:	GO:0015833	B	.	peptide transport
XR_134092.1 PREDICT: cyt c oxidase subunit 1-like (LOC100113910), miscRNA	XR_134092.1	1,00E-15	GLOS_LOC10011:	GO:0042886	B	.	amide transport
XR_134092.1 PREDICT: cyt c oxidase subunit 1-like (LOC100113910), miscRNA	XR_134092.1	1,00E-15	GLOS_LOC10011:	GO:0071705	B	.	nitrogen compound transport
XR_134092.1 PREDICT: cyt c oxidase subunit 1-like (LOC100113910), miscRNA	XR_134092.1	1,00E-15	GLOS_LOC10011:	GO:0046879	B	.	hormone secretion
XR_134092.1 PREDICT: cyt c oxidase subunit 1-like (LOC100113910), miscRNA	XR_134092.1	1,00E-15	GLOS_LOC10011:	GO:0009914	B	.	hormone transport
XR_134092.1 PREDICT: cyt c oxidase subunit 1-like (LOC100113910), miscRNA	XR_134092.1	1,00E-15	GLOS_LOC10011:	GO:0010817	B	.	regulation of hormone levels
XR_134092.1 PREDICT: cyt c oxidase subunit 1-like (LOC100113910), miscRNA	XR_134092.1	1,00E-15	GLOS_LOC10011:	GO:0023061	B	.	signal release
XR_134092.1 PREDICT: cyt c oxidase subunit 1-like (LOC100113910), miscRNA	XR_134092.1	1,00E-15	GLOS_LOC10011:	GO:0032940	B	.	secretion by cell
XR_134092.1 PREDICT: cyt c oxidase subunit 1-like (LOC100113910), miscRNA	XR_134092.1	1,00E-15	GLOS_LOC10011:	GO:0003001	B	.	generation of a signal involved in cell-cell signaling
XR_134092.1 PREDICT: cyt c oxidase subunit 1-like (LOC100113910), miscRNA	XR_134092.1	1,00E-15	GLOS_LOC10011:	GO:0007267	B	.	cell-cell signaling
XR_134092.1 PREDICT: cyt c oxidase subunit 1-like (LOC100113910), miscRNA	XR_134092.1	1,00E-15	GLOS_LOC10011:	GO:0030073	B	Refseq	insulin secretion
XR_134092.1 PREDICT: cyt c oxidase subunit 1-like (LOC100113910), miscRNA	XR_134092.1	1,00E-15	GLOS_LOC10011:	GO:0016023	C	.	cytoplasmic membrane-bounded vesicle
XR_134092.1 PREDICT: cyt c oxidase subunit 1-like (LOC100113910), miscRNA	XR_134092.1	1,00E-15	GLOS_LOC10011:	GO:0031410	C	.	cytoplasmic vesicle
XR_134092.1 PREDICT: cyt c oxidase subunit 1-like (LOC100113910), miscRNA	XR_134092.1	1,00E-15	GLOS_LOC10011:	GO:0031982	C	.	vesicle
XR_134092.1 PREDICT: cyt c oxidase subunit 1-like (LOC100113910), miscRNA	XR_134092.1	1,00E-15	GLOS_LOC10011:	GO:0031988	C	.	membrane-bounded vesicle
XR_134092.1 PREDICT: cyt c oxidase subunit 1-like (LOC100113910), miscRNA	XR_134092.1	1,00E-15	GLOS_LOC10011:	GO:0030133	C	Refseq	transport vesicle
XR_134092.1 PREDICT: cyt c oxidase subunit 1-like (LOC100113910), miscRNA	XR_134092.1	1,00E-15	GLOS_LOC10011:	GO:0031410	C	Refseq	cytoplasmic vesicle
XR_134092.1 PREDICT: cyt c oxidase subunit 1-like (LOC100113910), miscRNA	XR_134092.1	1,00E-15	GLOS_LOC10011:	GO:0043434	B	.	response to peptide hormone stimulus
XR_134092.1 PREDICT: cyt c oxidase subunit 1-like (LOC100113910), miscRNA	XR_134092.1	1,00E-15	GLOS_LOC10011:	GO:0009725	B	.	response to hormone stimulus
XR_134092.1 PREDICT: cyt c oxidase subunit 1-like (LOC100113910), miscRNA	XR_134092.1	1,00E-15	GLOS_LOC10011:	GO:1901652	B	.	response to peptide

XR_134092.1 PREDICT: cyt c oxidase subunit 1-like (LOC100113910), miscRNA	XR_134092.1	1,00E-15	GLOS_LOC10011:	GO:0032868	B	Refseq	response to insulin stimulus
XR_134092.1 PREDICT: cyt c oxidase subunit 1-like (LOC100113910), miscRNA	XR_134092.1	1,00E-15	GLOS_LOC10011:	GO:0033500	B	.	carbohydrate homeostasis
XR_134092.1 PREDICT: cyt c oxidase subunit 1-like (LOC100113910), miscRNA	XR_134092.1	1,00E-15	GLOS_LOC10011:	GO:0042593	B	Refseq	glucose homeostasis
XR_134092.1 PREDICT: cyt c oxidase subunit 1-like (LOC100113910), miscRNA	XR_134092.1	1,00E-15	GLOS_LOC10011:	GO:0006952	B	.	defense response
XR_134092.1 PREDICT: cyt c oxidase subunit 1-like (LOC100113910), miscRNA	XR_134092.1	1,00E-15	GLOS_LOC10011:	GO:0009617	B	.	response to bacterium
XR_134092.1 PREDICT: cyt c oxidase subunit 1-like (LOC100113910), miscRNA	XR_134092.1	1,00E-15	GLOS_LOC10011:	GO:0051707	B	.	response to other organism
XR_134092.1 PREDICT: cyt c oxidase subunit 1-like (LOC100113910), miscRNA	XR_134092.1	1,00E-15	GLOS_LOC10011:	GO:0009607	B	.	response to biotic stimulus
XR_134092.1 PREDICT: cyt c oxidase subunit 1-like (LOC100113910), miscRNA	XR_134092.1	1,00E-15	GLOS_LOC10011:	GO:0051704	B	.	multi-organism process
XR_134092.1 PREDICT: cyt c oxidase subunit 1-like (LOC100113910), miscRNA	XR_134092.1	1,00E-15	GLOS_LOC10011:	GO:0042742	B	Refseq	defense response to bacterium
XR_134092.1 PREDICT: cyt c oxidase subunit 1-like (LOC100113910), miscRNA	XR_134092.1	1,00E-15	GLOS_LOC10011:	GO:0051591	B	Refseq	response to cAMP
XM_001984293.1 Drosophila grimshawi GH16390 (DgriGH16390), mRNA	XM_001984293.1	3,00E-73	GLOS_DGRI_GH1	GO:0005262	M	Refseq	calcium channel activity
XM_001984293.1 Drosophila grimshawi GH16390 (DgriGH16390), mRNA	XM_001984293.1	3,00E-73	GLOS_DGRI_GH1	GO:0005515	M	Refseq	protein binding
XM_001984293.1 Drosophila grimshawi GH16390 (DgriGH16390), mRNA	XM_001984293.1	3,00E-73	GLOS_DGRI_GH1	GO:0005886	C	Refseq	plasma membrane
XM_001984293.1 Drosophila grimshawi GH16390 (DgriGH16390), mRNA	XM_001984293.1	3,00E-73	GLOS_DGRI_GH1	GO:0005887	C	Refseq	integral to plasma membrane
XM_001984293.1 Drosophila grimshawi GH16390 (DgriGH16390), mRNA	XM_001984293.1	3,00E-73	GLOS_DGRI_GH1	GO:0006816	B	Refseq	calcium ion transport
XM_001984293.1 Drosophila grimshawi GH16390 (DgriGH16390), mRNA	XM_001984293.1	3,00E-73	GLOS_DGRI_GH1	GO:0007411	B	Refseq	axon guidance
XM_001984293.1 Drosophila grimshawi GH16390 (DgriGH16390), mRNA	XM_001984293.1	3,00E-73	GLOS_DGRI_GH1	GO:0007596	B	Refseq	blood coagulation
XM_001984293.1 Drosophila grimshawi GH16390 (DgriGH16390), mRNA	XM_001984293.1	3,00E-73	GLOS_DGRI_GH1	GO:0007602	B	Refseq	phototransduction
XM_001984293.1 Drosophila grimshawi GH16390 (DgriGH16390), mRNA	XM_001984293.1	3,00E-73	GLOS_DGRI_GH1	GO:0010524	B	Refseq	positive regulation of calcium ion transport into cytosol
XM_001984293.1 Drosophila grimshawi GH16390 (DgriGH16390), mRNA	XM_001984293.1	3,00E-73	GLOS_DGRI_GH1	GO:0015279	M	Refseq	store-operated calcium channel activity
XM_001984293.1 Drosophila grimshawi GH16390 (DgriGH16390), mRNA	XM_001984293.1	3,00E-73	GLOS_DGRI_GH1	GO:0030168	B	Refseq	platelet activation
XM_001984293.1 Drosophila grimshawi GH16390 (DgriGH16390), mRNA	XM_001984293.1	3,00E-73	GLOS_DGRI_GH1	GO:0033198	B	Refseq	response to ATP
XM_001984293.1 Drosophila grimshawi GH16390 (DgriGH16390), mRNA	XM_001984293.1	3,00E-73	GLOS_DGRI_GH1	GO:0051592	B	Refseq	response to calcium ion
XM_001984293.1 Drosophila grimshawi GH16390 (DgriGH16390), mRNA	XM_001984293.1	3,00E-73	GLOS_DGRI_GH1	GO:0070588	B	Refseq	calcium ion transmembrane transport
XM_001984293.1 Drosophila grimshawi GH16390 (DgriGH16390), mRNA	XM_001984293.1	3,00E-73	GLOS_DGRI_GH1	GO:0070679	M	Refseq	inositol 1,4,5 trisphosphate binding
NP_001156901.1 uncharacterized prot. LOC100302525 [T. castaneum]	NP_001156901.1	3,00E-11	GLOS_MYEOV2.1	GO:0001525	B	Refseq	angiogenesis
NP_001156901.1 uncharacterized prot. LOC100302525 [T. castaneum]	NP_001156901.1	3,00E-11	GLOS_MYEOV2.1	GO:0005100	M	Refseq	Rho GTPase activator activity
NP_001156901.1 uncharacterized prot. LOC100302525 [T. castaneum]	NP_001156901.1	3,00E-11	GLOS_MYEOV2.1	GO:0005178	M	Refseq	integrin binding
NP_001156901.1 uncharacterized prot. LOC100302525 [T. castaneum]	NP_001156901.1	3,00E-11	GLOS_MYEOV2.1	GO:0005515	M	Refseq	protein binding
NP_001156901.1 uncharacterized prot. LOC100302525 [T. castaneum]	NP_001156901.1	3,00E-11	GLOS_MYEOV2.1	GO:0005783	C	Refseq	endoplasmic reticulum
NP_001156901.1 uncharacterized prot. LOC100302525 [T. castaneum]	NP_001156901.1	3,00E-11	GLOS_MYEOV2.1	GO:0005829	C	Refseq	cytosol
NP_001156901.1 uncharacterized prot. LOC100302525 [T. castaneum]	NP_001156901.1	3,00E-11	GLOS_MYEOV2.1	GO:0005886	C	Refseq	plasma membrane
NP_001156901.1 uncharacterized prot. LOC100302525 [T. castaneum]	NP_001156901.1	3,00E-11	GLOS_MYEOV2.1	GO:0005887	C	Refseq	integral to plasma membrane
NP_001156901.1 uncharacterized prot. LOC100302525 [T. castaneum]	NP_001156901.1	3,00E-11	GLOS_MYEOV2.1	GO:0006469	B	Refseq	negative regulation of protein kinase activity
NP_001156901.1 uncharacterized prot. LOC100302525 [T. castaneum]	NP_001156901.1	3,00E-11	GLOS_MYEOV2.1	GO:0007010	B	Refseq	cytoskeleton organization
NP_001156901.1 uncharacterized prot. LOC100302525 [T. castaneum]	NP_001156901.1	3,00E-11	GLOS_MYEOV2.1	GO:0009897	C	Refseq	external side of plasma membrane
NP_001156901.1 uncharacterized prot. LOC100302525 [T. castaneum]	NP_001156901.1	3,00E-11	GLOS_MYEOV2.1	GO:0016324	C	Refseq	apical plasma membrane
NP_001156901.1 uncharacterized prot. LOC100302525 [T. castaneum]	NP_001156901.1	3,00E-11	GLOS_MYEOV2.1	GO:0016337	B	Refseq	cell-cell adhesion
NP_001156901.1 uncharacterized prot. LOC100302525 [T. castaneum]	NP_001156901.1	3,00E-11	GLOS_MYEOV2.1	GO:0019901	M	Refseq	protein kinase binding
NP_001156901.1 uncharacterized prot. LOC100302525 [T. castaneum]	NP_001156901.1	3,00E-11	GLOS_MYEOV2.1	GO:0030336	B	Refseq	negative regulation of cell migration
NP_001156901.1 uncharacterized prot. LOC100302525 [T. castaneum]	NP_001156901.1	3,00E-11	GLOS_MYEOV2.1	GO:0030425	C	Refseq	dendrite
NP_001156901.1 uncharacterized prot. LOC100302525 [T. castaneum]	NP_001156901.1	3,00E-11	GLOS_MYEOV2.1	GO:0030426	C	Refseq	growth cone
NP_001156901.1 uncharacterized prot. LOC100302525 [T. castaneum]	NP_001156901.1	3,00E-11	GLOS_MYEOV2.1	GO:0031362	C	Refseq	anchored to external side of plasma membrane
NP_001156901.1 uncharacterized prot. LOC100302525 [T. castaneum]	NP_001156901.1	3,00E-11	GLOS_MYEOV2.1	GO:0034235	M	Refseq	GPI anchor binding
NP_001156901.1 uncharacterized prot. LOC100302525 [T. castaneum]	NP_001156901.1	3,00E-11	GLOS_MYEOV2.1	GO:0043547	B	Refseq	positive regulation of GTPase activity
NP_001156901.1 uncharacterized prot. LOC100302525 [T. castaneum]	NP_001156901.1	3,00E-11	GLOS_MYEOV2.1	GO:0045121	C	Refseq	membrane raft
NP_001156901.1 uncharacterized prot. LOC100302525 [T. castaneum]	NP_001156901.1	3,00E-11	GLOS_MYEOV2.1	GO:0046549	B	Refseq	retinal cone cell development
NP_001156901.1 uncharacterized prot. LOC100302525 [T. castaneum]	NP_001156901.1	3,00E-11	GLOS_MYEOV2.1	GO:0048041	B	Refseq	focal adhesion assembly
NP_001156901.1 uncharacterized prot. LOC100302525 [T. castaneum]	NP_001156901.1	3,00E-11	GLOS_MYEOV2.1	GO:0050771	B	Refseq	negative regulation of axonogenesis
NP_001156901.1 uncharacterized prot. LOC100302525 [T. castaneum]	NP_001156901.1	3,00E-11	GLOS_MYEOV2.1	GO:0050852	B	Refseq	T cell receptor signaling pathway
NP_001156901.1 uncharacterized prot. LOC100302525 [T. castaneum]	NP_001156901.1	3,00E-11	GLOS_MYEOV2.1	GO:0050860	B	Refseq	negative regulation of T cell receptor signaling pathway
NP_001156901.1 uncharacterized prot. LOC100302525 [T. castaneum]	NP_001156901.1	3,00E-11	GLOS_MYEOV2.1	GO:0050870	B	Refseq	positive regulation of T cell activation
NP_001156901.1 uncharacterized prot. LOC100302525 [T. castaneum]	NP_001156901.1	3,00E-11	GLOS_MYEOV2.1	GO:0051281	B	Refseq	positive regulation of release of sequestered calcium ion into cytosol
NP_995988.1 karst, isoform B [Drosophila melanogaster]	NP_995988.1	4,00E-17	GLOS_KST.1.1	GO:0000122	B	Refseq	negative regulation of transcription from RNA polymerase II promoter

NP_995988.1 karst, isoform B [Drosophila melanogaster]	NP_995988.1	4,00E-17	GLOS_KST.1.1	GO:0001501	B	Refseq	skeletal system development
NP_995988.1 karst, isoform B [Drosophila melanogaster]	NP_995988.1	4,00E-17	GLOS_KST.1.1	GO:0003700	M	Refseq	sequence-specific DNA binding transcription factor activity
NP_995988.1 karst, isoform B [Drosophila melanogaster]	NP_995988.1	4,00E-17	GLOS_KST.1.1	GO:0005515	M	Refseq	protein binding
NP_995988.1 karst, isoform B [Drosophila melanogaster]	NP_995988.1	4,00E-17	GLOS_KST.1.1	GO:0005634	C	Refseq	nucleus
NP_995988.1 karst, isoform B [Drosophila melanogaster]	NP_995988.1	4,00E-17	GLOS_KST.1.1	GO:0006366	B	Refseq	transcription from RNA polymerase II promoter
NP_995988.1 karst, isoform B [Drosophila melanogaster]	NP_995988.1	4,00E-17	GLOS_KST.1.1	GO:0006607	B	Refseq	NLS-bearing protein import into nucleus
NP_995988.1 karst, isoform B [Drosophila melanogaster]	NP_995988.1	4,00E-17	GLOS_KST.1.1	GO:0008270	M	Refseq	zinc ion binding
NP_995988.1 karst, isoform B [Drosophila melanogaster]	NP_995988.1	4,00E-17	GLOS_KST.1.1	GO:0032330	B	Refseq	regulation of chondrocyte differentiation
NP_995988.1 karst, isoform B [Drosophila melanogaster]	NP_995988.1	4,00E-17	GLOS_KST.1.1	GO:0043565	M	Refseq	sequence-specific DNA binding
XP_001969732.1 GG23787 [Drosophila erecta]	XP_001969732.1	0	GLOS_DERE_GG	GO:0005261	M	Refseq	cation channel activity
XP_001969732.1 GG23787 [Drosophila erecta]	XP_001969732.1	0	GLOS_DERE_GG	GO:0005262	M	Refseq	calcium channel activity
XP_001969732.1 GG23787 [Drosophila erecta]	XP_001969732.1	0	GLOS_DERE_GG	GO:0005515	M	Refseq	protein binding
XP_001969732.1 GG23787 [Drosophila erecta]	XP_001969732.1	0	GLOS_DERE_GG	GO:0005886	C	Refseq	plasma membrane
XP_001969732.1 GG23787 [Drosophila erecta]	XP_001969732.1	0	GLOS_DERE_GG	GO:0005887	C	Refseq	integral to plasma membrane
XP_001969732.1 GG23787 [Drosophila erecta]	XP_001969732.1	0	GLOS_DERE_GG	GO:0006816	B	Refseq	calcium ion transport
XP_001969732.1 GG23787 [Drosophila erecta]	XP_001969732.1	0	GLOS_DERE_GG	GO:0007411	B	Refseq	axon guidance
XP_001969732.1 GG23787 [Drosophila erecta]	XP_001969732.1	0	GLOS_DERE_GG	GO:0015279	M	Refseq	store-operated calcium channel activity
XP_001969732.1 GG23787 [Drosophila erecta]	XP_001969732.1	0	GLOS_DERE_GG	GO:0016323	C	Refseq	basolateral plasma membrane
XP_001969732.1 GG23787 [Drosophila erecta]	XP_001969732.1	0	GLOS_DERE_GG	GO:0030017	C	Refseq	sarcomere
XP_001969732.1 GG23787 [Drosophila erecta]	XP_001969732.1	0	GLOS_DERE_GG	GO:0043034	C	Refseq	costamere
XP_001969732.1 GG23787 [Drosophila erecta]	XP_001969732.1	0	GLOS_DERE_GG	GO:0043234	C	Refseq	protein complex
XP_001969732.1 GG23787 [Drosophila erecta]	XP_001969732.1	0	GLOS_DERE_GG	GO:0044325	M	Refseq	ion channel binding
XP_001969732.1 GG23787 [Drosophila erecta]	XP_001969732.1	0	GLOS_DERE_GG	GO:0045121	C	Refseq	membrane raft
XP_001969732.1 GG23787 [Drosophila erecta]	XP_001969732.1	0	GLOS_DERE_GG	GO:0046541	B	Refseq	saliva secretion
XP_001969732.1 GG23787 [Drosophila erecta]	XP_001969732.1	0	GLOS_DERE_GG	GO:0051281	B	Refseq	positive regulation of release of sequestered calcium ion into cytosol
XP_001969732.1 GG23787 [Drosophila erecta]	XP_001969732.1	0	GLOS_DERE_GG	GO:0051480	B	Refseq	cytosolic calcium ion homeostasis
XP_001969732.1 GG23787 [Drosophila erecta]	XP_001969732.1	0	GLOS_DERE_GG	GO:0051592	B	Refseq	response to calcium ion
XP_001969732.1 GG23787 [Drosophila erecta]	XP_001969732.1	0	GLOS_DERE_GG	GO:0070679	M	Refseq	inositol 1,4,5 trisphosphate binding
XP_001983976.1 GH16188 [Drosophila grimshawi]	XP_001983976.1	9,00E-42	GLOS_DGRI_GH1	GO:0005262	M	Refseq	calcium channel activity
XP_001983976.1 GH16188 [Drosophila grimshawi]	XP_001983976.1	9,00E-42	GLOS_DGRI_GH1	GO:0005515	M	Refseq	protein binding
XP_001983976.1 GH16188 [Drosophila grimshawi]	XP_001983976.1	9,00E-42	GLOS_DGRI_GH1	GO:0005886	C	Refseq	plasma membrane
XP_001983976.1 GH16188 [Drosophila grimshawi]	XP_001983976.1	9,00E-42	GLOS_DGRI_GH1	GO:0005887	C	Refseq	integral to plasma membrane
XP_001983976.1 GH16188 [Drosophila grimshawi]	XP_001983976.1	9,00E-42	GLOS_DGRI_GH1	GO:0006816	B	Refseq	calcium ion transport
XP_001983976.1 GH16188 [Drosophila grimshawi]	XP_001983976.1	9,00E-42	GLOS_DGRI_GH1	GO:0007411	B	Refseq	axon guidance
XP_001983976.1 GH16188 [Drosophila grimshawi]	XP_001983976.1	9,00E-42	GLOS_DGRI_GH1	GO:0007596	B	Refseq	blood coagulation
XP_001983976.1 GH16188 [Drosophila grimshawi]	XP_001983976.1	9,00E-42	GLOS_DGRI_GH1	GO:0007602	B	Refseq	phototransduction
XP_001983976.1 GH16188 [Drosophila grimshawi]	XP_001983976.1	9,00E-42	GLOS_DGRI_GH1	GO:0010524	B	Refseq	positive regulation of calcium ion transport into cytosol
XP_001983976.1 GH16188 [Drosophila grimshawi]	XP_001983976.1	9,00E-42	GLOS_DGRI_GH1	GO:0015279	M	Refseq	store-operated calcium channel activity
XP_001983976.1 GH16188 [Drosophila grimshawi]	XP_001983976.1	9,00E-42	GLOS_DGRI_GH1	GO:0030168	B	Refseq	platelet activation
XP_001983976.1 GH16188 [Drosophila grimshawi]	XP_001983976.1	9,00E-42	GLOS_DGRI_GH1	GO:0033198	B	Refseq	response to ATP
XP_001983976.1 GH16188 [Drosophila grimshawi]	XP_001983976.1	9,00E-42	GLOS_DGRI_GH1	GO:0051592	B	Refseq	response to calcium ion
XP_001983976.1 GH16188 [Drosophila grimshawi]	XP_001983976.1	9,00E-42	GLOS_DGRI_GH1	GO:0070588	B	Refseq	calcium ion transmembrane transport
XP_001983976.1 GH16188 [Drosophila grimshawi]	XP_001983976.1	9,00E-42	GLOS_DGRI_GH1	GO:0070679	M	Refseq	inositol 1,4,5 trisphosphate binding
XP_001969019.1 GG25189 [Drosophila erecta]	XP_001969019.1	1,00E-17	GLOS_DERE_GG	GO:0005261	M	Refseq	cation channel activity
XP_001969019.1 GG25189 [Drosophila erecta]	XP_001969019.1	1,00E-17	GLOS_DERE_GG	GO:0005262	M	Refseq	calcium channel activity
XP_001969019.1 GG25189 [Drosophila erecta]	XP_001969019.1	1,00E-17	GLOS_DERE_GG	GO:0005515	M	Refseq	protein binding
XP_001969019.1 GG25189 [Drosophila erecta]	XP_001969019.1	1,00E-17	GLOS_DERE_GG	GO:0005886	C	Refseq	plasma membrane
XP_001969019.1 GG25189 [Drosophila erecta]	XP_001969019.1	1,00E-17	GLOS_DERE_GG	GO:0005887	C	Refseq	integral to plasma membrane
XP_001969019.1 GG25189 [Drosophila erecta]	XP_001969019.1	1,00E-17	GLOS_DERE_GG	GO:0006816	B	Refseq	calcium ion transport
XP_001969019.1 GG25189 [Drosophila erecta]	XP_001969019.1	1,00E-17	GLOS_DERE_GG	GO:0007411	B	Refseq	axon guidance
XP_001969019.1 GG25189 [Drosophila erecta]	XP_001969019.1	1,00E-17	GLOS_DERE_GG	GO:0015279	M	Refseq	store-operated calcium channel activity
XP_001969019.1 GG25189 [Drosophila erecta]	XP_001969019.1	1,00E-17	GLOS_DERE_GG	GO:0016323	C	Refseq	basolateral plasma membrane
XP_001969019.1 GG25189 [Drosophila erecta]	XP_001969019.1	1,00E-17	GLOS_DERE_GG	GO:0030017	C	Refseq	sarcomere
XP_001969019.1 GG25189 [Drosophila erecta]	XP_001969019.1	1,00E-17	GLOS_DERE_GG	GO:0043034	C	Refseq	costamere

XP_001969019.1 GG25189 [Drosophila erecta]	XP_001969019.1	1,00E-17	GLOS_DERE_GG:	GO:0043234	C	Refseq	protein complex
XP_001969019.1 GG25189 [Drosophila erecta]	XP_001969019.1	1,00E-17	GLOS_DERE_GG:	GO:0044325	M	Refseq	ion channel binding
XP_001969019.1 GG25189 [Drosophila erecta]	XP_001969019.1	1,00E-17	GLOS_DERE_GG:	GO:0045121	C	Refseq	membrane raft
XP_001969019.1 GG25189 [Drosophila erecta]	XP_001969019.1	1,00E-17	GLOS_DERE_GG:	GO:0046541	B	Refseq	saliva secretion
XP_001969019.1 GG25189 [Drosophila erecta]	XP_001969019.1	1,00E-17	GLOS_DERE_GG:	GO:0051281	B	Refseq	positive regulation of release of sequestered calcium ion into cytosol
XP_001969019.1 GG25189 [Drosophila erecta]	XP_001969019.1	1,00E-17	GLOS_DERE_GG:	GO:0051480	B	Refseq	cytosolic calcium ion homeostasis
XP_001969019.1 GG25189 [Drosophila erecta]	XP_001969019.1	1,00E-17	GLOS_DERE_GG:	GO:0051592	B	Refseq	response to calcium ion
XP_001969019.1 GG25189 [Drosophila erecta]	XP_001969019.1	1,00E-17	GLOS_DERE_GG:	GO:0070679	M	Refseq	inositol 1,4,5 trisphosphate binding
NM_001278161.1 M. musc. hemoglobin, beta adult major chain (Hbb-b1), mRNA	NM_001278161.1	0	GLOS_HBB-B1.1.2	GO:0031090	C	.	organelle membrane
NM_001278161.1 M. musc. hemoglobin, beta adult major chain (Hbb-b1), mRNA	NM_001278161.1	0	GLOS_HBB-B1.1.2	GO:0044431	C	.	Golgi apparatus part
NM_001278161.1 M. musc. hemoglobin, beta adult major chain (Hbb-b1), mRNA	NM_001278161.1	0	GLOS_HBB-B1.1.2	GO:0005794	C	.	Golgi apparatus
NM_001278161.1 M. musc. hemoglobin, beta adult major chain (Hbb-b1), mRNA	NM_001278161.1	0	GLOS_HBB-B1.1.2	GO:0012505	C	.	endomembrane system
NM_001278161.1 M. musc. hemoglobin, beta adult major chain (Hbb-b1), mRNA	NM_001278161.1	0	GLOS_HBB-B1.1.2	GO:0000139	C	Refseq	Golgi membrane
NM_001278161.1 M. musc. hemoglobin, beta adult major chain (Hbb-b1), mRNA	NM_001278161.1	0	GLOS_HBB-B1.1.2	GO:0005975	B	Refseq	carbohydrate metabolic process
NM_001278161.1 M. musc. hemoglobin, beta adult major chain (Hbb-b1), mRNA	NM_001278161.1	0	GLOS_HBB-B1.1.2	GO:0006464	B	.	cellular protein modification process
NM_001278161.1 M. musc. hemoglobin, beta adult major chain (Hbb-b1), mRNA	NM_001278161.1	0	GLOS_HBB-B1.1.2	GO:0036211	B	.	protein modification process
NM_001278161.1 M. musc. hemoglobin, beta adult major chain (Hbb-b1), mRNA	NM_001278161.1	0	GLOS_HBB-B1.1.2	GO:0019538	B	.	protein metabolic process
NM_001278161.1 M. musc. hemoglobin, beta adult major chain (Hbb-b1), mRNA	NM_001278161.1	0	GLOS_HBB-B1.1.2	GO:0043412	B	.	macromolecule modification
NM_001278161.1 M. musc. hemoglobin, beta adult major chain (Hbb-b1), mRNA	NM_001278161.1	0	GLOS_HBB-B1.1.2	GO:0044267	B	.	cellular protein metabolic process
NM_001278161.1 M. musc. hemoglobin, beta adult major chain (Hbb-b1), mRNA	NM_001278161.1	0	GLOS_HBB-B1.1.2	GO:0051923	B	.	sulfation
NM_001278161.1 M. musc. hemoglobin, beta adult major chain (Hbb-b1), mRNA	NM_001278161.1	0	GLOS_HBB-B1.1.2	GO:0006790	B	.	sulfur compound metabolic process
NM_001278161.1 M. musc. hemoglobin, beta adult major chain (Hbb-b1), mRNA	NM_001278161.1	0	GLOS_HBB-B1.1.2	GO:0006477	B	Refseq	protein sulfation
NM_001278161.1 M. musc. hemoglobin, beta adult major chain (Hbb-b1), mRNA	NM_001278161.1	0	GLOS_HBB-B1.1.2	GO:0016782	M	.	transferase activity, transferring sulfur-containing groups
NM_001278161.1 M. musc. hemoglobin, beta adult major chain (Hbb-b1), mRNA	NM_001278161.1	0	GLOS_HBB-B1.1.2	GO:0016740	M	.	transferase activity
NM_001278161.1 M. musc. hemoglobin, beta adult major chain (Hbb-b1), mRNA	NM_001278161.1	0	GLOS_HBB-B1.1.2	GO:0003824	M	.	catalytic activity
NM_001278161.1 M. musc. hemoglobin, beta adult major chain (Hbb-b1), mRNA	NM_001278161.1	0	GLOS_HBB-B1.1.2	GO:0008146	M	Refseq	sulfotransferase activity
NM_001278161.1 M. musc. hemoglobin, beta adult major chain (Hbb-b1), mRNA	NM_001278161.1	0	GLOS_HBB-B1.1.2	GO:0016021	C	Refseq	integral to membrane
NM_001278161.1 M. musc. hemoglobin, beta adult major chain (Hbb-b1), mRNA	NM_001278161.1	0	GLOS_HBB-B1.1.2	GO:0006022	B	.	aminoglycan metabolic process
NM_001278161.1 M. musc. hemoglobin, beta adult major chain (Hbb-b1), mRNA	NM_001278161.1	0	GLOS_HBB-B1.1.2	GO:1901135	B	.	carbohydrate derivative metabolic process
NM_001278161.1 M. musc. hemoglobin, beta adult major chain (Hbb-b1), mRNA	NM_001278161.1	0	GLOS_HBB-B1.1.2	GO:1901564	B	.	organonitrogen compound metabolic process
NM_001278161.1 M. musc. hemoglobin, beta adult major chain (Hbb-b1), mRNA	NM_001278161.1	0	GLOS_HBB-B1.1.2	GO:0030203	B	Refseq	glycosaminoglycan metabolic process
NM_001278161.1 M. musc. hemoglobin, beta adult major chain (Hbb-b1), mRNA	NM_001278161.1	0	GLOS_HBB-B1.1.2	GO:0030203	B	.	glycosaminoglycan metabolic process
NM_001278161.1 M. musc. hemoglobin, beta adult major chain (Hbb-b1), mRNA	NM_001278161.1	0	GLOS_HBB-B1.1.2	GO:0043436	B	.	oxoacid metabolic process
NM_001278161.1 M. musc. hemoglobin, beta adult major chain (Hbb-b1), mRNA	NM_001278161.1	0	GLOS_HBB-B1.1.2	GO:0006082	B	.	organic acid metabolic process
NM_001278161.1 M. musc. hemoglobin, beta adult major chain (Hbb-b1), mRNA	NM_001278161.1	0	GLOS_HBB-B1.1.2	GO:0044281	B	.	small molecule metabolic process
NM_001278161.1 M. musc. hemoglobin, beta adult major chain (Hbb-b1), mRNA	NM_001278161.1	0	GLOS_HBB-B1.1.2	GO:0044710	B	.	single-organism metabolic process
NM_001278161.1 M. musc. hemoglobin, beta adult major chain (Hbb-b1), mRNA	NM_001278161.1	0	GLOS_HBB-B1.1.2	GO:0050654	B	.	chondroitin sulfate proteoglycan metabolic process
NM_001278161.1 M. musc. hemoglobin, beta adult major chain (Hbb-b1), mRNA	NM_001278161.1	0	GLOS_HBB-B1.1.2	GO:0006029	B	.	proteoglycan metabolic process
NM_001278161.1 M. musc. hemoglobin, beta adult major chain (Hbb-b1), mRNA	NM_001278161.1	0	GLOS_HBB-B1.1.2	GO:0009100	B	.	glycoprotein metabolic process
NM_001278161.1 M. musc. hemoglobin, beta adult major chain (Hbb-b1), mRNA	NM_001278161.1	0	GLOS_HBB-B1.1.2	GO:0030204	B	Refseq	chondroitin sulfate metabolic process
NM_001278161.1 M. musc. hemoglobin, beta adult major chain (Hbb-b1), mRNA	NM_001278161.1	0	GLOS_HBB-B1.1.2	GO:0006024	B	.	glycosaminoglycan biosynthetic process
NM_001278161.1 M. musc. hemoglobin, beta adult major chain (Hbb-b1), mRNA	NM_001278161.1	0	GLOS_HBB-B1.1.2	GO:0006023	B	.	aminoglycan biosynthetic process
NM_001278161.1 M. musc. hemoglobin, beta adult major chain (Hbb-b1), mRNA	NM_001278161.1	0	GLOS_HBB-B1.1.2	GO:1901137	B	.	carbohydrate derivative biosynthetic process
NM_001278161.1 M. musc. hemoglobin, beta adult major chain (Hbb-b1), mRNA	NM_001278161.1	0	GLOS_HBB-B1.1.2	GO:1901566	B	.	organonitrogen compound biosynthetic process
NM_001278161.1 M. musc. hemoglobin, beta adult major chain (Hbb-b1), mRNA	NM_001278161.1	0	GLOS_HBB-B1.1.2	GO:0030205	B	.	dermatan sulfate metabolic process
NM_001278161.1 M. musc. hemoglobin, beta adult major chain (Hbb-b1), mRNA	NM_001278161.1	0	GLOS_HBB-B1.1.2	GO:0032787	B	.	monocarboxylic acid metabolic process
NM_001278161.1 M. musc. hemoglobin, beta adult major chain (Hbb-b1), mRNA	NM_001278161.1	0	GLOS_HBB-B1.1.2	GO:0019752	B	.	carboxylic acid metabolic process
NM_001278161.1 M. musc. hemoglobin, beta adult major chain (Hbb-b1), mRNA	NM_001278161.1	0	GLOS_HBB-B1.1.2	GO:0050655	B	.	dermatan sulfate proteoglycan metabolic process
NM_001278161.1 M. musc. hemoglobin, beta adult major chain (Hbb-b1), mRNA	NM_001278161.1	0	GLOS_HBB-B1.1.2	GO:0044272	B	.	sulfur compound biosynthetic process
NM_001278161.1 M. musc. hemoglobin, beta adult major chain (Hbb-b1), mRNA	NM_001278161.1	0	GLOS_HBB-B1.1.2	GO:0072330	B	.	monocarboxylic acid biosynthetic process
NM_001278161.1 M. musc. hemoglobin, beta adult major chain (Hbb-b1), mRNA	NM_001278161.1	0	GLOS_HBB-B1.1.2	GO:0046394	B	.	carboxylic acid biosynthetic process
NM_001278161.1 M. musc. hemoglobin, beta adult major chain (Hbb-b1), mRNA	NM_001278161.1	0	GLOS_HBB-B1.1.2	GO:0016053	B	.	organic acid biosynthetic process
NM_001278161.1 M. musc. hemoglobin, beta adult major chain (Hbb-b1), mRNA	NM_001278161.1	0	GLOS_HBB-B1.1.2	GO:0044283	B	.	small molecule biosynthetic process
NM_001278161.1 M. musc. hemoglobin, beta adult major chain (Hbb-b1), mRNA	NM_001278161.1	0	GLOS_HBB-B1.1.2	GO:0044711	B	.	single-organism biosynthetic process

NM_001278161.1 M. musc. hemoglobin, beta adult major chain (Hbb-b1), mRNA	NM_001278161.1	0	GLOS_HBB-B1.1.2	GO:0050651	B	.	dermatan sulfate proteoglycan biosynthetic process
NM_001278161.1 M. musc. hemoglobin, beta adult major chain (Hbb-b1), mRNA	NM_001278161.1	0	GLOS_HBB-B1.1.2	GO:0030166	B	.	proteoglycan biosynthetic process
NM_001278161.1 M. musc. hemoglobin, beta adult major chain (Hbb-b1), mRNA	NM_001278161.1	0	GLOS_HBB-B1.1.2	GO:0009101	B	.	glycoprotein biosynthetic process
NM_001278161.1 M. musc. hemoglobin, beta adult major chain (Hbb-b1), mRNA	NM_001278161.1	0	GLOS_HBB-B1.1.2	GO:0030208	B	Refseq	dermatan sulfate biosynthetic process
NM_001278161.1 M. musc. hemoglobin, beta adult major chain (Hbb-b1), mRNA	NM_001278161.1	0	GLOS_HBB-B1.1.2	GO:0044281	B	Refseq	small molecule metabolic process
NP_649328.1 cytochrome c oxidase subunit VIII, isoform A [D. melanogaster] ref|NP_001262169.1| isoform B [D. melanogaster] ref|XP_002040826.1| GM22130 [D. sechellia] ref|XP_002085812.1| cytochrome c oxidase subunit VIII [D. simulans]	NP_649328.1	3,00E-29	GLOS_COVIII.1.4	GO:0000122	B	Refseq	negative regulation of transcription from RNA polymerase II promoter
NP_649328.1 cytochrome c oxidase subunit VIII, isoform A … Same as above	NP_649328.1	3,00E-29	GLOS_COVIII.1.4	GO:0001501	B	Refseq	skeletal system development
NP_649328.1 cytochrome c oxidase subunit VIII, isoform A … Same as above	NP_649328.1	3,00E-29	GLOS_COVIII.1.4	GO:0003700	M	Refseq	sequence-specific DNA binding transcription factor activity
NP_649328.1 cytochrome c oxidase subunit VIII, isoform A … Same as above	NP_649328.1	3,00E-29	GLOS_COVIII.1.4	GO:0005515	M	Refseq	protein binding
NP_649328.1 cytochrome c oxidase subunit VIII, isoform A … Same as above	NP_649328.1	3,00E-29	GLOS_COVIII.1.4	GO:0005634	C	Refseq	nucleus
NP_649328.1 cytochrome c oxidase subunit VIII, isoform A … Same as above	NP_649328.1	3,00E-29	GLOS_COVIII.1.4	GO:0006366	B	Refseq	transcription from RNA polymerase II promoter
NP_649328.1 cytochrome c oxidase subunit VIII, isoform A … Same as above	NP_649328.1	3,00E-29	GLOS_COVIII.1.4	GO:0006607	B	Refseq	NLS-bearing protein import into nucleus
NP_649328.1 cytochrome c oxidase subunit VIII, isoform A … Same as above	NP_649328.1	3,00E-29	GLOS_COVIII.1.4	GO:0008270	M	Refseq	zinc ion binding
NP_649328.1 cytochrome c oxidase subunit VIII, isoform A … Same as above	NP_649328.1	3,00E-29	GLOS_COVIII.1.4	GO:0032330	B	Refseq	regulation of chondrocyte differentiation
NP_649328.1 cytochrome c oxidase subunit VIII, isoform A … Same as above	NP_649328.1	3,00E-29	GLOS_COVIII.1.4	GO:0043565	M	Refseq	sequence-specific DNA binding
XP_001989100.1 GH11536[D. grim.]ref|XP_001997710.1|GH25231 [D.grimshawi]	XP_001989100.1	3,00E-09	GLOS_DGRI_GH1	GO:0005262	M	Refseq	calcium channel activity
XP_001989100.1 GH11536[D. grim.]ref|XP_001997710.1|GH25231 [D.grimshawi]	XP_001989100.1	3,00E-09	GLOS_DGRI_GH1	GO:0005515	M	Refseq	protein binding
XP_001989100.1 GH11536[D. grim.]ref|XP_001997710.1|GH25231 [D.grimshawi]	XP_001989100.1	3,00E-09	GLOS_DGRI_GH1	GO:0005886	C	Refseq	plasma membrane
XP_001989100.1 GH11536[D. grim.]ref|XP_001997710.1|GH25231 [D.grimshawi]	XP_001989100.1	3,00E-09	GLOS_DGRI_GH1	GO:0005887	C	Refseq	integral to plasma membrane
XP_001989100.1 GH11536[D. grim.]ref|XP_001997710.1|GH25231 [D.grimshawi]	XP_001989100.1	3,00E-09	GLOS_DGRI_GH1	GO:0006816	B	Refseq	calcium ion transport
XP_001989100.1 GH11536[D. grim.]ref|XP_001997710.1|GH25231 [D.grimshawi]	XP_001989100.1	3,00E-09	GLOS_DGRI_GH1	GO:0007411	B	Refseq	axon guidance
XP_001989100.1 GH11536[D. grim.]ref|XP_001997710.1|GH25231 [D.grimshawi]	XP_001989100.1	3,00E-09	GLOS_DGRI_GH1	GO:0007596	B	Refseq	blood coagulation
XP_001989100.1 GH11536[D. grim.]ref|XP_001997710.1|GH25231 [D.grimshawi]	XP_001989100.1	3,00E-09	GLOS_DGRI_GH1	GO:0007602	B	Refseq	phototransduction
XP_001989100.1 GH11536[D. grim.]ref|XP_001997710.1|GH25231 [D.grimshawi]	XP_001989100.1	3,00E-09	GLOS_DGRI_GH1	GO:0010524	B	Refseq	positive regulation of calcium ion transport into cytosol
XP_001989100.1 GH11536[D. grim.]ref|XP_001997710.1|GH25231 [D.grimshawi]	XP_001989100.1	3,00E-09	GLOS_DGRI_GH1	GO:0015279	M	Refseq	store-operated calcium channel activity
XP_001989100.1 GH11536[D. grim.]ref|XP_001997710.1|GH25231 [D.grimshawi]	XP_001989100.1	3,00E-09	GLOS_DGRI_GH1	GO:0030168	B	Refseq	platelet activation
XP_001989100.1 GH11536[D. grim.]ref|XP_001997710.1|GH25231 [D.grimshawi]	XP_001989100.1	3,00E-09	GLOS_DGRI_GH1	GO:0033198	B	Refseq	response to ATP
XP_001989100.1 GH11536[D. grim.]ref|XP_001997710.1|GH25231 [D.grimshawi]	XP_001989100.1	3,00E-09	GLOS_DGRI_GH1	GO:0051592	B	Refseq	response to calcium ion
XP_001989100.1 GH11536[D. grim.]ref|XP_001997710.1|GH25231 [D.grimshawi]	XP_001989100.1	3,00E-09	GLOS_DGRI_GH1	GO:0070588	B	Refseq	calcium ion transmembrane transport
XP_001989100.1 GH11536[D. grim.]ref|XP_001997710.1|GH25231 [D.grimshawi]	XP_001989100.1	3,00E-09	GLOS_DGRI_GH1	GO:0070679	M	Refseq	inositol 1,4,5 trisphosphate binding
XP_001986737.1 GH21531 [Drosophila grimshawi]	XP_001986737.1	1,00E-25	GLOS_DGRI_GH2	GO:0005262	M	Refseq	calcium channel activity
XP_001986737.1 GH21531 [Drosophila grimshawi]	XP_001986737.1	1,00E-25	GLOS_DGRI_GH2	GO:0005515	M	Refseq	protein binding
XP_001986737.1 GH21531 [Drosophila grimshawi]	XP_001986737.1	1,00E-25	GLOS_DGRI_GH2	GO:0005886	C	Refseq	plasma membrane
XP_001986737.1 GH21531 [Drosophila grimshawi]	XP_001986737.1	1,00E-25	GLOS_DGRI_GH2	GO:0005887	C	Refseq	integral to plasma membrane
XP_001986737.1 GH21531 [Drosophila grimshawi]	XP_001986737.1	1,00E-25	GLOS_DGRI_GH2	GO:0006816	B	Refseq	calcium ion transport
XP_001986737.1 GH21531 [Drosophila grimshawi]	XP_001986737.1	1,00E-25	GLOS_DGRI_GH2	GO:0007411	B	Refseq	axon guidance
XP_001986737.1 GH21531 [Drosophila grimshawi]	XP_001986737.1	1,00E-25	GLOS_DGRI_GH2	GO:0007596	B	Refseq	blood coagulation
XP_001986737.1 GH21531 [Drosophila grimshawi]	XP_001986737.1	1,00E-25	GLOS_DGRI_GH2	GO:0007602	B	Refseq	phototransduction
XP_001986737.1 GH21531 [Drosophila grimshawi]	XP_001986737.1	1,00E-25	GLOS_DGRI_GH2	GO:0010524	B	Refseq	positive regulation of calcium ion transport into cytosol
XP_001986737.1 GH21531 [Drosophila grimshawi]	XP_001986737.1	1,00E-25	GLOS_DGRI_GH2	GO:0015279	M	Refseq	store-operated calcium channel activity
XP_001986737.1 GH21531 [Drosophila grimshawi]	XP_001986737.1	1,00E-25	GLOS_DGRI_GH2	GO:0030168	B	Refseq	platelet activation
XP_001986737.1 GH21531 [Drosophila grimshawi]	XP_001986737.1	1,00E-25	GLOS_DGRI_GH2	GO:0033198	B	Refseq	response to ATP
XP_001986737.1 GH21531 [Drosophila grimshawi]	XP_001986737.1	1,00E-25	GLOS_DGRI_GH2	GO:0051592	B	Refseq	response to calcium ion
XP_001986737.1 GH21531 [Drosophila grimshawi]	XP_001986737.1	1,00E-25	GLOS_DGRI_GH2	GO:0070588	B	Refseq	calcium ion transmembrane transport
XP_001986737.1 GH21531 [Drosophila grimshawi]	XP_001986737.1	1,00E-25	GLOS_DGRI_GH2	GO:0070679	M	Refseq	inositol 1,4,5 trisphosphate binding
XP_001994910.1 GH17497 [Drosophila grimshawi]	XP_001994910.1	6,00E-53	GLOS_DGRI_GH1	GO:0005262	M	Refseq	calcium channel activity
XP_001994910.1 GH17497 [Drosophila grimshawi]	XP_001994910.1	6,00E-53	GLOS_DGRI_GH1	GO:0005515	M	Refseq	protein binding
XP_001994910.1 GH17497 [Drosophila grimshawi]	XP_001994910.1	6,00E-53	GLOS_DGRI_GH1	GO:0005886	C	Refseq	plasma membrane
XP_001994910.1 GH17497 [Drosophila grimshawi]	XP_001994910.1	6,00E-53	GLOS_DGRI_GH1	GO:0005887	C	Refseq	integral to plasma membrane
XP_001994910.1 GH17497 [Drosophila grimshawi]	XP_001994910.1	6,00E-53	GLOS_DGRI_GH1	GO:0006816	B	Refseq	calcium ion transport
XP_001994910.1 GH17497 [Drosophila grimshawi]	XP_001994910.1	6,00E-53	GLOS_DGRI_GH1	GO:0007411	B	Refseq	axon guidance

XP_001994910.1 GH17497 [Drosophila grimshawi]	XP_001994910.1	6,00E-53	GLOS_DGRI_GH1	GO:0007596	B	Refseq	blood coagulation
XP_001994910.1 GH17497 [Drosophila grimshawi]	XP_001994910.1	6,00E-53	GLOS_DGRI_GH1	GO:0007602	B	Refseq	phototransduction
XP_001994910.1 GH17497 [Drosophila grimshawi]	XP_001994910.1	6,00E-53	GLOS_DGRI_GH1	GO:0010524	B	Refseq	positive regulation of calcium ion transport into cytosol
XP_001994910.1 GH17497 [Drosophila grimshawi]	XP_001994910.1	6,00E-53	GLOS_DGRI_GH1	GO:0015279	M	Refseq	store-operated calcium channel activity
XP_001994910.1 GH17497 [Drosophila grimshawi]	XP_001994910.1	6,00E-53	GLOS_DGRI_GH1	GO:0030168	B	Refseq	platelet activation
XP_001994910.1 GH17497 [Drosophila grimshawi]	XP_001994910.1	6,00E-53	GLOS_DGRI_GH1	GO:0033198	B	Refseq	response to ATP
XP_001994910.1 GH17497 [Drosophila grimshawi]	XP_001994910.1	6,00E-53	GLOS_DGRI_GH1	GO:0051592	B	Refseq	response to calcium ion
XP_001994910.1 GH17497 [Drosophila grimshawi]	XP_001994910.1	6,00E-53	GLOS_DGRI_GH1	GO:0070588	B	Refseq	calcium ion transmembrane transport
XP_001994910.1 GH17497 [Drosophila grimshawi]	XP_001994910.1	6,00E-53	GLOS_DGRI_GH1	GO:0070679	M	Refseq	inositol 1,4,5 trisphosphate binding
XP_001983838.1 GH16119 [Drosophila grimshawi]	XP_001983838.1	2,00E-95	GLOS_DGRI_GH1	GO:0005262	M	Refseq	calcium channel activity
XP_001983838.1 GH16119 [Drosophila grimshawi]	XP_001983838.1	2,00E-95	GLOS_DGRI_GH1	GO:0005515	M	Refseq	protein binding
XP_001983838.1 GH16119 [Drosophila grimshawi]	XP_001983838.1	2,00E-95	GLOS_DGRI_GH1	GO:0005886	C	Refseq	plasma membrane
XP_001983838.1 GH16119 [Drosophila grimshawi]	XP_001983838.1	2,00E-95	GLOS_DGRI_GH1	GO:0005887	C	Refseq	integral to plasma membrane
XP_001983838.1 GH16119 [Drosophila grimshawi]	XP_001983838.1	2,00E-95	GLOS_DGRI_GH1	GO:0006816	B	Refseq	calcium ion transport
XP_001983838.1 GH16119 [Drosophila grimshawi]	XP_001983838.1	2,00E-95	GLOS_DGRI_GH1	GO:0007411	B	Refseq	axon guidance
XP_001983838.1 GH16119 [Drosophila grimshawi]	XP_001983838.1	2,00E-95	GLOS_DGRI_GH1	GO:0007596	B	Refseq	blood coagulation
XP_001983838.1 GH16119 [Drosophila grimshawi]	XP_001983838.1	2,00E-95	GLOS_DGRI_GH1	GO:0007602	B	Refseq	phototransduction
XP_001983838.1 GH16119 [Drosophila grimshawi]	XP_001983838.1	2,00E-95	GLOS_DGRI_GH1	GO:0010524	B	Refseq	positive regulation of calcium ion transport into cytosol
XP_001983838.1 GH16119 [Drosophila grimshawi]	XP_001983838.1	2,00E-95	GLOS_DGRI_GH1	GO:0015279	M	Refseq	store-operated calcium channel activity
XP_001983838.1 GH16119 [Drosophila grimshawi]	XP_001983838.1	2,00E-95	GLOS_DGRI_GH1	GO:0030168	B	Refseq	platelet activation
XP_001983838.1 GH16119 [Drosophila grimshawi]	XP_001983838.1	2,00E-95	GLOS_DGRI_GH1	GO:0033198	B	Refseq	response to ATP
XP_001983838.1 GH16119 [Drosophila grimshawi]	XP_001983838.1	2,00E-95	GLOS_DGRI_GH1	GO:0051592	B	Refseq	response to calcium ion
XP_001983838.1 GH16119 [Drosophila grimshawi]	XP_001983838.1	2,00E-95	GLOS_DGRI_GH1	GO:0070588	B	Refseq	calcium ion transmembrane transport
XP_001983838.1 GH16119 [Drosophila grimshawi]	XP_001983838.1	2,00E-95	GLOS_DGRI_GH1	GO:0070679	M	Refseq	inositol 1,4,5 trisphosphate binding
NP_524873.1 ferritin 1 heavy chain homologue, isoform A [D. melanogaster] ref|NP_733358.1 isoform B [D. melanogaster] ref|NP_733359.1| isoform C [D. melanogaster] ref|NP_733360.1| isoform D [D. melanogaster]	NP_524873.1	1,00E-38	GLOS_FER1HCH.	GO:0000122	B	Refseq	negative regulation of transcription from RNA polymerase II promoter
NP_524873.1 ferritin 1 heavy chain homologue, isoform A … Same as above	NP_524873.1	1,00E-38	GLOS_FER1HCH.	GO:0001501	B	Refseq	skeletal system development
NP_524873.1 ferritin 1 heavy chain homologue, isoform A … Same as above	NP_524873.1	1,00E-38	GLOS_FER1HCH.	GO:0003700	M	Refseq	sequence-specific DNA binding transcription factor activity
NP_524873.1 ferritin 1 heavy chain homologue, isoform A … Same as above	NP_524873.1	1,00E-38	GLOS_FER1HCH.	GO:0005515	M	Refseq	protein binding
NP_524873.1 ferritin 1 heavy chain homologue, isoform A … Same as above	NP_524873.1	1,00E-38	GLOS_FER1HCH.	GO:0005634	C	Refseq	nucleus
NP_524873.1 ferritin 1 heavy chain homologue, isoform A … Same as above	NP_524873.1	1,00E-38	GLOS_FER1HCH.	GO:0006366	B	Refseq	transcription from RNA polymerase II promoter
NP_524873.1 ferritin 1 heavy chain homologue, isoform A … Same as above	NP_524873.1	1,00E-38	GLOS_FER1HCH.	GO:0006607	B	Refseq	NLS-bearing protein import into nucleus
NP_524873.1 ferritin 1 heavy chain homologue, isoform A … Same as above	NP_524873.1	1,00E-38	GLOS_FER1HCH.	GO:0008270	M	Refseq	zinc ion binding
NP_524873.1 ferritin 1 heavy chain homologue, isoform A … Same as above	NP_524873.1	1,00E-38	GLOS_FER1HCH.	GO:0032330	B	Refseq	regulation of chondrocyte differentiation
NP_524873.1 ferritin 1 heavy chain homologue, isoform A … Same as above	NP_524873.1	1,00E-38	GLOS_FER1HCH.	GO:0043565	M	Refseq	sequence-specific DNA binding
XM_001987626.1 Drosophila grimshawi GH22044 (DgriGH22044), mRNA	XM_001987626.1	2,00E-59	GLOS_DGRI_GH2	GO:0005262	M	Refseq	calcium channel activity
XM_001987626.1 Drosophila grimshawi GH22044 (DgriGH22044), mRNA	XM_001987626.1	2,00E-59	GLOS_DGRI_GH2	GO:0005515	M	Refseq	protein binding
XM_001987626.1 Drosophila grimshawi GH22044 (DgriGH22044), mRNA	XM_001987626.1	2,00E-59	GLOS_DGRI_GH2	GO:0005886	C	Refseq	plasma membrane
XM_001987626.1 Drosophila grimshawi GH22044 (DgriGH22044), mRNA	XM_001987626.1	2,00E-59	GLOS_DGRI_GH2	GO:0005887	C	Refseq	integral to plasma membrane
XM_001987626.1 Drosophila grimshawi GH22044 (DgriGH22044), mRNA	XM_001987626.1	2,00E-59	GLOS_DGRI_GH2	GO:0006816	B	Refseq	calcium ion transport
XM_001987626.1 Drosophila grimshawi GH22044 (DgriGH22044), mRNA	XM_001987626.1	2,00E-59	GLOS_DGRI_GH2	GO:0007411	B	Refseq	axon guidance
XM_001987626.1 Drosophila grimshawi GH22044 (DgriGH22044), mRNA	XM_001987626.1	2,00E-59	GLOS_DGRI_GH2	GO:0007596	B	Refseq	blood coagulation
XM_001987626.1 Drosophila grimshawi GH22044 (DgriGH22044), mRNA	XM_001987626.1	2,00E-59	GLOS_DGRI_GH2	GO:0007602	B	Refseq	phototransduction
XM_001987626.1 Drosophila grimshawi GH22044 (DgriGH22044), mRNA	XM_001987626.1	2,00E-59	GLOS_DGRI_GH2	GO:0010524	B	Refseq	positive regulation of calcium ion transport into cytosol
XM_001987626.1 Drosophila grimshawi GH22044 (DgriGH22044), mRNA	XM_001987626.1	2,00E-59	GLOS_DGRI_GH2	GO:0015279	M	Refseq	store-operated calcium channel activity
XM_001987626.1 Drosophila grimshawi GH22044 (DgriGH22044), mRNA	XM_001987626.1	2,00E-59	GLOS_DGRI_GH2	GO:0030168	B	Refseq	platelet activation
XM_001987626.1 Drosophila grimshawi GH22044 (DgriGH22044), mRNA	XM_001987626.1	2,00E-59	GLOS_DGRI_GH2	GO:0033198	B	Refseq	response to ATP
XM_001987626.1 Drosophila grimshawi GH22044 (DgriGH22044), mRNA	XM_001987626.1	2,00E-59	GLOS_DGRI_GH2	GO:0051592	B	Refseq	response to calcium ion
XM_001987626.1 Drosophila grimshawi GH22044 (DgriGH22044), mRNA	XM_001987626.1	2,00E-59	GLOS_DGRI_GH2	GO:0070588	B	Refseq	calcium ion transmembrane transport
XM_001987626.1 Drosophila grimshawi GH22044 (DgriGH22044), mRNA	XM_001987626.1	2,00E-59	GLOS_DGRI_GH2	GO:0070679	M	Refseq	inositol 1,4,5 trisphosphate binding
XP_001977601.1 GG19134 [Drosophila erecta]	XP_001977601.1	1,00E-119	GLOS_DERE_GG	GO:0005261	M	Refseq	cation channel activity
XP_001977601.1 GG19134 [Drosophila erecta]	XP_001977601.1	1,00E-119	GLOS_DERE_GG	GO:0005262	M	Refseq	calcium channel activity
XP_001977601.1 GG19134 [Drosophila erecta]	XP_001977601.1	1,00E-119	GLOS_DERE_GG	GO:0005515	M	Refseq	protein binding

XP_001977601.1 GG19134 [Drosophila erecta]	XP_001977601.1	1,00E-119	GLOS_DERE_GG	GO:0005886	C	Refseq	plasma membrane
XP_001977601.1 GG19134 [Drosophila erecta]	XP_001977601.1	1,00E-119	GLOS_DERE_GG	GO:0005887	C	Refseq	integral to plasma membrane
XP_001977601.1 GG19134 [Drosophila erecta]	XP_001977601.1	1,00E-119	GLOS_DERE_GG	GO:0006816	B	Refseq	calcium ion transport
XP_001977601.1 GG19134 [Drosophila erecta]	XP_001977601.1	1,00E-119	GLOS_DERE_GG	GO:0007411	B	Refseq	axon guidance
XP_001977601.1 GG19134 [Drosophila erecta]	XP_001977601.1	1,00E-119	GLOS_DERE_GG	GO:0015279	M	Refseq	store-operated calcium channel activity
XP_001977601.1 GG19134 [Drosophila erecta]	XP_001977601.1	1,00E-119	GLOS_DERE_GG	GO:0016323	C	Refseq	basolateral plasma membrane
XP_001977601.1 GG19134 [Drosophila erecta]	XP_001977601.1	1,00E-119	GLOS_DERE_GG	GO:0030017	C	Refseq	sarcomere
XP_001977601.1 GG19134 [Drosophila erecta]	XP_001977601.1	1,00E-119	GLOS_DERE_GG	GO:0043034	C	Refseq	costamere
XP_001977601.1 GG19134 [Drosophila erecta]	XP_001977601.1	1,00E-119	GLOS_DERE_GG	GO:0043234	C	Refseq	protein complex
XP_001977601.1 GG19134 [Drosophila erecta]	XP_001977601.1	1,00E-119	GLOS_DERE_GG	GO:0044325	M	Refseq	ion channel binding
XP_001977601.1 GG19134 [Drosophila erecta]	XP_001977601.1	1,00E-119	GLOS_DERE_GG	GO:0045121	C	Refseq	membrane raft
XP_001977601.1 GG19134 [Drosophila erecta]	XP_001977601.1	1,00E-119	GLOS_DERE_GG	GO:0046541	B	Refseq	saliva secretion
XP_001977601.1 GG19134 [Drosophila erecta]	XP_001977601.1	1,00E-119	GLOS_DERE_GG	GO:0051281	B	Refseq	positive regulation of release of sequestered calcium ion into cytosol
XP_001977601.1 GG19134 [Drosophila erecta]	XP_001977601.1	1,00E-119	GLOS_DERE_GG	GO:0051480	B	Refseq	cytosolic calcium ion homeostasis
XP_001977601.1 GG19134 [Drosophila erecta]	XP_001977601.1	1,00E-119	GLOS_DERE_GG	GO:0051592	B	Refseq	response to calcium ion
XP_001977601.1 GG19134 [Drosophila erecta]	XP_001977601.1	1,00E-119	GLOS_DERE_GG	GO:0070679	M	Refseq	inositol 1,4,5 trisphosphate binding
XP_001977778.1 GG19229 [Drosophila erecta]	XP_001977778.1	9,00E-29	GLOS_DERE_GG	GO:0005261	M	Refseq	cation channel activity
XP_001977778.1 GG19229 [Drosophila erecta]	XP_001977778.1	9,00E-29	GLOS_DERE_GG	GO:0005262	M	Refseq	calcium channel activity
XP_001977778.1 GG19229 [Drosophila erecta]	XP_001977778.1	9,00E-29	GLOS_DERE_GG	GO:0005515	M	Refseq	protein binding
XP_001977778.1 GG19229 [Drosophila erecta]	XP_001977778.1	9,00E-29	GLOS_DERE_GG	GO:0005886	C	Refseq	plasma membrane
XP_001977778.1 GG19229 [Drosophila erecta]	XP_001977778.1	9,00E-29	GLOS_DERE_GG	GO:0005887	C	Refseq	integral to plasma membrane
XP_001977778.1 GG19229 [Drosophila erecta]	XP_001977778.1	9,00E-29	GLOS_DERE_GG	GO:0006816	B	Refseq	calcium ion transport
XP_001977778.1 GG19229 [Drosophila erecta]	XP_001977778.1	9,00E-29	GLOS_DERE_GG	GO:0007411	B	Refseq	axon guidance
XP_001977778.1 GG19229 [Drosophila erecta]	XP_001977778.1	9,00E-29	GLOS_DERE_GG	GO:0015279	M	Refseq	store-operated calcium channel activity
XP_001977778.1 GG19229 [Drosophila erecta]	XP_001977778.1	9,00E-29	GLOS_DERE_GG	GO:0016323	C	Refseq	basolateral plasma membrane
XP_001977778.1 GG19229 [Drosophila erecta]	XP_001977778.1	9,00E-29	GLOS_DERE_GG	GO:0030017	C	Refseq	sarcomere
XP_001977778.1 GG19229 [Drosophila erecta]	XP_001977778.1	9,00E-29	GLOS_DERE_GG	GO:0043034	C	Refseq	costamere
XP_001977778.1 GG19229 [Drosophila erecta]	XP_001977778.1	9,00E-29	GLOS_DERE_GG	GO:0043234	C	Refseq	protein complex
XP_001977778.1 GG19229 [Drosophila erecta]	XP_001977778.1	9,00E-29	GLOS_DERE_GG	GO:0044325	M	Refseq	ion channel binding
XP_001977778.1 GG19229 [Drosophila erecta]	XP_001977778.1	9,00E-29	GLOS_DERE_GG	GO:0045121	C	Refseq	membrane raft
XP_001977778.1 GG19229 [Drosophila erecta]	XP_001977778.1	9,00E-29	GLOS_DERE_GG	GO:0046541	B	Refseq	saliva secretion
XP_001977778.1 GG19229 [Drosophila erecta]	XP_001977778.1	9,00E-29	GLOS_DERE_GG	GO:0051281	B	Refseq	positive regulation of release of sequestered calcium ion into cytosol
XP_001977778.1 GG19229 [Drosophila erecta]	XP_001977778.1	9,00E-29	GLOS_DERE_GG	GO:0051480	B	Refseq	cytosolic calcium ion homeostasis
XP_001977778.1 GG19229 [Drosophila erecta]	XP_001977778.1	9,00E-29	GLOS_DERE_GG	GO:0051592	B	Refseq	response to calcium ion
XP_001977778.1 GG19229 [Drosophila erecta]	XP_001977778.1	9,00E-29	GLOS_DERE_GG	GO:0070679	M	Refseq	inositol 1,4,5 trisphosphate binding
XP_001994580.1 GH15379 [Drosophila grimshawi]	XP_001994580.1	8,00E-86	GLOS_DGRI_GH1	GO:0005262	M	Refseq	calcium channel activity
XP_001994580.1 GH15379 [Drosophila grimshawi]	XP_001994580.1	8,00E-86	GLOS_DGRI_GH1	GO:0005515	M	Refseq	protein binding
XP_001994580.1 GH15379 [Drosophila grimshawi]	XP_001994580.1	8,00E-86	GLOS_DGRI_GH1	GO:0005886	C	Refseq	plasma membrane
XP_001994580.1 GH15379 [Drosophila grimshawi]	XP_001994580.1	8,00E-86	GLOS_DGRI_GH1	GO:0005887	C	Refseq	integral to plasma membrane
XP_001994580.1 GH15379 [Drosophila grimshawi]	XP_001994580.1	8,00E-86	GLOS_DGRI_GH1	GO:0006816	B	Refseq	calcium ion transport
XP_001994580.1 GH15379 [Drosophila grimshawi]	XP_001994580.1	8,00E-86	GLOS_DGRI_GH1	GO:0007411	B	Refseq	axon guidance
XP_001994580.1 GH15379 [Drosophila grimshawi]	XP_001994580.1	8,00E-86	GLOS_DGRI_GH1	GO:0007596	B	Refseq	blood coagulation
XP_001994580.1 GH15379 [Drosophila grimshawi]	XP_001994580.1	8,00E-86	GLOS_DGRI_GH1	GO:0007602	B	Refseq	phototransduction
XP_001994580.1 GH15379 [Drosophila grimshawi]	XP_001994580.1	8,00E-86	GLOS_DGRI_GH1	GO:0010524	B	Refseq	positive regulation of calcium ion transport into cytosol
XP_001994580.1 GH15379 [Drosophila grimshawi]	XP_001994580.1	8,00E-86	GLOS_DGRI_GH1	GO:0015279	M	Refseq	store-operated calcium channel activity
XP_001994580.1 GH15379 [Drosophila grimshawi]	XP_001994580.1	8,00E-86	GLOS_DGRI_GH1	GO:0030168	B	Refseq	platelet activation
XP_001994580.1 GH15379 [Drosophila grimshawi]	XP_001994580.1	8,00E-86	GLOS_DGRI_GH1	GO:0033198	B	Refseq	response to ATP
XP_001994580.1 GH15379 [Drosophila grimshawi]	XP_001994580.1	8,00E-86	GLOS_DGRI_GH1	GO:0051592	B	Refseq	response to calcium ion
XP_001994580.1 GH15379 [Drosophila grimshawi]	XP_001994580.1	8,00E-86	GLOS_DGRI_GH1	GO:0070588	B	Refseq	calcium ion transmembrane transport
XP_001994580.1 GH15379 [Drosophila grimshawi]	XP_001994580.1	8,00E-86	GLOS_DGRI_GH1	GO:0070679	M	Refseq	inositol 1,4,5 trisphosphate binding
XP_003243964.1 PREDICTED: hypothetical prot LOC100568954 [A. pisum]	XP_003243964.1	2,00E-55	GLOS_LOC10056	GO:0000278	B	Refseq	mitotic cell cycle
XP_003243964.1 PREDICTED: hypothetical prot LOC100568954 [A. pisum]	XP_003243964.1	2,00E-55	GLOS_LOC10056	GO:0003677	M	Refseq	DNA binding
XP_003243964.1 PREDICTED: hypothetical prot LOC100568954 [A. pisum]	XP_003243964.1	2,00E-55	GLOS_LOC10056	GO:0003700	M	Refseq	sequence-specific DNA binding transcription factor activity
XP_003243964.1 PREDICTED: hypothetical prot LOC100568954 [A. pisum]	XP_003243964.1	2,00E-55	GLOS_LOC10056	GO:0003712	M	Refseq	transcription cofactor activity

XP_003243964.1 PREDICTED: hypothetical prot LOC100568954 [A. pisum]	XP_003243964.1	2,00E-55	GLOS_LOC10056	GO:0005654	C	Refseq	nucleoplasm
XP_003243964.1 PREDICTED: hypothetical prot LOC100568954 [A. pisum]	XP_003243964.1	2,00E-55	GLOS_LOC10056	GO:0005667	C	Refseq	transcription factor complex
XP_003243964.1 PREDICTED: hypothetical prot LOC100568954 [A. pisum]	XP_003243964.1	2,00E-55	GLOS_LOC10056	GO:0006351	B	Refseq	transcription, DNA-dependent
XP_003243964.1 PREDICTED: hypothetical prot LOC100568954 [A. pisum]	XP_003243964.1	2,00E-55	GLOS_LOC10056	GO:0006367	B	Refseq	transcription initiation from RNA polymerase II promoter
XP_003243964.1 PREDICTED: hypothetical prot LOC100568954 [A. pisum]	XP_003243964.1	2,00E-55	GLOS_LOC10056	GO:0007179	B	Refseq	transforming growth factor beta receptor signaling pathway
XP_003243964.1 PREDICTED: hypothetical prot LOC100568954 [A. pisum]	XP_003243964.1	2,00E-55	GLOS_LOC10056	GO:0007507	B	Refseq	heart development
XP_003243964.1 PREDICTED: hypothetical prot LOC100568954 [A. pisum]	XP_003243964.1	2,00E-55	GLOS_LOC10056	GO:0008134	M	Refseq	transcription factor binding
XP_003243964.1 PREDICTED: hypothetical prot LOC100568954 [A. pisum]	XP_003243964.1	2,00E-55	GLOS_LOC10056	GO:0010467	B	Refseq	gene expression
XP_003243964.1 PREDICTED: hypothetical prot LOC100568954 [A. pisum]	XP_003243964.1	2,00E-55	GLOS_LOC10056	GO:0019904	M	Refseq	protein domain specific binding
XP_003243964.1 PREDICTED: hypothetical prot LOC100568954 [A. pisum]	XP_003243964.1	2,00E-55	GLOS_LOC10056	GO:0045944	B	Refseq	positive regulation of transcription from RNA polymerase II promoter
NP_572344.2 CG14441, isoform A [D. melanogaster] ref|NP_001259288.1| CG14441, isoform B [D. melanogaster] ref|NP_001259289.1| CG14441, isoform C [D. melanogaster] ref|NP_001259290.1| CG14441, isoform D [D. melanogaster]	NP_572344.2	2,00E-31	GLOS_CG14441.1	GO:0000122	B	Refseq	negative regulation of transcription from RNA polymerase II promoter
NP_572344.2 CG14441, isoform A [Drosophila melanogaster] … Same as above	NP_572344.2	2,00E-31	GLOS_CG14441.1	GO:0001501	B	Refseq	skeletal system development
NP_572344.2 CG14441, isoform A [Drosophila melanogaster] … Same as above	NP_572344.2	2,00E-31	GLOS_CG14441.1	GO:0003700	M	Refseq	sequence-specific DNA binding transcription factor activity
NP_572344.2 CG14441, isoform A [Drosophila melanogaster] … Same as above	NP_572344.2	2,00E-31	GLOS_CG14441.1	GO:0005515	M	Refseq	protein binding
NP_572344.2 CG14441, isoform A [Drosophila melanogaster] … Same as above	NP_572344.2	2,00E-31	GLOS_CG14441.1	GO:0005634	C	Refseq	nucleus
NP_572344.2 CG14441, isoform A [Drosophila melanogaster] … Same as above	NP_572344.2	2,00E-31	GLOS_CG14441.1	GO:0006366	B	Refseq	transcription from RNA polymerase II promoter
NP_572344.2 CG14441, isoform A [Drosophila melanogaster] … Same as above	NP_572344.2	2,00E-31	GLOS_CG14441.1	GO:0006607	B	Refseq	NLS-bearing protein import into nucleus
NP_572344.2 CG14441, isoform A [Drosophila melanogaster] … Same as above	NP_572344.2	2,00E-31	GLOS_CG14441.1	GO:0008270	M	Refseq	zinc ion binding
NP_572344.2 CG14441, isoform A [Drosophila melanogaster] … Same as above	NP_572344.2	2,00E-31	GLOS_CG14441.1	GO:0032330	B	Refseq	regulation of chondrocyte differentiation
NP_572344.2 CG14441, isoform A [Drosophila melanogaster] … Same as above	NP_572344.2	2,00E-31	GLOS_CG14441.1	GO:0043565	M	Refseq	sequence-specific DNA binding
XP_004934030.1 PREDICTED: nuclear protein 1-like [Bombyx mori]	XP_004934030.1	3,00E-10	GLOS_LOC10174	GO:0000122	B	Refseq	negative regulation of transcription from RNA polymerase II promoter
XP_004934030.1 PREDICTED: nuclear protein 1-like [Bombyx mori]	XP_004934030.1	3,00E-10	GLOS_LOC10174	GO:0003674	M	Refseq	molecular_function
XP_004934030.1 PREDICTED: nuclear protein 1-like [Bombyx mori]	XP_004934030.1	3,00E-10	GLOS_LOC10174	GO:0003682	M	Refseq	chromatin binding
XP_004934030.1 PREDICTED: nuclear protein 1-like [Bombyx mori]	XP_004934030.1	3,00E-10	GLOS_LOC10174	GO:0000981	M	.	sequence-specific DNA binding RNA polymerase II transcription factor activity
XP_004934030.1 PREDICTED: nuclear protein 1-like [Bombyx mori]	XP_004934030.1	3,00E-10	GLOS_LOC10174	GO:0003700	M	.	sequence-specific DNA binding transcription factor activity
XP_004934030.1 PREDICTED: nuclear protein 1-like [Bombyx mori]	XP_004934030.1	3,00E-10	GLOS_LOC10174	GO:0003705	M	Refseq	RNA polymerase II distal enhancer sequence-specific DNA binding transcription factor activity
XP_004934030.1 PREDICTED: nuclear protein 1-like [Bombyx mori]	XP_004934030.1	3,00E-10	GLOS_LOC10174	GO:0003712	M	.	transcription cofactor activity
XP_004934030.1 PREDICTED: nuclear protein 1-like [Bombyx mori]	XP_004934030.1	3,00E-10	GLOS_LOC10174	GO:0003714	M	Refseq	transcription corepressor activity
XP_004934030.1 PREDICTED: nuclear protein 1-like [Bombyx mori]	XP_004934030.1	3,00E-10	GLOS_LOC10174	GO:0005634	C	Refseq	nucleus
XP_004934030.1 PREDICTED: nuclear protein 1-like [Bombyx mori]	XP_004934030.1	3,00E-10	GLOS_LOC10174	GO:0005654	C	Refseq	nucleoplasm
XP_004934030.1 PREDICTED: nuclear protein 1-like [Bombyx mori]	XP_004934030.1	3,00E-10	GLOS_LOC10174	GO:0007219	B	Refseq	Notch signaling pathway
XP_004934030.1 PREDICTED: nuclear protein 1-like [Bombyx mori]	XP_004934030.1	3,00E-10	GLOS_LOC10174	GO:0008150	B	Refseq	biological_process
XP_004934030.1 PREDICTED: nuclear protein 1-like [Bombyx mori]	XP_004934030.1	3,00E-10	GLOS_LOC10174	GO:0016055	B	Refseq	Wnt receptor signaling pathway
XP_004934030.1 PREDICTED: nuclear protein 1-like [Bombyx mori]	XP_004934030.1	3,00E-10	GLOS_LOC10174	GO:0070491	M	Refseq	repressing transcription factor binding
XP_001980400.1 GG18949 [Drosophila erecta]	XP_001980400.1	4,00E-54	GLOS_DERE_GG	GO:0005261	M	Refseq	cation channel activity
XP_001980400.1 GG18949 [Drosophila erecta]	XP_001980400.1	4,00E-54	GLOS_DERE_GG	GO:0005262	M	Refseq	calcium channel activity
XP_001980400.1 GG18949 [Drosophila erecta]	XP_001980400.1	4,00E-54	GLOS_DERE_GG	GO:0005515	M	Refseq	protein binding
XP_001980400.1 GG18949 [Drosophila erecta]	XP_001980400.1	4,00E-54	GLOS_DERE_GG	GO:0005886	C	Refseq	plasma membrane
XP_001980400.1 GG18949 [Drosophila erecta]	XP_001980400.1	4,00E-54	GLOS_DERE_GG	GO:0005887	C	Refseq	integral to plasma membrane
XP_001980400.1 GG18949 [Drosophila erecta]	XP_001980400.1	4,00E-54	GLOS_DERE_GG	GO:0006816	B	Refseq	calcium ion transport
XP_001980400.1 GG18949 [Drosophila erecta]	XP_001980400.1	4,00E-54	GLOS_DERE_GG	GO:0007411	B	Refseq	axon guidance
XP_001980400.1 GG18949 [Drosophila erecta]	XP_001980400.1	4,00E-54	GLOS_DERE_GG	GO:0015279	M	Refseq	store-operated calcium channel activity
XP_001980400.1 GG18949 [Drosophila erecta]	XP_001980400.1	4,00E-54	GLOS_DERE_GG	GO:0016323	C	Refseq	basolateral plasma membrane
XP_001980400.1 GG18949 [Drosophila erecta]	XP_001980400.1	4,00E-54	GLOS_DERE_GG	GO:0030017	C	Refseq	sarcomere
XP_001980400.1 GG18949 [Drosophila erecta]	XP_001980400.1	4,00E-54	GLOS_DERE_GG	GO:0043034	C	Refseq	costamere
XP_001980400.1 GG18949 [Drosophila erecta]	XP_001980400.1	4,00E-54	GLOS_DERE_GG	GO:0043234	C	Refseq	protein complex
XP_001980400.1 GG18949 [Drosophila erecta]	XP_001980400.1	4,00E-54	GLOS_DERE_GG	GO:0044325	M	Refseq	ion channel binding
XP_001980400.1 GG18949 [Drosophila erecta]	XP_001980400.1	4,00E-54	GLOS_DERE_GG	GO:0045121	C	Refseq	membrane raft
XP_001980400.1 GG18949 [Drosophila erecta]	XP_001980400.1	4,00E-54	GLOS_DERE_GG	GO:0046541	B	Refseq	saliva secretion
XP_001980400.1 GG18949 [Drosophila erecta]	XP_001980400.1	4,00E-54	GLOS_DERE_GG	GO:0051281	B	Refseq	positive regulation of release of sequestered calcium ion into cytosol

XP_001980400.1 GG18949 [Drosophila erecta]	XP_001980400.1	4,00E-54	GLOS_DERE_GG	GO:0051480	B	Refseq	cytosolic calcium ion homeostasis
XP_001980400.1 GG18949 [Drosophila erecta]	XP_001980400.1	4,00E-54	GLOS_DERE_GG	GO:0051592	B	Refseq	response to calcium ion
XP_001980400.1 GG18949 [Drosophila erecta]	XP_001980400.1	4,00E-54	GLOS_DERE_GG	GO:0070679	M	Refseq	inositol 1,4,5 trisphosphate binding
XP_003243964.1 PREDICTED: hypothetical prot LOC100568954 [A. pisum]	XP_003243964.1	2,00E-10	GLOS_LOC10056	GO:0000278	B	Refseq	mitotic cell cycle
XP_003243964.1 PREDICTED: hypothetical prot LOC100568954 [A. pisum]	XP_003243964.1	2,00E-10	GLOS_LOC10056	GO:0003677	M	Refseq	DNA binding
XP_003243964.1 PREDICTED: hypothetical prot LOC100568954 [A. pisum]	XP_003243964.1	2,00E-10	GLOS_LOC10056	GO:0003700	M	Refseq	sequence-specific DNA binding transcription factor activity
XP_003243964.1 PREDICTED: hypothetical prot LOC100568954 [A. pisum]	XP_003243964.1	2,00E-10	GLOS_LOC10056	GO:0003712	M	Refseq	transcription cofactor activity
XP_003243964.1 PREDICTED: hypothetical prot LOC100568954 [A. pisum]	XP_003243964.1	2,00E-10	GLOS_LOC10056	GO:0005654	C	Refseq	nucleoplasm
XP_003243964.1 PREDICTED: hypothetical prot LOC100568954 [A. pisum]	XP_003243964.1	2,00E-10	GLOS_LOC10056	GO:0005667	C	Refseq	transcription factor complex
XP_003243964.1 PREDICTED: hypothetical prot LOC100568954 [A. pisum]	XP_003243964.1	2,00E-10	GLOS_LOC10056	GO:0006351	B	Refseq	transcription, DNA-dependent
XP_003243964.1 PREDICTED: hypothetical prot LOC100568954 [A. pisum]	XP_003243964.1	2,00E-10	GLOS_LOC10056	GO:0006367	B	Refseq	transcription initiation from RNA polymerase II promoter
XP_003243964.1 PREDICTED: hypothetical prot LOC100568954 [A. pisum]	XP_003243964.1	2,00E-10	GLOS_LOC10056	GO:0007179	B	Refseq	transforming growth factor beta receptor signaling pathway
XP_003243964.1 PREDICTED: hypothetical prot LOC100568954 [A. pisum]	XP_003243964.1	2,00E-10	GLOS_LOC10056	GO:0007507	B	Refseq	heart development
XP_003243964.1 PREDICTED: hypothetical prot LOC100568954 [A. pisum]	XP_003243964.1	2,00E-10	GLOS_LOC10056	GO:0008134	M	Refseq	transcription factor binding
XP_003243964.1 PREDICTED: hypothetical prot LOC100568954 [A. pisum]	XP_003243964.1	2,00E-10	GLOS_LOC10056	GO:0010467	B	Refseq	gene expression
XP_003243964.1 PREDICTED: hypothetical prot LOC100568954 [A. pisum]	XP_003243964.1	2,00E-10	GLOS_LOC10056	GO:0019904	M	Refseq	protein domain specific binding
XP_003243964.1 PREDICTED: hypothetical prot LOC100568954 [A. pisum]	XP_003243964.1	2,00E-10	GLOS_LOC10056	GO:0045944	B	Refseq	positive regulation of transcription from RNA polymerase II promoter
XP_003103183.1 hypothetical protein CRE_26624 [Caenorhabditis remanei]	XP_003103183.1	3,00E-16	GLOS_CRE_2662	GO:0004888	M	.	transmembrane signaling receptor activity
XP_003103183.1 hypothetical protein CRE_26624 [Caenorhabditis remanei]	XP_003103183.1	3,00E-16	GLOS_CRE_2662	GO:0038023	M	.	signaling receptor activity
XP_003103183.1 hypothetical protein CRE_26624 [Caenorhabditis remanei]	XP_003103183.1	3,00E-16	GLOS_CRE_2662	GO:0004871	M	.	signal transducer activity
XP_003103183.1 hypothetical protein CRE_26624 [Caenorhabditis remanei]	XP_003103183.1	3,00E-16	GLOS_CRE_2662	GO:0060089	M	.	molecular transducer activity
XP_003103183.1 hypothetical protein CRE_26624 [Caenorhabditis remanei]	XP_003103183.1	3,00E-16	GLOS_CRE_2662	GO:0004872	M	.	receptor activity
XP_003103183.1 hypothetical protein CRE_26624 [Caenorhabditis remanei]	XP_003103183.1	3,00E-16	GLOS_CRE_2662	GO:0004930	M	Refseq	G-protein coupled receptor activity
XP_003103183.1 hypothetical protein CRE_26624 [Caenorhabditis remanei]	XP_003103183.1	3,00E-16	GLOS_CRE_2662	GO:0008528	M	.	G-protein coupled peptide receptor activity
XP_003103183.1 hypothetical protein CRE_26624 [Caenorhabditis remanei]	XP_003103183.1	3,00E-16	GLOS_CRE_2662	GO:0001653	M	.	peptide receptor activity
XP_003103183.1 hypothetical protein CRE_26624 [Caenorhabditis remanei]	XP_003103183.1	3,00E-16	GLOS_CRE_2662	GO:0004930	M	.	G-protein coupled receptor activity
XP_003103183.1 hypothetical protein CRE_26624 [Caenorhabditis remanei]	XP_003103183.1	3,00E-16	GLOS_CRE_2662	GO:0004948	M	Refseq	calcitonin receptor activity
XP_003103183.1 hypothetical protein CRE_26624 [Caenorhabditis remanei]	XP_003103183.1	3,00E-16	GLOS_CRE_2662	GO:0005887	C	Refseq	integral to plasma membrane
XP_003103183.1 hypothetical protein CRE_26624 [Caenorhabditis remanei]	XP_003103183.1	3,00E-16	GLOS_CRE_2662	GO:0007186	B	Refseq	G-protein coupled receptor signaling pathway
XP_003103183.1 hypothetical protein CRE_26624 [Caenorhabditis remanei]	XP_003103183.1	3,00E-16	GLOS_CRE_2662	GO:0007186	B	.	G-protein coupled receptor signaling pathway
XP_003103183.1 hypothetical protein CRE_26624 [Caenorhabditis remanei]	XP_003103183.1	3,00E-16	GLOS_CRE_2662	GO:0007218	B	Refseq	neuropeptide signaling pathway
XP_003103183.1 hypothetical protein CRE_26624 [Caenorhabditis remanei]	XP_003103183.1	3,00E-16	GLOS_CRE_2662	GO:0007623	B	Refseq	circadian rhythm
XP_003103183.1 hypothetical protein CRE_26624 [Caenorhabditis remanei]	XP_003103183.1	3,00E-16	GLOS_CRE_2662	GO:0030594	M	.	neurotransmitter receptor activity
XP_003103183.1 hypothetical protein CRE_26624 [Caenorhabditis remanei]	XP_003103183.1	3,00E-16	GLOS_CRE_2662	GO:0008188	M	Refseq	neuropeptide receptor activity
XP_003103183.1 hypothetical protein CRE_26624 [Caenorhabditis remanei]	XP_003103183.1	3,00E-16	GLOS_CRE_2662	GO:0010578	B	.	regulation of adenylate cyclase activity involved in G-protein coupled receptor signaling pathway
XP_003103183.1 hypothetical protein CRE_26624 [Caenorhabditis remanei]	XP_003103183.1	3,00E-16	GLOS_CRE_2662	GO:0045761	B	.	regulation of adenylate cyclase activity
XP_003103183.1 hypothetical protein CRE_26624 [Caenorhabditis remanei]	XP_003103183.1	3,00E-16	GLOS_CRE_2662	GO:0030817	B	.	regulation of cAMP biosynthetic process
XP_003103183.1 hypothetical protein CRE_26624 [Caenorhabditis remanei]	XP_003103183.1	3,00E-16	GLOS_CRE_2662	GO:0030802	B	.	regulation of cyclic nucleotide biosynthetic process
XP_003103183.1 hypothetical protein CRE_26624 [Caenorhabditis remanei]	XP_003103183.1	3,00E-16	GLOS_CRE_2662	GO:0030799	B	.	regulation of cyclic nucleotide metabolic process
XP_003103183.1 hypothetical protein CRE_26624 [Caenorhabditis remanei]	XP_003103183.1	3,00E-16	GLOS_CRE_2662	GO:0030808	B	.	regulation of nucleotide biosynthetic process
XP_003103183.1 hypothetical protein CRE_26624 [Caenorhabditis remanei]	XP_003103183.1	3,00E-16	GLOS_CRE_2662	GO:0030814	B	.	regulation of cAMP metabolic process
XP_003103183.1 hypothetical protein CRE_26624 [Caenorhabditis remanei]	XP_003103183.1	3,00E-16	GLOS_CRE_2662	GO:1900371	B	.	regulation of purine nucleotide biosynthetic process
XP_003103183.1 hypothetical protein CRE_26624 [Caenorhabditis remanei]	XP_003103183.1	3,00E-16	GLOS_CRE_2662	GO:0031279	B	.	regulation of cyclase activity
XP_003103183.1 hypothetical protein CRE_26624 [Caenorhabditis remanei]	XP_003103183.1	3,00E-16	GLOS_CRE_2662	GO:0051339	B	.	regulation of lyase activity
XP_003103183.1 hypothetical protein CRE_26624 [Caenorhabditis remanei]	XP_003103183.1	3,00E-16	GLOS_CRE_2662	GO:0007188	B	.	adenylate cyclase-modulating G-protein coupled receptor signaling pathway
XP_003103183.1 hypothetical protein CRE_26624 [Caenorhabditis remanei]	XP_003103183.1	3,00E-16	GLOS_CRE_2662	GO:0007187	B	.	G-protein coupled receptor signaling pathway, coupled to cyclic nucleotide second messenger
XP_003103183.1 hypothetical protein CRE_26624 [Caenorhabditis remanei]	XP_003103183.1	3,00E-16	GLOS_CRE_2662	GO:0045762	B	.	positive regulation of adenylate cyclase activity
XP_003103183.1 hypothetical protein CRE_26624 [Caenorhabditis remanei]	XP_003103183.1	3,00E-16	GLOS_CRE_2662	GO:0030819	B	.	positive regulation of cAMP biosynthetic process
XP_003103183.1 hypothetical protein CRE_26624 [Caenorhabditis remanei]	XP_003103183.1	3,00E-16	GLOS_CRE_2662	GO:0030804	B	.	positive regulation of cyclic nucleotide biosynthetic process
XP_003103183.1 hypothetical protein CRE_26624 [Caenorhabditis remanei]	XP_003103183.1	3,00E-16	GLOS_CRE_2662	GO:0030801	B	.	positive regulation of cyclic nucleotide metabolic process
XP_003103183.1 hypothetical protein CRE_26624 [Caenorhabditis remanei]	XP_003103183.1	3,00E-16	GLOS_CRE_2662	GO:0045981	B	.	positive regulation of nucleotide metabolic process
XP_003103183.1 hypothetical protein CRE_26624 [Caenorhabditis remanei]	XP_003103183.1	3,00E-16	GLOS_CRE_2662	GO:0045937	B	.	positive regulation of phosphate metabolic process

XP_003103183.1 hypothetical protein CRE_26624 [Caenorhabditis remanei]	XP_003103183.1	3,00E-16	GLOS_CRE_2662	GO:0010562	B	.	positive regulation of phosphorus metabolic process
XP_003103183.1 hypothetical protein CRE_26624 [Caenorhabditis remanei]	XP_003103183.1	3,00E-16	GLOS_CRE_2662	GO:0030810	B	.	positive regulation of nucleotide biosynthetic process
XP_003103183.1 hypothetical protein CRE_26624 [Caenorhabditis remanei]	XP_003103183.1	3,00E-16	GLOS_CRE_2662	GO:0030816	B	.	positive regulation of cAMP metabolic process
XP_003103183.1 hypothetical protein CRE_26624 [Caenorhabditis remanei]	XP_003103183.1	3,00E-16	GLOS_CRE_2662	GO:1900544	B	.	positive regulation of purine nucleotide metabolic process
XP_003103183.1 hypothetical protein CRE_26624 [Caenorhabditis remanei]	XP_003103183.1	3,00E-16	GLOS_CRE_2662	GO:1900373	B	.	positive regulation of purine nucleotide biosynthetic process
XP_003103183.1 hypothetical protein CRE_26624 [Caenorhabditis remanei]	XP_003103183.1	3,00E-16	GLOS_CRE_2662	GO:0031281	B	.	positive regulation of cyclase activity
XP_003103183.1 hypothetical protein CRE_26624 [Caenorhabditis remanei]	XP_003103183.1	3,00E-16	GLOS_CRE_2662	GO:0051349	B	.	positive regulation of lyase activity
XP_003103183.1 hypothetical protein CRE_26624 [Caenorhabditis remanei]	XP_003103183.1	3,00E-16	GLOS_CRE_2662	GO:0007189	B	.	adenylate cyclase-activating G-protein coupled receptor signaling pathway
XP_003103183.1 hypothetical protein CRE_26624 [Caenorhabditis remanei]	XP_003103183.1	3,00E-16	GLOS_CRE_2662	GO:0010579	B	Refseq	positive regulation of adenylate cyclase activity involved in G-protein coupled receptor signaling pathway
XP_003103183.1 hypothetical protein CRE_26624 [Caenorhabditis remanei]	XP_003103183.1	3,00E-16	GLOS_CRE_2662	GO:0016021	C	Refseq	integral to membrane
XP_003103183.1 hypothetical protein CRE_26624 [Caenorhabditis remanei]	XP_003103183.1	3,00E-16	GLOS_CRE_2662	GO:0009629	B	.	response to gravity
XP_003103183.1 hypothetical protein CRE_26624 [Caenorhabditis remanei]	XP_003103183.1	3,00E-16	GLOS_CRE_2662	GO:0042332	B	Refseq	gravitaxis
XP_003103183.1 hypothetical protein CRE_26624 [Caenorhabditis remanei]	XP_003103183.1	3,00E-16	GLOS_CRE_2662	GO:0048512	B	.	circadian behavior
XP_003103183.1 hypothetical protein CRE_26624 [Caenorhabditis remanei]	XP_003103183.1	3,00E-16	GLOS_CRE_2662	GO:0007622	B	.	rhythmic behavior
XP_003103183.1 hypothetical protein CRE_26624 [Caenorhabditis remanei]	XP_003103183.1	3,00E-16	GLOS_CRE_2662	GO:0044708	B	.	single-organism behavior
XP_003103183.1 hypothetical protein CRE_26624 [Caenorhabditis remanei]	XP_003103183.1	3,00E-16	GLOS_CRE_2662	GO:0007610	B	.	behavior
XP_003103183.1 hypothetical protein CRE_26624 [Caenorhabditis remanei]	XP_003103183.1	3,00E-16	GLOS_CRE_2662	GO:0007623	B	.	circadian rhythm
XP_003103183.1 hypothetical protein CRE_26624 [Caenorhabditis remanei]	XP_003103183.1	3,00E-16	GLOS_CRE_2662	GO:0042745	B	Refseq	circadian sleep/wake cycle
XP_003103183.1 hypothetical protein CRE_26624 [Caenorhabditis remanei]	XP_003103183.1	3,00E-16	GLOS_CRE_2662	GO:0022410	B	.	circadian sleep/wake cycle process
XP_003103183.1 hypothetical protein CRE_26624 [Caenorhabditis remanei]	XP_003103183.1	3,00E-16	GLOS_CRE_2662	GO:0042745	B	.	circadian sleep/wake cycle
XP_003103183.1 hypothetical protein CRE_26624 [Caenorhabditis remanei]	XP_003103183.1	3,00E-16	GLOS_CRE_2662	GO:0042752	B	.	regulation of circadian rhythm
XP_003103183.1 hypothetical protein CRE_26624 [Caenorhabditis remanei]	XP_003103183.1	3,00E-16	GLOS_CRE_2662	GO:0050795	B	.	regulation of behavior
XP_003103183.1 hypothetical protein CRE_26624 [Caenorhabditis remanei]	XP_003103183.1	3,00E-16	GLOS_CRE_2662	GO:0042749	B	Refseq	regulation of circadian sleep/wake cycle
XP_003103183.1 hypothetical protein CRE_26624 [Caenorhabditis remanei]	XP_003103183.1	3,00E-16	GLOS_CRE_2662	GO:0043005	C	Refseq	neuron projection
XP_003103183.1 hypothetical protein CRE_26624 [Caenorhabditis remanei]	XP_003103183.1	3,00E-16	GLOS_CRE_2662	GO:0044297	C	.	cell body
XP_003103183.1 hypothetical protein CRE_26624 [Caenorhabditis remanei]	XP_003103183.1	3,00E-16	GLOS_CRE_2662	GO:0043025	C	Refseq	neuronal cell body
XP_003103183.1 hypothetical protein CRE_26624 [Caenorhabditis remanei]	XP_003103183.1	3,00E-16	GLOS_CRE_2662	GO:0007626	B	.	locomotory behavior
XP_003103183.1 hypothetical protein CRE_26624 [Caenorhabditis remanei]	XP_003103183.1	3,00E-16	GLOS_CRE_2662	GO:0045475	B	Refseq	locomotor rhythm
XP_003103183.1 hypothetical protein CRE_26624 [Caenorhabditis remanei]	XP_003103183.1	3,00E-16	GLOS_CRE_2662	GO:0048512	B	Refseq	circadian behavior
XP_001996998.1 GH23427 [Drosophila grimshawi]	XP_001996998.1	2,00E-65	GLOS_DGRI_GH2	GO:0005262	M	Refseq	calcium channel activity
XP_001996998.1 GH23427 [Drosophila grimshawi]	XP_001996998.1	2,00E-65	GLOS_DGRI_GH2	GO:0005515	M	Refseq	protein binding
XP_001996998.1 GH23427 [Drosophila grimshawi]	XP_001996998.1	2,00E-65	GLOS_DGRI_GH2	GO:0005886	C	Refseq	plasma membrane
XP_001996998.1 GH23427 [Drosophila grimshawi]	XP_001996998.1	2,00E-65	GLOS_DGRI_GH2	GO:0005887	C	Refseq	integral to plasma membrane
XP_001996998.1 GH23427 [Drosophila grimshawi]	XP_001996998.1	2,00E-65	GLOS_DGRI_GH2	GO:0006816	B	Refseq	calcium ion transport
XP_001996998.1 GH23427 [Drosophila grimshawi]	XP_001996998.1	2,00E-65	GLOS_DGRI_GH2	GO:0007411	B	Refseq	axon guidance
XP_001996998.1 GH23427 [Drosophila grimshawi]	XP_001996998.1	2,00E-65	GLOS_DGRI_GH2	GO:0007596	B	Refseq	blood coagulation
XP_001996998.1 GH23427 [Drosophila grimshawi]	XP_001996998.1	2,00E-65	GLOS_DGRI_GH2	GO:0007602	B	Refseq	phototransduction
XP_001996998.1 GH23427 [Drosophila grimshawi]	XP_001996998.1	2,00E-65	GLOS_DGRI_GH2	GO:0010524	B	Refseq	positive regulation of calcium ion transport into cytosol
XP_001996998.1 GH23427 [Drosophila grimshawi]	XP_001996998.1	2,00E-65	GLOS_DGRI_GH2	GO:0015279	M	Refseq	store-operated calcium channel activity
XP_001996998.1 GH23427 [Drosophila grimshawi]	XP_001996998.1	2,00E-65	GLOS_DGRI_GH2	GO:0030168	B	Refseq	platelet activation
XP_001996998.1 GH23427 [Drosophila grimshawi]	XP_001996998.1	2,00E-65	GLOS_DGRI_GH2	GO:0033198	B	Refseq	response to ATP
XP_001996998.1 GH23427 [Drosophila grimshawi]	XP_001996998.1	2,00E-65	GLOS_DGRI_GH2	GO:0051592	B	Refseq	response to calcium ion
XP_001996998.1 GH23427 [Drosophila grimshawi]	XP_001996998.1	2,00E-65	GLOS_DGRI_GH2	GO:0070588	B	Refseq	calcium ion transmembrane transport
XP_001996998.1 GH23427 [Drosophila grimshawi]	XP_001996998.1	2,00E-65	GLOS_DGRI_GH2	GO:0070679	M	Refseq	inositol 1,4,5 trisphosphate binding
XP_001981879.1 GG12291 [Drosophila erecta]	XP_001981879.1	0	GLOS_DERE_GG	GO:0005261	M	Refseq	cation channel activity
XP_001981879.1 GG12291 [Drosophila erecta]	XP_001981879.1	0	GLOS_DERE_GG	GO:0005262	M	Refseq	calcium channel activity
XP_001981879.1 GG12291 [Drosophila erecta]	XP_001981879.1	0	GLOS_DERE_GG	GO:0005515	M	Refseq	protein binding
XP_001981879.1 GG12291 [Drosophila erecta]	XP_001981879.1	0	GLOS_DERE_GG	GO:0005886	C	Refseq	plasma membrane
XP_001981879.1 GG12291 [Drosophila erecta]	XP_001981879.1	0	GLOS_DERE_GG	GO:0005887	C	Refseq	integral to plasma membrane
XP_001981879.1 GG12291 [Drosophila erecta]	XP_001981879.1	0	GLOS_DERE_GG	GO:0006816	B	Refseq	calcium ion transport
XP_001981879.1 GG12291 [Drosophila erecta]	XP_001981879.1	0	GLOS_DERE_GG	GO:0007411	B	Refseq	axon guidance
XP_001981879.1 GG12291 [Drosophila erecta]	XP_001981879.1	0	GLOS_DERE_GG	GO:0015279	M	Refseq	store-operated calcium channel activity
XP_001981879.1 GG12291 [Drosophila erecta]	XP_001981879.1	0	GLOS_DERE_GG	GO:0016323	C	Refseq	basolateral plasma membrane

XP_001981879.1 GG12291 [Drosophila erecta]	XP_001981879.1	0	GLOS_DERE_GG	GO:0030017	C	Refseq	sarcomere
XP_001981879.1 GG12291 [Drosophila erecta]	XP_001981879.1	0	GLOS_DERE_GG	GO:0043034	C	Refseq	costamere
XP_001981879.1 GG12291 [Drosophila erecta]	XP_001981879.1	0	GLOS_DERE_GG	GO:0043234	C	Refseq	protein complex
XP_001981879.1 GG12291 [Drosophila erecta]	XP_001981879.1	0	GLOS_DERE_GG	GO:0044325	M	Refseq	ion channel binding
XP_001981879.1 GG12291 [Drosophila erecta]	XP_001981879.1	0	GLOS_DERE_GG	GO:0045121	C	Refseq	membrane raft
XP_001981879.1 GG12291 [Drosophila erecta]	XP_001981879.1	0	GLOS_DERE_GG	GO:0046541	B	Refseq	saliva secretion
XP_001981879.1 GG12291 [Drosophila erecta]	XP_001981879.1	0	GLOS_DERE_GG	GO:0051281	B	Refseq	positive regulation of release of sequestered calcium ion into cytosol
XP_001981879.1 GG12291 [Drosophila erecta]	XP_001981879.1	0	GLOS_DERE_GG	GO:0051480	B	Refseq	cytosolic calcium ion homeostasis
XP_001981879.1 GG12291 [Drosophila erecta]	XP_001981879.1	0	GLOS_DERE_GG	GO:0051592	B	Refseq	response to calcium ion
XP_001981879.1 GG12291 [Drosophila erecta]	XP_001981879.1	0	GLOS_DERE_GG	GO:0070679	M	Refseq	inositol 1,4,5 trisphosphate binding
NP_001262915.1 CG6454, isoform E [Drosophila melanogaster]	NP_001262915.1	0	GLOS_CG6454.1.:	GO:0000122	B	Refseq	negative regulation of transcription from RNA polymerase II promoter
NP_001262915.1 CG6454, isoform E [Drosophila melanogaster]	NP_001262915.1	0	GLOS_CG6454.1.:	GO:0001501	B	Refseq	skeletal system development
NP_001262915.1 CG6454, isoform E [Drosophila melanogaster]	NP_001262915.1	0	GLOS_CG6454.1.:	GO:0003700	M	Refseq	sequence-specific DNA binding transcription factor activity
NP_001262915.1 CG6454, isoform E [Drosophila melanogaster]	NP_001262915.1	0	GLOS_CG6454.1.:	GO:0005515	M	Refseq	protein binding
NP_001262915.1 CG6454, isoform E [Drosophila melanogaster]	NP_001262915.1	0	GLOS_CG6454.1.:	GO:0005634	C	Refseq	nucleus
NP_001262915.1 CG6454, isoform E [Drosophila melanogaster]	NP_001262915.1	0	GLOS_CG6454.1.:	GO:0006366	B	Refseq	transcription from RNA polymerase II promoter
NP_001262915.1 CG6454, isoform E [Drosophila melanogaster]	NP_001262915.1	0	GLOS_CG6454.1.:	GO:0006607	B	Refseq	NLS-bearing protein import into nucleus
NP_001262915.1 CG6454, isoform E [Drosophila melanogaster]	NP_001262915.1	0	GLOS_CG6454.1.:	GO:0008270	M	Refseq	zinc ion binding
NP_001262915.1 CG6454, isoform E [Drosophila melanogaster]	NP_001262915.1	0	GLOS_CG6454.1.:	GO:0032330	B	Refseq	regulation of chondrocyte differentiation
NP_001262915.1 CG6454, isoform E [Drosophila melanogaster]	NP_001262915.1	0	GLOS_CG6454.1.:	GO:0043565	M	Refseq	sequence-specific DNA binding
NP_001163207.1 focal adhesion kinase, isoform F [Drosophila melanogaster]	NP_001163207.1	0	GLOS_FAK.1.1	GO:0000122	B	Refseq	negative regulation of transcription from RNA polymerase II promoter
NP_001163207.1 focal adhesion kinase, isoform F [Drosophila melanogaster]	NP_001163207.1	0	GLOS_FAK.1.1	GO:0001501	B	Refseq	skeletal system development
NP_001163207.1 focal adhesion kinase, isoform F [Drosophila melanogaster]	NP_001163207.1	0	GLOS_FAK.1.1	GO:0003700	M	Refseq	sequence-specific DNA binding transcription factor activity
NP_001163207.1 focal adhesion kinase, isoform F [Drosophila melanogaster]	NP_001163207.1	0	GLOS_FAK.1.1	GO:0005515	M	Refseq	protein binding
NP_001163207.1 focal adhesion kinase, isoform F [Drosophila melanogaster]	NP_001163207.1	0	GLOS_FAK.1.1	GO:0005634	C	Refseq	nucleus
NP_001163207.1 focal adhesion kinase, isoform F [Drosophila melanogaster]	NP_001163207.1	0	GLOS_FAK.1.1	GO:0006366	B	Refseq	transcription from RNA polymerase II promoter
NP_001163207.1 focal adhesion kinase, isoform F [Drosophila melanogaster]	NP_001163207.1	0	GLOS_FAK.1.1	GO:0006607	B	Refseq	NLS-bearing protein import into nucleus
NP_001163207.1 focal adhesion kinase, isoform F [Drosophila melanogaster]	NP_001163207.1	0	GLOS_FAK.1.1	GO:0008270	M	Refseq	zinc ion binding
NP_001163207.1 focal adhesion kinase, isoform F [Drosophila melanogaster]	NP_001163207.1	0	GLOS_FAK.1.1	GO:0032330	B	Refseq	regulation of chondrocyte differentiation
NP_001163207.1 focal adhesion kinase, isoform F [Drosophila melanogaster]	NP_001163207.1	0	GLOS_FAK.1.1	GO:0043565	M	Refseq	sequence-specific DNA binding
NP_001027035.2 terribly reduced optic lobes, isoform W [D. melanogaster]	NP_001027035.2	0	GLOS_contig_008	GO:0000122	B	Refseq	negative regulation of transcription from RNA polymerase II promoter
NP_001027035.2 terribly reduced optic lobes, isoform W [D. melanogaster]	NP_001027035.2	0	GLOS_contig_008	GO:0001501	B	Refseq	skeletal system development
NP_001027035.2 terribly reduced optic lobes, isoform W [D. melanogaster]	NP_001027035.2	0	GLOS_contig_008	GO:0003700	M	Refseq	sequence-specific DNA binding transcription factor activity
NP_001027035.2 terribly reduced optic lobes, isoform W [D. melanogaster]	NP_001027035.2	0	GLOS_contig_008	GO:0005515	M	Refseq	protein binding
NP_001027035.2 terribly reduced optic lobes, isoform W [D. melanogaster]	NP_001027035.2	0	GLOS_contig_008	GO:0005634	C	Refseq	nucleus
NP_001027035.2 terribly reduced optic lobes, isoform W [D. melanogaster]	NP_001027035.2	0	GLOS_contig_008	GO:0006366	B	Refseq	transcription from RNA polymerase II promoter
NP_001027035.2 terribly reduced optic lobes, isoform W [D. melanogaster]	NP_001027035.2	0	GLOS_contig_008	GO:0006607	B	Refseq	NLS-bearing protein import into nucleus
NP_001027035.2 terribly reduced optic lobes, isoform W [D. melanogaster]	NP_001027035.2	0	GLOS_contig_008	GO:0008270	M	Refseq	zinc ion binding
NP_001027035.2 terribly reduced optic lobes, isoform W [D. melanogaster]	NP_001027035.2	0	GLOS_contig_008	GO:0032330	B	Refseq	regulation of chondrocyte differentiation
NP_001027035.2 terribly reduced optic lobes, isoform W [D. melanogaster]	NP_001027035.2	0	GLOS_contig_008	GO:0043565	M	Refseq	sequence-specific DNA binding
XP_005108944.1 PREDICTED: mucin-22-like [Aplysia californica]	XP_005108944.1	3,00E-06	GLOS_LOC10186	GO:0044772	B	.	mitotic cell cycle phase transition
XP_005108944.1 PREDICTED: mucin-22-like [Aplysia californica]	XP_005108944.1	3,00E-06	GLOS_LOC10186	GO:0044770	B	.	cell cycle phase transition
XP_005108944.1 PREDICTED: mucin-22-like [Aplysia californica]	XP_005108944.1	3,00E-06	GLOS_LOC10186	GO:0022402	B	.	cell cycle process
XP_005108944.1 PREDICTED: mucin-22-like [Aplysia californica]	XP_005108944.1	3,00E-06	GLOS_LOC10186	GO:0000278	B	.	mitotic cell cycle
XP_005108944.1 PREDICTED: mucin-22-like [Aplysia californica]	XP_005108944.1	3,00E-06	GLOS_LOC10186	GO:0000082	B	Refseq	G1/S transition of mitotic cell cycle
XP_005108944.1 PREDICTED: mucin-22-like [Aplysia californica]	XP_005108944.1	3,00E-06	GLOS_LOC10186	GO:0000278	B	Refseq	mitotic cell cycle
XP_005108944.1 PREDICTED: mucin-22-like [Aplysia californica]	XP_005108944.1	3,00E-06	GLOS_LOC10186	GO:0019787	M	.	small conjugating protein ligase activity
XP_005108944.1 PREDICTED: mucin-22-like [Aplysia californica]	XP_005108944.1	3,00E-06	GLOS_LOC10186	GO:0016881	M	.	acid-amino acid ligase activity
XP_005108944.1 PREDICTED: mucin-22-like [Aplysia californica]	XP_005108944.1	3,00E-06	GLOS_LOC10186	GO:0016879	M	.	ligase activity, forming carbon-nitrogen bonds
XP_005108944.1 PREDICTED: mucin-22-like [Aplysia californica]	XP_005108944.1	3,00E-06	GLOS_LOC10186	GO:0016874	M	.	ligase activity
XP_005108944.1 PREDICTED: mucin-22-like [Aplysia californica]	XP_005108944.1	3,00E-06	GLOS_LOC10186	GO:0004842	M	Refseq	ubiquitin-protein ligase activity
XP_005108944.1 PREDICTED: mucin-22-like [Aplysia californica]	XP_005108944.1	3,00E-06	GLOS_LOC10186	GO:0005515	M	Refseq	protein binding
XP_005108944.1 PREDICTED: mucin-22-like [Aplysia californica]	XP_005108944.1	3,00E-06	GLOS_LOC10186	GO:0005654	C	Refseq	nucleoplasm
XP_005108944.1 PREDICTED: mucin-22-like [Aplysia californica]	XP_005108944.1	3,00E-06	GLOS_LOC10186	GO:0005829	C	Refseq	cytosol

XP_005108944.1 PREDICTED: mucin-22-like [Aplysia californica]	XP_005108944.1	3,00E-06	GLOS_LOC10186:	GO:0007219	B	Refseq	Notch signaling pathway
XP_005108944.1 PREDICTED: mucin-22-like [Aplysia californica]	XP_005108944.1	3,00E-06	GLOS_LOC10186:	GO:0044403	B	.	symbiosis, encompassing mutualism through parasitism
XP_005108944.1 PREDICTED: mucin-22-like [Aplysia californica]	XP_005108944.1	3,00E-06	GLOS_LOC10186:	GO:0044419	B	.	interspecies interaction between organisms
XP_005108944.1 PREDICTED: mucin-22-like [Aplysia californica]	XP_005108944.1	3,00E-06	GLOS_LOC10186:	GO:0044764	B	.	multi-organism cellular process
XP_005108944.1 PREDICTED: mucin-22-like [Aplysia californica]	XP_005108944.1	3,00E-06	GLOS_LOC10186:	GO:0016032	B	Refseq	viral process
XP_005108944.1 PREDICTED: mucin-22-like [Aplysia californica]	XP_005108944.1	3,00E-06	GLOS_LOC10186:	GO:0032446	B	.	protein modification by small protein conjugation
XP_005108944.1 PREDICTED: mucin-22-like [Aplysia californica]	XP_005108944.1	3,00E-06	GLOS_LOC10186:	GO:0070647	B	.	protein modification by small protein conjugation or removal
XP_005108944.1 PREDICTED: mucin-22-like [Aplysia californica]	XP_005108944.1	3,00E-06	GLOS_LOC10186:	GO:0016567	B	Refseq	protein ubiquitination
XP_005108944.1 PREDICTED: mucin-22-like [Aplysia californica]	XP_005108944.1	3,00E-06	GLOS_LOC10186:	GO:0031461	C	.	cullin-RING ubiquitin ligase complex
XP_005108944.1 PREDICTED: mucin-22-like [Aplysia californica]	XP_005108944.1	3,00E-06	GLOS_LOC10186:	GO:0000151	C	.	ubiquitin ligase complex
XP_005108944.1 PREDICTED: mucin-22-like [Aplysia californica]	XP_005108944.1	3,00E-06	GLOS_LOC10186:	GO:0019005	C	Refseq	SCF ubiquitin ligase complex
XP_005108944.1 PREDICTED: mucin-22-like [Aplysia californica]	XP_005108944.1	3,00E-06	GLOS_LOC10186:	GO:0043161	B	.	proteasomal ubiquitin-dependent protein catabolic process
XP_005108944.1 PREDICTED: mucin-22-like [Aplysia californica]	XP_005108944.1	3,00E-06	GLOS_LOC10186:	GO:0006511	B	.	ubiquitin-dependent protein catabolic process
XP_005108944.1 PREDICTED: mucin-22-like [Aplysia californica]	XP_005108944.1	3,00E-06	GLOS_LOC10186:	GO:0019941	B	.	modification-dependent protein catabolic process
XP_005108944.1 PREDICTED: mucin-22-like [Aplysia californica]	XP_005108944.1	3,00E-06	GLOS_LOC10186:	GO:0043632	B	.	modification-dependent macromolecule catabolic process
XP_005108944.1 PREDICTED: mucin-22-like [Aplysia californica]	XP_005108944.1	3,00E-06	GLOS_LOC10186:	GO:0044265	B	.	cellular macromolecule catabolic process
XP_005108944.1 PREDICTED: mucin-22-like [Aplysia californica]	XP_005108944.1	3,00E-06	GLOS_LOC10186:	GO:0009057	B	.	macromolecule catabolic process
XP_005108944.1 PREDICTED: mucin-22-like [Aplysia californica]	XP_005108944.1	3,00E-06	GLOS_LOC10186:	GO:1901575	B	.	organic substance catabolic process
XP_005108944.1 PREDICTED: mucin-22-like [Aplysia californica]	XP_005108944.1	3,00E-06	GLOS_LOC10186:	GO:0009056	B	.	catabolic process
XP_005108944.1 PREDICTED: mucin-22-like [Aplysia californica]	XP_005108944.1	3,00E-06	GLOS_LOC10186:	GO:0044248	B	.	cellular catabolic process
XP_005108944.1 PREDICTED: mucin-22-like [Aplysia californica]	XP_005108944.1	3,00E-06	GLOS_LOC10186:	GO:0051603	B	.	proteolysis involved in cellular protein catabolic process
XP_005108944.1 PREDICTED: mucin-22-like [Aplysia californica]	XP_005108944.1	3,00E-06	GLOS_LOC10186:	GO:0006508	B	.	proteolysis
XP_005108944.1 PREDICTED: mucin-22-like [Aplysia californica]	XP_005108944.1	3,00E-06	GLOS_LOC10186:	GO:0044257	B	.	cellular protein catabolic process
XP_005108944.1 PREDICTED: mucin-22-like [Aplysia californica]	XP_005108944.1	3,00E-06	GLOS_LOC10186:	GO:0030163	B	.	protein catabolic process
XP_005108944.1 PREDICTED: mucin-22-like [Aplysia californica]	XP_005108944.1	3,00E-06	GLOS_LOC10186:	GO:0010498	B	.	proteasomal protein catabolic process
XP_005108944.1 PREDICTED: mucin-22-like [Aplysia californica]	XP_005108944.1	3,00E-06	GLOS_LOC10186:	GO:0031145	B	Refseq	anaphase-promoting complex-dependent proteasomal ubiquitin-dependent protein catabolic process
XP_005108944.1 PREDICTED: mucin-22-like [Aplysia californica]	XP_005108944.1	3,00E-06	GLOS_LOC10186:	GO:0031146	B	Refseq	SCF-dependent proteasomal ubiquitin-dependent protein catabolic process
XP_005108944.1 PREDICTED: mucin-22-like [Aplysia californica]	XP_005108944.1	3,00E-06	GLOS_LOC10186:	GO:0031467	C	Refseq	Cul7-RING ubiquitin ligase complex
XP_005108944.1 PREDICTED: mucin-22-like [Aplysia californica]	XP_005108944.1	3,00E-06	GLOS_LOC10186:	GO:0031519	C	Refseq	PcG protein complex
XP_005108944.1 PREDICTED: mucin-22-like [Aplysia californica]	XP_005108944.1	3,00E-06	GLOS_LOC10186:	GO:0010390	B	.	histone monoubiquitination
XP_005108944.1 PREDICTED: mucin-22-like [Aplysia californica]	XP_005108944.1	3,00E-06	GLOS_LOC10186:	GO:0006513	B	.	protein monoubiquitination
XP_005108944.1 PREDICTED: mucin-22-like [Aplysia californica]	XP_005108944.1	3,00E-06	GLOS_LOC10186:	GO:0016567	B	.	protein ubiquitination
XP_005108944.1 PREDICTED: mucin-22-like [Aplysia californica]	XP_005108944.1	3,00E-06	GLOS_LOC10186:	GO:0016574	B	.	histone ubiquitination
XP_005108944.1 PREDICTED: mucin-22-like [Aplysia californica]	XP_005108944.1	3,00E-06	GLOS_LOC10186:	GO:0016570	B	.	histone modification
XP_005108944.1 PREDICTED: mucin-22-like [Aplysia californica]	XP_005108944.1	3,00E-06	GLOS_LOC10186:	GO:0016569	B	.	covalent chromatin modification
XP_005108944.1 PREDICTED: mucin-22-like [Aplysia californica]	XP_005108944.1	3,00E-06	GLOS_LOC10186:	GO:0016568	B	.	chromatin modification
XP_005108944.1 PREDICTED: mucin-22-like [Aplysia californica]	XP_005108944.1	3,00E-06	GLOS_LOC10186:	GO:0006325	B	.	chromatin organization
XP_005108944.1 PREDICTED: mucin-22-like [Aplysia californica]	XP_005108944.1	3,00E-06	GLOS_LOC10186:	GO:0051276	B	.	chromosome organization
XP_005108944.1 PREDICTED: mucin-22-like [Aplysia californica]	XP_005108944.1	3,00E-06	GLOS_LOC10186:	GO:0033522	B	.	histone H2A ubiquitination
XP_005108944.1 PREDICTED: mucin-22-like [Aplysia californica]	XP_005108944.1	3,00E-06	GLOS_LOC10186:	GO:0035518	B	Refseq	histone H2A monoubiquitination
XP_005108944.1 PREDICTED: mucin-22-like [Aplysia californica]	XP_005108944.1	3,00E-06	GLOS_LOC10186:	GO:0051439	B	.	regulation of ubiquitin-protein ligase activity involved in mitotic cell cycle
XP_005108944.1 PREDICTED: mucin-22-like [Aplysia californica]	XP_005108944.1	3,00E-06	GLOS_LOC10186:	GO:0051438	B	.	regulation of ubiquitin-protein ligase activity
XP_005108944.1 PREDICTED: mucin-22-like [Aplysia californica]	XP_005108944.1	3,00E-06	GLOS_LOC10186:	GO:0031396	B	.	regulation of protein ubiquitination
XP_005108944.1 PREDICTED: mucin-22-like [Aplysia californica]	XP_005108944.1	3,00E-06	GLOS_LOC10186:	GO:0051340	B	.	regulation of ligase activity
XP_005108944.1 PREDICTED: mucin-22-like [Aplysia californica]	XP_005108944.1	3,00E-06	GLOS_LOC10186:	GO:0051443	B	.	positive regulation of ubiquitin-protein ligase activity
XP_005108944.1 PREDICTED: mucin-22-like [Aplysia californica]	XP_005108944.1	3,00E-06	GLOS_LOC10186:	GO:0031398	B	.	positive regulation of protein ubiquitination
XP_005108944.1 PREDICTED: mucin-22-like [Aplysia californica]	XP_005108944.1	3,00E-06	GLOS_LOC10186:	GO:0031401	B	.	positive regulation of protein modification process
XP_005108944.1 PREDICTED: mucin-22-like [Aplysia californica]	XP_005108944.1	3,00E-06	GLOS_LOC10186:	GO:0032270	B	.	positive regulation of cellular protein metabolic process
XP_005108944.1 PREDICTED: mucin-22-like [Aplysia californica]	XP_005108944.1	3,00E-06	GLOS_LOC10186:	GO:0051247	B	.	positive regulation of protein metabolic process
XP_005108944.1 PREDICTED: mucin-22-like [Aplysia californica]	XP_005108944.1	3,00E-06	GLOS_LOC10186:	GO:0051351	B	.	positive regulation of ligase activity
XP_005108944.1 PREDICTED: mucin-22-like [Aplysia californica]	XP_005108944.1	3,00E-06	GLOS_LOC10186:	GO:0051437	B	Refseq	positive regulation of ubiquitin-protein ligase activity involved in mitotic cell cycle
XP_005108944.1 PREDICTED: mucin-22-like [Aplysia californica]	XP_005108944.1	3,00E-06	GLOS_LOC10186:	GO:0051439	B	Refseq	regulation of ubiquitin-protein ligase activity involved in mitotic cell cycle
NP_609807.1 LSm7, isoform A [D. melanogaster] ref|NP_001246045.1| LSm7,	NP_609807.1	3,00E-54	GLOS_LSM7.1.1	GO:0000122	B	Refseq	negative regulation of transcription from RNA polymerase II promoter

isoform B [D. melanogaster] ref|XP_001976253.1| GG20109 [D. erecta] ref|XP_002038853.1| GM17159 [D. sechellia] ref|XP_002090522.1| GE13166 [D. yakuba] ref|XP_002079671.1| GD21898 [D. simulans]							
NP_609807.1 LSm7, isoform A [Drosophila melanogaster] … Same as above	NP_609807.1	3,00E-54	GLOS_LSM7.1.1	GO:0001501	B	Refseq	skeletal system development
NP_609807.1 LSm7, isoform A [Drosophila melanogaster] … Same as above	NP_609807.1	3,00E-54	GLOS_LSM7.1.1	GO:0003700	M	Refseq	sequence-specific DNA binding transcription factor activity
NP_609807.1 LSm7, isoform A [Drosophila melanogaster] … Same as above	NP_609807.1	3,00E-54	GLOS_LSM7.1.1	GO:0005515	M	Refseq	protein binding
NP_609807.1 LSm7, isoform A [Drosophila melanogaster] … Same as above	NP_609807.1	3,00E-54	GLOS_LSM7.1.1	GO:0005634	C	Refseq	nucleus
NP_609807.1 LSm7, isoform A [Drosophila melanogaster] … Same as above	NP_609807.1	3,00E-54	GLOS_LSM7.1.1	GO:0006366	B	Refseq	transcription from RNA polymerase II promoter
NP_609807.1 LSm7, isoform A [Drosophila melanogaster] … Same as above	NP_609807.1	3,00E-54	GLOS_LSM7.1.1	GO:0006607	B	Refseq	NLS-bearing protein import into nucleus
NP_609807.1 LSm7, isoform A [Drosophila melanogaster] … Same as above	NP_609807.1	3,00E-54	GLOS_LSM7.1.1	GO:0008270	M	Refseq	zinc ion binding
NP_609807.1 LSm7, isoform A [Drosophila melanogaster] … Same as above	NP_609807.1	3,00E-54	GLOS_LSM7.1.1	GO:0032330	B	Refseq	regulation of chondrocyte differentiation
NP_609807.1 LSm7, isoform A [Drosophila melanogaster] … Same as above	NP_609807.1	3,00E-54	GLOS_LSM7.1.1	GO:0043565	M	Refseq	sequence-specific DNA binding
XP_001993475.1 GH13046 [Drosophila grimshawi]	XP_001993475.1	1,00E-159	GLOS_DGRI_GH1	GO:0005262	M	Refseq	calcium channel activity
XP_001993475.1 GH13046 [Drosophila grimshawi]	XP_001993475.1	1,00E-159	GLOS_DGRI_GH1	GO:0005515	M	Refseq	protein binding
XP_001993475.1 GH13046 [Drosophila grimshawi]	XP_001993475.1	1,00E-159	GLOS_DGRI_GH1	GO:0005886	C	Refseq	plasma membrane
XP_001993475.1 GH13046 [Drosophila grimshawi]	XP_001993475.1	1,00E-159	GLOS_DGRI_GH1	GO:0005887	C	Refseq	integral to plasma membrane
XP_001993475.1 GH13046 [Drosophila grimshawi]	XP_001993475.1	1,00E-159	GLOS_DGRI_GH1	GO:0006816	B	Refseq	calcium ion transport
XP_001993475.1 GH13046 [Drosophila grimshawi]	XP_001993475.1	1,00E-159	GLOS_DGRI_GH1	GO:0007411	B	Refseq	axon guidance
XP_001993475.1 GH13046 [Drosophila grimshawi]	XP_001993475.1	1,00E-159	GLOS_DGRI_GH1	GO:0007596	B	Refseq	blood coagulation
XP_001993475.1 GH13046 [Drosophila grimshawi]	XP_001993475.1	1,00E-159	GLOS_DGRI_GH1	GO:0007602	B	Refseq	phototransduction
XP_001993475.1 GH13046 [Drosophila grimshawi]	XP_001993475.1	1,00E-159	GLOS_DGRI_GH1	GO:0010524	B	Refseq	positive regulation of calcium ion transport into cytosol
XP_001993475.1 GH13046 [Drosophila grimshawi]	XP_001993475.1	1,00E-159	GLOS_DGRI_GH1	GO:0015279	M	Refseq	store-operated calcium channel activity
XP_001993475.1 GH13046 [Drosophila grimshawi]	XP_001993475.1	1,00E-159	GLOS_DGRI_GH1	GO:0030168	B	Refseq	platelet activation
XP_001993475.1 GH13046 [Drosophila grimshawi]	XP_001993475.1	1,00E-159	GLOS_DGRI_GH1	GO:0033198	B	Refseq	response to ATP
XP_001993475.1 GH13046 [Drosophila grimshawi]	XP_001993475.1	1,00E-159	GLOS_DGRI_GH1	GO:0051592	B	Refseq	response to calcium ion
XP_001993475.1 GH13046 [Drosophila grimshawi]	XP_001993475.1	1,00E-159	GLOS_DGRI_GH1	GO:0070588	B	Refseq	calcium ion transmembrane transport
XP_001993475.1 GH13046 [Drosophila grimshawi]	XP_001993475.1	1,00E-159	GLOS_DGRI_GH1	GO:0070679	M	Refseq	inositol 1,4,5 trisphosphate binding
NP_995894.2 par-1, isoform X [Drosophila melanogaster]	NP_995894.2	0	GLOS_PAR-1.2.3	GO:0000122	B	Refseq	negative regulation of transcription from RNA polymerase II promoter
NP_995894.2 par-1, isoform X [Drosophila melanogaster]	NP_995894.2	0	GLOS_PAR-1.2.3	GO:0001501	B	Refseq	skeletal system development
NP_995894.2 par-1, isoform X [Drosophila melanogaster]	NP_995894.2	0	GLOS_PAR-1.2.3	GO:0003700	M	Refseq	sequence-specific DNA binding transcription factor activity
NP_995894.2 par-1, isoform X [Drosophila melanogaster]	NP_995894.2	0	GLOS_PAR-1.2.3	GO:0005515	M	Refseq	protein binding
NP_995894.2 par-1, isoform X [Drosophila melanogaster]	NP_995894.2	0	GLOS_PAR-1.2.3	GO:0005634	C	Refseq	nucleus
NP_995894.2 par-1, isoform X [Drosophila melanogaster]	NP_995894.2	0	GLOS_PAR-1.2.3	GO:0006366	B	Refseq	transcription from RNA polymerase II promoter
NP_995894.2 par-1, isoform X [Drosophila melanogaster]	NP_995894.2	0	GLOS_PAR-1.2.3	GO:0006607	B	Refseq	NLS-bearing protein import into nucleus
NP_995894.2 par-1, isoform X [Drosophila melanogaster]	NP_995894.2	0	GLOS_PAR-1.2.3	GO:0008270	M	Refseq	zinc ion binding
NP_995894.2 par-1, isoform X [Drosophila melanogaster]	NP_995894.2	0	GLOS_PAR-1.2.3	GO:0032330	B	Refseq	regulation of chondrocyte differentiation
NP_995894.2 par-1, isoform X [Drosophila melanogaster]	NP_995894.2	0	GLOS_PAR-1.2.3	GO:0043565	M	Refseq	sequence-specific DNA binding
NP_610536.1 electron transfer flavoprotein-ubiquinone oxidoreductase, isoform A [D. melanogaster] ref|NP_001246231.1| isoform B [D. melanogaster]	NP_610536.1	1,00E-14	GLOS_ETF-QO.1.	GO:0000122	B	Refseq	negative regulation of transcription from RNA polymerase II promoter
NP_610536.1 electron transfer flavoprotein-ubiquinone oxidor. … Same as above	NP_610536.1	1,00E-14	GLOS_ETF-QO.1.	GO:0001501	B	Refseq	skeletal system development
NP_610536.1 electron transfer flavoprotein-ubiquinone oxidor. … Same as above	NP_610536.1	1,00E-14	GLOS_ETF-QO.1.	GO:0003700	M	Refseq	sequence-specific DNA binding transcription factor activity
NP_610536.1 electron transfer flavoprotein-ubiquinone oxidor. … Same as above	NP_610536.1	1,00E-14	GLOS_ETF-QO.1.	GO:0005515	M	Refseq	protein binding
NP_610536.1 electron transfer flavoprotein-ubiquinone oxidor. … Same as above	NP_610536.1	1,00E-14	GLOS_ETF-QO.1.	GO:0005634	C	Refseq	nucleus
NP_610536.1 electron transfer flavoprotein-ubiquinone oxidor. … Same as above	NP_610536.1	1,00E-14	GLOS_ETF-QO.1.	GO:0006366	B	Refseq	transcription from RNA polymerase II promoter
NP_610536.1 electron transfer flavoprotein-ubiquinone oxidor. … Same as above	NP_610536.1	1,00E-14	GLOS_ETF-QO.1.	GO:0006607	B	Refseq	NLS-bearing protein import into nucleus
NP_610536.1 electron transfer flavoprotein-ubiquinone oxidor. … Same as above	NP_610536.1	1,00E-14	GLOS_ETF-QO.1.	GO:0008270	M	Refseq	zinc ion binding
NP_610536.1 electron transfer flavoprotein-ubiquinone oxidor. … Same as above	NP_610536.1	1,00E-14	GLOS_ETF-QO.1.	GO:0032330	B	Refseq	regulation of chondrocyte differentiation
NP_610536.1 electron transfer flavoprotein-ubiquinone oxidor. … Same as above	NP_610536.1	1,00E-14	GLOS_ETF-QO.1.	GO:0043565	M	Refseq	sequence-specific DNA binding
NP_723847.1 mitochondrial ribosomal protein S23 [Drosophila melanogaster]	NP_723847.1	1,00E-67	GLOS_MRPS23.1.	GO:0000122	B	Refseq	negative regulation of transcription from RNA polymerase II promoter
NP_723847.1 mitochondrial ribosomal protein S23 [Drosophila melanogaster]	NP_723847.1	1,00E-67	GLOS_MRPS23.1.	GO:0001501	B	Refseq	skeletal system development
NP_723847.1 mitochondrial ribosomal protein S23 [Drosophila melanogaster]	NP_723847.1	1,00E-67	GLOS_MRPS23.1.	GO:0003700	M	Refseq	sequence-specific DNA binding transcription factor activity
NP_723847.1 mitochondrial ribosomal protein S23 [Drosophila melanogaster]	NP_723847.1	1,00E-67	GLOS_MRPS23.1.	GO:0005515	M	Refseq	protein binding
NP_723847.1 mitochondrial ribosomal protein S23 [Drosophila melanogaster]	NP_723847.1	1,00E-67	GLOS_MRPS23.1.	GO:0005634	C	Refseq	nucleus
NP_723847.1 mitochondrial ribosomal protein S23 [Drosophila melanogaster]	NP_723847.1	1,00E-67	GLOS_MRPS23.1.	GO:0006366	B	Refseq	transcription from RNA polymerase II promoter

NP_723847.1 mitochondrial ribosomal protein S23 [Drosophila melanogaster]	NP_723847.1	1,00E-67	GLOS_MRPS23.1.	GO:0006607	B	Refseq	NLS-bearing protein import into nucleus
NP_723847.1 mitochondrial ribosomal protein S23 [Drosophila melanogaster]	NP_723847.1	1,00E-67	GLOS_MRPS23.1.	GO:0008270	M	Refseq	zinc ion binding
NP_723847.1 mitochondrial ribosomal protein S23 [Drosophila melanogaster]	NP_723847.1	1,00E-67	GLOS_MRPS23.1.	GO:0032330	B	Refseq	regulation of chondrocyte differentiation
NP_723847.1 mitochondrial ribosomal protein S23 [Drosophila melanogaster]	NP_723847.1	1,00E-67	GLOS_MRPS23.1.	GO:0043565	M	Refseq	sequence-specific DNA binding
ACE_HAEIX (sp|Q10715) Angiotensin-converting enzyme OS=H. irritans exigua	ACE_HAEIX	6,00E-52	GLOS_ACE.6.16	GO:0005615	C	UniProtKB-S	extracellular space
ACE_HAEIX (sp|Q10715) Angiotensin-converting enzyme OS=H. irritans exigua	ACE_HAEIX	6,00E-52	GLOS_ACE.6.16	GO:0016020	C	InterPro	membrane
ACE_HAEIX (sp|Q10715) Angiotensin-converting enzyme OS=H. irritans exigua	ACE_HAEIX	6,00E-52	GLOS_ACE.6.16	GO:0008238	M	.	exopeptidase activity
ACE_HAEIX (sp|Q10715) Angiotensin-converting enzyme OS=H. irritans exigua	ACE_HAEIX	6,00E-52	GLOS_ACE.6.16	GO:0070011	M	.	peptidase activity, acting on L-amino acid peptides
ACE_HAEIX (sp|Q10715) Angiotensin-converting enzyme OS=H. irritans exigua	ACE_HAEIX	6,00E-52	GLOS_ACE.6.16	GO:0008233	M	.	peptidase activity
ACE_HAEIX (sp|Q10715) Angiotensin-converting enzyme OS=H. irritans exigua	ACE_HAEIX	6,00E-52	GLOS_ACE.6.16	GO:0016787	M	.	hydrolase activity
ACE_HAEIX (sp|Q10715) Angiotensin-converting enzyme OS=H. irritans exigua	ACE_HAEIX	6,00E-52	GLOS_ACE.6.16	GO:0004180	M	UniProtKB-K	carboxypeptidase activity
ACE_HAEIX (sp|Q10715) Angiotensin-converting enzyme OS=H. irritans exigua	ACE_HAEIX	6,00E-52	GLOS_ACE.6.16	GO:0046872	M	UniProtKB-K	metal ion binding
ACE_HAEIX (sp|Q10715) Angiotensin-converting enzyme OS=H. irritans exigua	ACE_HAEIX	6,00E-52	GLOS_ACE.6.16	GO:0008237	M	UniProtKB-K	metallopeptidase activity
ACE_HAEIX (sp|Q10715) Angiotensin-converting enzyme OS=H. irritans exigua	ACE_HAEIX	6,00E-52	GLOS_ACE.6.16	GO:0008241	M	InterPro	peptidyl-dipeptidase activity
ACE_HAEIX (sp|Q10715) Angiotensin-converting enzyme OS=H. irritans exigua	ACE_HAEIX	6,00E-52	GLOS_ACE.6.16	GO:0006508	B	UniProtKB-K	proteolysis
[BBH] ADXH_DROME (sp|P37193) Adrenodoxin-like prot, mitoch OS=D. m.	ADXH_DROME	1,00E-79	GLOS_ADXH.1.1	GO:0044429	C	.	mitochondrial part
[BBH] ADXH_DROME (sp|P37193) Adrenodoxin-like prot, mitoch OS=D. m.	ADXH_DROME	1,00E-79	GLOS_ADXH.1.1	GO:0005739	C	.	mitochondrion
[BBH] ADXH_DROME (sp|P37193) Adrenodoxin-like prot, mitoch OS=D. m.	ADXH_DROME	1,00E-79	GLOS_ADXH.1.1	GO:0005759	C	UniProtKB-S	mitochondrial matrix
[BBH] ADXH_DROME (sp|P37193) Adrenodoxin-like prot, mitoch OS=D. m.	ADXH_DROME	1,00E-79	GLOS_ADXH.1.1	GO:0051536	M	.	iron-sulfur cluster binding
[BBH] ADXH_DROME (sp|P37193) Adrenodoxin-like prot, mitoch OS=D. m.	ADXH_DROME	1,00E-79	GLOS_ADXH.1.1	GO:0051540	M	.	metal cluster binding
[BBH] ADXH_DROME (sp|P37193) Adrenodoxin-like prot, mitoch OS=D. m.	ADXH_DROME	1,00E-79	GLOS_ADXH.1.1	GO:0051537	M	UniProtKB-K	2 iron, 2 sulfur cluster binding
[BBH] ADXH_DROME (sp|P37193) Adrenodoxin-like prot, mitoch OS=D. m.	ADXH_DROME	1,00E-79	GLOS_ADXH.1.1	GO:0009055	M	InterPro	electron carrier activity
[BBH] ADXH_DROME (sp|P37193) Adrenodoxin-like prot, mitoch OS=D. m.	ADXH_DROME	1,00E-79	GLOS_ADXH.1.1	GO:0046872	M	UniProtKB-K	metal ion binding
[BBH] ADXH_DROME (sp|P37193) Adrenodoxin-like prot, mitoch OS=D. m.	ADXH_DROME	1,00E-79	GLOS_ADXH.1.1	GO:0055114	B	UniProtKB-K	oxidation-reduction process
ARF6_XENLA (sp|P51645)ADP-ribosylation fact 6 OS=X.laevisGN=arf6 PE=2SV=2	ARF6_XENLA	5,00E-13	GLOS_ARF6.1.2	GO:0030139	C	UniProtKB	endocytic vesicle
ARF6_XENLA (sp|P51645)ADP-ribosylation fact 6 OS=X.laevisGN=arf6 PE=2SV=2	ARF6_XENLA	5,00E-13	GLOS_ARF6.1.2	GO:0005768	C	UniProtKB	endosome
ARF6_XENLA (sp|P51645)ADP-ribosylation fact 6 OS=X.laevisGN=arf6 PE=2SV=2	ARF6_XENLA	5,00E-13	GLOS_ARF6.1.2	GO:0044440	C	.	endosomal part
ARF6_XENLA (sp|P51645)ADP-ribosylation fact 6 OS=X.laevisGN=arf6 PE=2SV=2	ARF6_XENLA	5,00E-13	GLOS_ARF6.1.2	GO:0005768	C	.	endosome
ARF6_XENLA (sp|P51645)ADP-ribosylation fact 6 OS=X.laevisGN=arf6 PE=2SV=2	ARF6_XENLA	5,00E-13	GLOS_ARF6.1.2	GO:0010008	C	UniProtKB-S	endosome membrane
ARF6_XENLA (sp|P51645)ADP-ribosylation fact 6 OS=X.laevisGN=arf6 PE=2SV=2	ARF6_XENLA	5,00E-13	GLOS_ARF6.1.2	GO:0031253	C	.	cell projection membrane
ARF6_XENLA (sp|P51645)ADP-ribosylation fact 6 OS=X.laevisGN=arf6 PE=2SV=2	ARF6_XENLA	5,00E-13	GLOS_ARF6.1.2	GO:0030175	C	.	filopodium
ARF6_XENLA (sp|P51645)ADP-ribosylation fact 6 OS=X.laevisGN=arf6 PE=2SV=2	ARF6_XENLA	5,00E-13	GLOS_ARF6.1.2	GO:0031527	C	UniProtKB-S	filopodium membrane
ARF6_XENLA (sp|P51645)ADP-ribosylation fact 6 OS=X.laevisGN=arf6 PE=2SV=2	ARF6_XENLA	5,00E-13	GLOS_ARF6.1.2	GO:0005794	C	UniProtKB-S	Golgi apparatus
ARF6_XENLA (sp|P51645)ADP-ribosylation fact 6 OS=X.laevisGN=arf6 PE=2SV=2	ARF6_XENLA	5,00E-13	GLOS_ARF6.1.2	GO:0005886	C	UniProtKB	plasma membrane
ARF6_XENLA (sp|P51645)ADP-ribosylation fact 6 OS=X.laevisGN=arf6 PE=2SV=2	ARF6_XENLA	5,00E-13	GLOS_ARF6.1.2	GO:0032550	M	.	purine ribonucleoside binding
ARF6_XENLA (sp|P51645)ADP-ribosylation fact 6 OS=X.laevisGN=arf6 PE=2SV=2	ARF6_XENLA	5,00E-13	GLOS_ARF6.1.2	GO:0001883	M	.	purine nucleoside binding
ARF6_XENLA (sp|P51645)ADP-ribosylation fact 6 OS=X.laevisGN=arf6 PE=2SV=2	ARF6_XENLA	5,00E-13	GLOS_ARF6.1.2	GO:0001882	M	.	nucleoside binding
ARF6_XENLA (sp|P51645)ADP-ribosylation fact 6 OS=X.laevisGN=arf6 PE=2SV=2	ARF6_XENLA	5,00E-13	GLOS_ARF6.1.2	GO:0032549	M	.	ribonucleoside binding
ARF6_XENLA (sp|P51645)ADP-ribosylation fact 6 OS=X.laevisGN=arf6 PE=2SV=2	ARF6_XENLA	5,00E-13	GLOS_ARF6.1.2	GO:0032561	M	.	guanyl ribonucleotide binding
ARF6_XENLA (sp|P51645)ADP-ribosylation fact 6 OS=X.laevisGN=arf6 PE=2SV=2	ARF6_XENLA	5,00E-13	GLOS_ARF6.1.2	GO:0019001	M	.	guanyl nucleotide binding
ARF6_XENLA (sp|P51645)ADP-ribosylation fact 6 OS=X.laevisGN=arf6 PE=2SV=2	ARF6_XENLA	5,00E-13	GLOS_ARF6.1.2	GO:0017076	M	.	purine nucleotide binding
ARF6_XENLA (sp|P51645)ADP-ribosylation fact 6 OS=X.laevisGN=arf6 PE=2SV=2	ARF6_XENLA	5,00E-13	GLOS_ARF6.1.2	GO:0000166	M	.	nucleotide binding
ARF6_XENLA (sp|P51645)ADP-ribosylation fact 6 OS=X.laevisGN=arf6 PE=2SV=2	ARF6_XENLA	5,00E-13	GLOS_ARF6.1.2	GO:1901265	M	.	nucleoside phosphate binding
ARF6_XENLA (sp|P51645)ADP-ribosylation fact 6 OS=X.laevisGN=arf6 PE=2SV=2	ARF6_XENLA	5,00E-13	GLOS_ARF6.1.2	GO:0032555	M	.	purine ribonucleotide binding
ARF6_XENLA (sp|P51645)ADP-ribosylation fact 6 OS=X.laevisGN=arf6 PE=2SV=2	ARF6_XENLA	5,00E-13	GLOS_ARF6.1.2	GO:0032553	M	.	ribonucleotide binding
ARF6_XENLA (sp|P51645)ADP-ribosylation fact 6 OS=X.laevisGN=arf6 PE=2SV=2	ARF6_XENLA	5,00E-13	GLOS_ARF6.1.2	GO:0035639	M	.	purine ribonucleoside triphosphate binding
ARF6_XENLA (sp|P51645)ADP-ribosylation fact 6 OS=X.laevisGN=arf6 PE=2SV=2	ARF6_XENLA	5,00E-13	GLOS_ARF6.1.2	GO:0005525	M	UniProtKB-K	GTP binding
ARF6_XENLA (sp|P51645)ADP-ribosylation fact 6 OS=X.laevisGN=arf6 PE=2SV=2	ARF6_XENLA	5,00E-13	GLOS_ARF6.1.2	GO:0030154	B	UniProtKB-K	cell differentiation
ARF6_XENLA (sp|P51645)ADP-ribosylation fact 6 OS=X.laevisGN=arf6 PE=2SV=2	ARF6_XENLA	5,00E-13	GLOS_ARF6.1.2	GO:0007399	B	UniProtKB-K	nervous system development
ARF6_XENLA (sp|P51645)ADP-ribosylation fact 6 OS=X.laevisGN=arf6 PE=2SV=2	ARF6_XENLA	5,00E-13	GLOS_ARF6.1.2	GO:0015031	B	UniProtKB-K	protein transport
ARF6_XENLA (sp|P51645)ADP-ribosylation fact 6 OS=X.laevisGN=arf6 PE=2SV=2	ARF6_XENLA	5,00E-13	GLOS_ARF6.1.2	GO:0007264	B	InterPro	small GTPase mediated signal transduction
ARF6_XENLA (sp|P51645)ADP-ribosylation fact 6 OS=X.laevisGN=arf6 PE=2SV=2	ARF6_XENLA	5,00E-13	GLOS_ARF6.1.2	GO:0016192	B	UniProtKB-K	vesicle-mediated transport
[BBH] ATG13_DROME (sp|Q9VHR6) Autophagy-related prot 13 homolog OS=D.m.	ATG13_DROME	1,00E-171	GLOS_ATG13.1.1	GO:0005829	C	UniProtKB-S	cytosol
[BBH] ATG13_DROME (sp|Q9VHR6) Autophagy-related prot 13 homolog OS=D.m.	ATG13_DROME	1,00E-171	GLOS_ATG13.1.1	GO:0000407	C	UniProtKB-S	pre-autophagosomal structure

[BBH] ATG13_DROME (sp|Q9VHR6) Autophagy-related prot 13 homolog OS=D.m.	ATG13_DROME	1,00E-171	GLOS_ATG13.1.1	GO:0006914	B	FlyBase	autophagy
[BBH] ATG13_DROME (sp|Q9VHR6) Autophagy-related prot 13 homolog OS=D.m.	ATG13_DROME	1,00E-171	GLOS_ATG13.1.1	GO:0010506	B	FlyBase	regulation of autophagy
ATTA_GLOMM (sp|Q8WTD3) Attacin-A OS=G. m. morsitans PE=1 SV=1	ATTA_GLOMM	1,00E-112	GLOS_ATTA.5.7	GO:0005576	C	UniProtKB	extracellular region
ATTA_GLOMM (sp|Q8WTD3) Attacin-A OS=G. m. morsitans PE=1 SV=1	ATTA_GLOMM	1,00E-112	GLOS_ATTA.5.7	GO:0019730	B	.	antimicrobial humoral response
ATTA_GLOMM (sp|Q8WTD3) Attacin-A OS=G. m. morsitans PE=1 SV=1	ATTA_GLOMM	1,00E-112	GLOS_ATTA.5.7	GO:0006959	B	.	humoral immune response
ATTA_GLOMM (sp|Q8WTD3) Attacin-A OS=G. m. morsitans PE=1 SV=1	ATTA_GLOMM	1,00E-112	GLOS_ATTA.5.7	GO:0006955	B	.	immune response
ATTA_GLOMM (sp|Q8WTD3) Attacin-A OS=G. m. morsitans PE=1 SV=1	ATTA_GLOMM	1,00E-112	GLOS_ATTA.5.7	GO:0042742	B	.	defense response to bacterium
ATTA_GLOMM (sp|Q8WTD3) Attacin-A OS=G. m. morsitans PE=1 SV=1	ATTA_GLOMM	1,00E-112	GLOS_ATTA.5.7	GO:0019731	B	UniProtKB	antibacterial humoral response
ATTA_GLOMM (sp|Q8WTD3) Attacin-A OS=G. m. morsitans PE=1 SV=1	ATTA_GLOMM	1,00E-112	GLOS_ATTA.5.7	GO:0050829	B	UniProtKB	defense response to Gram-negative bacterium
ATTA_GLOMM (sp|Q8WTD3) Attacin-A OS=G. m. morsitans PE=1 SV=1	ATTA_GLOMM	1,00E-112	GLOS_ATTA.5.7	GO:0045087	B	UniProtKB-k	innate immune response
CAC1D_DROME (sp|Q24270) Voltage-depend. ca channel type D subunit alpha-1	CAC1D_DROME	0	GLOS_CAC1D.2.3	GO:0016324	C	FlyBase	apical plasma membrane
CAC1D_DROME (sp|Q24270) Voltage-depend. ca channel type D subunit alpha-1	CAC1D_DROME	0	GLOS_CAC1D.2.3	GO:0016323	C	FlyBase	basolateral plasma membrane
CAC1D_DROME (sp|Q24270) Voltage-depend. ca channel type D subunit alpha-1	CAC1D_DROME	0	GLOS_CAC1D.2.3	GO:0034704	C	.	calcium channel complex
CAC1D_DROME (sp|Q24270) Voltage-depend. ca channel type D subunit alpha-1	CAC1D_DROME	0	GLOS_CAC1D.2.3	GO:0034703	C	.	cation channel complex
CAC1D_DROME (sp|Q24270) Voltage-depend. ca channel type D subunit alpha-1	CAC1D_DROME	0	GLOS_CAC1D.2.3	GO:0034702	C	.	ion channel complex
CAC1D_DROME (sp|Q24270) Voltage-depend. ca channel type D subunit alpha-1	CAC1D_DROME	0	GLOS_CAC1D.2.3	GO:0005891	C	FlyBase	voltage-gated calcium channel complex
CAC1D_DROME (sp|Q24270) Voltage-depend. ca channel type D subunit alpha-1	CAC1D_DROME	0	GLOS_CAC1D.2.3	GO:0046872	M	UniProtKB-k	metal ion binding
CAC1D_DROME (sp|Q24270) Voltage-depend. ca channel type D subunit alpha-1	CAC1D_DROME	0	GLOS_CAC1D.2.3	GO:0022843	M	.	voltage-gated cation channel activity
CAC1D_DROME (sp|Q24270) Voltage-depend. ca channel type D subunit alpha-1	CAC1D_DROME	0	GLOS_CAC1D.2.3	GO:0005244	M	.	voltage-gated ion channel activity
CAC1D_DROME (sp|Q24270) Voltage-depend. ca channel type D subunit alpha-1	CAC1D_DROME	0	GLOS_CAC1D.2.3	GO:0022832	M	.	voltage-gated channel activity
CAC1D_DROME (sp|Q24270) Voltage-depend. ca channel type D subunit alpha-1	CAC1D_DROME	0	GLOS_CAC1D.2.3	GO:0005245	M	FlyBase	voltage-gated calcium channel activity
CAC1D_DROME (sp|Q24270) Voltage-depend. ca channel type D subunit alpha-1	CAC1D_DROME	0	GLOS_CAC1D.2.3	GO:0042044	B	.	fluid transport
CAC1D_DROME (sp|Q24270) Voltage-depend. ca channel type D subunit alpha-1	CAC1D_DROME	0	GLOS_CAC1D.2.3	GO:0042045	B	FlyBase	epithelial fluid transport
CAC1D_DROME (sp|Q24270) Voltage-depend. ca channel type D subunit alpha-1	CAC1D_DROME	0	GLOS_CAC1D.2.3	GO:0003012	B	.	muscle system process
CAC1D_DROME (sp|Q24270) Voltage-depend. ca channel type D subunit alpha-1	CAC1D_DROME	0	GLOS_CAC1D.2.3	GO:0006936	B	FlyBase	muscle contraction
CBPA1_DROPS (sp|Q29NC4) Zinc carboxypeptidase A 1 OS=D.p.pseudoob.	CBPA1_DROPS	1,00E-173	GLOS_CBPA1.1.1	GO:0005615	C	UniProtKB	extracellular space
CBPA1_DROPS (sp|Q29NC4) Zinc carboxypeptidase A 1 OS=D.p.pseudoob.	CBPA1_DROPS	1,00E-173	GLOS_CBPA1.1.1	GO:0004180	M	.	carboxypeptidase activity
CBPA1_DROPS (sp|Q29NC4) Zinc carboxypeptidase A 1 OS=D.p.pseudoob.	CBPA1_DROPS	1,00E-173	GLOS_CBPA1.1.1	GO:0008235	M	.	metalloexopeptidase activity
CBPA1_DROPS (sp|Q29NC4) Zinc carboxypeptidase A 1 OS=D.p.pseudoob.	CBPA1_DROPS	1,00E-173	GLOS_CBPA1.1.1	GO:0008237	M	.	metallopeptidase activity
CBPA1_DROPS (sp|Q29NC4) Zinc carboxypeptidase A 1 OS=D.p.pseudoob.	CBPA1_DROPS	1,00E-173	GLOS_CBPA1.1.1	GO:0004181	M	InterPro	metallocarboxypeptidase activity
CBPA1_DROPS (sp|Q29NC4) Zinc carboxypeptidase A 1 OS=D.p.pseudoob.	CBPA1_DROPS	1,00E-173	GLOS_CBPA1.1.1	GO:0008270	M	InterPro	zinc ion binding
CBPA1_DROPS (sp|Q29NC4) Zinc carboxypeptidase A 1 OS=D.p.pseudoob.	CBPA1_DROPS	1,00E-173	GLOS_CBPA1.1.1	GO:0006508	B	UniProtKB-k	proteolysis
CCNC_DROPS (sp|Q29AI1) Cyclin-C OS=D. p. pseudoobscura	CCNC_DROPS	1,00E-162	GLOS_CCNC.2.2	GO:0044451	C	.	nucleoplasm part
CCNC_DROPS (sp|Q29AI1) Cyclin-C OS=D. p. pseudoobscura	CCNC_DROPS	1,00E-162	GLOS_CCNC.2.2	GO:0005654	C	.	nucleoplasm
CCNC_DROPS (sp|Q29AI1) Cyclin-C OS=D. p. pseudoobscura	CCNC_DROPS	1,00E-162	GLOS_CCNC.2.2	GO:0055029	C	.	nuclear DNA-directed RNA polymerase complex
CCNC_DROPS (sp|Q29AI1) Cyclin-C OS=D. p. pseudoobscura	CCNC_DROPS	1,00E-162	GLOS_CCNC.2.2	GO:0000428	C	.	DNA-directed RNA polymerase complex
CCNC_DROPS (sp|Q29AI1) Cyclin-C OS=D. p. pseudoobscura	CCNC_DROPS	1,00E-162	GLOS_CCNC.2.2	GO:0030880	C	.	RNA polymerase complex
CCNC_DROPS (sp|Q29AI1) Cyclin-C OS=D. p. pseudoobscura	CCNC_DROPS	1,00E-162	GLOS_CCNC.2.2	GO:0016591	C	InterPro	DNA-directed RNA polymerase II, holoenzyme
CCNC_DROPS (sp|Q29AI1) Cyclin-C OS=D. p. pseudoobscura	CCNC_DROPS	1,00E-162	GLOS_CCNC.2.2	GO:0016592	C	UniProtKB	mediator complex
CCNC_DROPS (sp|Q29AI1) Cyclin-C OS=D. p. pseudoobscura	CCNC_DROPS	1,00E-162	GLOS_CCNC.2.2	GO:0001076	M	.	RNA polymerase II transcription factor binding transcription factor activity
CCNC_DROPS (sp|Q29AI1) Cyclin-C OS=D. p. pseudoobscura	CCNC_DROPS	1,00E-162	GLOS_CCNC.2.2	GO:0001104	M	UniProtKB	RNA polymerase II transcription cofactor activity
CCNC_DROPS (sp|Q29AI1) Cyclin-C OS=D. p. pseudoobscura	CCNC_DROPS	1,00E-162	GLOS_CCNC.2.2	GO:0071900	B	.	regulation of protein serine/threonine kinase activity
CCNC_DROPS (sp|Q29AI1) Cyclin-C OS=D. p. pseudoobscura	CCNC_DROPS	1,00E-162	GLOS_CCNC.2.2	GO:0000079	B	InterPro	regulation of cyclin-dependent protein serine/threonine kinase activity
[BBH] CSK2B_DROME (sp|P08182) Casein kinase II subunit beta OS=D. m.	CSK2B_DROME	1,00E-151	GLOS_CSK2B.1.1	GO:0005829	C	FlyBase	cytosol
[BBH] CSK2B_DROME (sp|P08182) Casein kinase II subunit beta OS=D. m.	CSK2B_DROME	1,00E-151	GLOS_CSK2B.1.1	GO:0005634	C	FlyBase	nucleus
[BBH] CSK2B_DROME (sp|P08182) Casein kinase II subunit beta OS=D. m.	CSK2B_DROME	1,00E-151	GLOS_CSK2B.1.1	GO:0005956	C	FlyBase	protein kinase CK2 complex
[BBH] CSK2B_DROME (sp|P08182) Casein kinase II subunit beta OS=D. m.	CSK2B_DROME	1,00E-151	GLOS_CSK2B.1.1	GO:0019207	M	.	kinase regulator activity
[BBH] CSK2B_DROME (sp|P08182) Casein kinase II subunit beta OS=D. m.	CSK2B_DROME	1,00E-151	GLOS_CSK2B.1.1	GO:0019887	M	FlyBase	protein kinase regulator activity
[BBH] CSK2B_DROME (sp|P08182) Casein kinase II subunit beta OS=D. m.	CSK2B_DROME	1,00E-151	GLOS_CSK2B.1.1	GO:0004672	M	.	protein kinase activity
[BBH] CSK2B_DROME (sp|P08182) Casein kinase II subunit beta OS=D. m.	CSK2B_DROME	1,00E-151	GLOS_CSK2B.1.1	GO:0016301	M	.	kinase activity
[BBH] CSK2B_DROME (sp|P08182) Casein kinase II subunit beta OS=D. m.	CSK2B_DROME	1,00E-151	GLOS_CSK2B.1.1	GO:0016772	M	.	transferase activity, transferring phosphorus-containing groups
[BBH] CSK2B_DROME (sp|P08182) Casein kinase II subunit beta OS=D. m.	CSK2B_DROME	1,00E-151	GLOS_CSK2B.1.1	GO:0016773	M	.	phosphotransferase activity, alcohol group as acceptor
[BBH] CSK2B_DROME (sp|P08182) Casein kinase II subunit beta OS=D. m.	CSK2B_DROME	1,00E-151	GLOS_CSK2B.1.1	GO:0004674	M	FlyBase	protein serine/threonine kinase activity
[BBH] CSK2B_DROME (sp|P08182) Casein kinase II subunit beta OS=D. m.	CSK2B_DROME	1,00E-151	GLOS_CSK2B.1.1	GO:0007562	B	.	eclosion

[BBH] CSK2B_DROME (sp|P08182) Casein kinase II subunit beta OS=D. m.	CSK2B_DROME	1,00E-151	GLOS_CSK2B.1.1	GO:0071684	B	.	organism emergence from protective structure
[BBH] CSK2B_DROME (sp|P08182) Casein kinase II subunit beta OS=D. m.	CSK2B_DROME	1,00E-151	GLOS_CSK2B.1.1	GO:0008062	B	FlyBase	eclosion rhythm
[BBH] CSK2B_DROME (sp|P08182) Casein kinase II subunit beta OS=D. m.	CSK2B_DROME	1,00E-151	GLOS_CSK2B.1.1	GO:0045475	B	FlyBase	locomotor rhythm
[BBH] CSK2B_DROME (sp|P08182) Casein kinase II subunit beta OS=D. m.	CSK2B_DROME	1,00E-151	GLOS_CSK2B.1.1	GO:0007420	B	.	brain development
[BBH] CSK2B_DROME (sp|P08182) Casein kinase II subunit beta OS=D. m.	CSK2B_DROME	1,00E-151	GLOS_CSK2B.1.1	GO:0007417	B	.	central nervous system development
[BBH] CSK2B_DROME (sp|P08182) Casein kinase II subunit beta OS=D. m.	CSK2B_DROME	1,00E-151	GLOS_CSK2B.1.1	GO:0016319	B	FlyBase	mushroom body development
[BBH] CSK2B_DROME (sp|P08182) Casein kinase II subunit beta OS=D. m.	CSK2B_DROME	1,00E-151	GLOS_CSK2B.1.1	GO:0022008	B	FlyBase	neurogenesis
[BBH] CSK2B_DROME (sp|P08182) Casein kinase II subunit beta OS=D. m.	CSK2B_DROME	1,00E-151	GLOS_CSK2B.1.1	GO:0016055	B	UniProtKB-K	Wnt receptor signaling pathway
[BBH] CYC_SARPE (sp|P12831) Cyt. c OS=S. peregrina PE=1 SV=2	CYC_SARPE	9,00E-74	GLOS_CYC.1.2	GO:0031970	C	.	organelle envelope lumen
[BBH] CYC_SARPE (sp|P12831) Cyt. c OS=S. peregrina PE=1 SV=2	CYC_SARPE	9,00E-74	GLOS_CYC.1.2	GO:0031967	C	.	organelle envelope
[BBH] CYC_SARPE (sp|P12831) Cyt. c OS=S. peregrina PE=1 SV=2	CYC_SARPE	9,00E-74	GLOS_CYC.1.2	GO:0031975	C	.	envelope
[BBH] CYC_SARPE (sp|P12831) Cyt. c OS=S. peregrina PE=1 SV=2	CYC_SARPE	9,00E-74	GLOS_CYC.1.2	GO:0005740	C	.	mitochondrial envelope
[BBH] CYC_SARPE (sp|P12831) Cyt. c OS=S. peregrina PE=1 SV=2	CYC_SARPE	9,00E-74	GLOS_CYC.1.2	GO:0005758	C	UniProtKB-S	mitochondrial intermembrane space
[BBH] CYC_SARPE (sp|P12831) Cyt. c OS=S. peregrina PE=1 SV=2	CYC_SARPE	9,00E-74	GLOS_CYC.1.2	GO:0070469	C	UniProtKB-K	respiratory chain
[BBH] CYC_SARPE (sp|P12831) Cyt. c OS=S. peregrina PE=1 SV=2	CYC_SARPE	9,00E-74	GLOS_CYC.1.2	GO:0009055	M	InterPro	electron carrier activity
[BBH] CYC_SARPE (sp|P12831) Cyt. c OS=S. peregrina PE=1 SV=2	CYC_SARPE	9,00E-74	GLOS_CYC.1.2	GO:0046906	M	.	tetrapyrrole binding
[BBH] CYC_SARPE (sp|P12831) Cyt. c OS=S. peregrina PE=1 SV=2	CYC_SARPE	9,00E-74	GLOS_CYC.1.2	GO:0020037	M	InterPro	heme binding
[BBH] CYC_SARPE (sp|P12831) Cyt. c OS=S. peregrina PE=1 SV=2	CYC_SARPE	9,00E-74	GLOS_CYC.1.2	GO:0005506	M	InterPro	iron ion binding
[BBH] CYC_SARPE (sp|P12831) Cyt. c OS=S. peregrina PE=1 SV=2	CYC_SARPE	9,00E-74	GLOS_CYC.1.2	GO:0055114	B	UniProtKB-K	oxidation-reduction process
[BBH] DHYS_DROME (sp|Q9VSF4) Prob deoxyhypusine synthase OS=D.m.	DHYS_DROME	0	GLOS_DHYS.1.1	GO:0016765	M	.	transferase activity, transferring alkyl or aryl (other than methyl) groups
[BBH] DHYS_DROME (sp|Q9VSF4) Prob deoxyhypusine synthase OS=D.m.	DHYS_DROME	0	GLOS_DHYS.1.1	GO:0034038	M	UniProtKB-E	deoxyhypusine synthase activity
[BBH] DHYS_DROME (sp|Q9VSF4) Prob deoxyhypusine synthase OS=D.m.	DHYS_DROME	0	GLOS_DHYS.1.1	GO:0042398	B	.	cellular modified amino acid biosynthetic process
[BBH] DHYS_DROME (sp|Q9VSF4) Prob deoxyhypusine synthase OS=D.m.	DHYS_DROME	0	GLOS_DHYS.1.1	GO:0006575	B	.	cellular modified amino acid metabolic process
[BBH] DHYS_DROME (sp|Q9VSF4) Prob deoxyhypusine synthase OS=D.m.	DHYS_DROME	0	GLOS_DHYS.1.1	GO:0006520	B	.	cellular amino acid metabolic process
[BBH] DHYS_DROME (sp|Q9VSF4) Prob deoxyhypusine synthase OS=D.m.	DHYS_DROME	0	GLOS_DHYS.1.1	GO:0008652	B	.	cellular amino acid biosynthetic process
[BBH] DHYS_DROME (sp|Q9VSF4) Prob deoxyhypusine synthase OS=D.m.	DHYS_DROME	0	GLOS_DHYS.1.1	GO:0046203	B	.	spermidine catabolic process
[BBH] DHYS_DROME (sp|Q9VSF4) Prob deoxyhypusine synthase OS=D.m.	DHYS_DROME	0	GLOS_DHYS.1.1	GO:0006598	B	.	polyamine catabolic process
[BBH] DHYS_DROME (sp|Q9VSF4) Prob deoxyhypusine synthase OS=D.m.	DHYS_DROME	0	GLOS_DHYS.1.1	GO:0006595	B	.	polyamine metabolic process
[BBH] DHYS_DROME (sp|Q9VSF4) Prob deoxyhypusine synthase OS=D.m.	DHYS_DROME	0	GLOS_DHYS.1.1	GO:0006576	B	.	cellular biogenic amine metabolic process
[BBH] DHYS_DROME (sp|Q9VSF4) Prob deoxyhypusine synthase OS=D.m.	DHYS_DROME	0	GLOS_DHYS.1.1	GO:0044106	B	.	cellular amine metabolic process
[BBH] DHYS_DROME (sp|Q9VSF4) Prob deoxyhypusine synthase OS=D.m.	DHYS_DROME	0	GLOS_DHYS.1.1	GO:0009308	B	.	amine metabolic process
[BBH] DHYS_DROME (sp|Q9VSF4) Prob deoxyhypusine synthase OS=D.m.	DHYS_DROME	0	GLOS_DHYS.1.1	GO:0042402	B	.	cellular biogenic amine catabolic process
[BBH] DHYS_DROME (sp|Q9VSF4) Prob deoxyhypusine synthase OS=D.m.	DHYS_DROME	0	GLOS_DHYS.1.1	GO:0009310	B	.	amine catabolic process
[BBH] DHYS_DROME (sp|Q9VSF4) Prob deoxyhypusine synthase OS=D.m.	DHYS_DROME	0	GLOS_DHYS.1.1	GO:1901565	B	.	organonitrogen compound catabolic process
[BBH] DHYS_DROME (sp|Q9VSF4) Prob deoxyhypusine synthase OS=D.m.	DHYS_DROME	0	GLOS_DHYS.1.1	GO:0008216	B	.	spermidine metabolic process
[BBH] DHYS_DROME (sp|Q9VSF4) Prob deoxyhypusine synthase OS=D.m.	DHYS_DROME	0	GLOS_DHYS.1.1	GO:0050983	B	UniProtKB-E	deoxyhypusine biosynthetic process from spermidine
[BBH] DHYS_DROME (sp|Q9VSF4) Prob deoxyhypusine synthase OS=D.m.	DHYS_DROME	0	GLOS_DHYS.1.1	GO:0018205	B	.	peptidyl-lysine modification
[BBH] DHYS_DROME (sp|Q9VSF4) Prob deoxyhypusine synthase OS=D.m.	DHYS_DROME	0	GLOS_DHYS.1.1	GO:0018193	B	.	peptidyl-amino acid modification
[BBH] DHYS_DROME (sp|Q9VSF4) Prob deoxyhypusine synthase OS=D.m.	DHYS_DROME	0	GLOS_DHYS.1.1	GO:0046516	B	.	hypusine metabolic process
[BBH] DHYS_DROME (sp|Q9VSF4) Prob deoxyhypusine synthase OS=D.m.	DHYS_DROME	0	GLOS_DHYS.1.1	GO:1901605	B	.	alpha-amino acid metabolic process
[BBH] DHYS_DROME (sp|Q9VSF4) Prob deoxyhypusine synthase OS=D.m.	DHYS_DROME	0	GLOS_DHYS.1.1	GO:1901607	B	.	alpha-amino acid biosynthetic process
[BBH] DHYS_DROME (sp|Q9VSF4) Prob deoxyhypusine synthase OS=D.m.	DHYS_DROME	0	GLOS_DHYS.1.1	GO:0008612	B	UniProtKB-K	peptidyl-lysine modification to hypusine
[BBH] DYIN_DROME (sp|Q24246) Cytoplas. dynein 1 intermediate chain D.m.	DYIN_DROME	0	GLOS_DYIN.1.1	GO:0030286	C	.	dynein complex
[BBH] DYIN_DROME (sp|Q24246) Cytoplas. dynein 1 intermediate chain D.m.	DYIN_DROME	0	GLOS_DYIN.1.1	GO:0005875	C	.	microtubule associated complex
[BBH] DYIN_DROME (sp|Q24246) Cytoplas. dynein 1 intermediate chain D.m.	DYIN_DROME	0	GLOS_DYIN.1.1	GO:0044430	C	.	cytoskeletal part
[BBH] DYIN_DROME (sp|Q24246) Cytoplas. dynein 1 intermediate chain D.m.	DYIN_DROME	0	GLOS_DYIN.1.1	GO:0005856	C	.	cytoskeleton
[BBH] DYIN_DROME (sp|Q24246) Cytoplas. dynein 1 intermediate chain D.m.	DYIN_DROME	0	GLOS_DYIN.1.1	GO:0015630	C	.	microtubule cytoskeleton
[BBH] DYIN_DROME (sp|Q24246) Cytoplas. dynein 1 intermediate chain D.m.	DYIN_DROME	0	GLOS_DYIN.1.1	GO:0005868	C	InterPro	cytoplasmic dynein complex
[BBH] DYIN_DROME (sp|Q24246) Cytoplas. dynein 1 intermediate chain D.m.	DYIN_DROME	0	GLOS_DYIN.1.1	GO:0005774	C	.	vacuolar membrane
[BBH] DYIN_DROME (sp|Q24246) Cytoplas. dynein 1 intermediate chain D.m.	DYIN_DROME	0	GLOS_DYIN.1.1	GO:0044437	C	.	vacuolar part
[BBH] DYIN_DROME (sp|Q24246) Cytoplas. dynein 1 intermediate chain D.m.	DYIN_DROME	0	GLOS_DYIN.1.1	GO:0005773	C	.	vacuole
[BBH] DYIN_DROME (sp|Q24246) Cytoplas. dynein 1 intermediate chain D.m.	DYIN_DROME	0	GLOS_DYIN.1.1	GO:0005764	C	.	lysosome
[BBH] DYIN_DROME (sp|Q24246) Cytoplas. dynein 1 intermediate chain D.m.	DYIN_DROME	0	GLOS_DYIN.1.1	GO:0000323	C	.	lytic vacuole
[BBH] DYIN_DROME (sp|Q24246) Cytoplas. dynein 1 intermediate chain D.m.	DYIN_DROME	0	GLOS_DYIN.1.1	GO:0005765	C	UniProtKB-S	lysosomal membrane

[BBH] DYIN_DROME (sp|Q24246) Cytoplas. dynein 1 intermediate chain D.m.	DYIN_DROME	0	GLOS_DYIN.1.1	GO:0005874	C	UniProtKB-K	microtubule
[BBH] DYIN_DROME (sp|Q24246) Cytoplas. dynein 1 intermediate chain D.m.	DYIN_DROME	0	GLOS_DYIN.1.1	GO:0005875	C	FlyBase	microtubule associated complex
[BBH] DYIN_DROME (sp|Q24246) Cytoplas. dynein 1 intermediate chain D.m.	DYIN_DROME	0	GLOS_DYIN.1.1	GO:0005635	C	.	nuclear envelope
[BBH] DYIN_DROME (sp|Q24246) Cytoplas. dynein 1 intermediate chain D.m.	DYIN_DROME	0	GLOS_DYIN.1.1	GO:0031965	C	UniProtKB-S	nuclear membrane
[BBH] DYIN_DROME (sp|Q24246) Cytoplas. dynein 1 intermediate chain D.m.	DYIN_DROME	0	GLOS_DYIN.1.1	GO:0017111	M	.	nucleoside-triphosphatase activity
[BBH] DYIN_DROME (sp|Q24246) Cytoplas. dynein 1 intermediate chain D.m.	DYIN_DROME	0	GLOS_DYIN.1.1	GO:0016462	M	.	pyrophosphatase activity
[BBH] DYIN_DROME (sp|Q24246) Cytoplas. dynein 1 intermediate chain D.m.	DYIN_DROME	0	GLOS_DYIN.1.1	GO:0016818	M	.	hydrolase act., acting on acid anhydrides, in phosphorus-containing anhydrides
[BBH] DYIN_DROME (sp|Q24246) Cytoplas. dynein 1 intermediate chain D.m.	DYIN_DROME	0	GLOS_DYIN.1.1	GO:0016817	M	.	hydrolase activity, acting on acid anhydrides
[BBH] DYIN_DROME (sp|Q24246) Cytoplas. dynein 1 intermediate chain D.m.	DYIN_DROME	0	GLOS_DYIN.1.1	GO:0003774	M	UniProtKB-K	motor activity
[BBH] DYIN_DROME (sp|Q24246) Cytoplas. dynein 1 intermediate chain D.m.	DYIN_DROME	0	GLOS_DYIN.1.1	GO:0010970	B	.	microtubule-based transport
[BBH] DYIN_DROME (sp|Q24246) Cytoplas. dynein 1 intermediate chain D.m.	DYIN_DROME	0	GLOS_DYIN.1.1	GO:0007018	B	.	microtubule-based movement
[BBH] DYIN_DROME (sp|Q24246) Cytoplas. dynein 1 intermediate chain D.m.	DYIN_DROME	0	GLOS_DYIN.1.1	GO:0007017	B	.	microtubule-based process
[BBH] DYIN_DROME (sp|Q24246) Cytoplas. dynein 1 intermediate chain D.m.	DYIN_DROME	0	GLOS_DYIN.1.1	GO:0030705	B	.	cytoskeleton-dependent intracellular transport
[BBH] DYIN_DROME (sp|Q24246) Cytoplas. dynein 1 intermediate chain D.m.	DYIN_DROME	0	GLOS_DYIN.1.1	GO:0008088	B	FlyBase	axon cargo transport
[BBH] DYIN_DROME (sp|Q24246) Cytoplas. dynein 1 intermediate chain D.m.	DYIN_DROME	0	GLOS_DYIN.1.1	GO:0051640	B	.	organelle localization
[BBH] DYIN_DROME (sp|Q24246) Cytoplas. dynein 1 intermediate chain D.m.	DYIN_DROME	0	GLOS_DYIN.1.1	GO:0051642	B	FlyBase	centrosome localization
[BBH] DYIN_DROME (sp|Q24246) Cytoplas. dynein 1 intermediate chain D.m.	DYIN_DROME	0	GLOS_DYIN.1.1	GO:0016482	B	FlyBase	cytoplasmic transport
[BBH] DYIN_DROME (sp|Q24246) Cytoplas. dynein 1 intermediate chain D.m.	DYIN_DROME	0	GLOS_DYIN.1.1	GO:0001754	B	FlyBase	eye photoreceptor cell differentiation
[BBH] DYIN_DROME (sp|Q24246) Cytoplas. dynein 1 intermediate chain D.m.	DYIN_DROME	0	GLOS_DYIN.1.1	GO:0034502	B	.	protein localization to chromosome
[BBH] DYIN_DROME (sp|Q24246) Cytoplas. dynein 1 intermediate chain D.m.	DYIN_DROME	0	GLOS_DYIN.1.1	GO:0034501	B	FlyBase	protein localization to kinetochore
[BBH] DYIN_DROME (sp|Q24246) Cytoplas. dynein 1 intermediate chain D.m.	DYIN_DROME	0	GLOS_DYIN.1.1	GO:0007349	B	.	cellularization
[BBH] DYIN_DROME (sp|Q24246) Cytoplas. dynein 1 intermediate chain D.m.	DYIN_DROME	0	GLOS_DYIN.1.1	GO:0007286	B	.	spermatid development
[BBH] DYIN_DROME (sp|Q24246) Cytoplas. dynein 1 intermediate chain D.m.	DYIN_DROME	0	GLOS_DYIN.1.1	GO:0002064	B	.	epithelial cell development
[BBH] DYIN_DROME (sp|Q24246) Cytoplas. dynein 1 intermediate chain D.m.	DYIN_DROME	0	GLOS_DYIN.1.1	GO:0030855	B	.	epithelial cell differentiation
[BBH] DYIN_DROME (sp|Q24246) Cytoplas. dynein 1 intermediate chain D.m.	DYIN_DROME	0	GLOS_DYIN.1.1	GO:0060429	B	.	epithelium development
[BBH] DYIN_DROME (sp|Q24246) Cytoplas. dynein 1 intermediate chain D.m.	DYIN_DROME	0	GLOS_DYIN.1.1	GO:0009888	B	.	tissue development
[BBH] DYIN_DROME (sp|Q24246) Cytoplas. dynein 1 intermediate chain D.m.	DYIN_DROME	0	GLOS_DYIN.1.1	GO:0007281	B	.	germ cell development
[BBH] DYIN_DROME (sp|Q24246) Cytoplas. dynein 1 intermediate chain D.m.	DYIN_DROME	0	GLOS_DYIN.1.1	GO:0022412	B	.	cellular process involved in reproduction in multicellular organism
[BBH] DYIN_DROME (sp|Q24246) Cytoplas. dynein 1 intermediate chain D.m.	DYIN_DROME	0	GLOS_DYIN.1.1	GO:0048610	B	.	cellular process involved in reproduction
[BBH] DYIN_DROME (sp|Q24246) Cytoplas. dynein 1 intermediate chain D.m.	DYIN_DROME	0	GLOS_DYIN.1.1	GO:0007276	B	.	gamete generation
[BBH] DYIN_DROME (sp|Q24246) Cytoplas. dynein 1 intermediate chain D.m.	DYIN_DROME	0	GLOS_DYIN.1.1	GO:0019953	B	.	sexual reproduction
[BBH] DYIN_DROME (sp|Q24246) Cytoplas. dynein 1 intermediate chain D.m.	DYIN_DROME	0	GLOS_DYIN.1.1	GO:0048515	B	.	spermatid differentiation
[BBH] DYIN_DROME (sp|Q24246) Cytoplas. dynein 1 intermediate chain D.m.	DYIN_DROME	0	GLOS_DYIN.1.1	GO:0007283	B	.	spermatogenesis
[BBH] DYIN_DROME (sp|Q24246) Cytoplas. dynein 1 intermediate chain D.m.	DYIN_DROME	0	GLOS_DYIN.1.1	GO:0048232	B	.	male gamete generation
[BBH] DYIN_DROME (sp|Q24246) Cytoplas. dynein 1 intermediate chain D.m.	DYIN_DROME	0	GLOS_DYIN.1.1	GO:0007291	B	FlyBase	sperm individualization
[BBH] DYIN_DROME (sp|Q24246) Cytoplas. dynein 1 intermediate chain D.m.	DYIN_DROME	0	GLOS_DYIN.1.1	GO:0000226	B	.	microtubule cytoskeleton organization
[BBH] DYIN_DROME (sp|Q24246) Cytoplas. dynein 1 intermediate chain D.m.	DYIN_DROME	0	GLOS_DYIN.1.1	GO:0007010	B	.	cytoskeleton organization
[BBH] DYIN_DROME (sp|Q24246) Cytoplas. dynein 1 intermediate chain D.m.	DYIN_DROME	0	GLOS_DYIN.1.1	GO:0071822	B	.	protein complex subunit organization
[BBH] DYIN_DROME (sp|Q24246) Cytoplas. dynein 1 intermediate chain D.m.	DYIN_DROME	0	GLOS_DYIN.1.1	GO:0043933	B	.	macromolecular complex subunit organization
[BBH] DYIN_DROME (sp|Q24246) Cytoplas. dynein 1 intermediate chain D.m.	DYIN_DROME	0	GLOS_DYIN.1.1	GO:0007051	B	FlyBase	spindle organization
GPL_GLOAU (sp|Q4TTV7) Lectizyme OS=G. austeni GN=Gpl PE=2 SV=1	GPL_GLOAU	7,00E-41	GLOS_GPL.10.22	GO:0005576	C	UniProtKB-S	extracellular region
GPL_GLOAU (sp|Q4TTV7) Lectizyme OS=G. austeni GN=Gpl PE=2 SV=1	GPL_GLOAU	7,00E-41	GLOS_GPL.10.22	GO:0004175	M	.	endopeptidase activity
GPL_GLOAU (sp|Q4TTV7) Lectizyme OS=G. austeni GN=Gpl PE=2 SV=1	GPL_GLOAU	7,00E-41	GLOS_GPL.10.22	GO:0008236	M	.	serine-type peptidase activity
GPL_GLOAU (sp|Q4TTV7) Lectizyme OS=G. austeni GN=Gpl PE=2 SV=1	GPL_GLOAU	7,00E-41	GLOS_GPL.10.22	GO:0017171	M	.	serine hydrolase activity
GPL_GLOAU (sp|Q4TTV7) Lectizyme OS=G. austeni GN=Gpl PE=2 SV=1	GPL_GLOAU	7,00E-41	GLOS_GPL.10.22	GO:0004252	M	InterPro	serine-type endopeptidase activity
GPL_GLOAU (sp|Q4TTV7) Lectizyme OS=G. austeni GN=Gpl PE=2 SV=1	GPL_GLOAU	7,00E-41	GLOS_GPL.10.22	GO:0006508	B	UniProtKB-K	proteolysis
[BBH] GPRK2_DROME (sp|P32866) G prot-coupled receptor kinase 2 D.m.	GPRK2_DROME	2,00E-43	GLOS_GPRK2.1.1	GO:0005737	C	FlyBase	cytoplasm
[BBH] GPRK2_DROME (sp|P32866) G prot-coupled receptor kinase 2 D.m.	GPRK2_DROME	2,00E-43	GLOS_GPRK2.1.1	GO:0005886	C	FlyBase	plasma membrane
[BBH] GPRK2_DROME (sp|P32866) G prot-coupled receptor kinase 2 D.m.	GPRK2_DROME	2,00E-43	GLOS_GPRK2.1.1	GO:0032559	M	.	adenyl ribonucleotide binding
[BBH] GPRK2_DROME (sp|P32866) G prot-coupled receptor kinase 2 D.m.	GPRK2_DROME	2,00E-43	GLOS_GPRK2.1.1	GO:0030554	M	.	adenyl nucleotide binding
[BBH] GPRK2_DROME (sp|P32866) G prot-coupled receptor kinase 2 D.m.	GPRK2_DROME	2,00E-43	GLOS_GPRK2.1.1	GO:0005524	M	UniProtKB-K	ATP binding
[BBH] GPRK2_DROME (sp|P32866) G prot-coupled receptor kinase 2 D.m.	GPRK2_DROME	2,00E-43	GLOS_GPRK2.1.1	GO:0004674	M	.	protein serine/threonine kinase activity
[BBH] GPRK2_DROME (sp|P32866) G prot-coupled receptor kinase 2 D.m.	GPRK2_DROME	2,00E-43	GLOS_GPRK2.1.1	GO:0004703	M	FlyBase	G-protein coupled receptor kinase activity
[BBH] GPRK2_DROME (sp|P32866) G prot-coupled receptor kinase 2 D.m.	GPRK2_DROME	2,00E-43	GLOS_GPRK2.1.1	GO:0030154	B	UniProtKB-K	cell differentiation

[BBH] GPRK2_DROME (sp|P32866) G prot-coupled receptor kinase 2 D.m.	GPRK2_DROME	2,00E-43	GLOS_GPRK2.1.1	GO:0050830	B	FlyBase	defense response to Gram-positive bacterium
[BBH] GPRK2_DROME (sp|P32866) G prot-coupled receptor kinase 2 D.m.	GPRK2_DROME	2,00E-43	GLOS_GPRK2.1.1	GO:0003002	B	.	regionalization
[BBH] GPRK2_DROME (sp|P32866) G prot-coupled receptor kinase 2 D.m.	GPRK2_DROME	2,00E-43	GLOS_GPRK2.1.1	GO:0007389	B	.	pattern specification process
[BBH] GPRK2_DROME (sp|P32866) G prot-coupled receptor kinase 2 D.m.	GPRK2_DROME	2,00E-43	GLOS_GPRK2.1.1	GO:0007476	B	.	imaginal disc-derived wing morphogenesis
[BBH] GPRK2_DROME (sp|P32866) G prot-coupled receptor kinase 2 D.m.	GPRK2_DROME	2,00E-43	GLOS_GPRK2.1.1	GO:0035114	B	.	imaginal disc-derived appendage morphogenesis
[BBH] GPRK2_DROME (sp|P32866) G prot-coupled receptor kinase 2 D.m.	GPRK2_DROME	2,00E-43	GLOS_GPRK2.1.1	GO:0035107	B	.	appendage morphogenesis
[BBH] GPRK2_DROME (sp|P32866) G prot-coupled receptor kinase 2 D.m.	GPRK2_DROME	2,00E-43	GLOS_GPRK2.1.1	GO:0048736	B	.	appendage development
[BBH] GPRK2_DROME (sp|P32866) G prot-coupled receptor kinase 2 D.m.	GPRK2_DROME	2,00E-43	GLOS_GPRK2.1.1	GO:0048737	B	.	imaginal disc-derived appendage development
[BBH] GPRK2_DROME (sp|P32866) G prot-coupled receptor kinase 2 D.m.	GPRK2_DROME	2,00E-43	GLOS_GPRK2.1.1	GO:0035120	B	.	post-embryonic appendage morphogenesis
[BBH] GPRK2_DROME (sp|P32866) G prot-coupled receptor kinase 2 D.m.	GPRK2_DROME	2,00E-43	GLOS_GPRK2.1.1	GO:0009886	B	.	post-embryonic morphogenesis
[BBH] GPRK2_DROME (sp|P32866) G prot-coupled receptor kinase 2 D.m.	GPRK2_DROME	2,00E-43	GLOS_GPRK2.1.1	GO:0009791	B	.	post-embryonic development
[BBH] GPRK2_DROME (sp|P32866) G prot-coupled receptor kinase 2 D.m.	GPRK2_DROME	2,00E-43	GLOS_GPRK2.1.1	GO:0007472	B	.	wing disc morphogenesis
[BBH] GPRK2_DROME (sp|P32866) G prot-coupled receptor kinase 2 D.m.	GPRK2_DROME	2,00E-43	GLOS_GPRK2.1.1	GO:0007560	B	.	imaginal disc morphogenesis
[BBH] GPRK2_DROME (sp|P32866) G prot-coupled receptor kinase 2 D.m.	GPRK2_DROME	2,00E-43	GLOS_GPRK2.1.1	GO:0048563	B	.	post-embryonic organ morphogenesis
[BBH] GPRK2_DROME (sp|P32866) G prot-coupled receptor kinase 2 D.m.	GPRK2_DROME	2,00E-43	GLOS_GPRK2.1.1	GO:0048569	B	.	post-embryonic organ development
[BBH] GPRK2_DROME (sp|P32866) G prot-coupled receptor kinase 2 D.m.	GPRK2_DROME	2,00E-43	GLOS_GPRK2.1.1	GO:0007444	B	.	imaginal disc development
[BBH] GPRK2_DROME (sp|P32866) G prot-coupled receptor kinase 2 D.m.	GPRK2_DROME	2,00E-43	GLOS_GPRK2.1.1	GO:0007552	B	.	metamorphosis
[BBH] GPRK2_DROME (sp|P32866) G prot-coupled receptor kinase 2 D.m.	GPRK2_DROME	2,00E-43	GLOS_GPRK2.1.1	GO:0048707	B	.	instar larval or pupal morphogenesis
[BBH] GPRK2_DROME (sp|P32866) G prot-coupled receptor kinase 2 D.m.	GPRK2_DROME	2,00E-43	GLOS_GPRK2.1.1	GO:0002165	B	.	instar larval or pupal development
[BBH] GPRK2_DROME (sp|P32866) G prot-coupled receptor kinase 2 D.m.	GPRK2_DROME	2,00E-43	GLOS_GPRK2.1.1	GO:0035220	B	.	wing disc development
[BBH] GPRK2_DROME (sp|P32866) G prot-coupled receptor kinase 2 D.m.	GPRK2_DROME	2,00E-43	GLOS_GPRK2.1.1	GO:0007474	B	FlyBase	imaginal disc-derived wing vein specification
[BBH] GPRK2_DROME (sp|P32866) G prot-coupled receptor kinase 2 D.m.	GPRK2_DROME	2,00E-43	GLOS_GPRK2.1.1	GO:0008589	B	.	regulation of smoothened signaling pathway
[BBH] GPRK2_DROME (sp|P32866) G prot-coupled receptor kinase 2 D.m.	GPRK2_DROME	2,00E-43	GLOS_GPRK2.1.1	GO:0045879	B	FlyBase	negative regulation of smoothened signaling pathway
[BBH] GPRK2_DROME (sp|P32866) G prot-coupled receptor kinase 2 D.m.	GPRK2_DROME	2,00E-43	GLOS_GPRK2.1.1	GO:0007292	B	.	female gamete generation
[BBH] GPRK2_DROME (sp|P32866) G prot-coupled receptor kinase 2 D.m.	GPRK2_DROME	2,00E-43	GLOS_GPRK2.1.1	GO:0048477	B	UniProtKB-K	oogenesis
[BBH] GPRK2_DROME (sp|P32866) G prot-coupled receptor kinase 2 D.m.	GPRK2_DROME	2,00E-43	GLOS_GPRK2.1.1	GO:0009967	B	.	positive regulation of signal transduction
[BBH] GPRK2_DROME (sp|P32866) G prot-coupled receptor kinase 2 D.m.	GPRK2_DROME	2,00E-43	GLOS_GPRK2.1.1	GO:0010647	B	.	positive regulation of cell communication
[BBH] GPRK2_DROME (sp|P32866) G prot-coupled receptor kinase 2 D.m.	GPRK2_DROME	2,00E-43	GLOS_GPRK2.1.1	GO:0023056	B	.	positive regulation of signaling
[BBH] GPRK2_DROME (sp|P32866) G prot-coupled receptor kinase 2 D.m.	GPRK2_DROME	2,00E-43	GLOS_GPRK2.1.1	GO:0043949	B	.	regulation of cAMP-mediated signaling
[BBH] GPRK2_DROME (sp|P32866) G prot-coupled receptor kinase 2 D.m.	GPRK2_DROME	2,00E-43	GLOS_GPRK2.1.1	GO:0043950	B	FlyBase	positive regulation of cAMP-mediated signaling
[BBH] GPRK2_DROME (sp|P32866) G prot-coupled receptor kinase 2 D.m.	GPRK2_DROME	2,00E-43	GLOS_GPRK2.1.1	GO:0002784	B	.	regulation of antimicrobial peptide production
[BBH] GPRK2_DROME (sp|P32866) G prot-coupled receptor kinase 2 D.m.	GPRK2_DROME	2,00E-43	GLOS_GPRK2.1.1	GO:0002700	B	.	regulation of production of molecular mediator of immune response
[BBH] GPRK2_DROME (sp|P32866) G prot-coupled receptor kinase 2 D.m.	GPRK2_DROME	2,00E-43	GLOS_GPRK2.1.1	GO:0002697	B	.	regulation of immune effector process
[BBH] GPRK2_DROME (sp|P32866) G prot-coupled receptor kinase 2 D.m.	GPRK2_DROME	2,00E-43	GLOS_GPRK2.1.1	GO:0002759	B	.	regulation of antimicrobial humoral response
[BBH] GPRK2_DROME (sp|P32866) G prot-coupled receptor kinase 2 D.m.	GPRK2_DROME	2,00E-43	GLOS_GPRK2.1.1	GO:0002831	B	.	regulation of response to biotic stimulus
[BBH] GPRK2_DROME (sp|P32866) G prot-coupled receptor kinase 2 D.m.	GPRK2_DROME	2,00E-43	GLOS_GPRK2.1.1	GO:0002920	B	.	regulation of humoral immune response
[BBH] GPRK2_DROME (sp|P32866) G prot-coupled receptor kinase 2 D.m.	GPRK2_DROME	2,00E-43	GLOS_GPRK2.1.1	GO:0043900	B	.	regulation of multi-organism process
[BBH] GPRK2_DROME (sp|P32866) G prot-coupled receptor kinase 2 D.m.	GPRK2_DROME	2,00E-43	GLOS_GPRK2.1.1	GO:0034248	B	.	regulation of cellular amide metabolic process
[BBH] GPRK2_DROME (sp|P32866) G prot-coupled receptor kinase 2 D.m.	GPRK2_DROME	2,00E-43	GLOS_GPRK2.1.1	GO:0002805	B	FlyBase	regulation of antimicrobial peptide biosynthetic process
[BBH] GPRK2_DROME (sp|P32866) G prot-coupled receptor kinase 2 D.m.	GPRK2_DROME	2,00E-43	GLOS_GPRK2.1.1	GO:0008592	B	FlyBase	regulation of Toll signaling pathway
[BBH] GPRK2_DROME (sp|P32866) G prot-coupled receptor kinase 2 D.m.	GPRK2_DROME	2,00E-43	GLOS_GPRK2.1.1	GO:0007224	B	FlyBase	smoothened signaling pathway
[BBH] GPRK2_DROME (sp|P32866) G prot-coupled receptor kinase 2 D.m.	GPRK2_DROME	2,00E-43	GLOS_GPRK2.1.1	GO:0023021	B	.	termination of signal transduction
[BBH] GPRK2_DROME (sp|P32866) G prot-coupled receptor kinase 2 D.m.	GPRK2_DROME	2,00E-43	GLOS_GPRK2.1.1	GO:0045744	B	.	negative regulation of G-protein coupled receptor protein signaling pathway
[BBH] GPRK2_DROME (sp|P32866) G prot-coupled receptor kinase 2 D.m.	GPRK2_DROME	2,00E-43	GLOS_GPRK2.1.1	GO:0008277	B	.	regulation of G-protein coupled receptor protein signaling pathway
[BBH] GPRK2_DROME (sp|P32866) G prot-coupled receptor kinase 2 D.m.	GPRK2_DROME	2,00E-43	GLOS_GPRK2.1.1	GO:0038032	B	InterPro	termination of G-protein coupled receptor signaling pathway
[BBH] HEM_DROME (sp|P55162) Membrane-associated prot. Hem D.m.	HEM_DROME	0	GLOS_HEM.1.1	GO:0016021	C	UniProtKB-K	integral to membrane
[BBH] HEM_DROME (sp|P55162) Membrane-associated prot. Hem D.m.	HEM_DROME	0	GLOS_HEM.1.1	GO:0005886	C	FlyBase	plasma membrane
[BBH] HEM_DROME (sp|P55162) Membrane-associated prot. Hem D.m.	HEM_DROME	0	GLOS_HEM.1.1	GO:0031209	C	FlyBase	SCAR complex
[BBH] HEM_DROME (sp|P55162) Membrane-associated prot. Hem D.m.	HEM_DROME	0	GLOS_HEM.1.1	GO:0007409	B	FlyBase	axonogenesis
[BBH] HEM_DROME (sp|P55162) Membrane-associated prot. Hem D.m.	HEM_DROME	0	GLOS_HEM.1.1	GO:0030031	B	FlyBase	cell projection assembly
[BBH] HEM_DROME (sp|P55162) Membrane-associated prot. Hem D.m.	HEM_DROME	0	GLOS_HEM.1.1	GO:0007417	B	FlyBase	central nervous system development
[BBH] HEM_DROME (sp|P55162) Membrane-associated prot. Hem D.m.	HEM_DROME	0	GLOS_HEM.1.1	GO:0022416	B	.	chaeta development
[BBH] HEM_DROME (sp|P55162) Membrane-associated prot. Hem D.m.	HEM_DROME	0	GLOS_HEM.1.1	GO:0008407	B	FlyBase	chaeta morphogenesis
[BBH] HEM_DROME (sp|P55162) Membrane-associated prot. Hem D.m.	HEM_DROME	0	GLOS_HEM.1.1	GO:0030036	B	.	actin cytoskeleton organization

[BBH] HEM_DROME (sp|P55162) Membrane-associated prot. Hem D.m.	HEM_DROME	0	GLOS_HEM.1.1	GO:0030029	B	.	actin filament-based process
[BBH] HEM_DROME (sp|P55162) Membrane-associated prot. Hem D.m.	HEM_DROME	0	GLOS_HEM.1.1	GO:0030865	B	.	cortical cytoskeleton organization
[BBH] HEM_DROME (sp|P55162) Membrane-associated prot. Hem D.m.	HEM_DROME	0	GLOS_HEM.1.1	GO:0030866	B	FlyBase	cortical actin cytoskeleton organization
[BBH] HEM_DROME (sp|P55162) Membrane-associated prot. Hem D.m.	HEM_DROME	0	GLOS_HEM.1.1	GO:0000768	B	.	syncytium formation by plasma membrane fusion
[BBH] HEM_DROME (sp|P55162) Membrane-associated prot. Hem D.m.	HEM_DROME	0	GLOS_HEM.1.1	GO:0006949	B	.	syncytium formation
[BBH] HEM_DROME (sp|P55162) Membrane-associated prot. Hem D.m.	HEM_DROME	0	GLOS_HEM.1.1	GO:0014902	B	.	myotube differentiation
[BBH] HEM_DROME (sp|P55162) Membrane-associated prot. Hem D.m.	HEM_DROME	0	GLOS_HEM.1.1	GO:0051146	B	.	striated muscle cell differentiation
[BBH] HEM_DROME (sp|P55162) Membrane-associated prot. Hem D.m.	HEM_DROME	0	GLOS_HEM.1.1	GO:0042692	B	.	muscle cell differentiation
[BBH] HEM_DROME (sp|P55162) Membrane-associated prot. Hem D.m.	HEM_DROME	0	GLOS_HEM.1.1	GO:0061061	B	.	muscle structure development
[BBH] HEM_DROME (sp|P55162) Membrane-associated prot. Hem D.m.	HEM_DROME	0	GLOS_HEM.1.1	GO:0007520	B	FlyBase	myoblast fusion
[BBH] HEM_DROME (sp|P55162) Membrane-associated prot. Hem D.m.	HEM_DROME	0	GLOS_HEM.1.1	GO:0050808	B	.	synapse organization
[BBH] HEM_DROME (sp|P55162) Membrane-associated prot. Hem D.m.	HEM_DROME	0	GLOS_HEM.1.1	GO:0007528	B	FlyBase	neuromuscular junction development
[BBH] HEM_DROME (sp|P55162) Membrane-associated prot. Hem D.m.	HEM_DROME	0	GLOS_HEM.1.1	GO:0016477	B	.	cell migration
[BBH] HEM_DROME (sp|P55162) Membrane-associated prot. Hem D.m.	HEM_DROME	0	GLOS_HEM.1.1	GO:0048870	B	.	cell motility
[BBH] HEM_DROME (sp|P55162) Membrane-associated prot. Hem D.m.	HEM_DROME	0	GLOS_HEM.1.1	GO:0051674	B	.	localization of cell
[BBH] HEM_DROME (sp|P55162) Membrane-associated prot. Hem D.m.	HEM_DROME	0	GLOS_HEM.1.1	GO:0001764	B	FlyBase	neuron migration
[BBH] HEM_DROME (sp|P55162) Membrane-associated prot. Hem D.m.	HEM_DROME	0	GLOS_HEM.1.1	GO:0006897	B	.	endocytosis
[BBH] HEM_DROME (sp|P55162) Membrane-associated prot. Hem D.m.	HEM_DROME	0	GLOS_HEM.1.1	GO:0016192	B	.	vesicle-mediated transport
[BBH] HEM_DROME (sp|P55162) Membrane-associated prot. Hem D.m.	HEM_DROME	0	GLOS_HEM.1.1	GO:0006909	B	FlyBase	phagocytosis
[BBH] HEM_DROME (sp|P55162) Membrane-associated prot. Hem D.m.	HEM_DROME	0	GLOS_HEM.1.1	GO:0008360	B	FlyBase	regulation of cell shape
[BBH] HS2ST_DROME (sp|P25722) Heparin sulfate O-sulfotransferase D.m.	HS2ST_DROME	0	GLOS_HS2ST.1.1	GO:0000139	C	UniProtKB-S	Golgi membrane
[BBH] HS2ST_DROME (sp|P25722) Heparin sulfate O-sulfotransferase D.m.	HS2ST_DROME	0	GLOS_HS2ST.1.1	GO:0016021	C	UniProtKB-K	integral to membrane
[BBH] HS2ST_DROME (sp|P25722) Heparin sulfate O-sulfotransferase D.m.	HS2ST_DROME	0	GLOS_HS2ST.1.1	GO:0034483	M	.	heparan sulfate sulfotransferase activity
[BBH] HS2ST_DROME (sp|P25722) Heparin sulfate O-sulfotransferase D.m.	HS2ST_DROME	0	GLOS_HS2ST.1.1	GO:0008146	M	.	sulfotransferase activity
[BBH] HS2ST_DROME (sp|P25722) Heparin sulfate O-sulfotransferase D.m.	HS2ST_DROME	0	GLOS_HS2ST.1.1	GO:0004394	M	FlyBase	heparan sulfate 2-O-sulfotransferase activity
[BBH] HS2ST_DROME (sp|P25722) Heparin sulfate O-sulfotransferase D.m.	HS2ST_DROME	0	GLOS_HS2ST.1.1	GO:0060541	B	.	respiratory system development
[BBH] HS2ST_DROME (sp|P25722) Heparin sulfate O-sulfotransferase D.m.	HS2ST_DROME	0	GLOS_HS2ST.1.1	GO:0007424	B	FlyBase	open tracheal system development
[BBH] HS2ST_DROME (sp|P25722) Heparin sulfate O-sulfotransferase D.m.	HS2ST_DROME	0	GLOS_HS2ST.1.1	GO:0007165	B	FlyBase	signal transduction
HYPB_HYPLI (sp|P35588) Hypodermin-B OS=H. lineatum PE=1 SV=1	HYPB_HYPLI	6,00E-31	GLOS_HYPB.1.2	GO:0005576	C	UniProtKB-S	extracellular region
HYPB_HYPLI (sp|P35588) Hypodermin-B OS=H. lineatum PE=1 SV=1	HYPB_HYPLI	6,00E-31	GLOS_HYPB.1.2	GO:0004252	M	InterPro	serine-type endopeptidase activity
HYPB_HYPLI (sp|P35588) Hypodermin-B OS=H. lineatum PE=1 SV=1	HYPB_HYPLI	6,00E-31	GLOS_HYPB.1.2	GO:0006508	B	UniProtKB-K	proteolysis
IMDH_DROME (sp|Q07152) Inosine-5'-monophosphate dehydrogenase D.m.	IMDH_DROME	0	GLOS_IMDH.2.2	GO:0005737	C	UniProtKB-S	cytoplasm
IMDH_DROME (sp|Q07152) Inosine-5'-monophosphate dehydrogenase D.m.	IMDH_DROME	0	GLOS_IMDH.2.2	GO:0030554	M	InterPro	adenyl nucleotide binding
IMDH_DROME (sp|Q07152) Inosine-5'-monophosphate dehydrogenase D.m.	IMDH_DROME	0	GLOS_IMDH.2.2	GO:0016616	M	.	oxidored. Act., acting on the CH-OH group of donors, NAD or NADP as acceptor
IMDH_DROME (sp|Q07152) Inosine-5'-monophosphate dehydrogenase D.m.	IMDH_DROME	0	GLOS_IMDH.2.2	GO:0016614	M	.	oxidoreductase activity, acting on CH-OH group of donors
IMDH_DROME (sp|Q07152) Inosine-5'-monophosphate dehydrogenase D.m.	IMDH_DROME	0	GLOS_IMDH.2.2	GO:0016491	M	.	oxidoreductase activity
IMDH_DROME (sp|Q07152) Inosine-5'-monophosphate dehydrogenase D.m.	IMDH_DROME	0	GLOS_IMDH.2.2	GO:0003938	M	UniProtKB-K	IMP dehydrogenase activity
IMDH_DROME (sp|Q07152) Inosine-5'-monophosphate dehydrogenase D.m.	IMDH_DROME	0	GLOS_IMDH.2.2	GO:0046872	M	UniProtKB-K	metal ion binding
IMDH_DROME (sp|Q07152) Inosine-5'-monophosphate dehydrogenase D.m.	IMDH_DROME	0	GLOS_IMDH.2.2	GO:0007411	B	FlyBase	axon guidance
IMDH_DROME (sp|Q07152) Inosine-5'-monophosphate dehydrogenase D.m.	IMDH_DROME	0	GLOS_IMDH.2.2	GO:0009152	B	.	purine ribonucleotide biosynthetic process
IMDH_DROME (sp|Q07152) Inosine-5'-monophosphate dehydrogenase D.m.	IMDH_DROME	0	GLOS_IMDH.2.2	GO:0006164	B	.	purine nucleotide biosynthetic process
IMDH_DROME (sp|Q07152) Inosine-5'-monophosphate dehydrogenase D.m.	IMDH_DROME	0	GLOS_IMDH.2.2	GO:0006163	B	.	purine nucleotide metabolic process
IMDH_DROME (sp|Q07152) Inosine-5'-monophosphate dehydrogenase D.m.	IMDH_DROME	0	GLOS_IMDH.2.2	GO:0009117	B	.	nucleotide metabolic process
IMDH_DROME (sp|Q07152) Inosine-5'-monophosphate dehydrogenase D.m.	IMDH_DROME	0	GLOS_IMDH.2.2	GO:0006753	B	.	nucleoside phosphate metabolic process
IMDH_DROME (sp|Q07152) Inosine-5'-monophosphate dehydrogenase D.m.	IMDH_DROME	0	GLOS_IMDH.2.2	GO:0006796	B	.	phosphate-containing compound metabolic process
IMDH_DROME (sp|Q07152) Inosine-5'-monophosphate dehydrogenase D.m.	IMDH_DROME	0	GLOS_IMDH.2.2	GO:0006793	B	.	phosphorus metabolic process
IMDH_DROME (sp|Q07152) Inosine-5'-monophosphate dehydrogenase D.m.	IMDH_DROME	0	GLOS_IMDH.2.2	GO:0019637	B	.	organophosphate metabolic process
IMDH_DROME (sp|Q07152) Inosine-5'-monophosphate dehydrogenase D.m.	IMDH_DROME	0	GLOS_IMDH.2.2	GO:0055086	B	.	nucleobase-containing small molecule metabolic process
IMDH_DROME (sp|Q07152) Inosine-5'-monophosphate dehydrogenase D.m.	IMDH_DROME	0	GLOS_IMDH.2.2	GO:0072521	B	.	purine-containing compound metabolic process
IMDH_DROME (sp|Q07152) Inosine-5'-monophosphate dehydrogenase D.m.	IMDH_DROME	0	GLOS_IMDH.2.2	GO:0009165	B	.	nucleotide biosynthetic process
IMDH_DROME (sp|Q07152) Inosine-5'-monophosphate dehydrogenase D.m.	IMDH_DROME	0	GLOS_IMDH.2.2	GO:1901293	B	.	nucleoside phosphate biosynthetic process
IMDH_DROME (sp|Q07152) Inosine-5'-monophosphate dehydrogenase D.m.	IMDH_DROME	0	GLOS_IMDH.2.2	GO:0090407	B	.	organophosphate biosynthetic process
IMDH_DROME (sp|Q07152) Inosine-5'-monophosphate dehydrogenase D.m.	IMDH_DROME	0	GLOS_IMDH.2.2	GO:0072522	B	.	purine-containing compound biosynthetic process
IMDH_DROME (sp|Q07152) Inosine-5'-monophosphate dehydrogenase D.m.	IMDH_DROME	0	GLOS_IMDH.2.2	GO:0009150	B	.	purine ribonucleotide metabolic process

IMDH_DROME (sp|Q07152) Inosine-5'-monophosphate dehydrogenase D.m.	IMDH_DROME	0	GLOS_IMDH.2.2	GO:0009259	B	.	ribonucleotide metabolic process
IMDH_DROME (sp|Q07152) Inosine-5'-monophosphate dehydrogenase D.m.	IMDH_DROME	0	GLOS_IMDH.2.2	GO:0019693	B	.	ribose phosphate metabolic process
IMDH_DROME (sp|Q07152) Inosine-5'-monophosphate dehydrogenase D.m.	IMDH_DROME	0	GLOS_IMDH.2.2	GO:0009260	B	.	ribonucleotide biosynthetic process
IMDH_DROME (sp|Q07152) Inosine-5'-monophosphate dehydrogenase D.m.	IMDH_DROME	0	GLOS_IMDH.2.2	GO:0046390	B	.	ribose phosphate biosynthetic process
IMDH_DROME (sp|Q07152) Inosine-5'-monophosphate dehydrogenase D.m.	IMDH_DROME	0	GLOS_IMDH.2.2	GO:0009168	B	.	purine ribonucleoside monophosphate biosynthetic process
IMDH_DROME (sp|Q07152) Inosine-5'-monophosphate dehydrogenase D.m.	IMDH_DROME	0	GLOS_IMDH.2.2	GO:0009127	B	.	purine nucleoside monophosphate biosynthetic process
IMDH_DROME (sp|Q07152) Inosine-5'-monophosphate dehydrogenase D.m.	IMDH_DROME	0	GLOS_IMDH.2.2	GO:0009124	B	.	nucleoside monophosphate biosynthetic process
IMDH_DROME (sp|Q07152) Inosine-5'-monophosphate dehydrogenase D.m.	IMDH_DROME	0	GLOS_IMDH.2.2	GO:0009123	B	.	nucleoside monophosphate metabolic process
IMDH_DROME (sp|Q07152) Inosine-5'-monophosphate dehydrogenase D.m.	IMDH_DROME	0	GLOS_IMDH.2.2	GO:0009126	B	.	purine nucleoside monophosphate metabolic process
IMDH_DROME (sp|Q07152) Inosine-5'-monophosphate dehydrogenase D.m.	IMDH_DROME	0	GLOS_IMDH.2.2	GO:0009156	B	.	ribonucleoside monophosphate biosynthetic process
IMDH_DROME (sp|Q07152) Inosine-5'-monophosphate dehydrogenase D.m.	IMDH_DROME	0	GLOS_IMDH.2.2	GO:0009161	B	.	ribonucleoside monophosphate metabolic process
IMDH_DROME (sp|Q07152) Inosine-5'-monophosphate dehydrogenase D.m.	IMDH_DROME	0	GLOS_IMDH.2.2	GO:0009167	B	.	purine ribonucleoside monophosphate metabolic process
IMDH_DROME (sp|Q07152) Inosine-5'-monophosphate dehydrogenase D.m.	IMDH_DROME	0	GLOS_IMDH.2.2	GO:0046037	B	.	GMP metabolic process
IMDH_DROME (sp|Q07152) Inosine-5'-monophosphate dehydrogenase D.m.	IMDH_DROME	0	GLOS_IMDH.2.2	GO:1901068	B	.	guanosine-containing compound metabolic process
IMDH_DROME (sp|Q07152) Inosine-5'-monophosphate dehydrogenase D.m.	IMDH_DROME	0	GLOS_IMDH.2.2	GO:0046128	B	.	purine ribonucleoside metabolic process
IMDH_DROME (sp|Q07152) Inosine-5'-monophosphate dehydrogenase D.m.	IMDH_DROME	0	GLOS_IMDH.2.2	GO:0009119	B	.	ribonucleoside metabolic process
IMDH_DROME (sp|Q07152) Inosine-5'-monophosphate dehydrogenase D.m.	IMDH_DROME	0	GLOS_IMDH.2.2	GO:0009116	B	.	nucleoside metabolic process
IMDH_DROME (sp|Q07152) Inosine-5'-monophosphate dehydrogenase D.m.	IMDH_DROME	0	GLOS_IMDH.2.2	GO:1901657	B	.	glycosyl compound metabolic process
IMDH_DROME (sp|Q07152) Inosine-5'-monophosphate dehydrogenase D.m.	IMDH_DROME	0	GLOS_IMDH.2.2	GO:0042278	B	.	purine nucleoside metabolic process
IMDH_DROME (sp|Q07152) Inosine-5'-monophosphate dehydrogenase D.m.	IMDH_DROME	0	GLOS_IMDH.2.2	GO:1901070	B	.	guanosine-containing compound biosynthetic process
IMDH_DROME (sp|Q07152) Inosine-5'-monophosphate dehydrogenase D.m.	IMDH_DROME	0	GLOS_IMDH.2.2	GO:0046129	B	.	purine ribonucleoside biosynthetic process
IMDH_DROME (sp|Q07152) Inosine-5'-monophosphate dehydrogenase D.m.	IMDH_DROME	0	GLOS_IMDH.2.2	GO:0042451	B	.	purine nucleoside biosynthetic process
IMDH_DROME (sp|Q07152) Inosine-5'-monophosphate dehydrogenase D.m.	IMDH_DROME	0	GLOS_IMDH.2.2	GO:0009163	B	.	nucleoside biosynthetic process
IMDH_DROME (sp|Q07152) Inosine-5'-monophosphate dehydrogenase D.m.	IMDH_DROME	0	GLOS_IMDH.2.2	GO:1901659	B	.	glycosyl compound biosynthetic process
IMDH_DROME (sp|Q07152) Inosine-5'-monophosphate dehydrogenase D.m.	IMDH_DROME	0	GLOS_IMDH.2.2	GO:0042455	B	.	ribonucleoside biosynthetic process
IMDH_DROME (sp|Q07152) Inosine-5'-monophosphate dehydrogenase D.m.	IMDH_DROME	0	GLOS_IMDH.2.2	GO:0006177	B	UniProtKB-	GMP biosynthetic process
IMDH_DROME (sp|Q07152) Inosine-5'-monophosphate dehydrogenase D.m.	IMDH_DROME	0	GLOS_IMDH.2.2	GO:0048477	B	FlyBase	oogenesis
IMDH_DROME (sp|Q07152) Inosine-5'-monophosphate dehydrogenase D.m.	IMDH_DROME	0	GLOS_IMDH.2.2	GO:0006909	B	FlyBase	phagocytosis
KPBB_DROME (sp|Q9VLS1)Prob phosphorylase b kinase regul subunit beta D.m.	KPBB_DROME	8,00E-44	GLOS_KPBB.4.7	GO:0005886	C	UniProtKB-	plasma membrane
KPBB_DROME (sp|Q9VLS1)Prob phosphorylase b kinase regul subunit beta D.m.	KPBB_DROME	8,00E-44	GLOS_KPBB.4.7	GO:0016798	M	.	hydrolase activity, acting on glycosyl bonds
KPBB_DROME (sp|Q9VLS1)Prob phosphorylase b kinase regul subunit beta D.m.	KPBB_DROME	8,00E-44	GLOS_KPBB.4.7	GO:0004553	M	InterPro	hydrolase activity, hydrolyzing O-glycosyl compounds
KPBB_DROME (sp|Q9VLS1)Prob phosphorylase b kinase regul subunit beta D.m.	KPBB_DROME	8,00E-44	GLOS_KPBB.4.7	GO:0006073	B	.	cellular glucan metabolic process
KPBB_DROME (sp|Q9VLS1)Prob phosphorylase b kinase regul subunit beta D.m.	KPBB_DROME	8,00E-44	GLOS_KPBB.4.7	GO:0044042	B	.	glucan metabolic process
KPBB_DROME (sp|Q9VLS1)Prob phosphorylase b kinase regul subunit beta D.m.	KPBB_DROME	8,00E-44	GLOS_KPBB.4.7	GO:0005976	B	.	polysaccharide metabolic process
KPBB_DROME (sp|Q9VLS1)Prob phosphorylase b kinase regul subunit beta D.m.	KPBB_DROME	8,00E-44	GLOS_KPBB.4.7	GO:0005975	B	.	carbohydrate metabolic process
KPBB_DROME (sp|Q9VLS1)Prob phosphorylase b kinase regul subunit beta D.m.	KPBB_DROME	8,00E-44	GLOS_KPBB.4.7	GO:0044264	B	.	cellular polysaccharide metabolic process
KPBB_DROME (sp|Q9VLS1)Prob phosphorylase b kinase regul subunit beta D.m.	KPBB_DROME	8,00E-44	GLOS_KPBB.4.7	GO:0044262	B	.	cellular carbohydrate metabolic process
KPBB_DROME (sp|Q9VLS1)Prob phosphorylase b kinase regul subunit beta D.m.	KPBB_DROME	8,00E-44	GLOS_KPBB.4.7	GO:0006112	B	.	energy reserve metabolic process
KPBB_DROME (sp|Q9VLS1)Prob phosphorylase b kinase regul subunit beta D.m.	KPBB_DROME	8,00E-44	GLOS_KPBB.4.7	GO:0015980	B	.	energy derivation by oxidation of organic compounds
KPBB_DROME (sp|Q9VLS1)Prob phosphorylase b kinase regul subunit beta D.m.	KPBB_DROME	8,00E-44	GLOS_KPBB.4.7	GO:0006091	B	.	generation of precursor metabolites and energy
KPBB_DROME (sp|Q9VLS1)Prob phosphorylase b kinase regul subunit beta D.m.	KPBB_DROME	8,00E-44	GLOS_KPBB.4.7	GO:0055114	B	.	oxidation-reduction process
KPBB_DROME (sp|Q9VLS1)Prob phosphorylase b kinase regul subunit beta D.m.	KPBB_DROME	8,00E-44	GLOS_KPBB.4.7	GO:0005977	B	UniProtKB-	glycogen metabolic process
[BBH] LAP1_DROME (sp|Q9V780) Prot lap1 OS=D.m. GN=Lap1 PE=2 SV=1	LAP1_DROME	0	GLOS_LAP1.1.1	GO:0070161	C	.	anchoring junction
[BBH] LAP1_DROME (sp|Q9V780) Prot lap1 OS=D.m. GN=Lap1 PE=2 SV=1	LAP1_DROME	0	GLOS_LAP1.1.1	GO:0030054	C	.	cell junction
[BBH] LAP1_DROME (sp|Q9V780) Prot lap1 OS=D.m. GN=Lap1 PE=2 SV=1	LAP1_DROME	0	GLOS_LAP1.1.1	GO:0005912	C	UniProtKB	adherens junction
[BBH] LAP1_DROME (sp|Q9V780) Prot lap1 OS=D.m. GN=Lap1 PE=2 SV=1	LAP1_DROME	0	GLOS_LAP1.1.1	GO:0007163	B	UniProtKB	establishment or maintenance of cell polarity
[BBH] LAP1_DROME (sp|Q9V780) Prot lap1 OS=D.m. GN=Lap1 PE=2 SV=1	LAP1_DROME	0	GLOS_LAP1.1.1	GO:0015031	B	UniProtKB	protein transport
M10L1_HUMAN (sp|Q9BXT6) Putative helicase Mov10l1 OS=H. sapiens	M10L1_HUMAN	3,00E-13	GLOS_M10L1.1.1	GO:0005622	C	UniProtKB	intracellular
M10L1_HUMAN (sp|Q9BXT6) Putative helicase Mov10l1 OS=H. sapiens	M10L1_HUMAN	3,00E-13	GLOS_M10L1.1.1	GO:0005524	M	UniProtKB	ATP binding
M10L1_HUMAN (sp|Q9BXT6) Putative helicase Mov10l1 OS=H. sapiens	M10L1_HUMAN	3,00E-13	GLOS_M10L1.1.1	GO:0003724	M	.	RNA helicase activity
M10L1_HUMAN (sp|Q9BXT6) Putative helicase Mov10l1 OS=H. sapiens	M10L1_HUMAN	3,00E-13	GLOS_M10L1.1.1	GO:0004386	M	.	helicase activity
M10L1_HUMAN (sp|Q9BXT6) Putative helicase Mov10l1 OS=H. sapiens	M10L1_HUMAN	3,00E-13	GLOS_M10L1.1.1	GO:0008026	M	.	ATP-dependent helicase activity
M10L1_HUMAN (sp|Q9BXT6) Putative helicase Mov10l1 OS=H. sapiens	M10L1_HUMAN	3,00E-13	GLOS_M10L1.1.1	GO:0042623	M	.	ATPase activity, coupled
M10L1_HUMAN (sp|Q9BXT6) Putative helicase Mov10l1 OS=H. sapiens	M10L1_HUMAN	3,00E-13	GLOS_M10L1.1.1	GO:0016887	M	.	ATPase activity

M10L1_HUMAN (sp|Q9BXT6) Putative helicase Mov10l1 OS=H. sapiens	M10L1_HUMAN	3,00E-13	GLOS_M10L1.1.1	GO:0070035	M	.	purine NTP-dependent helicase activity
M10L1_HUMAN (sp|Q9BXT6) Putative helicase Mov10l1 OS=H. sapiens	M10L1_HUMAN	3,00E-13	GLOS_M10L1.1.1	GO:0008186	M	.	RNA-dependent ATPase activity
M10L1_HUMAN (sp|Q9BXT6) Putative helicase Mov10l1 OS=H. sapiens	M10L1_HUMAN	3,00E-13	GLOS_M10L1.1.1	GO:0004004	M	UniProtKB	ATP-dependent RNA helicase activity
M10L1_HUMAN (sp|Q9BXT6) Putative helicase Mov10l1 OS=H. sapiens	M10L1_HUMAN	3,00E-13	GLOS_M10L1.1.1	GO:0000287	M	UniProtKB	magnesium ion binding
M10L1_HUMAN (sp|Q9BXT6) Putative helicase Mov10l1 OS=H. sapiens	M10L1_HUMAN	3,00E-13	GLOS_M10L1.1.1	GO:0003723	M	UniProtKB	RNA binding
M10L1_HUMAN (sp|Q9BXT6) Putative helicase Mov10l1 OS=H. sapiens	M10L1_HUMAN	3,00E-13	GLOS_M10L1.1.1	GO:0007281	B	UniProtKB	germ cell development
M10L1_HUMAN (sp|Q9BXT6) Putative helicase Mov10l1 OS=H. sapiens	M10L1_HUMAN	3,00E-13	GLOS_M10L1.1.1	GO:0007275	B	UniProtKB-K	multicellular organismal development
M10L1_HUMAN (sp|Q9BXT6) Putative helicase Mov10l1 OS=H. sapiens	M10L1_HUMAN	3,00E-13	GLOS_M10L1.1.1	GO:0007283	B	UniProtKB	spermatogenesis
MCM5_DROME (sp|Q9VGW6) DNA replication licensing factor Mcm5 D.m.	MCM5_DROME	0	GLOS_MCM5.4.5	GO:0042555	C	InterPro	MCM complex
MCM5_DROME (sp|Q9VGW6) DNA replication licensing factor Mcm5 D.m.	MCM5_DROME	0	GLOS_MCM5.4.5	GO:0005634	C	UniProtKB-S	nucleus
MCM5_DROME (sp|Q9VGW6) DNA replication licensing factor Mcm5 D.m.	MCM5_DROME	0	GLOS_MCM5.4.5	GO:0005524	M	UniProtKB-K	ATP binding
MCM5_DROME (sp|Q9VGW6) DNA replication licensing factor Mcm5 D.m.	MCM5_DROME	0	GLOS_MCM5.4.5	GO:0003677	M	UniProtKB-K	DNA binding
MCM5_DROME (sp|Q9VGW6) DNA replication licensing factor Mcm5 D.m.	MCM5_DROME	0	GLOS_MCM5.4.5	GO:0003678	M	InterPro	DNA helicase activity
MCM5_DROME (sp|Q9VGW6) DNA replication licensing factor Mcm5 D.m.	MCM5_DROME	0	GLOS_MCM5.4.5	GO:0006323	B	.	DNA packaging
MCM5_DROME (sp|Q9VGW6) DNA replication licensing factor Mcm5 D.m.	MCM5_DROME	0	GLOS_MCM5.4.5	GO:0071103	B	.	DNA conformation change
MCM5_DROME (sp|Q9VGW6) DNA replication licensing factor Mcm5 D.m.	MCM5_DROME	0	GLOS_MCM5.4.5	GO:0006259	B	.	DNA metabolic process
MCM5_DROME (sp|Q9VGW6) DNA replication licensing factor Mcm5 D.m.	MCM5_DROME	0	GLOS_MCM5.4.5	GO:0030261	B	FlyBase	chromosome condensation
MCM5_DROME (sp|Q9VGW6) DNA replication licensing factor Mcm5 D.m.	MCM5_DROME	0	GLOS_MCM5.4.5	GO:0032392	B	.	DNA geometric change
MCM5_DROME (sp|Q9VGW6) DNA replication licensing factor Mcm5 D.m.	MCM5_DROME	0	GLOS_MCM5.4.5	GO:0032508	B	GOC	DNA duplex unwinding
MCM5_DROME (sp|Q9VGW6) DNA replication licensing factor Mcm5 D.m.	MCM5_DROME	0	GLOS_MCM5.4.5	GO:0044786	B	.	cell cycle DNA replication
MCM5_DROME (sp|Q9VGW6) DNA replication licensing factor Mcm5 D.m.	MCM5_DROME	0	GLOS_MCM5.4.5	GO:0006261	B	.	DNA-dependent DNA replication
MCM5_DROME (sp|Q9VGW6) DNA replication licensing factor Mcm5 D.m.	MCM5_DROME	0	GLOS_MCM5.4.5	GO:0006260	B	.	DNA replication
MCM5_DROME (sp|Q9VGW6) DNA replication licensing factor Mcm5 D.m.	MCM5_DROME	0	GLOS_MCM5.4.5	GO:0042023	B	FlyBase	DNA endoreduplication
MCM5_DROME (sp|Q9VGW6) DNA replication licensing factor Mcm5 D.m.	MCM5_DROME	0	GLOS_MCM5.4.5	GO:0006270	B	InterPro	DNA replication initiation
MCM5_DROME (sp|Q9VGW6) DNA replication licensing factor Mcm5 D.m.	MCM5_DROME	0	GLOS_MCM5.4.5	GO:0051307	B	.	meiotic chromosome separation
MCM5_DROME (sp|Q9VGW6) DNA replication licensing factor Mcm5 D.m.	MCM5_DROME	0	GLOS_MCM5.4.5	GO:0051304	B	.	chromosome separation
MCM5_DROME (sp|Q9VGW6) DNA replication licensing factor Mcm5 D.m.	MCM5_DROME	0	GLOS_MCM5.4.5	GO:0007059	B	.	chromosome segregation
MCM5_DROME (sp|Q9VGW6) DNA replication licensing factor Mcm5 D.m.	MCM5_DROME	0	GLOS_MCM5.4.5	GO:0045132	B	.	meiotic chromosome segregation
MCM5_DROME (sp|Q9VGW6) DNA replication licensing factor Mcm5 D.m.	MCM5_DROME	0	GLOS_MCM5.4.5	GO:0007126	B	.	meiosis
MCM5_DROME (sp|Q9VGW6) DNA replication licensing factor Mcm5 D.m.	MCM5_DROME	0	GLOS_MCM5.4.5	GO:0051321	B	.	meiotic cell cycle
MCM5_DROME (sp|Q9VGW6) DNA replication licensing factor Mcm5 D.m.	MCM5_DROME	0	GLOS_MCM5.4.5	GO:0007131	B	.	reciprocal meiotic recombination
MCM5_DROME (sp|Q9VGW6) DNA replication licensing factor Mcm5 D.m.	MCM5_DROME	0	GLOS_MCM5.4.5	GO:0035825	B	.	reciprocal DNA recombination
MCM5_DROME (sp|Q9VGW6) DNA replication licensing factor Mcm5 D.m.	MCM5_DROME	0	GLOS_MCM5.4.5	GO:0006310	B	.	DNA recombination
MCM5_DROME (sp|Q9VGW6) DNA replication licensing factor Mcm5 D.m.	MCM5_DROME	0	GLOS_MCM5.4.5	GO:0007127	B	.	meiosis I
MCM5_DROME (sp|Q9VGW6) DNA replication licensing factor Mcm5 D.m.	MCM5_DROME	0	GLOS_MCM5.4.5	GO:0000712	B	FlyBase	resolution of meiotic recombination intermediates
[BBH] MED8_DROME (sp|A1ZBT5)Mediator of RNA polymerase II transcrip sub. 8	MED8_DROME	1,00E-113	GLOS_MED8.1.1	GO:0016592	C	UniProtKB	mediator complex
[BBH] MED8_DROME (sp|A1ZBT5)Mediator of RNA polymerase II transcrip sub. 8	MED8_DROME	1,00E-113	GLOS_MED8.1.1	GO:0001104	M	UniProtKB	RNA polymerase II transcription cofactor activity
[BBH] MED8_DROME (sp|A1ZBT5)Mediator of RNA polymerase II transcrip sub. 8	MED8_DROME	1,00E-113	GLOS_MED8.1.1	GO:0051726	B	FlyBase	regulation of cell cycle
[BBH] MED8_DROME (sp|A1ZBT5)Mediator of RNA polymerase II transcrip sub. 8	MED8_DROME	1,00E-113	GLOS_MED8.1.1	GO:0006367	B	FlyBase	transcription initiation from RNA polymerase II promoter
[BBH] MED9_DROPS (sp|Q290F0) Mediator of RNA polymerase II transcription subunit 9 OS=D. p. pseudoobscura	MED9_DROPS	1,00E-43	GLOS_MED9.1.1	GO:0016592	C	UniProtKB	mediator complex
[BBH] MED9_DROPS (sp|Q290F0) Mediator of RNA polymer. II … Same as above	MED9_DROPS	1,00E-43	GLOS_MED9.1.1	GO:0001104	M	UniProtKB	RNA polymerase II transcription cofactor activity
[BBH] MO25_DROME (sp|P91891) Protein Mo25 OS=D.m. GN=Mo25 PE=2 SV=2	MO25_DROME	0	GLOS_MO25.1.1	GO:0009792	B	.	embryo development ending in birth or egg hatching
[BBH] MO25_DROME (sp|P91891) Protein Mo25 OS=D.m. GN=Mo25 PE=2 SV=2	MO25_DROME	0	GLOS_MO25.1.1	GO:0001700	B	FlyBase	embryonic development via the syncytial blastoderm
[BBH] MP20_DROME (sp|P14318) Muscle-specific prot 20 OS=D.m.	MP20_DROME	1,00E-110	GLOS_MP20.2.4	GO:0008092	M	.	cytoskeletal protein binding
[BBH] MP20_DROME (sp|P14318) Muscle-specific prot 20 OS=D.m.	MP20_DROME	1,00E-110	GLOS_MP20.2.4	GO:0003779	M	FlyBase	actin binding
[BBH] MP20_DROME (sp|P14318) Muscle-specific prot 20 OS=D.m.	MP20_DROME	1,00E-110	GLOS_MP20.2.4	GO:0007155	B	FlyBase	cell adhesion
[BBH] MP20_DROME (sp|P14318) Muscle-specific prot 20 OS=D.m.	MP20_DROME	1,00E-110	GLOS_MP20.2.4	GO:0007520	B	FlyBase	myoblast fusion
[BBH] MP20_DROME (sp|P14318) Muscle-specific prot 20 OS=D.m.	MP20_DROME	1,00E-110	GLOS_MP20.2.4	GO:0008360	B	FlyBase	regulation of cell shape
NAA35_DROME (sp|Q9W1A2) N-alpha-acetyltransferase, 35 NatC auxiliary subunit homolog OS=D. melanogaster	NAA35_DROME	5,00E-70	GLOS_NAA35.3.3	GO:0005737	C	UniProtKB	cytoplasm
NAA35_DROME (sp|Q9W1A2) N-alpha-acetyltransferase, … Same as above	NADE_DROME	0	GLOS_NADE.1.1	GO:0005524	M	UniProtKB-K	ATP binding
NAA35_DROME (sp|Q9W1A2) N-alpha-acetyltransferase, … Same as above	NADE_DROME	0	GLOS_NADE.1.1	GO:0016810	M	InterPro	hydrolase activity, acting on carbon-nitrogen (but not peptide) bonds
NAA35_DROME (sp|Q9W1A2) N-alpha-acetyltransferase, … Same as above	NADE_DROME	0	GLOS_NADE.1.1	GO:0016884	M	.	carbon-nitrogen ligase activity, with glutamine as amido-N-donor

NAA35_DROME (sp|Q9W1A2) N-alpha-acetyltransferase, …	Same as above	NADE_DROME	0	GLOS_NADE.1.1	GO:0003952	M	UniProtKB-E	NAD+ synthase (glutamine-hydrolyzing) activity
NAA35_DROME (sp|Q9W1A2) N-alpha-acetyltransferase, …	Same as above	NADE_DROME	0	GLOS_NADE.1.1	GO:0019359	B	.	nicotinamide nucleotide biosynthetic process
NAA35_DROME (sp|Q9W1A2) N-alpha-acetyltransferase, …	Same as above	NADE_DROME	0	GLOS_NADE.1.1	GO:0019363	B	.	pyridine nucleotide biosynthetic process
NAA35_DROME (sp|Q9W1A2) N-alpha-acetyltransferase, …	Same as above	NADE_DROME	0	GLOS_NADE.1.1	GO:0009108	B	.	coenzyme biosynthetic process
NAA35_DROME (sp|Q9W1A2) N-alpha-acetyltransferase, …	Same as above	NADE_DROME	0	GLOS_NADE.1.1	GO:0006732	B	.	coenzyme metabolic process
NAA35_DROME (sp|Q9W1A2) N-alpha-acetyltransferase, …	Same as above	NADE_DROME	0	GLOS_NADE.1.1	GO:0051186	B	.	cofactor metabolic process
NAA35_DROME (sp|Q9W1A2) N-alpha-acetyltransferase, …	Same as above	NADE_DROME	0	GLOS_NADE.1.1	GO:0051188	B	.	cofactor biosynthetic process
NAA35_DROME (sp|Q9W1A2) N-alpha-acetyltransferase, …	Same as above	NADE_DROME	0	GLOS_NADE.1.1	GO:0019362	B	.	pyridine nucleotide metabolic process
NAA35_DROME (sp|Q9W1A2) N-alpha-acetyltransferase, …	Same as above	NADE_DROME	0	GLOS_NADE.1.1	GO:0006733	B	.	oxidoreduction coenzyme metabolic process
NAA35_DROME (sp|Q9W1A2) N-alpha-acetyltransferase, …	Same as above	NADE_DROME	0	GLOS_NADE.1.1	GO:0072524	B	.	pyridine-containing compound metabolic process
NAA35_DROME (sp|Q9W1A2) N-alpha-acetyltransferase, …	Same as above	NADE_DROME	0	GLOS_NADE.1.1	GO:0072525	B	.	pyridine-containing compound biosynthetic process
NAA35_DROME (sp|Q9W1A2) N-alpha-acetyltransferase, …	Same as above	NADE_DROME	0	GLOS_NADE.1.1	GO:0046496	B	.	nicotinamide nucleotide metabolic process
NAA35_DROME (sp|Q9W1A2) N-alpha-acetyltransferase, …	Same as above	NADE_DROME	0	GLOS_NADE.1.1	GO:0019674	B	.	NAD metabolic process
NAA35_DROME (sp|Q9W1A2) N-alpha-acetyltransferase, …	Same as above	NADE_DROME	0	GLOS_NADE.1.1	GO:0009435	B	UniProtKB-U	NAD biosynthetic process
[BBH] OAT_DROME (sp|Q9VW26) Ornithine aminotransferase, mitoch. OS=D.m.		OAT_DROME	0	GLOS_OAT.1.2	GO:0005759	C	UniProtKB	mitochondrial matrix
[BBH] OAT_DROME (sp|Q9VW26) Ornithine aminotransferase, mitoch. OS=D.m.		OAT_DROME	0	GLOS_OAT.1.2	GO:0008483	M	.	transaminase activity
[BBH] OAT_DROME (sp|Q9VW26) Ornithine aminotransferase, mitoch. OS=D.m.		OAT_DROME	0	GLOS_OAT.1.2	GO:0016769	M	.	transferase activity, transferring nitrogenous groups
[BBH] OAT_DROME (sp|Q9VW26) Ornithine aminotransferase, mitoch. OS=D.m.		OAT_DROME	0	GLOS_OAT.1.2	GO:0004587	M	UniProtKB	ornithine-oxo-acid transaminase activity
[BBH] OAT_DROME (sp|Q9VW26) Ornithine aminotransferase, mitoch. OS=D.m.		OAT_DROME	0	GLOS_OAT.1.2	GO:0048037	M	.	cofactor binding
[BBH] OAT_DROME (sp|Q9VW26) Ornithine aminotransferase, mitoch. OS=D.m.		OAT_DROME	0	GLOS_OAT.1.2	GO:0030170	M	InterPro	pyridoxal phosphate binding
[BBH] OAT_DROME (sp|Q9VW26) Ornithine aminotransferase, mitoch. OS=D.m.		OAT_DROME	0	GLOS_OAT.1.2	GO:0006561	B	.	proline biosynthetic process
[BBH] OAT_DROME (sp|Q9VW26) Ornithine aminotransferase, mitoch. OS=D.m.		OAT_DROME	0	GLOS_OAT.1.2	GO:0006560	B	.	proline metabolic process
[BBH] OAT_DROME (sp|Q9VW26) Ornithine aminotransferase, mitoch. OS=D.m.		OAT_DROME	0	GLOS_OAT.1.2	GO:0009064	B	.	glutamine family amino acid metabolic process
[BBH] OAT_DROME (sp|Q9VW26) Ornithine aminotransferase, mitoch. OS=D.m.		OAT_DROME	0	GLOS_OAT.1.2	GO:0009084	B	.	glutamine family amino acid biosynthetic process
[BBH] OAT_DROME (sp|Q9VW26) Ornithine aminotransferase, mitoch. OS=D.m.		OAT_DROME	0	GLOS_OAT.1.2	GO:0055129	B	UniProtKB-U	L-proline biosynthetic process
[BBH] OAT_DROME (sp|Q9VW26) Ornithine aminotransferase, mitoch. OS=D.m.		OAT_DROME	0	GLOS_OAT.1.2	GO:0022008	B	FlyBase	neurogenesis
[BBH] OAT_DROME (sp|Q9VW26) Ornithine aminotransferase, mitoch. OS=D.m.		OAT_DROME	0	GLOS_OAT.1.2	GO:0006591	B	UniProtKB	ornithine metabolic process
PDE6_DROVI (sp|B4LVU6) cGMP-specific 3',5'-cyclic phosphodiesterase D.m.		PDE6_DROVI	1,00E-117	GLOS_PDE6.1.2	GO:0016020	C	UniProtKB	membrane
PDE6_DROVI (sp|B4LVU6) cGMP-specific 3',5'-cyclic phosphodiesterase D.m.		PDE6_DROVI	1,00E-117	GLOS_PDE6.1.2	GO:0005886	C	UniProtKB-S	plasma membrane
PDE6_DROVI (sp|B4LVU6) cGMP-specific 3',5'-cyclic phosphodiesterase D.m.		PDE6_DROVI	1,00E-117	GLOS_PDE6.1.2	GO:0004114	M	.	3',5'-cyclic-nucleotide phosphodiesterase activity
PDE6_DROVI (sp|B4LVU6) cGMP-specific 3',5'-cyclic phosphodiesterase D.m.		PDE6_DROVI	1,00E-117	GLOS_PDE6.1.2	GO:0004112	M	.	cyclic-nucleotide phosphodiesterase activity
PDE6_DROVI (sp|B4LVU6) cGMP-specific 3',5'-cyclic phosphodiesterase D.m.		PDE6_DROVI	1,00E-117	GLOS_PDE6.1.2	GO:0008081	M	.	phosphoric diester hydrolase activity
PDE6_DROVI (sp|B4LVU6) cGMP-specific 3',5'-cyclic phosphodiesterase D.m.		PDE6_DROVI	1,00E-117	GLOS_PDE6.1.2	GO:0042578	M	.	phosphoric ester hydrolase activity
PDE6_DROVI (sp|B4LVU6) cGMP-specific 3',5'-cyclic phosphodiesterase D.m.		PDE6_DROVI	1,00E-117	GLOS_PDE6.1.2	GO:0016788	M	.	hydrolase activity, acting on ester bonds
PDE6_DROVI (sp|B4LVU6) cGMP-specific 3',5'-cyclic phosphodiesterase D.m.		PDE6_DROVI	1,00E-117	GLOS_PDE6.1.2	GO:0047555	M	UniProtKB	3',5'-cyclic-GMP phosphodiesterase activity
PDE6_DROVI (sp|B4LVU6) cGMP-specific 3',5'-cyclic phosphodiesterase D.m.		PDE6_DROVI	1,00E-117	GLOS_PDE6.1.2	GO:0046872	M	UniProtKB-K	metal ion binding
PDE6_DROVI (sp|B4LVU6) cGMP-specific 3',5'-cyclic phosphodiesterase D.m.		PDE6_DROVI	1,00E-117	GLOS_PDE6.1.2	GO:0009187	B	.	cyclic nucleotide metabolic process
PDE6_DROVI (sp|B4LVU6) cGMP-specific 3',5'-cyclic phosphodiesterase D.m.		PDE6_DROVI	1,00E-117	GLOS_PDE6.1.2	GO:0046068	B	UniProtKB	cGMP metabolic process
PDE6_DROVI (sp|B4LVU6) cGMP-specific 3',5'-cyclic phosphodiesterase D.m.		PDE6_DROVI	1,00E-117	GLOS_PDE6.1.2	GO:0007165	B	InterPro	signal transduction
[BBH] PNO1_DROPS (sp|Q29IG6) RNA-binding prot pno1 OS=D. p. pseudoobs.		PNO1_DROPS	1,00E-102	GLOS_PNO1.1.1	GO:0005730	C	UniProtKB	nucleolus
[BBH] PNO1_DROPS (sp|Q29IG6) RNA-binding prot pno1 OS=D. p. pseudoobs.		PNO1_DROPS	1,00E-102	GLOS_PNO1.1.1	GO:0003723	M	UniProtKB-K	RNA binding
[BBH] RALA_DROME (sp|P48555) Ras-related protein Ral-a OS=D.m.		RALA_DROME	1,00E-139	GLOS_RALA.1.1	GO:0005886	C	FlyBase	plasma membrane
[BBH] RALA_DROME (sp|P48555) Ras-related protein Ral-a OS=D.m.		RALA_DROME	1,00E-139	GLOS_RALA.1.1	GO:0005525	M	UniProtKB-K	GTP binding
[BBH] RALA_DROME (sp|P48555) Ras-related protein Ral-a OS=D.m.		RALA_DROME	1,00E-139	GLOS_RALA.1.1	GO:0003924	M	FlyBase	GTPase activity
[BBH] RALA_DROME (sp|P48555) Ras-related protein Ral-a OS=D.m.		RALA_DROME	1,00E-139	GLOS_RALA.1.1	GO:0007297	B	.	ovarian follicle cell migration
[BBH] RALA_DROME (sp|P48555) Ras-related protein Ral-a OS=D.m.		RALA_DROME	1,00E-139	GLOS_RALA.1.1	GO:0010631	B	.	epithelial cell migration
[BBH] RALA_DROME (sp|P48555) Ras-related protein Ral-a OS=D.m.		RALA_DROME	1,00E-139	GLOS_RALA.1.1	GO:0001667	B	.	ameboidal cell migration
[BBH] RALA_DROME (sp|P48555) Ras-related protein Ral-a OS=D.m.		RALA_DROME	1,00E-139	GLOS_RALA.1.1	GO:0090132	B	.	epithelium migration
[BBH] RALA_DROME (sp|P48555) Ras-related protein Ral-a OS=D.m.		RALA_DROME	1,00E-139	GLOS_RALA.1.1	GO:0090130	B	.	tissue migration
[BBH] RALA_DROME (sp|P48555) Ras-related protein Ral-a OS=D.m.		RALA_DROME	1,00E-139	GLOS_RALA.1.1	GO:0030707	B	.	ovarian follicle cell development
[BBH] RALA_DROME (sp|P48555) Ras-related protein Ral-a OS=D.m.		RALA_DROME	1,00E-139	GLOS_RALA.1.1	GO:0048477	B	.	oogenesis
[BBH] RALA_DROME (sp|P48555) Ras-related protein Ral-a OS=D.m.		RALA_DROME	1,00E-139	GLOS_RALA.1.1	GO:0007298	B	FlyBase	border follicle cell migration
[BBH] RALA_DROME (sp|P48555) Ras-related protein Ral-a OS=D.m.		RALA_DROME	1,00E-139	GLOS_RALA.1.1	GO:0016331	B	.	morphogenesis of embryonic epithelium
[BBH] RALA_DROME (sp|P48555) Ras-related protein Ral-a OS=D.m.		RALA_DROME	1,00E-139	GLOS_RALA.1.1	GO:0002009	B	.	morphogenesis of an epithelium

[BBH] RALA_DROME (sp|P48555) Ras-related protein Ral-a OS=D.m.	RALA_DROME	1,00E-139	GLOS_RALA.1.1	GO:0048729	B	.	tissue morphogenesis
[BBH] RALA_DROME (sp|P48555) Ras-related protein Ral-a OS=D.m.	RALA_DROME	1,00E-139	GLOS_RALA.1.1	GO:0048598	B	.	embryonic morphogenesis
[BBH] RALA_DROME (sp|P48555) Ras-related protein Ral-a OS=D.m.	RALA_DROME	1,00E-139	GLOS_RALA.1.1	GO:0001700	B	.	embryonic development via the syncytial blastoderm
[BBH] RALA_DROME (sp|P48555) Ras-related protein Ral-a OS=D.m.	RALA_DROME	1,00E-139	GLOS_RALA.1.1	GO:0007391	B	FlyBase	dorsal closure
[BBH] RALA_DROME (sp|P48555) Ras-related protein Ral-a OS=D.m.	RALA_DROME	1,00E-139	GLOS_RALA.1.1	GO:0045087	B	FlyBase	innate immune response
[BBH] RALA_DROME (sp|P48555) Ras-related protein Ral-a OS=D.m.	RALA_DROME	1,00E-139	GLOS_RALA.1.1	GO:0032873	B	.	negative regulation of stress-activated MAPK cascade
[BBH] RALA_DROME (sp|P48555) Ras-related protein Ral-a OS=D.m.	RALA_DROME	1,00E-139	GLOS_RALA.1.1	GO:0032872	B	.	regulation of stress-activated MAPK cascade
[BBH] RALA_DROME (sp|P48555) Ras-related protein Ral-a OS=D.m.	RALA_DROME	1,00E-139	GLOS_RALA.1.1	GO:0043408	B	.	regulation of MAPK cascade
[BBH] RALA_DROME (sp|P48555) Ras-related protein Ral-a OS=D.m.	RALA_DROME	1,00E-139	GLOS_RALA.1.1	GO:0010627	B	.	regulation of intracellular protein kinase cascade
[BBH] RALA_DROME (sp|P48555) Ras-related protein Ral-a OS=D.m.	RALA_DROME	1,00E-139	GLOS_RALA.1.1	GO:0070302	B	.	regulation of stress-activated protein kinase signaling cascade
[BBH] RALA_DROME (sp|P48555) Ras-related protein Ral-a OS=D.m.	RALA_DROME	1,00E-139	GLOS_RALA.1.1	GO:0080135	B	.	regulation of cellular response to stress
[BBH] RALA_DROME (sp|P48555) Ras-related protein Ral-a OS=D.m.	RALA_DROME	1,00E-139	GLOS_RALA.1.1	GO:0080134	B	.	regulation of response to stress
[BBH] RALA_DROME (sp|P48555) Ras-related protein Ral-a OS=D.m.	RALA_DROME	1,00E-139	GLOS_RALA.1.1	GO:0043409	B	.	negative regulation of MAPK cascade
[BBH] RALA_DROME (sp|P48555) Ras-related protein Ral-a OS=D.m.	RALA_DROME	1,00E-139	GLOS_RALA.1.1	GO:0010741	B	.	negative regulation of intracellular protein kinase cascade
[BBH] RALA_DROME (sp|P48555) Ras-related protein Ral-a OS=D.m.	RALA_DROME	1,00E-139	GLOS_RALA.1.1	GO:0070303	B	.	negative regulation of stress-activated protein kinase signaling cascade
[BBH] RALA_DROME (sp|P48555) Ras-related protein Ral-a OS=D.m.	RALA_DROME	1,00E-139	GLOS_RALA.1.1	GO:0046328	B	.	regulation of JNK cascade
[BBH] RALA_DROME (sp|P48555) Ras-related protein Ral-a OS=D.m.	RALA_DROME	1,00E-139	GLOS_RALA.1.1	GO:0046329	B	FlyBase	negative regulation of JNK cascade
[BBH] RALA_DROME (sp|P48555) Ras-related protein Ral-a OS=D.m.	RALA_DROME	1,00E-139	GLOS_RALA.1.1	GO:0001752	B	.	compound eye photoreceptor fate commitment
[BBH] RALA_DROME (sp|P48555) Ras-related protein Ral-a OS=D.m.	RALA_DROME	1,00E-139	GLOS_RALA.1.1	GO:0042706	B	.	eye photoreceptor cell fate commitment
[BBH] RALA_DROME (sp|P48555) Ras-related protein Ral-a OS=D.m.	RALA_DROME	1,00E-139	GLOS_RALA.1.1	GO:0046552	B	.	photoreceptor cell fate commitment
[BBH] RALA_DROME (sp|P48555) Ras-related protein Ral-a OS=D.m.	RALA_DROME	1,00E-139	GLOS_RALA.1.1	GO:0048663	B	.	neuron fate commitment
[BBH] RALA_DROME (sp|P48555) Ras-related protein Ral-a OS=D.m.	RALA_DROME	1,00E-139	GLOS_RALA.1.1	GO:0045165	B	.	cell fate commitment
[BBH] RALA_DROME (sp|P48555) Ras-related protein Ral-a OS=D.m.	RALA_DROME	1,00E-139	GLOS_RALA.1.1	GO:0001751	B	.	compound eye photoreceptor cell differentiation
[BBH] RALA_DROME (sp|P48555) Ras-related protein Ral-a OS=D.m.	RALA_DROME	1,00E-139	GLOS_RALA.1.1	GO:0001745	B	.	compound eye morphogenesis
[BBH] RALA_DROME (sp|P48555) Ras-related protein Ral-a OS=D.m.	RALA_DROME	1,00E-139	GLOS_RALA.1.1	GO:0048749	B	.	compound eye development
[BBH] RALA_DROME (sp|P48555) Ras-related protein Ral-a OS=D.m.	RALA_DROME	1,00E-139	GLOS_RALA.1.1	GO:0042067	B	.	establishment of ommatidial planar polarity
[BBH] RALA_DROME (sp|P48555) Ras-related protein Ral-a OS=D.m.	RALA_DROME	1,00E-139	GLOS_RALA.1.1	GO:0001736	B	.	establishment of planar polarity
[BBH] RALA_DROME (sp|P48555) Ras-related protein Ral-a OS=D.m.	RALA_DROME	1,00E-139	GLOS_RALA.1.1	GO:0007164	B	.	establishment of tissue polarity
[BBH] RALA_DROME (sp|P48555) Ras-related protein Ral-a OS=D.m.	RALA_DROME	1,00E-139	GLOS_RALA.1.1	GO:0001738	B	.	morphogenesis of a polarized epithelium
[BBH] RALA_DROME (sp|P48555) Ras-related protein Ral-a OS=D.m.	RALA_DROME	1,00E-139	GLOS_RALA.1.1	GO:0007163	B	.	establishment or maintenance of cell polarity
[BBH] RALA_DROME (sp|P48555) Ras-related protein Ral-a OS=D.m.	RALA_DROME	1,00E-139	GLOS_RALA.1.1	GO:0048056	B	.	R3/R4 cell differentiation
[BBH] RALA_DROME (sp|P48555) Ras-related protein Ral-a OS=D.m.	RALA_DROME	1,00E-139	GLOS_RALA.1.1	GO:0007464	B	FlyBase	R3/R4 cell fate commitment
[BBH] RALA_DROME (sp|P48555) Ras-related protein Ral-a OS=D.m.	RALA_DROME	1,00E-139	GLOS_RALA.1.1	GO:0007264	B	.	small GTPase mediated signal transduction
[BBH] RALA_DROME (sp|P48555) Ras-related protein Ral-a OS=D.m.	RALA_DROME	1,00E-139	GLOS_RALA.1.1	GO:0007265	B	FlyBase	Ras protein signal transduction
[BBH] RALA_DROME (sp|P48555) Ras-related protein Ral-a OS=D.m.	RALA_DROME	1,00E-139	GLOS_RALA.1.1	GO:0022604	B	FlyBase	regulation of cell morphogenesis
[BBH] RALA_DROME (sp|P48555) Ras-related protein Ral-a OS=D.m.	RALA_DROME	1,00E-139	GLOS_RALA.1.1	GO:0008593	B	FlyBase	regulation of Notch signaling pathway
RL10_DROME (sp|O61231) 60S ribosomal protein L10 OS=D.m.	RL10_DROME	5,00E-98	GLOS_RL10.1.9	GO:0043025	C	FlyBase	neuronal cell body
RL10_DROME (sp|O61231) 60S ribosomal protein L10 OS=D.m.	RL10_DROME	5,00E-98	GLOS_RL10.1.9	GO:0030529	C	.	ribonucleoprotein complex
RL10_DROME (sp|O61231) 60S ribosomal protein L10 OS=D.m.	RL10_DROME	5,00E-98	GLOS_RL10.1.9	GO:0005840	C	FlyBase	ribosome
RL10_DROME (sp|O61231) 60S ribosomal protein L10 OS=D.m.	RL10_DROME	5,00E-98	GLOS_RL10.1.9	GO:0005198	M	.	structural molecule activity
RL10_DROME (sp|O61231) 60S ribosomal protein L10 OS=D.m.	RL10_DROME	5,00E-98	GLOS_RL10.1.9	GO:0003735	M	FlyBase	structural constituent of ribosome
RL10_DROME (sp|O61231) 60S ribosomal protein L10 OS=D.m.	RL10_DROME	5,00E-98	GLOS_RL10.1.9	GO:0051297	B	.	centrosome organization
RL10_DROME (sp|O61231) 60S ribosomal protein L10 OS=D.m.	RL10_DROME	5,00E-98	GLOS_RL10.1.9	GO:0031023	B	.	microtubule organizing center organization
RL10_DROME (sp|O61231) 60S ribosomal protein L10 OS=D.m.	RL10_DROME	5,00E-98	GLOS_RL10.1.9	GO:0007098	B	.	centrosome cycle
RL10_DROME (sp|O61231) 60S ribosomal protein L10 OS=D.m.	RL10_DROME	5,00E-98	GLOS_RL10.1.9	GO:0051298	B	FlyBase	centrosome duplication
RL10_DROME (sp|O61231) 60S ribosomal protein L10 OS=D.m.	RL10_DROME	5,00E-98	GLOS_RL10.1.9	GO:0051231	B	.	spindle elongation
RL10_DROME (sp|O61231) 60S ribosomal protein L10 OS=D.m.	RL10_DROME	5,00E-98	GLOS_RL10.1.9	GO:0007051	B	.	spindle organization
RL10_DROME (sp|O61231) 60S ribosomal protein L10 OS=D.m.	RL10_DROME	5,00E-98	GLOS_RL10.1.9	GO:0007052	B	.	mitotic spindle organization
RL10_DROME (sp|O61231) 60S ribosomal protein L10 OS=D.m.	RL10_DROME	5,00E-98	GLOS_RL10.1.9	GO:0000022	B	FlyBase	mitotic spindle elongation
RL10_DROME (sp|O61231) 60S ribosomal protein L10 OS=D.m.	RL10_DROME	5,00E-98	GLOS_RL10.1.9	GO:0006412	B	InterPro	translation
RL11_DROME (sp|P46222) 60S ribosomal protein L11 OS=D.m.	RL11_DROME	1,00E-116	GLOS_RL11.3.4	GO:0005694	C	.	chromosome
RL11_DROME (sp|P46222) 60S ribosomal protein L11 OS=D.m.	RL11_DROME	1,00E-116	GLOS_RL11.3.4	GO:0000228	C	FlyBase	nuclear chromosome
RL11_DROME (sp|P46222) 60S ribosomal protein L11 OS=D.m.	RL11_DROME	1,00E-116	GLOS_RL11.3.4	GO:0005730	C	FlyBase	nucleolus
RL11_DROME (sp|P46222) 60S ribosomal protein L11 OS=D.m.	RL11_DROME	1,00E-116	GLOS_RL11.3.4	GO:0044427	C	.	chromosomal part

RL11_DROME (sp|P46222) 60S ribosomal protein L11 OS=D.m.	RL11_DROME	1,00E-116	GLOS_RL11.3.4	GO:0005700	C	.	polytene chromosome
RL11_DROME (sp|P46222) 60S ribosomal protein L11 OS=D.m.	RL11_DROME	1,00E-116	GLOS_RL11.3.4	GO:0005703	C	FlyBase	polytene chromosome puff
RL11_DROME (sp|P46222) 60S ribosomal protein L11 OS=D.m.	RL11_DROME	1,00E-116	GLOS_RL11.3.4	GO:0005840	C	FlyBase	ribosome
RL11_DROME (sp|P46222) 60S ribosomal protein L11 OS=D.m.	RL11_DROME	1,00E-116	GLOS_RL11.3.4	GO:0003723	M	.	RNA binding
RL11_DROME (sp|P46222) 60S ribosomal protein L11 OS=D.m.	RL11_DROME	1,00E-116	GLOS_RL11.3.4	GO:0019843	M	UniProtKB-k	rRNA binding
RL11_DROME (sp|P46222) 60S ribosomal protein L11 OS=D.m.	RL11_DROME	1,00E-116	GLOS_RL11.3.4	GO:0003735	M	FlyBase	structural constituent of ribosome
RL11_DROME (sp|P46222) 60S ribosomal protein L11 OS=D.m.	RL11_DROME	1,00E-116	GLOS_RL11.3.4	GO:0051298	B	FlyBase	centrosome duplication
RL11_DROME (sp|P46222) 60S ribosomal protein L11 OS=D.m.	RL11_DROME	1,00E-116	GLOS_RL11.3.4	GO:0000022	B	FlyBase	mitotic spindle elongation
RL11_DROME (sp|P46222) 60S ribosomal protein L11 OS=D.m.	RL11_DROME	1,00E-116	GLOS_RL11.3.4	GO:0006412	B	InterPro	translation
RL13A_DROME (sp|Q9VNE9) 60S ribosomal protein L13a OS=D.m.	RL13A_DROME	3,00E-62	GLOS_RL13A.4.1	GO:0044391	C	.	ribosomal subunit
RL13A_DROME (sp|Q9VNE9) 60S ribosomal protein L13a OS=D.m.	RL13A_DROME	3,00E-62	GLOS_RL13A.4.1	GO:0005840	C	.	ribosome
RL13A_DROME (sp|Q9VNE9) 60S ribosomal protein L13a OS=D.m.	RL13A_DROME	3,00E-62	GLOS_RL13A.4.1	GO:0015934	C	InterPro	large ribosomal subunit
RL13A_DROME (sp|Q9VNE9) 60S ribosomal protein L13a OS=D.m.	RL13A_DROME	3,00E-62	GLOS_RL13A.4.1	GO:0005840	C	FlyBase	ribosome
RL13A_DROME (sp|Q9VNE9) 60S ribosomal protein L13a OS=D.m.	RL13A_DROME	3,00E-62	GLOS_RL13A.4.1	GO:0003735	M	FlyBase	structural constituent of ribosome
RL13A_DROME (sp|Q9VNE9) 60S ribosomal protein L13a OS=D.m.	RL13A_DROME	3,00E-62	GLOS_RL13A.4.1	GO:0051298	B	FlyBase	centrosome duplication
RL13A_DROME (sp|Q9VNE9) 60S ribosomal protein L13a OS=D.m.	RL13A_DROME	3,00E-62	GLOS_RL13A.4.1	GO:0008407	B	FlyBase	chaeta morphogenesis
RL13A_DROME (sp|Q9VNE9) 60S ribosomal protein L13a OS=D.m.	RL13A_DROME	3,00E-62	GLOS_RL13A.4.1	GO:0007219	B	FlyBase	Notch signaling pathway
RL13A_DROME (sp|Q9VNE9) 60S ribosomal protein L13a OS=D.m.	RL13A_DROME	3,00E-62	GLOS_RL13A.4.1	GO:0006412	B	InterPro	translation
RL15_DROME (sp|O17445) 60S ribosomal protein L15 OS=D.m.	RL15_DROME	5,00E-86	GLOS_RL15.1.2	GO:0005840	C	UniProtKB-k	ribosome
RL15_DROME (sp|O17445) 60S ribosomal protein L15 OS=D.m.	RL15_DROME	5,00E-86	GLOS_RL15.1.2	GO:0003735	M	InterPro	structural constituent of ribosome
RL15_DROME (sp|O17445) 60S ribosomal protein L15 OS=D.m.	RL15_DROME	5,00E-86	GLOS_RL15.1.2	GO:0006412	B	InterPro	translation
RL18A_DROME (sp|P41093) 60S ribosomal prot L18a OS=D.m.	RL18A_DROME	6,00E-38	GLOS_RL18A.1.5	GO:0005840	C	UniProtKB-k	ribosome
RL18A_DROME (sp|P41093) 60S ribosomal prot L18a OS=D.m.	RL18A_DROME	6,00E-38	GLOS_RL18A.1.5	GO:0003735	M	InterPro	structural constituent of ribosome
RL18A_DROME (sp|P41093) 60S ribosomal prot L18a OS=D.m.	RL18A_DROME	6,00E-38	GLOS_RL18A.1.5	GO:0051298	B	FlyBase	centrosome duplication
RL18A_DROME (sp|P41093) 60S ribosomal prot L18a OS=D.m.	RL18A_DROME	6,00E-38	GLOS_RL18A.1.5	GO:0000022	B	FlyBase	mitotic spindle elongation
RL18A_DROME (sp|P41093) 60S ribosomal prot L18a OS=D.m.	RL18A_DROME	6,00E-38	GLOS_RL18A.1.5	GO:0006412	B	InterPro	translation
RL19_DROME (sp|P36241) 60S ribosomal protein L19 OS=D.m.	RL19_DROME	3,00E-76	GLOS_RL19.4.9	GO:0005840	C	FlyBase	ribosome
RL19_DROME (sp|P36241) 60S ribosomal protein L19 OS=D.m.	RL19_DROME	3,00E-76	GLOS_RL19.4.9	GO:0003735	M	FlyBase	structural constituent of ribosome
RL19_DROME (sp|P36241) 60S ribosomal protein L19 OS=D.m.	RL19_DROME	3,00E-76	GLOS_RL19.4.9	GO:0051298	B	FlyBase	centrosome duplication
RL19_DROME (sp|P36241) 60S ribosomal protein L19 OS=D.m.	RL19_DROME	3,00E-76	GLOS_RL19.4.9	GO:0000022	B	FlyBase	mitotic spindle elongation
RL19_DROME (sp|P36241) 60S ribosomal protein L19 OS=D.m.	RL19_DROME	3,00E-76	GLOS_RL19.4.9	GO:0006412	B	InterPro	translation
RL371_DROME (sp|Q9VXX8) Prob 60S ribosomal prot L37-A OS=D.m.	RL371_DROME	2,00E-42	GLOS_RL371.4.8	GO:0005840	C	UniProtKB-k	ribosome
RL371_DROME (sp|Q9VXX8) Prob 60S ribosomal prot L37-A OS=D.m.	RL371_DROME	2,00E-42	GLOS_RL371.4.8	GO:0046872	M	UniProtKB-k	metal ion binding
RL371_DROME (sp|Q9VXX8) Prob 60S ribosomal prot L37-A OS=D.m.	RL371_DROME	2,00E-42	GLOS_RL371.4.8	GO:0019843	M	UniProtKB-k	rRNA binding
RL371_DROME (sp|Q9VXX8) Prob 60S ribosomal prot L37-A OS=D.m.	RL371_DROME	2,00E-42	GLOS_RL371.4.8	GO:0003735	M	InterPro	structural constituent of ribosome
RL371_DROME (sp|Q9VXX8) Prob 60S ribosomal prot L37-A OS=D.m.	RL371_DROME	2,00E-42	GLOS_RL371.4.8	GO:0006412	B	InterPro	translation
RL3_DROME (sp|O16797) 60S ribosomal protein L3 OS=D.m.	RL3_DROME	1,00E-134	GLOS_RL3.4.5	GO:0005840	C	UniProtKB-k	ribosome
RL3_DROME (sp|O16797) 60S ribosomal protein L3 OS=D.m.	RL3_DROME	1,00E-134	GLOS_RL3.4.5	GO:0003735	M	InterPro	structural constituent of ribosome
RL3_DROME (sp|O16797) 60S ribosomal protein L3 OS=D.m.	RL3_DROME	1,00E-134	GLOS_RL3.4.5	GO:0051298	B	FlyBase	centrosome duplication
RL3_DROME (sp|O16797) 60S ribosomal protein L3 OS=D.m.	RL3_DROME	1,00E-134	GLOS_RL3.4.5	GO:0000022	B	FlyBase	mitotic spindle elongation
RL3_DROME (sp|O16797) 60S ribosomal protein L3 OS=D.m.	RL3_DROME	1,00E-134	GLOS_RL3.4.5	GO:0006412	B	InterPro	translation
RS12_DROME (sp|P80455) 40S ribosomal prot S12 OS=D.m.	RS12_DROME	1,00E-27	GLOS_RS12.2.4	GO:0005840	C	UniProtKB-k	ribosome
RS12_DROME (sp|P80455) 40S ribosomal prot S12 OS=D.m.	RS12_DROME	1,00E-27	GLOS_RS12.2.4	GO:0003735	M	InterPro	structural constituent of ribosome
RS12_DROME (sp|P80455) 40S ribosomal prot S12 OS=D.m.	RS12_DROME	1,00E-27	GLOS_RS12.2.4	GO:0006412	B	InterPro	translation
RS20_DROME (sp|P55828) 40S ribosomal prot S20 OS=D.m.	RS20_DROME	3,00E-70	GLOS_RS20.8.14	GO:0015935	C	UniProtKB	small ribosomal subunit
RS20_DROME (sp|P55828) 40S ribosomal prot S20 OS=D.m.	RS20_DROME	3,00E-70	GLOS_RS20.8.14	GO:0003723	M	InterPro	RNA binding
RS20_DROME (sp|P55828) 40S ribosomal prot S20 OS=D.m.	RS20_DROME	3,00E-70	GLOS_RS20.8.14	GO:0003735	M	UniProtKB	structural constituent of ribosome
RS20_DROME (sp|P55828) 40S ribosomal prot S20 OS=D.m.	RS20_DROME	3,00E-70	GLOS_RS20.8.14	GO:0006412	B	UniProtKB	translation
RS29_DROME (sp|Q9VH69) 40S ribosomal prot S29 OS=D.m.	RS29_DROME	1,00E-27	GLOS_RS29.8.12	GO:0005840	C	UniProtKB-k	ribosome
RS29_DROME (sp|Q9VH69) 40S ribosomal prot S29 OS=D.m.	RS29_DROME	1,00E-27	GLOS_RS29.8.12	GO:0046872	M	UniProtKB-k	metal ion binding
RS29_DROME (sp|Q9VH69) 40S ribosomal prot S29 OS=D.m.	RS29_DROME	1,00E-27	GLOS_RS29.8.12	GO:0003735	M	InterPro	structural constituent of ribosome
RS29_DROME (sp|Q9VH69) 40S ribosomal prot S29 OS=D.m.	RS29_DROME	1,00E-27	GLOS_RS29.8.12	GO:0048666	B	FlyBase	neuron development
RS29_DROME (sp|Q9VH69) 40S ribosomal prot S29 OS=D.m.	RS29_DROME	1,00E-27	GLOS_RS29.8.12	GO:0006412	B	InterPro	translation
[BBH] RS8_DROME (sp|Q8MLY8) 40S ribosomal prot S8 OS=D.m.	RS8_DROME	1,00E-125	GLOS_RS8.1.3	GO:0015935	C	.	small ribosomal subunit

[BBH] RS8_DROME (sp|Q8MLY8) 40S ribosomal prot S8 OS=D.m.	RS8_DROME	1,00E-125	GLOS_RS8.1.3	GO:0044445	C	.	cytosolic part
[BBH] RS8_DROME (sp|Q8MLY8) 40S ribosomal prot S8 OS=D.m.	RS8_DROME	1,00E-125	GLOS_RS8.1.3	GO:0005829	C	.	cytosol
[BBH] RS8_DROME (sp|Q8MLY8) 40S ribosomal prot S8 OS=D.m.	RS8_DROME	1,00E-125	GLOS_RS8.1.3	GO:0022626	C	.	cytosolic ribosome
[BBH] RS8_DROME (sp|Q8MLY8) 40S ribosomal prot S8 OS=D.m.	RS8_DROME	1,00E-125	GLOS_RS8.1.3	GO:0022627	C	RefGenome	cytosolic small ribosomal subunit
[BBH] RS8_DROME (sp|Q8MLY8) 40S ribosomal prot S8 OS=D.m.	RS8_DROME	1,00E-125	GLOS_RS8.1.3	GO:0003735	M	FlyBase	structural constituent of ribosome
[BBH] RS8_DROME (sp|Q8MLY8) 40S ribosomal prot S8 OS=D.m.	RS8_DROME	1,00E-125	GLOS_RS8.1.3	GO:0030490	B	.	maturation of SSU-rRNA
[BBH] RS8_DROME (sp|Q8MLY8) 40S ribosomal prot S8 OS=D.m.	RS8_DROME	1,00E-125	GLOS_RS8.1.3	GO:0006364	B	.	rRNA processing
[BBH] RS8_DROME (sp|Q8MLY8) 40S ribosomal prot S8 OS=D.m.	RS8_DROME	1,00E-125	GLOS_RS8.1.3	GO:0016072	B	.	rRNA metabolic process
[BBH] RS8_DROME (sp|Q8MLY8) 40S ribosomal prot S8 OS=D.m.	RS8_DROME	1,00E-125	GLOS_RS8.1.3	GO:0034660	B	.	ncRNA metabolic process
[BBH] RS8_DROME (sp|Q8MLY8) 40S ribosomal prot S8 OS=D.m.	RS8_DROME	1,00E-125	GLOS_RS8.1.3	GO:0034470	B	.	ncRNA processing
[BBH] RS8_DROME (sp|Q8MLY8) 40S ribosomal prot S8 OS=D.m.	RS8_DROME	1,00E-125	GLOS_RS8.1.3	GO:0006396	B	.	RNA processing
[BBH] RS8_DROME (sp|Q8MLY8) 40S ribosomal prot S8 OS=D.m.	RS8_DROME	1,00E-125	GLOS_RS8.1.3	GO:0042254	B	.	ribosome biogenesis
[BBH] RS8_DROME (sp|Q8MLY8) 40S ribosomal prot S8 OS=D.m.	RS8_DROME	1,00E-125	GLOS_RS8.1.3	GO:0022613	B	.	ribonucleoprotein complex biogenesis
[BBH] RS8_DROME (sp|Q8MLY8) 40S ribosomal prot S8 OS=D.m.	RS8_DROME	1,00E-125	GLOS_RS8.1.3	GO:0042274	B	.	ribosomal small subunit biogenesis
[BBH] RS8_DROME (sp|Q8MLY8) 40S ribosomal prot S8 OS=D.m.	RS8_DROME	1,00E-125	GLOS_RS8.1.3	GO:0000462	B	RefGenome	maturation of SSU-rRNA from tricistronic rRNA transcript (SSU-rRNA, 5.8S rRNA, LSU-rRNA)
[BBH] RS8_DROME (sp|Q8MLY8) 40S ribosomal prot S8 OS=D.m.	RS8_DROME	1,00E-125	GLOS_RS8.1.3	GO:0022008	B	FlyBase	neurogenesis
[BBH] RS8_DROME (sp|Q8MLY8) 40S ribosomal prot S8 OS=D.m.	RS8_DROME	1,00E-125	GLOS_RS8.1.3	GO:0006412	B	.	translation
[BBH] RS8_DROME (sp|Q8MLY8) 40S ribosomal prot S8 OS=D.m.	RS8_DROME	1,00E-125	GLOS_RS8.1.3	GO:0006414	B	RefGenome	translational elongation
RTBS_DROME (sp|Q95SX7)Prob RNA-direct. DNA polymera. from transposon BS	RTBS_DROME	1,00E-29	GLOS_RTBS.1.3	GO:0003723	M	InterPro	RNA binding
RTBS_DROME (sp|Q95SX7)Prob RNA-direct. DNA polymera. from transposon BS	RTBS_DROME	1,00E-29	GLOS_RTBS.1.3	GO:0034061	M	.	DNA polymerase activity
RTBS_DROME (sp|Q95SX7)Prob RNA-direct. DNA polymera. from transposon BS	RTBS_DROME	1,00E-29	GLOS_RTBS.1.3	GO:0016779	M	.	nucleotidyltransferase activity
RTBS_DROME (sp|Q95SX7)Prob RNA-direct. DNA polymera. from transposon BS	RTBS_DROME	1,00E-29	GLOS_RTBS.1.3	GO:0003964	M	UniProtKB	RNA-directed DNA polymerase activity
RTBS_DROME (sp|Q95SX7)Prob RNA-direct. DNA polymera. from transposon BS	RTBS_DROME	1,00E-29	GLOS_RTBS.1.3	GO:0006278	B	InterPro	RNA-dependent DNA replication
RTBS_DROME (sp|Q95SX7)Prob RNA-direct. DNA polymera. from transposon BS	RTBS_DROME	1,00E-29	GLOS_RTBS.1.3	GO:0032196	B	.	transposition
RTBS_DROME (sp|Q95SX7)Prob RNA-direct. DNA polymera. from transposon BS	RTBS_DROME	1,00E-29	GLOS_RTBS.1.3	GO:0006313	B	UniProtKB	transposition, DNA-mediated
SLU7_CHICK (sp|Q5ZIG2) Pre-mRNA-splicing factor SLU7 OS=G. gallus	SLU7_CHICK	9,00E-12	GLOS_SLU7.5.13	GO:0005681	C	UniProtKB-k	spliceosomal complex
SLU7_CHICK (sp|Q5ZIG2) Pre-mRNA-splicing factor SLU7 OS=G. gallus	SLU7_CHICK	9,00E-12	GLOS_SLU7.5.13	GO:0003676	M	InterPro	nucleic acid binding
SLU7_CHICK (sp|Q5ZIG2) Pre-mRNA-splicing factor SLU7 OS=G. gallus	SLU7_CHICK	9,00E-12	GLOS_SLU7.5.13	GO:0008270	M	InterPro	zinc ion binding
SLU7_CHICK (sp|Q5ZIG2) Pre-mRNA-splicing factor SLU7 OS=G. gallus	SLU7_CHICK	9,00E-12	GLOS_SLU7.5.13	GO:0016071	B	.	mRNA metabolic process
SLU7_CHICK (sp|Q5ZIG2) Pre-mRNA-splicing factor SLU7 OS=G. gallus	SLU7_CHICK	9,00E-12	GLOS_SLU7.5.13	GO:0006397	B	UniProtKB-k	mRNA processing
SLU7_CHICK (sp|Q5ZIG2) Pre-mRNA-splicing factor SLU7 OS=G. gallus	SLU7_CHICK	9,00E-12	GLOS_SLU7.5.13	GO:0008380	B	UniProtKB-k	RNA splicing
BBH] SMCO4_DROME (sp|Q8SY72) Single-pass membrane and coiled-coil domain-containing protein 4 homolog OS=D. melanogaster	SMCO4_DROME	1,00E-20	GLOS_SMCO4.2.2	GO:0016021	C	UniProtKB-k	integral to membrane
SMDT1_DROME (sp|Q7JX57) Protein SMDT1 homolog, mitochondrial OS=D.m.	SMDT1_DROME	3,00E-20	GLOS_SMDT1.1.1	GO:0016021	C	UniProtKB-k	integral to membrane
SMDT1_DROME (sp|Q7JX57) Protein SMDT1 homolog, mitochondrial OS=D.m.	SMDT1_DROME	3,00E-20	GLOS_SMDT1.1.1	GO:0005739	C	UniProtKB-S	mitochondrion
[BBH] SUI1_ANOGA (sp|P42678) Protein transl. fact. SUI1 homolog A. gambiae	SUI1_ANOGA	1,00E-68	GLOS_SUI1.1.2	GO:0008135	M	.	translation factor activity, nucleic acid binding
[BBH] SUI1_ANOGA (sp|P42678) Protein transl. fact. SUI1 homolog A. gambiae	SUI1_ANOGA	1,00E-68	GLOS_SUI1.1.2	GO:0003743	M	InterPro	translation initiation factor activity
[BBH] SUI1_ANOGA (sp|P42678) Protein transl. fact. SUI1 homolog A. gambiae	SUI1_ANOGA	1,00E-68	GLOS_SUI1.1.2	GO:0010608	B	.	posttranscriptional regulation of gene expression
[BBH] SUI1_ANOGA (sp|P42678) Protein transl. fact. SUI1 homolog A. gambiae	SUI1_ANOGA	1,00E-68	GLOS_SUI1.1.2	GO:0006417	B	UniProtKB-k	regulation of translation
[BBH] SXL_MUSDO (sp|O17310) Sex-lethal homolog OS=M. domestica	SXL_MUSDO	1,00E-163	GLOS_SXL.1.2	GO:0005634	C	UniProtKB-S	nucleus
[BBH] SXL_MUSDO (sp|O17310) Sex-lethal homolog OS=M. domestica	SXL_MUSDO	1,00E-163	GLOS_SXL.1.2	GO:0000166	M	InterPro	nucleotide binding
[BBH] SXL_MUSDO (sp|O17310) Sex-lethal homolog OS=M. domestica	SXL_MUSDO	1,00E-163	GLOS_SXL.1.2	GO:0003723	M	UniProtKB-k	RNA binding
[BBH] T23O_DROWI (sp|B4MSH7) Tryptophan 2,3-dioxygenase D. willistoni	T23O_DROWI	0	GLOS_T23O.1.1	GO:0020037	M	UniProtKB	heme binding
[BBH] T23O_DROWI (sp|B4MSH7) Tryptophan 2,3-dioxygenase D. willistoni	T23O_DROWI	0	GLOS_T23O.1.1	GO:0046872	M	UniProtKB-k	metal ion binding
[BBH] T23O_DROWI (sp|B4MSH7) Tryptophan 2,3-dioxygenase D. willistoni	T23O_DROWI	0	GLOS_T23O.1.1	GO:0016702	M	.	oxidoreductase activity, acting on single donors with incorporation of molecular oxygen, incorporation of two atoms of oxygen
[BBH] T23O_DROWI (sp|B4MSH7) Tryptophan 2,3-dioxygenase D. willistoni	T23O_DROWI	0	GLOS_T23O.1.1	GO:0016701	M	.	oxidored. Act., acting on single donors with incorporation of molecular oxygen
[BBH] T23O_DROWI (sp|B4MSH7) Tryptophan 2,3-dioxygenase D. willistoni	T23O_DROWI	0	GLOS_T23O.1.1	GO:0051213	M	.	dioxygenase activity
[BBH] T23O_DROWI (sp|B4MSH7) Tryptophan 2,3-dioxygenase D. willistoni	T23O_DROWI	0	GLOS_T23O.1.1	GO:0004833	M	UniProtKB	tryptophan 2,3-dioxygenase activity
[BBH] T23O_DROWI (sp|B4MSH7) Tryptophan 2,3-dioxygenase D. willistoni	T23O_DROWI	0	GLOS_T23O.1.1	GO:0006726	B	.	eye pigment biosynthetic process
[BBH] T23O_DROWI (sp|B4MSH7) Tryptophan 2,3-dioxygenase D. willistoni	T23O_DROWI	0	GLOS_T23O.1.1	GO:0042441	B	.	eye pigment metabolic process
[BBH] T23O_DROWI (sp|B4MSH7) Tryptophan 2,3-dioxygenase D. willistoni	T23O_DROWI	0	GLOS_T23O.1.1	GO:0043324	B	.	pigment metabolic process involved in developmental pigmentation
[BBH] T23O_DROWI (sp|B4MSH7) Tryptophan 2,3-dioxygenase D. willistoni	T23O_DROWI	0	GLOS_T23O.1.1	GO:0043474	B	.	pigment metabolic process involved in pigmentation

[BBH] T23O_DROWI (sp|B4MSH7) Tryptophan 2,3-dioxygenase D. willistoni	T23O_DROWI	0	GLOS_T23O.1.1	GO:0042440	B	.	pigment metabolic process
[BBH] T23O_DROWI (sp|B4MSH7) Tryptophan 2,3-dioxygenase D. willistoni	T23O_DROWI	0	GLOS_T23O.1.1	GO:0043473	B	.	pigmentation
[BBH] T23O_DROWI (sp|B4MSH7) Tryptophan 2,3-dioxygenase D. willistoni	T23O_DROWI	0	GLOS_T23O.1.1	GO:0048066	B	.	developmental pigmentation
[BBH] T23O_DROWI (sp|B4MSH7) Tryptophan 2,3-dioxygenase D. willistoni	T23O_DROWI	0	GLOS_T23O.1.1	GO:0048069	B	.	eye pigmentation
[BBH] T23O_DROWI (sp|B4MSH7) Tryptophan 2,3-dioxygenase D. willistoni	T23O_DROWI	0	GLOS_T23O.1.1	GO:0046148	B	.	pigment biosynthetic process
[BBH] T23O_DROWI (sp|B4MSH7) Tryptophan 2,3-dioxygenase D. willistoni	T23O_DROWI	0	GLOS_T23O.1.1	GO:0008055	B	.	ocellus pigment biosynthetic process
[BBH] T23O_DROWI (sp|B4MSH7) Tryptophan 2,3-dioxygenase D. willistoni	T23O_DROWI	0	GLOS_T23O.1.1	GO:0044550	B	.	secondary metabolite biosynthetic process
[BBH] T23O_DROWI (sp|B4MSH7) Tryptophan 2,3-dioxygenase D. willistoni	T23O_DROWI	0	GLOS_T23O.1.1	GO:0019748	B	.	secondary metabolic process
[BBH] T23O_DROWI (sp|B4MSH7) Tryptophan 2,3-dioxygenase D. willistoni	T23O_DROWI	0	GLOS_T23O.1.1	GO:0046158	B	.	ocellus pigment metabolic process
[BBH] T23O_DROWI (sp|B4MSH7) Tryptophan 2,3-dioxygenase D. willistoni	T23O_DROWI	0	GLOS_T23O.1.1	GO:0033060	B	.	ocellus pigmentation
[BBH] T23O_DROWI (sp|B4MSH7) Tryptophan 2,3-dioxygenase D. willistoni	T23O_DROWI	0	GLOS_T23O.1.1	GO:0046152	B	.	ommochrome metabolic process
[BBH] T23O_DROWI (sp|B4MSH7) Tryptophan 2,3-dioxygenase D. willistoni	T23O_DROWI	0	GLOS_T23O.1.1	GO:0006727	B	UniProtKB-U	ommochrome biosynthetic process
[BBH] T23O_DROWI (sp|B4MSH7) Tryptophan 2,3-dioxygenase D. willistoni	T23O_DROWI	0	GLOS_T23O.1.1	GO:0006569	B	.	tryptophan catabolic process
[BBH] T23O_DROWI (sp|B4MSH7) Tryptophan 2,3-dioxygenase D. willistoni	T23O_DROWI	0	GLOS_T23O.1.1	GO:0006568	B	.	tryptophan metabolic process
[BBH] T23O_DROWI (sp|B4MSH7) Tryptophan 2,3-dioxygenase D. willistoni	T23O_DROWI	0	GLOS_T23O.1.1	GO:0006586	B	.	indolalkylamine metabolic process
[BBH] T23O_DROWI (sp|B4MSH7) Tryptophan 2,3-dioxygenase D. willistoni	T23O_DROWI	0	GLOS_T23O.1.1	GO:0042430	B	.	indole-containing compound metabolic process
[BBH] T23O_DROWI (sp|B4MSH7) Tryptophan 2,3-dioxygenase D. willistoni	T23O_DROWI	0	GLOS_T23O.1.1	GO:0009072	B	.	aromatic amino acid family metabolic process
[BBH] T23O_DROWI (sp|B4MSH7) Tryptophan 2,3-dioxygenase D. willistoni	T23O_DROWI	0	GLOS_T23O.1.1	GO:0009074	B	.	aromatic amino acid family catabolic process
[BBH] T23O_DROWI (sp|B4MSH7) Tryptophan 2,3-dioxygenase D. willistoni	T23O_DROWI	0	GLOS_T23O.1.1	GO:0009063	B	.	cellular amino acid catabolic process
[BBH] T23O_DROWI (sp|B4MSH7) Tryptophan 2,3-dioxygenase D. willistoni	T23O_DROWI	0	GLOS_T23O.1.1	GO:0046395	B	.	carboxylic acid catabolic process
[BBH] T23O_DROWI (sp|B4MSH7) Tryptophan 2,3-dioxygenase D. willistoni	T23O_DROWI	0	GLOS_T23O.1.1	GO:0016054	B	.	organic acid catabolic process
[BBH] T23O_DROWI (sp|B4MSH7) Tryptophan 2,3-dioxygenase D. willistoni	T23O_DROWI	0	GLOS_T23O.1.1	GO:0044282	B	.	small molecule catabolic process
[BBH] T23O_DROWI (sp|B4MSH7) Tryptophan 2,3-dioxygenase D. willistoni	T23O_DROWI	0	GLOS_T23O.1.1	GO:0044712	B	.	single-organism catabolic process
[BBH] T23O_DROWI (sp|B4MSH7) Tryptophan 2,3-dioxygenase D. willistoni	T23O_DROWI	0	GLOS_T23O.1.1	GO:0019439	B	.	aromatic compound catabolic process
[BBH] T23O_DROWI (sp|B4MSH7) Tryptophan 2,3-dioxygenase D. willistoni	T23O_DROWI	0	GLOS_T23O.1.1	GO:1901361	B	.	organic cyclic compound catabolic process
[BBH] T23O_DROWI (sp|B4MSH7) Tryptophan 2,3-dioxygenase D. willistoni	T23O_DROWI	0	GLOS_T23O.1.1	GO:0046218	B	.	indolalkylamine catabolic process
[BBH] T23O_DROWI (sp|B4MSH7) Tryptophan 2,3-dioxygenase D. willistoni	T23O_DROWI	0	GLOS_T23O.1.1	GO:0042436	B	.	indole-containing compound catabolic process
[BBH] T23O_DROWI (sp|B4MSH7) Tryptophan 2,3-dioxygenase D. willistoni	T23O_DROWI	0	GLOS_T23O.1.1	GO:0044270	B	.	cellular nitrogen compound catabolic process
[BBH] T23O_DROWI (sp|B4MSH7) Tryptophan 2,3-dioxygenase D. willistoni	T23O_DROWI	0	GLOS_T23O.1.1	GO:0046700	B	.	heterocycle catabolic process
[BBH] T23O_DROWI (sp|B4MSH7) Tryptophan 2,3-dioxygenase D. willistoni	T23O_DROWI	0	GLOS_T23O.1.1	GO:1901606	B	.	alpha-amino acid catabolic process
[BBH] T23O_DROWI (sp|B4MSH7) Tryptophan 2,3-dioxygenase D. willistoni	T23O_DROWI	0	GLOS_T23O.1.1	GO:0070189	B	.	kynurenine metabolic process
[BBH] T23O_DROWI (sp|B4MSH7) Tryptophan 2,3-dioxygenase D. willistoni	T23O_DROWI	0	GLOS_T23O.1.1	GO:0042180	B	.	cellular ketone metabolic process
[BBH] T23O_DROWI (sp|B4MSH7) Tryptophan 2,3-dioxygenase D. willistoni	T23O_DROWI	0	GLOS_T23O.1.1	GO:0042537	B	.	benzene-containing compound metabolic process
[BBH] T23O_DROWI (sp|B4MSH7) Tryptophan 2,3-dioxygenase D. willistoni	T23O_DROWI	0	GLOS_T23O.1.1	GO:0019441	B	UniProtKB	tryptophan catabolic process to kynurenine
TBA1_DROME (sp|P06603) Tubulin alpha-1 chain OS=D.m.	TBA1_DROME	0	GLOS_TBA1.1.1	GO:0005876	C	.	spindle microtubule
TBA1_DROME (sp|P06603) Tubulin alpha-1 chain OS=D.m.	TBA1_DROME	0	GLOS_TBA1.1.1	GO:0005874	C	.	microtubule
TBA1_DROME (sp|P06603) Tubulin alpha-1 chain OS=D.m.	TBA1_DROME	0	GLOS_TBA1.1.1	GO:0005819	C	.	spindle
TBA1_DROME (sp|P06603) Tubulin alpha-1 chain OS=D.m.	TBA1_DROME	0	GLOS_TBA1.1.1	GO:0005881	C	.	cytoplasmic microtubule
TBA1_DROME (sp|P06603) Tubulin alpha-1 chain OS=D.m.	TBA1_DROME	0	GLOS_TBA1.1.1	GO:0005818	C	.	aster
TBA1_DROME (sp|P06603) Tubulin alpha-1 chain OS=D.m.	TBA1_DROME	0	GLOS_TBA1.1.1	GO:0000235	C	FlyBase	astral microtubule
TBA1_DROME (sp|P06603) Tubulin alpha-1 chain OS=D.m.	TBA1_DROME	0	GLOS_TBA1.1.1	GO:0005815	C	.	microtubule organizing center
TBA1_DROME (sp|P06603) Tubulin alpha-1 chain OS=D.m.	TBA1_DROME	0	GLOS_TBA1.1.1	GO:0005813	C	FlyBase	centrosome
TBA1_DROME (sp|P06603) Tubulin alpha-1 chain OS=D.m.	TBA1_DROME	0	GLOS_TBA1.1.1	GO:0005525	M	UniProtKB-K	GTP binding
TBA1_DROME (sp|P06603) Tubulin alpha-1 chain OS=D.m.	TBA1_DROME	0	GLOS_TBA1.1.1	GO:0003924	M	InterPro	GTPase activity
TBA1_DROME (sp|P06603) Tubulin alpha-1 chain OS=D.m.	TBA1_DROME	0	GLOS_TBA1.1.1	GO:0005200	M	FlyBase	structural constituent of cytoskeleton
TBA1_DROME (sp|P06603) Tubulin alpha-1 chain OS=D.m.	TBA1_DROME	0	GLOS_TBA1.1.1	GO:0019730	B	FlyBase	antimicrobial humoral response
TBA1_DROME (sp|P06603) Tubulin alpha-1 chain OS=D.m.	TBA1_DROME	0	GLOS_TBA1.1.1	GO:0045841	B	.	negative regulation of mitotic metaphase/anaphase transition
TBA1_DROME (sp|P06603) Tubulin alpha-1 chain OS=D.m.	TBA1_DROME	0	GLOS_TBA1.1.1	GO:0030071	B	.	regulation of mitotic metaphase/anaphase transition
TBA1_DROME (sp|P06603) Tubulin alpha-1 chain OS=D.m.	TBA1_DROME	0	GLOS_TBA1.1.1	GO:0007088	B	.	regulation of mitosis
TBA1_DROME (sp|P06603) Tubulin alpha-1 chain OS=D.m.	TBA1_DROME	0	GLOS_TBA1.1.1	GO:0007346	B	.	regulation of mitotic cell cycle
TBA1_DROME (sp|P06603) Tubulin alpha-1 chain OS=D.m.	TBA1_DROME	0	GLOS_TBA1.1.1	GO:0010564	B	.	regulation of cell cycle process
TBA1_DROME (sp|P06603) Tubulin alpha-1 chain OS=D.m.	TBA1_DROME	0	GLOS_TBA1.1.1	GO:0051783	B	.	regulation of nuclear division
TBA1_DROME (sp|P06603) Tubulin alpha-1 chain OS=D.m.	TBA1_DROME	0	GLOS_TBA1.1.1	GO:0033043	B	.	regulation of organelle organization
TBA1_DROME (sp|P06603) Tubulin alpha-1 chain OS=D.m.	TBA1_DROME	0	GLOS_TBA1.1.1	GO:1901990	B	.	regulation of mitotic cell cycle phase transition

TBA1_DROME (sp|P06603) Tubulin alpha-1 chain OS=D.m.	TBA1_DROME	0	GLOS_TBA1.1.1	GO:1901987	B	.	regulation of cell cycle phase transition
TBA1_DROME (sp|P06603) Tubulin alpha-1 chain OS=D.m.	TBA1_DROME	0	GLOS_TBA1.1.1	GO:1902099	B	.	regulation of metaphase/anaphase transition of cell cycle
TBA1_DROME (sp|P06603) Tubulin alpha-1 chain OS=D.m.	TBA1_DROME	0	GLOS_TBA1.1.1	GO:0045839	B	.	negative regulation of mitosis
TBA1_DROME (sp|P06603) Tubulin alpha-1 chain OS=D.m.	TBA1_DROME	0	GLOS_TBA1.1.1	GO:0010948	B	.	negative regulation of cell cycle process
TBA1_DROME (sp|P06603) Tubulin alpha-1 chain OS=D.m.	TBA1_DROME	0	GLOS_TBA1.1.1	GO:0045786	B	.	negative regulation of cell cycle
TBA1_DROME (sp|P06603) Tubulin alpha-1 chain OS=D.m.	TBA1_DROME	0	GLOS_TBA1.1.1	GO:0051784	B	.	negative regulation of nuclear division
TBA1_DROME (sp|P06603) Tubulin alpha-1 chain OS=D.m.	TBA1_DROME	0	GLOS_TBA1.1.1	GO:0010639	B	.	negative regulation of organelle organization
TBA1_DROME (sp|P06603) Tubulin alpha-1 chain OS=D.m.	TBA1_DROME	0	GLOS_TBA1.1.1	GO:1901991	B	.	negative regulation of mitotic cell cycle phase transition
TBA1_DROME (sp|P06603) Tubulin alpha-1 chain OS=D.m.	TBA1_DROME	0	GLOS_TBA1.1.1	GO:1901988	B	.	negative regulation of cell cycle phase transition
TBA1_DROME (sp|P06603) Tubulin alpha-1 chain OS=D.m.	TBA1_DROME	0	GLOS_TBA1.1.1	GO:1902100	B	.	negative regulation of metaphase/anaphase transition of cell cycle
TBA1_DROME (sp|P06603) Tubulin alpha-1 chain OS=D.m.	TBA1_DROME	0	GLOS_TBA1.1.1	GO:0071173	B	.	spindle assembly checkpoint
TBA1_DROME (sp|P06603) Tubulin alpha-1 chain OS=D.m.	TBA1_DROME	0	GLOS_TBA1.1.1	GO:0031577	B	.	spindle checkpoint
TBA1_DROME (sp|P06603) Tubulin alpha-1 chain OS=D.m.	TBA1_DROME	0	GLOS_TBA1.1.1	GO:0000075	B	.	cell cycle checkpoint
TBA1_DROME (sp|P06603) Tubulin alpha-1 chain OS=D.m.	TBA1_DROME	0	GLOS_TBA1.1.1	GO:0071174	B	.	mitotic spindle checkpoint
TBA1_DROME (sp|P06603) Tubulin alpha-1 chain OS=D.m.	TBA1_DROME	0	GLOS_TBA1.1.1	GO:0007093	B	.	mitotic cell cycle checkpoint
TBA1_DROME (sp|P06603) Tubulin alpha-1 chain OS=D.m.	TBA1_DROME	0	GLOS_TBA1.1.1	GO:0007094	B	FlyBase	mitotic spindle assembly checkpoint
TBA1_DROME (sp|P06603) Tubulin alpha-1 chain OS=D.m.	TBA1_DROME	0	GLOS_TBA1.1.1	GO:0007052	B	FlyBase	mitotic spindle organization
TBA1_DROME (sp|P06603) Tubulin alpha-1 chain OS=D.m.	TBA1_DROME	0	GLOS_TBA1.1.1	GO:0043623	B	.	cellular protein complex assembly
TBA1_DROME (sp|P06603) Tubulin alpha-1 chain OS=D.m.	TBA1_DROME	0	GLOS_TBA1.1.1	GO:0006461	B	.	protein complex assembly
TBA1_DROME (sp|P06603) Tubulin alpha-1 chain OS=D.m.	TBA1_DROME	0	GLOS_TBA1.1.1	GO:0065003	B	.	macromolecular complex assembly
TBA1_DROME (sp|P06603) Tubulin alpha-1 chain OS=D.m.	TBA1_DROME	0	GLOS_TBA1.1.1	GO:0070271	B	.	protein complex biogenesis
TBA1_DROME (sp|P06603) Tubulin alpha-1 chain OS=D.m.	TBA1_DROME	0	GLOS_TBA1.1.1	GO:0034622	B	.	cellular macromolecular complex assembly
TBA1_DROME (sp|P06603) Tubulin alpha-1 chain OS=D.m.	TBA1_DROME	0	GLOS_TBA1.1.1	GO:0051258	B	InterPro	protein polymerization
TRYP_SARBU (sp|P51588) Trypsin OS=S. bullata PE=1 SV=1	TRYP_SARBU	1,00E-26	GLOS_TRYP.9.26	GO:0005615	C	UniProtKB-S	extracellular space
TRYP_SARBU (sp|P51588) Trypsin OS=S. bullata PE=1 SV=1	TRYP_SARBU	1,00E-26	GLOS_TRYP.9.26	GO:0004252	M	InterPro	serine-type endopeptidase activity
TRYP_SARBU (sp|P51588) Trypsin OS=S. bullata PE=1 SV=1	TRYP_SARBU	1,00E-26	GLOS_TRYP.9.26	GO:0007586	B	UniProtKB-K	digestion
TRYP_SARBU (sp|P51588) Trypsin OS=S. bullata PE=1 SV=1	TRYP_SARBU	1,00E-26	GLOS_TRYP.9.26	GO:0006508	B	UniProtKB-K	proteolysis
TSEP_GLOMM (sp|Q95P09) Protein TsetseEP G. m.morsitans PE=1 SV=1	TSEP_GLOMM	6,00E-52	GLOS_TSEP.9.20	GO:0005576	C	UniProtKB-S	extracellular region
TSEP_GLOPP (sp|Q8T4N5) Protein TsetseEP G. p. palpalis PE=4 SV=1	TSEP_GLOPP	3,00E-36	GLOS_TSEP.10.2(	GO:0005576	C	UniProtKB-S	extracellular region
UBB_PONPY (sp|P0CG60) Polyubiquitin-B OS=P. pygmaeus	UBB_PONPY	8,00E-92	GLOS_UBB.3.7	GO:0005737	C	UniProtKB-S	cytoplasm
UBB_PONPY (sp|P0CG60) Polyubiquitin-B OS=P. pygmaeus	UBB_PONPY	8,00E-92	GLOS_UBB.3.7	GO:0005634	C	UniProtKB-S	nucleus
[BBH] Y2666_DROME (sp|Q0KHT7) Probable serine/threonine-protein kinase	Y2666_DROME	1,00E-166	GLOS_Y2666.1.1	GO:0005524	M	UniProtKB-K	ATP binding
[BBH] Y2666_DROME (sp|Q0KHT7) Probable serine/threonine-protein kinase	Y2666_DROME	1,00E-166	GLOS_Y2666.1.1	GO:0004674	M	FlyBase	protein serine/threonine kinase activity
[BBH] Y2666_DROME (sp|Q0KHT7) Probable serine/threonine-protein kinase	Y2666_DROME	1,00E-166	GLOS_Y2666.1.1	GO:0007155	B	FlyBase	cell adhesion
[BBH] Y2666_DROME (sp|Q0KHT7) Probable serine/threonine-protein kinase	Y2666_DROME	1,00E-166	GLOS_Y2666.1.1	GO:0007478	B	.	leg disc morphogenesis
[BBH] Y2666_DROME (sp|Q0KHT7) Probable serine/threonine-protein kinase	Y2666_DROME	1,00E-166	GLOS_Y2666.1.1	GO:0035218	B	.	leg disc development
[BBH] Y2666_DROME (sp|Q0KHT7) Probable serine/threonine-protein kinase	Y2666_DROME	1,00E-166	GLOS_Y2666.1.1	GO:0007480	B	FlyBase	imaginal disc-derived leg morphogenesis
[BBH] Y2666_DROME (sp|Q0KHT7) Probable serine/threonine-protein kinase	Y2666_DROME	1,00E-166	GLOS_Y2666.1.1	GO:0007476	B	FlyBase	imaginal disc-derived wing morphogenesis
[BBH] Y2666_DROME (sp|Q0KHT7) Probable serine/threonine-protein kinase	Y2666_DROME	1,00E-166	GLOS_Y2666.1.1	GO:0002009	B	FlyBase	morphogenesis of an epithelium
[BBH] Y2666_DROME (sp|Q0KHT7) Probable serine/threonine-protein kinase	Y2666_DROME	1,00E-166	GLOS_Y2666.1.1	GO:0008360	B	FlyBase	regulation of cell shape
XP_001979918.1 GG16851 [Drosophila erecta]	XP_001979918.1	1,00E-16	GLOS_DERE_GG	GO:0005216	M	.	ion channel activity
XP_001979918.1 GG16851 [Drosophila erecta]	XP_001979918.1	1,00E-16	GLOS_DERE_GG	GO:0015075	M	.	ion transmembrane transporter activity
XP_001979918.1 GG16851 [Drosophila erecta]	XP_001979918.1	1,00E-16	GLOS_DERE_GG	GO:0022891	M	.	substrate-specific transmembrane transporter activity
XP_001979918.1 GG16851 [Drosophila erecta]	XP_001979918.1	1,00E-16	GLOS_DERE_GG	GO:0022857	M	.	transmembrane transporter activity
XP_001979918.1 GG16851 [Drosophila erecta]	XP_001979918.1	1,00E-16	GLOS_DERE_GG	GO:0005215	M	.	transporter activity
XP_001979918.1 GG16851 [Drosophila erecta]	XP_001979918.1	1,00E-16	GLOS_DERE_GG	GO:0003674	M	.	molecular_function
XP_001979918.1 GG16851 [Drosophila erecta]	XP_001979918.1	1,00E-16	GLOS_DERE_GG	GO:0022892	M	.	substrate-specific transporter activity
XP_001979918.1 GG16851 [Drosophila erecta]	XP_001979918.1	1,00E-16	GLOS_DERE_GG	GO:0022838	M	.	substrate-specific channel activity
XP_001979918.1 GG16851 [Drosophila erecta]	XP_001979918.1	1,00E-16	GLOS_DERE_GG	GO:0015267	M	.	channel activity
XP_001979918.1 GG16851 [Drosophila erecta]	XP_001979918.1	1,00E-16	GLOS_DERE_GG	GO:0022803	M	.	passive transmembrane transporter activity
XP_001979918.1 GG16851 [Drosophila erecta]	XP_001979918.1	1,00E-16	GLOS_DERE_GG	GO:0008324	M	.	cation transmembrane transporter activity
XP_001979918.1 GG16851 [Drosophila erecta]	XP_001979918.1	1,00E-16	GLOS_DERE_GG	GO:0005261	M	Refseq	cation channel activity
XP_001979918.1 GG16851 [Drosophila erecta]	XP_001979918.1	1,00E-16	GLOS_DERE_GG	GO:0005261	M	.	cation channel activity
XP_001979918.1 GG16851 [Drosophila erecta]	XP_001979918.1	1,00E-16	GLOS_DERE_GG	GO:0015085	M	.	calcium ion transmembrane transporter activity

XP_001979918.1 GG16851 [Drosophila erecta]	XP_001979918.1	1,00E-16	GLOS_DERE_GG	GO:0046873	M	.	metal ion transmembrane transporter activity
XP_001979918.1 GG16851 [Drosophila erecta]	XP_001979918.1	1,00E-16	GLOS_DERE_GG	GO:0022890	M	.	inorganic cation transmembrane transporter activity
XP_001979918.1 GG16851 [Drosophila erecta]	XP_001979918.1	1,00E-16	GLOS_DERE_GG	GO:0072509	M	.	divalent inorganic cation transmembrane transporter activity
XP_001979918.1 GG16851 [Drosophila erecta]	XP_001979918.1	1,00E-16	GLOS_DERE_GG	GO:0005262	M	Refseq	calcium channel activity
XP_001979918.1 GG16851 [Drosophila erecta]	XP_001979918.1	1,00E-16	GLOS_DERE_GG	GO:0005488	M	.	binding
XP_001979918.1 GG16851 [Drosophila erecta]	XP_001979918.1	1,00E-16	GLOS_DERE_GG	GO:0005515	M	Refseq	protein binding
XP_001979918.1 GG16851 [Drosophila erecta]	XP_001979918.1	1,00E-16	GLOS_DERE_GG	GO:0016020	C	.	membrane
XP_001979918.1 GG16851 [Drosophila erecta]	XP_001979918.1	1,00E-16	GLOS_DERE_GG	GO:0005575	C	.	cellular_component
XP_001979918.1 GG16851 [Drosophila erecta]	XP_001979918.1	1,00E-16	GLOS_DERE_GG	GO:0044464	C	.	cell part
XP_001979918.1 GG16851 [Drosophila erecta]	XP_001979918.1	1,00E-16	GLOS_DERE_GG	GO:0005623	C	.	cell
XP_001979918.1 GG16851 [Drosophila erecta]	XP_001979918.1	1,00E-16	GLOS_DERE_GG	GO:0071944	C	.	cell periphery
XP_001979918.1 GG16851 [Drosophila erecta]	XP_001979918.1	1,00E-16	GLOS_DERE_GG	GO:0005886	C	Refseq	plasma membrane
XP_001979918.1 GG16851 [Drosophila erecta]	XP_001979918.1	1,00E-16	GLOS_DERE_GG	GO:0016021	C	.	integral to membrane
XP_001979918.1 GG16851 [Drosophila erecta]	XP_001979918.1	1,00E-16	GLOS_DERE_GG	GO:0031224	C	.	intrinsic to membrane
XP_001979918.1 GG16851 [Drosophila erecta]	XP_001979918.1	1,00E-16	GLOS_DERE_GG	GO:0044425	C	.	membrane part
XP_001979918.1 GG16851 [Drosophila erecta]	XP_001979918.1	1,00E-16	GLOS_DERE_GG	GO:0031226	C	.	intrinsic to plasma membrane
XP_001979918.1 GG16851 [Drosophila erecta]	XP_001979918.1	1,00E-16	GLOS_DERE_GG	GO:0044459	C	.	plasma membrane part
XP_001979918.1 GG16851 [Drosophila erecta]	XP_001979918.1	1,00E-16	GLOS_DERE_GG	GO:0005886	C	.	plasma membrane
XP_001979918.1 GG16851 [Drosophila erecta]	XP_001979918.1	1,00E-16	GLOS_DERE_GG	GO:0005887	C	Refseq	integral to plasma membrane
XP_001979918.1 GG16851 [Drosophila erecta]	XP_001979918.1	1,00E-16	GLOS_DERE_GG	GO:0070838	B	.	divalent metal ion transport
XP_001979918.1 GG16851 [Drosophila erecta]	XP_001979918.1	1,00E-16	GLOS_DERE_GG	GO:0030001	B	.	metal ion transport
XP_001979918.1 GG16851 [Drosophila erecta]	XP_001979918.1	1,00E-16	GLOS_DERE_GG	GO:0006812	B	.	cation transport
XP_001979918.1 GG16851 [Drosophila erecta]	XP_001979918.1	1,00E-16	GLOS_DERE_GG	GO:0006811	B	.	ion transport
XP_001979918.1 GG16851 [Drosophila erecta]	XP_001979918.1	1,00E-16	GLOS_DERE_GG	GO:0044765	B	.	single-organism transport
XP_001979918.1 GG16851 [Drosophila erecta]	XP_001979918.1	1,00E-16	GLOS_DERE_GG	GO:0006810	B	.	transport
XP_001979918.1 GG16851 [Drosophila erecta]	XP_001979918.1	1,00E-16	GLOS_DERE_GG	GO:0051234	B	.	establishment of localization
XP_001979918.1 GG16851 [Drosophila erecta]	XP_001979918.1	1,00E-16	GLOS_DERE_GG	GO:0008150	B	.	biological_process
XP_001979918.1 GG16851 [Drosophila erecta]	XP_001979918.1	1,00E-16	GLOS_DERE_GG	GO:0051179	B	.	localization
XP_001979918.1 GG16851 [Drosophila erecta]	XP_001979918.1	1,00E-16	GLOS_DERE_GG	GO:0044699	B	.	single-organism process
XP_001979918.1 GG16851 [Drosophila erecta]	XP_001979918.1	1,00E-16	GLOS_DERE_GG	GO:0072511	B	.	divalent inorganic cation transport
XP_001979918.1 GG16851 [Drosophila erecta]	XP_001979918.1	1,00E-16	GLOS_DERE_GG	GO:0006816	B	Refseq	calcium ion transport
XP_001979918.1 GG16851 [Drosophila erecta]	XP_001979918.1	1,00E-16	GLOS_DERE_GG	GO:0097485	B	.	neuron projection guidance
XP_001979918.1 GG16851 [Drosophila erecta]	XP_001979918.1	1,00E-16	GLOS_DERE_GG	GO:0006928	B	.	cellular component movement
XP_001979918.1 GG16851 [Drosophila erecta]	XP_001979918.1	1,00E-16	GLOS_DERE_GG	GO:0044763	B	.	single-organism cellular process
XP_001979918.1 GG16851 [Drosophila erecta]	XP_001979918.1	1,00E-16	GLOS_DERE_GG	GO:0009987	B	.	cellular process
XP_001979918.1 GG16851 [Drosophila erecta]	XP_001979918.1	1,00E-16	GLOS_DERE_GG	GO:0006935	B	.	chemotaxis
XP_001979918.1 GG16851 [Drosophila erecta]	XP_001979918.1	1,00E-16	GLOS_DERE_GG	GO:0042221	B	.	response to chemical stimulus
XP_001979918.1 GG16851 [Drosophila erecta]	XP_001979918.1	1,00E-16	GLOS_DERE_GG	GO:0050896	B	.	response to stimulus
XP_001979918.1 GG16851 [Drosophila erecta]	XP_001979918.1	1,00E-16	GLOS_DERE_GG	GO:0042330	B	.	taxis
XP_001979918.1 GG16851 [Drosophila erecta]	XP_001979918.1	1,00E-16	GLOS_DERE_GG	GO:0009605	B	.	response to external stimulus
XP_001979918.1 GG16851 [Drosophila erecta]	XP_001979918.1	1,00E-16	GLOS_DERE_GG	GO:0040011	B	.	locomotion
XP_001979918.1 GG16851 [Drosophila erecta]	XP_001979918.1	1,00E-16	GLOS_DERE_GG	GO:0048812	B	.	neuron projection morphogenesis
XP_001979918.1 GG16851 [Drosophila erecta]	XP_001979918.1	1,00E-16	GLOS_DERE_GG	GO:0048858	B	.	cell projection morphogenesis
XP_001979918.1 GG16851 [Drosophila erecta]	XP_001979918.1	1,00E-16	GLOS_DERE_GG	GO:0030030	B	.	cell projection organization
XP_001979918.1 GG16851 [Drosophila erecta]	XP_001979918.1	1,00E-16	GLOS_DERE_GG	GO:0016043	B	.	cellular component organization
XP_001979918.1 GG16851 [Drosophila erecta]	XP_001979918.1	1,00E-16	GLOS_DERE_GG	GO:0071840	B	.	cellular component organization or biogenesis
XP_001979918.1 GG16851 [Drosophila erecta]	XP_001979918.1	1,00E-16	GLOS_DERE_GG	GO:0032990	B	.	cell part morphogenesis
XP_001979918.1 GG16851 [Drosophila erecta]	XP_001979918.1	1,00E-16	GLOS_DERE_GG	GO:0032989	B	.	cellular component morphogenesis
XP_001979918.1 GG16851 [Drosophila erecta]	XP_001979918.1	1,00E-16	GLOS_DERE_GG	GO:0009653	B	.	anatomical structure morphogenesis
XP_001979918.1 GG16851 [Drosophila erecta]	XP_001979918.1	1,00E-16	GLOS_DERE_GG	GO:0032502	B	.	developmental process
XP_001979918.1 GG16851 [Drosophila erecta]	XP_001979918.1	1,00E-16	GLOS_DERE_GG	GO:0048856	B	.	anatomical structure development
XP_001979918.1 GG16851 [Drosophila erecta]	XP_001979918.1	1,00E-16	GLOS_DERE_GG	GO:0044767	B	.	single-organism developmental process
XP_001979918.1 GG16851 [Drosophila erecta]	XP_001979918.1	1,00E-16	GLOS_DERE_GG	GO:0048869	B	.	cellular developmental process
XP_001979918.1 GG16851 [Drosophila erecta]	XP_001979918.1	1,00E-16	GLOS_DERE_GG	GO:0000902	B	.	cell morphogenesis

XP_001979918.1 GG16851 [Drosophila erecta]	XP_001979918.1	1,00E-16	GLOS_DERE_GG	GO:0031175	B	.	neuron projection development
XP_001979918.1 GG16851 [Drosophila erecta]	XP_001979918.1	1,00E-16	GLOS_DERE_GG	GO:0048666	B	.	neuron development
XP_001979918.1 GG16851 [Drosophila erecta]	XP_001979918.1	1,00E-16	GLOS_DERE_GG	GO:0048468	B	.	cell development
XP_001979918.1 GG16851 [Drosophila erecta]	XP_001979918.1	1,00E-16	GLOS_DERE_GG	GO:0030154	B	.	cell differentiation
XP_001979918.1 GG16851 [Drosophila erecta]	XP_001979918.1	1,00E-16	GLOS_DERE_GG	GO:0030182	B	.	neuron differentiation
XP_001979918.1 GG16851 [Drosophila erecta]	XP_001979918.1	1,00E-16	GLOS_DERE_GG	GO:0048699	B	.	generation of neurons
XP_001979918.1 GG16851 [Drosophila erecta]	XP_001979918.1	1,00E-16	GLOS_DERE_GG	GO:0022008	B	.	neurogenesis
XP_001979918.1 GG16851 [Drosophila erecta]	XP_001979918.1	1,00E-16	GLOS_DERE_GG	GO:0007399	B	.	nervous system development
XP_001979918.1 GG16851 [Drosophila erecta]	XP_001979918.1	1,00E-16	GLOS_DERE_GG	GO:0048731	B	.	system development
XP_001979918.1 GG16851 [Drosophila erecta]	XP_001979918.1	1,00E-16	GLOS_DERE_GG	GO:0007275	B	.	multicellular organismal development
XP_001979918.1 GG16851 [Drosophila erecta]	XP_001979918.1	1,00E-16	GLOS_DERE_GG	GO:0044707	B	.	single-multicellular organism process
XP_001979918.1 GG16851 [Drosophila erecta]	XP_001979918.1	1,00E-16	GLOS_DERE_GG	GO:0032501	B	.	multicellular organismal process
XP_001979918.1 GG16851 [Drosophila erecta]	XP_001979918.1	1,00E-16	GLOS_DERE_GG	GO:0007409	B	.	axonogenesis
XP_001979918.1 GG16851 [Drosophila erecta]	XP_001979918.1	1,00E-16	GLOS_DERE_GG	GO:0048667	B	.	cell morphogenesis involved in neuron differentiation
XP_001979918.1 GG16851 [Drosophila erecta]	XP_001979918.1	1,00E-16	GLOS_DERE_GG	GO:0000904	B	.	cell morphogenesis involved in differentiation
XP_001979918.1 GG16851 [Drosophila erecta]	XP_001979918.1	1,00E-16	GLOS_DERE_GG	GO:0061564	B	.	axon development
XP_001979918.1 GG16851 [Drosophila erecta]	XP_001979918.1	1,00E-16	GLOS_DERE_GG	GO:0007411	B	Refseq	axon guidance
XP_001979918.1 GG16851 [Drosophila erecta]	XP_001979918.1	1,00E-16	GLOS_DERE_GG	GO:0005262	M	.	calcium channel activity
XP_001979918.1 GG16851 [Drosophila erecta]	XP_001979918.1	1,00E-16	GLOS_DERE_GG	GO:0015276	M	.	ligand-gated ion channel activity
XP_001979918.1 GG16851 [Drosophila erecta]	XP_001979918.1	1,00E-16	GLOS_DERE_GG	GO:0022834	M	.	ligand-gated channel activity
XP_001979918.1 GG16851 [Drosophila erecta]	XP_001979918.1	1,00E-16	GLOS_DERE_GG	GO:0022836	M	.	gated channel activity
XP_001979918.1 GG16851 [Drosophila erecta]	XP_001979918.1	1,00E-16	GLOS_DERE_GG	GO:0022839	M	.	ion gated channel activity
XP_001979918.1 GG16851 [Drosophila erecta]	XP_001979918.1	1,00E-16	GLOS_DERE_GG	GO:0015279	M	Refseq	store-operated calcium channel activity
XP_001979918.1 GG16851 [Drosophila erecta]	XP_001979918.1	1,00E-16	GLOS_DERE_GG	GO:0016323	C	Refseq	basolateral plasma membrane
XP_001979918.1 GG16851 [Drosophila erecta]	XP_001979918.1	1,00E-16	GLOS_DERE_GG	GO:0044449	C	.	contractile fiber part
XP_001979918.1 GG16851 [Drosophila erecta]	XP_001979918.1	1,00E-16	GLOS_DERE_GG	GO:0044422	C	.	organelle part
XP_001979918.1 GG16851 [Drosophila erecta]	XP_001979918.1	1,00E-16	GLOS_DERE_GG	GO:0043226	C	.	organelle
XP_001979918.1 GG16851 [Drosophila erecta]	XP_001979918.1	1,00E-16	GLOS_DERE_GG	GO:0044444	C	.	cytoplasmic part
XP_001979918.1 GG16851 [Drosophila erecta]	XP_001979918.1	1,00E-16	GLOS_DERE_GG	GO:0044424	C	.	intracellular part
XP_001979918.1 GG16851 [Drosophila erecta]	XP_001979918.1	1,00E-16	GLOS_DERE_GG	GO:0005622	C	.	intracellular
XP_001979918.1 GG16851 [Drosophila erecta]	XP_001979918.1	1,00E-16	GLOS_DERE_GG	GO:0005737	C	.	cytoplasm
XP_001979918.1 GG16851 [Drosophila erecta]	XP_001979918.1	1,00E-16	GLOS_DERE_GG	GO:0043292	C	.	contractile fiber
XP_001979918.1 GG16851 [Drosophila erecta]	XP_001979918.1	1,00E-16	GLOS_DERE_GG	GO:0043232	C	.	intracellular non-membrane-bounded organelle
XP_001979918.1 GG16851 [Drosophila erecta]	XP_001979918.1	1,00E-16	GLOS_DERE_GG	GO:0043228	C	.	non-membrane-bounded organelle
XP_001979918.1 GG16851 [Drosophila erecta]	XP_001979918.1	1,00E-16	GLOS_DERE_GG	GO:0043229	C	.	intracellular organelle
XP_001979918.1 GG16851 [Drosophila erecta]	XP_001979918.1	1,00E-16	GLOS_DERE_GG	GO:0030016	C	.	myofibril
XP_001979918.1 GG16851 [Drosophila erecta]	XP_001979918.1	1,00E-16	GLOS_DERE_GG	GO:0030017	C	Refseq	sarcomere
XP_001979918.1 GG16851 [Drosophila erecta]	XP_001979918.1	1,00E-16	GLOS_DERE_GG	GO:0043034	C	Refseq	costamere
XP_001979918.1 GG16851 [Drosophila erecta]	XP_001979918.1	1,00E-16	GLOS_DERE_GG	GO:0032991	C	.	macromolecular complex
XP_001979918.1 GG16851 [Drosophila erecta]	XP_001979918.1	1,00E-16	GLOS_DERE_GG	GO:0043234	C	Refseq	protein complex
XP_001979918.1 GG16851 [Drosophila erecta]	XP_001979918.1	1,00E-16	GLOS_DERE_GG	GO:0005515	M	.	protein binding
XP_001979918.1 GG16851 [Drosophila erecta]	XP_001979918.1	1,00E-16	GLOS_DERE_GG	GO:0044325	M	Refseq	ion channel binding
XP_001979918.1 GG16851 [Drosophila erecta]	XP_001979918.1	1,00E-16	GLOS_DERE_GG	GO:0045121	C	Refseq	membrane raft
XP_001979918.1 GG16851 [Drosophila erecta]	XP_001979918.1	1,00E-16	GLOS_DERE_GG	GO:0007589	B	.	body fluid secretion
XP_001979918.1 GG16851 [Drosophila erecta]	XP_001979918.1	1,00E-16	GLOS_DERE_GG	GO:0046903	B	.	secretion
XP_001979918.1 GG16851 [Drosophila erecta]	XP_001979918.1	1,00E-16	GLOS_DERE_GG	GO:0050878	B	.	regulation of body fluid levels
XP_001979918.1 GG16851 [Drosophila erecta]	XP_001979918.1	1,00E-16	GLOS_DERE_GG	GO:0065008	B	.	regulation of biological quality
XP_001979918.1 GG16851 [Drosophila erecta]	XP_001979918.1	1,00E-16	GLOS_DERE_GG	GO:0065007	B	.	biological regulation
XP_001979918.1 GG16851 [Drosophila erecta]	XP_001979918.1	1,00E-16	GLOS_DERE_GG	GO:0022600	B	.	digestive system process
XP_001979918.1 GG16851 [Drosophila erecta]	XP_001979918.1	1,00E-16	GLOS_DERE_GG	GO:0003008	B	.	system process
XP_001979918.1 GG16851 [Drosophila erecta]	XP_001979918.1	1,00E-16	GLOS_DERE_GG	GO:0007586	B	.	digestion
XP_001979918.1 GG16851 [Drosophila erecta]	XP_001979918.1	1,00E-16	GLOS_DERE_GG	GO:0032941	B	.	secretion by tissue
XP_001979918.1 GG16851 [Drosophila erecta]	XP_001979918.1	1,00E-16	GLOS_DERE_GG	GO:0046541	B	Refseq	saliva secretion
XP_001979918.1 GG16851 [Drosophila erecta]	XP_001979918.1	1,00E-16	GLOS_DERE_GG	GO:0010524	B	.	positive regulation of calcium ion transport into cytosol

XP_001979918.1 GG16851 [Drosophila erecta]	XP_001979918.1	1,00E-16	GLOS_DERE_GG	GO:0010522	B	.	regulation of calcium ion transport into cytosol
XP_001979918.1 GG16851 [Drosophila erecta]	XP_001979918.1	1,00E-16	GLOS_DERE_GG	GO:0032386	B	.	regulation of intracellular transport
XP_001979918.1 GG16851 [Drosophila erecta]	XP_001979918.1	1,00E-16	GLOS_DERE_GG	GO:0051049	B	.	regulation of transport
XP_001979918.1 GG16851 [Drosophila erecta]	XP_001979918.1	1,00E-16	GLOS_DERE_GG	GO:0032879	B	.	regulation of localization
XP_001979918.1 GG16851 [Drosophila erecta]	XP_001979918.1	1,00E-16	GLOS_DERE_GG	GO:0050789	B	.	regulation of biological process
XP_001979918.1 GG16851 [Drosophila erecta]	XP_001979918.1	1,00E-16	GLOS_DERE_GG	GO:0060341	B	.	regulation of cellular localization
XP_001979918.1 GG16851 [Drosophila erecta]	XP_001979918.1	1,00E-16	GLOS_DERE_GG	GO:0050794	B	.	regulation of cellular process
XP_001979918.1 GG16851 [Drosophila erecta]	XP_001979918.1	1,00E-16	GLOS_DERE_GG	GO:0051924	B	.	regulation of calcium ion transport
XP_001979918.1 GG16851 [Drosophila erecta]	XP_001979918.1	1,00E-16	GLOS_DERE_GG	GO:0010959	B	.	regulation of metal ion transport
XP_001979918.1 GG16851 [Drosophila erecta]	XP_001979918.1	1,00E-16	GLOS_DERE_GG	GO:0043269	B	.	regulation of ion transport
XP_001979918.1 GG16851 [Drosophila erecta]	XP_001979918.1	1,00E-16	GLOS_DERE_GG	GO:2000021	B	.	regulation of ion homeostasis
XP_001979918.1 GG16851 [Drosophila erecta]	XP_001979918.1	1,00E-16	GLOS_DERE_GG	GO:0032844	B	.	regulation of homeostatic process
XP_001979918.1 GG16851 [Drosophila erecta]	XP_001979918.1	1,00E-16	GLOS_DERE_GG	GO:0032388	B	.	positive regulation of intracellular transport
XP_001979918.1 GG16851 [Drosophila erecta]	XP_001979918.1	1,00E-16	GLOS_DERE_GG	GO:0051050	B	.	positive regulation of transport
XP_001979918.1 GG16851 [Drosophila erecta]	XP_001979918.1	1,00E-16	GLOS_DERE_GG	GO:0048518	B	.	positive regulation of biological process
XP_001979918.1 GG16851 [Drosophila erecta]	XP_001979918.1	1,00E-16	GLOS_DERE_GG	GO:0032846	B	.	positive regulation of homeostatic process
XP_001979918.1 GG16851 [Drosophila erecta]	XP_001979918.1	1,00E-16	GLOS_DERE_GG	GO:0048522	B	.	positive regulation of cellular process
XP_001979918.1 GG16851 [Drosophila erecta]	XP_001979918.1	1,00E-16	GLOS_DERE_GG	GO:0051928	B	.	positive regulation of calcium ion transport
XP_001979918.1 GG16851 [Drosophila erecta]	XP_001979918.1	1,00E-16	GLOS_DERE_GG	GO:0043270	B	.	positive regulation of ion transport
XP_001979918.1 GG16851 [Drosophila erecta]	XP_001979918.1	1,00E-16	GLOS_DERE_GG	GO:0051279	B	.	regulation of release of sequestered calcium ion into cytosol
XP_001979918.1 GG16851 [Drosophila erecta]	XP_001979918.1	1,00E-16	GLOS_DERE_GG	GO:0051281	B	Refseq	positive regulation of release of sequestered calcium ion into cytosol
XP_001979918.1 GG16851 [Drosophila erecta]	XP_001979918.1	1,00E-16	GLOS_DERE_GG	GO:0006874	B	.	cellular calcium ion homeostasis
XP_001979918.1 GG16851 [Drosophila erecta]	XP_001979918.1	1,00E-16	GLOS_DERE_GG	GO:0006875	B	.	cellular metal ion homeostasis
XP_001979918.1 GG16851 [Drosophila erecta]	XP_001979918.1	1,00E-16	GLOS_DERE_GG	GO:0030003	B	.	cellular cation homeostasis
XP_001979918.1 GG16851 [Drosophila erecta]	XP_001979918.1	1,00E-16	GLOS_DERE_GG	GO:0006873	B	.	cellular ion homeostasis
XP_001979918.1 GG16851 [Drosophila erecta]	XP_001979918.1	1,00E-16	GLOS_DERE_GG	GO:0050801	B	.	ion homeostasis
XP_001979918.1 GG16851 [Drosophila erecta]	XP_001979918.1	1,00E-16	GLOS_DERE_GG	GO:0048878	B	.	chemical homeostasis
XP_001979918.1 GG16851 [Drosophila erecta]	XP_001979918.1	1,00E-16	GLOS_DERE_GG	GO:0042592	B	.	homeostatic process
XP_001979918.1 GG16851 [Drosophila erecta]	XP_001979918.1	1,00E-16	GLOS_DERE_GG	GO:0055082	B	.	cellular chemical homeostasis
XP_001979918.1 GG16851 [Drosophila erecta]	XP_001979918.1	1,00E-16	GLOS_DERE_GG	GO:0019725	B	.	cellular homeostasis
XP_001979918.1 GG16851 [Drosophila erecta]	XP_001979918.1	1,00E-16	GLOS_DERE_GG	GO:0055080	B	.	cation homeostasis
XP_001979918.1 GG16851 [Drosophila erecta]	XP_001979918.1	1,00E-16	GLOS_DERE_GG	GO:0055065	B	.	metal ion homeostasis
XP_001979918.1 GG16851 [Drosophila erecta]	XP_001979918.1	1,00E-16	GLOS_DERE_GG	GO:0055074	B	.	calcium ion homeostasis
XP_001979918.1 GG16851 [Drosophila erecta]	XP_001979918.1	1,00E-16	GLOS_DERE_GG	GO:0072507	B	.	divalent inorganic cation homeostasis
XP_001979918.1 GG16851 [Drosophila erecta]	XP_001979918.1	1,00E-16	GLOS_DERE_GG	GO:0072503	B	.	cellular divalent inorganic cation homeostasis
XP_001979918.1 GG16851 [Drosophila erecta]	XP_001979918.1	1,00E-16	GLOS_DERE_GG	GO:0051480	B	Refseq	cytosolic calcium ion homeostasis
XP_001979918.1 GG16851 [Drosophila erecta]	XP_001979918.1	1,00E-16	GLOS_DERE_GG	GO:0010038	B	.	response to metal ion
XP_001979918.1 GG16851 [Drosophila erecta]	XP_001979918.1	1,00E-16	GLOS_DERE_GG	GO:0010035	B	.	response to inorganic substance
XP_001979918.1 GG16851 [Drosophila erecta]	XP_001979918.1	1,00E-16	GLOS_DERE_GG	GO:0051592	B	Refseq	response to calcium ion
XP_001979918.1 GG16851 [Drosophila erecta]	XP_001979918.1	1,00E-16	GLOS_DERE_GG	GO:0043168	M	.	anion binding
XP_001979918.1 GG16851 [Drosophila erecta]	XP_001979918.1	1,00E-16	GLOS_DERE_GG	GO:0043167	M	.	ion binding
XP_001979918.1 GG16851 [Drosophila erecta]	XP_001979918.1	1,00E-16	GLOS_DERE_GG	GO:0043178	M	.	alcohol binding
XP_001979918.1 GG16851 [Drosophila erecta]	XP_001979918.1	1,00E-16	GLOS_DERE_GG	GO:0036094	M	.	small molecule binding
XP_001979918.1 GG16851 [Drosophila erecta]	XP_001979918.1	1,00E-16	GLOS_DERE_GG	GO:0070679	M	Refseq	inositol 1,4,5 trisphosphate binding
NP_001033997.1 CG43897, isoform H [Drosophila melanogaster]	NP_001033997.1	1,00E-44	GLOS_contig_009	GO:0006357	B	.	regulation of transcription from RNA polymerase II promoter
NP_001033997.1 CG43897, isoform H [Drosophila melanogaster]	NP_001033997.1	1,00E-44	GLOS_contig_009	GO:0006355	B	.	regulation of transcription, DNA-dependent
NP_001033997.1 CG43897, isoform H [Drosophila melanogaster]	NP_001033997.1	1,00E-44	GLOS_contig_009	GO:0010468	B	.	regulation of gene expression
NP_001033997.1 CG43897, isoform H [Drosophila melanogaster]	NP_001033997.1	1,00E-44	GLOS_contig_009	GO:0060255	B	.	regulation of macromolecule metabolic process
NP_001033997.1 CG43897, isoform H [Drosophila melanogaster]	NP_001033997.1	1,00E-44	GLOS_contig_009	GO:0019222	B	.	regulation of metabolic process
NP_001033997.1 CG43897, isoform H [Drosophila melanogaster]	NP_001033997.1	1,00E-44	GLOS_contig_009	GO:2000112	B	.	regulation of cellular macromolecule biosynthetic process
NP_001033997.1 CG43897, isoform H [Drosophila melanogaster]	NP_001033997.1	1,00E-44	GLOS_contig_009	GO:0010556	B	.	regulation of macromolecule biosynthetic process
NP_001033997.1 CG43897, isoform H [Drosophila melanogaster]	NP_001033997.1	1,00E-44	GLOS_contig_009	GO:0009889	B	.	regulation of biosynthetic process
NP_001033997.1 CG43897, isoform H [Drosophila melanogaster]	NP_001033997.1	1,00E-44	GLOS_contig_009	GO:0031326	B	.	regulation of cellular biosynthetic process
NP_001033997.1 CG43897, isoform H [Drosophila melanogaster]	NP_001033997.1	1,00E-44	GLOS_contig_009	GO:0031323	B	.	regulation of cellular metabolic process

NP_001033997.1 CG43897, isoform H [Drosophila melanogaster]	NP_001033997.1	1,00E-44	GLOS_contig_009	GO:2001141	B	.	regulation of RNA biosynthetic process
NP_001033997.1 CG43897, isoform H [Drosophila melanogaster]	NP_001033997.1	1,00E-44	GLOS_contig_009	GO:0051252	B	.	regulation of RNA metabolic process
NP_001033997.1 CG43897, isoform H [Drosophila melanogaster]	NP_001033997.1	1,00E-44	GLOS_contig_009	GO:0019219	B	.	regulation of nucleobase-containing compound metabolic process
NP_001033997.1 CG43897, isoform H [Drosophila melanogaster]	NP_001033997.1	1,00E-44	GLOS_contig_009	GO:0051171	B	.	regulation of nitrogen compound metabolic process
NP_001033997.1 CG43897, isoform H [Drosophila melanogaster]	NP_001033997.1	1,00E-44	GLOS_contig_009	GO:0080090	B	.	regulation of primary metabolic process
NP_001033997.1 CG43897, isoform H [Drosophila melanogaster]	NP_001033997.1	1,00E-44	GLOS_contig_009	GO:0045892	B	.	negative regulation of transcription, DNA-dependent
NP_001033997.1 CG43897, isoform H [Drosophila melanogaster]	NP_001033997.1	1,00E-44	GLOS_contig_009	GO:0010629	B	.	negative regulation of gene expression
NP_001033997.1 CG43897, isoform H [Drosophila melanogaster]	NP_001033997.1	1,00E-44	GLOS_contig_009	GO:0010605	B	.	negative regulation of macromolecule metabolic process
NP_001033997.1 CG43897, isoform H [Drosophila melanogaster]	NP_001033997.1	1,00E-44	GLOS_contig_009	GO:0009892	B	.	negative regulation of metabolic process
NP_001033997.1 CG43897, isoform H [Drosophila melanogaster]	NP_001033997.1	1,00E-44	GLOS_contig_009	GO:0048519	B	.	negative regulation of biological process
NP_001033997.1 CG43897, isoform H [Drosophila melanogaster]	NP_001033997.1	1,00E-44	GLOS_contig_009	GO:0051253	B	.	negative regulation of RNA metabolic process
NP_001033997.1 CG43897, isoform H [Drosophila melanogaster]	NP_001033997.1	1,00E-44	GLOS_contig_009	GO:0045934	B	.	negative regulation of nucleobase-containing compound metabolic process
NP_001033997.1 CG43897, isoform H [Drosophila melanogaster]	NP_001033997.1	1,00E-44	GLOS_contig_009	GO:0031324	B	.	negative regulation of cellular metabolic process
NP_001033997.1 CG43897, isoform H [Drosophila melanogaster]	NP_001033997.1	1,00E-44	GLOS_contig_009	GO:0048523	B	.	negative regulation of cellular process
NP_001033997.1 CG43897, isoform H [Drosophila melanogaster]	NP_001033997.1	1,00E-44	GLOS_contig_009	GO:0051172	B	.	negative regulation of nitrogen compound metabolic process
NP_001033997.1 CG43897, isoform H [Drosophila melanogaster]	NP_001033997.1	1,00E-44	GLOS_contig_009	GO:2000113	B	.	negative regulation of cellular macromolecule biosynthetic process
NP_001033997.1 CG43897, isoform H [Drosophila melanogaster]	NP_001033997.1	1,00E-44	GLOS_contig_009	GO:0010558	B	.	negative regulation of macromolecule biosynthetic process
NP_001033997.1 CG43897, isoform H [Drosophila melanogaster]	NP_001033997.1	1,00E-44	GLOS_contig_009	GO:0009890	B	.	negative regulation of biosynthetic process
NP_001033997.1 CG43897, isoform H [Drosophila melanogaster]	NP_001033997.1	1,00E-44	GLOS_contig_009	GO:0031327	B	.	negative regulation of cellular biosynthetic process
NP_001033997.1 CG43897, isoform H [Drosophila melanogaster]	NP_001033997.1	1,00E-44	GLOS_contig_009	GO:0000122	B	Refseq	negative regulation of transcription from RNA polymerase II promoter
NP_001033997.1 CG43897, isoform H [Drosophila melanogaster]	NP_001033997.1	1,00E-44	GLOS_contig_009	GO:0001501	B	Refseq	skeletal system development
NP_001033997.1 CG43897, isoform H [Drosophila melanogaster]	NP_001033997.1	1,00E-44	GLOS_contig_009	GO:0001071	M	.	nucleic acid binding transcription factor activity
NP_001033997.1 CG43897, isoform H [Drosophila melanogaster]	NP_001033997.1	1,00E-44	GLOS_contig_009	GO:0003700	M	Refseq	sequence-specific DNA binding transcription factor activity
NP_001033997.1 CG43897, isoform H [Drosophila melanogaster]	NP_001033997.1	1,00E-44	GLOS_contig_009	GO:0005515	M	Refseq	protein binding
NP_001033997.1 CG43897, isoform H [Drosophila melanogaster]	NP_001033997.1	1,00E-44	GLOS_contig_009	GO:0043231	C	.	intracellular membrane-bounded organelle
NP_001033997.1 CG43897, isoform H [Drosophila melanogaster]	NP_001033997.1	1,00E-44	GLOS_contig_009	GO:0043227	C	.	membrane-bounded organelle
NP_001033997.1 CG43897, isoform H [Drosophila melanogaster]	NP_001033997.1	1,00E-44	GLOS_contig_009	GO:0005634	C	Refseq	nucleus
NP_001033997.1 CG43897, isoform H [Drosophila melanogaster]	NP_001033997.1	1,00E-44	GLOS_contig_009	GO:0006351	B	.	transcription, DNA-dependent
NP_001033997.1 CG43897, isoform H [Drosophila melanogaster]	NP_001033997.1	1,00E-44	GLOS_contig_009	GO:0032774	B	.	RNA biosynthetic process
NP_001033997.1 CG43897, isoform H [Drosophila melanogaster]	NP_001033997.1	1,00E-44	GLOS_contig_009	GO:0009059	B	.	macromolecule biosynthetic process
NP_001033997.1 CG43897, isoform H [Drosophila melanogaster]	NP_001033997.1	1,00E-44	GLOS_contig_009	GO:0043170	B	.	macromolecule metabolic process
NP_001033997.1 CG43897, isoform H [Drosophila melanogaster]	NP_001033997.1	1,00E-44	GLOS_contig_009	GO:0071704	B	.	organic substance metabolic process
NP_001033997.1 CG43897, isoform H [Drosophila melanogaster]	NP_001033997.1	1,00E-44	GLOS_contig_009	GO:0008152	B	.	metabolic process
NP_001033997.1 CG43897, isoform H [Drosophila melanogaster]	NP_001033997.1	1,00E-44	GLOS_contig_009	GO:1901576	B	.	organic substance biosynthetic process
NP_001033997.1 CG43897, isoform H [Drosophila melanogaster]	NP_001033997.1	1,00E-44	GLOS_contig_009	GO:0009058	B	.	biosynthetic process
NP_001033997.1 CG43897, isoform H [Drosophila melanogaster]	NP_001033997.1	1,00E-44	GLOS_contig_009	GO:0016070	B	.	RNA metabolic process
NP_001033997.1 CG43897, isoform H [Drosophila melanogaster]	NP_001033997.1	1,00E-44	GLOS_contig_009	GO:0044260	B	.	cellular macromolecule metabolic process
NP_001033997.1 CG43897, isoform H [Drosophila melanogaster]	NP_001033997.1	1,00E-44	GLOS_contig_009	GO:0044237	B	.	cellular metabolic process
NP_001033997.1 CG43897, isoform H [Drosophila melanogaster]	NP_001033997.1	1,00E-44	GLOS_contig_009	GO:0090304	B	.	nucleic acid metabolic process
NP_001033997.1 CG43897, isoform H [Drosophila melanogaster]	NP_001033997.1	1,00E-44	GLOS_contig_009	GO:0006139	B	.	nucleobase-containing compound metabolic process
NP_001033997.1 CG43897, isoform H [Drosophila melanogaster]	NP_001033997.1	1,00E-44	GLOS_contig_009	GO:0006725	B	.	cellular aromatic compound metabolic process
NP_001033997.1 CG43897, isoform H [Drosophila melanogaster]	NP_001033997.1	1,00E-44	GLOS_contig_009	GO:0034641	B	.	cellular nitrogen compound metabolic process
NP_001033997.1 CG43897, isoform H [Drosophila melanogaster]	NP_001033997.1	1,00E-44	GLOS_contig_009	GO:0006807	B	.	nitrogen compound metabolic process
NP_001033997.1 CG43897, isoform H [Drosophila melanogaster]	NP_001033997.1	1,00E-44	GLOS_contig_009	GO:0044238	B	.	primary metabolic process
NP_001033997.1 CG43897, isoform H [Drosophila melanogaster]	NP_001033997.1	1,00E-44	GLOS_contig_009	GO:0046483	B	.	heterocycle metabolic process
NP_001033997.1 CG43897, isoform H [Drosophila melanogaster]	NP_001033997.1	1,00E-44	GLOS_contig_009	GO:1901360	B	.	organic cyclic compound metabolic process
NP_001033997.1 CG43897, isoform H [Drosophila melanogaster]	NP_001033997.1	1,00E-44	GLOS_contig_009	GO:0034654	B	.	nucleobase-containing compound biosynthetic process
NP_001033997.1 CG43897, isoform H [Drosophila melanogaster]	NP_001033997.1	1,00E-44	GLOS_contig_009	GO:0018130	B	.	heterocycle biosynthetic process
NP_001033997.1 CG43897, isoform H [Drosophila melanogaster]	NP_001033997.1	1,00E-44	GLOS_contig_009	GO:0044249	B	.	cellular biosynthetic process
NP_001033997.1 CG43897, isoform H [Drosophila melanogaster]	NP_001033997.1	1,00E-44	GLOS_contig_009	GO:0019438	B	.	aromatic compound biosynthetic process
NP_001033997.1 CG43897, isoform H [Drosophila melanogaster]	NP_001033997.1	1,00E-44	GLOS_contig_009	GO:0044271	B	.	cellular nitrogen compound biosynthetic process
NP_001033997.1 CG43897, isoform H [Drosophila melanogaster]	NP_001033997.1	1,00E-44	GLOS_contig_009	GO:1901362	B	.	organic cyclic compound biosynthetic process
NP_001033997.1 CG43897, isoform H [Drosophila melanogaster]	NP_001033997.1	1,00E-44	GLOS_contig_009	GO:0034645	B	.	cellular macromolecule biosynthetic process
NP_001033997.1 CG43897, isoform H [Drosophila melanogaster]	NP_001033997.1	1,00E-44	GLOS_contig_009	GO:0010467	B	.	gene expression

NP_001033997.1 CG43897, isoform H [Drosophila melanogaster]	NP_001033997.1	1,00E-44	GLOS_contig_009	GO:0006366	B	Refseq	transcription from RNA polymerase II promoter
NP_001033997.1 CG43897, isoform H [Drosophila melanogaster]	NP_001033997.1	1,00E-44	GLOS_contig_009	GO:0006606	B	.	protein import into nucleus
NP_001033997.1 CG43897, isoform H [Drosophila melanogaster]	NP_001033997.1	1,00E-44	GLOS_contig_009	GO:0006886	B	.	intracellular protein transport
NP_001033997.1 CG43897, isoform H [Drosophila melanogaster]	NP_001033997.1	1,00E-44	GLOS_contig_009	GO:0015031	B	.	protein transport
NP_001033997.1 CG43897, isoform H [Drosophila melanogaster]	NP_001033997.1	1,00E-44	GLOS_contig_009	GO:0045184	B	.	establishment of protein localization
NP_001033997.1 CG43897, isoform H [Drosophila melanogaster]	NP_001033997.1	1,00E-44	GLOS_contig_009	GO:0008104	B	.	protein localization
NP_001033997.1 CG43897, isoform H [Drosophila melanogaster]	NP_001033997.1	1,00E-44	GLOS_contig_009	GO:0033036	B	.	macromolecule localization
NP_001033997.1 CG43897, isoform H [Drosophila melanogaster]	NP_001033997.1	1,00E-44	GLOS_contig_009	GO:0071702	B	.	organic substance transport
NP_001033997.1 CG43897, isoform H [Drosophila melanogaster]	NP_001033997.1	1,00E-44	GLOS_contig_009	GO:0046907	B	.	intracellular transport
NP_001033997.1 CG43897, isoform H [Drosophila melanogaster]	NP_001033997.1	1,00E-44	GLOS_contig_009	GO:0051649	B	.	establishment of localization in cell
NP_001033997.1 CG43897, isoform H [Drosophila melanogaster]	NP_001033997.1	1,00E-44	GLOS_contig_009	GO:0051641	B	.	cellular localization
NP_001033997.1 CG43897, isoform H [Drosophila melanogaster]	NP_001033997.1	1,00E-44	GLOS_contig_009	GO:0034613	B	.	cellular protein localization
NP_001033997.1 CG43897, isoform H [Drosophila melanogaster]	NP_001033997.1	1,00E-44	GLOS_contig_009	GO:0070727	B	.	cellular macromolecule localization
NP_001033997.1 CG43897, isoform H [Drosophila melanogaster]	NP_001033997.1	1,00E-44	GLOS_contig_009	GO:0017038	B	.	protein import
NP_001033997.1 CG43897, isoform H [Drosophila melanogaster]	NP_001033997.1	1,00E-44	GLOS_contig_009	GO:0051170	B	.	nuclear import
NP_001033997.1 CG43897, isoform H [Drosophila melanogaster]	NP_001033997.1	1,00E-44	GLOS_contig_009	GO:0006913	B	.	nucleocytoplasmic transport
NP_001033997.1 CG43897, isoform H [Drosophila melanogaster]	NP_001033997.1	1,00E-44	GLOS_contig_009	GO:0016482	B	.	cytoplasmic transport
NP_001033997.1 CG43897, isoform H [Drosophila melanogaster]	NP_001033997.1	1,00E-44	GLOS_contig_009	GO:0051169	B	.	nuclear transport
NP_001033997.1 CG43897, isoform H [Drosophila melanogaster]	NP_001033997.1	1,00E-44	GLOS_contig_009	GO:0072594	B	.	establishment of protein localization to organelle
NP_001033997.1 CG43897, isoform H [Drosophila melanogaster]	NP_001033997.1	1,00E-44	GLOS_contig_009	GO:0033365	B	.	protein localization to organelle
NP_001033997.1 CG43897, isoform H [Drosophila melanogaster]	NP_001033997.1	1,00E-44	GLOS_contig_009	GO:0034504	B	.	protein localization to nucleus
NP_001033997.1 CG43897, isoform H [Drosophila melanogaster]	NP_001033997.1	1,00E-44	GLOS_contig_009	GO:0044744	B	.	protein targeting to nucleus
NP_001033997.1 CG43897, isoform H [Drosophila melanogaster]	NP_001033997.1	1,00E-44	GLOS_contig_009	GO:0006605	B	.	protein targeting
NP_001033997.1 CG43897, isoform H [Drosophila melanogaster]	NP_001033997.1	1,00E-44	GLOS_contig_009	GO:0006607	B	Refseq	NLS-bearing protein import into nucleus
NP_001033997.1 CG43897, isoform H [Drosophila melanogaster]	NP_001033997.1	1,00E-44	GLOS_contig_009	GO:0046914	M	.	transition metal ion binding
NP_001033997.1 CG43897, isoform H [Drosophila melanogaster]	NP_001033997.1	1,00E-44	GLOS_contig_009	GO:0046872	M	.	metal ion binding
NP_001033997.1 CG43897, isoform H [Drosophila melanogaster]	NP_001033997.1	1,00E-44	GLOS_contig_009	GO:0043169	M	.	cation binding
NP_001033997.1 CG43897, isoform H [Drosophila melanogaster]	NP_001033997.1	1,00E-44	GLOS_contig_009	GO:0008270	M	Refseq	zinc ion binding
NP_001033997.1 CG43897, isoform H [Drosophila melanogaster]	NP_001033997.1	1,00E-44	GLOS_contig_009	GO:0045595	B	.	regulation of cell differentiation
NP_001033997.1 CG43897, isoform H [Drosophila melanogaster]	NP_001033997.1	1,00E-44	GLOS_contig_009	GO:0050793	B	.	regulation of developmental process
NP_001033997.1 CG43897, isoform H [Drosophila melanogaster]	NP_001033997.1	1,00E-44	GLOS_contig_009	GO:0061035	B	.	regulation of cartilage development
NP_001033997.1 CG43897, isoform H [Drosophila melanogaster]	NP_001033997.1	1,00E-44	GLOS_contig_009	GO:2000026	B	.	regulation of multicellular organismal development
NP_001033997.1 CG43897, isoform H [Drosophila melanogaster]	NP_001033997.1	1,00E-44	GLOS_contig_009	GO:0051239	B	.	regulation of multicellular organismal process
NP_001033997.1 CG43897, isoform H [Drosophila melanogaster]	NP_001033997.1	1,00E-44	GLOS_contig_009	GO:0032330	B	Refseq	regulation of chondrocyte differentiation
NP_001033997.1 CG43897, isoform H [Drosophila melanogaster]	NP_001033997.1	1,00E-44	GLOS_contig_009	GO:0003677	M	.	DNA binding
NP_001033997.1 CG43897, isoform H [Drosophila melanogaster]	NP_001033997.1	1,00E-44	GLOS_contig_009	GO:0003676	M	.	nucleic acid binding
NP_001033997.1 CG43897, isoform H [Drosophila melanogaster]	NP_001033997.1	1,00E-44	GLOS_contig_009	GO:0097159	M	.	organic cyclic compound binding
NP_001033997.1 CG43897, isoform H [Drosophila melanogaster]	NP_001033997.1	1,00E-44	GLOS_contig_009	GO:1901363	M	.	heterocyclic compound binding
NP_001033997.1 CG43897, isoform H [Drosophila melanogaster]	NP_001033997.1	1,00E-44	GLOS_contig_009	GO:0043565	M	Refseq	sequence-specific DNA binding
XP_001992251.1 GH24648 [Drosophila grimshawi]	XP_001992251.1	0	GLOS_DGRI_GH2	GO:0005262	M	Refseq	calcium channel activity
XP_001992251.1 GH24648 [Drosophila grimshawi]	XP_001992251.1	0	GLOS_DGRI_GH2	GO:0005515	M	Refseq	protein binding
XP_001992251.1 GH24648 [Drosophila grimshawi]	XP_001992251.1	0	GLOS_DGRI_GH2	GO:0005886	C	Refseq	plasma membrane
XP_001992251.1 GH24648 [Drosophila grimshawi]	XP_001992251.1	0	GLOS_DGRI_GH2	GO:0005887	C	Refseq	integral to plasma membrane
XP_001992251.1 GH24648 [Drosophila grimshawi]	XP_001992251.1	0	GLOS_DGRI_GH2	GO:0006816	B	Refseq	calcium ion transport
XP_001992251.1 GH24648 [Drosophila grimshawi]	XP_001992251.1	0	GLOS_DGRI_GH2	GO:0007411	B	Refseq	axon guidance
XP_001992251.1 GH24648 [Drosophila grimshawi]	XP_001992251.1	0	GLOS_DGRI_GH2	GO:0007599	B	.	hemostasis
XP_001992251.1 GH24648 [Drosophila grimshawi]	XP_001992251.1	0	GLOS_DGRI_GH2	GO:0050817	B	.	coagulation
XP_001992251.1 GH24648 [Drosophila grimshawi]	XP_001992251.1	0	GLOS_DGRI_GH2	GO:0042060	B	.	wound healing
XP_001992251.1 GH24648 [Drosophila grimshawi]	XP_001992251.1	0	GLOS_DGRI_GH2	GO:0009611	B	.	response to wounding
XP_001992251.1 GH24648 [Drosophila grimshawi]	XP_001992251.1	0	GLOS_DGRI_GH2	GO:0006950	B	.	response to stress
XP_001992251.1 GH24648 [Drosophila grimshawi]	XP_001992251.1	0	GLOS_DGRI_GH2	GO:0007596	B	Refseq	blood coagulation
XP_001992251.1 GH24648 [Drosophila grimshawi]	XP_001992251.1	0	GLOS_DGRI_GH2	GO:0007165	B	.	signal transduction
XP_001992251.1 GH24648 [Drosophila grimshawi]	XP_001992251.1	0	GLOS_DGRI_GH2	GO:0051716	B	.	cellular response to stimulus
XP_001992251.1 GH24648 [Drosophila grimshawi]	XP_001992251.1	0	GLOS_DGRI_GH2	GO:0007154	B	.	cell communication

XP_001992251.1 GH24648 [Drosophila grimshawi]	XP_001992251.1	0	GLOS_DGRI_GH2	GO:0044700	B	.	single organism signaling
XP_001992251.1 GH24648 [Drosophila grimshawi]	XP_001992251.1	0	GLOS_DGRI_GH2	GO:0023052	B	.	signaling
XP_001992251.1 GH24648 [Drosophila grimshawi]	XP_001992251.1	0	GLOS_DGRI_GH2	GO:0009583	B	.	detection of light stimulus
XP_001992251.1 GH24648 [Drosophila grimshawi]	XP_001992251.1	0	GLOS_DGRI_GH2	GO:0009416	B	.	response to light stimulus
XP_001992251.1 GH24648 [Drosophila grimshawi]	XP_001992251.1	0	GLOS_DGRI_GH2	GO:0009314	B	.	response to radiation
XP_001992251.1 GH24648 [Drosophila grimshawi]	XP_001992251.1	0	GLOS_DGRI_GH2	GO:0009628	B	.	response to abiotic stimulus
XP_001992251.1 GH24648 [Drosophila grimshawi]	XP_001992251.1	0	GLOS_DGRI_GH2	GO:0009581	B	.	detection of external stimulus
XP_001992251.1 GH24648 [Drosophila grimshawi]	XP_001992251.1	0	GLOS_DGRI_GH2	GO:0051606	B	.	detection of stimulus
XP_001992251.1 GH24648 [Drosophila grimshawi]	XP_001992251.1	0	GLOS_DGRI_GH2	GO:0009582	B	.	detection of abiotic stimulus
XP_001992251.1 GH24648 [Drosophila grimshawi]	XP_001992251.1	0	GLOS_DGRI_GH2	GO:0007602	B	Refseq	phototransduction
XP_001992251.1 GH24648 [Drosophila grimshawi]	XP_001992251.1	0	GLOS_DGRI_GH2	GO:0010524	B	Refseq	positive regulation of calcium ion transport into cytosol
XP_001992251.1 GH24648 [Drosophila grimshawi]	XP_001992251.1	0	GLOS_DGRI_GH2	GO:0015279	M	Refseq	store-operated calcium channel activity
XP_001992251.1 GH24648 [Drosophila grimshawi]	XP_001992251.1	0	GLOS_DGRI_GH2	GO:0001775	B	.	cell activation
XP_001992251.1 GH24648 [Drosophila grimshawi]	XP_001992251.1	0	GLOS_DGRI_GH2	GO:0007596	B	.	blood coagulation
XP_001992251.1 GH24648 [Drosophila grimshawi]	XP_001992251.1	0	GLOS_DGRI_GH2	GO:0030168	B	Refseq	platelet activation
XP_001992251.1 GH24648 [Drosophila grimshawi]	XP_001992251.1	0	GLOS_DGRI_GH2	GO:0014074	B	.	response to purine-containing compound
XP_001992251.1 GH24648 [Drosophila grimshawi]	XP_001992251.1	0	GLOS_DGRI_GH2	GO:0010243	B	.	response to organonitrogen compound
XP_001992251.1 GH24648 [Drosophila grimshawi]	XP_001992251.1	0	GLOS_DGRI_GH2	GO:0009719	B	.	response to endogenous stimulus
XP_001992251.1 GH24648 [Drosophila grimshawi]	XP_001992251.1	0	GLOS_DGRI_GH2	GO:0010033	B	.	response to organic substance
XP_001992251.1 GH24648 [Drosophila grimshawi]	XP_001992251.1	0	GLOS_DGRI_GH2	GO:1901698	B	.	response to nitrogen compound
XP_001992251.1 GH24648 [Drosophila grimshawi]	XP_001992251.1	0	GLOS_DGRI_GH2	GO:0014070	B	.	response to organic cyclic compound
XP_001992251.1 GH24648 [Drosophila grimshawi]	XP_001992251.1	0	GLOS_DGRI_GH2	GO:0046683	B	.	response to organophosphorus
XP_001992251.1 GH24648 [Drosophila grimshawi]	XP_001992251.1	0	GLOS_DGRI_GH2	GO:1901700	B	.	response to oxygen-containing compound
XP_001992251.1 GH24648 [Drosophila grimshawi]	XP_001992251.1	0	GLOS_DGRI_GH2	GO:0033198	B	Refseq	response to ATP
XP_001992251.1 GH24648 [Drosophila grimshawi]	XP_001992251.1	0	GLOS_DGRI_GH2	GO:0051592	B	Refseq	response to calcium ion
XP_001992251.1 GH24648 [Drosophila grimshawi]	XP_001992251.1	0	GLOS_DGRI_GH2	GO:0006816	B	.	calcium ion transport
XP_001992251.1 GH24648 [Drosophila grimshawi]	XP_001992251.1	0	GLOS_DGRI_GH2	GO:0034220	B	.	ion transmembrane transport
XP_001992251.1 GH24648 [Drosophila grimshawi]	XP_001992251.1	0	GLOS_DGRI_GH2	GO:0055085	B	.	transmembrane transport
XP_001992251.1 GH24648 [Drosophila grimshawi]	XP_001992251.1	0	GLOS_DGRI_GH2	GO:0070588	B	Refseq	calcium ion transmembrane transport
XP_001992251.1 GH24648 [Drosophila grimshawi]	XP_001992251.1	0	GLOS_DGRI_GH2	GO:0070679	M	Refseq	inositol 1,4,5 trisphosphate binding
XP_001986241.1 GH21252 [Drosophila grimshawi]	XP_001986241.1	1,00E-102	GLOS_DGRI_GH2	GO:0005262	M	Refseq	calcium channel activity
XP_001986241.1 GH21252 [Drosophila grimshawi]	XP_001986241.1	1,00E-102	GLOS_DGRI_GH2	GO:0005515	M	Refseq	protein binding
XP_001986241.1 GH21252 [Drosophila grimshawi]	XP_001986241.1	1,00E-102	GLOS_DGRI_GH2	GO:0005886	C	Refseq	plasma membrane
XP_001986241.1 GH21252 [Drosophila grimshawi]	XP_001986241.1	1,00E-102	GLOS_DGRI_GH2	GO:0005887	C	Refseq	integral to plasma membrane
XP_001986241.1 GH21252 [Drosophila grimshawi]	XP_001986241.1	1,00E-102	GLOS_DGRI_GH2	GO:0006816	B	Refseq	calcium ion transport
XP_001986241.1 GH21252 [Drosophila grimshawi]	XP_001986241.1	1,00E-102	GLOS_DGRI_GH2	GO:0007411	B	Refseq	axon guidance
XP_001986241.1 GH21252 [Drosophila grimshawi]	XP_001986241.1	1,00E-102	GLOS_DGRI_GH2	GO:0007596	B	Refseq	blood coagulation
XP_001986241.1 GH21252 [Drosophila grimshawi]	XP_001986241.1	1,00E-102	GLOS_DGRI_GH2	GO:0007602	B	Refseq	phototransduction
XP_001986241.1 GH21252 [Drosophila grimshawi]	XP_001986241.1	1,00E-102	GLOS_DGRI_GH2	GO:0010524	B	Refseq	positive regulation of calcium ion transport into cytosol
XP_001986241.1 GH21252 [Drosophila grimshawi]	XP_001986241.1	1,00E-102	GLOS_DGRI_GH2	GO:0015279	M	Refseq	store-operated calcium channel activity
XP_001986241.1 GH21252 [Drosophila grimshawi]	XP_001986241.1	1,00E-102	GLOS_DGRI_GH2	GO:0030168	B	Refseq	platelet activation
XP_001986241.1 GH21252 [Drosophila grimshawi]	XP_001986241.1	1,00E-102	GLOS_DGRI_GH2	GO:0033198	B	Refseq	response to ATP
XP_001986241.1 GH21252 [Drosophila grimshawi]	XP_001986241.1	1,00E-102	GLOS_DGRI_GH2	GO:0051592	B	Refseq	response to calcium ion
XP_001986241.1 GH21252 [Drosophila grimshawi]	XP_001986241.1	1,00E-102	GLOS_DGRI_GH2	GO:0070588	B	Refseq	calcium ion transmembrane transport
XP_001986241.1 GH21252 [Drosophila grimshawi]	XP_001986241.1	1,00E-102	GLOS_DGRI_GH2	GO:0070679	M	Refseq	inositol 1,4,5 trisphosphate binding
XP_001990647.1 GH19473 [Drosophila grimshawi]	XP_001990647.1	1,00E-100	GLOS_DGRI_GH1	GO:0005262	M	Refseq	calcium channel activity
XP_001990647.1 GH19473 [Drosophila grimshawi]	XP_001990647.1	1,00E-100	GLOS_DGRI_GH1	GO:0005515	M	Refseq	protein binding
XP_001990647.1 GH19473 [Drosophila grimshawi]	XP_001990647.1	1,00E-100	GLOS_DGRI_GH1	GO:0005886	C	Refseq	plasma membrane
XP_001990647.1 GH19473 [Drosophila grimshawi]	XP_001990647.1	1,00E-100	GLOS_DGRI_GH1	GO:0005887	C	Refseq	integral to plasma membrane
XP_001990647.1 GH19473 [Drosophila grimshawi]	XP_001990647.1	1,00E-100	GLOS_DGRI_GH1	GO:0006816	B	Refseq	calcium ion transport
XP_001990647.1 GH19473 [Drosophila grimshawi]	XP_001990647.1	1,00E-100	GLOS_DGRI_GH1	GO:0007411	B	Refseq	axon guidance
XP_001990647.1 GH19473 [Drosophila grimshawi]	XP_001990647.1	1,00E-100	GLOS_DGRI_GH1	GO:0007596	B	Refseq	blood coagulation
XP_001990647.1 GH19473 [Drosophila grimshawi]	XP_001990647.1	1,00E-100	GLOS_DGRI_GH1	GO:0007602	B	Refseq	phototransduction
XP_001990647.1 GH19473 [Drosophila grimshawi]	XP_001990647.1	1,00E-100	GLOS_DGRI_GH1	GO:0010524	B	Refseq	positive regulation of calcium ion transport into cytosol

XP_001990647.1 GH19473 [Drosophila grimshawi]	XP_001990647.1	1,00E-100	GLOS_DGRI_GH1	GO:0015279	M	Refseq	store-operated calcium channel activity
XP_001990647.1 GH19473 [Drosophila grimshawi]	XP_001990647.1	1,00E-100	GLOS_DGRI_GH1	GO:0030168	B	Refseq	platelet activation
XP_001990647.1 GH19473 [Drosophila grimshawi]	XP_001990647.1	1,00E-100	GLOS_DGRI_GH1	GO:0033198	B	Refseq	response to ATP
XP_001990647.1 GH19473 [Drosophila grimshawi]	XP_001990647.1	1,00E-100	GLOS_DGRI_GH1	GO:0051592	B	Refseq	response to calcium ion
XP_001990647.1 GH19473 [Drosophila grimshawi]	XP_001990647.1	1,00E-100	GLOS_DGRI_GH1	GO:0070588	B	Refseq	calcium ion transmembrane transport
XP_001990647.1 GH19473 [Drosophila grimshawi]	XP_001990647.1	1,00E-100	GLOS_DGRI_GH1	GO:0070679	M	Refseq	inositol 1,4,5 trisphosphate binding
XP_001996857.1 GH23671 [Drosophila grimshawi]	XP_001996857.1	7,00E-82	GLOS_DGRI_GH2	GO:0005262	M	Refseq	calcium channel activity
XP_001996857.1 GH23671 [Drosophila grimshawi]	XP_001996857.1	7,00E-82	GLOS_DGRI_GH2	GO:0005515	M	Refseq	protein binding
XP_001996857.1 GH23671 [Drosophila grimshawi]	XP_001996857.1	7,00E-82	GLOS_DGRI_GH2	GO:0005886	C	Refseq	plasma membrane
XP_001996857.1 GH23671 [Drosophila grimshawi]	XP_001996857.1	7,00E-82	GLOS_DGRI_GH2	GO:0005887	C	Refseq	integral to plasma membrane
XP_001996857.1 GH23671 [Drosophila grimshawi]	XP_001996857.1	7,00E-82	GLOS_DGRI_GH2	GO:0006816	B	Refseq	calcium ion transport
XP_001996857.1 GH23671 [Drosophila grimshawi]	XP_001996857.1	7,00E-82	GLOS_DGRI_GH2	GO:0007411	B	Refseq	axon guidance
XP_001996857.1 GH23671 [Drosophila grimshawi]	XP_001996857.1	7,00E-82	GLOS_DGRI_GH2	GO:0007596	B	Refseq	blood coagulation
XP_001996857.1 GH23671 [Drosophila grimshawi]	XP_001996857.1	7,00E-82	GLOS_DGRI_GH2	GO:0007602	B	Refseq	phototransduction
XP_001996857.1 GH23671 [Drosophila grimshawi]	XP_001996857.1	7,00E-82	GLOS_DGRI_GH2	GO:0010524	B	Refseq	positive regulation of calcium ion transport into cytosol
XP_001996857.1 GH23671 [Drosophila grimshawi]	XP_001996857.1	7,00E-82	GLOS_DGRI_GH2	GO:0015279	M	Refseq	store-operated calcium channel activity
XP_001996857.1 GH23671 [Drosophila grimshawi]	XP_001996857.1	7,00E-82	GLOS_DGRI_GH2	GO:0030168	B	Refseq	platelet activation
XP_001996857.1 GH23671 [Drosophila grimshawi]	XP_001996857.1	7,00E-82	GLOS_DGRI_GH2	GO:0033198	B	Refseq	response to ATP
XP_001996857.1 GH23671 [Drosophila grimshawi]	XP_001996857.1	7,00E-82	GLOS_DGRI_GH2	GO:0051592	B	Refseq	response to calcium ion
XP_001996857.1 GH23671 [Drosophila grimshawi]	XP_001996857.1	7,00E-82	GLOS_DGRI_GH2	GO:0070588	B	Refseq	calcium ion transmembrane transport
XP_001996857.1 GH23671 [Drosophila grimshawi]	XP_001996857.1	7,00E-82	GLOS_DGRI_GH2	GO:0070679	M	Refseq	inositol 1,4,5 trisphosphate binding
NP_001247254.1 unkempt, isoform D [Drosophila melanogaster]	NP_001247254.1	0	GLOS_UNK.1.1	GO:0000122	B	Refseq	negative regulation of transcription from RNA polymerase II promoter
NP_001247254.1 unkempt, isoform D [Drosophila melanogaster]	NP_001247254.1	0	GLOS_UNK.1.1	GO:0001501	B	Refseq	skeletal system development
NP_001247254.1 unkempt, isoform D [Drosophila melanogaster]	NP_001247254.1	0	GLOS_UNK.1.1	GO:0003700	M	Refseq	sequence-specific DNA binding transcription factor activity
NP_001247254.1 unkempt, isoform D [Drosophila melanogaster]	NP_001247254.1	0	GLOS_UNK.1.1	GO:0005515	M	Refseq	protein binding
NP_001247254.1 unkempt, isoform D [Drosophila melanogaster]	NP_001247254.1	0	GLOS_UNK.1.1	GO:0005634	C	Refseq	nucleus
NP_001247254.1 unkempt, isoform D [Drosophila melanogaster]	NP_001247254.1	0	GLOS_UNK.1.1	GO:0006366	B	Refseq	transcription from RNA polymerase II promoter
NP_001247254.1 unkempt, isoform D [Drosophila melanogaster]	NP_001247254.1	0	GLOS_UNK.1.1	GO:0006607	B	Refseq	NLS-bearing protein import into nucleus
NP_001247254.1 unkempt, isoform D [Drosophila melanogaster]	NP_001247254.1	0	GLOS_UNK.1.1	GO:0008270	M	Refseq	zinc ion binding
NP_001247254.1 unkempt, isoform D [Drosophila melanogaster]	NP_001247254.1	0	GLOS_UNK.1.1	GO:0032330	B	Refseq	regulation of chondrocyte differentiation
NP_001247254.1 unkempt, isoform D [Drosophila melanogaster]	NP_001247254.1	0	GLOS_UNK.1.1	GO:0043565	M	Refseq	sequence-specific DNA binding
XP_001015327.1 Leucine Rich Repeat family protein [T. thermophila]	XP_001015327.1	9,00E-06	GLOS_TTHERM_(	GO:0017111	M	.	nucleoside-triphosphatase activity
XP_001015327.1 Leucine Rich Repeat family protein [T. thermophila]	XP_001015327.1	9,00E-06	GLOS_TTHERM_(	GO:0016462	M	.	pyrophosphatase activity
XP_001015327.1 Leucine Rich Repeat family protein [T. thermophila]	XP_001015327.1	9,00E-06	GLOS_TTHERM_(	GO:0016818	M	.	hydrolase activity, acting on acid anhydrides, in phosphorus-containing anhydrides
XP_001015327.1 Leucine Rich Repeat family protein [T. thermophila]	XP_001015327.1	9,00E-06	GLOS_TTHERM_(	GO:0016817	M	.	hydrolase activity, acting on acid anhydrides
XP_001015327.1 Leucine Rich Repeat family protein [T. thermophila]	XP_001015327.1	9,00E-06	GLOS_TTHERM_(	GO:0016787	M	.	hydrolase activity
XP_001015327.1 Leucine Rich Repeat family protein [T. thermophila]	XP_001015327.1	9,00E-06	GLOS_TTHERM_(	GO:0003824	M	.	catalytic activity
XP_001015327.1 Leucine Rich Repeat family protein [T. thermophila]	XP_001015327.1	9,00E-06	GLOS_TTHERM_(	GO:0003924	M	Refseq	GTPase activity
XP_001015327.1 Leucine Rich Repeat family protein [T. thermophila]	XP_001015327.1	9,00E-06	GLOS_TTHERM_(	GO:0005515	M	Refseq	protein binding
XP_001015327.1 Leucine Rich Repeat family protein [T. thermophila]	XP_001015327.1	9,00E-06	GLOS_TTHERM_(	GO:0032550	M	.	purine ribonucleoside binding
XP_001015327.1 Leucine Rich Repeat family protein [T. thermophila]	XP_001015327.1	9,00E-06	GLOS_TTHERM_(	GO:0001883	M	.	purine nucleoside binding
XP_001015327.1 Leucine Rich Repeat family protein [T. thermophila]	XP_001015327.1	9,00E-06	GLOS_TTHERM_(	GO:0001882	M	.	nucleoside binding
XP_001015327.1 Leucine Rich Repeat family protein [T. thermophila]	XP_001015327.1	9,00E-06	GLOS_TTHERM_(	GO:0097367	M	.	carbohydrate derivative binding
XP_001015327.1 Leucine Rich Repeat family protein [T. thermophila]	XP_001015327.1	9,00E-06	GLOS_TTHERM_(	GO:0032549	M	.	ribonucleoside binding
XP_001015327.1 Leucine Rich Repeat family protein [T. thermophila]	XP_001015327.1	9,00E-06	GLOS_TTHERM_(	GO:0032561	M	.	guanyl ribonucleotide binding
XP_001015327.1 Leucine Rich Repeat family protein [T. thermophila]	XP_001015327.1	9,00E-06	GLOS_TTHERM_(	GO:0019001	M	.	guanyl nucleotide binding
XP_001015327.1 Leucine Rich Repeat family protein [T. thermophila]	XP_001015327.1	9,00E-06	GLOS_TTHERM_(	GO:0017076	M	.	purine nucleotide binding
XP_001015327.1 Leucine Rich Repeat family protein [T. thermophila]	XP_001015327.1	9,00E-06	GLOS_TTHERM_(	GO:0000166	M	.	nucleotide binding
XP_001015327.1 Leucine Rich Repeat family protein [T. thermophila]	XP_001015327.1	9,00E-06	GLOS_TTHERM_(	GO:1901265	M	.	nucleoside phosphate binding
XP_001015327.1 Leucine Rich Repeat family protein [T. thermophila]	XP_001015327.1	9,00E-06	GLOS_TTHERM_(	GO:0032555	M	.	purine ribonucleotide binding
XP_001015327.1 Leucine Rich Repeat family protein [T. thermophila]	XP_001015327.1	9,00E-06	GLOS_TTHERM_(	GO:0032553	M	.	ribonucleotide binding
XP_001015327.1 Leucine Rich Repeat family protein [T. thermophila]	XP_001015327.1	9,00E-06	GLOS_TTHERM_(	GO:0035639	M	.	purine ribonucleoside triphosphate binding
XP_001015327.1 Leucine Rich Repeat family protein [T. thermophila]	XP_001015327.1	9,00E-06	GLOS_TTHERM_(	GO:0005525	M	Refseq	GTP binding
XP_001015327.1 Leucine Rich Repeat family protein [T. thermophila]	XP_001015327.1	9,00E-06	GLOS_TTHERM_(	GO:0005829	C	Refseq	cytosol

XP_001015327.1 Leucine Rich Repeat family protein [T. thermophila]	XP_001015327.1	9,00E-06	GLOS_TTHERM_(	GO:0030334	B	.	regulation of cell migration
XP_001015327.1 Leucine Rich Repeat family protein [T. thermophila]	XP_001015327.1	9,00E-06	GLOS_TTHERM_(	GO:2000145	B	.	regulation of cell motility
XP_001015327.1 Leucine Rich Repeat family protein [T. thermophila]	XP_001015327.1	9,00E-06	GLOS_TTHERM_(	GO:0040012	B	.	regulation of locomotion
XP_001015327.1 Leucine Rich Repeat family protein [T. thermophila]	XP_001015327.1	9,00E-06	GLOS_TTHERM_(	GO:0051270	B	.	regulation of cellular component movement
XP_001015327.1 Leucine Rich Repeat family protein [T. thermophila]	XP_001015327.1	9,00E-06	GLOS_TTHERM_(	GO:2000146	B	.	negative regulation of cell motility
XP_001015327.1 Leucine Rich Repeat family protein [T. thermophila]	XP_001015327.1	9,00E-06	GLOS_TTHERM_(	GO:0040013	B	.	negative regulation of locomotion
XP_001015327.1 Leucine Rich Repeat family protein [T. thermophila]	XP_001015327.1	9,00E-06	GLOS_TTHERM_(	GO:0051271	B	.	negative regulation of cellular component movement
XP_001015327.1 Leucine Rich Repeat family protein [T. thermophila]	XP_001015327.1	9,00E-06	GLOS_TTHERM_(	GO:0030336	B	Refseq	negative regulation of cell migration
XP_001015327.1 Leucine Rich Repeat family protein [T. thermophila]	XP_001015327.1	9,00E-06	GLOS_TTHERM_(	GO:0030496	C	Refseq	midbody
XP_001015327.1 Leucine Rich Repeat family protein [T. thermophila]	XP_001015327.1	9,00E-06	GLOS_TTHERM_(	GO:0030036	B	.	actin cytoskeleton organization
XP_001015327.1 Leucine Rich Repeat family protein [T. thermophila]	XP_001015327.1	9,00E-06	GLOS_TTHERM_(	GO:0007010	B	.	cytoskeleton organization
XP_001015327.1 Leucine Rich Repeat family protein [T. thermophila]	XP_001015327.1	9,00E-06	GLOS_TTHERM_(	GO:0006996	B	.	organelle organization
XP_001015327.1 Leucine Rich Repeat family protein [T. thermophila]	XP_001015327.1	9,00E-06	GLOS_TTHERM_(	GO:0030029	B	.	actin filament-based process
XP_001015327.1 Leucine Rich Repeat family protein [T. thermophila]	XP_001015327.1	9,00E-06	GLOS_TTHERM_(	GO:0031532	B	Refseq	actin cytoskeleton reorganization
XP_001015327.1 Leucine Rich Repeat family protein [T. thermophila]	XP_001015327.1	9,00E-06	GLOS_TTHERM_(	GO:0001934	B	.	positive regulation of protein phosphorylation
XP_001015327.1 Leucine Rich Repeat family protein [T. thermophila]	XP_001015327.1	9,00E-06	GLOS_TTHERM_(	GO:0001932	B	.	regulation of protein phosphorylation
XP_001015327.1 Leucine Rich Repeat family protein [T. thermophila]	XP_001015327.1	9,00E-06	GLOS_TTHERM_(	GO:0031399	B	.	regulation of protein modification process
XP_001015327.1 Leucine Rich Repeat family protein [T. thermophila]	XP_001015327.1	9,00E-06	GLOS_TTHERM_(	GO:0032268	B	.	regulation of cellular protein metabolic process
XP_001015327.1 Leucine Rich Repeat family protein [T. thermophila]	XP_001015327.1	9,00E-06	GLOS_TTHERM_(	GO:0051246	B	.	regulation of protein metabolic process
XP_001015327.1 Leucine Rich Repeat family protein [T. thermophila]	XP_001015327.1	9,00E-06	GLOS_TTHERM_(	GO:0042325	B	.	regulation of phosphorylation
XP_001015327.1 Leucine Rich Repeat family protein [T. thermophila]	XP_001015327.1	9,00E-06	GLOS_TTHERM_(	GO:0019220	B	.	regulation of phosphate metabolic process
XP_001015327.1 Leucine Rich Repeat family protein [T. thermophila]	XP_001015327.1	9,00E-06	GLOS_TTHERM_(	GO:0051174	B	.	regulation of phosphorus metabolic process
XP_001015327.1 Leucine Rich Repeat family protein [T. thermophila]	XP_001015327.1	9,00E-06	GLOS_TTHERM_(	GO:0031401	B	.	positive regulation of protein modification process
XP_001015327.1 Leucine Rich Repeat family protein [T. thermophila]	XP_001015327.1	9,00E-06	GLOS_TTHERM_(	GO:0032270	B	.	positive regulation of cellular protein metabolic process
XP_001015327.1 Leucine Rich Repeat family protein [T. thermophila]	XP_001015327.1	9,00E-06	GLOS_TTHERM_(	GO:0031325	B	.	positive regulation of cellular metabolic process
XP_001015327.1 Leucine Rich Repeat family protein [T. thermophila]	XP_001015327.1	9,00E-06	GLOS_TTHERM_(	GO:0009893	B	.	positive regulation of metabolic process
XP_001015327.1 Leucine Rich Repeat family protein [T. thermophila]	XP_001015327.1	9,00E-06	GLOS_TTHERM_(	GO:0051247	B	.	positive regulation of protein metabolic process
XP_001015327.1 Leucine Rich Repeat family protein [T. thermophila]	XP_001015327.1	9,00E-06	GLOS_TTHERM_(	GO:0010604	B	.	positive regulation of macromolecule metabolic process
XP_001015327.1 Leucine Rich Repeat family protein [T. thermophila]	XP_001015327.1	9,00E-06	GLOS_TTHERM_(	GO:0042327	B	.	positive regulation of phosphorylation
XP_001015327.1 Leucine Rich Repeat family protein [T. thermophila]	XP_001015327.1	9,00E-06	GLOS_TTHERM_(	GO:0045937	B	.	positive regulation of phosphate metabolic process
XP_001015327.1 Leucine Rich Repeat family protein [T. thermophila]	XP_001015327.1	9,00E-06	GLOS_TTHERM_(	GO:0010562	B	.	positive regulation of phosphorus metabolic process
XP_001015327.1 Leucine Rich Repeat family protein [T. thermophila]	XP_001015327.1	9,00E-06	GLOS_TTHERM_(	GO:0031952	B	.	regulation of protein autophosphorylation
XP_001015327.1 Leucine Rich Repeat family protein [T. thermophila]	XP_001015327.1	9,00E-06	GLOS_TTHERM_(	GO:0031954	B	Refseq	positive regulation of protein autophosphorylation
XP_001015327.1 Leucine Rich Repeat family protein [T. thermophila]	XP_001015327.1	9,00E-06	GLOS_TTHERM_(	GO:0007265	B	.	Ras protein signal transduction
XP_001015327.1 Leucine Rich Repeat family protein [T. thermophila]	XP_001015327.1	9,00E-06	GLOS_TTHERM_(	GO:0007264	B	.	small GTPase mediated signal transduction
XP_001015327.1 Leucine Rich Repeat family protein [T. thermophila]	XP_001015327.1	9,00E-06	GLOS_TTHERM_(	GO:0035556	B	.	intracellular signal transduction
XP_001015327.1 Leucine Rich Repeat family protein [T. thermophila]	XP_001015327.1	9,00E-06	GLOS_TTHERM_(	GO:0032486	B	Refseq	Rap protein signal transduction
XP_001015327.1 Leucine Rich Repeat family protein [T. thermophila]	XP_001015327.1	9,00E-06	GLOS_TTHERM_(	GO:0034613	B	Refseq	cellular protein localization
XP_001015327.1 Leucine Rich Repeat family protein [T. thermophila]	XP_001015327.1	9,00E-06	GLOS_TTHERM_(	GO:0045184	B	Refseq	establishment of protein localization
XP_001015327.1 Leucine Rich Repeat family protein [T. thermophila]	XP_001015327.1	9,00E-06	GLOS_TTHERM_(	GO:0032872	B	.	regulation of stress-activated MAPK cascade
XP_001015327.1 Leucine Rich Repeat family protein [T. thermophila]	XP_001015327.1	9,00E-06	GLOS_TTHERM_(	GO:0043408	B	.	regulation of MAPK cascade
XP_001015327.1 Leucine Rich Repeat family protein [T. thermophila]	XP_001015327.1	9,00E-06	GLOS_TTHERM_(	GO:0010627	B	.	regulation of intracellular protein kinase cascade
XP_001015327.1 Leucine Rich Repeat family protein [T. thermophila]	XP_001015327.1	9,00E-06	GLOS_TTHERM_(	GO:0009966	B	.	regulation of signal transduction
XP_001015327.1 Leucine Rich Repeat family protein [T. thermophila]	XP_001015327.1	9,00E-06	GLOS_TTHERM_(	GO:0010646	B	.	regulation of cell communication
XP_001015327.1 Leucine Rich Repeat family protein [T. thermophila]	XP_001015327.1	9,00E-06	GLOS_TTHERM_(	GO:0023051	B	.	regulation of signaling
XP_001015327.1 Leucine Rich Repeat family protein [T. thermophila]	XP_001015327.1	9,00E-06	GLOS_TTHERM_(	GO:0048583	B	.	regulation of response to stimulus
XP_001015327.1 Leucine Rich Repeat family protein [T. thermophila]	XP_001015327.1	9,00E-06	GLOS_TTHERM_(	GO:0070302	B	.	regulation of stress-activated protein kinase signaling cascade
XP_001015327.1 Leucine Rich Repeat family protein [T. thermophila]	XP_001015327.1	9,00E-06	GLOS_TTHERM_(	GO:0080135	B	.	regulation of cellular response to stress
XP_001015327.1 Leucine Rich Repeat family protein [T. thermophila]	XP_001015327.1	9,00E-06	GLOS_TTHERM_(	GO:0080134	B	.	regulation of response to stress
XP_001015327.1 Leucine Rich Repeat family protein [T. thermophila]	XP_001015327.1	9,00E-06	GLOS_TTHERM_(	GO:0046328	B	Refseq	regulation of JNK cascade
XP_001015327.1 Leucine Rich Repeat family protein [T. thermophila]	XP_001015327.1	9,00E-06	GLOS_TTHERM_(	GO:0010769	B	.	regulation of cell morphogenesis involved in differentiation
XP_001015327.1 Leucine Rich Repeat family protein [T. thermophila]	XP_001015327.1	9,00E-06	GLOS_TTHERM_(	GO:0022604	B	.	regulation of cell morphogenesis
XP_001015327.1 Leucine Rich Repeat family protein [T. thermophila]	XP_001015327.1	9,00E-06	GLOS_TTHERM_(	GO:0022603	B	.	regulation of anatomical structure morphogenesis
XP_001015327.1 Leucine Rich Repeat family protein [T. thermophila]	XP_001015327.1	9,00E-06	GLOS_TTHERM_(	GO:0051128	B	.	regulation of cellular component organization

XP_001015327.1 Leucine Rich Repeat family protein [T. thermophila]	XP_001015327.1	9,00E-06	GLOS_TTHERM_(	GO:0060284	B	.	regulation of cell development
XP_001015327.1 Leucine Rich Repeat family protein [T. thermophila]	XP_001015327.1	9,00E-06	GLOS_TTHERM_(	GO:0050773	B	.	regulation of dendrite development
XP_001015327.1 Leucine Rich Repeat family protein [T. thermophila]	XP_001015327.1	9,00E-06	GLOS_TTHERM_(	GO:0010975	B	.	regulation of neuron projection development
XP_001015327.1 Leucine Rich Repeat family protein [T. thermophila]	XP_001015327.1	9,00E-06	GLOS_TTHERM_(	GO:0031344	B	.	regulation of cell projection organization
XP_001015327.1 Leucine Rich Repeat family protein [T. thermophila]	XP_001015327.1	9,00E-06	GLOS_TTHERM_(	GO:0045664	B	.	regulation of neuron differentiation
XP_001015327.1 Leucine Rich Repeat family protein [T. thermophila]	XP_001015327.1	9,00E-06	GLOS_TTHERM_(	GO:0050767	B	.	regulation of neurogenesis
XP_001015327.1 Leucine Rich Repeat family protein [T. thermophila]	XP_001015327.1	9,00E-06	GLOS_TTHERM_(	GO:0051960	B	.	regulation of nervous system development
XP_001015327.1 Leucine Rich Repeat family protein [T. thermophila]	XP_001015327.1	9,00E-06	GLOS_TTHERM_(	GO:0048814	B	Refseq	regulation of dendrite morphogenesis
XP_001015327.1 Leucine Rich Repeat family protein [T. thermophila]	XP_001015327.1	9,00E-06	GLOS_TTHERM_(	GO:0005768	C	.	endosome
XP_001015327.1 Leucine Rich Repeat family protein [T. thermophila]	XP_001015327.1	9,00E-06	GLOS_TTHERM_(	GO:0055037	C	Refseq	recycling endosome
XP_001015327.1 Leucine Rich Repeat family protein [T. thermophila]	XP_001015327.1	9,00E-06	GLOS_TTHERM_(	GO:0010008	C	.	endosome membrane
XP_001015327.1 Leucine Rich Repeat family protein [T. thermophila]	XP_001015327.1	9,00E-06	GLOS_TTHERM_(	GO:0031090	C	.	organelle membrane
XP_001015327.1 Leucine Rich Repeat family protein [T. thermophila]	XP_001015327.1	9,00E-06	GLOS_TTHERM_(	GO:0044440	C	.	endosomal part
XP_001015327.1 Leucine Rich Repeat family protein [T. thermophila]	XP_001015327.1	9,00E-06	GLOS_TTHERM_(	GO:0044446	C	.	intracellular organelle part
XP_001015327.1 Leucine Rich Repeat family protein [T. thermophila]	XP_001015327.1	9,00E-06	GLOS_TTHERM_(	GO:0055037	C	.	recycling endosome
XP_001015327.1 Leucine Rich Repeat family protein [T. thermophila]	XP_001015327.1	9,00E-06	GLOS_TTHERM_(	GO:0055038	C	Refseq	recycling endosome membrane
XP_001977467.1 GG19064 [Drosophila erecta]	XP_001977467.1	3,00E-38	GLOS_DERE_GG	GO:0005261	M	Refseq	cation channel activity
XP_001977467.1 GG19064 [Drosophila erecta]	XP_001977467.1	3,00E-38	GLOS_DERE_GG	GO:0005262	M	Refseq	calcium channel activity
XP_001977467.1 GG19064 [Drosophila erecta]	XP_001977467.1	3,00E-38	GLOS_DERE_GG	GO:0005515	M	Refseq	protein binding
XP_001977467.1 GG19064 [Drosophila erecta]	XP_001977467.1	3,00E-38	GLOS_DERE_GG	GO:0005886	C	Refseq	plasma membrane
XP_001977467.1 GG19064 [Drosophila erecta]	XP_001977467.1	3,00E-38	GLOS_DERE_GG	GO:0005887	C	Refseq	integral to plasma membrane
XP_001977467.1 GG19064 [Drosophila erecta]	XP_001977467.1	3,00E-38	GLOS_DERE_GG	GO:0006816	B	Refseq	calcium ion transport
XP_001977467.1 GG19064 [Drosophila erecta]	XP_001977467.1	3,00E-38	GLOS_DERE_GG	GO:0007411	B	Refseq	axon guidance
XP_001977467.1 GG19064 [Drosophila erecta]	XP_001977467.1	3,00E-38	GLOS_DERE_GG	GO:0015279	M	Refseq	store-operated calcium channel activity
XP_001977467.1 GG19064 [Drosophila erecta]	XP_001977467.1	3,00E-38	GLOS_DERE_GG	GO:0016323	C	Refseq	basolateral plasma membrane
XP_001977467.1 GG19064 [Drosophila erecta]	XP_001977467.1	3,00E-38	GLOS_DERE_GG	GO:0030017	C	Refseq	sarcomere
XP_001977467.1 GG19064 [Drosophila erecta]	XP_001977467.1	3,00E-38	GLOS_DERE_GG	GO:0043034	C	Refseq	costamere
XP_001977467.1 GG19064 [Drosophila erecta]	XP_001977467.1	3,00E-38	GLOS_DERE_GG	GO:0043234	C	Refseq	protein complex
XP_001977467.1 GG19064 [Drosophila erecta]	XP_001977467.1	3,00E-38	GLOS_DERE_GG	GO:0044325	M	Refseq	ion channel binding
XP_001977467.1 GG19064 [Drosophila erecta]	XP_001977467.1	3,00E-38	GLOS_DERE_GG	GO:0045121	C	Refseq	membrane raft
XP_001977467.1 GG19064 [Drosophila erecta]	XP_001977467.1	3,00E-38	GLOS_DERE_GG	GO:0046541	B	Refseq	saliva secretion
XP_001977467.1 GG19064 [Drosophila erecta]	XP_001977467.1	3,00E-38	GLOS_DERE_GG	GO:0051281	B	Refseq	positive regulation of release of sequestered calcium ion into cytosol
XP_001977467.1 GG19064 [Drosophila erecta]	XP_001977467.1	3,00E-38	GLOS_DERE_GG	GO:0051480	B	Refseq	cytosolic calcium ion homeostasis
XP_001977467.1 GG19064 [Drosophila erecta]	XP_001977467.1	3,00E-38	GLOS_DERE_GG	GO:0051592	B	Refseq	response to calcium ion
XP_001977467.1 GG19064 [Drosophila erecta]	XP_001977467.1	3,00E-38	GLOS_DERE_GG	GO:0070679	M	Refseq	inositol 1,4,5 trisphosphate binding
XP_004929373.1 PREDICT: uncharact prot LOC101742112 [B. mori]	XP_004929373.1	4,00E-06	GLOS_LOC10174:	GO:0000122	B	Refseq	negative regulation of transcription from RNA polymerase II promoter
XP_004929373.1 PREDICT: uncharact prot LOC101742112 [B. mori]	XP_004929373.1	4,00E-06	GLOS_LOC10174:	GO:0003674	M	Refseq	molecular_function
XP_004929373.1 PREDICT: uncharact prot LOC101742112 [B. mori]	XP_004929373.1	4,00E-06	GLOS_LOC10174:	GO:0003682	M	Refseq	chromatin binding
XP_004929373.1 PREDICT: uncharact prot LOC101742112 [B. mori]	XP_004929373.1	4,00E-06	GLOS_LOC10174:	GO:0000981	M	.	sequence-specific DNA binding RNA polymerase II transcription factor activity
XP_004929373.1 PREDICT: uncharact prot LOC101742112 [B. mori]	XP_004929373.1	4,00E-06	GLOS_LOC10174:	GO:0003700	M	.	sequence-specific DNA binding transcription factor activity
XP_004929373.1 PREDICT: uncharact prot LOC101742112 [B. mori]	XP_004929373.1	4,00E-06	GLOS_LOC10174:	GO:0003705	M	Refseq	RNA polymerase II distal enhancer sequence-specific DNA binding transcription factor activity
XP_004929373.1 PREDICT: uncharact prot LOC101742112 [B. mori]	XP_004929373.1	4,00E-06	GLOS_LOC10174:	GO:0003712	M	.	transcription cofactor activity
XP_004929373.1 PREDICT: uncharact prot LOC101742112 [B. mori]	XP_004929373.1	4,00E-06	GLOS_LOC10174:	GO:0000989	M	.	transcription factor binding transcription factor activity
XP_004929373.1 PREDICT: uncharact prot LOC101742112 [B. mori]	XP_004929373.1	4,00E-06	GLOS_LOC10174:	GO:0000988	M	.	protein binding transcription factor activity
XP_004929373.1 PREDICT: uncharact prot LOC101742112 [B. mori]	XP_004929373.1	4,00E-06	GLOS_LOC10174:	GO:0003714	M	Refseq	transcription corepressor activity
XP_004929373.1 PREDICT: uncharact prot LOC101742112 [B. mori]	XP_004929373.1	4,00E-06	GLOS_LOC10174:	GO:0005634	C	Refseq	nucleus
XP_004929373.1 PREDICT: uncharact prot LOC101742112 [B. mori]	XP_004929373.1	4,00E-06	GLOS_LOC10174:	GO:0044428	C	.	nuclear part
XP_004929373.1 PREDICT: uncharact prot LOC101742112 [B. mori]	XP_004929373.1	4,00E-06	GLOS_LOC10174:	GO:0005634	C	.	nucleus
XP_004929373.1 PREDICT: uncharact prot LOC101742112 [B. mori]	XP_004929373.1	4,00E-06	GLOS_LOC10174:	GO:0031981	C	.	nuclear lumen
XP_004929373.1 PREDICT: uncharact prot LOC101742112 [B. mori]	XP_004929373.1	4,00E-06	GLOS_LOC10174:	GO:0070013	C	.	intracellular organelle lumen
XP_004929373.1 PREDICT: uncharact prot LOC101742112 [B. mori]	XP_004929373.1	4,00E-06	GLOS_LOC10174:	GO:0043233	C	.	organelle lumen
XP_004929373.1 PREDICT: uncharact prot LOC101742112 [B. mori]	XP_004929373.1	4,00E-06	GLOS_LOC10174:	GO:0031974	C	.	membrane-enclosed lumen
XP_004929373.1 PREDICT: uncharact prot LOC101742112 [B. mori]	XP_004929373.1	4,00E-06	GLOS_LOC10174:	GO:0005654	C	Refseq	nucleoplasm

XP_004929373.1 PREDICT: uncharact prot LOC101742112 [B. mori]	XP_004929373.1	4,00E-06	GLOS_LOC10174:	GO:0007166	B	.	cell surface receptor signaling pathway
XP_004929373.1 PREDICT: uncharact prot LOC101742112 [B. mori]	XP_004929373.1	4,00E-06	GLOS_LOC10174:	GO:0007219	B	Refseq	Notch signaling pathway
XP_004929373.1 PREDICT: uncharact prot LOC101742112 [B. mori]	XP_004929373.1	4,00E-06	GLOS_LOC10174:	GO:0008150	B	Refseq	biological_process
XP_004929373.1 PREDICT: uncharact prot LOC101742112 [B. mori]	XP_004929373.1	4,00E-06	GLOS_LOC10174:	GO:0016055	B	Refseq	Wnt receptor signaling pathway
XP_004929373.1 PREDICT: uncharact prot LOC101742112 [B. mori]	XP_004929373.1	4,00E-06	GLOS_LOC10174:	GO:0008134	M	.	transcription factor binding
XP_004929373.1 PREDICT: uncharact prot LOC101742112 [B. mori]	XP_004929373.1	4,00E-06	GLOS_LOC10174:	GO:0070491	M	Refseq	repressing transcription factor binding
NP_001097534.1 ankyrin 2, isoform E [Drosophila melanogaster]	NP_001097534.1	0	GLOS_ANK2.1.1	GO:0000122	B	Refseq	negative regulation of transcription from RNA polymerase II promoter
NP_001097534.1 ankyrin 2, isoform E [Drosophila melanogaster]	NP_001097534.1	0	GLOS_ANK2.1.1	GO:0001501	B	Refseq	skeletal system development
NP_001097534.1 ankyrin 2, isoform E [Drosophila melanogaster]	NP_001097534.1	0	GLOS_ANK2.1.1	GO:0003700	M	Refseq	sequence-specific DNA binding transcription factor activity
NP_001097534.1 ankyrin 2, isoform E [Drosophila melanogaster]	NP_001097534.1	0	GLOS_ANK2.1.1	GO:0005515	M	Refseq	protein binding
NP_001097534.1 ankyrin 2, isoform E [Drosophila melanogaster]	NP_001097534.1	0	GLOS_ANK2.1.1	GO:0005634	C	Refseq	nucleus
NP_001097534.1 ankyrin 2, isoform E [Drosophila melanogaster]	NP_001097534.1	0	GLOS_ANK2.1.1	GO:0006366	B	Refseq	transcription from RNA polymerase II promoter
NP_001097534.1 ankyrin 2, isoform E [Drosophila melanogaster]	NP_001097534.1	0	GLOS_ANK2.1.1	GO:0006607	B	Refseq	NLS-bearing protein import into nucleus
NP_001097534.1 ankyrin 2, isoform E [Drosophila melanogaster]	NP_001097534.1	0	GLOS_ANK2.1.1	GO:0008270	M	Refseq	zinc ion binding
NP_001097534.1 ankyrin 2, isoform E [Drosophila melanogaster]	NP_001097534.1	0	GLOS_ANK2.1.1	GO:0032330	B	Refseq	regulation of chondrocyte differentiation
NP_001097534.1 ankyrin 2, isoform E [Drosophila melanogaster]	NP_001097534.1	0	GLOS_ANK2.1.1	GO:0043565	M	Refseq	sequence-specific DNA binding
XP_001664151.1 RP58 protein, putative [Aedes aegypti]	XP_001664151.1	1,00E-26	GLOS_AAEL_AAE	GO:0030674	M	.	protein binding, bridging
XP_001664151.1 RP58 protein, putative [Aedes aegypti]	XP_001664151.1	1,00E-26	GLOS_AAEL_AAE	GO:0060090	M	.	binding, bridging
XP_001664151.1 RP58 protein, putative [Aedes aegypti]	XP_001664151.1	1,00E-26	GLOS_AAEL_AAE	GO:0035591	M	.	signaling adaptor activity
XP_001664151.1 RP58 protein, putative [Aedes aegypti]	XP_001664151.1	1,00E-26	GLOS_AAEL_AAE	GO:0005070	M	Refseq	SH3/SH2 adaptor activity
XP_001664151.1 RP58 protein, putative [Aedes aegypti]	XP_001664151.1	1,00E-26	GLOS_AAEL_AAE	GO:0005515	M	Refseq	protein binding
XP_001664151.1 RP58 protein, putative [Aedes aegypti]	XP_001664151.1	1,00E-26	GLOS_AAEL_AAE	GO:0005634	C	Refseq	nucleus
XP_001664151.1 RP58 protein, putative [Aedes aegypti]	XP_001664151.1	1,00E-26	GLOS_AAEL_AAE	GO:0005737	C	Refseq	cytoplasm
XP_001664151.1 RP58 protein, putative [Aedes aegypti]	XP_001664151.1	1,00E-26	GLOS_AAEL_AAE	GO:0007049	B	Refseq	cell cycle
XP_001664151.1 RP58 protein, putative [Aedes aegypti]	XP_001664151.1	1,00E-26	GLOS_AAEL_AAE	GO:0007165	B	Refseq	signal transduction
XP_001664151.1 RP58 protein, putative [Aedes aegypti]	XP_001664151.1	1,00E-26	GLOS_AAEL_AAE	GO:0007417	B	Refseq	central nervous system development
XP_001664151.1 RP58 protein, putative [Aedes aegypti]	XP_001664151.1	1,00E-26	GLOS_AAEL_AAE	GO:0048513	B	.	organ development
XP_001664151.1 RP58 protein, putative [Aedes aegypti]	XP_001664151.1	1,00E-26	GLOS_AAEL_AAE	GO:0072358	B	.	cardiovascular system development
XP_001664151.1 RP58 protein, putative [Aedes aegypti]	XP_001664151.1	1,00E-26	GLOS_AAEL_AAE	GO:0072359	B	.	circulatory system development
XP_001664151.1 RP58 protein, putative [Aedes aegypti]	XP_001664151.1	1,00E-26	GLOS_AAEL_AAE	GO:0007507	B	Refseq	heart development
XP_001664151.1 RP58 protein, putative [Aedes aegypti]	XP_001664151.1	1,00E-26	GLOS_AAEL_AAE	GO:0009790	B	.	embryo development
XP_001664151.1 RP58 protein, putative [Aedes aegypti]	XP_001664151.1	1,00E-26	GLOS_AAEL_AAE	GO:0009792	B	Refseq	embryo development ending in birth or egg hatching
XP_001664151.1 RP58 protein, putative [Aedes aegypti]	XP_001664151.1	1,00E-26	GLOS_AAEL_AAE	GO:0010212	B	Refseq	response to ionizing radiation
XP_001664151.1 RP58 protein, putative [Aedes aegypti]	XP_001664151.1	1,00E-26	GLOS_AAEL_AAE	GO:0019904	M	.	protein domain specific binding
XP_001664151.1 RP58 protein, putative [Aedes aegypti]	XP_001664151.1	1,00E-26	GLOS_AAEL_AAE	GO:0017124	M	Refseq	SH3 domain binding
XP_001664151.1 RP58 protein, putative [Aedes aegypti]	XP_001664151.1	1,00E-26	GLOS_AAEL_AAE	GO:0042802	M	Refseq	identical protein binding
XP_001664151.1 RP58 protein, putative [Aedes aegypti]	XP_001664151.1	1,00E-26	GLOS_AAEL_AAE	GO:0051726	B	.	regulation of cell cycle
XP_001664151.1 RP58 protein, putative [Aedes aegypti]	XP_001664151.1	1,00E-26	GLOS_AAEL_AAE	GO:0045786	B	Refseq	negative regulation of cell cycle
XP_001664151.1 RP58 protein, putative [Aedes aegypti]	XP_001664151.1	1,00E-26	GLOS_AAEL_AAE	GO:0048471	C	Refseq	perinuclear region of cytoplasm
XP_001664151.1 RP58 protein, putative [Aedes aegypti]	XP_001664151.1	1,00E-26	GLOS_AAEL_AAE	GO:0051059	M	Refseq	NF-kappaB binding
XP_001664151.1 RP58 protein, putative [Aedes aegypti]	XP_001664151.1	1,00E-26	GLOS_AAEL_AAE	GO:0072331	B	.	signal transduction by p53 class mediator
XP_001664151.1 RP58 protein, putative [Aedes aegypti]	XP_001664151.1	1,00E-26	GLOS_AAEL_AAE	GO:0097193	B	.	intrinsic apoptotic signaling pathway
XP_001664151.1 RP58 protein, putative [Aedes aegypti]	XP_001664151.1	1,00E-26	GLOS_AAEL_AAE	GO:0097190	B	.	apoptotic signaling pathway
XP_001664151.1 RP58 protein, putative [Aedes aegypti]	XP_001664151.1	1,00E-26	GLOS_AAEL_AAE	GO:0006915	B	.	apoptotic process
XP_001664151.1 RP58 protein, putative [Aedes aegypti]	XP_001664151.1	1,00E-26	GLOS_AAEL_AAE	GO:0012501	B	.	programmed cell death
XP_001664151.1 RP58 protein, putative [Aedes aegypti]	XP_001664151.1	1,00E-26	GLOS_AAEL_AAE	GO:0008219	B	.	cell death
XP_001664151.1 RP58 protein, putative [Aedes aegypti]	XP_001664151.1	1,00E-26	GLOS_AAEL_AAE	GO:0016265	B	.	death
XP_001664151.1 RP58 protein, putative [Aedes aegypti]	XP_001664151.1	1,00E-26	GLOS_AAEL_AAE	GO:0072332	B	Refseq	intrinsic apoptotic signaling pathway by p53 class mediator
NM_176552.2 D.m. eIF4E-Homologous prot (4EHP), transcript variant A, mRNA	NM_176552.2	5,00E-32	GLOS_4EHP.1.1	GO:0000122	B	Refseq	negative regulation of transcription from RNA polymerase II promoter
NM_176552.2 D.m. eIF4E-Homologous prot (4EHP), transcript variant A, mRNA	NM_176552.2	5,00E-32	GLOS_4EHP.1.1	GO:0001501	B	Refseq	skeletal system development
NM_176552.2 D.m. eIF4E-Homologous prot (4EHP), transcript variant A, mRNA	NM_176552.2	5,00E-32	GLOS_4EHP.1.1	GO:0003700	M	Refseq	sequence-specific DNA binding transcription factor activity
NM_176552.2 D.m. eIF4E-Homologous prot (4EHP), transcript variant A, mRNA	NM_176552.2	5,00E-32	GLOS_4EHP.1.1	GO:0005515	M	Refseq	protein binding
NM_176552.2 D.m. eIF4E-Homologous prot (4EHP), transcript variant A, mRNA	NM_176552.2	5,00E-32	GLOS_4EHP.1.1	GO:0005634	C	Refseq	nucleus
NM_176552.2 D.m. eIF4E-Homologous prot (4EHP), transcript variant A, mRNA	NM_176552.2	5,00E-32	GLOS_4EHP.1.1	GO:0006366	B	Refseq	transcription from RNA polymerase II promoter

NM_176552.2 D.m. eIF4E-Homologous prot (4EHP), transcript variant A, mRNA	NM_176552.2	5,00E-32	GLOS_4EHP.1.1	GO:0006607	B	Refseq	NLS-bearing protein import into nucleus
NM_176552.2 D.m. eIF4E-Homologous prot (4EHP), transcript variant A, mRNA	NM_176552.2	5,00E-32	GLOS_4EHP.1.1	GO:0008270	M	Refseq	zinc ion binding
NM_176552.2 D.m. eIF4E-Homologous prot (4EHP), transcript variant A, mRNA	NM_176552.2	5,00E-32	GLOS_4EHP.1.1	GO:0032330	B	Refseq	regulation of chondrocyte differentiation
NM_176552.2 D.m. eIF4E-Homologous prot (4EHP), transcript variant A, mRNA	NM_176552.2	5,00E-32	GLOS_4EHP.1.1	GO:0043565	M	Refseq	sequence-specific DNA binding
NP_649919.1 CG9427, isoform A [Drosophila melanogaster]	NP_649919.1	3,00E-32	GLOS_CG9427.2.:	GO:0000122	B	Refseq	negative regulation of transcription from RNA polymerase II promoter
NP_649919.1 CG9427, isoform A [Drosophila melanogaster]	NP_649919.1	3,00E-32	GLOS_CG9427.2.:	GO:0001501	B	Refseq	skeletal system development
NP_649919.1 CG9427, isoform A [Drosophila melanogaster]	NP_649919.1	3,00E-32	GLOS_CG9427.2.:	GO:0003700	M	Refseq	sequence-specific DNA binding transcription factor activity
NP_649919.1 CG9427, isoform A [Drosophila melanogaster]	NP_649919.1	3,00E-32	GLOS_CG9427.2.:	GO:0005515	M	Refseq	protein binding
NP_649919.1 CG9427, isoform A [Drosophila melanogaster]	NP_649919.1	3,00E-32	GLOS_CG9427.2.:	GO:0005634	C	Refseq	nucleus
NP_649919.1 CG9427, isoform A [Drosophila melanogaster]	NP_649919.1	3,00E-32	GLOS_CG9427.2.:	GO:0006366	B	Refseq	transcription from RNA polymerase II promoter
NP_649919.1 CG9427, isoform A [Drosophila melanogaster]	NP_649919.1	3,00E-32	GLOS_CG9427.2.:	GO:0006607	B	Refseq	NLS-bearing protein import into nucleus
NP_649919.1 CG9427, isoform A [Drosophila melanogaster]	NP_649919.1	3,00E-32	GLOS_CG9427.2.:	GO:0008270	M	Refseq	zinc ion binding
NP_649919.1 CG9427, isoform A [Drosophila melanogaster]	NP_649919.1	3,00E-32	GLOS_CG9427.2.:	GO:0032330	B	Refseq	regulation of chondrocyte differentiation
NP_649919.1 CG9427, isoform A [Drosophila melanogaster]	NP_649919.1	3,00E-32	GLOS_CG9427.2.:	GO:0043565	M	Refseq	sequence-specific DNA binding
XP_001987803.1 GH22112 [Drosophila grimshawi]	XP_001987803.1	1,00E-124	GLOS_DGRI_GH2	GO:0005262	M	Refseq	calcium channel activity
XP_001987803.1 GH22112 [Drosophila grimshawi]	XP_001987803.1	1,00E-124	GLOS_DGRI_GH2	GO:0005515	M	Refseq	protein binding
XP_001987803.1 GH22112 [Drosophila grimshawi]	XP_001987803.1	1,00E-124	GLOS_DGRI_GH2	GO:0005886	C	Refseq	plasma membrane
XP_001987803.1 GH22112 [Drosophila grimshawi]	XP_001987803.1	1,00E-124	GLOS_DGRI_GH2	GO:0005887	C	Refseq	integral to plasma membrane
XP_001987803.1 GH22112 [Drosophila grimshawi]	XP_001987803.1	1,00E-124	GLOS_DGRI_GH2	GO:0006816	B	Refseq	calcium ion transport
XP_001987803.1 GH22112 [Drosophila grimshawi]	XP_001987803.1	1,00E-124	GLOS_DGRI_GH2	GO:0007411	B	Refseq	axon guidance
XP_001987803.1 GH22112 [Drosophila grimshawi]	XP_001987803.1	1,00E-124	GLOS_DGRI_GH2	GO:0007596	B	Refseq	blood coagulation
XP_001987803.1 GH22112 [Drosophila grimshawi]	XP_001987803.1	1,00E-124	GLOS_DGRI_GH2	GO:0007602	B	Refseq	phototransduction
XP_001987803.1 GH22112 [Drosophila grimshawi]	XP_001987803.1	1,00E-124	GLOS_DGRI_GH2	GO:0010524	B	Refseq	positive regulation of calcium ion transport into cytosol
XP_001987803.1 GH22112 [Drosophila grimshawi]	XP_001987803.1	1,00E-124	GLOS_DGRI_GH2	GO:0015279	M	Refseq	store-operated calcium channel activity
XP_001987803.1 GH22112 [Drosophila grimshawi]	XP_001987803.1	1,00E-124	GLOS_DGRI_GH2	GO:0030168	B	Refseq	platelet activation
XP_001987803.1 GH22112 [Drosophila grimshawi]	XP_001987803.1	1,00E-124	GLOS_DGRI_GH2	GO:0033198	B	Refseq	response to ATP
XP_001987803.1 GH22112 [Drosophila grimshawi]	XP_001987803.1	1,00E-124	GLOS_DGRI_GH2	GO:0051592	B	Refseq	response to calcium ion
XP_001987803.1 GH22112 [Drosophila grimshawi]	XP_001987803.1	1,00E-124	GLOS_DGRI_GH2	GO:0070588	B	Refseq	calcium ion transmembrane transport
XP_001987803.1 GH22112 [Drosophila grimshawi]	XP_001987803.1	1,00E-124	GLOS_DGRI_GH2	GO:0070679	M	Refseq	inositol 1,4,5 trisphosphate binding
NP_608658.1 lethal (2) s5379, isoform A [D. melanogaster] ref|NP_001014460.1| isoform D [D. melanogaster] ref|NP_001027220.1| isoform E [D. melanogaster]	NP_608658.1	1,00E-146	GLOS_L_2_S5379	GO:0000122	B	Refseq	negative regulation of transcription from RNA polymerase II promoter
NP_608658.1 lethal (2) s5379, isoform A [D.m. … Same as above	NP_608658.1	1,00E-146	GLOS_L_2_S5379	GO:0001501	B	Refseq	skeletal system development
NP_608658.1 lethal (2) s5379, isoform A [D.m. … Same as above	NP_608658.1	1,00E-146	GLOS_L_2_S5379	GO:0003700	M	Refseq	sequence-specific DNA binding transcription factor activity
NP_608658.1 lethal (2) s5379, isoform A [D.m. … Same as above	NP_608658.1	1,00E-146	GLOS_L_2_S5379	GO:0005515	M	Refseq	protein binding
NP_608658.1 lethal (2) s5379, isoform A [D.m. … Same as above	NP_608658.1	1,00E-146	GLOS_L_2_S5379	GO:0005634	C	Refseq	nucleus
NP_608658.1 lethal (2) s5379, isoform A [D.m. … Same as above	NP_608658.1	1,00E-146	GLOS_L_2_S5379	GO:0006366	B	Refseq	transcription from RNA polymerase II promoter
NP_608658.1 lethal (2) s5379, isoform A [D.m. … Same as above	NP_608658.1	1,00E-146	GLOS_L_2_S5379	GO:0006607	B	Refseq	NLS-bearing protein import into nucleus
NP_608658.1 lethal (2) s5379, isoform A [D.m. … Same as above	NP_608658.1	1,00E-146	GLOS_L_2_S5379	GO:0008270	M	Refseq	zinc ion binding
NP_608658.1 lethal (2) s5379, isoform A [D.m. … Same as above	NP_608658.1	1,00E-146	GLOS_L_2_S5379	GO:0032330	B	Refseq	regulation of chondrocyte differentiation
NP_608658.1 lethal (2) s5379, isoform A [D.m. … Same as above	NP_608658.1	1,00E-146	GLOS_L_2_S5379	GO:0043565	M	Refseq	sequence-specific DNA binding
NP_001137703.1 CG42351, isoform C [Drosophila melanogaster]	NP_001137703.1	6,00E-25	GLOS_CG42351.1	GO:0000122	B	Refseq	negative regulation of transcription from RNA polymerase II promoter
NP_001137703.1 CG42351, isoform C [Drosophila melanogaster]	NP_001137703.1	6,00E-25	GLOS_CG42351.1	GO:0001501	B	Refseq	skeletal system development
NP_001137703.1 CG42351, isoform C [Drosophila melanogaster]	NP_001137703.1	6,00E-25	GLOS_CG42351.1	GO:0003700	M	Refseq	sequence-specific DNA binding transcription factor activity
NP_001137703.1 CG42351, isoform C [Drosophila melanogaster]	NP_001137703.1	6,00E-25	GLOS_CG42351.1	GO:0005515	M	Refseq	protein binding
NP_001137703.1 CG42351, isoform C [Drosophila melanogaster]	NP_001137703.1	6,00E-25	GLOS_CG42351.1	GO:0005634	C	Refseq	nucleus
NP_001137703.1 CG42351, isoform C [Drosophila melanogaster]	NP_001137703.1	6,00E-25	GLOS_CG42351.1	GO:0006366	B	Refseq	transcription from RNA polymerase II promoter
NP_001137703.1 CG42351, isoform C [Drosophila melanogaster]	NP_001137703.1	6,00E-25	GLOS_CG42351.1	GO:0006607	B	Refseq	NLS-bearing protein import into nucleus
NP_001137703.1 CG42351, isoform C [Drosophila melanogaster]	NP_001137703.1	6,00E-25	GLOS_CG42351.1	GO:0008270	M	Refseq	zinc ion binding
NP_001137703.1 CG42351, isoform C [Drosophila melanogaster]	NP_001137703.1	6,00E-25	GLOS_CG42351.1	GO:0032330	B	Refseq	regulation of chondrocyte differentiation
NP_001137703.1 CG42351, isoform C [Drosophila melanogaster]	NP_001137703.1	6,00E-25	GLOS_CG42351.1	GO:0043565	M	Refseq	sequence-specific DNA binding
XP_001991031.1 GH12294 [Drosophila grimshawi]	XP_001991031.1	1,00E-30	GLOS_DGRI_GH1	GO:0005262	M	Refseq	calcium channel activity
XP_001991031.1 GH12294 [Drosophila grimshawi]	XP_001991031.1	1,00E-30	GLOS_DGRI_GH1	GO:0005515	M	Refseq	protein binding
XP_001991031.1 GH12294 [Drosophila grimshawi]	XP_001991031.1	1,00E-30	GLOS_DGRI_GH1	GO:0005886	C	Refseq	plasma membrane
XP_001991031.1 GH12294 [Drosophila grimshawi]	XP_001991031.1	1,00E-30	GLOS_DGRI_GH1	GO:0005887	C	Refseq	integral to plasma membrane

XP_001991031.1 GH12294 [Drosophila grimshawi]	XP_001991031.1	1,00E-30	GLOS_DGRI_GH1	GO:0006816	B	Refseq	calcium ion transport
XP_001991031.1 GH12294 [Drosophila grimshawi]	XP_001991031.1	1,00E-30	GLOS_DGRI_GH1	GO:0007411	B	Refseq	axon guidance
XP_001991031.1 GH12294 [Drosophila grimshawi]	XP_001991031.1	1,00E-30	GLOS_DGRI_GH1	GO:0007596	B	Refseq	blood coagulation
XP_001991031.1 GH12294 [Drosophila grimshawi]	XP_001991031.1	1,00E-30	GLOS_DGRI_GH1	GO:0007602	B	Refseq	phototransduction
XP_001991031.1 GH12294 [Drosophila grimshawi]	XP_001991031.1	1,00E-30	GLOS_DGRI_GH1	GO:0010524	B	Refseq	positive regulation of calcium ion transport into cytosol
XP_001991031.1 GH12294 [Drosophila grimshawi]	XP_001991031.1	1,00E-30	GLOS_DGRI_GH1	GO:0015279	M	Refseq	store-operated calcium channel activity
XP_001991031.1 GH12294 [Drosophila grimshawi]	XP_001991031.1	1,00E-30	GLOS_DGRI_GH1	GO:0030168	B	Refseq	platelet activation
XP_001991031.1 GH12294 [Drosophila grimshawi]	XP_001991031.1	1,00E-30	GLOS_DGRI_GH1	GO:0033198	B	Refseq	response to ATP
XP_001991031.1 GH12294 [Drosophila grimshawi]	XP_001991031.1	1,00E-30	GLOS_DGRI_GH1	GO:0051592	B	Refseq	response to calcium ion
XP_001991031.1 GH12294 [Drosophila grimshawi]	XP_001991031.1	1,00E-30	GLOS_DGRI_GH1	GO:0070588	B	Refseq	calcium ion transmembrane transport
XP_001991031.1 GH12294 [Drosophila grimshawi]	XP_001991031.1	1,00E-30	GLOS_DGRI_GH1	GO:0070679	M	Refseq	inositol 1,4,5 trisphosphate binding
XP_001975622.1 GG20463 [D.erecta]ref|XP_002081512.1|GD11059 [D.simulans]	XP_001975622.1	4,00E-53	GLOS_DERE_GG	GO:0005261	M	Refseq	cation channel activity
XP_001975622.1 GG20463 [D.erecta]ref|XP_002081512.1|GD11059 [D.simulans]	XP_001975622.1	4,00E-53	GLOS_DERE_GG	GO:0005262	M	Refseq	calcium channel activity
XP_001975622.1 GG20463 [D.erecta]ref|XP_002081512.1|GD11059 [D.simulans]	XP_001975622.1	4,00E-53	GLOS_DERE_GG	GO:0005515	M	Refseq	protein binding
XP_001975622.1 GG20463 [D.erecta]ref|XP_002081512.1|GD11059 [D.simulans]	XP_001975622.1	4,00E-53	GLOS_DERE_GG	GO:0005886	C	Refseq	plasma membrane
XP_001975622.1 GG20463 [D.erecta]ref|XP_002081512.1|GD11059 [D.simulans]	XP_001975622.1	4,00E-53	GLOS_DERE_GG	GO:0005887	C	Refseq	integral to plasma membrane
XP_001975622.1 GG20463 [D.erecta]ref|XP_002081512.1|GD11059 [D.simulans]	XP_001975622.1	4,00E-53	GLOS_DERE_GG	GO:0006816	B	Refseq	calcium ion transport
XP_001975622.1 GG20463 [D.erecta]ref|XP_002081512.1|GD11059 [D.simulans]	XP_001975622.1	4,00E-53	GLOS_DERE_GG	GO:0007411	B	Refseq	axon guidance
XP_001975622.1 GG20463 [D.erecta]ref|XP_002081512.1|GD11059 [D.simulans]	XP_001975622.1	4,00E-53	GLOS_DERE_GG	GO:0015279	M	Refseq	store-operated calcium channel activity
XP_001975622.1 GG20463 [D.erecta]ref|XP_002081512.1|GD11059 [D.simulans]	XP_001975622.1	4,00E-53	GLOS_DERE_GG	GO:0016323	C	Refseq	basolateral plasma membrane
XP_001975622.1 GG20463 [D.erecta]ref|XP_002081512.1|GD11059 [D.simulans]	XP_001975622.1	4,00E-53	GLOS_DERE_GG	GO:0030017	C	Refseq	sarcomere
XP_001975622.1 GG20463 [D.erecta]ref|XP_002081512.1|GD11059 [D.simulans]	XP_001975622.1	4,00E-53	GLOS_DERE_GG	GO:0043034	C	Refseq	costamere
XP_001975622.1 GG20463 [D.erecta]ref|XP_002081512.1|GD11059 [D.simulans]	XP_001975622.1	4,00E-53	GLOS_DERE_GG	GO:0043234	C	Refseq	protein complex
XP_001975622.1 GG20463 [D.erecta]ref|XP_002081512.1|GD11059 [D.simulans]	XP_001975622.1	4,00E-53	GLOS_DERE_GG	GO:0044325	M	Refseq	ion channel binding
XP_001975622.1 GG20463 [D.erecta]ref|XP_002081512.1|GD11059 [D.simulans]	XP_001975622.1	4,00E-53	GLOS_DERE_GG	GO:0045121	C	Refseq	membrane raft
XP_001975622.1 GG20463 [D.erecta]ref|XP_002081512.1|GD11059 [D.simulans]	XP_001975622.1	4,00E-53	GLOS_DERE_GG	GO:0046541	B	Refseq	saliva secretion
XP_001975622.1 GG20463 [D.erecta]ref|XP_002081512.1|GD11059 [D.simulans]	XP_001975622.1	4,00E-53	GLOS_DERE_GG	GO:0051281	B	Refseq	positive regulation of release of sequestered calcium ion into cytosol
XP_001975622.1 GG20463 [D.erecta]ref|XP_002081512.1|GD11059 [D.simulans]	XP_001975622.1	4,00E-53	GLOS_DERE_GG	GO:0051480	B	Refseq	cytosolic calcium ion homeostasis
XP_001975622.1 GG20463 [D.erecta]ref|XP_002081512.1|GD11059 [D.simulans]	XP_001975622.1	4,00E-53	GLOS_DERE_GG	GO:0051592	B	Refseq	response to calcium ion
XP_001975622.1 GG20463 [D.erecta]ref|XP_002081512.1|GD11059 [D.simulans]	XP_001975622.1	4,00E-53	GLOS_DERE_GG	GO:0070679	M	Refseq	inositol 1,4,5 trisphosphate binding
XP_001661092.1 AMP dependent ligase [Aedes aegypti]	XP_001661092.1	1,00E-128	GLOS_AAEL_AAE	GO:0005070	M	Refseq	SH3/SH2 adaptor activity
XP_001661092.1 AMP dependent ligase [Aedes aegypti]	XP_001661092.1	1,00E-128	GLOS_AAEL_AAE	GO:0005515	M	Refseq	protein binding
XP_001661092.1 AMP dependent ligase [Aedes aegypti]	XP_001661092.1	1,00E-128	GLOS_AAEL_AAE	GO:0005634	C	Refseq	nucleus
XP_001661092.1 AMP dependent ligase [Aedes aegypti]	XP_001661092.1	1,00E-128	GLOS_AAEL_AAE	GO:0005737	C	Refseq	cytoplasm
XP_001661092.1 AMP dependent ligase [Aedes aegypti]	XP_001661092.1	1,00E-128	GLOS_AAEL_AAE	GO:0007049	B	Refseq	cell cycle
XP_001661092.1 AMP dependent ligase [Aedes aegypti]	XP_001661092.1	1,00E-128	GLOS_AAEL_AAE	GO:0007165	B	Refseq	signal transduction
XP_001661092.1 AMP dependent ligase [Aedes aegypti]	XP_001661092.1	1,00E-128	GLOS_AAEL_AAE	GO:0007417	B	Refseq	central nervous system development
XP_001661092.1 AMP dependent ligase [Aedes aegypti]	XP_001661092.1	1,00E-128	GLOS_AAEL_AAE	GO:0007507	B	Refseq	heart development
XP_001661092.1 AMP dependent ligase [Aedes aegypti]	XP_001661092.1	1,00E-128	GLOS_AAEL_AAE	GO:0009792	B	Refseq	embryo development ending in birth or egg hatching
XP_001661092.1 AMP dependent ligase [Aedes aegypti]	XP_001661092.1	1,00E-128	GLOS_AAEL_AAE	GO:0010212	B	Refseq	response to ionizing radiation
XP_001661092.1 AMP dependent ligase [Aedes aegypti]	XP_001661092.1	1,00E-128	GLOS_AAEL_AAE	GO:0017124	M	Refseq	SH3 domain binding
XP_001661092.1 AMP dependent ligase [Aedes aegypti]	XP_001661092.1	1,00E-128	GLOS_AAEL_AAE	GO:0042802	M	Refseq	identical protein binding
XP_001661092.1 AMP dependent ligase [Aedes aegypti]	XP_001661092.1	1,00E-128	GLOS_AAEL_AAE	GO:0045786	B	Refseq	negative regulation of cell cycle
XP_001661092.1 AMP dependent ligase [Aedes aegypti]	XP_001661092.1	1,00E-128	GLOS_AAEL_AAE	GO:0048471	C	Refseq	perinuclear region of cytoplasm
XP_001661092.1 AMP dependent ligase [Aedes aegypti]	XP_001661092.1	1,00E-128	GLOS_AAEL_AAE	GO:0051059	M	Refseq	NF-kappaB binding
XP_001661092.1 AMP dependent ligase [Aedes aegypti]	XP_001661092.1	1,00E-128	GLOS_AAEL_AAE	GO:0072332	B	Refseq	intrinsic apoptotic signaling pathway by p53 class mediator
NP_609315.1 CG13123, isoform A [Drosophila melanogaster]	NP_609315.1	3,00E-42	GLOS_CG13123.1	GO:0000122	B	Refseq	negative regulation of transcription from RNA polymerase II promoter
NP_609315.1 CG13123, isoform A [Drosophila melanogaster]	NP_609315.1	3,00E-42	GLOS_CG13123.1	GO:0001501	B	Refseq	skeletal system development
NP_609315.1 CG13123, isoform A [Drosophila melanogaster]	NP_609315.1	3,00E-42	GLOS_CG13123.1	GO:0003700	M	Refseq	sequence-specific DNA binding transcription factor activity
NP_609315.1 CG13123, isoform A [Drosophila melanogaster]	NP_609315.1	3,00E-42	GLOS_CG13123.1	GO:0005515	M	Refseq	protein binding
NP_609315.1 CG13123, isoform A [Drosophila melanogaster]	NP_609315.1	3,00E-42	GLOS_CG13123.1	GO:0005634	C	Refseq	nucleus
NP_609315.1 CG13123, isoform A [Drosophila melanogaster]	NP_609315.1	3,00E-42	GLOS_CG13123.1	GO:0006366	B	Refseq	transcription from RNA polymerase II promoter
NP_609315.1 CG13123, isoform A [Drosophila melanogaster]	NP_609315.1	3,00E-42	GLOS_CG13123.1	GO:0006607	B	Refseq	NLS-bearing protein import into nucleus
NP_609315.1 CG13123, isoform A [Drosophila melanogaster]	NP_609315.1	3,00E-42	GLOS_CG13123.1	GO:0008270	M	Refseq	zinc ion binding

NP_609315.1 CG13123, isoform A [Drosophila melanogaster]	NP_609315.1	3,00E-42	GLOS_CG13123.1	GO:0032330	B	Refseq	regulation of chondrocyte differentiation
NP_609315.1 CG13123, isoform A [Drosophila melanogaster]	NP_609315.1	3,00E-42	GLOS_CG13123.1	GO:0043565	M	Refseq	sequence-specific DNA binding
NP_001260550.1 ribosomal prot L30, isoform D [D. melanogaster]	NP_001260550.1	1,00E-28	GLOS_RPL30.3.3	GO:0000122	B	Refseq	negative regulation of transcription from RNA polymerase II promoter
NP_001260550.1 ribosomal prot L30, isoform D [D. melanogaster]	NP_001260550.1	1,00E-28	GLOS_RPL30.3.3	GO:0001501	B	Refseq	skeletal system development
NP_001260550.1 ribosomal prot L30, isoform D [D. melanogaster]	NP_001260550.1	1,00E-28	GLOS_RPL30.3.3	GO:0003700	M	Refseq	sequence-specific DNA binding transcription factor activity
NP_001260550.1 ribosomal prot L30, isoform D [D. melanogaster]	NP_001260550.1	1,00E-28	GLOS_RPL30.3.3	GO:0005515	M	Refseq	protein binding
NP_001260550.1 ribosomal prot L30, isoform D [D. melanogaster]	NP_001260550.1	1,00E-28	GLOS_RPL30.3.3	GO:0005634	C	Refseq	nucleus
NP_001260550.1 ribosomal prot L30, isoform D [D. melanogaster]	NP_001260550.1	1,00E-28	GLOS_RPL30.3.3	GO:0006366	B	Refseq	transcription from RNA polymerase II promoter
NP_001260550.1 ribosomal prot L30, isoform D [D. melanogaster]	NP_001260550.1	1,00E-28	GLOS_RPL30.3.3	GO:0006607	B	Refseq	NLS-bearing protein import into nucleus
NP_001260550.1 ribosomal prot L30, isoform D [D. melanogaster]	NP_001260550.1	1,00E-28	GLOS_RPL30.3.3	GO:0008270	M	Refseq	zinc ion binding
NP_001260550.1 ribosomal prot L30, isoform D [D. melanogaster]	NP_001260550.1	1,00E-28	GLOS_RPL30.3.3	GO:0032330	B	Refseq	regulation of chondrocyte differentiation
NP_001260550.1 ribosomal prot L30, isoform D [D. melanogaster]	NP_001260550.1	1,00E-28	GLOS_RPL30.3.3	GO:0043565	M	Refseq	sequence-specific DNA binding
XP_001976256.1 GG22769 [Drosophila erecta]	XP_001976256.1	0	GLOS_DERE_GG	GO:0005261	M	Refseq	cation channel activity
XP_001976256.1 GG22769 [Drosophila erecta]	XP_001976256.1	0	GLOS_DERE_GG	GO:0005262	M	Refseq	calcium channel activity
XP_001976256.1 GG22769 [Drosophila erecta]	XP_001976256.1	0	GLOS_DERE_GG	GO:0005515	M	Refseq	protein binding
XP_001976256.1 GG22769 [Drosophila erecta]	XP_001976256.1	0	GLOS_DERE_GG	GO:0005886	C	Refseq	plasma membrane
XP_001976256.1 GG22769 [Drosophila erecta]	XP_001976256.1	0	GLOS_DERE_GG	GO:0005887	C	Refseq	integral to plasma membrane
XP_001976256.1 GG22769 [Drosophila erecta]	XP_001976256.1	0	GLOS_DERE_GG	GO:0006816	B	Refseq	calcium ion transport
XP_001976256.1 GG22769 [Drosophila erecta]	XP_001976256.1	0	GLOS_DERE_GG	GO:0007411	B	Refseq	axon guidance
XP_001976256.1 GG22769 [Drosophila erecta]	XP_001976256.1	0	GLOS_DERE_GG	GO:0015279	M	Refseq	store-operated calcium channel activity
XP_001976256.1 GG22769 [Drosophila erecta]	XP_001976256.1	0	GLOS_DERE_GG	GO:0016323	C	Refseq	basolateral plasma membrane
XP_001976256.1 GG22769 [Drosophila erecta]	XP_001976256.1	0	GLOS_DERE_GG	GO:0030017	C	Refseq	sarcomere
XP_001976256.1 GG22769 [Drosophila erecta]	XP_001976256.1	0	GLOS_DERE_GG	GO:0043034	C	Refseq	costamere
XP_001976256.1 GG22769 [Drosophila erecta]	XP_001976256.1	0	GLOS_DERE_GG	GO:0043234	C	Refseq	protein complex
XP_001976256.1 GG22769 [Drosophila erecta]	XP_001976256.1	0	GLOS_DERE_GG	GO:0044325	M	Refseq	ion channel binding
XP_001976256.1 GG22769 [Drosophila erecta]	XP_001976256.1	0	GLOS_DERE_GG	GO:0045121	C	Refseq	membrane raft
XP_001976256.1 GG22769 [Drosophila erecta]	XP_001976256.1	0	GLOS_DERE_GG	GO:0046541	B	Refseq	saliva secretion
XP_001976256.1 GG22769 [Drosophila erecta]	XP_001976256.1	0	GLOS_DERE_GG	GO:0051281	B	Refseq	positive regulation of release of sequestered calcium ion into cytosol
XP_001976256.1 GG22769 [Drosophila erecta]	XP_001976256.1	0	GLOS_DERE_GG	GO:0051480	B	Refseq	cytosolic calcium ion homeostasis
XP_001976256.1 GG22769 [Drosophila erecta]	XP_001976256.1	0	GLOS_DERE_GG	GO:0051592	B	Refseq	response to calcium ion
XP_001976256.1 GG22769 [Drosophila erecta]	XP_001976256.1	0	GLOS_DERE_GG	GO:0070679	M	Refseq	inositol 1,4,5 trisphosphate binding
XP_001989447.1 GH18800 [Drosophila grimshawi]	XP_001989447.1	0	GLOS_DGRI_GH1	GO:0005262	M	Refseq	calcium channel activity
XP_001989447.1 GH18800 [Drosophila grimshawi]	XP_001989447.1	0	GLOS_DGRI_GH1	GO:0005515	M	Refseq	protein binding
XP_001989447.1 GH18800 [Drosophila grimshawi]	XP_001989447.1	0	GLOS_DGRI_GH1	GO:0005886	C	Refseq	plasma membrane
XP_001989447.1 GH18800 [Drosophila grimshawi]	XP_001989447.1	0	GLOS_DGRI_GH1	GO:0005887	C	Refseq	integral to plasma membrane
XP_001989447.1 GH18800 [Drosophila grimshawi]	XP_001989447.1	0	GLOS_DGRI_GH1	GO:0006816	B	Refseq	calcium ion transport
XP_001989447.1 GH18800 [Drosophila grimshawi]	XP_001989447.1	0	GLOS_DGRI_GH1	GO:0007411	B	Refseq	axon guidance
XP_001989447.1 GH18800 [Drosophila grimshawi]	XP_001989447.1	0	GLOS_DGRI_GH1	GO:0007596	B	Refseq	blood coagulation
XP_001989447.1 GH18800 [Drosophila grimshawi]	XP_001989447.1	0	GLOS_DGRI_GH1	GO:0007602	B	Refseq	phototransduction
XP_001989447.1 GH18800 [Drosophila grimshawi]	XP_001989447.1	0	GLOS_DGRI_GH1	GO:0010524	B	Refseq	positive regulation of calcium ion transport into cytosol
XP_001989447.1 GH18800 [Drosophila grimshawi]	XP_001989447.1	0	GLOS_DGRI_GH1	GO:0015279	M	Refseq	store-operated calcium channel activity
XP_001989447.1 GH18800 [Drosophila grimshawi]	XP_001989447.1	0	GLOS_DGRI_GH1	GO:0030168	B	Refseq	platelet activation
XP_001989447.1 GH18800 [Drosophila grimshawi]	XP_001989447.1	0	GLOS_DGRI_GH1	GO:0033198	B	Refseq	response to ATP
XP_001989447.1 GH18800 [Drosophila grimshawi]	XP_001989447.1	0	GLOS_DGRI_GH1	GO:0051592	B	Refseq	response to calcium ion
XP_001989447.1 GH18800 [Drosophila grimshawi]	XP_001989447.1	0	GLOS_DGRI_GH1	GO:0070588	B	Refseq	calcium ion transmembrane transport
XP_001989447.1 GH18800 [Drosophila grimshawi]	XP_001989447.1	0	GLOS_DGRI_GH1	GO:0070679	M	Refseq	inositol 1,4,5 trisphosphate binding
XP_003245892.1 PREDICT: hypothet prot LOC100575767 [A. pisum]	XP_003245892.1	1,00E-23	GLOS_LOC10057	GO:0007049	B	.	cell cycle
XP_003245892.1 PREDICT: hypothet prot LOC100575767 [A. pisum]	XP_003245892.1	1,00E-23	GLOS_LOC10057	GO:0000278	B	Refseq	mitotic cell cycle
XP_003245892.1 PREDICT: hypothet prot LOC100575767 [A. pisum]	XP_003245892.1	1,00E-23	GLOS_LOC10057	GO:0003677	M	Refseq	DNA binding
XP_003245892.1 PREDICT: hypothet prot LOC100575767 [A. pisum]	XP_003245892.1	1,00E-23	GLOS_LOC10057	GO:0003700	M	Refseq	sequence-specific DNA binding transcription factor activity
XP_003245892.1 PREDICT: hypothet prot LOC100575767 [A. pisum]	XP_003245892.1	1,00E-23	GLOS_LOC10057	GO:0003712	M	Refseq	transcription cofactor activity
XP_003245892.1 PREDICT: hypothet prot LOC100575767 [A. pisum]	XP_003245892.1	1,00E-23	GLOS_LOC10057	GO:0005654	C	Refseq	nucleoplasm
XP_003245892.1 PREDICT: hypothet prot LOC100575767 [A. pisum]	XP_003245892.1	1,00E-23	GLOS_LOC10057	GO:0043234	C	.	protein complex
XP_003245892.1 PREDICT: hypothet prot LOC100575767 [A. pisum]	XP_003245892.1	1,00E-23	GLOS_LOC10057	GO:0005667	C	Refseq	transcription factor complex

XP_003245892.1 PREDICT: hypothet prot LOC100575767 [A. pisum]	XP_003245892.1	1,00E-23	GLOS_LOC10057	GO:0006351	B	Refseq	transcription, DNA-dependent
XP_003245892.1 PREDICT: hypothet prot LOC100575767 [A. pisum]	XP_003245892.1	1,00E-23	GLOS_LOC10057	GO:0006352	B	.	DNA-dependent transcription, initiation
XP_003245892.1 PREDICT: hypothet prot LOC100575767 [A. pisum]	XP_003245892.1	1,00E-23	GLOS_LOC10057	GO:0006366	B	.	transcription from RNA polymerase II promoter
XP_003245892.1 PREDICT: hypothet prot LOC100575767 [A. pisum]	XP_003245892.1	1,00E-23	GLOS_LOC10057	GO:0006367	B	Refseq	transcription initiation from RNA polymerase II promoter
XP_003245892.1 PREDICT: hypothet prot LOC100575767 [A. pisum]	XP_003245892.1	1,00E-23	GLOS_LOC10057	GO:0007178	B	.	transmembrane receptor protein serine/threonine kinase signaling pathway
XP_003245892.1 PREDICT: hypothet prot LOC100575767 [A. pisum]	XP_003245892.1	1,00E-23	GLOS_LOC10057	GO:0007167	B	.	enzyme linked receptor protein signaling pathway
XP_003245892.1 PREDICT: hypothet prot LOC100575767 [A. pisum]	XP_003245892.1	1,00E-23	GLOS_LOC10057	GO:0071560	B	.	cellular response to transforming growth factor beta stimulus
XP_003245892.1 PREDICT: hypothet prot LOC100575767 [A. pisum]	XP_003245892.1	1,00E-23	GLOS_LOC10057	GO:0071363	B	.	cellular response to growth factor stimulus
XP_003245892.1 PREDICT: hypothet prot LOC100575767 [A. pisum]	XP_003245892.1	1,00E-23	GLOS_LOC10057	GO:0070848	B	.	response to growth factor stimulus
XP_003245892.1 PREDICT: hypothet prot LOC100575767 [A. pisum]	XP_003245892.1	1,00E-23	GLOS_LOC10057	GO:0071310	B	.	cellular response to organic substance
XP_003245892.1 PREDICT: hypothet prot LOC100575767 [A. pisum]	XP_003245892.1	1,00E-23	GLOS_LOC10057	GO:0070887	B	.	cellular response to chemical stimulus
XP_003245892.1 PREDICT: hypothet prot LOC100575767 [A. pisum]	XP_003245892.1	1,00E-23	GLOS_LOC10057	GO:0071495	B	.	cellular response to endogenous stimulus
XP_003245892.1 PREDICT: hypothet prot LOC100575767 [A. pisum]	XP_003245892.1	1,00E-23	GLOS_LOC10057	GO:0071559	B	.	response to transforming growth factor beta stimulus
XP_003245892.1 PREDICT: hypothet prot LOC100575767 [A. pisum]	XP_003245892.1	1,00E-23	GLOS_LOC10057	GO:0007179	B	Refseq	transforming growth factor beta receptor signaling pathway
XP_003245892.1 PREDICT: hypothet prot LOC100575767 [A. pisum]	XP_003245892.1	1,00E-23	GLOS_LOC10057	GO:0007507	B	Refseq	heart development
XP_003245892.1 PREDICT: hypothet prot LOC100575767 [A. pisum]	XP_003245892.1	1,00E-23	GLOS_LOC10057	GO:0008134	M	Refseq	transcription factor binding
XP_003245892.1 PREDICT: hypothet prot LOC100575767 [A. pisum]	XP_003245892.1	1,00E-23	GLOS_LOC10057	GO:0010467	B	Refseq	gene expression
XP_003245892.1 PREDICT: hypothet prot LOC100575767 [A. pisum]	XP_003245892.1	1,00E-23	GLOS_LOC10057	GO:0019904	M	Refseq	protein domain specific binding
XP_003245892.1 PREDICT: hypothet prot LOC100575767 [A. pisum]	XP_003245892.1	1,00E-23	GLOS_LOC10057	GO:0045893	B	.	positive regulation of transcription, DNA-dependent
XP_003245892.1 PREDICT: hypothet prot LOC100575767 [A. pisum]	XP_003245892.1	1,00E-23	GLOS_LOC10057	GO:0010557	B	.	positive regulation of macromolecule biosynthetic process
XP_003245892.1 PREDICT: hypothet prot LOC100575767 [A. pisum]	XP_003245892.1	1,00E-23	GLOS_LOC10057	GO:0009891	B	.	positive regulation of biosynthetic process
XP_003245892.1 PREDICT: hypothet prot LOC100575767 [A. pisum]	XP_003245892.1	1,00E-23	GLOS_LOC10057	GO:0010628	B	.	positive regulation of gene expression
XP_003245892.1 PREDICT: hypothet prot LOC100575767 [A. pisum]	XP_003245892.1	1,00E-23	GLOS_LOC10057	GO:0031328	B	.	positive regulation of cellular biosynthetic process
XP_003245892.1 PREDICT: hypothet prot LOC100575767 [A. pisum]	XP_003245892.1	1,00E-23	GLOS_LOC10057	GO:0051254	B	.	positive regulation of RNA metabolic process
XP_003245892.1 PREDICT: hypothet prot LOC100575767 [A. pisum]	XP_003245892.1	1,00E-23	GLOS_LOC10057	GO:0045935	B	.	positive regulation of nucleobase-containing compound metabolic process
XP_003245892.1 PREDICT: hypothet prot LOC100575767 [A. pisum]	XP_003245892.1	1,00E-23	GLOS_LOC10057	GO:0051173	B	.	positive regulation of nitrogen compound metabolic process
XP_003245892.1 PREDICT: hypothet prot LOC100575767 [A. pisum]	XP_003245892.1	1,00E-23	GLOS_LOC10057	GO:0045944	B	Refseq	positive regulation of transcription from RNA polymerase II promoter
NP_611636.1 GlcT-1 [Drosophila melanogaster]	NP_611636.1	0	GLOS_GLCT-1.1.1	GO:0000122	B	Refseq	negative regulation of transcription from RNA polymerase II promoter
NP_611636.1 GlcT-1 [Drosophila melanogaster]	NP_611636.1	0	GLOS_GLCT-1.1.1	GO:0001501	B	Refseq	skeletal system development
NP_611636.1 GlcT-1 [Drosophila melanogaster]	NP_611636.1	0	GLOS_GLCT-1.1.1	GO:0003700	M	Refseq	sequence-specific DNA binding transcription factor activity
NP_611636.1 GlcT-1 [Drosophila melanogaster]	NP_611636.1	0	GLOS_GLCT-1.1.1	GO:0005515	M	Refseq	protein binding
NP_611636.1 GlcT-1 [Drosophila melanogaster]	NP_611636.1	0	GLOS_GLCT-1.1.1	GO:0005634	C	Refseq	nucleus
NP_611636.1 GlcT-1 [Drosophila melanogaster]	NP_611636.1	0	GLOS_GLCT-1.1.1	GO:0006366	B	Refseq	transcription from RNA polymerase II promoter
NP_611636.1 GlcT-1 [Drosophila melanogaster]	NP_611636.1	0	GLOS_GLCT-1.1.1	GO:0006607	B	Refseq	NLS-bearing protein import into nucleus
NP_611636.1 GlcT-1 [Drosophila melanogaster]	NP_611636.1	0	GLOS_GLCT-1.1.1	GO:0008270	M	Refseq	zinc ion binding
NP_611636.1 GlcT-1 [Drosophila melanogaster]	NP_611636.1	0	GLOS_GLCT-1.1.1	GO:0032330	B	Refseq	regulation of chondrocyte differentiation
NP_611636.1 GlcT-1 [Drosophila melanogaster]	NP_611636.1	0	GLOS_GLCT-1.1.1	GO:0043565	M	Refseq	sequence-specific DNA binding
XP_001992555.1 GH24143 [Drosophila grimshawi]	XP_001992555.1	1,00E-106	GLOS_DGRI_GH2	GO:0005262	M	Refseq	calcium channel activity
XP_001992555.1 GH24143 [Drosophila grimshawi]	XP_001992555.1	1,00E-106	GLOS_DGRI_GH2	GO:0005515	M	Refseq	protein binding
XP_001992555.1 GH24143 [Drosophila grimshawi]	XP_001992555.1	1,00E-106	GLOS_DGRI_GH2	GO:0005886	C	Refseq	plasma membrane
XP_001992555.1 GH24143 [Drosophila grimshawi]	XP_001992555.1	1,00E-106	GLOS_DGRI_GH2	GO:0005887	C	Refseq	integral to plasma membrane
XP_001992555.1 GH24143 [Drosophila grimshawi]	XP_001992555.1	1,00E-106	GLOS_DGRI_GH2	GO:0006816	B	Refseq	calcium ion transport
XP_001992555.1 GH24143 [Drosophila grimshawi]	XP_001992555.1	1,00E-106	GLOS_DGRI_GH2	GO:0007411	B	Refseq	axon guidance
XP_001992555.1 GH24143 [Drosophila grimshawi]	XP_001992555.1	1,00E-106	GLOS_DGRI_GH2	GO:0007596	B	Refseq	blood coagulation
XP_001992555.1 GH24143 [Drosophila grimshawi]	XP_001992555.1	1,00E-106	GLOS_DGRI_GH2	GO:0007602	B	Refseq	phototransduction
XP_001992555.1 GH24143 [Drosophila grimshawi]	XP_001992555.1	1,00E-106	GLOS_DGRI_GH2	GO:0010524	B	Refseq	positive regulation of calcium ion transport into cytosol
XP_001992555.1 GH24143 [Drosophila grimshawi]	XP_001992555.1	1,00E-106	GLOS_DGRI_GH2	GO:0015279	M	Refseq	store-operated calcium channel activity
XP_001992555.1 GH24143 [Drosophila grimshawi]	XP_001992555.1	1,00E-106	GLOS_DGRI_GH2	GO:0030168	B	Refseq	platelet activation
XP_001992555.1 GH24143 [Drosophila grimshawi]	XP_001992555.1	1,00E-106	GLOS_DGRI_GH2	GO:0033198	B	Refseq	response to ATP
XP_001992555.1 GH24143 [Drosophila grimshawi]	XP_001992555.1	1,00E-106	GLOS_DGRI_GH2	GO:0051592	B	Refseq	response to calcium ion
XP_001992555.1 GH24143 [Drosophila grimshawi]	XP_001992555.1	1,00E-106	GLOS_DGRI_GH2	GO:0070588	B	Refseq	calcium ion transmembrane transport
XP_001992555.1 GH24143 [Drosophila grimshawi]	XP_001992555.1	1,00E-106	GLOS_DGRI_GH2	GO:0070679	M	Refseq	inositol 1,4,5 trisphosphate binding
NM_001272605.1 D. retinal degeneration B (rdgB), transcript variant I, mRNA	NM_001272605.1	3,00E-09	GLOS_RDGB.1.2	GO:0000122	B	Refseq	negative regulation of transcription from RNA polymerase II promoter
NM_001272605.1 D. retinal degeneration B (rdgB), transcript variant I, mRNA	NM_001272605.1	3,00E-09	GLOS_RDGB.1.2	GO:0001501	B	Refseq	skeletal system development

NM_001272605.1 D. retinal degeneration B (rdgB), transcript variant I, mRNA	NM_001272605.1	3,00E-09	GLOS_RDGB.1.2	GO:0003700	M	Refseq	sequence-specific DNA binding transcription factor activity
NM_001272605.1 D. retinal degeneration B (rdgB), transcript variant I, mRNA	NM_001272605.1	3,00E-09	GLOS_RDGB.1.2	GO:0005515	M	Refseq	protein binding
NM_001272605.1 D. retinal degeneration B (rdgB), transcript variant I, mRNA	NM_001272605.1	3,00E-09	GLOS_RDGB.1.2	GO:0005634	C	Refseq	nucleus
NM_001272605.1 D. retinal degeneration B (rdgB), transcript variant I, mRNA	NM_001272605.1	3,00E-09	GLOS_RDGB.1.2	GO:0006366	B	Refseq	transcription from RNA polymerase II promoter
NM_001272605.1 D. retinal degeneration B (rdgB), transcript variant I, mRNA	NM_001272605.1	3,00E-09	GLOS_RDGB.1.2	GO:0006607	B	Refseq	NLS-bearing protein import into nucleus
NM_001272605.1 D. retinal degeneration B (rdgB), transcript variant I, mRNA	NM_001272605.1	3,00E-09	GLOS_RDGB.1.2	GO:0008270	M	Refseq	zinc ion binding
NM_001272605.1 D. retinal degeneration B (rdgB), transcript variant I, mRNA	NM_001272605.1	3,00E-09	GLOS_RDGB.1.2	GO:0032330	B	Refseq	regulation of chondrocyte differentiation
NM_001272605.1 D. retinal degeneration B (rdgB), transcript variant I, mRNA	NM_001272605.1	3,00E-09	GLOS_RDGB.1.2	GO:0043565	M	Refseq	sequence-specific DNA binding
NM_143402.3 Drosophila melanogaster Slu7 (Slu7), mRNA	NM_143402.3	8,00E-12	GLOS_SLU7.6.13	GO:0000122	B	Refseq	negative regulation of transcription from RNA polymerase II promoter
NM_143402.3 Drosophila melanogaster Slu7 (Slu7), mRNA	NM_143402.3	8,00E-12	GLOS_SLU7.6.13	GO:0001501	B	Refseq	skeletal system development
NM_143402.3 Drosophila melanogaster Slu7 (Slu7), mRNA	NM_143402.3	8,00E-12	GLOS_SLU7.6.13	GO:0003700	M	Refseq	sequence-specific DNA binding transcription factor activity
NM_143402.3 Drosophila melanogaster Slu7 (Slu7), mRNA	NM_143402.3	8,00E-12	GLOS_SLU7.6.13	GO:0005515	M	Refseq	protein binding
NM_143402.3 Drosophila melanogaster Slu7 (Slu7), mRNA	NM_143402.3	8,00E-12	GLOS_SLU7.6.13	GO:0005634	C	Refseq	nucleus
NM_143402.3 Drosophila melanogaster Slu7 (Slu7), mRNA	NM_143402.3	8,00E-12	GLOS_SLU7.6.13	GO:0006366	B	Refseq	transcription from RNA polymerase II promoter
NM_143402.3 Drosophila melanogaster Slu7 (Slu7), mRNA	NM_143402.3	8,00E-12	GLOS_SLU7.6.13	GO:0006607	B	Refseq	NLS-bearing protein import into nucleus
NM_143402.3 Drosophila melanogaster Slu7 (Slu7), mRNA	NM_143402.3	8,00E-12	GLOS_SLU7.6.13	GO:0008270	M	Refseq	zinc ion binding
NM_143402.3 Drosophila melanogaster Slu7 (Slu7), mRNA	NM_143402.3	8,00E-12	GLOS_SLU7.6.13	GO:0032330	B	Refseq	regulation of chondrocyte differentiation
NM_143402.3 Drosophila melanogaster Slu7 (Slu7), mRNA	NM_143402.3	8,00E-12	GLOS_SLU7.6.13	GO:0043565	M	Refseq	sequence-specific DNA binding
NP_651825.3 dj-1beta [Drosophila melanogaster]	NP_651825.3	1,00E-31	GLOS_DJ-1BETA.	GO:0000122	B	Refseq	negative regulation of transcription from RNA polymerase II promoter
NP_651825.3 dj-1beta [Drosophila melanogaster]	NP_651825.3	1,00E-31	GLOS_DJ-1BETA.	GO:0001501	B	Refseq	skeletal system development
NP_651825.3 dj-1beta [Drosophila melanogaster]	NP_651825.3	1,00E-31	GLOS_DJ-1BETA.	GO:0003700	M	Refseq	sequence-specific DNA binding transcription factor activity
NP_651825.3 dj-1beta [Drosophila melanogaster]	NP_651825.3	1,00E-31	GLOS_DJ-1BETA.	GO:0005515	M	Refseq	protein binding
NP_651825.3 dj-1beta [Drosophila melanogaster]	NP_651825.3	1,00E-31	GLOS_DJ-1BETA.	GO:0005634	C	Refseq	nucleus
NP_651825.3 dj-1beta [Drosophila melanogaster]	NP_651825.3	1,00E-31	GLOS_DJ-1BETA.	GO:0006366	B	Refseq	transcription from RNA polymerase II promoter
NP_651825.3 dj-1beta [Drosophila melanogaster]	NP_651825.3	1,00E-31	GLOS_DJ-1BETA.	GO:0006607	B	Refseq	NLS-bearing protein import into nucleus
NP_651825.3 dj-1beta [Drosophila melanogaster]	NP_651825.3	1,00E-31	GLOS_DJ-1BETA.	GO:0008270	M	Refseq	zinc ion binding
NP_651825.3 dj-1beta [Drosophila melanogaster]	NP_651825.3	1,00E-31	GLOS_DJ-1BETA.	GO:0032330	B	Refseq	regulation of chondrocyte differentiation
NP_651825.3 dj-1beta [Drosophila melanogaster]	NP_651825.3	1,00E-31	GLOS_DJ-1BETA.	GO:0043565	M	Refseq	sequence-specific DNA binding
XP_001978194.1 GG17833 [Drosophila erecta]	XP_001978194.1	5,00E-48	GLOS_DERE_GG	GO:0005261	M	Refseq	cation channel activity
XP_001978194.1 GG17833 [Drosophila erecta]	XP_001978194.1	5,00E-48	GLOS_DERE_GG	GO:0005262	M	Refseq	calcium channel activity
XP_001978194.1 GG17833 [Drosophila erecta]	XP_001978194.1	5,00E-48	GLOS_DERE_GG	GO:0005515	M	Refseq	protein binding
XP_001978194.1 GG17833 [Drosophila erecta]	XP_001978194.1	5,00E-48	GLOS_DERE_GG	GO:0005886	C	Refseq	plasma membrane
XP_001978194.1 GG17833 [Drosophila erecta]	XP_001978194.1	5,00E-48	GLOS_DERE_GG	GO:0005887	C	Refseq	integral to plasma membrane
XP_001978194.1 GG17833 [Drosophila erecta]	XP_001978194.1	5,00E-48	GLOS_DERE_GG	GO:0006816	B	Refseq	calcium ion transport
XP_001978194.1 GG17833 [Drosophila erecta]	XP_001978194.1	5,00E-48	GLOS_DERE_GG	GO:0007411	B	Refseq	axon guidance
XP_001978194.1 GG17833 [Drosophila erecta]	XP_001978194.1	5,00E-48	GLOS_DERE_GG	GO:0015279	M	Refseq	store-operated calcium channel activity
XP_001978194.1 GG17833 [Drosophila erecta]	XP_001978194.1	5,00E-48	GLOS_DERE_GG	GO:0016323	C	Refseq	basolateral plasma membrane
XP_001978194.1 GG17833 [Drosophila erecta]	XP_001978194.1	5,00E-48	GLOS_DERE_GG	GO:0030017	C	Refseq	sarcomere
XP_001978194.1 GG17833 [Drosophila erecta]	XP_001978194.1	5,00E-48	GLOS_DERE_GG	GO:0043034	C	Refseq	costamere
XP_001978194.1 GG17833 [Drosophila erecta]	XP_001978194.1	5,00E-48	GLOS_DERE_GG	GO:0043234	C	Refseq	protein complex
XP_001978194.1 GG17833 [Drosophila erecta]	XP_001978194.1	5,00E-48	GLOS_DERE_GG	GO:0044325	M	Refseq	ion channel binding
XP_001978194.1 GG17833 [Drosophila erecta]	XP_001978194.1	5,00E-48	GLOS_DERE_GG	GO:0045121	C	Refseq	membrane raft
XP_001978194.1 GG17833 [Drosophila erecta]	XP_001978194.1	5,00E-48	GLOS_DERE_GG	GO:0046541	B	Refseq	saliva secretion
XP_001978194.1 GG17833 [Drosophila erecta]	XP_001978194.1	5,00E-48	GLOS_DERE_GG	GO:0051281	B	Refseq	positive regulation of release of sequestered calcium ion into cytosol
XP_001978194.1 GG17833 [Drosophila erecta]	XP_001978194.1	5,00E-48	GLOS_DERE_GG	GO:0051480	B	Refseq	cytosolic calcium ion homeostasis
XP_001978194.1 GG17833 [Drosophila erecta]	XP_001978194.1	5,00E-48	GLOS_DERE_GG	GO:0051592	B	Refseq	response to calcium ion
XP_001978194.1 GG17833 [Drosophila erecta]	XP_001978194.1	5,00E-48	GLOS_DERE_GG	GO:0070679	M	Refseq	inositol 1,4,5 trisphosphate binding
XP_001980400.1 GG18949 [Drosophila erecta]	XP_001980400.1	2,00E-59	GLOS_DERE_GG	GO:0005261	M	Refseq	cation channel activity
XP_001980400.1 GG18949 [Drosophila erecta]	XP_001980400.1	2,00E-59	GLOS_DERE_GG	GO:0005262	M	Refseq	calcium channel activity
XP_001980400.1 GG18949 [Drosophila erecta]	XP_001980400.1	2,00E-59	GLOS_DERE_GG	GO:0005515	M	Refseq	protein binding
XP_001980400.1 GG18949 [Drosophila erecta]	XP_001980400.1	2,00E-59	GLOS_DERE_GG	GO:0005886	C	Refseq	plasma membrane
XP_001980400.1 GG18949 [Drosophila erecta]	XP_001980400.1	2,00E-59	GLOS_DERE_GG	GO:0005887	C	Refseq	integral to plasma membrane
XP_001980400.1 GG18949 [Drosophila erecta]	XP_001980400.1	2,00E-59	GLOS_DERE_GG	GO:0006816	B	Refseq	calcium ion transport
XP_001980400.1 GG18949 [Drosophila erecta]	XP_001980400.1	2,00E-59	GLOS_DERE_GG	GO:0007411	B	Refseq	axon guidance

XP_001980400.1 GG18949 [Drosophila erecta]	XP_001980400.1	2,00E-59	GLOS_DERE_GG	GO:0015279	M	Refseq	store-operated calcium channel activity
XP_001980400.1 GG18949 [Drosophila erecta]	XP_001980400.1	2,00E-59	GLOS_DERE_GG	GO:0016323	C	Refseq	basolateral plasma membrane
XP_001980400.1 GG18949 [Drosophila erecta]	XP_001980400.1	2,00E-59	GLOS_DERE_GG	GO:0030017	C	Refseq	sarcomere
XP_001980400.1 GG18949 [Drosophila erecta]	XP_001980400.1	2,00E-59	GLOS_DERE_GG	GO:0043034	C	Refseq	costamere
XP_001980400.1 GG18949 [Drosophila erecta]	XP_001980400.1	2,00E-59	GLOS_DERE_GG	GO:0043234	C	Refseq	protein complex
XP_001980400.1 GG18949 [Drosophila erecta]	XP_001980400.1	2,00E-59	GLOS_DERE_GG	GO:0044325	M	Refseq	ion channel binding
XP_001980400.1 GG18949 [Drosophila erecta]	XP_001980400.1	2,00E-59	GLOS_DERE_GG	GO:0045121	C	Refseq	membrane raft
XP_001980400.1 GG18949 [Drosophila erecta]	XP_001980400.1	2,00E-59	GLOS_DERE_GG	GO:0046541	B	Refseq	saliva secretion
XP_001980400.1 GG18949 [Drosophila erecta]	XP_001980400.1	2,00E-59	GLOS_DERE_GG	GO:0051281	B	Refseq	positive regulation of release of sequestered calcium ion into cytosol
XP_001980400.1 GG18949 [Drosophila erecta]	XP_001980400.1	2,00E-59	GLOS_DERE_GG	GO:0051480	B	Refseq	cytosolic calcium ion homeostasis
XP_001980400.1 GG18949 [Drosophila erecta]	XP_001980400.1	2,00E-59	GLOS_DERE_GG	GO:0051592	B	Refseq	response to calcium ion
XP_001980400.1 GG18949 [Drosophila erecta]	XP_001980400.1	2,00E-59	GLOS_DERE_GG	GO:0070679	M	Refseq	inositol 1,4,5 trisphosphate binding
XP_001988793.1 GH11353 [Drosophila grimshawi]	XP_001988793.1	3,00E-06	GLOS_DGRI_GH1	GO:0005262	M	Refseq	calcium channel activity
XP_001988793.1 GH11353 [Drosophila grimshawi]	XP_001988793.1	3,00E-06	GLOS_DGRI_GH1	GO:0005515	M	Refseq	protein binding
XP_001988793.1 GH11353 [Drosophila grimshawi]	XP_001988793.1	3,00E-06	GLOS_DGRI_GH1	GO:0005886	C	Refseq	plasma membrane
XP_001988793.1 GH11353 [Drosophila grimshawi]	XP_001988793.1	3,00E-06	GLOS_DGRI_GH1	GO:0005887	C	Refseq	integral to plasma membrane
XP_001988793.1 GH11353 [Drosophila grimshawi]	XP_001988793.1	3,00E-06	GLOS_DGRI_GH1	GO:0006816	B	Refseq	calcium ion transport
XP_001988793.1 GH11353 [Drosophila grimshawi]	XP_001988793.1	3,00E-06	GLOS_DGRI_GH1	GO:0007411	B	Refseq	axon guidance
XP_001988793.1 GH11353 [Drosophila grimshawi]	XP_001988793.1	3,00E-06	GLOS_DGRI_GH1	GO:0007596	B	Refseq	blood coagulation
XP_001988793.1 GH11353 [Drosophila grimshawi]	XP_001988793.1	3,00E-06	GLOS_DGRI_GH1	GO:0007602	B	Refseq	phototransduction
XP_001988793.1 GH11353 [Drosophila grimshawi]	XP_001988793.1	3,00E-06	GLOS_DGRI_GH1	GO:0010524	B	Refseq	positive regulation of calcium ion transport into cytosol
XP_001988793.1 GH11353 [Drosophila grimshawi]	XP_001988793.1	3,00E-06	GLOS_DGRI_GH1	GO:0015279	M	Refseq	store-operated calcium channel activity
XP_001988793.1 GH11353 [Drosophila grimshawi]	XP_001988793.1	3,00E-06	GLOS_DGRI_GH1	GO:0030168	B	Refseq	platelet activation
XP_001988793.1 GH11353 [Drosophila grimshawi]	XP_001988793.1	3,00E-06	GLOS_DGRI_GH1	GO:0033198	B	Refseq	response to ATP
XP_001988793.1 GH11353 [Drosophila grimshawi]	XP_001988793.1	3,00E-06	GLOS_DGRI_GH1	GO:0051592	B	Refseq	response to calcium ion
XP_001988793.1 GH11353 [Drosophila grimshawi]	XP_001988793.1	3,00E-06	GLOS_DGRI_GH1	GO:0070588	B	Refseq	calcium ion transmembrane transport
XP_001988793.1 GH11353 [Drosophila grimshawi]	XP_001988793.1	3,00E-06	GLOS_DGRI_GH1	GO:0070679	M	Refseq	inositol 1,4,5 trisphosphate binding
NP_724910.1 CAP, isoform C [Drosophila melanogaster]	NP_724910.1	0	GLOS_CAP.1.1	GO:0000122	B	Refseq	negative regulation of transcription from RNA polymerase II promoter
NP_724910.1 CAP, isoform C [Drosophila melanogaster]	NP_724910.1	0	GLOS_CAP.1.1	GO:0001501	B	Refseq	skeletal system development
NP_724910.1 CAP, isoform C [Drosophila melanogaster]	NP_724910.1	0	GLOS_CAP.1.1	GO:0003700	M	Refseq	sequence-specific DNA binding transcription factor activity
NP_724910.1 CAP, isoform C [Drosophila melanogaster]	NP_724910.1	0	GLOS_CAP.1.1	GO:0005515	M	Refseq	protein binding
NP_724910.1 CAP, isoform C [Drosophila melanogaster]	NP_724910.1	0	GLOS_CAP.1.1	GO:0005634	C	Refseq	nucleus
NP_724910.1 CAP, isoform C [Drosophila melanogaster]	NP_724910.1	0	GLOS_CAP.1.1	GO:0006366	B	Refseq	transcription from RNA polymerase II promoter
NP_724910.1 CAP, isoform C [Drosophila melanogaster]	NP_724910.1	0	GLOS_CAP.1.1	GO:0006607	B	Refseq	NLS-bearing protein import into nucleus
NP_724910.1 CAP, isoform C [Drosophila melanogaster]	NP_724910.1	0	GLOS_CAP.1.1	GO:0008270	M	Refseq	zinc ion binding
NP_724910.1 CAP, isoform C [Drosophila melanogaster]	NP_724910.1	0	GLOS_CAP.1.1	GO:0032330	B	Refseq	regulation of chondrocyte differentiation
NP_724910.1 CAP, isoform C [Drosophila melanogaster]	NP_724910.1	0	GLOS_CAP.1.1	GO:0043565	M	Refseq	sequence-specific DNA binding
XP_001972666.1 GG15649 [Drosophila erecta]	XP_001972666.1	3,00E-09	GLOS_DERE_GG	GO:0005261	M	Refseq	cation channel activity
XP_001972666.1 GG15649 [Drosophila erecta]	XP_001972666.1	3,00E-09	GLOS_DERE_GG	GO:0005262	M	Refseq	calcium channel activity
XP_001972666.1 GG15649 [Drosophila erecta]	XP_001972666.1	3,00E-09	GLOS_DERE_GG	GO:0005515	M	Refseq	protein binding
XP_001972666.1 GG15649 [Drosophila erecta]	XP_001972666.1	3,00E-09	GLOS_DERE_GG	GO:0005886	C	Refseq	plasma membrane
XP_001972666.1 GG15649 [Drosophila erecta]	XP_001972666.1	3,00E-09	GLOS_DERE_GG	GO:0005887	C	Refseq	integral to plasma membrane
XP_001972666.1 GG15649 [Drosophila erecta]	XP_001972666.1	3,00E-09	GLOS_DERE_GG	GO:0006816	B	Refseq	calcium ion transport
XP_001972666.1 GG15649 [Drosophila erecta]	XP_001972666.1	3,00E-09	GLOS_DERE_GG	GO:0007411	B	Refseq	axon guidance
XP_001972666.1 GG15649 [Drosophila erecta]	XP_001972666.1	3,00E-09	GLOS_DERE_GG	GO:0015279	M	Refseq	store-operated calcium channel activity
XP_001972666.1 GG15649 [Drosophila erecta]	XP_001972666.1	3,00E-09	GLOS_DERE_GG	GO:0016323	C	Refseq	basolateral plasma membrane
XP_001972666.1 GG15649 [Drosophila erecta]	XP_001972666.1	3,00E-09	GLOS_DERE_GG	GO:0030017	C	Refseq	sarcomere
XP_001972666.1 GG15649 [Drosophila erecta]	XP_001972666.1	3,00E-09	GLOS_DERE_GG	GO:0043034	C	Refseq	costamere
XP_001972666.1 GG15649 [Drosophila erecta]	XP_001972666.1	3,00E-09	GLOS_DERE_GG	GO:0043234	C	Refseq	protein complex
XP_001972666.1 GG15649 [Drosophila erecta]	XP_001972666.1	3,00E-09	GLOS_DERE_GG	GO:0044325	M	Refseq	ion channel binding
XP_001972666.1 GG15649 [Drosophila erecta]	XP_001972666.1	3,00E-09	GLOS_DERE_GG	GO:0045121	C	Refseq	membrane raft
XP_001972666.1 GG15649 [Drosophila erecta]	XP_001972666.1	3,00E-09	GLOS_DERE_GG	GO:0046541	B	Refseq	saliva secretion
XP_001972666.1 GG15649 [Drosophila erecta]	XP_001972666.1	3,00E-09	GLOS_DERE_GG	GO:0051281	B	Refseq	positive regulation of release of sequestered calcium ion into cytosol
XP_001972666.1 GG15649 [Drosophila erecta]	XP_001972666.1	3,00E-09	GLOS_DERE_GG	GO:0051480	B	Refseq	cytosolic calcium ion homeostasis

XP_001972666.1 GG15649 [Drosophila erecta]	XP_001972666.1	3,00E-09	GLOS_DERE_GG	GO:0051592	B	Refseq	response to calcium ion
XP_001972666.1 GG15649 [Drosophila erecta]	XP_001972666.1	3,00E-09	GLOS_DERE_GG	GO:0070679	M	Refseq	inositol 1,4,5 trisphosphate binding
XP_001988793.1 GH11353 [Drosophila grimshawi]	XP_001988793.1	2,00E-08	GLOS_DGRI_GH1	GO:0005262	M	Refseq	calcium channel activity
XP_001988793.1 GH11353 [Drosophila grimshawi]	XP_001988793.1	2,00E-08	GLOS_DGRI_GH1	GO:0005515	M	Refseq	protein binding
XP_001988793.1 GH11353 [Drosophila grimshawi]	XP_001988793.1	2,00E-08	GLOS_DGRI_GH1	GO:0005886	C	Refseq	plasma membrane
XP_001988793.1 GH11353 [Drosophila grimshawi]	XP_001988793.1	2,00E-08	GLOS_DGRI_GH1	GO:0005887	C	Refseq	integral to plasma membrane
XP_001988793.1 GH11353 [Drosophila grimshawi]	XP_001988793.1	2,00E-08	GLOS_DGRI_GH1	GO:0006816	B	Refseq	calcium ion transport
XP_001988793.1 GH11353 [Drosophila grimshawi]	XP_001988793.1	2,00E-08	GLOS_DGRI_GH1	GO:0007411	B	Refseq	axon guidance
XP_001988793.1 GH11353 [Drosophila grimshawi]	XP_001988793.1	2,00E-08	GLOS_DGRI_GH1	GO:0007596	B	Refseq	blood coagulation
XP_001988793.1 GH11353 [Drosophila grimshawi]	XP_001988793.1	2,00E-08	GLOS_DGRI_GH1	GO:0007602	B	Refseq	phototransduction
XP_001988793.1 GH11353 [Drosophila grimshawi]	XP_001988793.1	2,00E-08	GLOS_DGRI_GH1	GO:0010524	B	Refseq	positive regulation of calcium ion transport into cytosol
XP_001988793.1 GH11353 [Drosophila grimshawi]	XP_001988793.1	2,00E-08	GLOS_DGRI_GH1	GO:0015279	M	Refseq	store-operated calcium channel activity
XP_001988793.1 GH11353 [Drosophila grimshawi]	XP_001988793.1	2,00E-08	GLOS_DGRI_GH1	GO:0030168	B	Refseq	platelet activation
XP_001988793.1 GH11353 [Drosophila grimshawi]	XP_001988793.1	2,00E-08	GLOS_DGRI_GH1	GO:0033198	B	Refseq	response to ATP
XP_001988793.1 GH11353 [Drosophila grimshawi]	XP_001988793.1	2,00E-08	GLOS_DGRI_GH1	GO:0051592	B	Refseq	response to calcium ion
XP_001988793.1 GH11353 [Drosophila grimshawi]	XP_001988793.1	2,00E-08	GLOS_DGRI_GH1	GO:0070588	B	Refseq	calcium ion transmembrane transport
XP_001988793.1 GH11353 [Drosophila grimshawi]	XP_001988793.1	2,00E-08	GLOS_DGRI_GH1	GO:0070679	M	Refseq	inositol 1,4,5 trisphosphate binding
NP_610629.1 CG7712, isoform A [D. melanogaster] ref|NP_001260877.1| CG7712, isoform B [D. melanogaster] ref|XP_001960503.1| GF13389 [D. ananassae] ref|XP_002076282.1| GD15258 [D. simulans] ref|XP_002076286.1| GD15256 [D. simulans]	NP_610629.1	6,00E-76	GLOS_CG7712.1.	GO:0000122	B	Refseq	negative regulation of transcription from RNA polymerase II promoter
NP_610629.1 CG7712, isoform A [D. m.] … Same as above	NP_610629.1	6,00E-76	GLOS_CG7712.1.	GO:0001501	B	Refseq	skeletal system development
NP_610629.1 CG7712, isoform A [D. m.] … Same as above	NP_610629.1	6,00E-76	GLOS_CG7712.1.	GO:0003700	M	Refseq	sequence-specific DNA binding transcription factor activity
NP_610629.1 CG7712, isoform A [D. m.] … Same as above	NP_610629.1	6,00E-76	GLOS_CG7712.1.	GO:0005515	M	Refseq	protein binding
NP_610629.1 CG7712, isoform A [D. m.] … Same as above	NP_610629.1	6,00E-76	GLOS_CG7712.1.	GO:0005634	C	Refseq	nucleus
NP_610629.1 CG7712, isoform A [D. m.] … Same as above	NP_610629.1	6,00E-76	GLOS_CG7712.1.	GO:0006366	B	Refseq	transcription from RNA polymerase II promoter
NP_610629.1 CG7712, isoform A [D. m.] … Same as above	NP_610629.1	6,00E-76	GLOS_CG7712.1.	GO:0006607	B	Refseq	NLS-bearing protein import into nucleus
NP_610629.1 CG7712, isoform A [D. m.] … Same as above	NP_610629.1	6,00E-76	GLOS_CG7712.1.	GO:0008270	M	Refseq	zinc ion binding
NP_610629.1 CG7712, isoform A [D. m.] … Same as above	NP_610629.1	6,00E-76	GLOS_CG7712.1.	GO:0032330	B	Refseq	regulation of chondrocyte differentiation
NP_610629.1 CG7712, isoform A [D. m.] … Same as above	NP_610629.1	6,00E-76	GLOS_CG7712.1.	GO:0043565	M	Refseq	sequence-specific DNA binding
NP_649070.1 ribosomal protein L26, isoform A [D. melanogaster] ref|NP_001262025.1| isoform B [D. melanogaster] ref|XP_001958190.1| GF23649 [D. ananassae] ref|XP_002042631.1| GM15002 [D. sechellia] ref|XP_002095681.1| GE19578 [D. yakuba] ref|XP_002095689.1| GE19574 [D. yakuba] ref|XP_002085421.1| GD14780 [D. simulans]	NP_649070.1	2,00E-77	GLOS_RPL26.1.3	GO:0000122	B	Refseq	negative regulation of transcription from RNA polymerase II promoter
NP_649070.1 ribosomal protein L26, isoform A [D. m.] ….Same as above	NP_649070.1	2,00E-77	GLOS_RPL26.1.3	GO:0001501	B	Refseq	skeletal system development
NP_649070.1 ribosomal protein L26, isoform A [D. m.] ….Same as above	NP_649070.1	2,00E-77	GLOS_RPL26.1.3	GO:0003700	M	Refseq	sequence-specific DNA binding transcription factor activity
NP_649070.1 ribosomal protein L26, isoform A [D. m.] ….Same as above	NP_649070.1	2,00E-77	GLOS_RPL26.1.3	GO:0005515	M	Refseq	protein binding
NP_649070.1 ribosomal protein L26, isoform A [D. m.] ….Same as above	NP_649070.1	2,00E-77	GLOS_RPL26.1.3	GO:0005634	C	Refseq	nucleus
NP_649070.1 ribosomal protein L26, isoform A [D. m.] ….Same as above	NP_649070.1	2,00E-77	GLOS_RPL26.1.3	GO:0006366	B	Refseq	transcription from RNA polymerase II promoter
NP_649070.1 ribosomal protein L26, isoform A [D. m.] ….Same as above	NP_649070.1	2,00E-77	GLOS_RPL26.1.3	GO:0006607	B	Refseq	NLS-bearing protein import into nucleus
NP_649070.1 ribosomal protein L26, isoform A [D. m.] ….Same as above	NP_649070.1	2,00E-77	GLOS_RPL26.1.3	GO:0008270	M	Refseq	zinc ion binding
NP_649070.1 ribosomal protein L26, isoform A [D. m.] ….Same as above	NP_649070.1	2,00E-77	GLOS_RPL26.1.3	GO:0032330	B	Refseq	regulation of chondrocyte differentiation
NP_649070.1 ribosomal protein L26, isoform A [D. m.] ….Same as above	NP_649070.1	2,00E-77	GLOS_RPL26.1.3	GO:0043565	M	Refseq	sequence-specific DNA binding
XP_001985481.1 GH17085 [Drosophila grimshawi]	XP_001985481.1	1,00E-50	GLOS_DGRI_GH1	GO:0005262	M	Refseq	calcium channel activity
XP_001985481.1 GH17085 [Drosophila grimshawi]	XP_001985481.1	1,00E-50	GLOS_DGRI_GH1	GO:0005515	M	Refseq	protein binding
XP_001985481.1 GH17085 [Drosophila grimshawi]	XP_001985481.1	1,00E-50	GLOS_DGRI_GH1	GO:0005886	C	Refseq	plasma membrane
XP_001985481.1 GH17085 [Drosophila grimshawi]	XP_001985481.1	1,00E-50	GLOS_DGRI_GH1	GO:0005887	C	Refseq	integral to plasma membrane
XP_001985481.1 GH17085 [Drosophila grimshawi]	XP_001985481.1	1,00E-50	GLOS_DGRI_GH1	GO:0006816	B	Refseq	calcium ion transport
XP_001985481.1 GH17085 [Drosophila grimshawi]	XP_001985481.1	1,00E-50	GLOS_DGRI_GH1	GO:0007411	B	Refseq	axon guidance
XP_001985481.1 GH17085 [Drosophila grimshawi]	XP_001985481.1	1,00E-50	GLOS_DGRI_GH1	GO:0007596	B	Refseq	blood coagulation
XP_001985481.1 GH17085 [Drosophila grimshawi]	XP_001985481.1	1,00E-50	GLOS_DGRI_GH1	GO:0007602	B	Refseq	phototransduction
XP_001985481.1 GH17085 [Drosophila grimshawi]	XP_001985481.1	1,00E-50	GLOS_DGRI_GH1	GO:0010524	B	Refseq	positive regulation of calcium ion transport into cytosol
XP_001985481.1 GH17085 [Drosophila grimshawi]	XP_001985481.1	1,00E-50	GLOS_DGRI_GH1	GO:0015279	M	Refseq	store-operated calcium channel activity

XP_001985481.1 GH17085 [Drosophila grimshawi]	XP_001985481.1	1,00E-50	GLOS_DGRI_GH1	GO:0030168	B	Refseq	platelet activation
XP_001985481.1 GH17085 [Drosophila grimshawi]	XP_001985481.1	1,00E-50	GLOS_DGRI_GH1	GO:0033198	B	Refseq	response to ATP
XP_001985481.1 GH17085 [Drosophila grimshawi]	XP_001985481.1	1,00E-50	GLOS_DGRI_GH1	GO:0051592	B	Refseq	response to calcium ion
XP_001985481.1 GH17085 [Drosophila grimshawi]	XP_001985481.1	1,00E-50	GLOS_DGRI_GH1	GO:0070588	B	Refseq	calcium ion transmembrane transport
XP_001985481.1 GH17085 [Drosophila grimshawi]	XP_001985481.1	1,00E-50	GLOS_DGRI_GH1	GO:0070679	M	Refseq	inositol 1,4,5 trisphosphate binding
XP_001986849.1 GH21600 [Drosophila grimshawi]	XP_001986849.1	1,00E-136	GLOS_DGRI_GH2	GO:0005262	M	Refseq	calcium channel activity
XP_001986849.1 GH21600 [Drosophila grimshawi]	XP_001986849.1	1,00E-136	GLOS_DGRI_GH2	GO:0005515	M	Refseq	protein binding
XP_001986849.1 GH21600 [Drosophila grimshawi]	XP_001986849.1	1,00E-136	GLOS_DGRI_GH2	GO:0005886	C	Refseq	plasma membrane
XP_001986849.1 GH21600 [Drosophila grimshawi]	XP_001986849.1	1,00E-136	GLOS_DGRI_GH2	GO:0005887	C	Refseq	integral to plasma membrane
XP_001986849.1 GH21600 [Drosophila grimshawi]	XP_001986849.1	1,00E-136	GLOS_DGRI_GH2	GO:0006816	B	Refseq	calcium ion transport
XP_001986849.1 GH21600 [Drosophila grimshawi]	XP_001986849.1	1,00E-136	GLOS_DGRI_GH2	GO:0007411	B	Refseq	axon guidance
XP_001986849.1 GH21600 [Drosophila grimshawi]	XP_001986849.1	1,00E-136	GLOS_DGRI_GH2	GO:0007596	B	Refseq	blood coagulation
XP_001986849.1 GH21600 [Drosophila grimshawi]	XP_001986849.1	1,00E-136	GLOS_DGRI_GH2	GO:0007602	B	Refseq	phototransduction
XP_001986849.1 GH21600 [Drosophila grimshawi]	XP_001986849.1	1,00E-136	GLOS_DGRI_GH2	GO:0010524	B	Refseq	positive regulation of calcium ion transport into cytosol
XP_001986849.1 GH21600 [Drosophila grimshawi]	XP_001986849.1	1,00E-136	GLOS_DGRI_GH2	GO:0015279	M	Refseq	store-operated calcium channel activity
XP_001986849.1 GH21600 [Drosophila grimshawi]	XP_001986849.1	1,00E-136	GLOS_DGRI_GH2	GO:0030168	B	Refseq	platelet activation
XP_001986849.1 GH21600 [Drosophila grimshawi]	XP_001986849.1	1,00E-136	GLOS_DGRI_GH2	GO:0033198	B	Refseq	response to ATP
XP_001986849.1 GH21600 [Drosophila grimshawi]	XP_001986849.1	1,00E-136	GLOS_DGRI_GH2	GO:0051592	B	Refseq	response to calcium ion
XP_001986849.1 GH21600 [Drosophila grimshawi]	XP_001986849.1	1,00E-136	GLOS_DGRI_GH2	GO:0070588	B	Refseq	calcium ion transmembrane transport
XP_001986849.1 GH21600 [Drosophila grimshawi]	XP_001986849.1	1,00E-136	GLOS_DGRI_GH2	GO:0070679	M	Refseq	inositol 1,4,5 trisphosphate binding
NP_573149.1 CG9673 [Drosophila melanogaster]	NP_573149.1	3,00E-19	GLOS_CG9673.1.:	GO:0000122	B	Refseq	negative regulation of transcription from RNA polymerase II promoter
NP_573149.1 CG9673 [Drosophila melanogaster]	NP_573149.1	3,00E-19	GLOS_CG9673.1.:	GO:0001501	B	Refseq	skeletal system development
NP_573149.1 CG9673 [Drosophila melanogaster]	NP_573149.1	3,00E-19	GLOS_CG9673.1.:	GO:0003700	M	Refseq	sequence-specific DNA binding transcription factor activity
NP_573149.1 CG9673 [Drosophila melanogaster]	NP_573149.1	3,00E-19	GLOS_CG9673.1.:	GO:0005515	M	Refseq	protein binding
NP_573149.1 CG9673 [Drosophila melanogaster]	NP_573149.1	3,00E-19	GLOS_CG9673.1.:	GO:0005634	C	Refseq	nucleus
NP_573149.1 CG9673 [Drosophila melanogaster]	NP_573149.1	3,00E-19	GLOS_CG9673.1.:	GO:0006366	B	Refseq	transcription from RNA polymerase II promoter
NP_573149.1 CG9673 [Drosophila melanogaster]	NP_573149.1	3,00E-19	GLOS_CG9673.1.:	GO:0006607	B	Refseq	NLS-bearing protein import into nucleus
NP_573149.1 CG9673 [Drosophila melanogaster]	NP_573149.1	3,00E-19	GLOS_CG9673.1.:	GO:0008270	M	Refseq	zinc ion binding
NP_573149.1 CG9673 [Drosophila melanogaster]	NP_573149.1	3,00E-19	GLOS_CG9673.1.:	GO:0032330	B	Refseq	regulation of chondrocyte differentiation
NP_573149.1 CG9673 [Drosophila melanogaster]	NP_573149.1	3,00E-19	GLOS_CG9673.1.:	GO:0043565	M	Refseq	sequence-specific DNA binding
XM_002591829.1 B. floridae hypothetical protein, mRNA	XM_002591829.1	1,00E-06	GLOS_BRAFLDR/	GO:0005737	C	Refseq	cytoplasm
XM_002591829.1 B. floridae hypothetical protein, mRNA	XM_002591829.1	1,00E-06	GLOS_BRAFLDR/	GO:0005856	C	Refseq	cytoskeleton
XM_002591829.1 B. floridae hypothetical protein, mRNA	XM_002591829.1	1,00E-06	GLOS_BRAFLDR/	GO:0005924	C	.	cell-substrate adherens junction
XM_002591829.1 B. floridae hypothetical protein, mRNA	XM_002591829.1	1,00E-06	GLOS_BRAFLDR/	GO:0005912	C	.	adherens junction
XM_002591829.1 B. floridae hypothetical protein, mRNA	XM_002591829.1	1,00E-06	GLOS_BRAFLDR/	GO:0070161	C	.	anchoring junction
XM_002591829.1 B. floridae hypothetical protein, mRNA	XM_002591829.1	1,00E-06	GLOS_BRAFLDR/	GO:0030054	C	.	cell junction
XM_002591829.1 B. floridae hypothetical protein, mRNA	XM_002591829.1	1,00E-06	GLOS_BRAFLDR/	GO:0030055	C	.	cell-substrate junction
XM_002591829.1 B. floridae hypothetical protein, mRNA	XM_002591829.1	1,00E-06	GLOS_BRAFLDR/	GO:0005925	C	Refseq	focal adhesion
XM_002591829.1 B. floridae hypothetical protein, mRNA	XM_002591829.1	1,00E-06	GLOS_BRAFLDR/	GO:0008270	M	Refseq	zinc ion binding
XP_001983838.1 GH16119 [Drosophila grimshawi]	XP_001983838.1	2,00E-66	GLOS_DGRI_GH1	GO:0005262	M	Refseq	calcium channel activity
XP_001983838.1 GH16119 [Drosophila grimshawi]	XP_001983838.1	2,00E-66	GLOS_DGRI_GH1	GO:0005515	M	Refseq	protein binding
XP_001983838.1 GH16119 [Drosophila grimshawi]	XP_001983838.1	2,00E-66	GLOS_DGRI_GH1	GO:0005886	C	Refseq	plasma membrane
XP_001983838.1 GH16119 [Drosophila grimshawi]	XP_001983838.1	2,00E-66	GLOS_DGRI_GH1	GO:0005887	C	Refseq	integral to plasma membrane
XP_001983838.1 GH16119 [Drosophila grimshawi]	XP_001983838.1	2,00E-66	GLOS_DGRI_GH1	GO:0006816	B	Refseq	calcium ion transport
XP_001983838.1 GH16119 [Drosophila grimshawi]	XP_001983838.1	2,00E-66	GLOS_DGRI_GH1	GO:0007411	B	Refseq	axon guidance
XP_001983838.1 GH16119 [Drosophila grimshawi]	XP_001983838.1	2,00E-66	GLOS_DGRI_GH1	GO:0007596	B	Refseq	blood coagulation
XP_001983838.1 GH16119 [Drosophila grimshawi]	XP_001983838.1	2,00E-66	GLOS_DGRI_GH1	GO:0007602	B	Refseq	phototransduction
XP_001983838.1 GH16119 [Drosophila grimshawi]	XP_001983838.1	2,00E-66	GLOS_DGRI_GH1	GO:0010524	B	Refseq	positive regulation of calcium ion transport into cytosol
XP_001983838.1 GH16119 [Drosophila grimshawi]	XP_001983838.1	2,00E-66	GLOS_DGRI_GH1	GO:0015279	M	Refseq	store-operated calcium channel activity
XP_001983838.1 GH16119 [Drosophila grimshawi]	XP_001983838.1	2,00E-66	GLOS_DGRI_GH1	GO:0030168	B	Refseq	platelet activation
XP_001983838.1 GH16119 [Drosophila grimshawi]	XP_001983838.1	2,00E-66	GLOS_DGRI_GH1	GO:0033198	B	Refseq	response to ATP
XP_001983838.1 GH16119 [Drosophila grimshawi]	XP_001983838.1	2,00E-66	GLOS_DGRI_GH1	GO:0051592	B	Refseq	response to calcium ion
XP_001983838.1 GH16119 [Drosophila grimshawi]	XP_001983838.1	2,00E-66	GLOS_DGRI_GH1	GO:0070588	B	Refseq	calcium ion transmembrane transport
XP_001983838.1 GH16119 [Drosophila grimshawi]	XP_001983838.1	2,00E-66	GLOS_DGRI_GH1	GO:0070679	M	Refseq	inositol 1,4,5 trisphosphate binding

XP_001983716.1 GH16040 [Drosophila grimshawi]	XP_001983716.1	1,00E-144	GLOS_DGRI_GH1	GO:0005262	M	Refseq	calcium channel activity
XP_001983716.1 GH16040 [Drosophila grimshawi]	XP_001983716.1	1,00E-144	GLOS_DGRI_GH1	GO:0005515	M	Refseq	protein binding
XP_001983716.1 GH16040 [Drosophila grimshawi]	XP_001983716.1	1,00E-144	GLOS_DGRI_GH1	GO:0005886	C	Refseq	plasma membrane
XP_001983716.1 GH16040 [Drosophila grimshawi]	XP_001983716.1	1,00E-144	GLOS_DGRI_GH1	GO:0005887	C	Refseq	integral to plasma membrane
XP_001983716.1 GH16040 [Drosophila grimshawi]	XP_001983716.1	1,00E-144	GLOS_DGRI_GH1	GO:0006816	B	Refseq	calcium ion transport
XP_001983716.1 GH16040 [Drosophila grimshawi]	XP_001983716.1	1,00E-144	GLOS_DGRI_GH1	GO:0007411	B	Refseq	axon guidance
XP_001983716.1 GH16040 [Drosophila grimshawi]	XP_001983716.1	1,00E-144	GLOS_DGRI_GH1	GO:0007596	B	Refseq	blood coagulation
XP_001983716.1 GH16040 [Drosophila grimshawi]	XP_001983716.1	1,00E-144	GLOS_DGRI_GH1	GO:0007602	B	Refseq	phototransduction
XP_001983716.1 GH16040 [Drosophila grimshawi]	XP_001983716.1	1,00E-144	GLOS_DGRI_GH1	GO:0010524	B	Refseq	positive regulation of calcium ion transport into cytosol
XP_001983716.1 GH16040 [Drosophila grimshawi]	XP_001983716.1	1,00E-144	GLOS_DGRI_GH1	GO:0015279	M	Refseq	store-operated calcium channel activity
XP_001983716.1 GH16040 [Drosophila grimshawi]	XP_001983716.1	1,00E-144	GLOS_DGRI_GH1	GO:0030168	B	Refseq	platelet activation
XP_001983716.1 GH16040 [Drosophila grimshawi]	XP_001983716.1	1,00E-144	GLOS_DGRI_GH1	GO:0033198	B	Refseq	response to ATP
XP_001983716.1 GH16040 [Drosophila grimshawi]	XP_001983716.1	1,00E-144	GLOS_DGRI_GH1	GO:0051592	B	Refseq	response to calcium ion
XP_001983716.1 GH16040 [Drosophila grimshawi]	XP_001983716.1	1,00E-144	GLOS_DGRI_GH1	GO:0070588	B	Refseq	calcium ion transmembrane transport
XP_001983716.1 GH16040 [Drosophila grimshawi]	XP_001983716.1	1,00E-144	GLOS_DGRI_GH1	GO:0070679	M	Refseq	inositol 1,4,5 trisphosphate binding
[BBH] 41_DROME (sp|Q9V8R9) Prot 4.1 homolog OS=D. m.	41_DROME	0	GLOS_41.1.1	GO:0005856	C	InterPro	cytoskeleton
[BBH] 41_DROME (sp|Q9V8R9) Prot 4.1 homolog OS=D. m.	41_DROME	0	GLOS_41.1.1	GO:0005886	C	FlyBase	plasma membrane
[BBH] 41_DROME (sp|Q9V8R9) Prot 4.1 homolog OS=D. m.	41_DROME	0	GLOS_41.1.1	GO:0005918	C	.	septate junction
[BBH] 41_DROME (sp|Q9V8R9) Prot 4.1 homolog OS=D. m.	41_DROME	0	GLOS_41.1.1	GO:0070160	C	.	occluding junction
[BBH] 41_DROME (sp|Q9V8R9) Prot 4.1 homolog OS=D. m.	41_DROME	0	GLOS_41.1.1	GO:0005911	C	.	cell-cell junction
[BBH] 41_DROME (sp|Q9V8R9) Prot 4.1 homolog OS=D. m.	41_DROME	0	GLOS_41.1.1	GO:0043296	C	.	apical junction complex
[BBH] 41_DROME (sp|Q9V8R9) Prot 4.1 homolog OS=D. m.	41_DROME	0	GLOS_41.1.1	GO:0005920	C	FlyBase	smooth septate junction
[BBH] 41_DROME (sp|Q9V8R9) Prot 4.1 homolog OS=D. m.	41_DROME	0	GLOS_41.1.1	GO:0008092	M	FlyBase	cytoskeletal protein binding
[BBH] 41_DROME (sp|Q9V8R9) Prot 4.1 homolog OS=D. m.	41_DROME	0	GLOS_41.1.1	GO:0005198	M	InterPro	structural molecule activity
[BBH] 41_DROME (sp|Q9V8R9) Prot 4.1 homolog OS=D. m.	41_DROME	0	GLOS_41.1.1	GO:0007525	B	.	somatic muscle development
[BBH] 41_DROME (sp|Q9V8R9) Prot 4.1 homolog OS=D. m.	41_DROME	0	GLOS_41.1.1	GO:0061061	B	.	muscle structure development
[BBH] 41_DROME (sp|Q9V8R9) Prot 4.1 homolog OS=D. m.	41_DROME	0	GLOS_41.1.1	GO:0007527	B	FlyBase	adult somatic muscle development
[BBH] 41_DROME (sp|Q9V8R9) Prot 4.1 homolog OS=D. m.	41_DROME	0	GLOS_41.1.1	GO:0007155	B	.	cell adhesion
[BBH] 41_DROME (sp|Q9V8R9) Prot 4.1 homolog OS=D. m.	41_DROME	0	GLOS_41.1.1	GO:0022610	B	.	biological adhesion
[BBH] 41_DROME (sp|Q9V8R9) Prot 4.1 homolog OS=D. m.	41_DROME	0	GLOS_41.1.1	GO:0003007	B	.	heart morphogenesis
[BBH] 41_DROME (sp|Q9V8R9) Prot 4.1 homolog OS=D. m.	41_DROME	0	GLOS_41.1.1	GO:0009887	B	.	organ morphogenesis
[BBH] 41_DROME (sp|Q9V8R9) Prot 4.1 homolog OS=D. m.	41_DROME	0	GLOS_41.1.1	GO:0007507	B	.	heart development
[BBH] 41_DROME (sp|Q9V8R9) Prot 4.1 homolog OS=D. m.	41_DROME	0	GLOS_41.1.1	GO:0061343	B	FlyBase	cell adhesion involved in heart morphogenesis
[BBH] 41_DROME (sp|Q9V8R9) Prot 4.1 homolog OS=D. m.	41_DROME	0	GLOS_41.1.1	GO:0040003	B	.	chitin-based cuticle development
[BBH] 41_DROME (sp|Q9V8R9) Prot 4.1 homolog OS=D. m.	41_DROME	0	GLOS_41.1.1	GO:0042335	B	.	cuticle development
[BBH] 41_DROME (sp|Q9V8R9) Prot 4.1 homolog OS=D. m.	41_DROME	0	GLOS_41.1.1	GO:0008362	B	FlyBase	chitin-based embryonic cuticle biosynthetic process
[BBH] 41_DROME (sp|Q9V8R9) Prot 4.1 homolog OS=D. m.	41_DROME	0	GLOS_41.1.1	GO:0016331	B	.	morphogenesis of embryonic epithelium
[BBH] 41_DROME (sp|Q9V8R9) Prot 4.1 homolog OS=D. m.	41_DROME	0	GLOS_41.1.1	GO:0002009	B	.	morphogenesis of an epithelium
[BBH] 41_DROME (sp|Q9V8R9) Prot 4.1 homolog OS=D. m.	41_DROME	0	GLOS_41.1.1	GO:0048729	B	.	tissue morphogenesis
[BBH] 41_DROME (sp|Q9V8R9) Prot 4.1 homolog OS=D. m.	41_DROME	0	GLOS_41.1.1	GO:0009888	B	.	tissue development
[BBH] 41_DROME (sp|Q9V8R9) Prot 4.1 homolog OS=D. m.	41_DROME	0	GLOS_41.1.1	GO:0060429	B	.	epithelium development
[BBH] 41_DROME (sp|Q9V8R9) Prot 4.1 homolog OS=D. m.	41_DROME	0	GLOS_41.1.1	GO:0048598	B	.	embryonic morphogenesis
[BBH] 41_DROME (sp|Q9V8R9) Prot 4.1 homolog OS=D. m.	41_DROME	0	GLOS_41.1.1	GO:0001700	B	.	embryonic development via the syncytial blastoderm
[BBH] 41_DROME (sp|Q9V8R9) Prot 4.1 homolog OS=D. m.	41_DROME	0	GLOS_41.1.1	GO:0009792	B	.	embryo development ending in birth or egg hatching
[BBH] 41_DROME (sp|Q9V8R9) Prot 4.1 homolog OS=D. m.	41_DROME	0	GLOS_41.1.1	GO:0007391	B	FlyBase	dorsal closure
[BBH] 41_DROME (sp|Q9V8R9) Prot 4.1 homolog OS=D. m.	41_DROME	0	GLOS_41.1.1	GO:0021782	B	.	glial cell development
[BBH] 41_DROME (sp|Q9V8R9) Prot 4.1 homolog OS=D. m.	41_DROME	0	GLOS_41.1.1	GO:0010001	B	.	glial cell differentiation
[BBH] 41_DROME (sp|Q9V8R9) Prot 4.1 homolog OS=D. m.	41_DROME	0	GLOS_41.1.1	GO:0042063	B	.	gliogenesis
[BBH] 41_DROME (sp|Q9V8R9) Prot 4.1 homolog OS=D. m.	41_DROME	0	GLOS_41.1.1	GO:0060856	B	.	establishment of blood-brain barrier
[BBH] 41_DROME (sp|Q9V8R9) Prot 4.1 homolog OS=D. m.	41_DROME	0	GLOS_41.1.1	GO:0060857	B	FlyBase	establishment of glial blood-brain barrier
[BBH] 41_DROME (sp|Q9V8R9) Prot 4.1 homolog OS=D. m.	41_DROME	0	GLOS_41.1.1	GO:0007163	B	FlyBase	establishment or maintenance of cell polarity
[BBH] 41_DROME (sp|Q9V8R9) Prot 4.1 homolog OS=D. m.	41_DROME	0	GLOS_41.1.1	GO:0003013	B	.	circulatory system process
[BBH] 41_DROME (sp|Q9V8R9) Prot 4.1 homolog OS=D. m.	41_DROME	0	GLOS_41.1.1	GO:0003015	B	FlyBase	heart process
[BBH] 41_DROME (sp|Q9V8R9) Prot 4.1 homolog OS=D. m.	41_DROME	0	GLOS_41.1.1	GO:0009954	B	.	proximal/distal pattern formation

[BBH] 41_DROME (sp|Q9V8R9) Prot 4.1 homolog OS=D. m.	41_DROME	0	GLOS_41.1.1	GO:0003002	B	.	regionalization
[BBH] 41_DROME (sp|Q9V8R9) Prot 4.1 homolog OS=D. m.	41_DROME	0	GLOS_41.1.1	GO:0007389	B	.	pattern specification process
[BBH] 41_DROME (sp|Q9V8R9) Prot 4.1 homolog OS=D. m.	41_DROME	0	GLOS_41.1.1	GO:0035317	B	.	imaginal disc-derived wing hair organization
[BBH] 41_DROME (sp|Q9V8R9) Prot 4.1 homolog OS=D. m.	41_DROME	0	GLOS_41.1.1	GO:0035316	B	.	non-sensory hair organization
[BBH] 41_DROME (sp|Q9V8R9) Prot 4.1 homolog OS=D. m.	41_DROME	0	GLOS_41.1.1	GO:0035315	B	.	hair cell differentiation
[BBH] 41_DROME (sp|Q9V8R9) Prot 4.1 homolog OS=D. m.	41_DROME	0	GLOS_41.1.1	GO:0009913	B	.	epidermal cell differentiation
[BBH] 41_DROME (sp|Q9V8R9) Prot 4.1 homolog OS=D. m.	41_DROME	0	GLOS_41.1.1	GO:0030855	B	.	epithelial cell differentiation
[BBH] 41_DROME (sp|Q9V8R9) Prot 4.1 homolog OS=D. m.	41_DROME	0	GLOS_41.1.1	GO:0008544	B	.	epidermis development
[BBH] 41_DROME (sp|Q9V8R9) Prot 4.1 homolog OS=D. m.	41_DROME	0	GLOS_41.1.1	GO:0043588	B	.	skin development
[BBH] 41_DROME (sp|Q9V8R9) Prot 4.1 homolog OS=D. m.	41_DROME	0	GLOS_41.1.1	GO:0007476	B	.	imaginal disc-derived wing morphogenesis
[BBH] 41_DROME (sp|Q9V8R9) Prot 4.1 homolog OS=D. m.	41_DROME	0	GLOS_41.1.1	GO:0035114	B	.	imaginal disc-derived appendage morphogenesis
[BBH] 41_DROME (sp|Q9V8R9) Prot 4.1 homolog OS=D. m.	41_DROME	0	GLOS_41.1.1	GO:0035107	B	.	appendage morphogenesis
[BBH] 41_DROME (sp|Q9V8R9) Prot 4.1 homolog OS=D. m.	41_DROME	0	GLOS_41.1.1	GO:0048736	B	.	appendage development
[BBH] 41_DROME (sp|Q9V8R9) Prot 4.1 homolog OS=D. m.	41_DROME	0	GLOS_41.1.1	GO:0048737	B	.	imaginal disc-derived appendage development
[BBH] 41_DROME (sp|Q9V8R9) Prot 4.1 homolog OS=D. m.	41_DROME	0	GLOS_41.1.1	GO:0035120	B	.	post-embryonic appendage morphogenesis
[BBH] 41_DROME (sp|Q9V8R9) Prot 4.1 homolog OS=D. m.	41_DROME	0	GLOS_41.1.1	GO:0009886	B	.	post-embryonic morphogenesis
[BBH] 41_DROME (sp|Q9V8R9) Prot 4.1 homolog OS=D. m.	41_DROME	0	GLOS_41.1.1	GO:0009791	B	.	post-embryonic development
[BBH] 41_DROME (sp|Q9V8R9) Prot 4.1 homolog OS=D. m.	41_DROME	0	GLOS_41.1.1	GO:0007472	B	.	wing disc morphogenesis
[BBH] 41_DROME (sp|Q9V8R9) Prot 4.1 homolog OS=D. m.	41_DROME	0	GLOS_41.1.1	GO:0007560	B	.	imaginal disc morphogenesis
[BBH] 41_DROME (sp|Q9V8R9) Prot 4.1 homolog OS=D. m.	41_DROME	0	GLOS_41.1.1	GO:0048563	B	.	post-embryonic organ morphogenesis
[BBH] 41_DROME (sp|Q9V8R9) Prot 4.1 homolog OS=D. m.	41_DROME	0	GLOS_41.1.1	GO:0048569	B	.	post-embryonic organ development
[BBH] 41_DROME (sp|Q9V8R9) Prot 4.1 homolog OS=D. m.	41_DROME	0	GLOS_41.1.1	GO:0007444	B	.	imaginal disc development
[BBH] 41_DROME (sp|Q9V8R9) Prot 4.1 homolog OS=D. m.	41_DROME	0	GLOS_41.1.1	GO:0007552	B	.	metamorphosis
[BBH] 41_DROME (sp|Q9V8R9) Prot 4.1 homolog OS=D. m.	41_DROME	0	GLOS_41.1.1	GO:0048707	B	.	instar larval or pupal morphogenesis
[BBH] 41_DROME (sp|Q9V8R9) Prot 4.1 homolog OS=D. m.	41_DROME	0	GLOS_41.1.1	GO:0002165	B	.	instar larval or pupal development
[BBH] 41_DROME (sp|Q9V8R9) Prot 4.1 homolog OS=D. m.	41_DROME	0	GLOS_41.1.1	GO:0035220	B	.	wing disc development
[BBH] 41_DROME (sp|Q9V8R9) Prot 4.1 homolog OS=D. m.	41_DROME	0	GLOS_41.1.1	GO:0035321	B	FlyBase	maintenance of imaginal disc-derived wing hair orientation
[BBH] 41_DROME (sp|Q9V8R9) Prot 4.1 homolog OS=D. m.	41_DROME	0	GLOS_41.1.1	GO:0090150	B	.	establishment of protein localization to membrane
[BBH] 41_DROME (sp|Q9V8R9) Prot 4.1 homolog OS=D. m.	41_DROME	0	GLOS_41.1.1	GO:0072657	B	.	protein localization to membrane
[BBH] 41_DROME (sp|Q9V8R9) Prot 4.1 homolog OS=D. m.	41_DROME	0	GLOS_41.1.1	GO:0044802	B	.	single-organism membrane organization
[BBH] 41_DROME (sp|Q9V8R9) Prot 4.1 homolog OS=D. m.	41_DROME	0	GLOS_41.1.1	GO:0061024	B	.	membrane organization
[BBH] 41_DROME (sp|Q9V8R9) Prot 4.1 homolog OS=D. m.	41_DROME	0	GLOS_41.1.1	GO:0006612	B	FlyBase	protein targeting to membrane
[BBH] 41_DROME (sp|Q9V8R9) Prot 4.1 homolog OS=D. m.	41_DROME	0	GLOS_41.1.1	GO:0035150	B	.	regulation of tube size
[BBH] 41_DROME (sp|Q9V8R9) Prot 4.1 homolog OS=D. m.	41_DROME	0	GLOS_41.1.1	GO:0090066	B	.	regulation of anatomical structure size
[BBH] 41_DROME (sp|Q9V8R9) Prot 4.1 homolog OS=D. m.	41_DROME	0	GLOS_41.1.1	GO:0035152	B	.	regulation of tube architecture, open tracheal system
[BBH] 41_DROME (sp|Q9V8R9) Prot 4.1 homolog OS=D. m.	41_DROME	0	GLOS_41.1.1	GO:0007424	B	.	open tracheal system development
[BBH] 41_DROME (sp|Q9V8R9) Prot 4.1 homolog OS=D. m.	41_DROME	0	GLOS_41.1.1	GO:0060541	B	.	respiratory system development
[BBH] 41_DROME (sp|Q9V8R9) Prot 4.1 homolog OS=D. m.	41_DROME	0	GLOS_41.1.1	GO:0035151	B	FlyBase	regulation of tube size, open tracheal system
[BBH] 41_DROME (sp|Q9V8R9) Prot 4.1 homolog OS=D. m.	41_DROME	0	GLOS_41.1.1	GO:0022612	B	.	gland morphogenesis
[BBH] 41_DROME (sp|Q9V8R9) Prot 4.1 homolog OS=D. m.	41_DROME	0	GLOS_41.1.1	GO:0048732	B	.	gland development
[BBH] 41_DROME (sp|Q9V8R9) Prot 4.1 homolog OS=D. m.	41_DROME	0	GLOS_41.1.1	GO:0007431	B	.	salivary gland development
[BBH] 41_DROME (sp|Q9V8R9) Prot 4.1 homolog OS=D. m.	41_DROME	0	GLOS_41.1.1	GO:0035272	B	.	exocrine system development
[BBH] 41_DROME (sp|Q9V8R9) Prot 4.1 homolog OS=D. m.	41_DROME	0	GLOS_41.1.1	GO:0007435	B	FlyBase	salivary gland morphogenesis
[BBH] 41_DROME (sp|Q9V8R9) Prot 4.1 homolog OS=D. m.	41_DROME	0	GLOS_41.1.1	GO:0007043	B	.	cell-cell junction assembly
[BBH] 41_DROME (sp|Q9V8R9) Prot 4.1 homolog OS=D. m.	41_DROME	0	GLOS_41.1.1	GO:0034329	B	.	cell junction assembly
[BBH] 41_DROME (sp|Q9V8R9) Prot 4.1 homolog OS=D. m.	41_DROME	0	GLOS_41.1.1	GO:0022607	B	.	cellular component assembly
[BBH] 41_DROME (sp|Q9V8R9) Prot 4.1 homolog OS=D. m.	41_DROME	0	GLOS_41.1.1	GO:0044085	B	.	cellular component biogenesis
[BBH] 41_DROME (sp|Q9V8R9) Prot 4.1 homolog OS=D. m.	41_DROME	0	GLOS_41.1.1	GO:0034330	B	.	cell junction organization
[BBH] 41_DROME (sp|Q9V8R9) Prot 4.1 homolog OS=D. m.	41_DROME	0	GLOS_41.1.1	GO:0045216	B	.	cell-cell junction organization
[BBH] 41_DROME (sp|Q9V8R9) Prot 4.1 homolog OS=D. m.	41_DROME	0	GLOS_41.1.1	GO:0043297	B	.	apical junction assembly
[BBH] 41_DROME (sp|Q9V8R9) Prot 4.1 homolog OS=D. m.	41_DROME	0	GLOS_41.1.1	GO:0019991	B	FlyBase	septate junction assembly
ACE_HAEIX (sp|Q10715) Angiotensin-converting enzyme H. irritans exigua	ACE_HAEIX	6,00E-90	GLOS_ACE.7.16	GO:0044421	C	.	extracellular region part
ACE_HAEIX (sp|Q10715) Angiotensin-converting enzyme H. irritans exigua	ACE_HAEIX	6,00E-90	GLOS_ACE.7.16	GO:0005576	C	.	extracellular region
ACE_HAEIX (sp|Q10715) Angiotensin-converting enzyme H. irritans exigua	ACE_HAEIX	6,00E-90	GLOS_ACE.7.16	GO:0005615	C	UniProtKB-S	extracellular space

ACE_HAEIX (sp|Q10715) Angiotensin-converting enzyme H. irritans exigua	ACE_HAEIX	6,00E-90	GLOS_ACE.7.16	GO:0016020	C	InterPro	membrane
ACE_HAEIX (sp|Q10715) Angiotensin-converting enzyme H. irritans exigua	ACE_HAEIX	6,00E-90	GLOS_ACE.7.16	GO:0008238	M	.	exopeptidase activity
ACE_HAEIX (sp|Q10715) Angiotensin-converting enzyme H. irritans exigua	ACE_HAEIX	6,00E-90	GLOS_ACE.7.16	GO:0070011	M	.	peptidase activity, acting on L-amino acid peptides
ACE_HAEIX (sp|Q10715) Angiotensin-converting enzyme H. irritans exigua	ACE_HAEIX	6,00E-90	GLOS_ACE.7.16	GO:0008233	M	.	peptidase activity
ACE_HAEIX (sp|Q10715) Angiotensin-converting enzyme H. irritans exigua	ACE_HAEIX	6,00E-90	GLOS_ACE.7.16	GO:0004180	M	UniProtKB-k	carboxypeptidase activity
ACE_HAEIX (sp|Q10715) Angiotensin-converting enzyme H. irritans exigua	ACE_HAEIX	6,00E-90	GLOS_ACE.7.16	GO:0046872	M	UniProtKB-k	metal ion binding
ACE_HAEIX (sp|Q10715) Angiotensin-converting enzyme H. irritans exigua	ACE_HAEIX	6,00E-90	GLOS_ACE.7.16	GO:0008237	M	UniProtKB-k	metallopeptidase activity
ACE_HAEIX (sp|Q10715) Angiotensin-converting enzyme H. irritans exigua	ACE_HAEIX	6,00E-90	GLOS_ACE.7.16	GO:0008241	M	InterPro	peptidyl-dipeptidase activity
ACE_HAEIX (sp|Q10715) Angiotensin-converting enzyme H. irritans exigua	ACE_HAEIX	6,00E-90	GLOS_ACE.7.16	GO:0019538	B	.	protein metabolic process
ACE_HAEIX (sp|Q10715) Angiotensin-converting enzyme H. irritans exigua	ACE_HAEIX	6,00E-90	GLOS_ACE.7.16	GO:0006508	B	UniProtKB-k	proteolysis
[BBH] ANXB9_DROME (sp|P22464) Annexin-B9 OS=D. m.	ANXB9_DROME	0	GLOS_ANXB9.1.1	GO:0005938	C	FlyBase	cell cortex
[BBH] ANXB9_DROME (sp|P22464) Annexin-B9 OS=D. m.	ANXB9_DROME	0	GLOS_ANXB9.1.1	GO:0012505	C	FlyBase	endomembrane system
[BBH] ANXB9_DROME (sp|P22464) Annexin-B9 OS=D. m.	ANXB9_DROME	0	GLOS_ANXB9.1.1	GO:0008092	M	.	cytoskeletal protein binding
[BBH] ANXB9_DROME (sp|P22464) Annexin-B9 OS=D. m.	ANXB9_DROME	0	GLOS_ANXB9.1.1	GO:0003779	M	FlyBase	actin binding
[BBH] ANXB9_DROME (sp|P22464) Annexin-B9 OS=D. m.	ANXB9_DROME	0	GLOS_ANXB9.1.1	GO:0005509	M	InterPro	calcium ion binding
[BBH] ANXB9_DROME (sp|P22464) Annexin-B9 OS=D. m.	ANXB9_DROME	0	GLOS_ANXB9.1.1	GO:0005543	M	.	phospholipid binding
[BBH] ANXB9_DROME (sp|P22464) Annexin-B9 OS=D. m.	ANXB9_DROME	0	GLOS_ANXB9.1.1	GO:0008289	M	.	lipid binding
[BBH] ANXB9_DROME (sp|P22464) Annexin-B9 OS=D. m.	ANXB9_DROME	0	GLOS_ANXB9.1.1	GO:0005544	M	UniProtKB-k	calcium-dependent phospholipid binding
[BBH] ANXB9_DROME (sp|P22464) Annexin-B9 OS=D. m.	ANXB9_DROME	0	GLOS_ANXB9.1.1	GO:0008105	B	.	asymmetric protein localization
[BBH] ANXB9_DROME (sp|P22464) Annexin-B9 OS=D. m.	ANXB9_DROME	0	GLOS_ANXB9.1.1	GO:0061467	B	FlyBase	basolateral protein localization
[BBH] ANXB9_DROME (sp|P22464) Annexin-B9 OS=D. m.	ANXB9_DROME	0	GLOS_ANXB9.1.1	GO:0007163	B	.	establishment or maintenance of cell polarity
[BBH] ANXB9_DROME (sp|P22464) Annexin-B9 OS=D. m.	ANXB9_DROME	0	GLOS_ANXB9.1.1	GO:0030011	B	FlyBase	maintenance of cell polarity
[BBH] ANXB9_DROME (sp|P22464) Annexin-B9 OS=D. m.	ANXB9_DROME	0	GLOS_ANXB9.1.1	GO:0010796	B	.	regulation of multivesicular body size
[BBH] ANXB9_DROME (sp|P22464) Annexin-B9 OS=D. m.	ANXB9_DROME	0	GLOS_ANXB9.1.1	GO:0051036	B	.	regulation of endosome size
[BBH] ANXB9_DROME (sp|P22464) Annexin-B9 OS=D. m.	ANXB9_DROME	0	GLOS_ANXB9.1.1	GO:0032535	B	.	regulation of cellular component size
[BBH] ANXB9_DROME (sp|P22464) Annexin-B9 OS=D. m.	ANXB9_DROME	0	GLOS_ANXB9.1.1	GO:0032509	B	.	endosome transport via multivesicular body sorting pathway
[BBH] ANXB9_DROME (sp|P22464) Annexin-B9 OS=D. m.	ANXB9_DROME	0	GLOS_ANXB9.1.1	GO:0016197	B	.	endosomal transport
[BBH] ANXB9_DROME (sp|P22464) Annexin-B9 OS=D. m.	ANXB9_DROME	0	GLOS_ANXB9.1.1	GO:0010797	B	FlyBase	regulation of multivesicular body size involved in endosome transport
[BBH] ANXB9_DROME (sp|P22464) Annexin-B9 OS=D. m.	ANXB9_DROME	0	GLOS_ANXB9.1.1	GO:0007450	B	.	dorsal/ventral pattern formation, imaginal disc
[BBH] ANXB9_DROME (sp|P22464) Annexin-B9 OS=D. m.	ANXB9_DROME	0	GLOS_ANXB9.1.1	GO:0007447	B	.	imaginal disc pattern formation
[BBH] ANXB9_DROME (sp|P22464) Annexin-B9 OS=D. m.	ANXB9_DROME	0	GLOS_ANXB9.1.1	GO:0009953	B	.	dorsal/ventral pattern formation
[BBH] ANXB9_DROME (sp|P22464) Annexin-B9 OS=D. m.	ANXB9_DROME	0	GLOS_ANXB9.1.1	GO:0035222	B	.	wing disc pattern formation
[BBH] ANXB9_DROME (sp|P22464) Annexin-B9 OS=D. m.	ANXB9_DROME	0	GLOS_ANXB9.1.1	GO:0048190	B	FlyBase	wing disc dorsal/ventral pattern formation
APC10_DROME (sp|Q9V831) Anaphase-promoting complex subunit 10 D.m.	APC10_DROME	1,00E-63	GLOS_APC10.1.3	GO:0000152	C	.	nuclear ubiquitin ligase complex
APC10_DROME (sp|Q9V831) Anaphase-promoting complex subunit 10 D.m.	APC10_DROME	1,00E-63	GLOS_APC10.1.3	GO:0000151	C	.	ubiquitin ligase complex
APC10_DROME (sp|Q9V831) Anaphase-promoting complex subunit 10 D.m.	APC10_DROME	1,00E-63	GLOS_APC10.1.3	GO:0031461	C	.	cullin-RING ubiquitin ligase complex
APC10_DROME (sp|Q9V831) Anaphase-promoting complex subunit 10 D.m.	APC10_DROME	1,00E-63	GLOS_APC10.1.3	GO:0005680	C	InterPro	anaphase-promoting complex
APC10_DROME (sp|Q9V831) Anaphase-promoting complex subunit 10 D.m.	APC10_DROME	1,00E-63	GLOS_APC10.1.3	GO:0043161	B	.	proteasomal ubiquitin-dependent protein catabolic process
APC10_DROME (sp|Q9V831) Anaphase-promoting complex subunit 10 D.m.	APC10_DROME	1,00E-63	GLOS_APC10.1.3	GO:0006511	B	.	ubiquitin-dependent protein catabolic process
APC10_DROME (sp|Q9V831) Anaphase-promoting complex subunit 10 D.m.	APC10_DROME	1,00E-63	GLOS_APC10.1.3	GO:0019941	B	.	modification-dependent protein catabolic process
APC10_DROME (sp|Q9V831) Anaphase-promoting complex subunit 10 D.m.	APC10_DROME	1,00E-63	GLOS_APC10.1.3	GO:0043632	B	.	modification-dependent macromolecule catabolic process
APC10_DROME (sp|Q9V831) Anaphase-promoting complex subunit 10 D.m.	APC10_DROME	1,00E-63	GLOS_APC10.1.3	GO:0044265	B	.	cellular macromolecule catabolic process
APC10_DROME (sp|Q9V831) Anaphase-promoting complex subunit 10 D.m.	APC10_DROME	1,00E-63	GLOS_APC10.1.3	GO:0009057	B	.	macromolecule catabolic process
APC10_DROME (sp|Q9V831) Anaphase-promoting complex subunit 10 D.m.	APC10_DROME	1,00E-63	GLOS_APC10.1.3	GO:1901575	B	.	organic substance catabolic process
APC10_DROME (sp|Q9V831) Anaphase-promoting complex subunit 10 D.m.	APC10_DROME	1,00E-63	GLOS_APC10.1.3	GO:0009056	B	.	catabolic process
APC10_DROME (sp|Q9V831) Anaphase-promoting complex subunit 10 D.m.	APC10_DROME	1,00E-63	GLOS_APC10.1.3	GO:0044248	B	.	cellular catabolic process
APC10_DROME (sp|Q9V831) Anaphase-promoting complex subunit 10 D.m.	APC10_DROME	1,00E-63	GLOS_APC10.1.3	GO:0051603	B	.	proteolysis involved in cellular protein catabolic process
APC10_DROME (sp|Q9V831) Anaphase-promoting complex subunit 10 D.m.	APC10_DROME	1,00E-63	GLOS_APC10.1.3	GO:0006508	B	.	proteolysis
APC10_DROME (sp|Q9V831) Anaphase-promoting complex subunit 10 D.m.	APC10_DROME	1,00E-63	GLOS_APC10.1.3	GO:0044257	B	.	cellular protein catabolic process
APC10_DROME (sp|Q9V831) Anaphase-promoting complex subunit 10 D.m.	APC10_DROME	1,00E-63	GLOS_APC10.1.3	GO:0030163	B	.	protein catabolic process
APC10_DROME (sp|Q9V831) Anaphase-promoting complex subunit 10 D.m.	APC10_DROME	1,00E-63	GLOS_APC10.1.3	GO:0044267	B	.	cellular protein metabolic process
APC10_DROME (sp|Q9V831) Anaphase-promoting complex subunit 10 D.m.	APC10_DROME	1,00E-63	GLOS_APC10.1.3	GO:0010498	B	.	proteasomal protein catabolic process
APC10_DROME (sp|Q9V831) Anaphase-promoting complex subunit 10 D.m.	APC10_DROME	1,00E-63	GLOS_APC10.1.3	GO:0031145	B	InterPro	anaphase-promoting complex-dependent proteasomal ubiquitin-dependent protein catabolic process

APC10_DROME (sp|Q9V831) Anaphase-promoting complex subunit 10 D.m.	APC10_DROME	1,00E-63	GLOS_APC10.1.3	GO:0051301	B	UniProtKB-K	cell division
APC10_DROME (sp|Q9V831) Anaphase-promoting complex subunit 10 D.m.	APC10_DROME	1,00E-63	GLOS_APC10.1.3	GO:0000278	B	.	mitotic cell cycle
APC10_DROME (sp|Q9V831) Anaphase-promoting complex subunit 10 D.m.	APC10_DROME	1,00E-63	GLOS_APC10.1.3	GO:0007113	B	FlyBase	endomitotic cell cycle
APC10_DROME (sp|Q9V831) Anaphase-promoting complex subunit 10 D.m.	APC10_DROME	1,00E-63	GLOS_APC10.1.3	GO:0000280	B	.	nuclear division
APC10_DROME (sp|Q9V831) Anaphase-promoting complex subunit 10 D.m.	APC10_DROME	1,00E-63	GLOS_APC10.1.3	GO:0048285	B	.	organelle fission
APC10_DROME (sp|Q9V831) Anaphase-promoting complex subunit 10 D.m.	APC10_DROME	1,00E-63	GLOS_APC10.1.3	GO:0022402	B	.	cell cycle process
APC10_DROME (sp|Q9V831) Anaphase-promoting complex subunit 10 D.m.	APC10_DROME	1,00E-63	GLOS_APC10.1.3	GO:0007067	B	FlyBase	mitosis
APC10_DROME (sp|Q9V831) Anaphase-promoting complex subunit 10 D.m.	APC10_DROME	1,00E-63	GLOS_APC10.1.3	GO:0022008	B	FlyBase	neurogenesis
APC10_DROME (sp|Q9V831) Anaphase-promoting complex subunit 10 D.m.	APC10_DROME	1,00E-63	GLOS_APC10.1.3	GO:0007088	B	.	regulation of mitosis
APC10_DROME (sp|Q9V831) Anaphase-promoting complex subunit 10 D.m.	APC10_DROME	1,00E-63	GLOS_APC10.1.3	GO:0007346	B	.	regulation of mitotic cell cycle
APC10_DROME (sp|Q9V831) Anaphase-promoting complex subunit 10 D.m.	APC10_DROME	1,00E-63	GLOS_APC10.1.3	GO:0010564	B	.	regulation of cell cycle process
APC10_DROME (sp|Q9V831) Anaphase-promoting complex subunit 10 D.m.	APC10_DROME	1,00E-63	GLOS_APC10.1.3	GO:0051783	B	.	regulation of nuclear division
APC10_DROME (sp|Q9V831) Anaphase-promoting complex subunit 10 D.m.	APC10_DROME	1,00E-63	GLOS_APC10.1.3	GO:0033043	B	.	regulation of organelle organization
APC10_DROME (sp|Q9V831) Anaphase-promoting complex subunit 10 D.m.	APC10_DROME	1,00E-63	GLOS_APC10.1.3	GO:1901990	B	.	regulation of mitotic cell cycle phase transition
APC10_DROME (sp|Q9V831) Anaphase-promoting complex subunit 10 D.m.	APC10_DROME	1,00E-63	GLOS_APC10.1.3	GO:1901987	B	.	regulation of cell cycle phase transition
APC10_DROME (sp|Q9V831) Anaphase-promoting complex subunit 10 D.m.	APC10_DROME	1,00E-63	GLOS_APC10.1.3	GO:1902099	B	.	regulation of metaphase/anaphase transition of cell cycle
APC10_DROME (sp|Q9V831) Anaphase-promoting complex subunit 10 D.m.	APC10_DROME	1,00E-63	GLOS_APC10.1.3	GO:0030071	B	InterPro	regulation of mitotic metaphase/anaphase transition
CAC1D_DROME (sp|Q24270) Voltage-dependent calcium channel type D subunit alpha-1 OS=D. melanogaster	CAC1D_DROME	0	GLOS_CAC1D.3.3	GO:0045177	C	.	apical part of cell
CAC1D_DROME (sp|Q24270) Voltage-dependent calcium … Same as above	CAC1D_DROME	0	GLOS_CAC1D.3.3	GO:0016324	C	FlyBase	apical plasma membrane
CAC1D_DROME (sp|Q24270) Voltage-dependent calcium … Same as above	CAC1D_DROME	0	GLOS_CAC1D.3.3	GO:0016323	C	FlyBase	basolateral plasma membrane
CAC1D_DROME (sp|Q24270) Voltage-dependent calcium … Same as above	CAC1D_DROME	0	GLOS_CAC1D.3.3	GO:0034704	C	.	calcium channel complex
CAC1D_DROME (sp|Q24270) Voltage-dependent calcium … Same as above	CAC1D_DROME	0	GLOS_CAC1D.3.3	GO:0034703	C	.	cation channel complex
CAC1D_DROME (sp|Q24270) Voltage-dependent calcium … Same as above	CAC1D_DROME	0	GLOS_CAC1D.3.3	GO:0034702	C	.	ion channel complex
CAC1D_DROME (sp|Q24270) Voltage-dependent calcium … Same as above	CAC1D_DROME	0	GLOS_CAC1D.3.3	GO:0005891	C	FlyBase	voltage-gated calcium channel complex
CAC1D_DROME (sp|Q24270) Voltage-dependent calcium … Same as above	CAC1D_DROME	0	GLOS_CAC1D.3.3	GO:0046872	M	UniProtKB-K	metal ion binding
CAC1D_DROME (sp|Q24270) Voltage-dependent calcium … Same as above	CAC1D_DROME	0	GLOS_CAC1D.3.3	GO:0022843	M	.	voltage-gated cation channel activity
CAC1D_DROME (sp|Q24270) Voltage-dependent calcium … Same as above	CAC1D_DROME	0	GLOS_CAC1D.3.3	GO:0005244	M	.	voltage-gated ion channel activity
CAC1D_DROME (sp|Q24270) Voltage-dependent calcium … Same as above	CAC1D_DROME	0	GLOS_CAC1D.3.3	GO:0022832	M	.	voltage-gated channel activity
CAC1D_DROME (sp|Q24270) Voltage-dependent calcium … Same as above	CAC1D_DROME	0	GLOS_CAC1D.3.3	GO:0005245	M	FlyBase	voltage-gated calcium channel activity
CAC1D_DROME (sp|Q24270) Voltage-dependent calcium … Same as above	CAC1D_DROME	0	GLOS_CAC1D.3.3	GO:0042044	B	.	fluid transport
CAC1D_DROME (sp|Q24270) Voltage-dependent calcium … Same as above	CAC1D_DROME	0	GLOS_CAC1D.3.3	GO:0042045	B	FlyBase	epithelial fluid transport
CAC1D_DROME (sp|Q24270) Voltage-dependent calcium … Same as above	CAC1D_DROME	0	GLOS_CAC1D.3.3	GO:0003012	B	.	muscle system process
CAC1D_DROME (sp|Q24270) Voltage-dependent calcium … Same as above	CAC1D_DROME	0	GLOS_CAC1D.3.3	GO:0006936	B	FlyBase	muscle contraction
[BBH] CADF_DROME (sp|P45594) Cofilin/actin-depolymerizing fact homolog D.m.	CADF_DROME	1,00E-103	GLOS_CADF.2.7	GO:0005856	C	.	cytoskeleton
[BBH] CADF_DROME (sp|P45594) Cofilin/actin-depolymerizing fact homolog D.m.	CADF_DROME	1,00E-103	GLOS_CADF.2.7	GO:0015629	C	InterPro	actin cytoskeleton
[BBH] CADF_DROME (sp|P45594) Cofilin/actin-depolymerizing fact homolog D.m.	CADF_DROME	1,00E-103	GLOS_CADF.2.7	GO:0005737	C	UniProtKB-K	cytoplasm
[BBH] CADF_DROME (sp|P45594) Cofilin/actin-depolymerizing fact homolog D.m.	CADF_DROME	1,00E-103	GLOS_CADF.2.7	GO:0044430	C	.	cytoskeletal part
[BBH] CADF_DROME (sp|P45594) Cofilin/actin-depolymerizing fact homolog D.m.	CADF_DROME	1,00E-103	GLOS_CADF.2.7	GO:0015630	C	.	microtubule cytoskeleton
[BBH] CADF_DROME (sp|P45594) Cofilin/actin-depolymerizing fact homolog D.m.	CADF_DROME	1,00E-103	GLOS_CADF.2.7	GO:0005875	C	FlyBase	microtubule associated complex
[BBH] CADF_DROME (sp|P45594) Cofilin/actin-depolymerizing fact homolog D.m.	CADF_DROME	1,00E-103	GLOS_CADF.2.7	GO:0034399	C	.	nuclear periphery
[BBH] CADF_DROME (sp|P45594) Cofilin/actin-depolymerizing fact homolog D.m.	CADF_DROME	1,00E-103	GLOS_CADF.2.7	GO:0016363	C	UniProtKB-S	nuclear matrix
[BBH] CADF_DROME (sp|P45594) Cofilin/actin-depolymerizing fact homolog D.m.	CADF_DROME	1,00E-103	GLOS_CADF.2.7	GO:0003779	M	FlyBase	actin binding
[BBH] CADF_DROME (sp|P45594) Cofilin/actin-depolymerizing fact homolog D.m.	CADF_DROME	1,00E-103	GLOS_CADF.2.7	GO:0008154	B	.	actin polymerization or depolymerization
[BBH] CADF_DROME (sp|P45594) Cofilin/actin-depolymerizing fact homolog D.m.	CADF_DROME	1,00E-103	GLOS_CADF.2.7	GO:0007015	B	.	actin filament organization
[BBH] CADF_DROME (sp|P45594) Cofilin/actin-depolymerizing fact homolog D.m.	CADF_DROME	1,00E-103	GLOS_CADF.2.7	GO:0071822	B	.	protein complex subunit organization
[BBH] CADF_DROME (sp|P45594) Cofilin/actin-depolymerizing fact homolog D.m.	CADF_DROME	1,00E-103	GLOS_CADF.2.7	GO:0043933	B	.	macromolecular complex subunit organization
[BBH] CADF_DROME (sp|P45594) Cofilin/actin-depolymerizing fact homolog D.m.	CADF_DROME	1,00E-103	GLOS_CADF.2.7	GO:0051261	B	.	protein depolymerization
[BBH] CADF_DROME (sp|P45594) Cofilin/actin-depolymerizing fact homolog D.m.	CADF_DROME	1,00E-103	GLOS_CADF.2.7	GO:0043624	B	.	cellular protein complex disassembly
[BBH] CADF_DROME (sp|P45594) Cofilin/actin-depolymerizing fact homolog D.m.	CADF_DROME	1,00E-103	GLOS_CADF.2.7	GO:0043241	B	.	protein complex disassembly
[BBH] CADF_DROME (sp|P45594) Cofilin/actin-depolymerizing fact homolog D.m.	CADF_DROME	1,00E-103	GLOS_CADF.2.7	GO:0032984	B	.	macromolecular complex disassembly
[BBH] CADF_DROME (sp|P45594) Cofilin/actin-depolymerizing fact homolog D.m.	CADF_DROME	1,00E-103	GLOS_CADF.2.7	GO:0022411	B	.	cellular component disassembly
[BBH] CADF_DROME (sp|P45594) Cofilin/actin-depolymerizing fact homolog D.m.	CADF_DROME	1,00E-103	GLOS_CADF.2.7	GO:0030042	B	InterPro	actin filament depolymerization
[BBH] CADF_DROME (sp|P45594) Cofilin/actin-depolymerizing fact homolog D.m.	CADF_DROME	1,00E-103	GLOS_CADF.2.7	GO:0051258	B	.	protein polymerization

[BBH] CADF_DROME (sp|P45594) Cofilin/actin-depolymerizing fact homolog D.m.	CADF_DROME	1,00E-103	GLOS_CADF.2.7	GO:0043623	B	.	cellular protein complex assembly
[BBH] CADF_DROME (sp|P45594) Cofilin/actin-depolymerizing fact homolog D.m.	CADF_DROME	1,00E-103	GLOS_CADF.2.7	GO:0006461	B	.	protein complex assembly
[BBH] CADF_DROME (sp|P45594) Cofilin/actin-depolymerizing fact homolog D.m.	CADF_DROME	1,00E-103	GLOS_CADF.2.7	GO:0065003	B	.	macromolecular complex assembly
[BBH] CADF_DROME (sp|P45594) Cofilin/actin-depolymerizing fact homolog D.m.	CADF_DROME	1,00E-103	GLOS_CADF.2.7	GO:0070271	B	.	protein complex biogenesis
[BBH] CADF_DROME (sp|P45594) Cofilin/actin-depolymerizing fact homolog D.m.	CADF_DROME	1,00E-103	GLOS_CADF.2.7	GO:0034622	B	.	cellular macromolecular complex assembly
[BBH] CADF_DROME (sp|P45594) Cofilin/actin-depolymerizing fact homolog D.m.	CADF_DROME	1,00E-103	GLOS_CADF.2.7	GO:0030041	B	FlyBase	actin filament polymerization
[BBH] CADF_DROME (sp|P45594) Cofilin/actin-depolymerizing fact homolog D.m.	CADF_DROME	1,00E-103	GLOS_CADF.2.7	GO:0007409	B	FlyBase	axonogenesis
[BBH] CADF_DROME (sp|P45594) Cofilin/actin-depolymerizing fact homolog D.m.	CADF_DROME	1,00E-103	GLOS_CADF.2.7	GO:0007297	B	.	ovarian follicle cell migration
[BBH] CADF_DROME (sp|P45594) Cofilin/actin-depolymerizing fact homolog D.m.	CADF_DROME	1,00E-103	GLOS_CADF.2.7	GO:0010631	B	.	epithelial cell migration
[BBH] CADF_DROME (sp|P45594) Cofilin/actin-depolymerizing fact homolog D.m.	CADF_DROME	1,00E-103	GLOS_CADF.2.7	GO:0001667	B	.	ameboidal cell migration
[BBH] CADF_DROME (sp|P45594) Cofilin/actin-depolymerizing fact homolog D.m.	CADF_DROME	1,00E-103	GLOS_CADF.2.7	GO:0016477	B	.	cell migration
[BBH] CADF_DROME (sp|P45594) Cofilin/actin-depolymerizing fact homolog D.m.	CADF_DROME	1,00E-103	GLOS_CADF.2.7	GO:0048870	B	.	cell motility
[BBH] CADF_DROME (sp|P45594) Cofilin/actin-depolymerizing fact homolog D.m.	CADF_DROME	1,00E-103	GLOS_CADF.2.7	GO:0051674	B	.	localization of cell
[BBH] CADF_DROME (sp|P45594) Cofilin/actin-depolymerizing fact homolog D.m.	CADF_DROME	1,00E-103	GLOS_CADF.2.7	GO:0090132	B	.	epithelium migration
[BBH] CADF_DROME (sp|P45594) Cofilin/actin-depolymerizing fact homolog D.m.	CADF_DROME	1,00E-103	GLOS_CADF.2.7	GO:0090130	B	.	tissue migration
[BBH] CADF_DROME (sp|P45594) Cofilin/actin-depolymerizing fact homolog D.m.	CADF_DROME	1,00E-103	GLOS_CADF.2.7	GO:0022412	B	.	cellular process involved in reproduction in multicellular organism
[BBH] CADF_DROME (sp|P45594) Cofilin/actin-depolymerizing fact homolog D.m.	CADF_DROME	1,00E-103	GLOS_CADF.2.7	GO:0048610	B	.	cellular process involved in reproduction
[BBH] CADF_DROME (sp|P45594) Cofilin/actin-depolymerizing fact homolog D.m.	CADF_DROME	1,00E-103	GLOS_CADF.2.7	GO:0000003	B	.	reproduction
[BBH] CADF_DROME (sp|P45594) Cofilin/actin-depolymerizing fact homolog D.m.	CADF_DROME	1,00E-103	GLOS_CADF.2.7	GO:0032504	B	.	multicellular organism reproduction
[BBH] CADF_DROME (sp|P45594) Cofilin/actin-depolymerizing fact homolog D.m.	CADF_DROME	1,00E-103	GLOS_CADF.2.7	GO:0030707	B	.	ovarian follicle cell development
[BBH] CADF_DROME (sp|P45594) Cofilin/actin-depolymerizing fact homolog D.m.	CADF_DROME	1,00E-103	GLOS_CADF.2.7	GO:0003006	B	.	developmental process involved in reproduction
[BBH] CADF_DROME (sp|P45594) Cofilin/actin-depolymerizing fact homolog D.m.	CADF_DROME	1,00E-103	GLOS_CADF.2.7	GO:0022414	B	.	reproductive process
[BBH] CADF_DROME (sp|P45594) Cofilin/actin-depolymerizing fact homolog D.m.	CADF_DROME	1,00E-103	GLOS_CADF.2.7	GO:0048477	B	.	oogenesis
[BBH] CADF_DROME (sp|P45594) Cofilin/actin-depolymerizing fact homolog D.m.	CADF_DROME	1,00E-103	GLOS_CADF.2.7	GO:0007292	B	.	female gamete generation
[BBH] CADF_DROME (sp|P45594) Cofilin/actin-depolymerizing fact homolog D.m.	CADF_DROME	1,00E-103	GLOS_CADF.2.7	GO:0007276	B	.	gamete generation
[BBH] CADF_DROME (sp|P45594) Cofilin/actin-depolymerizing fact homolog D.m.	CADF_DROME	1,00E-103	GLOS_CADF.2.7	GO:0044702	B	.	single organism reproductive process
[BBH] CADF_DROME (sp|P45594) Cofilin/actin-depolymerizing fact homolog D.m.	CADF_DROME	1,00E-103	GLOS_CADF.2.7	GO:0048609	B	.	multicellular organismal reproductive process
[BBH] CADF_DROME (sp|P45594) Cofilin/actin-depolymerizing fact homolog D.m.	CADF_DROME	1,00E-103	GLOS_CADF.2.7	GO:0019953	B	.	sexual reproduction
[BBH] CADF_DROME (sp|P45594) Cofilin/actin-depolymerizing fact homolog D.m.	CADF_DROME	1,00E-103	GLOS_CADF.2.7	GO:0007298	B	FlyBase	border follicle cell migration
[BBH] CADF_DROME (sp|P45594) Cofilin/actin-depolymerizing fact homolog D.m.	CADF_DROME	1,00E-103	GLOS_CADF.2.7	GO:0000912	B	.	assembly of actomyosin apparatus involved in cytokinesis
[BBH] CADF_DROME (sp|P45594) Cofilin/actin-depolymerizing fact homolog D.m.	CADF_DROME	1,00E-103	GLOS_CADF.2.7	GO:0031032	B	.	actomyosin structure organization
[BBH] CADF_DROME (sp|P45594) Cofilin/actin-depolymerizing fact homolog D.m.	CADF_DROME	1,00E-103	GLOS_CADF.2.7	GO:0032506	B	.	cytokinetic process
[BBH] CADF_DROME (sp|P45594) Cofilin/actin-depolymerizing fact homolog D.m.	CADF_DROME	1,00E-103	GLOS_CADF.2.7	GO:0000910	B	.	cytokinesis
[BBH] CADF_DROME (sp|P45594) Cofilin/actin-depolymerizing fact homolog D.m.	CADF_DROME	1,00E-103	GLOS_CADF.2.7	GO:0051301	B	.	cell division
[BBH] CADF_DROME (sp|P45594) Cofilin/actin-depolymerizing fact homolog D.m.	CADF_DROME	1,00E-103	GLOS_CADF.2.7	GO:0000915	B	FlyBase	cytokinesis, actomyosin contractile ring assembly
[BBH] CADF_DROME (sp|P45594) Cofilin/actin-depolymerizing fact homolog D.m.	CADF_DROME	1,00E-103	GLOS_CADF.2.7	GO:0001736	B	.	establishment of planar polarity
[BBH] CADF_DROME (sp|P45594) Cofilin/actin-depolymerizing fact homolog D.m.	CADF_DROME	1,00E-103	GLOS_CADF.2.7	GO:0007164	B	.	establishment of tissue polarity
[BBH] CADF_DROME (sp|P45594) Cofilin/actin-depolymerizing fact homolog D.m.	CADF_DROME	1,00E-103	GLOS_CADF.2.7	GO:0001738	B	.	morphogenesis of a polarized epithelium
[BBH] CADF_DROME (sp|P45594) Cofilin/actin-depolymerizing fact homolog D.m.	CADF_DROME	1,00E-103	GLOS_CADF.2.7	GO:0001737	B	FlyBase	establishment of imaginal disc-derived wing hair orientation
[BBH] CADF_DROME (sp|P45594) Cofilin/actin-depolymerizing fact homolog D.m.	CADF_DROME	1,00E-103	GLOS_CADF.2.7	GO:0001745	B	.	compound eye morphogenesis
[BBH] CADF_DROME (sp|P45594) Cofilin/actin-depolymerizing fact homolog D.m.	CADF_DROME	1,00E-103	GLOS_CADF.2.7	GO:0048592	B	.	eye morphogenesis
[BBH] CADF_DROME (sp|P45594) Cofilin/actin-depolymerizing fact homolog D.m.	CADF_DROME	1,00E-103	GLOS_CADF.2.7	GO:0001654	B	.	eye development
[BBH] CADF_DROME (sp|P45594) Cofilin/actin-depolymerizing fact homolog D.m.	CADF_DROME	1,00E-103	GLOS_CADF.2.7	GO:0007423	B	.	sensory organ development
[BBH] CADF_DROME (sp|P45594) Cofilin/actin-depolymerizing fact homolog D.m.	CADF_DROME	1,00E-103	GLOS_CADF.2.7	GO:0048749	B	.	compound eye development
[BBH] CADF_DROME (sp|P45594) Cofilin/actin-depolymerizing fact homolog D.m.	CADF_DROME	1,00E-103	GLOS_CADF.2.7	GO:0042067	B	FlyBase	establishment of ommatidial planar polarity
[BBH] CADF_DROME (sp|P45594) Cofilin/actin-depolymerizing fact homolog D.m.	CADF_DROME	1,00E-103	GLOS_CADF.2.7	GO:0008406	B	.	gonad development
[BBH] CADF_DROME (sp|P45594) Cofilin/actin-depolymerizing fact homolog D.m.	CADF_DROME	1,00E-103	GLOS_CADF.2.7	GO:0048608	B	.	reproductive structure development
[BBH] CADF_DROME (sp|P45594) Cofilin/actin-depolymerizing fact homolog D.m.	CADF_DROME	1,00E-103	GLOS_CADF.2.7	GO:0061458	B	.	reproductive system development
[BBH] CADF_DROME (sp|P45594) Cofilin/actin-depolymerizing fact homolog D.m.	CADF_DROME	1,00E-103	GLOS_CADF.2.7	GO:0045137	B	.	development of primary sexual characteristics
[BBH] CADF_DROME (sp|P45594) Cofilin/actin-depolymerizing fact homolog D.m.	CADF_DROME	1,00E-103	GLOS_CADF.2.7	GO:0007548	B	.	sex differentiation
[BBH] CADF_DROME (sp|P45594) Cofilin/actin-depolymerizing fact homolog D.m.	CADF_DROME	1,00E-103	GLOS_CADF.2.7	GO:0046545	B	.	development of primary female sexual characteristics
[BBH] CADF_DROME (sp|P45594) Cofilin/actin-depolymerizing fact homolog D.m.	CADF_DROME	1,00E-103	GLOS_CADF.2.7	GO:0046660	B	.	female sex differentiation
[BBH] CADF_DROME (sp|P45594) Cofilin/actin-depolymerizing fact homolog D.m.	CADF_DROME	1,00E-103	GLOS_CADF.2.7	GO:0008585	B	FlyBase	female gonad development
[BBH] CADF_DROME (sp|P45594) Cofilin/actin-depolymerizing fact homolog D.m.	CADF_DROME	1,00E-103	GLOS_CADF.2.7	GO:0035285	B	.	appendage segmentation

[BBH] CADF_DROME (sp|P45594) Cofilin/actin-depolymerizing fact homolog D.m.	CADF_DROME	1,00E-103	GLOS_CADF.2.7	GO:0035282	B	.	segmentation
[BBH] CADF_DROME (sp|P45594) Cofilin/actin-depolymerizing fact homolog D.m.	CADF_DROME	1,00E-103	GLOS_CADF.2.7	GO:0007480	B	.	imaginal disc-derived leg morphogenesis
[BBH] CADF_DROME (sp|P45594) Cofilin/actin-depolymerizing fact homolog D.m.	CADF_DROME	1,00E-103	GLOS_CADF.2.7	GO:0007478	B	.	leg disc morphogenesis
[BBH] CADF_DROME (sp|P45594) Cofilin/actin-depolymerizing fact homolog D.m.	CADF_DROME	1,00E-103	GLOS_CADF.2.7	GO:0035218	B	.	leg disc development
[BBH] CADF_DROME (sp|P45594) Cofilin/actin-depolymerizing fact homolog D.m.	CADF_DROME	1,00E-103	GLOS_CADF.2.7	GO:0036011	B	FlyBase	imaginal disc-derived leg segmentation
[BBH] CADF_DROME (sp|P45594) Cofilin/actin-depolymerizing fact homolog D.m.	CADF_DROME	1,00E-103	GLOS_CADF.2.7	GO:0007420	B	.	brain development
[BBH] CADF_DROME (sp|P45594) Cofilin/actin-depolymerizing fact homolog D.m.	CADF_DROME	1,00E-103	GLOS_CADF.2.7	GO:0007417	B	.	central nervous system development
[BBH] CADF_DROME (sp|P45594) Cofilin/actin-depolymerizing fact homolog D.m.	CADF_DROME	1,00E-103	GLOS_CADF.2.7	GO:0016319	B	FlyBase	mushroom body development
[BBH] CADF_DROME (sp|P45594) Cofilin/actin-depolymerizing fact homolog D.m.	CADF_DROME	1,00E-103	GLOS_CADF.2.7	GO:0060491	B	.	regulation of cell projection assembly
[BBH] CADF_DROME (sp|P45594) Cofilin/actin-depolymerizing fact homolog D.m.	CADF_DROME	1,00E-103	GLOS_CADF.2.7	GO:0044087	B	.	regulation of cellular component biogenesis
[BBH] CADF_DROME (sp|P45594) Cofilin/actin-depolymerizing fact homolog D.m.	CADF_DROME	1,00E-103	GLOS_CADF.2.7	GO:0010591	B	FlyBase	regulation of lamellipodium assembly
[BBH] CADF_DROME (sp|P45594) Cofilin/actin-depolymerizing fact homolog D.m.	CADF_DROME	1,00E-103	GLOS_CADF.2.7	GO:0042051	B	.	compound eye photoreceptor development
[BBH] CADF_DROME (sp|P45594) Cofilin/actin-depolymerizing fact homolog D.m.	CADF_DROME	1,00E-103	GLOS_CADF.2.7	GO:0042462	B	.	eye photoreceptor cell development
[BBH] CADF_DROME (sp|P45594) Cofilin/actin-depolymerizing fact homolog D.m.	CADF_DROME	1,00E-103	GLOS_CADF.2.7	GO:0042461	B	.	photoreceptor cell development
[BBH] CADF_DROME (sp|P45594) Cofilin/actin-depolymerizing fact homolog D.m.	CADF_DROME	1,00E-103	GLOS_CADF.2.7	GO:0046530	B	.	photoreceptor cell differentiation
[BBH] CADF_DROME (sp|P45594) Cofilin/actin-depolymerizing fact homolog D.m.	CADF_DROME	1,00E-103	GLOS_CADF.2.7	GO:0001754	B	.	eye photoreceptor cell differentiation
[BBH] CADF_DROME (sp|P45594) Cofilin/actin-depolymerizing fact homolog D.m.	CADF_DROME	1,00E-103	GLOS_CADF.2.7	GO:0001751	B	.	compound eye photoreceptor cell differentiation
[BBH] CADF_DROME (sp|P45594) Cofilin/actin-depolymerizing fact homolog D.m.	CADF_DROME	1,00E-103	GLOS_CADF.2.7	GO:0042052	B	FlyBase	rhabdomere development
CBPA1_DROME (sp|Q9VL86) Zinc carboxypeptidase A 1 OS=D.m.	CBPA1_DROME	1,00E-27	GLOS_CBPA1.2.1	GO:0005615	C	UniProtKB	extracellular space
CBPA1_DROME (sp|Q9VL86) Zinc carboxypeptidase A 1 OS=D.m.	CBPA1_DROME	1,00E-27	GLOS_CBPA1.2.1	GO:0004180	M	.	carboxypeptidase activity
CBPA1_DROME (sp|Q9VL86) Zinc carboxypeptidase A 1 OS=D.m.	CBPA1_DROME	1,00E-27	GLOS_CBPA1.2.1	GO:0008235	M	.	metalloexopeptidase activity
CBPA1_DROME (sp|Q9VL86) Zinc carboxypeptidase A 1 OS=D.m.	CBPA1_DROME	1,00E-27	GLOS_CBPA1.2.1	GO:0008237	M	.	metallopeptidase activity
CBPA1_DROME (sp|Q9VL86) Zinc carboxypeptidase A 1 OS=D.m.	CBPA1_DROME	1,00E-27	GLOS_CBPA1.2.1	GO:0004181	M	InterPro	metallocarboxypeptidase activity
CBPA1_DROME (sp|Q9VL86) Zinc carboxypeptidase A 1 OS=D.m.	CBPA1_DROME	1,00E-27	GLOS_CBPA1.2.1	GO:0008270	M	InterPro	zinc ion binding
CBPA1_DROME (sp|Q9VL86) Zinc carboxypeptidase A 1 OS=D.m.	CBPA1_DROME	1,00E-27	GLOS_CBPA1.2.1	GO:0022008	B	FlyBase	neurogenesis
CBPA1_DROME (sp|Q9VL86) Zinc carboxypeptidase A 1 OS=D.m.	CBPA1_DROME	1,00E-27	GLOS_CBPA1.2.1	GO:0006508	B	UniProtKB-k	proteolysis
[BBH] CP6U1_DROME (sp|Q9V979) Prob cyto P450 6u1 OS=D.m.	CP6U1_DROME	1,00E-122	GLOS_CP6U1.1.2	GO:0044432	C	.	endoplasmic reticulum part
[BBH] CP6U1_DROME (sp|Q9V979) Prob cyto P450 6u1 OS=D.m.	CP6U1_DROME	1,00E-122	GLOS_CP6U1.1.2	GO:0005783	C	.	endoplasmic reticulum
[BBH] CP6U1_DROME (sp|Q9V979) Prob cyto P450 6u1 OS=D.m.	CP6U1_DROME	1,00E-122	GLOS_CP6U1.1.2	GO:0042175	C	.	nuclear outer membrane-endoplasmic reticulum membrane network
[BBH] CP6U1_DROME (sp|Q9V979) Prob cyto P450 6u1 OS=D.m.	CP6U1_DROME	1,00E-122	GLOS_CP6U1.1.2	GO:0012505	C	.	endomembrane system
[BBH] CP6U1_DROME (sp|Q9V979) Prob cyto P450 6u1 OS=D.m.	CP6U1_DROME	1,00E-122	GLOS_CP6U1.1.2	GO:0005789	C	UniProtKB-S	endoplasmic reticulum membrane
[BBH] CP6U1_DROME (sp|Q9V979) Prob cyto P450 6u1 OS=D.m.	CP6U1_DROME	1,00E-122	GLOS_CP6U1.1.2	GO:0009055	M	InterPro	electron carrier activity
[BBH] CP6U1_DROME (sp|Q9V979) Prob cyto P450 6u1 OS=D.m.	CP6U1_DROME	1,00E-122	GLOS_CP6U1.1.2	GO:0046906	M	.	tetrapyrrole binding
[BBH] CP6U1_DROME (sp|Q9V979) Prob cyto P450 6u1 OS=D.m.	CP6U1_DROME	1,00E-122	GLOS_CP6U1.1.2	GO:0020037	M	InterPro	heme binding
[BBH] CP6U1_DROME (sp|Q9V979) Prob cyto P450 6u1 OS=D.m.	CP6U1_DROME	1,00E-122	GLOS_CP6U1.1.2	GO:0005506	M	InterPro	iron ion binding
[BBH] CP6U1_DROME (sp|Q9V979) Prob cyto P450 6u1 OS=D.m.	CP6U1_DROME	1,00E-122	GLOS_CP6U1.1.2	GO:0016491	M	.	oxidoreductase activity
[BBH] CP6U1_DROME (sp|Q9V979) Prob cyto P450 6u1 OS=D.m.	CP6U1_DROME	1,00E-122	GLOS_CP6U1.1.2	GO:0004497	M	UniProtKB-k	monooxygenase activity
[BBH] CP6U1_DROME (sp|Q9V979) Prob cyto P450 6u1 OS=D.m.	CP6U1_DROME	1,00E-122	GLOS_CP6U1.1.2	GO:0016705	M	InterPro	oxidoreductase activity, acting on paired donors, with incorporation or reduction of molecular oxygen
CSN4_DROME (sp|Q9V345) COP9 signalosome complex subunit 4 D.m.	CSN4_DROME	1,00E-38	GLOS_CSN4.2.4	GO:0008180	C	UniProtKB	COP9 signalosome
CSN4_DROME (sp|Q9V345) COP9 signalosome complex subunit 4 D.m.	CSN4_DROME	1,00E-38	GLOS_CSN4.2.4	GO:0005737	C	UniProtKB-S	cytoplasm
CSN4_DROME (sp|Q9V345) COP9 signalosome complex subunit 4 D.m.	CSN4_DROME	1,00E-38	GLOS_CSN4.2.4	GO:0008641	M	.	small protein activating enzyme activity
CSN4_DROME (sp|Q9V345) COP9 signalosome complex subunit 4 D.m.	CSN4_DROME	1,00E-38	GLOS_CSN4.2.4	GO:0019781	M	UniProtKB	NEDD8 activating enzyme activity
CSN4_DROME (sp|Q9V345) COP9 signalosome complex subunit 4 D.m.	CSN4_DROME	1,00E-38	GLOS_CSN4.2.4	GO:0000975	M	.	regulatory region DNA binding
CSN4_DROME (sp|Q9V345) COP9 signalosome complex subunit 4 D.m.	CSN4_DROME	1,00E-38	GLOS_CSN4.2.4	GO:0001067	M	.	regulatory region nucleic acid binding
CSN4_DROME (sp|Q9V345) COP9 signalosome complex subunit 4 D.m.	CSN4_DROME	1,00E-38	GLOS_CSN4.2.4	GO:0044212	M	FlyBase	transcription regulatory region DNA binding
CSN4_DROME (sp|Q9V345) COP9 signalosome complex subunit 4 D.m.	CSN4_DROME	1,00E-38	GLOS_CSN4.2.4	GO:0001751	B	FlyBase	compound eye photoreceptor cell differentiation
CSN4_DROME (sp|Q9V345) COP9 signalosome complex subunit 4 D.m.	CSN4_DROME	1,00E-38	GLOS_CSN4.2.4	GO:0045168	B	.	cell-cell signaling involved in cell fate commitment
CSN4_DROME (sp|Q9V345) COP9 signalosome complex subunit 4 D.m.	CSN4_DROME	1,00E-38	GLOS_CSN4.2.4	GO:0007267	B	.	cell-cell signaling
CSN4_DROME (sp|Q9V345) COP9 signalosome complex subunit 4 D.m.	CSN4_DROME	1,00E-38	GLOS_CSN4.2.4	GO:0045165	B	.	cell fate commitment
CSN4_DROME (sp|Q9V345) COP9 signalosome complex subunit 4 D.m.	CSN4_DROME	1,00E-38	GLOS_CSN4.2.4	GO:0046331	B	FlyBase	lateral inhibition
CSN4_DROME (sp|Q9V345) COP9 signalosome complex subunit 4 D.m.	CSN4_DROME	1,00E-38	GLOS_CSN4.2.4	GO:0031572	B	.	G2 DNA damage checkpoint
CSN4_DROME (sp|Q9V345) COP9 signalosome complex subunit 4 D.m.	CSN4_DROME	1,00E-38	GLOS_CSN4.2.4	GO:0000077	B	.	DNA damage checkpoint
CSN4_DROME (sp|Q9V345) COP9 signalosome complex subunit 4 D.m.	CSN4_DROME	1,00E-38	GLOS_CSN4.2.4	GO:0006974	B	.	cellular response to DNA damage stimulus

CSN4_DROME (sp|Q9V345) COP9 signalosome complex subunit 4 D.m.	CSN4_DROME	1,00E-38	GLOS_CSN4.2.4	GO:0033554	B	.	cellular response to stress
CSN4_DROME (sp|Q9V345) COP9 signalosome complex subunit 4 D.m.	CSN4_DROME	1,00E-38	GLOS_CSN4.2.4	GO:0031570	B	.	DNA integrity checkpoint
CSN4_DROME (sp|Q9V345) COP9 signalosome complex subunit 4 D.m.	CSN4_DROME	1,00E-38	GLOS_CSN4.2.4	GO:0000075	B	.	cell cycle checkpoint
CSN4_DROME (sp|Q9V345) COP9 signalosome complex subunit 4 D.m.	CSN4_DROME	1,00E-38	GLOS_CSN4.2.4	GO:0044773	B	.	mitotic DNA damage checkpoint
CSN4_DROME (sp|Q9V345) COP9 signalosome complex subunit 4 D.m.	CSN4_DROME	1,00E-38	GLOS_CSN4.2.4	GO:0044774	B	.	mitotic DNA integrity checkpoint
CSN4_DROME (sp|Q9V345) COP9 signalosome complex subunit 4 D.m.	CSN4_DROME	1,00E-38	GLOS_CSN4.2.4	GO:0007093	B	.	mitotic cell cycle checkpoint
CSN4_DROME (sp|Q9V345) COP9 signalosome complex subunit 4 D.m.	CSN4_DROME	1,00E-38	GLOS_CSN4.2.4	GO:1901991	B	.	negative regulation of mitotic cell cycle phase transition
CSN4_DROME (sp|Q9V345) COP9 signalosome complex subunit 4 D.m.	CSN4_DROME	1,00E-38	GLOS_CSN4.2.4	GO:1901988	B	.	negative regulation of cell cycle phase transition
CSN4_DROME (sp|Q9V345) COP9 signalosome complex subunit 4 D.m.	CSN4_DROME	1,00E-38	GLOS_CSN4.2.4	GO:0010948	B	.	negative regulation of cell cycle process
CSN4_DROME (sp|Q9V345) COP9 signalosome complex subunit 4 D.m.	CSN4_DROME	1,00E-38	GLOS_CSN4.2.4	GO:0007095	B	FlyBase	mitotic G2 DNA damage checkpoint
CSN4_DROME (sp|Q9V345) COP9 signalosome complex subunit 4 D.m.	CSN4_DROME	1,00E-38	GLOS_CSN4.2.4	GO:0031330	B	.	negative regulation of cellular catabolic process
CSN4_DROME (sp|Q9V345) COP9 signalosome complex subunit 4 D.m.	CSN4_DROME	1,00E-38	GLOS_CSN4.2.4	GO:0009895	B	.	negative regulation of catabolic process
CSN4_DROME (sp|Q9V345) COP9 signalosome complex subunit 4 D.m.	CSN4_DROME	1,00E-38	GLOS_CSN4.2.4	GO:0009894	B	.	regulation of catabolic process
CSN4_DROME (sp|Q9V345) COP9 signalosome complex subunit 4 D.m.	CSN4_DROME	1,00E-38	GLOS_CSN4.2.4	GO:0031329	B	.	regulation of cellular catabolic process
CSN4_DROME (sp|Q9V345) COP9 signalosome complex subunit 4 D.m.	CSN4_DROME	1,00E-38	GLOS_CSN4.2.4	GO:0032434	B	.	regulation of proteasomal ubiquitin-dependent protein catabolic process
CSN4_DROME (sp|Q9V345) COP9 signalosome complex subunit 4 D.m.	CSN4_DROME	1,00E-38	GLOS_CSN4.2.4	GO:0061136	B	.	regulation of proteasomal protein catabolic process
CSN4_DROME (sp|Q9V345) COP9 signalosome complex subunit 4 D.m.	CSN4_DROME	1,00E-38	GLOS_CSN4.2.4	GO:0030162	B	.	regulation of proteolysis
CSN4_DROME (sp|Q9V345) COP9 signalosome complex subunit 4 D.m.	CSN4_DROME	1,00E-38	GLOS_CSN4.2.4	GO:0042176	B	.	regulation of protein catabolic process
CSN4_DROME (sp|Q9V345) COP9 signalosome complex subunit 4 D.m.	CSN4_DROME	1,00E-38	GLOS_CSN4.2.4	GO:1901799	B	.	negative regulation of proteasomal protein catabolic process
CSN4_DROME (sp|Q9V345) COP9 signalosome complex subunit 4 D.m.	CSN4_DROME	1,00E-38	GLOS_CSN4.2.4	GO:0042177	B	.	negative regulation of protein catabolic process
CSN4_DROME (sp|Q9V345) COP9 signalosome complex subunit 4 D.m.	CSN4_DROME	1,00E-38	GLOS_CSN4.2.4	GO:0051248	B	.	negative regulation of protein metabolic process
CSN4_DROME (sp|Q9V345) COP9 signalosome complex subunit 4 D.m.	CSN4_DROME	1,00E-38	GLOS_CSN4.2.4	GO:0045861	B	.	negative regulation of proteolysis
CSN4_DROME (sp|Q9V345) COP9 signalosome complex subunit 4 D.m.	CSN4_DROME	1,00E-38	GLOS_CSN4.2.4	GO:0032269	B	.	negative regulation of cellular protein metabolic process
CSN4_DROME (sp|Q9V345) COP9 signalosome complex subunit 4 D.m.	CSN4_DROME	1,00E-38	GLOS_CSN4.2.4	GO:0032435	B	FlyBase	negative regulation of proteasomal ubiquitin-dependent protein catabolic process
CSN4_DROME (sp|Q9V345) COP9 signalosome complex subunit 4 D.m.	CSN4_DROME	1,00E-38	GLOS_CSN4.2.4	GO:0048477	B	UniProtKB-K	oogenesis
CSN4_DROME (sp|Q9V345) COP9 signalosome complex subunit 4 D.m.	CSN4_DROME	1,00E-38	GLOS_CSN4.2.4	GO:0070646	B	.	protein modification by small protein removal
CSN4_DROME (sp|Q9V345) COP9 signalosome complex subunit 4 D.m.	CSN4_DROME	1,00E-38	GLOS_CSN4.2.4	GO:0070647	B	.	protein modification by small protein conjugation or removal
CSN4_DROME (sp|Q9V345) COP9 signalosome complex subunit 4 D.m.	CSN4_DROME	1,00E-38	GLOS_CSN4.2.4	GO:0006464	B	.	cellular protein modification process
CSN4_DROME (sp|Q9V345) COP9 signalosome complex subunit 4 D.m.	CSN4_DROME	1,00E-38	GLOS_CSN4.2.4	GO:0036211	B	.	protein modification process
CSN4_DROME (sp|Q9V345) COP9 signalosome complex subunit 4 D.m.	CSN4_DROME	1,00E-38	GLOS_CSN4.2.4	GO:0043412	B	.	macromolecule modification
CSN4_DROME (sp|Q9V345) COP9 signalosome complex subunit 4 D.m.	CSN4_DROME	1,00E-38	GLOS_CSN4.2.4	GO:0000338	B	UniProtKB	protein deneddylation
CSN4_DROME (sp|Q9V345) COP9 signalosome complex subunit 4 D.m.	CSN4_DROME	1,00E-38	GLOS_CSN4.2.4	GO:0031647	B	.	regulation of protein stability
CSN4_DROME (sp|Q9V345) COP9 signalosome complex subunit 4 D.m.	CSN4_DROME	1,00E-38	GLOS_CSN4.2.4	GO:0010608	B	.	posttranscriptional regulation of gene expression
CSN4_DROME (sp|Q9V345) COP9 signalosome complex subunit 4 D.m.	CSN4_DROME	1,00E-38	GLOS_CSN4.2.4	GO:0050821	B	FlyBase	protein stabilization
[BBH] DBNL_DROME (sp|Q9VU84) Drebrin-like protein OS=D.m.	DBNL_DROME	0	GLOS_DBNL.1.1	GO:0005737	C	UniProtKB-K	cytoplasm
[BBH] DBNL_DROME (sp|Q9VU84) Drebrin-like protein OS=D.m.	DBNL_DROME	0	GLOS_DBNL.1.1	GO:0005856	C	UniProtKB	cytoskeleton
[BBH] DBNL_DROME (sp|Q9VU84) Drebrin-like protein OS=D.m.	DBNL_DROME	0	GLOS_DBNL.1.1	GO:0003779	M	.	actin binding
[BBH] DBNL_DROME (sp|Q9VU84) Drebrin-like protein OS=D.m.	DBNL_DROME	0	GLOS_DBNL.1.1	GO:0032403	M	.	protein complex binding
[BBH] DBNL_DROME (sp|Q9VU84) Drebrin-like protein OS=D.m.	DBNL_DROME	0	GLOS_DBNL.1.1	GO:0051015	M	FlyBase	actin filament binding
[BBH] DBNL_DROME (sp|Q9VU84) Drebrin-like protein OS=D.m.	DBNL_DROME	0	GLOS_DBNL.1.1	GO:1901981	M	.	phosphatidylinositol phosphate binding
[BBH] DBNL_DROME (sp|Q9VU84) Drebrin-like protein OS=D.m.	DBNL_DROME	0	GLOS_DBNL.1.1	GO:0035091	M	.	phosphatidylinositol binding
[BBH] DBNL_DROME (sp|Q9VU84) Drebrin-like protein OS=D.m.	DBNL_DROME	0	GLOS_DBNL.1.1	GO:0005547	M	FlyBase	phosphatidylinositol-3,4,5-trisphosphate binding
[BBH] DBNL_DROME (sp|Q9VU84) Drebrin-like protein OS=D.m.	DBNL_DROME	0	GLOS_DBNL.1.1	GO:0005546	M	FlyBase	phosphatidylinositol-4,5-bisphosphate binding
[BBH] DBNL_DROME (sp|Q9VU84) Drebrin-like protein OS=D.m.	DBNL_DROME	0	GLOS_DBNL.1.1	GO:0072341	M	.	modified amino acid binding
[BBH] DBNL_DROME (sp|Q9VU84) Drebrin-like protein OS=D.m.	DBNL_DROME	0	GLOS_DBNL.1.1	GO:0016597	M	.	amino acid binding
[BBH] DBNL_DROME (sp|Q9VU84) Drebrin-like protein OS=D.m.	DBNL_DROME	0	GLOS_DBNL.1.1	GO:0031406	M	.	carboxylic acid binding
[BBH] DBNL_DROME (sp|Q9VU84) Drebrin-like protein OS=D.m.	DBNL_DROME	0	GLOS_DBNL.1.1	GO:0043177	M	.	organic acid binding
[BBH] DBNL_DROME (sp|Q9VU84) Drebrin-like protein OS=D.m.	DBNL_DROME	0	GLOS_DBNL.1.1	GO:0001786	M	FlyBase	phosphatidylserine binding
[BBH] DBNL_DROME (sp|Q9VU84) Drebrin-like protein OS=D.m.	DBNL_DROME	0	GLOS_DBNL.1.1	GO:0022416	B	FlyBase	chaeta development
EIF1A_DROME (sp|Q8IQ13) Probable RNA-binding protein EIF1AD D.m.	EIF1A_DROME	1,00E-51	GLOS_EIF1A.1.1	GO:0008135	M	.	translation factor activity, nucleic acid binding
EIF1A_DROME (sp|Q8IQ13) Probable RNA-binding protein EIF1AD D.m.	EIF1A_DROME	1,00E-51	GLOS_EIF1A.1.1	GO:0003723	M	.	RNA binding
EIF1A_DROME (sp|Q8IQ13) Probable RNA-binding protein EIF1AD D.m.	EIF1A_DROME	1,00E-51	GLOS_EIF1A.1.1	GO:0003743	M	InterPro	translation initiation factor activity
[BBH] ENA_DROME (sp|Q8T4F7) Prot enabled OS=D.m. GN=ena PE=1 SV=4	ENA_DROME	1,00E-108	GLOS_ENA.2.3	GO:0005912	C	FlyBase	adherens junction
[BBH] ENA_DROME (sp|Q8T4F7) Prot enabled OS=D.m. GN=ena PE=1 SV=4	ENA_DROME	1,00E-108	GLOS_ENA.2.3	GO:0043005	C	.	neuron projection

[BBH] ENA_DROME (sp|Q8T4F7) Prot enabled OS=D.m. GN=ena PE=1 SV=4	ENA_DROME	1,00E-108	GLOS_ENA.2.3	GO:0042995	C	.	cell projection
[BBH] ENA_DROME (sp|Q8T4F7) Prot enabled OS=D.m. GN=ena PE=1 SV=4	ENA_DROME	1,00E-108	GLOS_ENA.2.3	GO:0097458	C	.	neuron part
[BBH] ENA_DROME (sp|Q8T4F7) Prot enabled OS=D.m. GN=ena PE=1 SV=4	ENA_DROME	1,00E-108	GLOS_ENA.2.3	GO:0030424	C	FlyBase	axon
[BBH] ENA_DROME (sp|Q8T4F7) Prot enabled OS=D.m. GN=ena PE=1 SV=4	ENA_DROME	1,00E-108	GLOS_ENA.2.3	GO:0005911	C	FlyBase	cell-cell junction
[BBH] ENA_DROME (sp|Q8T4F7) Prot enabled OS=D.m. GN=ena PE=1 SV=4	ENA_DROME	1,00E-108	GLOS_ENA.2.3	GO:0005856	C	UniProtKB-S	cytoskeleton
[BBH] ENA_DROME (sp|Q8T4F7) Prot enabled OS=D.m. GN=ena PE=1 SV=4	ENA_DROME	1,00E-108	GLOS_ENA.2.3	GO:0005829	C	Reactome	cytosol
[BBH] ENA_DROME (sp|Q8T4F7) Prot enabled OS=D.m. GN=ena PE=1 SV=4	ENA_DROME	1,00E-108	GLOS_ENA.2.3	GO:0044463	C	.	cell projection part
[BBH] ENA_DROME (sp|Q8T4F7) Prot enabled OS=D.m. GN=ena PE=1 SV=4	ENA_DROME	1,00E-108	GLOS_ENA.2.3	GO:0030175	C	.	filopodium
[BBH] ENA_DROME (sp|Q8T4F7) Prot enabled OS=D.m. GN=ena PE=1 SV=4	ENA_DROME	1,00E-108	GLOS_ENA.2.3	GO:0032433	C	FlyBase	filopodium tip
[BBH] ENA_DROME (sp|Q8T4F7) Prot enabled OS=D.m. GN=ena PE=1 SV=4	ENA_DROME	1,00E-108	GLOS_ENA.2.3	GO:0031252	C	.	cell leading edge
[BBH] ENA_DROME (sp|Q8T4F7) Prot enabled OS=D.m. GN=ena PE=1 SV=4	ENA_DROME	1,00E-108	GLOS_ENA.2.3	GO:0030027	C	UniProtKB	lamellipodium
[BBH] ENA_DROME (sp|Q8T4F7) Prot enabled OS=D.m. GN=ena PE=1 SV=4	ENA_DROME	1,00E-108	GLOS_ENA.2.3	GO:0003779	M	FlyBase	actin binding
[BBH] ENA_DROME (sp|Q8T4F7) Prot enabled OS=D.m. GN=ena PE=1 SV=4	ENA_DROME	1,00E-108	GLOS_ENA.2.3	GO:0017124	M	UniProtKB	SH3 domain binding
[BBH] ENA_DROME (sp|Q8T4F7) Prot enabled OS=D.m. GN=ena PE=1 SV=4	ENA_DROME	1,00E-108	GLOS_ENA.2.3	GO:0007015	B	FlyBase	actin filament organization
[BBH] ENA_DROME (sp|Q8T4F7) Prot enabled OS=D.m. GN=ena PE=1 SV=4	ENA_DROME	1,00E-108	GLOS_ENA.2.3	GO:0007411	B	FlyBase	axon guidance
[BBH] ENA_DROME (sp|Q8T4F7) Prot enabled OS=D.m. GN=ena PE=1 SV=4	ENA_DROME	1,00E-108	GLOS_ENA.2.3	GO:0007298	B	FlyBase	border follicle cell migration
[BBH] ENA_DROME (sp|Q8T4F7) Prot enabled OS=D.m. GN=ena PE=1 SV=4	ENA_DROME	1,00E-108	GLOS_ENA.2.3	GO:0048749	B	FlyBase	compound eye development
[BBH] ENA_DROME (sp|Q8T4F7) Prot enabled OS=D.m. GN=ena PE=1 SV=4	ENA_DROME	1,00E-108	GLOS_ENA.2.3	GO:0007300	B	.	ovarian nurse cell to oocyte transport
[BBH] ENA_DROME (sp|Q8T4F7) Prot enabled OS=D.m. GN=ena PE=1 SV=4	ENA_DROME	1,00E-108	GLOS_ENA.2.3	GO:0007303	B	FlyBase	cytoplasmic transport, nurse cell to oocyte
[BBH] ENA_DROME (sp|Q8T4F7) Prot enabled OS=D.m. GN=ena PE=1 SV=4	ENA_DROME	1,00E-108	GLOS_ENA.2.3	GO:0016358	B	.	dendrite development
[BBH] ENA_DROME (sp|Q8T4F7) Prot enabled OS=D.m. GN=ena PE=1 SV=4	ENA_DROME	1,00E-108	GLOS_ENA.2.3	GO:0048813	B	FlyBase	dendrite morphogenesis
[BBH] ENA_DROME (sp|Q8T4F7) Prot enabled OS=D.m. GN=ena PE=1 SV=4	ENA_DROME	1,00E-108	GLOS_ENA.2.3	GO:0002064	B	.	epithelial cell development
[BBH] ENA_DROME (sp|Q8T4F7) Prot enabled OS=D.m. GN=ena PE=1 SV=4	ENA_DROME	1,00E-108	GLOS_ENA.2.3	GO:0003382	B	FlyBase	epithelial cell morphogenesis
[BBH] ENA_DROME (sp|Q8T4F7) Prot enabled OS=D.m. GN=ena PE=1 SV=4	ENA_DROME	1,00E-108	GLOS_ENA.2.3	GO:0048859	B	.	formation of anatomical boundary
[BBH] ENA_DROME (sp|Q8T4F7) Prot enabled OS=D.m. GN=ena PE=1 SV=4	ENA_DROME	1,00E-108	GLOS_ENA.2.3	GO:0048646	B	.	anatomical structure formation involved in morphogenesis
[BBH] ENA_DROME (sp|Q8T4F7) Prot enabled OS=D.m. GN=ena PE=1 SV=4	ENA_DROME	1,00E-108	GLOS_ENA.2.3	GO:0007386	B	.	compartment pattern specification
[BBH] ENA_DROME (sp|Q8T4F7) Prot enabled OS=D.m. GN=ena PE=1 SV=4	ENA_DROME	1,00E-108	GLOS_ENA.2.3	GO:0009952	B	.	anterior/posterior pattern specification
[BBH] ENA_DROME (sp|Q8T4F7) Prot enabled OS=D.m. GN=ena PE=1 SV=4	ENA_DROME	1,00E-108	GLOS_ENA.2.3	GO:0060288	B	FlyBase	formation of a compartment boundary
[BBH] ENA_DROME (sp|Q8T4F7) Prot enabled OS=D.m. GN=ena PE=1 SV=4	ENA_DROME	1,00E-108	GLOS_ENA.2.3	GO:0007390	B	FlyBase	germ-band shortening
[BBH] ENA_DROME (sp|Q8T4F7) Prot enabled OS=D.m. GN=ena PE=1 SV=4	ENA_DROME	1,00E-108	GLOS_ENA.2.3	GO:0035262	B	FlyBase	gonad morphogenesis
[BBH] ENA_DROME (sp|Q8T4F7) Prot enabled OS=D.m. GN=ena PE=1 SV=4	ENA_DROME	1,00E-108	GLOS_ENA.2.3	GO:0008258	B	FlyBase	head involution
[BBH] ENA_DROME (sp|Q8T4F7) Prot enabled OS=D.m. GN=ena PE=1 SV=4	ENA_DROME	1,00E-108	GLOS_ENA.2.3	GO:0031346	B	.	positive regulation of cell projection organization
[BBH] ENA_DROME (sp|Q8T4F7) Prot enabled OS=D.m. GN=ena PE=1 SV=4	ENA_DROME	1,00E-108	GLOS_ENA.2.3	GO:0051130	B	.	positive regulation of cellular component organization
[BBH] ENA_DROME (sp|Q8T4F7) Prot enabled OS=D.m. GN=ena PE=1 SV=4	ENA_DROME	1,00E-108	GLOS_ENA.2.3	GO:0051489	B	.	regulation of filopodium assembly
[BBH] ENA_DROME (sp|Q8T4F7) Prot enabled OS=D.m. GN=ena PE=1 SV=4	ENA_DROME	1,00E-108	GLOS_ENA.2.3	GO:0051491	B	FlyBase	positive regulation of filopodium assembly
[BBH] ENA_DROME (sp|Q8T4F7) Prot enabled OS=D.m. GN=ena PE=1 SV=4	ENA_DROME	1,00E-108	GLOS_ENA.2.3	GO:0030832	B	.	regulation of actin filament length
[BBH] ENA_DROME (sp|Q8T4F7) Prot enabled OS=D.m. GN=ena PE=1 SV=4	ENA_DROME	1,00E-108	GLOS_ENA.2.3	GO:0032956	B	.	regulation of actin cytoskeleton organization
[BBH] ENA_DROME (sp|Q8T4F7) Prot enabled OS=D.m. GN=ena PE=1 SV=4	ENA_DROME	1,00E-108	GLOS_ENA.2.3	GO:0032970	B	.	regulation of actin filament-based process
[BBH] ENA_DROME (sp|Q8T4F7) Prot enabled OS=D.m. GN=ena PE=1 SV=4	ENA_DROME	1,00E-108	GLOS_ENA.2.3	GO:0051493	B	.	regulation of cytoskeleton organization
[BBH] ENA_DROME (sp|Q8T4F7) Prot enabled OS=D.m. GN=ena PE=1 SV=4	ENA_DROME	1,00E-108	GLOS_ENA.2.3	GO:0008064	B	FlyBase	regulation of actin polymerization or depolymerization
[BBH] ENA_DROME (sp|Q8T4F7) Prot enabled OS=D.m. GN=ena PE=1 SV=4	ENA_DROME	1,00E-108	GLOS_ENA.2.3	GO:0008360	B	FlyBase	regulation of cell shape
[BBH] ENA_DROME (sp|Q8T4F7) Prot enabled OS=D.m. GN=ena PE=1 SV=4	ENA_DROME	1,00E-108	GLOS_ENA.2.3	GO:0010591	B	FlyBase	regulation of lamellipodium assembly
[BBH] ENA_DROME (sp|Q8T4F7) Prot enabled OS=D.m. GN=ena PE=1 SV=4	ENA_DROME	1,00E-108	GLOS_ENA.2.3	GO:0007391	B	.	dorsal closure
[BBH] ENA_DROME (sp|Q8T4F7) Prot enabled OS=D.m. GN=ena PE=1 SV=4	ENA_DROME	1,00E-108	GLOS_ENA.2.3	GO:0007396	B	FlyBase	suture of dorsal opening
EXOC3_DROME (sp|Q9V8K2) Exocyst complex component 3 D.m.	EXOC3_DROME	0	GLOS_EXOC3.2.3	GO:0044448	C	.	cell cortex part
EXOC3_DROME (sp|Q9V8K2) Exocyst complex component 3 D.m.	EXOC3_DROME	0	GLOS_EXOC3.2.3	GO:0005938	C	.	cell cortex
EXOC3_DROME (sp|Q9V8K2) Exocyst complex component 3 D.m.	EXOC3_DROME	0	GLOS_EXOC3.2.3	GO:0000145	C	FlyBase	exocyst
EXOC3_DROME (sp|Q9V8K2) Exocyst complex component 3 D.m.	EXOC3_DROME	0	GLOS_EXOC3.2.3	GO:0016028	C	FlyBase	rhabdomere
EXOC3_DROME (sp|Q9V8K2) Exocyst complex component 3 D.m.	EXOC3_DROME	0	GLOS_EXOC3.2.3	GO:0005913	C	.	cell-cell adherens junction
EXOC3_DROME (sp|Q9V8K2) Exocyst complex component 3 D.m.	EXOC3_DROME	0	GLOS_EXOC3.2.3	GO:0005915	C	FlyBase	zonula adherens
EXOC3_DROME (sp|Q9V8K2) Exocyst complex component 3 D.m.	EXOC3_DROME	0	GLOS_EXOC3.2.3	GO:0007298	B	FlyBase	border follicle cell migration
EXOC3_DROME (sp|Q9V8K2) Exocyst complex component 3 D.m.	EXOC3_DROME	0	GLOS_EXOC3.2.3	GO:0002164	B	.	larval development
EXOC3_DROME (sp|Q9V8K2) Exocyst complex component 3 D.m.	EXOC3_DROME	0	GLOS_EXOC3.2.3	GO:0002168	B	FlyBase	instar larval development
EXOC3_DROME (sp|Q9V8K2) Exocyst complex component 3 D.m.	EXOC3_DROME	0	GLOS_EXOC3.2.3	GO:0001895	B	.	retina homeostasis

EXOC3_DROME (sp|Q9V8K2) Exocyst complex component 3 D.m.	EXOC3_DROME	0	GLOS_EXOC3.2.3	GO:0001894	B	.	tissue homeostasis
EXOC3_DROME (sp|Q9V8K2) Exocyst complex component 3 D.m.	EXOC3_DROME	0	GLOS_EXOC3.2.3	GO:0060249	B	.	anatomical structure homeostasis
EXOC3_DROME (sp|Q9V8K2) Exocyst complex component 3 D.m.	EXOC3_DROME	0	GLOS_EXOC3.2.3	GO:0048871	B	.	multicellular organismal homeostasis
EXOC3_DROME (sp|Q9V8K2) Exocyst complex component 3 D.m.	EXOC3_DROME	0	GLOS_EXOC3.2.3	GO:0045494	B	FlyBase	photoreceptor cell maintenance
EXOC3_DROME (sp|Q9V8K2) Exocyst complex component 3 D.m.	EXOC3_DROME	0	GLOS_EXOC3.2.3	GO:0008594	B	FlyBase	photoreceptor cell morphogenesis
EXOC3_DROME (sp|Q9V8K2) Exocyst complex component 3 D.m.	EXOC3_DROME	0	GLOS_EXOC3.2.3	GO:0007009	B	FlyBase	plasma membrane organization
EXOC3_DROME (sp|Q9V8K2) Exocyst complex component 3 D.m.	EXOC3_DROME	0	GLOS_EXOC3.2.3	GO:0017157	B	.	regulation of exocytosis
EXOC3_DROME (sp|Q9V8K2) Exocyst complex component 3 D.m.	EXOC3_DROME	0	GLOS_EXOC3.2.3	GO:0051046	B	.	regulation of secretion
EXOC3_DROME (sp|Q9V8K2) Exocyst complex component 3 D.m.	EXOC3_DROME	0	GLOS_EXOC3.2.3	GO:0060627	B	.	regulation of vesicle-mediated transport
EXOC3_DROME (sp|Q9V8K2) Exocyst complex component 3 D.m.	EXOC3_DROME	0	GLOS_EXOC3.2.3	GO:0051047	B	.	positive regulation of secretion
EXOC3_DROME (sp|Q9V8K2) Exocyst complex component 3 D.m.	EXOC3_DROME	0	GLOS_EXOC3.2.3	GO:0045921	B	FlyBase	positive regulation of exocytosis
EXOC3_DROME (sp|Q9V8K2) Exocyst complex component 3 D.m.	EXOC3_DROME	0	GLOS_EXOC3.2.3	GO:0015031	B	FlyBase	protein transport
EXOC3_DROME (sp|Q9V8K2) Exocyst complex component 3 D.m.	EXOC3_DROME	0	GLOS_EXOC3.2.3	GO:0044091	B	.	membrane biogenesis
EXOC3_DROME (sp|Q9V8K2) Exocyst complex component 3 D.m.	EXOC3_DROME	0	GLOS_EXOC3.2.3	GO:0042052	B	.	rhabdomere development
EXOC3_DROME (sp|Q9V8K2) Exocyst complex component 3 D.m.	EXOC3_DROME	0	GLOS_EXOC3.2.3	GO:0045313	B	FlyBase	rhabdomere membrane biogenesis
EXOC3_DROME (sp|Q9V8K2) Exocyst complex component 3 D.m.	EXOC3_DROME	0	GLOS_EXOC3.2.3	GO:0006904	B	.	vesicle docking involved in exocytosis
EXOC3_DROME (sp|Q9V8K2) Exocyst complex component 3 D.m.	EXOC3_DROME	0	GLOS_EXOC3.2.3	GO:0048278	B	.	vesicle docking
EXOC3_DROME (sp|Q9V8K2) Exocyst complex component 3 D.m.	EXOC3_DROME	0	GLOS_EXOC3.2.3	GO:0022406	B	.	membrane docking
EXOC3_DROME (sp|Q9V8K2) Exocyst complex component 3 D.m.	EXOC3_DROME	0	GLOS_EXOC3.2.3	GO:0016192	B	.	vesicle-mediated transport
EXOC3_DROME (sp|Q9V8K2) Exocyst complex component 3 D.m.	EXOC3_DROME	0	GLOS_EXOC3.2.3	GO:0006887	B	.	exocytosis
EXOC3_DROME (sp|Q9V8K2) Exocyst complex component 3 D.m.	EXOC3_DROME	0	GLOS_EXOC3.2.3	GO:0032940	B	.	secretion by cell
EXOC3_DROME (sp|Q9V8K2) Exocyst complex component 3 D.m.	EXOC3_DROME	0	GLOS_EXOC3.2.3	GO:0016079	B	.	synaptic vesicle exocytosis
EXOC3_DROME (sp|Q9V8K2) Exocyst complex component 3 D.m.	EXOC3_DROME	0	GLOS_EXOC3.2.3	GO:0048489	B	.	synaptic vesicle transport
EXOC3_DROME (sp|Q9V8K2) Exocyst complex component 3 D.m.	EXOC3_DROME	0	GLOS_EXOC3.2.3	GO:0097480	B	.	establishment of synaptic vesicle localization
EXOC3_DROME (sp|Q9V8K2) Exocyst complex component 3 D.m.	EXOC3_DROME	0	GLOS_EXOC3.2.3	GO:0051650	B	.	establishment of vesicle localization
EXOC3_DROME (sp|Q9V8K2) Exocyst complex component 3 D.m.	EXOC3_DROME	0	GLOS_EXOC3.2.3	GO:0051656	B	.	establishment of organelle localization
EXOC3_DROME (sp|Q9V8K2) Exocyst complex component 3 D.m.	EXOC3_DROME	0	GLOS_EXOC3.2.3	GO:0051640	B	.	organelle localization
EXOC3_DROME (sp|Q9V8K2) Exocyst complex component 3 D.m.	EXOC3_DROME	0	GLOS_EXOC3.2.3	GO:0051648	B	.	vesicle localization
EXOC3_DROME (sp|Q9V8K2) Exocyst complex component 3 D.m.	EXOC3_DROME	0	GLOS_EXOC3.2.3	GO:0097479	B	.	synaptic vesicle localization
EXOC3_DROME (sp|Q9V8K2) Exocyst complex component 3 D.m.	EXOC3_DROME	0	GLOS_EXOC3.2.3	GO:0007268	B	.	synaptic transmission
EXOC3_DROME (sp|Q9V8K2) Exocyst complex component 3 D.m.	EXOC3_DROME	0	GLOS_EXOC3.2.3	GO:0019226	B	.	transmission of nerve impulse
EXOC3_DROME (sp|Q9V8K2) Exocyst complex component 3 D.m.	EXOC3_DROME	0	GLOS_EXOC3.2.3	GO:0035637	B	.	multicellular organismal signaling
EXOC3_DROME (sp|Q9V8K2) Exocyst complex component 3 D.m.	EXOC3_DROME	0	GLOS_EXOC3.2.3	GO:0050877	B	.	neurological system process
EXOC3_DROME (sp|Q9V8K2) Exocyst complex component 3 D.m.	EXOC3_DROME	0	GLOS_EXOC3.2.3	GO:0007269	B	.	neurotransmitter secretion
EXOC3_DROME (sp|Q9V8K2) Exocyst complex component 3 D.m.	EXOC3_DROME	0	GLOS_EXOC3.2.3	GO:0001505	B	.	regulation of neurotransmitter levels
EXOC3_DROME (sp|Q9V8K2) Exocyst complex component 3 D.m.	EXOC3_DROME	0	GLOS_EXOC3.2.3	GO:0006836	B	.	neurotransmitter transport
EXOC3_DROME (sp|Q9V8K2) Exocyst complex component 3 D.m.	EXOC3_DROME	0	GLOS_EXOC3.2.3	GO:0023061	B	.	signal release
EXOC3_DROME (sp|Q9V8K2) Exocyst complex component 3 D.m.	EXOC3_DROME	0	GLOS_EXOC3.2.3	GO:0003001	B	.	generation of a signal involved in cell-cell signaling
EXOC3_DROME (sp|Q9V8K2) Exocyst complex component 3 D.m.	EXOC3_DROME	0	GLOS_EXOC3.2.3	GO:0016081	B	FlyBase	synaptic vesicle docking involved in exocytosis
EXOC3_DROME (sp|Q9V8K2) Exocyst complex component 3 D.m.	EXOC3_DROME	0	GLOS_EXOC3.2.3	GO:0006903	B	.	vesicle targeting
EXOC3_DROME (sp|Q9V8K2) Exocyst complex component 3 D.m.	EXOC3_DROME	0	GLOS_EXOC3.2.3	GO:0016080	B	FlyBase	synaptic vesicle targeting
[BBH] FKB12_DROME (sp|P48375) 12 kDa FK506-binding prot D.m.	FKB12_DROME	7,00E-71	GLOS_FKB12.2.3	GO:0005737	C	UniProtKB-S	cytoplasm
[BBH] FKB12_DROME (sp|P48375) 12 kDa FK506-binding prot D.m.	FKB12_DROME	7,00E-71	GLOS_FKB12.2.3	GO:0016020	C	RefGenome	membrane
[BBH] FKB12_DROME (sp|P48375) 12 kDa FK506-binding prot D.m.	FKB12_DROME	7,00E-71	GLOS_FKB12.2.3	GO:0005527	M	.	macrolide binding
[BBH] FKB12_DROME (sp|P48375) 12 kDa FK506-binding prot D.m.	FKB12_DROME	7,00E-71	GLOS_FKB12.2.3	GO:0008144	M	.	drug binding
[BBH] FKB12_DROME (sp|P48375) 12 kDa FK506-binding prot D.m.	FKB12_DROME	7,00E-71	GLOS_FKB12.2.3	GO:0005528	M	RefGenome	FK506 binding
[BBH] FKB12_DROME (sp|P48375) 12 kDa FK506-binding prot D.m.	FKB12_DROME	7,00E-71	GLOS_FKB12.2.3	GO:0016859	M	.	cis-trans isomerase activity
[BBH] FKB12_DROME (sp|P48375) 12 kDa FK506-binding prot D.m.	FKB12_DROME	7,00E-71	GLOS_FKB12.2.3	GO:0016853	M	.	isomerase activity
[BBH] FKB12_DROME (sp|P48375) 12 kDa FK506-binding prot D.m.	FKB12_DROME	7,00E-71	GLOS_FKB12.2.3	GO:0003755	M	RefGenome	peptidyl-prolyl cis-trans isomerase activity
[BBH] FKB12_DROME (sp|P48375) 12 kDa FK506-binding prot D.m.	FKB12_DROME	7,00E-71	GLOS_FKB12.2.3	GO:0006457	B	UniProtKB-k	protein folding
[BBH] FKB12_DROME (sp|P48375) 12 kDa FK506-binding prot D.m.	FKB12_DROME	7,00E-71	GLOS_FKB12.2.3	GO:0006974	B	FlyBase	cellular response to DNA damage stimulus
GPL_GLOFF (sp|Q8MUG0) Lectizyme OS=G. f. fuscipes GN=Gpl PE=2 SV=1	GPL_GLOFF	4,00E-11	GLOS_GPL.12.22	GO:0005576	C	UniProtKB-S	extracellular region
GPL_GLOFF (sp|Q8MUG0) Lectizyme OS=G. f. fuscipes GN=Gpl PE=2 SV=1	GPL_GLOFF	4,00E-11	GLOS_GPL.12.22	GO:0004175	M	.	endopeptidase activity
GPL_GLOFF (sp|Q8MUG0) Lectizyme OS=G. f. fuscipes GN=Gpl PE=2 SV=1	GPL_GLOFF	4,00E-11	GLOS_GPL.12.22	GO:0008236	M	.	serine-type peptidase activity

GPL_GLOFF (sp|Q8MUG0) Lectizyme OS=G. f. fuscipes GN=Gpl PE=2 SV=1	GPL_GLOFF	4,00E-11	GLOS_GPL.12.22	GO:0017171	M	.	serine hydrolase activity
GPL_GLOFF (sp|Q8MUG0) Lectizyme OS=G. f. fuscipes GN=Gpl PE=2 SV=1	GPL_GLOFF	4,00E-11	GLOS_GPL.12.22	GO:0004252	M	InterPro	serine-type endopeptidase activity
GPL_GLOFF (sp|Q8MUG0) Lectizyme OS=G. f. fuscipes GN=Gpl PE=2 SV=1	GPL_GLOFF	4,00E-11	GLOS_GPL.12.22	GO:0006508	B	UniProtKB-K	proteolysis
GSTT1_MUSDO (sp|P28338) Glutathione S-transferase 1 OS=M. domestica	GSTT1_MUSDO	3,00E-80	GLOS_GSTT1.3.5	GO:0016765	M	.	transferase activity, transferring alkyl or aryl (other than methyl) groups
GSTT1_MUSDO (sp|P28338) Glutathione S-transferase 1 OS=M. domestica	GSTT1_MUSDO	3,00E-80	GLOS_GSTT1.3.5	GO:0016740	M	.	transferase activity
GSTT1_MUSDO (sp|P28338) Glutathione S-transferase 1 OS=M. domestica	GSTT1_MUSDO	3,00E-80	GLOS_GSTT1.3.5	GO:0004364	M	UniProtKB-E	glutathione transferase activity
ISK1_HORSE (sp|P81634) Pancreatic secretory trypsin inhibitor E. caballus	ISK1_HORSE	4,00E-06	GLOS_ISK1.5.5	GO:0005576	C	UniProtKB-S	extracellular region
ISK1_HORSE (sp|P81634) Pancreatic secretory trypsin inhibitor E. caballus	ISK1_HORSE	4,00E-06	GLOS_ISK1.5.5	GO:0004866	M	.	endopeptidase inhibitor activity
ISK1_HORSE (sp|P81634) Pancreatic secretory trypsin inhibitor E. caballus	ISK1_HORSE	4,00E-06	GLOS_ISK1.5.5	GO:0030414	M	.	peptidase inhibitor activity
ISK1_HORSE (sp|P81634) Pancreatic secretory trypsin inhibitor E. caballus	ISK1_HORSE	4,00E-06	GLOS_ISK1.5.5	GO:0004857	M	.	enzyme inhibitor activity
ISK1_HORSE (sp|P81634) Pancreatic secretory trypsin inhibitor E. caballus	ISK1_HORSE	4,00E-06	GLOS_ISK1.5.5	GO:0030234	M	.	enzyme regulator activity
ISK1_HORSE (sp|P81634) Pancreatic secretory trypsin inhibitor E. caballus	ISK1_HORSE	4,00E-06	GLOS_ISK1.5.5	GO:0061134	M	.	peptidase regulator activity
ISK1_HORSE (sp|P81634) Pancreatic secretory trypsin inhibitor E. caballus	ISK1_HORSE	4,00E-06	GLOS_ISK1.5.5	GO:0061135	M	.	endopeptidase regulator activity
ISK1_HORSE (sp|P81634) Pancreatic secretory trypsin inhibitor E. caballus	ISK1_HORSE	4,00E-06	GLOS_ISK1.5.5	GO:0004867	M	UniProtKB-K	serine-type endopeptidase inhibitor activity
ISK1_HORSE (sp|P81634) Pancreatic secretory trypsin inhibitor E. caballus	ISK1_HORSE	4,00E-06	GLOS_ISK1.5.5	GO:0034766	B	.	negative regulation of ion transmembrane transport
ISK1_HORSE (sp|P81634) Pancreatic secretory trypsin inhibitor E. caballus	ISK1_HORSE	4,00E-06	GLOS_ISK1.5.5	GO:0034763	B	.	negative regulation of transmembrane transport
ISK1_HORSE (sp|P81634) Pancreatic secretory trypsin inhibitor E. caballus	ISK1_HORSE	4,00E-06	GLOS_ISK1.5.5	GO:0034762	B	.	regulation of transmembrane transport
ISK1_HORSE (sp|P81634) Pancreatic secretory trypsin inhibitor E. caballus	ISK1_HORSE	4,00E-06	GLOS_ISK1.5.5	GO:0051051	B	.	negative regulation of transport
ISK1_HORSE (sp|P81634) Pancreatic secretory trypsin inhibitor E. caballus	ISK1_HORSE	4,00E-06	GLOS_ISK1.5.5	GO:0034765	B	.	regulation of ion transmembrane transport
ISK1_HORSE (sp|P81634) Pancreatic secretory trypsin inhibitor E. caballus	ISK1_HORSE	4,00E-06	GLOS_ISK1.5.5	GO:0043271	B	.	negative regulation of ion transport
ISK1_HORSE (sp|P81634) Pancreatic secretory trypsin inhibitor E. caballus	ISK1_HORSE	4,00E-06	GLOS_ISK1.5.5	GO:0051926	B	.	negative regulation of calcium ion transport
ISK1_HORSE (sp|P81634) Pancreatic secretory trypsin inhibitor E. caballus	ISK1_HORSE	4,00E-06	GLOS_ISK1.5.5	GO:0090279	B	.	regulation of calcium ion import
ISK1_HORSE (sp|P81634) Pancreatic secretory trypsin inhibitor E. caballus	ISK1_HORSE	4,00E-06	GLOS_ISK1.5.5	GO:0090281	B	Ensembl	negative regulation of calcium ion import
ISK1_HORSE (sp|P81634) Pancreatic secretory trypsin inhibitor E. caballus	ISK1_HORSE	4,00E-06	GLOS_ISK1.5.5	GO:0009968	B	.	negative regulation of signal transduction
ISK1_HORSE (sp|P81634) Pancreatic secretory trypsin inhibitor E. caballus	ISK1_HORSE	4,00E-06	GLOS_ISK1.5.5	GO:0010648	B	.	negative regulation of cell communication
ISK1_HORSE (sp|P81634) Pancreatic secretory trypsin inhibitor E. caballus	ISK1_HORSE	4,00E-06	GLOS_ISK1.5.5	GO:0023057	B	.	negative regulation of signaling
ISK1_HORSE (sp|P81634) Pancreatic secretory trypsin inhibitor E. caballus	ISK1_HORSE	4,00E-06	GLOS_ISK1.5.5	GO:0048585	B	.	negative regulation of response to stimulus
ISK1_HORSE (sp|P81634) Pancreatic secretory trypsin inhibitor E. caballus	ISK1_HORSE	4,00E-06	GLOS_ISK1.5.5	GO:0010749	B	.	regulation of nitric oxide mediated signal transduction
ISK1_HORSE (sp|P81634) Pancreatic secretory trypsin inhibitor E. caballus	ISK1_HORSE	4,00E-06	GLOS_ISK1.5.5	GO:0010751	B	Ensembl	negative regulation of nitric oxide mediated signal transduction
ISK1_HORSE (sp|P81634) Pancreatic secretory trypsin inhibitor E. caballus	ISK1_HORSE	4,00E-06	GLOS_ISK1.5.5	GO:0001933	B	.	negative regulation of protein phosphorylation
ISK1_HORSE (sp|P81634) Pancreatic secretory trypsin inhibitor E. caballus	ISK1_HORSE	4,00E-06	GLOS_ISK1.5.5	GO:0031400	B	.	negative regulation of protein modification process
ISK1_HORSE (sp|P81634) Pancreatic secretory trypsin inhibitor E. caballus	ISK1_HORSE	4,00E-06	GLOS_ISK1.5.5	GO:0042326	B	.	negative regulation of phosphorylation
ISK1_HORSE (sp|P81634) Pancreatic secretory trypsin inhibitor E. caballus	ISK1_HORSE	4,00E-06	GLOS_ISK1.5.5	GO:0045936	B	.	negative regulation of phosphate metabolic process
ISK1_HORSE (sp|P81634) Pancreatic secretory trypsin inhibitor E. caballus	ISK1_HORSE	4,00E-06	GLOS_ISK1.5.5	GO:0010563	B	.	negative regulation of phosphorus metabolic process
ISK1_HORSE (sp|P81634) Pancreatic secretory trypsin inhibitor E. caballus	ISK1_HORSE	4,00E-06	GLOS_ISK1.5.5	GO:0050730	B	.	regulation of peptidyl-tyrosine phosphorylation
ISK1_HORSE (sp|P81634) Pancreatic secretory trypsin inhibitor E. caballus	ISK1_HORSE	4,00E-06	GLOS_ISK1.5.5	GO:0050732	B	Ensembl	negative regulation of peptidyl-tyrosine phosphorylation
ISK1_HORSE (sp|P81634) Pancreatic secretory trypsin inhibitor E. caballus	ISK1_HORSE	4,00E-06	GLOS_ISK1.5.5	GO:0010951	B	.	negative regulation of endopeptidase activity
ISK1_HORSE (sp|P81634) Pancreatic secretory trypsin inhibitor E. caballus	ISK1_HORSE	4,00E-06	GLOS_ISK1.5.5	GO:0010466	B	.	negative regulation of peptidase activity
ISK1_HORSE (sp|P81634) Pancreatic secretory trypsin inhibitor E. caballus	ISK1_HORSE	4,00E-06	GLOS_ISK1.5.5	GO:0051346	B	.	negative regulation of hydrolase activity
ISK1_HORSE (sp|P81634) Pancreatic secretory trypsin inhibitor E. caballus	ISK1_HORSE	4,00E-06	GLOS_ISK1.5.5	GO:0043086	B	.	negative regulation of catalytic activity
ISK1_HORSE (sp|P81634) Pancreatic secretory trypsin inhibitor E. caballus	ISK1_HORSE	4,00E-06	GLOS_ISK1.5.5	GO:0044092	B	.	negative regulation of molecular function
ISK1_HORSE (sp|P81634) Pancreatic secretory trypsin inhibitor E. caballus	ISK1_HORSE	4,00E-06	GLOS_ISK1.5.5	GO:0065009	B	.	regulation of molecular function
ISK1_HORSE (sp|P81634) Pancreatic secretory trypsin inhibitor E. caballus	ISK1_HORSE	4,00E-06	GLOS_ISK1.5.5	GO:0050790	B	.	regulation of catalytic activity
ISK1_HORSE (sp|P81634) Pancreatic secretory trypsin inhibitor E. caballus	ISK1_HORSE	4,00E-06	GLOS_ISK1.5.5	GO:0051336	B	.	regulation of hydrolase activity
ISK1_HORSE (sp|P81634) Pancreatic secretory trypsin inhibitor E. caballus	ISK1_HORSE	4,00E-06	GLOS_ISK1.5.5	GO:0052547	B	.	regulation of peptidase activity
ISK1_HORSE (sp|P81634) Pancreatic secretory trypsin inhibitor E. caballus	ISK1_HORSE	4,00E-06	GLOS_ISK1.5.5	GO:0052548	B	.	regulation of endopeptidase activity
ISK1_HORSE (sp|P81634) Pancreatic secretory trypsin inhibitor E. caballus	ISK1_HORSE	4,00E-06	GLOS_ISK1.5.5	GO:1900003	B	.	regulation of serine-type endopeptidase activity
ISK1_HORSE (sp|P81634) Pancreatic secretory trypsin inhibitor E. caballus	ISK1_HORSE	4,00E-06	GLOS_ISK1.5.5	GO:1900004	B	Ensembl	negative regulation of serine-type endopeptidase activity
ISK1_HORSE (sp|P81634) Pancreatic secretory trypsin inhibitor E. caballus	ISK1_HORSE	4,00E-06	GLOS_ISK1.5.5	GO:0017158	B	.	regulation of calcium ion-dependent exocytosis
ISK1_HORSE (sp|P81634) Pancreatic secretory trypsin inhibitor E. caballus	ISK1_HORSE	4,00E-06	GLOS_ISK1.5.5	GO:2000241	B	.	regulation of reproductive process
ISK1_HORSE (sp|P81634) Pancreatic secretory trypsin inhibitor E. caballus	ISK1_HORSE	4,00E-06	GLOS_ISK1.5.5	GO:0060046	B	Ensembl	regulation of acrosome reaction
ISK1_HORSE (sp|P81634) Pancreatic secretory trypsin inhibitor E. caballus	ISK1_HORSE	4,00E-06	GLOS_ISK1.5.5	GO:2001256	B	Ensembl	regulation of store-operated calcium entry
KPBB_DROME (sp|Q9VLS1) Probable phosphorylase b kinase regulatory subunit beta OS=D. melanogaster	KPBB_DROME	2,00E-57	GLOS_KPBB.5.7	GO:0005886	C	UniProtKB-S	plasma membrane

KPBB_DROME (sp|Q9VLS1) Prob phosphorylase b kinase … Same as above	KPBB_DROME	2,00E-57	GLOS_KPBB.5.7	GO:0016798	M	.	hydrolase activity, acting on glycosyl bonds
KPBB_DROME (sp|Q9VLS1) Prob phosphorylase b kinase … Same as above	KPBB_DROME	2,00E-57	GLOS_KPBB.5.7	GO:0004553	M	InterPro	hydrolase activity, hydrolyzing O-glycosyl compounds
KPBB_DROME (sp|Q9VLS1) Prob phosphorylase b kinase … Same as above	KPBB_DROME	2,00E-57	GLOS_KPBB.5.7	GO:0006073	B	.	cellular glucan metabolic process
KPBB_DROME (sp|Q9VLS1) Prob phosphorylase b kinase … Same as above	KPBB_DROME	2,00E-57	GLOS_KPBB.5.7	GO:0044042	B	.	glucan metabolic process
KPBB_DROME (sp|Q9VLS1) Prob phosphorylase b kinase … Same as above	KPBB_DROME	2,00E-57	GLOS_KPBB.5.7	GO:0005976	B	.	polysaccharide metabolic process
KPBB_DROME (sp|Q9VLS1) Prob phosphorylase b kinase … Same as above	KPBB_DROME	2,00E-57	GLOS_KPBB.5.7	GO:0005975	B	.	carbohydrate metabolic process
KPBB_DROME (sp|Q9VLS1) Prob phosphorylase b kinase … Same as above	KPBB_DROME	2,00E-57	GLOS_KPBB.5.7	GO:0044264	B	.	cellular polysaccharide metabolic process
KPBB_DROME (sp|Q9VLS1) Prob phosphorylase b kinase … Same as above	KPBB_DROME	2,00E-57	GLOS_KPBB.5.7	GO:0044262	B	.	cellular carbohydrate metabolic process
KPBB_DROME (sp|Q9VLS1) Prob phosphorylase b kinase … Same as above	KPBB_DROME	2,00E-57	GLOS_KPBB.5.7	GO:0006112	B	.	energy reserve metabolic process
KPBB_DROME (sp|Q9VLS1) Prob phosphorylase b kinase … Same as above	KPBB_DROME	2,00E-57	GLOS_KPBB.5.7	GO:0015980	B	.	energy derivation by oxidation of organic compounds
KPBB_DROME (sp|Q9VLS1) Prob phosphorylase b kinase … Same as above	KPBB_DROME	2,00E-57	GLOS_KPBB.5.7	GO:0006091	B	.	generation of precursor metabolites and energy
KPBB_DROME (sp|Q9VLS1) Prob phosphorylase b kinase … Same as above	KPBB_DROME	2,00E-57	GLOS_KPBB.5.7	GO:0055114	B	.	oxidation-reduction process
KPBB_DROME (sp|Q9VLS1) Prob phosphorylase b kinase … Same as above	KPBB_DROME	2,00E-57	GLOS_KPBB.5.7	GO:0044710	B	.	single-organism metabolic process
KPBB_DROME (sp|Q9VLS1) Prob phosphorylase b kinase … Same as above	KPBB_DROME	2,00E-57	GLOS_KPBB.5.7	GO:0005977	B	UniProtKB-U	glycogen metabolic process
KTU_DROWI (sp|B4MPK3) Prot kintoun OS=D. willistoni GN=Ppi20 PE=3 SV=1	KTU_DROWI	1,00E-135	GLOS_KTU.1.2	GO:0005737	C	UniProtKB-S	cytoplasm
KTU_DROWI (sp|B4MPK3) Prot kintoun OS=D. willistoni GN=Ppi20 PE=3 SV=1	KTU_DROWI	1,00E-135	GLOS_KTU.1.2	GO:0016709	M	.	oxidoreductase activity, acting on paired donors, with incorporation or reduction of of molecular oxygen, NAD(P)H as 1 donor, and incorporation of 1 atom of oxygen
KTU_DROWI (sp|B4MPK3) Prot kintoun OS=D. willistoni GN=Ppi20 PE=3 SV=1	KTU_DROWI	1,00E-135	GLOS_KTU.1.2	GO:0004497	M	.	monooxygenase activity
KTU_DROWI (sp|B4MPK3) Prot kintoun OS=D. willistoni GN=Ppi20 PE=3 SV=1	KTU_DROWI	1,00E-135	GLOS_KTU.1.2	GO:0016705	M	.	oxidoreductase activity, acting on paired donors, with incorporation or reduction of molecular oxygen
KTU_DROWI (sp|B4MPK3) Prot kintoun OS=D. willistoni GN=Ppi20 PE=3 SV=1	KTU_DROWI	1,00E-135	GLOS_KTU.1.2	GO:0004502	M	InterPro	kynurenine 3-monooxygenase activity
KTU_DROWI (sp|B4MPK3) Prot kintoun OS=D. willistoni GN=Ppi20 PE=3 SV=1	KTU_DROWI	1,00E-135	GLOS_KTU.1.2	GO:0055114	B	GOC	oxidation-reduction process
KTU_DROWI (sp|B4MPK3) Prot kintoun OS=D. willistoni GN=Ppi20 PE=3 SV=1	KTU_DROWI	1,00E-135	GLOS_KTU.1.2	GO:0043650	B	.	dicarboxylic acid biosynthetic process
KTU_DROWI (sp|B4MPK3) Prot kintoun OS=D. willistoni GN=Ppi20 PE=3 SV=1	KTU_DROWI	1,00E-135	GLOS_KTU.1.2	GO:0043648	B	.	dicarboxylic acid metabolic process
KTU_DROWI (sp|B4MPK3) Prot kintoun OS=D. willistoni GN=Ppi20 PE=3 SV=1	KTU_DROWI	1,00E-135	GLOS_KTU.1.2	GO:0019752	B	.	carboxylic acid metabolic process
KTU_DROWI (sp|B4MPK3) Prot kintoun OS=D. willistoni GN=Ppi20 PE=3 SV=1	KTU_DROWI	1,00E-135	GLOS_KTU.1.2	GO:0043436	B	.	oxoacid metabolic process
KTU_DROWI (sp|B4MPK3) Prot kintoun OS=D. willistoni GN=Ppi20 PE=3 SV=1	KTU_DROWI	1,00E-135	GLOS_KTU.1.2	GO:0006082	B	.	organic acid metabolic process
KTU_DROWI (sp|B4MPK3) Prot kintoun OS=D. willistoni GN=Ppi20 PE=3 SV=1	KTU_DROWI	1,00E-135	GLOS_KTU.1.2	GO:0044281	B	.	small molecule metabolic process
KTU_DROWI (sp|B4MPK3) Prot kintoun OS=D. willistoni GN=Ppi20 PE=3 SV=1	KTU_DROWI	1,00E-135	GLOS_KTU.1.2	GO:0046394	B	.	carboxylic acid biosynthetic process
KTU_DROWI (sp|B4MPK3) Prot kintoun OS=D. willistoni GN=Ppi20 PE=3 SV=1	KTU_DROWI	1,00E-135	GLOS_KTU.1.2	GO:0016053	B	.	organic acid biosynthetic process
KTU_DROWI (sp|B4MPK3) Prot kintoun OS=D. willistoni GN=Ppi20 PE=3 SV=1	KTU_DROWI	1,00E-135	GLOS_KTU.1.2	GO:0044283	B	.	small molecule biosynthetic process
KTU_DROWI (sp|B4MPK3) Prot kintoun OS=D. willistoni GN=Ppi20 PE=3 SV=1	KTU_DROWI	1,00E-135	GLOS_KTU.1.2	GO:0044711	B	.	single-organism biosynthetic process
KTU_DROWI (sp|B4MPK3) Prot kintoun OS=D. willistoni GN=Ppi20 PE=3 SV=1	KTU_DROWI	1,00E-135	GLOS_KTU.1.2	GO:0046874	B	.	quinolinate metabolic process
KTU_DROWI (sp|B4MPK3) Prot kintoun OS=D. willistoni GN=Ppi20 PE=3 SV=1	KTU_DROWI	1,00E-135	GLOS_KTU.1.2	GO:0072524	B	.	pyridine-containing compound metabolic process
KTU_DROWI (sp|B4MPK3) Prot kintoun OS=D. willistoni GN=Ppi20 PE=3 SV=1	KTU_DROWI	1,00E-135	GLOS_KTU.1.2	GO:1901564	B	.	organonitrogen compound metabolic process
KTU_DROWI (sp|B4MPK3) Prot kintoun OS=D. willistoni GN=Ppi20 PE=3 SV=1	KTU_DROWI	1,00E-135	GLOS_KTU.1.2	GO:0072525	B	.	pyridine-containing compound biosynthetic process
KTU_DROWI (sp|B4MPK3) Prot kintoun OS=D. willistoni GN=Ppi20 PE=3 SV=1	KTU_DROWI	1,00E-135	GLOS_KTU.1.2	GO:1901566	B	.	organonitrogen compound biosynthetic process
KTU_DROWI (sp|B4MPK3) Prot kintoun OS=D. willistoni GN=Ppi20 PE=3 SV=1	KTU_DROWI	1,00E-135	GLOS_KTU.1.2	GO:0019805	B	InterPro	quinolinate biosynthetic process
KTU_DROWI (sp|B4MPK3) Prot kintoun OS=D. willistoni GN=Ppi20 PE=3 SV=1	KTU_DROWI	1,00E-135	GLOS_KTU.1.2	GO:0006568	B	.	tryptophan metabolic process
KTU_DROWI (sp|B4MPK3) Prot kintoun OS=D. willistoni GN=Ppi20 PE=3 SV=1	KTU_DROWI	1,00E-135	GLOS_KTU.1.2	GO:0006586	B	.	indolalkylamine metabolic process
KTU_DROWI (sp|B4MPK3) Prot kintoun OS=D. willistoni GN=Ppi20 PE=3 SV=1	KTU_DROWI	1,00E-135	GLOS_KTU.1.2	GO:0006576	B	.	cellular biogenic amine metabolic process
KTU_DROWI (sp|B4MPK3) Prot kintoun OS=D. willistoni GN=Ppi20 PE=3 SV=1	KTU_DROWI	1,00E-135	GLOS_KTU.1.2	GO:0044106	B	.	cellular amine metabolic process
KTU_DROWI (sp|B4MPK3) Prot kintoun OS=D. willistoni GN=Ppi20 PE=3 SV=1	KTU_DROWI	1,00E-135	GLOS_KTU.1.2	GO:0009308	B	.	amine metabolic process
KTU_DROWI (sp|B4MPK3) Prot kintoun OS=D. willistoni GN=Ppi20 PE=3 SV=1	KTU_DROWI	1,00E-135	GLOS_KTU.1.2	GO:0042430	B	.	indole-containing compound metabolic process
KTU_DROWI (sp|B4MPK3) Prot kintoun OS=D. willistoni GN=Ppi20 PE=3 SV=1	KTU_DROWI	1,00E-135	GLOS_KTU.1.2	GO:0009072	B	.	aromatic amino acid family metabolic process
KTU_DROWI (sp|B4MPK3) Prot kintoun OS=D. willistoni GN=Ppi20 PE=3 SV=1	KTU_DROWI	1,00E-135	GLOS_KTU.1.2	GO:0006520	B	.	cellular amino acid metabolic process
KTU_DROWI (sp|B4MPK3) Prot kintoun OS=D. willistoni GN=Ppi20 PE=3 SV=1	KTU_DROWI	1,00E-135	GLOS_KTU.1.2	GO:1901605	B	.	alpha-amino acid metabolic process
KTU_DROWI (sp|B4MPK3) Prot kintoun OS=D. willistoni GN=Ppi20 PE=3 SV=1	KTU_DROWI	1,00E-135	GLOS_KTU.1.2	GO:0009074	B	.	aromatic amino acid family catabolic process
KTU_DROWI (sp|B4MPK3) Prot kintoun OS=D. willistoni GN=Ppi20 PE=3 SV=1	KTU_DROWI	1,00E-135	GLOS_KTU.1.2	GO:0009063	B	.	cellular amino acid catabolic process
KTU_DROWI (sp|B4MPK3) Prot kintoun OS=D. willistoni GN=Ppi20 PE=3 SV=1	KTU_DROWI	1,00E-135	GLOS_KTU.1.2	GO:0046395	B	.	carboxylic acid catabolic process
KTU_DROWI (sp|B4MPK3) Prot kintoun OS=D. willistoni GN=Ppi20 PE=3 SV=1	KTU_DROWI	1,00E-135	GLOS_KTU.1.2	GO:0016054	B	.	organic acid catabolic process
KTU_DROWI (sp|B4MPK3) Prot kintoun OS=D. willistoni GN=Ppi20 PE=3 SV=1	KTU_DROWI	1,00E-135	GLOS_KTU.1.2	GO:0044282	B	.	small molecule catabolic process
KTU_DROWI (sp|B4MPK3) Prot kintoun OS=D. willistoni GN=Ppi20 PE=3 SV=1	KTU_DROWI	1,00E-135	GLOS_KTU.1.2	GO:0044712	B	.	single-organism catabolic process
KTU_DROWI (sp|B4MPK3) Prot kintoun OS=D. willistoni GN=Ppi20 PE=3 SV=1	KTU_DROWI	1,00E-135	GLOS_KTU.1.2	GO:1901565	B	.	organonitrogen compound catabolic process

KTU_DROWI (sp|B4MPK3) Prot kintoun OS=D. willistoni GN=Ppi20 PE=3 SV=1	KTU_DROWI	1,00E-135	GLOS_KTU.1.2	GO:0019439	B	.	aromatic compound catabolic process
KTU_DROWI (sp|B4MPK3) Prot kintoun OS=D. willistoni GN=Ppi20 PE=3 SV=1	KTU_DROWI	1,00E-135	GLOS_KTU.1.2	GO:1901361	B	.	organic cyclic compound catabolic process
KTU_DROWI (sp|B4MPK3) Prot kintoun OS=D. willistoni GN=Ppi20 PE=3 SV=1	KTU_DROWI	1,00E-135	GLOS_KTU.1.2	GO:0046218	B	.	indolalkylamine catabolic process
KTU_DROWI (sp|B4MPK3) Prot kintoun OS=D. willistoni GN=Ppi20 PE=3 SV=1	KTU_DROWI	1,00E-135	GLOS_KTU.1.2	GO:0042402	B	.	cellular biogenic amine catabolic process
KTU_DROWI (sp|B4MPK3) Prot kintoun OS=D. willistoni GN=Ppi20 PE=3 SV=1	KTU_DROWI	1,00E-135	GLOS_KTU.1.2	GO:0009310	B	.	amine catabolic process
KTU_DROWI (sp|B4MPK3) Prot kintoun OS=D. willistoni GN=Ppi20 PE=3 SV=1	KTU_DROWI	1,00E-135	GLOS_KTU.1.2	GO:0042436	B	.	indole-containing compound catabolic process
KTU_DROWI (sp|B4MPK3) Prot kintoun OS=D. willistoni GN=Ppi20 PE=3 SV=1	KTU_DROWI	1,00E-135	GLOS_KTU.1.2	GO:0044270	B	.	cellular nitrogen compound catabolic process
KTU_DROWI (sp|B4MPK3) Prot kintoun OS=D. willistoni GN=Ppi20 PE=3 SV=1	KTU_DROWI	1,00E-135	GLOS_KTU.1.2	GO:0046700	B	.	heterocycle catabolic process
KTU_DROWI (sp|B4MPK3) Prot kintoun OS=D. willistoni GN=Ppi20 PE=3 SV=1	KTU_DROWI	1,00E-135	GLOS_KTU.1.2	GO:1901606	B	.	alpha-amino acid catabolic process
KTU_DROWI (sp|B4MPK3) Prot kintoun OS=D. willistoni GN=Ppi20 PE=3 SV=1	KTU_DROWI	1,00E-135	GLOS_KTU.1.2	GO:0006569	B	InterPro	tryptophan catabolic process
LECA_SARPE (sp|P05047) Lectin subunit alpha OS=S. peregrina PE=1 SV=1	LECA_SARPE	1,00E-31	GLOS_LECA.7.13	GO:0030246	M	InterPro	carbohydrate binding
MEX1_DROME (sp|P23487) Protein midgut expression 1 OS=D. m.	MEX1_DROME	1,00E-13	GLOS_MEX1.3.5	GO:0016021	C	InterPro	integral to membrane
MEX1_DROME (sp|P23487) Protein midgut expression 1 OS=D. m.	MEX1_DROME	1,00E-13	GLOS_MEX1.3.5	GO:0022900	B	InterPro	electron transport chain
MEX1_DROME (sp|P23487) Protein midgut expression 1 OS=D. m.	MEX1_DROME	1,00E-13	GLOS_MEX1.3.5	GO:0007275	B	UniProtKB-K	multicellular organismal development
[BBH] MYSP1_DROME (sp|P35415) Paramyosin, long form D. m.	MYSP1_DROME	0	GLOS_MYSP1.1.1	GO:0030016	C	UniProtKB-S	myofibril
[BBH] MYSP1_DROME (sp|P35415) Paramyosin, long form D. m.	MYSP1_DROME	0	GLOS_MYSP1.1.1	GO:0016459	C	.	myosin complex
[BBH] MYSP1_DROME (sp|P35415) Paramyosin, long form D. m.	MYSP1_DROME	0	GLOS_MYSP1.1.1	GO:0015629	C	.	actin cytoskeleton
[BBH] MYSP1_DROME (sp|P35415) Paramyosin, long form D. m.	MYSP1_DROME	0	GLOS_MYSP1.1.1	GO:0032982	C	UniProtKB-K	myosin filament
[BBH] MYSP1_DROME (sp|P35415) Paramyosin, long form D. m.	MYSP1_DROME	0	GLOS_MYSP1.1.1	GO:0003774	M	FlyBase	motor activity
[BBH] MYSP1_DROME (sp|P35415) Paramyosin, long form D. m.	MYSP1_DROME	0	GLOS_MYSP1.1.1	GO:0007498	B	FlyBase	mesoderm development
[BBH] MYSP1_DROME (sp|P35415) Paramyosin, long form D. m.	MYSP1_DROME	0	GLOS_MYSP1.1.1	GO:0010927	B	.	cellular component assembly involved in morphogenesis
[BBH] MYSP1_DROME (sp|P35415) Paramyosin, long form D. m.	MYSP1_DROME	0	GLOS_MYSP1.1.1	GO:0070925	B	.	organelle assembly
[BBH] MYSP1_DROME (sp|P35415) Paramyosin, long form D. m.	MYSP1_DROME	0	GLOS_MYSP1.1.1	GO:0055002	B	.	striated muscle cell development
[BBH] MYSP1_DROME (sp|P35415) Paramyosin, long form D. m.	MYSP1_DROME	0	GLOS_MYSP1.1.1	GO:0055001	B	.	muscle cell development
[BBH] MYSP1_DROME (sp|P35415) Paramyosin, long form D. m.	MYSP1_DROME	0	GLOS_MYSP1.1.1	GO:0042692	B	.	muscle cell differentiation
[BBH] MYSP1_DROME (sp|P35415) Paramyosin, long form D. m.	MYSP1_DROME	0	GLOS_MYSP1.1.1	GO:0051146	B	.	striated muscle cell differentiation
[BBH] MYSP1_DROME (sp|P35415) Paramyosin, long form D. m.	MYSP1_DROME	0	GLOS_MYSP1.1.1	GO:0030239	B	FlyBase	myofibril assembly
[BBH] N2B_HAEIR (sp|P46441) Putative ATPase N2B OS=H. irritans PE=2 SV=1	N2B_HAEIR	0	GLOS_N2B.1.1	GO:0032559	M	.	adenyl ribonucleotide binding
[BBH] N2B_HAEIR (sp|P46441) Putative ATPase N2B OS=H. irritans PE=2 SV=1	N2B_HAEIR	0	GLOS_N2B.1.1	GO:0030554	M	.	adenyl nucleotide binding
[BBH] N2B_HAEIR (sp|P46441) Putative ATPase N2B OS=H. irritans PE=2 SV=1	N2B_HAEIR	0	GLOS_N2B.1.1	GO:0005524	M	UniProtKB-K	ATP binding
POL1_BPMV (sp|Q9YJU5) RNA1 polyprotein OS=Bean-pod mottle virus	POL1_BPMV	2,00E-06	GLOS_POL1.1.1	GO:0033643	C	.	host cell part
POL1_BPMV (sp|Q9YJU5) RNA1 polyprotein OS=Bean-pod mottle virus	POL1_BPMV	2,00E-06	GLOS_POL1.1.1	GO:0044217	C	.	other organism part
POL1_BPMV (sp|Q9YJU5) RNA1 polyprotein OS=Bean-pod mottle virus	POL1_BPMV	2,00E-06	GLOS_POL1.1.1	GO:0044215	C	.	other organism
POL1_BPMV (sp|Q9YJU5) RNA1 polyprotein OS=Bean-pod mottle virus	POL1_BPMV	2,00E-06	GLOS_POL1.1.1	GO:0043245	C	.	extraorganismal space
POL1_BPMV (sp|Q9YJU5) RNA1 polyprotein OS=Bean-pod mottle virus	POL1_BPMV	2,00E-06	GLOS_POL1.1.1	GO:0043657	C	.	host cell
POL1_BPMV (sp|Q9YJU5) RNA1 polyprotein OS=Bean-pod mottle virus	POL1_BPMV	2,00E-06	GLOS_POL1.1.1	GO:0044216	C	.	other organism cell
POL1_BPMV (sp|Q9YJU5) RNA1 polyprotein OS=Bean-pod mottle virus	POL1_BPMV	2,00E-06	GLOS_POL1.1.1	GO:0018995	C	.	host
POL1_BPMV (sp|Q9YJU5) RNA1 polyprotein OS=Bean-pod mottle virus	POL1_BPMV	2,00E-06	GLOS_POL1.1.1	GO:0044218	C	.	other organism cell membrane
POL1_BPMV (sp|Q9YJU5) RNA1 polyprotein OS=Bean-pod mottle virus	POL1_BPMV	2,00E-06	GLOS_POL1.1.1	GO:0044279	C	.	other organism membrane
POL1_BPMV (sp|Q9YJU5) RNA1 polyprotein OS=Bean-pod mottle virus	POL1_BPMV	2,00E-06	GLOS_POL1.1.1	GO:0033644	C	UniProtKB-S	host cell membrane
POL1_BPMV (sp|Q9YJU5) RNA1 polyprotein OS=Bean-pod mottle virus	POL1_BPMV	2,00E-06	GLOS_POL1.1.1	GO:0016021	C	UniProtKB-K	integral to membrane
POL1_BPMV (sp|Q9YJU5) RNA1 polyprotein OS=Bean-pod mottle virus	POL1_BPMV	2,00E-06	GLOS_POL1.1.1	GO:0005524	M	UniProtKB-K	ATP binding
POL1_BPMV (sp|Q9YJU5) RNA1 polyprotein OS=Bean-pod mottle virus	POL1_BPMV	2,00E-06	GLOS_POL1.1.1	GO:0008234	M	.	cysteine-type peptidase activity
POL1_BPMV (sp|Q9YJU5) RNA1 polyprotein OS=Bean-pod mottle virus	POL1_BPMV	2,00E-06	GLOS_POL1.1.1	GO:0004197	M	InterPro	cysteine-type endopeptidase activity
POL1_BPMV (sp|Q9YJU5) RNA1 polyprotein OS=Bean-pod mottle virus	POL1_BPMV	2,00E-06	GLOS_POL1.1.1	GO:0003723	M	InterPro	RNA binding
POL1_BPMV (sp|Q9YJU5) RNA1 polyprotein OS=Bean-pod mottle virus	POL1_BPMV	2,00E-06	GLOS_POL1.1.1	GO:0004386	M	.	helicase activity
POL1_BPMV (sp|Q9YJU5) RNA1 polyprotein OS=Bean-pod mottle virus	POL1_BPMV	2,00E-06	GLOS_POL1.1.1	GO:0003724	M	InterPro	RNA helicase activity
POL1_BPMV (sp|Q9YJU5) RNA1 polyprotein OS=Bean-pod mottle virus	POL1_BPMV	2,00E-06	GLOS_POL1.1.1	GO:0034062	M	.	RNA polymerase activity
POL1_BPMV (sp|Q9YJU5) RNA1 polyprotein OS=Bean-pod mottle virus	POL1_BPMV	2,00E-06	GLOS_POL1.1.1	GO:0016779	M	.	nucleotidyltransferase activity
POL1_BPMV (sp|Q9YJU5) RNA1 polyprotein OS=Bean-pod mottle virus	POL1_BPMV	2,00E-06	GLOS_POL1.1.1	GO:0016772	M	.	transferase activity, transferring phosphorus-containing groups
POL1_BPMV (sp|Q9YJU5) RNA1 polyprotein OS=Bean-pod mottle virus	POL1_BPMV	2,00E-06	GLOS_POL1.1.1	GO:0003968	M	UniProtKB-K	RNA-directed RNA polymerase activity
POL1_BPMV (sp|Q9YJU5) RNA1 polyprotein OS=Bean-pod mottle virus	POL1_BPMV	2,00E-06	GLOS_POL1.1.1	GO:0006508	B	UniProtKB-K	proteolysis
POL1_BPMV (sp|Q9YJU5) RNA1 polyprotein OS=Bean-pod mottle virus	POL1_BPMV	2,00E-06	GLOS_POL1.1.1	GO:0018143	B	.	nucleic acid-protein covalent cross-linking
POL1_BPMV (sp|Q9YJU5) RNA1 polyprotein OS=Bean-pod mottle virus	POL1_BPMV	2,00E-06	GLOS_POL1.1.1	GO:0018144	B	UniProtKB-K	RNA-protein covalent cross-linking

POL1_BPMV (sp|Q9YJU5) RNA1 polyprotein OS=Bean-pod mottle virus	POL1_BPMV	2,00E-06	GLOS_POL1.1.1	GO:0006351	B	InterPro	transcription, DNA-dependent
POL1_BPMV (sp|Q9YJU5) RNA1 polyprotein OS=Bean-pod mottle virus	POL1_BPMV	2,00E-06	GLOS_POL1.1.1	GO:0016032	B	.	viral process
POL1_BPMV (sp|Q9YJU5) RNA1 polyprotein OS=Bean-pod mottle virus	POL1_BPMV	2,00E-06	GLOS_POL1.1.1	GO:0044403	B	.	symbiosis, encompassing mutualism through parasitism
POL1_BPMV (sp|Q9YJU5) RNA1 polyprotein OS=Bean-pod mottle virus	POL1_BPMV	2,00E-06	GLOS_POL1.1.1	GO:0044419	B	.	interspecies interaction between organisms
POL1_BPMV (sp|Q9YJU5) RNA1 polyprotein OS=Bean-pod mottle virus	POL1_BPMV	2,00E-06	GLOS_POL1.1.1	GO:0051704	B	.	multi-organism process
POL1_BPMV (sp|Q9YJU5) RNA1 polyprotein OS=Bean-pod mottle virus	POL1_BPMV	2,00E-06	GLOS_POL1.1.1	GO:0044764	B	.	multi-organism cellular process
POL1_BPMV (sp|Q9YJU5) RNA1 polyprotein OS=Bean-pod mottle virus	POL1_BPMV	2,00E-06	GLOS_POL1.1.1	GO:0019058	B	.	viral life cycle
POL1_BPMV (sp|Q9YJU5) RNA1 polyprotein OS=Bean-pod mottle virus	POL1_BPMV	2,00E-06	GLOS_POL1.1.1	GO:0019079	B	InterPro	viral genome replication
PURA_DROWI (sp|B4NFS5) Adenylosuccinate synthetase OS=D. willistoni	PURA_DROWI	1,00E-154	GLOS_PURA.1.3	GO:0005737	C	UniProtKB-S	cytoplasm
PURA_DROWI (sp|B4NFS5) Adenylosuccinate synthetase OS=D. willistoni	PURA_DROWI	1,00E-154	GLOS_PURA.1.3	GO:0016879	M	.	ligase activity, forming carbon-nitrogen bonds
PURA_DROWI (sp|B4NFS5) Adenylosuccinate synthetase OS=D. willistoni	PURA_DROWI	1,00E-154	GLOS_PURA.1.3	GO:0016874	M	.	ligase activity
PURA_DROWI (sp|B4NFS5) Adenylosuccinate synthetase OS=D. willistoni	PURA_DROWI	1,00E-154	GLOS_PURA.1.3	GO:0004019	M	UniProtKB-H	adenylosuccinate synthase activity
PURA_DROWI (sp|B4NFS5) Adenylosuccinate synthetase OS=D. willistoni	PURA_DROWI	1,00E-154	GLOS_PURA.1.3	GO:0005525	M	UniProtKB-H	GTP binding
PURA_DROWI (sp|B4NFS5) Adenylosuccinate synthetase OS=D. willistoni	PURA_DROWI	1,00E-154	GLOS_PURA.1.3	GO:0000287	M	UniProtKB-H	magnesium ion binding
PURA_DROWI (sp|B4NFS5) Adenylosuccinate synthetase OS=D. willistoni	PURA_DROWI	1,00E-154	GLOS_PURA.1.3	GO:0006167	B	.	AMP biosynthetic process
PURA_DROWI (sp|B4NFS5) Adenylosuccinate synthetase OS=D. willistoni	PURA_DROWI	1,00E-154	GLOS_PURA.1.3	GO:0009152	B	.	purine ribonucleotide biosynthetic process
PURA_DROWI (sp|B4NFS5) Adenylosuccinate synthetase OS=D. willistoni	PURA_DROWI	1,00E-154	GLOS_PURA.1.3	GO:0006164	B	.	purine nucleotide biosynthetic process
PURA_DROWI (sp|B4NFS5) Adenylosuccinate synthetase OS=D. willistoni	PURA_DROWI	1,00E-154	GLOS_PURA.1.3	GO:0006163	B	.	purine nucleotide metabolic process
PURA_DROWI (sp|B4NFS5) Adenylosuccinate synthetase OS=D. willistoni	PURA_DROWI	1,00E-154	GLOS_PURA.1.3	GO:0009117	B	.	nucleotide metabolic process
PURA_DROWI (sp|B4NFS5) Adenylosuccinate synthetase OS=D. willistoni	PURA_DROWI	1,00E-154	GLOS_PURA.1.3	GO:0006753	B	.	nucleoside phosphate metabolic process
PURA_DROWI (sp|B4NFS5) Adenylosuccinate synthetase OS=D. willistoni	PURA_DROWI	1,00E-154	GLOS_PURA.1.3	GO:0006796	B	.	phosphate-containing compound metabolic process
PURA_DROWI (sp|B4NFS5) Adenylosuccinate synthetase OS=D. willistoni	PURA_DROWI	1,00E-154	GLOS_PURA.1.3	GO:0006793	B	.	phosphorus metabolic process
PURA_DROWI (sp|B4NFS5) Adenylosuccinate synthetase OS=D. willistoni	PURA_DROWI	1,00E-154	GLOS_PURA.1.3	GO:0019637	B	.	organophosphate metabolic process
PURA_DROWI (sp|B4NFS5) Adenylosuccinate synthetase OS=D. willistoni	PURA_DROWI	1,00E-154	GLOS_PURA.1.3	GO:0055086	B	.	nucleobase-containing small molecule metabolic process
PURA_DROWI (sp|B4NFS5) Adenylosuccinate synthetase OS=D. willistoni	PURA_DROWI	1,00E-154	GLOS_PURA.1.3	GO:0072521	B	.	purine-containing compound metabolic process
PURA_DROWI (sp|B4NFS5) Adenylosuccinate synthetase OS=D. willistoni	PURA_DROWI	1,00E-154	GLOS_PURA.1.3	GO:0009165	B	.	nucleotide biosynthetic process
PURA_DROWI (sp|B4NFS5) Adenylosuccinate synthetase OS=D. willistoni	PURA_DROWI	1,00E-154	GLOS_PURA.1.3	GO:1901293	B	.	nucleoside phosphate biosynthetic process
PURA_DROWI (sp|B4NFS5) Adenylosuccinate synthetase OS=D. willistoni	PURA_DROWI	1,00E-154	GLOS_PURA.1.3	GO:0090407	B	.	organophosphate biosynthetic process
PURA_DROWI (sp|B4NFS5) Adenylosuccinate synthetase OS=D. willistoni	PURA_DROWI	1,00E-154	GLOS_PURA.1.3	GO:0072522	B	.	purine-containing compound biosynthetic process
PURA_DROWI (sp|B4NFS5) Adenylosuccinate synthetase OS=D. willistoni	PURA_DROWI	1,00E-154	GLOS_PURA.1.3	GO:0009150	B	.	purine ribonucleotide metabolic process
PURA_DROWI (sp|B4NFS5) Adenylosuccinate synthetase OS=D. willistoni	PURA_DROWI	1,00E-154	GLOS_PURA.1.3	GO:0009259	B	.	ribonucleotide metabolic process
PURA_DROWI (sp|B4NFS5) Adenylosuccinate synthetase OS=D. willistoni	PURA_DROWI	1,00E-154	GLOS_PURA.1.3	GO:0019693	B	.	ribose phosphate metabolic process
PURA_DROWI (sp|B4NFS5) Adenylosuccinate synthetase OS=D. willistoni	PURA_DROWI	1,00E-154	GLOS_PURA.1.3	GO:1901135	B	.	carbohydrate derivative metabolic process
PURA_DROWI (sp|B4NFS5) Adenylosuccinate synthetase OS=D. willistoni	PURA_DROWI	1,00E-154	GLOS_PURA.1.3	GO:0009260	B	.	ribonucleotide biosynthetic process
PURA_DROWI (sp|B4NFS5) Adenylosuccinate synthetase OS=D. willistoni	PURA_DROWI	1,00E-154	GLOS_PURA.1.3	GO:0046390	B	.	ribose phosphate biosynthetic process
PURA_DROWI (sp|B4NFS5) Adenylosuccinate synthetase OS=D. willistoni	PURA_DROWI	1,00E-154	GLOS_PURA.1.3	GO:1901137	B	.	carbohydrate derivative biosynthetic process
PURA_DROWI (sp|B4NFS5) Adenylosuccinate synthetase OS=D. willistoni	PURA_DROWI	1,00E-154	GLOS_PURA.1.3	GO:0009168	B	.	purine ribonucleoside monophosphate biosynthetic process
PURA_DROWI (sp|B4NFS5) Adenylosuccinate synthetase OS=D. willistoni	PURA_DROWI	1,00E-154	GLOS_PURA.1.3	GO:0009127	B	.	purine nucleoside monophosphate biosynthetic process
PURA_DROWI (sp|B4NFS5) Adenylosuccinate synthetase OS=D. willistoni	PURA_DROWI	1,00E-154	GLOS_PURA.1.3	GO:0009124	B	.	nucleoside monophosphate biosynthetic process
PURA_DROWI (sp|B4NFS5) Adenylosuccinate synthetase OS=D. willistoni	PURA_DROWI	1,00E-154	GLOS_PURA.1.3	GO:0009123	B	.	nucleoside monophosphate metabolic process
PURA_DROWI (sp|B4NFS5) Adenylosuccinate synthetase OS=D. willistoni	PURA_DROWI	1,00E-154	GLOS_PURA.1.3	GO:0009126	B	.	purine nucleoside monophosphate metabolic process
PURA_DROWI (sp|B4NFS5) Adenylosuccinate synthetase OS=D. willistoni	PURA_DROWI	1,00E-154	GLOS_PURA.1.3	GO:0009156	B	.	ribonucleoside monophosphate biosynthetic process
PURA_DROWI (sp|B4NFS5) Adenylosuccinate synthetase OS=D. willistoni	PURA_DROWI	1,00E-154	GLOS_PURA.1.3	GO:0009161	B	.	ribonucleoside monophosphate metabolic process
PURA_DROWI (sp|B4NFS5) Adenylosuccinate synthetase OS=D. willistoni	PURA_DROWI	1,00E-154	GLOS_PURA.1.3	GO:0009167	B	.	purine ribonucleoside monophosphate metabolic process
PURA_DROWI (sp|B4NFS5) Adenylosuccinate synthetase OS=D. willistoni	PURA_DROWI	1,00E-154	GLOS_PURA.1.3	GO:0046033	B	.	AMP metabolic process
PURA_DROWI (sp|B4NFS5) Adenylosuccinate synthetase OS=D. willistoni	PURA_DROWI	1,00E-154	GLOS_PURA.1.3	GO:0046128	B	.	purine ribonucleoside metabolic process
PURA_DROWI (sp|B4NFS5) Adenylosuccinate synthetase OS=D. willistoni	PURA_DROWI	1,00E-154	GLOS_PURA.1.3	GO:0009119	B	.	ribonucleoside metabolic process
PURA_DROWI (sp|B4NFS5) Adenylosuccinate synthetase OS=D. willistoni	PURA_DROWI	1,00E-154	GLOS_PURA.1.3	GO:0009116	B	.	nucleoside metabolic process
PURA_DROWI (sp|B4NFS5) Adenylosuccinate synthetase OS=D. willistoni	PURA_DROWI	1,00E-154	GLOS_PURA.1.3	GO:1901657	B	.	glycosyl compound metabolic process
PURA_DROWI (sp|B4NFS5) Adenylosuccinate synthetase OS=D. willistoni	PURA_DROWI	1,00E-154	GLOS_PURA.1.3	GO:0042278	B	.	purine nucleoside metabolic process
PURA_DROWI (sp|B4NFS5) Adenylosuccinate synthetase OS=D. willistoni	PURA_DROWI	1,00E-154	GLOS_PURA.1.3	GO:0046129	B	.	purine ribonucleoside biosynthetic process
PURA_DROWI (sp|B4NFS5) Adenylosuccinate synthetase OS=D. willistoni	PURA_DROWI	1,00E-154	GLOS_PURA.1.3	GO:0042451	B	.	purine nucleoside biosynthetic process
PURA_DROWI (sp|B4NFS5) Adenylosuccinate synthetase OS=D. willistoni	PURA_DROWI	1,00E-154	GLOS_PURA.1.3	GO:0009163	B	.	nucleoside biosynthetic process
PURA_DROWI (sp|B4NFS5) Adenylosuccinate synthetase OS=D. willistoni	PURA_DROWI	1,00E-154	GLOS_PURA.1.3	GO:1901659	B	.	glycosyl compound biosynthetic process

PURA_DROWI (sp|B4NFS5) Adenylosuccinate synthetase OS=D. willistoni	PURA_DROWI	1,00E-154	GLOS_PURA.1.3	GO:0042455	B	.	ribonucleoside biosynthetic process
PURA_DROWI (sp|B4NFS5) Adenylosuccinate synthetase OS=D. willistoni	PURA_DROWI	1,00E-154	GLOS_PURA.1.3	GO:0044208	B	UniProtKB-U	'de novo' AMP biosynthetic process
RL10_DROME (sp|O61231) 60S ribosomal protein L10 OS=D. m.	RL10_DROME	3,00E-75	GLOS_RL10.2.9	GO:0044297	C	.	cell body
RL10_DROME (sp|O61231) 60S ribosomal protein L10 OS=D. m.	RL10_DROME	3,00E-75	GLOS_RL10.2.9	GO:0043025	C	FlyBase	neuronal cell body
RL10_DROME (sp|O61231) 60S ribosomal protein L10 OS=D. m.	RL10_DROME	3,00E-75	GLOS_RL10.2.9	GO:0030529	C	.	ribonucleoprotein complex
RL10_DROME (sp|O61231) 60S ribosomal protein L10 OS=D. m.	RL10_DROME	3,00E-75	GLOS_RL10.2.9	GO:0005840	C	FlyBase	ribosome
RL10_DROME (sp|O61231) 60S ribosomal protein L10 OS=D. m.	RL10_DROME	3,00E-75	GLOS_RL10.2.9	GO:0005198	M	.	structural molecule activity
RL10_DROME (sp|O61231) 60S ribosomal protein L10 OS=D. m.	RL10_DROME	3,00E-75	GLOS_RL10.2.9	GO:0003735	M	FlyBase	structural constituent of ribosome
RL10_DROME (sp|O61231) 60S ribosomal protein L10 OS=D. m.	RL10_DROME	3,00E-75	GLOS_RL10.2.9	GO:0051297	B	.	centrosome organization
RL10_DROME (sp|O61231) 60S ribosomal protein L10 OS=D. m.	RL10_DROME	3,00E-75	GLOS_RL10.2.9	GO:0031023	B	.	microtubule organizing center organization
RL10_DROME (sp|O61231) 60S ribosomal protein L10 OS=D. m.	RL10_DROME	3,00E-75	GLOS_RL10.2.9	GO:0000226	B	.	microtubule cytoskeleton organization
RL10_DROME (sp|O61231) 60S ribosomal protein L10 OS=D. m.	RL10_DROME	3,00E-75	GLOS_RL10.2.9	GO:0007017	B	.	microtubule-based process
RL10_DROME (sp|O61231) 60S ribosomal protein L10 OS=D. m.	RL10_DROME	3,00E-75	GLOS_RL10.2.9	GO:0007098	B	.	centrosome cycle
RL10_DROME (sp|O61231) 60S ribosomal protein L10 OS=D. m.	RL10_DROME	3,00E-75	GLOS_RL10.2.9	GO:0051298	B	FlyBase	centrosome duplication
RL10_DROME (sp|O61231) 60S ribosomal protein L10 OS=D. m.	RL10_DROME	3,00E-75	GLOS_RL10.2.9	GO:0051231	B	.	spindle elongation
RL10_DROME (sp|O61231) 60S ribosomal protein L10 OS=D. m.	RL10_DROME	3,00E-75	GLOS_RL10.2.9	GO:0007051	B	.	spindle organization
RL10_DROME (sp|O61231) 60S ribosomal protein L10 OS=D. m.	RL10_DROME	3,00E-75	GLOS_RL10.2.9	GO:0007052	B	.	mitotic spindle organization
RL10_DROME (sp|O61231) 60S ribosomal protein L10 OS=D. m.	RL10_DROME	3,00E-75	GLOS_RL10.2.9	GO:0000022	B	FlyBase	mitotic spindle elongation
RL10_DROME (sp|O61231) 60S ribosomal protein L10 OS=D. m.	RL10_DROME	3,00E-75	GLOS_RL10.2.9	GO:0006412	B	InterPro	translation
RL13_DROME (sp|P41126) 60S ribosomal protein L13 OS=D. m.	RL13_DROME	4,00E-68	GLOS_RL13.3.12	GO:0005840	C	FlyBase	ribosome
RL13_DROME (sp|P41126) 60S ribosomal protein L13 OS=D. m.	RL13_DROME	4,00E-68	GLOS_RL13.3.12	GO:0003735	M	FlyBase	structural constituent of ribosome
RL13_DROME (sp|P41126) 60S ribosomal protein L13 OS=D. m.	RL13_DROME	4,00E-68	GLOS_RL13.3.12	GO:0051298	B	FlyBase	centrosome duplication
RL13_DROME (sp|P41126) 60S ribosomal protein L13 OS=D. m.	RL13_DROME	4,00E-68	GLOS_RL13.3.12	GO:0000022	B	FlyBase	mitotic spindle elongation
RL13_DROME (sp|P41126) 60S ribosomal protein L13 OS=D. m.	RL13_DROME	4,00E-68	GLOS_RL13.3.12	GO:0006412	B	InterPro	translation
RL23_DROME (sp|P48159) 60S ribosomal protein L23 OS=D. m.	RL23_DROME	2,00E-76	GLOS_RL23.7.16	GO:0005840	C	UniProtKB-K	ribosome
RL23_DROME (sp|P48159) 60S ribosomal protein L23 OS=D. m.	RL23_DROME	2,00E-76	GLOS_RL23.7.16	GO:0003735	M	InterPro	structural constituent of ribosome
RL23_DROME (sp|P48159) 60S ribosomal protein L23 OS=D. m.	RL23_DROME	2,00E-76	GLOS_RL23.7.16	GO:0000022	B	FlyBase	mitotic spindle elongation
RL23_DROME (sp|P48159) 60S ribosomal protein L23 OS=D. m.	RL23_DROME	2,00E-76	GLOS_RL23.7.16	GO:0022008	B	FlyBase	neurogenesis
RL23_DROME (sp|P48159) 60S ribosomal protein L23 OS=D. m.	RL23_DROME	2,00E-76	GLOS_RL23.7.16	GO:0006412	B	InterPro	translation
RL28_DROME (sp|Q9VZS5) 60S ribosomal protein L28 OS=D.m.	RL28_DROME	5,00E-40	GLOS_RL28.2.6	GO:0005840	C	UniProtKB-K	ribosome
RL28_DROME (sp|Q9VZS5) 60S ribosomal protein L28 OS=D.m.	RL28_DROME	5,00E-40	GLOS_RL28.2.6	GO:0003735	M	InterPro	structural constituent of ribosome
RL28_DROME (sp|Q9VZS5) 60S ribosomal protein L28 OS=D.m.	RL28_DROME	5,00E-40	GLOS_RL28.2.6	GO:0051298	B	FlyBase	centrosome duplication
RL28_DROME (sp|Q9VZS5) 60S ribosomal protein L28 OS=D.m.	RL28_DROME	5,00E-40	GLOS_RL28.2.6	GO:0000022	B	FlyBase	mitotic spindle elongation
RL28_DROME (sp|Q9VZS5) 60S ribosomal protein L28 OS=D.m.	RL28_DROME	5,00E-40	GLOS_RL28.2.6	GO:0022008	B	FlyBase	neurogenesis
RL28_DROME (sp|Q9VZS5) 60S ribosomal protein L28 OS=D.m.	RL28_DROME	5,00E-40	GLOS_RL28.2.6	GO:0006412	B	InterPro	translation
RL38_DROYA (sp|Q6XIM7) 60S ribosomal prot L38 OS=D. yakuba	RL38_DROYA	4,00E-29	GLOS_RL38.2.3	GO:0005840	C	UniProtKB-K	ribosome
RL38_DROYA (sp|Q6XIM7) 60S ribosomal prot L38 OS=D. yakuba	RL38_DROYA	4,00E-29	GLOS_RL38.2.3	GO:0003735	M	InterPro	structural constituent of ribosome
RL38_DROYA (sp|Q6XIM7) 60S ribosomal prot L38 OS=D. yakuba	RL38_DROYA	4,00E-29	GLOS_RL38.2.3	GO:0006412	B	InterPro	translation
RPA2_DROME (sp|P20028) DNA-directed RNA polymerase I subunit RPA2 D.m.	RPA2_DROME	1,00E-17	GLOS_RPA2.1.1	GO:0044452	C	.	nucleolar part
RPA2_DROME (sp|P20028) DNA-directed RNA polymerase I subunit RPA2 D.m.	RPA2_DROME	1,00E-17	GLOS_RPA2.1.1	GO:0005730	C	.	nucleolus
RPA2_DROME (sp|P20028) DNA-directed RNA polymerase I subunit RPA2 D.m.	RPA2_DROME	1,00E-17	GLOS_RPA2.1.1	GO:0055029	C	.	nuclear DNA-directed RNA polymerase complex
RPA2_DROME (sp|P20028) DNA-directed RNA polymerase I subunit RPA2 D.m.	RPA2_DROME	1,00E-17	GLOS_RPA2.1.1	GO:0000428	C	.	DNA-directed RNA polymerase complex
RPA2_DROME (sp|P20028) DNA-directed RNA polymerase I subunit RPA2 D.m.	RPA2_DROME	1,00E-17	GLOS_RPA2.1.1	GO:0030880	C	.	RNA polymerase complex
RPA2_DROME (sp|P20028) DNA-directed RNA polymerase I subunit RPA2 D.m.	RPA2_DROME	1,00E-17	GLOS_RPA2.1.1	GO:0005736	C	RefGenome	DNA-directed RNA polymerase I complex
RPA2_DROME (sp|P20028) DNA-directed RNA polymerase I subunit RPA2 D.m.	RPA2_DROME	1,00E-17	GLOS_RPA2.1.1	GO:0003677	M	InterPro	DNA binding
RPA2_DROME (sp|P20028) DNA-directed RNA polymerase I subunit RPA2 D.m.	RPA2_DROME	1,00E-17	GLOS_RPA2.1.1	GO:0003899	M	UniProtKB-K	DNA-directed RNA polymerase activity
RPA2_DROME (sp|P20028) DNA-directed RNA polymerase I subunit RPA2 D.m.	RPA2_DROME	1,00E-17	GLOS_RPA2.1.1	GO:0046872	M	UniProtKB-K	metal ion binding
RPA2_DROME (sp|P20028) DNA-directed RNA polymerase I subunit RPA2 D.m.	RPA2_DROME	1,00E-17	GLOS_RPA2.1.1	GO:0032549	M	InterPro	ribonucleoside binding
RPA2_DROME (sp|P20028) DNA-directed RNA polymerase I subunit RPA2 D.m.	RPA2_DROME	1,00E-17	GLOS_RPA2.1.1	GO:0022008	B	FlyBase	neurogenesis
RPA2_DROME (sp|P20028) DNA-directed RNA polymerase I subunit RPA2 D.m.	RPA2_DROME	1,00E-17	GLOS_RPA2.1.1	GO:0006360	B	GOC	transcription from RNA polymerase I promoter
[BBH] RPB2_DROME (sp|P08266)DNA-directed RNA polymeraseII subunit RPB2	RPB2_DROME	0	GLOS_RPB2.1.1	GO:0044451	C	.	nucleoplasm part
[BBH] RPB2_DROME (sp|P08266)DNA-directed RNA polymeraseII subunit RPB2	RPB2_DROME	0	GLOS_RPB2.1.1	GO:0005654	C	.	nucleoplasm
[BBH] RPB2_DROME (sp|P08266)DNA-directed RNA polymeraseII subunit RPB2	RPB2_DROME	0	GLOS_RPB2.1.1	GO:0016591	C	.	DNA-directed RNA polymerase II, holoenzyme
[BBH] RPB2_DROME (sp|P08266)DNA-directed RNA polymeraseII subunit RPB2	RPB2_DROME	0	GLOS_RPB2.1.1	GO:0005665	C	UniProtKB	DNA-directed RNA polymerase II, core complex

[BBH] RPB2_DROME (sp|P08266)DNA-directed RNA polymeraseII subunit RPB2	RPB2_DROME	0	GLOS_RPB2.1.1	GO:0003677	M	InterPro	DNA binding
[BBH] RPB2_DROME (sp|P08266)DNA-directed RNA polymeraseII subunit RPB2	RPB2_DROME	0	GLOS_RPB2.1.1	GO:0003899	M	UniProtKB	DNA-directed RNA polymerase activity
[BBH] RPB2_DROME (sp|P08266)DNA-directed RNA polymeraseII subunit RPB2	RPB2_DROME	0	GLOS_RPB2.1.1	GO:0046872	M	UniProtKB-K	metal ion binding
[BBH] RPB2_DROME (sp|P08266)DNA-directed RNA polymeraseII subunit RPB2	RPB2_DROME	0	GLOS_RPB2.1.1	GO:0032549	M	InterPro	ribonucleoside binding
[BBH] RPB2_DROME (sp|P08266)DNA-directed RNA polymeraseII subunit RPB2	RPB2_DROME	0	GLOS_RPB2.1.1	GO:0006366	B	UniProtKB	transcription from RNA polymerase II promoter
RS23_DROME (sp|Q8T3U2) 40S ribosomal protein S23 OS=D.m.	RS23_DROME	5,00E-87	GLOS_RS23.3.4	GO:0005840	C	FlyBase	ribosome
RS23_DROME (sp|Q8T3U2) 40S ribosomal protein S23 OS=D.m.	RS23_DROME	5,00E-87	GLOS_RS23.3.4	GO:0044391	C	.	ribosomal subunit
RS23_DROME (sp|Q8T3U2) 40S ribosomal protein S23 OS=D.m.	RS23_DROME	5,00E-87	GLOS_RS23.3.4	GO:0005840	C	.	ribosome
RS23_DROME (sp|Q8T3U2) 40S ribosomal protein S23 OS=D.m.	RS23_DROME	5,00E-87	GLOS_RS23.3.4	GO:0015935	C	InterPro	small ribosomal subunit
RS23_DROME (sp|Q8T3U2) 40S ribosomal protein S23 OS=D.m.	RS23_DROME	5,00E-87	GLOS_RS23.3.4	GO:0003735	M	FlyBase	structural constituent of ribosome
RS23_DROME (sp|Q8T3U2) 40S ribosomal protein S23 OS=D.m.	RS23_DROME	5,00E-87	GLOS_RS23.3.4	GO:0051298	B	FlyBase	centrosome duplication
RS23_DROME (sp|Q8T3U2) 40S ribosomal protein S23 OS=D.m.	RS23_DROME	5,00E-87	GLOS_RS23.3.4	GO:0006412	B	InterPro	translation
RS29_DROME (sp|Q9VH69) 40S ribosomal protein S29 OS=D.m.	RS29_DROME	4,00E-37	GLOS_RS29.9.12	GO:0005840	C	UniProtKB-K	ribosome
RS29_DROME (sp|Q9VH69) 40S ribosomal protein S29 OS=D.m.	RS29_DROME	4,00E-37	GLOS_RS29.9.12	GO:0046872	M	UniProtKB-K	metal ion binding
RS29_DROME (sp|Q9VH69) 40S ribosomal protein S29 OS=D.m.	RS29_DROME	4,00E-37	GLOS_RS29.9.12	GO:0003735	M	InterPro	structural constituent of ribosome
RS29_DROME (sp|Q9VH69) 40S ribosomal protein S29 OS=D.m.	RS29_DROME	4,00E-37	GLOS_RS29.9.12	GO:0048666	B	FlyBase	neuron development
RS29_DROME (sp|Q9VH69) 40S ribosomal protein S29 OS=D.m.	RS29_DROME	4,00E-37	GLOS_RS29.9.12	GO:0006412	B	InterPro	translation
[BBH] RT16_DROME (sp|Q9V6Y3) Prob 28S ribosomal prot S16, mitoch D.m.	RT16_DROME	7,00E-67	GLOS_RT16.1.1	GO:0000314	C	.	organellar small ribosomal subunit
[BBH] RT16_DROME (sp|Q9V6Y3) Prob 28S ribosomal prot S16, mitoch D.m.	RT16_DROME	7,00E-67	GLOS_RT16.1.1	GO:0015935	C	.	small ribosomal subunit
[BBH] RT16_DROME (sp|Q9V6Y3) Prob 28S ribosomal prot S16, mitoch D.m.	RT16_DROME	7,00E-67	GLOS_RT16.1.1	GO:0000313	C	.	organellar ribosome
[BBH] RT16_DROME (sp|Q9V6Y3) Prob 28S ribosomal prot S16, mitoch D.m.	RT16_DROME	7,00E-67	GLOS_RT16.1.1	GO:0044429	C	.	mitochondrial part
[BBH] RT16_DROME (sp|Q9V6Y3) Prob 28S ribosomal prot S16, mitoch D.m.	RT16_DROME	7,00E-67	GLOS_RT16.1.1	GO:0005739	C	.	mitochondrion
[BBH] RT16_DROME (sp|Q9V6Y3) Prob 28S ribosomal prot S16, mitoch D.m.	RT16_DROME	7,00E-67	GLOS_RT16.1.1	GO:0005761	C	.	mitochondrial ribosome
[BBH] RT16_DROME (sp|Q9V6Y3) Prob 28S ribosomal prot S16, mitoch D.m.	RT16_DROME	7,00E-67	GLOS_RT16.1.1	GO:0005759	C	.	mitochondrial matrix
[BBH] RT16_DROME (sp|Q9V6Y3) Prob 28S ribosomal prot S16, mitoch D.m.	RT16_DROME	7,00E-67	GLOS_RT16.1.1	GO:0005763	C	FlyBase	mitochondrial small ribosomal subunit
[BBH] RT16_DROME (sp|Q9V6Y3) Prob 28S ribosomal prot S16, mitoch D.m.	RT16_DROME	7,00E-67	GLOS_RT16.1.1	GO:0003735	M	FlyBase	structural constituent of ribosome
[BBH] RT16_DROME (sp|Q9V6Y3) Prob 28S ribosomal prot S16, mitoch D.m.	RT16_DROME	7,00E-67	GLOS_RT16.1.1	GO:0006412	B	FlyBase	translation
SETMR_HUMAN (sp|Q53H47) Histone-lysine N-methyltransferase SETMAR	SETMR_HUMAN	1,00E-12	GLOS_SETMR.1.1	GO:0005694	C	UniProtKB-S	chromosome
SETMR_HUMAN (sp|Q53H47) Histone-lysine N-methyltransferase SETMAR	SETMR_HUMAN	1,00E-12	GLOS_SETMR.1.1	GO:0005634	C	UniProtKB	nucleus
SETMR_HUMAN (sp|Q53H47) Histone-lysine N-methyltransferase SETMAR	SETMR_HUMAN	1,00E-12	GLOS_SETMR.1.1	GO:0004518	M	.	nuclease activity
SETMR_HUMAN (sp|Q53H47) Histone-lysine N-methyltransferase SETMAR	SETMR_HUMAN	1,00E-12	GLOS_SETMR.1.1	GO:0016788	M	.	hydrolase activity, acting on ester bonds
SETMR_HUMAN (sp|Q53H47) Histone-lysine N-methyltransferase SETMAR	SETMR_HUMAN	1,00E-12	GLOS_SETMR.1.1	GO:0004519	M	UniProtKB	endonuclease activity
SETMR_HUMAN (sp|Q53H47) Histone-lysine N-methyltransferase SETMAR	SETMR_HUMAN	1,00E-12	GLOS_SETMR.1.1	GO:0016279	M	.	protein-lysine N-methyltransferase activity
SETMR_HUMAN (sp|Q53H47) Histone-lysine N-methyltransferase SETMAR	SETMR_HUMAN	1,00E-12	GLOS_SETMR.1.1	GO:0008276	M	.	protein methyltransferase activity
SETMR_HUMAN (sp|Q53H47) Histone-lysine N-methyltransferase SETMAR	SETMR_HUMAN	1,00E-12	GLOS_SETMR.1.1	GO:0008168	M	.	methyltransferase activity
SETMR_HUMAN (sp|Q53H47) Histone-lysine N-methyltransferase SETMAR	SETMR_HUMAN	1,00E-12	GLOS_SETMR.1.1	GO:0016741	M	.	transferase activity, transferring one-carbon groups
SETMR_HUMAN (sp|Q53H47) Histone-lysine N-methyltransferase SETMAR	SETMR_HUMAN	1,00E-12	GLOS_SETMR.1.1	GO:0016278	M	.	lysine N-methyltransferase activity
SETMR_HUMAN (sp|Q53H47) Histone-lysine N-methyltransferase SETMAR	SETMR_HUMAN	1,00E-12	GLOS_SETMR.1.1	GO:0008170	M	.	N-methyltransferase activity
SETMR_HUMAN (sp|Q53H47) Histone-lysine N-methyltransferase SETMAR	SETMR_HUMAN	1,00E-12	GLOS_SETMR.1.1	GO:0008757	M	.	S-adenosylmethionine-dependent methyltransferase activity
SETMR_HUMAN (sp|Q53H47) Histone-lysine N-methyltransferase SETMAR	SETMR_HUMAN	1,00E-12	GLOS_SETMR.1.1	GO:0042054	M	.	histone methyltransferase activity
SETMR_HUMAN (sp|Q53H47) Histone-lysine N-methyltransferase SETMAR	SETMR_HUMAN	1,00E-12	GLOS_SETMR.1.1	GO:0018024	M	UniProtKB-E	histone-lysine N-methyltransferase activity
SETMR_HUMAN (sp|Q53H47) Histone-lysine N-methyltransferase SETMAR	SETMR_HUMAN	1,00E-12	GLOS_SETMR.1.1	GO:0043566	M	UniProtKB	structure-specific DNA binding
SETMR_HUMAN (sp|Q53H47) Histone-lysine N-methyltransferase SETMAR	SETMR_HUMAN	1,00E-12	GLOS_SETMR.1.1	GO:0004803	M	UniProtKB	transposase activity
SETMR_HUMAN (sp|Q53H47) Histone-lysine N-methyltransferase SETMAR	SETMR_HUMAN	1,00E-12	GLOS_SETMR.1.1	GO:0008270	M	InterPro	zinc ion binding
SETMR_HUMAN (sp|Q53H47) Histone-lysine N-methyltransferase SETMAR	SETMR_HUMAN	1,00E-12	GLOS_SETMR.1.1	GO:0006308	B	.	DNA catabolic process
SETMR_HUMAN (sp|Q53H47) Histone-lysine N-methyltransferase SETMAR	SETMR_HUMAN	1,00E-12	GLOS_SETMR.1.1	GO:0006259	B	.	DNA metabolic process
SETMR_HUMAN (sp|Q53H47) Histone-lysine N-methyltransferase SETMAR	SETMR_HUMAN	1,00E-12	GLOS_SETMR.1.1	GO:0034655	B	.	nucleobase-containing compound catabolic process
SETMR_HUMAN (sp|Q53H47) Histone-lysine N-methyltransferase SETMAR	SETMR_HUMAN	1,00E-12	GLOS_SETMR.1.1	GO:0090305	B	.	nucleic acid phosphodiester bond hydrolysis
SETMR_HUMAN (sp|Q53H47) Histone-lysine N-methyltransferase SETMAR	SETMR_HUMAN	1,00E-12	GLOS_SETMR.1.1	GO:0000737	B	UniProtKB	DNA catabolic process, endonucleolytic
SETMR_HUMAN (sp|Q53H47) Histone-lysine N-methyltransferase SETMAR	SETMR_HUMAN	1,00E-12	GLOS_SETMR.1.1	GO:0000738	B	.	DNA catabolic process, exonucleolytic
SETMR_HUMAN (sp|Q53H47) Histone-lysine N-methyltransferase SETMAR	SETMR_HUMAN	1,00E-12	GLOS_SETMR.1.1	GO:0006302	B	.	double-strand break repair
SETMR_HUMAN (sp|Q53H47) Histone-lysine N-methyltransferase SETMAR	SETMR_HUMAN	1,00E-12	GLOS_SETMR.1.1	GO:0006281	B	.	DNA repair
SETMR_HUMAN (sp|Q53H47) Histone-lysine N-methyltransferase SETMAR	SETMR_HUMAN	1,00E-12	GLOS_SETMR.1.1	GO:0000729	B	UniProtKB	DNA double-strand break processing
SETMR_HUMAN (sp|Q53H47) Histone-lysine N-methyltransferase SETMAR	SETMR_HUMAN	1,00E-12	GLOS_SETMR.1.1	GO:0015074	B	InterPro	DNA integration

SETMR_HUMAN (sp|Q53H47) Histone-lysine N-methyltransferase SETMAR	SETMR_HUMAN	1,00E-12	GLOS_SETMR.1.1	GO:0071156	B	.	regulation of cell cycle arrest
SETMR_HUMAN (sp|Q53H47) Histone-lysine N-methyltransferase SETMAR	SETMR_HUMAN	1,00E-12	GLOS_SETMR.1.1	GO:0071157	B	UniProtKB	negative regulation of cell cycle arrest
SETMR_HUMAN (sp|Q53H47) Histone-lysine N-methyltransferase SETMAR	SETMR_HUMAN	1,00E-12	GLOS_SETMR.1.1	GO:0010639	B	.	negative regulation of organelle organization
SETMR_HUMAN (sp|Q53H47) Histone-lysine N-methyltransferase SETMAR	SETMR_HUMAN	1,00E-12	GLOS_SETMR.1.1	GO:0051129	B	.	negative regulation of cellular component organization
SETMR_HUMAN (sp|Q53H47) Histone-lysine N-methyltransferase SETMAR	SETMR_HUMAN	1,00E-12	GLOS_SETMR.1.1	GO:0033044	B	.	regulation of chromosome organization
SETMR_HUMAN (sp|Q53H47) Histone-lysine N-methyltransferase SETMAR	SETMR_HUMAN	1,00E-12	GLOS_SETMR.1.1	GO:2001251	B	UniProtKB	negative regulation of chromosome organization
SETMR_HUMAN (sp|Q53H47) Histone-lysine N-methyltransferase SETMAR	SETMR_HUMAN	1,00E-12	GLOS_SETMR.1.1	GO:2000781	B	.	positive regulation of double-strand break repair
SETMR_HUMAN (sp|Q53H47) Histone-lysine N-methyltransferase SETMAR	SETMR_HUMAN	1,00E-12	GLOS_SETMR.1.1	GO:0045739	B	.	positive regulation of DNA repair
SETMR_HUMAN (sp|Q53H47) Histone-lysine N-methyltransferase SETMAR	SETMR_HUMAN	1,00E-12	GLOS_SETMR.1.1	GO:0006282	B	.	regulation of DNA repair
SETMR_HUMAN (sp|Q53H47) Histone-lysine N-methyltransferase SETMAR	SETMR_HUMAN	1,00E-12	GLOS_SETMR.1.1	GO:0051052	B	.	regulation of DNA metabolic process
SETMR_HUMAN (sp|Q53H47) Histone-lysine N-methyltransferase SETMAR	SETMR_HUMAN	1,00E-12	GLOS_SETMR.1.1	GO:2001020	B	.	regulation of response to DNA damage stimulus
SETMR_HUMAN (sp|Q53H47) Histone-lysine N-methyltransferase SETMAR	SETMR_HUMAN	1,00E-12	GLOS_SETMR.1.1	GO:0051054	B	.	positive regulation of DNA metabolic process
SETMR_HUMAN (sp|Q53H47) Histone-lysine N-methyltransferase SETMAR	SETMR_HUMAN	1,00E-12	GLOS_SETMR.1.1	GO:2001022	B	.	positive regulation of response to DNA damage stimulus
SETMR_HUMAN (sp|Q53H47) Histone-lysine N-methyltransferase SETMAR	SETMR_HUMAN	1,00E-12	GLOS_SETMR.1.1	GO:0048584	B	.	positive regulation of response to stimulus
SETMR_HUMAN (sp|Q53H47) Histone-lysine N-methyltransferase SETMAR	SETMR_HUMAN	1,00E-12	GLOS_SETMR.1.1	GO:2000779	B	.	regulation of double-strand break repair
SETMR_HUMAN (sp|Q53H47) Histone-lysine N-methyltransferase SETMAR	SETMR_HUMAN	1,00E-12	GLOS_SETMR.1.1	GO:2001032	B	.	regulation of double-strand break repair via nonhomologous end joining
SETMR_HUMAN (sp|Q53H47) Histone-lysine N-methyltransferase SETMAR	SETMR_HUMAN	1,00E-12	GLOS_SETMR.1.1	GO:2001034	B	UniProtKB	positive regulation of double-strand break repair via nonhomologous end joining
SETMR_HUMAN (sp|Q53H47) Histone-lysine N-methyltransferase SETMAR	SETMR_HUMAN	1,00E-12	GLOS_SETMR.1.1	GO:0006310	B	.	DNA recombination
SETMR_HUMAN (sp|Q53H47) Histone-lysine N-methyltransferase SETMAR	SETMR_HUMAN	1,00E-12	GLOS_SETMR.1.1	GO:0032196	B	.	transposition
SETMR_HUMAN (sp|Q53H47) Histone-lysine N-methyltransferase SETMAR	SETMR_HUMAN	1,00E-12	GLOS_SETMR.1.1	GO:0006313	B	UniProtKB	transposition, DNA-mediated
TERA_DROME (sp|Q7KN62)Transitional endoplasmic reticulum ATPase TER94	TERA_DROME	0	GLOS_TERA.1.2	GO:0045169	C	FlyBase	fusome
TERA_DROME (sp|Q7KN62)Transitional endoplasmic reticulum ATPase TER94	TERA_DROME	0	GLOS_TERA.1.2	GO:0005811	C	FlyBase	lipid particle
TERA_DROME (sp|Q7KN62)Transitional endoplasmic reticulum ATPase TER94	TERA_DROME	0	GLOS_TERA.1.2	GO:0005875	C	FlyBase	microtubule associated complex
TERA_DROME (sp|Q7KN62)Transitional endoplasmic reticulum ATPase TER94	TERA_DROME	0	GLOS_TERA.1.2	GO:0005634	C	UniProtKB-S	nucleus
TERA_DROME (sp|Q7KN62)Transitional endoplasmic reticulum ATPase TER94	TERA_DROME	0	GLOS_TERA.1.2	GO:0000502	C	FlyBase	proteasome complex
TERA_DROME (sp|Q7KN62)Transitional endoplasmic reticulum ATPase TER94	TERA_DROME	0	GLOS_TERA.1.2	GO:0005524	M	UniProtKB-K	ATP binding
TERA_DROME (sp|Q7KN62)Transitional endoplasmic reticulum ATPase TER94	TERA_DROME	0	GLOS_TERA.1.2	GO:0017111	M	InterPro	nucleoside-triphosphatase activity
TERA_DROME (sp|Q7KN62)Transitional endoplasmic reticulum ATPase TER94	TERA_DROME	0	GLOS_TERA.1.2	GO:0009154	B	.	purine ribonucleotide catabolic process
TERA_DROME (sp|Q7KN62)Transitional endoplasmic reticulum ATPase TER94	TERA_DROME	0	GLOS_TERA.1.2	GO:0006195	B	.	purine nucleotide catabolic process
TERA_DROME (sp|Q7KN62)Transitional endoplasmic reticulum ATPase TER94	TERA_DROME	0	GLOS_TERA.1.2	GO:0009166	B	.	nucleotide catabolic process
TERA_DROME (sp|Q7KN62)Transitional endoplasmic reticulum ATPase TER94	TERA_DROME	0	GLOS_TERA.1.2	GO:1901292	B	.	nucleoside phosphate catabolic process
TERA_DROME (sp|Q7KN62)Transitional endoplasmic reticulum ATPase TER94	TERA_DROME	0	GLOS_TERA.1.2	GO:0046434	B	.	organophosphate catabolic process
TERA_DROME (sp|Q7KN62)Transitional endoplasmic reticulum ATPase TER94	TERA_DROME	0	GLOS_TERA.1.2	GO:0072523	B	.	purine-containing compound catabolic process
TERA_DROME (sp|Q7KN62)Transitional endoplasmic reticulum ATPase TER94	TERA_DROME	0	GLOS_TERA.1.2	GO:0009261	B	.	ribonucleotide catabolic process
TERA_DROME (sp|Q7KN62)Transitional endoplasmic reticulum ATPase TER94	TERA_DROME	0	GLOS_TERA.1.2	GO:1901136	B	.	carbohydrate derivative catabolic process
TERA_DROME (sp|Q7KN62)Transitional endoplasmic reticulum ATPase TER94	TERA_DROME	0	GLOS_TERA.1.2	GO:0009169	B	.	purine ribonucleoside monophosphate catabolic process
TERA_DROME (sp|Q7KN62)Transitional endoplasmic reticulum ATPase TER94	TERA_DROME	0	GLOS_TERA.1.2	GO:0009128	B	.	purine nucleoside monophosphate catabolic process
TERA_DROME (sp|Q7KN62)Transitional endoplasmic reticulum ATPase TER94	TERA_DROME	0	GLOS_TERA.1.2	GO:0009125	B	.	nucleoside monophosphate catabolic process
TERA_DROME (sp|Q7KN62)Transitional endoplasmic reticulum ATPase TER94	TERA_DROME	0	GLOS_TERA.1.2	GO:0009158	B	.	ribonucleoside monophosphate catabolic process
TERA_DROME (sp|Q7KN62)Transitional endoplasmic reticulum ATPase TER94	TERA_DROME	0	GLOS_TERA.1.2	GO:0009207	B	.	purine ribonucleoside triphosphate catabolic process
TERA_DROME (sp|Q7KN62)Transitional endoplasmic reticulum ATPase TER94	TERA_DROME	0	GLOS_TERA.1.2	GO:0009146	B	.	purine nucleoside triphosphate catabolic process
TERA_DROME (sp|Q7KN62)Transitional endoplasmic reticulum ATPase TER94	TERA_DROME	0	GLOS_TERA.1.2	GO:0009143	B	.	nucleoside triphosphate catabolic process
TERA_DROME (sp|Q7KN62)Transitional endoplasmic reticulum ATPase TER94	TERA_DROME	0	GLOS_TERA.1.2	GO:0009141	B	.	nucleoside triphosphate metabolic process
TERA_DROME (sp|Q7KN62)Transitional endoplasmic reticulum ATPase TER94	TERA_DROME	0	GLOS_TERA.1.2	GO:0009144	B	.	purine nucleoside triphosphate metabolic process
TERA_DROME (sp|Q7KN62)Transitional endoplasmic reticulum ATPase TER94	TERA_DROME	0	GLOS_TERA.1.2	GO:0009203	B	.	ribonucleoside triphosphate catabolic process
TERA_DROME (sp|Q7KN62)Transitional endoplasmic reticulum ATPase TER94	TERA_DROME	0	GLOS_TERA.1.2	GO:0009199	B	.	ribonucleoside triphosphate metabolic process
TERA_DROME (sp|Q7KN62)Transitional endoplasmic reticulum ATPase TER94	TERA_DROME	0	GLOS_TERA.1.2	GO:0009205	B	.	purine ribonucleoside triphosphate metabolic process
TERA_DROME (sp|Q7KN62)Transitional endoplasmic reticulum ATPase TER94	TERA_DROME	0	GLOS_TERA.1.2	GO:0046034	B	.	ATP metabolic process
TERA_DROME (sp|Q7KN62)Transitional endoplasmic reticulum ATPase TER94	TERA_DROME	0	GLOS_TERA.1.2	GO:0046130	B	.	purine ribonucleoside catabolic process
TERA_DROME (sp|Q7KN62)Transitional endoplasmic reticulum ATPase TER94	TERA_DROME	0	GLOS_TERA.1.2	GO:0006152	B	.	purine nucleoside catabolic process
TERA_DROME (sp|Q7KN62)Transitional endoplasmic reticulum ATPase TER94	TERA_DROME	0	GLOS_TERA.1.2	GO:0009164	B	.	nucleoside catabolic process
TERA_DROME (sp|Q7KN62)Transitional endoplasmic reticulum ATPase TER94	TERA_DROME	0	GLOS_TERA.1.2	GO:1901658	B	.	glycosyl compound catabolic process
TERA_DROME (sp|Q7KN62)Transitional endoplasmic reticulum ATPase TER94	TERA_DROME	0	GLOS_TERA.1.2	GO:0042454	B	.	ribonucleoside catabolic process
TERA_DROME (sp|Q7KN62)Transitional endoplasmic reticulum ATPase TER94	TERA_DROME	0	GLOS_TERA.1.2	GO:0006200	B	FlyBase	ATP catabolic process

TERA_DROME (sp|Q7KN62)Transitional endoplasmic reticulum ATPase TER94	TERA_DROME	0	GLOS_TERA.1.2	GO:0048813	B	FlyBase	dendrite morphogenesis
TERA_DROME (sp|Q7KN62)Transitional endoplasmic reticulum ATPase TER94	TERA_DROME	0	GLOS_TERA.1.2	GO:0048284	B	.	organelle fusion
TERA_DROME (sp|Q7KN62)Transitional endoplasmic reticulum ATPase TER94	TERA_DROME	0	GLOS_TERA.1.2	GO:0090174	B	.	organelle membrane fusion
TERA_DROME (sp|Q7KN62)Transitional endoplasmic reticulum ATPase TER94	TERA_DROME	0	GLOS_TERA.1.2	GO:0044801	B	.	single-organism membrane fusion
TERA_DROME (sp|Q7KN62)Transitional endoplasmic reticulum ATPase TER94	TERA_DROME	0	GLOS_TERA.1.2	GO:0061025	B	.	membrane fusion
TERA_DROME (sp|Q7KN62)Transitional endoplasmic reticulum ATPase TER94	TERA_DROME	0	GLOS_TERA.1.2	GO:0016320	B	FlyBase	endoplasmic reticulum membrane fusion
TERA_DROME (sp|Q7KN62)Transitional endoplasmic reticulum ATPase TER94	TERA_DROME	0	GLOS_TERA.1.2	GO:0007029	B	FlyBase	endoplasmic reticulum organization
TERA_DROME (sp|Q7KN62)Transitional endoplasmic reticulum ATPase TER94	TERA_DROME	0	GLOS_TERA.1.2	GO:0030433	B	FlyBase	ER-associated protein catabolic process
TERA_DROME (sp|Q7KN62)Transitional endoplasmic reticulum ATPase TER94	TERA_DROME	0	GLOS_TERA.1.2	GO:0007030	B	FlyBase	Golgi organization
TERA_DROME (sp|Q7KN62)Transitional endoplasmic reticulum ATPase TER94	TERA_DROME	0	GLOS_TERA.1.2	GO:0000226	B	FlyBase	microtubule cytoskeleton organization
TERA_DROME (sp|Q7KN62)Transitional endoplasmic reticulum ATPase TER94	TERA_DROME	0	GLOS_TERA.1.2	GO:0048477	B	UniProtKB-k	oogenesis
TERA_DROME (sp|Q7KN62)Transitional endoplasmic reticulum ATPase TER94	TERA_DROME	0	GLOS_TERA.1.2	GO:0031331	B	.	positive regulation of cellular catabolic process
TERA_DROME (sp|Q7KN62)Transitional endoplasmic reticulum ATPase TER94	TERA_DROME	0	GLOS_TERA.1.2	GO:0009896	B	.	positive regulation of catabolic process
TERA_DROME (sp|Q7KN62)Transitional endoplasmic reticulum ATPase TER94	TERA_DROME	0	GLOS_TERA.1.2	GO:1901800	B	.	positive regulation of proteasomal protein catabolic process
TERA_DROME (sp|Q7KN62)Transitional endoplasmic reticulum ATPase TER94	TERA_DROME	0	GLOS_TERA.1.2	GO:0045732	B	.	positive regulation of protein catabolic process
TERA_DROME (sp|Q7KN62)Transitional endoplasmic reticulum ATPase TER94	TERA_DROME	0	GLOS_TERA.1.2	GO:0045862	B	.	positive regulation of proteolysis
TERA_DROME (sp|Q7KN62)Transitional endoplasmic reticulum ATPase TER94	TERA_DROME	0	GLOS_TERA.1.2	GO:0032436	B	FlyBase	positive regulation of proteasomal ubiquitin-dependent protein catabolic process
TERA_DROME (sp|Q7KN62)Transitional endoplasmic reticulum ATPase TER94	TERA_DROME	0	GLOS_TERA.1.2	GO:0042981	B	.	regulation of apoptotic process
TERA_DROME (sp|Q7KN62)Transitional endoplasmic reticulum ATPase TER94	TERA_DROME	0	GLOS_TERA.1.2	GO:0043067	B	.	regulation of programmed cell death
TERA_DROME (sp|Q7KN62)Transitional endoplasmic reticulum ATPase TER94	TERA_DROME	0	GLOS_TERA.1.2	GO:0010941	B	.	regulation of cell death
TERA_DROME (sp|Q7KN62)Transitional endoplasmic reticulum ATPase TER94	TERA_DROME	0	GLOS_TERA.1.2	GO:1901214	B	.	regulation of neuron death
TERA_DROME (sp|Q7KN62)Transitional endoplasmic reticulum ATPase TER94	TERA_DROME	0	GLOS_TERA.1.2	GO:0043523	B	FlyBase	regulation of neuron apoptotic process
TERA_DROME (sp|Q7KN62)Transitional endoplasmic reticulum ATPase TER94	TERA_DROME	0	GLOS_TERA.1.2	GO:0045995	B	.	regulation of embryonic development
TERA_DROME (sp|Q7KN62)Transitional endoplasmic reticulum ATPase TER94	TERA_DROME	0	GLOS_TERA.1.2	GO:0060281	B	.	regulation of oocyte development
TERA_DROME (sp|Q7KN62)Transitional endoplasmic reticulum ATPase TER94	TERA_DROME	0	GLOS_TERA.1.2	GO:0007317	B	FlyBase	regulation of pole plasm oskar mRNA localization
TERA_DROME (sp|Q7KN62)Transitional endoplasmic reticulum ATPase TER94	TERA_DROME	0	GLOS_TERA.1.2	GO:0006810	B	UniProtKB-k	transport
TMEDA_DROVI (sp|B4MGF8)Transmemb. emp24 domain-containing protein bai	TMEDA_DROVI	1,00E-34	GLOS_TMEDA.2.3	GO:0016021	C	UniProtKB-k	integral to membrane
TMEDA_DROVI (sp|B4MGF8)Transmemb. emp24 domain-containing protein bai	TMEDA_DROVI	1,00E-34	GLOS_TMEDA.2.3	GO:0009953	B	UniProtKB	dorsal/ventral pattern formation
TMEDA_DROVI (sp|B4MGF8)Transmemb. emp24 domain-containing protein bai	TMEDA_DROVI	1,00E-34	GLOS_TMEDA.2.3	GO:0006810	B	InterPro	transport
TNNC3_DROME (sp|P47949) Troponin C, isoform 3 OS=D.m.	TNNC3_DROME	1,00E-62	GLOS_TNNC3.3.4	GO:0005509	M	InterPro	calcium ion binding
TPM1_DROME (sp|P06754) Tropomyosin-1, isoforms 9A/A/B D.m.	TPM1_DROME	9,00E-51	GLOS_TPM1.2.3	GO:0070864	C	.	sperm individualization complex
TPM1_DROME (sp|P06754) Tropomyosin-1, isoforms 9A/A/B D.m.	TPM1_DROME	9,00E-51	GLOS_TPM1.2.3	GO:0070865	C	FlyBase	investment cone
TPM1_DROME (sp|P06754) Tropomyosin-1, isoforms 9A/A/B D.m.	TPM1_DROME	9,00E-51	GLOS_TPM1.2.3	GO:0030017	C	FlyBase	sarcomere
TPM1_DROME (sp|P06754) Tropomyosin-1, isoforms 9A/A/B D.m.	TPM1_DROME	9,00E-51	GLOS_TPM1.2.3	GO:0051015	M	FlyBase	actin filament binding
TPM1_DROME (sp|P06754) Tropomyosin-1, isoforms 9A/A/B D.m.	TPM1_DROME	9,00E-51	GLOS_TPM1.2.3	GO:0048813	B	FlyBase	dendrite morphogenesis
TPM1_DROME (sp|P06754) Tropomyosin-1, isoforms 9A/A/B D.m.	TPM1_DROME	9,00E-51	GLOS_TPM1.2.3	GO:0006936	B	FlyBase	muscle contraction
TPM1_DROME (sp|P06754) Tropomyosin-1, isoforms 9A/A/B D.m.	TPM1_DROME	9,00E-51	GLOS_TPM1.2.3	GO:0019094	B	.	pole plasm mRNA localization
TPM1_DROME (sp|P06754) Tropomyosin-1, isoforms 9A/A/B D.m.	TPM1_DROME	9,00E-51	GLOS_TPM1.2.3	GO:0007316	B	.	pole plasm RNA localization
TPM1_DROME (sp|P06754) Tropomyosin-1, isoforms 9A/A/B D.m.	TPM1_DROME	9,00E-51	GLOS_TPM1.2.3	GO:0006403	B	.	RNA localization
TPM1_DROME (sp|P06754) Tropomyosin-1, isoforms 9A/A/B D.m.	TPM1_DROME	9,00E-51	GLOS_TPM1.2.3	GO:0007315	B	.	pole plasm assembly
TPM1_DROME (sp|P06754) Tropomyosin-1, isoforms 9A/A/B D.m.	TPM1_DROME	9,00E-51	GLOS_TPM1.2.3	GO:0007028	B	.	cytoplasm organization
TPM1_DROME (sp|P06754) Tropomyosin-1, isoforms 9A/A/B D.m.	TPM1_DROME	9,00E-51	GLOS_TPM1.2.3	GO:0007314	B	.	oocyte anterior/posterior axis specification
TPM1_DROME (sp|P06754) Tropomyosin-1, isoforms 9A/A/B D.m.	TPM1_DROME	9,00E-51	GLOS_TPM1.2.3	GO:0007309	B	.	oocyte axis specification
TPM1_DROME (sp|P06754) Tropomyosin-1, isoforms 9A/A/B D.m.	TPM1_DROME	9,00E-51	GLOS_TPM1.2.3	GO:0009798	B	.	axis specification
TPM1_DROME (sp|P06754) Tropomyosin-1, isoforms 9A/A/B D.m.	TPM1_DROME	9,00E-51	GLOS_TPM1.2.3	GO:0007308	B	.	oocyte construction
TPM1_DROME (sp|P06754) Tropomyosin-1, isoforms 9A/A/B D.m.	TPM1_DROME	9,00E-51	GLOS_TPM1.2.3	GO:0048469	B	.	cell maturation
TPM1_DROME (sp|P06754) Tropomyosin-1, isoforms 9A/A/B D.m.	TPM1_DROME	9,00E-51	GLOS_TPM1.2.3	GO:0021700	B	.	developmental maturation
TPM1_DROME (sp|P06754) Tropomyosin-1, isoforms 9A/A/B D.m.	TPM1_DROME	9,00E-51	GLOS_TPM1.2.3	GO:0048599	B	.	oocyte development
TPM1_DROME (sp|P06754) Tropomyosin-1, isoforms 9A/A/B D.m.	TPM1_DROME	9,00E-51	GLOS_TPM1.2.3	GO:0007281	B	.	germ cell development
TPM1_DROME (sp|P06754) Tropomyosin-1, isoforms 9A/A/B D.m.	TPM1_DROME	9,00E-51	GLOS_TPM1.2.3	GO:0009994	B	.	oocyte differentiation
TPM1_DROME (sp|P06754) Tropomyosin-1, isoforms 9A/A/B D.m.	TPM1_DROME	9,00E-51	GLOS_TPM1.2.3	GO:0009948	B	.	anterior/posterior axis specification
TPM1_DROME (sp|P06754) Tropomyosin-1, isoforms 9A/A/B D.m.	TPM1_DROME	9,00E-51	GLOS_TPM1.2.3	GO:0008358	B	.	maternal determination of anterior/posterior axis, embryo
TPM1_DROME (sp|P06754) Tropomyosin-1, isoforms 9A/A/B D.m.	TPM1_DROME	9,00E-51	GLOS_TPM1.2.3	GO:0008595	B	.	anterior/posterior axis specification, embryo
TPM1_DROME (sp|P06754) Tropomyosin-1, isoforms 9A/A/B D.m.	TPM1_DROME	9,00E-51	GLOS_TPM1.2.3	GO:0000578	B	.	embryonic axis specification

TPM1_DROME (sp|P06754) Tropomyosin-1, isoforms 9A/A/B D.m.	TPM1_DROME	9,00E-51	GLOS_TPM1.2.3	GO:0009880	B	.	embryonic pattern specification
TPM1_DROME (sp|P06754) Tropomyosin-1, isoforms 9A/A/B D.m.	TPM1_DROME	9,00E-51	GLOS_TPM1.2.3	GO:0007351	B	.	tripartite regional subdivision
TPM1_DROME (sp|P06754) Tropomyosin-1, isoforms 9A/A/B D.m.	TPM1_DROME	9,00E-51	GLOS_TPM1.2.3	GO:0007350	B	.	blastoderm segmentation
TPM1_DROME (sp|P06754) Tropomyosin-1, isoforms 9A/A/B D.m.	TPM1_DROME	9,00E-51	GLOS_TPM1.2.3	GO:0060811	B	.	intracellular mRNA localization involved in anterior/posterior axis specification
TPM1_DROME (sp|P06754) Tropomyosin-1, isoforms 9A/A/B D.m.	TPM1_DROME	9,00E-51	GLOS_TPM1.2.3	GO:0060810	B	.	intracellular mRNA localization involved in pattern specification process
TPM1_DROME (sp|P06754) Tropomyosin-1, isoforms 9A/A/B D.m.	TPM1_DROME	9,00E-51	GLOS_TPM1.2.3	GO:0008298	B	.	intracellular mRNA localization
TPM1_DROME (sp|P06754) Tropomyosin-1, isoforms 9A/A/B D.m.	TPM1_DROME	9,00E-51	GLOS_TPM1.2.3	GO:0045451	B	FlyBase	pole plasm oskar mRNA localization
TPM1_DROME (sp|P06754) Tropomyosin-1, isoforms 9A/A/B D.m.	TPM1_DROME	9,00E-51	GLOS_TPM1.2.3	GO:0010591	B	FlyBase	regulation of lamellipodium assembly
TRYA4_LUCCU (sp|P35044) Trypsin alpha-4 OS=L cuprina PE=3 SV=1	TRYA4_LUCCU	1,00E-65	GLOS_TRYA4.2.6	GO:0005615	C	UniProtKB-S	extracellular space
TRYA4_LUCCU (sp|P35044) Trypsin alpha-4 OS=L cuprina PE=3 SV=1	TRYA4_LUCCU	1,00E-65	GLOS_TRYA4.2.6	GO:0004252	M	InterPro	serine-type endopeptidase activity
TRYA4_LUCCU (sp|P35044) Trypsin alpha-4 OS=L cuprina PE=3 SV=1	TRYA4_LUCCU	1,00E-65	GLOS_TRYA4.2.6	GO:0006508	B	UniProtKB-K	proteolysis
TRYA_DROME (sp|P04814) Trypsin alpha D.m. GN=alphaTry PE=2 SV=1	TRYA_DROME	9,00E-77	GLOS_TRYA.3.5	GO:0005615	C	UniProtKB-S	extracellular space
TRYA_DROME (sp|P04814) Trypsin alpha D.m. GN=alphaTry PE=2 SV=1	TRYA_DROME	9,00E-77	GLOS_TRYA.3.5	GO:0004252	M	FlyBase	serine-type endopeptidase activity
TRYA_DROME (sp|P04814) Trypsin alpha D.m. GN=alphaTry PE=2 SV=1	TRYA_DROME	9,00E-77	GLOS_TRYA.3.5	GO:0006508	B	FlyBase	proteolysis
TRYDG_DROER (sp|P54626) Trypsin delta/gamma OS=D. erecta	TRYDG_DROER	5,00E-60	GLOS_TRYDG.2.5	GO:0005615	C	UniProtKB-S	extracellular space
TRYDG_DROER (sp|P54626) Trypsin delta/gamma OS=D. erecta	TRYDG_DROER	5,00E-60	GLOS_TRYDG.2.5	GO:0004252	M	InterPro	serine-type endopeptidase activity
TRYDG_DROER (sp|P54626) Trypsin delta/gamma OS=D. erecta	TRYDG_DROER	5,00E-60	GLOS_TRYDG.2.5	GO:0006508	B	UniProtKB-K	proteolysis
[BBH] TSR1_DROME (sp|Q9VP47) Pre-rRNA-processing protein TSR1 homolog	TSR1_DROME	0	GLOS_TSR1.1.1	GO:0005730	C	UniProtKB	nucleolus
[BBH] TSR1_DROME (sp|Q9VP47) Pre-rRNA-processing protein TSR1 homolog	TSR1_DROME	0	GLOS_TSR1.1.1	GO:0022008	B	FlyBase	neurogenesis
[BBH] TSR1_DROME (sp|Q9VP47) Pre-rRNA-processing protein TSR1 homolog	TSR1_DROME	0	GLOS_TSR1.1.1	GO:0022618	B	.	ribonucleoprotein complex assembly
[BBH] TSR1_DROME (sp|Q9VP47) Pre-rRNA-processing protein TSR1 homolog	TSR1_DROME	0	GLOS_TSR1.1.1	GO:0071826	B	.	ribonucleoprotein complex subunit organization
[BBH] TSR1_DROME (sp|Q9VP47) Pre-rRNA-processing protein TSR1 homolog	TSR1_DROME	0	GLOS_TSR1.1.1	GO:0022613	B	.	ribonucleoprotein complex biogenesis
[BBH] TSR1_DROME (sp|Q9VP47) Pre-rRNA-processing protein TSR1 homolog	TSR1_DROME	0	GLOS_TSR1.1.1	GO:0042254	B	.	ribosome biogenesis
[BBH] TSR1_DROME (sp|Q9VP47) Pre-rRNA-processing protein TSR1 homolog	TSR1_DROME	0	GLOS_TSR1.1.1	GO:0042255	B	UniProtKB	ribosome assembly
TTI_GLOMM (sp|O97373) Tsetse thrombin inhibitor OS=G. m. morsitans	TTI_GLOMM	4,00E-19	GLOS_TTI.5.16	GO:0005576	C	UniProtKB-S	extracellular region
TTI_GLOMM (sp|O97373) Tsetse thrombin inhibitor OS=G. m. morsitans	TTI_GLOMM	4,00E-19	GLOS_TTI.5.16	GO:0004867	M	UniProtKB-K	serine-type endopeptidase inhibitor activity
[BBH] U0468_DROME (sp|Q9VKV8) UPF0468 protein CG5343 D.m.	U0468_DROME	1,00E-138	GLOS_U0468.1.1	GO:0005681	C	.	spliceosomal complex
[BBH] U0468_DROME (sp|Q9VKV8) UPF0468 protein CG5343 D.m.	U0468_DROME	1,00E-138	GLOS_U0468.1.1	GO:0071013	C	FlyBase	catalytic step 2 spliceosome
[BBH] U0468_DROME (sp|Q9VKV8) UPF0468 protein CG5343 D.m.	U0468_DROME	1,00E-138	GLOS_U0468.1.1	GO:0071011	C	FlyBase	precatalytic spliceosome
[BBH] U0468_DROME (sp|Q9VKV8) UPF0468 protein CG5343 D.m.	U0468_DROME	1,00E-138	GLOS_U0468.1.1	GO:0048813	B	FlyBase	dendrite morphogenesis
[BBH] U0468_DROME (sp|Q9VKV8) UPF0468 protein CG5343 D.m.	U0468_DROME	1,00E-138	GLOS_U0468.1.1	GO:0000377	B	.	RNA splicing, via transesterification react. with bulged adenosine as nucleophile
[BBH] U0468_DROME (sp|Q9VKV8) UPF0468 protein CG5343 D.m.	U0468_DROME	1,00E-138	GLOS_U0468.1.1	GO:0000375	B	.	RNA splicing, via transesterification reactions
[BBH] U0468_DROME (sp|Q9VKV8) UPF0468 protein CG5343 D.m.	U0468_DROME	1,00E-138	GLOS_U0468.1.1	GO:0008380	B	.	RNA splicing
[BBH] U0468_DROME (sp|Q9VKV8) UPF0468 protein CG5343 D.m.	U0468_DROME	1,00E-138	GLOS_U0468.1.1	GO:0006396	B	.	RNA processing
[BBH] U0468_DROME (sp|Q9VKV8) UPF0468 protein CG5343 D.m.	U0468_DROME	1,00E-138	GLOS_U0468.1.1	GO:0006397	B	.	mRNA processing
[BBH] U0468_DROME (sp|Q9VKV8) UPF0468 protein CG5343 D.m.	U0468_DROME	1,00E-138	GLOS_U0468.1.1	GO:0016071	B	.	mRNA metabolic process
[BBH] U0468_DROME (sp|Q9VKV8) UPF0468 protein CG5343 D.m.	U0468_DROME	1,00E-138	GLOS_U0468.1.1	GO:0000398	B	FlyBase	mRNA splicing, via spliceosome
[BBH] U0468_DROME (sp|Q9VKV8) UPF0468 protein CG5343 D.m.	U0468_DROME	1,00E-138	GLOS_U0468.1.1	GO:0007517	B	FlyBase	muscle organ development
NP_001260747.1 missing-in-metastasis, isoform K [D.m.]	NP_001260747.1	0	GLOS_MIM.2.2	GO:0006357	B	.	regulation of transcription from RNA polymerase II promoter
NP_001260747.1 missing-in-metastasis, isoform K [D.m.]	NP_001260747.1	0	GLOS_MIM.2.2	GO:0006355	B	.	regulation of transcription, DNA-dependent
NP_001260747.1 missing-in-metastasis, isoform K [D.m.]	NP_001260747.1	0	GLOS_MIM.2.2	GO:0010468	B	.	regulation of gene expression
NP_001260747.1 missing-in-metastasis, isoform K [D.m.]	NP_001260747.1	0	GLOS_MIM.2.2	GO:0060255	B	.	regulation of macromolecule metabolic process
NP_001260747.1 missing-in-metastasis, isoform K [D.m.]	NP_001260747.1	0	GLOS_MIM.2.2	GO:0019222	B	.	regulation of metabolic process
NP_001260747.1 missing-in-metastasis, isoform K [D.m.]	NP_001260747.1	0	GLOS_MIM.2.2	GO:0050789	B	.	regulation of biological process
NP_001260747.1 missing-in-metastasis, isoform K [D.m.]	NP_001260747.1	0	GLOS_MIM.2.2	GO:0065007	B	.	biological regulation
NP_001260747.1 missing-in-metastasis, isoform K [D.m.]	NP_001260747.1	0	GLOS_MIM.2.2	GO:0008150	B	.	biological_process
NP_001260747.1 missing-in-metastasis, isoform K [D.m.]	NP_001260747.1	0	GLOS_MIM.2.2	GO:2000112	B	.	regulation of cellular macromolecule biosynthetic process
NP_001260747.1 missing-in-metastasis, isoform K [D.m.]	NP_001260747.1	0	GLOS_MIM.2.2	GO:0010556	B	.	regulation of macromolecule biosynthetic process
NP_001260747.1 missing-in-metastasis, isoform K [D.m.]	NP_001260747.1	0	GLOS_MIM.2.2	GO:0009889	B	.	regulation of biosynthetic process
NP_001260747.1 missing-in-metastasis, isoform K [D.m.]	NP_001260747.1	0	GLOS_MIM.2.2	GO:0031326	B	.	regulation of cellular biosynthetic process
NP_001260747.1 missing-in-metastasis, isoform K [D.m.]	NP_001260747.1	0	GLOS_MIM.2.2	GO:0031323	B	.	regulation of cellular metabolic process
NP_001260747.1 missing-in-metastasis, isoform K [D.m.]	NP_001260747.1	0	GLOS_MIM.2.2	GO:0050794	B	.	regulation of cellular process
NP_001260747.1 missing-in-metastasis, isoform K [D.m.]	NP_001260747.1	0	GLOS_MIM.2.2	GO:2001141	B	.	regulation of RNA biosynthetic process
NP_001260747.1 missing-in-metastasis, isoform K [D.m.]	NP_001260747.1	0	GLOS_MIM.2.2	GO:0051252	B	.	regulation of RNA metabolic process

NP_001260747.1 missing-in-metastasis, isoform K [D.m.]	NP_001260747.1	0	GLOS_MIM.2.2	GO:0019219	B	.	regulation of nucleobase-containing compound metabolic process
NP_001260747.1 missing-in-metastasis, isoform K [D.m.]	NP_001260747.1	0	GLOS_MIM.2.2	GO:0051171	B	.	regulation of nitrogen compound metabolic process
NP_001260747.1 missing-in-metastasis, isoform K [D.m.]	NP_001260747.1	0	GLOS_MIM.2.2	GO:0080090	B	.	regulation of primary metabolic process
NP_001260747.1 missing-in-metastasis, isoform K [D.m.]	NP_001260747.1	0	GLOS_MIM.2.2	GO:0045892	B	.	negative regulation of transcription, DNA-dependent
NP_001260747.1 missing-in-metastasis, isoform K [D.m.]	NP_001260747.1	0	GLOS_MIM.2.2	GO:0010629	B	.	negative regulation of gene expression
NP_001260747.1 missing-in-metastasis, isoform K [D.m.]	NP_001260747.1	0	GLOS_MIM.2.2	GO:0010605	B	.	negative regulation of macromolecule metabolic process
NP_001260747.1 missing-in-metastasis, isoform K [D.m.]	NP_001260747.1	0	GLOS_MIM.2.2	GO:0009892	B	.	negative regulation of metabolic process
NP_001260747.1 missing-in-metastasis, isoform K [D.m.]	NP_001260747.1	0	GLOS_MIM.2.2	GO:0048519	B	.	negative regulation of biological process
NP_001260747.1 missing-in-metastasis, isoform K [D.m.]	NP_001260747.1	0	GLOS_MIM.2.2	GO:0051253	B	.	negative regulation of RNA metabolic process
NP_001260747.1 missing-in-metastasis, isoform K [D.m.]	NP_001260747.1	0	GLOS_MIM.2.2	GO:0045934	B	.	negative regulation of nucleobase-containing compound metabolic process
NP_001260747.1 missing-in-metastasis, isoform K [D.m.]	NP_001260747.1	0	GLOS_MIM.2.2	GO:0031324	B	.	negative regulation of cellular metabolic process
NP_001260747.1 missing-in-metastasis, isoform K [D.m.]	NP_001260747.1	0	GLOS_MIM.2.2	GO:0048523	B	.	negative regulation of cellular process
NP_001260747.1 missing-in-metastasis, isoform K [D.m.]	NP_001260747.1	0	GLOS_MIM.2.2	GO:0051172	B	.	negative regulation of nitrogen compound metabolic process
NP_001260747.1 missing-in-metastasis, isoform K [D.m.]	NP_001260747.1	0	GLOS_MIM.2.2	GO:2000113	B	.	negative regulation of cellular macromolecule biosynthetic process
NP_001260747.1 missing-in-metastasis, isoform K [D.m.]	NP_001260747.1	0	GLOS_MIM.2.2	GO:0010558	B	.	negative regulation of macromolecule biosynthetic process
NP_001260747.1 missing-in-metastasis, isoform K [D.m.]	NP_001260747.1	0	GLOS_MIM.2.2	GO:0009890	B	.	negative regulation of biosynthetic process
NP_001260747.1 missing-in-metastasis, isoform K [D.m.]	NP_001260747.1	0	GLOS_MIM.2.2	GO:0031327	B	.	negative regulation of cellular biosynthetic process
NP_001260747.1 missing-in-metastasis, isoform K [D.m.]	NP_001260747.1	0	GLOS_MIM.2.2	GO:0000122	B	Refseq	negative regulation of transcription from RNA polymerase II promoter
NP_001260747.1 missing-in-metastasis, isoform K [D.m.]	NP_001260747.1	0	GLOS_MIM.2.2	GO:0048731	B	.	system development
NP_001260747.1 missing-in-metastasis, isoform K [D.m.]	NP_001260747.1	0	GLOS_MIM.2.2	GO:0048856	B	.	anatomical structure development
NP_001260747.1 missing-in-metastasis, isoform K [D.m.]	NP_001260747.1	0	GLOS_MIM.2.2	GO:0032502	B	.	developmental process
NP_001260747.1 missing-in-metastasis, isoform K [D.m.]	NP_001260747.1	0	GLOS_MIM.2.2	GO:0007275	B	.	multicellular organismal development
NP_001260747.1 missing-in-metastasis, isoform K [D.m.]	NP_001260747.1	0	GLOS_MIM.2.2	GO:0044707	B	.	single-multicellular organism process
NP_001260747.1 missing-in-metastasis, isoform K [D.m.]	NP_001260747.1	0	GLOS_MIM.2.2	GO:0032501	B	.	multicellular organismal process
NP_001260747.1 missing-in-metastasis, isoform K [D.m.]	NP_001260747.1	0	GLOS_MIM.2.2	GO:0044699	B	.	single-organism process
NP_001260747.1 missing-in-metastasis, isoform K [D.m.]	NP_001260747.1	0	GLOS_MIM.2.2	GO:0001501	B	Refseq	skeletal system development
NP_001260747.1 missing-in-metastasis, isoform K [D.m.]	NP_001260747.1	0	GLOS_MIM.2.2	GO:0001071	M	.	nucleic acid binding transcription factor activity
NP_001260747.1 missing-in-metastasis, isoform K [D.m.]	NP_001260747.1	0	GLOS_MIM.2.2	GO:0003674	M	.	molecular_function
NP_001260747.1 missing-in-metastasis, isoform K [D.m.]	NP_001260747.1	0	GLOS_MIM.2.2	GO:0003700	M	Refseq	sequence-specific DNA binding transcription factor activity
NP_001260747.1 missing-in-metastasis, isoform K [D.m.]	NP_001260747.1	0	GLOS_MIM.2.2	GO:0005488	M	.	binding
NP_001260747.1 missing-in-metastasis, isoform K [D.m.]	NP_001260747.1	0	GLOS_MIM.2.2	GO:0005515	M	Refseq	protein binding
NP_001260747.1 missing-in-metastasis, isoform K [D.m.]	NP_001260747.1	0	GLOS_MIM.2.2	GO:0043231	C	.	intracellular membrane-bounded organelle
NP_001260747.1 missing-in-metastasis, isoform K [D.m.]	NP_001260747.1	0	GLOS_MIM.2.2	GO:0043227	C	.	membrane-bounded organelle
NP_001260747.1 missing-in-metastasis, isoform K [D.m.]	NP_001260747.1	0	GLOS_MIM.2.2	GO:0043226	C	.	organelle
NP_001260747.1 missing-in-metastasis, isoform K [D.m.]	NP_001260747.1	0	GLOS_MIM.2.2	GO:0005575	C	.	cellular_component
NP_001260747.1 missing-in-metastasis, isoform K [D.m.]	NP_001260747.1	0	GLOS_MIM.2.2	GO:0043229	C	.	intracellular organelle
NP_001260747.1 missing-in-metastasis, isoform K [D.m.]	NP_001260747.1	0	GLOS_MIM.2.2	GO:0044424	C	.	intracellular part
NP_001260747.1 missing-in-metastasis, isoform K [D.m.]	NP_001260747.1	0	GLOS_MIM.2.2	GO:0044464	C	.	cell part
NP_001260747.1 missing-in-metastasis, isoform K [D.m.]	NP_001260747.1	0	GLOS_MIM.2.2	GO:0005623	C	.	cell
NP_001260747.1 missing-in-metastasis, isoform K [D.m.]	NP_001260747.1	0	GLOS_MIM.2.2	GO:0005622	C	.	intracellular
NP_001260747.1 missing-in-metastasis, isoform K [D.m.]	NP_001260747.1	0	GLOS_MIM.2.2	GO:0005634	C	Refseq	nucleus
NP_001260747.1 missing-in-metastasis, isoform K [D.m.]	NP_001260747.1	0	GLOS_MIM.2.2	GO:0006351	B	.	transcription, DNA-dependent
NP_001260747.1 missing-in-metastasis, isoform K [D.m.]	NP_001260747.1	0	GLOS_MIM.2.2	GO:0032774	B	.	RNA biosynthetic process
NP_001260747.1 missing-in-metastasis, isoform K [D.m.]	NP_001260747.1	0	GLOS_MIM.2.2	GO:0009059	B	.	macromolecule biosynthetic process
NP_001260747.1 missing-in-metastasis, isoform K [D.m.]	NP_001260747.1	0	GLOS_MIM.2.2	GO:0043170	B	.	macromolecule metabolic process
NP_001260747.1 missing-in-metastasis, isoform K [D.m.]	NP_001260747.1	0	GLOS_MIM.2.2	GO:0071704	B	.	organic substance metabolic process
NP_001260747.1 missing-in-metastasis, isoform K [D.m.]	NP_001260747.1	0	GLOS_MIM.2.2	GO:0008152	B	.	metabolic process
NP_001260747.1 missing-in-metastasis, isoform K [D.m.]	NP_001260747.1	0	GLOS_MIM.2.2	GO:1901576	B	.	organic substance biosynthetic process
NP_001260747.1 missing-in-metastasis, isoform K [D.m.]	NP_001260747.1	0	GLOS_MIM.2.2	GO:0009058	B	.	biosynthetic process
NP_001260747.1 missing-in-metastasis, isoform K [D.m.]	NP_001260747.1	0	GLOS_MIM.2.2	GO:0016070	B	.	RNA metabolic process
NP_001260747.1 missing-in-metastasis, isoform K [D.m.]	NP_001260747.1	0	GLOS_MIM.2.2	GO:0044260	B	.	cellular macromolecule metabolic process
NP_001260747.1 missing-in-metastasis, isoform K [D.m.]	NP_001260747.1	0	GLOS_MIM.2.2	GO:0044237	B	.	cellular metabolic process
NP_001260747.1 missing-in-metastasis, isoform K [D.m.]	NP_001260747.1	0	GLOS_MIM.2.2	GO:0009987	B	.	cellular process
NP_001260747.1 missing-in-metastasis, isoform K [D.m.]	NP_001260747.1	0	GLOS_MIM.2.2	GO:0090304	B	.	nucleic acid metabolic process

NP_001260747.1 missing-in-metastasis, isoform K [D.m.]	NP_001260747.1	0	GLOS_MIM.2.2	GO:0006139	B	.	nucleobase-containing compound metabolic process
NP_001260747.1 missing-in-metastasis, isoform K [D.m.]	NP_001260747.1	0	GLOS_MIM.2.2	GO:0006725	B	.	cellular aromatic compound metabolic process
NP_001260747.1 missing-in-metastasis, isoform K [D.m.]	NP_001260747.1	0	GLOS_MIM.2.2	GO:0034641	B	.	cellular nitrogen compound metabolic process
NP_001260747.1 missing-in-metastasis, isoform K [D.m.]	NP_001260747.1	0	GLOS_MIM.2.2	GO:0006807	B	.	nitrogen compound metabolic process
NP_001260747.1 missing-in-metastasis, isoform K [D.m.]	NP_001260747.1	0	GLOS_MIM.2.2	GO:0044238	B	.	primary metabolic process
NP_001260747.1 missing-in-metastasis, isoform K [D.m.]	NP_001260747.1	0	GLOS_MIM.2.2	GO:0046483	B	.	heterocycle metabolic process
NP_001260747.1 missing-in-metastasis, isoform K [D.m.]	NP_001260747.1	0	GLOS_MIM.2.2	GO:1901360	B	.	organic cyclic compound metabolic process
NP_001260747.1 missing-in-metastasis, isoform K [D.m.]	NP_001260747.1	0	GLOS_MIM.2.2	GO:0034654	B	.	nucleobase-containing compound biosynthetic process
NP_001260747.1 missing-in-metastasis, isoform K [D.m.]	NP_001260747.1	0	GLOS_MIM.2.2	GO:0018130	B	.	heterocycle biosynthetic process
NP_001260747.1 missing-in-metastasis, isoform K [D.m.]	NP_001260747.1	0	GLOS_MIM.2.2	GO:0044249	B	.	cellular biosynthetic process
NP_001260747.1 missing-in-metastasis, isoform K [D.m.]	NP_001260747.1	0	GLOS_MIM.2.2	GO:0019438	B	.	aromatic compound biosynthetic process
NP_001260747.1 missing-in-metastasis, isoform K [D.m.]	NP_001260747.1	0	GLOS_MIM.2.2	GO:0044271	B	.	cellular nitrogen compound biosynthetic process
NP_001260747.1 missing-in-metastasis, isoform K [D.m.]	NP_001260747.1	0	GLOS_MIM.2.2	GO:1901362	B	.	organic cyclic compound biosynthetic process
NP_001260747.1 missing-in-metastasis, isoform K [D.m.]	NP_001260747.1	0	GLOS_MIM.2.2	GO:0034645	B	.	cellular macromolecule biosynthetic process
NP_001260747.1 missing-in-metastasis, isoform K [D.m.]	NP_001260747.1	0	GLOS_MIM.2.2	GO:0010467	B	.	gene expression
NP_001260747.1 missing-in-metastasis, isoform K [D.m.]	NP_001260747.1	0	GLOS_MIM.2.2	GO:0006366	B	Refseq	transcription from RNA polymerase II promoter
NP_001260747.1 missing-in-metastasis, isoform K [D.m.]	NP_001260747.1	0	GLOS_MIM.2.2	GO:0006606	B	.	protein import into nucleus
NP_001260747.1 missing-in-metastasis, isoform K [D.m.]	NP_001260747.1	0	GLOS_MIM.2.2	GO:0006886	B	.	intracellular protein transport
NP_001260747.1 missing-in-metastasis, isoform K [D.m.]	NP_001260747.1	0	GLOS_MIM.2.2	GO:0015031	B	.	protein transport
NP_001260747.1 missing-in-metastasis, isoform K [D.m.]	NP_001260747.1	0	GLOS_MIM.2.2	GO:0045184	B	.	establishment of protein localization
NP_001260747.1 missing-in-metastasis, isoform K [D.m.]	NP_001260747.1	0	GLOS_MIM.2.2	GO:0051234	B	.	establishment of localization
NP_001260747.1 missing-in-metastasis, isoform K [D.m.]	NP_001260747.1	0	GLOS_MIM.2.2	GO:0051179	B	.	localization
NP_001260747.1 missing-in-metastasis, isoform K [D.m.]	NP_001260747.1	0	GLOS_MIM.2.2	GO:0008104	B	.	protein localization
NP_001260747.1 missing-in-metastasis, isoform K [D.m.]	NP_001260747.1	0	GLOS_MIM.2.2	GO:0033036	B	.	macromolecule localization
NP_001260747.1 missing-in-metastasis, isoform K [D.m.]	NP_001260747.1	0	GLOS_MIM.2.2	GO:0071702	B	.	organic substance transport
NP_001260747.1 missing-in-metastasis, isoform K [D.m.]	NP_001260747.1	0	GLOS_MIM.2.2	GO:0006810	B	.	transport
NP_001260747.1 missing-in-metastasis, isoform K [D.m.]	NP_001260747.1	0	GLOS_MIM.2.2	GO:0046907	B	.	intracellular transport
NP_001260747.1 missing-in-metastasis, isoform K [D.m.]	NP_001260747.1	0	GLOS_MIM.2.2	GO:0051649	B	.	establishment of localization in cell
NP_001260747.1 missing-in-metastasis, isoform K [D.m.]	NP_001260747.1	0	GLOS_MIM.2.2	GO:0051641	B	.	cellular localization
NP_001260747.1 missing-in-metastasis, isoform K [D.m.]	NP_001260747.1	0	GLOS_MIM.2.2	GO:0044763	B	.	single-organism cellular process
NP_001260747.1 missing-in-metastasis, isoform K [D.m.]	NP_001260747.1	0	GLOS_MIM.2.2	GO:0034613	B	.	cellular protein localization
NP_001260747.1 missing-in-metastasis, isoform K [D.m.]	NP_001260747.1	0	GLOS_MIM.2.2	GO:0070727	B	.	cellular macromolecule localization
NP_001260747.1 missing-in-metastasis, isoform K [D.m.]	NP_001260747.1	0	GLOS_MIM.2.2	GO:0017038	B	.	protein import
NP_001260747.1 missing-in-metastasis, isoform K [D.m.]	NP_001260747.1	0	GLOS_MIM.2.2	GO:0044765	B	.	single-organism transport
NP_001260747.1 missing-in-metastasis, isoform K [D.m.]	NP_001260747.1	0	GLOS_MIM.2.2	GO:0051170	B	.	nuclear import
NP_001260747.1 missing-in-metastasis, isoform K [D.m.]	NP_001260747.1	0	GLOS_MIM.2.2	GO:0006913	B	.	nucleocytoplasmic transport
NP_001260747.1 missing-in-metastasis, isoform K [D.m.]	NP_001260747.1	0	GLOS_MIM.2.2	GO:0016482	B	.	cytoplasmic transport
NP_001260747.1 missing-in-metastasis, isoform K [D.m.]	NP_001260747.1	0	GLOS_MIM.2.2	GO:0051169	B	.	nuclear transport
NP_001260747.1 missing-in-metastasis, isoform K [D.m.]	NP_001260747.1	0	GLOS_MIM.2.2	GO:0072594	B	.	establishment of protein localization to organelle
NP_001260747.1 missing-in-metastasis, isoform K [D.m.]	NP_001260747.1	0	GLOS_MIM.2.2	GO:0033365	B	.	protein localization to organelle
NP_001260747.1 missing-in-metastasis, isoform K [D.m.]	NP_001260747.1	0	GLOS_MIM.2.2	GO:0034504	B	.	protein localization to nucleus
NP_001260747.1 missing-in-metastasis, isoform K [D.m.]	NP_001260747.1	0	GLOS_MIM.2.2	GO:0044744	B	.	protein targeting to nucleus
NP_001260747.1 missing-in-metastasis, isoform K [D.m.]	NP_001260747.1	0	GLOS_MIM.2.2	GO:0006605	B	.	protein targeting
NP_001260747.1 missing-in-metastasis, isoform K [D.m.]	NP_001260747.1	0	GLOS_MIM.2.2	GO:0006607	B	Refseq	NLS-bearing protein import into nucleus
NP_001260747.1 missing-in-metastasis, isoform K [D.m.]	NP_001260747.1	0	GLOS_MIM.2.2	GO:0046914	M	.	transition metal ion binding
NP_001260747.1 missing-in-metastasis, isoform K [D.m.]	NP_001260747.1	0	GLOS_MIM.2.2	GO:0046872	M	.	metal ion binding
NP_001260747.1 missing-in-metastasis, isoform K [D.m.]	NP_001260747.1	0	GLOS_MIM.2.2	GO:0043169	M	.	cation binding
NP_001260747.1 missing-in-metastasis, isoform K [D.m.]	NP_001260747.1	0	GLOS_MIM.2.2	GO:0043167	M	.	ion binding
NP_001260747.1 missing-in-metastasis, isoform K [D.m.]	NP_001260747.1	0	GLOS_MIM.2.2	GO:0008270	M	Refseq	zinc ion binding
NP_001260747.1 missing-in-metastasis, isoform K [D.m.]	NP_001260747.1	0	GLOS_MIM.2.2	GO:0045595	B	.	regulation of cell differentiation
NP_001260747.1 missing-in-metastasis, isoform K [D.m.]	NP_001260747.1	0	GLOS_MIM.2.2	GO:0050793	B	.	regulation of developmental process
NP_001260747.1 missing-in-metastasis, isoform K [D.m.]	NP_001260747.1	0	GLOS_MIM.2.2	GO:0061035	B	.	regulation of cartilage development
NP_001260747.1 missing-in-metastasis, isoform K [D.m.]	NP_001260747.1	0	GLOS_MIM.2.2	GO:2000026	B	.	regulation of multicellular organismal development
NP_001260747.1 missing-in-metastasis, isoform K [D.m.]	NP_001260747.1	0	GLOS_MIM.2.2	GO:0051239	B	.	regulation of multicellular organismal process

NP_001260747.1 missing-in-metastasis, isoform K [D.m.]	NP_001260747.1	0	GLOS_MIM.2.2	GO:0032330	B	Refseq	regulation of chondrocyte differentiation
NP_001260747.1 missing-in-metastasis, isoform K [D.m.]	NP_001260747.1	0	GLOS_MIM.2.2	GO:0003677	M	.	DNA binding
NP_001260747.1 missing-in-metastasis, isoform K [D.m.]	NP_001260747.1	0	GLOS_MIM.2.2	GO:0003676	M	.	nucleic acid binding
NP_001260747.1 missing-in-metastasis, isoform K [D.m.]	NP_001260747.1	0	GLOS_MIM.2.2	GO:0097159	M	.	organic cyclic compound binding
NP_001260747.1 missing-in-metastasis, isoform K [D.m.]	NP_001260747.1	0	GLOS_MIM.2.2	GO:1901363	M	.	heterocyclic compound binding
NP_001260747.1 missing-in-metastasis, isoform K [D.m.]	NP_001260747.1	0	GLOS_MIM.2.2	GO:0043565	M	Refseq	sequence-specific DNA binding
NP_609681.1 CG8997 [Drosophila melanogaster]	NP_609681.1	1,00E-58	GLOS_CG8997.1.:	GO:0000122	B	Refseq	negative regulation of transcription from RNA polymerase II promoter
NP_609681.1 CG8997 [Drosophila melanogaster]	NP_609681.1	1,00E-58	GLOS_CG8997.1.:	GO:0001501	B	Refseq	skeletal system development
NP_609681.1 CG8997 [Drosophila melanogaster]	NP_609681.1	1,00E-58	GLOS_CG8997.1.:	GO:0003700	M	Refseq	sequence-specific DNA binding transcription factor activity
NP_609681.1 CG8997 [Drosophila melanogaster]	NP_609681.1	1,00E-58	GLOS_CG8997.1.:	GO:0005515	M	Refseq	protein binding
NP_609681.1 CG8997 [Drosophila melanogaster]	NP_609681.1	1,00E-58	GLOS_CG8997.1.:	GO:0005634	C	Refseq	nucleus
NP_609681.1 CG8997 [Drosophila melanogaster]	NP_609681.1	1,00E-58	GLOS_CG8997.1.:	GO:0006366	B	Refseq	transcription from RNA polymerase II promoter
NP_609681.1 CG8997 [Drosophila melanogaster]	NP_609681.1	1,00E-58	GLOS_CG8997.1.:	GO:0006607	B	Refseq	NLS-bearing protein import into nucleus
NP_609681.1 CG8997 [Drosophila melanogaster]	NP_609681.1	1,00E-58	GLOS_CG8997.1.:	GO:0008270	M	Refseq	zinc ion binding
NP_609681.1 CG8997 [Drosophila melanogaster]	NP_609681.1	1,00E-58	GLOS_CG8997.1.:	GO:0032330	B	Refseq	regulation of chondrocyte differentiation
NP_609681.1 CG8997 [Drosophila melanogaster]	NP_609681.1	1,00E-58	GLOS_CG8997.1.:	GO:0043565	M	Refseq	sequence-specific DNA binding
NP_001260901.1 CG9003, isoform F [Drosophila melanogaster]	NP_001260901.1	0	GLOS_CG9003.1.:	GO:0000122	B	Refseq	negative regulation of transcription from RNA polymerase II promoter
NP_001260901.1 CG9003, isoform F [Drosophila melanogaster]	NP_001260901.1	0	GLOS_CG9003.1.:	GO:0001501	B	Refseq	skeletal system development
NP_001260901.1 CG9003, isoform F [Drosophila melanogaster]	NP_001260901.1	0	GLOS_CG9003.1.:	GO:0003700	M	Refseq	sequence-specific DNA binding transcription factor activity
NP_001260901.1 CG9003, isoform F [Drosophila melanogaster]	NP_001260901.1	0	GLOS_CG9003.1.:	GO:0005515	M	Refseq	protein binding
NP_001260901.1 CG9003, isoform F [Drosophila melanogaster]	NP_001260901.1	0	GLOS_CG9003.1.:	GO:0005634	C	Refseq	nucleus
NP_001260901.1 CG9003, isoform F [Drosophila melanogaster]	NP_001260901.1	0	GLOS_CG9003.1.:	GO:0006366	B	Refseq	transcription from RNA polymerase II promoter
NP_001260901.1 CG9003, isoform F [Drosophila melanogaster]	NP_001260901.1	0	GLOS_CG9003.1.:	GO:0006607	B	Refseq	NLS-bearing protein import into nucleus
NP_001260901.1 CG9003, isoform F [Drosophila melanogaster]	NP_001260901.1	0	GLOS_CG9003.1.:	GO:0008270	M	Refseq	zinc ion binding
NP_001260901.1 CG9003, isoform F [Drosophila melanogaster]	NP_001260901.1	0	GLOS_CG9003.1.:	GO:0032330	B	Refseq	regulation of chondrocyte differentiation
NP_001260901.1 CG9003, isoform F [Drosophila melanogaster]	NP_001260901.1	0	GLOS_CG9003.1.:	GO:0043565	M	Refseq	sequence-specific DNA binding
NP_001188535.1 moody, isoform C / ref|NP_001188536.1| moody, isoform D	NP_001188535.1	1,00E-178	GLOS_MOODY.1..	GO:0000122	B	Refseq	negative regulation of transcription from RNA polymerase II promoter
NP_001188535.1 moody, isoform C / ref|NP_001188536.1| moody, isoform D	NP_001188535.1	1,00E-178	GLOS_MOODY.1..	GO:0001501	B	Refseq	skeletal system development
NP_001188535.1 moody, isoform C / ref|NP_001188536.1| moody, isoform D	NP_001188535.1	1,00E-178	GLOS_MOODY.1..	GO:0003700	M	Refseq	sequence-specific DNA binding transcription factor activity
NP_001188535.1 moody, isoform C / ref|NP_001188536.1| moody, isoform D	NP_001188535.1	1,00E-178	GLOS_MOODY.1..	GO:0005515	M	Refseq	protein binding
NP_001188535.1 moody, isoform C / ref|NP_001188536.1| moody, isoform D	NP_001188535.1	1,00E-178	GLOS_MOODY.1..	GO:0005634	C	Refseq	nucleus
NP_001188535.1 moody, isoform C / ref|NP_001188536.1| moody, isoform D	NP_001188535.1	1,00E-178	GLOS_MOODY.1..	GO:0006366	B	Refseq	transcription from RNA polymerase II promoter
NP_001188535.1 moody, isoform C / ref|NP_001188536.1| moody, isoform D	NP_001188535.1	1,00E-178	GLOS_MOODY.1..	GO:0006607	B	Refseq	NLS-bearing protein import into nucleus
NP_001188535.1 moody, isoform C / ref|NP_001188536.1| moody, isoform D	NP_001188535.1	1,00E-178	GLOS_MOODY.1..	GO:0008270	M	Refseq	zinc ion binding
NP_001188535.1 moody, isoform C / ref|NP_001188536.1| moody, isoform D	NP_001188535.1	1,00E-178	GLOS_MOODY.1..	GO:0032330	B	Refseq	regulation of chondrocyte differentiation
NP_001188535.1 moody, isoform C / ref|NP_001188536.1| moody, isoform D	NP_001188535.1	1,00E-178	GLOS_MOODY.1..	GO:0043565	M	Refseq	sequence-specific DNA binding
XP_001983231.1 GH15701 [Drosophila grimshawi]	XP_001983231.1	6,00E-07	GLOS_DGRI_GH1	GO:0005261	M	.	cation channel activity
XP_001983231.1 GH15701 [Drosophila grimshawi]	XP_001983231.1	6,00E-07	GLOS_DGRI_GH1	GO:0005216	M	.	ion channel activity
XP_001983231.1 GH15701 [Drosophila grimshawi]	XP_001983231.1	6,00E-07	GLOS_DGRI_GH1	GO:0015075	M	.	ion transmembrane transporter activity
XP_001983231.1 GH15701 [Drosophila grimshawi]	XP_001983231.1	6,00E-07	GLOS_DGRI_GH1	GO:0022891	M	.	substrate-specific transmembrane transporter activity
XP_001983231.1 GH15701 [Drosophila grimshawi]	XP_001983231.1	6,00E-07	GLOS_DGRI_GH1	GO:0022857	M	.	transmembrane transporter activity
XP_001983231.1 GH15701 [Drosophila grimshawi]	XP_001983231.1	6,00E-07	GLOS_DGRI_GH1	GO:0005215	M	.	transporter activity
XP_001983231.1 GH15701 [Drosophila grimshawi]	XP_001983231.1	6,00E-07	GLOS_DGRI_GH1	GO:0022892	M	.	substrate-specific transporter activity
XP_001983231.1 GH15701 [Drosophila grimshawi]	XP_001983231.1	6,00E-07	GLOS_DGRI_GH1	GO:0022838	M	.	substrate-specific channel activity
XP_001983231.1 GH15701 [Drosophila grimshawi]	XP_001983231.1	6,00E-07	GLOS_DGRI_GH1	GO:0015267	M	.	channel activity
XP_001983231.1 GH15701 [Drosophila grimshawi]	XP_001983231.1	6,00E-07	GLOS_DGRI_GH1	GO:0022803	M	.	passive transmembrane transporter activity
XP_001983231.1 GH15701 [Drosophila grimshawi]	XP_001983231.1	6,00E-07	GLOS_DGRI_GH1	GO:0008324	M	.	cation transmembrane transporter activity
XP_001983231.1 GH15701 [Drosophila grimshawi]	XP_001983231.1	6,00E-07	GLOS_DGRI_GH1	GO:0015085	M	.	calcium ion transmembrane transporter activity
XP_001983231.1 GH15701 [Drosophila grimshawi]	XP_001983231.1	6,00E-07	GLOS_DGRI_GH1	GO:0046873	M	.	metal ion transmembrane transporter activity
XP_001983231.1 GH15701 [Drosophila grimshawi]	XP_001983231.1	6,00E-07	GLOS_DGRI_GH1	GO:0022890	M	.	inorganic cation transmembrane transporter activity
XP_001983231.1 GH15701 [Drosophila grimshawi]	XP_001983231.1	6,00E-07	GLOS_DGRI_GH1	GO:0072509	M	.	divalent inorganic cation transmembrane transporter activity
XP_001983231.1 GH15701 [Drosophila grimshawi]	XP_001983231.1	6,00E-07	GLOS_DGRI_GH1	GO:0005262	M	Refseq	calcium channel activity
XP_001983231.1 GH15701 [Drosophila grimshawi]	XP_001983231.1	6,00E-07	GLOS_DGRI_GH1	GO:0005515	M	Refseq	protein binding
XP_001983231.1 GH15701 [Drosophila grimshawi]	XP_001983231.1	6,00E-07	GLOS_DGRI_GH1	GO:0016020	C	.	membrane

XP_001983231.1 GH15701 [Drosophila grimshawi]	XP_001983231.1	6,00E-07	GLOS_DGRI_GH1	GO:0071944	C	.	cell periphery
XP_001983231.1 GH15701 [Drosophila grimshawi]	XP_001983231.1	6,00E-07	GLOS_DGRI_GH1	GO:0005886	C	Refseq	plasma membrane
XP_001983231.1 GH15701 [Drosophila grimshawi]	XP_001983231.1	6,00E-07	GLOS_DGRI_GH1	GO:0016021	C	.	integral to membrane
XP_001983231.1 GH15701 [Drosophila grimshawi]	XP_001983231.1	6,00E-07	GLOS_DGRI_GH1	GO:0031224	C	.	intrinsic to membrane
XP_001983231.1 GH15701 [Drosophila grimshawi]	XP_001983231.1	6,00E-07	GLOS_DGRI_GH1	GO:0044425	C	.	membrane part
XP_001983231.1 GH15701 [Drosophila grimshawi]	XP_001983231.1	6,00E-07	GLOS_DGRI_GH1	GO:0031226	C	.	intrinsic to plasma membrane
XP_001983231.1 GH15701 [Drosophila grimshawi]	XP_001983231.1	6,00E-07	GLOS_DGRI_GH1	GO:0044459	C	.	plasma membrane part
XP_001983231.1 GH15701 [Drosophila grimshawi]	XP_001983231.1	6,00E-07	GLOS_DGRI_GH1	GO:0005886	C	.	plasma membrane
XP_001983231.1 GH15701 [Drosophila grimshawi]	XP_001983231.1	6,00E-07	GLOS_DGRI_GH1	GO:0005887	C	Refseq	integral to plasma membrane
XP_001983231.1 GH15701 [Drosophila grimshawi]	XP_001983231.1	6,00E-07	GLOS_DGRI_GH1	GO:0070838	B	.	divalent metal ion transport
XP_001983231.1 GH15701 [Drosophila grimshawi]	XP_001983231.1	6,00E-07	GLOS_DGRI_GH1	GO:0030001	B	.	metal ion transport
XP_001983231.1 GH15701 [Drosophila grimshawi]	XP_001983231.1	6,00E-07	GLOS_DGRI_GH1	GO:0006812	B	.	cation transport
XP_001983231.1 GH15701 [Drosophila grimshawi]	XP_001983231.1	6,00E-07	GLOS_DGRI_GH1	GO:0006811	B	.	ion transport
XP_001983231.1 GH15701 [Drosophila grimshawi]	XP_001983231.1	6,00E-07	GLOS_DGRI_GH1	GO:0072511	B	.	divalent inorganic cation transport
XP_001983231.1 GH15701 [Drosophila grimshawi]	XP_001983231.1	6,00E-07	GLOS_DGRI_GH1	GO:0006816	B	Refseq	calcium ion transport
XP_001983231.1 GH15701 [Drosophila grimshawi]	XP_001983231.1	6,00E-07	GLOS_DGRI_GH1	GO:0097485	B	.	neuron projection guidance
XP_001983231.1 GH15701 [Drosophila grimshawi]	XP_001983231.1	6,00E-07	GLOS_DGRI_GH1	GO:0006928	B	.	cellular component movement
XP_001983231.1 GH15701 [Drosophila grimshawi]	XP_001983231.1	6,00E-07	GLOS_DGRI_GH1	GO:0006935	B	.	chemotaxis
XP_001983231.1 GH15701 [Drosophila grimshawi]	XP_001983231.1	6,00E-07	GLOS_DGRI_GH1	GO:0042221	B	.	response to chemical stimulus
XP_001983231.1 GH15701 [Drosophila grimshawi]	XP_001983231.1	6,00E-07	GLOS_DGRI_GH1	GO:0050896	B	.	response to stimulus
XP_001983231.1 GH15701 [Drosophila grimshawi]	XP_001983231.1	6,00E-07	GLOS_DGRI_GH1	GO:0042330	B	.	taxis
XP_001983231.1 GH15701 [Drosophila grimshawi]	XP_001983231.1	6,00E-07	GLOS_DGRI_GH1	GO:0009605	B	.	response to external stimulus
XP_001983231.1 GH15701 [Drosophila grimshawi]	XP_001983231.1	6,00E-07	GLOS_DGRI_GH1	GO:0040011	B	.	locomotion
XP_001983231.1 GH15701 [Drosophila grimshawi]	XP_001983231.1	6,00E-07	GLOS_DGRI_GH1	GO:0048812	B	.	neuron projection morphogenesis
XP_001983231.1 GH15701 [Drosophila grimshawi]	XP_001983231.1	6,00E-07	GLOS_DGRI_GH1	GO:0048858	B	.	cell projection morphogenesis
XP_001983231.1 GH15701 [Drosophila grimshawi]	XP_001983231.1	6,00E-07	GLOS_DGRI_GH1	GO:0030030	B	.	cell projection organization
XP_001983231.1 GH15701 [Drosophila grimshawi]	XP_001983231.1	6,00E-07	GLOS_DGRI_GH1	GO:0016043	B	.	cellular component organization
XP_001983231.1 GH15701 [Drosophila grimshawi]	XP_001983231.1	6,00E-07	GLOS_DGRI_GH1	GO:0071840	B	.	cellular component organization or biogenesis
XP_001983231.1 GH15701 [Drosophila grimshawi]	XP_001983231.1	6,00E-07	GLOS_DGRI_GH1	GO:0032990	B	.	cell part morphogenesis
XP_001983231.1 GH15701 [Drosophila grimshawi]	XP_001983231.1	6,00E-07	GLOS_DGRI_GH1	GO:0032989	B	.	cellular component morphogenesis
XP_001983231.1 GH15701 [Drosophila grimshawi]	XP_001983231.1	6,00E-07	GLOS_DGRI_GH1	GO:0009653	B	.	anatomical structure morphogenesis
XP_001983231.1 GH15701 [Drosophila grimshawi]	XP_001983231.1	6,00E-07	GLOS_DGRI_GH1	GO:0044767	B	.	single-organism developmental process
XP_001983231.1 GH15701 [Drosophila grimshawi]	XP_001983231.1	6,00E-07	GLOS_DGRI_GH1	GO:0048869	B	.	cellular developmental process
XP_001983231.1 GH15701 [Drosophila grimshawi]	XP_001983231.1	6,00E-07	GLOS_DGRI_GH1	GO:0000902	B	.	cell morphogenesis
XP_001983231.1 GH15701 [Drosophila grimshawi]	XP_001983231.1	6,00E-07	GLOS_DGRI_GH1	GO:0031175	B	.	neuron projection development
XP_001983231.1 GH15701 [Drosophila grimshawi]	XP_001983231.1	6,00E-07	GLOS_DGRI_GH1	GO:0048666	B	.	neuron development
XP_001983231.1 GH15701 [Drosophila grimshawi]	XP_001983231.1	6,00E-07	GLOS_DGRI_GH1	GO:0048468	B	.	cell development
XP_001983231.1 GH15701 [Drosophila grimshawi]	XP_001983231.1	6,00E-07	GLOS_DGRI_GH1	GO:0030154	B	.	cell differentiation
XP_001983231.1 GH15701 [Drosophila grimshawi]	XP_001983231.1	6,00E-07	GLOS_DGRI_GH1	GO:0030182	B	.	neuron differentiation
XP_001983231.1 GH15701 [Drosophila grimshawi]	XP_001983231.1	6,00E-07	GLOS_DGRI_GH1	GO:0048699	B	.	generation of neurons
XP_001983231.1 GH15701 [Drosophila grimshawi]	XP_001983231.1	6,00E-07	GLOS_DGRI_GH1	GO:0022008	B	.	neurogenesis
XP_001983231.1 GH15701 [Drosophila grimshawi]	XP_001983231.1	6,00E-07	GLOS_DGRI_GH1	GO:0007399	B	.	nervous system development
XP_001983231.1 GH15701 [Drosophila grimshawi]	XP_001983231.1	6,00E-07	GLOS_DGRI_GH1	GO:0007409	B	.	axonogenesis
XP_001983231.1 GH15701 [Drosophila grimshawi]	XP_001983231.1	6,00E-07	GLOS_DGRI_GH1	GO:0048667	B	.	cell morphogenesis involved in neuron differentiation
XP_001983231.1 GH15701 [Drosophila grimshawi]	XP_001983231.1	6,00E-07	GLOS_DGRI_GH1	GO:0000904	B	.	cell morphogenesis involved in differentiation
XP_001983231.1 GH15701 [Drosophila grimshawi]	XP_001983231.1	6,00E-07	GLOS_DGRI_GH1	GO:0061564	B	.	axon development
XP_001983231.1 GH15701 [Drosophila grimshawi]	XP_001983231.1	6,00E-07	GLOS_DGRI_GH1	GO:0007411	B	Refseq	axon guidance
XP_001983231.1 GH15701 [Drosophila grimshawi]	XP_001983231.1	6,00E-07	GLOS_DGRI_GH1	GO:0007599	B	.	hemostasis
XP_001983231.1 GH15701 [Drosophila grimshawi]	XP_001983231.1	6,00E-07	GLOS_DGRI_GH1	GO:0050878	B	.	regulation of body fluid levels
XP_001983231.1 GH15701 [Drosophila grimshawi]	XP_001983231.1	6,00E-07	GLOS_DGRI_GH1	GO:0065008	B	.	regulation of biological quality
XP_001983231.1 GH15701 [Drosophila grimshawi]	XP_001983231.1	6,00E-07	GLOS_DGRI_GH1	GO:0050817	B	.	coagulation
XP_001983231.1 GH15701 [Drosophila grimshawi]	XP_001983231.1	6,00E-07	GLOS_DGRI_GH1	GO:0042060	B	.	wound healing
XP_001983231.1 GH15701 [Drosophila grimshawi]	XP_001983231.1	6,00E-07	GLOS_DGRI_GH1	GO:0009611	B	.	response to wounding
XP_001983231.1 GH15701 [Drosophila grimshawi]	XP_001983231.1	6,00E-07	GLOS_DGRI_GH1	GO:0006950	B	.	response to stress

XP_001983231.1 GH15701 [Drosophila grimshawi]	XP_001983231.1	6,00E-07	GLOS_DGRI_GH1	GO:0007596	B	Refseq	blood coagulation
XP_001983231.1 GH15701 [Drosophila grimshawi]	XP_001983231.1	6,00E-07	GLOS_DGRI_GH1	GO:0007165	B	.	signal transduction
XP_001983231.1 GH15701 [Drosophila grimshawi]	XP_001983231.1	6,00E-07	GLOS_DGRI_GH1	GO:0051716	B	.	cellular response to stimulus
XP_001983231.1 GH15701 [Drosophila grimshawi]	XP_001983231.1	6,00E-07	GLOS_DGRI_GH1	GO:0007154	B	.	cell communication
XP_001983231.1 GH15701 [Drosophila grimshawi]	XP_001983231.1	6,00E-07	GLOS_DGRI_GH1	GO:0044700	B	.	single organism signaling
XP_001983231.1 GH15701 [Drosophila grimshawi]	XP_001983231.1	6,00E-07	GLOS_DGRI_GH1	GO:0023052	B	.	signaling
XP_001983231.1 GH15701 [Drosophila grimshawi]	XP_001983231.1	6,00E-07	GLOS_DGRI_GH1	GO:0009583	B	.	detection of light stimulus
XP_001983231.1 GH15701 [Drosophila grimshawi]	XP_001983231.1	6,00E-07	GLOS_DGRI_GH1	GO:0009416	B	.	response to light stimulus
XP_001983231.1 GH15701 [Drosophila grimshawi]	XP_001983231.1	6,00E-07	GLOS_DGRI_GH1	GO:0009314	B	.	response to radiation
XP_001983231.1 GH15701 [Drosophila grimshawi]	XP_001983231.1	6,00E-07	GLOS_DGRI_GH1	GO:0009628	B	.	response to abiotic stimulus
XP_001983231.1 GH15701 [Drosophila grimshawi]	XP_001983231.1	6,00E-07	GLOS_DGRI_GH1	GO:0009581	B	.	detection of external stimulus
XP_001983231.1 GH15701 [Drosophila grimshawi]	XP_001983231.1	6,00E-07	GLOS_DGRI_GH1	GO:0051606	B	.	detection of stimulus
XP_001983231.1 GH15701 [Drosophila grimshawi]	XP_001983231.1	6,00E-07	GLOS_DGRI_GH1	GO:0009582	B	.	detection of abiotic stimulus
XP_001983231.1 GH15701 [Drosophila grimshawi]	XP_001983231.1	6,00E-07	GLOS_DGRI_GH1	GO:0007602	B	Refseq	phototransduction
XP_001983231.1 GH15701 [Drosophila grimshawi]	XP_001983231.1	6,00E-07	GLOS_DGRI_GH1	GO:0010522	B	.	regulation of calcium ion transport into cytosol
XP_001983231.1 GH15701 [Drosophila grimshawi]	XP_001983231.1	6,00E-07	GLOS_DGRI_GH1	GO:0032386	B	.	regulation of intracellular transport
XP_001983231.1 GH15701 [Drosophila grimshawi]	XP_001983231.1	6,00E-07	GLOS_DGRI_GH1	GO:0051049	B	.	regulation of transport
XP_001983231.1 GH15701 [Drosophila grimshawi]	XP_001983231.1	6,00E-07	GLOS_DGRI_GH1	GO:0032879	B	.	regulation of localization
XP_001983231.1 GH15701 [Drosophila grimshawi]	XP_001983231.1	6,00E-07	GLOS_DGRI_GH1	GO:0060341	B	.	regulation of cellular localization
XP_001983231.1 GH15701 [Drosophila grimshawi]	XP_001983231.1	6,00E-07	GLOS_DGRI_GH1	GO:0051924	B	.	regulation of calcium ion transport
XP_001983231.1 GH15701 [Drosophila grimshawi]	XP_001983231.1	6,00E-07	GLOS_DGRI_GH1	GO:0010959	B	.	regulation of metal ion transport
XP_001983231.1 GH15701 [Drosophila grimshawi]	XP_001983231.1	6,00E-07	GLOS_DGRI_GH1	GO:0043269	B	.	regulation of ion transport
XP_001983231.1 GH15701 [Drosophila grimshawi]	XP_001983231.1	6,00E-07	GLOS_DGRI_GH1	GO:2000021	B	.	regulation of ion homeostasis
XP_001983231.1 GH15701 [Drosophila grimshawi]	XP_001983231.1	6,00E-07	GLOS_DGRI_GH1	GO:0032844	B	.	regulation of homeostatic process
XP_001983231.1 GH15701 [Drosophila grimshawi]	XP_001983231.1	6,00E-07	GLOS_DGRI_GH1	GO:0032388	B	.	positive regulation of intracellular transport
XP_001983231.1 GH15701 [Drosophila grimshawi]	XP_001983231.1	6,00E-07	GLOS_DGRI_GH1	GO:0051050	B	.	positive regulation of transport
XP_001983231.1 GH15701 [Drosophila grimshawi]	XP_001983231.1	6,00E-07	GLOS_DGRI_GH1	GO:0048518	B	.	positive regulation of biological process
XP_001983231.1 GH15701 [Drosophila grimshawi]	XP_001983231.1	6,00E-07	GLOS_DGRI_GH1	GO:0032846	B	.	positive regulation of homeostatic process
XP_001983231.1 GH15701 [Drosophila grimshawi]	XP_001983231.1	6,00E-07	GLOS_DGRI_GH1	GO:0048522	B	.	positive regulation of cellular process
XP_001983231.1 GH15701 [Drosophila grimshawi]	XP_001983231.1	6,00E-07	GLOS_DGRI_GH1	GO:0051928	B	.	positive regulation of calcium ion transport
XP_001983231.1 GH15701 [Drosophila grimshawi]	XP_001983231.1	6,00E-07	GLOS_DGRI_GH1	GO:0043270	B	.	positive regulation of ion transport
XP_001983231.1 GH15701 [Drosophila grimshawi]	XP_001983231.1	6,00E-07	GLOS_DGRI_GH1	GO:0010524	B	Refseq	positive regulation of calcium ion transport into cytosol
XP_001983231.1 GH15701 [Drosophila grimshawi]	XP_001983231.1	6,00E-07	GLOS_DGRI_GH1	GO:0005262	M	.	calcium channel activity
XP_001983231.1 GH15701 [Drosophila grimshawi]	XP_001983231.1	6,00E-07	GLOS_DGRI_GH1	GO:0015276	M	.	ligand-gated ion channel activity
XP_001983231.1 GH15701 [Drosophila grimshawi]	XP_001983231.1	6,00E-07	GLOS_DGRI_GH1	GO:0022834	M	.	ligand-gated channel activity
XP_001983231.1 GH15701 [Drosophila grimshawi]	XP_001983231.1	6,00E-07	GLOS_DGRI_GH1	GO:0022836	M	.	gated channel activity
XP_001983231.1 GH15701 [Drosophila grimshawi]	XP_001983231.1	6,00E-07	GLOS_DGRI_GH1	GO:0022839	M	.	ion gated channel activity
XP_001983231.1 GH15701 [Drosophila grimshawi]	XP_001983231.1	6,00E-07	GLOS_DGRI_GH1	GO:0015279	M	Refseq	store-operated calcium channel activity
XP_001983231.1 GH15701 [Drosophila grimshawi]	XP_001983231.1	6,00E-07	GLOS_DGRI_GH1	GO:0001775	B	.	cell activation
XP_001983231.1 GH15701 [Drosophila grimshawi]	XP_001983231.1	6,00E-07	GLOS_DGRI_GH1	GO:0007596	B	.	blood coagulation
XP_001983231.1 GH15701 [Drosophila grimshawi]	XP_001983231.1	6,00E-07	GLOS_DGRI_GH1	GO:0030168	B	Refseq	platelet activation
XP_001983231.1 GH15701 [Drosophila grimshawi]	XP_001983231.1	6,00E-07	GLOS_DGRI_GH1	GO:0014074	B	.	response to purine-containing compound
XP_001983231.1 GH15701 [Drosophila grimshawi]	XP_001983231.1	6,00E-07	GLOS_DGRI_GH1	GO:0010243	B	.	response to organonitrogen compound
XP_001983231.1 GH15701 [Drosophila grimshawi]	XP_001983231.1	6,00E-07	GLOS_DGRI_GH1	GO:0009719	B	.	response to endogenous stimulus
XP_001983231.1 GH15701 [Drosophila grimshawi]	XP_001983231.1	6,00E-07	GLOS_DGRI_GH1	GO:0010033	B	.	response to organic substance
XP_001983231.1 GH15701 [Drosophila grimshawi]	XP_001983231.1	6,00E-07	GLOS_DGRI_GH1	GO:1901698	B	.	response to nitrogen compound
XP_001983231.1 GH15701 [Drosophila grimshawi]	XP_001983231.1	6,00E-07	GLOS_DGRI_GH1	GO:0014070	B	.	response to organic cyclic compound
XP_001983231.1 GH15701 [Drosophila grimshawi]	XP_001983231.1	6,00E-07	GLOS_DGRI_GH1	GO:0046683	B	.	response to organophosphorus
XP_001983231.1 GH15701 [Drosophila grimshawi]	XP_001983231.1	6,00E-07	GLOS_DGRI_GH1	GO:1901700	B	.	response to oxygen-containing compound
XP_001983231.1 GH15701 [Drosophila grimshawi]	XP_001983231.1	6,00E-07	GLOS_DGRI_GH1	GO:0033198	B	Refseq	response to ATP
XP_001983231.1 GH15701 [Drosophila grimshawi]	XP_001983231.1	6,00E-07	GLOS_DGRI_GH1	GO:0010038	B	.	response to metal ion
XP_001983231.1 GH15701 [Drosophila grimshawi]	XP_001983231.1	6,00E-07	GLOS_DGRI_GH1	GO:0010035	B	.	response to inorganic substance
XP_001983231.1 GH15701 [Drosophila grimshawi]	XP_001983231.1	6,00E-07	GLOS_DGRI_GH1	GO:0051592	B	Refseq	response to calcium ion
XP_001983231.1 GH15701 [Drosophila grimshawi]	XP_001983231.1	6,00E-07	GLOS_DGRI_GH1	GO:0006816	B	.	calcium ion transport

XP_001983231.1 GH15701 [Drosophila grimshawi]	XP_001983231.1	6,00E-07	GLOS_DGRI_GH1	GO:0034220	B	.	ion transmembrane transport
XP_001983231.1 GH15701 [Drosophila grimshawi]	XP_001983231.1	6,00E-07	GLOS_DGRI_GH1	GO:0055085	B	.	transmembrane transport
XP_001983231.1 GH15701 [Drosophila grimshawi]	XP_001983231.1	6,00E-07	GLOS_DGRI_GH1	GO:0070588	B	Refseq	calcium ion transmembrane transport
XP_001983231.1 GH15701 [Drosophila grimshawi]	XP_001983231.1	6,00E-07	GLOS_DGRI_GH1	GO:0043168	M	.	anion binding
XP_001983231.1 GH15701 [Drosophila grimshawi]	XP_001983231.1	6,00E-07	GLOS_DGRI_GH1	GO:0043178	M	.	alcohol binding
XP_001983231.1 GH15701 [Drosophila grimshawi]	XP_001983231.1	6,00E-07	GLOS_DGRI_GH1	GO:0036094	M	.	small molecule binding
XP_001983231.1 GH15701 [Drosophila grimshawi]	XP_001983231.1	6,00E-07	GLOS_DGRI_GH1	GO:0070679	M	Refseq	inositol 1,4,5 trisphosphate binding
XP_003245892.1 PREDICTED: hypoth protein LOC100575767 [A. pisum]	XP_003245892.1	5,00E-16	GLOS_LOC10057!	GO:0007049	B	.	cell cycle
XP_003245892.1 PREDICTED: hypoth protein LOC100575767 [A. pisum]	XP_003245892.1	5,00E-16	GLOS_LOC10057!	GO:0000278	B	Refseq	mitotic cell cycle
XP_003245892.1 PREDICTED: hypoth protein LOC100575767 [A. pisum]	XP_003245892.1	5,00E-16	GLOS_LOC10057!	GO:0003677	M	Refseq	DNA binding
XP_003245892.1 PREDICTED: hypoth protein LOC100575767 [A. pisum]	XP_003245892.1	5,00E-16	GLOS_LOC10057!	GO:0003700	M	Refseq	sequence-specific DNA binding transcription factor activity
XP_003245892.1 PREDICTED: hypoth protein LOC100575767 [A. pisum]	XP_003245892.1	5,00E-16	GLOS_LOC10057!	GO:0000989	M	.	transcription factor binding transcription factor activity
XP_003245892.1 PREDICTED: hypoth protein LOC100575767 [A. pisum]	XP_003245892.1	5,00E-16	GLOS_LOC10057!	GO:0000988	M	.	protein binding transcription factor activity
XP_003245892.1 PREDICTED: hypoth protein LOC100575767 [A. pisum]	XP_003245892.1	5,00E-16	GLOS_LOC10057!	GO:0003712	M	Refseq	transcription cofactor activity
XP_003245892.1 PREDICTED: hypoth protein LOC100575767 [A. pisum]	XP_003245892.1	5,00E-16	GLOS_LOC10057!	GO:0044428	C	.	nuclear part
XP_003245892.1 PREDICTED: hypoth protein LOC100575767 [A. pisum]	XP_003245892.1	5,00E-16	GLOS_LOC10057!	GO:0044446	C	.	intracellular organelle part
XP_003245892.1 PREDICTED: hypoth protein LOC100575767 [A. pisum]	XP_003245892.1	5,00E-16	GLOS_LOC10057!	GO:0044422	C	.	organelle part
XP_003245892.1 PREDICTED: hypoth protein LOC100575767 [A. pisum]	XP_003245892.1	5,00E-16	GLOS_LOC10057!	GO:0005634	C	.	nucleus
XP_003245892.1 PREDICTED: hypoth protein LOC100575767 [A. pisum]	XP_003245892.1	5,00E-16	GLOS_LOC10057!	GO:0031981	C	.	nuclear lumen
XP_003245892.1 PREDICTED: hypoth protein LOC100575767 [A. pisum]	XP_003245892.1	5,00E-16	GLOS_LOC10057!	GO:0070013	C	.	intracellular organelle lumen
XP_003245892.1 PREDICTED: hypoth protein LOC100575767 [A. pisum]	XP_003245892.1	5,00E-16	GLOS_LOC10057!	GO:0043233	C	.	organelle lumen
XP_003245892.1 PREDICTED: hypoth protein LOC100575767 [A. pisum]	XP_003245892.1	5,00E-16	GLOS_LOC10057!	GO:0031974	C	.	membrane-enclosed lumen
XP_003245892.1 PREDICTED: hypoth protein LOC100575767 [A. pisum]	XP_003245892.1	5,00E-16	GLOS_LOC10057!	GO:0005654	C	Refseq	nucleoplasm
XP_003245892.1 PREDICTED: hypoth protein LOC100575767 [A. pisum]	XP_003245892.1	5,00E-16	GLOS_LOC10057!	GO:0043234	C	.	protein complex
XP_003245892.1 PREDICTED: hypoth protein LOC100575767 [A. pisum]	XP_003245892.1	5,00E-16	GLOS_LOC10057!	GO:0032991	C	.	macromolecular complex
XP_003245892.1 PREDICTED: hypoth protein LOC100575767 [A. pisum]	XP_003245892.1	5,00E-16	GLOS_LOC10057!	GO:0005667	C	Refseq	transcription factor complex
XP_003245892.1 PREDICTED: hypoth protein LOC100575767 [A. pisum]	XP_003245892.1	5,00E-16	GLOS_LOC10057!	GO:0006351	B	Refseq	transcription, DNA-dependent
XP_003245892.1 PREDICTED: hypoth protein LOC100575767 [A. pisum]	XP_003245892.1	5,00E-16	GLOS_LOC10057!	GO:0006352	B	.	DNA-dependent transcription, initiation
XP_003245892.1 PREDICTED: hypoth protein LOC100575767 [A. pisum]	XP_003245892.1	5,00E-16	GLOS_LOC10057!	GO:0006366	B	.	transcription from RNA polymerase II promoter
XP_003245892.1 PREDICTED: hypoth protein LOC100575767 [A. pisum]	XP_003245892.1	5,00E-16	GLOS_LOC10057!	GO:0006367	B	Refseq	transcription initiation from RNA polymerase II promoter
XP_003245892.1 PREDICTED: hypoth protein LOC100575767 [A. pisum]	XP_003245892.1	5,00E-16	GLOS_LOC10057!	GO:0007178	B	.	transmembrane receptor protein serine/threonine kinase signaling pathway
XP_003245892.1 PREDICTED: hypoth protein LOC100575767 [A. pisum]	XP_003245892.1	5,00E-16	GLOS_LOC10057!	GO:0007167	B	.	enzyme linked receptor protein signaling pathway
XP_003245892.1 PREDICTED: hypoth protein LOC100575767 [A. pisum]	XP_003245892.1	5,00E-16	GLOS_LOC10057!	GO:0007166	B	.	cell surface receptor signaling pathway
XP_003245892.1 PREDICTED: hypoth protein LOC100575767 [A. pisum]	XP_003245892.1	5,00E-16	GLOS_LOC10057!	GO:0071560	B	.	cellular response to transforming growth factor beta stimulus
XP_003245892.1 PREDICTED: hypoth protein LOC100575767 [A. pisum]	XP_003245892.1	5,00E-16	GLOS_LOC10057!	GO:0071363	B	.	cellular response to growth factor stimulus
XP_003245892.1 PREDICTED: hypoth protein LOC100575767 [A. pisum]	XP_003245892.1	5,00E-16	GLOS_LOC10057!	GO:0070848	B	.	response to growth factor stimulus
XP_003245892.1 PREDICTED: hypoth protein LOC100575767 [A. pisum]	XP_003245892.1	5,00E-16	GLOS_LOC10057!	GO:0071310	B	.	cellular response to organic substance
XP_003245892.1 PREDICTED: hypoth protein LOC100575767 [A. pisum]	XP_003245892.1	5,00E-16	GLOS_LOC10057!	GO:0070887	B	.	cellular response to chemical stimulus
XP_003245892.1 PREDICTED: hypoth protein LOC100575767 [A. pisum]	XP_003245892.1	5,00E-16	GLOS_LOC10057!	GO:0071495	B	.	cellular response to endogenous stimulus
XP_003245892.1 PREDICTED: hypoth protein LOC100575767 [A. pisum]	XP_003245892.1	5,00E-16	GLOS_LOC10057!	GO:0071559	B	.	response to transforming growth factor beta stimulus
XP_003245892.1 PREDICTED: hypoth protein LOC100575767 [A. pisum]	XP_003245892.1	5,00E-16	GLOS_LOC10057!	GO:0007179	B	Refseq	transforming growth factor beta receptor signaling pathway
XP_003245892.1 PREDICTED: hypoth protein LOC100575767 [A. pisum]	XP_003245892.1	5,00E-16	GLOS_LOC10057!	GO:0048513	B	.	organ development
XP_003245892.1 PREDICTED: hypoth protein LOC100575767 [A. pisum]	XP_003245892.1	5,00E-16	GLOS_LOC10057!	GO:0072358	B	.	cardiovascular system development
XP_003245892.1 PREDICTED: hypoth protein LOC100575767 [A. pisum]	XP_003245892.1	5,00E-16	GLOS_LOC10057!	GO:0072359	B	.	circulatory system development
XP_003245892.1 PREDICTED: hypoth protein LOC100575767 [A. pisum]	XP_003245892.1	5,00E-16	GLOS_LOC10057!	GO:0007507	B	Refseq	heart development
XP_003245892.1 PREDICTED: hypoth protein LOC100575767 [A. pisum]	XP_003245892.1	5,00E-16	GLOS_LOC10057!	GO:0005515	M	.	protein binding
XP_003245892.1 PREDICTED: hypoth protein LOC100575767 [A. pisum]	XP_003245892.1	5,00E-16	GLOS_LOC10057!	GO:0008134	M	Refseq	transcription factor binding
XP_003245892.1 PREDICTED: hypoth protein LOC100575767 [A. pisum]	XP_003245892.1	5,00E-16	GLOS_LOC10057!	GO:0010467	B	Refseq	gene expression
XP_003245892.1 PREDICTED: hypoth protein LOC100575767 [A. pisum]	XP_003245892.1	5,00E-16	GLOS_LOC10057!	GO:0019904	M	Refseq	protein domain specific binding
XP_003245892.1 PREDICTED: hypoth protein LOC100575767 [A. pisum]	XP_003245892.1	5,00E-16	GLOS_LOC10057!	GO:0045893	B	.	positive regulation of transcription, DNA-dependent
XP_003245892.1 PREDICTED: hypoth protein LOC100575767 [A. pisum]	XP_003245892.1	5,00E-16	GLOS_LOC10057!	GO:0010557	B	.	positive regulation of macromolecule biosynthetic process
XP_003245892.1 PREDICTED: hypoth protein LOC100575767 [A. pisum]	XP_003245892.1	5,00E-16	GLOS_LOC10057!	GO:0009891	B	.	positive regulation of biosynthetic process
XP_003245892.1 PREDICTED: hypoth protein LOC100575767 [A. pisum]	XP_003245892.1	5,00E-16	GLOS_LOC10057!	GO:0009893	B	.	positive regulation of metabolic process
XP_003245892.1 PREDICTED: hypoth protein LOC100575767 [A. pisum]	XP_003245892.1	5,00E-16	GLOS_LOC10057!	GO:0010604	B	.	positive regulation of macromolecule metabolic process

XP_003245892.1 PREDICTED: hypoth protein LOC100575767 [A. pisum]	XP_003245892.1	5,00E-16	GLOS_LOC10057!	GO:0010628	B	.	positive regulation of gene expression
XP_003245892.1 PREDICTED: hypoth protein LOC100575767 [A. pisum]	XP_003245892.1	5,00E-16	GLOS_LOC10057!	GO:0031328	B	.	positive regulation of cellular biosynthetic process
XP_003245892.1 PREDICTED: hypoth protein LOC100575767 [A. pisum]	XP_003245892.1	5,00E-16	GLOS_LOC10057!	GO:0031325	B	.	positive regulation of cellular metabolic process
XP_003245892.1 PREDICTED: hypoth protein LOC100575767 [A. pisum]	XP_003245892.1	5,00E-16	GLOS_LOC10057!	GO:0051254	B	.	positive regulation of RNA metabolic process
XP_003245892.1 PREDICTED: hypoth protein LOC100575767 [A. pisum]	XP_003245892.1	5,00E-16	GLOS_LOC10057!	GO:0045935	B	.	positive regulation of nucleobase-containing compound metabolic process
XP_003245892.1 PREDICTED: hypoth protein LOC100575767 [A. pisum]	XP_003245892.1	5,00E-16	GLOS_LOC10057!	GO:0051173	B	.	positive regulation of nitrogen compound metabolic process
XP_003245892.1 PREDICTED: hypoth protein LOC100575767 [A. pisum]	XP_003245892.1	5,00E-16	GLOS_LOC10057!	GO:0045944	B	Refseq	positive regulation of transcription from RNA polymerase II promoter
XP_001985069.1 GH16853 [Drosophila grimshawi]	XP_001985069.1	0	GLOS_DGRI_GH1	GO:0005262	M	Refseq	calcium channel activity
XP_001985069.1 GH16853 [Drosophila grimshawi]	XP_001985069.1	0	GLOS_DGRI_GH1	GO:0005515	M	Refseq	protein binding
XP_001985069.1 GH16853 [Drosophila grimshawi]	XP_001985069.1	0	GLOS_DGRI_GH1	GO:0005886	C	Refseq	plasma membrane
XP_001985069.1 GH16853 [Drosophila grimshawi]	XP_001985069.1	0	GLOS_DGRI_GH1	GO:0005887	C	Refseq	integral to plasma membrane
XP_001985069.1 GH16853 [Drosophila grimshawi]	XP_001985069.1	0	GLOS_DGRI_GH1	GO:0006816	B	Refseq	calcium ion transport
XP_001985069.1 GH16853 [Drosophila grimshawi]	XP_001985069.1	0	GLOS_DGRI_GH1	GO:0007411	B	Refseq	axon guidance
XP_001985069.1 GH16853 [Drosophila grimshawi]	XP_001985069.1	0	GLOS_DGRI_GH1	GO:0007596	B	Refseq	blood coagulation
XP_001985069.1 GH16853 [Drosophila grimshawi]	XP_001985069.1	0	GLOS_DGRI_GH1	GO:0007602	B	Refseq	phototransduction
XP_001985069.1 GH16853 [Drosophila grimshawi]	XP_001985069.1	0	GLOS_DGRI_GH1	GO:0010524	B	Refseq	positive regulation of calcium ion transport into cytosol
XP_001985069.1 GH16853 [Drosophila grimshawi]	XP_001985069.1	0	GLOS_DGRI_GH1	GO:0015279	M	Refseq	store-operated calcium channel activity
XP_001985069.1 GH16853 [Drosophila grimshawi]	XP_001985069.1	0	GLOS_DGRI_GH1	GO:0030168	B	Refseq	platelet activation
XP_001985069.1 GH16853 [Drosophila grimshawi]	XP_001985069.1	0	GLOS_DGRI_GH1	GO:0033198	B	Refseq	response to ATP
XP_001985069.1 GH16853 [Drosophila grimshawi]	XP_001985069.1	0	GLOS_DGRI_GH1	GO:0051592	B	Refseq	response to calcium ion
XP_001985069.1 GH16853 [Drosophila grimshawi]	XP_001985069.1	0	GLOS_DGRI_GH1	GO:0070588	B	Refseq	calcium ion transmembrane transport
XP_001985069.1 GH16853 [Drosophila grimshawi]	XP_001985069.1	0	GLOS_DGRI_GH1	GO:0070679	M	Refseq	inositol 1,4,5 trisphosphate binding
XP_003090483.1 hypothetical protein CRE_20617 [C. remanei]	XP_003090483.1	4,00E-17	GLOS_CRE_2061	GO:0004888	M	.	transmembrane signaling receptor activity
XP_003090483.1 hypothetical protein CRE_20617 [C. remanei]	XP_003090483.1	4,00E-17	GLOS_CRE_2061	GO:0038023	M	.	signaling receptor activity
XP_003090483.1 hypothetical protein CRE_20617 [C. remanei]	XP_003090483.1	4,00E-17	GLOS_CRE_2061	GO:0004871	M	.	signal transducer activity
XP_003090483.1 hypothetical protein CRE_20617 [C. remanei]	XP_003090483.1	4,00E-17	GLOS_CRE_2061	GO:0060089	M	.	molecular transducer activity
XP_003090483.1 hypothetical protein CRE_20617 [C. remanei]	XP_003090483.1	4,00E-17	GLOS_CRE_2061	GO:0004872	M	.	receptor activity
XP_003090483.1 hypothetical protein CRE_20617 [C. remanei]	XP_003090483.1	4,00E-17	GLOS_CRE_2061	GO:0004930	M	Refseq	G-protein coupled receptor activity
XP_003090483.1 hypothetical protein CRE_20617 [C. remanei]	XP_003090483.1	4,00E-17	GLOS_CRE_2061	GO:0008528	M	.	G-protein coupled peptide receptor activity
XP_003090483.1 hypothetical protein CRE_20617 [C. remanei]	XP_003090483.1	4,00E-17	GLOS_CRE_2061	GO:0001653	M	.	peptide receptor activity
XP_003090483.1 hypothetical protein CRE_20617 [C. remanei]	XP_003090483.1	4,00E-17	GLOS_CRE_2061	GO:0004930	M	.	G-protein coupled receptor activity
XP_003090483.1 hypothetical protein CRE_20617 [C. remanei]	XP_003090483.1	4,00E-17	GLOS_CRE_2061	GO:0004948	M	Refseq	calcitonin receptor activity
XP_003090483.1 hypothetical protein CRE_20617 [C. remanei]	XP_003090483.1	4,00E-17	GLOS_CRE_2061	GO:0005887	C	Refseq	integral to plasma membrane
XP_003090483.1 hypothetical protein CRE_20617 [C. remanei]	XP_003090483.1	4,00E-17	GLOS_CRE_2061	GO:0007186	B	Refseq	G-protein coupled receptor signaling pathway
XP_003090483.1 hypothetical protein CRE_20617 [C. remanei]	XP_003090483.1	4,00E-17	GLOS_CRE_2061	GO:0007186	B	.	G-protein coupled receptor signaling pathway
XP_003090483.1 hypothetical protein CRE_20617 [C. remanei]	XP_003090483.1	4,00E-17	GLOS_CRE_2061	GO:0007218	B	Refseq	neuropeptide signaling pathway
XP_003090483.1 hypothetical protein CRE_20617 [C. remanei]	XP_003090483.1	4,00E-17	GLOS_CRE_2061	GO:0048511	B	.	rhythmic process
XP_003090483.1 hypothetical protein CRE_20617 [C. remanei]	XP_003090483.1	4,00E-17	GLOS_CRE_2061	GO:0007623	B	Refseq	circadian rhythm
XP_003090483.1 hypothetical protein CRE_20617 [C. remanei]	XP_003090483.1	4,00E-17	GLOS_CRE_2061	GO:0030594	M	.	neurotransmitter receptor activity
XP_003090483.1 hypothetical protein CRE_20617 [C. remanei]	XP_003090483.1	4,00E-17	GLOS_CRE_2061	GO:0008188	M	Refseq	neuropeptide receptor activity
XP_003090483.1 hypothetical protein CRE_20617 [C. remanei]	XP_003090483.1	4,00E-17	GLOS_CRE_2061	GO:0010578	B	.	regulation of adenylate cyclase activity involved in G-protein coupled receptor signaling pathway
XP_003090483.1 hypothetical protein CRE_20617 [C. remanei]	XP_003090483.1	4,00E-17	GLOS_CRE_2061	GO:0045761	B	.	regulation of adenylate cyclase activity
XP_003090483.1 hypothetical protein CRE_20617 [C. remanei]	XP_003090483.1	4,00E-17	GLOS_CRE_2061	GO:0030817	B	.	regulation of cAMP biosynthetic process
XP_003090483.1 hypothetical protein CRE_20617 [C. remanei]	XP_003090483.1	4,00E-17	GLOS_CRE_2061	GO:0030802	B	.	regulation of cyclic nucleotide biosynthetic process
XP_003090483.1 hypothetical protein CRE_20617 [C. remanei]	XP_003090483.1	4,00E-17	GLOS_CRE_2061	GO:0030799	B	.	regulation of cyclic nucleotide metabolic process
XP_003090483.1 hypothetical protein CRE_20617 [C. remanei]	XP_003090483.1	4,00E-17	GLOS_CRE_2061	GO:0006140	B	.	regulation of nucleotide metabolic process
XP_003090483.1 hypothetical protein CRE_20617 [C. remanei]	XP_003090483.1	4,00E-17	GLOS_CRE_2061	GO:0019220	B	.	regulation of phosphate metabolic process
XP_003090483.1 hypothetical protein CRE_20617 [C. remanei]	XP_003090483.1	4,00E-17	GLOS_CRE_2061	GO:0051174	B	.	regulation of phosphorus metabolic process
XP_003090483.1 hypothetical protein CRE_20617 [C. remanei]	XP_003090483.1	4,00E-17	GLOS_CRE_2061	GO:0030808	B	.	regulation of nucleotide biosynthetic process
XP_003090483.1 hypothetical protein CRE_20617 [C. remanei]	XP_003090483.1	4,00E-17	GLOS_CRE_2061	GO:0030814	B	.	regulation of cAMP metabolic process
XP_003090483.1 hypothetical protein CRE_20617 [C. remanei]	XP_003090483.1	4,00E-17	GLOS_CRE_2061	GO:1900542	B	.	regulation of purine nucleotide metabolic process
XP_003090483.1 hypothetical protein CRE_20617 [C. remanei]	XP_003090483.1	4,00E-17	GLOS_CRE_2061	GO:1900371	B	.	regulation of purine nucleotide biosynthetic process
XP_003090483.1 hypothetical protein CRE_20617 [C. remanei]	XP_003090483.1	4,00E-17	GLOS_CRE_2061	GO:0031279	B	.	regulation of cyclase activity

XP_003090483.1 hypothetical protein CRE_20617 [C. remanei]	XP_003090483.1	4,00E-17	GLOS_CRE_2061	GO:0050790	B	.	regulation of catalytic activity
XP_003090483.1 hypothetical protein CRE_20617 [C. remanei]	XP_003090483.1	4,00E-17	GLOS_CRE_2061	GO:0065009	B	.	regulation of molecular function
XP_003090483.1 hypothetical protein CRE_20617 [C. remanei]	XP_003090483.1	4,00E-17	GLOS_CRE_2061	GO:0051339	B	.	regulation of lyase activity
XP_003090483.1 hypothetical protein CRE_20617 [C. remanei]	XP_003090483.1	4,00E-17	GLOS_CRE_2061	GO:0007188	B	.	adenylate cyclase-modulating G-protein coupled receptor signaling pathway
XP_003090483.1 hypothetical protein CRE_20617 [C. remanei]	XP_003090483.1	4,00E-17	GLOS_CRE_2061	GO:0007187	B	.	G-protein coupled receptor signaling pathway, coupled to cyclic nucleotide second messenger
XP_003090483.1 hypothetical protein CRE_20617 [C. remanei]	XP_003090483.1	4,00E-17	GLOS_CRE_2061	GO:0045762	B	.	positive regulation of adenylate cyclase activity
XP_003090483.1 hypothetical protein CRE_20617 [C. remanei]	XP_003090483.1	4,00E-17	GLOS_CRE_2061	GO:0030819	B	.	positive regulation of cAMP biosynthetic process
XP_003090483.1 hypothetical protein CRE_20617 [C. remanei]	XP_003090483.1	4,00E-17	GLOS_CRE_2061	GO:0030804	B	.	positive regulation of cyclic nucleotide biosynthetic process
XP_003090483.1 hypothetical protein CRE_20617 [C. remanei]	XP_003090483.1	4,00E-17	GLOS_CRE_2061	GO:0030801	B	.	positive regulation of cyclic nucleotide metabolic process
XP_003090483.1 hypothetical protein CRE_20617 [C. remanei]	XP_003090483.1	4,00E-17	GLOS_CRE_2061	GO:0045981	B	.	positive regulation of nucleotide metabolic process
XP_003090483.1 hypothetical protein CRE_20617 [C. remanei]	XP_003090483.1	4,00E-17	GLOS_CRE_2061	GO:0045937	B	.	positive regulation of phosphate metabolic process
XP_003090483.1 hypothetical protein CRE_20617 [C. remanei]	XP_003090483.1	4,00E-17	GLOS_CRE_2061	GO:0010562	B	.	positive regulation of phosphorus metabolic process
XP_003090483.1 hypothetical protein CRE_20617 [C. remanei]	XP_003090483.1	4,00E-17	GLOS_CRE_2061	GO:0030810	B	.	positive regulation of nucleotide biosynthetic process
XP_003090483.1 hypothetical protein CRE_20617 [C. remanei]	XP_003090483.1	4,00E-17	GLOS_CRE_2061	GO:0030816	B	.	positive regulation of cAMP metabolic process
XP_003090483.1 hypothetical protein CRE_20617 [C. remanei]	XP_003090483.1	4,00E-17	GLOS_CRE_2061	GO:1900544	B	.	positive regulation of purine nucleotide metabolic process
XP_003090483.1 hypothetical protein CRE_20617 [C. remanei]	XP_003090483.1	4,00E-17	GLOS_CRE_2061	GO:1900373	B	.	positive regulation of purine nucleotide biosynthetic process
XP_003090483.1 hypothetical protein CRE_20617 [C. remanei]	XP_003090483.1	4,00E-17	GLOS_CRE_2061	GO:0031281	B	.	positive regulation of cyclase activity
XP_003090483.1 hypothetical protein CRE_20617 [C. remanei]	XP_003090483.1	4,00E-17	GLOS_CRE_2061	GO:0043085	B	.	positive regulation of catalytic activity
XP_003090483.1 hypothetical protein CRE_20617 [C. remanei]	XP_003090483.1	4,00E-17	GLOS_CRE_2061	GO:0044093	B	.	positive regulation of molecular function
XP_003090483.1 hypothetical protein CRE_20617 [C. remanei]	XP_003090483.1	4,00E-17	GLOS_CRE_2061	GO:0051349	B	.	positive regulation of lyase activity
XP_003090483.1 hypothetical protein CRE_20617 [C. remanei]	XP_003090483.1	4,00E-17	GLOS_CRE_2061	GO:0007189	B	.	adenylate cyclase-activating G-protein coupled receptor signaling pathway
XP_003090483.1 hypothetical protein CRE_20617 [C. remanei]	XP_003090483.1	4,00E-17	GLOS_CRE_2061	GO:0010579	B	Refseq	positive regulation of adenylate cyclase activity involved in G-protein coupled receptor signaling pathway
XP_003090483.1 hypothetical protein CRE_20617 [C. remanei]	XP_003090483.1	4,00E-17	GLOS_CRE_2061	GO:0016021	C	Refseq	integral to membrane
XP_003090483.1 hypothetical protein CRE_20617 [C. remanei]	XP_003090483.1	4,00E-17	GLOS_CRE_2061	GO:0009629	B	.	response to gravity
XP_003090483.1 hypothetical protein CRE_20617 [C. remanei]	XP_003090483.1	4,00E-17	GLOS_CRE_2061	GO:0042332	B	Refseq	gravitaxis
XP_003090483.1 hypothetical protein CRE_20617 [C. remanei]	XP_003090483.1	4,00E-17	GLOS_CRE_2061	GO:0048512	B	.	circadian behavior
XP_003090483.1 hypothetical protein CRE_20617 [C. remanei]	XP_003090483.1	4,00E-17	GLOS_CRE_2061	GO:0007622	B	.	rhythmic behavior
XP_003090483.1 hypothetical protein CRE_20617 [C. remanei]	XP_003090483.1	4,00E-17	GLOS_CRE_2061	GO:0044708	B	.	single-organism behavior
XP_003090483.1 hypothetical protein CRE_20617 [C. remanei]	XP_003090483.1	4,00E-17	GLOS_CRE_2061	GO:0007610	B	.	behavior
XP_003090483.1 hypothetical protein CRE_20617 [C. remanei]	XP_003090483.1	4,00E-17	GLOS_CRE_2061	GO:0007623	B	.	circadian rhythm
XP_003090483.1 hypothetical protein CRE_20617 [C. remanei]	XP_003090483.1	4,00E-17	GLOS_CRE_2061	GO:0042745	B	Refseq	circadian sleep/wake cycle
XP_003090483.1 hypothetical protein CRE_20617 [C. remanei]	XP_003090483.1	4,00E-17	GLOS_CRE_2061	GO:0022410	B	.	circadian sleep/wake cycle process
XP_003090483.1 hypothetical protein CRE_20617 [C. remanei]	XP_003090483.1	4,00E-17	GLOS_CRE_2061	GO:0042745	B	.	circadian sleep/wake cycle
XP_003090483.1 hypothetical protein CRE_20617 [C. remanei]	XP_003090483.1	4,00E-17	GLOS_CRE_2061	GO:0042752	B	.	regulation of circadian rhythm
XP_003090483.1 hypothetical protein CRE_20617 [C. remanei]	XP_003090483.1	4,00E-17	GLOS_CRE_2061	GO:0050795	B	.	regulation of behavior
XP_003090483.1 hypothetical protein CRE_20617 [C. remanei]	XP_003090483.1	4,00E-17	GLOS_CRE_2061	GO:0048583	B	.	regulation of response to stimulus
XP_003090483.1 hypothetical protein CRE_20617 [C. remanei]	XP_003090483.1	4,00E-17	GLOS_CRE_2061	GO:0042749	B	Refseq	regulation of circadian sleep/wake cycle
XP_003090483.1 hypothetical protein CRE_20617 [C. remanei]	XP_003090483.1	4,00E-17	GLOS_CRE_2061	GO:0042995	C	.	cell projection
XP_003090483.1 hypothetical protein CRE_20617 [C. remanei]	XP_003090483.1	4,00E-17	GLOS_CRE_2061	GO:0097458	C	.	neuron part
XP_003090483.1 hypothetical protein CRE_20617 [C. remanei]	XP_003090483.1	4,00E-17	GLOS_CRE_2061	GO:0043005	C	Refseq	neuron projection
XP_003090483.1 hypothetical protein CRE_20617 [C. remanei]	XP_003090483.1	4,00E-17	GLOS_CRE_2061	GO:0044297	C	.	cell body
XP_003090483.1 hypothetical protein CRE_20617 [C. remanei]	XP_003090483.1	4,00E-17	GLOS_CRE_2061	GO:0043025	C	Refseq	neuronal cell body
XP_003090483.1 hypothetical protein CRE_20617 [C. remanei]	XP_003090483.1	4,00E-17	GLOS_CRE_2061	GO:0007626	B	.	locomotory behavior
XP_003090483.1 hypothetical protein CRE_20617 [C. remanei]	XP_003090483.1	4,00E-17	GLOS_CRE_2061	GO:0045475	B	Refseq	locomotor rhythm
XP_003090483.1 hypothetical protein CRE_20617 [C. remanei]	XP_003090483.1	4,00E-17	GLOS_CRE_2061	GO:0048512	B	Refseq	circadian behavior
XP_001970957.1 GG10935 [Drosophila erecta]	XP_001970957.1	5,00E-06	GLOS_DERE_GG	GO:0005261	M	Refseq	cation channel activity
XP_001970957.1 GG10935 [Drosophila erecta]	XP_001970957.1	5,00E-06	GLOS_DERE_GG	GO:0005262	M	Refseq	calcium channel activity
XP_001970957.1 GG10935 [Drosophila erecta]	XP_001970957.1	5,00E-06	GLOS_DERE_GG	GO:0005515	M	Refseq	protein binding
XP_001970957.1 GG10935 [Drosophila erecta]	XP_001970957.1	5,00E-06	GLOS_DERE_GG	GO:0005886	C	Refseq	plasma membrane
XP_001970957.1 GG10935 [Drosophila erecta]	XP_001970957.1	5,00E-06	GLOS_DERE_GG	GO:0005887	C	Refseq	integral to plasma membrane
XP_001970957.1 GG10935 [Drosophila erecta]	XP_001970957.1	5,00E-06	GLOS_DERE_GG	GO:0006816	B	Refseq	calcium ion transport
XP_001970957.1 GG10935 [Drosophila erecta]	XP_001970957.1	5,00E-06	GLOS_DERE_GG	GO:0007411	B	Refseq	axon guidance

XP_001970957.1 GG10935 [Drosophila erecta]	XP_001970957.1	5,00E-06	GLOS_DERE_GG	GO:0015279	M	Refseq	store-operated calcium channel activity
XP_001970957.1 GG10935 [Drosophila erecta]	XP_001970957.1	5,00E-06	GLOS_DERE_GG	GO:0016323	C	Refseq	basolateral plasma membrane
XP_001970957.1 GG10935 [Drosophila erecta]	XP_001970957.1	5,00E-06	GLOS_DERE_GG	GO:0044449	C	.	contractile fiber part
XP_001970957.1 GG10935 [Drosophila erecta]	XP_001970957.1	5,00E-06	GLOS_DERE_GG	GO:0044444	C	.	cytoplasmic part
XP_001970957.1 GG10935 [Drosophila erecta]	XP_001970957.1	5,00E-06	GLOS_DERE_GG	GO:0005737	C	.	cytoplasm
XP_001970957.1 GG10935 [Drosophila erecta]	XP_001970957.1	5,00E-06	GLOS_DERE_GG	GO:0043292	C	.	contractile fiber
XP_001970957.1 GG10935 [Drosophila erecta]	XP_001970957.1	5,00E-06	GLOS_DERE_GG	GO:0043232	C	.	intracellular non-membrane-bounded organelle
XP_001970957.1 GG10935 [Drosophila erecta]	XP_001970957.1	5,00E-06	GLOS_DERE_GG	GO:0043228	C	.	non-membrane-bounded organelle
XP_001970957.1 GG10935 [Drosophila erecta]	XP_001970957.1	5,00E-06	GLOS_DERE_GG	GO:0030016	C	.	myofibril
XP_001970957.1 GG10935 [Drosophila erecta]	XP_001970957.1	5,00E-06	GLOS_DERE_GG	GO:0030017	C	Refseq	sarcomere
XP_001970957.1 GG10935 [Drosophila erecta]	XP_001970957.1	5,00E-06	GLOS_DERE_GG	GO:0043034	C	Refseq	costamere
XP_001970957.1 GG10935 [Drosophila erecta]	XP_001970957.1	5,00E-06	GLOS_DERE_GG	GO:0043234	C	Refseq	protein complex
XP_001970957.1 GG10935 [Drosophila erecta]	XP_001970957.1	5,00E-06	GLOS_DERE_GG	GO:0044325	M	Refseq	ion channel binding
XP_001970957.1 GG10935 [Drosophila erecta]	XP_001970957.1	5,00E-06	GLOS_DERE_GG	GO:0045121	C	Refseq	membrane raft
XP_001970957.1 GG10935 [Drosophila erecta]	XP_001970957.1	5,00E-06	GLOS_DERE_GG	GO:0007589	B	.	body fluid secretion
XP_001970957.1 GG10935 [Drosophila erecta]	XP_001970957.1	5,00E-06	GLOS_DERE_GG	GO:0046903	B	.	secretion
XP_001970957.1 GG10935 [Drosophila erecta]	XP_001970957.1	5,00E-06	GLOS_DERE_GG	GO:0022600	B	.	digestive system process
XP_001970957.1 GG10935 [Drosophila erecta]	XP_001970957.1	5,00E-06	GLOS_DERE_GG	GO:0003008	B	.	system process
XP_001970957.1 GG10935 [Drosophila erecta]	XP_001970957.1	5,00E-06	GLOS_DERE_GG	GO:0007586	B	.	digestion
XP_001970957.1 GG10935 [Drosophila erecta]	XP_001970957.1	5,00E-06	GLOS_DERE_GG	GO:0032941	B	.	secretion by tissue
XP_001970957.1 GG10935 [Drosophila erecta]	XP_001970957.1	5,00E-06	GLOS_DERE_GG	GO:0046541	B	Refseq	saliva secretion
XP_001970957.1 GG10935 [Drosophila erecta]	XP_001970957.1	5,00E-06	GLOS_DERE_GG	GO:0010524	B	.	positive regulation of calcium ion transport into cytosol
XP_001970957.1 GG10935 [Drosophila erecta]	XP_001970957.1	5,00E-06	GLOS_DERE_GG	GO:0051279	B	.	regulation of release of sequestered calcium ion into cytosol
XP_001970957.1 GG10935 [Drosophila erecta]	XP_001970957.1	5,00E-06	GLOS_DERE_GG	GO:0051281	B	Refseq	positive regulation of release of sequestered calcium ion into cytosol
XP_001970957.1 GG10935 [Drosophila erecta]	XP_001970957.1	5,00E-06	GLOS_DERE_GG	GO:0006874	B	.	cellular calcium ion homeostasis
XP_001970957.1 GG10935 [Drosophila erecta]	XP_001970957.1	5,00E-06	GLOS_DERE_GG	GO:0006875	B	.	cellular metal ion homeostasis
XP_001970957.1 GG10935 [Drosophila erecta]	XP_001970957.1	5,00E-06	GLOS_DERE_GG	GO:0030003	B	.	cellular cation homeostasis
XP_001970957.1 GG10935 [Drosophila erecta]	XP_001970957.1	5,00E-06	GLOS_DERE_GG	GO:0006873	B	.	cellular ion homeostasis
XP_001970957.1 GG10935 [Drosophila erecta]	XP_001970957.1	5,00E-06	GLOS_DERE_GG	GO:0050801	B	.	ion homeostasis
XP_001970957.1 GG10935 [Drosophila erecta]	XP_001970957.1	5,00E-06	GLOS_DERE_GG	GO:0048878	B	.	chemical homeostasis
XP_001970957.1 GG10935 [Drosophila erecta]	XP_001970957.1	5,00E-06	GLOS_DERE_GG	GO:0042592	B	.	homeostatic process
XP_001970957.1 GG10935 [Drosophila erecta]	XP_001970957.1	5,00E-06	GLOS_DERE_GG	GO:0055082	B	.	cellular chemical homeostasis
XP_001970957.1 GG10935 [Drosophila erecta]	XP_001970957.1	5,00E-06	GLOS_DERE_GG	GO:0019725	B	.	cellular homeostasis
XP_001970957.1 GG10935 [Drosophila erecta]	XP_001970957.1	5,00E-06	GLOS_DERE_GG	GO:0055080	B	.	cation homeostasis
XP_001970957.1 GG10935 [Drosophila erecta]	XP_001970957.1	5,00E-06	GLOS_DERE_GG	GO:0055065	B	.	metal ion homeostasis
XP_001970957.1 GG10935 [Drosophila erecta]	XP_001970957.1	5,00E-06	GLOS_DERE_GG	GO:0055074	B	.	calcium ion homeostasis
XP_001970957.1 GG10935 [Drosophila erecta]	XP_001970957.1	5,00E-06	GLOS_DERE_GG	GO:0072507	B	.	divalent inorganic cation homeostasis
XP_001970957.1 GG10935 [Drosophila erecta]	XP_001970957.1	5,00E-06	GLOS_DERE_GG	GO:0072503	B	.	cellular divalent inorganic cation homeostasis
XP_001970957.1 GG10935 [Drosophila erecta]	XP_001970957.1	5,00E-06	GLOS_DERE_GG	GO:0051480	B	Refseq	cytosolic calcium ion homeostasis
XP_001970957.1 GG10935 [Drosophila erecta]	XP_001970957.1	5,00E-06	GLOS_DERE_GG	GO:0051592	B	Refseq	response to calcium ion
XP_001970957.1 GG10935 [Drosophila erecta]	XP_001970957.1	5,00E-06	GLOS_DERE_GG	GO:0070679	M	Refseq	inositol 1,4,5 trisphosphate binding
XP_001949504.2 PREDICT: hypoth prot LOC100162931 [A. pisum]	XP_001949504.2	1,00E-15	GLOS_LOC10016:	GO:0000278	B	Refseq	mitotic cell cycle
XP_001949504.2 PREDICT: hypoth prot LOC100162931 [A. pisum]	XP_001949504.2	1,00E-15	GLOS_LOC10016:	GO:0003677	M	Refseq	DNA binding
XP_001949504.2 PREDICT: hypoth prot LOC100162931 [A. pisum]	XP_001949504.2	1,00E-15	GLOS_LOC10016:	GO:0003700	M	Refseq	sequence-specific DNA binding transcription factor activity
XP_001949504.2 PREDICT: hypoth prot LOC100162931 [A. pisum]	XP_001949504.2	1,00E-15	GLOS_LOC10016:	GO:0003712	M	Refseq	transcription cofactor activity
XP_001949504.2 PREDICT: hypoth prot LOC100162931 [A. pisum]	XP_001949504.2	1,00E-15	GLOS_LOC10016:	GO:0005654	C	Refseq	nucleoplasm
XP_001949504.2 PREDICT: hypoth prot LOC100162931 [A. pisum]	XP_001949504.2	1,00E-15	GLOS_LOC10016:	GO:0005667	C	Refseq	transcription factor complex
XP_001949504.2 PREDICT: hypoth prot LOC100162931 [A. pisum]	XP_001949504.2	1,00E-15	GLOS_LOC10016:	GO:0006351	B	Refseq	transcription, DNA-dependent
XP_001949504.2 PREDICT: hypoth prot LOC100162931 [A. pisum]	XP_001949504.2	1,00E-15	GLOS_LOC10016:	GO:0006367	B	Refseq	transcription initiation from RNA polymerase II promoter
XP_001949504.2 PREDICT: hypoth prot LOC100162931 [A. pisum]	XP_001949504.2	1,00E-15	GLOS_LOC10016:	GO:0007179	B	Refseq	transforming growth factor beta receptor signaling pathway
XP_001949504.2 PREDICT: hypoth prot LOC100162931 [A. pisum]	XP_001949504.2	1,00E-15	GLOS_LOC10016:	GO:0007507	B	Refseq	heart development
XP_001949504.2 PREDICT: hypoth prot LOC100162931 [A. pisum]	XP_001949504.2	1,00E-15	GLOS_LOC10016:	GO:0008134	M	Refseq	transcription factor binding
XP_001949504.2 PREDICT: hypoth prot LOC100162931 [A. pisum]	XP_001949504.2	1,00E-15	GLOS_LOC10016:	GO:0010467	B	Refseq	gene expression
XP_001949504.2 PREDICT: hypoth prot LOC100162931 [A. pisum]	XP_001949504.2	1,00E-15	GLOS_LOC10016:	GO:0019904	M	Refseq	protein domain specific binding

XP_001949504.2 PREDICT: hypoth prot LOC100162931 [A. pisum]	XP_001949504.2	1,00E-15	GLOS_LOC10016:	GO:0045944	B	Refseq	positive regulation of transcription from RNA polymerase II promoter
XP_001949504.2 PREDICT: hypoth prot LOC100162931 [A. pisum]	XP_001949504.2	4,00E-19	GLOS_LOC10016:	GO:0000278	B	Refseq	mitotic cell cycle
XP_001949504.2 PREDICT: hypoth prot LOC100162931 [A. pisum]	XP_001949504.2	4,00E-19	GLOS_LOC10016:	GO:0003677	M	Refseq	DNA binding
XP_001949504.2 PREDICT: hypoth prot LOC100162931 [A. pisum]	XP_001949504.2	4,00E-19	GLOS_LOC10016:	GO:0003700	M	Refseq	sequence-specific DNA binding transcription factor activity
XP_001949504.2 PREDICT: hypoth prot LOC100162931 [A. pisum]	XP_001949504.2	4,00E-19	GLOS_LOC10016:	GO:0003712	M	Refseq	transcription cofactor activity
XP_001949504.2 PREDICT: hypoth prot LOC100162931 [A. pisum]	XP_001949504.2	4,00E-19	GLOS_LOC10016:	GO:0005654	C	Refseq	nucleoplasm
XP_001949504.2 PREDICT: hypoth prot LOC100162931 [A. pisum]	XP_001949504.2	4,00E-19	GLOS_LOC10016:	GO:0005667	C	Refseq	transcription factor complex
XP_001949504.2 PREDICT: hypoth prot LOC100162931 [A. pisum]	XP_001949504.2	4,00E-19	GLOS_LOC10016:	GO:0006351	B	Refseq	transcription, DNA-dependent
XP_001949504.2 PREDICT: hypoth prot LOC100162931 [A. pisum]	XP_001949504.2	4,00E-19	GLOS_LOC10016:	GO:0006367	B	Refseq	transcription initiation from RNA polymerase II promoter
XP_001949504.2 PREDICT: hypoth prot LOC100162931 [A. pisum]	XP_001949504.2	4,00E-19	GLOS_LOC10016:	GO:0007179	B	Refseq	transforming growth factor beta receptor signaling pathway
XP_001949504.2 PREDICT: hypoth prot LOC100162931 [A. pisum]	XP_001949504.2	4,00E-19	GLOS_LOC10016:	GO:0007507	B	Refseq	heart development
XP_001949504.2 PREDICT: hypoth prot LOC100162931 [A. pisum]	XP_001949504.2	4,00E-19	GLOS_LOC10016:	GO:0008134	M	Refseq	transcription factor binding
XP_001949504.2 PREDICT: hypoth prot LOC100162931 [A. pisum]	XP_001949504.2	4,00E-19	GLOS_LOC10016:	GO:0010467	B	Refseq	gene expression
XP_001949504.2 PREDICT: hypoth prot LOC100162931 [A. pisum]	XP_001949504.2	4,00E-19	GLOS_LOC10016:	GO:0019904	M	Refseq	protein domain specific binding
XP_001949504.2 PREDICT: hypoth prot LOC100162931 [A. pisum]	XP_001949504.2	4,00E-19	GLOS_LOC10016:	GO:0045944	B	Refseq	positive regulation of transcription from RNA polymerase II promoter
NP_001246163.1 down syndrome cell adh. molecule, isoform BY, partial [D.m.]	NP_001246163.1	0	GLOS_contig_09	GO:0000122	B	Refseq	negative regulation of transcription from RNA polymerase II promoter
NP_001246163.1 down syndrome cell adh. molecule, isoform BY, partial [D.m.]	NP_001246163.1	0	GLOS_contig_09	GO:0001501	B	Refseq	skeletal system development
NP_001246163.1 down syndrome cell adh. molecule, isoform BY, partial [D.m.]	NP_001246163.1	0	GLOS_contig_09	GO:0003700	M	Refseq	sequence-specific DNA binding transcription factor activity
NP_001246163.1 down syndrome cell adh. molecule, isoform BY, partial [D.m.]	NP_001246163.1	0	GLOS_contig_09	GO:0005515	M	Refseq	protein binding
NP_001246163.1 down syndrome cell adh. molecule, isoform BY, partial [D.m.]	NP_001246163.1	0	GLOS_contig_09	GO:0005634	C	Refseq	nucleus
NP_001246163.1 down syndrome cell adh. molecule, isoform BY, partial [D.m.]	NP_001246163.1	0	GLOS_contig_09	GO:0006366	B	Refseq	transcription from RNA polymerase II promoter
NP_001246163.1 down syndrome cell adh. molecule, isoform BY, partial [D.m.]	NP_001246163.1	0	GLOS_contig_09	GO:0006607	B	Refseq	NLS-bearing protein import into nucleus
NP_001246163.1 down syndrome cell adh. molecule, isoform BY, partial [D.m.]	NP_001246163.1	0	GLOS_contig_09	GO:0008270	M	Refseq	zinc ion binding
NP_001246163.1 down syndrome cell adh. molecule, isoform BY, partial [D.m.]	NP_001246163.1	0	GLOS_contig_09	GO:0032330	B	Refseq	regulation of chondrocyte differentiation
NP_001246163.1 down syndrome cell adh. molecule, isoform BY, partial [D.m.]	NP_001246163.1	0	GLOS_contig_09	GO:0043565	M	Refseq	sequence-specific DNA binding
XP_001990298.1 GH18314 [Drosophila grimshawi]	XP_001990298.1	0	GLOS_DGRI_GH1	GO:0005262	M	Refseq	calcium channel activity
XP_001990298.1 GH18314 [Drosophila grimshawi]	XP_001990298.1	0	GLOS_DGRI_GH1	GO:0005515	M	Refseq	protein binding
XP_001990298.1 GH18314 [Drosophila grimshawi]	XP_001990298.1	0	GLOS_DGRI_GH1	GO:0005886	C	Refseq	plasma membrane
XP_001990298.1 GH18314 [Drosophila grimshawi]	XP_001990298.1	0	GLOS_DGRI_GH1	GO:0005887	C	Refseq	integral to plasma membrane
XP_001990298.1 GH18314 [Drosophila grimshawi]	XP_001990298.1	0	GLOS_DGRI_GH1	GO:0006816	B	Refseq	calcium ion transport
XP_001990298.1 GH18314 [Drosophila grimshawi]	XP_001990298.1	0	GLOS_DGRI_GH1	GO:0007411	B	Refseq	axon guidance
XP_001990298.1 GH18314 [Drosophila grimshawi]	XP_001990298.1	0	GLOS_DGRI_GH1	GO:0007596	B	Refseq	blood coagulation
XP_001990298.1 GH18314 [Drosophila grimshawi]	XP_001990298.1	0	GLOS_DGRI_GH1	GO:0007602	B	Refseq	phototransduction
XP_001990298.1 GH18314 [Drosophila grimshawi]	XP_001990298.1	0	GLOS_DGRI_GH1	GO:0010524	B	Refseq	positive regulation of calcium ion transport into cytosol
XP_001990298.1 GH18314 [Drosophila grimshawi]	XP_001990298.1	0	GLOS_DGRI_GH1	GO:0015279	M	Refseq	store-operated calcium channel activity
XP_001990298.1 GH18314 [Drosophila grimshawi]	XP_001990298.1	0	GLOS_DGRI_GH1	GO:0030168	B	Refseq	platelet activation
XP_001990298.1 GH18314 [Drosophila grimshawi]	XP_001990298.1	0	GLOS_DGRI_GH1	GO:0033198	B	Refseq	response to ATP
XP_001990298.1 GH18314 [Drosophila grimshawi]	XP_001990298.1	0	GLOS_DGRI_GH1	GO:0051592	B	Refseq	response to calcium ion
XP_001990298.1 GH18314 [Drosophila grimshawi]	XP_001990298.1	0	GLOS_DGRI_GH1	GO:0070588	B	Refseq	calcium ion transmembrane transport
XP_001990298.1 GH18314 [Drosophila grimshawi]	XP_001990298.1	0	GLOS_DGRI_GH1	GO:0070679	M	Refseq	inositol 1,4,5 trisphosphate binding
XP_001988193.1 GH11034 [Drosophila grimshawi]	XP_001988193.1	0	GLOS_DGRI_GH1	GO:0005262	M	Refseq	calcium channel activity
XP_001988193.1 GH11034 [Drosophila grimshawi]	XP_001988193.1	0	GLOS_DGRI_GH1	GO:0005515	M	Refseq	protein binding
XP_001988193.1 GH11034 [Drosophila grimshawi]	XP_001988193.1	0	GLOS_DGRI_GH1	GO:0005886	C	Refseq	plasma membrane
XP_001988193.1 GH11034 [Drosophila grimshawi]	XP_001988193.1	0	GLOS_DGRI_GH1	GO:0005887	C	Refseq	integral to plasma membrane
XP_001988193.1 GH11034 [Drosophila grimshawi]	XP_001988193.1	0	GLOS_DGRI_GH1	GO:0006816	B	Refseq	calcium ion transport
XP_001988193.1 GH11034 [Drosophila grimshawi]	XP_001988193.1	0	GLOS_DGRI_GH1	GO:0007411	B	Refseq	axon guidance
XP_001988193.1 GH11034 [Drosophila grimshawi]	XP_001988193.1	0	GLOS_DGRI_GH1	GO:0007596	B	Refseq	blood coagulation
XP_001988193.1 GH11034 [Drosophila grimshawi]	XP_001988193.1	0	GLOS_DGRI_GH1	GO:0007602	B	Refseq	phototransduction
XP_001988193.1 GH11034 [Drosophila grimshawi]	XP_001988193.1	0	GLOS_DGRI_GH1	GO:0010524	B	Refseq	positive regulation of calcium ion transport into cytosol
XP_001988193.1 GH11034 [Drosophila grimshawi]	XP_001988193.1	0	GLOS_DGRI_GH1	GO:0015279	M	Refseq	store-operated calcium channel activity
XP_001988193.1 GH11034 [Drosophila grimshawi]	XP_001988193.1	0	GLOS_DGRI_GH1	GO:0030168	B	Refseq	platelet activation
XP_001988193.1 GH11034 [Drosophila grimshawi]	XP_001988193.1	0	GLOS_DGRI_GH1	GO:0033198	B	Refseq	response to ATP
XP_001988193.1 GH11034 [Drosophila grimshawi]	XP_001988193.1	0	GLOS_DGRI_GH1	GO:0051592	B	Refseq	response to calcium ion
XP_001988193.1 GH11034 [Drosophila grimshawi]	XP_001988193.1	0	GLOS_DGRI_GH1	GO:0070588	B	Refseq	calcium ion transmembrane transport

XP_001988193.1 GH11034 [Drosophila grimshawi]	XP_001988193.1	0	GLOS_DGRI_GH1	GO:0070679	M	Refseq	inositol 1,4,5 trisphosphate binding
XP_003740993.1 PREDICT: uncharact prot LOC100904700 [M. occidentalis]	XP_003740993.1	5,00E-65	GLOS_LOC10090	GO:0004175	M	.	endopeptidase activity
XP_003740993.1 PREDICT: uncharact prot LOC100904700 [M. occidentalis]	XP_003740993.1	5,00E-65	GLOS_LOC10090	GO:0070011	M	.	peptidase activity, acting on L-amino acid peptides
XP_003740993.1 PREDICT: uncharact prot LOC100904700 [M. occidentalis]	XP_003740993.1	5,00E-65	GLOS_LOC10090	GO:0008233	M	.	peptidase activity
XP_003740993.1 PREDICT: uncharact prot LOC100904700 [M. occidentalis]	XP_003740993.1	5,00E-65	GLOS_LOC10090	GO:0016787	M	.	hydrolase activity
XP_003740993.1 PREDICT: uncharact prot LOC100904700 [M. occidentalis]	XP_003740993.1	5,00E-65	GLOS_LOC10090	GO:0003824	M	.	catalytic activity
XP_003740993.1 PREDICT: uncharact prot LOC100904700 [M. occidentalis]	XP_003740993.1	5,00E-65	GLOS_LOC10090	GO:0008237	M	.	metallopeptidase activity
XP_003740993.1 PREDICT: uncharact prot LOC100904700 [M. occidentalis]	XP_003740993.1	5,00E-65	GLOS_LOC10090	GO:0004222	M	Refseq	metalloendopeptidase activity
XP_003740993.1 PREDICT: uncharact prot LOC100904700 [M. occidentalis]	XP_003740993.1	5,00E-65	GLOS_LOC10090	GO:0008236	M	.	serine-type peptidase activity
XP_003740993.1 PREDICT: uncharact prot LOC100904700 [M. occidentalis]	XP_003740993.1	5,00E-65	GLOS_LOC10090	GO:0017171	M	.	serine hydrolase activity
XP_003740993.1 PREDICT: uncharact prot LOC100904700 [M. occidentalis]	XP_003740993.1	5,00E-65	GLOS_LOC10090	GO:0004252	M	Refseq	serine-type endopeptidase activity
XP_003740993.1 PREDICT: uncharact prot LOC100904700 [M. occidentalis]	XP_003740993.1	5,00E-65	GLOS_LOC10090	GO:0019538	B	.	protein metabolic process
XP_003740993.1 PREDICT: uncharact prot LOC100904700 [M. occidentalis]	XP_003740993.1	5,00E-65	GLOS_LOC10090	GO:0006508	B	Refseq	proteolysis
XP_003247848.1 PREDICTED: hypoth prot LOC100574925 [A. pisum]	XP_003247848.1	5,00E-43	GLOS_LOC10057	GO:0000278	B	Refseq	mitotic cell cycle
XP_003247848.1 PREDICTED: hypoth prot LOC100574925 [A. pisum]	XP_003247848.1	5,00E-43	GLOS_LOC10057	GO:0003677	M	Refseq	DNA binding
XP_003247848.1 PREDICTED: hypoth prot LOC100574925 [A. pisum]	XP_003247848.1	5,00E-43	GLOS_LOC10057	GO:0003700	M	Refseq	sequence-specific DNA binding transcription factor activity
XP_003247848.1 PREDICTED: hypoth prot LOC100574925 [A. pisum]	XP_003247848.1	5,00E-43	GLOS_LOC10057	GO:0003712	M	Refseq	transcription cofactor activity
XP_003247848.1 PREDICTED: hypoth prot LOC100574925 [A. pisum]	XP_003247848.1	5,00E-43	GLOS_LOC10057	GO:0005654	C	Refseq	nucleoplasm
XP_003247848.1 PREDICTED: hypoth prot LOC100574925 [A. pisum]	XP_003247848.1	5,00E-43	GLOS_LOC10057	GO:0005667	C	Refseq	transcription factor complex
XP_003247848.1 PREDICTED: hypoth prot LOC100574925 [A. pisum]	XP_003247848.1	5,00E-43	GLOS_LOC10057	GO:0006351	B	Refseq	transcription, DNA-dependent
XP_003247848.1 PREDICTED: hypoth prot LOC100574925 [A. pisum]	XP_003247848.1	5,00E-43	GLOS_LOC10057	GO:0006367	B	Refseq	transcription initiation from RNA polymerase II promoter
XP_003247848.1 PREDICTED: hypoth prot LOC100574925 [A. pisum]	XP_003247848.1	5,00E-43	GLOS_LOC10057	GO:0007179	B	Refseq	transforming growth factor beta receptor signaling pathway
XP_003247848.1 PREDICTED: hypoth prot LOC100574925 [A. pisum]	XP_003247848.1	5,00E-43	GLOS_LOC10057	GO:0007507	B	Refseq	heart development
XP_003247848.1 PREDICTED: hypoth prot LOC100574925 [A. pisum]	XP_003247848.1	5,00E-43	GLOS_LOC10057	GO:0008134	M	Refseq	transcription factor binding
XP_003247848.1 PREDICTED: hypoth prot LOC100574925 [A. pisum]	XP_003247848.1	5,00E-43	GLOS_LOC10057	GO:0010467	B	Refseq	gene expression
XP_003247848.1 PREDICTED: hypoth prot LOC100574925 [A. pisum]	XP_003247848.1	5,00E-43	GLOS_LOC10057	GO:0019904	M	Refseq	protein domain specific binding
XP_003247848.1 PREDICTED: hypoth prot LOC100574925 [A. pisum]	XP_003247848.1	5,00E-43	GLOS_LOC10057	GO:0045944	B	Refseq	positive regulation of transcription from RNA polymerase II promoter
NP_610599.3 Git [Drosophila melanogaster]	NP_610599.3	0	GLOS_GIT.2.2	GO:0000122	B	Refseq	negative regulation of transcription from RNA polymerase II promoter
NP_610599.3 Git [Drosophila melanogaster]	NP_610599.3	0	GLOS_GIT.2.2	GO:0001501	B	Refseq	skeletal system development
NP_610599.3 Git [Drosophila melanogaster]	NP_610599.3	0	GLOS_GIT.2.2	GO:0003700	M	Refseq	sequence-specific DNA binding transcription factor activity
NP_610599.3 Git [Drosophila melanogaster]	NP_610599.3	0	GLOS_GIT.2.2	GO:0005515	M	Refseq	protein binding
NP_610599.3 Git [Drosophila melanogaster]	NP_610599.3	0	GLOS_GIT.2.2	GO:0005634	C	Refseq	nucleus
NP_610599.3 Git [Drosophila melanogaster]	NP_610599.3	0	GLOS_GIT.2.2	GO:0006366	B	Refseq	transcription from RNA polymerase II promoter
NP_610599.3 Git [Drosophila melanogaster]	NP_610599.3	0	GLOS_GIT.2.2	GO:0006607	B	Refseq	NLS-bearing protein import into nucleus
NP_610599.3 Git [Drosophila melanogaster]	NP_610599.3	0	GLOS_GIT.2.2	GO:0008270	M	Refseq	zinc ion binding
NP_610599.3 Git [Drosophila melanogaster]	NP_610599.3	0	GLOS_GIT.2.2	GO:0032330	B	Refseq	regulation of chondrocyte differentiation
NP_610599.3 Git [Drosophila melanogaster]	NP_610599.3	0	GLOS_GIT.2.2	GO:0043565	M	Refseq	sequence-specific DNA binding
NP_001096850.2 dpr7, isoform F [Drosophila melanogaster]	NP_001096850.2	1,00E-131	GLOS_DPR7.1.1	GO:0000122	B	Refseq	negative regulation of transcription from RNA polymerase II promoter
NP_001096850.2 dpr7, isoform F [Drosophila melanogaster]	NP_001096850.2	1,00E-131	GLOS_DPR7.1.1	GO:0001501	B	Refseq	skeletal system development
NP_001096850.2 dpr7, isoform F [Drosophila melanogaster]	NP_001096850.2	1,00E-131	GLOS_DPR7.1.1	GO:0003700	M	Refseq	sequence-specific DNA binding transcription factor activity
NP_001096850.2 dpr7, isoform F [Drosophila melanogaster]	NP_001096850.2	1,00E-131	GLOS_DPR7.1.1	GO:0005515	M	Refseq	protein binding
NP_001096850.2 dpr7, isoform F [Drosophila melanogaster]	NP_001096850.2	1,00E-131	GLOS_DPR7.1.1	GO:0005634	C	Refseq	nucleus
NP_001096850.2 dpr7, isoform F [Drosophila melanogaster]	NP_001096850.2	1,00E-131	GLOS_DPR7.1.1	GO:0006366	B	Refseq	transcription from RNA polymerase II promoter
NP_001096850.2 dpr7, isoform F [Drosophila melanogaster]	NP_001096850.2	1,00E-131	GLOS_DPR7.1.1	GO:0006607	B	Refseq	NLS-bearing protein import into nucleus
NP_001096850.2 dpr7, isoform F [Drosophila melanogaster]	NP_001096850.2	1,00E-131	GLOS_DPR7.1.1	GO:0008270	M	Refseq	zinc ion binding
NP_001096850.2 dpr7, isoform F [Drosophila melanogaster]	NP_001096850.2	1,00E-131	GLOS_DPR7.1.1	GO:0032330	B	Refseq	regulation of chondrocyte differentiation
NP_001096850.2 dpr7, isoform F [Drosophila melanogaster]	NP_001096850.2	1,00E-131	GLOS_DPR7.1.1	GO:0043565	M	Refseq	sequence-specific DNA binding
XP_004921914.1 PREDICTED: uncharact prot LOC101743910 [B. mori]	XP_004921914.1	2,00E-09	GLOS_LOC10174	GO:0000122	B	Refseq	negative regulation of transcription from RNA polymerase II promoter
XP_004921914.1 PREDICTED: uncharact prot LOC101743910 [B. mori]	XP_004921914.1	2,00E-09	GLOS_LOC10174	GO:0003674	M	Refseq	molecular_function
XP_004921914.1 PREDICTED: uncharact prot LOC101743910 [B. mori]	XP_004921914.1	2,00E-09	GLOS_LOC10174	GO:0003682	M	Refseq	chromatin binding
XP_004921914.1 PREDICTED: uncharact prot LOC101743910 [B. mori]	XP_004921914.1	2,00E-09	GLOS_LOC10174	GO:0000981	M	.	sequence-specific DNA binding RNA polymerase II transcription factor activity
XP_004921914.1 PREDICTED: uncharact prot LOC101743910 [B. mori]	XP_004921914.1	2,00E-09	GLOS_LOC10174	GO:0003700	M	.	sequence-specific DNA binding transcription factor activity
XP_004921914.1 PREDICTED: uncharact prot LOC101743910 [B. mori]	XP_004921914.1	2,00E-09	GLOS_LOC10174	GO:0003705	M	Refseq	RNA polymerase II distal enhancer sequence-specific DNA binding transcription factor activity

XP_004921914.1 PREDICTED: uncharact prot LOC101743910 [B. mori]	XP_004921914.1	2,00E-09	GLOS_LOC10174	GO:0003712	M	.	transcription cofactor activity
XP_004921914.1 PREDICTED: uncharact prot LOC101743910 [B. mori]	XP_004921914.1	2,00E-09	GLOS_LOC10174	GO:0003714	M	Refseq	transcription corepressor activity
XP_004921914.1 PREDICTED: uncharact prot LOC101743910 [B. mori]	XP_004921914.1	2,00E-09	GLOS_LOC10174	GO:0005634	C	Refseq	nucleus
XP_004921914.1 PREDICTED: uncharact prot LOC101743910 [B. mori]	XP_004921914.1	2,00E-09	GLOS_LOC10174	GO:0005654	C	Refseq	nucleoplasm
XP_004921914.1 PREDICTED: uncharact prot LOC101743910 [B. mori]	XP_004921914.1	2,00E-09	GLOS_LOC10174	GO:0007219	B	Refseq	Notch signaling pathway
XP_004921914.1 PREDICTED: uncharact prot LOC101743910 [B. mori]	XP_004921914.1	2,00E-09	GLOS_LOC10174	GO:0008150	B	Refseq	biological_process
XP_004921914.1 PREDICTED: uncharact prot LOC101743910 [B. mori]	XP_004921914.1	2,00E-09	GLOS_LOC10174	GO:0016055	B	Refseq	Wnt receptor signaling pathway
XP_004921914.1 PREDICTED: uncharact prot LOC101743910 [B. mori]	XP_004921914.1	2,00E-09	GLOS_LOC10174	GO:0008134	M	.	transcription factor binding
XP_004921914.1 PREDICTED: uncharact prot LOC101743910 [B. mori]	XP_004921914.1	2,00E-09	GLOS_LOC10174	GO:0070491	M	Refseq	repressing transcription factor binding
XP_004921914.1 PREDICTED: uncharact prot LOC101743910 [B. mori]	XP_004921914.1	1,00E-08	GLOS_LOC10174	GO:0000122	B	Refseq	negative regulation of transcription from RNA polymerase II promoter
XP_004921914.1 PREDICTED: uncharact prot LOC101743910 [B. mori]	XP_004921914.1	1,00E-08	GLOS_LOC10174	GO:0003674	M	Refseq	molecular_function
XP_004921914.1 PREDICTED: uncharact prot LOC101743910 [B. mori]	XP_004921914.1	1,00E-08	GLOS_LOC10174	GO:0003682	M	Refseq	chromatin binding
XP_004921914.1 PREDICTED: uncharact prot LOC101743910 [B. mori]	XP_004921914.1	1,00E-08	GLOS_LOC10174	GO:0003705	M	Refseq	RNA polymerase II distal enhancer sequence-specific DNA binding transcription factor activity
XP_004921914.1 PREDICTED: uncharact prot LOC101743910 [B. mori]	XP_004921914.1	1,00E-08	GLOS_LOC10174	GO:0003714	M	Refseq	transcription corepressor activity
XP_004921914.1 PREDICTED: uncharact prot LOC101743910 [B. mori]	XP_004921914.1	1,00E-08	GLOS_LOC10174	GO:0005634	C	Refseq	nucleus
XP_004921914.1 PREDICTED: uncharact prot LOC101743910 [B. mori]	XP_004921914.1	1,00E-08	GLOS_LOC10174	GO:0005654	C	Refseq	nucleoplasm
XP_004921914.1 PREDICTED: uncharact prot LOC101743910 [B. mori]	XP_004921914.1	1,00E-08	GLOS_LOC10174	GO:0007219	B	Refseq	Notch signaling pathway
XP_004921914.1 PREDICTED: uncharact prot LOC101743910 [B. mori]	XP_004921914.1	1,00E-08	GLOS_LOC10174	GO:0008150	B	Refseq	biological_process
XP_004921914.1 PREDICTED: uncharact prot LOC101743910 [B. mori]	XP_004921914.1	1,00E-08	GLOS_LOC10174	GO:0016055	B	Refseq	Wnt receptor signaling pathway
XP_004921914.1 PREDICTED: uncharact prot LOC101743910 [B. mori]	XP_004921914.1	1,00E-08	GLOS_LOC10174	GO:0070491	M	Refseq	repressing transcription factor binding
NP_610760.1 CG8841, isoform A [D. melanogaster] ref|NP_725131.1| CG8841, isoform B [D. melanogaster] ref|NP_725132.1| CG8841, isoform C [D. melano.]	NP_610760.1	0	GLOS_CG8841.1.	GO:0000122	B	Refseq	negative regulation of transcription from RNA polymerase II promoter
NP_610760.1 CG8841, isoform A [D. m.] … Same as above	NP_610760.1	0	GLOS_CG8841.1.	GO:0001501	B	Refseq	skeletal system development
NP_610760.1 CG8841, isoform A [D. m.] … Same as above	NP_610760.1	0	GLOS_CG8841.1.	GO:0003700	M	Refseq	sequence-specific DNA binding transcription factor activity
NP_610760.1 CG8841, isoform A [D. m.] … Same as above	NP_610760.1	0	GLOS_CG8841.1.	GO:0005515	M	Refseq	protein binding
NP_610760.1 CG8841, isoform A [D. m.] … Same as above	NP_610760.1	0	GLOS_CG8841.1.	GO:0005634	C	Refseq	nucleus
NP_610760.1 CG8841, isoform A [D. m.] … Same as above	NP_610760.1	0	GLOS_CG8841.1.	GO:0006366	B	Refseq	transcription from RNA polymerase II promoter
NP_610760.1 CG8841, isoform A [D. m.] … Same as above	NP_610760.1	0	GLOS_CG8841.1.	GO:0006607	B	Refseq	NLS-bearing protein import into nucleus
NP_610760.1 CG8841, isoform A [D. m.] … Same as above	NP_610760.1	0	GLOS_CG8841.1.	GO:0008270	M	Refseq	zinc ion binding
NP_610760.1 CG8841, isoform A [D. m.] … Same as above	NP_610760.1	0	GLOS_CG8841.1.	GO:0032330	B	Refseq	regulation of chondrocyte differentiation
NP_610760.1 CG8841, isoform A [D. m.] … Same as above	NP_610760.1	0	GLOS_CG8841.1.	GO:0043565	M	Refseq	sequence-specific DNA binding
NP_001260788.1 CG1399, isoform F [Drosophila melanogaster]	NP_001260788.1	3,00E-06	GLOS_CG1399.1.	GO:0000122	B	Refseq	negative regulation of transcription from RNA polymerase II promoter
NP_001260788.1 CG1399, isoform F [Drosophila melanogaster]	NP_001260788.1	3,00E-06	GLOS_CG1399.1.	GO:0001501	B	Refseq	skeletal system development
NP_001260788.1 CG1399, isoform F [Drosophila melanogaster]	NP_001260788.1	3,00E-06	GLOS_CG1399.1.	GO:0003700	M	Refseq	sequence-specific DNA binding transcription factor activity
NP_001260788.1 CG1399, isoform F [Drosophila melanogaster]	NP_001260788.1	3,00E-06	GLOS_CG1399.1.	GO:0005515	M	Refseq	protein binding
NP_001260788.1 CG1399, isoform F [Drosophila melanogaster]	NP_001260788.1	3,00E-06	GLOS_CG1399.1.	GO:0005634	C	Refseq	nucleus
NP_001260788.1 CG1399, isoform F [Drosophila melanogaster]	NP_001260788.1	3,00E-06	GLOS_CG1399.1.	GO:0006366	B	Refseq	transcription from RNA polymerase II promoter
NP_001260788.1 CG1399, isoform F [Drosophila melanogaster]	NP_001260788.1	3,00E-06	GLOS_CG1399.1.	GO:0006607	B	Refseq	NLS-bearing protein import into nucleus
NP_001260788.1 CG1399, isoform F [Drosophila melanogaster]	NP_001260788.1	3,00E-06	GLOS_CG1399.1.	GO:0008270	M	Refseq	zinc ion binding
NP_001260788.1 CG1399, isoform F [Drosophila melanogaster]	NP_001260788.1	3,00E-06	GLOS_CG1399.1.	GO:0032330	B	Refseq	regulation of chondrocyte differentiation
NP_001260788.1 CG1399, isoform F [Drosophila melanogaster]	NP_001260788.1	3,00E-06	GLOS_CG1399.1.	GO:0043565	M	Refseq	sequence-specific DNA binding
XP_003816862.1 PREDICT: uncharact prot LOC100991169 [P paniscus]	XP_003816862.1	2,00E-07	GLOS_LOC10099	GO:0007165	B	Refseq	signal transduction
NP_001262986.1 CG5447, isoform B [Drosophila melanogaster]	NP_001262986.1	1,00E-82	GLOS_CG5447.1.	GO:0000122	B	Refseq	negative regulation of transcription from RNA polymerase II promoter
NP_001262986.1 CG5447, isoform B [Drosophila melanogaster]	NP_001262986.1	1,00E-82	GLOS_CG5447.1.	GO:0001501	B	Refseq	skeletal system development
NP_001262986.1 CG5447, isoform B [Drosophila melanogaster]	NP_001262986.1	1,00E-82	GLOS_CG5447.1.	GO:0003700	M	Refseq	sequence-specific DNA binding transcription factor activity
NP_001262986.1 CG5447, isoform B [Drosophila melanogaster]	NP_001262986.1	1,00E-82	GLOS_CG5447.1.	GO:0005515	M	Refseq	protein binding
NP_001262986.1 CG5447, isoform B [Drosophila melanogaster]	NP_001262986.1	1,00E-82	GLOS_CG5447.1.	GO:0005634	C	Refseq	nucleus
NP_001262986.1 CG5447, isoform B [Drosophila melanogaster]	NP_001262986.1	1,00E-82	GLOS_CG5447.1.	GO:0006366	B	Refseq	transcription from RNA polymerase II promoter
NP_001262986.1 CG5447, isoform B [Drosophila melanogaster]	NP_001262986.1	1,00E-82	GLOS_CG5447.1.	GO:0006607	B	Refseq	NLS-bearing protein import into nucleus
NP_001262986.1 CG5447, isoform B [Drosophila melanogaster]	NP_001262986.1	1,00E-82	GLOS_CG5447.1.	GO:0008270	M	Refseq	zinc ion binding
NP_001262986.1 CG5447, isoform B [Drosophila melanogaster]	NP_001262986.1	1,00E-82	GLOS_CG5447.1.	GO:0032330	B	Refseq	regulation of chondrocyte differentiation
NP_001262986.1 CG5447, isoform B [Drosophila melanogaster]	NP_001262986.1	1,00E-82	GLOS_CG5447.1.	GO:0043565	M	Refseq	sequence-specific DNA binding
NP_650802.1 CG3734 [Drosophila melanogaster]	NP_650802.1	4,00E-45	GLOS_CG3734.2.	GO:0000122	B	Refseq	negative regulation of transcription from RNA polymerase II promoter

NP_650802.1 CG3734 [Drosophila melanogaster]	NP_650802.1	4,00E-45	GLOS_CG3734.2.:	GO:0001501	B	Refseq	skeletal system development
NP_650802.1 CG3734 [Drosophila melanogaster]	NP_650802.1	4,00E-45	GLOS_CG3734.2.:	GO:0003700	M	Refseq	sequence-specific DNA binding transcription factor activity
NP_650802.1 CG3734 [Drosophila melanogaster]	NP_650802.1	4,00E-45	GLOS_CG3734.2.:	GO:0005515	M	Refseq	protein binding
NP_650802.1 CG3734 [Drosophila melanogaster]	NP_650802.1	4,00E-45	GLOS_CG3734.2.:	GO:0005634	C	Refseq	nucleus
NP_650802.1 CG3734 [Drosophila melanogaster]	NP_650802.1	4,00E-45	GLOS_CG3734.2.:	GO:0006366	B	Refseq	transcription from RNA polymerase II promoter
NP_650802.1 CG3734 [Drosophila melanogaster]	NP_650802.1	4,00E-45	GLOS_CG3734.2.:	GO:0006607	B	Refseq	NLS-bearing protein import into nucleus
NP_650802.1 CG3734 [Drosophila melanogaster]	NP_650802.1	4,00E-45	GLOS_CG3734.2.:	GO:0008270	M	Refseq	zinc ion binding
NP_650802.1 CG3734 [Drosophila melanogaster]	NP_650802.1	4,00E-45	GLOS_CG3734.2.:	GO:0032330	B	Refseq	regulation of chondrocyte differentiation
NP_650802.1 CG3734 [Drosophila melanogaster]	NP_650802.1	4,00E-45	GLOS_CG3734.2.:	GO:0043565	M	Refseq	sequence-specific DNA binding
NP_001163401.1 CG42526 [Drosophila melanogaster]	NP_001163401.1	5,00E-16	GLOS_CG42526.1	GO:0000122	B	Refseq	negative regulation of transcription from RNA polymerase II promoter
NP_001163401.1 CG42526 [Drosophila melanogaster]	NP_001163401.1	5,00E-16	GLOS_CG42526.1	GO:0001501	B	Refseq	skeletal system development
NP_001163401.1 CG42526 [Drosophila melanogaster]	NP_001163401.1	5,00E-16	GLOS_CG42526.1	GO:0003700	M	Refseq	sequence-specific DNA binding transcription factor activity
NP_001163401.1 CG42526 [Drosophila melanogaster]	NP_001163401.1	5,00E-16	GLOS_CG42526.1	GO:0005515	M	Refseq	protein binding
NP_001163401.1 CG42526 [Drosophila melanogaster]	NP_001163401.1	5,00E-16	GLOS_CG42526.1	GO:0005634	C	Refseq	nucleus
NP_001163401.1 CG42526 [Drosophila melanogaster]	NP_001163401.1	5,00E-16	GLOS_CG42526.1	GO:0006366	B	Refseq	transcription from RNA polymerase II promoter
NP_001163401.1 CG42526 [Drosophila melanogaster]	NP_001163401.1	5,00E-16	GLOS_CG42526.1	GO:0006607	B	Refseq	NLS-bearing protein import into nucleus
NP_001163401.1 CG42526 [Drosophila melanogaster]	NP_001163401.1	5,00E-16	GLOS_CG42526.1	GO:0008270	M	Refseq	zinc ion binding
NP_001163401.1 CG42526 [Drosophila melanogaster]	NP_001163401.1	5,00E-16	GLOS_CG42526.1	GO:0032330	B	Refseq	regulation of chondrocyte differentiation
NP_001163401.1 CG42526 [Drosophila melanogaster]	NP_001163401.1	5,00E-16	GLOS_CG42526.1	GO:0043565	M	Refseq	sequence-specific DNA binding
NP_001097089.2 no mechanoreceptor potential C, isoform D [D.m.]	NP_001097089.2	3,00E-15	GLOS_NOMPC.1.:	GO:0000122	B	Refseq	negative regulation of transcription from RNA polymerase II promoter
NP_001097089.2 no mechanoreceptor potential C, isoform D [D.m.]	NP_001097089.2	3,00E-15	GLOS_NOMPC.1.:	GO:0001501	B	Refseq	skeletal system development
NP_001097089.2 no mechanoreceptor potential C, isoform D [D.m.]	NP_001097089.2	3,00E-15	GLOS_NOMPC.1.:	GO:0003700	M	Refseq	sequence-specific DNA binding transcription factor activity
NP_001097089.2 no mechanoreceptor potential C, isoform D [D.m.]	NP_001097089.2	3,00E-15	GLOS_NOMPC.1.:	GO:0005515	M	Refseq	protein binding
NP_001097089.2 no mechanoreceptor potential C, isoform D [D.m.]	NP_001097089.2	3,00E-15	GLOS_NOMPC.1.:	GO:0005634	C	Refseq	nucleus
NP_001097089.2 no mechanoreceptor potential C, isoform D [D.m.]	NP_001097089.2	3,00E-15	GLOS_NOMPC.1.:	GO:0006366	B	Refseq	transcription from RNA polymerase II promoter
NP_001097089.2 no mechanoreceptor potential C, isoform D [D.m.]	NP_001097089.2	3,00E-15	GLOS_NOMPC.1.:	GO:0006607	B	Refseq	NLS-bearing protein import into nucleus
NP_001097089.2 no mechanoreceptor potential C, isoform D [D.m.]	NP_001097089.2	3,00E-15	GLOS_NOMPC.1.:	GO:0008270	M	Refseq	zinc ion binding
NP_001097089.2 no mechanoreceptor potential C, isoform D [D.m.]	NP_001097089.2	3,00E-15	GLOS_NOMPC.1.:	GO:0032330	B	Refseq	regulation of chondrocyte differentiation
NP_001097089.2 no mechanoreceptor potential C, isoform D [D.m.]	NP_001097089.2	3,00E-15	GLOS_NOMPC.1.:	GO:0043565	M	Refseq	sequence-specific DNA binding
NM_001272673.1 D. m. cabeza (caz), transcript variant E, mRNA	NM_001272673.1	6,00E-06	GLOS_CAZ.2.2	GO:0000122	B	Refseq	negative regulation of transcription from RNA polymerase II promoter
NM_001272673.1 D. m. cabeza (caz), transcript variant E, mRNA	NM_001272673.1	6,00E-06	GLOS_CAZ.2.2	GO:0001501	B	Refseq	skeletal system development
NM_001272673.1 D. m. cabeza (caz), transcript variant E, mRNA	NM_001272673.1	6,00E-06	GLOS_CAZ.2.2	GO:0003700	M	Refseq	sequence-specific DNA binding transcription factor activity
NM_001272673.1 D. m. cabeza (caz), transcript variant E, mRNA	NM_001272673.1	6,00E-06	GLOS_CAZ.2.2	GO:0005515	M	Refseq	protein binding
NM_001272673.1 D. m. cabeza (caz), transcript variant E, mRNA	NM_001272673.1	6,00E-06	GLOS_CAZ.2.2	GO:0005634	C	Refseq	nucleus
NM_001272673.1 D. m. cabeza (caz), transcript variant E, mRNA	NM_001272673.1	6,00E-06	GLOS_CAZ.2.2	GO:0006366	B	Refseq	transcription from RNA polymerase II promoter
NM_001272673.1 D. m. cabeza (caz), transcript variant E, mRNA	NM_001272673.1	6,00E-06	GLOS_CAZ.2.2	GO:0006607	B	Refseq	NLS-bearing protein import into nucleus
NM_001272673.1 D. m. cabeza (caz), transcript variant E, mRNA	NM_001272673.1	6,00E-06	GLOS_CAZ.2.2	GO:0008270	M	Refseq	zinc ion binding
NM_001272673.1 D. m. cabeza (caz), transcript variant E, mRNA	NM_001272673.1	6,00E-06	GLOS_CAZ.2.2	GO:0032330	B	Refseq	regulation of chondrocyte differentiation
NM_001272673.1 D. m. cabeza (caz), transcript variant E, mRNA	NM_001272673.1	6,00E-06	GLOS_CAZ.2.2	GO:0043565	M	Refseq	sequence-specific DNA binding
NP_611517.1 CG17999 [Drosophila melanogaster]	NP_611517.1	1,00E-143	GLOS_CG17999.1	GO:0000122	B	Refseq	negative regulation of transcription from RNA polymerase II promoter
NP_611517.1 CG17999 [Drosophila melanogaster]	NP_611517.1	1,00E-143	GLOS_CG17999.1	GO:0001501	B	Refseq	skeletal system development
NP_611517.1 CG17999 [Drosophila melanogaster]	NP_611517.1	1,00E-143	GLOS_CG17999.1	GO:0003700	M	Refseq	sequence-specific DNA binding transcription factor activity
NP_611517.1 CG17999 [Drosophila melanogaster]	NP_611517.1	1,00E-143	GLOS_CG17999.1	GO:0005515	M	Refseq	protein binding
NP_611517.1 CG17999 [Drosophila melanogaster]	NP_611517.1	1,00E-143	GLOS_CG17999.1	GO:0005634	C	Refseq	nucleus
NP_611517.1 CG17999 [Drosophila melanogaster]	NP_611517.1	1,00E-143	GLOS_CG17999.1	GO:0006366	B	Refseq	transcription from RNA polymerase II promoter
NP_611517.1 CG17999 [Drosophila melanogaster]	NP_611517.1	1,00E-143	GLOS_CG17999.1	GO:0006607	B	Refseq	NLS-bearing protein import into nucleus
NP_611517.1 CG17999 [Drosophila melanogaster]	NP_611517.1	1,00E-143	GLOS_CG17999.1	GO:0008270	M	Refseq	zinc ion binding
NP_611517.1 CG17999 [Drosophila melanogaster]	NP_611517.1	1,00E-143	GLOS_CG17999.1	GO:0032330	B	Refseq	regulation of chondrocyte differentiation
NP_611517.1 CG17999 [Drosophila melanogaster]	NP_611517.1	1,00E-143	GLOS_CG17999.1	GO:0043565	M	Refseq	sequence-specific DNA binding
NP_611517.1 CG17999 [Drosophila melanogaster]	NP_611517.1	1,00E-154	GLOS_CG17999.2	GO:0000122	B	Refseq	negative regulation of transcription from RNA polymerase II promoter
NP_611517.1 CG17999 [Drosophila melanogaster]	NP_611517.1	1,00E-154	GLOS_CG17999.2	GO:0001501	B	Refseq	skeletal system development
NP_611517.1 CG17999 [Drosophila melanogaster]	NP_611517.1	1,00E-154	GLOS_CG17999.2	GO:0003700	M	Refseq	sequence-specific DNA binding transcription factor activity
NP_611517.1 CG17999 [Drosophila melanogaster]	NP_611517.1	1,00E-154	GLOS_CG17999.2	GO:0005515	M	Refseq	protein binding
NP_611517.1 CG17999 [Drosophila melanogaster]	NP_611517.1	1,00E-154	GLOS_CG17999.2	GO:0005634	C	Refseq	nucleus

NP_611517.1 CG17999 [Drosophila melanogaster]	NP_611517.1	1,00E-154	GLOS_CG17999.2	GO:0006366	B	Refseq	transcription from RNA polymerase II promoter
NP_611517.1 CG17999 [Drosophila melanogaster]	NP_611517.1	1,00E-154	GLOS_CG17999.2	GO:0006607	B	Refseq	NLS-bearing protein import into nucleus
NP_611517.1 CG17999 [Drosophila melanogaster]	NP_611517.1	1,00E-154	GLOS_CG17999.2	GO:0008270	M	Refseq	zinc ion binding
NP_611517.1 CG17999 [Drosophila melanogaster]	NP_611517.1	1,00E-154	GLOS_CG17999.2	GO:0032330	B	Refseq	regulation of chondrocyte differentiation
NP_611517.1 CG17999 [Drosophila melanogaster]	NP_611517.1	1,00E-154	GLOS_CG17999.2	GO:0043565	M	Refseq	sequence-specific DNA binding
NR_004370.1 D. m. ribosomal RNA (CR41602), rRNA	NR_004370.1	1,00E-39	GLOS_CR41602.2	GO:0000122	B	Refseq	negative regulation of transcription from RNA polymerase II promoter
NR_004370.1 D. m. ribosomal RNA (CR41602), rRNA	NR_004370.1	1,00E-39	GLOS_CR41602.2	GO:0001501	B	Refseq	skeletal system development
NR_004370.1 D. m. ribosomal RNA (CR41602), rRNA	NR_004370.1	1,00E-39	GLOS_CR41602.2	GO:0003700	M	Refseq	sequence-specific DNA binding transcription factor activity
NR_004370.1 D. m. ribosomal RNA (CR41602), rRNA	NR_004370.1	1,00E-39	GLOS_CR41602.2	GO:0005515	M	Refseq	protein binding
NR_004370.1 D. m. ribosomal RNA (CR41602), rRNA	NR_004370.1	1,00E-39	GLOS_CR41602.2	GO:0005634	C	Refseq	nucleus
NR_004370.1 D. m. ribosomal RNA (CR41602), rRNA	NR_004370.1	1,00E-39	GLOS_CR41602.2	GO:0006366	B	Refseq	transcription from RNA polymerase II promoter
NR_004370.1 D. m. ribosomal RNA (CR41602), rRNA	NR_004370.1	1,00E-39	GLOS_CR41602.2	GO:0006607	B	Refseq	NLS-bearing protein import into nucleus
NR_004370.1 D. m. ribosomal RNA (CR41602), rRNA	NR_004370.1	1,00E-39	GLOS_CR41602.2	GO:0008270	M	Refseq	zinc ion binding
NR_004370.1 D. m. ribosomal RNA (CR41602), rRNA	NR_004370.1	1,00E-39	GLOS_CR41602.2	GO:0032330	B	Refseq	regulation of chondrocyte differentiation
NR_004370.1 D. m. ribosomal RNA (CR41602), rRNA	NR_004370.1	1,00E-39	GLOS_CR41602.2	GO:0043565	M	Refseq	sequence-specific DNA binding
XP_001652845.1 60S ribos prot L21 [A aeg.]ref|XP_001652846.1| 60S ribos prot L21	XP_001652845.1	2,00E-46	GLOS_AAEL_AAE	GO:0030674	M	.	protein binding, bridging
XP_001652845.1 60S ribos prot L21 [A aeg.]ref|XP_001652846.1| 60S ribos prot L21	XP_001652845.1	2,00E-46	GLOS_AAEL_AAE	GO:0060090	M	.	binding, bridging
XP_001652845.1 60S ribos prot L21 [A aeg.]ref|XP_001652846.1| 60S ribos prot L21	XP_001652845.1	2,00E-46	GLOS_AAEL_AAE	GO:0035591	M	.	signaling adaptor activity
XP_001652845.1 60S ribos prot L21 [A aeg.]ref|XP_001652846.1| 60S ribos prot L21	XP_001652845.1	2,00E-46	GLOS_AAEL_AAE	GO:0005070	M	Refseq	SH3/SH2 adaptor activity
XP_001652845.1 60S ribos prot L21 [A aeg.]ref|XP_001652846.1| 60S ribos prot L21	XP_001652845.1	2,00E-46	GLOS_AAEL_AAE	GO:0005515	M	Refseq	protein binding
XP_001652845.1 60S ribos prot L21 [A aeg.]ref|XP_001652846.1| 60S ribos prot L21	XP_001652845.1	2,00E-46	GLOS_AAEL_AAE	GO:0005634	C	Refseq	nucleus
XP_001652845.1 60S ribos prot L21 [A aeg.]ref|XP_001652846.1| 60S ribos prot L21	XP_001652845.1	2,00E-46	GLOS_AAEL_AAE	GO:0005737	C	Refseq	cytoplasm
XP_001652845.1 60S ribos prot L21 [A aeg.]ref|XP_001652846.1| 60S ribos prot L21	XP_001652845.1	2,00E-46	GLOS_AAEL_AAE	GO:0007049	B	Refseq	cell cycle
XP_001652845.1 60S ribos prot L21 [A aeg.]ref|XP_001652846.1| 60S ribos prot L21	XP_001652845.1	2,00E-46	GLOS_AAEL_AAE	GO:0007165	B	Refseq	signal transduction
XP_001652845.1 60S ribos prot L21 [A aeg.]ref|XP_001652846.1| 60S ribos prot L21	XP_001652845.1	2,00E-46	GLOS_AAEL_AAE	GO:0007417	B	Refseq	central nervous system development
XP_001652845.1 60S ribos prot L21 [A aeg.]ref|XP_001652846.1| 60S ribos prot L21	XP_001652845.1	2,00E-46	GLOS_AAEL_AAE	GO:0007507	B	Refseq	heart development
XP_001652845.1 60S ribos prot L21 [A aeg.]ref|XP_001652846.1| 60S ribos prot L21	XP_001652845.1	2,00E-46	GLOS_AAEL_AAE	GO:0009790	B	.	embryo development
XP_001652845.1 60S ribos prot L21 [A aeg.]ref|XP_001652846.1| 60S ribos prot L21	XP_001652845.1	2,00E-46	GLOS_AAEL_AAE	GO:0009792	B	Refseq	embryo development ending in birth or egg hatching
XP_001652845.1 60S ribos prot L21 [A aeg.]ref|XP_001652846.1| 60S ribos prot L21	XP_001652845.1	2,00E-46	GLOS_AAEL_AAE	GO:0010212	B	Refseq	response to ionizing radiation
XP_001652845.1 60S ribos prot L21 [A aeg.]ref|XP_001652846.1| 60S ribos prot L21	XP_001652845.1	2,00E-46	GLOS_AAEL_AAE	GO:0019904	M	.	protein domain specific binding
XP_001652845.1 60S ribos prot L21 [A aeg.]ref|XP_001652846.1| 60S ribos prot L21	XP_001652845.1	2,00E-46	GLOS_AAEL_AAE	GO:0017124	M	Refseq	SH3 domain binding
XP_001652845.1 60S ribos prot L21 [A aeg.]ref|XP_001652846.1| 60S ribos prot L21	XP_001652845.1	2,00E-46	GLOS_AAEL_AAE	GO:0042802	M	Refseq	identical protein binding
XP_001652845.1 60S ribos prot L21 [A aeg.]ref|XP_001652846.1| 60S ribos prot L21	XP_001652845.1	2,00E-46	GLOS_AAEL_AAE	GO:0051726	B	.	regulation of cell cycle
XP_001652845.1 60S ribos prot L21 [A aeg.]ref|XP_001652846.1| 60S ribos prot L21	XP_001652845.1	2,00E-46	GLOS_AAEL_AAE	GO:0045786	B	Refseq	negative regulation of cell cycle
XP_001652845.1 60S ribos prot L21 [A aeg.]ref|XP_001652846.1| 60S ribos prot L21	XP_001652845.1	2,00E-46	GLOS_AAEL_AAE	GO:0048471	C	Refseq	perinuclear region of cytoplasm
XP_001652845.1 60S ribos prot L21 [A aeg.]ref|XP_001652846.1| 60S ribos prot L21	XP_001652845.1	2,00E-46	GLOS_AAEL_AAE	GO:0051059	M	Refseq	NF-kappaB binding
XP_001652845.1 60S ribos prot L21 [A aeg.]ref|XP_001652846.1| 60S ribos prot L21	XP_001652845.1	2,00E-46	GLOS_AAEL_AAE	GO:0072331	B	.	signal transduction by p53 class mediator
XP_001652845.1 60S ribos prot L21 [A aeg.]ref|XP_001652846.1| 60S ribos prot L21	XP_001652845.1	2,00E-46	GLOS_AAEL_AAE	GO:0035556	B	.	intracellular signal transduction
XP_001652845.1 60S ribos prot L21 [A aeg.]ref|XP_001652846.1| 60S ribos prot L21	XP_001652845.1	2,00E-46	GLOS_AAEL_AAE	GO:0097193	B	.	intrinsic apoptotic signaling pathway
XP_001652845.1 60S ribos prot L21 [A aeg.]ref|XP_001652846.1| 60S ribos prot L21	XP_001652845.1	2,00E-46	GLOS_AAEL_AAE	GO:0097190	B	.	apoptotic signaling pathway
XP_001652845.1 60S ribos prot L21 [A aeg.]ref|XP_001652846.1| 60S ribos prot L21	XP_001652845.1	2,00E-46	GLOS_AAEL_AAE	GO:0006915	B	.	apoptotic process
XP_001652845.1 60S ribos prot L21 [A aeg.]ref|XP_001652846.1| 60S ribos prot L21	XP_001652845.1	2,00E-46	GLOS_AAEL_AAE	GO:0012501	B	.	programmed cell death
XP_001652845.1 60S ribos prot L21 [A aeg.]ref|XP_001652846.1| 60S ribos prot L21	XP_001652845.1	2,00E-46	GLOS_AAEL_AAE	GO:0008219	B	.	cell death
XP_001652845.1 60S ribos prot L21 [A aeg.]ref|XP_001652846.1| 60S ribos prot L21	XP_001652845.1	2,00E-46	GLOS_AAEL_AAE	GO:0016265	B	.	death
XP_001652845.1 60S ribos prot L21 [A aeg.]ref|XP_001652846.1| 60S ribos prot L21	XP_001652845.1	2,00E-46	GLOS_AAEL_AAE	GO:0072332	B	Refseq	intrinsic apoptotic signaling pathway by p53 class mediator
XP_001648627.1 clathrin coat assembly protein ap17 [A. aegypti] ref|XP_001648628.1| clathrin coat assembly protein ap17 [A. aegypti] ref|XP_321389.2| AGAP001703-PA [A. gambiae str. PEST] ref|XP_001851131.1| clathrin coat assembly protein AP17 [C. quinquefasciatus] ref|XP_004533559.1| PREDICTED: AP-2 complex subunit sigma-like [C. capitata]	XP_001648627.1	7,00E-98	GLOS_AAEL_AAE	GO:0005070	M	Refseq	SH3/SH2 adaptor activity
XP_001648627.1 clathrin coat assembly protein ap17 … Same as above	XP_001648627.1	7,00E-98	GLOS_AAEL_AAE	GO:0005515	M	Refseq	protein binding
XP_001648627.1 clathrin coat assembly protein ap17 … Same as above	XP_001648627.1	7,00E-98	GLOS_AAEL_AAE	GO:0005634	C	Refseq	nucleus
XP_001648627.1 clathrin coat assembly protein ap17 … Same as above	XP_001648627.1	7,00E-98	GLOS_AAEL_AAE	GO:0005737	C	Refseq	cytoplasm
XP_001648627.1 clathrin coat assembly protein ap17 … Same as above	XP_001648627.1	7,00E-98	GLOS_AAEL_AAE	GO:0007049	B	Refseq	cell cycle

XP_001648627.1 clathrin coat assembly protein ap17 … Same as above	XP_001648627.1	7,00E-98	GLOS_AAEL_AAE	GO:0007165	B	Refseq	signal transduction
XP_001648627.1 clathrin coat assembly protein ap17 … Same as above	XP_001648627.1	7,00E-98	GLOS_AAEL_AAE	GO:0007417	B	Refseq	central nervous system development
XP_001648627.1 clathrin coat assembly protein ap17 … Same as above	XP_001648627.1	7,00E-98	GLOS_AAEL_AAE	GO:0007507	B	Refseq	heart development
XP_001648627.1 clathrin coat assembly protein ap17 … Same as above	XP_001648627.1	7,00E-98	GLOS_AAEL_AAE	GO:0009792	B	Refseq	embryo development ending in birth or egg hatching
XP_001648627.1 clathrin coat assembly protein ap17 … Same as above	XP_001648627.1	7,00E-98	GLOS_AAEL_AAE	GO:0010212	B	Refseq	response to ionizing radiation
XP_001648627.1 clathrin coat assembly protein ap17 … Same as above	XP_001648627.1	7,00E-98	GLOS_AAEL_AAE	GO:0017124	M	Refseq	SH3 domain binding
XP_001648627.1 clathrin coat assembly protein ap17 … Same as above	XP_001648627.1	7,00E-98	GLOS_AAEL_AAE	GO:0042802	M	Refseq	identical protein binding
XP_001648627.1 clathrin coat assembly protein ap17 … Same as above	XP_001648627.1	7,00E-98	GLOS_AAEL_AAE	GO:0045786	B	Refseq	negative regulation of cell cycle
XP_001648627.1 clathrin coat assembly protein ap17 … Same as above	XP_001648627.1	7,00E-98	GLOS_AAEL_AAE	GO:0048471	C	Refseq	perinuclear region of cytoplasm
XP_001648627.1 clathrin coat assembly protein ap17 … Same as above	XP_001648627.1	7,00E-98	GLOS_AAEL_AAE	GO:0051059	M	Refseq	NF-kappaB binding
XP_001648627.1 clathrin coat assembly protein ap17 … Same as above	XP_001648627.1	7,00E-98	GLOS_AAEL_AAE	GO:0072332	B	Refseq	intrinsic apoptotic signaling pathway by p53 class mediator
XP_001949504.2 PREDICTED: hypoth prot LOC100162931 [A. pisum]	XP_001949504.2	1,00E-08	GLOS_LOC10016:	GO:0000278	B	Refseq	mitotic cell cycle
XP_001949504.2 PREDICTED: hypoth prot LOC100162931 [A. pisum]	XP_001949504.2	1,00E-08	GLOS_LOC10016:	GO:0003677	M	Refseq	DNA binding
XP_001949504.2 PREDICTED: hypoth prot LOC100162931 [A. pisum]	XP_001949504.2	1,00E-08	GLOS_LOC10016:	GO:0003700	M	Refseq	sequence-specific DNA binding transcription factor activity
XP_001949504.2 PREDICTED: hypoth prot LOC100162931 [A. pisum]	XP_001949504.2	1,00E-08	GLOS_LOC10016:	GO:0003712	M	Refseq	transcription cofactor activity
XP_001949504.2 PREDICTED: hypoth prot LOC100162931 [A. pisum]	XP_001949504.2	1,00E-08	GLOS_LOC10016:	GO:0005654	C	Refseq	nucleoplasm
XP_001949504.2 PREDICTED: hypoth prot LOC100162931 [A. pisum]	XP_001949504.2	1,00E-08	GLOS_LOC10016:	GO:0005667	C	Refseq	transcription factor complex
XP_001949504.2 PREDICTED: hypoth prot LOC100162931 [A. pisum]	XP_001949504.2	1,00E-08	GLOS_LOC10016:	GO:0006351	B	Refseq	transcription, DNA-dependent
XP_001949504.2 PREDICTED: hypoth prot LOC100162931 [A. pisum]	XP_001949504.2	1,00E-08	GLOS_LOC10016:	GO:0006367	B	Refseq	transcription initiation from RNA polymerase II promoter
XP_001949504.2 PREDICTED: hypoth prot LOC100162931 [A. pisum]	XP_001949504.2	1,00E-08	GLOS_LOC10016:	GO:0007179	B	Refseq	transforming growth factor beta receptor signaling pathway
XP_001949504.2 PREDICTED: hypoth prot LOC100162931 [A. pisum]	XP_001949504.2	1,00E-08	GLOS_LOC10016:	GO:0007507	B	Refseq	heart development
XP_001949504.2 PREDICTED: hypoth prot LOC100162931 [A. pisum]	XP_001949504.2	1,00E-08	GLOS_LOC10016:	GO:0008134	M	Refseq	transcription factor binding
XP_001949504.2 PREDICTED: hypoth prot LOC100162931 [A. pisum]	XP_001949504.2	1,00E-08	GLOS_LOC10016:	GO:0010467	B	Refseq	gene expression
XP_001949504.2 PREDICTED: hypoth prot LOC100162931 [A. pisum]	XP_001949504.2	1,00E-08	GLOS_LOC10016:	GO:0019904	M	Refseq	protein domain specific binding
XP_001949504.2 PREDICTED: hypoth prot LOC100162931 [A. pisum]	XP_001949504.2	1,00E-08	GLOS_LOC10016:	GO:0045944	B	Refseq	positive regulation of transcription from RNA polymerase II promoter
XP_001996272.1 GH22284 [Drosophila grimshawi]	XP_001996272.1	1,00E-126	GLOS_DGRI_GH2	GO:0005262	M	Refseq	calcium channel activity
XP_001996272.1 GH22284 [Drosophila grimshawi]	XP_001996272.1	1,00E-126	GLOS_DGRI_GH2	GO:0005515	M	Refseq	protein binding
XP_001996272.1 GH22284 [Drosophila grimshawi]	XP_001996272.1	1,00E-126	GLOS_DGRI_GH2	GO:0005886	C	Refseq	plasma membrane
XP_001996272.1 GH22284 [Drosophila grimshawi]	XP_001996272.1	1,00E-126	GLOS_DGRI_GH2	GO:0005887	C	Refseq	integral to plasma membrane
XP_001996272.1 GH22284 [Drosophila grimshawi]	XP_001996272.1	1,00E-126	GLOS_DGRI_GH2	GO:0006816	B	Refseq	calcium ion transport
XP_001996272.1 GH22284 [Drosophila grimshawi]	XP_001996272.1	1,00E-126	GLOS_DGRI_GH2	GO:0007411	B	Refseq	axon guidance
XP_001996272.1 GH22284 [Drosophila grimshawi]	XP_001996272.1	1,00E-126	GLOS_DGRI_GH2	GO:0007596	B	Refseq	blood coagulation
XP_001996272.1 GH22284 [Drosophila grimshawi]	XP_001996272.1	1,00E-126	GLOS_DGRI_GH2	GO:0007602	B	Refseq	phototransduction
XP_001996272.1 GH22284 [Drosophila grimshawi]	XP_001996272.1	1,00E-126	GLOS_DGRI_GH2	GO:0010524	B	Refseq	positive regulation of calcium ion transport into cytosol
XP_001996272.1 GH22284 [Drosophila grimshawi]	XP_001996272.1	1,00E-126	GLOS_DGRI_GH2	GO:0015279	M	Refseq	store-operated calcium channel activity
XP_001996272.1 GH22284 [Drosophila grimshawi]	XP_001996272.1	1,00E-126	GLOS_DGRI_GH2	GO:0030168	B	Refseq	platelet activation
XP_001996272.1 GH22284 [Drosophila grimshawi]	XP_001996272.1	1,00E-126	GLOS_DGRI_GH2	GO:0033198	B	Refseq	response to ATP
XP_001996272.1 GH22284 [Drosophila grimshawi]	XP_001996272.1	1,00E-126	GLOS_DGRI_GH2	GO:0051592	B	Refseq	response to calcium ion
XP_001996272.1 GH22284 [Drosophila grimshawi]	XP_001996272.1	1,00E-126	GLOS_DGRI_GH2	GO:0070588	B	Refseq	calcium ion transmembrane transport
XP_001996272.1 GH22284 [Drosophila grimshawi]	XP_001996272.1	1,00E-126	GLOS_DGRI_GH2	GO:0070679	M	Refseq	inositol 1,4,5 trisphosphate binding
XP_001984835.1 GH16696 [Drosophila grimshawi]	XP_001984835.1	4,00E-56	GLOS_DGRI_GH1	GO:0005262	M	Refseq	calcium channel activity
XP_001984835.1 GH16696 [Drosophila grimshawi]	XP_001984835.1	4,00E-56	GLOS_DGRI_GH1	GO:0005515	M	Refseq	protein binding
XP_001984835.1 GH16696 [Drosophila grimshawi]	XP_001984835.1	4,00E-56	GLOS_DGRI_GH1	GO:0005886	C	Refseq	plasma membrane
[truncated: 945,650 more chars]
