# Supplementary material for: RNA-seq de novo Assembly Reveals Differential Gene Expression in Glossina palpalis gambiensis Infected with Trypanosoma brucei gambiense vs. Non-Infected and Self-Cured Flies
Source: Front Microbiol. 2015 Nov 13;6:1259. doi: 10.3389/fmicb.2015.01259 (PMC4643127; doi:10.3389/fmicb.2015.01259)
Supplement: Supplementary file 2 [file Table2.PDF]

Supplementary Table S2: significantly differentially expressed genes (p<0.05) between tsetse flies 3 days post-infected bloodmeal (S3) and tsetse flies 3 days post-noninfected bloodmeal (NS3)

id	Base MeanA (S3)	Base MeanB (NS3)	Fold Change (NS3/N3)	pval	Best hit description
GLOS_UCP2.1.1	1.02803511192406	660.938157321403	642.913991609099	5.61591653535498e-23	NM_011671.4 Mus musculus uncoupling protein 2 (mitochondrial, proton carrier) (Ucp2), mRNA
GLOS_HBB2.2.2	34.0491011393587	3586.30584993997	105.327475026776	1.5964126199101e-143	HBB2_MOUSE (sp|P02089) Hemoglobin subunit beta-2 OS=Mus musculus GN=Hbb-b2 PE=1 SV=2
GLOS_ALAS2.1.1	23.1023519746178	1679.97992287524	72.7189995512581	2.78209207769622e-14	NM_009653.3 Mus musculus aminolevulinic acid synthase 2, erythroid (Alas2), transcript variant 1, mRNA
GLOS_HBB-BT.1.1	238.560690215365	15233.6545186029	63.856515945064	1.73352399267563e-123	NM_008220.5 Mus musculus hemoglobin, beta adult t chain (Hbb-bt), mRNA
GLOS_HBA1.1.1	17.0426324230005	1027.07818240203	60.2652311514912	1.35575088417974e-55	NM_013096.1 Rattus norvegicus hemoglobin, alpha 1 (Hba1), mRNA
GLOS_HBA-A1.6.6	239.139309521643	14358.7450240548	60.0434326450847	5.69969983599993e-30	NM_008218.2 Mus musculus hemoglobin alpha, adult chain 1 (Hba-a1), mRNA
GLOS_HBB-B1.1.2	1015.07238994343	60533.3647116829	59.6345298240815	4.72731072973197e-82	NM_001278161.1 Mus musculus hemoglobin, beta adult major chain (Hbb-b1), mRNA
GLOS_HBA.5.5	68.2428571052869	3895.95387133713	57.0895480727946	1.7309356516213e-35	HBA_MOUSE (sp|P01942) Hemoglobin subunit alpha OS=Mus musculus GN=Hba PE=1 SV=2
GLOS_HBA-A1.1.6	1649.32796556243	93894.0323458087	56.9286608281029	1.94986314296189e-45	NM_008218.2 Mus musculus hemoglobin alpha, adult chain 1 (Hba-a1), mRNA
GLOS_HBB-B1.2.2	40.0726569843336	2226.83424384574	55.5699175304578	9.51695031757584e-106	NM_001278161.1 Mus musculus hemoglobin, beta adult major chain (Hbb-b1), mRNA
GLOS_HBA-A1.5.6	166.458838608879	9158.45491500204	55.0193368615365	4.25064097445301e-34	NM_008218.2 Mus musculus hemoglobin alpha, adult chain 1 (Hba-a1), mRNA
GLOS_HBA-A1.3.6	122.057058936995	6317.59102663229	51.7593253651426	2.33827304873888e-56	NM_008218.2 Mus musculus hemoglobin alpha, adult chain 1 (Hba-a1), mRNA
GLOS_OAZ1.1.1	8.00729865553824	391.017762166672	48.8326686673819	1.03026645730061e-23	NM_008753.4 Mus musculus ornithine decarboxylase antizyme 1 (Oaz1), mRNA
GLOS_HBA-A1.2.6	184.240196490664	8977.75745167667	48.7285490499984	2.91974743838933e-51	NM_008218.2 Mus musculus hemoglobin alpha, adult chain 1 (Hba-a1), mRNA
GLOS_LOC100862446.1.1	47.4858850076562	1492.98307413747	31.4405654205825	1.22176813829409e-08	XM_003689208.2: M. musculus ferritin light chain 1-like, transcript vari. 1 (LOC100862446), mRNA
GLOS_DANA_GF24496.1.1	26.4034395502442	167.418212380957	6.34077283993132	3.6754496479536e-09	XP_001956622.1 GF24496 [Drosophila ananassae]
GLOS_DERE_GG19064.1.3	844.201910962541	3700.70083390462	4.38366791859682	1.75935021587626e-08	XP_001977467.1 GG19064 [Drosophila erecta]
GLOS_contig_014921	57.5492538870426	248.358503331181	4.31558163757706	0.000657085657652888	
GLOS_contig_011799	18.3599771880636	65.0189432533506	3.54134117855122	0.00386155413116617	
GLOS_HBA-A1.4.6	3459.86216935288	11993.0684716478	3.46634284390898	1.61787285411059e-11	NM_008218.2 Mus musculus hemoglobin alpha, adult chain 1 (Hba-a1), mRNA
GLOS_contig_014759	559.204083232148	1712.54542046741	3.06246944866543	5.88546782586107e-15	
GLOS_contig_014175	33.346539387216	99.2815568777615	2.97726716781361	0.00183561266210138	
GLOS_contig_012370	29.162071526235	81.5592168323841	2.7967566281775	0.00691207421792005	
GLOS_contig_003540	44.4741070851688	121.12910680077	2.72358715530376	0.00152513453061895	
GLOS_contig_013097	47.3773938877291	124.024640528813	2.6178020855836	0.00200142179043248	
GLOS_DVIR_GJ19325.1.1	1473.14241722021	3747.80747971467	2.5440903987998	0.00518376763604483	XP_002055362.1 GJ19325 [Drosophila virilis]
GLOS_CBPA1.4.11	81.932786920081	203.949739571569	2.48923229928095	0.00315948690991365	CBPA1_DROPS (sp|Q29NC4)Zinc carboxypeptidase A 1; D.p. peseudoob. GN=GA14587 PE=3 SV=1
GLOS_contig_011969	237.00072161334	588.605118083237	2.48355833719161	0.00251627369510623	
GLOS_contig_013203	172.337739627284	421.242691000256	2.44428580710923	0.00133066621623837	
GLOS_contig_011645	53.8349142124124	130.664506668543	2.42713318262178	0.00288667979879937	
GLOS_contig_002688	63.5366460253751	151.425209491693	2.38327357461106	0.00200256075772496	
GLOS_contig_013996	49.5419552315043	117.963784867682	2.38108860089291	0.00540152771795396	
GLOS_TTI.3.16	55.7101659030487	131.767223662815	2.36522762994688	0.00367641601750226	TTI_GLOMM (sp|O97373) Tsetse thrombin inhibitor OS=G. m. morsitans GN=TTI PE=1 SV=1
GLOS_LOC101236672.1.1	65.8820259023621	155.613898946981	2.36200840541247	0.00187822650745175	XP_004206943.1 PRED.: RNA-directed DNA polymerase from mobile element jockey-like
GLOS_contig_016710	71.6524358008404	166.109186759298	2.31826294392841	0.00176954977356873	
GLOS_contig_016335	60.7780140493842	139.81133479069	2.3003603684235	0.00381949165099124	
GLOS_contig_015170	88.8035593437681	192.581607566464	2.16862487257926	0.00200442331589017	
GLOS_contig_011158	71.5077809742709	154.289003430908	2.15765335364584	0.00510216409625533	

GLOS_contig_005593	70.8052192221282	150.592280702465	2.12685282747351	0.00621042377006465	
GLOS_DMOJ_GI19764.1.1	4392.65132109607	9306.60131370561	2.11867517665468	0.00174948675005818	XP_002004165.1 GI19764 [Drosophila mojavensis]
GLOS_SCNA.2.3	69.9218389367736	147.649137291178	2.11163120902311	0.00698439560381988	SCNA_DROME (sp|P35500) Sodium channel protein para OS=D. melanogaster GN=para PE=2 SV=3
GLOS_DWIL_GK24194.1.1	128.860765002164	271.737257397649	2.10876644565229	0.00205137196233883	XP_002067109.1 GK24194 [Drosophila willistoni]
GLOS_DVIR_GJ18228.1.1	2680.62274030071	5619.94148567728	2.0965059354256	0.000228309451064939	XP_002059296.1 GJ18228 [Drosophila virilis]
GLOS_DPER_GL10214.1.1	1916.91725826372	4009.71550353036	2.09175199724698	0.00194283485433695	XP_002025973.1 GL10214 [Drosophila persimilis]
GLOS_contig_011473	75.1343419096787	156.462697630624	2.08243918365203	0.00640185366985821	
GLOS_contig_012062	96.3976059003026	199.697570538624	2.07160300998717	0.0028127113653554	XP_004521238.1 PREDICTED: type-2 histone deacetylase 2-like isoform X1 [Ceratitis capitata]
GLOS_FDL.1.2	19162.9162613881	39666.9126701652	2.06998309281825	2.96913496660919e-05	FDL_DROME (sp|Q8WSF3) Probable beta-hexosaminidase fdl OS=D. melanog. GN=fdl PE=1 SV=1
GLOS_LOC101459419.1.2	12484.8728819401	25683.3693738205	2.05715906094427	0.00165314522541183	XP_004533256.1 PREDICTED: zinc carboxypeptidase A 1-like [Ceratitis capitata]
GLOS_DSEC_GM17695.1.1	2815.99264066014	5749.34457640741	2.04167599495559	9.17915907915511e-05	XP_002036375.1 GM17695 [Drosophila sechellia]
GLOS_LOC101455604.4.10	4147.78762381539	8446.31327951755	2.03634179122896	4.61981036388972e-06	XP_004520096.1 PREDICTED: serine protease SP24D-like [Ceratitis capitata]
GLOS_CBPA1.11.11	12571.5016896689	25447.6240672034	2.024231050147	0.00210256331643669	CBPA1_DROPS (sp|Q29NC4) Zinc carboxypeptidase A 1 OS=D.p. p. GN=GA14587 PE=3 SV=1
GLOS_DPSE_GA26760.1.1	3220.20637766708	6488.24862281397	2.01485490737848	1.89541080113992e-05	XP_001358028.1 GA26760 [Drosophila pseudoobscura pseudoobscura]
GLOS_DMOJ_GI20641.1.1	193.063809073039	388.035184686874	2.00988049779995	0.00119313775126797	XP_002005755.1 GI20641 [Drosophila mojavensis]
GLOS_contig_006296	86.8559802398471	173.018841104072	1.99201990037176	0.0075020928609857	
GLOS_LOC101449871.1.1	154.272333147126	307.181937488404	1.99116673237482	0.00656806123302449	XP_004526049.1 PREDICTED: bromodomain-containing protein DDB_G0280777-like [C. capitata]
GLOS_LOC101455604.5.10	8916.47195968791	17638.7182236062	1.9782172033235	2.86174923794195e-05	XP_004520096.1 PREDICTED: serine protease SP24D-like [Ceratitis capitata]
GLOS_contig_012200	108.083080523829	213.548619017	1.97578212965461	0.00410867307117775	
GLOS_DMOJ_GI20119.1.1	241.179928419554	473.656131819232	1.96391190147161	0.000342944250744146	XP_002003981.1 GI20119 [Drosophila mojavensis]
GLOS_DVIR_GJ13580.1.1	170.39542157813	333.813057239713	1.95904945184605	0.00745743605526405	XP_002047702.1 GJ13580 [Drosophila virilis]
GLOS_contig_001797	384.557127434519	724.347028294272	1.88358757807086	8.72990948920099e-05	
GLOS_MLC_9020.1.1	1932.46698844322	3639.05929015123	1.88311588860974	0.00371731223656814	YP_004400609.1 transmembrane protein [Mycoplasma mycoides subsp. capri LC str. 95010] ref|WP_013729997.1| transmembrane protein [Mycoplasma mycoides]
GLOS_LOC101456285.1.1	32755.6634808884	60800.4453796509	1.85618115826368	0.000109704765508011	XP_004529473.1 PREDICTED: protein FAM188A homolog [Ceratitis capitata]
GLOS_HYPA.1.1	547.957834143097	1016.09092786286	1.85432320618582	0.00208247288312894	HYPA_HYPLI (sp|P35587) Hypodermin-A OS=Hypoderma lineatum PE=1 SV=2
GLOS_TRYDG.5.5	6387.2504957371	11727.4656504439	1.83607416967142	0.00106976625124384	TRYDG_DROER (sp|P54626) Trypsin delta/gamma OS=Drosophila erecta GN=deltaTry PE=3 SV=1
GLOS_LOC101456419.1.1	9446.58696248384	17269.890410349	1.82816190428719	6.47862902956799e-07	XP_004535276.1 PREDICTED: inner centromere protein-like isoform X1 [Ceratitis capitata]
GLOS_LOC101448839.1.1	3621.20220845957	6616.91400412136	1.82726995710525	0.00400927644056044	XP_004518567.1 PREDICTED: protein timeless-like isoform X1 [Ceratitis capitata] ref|XP_004518568.1| PREDICTED: protein timeless-like isoform X2 [Ceratitis capitata]
GLOS_contig_014288	218.780138197079	398.903174350403	1.82330616315394	0.00176000133039683	
GLOS_contig_014690	1068.4943838952	1942.20678858436	1.81770425550019	0.00407800621054055	
GLOS_contig_009448	647.883700130051	1176.77405473761	1.81633533071042	4.0676486590503e-05	
GLOS_LOC101449088.1.1	3335.73918175923	6030.18105224396	1.80774956424012	0.00630412911054168	XP_004523175.1 PREDICTED: lysM and putative peptidoglycan-binding domain-containing protein 1-like isoform X1 [Ceratitis capitata]
GLOS_DANA_GF17961.1.1	18652.1333903199	33654.7025886728	1.80433529422103	0.00774204756590883	XP_001953827.1 GF17961 [Drosophila ananassae]
GLOS_LOC101450530.4.4	870.99296101468	1562.96111872778	1.7944589551068	2.64798774988597e-05	XP_004521927.1 PREDICTED: zinc carboxypeptidase A 1-like [Ceratitis capitata]
GLOS_contig_015454	521.440642418159	933.32608039004	1.78989899226454	0.000127289748518986	
GLOS_LOC101458352.1.1	381.31291594624	680.937586547714	1.78577110313178	0.000350390864373693	XP_004533341.1 PRED: ATP-binding cassette sub-family G member 1-like isoform X1 [C. capitata] ref|XP_004533342.1| PREDICTED: ATP-binding cassette sub-family G member 1-like isoform X2
GLOS_contig_004199	146.678286590592	261.551191858887	1.78316230669457	0.00735835936636884	

GLOS_DMOJ_GI21769.1.1	959.078507280368	1708.92852854609	1.78184425526545	2.5172430392605e-05	XP_002010850.1 GI21769 [Drosophila mojavensis]
GLOS_CG9517.1.1	177.028499381258	314.797561467091	1.77823097731358	0.00486457755138296	NP_727805.2 CG9517, isoform B [Drosophila melanogaster]
GLOS_contig_012510	626.40871495434	1113.70710849722	1.77792403251988	0.00388020535718178	
GLOS_contig_014875	161.809503616314	285.255038093324	1.76290657667257	0.00671369314256551	
GLOS_DPER_GL24111.1.1	654.702857521158	1153.64970031682	1.76209663218025	9.95933404201514e-05	XP_002013379.1 GL24111 [Drosophila persimilis]
GLOS_contig_016571	169.925293391779	299.026737099628	1.75975413154176	0.00599832024922276	
GLOS_LOC101450510.1.1	2058.95673197129	3615.45932023306	1.75596663304894	0.00379085646845137	XP_004535333.1 PREDICTED: uncharacterized protein LOC101450510 isoform X1 [C. capitata]
GLOS_contig_008438	471.537050120231	820.399783661288	1.73984161679789	0.00038465304063295	
GLOS_DWIL_GK11338.1.1	4017.78080598693	6986.23425835939	1.73882911878844	0.00320957685303684	XP_002069867.1 GK11338 [Drosophila willistoni]
GLOS_LOC101462349.8.8	222.442862839129	384.711163809643	1.72948306319842	0.00377821752917716	XP_004529335.1 PRED: low quality prot.: activating signal cointegrator 1 complex subunit 3-like
GLOS_DMOJ_GI10981.2.2	16938.1956686368	29276.1559025399	1.72841053883611	4.21182762214221e-06	XP_002011642.1 GI10981 [Drosophila mojavensis]
GLOS_CG10252.1.2	536.520244411301	925.290144876897	1.7246136646571	0.00031879989124324	NP_651171.1 CG10252 [Drosophila melanogaster] ref|XP_002032372.1| GM23550 [Drosophila sechellia] ref|XP_002104582.1| GD18365 [Drosophila simulans]
GLOS_DVIR_GJ22731.1.1	364.611208208958	628.151443877526	1.72279795501386	0.00100108923360995	XP_002054581.1 GJ22731 [Drosophila virilis]
GLOS_contig_011904	6662.95851640008	11349.2019887008	1.70332772758018	0.000348744455265973	
GLOS_DWIL_GK23174.1.1	3354.73432250256	5690.90730732057	1.69638092326646	0.00111234728135009	XP_002074627.1 GK23174 [Drosophila willistoni]
GLOS_contig_008758	279.377333713251	473.243514564458	1.69392236755394	0.00309363118626525	
GLOS_LOC101458418.1.1	329.714890490887	558.031532907587	1.69246688275673	0.0019065689881362	XP_004535088.1 PREDICTED: inositol polyphosphate multikinase-like [Ceratitis capitata]
GLOS_contig_015681	228.936546870454	385.686921648601	1.68468917226591	0.00544097939894591	
GLOS_LOC101453231.2.2	320.121649797852	537.254960190023	1.67828374160037	0.00238219264141738	XP_004537792.1 PREDICTED: LYR motif-containing protein 5-like [Ceratitis capitata]
GLOS_DSIM_GD14429.1.1	267.133952164152	446.850443229364	1.67275795386271	0.00422802878732598	XP_002084740.1 GD14429 [Drosophila simulans]
GLOS_DWIL_GK12510.4.4	1736.84048541497	2902.86178274489	1.67134622155662	7.24059086720181e-05	XP_002067829.1 GK12510 [Drosophila willistoni]
GLOS_LOC101459622.6.22	7690.17172150417	12813.6471561631	1.66623680461283	2.07424058890951e-05	XP_004537835.1 PREDICTED: zinc metalloproteinase nas-4-like [Ceratitis capitata]
GLOS_DWIL_GK25686.1.1	1060.07383314066	1762.61107958316	1.66272482583701	0.000181273715239916	XP_002071245.1 GK25686 [Drosophila willistoni]
GLOS_LOC101455624.1.2	2482.41875566805	4127.34505794524	1.662630468176	4.92258077207646e-05	XP_004527398.1 PREDICTED: synaptic vesicle glycoprotein 2A-like isoform X2 [Ceratitis capitata]
GLOS_LOC101450530.2.4	298.94616454645	495.638335177237	1.65795181192306	0.00381856565597276	XP_004521927.1 PREDICTED: zinc carboxypeptidase A 1-like [Ceratitis capitata]
GLOS_LOC101456777.3.6	2362.23694842169	3911.96466946101	1.6560424525045	0.00522508156611119	XP_004534236.1 PREDICTED: uncharacterized protein LOC101456777 [Ceratitis capitata]
GLOS_CG10252.2.2	1678.96082895175	2773.18890380972	1.65172936496747	0.000102662261343883	NP_651171.1 CG10252 [D. melanogaster] ref|XP_002032372.1| GM23550 [Drosophila sechellia] ref|XP_002104582.1| GD18365 [Drosophila simulans]
GLOS_contig_007302	586.273920827893	962.18619830399	1.64118880973805	0.00198699084046708	
GLOS_contig_007483	2273.59349523043	3692.18062594432	1.62394053012987	0.00365042546364229	
GLOS_LOC101453541.1.1	706.430086477143	1140.10017983231	1.61388961435351	0.00673376073419366	XP_004537158.1 PREDICTED: transcription factor E2f-like [Ceratitis capitata]
GLOS_LOC101459622.15.22	9584.8959815141	15468.1196584581	1.6138015152476	0.000404083185498932	XP_004537835.1 PREDICTED: zinc metalloproteinase nas-4-like [Ceratitis capitata]
GLOS_LOC101454883.1.1	7033.52720961058	11259.7140820169	1.60086308710538	0.00388166495870227	XP_004534042.1 PREDICTED: protein rolling stone-like [Ceratitis capitata]
GLOS_DERE_GG21392.1.1	820.913811238307	1307.26399256759	1.59244974889099	0.000946854920198417	XP_001973896.1 GG21392 [Drosophila erecta]
GLOS_LOC101454308.1.2	394.496553868041	625.438173267723	1.58540845828765	0.00469827378751049	XM_004518198.1 PRED.: C. capit. prot. CLEC16A-like (LOC101454308), transcript variant X5, mRNA
GLOS_LOC101453821.1.1	5254.80521820243	8284.50290880434	1.57655756299152	0.00016712537249433	XP_004530529.1 PREDICTED: 60S ribosomal export protein NMD3-like [Ceratitis capitata]
GLOS_LOC101453078.1.2	1736.76322878528	2734.84868999092	1.57468136396676	0.000430749145711803	XP_004527472.1 PREDICTED: synaptic vesicle glycoprotein 2B-like [Ceratitis capitata]
GLOS_contig_004530	3264.55128125161	5082.23351149388	1.55679389712187	0.000344181795760686	XP_004527397.1 PREDICTED: synaptic vesicle glycoprotein 2A-like isoform X1 [Ceratitis capitata]
GLOS_DWIL_GK18237.1.1	1458.47080627131	2268.84809755393	1.55563490732764	0.00344295707634937	XP_002066329.1 GK18237 [Drosophila willistoni]
GLOS_contig_009455	6876.05635527627	10686.9235473039	1.55422279794194	0.000235451374881496	

GLOS_LOC101455624.2.2	3082.76495575786	4786.68131860089	1.55272341138449	0.000380300427919402	XP_004527398.1 PREDICTED: synaptic vesicle glycoprotein 2A-like isoform X2 [Ceratitis capitata]
GLOS_LOC101458193.1.1	961.103674852341	1488.76360756346	1.54901458241972	0.0026646622175852	XP_004521696.1 PREDICTED: uncharacterized protein LOC101458193 [Ceratitis capitata]
GLOS_CP9F2.9.9	551.269111989894	847.491130350614	1.53734557572337	0.0050260720208538	CP9F2_DROME (sp|Q9VG82) Probable cyt. P450 9f2 OS=D. melanogaster GN=Cyp9f2 PE=2 SV=1
GLOS_LOC101742112.3.5	1347.90788131509	2070.07049955629	1.53576555805625	0.00122533347664656	XP_004929373.1 PREDICTED: uncharacterized protein LOC101742112 [Bombyx mori]
GLOS_LOC101459622.10.22	14572.6754068339	22364.8222440496	1.53470942154942	0.000301791087764434	XP_004537835.1 PREDICTED: zinc metalloproteinase nas-4-like [Ceratitis capitata]
GLOS_LOC101454485.7.7	4295.14283056102	6584.57837933067	1.53302896762355	0.000459664616130264	XP_004537801.1 PREDICTED: zinc metalloproteinase nas-4-like [Ceratitis capitata]
GLOS_CRY1.1.1	681.431770431184	1043.49197816079	1.53132275811049	0.00355017343048748	[BBH] CRY1_DROME (sp|O77059) Cryptochrome-1 OS=D. melanogaster GN=cry PE=1 SV=1
GLOS_LOC101454485.3.7	30233.9308423988	46253.0111900143	1.52983783124724	0.00030285334707867	XP_004537801.1 PREDICTED: zinc metalloproteinase nas-4-like [Ceratitis capitata]
GLOS_LOC101457078.1.1	1317.86769055827	2004.87698746438	1.52130369522535	0.0015453395767433	XP_004525658.1 PREDICTED: heat shock 70 kDa protein 14-like [Ceratitis capitata]
GLOS_DYAK_GE15912.4.4	3814.7358138806	5792.82471067626	1.51853889582551	0.000642487782273917	XP_002100904.1 GE15912 [Drosophila yakuba]
GLOS_LOC101459622.18.22	1142.6575667803	1733.7586559489	1.51730378930073	0.0020057694229947	XP_004537835.1 PREDICTED: zinc metalloproteinase nas-4-like [Ceratitis capitata]
GLOS_LOC101459622.19.22	2441.45745734359	3698.78057668049	1.51498874803451	0.000964621034878694	XP_004537835.1 PREDICTED: zinc metalloproteinase nas-4-like [Ceratitis capitata]
GLOS_DWIL_GK15016.1.2	7784.0013723945	11790.7384239766	1.5147400237867	0.000533143789740526	XP_002064786.1 GK15016 [Drosophila willistoni]
GLOS_LOC101453261.1.1	1781.45431811031	2693.13684561165	1.5117630680917	0.00132988593406528	XP_004527473.1 PREDICTED: synaptic vesicle glycoprotein 2A-like [Ceratitis capitata]
GLOS_contig_003328	2597.15562637571	3925.12513686483	1.51131687951341	0.0009734139991137	XP_004527397.1 PREDICTED: synaptic vesicle glycoprotein 2A-like isoform X1 [Ceratitis capitata]
GLOS_LOC101454485.5.7	3780.77942069687	5697.44618515433	1.50695016851953	0.000847697269975421	XP_004537801.1 PREDICTED: zinc metalloproteinase nas-4-like [Ceratitis capitata]
GLOS_CG34402.1.1	1602.83461563679	2415.13242392548	1.50678828642965	0.00162863525309745	NP_001097756.1 CG34402, isoform C [Drosophila melanogaster]
GLOS_LOC101463325.1.1	70961.0232275734	106614.860871125	1.50244255257157	0.000515512244632668	XP_004517889.1 PREDICTED: trypsin-like [Ceratitis capitata]
GLOS_LOC101459622.16.22	1590.73062785949	2385.63799157	1.49971211328228	0.005401727811479	XP_004537835.1 PREDICTED: zinc metalloproteinase nas-4-like [Ceratitis capitata]
GLOS_LOC101450530.3.4	4117.14810137159	6160.44015460237	1.49628820798311	0.000994901843099503	XP_004521927.1 PREDICTED: zinc carboxypeptidase A 1-like [Ceratitis capitata]
GLOS_LOC101459622.13.22	171830.817706738	256803.103759313	1.494511329147	0.000596041736023671	XP_004537835.1 PREDICTED: zinc metalloproteinase nas-4-like [Ceratitis capitata]
GLOS_RL23.14.16	685.001455279245	1021.40637982157	1.49110103628202	0.00637780408092429	RL23_DROME (sp|P48159) 60S ribosomal protein L23 OS=D. melanogaster GN=RpL23 PE=1 SV=2
GLOS_LOC101450347.1.1	632.55621324518	942.504648301015	1.48999350344805	0.0072641420872873	XP_004521926.1 PREDICTED: zinc carboxypeptidase A 1-like [Ceratitis capitata]
GLOS_DPER_GL21241.1.4	5563.52018092036	8288.31600839812	1.4897611114672	0.00211829962211599	XP_002023222.1 GL21241 [Drosophila persimilis]
GLOS_LOC101459622.21.22	13719.3809541782	20423.1305050311	1.48863353042261	0.000795569373887038	XP_004537835.1 PREDICTED: zinc metalloproteinase nas-4-like [Ceratitis capitata]
GLOS_TB11.02.4440.1.1	8050.12866550416	11979.6901577376	1.48813648272134	0.000990966693429187	XP_828733.1 aminopeptidase [Trypanosoma brucei brucei strain 927/4 GUTat10.1]
GLOS_contig_008190	675.769851652633	1002.29566468246	1.4831908559272	0.0070401397928225	
GLOS_DYAK_GE18501.1.1	704.063994219451	1044.23738186322	1.48315691533253	0.00678790491471612	XP_002088313.1 GE18501 [Drosophila yakuba]
GLOS_DWIL_GK24139.1.1	72816.2949230257	107341.056975705	1.4741351106806	0.00380984252534041	XP_002067211.1 GK24139 [Drosophila willistoni]
GLOS_LOC101455387.1.1	1515.99934777764	2230.33331489658	1.4711967509525	0.00318264266900141	XP_004526841.1 PREDICTED: proton-coupled folate transporter-like isoform X1 [C. capitata] ref|XP_004526842.1| PREDICTED: proton-coupled folate transporter-like
GLOS_TRYT.2.4	213498.66488243	314065.551247621	1.47104222605126	0.00632090284505333	TRYT_DROME (sp|P42278) Trypsin theta OS=Drosophila melanogaster GN=thetaTry PE=2 SV=2
GLOS_DSIM_MCT1.1.1	2106.20985157479	3097.67286980904	1.47073325456765	0.00241443812144802	XP_002105969.1 Mct1 [Drosophila simulans]
GLOS_SCRAMB1.1.1	1723.94122792991	2534.32588494275	1.47007673108784	0.0028593065542354	NP_729600.1 scramblase 1, isoform C [Drosophila melanogaster]
GLOS_DANA_GF23835.1.3	2967.65099665784	4356.69624111853	1.46806219667509	0.00195621062690214	XP_001957802.1 GF23835 [Drosophila ananassae]
GLOS_DWIL_GK18649.1.3	3040.13519771146	4460.33288067541	1.46714951494034	0.00194158487057045	XP_002070831.1 GK18649 [Drosophila willistoni]
GLOS_contig_010920	1671.04130903543	2443.59627581412	1.4623194906083	0.0034567487713553	
GLOS_CG15097.1.5	1321.28745952499	1931.060353766	1.46149904008037	0.00444689608191625	NP_001188973.1 CG15097, isoform C [Drosophila melanogaster]
GLOS_DANA_GF23835.2.3	4195.35635834587	6126.19292953732	1.46023183879253	0.00191197145011606	XP_001957802.1 GF23835 [Drosophila ananassae]
GLOS_DWIL_GK21356.4.5	953.72661053566	1390.98920932001	1.45847792643508	0.00628095525149407	XP_002063538.1 GK21356 [Drosophila willistoni]
GLOS_LOC101462766.13.14	7800.4042438842	11374.9482450453	1.45825112255735	0.0015854225637485	XP_004529420.1 PRED.: prob. isoaspartyl peptidase/L-asparaginase GA20639-like [C. capitata]

GLOS_LOC101456506.1.1	1291.71213589975	1878.96431083531	1.45463084120248	0.0050302138793932	XP_004523124.1 PRED.: myosin regulatory light chain LC-2, mantle muscle-like isoform X2
GLOS_DSEC_GM15320.1.1	6153.62778649071	8917.49226380299	1.44914391529821	0.00195980071579452	XP_002035771.1 GM15320 [Drosophila sechellia]
GLOS_LOC101462766.9.14	1055.65693171388	1529.43621452611	1.44880042803588	0.00673073219814152	XP_004529420.1 PRED.: prob. isoaspartyl peptidase/L-asparaginase GA20639-like [C. capitata]
GLOS_LOC101459622.5.22	1167.92448009869	1691.64236992958	1.44841759784557	0.00604748403246016	XP_004537835.1 PREDICTED: zinc metalloproteinase nas-4-like [Ceratitis capitata]
GLOS_LOC101459622.9.22	6566.73153876182	9497.58583087571	1.44631857946587	0.00218365608320994	XP_004537835.1 PREDICTED: zinc metalloproteinase nas-4-like [Ceratitis capitata]
GLOS_LOC101455574.1.1	2151.36580803139	3107.1923997824	1.44428826942529	0.00384657349519793	XP_004530363.1 PREDICTED: ATP-binding cassette sub-family G member 1-like isoform X1 [Ceratitis capitata] ref|XP_004530364.1| PREDICTED: ATP-binding cassette sub-family G
GLOS_LOC101460605.2.3	1620.57980981193	2338.65830491527	1.44309974168238	0.0048860470764701	XM_004525528.1 PREDICTED: Ceratitis capitata phospholipid scramblase 1-like (LOC101460605), transcript variant X2, mRNA
GLOS_LOC101460605.3.3	1955.27476970993	2814.76561341894	1.43957547912129	0.00438332747136556	XM_004525528.1 PREDICTED: Ceratitis capitata phospholipid scramblase 1-like (LOC101460605), transcript variant X2, mRNA
GLOS_LOC101462766.8.14	6363.35713959439	9147.34407065291	1.43750285737317	0.00652542950308728	XP_004529420.1 PRED.: prob. isoaspartyl peptidase/L-asparaginase GA20639-like [C. capitata]
GLOS_AAEL_AAEL000797.1.1	1994.4226216475	2863.80742367744	1.43590801297259	0.00472645936447839	XP_001651282.1 dimethylaniline monooxygenase [Aedes aegypti]
GLOS_DWIL_GK22031.1.1	2088.20625039838	2995.30485913522	1.43439129088124	0.00463294500309518	XP_002063649.1 GK22031 [Drosophila willistoni]
GLOS_DANA_GF22379.1.1	5563.30353051887	7973.3294522032	1.43320050909743	0.0027536289298296	XP_001965577.1 GF22379 [Drosophila ananassae]
GLOS_LTV1.1.1	1777.93624829483	2547.232915371	1.43269080531655	0.00533876444680376	[BBH] LTV1_DROME (sp|Q7KN79) Protein LTV1 homolog; D. melanogaster GN=CG7686 PE=1 SV=1
GLOS_CG9427.3.3	4220.3855770437	6042.83945196602	1.43182165270286	0.00319651895208208	NP_649919.1 CG9427, isoform A [Drosophila melanogaster]
GLOS_LOC101462766.10.14	11733.0800470377	16794.3818328987	1.43137025960535	0.00243441935186181	XP_004529420.1 PRED.: prob. isoaspartyl peptidase/L-asparaginase GA20639-like [C. capitata]
GLOS_LOC101451574.1.1	1244.21606062093	1779.97610633669	1.43060049027851	0.00721965808526602	XP_004523277.1 PREDICTED: WD repeat-containing protein 81-like isoform X1 [Ceratitis capitata]
GLOS_DVIR_GJ21497.1.3	76232.0432466473	108979.930028448	1.42958164817708	0.00226219941350424	XP_002049270.1 GJ21497 [Drosophila virilis]
GLOS_LOC101462766.14.14	4105.20981263993	5867.85466929476	1.42936778803063	0.00336015556687099	XP_004529420.1 PRED.: prob. isoaspartyl peptidase/L-asparaginase GA20639-like [C. capitata]
GLOS_LOC101462766.7.14	3072.2574321276	4389.18190790358	1.4286504320909	0.0038987748708074	XP_004529420.1 PRED.: prob. isoaspartyl peptidase/L-asparaginase GA20639-like [C. capitata]
GLOS_LOC101461462.1.1	1321.56657890696	1887.59560805652	1.42830156133164	0.00726071126253865	XP_004533269.1 PREDICTED: uncharacterized protein LOC101461462 [Ceratitis capitata]
GLOS_SSRP1.1.1	1831.08405208103	2614.10046065609	1.42762450346567	0.00576418179164445	[BBH] SSRP1_DROPS (sp|Q293F6) FACT complex subunit Ssrp1 OS=D. p. p. GN=Ssrp PE=3 SV=2
GLOS_IMDH.2.2	8087.32434095977	11514.5609654883	1.42377880248608	0.00806412944440417	IMDH_DROME (sp|Q07152) Inosine-5'-monophosphate dehydrogenase OS=D. melanogaster
GLOS_DWIL_GK15974.1.7	7172.28038339173	10208.6536082256	1.42334837213906	0.00319285716848987	XP_002075290.1 GK15974 [Drosophila willistoni]
GLOS_contig_014089	1535.32522293552	2184.59148000616	1.42288516294239	0.00732745554919626	
GLOS_LOC101462766.1.14	7498.11556700071	10625.7234796649	1.4171191927781	0.00356163899276905	XP_004529420.1 PRED.: prob. isoaspartyl peptidase/L-asparaginase GA20639-like [C. capitata]
GLOS_LOC101462766.5.14	6944.47017248196	9839.68152899456	1.41690889075816	0.00359640015093906	XP_004529420.1 PRED.: prob. isoaspartyl peptidase/L-asparaginase GA20639-like [C. capitata]
GLOS_LOC101462766.6.14	4495.91443836529	6365.42913159055	1.41582523841468	0.00423446078297998	XP_004529420.1 PRED.: prob. isoaspartyl peptidase/L-asparaginase GA20639-like [C. capitata]
GLOS_DVIR_GJ22461.1.1	3376.52918814492	4779.93757614489	1.41563638570855	0.00511078880381684	XP_002051013.1 GJ22461 [Drosophila virilis]
GLOS_LOC101461187.1.1	2025.7236129204	2863.57755087596	1.41360723279898	0.00655696561398106	XP_004519566.1 PREDICTED: myb-like protein Q-like [Ceratitis capitata]
GLOS_U183.1.1	1614.77849726158	2281.19150606295	1.4126962366241	0.00781344924681374	[BBH] U183_DROME (sp|Q9VSH9) UPF0183 protein CG7083; D. melanog. GN=CG7083 PE=2 SV=1
GLOS_LOC101455432.1.1	2244.19372908364	3169.92655957514	1.41250129990761	0.0064049806230512	XP_004521067.1 PREDICTED: uncharacterized protein LOC101455432 isoform X3 [C. capitata]
GLOS_LOC101453673.1.2	3579.45516702179	5039.79165197155	1.40797730850329	0.0055172694562358	XP_004518597.1 PRED.: putative uncharact. prot. DDB_G0279653-like isoform X3 [C. capitata]
GLOS_DANA_GF17838.2.3	3346.13262137644	4705.43901832175	1.40623207468273	0.00592736743156315	XP_001953586.1 GF17838 [Drosophila ananassae]
GLOS_LOC101456159.4.4	10357.1870496745	14556.9484249244	1.40549247156657	0.00424504732245027	XP_004524646.1 PREDICTED: retinoid-inducible serine carboxypeptidase-like [Ceratitis capitata]
GLOS_contig_013569	5877.41707579769	8260.28208766381	1.40542724484849	0.00456113073908722	
GLOS_DYAK_GE15912.2.4	26244.9468690087	36877.1714664814	1.40511511227434	0.0037664324819367	XP_002100904.1 GE15912 [Drosophila yakuba]
GLOS_DWIL_GK15974.3.7	9254.12741431418	12984.2941700548	1.40308141316174	0.00443133587608562	XP_002075290.1 GK15974 [Drosophila willistoni]
GLOS_DANA_GF15448.2.3	17282.9148955213	24189.3624437447	1.39961126869942	0.00434632498910172	XP_001962412.1 GF15448 [Drosophila ananassae]

GLOS_DWIL_GK10932.1.1	46567.3400907905	65155.8729777242	1.39917532009972	0.00408109176019396	XP_002070627.1 GK10932 [Drosophila willistoni]
GLOS_DMOJ_GI12593.1.5	2627.80040987428	3675.00507525366	1.39850997109384	0.00729124516659225	XP_002002467.1 GI12593 [Drosophila mojavensis]
GLOS_ELP2.1.1	9708.13132328272	13574.9373662326	1.39830590606827	0.00471842146104449	[BBH] ELP2_DROME (sp|Q7K4B3) Prob. elongator complex protein 2; D. m. GN=Elp2 PE=1 SV=1
GLOS_DMOJ_GI18413.2.2	59761.8497796359	83442.7714075478	1.39625483005014	0.00432172750724075	XP_002006760.1 GI18413 [Drosophila mojavensis]
GLOS_DANA_GF23835.3.3	4441.94505959748	6166.93671035732	1.38834150977008	0.00704370738434363	XP_001957802.1 GF23835 [Drosophila ananassae]
GLOS_DVIR_GJ21499.1.1	255581.551775154	354621.248421349	1.38750722013506	0.00485362414986916	XP_002049272.1 GJ21499 [Drosophila virilis] ref|XP_002049273.1| Trypsin-1 [D. virilis]
GLOS_TRYT.4.4	54462.2962641827	75445.4288939177	1.38527814780249	0.00543528687520234	TRYT_DROER (sp|P54628) Trypsin theta OS=Drosophila erecta GN=thetaTry PE=3 SV=1
GLOS_DMOJ_GI19297.1.1	6115.30084585802	8466.381957261	1.3844587814507	0.00681631378262726	XP_002005009.1 GI19297 [Drosophila mojavensis]
GLOS_DVIR_GJ21498.1.1	193420.781208067	266386.846716062	1.37724005172693	0.00600726693151056	XP_002049271.1 GJ21498 [Drosophila virilis]
GLOS_DMOJ_GI18413.1.2	301792.209040569	415103.786221563	1.37546223456604	0.00614842586981586	XP_002006760.1 GI18413 [Drosophila mojavensis]
GLOS_TRYA.1.5	43558.9299903988	59812.1329960443	1.37313136500892	0.00660753745054787	TRYA_DROER (sp|P54624) Trypsin alpha OS=Drosophila erecta GN=alphaTry PE=3 SV=1
GLOS_TRYA4.3.6	68934.0394778136	94618.0216007194	1.37258780012699	0.00670767051788875	TRYA4_LUCCU (sp|P35044) Trypsin alpha-4 OS=Lucilia cuprina PE=3 SV=1
GLOS_DWIL_GK19454.1.1	61030.098258333	83727.5633306928	1.37190608765341	0.00679964693974115	XP_002062897.1 GK19454 [Drosophila willistoni]
GLOS_DVIR_GJ21497.2.3	116330.887780524	159479.73028639	1.37091475298695	0.00710188513671721	XP_002049270.1 GJ21497 [Drosophila virilis]
GLOS_LOC101455430.10.10	285833.411900285	390490.107147733	1.36614577194341	0.00735053466585773	XP_004520095.1 PREDICTED: serine protease SP24D-like [Ceratitis capitata]
GLOS_DANA_GF15448.3.3	233128.441177959	317437.570733716	1.36164240248747	0.00808188750451576	XP_001962412.1 GF15448 [Drosophila ananassae]
GLOS_LOC101448504.1.1	84032.5352209117	61178.0530597941	0.728028172647227	0.00686627055509191	XP_004526797.1 PREDICTED: arginine kinase-like isoform X1 [Ceratitis capitata]
GLOS_DMOJ_GI19420.1.1	15900.5120991242	11514.5109491298	0.724159755192036	0.00660797208012198	XP_002004759.1 GI19420 [Drosophila mojavensis]
GLOS_PCKG.1.2	18043.2680067884	12966.1159280057	0.71861238901553	0.00568246041749271	[BBH] PCKG_DROME (sp|P20007) Phosphoenolpyruvate carboxykinase [GTP] OS=Drosophila melanogaster [illegible]
GLOS_KCC2A.2.2	6202.57467557491	4438.09531500162	0.715524688880954	0.00783731224237149	[BBH] KCC2A_DROME (sp|Q00168) Calcium/calmodulin-dependent protein kinase type II alpha chain OS=Drosophila melanogaster GN=CaMKII PE=1 SV=1
GLOS_CP6W1.1.1	6272.66714277372	4488.09749453972	0.715500662219917	0.00616470358715159	CP6W1_DROME (sp|Q9V9L1) Probable cyt. P450 6w1 OS=D. melanogaster GN=Cyp6w1 PE=2 SV=1
GLOS_DPSE_GA22522.1.1	3879.16043756937	2774.51331799074	0.715235516200823	0.00718933579315652	XP_002135879.1 GA22522 [Drosophila pseudoobscura pseudoobscura]
GLOS_LOC101458435.2.2	38534.493695001	27513.9189688131	0.714007537936912	0.00415329053707766	XP_004520550.1 PREDICTED: uncharacterized protein LOC101458435 isoform X2 [C. capitata]
GLOS_DWIL_GK11147.2.2	21042.2952603187	14995.4734238607	0.712634873636576	0.0042635111184328	XP_002070226.1 GK11147 [Drosophila willistoni]
GLOS_DANA_GF16109.1.1	5191.54176682753	3682.81835090995	0.709388177215891	0.00525549357747551	XP_001955699.1 GF16109 [Drosophila ananassae]
GLOS_QC.1.1	2639.70286673766	1868.06410004791	0.707679687584155	0.0076738706046616	NP_729109.1 glutaminyl cyclase [Drosophila melanogaster]
GLOS_DANA_GF12918.1.1	45812.5811682666	32415.3905676527	0.70756525262336	0.00343286284472303	XP_001959526.1 GF12918 [Drosophila ananassae]
GLOS_DWIL_GK25258.2.2	2911.79091487346	2058.3849696629	0.706913727613013	0.00684483775983255	XP_002071201.1 GK25258 [Drosophila willistoni]
GLOS_LOC101451482.1.1	5349.5698644107	3771.779670149	0.705062232244442	0.00443757120257122	XP_004536327.1 PREDICTED: uncharacterized protein LOC101451482 [Ceratitis capitata]
GLOS_contig_007349	12747.2891053997	8934.37926838389	0.700884650415538	0.00306082435094379	
GLOS_DYAK_GE11964.2.2	2575.28776964334	1803.81460600613	0.700432249657267	0.00593312379612633	XP_002091923.1 GE11964 [Drosophila yakuba]
GLOS_LOC100575767.7.33	2229.1348394712	1554.25816631419	0.697247263284841	0.00619446918712815	XP_003245892.1 PREDICTED: hypothetical protein LOC100575767 [Acyrthosiphon pisum]
GLOS_CP6G1.1.1	19952.7345459687	13866.6620265615	0.694975517998012	0.00221397902840682	CP6G1_DROME (sp|Q9V674) Cytochrome P450 6g1 OS=Drosophila melanogaster GN=Cyp6g1 PE=2 SV=1
GLOS_DMOJ_GI19977.2.3	9026.7445794759	6263.71130525266	0.693905898201048	0.00236075319872078	XP_002004520.1 GI19977 [Drosophila mojavensis]
GLOS_LOC101459752.1.1	3282.35828073051	2269.15683849244	0.691319059169685	0.00357181865111425	XP_004520747.1 PRED.: prob. 3-hydroxyisobutyrate DHase, mitochondrial-like [C. capitata]
GLOS_DPER_GL19505.1.1	1984.16298290896	1371.31535359672	0.691130398767062	0.00546392669236745	XP_002015053.1 GL19505 [Drosophila persimilis]
GLOS_LOC101449625.1.1	1598.13333377328	1102.26732129972	0.689721750998843	0.0062080282001497	XP_004524436.1 PRED.: glyoxalase domain-containing protein 4-like isoform X1 [C. capitata]
GLOS_contig_013557	1758.05739542778	1211.09245365999	0.688881066573653	0.00565858549108744	
GLOS_DMOJ_GI21244.4.5	10714.7828306332	7374.59164720393	0.68826328669212	0.00677314437764893	XP_002006762.1 GI21244 [Drosophila mojavensis]
GLOS_AAEL_AAEL012043.1.1	3594.41575578547	2472.37656292095	0.687838227656742	0.00297247417027868	XP_001655838.1 secreted modular calcium-binding protein [Aedes aegypti]

GLOS_DMOJ_GI20590.1.1	6650.19339163205	4573.08412723055	0.687661825441779	0.00214910075943323	XP_002005661.1 GI20590 [Drosophila mojavensis]
GLOS_contig_008697	13532.0967497598	9297.29193595892	0.687054793347083	0.00163901192879823	
GLOS_DMOJ_GI19977.3.3	10726.8861547338	7345.03517927595	0.684731344522992	0.00159148144597895	XP_002004520.1 GI19977 [Drosophila mojavensis]
GLOS_COX1.6.10	28261.999391576	19347.3452170773	0.684571001117641	0.00136758490273156	COX1_DROYA (sp|P00400) Cytochrome c oxidase subunit 1 OS=D. yakuba GN=mt:CoI PE=3 SV=2
GLOS_LOC101458435.1.2	44955.6197254292	30774.8627138754	0.684560971505584	0.00135671809777901	XP_004520550.1 PREDICTED: uncharacterized protein LOC101458435 isoform X2 [C. capitata]
GLOS_LOC101459846.1.2	2433.4912516111	1664.43993397936	0.68397202286115	0.00339241468627918	XP_004526015.1 PREDICTED: alaserpin-like [Ceratitis capitata]
GLOS_CADF.2.7	1418.78016689574	968.056615232149	0.682316145812947	0.00580080630991777	[BBH] CADF_DROME (sp|P45594) Cofilin/actin-depolymer. fact homolog; D.m. GN=tsr PE=2 SV=1
GLOS_LOC101459287.3.5	8202.73647263065	5566.68405244405	0.678637436545465	0.00635438478715538	XP_004526285.1 PREDICTED: cytosolic non-specific dipeptidase-like isoform X1 [C. capitata] ref|XP_004526286.1| PRED.: cytosol. non-specific dipeptid.-like isoform X2 ref|XP_004526287.1|
GLOS_contig_000022	3261.71013159117	2211.13555600572	0.677906823966314	0.00742983024471662	
GLOS_LOC101459287.2.5	8270.42668386513	5589.35635554155	0.675824424687289	0.00810162988865942	XP_004526285.1 PRED.: cytosolic non-specific dipeptidase-like isoform X1 [C. capitata] ref|XP_004526286.1| PREDICTED: cytosolic non-specific dipeptidase-like isoform X2
GLOS_DMOJ_GI13574.3.3	2761.53242132527	1860.29747140476	0.67364679735029	0.00217425564448553	XP_002008581.1 GI13574 [Drosophila mojavensis]
GLOS_LOC101458811.1.1	9396.30462260173	6328.67350053794	0.673527919190172	0.00335285296504495	XP_004526962.1 PREDICTED: pyridoxal kinase-like [Ceratitis capitata]
GLOS_DVIR_GJ15522.2.3	1091.57602348911	734.976969541586	0.673317252968151	0.00632855607444086	XP_002060062.1 GJ15522 [Drosophila virilis]
GLOS_AAEL_AAEL014871.1.1	2410.16698818023	1618.42061660421	0.671497296469979	0.00228477168305057	XP_001649874.1 methylenetetrahydrofolate dehydrogenase [Aedes aegypti]
GLOS_DANA_GF14653.1.2	2955.50558411913	1982.60864467187	0.67081877812211	0.00188151288317968	XP_001961999.1 GF14653 [Drosophila ananassae]
GLOS_DWIL_GK11147.1.2	25172.0023581664	16874.9753857659	0.670386691756023	0.000752659918172159	XP_002070226.1 GK11147 [Drosophila willistoni]
GLOS_LOC101457705.1.2	5855.36740501701	3916.64388163799	0.668898057239264	0.00305740395559177	XP_004534337.1 PREDICTED: acyl-CoA Delta(11) desaturase-like [Ceratitis capitata]
GLOS_LOC101451961.1.2	888.541885330674	593.17468500447	0.667582130676617	0.00727385776585012	XP_004534769.1 PREDICTED: solute carrier family 12 member 6-like isoform X5 [C. capitata]
GLOS_ARP3.1.1	1686.13110129974	1124.22596048363	0.666748842730574	0.00278306765368257	[BBH] ARP3_DROME (sp|P32392) Actin-related protein 3 OS=D. melanog. GN=Arp66B PE=2 SV=3
GLOS_ARC1.1.1	112908.784502967	75243.2974322697	0.666407824364567	0.00216101432952218	NP_610955.1 Activity-regulated cytoskeleton associated protein 1 [Drosophila melanogaster]
GLOS_AGAP_AGAP009641.2.	1268.67685028365	844.516247150498	0.665666948176503	0.0038291476045624	XP_318675.2 AGAP009641-PA [Anopheles gambiae str. PEST]
GLOS_DMOJ_GI24323.1.1	6094.20721461435	4040.88252507387	0.663069433442228	0.000768096707752545	XP_001999095.1 GI24323 [Drosophila mojavensis]
GLOS_RUVB1.1.1	1364.22197855048	904.3943072837	0.662937792751766	0.00659774115652746	[BBH] RUVB1_DROPS (sp|Q29AK9) RuvB-like helicase 1; D. p. pseudoobscura GN=pont PE=3 SV=1
GLOS_DSEC_GM25313.1.1	13144.0116940769	8712.6615044239	0.662861667138509	0.000583288717045948	XP_002030210.1 GM25313 [Drosophila sechellia]
GLOS_contig_011571	5527.78683689475	3663.16805936076	0.662682583002522	0.000863156346505391	
GLOS_contig_010890	4214.08290181676	2790.70397255761	0.662232812590016	0.000998031627836644	
GLOS_DERE_GG17809.1.1	8564.04199588594	5663.18982180445	0.661275344577359	0.000645143171738742	XP_001978234.1 GG17809 [D. erecta] ref|XP_002042722.1| GM17637 [D. sechellia] ref|XP_002100509.1| GE17105 [Drosophila yakuba]
GLOS_contig_002114	1413.13896049789	934.198924582829	0.661080722205609	0.00286184274605981	
GLOS_LOC100869906.2.4	1054.19493212225	695.47873476559	0.659724984036385	0.00446738758235346	XP_003693410.1 PREDICTED: TRAF-interacting protein-like, partial [Apis florea]
GLOS_SEL.1.1	2165.18460950846	1427.63134443647	0.659357792479679	0.00715064716776189	NP_610547.1 seele [D. melanogaster] ref|XP_002080855.1| GD26011 [D. simulans]
GLOS_contig_003122	943.508064720171	621.265353706849	0.65846321503474	0.00493429532409544	
GLOS_DWIL_GK10672.1.1	20180.2442614391	13278.6912229197	0.658004484529108	0.00042315193839155	XP_002068050.1 GK10672 [Drosophila willistoni]
GLOS_LOC101450586.4.9	9570.06426441269	6269.23306583876	0.655087875339727	0.000463297472321404	XP_004521992.1 PREDICTED: uncharacterized protein LOC101450586 [Ceratitis capitata]
GLOS_RPL26.1.3	57784.2909629084	37725.5484158278	0.652868587416669	0.00122419264691727	NP_649070.1 ribosomal protein L26, isoform A [D. m.] ref|NP_001262025.1| ribosomal prot. L26, isoform B [D.m.] ref|XP_001958190.1| GF23649 [D. ananassae] ref|XP_002042631.1| GM15002 [D. sechellia] ref|XP_002095681.1| GE19578 [D. yakuba] ref|XP_002095689.1| GE19574 [D. yakuba] ref|XP_002085421.1| GD14780 [D. simulans]
GLOS_RIR2.2.2	763.39546000314	497.208973389207	0.651312457880169	0.00589338409821024	[BBH] RIR2_DROME (sp|P48592) Ribonucleoside-diphosphate reductase subunit M2

GLOS_DVIR_GJ17549.1.1	4296.86783453196	2796.28921272136	0.650773847463695	0.000567613931115643	XP_002052446.1 GJ17549 [Drosophila virilis]
GLOS_DVIR_GJ16799.1.1	3386.93874276479	2200.27429795177	0.64963510268735	0.000668793832292375	XP_002056942.1 GJ16799 [Drosophila virilis]
GLOS_LOC101459287.4.5	6607.61491677986	4291.60419866764	0.64949369064611	0.0040274746477086	XP_004526285.1 PRED.: cytosolic non-specific dipeptidase-like isoform X1 [C. capitata] ref| XP_004526286.1| PRED.: cytosolic non-specific dipeptidase-like isoform X2 [C. capitata] ref| XP_004526287.1| PRED.: cytosolic non-specific dipeptidase-like isoform X3 [C. capitata]
GLOS_DSIM_CRQ.1.1	1592.14594163494	1034.05131632446	0.649470183155829	0.00353908576562937	XP_002077642.1 crq [Drosophila simulans]
GLOS_contig_006420	655.348543185954	425.59777367937	0.64942201841228	0.00736986040996324	
GLOS_contig_007005	755.083400368525	489.656828988177	0.648480457588123	0.00558729939379849	
GLOS_LOC101456384.1.1	16852.6744755181	10924.9358421471	0.648261251234498	0.00256017017102657	XP_004523400.1 PREDICTED: endoplasmin-like [Ceratitis capitata]
GLOS_contig_012940	2850.62036110577	1846.20068023048	0.647648738295804	0.000774915459115606	
GLOS_PMP3.2.2	20992.2729866297	13567.5958553933	0.646313806229309	0.000239377749942133	NP_001161922.1 peritrophic matrix protein 3 precursor [Tribolium castaneum]
GLOS_LOC101460522.2.3	2027.90329375175	1310.15183201595	0.64606228317332	0.000975436804328596	XP_004537383.1 PREDICTED: uncharacterized protein LOC101460522 [Ceratitis capitata]
GLOS_LOC101450402.4.11	65771.9659025563	42324.8144924155	0.643508429641914	0.000480315753371348	XP_004521991.1 PREDICTED: uncharacterized protein LOC101450402 [Ceratitis capitata]
GLOS_DGRI_GH23671.1.1	6271.67493953008	4033.65547284081	0.643154422340492	0.000305930534596176	XP_001996857.1 GH23671 [Drosophila grimshawi]
GLOS_contig_012845	1399.08739361667	899.325079355941	0.642794069519249	0.00154403931396098	
GLOS_LOC101457705.2.2	4013.45068778429	2565.87522795736	0.639318986967272	0.000352932514249359	XP_004534337.1 PREDICTED: acyl-CoA Delta(11) desaturase-like [Ceratitis capitata]
GLOS_ANXB9.1.1	8985.45780779166	5740.1155108029	0.638822821673638	0.00226798511608827	[BBH] ANXB9_DROME (sp|P22464) Annexin-B9 OS=Drosophila melanogaster GN=AnnIX PE=2 SV=2
GLOS_contig_005736	3202.52824985045	2045.09706176871	0.638588297188076	0.000447197898019075	
GLOS_LOC101453313.1.1	31675.0798968652	20053.1336071916	0.633088651156844	0.00271517548753162	XP_004524284.1 PREDICTED: pre-mRNA-processing factor 39-like [Ceratitis capitata]
GLOS_LOC101459058.1.1	1156.0943506486	731.470685546115	0.632708468072473	0.00157640878452394	XP_004533346.1 PRED.: ATP-binding cassette sub-family G member 1-like isoform X1 [C. capitata] ref|XP_004533347.1| PRED.: ATP-binding cassette sub-family G member 1-like isoform X2
GLOS_DANA_GF17298.1.1	10217.5797390681	6449.68382379813	0.631234009276875	0.00266690614215954	XP_001953259.1 GF17298 [Drosophila ananassae]
GLOS_LOC101450586.9.9	46512.0642212055	29353.100823697	0.631085747648124	0.00020186808477454	XP_004521992.1 PREDICTED: uncharacterized protein LOC101450586 [Ceratitis capitata]
GLOS_DGRI_GH18314.1.1	786.554688065105	494.147046437338	0.628242452731317	0.00273119890969632	XP_001990298.1 GH18314 [Drosophila grimshawi]
GLOS_LOC101456970.1.1	1280.09898868951	803.907119765498	0.628003870691664	0.00104560391423127	XP_004536181.1 PREDICTED: UHRF1-binding protein 1-like isoform X2 [Ceratitis capitata]
GLOS_DPER_GL15114.1.3	5804.63764035944	3638.05996813817	0.626750573169783	0.000147052841776522	XP_002029322.1 GL15114 [Drosophila persimilis]
GLOS_DWIL_GK14328.2.2	5346.85265719612	3341.54067435601	0.624954695517702	0.000143258684215402	XP_002073839.1 GK14328 [Drosophila willistoni]
GLOS_LOC101461359.1.1	7734.25788624908	4831.23964301982	0.624654584069325	0.000109144787853719	XP_004535853.1 PRED.: endoplasmic reticulum metallopeptidase 1-like isoform X1 [C. capitata] ref|XP_004535854.1| PRED.: endoplasmic reticulum metallopeptidase 1-like isoform X2 [C. c.] ref|XP_004535855.1| PRED.: endoplasmic reticulum metallopeptidase 1-like isoform X3 [C. c.] ref|XP_004535856.1| PRED.: endoplasmic reticulum metallopeptidase 1-like isoform X4 [C. c.]
GLOS_DSIM_GD12510.1.1	630.98572253362	393.540594043503	0.623691757181613	0.00387859010255041	XP_002085063.1 GD12510 [Drosophila simulans]
GLOS_DWIL_GK21639.2.5	536.070828605655	334.265108562997	0.62354653662546	0.00584767523794465	XP_002062967.1 GK21639 [Drosophila willistoni]
GLOS_contig_009266	511.521928365342	318.58950356202	0.622826678379417	0.00607367749511765	
GLOS_LOC101453846.2.2	454.856054763654	283.208303048923	0.622632808957725	0.00782885824778485	XP_004536888.1 PREDICTED: uncharacterized protein LOC101453846 [Ceratitis capitata]
GLOS_LOC101455051.1.1	463.819061117832	288.293400871097	0.621564366449909	0.00718989492619537	XP_004529560.1 PREDICTED: ras-related protein Ral-a-like isoform X3 [Ceratitis capitata]
GLOS_LOC101453326.1.1	2250.16533805767	1396.58214376344	0.620657566865271	0.000279138623949065	XP_004527026.1 PREDICTED: glycine cleavage system H protein, mitochondrial-like [C. capitata]
GLOS_Y816.1.1	6730.04906410921	4165.18464808741	0.618893652692666	8.519101605528e-05	[BBH] Y816_DROME (sp|Q9VAF0) Uncharacterized protein CG7816; D.m.r GN=CG7816 PE=2 SV=1
GLOS_LOC101458997.1.1	4070.14746403786	2508.14681651472	0.616229961856585	0.000109766238931392	XP_004530196.1 PREDICTED: protein yellow-like [Ceratitis capitata]
GLOS_DGRI_GH14440.1.1	12895.8679534397	7945.24551511041	0.61610785282515	5.38279739332277e-05	XP_001985553.1 GH14440 [Drosophila grimshawi]
GLOS_contig_010349	1651.56551799622	1016.31310638467	0.615363481079284	0.000408126526326693	

GLOS_LOC101463338.1.1	1180.03339709239	726.115799518897	0.615334956881771	0.00376328912033541	XP_004523823.1 PREDICTED: 2-aminoethanethiol dioxygenase-like [Ceratitis capitata]
GLOS_LOC101460408.1.1	984.758672697765	605.811927227673	0.615188212121088	0.00110157412884986	XP_004537752.1 PREDICTED: methylenetetrahydrofolate reductase-like [Ceratitis capitata]
GLOS_WDR1.1.1	3614.47509534737	2220.74164839578	0.614402254771203	0.000871459813884276	[BBH] WDR1_DROME (sp|Q9VU68) Actin-interacting protein 1 OS=D. melanog. GN=flr PE=2 SV=1
GLOS_DVIR_GJ17584.1.1	473.882429997218	291.030235654997	0.614140169021893	0.00570367402102769	XP_002052520.1 GJ17584 [Drosophila virilis]
GLOS_contig_002429	527.500361969776	323.40481317915	0.613089272529599	0.00432772794446455	
GLOS_DWIL_GK21058.1.1	643.120612962792	392.985147739001	0.611059791612895	0.0025180263321501	XP_002069265.1 GK21058 [Drosophila willistoni]
GLOS_LOC101456617.4.5	1101.2468526502	672.330334835655	0.610517372392632	0.000711778142916217	XM_004521817.1 PRED.: C. capitata 60S ribosomal protein L27a-like (LOC101456617), mRNA
GLOS_LOC101450103.1.1	8028.64249454219	4895.81326655947	0.609793407775674	0.0010274961054689	XP_004518575.1 PREDICTED: protein disulfide-isomerase A6-like [Ceratitis capitata]
GLOS_DANA_GF16603.1.1	1210.37341961189	735.524721566405	0.607684132556591	0.000503070026626651	XP_001954716.1 GF16603 [Drosophila ananassae]
GLOS_LOC100933241.1.14	679.174169293419	412.690743251122	0.607636099706893	0.00201807798843903	XM_003771971.1 PREDICTED: S. harrisii uncharacterized LOC100933241 (LOC100933241), mRNA
GLOS_LOC101452929.1.1	1303.66620779571	792.079242157426	0.607578256934881	0.00114471553908787	XP_004537708.1 PREDICTED: adenosine kinase-like isoform X1 [Ceratitis capitata]
GLOS_LOC101461034.1.1	15304.4700438015	9295.61886543583	0.607379336810206	3.06202826001761e-05	XP_004530393.1 PREDICTED: acid trehalase-like protein 1-like [Ceratitis capitata]
GLOS_DWIL_GK21065.1.1	638.502180622103	387.797136270659	0.607354443007261	0.00706959817419262	XP_002069253.1 GK21065 [Drosophila willistoni]
GLOS_LOC100499562.2.3	1662.46558134478	1009.55445670435	0.607263373168733	0.00025410810050592	NM_001195093.1 Macaca mulatta MTRNR2-like (LOC100499562), mRNA
GLOS_DPSE_GA24742.1.1	1134.09729041559	688.632559998473	0.607207658300747	0.00321681622418798	XP_002138392.1 GA24742 [Drosophila pseudoobscura pseudoobscura]
GLOS_contig_011776	805.007705047158	488.347803366518	0.606637427573331	0.00135267850173262	
GLOS_DERE_GG22534.1.1	2402.76395042807	1451.02596839735	0.603898675997216	0.000120505054120058	XP_001975829.1 GG22534 [Drosophila erecta]
GLOS_RN181.1.1	399.182052567248	240.965057874295	0.603647023518675	0.00656296289697629	[BBH] RN181_DROME (sp|Q9VE61) E3 ubiquitin-protein ligase RNF181 homolog
GLOS_AGAP_AGAP004677.1.	3657.59076474443	2197.0777175649	0.60068986906424	5.57107471655101e-05	XP_314619.3 AGAP004677-PB [Anopheles gambiae str. PEST]
GLOS_LOC101460522.3.3	4331.82595755378	2595.85393275999	0.599251668510222	0.000333167576967316	XP_004537383.1 PREDICTED: uncharacterized protein LOC101460522 [Ceratitis capitata]
GLOS_LOC101450058.3.3	44645.131081894	26605.7149241999	0.595937659481751	1.19056375813528e-05	XP_004527004.1 PREDICTED: uncharacterized protein LOC101450058 [Ceratitis capitata]
GLOS_ENDOU.1.1	935.123677672271	556.111757018501	0.594693269239836	0.000542681324455835	[BBH] ENDOU_DROME (sp|Q9VZ49) Poly(U)-specific endoribonuclease homolog
GLOS_DWIL_GK21639.1.5	15983.8474032007	9483.01149886385	0.593287164200838	0.00496000255439412	XP_002062967.1 GK21639 [Drosophila willistoni]
GLOS_LOC101459846.2.2	1420.61925487973	838.558305135536	0.590276601035179	0.000166420139067953	XP_004526015.1 PREDICTED: alaserpin-like [Ceratitis capitata]
GLOS_DGRI_GH21600.3.3	29652.2230752023	17483.0331437208	0.589602779507671	8.78790299968964e-06	XP_001986849.1 GH21600 [Drosophila grimshawi]
GLOS_contig_010763	477.204229953549	280.344509109708	0.587472808313113	0.00277766714920092	
GLOS_DMOJ_GI24237.1.1	3123.56581609145	1832.22315393184	0.586580613890993	3.15041272195749e-05	XP_001998941.1 GI24237 [Drosophila mojavensis]
GLOS_GSTT1.2.5	11378.5249556232	6669.0302418808	0.586106746515067	0.00177525827269329	[BBH] GSTT1_LUCCU (sp|P42860) Glutathione S-transferase 1-1 OS=L. cuprina GN=GST1 PE=1 SV=2
GLOS_LOC101454487.2.2	30380.9122307767	17783.0447769442	0.585336103203955	6.30269111614997e-06	XM_004517917.1 PREDICTED: Ceratitis capitata serine/threonine-protein kinase PAK 1-like (LOC101454487), transcript variant X4, mRNA
GLOS_DERE_GG10957.1.2	597.639183146404	349.250613824474	0.584383727964031	0.00129359531922334	XP_001983080.1 GG10957 [Drosophila erecta]
GLOS_LOC101450402.8.11	752.453971893166	438.600023474103	0.582892827810571	0.00067034338835718	XP_004521991.1 PREDICTED: uncharacterized protein LOC101450402 [Ceratitis capitata]
GLOS_contig_006689	862.84626858734	501.603971471882	0.581336432378751	0.000431291652636083	
GLOS_LOC101448709.1.1	423.240112240505	245.891456752325	0.580973895528592	0.00305048694520835	XP_004535023.1 PREDICTED: uncharacterized protein LOC101448709 [Ceratitis capitata]
GLOS_CG15097.3.5	742.798594058017	430.072121234116	0.578988873531073	0.000543926185809841	NP_001188973.1 CG15097, isoform C [Drosophila melanogaster]
GLOS_LOC101453125.1.2	710.449187130849	409.343158199794	0.576175137665971	0.000596125787276521	XP_004519981.1 PRED.: histone-lysine N-methyltransferase Suv4-20-like isoform X1 [C. capitata] ref|XP_004519982.1| PRED.: histone-lysine N-methyltransferase Suv4-20-like isoform X2 [C.c.]
GLOS_LOC101459395.1.1	3420.47695463312	1968.57533307526	0.575526559361487	0.00110409121151624	XP_004525673.1 PREDICTED: gamma-glutamyl hydrolase-like [Ceratitis capitata]
GLOS_contig_010675	360.209758108123	206.591354988983	0.573530700761781	0.00404571778060828	
GLOS_LOC101454317.2.2	313.751908212391	179.769796504702	0.572967978199542	0.00603728910349934	XP_004520432.1 PREDICTED: probable small nuclear ribonucleoprotein G-like [Ceratitis capitata]
GLOS_CP305.1.2	30250.4944353587	17329.2649587952	0.572858899738832	0.00159001496528151	CP305_DROME (sp|Q9VW43) Prob. cyt. P450 305a1 OS=D. melanogaster GN=Cyp305a1 PE=2 SV=1

GLOS_GSTT1.4.5	8164.08931969294	4676.38701766967	0.572799590321674	0.000835610852598589	GSTT1_LUCCU (sp|P42860) Glutathione S-transferase 1-1 OS=Lucilia cuprina GN=GST1 PE=1 SV=2
GLOS_LOC101450402.5.11	50434.0556158968	28853.8465592685	0.572110376746577	2.41983737921118e-06	XP_004521991.1 PREDICTED: uncharacterized protein LOC101450402 [Ceratitis capitata]
GLOS_DWIL_GK14328.1.2	2714.71786357952	1552.43360979159	0.571858177462542	0.000968795887129339	XP_002073839.1 GK14328 [Drosophila willistoni]
GLOS_EXPA.1.1	533.575532847331	305.024113183102	0.571660607365925	0.00690741258701729	[BBH] EXPA_DROME (sp|Q07436) Protein expanded OS=D. melanogaster GN=ex PE=1 SV=3
GLOS_DMOJ_GI19977.1.3	19122.3494454531	10923.7807092002	0.571257247461169	0.000331576479318051	XP_002004520.1 GI19977 [Drosophila mojavensis]
GLOS_CALR.1.1	18348.0463865371	10455.4914826222	0.569842219839485	0.00215052653936987	[BBH] CALR_DROME (sp|P29413) Calreticulin OS=Drosophila melanogaster GN=Crc PE=1 SV=2
GLOS_DWIL_GK21639.3.5	48629.5857773951	27708.773333545	0.569792501634369	0.00766211191518634	XP_002062967.1 GK21639 [Drosophila willistoni]
GLOS_LOC101450402.2.11	6078.18309070882	3462.64477297754	0.569684183793439	0.0010474949981356	XP_004521991.1 PREDICTED: uncharacterized protein LOC101450402 [Ceratitis capitata]
GLOS_DPSE_GA11668.1.3	104201.171644554	58932.5471773295	0.565565110710628	1.34080091745469e-06	XP_001360538.1 GA11668 [Drosophila pseudoobscura pseudoobscura]
GLOS_EAST.2.2	326.232984382049	184.021965537647	0.564081421399562	0.0042898234428673	[BBH] EAST_DROME (sp|P13582) Serine protease easter OS=D. melanogaster GN=ea PE=1 SV=3
GLOS_GCH1.1.1	316.293557948528	178.262637870389	0.563598699343092	0.00447938513843822	[BBH] GCH1_DROME (sp|P48596) GTP cyclohydrolase 1 OS=D. melanogaster GN=Pu PE=2 SV=3
GLOS_contig_003408	519.508514640176	291.236544282383	0.560600136619706	0.00461382173818798	
GLOS_LOC101462532.1.1	2757.71451974712	1545.80961354627	0.560540114822336	0.000110109348645582	XP_004529143.1 PREDICTED: adenylosuccinate lyase-like [Ceratitis capitata]
GLOS_contig_008620	277.502082022615	155.39172042518	0.55996596239057	0.00622172039741895	
GLOS_LOC101462140.1.1	5318.98721805424	2975.07507577237	0.55933111959248	2.94362964701832e-06	XP_004518749.1 PREDICTED: probable chitinase 3-like [Ceratitis capitata]
GLOS_DVIR_GJ22515.1.1	11030.146986157	6153.28138821635	0.557860325518674	0.0013829643979209	XM_002060180.1 Drosophila virilis GJ22515 (DvirGJ22515), mRNA
GLOS_LOC101458246.1.1	552.948093821381	307.713338283759	0.556495884011782	0.000629513627243667	XP_004519269.1 PREDICTED: uncharacterized protein LOC101458246 isoform X2 [C. capitata]
GLOS_LOC101450402.10.11	73651.5245845189	40918.354075544	0.555566966283068	6.58334641435792e-07	XP_004521991.1 PREDICTED: uncharacterized protein LOC101450402 [Ceratitis capitata]
GLOS_LOC101461076.2.2	328.738470411543	182.324368170362	0.554618289554346	0.00311678200458136	XP_004521971.1 PREDICTED: epidermal growth factor-like protein 6-like [Ceratitis capitata]
GLOS_DPER_GL12526.1.1	3304.16892953717	1830.90595269545	0.554119959281838	3.83104114205313e-05	XP_002019682.1 GL12526 [Drosophila persimilis]
GLOS_LOC101459623.1.5	647.904412510756	357.651556909147	0.552012843257506	0.000327598802828511	XP_004518098.1 PREDICTED: exonuclease 3'-5' domain-containing protein 2-like [C. capitata]
GLOS_DSEC_GM18382.1.1	1119.99410850179	614.276349890003	0.548463911753711	0.00157490346898844	XP_002037661.1 GM18382 [Drosophila sechellia]
GLOS_LOC101459847.1.2	2426.20196603364	1329.62707339154	0.548028190565361	3.11988820700517e-05	XP_004527242.1 PRED.: uncharacterized protein LOC101459847 isoform X1 [C. capitata] ref|XP_004527243.1| PRED.: uncharacterized protein LOC101459847 isoform X2 [C. capitata] ref|XP_004527244.1| PRED.: uncharacterized protein LOC101459847 isoform X3 [C. capitata] ref|XP_004527245.1| PRED.: uncharacterized protein LOC101459847 isoform X4 [C. capitata]
GLOS_LOC101455374.1.1	2139.80920507014	1169.80861509486	0.546688280582714	0.000703557006582868	XP_004519344.1 PREDICTED: sphingolipid delta(4)-desaturase DES1-like [Ceratitis capitata]
GLOS_LOC101462958.2.2	4823.08208622691	2635.51856075981	0.546438670054147	1.36243113722972e-06	XP_004536658.1 PREDICTED: chymotrypsin-2-like [Ceratitis capitata]
GLOS_LOC101462601.1.5	8394.79788818887	4561.90739662852	0.54342075382743	5.1187043275306e-07	XP_004536656.1 PREDICTED: chymotrypsin-1-like [Ceratitis capitata]
GLOS_MVL.1.1	6559.63259831202	3542.9671007157	0.540116698247309	0.00260543802074638	[BBH] MVL_DROME (sp|P49283) Protein Malvolio OS=D. melanogaster GN=Mvl PE=2 SV=2
GLOS_contig_015559	231.080395833525	123.738982429355	0.53548022532599	0.00627617653566565	
GLOS_contig_009639	420.791502298358	225.162493718003	0.535092777511354	0.000824404291584316	
GLOS_LOC101169087.1.1	385.621326253086	206.258087206282	0.534872096443426	0.00110276923699278	XP_004085317.1 PREDICTED: uncharacterized protein LOC101169087, partial [Oryzias latipes]
GLOS_LOC101449710.1.1	548.185006654122	290.839796922025	0.530550440803154	0.000240928554241678	XP_004529622.1 PREDICTED: protein xmas-2-like [Ceratitis capitata]
GLOS_LOC101450592.2.2	381.292203565535	201.25233885618	0.527816558991324	0.000912859206120394	XP_004523930.1 PREDICTED: sialin-like [Ceratitis capitata]
GLOS_LOC101458989.1.1	44558.1639558273	23474.7263397457	0.526833339969245	2.34645692395909e-07	XP_004527418.1 PRED.: type I inositol 3,4-bisphosphate 4-phosphatase-like isof. X4 [C. capitata]
GLOS_LOC101462958.1.2	6661.05762311233	3499.22439118644	0.525325644841285	1.80855136502965e-07	XP_004536658.1 PREDICTED: chymotrypsin-2-like [Ceratitis capitata]
GLOS_contig_000213	474.244067063642	248.065150952041	0.523074864147433	0.000317321531267365	
GLOS_MYSN.1.1	13072.4772759905	6830.73818028289	0.522528212217935	6.89401567937574e-07	[BBH] MYSN_DROME (sp|Q99323) Myosin heavy chain, non-muscle OS=D. m. GN=zip PE=1 SV=2
GLOS_DGRI_GH21360.1.1	4055.78094590639	2114.8606221169	0.521443502576609	2.49294200400405e-07	XP_001986425.1 GH21360 [Drosophila grimshawi]
GLOS_contig_003620	218.330722391432	113.822705095637	0.521331601200728	0.00564562161627442	

GLOS_contig_008318	252.705296890572	131.656134401915	0.520986841280676	0.00338651663686202	
GLOS_LOC100170569.1.1	446.233973095196	231.738880280076	0.519321464192147	0.00389750246039228	NP_001123818.2 uncharacterized protein LOC100170569 [Xenopus (Silurana) tropicalis]
GLOS_DWIL_GK14466.1.1	253.154712696219	130.934294873587	0.517210576406318	0.00287751039115985	XP_002074103.1 GK14466 [Drosophila willistoni]
GLOS_LOC101453109.1.1	1529.52883960157	786.224213788632	0.514030329753991	7.12712756997903e-05	XP_004535897.1 PREDICTED: protein ETHE1, mitochondrial-like [Ceratitis capitata]
GLOS_contig_011297	364.879805481393	184.950113693362	0.506879555719325	0.000516785633244424	
GLOS_CG15097.2.5	1260.8812728132	638.916519894887	0.50672219000396	0.000734651705228922	NP_001188973.1 CG15097, isoform C [Drosophila melanogaster]
GLOS_PTER.1.1	2191.65018620081	1102.56836795855	0.503076802539291	1.14906650140909e-05	[BBH] PTER_DROGR (sp|B4J340) Phosphotriesterase-related protein; D. g. GN=GH16075 PE=3 SV=1
GLOS_LOC101452090.1.1	1041.31079412476	520.270329567388	0.499630208870239	3.58484259669135e-06	XP_004517444.1 PREDICTED: inositol-3-phosphate synthase-like [Ceratitis capitata]
GLOS_contig_006679	196.705821334385	98.1947097779036	0.499195748818131	0.0046750813163976	
GLOS_LOC101449597.1.1	421.74194894223	210.33568740067	0.498730771098801	0.00380153014488157	XP_004535877.1 PREDICTED: uncharacterized protein LOC101449597 isoform X2 [C. capitata]
GLOS_LOC101450058.1.3	13943.5646041526	6949.09726850578	0.498373082191353	8.10935774884838e-09	XP_004527004.1 PREDICTED: uncharacterized protein LOC101450058 [Ceratitis capitata]
GLOS_DPSE_GA30464.1.1	6983.20377680543	3453.98221730255	0.494612834981972	6.09222713465808e-07	XP_004444371.1 GA30464 [Drosophila pseudoobscura pseudoobscura]
GLOS_LOC101450402.9.11	191.420990948195	94.5455967327034	0.493914467083135	0.00432919568545611	XP_004521991.1 PREDICTED: uncharacterized protein LOC101450402 [Ceratitis capitata]
GLOS_LOC101455841.1.3	1195.91386168643	585.757675373487	0.48979921893996	0.00194575302653326	XP_004521777.1 PREDICTED: CD109 antigen-like isoform X5 [Ceratitis capitata]
GLOS_DMOJ_GI12536.1.1	8731.58607753245	4253.22503211538	0.487107954310787	4.30598155866905e-09	XP_002007161.1 GI12536 [Drosophila mojavensis]
GLOS_LOC101449626.1.1	1634.1661777232	792.586597443635	0.485009791689549	8.30503327576169e-06	XP_004524798.1 PRED.: microsomal triglyceride transfer protein large subunit-like [C. capitata]
GLOS_DANA_GF12088.1.1	1419.19868004951	684.546784189354	0.482347393506224	3.30655197237735e-07	XP_001959348.1 GF12088 [Drosophila ananassae]
GLOS_LOC101462636.1.1	455.971868614801	217.118382590129	0.476166179395483	0.00354228415871589	XP_004527440.1 PREDICTED: sepiapterin reductase-like [Ceratitis capitata]
GLOS_LOC101456499.1.1	153.368240481067	72.9837049091536	0.475872349322305	0.00636969258815043	XP_004520728.1 PREDICTED: probable salivary secreted peptide-like [Ceratitis capitata]
GLOS_LOC101448305.1.1	269.603274487004	126.666255946227	0.469824619850206	0.000614773407312931	XP_004531060.1 PREDICTED: protoporphyrinogen oxidase-like [Ceratitis capitata]
GLOS_LOC101461145.1.1	623.484715771075	291.220674387969	0.467085506703738	7.36709777979632e-06	XP_004523994.1 PREDICTED: stress response protein NST1-like [Ceratitis capitata]
GLOS_contig_010028	211.279131434533	98.3534087220471	0.465514071618203	0.00156303180154983	
GLOS_contig_002748	1697.86292990108	787.144186329614	0.463608794601266	0.00381904311100171	
GLOS_LOC101457248.1.1	221.053190660781	102.431008916435	0.463377201705363	0.00116539939615791	XP_004521782.1 PRED.: Na- and chloride-depend. neutral and basic amino acid transp. B(0)-like
GLOS_HH.1.1	328.723019085606	152.162918914435	0.462890975319283	0.000287402441865948	[BBH] HH_DROWI (sp|B4NJP3) Protein hedgehog OS=Drosophila willistoni GN=hh PE=3 SV=1
GLOS_contig_010994	518.521904289661	236.736934350496	0.456561106468219	0.00104242802932504	
GLOS_LOC101457813.1.1	2524.74375273538	1132.04837442476	0.448381493447904	1.51212863751437e-07	XP_004530636.1 PRED.: bifunctional purine biosynthesis prot. PURH-like isoform X1 [. capitata] ref|XP_004530637.1|PRED.: bifunctional purine biosynth. prot. PURH-like isoform X2 [C. capit.] ref|XP_004530638.1| PRED.: bifunctional purine biosynth. Prot. PURH-like isoform X3 [C. capit.]
GLOS_DVIR_GJ13129.1.1	13858.5677606007	6206.17958281754	0.447822580949629	3.24019154642523e-11	XP_002046879.1 GJ13129 [Drosophila virilis]
GLOS_contig_006942	198.798055264875	88.4053915995005	0.444699478984896	0.00114421757412338	
GLOS_6PGD.1.1	221.704137380344	98.5279775606049	0.444411993049889	0.0011992931770726	[BBH] 6PGD_CERCA (sp|P41570) 6-phosphogluconate dehydrogenase, decarboxylating
GLOS_LOC101456017.3.4	3524.09611607563	1547.40381593234	0.439092398437616	1.86206614571317e-10	XP_004520628.1 PREDICTED: uncharacterized protein LOC101456017 isoform X1 [C. capitata]
GLOS_LOC101456017.2.4	3144.82908008207	1376.9313708792	0.43783981126353	2.54472135439167e-10	XP_004520628.1 PREDICTED: uncharacterized protein LOC101456017 isoform X1 [C. capitata]
GLOS_LSD1.1.1	4868.74893195455	2131.43984842939	0.437779782489982	8.98925622032185e-05	[BBH] LSD1_DROME (sp|Q9VCI3) Lipid storage droplets surface-binding protein 1
GLOS_DMOJ_GI22614.1.1	205.198699502212	89.4922386993584	0.436124784983805	0.00524265169663149	XP_002000268.1 GI22614 [Drosophila mojavensis]
GLOS_LOC101462125.1.1	445.769105963612	190.804660727107	0.428034734068542	7.02131523046711e-06	XP_004534627.1 PREDICTED: uncharacterized protein LOC101462125 [Ceratitis capitata]
GLOS_LOC101452734.1.1	299.018491959735	126.951914045685	0.424562083815139	6.92338002049958e-05	XP_004533488.1 PREDICTED: alpha-N-acetylgalactosaminidase-like [Ceratitis capitata]
GLOS_contig_007723	634.338425141827	264.811733158461	0.417461283540018	3.14048669643259e-07	
GLOS_contig_009034	411.048345723986	171.035585637328	0.416096032052095	6.03928727724341e-06	
GLOS_contig_013166	232.5785591318	96.4336328329609	0.41462821505534	0.000205935382801171	

GLOS_contig_007102	456.658979041006	188.662706316219	0.413136968668426	2.55895354731225e-06	
GLOS_contig_013915	397.720052975615	164.062451714897	0.41250736664504	0.000208759482696926	
GLOS_contig_013881	212.901237178673	87.334414394057	0.410210929496682	0.000290238928464766	
GLOS_LOC101457953.3.5	257.158362023988	104.715792377051	0.407203528412904	0.00645109969788653	XP_004518274.1 PREDICTED: serine protease easter-like [Ceratitis capitata]
GLOS_CG15097.4.5	968.759526712626	394.183084099759	0.406894666045117	7.15905728089207e-09	NP_001188973.1 CG15097, isoform C [Drosophila melanogaster]
GLOS_DANA_GF23598.1.1	20538.7894935266	8344.71711764943	0.406290600538046	4.9057191708041e-05	XP_001958284.1 GF23598 [Drosophila ananassae]
GLOS_contig_010153	625.721604528135	249.873837580226	0.39933707861767	1.1010688375962e-07	
GLOS_PAPSS.1.1	15169.4292186648	6030.03389117879	0.397512246786405	6.83104367670998e-06	NP_730457.1 PAPS synthetase, isoform A [D. melanogaster] ref|NP_730458.1| PAPS synthetase, isoform B [D. melanogaster] ref|NP_730459.1| PAPS synthetase, isoform C [D. melanogaster] ref|NP_001262072.1| PAPS synthetase, isoform G [D. melanogaster]
GLOS_DGRI_GH24075.1.1	4345.16510425005	1719.2497288549	0.395669597726743	4.73432150151291e-13	XP_001992691.1 GH24075 [Drosophila grimshawi]
GLOS_LOC101456017.1.4	1346.91600990981	528.750622124132	0.39256391507258	1.76483875199428e-10	XP_004520628.1 PREDICTED: uncharacterized protein LOC101456017 isoform X1 [C. capitata]
GLOS_contig_012452	2769.98946762482	1086.05165855361	0.392077901828582	0.000187794011695758	
GLOS_LOC101449850.3.3	1249.2993600932	488.380024490397	0.390923136672353	0.000461806542407604	XP_004519690.1 PREDICTED: uncharacterized protein LOC101449850 isoform X1 [C. capitata] ref|XP_004519691.1| PRED.: uncharacterized protein LOC101449850 isoform X2 [C. capitata]
GLOS_LOC101454791.1.1	113.817326715664	44.1630210637144	0.388016678462685	0.00322637739129391	XP_004523031.1 PREDICTED: enkurin-like [Ceratitis capitata]
GLOS_DERE_GG24413.1.1	315.699487316312	122.438132422428	0.387831267840333	0.000832637785118773	XP_001968601.1 GG24413 [Drosophila erecta]
GLOS_contig_013650	213.335201658382	82.328666043955	0.385912242348967	0.000790370512868105	
GLOS_LOC101457066.1.1	412.241748043184	158.906180035384	0.385468431544535	0.000130002906420229	XP_004523041.1 PREDICTED: phosphate-regulating neutral endopeptidase-like [C. capitata]
GLOS_DWIL_GK24277.1.1	1148.83070666825	434.340160161476	0.378071510136699	6.85947859015019e-05	XP_002066964.1 GK24277 [Drosophila willistoni]
GLOS_DANA_GF14653.2.2	188.032124633346	70.9369698647527	0.377259843247937	0.000189631970336087	XP_001961999.1 GF14653 [Drosophila ananassae]
GLOS_contig_009651	489.540651296638	182.744679704817	0.373298273025508	1.40393195689717e-07	
GLOS_DSEC_GM25119.2.2	874.996610342449	326.32439241634	0.372943607505663	8.09447656745353e-10	XP_002029827.1 GM25119 [Drosophila sechellia]
GLOS_contig_003537	125.487350013253	45.4561767909588	0.362237124189475	0.00648501670941759	
GLOS_LOC101460827.2.2	3437.84932595558	1229.98916589203	0.357778671859083	0.000258512852080453	XP_004518643.1 PREDICTED: mucin-5AC-like [Ceratitis capitata]
GLOS_DMOJ_GI24442.1.1	6209.45990380944	2219.84524002889	0.357494093595329	0.00533197661305151	XP_001999308.1 GI24442 [Drosophila mojavensis]
GLOS_DGRI_GH19078.1.1	1187.80300112737	417.665233147445	0.351628369983094	0.00345244759205761	XP_001993820.1 GH19078 [Drosophila grimshawi]
GLOS_DERE_GG19064.2.3	196.995130987524	69.1600230253956	0.351074783821818	0.000466324745946972	XP_001977467.1 GG19064 [Drosophila erecta]
GLOS_contig_007084	75.6560851286095	26.5517702792374	0.350953531815735	0.00786238514884161	
GLOS_LOC101460687.1.1	3414.14304491594	1197.74972313792	0.350820017609255	6.30975966874112e-13	XP_004531215.1 PREDICTED: collagen alpha-1(IV) chain-like [Ceratitis capitata]
GLOS_DWIL_GK12510.1.4	4699.42330208811	1631.95378585925	0.347266819980681	1.56318404588744e-16	XP_002067829.1 GK12510 [Drosophila willistoni]
GLOS_contig_006723	116.343525125863	40.323469285542	0.3465897156023	0.0011207882945763	
GLOS_H12.1.1	508.995729955143	175.866765148872	0.345517171950286	1.14472056391666e-08	[BBH] H12_DROVI (sp|Q94555) Histone H1.2 OS=Drosophila virilis GN=His1.2 PE=3 SV=1
GLOS_DPER_GL10892.1.1	1052.30389726731	355.200380224705	0.33754543829697	0.00548230746492533	XP_002015682.1 GL10892 [Drosophila persimilis]
GLOS_LOC101457181.4.5	25283.1929111136	8470.94911750698	0.335042696042693	2.14480702279639e-07	XP_004519837.1 PREDICTED: laccase-2-like [Ceratitis capitata]
GLOS_LOC101457181.2.5	17482.0137780639	5829.73422732252	0.333470405717077	3.99486478033577e-06	XP_004519837.1 PREDICTED: laccase-2-like [Ceratitis capitata]
GLOS_DMOJ_GI19892.1.1	605.269393409581	200.832027321725	0.331806018127573	5.95788537898864e-10	XP_002004358.1 GI19892 [Drosophila mojavensis]
GLOS_LOC101462986.1.1	253.733332002497	83.9868293427294	0.331004321268689	0.000322332555581486	XP_004524787.1 PREDICTED: serine protease easter-like [Ceratitis capitata]
GLOS_DWIL_GK13726.1.1	1746.61980569599	575.706744604772	0.329611941149016	1.06242229124892e-05	XP_002072669.1 GK13726 [Drosophila willistoni]
GLOS_DERE_GG19064.3.3	199.428289603733	65.2093819863228	0.326981603843139	0.00142156866726784	XP_001977467.1 GG19064 [Drosophila erecta]
GLOS_contig_009600	1052.99133953188	341.992303137634	0.324781686513842	0.00410398570333554	

GLOS_LOC101460827.1.2	2004.28412777461	643.200428716661	0.320912798641387	0.00132851234017815	XP_004518643.1 PREDICTED: mucin-5AC-like [Ceratitis capitata]
GLOS_contig_006277	514.177330276173	164.967035696515	0.320836851379481	0.000939551544089095	
GLOS_LOC101462556.1.1	603.750517730601	189.583160192252	0.314009105789032	2.85274369788388e-05	XP_004521288.1 PREDICTED: MD-2-related lipid-recognition protein-like [Ceratitis capitata]
GLOS_AAEL_AAEL007969.1.1	180.277971924305	56.3323420692197	0.312474904548364	0.0038269305841737	XP_001658781.1 serine protease [Aedes aegypti]
GLOS_LOC101457181.3.5	4844.58863773122	1509.74552623831	0.311635442993017	2.97769751603337e-07	XP_004519837.1 PREDICTED: laccase-2-like [Ceratitis capitata]
GLOS_contig_007364	215.768360274591	65.7566526760929	0.304755769531778	3.38423753134657e-06	
GLOS_CPIPJ_CPIJ000864.1.1	3882.15216678788	1175.69490191743	0.302846166612322	7.40157594891923e-09	XP_001842487.1 multicopper oxidase [Culex quinquefasciatus]
GLOS_contig_008738	810.834991032983	245.241272416386	0.30245521607788	0.00236548906253506	
GLOS_LOC101457181.1.5	73109.9421332135	21931.6982826563	0.299982432521894	4.65153903810918e-05	XP_004519837.1 PREDICTED: laccase-2-like [Ceratitis capitata]
GLOS_DSEC_GM25119.1.2	698.856752301313	209.193536337886	0.299336789190373	0.000385302554161839	XP_002029827.1 GM25119 [Drosophila sechellia]
GLOS_LOC101460835.1.1	1793.0105892332	524.562414003894	0.292559573910954	1.00467942761097e-16	XP_004522056.1 PREDICTED: counting factor associated protein D-like [Ceratitis capitata]
GLOS_DVIR_GJ10540.1.1	969.451898193598	281.439531824298	0.290307886702487	5.5844762861934e-05	XP_002055855.1 GJ10540 [Drosophila virilis]
GLOS_DWIL_GK11657.1.1	448.744720179457	128.276809561759	0.285856977905967	9.4893314562301e-10	XP_002069687.1 GK11657 [Drosophila willistoni]
GLOS_DGRI_GH14582.1.1	354.475511916287	98.7184162935771	0.278491497931429	2.70046957060934e-09	XP_001994707.1 GH14582 [Drosophila grimshawi]
GLOS_LOC101460389.1.1	4055.61031764435	1123.96482922837	0.277138270493702	1.00349488622624e-11	XP_004531213.1 PREDICTED: collagen alpha-1(IV) chain-like isoform X1 [Ceratitis capitata] ref|XP_004531214.1| PREDICTED: collagen alpha-1(IV) chain-like isoform X2 [C. capitata]
GLOS_contig_009922	114.395946021942	31.6209982069968	0.276417122342181	0.000357591463226881	
GLOS_LOC101460221.1.1	537.222806163444	148.315672856581	0.276078511848318	9.37790639216183e-06	XP_004536554.1 PREDICTED: uncharacterized protein LOC101460221 [Ceratitis capitata]
GLOS_DVIR_GJ16564.1.2	315.756363403659	86.0018245983017	0.272367668766054	0.000214823801685928	XP_002057045.1 GJ16564 [Drosophila virilis]
GLOS_contig_007422	757.645762485367	205.369854454128	0.271063159886801	1.02741547300718e-09	
GLOS_contig_011540	72.8974531526187	19.753205195364	0.270972500973505	0.00182979738588218	
GLOS_LOC101452450.1.1	255.086840474202	69.0489337644952	0.270687949390625	4.80601075901305e-06	XP_004536688.1 PREDICTED: uncharacterized protein LOC101452450 [Ceratitis capitata]
GLOS_LOC101456392.1.1	593.945555852479	160.167595973799	0.269667134294681	0.00014398644573015	XP_004525823.1 PREDICTED: fibroin heavy chain-like [Ceratitis capitata]
GLOS_CYTA.1.2	733.566990431405	190.860446025082	0.260181344736952	3.41504796549947e-06	CYTA_SARPE (sp|P31727) Sarcocystatin-A OS=Sarcophaga peregrina PE=1 SV=1
GLOS_DVIR_GJ16564.2.2	258.424091756471	67.176767558652	0.259947774613665	0.00134394950452721	XP_002057045.1 GJ16564 [Drosophila virilis]
GLOS_DMOJ_GI22128.1.1	584.367766485381	141.905679518334	0.242836254251002	3.6498669428162e-14	XP_002003524.1 GI22128 [Drosophila mojavensis]
GLOS_LOC101457181.5.5	34357.2827028309	8198.37988690909	0.238621312337765	0.000924739690679602	XP_004519837.1 PREDICTED: laccase-2-like [Ceratitis capitata]
GLOS_DGRI_GH21074.1.2	278.183931394053	63.9638359423215	0.229933611268566	0.00420329025629875	XP_001985908.1 GH21074 [Drosophila grimshawi]
GLOS_LOC101462302.2.2	470.137187670713	103.803514115751	0.220794093379517	0.000308718027288675	XP_004534628.1 PREDICTED: uncharacterized protein LOC101462302 [Ceratitis capitata]
GLOS_DVIR_GJ10399.1.1	470.493563682369	103.787644221336	0.220593122271495	6.2364571278804e-11	XP_002056122.1 GJ10399 [Drosophila virilis]
GLOS_contig_009795	2743.2970502598	590.532588252006	0.215263814830436	0.000723735490852312	NR_076090.1 Proteus mirabilis HI4320 strain HI4320 23S ribosomal RNA, complete sequence
GLOS_CEC.2.2	761.31341634382	160.373423266136	0.210653614954434	0.00750973127074519	[BBH] CEC_GLOMM (sp|P83403) Cecropin OS=Glossina morsitans morsitans PE=1 SV=2
GLOS_DGRI_GH22644.1.1	434.047467633443	90.8647438986745	0.209342873013627	1.90683844534625e-08	XP_001995415.1 GH22644 [Drosophila grimshawi]
GLOS_DWIL_GK13707.1.1	21399.8762536282	4446.24811579626	0.207769804979243	5.30260452180381e-18	XP_002072631.1 GK13707 [Drosophila willistoni]
GLOS_LOC100876965.1.1	59.5691604042483	11.8201833283897	0.198427898734439	0.000877576329678246	XP_003708657.1 PREDICTED: chymotrypsin inhibitor-like [Megachile rotundata]
GLOS_contig_007475	1466.14770235066	285.46134672071	0.194701629490012	0.00377701352275518	
GLOS_DERE_GG14514.1.1	17921.1790496845	3460.37585941134	0.193088627138752	3.96788888405569e-39	XP_001971281.1 GG14514 [Drosophila erecta]
GLOS_contig_011331	1106.38702820982	212.327118482145	0.191910346983822	5.34363981109245e-19	
GLOS_contig_005205	1246.73699797636	239.005847916698	0.191705105651505	3.12249998415268e-13	
GLOS_LOC101453761.1.3	617.50258468751	117.59108301647	0.190430106581622	0.000423564564304709	XP_004531360.1 PREDICTED: protease inhibitor-like [Ceratitis capitata]
GLOS_contig_001698	1512.89486189953	283.303522415409	0.187259227028972	0.00339965701583362	

GLOS_contig_013629	2402.16987979586	436.093542826736	0.181541508156699	1.00059531356115e-32	
GLOS_contig_010960	158.436088627403	28.5508956403953	0.180204496890472	4.92587679156104e-08	
GLOS_contig_013214	769.4913432614	138.320046050791	0.179755168478618	4.15535347001399e-16	
GLOS_contig_008882	194.200335304891	34.5641416182835	0.177981884346484	2.27643926409959e-09	
GLOS_LOC101463381.2.3	1340.53081699841	233.143125268221	0.173918512213134	2.41814140473641e-28	XP_004534544.1 PREDICTED: uncharacterized protein LOC101463381 [Ceratitis capitata]
GLOS_LOC101463381.3.3	998.541642306548	170.250266531343	0.170498914935664	3.40183769877174e-17	XP_004534544.1 PREDICTED: uncharacterized protein LOC101463381 [Ceratitis capitata]
GLOS_contig_013869	211.821587034169	35.6033790348983	0.16808191994688	6.91390836460967e-05	
GLOS_CECC.1.1	679.949058458847	104.628267290247	0.153876626474628	2.76349434339453e-16	CECC_DROYA (sp|P84226) Cecropin-C OS=Drosophila yakuba GN=CecC PE=2 SV=1
GLOS_C4AC3.1.1	430.74111900305	63.2261265195792	0.146784515641126	2.07420268810219e-07	[BBH] C4AC3_DROME (sp|Q9VMS7) Probable cytochrome P450 4ac3 OS=D. melanogaster
GLOS_LOC101461912.1.1	222.479026545771	32.6126259403686	0.146587417460041	8.0940403159832e-12	XP_004526501.1 PREDICTED: lysozyme-like [Ceratitis capitata]
GLOS_DVIR_GJ17669.1.1	33.5273579204278	4.9105289836159	0.146463344808449	0.00659590404718084	XP_002052653.1 GJ17669 [Drosophila virilis]
GLOS_contig_013149	124.712460847825	17.8175594118635	0.142869119017742	0.000123891117495491	
GLOS_LOC101457953.2.5	1048.79142034496	149.323170484367	0.142376422601982	4.61873831622467e-06	XP_004518274.1 PREDICTED: serine protease easter-like [Ceratitis capitata]
GLOS_DWIL_GK14123.1.1	379.525442994826	51.8103848312305	0.136513600833704	9.47808915533087e-18	XP_002073449.1 GK14123 [Drosophila willistoni]
GLOS_contig_013212	484.741400422737	63.2419964139936	0.130465432411676	1.64173148416937e-21	
GLOS_contig_007169	800.053609075517	98.7025463991627	0.123369915815037	1.44780046208242e-05	
GLOS_contig_007229	453.306276432799	55.4200638079197	0.122257437607166	1.46432297328434e-21	
GLOS_CI.1.1	1339.43078631156	163.56279070837	0.122113656323204	0.00013917335658182	XP_003401252.1 PREDICTED: chymotrypsin inhibitor-like isoform 1 [Bombus terrestris] ref|XP_003401253.1| PREDICTED: chymotrypsin inhibitor-like isoform 2 [Bombus terrestris]
GLOS_contig_010457	173.877327686968	19.7373353009497	0.11351298966386	0.00721679888395249	
GLOS_LOC101449850.2.3	366.884260672661	41.3309669133282	0.112653965688117	1.11561599024693e-19	XP_004519690.1 PREDICTED: uncharacterized protein LOC101449850 isoform X1 [C. capitata] ref|XP_004519691.1| PRED.: uncharacterized protein LOC101449850 isoform X2 [C. capitata]
GLOS_contig_011996	2247.94916168131	235.904005561267	0.104941877504395	2.71157770606034e-48	
GLOS_contig_008577	366.842835911251	37.364455979841	0.101854124769879	9.66899941650231e-06	
GLOS_contig_003065	1035.55583555222	104.779271954708	0.101181673027639	5.34547030937095e-16	
GLOS_LOC101453038.1.1	1302.20420820408	130.307674711745	0.100067004768367	1.81753137364513e-42	XP_004533218.1 PREDICTED: uncharacterized protein LOC101453038 [Ceratitis capitata]
GLOS_contig_009648	363.407615618589	35.635118823727	0.0980582610055355	0.000268315114479129	
GLOS_contig_008229	178.004919460602	16.8100617840774	0.0944359393831131	6.89163832781983e-13	
GLOS_contig_011033	4742.54849807962	442.915672084706	0.0933919120203102	3.73085382381941e-09	
GLOS_contig_014768	7863.88235463041	728.075490811544	0.092584738425396	1.23934802566868e-13	
GLOS_contig_008466	246.893462230685	22.6011292401646	0.0915420320812191	0.000581791786507142	
GLOS_LOC101449850.1.3	507.234230439201	41.3468368077425	0.0815142873381028	2.05738804680884e-14	XP_004519690.1 PREDICTED: uncharacterized protein LOC101449850 isoform X1 [C. capitata] ref|XP_004519691.1| PRED.: uncharacterized protein LOC101449850 isoform X2 [C. capitata]
GLOS_TB927.1.2370.1.2	301.756411555021	24.5209051292508	0.0812605936122081	2.01105803786959e-08	XM_001218936.1 Trypanosoma brucei brucei strain 927/4 GUTat10.1 beta tubulin partial mRNA
GLOS_contig_010547	348.436504745375	26.3613315462653	0.0756560555144163	4.6837313195658e-10	
GLOS_contig_010922	659.538272101702	49.3750780412029	0.0748630976089727	9.93600511203819e-27	
GLOS_LOC101457953.1.5	467.559374227934	28.6143752180527	0.0611994471617701	5.22879204424478e-07	XP_004518274.1 PREDICTED: serine protease easter-like [Ceratitis capitata]
GLOS_LOC101457953.5.5	535.735164974703	31.6209982069968	0.059023562898825	0.000871377171821999	XP_004518274.1 PREDICTED: serine protease easter-like [Ceratitis capitata]
GLOS_LOC101461009.2.2	2098.5697828788	112.474245405467	0.0535956661165568	9.70939962336596e-05	XP_004521535.1 PREDICTED: serine proteinase stubble-like [Ceratitis capitata]
GLOS_contig_000973	1216.07643131349	57.8712404923616	0.0475884895078962	1.14307596991894e-07	
GLOS_LOC101462454.1.1	397.03294254941	18.7298376731635	0.0471745179452776	6.59904352340678e-08	XP_004527439.1 PREDICTED: sepiapterin reductase-like [Ceratitis capitata]

GLOS_contig_007486	1413.23166845352	66.0264408811368	0.0467201820869106	4.07571915212763e-08	
GLOS_OB99B.1.1	1608.42913621846	73.7690240151389	0.0458640187211329	1.42665232956433e-23	[BBH] OB99B_DROME (sp|Q9VAI6) General odorant-binding protein 99b
GLOS_DGRI_GH13991.1.1	351.334530493168	15.7708243674625	0.0448883414486046	3.13610494577602e-16	XP_001996119.1 GH13991 [Drosophila grimshawi]
GLOS_TB927.6.4540.1.1	22.1828079826208	0.975757838957441	0.0439871201031853	0.0027299276760477	XM_840478.1 T.b.b. strain 927/4 GUTat10.1 3-hydroxy-3-methylglutaryl-CoA reduct. partial mRNA
GLOS_contig_008334	1064.20109307593	41.188137863599	0.0387033410617447	0.00013917574238261	
GLOS_contig_008626	742.106222577045	28.4398063794949	0.0383230938028448	2.97425317660788e-06	
GLOS_contig_013033	2120.00794067114	70.7941408150236	0.0333933375705243	1.72197104394716e-63	
GLOS_contig_008085	754.189497973637	22.6328690289933	0.0300095255765342	7.81545358570288e-07	
GLOS_DANA_GF14647.1.1	757.345930722694	22.6011292401646	0.0298425439727359	2.92500132201027e-07	XP_001962018.1 GF14647 [Drosophila ananassae]
GLOS_contig_009850	2347.22374911034	67.6605586707647	0.0288257813923406	2.84164738630856e-07	
GLOS_contig_011544	2533.32407780407	67.8192576149082	0.0267708573921167	1.06868172497578e-31	
GLOS_LOC101450467.1.1	289.4252512667	6.90965434477382	0.0238737094104022	1.78259467524369e-27	XP_004520408.1 PREDICTED: membrane-bound alkaline phosphatase-like [Ceratitis capitata]
GLOS_TRF.1.1	5009.46020694915	116.107488556253	0.0231776446482574	2.242862806534e-14	[BBH] TRF_SARPE (sp|Q26643) Transferrin OS=Sarcophaga peregrina PE=1 SV=1
GLOS_RL402.2.2	48.4054289996532	0.991627733371788	0.0204858784203502	4.95959852861046e-07	RL402_TRYCR (sp|P0CH27) Ubiquitin-60S ribosomal protein L40 OS=Trypanosoma cruzi PE=2 SV=1
GLOS_TB11.01.7800.1.1	62.2916286735968	0.991627733371788	0.0159191171347893	2.13516041758806e-07	XM_824577.1 Tbb strain 927/4 GUTat10.1 nucleos. diphosph. kinase(Tb11.01.7800)partial mRNA
GLOS_contig_009842	442.431854681343	5.93389650581638	0.0134120010641869	2.13424489498341e-46	
GLOS_TB927.6.4980.2.2	90.7149747410467	0.975757838957441	0.0107563039260367	4.07871447526485e-13	XM_840522.1 T.b.b. strain 927/4 GUTat10.1 40S ribosomal protein S14 partial mRNA
GLOS_EF1A2.1.2	434.419294971038	2.97488320011536	0.00684795365802915	5.30080997495085e-15	[BBH] EF1A2_TRYB2 (sp|P86939) Elongation factor 1-alpha 2 OS=Tbb GN=Tb10.70.5670 PE=1 SV=1
GLOS_TB927.6.3800.1.1	148.93062667359	0.991627733371788	0.00665831975276068	5.73762235406834e-17	XM_840405.1 Tbb strain 927/4 GUTat10.1 heat shock 70 kDa prot., mitoch. precurs partial mRNA
GLOS_PRO2.1.2	430.9373888622	1.98325546674358	0.00460218936207867	9.58066311057550e-11	PRO2_TRYBB (sp|P14044) Duplicate procyclin OS=Trypanosoma brucei brucei PE=4 SV=1
GLOS_TB927.3.5050.1.1	382.712778395758	0.991627733371788	0.00259104944843613	3.14965030012817e-11	XM_839002.1 Trypanosoma brucei 60S ribosomal protein L4 (Tb927.3.5050) partial mRNA
GLOS_GBLP.3.4	534.350422012759	0.991627733371788	0.00185576298346801	3.08516340737475e-24	[BBH] GBLP_TRYBR (sp|P69104)Guanine nucleot.-binding prot. subunit beta-like prot. Tbr PE=2SV=1
GLOS_TB10.26.1080.1.1	655.343282131187	0.991627733371788	0.00151314244062592	1.39888474834812e-15	XM_818214.1 Tbb strain 927/4 GUTat10.1 heat shock protein 83 (Tb10.26.1080) partial mRNA
GLOS_ACT1.1.1	30.1901066381591	0	0,00	1.36958874709015e-05	[BBH] ACT1_TRYBB (sp|P12432) Actin A OS=Trypanosoma brucei brucei PE=3 SV=1
GLOS_ALF.1.1	41.3176743361119	0	0,00	2.19713811992146e-07	[BBH] ALF_TRYBB (sp|P07752) Fructose-bisphosphate aldolase, glycosomal Tbb GN=ALD PE=1 SV=2
GLOS_CALM.1.1	50.3168443969318	0	0,00	8.00261111556065e-09	[BBH] CALM_TRYBG (sp|P69098) Calmodulin OS=Trypanosoma brucei gambiense PE=3 SV=2
GLOS_CC2H2.1.1	20.0905740521303	0	0,00	0.00061708615088037	[BBH] CC2H2_TRYBB (sp|P54665) Cell division control prot. 2 homolog 2; Tbb GN=CRK2 PE=3 SV=1
GLOS_CH60.1.3	96.6300394660945	0	0,00	6.19404320832783e-16	[BBH] CH60_TRYBB (sp|Q37683) Chaperonin HSP60, mitochondrial OS=Tbb GN=HSP60 PE=2 SV=2
GLOS_CLP.1.1	16.1230884310036	0	0,00	0.00282541529726647	[BBH] CLP_TRYBB (sp|P31543) Heat shock protein 100 OS=Tbb GN=HSP100 PE=3 SV=1
GLOS_contig_000050	36.1774987764916	0	0,00	1.45165037040756e-06	
GLOS_contig_000389	35.294118491137	0	0,00	2.05654076416339e-06	
GLOS_contig_000427	17.18728724957	0	0,00	0.00189992795526782	
GLOS_contig_000441	38.1974052936973	0	0,00	6.86290003011148e-07	
GLOS_contig_000530	21.1186091640544	0	0,00	0.000419044355280004	
GLOS_contig_000567	34.1937559659282	0	0,00	3.05321856536068e-06	
GLOS_contig_000712	15.0950533190795	0	0,00	0.00417062745104592	
GLOS_contig_000809	25.1945859051083	0	0,00	9.02751868220559e-05	
GLOS_contig_000926	28.0617090010262	0	0,00	0.000337508122349936	
GLOS_contig_001171	22.0381531560514	0	0,00	0.00114193162349596	
GLOS_contig_001756	40.2534755175454	0	0,00	3.22644930696384e-07	
GLOS_contig_001921	63.428154905448	0	0,00	7.01964542689587e-11	

GLOS_contig_004527	32.1738494487225	0	0,00	6.48502277463704e-06	
GLOS_contig_005069	19.0987026468487	0	0,00	0.000902028346384345	
GLOS_contig_007423	84.2936181230058	0	0,00	4.88690087083086e-07	
GLOS_contig_008528	410.898429842649	0	0,00	2.67620830053071e-07	NR_077014.1 E. fergusonii ATCC 35469 strain ATCC 35469 23S ribosomal RNA, complete sequence
GLOS_COPB.1.2	37.3501887149851	0	0,00	9.63892803228974e-07	[BBH] COPB_TRYBB (sp|Q9NFU6) Coatomer subunit beta OS=Tbb PE=3 SV=1
GLOS_CRAM.1.1	69.2347285105686	0	0,00	1.40314670398059e-06	CRAM_TRYBB (sp|Q03650) Cysteine-rich, acidic integral membrane prot.; Tbb GN=CRAM PE=2 SV=1
GLOS_DCOR.1.1	30.2624340514438	0	0,00	1.34845286515237e-05	[BBH] DCOR_TRYBB (sp|P07805) Ornithine decarboxylase OS=Tbb PE=1 SV=2
GLOS_DWIL_GK10999.1.1	32.3546679819343	0	0,00	6.23853162604361e-06	XP_002070501.1 GK10999 [Drosophila willistoni]
GLOS_ERF1.1.2	20.2352288786998	0	0,00	0.000597952029826168	[BBH] ERF1_TRYBB (sp)Q9NAX8) Eukary. pept. chain release fact. subunit 1; Tbb GN=ERF1 PE=3 SV=1
GLOS_GPDA.1.2	187.794430012787	0	0,00	3.69195308825051e-10	[BBH] GPDA_TRYBR (sp|Q26756) Glycerol-3-phosphate dehydrogenase [NAD()], glycosomal.
GLOS_GSK3B.1.1	21.1547728706968	0	0,00	0.000415757696464543	[BBH] GSK3B_TRYB2 (sp|Q388M1) Glycogen synthase kinase 3; Tbb (strain 927/4 GUTat10.1)
GLOS_HPRT.1.1	19.0987026468487	0	0,00	0.000902028346384345	[BBH] HPRT_TRYBB (sp|Q07010) Hypoxanthine-guanine phosphoribosyltransferase
GLOS_HSP70.1.1	16.159252137646	0	0,00	0.00280318659597305	XM_946483.1 Tbb strain 927/4 GUTat10.1 heat shock protein 78 (hsp70) partial mRNA
GLOS_IF4A.1.2	104.60117441499	0	0,00	3.9747413038092e-14	[BBH] IF4A_TRYB2 (sp|Q38F76) Prob. eukaryotic initiation factor 4A
GLOS_KPYK1.1.1	14.1755093270826	0	0,00	0.00601463776233708	[BBH] KPYK1_TRYBB (sp|P30615) Pyruvate kinase 1 OS=Tbb GN=PYK1 PE=3 SV=1
GLOS_LOC100843429.1.1	196.199529441392	0	0,00	2.89061045397666e-19	XM_003581200.1 PREDICTED: B. distachyon uncharacteri. LOC100843429 (LOC100843429), mRNA
GLOS_NDUS2.1.1	124.800239587048	0	0,00	2.51739833300038e-19	NDUS2_TRYBB (sp|P21301) NADH-ubiquinone oxidoreductase 49 kDa subunit homolog
GLOS_NOG1.1.2	27.1783287156716	0	0,00	4.25270484194831e-05	[BBH] NOG1_TRYBB (sp|Q9U6A9) Nucleolar GTP-binding protein 1; Tbb GN=NOG1 PE=1 SV=1
GLOS_P320.1.1	19.0987026468487	0	0,00	0.000902028346384345	[BBH] P320_TRYBB (sp|P21787) Microtubule-associated protein P320 (Fragment)
GLOS_PFR1.1.1	115.728742112943	0	0,00	5.32204646241726e-18	[BBH] PFR1_TRYBB (sp|P22225) 69 kDa paraflagellar rod protein OS=Tbb GN=PFRA PE=2 SV=1
GLOS_PGKE.1.1	62.3277923802392	0	0,00	2.19654576070157e-10	[BBH] PGKE_TRYBB (sp|P08893) Phosphoglycerate kinase, cytosolic OS=Tbb PE=3 SV=1
GLOS_PRO2.2.2	53.1478037862074	0	0,00	1.8966211367663e-05	PRO2_TRYBB (sp|P14044) Duplicate procyclin OS=Trypanosoma brucei brucei PE=4 SV=1
GLOS_RIR2.1.2	47.1965753545173	0	0,00	3.93529853458239e-07	[BBH] RIR2_TRYBB (sp|O15910) Ribonucleoside-diphosphate reductase small chain
GLOS_RL27A.1.6	193.709494737835	0	0,00	7.92052299576191e-09	[BBH] RL27A_TRYBB (sp|O15883) 60S ribosomal protein L27a OS=Tbb GN=RPL27A PE=2 SV=1
GLOS_RL30.1.1	71.4716172676286	0	0,00	3.98830779419933e-12	RL30_TRYBB (sp|P49153) 60S ribosomal protein L30 OS=Tbb GN=RPL30 PE=3 SV=1
GLOS_RL40.2.8	38.3058964136244	0	0,00	6.70640609230663e-07	RL40_TRYBB (sp|P21899) Ubiquitin-60S ribosomal protein L40 OS=T. b. brucei PE=1 SV=2
GLOS_RL40.3.8	79.3704248032397	0	0,00	2.53209439055001e-09	RL40_TRYBB (sp|P21899) Ubiquitin-60S ribosomal protein L40 OS=T. b. brucei PE=1 SV=2
GLOS_RL402.1.2	77.4951731126034	0	0,00	4.74511935817587e-13	RL402_TRYCR (sp|P0CH27) Ubiquitin-60S ribosomal protein L40 OS=Trypanosoma cruzi PE=2 SV=1
GLOS_RNA45S5.1.2	141.336580117055	0	0,00	3.06305858108841e-06	NR_046235.1 Homo sapiens RNA, 45S pre-ribosomal 5 (RNA45S5), ribosomal RNA
GLOS_RPB1B.1.1	19.0625389402063	0	0,00	0.000909184655185172	[BBH] RPB1B_TRYBB (sp|P17545) DNA-directed RNA polymerase II subunit RPB1-B
GLOS_RPOA.1.1	1094.51514215995	0	0,00	1.01762433920799e-08	[BBH] RPOA_LDVP (sp|Q83017) Replicase polyprot. 1ab OS=Lactate dehydrogenase elevating virus
GLOS_RS12.1.4	105.303736167133	0	0,00	1.68785809945514e-06	[BBH] RS12_TRYBB (sp|Q03253) 40S ribosomal protein S12 OS=Tbb GN=RPS12 PE=2 SV=2
GLOS_SMP_112590.1.1	28.0255452943838	0	0,00	0.00189421247233838	XM_002581801.1 S. mansoni conserved hypothetical protein (Smp_112590) mRNA, partial cds
GLOS_TB09.160.0430.1.1	38.2335690003397	0	0,00	6.8103005804718e-07	XM_798344.1 Tbb strain 927/4 GUTat10.1 hypothetical protein (Tb09.160.0430) partial mRNA
GLOS_TB09.160.0710.1.1	150.733550950941	0	0,00	1.34050723341789e-11	XM_798407.1 Tbb strain 927/4 GUTat10.1 60S ribosomal prot. L35 (Tb09.160.0710) partial mRNA
GLOS_TB09.160.0810.1.1	25.0860947851812	0	0,00	9.24236372795687e-05	XM_798420.1 Tbb strain 927/4 GUTat10.1 kynureninase (Tb09.160.0810) partial mRNA
GLOS_TB09.160.0815.1.1	66.2591142947235	0	0,00	2.5010096106972e-07	XP_803515.1 60S ribosomal protein L38 [Trypanosoma brucei]
GLOS_TB09.160.0930.1.1	21.2271002839815	0	0,00	0.0004092721885638	XM_798434.1 Tbb strain 927/4 GUTat10.1 protein kinase (Tb09.160.0930) partial mRNA
GLOS_TB09.160.1160.1.1	85.7194540079959	0	0,00	2.74492771674057e-14	XM_798466.1 Tbb strain 927/4 GUTat10.1 hypothetical protein (Tb09.160.1160) partial mRNA
GLOS_TB09.160.1200.1.1	14.1393456204402	0	0,00	0.00606230038830442	XP_803563.1 mitotubule-associated protein Gb4 [Tbb strain 927/4 GUTat10.1]
GLOS_TB09.160.1520.1.1	50.1360258637199	0	0,00	2.64832682165044e-05	XM_798500.1 Tbb strain 927/4 GUTat10.1 hypothetical protein (Tb09.160.1520) partial mRNA

GLOS_TB09.160.1780.1.1	30.2624340514438	0	0,00	1.34845286515237e-05	XM_798524.1 Tbb strain 927/4 GUTat10.1 protein kinase (Tb09.160.1780) partial mRNA
GLOS_TB09.160.1820.1.1	31.0373232168713	0	0,00	0.0011112312656724	XP_803621.1 cytochrome c oxidase subunit V [Tbb strain 927/4 GUTat10.1]
GLOS_TB09.160.2550.1.1	285.524832004091	0	0,00	1.02830615765409e-15	XP_803698.1 ribosomal protein S7 [Trypanosoma brucei brucei strain 927/4 GUTat10.1]
GLOS_TB09.160.2770.1.1	18.106831241567	0	0,00	0.0013191789443401	XP_803723.1 fatty acyl CoA synthetase 1 [Trypanosoma brucei brucei strain 927/4 GUTat10.1]
GLOS_TB09.160.2810.1.2	19.1710300601334	0	0,00	0.000887909871470473	XP_803725.1 fatty acyl CoA synthetase 3 [Trypanosoma brucei brucei strain 927/4 GUTat10.1]
GLOS_TB09.160.2810.2.2	19.134866353491	0	0,00	0.000894936973946279	XP_803725.1 fatty acyl CoA synthetase 3 [Trypanosoma brucei brucei strain 927/4 GUTat10.1]
GLOS_TB09.160.3530.1.1	47.5220487142986	0	0,00	2.30230896844926e-08	XM_798709.1 Tbb strain 927/4 GUTat10.1 hypothetical protein (Tb09.160.3530) partial mRNA
GLOS_TB09.160.3590.1.1	53.400949732704	0	0,00	2.63038935941488e-09	XM_798715.1 Tbb strain 927/4 GUTat10.1 cAMP-specific phosphodiesterase partial mRNA
GLOS_TB09.160.3670.1.1	14.211673033725	0	0,00	0.00596742626752826	XP_803820.1 ribosomal protein S6 [Trypanosoma brucei brucei strain 927/4 GUTat10.1]
GLOS_TB09.160.3780.1.1	26.0779661904628	0	0,00	9.76903094824833e-05	XM_798741.1 Tbb strain 927/4 GUTat10.1 hypothetical protein (Tb09.160.3780) partial mRNA
GLOS_TB09.160.4200.1.1	121.752297957918	0	0,00	2.16773464490887e-17	XM_821871.1 Tbb strain 927/4 GUTat10.1 60S acid. ribosomalprot (Tb09.160.4200) partial mRNA
GLOS_TB09.160.4240.1.1	33.2018845606465	0	0,00	4.43137317533808e-06	XM_821875.1 Tbb GUTat10.1 nucleosome assembly prot.-like prot. (Tb09.160.4240) partial mRNA
GLOS_TB09.160.4300.1.1	33.346539387216	0	0,00	4.29630721675298e-06	XM_821880.1 Tbb GUTat10.1 farnesyl pyrophosphate synthetase (Tb09.160.4300) partial mRNA
GLOS_TB09.160.4310.1.1	80.3622962085214	0	0,00	5.9118123587722e-09	XP_826974.1 glutamate dehydrogenase [Trypanosoma brucei brucei strain 927/4 GUTat10.1]
GLOS_TB09.160.4380.1.1	18.2153223614941	0	0,00	0.00128833644925981	XP_826981.1 succinate dehydrogenase [Trypanosoma brucei brucei strain 927/4 GUTat10.1]
GLOS_TB09.160.4460.1.1	108.496332622832	0	0,00	7.80283179867946e-10	XM_821897.1 Tbb strain 927/4 GUTat10.1 hypothetical protein (Tb09.160.4460) partial mRNA
GLOS_TB09.160.4560.1.1	137.766895268994	0	0,00	2.93382464734714e-14	XM_821905.1 Tbb strain 927/4 GUTat10.1 arginine kinase (Tb09.160.4560) partial mRNA
GLOS_TB09.160.4580.1.1	32.2100131553648	0	0,00	6.43488331005283e-06	XM_821907.1 Tbb strain 927/4 GUTat10.1 hypothetical protein (Tb09.160.4580) partial mRNA
GLOS_TB09.160.4600.1.1	31.1458143367984	0	0,00	9.49548498458617e-06	XM_821909.1 Tbb strain 927/4 GUTat10.1 ABC transporter (Tb09.160.4600) partial mRNA
GLOS_TB09.160.5060.1.1	32.0653583287953	0	0,00	0.000317981322930628	XM_821961.1 Tbb strain 927/4 GUTat10.1 hypothetical protein (Tb09.160.5060) partial mRNA
GLOS_TB09.160.5480.1.1	41.426165456039	0	0,00	2.14734531495523e-07	XM_822007.1 Tbb strain 927/4 GUTat10.1 adenosine transporter (Tb09.160.5480) partial mRNA
GLOS_TB09.160.5590.1.1	239.661052740574	0	0,00	1.78334388755011e-07	XM_822018.1 Tbb strain 927/4 GUTat10.1 60S ribosomal prot. L11 (Tb09.160.5590) partial mRNA
GLOS_TB09.211.0040.1.1	24.0942233798995	0	0,00	0.000134764342551207	XM_822050.1 Tbb strain 927/4 GUTat10.1 hypothetical protein (Tb09.211.0040) partial mRNA
GLOS_TB09.211.0120.1.2	108.351677796263	0	0,00	4.56438470318792e-07	XP_827151.1 nascent polypeptide associated complex subunit [Trypanosoma brucei] ref|XP_827152.1| nascent polypeptide associated complex subunit [Trypanosoma brucei]
GLOS_TB09.211.0120.2.2	33.2018845606465	0	0,00	4.43137317533808e-06	XP_827151.1 nascent polypeptide associated complex subunit [Trypanosoma brucei] ref|XP_827152.1| nascent polypeptide associated complex subunit [Trypanosoma brucei]
GLOS_TB09.211.0320.1.1	21.2271002839815	0	0,00	0.0004092721885638	XM_822080.1 Tbb strain 927/4 GUTat10.1 hypothetical protein (Tb09.211.0320) partial mRNA
GLOS_TB09.211.0340.1.1	260.113263859128	0	0,00	4.81431815966296e-11	XM_822082.1 Tbb strain 927/4 GUTat10.1 60S ribosomal prot. L10 (Tb09.211.0340) partial mRNA
GLOS_TB09.211.0560.1.1	58.1071608126158	0	0,00	0.000108381973212401	XM_822105.1 Tbb strain 927/4 GUTat10.1 hypothetical protein (Tb09.211.0560) partial mRNA
GLOS_TB09.211.0610.1.1	19.1710300601334	0	0,00	0.000887909871470473	XM_822111.1 Tbb strain 927/4 GUTat10.1 hypothetical protein (Tb09.211.0610) partial mRNA
GLOS_TB09.211.0930.1.1	30.1177792248743	0	0,00	1.39109013062701e-05	XM_822144.1 Tbb strain 927/4 GUTat10.1 polyadenylate-binding protein 1 partial mRNA
GLOS_TB09.211.0960.1.1	32.3546679819343	0	0,00	6.23853162604361e-06	XP_827240.1 protein kinase [Trypanosoma brucei brucei strain 927/4 GUTat10.1]
GLOS_TB09.211.1000.1.1	53.3286223194192	0	0,00	2.67019621177704e-09	XM_822151.1 Trypanosoma brucei brucei strain 927/4 GUTat10.1 phosphatidylcholine:ceramide cholinephosphotransferase 2 (Tb09.211.1000) partial mRNA
GLOS_TB09.211.1070.1.1	55.7101659030487	0	0,00	1.19207799926778e-09	XM_822158.1 Tbb strain 927/4 GUTat10.1 hypothetical protein (Tb09.211.1070) partial mRNA
GLOS_TB09.211.1190.1.1	31.109650630156	0	0,00	1.61172182692142e-05	XM_822171.1 Trypanosoma brucei brucei strain 927/4 GUTat10.1 minichromosome maintenance complex subunit (Tb09.211.1190) partial mRNA
GLOS_TB09.211.1230.1.1	26.1141298971052	0	0,00	6.29225505279159e-05	XM_822175.1 Tbb strain 927/4 GUTat10.1 hypothetical protein (Tb09.211.1230) partial mRNA
GLOS_TB09.211.1240.1.1	21.1547728706968	0	0,00	0.000415757696464543	XM_822176.1 Tbb strain 927/4 GUTat10.1 hypothetical protein (Tb09.211.1240) partial mRNA
GLOS_TB09.211.1620.1.1	43.1567623201058	0	0,00	3.67107325072828e-06	XP_827306.1 hypothetical protein [Trypanosoma brucei brucei strain 927/4 GUTat10.1]

GLOS_TB09.211.1690.1.1	30.1539429315167	0	0,00	1.38029332263529e-05	XM_822222.1 Tbb strain 927/4 GUTat10.1 hypothetical protein (Tb09.211.1690) partial mRNA
GLOS_TB09.211.1750.1.1	21.082445457412	0	0,00	0.000422360679473223	XM_822228.1 Trypanosoma brucei brucei strain 927/4 GUTat10.1 mitochondrial carrier protein (Tb09.211.1750) partial mRNA. nuclear gene for mitochondrial product
GLOS_TB09.211.1950.1.1	28.0978727076686	0	0,00	2.96709423828632e-05	XM_822248.1 Tbb strain 927/4 GUTat10.1 hypothetical protein (Tb09.211.1950) partial mRNA
GLOS_TB09.211.2150.1.1	174.827774330841	0	0,00	1.52167932567464e-11	XM_822265.1 Tbb strain 927/4 GUTat10.1 poly(A)-binding prot. 1 (Tb09.211.2150) partial mRNA
GLOS_TB09.211.2570.1.1	35.1856273712099	0	0,00	2.10474362719246e-06	XM_822308.1 Tbb strain 927/4 GUTat10.1 t-complex prot. 1 subunit eta partial mRNA
GLOS_TB09.211.2630.1.2	68.5321667584259	0	0,00	1.14685648065048e-11	XP_827407.1 60S ribosomal protein L23 [Tbb strain 927/4 GUTat10.1] ref|XP_827408.1| 60S ribosomal protein L23 [Trypanosoma brucei brucei strain 927/4 GUTat10.1]
GLOS_TB09.211.2630.2.2	97.5857471647338	0	0,00	3.11971949428316e-15	XP_827407.1 60S ribosomal protein L23 [Tbb strain 927/4 GUTat10.1] ref|XP_827408.1| 60S ribosomal protein L23 [Trypanosoma brucei brucei strain 927/4 GUTat10.1]
GLOS_TB09.211.2700.1.1	34.3384107924977	0	0,00	2.96026500676158e-06	XM_822323.1 Tbb strain 927/4 GUTat10.1 hypothetical protein (Tb09.211.2700) partial mRNA
GLOS_TB09.211.2740.1.1	206.44371685399	0	0,00	1.21239933087661e-29	XP_827420.1 Gim5B protein [Trypanosoma brucei brucei strain 927/4 GUTat10.1]
GLOS_TB09.211.2880.1.1	32.2100131553648	0	0,00	6.43488331005283e-06	XM_822340.1 Tbb strain 927/4 GUTat10.1 hypothetical protein (Tb09.211.2880) partial mRNA
GLOS_TB09.211.2900.1.1	47.3050664744444	0	0,00	2.40931095393544e-08	XM_822342.1 Tbb strain 927/4 GUTat10.1 hypothetical protein (Tb09.211.2900) partial mRNA
GLOS_TB09.211.3280.1.1	220.598513800368	0	0,00	2.74853164155875e-07	XM_822379.1 Tbb GUTat10.1 60S ribosomal protein L31 (Tb09.211.3280) partial mRNA
GLOS_TB09.211.3330.1.1	80.7600969815875	0	0,00	1.56641818761274e-13	XM_822384.1 Tbb strain 927/4 GUTat10.1 cystathione gamma lyase partial mRNA
GLOS_TB09.211.3510.1.1	55.3123651299826	0	0,00	1.29422456743024e-09	XM_822401.1 Tbb GUTat10.1 ATP-dependent DEAD/H RNA helicase (Tb09.211.3510) partial mRNA
GLOS_TB09.211.3550.1.1	237.336385244292	0	0,00	1.0241733419489e-16	XM_822405.1 Tbb strain 927/4 GUTat10.1 glycerol kinase glycosomal partial mRNA
GLOS_TB09.211.3610.1.1	40.2173118109031	0	0,00	3.25128880335159e-07	XM_822409.1 Tbb GUTat10.1 ubiquitin-activating enzyme E1 (Tb09.211.3610) partial mRNA
GLOS_TB09.211.4070.1.1	39.1169492856943	0	0,00	2.90995178261476e-05	XM_822456.1 Tbb strain 927/4 GUTat10.1 hypothetical protein (Tb09.211.4070) partial mRNA
GLOS_TB09.211.4360.1.1	28.134036414311	0	0,00	2.94399465636344e-05	XM_822482.1 Tbb strain 927/4 GUTat10.1 hypothetical protein (Tb09.211.4360) partial mRNA
GLOS_TB09.211.4513.1.1	49.1803181650806	0	0,00	2.01498569262621e-06	XM_822500.1 Tbb strain 927/4 GUTat10.1 kinetoplastid membrane protein KMP-11 partial mRNA. nuclear gene for plastid product
GLOS_TB09.211.4550.1.1	123.699877061839	0	0,00	2.5682762741824e-14	XM_822504.1 Tbb GUTat10.1 60S ribosomal protein L12 (Tb09.211.4550) partial mRNA
GLOS_TB09.211.4700.1.1	37.3140250083427	0	0,00	9.71332393538796e-07	XP_827610.1 reiske iron-sulfur protein mitochondrial precursor [Trypanosoma brucei]
GLOS_TB09.211.4760.1.1	42.3818731546783	0	0,00	1.49704427272077e-07	XM_822523.1 Tbb strain 927/4 GUTat10.1 metacaspase 5 (Tb09.211.4760) partial mRNA
GLOS_TB09.211.4940.1.1	36.2498261897763	0	0,00	1.42942087176386e-06	XP_827634.1 hypothetical protein [Trypanosoma brucei brucei strain 927/4 GUTat10.1]
GLOS_TB09.244.2170.1.1	62.4724472068086	0	0,00	9.96646964497539e-11	XM_822625.1 Tbb strain 927/4 GUTat10.1 hypothetical protein (Tb09.244.2170) partial mRNA
GLOS_TB09.244.2570.1.1	32.0653583287953	0	0,00	0.000317981322930628	XM_822590.1 Tbb GUTat10.1 calcium motive p-type ATPase (Tb09.244.2570) partial mRNA
GLOS_TB09.244.2590.1.1	137.839222682279	0	0,00	2.36648718152184e-17	XP_827681.1 60S ribosomal protein L32 [Trypanosoma brucei brucei strain 927/4 GUTat10.1]
GLOS_TB09.244.2630.1.1	152.159386835931	0	0,00	6.68192983471631e-24	XM_822584.1 Tbb GUTat10.1 40S ribosomal protein S6 (Tb09.244.2630) partial mRNA
GLOS_TB09.244.2660.1.1	27.1783287156716	0	0,00	4.25270484194831e-05	XM_822578.1 Tbb strain 927/4 GUTat10.1 hypothetical protein (Tb09.244.2660) partial mRNA
GLOS_TB09.244.2720.1.1	219.15722658944	0	0,00	1.75770936235787e-14	XM_822570.1 Tbb strain 927/4 GUTat10.1 ribosomal protein L15 (Tb09.244.2720) partial mRNA
GLOS_TB09.244.2725.1.1	48.3692652930108	0	0,00	1.64680064138597e-08	XM_822571.1 Tbb strain 927/4 GUTat10.1 ribosomal protein L36 (Tb09.244.2725) partial mRNA
GLOS_TB09.244.2730.1.1	366.51736255147	0	0,00	2.20601343053567e-11	XM_822569.1 Tbb strain 927/4 GUTat10.1 60S ribosomal prot. L5 (Tb09.244.2730) partial mRNA
GLOS_TB09.V1.0150.1.1	33.2018845606465	0	0,00	4.43137317533808e-06	XM_798607.1 Tbb strain 927/4 GUTat10.1 hypothetical protein (Tb09.v1.0150) partial mRNA
GLOS_TB09.V1.0380.1.1	34.3384107924977	0	0,00	2.96026500676158e-06	XM_822031.1 Tbb strain 927/4 GUTat10.1 spermidine synthase (Tb09.v1.0380) partial mRNA
GLOS_TB09.V1.0420.1.1	42.3457094480359	0	0,00	1.50851248097151e-07	XP_827128.1 hypothetical protein [Trypanosoma brucei brucei strain 927/4 GUTat10.1]
GLOS_TB09.V2.0030.1.1	16.1954158442883	0	0,00	0.00278116418906016	XM_821914.1 Tbb strain 927/4 GUTat10.1 hypothetical protein (Tb09.v2.0030) partial mRNA
GLOS_TB10.05.0010.1.1	21.082445457412	0	0,00	0.000422360679473223	XP_827840.1 diphosphomevalonate decarboxylase [Tbb strain 927/4 GUTat10.1]
GLOS_TB10.05.0080.1.1	19.1710300601334	0	0,00	0.000887909871470473	XM_822754.1 Tbb strain 927/4 GUTat10.1 glucosidase (Tb10.05.0080) partial mRNA

GLOS_TB10.05.0110.1.1	25.0137673718964	0	0,00	0.00335534660432802	XM_822757.1 Tbb GUTat10.1 serine/threonine prot phosphatase type 5 partial mRNA
GLOS_TB10.05.0220.1.1	342.190705605779	0	0,00	3.60618486150839e-25	XM_822741.1 Tbb GUTat10.1 60S ribosomal protein L10a (Tb10.05.0220) partial mRNA
GLOS_TB10.100.0070.1.1	22.1828079826208	0	0,00	0.000282477536862942	XP_822278.1 ATP synthase F1 subunit gamma [Tbb strain 927/4 GUTat10.1]
GLOS_TB10.100.0080.1.1	137.658404149067	0	0,00	4.5656941039466e-11	XM_817186.1 Tbb GUTat10.1 40S ribosomal protein S6 (Tb10.100.0080) partial mRNA
GLOS_TB10.100.0155.1.1	52.3367509141375	0	0,00	3.83823564900603e-09	XP_822287.1 60S ribosomal protein L32 [Trypanosoma brucei brucei strain 927/4 GUTat10.1]
GLOS_TB10.100.0160.1.1	68.2066933986445	0	0,00	4.48908039416458e-06	XP_822288.1 cytochrome C oxidase subunit VI [Tbb strain 927/4 GUTat10.1]
GLOS_TB10.26.0100.1.1	19.0625389402063	0	0,00	0.000909184655185172	XP_823384.1 hypothetical protein [Trypanosoma brucei brucei strain 927/4 GUTat10.1]
GLOS_TB10.26.0140.1.1	31.2543054567255	0	0,00	9.27668759581189e-06	XM_818290.1 Tbb GUTat10.1 pumilio RNA-binding protein (Tb10.26.0140) partial mRNA
GLOS_TB10.26.0370.1.1	285.452504590806	0	0,00	2.03465102664625e-14	XP_823361.1 40S ribosomal protein S3 [Trypanosoma brucei brucei strain 927/4 GUTat10.1]
GLOS_TB10.26.0510.1.1	53.4732771459887	0	0,00	2.59121231931225e-09	XM_818259.1 Tbb strain 927/4 GUTat10.1 CYC2-like cyclin (Tb10.26.0510) partial mRNA
GLOS_TB10.26.0560.1.1	144.926977345821	0	0,00	5.04532241969599e-20	XM_818254.1 Tbb GUTat10.1 60S ribosomal protein L6 (Tb10.26.0560) partial mRNA
GLOS_TB10.26.0680.1.1	30.1901066381591	0	0,00	1.36958874709015e-05	XP_823339.1 hypothetical protein [Trypanosoma brucei brucei strain 927/4 GUTat10.1]
GLOS_TB10.26.0790.1.1	52.1920960875681	0	0,00	1.53134963098513e-06	XM_818238.1 Tbb GUTat10.1 procyclic form surface glycoprotein (Tb10.26.0790) partial mRNA
GLOS_TB10.26.0880.1.1	31.109650630156	0	0,00	1.61172182692142e-05	XM_818231.1 Tbb strain 927/4 GUTat10.1 hypothetical protein (Tb10.26.0880) partial mRNA
GLOS_TB10.389.0070.1.1	36.2136624831339	0	0,00	1.4404890361234e-06	XM_822728.1 Tbb strain 927/4 GUTat10.1 elongation factor TU (Tb10.389.0070) partial mRNA
GLOS_TB10.389.0430.1.1	58.1433245192582	0	0,00	3.06364638547092e-05	XM_822697.1 Tbb GUTat10.1 receptor-type adenylate cyclase GRESAG 4 partial mRNA
GLOS_TB10.389.0510.1.1	25.1222584918235	0	0,00	9.17011903466956e-05	XP_827786.1 3' 5'-cyclic nucleotide phosphodiesterase [Trypanosoma brucei TREU927]
GLOS_TB10.389.0570.1.1	24.2027144998266	0	0,00	0.000131628791765919	XM_822687.1 Tbb strain 927/4 GUTat10.1 hypothetical protein (Tb10.389.0570) partial mRNA
GLOS_TB10.389.0630.1.1	24.238878206469	0	0,00	0.00013060190031178	XM_822682.1 Tbb strain 927/4 GUTat10.1 prolyl-tRNA synthetase (Tb10.389.0630) partial mRNA
GLOS_TB10.389.0680.1.1	17.1511235429277	0	0,00	0.00191497267072198	XP_823487.1 hypothetical protein [Trypanosoma brucei brucei strain 927/4 GUTat10.1]
GLOS_TB10.389.0690.1.1	35.2217910778523	0	0,00	2.08854072568386e-06	XP_823486.1 mitochondrial carrier protein [Trypanosoma brucei brucei strain 927/4 GUTat10.1]
GLOS_TB10.389.0730.1.1	22.0743168626937	0	0,00	0.000289224804544453	XP_823483.1 cholinephosphate cytidylyltransferase A [Trypanosoma brucei TREU927]
GLOS_TB10.389.0880.1.1	30.1901066381591	0	0,00	1.36958874709015e-05	XM_818383.1 Tbb strain 927/4 GUTat10.1 heat shock protein (Tb10.389.0880) partial mRNA
GLOS_TB10.389.0890.1.1	55.059219183486	0	0,00	0.000425612250809484	XM_818382.1 Tbb GUTat10.1 pyruvate dehydrogenase E1 component subunit alpha partial mRNA
GLOS_TB10.389.0910.1.2	131.779503130662	0	0,00	5.1364392016508e-16	XM_818380.1 Tbb strain 927/4 GUTat10.1 60S ribosomal prot. L34 (Tb10.389.0910) partial mRNA
GLOS_TB10.389.0910.2.2	112.572309363886	0	0,00	1.55141755360111e-11	XM_818380.1 Tbb GUTat10.1 60S ribosomal protein L34 (Tb10.389.0910) partial mRNA
GLOS_TB10.389.1180.1.1	67.3594768199323	0	0,00	7.08814540633374e-11	XM_818362.1 Tbb strain 927/4 GUTat10.1 P-type H+ATPase (Tb10.389.1180) partial mRNA
GLOS_TB10.389.1480.1.1	26.0779661904628	0	0,00	9.76903094824833e-05	XM_818338.1 Tbb GUTat10.1 cytosolic nonspecific dipeptidase (Tb10.389.1480) partial mRNA
GLOS_TB10.389.1810.1.1	42.1648909148241	0	0,00	1.62070436675388e-06	XP_823403.1 kynurenine aminotransferase [Trypanosoma brucei brucei strain 927/4 GUTat10.1]
GLOS_TB10.406.0450.1.1	140.148438852624	0	0,00	3.21564337738346e-22	XM_818174.1 Tbb GUTat10.1 histone H2B partial mRNA ref|XM_818176.1| Tbb GUTat10.1 histone H2B partial mRNA ref|XM_818177.1| Tbb GUTat10.1 histone H2B partial mRNA ref|XM_818178.1| Tbb GUTat10.1 histone H2B partial mRNA ref|XM_818179.1| Tbb GUTat10.1 histone H2B partial mRNA ref|XM_818181.1| Tbb GUTat10.1 histone H2B partial mRNA ref|XM_818182.1| Tbb GUTat10.1 histone H2B partial mRNA ref|XM_818184.1| Tbb GUTat10.1 histone H2B partial mRNA
GLOS_TB10.406.0650.1.1	47.4858850076562	0	0,00	2.31978392115287e-08	XM_818156.1 Tbb GUTat10.1 microtubule-associated protein (Tb10.406.0650) partial mRNA
GLOS_TB10.61.0540.1.1	52.2644235008528	0	0,00	3.8964579424045e-09	XM_822953.1 Tbb strain 927/4 GUTat10.1 hypothetical protein (Tb10.61.0540) partial mRNA
GLOS_TB10.61.0980.1.1	206.603823006497	0	0,00	1.57505082585526e-07	XM_822919.1 Tbb GUTat10.1 glycosomal malate dehydrogenase (Tb10.61.0980) partial mRNA
GLOS_TB10.61.1260.1.1	26.1502936037476	0	0,00	6.24316458047021e-05	XM_822905.1 Tbb strain 927/4 GUTat10.1 hypothetical protein (Tb10.61.1260) partial mRNA
GLOS_TB10.61.1330.1.1	37.241697595058	0	0,00	9.86399057604768e-07	XM_822898.1 Tbb GUTat10.1 nucleosome assembly prot. (Tb10.61.1330) partial mRNA
GLOS_TB10.61.1390.1.2	35.3302821977794	0	0,00	2.04074126177174e-06	XP_827985.1 40S ribosomal protein S13 [Tbb strain 927/4 GUTat10.1]

<table>
<tr><td></td><td></td><td></td><td></td><td></td><td>ref|XP_951734.1| 40S ribosomal protein S13 [Tbb< strain 927/4 GUTat10.1]</td></tr>
<tr><td>GLOS_TB10.61.1390.2.2</td><td>142.204509076472</td><td>0</td><td>0,00</td><td>1.66282676606568e-22</td><td>XP_827985.1 40S ribosomal protein S13 [Tbb strain 927/4 GUTat10.1] ref|XP_951734.1| 40S ribosomal protein S13 [Tbb strain 927/4 GUTat10.1]</td></tr>
<tr><td>GLOS_TB10.61.1790.1.1</td><td>20.1629014654151</td><td>0</td><td>0,00</td><td>0.00060743267903454</td><td>XP_827964.1 hypothetical protein [Trypanosoma brucei brucei strain 927/4 GUTat10.1]</td></tr>
<tr><td>GLOS_TB10.61.1820.1.1</td><td>61.3359209749575</td><td>0</td><td>0,00</td><td>1.46916959309323e-10</td><td>XM_822868.1 Tbb GUTat10.1 mitochondrial carrier protein partial mRNA. nuclear gene for mitochondrial product ref|XM_822869.1| Tbb GUTat10.1 mitochondrial carrier protein partial mRNA. nuclear gene for mitochondrial product</td></tr>
<tr><td>GLOS_TB10.61.1840.1.1</td><td>23.1023519746178</td><td>0</td><td>0,00</td><td>0.000196601085738671</td><td>XP_827959.1 hypothetical protein [Trypanosoma brucei brucei strain 927/4 GUTat10.1]</td></tr>
<tr><td>GLOS_TB10.61.1870.1.1</td><td>28.0978727076686</td><td>0</td><td>0,00</td><td>2.96709423828632e-05</td><td>XM_822864.1 Tbb strain 927/4 GUTat10.1 aminopeptidase (Tb10.61.1870) partial mRNA</td></tr>
<tr><td>GLOS_TB10.61.1920.1.1</td><td>22.0381531560514</td><td>0</td><td>0,00</td><td>0.00114193162349596</td><td>XM_822860.1 Tbb strain 927/4 GUTat10.1 fibrillarin (Tb10.61.1920) partial mRNA</td></tr>
<tr><td>GLOS_TB10.61.1940.1.1</td><td>24.0218959666147</td><td>0</td><td>0,00</td><td>0.00250078634343649</td><td>XM_822858.1 Tbb strain 927/4 GUTat10.1 chaperone protein DnaJ (Tb10.61.1940) partial mRNA</td></tr>
<tr><td>GLOS_TB10.61.1960.1.2</td><td>119.5515729075</td><td>0</td><td>0,00</td><td>3.50247323763732e-10</td><td>XM_822856.1 Tbb strain 927/4 GUTat10.1 40S ribosomal prot. S2 (Tb10.61.1960) partial mRNA</td></tr>
<tr><td>GLOS_TB10.61.1960.2.2</td><td>184.260908871369</td><td>0</td><td>0,00</td><td>1.68201489439681e-26</td><td>XM_822856.1 Tbb strain 927/4 GUTat10.1 40S ribosomal prot. S2 (Tb10.61.1960) partial mRNA</td></tr>
<tr><td>GLOS_TB10.61.1970.1.1</td><td>20.1267377587727</td><td>0</td><td>0,00</td><td>0.000612237591608327</td><td>XM_822855.1 Tbb GUTat10.1 hypothetical protein (Tb10.61.1970) partial mRNA</td></tr>
<tr><td>GLOS_TB10.61.2090.1.2</td><td>91.670682439686</td><td>0</td><td>0,00</td><td>3.44711812993132e-15</td><td>XM_822844.1 Tbb GUTat10.1 60S ribosomal protein L17 (Tb10.61.2090) partial mRNA</td></tr>
<tr><td>GLOS_TB10.61.2090.2.2</td><td>34.1575922592858</td><td>0</td><td>0,00</td><td>3.07695129569848e-06</td><td>XP_827937.1 60S ribosomal protein L17 [Tbb GUTat10.1] ref|XP_828557.1| 60S ribosomal protein L17 [Trypanosoma brucei brucei strain 927/4 GUTat10.1]</td></tr>
<tr><td>GLOS_TB10.61.2180.1.1</td><td>30.3347614647286</td><td>0</td><td>0,00</td><td>1.32767571453268e-05</td><td>XM_822840.1 Tbb GUTat10.1 proteasome regulatory non-ATPase subunit 8 partial mRNA</td></tr>
<tr><td>GLOS_TB10.61.2210.1.1</td><td>21.082445457412</td><td>0</td><td>0,00</td><td>0.000422360679473223</td><td>XM_822837.1 Tbb strain 927/4 GUTat10.1 hypothetical protein (Tb10.61.2210) partial mRNA</td></tr>
<tr><td>GLOS_TB10.61.2220.1.1</td><td>28.2063638275957</td><td>0</td><td>0,00</td><td>2.8983908845462e-05</td><td>XM_822836.1 Tbb strain 927/4 GUTat10.1 hypothetical protein (Tb10.61.2220) partial mRNA</td></tr>
<tr><td>GLOS_TB10.61.2270.1.1</td><td>17.1511235429277</td><td>0</td><td>0,00</td><td>0.00191497267072198</td><td>XM_822833.1 Tbb strain 927/4 GUTat10.1 hypothetical protein (Tb10.61.2270) partial mRNA</td></tr>
<tr><td>GLOS_TB10.61.2300.1.1</td><td>18.1429949482093</td><td>0</td><td>0,00</td><td>0.00130880372149336</td><td>XM_822830.1 Tbb strain 927/4 GUTat10.1 hypothetical protein (Tb10.61.2300) partial mRNA</td></tr>
<tr><td>GLOS_TB10.61.2850.1.1</td><td>14.1031819137979</td><td>0</td><td>0,00</td><td>0.00611041919132889</td><td>XM_822793.1 Tbb strain 927/4 GUTat10.1 hypothetical protein (Tb10.61.2850) partial mRNA</td></tr>
<tr><td>GLOS_TB10.61.2880.1.1</td><td>45.9877217093813</td><td>0</td><td>0,00</td><td>0.00281119292790344</td><td>XP_827883.1 aconitase [Trypanosoma brucei brucei strain 927/4 GUTat10.1]</td></tr>
<tr><td>GLOS_TB10.61.3120.1.1</td><td>58.3603067591124</td><td>0</td><td>0,00</td><td>4.31850142889001e-10</td><td>XM_822770.1 Tbb strain 927/4 GUTat10.1 hypothetical protein (Tb10.61.3120) partial mRNA</td></tr>
<tr><td>GLOS_TB10.6K15.0020.1.3</td><td>95.2765309943891</td><td>0</td><td>0,00</td><td>2.2083980155213e-06</td><td>XM_818154.1 Tbb GUTat10.1 EP1 procyclin precursor (Tb10.6k15.0020) partial mRNA</td></tr>
<tr><td>GLOS_TB10.6K15.0020.2.3</td><td>103.428484476497</td><td>0</td><td>0,00</td><td>1.17157680362881e-08</td><td>XM_818154.1 Tbb GUTat10.1 EP1 procyclin precursor (Tb10.6k15.0020) partial mRNA</td></tr>
<tr><td>GLOS_TB10.6K15.0020.3.3</td><td>482.876339003271</td><td>0</td><td>0,00</td><td>3.47128115006587e-11</td><td>XM_818154.1 Tbb GUTat10.1 EP1 procyclin precursor (Tb10.6k15.0020) partial mRNA</td></tr>
<tr><td>GLOS_TB10.6K15.0240.1.1</td><td>38.2697327069821</td><td>0</td><td>0,00</td><td>6.75813722995807e-07</td><td>XM_818133.1 Tbb strain 927/4 GUTat10.1 hypothetical protein (Tb10.6k15.0240) partial mRNA</td></tr>
<tr><td>GLOS_TB10.6K15.0280.1.1</td><td>35.1494636645675</td><td>0</td><td>0,00</td><td>2.12108345743693e-06</td><td>XM_818129.1 Tbb GUTat10.1 hypothetical protein (Tb10.6k15.0280) partial mRNA</td></tr>
<tr><td>GLOS_TB10.6K15.0380.1.1</td><td>27.9893815877415</td><td>0</td><td>0,00</td><td>0.00638397583964189</td><td>XM_818120.1 Tbb strain 927/4 GUTat10.1 hypothetical protein (Tb10.6k15.0380) partial mRNA</td></tr>
<tr><td>GLOS_TB10.6K15.0410.1.1</td><td>221.104805693361</td><td>0</td><td>0,00</td><td>3.25843734851555e-13</td><td>XM_818117.1 Tbb GUTat10.1 60S ribosomal protein L18 (Tb10.6k15.0410) partial mRNA</td></tr>
<tr><td>GLOS_TB10.6K15.0460.1.1</td><td>21.082445457412</td><td>0</td><td>0,00</td><td>0.000422360679473223</td><td>XP_823206.1 chaperone protein DnaJ [Trypanosoma brucei brucei strain 927/4 GUTat10.1]</td></tr>
<tr><td>GLOS_TB10.6K15.0520.1.1</td><td>217.912209237662</td><td>0</td><td>0,00</td><td>2.29356313042257e-10</td><td>XM_818108.1 Tbb strain 927/4 GUTat10.1 hypothetical protein (Tb10.6k15.0520) partial mRNA</td></tr>
<tr><td>GLOS_TB10.6K15.0690.1.1</td><td>30.081615518232</td><td>0</td><td>0,00</td><td>0.000113200152092862</td><td>XP_823188.1 hypothetical protein [Trypanosoma brucei brucei strain 927/4 GUTat10.1]</td></tr>
<tr><td>GLOS_TB10.6K15.1220.1.1</td><td>36.3221536030611</td><td>0</td><td>0,00</td><td>1.40756070683505e-06</td><td>XM_818050.1 Tbb GUTat10.1 isoleucyl-tRNA synthetase (Tb10.6k15.1220) partial mRNA</td></tr>
<tr><td>GLOS_TB10.6K15.1350.1.1</td><td>54.428984844628</td><td>0</td><td>0,00</td><td>1.81719040389825e-09</td><td>XM_818039.1 Tbb strain 927/4 GUTat10.1 pteridine transporter (Tb10.6k15.1350) partial mRNA</td></tr>
<tr><td>GLOS_TB10.6K15.1510.1.1</td><td>31.2904691633679</td><td>0</td><td>0,00</td><td>9.2049907479382e-06</td><td>XP_823117.1 hypothetical protein [Trypanosoma brucei brucei strain 927/4 GUTat10.1]</td></tr>
<tr><td>GLOS_TB10.6K15.1520.1.1</td><td>22.1466442759785</td><td>0</td><td>0,00</td><td>0.000284706581109359</td><td>XM_818023.1 Tbb strain 927/4 GUTat10.1 small GTPase (Tb10.6k15.1520) partial mRNA</td></tr>
<tr><td>GLOS_TB10.6K15.1820.1.1</td><td>31.1458143367984</td><td>0</td><td>0,00</td><td>9.49548498458617e-06</td><td>XM_818004.1 Tbb GUTat10.1 hypothetical protein (Tb10.6k15.1820) partial mRNA</td></tr>
<tr><td>GLOS_TB10.6K15.2050.1.1</td><td>96.3768935195979</td><td>0</td><td>0,00</td><td>4.95820827408446e-08</td><td>XP_823077.1 40S ribosomal protein S12 [Trypanosoma brucei brucei strain 927/4 GUTat10.1]</td></tr>
</table>

GLOS_TB10.6K15.2180.1.1	32.2461768620072	0	0,00	6.38516834635607e-06	XP_823066.1 cytochrome c oxidase subunit IX [Tbb strain 927/4 GUTat10.1]
GLOS_TB10.6K15.2250.1.1	46.3855224824474	0	0,00	3.4213345343636e-08	XM_817968.1 Tbb GUTat10.1 eukaryotic translation initiation factor 3 subunit 8 partial mRNA
GLOS_TB10.6K15.2330.1.1	38.1974052936973	0	0,00	6.86290003011148e-07	XP_823053.1 t-complex protein 1 subunit theta [Tbb strain 927/4 GUTat10.1]
GLOS_TB10.6K15.2510.1.1	26.0056387771781	0	0,00	0.00430058047112174	XM_817944.1 Tbb strain 927/4 GUTat10.1 hypothetical protein (Tb10.6k15.2510) partial mRNA
GLOS_TB10.6K15.2620.1.1	55.3846925432673	0	0,00	1.27498365293531e-09	XP_823027.1 2,3-bisphosphoglycerate-independent phosphoglycerate mutase [Tbb GUTat10.1]
GLOS_TB10.6K15.2660.1.1	31.0373232168713	0	0,00	0.0011112312656724	XM_817930.1 Tbb strain 927/4 GUTat10.1 hypothetical protein (Tb10.6k15.2660) partial mRNA
GLOS_TB10.6K15.2690.1.1	22.0743168626937	0	0,00	0.000289224804544453	XM_817927.1 Tbb strain 927/4 GUTat10.1 hypothetical protein (Tb10.6k15.2690) partial mRNA
GLOS_TB10.6K15.2900.1.1	43.2290897333905	0	0,00	1.06836980806488e-07	XP_823004.1 ABC transporter [Trypanosoma brucei brucei strain 927/4 GUTat10.1]
GLOS_TB10.6K15.3080.1.1	66.4760965345778	0	0,00	2.37060509551536e-11	XM_817898.1 Tbb GUTat10.1 dihydrolipoamide acetyltransferase precursor partial mRNA
GLOS_TB10.6K15.3250.1.1	70.3712547424198	0	0,00	8.42858954051497e-11	XM_817883.1 Tbb GUTat10.1 succinyl-CoA ligase [GDP-forming] beta-chain partial mRNA
GLOS_TB10.6K15.3350.1.1	149.958661785514	0	0,00	3.46807823415872e-20	XM_817875.1 Tbb GUTat10.1 40S ribosomal protein S24e (Tb10.6k15.3350) partial mRNA
GLOS_TB10.6K15.3460.1.1	63.2835000788785	0	0,00	2.37635219842984e-08	XP_822958.1 hypothetical protein [Trypanosoma brucei brucei strain 927/4 GUTat10.1]
GLOS_TB10.6K15.3510.1.1	80.1091502620248	0	0,00	0.000123849590002095	XM_817860.1 Tbb GUTat10.1 cysteine-rich acidic integral membrane prot precursor partial mRNA
GLOS_TB10.6K15.3800.1.1	68.6044941717106	0	0,00	1.13017442439167e-11	XM_817839.1 Tbb GUTat10.1 dipeptidyl-peptidase 8-like serine peptidase partial mRNA
GLOS_TB10.6K15.3820.1.1	91.4175364931894	0	0,00	2.10229119517782e-09	XM_817837.1 Tbb GUTat10.1 sterol 24-c-methyltransferase (Tb10.6k15.3820) partial mRNA
GLOS_TB10.70.0010.1.1	50.2083532770047	0	0,00	3.18129468393093e-07	XM_817822.1 Tbb strain 927/4 GUTat10.1 hypothetical protein (Tb10.70.0010) partial mRNA
GLOS_TB10.70.0440.1.1	23.2470068011873	0	0,00	0.000190523711257254	XM_817788.1 Tbb strain 927/4 GUTat10.1 hypothetical protein (Tb10.70.0440) partial mRNA
GLOS_TB10.70.0465.1.1	55.348528836625	0	0,00	1.28456590875426e-09	XM_817786.1 Tbb GUTat10.1 60S ribosomal proteins L37 (Tb10.70.0465) partial mRNA
GLOS_TB10.70.0730.1.1	28.134036414311	0	0,00	2.94399465636344e-05	XP_822855.1 hypothetical protein [Trypanosoma brucei brucei strain 927/4 GUTat10.1]
GLOS_TB10.70.0800.1.2	55.2400377166979	0	0,00	8.86450756124198e-08	XM_817756.1 Tbb GUTat10.1 universal minicircle sequence binding prot (UMSBP) partial mRNA
GLOS_TB10.70.0800.2.2	75.3667754754706	0	0,00	9.8856073565468e-10	XM_817756.1 Tbb GUTat10.1 universal minicircle sequence binding prot (UMSBP) partial mRNA
GLOS_TB10.70.0830.1.1	35.1494636645675	0	0,00	2.12108345743693e-06	XP_822847.1 clathrin heavy chain [Trypanosoma brucei brucei strain 927/4 GUTat10.1]
GLOS_TB10.70.0960.1.1	74.5918863100431	0	0,00	1.34313715310039e-12	XP_822838.1 protein kinase [Trypanosoma brucei brucei strain 927/4 GUTat10.1]
GLOS_TB10.70.1100.1.1	25.0499310785388	0	0,00	0.000603329057270972	XM_817735.1 Tbb GUTat10.1 translation elongation factor 1-beta (Tb10.70.1100) partial mRNA
GLOS_TB10.70.1120.1.1	25.1584221984659	0	0,00	9.09850605384415e-05	XM_817733.1 Tbb GUTat10.1 hypothetical protein (Tb10.70.1120) partial mRNA
GLOS_TB10.70.1130.1.1	22.1828079826208	0	0,00	0.000282477536862942	XM_817732.1 Tbb strain 927/4 GUTat10.1 hypothetical protein (Tb10.70.1130) partial mRNA
GLOS_TB10.70.1190.1.1	23.030024561333	0	0,00	0.00175702719966662	XP_822821.1 valosin-containing protein homolog [Trypanosoma brucei TREU927]
GLOS_TB10.70.1540.1.2	106.657244638838	0	0,00	1.2315993342603e-16	XM_817699.1 Tbb strain 927/4 GUTat10.1 60S ribosomal protein L24 partial mRNA
GLOS_TB10.70.1540.2.2	39.3339315255485	0	0,00	4.59728931614666e-07	XM_817699.1 Tbb GUTat10.1 60S ribosomal protein L24 (Tb10.70.1540) partial mRNA
GLOS_TB10.70.1660.1.1	24.3112056197537	0	0,00	0.000128575024138409	XP_822783.1 hypothetical protein [Trypanosoma brucei brucei strain 927/4 GUTat10.1]
GLOS_TB10.70.1690.1.2	102.653595311069	0	0,00	7.90251029207624e-17	XM_817688.1 Tbb GUTat10.1 40S ribosomal protein S10 (Tb10.70.1690) partial mRNA
GLOS_TB10.70.1690.2.2	43.1205986134634	0	0,00	3.39741817331257e-05	XM_817688.1 Tbb GUTat10.1 40S ribosomal protein S10 (Tb10.70.1690) partial mRNA
GLOS_TB10.70.1740.1.1	91.6345187330437	0	0,00	3.47158079371278e-15	XM_817685.1 Tbb GUTat10.1 40S ribosomal protein S18 (Tb10.70.1740) partial mRNA
GLOS_TB10.70.1890.1.1	25.1584221984659	0	0,00	9.09850605384415e-05	XP_822767.1 hypothetical protein [Trypanosoma brucei brucei strain 927/4 GUTat10.1]
GLOS_TB10.70.1930.1.1	35.0409725446404	0	0,00	0.000914263354952999	XM_817671.1 Tbb GUTat10.1 hypothetical protein (Tb10.70.1930) partial mRNA
GLOS_TB10.70.2170.1.1	105.267572460491	0	0,00	4.61461004653562e-06	XM_817657.1 Tbb GUTat10.1 ubiquitin/ribosomal protein S27a (Tb10.70.2170) partial mRNA
GLOS_TB10.70.2320.1.1	50.2083532770047	0	0,00	3.18129468393093e-07	XP_822735.1 hypothetical protein [Trypanosoma brucei brucei strain 927/4 GUTat10.1]
GLOS_TB10.70.2460.1.1	30.1177792248743	0	0,00	1.39109013062701e-05	XP_822722.1 hypothetical protein [Trypanosoma brucei brucei strain 927/4 GUTat10.1]
GLOS_TB10.70.2660.1.1	136.449550503931	0	0,00	1.63344241844457e-07	XP_822703.1 elongation factor 2 [Tbb GUTat10.1] ref|XP_822704.1| elongation factor 2 [Tbb]
GLOS_TB10.70.2970.1.1	34.1575922592858	0	0,00	3.07695129569848e-06	XP_822676.1 hypothetical protein [Trypanosoma brucei brucei strain 927/4 GUTat10.1]
GLOS_TB10.70.3120.1.1	16.0145973110765	0	0,00	0.00508109428667914	XP_822663.1 hypothetical protein [Trypanosoma brucei brucei strain 927/4 GUTat10.1]

GLOS_TB10.70.3150.1.1	34.3384107924977	0	0,00	2.96026500676158e-06	XP_822661.1 hypothetical protein [Trypanosoma brucei brucei strain 927/4 GUTat10.1]
GLOS_TB10.70.3160.1.2	56.2680728286219	0	0,00	9.82964564973082e-09	XM_817567.1 Tbb GUTat10.1 60S ribosomal protein L30 (Tb10.70.3160) partial mRNA
GLOS_TB10.70.3160.2.2	42.0925635015393	0	0,00	0.000124015874264669	XM_817567.1 Tbb GUTat10.1 60S ribosomal protein L30 (Tb10.70.3160) partial mRNA
GLOS_TB10.70.3190.1.1	23.030024561333	0	0,00	0.00175702719966662	XM_817563.1 Tbb strain 927/4 GUTat10.1 hypothetical protein (Tb10.70.3190) partial mRNA
GLOS_TB10.70.3290.1.1	29.3067263528045	0	0,00	1.94591649205139e-05	XP_822647.1 ATP-dependent DEAD-box RNA helicase [Trypanosoma brucei TREU927]
GLOS_TB10.70.3370.1.1	359.052519495567	0	0,00	5.13188065696772e-19	XM_817548.1 Tbb GUTat10.1 40S ribosomal protein S3a (Tb10.70.3370) partial mRNA
GLOS_TB10.70.3510.1.1	97.5495834580914	0	0,00	2.5997338116516e-13	XP_822632.1 60S ribosomal protein L18a [Tbb GUTat10.1] ref|XP_829469.1| ribosomal prot L18
GLOS_TB10.70.3660.1.1	36.4306447229882	0	0,00	1.37544853211616e-06	XM_817527.1 Tbb GUTat10.1 proteasome activator protein PA26 (Tb10.70.3660) partial mRNA
GLOS_TB10.70.4060.1.1	63.3196637855208	0	0,00	8.69937212253629e-10	XM_817496.1 Tbb GUTat10.1 60S acidic ribosomal protein P2 (Tb10.70.4060) partial mRNA
GLOS_TB10.70.4155.1.2	35.1856273712099	0	0,00	2.10474362719246e-06	XP_822579.1 60S ribosomal protein L38 [Trypanosoma brucei brucei strain 927/4 GUTat10.1]
GLOS_TB10.70.4155.2.2	79.5874070430939	0	0,00	2.30366195853494e-13	XP_822579.1 60S ribosomal protein L38 [Trypanosoma brucei brucei strain 927/4 GUTat10.1]
GLOS_TB10.70.4280.1.1	25.2307496117506	0	0,00	8.95715088102793e-05	XP_822573.1 delta-1-pyrroline-5-carboxylate dehydrogenase [Trypanosoma brucei]
GLOS_TB10.70.4540.1.1	18.0345038282822	0	0,00	0.00138690256028253	XM_817461.1 Tbb strain 927/4 GUTat10.1 hypothetical protein (Tb10.70.4540) partial mRNA
GLOS_TB10.70.4590.1.1	34.2299196725706	0	0,00	3.02968537743753e-06	XP_822550.1 hypothetical protein [Trypanosoma brucei brucei strain 927/4 GUTat10.1]
GLOS_TB10.70.4720.1.1	20.1267377587727	0	0,00	0.000612237591608327	XM_817450.1 Tbb GUTat10.1 importin subunit beta-1 (Tb10.70.4720) partial mRNA
GLOS_TB10.70.4740.1.1	145.955012457745	0	0,00	1.96174367038723e-21	XM_817449.1 Tbb strain 927/4 GUTat10.1 enolase (Tb10.70.4740) partial mRNA
GLOS_TB10.70.4800.1.2	41.3538380427542	0	0,00	2.18040369926485e-07	XM_817444.1 Tbb strain 927/4 GUTat10.1 ribosomal protein S25 (Tb10.70.4800) partial mRNA
GLOS_TB10.70.4800.2.2	37.0247153552038	0	0,00	0.0015114259308321	XM_817444.1 Tbb strain 927/4 GUTat10.1 ribosomal protein S25 (Tb10.70.4800) partial mRNA
GLOS_TB10.70.4880.1.1	15.0588896124372	0	0,00	0.00420379489573978	XM_817437.1 Tbb GUTat10.1 eukaryotic translation initiation factor 5 partial mRNA
GLOS_TB10.70.4930.1.1	25.1584221984659	0	0,00	9.09850605384415e-05	XM_817433.1 Tbb strain 927/4 GUTat10.1 hypothetical protein (Tb10.70.4930) partial mRNA
GLOS_TB10.70.5100.1.1	26.2949484303171	0	0,00	6.0510304148871e-05	XM_817417.1 Tbb GUTat10.1 lysosomal alpha-mannosidase precursor partial mRNA
GLOS_TB10.70.5110.1.1	283.396434366958	0	0,00	1.16897120072656e-13	XM_817416.1 Trypanosoma brucei brucei strain 927/4 GUTat10.1 mitochondrial malate dehydrogenase (Tb10.70.5110) partial mRNA. nuclear gene for mitochondrial product
GLOS_TB10.70.5150.1.1	23.2108430945449	0	0,00	0.000192022919835166	XP_822506.1 adenylate kinase [Trypanosoma brucei brucei strain 927/4 GUTat10.1]
GLOS_TB10.70.5360.1.1	16.0145973110765	0	0,00	0.00508109428667914	XM_817398.1 Tbb strain 927/4 GUTat10.1 La protein (Tb10.70.5360) partial mRNA
GLOS_TB10.70.5500.1.1	32.1015220354377	0	0,00	3.57682577449666e-05	XM_817385.1 Tbb GUTat10.1 hypothetical protein (Tb10.70.5500) partial mRNA
GLOS_TB10.70.5560.1.1	30.1539429315167	0	0,00	1.38029332263529e-05	XM_817380.1 Tbb GUTat10.1 hypothetical protein (Tb10.70.5560) partial mRNA
GLOS_TB10.70.5590.1.1	24.3112056197537	0	0,00	0.000128575024138409	XM_817377.1 Tbb strain 927/4 GUTat10.1 hypothetical protein (Tb10.70.5590) partial mRNA
GLOS_TB10.70.5820.1.1	458.585845764222	0	0,00	1.79025648669898e-19	XP_822456.1 hexokinase [Trypanosoma brucei brucei strain 927/4 GUTat10.1]
GLOS_TB10.70.5840.1.1	28.0978727076686	0	0,00	2.96709423828632e-05	XM_817361.1 Tbb strain 927/4 GUTat10.1 major vault protein (Tb10.70.5840) partial mRNA
GLOS_TB10.70.6300.1.1	29.2343989395198	0	0,00	1.97642511792219e-05	XM_817326.1 Tbb GUTat10.1 dual specificity protein phosphatase (Tb10.70.6300) partial mRNA
GLOS_TB10.70.6325.1.1	56.6297098950456	0	0,00	8.43096831302367e-10	XM_817323.1 Tbb strain 927/4 GUTat10.1 ribosomal protein L36 (Tb10.70.6325) partial mRNA
GLOS_TB10.70.6340.1.1	41.3538380427542	0	0,00	2.18040369926485e-07	XM_817321.1 Tbb strain 927/4 GUTat10.1 ATPase subunit 9 (Tb10.70.6340) partial mRNA
GLOS_TB10.70.6450.1.1	21.2632639906239	0	0,00	0.000406072754494131	XM_817315.1 Tbb GUTat10.1 transcriptional regulatory prot NOT1 (Tb10.70.6450) partial mRNA
GLOS_TB10.70.6470.1.1	41.1368558029	0	0,00	1.05494970785651e-05	XM_817314.1 Tbb GUTat10.1 methionyl-tRNA synthetase (Tb10.70.6470) partial mRNA
GLOS_TB10.70.6480.1.1	26.1502936037476	0	0,00	6.24316458047021e-05	XM_817313.1 Tbb strain 927/4 GUTat10.1 hypothetical protein (Tb10.70.6480) partial mRNA
GLOS_TB10.70.6880.1.1	38.3782238269092	0	0,00	6.60422509531013e-07	XM_817286.1 Tbb strain 927/4 GUTat10.1 katanin (Tb10.70.6880) partial mRNA
GLOS_TB10.70.6920.1.1	30.2624340514438	0	0,00	1.34845286515237e-05	XP_822375.1 hypothetical protein [Trypanosoma brucei brucei strain 927/4 GUTat10.1]
GLOS_TB10.70.7010.1.1	312.775488133047	0	0,00	3.80760777873237e-18	XP_822368.1 60S ribosomal protein L9 [Trypanosoma brucei brucei strain 927/4 GUTat10.1]
GLOS_TB10.70.7030.1.1	223.414021863706	0	0,00	7.3978582078044e-22	XP_822366.1 40S ribosomal protein S23 [Tbb] ref|XP_822367.1| 40S ribosomal protein S23
GLOS_TB10.70.7050.1.1	28.3148549475228	0	0,00	2.83144579286955e-05	XP_822364.1 t-complex protein 1 subunit delta [Tbb strain 927/4 GUTat10.1]

GLOS_TB10.70.7100.1.1	29.2705626461621	0	0,00	1.96110526903119e-05	XM_817268.1 Tbb GUTat10.1 serine carboxypeptidase III precursor (Tb10.70.7100) partial mRNA
GLOS_TB10.70.7220.1.1	26.2226210170323	0	0,00	6.14626006604988e-05	XM_817258.1 Tbb strain 927/4 GUTat10.1 hypothetical protein (Tb10.70.7220) partial mRNA
GLOS_TB10.70.7730.1.1	31.2181417500831	0	0,00	9.34899842159307e-06	XM_817219.1 Tbb GUTat10.1 ATP-dependent DEAD/H RNA helicase (Tb10.70.7730) partial mRNA
GLOS_TB10.70.7760.1.1	48.296937879726	0	0,00	1.67191971253266e-08	XP_822310.1 hypothetical protein [Trypanosoma brucei brucei strain 927/4 GUTat10.1]
GLOS_TB11.01.0355.1.1	124.22162028077	0	0,00	1.94913859492559e-05	XM_823801.1 Tbb strain 927/4 GUTat10.1 ribosomal protein S26 (Tb11.01.0355) partial mRNA
GLOS_TB11.01.0700.1.1	34.3384107924977	0	0,00	2.96026500676158e-06	XP_828962.1 ribose 5-phosphate isomerase [Trypanosoma brucei brucei strain 927/4 GUTat10.1]
GLOS_TB11.01.0720.1.1	32.1376857420801	0	0,00	6.5355906819989e-06	XM_823871.1 Tbb strain 927/4 GUTat10.1 cation transporter (Tb11.01.0720) partial mRNA
GLOS_TB11.01.0960.1.1	30.1177792248743	0	0,00	1.39109013062701e-05	XM_823894.1 Tbb GUTat10.1 proteasome regulatory non-ATPase subunit 2 partial mRNA
GLOS_TB11.01.1290.1.1	35.0771362512828	0	0,00	0.000195575806691878	XP_829017.1 hypothetical protein [Trypanosoma brucei brucei strain 927/4 GUTat10.1]
GLOS_TB11.01.1350.1.1	87.5223782853474	0	0,00	1.42178309490539e-14	XM_823930.1 Tbb GUTat10.1 S-adenosylhomocysteine hydrolase partial mRNA
GLOS_TB11.01.1465.1.1	67.5041316465018	0	0,00	1.64844712328036e-11	XM_823941.1 Tbb nascent polypeptide associated complex subunit alpha partial mRNA
GLOS_TB11.01.1475.1.3	39.0446218724095	0	0,00	0.000765301112763942	XP_829036.1 40S ribosomal protein S27 [Trypanosoma brucei brucei strain 927/4 GUTat10.1]
GLOS_TB11.01.1475.2.3	33.0933934407194	0	0,00	6.93900465780348e-05	XP_829036.1 40S ribosomal protein S27 [Trypanosoma brucei brucei strain 927/4 GUTat10.1]
GLOS_TB11.01.1475.3.3	43.1929260267481	0	0,00	1.68035944509075e-07	XP_829036.1 40S ribosomal protein S27 [Trypanosoma brucei brucei strain 927/4 GUTat10.1]
GLOS_TB11.01.1625.1.1	27.0336738891022	0	0,00	0.00137926524453827	XP_829050.1 hypothetical protein [Trypanosoma brucei brucei strain 927/4 GUTat10.1]
GLOS_TB11.01.1740.1.1	47.4135575943715	0	0,00	2.35516057044345e-08	XM_823969.1 Tbb GUTat10.1 2-oxoglutarate dehydrogenase E1 component partial mRNA
GLOS_TB11.01.1790.1.2	21.1547728706968	0	0,00	0.000415757696464543	XM_823974.1 Tbb strain 927/4 GUTat10.1 60S ribosomal prot. L29 (Tb11.01.1790) partial mRNA
GLOS_TB11.01.1790.2.2	55.2038740100555	0	0,00	1.18085574885332e-06	XM_823974.1 Tbb GUTat10.1 60S ribosomal protein L29 (Tb11.01.1790) partial mRNA
GLOS_TB11.01.1880.1.1	22.0743168626937	0	0,00	0.000289224804544453	XM_823983.1 Tbb strain 927/4 GUTat10.1 hypothetical protein (Tb11.01.1880) partial mRNA
GLOS_TB11.01.1910.1.1	42.1648909148241	0	0,00	1.62070436675388e-06	XM_823986.1 Tbb strain 927/4 GUTat10.1 hypothetical protein (Tb11.01.1910) partial mRNA
GLOS_TB11.01.2310.1.1	39.2977678189061	0	0,00	4.63268452420724e-07	XM_824025.1 Tbb strain 927/4 GUTat10.1 hypothetical protein (Tb11.01.2310) partial mRNA
GLOS_TB11.01.2330.1.1	29.162071526235	0	0,00	2.00746299740489e-05	XM_824027.1 Tbb strain 927/4 GUTat10.1 hypothetical protein (Tb11.01.2330) partial mRNA
GLOS_TB11.01.2490.1.1	24.1303870865418	0	0,00	0.000133709923111234	XM_824043.1 Tbb GUTat10.1 hypothetical protein (Tb11.01.2490) partial mRNA
GLOS_TB11.01.2680.1.1	346.462952205982	0	0,00	1.60466872425768e-11	XM_824062.1 Tbb GUTat10.1 40S ribosomal protein SA (Tb11.01.2680) partial mRNA
GLOS_TB11.01.2740.1.1	26.1502936037476	0	0,00	6.24316458047021e-05	XM_824068.1 Tbb strain 927/4 GUTat10.1 hypothetical protein (Tb11.01.2740) partial mRNA
GLOS_TB11.01.2800.1.1	33.3103756805736	0	0,00	4.3296465103686e-06	XM_824073.1 Tbb strain 927/4 GUTat10.1 hypothetical protein (Tb11.01.2800) partial mRNA
GLOS_TB11.01.2880.1.1	14.0670182071555	0	0,00	0.00615899928204462	XP_829173.1 hypothetical protein [Trypanosoma brucei brucei strain 927/4 GUTat10.1]
GLOS_TB11.01.3020.1.1	45.2128325439539	0	0,00	5.1264283027574e-08	XM_824095.1 Tbb GUTat10.1 40S ribosomal protein L14 (Tb11.01.3020) partial mRNA
GLOS_TB11.01.3110.1.1	277.104281249549	0	0,00	5.50477987528824e-40	XM_824105.1 Tbb strain 927/4 GUTat10.1 heat shock protein 70 (Tb11.01.3110) partial mRNA
GLOS_TB11.01.3290.1.1	21.1547728706968	0	0,00	0.000415757696464543	XM_824118.1 Tbb strain 927/4 GUTat10.1 hypothetical protein (Tb11.01.3290) partial mRNA
GLOS_TB11.01.3320.1.1	22.1828079826208	0	0,00	0.000282477536862942	XM_824120.1 Tbb strain 927/4 GUTat10.1 trichohyalin (Tb11.01.3320) partial mRNA
GLOS_TB11.01.3370.1.1	28.134036414311	0	0,00	2.94399465636344e-05	XM_824124.1 Tbb GUTat10.1 glycosomal membrane protein (Tb11.01.3370) partial mRNA
GLOS_TB11.01.3420.1.1	24.2027144998266	0	0,00	0.000131628791765919	XP_829222.1 eukaryotic translation initiation factor [Trypanosoma brucei TREU927]
GLOS_TB11.01.3490.1.1	17.1511235429277	0	0,00	0.00191497267072198	XP_829225.1 hypothetical protein [Trypanosoma brucei brucei strain 927/4 GUTat10.1]
GLOS_TB11.01.3550.1.1	36.3221536030611	0	0,00	1.40756070683505e-06	XM_824138.1 Tbb strain 927/4 GUTat10.1 2-oxoglutarate dehydrogenase E2 component dihydrolipoamide succinyltransferase (Tb11.01.3550) partial mRNA
GLOS_TB11.01.3610.1.1	66.5484239478625	0	0,00	2.33599951794366e-11	XP_829237.1 membrane-bound acid phosphatase [Tbb strain 927/4 GUTat10.1]
GLOS_TB11.01.3675.1.2	89.5061210959108	0	0,00	4.31166546586206e-13	XP_829244.1 40S ribosomal protein S17 [Tbb strain 927/4 GUTat10.1] ref|XP_829245.1| 40S ribosomal protein S17 [Trypanosoma brucei brucei strain 927/4 GUTat10.1]
GLOS_TB11.01.3675.2.2	92.5178990183982	0	0,00	5.90370714166526e-13	XP_829244.1 40S ribosomal protein S17 [Tbb strain 927/4 GUTat10.1] ref|XP_829245.1| 40S ribosomal protein S17 [Trypanosoma brucei brucei strain 927/4 GUTat10.1]

GLOS_TB11.01.3740.1.1	29.2705626461621	0	0,00	1.96110526903119e-05	XM_824159.1 Tbb GUTat10.1 coatomer subunit gamma (Tb11.01.3740) partial mRNA
GLOS_TB11.01.3860.1.1	25.1945859051083	0	0,00	9.02751868220559e-05	XP_829266.1 hypothetical protein [Trypanosoma brucei brucei strain 927/4 GUTat10.1]
GLOS_TB11.01.3915.1.1	18.1429949482093	0	0,00	0.00130880372149336	XP_829272.1 hypothetical protein [Trypanosoma brucei brucei strain 927/4 GUTat10.1]
GLOS_TB11.01.4030.1.1	26.2587847236747	0	0,00	6.0984378896027e-05	XM_824190.1 Tbb strain 927/4 GUTat10.1 hypothetical protein (Tb11.01.4030) partial mRNA
GLOS_TB11.01.4130.1.1	34.1937559659282	0	0,00	3.05321856536068e-06	XM_824200.1 Tbb strain 927/4 GUTat10.1 protein kinase (Tb11.01.4130) partial mRNA
GLOS_TB11.01.4140.1.1	39.2616041122637	0	0,00	4.66837439827811e-07	XP_829294.1 hypothetical protein [Trypanosoma brucei brucei strain 927/4 GUTat10.1]
GLOS_TB11.01.4370.1.1	23.0661882679754	0	0,00	0.000198154433233135	XM_824222.1 Tbb strain 927/4 GUTat10.1 hypothetical protein (Tb11.01.4370) partial mRNA
GLOS_TB11.01.4480.1.1	25.266913318393	0	0,00	8.88739667539241e-05	XM_824233.1 Tbb strain 927/4 GUTat10.1 hypothetical protein (Tb11.01.4480) partial mRNA
GLOS_TB11.01.4702.1.1	49.2526455783654	0	0,00	1.16960730368483e-08	XP_829371.1 cytochrome c oxidase subunit 10 [Tbb strain 927/4 GUTat10.1]
GLOS_TB11.01.4740.1.1	37.3501887149851	0	0,00	9.63892803228974e-07	XM_824282.1 Tbb GUTat10.1 hypothetical protein (Tb11.01.4740) partial mRNA
GLOS_TB11.01.4750.1.1	46.2770313625203	0	0,00	3.50014716308022e-08	XM_824283.1 Tbb GUTat10.1 elongation factor 1 gamma (Tb11.01.4750) partial mRNA
GLOS_TB11.01.4830.1.1	49.1441544584383	0	0,00	1.6534436318891e-05	XM_824289.1 Tbb GUTat10.1 eukaryotic translation initiation factor 2 gamma partial mRNA
GLOS_TB11.01.4850.1.1	26.18645731039	0	0,00	6.19450094774168e-05	XM_824291.1 Tbb strain 927/4 GUTat10.1 hypothetical protein (Tb11.01.4850) partial mRNA
GLOS_TB11.01.4860.1.1	46.5663410156593	0	0,00	3.29416935383292e-08	XP_829385.1 guide RNA-binding protein [Trypanosoma brucei brucei strain 927/4 GUTat10.1]
GLOS_TB11.01.5100.1.1	43.3014171466752	0	0,00	1.05218537223908e-07	XM_824313.1 Tbb GUTat10.1 paraflagellar rod component (Tb11.01.5100) partial mRNA
GLOS_TB11.01.5120.1.1	22.2189716892632	0	0,00	0.000280268272706973	XM_824315.1 Tbb strain 927/4 GUTat10.1 hypothetical protein (Tb11.01.5120) partial mRNA
GLOS_TB11.01.5310.1.1	48.296937879726	0	0,00	1.67191971253266e-08	XM_824337.1 Tbb GUTat10.1 receptor-type adenylate cyclase GRESAG 4 partial mRNA
GLOS_TB11.01.5355.1.1	34.2299196725706	0	0,00	3.02968537743753e-06	XM_824342.1 Tbb strain 927/4 GUTat10.1 hypothetical protein (Tb11.01.5355) partial mRNA
GLOS_TB11.01.5590.1.1	214.161705856389	0	0,00	3.86917747133708e-15	XM_824365.1 Tbb strain 927/4 GUTat10.1 hypothetical protein (Tb11.01.5590) partial mRNA
GLOS_TB11.01.5680.1.1	16.2677432575731	0	0,00	0.00273772947670125	XM_824372.1 Tbb strain 927/4 GUTat10.1 hypothetical protein (Tb11.01.5680) partial mRNA
GLOS_TB11.01.5690.1.1	24.0942233798995	0	0,00	0.000134764342551207	XM_824373.1 Tbb strain 927/4 GUTat10.1 hypothetical protein (Tb11.01.5690) partial mRNA
GLOS_TB11.01.5710.1.1	157.893633027767	0	0,00	2.85744073046411e-15	XP_829468.1 phenylalanyl-tRNA synthetase alpha subunit [Trypanosoma brucei TREU927]
GLOS_TB11.01.5780.1.1	30.1177792248743	0	0,00	1.39109013062701e-05	XM_824382.1 Tbb strain 927/4 GUTat10.1 hypothetical protein (Tb11.01.5780) partial mRNA
GLOS_TB11.01.5860.1.1	48.296937879726	0	0,00	1.67191971253266e-08	XP_829481.1 t-complex protein 1 subunit epsilon [Trypanosoma brucei TREU927]
GLOS_TB11.01.5930.1.1	16.159252137646	0	0,00	0.00280318659597305	XM_824396.1 Tbb strain 927/4 GUTat10.1 hypothetical protein (Tb11.01.5930) partial mRNA
GLOS_TB11.01.6360.1.1	34.1575922592858	0	0,00	3.07695129569848e-06	XM_824438.1 Tbb strain 927/4 GUTat10.1 metalloprotease (Tb11.01.6360) partial mRNA
GLOS_TB11.01.6610.1.1	35.294118491137	0	0,00	2.05654076416339e-06	XM_824462.1 Tbb strain 927/4 GUTat10.1 hypothetical protein (Tb11.01.6610) partial mRNA
GLOS_TB11.01.6660.1.1	16.0507610177189	0	0,00	0.00287050052632492	XM_824467.1 Tbb GUTat10.1 iron superoxide dismutase (Tb11.01.6660) partial mRNA
GLOS_TB11.01.7010.1.1	34.2299196725706	0	0,00	3.02968537743753e-06	XM_824502.1 Tbb strain 927/4 GUTat10.1 hypothetical protein (Tb11.01.7010) partial mRNA
GLOS_TB11.01.7460.1.1	32.1738494487225	0	0,00	6.48502277463704e-06	XP_829638.1 hypothetical protein [Trypanosoma brucei brucei strain 927/4 GUTat10.1]
GLOS_TB11.01.7500.1.1	54.1758388981314	0	0,00	5.20304582108766e-06	XM_824548.1 Tbb strain 927/4 GUTat10.1 amino acid transporter (Tb11.01.7500) partial mRNA
GLOS_TB11.01.7535.1.1	186.983377140717	0	0,00	7.58776659871259e-14	XP_829645.1 60S ribosomal protein L27 [Trypanosoma brucei brucei strain 927/4 GUTat10.1] ref|XP_829647.1| 60S ribosomal protein L27 [Tbb strain 927/4 GUTat10.1]
GLOS_TB11.01.7620.1.1	22.2189716892632	0	0,00	0.000280268272706973	XM_824562.1 Tbb strain 927/4 GUTat10.1 hypothetical protein (Tb11.01.7620) partial mRNA
GLOS_TB11.01.7630.1.1	17.0426324230005	0	0,00	0.00196095109746437	XM_824563.1 Tbb strain 927/4 GUTat10.1 hypothetical protein (Tb11.01.7630) partial mRNA
GLOS_TB11.01.7880.1.1	27.2506561289564	0	0,00	4.18681751684288e-05	XM_824584.1 Tbb GUTat10.1 microtubule-associated protein (Tb11.01.7880) partial mRNA
GLOS_TB11.01.8225.1.1	26.0779661904628	0	0,00	9.76903094824833e-05	XP_829708.1 hypothetical protein [Trypanosoma brucei brucei strain 927/4 GUTat10.1]
GLOS_TB11.01.8470.1.1	23.0661882679754	0	0,00	0.000198154433233135	XM_824637.1 Tbb GUTat10.1 dihydrolipoyl dehydrogenase (Tb11.01.8470) partial mRNA
GLOS_TB11.01.8510.1.1	22.1828079826208	0	0,00	0.000282477536862942	XM_824640.1 Tbb GUTat10.1 t-complex protein 1 subunit alpha (Tb11.01.8510) partial mRNA
GLOS_TB11.01.8520.1.1	23.1023519746178	0	0,00	0.000196601085738671	XM_824641.1 Tbb GUTat10.1 glucosamine-6-phosphate isomerase (Tb11.01.8520) partial mRNA
GLOS_TB11.01.8770.1.1	83.193255597797	0	0,00	1.58335555002121e-05	XP_829756.1 hypothetical protein [Trypanosoma brucei brucei strain 927/4 GUTat10.1]

GLOS_TB11.02.0010.1.1	100.308215434082	0	0,00	1.08527962628636e-06	XP_828324.1 hypothetical protein [Trypanosoma brucei brucei strain 927/4 GUTat10.1]
GLOS_TB11.02.0210.1.1	71.6524358008404	0	0,00	3.8454761157108e-12	XM_823251.1 Tbb strain 927/4 GUTat10.1 hypothetical protein (Tb11.02.0210) partial mRNA
GLOS_TB11.02.0250.1.1	40.1449843976183	0	0,00	5.10623385072083e-06	XP_828348.1 heat shock protein mitochondrial precursor [Trypanosoma brucei TREU927]
GLOS_TB11.02.0445.1.1	42.3457094480359	0	0,00	1.50851248097151e-07	XM_823282.1 Tbb strain 927/4 GUTat10.1 hypothetical protein (Tb11.02.0445) partial mRNA
GLOS_TB11.02.0530.1.1	20.1267377587727	0	0,00	0.000612237591608327	XM_823292.1 Tbb GUTat10.1 phosphoribosylpyrophosphate synthetase partial mRNA
GLOS_TB11.02.0740.1.2	41.0283646829729	0	0,00	0.00122456306282294	XM_823312.1 Tbb GUTat10.1 60S ribosomal protein L44 (Tb11.02.0740) partial mRNA
GLOS_TB11.02.0740.2.2	67.3594768199323	0	0,00	7.08814540633374e-11	XM_823312.1 Tbb GUTat10.1 60S ribosomal protein L44 (Tb11.02.0740) partial mRNA
GLOS_TB11.02.0750.1.1	30.2985977580862	0	0,00	1.33801986623624e-05	XP_828406.1 t-complex protein 1 subunit zeta [Tbb strain 927/4 GUTat10.1]
GLOS_TB11.02.0760.1.1	21.082445457412	0	0,00	0.000422360679473223	XP_828407.1 dynein heavy chain [Trypanosoma brucei brucei strain 927/4 GUTat10.1]
GLOS_TB11.02.0780.1.1	30.0454518115896	0	0,00	0.000794848907841266	XM_823316.1 Tbb GUTat10.1 squalene monooxygenase (Tb11.02.0780) partial mRNA
GLOS_TB11.02.0790.1.1	42.0925635015393	0	0,00	0.000124015874264669	XM_823317.1 Tbb strain 927/4 GUTat10.1 kinesin (Tb11.02.0790) partial mRNA
GLOS_TB11.02.0980.1.1	24.1665507931842	0	0,00	0.000132664769601763	XM_823338.1 Tbb strain 927/4 GUTat10.1 hypothetical protein (Tb11.02.0980) partial mRNA
GLOS_TB11.02.1070.1.1	64.4923537240144	0	0,00	4.83809989674352e-11	XP_828438.1 aminopeptidase [Trypanosoma brucei brucei strain 927/4 GUTat10.1]
GLOS_TB11.02.1085.1.1	296.022165363185	0	0,00	5.19717011447107e-30	XP_828440.1 40s ribosomal protein S4 [Tbb] ref|XP_828441.1| 40S ribosomal protein S4 [Tbb]
GLOS_TB11.02.1100.1.1	108.351677796263	0	0,00	4.56438470318792e-07	XM_823349.1 Tbb GUTat10.1 nucleobase/nucleoside transporter 8.1 partial mRNA
GLOS_TB11.02.1190.1.1	17.1149598362853	0	0,00	0.00193015710508488	XM_823359.1 Tbb GUTat10.1 hypothetical protein (Tb11.02.1190) partial mRNA
GLOS_TB11.02.1210.1.1	15.1673807323643	0	0,00	0.00410522409475963	XP_828454.1 leucyl-tRNA synthetase [Trypanosoma brucei brucei strain 927/4 GUTat10.1]
GLOS_TB11.02.1470.1.1	22.2189716892632	0	0,00	0.000280268272706973	XM_823383.1 Tbbstrain 927/4 GUTat10.1 hypothetical protein (Tb11.02.1470) partial mRNA
GLOS_TB11.02.1480.1.1	38.3782238269092	0	0,00	6.60422509531013e-07	XM_823384.1 Tbb strain 927/4 GUTat10.1 mitochondrial processing peptidase subunit alpha (Tb11.02.1480) partial mRNA. nuclear gene for mitochondrial product
GLOS_TB11.02.1680.1.1	29.162071526235	0	0,00	2.00746299740489e-05	XM_823403.1 Tbb strain 927/4 GUTat10.1 lectin (Tb11.02.1680) partial mRNA
GLOS_TB11.02.2430.1.1	85.5747991814264	0	0,00	2.8244090091793e-14	XM_823464.1 Tbb GUTat10.1 60S ribosomal protein L17 (Tb11.02.2430) partial mRNA
GLOS_TB11.02.2510.1.1	25.0499310785388	0	0,00	0.000603329057270972	XP_828565.1 guanine nucleotide-binding subunit beta-like protein [Tbb GUTat10.1]
GLOS_TB11.02.2700.1.1	26.18645731039	0	0,00	6.19450094774168e-05	XP_828580.1 fumarate hydratase class I [Trypanosoma brucei brucei strain 927/4 GUTat10.1]
GLOS_TB11.02.2940.1.1	19.0625389402063	0	0,00	0.000909184655185172	XP_828602.1 ubiquitin carboxyl-terminal hydrolase [Trypanosoma brucei TREU927]
GLOS_TB11.02.2950.1.1	30.081615518232	0	0,00	0.000113200152092862	XM_823510.1 Tbb strain 927/4 GUTat10.1 ATPase subunit 9 (Tb11.02.2950) partial mRNA
GLOS_TB11.02.3020.1.1	23.1385156812602	0	0,00	0.000195061474162675	XM_823517.1 Tbb strain 927/4 GUTat10.1 sugar transporter (Tb11.02.3020) partial mRNA
GLOS_TB11.02.3120.1.1	48.2607741730837	0	0,00	1.68463235702496e-08	XM_823526.1 Tbb strain 927/4 GUTat10.1 malic enzyme (Tb11.02.3120) partial mRNA
GLOS_TB11.02.3210.1.1	65.4842251292961	0	0,00	3.38580974726294e-11	XM_823534.1 Tbb GUTat10.1 triosephosphate isomerase (Tb11.02.3210) partial mRNA
GLOS_TB11.02.3310.1.1	18.2514860681365	0	0,00	0.0012782424453452	XP_828635.1 hypothetical protein [Trypanosoma brucei brucei strain 927/4 GUTat10.1]
GLOS_TB11.02.3570.1.1	36.2859898964187	0	0,00	1.41844503881037e-06	XP_828657.1 hypothetical protein [Trypanosoma brucei brucei strain 927/4 GUTat10.1]
GLOS_TB11.02.3610.1.1	73.4915237848343	0	0,00	1.95309278059608e-12	XM_823567.1 Tbb strain 927/4 GUTat10.1 hypothetical protein (Tb11.02.3610) partial mRNA
GLOS_TB11.02.3740.1.1	46.2047039492356	0	0,00	1.42560152339641e-07	XM_823576.1 Tbb GUTat10.1 receptor-type adenylate cyclase GRESAG 4 partial mRNA
GLOS_TB11.02.3770.1.2	71.4354535609862	0	0,00	4.01753625833898e-12	XM_823578.1 Tbb GUTat10.1 hypothetical protein (Tb11.02.3770) partial mRNA
GLOS_TB11.02.3770.2.2	33.057229734077	0	0,00	0.000476182210968268	XM_823578.1 Tbbstrain 927/4 GUTat10.1 hypothetical protein (Tb11.02.3770) partial mRNA
GLOS_TB11.02.3860.1.1	24.1303870865418	0	0,00	0.000133709923111234	XP_828679.1 hypothetical protein [Trypanosoma brucei brucei strain 927/4 GUTat10.1]
GLOS_TB11.02.3880.1.1	24.1665507931842	0	0,00	0.000132664769601763	XM_823587.1 Tbb strain 927/4 GUTat10.1 hypothetical protein (Tb11.02.3880) partial mRNA
GLOS_TB11.02.3990.1.1	22.1104805693361	0	0,00	0.000286955603565101	XM_823594.1 Tbb GUTat10.1 S-phase kinase-associated protein (Tb11.02.3990) partial mRNA
GLOS_TB11.02.4000.1.1	74.3387403635465	0	0,00	6.8314453305449e-09	XP_828688.1 40S ribosomal protein S15a [Tbb strain 927/4 GUTat10.1] ref|XP_844028.1| 40S ribosomal protein S15a [Trypanosoma brucei brucei strain 927/4 GUTat10.1]
GLOS_TB11.02.4040.1.1	29.1259078195926	0	0,00	2.02318354289899e-05	XP_828691.1 protein transport protein Sec31 [Tbb strain 927/4 GUTat10.1]

GLOS_TB11.02.4050.1.1	173.944394045486	0	0,00	2.03669385776966e-14	XM_823599.1 Tbb GUTat10.1 60S ribosomal protein L28 (Tb11.02.4050) partial mRNA
GLOS_TB11.02.4100.1.1	35.2579547844946	0	0,00	2.07247351372334e-06	XM_823604.1 Tbb GUTat10.1 pretranslocation prot? subunit alpha (Tb11.02.4100) partial mRNA
GLOS_TB11.02.4150.1.1	264.478550253321	0	0,00	6.76669710955771e-17	XP_828702.1 pyruvate phosphate dikinase [Trypanosoma brucei brucei strain 927/4 GUTat10.1]
GLOS_TB11.02.4170.1.2	179.931786183819	0	0,00	2.34259469351803e-13	XM_823611.1 Tbb strain 927/4 GUTat10.1 40S ribosomal prot. S5 (Tb11.02.4170) partial mRNA
GLOS_TB11.02.4170.2.2	40.3619666374725	0	0,00	3.15315130291203e-07	XM_823611.1 Tbb strain 927/4 GUTat10.1 40S ribosomal prot. S5 (Tb11.02.4170) partial mRNA
GLOS_TB11.02.4290.1.1	58.4326341723971	0	0,00	4.25462251818947e-10	XM_823624.1 Tbb strain 927/4 GUTat10.1 hypothetical protein (Tb11.02.4290) partial mRNA
GLOS_TB11.02.4300.1.1	61.4082483882422	0	0,00	1.44752889732038e-10	XM_823625.1 Tbb strain 927/4 GUTat10.1 hypothetical protein (Tb11.02.4300) partial mRNA
GLOS_TB11.02.4320.1.1	16.1954158442883	0	0,00	0.00278116418906016	XM_823627.1 Tbb strain 927/4 GUTat10.1 hypothetical protein (Tb11.02.4320) partial mRNA
GLOS_TB11.02.4350.1.1	159.588066185192	0	0,00	1.61435784461903e-08	XM_823631.1 Tbb strain 927/4 GUTat10.1 40S ribosomal prot. S21 (Tb11.02.4350) partial mRNA
GLOS_TB11.02.4400.1.1	21.1186091640544	0	0,00	0.000419044355280004	XM_823636.1 Tbbstrain 927/4 GUTat10.1 hypothetical protein (Tb11.02.4400) partial mRNA
GLOS_TB11.02.4420.1.1	47.4497213010138	0	0,00	2.33740071335457e-08	XM_823638.1 Tbb GUTat10.1 hypothetical protein (Tb11.02.4420) partial mRNA
GLOS_TB11.02.4520.1.1	17.0426324230005	0	0,00	0.00196095109746437	XM_823649.1 Tbb GUTat10.1 amino acid permease/transporter (Tb11.02.4520) partial mRNA
GLOS_TB11.02.4810.1.1	39.2616041122637	0	0,00	4.66837439827811e-07	XM_823677.1 Tbb strain 927/4 GUTat10.1 hypothetical protein (Tb11.02.4810) partial mRNA
GLOS_TB11.02.4910.1.1	23.0661882679754	0	0,00	0.000198154433233135	XP_828778.1 acidocalcisomal pyrophosphatase [Tbb strain 927/4 GUTat10.1]
GLOS_TB11.02.5120.1.1	29.162071526235	0	0,00	2.00746299740489e-05	XM_823706.1 Tbbstrain 927/4 GUTat10.1 hypothetical protein (Tb11.02.5120) partial mRNA
GLOS_TB11.02.5190.1.1	40.0726569843336	0	0,00	0.000272249652233953	XP_828807.1 pantothenate kinase subunit [Trypanosoma brucei brucei strain 927/4 GUTat10.1]
GLOS_TB11.02.5270.1.1	25.1584221984659	0	0,00	9.09850605384415e-05	XP_828815.1 haloacid dehalogenase hydrolase [Tbb strain 927/4 GUTat10.1]
GLOS_TB11.02.5400.1.1	43.1567623201058	0	0,00	3.67107325072828e-06	XM_823735.1 Tbb GUTat10.1 cystathionine beta-synthase (Tb11.02.5400) partial mRNA
GLOS_TB11.02.5490.1.1	122.635678243273	0	0,00	3.62204769380691e-12	XM_823744.1 Tbb strain 927/4 GUTat10.1 hypothetical protein (Tb11.02.5490) partial mRNA
GLOS_TB11.02.5500.1.1	56.2319091219795	0	0,00	2.13484770534938e-07	XM_823745.1 Tbb GUTat10.1 glucose-regulated protein 78 (Tb11.02.5500) partial mRNA
GLOS_TB11.02.5570.1.1	21.1186091640544	0	0,00	0.000419044355280004	XM_823752.1 Tbb strain 927/4 GUTat10.1 hypothetical protein (Tb11.02.5570) partial mRNA
GLOS_TB11.02.5590.1.1	36.2859898964187	0	0,00	1.41844503881037e-06	XM_823754.1 Tbb strain 927/4 GUTat10.1 hypothetical protein (Tb11.02.5590) partial mRNA
GLOS_TB11.02.5770.1.1	41.1006920962576	0	0,00	8.1453765059596e-05	XM_823772.1 Tbb mitochond. RNA-binding prot. partial mRNA. nuclear gene for mitocho product
GLOS_TB11.03.0030.1.1	33.1295571473618	0	0,00	4.50064134748734e-06	XM_823107.1 Tbb strain 927/4 GUTat10.1 ABC transporter (Tb11.03.0030) partial mRNA
GLOS_TB11.03.0090.1.1	86.4220157601386	0	0,00	3.86834462098995e-10	XM_823102.1 Tbb strain 927/4 GUTat10.1 ribokinase (Tb11.03.0090) partial mRNA
GLOS_TB11.03.0140.1.1	24.0942233798995	0	0,00	0.000134764342551207	XM_823098.1 Tbb strain 927/4 GUTat10.1 nucleoporin (Tb11.03.0140) partial mRNA
GLOS_TB11.03.0230.1.1	32.2823405686496	0	0,00	6.33587398078003e-06	XP_828183.1 isocitrate dehydrogenase [Trypanosoma brucei brucei strain 927/4 GUTat10.1]
GLOS_TB11.03.0240.1.1	25.1945859051083	0	0,00	9.02751868220559e-05	XM_823089.1 Tbb strain 927/4 GUTat10.1 hypothetical protein (Tb11.03.0240) partial mRNA
GLOS_TB11.03.0250.1.1	42.1648909148241	0	0,00	1.62070436675388e-06	XM_823088.1 Tbb strain 927/4 GUTat10.1 cyclophilin A (Tb11.03.0250) partial mRNA
GLOS_TB11.03.0300.1.1	27.214492422314	0	0,00	4.21961822732082e-05	XM_823084.1 Tbb strain 927/4 GUTat10.1 hypothetical protein (Tb11.03.0300) partial mRNA
GLOS_TB11.03.0390.1.1	52.2282597942104	0	0,00	1.03501741411838e-07	XP_828169.1 protein phosphatase 2C [Trypanosoma brucei brucei strain 927/4 GUTat10.1]
GLOS_TB11.03.0400.1.1	23.2831705078296	0	0,00	0.00018903770691066	XM_823075.1 Tbb strain 927/4 GUTat10.1 DNA repair protein (Tb11.03.0400) partial mRNA
GLOS_TB11.03.0410.1.1	124.655584760478	0	0,00	1.6050143326939e-12	XP_828167.1 eukaryotic translation initiation factor 5a [Trypanosoma brucei]
GLOS_TB11.03.0475.1.1	26.1141298971052	0	0,00	6.29225505279159e-05	XP_828152.1 hypothetical protein [Trypanosoma brucei brucei strain 927/4 GUTat10.1]
GLOS_TB11.03.0530.1.1	31.3627965766526	0	0,00	9.06341635172902e-06	XM_823054.1 Tbb strain 927/4 GUTat10.1 hypothetical protein (Tb11.03.0530) partial mRNA
GLOS_TB11.03.0900.1.1	19.134866353491	0	0,00	0.000894936973946279	XM_823023.1 Tbb strain 927/4 GUTat10.1 hypothetical protein (Tb11.03.0900) partial mRNA
GLOS_TB11.03.0940.1.1	21.1547728706968	0	0,00	0.000415757696464543	XP_828111.1 elongation factor [Trypanosoma brucei brucei strain 927/4 GUTat10.1]
GLOS_TB11.18.0002.1.1	19.0625389402063	0	0,00	0.000909184655185172	XM_823219.1 Tbb strain 927/4 GUTat10.1 hypothetical protein (Tb11.18.0002) partial mRNA
GLOS_TB11.18.0005.1.1	29.2705626461621	0	0,00	1.96110526903119e-05	XM_823216.1 Tbb strain 927/4 GUTat10.1 hypothetical protein (Tb11.18.0005) partial mRNA
GLOS_TB11.22.0004.1.1	28.2063638275957	0	0,00	2.8983908845462e-05	XM_823228.1 Tbb strain 927/4 GUTat10.1 hypothetical protein (Tb11.22.0004) partial mRNA
GLOS_TB11.22.0005.1.1	20.1267377587727	0	0,00	0.000612237591608327	XP_828320.1 phenylalanyl-tRNA synthetase [Trypanosoma brucei brucei strain 927/4 GUTat10.1]

GLOS_TB11.27.0001.1.1	55.3123651299826	0	0,00	1.29422456743024e-09	XM_823146.1 Tbb GUTat10.1 receptor-type adenylate cyclase GRESAG 4 partial mRNA
GLOS_TB11.39.0004.1.1	20.1629014654151	0	0,00	0.00060743267903454	XM_823178.1 Tbb GUTat10.1 hypothetical protein (Tb11.39.0004) partial mRNA
GLOS_TB11.39.0005.1.1	39.1531129923366	0	0,00	2.18006865414666e-06	XM_823177.1 Tbb strain 927/4 GUTat10.1 hypothetical protein (Tb11.39.0005) partial mRNA
GLOS_TB11.42.0003.1.1	46.1685402425932	0	0,00	2.69491388190795e-06	XP_828274.1 t-complex protein 1 subunit beta [T. brucei brucei strain 927/4 GUTat10.1]
GLOS_TB11.46.0002.1.1	304.783640803446	0	0,00	1.65612732751334e-07	XP_828289.1 60S acidic ribosomal subunit protein 60S acidic ribosomal subunit protein [Tbb]
GLOS_TB11.46.0009.1.1	44.4379433785264	0	0,00	7.06837259822957e-08	XM_823189.1 Tbb strain 927/4 GUTat10.1 hypothetical protein (Tb11.46.0009) partial mRNA
GLOS_TB11.47.0004.1.1	77.350518286034	0	0,00	6.35906815117475e-09	XP_828236.1 2-oxoglutarate dehydrogenase subunit [Trypanosoma brucei TREU927]
GLOS_TB11.47.0006.1.1	27.1783287156716	0	0,00	4.25270484194831e-05	XP_828234.1 hypothetical protein [Trypanosoma brucei brucei strain 927/4 GUTat10.1]
GLOS_TB11.47.0022.1.1	21.1186091640544	0	0,00	0.000419044355280004	XP_828218.1 hypothetical protein [Trypanosoma brucei brucei strain 927/4 GUTat10.1]
GLOS_TB11.47.0036.1.1	14.0308545005131	0	0,00	0.00620804583676025	XP_828203.1 calpain, partial [Trypanosoma brucei brucei strain 927/4 GUTat10.1]
GLOS_TB11.50.0001.1.1	16.0869247243612	0	0,00	0.00284785252194525	XM_823072.1 Tbb strain 927/4 GUTat10.1 hypothetical protein (Tb11.50.0001) partial mRNA
GLOS_TB11.50.0005.1.1	160.724592417043	0	0,00	1.3417138970082e-10	XM_823067.1 Tbb GUTat10.1 60S ribosomal protein L21E (Tb11.50.0005) partial mRNA
GLOS_TB11.50.0007.1.1	60.3440495696758	0	0,00	2.10352417713603e-10	XP_828164.1 dynein light chain [Trypanosoma brucei brucei strain 927/4 GUTat10.1]
GLOS_TB11.52.0003.1.1	14.1393456204402	0	0,00	0.00606230038830442	XP_829346.1 oligopeptidase b [Trypanosoma brucei brucei strain 927/4 GUTat10.1]
GLOS_TB11.52.0008.1.1	27.3591472488835	0	0,00	4.09010380976341e-05	XM_824256.1 Tbb GUTat10.1 hypothetical protein (Tb11.52.0008) partial mRNA
GLOS_TB927.1.1240.1.1	16.1954158442883	0	0,00	0.00278116418906016	XM_001218825.1 Tbb cytidine triphosphate synthase, putative (Tb927.1.1240) partial mRNA
GLOS_TB927.1.1670.1.1	18.1791586548517	0	0,00	0.00129852321745928	XM_001218868.1 T. brucei hypothetical protein, conserved (Tb927.1.1670) partial mRNA
GLOS_TB927.1.1710.1.1	32.2100131553648	0	0,00	6.43488331005283e-06	XM_001218872.1 T. brucei hypothetical protein, conserved (Tb927.1.1710) partial mRNA
GLOS_TB927.1.2100.1.1	158.255270094191	0	0,00	9.68722849554063e-25	XM_001218909.1 Trypanosoma brucei brucei strain 927/4 GUTat10.1 calpain-like cysteine peptidase. cysteine peptidase, Clan CA, family C2 partial mRNA
GLOS_TB927.1.2230.1.1	28.4233460674499	0	0,00	2.76620973964519e-05	XM_001218922.1 Tbb calpain-like protein fragment, putative (Tb927.1.2230) partial mRNA
GLOS_TB927.1.230.1.1	75.3667754754706	0	0,00	9.8856073565468e-10	XM_841300.1 Trypanosoma brucei hypothetical protein, unlikely (Tb927.1.230) partial mRNA
GLOS_TB927.1.2370.2.2	1295.25058625758	0	0,00	1.5929198771444e-23	XM_001218936.1 Trypanosoma brucei brucei strain 927/4 GUTat10.1 beta tubulin partial mRNA
GLOS_TB927.1.2430.1.1	352.868857498085	0	0,00	1.1157458347829e-48	XM_001218941.1 Trypanosoma brucei brucei strain 927/4 GUTat10.1 histone H3 partial mRNA
GLOS_TB927.1.2820.1.1	54.5013122579128	0	0,00	1.79017429112768e-09	XP_001218979.1 pteridine transporter [Trypanosoma brucei brucei strain 927/4 GUTat10.1]
GLOS_TB927.1.3180.1.1	270.321287565084	0	0,00	2.38672406808346e-13	XP_001219016.1 40S ribosomal protein S11 [Trypanosoma brucei brucei strain 927/4 GUTat10.1]
GLOS_TB927.1.3950.1.1	17.1149598362853	0	0,00	0.00193015710508488	XP_001219089.1 alanine aminotransferase [Trypanosoma brucei brucei strain 927/4 GUTat10.1]
GLOS_TB927.1.4370.1.1	21.1909365773391	0	0,00	0.000412500405600322	XM_001219129.1 T. brucei hypothetical protein, conserved (Tb927.1.4370) partial mRNA
GLOS_TB927.1.4740.1.1	21.082445457412	0	0,00	0.000422360679473223	XM_001219166.1 T. brucei hypothetical protein, conserved (Tb927.1.4740) partial mRNA
GLOS_TB927.1.540.1.2	38.2697327069821	0	0,00	6.75813722995807e-07	XM_001218756.1 Tbb DNA-directed RNA polymerase III, putative (Tb927.1.540) partial mRNA
GLOS_TB927.1.540.2.2	114.773034414304	0	0,00	1.33154952493496e-18	XM_001218756.1 Tbb DNA-directed RNA polymerase III, putative (Tb927.1.540) partial mRNA
GLOS_TB927.1.600.1.1	99.750308508509	0	0,00	2.16215710166491e-16	XM_001218762.1 Tbb phosphate-repressible phosphate permease, putative partial mRNA
GLOS_TB927.1.860.1.1	29.0535804063079	0	0,00	0.000537020484942746	XM_001218788.1 T. brucei hypothetical protein, conserved (Tb927.1.860) partial mRNA
GLOS_TB927.2.240.1.1	88.3334311574173	0	0,00	1.30038538929062e-07	XP_951477.1 retrotransposon hot spot (RHS) protein [Trypanosoma brucei TREU927]
GLOS_TB927.2.2510.1.1	26.2226210170323	0	0,00	6.14626006604988e-05	XP_951552.1 hypothetical protein [Tbb] ref|XP_951553.1| hypothetical protein [Tbb]
GLOS_TB927.2.3460.1.1	14.1755093270826	0	0,00	0.00601463776233708	XM_946502.1 Tbb GUTat10.1 D-alanyl-glycyl endopeptidase (Tb927.2.3460) partial mRNA
GLOS_TB927.2.380.1.1	43.1567623201058	0	0,00	3.67107325072828e-06	XP_951481.1 retrotransposon hot spot (RHS) protein [Trypanosoma brucei TREU927]
GLOS_TB927.2.3800.1.1	36.4668084296305	0	0,00	1.36492205869126e-06	XM_946512.1 Tbb strain 927/4 GUTat10.1 hypothetical protein (Tb927.2.3800) partial mRNA
GLOS_TB927.2.400.1.1	73.3107052516225	0	0,00	3.77706540309287e-08	XP_951482.1 retrotransposon hot spot (RHS) protein [Trypanosoma brucei TREU927]
GLOS_TB927.2.4130.1.1	19.0263752335639	0	0,00	0.00231303233548431	XM_946526.1 Tbb GUTat10.1 enoyl-CoA hydratase/Enoyl-CoA isomerase/3-hydroxyacyl-CoA dehydrogenase (Tb927.2.4130) partial mRNA

GLOS_TB927.2.4210.1.1	272.847485975283	0	0,00	1.83984216030799e-28	XM_946531.1 Tbb GUTat10.1 glycosomal phosphoenolpyruvate carboxykinase partial mRNA
GLOS_TB927.2.4230.1.1	22.1828079826208	0	0,00	0.000282477536862942	XM_946532.1 Tbb strain 927/4 GUTat10.1 NUP-1 protein (Tb927.2.4230) partial mRNA
GLOS_TB927.2.4330.1.1	16.0507610177189	0	0,00	0.00287050052632492	XP_951628.1 paraflagellar rod protein [Trypanosoma brucei brucei strain 927/4 GUTat10.1]
GLOS_TB927.2.4370.1.1	100.88683474036	0	0,00	1.49578765923147e-16	XP_951630.1 trypanothione synthetase [Trypanosoma brucei brucei strain 927/4 GUTat10.1]
GLOS_TB927.2.4580.1.1	23.1023519746178	0	0,00	0.000196601085738671	XM_946554.1 Tbb strain 927/4 GUTat10.1 hypothetical protein (Tb927.2.4580) partial mRNA
GLOS_TB927.2.470.1.2	37.1693701817733	0	0,00	1.00171947649774e-06	XM_946391.1 Tbb GUTat10.1 retrotransposon hot spot (RHS) protein (Tb927.2.470) partial mRNA
GLOS_TB927.2.470.2.2	31.0011595102289	0	0,00	0.00384961714161903	XM_946391.1 Tbb GUTat10.1 retrotransposon hot spot (RHS) protein (Tb927.2.470) partial mRNA
GLOS_TB927.2.4700.1.1	38.1612415870549	0	0,00	7.932136122615e-07	XM_946561.1 Tbb strain 927/4 GUTat10.1 hypothetical protein (Tb927.2.4700) partial mRNA
GLOS_TB927.2.4710.1.1	61.4444120948846	0	0,00	1.43683497607454e-10	XM_946562.1 Tbb strain 927/4 GUTat10.1 RNA-binding protein (Tb927.2.4710) partial mRNA
GLOS_TB927.2.4850.1.1	38.4867149468363	0	0,00	6.45410014893422e-07	XM_946572.1 Tbb strain 927/4 GUTat10.1 hypothetical protein (Tb927.2.4850) partial mRNA
GLOS_TB927.2.5360.1.1	49.1441544584383	0	0,00	1.6534436318891e-05	XM_946615.1 Tbb strain 927/4 GUTat10.1 hypothetical protein (Tb927.2.5360) partial mRNA
GLOS_TB927.2.5500.1.1	46.2408676558779	0	0,00	3.52684793527727e-08	XM_946619.1 Tbb strain 927/4 GUTat10.1 hypothetical protein (Tb927.2.5500) partial mRNA
GLOS_TB927.2.5810.1.1	22.1104805693361	0	0,00	0.000286955603565101	XM_946633.1 Tbb strain 927/4 GUTat10.1 hypothetical protein (Tb927.2.5810) partial mRNA
GLOS_TB927.2.6090.1.1	32.1738494487225	0	0,00	6.48502277463704e-06	XM_946650.1 Tbb GUTat10.1 60S ribosomal protein L44 (Tb927.2.6090) partial mRNA
GLOS_TB927.2.900.1.1	40.3981303441149	0	0,00	3.12911949774908e-07	XM_946397.1 Tbb strain 927/4 GUTat10.1 hypothetical protein (Tb927.2.900) partial mRNA
GLOS_TB927.3.1010.1.1	46.349358775805	0	0,00	3.44739231712461e-08	XM_838604.1 Trypanosoma brucei hypothetical protein, conserved (Tb927.3.1010) partial mRNA
GLOS_TB927.3.1110.1.1	14.1393456204402	0	0,00	0.00606230038830442	XM_838612.1 Trypanosoma brucei hypothetical protein, conserved (Tb927.3.1110) partial mRNA
GLOS_TB927.3.1120.1.1	45.3936510771657	0	0,00	4.93513853266323e-08	XP_843706.1 GTP-binding nuclear protein rtb2 [Tbb strain 927/4 GUTat10.1]
GLOS_TB927.3.1210.1.1	25.1584221984659	0	0,00	9.09850605384415e-05	XM_838622.1 Tbb protein transport protein Sec24C, putative (Tb927.3.1210) partial mRNA
GLOS_TB927.3.1370.1.2	37.241697595058	0	0,00	9.86399057604768e-07	XM_838638.1 T. brucei 40S ribosomal protein S25, putative (Tb927.3.1370) partial mRNA
GLOS_TB927.3.1370.2.2	47.268902767802	0	0,00	2.42765555057836e-08	XM_838638.1 T.brucei 40S ribosomal protein S25, putative (Tb927.3.1370) partial mRNA
GLOS_TB927.3.1380.1.1	105.882355473411	0	0,00	2.72439367503336e-17	XM_838639.1 Tbb ATP synthase beta chain, mitochondrial precursor partial mRNA
GLOS_TB927.3.1410.1.1	39.189276698979	0	0,00	4.74064871430891e-07	XP_843735.1 cytochrome c oxidase VII [Trypanosoma brucei brucei strain 927/4 GUTat10.1]
GLOS_TB927.3.1690.1.1	20.1990651720575	0	0,00	0.000602670970874725	XP_843763.1 hypothetical protein [Trypanosoma brucei brucei strain 927/4 GUTat10.1]
GLOS_TB927.3.1790.1.1	58.1433245192582	0	0,00	3.06364638547092e-05	XM_838680.1 Tbb pyruvate dehydrogenase E1 beta subunit, putative (Tb927.3.1790) partial mRNA
GLOS_TB927.3.1840.1.1	21.1547728706968	0	0,00	0.000415757696464543	XP_843778.1 3-oxo-5-alpha-steroid 4-dehydrogenase [Trypanosoma brucei TREU927]
GLOS_TB927.3.1940.1.1	19.2071937667758	0	0,00	0.000880946380186712	XP_843788.1 hypothetical protein [Trypanosoma brucei brucei strain 927/4 GUTat10.1]
GLOS_TB927.3.2100.1.1	22.1466442759785	0	0,00	0.000284706581109359	XM_838711.1 Trypanosoma brucei hypothetical protein, conserved (Tb927.3.2100) partial mRNA
GLOS_TB927.3.2180.1.1	25.0137673718964	0	0,00	0.00335534660432802	XP_843812.1 hypothetical protein [Trypanosoma brucei brucei strain 927/4 GUTat10.1]
GLOS_TB927.3.2230.1.1	59.3160144577517	0	0,00	3.03576342966866e-10	XM_838724.1 Tbb succinyl-CoA synthetase alpha subunit, putative (Tb927.3.2230) partial mRNA
GLOS_TB927.3.2490.1.1	27.1783287156716	0	0,00	4.25270484194831e-05	XM_838750.1 Trypanosoma brucei hypothetical protein, conserved (Tb927.3.2490) partial mRNA
GLOS_TB927.3.2600.1.1	38.2335690003397	0	0,00	6.8103005804718e-07	XM_838760.1 Tbb ATP-dependent DEAD/H RNA helicase, putative (Tb927.3.2600) partial mRNA
GLOS_TB927.3.2880.1.1	17.18728724957	0	0,00	0.00189992795526782	XP_843881.1 hypothetical protein [Trypanosoma brucei brucei strain 927/4 GUTat10.1]
GLOS_TB927.3.2900.1.1	26.1141298971052	0	0,00	6.29225505279159e-05	XM_838790.1 Tbb elongation initiation factor 2 alpha subunit, putative partial mRNA
GLOS_TB927.3.3130.1.1	38.2335690003397	0	0,00	6.8103005804718e-07	XM_838813.1 Trypanosoma brucei hypothetical protein, conserved (Tb927.3.3130) partial mRNA
GLOS_TB927.3.3270.1.1	32.1376857420801	0	0,00	6.5355906819989e-06	XM_838827.1 T. brucei ATP-dependent phosphofructokinase (Tb927.3.3270) partial mRNA
GLOS_TB927.3.3310.1.1	215.639156773959	0	0,00	1.15987382910896e-07	XM_838831.1 T. brucei 60S ribosomal protein L13, putative (Tb927.3.3310) partial mRNA
GLOS_TB927.3.3410.1.1	29.162071526235	0	0,00	2.00746299740489e-05	XP_843934.1 aspartyl aminopeptidase [Trypanosoma brucei brucei strain 927/4 GUTat10.1]
GLOS_TB927.3.3460.1.1	37.4586798349122	0	0,00	9.41942330339214e-07	XM_838845.1 Trypanosoma brucei hypothetical protein, conserved (Tb927.3.3460) partial mRNA
GLOS_TB927.3.3490.1.1	24.3835330330384	0	0,00	0.000126583445621854	XM_838848.1 T. brucei high mobility group protein, putative (Tb927.3.3490) partial mRNA
GLOS_TB927.3.3580.1.1	43.084434906821	0	0,00	0.000179834032123999	XM_838857.1 Tbb lipophosphoglycan biosynthetic protein, putative (Tb927.3.3580) partial mRNA

GLOS_TB927.3.3690.1.1	35.294118491137	0	0,00	2.05654076416339e-06	XM_838868.1 Tbb flagellar radial spoke protein-like, putative (Tb927.3.3690) partial mRNA
GLOS_TB927.3.3750.1.1	28.134036414311	0	0,00	2.94399465636344e-05	XM_838874.1 Trypanosoma brucei hypothetical protein, conserved (Tb927.3.3750) partial mRNA
GLOS_TB927.3.3770.1.1	23.1385156812602	0	0,00	0.000195061474162675	XP_843969.1 hypothetical protein [Tbb] ref|XP_843971.1| hypothetical protein [Tbb]
GLOS_TB927.3.3900.1.1	34.4107382057824	0	0,00	2.91494966656957e-06	XM_838889.1 Tbb carnitine O-palmitoyltransferase II, putative (Tb927.3.3900) partial mRNA
GLOS_TB927.3.4040.1.1	22.1828079826208	0	0,00	0.000282477536862942	XM_838903.1 Trypanosoma brucei hypothetical protein, conserved (Tb927.3.4040) partial mRNA
GLOS_TB927.3.4080.1.1	72.535816086195	0	0,00	2.75996549412034e-12	XM_838907.1 Trypanosoma brucei hypothetical protein, conserved (Tb927.3.4080) partial mRNA
GLOS_TB927.3.4160.1.1	29.2705626461621	0	0,00	1.96110526903119e-05	XM_838915.1 Trypanosoma brucei hypothetical protein, conserved (Tb927.3.4160) partial mRNA
GLOS_TB927.3.4290.1.1	146.316649524168	0	0,00	4.46900693504577e-23	XM_838928.1 Trypanosoma brucei 73 kDa paraflagellar rod protein (Tb927.3.4290) partial mRNA
GLOS_TB927.3.4500.1.1	141.40890753034	0	0,00	6.27514235732047e-07	XP_844042.1 fumarate hydratase [Trypanosoma brucei brucei strain 927/4 GUTat10.1]
GLOS_TB927.3.4650.1.1	24.1303870865418	0	0,00	0.000133709923111234	XM_838964.1 Trypanosoma brucei C-8 sterol isomerase, putative (Tb927.3.4650) partial mRNA
GLOS_TB927.3.4750.1.1	50.3530081035742	0	0,00	7.94244630802683e-09	XP_844067.1 aminopeptidase [Tbb] ref|XP_844071.1| aminopeptidase [Tbb]
GLOS_TB927.3.4760.1.1	34.3022470858553	0	0,00	2.98321038660061e-06	XM_838975.1 Trypanosoma brucei brucei strain 927/4 GUTat10.1 dynamin partial mRNA
GLOS_TB927.3.4840.1.1	24.0218959666147	0	0,00	0.00250078634343649	XP_844076.1 ubiquitin hydrolase [Trypanosoma brucei brucei strain 927/4 GUTat10.1]
GLOS_TB927.3.5180.1.1	16.0145973110765	0	0,00	0.00508109428667914	XP_844108.1 cofilin/actin depolymerizing factor [Trypanosoma brucei TREU927]
GLOS_TB927.3.5520.1.1	33.2742119739313	0	0,00	4.36326885814757e-06	XM_839049.1 Tbb 26S proteasome regulatory non-ATPase subunit (Tb927.3.5520) partial mRNA
GLOS_TB927.3.720.1.1	30.081615518232	0	0,00	0.000113200152092862	XM_838575.1 Trypanosoma brucei zinc finger protein 2, putative (Tb927.3.720) partial mRNA
GLOS_TB927.3.740.1.1	29.2705626461621	0	0,00	1.96110526903119e-05	XM_838577.1 Trypanosoma brucei hypothetical protein, conserved (Tb927.3.740) partial mRNA
GLOS_TB927.3.860.1.1	19.2433574734181	0	0,00	0.000874045848858602	XP_843682.1 acyl carrier protein, mitochondrial precursor [Trypanosoma brucei TREU927]
GLOS_TB927.3.930.1.1	43.4822356798871	0	0,00	1.01286520014078e-07	XM_838596.1 Trypanosoma brucei dynein heavy chain, putative (Tb927.3.930) partial mRNA
GLOS_TB927.4.1080.1.1	22.1104805693361	0	0,00	0.000286955603565101	XM_839165.1 Tbb GUTat10.1 V-type ATPase, A subunit, putative (Tb927.4.1080) partial mRNA
GLOS_TB927.4.1120.1.1	17.1511235429277	0	0,00	0.00191497267072198	XP_844262.1 hypothetical protein [Trypanosoma brucei brucei strain 927/4 GUTat10.1]
GLOS_TB927.4.1300.1.1	79.3704248032397	0	0,00	2.53209439055001e-09	XP_844280.1 hypothetical protein [Trypanosoma brucei brucei strain 927/4 GUTat10.1]
GLOS_TB927.4.1350.1.1	23.1746793879025	0	0,00	0.000193535463406783	XP_844285.1 hydroxyacylglutathione hydrolase [Tbb strain 927/4 GUTat10.1]
GLOS_TB927.4.1630.1.1	20.1267377587727	0	0,00	0.000612237591608327	XM_839220.1 Tbb GUTat10.1 ribosomal RNA processing protein 6 (Tb927.4.1630) partial mRNA
GLOS_TB927.4.1790.1.1	1748.7840352014	0	0,00	6.32618607277007e-20	XM_839236.1 Tbb GUTat10.1 ribosomal protein L3, putative (Tb927.4.1790) partial mRNA
GLOS_TB927.4.1850.1.1	24.1665507931842	0	0,00	0.000132664769601763	XM_839242.1 Tbb GUTat10.1 hypothetical protein, conserved (Tb927.4.1850) partial mRNA
GLOS_TB927.4.1860.1.2	63.3558274921632	0	0,00	7.12424686616413e-11	XM_839243.1 Tbb GUTat10.1 ribosomal protein S19, putative (Tb927.4.1860) partial mRNA
GLOS_TB927.4.1860.2.2	108.785642275971	0	0,00	1.00367390399703e-17	XP_844336.1 ribosomal protein S19 [Trypanosoma brucei brucei strain 927/4 GUTat10.1]
GLOS_TB927.4.2000.1.1	29.162071526235	0	0,00	2.00746299740489e-05	XM_839257.1 Tbb GUTat10.1 RuvB-like DNA helicase, putative (Tb927.4.2000) partial mRNA
GLOS_TB927.4.2030.1.1	28.2425275342381	0	0,00	2.87588295365255e-05	XM_839260.1 Tbb GUTat10.1 hypothetical protein, conserved (Tb927.4.2030) partial mRNA
GLOS_TB927.4.2060.1.1	19.0987026468487	0	0,00	0.000902028346384345	XM_839263.1 Tbb GUTat10.1 hypothetical protein, conserved (Tb927.4.2060) partial mRNA
GLOS_TB927.4.2080.1.1	17.1149598362853	0	0,00	0.00193015710508488	XM_839265.1 Tbb GUTat10.1 hypothetical protein, conserved (Tb927.4.2080) partial mRNA
GLOS_TB927.4.2180.1.1	153.962311113283	0	0,00	4.03258142552164e-19	XM_839275.1 Tbb GUTat10.1 60S ribosomal protein L35A, putative (Tb927.4.2180) partial mRNA
GLOS_TB927.4.2410.1.1	26.0779661904628	0	0,00	9.76903094824833e-05	XM_839298.1 Tbb GUTat10.1 hypothetical protein, conserved (Tb927.4.2410) partial mRNA
GLOS_TB927.4.2530.1.1	37.241697595058	0	0,00	9.86399057604768e-07	XM_839310.1 Tbb GUTat10.1 hypothetical protein, conserved (Tb927.4.2530) partial mRNA
GLOS_TB927.4.2740.1.1	30.1539429315167	0	0,00	1.38029332263529e-05	XM_839331.1 Tbb GUTat10.1 hypothetical protein, conserved (Tb927.4.2740) partial mRNA
GLOS_TB927.4.2890.1.1	18.0706675349246	0	0,00	0.00132964988055519	XM_839346.1 Tbb GUTat10.1 hypothetical protein, conserved (Tb927.4.2890) partial mRNA
GLOS_TB927.4.3060.1.1	60.2355584497487	0	0,00	3.73924035319405e-07	XM_839363.1 Tbb GUTat10.1 hypothetical protein, conserved (Tb927.4.3060) partial mRNA
GLOS_TB927.4.3450.1.1	37.1332064751309	0	0,00	7.27429135211843e-06	XP_844495.1 hypothetical protein [Trypanosoma brucei brucei strain 927/4 GUTat10.1]
GLOS_TB927.4.3500.1.1	100.308215434082	0	0,00	1.08527962628636e-06	XM_839407.1 Tbb GUTat10.1 hypothetical protein, conserved (Tb927.4.3500) partial mRNA
GLOS_TB927.4.3550.1.1	257.897087482773	0	0,00	1.14992599613924e-37	XM_839412.1 Tbb GUTat10.1 60S ribosomal protein L13a, putative (Tb927.4.3550) partial mRNA

GLOS_TB927.4.3590.1.1	37.3501887149851	0	0,00	9.63892803228974e-07	XM_839416.1 Tbb GUTat10.1 translation elongation factor 1-beta, putative partial mRNA
GLOS_TB927.4.3620.1.1	44.2932885519569	0	0,00	7.28682717232413e-08	XM_839419.1 Tbb GUTat10.1 serine/threonine-protein phosphatase PP1, putative partial mRNA
GLOS_TB927.4.3740.1.1	34.1214285526435	0	0,00	1.06536966934273e-05	XM_839431.1 Tbb GUTat10.1 hypothetical protein, conserved (Tb927.4.3740) partial mRNA
GLOS_TB927.4.3920.1.1	29.1259078195926	0	0,00	2.02318354289899e-05	XM_839449.1 Tbb GUTat10.1 hypothetical protein, conserved (Tb927.4.3920) partial mRNA
GLOS_TB927.4.3950.1.1	53.5094408526311	0	0,00	2.57185667958856e-09	XM_839452.1 Tbb GUTat10.1 cytoskeleton-associated protein CAP5.5, putative partial mRNA
GLOS_TB927.4.4040.1.1	17.0787961296429	0	0,00	0.00194548274562265	XM_839461.1 Tbb GUTat10.1 hypothetical protein, conserved (Tb927.4.4040) partial mRNA
GLOS_TB927.4.4050.1.1	23.1023519746178	0	0,00	0.000196601085738671	XM_839462.1 Tbb GUTat10.1 ABC transporter, putative (Tb927.4.4050) partial mRNA
GLOS_TB927.4.4160.1.1	23.1746793879025	0	0,00	0.000193535463406783	XM_839473.1 Tbb GUTat10.1 hypothetical protein, conserved (Tb927.4.4160) partial mRNA
GLOS_TB927.4.4310.1.1	20.1629014654151	0	0,00	0.00060743267903454	XM_839488.1 Tbb GUTat10.1 hypothetical protein, conserved (Tb927.4.4310) partial mRNA
GLOS_TB927.4.4450.1.1	45.2489962505962	0	0,00	5.08754417316258e-08	XM_839501.1 Tbb GUTat10.1 receptor-type adenylate cyclase GRESAG 4, putative partial mRNA
GLOS_TB927.4.4550.1.1	35.1494636645675	0	0,00	2.12108345743693e-06	XM_839511.1 Tbb GUTat10.1 hypothetical protein, conserved (Tb927.4.4550) partial mRNA
GLOS_TB927.4.4570.1.1	33.1657208540041	0	0,00	4.46586043940135e-06	XM_839513.1 Tbb GUTat10.1 hypothetical protein, conserved (Tb927.4.4570) partial mRNA
GLOS_TB927.4.4610.1.1	43.37374455996	0	0,00	1.03626399389373e-07	XM_839517.1 Tbb GUTat10.1 hypothetical protein, conserved (Tb927.4.4610) partial mRNA
GLOS_TB927.4.4620.1.1	28.3148549475228	0	0,00	2.83144579286955e-05	XP_844611.1 cytochrome c oxidase VIII (COX VIII) [Trypanosoma brucei TREU927]
GLOS_TB927.4.4730.1.1	33.2380482672889	0	0,00	4.39717686839511e-06	XM_839529.1 Tbb GUTat10.1 amino acid transporter, putative (Tb927.4.4730) partial mRNA
GLOS_TB927.4.4990.1.1	34.1575922592858	0	0,00	3.07695129569848e-06	XP_844648.1 ubiquinol-cytochrome C reductase [Tbb strain 927/4 GUTat10.1]
GLOS_TB927.4.5060.1.1	28.0978727076686	0	0,00	2.96709423828632e-05	XM_839561.1 Tbb strain 927/4 GUTat10.1 hypothetical protein (Tb927.4.5060) partial mRNA
GLOS_TB927.4.760.1.1	22.2551353959056	0	0,00	0.000278078592691657	XM_839133.1 Tbb GUTat10.1 gamma-adaptin 1, putative (Tb927.4.760) partial mRNA
GLOS_TB927.5.1000.1.1	43.1205986134634	0	0,00	3.39741817331257e-05	XP_844774.1 ubiquitin-conjugating enzyme E2 [T. brucei brucei strain 927/4 GUTat10.1]
GLOS_TB927.5.1060.1.1	57.2237805272612	0	0,00	4.63488680988762e-07	XM_839687.1 Tbb GUTat10.1 mitochondrial processing peptidase, beta subunit partial mRNA
GLOS_TB927.5.1090.1.1	26.18645731039	0	0,00	6.19450094774168e-05	XP_844783.1 threonyl-tRNA synthetase [Trypanosoma brucei brucei strain 927/4 GUTat10.1]
GLOS_TB927.5.1110.1.1	264.189240600182	0	0,00	8.30705437502516e-12	XP_844785.1 60S ribosomal protein L2 [Tbb] ref|XP_829685.1| 60S ribosomal protein L2 [Tbb]
GLOS_TB927.5.1250.1.1	23.1746793879025	0	0,00	0.000193535463406783	XM_839706.1 T. brucei brucei strain 927/4 GUTat10.1 hypothetical protein partial mRNA
GLOS_TB927.5.1470.1.1	20.1267377587727	0	0,00	0.000612237591608327	XP_844821.1 NADH-cytochrome b5 reductase [T. brucei brucei strain 927/4 GUTat10.1]
GLOS_TB927.5.1570.1.1	15.0950533190795	0	0,00	0.00417062745104592	XM_839738.1 T. brucei brucei strain 927/4 GUTat10.1 hypothetical protein partial mRNA
GLOS_TB927.5.1660.1.1	21.082445457412	0	0,00	0.000422360679473223	XM_839747.1 T. brucei brucei strain 927/4 GUTat10.1 protein phosphatase 2C partial mRNA
GLOS_TB927.5.1710.1.1	46.349358775805	0	0,00	3.44739231712461e-08	XM_839752.1 Tbb GUTat10.1 ribonucleoprotein p18, mitochondrial precursor partial mRNA
GLOS_TB927.5.1780.1.1	39.2254404056214	0	0,00	4.7043615745198e-07	XM_839759.1 T. brucei brucei strain 927/4 GUTat10.1 hypothetical protein partial mRNA
GLOS_TB927.5.1790.1.1	19.0625389402063	0	0,00	0.000909184655185172	XP_844853.1 hypothetical protein [Trypanosoma brucei brucei strain 927/4 GUTat10.1]
GLOS_TB927.5.1810.1.1	209.941074288766	0	0,00	3.5755937159948e-11	XM_839762.1 Tbb GUTat10.1 lysosomal/endosomal membrane protein p67 partial mRNA
GLOS_TB927.5.2100.1.1	27.0698375957445	0	0,00	0.000193176957744829	XM_839791.1 T. brucei brucei strain 927/4 GUTat10.1 hypothetical protein partial mRNA
GLOS_TB927.5.2260.1.2	29.2343989395198	0	0,00	1.97642511792219e-05	XM_839807.1 T. brucei brucei strain 927/4 GUTat10.1 hypothetical protein partial mRNA
GLOS_TB927.5.2260.2.2	295.515873470192	0	0,00	9.92873283890846e-15	XM_839807.1 T. brucei brucei strain 927/4 GUTat10.1 hypothetical protein partial mRNA
GLOS_TB927.5.2320.1.1	39.4424226454756	0	0,00	4.4928456993471e-07	XP_844906.1 hypothetical protein [Trypanosoma brucei brucei strain 927/4 GUTat10.1]
GLOS_TB927.5.2430.1.1	42.1648909148241	0	0,00	1.62070436675388e-06	XM_839824.1 Tbb strain 927/4 GUTat10.1 membrane transporter protein partial mRNA
GLOS_TB927.5.2570.1.1	25.2307496117506	0	0,00	8.95715088102793e-05	XM_839836.1 Tbb strain 927/4 GUTat10.1 translation initiation factor partial mRNA
GLOS_TB927.5.2850.1.1	28.2063638275957	0	0,00	2.8983908845462e-05	XM_839864.1 T. brucei brucei strain 927/4 GUTat10.1 hypothetical protein partial mRNA
GLOS_TB927.5.2930.1.1	29.1982352328774	0	0,00	1.99187727703837e-05	XM_839872.1 T. brucei brucei strain 927/4 GUTat10.1 hypothetical protein partial mRNA
GLOS_TB927.5.2960.1.1	28.134036414311	0	0,00	2.94399465636344e-05	XP_844968.1 phosphoribosylpyrophosphate synthetase [Trypanosoma brucei TREU927]
GLOS_TB927.5.3120.1.1	27.2506561289564	0	0,00	4.18681751684288e-05	XM_839891.1 Tbb strain 927/4 GUTat10.1 translation initiation factor partial mRNA
GLOS_TB927.5.3160.1.1	43.2290897333905	0	0,00	1.06836980806488e-07	XM_839895.1 Trypanosoma brucei brucei strain 927/4 GUTat10.1 protein kinase partial mRNA

GLOS_TB927.5.320.1.1	108.532496329475	0	0,00	7.95707651665381e-11	XM_839613.1 Tbb GUTat10.1 receptor-type adenylate cyclase GRESAG 4 partial mRNA
GLOS_TB927.5.3240.1.1	21.082445457412	0	0,00	0.000422360679473223	XM_839903.1 T. brucei brucei strain 927/4 GUTat10.1 hypothetical protein partial mRNA
GLOS_TB927.5.340.1.1	35.294118491137	0	0,00	2.05654076416339e-06	XP_844708.1 expression site-associated gene (ESAG) protein [Trypanosoma brucei TREU927]
GLOS_TB927.5.3590.1.1	17.2596146628548	0	0,00	0.00187025181992498	XM_839936.1 T. brucei brucei strain 927/4 GUTat10.1 hypothetical protein partial mRNA
GLOS_TB927.5.360.1.1	35.2579547844946	0	0,00	2.07247351372334e-06	XM_839617.1 Tbbstrain 927/4 GUTat10.1 75 kDa invariant surface glycoprotein partial mRNA
GLOS_TB927.5.3800.1.1	107.685279750763	0	0,00	1.43533126039985e-17	XP_845050.1 glutamine hydrolysing (not ammonia-dependent) carbomoyl phosphate synth. [Tbb]
GLOS_TB927.5.3960.1.1	25.0499310785388	0	0,00	0.000603329057270972	XM_839973.1 Tbb strain 927/4 GUTat10.1 arginine N-methyltransferase partial mRNA
GLOS_TB927.5.4020.1.1	33.2742119739313	0	0,00	4.36326885814757e-06	XM_839979.1 Tbb GUTat10.1 hypothetical protein Tb927.5.4020 partial mRNA
GLOS_TB927.5.4190.1.3	32.2461768620072	0	0,00	6.38516834635607e-06	XM_839996.1 Trypanosoma brucei brucei strain 927/4 GUTat10.1 histone H4 partial mRNA
GLOS_TB927.5.4190.2.3	21.1547728706968	0	0,00	0.000415757696464543	XM_839996.1 Trypanosoma brucei brucei strain 927/4 GUTat10.1 histone H4 partial mRNA
GLOS_TB927.5.4190.3.3	33.3103756805736	0	0,00	4.3296465103686e-06	XM_839996.1 Trypanosoma brucei brucei strain 927/4 GUTat10.1 histone H4 partial mRNA
GLOS_TB927.5.440.1.1	18.1429949482093	0	0,00	0.00130880372149336	XM_839625.1 T. brucei brucei strain 927/4 GUTat10.1 hypothetical protein partial mRNA
GLOS_TB927.5.4420.1.1	38.4505512401939	0	0,00	6.50372723324067e-07	XM_840019.1 T. brucei brucei strain 927/4 GUTat10.1 nucleolar RNA helicase II partial mRNA
GLOS_TB927.5.4460.1.1	18.1791586548517	0	0,00	0.00129852321745928	XM_840023.1 T. brucei brucei strain 927/4 GUTat10.1 major vault protein partial mRNA
GLOS_TB927.5.4480.1.1	30.1177792248743	0	0,00	1.39109013062701e-05	XM_840025.1 Tbb strain 927/4 GUTat10.1 paraflagellar rod component Par4 partial mRNA
GLOS_TB927.5.510.1.1	25.1584221984659	0	0,00	9.09850605384415e-05	XP_844725.1 hypothetical protein [Trypanosoma brucei brucei strain 927/4 GUTat10.1]
GLOS_TB927.5.700.1.1	28.3871823608076	0	0,00	2.78776817396476e-05	XM_839651.1 T. brucei brucei strain 927/4 GUTat10.1 hypothetical protein partial mRNA
GLOS_TB927.5.860.1.1	28.2425275342381	0	0,00	2.87588295365255e-05	XM_839667.1 T. brucei brucei strain 927/4 GUTat10.1 hypothetical protein partial mRNA
GLOS_TB927.5.900.1.1	48.0799556398718	0	0,00	0.000229998727133559	XP_844764.1 oligosaccharyl transferase subunit [Trypanosoma brucei TREU927]
GLOS_TB927.5.930.1.1	112.355327124032	0	0,00	5.39214107954569e-07	XM_839674.1 Tbb strain 927/4 GUTat10.1 NADH-dependent fumarate reductase partial mRNA
GLOS_TB927.6.1020.1.1	261.756081983972	0	0,00	2.86377500688664e-27	XM_840131.1 Tbb strain 927/4 GUTat10.1 cysteine peptidase precursor partial mRNA
GLOS_TB927.6.1090.1.1	28.3148549475228	0	0,00	2.83144579286955e-05	XP_845231.1 proteasome regulatory ATPase subunit 3 [Trypanosoma brucei TREU927]
GLOS_TB927.6.1520.1.1	67.2871494066476	0	0,00	4.94817721551329e-08	XM_840181.1 Trypanosoma brucei brucei strain 927/4 GUTat10.1 aquaporin 3 partial mRNA
GLOS_TB927.6.2010.1.1	26.1141298971052	0	0,00	6.29225505279159e-05	XM_840229.1 T. brucei brucei strain 927/4 GUTat10.1 hypothetical protein partial mRNA
GLOS_TB927.6.2100.1.2	43.1205986134634	0	0,00	3.39741817331257e-05	XP_845331.1 40S ribosomal protein S30 [Trypanosoma brucei brucei strain 927/4 GUTat10.1]
GLOS_TB927.6.2100.2.2	26.3311121369594	0	0,00	6.00403368018993e-05	XP_845331.1 40S ribosomal protein S30 [Trypanosoma brucei brucei strain 927/4 GUTat10.1]
GLOS_TB927.6.2230.1.1	18.106831241567	0	0,00	0.0013191789443401	XM_840251.1 T. brucei brucei strain 927/4 GUTat10.1 hypothetical protein partial mRNA
GLOS_TB927.6.2290.1.1	31.1458143367984	0	0,00	9.49548498458617e-06	XM_840257.1 T. brucei brucei strain 927/4 GUTat10.1 hypothetical protein partial mRNA
GLOS_TB927.6.2740.1.1	48.3692652930108	0	0,00	1.64680064138597e-08	XP_845395.1 pyridoxal kinase [Trypanosoma brucei brucei strain 927/4 GUTat10.1]
GLOS_TB927.6.2790.1.1	225.325437260984	0	0,00	3.58014869223803e-18	XP_845400.1 L-threonine 3-dehydrogenase [Trypanosoma brucei brucei strain 927/4 GUTat10.1]
GLOS_TB927.6.3090.1.1	33.2018845606465	0	0,00	4.43137317533808e-06	XM_840337.1 T. brucei brucei strain 927/4 GUTat10.1 hypothetical protein partial mRNA
GLOS_TB927.6.3500.1.1	26.2949484303171	0	0,00	6.0510304148871e-05	XP_845468.1 endosomal trafficking protein RME-8 [Trypanosoma brucei TREU927]
GLOS_TB927.6.3650.1.1	22.0743168626937	0	0,00	0.000289224804544453	XP_845483.1 ADP-ribosylation factor [Trypanosoma brucei brucei strain 927/4 GUTat10.1]
GLOS_TB927.6.3840.1.1	24.2750419131113	0	0,00	0.000129584006901138	XM_840409.1 Tbb strain 927/4 GUTat10.1 reticulon domain protein partial mRNA
GLOS_TB927.6.3950.1.1	26.2949484303171	0	0,00	6.0510304148871e-05	XP_845513.1 hypothetical protein [Trypanosoma brucei brucei strain 927/4 GUTat10.1]
GLOS_TB927.6.4130.1.1	20.0182466388456	0	0,00	0.00345370030582204	XP_845531.1 hypothetical protein [Trypanosoma brucei brucei strain 927/4 GUTat10.1]
GLOS_TB927.6.4140.1.1	41.3538380427542	0	0,00	2.18040369926485e-07	XP_845532.1 hypothetical protein [Trypanosoma brucei brucei strain 927/4 GUTat10.1]
GLOS_TB927.6.4210.1.1	20.0905740521303	0	0,00	0.00061708615088037	XM_840446.1 T. brucei brucei strain 927/4 GUTat10.1 aldehyde dehydrogenase partial mRNA
GLOS_TB927.6.4300.1.1	257.571614122991	0	0,00	1.53327449306262e-20	XP_845547.1 glyceraldehyde 3-phosphate dehydrogenase, glycosomal [Tbb GUTat10.1]
GLOS_TB927.6.4370.1.1	15.0950533190795	0	0,00	0.00417062745104592	XP_845554.1 eukaryotic translation initiation factor 3 subunit 7-like protein [Tbb GUTat10.1]
GLOS_TB927.6.4440.1.1	81.5711498536573	0	0,00	1.14503332024045e-13	XM_840468.1 T. brucei brucei strain 927/4 GUTat10.1 hypothetical protein partial mRNA

GLOS_TB927.6.4480.1.1	26.1141298971052	0	0,00	6.29225505279159e-05	XM_840472.1 T. brucei brucei strain 927/4 GUTat10.1 valyl-tRNA synthetase partial mRNA
GLOS_TB927.6.4490.1.1	39.0084581657672	0	0,00	0.0022504620723734	XP_845566.1 hypothetical protein [Trypanosoma brucei brucei strain 927/4 GUTat10.1]
GLOS_TB927.6.4750.1.1	30.081615518232	0	0,00	0.000113200152092862	XM_840499.1 T. brucei brucei strain 927/4 GUTat10.1 hypothetical protein partial mRNA
GLOS_TB927.6.4840.1.1	129.651105493529	0	0,00	9.99680813004698e-12	XM_840508.1 Tbb strain 927/4 GUTat10.1 S-adenosylmethionine synthetase partial mRNA
GLOS_TB927.6.4980.1.2	81.3180039071607	0	0,00	1.03998731650396e-07	XM_840522.1 Tbb strain 927/4 GUTat10.1 40S ribosomal protein S14 partial mRNA
GLOS_TB927.6.5070.1.1	25.266913318393	0	0,00	8.88739667539241e-05	XM_840531.1 T. brucei brucei strain 927/4 GUTat10.1 hypothetical protein partial mRNA
GLOS_TB927.6.5080.1.1	28.0978727076686	0	0,00	2.96709423828632e-05	XM_840532.1 T. brucei brucei strain 927/4 GUTat10.1 hypothetical protein partial mRNA
GLOS_TB927.6.5090.1.1	28.2425275342381	0	0,00	2.87588295365255e-05	XM_840533.1 T. brucei brucei strain 927/4 GUTat10.1 hypothetical protein partial mRNA
GLOS_TB927.6.5120.1.2	35.2217910778523	0	0,00	2.08854072568386e-06	XM_840536.1 Tbb strain 927/4 GUTat10.1 60S acidic ribosomal protein P2 partial mRNA
GLOS_TB927.6.5120.2.2	39.0084581657672	0	0,00	0.0022504620723734	XM_840536.1 Tbb strain 927/4 GUTat10.1 60S acidic ribosomal protein P2 partial mRNA
GLOS_TB927.6.520.1.3	148.858299260305	0	0,00	1.22630117989292e-15	XM_840082.1 Trypanosoma brucei brucei strain 927/4 GUTat10.1 EP3-2 procyclin partial mRNA
GLOS_TB927.6.520.2.3	53.2562949061345	0	0,00	1.02114036517936e-08	XM_840082.1 Trypanosoma brucei brucei strain 927/4 GUTat10.1 EP3-2 procyclin partial mRNA
GLOS_TB927.6.520.3.3	177.586406306831	0	0,00	5.90783118870252e-08	XM_840082.1 Trypanosoma brucei brucei strain 927/4 GUTat10.1 EP3-2 procyclin partial mRNA
GLOS_TB927.6.660.1.1	46.1323765359508	0	0,00	2.34444083070287e-05	XM_840096.1 T. brucei brucei strain 927/4 GUTat10.1 hypothetical protein partial mRNA
GLOS_TB927.6.700.1.1	23.0661882679754	0	0,00	0.000198154433233135	XP_845193.1 alanyl-tRNA synthetase [Trypanosoma brucei brucei strain 927/4 GUTat10.1]
GLOS_TB927.6.720.1.1	155.837562803919	0	0,00	9.08919633903375e-14	XM_840102.1 T. brucei brucei strain 927/4 GUTat10.1 40S ribosomal protein L14 partial mRNA
GLOS_TB927.6.790.1.2	31.109650630156	0	0,00	1.61172182692142e-05	XM_840109.1 Tbb strain 927/4 GUTat10.1 receptor-type adenylate cyclase GRESAG 4 partial mRNA
GLOS_TB927.6.790.2.2	14.0670182071555	0	0,00	0.00615899928204462	XP_845202.1 receptor-type adenylate cyclase GRESAG 4 [Trypanosoma brucei TREU927]
GLOS_TB927.6.950.1.1	44.2932885519569	0	0,00	7.28682717232413e-08	XM_840124.1 Tbbstrain 927/4 GUTat10.1 cysteinyl-tRNA synthetase partial mRNA
GLOS_TB927.7.1050.1.2	52.3005872074952	0	0,00	3.8672302974418e-09	XM_840636.1 Tbb GUTat10.1 40S ribosomal protein S16, putative (Tb927.7.1050) partial mRNA
GLOS_TB927.7.1050.2.2	58.32414305247	0	0,00	4.35082063289626e-10	XM_840636.1 Tbb GUTat10.1 40S ribosomal protein S16, putative (Tb927.7.1050) partial mRNA
GLOS_TB927.7.1110.1.1	16.2315795509307	0	0,00	0.00275934587446463	XP_845735.1 asparagine synthetase a [Trypanosoma brucei brucei strain 927/4 GUTat10.1]
GLOS_TB927.7.1290.1.1	18.2514860681365	0	0,00	0.0012782424453452	XM_840660.1 Tbb GUTat10.1 hypothetical protein, conserved (Tb927.7.1290) partial mRNA
GLOS_TB927.7.1320.1.1	26.0779661904628	0	0,00	9.76903094824833e-05	XP_845756.1 10 kDa heat shock protein [Tbb] ref|XP_845758.1| 10 kDa heat shock protein [Tbb]
GLOS_TB927.7.1470.1.1	17.0426324230005	0	0,00	0.00196095109746437	XM_840678.1 Tbb GUTat10.1 ATPase subunit 9, putative (Tb927.7.1470) partial mRNA
GLOS_TB927.7.1740.1.1	294.994130251261	0	0,00	6.89194343530893e-29	XM_840705.1 Tbb GUTat10.1 60S ribosomal protein L7, putative (Tb927.7.1740) partial mRNA
GLOS_TB927.7.1790.1.1	30.1901066381591	0	0,00	1.36958874709015e-05	XP_845803.1 adenine phosphoribosyltransferase [T. brucei brucei strain 927/4 GUTat10.1]
GLOS_TB927.7.190.1.1	20.0182466388456	0	0,00	0.00345370030582204	XP_845643.1 thimet oligopeptidase A [Trypanosoma brucei brucei strain 927/4 GUTat10.1]
GLOS_TB927.7.210.1.1	21.1186091640544	0	0,00	0.000419044355280004	XP_845645.1 proline oxidase [Trypanosoma brucei brucei strain 927/4 GUTat10.1]
GLOS_TB927.7.2170.1.1	21.1547728706968	0	0,00	0.000415757696464543	XM_840747.1 Tbb GUTat10.1 hypothetical protein, conserved (Tb927.7.2170) partial mRNA
GLOS_TB927.7.2190.1.1	34.2660833792129	0	0,00	3.00634991693261e-06	XM_840749.1 Tbb GUTat10.1 hypothetical protein, conserved (Tb927.7.2190) partial mRNA
GLOS_TB927.7.230.1.1	86.6389979999929	0	0,00	1.96808274120208e-14	XM_840554.1 Tbb GUTat10.1 40S ribosomal protein S33, putative (Tb927.7.230) partial mRNA
GLOS_TB927.7.2300.1.1	27.1421650090293	0	0,00	4.28608010316753e-05	XM_840760.1 Tbb GUTat10.1 hypothetical protein, conserved (Tb927.7.2300) partial mRNA
GLOS_TB927.7.2340.1.1	210.519693595044	0	0,00	1.02966781107285e-31	XM_840764.1 Tbb GUTat10.1 40S ribosomal protein S15, putative (Tb927.7.2340) partial mRNA
GLOS_TB927.7.2360.1.1	20.054410345488	0	0,00	0.000621978804179148	XM_840766.1 Tbb GUTat10.1 N-acetyltransferase, putative (Tb927.7.2360) partial mRNA
GLOS_TB927.7.2390.1.1	18.2876497747788	0	0,00	0.00126824024544669	XM_840769.1 Tbb GUTat10.1 hypothetical protein, conserved (Tb927.7.2390) partial mRNA
GLOS_TB927.7.2500.1.1	32.2100131553648	0	0,00	6.43488331005283e-06	XM_840780.1 Tbb GUTat10.1 proteasome regulatory ATPase subunit 1 partial mRNA
GLOS_TB927.7.2550.1.1	27.1421650090293	0	0,00	4.28608010316753e-05	XP_845878.1 proteasome regulatory ATPase subunit 5 [Trypanosoma brucei TREU927]
GLOS_TB927.7.2650.1.1	75.6560851286095	0	0,00	9.30903492137891e-13	XP_845888.1 hypothetical protein [Trypanosoma brucei brucei strain 927/4 GUTat10.1]
GLOS_TB927.7.2820.1.2	38.2697327069821	0	0,00	6.75813722995807e-07	XP_845905.1 histone H2A [Tbb] ref|XP_845906.1| histone H2A [Tbb] ref|XP_845907.1| histone H2A [Tbb] ref|XP_845908.1| histone H2A [Tbb] ref|XP_845909.1| histone H2A [Tbb]

					ref|XP_845910.1| histone H2A [Tbb] ref|XP_845911.1| histone H2A [Tbb] ref|XP_845912.1| histone H2A [Tbb] ref|XP_845913.1| histone H2A [Tbb] ref|XP_845914.1| histone H2A [Tbb] ref|XP_845915.1| histone H2A [Tbb] ref|XP_845916.1| histone H2A [Tbb] ref|XP_845917.1| histone H2A [Tbb]
GLOS_TB927.7.2820.2.2	87.486214578705	0	0,00	1.39551347824601e-12	XP_845905.1 histone H2A [Tbb] ref|XP_845906.1| histone H2A [Tbb] ref|XP_845907.1| histone H2A [Tbb] ref|XP_845908.1| histone H2A [Tbb] ref|XP_845909.1| histone H2A [Tbb] ref|XP_845910.1| histone H2A [Tbb] ref|XP_845911.1| histone H2A [Tbb] ref|XP_845912.1| histone H2A [Tbb] ref|XP_845913.1| histone H2A [Tbb] ref|XP_845914.1| histone H2A [Tbb] ref|XP_845915.1| histone H2A [Tbb] ref|XP_845916.1| histone H2A [Tbb] ref|XP_845917.1| histone H2A [Trypanosoma brucei brucei strain 927/4 GUTat10.1]
GLOS_TB927.7.2980.1.1	52.9308215463532	0	0,00	0.00568297388835002	XP_845921.1 hypothetical protein [Trypanosoma brucei brucei strain 927/4 GUTat10.1]
GLOS_TB927.7.3550.1.2	63.3558274921632	0	0,00	7.12424686616413e-11	XP_845978.1 hypothetical protein [Trypanosoma brucei brucei strain 927/4 GUTat10.1]
GLOS_TB927.7.3550.2.2	19.2071937667758	0	0,00	0.000880946380186712	XP_845978.1 hypothetical protein [Trypanosoma brucei brucei strain 927/4 GUTat10.1]
GLOS_TB927.7.3620.1.1	28.2063638275957	0	0,00	2.8983908845462e-05	XM_840892.1 Tbb GUTat10.1 tyrosyl-tRNA synthetase, putative (Tb927.7.3620) partial mRNA
GLOS_TB927.7.3630.1.1	20.1267377587727	0	0,00	0.000612237591608327	XM_840893.1 Tbb GUTat10.1 TPR-repeat-containing chaperone prot. DNAJ, putative partial mRNA
GLOS_TB927.7.3680.1.2	57.4407627671155	0	0,00	6.10046459721529e-10	XM_840898.1 Tbb GUTat10.1 ubiquitin/ribosomal protein S27a, putative partial mRNA
GLOS_TB927.7.3680.2.2	72.5719797928374	0	0,00	2.73993620968143e-12	XM_840898.1 Tbb GUTat10.1 ubiquitin/ribosomal protein S27a, putative partial mRNA
GLOS_TB927.7.3740.1.1	27.1783287156716	0	0,00	4.25270484194831e-05	XM_840904.1 Tbb GUTat10.1 hypothetical protein, conserved (Tb927.7.3740) partial mRNA
GLOS_TB927.7.3940.1.1	18.1429949482093	0	0,00	0.00130880372149336	XM_840924.1 Tbb GUTat10.1 mitochondrial carrier prot., putative (Tb927.7.3940) partial mRNA
GLOS_TB927.7.3980.1.1	23.0661882679754	0	0,00	0.000198154433233135	XM_840928.1 Tbb GUTat10.1 immunodominant antigen, putative (Tb927.7.3980) partial mRNA
GLOS_TB927.7.4070.1.1	50.4614992235013	0	0,00	7.76482545157202e-09	XP_846030.1 calpain-like cysteine peptidase [T. brucei brucei strain 927/4 GUTat10.1]
GLOS_TB927.7.4120.1.1	28.0617090010262	0	0,00	0.000337508122349936	XM_840942.1 Tbb GUTat10.1 hypothetical protein, conserved (Tb927.7.4120) partial mRNA
GLOS_TB927.7.4180.1.1	19.2071937667758	0	0,00	0.000880946380186712	XM_840948.1 Tbb GUTat10.1 fatty acid elongase, putative (Tb927.7.4180) partial mRNA
GLOS_TB927.7.4270.1.1	22.2551353959056	0	0,00	0.000278078592691657	XM_840957.1 Tbb GUTat10.1 hypothetical protein, conserved (Tb927.7.4270) partial mRNA
GLOS_TB927.7.4390.1.1	123.519058528627	0	0,00	3.60259114161553e-09	XP_846062.1 threonine synthase [Trypanosoma brucei brucei strain 927/4 GUTat10.1]
GLOS_TB927.7.4450.1.1	15.0588896124372	0	0,00	0.00420379489573978	XM_840975.1 Tbb GUTat10.1 hypothetical protein, conserved (Tb927.7.4450) partial mRNA
GLOS_TB927.7.4500.1.1	24.1665507931842	0	0,00	0.000132664769601763	XM_840980.1 Tbb GUTat10.1 hypothetical protein, conserved (Tb927.7.4500) partial mRNA
GLOS_TB927.7.4520.1.1	18.1791586548517	0	0,00	0.00129852321745928	XM_840982.1 Tbb GUTat10.1 hypothetical protein, conserved (Tb927.7.4520) partial mRNA
GLOS_TB927.7.4570.1.1	38.0889141737702	0	0,00	0.000124370195795884	XM_840987.1 Tbb GUTat10.1 nucleoside hydrolase, putative (Tb927.7.4570) partial mRNA
GLOS_TB927.7.4900.1.1	34.4107382057824	0	0,00	2.91494966656957e-06	XM_841020.1 TbbGUTat10.1 5'-3' exonuclease XRNA, putative (Tb927.7.4900) partial mRNA
GLOS_TB927.7.4910.1.1	25.1584221984659	0	0,00	9.09850605384415e-05	XM_841021.1 Tbb GUTat10.1 hypothetical protein, conserved (Tb927.7.4910) partial mRNA
GLOS_TB927.7.5000.1.1	252.322947443444	0	0,00	8.98529045277903e-15	XM_841030.1 Tbb GUTat10.1 60S ribosomal protein L19, putative (Tb927.7.5000) partial mRNA
GLOS_TB927.7.5180.1.1	127.703526389608	0	0,00	1.05790787315957e-13	XM_841048.1 Tbb GUTat10.1 60S ribosomal protein L23a, putative (Tb927.7.5180) partial mRNA
GLOS_TB927.7.5210.1.1	27.1060013023869	0	0,00	4.31974678212813e-05	XP_846144.1 hypothetical protein [Trypanosoma brucei brucei strain 927/4 GUTat10.1]
GLOS_TB927.7.5230.1.1	24.238878206469	0	0,00	0.00013060190031178	XM_841053.1 Tbb strain 927/4 GUTat10.1 lanosterol synthase (Tb927.7.5230) partial mRNA
GLOS_TB927.7.5280.1.1	36.1413350698492	0	0,00	2.99276009920107e-06	XM_841058.1 Tbb GUTat10.1 hypothetical protein, conserved (Tb927.7.5280) partial mRNA
GLOS_TB927.7.5940.1.1	128.912380034744	0	0,00	1.23053816944329e-20	XM_841124.1 Tbb GUTat10.1 hypothetical protein, conserved (Tb927.7.5940) partial mRNA
GLOS_TB927.7.6050.1.1	44.1124700187451	0	0,00	5.47605437010252e-05	XM_841135.1 Tbb GUTat10.1 receptor-type adenylate cyclase GRESAG 4, putative partial mRNA
GLOS_TB927.7.6090.1.1	29.1259078195926	0	0,00	2.02318354289899e-05	XM_841139.1 Tbb GUTat10.1 hypothetical protein, conserved (Tb927.7.6090) partial mRNA
GLOS_TB927.7.610.1.1	16.1954158442883	0	0,00	0.00278116418906016	XM_840592.1 Tbb strain 927/4 GUTat10.1 DNA ligase, putative (Tb927.7.610) partial mRNA
GLOS_TB927.7.6260.1.1	32.2823405686496	0	0,00	6.33587398078003e-06	XM_841156.1 Tbb GUTat10.1 hypothetical protein, conserved (Tb927.7.6260) partial mRNA

GLOS_TB927.7.6420.1.1	20.054410345488	0	0,00	0.000621978804179148	XM_841172.1 Tbb GUTat10.1 hypothetical protein, conserved (Tb927.7.6420) partial mRNA
GLOS_TB927.7.6770.1.1	19.0987026468487	0	0,00	0.000902028346384345	XP_846297.1 hypothetical protein [Trypanosoma brucei brucei strain 927/4 GUTat10.1]
GLOS_TB927.7.6850.1.1	102.436613071215	0	0,00	6.55492116469789e-09	XM_841212.1 Trypanosoma brucei brucei strain 927/4 GUTat10.1 trans-sialidase partial mRNA
GLOS_TB927.7.6900.1.1	31.1819780434408	0	0,00	9.42192895524018e-06	XM_841217.1 Tbb double-strand-break repair prot rad21 homolog, putat partial mRNA
GLOS_TB927.7.6970.1.1	25.1584221984659	0	0,00	9.09850605384415e-05	XM_841224.1 Tbb GUTat10.1 paraflagellar rod protein, putative (Tb927.7.6970) partial mRNA
GLOS_TB927.7.7090.1.1	56.3404002419067	0	0,00	8.94983307693217e-10	XM_841236.1 Tbb GUTat10.1 hypothetical protein, conserved (Tb927.7.7090) partial mRNA
GLOS_TB927.7.710.1.1	50.2806806902894	0	0,00	8.06326169551396e-09	XM_840602.1 Tbb GUTat10.1 heat shock 70 kDa protein, putative (Tb927.7.710) partial mRNA
GLOS_TB927.7.7110.1.1	30.1901066381591	0	0,00	1.36958874709015e-05	XM_841238.1 Tbb GUTat10.1 leucine-rich repeat protein (LRRP), putative partial mRNA
GLOS_TB927.7.7420.1.1	100.452870260652	0	0,00	1.79288220768985e-09	XM_841269.1 Tbb ATP synthase alpha chain, mitochondrial precursor partial mRNA
GLOS_TB927.7.7470.1.1	104.528847001706	0	0,00	3.12259302108394e-11	XM_841274.1 Tbbreceptor-type adenylate cyclase GRESAG 4, putative partial mRNA
GLOS_TB927.8.1110.1.1	228.988161903034	0	0,00	4.70049075718207e-34	XM_841855.1 Tbb GUTat10.1 40S ribosomal protein S9, putative (Tb927.8.1110) partial mRNA
GLOS_TB927.8.1330.1.1	344.515373102061	0	0,00	4.18894056042778e-12	XM_841876.1 Tbb GUTat10.1 60S ribosomal protein L7a, putative (Tb927.8.1330) partial mRNA
GLOS_TB927.8.1500.1.1	40.1811481042607	0	0,00	3.27633473930504e-07	XM_841891.1 Tbb GUTat10.1 hypothetical protein, conserved (Tb927.8.1500) partial mRNA
GLOS_TB927.8.1510.1.1	20.1267377587727	0	0,00	0.000612237591608327	XM_841892.1 Tbb GUTat10.1 ATP-dependent DEAD/H RNA helicase, putative partial mRNA
GLOS_TB927.8.1550.1.1	45.1405051306691	0	0,00	1.37002034676951e-05	XM_841896.1 Tbb GUTat10.1 hypothetical protein, conserved (Tb927.8.1550) partial mRNA
GLOS_TB927.8.1600.1.1	23.2108430945449	0	0,00	0.000192022919835166	XM_841901.1 Tbb GUTat10.1 lysyl-tRNA synthetase, putative (Tb927.8.1600) partial mRNA
GLOS_TB927.8.1620.1.1	174.197539991983	0	0,00	1.66867674444901e-26	XP_846996.1 major surface protease gp63 [Tbb] ref|XP_846997.1| major surface protease gp63
GLOS_TB927.8.1790.1.1	22.0381531560514	0	0,00	0.00114193162349596	XM_841919.1 Tbb GUTat10.1 hypothetical protein, conserved (Tb927.8.1790) partial mRNA
GLOS_TB927.8.1830.1.1	25.0137673718964	0	0,00	0.00335534660432802	XM_841923.1 Tbb GUTat10.1 tRNA-methyl transferase, putative (Tb927.8.1830) partial mRNA
GLOS_TB927.8.1870.1.1	41.2815106294695	0	0,00	2.21401097691727e-07	XM_841927.1 Tbb GUTat10.1 Golgi/lysosome glycoprotein 1 (Tb927.8.1870) partial mRNA
GLOS_TB927.8.1890.1.1	57.3322716471883	0	0,00	6.23859352680011e-10	XP_847022.1 cytochrome c1, heme protein, mitochondrial precursor [Tbb1]
GLOS_TB927.8.1990.1.1	37.0247153552038	0	0,00	0.0015114259308321	XP_847032.1 tryparedoxin peroxidase [Trypanosoma brucei brucei strain 927/4 GUTat10.1]
GLOS_TB927.8.2030.1.1	30.1539429315167	0	0,00	1.38029332263529e-05	XM_841943.1 Tbb GUTat10.1 hypothetical protein, conserved (Tb927.8.2030) partial mRNA
GLOS_TB927.8.2160.1.1	24.3473693263961	0	0,00	0.000127574865559671	XM_841956.1 Tbb GUTat10.1 multidrug resistance protein A (Tb927.8.2160) partial mRNA
GLOS_TB927.8.2470.1.1	16.159252137646	0	0,00	0.00280318659597305	XP_847080.1 hypothetical protein [Trypanosoma brucei brucei strain 927/4 GUTat10.1]
GLOS_TB927.8.2520.1.1	14.0670182071555	0	0,00	0.00615899928204462	XM_841992.1 Tbb GUTat10.1 acetyl-CoA synthetase, putative (Tb927.8.2520) partial mRNA
GLOS_TB927.8.2540.1.1	44.2932885519569	0	0,00	7.28682717232413e-08	XM_841994.1 Tbb GUTat10.1 3-ketoacyl-CoA thiolase, putative (Tb927.8.2540) partial mRNA
GLOS_TB927.8.2630.1.1	22.2189716892632	0	0,00	0.000280268272706973	XP_847096.1 kinesin [Trypanosoma brucei brucei strain 927/4 GUTat10.1]
GLOS_TB927.8.2640.1.1	43.4822356798871	0	0,00	1.01286520014078e-07	XP_847097.1 ubiquitin-activating enzyme E1 [Trypanosoma brucei brucei strain 927/4 GUTat10.1]
GLOS_TB927.8.2910.1.1	51.2002246822864	0	0,00	7.35181309760801e-07	XM_842029.1 Tbb mannosyl-oligosaccharide 1,2-alpha-mannosidase IB, putative partial mRNA
GLOS_TB927.8.3060.1.1	30.2624340514438	0	0,00	1.34845286515237e-05	XM_842044.1 Tbb GUTat10.1 cytosolic leucyl aminopeptidase, putative partial mRNA
GLOS_TB927.8.3100.1.1	22.0381531560514	0	0,00	0.00114193162349596	XM_842048.1 Tbb strain 927/4 GUTat10.1 coronin, putative (Tb927.8.3100) partial mRNA
GLOS_TB927.8.3150.1.1	42.2010546214664	0	0,00	1.55533924353495e-07	XM_842053.1 Tbb GUTat10.1 t-complex protein 1 gamma subunit, putative partial mRNA
GLOS_TB927.8.3380.1.1	34.1214285526435	0	0,00	1.06536966934273e-05	XP_847169.1 electron transfer protein [Trypanosoma brucei brucei strain 927/4 GUTat10.1]
GLOS_TB927.8.3690.1.1	28.2425275342381	0	0,00	2.87588295365255e-05	XP_847200.1 isocitrate dehydrogenase [Trypanosoma brucei brucei strain 927/4 GUTat10.1]
GLOS_TB927.8.3820.1.1	23.1746793879025	0	0,00	0.000193535463406783	XM_842120.1 Tbb GUTat10.1 hypothetical protein, conserved (Tb927.8.3820) partial mRNA
GLOS_TB927.8.3840.1.1	20.1267377587727	0	0,00	0.000612237591608327	XM_842122.1 Tbb GUTat10.1 hypothetical protein, conserved (Tb927.8.3840) partial mRNA
GLOS_TB927.8.4010.1.1	34.1575922592858	0	0,00	3.07695129569848e-06	XM_842139.1 Tbb GUTat10.1 flagellum-adhesion glycoprotein (Tb927.8.4010) partial mRNA
GLOS_TB927.8.4050.1.1	88.405758570702	0	0,00	2.21033288390312e-09	XM_842143.1 Tbb GUTat10.1 hypothetical protein, conserved (Tb927.8.4050) partial mRNA
GLOS_TB927.8.4330.1.1	20.1990651720575	0	0,00	0.000602670970874725	XP_847264.1 small GTP-binding protein Rab11 [Tbb brucei brucei strain 927/4 GUTat10.1]
GLOS_TB927.8.4400.1.1	18.2153223614941	0	0,00	0.00128833644925981	XM_842178.1 Tbb GUTat10.1 hypothetical protein, conserved (Tb927.8.4400) partial mRNA

GLOS_TB927.8.4700.1.1	112.93394643031	0	0,00	2.5300932051349e-18	XM_842208.1 Tbb GUTat10.1 amino acid transporter, putative (Tb927.8.4700) partial mRNA
GLOS_TB927.8.4780.1.1	25.1584221984659	0	0,00	9.09850605384415e-05	XP_847308.1 hypothetical protein [Trypanosoma brucei brucei strain 927/4 GUTat10.1]
GLOS_TB927.8.5070.1.1	27.0698375957445	0	0,00	0.000193176957744829	XM_842244.1 Tbb GUTat10.1 hypothetical protein, conserved (Tb927.8.5070) partial mRNA
GLOS_TB927.8.5120.1.1	26.0056387771781	0	0,00	0.00430058047112174	XP_847342.1 cytochrome c [Trypanosoma brucei brucei strain 927/4 GUTat10.1]
GLOS_TB927.8.5260.1.3	38.3058964136244	0	0,00	6.70640609230663e-07	XM_842263.1 Tbb GUTat10.1 60S ribosomal protein L39, putative (Tb927.8.5260) partial mRNA
GLOS_TB927.8.5260.2.3	56.2319091219795	0	0,00	2.13484770534938e-07	XM_842263.1 Tbb GUTat10.1 60S ribosomal protein L39, putative (Tb927.8.5260) partial mRNA
GLOS_TB927.8.5260.3.3	38.0889141737702	0	0,00	0.000124370195795884	XM_842263.1 Tbb GUTat10.1 60S ribosomal protein L39, putative (Tb927.8.5260) partial mRNA
GLOS_TB927.8.5470.1.1	60.271722156391	0	0,00	2.63968797441221e-08	XM_842284.1 Tbb GUTat10.1 flagellar calcium-binding protein (Tb927.8.5470) partial mRNA
GLOS_TB927.8.5600.1.1	16.159252137646	0	0,00	0.00280318659597305	XP_847390.1 transaldolase [Trypanosoma brucei brucei strain 927/4 GUTat10.1]
GLOS_TB927.8.5640.1.1	30.1177792248743	0	0,00	1.39109013062701e-05	XP_847394.1 hypothetical protein [Trypanosoma brucei brucei strain 927/4 GUTat10.1]
GLOS_TB927.8.5780.1.1	44.4379433785264	0	0,00	7.06837259822957e-08	XM_842315.1 Tbb GUTat10.1 protein tyrosine phosphatase, putative partial mRNA
GLOS_TB927.8.6000.1.1	44.2571248453146	0	0,00	7.34256508430987e-08	XM_842337.1 Tbb GUTat10.1 fatty acid desaturase, putative (Tb927.8.6000) partial mRNA
GLOS_TB927.8.6060.1.1	76.3224831741099	0	0,00	3.31656972088321e-08	XM_842343.1 Tbb GUTat10.1 2-amino-3-ketobutyrate coenzyme A ligase, putative partial mRNA
GLOS_TB927.8.6110.1.1	43.0482712001786	0	0,00	0.000649922733095283	XP_847441.1 hypothetical protein [Trypanosoma brucei brucei strain 927/4 GUTat10.1]
GLOS_TB927.8.6160.1.1	295.066457664546	0	0,00	6.22626536344739e-33	XM_842353.1 Tbb GUTat10.1 40S ribosomal protein S8, putative (Tb927.8.6160) partial mRNA
GLOS_TB927.8.6170.1.1	110.190765780257	0	0,00	2.92816537494625e-05	XM_842354.1 Tbb GUTat10.1 transketolase, putative (Tb927.8.6170) partial mRNA
GLOS_TB927.8.6180.1.2	81.4264950270878	0	0,00	4.12041836804767e-11	XM_842355.1 Tbb GUTat10.1 60S ribosomal protein L26, putative (Tb927.8.6180) partial mRNA
GLOS_TB927.8.6180.2.2	83.4464015442936	0	0,00	1.36421469672968e-11	XP_847448.1 60S ribosomal protein L26 [Trypanosoma brucei brucei strain 927/4 GUTat10.1]
GLOS_TB927.8.6210.1.1	15.0950533190795	0	0,00	0.00417062745104592	XM_842358.1 Tbb GUTat10.1 phosphatidylinositol 3-kinase, putative partial mRNA
GLOS_TB927.8.6240.1.1	31.2543054567255	0	0,00	9.27668759581189e-06	XP_847454.1 hypothetical protein [Trypanosoma brucei brucei strain 927/4 GUTat10.1]
GLOS_TB927.8.6440.1.1	30.1901066381591	0	0,00	1.36958874709015e-05	XM_842381.1 Tbb GUTat10.1 RNA-binding protein, putative (Tb927.8.6440) partial mRNA
GLOS_TB927.8.6450.1.1	31.3266328700102	0	0,00	9.13390220526645e-06	XP_847475.1 inhibitor of cysteine peptidase [Trypanosoma brucei brucei strain 927/4 GUTat10.1]
GLOS_TB927.8.650.1.1	21.1186091640544	0	0,00	0.000419044355280004	XP_846902.1 cation-transporting ATPase [Trypanosoma brucei brucei strain 927/4 GUTat10.1]
GLOS_TB927.8.6580.1.1	48.4054289996532	0	0,00	1.63439247154829e-08	XM_842395.1 Tbb GUTat10.1 succinate dehydrogenase flavoprotein, putative partial mRNA
GLOS_TB927.8.6640.1.1	18.2153223614941	0	0,00	0.00128833644925981	XM_842401.1 Tbb GUTat10.1 hypothetical protein, conserved (Tb927.8.6640) partial mRNA
GLOS_TB927.8.6660.1.1	38.2697327069821	0	0,00	6.75813722995807e-07	XM_842403.1 Tbb GUTat10.1 hypothetical protein, conserved (Tb927.8.6660) partial mRNA
GLOS_TB927.8.6750.1.1	35.2217910778523	0	0,00	2.08854072568386e-06	XM_842412.1 Tbb GUTat10.1 translationally controlled tumor prot. (TCTP), putative partial mRNA
GLOS_TB927.8.6970.1.1	55.4931836631945	0	0,00	1.24668894831255e-09	XM_842434.1 Tbb GUTat10.1 3-methylcrotonyl-CoA carboxylase, putative partial mRNA
GLOS_TB927.8.7020.1.1	28.2425275342381	0	0,00	2.87588295365255e-05	XP_847532.1 peptidase [Trypanosoma brucei brucei strain 927/4 GUTat10.1]
GLOS_TB927.8.7100.1.1	42.2733820347512	0	0,00	1.53173339567489e-07	XM_842447.1 Tbb GUTat10.1 acetyl-CoA carboxylase, putative (Tb927.8.7100) partial mRNA
GLOS_TB927.8.7120.1.1	53.5456045592734	0	0,00	2.55265455714277e-09	XM_842449.1 Tbb GUTat10.1 farnesyltransferase, putative (Tb927.8.7120) partial mRNA
GLOS_TB927.8.7150.1.1	71.2546350277743	0	0,00	6.7005860742428e-07	XM_842452.1 Tbb UDP-Gal or UDP-GlcNAc-dependent glycosyltransferase, putative partial mRNA
GLOS_TB927.8.7410.1.1	49.3249729916501	0	0,00	1.15204997275643e-08	XM_842477.1 Tbb strain 927/4 GUTat10.1 calreticulin, putative (Tb927.8.7410) partial mRNA
GLOS_TB927.8.7490.1.1	20.1990651720575	0	0,00	0.000602670970874725	XM_842485.1 Tbb GUTat10.1 hypothetical protein, conserved (Tb927.8.7490) partial mRNA
GLOS_TB927.8.760.1.1	99.46099885537	0	0,00	8.67413211429541e-10	XM_841820.1 Tbb GUTat10.1 nucleolar RNA-binding protein (Tb927.8.760) partial mRNA
GLOS_TB927.8.7940.1.1	32.3908316885767	0	0,00	6.19047602323976e-06	XM_842526.1 Tbb GUTat10.1 receptor-type adenylate cyclase GRESAG 4, putative partial mRNA
GLOS_TB927.8.7950.1.1	18.1791586548517	0	0,00	0.00129852321745928	XM_842527.1 Tbb GUTat10.1 hypothetical protein, conserved (Tb927.8.7950) partial mRNA
GLOS_TB927.8.7980.1.1	91.5983550264013	0	0,00	3.49622425301603e-15	XM_842530.1 Tbb vacuolar-type proton translocating pyrophosphatase 1 partial mRNA
GLOS_TB927.8.8200.1.1	25.3392407316777	0	0,00	8.74970546572211e-05	XP_847643.1 hypothetical protein [Trypanosoma brucei brucei strain 927/4 GUTat10.1]
GLOS_TB927.8.8240.1.1	26.18645731039	0	0,00	6.19450094774168e-05	XM_842554.1 Tbb GUTat10.1 amino acid transporter, putative (Tb927.8.8240) partial mRNA
GLOS_TB927.8.8250.1.1	20.0182466388456	0	0,00	0.00345370030582204	XM_842555.1 Tbb GUTat10.1 amino acid transporter, putative (Tb927.8.8250) partial mRNA

GLOS_TB927.8.8300.1.1	41.2453469228271	0	0,00	2.23102349759137e-07	XP_847653.1 amino acid transporter [Trypanosoma brucei brucei strain 927/4 GUTat10.1]
GLOS_TBA.1.1	1840.60463352242	0	0,00	2.25548482612608e-34	[BBH] TBA_TRYBR (sp|P04106) Tubulin alpha chain OS=T. brucei rhodesiense PE=3 SV=1
GLOS_TC00.1047053507017.	25.3030770250354	0	0,00	8.81825015345488e-05	XP_813091.1 ubiquitin hydrolase [Trypanosoma cruzi strain CL Brener]
GLOS_TC00.1047053508475.	82.4545301390119	0	0,00	3.44687281068132e-12	XP_804279.1 ribosomal protein S20 [T. cruzi strain CL Brener] ref|XP_809988.1| ribosomal protein S20 [T. cruzi strain CL Brener] ref|XP_809990.1| ribosomal protein S20 [T. cruzi]
GLOS_TC00.1047053508475.	52.1197686742833	0	0,00	5.89472984520294e-05	XP_804279.1 ribosomal protein S20 [T. cruzi strain CL Brener] ref|XP_809988.1| ribosomal prot. S20 [T. cruzi strain CL Brener] ref|XP_809990.1| ribosomal prot. S20 [T. cruzi strain CL Brener]
GLOS_TC00.1047053508823.	75.2582843555435	0	0,00	8.97408017858243e-07	XP_809984.1 hypothetical protein [Trypanosoma cruzi strain CL Brener]
GLOS_TC00.1047053511281.	47.4135575943715	0	0,00	2.35516057044345e-08	XP_811198.1 transporter [Trypanosoma cruzi strain CL Brener]
GLOS_TC00.1047053511805.	117.784812336791	0	0,00	4.86192262441854e-19	XP_804510.1 ribosomal protein S29 [T. cruzi] ref|XP_806920.1| ribosomal protein S29 [T. cruzi] ref|XP_808328.1| ribosomal protein S29 [T. cruzi strain CL Brener]
GLOS_TDX.1.1	93.7267526635341	0	0,00	1.70826236375548e-15	[BBH] TDX_TRYBR (sp|Q26695) Thioredoxin peroxidase OS=T. brucei rhodesiense PE=2 SV=1
GLOS_TH2A.1.1	410.919142223354	0	0,00	1.30490932091876e-13	[BBH] TH2A_TRYBB (sp|Q06222) Glucose transporter 2A OS=T. brucei brucei GN=THT2A PE=2 SV=1
GLOS_TRCOIV.1.1	89.7954307490498	0	0,00	6.72648600623979e-15	XM_001219103.1 Tbb cytochrome C oxidase subunit IV (PMID:12467979) (trCOIV) partial mRNA
GLOS_TULP4.1.1	85.4146930289193	0	0,00	9.62525855541802e-06	XM_005390525.1 PREDIT: C. lanigera tubby like protein 4 (Tulp4), transcript variant X4, mRNA
GLOS_TVAG_157670.1.1	61.5529032148117	0	0,00	1.40525066315727e-10	XP_001288661.1 hypothetical protein [Trichomonas vaginalis G3]
GLOS_TYPX.1.1	50.2445169836471	0	0,00	1.04783107522699e-08	[BBH] TYPX_TRYBB (sp|O77404) Tryparedoxin OS=Trypanosoma brucei brucei PE=1 SV=1
GLOS_TYTR.1.1	28.134036414311	0	0,00	2.94399465636344e-05	[BBH] TYTR_TRYBB (sp|P39051) Trypanothione reductase OS=T. b. brucei GN=TPR PE=1 SV=1

GN=Pepck PE=2 SV=2
