# Supplementary material for: RNA-seq de novo Assembly Reveals Differential Gene Expression in Glossina palpalis gambiensis Infected with Trypanosoma brucei gambiense vs. Non-Infected and Self-Cured Flies
Source: Front Microbiol. 2015 Nov 13;6:1259. doi: 10.3389/fmicb.2015.01259 (PMC4643127; doi:10.3389/fmicb.2015.01259)
Supplement: Supplementary file 3 [file Table3.PDF]

Supplementary Table S3: significantly differentially expressed genes (p<0.05) between infected tsetse flies (I10) and self-cured tsetse flies (NI10) 10 days post-infected bloodmeal

id	Base Mean A (I10)	Base Mean B (NI10)	Fold Change (NI10/I10)	pval	Best hit description
GLOS_contig_011291	66.1799029791024	1075.45081088531	16.2504138337119	4.43026976130682e-05	
GLOS_contig_003856	36.7047326033613	272.13726931621	7.41422835733418	1.26744137482751e-06	
GLOS_RS25.5.10	44.5773076305744	300.900363824775	6.75007935244603	4.48702180252512e-06	RS25_DROME (sp|P48588) 40S ribosomal protein S25 OS=D. melanogaster GN=RpS25 PE=1 SV=3
GLOS_contig_009185	110.893445312142	563.70330475056	5.08328786398378	0.000257461103283607	
GLOS_MLC_9020.1.1	318.820072240346	1124.69330119024	3.52767406796891	1.21861134165654e-05	YP_004400609.1 transmembrane protein [M. mycoides subsp. capri LC str. 95010] ref|WP_013729997.1| transmembrane protein [Mycoplasma mycoides]
GLOS_contig_002594	97.314377040103	305.563241416699	3.13995989812253	8.83306666234984e-05	
GLOS_LOC101461327.1.1	631.402907827938	1439.79202957998	2.28030630161801	3.47606825701985e-05	XP_004526299.1 PREDICTED: uncharacterized protein LOC101461327 [Ceratitis capitata]
GLOS_contig_009515	572.01989765826	1249.10870001645	2.18368050679717	6.01414375263381e-07	
GLOS_HYPB.1.2	4304.31511632924	8966.50530287255	2.08314332490584	9.26129492140137e-13	HYPB_HYPLI (sp|P35588) Hypodermin-B OS=Hypoderma lineatum PE=1 SV=1
GLOS_contig_011841	5629.06348875289	11187.830878368	1.98751193706053	3.8505647347517e-08	
GLOS_DPER_GL24331.1.3	1084.18595501592	2087.80998586621	1.92569362866867	4.94944175616519e-06	XP_002022319.1 GL24331 [Drosophila persimilis]
GLOS_PMAR_PMAR029216.1.2	1356.38415519895	2415.73855022174	1.78101354322249	4.6203380556504e-06	XP_002782358.1 conserved hypothetical protein [Perkinsus marinus ATCC 50983]
GLOS_DSIM_GD20325.1.4	8596.20811546765	15297.9357693153	1.77961440251648	3.5027022923684e-06	XP_002103263.1 GD20325 [Drosophila simulans]
GLOS_DMOJ_GI19764.1.1	7677.09564414956	12872.8615768816	1.67678796429892	1.49115093629744e-07	XP_002004165.1 GI19764 [Drosophila mojavensis]
GLOS_LOC101461142.7.9	6823.29226759137	10238.5963182963	1.50053609266111	4.51035760495085e-05	XP_004523338.1 PREDICTED: acyl-CoA Delta(11) desaturase-like isoform X3 [Ceratitis capitata] ref|XP_004523339.1| PREDIC.: acyl-CoA Delta(11) desaturase-like isoform X4 [C. capitata]
GLOS_DVIR_GJ18228.1.1	4751.38789169123	7095.38556673467	1.49332905005344	9.50243181621113e-05	XP_002059296.1 GJ18228 [Drosophila virilis]
GLOS_GPL.16.22	88429.6303085163	127653.433076573	1.44355950184583	5.82375939244455e-05	GPL_GLOFF (sp|Q8MUG0) Lectizyme OS=Glossina fuscipes fuscipes GN=Gpl PE=2 SV=1
GLOS_GPL.11.22	28498.3971841483	40606.4755944312	1.4248687507597	0.00012059383366798	GPL_GLOAU (sp|Q4TTV7) Lectizyme OS=Glossina austeni GN=Gpl PE=2 SV=1
GLOS_FDL.1.2	34480.1168668637	48644.5775796369	1.41080083247588	0.00018565141918138	FDL_DROME (sp|Q8WSF3) Prob. beta-hexosaminidase fdl OS=D. melanogaster GN=fdl PE=1 SV=1
GLOS_contig_008288	26845.2015214735	17851.2209637476	0.664968782203716	1.28906765065872e-05	
GLOS_RL31.2.3	5894.08826140414	3872.98900053312	0.657097218223614	7.80155465386826e-05	[BBH] RL31_DROME (sp|Q9V597) 60S ribosomal prot. L31 OS=D. melanog. GN=RpL31 PE=1 SV=1
GLOS_ND4.5.6	12286.9040471224	7978.02012351669	0.649310851042671	0.000237924224057469	YP_007026246.1 NADH dehydrogenase subunit 4 (mitochondrion) [Chrysomya bezziana]
GLOS_LOC101450586.2.9	7835.25071768231	4885.13778681407	0.623481999853491	0.000111144188392274	XP_004521992.1 PREDICTED: uncharacterized protein LOC101450586 [Ceratitis capitata]
GLOS_DWIL_GK13541.1.5	20239.3208727909	12499.6232966988	0.617591043457534	4.38949267245494e-07	XP_002072711.1 GK13541 [Drosophila willistoni]
GLOS_TRYDG.5.5	25628.5380217249	15491.6912014113	0.604470344281022	1.33463685185297e-07	TRYDG_DROER (sp|P54626) Trypsin delta/gamma OS=Drosophila erecta GN=deltaTry PE=3 SV=1
GLOS_LECA.9.13	3360.64221577791	1983.19334822931	0.590123321940789	7.17213675986716e-05	LECA_SARPE (sp|P05047) Lectin subunit alpha OS=Sarcophaga peregrina PE=1 SV=1
GLOS_contig_012866	10875.6581580806	6382.59483567672	0.586869754722337	1.07353336567137e-07	
GLOS_DGRI_GH11353.5.6	15691.214726566	9178.99071801695	0.584976426488925	3.40029140237571e-06	XP_001988793.1 GH11353 [Drosophila grimshawi]
GLOS_contig_000114	1389.96317177497	798.146782154513	0.574221532168572	0.000157910596672976	XP_004529509.1 PREDICTED: uncharacterized protein LOC101461922 isoform X1 [C. capitata]
GLOS_contig_010890	2142.86982053672	1223.47540461536	0.570951810926583	1.3087888943788e-05	
GLOS_ND1.2.4	9129.97403348242	5081.89604551235	0.556616703056928	8.57122376460978e-09	YP_007026263.1 NADH dehydrogenase subunit 1 (mitochondrion) [Chrysomya megacephala]
GLOS_TTI.10.16	988.992985772889	513.944729723846	0.519664686319492	8.2998273940799e-05	TTI_GLOMM (sp|O97373) Tsetse thrombin inhibitor OS=G. m. morsitans GN=TTI PE=1 SV=1
GLOS_MTRNR2L10.1.3	875.376537213099	451.024903089773	0.515235311795862	0.000136482608955043	NM_001193606.1 Pan troglodytes MTRNR2-like 10 (MTRNR2L10), mRNA
GLOS_LECA.3.13	8003.52181333989	3988.45786936398	0.498337852058604	1.95803093709127e-10	LECA_SARPE (sp|P05047) Lectin subunit alpha OS=Sarcophaga peregrina PE=1 SV=1
GLOS_ND3.1.1	1793.27110028323	869.653030740837	0.484953463312649	2.15284599516988e-07	YP_007517102.1 NADH dehydrogenase subunit 3 (mitochondrion) [Procecidochares utilis]
GLOS_LOC101462034.1.2	1260.99555992134	595.650610387858	0.472365351092127	9.24039234832033e-06	XP_004522257.1 PREDICTED: uncharacterized protein LOC101462034 [Ceratitis capitata]

GLOS_LECA.5.13	2385.92471108107	1113.98121408331	0.466897052077792	3.58354838365319e-08	LECA_SARPE (sp|P05047) Lectin subunit alpha OS=Sarcophaga peregrina PE=1 SV=1
GLOS_LECA.4.13	1427.78773863655	656.680223276177	0.459928465209587	1.27373944149782e-06	LECA_SARPE (sp|P05047) Lectin subunit alpha OS=Sarcophaga peregrina PE=1 SV=1
GLOS_contig_010976	542.097340517513	237.783008300625	0.43863526073311	0.000129319498618887	
GLOS_LOC101461571.2.2	2911.77937141102	1267.16399402137	0.435185442435262	1.2816461916788e-11	XP_004531314.1 PREDICTED: lysosomal aspartic protease-like [Ceratitis capitata]
GLOS_DWIL_GK15974.5.7	966.892338346344	420.27497369206	0.434665739942513	1.88485818294312e-05	XP_002075290.1 GK15974 [Drosophila willistoni]
GLOS_LECA.6.13	1794.03836512989	740.579040925993	0.412800002118335	1.34257975416511e-06	LECA_SARPE (sp|P05047) Lectin subunit alpha OS=Sarcophaga peregrina PE=1 SV=1
GLOS_LECA.10.13	3842.80835067959	1551.51344992415	0.403744685745197	9.95315910202037e-05	LECA_SARPE (sp|P05047) Lectin subunit alpha OS=Sarcophaga peregrina PE=1 SV=1
GLOS_DWIL_GK12502.1.1	905.983524502461	358.539663487707	0.395746339520474	2.39016468283814e-07	XP_002067848.1 GK12502 [Drosophila willistoni]
GLOS_contig_005481	529.282802400721	204.235352347757	0.385871884409217	1.48801808857524e-05	
GLOS_contig_004072	501.517079078372	191.784084659722	0.382407883321062	2.1848168208646e-05	
GLOS_GST.1.1	1624.86702655948	583.497779336584	0.359104942003834	3.14192118014464e-12	[BBH] GST_MUSDO (sp|P46437) Glutathione S-transferase OS=Musca domestica PE=2 SV=1
GLOS_DERE_GG10538.1.1	420.925460320751	149.708705379148	0.355665597574134	2.89282802523823e-05	XP_001970283.1 GG10538 [Drosophila erecta]
GLOS_contig_000837	2146.61510599774	678.104397490043	0.315894729146082	5.85515042400718e-17	WP_021127132.1 hypothetical protein, partial [[Clostridium] sordellii]
GLOS_contig_011756	1584.50583452085	263.496435342702	0.166295654835745	8.56758493573187e-09	
GLOS_DMOJ_GI24301.1.1	467.956036485454	46.8685139314539	0.100155805839061	3.60577900348658e-16	XP_001999054.1 GI24301 [Drosophila mojavensis]
GLOS_CG34109.1.1	384.620705805675	1.12581780517392	0.00292708579694283	1.3089973692447e-31	NP_001036351.2 CG34109 [Drosophila melanogaster]
