# Supplementary material for: RNA-seq de novo Assembly Reveals Differential Gene Expression in Glossina palpalis gambiensis Infected with Trypanosoma brucei gambiense vs. Non-Infected and Self-Cured Flies
Source: Front Microbiol. 2015 Nov 13;6:1259. doi: 10.3389/fmicb.2015.01259 (PMC4643127; doi:10.3389/fmicb.2015.01259)
Supplement: Supplementary file 4 [file Table4.PDF]

Supplementary Table S4: significantly differentially expressed genes (p<0.05) between infected tsetse flies(I20) and self-cured tsetse flies (NI20) 20 days post-infected bloodmeal

id	Base Mean A (I20)	Base Mean B (NI20)	FoldChange (NI20/I20)	pval	Best Hit Description
GLOS_contig_011116	11.312042659689	164.932867273743	14.5802904246012	0.00193000186862642	
GLOS_BBPLV_GP1.2.6	8866.26769809143	90548.2999818874	10.2126738177981	5.94208417179274e-07	YP_001285409.1 polyprotein [Brevicoryne brassicae picorna-like virus]
GLOS_DANA_GF24494.3.12	28.0310709980833	251.667016804859	8.97814488865115	0.0038367734375335	XP_001956624.1 GF24494 [Drosophila ananassae]
GLOS_LOC100116763.1.1	44.5324650275077	331.461237282362	7.44313698057403	0.000869493535494474	XP_001601177.1 PREDICTED: hypothetical protein LOC100116763 [Nasonia vitripennis]
GLOS_LOC101462313.1.1	347.692539080283	2517.50670187853	7.2406118018461	0.00297932516805255	XP_004517510.1 PREDICTED: uncharacterized protein LOC101462313 [Ceratitis capitata]
GLOS_AAEL_AAEL008876.1.1	16.086060088262	103.90018464478	6.45902005057135	7.74059722867862e-06	XP_001653478.1 deoxyribonuclease I, putative [Aedes aegypti]
GLOS_contig_006893	26.2195493983909	164.764386733643	6.2840281589185	9.2099616672897e-08	
GLOS_PMAR_PMAR029216.2.	160.975563153697	705.72414939665	4.38404522755319	0.00415333669013639	XP_002782358.1 conserved hypothetical protein [Perkinsus marinus ATCC 50983]
GLOS_contig_005917	169.222437946612	582.665507509798	3.4431929629428	1.66232226472372e-11	
GLOS_SLU7.13.13	36.0878906024003	108.340293659497	3.00212320119068	0.00248303759345507	SLU7_DROME (sp|Q9VAQ7) Pre-mRNA-splicing factor Slu7 OS=D. melanog. GN=Slu7 PE=1 SV=2
GLOS_contig_001185	320.302080775927	867.888019025374	2.70959219784938	1.81319726822662e-10	
GLOS_DANA_GF24494.12.12	158.578587101021	429.05467628124	2.7056280682329	1.00043572372948e-06	XP_001956624.1 GF24494 [Drosophila ananassae]
GLOS_CG15097.4.5	351.255778249336	864.4529243807	2.46103545595505	4.83481897235792e-09	NP_001188973.1 CG15097, isoform C [Drosophila melanogaster]
GLOS_LOC101459622.12.22	805.760643237546	1837.58114721091	2.28055460716909	1.64308143782593e-10	XP_004537835.1 PREDICTED: zinc metalloproteinase nas-4-like [Ceratitis capitata]
GLOS_LOC101454485.4.7	682.331961029118	1384.7574678426	2.02944834322879	0.000185385381479463	XP_004537801.1 PREDICTED: zinc metalloproteinase nas-4-like [Ceratitis capitata]
GLOS_contig_014009	1394.35632882097	2816.45854091414	2.01989870358007	0.000773652881585381	NR_076264.1 Sodalis glossinidius strain morsitans 23S ribosomal RNA, complete sequence
GLOS_LOC101459622.8.22	457.878048968665	909.889986199675	1.98718848446466	2.62422353998999e-06	XP_004537835.1 PREDICTED: zinc metalloproteinase nas-4-like [Ceratitis capitata]
GLOS_contig_002220	164.551092555932	324.753659662555	1.97357340275434	0.000973055774373687	
GLOS_DWIL_GK11188.1.1	1002.27851189422	1911.63597826633	1.9072901948716	0.000152357411289578	XP_002070141.1 GK11188 [Drosophila willistoni]
GLOS_LOC101454382.1.4	203.130986805307	367.255199586597	1.80797231068738	0.00287562078754078	XP_004522484.1 PREDICTED: uncharacterized protein LOC101454382 [Ceratitis capitata]
GLOS_LOC101453268.1.1	733.279844573071	1317.63865326042	1.7969110470063	0.00154731999882076	XP_004529547.1 PREDICTED: period circadian protein-like [Ceratitis capitata]
GLOS_LOC101459622.17.22	629.489818524357	1129.24474296822	1.79390469827675	3.19935110156998e-05	XP_004537835.1 PREDICTED: zinc metalloproteinase nas-4-like [Ceratitis capitata]
GLOS_DVIR_GJ22718.8.10	1878.68849751977	3274.11337982014	1.74276543670896	1.46968214281635e-06	XP_002054598.1 GJ22718 [Drosophila virilis]
GLOS_LOC101459622.1.22	1301.8231871912	2242.42720596867	1.72252824195496	7.00372407136869e-06	XP_004537835.1 PREDICTED: zinc metalloproteinase nas-4-like [Ceratitis capitata]
GLOS_LOC101459622.7.22	1175.45958827426	2001.84009232425	1.70302757516592	1.12298133465465e-05	XP_004537835.1 PREDICTED: zinc metalloproteinase nas-4-like [Ceratitis capitata]
GLOS_contig_013569	1904.62296413526	3236.63852469528	1.69935918323067	0.00390531636716592	
GLOS_contig_011873	345.15302163616	585.60689827529	1.69665876166833	0.00179940800791437	
GLOS_LRRX1.1.4	620.895058264207	1049.97909625204	1.69107336622615	0.000172014141451088	LRRX1_DICDI (sp|Q54G05) Putative leucine-rich repeat-containing protein DDB_G0290503 OS=Dictyostelium discoideum GN=DDB_G0290503 PE=4 SV=1
GLOS_DWIL_GK21356.5.5	1226.64803945797	2055.63075470154	1.6758113889049	0.00430264427352376	XP_002063538.1 GK21356 [Drosophila willistoni]
GLOS_CG15097.2.5	485.976568440675	812.869772295177	1.67265219165481	0.000656724636825291	NP_001188973.1 CG15097, isoform C [Drosophila melanogaster]
GLOS_PCNPT3_05895.1.1	310.706532121631	518.195773945974	1.66779813223598	0.0028943907659419	YP_007639959.1 type IV pilus assembly FimV-related transmembr prot [Psychromonas sp.] ref|WP_015465000.1| type IV pilus assembly FimV-related transmembrprot [Psychromonas]
GLOS_LOC101460048.2.4	840.792587205057	1382.57700227222	1.64437344395263	0.00215181083496741	XP_004537921.1 PREDICTED: probable RNA helicase armi-like isoform X2 [Ceratitis capitata]
GLOS_contig_006840	999.976563435839	1615.72265782309	1.61576052569832	0.000187907198503483	
GLOS_contig_009795	964.299360985012	1553.82835293299	1.61135474708371	0.0032436180494037	NR_076090.1 Proteus mirabilis HI4320 strain HI4320 23S ribosomal RNA, complete sequence
GLOS_DSEC_GM10148.1.1	1188.91574911369	1907.11439174623	1.60407866845732	0.00339923407469363	XP_002041441.1 GM10148 [Drosophila sechellia]
GLOS_contig_005746	359.795380268742	573.885986546386	1.59503433901162	0.0047435601038367	

GLOS_contig_002129	575.899745498387	916.665347293561	1.59170993642352	0.00115389625299365	
GLOS_contig_009274	1037.42077234321	1647.60704116411	1.58817625893751	0.000243046110998284	
GLOS_DANA_GF11702.1.1	533.526687537807	845.489992400115	1.58471921302006	0.00180389429870222	XP_001960091.1 GF11702 [Drosophila ananassae]
GLOS_AGAP_AGAP009641.2.2	563.369280135583	882.780716663389	1.56696637141971	0.00184677036355155	XP_318675.2 AGAP009641-PA [Anopheles gambiae str. PEST]
GLOS_LOC101459449.2.2	2643.66067850828	4018.1603125715	1.51992286500202	0.000243877769797144	XM_004521213.1 PREDIC: C. capitata eukaryotic translation initiat fact 3 subunit D-1-like, mRNA
GLOS_contig_013399	1218.57593096646	1804.26191651437	1.48063150655158	0.00144015759711973	NR_074525.1 Sodalis glossinidius str. 'morsitans' 16S ribosomal RNA, complete sequence
GLOS_DVIR_GJ20262.2.2	2385.46069365735	3475.5431325701	1.45696935682535	0.000800915805531803	XP_002050345.1 GJ20262 [Drosophila virilis]
GLOS_DVIR_GJ17492.1.1	1437.36999947528	2036.26699074705	1.41666167478826	0.00402122800300775	XP_002052322.1 GJ17492 [Drosophila virilis]
GLOS_DPSE_GA13007.1.1	5650.3367367767	7782.63609777853	1.37737562561948	0.00191863062365207	XP_001357586.2 GA13007 [Drosophila pseudoobscura pseudoobscura]
GLOS_DVIR_GJ12604.2.15	4596.9448666163	6319.8640430549	1.37479657172978	0.00249845471177484	XP_002046266.1 GJ12604 [Drosophila virilis]
GLOS_LOC101459895.9.9	33636.5530632153	46159.7404600397	1.37230887996427	0.0013643287655118	XP_004524083.1 PREDICTED: putative serine protease K12H4.7-like [Ceratitis capitata]
GLOS_contig_006678	3218.94903756889	4404.02030720765	1.36815471627776	0.00444035797563997	
GLOS_DVIR_GJ12156.1.1	13666.2918141108	18341.5914933477	1.34210448180314	0.00336842086268634	XP_002047025.1 GJ12156 [Drosophila virilis]
GLOS_LOC101450759.15.17	101392.560538357	76275.2604366462	0.752276695959281	0.00464641553680286	XP_004520156.1 PREDICTED: transmembrane protease serine 9-like [Ceratitis capitata]
GLOS_LOC101450402.3.11	35387.2262132498	26492.8664311823	0.74865620355581	0.00409542199397407	XP_004521991.1 PREDICTED: uncharacterized protein LOC101450402 [Ceratitis capitata]
GLOS_DVIR_GJ20013.2.2	7801.68216838096	5745.63718284794	0.736461324473603	0.00326458402309942	XP_002050776.1 GJ20013 [Drosophila virilis]
GLOS_HSP83.1.1	12643.9536104249	9049.70052590384	0.715733448946095	0.00244343192537794	[BBH] HSP83_DROAV (sp|O02192) Heat shock protein 83 OS=D. auraria GN=Hsp83 PE=3 SV=1
GLOS_LOC101459395.1.1	2507.53605621481	1756.30742804482	0.700411634637076	0.00512305352368076	XP_004525673.1 PREDICTED: gamma-glutamyl hydrolase-like [Ceratitis capitata]
GLOS_TB10.6K15.1500.1.1	10179.8555547676	7127.16060683668	0.700123942672132	0.00208537731057207	XM_818025.1 T. b. brucei strain 927/4 GUTat10.1 hypothet. Prot. (Tb10.6k15.1500) partial mRNA
GLOS_DWIL_GK25258.2.2	1640.97912290608	1147.8726701588	0.699504737224187	0.00476710193123178	XP_002071201.1 GK25258 [Drosophila willistoni]
GLOS_LOC101461063.16.16	1158.09230279855	807.629955622835	0.697379607541804	0.00603164346773595	XP_004518012.1 PREDICTED: probable fatty acid-binding protein-like [Ceratitis capitata]
GLOS_LOC101461063.7.16	1470.48010536161	1005.91496718506	0.684072476409124	0.00221477780795729	XP_004518012.1 PREDICTED: probable fatty acid-binding protein-like [Ceratitis capitata]
GLOS_LOC101450402.1.11	1912.20464576808	1300.24417929332	0.679971247936722	0.00196015697124694	XP_004521991.1 PREDICTED: uncharacterized protein LOC101450402 [Ceratitis capitata]
GLOS_LOC101461034.1.1	6698.07505631063	4520.03832297487	0.674826466555684	0.000173932896274394	XP_004530393.1 PREDICTED: acid trehalase-like protein 1-like [Ceratitis capitata]
GLOS_LOC101450402.11.11	2044.99130764506	1371.93084467763	0.670873680268839	0.000878445559575027	XP_004521991.1 PREDICTED: uncharacterized protein LOC101450402 [Ceratitis capitata]
GLOS_LOC101456033.1.1	1049.08070047014	701.789482002234	0.668956622391139	0.00441316010145174	XP_004525558.1 PREDICTED: platelet binding protein GspB-like [Ceratitis capitata]
GLOS_LOC101463546.1.1	22942.2243182338	15291.1898888565	0.666508603383477	4.33940084351878e-05	XM_004533853.1 PREDICTED: C. capitata 60S ribosomal protein L24-like (LOC101463546), mRNA
GLOS_TM120.1.1	2353.58594649691	1557.79789020954	0.661882729427481	0.000373153509160491	[BBH] TM120_DROME (sp|Q9U1M2) Transmembrane protein 120 homolog OS=D. melanogaster
GLOS_LOC101459622.11.22	24050.5093642372	15900.6378296145	0.661135179667279	0.00177683700936054	XP_004537835.1 PREDICTED: zinc metalloproteinase nas-4-like [Ceratitis capitata]
GLOS_contig_009491	3080.90857450226	2024.30003355303	0.657046447371477	0.00175609071528915	
GLOS_LOC101450039.1.1	16810.275989429	11001.1560494839	0.654430424366731	2.34216485708477e-05	XP_004521989.1 PREDICTED: uncharacterized protein LOC101450039 [Ceratitis capitata]
GLOS_LOC101454918.1.1	3298.3463247061	2156.38228557822	0.653776793972769	0.000185985346955083	XP_004527119.1 PREDICTED: uncharacterized protein LOC101454918 [Ceratitis capitata]
GLOS_LOC101461063.10.16	708.468773066393	461.874334228037	0.651933228092685	0.00468676602525856	XP_004518012.1 PREDICTED: probable fatty acid-binding protein-like [Ceratitis capitata]
GLOS_DPER_GL27219.2.2	1505.19938742585	976.828582500636	0.64896955889092	0.000788999482527074	XP_002023790.1 GL27219 [Drosophila persimilis]
GLOS_RL7.1.5	2437.11663836896	1580.03330516526	0.648320757525455	0.000215620236510678	RL7_DROME (sp|P32100) 60S ribosomal protein L7 OS=D. melanogaster GN=RpL7 PE=1 SV=2
GLOS_DSEC_GM15369.1.1	1053.74440141722	680.255033928303	0.645559808444439	0.00164911072522808	XP_002041100.1 GM15369 [Drosophila sechellia]
GLOS_LOC101450402.7.11	15185.5268195211	9756.61186424194	0.642494131431762	1.01976615201331e-05	XP_004521991.1 PREDICTED: uncharacterized protein LOC101450402 [Ceratitis capitata]
GLOS_LOC101461359.1.1	6189.80999778463	3970.13642767891	0.641398755228326	2.10505437063587e-05	XP_004535853.1 PREDIC: endoplasmic reticulum metallopeptidase 1-like isoform X1 [C. capitata]<br>ref|XP_004535854.1| PREDIC: endoplasmic reticulum metallopeptidase 1-like isoform X2<br>ref|XP_004535855.1| PREDICTED: endoplasmic reticulum metallopeptidase 1-like isoform X3<br>ref|XP_004535856.1| PREDIC: endoplasmic reticulum metallopeptidase 1-like isoform X4 [C.c.]

GLOS_DPER_GL10349.1.1	81155.3701118601	51767.9935558294	0.637887468007049	1.24097112308459e-05	XM_002016687.1 Drosophila persimilis GL10349 (DperGL10349), mRNA
GLOS_LOC101450586.3.9	28333.4047969086	17973.2426634074	0.63434814108075	3.86878077963694e-06	XP_004521992.1 PREDICTED: uncharacterized protein LOC101450586 [Ceratitis capitata]
GLOS_TB927.8.2530.1.1	894.275342403545	565.474383964935	0.632326932379807	0.00144289948703008	XP_847086.1 hypothetical protein [Trypanosoma brucei brucei strain 927/4 GUTat10.1]
GLOS_LOC101453488.1.1	1313.15516452767	829.830500696422	0.631936364500311	0.000401681001612761	XP_004518517.1 PREDIC: thiomorpholine-carboxylate DHasease-like isoform X1 [C. capitata]
GLOS_DMOJ_GI16517.2.2	5500.66422032068	3447.89355153138	0.626814037983648	0.000455452156364293	XP_002001579.1 GI16517 [Drosophila mojavensis]
GLOS_DVIR_GJ12604.6.15	9337.47972336939	5763.0649137482	0.617197047220856	0.000457691701970282	XP_002046266.1 GJ12604 [Drosophila virilis]
GLOS_LOC101457181.5.5	11790.1166064332	7254.71183423206	0.615321465970366	0.000131661011783457	XP_004519837.1 PREDICTED: laccase-2-like [Ceratitis capitata]
GLOS_C12A2.3.4	2193.30615427768	1347.31943889005	0.614286991472815	6.18583616566853e-05	C12A2_MUSDO (sp|O18635) Cyt. P450 CYP12A2 OS=Musca domestica GN=CYP12A2 PE=2 SV=1
GLOS_LOC101450402.5.11	24562.6178033084	14995.9595853421	0.610519599556783	1.03218051509581e-06	XP_004521991.1 PREDICTED: uncharacterized protein LOC101450402 [Ceratitis capitata]
GLOS_contig_011571	3391.63968709026	2064.40636308457	0.608675022568704	9.75920276088882e-06	
GLOS_DANA_GF11880.1.1	4849.85912098477	2934.09519155724	0.604985653884617	0.00010152226761666	XP_001965963.1 GF11880 [Drosophila ananassae]
GLOS_LOC101457181.3.5	1802.2091910683	1078.38960248588	0.598370937086741	0.00359059113453773	XP_004519837.1 PREDICTED: laccase-2-like [Ceratitis capitata]
GLOS_LOC101450586.9.9	18887.1477459254	11190.0961401491	0.592471467406357	2.49774234653054e-07	XP_004521992.1 PREDICTED: uncharacterized protein LOC101450586 [Ceratitis capitata]
GLOS_DANA_GF16453.1.1	3201.49736759648	1889.3229983856	0.590137295600531	4.13850637725955e-06	XP_001955025.1 GF16453 [Drosophila ananassae]
GLOS_LOC101450402.6.11	32451.2016091719	19075.3416540082	0.587816188865463	0.000446848224762399	XP_004521991.1 PREDICTED: uncharacterized protein LOC101450402 [Ceratitis capitata]
GLOS_LOC101454505.3.12	8857.13841801399	5131.74786346744	0.579391178197045	1.01741547379835e-05	XP_004525358.1 PREDICTED: acyl-CoA-binding protein homolog isoform X1 [Ceratitis capitata] ref|XP_004525359.1| PREDICT: acyl-CoA-binding protein homolog isoform X2 [C. capitata]
GLOS_ARC1.1.1	52718.9124072766	30490.7844376089	0.578365202264688	3.33501654152202e-08	NP_610955.1 Activity-regulated cytoskeleton associated protein 1 [Drosophila melanogaster]
GLOS_RPL26.1.3	21956.0501539691	12696.8397953756	0.578284331942118	4.22307050307335e-08	NP_649070.1 ribosomal prot. L26, isoform A [D. melanogaster] ref|NP_001262025.1| ribosomal protein L26, isoform B [D. melanogaster] ref|XP_001958190.1| GF23649 [D. ananassae] ref|XP_002042631.1| GM15002 [D. sechellia] ref|XP_002095681.1| GE19578 [D. yakuba] ref|XP_002095689.1| GE19574 [D. yakuba] ref|XP_002085421.1| GD14780 [Drosophila simulans]
GLOS_SODC.1.5	2617.47035536584	1513.28436525522	0.578147661597379	0.000843701857114995	SODC_CHYAM (sp|Q07182) Superoxide dismutase [Cu-Zn] OS=C. amoena GN=Sod PE=3 SV=2
GLOS_LOC101455604.7.10	7256.57529142334	4107.40210730225	0.566024872939285	8.34112405476911e-08	XP_004520096.1 PREDICTED: serine protease SP24D-like [Ceratitis capitata]
GLOS_contig_012940	1277.65272837825	722.561048590859	0.565537905991106	0.00155224224595672	
GLOS_CPIPJ_CPIJ000864.1.1	1474.91388176654	833.931692340752	0.565410430161477	0.000114123847496286	XP_001842487.1 multicopper oxidase [Culex quinquefasciatus]
GLOS_DVIR_GJ12604.1.15	1213.6992415	681.991972481892	0.561911838750946	2.64462496557353e-05	XP_002046266.1 GJ12604 [Drosophila virilis]
GLOS_USO1.1.1	1738.24805974645	973.424445157721	0.560003182341943	4.31434708394157e-06	USO1_YEAST (sp|P25386) Intracellular protein transport protein USO1 OS=S. cerevisiae
GLOS_DWIL_GK13541.5.5	5937.45526588436	3315.00572297801	0.558320959826929	6.03489389654549e-08	XP_002072711.1 GK13541 [Drosophila willistoni]
GLOS_LOC101450402.4.11	27612.0839006141	15267.7128521753	0.552935914113884	3.53377298462745e-09	XP_004521991.1 PREDICTED: uncharacterized protein LOC101450402 [Ceratitis capitata]
GLOS_contig_007349	8155.08835773521	4488.65742496396	0.550411869015056	4.61442715397888e-07	
GLOS_LOC101454212.1.1	433.292256214551	237.18102001131	0.547392704599516	0.00109683052831384	XP_004525356.1 PREDICTED: acyl-CoA-binding protein homolog isoform X1 [Ceratitis capitata] ref|XP_004525357.1| PREDICTED: acyl-CoA-binding protein homolog isoform X2 [C. capitata]
GLOS_DPSE_GA11668.1.3	43673.2595981549	23805.1525188168	0.545073867575995	1.54489226977575e-09	XP_001360538.1 GA11668 [Drosophila pseudoobscura pseudoobscura]
GLOS_LOC101450586.4.9	3450.27492295893	1871.67695342406	0.542471830569062	0.00113740113516192	XP_004521992.1 PREDICTED: uncharacterized protein LOC101450586 [Ceratitis capitata]
GLOS_LOC101450402.2.11	3222.46176428679	1743.22532130504	0.540960746415824	0.000647224156426735	XP_004521991.1 PREDICTED: uncharacterized protein LOC101450402 [Ceratitis capitata]
GLOS_contig_007723	374.25997394591	202.157764353126	0.540153311671911	0.00150073528265576	
GLOS_DWIL_GK13541.3.5	21901.4895747062	11743.4135671879	0.536192459747132	5.98088600809401e-06	XP_002072711.1 GK13541 [Drosophila willistoni]
GLOS_contig_007993	271.636211013573	143.56298715954	0.528511963202014	0.00546561232272124	
GLOS_LOC101450402.8.11	641.019495493016	336.406787917381	0.524799620421289	6.4002498422202e-05	XP_004521991.1 PREDICTED: uncharacterized protein LOC101450402 [Ceratitis capitata]

GLOS_DPSE_GA11668.2.3	2589.90513076008	1346.76186411716	0.520004322985344	2.38194092093176e-08	XP_001360538.1 GA11668 [Drosophila pseudoobscura pseudoobscura]
GLOS_contig_011049	9980.31373911611	5160.96394622946	0.517114399520524	1.26153591147918e-07	
GLOS_DWIL_GK13541.2.5	114912.974646858	59393.4196068733	0.516855644799003	2.12096210618517e-11	XP_002072711.1 GK13541 [Drosophila willistoni]
GLOS_DWIL_GK13541.4.5	4525.40805096965	2324.95751049924	0.51375643573204	0.000780196426233256	XP_002072711.1 GK13541 [Drosophila willistoni]
GLOS_TVAG_198570.1.2	1670.63330745084	838.776545909748	0.50207100634765	0.00124699471074599	XP_001582404.1 viral A-type inclusion protein [Trichomonas vaginalis G3]
GLOS_LOC101462178.1.1	12783.8514617528	6292.2105494358	0.492199910821952	9.03325335907833e-09	XP_004531052.1 PREDICTED: eukaryotic translation initiation factor 2D-like [Ceratitis capitata]
GLOS_LOC101450402.10.11	30785.4299979175	15111.2698390905	0.490857845419495	1.04575491541262e-09	XP_004521991.1 PREDICTED: uncharacterized protein LOC101450402 [Ceratitis capitata]
GLOS_TRYDG.4.5	205.385421466533	100.193956103796	0.48783382670674	0.00543078563896541	TRYDG_DROME (sp|P42276) Trypsin delta/gamma OS=D. melanogaster GN=deltaTry PE=2 SV=2
GLOS_contig_001123	254.834445314063	123.231035007325	0.483572912819743	0.00162175255382232	
GLOS_contig_008231	532.755823685815	248.834148266136	0.467069785449933	0.0032657927663446	
GLOS_LOC101460475.2.2	622.844475465759	282.118509080543	0.452951772381985	0.000633028469011932	XP_004523332.1 PREDICTED: venom carboxylesterase-6-like [Ceratitis capitata]
GLOS_contig_014337	370.909723296239	165.730618641181	0.446821984520517	0.000121192104089843	
GLOS_contig_014759	1251.90666981577	555.724718513612	0.443902674146941	0.000288964349552509	
GLOS_DWIL_GK12510.2.4	347.522418568463	152.677512912966	0.439331406422311	0.00196551313681258	XP_002067829.1 GK12510 [Drosophila willistoni]
GLOS_TTI.4.16	9152.82847843386	3778.11224082178	0.412780841433211	0.00585842719799519	TTI_GLOMM (sp|O97373) Tsetse thrombin inhibitor OS=G. m. morsitans GN=TTI PE=1 SV=1
GLOS_contig_006723	316.338796552901	129.073077785617	0.408021650180466	5.71115816751612e-05	
GLOS_contig_009631	396.706294309873	160.523715560785	0.404641211554354	0.00149913882337999	
GLOS_LOC101459427.1.1	334.67929130239	131.11015124709	0.391748622201512	1.82086655837686e-05	XP_004534903.1 PREDICT: solute carrier family 2, facilitated glucose transporter member 1-like
GLOS_contig_007356	305.857421775537	119.257585150404	0.389912346929821	2.88401692679512e-05	
GLOS_contig_001176	133.992794346908	51.3488443615319	0.383220938199031	0.0032685137256753	
GLOS_TB927.8.3880.1.1	823.44059061653	307.893628327332	0.373911162305959	5.19217451745766e-10	XM_842126.1 T. b. brucei strain 927/4 GUTat10.1 hypothetical prot., conserved partial mRNA
GLOS_DPER_GL27219.1.2	548.841883774077	204.762194038418	0.373080481085706	1.06271898492339e-07	XP_002023790.1 GL27219 [Drosophila persimilis]
GLOS_contig_007915	108.160999769308	38.3615658172078	0.354670961797947	0.00469197001943204	
GLOS_TB11.01.6590.1.1	6447.56977261086	2236.92799314113	0.346941261906704	0.00116864798839945	XM_824460.1 T. b. brucei strain 927/4 GUTat10.1 hypothetical protein partial mRNA
GLOS_contig_014405	300.177643547118	82.3986501627943	0.274499623586593	6.84531977243797e-08	
GLOS_TVAG_012450.1.1	120.358868552065	25.8080328387344	0.214425685030184	0.000522857730630966	XP_001323102.1 viral A-type inclusion protein [Trichomonas vaginalis G3]
GLOS_contig_005292	144.351562409601	30.0138341294604	0.207921782268593	0.00332002031236257	
GLOS_TB10.100.0120.1.1	306.810696372533	62.3668329184616	0.203274636953124	1.07785977227751e-10	XM_817190.1 T. b. brucei strain 927/4 GUTat10.1 proteasome subunit alpha 5 partial mRNA
GLOS_LOC101455536.1.2	854.418868556306	166.766590312983	0.195181305622107	6.89448872571521e-05	XP_004536445.1 PREDICTED: protein halfway-like [Ceratitis capitata]
GLOS_LRRX1.3.4	146.115570212144	25.1070659569467	0.171830188394666	0.000898147419238198	LRRX1_DICDI (sp|Q54G05) Putative leucine-rich repeat-containing protein DDB_G0290503
GLOS_PTSG_02057.1.1	374.355001540209	58.6606043774774	0.15669779790875	1.86507720185737e-08	XM_004997989.1 Salpingoeca sp. ATCC 50818 hypothet. Prot. (PTSG_02057) mRNA, complete cds
GLOS_TB927.8.3750.1.1	519.0943187706	80.830192149305	0.155713883251005	8.67310471092905e-11	XM_842113.1 Tbb strain 927/4 GUTat10.1 nucleolar protein, putative (Tb927.8.3750) partial mRNA
GLOS_TTI.1.16	1304.64014297463	185.258950555399	0.142000038518669	0.000138538181827885	TTI_GLOMM (sp|O97373) Tsetse thrombin inhibitor OS=G. m. morsitans GN=TTI PE=1 SV=1
GLOS_TVAG_087170.1.1	108.801612463034	14.356298715954	0.131949319416857	2.23256143570634e-06	XP_001318162.1 viral A-type inclusion protein [Trichomonas vaginalis G3]
GLOS_LRRX1.4.4	85.7146794053444	9.51535772733135	0.111011996933842	6.92573275772957e-06	LRRX1_DICDI (sp|Q54G05) Putative leucine-rich repeat-containing protein DDB_G0290503
GLOS_TB927.3.4650.1.1	345.75841076592	14.7571306898593	0.0426804677207113	5.90800240017656e-10	XM_838964.1 Trypanosoma brucei C-8 sterol isomerase, putative (Tb927.3.4650) partial mRNA
GLOS_TB927.8.3460.1.1	333.077759568073	13.9554667420487	0.0418985247173087	2.38717206412522e-12	XM_842084.1 Tbb strain 927/4 GUTat10.1 hypothetical prot., conserved partial mRNA
GLOS_TB927.6.4980.2.2	890.894513979496	11.318123464812	0.0127042239987039	4.43204537101996e-11	XM_840522.1 T. b. brucei strain 927/4 GUTat10.1 40S ribosomal protein S14 partial mRNA
GLOS_HSP70.1.1	378.808712621918	4.40719542277185	0.011634356011158	4.36864419977951e-13	XM_946483.1 T. b. brucei strain 927/4 GUTat10.1 heat shock protein 78 (hsp70) partial mRNA
GLOS_TB10.389.0570.1.1	331.26623796838	3.17178590911045	0.00957473338835454	2.06132305538982e-18	XM_822687.1 Tbb strain 927/4 GUTat10.1 hypothetical protein (Tb10.389.0570) partial mRNA
GLOS_TB11.02.0740.1.2	320.199408738033	2.06998705341749	0.00646468106101664	2.93768986010744e-15	XM_823312.1 Tbb GUTat10.1 60S ribosomal protein L44 (Tb11.02.0740) partial mRNA

GLOS_TB10.70.3660.1.1	333.93600657077	2.06998705341749	0.00619875369138669	2.3688957082742e-27	XM_817527.1 T. GUTat10.1 proteasome activator protein PA26 (Tb10.70.3660) partial mRNA
GLOS_TB927.1.2370.1.2	4084.01640276038	24.0052671012538	0.00587785766115647	7.34493349151939e-24	XM_001218936.1 Trypanosoma brucei brucei strain 927/4 GUTat10.1 beta tubulin partial mRNA
GLOS_CRAM.1.1	558.192218999031	3.17178590911045	0.00568224672640225	1.05692998254503e-30	CRAM_TRYBB (sp|Q03650) Cysteine-rich, acidic integral membrane protein OS=Tbb
GLOS_TB927.3.2880.1.1	393.356043660202	2.20359771138592	0.00560204361138401	4.29624287407086e-44	XP_843881.1 hypothetical protein [Trypanosoma brucei brucei strain 927/4 GUTat10.1]
GLOS_TB11.01.7190.1.1	446.693258813235	2.20359771138592	0.00493313401961873	3.93333867348152e-09	XP_829613.1 NADH-cytochrome b5 reductase [T. b.i strain 927/4 GUTat10.1]
GLOS_TB927.3.5520.1.1	553.513229164757	2.20359771138592	0.00398111119170741	9.27221240161892e-20	XM_839049.1 Tbb 26S proteasome regulatory non-ATPase subunit (Tb927.3.5520) partial mRNA
GLOS_TB927.5.2260.1.2	246.852718627267	0.968188197724528	0.0039221289646254	1.14774615589718e-22	XM_839807.1 T. b. brucei strain 927/4 GUTat10.1 hypothetical protein partial mRNA
GLOS_TB927.6.2100.2.2	291.139970225433	1.10179885569296	0.00378443006241921	8.03924894752137e-19	XP_845331.1 40S ribosomal protein S30 [Trypanosoma brucei brucei strain 927/4 GUTat10.1]
GLOS_TB11.02.3860.1.1	589.035599990952	2.20359771138592	0.00374102636821913	1.28140535076e-17	XP_828679.1 hypothetical protein [Trypanosoma brucei brucei strain 927/4 GUTat10.1]
GLOS_TB11.02.5400.1.1	299.734730485583	1.10179885569296	0.00367591321135192	2.19110105136734e-30	XM_823735.1 Tbb GUTat10.1 cystathionine beta-synthase (Tb11.02.5400) partial mRNA
GLOS_TB11.50.0001.1.1	309.425306734178	1.10179885569296	0.00356079102682929	8.9489854430512e-11	XM_823072.1 Tbb strain 927/4 GUTat10.1 hypothetical protein (Tb11.50.0001) partial mRNA
GLOS_TB10.61.2180.1.1	311.727255192554	1.10179885569296	0.00353449638214143	3.51591394477038e-12	XM_822840.1 Tbb GUTat10.1 proteasome regulatory non-ATPase subunit 8 partial mRNA
GLOS_TB927.5.4190.1.3	313.886662259482	1.10179885569296	0.00351018054657618	1.21829490708135e-37	XM_839996.1 Trypanosoma brucei brucei strain 927/4 GUTat10.1 histone H4 partial mRNA
GLOS_TB11.18.0002.1.1	316.453758823978	1.10179885569296	0.00348170569939673	1.10127143419746e-16	XM_823219.1 Tbb strain 927/4 GUTat10.1 hypothetical protein (Tb11.18.0002) partial mRNA
GLOS_TB927.6.3840.1.1	651.030378088474	2.20359771138592	0.00338478477433884	1.2860475623907e-47	XM_840409.1 T. b. brucei strain 927/4 GUTat10.1 reticulon domain protein partial mRNA
GLOS_TB11.02.0760.1.1	651.370619112115	2.20359771138592	0.00338301674458337	1.12256874705981e-30	XP_828407.1 dynein heavy chain [Trypanosoma brucei brucei strain 927/4 GUTat10.1]
GLOS_TB11.03.0475.1.1	326.567313457329	1.10179885569296	0.00337387978003172	6.95567304039185e-33	XP_828152.1 hypothetical protein [Trypanosoma brucei brucei strain 927/4 GUTat10.1]
GLOS_TB10.6K15.0280.1.1	336.040255396954	1.10179885569296	0.00327877043895066	5.80415112328165e-21	XM_818129.1 Tbb strain 927/4 GUTat10.1 hypothetical protein (Tb10.6k15.0280) partial mRNA
GLOS_TB09.211.0040.1.1	691.817193201926	2.20359771138592	0.00318523120419579	4.21995141375279e-44	XM_822050.1 Tbb strain 927/4 GUTat10.1 hypothetical protein (Tb09.211.0040) partial mRNA
GLOS_TB927.5.3590.1.1	348.871092429843	1.10179885569296	0.00315818329348835	4.92558089320629e-40	XM_839936.1 T. b. brucei strain 927/4 GUTat10.1 hypothetical protein partial mRNA
GLOS_TB927.8.5470.1.1	351.635888626532	1.10179885569296	0.00313335154723973	1.8590204152518e-24	XM_842284.1 Tbb GUTat10.1 flagellar calcium-binding protein (Tb927.8.5470) partial mRNA
GLOS_TB927.8.710.1.1	355.721779564228	1.10179885569296	0.00309736124968987	4.29052420854272e-14	XM_841815.1 Tbb GUTat10.1 RNA-binding protein, putative (Tb927.8.710) partial mRNA
GLOS_TB927.6.2230.1.1	355.7493586846	1.10179885569296	0.00309712112979462	7.913386978193e-28	XM_840251.1 T. b. brucei strain 927/4 GUTat10.1 hypothetical protein partial mRNA
GLOS_TB927.2.4330.1.1	315.547998024131	0.968188197724528	0.00306827552000658	6.15170402071892e-17	XP_951628.1 paraflagellar rod protein [Trypanosoma brucei brucei strain 927/4 GUTat10.1]
GLOS_TB10.389.0510.1.1	359.835249622297	1.10179885569296	0.00306195364920327	1.57628095540002e-15	XP_827786.1 3' 5'-cyclic nucleotide phosphodiesterase [Trypanosoma brucei TREU927]
GLOS_TC00.1047053507017.120.1.1	362.259804795344	1.10179885569296	0.00304146041351569	2.24219203734048e-18	XP_813091.1 ubiquitin hydrolase [Trypanosoma cruzi strain CL Brener]
GLOS_TB11.02.2510.1.1	321.010141943581	0.968188197724528	0.00301606731756996	6.08519554254952e-36	XP_828565.1 guanine nucleotide-binding subunit beta-like protein [Tbb]
GLOS_TB927.4.3920.1.1	365.392421136045	1.10179885569296	0.00301538508179055	5.87105574262952e-15	XM_839449.1 Tbb GUTat10.1 hypothetical protein, conserved (Tb927.4.3920) partial mRNA
GLOS_TB09.211.0120.2.2	366.938794629618	1.10179885569296	0.00300267748141784	9.55530361764948e-39	XP_827151.1 nascent polypeptide associated complex subunit [Trypanosoma brucei] ref|XP_827152.1| nascent polypeptide associated complex subunit [Trypanosoma brucei]
GLOS_TB927.7.2160.1.1	367.40164236793	1.10179885569296	0.00299889474797061	4.69587218447747e-21	XM_840746.1 Tbb GUTat10.1 hypothetical protein, conserved (Tb927.7.2160) partial mRNA
GLOS_TB11.01.1625.1.1	367.6192766769	1.10179885569296	0.00299711937211968	1.25452430983402e-12	XP_829050.1 hypothetical protein [Trypanosoma brucei brucei strain 927/4 GUTat10.1]
GLOS_TB11.02.5270.1.1	369.213163967623	1.10179885569296	0.00298418085599348	2.06155061049176e-20	XP_828815.1 haloacid dehalogenase hydrolase [T. brucei brucei strain 927/4 GUTat10.1]
GLOS_TB927.5.1470.1.1	370.949592649794	1.10179885569296	0.002970211795685	1.94505990906777e-14	XP_844821.1 NADH-cytochrome b5 reductase [T. brucei brucei strain 927/4 GUTat10.1]
GLOS_TB927.5.3510.1.1	371.052264687687	1.10179885569296	0.00296938992306203	7.4407013734721e-44	XM_839928.1 Tbb GUTat10.1 structural maintenance of chromosome 3 partial mRNA
GLOS_TB927.6.2290.1.1	373.666875049332	1.10179885569296	0.00294861259925033	5.88084330247529e-14	XM_840257.1 T. brucei brucei strain 927/4 GUTat10.1 hypothetical protein partial mRNA
GLOS_TB927.8.3820.1.1	379.204111886303	1.10179885569296	0.00290555619297534	2.8485314830335e-36	XM_842120.1 Tbb GUTat10.1 hypothetical protein, conserved (Tb927.8.3820) partial mRNA
GLOS_TB927.7.3980.1.1	380.940540568474	1.10179885569296	0.00289231189216238	6.6234720703183e-23	XM_840928.1 Tbb strain 927/4 GUTat10.1 immunodominant antigen, putative partial mRNA
GLOS_TB10.70.7730.1.1	716.367762392073	2.06998705341749	0.00288955919303997	6.46777271084839e-22	XM_817219.1 Tbb strain 927/4 GUTat10.1 ATP-dependent DEAD/H RNA helicase partial mRNA
GLOS_TB11.03.0030.1.1	386.007285223538	1.10179885569296	0.00285434730863928	1.54449349468977e-18	XM_823107.1 Tbb strain 927/4 GUTat10.1 ABC transporter (Tb11.03.0030) partial mRNA
GLOS_TB927.3.5180.1.1	386.940625143757	1.10179885569296	0.00284746233426283	3.82160381540255e-38	XP_844108.1 cofilin/actin depolymerizing factor [Trypanosoma brucei TREU927]

GLOS_TB927.6.4370.1.1	389.242573602133	1.10179885569296	0.00283062267700237	8.79197928881031e-46	XP_845554.1 eukaryotic translation initiation factor 3 subunit 7-like protein [Tbb]
GLOS_TB11.01.5680.1.1	391.912342204522	1.10179885569296	0.00281134002949563	4.94703533127747e-46	XM_824372.1 Tbb strain 927/4 GUTat10.1 hypothetical protein (Tb11.01.5680) partial mRNA
GLOS_TB10.61.2300.1.1	397.68714802724	1.10179885569296	0.00277051662634441	2.18506998650759e-28	XM_822830.1 Tbb strain 927/4 GUTat10.1 hypothetical protein (Tb10.61.2300) partial mRNA
GLOS_TB927.1.1670.1.1	397.68714802724	1.10179885569296	0.00277051662634441	2.18506998650759e-28	XM_001218868.1 T. brucei hypothetical protein, conserved (Tb927.1.1670) partial mRNA
GLOS_TB927.3.4040.1.1	400.159216997437	1.10179885569296	0.00275340116856541	1.24849084308147e-24	XM_838903.1 Trypanosoma brucei hypothetical protein, conserved (Tb927.3.4040) partial mRNA
GLOS_TB09.211.2880.1.1	400.669578532899	1.10179885569296	0.00274989396431677	1.52760013380954e-12	XM_822340.1 Tbb strain 927/4 GUTat10.1 hypothetical protein (Tb09.211.2880) partial mRNA
GLOS_TB927.5.860.1.1	752.851052258858	2.06998705341749	0.00274953066374377	1.00254472746215e-43	XM_839667.1 T. brucei brucei strain 927/4 GUTat10.1 hypothetical protein partial mRNA
GLOS_TB927.4.4310.1.1	352.664256141049	0.968188197724528	0.00274535391910344	3.12619564315293e-25	XM_839488.1 Tbb GUTat10.1 hypothetical protein, conserved (Tb927.4.4310) partial mRNA
GLOS_TB927.4.1660.1.1	353.957771761685	0.968188197724528	0.00273532120203422	2.84487779360599e-11	XM_839223.1 Tbb GUTat10.1 mitochondrial carrier protein, putative partial mRNA
GLOS_TB927.7.6770.1.1	407.603003028399	1.10179885569296	0.0027031176107802	3.3848693410588e-33	XP_846297.1 hypothetical protein [Trypanosoma brucei brucei strain 927/4 GUTat10.1]
GLOS_TB927.8.5600.1.1	359.882763419447	0.968188197724528	0.00269028777184334	4.74247905203811e-12	XP_847390.1 transaldolase [Trypanosoma brucei brucei strain 927/4 GUTat10.1]
GLOS_TB10.389.0730.1.1	413.037567827477	1.10179885569296	0.0026675511902907	1.09807034236102e-29	XP_823483.1 cholinephosphate cytidylyltransferase A [Trypanosoma brucei TREU927]
GLOS_TB09.211.0560.1.1	1262.98279062529	3.30539656707889	0.00261713508023526	3.63984738197992e-36	XM_822105.1 Tbb strain 927/4 GUTat10.1 hypothetical protein (Tb09.211.0560) partial mRNA
GLOS_TB11.01.8520.1.1	421.482142252584	1.10179885569296	0.00261410566484376	1.11902192831605e-20	XM_824641.1 Tbb GUTat10.1 glucosamine-6-phosphate isomerase (Tb11.01.8520) partial mRNA
GLOS_TB10.70.1190.1.1	422.585602684623	1.10179885569296	0.00260727968178139	5.69777996870052e-30	XP_822821.1 valosin-containing protein homolog [Trypanosoma brucei TREU927]
GLOS_TB927.6.950.1.1	798.186606048318	2.06998705341749	0.00259336230116117	1.71647440819287e-22	XM_840124.1 T. brucei brucei strain 927/4 GUTat10.1 cysteinyl-tRNA synthetase partial mRNA
GLOS_contig_000050	373.817060884375	0.968188197724528	0.00259000537705259	1.39161652130875e-27	
GLOS_TB927.7.6910.1.1	375.628582484068	0.968188197724528	0.00257751471233048	1.40886840295492e-26	XM_841218.1 Tbb GUTat10.1 hypothetical protein, conserved (Tb927.7.6910) partial mRNA
GLOS_TB09.160.4460.1.1	428.19028799552	1.10179885569296	0.00257315237309747	4.63466973328308e-21	XM_821897.1 Tbb GUTat10.1 hypothetical protein (Tb09.160.4460) partial mRNA
GLOS_TB11.39.0004.1.1	428.360408507341	1.10179885569296	0.00257213046259872	6.58578636915171e-17	XM_823178.1 Tbb strain 927/4 GUTat10.1 hypothetical protein (Tb11.39.0004) partial mRNA
GLOS_TB10.61.1970.1.1	376.609436201435	0.968188197724528	0.00257080175018949	2.3548231700753e-41	XM_822855.1 Tbb strain 927/4 GUTat10.1 hypothetical protein (Tb10.61.1970) partial mRNA
GLOS_TB11.02.0530.1.1	377.26998357194	0.968188197724528	0.00256630063319074	8.46055101313432e-36	XM_823292.1 Tbb GUTat10.1 phosphoribosylpyrophosphate synthetase partial mRNA
GLOS_TB927.7.4450.1.1	381.015633485996	0.968188197724528	0.00254107210474899	1.10435321342238e-34	XM_840975.1 Tbb GUTat10.1 hypothetical protein, conserved (Tb927.7.4450) partial mRNA
GLOS_TB11.01.2310.1.1	434.190372570803	1.10179885569296	0.00253759393412919	5.21951672468385e-32	XM_824025.1 Tbb strain 927/4 GUTat10.1 hypothetical protein (Tb11.01.2310) partial mRNA
GLOS_TB10.61.1790.1.1	382.534427859197	0.968188197724528	0.00253098316703902	4.57802147928635e-45	XP_827964.1 hypothetical protein [Trypanosoma brucei brucei strain 927/4 GUTat10.1]
GLOS_TB11.01.3320.1.1	436.124500885166	1.10179885569296	0.00252634019289613	2.33631194824974e-32	XM_824120.1 T. b. brucei strain 927/4 GUTat10.1 trichohyalin (Tb11.01.3320) partial mRNA
GLOS_TB927.4.760.1.1	384.591162888231	0.968188197724528	0.00251744785411489	2.81251137233947e-45	XM_839133.1 Tbb strain 927/4 GUTat10.1 gamma-adaptin 1, putative partial mRNA
GLOS_TB927.8.8300.1.1	440.605791087248	1.10179885569296	0.00250064542495944	1.95145856221268e-42	XP_847653.1 amino acid transporter [Trypanosoma brucei brucei strain 927/4 GUTat10.1]
GLOS_TB927.8.8240.1.1	443.880948819398	1.10179885569296	0.0024821945132438	1.22331214526899e-14	XM_842554.1 Tbb GUTat10.1 amino acid transporter, putative (Tb927.8.8240) partial mRNA
GLOS_TB10.6K15.3460.1.1	890.241611052586	2.20359771138592	0.00247528051264698	2.67286108365039e-30	XP_822958.1 hypothetical protein [Trypanosoma brucei brucei strain 927/4 GUTat10.1]
GLOS_TB927.3.1210.1.1	449.323158062069	1.10179885569296	0.00245213013378838	5.74102763792341e-52	XM_838622.1 T. brucei protein transport protein Sec24C, putative (Tb927.3.1210) partial mRNA
GLOS_TB927.8.4010.1.1	450.589094562334	1.10179885569296	0.00244524083913562	6.3333184176948e-15	XM_842139.1 Tbb GUTat10.1 flagellum-adhesion glycoprotein (Tb927.8.4010) partial mRNA
GLOS_TB927.5.1250.1.1	403.434374729587	0.968188197724528	0.00239986540158727	4.98160254798079e-10	XM_839706.1 T. b. brucei strain 927/4 GUTat10.1 hypothetical protein partial mRNA
GLOS_TB11.02.0980.1.1	465.401573706737	1.10179885569296	0.00236741540626426	3.07717407975507e-17	XM_823338.1 Tbb strain 927/4 GUTat10.1 hypothetical protein (Tb11.02.0980) partial mRNA
GLOS_TB927.5.4460.1.1	471.61929259099	1.10179885569296	0.00233620395306537	1.23225651959268e-15	XM_840023.1 T. b. brucei strain 927/4 GUTat10.1 major vault protein partial mRNA
GLOS_TB09.211.4513.1.1	950.349774632895	2.20359771138592	0.00231872282206532	7.10741364642572e-54	XM_822500.1 Trypanosoma brucei brucei strain 927/4 GUTat10.1 kinetoplastid membrane protein KMP-11 partial mRNA. nuclear gene for plastid product
GLOS_TB927.4.2080.1.1	476.448468260306	1.10179885569296	0.00231252470957887	2.34196248657373e-53	XM_839265.1 Tbb GUTat10.1 hypothetical protein, conserved (Tb927.4.2080) partial mRNA
GLOS_TB927.6.5080.1.1	422.462995969952	0.968188197724528	0.00229177042003791	1.24953016896237e-28	XM_840532.1 T. brucei brucei strain 927/4 GUTat10.1 hypothetical protein partial mRNA
GLOS_TB927.5.1090.1.1	483.564303500811	1.10179885569296	0.00227849501651876	5.70779374135242e-12	XP_844783.1 threonyl-tRNA synthetase [Trypanosoma brucei brucei strain 927/4 GUTat10.1]
GLOS_TB927.8.5260.3.3	427.747374933986	0.968188197724528	0.00226345795312934	1.78837418734174e-14	XM_842263.1 Tbb GUTat10.1 60S ribosomal protein L39, putative (Tb927.8.5260) partial mRNA

GLOS_TB10.6K15.2620.1.1	488.365900049756	1.10179885569296	0.00225609293273897	3.83270681377467e-18	XP_823027.1 2,3-bisphosphoglycerate-independent phosphoglycerate mutase [Tbb]
GLOS_TB11.02.4300.1.1	1359.09610744689	3.03817525114202	0.00223543812280453	1.56018510459927e-28	XM_823625.1 Tbb strain 927/4 GUTat10.1 hypothetical protein (Tb11.02.4300) partial mRNA
GLOS_TB927.8.1870.1.1	927.357869169504	2.06998705341749	0.00223213402531567	7.80241265535546e-36	XM_841927.1 Tbb GUTat10.1 Golgi/lysosome glycoprotein 1 (Tb927.8.1870) partial mRNA
GLOS_TB10.6K15.2900.1.1	493.828043969205	1.10179885569296	0.00223113869118715	2.04197849980447e-28	XP_823004.1 ABC transporter [Trypanosoma brucei brucei strain 927/4 GUTat10.1]
GLOS_ALF.1.1	987.70360173556	2.20359771138592	0.00223103136154797	7.86817992887431e-28	[BBH] ALF_TRYBB (sp|P07752) Fructose-bisphosphate aldolase, glycosomal OS=Tbb
GLOS_TB09.160.4580.1.1	495.204296950958	1.10179885569296	0.00222493799524134	1.58253768491766e-56	XM_821907.1 Tbb strain 927/4 GUTat10.1 hypothetical protein (Tb09.160.4580) partial mRNA
GLOS_TB927.4.4550.1.1	495.639565568898	1.10179885569296	0.00222298406388988	1.22846124004688e-27	XM_839511.1 Tbb GUTat10.1 hypothetical protein, conserved (Tb927.4.4550) partial mRNA
GLOS_TB09.211.1390.1.1	496.960660309906	1.10179885569296	0.00221707459702319	2.06055572660638e-23	XM_822191.1 Tbb strain 927/4 GUTat10.1 hypothetical protein (Tb09.211.1390) partial mRNA
GLOS_TB09.211.3960.1.1	437.322988911504	0.968188197724528	0.00221389733051616	3.03137327013384e-25	XM_822445.1 Tbb strain 927/4 GUTat10.1 hypothetical protein (Tb09.211.3960) partial mRNA
GLOS_TB09.211.4940.1.1	499.167581173983	1.10179885569296	0.00220727246168844	8.49929531013265e-49	XP_827634.1 hypothetical protein [Trypanosoma brucei brucei strain 927/4 GUTat10.1]
GLOS_TB09.160.2810.2.2	504.629725093433	1.10179885569296	0.00218338080557772	1.84143831557279e-57	XP_803725.1 fatty acyl CoA synthetase 3 [Trypanosoma brucei brucei strain 927/4 GUTat10.1]
GLOS_TB09.160.2770.1.1	444.521561513124	0.968188197724528	0.00217804552478596	2.53156022516579e-30	XP_803723.1 fatty acyl CoA synthetase 1 [Trypanosoma brucei brucei strain 927/4 GUTat10.1]
GLOS_TB10.61.1960.1.2	1023.71639942044	2.20359771138592	0.00215254704587467	2.41668173569368e-27	XM_822856.1 Tbb strain 927/4 GUTat10.1 40S ribosomal prot. S2 (Tb10.61.1960) partial mRNA
GLOS_TB10.70.4930.1.1	457.032092199151	0.968188197724528	0.00211842497332253	9.52292084314263e-32	XM_817433.1 Tbb strain 927/4 GUTat10.1 hypothetical protein (Tb10.70.4930) partial mRNA
GLOS_TB09.211.1240.1.1	457.862760081476	0.968188197724528	0.00211458166537117	1.67293012923432e-22	XM_822176.1 Tbb strain 927/4 GUTat10.1 hypothetical protein (Tb09.211.1240) partial mRNA
GLOS_TB10.70.3510.1.1	1059.78435534606	2.20359771138592	0.00207928877254124	4.40543680579055e-50	XP_822632.1 60S ribosomal protein L18a [Trypanosoma brucei brucei strain 927/4 GUTat10.1] ref|XP_829469.1| ribosomal prot. L18 [Trypanosoma brucei brucei strain 927/4 GUTat10.1]
GLOS_TB927.5.1660.1.1	465.741814730378	0.968188197724528	0.00207880883163781	3.05812899212211e-12	XM_839747.1 T. b. brucei strain 927/4 GUTat10.1 protein phosphatase 2C partial mRNA
GLOS_TB11.01.3550.1.1	535.077706820969	1.10179885569296	0.00205913803107034	3.51259557386311e-19	XM_824138.1 Trypanosoma brucei brucei strain 927/4 GUTat10.1 2-oxoglutarate dehydrogenase E2 component dihydrolipoamide succinyltransferase partial mRNA
GLOS_TB10.70.5840.1.1	546.464842398179	1.10179885569296	0.00201623008510059	6.99082894898525e-31	XM_817361.1 Tbb strain 927/4 GUTat10.1 major vault protein (Tb10.70.5840) partial mRNA
GLOS_TB927.7.5230.1.1	547.267931160132	1.10179885569296	0.00201327136665454	2.33287493499372e-15	XM_841053.1 Tbb strain 927/4 GUTat10.1 lanosterol synthase (Tb927.7.5230) partial mRNA
GLOS_TB10.70.2660.1.1	2612.36838714681	5.24177296252794	0.00200652135752298	6.16735194880395e-52	XP_822703.1 elongation factor 2 [Trypanosoma brucei brucei strain 927/4 GUTat10.1] ref|XP_822704.1| elongation factor 2 [Trypanosoma brucei brucei strain 927/4 GUTat10.1]
GLOS_TB927.3.3270.1.1	487.187346700196	0.968188197724528	0.00198730160847205	1.24808246415334e-11	XM_838827.1 T. brucei ATP-dependent phosphofructokinase (Tb927.3.3270) partial mRNA
GLOS_TB10.70.6880.1.1	569.646803050168	1.10179885569296	0.00193417895052406	5.30502312754467e-21	XM_817286.1 T. b. brucei strain 927/4 GUTat10.1 katanin (Tb10.70.6880) partial mRNA
GLOS_TB927.3.4760.1.1	571.778630996724	1.10179885569296	0.00192696752897587	8.040209306266e-28	XM_838975.1 Trypanosoma brucei brucei strain 927/4 GUTat10.1 dynamin partial mRNA
GLOS_TB10.70.4060.1.1	571.94110706495	1.10179885569296	0.00192642011927959	6.89565360387975e-10	XM_817496.1 Tbb GUTat10.1 60S acidic ribosomal protein P2 (Tb10.70.4060) partial mRNA
GLOS_TB11.01.5710.1.1	1578.57830244658	3.03817525114202	0.00192462752492751	9.49089871504146e-31	XP_829468.1 phenylalanyl-tRNA synthetase alpha subunit [Trypanosoma brucei TREU927]
GLOS_TB11.03.0240.1.1	517.112676659087	0.968188197724528	0.00187229638998546	3.64645288460587e-45	XM_823089.1 Tbb strain 927/4 GUTat10.1 hypothetical protein (Tb11.03.0240) partial mRNA
GLOS_TB11.52.0008.1.1	588.968151517025	1.10179885569296	0.00187072739477512	1.39239446740934e-49	XM_824256.1 Tbb strain 927/4 GUTat10.1 hypothetical protein (Tb11.52.0008) partial mRNA
GLOS_TB927.8.2030.1.1	589.648633564306	1.10179885569296	0.00186856848803806	2.60222946638238e-21	XM_841943.1 Tbb GUTat10.1 hypothetical protein, conserved (Tb927.8.2030) partial mRNA
GLOS_TB10.70.1520.1.1	519.822314615032	0.968188197724528	0.00186253681402951	8.14470866964985e-13	XP_822794.1 hypothetical protein [Trypanosoma brucei brucei strain 927/4 GUTat10.1]
GLOS_TB11.01.0355.1.1	1187.5717210419	2.20359771138592	0.00185554916165621	7.97315364114174e-23	XM_823801.1 Tbb strain 927/4 GUTat10.1 ribosomal protein S26 (Tb11.01.0355) partial mRNA
GLOS_TB10.70.5820.1.1	1200.79031289558	2.20359771138592	0.00183512282512688	1.66820265601463e-20	XP_822456.1 hexokinase [Trypanosoma brucei brucei strain 927/4 GUTat10.1]
GLOS_TB10.100.0155.1.1	601.57370979735	1.10179885569296	0.00183152760459582	5.20661875973765e-30	XP_822287.1 60S ribosomal protein L32 [Trypanosoma brucei brucei strain 927/4 GUTat10.1]
GLOS_TB927.8.4780.1.1	605.394452628928	1.10179885569296	0.00181996853606503	4.81019675647883e-39	XP_847308.1 hypothetical protein [Trypanosoma brucei brucei strain 927/4 GUTat10.1]
GLOS_TB927.7.6900.1.1	541.833366361055	0.968188197724528	0.00178687444855393	4.16352695216724e-16	XM_841217.1 Tbb double-strand-break repair protein rad21 homolog, putative partial mRNA
GLOS_TB927.7.5210.1.1	546.567514436073	0.968188197724528	0.00177139726045275	1.57743139265578e-61	XP_846144.1 hypothetical protein [Trypanosoma brucei brucei strain 927/4 GUTat10.1]
GLOS_TB927.8.650.1.1	549.897830408966	0.968188197724528	0.0017606692446931	7.88763331097183e-62	XP_846902.1 cation-transporting ATPase [Trypanosoma brucei brucei strain 927/4 GUTat10.1]
GLOS_TB11.03.0390.1.1	1253.50984868567	2.20359771138592	0.00175794208054802	3.12121338767177e-43	XP_828169.1 protein phosphatase 2C [Trypanosoma brucei brucei strain 927/4 GUTat10.1]

GLOS_TB927.3.3130.1.1	636.000264635101	1.10179885569296	0.00173238741704787	6.66216713008797e-70	XM_838813.1 T. brucei hypothetical protein, conserved (Tb927.3.3130) partial mRNA
GLOS_RL30.1.1	641.965125646417	1.10179885569296	0.00171629082589731	3.16645373578856e-18	RL30_TRYBB (sp|P49153) 60S ribosomal protein L30 OS=T. brucei brucei GN=RPL30 PE=3 SV=1
GLOS_TB927.8.5780.1.1	565.778546421441	0.968188197724528	0.00171124939934244	1.14874878224584e-20	XM_842315.1 Tbb GUTat10.1 prot. tyrosine phosphatase, putative (Tb927.8.5780) partial mRNA
GLOS_TB11.02.4520.1.1	596.554478939435	0.968188197724528	0.00162296694083295	4.27384865228412e-28	XM_823649.1 Tbb GUTat10.1 amino acid permease/transporter (Tb11.02.4520) partial mRNA
GLOS_TB927.5.510.1.1	597.037261354525	0.968188197724528	0.00162165456060139	2.37144926857565e-12	XP_844725.1 hypothetical protein [Trypanosoma brucei brucei strain 927/4 GUTat10.1]
GLOS_PFR1.1.1	2078.37885187069	3.30539656707889	0.00159037249830741	8.77691950606827e-32	[BBH] PFR1_TRYBB (sp|P22225) 69 kDa paraflagellar rod protein OS=Tbb GN=PFRA PE=2 SV=1
GLOS_TB09.211.1000.1.1	693.450949846203	1.10179885569296	0.00158886343141836	3.51604952754287e-19	XM_822151.1 Tbb phosphatidylcholine:ceramide cholinephosphotransferase 2 partial mRNA
GLOS_TB927.8.4700.1.1	1321.04650940975	2.06998705341749	0.00156692973235467	4.83352413668023e-22	XM_842208.1 Tbb GUTat10.1 amino acid transporter, putative (Tb927.8.4700) partial mRNA
GLOS_TB09.160.1160.1.1	2033.84638684318	3.17178590911045	0.00155950121387167	3.28229069002547e-32	XM_798466.1 Tbb strain 927/4 GUTat10.1 hypothetical protein (Tb09.160.1160) partial mRNA
GLOS_TB927.4.1300.1.1	1351.55729382163	2.06998705341749	0.00153155701417914	2.48037933184896e-33	XP_844280.1 hypothetical protein [Trypanosoma brucei brucei strain 927/4 GUTat10.1]
GLOS_TB11.02.0740.2.2	632.982610565477	0.968188197724528	0.00152956523854517	3.44693680654102e-24	XM_823312.1 Tbb GUTat10.1 60S ribosomal protein L44 (Tb11.02.0740) partial mRNA
GLOS_TB09.160.0430.1.1	724.179368567048	1.10179885569296	0.0015214446910758	5.10438866511837e-29	XM_798344.1 Tbb strain 927/4 GUTat10.1 hypothetical protein (Tb09.160.0430) partial mRNA
GLOS_TB927.7.1050.2.2	636.850867194203	0.968188197724528	0.00152027460053577	1.541796464103e-24	XM_840636.1 Tbb GUTat10.1 40S ribosomal protein S16, putative (Tb927.7.1050) partial mRNA
GLOS_TB11.01.5310.1.1	638.369661567404	0.968188197724528	0.00151665759827513	8.1068252220984e-28	XM_824337.1 Tbb receptor-type adenylate cyclase GRESAG 4 (Tb11.01.5310) partial mRNA
GLOS_TB927.8.6240.1.1	728.340352422266	1.10179885569296	0.00151275272889752	8.07824784127515e-26	XP_847454.1 hypothetical protein [Trypanosoma brucei brucei strain 927/4 GUTat10.1]
GLOS_TB10.6K15.0240.1.1	648.748364306875	0.968188197724528	0.00149239404828241	2.30795746071551e-22	XM_818133.1 Tbb strain 927/4 GUTat10.1 hypothetical protein (Tb10.6k15.0240) partial mRNA
GLOS_TB10.100.0080.1.1	1495.70351215544	2.20359771138592	0.00147328510863115	2.95743611478224e-26	XM_817186.1 Tbb strain 927/4 GUTat10.1 40S ribosomal prot. S6 (Tb10.100.0080) partial mRNA
GLOS_TB927.5.2430.1.1	747.906914318464	1.10179885569296	0.00147317645364595	2.4566430013695e-56	XM_839824.1 Tbb strain 927/4 GUTat10.1 membrane transporter protein partial mRNA
GLOS_TB927.4.5060.1.1	764.353150107145	1.10179885569296	0.00144147879228144	2.43600554195943e-25	XM_839561.1 Tbb strain 927/4 GUTat10.1 hypothetical protein (Tb927.4.5060) partial mRNA
GLOS_TC00.1047053508475.10.1.2	766.777705280192	1.10179885569296	0.00143692082869095	6.97259969319476e-28	XP_804279.1 ribosomal protein S20 [T. cruzi strain CL Brener] ref|XP_809988.1| ribosomal protein S20 [T. cruzi strain CL Brener] ref|XP_809990.1| ribosomal protein S20 [T. cruzi]
GLOS_TB11.02.4100.1.1	771.409181317317	1.10179885569296	0.00142829367653033	7.01703127041758e-51	XM_823604.1 Tbb GUTat10.1 pretranslocation prot. subunit alpha (Tb11.02.4100) partial mRNA
GLOS_TB927.8.6580.1.1	774.779366643765	1.10179885569296	0.00142208079245295	1.31931443677121e-18	XM_842395.1 Tbb GUTat10.1 succinate dehydrogenase flavoprotein, putative partial mRNA
GLOS_TB927.6.1520.1.1	1555.53888318604	2.20359771138592	0.00141661371194563	1.40401556202627e-28	XM_840181.1 Trypanosoma brucei brucei strain 927/4 GUTat10.1 aquaporin 3 partial mRNA
GLOS_TB927.2.900.1.1	687.240875405545	0.968188197724528	0.00140880473262478	8.97720388821115e-66	XM_946397.1 Tbb strain 927/4 GUTat10.1 hypothetical protein (Tb927.2.900) partial mRNA
GLOS_TB10.61.0540.1.1	789.081484252707	1.10179885569296	0.00139630554978287	3.25820990813103e-30	XM_822953.1 Tbb strain 927/4 GUTat10.1 hypothetical protein (Tb10.61.0540) partial mRNA
GLOS_TB11.02.2430.1.1	804.819658873733	1.10179885569296	0.001369000922809	1.75389217794033e-20	XM_823464.1 Tbb strain 927/4 GUTat10.1 60S ribosomal prot. L17 (Tb11.02.2430) partial mRNA
GLOS_TB11.02.4050.1.1	1615.83710195494	2.20359771138592	0.00136374991558238	2.93783054449695e-27	XM_823599.1 Tbb strain 927/4 GUTat10.1 60S ribosomal prot. L28 (Tb11.02.4050) partial mRNA
GLOS_TB927.6.660.1.1	808.585243464567	1.10179885569296	0.00136262548024257	5.26534231967521e-14	XM_840096.1 T. brucei brucei strain 927/4 GUTat10.1 hypothetical protein partial mRNA
GLOS_TB10.389.0890.1.1	712.642047154794	0.968188197724528	0.00135858977391244	6.90632176594088e-15	XM_818382.1 Tbb pyruvate dehydrogenase E1 component subunit alpha partial mRNA
GLOS_TB927.7.4570.1.1	814.122480301538	1.10179885569296	0.00135335761184837	6.04990836311874e-20	XM_840987.1 Tbb GUTat10.1 nucleoside hydrolase, putative (Tb927.7.4570) partial mRNA
GLOS_TB927.4.2530.1.1	816.772314227149	1.10179885569296	0.00134896694770502	4.85667117670003e-37	XM_839310.1 Tbb GUTat10.1 hypothetical protein, conserved (Tb927.4.2530) partial mRNA
GLOS_TB927.7.4180.1.1	723.566334993693	0.968188197724528	0.0013380780046006	1.04832490143116e-25	XM_840948.1 Tbb GUTat10.1 fatty acid elongase, putative (Tb927.7.4180) partial mRNA
GLOS_TB927.3.2600.1.1	828.452177030851	1.10179885569296	0.00132994865152238	4.12062612768155e-48	XM_838760.1 Tbb ATP-dependent DEAD/H RNA helicase, putative (Tb927.3.2600) partial mRNA
GLOS_TB10.389.1810.1.1	729.463747531082	0.968188197724528	0.00132726019764714	3.96901733663612e-19	XP_823403.1 kynurenine aminotransferase [Trypanosoma brucei brucei strain 927/4 GUTat10.1]
GLOS_TB09.211.3280.1.1	2411.40145319802	3.17178590911045	0.00131532885364401	1.57050386161006e-25	XM_822379.1 Tbb GUTat10.1 60S ribosomal protein L31 (Tb09.211.3280) partial mRNA
GLOS_TB10.70.1690.1.2	841.690703561309	1.10179885569296	0.00130903056316424	3.83847792403006e-23	XM_817688.1 Tbb GUTat10.1 40S ribosomal protein S10 (Tb10.70.1690) partial mRNA
GLOS_TB09.211.0340.1.1	2425.2882368658	3.17178590911045	0.00130779750666228	2.79065631821474e-31	XM_822082.1 Tbb GUTat10.1 60S ribosomal protein L10 (Tb09.211.0340) partial mRNA
GLOS_DWIL_GK10999.1.1	742.211847202856	0.968188197724528	0.0013044634107813	7.48317433098429e-11	XP_002070501.1 GK10999 [Drosophila willistoni]
GLOS_TB927.2.4370.1.1	1604.21239739198	2.06998705341749	0.0012903447553346	7.41567131111023e-35	XP_951630.1 trypanothione synthetase [Trypanosoma brucei brucei strain 927/4 GUTat10.1]
GLOS_TB927.1.2820.1.1	2357.35617687733	3.03817525114202	0.0012888061977832	1.8989527250954e-39	XP_001218979.1 pteridine transporter [Trypanosoma brucei brucei strain 927/4 GUTat10.1]

GLOS_RL40.3.8	758.060338305371	0.968188197724528	0.00127719146986227	7.51281469675909e-23	RL40_TRYBB (sp|P21899) Ubiquitin-60S ribosomal protein L40 OS=T. b. brucei PE=1 SV=2
GLOS_TB09.244.2630.1.1	1733.23347467813	2.20359771138592	0.00127137961710274	1.36544051601493e-36	XM_822584.1 Tbb GUTat10.1 40S ribosomal protein S6 (Tb09.244.2630) partial mRNA
GLOS_TB09.211.4700.1.1	867.842804485773	1.10179885569296	0.00126958344298979	1.61104899148934e-41	XP_827610.1 reiske iron-sulfur protein mitochondrial precursor [Trypanosoma brucei]
GLOS_TB927.8.3150.1.1	879.617694883774	1.10179885569296	0.00125258832570273	8.75922384082356e-37	XM_842053.1 Tbb 1 t-complex protein 1 gamma subunit, putative (Tb927.8.3150) partial mRNA
GLOS_TB927.5.320.1.1	905.319238303109	1.10179885569296	0.00121702799308466	3.32122112900982e-23	XM_839613.1 Tbb GUTat10.1 receptor-type adenylate cyclase GRESAG 4 partial mRNA
GLOS_TB927.5.900.1.1	906.034943914357	1.10179885569296	0.00121606662424392	5.54563276904436e-42	XP_844764.1 oligosaccharyl transferase subunit [Trypanosoma brucei TREU927]
GLOS_TB927.8.6180.2.2	948.210302242745	1.10179885569296	0.00116197730934471	5.73134131323788e-21	XP_847448.1 60S ribosomal protein L26 [Trypanosoma brucei brucei strain 927/4 GUTat10.1]
GLOS_TB10.100.0160.1.1	948.823335816099	1.10179885569296	0.00116122655725503	1.06240137229359e-22	XP_822288.1 cytochrome C oxidase subunit VI [T. brucei brucei strain 927/4 GUTat10.1]
GLOS_TB927.5.440.1.1	850.803469800515	0.968188197724528	0.00113796926327949	1.343933698115e-42	XM_839625.1 T. brucei brucei strain 927/4 GUTat10.1 hypothetical protein partial mRNA
GLOS_TB927.3.930.1.1	852.369777970866	0.968188197724528	0.00113587813968414	1.11663461107047e-39	XM_838596.1 Trypanosoma brucei dynein heavy chain, putative (Tb927.3.930) partial mRNA
GLOS_TB927.1.2100.1.1	976.711865422734	1.10179885569296	0.00112806948978355	3.411695733076e-31	XM_001218909.1 Trypanosoma brucei brucei strain 927/4 GUTat10.1 calpain-like cysteine peptidase. cysteine peptidase, Clan CA, family C2 partial mRNA
GLOS_TB927.1.230.1.1	979.081262355037	1.10179885569296	0.00112533953825523	6.46380401478816e-20	XM_841300.1 Trypanosoma brucei hypothetical protein, unlikely (Tb927.1.230) partial mRNA
GLOS_TB927.7.230.1.1	881.504309400987	0.968188197724528	0.00109833631826763	2.80917552283085e-44	XM_840554.1 Tbb GUTat10.1 40S ribosomal protein S33, putative (Tb927.7.230) partial mRNA
GLOS_TB11.02.3210.1.1	884.411646989124	0.968188197724528	0.0010947257434031	3.96168395321522e-23	XM_823534.1 Tbb GUTat10.1 triosephosphate isomerase (Tb11.02.3210) partial mRNA
GLOS_NDUS2.1.1	1019.45274352733	1.10179885569296	0.00108077482030282	3.0228230248306e-21	NDUS2_TRYBB (sp|P21301) NADH-ubiquinone oxidoreductase 49 kDa subunit homolog OS=Tbb
GLOS_TB10.389.0880.1.1	1022.64051810877	1.10179885569296	0.00107740582949968	2.0528311433661e-32	XM_818383.1 Tbb strain 927/4 GUTat10.1 heat shock protein (Tb10.389.0880) partial mRNA
GLOS_TB927.8.7410.1.1	1024.91488744678	1.10179885569296	0.00107501497850003	3.76584627678435e-26	XM_842477.1 Tbb strain 927/4 GUTat10.1 calreticulin, putative (Tb927.8.7410) partial mRNA
GLOS_TB10.6K15.2050.1.1	1025.54785569691	1.10179885569296	0.00107435147913623	5.88840831404381e-20	XP_823077.1 40S ribosomal protein S12 [Trypanosoma brucei brucei strain 927/4 GUTat10.1]
GLOS_contig_001921	906.818097999532	0.968188197724528	0.00106767630670405	1.33426130124957e-40	
GLOS_EF1A2.1.2	8686.0678833853	9.24813641139449	0.00106470920277797	1.00723001107548e-145	[BBH] EF1A2_TRYB2 (sp|P86939) Elongation factor 1-alpha 2 OS=Tbb
GLOS_LOC100843429.1.1	2074.78638644568	2.20359771138592	0.00106208413829093	1.10965284507998e-160	XM_003581200.1 PREDICT: B. distachyon uncharacteri LOC100843429 (LOC100843429), mRNA
GLOS_TB10.70.0800.1.2	1041.3288983255	1.10179885569296	0.00105806998870837	4.75983615628678e-92	XM_817756.1 Tbb universal minicircle sequence binding protein (UMSBP) partial mRNA
GLOS_TB10.61.3120.1.1	1045.08683847274	1.10179885569296	0.00105426536353965	7.48372045733926e-24	XM_822770.1 Tbb strain 927/4 GUTat10.1 hypothetical protein (Tb10.61.3120) partial mRNA
GLOS_TB09.211.1070.1.1	923.264333788213	0.968188197724528	0.00104865764038776	6.61006578740667e-23	XM_822158.1 Tbb strain 927/4 GUTat10.1 hypothetical protein (Tb09.211.1070) partial mRNA
GLOS_TDX.1.1	931.028426166038	0.968188197724528	0.00103991260687014	3.96623574679091e-31	[BBH] TDX_TRYBR (sp|Q26695) Thioredoxin peroxidase OS=T. brucei rhodesiense PE=2 SV=1
GLOS_TB11.46.0009.1.1	1060.64260234876	1.10179885569296	0.00103880313052961	1.33278813629888e-61	XM_823189.1 Tbb strain 927/4 GUTat10.1 hypothetical protein (Tb11.46.0009) partial mRNA
GLOS_TB09.244.2170.1.1	1084.29505518265	1.10179885569296	0.00101614302345717	8.90372604569981e-90	XM_822625.1 Tbb strain 927/4 GUTat10.1 hypothetical protein (Tb09.244.2170) partial mRNA
GLOS_TB10.70.2320.1.1	956.634941991074	0.968188197724528	0.00101207697443019	1.20300031120289e-26	XP_822735.1 hypothetical protein [Trypanosoma brucei brucei strain 927/4 GUTat10.1]
GLOS_TC00.1047053511281.20.1.1	1089.83693780921	1.10179885569296	0.00101097587856381	1.30695849118595e-19	XP_811198.1 transporter [Trypanosoma cruzi strain CL Brener]
GLOS_TB927.8.1890.1.1	962.662605686729	0.968188197724528	0.00100573990513931	4.59036480635872e-45	XP_847022.1 cytochrome c1, heme protein, mitochondrial precursor [Tbb]
GLOS_TB927.2.400.1.1	1102.98043674537	1.10179885569296	0.000998928737978439	4.19855213248693e-16	XP_951482.1 retrotransposon hot spot (RHS) protein [Trypanosoma brucei TREU927]
GLOS_TB927.3.3580.1.1	1112.27096793999	1.10179885569296	0.000990584927100609	4.32922426620866e-38	XM_838857.1 Tbb lipophosphoglycan biosynthetic prot., putative (Tb927.3.3580) partial mRNA
GLOS_TB10.70.0800.2.2	1138.36791062371	1.10179885569296	0.000967875890922896	4.32295012589381e-35	XM_817756.1 Tbb universal minicircle sequence binding protein (UMSBP) partial mRNA
GLOS_TB927.4.1790.1.1	15719.5262376107	15.0243520057962	0.000955776387830871	4.02489541589326e-28	XM_839236.1 Tbb GUTat10.1 ribosomal protein L3, putative (Tb927.4.1790) partial mRNA
GLOS_GBLP.3.4	4535.71660219834	4.27358476480341	0.000942207183476172	4.2604573107577e-37	[BBH] GBLP_TRYBR (sp|P69104) Guanine nucleotide-binding prot. subunit beta-like prot. OS=Tbb
GLOS_TB10.389.0910.1.2	1182.62758310151	1.10179885569296	0.000931653270595493	3.60206038122734e-26	XM_818380.1 Tbb GUTat10.1 60S ribosomal protein L34 (Tb10.389.0910) partial mRNA
GLOS_TB09.211.3510.1.1	1196.0239399106	1.10179885569296	0.00092121806171816	1.05414023750057e-37	XM_822401.1 Tbb ATP-dependent DEAD/H RNA helicase (Tb09.211.3510) partial mRNA
GLOS_TB927.1.600.1.1	1212.28776495428	1.10179885569296	0.000908859173163821	1.77553204865235e-85	XM_001218762.1 Tbb phosphate-repressible phosphate permease, putative partial mRNA
GLOS_TB11.02.5500.1.1	1254.93361546457	1.10179885569296	0.000877973816395921	7.08414483437228e-66	XM_823745.1 Tbb GUTat10.1 glucose-regulated protein 78 (Tb11.02.5500) partial mRNA
GLOS_TB09.160.5480.1.1	1259.42719589984	1.10179885569296	0.000874841244718197	8.88064840426781e-26	XM_822007.1 Tbb GUTat10.1 adenosine transporter (Tb09.160.5480) partial mRNA

GLOS_TB927.1.2430.1.1	4749.87454888044	4.13997410683498	0.000871596515703932	5.54563962447183e-51	XM_001218941.1 Trypanosoma brucei brucei strain 927/4 GUTat10.1 histone H3 partial mRNA
GLOS_PRO2.1.2	3824.00025052017	3.30539656707889	0.000864381890829964	2.61334905820484e-38	PRO2_TRYBB (sp|P14044) Duplicate procyclin OS=Trypanosoma brucei brucei PE=4 SV=1
GLOS_TB09.160.2550.1.1	2397.99280615574	2.06998705341749	0.000863216540142969	4.69092577570015e-35	XP_803698.1 ribosomal protein S7 [Trypanosoma brucei brucei strain 927/4 GUTat10.1]
GLOS_TB927.7.4390.1.1	1135.60011577302	0.968188197724528	0.000852578459861699	2.21783183965187e-08	XP_846062.1 threonine synthase [Trypanosoma brucei brucei strain 927/4 GUTat10.1]
GLOS_TB11.02.3770.1.2	1295.51508650224	1.10179885569296	0.000850471651910832	1.82558041078527e-28	XM_823578.1 Tbb strain 927/4 GUTat10.1 hypothetical protein (Tb11.02.3770) partial mRNA
GLOS_TB09.160.4560.1.1	2448.83037321821	2.06998705341749	0.000845296218168493	2.61656062068294e-23	XM_821905.1 Tbb strain 927/4 GUTat10.1 arginine kinase (Tb09.160.4560) partial mRNA
GLOS_TB927.7.5940.1.1	1307.44016273528	1.10179885569296	0.000842714555584632	1.53915060488459e-33	XM_841124.1 Tbb GUTat10.1 hypothetical protein, conserved (Tb927.7.5940) partial mRNA
GLOS_TB927.1.3180.1.1	2644.92061770053	2.20359771138592	0.000833143231837979	3.00816795726219e-38	XP_001219016.1 40S ribosomal protein S11 [Trypanosoma brucei brucei strain 927/4 GUTat10.1]
GLOS_TB927.8.1110.1.1	2523.37090556572	2.06998705341749	0.000820326115693806	2.27678948631097e-44	XM_841855.1 Tbb GUTat10.1 40S ribosomal protein S9, putative (Tb927.8.1110) partial mRNA
GLOS_TB11.02.0445.1.1	1390.30730858282	1.10179885569296	0.000792485840282358	2.36538851422435e-25	XM_823282.1 Tbb strain 927/4 GUTat10.1 hypothetical protein (Tb11.02.0445) partial mRNA
GLOS_TB11.02.4150.1.1	2639.65617341327	2.06998705341749	0.000784188135661942	4.36383512864248e-37	XP_828702.1 pyruvate phosphate dikinase [Trypanosoma brucei brucei strain 927/4 GUTat10.1]
GLOS_TB10.70.7100.1.1	1243.25375266087	0.968188197724528	0.000778753489102579	5.70750765052631e-54	XM_817268.1 Tbb GUTat10.1 serine carboxypeptidase III precursor (Tb10.70.7100) partial mRNA
GLOS_TB927.6.3800.1.1	2855.47545586002	2.20359771138592	0.000771709561314452	3.07524983320267e-55	XM_840405.1 Tbb heat shock 70 kDa protein, mitochondrial precursor partial mRNA
GLOS_TB927.2.4710.1.1	1264.13376485448	0.968188197724528	0.000765890623794847	3.42812558766045e-40	XM_946562.1 Tbb strain 927/4 GUTat10.1 RNA-binding protein (Tb927.2.4710) partial mRNA
GLOS_TB927.6.720.1.1	1439.12336418022	1.10179885569296	0.000765604174816931	3.94186968578911e-24	XM_840102.1 Tbb strain 927/4 GUTat10.1 40S ribosomal protein L14 partial mRNA
GLOS_TB11.02.1085.1.1	2887.5924177958	2.20359771138592	0.000763126297813181	9.57671775121072e-48	XP_828440.1 40s ribosomal protein S4 [Trypanosoma brucei brucei strain 927/4 GUTat10.1] ref|XP_828441.1| 40S ribosomal protein S4 [T. brucei brucei strain 927/4 GUTat10.1]
GLOS_TB10.70.7010.1.1	2832.97397725531	2.06998705341749	0.000730676338729721	7.30413820996779e-50	XP_822368.1 60S ribosomal protein L9 [Trypanosoma brucei brucei strain 927/4 GUTat10.1]
GLOS_TB10.61.0980.1.1	1543.95404797663	1.10179885569296	0.000713621533708779	3.65043434340473e-23	XM_822919.1 Tbb GUTat10.1 glycosomal malate dehydrogenase (Tb10.61.0980) partial mRNA
GLOS_TB11.02.0010.1.1	1581.0227922964	1.10179885569296	0.000696889925345493	3.6502440988471e-27	XP_828324.1 hypothetical protein [Trypanosoma brucei brucei strain 927/4 GUTat10.1]
GLOS_TB11.47.0004.1.1	1394.13569585799	0.968188197724528	0.000694471994800101	7.17761662315631e-48	XP_828236.1 2-oxoglutarate dehydrogenase subunit [Trypanosoma brucei TREU927]
GLOS_GPDA.1.2	3038.3886249778	2.06998705341749	0.000681277910403121	7.43053635477231e-17	[BBH] GPDA_TRYBR (sp|Q26756) Glycerol-3-phosphate dehydrogenase [NAD()], glycosomal Tbb
GLOS_TB927.2.240.1.1	1635.40366385114	1.10179885569296	0.000673716758771583	4.07279233655417e-39	XP_951477.1 retrotransposon hot spot (RHS) protein [Trypanosoma brucei TREU927]
GLOS_TB11.46.0002.1.1	3288.68962050305	2.20359771138592	0.000670053415089033	8.92660167344186e-29	XP_828289.1 60S acidic ribosomal subunit protein [Trypanosoma brucei TREU927] ref|XP_828290.1| 60S acidic ribosomal subunit protein [Trypanosoma brucei TREU927]
GLOS_TB10.70.4740.1.1	1729.12000462006	1.10179885569296	0.000637202075477151	4.06339716396299e-37	XM_817449.1 T. brucei brucei strain 927/4 GUTat10.1 enolase (Tb10.70.4740) partial mRNA
GLOS_TB10.70.3370.1.1	3313.17274121927	2.06998705341749	0.000624774865392537	1.13338339432224e-29	XM_817548.1 Tbb strain 927/4 GUTat10.1 40S ribosomal prot. S3a (Tb10.70.3370) partial mRNA
GLOS_CH60.1.3	1764.91516799597	1.10179885569296	0.000624278648442937	8.84756271353425e-43	[BBH] CH60_TRYBB (sp|Q37683) Chaperonin HSP60, mitochondrial OS=Tbb
GLOS_TB10.70.5110.1.1	3338.04361675628	2.06998705341749	0.000620119833972987	1.18952411693691e-27	XM_817416.1 Trypanosoma brucei brucei strain 927/4 GUTat10.1 mitochondrial malate dehydrogenase (Tb10.70.5110) partial mRNA. nuclear gene for mitochondrial product
GLOS_TB11.01.8770.1.1	1593.51338830565	0.968188197724528	0.000607580836678117	1.32436324297485e-35	XP_829756.1 hypothetical protein [Trypanosoma brucei brucei strain 927/4 GUTat10.1]
GLOS_TB11.01.7535.1.1	1816.14048987922	1.10179885569296	0.000606670498143144	1.86961816688982e-20	XP_829645.1 60S ribosomal protein L27 [Trypanosoma brucei brucei strain 927/4 GUTat10.1] ref|XP_829647.1| 60S ribosomal protein L27 [T. brucei brucei strain 927/4 GUTat10.1]
GLOS_TB11.02.1100.1.1	1604.46525526492	0.968188197724528	0.00060343357049802	8.82110186762869e-65	XM_823349.1 Tbb nucleobase/nucleoside transporter 8.1 (Tb11.02.1100) partial mRNA
GLOS_TB10.6K15.3350.1.1	1616.37504261078	0.968188197724528	0.000598987346501408	1.24340497719524e-27	XM_817875.1 T. GUTat10.1 40S ribosomal protein S24e (Tb10.6k15.3350) partial mRNA
GLOS_TB927.1.2370.2.2	21694.2894639362	12.9543649523787	0.000597132483823154	9.4854985237505e-40	XM_001218936.1 Trypanosoma brucei brucei strain 927/4 GUTat10.1 beta tubulin partial mRNA
GLOS_TRCOIV.1.1	1858.47367848625	1.10179885569296	0.000592851471854254	4.89887198924919e-24	XM_001219103.1 Tbb cyt C oxidase subunit IV (PMID:12467979) (trCOIV) partial mRNA
GLOS_TB09.244.2590.1.1	1658.72816589458	0.968188197724528	0.000583693107545665	9.08636957648908e-27	XP_827681.1 60S ribosomal protein L32 [Trypanosoma brucei brucei strain 927/4 GUTat10.1]
GLOS_TB09.211.3550.1.1	3663.19180922429	2.06998705341749	0.000565077440991501	2.70454208900581e-18	XM_822405.1 Tbb GUTat10.1 glycerol kinase glycosomal (Tb09.211.3550) partial mRNA
GLOS_TB927.6.2790.1.1	2016.72431479681	1.10179885569296	0.00054633092267942	8.75192106655322e-24	XP_845400.1 L-threonine 3-dehydrogenase [Trypanosoma brucei brucei strain 927/4 GUTat10.1]
GLOS_TB927.3.4290.1.1	2078.64399997681	1.10179885569296	0.00053005654441321	7.94543526845588e-29	XM_838928.1 T. brucei 73 kDa paraflagellar rod protein (Tb927.3.4290) partial mRNA

<table>
<tr><td>GLOS_TB09.211.2740.1.1</td><td>2115.57484869472</td><td>1.10179885569296</td><td>0.000520803533078849</td><td>1.15185319854177e-18</td><td>XP_827420.1 Gim5B protein [Trypanosoma brucei brucei strain 927/4 GUTat10.1]</td></tr>
<tr><td>GLOS_TB927.5.930.1.1</td><td>1900.84209065724</td><td>0.968188197724528</td><td>0.000509346990201467</td><td>1.43599433549081e-49</td><td>XM_839674.1 Tbb strain 927/4 GUTat10.1 NADH-dependent fumarate reductase partial mRNA</td></tr>
<tr><td>GLOS_TB10.6K15.0410.1.1</td><td>2181.1252215177</td><td>1.10179885569296</td><td>0.000505151581772226</td><td>1.27563997606224e-28</td><td>XM_818117.1 Tbb GUTat10.1 60S ribosomal protein L18 (Tb10.6k15.0410) partial mRNA</td></tr>
<tr><td>GLOS_TB09.160.5590.1.1</td><td>2228.73514464516</td><td>1.10179885569296</td><td>0.000494360605539057</td><td>4.31766948270827e-22</td><td>XM_822018.1 Tbb GUTat10.1 60S ribosomal protein L11 (Tb09.160.5590) partial mRNA</td></tr>
<tr><td>GLOS_TB10.26.1080.1.1</td><td>13406.7115217229</td><td>6.47718247618934</td><td>0.000483129846248601</td><td>5.30765963182577e-51</td><td>XM_818214.1 Tbb strain 927/4 GUTat10.1 heat shock protein 83 (Tb10.26.1080) partial mRNA</td></tr>
<tr><td>GLOS_TB10.6K15.0520.1.1</td><td>2370.31426641448</td><td>1.10179885569296</td><td>0.000464832394296654</td><td>1.70440960631013e-22</td><td>XM_818108.1 Tbb strain 927/4 GUTat10.1 hypothetical protein (Tb10.6k15.0520) partial mRNA</td></tr>
<tr><td>GLOS_TB927.3.1380.1.1</td><td>2089.60351137968</td><td>0.968188197724528</td><td>0.000463335839766691</td><td>1.1934058509551e-64</td><td>XM_838639.1 Tbb ATP synthase beta chain, mitochond. precursor (Tb927.3.1380) partial mRNA</td></tr>
<tr><td>GLOS_TB11.01.3110.1.1</td><td>4766.23804730801</td><td>2.20359771138592</td><td>0.000462334799377158</td><td>2.25008124257086e-38</td><td>XM_824105.1 Tbb strain 927/4 GUTat10.1 heat shock protein 70 (Tb11.01.3110) partial mRNA</td></tr>
<tr><td>GLOS_TB927.6.4840.1.1</td><td>2527.82461664743</td><td>1.10179885569296</td><td>0.0004358683938897</td><td>2.52218624533298e-42</td><td>XM_840508.1 Tbb strain 927/4 GUTat10.1 S-adenosylmethionine synthetase partial mRNA</td></tr>
<tr><td>GLOS_TB10.70.7030.1.1</td><td>2545.27928527385</td><td>1.10179885569296</td><td>0.000432879355152736</td><td>1.28563669411633e-40</td><td>XP_822366.1 40S ribosomal protein S23 [Trypanosoma brucei brucei strain 927/4 GUTat10.1]
ref|XP_822367.1| 40S ribosomal protein S23 [T. b. brucei strain 927/4 GUTat10.1]</td></tr>
<tr><td>GLOS_TB927.3.3310.1.1</td><td>2354.11324405514</td><td>0.968188197724528</td><td>0.000411275116084368</td><td>2.60267561437276e-27</td><td>XM_838831.1 Tbb 60S ribosomal protein L13, putative (Tb927.3.3310) partial mRNA</td></tr>
<tr><td>GLOS_TB10.26.0370.1.1</td><td>2768.89488500839</td><td>1.10179885569296</td><td>0.000397920073332659</td><td>3.13442952321168e-36</td><td>XP_823361.1 40S ribosomal protein S3 [Trypanosoma brucei brucei strain 927/4 GUTat10.1]</td></tr>
<tr><td>GLOS_TB927.7.1740.1.1</td><td>3039.725008606</td><td>1.10179885569296</td><td>0.000362466622004811</td><td>1.53503435404491e-24</td><td>XM_840705.1 Tbb GUTat10.1 60S ribosomal protein L7, putative (Tb927.7.1740) partial mRNA</td></tr>
<tr><td>GLOS_RNA45S5.1.2</td><td>2694.07526718599</td><td>0.968188197724528</td><td>0.000359376818278659</td><td>2.28434641003114e-190</td><td>NR_046235.1 Homo sapiens RNA, 45S pre-ribosomal 5 (RNA45S5), ribosomal RNA</td></tr>
<tr><td>GLOS_TBA.1.1</td><td>27162.0123473639</td><td>9.24813641139449</td><td>0.000340480531895938</td><td>2.07521083212209e-39</td><td>[BBH] TBA_TRYBR (sp|P04106) Tubulin alpha chain OS=T. brucei rhodesiense PE=3 SV=1</td></tr>
<tr><td>GLOS_TB10.05.0220.1.1</td><td>3370.85634962653</td><td>1.10179885569296</td><td>0.000326860222274092</td><td>1.60528887100678e-34</td><td>XM_822741.1 Tbb GUTat10.1 60S ribosomal protein L10a (Tb10.05.0220) partial mRNA</td></tr>
<tr><td>GLOS_TB09.244.2730.1.1</td><td>3590.59604828874</td><td>1.10179885569296</td><td>0.000306856811759171</td><td>5.03982573546022e-27</td><td>XM_822569.1 Tbb GUTat10.1 60S ribosomal protein L5 (Tb09.244.2730) partial mRNA</td></tr>
<tr><td>GLOS_TB927.6.520.3.3</td><td>17280.2621166101</td><td>5.10816230455951</td><td>0.000295606760481339</td><td>4.3335084899751e-34</td><td>XM_840082.1 Trypanosoma brucei brucei strain 927/4 GUTat10.1 EP3-2 procyclin partial mRNA</td></tr>
<tr><td>GLOS_TH2A.1.1</td><td>3780.73836778252</td><td>1.10179885569296</td><td>0.00029142425328394</td><td>7.63737735953985e-23</td><td>[BBH] TH2A_TRYBB (sp|Q06222) Glucose transporter 2A OS=T. b. brucei GN=THT2A PE=2 SV=1</td></tr>
<tr><td>GLOS_TB927.3.5050.1.1</td><td>3957.91030750597</td><td>1.10179885569296</td><td>0.000278378934864557</td><td>4.07551744384523e-35</td><td>XM_839002.1 Trypanosoma brucei 60S ribosomal protein L4 (Tb927.3.5050) partial mRNA</td></tr>
<tr><td>GLOS_TB927.6.1020.1.1</td><td>3589.95843424902</td><td>0.968188197724528</td><td>0.000269693428338276</td><td>2.84922426571942e-49</td><td>XM_840131.1 Tbb strain 927/4 GUTat10.1 cysteine peptidase precursor partial mRNA</td></tr>
<tr><td>GLOS_TB927.5.2260.2.2</td><td>3906.76472432982</td><td>0.968188197724528</td><td>0.000247823523053494</td><td>5.33109662595934e-33</td><td>XM_839807.1 T. brucei brucei strain 927/4 GUTat10.1 hypothetical protein partial mRNA</td></tr>
<tr><td>GLOS_TB927.6.520.1.3</td><td>4722.37841988419</td><td>1.10179885569296</td><td>0.000233314393241697</td><td>1.53903698057838e-31</td><td>XM_840082.1 T. brucei brucei strain 927/4 GUTat10.1 EP3-2 procyclin partial mRNA</td></tr>
<tr><td>GLOS_ACT1.1.1</td><td>529.030108448537</td><td>0</td><td>0</td><td>1.70480690429143e-19</td><td>[BBH] ACT1_TRYBB (sp|P12432) Actin A OS=Trypanosoma brucei brucei PE=3 SV=1</td></tr>
<tr><td>GLOS_CALM.1.1</td><td>805.72541967358</td><td>0</td><td>0</td><td>3.56205792818271e-22</td><td>[BBH] CALM_TRYBG (sp|P69098) Calmodulin OS=Trypanosoma brucei gambiense PE=3 SV=2</td></tr>
<tr><td>GLOS_CC2H2.1.1</td><td>321.813230705533</td><td>0</td><td>0</td><td>1.40842724394505e-12</td><td>[BBH] CC2H2_TRYBB (sp|P54665) Cell division control protein 2 homolog 2 OS=Tbb</td></tr>
<tr><td>GLOS_CLP.1.1</td><td>489.018802976666</td><td>0</td><td>0</td><td>1.93093635697776e-08</td><td>[BBH] CLP_TRYBB (sp|P31543) Heat shock protein 100 OS=T. b. brucei GN=HSP100 PE=3 SV=1</td></tr>
<tr><td>GLOS_contig_000059</td><td>401.745459844565</td><td>0</td><td>0</td><td>8.32804919018073e-12</td><td></td></tr>
<tr><td>GLOS_contig_000389</td><td>471.591713470618</td><td>0</td><td>0</td><td>5.85090412906252e-12</td><td></td></tr>
<tr><td>GLOS_contig_000427</td><td>313.906596936259</td><td>0</td><td>0</td><td>6.16796236170678e-15</td><td></td></tr>
<tr><td>GLOS_contig_000441</td><td>447.844233042423</td><td>0</td><td>0</td><td>1.3710950761396e-11</td><td></td></tr>
<tr><td>GLOS_contig_000530</td><td>322.28372288744</td><td>0</td><td>0</td><td>2.79097105358027e-41</td><td></td></tr>
<tr><td>GLOS_contig_000567</td><td>370.19401768499</td><td>0</td><td>0</td><td>3.68299782584154e-32</td><td></td></tr>
<tr><td>GLOS_contig_000712</td><td>306.537903822819</td><td>0</td><td>0</td><td>6.21401711921335e-16</td><td></td></tr>
<tr><td>GLOS_contig_000809</td><td>333.643279344278</td><td>0</td><td>0</td><td>1.48512353321619e-42</td><td></td></tr>
<tr><td>GLOS_contig_000926</td><td>383.855522600205</td><td>0</td><td>0</td><td>7.70219114255184e-37</td><td></td></tr>
<tr><td>GLOS_contig_001171</td><td>347.107084627301</td><td>0</td><td>0</td><td>3.5748670735612e-29</td><td></td></tr>
<tr><td>GLOS_contig_001756</td><td>426.473793990127</td><td>0</td><td>0</td><td>5.89971100888679e-15</td><td></td></tr>
<tr><td>GLOS_contig_004073</td><td>379.912173053957</td><td>0</td><td>0</td><td>2.59055699223215e-24</td><td></td></tr>
<tr><td>GLOS_contig_004527</td><td>557.776885057868</td><td>0</td><td>0</td><td>2.55598753980362e-42</td><td></td></tr>
</table>

GLOS_contig_005069	385.959771426389	0	0	2.14447024435434e-27	
GLOS_contig_007423	1183.75862265392	0	0	1.13279532450426e-40	
GLOS_COPB.1.2	422.925843708264	0	0	6.08811421440257e-20	[BBH] COPB_TRYBB (sp|Q9NFU6) Coatomer subunit beta OS=T. brucei brucei PE=3 SV=1
GLOS_DCOR.1.1	388.139113170094	0	0	2.48770738130952e-30	[BBH] DCOR_TRYBB (sp|P07805) Ornithine decarboxylase OS=T. brucei brucei PE=1 SV=2
GLOS_ERF1.1.2	363.608478656725	0	0	2.62633256269927e-33	[BBH] ERF1_TRYBB (sp|Q9NAX8) Eukaryotic peptide chain release factor subunit 1 OS=Tbb
GLOS_GSK3B.1.1	368.430009882447	0	0	1.1411277422776e-23	[BBH] GSK3B_TRYB2 (sp|Q388M1) Glycogen synthase kinase 3 OS=Tbb
GLOS_HPRT.1.1	361.796957057033	0	0	1.03026489736989e-34	[BBH] HPRT_TRYBB (sp|Q07010) Hypoxanthine-guanine phosphoribosyltransferase OS=Tbb
GLOS_IF4A.1.2	1892.37758155535	0	0	2.535335269655e-79	[BBH] IF4A_TRYB2 (sp|Q38F76) Probable eukaryotic initiation factor 4A OS=Tbb
GLOS_KPYK1.1.1	416.660611026861	0	0	6.1893009037862e-30	[BBH] KPYK1_TRYBB (sp|P30615) Pyruvate kinase 1 OS=T. b. brucei GN=PYK1 PE=3 SV=1
GLOS_NOG1.1.2	397.884847659433	0	0	1.52062958343928e-49	[BBH] NOG1_TRYBB (sp|Q9U6A9) Nucleolar GTP-binding protein 1 OS=Tbb GN=NOG1 PE=1 SV=1
GLOS_P320.1.1	570.627656767536	0	0	1.44454946380463e-28	[BBH] P320_TRYBB (sp|P21787) Microtubule-associated protein P320 (Fragment) OS=Tbb
GLOS_PGKE.1.1	688.779604455524	0	0	9.48750615926845e-37	[BBH] PGKE_TRYBB (sp|P08893) Phosphoglycerate kinase, cytosolic OS=T. b. brucei PE=3 SV=1
GLOS_PRO2.2.2	1083.84449767752	0	0	9.13775675931722e-32	PRO2_TRYBB (sp|P14044) Duplicate procyclin OS=Trypanosoma brucei brucei PE=4 SV=1
GLOS_RIR2.1.2	628.386358092318	0	0	2.26961626629818e-72	[BBH] RIR2_TRYBB (sp|O15910) Ribonucleoside-diphosphate reductase small chain OS=Tbb
GLOS_RL27A.1.6	1971.49143304524	0	0	2.83838054715411e-38	[BBH] RL27A_TRYBB (sp|O15883) 60S ribosomal protein L27a OS=Tbb GN=RPL27A PE=2 SV=1
GLOS_RL40.2.8	402.658865088006	0	0	4.71583811016029e-50	RL40_TRYBB (sp|P21899) Ubiquitin-60S ribosomal protein L40 OS=T. brucei brucei PE=1 SV=2
GLOS_RL402.1.2	815.593760877589	0	0	4.76899463412765e-27	RL402_TRYCR (sp|P0CH27) Ubiquitin-60S ribosomal protein L40 OS=T. cruzi PE=2 SV=1
GLOS_RL402.2.2	564.069696859642	0	0	5.08727219321557e-51	RL402_TRYCR (sp|P0CH27) Ubiquitin-60S ribosomal protein L40 OS=T. cruzi PE=2 SV=1
GLOS_RPB1B.1.1	406.301842964168	0	0	1.85995754919627e-17	[BBH] RPB1B_TRYBB (sp|P17545) DNA-directed RNA polymerase II subunit RPB1-B OS=Tbb
GLOS_RS12.1.4	1106.83640314092	0	0	3.77217712079409e-42	[BBH] RS12_TRYBB (sp|Q03253) 40S ribosomal protein S12 OS=T. b. brucei GN=RPS12 PE=2 SV=2
GLOS_SMP_112590.1.1	616.481216536052	0	0	2.05048488169034e-24	XM_002581801.1 S. mansoni conserved hypothetical protein (Smp_112590) mRNA, partial cds
GLOS_TB09.160.0710.1.1	1537.30106047444	0	0	2.21292690898159e-34	XM_798407.1 Tbb GUTat10.1 60S ribosomal protein L35 (Tb09.160.0710) partial mRNA
GLOS_TB09.160.0810.1.1	417.641464744229	0	0	6.48090558344358e-44	XM_798420.1 Tbb strain 927/4 GUTat10.1 kynureninase (Tb09.160.0810) partial mRNA
GLOS_TB09.160.0815.1.1	736.171893274019	0	0	3.82806749442615e-21	XP_803515.1 60S ribosomal protein L38 [Trypanosoma brucei]
GLOS_TB09.160.0930.1.1	343.211248878202	0	0	7.79605946746845e-15	XM_798434.1 Tbb strain 927/4 GUTat10.1 protein kinase (Tb09.160.0930) partial mRNA
GLOS_TB09.160.1200.1.1	327.983435792636	0	0	1.90847228153382e-14	XP_803563.1 mitotubule-associated protein Gb4 [T. brucei brucei strain 927/4 GUTat10.1]
GLOS_TB09.160.1520.1.1	354.673477372934	0	0	2.06206106221301e-44	XM_798500.1 Tbb strain 927/4 GUTat10.1 hypothetical protein (Tb09.160.1520) partial mRNA
GLOS_TB09.160.1780.1.1	490.327607484491	0	0	1.23391072239578e-33	XM_798524.1 Tbb strain 927/4 GUTat10.1 protein kinase (Tb09.160.1780) partial mRNA
GLOS_TB09.160.1820.1.1	722.830694705667	0	0	2.14119600098021e-24	XP_803621.1 cytochrome c oxidase subunit V [T. brucei brucei strain 927/4 GUTat10.1]
GLOS_TB09.160.2810.1.2	478.695258477939	0	0	4.21891564195764e-19	XP_803725.1 fatty acyl CoA synthetase 3 [Trypanosoma brucei brucei strain 927/4 GUTat10.1]
GLOS_TB09.160.3530.1.1	686.109835853135	0	0	4.18608246361453e-31	XM_798709.1 Tbb strain 927/4 GUTat10.1 hypothetical protein (Tb09.160.3530) partial mRNA
GLOS_TB09.160.3590.1.1	934.378676815709	0	0	5.96262568483174e-24	XM_798715.1 Tbb GUTat10.1 cAMP-specific phosphodiesterase (Tb09.160.3590) partial mRNA
GLOS_TB09.160.3670.1.1	364.711939088764	0	0	5.89063000043734e-46	XP_803820.1 ribosomal protein S6 [Trypanosoma brucei brucei strain 927/4 GUTat10.1]
GLOS_TB09.160.3780.1.1	318.360308017969	0	0	9.99756286527386e-11	XM_798741.1 Tbb strain 927/4 GUTat10.1 hypothetical protein (Tb09.160.3780) partial mRNA
GLOS_TB09.160.3820.1.1	376.439315689615	0	0	3.2181700717818e-47	XM_798745.1 Tbb strain 927/4 GUTat10.1 hypothetical protein (Tb09.160.3820) partial mRNA
GLOS_TB09.160.4180.1.1	340.201239252172	0	0	9.81346153718421e-19	XP_826963.1 inositol/phosphatidylinositol phosphatase [Trypanosoma brucei TREU927]
GLOS_TB09.160.4200.1.1	1143.25689032336	0	0	4.40079289329377e-22	XM_821871.1 Tbb GUTat10.1 60S acidic ribosomalprotein (Tb09.160.4200) partial mRNA
GLOS_TB09.160.4240.1.1	473.533486228575	0	0	7.50699521362465e-38	XM_821875.1 Tbb GUTat10.1 nucleosome assembly prot-like prot. (Tb09.160.4240) partial mRNA
GLOS_TB09.160.4300.1.1	368.920436741131	0	0	2.73847294604835e-28	XM_821880.1 Tbb GUTat10.1 farnesyl pyrophosphate synthetase (Tb09.160.4300) partial mRNA
GLOS_TB09.160.4310.1.1	1232.88734015458	0	0	2.33436799081032e-25	XP_826974.1 glutamate dehydrogenase [Trypanosoma brucei brucei strain 927/4 GUTat10.1]
GLOS_TB09.160.4380.1.1	606.537782414521	0	0	4.84151254646474e-16	XP_826981.1 succinate dehydrogenase [Trypanosoma brucei brucei strain 927/4 GUTat10.1]

GLOS_TB09.160.4600.1.1	408.651305219694	0	0	1.79371971869328e-15	XM_821909.1 Tbb strain 927/4 GUTat10.1 ABC transporter (Tb09.160.4600) partial mRNA
GLOS_TB09.160.5060.1.1	477.15652942796	0	0	1.74941841722282e-35	XM_821961.1 Tbb strain 927/4 GUTat10.1 hypothetical protein (Tb09.160.5060) partial mRNA
GLOS_TB09.211.0120.1.2	1083.76940476	0	0	5.56408994171711e-28	XP_827151.1 nascent polypeptide associated complex subunit [Trypanosoma brucei]<br>ref|XP_827152.1| nascent polypeptide associated complex subunit [Trypanosoma brucei]
GLOS_TB09.211.0140.1.1	383.562795373714	0	0	5.21248146944291e-48	XM_822060.1 Tbb strain 927/4 GUTat10.1 chaperone prot. DnaJ (Tb09.211.0140) partial mRNA
GLOS_TB09.211.0320.1.1	365.760241280058	0	0	1.71247388601775e-18	XM_822080.1 Tbb strain 927/4 GUTat10.1 hypothetical protein (Tb09.211.0320) partial mRNA
GLOS_TB09.211.0610.1.1	400.91479196224	0	0	7.22702049393427e-15	XM_822111.1 Tbb GUTat10.1 hypothetical protein (Tb09.211.0610) partial mRNA
GLOS_TB09.211.0930.1.1	658.786826022705	0	0	5.36245885925394e-27	XM_822144.1 Tbb GUTat10.1 polyadenylate-binding protein 1 (Tb09.211.0930) partial mRNA
GLOS_TB09.211.0960.1.1	325.266153393098	0	0	5.82082312178506e-15	XP_827240.1 protein kinase [Trypanosoma brucei brucei strain 927/4 GUTat10.1]
GLOS_TB09.211.1190.1.1	343.068707486753	0	0	5.65009156682346e-41	XM_822171.1 Tbb GUTat10.1 minichromosome maintenance complex subunit partial mRNA
GLOS_TB09.211.1230.1.1	445.182108883629	0	0	1.25398828042474e-29	XM_822175.1 Tbb GUTat10.1 hypothetical protein (Tb09.211.1230) partial mRNA
GLOS_TB09.211.1620.1.1	442.24719217512	0	0	3.43200225536776e-54	XP_827306.1 hypothetical protein [Trypanosoma brucei brucei strain 927/4 GUTat10.1]
GLOS_TB09.211.1690.1.1	523.180209708297	0	0	3.48245085962238e-23	XM_822222.1 Tbb GUTat10.1 hypothetical protein (Tb09.211.1690) partial mRNA
GLOS_TB09.211.1750.1.1	611.604527069586	0	0	1.26336790394544e-14	XM_822228.1 Trypanosoma brucei brucei strain 927/4 GUTat10.1 mitochondrial carrier<br>protein (Tb09.211.1750) partial mRNA. nuclear gene for mitochondrial product
GLOS_TB09.211.1950.1.1	386.007285223538	0	0	2.12439655335132e-20	XM_822248.1 Tbb strain 927/4 GUTat10.1 hypothetical protein (Tb09.211.1950) partial mRNA
GLOS_TB09.211.2150.1.1	3666.86071908524	0	0	1.77047839079788e-58	XM_822265.1 Tbb strain 927/4 GUTat10.1 poly(A)-binding prot. 1 (Tb09.211.2150) partial mRNA
GLOS_TB09.211.2160.1.1	334.719160655945	0	0	5.19731858671496e-29	XM_822266.1 Tbb strain 927/4 GUTat10.1 hypothetical protein (Tb09.211.2160) partial mRNA
GLOS_TB09.211.2570.1.1	640.161248490319	0	0	2.33631818274226e-63	XM_822308.1 Tbb GUTat10.1 t-complex protein 1 subunit eta (Tb09.211.2570) partial mRNA
GLOS_TB09.211.2630.1.2	605.734693652568	0	0	4.67164375386835e-28	XP_827407.1 60S ribosomal protein L23 [Trypanosoma brucei brucei strain 927/4 GUTat10.1]<br>ref|XP_827408.1| 60S ribosomal protein L23 [T. brucei brucei strain 927/4 GUTat10.1]
GLOS_TB09.211.2630.2.2	724.717309222881	0	0	8.13179735331247e-29	XP_827407.1 60S ribosomal protein L23 [Trypanosoma brucei brucei strain 927/4 GUTat10.1]<br>ref|XP_827408.1| 60S ribosomal protein L23 [T. brucei brucei strain 927/4 GUTat10.1]
GLOS_TB09.211.2700.1.1	631.491395312647	0	0	1.41535785232739e-35	XM_822323.1 Tbb strain 927/4 GUTat10.1 hypothetical protein (Tb09.211.2700) partial mRNA
GLOS_TB09.211.2900.1.1	653.079468673914	0	0	3.80774927434674e-19	XM_822342.1 Tbb strain 927/4 GUTat10.1 hypothetical protein (Tb09.211.2900) partial mRNA
GLOS_TB09.211.3330.1.1	845.851687416527	0	0	4.49647769379833e-23	XM_822384.1 Tbb GUTat10.1 cystathione gamma lyase (Tb09.211.3330) partial mRNA
GLOS_TB09.211.3500.1.1	389.903120972637	0	0	1.23961456888981e-42	XP_827493.1 hypothetical protein [Trypanosoma brucei brucei strain 927/4 GUTat10.1]
GLOS_TB09.211.3610.1.1	651.540739623935	0	0	4.64150475967656e-29	XM_822409.1 Tbb GUTat10.1 ubiquitin-activating enzyme E1 (Tb09.211.3610) partial mRNA
GLOS_TB09.211.4070.1.1	830.718901925261	0	0	4.0655083858192e-18	XM_822456.1 Tbb strain 927/4 GUTat10.1 hypothetical protein (Tb09.211.4070) partial mRNA
GLOS_TB09.211.4360.1.1	389.903120972637	0	0	1.23961456888981e-42	XM_822482.1 Tbb strain 927/4 GUTat10.1 hypothetical protein (Tb09.211.4360) partial mRNA
GLOS_TB09.211.4470.1.1	368.607774837862	0	0	2.69097341005232e-46	XP_827586.1 ADP-ribosylation factor [Trypanosoma brucei brucei strain 927/4 GUTat10.1]<br>ref|XP_827587.1| ADP-ribosylation factor [T. brucei brucei strain 927/4 GUTat10.1]<br>ref|XP_827588.1| ADP-ribosylation factor [T. brucei brucei strain 927/4 GUTat10.1]
GLOS_TB09.211.4550.1.1	1393.1747768174	0	0	1.01341779293047e-32	XM_822504.1 Tbb GUTat10.1 60S ribosomal protein L12 (Tb09.211.4550) partial mRNA
GLOS_TB09.211.4760.1.1	680.062237480702	0	0	5.1437432012731e-29	XM_822523.1 Tbb strain 927/4 GUTat10.1 metacaspase 5 (Tb09.211.4760) partial mRNA
GLOS_TB09.244.2570.1.1	732.453822480335	0	0	1.5977312474628e-29	XM_822590.1 Tbb GUTat10.1 calcium motive p-type ATPase (Tb09.244.2570) partial mRNA
GLOS_TB09.244.2660.1.1	436.61492774385	0	0	6.18944712647057e-42	XM_822578.1 Tbb strain 927/4 GUTat10.1 hypothetical protein (Tb09.244.2660) partial mRNA
GLOS_TB09.244.2720.1.1	2207.7125910601	0	0	1.41014674814952e-30	XM_822570.1 Tbb GUTat10.1 ribosomal protein L15 (Tb09.244.2720) partial mRNA
GLOS_TB09.244.2725.1.1	559.90106856083	0	0	4.89560853560191e-18	XM_822571.1 Tbb strain 927/4 GUTat10.1 ribosomal protein L36 (Tb09.244.2725) partial mRNA
GLOS_TB09.V1.0150.1.1	704.5652928737	0	0	4.01455651229244e-20	XM_798607.1 Tbb strain 927/4 GUTat10.1 hypothetical protein (Tb09.v1.0150) partial mRNA
GLOS_TB09.V1.0380.1.1	510.424465592929	0	0	4.49963175671889e-21	XM_822031.1 Tbb strain 927/4 GUTat10.1 spermidine synthase (Tb09.v1.0380) partial mRNA

GLOS_TB09.V1.0420.1.1	535.057772144191	0	0	8.47950901640774e-45	XP_827128.1 hypothetical protein [Trypanosoma brucei brucei strain 927/4 GUTat10.1]
GLOS_TB09.V2.0030.1.1	341.13457917239	0	0	2.017479646338e-40	XM_821914.1 Tbb strain 927/4 GUTat10.1 hypothetical protein (Tb09.v2.0030) partial mRNA
GLOS_TB10.05.0010.1.1	346.786778280437	0	0	1.99530512472048e-18	XP_827840.1 diphosphomevalonate decarboxylase [T. brucei brucei strain 927/4 GUTat10.1]
GLOS_TB10.05.0080.1.1	520.395478834831	0	0	4.62520038653491e-62	XM_822754.1 T. b. brucei strain 927/4 GUTat10.1 glucosidase (Tb10.05.0080) partial mRNA
GLOS_TB10.05.0110.1.1	510.970050692356	0	0	4.00627619531178e-61	XM_822757.1 Tbb serine/threonine protein phosphatase type 5 (Tb10.05.0110) partial mRNA
GLOS_TB10.100.0070.1.1	361.259016401199	0	0	2.83462354285661e-42	XP_822278.1 ATP synthase F1 subunit gamma [T. brucei brucei strain 927/4 GUTat10.1]
GLOS_TB10.26.0100.1.1	583.573456071502	0	0	2.29024917960063e-15	XP_823384.1 hypothetical protein [Trypanosoma brucei brucei strain 927/4 GUTat10.1]
GLOS_TB10.26.0140.1.1	496.838053595235	0	0	1.18064192788732e-24	XM_818290.1 Tbb GUTat10.1 pumilio RNA-binding protein (Tb10.26.0140) partial mRNA
GLOS_TB10.26.0510.1.1	860.759194155229	0	0	1.05355651139416e-19	XM_818259.1 Tbb strain 927/4 GUTat10.1 CYC2-like cyclin (Tb10.26.0510) partial mRNA
GLOS_TB10.26.0560.1.1	1647.93412921394	0	0	1.54181062123259e-32	XM_818254.1 Tbb GUTat10.1 60S ribosomal protein L6 (Tb10.26.0560) partial mRNA
GLOS_TB10.26.0680.1.1	517.160190456237	0	0	2.3589090995888e-35	XP_823339.1 hypothetical protein [Trypanosoma brucei brucei strain 927/4 GUTat10.1]
GLOS_TB10.26.0790.1.1	1236.90578261835	0	0	1.26479666361262e-31	XM_818238.1 Tbb GUTat10.1 procyclic form surface glycoprotein (Tb10.26.0790) partial mRNA
GLOS_TB10.26.0880.1.1	480.847021101272	0	0	1.08086714860525e-13	XM_818231.1 Tbb strain 927/4 GUTat10.1 hypothetical protein (Tb10.26.0880) partial mRNA
GLOS_TB10.389.0070.1.1	892.053132652279	0	0	1.53317045406077e-33	XM_822728.1 Tbb GUTat10.1 elongation factor TU (Tb10.389.0070) partial mRNA
GLOS_TB10.389.0430.1.1	1206.66779075619	0	0	2.75368215341431e-26	XM_822697.1 Tbb receptor-type adenylate cyclase GRESAG 4 (Tb10.389.0430) partial mRNA
GLOS_TB10.389.0630.1.1	639.493056676221	0	0	2.52254010141353e-21	XM_822682.1 Tbb GUTat10.1 prolyl-tRNA synthetase (Tb10.389.0630) partial mRNA
GLOS_TB10.389.0680.1.1	306.565482943191	0	0	1.11005152264024e-35	XP_823487.1 hypothetical protein [Trypanosoma brucei brucei strain 927/4 GUTat10.1]
GLOS_TB10.389.0690.1.1	439.474751534837	0	0	1.55455907972214e-17	XP_823486.1 mitochondrial carrier protein [Trypanosoma brucei brucei strain 927/4 GUTat10.1]
GLOS_TB10.389.0910.2.2	1003.78966182382	0	0	1.39526608557279e-35	XM_818380.1 Tbb GUTat10.1 60S ribosomal protein L34 (Tb10.389.0910) partial mRNA
GLOS_TB10.389.1180.1.1	1404.48681947709	0	0	1.1131670067657e-35	XM_818362.1 Tbb strain 927/4 GUTat10.1 P-type H+ATPase (Tb10.389.1180) partial mRNA
GLOS_TB10.389.1480.1.1	801.10158808005	0	0	1.83277919694639e-30	XM_818338.1 Tbb GUTat10.1 cytosolic nonspecific dipeptidase (Tb10.389.1480) partial mRNA
GLOS_TB10.406.0450.1.1	1361.06545932522	0	0	9.51165737307411e-82	XM_818174.1 Tbb strain 927/4 GUTat10.1 histone H2B (Tb10.406.0450) partial mRNA ref|XM_818176.1| Tbb histone H2B (Tb10.406.0430) partial mRNA ref|XM_818177.1| Tbb histone H2B (Tb10.406.0420) partial mRNA ref|XM_818178.1| Tbb histone H2B (Tb10.406.0410) partial mRNA ref|XM_818179.1| Tbb histone H2B (Tb10.406.0400) part. mRNA ref|XM_818181.1| Tbb histone H2B (Tb10.406.0380) partial mRNA ref|XM_818182.1| Tbb histone H2B (Tb10.406.0370) partial mRNA ref|XM_818184.1| Tbb histone H2B (Tb10.406.0350) partial mRNA
GLOS_TB10.406.0650.1.1	788.468450679352	0	0	4.52032648506049e-29	XM_818156.1 Tbb GUTat10.1 microtubule-associated protein (Tb10.406.0650) partial mRNA
GLOS_TB10.61.0180.1.1	391.414270902244	0	0	6.54826332452674e-12	XM_822986.1 Tbb GUTat10.1 peptidylprolyl isomerase-like protein (Tb10.61.0180) partial mRNA
GLOS_TB10.61.1260.1.1	544.653320798487	0	0	4.92578104596384e-34	XM_822905.1 Tbb GUTat10.1 hypothetical protein (Tb10.61.1260) partial mRNA
GLOS_TB10.61.1330.1.1	709.074162196153	0	0	6.18737858705753e-32	XM_822898.1 Tbb GUTat10.1 nucleosome assembly protein (Tb10.61.1330) partial mRNA
GLOS_TB10.61.1390.1.2	340.569059396185	0	0	3.30114656705591e-21	XP_827985.1 40S ribosomal protein S13 [Tbb strain 927/4 GUTat10.1] ref|XP_951734.1| 40S ribosomal protein S13 [Trypanosoma brucei brucei strain 927/4 GUTat10.1]
GLOS_TB10.61.1390.2.2	1255.55893927111	0	0	1.50048925261142e-27	XP_827985.1 40S ribosomal protein S13 [Tbb strain 927/4 GUTat10.1] ref|XP_951734.1| 40S ribosomal protein S13 [Trypanosoma brucei brucei strain 927/4 GUTat10.1]
GLOS_TB10.61.1820.1.1	1132.88583202749	0	0	3.45226862513918e-44	XM_822868.1 Tbb strain 927/4 GUTat10.1 mitochondrial carrier protein (Tb10.61.1820) partial mRNA. nuclear gene for mitochondrial product ref|XM_822869.1| Tbb GUTat10.1 mitochond. carrier prot. (Tb10.61.1810) partial mRNA. nuclear gene for mitochondrial product
GLOS_TB10.61.1840.1.1	339.710812393488	0	0	4.27000614607731e-16	XP_827959.1 hypothetical protein [Trypanosoma brucei brucei strain 927/4 GUTat10.1]
GLOS_TB10.61.1870.1.1	552.26722734127	0	0	5.47740430533809e-34	XM_822864.1 Tbb strain 927/4 GUTat10.1 aminopeptidase (Tb10.61.1870) partial mRNA

GLOS_TB10.61.1920.1.1	410.395378345459	0	0	7.19437223727267e-51	XM_822860.1 T. b. brucei strain 927/4 GUTat10.1 fibrillarin (Tb10.61.1920) partial mRNA
GLOS_TB10.61.1940.1.1	344.069495880899	0	0	3.40250598046785e-19	XM_822858.1 Tbb strain 927/4 GUTat10.1 chaperone protein DnaJ (Tb10.61.1940) partial mRNA
GLOS_TB10.61.1960.2.2	1720.10226597515	0	0	3.73151545196267e-26	XM_822856.1 Tbb GUTat10.1 40S ribosomal protein S2 (Tb10.61.1960) partial mRNA
GLOS_TB10.61.2090.1.2	913.348478787054	0	0	1.75418059841999e-23	XM_822844.1 Tbb GUTat10.1 60S ribosomal protein L17 (Tb10.61.2090) partial mRNA
GLOS_TB10.61.2090.2.2	302.056613620737	0	0	7.59505657446722e-13	XP_827937.1 60S ribosomal protein L17 [Tbb strain 927/4 GUTat10.1] ref|XP_828557.1| 60S ribosomal protein L17 [Trypanosoma brucei brucei strain 927/4 GUTat10.1]
GLOS_TB10.61.2210.1.1	498.649575194928	0	0	4.15133069001321e-24	XM_822837.1 Tbb strain 927/4 GUTat10.1 hypothetical protein (Tb10.61.2210) partial mRNA
GLOS_TB10.61.2220.1.1	931.831514927991	0	0	4.30200282991466e-22	XM_822836.1 Tbb strain 927/4 GUTat10.1 hypothetical protein (Tb10.61.2220) partial mRNA
GLOS_TB10.61.2270.1.1	343.286341795723	0	0	2.80856231477939e-20	XM_822833.1 Tbb strain 927/4 GUTat10.1 hypothetical protein (Tb10.61.2270) partial mRNA
GLOS_TB10.61.2850.1.1	316.161031597486	0	0	1.16483891939731e-23	XM_822793.1 Tbb strain 927/4 GUTat10.1 hypothetical protein (Tb10.61.2850) partial mRNA
GLOS_TB10.61.2880.1.1	1126.64517981245	0	0	1.55941058343913e-11	XP_827883.1 aconitase [Trypanosoma brucei brucei strain 927/4 GUTat10.1]
GLOS_TB10.6K15.0020.1.3	2383.08529941704	0	0	5.2586354854827e-42	XM_818154.1 Tbb GUTat10.1 EP1 procyclin precursor (Tb10.6k15.0020) partial mRNA
GLOS_TB10.6K15.0020.2.3	1950.41372121944	0	0	9.05030886383741e-41	XM_818154.1 Tbb GUTat10.1 EP1 procyclin precursor (Tb10.6k15.0020) partial mRNA
GLOS_TB10.6K15.0020.3.3	2350.28785543398	0	0	3.68251791360689e-62	XM_818154.1 Tbb GUTat10.1 EP1 procyclin precursor (Tb10.6k15.0020) partial mRNA
GLOS_TB10.6K15.0380.1.1	549.720065453551	0	0	1.10797148967328e-28	XM_818120.1 Tbb strain 927/4 GUTat10.1 hypothetical protein (Tb10.6k15.0380) partial mRNA
GLOS_TB10.6K15.0460.1.1	465.892000565421	0	0	2.14662006543242e-21	XP_823206.1 chaperone protein DnaJ [Trypanosoma brucei brucei strain 927/4 GUTat10.1]
GLOS_TB10.6K15.0690.1.1	592.563615596038	0	0	5.53402008577482e-29	XP_823188.1 hypothetical protein [Trypanosoma brucei brucei strain 927/4 GUTat10.1]
GLOS_TB10.6K15.0810.1.1	479.846232707127	0	0	5.15697002139332e-20	XP_823180.1 hypothetical protein [Trypanosoma brucei brucei strain 927/4 GUTat10.1]
GLOS_TB10.6K15.1220.1.1	808.648046148905	0	0	8.20945418355577e-69	XM_818050.1 Tbb GUTat10.1 isoleucyl-tRNA synthetase (Tb10.6k15.1220) partial mRNA
GLOS_TB10.6K15.1350.1.1	884.957232088552	0	0	5.29209208614326e-53	XM_818039.1 Tbb strain 927/4 GUTat10.1 pteridine transporter (Tb10.6k15.1350) partial mRNA
GLOS_TB10.6K15.1510.1.1	385.12145910047	0	0	1.82120913535352e-10	XP_823117.1 hypothetical protein [Trypanosoma brucei brucei strain 927/4 GUTat10.1]
GLOS_TB10.6K15.1520.1.1	332.49230511509	0	0	3.0558962373827e-39	XM_818023.1 Tbb strain 927/4 GUTat10.1 small GTPase (Tb10.6k15.1520) partial mRNA
GLOS_TB10.6K15.1820.1.1	530.283754715618	0	0	9.5988255752072e-45	XM_818004.1 Tbb strain 927/4 GUTat10.1 hypothetical protein (Tb10.6k15.1820) partial mRNA
GLOS_TB10.6K15.2180.1.1	499.405150159731	0	0	2.55945238284475e-15	XP_823066.1 cytochrome c oxidase subunit IX [T. brucei brucei strain 927/4 GUTat10.1]
GLOS_TB10.6K15.2250.1.1	642.850951769486	0	0	5.54474385929245e-35	XM_817968.1 Tbb eukaryotic translat. Initiati. fact. 3 subunit 8 (Tb10.6k15.2250) partial mRNA
GLOS_TB10.6K15.2290.1.1	331.218724171231	0	0	2.10178801374501e-33	XM_817964.1 Tbb GUTat10.1 protein disulfide isomerase (Tb10.6k15.2290) partial mRNA
GLOS_TB10.6K15.2330.1.1	616.603823250723	0	0	4.98565958361289e-25	XP_823053.1 t-complex protein 1 subunit theta [T. brucei brucei strain 927/4 GUTat10.1]
GLOS_TB10.6K15.2510.1.1	409.537131342763	0	0	5.26958103119322e-36	XM_817944.1 Tbb strain 927/4 GUTat10.1 hypothetical protein (Tb10.6k15.2510) partial mRNA
GLOS_TB10.6K15.2660.1.1	610.018284222458	0	0	2.31214172281683e-25	XM_817930.1 Tbb strain 927/4 GUTat10.1 hypothetical protein (Tb10.6k15.2660) partial mRNA
GLOS_TB10.6K15.2690.1.1	359.957856336968	0	0	7.14878820698066e-18	XM_817927.1 Tbb strain 927/4 GUTat10.1 hypothetical protein (Tb10.6k15.2690) partial mRNA
GLOS_TB10.6K15.3080.1.1	896.336723222168	0	0	4.22022746949174e-31	XM_817898.1 Tbb dihydrolipoamide acetyltransferase precursor (Tb10.6k15.3080) partial mRNA
GLOS_TB10.6K15.3250.1.1	926.507266610402	0	0	4.2138683669618e-96	XM_817883.1 Tbb succinyl-CoA ligase [GDP-forming] beta-chain (Tb10.6k15.3250) partial mRNA
GLOS_TB10.6K15.3510.1.1	608.281855540287	0	0	1.33731727133809e-32	XM_817860.1 Trypanosoma brucei brucei strain 927/4 GUTat10.1 cysteine-rich acidic integral membrane protein precursor (Tb10.6k15.3510) partial mRNA
GLOS_TB10.6K15.3800.1.1	675.873674505112	0	0	1.34364689928524e-22	XM_817839.1 Tbb dipeptidyl-peptidase 8-like serine peptidase (Tb10.6k15.3800) partial mRNA
GLOS_TB10.6K15.3820.1.1	706.527000308435	0	0	3.87257183498673e-28	XM_817837.1 Tbb GUTat10.1 sterol 24-c-methyltransferase (Tb10.6k15.3820) partial mRNA
GLOS_TB10.70.0010.1.1	664.316418416081	0	0	9.52919490422418e-19	XM_817822.1 Tbb strain 927/4 GUTat10.1 hypothetical protein (Tb10.70.0010) partial mRNA
GLOS_TB10.70.0440.1.1	472.675239225879	0	0	1.00685584646843e-28	XM_817788.1 Tbb strain 927/4 GUTat10.1 hypothetical protein (Tb10.70.0440) partial mRNA
GLOS_TB10.70.0465.1.1	493.460223825192	0	0	1.36288027092049e-27	XM_817786.1 Tbb GUTat10.1 60S ribosomal proteins L37 (Tb10.70.0465) partial mRNA
GLOS_TB10.70.0730.1.1	382.289214429855	0	0	4.87744578517148e-43	XP_822855.1 hypothetical protein [Trypanosoma brucei brucei strain 927/4 GUTat10.1]
GLOS_TB10.70.0830.1.1	775.835313278654	0	0	9.77160769921249e-28	XP_822847.1 clathrin heavy chain [Trypanosoma brucei brucei strain 927/4 GUTat10.1]

GLOS_TB10.70.0960.1.1	693.158222619712	0	0	2.11283010168548e-23	XP_822838.1 protein kinase [Trypanosoma brucei brucei strain 927/4 GUTat10.1]
GLOS_TB10.70.1100.1.1	519.462138914613	0	0	4.61054261353442e-40	XM_817735.1 Tbb GUTat10.1 translation elongation factor 1-beta (Tb10.70.1100) partial mRNA
GLOS_TB10.70.1120.1.1	337.879356117018	0	0	5.23136512094107e-43	XM_817733.1 Tbb strain 927/4 GUTat10.1 hypothetical protein (Tb10.70.1120) partial mRNA
GLOS_TB10.70.1130.1.1	437.350568031876	0	0	1.09363995824421e-53	XM_817732.1 Tbb strain 927/4 GUTat10.1 hypothetical protein (Tb10.70.1130) partial mRNA
GLOS_TB10.70.1540.1.2	1011.10319669652	0	0	3.39873017925452e-21	XM_817699.1 Tbb GUTat10.1 60S ribosomal protein L24 (Tb10.70.1540) partial mRNA
GLOS_TB10.70.1540.2.2	298.971511077185	0	0	5.93650868639444e-12	XM_817699.1 Tbb GUTat10.1 60S ribosomal protein L24 (Tb10.70.1540) partial mRNA
GLOS_TB10.70.1660.1.1	393.205857825159	0	0	3.18219493760251e-24	XP_822783.1 hypothetical protein [Trypanosoma brucei brucei strain 927/4 GUTat10.1]
GLOS_TB10.70.1690.2.2	517.943344541412	0	0	1.32933560826529e-33	XM_817688.1 Tbb GUTat10.1 40S ribosomal protein S10 (Tb10.70.1690) partial mRNA
GLOS_TB10.70.1740.1.1	898.903819786664	0	0	7.62035264236729e-23	XM_817685.1 Tbb GUTat10.1 40S ribosomal protein S18 (Tb10.70.1740) partial mRNA
GLOS_TB10.70.1890.1.1	454.655050823253	0	0	2.95972234961522e-22	XP_822767.1 hypothetical protein [Trypanosoma brucei brucei strain 927/4 GUTat10.1]
GLOS_TB10.70.1930.1.1	461.778530507352	0	0	8.8306980273764e-20	XM_817671.1 Tbb strain 927/4 GUTat10.1 hypothetical protein (Tb10.70.1930) partial mRNA
GLOS_TB10.70.2170.1.1	1047.6815141576	0	0	5.01989036279771e-25	XM_817657.1 Tbb GUTat10.1 ubiquitin/ribosomal protein S27a (Tb10.70.2170) partial mRNA
GLOS_TB10.70.2300.1.1	362.702717856879	0	0	5.36969322770013e-34	XM_817644.1 Tbb strain 927/4 GUTat10.1 chaperone protein DnaJ (Tb10.70.2300) partial mRNA
GLOS_TB10.70.2460.1.1	442.437247363718	0	0	4.8226494583674e-18	XP_822722.1 hypothetical protein [Trypanosoma brucei brucei strain 927/4 GUTat10.1]
GLOS_TB10.70.2970.1.1	529.377993915772	0	0	1.69684735701837e-45	XP_822676.1 hypothetical protein [Trypanosoma brucei brucei strain 927/4 GUTat10.1]
GLOS_TB10.70.3120.1.1	352.78686285572	0	0	8.47106563844441e-29	XP_822663.1 hypothetical protein [Trypanosoma brucei brucei strain 927/4 GUTat10.1]
GLOS_TB10.70.3150.1.1	548.644184141885	0	0	7.83472445740591e-36	XP_822661.1 hypothetical protein [Trypanosoma brucei brucei strain 927/4 GUTat10.1]
GLOS_TB10.70.3160.1.2	560.711801766377	0	0	6.10814238421487e-26	XM_817567.1 Tbb GUTat10.1 60S ribosomal protein L30 (Tb10.70.3160) partial mRNA
GLOS_TB10.70.3160.2.2	395.187499936672	0	0	1.07606133859762e-18	XM_817567.1 Tbb GUTat10.1 60S ribosomal protein L30 (Tb10.70.3160) partial mRNA
GLOS_TB10.70.3190.1.1	332.690004747282	0	0	2.08466867712137e-42	XM_817563.1 Tbb strain 927/4 GUTat10.1 hypothetical protein (Tb10.70.3190) partial mRNA
GLOS_TB10.70.3290.1.1	944.982658307744	0	0	9.25349285649681e-32	XP_822647.1 ATP-dependent DEAD-box RNA helicase [Trypanosoma brucei TREU927]
GLOS_TB10.70.3950.1.1	338.049476628838	0	0	4.41471959246717e-36	XM_817506.1 Tbb strain 927/4 GUTat10.1 hypothetical protein (Tb10.70.3950) partial mRNA
GLOS_TB10.70.4155.1.2	316.208545394636	0	0	5.20986144512758e-17	XP_822579.1 60S ribosomal protein L38 [Trypanosoma brucei brucei strain 927/4 GUTat10.1]
GLOS_TB10.70.4155.2.2	971.589962526926	0	0	6.38831027564359e-22	XP_822579.1 60S ribosomal protein L38 [Trypanosoma brucei brucei strain 927/4 GUTat10.1]
GLOS_TB10.70.4280.1.1	841.425555455189	0	0	1.56790732609342e-38	XP_822573.1 delta-1-pyrroline-5-carboxylate dehydrogenase [Trypanosoma brucei]
GLOS_TB10.70.4540.1.1	379.884593933585	0	0	4.48281614598317e-14	XM_817461.1 Tbb strain 927/4 GUTat10.1 hypothetical protein (Tb10.70.4540) partial mRNA
GLOS_TB10.70.4590.1.1	713.472715037119	0	0	2.38140944093973e-14	XP_822550.1 hypothetical protein [Trypanosoma brucei brucei strain 927/4 GUTat10.1]
GLOS_TB10.70.4720.1.1	709.71477488988	0	0	1.82314930624384e-59	XM_817450.1 Tbb GUTat10.1 importin subunit beta-1 (Tb10.70.4720) partial mRNA
GLOS_TB10.70.4800.1.2	599.687095280136	0	0	5.1176221355634e-26	XM_817444.1 Tbb strain 927/4 GUTat10.1 ribosomal protein S25 (Tb10.70.4800) partial mRNA
GLOS_TB10.70.4800.2.2	403.216740420617	0	0	2.05227138729629e-16	XM_817444.1 Tbb strain 927/4 GUTat10.1 ribosomal protein S25 (Tb10.70.4800) partial mRNA
GLOS_TB10.70.4880.1.1	355.674265767079	0	0	1.27762750579402e-20	XM_817437.1 Tbb eukaryotic translation initiation factor 5 (Tb10.70.4880) partial mRNA
GLOS_TB10.70.5020.1.1	327.445495136803	0	0	2.5777522056488e-16	XM_817424.1 Tbb strain 927/4 GUTat10.1 hypothetical protein (Tb10.70.5020) partial mRNA
GLOS_TB10.70.5100.1.1	337.606563567304	0	0	2.22850471565623e-20	XM_817417.1 Tbb lysosomal alpha-mannosidase precursor (Tb10.70.5100) partial mRNA
GLOS_TB10.70.5150.1.1	448.805152083014	0	0	5.6958062311563e-28	XP_822506.1 adenylate kinase [Trypanosoma brucei brucei strain 927/4 GUTat10.1]
GLOS_TB10.70.5360.1.1	394.582110806911	0	0	3.94533287706312e-49	XM_817398.1 Tbb strain 927/4 GUTat10.1 La protein (Tb10.70.5360) partial mRNA
GLOS_TB10.70.5500.1.1	398.640422624236	0	0	2.76938906829312e-22	XM_817385.1 Tbb strain 927/4 GUTat10.1 hypothetical protein (Tb10.70.5500) partial mRNA
GLOS_TB10.70.5560.1.1	472.817780617327	0	0	5.43986293774445e-15	XM_817380.1 Tbb strain 927/4 GUTat10.1 hypothetical protein (Tb10.70.5560) partial mRNA
GLOS_TB10.70.5590.1.1	339.615784799189	0	0	8.54729704014891e-31	XM_817377.1 Tbb strain 927/4 GUTat10.1 hypothetical protein (Tb10.70.5590) partial mRNA
GLOS_TB10.70.6300.1.1	399.838910650574	0	0	2.9807999603811e-18	XM_817326.1 Tbb dual specificity protein phosphatase (Tb10.70.6300) partial mRNA
GLOS_TB10.70.6325.1.1	601.130796735816	0	0	1.18428950866364e-23	XM_817323.1 Tbb GUTat10.1 ribosomal protein L36 (Tb10.70.6325) partial mRNA
GLOS_TB10.70.6340.1.1	990.2308289465	0	0	4.59338296592086e-56	XM_817321.1 Tbb strain 927/4 GUTat10.1 ATPase subunit 9 (Tb10.70.6340) partial mRNA

GLOS_TB10.70.6450.1.1	568.448315023831	0	0 9.4269602309486e-27	XM_817315.1 Tbb transcriptional regulatory protein NOT1 (Tb10.70.6450) partial mRNA
GLOS_TB10.70.6470.1.1	894.525201622476	0	0 1.51530198198906e-31	XM_817314.1 Tbb GUTat10.1 methionyl-tRNA synthetase (Tb10.70.6470) partial mRNA
GLOS_TB10.70.6480.1.1	716.122548962731	0	0 3.85752764406045e-24	XM_817313.1 Tbb GUTat10.1 hypothetical protein (Tb10.70.6480) partial mRNA
GLOS_TB10.70.6920.1.1	560.146281990172	0	0 6.91439279373047e-19	XP_822375.1 hypothetical protein [Trypanosoma brucei brucei strain 927/4 GUTat10.1]
GLOS_TB10.70.7050.1.1	733.850010138865	0	0 3.70636733846009e-32	XP_822364.1 t-complex protein 1 subunit delta [T. brucei brucei strain 927/4 GUTat10.1]
GLOS_TB10.70.7220.1.1	382.431755821303	0	0 1.32073423728318e-16	XM_817258.1 Tbb strain 927/4 GUTat10.1 hypothetical protein (Tb10.70.7220) partial mRNA
GLOS_TB10.70.7760.1.1	849.917643677446	0	0 1.52002237719712e-27	XP_822310.1 hypothetical protein [Trypanosoma brucei brucei strain 927/4 GUTat10.1]
GLOS_TB11.01.0700.1.1	413.79314279228	0	0 4.41710099040914e-17	XP_828962.1 ribose 5-phosphate isomerase [T. brucei brucei strain 927/4 GUTat10.1]
GLOS_TB11.01.0720.1.1	446.455689827488	0	0 5.72327342957202e-33	XM_823871.1 Tbb strain 927/4 GUTat10.1 cation transporter (Tb11.01.0720) partial mRNA
GLOS_TB11.01.0725.1.1	390.365968710949	0	0 2.59908265847471e-24	XM_823872.1 Tbb strain 927/4 GUTat10.1 cation transporter (Tb11.01.0725) partial mRNA
GLOS_TB11.01.0960.1.1	422.312810134909	0	0 4.58364274866013e-17	XM_823894.1 Tbb proteasome regulatory non-ATPase subunit 2 (Tb11.01.0960) partial mRNA
GLOS_TB11.01.1000.1.1	390.686275057813	0	0 1.90291704498363e-39	XM_823896.1 Tbb GUTat10.1 hypothetical protein (Tb11.01.1000) partial mRNA
GLOS_TB11.01.1290.1.1	739.870029390925	0	0 3.42347951793801e-24	XP_829017.1 hypothetical protein [Trypanosoma brucei brucei strain 927/4 GUTat10.1]
GLOS_TB11.01.1350.1.1	1439.98925562651	0	0 2.43750358918847e-45	XM_823930.1 Tbb GUTat10.1 S-adenosylhomocysteine hydrolase (Tb11.01.1350) partial mRNA
GLOS_TB11.01.1465.1.1	703.094012297648	0	0 2.9524946355634e-16	XM_823941.1 Trypanosoma brucei brucei strain 927/4 GUTat10.1 nascent polypeptide associated complex subunit alpha (Tb11.01.1465) partial mRNA
GLOS_TB11.01.1475.1.3	437.445595626175	0	0 1.81093523056787e-28	XP_829036.1 40S ribosomal protein S27 [Trypanosoma brucei brucei strain 927/4 GUTat10.1]
GLOS_TB11.01.1475.2.3	373.42166161999	0	0 1.27452996405609e-14	XP_829036.1 40S ribosomal protein S27 [Trypanosoma brucei brucei strain 927/4 GUTat10.1]
GLOS_TB11.01.1475.3.3	303.132494932404	0	0 2.53059162597836e-10	XP_829036.1 40S ribosomal protein S27 [Trypanosoma brucei brucei strain 927/4 GUTat10.1]
GLOS_TB11.01.1740.1.1	929.802359019329	0	0 8.19698267222308e-28	XM_823969.1 Tbb 2-oxoglutarate dehydrogenase E1 component (Tb11.01.1740) partial mRNA
GLOS_TB11.01.1790.1.2	214.265264509581	0	0 2.54608675746026e-13	XM_823974.1 Tbb GUTat10.1 60S ribosomal protein L29 (Tb11.01.1790) partial mRNA
GLOS_TB11.01.1790.2.2	531.992604277417	0	0 5.58743050967734e-20	XM_823974.1 Tbb GUTat10.1 60S ribosomal protein L29 (Tb11.01.1790) partial mRNA
GLOS_TB11.01.1880.1.1	356.192271746135	0	0 5.07016317702319e-45	XM_823983.1 Tbb GUTat10.1 hypothetical protein (Tb11.01.1880) partial mRNA
GLOS_TB11.01.1910.1.1	534.349710976537	0	0 1.90448749558716e-63	XM_823986.1 Tbb strain 927/4 GUTat10.1 hypothetical protein (Tb11.01.1910) partial mRNA
GLOS_TB11.01.2330.1.1	546.757569624671	0	0 6.57154594133441e-28	XM_824027.1 Tbb strain 927/4 GUTat10.1 hypothetical protein (Tb11.01.2330) partial mRNA
GLOS_TB11.01.2490.1.1	443.465614878235	0	0 2.1400150327473e-18	XM_824043.1 Tbb strain 927/4 GUTat10.1 hypothetical protein (Tb11.01.2490) partial mRNA
GLOS_TB11.01.2680.1.1	3402.15028812358	0	0 5.61992315222552e-43	XM_824062.1 Tbb GUTat10.1 40S ribosomal protein SA (Tb11.01.2680) partial mRNA
GLOS_TB11.01.2740.1.1	304.235955364442	0	0 5.3339040260324e-14	XM_824068.1 Tbb strain 927/4 GUTat10.1 hypothetical protein (Tb11.01.2740) partial mRNA
GLOS_TB11.01.2800.1.1	416.80315241831	0	0 2.93103614220179e-14	XM_824073.1 Tbb strain 927/4 GUTat10.1 hypothetical protein (Tb11.01.2800) partial mRNA
GLOS_TB11.01.2880.1.1	484.307588232431	0	0 1.72209571986197e-58	XP_829173.1 hypothetical protein [Trypanosoma brucei brucei strain 927/4 GUTat10.1]
GLOS_TB11.01.3020.1.1	562.47580956892	0	0 1.1645302858132e-31	XM_824095.1 Tbb GUTat10.1 40S ribosomal protein L14 (Tb11.01.3020) partial mRNA
GLOS_TB11.01.3290.1.1	578.194049513169	0	0 5.28663021203655e-36	XM_824118.1 Tbb GUTat10.1 hypothetical protein (Tb11.01.3290) partial mRNA
GLOS_TB11.01.3350.1.1	466.777826688489	0	0 2.24077315883752e-53	XM_824122.1 Tbb cyclin dependent kinase-binding protein (Tb11.01.3350) partial mRNA
GLOS_TB11.01.3370.1.1	470.863717626186	0	0 1.61003900798604e-29	XM_824124.1 Tbb GUTat10.1 glycosomal membrane protein (Tb11.01.3370) partial mRNA
GLOS_TB11.01.3420.1.1	448.417397262223	0	0 8.22631278848926e-55	XP_829222.1 eukaryotic translation initiation factor [Trypanosoma brucei TREU927]
GLOS_TB11.01.3490.1.1	312.46289548058	0	0 2.88913100214115e-17	XP_829225.1 hypothetical protein [Trypanosoma brucei brucei strain 927/4 GUTat10.1]
GLOS_TB11.01.3560.1.1	481.752781901118	0	0 1.34961011272322e-13	XM_824139.1 Tbb GUTat10.1 vacuolar ATP synthase subunit B (Tb11.01.3560) partial mRNA
GLOS_TB11.01.3610.1.1	655.994450705645	0	0 8.51630395322227e-23	XP_829237.1 membrane-bound acid phosphatase [T. brucei brucei strain 927/4 GUTat10.1]
GLOS_TB11.01.3675.1.2	777.156408019663	0	0 3.25093929638627e-25	XP_829244.1 40S ribosomal protein S17 [Tbb strain 927/4 GUTat10.1] ref|XP_829245.1| 40S ribosomal protein S17 [Trypanosoma brucei brucei strain 927/4 GUTat10.1]
GLOS_TB11.01.3675.2.2	765.088790395171	0	0 3.39109250177842e-31	XP_829244.1 40S ribosomal protein S17 [Tbb strain 927/4 GUTat10.1] ref|XP_829245.1|

					40S ribosomal protein S17 [Trypanosoma brucei brucei strain 927/4 GUTat10.1]
GLOS_TB11.01.3740.1.1	378.298351086457	0	0	1.20557071066298e-39	XM_824159.1 Tbb GUTat10.1 coatomer subunit gamma (Tb11.01.3740) partial mRNA
GLOS_TB11.01.3860.1.1	475.072215278554	0	0	7.30310477009966e-20	XP_829266.1 hypothetical protein [Trypanosoma brucei brucei strain 927/4 GUTat10.1]
GLOS_TB11.01.3915.1.1	453.061163532531	0	0	2.63960037471088e-15	XP_829272.1 hypothetical protein [Trypanosoma brucei brucei strain 927/4 GUTat10.1]
GLOS_TB11.01.4030.1.1	459.061248107813	0	0	2.0751208068882e-20	XM_824190.1 Tbb strain 927/4 GUTat10.1 hypothetical protein (Tb11.01.4030) partial mRNA
GLOS_TB11.01.4130.1.1	377.044704819375	0	0	4.63686586598366e-14	XM_824200.1 Tbb strain 927/4 GUTat10.1 protein kinase (Tb11.01.4130) partial mRNA
GLOS_TB11.01.4140.1.1	527.123559254545	0	0	1.6259918124486e-30	XP_829294.1 hypothetical protein [Trypanosoma brucei brucei strain 927/4 GUTat10.1]
GLOS_TB11.01.4370.1.1	382.139028594812	0	0	5.85039591371523e-20	XM_824222.1 Tbb strain 927/4 GUTat10.1 hypothetical protein (Tb11.01.4370) partial mRNA
GLOS_TB11.01.4480.1.1	336.115348314475	0	0	1.34443557326814e-35	XM_824233.1 Tbb strain 927/4 GUTat10.1 hypothetical protein (Tb11.01.4480) partial mRNA
GLOS_TB11.01.4702.1.1	653.399775020777	0	0	7.04245598426349e-24	XP_829371.1 cytochrome c oxidase subunit 10 [T. brucei brucei strain 927/4 GUTat10.1]
GLOS_TB11.01.4740.1.1	751.134558253465	0	0	3.80132343100437e-33	XM_824282.1 Tbb strain 927/4 GUTat10.1 hypothetical protein (Tb11.01.4740) partial mRNA
GLOS_TB11.01.4750.1.1	846.52752367422	0	0	3.23165058779782e-91	XM_824283.1 Tbb GUTat10.1 elongation factor 1 gamma (Tb11.01.4750) partial mRNA
GLOS_TB11.01.4830.1.1	667.899592261911	0	0	7.64432049395892e-64	XM_824289.1 Tbb eukaryotic translation initiation fact. 2 gamma (Tb11.01.4830) partial mRNA
GLOS_TB11.01.4850.1.1	459.108761904963	0	0	1.45016338730329e-16	XM_824291.1 Tbb strain 927/4 GUTat10.1 hypothetical protein (Tb11.01.4850) partial mRNA
GLOS_TB11.01.4860.1.1	604.876446649872	0	0	2.08992355551455e-23	XP_829385.1 guide RNA-binding protein [Trypanosoma brucei brucei strain 927/4 GUTat10.1]
GLOS_TB11.01.5100.1.1	745.257080392853	0	0	5.99845091367729e-27	XM_824313.1 Tbb GUTat10.1 paraflagellar rod component (Tb11.01.5100) partial mRNA
GLOS_TB11.01.5120.1.1	386.742925511564	0	0	7.97516461638166e-26	XM_824315.1 Tbb strain 927/4 GUTat10.1 hypothetical protein (Tb11.01.5120) partial mRNA
GLOS_TB11.01.5355.1.1	393.225792501936	0	0	9.98413625789788e-12	XM_824342.1 Tbb strain 927/4 GUTat10.1 hypothetical protein (Tb11.01.5355) partial mRNA
GLOS_TB11.01.5590.1.1	2898.57650966503	0	0	5.65708262389385e-39	XM_824365.1 Tbb strain 927/4 GUTat10.1 hypothetical protein (Tb11.01.5590) partial mRNA
GLOS_TB11.01.5690.1.1	328.276163019128	0	0	4.13167105700678e-12	XM_824373.1 Tbb strain 927/4 GUTat10.1 hypothetical protein (Tb11.01.5690) partial mRNA
GLOS_TB11.01.5780.1.1	578.779503966152	0	0	5.21158757340898e-26	XM_824382.1 Tbb strain 927/4 GUTat10.1 hypothetical protein (Tb11.01.5780) partial mRNA
GLOS_TB11.01.5860.1.1	813.319391539585	0	0	3.67002593391919e-35	XP_829481.1 t-complex protein 1 subunit epsilon [Trypanosoma brucei TREU927]
GLOS_TB11.01.5930.1.1	533.729032959588	0	0	4.08658499795442e-16	XM_824396.1 Tbb strain 927/4 GUTat10.1 hypothetical protein (Tb11.01.5930) partial mRNA
GLOS_TB11.01.6360.1.1	327.180347030684	0	0	7.9887290926912e-42	XM_824438.1 Tbb strain 927/4 GUTat10.1 metalloprotease (Tb11.01.6360) partial mRNA
GLOS_TB11.01.6610.1.1	496.44265433085	0	0	5.85154135389404e-15	XM_824462.1 Tbb strain 927/4 GUTat10.1 hypothetical protein (Tb11.01.6610) partial mRNA
GLOS_TB11.01.6660.1.1	543.652532404342	0	0	4.46141800313222e-64	XM_824467.1 Tbb GUTat10.1 iron superoxide dismutase (Tb11.01.6660) partial mRNA
GLOS_TB11.01.7010.1.1	643.831805486854	0	0	4.80774199692442e-45	XM_824502.1 Tbb strain 927/4 GUTat10.1 hypothetical protein (Tb11.01.7010) partial mRNA
GLOS_TB11.01.7460.1.1	336.870923279278	0	0	4.24164362892329e-16	XP_829638.1 hypothetical protein [Trypanosoma brucei brucei strain 927/4 GUTat10.1]
GLOS_TB11.01.7500.1.1	696.270904283635	0	0	1.06144290818005e-35	XM_824548.1 Tbb GUTat10.1 amino acid transporter (Tb11.01.7500) partial mRNA
GLOS_TB11.01.7620.1.1	430.274602144927	0	0	1.54059719890166e-48	XM_824562.1 Tbb strain 927/4 GUTat10.1 hypothetical protein (Tb11.01.7620) partial mRNA
GLOS_TB11.01.7630.1.1	355.211418028767	0	0	4.11534922661877e-36	XM_824563.1 Tbb strain 927/4 GUTat10.1 hypothetical protein (Tb11.01.7630) partial mRNA
GLOS_TB11.01.7800.1.1	583.308307965383	0	0	8.96642646310449e-25	XM_824577.1 Tbb GUTat10.1 nucleoside diphosphate kinase (Tb11.01.7800) partial mRNA
GLOS_TB11.01.7880.1.1	647.815024386657	0	0	1.34645958083316e-17	XM_824584.1 Tbb GUTat10.1 microtubule-associated protein (Tb11.01.7880) partial mRNA
GLOS_TB11.01.8225.1.1	522.982510076105	0	0	4.10666602883583e-18	XP_829708.1 hypothetical protein [Trypanosoma brucei brucei strain 927/4 GUTat10.1]
GLOS_TB11.01.8470.1.1	399.526248747305	0	0	1.08532700958409e-49	XM_824637.1 Tbb GUTat10.1 dihydrolipoyl dehydrogenase (Tb11.01.8470) partial mRNA
GLOS_TB11.01.8510.1.1	600.497828485684	0	0	4.78064992659235e-40	XM_824640.1 Tbb GUTat10.1 t-complex protein 1 subunit alpha (Tb11.01.8510) partial mRNA
GLOS_TB11.01.8820.1.1	964.058793345259	0	0	8.40272239209531e-52	XM_824667.1 Tbb expression site-associated gene (ESAG) protein (Tb11.01.8820) partial mRNA
GLOS_TB11.02.0070.1.1	341.644940707851	0	0	2.73644502312158e-16	XM_823237.1 Tbb strain 927/4 GUTat10.1 aminopeptidase (Tb11.02.0070) partial mRNA
GLOS_TB11.02.0210.1.1	1415.1107356459	0	0	6.27246145186721e-33	XM_823251.1 Tbb strain 927/4 GUTat10.1 hypothetical protein (Tb11.02.0210) partial mRNA
GLOS_TB11.02.0250.1.1	937.566451397154	0	0	4.43568977670933e-38	XP_828348.1 heat shock protein mitochondrial precursor [Trypanosoma brucei TREU927]
GLOS_TB11.02.0750.1.1	561.842841318787	0	0	8.51938047339581e-65	XP_828406.1 t-complex protein 1 subunit zeta [T. brucei brucei strain 927/4 GUTat10.1]

GLOS_TB11.02.0780.1.1	393.185923148381	0	0	5.33347639660975e-49	XM_823316.1 Tbb GUTat10.1 squalene monooxygenase (Tb11.02.0780) partial mRNA
GLOS_TB11.02.0790.1.1	563.844418107078	0	0	2.12365311022101e-22	XM_823317.1 Tbb strain 927/4 GUTat10.1 kinesin (Tb11.02.0790) partial mRNA
GLOS_TB11.02.0815.1.1	734.067644447835	0	0	8.99494763322937e-24	XP_828413.1 ubiquitin-conjugating enzyme [Trypanosoma brucei brucei strain 927/4 GUTat10.1]
GLOS_TB11.02.1070.1.1	952.493892812634	0	0	1.39826283621093e-21	XP_828438.1 aminopeptidase [Trypanosoma brucei brucei strain 927/4 GUTat10.1]
GLOS_TB11.02.1190.1.1	327.493008933953	0	0	1.25993508574481e-12	XM_823359.1 Tbb GUTat10.1 hypothetical protein (Tb11.02.1190) partial mRNA
GLOS_TB11.02.1210.1.1	418.669832258746	0	0	2.2077588475578e-45	XP_828454.1 leucyl-tRNA synthetase [Trypanosoma brucei brucei strain 927/4 GUTat10.1]
GLOS_TB11.02.1470.1.1	398.320116277373	0	0	9.20494774302033e-16	XM_823383.1 Tbb strain 927/4 GUTat10.1 hypothetical protein (Tb11.02.1470) partial mRNA
GLOS_TB11.02.1480.1.1	875.891979646495	0	0	3.80538761654814e-25	XM_823384.1 Tbb strain 927/4 GUTat10.1 mitochondrial processing peptidase subunit alpha (Tb11.02.1480) partial mRNA. nuclear gene for mitochondrial product
GLOS_TB11.02.1680.1.1	456.937064604852	0	0	1.08390470719206e-55	XM_823403.1 T. brucei brucei strain 927/4 GUTat10.1 lectin (Tb11.02.1680) partial mRNA
GLOS_TB11.02.1990.1.1	319.6614680822	0	0	4.6971795623484e-21	XP_828519.1 ferric reductase [Trypanosoma brucei brucei strain 927/4 GUTat10.1]
GLOS_TB11.02.2700.1.1	366.863701712097	0	0	1.09330893067133e-26	XP_828580.1 fumarate hydratase class I [Trypanosoma brucei brucei strain 927/4 GUTat10.1]
GLOS_TB11.02.2940.1.1	385.762071794197	0	0	5.45506065175466e-19	XP_828602.1 ubiquitin carboxyl-terminal hydrolase [Trypanosoma brucei TREU927]
GLOS_TB11.02.2950.1.1	469.930377705968	0	0	4.11797294448998e-18	XM_823510.1 Tbb strain 927/4 GUTat10.1 ATPase subunit 9 (Tb11.02.2950) partial mRNA
GLOS_TB11.02.3020.1.1	432.746671115123	0	0	4.79025459028486e-41	XM_823517.1 Tbb strain 927/4 GUTat10.1 sugar transporter (Tb11.02.3020) partial mRNA
GLOS_TB11.02.3120.1.1	600.940741547218	0	0	1.48996839672009e-62	XM_823526.1 Tbb strain 927/4 GUTat10.1 malic enzyme (Tb11.02.3120) partial mRNA
GLOS_TB11.02.3310.1.1	380.184965603671	0	0	1.25810319819597e-47	XP_828635.1 hypothetical protein [Trypanosoma brucei brucei strain 927/4 GUTat10.1]
GLOS_TB11.02.3570.1.1	404.395293770177	0	0	3.82188112598561e-31	XP_828657.1 hypothetical protein [Trypanosoma brucei brucei strain 927/4 GUTat10.1]
GLOS_TB11.02.3610.1.1	1057.70768564025	0	0	5.17045505376153e-98	XM_823567.1 Tbb strain 927/4 GUTat10.1 hypothetical protein (Tb11.02.3610) partial mRNA
GLOS_TB11.02.3740.1.1	1417.33759118676	0	0	5.38852322602379e-31	XM_823576.1 Tbb receptor-type adenylate cyclase GRESAG 4 (Tb11.02.3740) partial mRNA
GLOS_TB11.02.3770.2.2	826.585497190415	0	0	2.10866484973188e-25	XM_823578.1 Tbb strain 927/4 GUTat10.1 hypothetical protein (Tb11.02.3770) partial mRNA
GLOS_TB11.02.3880.1.1	419.227707591357	0	0	4.27306634862403e-16	XM_823587.1 Tbb strain 927/4 GUTat10.1 hypothetical protein (Tb11.02.3880) partial mRNA
GLOS_TB11.02.3990.1.1	476.788709283947	0	0	1.59805654114485e-31	XM_823594.1 Tbb GUTat10.1 S-phase kinase-associated protein (Tb11.02.3990) partial mRNA
GLOS_TB11.02.4000.1.1	869.191478347153	0	0	6.89397859339507e-59	XP_828688.1 40S ribosomal protein S15a [Tbb strain 927/4 GUTat10.1] ref|XP_844028.1| 40S ribosomal protein S15a [Trypanosoma brucei brucei strain 927/4 GUTat10.1]
GLOS_TB11.02.4040.1.1	453.749290023407	0	0	1.62957993433109e-22	XP_828691.1 protein transport protein Sec31 [T. brucei brucei strain 927/4 GUTat10.1]
GLOS_TB11.02.4120.1.1	348.871092429843	0	0	1.14678424753129e-42	XM_823606.1 Tbb GUTat10.1 hypothetical protein (Tb11.02.4120) partial mRNA
GLOS_TB11.02.4170.1.2	1854.68815921864	0	0	4.4456987374188e-39	XM_823611.1 Tbb GUTat10.1 40S ribosomal protein S5 (Tb11.02.4170) partial mRNA
GLOS_TB11.02.4170.2.2	365.222300624225	0	0	5.29499894297257e-21	XM_823611.1 Tbb GUTat10.1 40S ribosomal protein S5 (Tb11.02.4170) partial mRNA
GLOS_TB11.02.4290.1.1	666.693459791979	0	0	6.91878274630354e-24	XM_823624.1 Tbb strain 927/4 GUTat10.1 hypothetical protein (Tb11.02.4290) partial mRNA
GLOS_TB11.02.4320.1.1	375.553489566546	0	0	2.67388279316436e-20	XM_823627.1 Tbb strain 927/4 GUTat10.1 hypothetical protein (Tb11.02.4320) partial mRNA
GLOS_TB11.02.4350.1.1	1437.97238995103	0	0	1.40676381323204e-25	XM_823631.1 Tbb GUTat10.1 40S ribosomal protein S21 (Tb11.02.4350) partial mRNA
GLOS_TB11.02.4400.1.1	352.003708770545	0	0	6.67663481041933e-31	XM_823636.1 Tbb strain 927/4 GUTat10.1 hypothetical protein (Tb11.02.4400) partial mRNA
GLOS_TB11.02.4420.1.1	881.31425421239	0	0	4.35530687678578e-94	XM_823638.1 Tbb strain 927/4 GUTat10.1 hypothetical protein (Tb11.02.4420) partial mRNA
GLOS_TB11.02.4810.1.1	390.290875793428	0	0	6.61396369702702e-18	XM_823677.1 Tbb strain 927/4 GUTat10.1 hypothetical protein (Tb11.02.4810) partial mRNA
GLOS_TB11.02.4910.1.1	329.161989142197	0	0	4.16247061129364e-31	XP_828778.1 acidocalcisomal pyrophosphatase [T. brucei brucei strain 927/4 GUTat10.1]
GLOS_TB11.02.5120.1.1	386.375105367551	0	0	7.44046372439228e-23	XM_823706.1 Tbb GUTat10.1 hypothetical protein (Tb11.02.5120) partial mRNA
GLOS_TB11.02.5190.1.1	955.946815500198	0	0	3.32794496405222e-23	XP_828807.1 pantothenate kinase subunit [Trypanosoma brucei brucei strain 927/4 GUTat10.1]
GLOS_TB11.02.5490.1.1	1304.42250866566	0	0	4.94238404567299e-24	XM_823744.1 Tbb strain 927/4 GUTat10.1 hypothetical protein (Tb11.02.5490) partial mRNA
GLOS_TB11.02.5570.1.1	362.750231654028	0	0	7.54109804958954e-24	XM_823752.1 Tbb strain 927/4 GUTat10.1 hypothetical protein (Tb11.02.5570) partial mRNA
GLOS_TB11.02.5590.1.1	478.034711107435	0	0	2.02588634383427e-20	XM_823754.1 Tbb strain 927/4 GUTat10.1 hypothetical protein (Tb11.02.5590) partial mRNA

GLOS_TB11.02.5770.1.1	870.022146229478	0	0	1.76048852571958e-46	XM_823772.1 Tbb strain 927/4 GUTat10.1 mitochondrial RNA-binding protein (Tb11.02.5770) partial mRNA. nuclear gene for mitochondrial product
GLOS_TB11.03.0090.1.1	1101.88462075693	0	0	2.63485535672307e-25	XM_823102.1 T. brucei brucei strain 927/4 GUTat10.1 ribokinase (Tb11.03.0090) partial mRNA
GLOS_TB11.03.0140.1.1	448.975272594834	0	0	2.35787416311018e-22	XM_823098.1 Tbb strain 927/4 GUTat10.1 nucleoporin (Tb11.03.0140) partial mRNA
GLOS_TB11.03.0230.1.1	532.230173263165	0	0	1.40220595056907e-09	XP_828183.1 isocitrate dehydrogenase [Trypanosoma brucei brucei strain 927/4 GUTat10.1]
GLOS_TB11.03.0250.1.1	738.924399237524	0	0	3.05139214238781e-82	XM_823088.1 Tbb strain 927/4 GUTat10.1 cyclophilin A (Tb11.03.0250) partial mRNA
GLOS_TB11.03.0300.1.1	552.880260914624	0	0	1.16742523415206e-40	XM_823084.1 Tbb strain 927/4 GUTat10.1 hypothetical protein (Tb11.03.0300) partial mRNA
GLOS_TB11.03.0400.1.1	352.5217147496	0	0	1.42929845899397e-44	XM_823075.1 Tbb strain 927/4 GUTat10.1 DNA repair protein (Tb11.03.0400) partial mRNA
GLOS_TB11.03.0410.1.1	1328.22514733459	0	0	3.74566864647199e-35	XP_828167.1 eukaryotic translation initiation factor 5a [Trypanosoma brucei]
GLOS_TB11.03.0530.1.1	883.5809791068	0	0	2.86724639776862e-29	XM_823054.1 Tbb strain 927/4 GUTat10.1 hypothetical protein (Tb11.03.0530) partial mRNA
GLOS_TB11.03.0900.1.1	367.674434917644	0	0	2.82851746721854e-46	XM_823023.1 Tbb strain 927/4 GUTat10.1 hypothetical protein (Tb11.03.0900) partial mRNA
GLOS_TB11.03.0940.1.1	319.416254652858	0	0	2.93014646108033e-19	XP_828111.1 elongation factor [Trypanosoma brucei brucei strain 927/4 GUTat10.1]
GLOS_TB11.18.0005.1.1	462.806898021869	0	0	3.61989090703218e-20	XM_823216.1 Tbb strain 927/4 GUTat10.1 hypothetical protein (Tb11.18.0005) partial mRNA
GLOS_TB11.22.0004.1.1	325.001005286978	0	0	1.36389272087286e-41	XM_823228.1 Tbb strain 927/4 GUTat10.1 hypothetical protein (Tb11.22.0004) partial mRNA
GLOS_TB11.22.0005.1.1	346.343865218903	0	0	1.59004602425149e-12	XP_828320.1 phenylalanyl-tRNA synthetase [Trypanosoma brucei brucei strain 927/4 GUTat10.1]
GLOS_TB11.27.0001.1.1	2274.91665520414	0	0	7.42689392898002e-32	XM_823146.1 Tbb receptor-type adenylate cyclase GRESAG 4 (Tb11.27.0001) partial mRNA
GLOS_TB11.39.0005.1.1	709.291796505123	0	0	2.15640630827373e-23	XM_823177.1 Tbb GUTat10.1 hypothetical protein (Tb11.39.0005) partial mRNA
GLOS_TB11.42.0003.1.1	749.595829203486	0	0	1.10922122011149e-55	XP_828274.1 t-complex protein 1 subunit beta [T. brucei brucei strain 927/4 GUTat10.1]
GLOS_TB11.47.0006.1.1	440.530698169727	0	0	1.08717976813428e-30	XP_828234.1 hypothetical protein [Trypanosoma brucei brucei strain 927/4 GUTat10.1]
GLOS_TB11.47.0022.1.1	343.191314201424	0	0	1.29433681534979e-43	XP_828218.1 hypothetical protein [Trypanosoma brucei brucei strain 927/4 GUTat10.1]
GLOS_TB11.47.0036.1.1	440.700818681547	0	0	4.08687579751949e-24	XP_828203.1 calpain, partial [Trypanosoma brucei brucei strain 927/4 GUTat10.1]
GLOS_TB11.50.0005.1.1	1567.38122205796	0	0	7.71040503824869e-23	XM_823067.1 Tbb GUTat10.1 60S ribosomal protein L21E (Tb11.50.0005) partial mRNA
GLOS_TB11.50.0007.1.1	1076.15549821722	0	0	6.17257192026418e-28	XP_828164.1 dynein light chain [Trypanosoma brucei brucei strain 927/4 GUTat10.1]
GLOS_TB11.52.0003.1.1	587.864691084986	0	0	1.93399028722518e-37	XP_829346.1 oligopeptidase b [Trypanosoma brucei brucei strain 927/4 GUTat10.1]
GLOS_TB927.1.1240.1.1	326.64240637485	0	0	9.5635731589306e-42	XM_001218825.1 Tbb cytidine triphosphate synthase, putative (Tb927.1.1240) partial mRNA
GLOS_TB927.1.1710.1.1	414.406176365635	0	0	4.9085870631261e-20	XM_001218872.1 T. brucei hypothetical protein, conserved (Tb927.1.1710) partial mRNA
GLOS_TB927.1.2230.1.1	582.307519571238	0	0	3.63299524363963e-39	XM_001218922.1 T. b. calpain-like protein fragment, putative (Tb927.1.2230) partial mRNA
GLOS_TB927.1.2250.1.1	435.341346799991	0	0	5.51883378378475e-37	XM_001218924.1 T. brucei hypothetical protein, unlikely (Tb927.1.2250) partial mRNA
GLOS_TB927.1.3070.1.1	334.228733797261	0	0	9.44501636071031e-24	XM_001219004.1 T. brucei hypothetical protein, conserved (Tb927.1.3070) partial mRNA
GLOS_TB927.1.3310.1.1	369.533470314486	0	0	1.75530070914364e-36	XM_001219028.1 T. brucei hypothetical protein, conserved (Tb927.1.3310) partial mRNA
GLOS_TB927.1.3950.1.1	364.759452885913	0	0	2.32430031892927e-36	XP_001219089.1 alanine aminotransferase [Trypanosoma brucei brucei strain 927/4 GUTat10.1]
GLOS_TB927.1.4370.1.1	343.654161939736	0	0	4.47945956787265e-23	XM_001219129.1 T. brucei hypothetical protein, conserved (Tb927.1.4370) partial mRNA
GLOS_TB927.1.4740.1.1	334.719160655945	0	0	5.19731858671496e-29	XM_001219166.1 T. brucei hypothetical protein, conserved (Tb927.1.4740) partial mRNA
GLOS_TB927.1.540.1.2	645.880896072294	0	0	1.74208345306904e-17	XM_001218756.1 Tbb DNA-directed RNA polymerase III, putative (Tb927.1.540) partial mRNA
GLOS_TB927.1.540.2.2	1682.84346646678	0	0	1.48815352029711e-29	XM_001218756.1 Tbb DNA-directed RNA polymerase III, putative (Tb927.1.540) partial mRNA
GLOS_TB927.1.860.1.1	339.172871737655	0	0	2.95111530602461e-18	XM_001218788.1 T. brucei hypothetical protein, conserved (Tb927.1.860) partial mRNA
GLOS_TB927.2.2510.1.1	688.752025335152	0	0	7.46097066874278e-25	XP_951552.1 hypothetical protein [Tbb strain 927/4 GUTat10.1] ref|XP_951553.1| hypothetical protein [Trypanosoma brucei brucei strain 927/4 GUTat10.1]
GLOS_TB927.2.3460.1.1	426.133552966486	0	0	5.17175472640749e-22	XM_946502.1 Tbb GUTat10.1 D-alanyl-glycyl endopeptidase (Tb927.2.3460) partial mRNA
GLOS_TB927.2.3780.1.1	334.038678608663	0	0	1.68625076738733e-42	XM_946511.1 Tbb GUTat10.1 translation initiation factor IF-2 (Tb927.2.3780) partial mRNA
GLOS_TB927.2.380.1.1	708.939265248299	0	0	1.70805635104833e-79	XP_951481.1 retrotransposon hot spot (RHS) protein [Trypanosoma brucei TREU927]

GLOS_TB927.2.3800.1.1	491.478581713679	0	0	7.82606490143965e-36	XM_946512.1 Tbb GUTat10.1 hypothetical protein (Tb927.2.3800) partial mRNA
GLOS_TB927.2.4060.1.1	336.775895684979	0	0	4.95464138624796e-31	XM_946523.1 Tbb GUTat10.1 dynein intermediate chain (Tb927.2.4060) partial mRNA
GLOS_TB927.2.4130.1.1	338.069411305616	0	0	2.90958261283008e-13	XM_946526.1 Tbb strain 927/4 GUTat10.1 enoyl-CoA hydratase/Enoyl-CoA isomerase/3-hydroxyacyl-CoA dehydrogenase (Tb927.2.4130) partial mRNA
GLOS_TB927.2.4210.1.1	1735.95540286725	0	0	3.48343942032609e-17	XM_946531.1 Tbb glycosomal phosphoenolpyruvate carboxykinase (Tb927.2.4210) partial mRNA
GLOS_TB927.2.4230.1.1	461.288103648668	0	0	1.20676489351185e-17	XM_946532.1 Tbb strain 927/4 GUTat10.1 NUP-1 protein (Tb927.2.4230) partial mRNA
GLOS_TB927.2.4580.1.1	547.568302830218	0	0	1.93955560617738e-46	XM_946554.1 Tbb strain 927/4 GUTat10.1 hypothetical protein (Tb927.2.4580) partial mRNA
GLOS_TB927.2.4610.1.1	344.164523475198	0	0	1.13668295645311e-11	XP_951649.1 branched-chain amino acid aminotransferase [Trypanosoma brucei TREU927]
GLOS_TB927.2.470.1.2	719.555536973518	0	0	1.79256653404514e-49	XM_946391.1 Tbb GUTat10.1 retrotransposon hot spot (RHS) protein (Tb927.2.470) partial mRNA
GLOS_TB927.2.470.2.2	572.541850405122	0	0	3.86465876521897e-67	XM_946391.1 Tbb GUTat10.1 retrotransposon hot spot (RHS) protein (Tb927.2.470) partial mRNA
GLOS_TB927.2.4700.1.1	788.243171926788	0	0	2.10965422908985e-18	XM_946561.1 Tbb strain 927/4 GUTat10.1 hypothetical protein (Tb927.2.4700) partial mRNA
GLOS_TB927.2.4850.1.1	437.860929567337	0	0	3.45749253094489e-24	XM_946572.1 Tbb strain 927/4 GUTat10.1 hypothetical protein (Tb927.2.4850) partial mRNA
GLOS_TB927.2.5360.1.1	622.631486946378	0	0	1.22535930227578e-56	XM_946615.1 Tbb strain 927/4 GUTat10.1 hypothetical protein (Tb927.2.5360) partial mRNA
GLOS_TB927.2.5500.1.1	776.250647219817	0	0	2.1571505626463e-25	XM_946619.1 Tbb strain 927/4 GUTat10.1 hypothetical protein (Tb927.2.5500) partial mRNA
GLOS_TB927.2.5810.1.1	317.849946482508	0	0	9.14213969353597e-22	XM_946633.1 Tbb strain 927/4 GUTat10.1 hypothetical protein (Tb927.2.5810) partial mRNA
GLOS_TB927.2.6090.1.1	471.223893326605	0	0	3.06201861959093e-11	XM_946650.1 Tbb GUTat10.1 60S ribosomal protein L44 (Tb927.2.6090) partial mRNA
GLOS_TB927.3.1010.1.1	893.912168049121	0	0	1.00924952815533e-28	XM_838604.1 Trypanosoma brucei hypothetical protein, conserved (Tb927.3.1010) partial mRNA
GLOS_TB927.3.1110.1.1	366.618488282755	0	0	2.04146677104804e-24	XM_838612.1 Trypanosoma brucei hypothetical protein, conserved (Tb927.3.1110) partial mRNA
GLOS_TB927.3.1120.1.1	915.167644830341	0	0	1.64776976657946e-49	XP_843706.1 GTP-binding nuclear protein rtb2 [T. brucei brucei strain 927/4 GUTat10.1]
GLOS_TB927.3.1370.1.2	463.439866272002	0	0	1.07715985569187e-12	XM_838638.1 T. brucei 40S ribosomal protein S25, putative (Tb927.3.1370) partial mRNA
GLOS_TB927.3.1370.2.2	412.274348419079	0	0	6.60236803128131e-15	XM_838638.1 T. brucei 40S ribosomal protein S25, putative (Tb927.3.1370) partial mRNA
GLOS_TB927.3.1410.1.1	765.081145951576	0	0	1.95487123515187e-15	XP_843735.1 cytochrome c oxidase VII [Trypanosoma brucei brucei strain 927/4 GUTat10.1]
GLOS_TB927.3.1690.1.1	377.44010408376	0	0	7.99448809647685e-28	XP_843763.1 hypothetical protein [Trypanosoma brucei brucei strain 927/4 GUTat10.1]
GLOS_TB927.3.1790.1.1	787.345055570536	0	0	2.41717213921018e-40	XM_838680.1 Tbb pyruvate dehydrogenase E1 beta subunit, putat. (Tb927.3.1790) partial mRNA
GLOS_TB927.3.1840.1.1	1261.33374509383	0	0	3.65324967281034e-23	XP_843778.1 3-oxo-5-alpha-steroid 4-dehydrogenase [Trypanosoma brucei TREU927]
GLOS_TB927.3.1940.1.1	362.137198080673	0	0	1.78392520747833e-19	XP_843788.1 hypothetical protein [Trypanosoma brucei brucei strain 927/4 GUTat10.1]
GLOS_TB927.3.2100.1.1	418.567160220853	0	0	3.0349455211601e-17	XM_838711.1 Trypanosoma brucei hypothetical protein, conserved (Tb927.3.2100) partial mRNA
GLOS_TB927.3.2180.1.1	405.961601940527	0	0	1.23471677947225e-27	XP_843812.1 hypothetical protein [Trypanosoma brucei brucei strain 927/4 GUTat10.1]
GLOS_TB927.3.2230.1.1	803.696263764917	0	0	3.77655532987561e-29	XM_838724.1 Tbb succinyl-CoA synthetase alpha subunit, putative (Tb927.3.2230) partial mRNA
GLOS_TB927.3.2490.1.1	407.868151134519	0	0	4.34424667060932e-16	XM_838750.1 Trypanosoma brucei hypothetical protein, conserved (Tb927.3.2490) partial mRNA
GLOS_TB927.3.2900.1.1	428.463080545234	0	0	8.64214015823622e-51	XM_838790.1 Tbb elongat. initiation fact. 2 alpha subunit, putative (Tb927.3.2900) partial mRNA
GLOS_TB927.3.3410.1.1	337.361350137962	0	0	9.13841760973053e-19	XP_843934.1 aspartyl aminopeptidase [Trypanosoma brucei brucei strain 927/4 GUTat10.1]
GLOS_TB927.3.3460.1.1	559.363127904996	0	0	1.70594781172658e-19	XM_838845.1 Trypanosoma brucei hypothetical protein, conserved (Tb927.3.3460) partial mRNA
GLOS_TB927.3.3490.1.1	399.621276341604	0	0	5.44137864844368e-31	XM_838848.1 T. brucei high mobility group protein, putative (Tb927.3.3490) partial mRNA
GLOS_TB927.3.3690.1.1	408.556277625395	0	0	2.26575490668904e-25	XM_838868.1 Tbb flagellar radial spoke protein-like, putative (Tb927.3.3690) partial mRNA
GLOS_TB927.3.3750.1.1	508.545495519309	0	0	6.65629955110795e-61	XM_838874.1 Trypanosoma brucei hypothetical protein, conserved (Tb927.3.3750) partial mRNA
GLOS_TB927.3.3770.1.1	311.434527966063	0	0	8.37512613108039e-17	XP_843969.1 hypothetical protein [Trypanosoma brucei brucei strain 927/4 GUTat10.1] ref|XP_843971.1| hypothetical protein [Trypanosoma brucei brucei strain 927/4 GUTat10.1]
GLOS_TB927.3.3900.1.1	410.170099592895	0	0	8.20187446712137e-18	XM_838889.1 Tbb carnitine O-palmitoyltransferase II, putative (Tb927.3.3900) partial mRNA
GLOS_TB927.3.4080.1.1	930.082796012637	0	0	5.96040482357678e-98	XM_838907.1 Trypanosoma brucei hypothetical protein, conserved (Tb927.3.4080) partial mRNA
GLOS_TB927.3.4160.1.1	373.987181396196	0	0	1.4728912425382e-22	XM_838915.1 Trypanosoma brucei hypothetical protein, conserved (Tb927.3.4160) partial mRNA

GLOS_TB927.3.4500.1.1	1839.96306322494	0	0 1.19341595936961e-23	XP_844042.1 fumarate hydratase [Trypanosoma brucei brucei strain 927/4 GUTat10.1]
GLOS_TB927.3.4750.1.1	789.742031623211	0	0 9.40955430726733e-31	XP_844067.1 aminopeptidase [Tbb] ref|XP_844071.1| aminopeptidase [Tbb]
GLOS_TB927.3.4840.1.1	374.355001540209	0	0 1.40304429346588e-25	XP_844076.1 ubiquitin hydrolase [Trypanosoma brucei brucei strain 927/4 GUTat10.1]
GLOS_TB927.3.720.1.1	502.225104597163	0	0 3.34105621216166e-29	XM_838575.1 Trypanosoma brucei zinc finger protein 2, putative (Tb927.3.720) partial mRNA
GLOS_TB927.3.740.1.1	585.922918327028	0	0 3.03498675765419e-14	XM_838577.1 Trypanosoma brucei hypothetical protein, conserved (Tb927.3.740) partial mRNA
GLOS_TB927.3.860.1.1	463.862844656758	0	0 4.73904049789792e-36	XP_843682.1 acyl carrier protein, mitochondrial precursor [Trypanosoma brucei TREU927]
GLOS_TB927.4.1080.1.1	414.706548035721	0	0 2.8923125274129e-51	XM_839165.1 Tbb GUTat10.1 V-type ATPase, A subunit, putative (Tb927.4.1080) partial mRNA
GLOS_TB927.4.1120.1.1	413.915749506951	0	0 1.29716863508433e-17	XP_844262.1 hypothetical protein [Trypanosoma brucei brucei strain 927/4 GUTat10.1]
GLOS_TB927.4.1350.1.1	458.938641393142	0	0 8.35182327187135e-20	XP_844285.1 hydroxyacylglutathione hydrolase [T. brucei brucei strain 927/4 GUTat10.1]
GLOS_TB927.4.1630.1.1	385.64710952312	0	0 3.80306737703652e-48	XM_839220.1 Tbb GUTat10.1 ribosomal RNA processing protein 6 (Tb927.4.1630) partial mRNA
GLOS_TB927.4.1850.1.1	319.048434508845	0	0 5.38626776651083e-17	XM_839242.1 Tbb GUTat10.1 hypothetical protein, conserved (Tb927.4.1850) partial mRNA
GLOS_TB927.4.1860.1.2	586.365831388562	0	0 7.22047901163224e-18	XM_839243.1 Tbb GUTat10.1 ribosomal protein S19, putative (Tb927.4.1860) partial mRNA
GLOS_TB927.4.1860.2.2	1176.06962319361	0	0 1.38989729035178e-35	XP_844336.1 ribosomal protein S19 [Trypanosoma brucei brucei strain 927/4 GUTat10.1]
GLOS_TB927.4.2000.1.1	319.784074796871	0	0 4.68205070495786e-22	XM_839257.1 Tbb GUTat10.1 RuvB-like DNA helicase, putative (Tb927.4.2000) partial mRNA
GLOS_TB927.4.2030.1.1	469.760257194148	0	0 8.7377995206122e-22	XM_839260.1 Tbb GUTat10.1 hypothetical protein, conserved (Tb927.4.2030) partial mRNA
GLOS_TB927.4.2060.1.1	362.334897712866	0	0 3.78414284788445e-29	XM_839263.1 Tbb GUTat10.1 hypothetical protein, conserved (Tb927.4.2060) partial mRNA
GLOS_TB927.4.2180.1.1	1244.27447573179	0	0 8.248140642021e-31	XM_839275.1 Tbb GUTat10.1 60S ribosomal protein L35A, putative (Tb927.4.2180) partial mRNA
GLOS_TB927.4.2410.1.1	514.933334915382	0	0 1.94540436423473e-43	XM_839298.1 Tb GUTat10.1 hypothetical protein, conserved (Tb927.4.2410) partial mRNA
GLOS_TB927.4.2740.1.1	703.809717908897	0	0 7.12154299044325e-29	XM_839331.1 Tbb GUTat10.1 hypothetical protein, conserved (Tb927.4.2740) partial mRNA
GLOS_TB927.4.2890.1.1	317.849946482508	0	0 9.14213969353597e-22	XM_839346.1 Tbb GUTat10.1 hypothetical protein, conserved (Tb927.4.2890) partial mRNA
GLOS_TB927.4.3060.1.1	896.091509792826	0	0 5.98418756857923e-30	XM_839363.1 Tbb GUTat10.1 hypothetical protein, conserved (Tb927.4.3060) partial mRNA
GLOS_TB927.4.3450.1.1	438.2762635085	0	0 7.20171782317088e-21	XP_844495.1 hypothetical protein [Trypanosoma brucei brucei strain 927/4 GUTat10.1]
GLOS_TB927.4.3500.1.1	594.517678587178	0	0 8.80820960755157e-17	XM_839407.1 Tbb GUTat10.1 hypothetical protein, conserved (Tb927.4.3500) partial mRNA
GLOS_TB927.4.3550.1.1	2775.6306098717	0	0 9.7644640713637e-43	XM_839412.1 Tbb GUTat10.1 60S ribosomal protein L13a, putative (Tb927.4.3550) partial mRNA
GLOS_TB927.4.3590.1.1	537.577354911538	0	0 2.11737886656428e-30	XM_839416.1 Tbb translation elongation factor 1-beta, putative (Tb927.4.3590) partial mRNA
GLOS_TB927.4.3620.1.1	500.338490079949	0	0 6.86960449468101e-23	XM_839419.1 Tbb serine/threonine-protein phosphatase PP1, putative partial mRNA
GLOS_TB927.4.3740.1.1	494.073257398547	0	0 5.61887357475087e-33	XM_839431.1 Tbb GUTat10.1 hypothetical protein, conserved (Tb927.4.3740) partial mRNA
GLOS_TB927.4.3950.1.1	1073.7033639238	0	0 2.64695593506773e-21	XM_839452.1 Tbb cytoskeleton-associated prot. CAP5.5, putative (Tb927.4.3950) partial mRNA
GLOS_TB927.4.4040.1.1	317.482126338495	0	0 5.20479434457952e-19	XM_839461.1 Tbb GUTat10.1 hypothetical protein, conserved (Tb927.4.4040) partial mRNA
GLOS_TB927.4.4050.1.1	330.605690597876	0	0 1.69769879315893e-25	XM_839462.1 Tbb GUTat10.1 ABC transporter, putative (Tb927.4.4050) partial mRNA
GLOS_TB927.4.4160.1.1	381.015633485996	0	0 3.63366244085443e-37	XM_839473.1 Tbb GUTat10.1 hypothetical protein, conserved (Tb927.4.4160) partial mRNA
GLOS_TB927.4.4450.1.1	644.349811465909	0	0 8.17870118227943e-74	XM_839501.1 Tbb receptor-type adenylate cyclase GRESAG 4, putat. (Tb927.4.4450) partial mRNA
GLOS_TB927.4.4570.1.1	494.536105136859	0	0 2.61234862707309e-22	XM_839513.1 Tbb GUTat10.1 hypothetical protein, conserved (Tb927.4.4570) partial mRNA
GLOS_TB927.4.4610.1.1	616.944064274364	0	0 1.01230345767025e-18	XM_839517.1 Tbb GUTat10.1 hypothetical protein, conserved (Tb927.4.4610) partial mRNA
GLOS_TB927.4.4620.1.1	475.364942505045	0	0 3.74835109864058e-17	XP_844611.1 cytochrome c oxidase VIII (COX VIII) [Trypanosoma brucei TREU927]
GLOS_TB927.4.4730.1.1	943.293743422723	0	0 8.48399910599984e-33	XM_839529.1 Tbb GUTat10.1 amino acid transporter, putative (Tb927.4.4730) partial mRNA
GLOS_TB927.4.4990.1.1	414.944117021468	0	0 5.59181801746295e-18	XP_844648.1 ubiquinol-cytochrome C reductase [T. brucei brucei strain 927/4 GUTat10.1]
GLOS_TB927.4.590.1.1	361.259016401199	0	0 2.83462354285661e-42	XM_839116.1 Tbb GUTat10.1 hypothetical protein, conserved (Tb927.4.590) partial mRNA
GLOS_TB927.5.1000.1.1	835.425470879907	0	0 6.67758509482637e-31	XP_844774.1 ubiquitin-conjugating enzyme E2 [T. brucei brucei strain 927/4 GUTat10.1]
GLOS_TB927.5.1060.1.1	927.990837419636	0	0 3.66116897774113e-28	XM_839687.1 Tbb GUTat10.1 mitochondrial processing peptidase, beta subunit partial mRNA
GLOS_TB927.5.1110.1.1	2981.17850740645	0	0 1.18745244202525e-38	XP_844785.1 60S ribosomal protein L2 [Trypanosoma brucei brucei strain 927/4 GUTat10.1]

					ref|XP_829685.1| 60S ribosomal protein L2 [T. brucei brucei strain 927/4 GUTat10.1]
GLOS_TB927.5.1210.1.1	385.591951282376	0	0	3.92803236636082e-24	XM_839702.1 T. b. brucei strain 927/4 GUTat10.1 short-chain dehydrogenase partial mRNA
GLOS_TB927.5.1570.1.1	463.174718165882	0	0	4.0922618616248e-22	XM_839738.1 T. brucei brucei strain 927/4 GUTat10.1 hypothetical protein partial mRNA
GLOS_TB927.5.1710.1.1	909.930779663456	0	0	3.06665365105546e-68	XM_839752.1 Tbb GUTat10.1 ribonucleoprotein p18, mitochondrial precursor partial mRNA
GLOS_TB927.5.1720.1.1	425.718219025324	0	0	8.88354509108269e-26	XM_839753.1 T. brucei brucei strain 927/4 GUTat10.1 hypothetical protein partial mRNA
GLOS_TB927.5.1780.1.1	662.259683387047	0	0	2.82520834599248e-18	XM_839759.1 T. brucei brucei strain 927/4 GUTat10.1 hypothetical protein partial mRNA
GLOS_TB927.5.1790.1.1	335.700014373313	0	0	8.89641732060429e-43	XP_844853.1 hypothetical protein [Trypanosoma brucei brucei strain 927/4 GUTat10.1]
GLOS_TB927.5.1810.1.1	2837.64532264599	0	0	2.16346825050982e-45	XM_839762.1 Tbb GUTat10.1 lysosomal/endosomal membrane protein p67 partial mRNA
GLOS_TB927.5.2100.1.1	376.874584307555	0	0	3.53458360768797e-17	XM_839791.1 T. brucei brucei strain 927/4 GUTat10.1 hypothetical protein partial mRNA
GLOS_TB927.5.2320.1.1	771.429115994094	0	0	1.86567915291619e-29	XP_844906.1 hypothetical protein [Trypanosoma brucei brucei strain 927/4 GUTat10.1]
GLOS_TB927.5.2570.1.1	457.862760081476	0	0	1.59313790462331e-24	XM_839836.1 Tbb strain 927/4 GUTat10.1 translation initiation factor partial mRNA
GLOS_TB927.5.2850.1.1	297.480295824356	0	0	1.93045008047566e-18	XM_839864.1 T. brucei brucei strain 927/4 GUTat10.1 hypothetical protein partial mRNA
GLOS_TB927.5.2930.1.1	512.849020765976	0	0	7.98090982864959e-24	XM_839872.1 T. brucei brucei strain 927/4 GUTat10.1 hypothetical protein partial mRNA
GLOS_TB927.5.2940.1.1	455.097963884788	0	0	9.1004676801061e-34	XM_839873.1 Tbb strain 927/4 GUTat10.1 stress-induced protein sti1 partial mRNA
GLOS_TB927.5.2960.1.1	390.781302652112	0	0	1.40399704173594e-20	XP_844968.1 phosphoribosylpyrophosphate synthetase [Trypanosoma brucei TREU927]
GLOS_TB927.5.3120.1.1	445.154529763257	0	0	1.60770795190503e-17	XM_839891.1 Tbb strain 927/4 GUTat10.1 translation initiation factor partial mRNA
GLOS_TB927.5.3160.1.1	444.323861880932	0	0	6.01249068788316e-23	XM_839895.1 Trypanosoma brucei brucei strain 927/4 GUTat10.1 protein kinase partial mRNA
GLOS_TB927.5.3240.1.1	353.249710594032	0	0	1.76040761239188e-17	XM_839903.1 T. brucei brucei strain 927/4 GUTat10.1 hypothetical protein partial mRNA
GLOS_TB927.5.340.1.1	392.007369798821	0	0	4.98308703991863e-31	XP_844708.1 expression site-associated gene (ESAG) protein [Trypanosoma brucei TREU927]
GLOS_TB927.5.360.1.1	512.140959598322	0	0	9.80834552048823e-33	XM_839617.1 Tbb GUTat10.1 75 kDa invariant surface glycoprotein partial mRNA
GLOS_TB927.5.3800.1.1	2322.60167124912	0	0	3.22012243816527e-26	XP_845050.1 glutamine hydrolysing (not ammonia-dependent) carbomoyl phosphate synthase
GLOS_TB927.5.3960.1.1	334.889281167765	0	0	2.46043591417922e-21	XM_839973.1 Tbb strain 927/4 GUTat10.1 arginine N-methyltransferase partial mRNA
GLOS_TB927.5.4020.1.1	716.940926611872	0	0	2.73651699344459e-80	XM_839979.1 Tbb strain 927/4 GUTat10.1 hypothetical protein Tb927.5.4020 partial mRNA
GLOS_TB927.5.4190.2.3	333.152852485594	0	0	7.35734658330179e-34	XM_839996.1 Trypanosoma brucei brucei strain 927/4 GUTat10.1 histone H4 partial mRNA
GLOS_TB927.5.4190.3.3	395.139986139522	0	0	1.72716982169115e-24	XM_839996.1 Trypanosoma brucei brucei strain 927/4 GUTat10.1 histone H4 partial mRNA
GLOS_TB927.5.4420.1.1	584.316740803123	0	0	1.42389854681981e-54	XM_840019.1 Tbb strain 927/4 GUTat10.1 nucleolar RNA helicase II partial mRNA
GLOS_TB927.5.4480.1.1	507.244335455078	0	0	1.86061270881398e-31	XM_840025.1 Tbb strain 927/4 GUTat10.1 paraflagellar rod component Par4 partial mRNA
GLOS_TB927.5.700.1.1	434.388072202995	0	0	2.20385764828214e-53	XM_839651.1 T. brucei brucei strain 927/4 GUTat10.1 hypothetical protein partial mRNA
GLOS_TB927.6.1090.1.1	310.113433225054	0	0	1.25165300254448e-20	XP_845231.1 proteasome regulatory ATPase subunit 3 [Trypanosoma brucei TREU927]
GLOS_TB927.6.1480.1.1	680.259937112895	0	0	3.28192157193141e-37	XP_845270.1 hypothetical protein [Trypanosoma brucei brucei strain 927/4 GUTat10.1]
GLOS_TB927.6.2010.1.1	400.677222976493	0	0	8.33877227067506e-50	XM_840229.1 T. brucei brucei strain 927/4 GUTat10.1 hypothetical protein partial mRNA
GLOS_TB927.6.2100.1.2	389.752935137594	0	0	4.20481046386436e-20	XP_845331.1 40S ribosomal protein S30 [Trypanosoma brucei brucei strain 927/4 GUTat10.1]
GLOS_TB927.6.2740.1.1	312.537988398101	0	0	1.6323784259631e-25	XP_845395.1 pyridoxal kinase [Trypanosoma brucei brucei strain 927/4 GUTat10.1]
GLOS_TB927.6.3090.1.1	403.53704676748	0	0	2.56288984170925e-23	XM_840337.1 T. brucei brucei strain 927/4 GUTat10.1 hypothetical protein partial mRNA
GLOS_TB927.6.3500.1.1	485.063163197234	0	0	6.78606438735855e-29	XP_845468.1 endosomal trafficking protein RME-8 [Trypanosoma brucei TREU927]
GLOS_TB927.6.3650.1.1	431.322904336221	0	0	3.08654377741019e-19	XP_845483.1 ADP-ribosylation factor [Trypanosoma brucei brucei strain 927/4 GUTat10.1]
GLOS_TB927.6.3950.1.1	332.53981891224	0	0	7.8562280447185e-26	XP_845513.1 hypothetical protein [Trypanosoma brucei brucei strain 927/4 GUTat10.1]
GLOS_TB927.6.4000.1.1	486.01643779423	0	0	1.92987677915577e-22	XM_840425.1 T. brucei brucei strain 927/4 GUTat10.1 hypothetical protein partial mRNA
GLOS_TB927.6.4130.1.1	335.427221823599	0	0	1.55029995189939e-18	XP_845531.1 hypothetical protein [Trypanosoma brucei brucei strain 927/4 GUTat10.1]
GLOS_TB927.6.4140.1.1	624.020030161313	0	0	3.23684066366281e-20	XP_845532.1 hypothetical protein [Trypanosoma brucei brucei strain 927/4 GUTat10.1]
GLOS_TB927.6.4210.1.1	344.437316024911	0	0	9.76602916909014e-22	XM_840446.1 T. brucei brucei strain 927/4 GUTat10.1 aldehyde dehydrogenase partial mRNA

GLOS_TB927.6.4300.1.1	2637.20868490944	0	0	1.73040517419679e-20	XP_845547.1 glyceraldehyde 3-phosphate dehydrogenase, glycosomal [Tbb]
GLOS_TB927.6.4440.1.1	1051.03940925087	0	0	6.62650860123853e-34	XM_840468.1 T. brucei brucei strain 927/4 GUTat10.1 hypothetical protein partial mRNA
GLOS_TB927.6.4480.1.1	399.158428603292	0	0	1.14674900714896e-49	XM_840472.1 T. brucei brucei strain 927/4 GUTat10.1 valyl-tRNA synthetase partial mRNA
GLOS_TB927.6.4490.1.1	449.900968071458	0	0	5.96622233236162e-12	XP_845566.1 hypothetical protein [Trypanosoma brucei brucei strain 927/4 GUTat10.1]
GLOS_TB927.6.4540.1.1	392.205069431013	0	0	6.18137044667504e-49	XM_840478.1 Tbb GUTat10.1 3-hydroxy-3-methylglutaryl-CoA reductase partial mRNA
GLOS_TB927.6.4750.1.1	341.249541443466	0	0	2.71772772880099e-09	XM_840499.1 T. brucei brucei strain 927/4 GUTat10.1 hypothetical protein partial mRNA
GLOS_TB927.6.4980.1.2	723.764034625885	0	0	9.03552814635618e-35	XM_840522.1 Tbb strain 927/4 GUTat10.1 40S ribosomal protein S14 partial mRNA
GLOS_TB927.6.5070.1.1	584.629402706392	0	0	5.61346753016334e-22	XM_840531.1 T. brucei brucei strain 927/4 GUTat10.1 hypothetical protein partial mRNA
GLOS_TB927.6.5090.1.1	368.137282655956	0	0	3.16919476169587e-30	XM_840533.1 T. brucei brucei strain 927/4 GUTat10.1 hypothetical protein partial mRNA
GLOS_TB927.6.5120.1.2	353.447410226224	0	0	1.09493923823728e-25	XM_840536.1 Tbb strain 927/4 GUTat10.1 60S acidic ribosomal protein P2 partial mRNA
GLOS_TB927.6.5120.2.2	335.890069561911	0	0	2.62583957225722e-12	XM_840536.1 Tbb strain 927/4 GUTat10.1 60S acidic ribosomal protein P2 partial mRNA
GLOS_TB927.6.520.2.3	1232.06431671585	0	0	5.58391652233859e-51	XM_840082.1 Trypanosoma brucei brucei strain 927/4 GUTat10.1 EP3-2 procyclin partial mRNA
GLOS_TB927.6.700.1.1	534.247038938644	0	0	6.91861340147401e-27	XP_845193.1 alanyl-tRNA synthetase [Trypanosoma brucei brucei strain 927/4 GUTat10.1]
GLOS_TB927.6.790.1.2	1022.10257745294	0	0	8.52875717483349e-37	XM_840109.1 Tbb GUTat10.1 receptor-type adenylate cyclase GRESAG 4 partial mRNA
GLOS_TB927.6.790.2.2	448.124670035732	0	0	9.3833045962166e-55	XP_845202.1 receptor-type adenylate cyclase GRESAG 4 [Trypanosoma brucei TREU927]
GLOS_TB927.7.1050.1.2	680.87297068625	0	0	3.23215354561429e-43	XM_840636.1 Tbb GUTat10.1 40S ribosomal protein S16, putative (Tb927.7.1050) partial mRNA
GLOS_TB927.7.1110.1.1	361.654415665584	0	0	1.50241446263671e-45	XP_845735.1 asparagine synthetase a [Trypanosoma brucei brucei strain 927/4 GUTat10.1]
GLOS_TB927.7.1290.1.1	332.369698400419	0	0	7.92959740066052e-37	XM_840660.1 Tbb GUTat10.1 hypothetical protein, conserved (Tb927.7.1290) partial mRNA
GLOS_TB927.7.1320.1.1	540.096937678884	0	0	2.63436303047648e-22	XP_845756.1 10 kDa heat shock protein [Trypanosoma brucei brucei strain 927/4 GUTat10.1] ref|XP_845758.1| 10 kDa heat shock protein [T. brucei brucei strain 927/4 GUTat10.1]
GLOS_TB927.7.1470.1.1	480.534359198003	0	0	6.30188436114515e-31	XM_840678.1 Tbb GUTat10.1 ATPase subunit 9, putative (Tb927.7.1470) partial mRNA
GLOS_TB927.7.1790.1.1	437.172803076461	0	0	5.79689626435998e-16	XP_845803.1 adenine phosphoribosyltransferase [T. brucei brucei strain 927/4 GUTat10.1]
GLOS_TB927.7.190.1.1	471.919664261075	0	0	3.10467208101045e-57	XP_845643.1 thimet oligopeptidase A [Trypanosoma brucei brucei strain 927/4 GUTat10.1]
GLOS_TB927.7.210.1.1	411.491194333904	0	0	1.76912222902175e-15	XP_845645.1 proline oxidase [Trypanosoma brucei brucei strain 927/4 GUTat10.1]
GLOS_TB927.7.2170.1.1	474.834646292806	0	0	1.72253427425519e-57	XM_840747.1 Tbb GUTat10.1 hypothetical protein, conserved (Tb927.7.2170) partial mRNA
GLOS_TB927.7.2190.1.1	402.386072538292	0	0	6.19045960167358e-22	XM_840749.1 Tbb GUTat10.1 hypothetical protein, conserved (Tb927.7.2190) partial mRNA
GLOS_TB927.7.2240.1.1	354.230564311399	0	0	3.8803819195241e-24	XM_840754.1 Tbb GUTat10.1 hypothetical protein, conserved (Tb927.7.2240) partial mRNA
GLOS_TB927.7.2300.1.1	334.133706202962	0	0	1.37470089593777e-42	XM_840760.1 Tbb GUTat10.1 hypothetical protein, conserved (Tb927.7.2300) partial mRNA
GLOS_TB927.7.2340.1.1	2190.10773659864	0	0	4.12229348581492e-28	XM_840764.1 Tbb GUTat10.1 40S ribosomal protein S15, putative (Tb927.7.2340) partial mRNA
GLOS_TB927.7.2360.1.1	318.878313997025	0	0	1.95029031588223e-22	XM_840766.1 Tbb GUTat10.1 N-acetyltransferase, putative (Tb927.7.2360) partial mRNA
GLOS_TB927.7.2390.1.1	406.064273978421	0	0	2.36517613098042e-50	XM_840769.1 Tbb GUTat10.1 hypothetical protein, conserved (Tb927.7.2390) partial mRNA
GLOS_TB927.7.2500.1.1	396.876414821693	0	0	1.11396529777532e-17	XM_840780.1 Tbb GUTat10.1 proteasome regulat. ATPase subunit 1 (Tb927.7.2500) partial mRNA
GLOS_TB927.7.2550.1.1	471.694385508511	0	0	5.55172130110584e-22	XP_845878.1 proteasome regulatory ATPase subunit 5 [Trypanosoma brucei TREU927]
GLOS_TB927.7.2570.1.1	341.597426910702	0	0	8.32774087517347e-22	XM_840787.1 Tbb GUTat10.1 hypothetical protein, conserved (Tb927.7.2570) partial mRNA
GLOS_TB927.7.2650.1.1	1558.54889281207	0	0	6.11555575898421e-30	XP_845888.1 hypothetical protein [Trypanosoma brucei brucei strain 927/4 GUTat10.1]
GLOS_TB927.7.2700.1.1	371.902867246789	0	0	9.80661980698865e-13	XP_845893.1 NADH-cytochrome b5 reductase [T. brucei brucei strain 927/4 GUTat10.1]
GLOS_TB927.7.2820.1.2	495.346838342406	0	0	1.68343207128508e-36	XP_845905.1 histone H2A [Trypanosoma brucei brucei strain 927/4 GUTat10.1] ref|XP_845906.1| histone H2A [Tbb] ref|XP_845907.1| histone H2A [Tbb] ref|XP_845908.1| histone H2A [Tbb] ref|XP_845909.1| histone H2A [Tbb] ref|XP_845910.1| histone H2A [Tbb] ref|XP_845911.1| histone H2A [Tbb] ref|XP_845912.1| histone H2A [Tbb] ref|XP_845913.1| histone H2A [Tbb] ref|XP_845914.1| histone H2A [Tbb] ref|XP_845915.1| histone H2A [Tbb] ref|XP_845916.1|

					histone H2A [Tbb] ref|XP_845917.1| histone H2A [Tbb]
GLOS_TB927.7.2820.2.2	1145.32126979599	0	0	7.598376853709e-39	XP_845905.1 histone H2A [Tbb] ref|XP_845906.1| histone H2A [Tbb] ref|XP_845907.1| histone H2A [Tbb] ref|XP_845908.1| histone H2A [Tbb] ref|XP_845909.1| histone H2A [Tbb] ref|XP_845910.1| histone H2A [Tbb] ref|XP_845911.1| histone H2A [Tbb] ref|XP_845912.1| histone H2A [Tbb] ref|XP_845913.1| histone H2A [Tbb] ref|XP_845914.1| histone H2A [Tbb] ref|XP_845915.1| histone H2A [Tbb] ref|XP_845916.1| histone H2A [Tbb] ref|XP_845917.1| histone H2A [Tbb]
GLOS_TB927.7.290.1.1	336.285468826295	0	0	3.10036468510998e-25	XM_840560.1 Tbb leucine-rich repeat protein (LRRP), putative (Tb927.7.290) partial mRNA
GLOS_TB927.7.2980.1.1	514.245208424506	0	0	1.24861842996325e-26	XP_845921.1 hypothetical protein [Trypanosoma brucei brucei strain 927/4 GUTat10.1]
GLOS_TB927.7.3550.1.2	1157.92682807632	0	0	2.06758336800399e-31	XP_845978.1 hypothetical protein [Trypanosoma brucei brucei strain 927/4 GUTat10.1]
GLOS_TB927.7.3550.2.2	396.753808107022	0	0	4.04042862125139e-17	XP_845978.1 hypothetical protein [Trypanosoma brucei brucei strain 927/4 GUTat10.1]
GLOS_TB927.7.3620.1.1	461.683502913053	0	0	7.40524526491172e-33	XM_840892.1 Tbb GUTat10.1 tyrosyl-tRNA synthetase, putative (Tb927.7.3620) partial mRNA
GLOS_TB927.7.3630.1.1	440.380512334684	0	0	2.29928914392385e-17	XM_840893.1 Tbb TPR-repeat-containing chaperone protein DNAJ, putative partial mRNA
GLOS_TB927.7.3680.1.2	456.343965708275	0	0	4.42542466799882e-21	XM_840898.1 Tbb ubiquitin/ribosomal protein S27a, putative (Tb927.7.3680) partial mRNA
GLOS_TB927.7.3680.2.2	751.992805256161	0	0	1.46615183928132e-39	XM_840898.1 Tbb ubiquitin/ribosomal protein S27a, putative (Tb927.7.3680) partial mRNA
GLOS_TB927.7.3740.1.1	472.505118714058	0	0	7.11710767242044e-37	XM_840904.1 Tbb GUTat10.1 hypothetical protein, conserved (Tb927.7.3740) partial mRNA
GLOS_TB927.7.3940.1.1	424.274517569644	0	0	6.27702475516334e-30	XM_840924.1 Tbb GUTat10.1 mitochondrial carrier prot., putative (Tb927.7.3940) partial mRNA
GLOS_TB927.7.4070.1.1	951.200377191997	0	0	8.53824890073213e-30	XP_846030.1 calpain-like cysteine peptidase [T. brucei brucei strain 927/4 GUTat10.1]
GLOS_TB927.7.4120.1.1	384.195763623846	0	0	6.13950929730107e-21	XM_840942.1 Tbb GUTat10.1 hypothetical protein, conserved (Tb927.7.4120) partial mRNA
GLOS_TB927.7.4270.1.1	323.774938140269	0	0	9.28452480940303e-24	XM_840957.1 Tbb GUTat10.1 hypothetical protein, conserved (Tb927.7.4270) partial mRNA
GLOS_TB927.7.4500.1.1	360.938710054336	0	0	1.27612359490207e-24	XM_840980.1 Tbb GUTat10.1 hypothetical protein, conserved (Tb927.7.4500) partial mRNA
GLOS_TB927.7.4520.1.1	494.045678278175	0	0	6.71418565782828e-20	XM_840982.1 Tbb GUTat10.1 hypothetical protein, conserved (Tb927.7.4520) partial mRNA
GLOS_TB927.7.4900.1.1	687.240875405545	0	0	8.79465058073322e-69	XM_841020.1 Tbb GUTat10.1 5'-3' exonuclease XRNA, putative (Tb927.7.4900) partial mRNA
GLOS_TB927.7.4910.1.1	352.759283735348	0	0	3.38868060472716e-15	XM_841021.1 Tbb GUTat10.1 hypothetical protein, conserved (Tb927.7.4910) partial mRNA
GLOS_TB927.7.4970.1.1	405.914088143378	0	0	6.34648597977912e-39	XP_846120.1 glutamine synthetase [Trypanosoma brucei brucei strain 927/4 GUTat10.1]
GLOS_TB927.7.5000.1.1	2335.50760119954	0	0	7.52491612186159e-30	XM_841030.1 Tbb GUTat10.1 60S ribosomal protein L19, putative (Tb927.7.5000) partial mRNA
GLOS_TB927.7.5180.1.1	1478.7392703877	0	0	1.14476766893148e-32	XM_841048.1 Tbb GUTat10.1 60S ribosomal protein L23a, putative (Tb927.7.5180) partial mRNA
GLOS_TB927.7.5280.1.1	499.800549424116	0	0	2.25653914587581e-25	XM_841058.1 Tbb GUTat10.1 hypothetical protein, conserved (Tb927.7.5280) partial mRNA
GLOS_TB927.7.5470.1.1	479.94890474502	0	0	4.34205297664754e-49	XM_841077.1 Tbb GUTat10.1 hypothetical protein, conserved (Tb927.7.5470) partial mRNA
GLOS_TB927.7.6050.1.1	716.197641880252	0	0	5.05972467212877e-29	XM_841135.1 Tbb receptor-type adenylate cyclase GRESAG 4, putative partial mRNA
GLOS_TB927.7.6090.1.1	626.377136860433	0	0	2.90724529720123e-55	XM_841139.1 Tbb GUTat10.1 hypothetical protein, conserved (Tb927.7.6090) partial mRNA
GLOS_TB927.7.610.1.1	331.191145050859	0	0	4.17748976580523e-16	XM_840592.1 Tbb strain 927/4 GUTat10.1 DNA ligase, putative (Tb927.7.610) partial mRNA
GLOS_TB927.7.6260.1.1	404.735534793818	0	0	4.84853067933282e-19	XM_841156.1 Tbb GUTat10.1 hypothetical protein, conserved (Tb927.7.6260) partial mRNA
GLOS_TB927.7.6420.1.1	362.334897712866	0	0	3.78414284788445e-29	XM_841172.1 Tbb GUTat10.1 hypothetical protein, conserved (Tb927.7.6420) partial mRNA
GLOS_TB927.7.6790.1.1	448.900179677313	0	0	2.39436316775919e-17	XM_841206.1 Tbb GUTat10.1 hypothetical protein, conserved (Tb927.7.6790) partial mRNA
GLOS_TB927.7.6850.1.1	1626.91157562888	0	0	7.35920996108031e-51	XM_841212.1 Trypanosoma brucei brucei strain 927/4 GUTat10.1 trans-sialidase partial mRNA
GLOS_TB927.7.6970.1.1	473.703606740396	0	0	2.17401399597356e-29	XM_841224.1 Tbb GUTat10.1 paraflagellar rod protein, putative (Tb927.7.6970) partial mRNA
GLOS_TB927.7.7050.1.1	364.466725659422	0	0	6.11700648700762e-46	XM_841232.1 Tbb GUTat10.1 hypothetical protein, conserved (Tb927.7.7050) partial mRNA
GLOS_TB927.7.7090.1.1	1431.48187851706	0	0	3.11732434135139e-22	XM_841236.1 Tbb GUTat10.1 hypothetical protein, conserved (Tb927.7.7090) partial mRNA
GLOS_TB927.7.710.1.1	801.10158808005	0	0	1.83277919694639e-30	XM_840602.1 Tbb GUTat10.1 heat shock 70 kDa protein, putative (Tb927.7.710) partial mRNA
GLOS_TB927.7.7110.1.1	320.567228882046	0	0	1.02318158528246e-20	XM_841238.1 Tbb leucine-rich repeat protein (LRRP), putative (Tb927.7.7110) partial mRNA

GLOS_TB927.7.7420.1.1	1722.09155253026	0	0	3.66558896358838e-35 XM_841269.1 Tbb ATP synthase alpha chain, mitochondrial precursor partial mRNA
GLOS_TB927.7.7470.1.1	1222.85652288235	0	0	6.8071968258323e-41 XM_841274.1 Tbb receptor-type adenylate cyclase GRESAG 4, putative partial mRNA
GLOS_TB927.8.1330.1.1	3714.65305295771	0	0	1.13642130598919e-33 XM_841876.1 Tbb GUTat10.1 60S ribosomal protein L7a, putative (Tb927.8.1330) partial mRNA
GLOS_TB927.8.1500.1.1	398.422788315266	0	0	2.40364081469964e-41 XM_841891.1 Tbb GUTat10.1 hypothetical protein, conserved (Tb927.8.1500) partial mRNA
GLOS_TB927.8.1510.1.1	408.508763828245	0	0	5.98561203321621e-35 XM_841892.1 Tbb ATP-dependent DEAD/H RNA helicase, putative (Tb927.8.1510) partial mRNA
GLOS_TB927.8.1550.1.1	835.010136938744	0	0	6.92953302685236e-34 XM_841896.1 Tbb GUTat10.1 hypothetical protein, conserved (Tb927.8.1550) partial mRNA
GLOS_TB927.8.1600.1.1	312.905808542114	0	0	3.71891862779915e-30 XM_841901.1 Tbb GUTat10.1 lysyl-tRNA synthetase, putative (Tb927.8.1600) partial mRNA
GLOS_TB927.8.1620.1.1	1486.21827998263	0	0	1.99428220280222e-80 XP_846996.1 major surface protease gp63 [Trypanosoma brucei brucei strain 927/4 GUTat10.1] ref|XP_846997.1| major surface protease gp63 [T. brucei brucei strain 927/4 GUTat10.1]
GLOS_TB927.8.1790.1.1	316.481337944349	0	0	1.24445390025217e-40 XM_841919.1 Tbb GUTat10.1 hypothetical protein, conserved (Tb927.8.1790) partial mRNA
GLOS_TB927.8.1830.1.1	378.686105907247	0	0	8.54506087022662e-17 XM_841923.1 Tbb GUTat10.1 tRNA-methyl transferase, putative (Tb927.8.1830) partial mRNA
GLOS_TB927.8.1990.1.1	507.612155599091	0	0	4.01679835520041e-35 XP_847032.1 tryparedoxin peroxidase [Trypanosoma brucei brucei strain 927/4 GUTat10.1]
GLOS_TB927.8.2160.1.1	501.074130367975	0	0	1.22694352155263e-27 XM_841956.1 Tbb GUTat10.1 multidrug resistance protein A (Tb927.8.2160) partial mRNA
GLOS_TB927.8.2320.1.1	339.833419108159	0	0	1.07116962594331e-16 XM_841972.1 Tbb GUTat10.1 hypothetical protein (Tb927.8.2320) partial mRNA
GLOS_TB927.8.2470.1.1	332.096905850705	0	0	6.59784333643045e-16 XP_847080.1 hypothetical protein [Trypanosoma brucei brucei strain 927/4 GUTat10.1]
GLOS_TB927.8.2520.1.1	636.53056084734	0	0	6.93091836074391e-21 XM_841992.1 Tbb GUTat10.1 acetyl-CoA synthetase, putative (Tb927.8.2520) partial mRNA
GLOS_TB927.8.2540.1.1	773.608457737799	0	0	5.87293668610439e-31 XM_841994.1 Tbb GUTat10.1 3-ketoacyl-CoA thiolase, putative (Tb927.8.2540) partial mRNA
GLOS_TB927.8.2630.1.1	431.983451706726	0	0	6.59980482453956e-18 XP_847096.1 kinesin [Trypanosoma brucei brucei strain 927/4 GUTat10.1]
GLOS_TB927.8.2640.1.1	614.45206062739	0	0	1.50798311427902e-36 XP_847097.1 ubiquitin-activating enzyme E1 [T. brucei brucei strain 927/4 GUTat10.1]
GLOS_TB927.8.2910.1.1	827.906591931423	0	0	2.41232267440534e-23 XM_842029.1 Tbb strain 927/4 GUTat10.1 mannosyl-oligosaccharide 1,2-alpha-mannosidase IB,
GLOS_TB927.8.3060.1.1	467.390860261844	0	0	9.18638339612295e-57 XM_842044.1 Tbb cytosolic leucyl aminopeptidase, putative (Tb927.8.3060) partial mRNA
GLOS_TB927.8.3100.1.1	342.087853769386	0	0	3.21571354279603e-26 XM_842048.1 Tbb strain 927/4 GUTat10.1 coronin, putative (Tb927.8.3100) partial mRNA
GLOS_TB927.8.3380.1.1	557.571540982082	0	0	2.66388488212171e-12 XP_847169.1 electron transfer protein [Trypanosoma brucei brucei strain 927/4 GUTat10.1]
GLOS_TB927.8.3690.1.1	452.155402732685	0	0	1.9579748770221e-15 XP_847200.1 isocitrate dehydrogenase [Trypanosoma brucei brucei strain 927/4 GUTat10.1]
GLOS_TB927.8.3840.1.1	345.070284275044	0	0	1.4392283739914e-11 XM_842122.1 Tbb GUTat10.1 hypothetical protein, conserved (Tb927.8.3840) partial mRNA
GLOS_TB927.8.4050.1.1	1153.41795875386	0	0	1.28280711242783e-23 XM_842143.1 Tbb GUTat10.1 hypothetical protein, conserved (Tb927.8.4050) partial mRNA
GLOS_TB927.8.4330.1.1	417.716557661751	0	0	1.28971504197843e-51 XP_847264.1 small GTP-binding protein Rab11 [T. brucei brucei strain 927/4 GUTat10.1]
GLOS_TB927.8.4400.1.1	323.454631793405	0	0	2.5026665567427e-15 XM_842178.1 Tbb GUTat10.1 hypothetical protein, conserved (Tb927.8.4400) partial mRNA
GLOS_TB927.8.5070.1.1	323.407117996256	0	0	1.17613422444567e-20 XM_842244.1 Tbb GUTat10.1 hypothetical protein, conserved (Tb927.8.5070) partial mRNA
GLOS_TB927.8.5120.1.1	702.923891785828	0	0	3.93082486974211e-18 XP_847342.1 cytochrome c [Trypanosoma brucei brucei strain 927/4 GUTat10.1]
GLOS_TB927.8.5260.1.3	331.218724171231	0	0	2.10178801374501e-33 XM_842263.1 Tbb GUTat10.1 60S ribosomal protein L39, putative (Tb927.8.5260) partial mRNA
GLOS_TB927.8.5260.2.3	564.627572192253	0	0	1.21694367715489e-21 XM_842263.1 Tbb GUTat10.1 60S ribosomal protein L39, putative (Tb927.8.5260) partial mRNA
GLOS_TB927.8.5640.1.1	515.831451271634	0	0	8.60287550621422e-14 XP_847394.1 hypothetical protein [Trypanosoma brucei brucei strain 927/4 GUTat10.1]
GLOS_TB927.8.6000.1.1	963.683328757652	0	0	1.26996434402656e-23 XM_842337.1 Tbb GUTat10.1 fatty acid desaturase, putative (Tb927.8.6000) partial mRNA
GLOS_TB927.8.6010.1.1	346.051137992411	0	0	7.64653531541623e-15 XM_842338.1 Tbb hypothetical predicted multi-pass transmembrane protein partial mRNA
GLOS_TB927.8.6060.1.1	1429.41749904444	0	0	8.12372076200384e-16 XM_842343.1 Tbb 2-amino-3-ketobutyrate coenzyme A ligase, putative partial mRNA
GLOS_TB927.8.6110.1.1	1154.01105765044	0	0	7.94898414611758e-35 XP_847441.1 hypothetical protein [Trypanosoma brucei brucei strain 927/4 GUTat10.1]
GLOS_TB927.8.6160.1.1	2681.81924150848	0	0	2.87707017064143e-49 XM_842353.1 Tbb GUTat10.1 40S ribosomal protein S8, putative (Tb927.8.6160) partial mRNA
GLOS_TB927.8.6170.1.1	1298.58025436901	0	0	2.2741249849448e-43 XM_842354.1 Tbb GUTat10.1 transketolase, putative (Tb927.8.6170) partial mRNA
GLOS_TB927.8.6180.1.2	886.305905949933	0	0	3.93858541808985e-73 XM_842355.1 Tbb GUTat10.1 60S ribosomal protein L26, putative (Tb927.8.6180) partial mRNA
GLOS_TB927.8.6210.1.1	335.700014373313	0	0	8.89641732060429e-43 XM_842358.1 Tbb GUTat10.1 phosphatidylinositol 3-kinase, putative partial mRNA

GLOS_TB927.8.6440.1.1	345.418169742279	0	0	5.0877565960148e-32	XM_842381.1 Tbb GUTat10.1 RNA-binding protein, putative (Tb927.8.6440) partial mRNA
GLOS_TB927.8.6450.1.1	439.059417593675	0	0	5.45736663136047e-20	XP_847475.1 inhibitor of cysteine peptidase [T. brucei brucei strain 927/4 GUTat10.1]
GLOS_TB927.8.6640.1.1	327.057740316013	0	0	8.14539529461031e-42	XM_842401.1 Tbb GUTat10.1 hypothetical protein, conserved (Tb927.8.6640) partial mRNA
GLOS_TB927.8.6660.1.1	567.645226261878	0	0	1.47250881219205e-66	XM_842403.1 Tbb GUTat10.1 hypothetical protein, conserved (Tb927.8.6660) partial mRNA
GLOS_TB927.8.6750.1.1	483.96734720879	0	0	2.15343329754537e-58	XM_842412.1 Tbb translationally controlled tumor protein (TCTP), putative partial mRNA
GLOS_TB927.8.6890.1.1	540.634878334717	0	0	1.96589200728591e-20	XP_847519.1 hypothetical protein [Trypanosoma brucei brucei strain 927/4 GUTat10.1]
GLOS_TB927.8.6970.1.1	700.827287403238	0	0	1.23838606599155e-54	XM_842434.1 Tbb 3-methylcrotonyl-CoA carboxylase, putative (Tb927.8.6970) partial mRNA
GLOS_TB927.8.7020.1.1	394.621980160466	0	0	6.56006055866621e-13	XP_847532.1 peptidase [Trypanosoma brucei brucei strain 927/4 GUTat10.1]
GLOS_TB927.8.7100.1.1	914.602125054135	0	0	2.11880848858532e-37	XM_842447.1 Tbb GUTat10.1 acetyl-CoA carboxylase, putative (Tb927.8.7100) partial mRNA
GLOS_TB927.8.7120.1.1	515.811516594856	0	0	1.73685019797501e-24	XM_842449.1 Tbb GUTat10.1 farnesyltransferase, putative (Tb927.8.7120) partial mRNA
GLOS_TB927.8.7150.1.1	903.317661514818	0	0	1.03493168902166e-45	XM_842452.1 Tbb UDP-Gal or UDP-GlcNAc-depend. glycosyltransferase, putative partial mRNA
GLOS_TB927.8.7490.1.1	405.471175081844	0	0	1.44911726358551e-23	XM_842485.1 Tbb GUTat10.1 hypothetical protein, conserved (Tb927.8.7490) partial mRNA
GLOS_TB927.8.760.1.1	1348.37716368378	0	0	1.04706328069843e-44	XM_841820.1 Tbb GUTat10.1 nucleolar RNA-binding protein (Tb927.8.760) partial mRNA
GLOS_TB927.8.7940.1.1	399.423576709411	0	0	3.34784215426198e-21	XM_842526.1 Tbb receptor-type adenylate cyclase GRESAG 4, putative partial mRNA
GLOS_TB927.8.7950.1.1	351.268068482519	0	0	3.48738600553059e-23	XM_842527.1 Tbb GUTat10.1 hypothetical protein, conserved (Tb927.8.7950) partial mRNA
GLOS_TB927.8.7980.1.1	1943.7132199201	0	0	2.03108214425487e-62	XM_842530.1 Tbb vacuolar-type proton translocating pyrophosphatase 1; partial mRNA
GLOS_TB927.8.8200.1.1	356.409906055105	0	0	8.78332465496566e-27	XP_847643.1 hypothetical protein [Trypanosoma brucei brucei strain 927/4 GUTat10.1]
GLOS_TB927.8.8250.1.1	452.871108343933	0	0	1.37642903088079e-42	XM_842555.1 Tbb GUTat10.1 amino acid transporter, putative (Tb927.8.8250) partial mRNA
GLOS_TB927.8.890.1.1	394.649559280838	0	0	5.03476193376032e-21	XP_846926.1 small GTP-binding protein Rab1 [Trypanosoma brucei brucei strain 927/4 GUTat10.1]
GLOS_TC00.1047053508475.1	605.904814164389	0	0	8.83387356333197e-24	XP_804279.1 ribosomal protein S20 [T. cruzi] ref|XP_809988.1| ribosomal protein S20 [T. cruzi] ref|XP_809990.1| ribosomal protein S20 [T. cruzi strain CL Brener]
GLOS_TC00.1047053508823.7	1347.48369311711	0	0	3.45075324217287e-21	XP_809984.1 hypothetical protein [Trypanosoma cruzi strain CL Brener]
GLOS_TC00.1047053511805.1	1070.50329910917	0	0	5.40866300060559e-36	XP_804510.1 ribosomal protein S29 [T. crui] ref|XP_806920.1| ribosomal protein S29 [T. cruzi] ref|XP_808328.1| ribosomal protein S29 [Trypanosoma cruzi strain CL Brener]
GLOS_TVAG_157670.1.1	590.486945890226	0	0	5.77539584780519e-64	XP_001288661.1 hypothetical protein [Trichomonas vaginalis G3]
GLOS_TYPX.1.1	642.210339075759	0	0	7.53913170675669e-21	[BBH] TYPX_TRYBB (sp|O77404) Tryparedoxin OS=Trypanosoma brucei brucei PE=1 SV=1
GLOS_TYTR.1.1	546.58744911285	0	0	2.56915781329862e-34	[BBH] TYTR_TRYBB (sp|P39051) Trypanothione reductase OS=T. b. brucei GN=TPR PE=1 SV=1
