# Supplementary material for: RNA-seq de novo Assembly Reveals Differential Gene Expression in Glossina palpalis gambiensis Infected with Trypanosoma brucei gambiense vs. Non-Infected and Self-Cured Flies
Source: Front Microbiol. 2015 Nov 13;6:1259. doi: 10.3389/fmicb.2015.01259 (PMC4643127; doi:10.3389/fmicb.2015.01259)
Supplement: Supplementary file 5 [file Table5.PDF]

Supplementary Table S5: SNP found in the differentially expressed genes from 3 days tsetse flies samples

Best hit description	Name	Alleles	Type	Position
[BBH] 6PGD_CERCA (sp|P41570) 6-phosphogluconate dehydrogenase, decarboxylating OS=Ceratitis capitata GN=Pgd PE=2 SV=1	GLOS_6PGD.1.1	A/G	SNP	443
		A/G	SNP	535
		T/C	SNP	816
		G/A	SNP	876
		G/A	SNP	978
		G/A	SNP	1074
		T/C	SNP	1098
		G/T	SNP	1102
		C/G	SNP	1128
		G/A	SNP	1136
		G/T	SNP	1448
XP_001651282.1 dimethylaniline monooxygenase [Aedes aegypti]	GLOS_AAEL_AAEL000797.1.1	C/G	SNP	169
		A/G	SNP	226
		C/T	SNP	248
		A/G	SNP	279
		C/T	SNP	324
		C/T	SNP	387
		T/A	SNP	568
		A/G	SNP	596
		A/T	SNP	599
		A/G	SNP	681
		A/G	SNP	693
		G/C	SNP	697
		A/G	SNP	699
		G/A	SNP	706
		C/T	SNP	732
		G/C	SNP	780
		G/A	SNP	822
		C/G	SNP	843
		C/T	SNP	852
		C/G	SNP	860
		T/C	SNP	865
		T/C	SNP	869

		A/G	SNP	910
		T/C	SNP	1020
		G/A	SNP	1086
		G/A	SNP	1146
		T/C	SNP	1284
		G/A	SNP	1441
		G/A	SNP	1449
		A/G	SNP	1563
		T/C	SNP	1595
		T/C	SNP	1669
XP_001658781.1 serine protease [Aedes aegypti]	GLOS_AAEL_AAEL007969.1.1	A/G	SNP	199
		C/A	SNP	257
		G/A	SNP	344
		T/A	SNP	440
		G/A	SNP	442
		T/C	SNP	443
		G/T	SNP	445
		G/C	SNP	571
		T/C	SNP	583
		C/A	SNP	615
		C/T	SNP	621
		C/G	SNP	715
		C/T	SNP	742
		C/T	SNP	766
		A/T	SNP	767
		A/T	SNP	788
		A/G	SNP	794
		C/T	SNP	865
		G/A	SNP	926
		T/G	SNP	964
		T/C	SNP	1017
		C/G	SNP	1237
		C/T	SNP	1272
		T/C	SNP	1308
		T/A	SNP	1374
		C/G	SNP	1439

		T/C	SNP	1494
		T/C	SNP	1602
		G/A	SNP	1608
		T/A	SNP	1632
		C/G	SNP	1678
		G/C	SNP	1894
		C/A	SNP	1898
XP_001655838.1 secreted modular calcium-binding protein [A. aegypti]	GLOS_AAEL_AAEL012043.1.1	T/C	SNP	53
		T/C	SNP	245
		T/A	SNP	374
		A/C	SNP	383
		A/C	SNP	384
		T/C	SNP	513
		T/C	SNP	541
		A/G	SNP	2715
		T/C	SNP	2730
		C/T	SNP	2746
		G/A	SNP	2758
		A/AT	INSERTION	2763
		C/CA	INSERTION	2800
		C/T	SNP	2819
		G/C	SNP	2840
		T/C	SNP	3001
		T/C	SNP	3132
		T/TA	INSERTION	3306
XP_001649874.1 methylenetetrahydrofolate dehydrogenase [Aedes aegypti]	GLOS_AAEL_AAEL014871.1.1	A/T	SNP	16
		A/G	SNP	49
		G/C	SNP	193
		C/A	SNP	295
		G/C	SNP	328
		G/A	SNP	343
		G/A	SNP	370
		C/T	SNP	373
		A/T	SNP	415
		C/A	SNP	694
		C/G	SNP	747

		A/G	SNP	775
		C/G	SNP	796
		C/T	SNP	909
		C/T	SNP	969
		C/T	SNP	1000
		C/T	SNP	1004
		G/A	SNP	1038
		C/G	SNP	1049
		A/G	SNP	1114
XP_314619.3 AGAP004677-PB [Anopheles gambiae str. PEST]	GLOS_AGAP_AGAP004677.1.1	CA/C	DELETION	115
		C/A	SNP	162
		C/T	SNP	205
		G/A	SNP	211
		A/G	SNP	258
		C/T	SNP	290
		C/T	SNP	299
		A/T	SNP	308
		CT/CTT/C	INSERTION	317
		C/G	SNP	334
		A/T	SNP	335
		A/G	SNP	400
		A/C	SNP	413
		C/T	SNP	516
		G/C	SNP	540
		T/C	SNP	561
		T/G	SNP	645
		T/G	SNP	861
		C/T	SNP	915
		T/A	SNP	921
		G/T	SNP	1041
XP_318675.2 AGAP009641-PA [Anopheles gambiae str. PEST]	GLOS_AGAP_AGAP009641.2.2	C/T	SNP	836
		A/C	SNP	920
		A/C	SNP	1149
		T/C	SNP	1161
		C/T	SNP	1173
		G/C	SNP	1669

		T/C	SNP	2514
		C/T	SNP	2630
		A/C	SNP	2693
[BBH] ALF_TRYBB (sp|P07752) Fructose-bisphosphate aldolase, glycosomal OS=Trypa OS=Trypanosoma brucei brucei GN=ALD PE=1 SV=2	GLOS_ALF.1.1	AGT/A	DELETION	1667
[BBH] ANXB9_DROME (sp|P22464) Annexin-B9 D. melan. GN=AnnIX PE=2 SV=2	GLOS_ANXB9.1.1	T/A	SNP	38
		T/C	SNP	39
		C/G	SNP	43
		A/C	SNP	46
		C/T	SNP	158
		A/T	SNP	170
		C/T	SNP	188
		G/A	SNP	233
		G/T	SNP	383
		C/T	SNP	440
		A/G	SNP	821
		T/C	SNP	1014
NP_610955.1 Activity-regulated cytoskeleton associated protein 1 [D. melanog.]	GLOS_ARC1.1.1	A/C	SNP	1713
		G/A	SNP	1728
		A/AG	INSERTION	2254
		T/A	SNP	2255
		A/G	SNP	2258
		T/A	SNP	2260
[BBH] ARP3_DROME (sp|P32392) Actin-related protein 3 OS=D. melanogaster	GLOS_ARP3.1.1	G/A	SNP	225
[BBH] C4AC3_DROME (sp|Q9VMS7) Probable cytochrome P450 4ac3 OS=D.m.	GLOS_C4AC3.1.1	T/G	SNP	158
		T/A	SNP	257
		T/C	SNP	359
		C/T	SNP	395
		A/C	SNP	432
		T/C	SNP	699
		T/A	SNP	871
		T/G	SNP	900
		T/C	SNP	904
		A/C	SNP	936
		A/G	SNP	982
		C/T	SNP	985

C/T	SNP	988
C/G	SNP	991
A/G	SNP	994
A/G	SNP	995
A/G	SNP	1005
G/T	SNP	1009
C/T	SNP	1017
C/T	SNP	1018
A/G	SNP	1023
A/C	SNP	1030
C/T	SNP	1033
A/G	SNP	1053
A/G	SNP	1078
G/A	SNP	1116
G/C	SNP	1162
A/G	SNP	1201
T/A	SNP	1205
C/T	SNP	1264
G/A	SNP	1302
C/A	SNP	1372
A/G	SNP	1420
T/A	SNP	1435
C/A	SNP	1449
A/G	SNP	1493
T/C	SNP	1518
A/T	SNP	1608
A/T	SNP	1657
T/G	SNP	1737
C/G	SNP	2256
G/A	SNP	2258
C/T	SNP	2310
C/G	SNP	2392
T/C	SNP	2428
A/C	SNP	2455
C/T	SNP	2461
T/C	SNP	2473

[BBH] CADF_DROME (sp|P45594) Cofilin/actin-depolymerizing factor homolog;D.m.	GLOS_CADF.2.7	A/T	SNP	584
[BBH] CALM_TRYBG (sp|P69098) Calmodulin OS=T. b. gambiense PE=3 SV=2	GLOS_CALM.1.1	A/G	SNP	448
		G/A	SNP	495
		A/G	SNP	840
		T/A	SNP	966
		C/T	SNP	1094
		C/T	SNP	1127
		A/C	SNP	1128
[BBH] CALR_DROME (sp|P29413) Calreticulin OS=D. m. GN=Crc PE=1 SV=2	GLOS_CALR.1.1	C/A	SNP	210
		C/CA	INSERTION	210
		G/T	SNP	283
		A/G	SNP	354
		C/A	SNP	514
		C/A	SNP	901
		T/A	SNP	904
		C/T	SNP	1561
		A/G	SNP	1576
CBPA1_DROPS (sp|Q29NC4) Zinc carboxypeptidase A 1 OS=D. p. pseudoobscura	GLOS_CBPA1.11.11	A/C	SNP	167
		G/C	SNP	691
		A/G	SNP	692
		C/T	SNP	721
		A/T	SNP	733
		T/C	SNP	734
		G/A	SNP	879
		G/GA	INSERTION	879
		C/T	SNP	882
		G/T	SNP	912
		A/G	SNP	913
		T/G	SNP	990
		G/C	SNP	991
		GC/G	DELETION	991
		G/A	SNP	1060
		T/G	SNP	1186
		T/G	SNP	1196
		G/T	SNP	1200
CBPA1_DROPS (sp|Q29NC4) Zinc carboxypeptidase A 1 OS=D. p. pseudoobscura	GLOS_CBPA1.4.11	T/A	SNP	1252

		A/T	SNP	2263
		A/C	SNP	2264
		T/G	SNP	2266
		A/T	SNP	2267
		A/C	SNP	2269
		TA/T	DELETION	2270
		A/T	SNP	2273
[BBH] CC2H2_TRYBB (sp|P54665) Cell division control protein 2 homolog 2; Tbb	GLOS_CC2H2.1.1	C/CT	INSERTION	302
		A/T	SNP	2298
		G/GT	INSERTION	2763
		C/T	SNP	2771
[BBH] CEC_GLOMM (sp|P83403) Cecropin OS=G. m. morsitans PE=1 SV=2	GLOS_CEC.2.2	G/A	SNP	30
		A/C	SNP	63
		A/G	SNP	125
		C/CA	INSERTION	160
		G/C	SNP	209
		T/C	SNP	213
		C/T	SNP	298
		A/G	SNP	338
CECC_DROYA (sp|P84226) Cecropin-C OS=D. yakuba GN=CecC PE=2 SV=1	GLOS_CECC.1.1	A/G	SNP	150
		A/G	SNP	180
		A/T	SNP	225
		T/C	SNP	256
		A/T	SNP	288
		A/T	SNP	296
		CCG/C	DELETION	297
		C/T	SNP	297
		CG/C	DELETION	298
		T/A	SNP	302
NP_651171.1 CG10252 [Drosophila melanogaster] ref|XP_002032372.1| GM23550 [D. sechellia] ref|XP_002104582.1| GD18365 [D. simulans]	GLOS_CG10252.1.2	G/C	SNP	141
		G/A	SNP	15
		G/A	SNP	64
		T/A	SNP	1278
NP_001188973.1 CG15097, isoform C [Drosophila melanogaster]	GLOS_CG15097.1.5	T/C	SNP	21
		A/C	SNP	159

		C/G	SNP	188
		C/A	SNP	258
		C/G	SNP	420
		C/T	SNP	499
		C/A	SNP	532
		T/A	SNP	537
		T/C	SNP	539
		C/T	SNP	960
		C/G	SNP	1187
		A/G	SNP	1223
		C/T	SNP	1248
		A/G	SNP	1249
		C/T	SNP	1251
		A/G	SNP	1357
		A/G	SNP	1362
		A/C	SNP	1366
		A/C	SNP	1369
		A/T	SNP	1378
		C/T	SNP	1476
		A/G	SNP	1480
		C/T	SNP	1510
		C/T	SNP	2717
		A/T	SNP	2728
		C/T	SNP	2852
NP_001188973.1 CG15097, isoform C [Drosophila melanogaster]	GLOS_CG15097.2.5	A/G	SNP	1652
		G/T	SNP	1777
		T/A	SNP	1819
		T/C	SNP	2161
		G/A	SNP	2187
		T/A	SNP	2248
		T/A	SNP	2709
		A/T	SNP	2748
		C/T	SNP	3672
		A/G	SNP	3679
		T/C	SNP	3713
		CT/C	DELETION	3771

		G/T	SNP	3809
		C/T	SNP	3836
		G/A	SNP	3852
		G/A	SNP	3869
		T/C	SNP	3877
		T/C	SNP	4000
NP_001188973.1 CG15097, isoform C [Drosophila melanogaster]	GLOS_CG15097.3.5	C/G	SNP	1774
		T/A	SNP	1885
		T/G	SNP	1897
NP_001188973.1 CG15097, isoform C [Drosophila melanogaster]	GLOS_CG15097.4.5	A/G	SNP	669
		G/T	SNP	726
		T/A	SNP	3518
		C/G	SNP	3767
		C/CT	INSERTION	4386
NP_001097756.1 CG34402, isoform C [Drosophila melanogaster]	GLOS_CG34402.1.1	GGAA/G	DELETION	178
		T/C	SNP	932
		G/GA	INSERTION	948
		A/T	SNP	992
		A/G	SNP	1005
		T/C	SNP	1006
		G/A	SNP	1057
		T/C	SNP	1323
		T/C	SNP	1391
		A/G	SNP	1684
		C/T	SNP	1789
		T/C	SNP	1808
		G/A	SNP	1841
		A/T	SNP	2153
		C/T	SNP	2481
NP_649919.1 CG9427, isoform A [Drosophila melanogaster]	GLOS_CG9427.3.3	A/C/T	SNP	366
		G/T	SNP	393
		T/C	SNP	427
NP_727805.2 CG9517, isoform B [Drosophila melanogaster]	GLOS_CG9517.1.1	C/T	SNP	50
		G/A	SNP	56
		G/C	SNP	217
		T/C	SNP	555

		C/T	SNP	596
		T/A	SNP	855
		C/T	SNP	1209
		C/G	SNP	1291
		T/C	SNP	1537
		G/T	SNP	1633
		T/A	SNP	1765
		T/C	SNP	2461
		A/G	SNP	2585
		T/G	SNP	2802
		G/A	SNP	2820
[BBH] CH60_TRYBB (sp|Q37683) Chaperonin HSP60, mitochondrial OS=T.b. brucei	GLOS_CH60.1.3	C/T	SNP	1684
XP_003401252.1 PREDICTED: chymotrypsin inhibitor-like isoform 1 [B. terrestris] ref|XP_003401253.1| PREDIC: chymotrypsin inhibitor-like isoform 2 [B. terrestris]	GLOS_CI.1.1	G/A	SNP	157
		A/C	SNP	176
		A/G	SNP	197
		A/C	SNP	209
		C/T	SNP	234
		T/C	SNP	242
		G/T	SNP	245
		C/G	SNP	270
		C/T	SNP	275
		C/T	SNP	314
		A/T	SNP	464
		T/G	SNP	466
[BBH] CLP_TRYBB (sp|P31543) Heat shock protein 100 OS=T. b. brucei	GLOS_CLP.1.1	T/C	SNP	1674
		T/C	SNP	2424
	GLOS_contig_000022	A/T	SNP	1311
		G/A	SNP	1362
		A/T	SNP	1431
		C/G	SNP	1463
		C/G	SNP	1466
		A/C	SNP	1487
		T/A	SNP	1511
		A/C	SNP	1532
		G/T	SNP	1590

	T/C	SNP	1609
	T/G	SNP	1748
GLOS_contig_000050	G/GA	INSERTION	145
	C/T	SNP	825
GLOS_contig_000213	T/A	SNP	8
	G/C	SNP	94
	GA/G	DELETION	95
	C/G	SNP	109
	G/T	SNP	123
	G/A	SNP	125
	A/AAG	INSERTION	133
	A/G	SNP	303
	C/T	SNP	319
	G/A	SNP	533
	T/C	SNP	594
	T/G	SNP	622
	T/A	SNP	632
	TA/T	DELETION	659
	A/T	SNP	921
	C/T	SNP	927
	A/C	SNP	950
	A/T	SNP	968
	C/T	SNP	997
	A/C	SNP	1128
	G/A	SNP	1131
	C/T	SNP	1202
	G/T	SNP	1227
	G/A	SNP	1228
	A/T	SNP	1263
	C/CA	INSERTION	1268
	C/A	SNP	1322
	C/A	SNP	1344
	T/A	SNP	1646
	C/G	SNP	1669
	A/T	SNP	1726
	C/T	SNP	2080

GLOS_contig_000389	T/C	SNP	928
	T/TTG/TTG	INSERTION	1191
	AT/A	DELETION	1266
GLOS_contig_000427	A/G	SNP	146
	A/G	SNP	221
	A/G	SNP	790
	GC/G	DELETION	1031
GLOS_contig_000441	A/T	SNP	92
	A/G	SNP	304
	C/T	SNP	331
	G/T	SNP	359
	A/T	SNP	364
	A/G	SNP	366
	A/G	SNP	473
	G/T	SNP	483
	T/TC	INSERTION	909
	C/T	SNP	1343
	GA/G	DELETION	1360
	C/T	SNP	1368
	A/G	SNP	1470
	A/G	SNP	1480
	A/T	SNP	1506
	A/G	SNP	1511
	C/T	SNP	1918
	C/T	SNP	1920
	A/T	SNP	1922
	C/T	SNP	1951
	A/G	SNP	1952
	C/T	SNP	1955
	A/G	SNP	1970
	C/T	SNP	2012
	G/T	SNP	2022
	A/T	SNP	2082
	G/A	SNP	2097
	G/A	SNP	2146
	T/A	SNP	2161

	A/G	SNP	2173
	G/A	SNP	2253
	G/A	SNP	2302
	T/C	SNP	2441
	C/A	SNP	2500
	C/T	SNP	2549
	C/G	SNP	3128
	C/T	SNP	3146
	C/T	SNP	3374
	A/C	SNP	3750
GLOS_contig_000530	G/A	SNP	325
	C/CT	INSERTION	564
	C/CT	INSERTION	1086
	TAA/T	DELETION	1239
GLOS_contig_000567	T/G	SNP	47
	T/G	SNP	48
	T/TG	INSERTION	117
	T/TG	INSERTION	118
	G/A	SNP	162
	T/C	SNP	171
	A/G	SNP	172
	A/G	SNP	379
	A/AT	INSERTION	385
	C/G	SNP	444
	G/A	SNP	643
	C/T	SNP	734
	G/T	SNP	758
	T/C	SNP	792
	C/T	SNP	839
	G/T	SNP	909
	A/G	SNP	1171
	A/T	SNP	1518
	A/G	SNP	1648
	A/G	SNP	1659
	G/A	SNP	1694
	C/A	SNP	1746

GLOS_contig_000712	A/C	SNP	601
GLOS_contig_000809	A/ATT	INSERTION	371
	A/T	SNP	379
	C/CT	INSERTION	519
	C/CA	INSERTION	662
	A/T	SNP	858
GLOS_contig_000926	A/G	SNP	268
	G/A	SNP	925
	T/C	SNP	1248
GLOS_contig_000973	G/C	SNP	69
	T/A	SNP	79
	C/CA	INSERTION	525
	A/AT	INSERTION	534
GLOS_contig_001171	TA/T	DELETION	214
	A/AT	INSERTION	372
	G/A	SNP	509
	AT/A	DELETION	529
	A/G	SNP	559
	A/G	SNP	672
	A/G	SNP	962
	G/A	SNP	965
	CT/C	DELETION	2060
	G/T	SNP	2595
	G/C	SNP	2752
GLOS_contig_001698	T/G	SNP	39
	A/G	SNP	40
	C/T	SNP	267
	T/A	SNP	411
GLOS_contig_001756	C/CT	INSERTION	114
	G/A	SNP	228
	A/G	SNP	271
	TTTCTC/T	DELETION	347
	GA/G	DELETION	1354
	T/C	SNP	2372
GLOS_contig_001921	GAA/G/GA	DELETION	620
	A/G	SNP	852

	A/G	SNP	908
	G/A	SNP	1289
	G/A	SNP	1377
	C/T	SNP	1622
GLOS_contig_002114	C/T	SNP	188
	G/T	SNP	236
	C/G	SNP	257
	G/A	SNP	289
	C/G	SNP	324
	T/G	SNP	334
	C/T	SNP	376
	G/C	SNP	378
	A/G	SNP	387
	G/T	SNP	391
	G/T	SNP	393
	A/T	SNP	400
	T/C	SNP	424
	C/T	SNP	427
	C/A	SNP	451
	T/C	SNP	456
	A/G	SNP	573
	T/C	SNP	587
	G/A	SNP	615
	G/A	SNP	618
	T/A	SNP	619
	A/G	SNP	658
	A/G	SNP	664
	T/C	SNP	722
	C/T	SNP	798
	T/A	SNP	802
	A/C	SNP	831
	G/A	SNP	841
	G/A	SNP	877
	G/A	SNP	919
	C/T	SNP	935
	C/A	SNP	940

C/T	SNP	979
A/T	SNP	1063
C/A	SNP	1077
G/A	SNP	1131
GT/G	DELETION	1145
G/A	SNP	1195
G/A	SNP	1224
A/G	SNP	1242
T/C	SNP	1243
C/G	SNP	1478
G/A	SNP	1496
A/T	SNP	1511
G/A	SNP	1616
G/A	SNP	1647
G/C	SNP	1731
G/A	SNP	1732
G/GT	INSERTION	1809
G/T	SNP	1916
G/A	SNP	1938
G/T	SNP	1959
G/A	SNP	1966
T/C	SNP	1971
G/A	SNP	2003
T/C	SNP	2011
G/A	SNP	2021
C/T	SNP	2033
G/C	SNP	2047
C/T	SNP	2085
G/C	SNP	2086
G/A	SNP	2119
G/A	SNP	2125
A/C	SNP	2179
CTATT/C	DELETION	2181
T/TGG	INSERTION	2197
T/C	SNP	2289
C/A	SNP	2295

	C/T	SNP	2304
	A/C/T	SNP	2331
	A/G	SNP	2333
	G/A	SNP	2361
	G/A	SNP	2392
	G/A	SNP	2409
	G/T	SNP	2414
	G/A	SNP	2421
	G/C	SNP	2435
	A/G	SNP	2501
	C/T	SNP	2543
	C/T	SNP	2546
	G/A	SNP	2571
	G/A	SNP	2620
	A/C	SNP	2628
	A/G	SNP	2630
	G/A	SNP	2651
	A/G	SNP	2663
	T/C	SNP	2680
	G/C	SNP	2684
GLOS_contig_002429	T/C	SNP	199
	T/C	SNP	216
	A/G	SNP	474
	A/T	SNP	486
	C/T	SNP	636
	C/T	SNP	641
	T/C	SNP	647
	AG/A	DELETION	743
	A/G	SNP	817
	G/A	SNP	819
	G/A	SNP	850
	C/T	SNP	854
	A/G	SNP	860
	G/A	SNP	918
	T/G	SNP	979
	A/T	SNP	987

C/T	SNP	1195
T/C	SNP	1258
A/C	SNP	1261
C/T	SNP	1347
AT/A	DELETION	1393
A/G	SNP	1471
A/G	SNP	1523
C/CT	INSERTION	1723
A/G	SNP	1812
CT/C	DELETION	1997
G/A	SNP	2125
T/C	SNP	2254
C/T	SNP	2273
C/T	SNP	2281
T/G	SNP	2320
T/C	SNP	2324
A/G	SNP	2410
T/C	SNP	2411
A/T	SNP	2550
T/C	SNP	2581
A/T	SNP	2605
T/TA	INSERTION	2656
A/AT	INSERTION	2662
A/T	SNP	2663
A/G	SNP	2680
G/A/T	SNP	2853
G/A/T	SNP	2854
A/G	SNP	2868
A/G	SNP	2923
C/T	SNP	3010
G/A	SNP	3101
C/T	SNP	3138
G/T	SNP	3146
T/C	SNP	3156
A/G	SNP	3167
G/C	SNP	3206

	G/T	SNP	3233
	G/A	SNP	3333
	G/T	SNP	3371
	T/G	SNP	3433
	G/A	SNP	3518
	A/T	SNP	3524
	T/C	SNP	3539
	G/A	SNP	3591
	T/G	SNP	3592
	C/T	SNP	3709
	C/A	SNP	3741
	T/C	SNP	3756
	C/T	SNP	3776
	C/G	SNP	3813
	T/A	SNP	3944
GLOS_contig_002688	G/C	SNP	151
	C/A	SNP	281
	A/G	SNP	328
	C/T	SNP	359
	T/G	SNP	390
	C/T	SNP	431
	T/G	SNP	528
	G/C	SNP	664
	C/T	SNP	736
	A/G	SNP	962
	C/A	SNP	987
	G/C	SNP	1041
	C/T	SNP	1072
	C/T	SNP	1082
	T/C	SNP	1089
	A/G	SNP	1139
	A/G	SNP	1488
	G/T	SNP	1618
	T/C	SNP	1685
	G/A	SNP	1735
	C/A	SNP	1778

		C/T	SNP	1829
		G/GA	INSERTION	1901
		TA/T	DELETION	1982
	GLOS_contig_002748	G/C	SNP	130
		A/C	SNP	206
		C/T	SNP	686
		T/C	SNP	707
		G/A	SNP	795
		G/A	SNP	992
		T/TA	INSERTION	1171
		C/T	SNP	1269
		G/A	SNP	1418
		C/T	SNP	1462
		T/C	SNP	1463
		G/A	SNP	1470
		C/A	SNP	1501
		A/G	SNP	1519
		G/C	SNP	1520
		A/G	SNP	1534
		G/T	SNP	1590
		C/T	SNP	1690
		C/G	SNP	1726
		C/G	SNP	1922
		ATG/A	DELETION	2058
		A/G	SNP	566
		G/A	SNP	569
		G/T	SNP	630
		C/A	SNP	633
		T/TA	INSERTION	211
XP_004527397.1 PREDICT.: synaptic vesicle glycoprotein 2A-like isoform X1 [C. c.]	GLOS_contig_003328	A/T	SNP	643
		G/C	SNP	889
		G/A	SNP	892
		A/T	SNP	893
		C/T	SNP	898
		A/C	SNP	1362
		T/C	SNP	1411

	A/T	SNP	1542
	C/T	SNP	1549
	G/C	SNP	1557
	T/TG	INSERTION	1691
GLOS_contig_003408	A/T	SNP	246
	T/G	SNP	344
	T/G	SNP	348
	A/T	SNP	356
	C/G	SNP	360
	C/A	SNP	504
GLOS_contig_003540	A/C	SNP	42
	A/G	SNP	102
	T/C	SNP	124
	A/C	SNP	138
	G/A	SNP	196
	G/C	SNP	541
	G/A	SNP	569
	G/T	SNP	659
GLOS_contig_003620	A/T	SNP	90
	A/C	SNP	91
	C/CA	INSERTION	157
	A/T	SNP	168
	TTTA/T	DELETION	540
	A/C	SNP	544
	C/G	SNP	559
	A/C	SNP	619
	A/G	SNP	1061
	A/G	SNP	1258
	G/A	SNP	1326
GLOS_contig_004199	C/G	SNP	191
	C/A	SNP	233
	G/A	SNP	1019
	G/A	SNP	1893
	T/C	SNP	1931
	A/G	SNP	2362
GLOS_contig_004527	A/G	SNP	150

		G/A	SNP	470
		A/G	SNP	591
		G/C	SNP	623
		G/A	SNP	686
		G/T	SNP	836
		A/G	SNP	1446
XP_004527397.1 PRED: synaptic vesicle glycoprot. 2A-like isoform X1 [C. capitata]	GLOS_contig_004530	A/T	SNP	865
		AT/A	DELETION	865
		G/A	SNP	867
		T/G	SNP	868
		A/T	SNP	869
		G/T	SNP	3401
		G/C	SNP	3402
		A/T	SNP	3403
	GLOS_contig_005205	G/GT	INSERTION	277
		A/G	SNP	444
		C/T	SNP	476
		T/C	SNP	636
	GLOS_contig_005593	T/A	SNP	14
		G/A	SNP	73
		A/G	SNP	216
		C/T	SNP	223
		G/A	SNP	234
		G/C	SNP	249
		T/C	SNP	275
		A/G	SNP	280
		A/G	SNP	282
		A/T	SNP	305
		A/T	SNP	325
		A/T	SNP	337
		C/T	SNP	417
		T/C	SNP	435
		T/G	SNP	491
		T/C	SNP	502
		G/A	SNP	506
		G/A	SNP	521

C/T	SNP	568
G/A	SNP	591
G/GA	INSERTION	598
G/T	SNP	598
T/A	SNP	639
G/A	SNP	677
C/A	SNP	738
C/T	SNP	770
G/A	SNP	809
A/C	SNP	816
G/A	SNP	856
A/C	SNP	861
G/A	SNP	889
C/A	SNP	1174
G/A	SNP	1175
T/C	SNP	1216
A/G	SNP	1240
T/C	SNP	1249
G/A	SNP	1270
A/T	SNP	1281
A/G	SNP	1295
T/C	SNP	1342
T/A	SNP	1344
C/T	SNP	1345
C/T	SNP	1348
T/TG	INSERTION	1355
G/C	SNP	1392
A/T	SNP	1439
A/G	SNP	1441
A/G	SNP	1445
G/A	SNP	1476
A/T	SNP	65
A/T	SNP	121
G/GTTTTT	INSERTION	277
C/G	SNP	339
T/TC	INSERTION	361

GLOS_contig_006296	T/G	SNP	838
	C/T	SNP	983
GLOS_contig_006420	A/C	SNP	39
	G/C	SNP	142
	T/A	SNP	671
	A/C	SNP	686
	C/T	SNP	753
	T/G	SNP	1007
	A/G	SNP	1027
	C/A	SNP	1052
	C/A	SNP	1123
	G/A	SNP	1133
	G/A	SNP	1277
	G/A	SNP	1322
GLOS_contig_006679	A/G	SNP	98
GLOS_contig_006689	A/C	SNP	45
	C/G	SNP	58
	A/G	SNP	62
	A/G	SNP	185
GLOS_contig_006723	G/T	SNP	239
	C/T	SNP	411
	T/C	SNP	427
	A/AT	INSERTION	636
GLOS_contig_006942	G/C	SNP	45
	C/CT	INSERTION	242
	C/T	SNP	269
	C/T	SNP	504
	A/C	SNP	579
	A/G	SNP	607
	A/G	SNP	761
	C/T	SNP	764
	A/AT	INSERTION	1010
	A/T	SNP	1064
	T/C	SNP	1068
	C/T	SNP	1084
	G/T	SNP	1133

	C/T	SNP	1153
	C/T	SNP	1244
	G/GT	INSERTION	1299
	C/T	SNP	1348
GLOS_contig_007005	C/CT	INSERTION	104
	C/CA	INSERTION	1248
	G/A	SNP	1266
	G/A	SNP	1600
	C/T	SNP	1601
	C/CA	INSERTION	1623
	G/T	SNP	1637
	C/T	SNP	1644
	A/G	SNP	1878
	T/C	SNP	3727
	G/GT	INSERTION	3748
GLOS_contig_007084	A/G	SNP	1253
	A/G	SNP	1254
GLOS_contig_007102	A/G	SNP	696
	G/A	SNP	797
	T/C	SNP	1005
	C/T	SNP	1030
	TTCTC/T/T	DELETION	1095
	T/A	SNP	1130
	C/A	SNP	1566
GLOS_contig_007169	G/T	SNP	38
	C/T	SNP	39
	C/A	SNP	40
	A/C	SNP	129
	T/A	SNP	158
	G/A	SNP	208
GLOS_contig_007302	T/G	SNP	536
	G/T	SNP	538
GLOS_contig_007349	G/A	SNP	161
	TA/T	DELETION	221
	A/AT	INSERTION	479
	G/GGT	INSERTION	548

	T/A	SNP	574
	C/T	SNP	612
	A/T	SNP	614
	A/G	SNP	619
	A/T	SNP	622
	A/C	SNP	1024
	G/T	SNP	1806
	CA/C	DELETION	1840
	T/C	SNP	1905
GLOS_contig_007422	C/T	SNP	217
	A/G	SNP	222
	A/G	SNP	233
	T/C	SNP	349
	G/A	SNP	350
	A/T	SNP	426
	T/C	SNP	428
GLOS_contig_007423	A/G	SNP	433
	G/T	SNP	451
	C/T	SNP	529
GLOS_contig_007475	T/C	SNP	286
	A/C	SNP	635
	C/A	SNP	636
	A/T	SNP	809
	C/G	SNP	851
	A/G	SNP	878
	G/T	SNP	890
	G/A	SNP	893
GLOS_contig_007483	T/C	SNP	343
	A/T	SNP	344
	A/AT	INSERTION	432
	C/G	SNP	434
	T/A	SNP	437
	T/G	SNP	440
	T/C	SNP	441
	A/T	SNP	594
	T/C	SNP	597

	T/TC	INSERTION	602
	C/T	SNP	605
	A/G	SNP	645
	A/T	SNP	803
GLOS_contig_007486	A/G	SNP	268
	A/G	SNP	506
	C/T	SNP	542
GLOS_contig_008085	G/A	SNP	27
	A/C	SNP	65
	A/G	SNP	184
	A/T	SNP	191
	A/G	SNP	295
	T/A	SNP	397
	T/G	SNP	581
	T/A	SNP	674
	A/G	SNP	709
GLOS_contig_008438	A/T	SNP	2
	T/A	SNP	343
	T/C	SNP	471
	CAA/C/CA	DELETION	545
	G/A	SNP	587
	C/T	SNP	606
	A/G	SNP	613
	C/T	SNP	678
	T/A	SNP	728
	C/T	SNP	755
	C/G	SNP	1062
	G/C	SNP	1173
	T/TA	INSERTION	1419
	A/G	SNP	1501
	A/G	SNP	1528
GLOS_contig_008466	G/T	SNP	258
	C/T	SNP	339
	T/C	SNP	345
	A/C	SNP	413
	A/G	SNP	444

		T/A	SNP	474
		C/G	SNP	551
		G/A	SNP	568
		T/A	SNP	570
		T/C	SNP	651
		A/G	SNP	657
		C/A	SNP	732
NR_077014.1 Escherichia fergusonii ATCC 35469 strain ATCC 35469 23S ribosomal RNA, complete sequence	GLOS_contig_008528	G/A	SNP	64
		A/G	SNP	105
		C/A	SNP	115
		T/C	SNP	148
		A/T	SNP	362
		T/A	SNP	367
	GLOS_contig_008620	C/T	SNP	1529
	GLOS_contig_008697	A/G	SNP	50
		T/C	SNP	495
	GLOS_contig_008738	A/C	SNP	1047
		T/A	SNP	1335
		C/T	SNP	1337
		C/T	SNP	1454
		C/T	SNP	2139
		A/T	SNP	2141
		C/T	SNP	2142
		C/T	SNP	2181
	GLOS_contig_008758	C/G	SNP	48
		G/A	SNP	143
		T/A	SNP	318
		T/C	SNP	374
		A/T	SNP	494
		G/A	SNP	502
		G/A	SNP	512
		C/A	SNP	523
		A/G	SNP	531
		A/G	SNP	543
		C/T	SNP	793

	T/A	SNP	1037
	A/T	SNP	1328
	G/A	SNP	1365
GLOS_contig_009034	G/GT	INSERTION	81
	A/G	SNP	121
	C/A	SNP	127
	G/C	SNP	135
	G/A	SNP	138
GLOS_contig_009266	G/A	SNP	141
	CA/C	DELETION	149
	G/A	SNP	290
	C/A	SNP	525
	TG/T	DELETION	529
	T/C	SNP	560
	C/G	SNP	738
	T/A	SNP	948
	A/T	SNP	1033
	T/C	SNP	1076
	A/G	SNP	1527
	A/G	SNP	1558
	G/A	SNP	1685
	G/A	SNP	1757
	C/T	SNP	1791
	A/G	SNP	1801
	T/C	SNP	1855
	T/G	SNP	1969
	G/A	SNP	2101
	A/G	SNP	2252
	G/A	SNP	2291
	C/A	SNP	2429
GLOS_contig_009448	A/G	SNP	36
	A/G	SNP	120
	A/AG	INSERTION	195
GLOS_contig_009455	G/T	SNP	512
	C/G	SNP	515
GLOS_contig_009600	G/T	SNP	1385

	A/T	SNP	1392
	C/T	SNP	1457
	C/T	SNP	1492
GLOS_contig_009639	G/A	SNP	188
	G/A	SNP	204
	T/C	SNP	314
	G/A	SNP	390
	A/G	SNP	404
	G/T	SNP	441
	A/T	SNP	507
	G/C	SNP	570
	A/G	SNP	658
	C/T	SNP	662
	T/G	SNP	707
	C/T	SNP	710
	G/A	SNP	824
	A/G	SNP	921
	C/T	SNP	1002
	G/A	SNP	1094
	G/T	SNP	1265
	G/A	SNP	1313
	G/A	SNP	1325
	C/A	SNP	1361
	G/A	SNP	1363
	G/A	SNP	1365
	T/C	SNP	1367
	C/CT	INSERTION	1438
	G/A	SNP	1464
	G/T	SNP	1506
	G/A	SNP	1511
GLOS_contig_009648	C/G	SNP	826
GLOS_contig_009651	T/A	SNP	481
	G/A	SNP	865
	AAC/A	DELETION	1152
	G/T	SNP	1478
	T/C	SNP	1547

		A/G	SNP	2321
		G/A	SNP	2429
		G/GA	INSERTION	2475
		C/T	SNP	2536
NR_076090.1 P. mirabilis HI4320 strain HI4320 23S rib. RNA, complete sequence	GLOS_contig_009795	C/T	SNP	476
		C/T	SNP	495
		A/T	SNP	524
		C/T	SNP	537
		C/T	SNP	617
		C/T	SNP	633
		GT/G	DELETION	634
		A/G	SNP	640
		T/A	SNP	649
		A/T	SNP	675
		A/G	SNP	685
		G/T	SNP	768
		A/C/T	SNP	795
		A/G	SNP	797
		C/G	SNP	869
		T/A	SNP	876
		A/G	SNP	898
		G/T	SNP	906
		C/T	SNP	924
		A/G	SNP	937
		A/G	SNP	995
		C/T	SNP	1017
		A/G	SNP	1019
		A/G	SNP	1039
		A/G	SNP	1040
		A/T	SNP	1056
		A/G	SNP	1063
		G/T	SNP	1065
		T/C	SNP	1067
		C/T	SNP	1068
		C/T	SNP	1142
		C/G	SNP	1193

	A/T	SNP	1197
	T/C	SNP	1200
	A/G	SNP	1242
	T/C	SNP	1251
	T/A	SNP	1298
	A/G	SNP	1310
	A/T	SNP	1316
	T/C	SNP	1556
	C/T	SNP	1751
	G/A	SNP	1767
	C/T	SNP	1768
	T/C	SNP	1865
	C/T	SNP	1891
	C/T	SNP	1903
	A/G	SNP	1917
	A/G	SNP	1936
	A/G	SNP	2183
	G/T	SNP	2320
	A/C	SNP	2322
	T/G	SNP	2336
	C/A	SNP	2338
	A/T	SNP	2350
GLOS_contig_009842	G/A	SNP	310
	G/A	SNP	962
	T/A	SNP	1003
	A/T	SNP	1147
	T/G	SNP	1204
	A/G	SNP	1205
	G/A	SNP	1401
	G/A	SNP	1480
	G/T	SNP	1732
	G/A	SNP	1808
	A/C	SNP	1911
	C/G	SNP	1922
	C/A	SNP	1942
	A/G	SNP	2101

	C/T	SNP	2231
	A/G	SNP	2318
	T/C	SNP	2471
	G/A	SNP	2553
	C/T	SNP	2567
	A/G	SNP	2850
	A/G	SNP	2851
	G/A	SNP	2872
	A/G	SNP	2925
	G/C	SNP	2933
	C/G	SNP	2938
GLOS_contig_009922	T/A	SNP	357
	C/T	SNP	369
	G/A	SNP	381
	A/G	SNP	383
	G/T	SNP	476
	T/C	SNP	599
	T/G	SNP	674
	C/T	SNP	902
	G/C	SNP	925
	C/A	SNP	1172
	C/G	SNP	1719
GLOS_contig_010028	A/C	SNP	13
	G/A	SNP	93
	A/G	SNP	104
	T/C	SNP	114
	A/C	SNP	154
	C/T	SNP	178
	A/G	SNP	257
	C/T	SNP	382
	A/G	SNP	417
	G/A	SNP	441
	T/A	SNP	445
	C/T	SNP	503
	G/GT	INSERTION	506
	A/T	SNP	527

	C/T	SNP	638
	G/C	SNP	675
	C/G	SNP	691
	G/A	SNP	699
	C/T	SNP	753
	A/G	SNP	768
	A/G	SNP	881
	G/GCT	INSERTION	1025
	A/T	SNP	1089
	G/A	SNP	1119
	C/T	SNP	1133
	T/A	SNP	1154
	A/G	SNP	1250
	T/A	SNP	1316
	T/C	SNP	1371
	T/C	SNP	1381
	A/G	SNP	1385
	T/C	SNP	1420
	A/C/T	SNP	1433
	T/A	SNP	1445
	C/A	SNP	1446
	C/T	SNP	1490
	A/G	SNP	1522
	G/A	SNP	1549
	C/A	SNP	1566
	A/T	SNP	1620
	G/A	SNP	1634
	C/T	SNP	1666
GLOS_contig_010153	GA/G	DELETION	734
	G/A	SNP	741
	T/C	SNP	756
	G/A	SNP	785
	A/G	SNP	885
	A/G	SNP	972
	G/A	SNP	984
	C/T	SNP	1089

GLOS_contig_010349	T/G	SNP	819
	G/T	SNP	2508
	T/C	SNP	5199
	A/G	SNP	5248
	G/A	SNP	5301
	A/G	SNP	5363
	C/T	SNP	5423
	T/C	SNP	5772
GLOS_contig_010675	G/T	SNP	351
	C/T	SNP	389
	C/T	SNP	485
	C/A	SNP	486
	C/T	SNP	754
	G/T	SNP	844
	C/A	SNP	869
	G/C	SNP	896
	C/G	SNP	924
	G/A	SNP	940
	C/T	SNP	962
	C/A	SNP	1052
	G/A	SNP	1107
	T/C	SNP	1148
	C/T	SNP	1569
	G/A	SNP	1631
	T/C	SNP	1632
GLOS_contig_010763	T/C	SNP	15
	AG/A	DELETION	131
	G/GAAA	INSERTION	132
	A/T	SNP	133
	C/T	SNP	153
	A/G	SNP	193
	G/T	SNP	263
	G/A	SNP	276
	C/T	SNP	282
	A/G	SNP	305
	G/A	SNP	317

	G/A	SNP	399
	G/T	SNP	411
	A/G	SNP	419
	T/C	SNP	474
	A/C	SNP	494
	G/A	SNP	513
	A/G	SNP	637
	T/G	SNP	670
	C/T	SNP	772
	T/C	SNP	784
	A/T	SNP	893
	T/G	SNP	894
	C/T	SNP	962
	A/AT	INSERTION	1061
	G/C	SNP	1075
	A/G	SNP	1115
	C/A	SNP	1162
	T/G	SNP	1199
	C/G	SNP	1230
	A/T	SNP	1279
	T/C	SNP	1308
	C/T	SNP	1329
	C/T	SNP	1376
	C/T	SNP	1394
	T/C	SNP	1400
	C/T	SNP	1437
	T/C	SNP	1460
	A/G	SNP	1499
	C/CA	INSERTION	1501
	G/T	SNP	1536
	AAC/A	DELETION	1553
	T/TA	INSERTION	1613
	T/A	SNP	1735
GLOS_contig_010890	T/G	SNP	1697
	G/A	SNP	1765
	G/T	SNP	1796

	T/C	SNP	2065
	T/A	SNP	2077
GLOS_contig_010920	AT/A	DELETION	291
	T/TA	INSERTION	292
	T/G	SNP	293
	G/A	SNP	299
	T/A	SNP	300
	C/G	SNP	301
	A/C	SNP	496
GLOS_contig_011158	A/G	SNP	336
	C/CT	INSERTION	857
GLOS_contig_011297	G/A	SNP	157
	A/G	SNP	433
	T/C	SNP	462
	TA/T	DELETION	539
	C/T	SNP	620
GLOS_contig_011331	C/T	SNP	43
	A/C	SNP	66
	A/G	SNP	92
	C/T	SNP	100
	G/C	SNP	154
	A/G	SNP	187
	T/G	SNP	292
	C/A	SNP	665
GLOS_contig_011473	C/T	SNP	268
	T/C	SNP	492
	T/C	SNP	533
GLOS_contig_011540	G/A	SNP	171
	C/A	SNP	172
	T/C	SNP	243
	G/T	SNP	312
	GT/G	DELETION	329
GLOS_contig_011544	G/A	SNP	36
	C/T	SNP	64
	C/T	SNP	114
	C/A	SNP	144

	T/C	SNP	184
	A/T	SNP	223
	A/C/G	SNP	306
	G/A	SNP	307
	A/C	SNP	312
	G/A	SNP	337
	T/G	SNP	351
	G/T	SNP	449
	G/C	SNP	500
	G/A	SNP	555
	G/A	SNP	558
	C/T	SNP	604
	A/G	SNP	606
	G/A	SNP	666
	A/G	SNP	721
	C/T	SNP	725
GLOS_contig_011571	C/G	SNP	516
	A/G	SNP	547
	G/A	SNP	562
	A/C	SNP	576
	T/A	SNP	1234
	A/T	SNP	1294
	G/A	SNP	1296
GLOS_contig_011776	A/G	SNP	331
	C/A	SNP	347
	C/G	SNP	654
	T/C	SNP	786
	G/A	SNP	854
	T/G	SNP	947
	T/C	SNP	964
	G/A	SNP	1005
	G/C	SNP	1123
	C/A	SNP	1133
	C/A	SNP	1134
	C/T	SNP	1138
	T/C	SNP	1318

	GLOS_contig_011904	T/A	SNP	399
		A/G	SNP	414
		T/A	SNP	418
		C/T	SNP	480
	GLOS_contig_011969	G/A	SNP	49
		A/T	SNP	56
		A/G	SNP	74
		A/G	SNP	102
		C/T	SNP	106
		T/A	SNP	134
		C/T	SNP	155
		G/C	SNP	481
		C/T	SNP	529
		T/C	SNP	616
		C/G	SNP	624
		C/T	SNP	631
		G/C	SNP	777
	GLOS_contig_011996	T/A	SNP	56
		A/G	SNP	79
		G/T	SNP	101
		A/G	SNP	586
		G/A	SNP	591
		A/G	SNP	598
		A/G	SNP	601
		C/T	SNP	631
		CA/C	DELETION	654
		A/G	SNP	678
		A/G	SNP	706
		A/G	SNP	710
		G/A	SNP	713
XP_004521238.1 PRED.: type-2 histone deacetylase 2-like isoform X1 [C. capitata]	GLOS_contig_012062	A/C	SNP	337
		G/T	SNP	349
		G/A	SNP	360
		C/T	SNP	379
		G/T	SNP	453
		T/C	SNP	619

GLOS_contig_012452	G/T	SNP	110
	T/C	SNP	215
	C/T	SNP	298
	G/A	SNP	359
	C/G	SNP	397
	T/C	SNP	433
	C/A	SNP	487
	C/A	SNP	692
	G/T	SNP	770
	G/A	SNP	814
GLOS_contig_012510	G/T	SNP	397
	A/C	SNP	430
	A/G	SNP	894
	C/T	SNP	895
	C/T	SNP	921
	C/A	SNP	962
	A/C	SNP	974
GLOS_contig_012845	T/C	SNP	319
	T/C	SNP	320
	A/T	SNP	382
GLOS_contig_012940	A/G	SNP	301
	A/G	SNP	304
	A/T	SNP	305
	T/A	SNP	306
GLOS_contig_013033	G/A	SNP	247
	T/G	SNP	282
	T/G	SNP	312
	T/C	SNP	627
	G/A	SNP	702
	T/C	SNP	708
	C/T	SNP	717
	C/G	SNP	730
	C/T	SNP	771
	G/A	SNP	775
GLOS_contig_013097	C/T	SNP	59
	T/C	SNP	135

	A/G	SNP	137
	AT/A	DELETION	142
	T/A	SNP	311
	A/G	SNP	316
	C/T	SNP	321
	C/CA	INSERTION	364
	C/T	SNP	397
	C/T	SNP	587
	A/AT	INSERTION	624
	T/G	SNP	628
	C/G	SNP	641
	C/T	SNP	664
	T/C	SNP	697
	G/T	SNP	699
	A/G	SNP	807
	A/G	SNP	813
	C/T	SNP	825
	T/G	SNP	903
	T/C	SNP	950
	A/G	SNP	962
	G/T	SNP	973
GLOS_contig_013166	C/T	SNP	118
	G/A	SNP	330
	T/C	SNP	341
	G/T	SNP	700
	T/A	SNP	1044
	G/GT	INSERTION	1270
	G/A	SNP	1324
	T/C	SNP	1441
GLOS_contig_013203	G/A	SNP	293
	G/A	SNP	371
	C/G	SNP	547
	A/T	SNP	571
	C/CT	INSERTION	661
	A/G	SNP	675
	G/T	SNP	840

	A/C	SNP	903
	G/C	SNP	931
	C/T	SNP	1026
	A/G	SNP	1421
GLOS_contig_013214	C/A	SNP	33
	A/G	SNP	264
	T/C	SNP	271
	G/C	SNP	404
	T/G	SNP	461
	T/C	SNP	467
	T/C	SNP	566
	G/C/T	SNP	656
	A/G	SNP	658
	G/T	SNP	737
	A/G	SNP	793
	G/A	SNP	809
GLOS_contig_013557	T/A	SNP	33
	G/A/T	SNP	35
	T/A	SNP	36
	A/G	SNP	37
	G/C	SNP	38
	G/A	SNP	273
	G/A	SNP	304
GLOS_contig_013569	G/C	SNP	133
	T/C	SNP	1040
	G/A	SNP	1297
	C/A	SNP	1298
	A/G	SNP	35
	T/A	SNP	158
	T/C	SNP	188
	C/T	SNP	215
	T/C	SNP	323
	G/A	SNP	370
	C/G	SNP	372
GLOS_contig_013650	G/A	SNP	768
	C/T	SNP	788

	C/T	SNP	844
	T/G	SNP	857
GLOS_contig_013881	C/T	SNP	21
	A/G	SNP	163
GLOS_contig_013915	C/CA	INSERTION	181
	G/T	SNP	238
	G/GAA/GA	INSERTION	279
	A/G	SNP	331
	A/G	SNP	337
GLOS_contig_013996	A/C	SNP	814
	C/T	SNP	826
GLOS_contig_014089	C/A	SNP	33
	A/C	SNP	122
	C/T	SNP	123
	CG/C	DELETION	125
	G/A	SNP	128
	T/TC	INSERTION	129
	T/A	SNP	181
	A/G	SNP	188
	T/G	SNP	296
	T/A	SNP	297
	A/G	SNP	444
	G/C	SNP	837
GLOS_contig_014288	C/T	SNP	65
	A/G	SNP	137
	C/A	SNP	212
	C/T	SNP	216
	G/T	SNP	217
	T/A	SNP	231
	G/A	SNP	358
	A/G	SNP	423
	G/C	SNP	437
	A/T	SNP	439
	T/A	SNP	514
	T/C	SNP	523
	A/G	SNP	556

	A/G	SNP	601
	T/C	SNP	624
	T/C	SNP	637
	A/G	SNP	918
	A/G	SNP	929
	A/G	SNP	955
	G/A	SNP	957
	T/TA	INSERTION	1003
	A/C	SNP	1030
	T/C	SNP	1061
	T/C	SNP	1066
	T/C	SNP	1071
	T/C	SNP	1161
	T/C	SNP	1177
	T/C	SNP	1185
	T/C	SNP	1186
	T/C	SNP	1196
	T/C	SNP	1206
	T/C	SNP	1236
	C/T	SNP	1327
	C/A	SNP	1346
	G/A	SNP	1362
	G/T	SNP	1424
	C/A	SNP	1428
	A/G	SNP	1444
	A/G	SNP	1484
	A/T	SNP	1535
	G/A	SNP	1559
	T/C	SNP	1616
	T/C	SNP	1617
	A/G	SNP	1648
	T/C	SNP	1656
	T/C	SNP	1681
GLOS_contig_014690	C/T	SNP	737
	C/T	SNP	747
	C/T	SNP	848

	G/A	SNP	861
	A/G	SNP	893
	G/T	SNP	1615
GLOS_contig_014768	A/T	SNP	51
	T/C	SNP	97
	T/C	SNP	142
	A/G	SNP	145
	A/AC	INSERTION	159
	T/A/C	SNP	160
	T/TA	INSERTION	160
	T/G	SNP	171
	T/A	SNP	182
	C/T	SNP	188
	C/G	SNP	191
	C/T	SNP	232
	T/C	SNP	362
	G/A	SNP	483
GLOS_contig_014921	C/T	SNP	284
	A/T	SNP	361
	T/A	SNP	412
	T/C	SNP	464
	C/T	SNP	471
	G/A	SNP	508
	A/G	SNP	556
	T/G	SNP	594
	G/T	SNP	679
	G/A	SNP	729
	T/C	SNP	892
	C/T	SNP	893
	G/A	SNP	978
	T/G	SNP	990
	G/C	SNP	1054
	T/A	SNP	1117
	A/G	SNP	1308
	G/A	SNP	1370
	C/T	SNP	1683

GLOS_contig_015170	T/A	SNP	14
	A/G	SNP	35
	T/C	SNP	692
	C/T	SNP	891
	A/T	SNP	942
	T/A	SNP	944
	T/G	SNP	947
	T/A	SNP	972
	A/C	SNP	1027
	G/A	SNP	1032
GLOS_contig_015454	G/A	SNP	261
	T/C	SNP	282
	T/G	SNP	313
	T/C	SNP	380
	C/T	SNP	1002
	C/T	SNP	1003
	T/C	SNP	1175
	C/T	SNP	1197
	GTA/G	DELETION	1513
	A/T	SNP	1818
	G/A	SNP	1906
	T/TA	INSERTION	2003
	A/G	SNP	2026
	A/G	SNP	2030
	C/G	SNP	2035
	T/C	SNP	2060
	C/G	SNP	2079
	A/G	SNP	2124
	G/A	SNP	2231
	C/T	SNP	2290
	A/T	SNP	2502
	A/T	SNP	2549
	T/C	SNP	2608
	G/A	SNP	2623
	C/T	SNP	2624
	G/A	SNP	2679

	G/A	SNP	2756
	A/G	SNP	3080
	A/G	SNP	3100
	C/CA	INSERTION	3105
	A/C	SNP	3130
	A/G	SNP	3136
	C/G	SNP	3141
	C/G	SNP	3191
	A/G	SNP	3208
	C/T	SNP	3210
	A/AT	INSERTION	3215
	A/C	SNP	3216
	A/C	SNP	3218
	A/G	SNP	3358
	A/G	SNP	3376
	G/GA	INSERTION	3547
GLOS_contig_015559	C/G	SNP	370
	T/C	SNP	419
	G/GT	INSERTION	471
	A/G	SNP	1202
GLOS_contig_015681	G/A	SNP	295
	T/A	SNP	392
GLOS_contig_016335	T/C	SNP	1145
	C/T	SNP	1168
	A/G	SNP	1231
	G/A	SNP	1238
	T/C	SNP	1260
	T/A	SNP	1283
	C/T	SNP	1310
	TTG/T/TT(	DELETION	1503
	G/C	SNP	1545
GLOS_contig_016571	G/A	SNP	1877
GLOS_contig_016710	C/T	SNP	80
	G/A	SNP	102
	A/G	SNP	327
	G/A	SNP	514

		C/CT	INSERTION	538
		G/A	SNP	545
		C/T	SNP	566
		C/T	SNP	567
		A/G	SNP	631
		G/C	SNP	697
		T/G	SNP	714
		G/A	SNP	759
		A/G	SNP	840
		T/G	SNP	869
		G/T	SNP	885
		C/A	SNP	959
		C/T	SNP	970
		T/A	SNP	1004
		A/G	SNP	1049
		C/A	SNP	1050
		T/G	SNP	1165
[BBH] COPB_TRYBB (sp|Q9NFU6) Coatomer subunit beta OS=Trypanosoma brucei bru	GLOS_COPB.1.2	C/CT	INSERTION	418
		GT/G	DELETION	517
		A/T	SNP	1026
		G/A	SNP	1406
		G/A	SNP	2252
		A/C	SNP	2825
COX1_DROYA (sp|P00400) Cytochrome c oxidase subunit 1 OS=Drosophila yakuba GN	GLOS_COX1.6.10	C/A/T	SNP	1043
		C/A	SNP	1083
		C/CA	INSERTION	1799
		G/GA	INSERTION	1800
		C/G	SNP	1804
		T/G	SNP	1807
		A/T	SNP	1920
		A/C	SNP	1940
		A/G	SNP	1941
		A/T	SNP	2011
		A/T	SNP	2046
		T/A	SNP	2047
		A/T	SNP	2087

		T/A	SNP	2115
		C/T	SNP	2877
		A/C	SNP	3067
		C/T	SNP	3390
		A/T	SNP	3393
		G/T	SNP	3428
		T/C	SNP	3493
		C/T	SNP	3519
		A/C	SNP	3582
		A/AG	INSERTION	3787
		A/T	SNP	3788
		A/C	SNP	3790
		T/G	SNP	3792
		T/A	SNP	3793
		C/A	SNP	3796
		T/A	SNP	3872
CP305_DROME (sp|Q9VW43) Probable cytochrome P450 305a1 OS=Drosophila melan	GLOS_CP305.1.2	A/G	SNP	746
		G/A	SNP	747
		T/C	SNP	1762
		A/G	SNP	1779
CP6G1_DROME (sp|Q9V674) Cyt. P450 6g1 OS=D. melanogaster	GLOS_CP6G1.1.1	C/A	SNP	1713
		C/A	SNP	1753
		TG/T	DELETION	1767
		G/A	SNP	1770
		C/A	SNP	1873
CP6W1_DROME (sp|Q9V9L1) Probable cyt. P450 6w1 OS=D. melanogaster	GLOS_CP6W1.1.1	T/C	SNP	1716
		C/A	SNP	1757
		T/C	SNP	2239
		AT/A	DELETION	2307
		C/G	SNP	2313
		T/A	SNP	2616
		T/A	SNP	2618
CP9F2_DROME (sp|Q9VG82) Probable cyt. P450 9f2 OS=D. melanogaster	GLOS_CP9F2.9.9	A/C	SNP	98
		T/C	SNP	130
		G/A	SNP	190
		A/G	SNP	211

		G/A	SNP	250
		A/G	SNP	279
		G/T	SNP	322
		A/C	SNP	428
		C/T	SNP	433
		T/C	SNP	436
		C/T	SNP	661
		A/T	SNP	788
		T/C	SNP	806
XP_001842487.1 multicopper oxidase [Culex quinquefasciatus]	GLOS_CPIPJ_CPIJ000864.1.1	A/T	SNP	56
		T/G	SNP	125
		T/C	SNP	126
		T/A	SNP	742
		T/C	SNP	917
		G/C	SNP	921
		A/G	SNP	922
		T/A	SNP	1092
		A/G	SNP	1095
		C/T	SNP	1141
		T/G	SNP	2002
CRAM_TRYBB (sp|Q03650) Cysteine-rich, acidic integral membrane prot. OS=Tbb	GLOS_CRAM.1.1	G/A	SNP	24
		A/G	SNP	33
		T/C	SNP	59
		A/G	SNP	60
		A/G	SNP	69
		T/C	SNP	95
		G/A	SNP	141
		G/A	SNP	168
[BBH] CRY1_DROME (sp|O77059) Cryptochrome-1 OS=D. m. GN=cry PE=1 SV=1	GLOS_CRY1.1.1	G/C	SNP	70
		CT/C	DELETION	90
		C/G	SNP	111
		C/T	SNP	245
		C/T	SNP	336
		T/C	SNP	359
		A/G	SNP	443
		A/G	SNP	578

		C/A/T	SNP	584
		A/T	SNP	647
		G/A	SNP	818
		T/C	SNP	1337
		C/T	SNP	1358
		G/A	SNP	1544
		A/T	SNP	1669
		T/C	SNP	1700
		G/A	SNP	1712
		A/G	SNP	1809
		T/C	SNP	1816
CYTA_SARPE (sp|P31727) Sarcocystatin-A OS=Sarcophaga peregrina PE=1 SV=1	GLOS_CYTA.1.2	G/A	SNP	65
		T/G	SNP	196
		T/C	SNP	406
		G/A	SNP	417
		C/G	SNP	522
		G/T	SNP	640
XP_001959348.1 GF12088 [Drosophila ananassae]	GLOS_DANA_GF12088.1.1	C/CAT	INSERTION	339
		A/G	SNP	412
		C/T	SNP	451
		G/A	SNP	456
		T/G	SNP	273
		T/A	SNP	344
		C/G	SNP	379
		C/T	SNP	391
		T/C	SNP	415
		C/T	SNP	448
		G/C	SNP	520
		A/T	SNP	540
		G/C	SNP	675
		A/G	SNP	760
		A/C	SNP	801
		G/T	SNP	818
		C/A	SNP	867
		C/T	SNP	872
		C/T	SNP	877

		C/T	SNP	925
		A/G	SNP	926
		G/A	SNP	940
		C/T	SNP	976
		T/C	SNP	1012
		T/C	SNP	1020
		T/C	SNP	1028
		T/A	SNP	1044
		G/T	SNP	1080
		C/T	SNP	1188
		C/T	SNP	1248
		C/T	SNP	1286
		C/T	SNP	1407
		A/G	SNP	1491
		A/G	SNP	1516
		A/T	SNP	1517
		C/A	SNP	1696
		T/C	SNP	1721
		G/A	SNP	1752
		C/G	SNP	1845
		G/C	SNP	1956
		G/A	SNP	2061
		A/T	SNP	2709
		T/A	SNP	2710
		T/C	SNP	2712
		G/T	SNP	2713
		T/C	SNP	2768
		A/AC	INSERTION	2860
		T/TAG	INSERTION	3000
		T/C	SNP	3003
		A/G	SNP	3005
		A/G	SNP	3008
XP_001962018.1 GF14647 [Drosophila ananassae]	GLOS_DANA_GF14647.1.1	T/C	SNP	169
		C/T	SNP	233
		A/G	SNP	334
		T/C	SNP	400

		T/C	SNP	433
		C/T	SNP	610
		C/T	SNP	706
		C/T	SNP	710
		T/C	SNP	1033
		C/T	SNP	1101
		C/T	SNP	1150
		C/A	SNP	1221
		A/G	SNP	1281
		A/T	SNP	1391
		T/C	SNP	1399
XP_001961999.1 GF14653 [Drosophila ananassae]	GLOS_DANA_GF14653.1.2	A/T	SNP	11
		A/G	SNP	92
		A/C	SNP	93
		C/G	SNP	162
		T/C	SNP	318
		T/C	SNP	419
		G/T	SNP	554
XP_001962412.1 GF15448 [Drosophila ananassae]	GLOS_DANA_GF15448.2.3	T/A	SNP	367
		CA/C	DELETION	368
		C/G	SNP	374
		G/C	SNP	375
		G/C	SNP	445
		GTC/G	DELETION	511
		T/A/G	SNP	514
		G/GT	INSERTION	654
		A/AGC	INSERTION	655
		T/G	SNP	731
		T/C	SNP	732
		T/A	SNP	746
		G/GT	INSERTION	800
		GC/G	DELETION	869
		T/G	SNP	871
		T/C	SNP	939
		C/G	SNP	1007
		TC/T	DELETION	1014

T/G	SNP	1016
A/G	SNP	1209
A/T	SNP	1216
G/C	SNP	1635
T/C	SNP	1639
C/T	SNP	1701
T/A	SNP	1739
A/G	SNP	1742
G/A	SNP	1743
G/T	SNP	1744
C/T	SNP	34
C/A	SNP	87
G/A	SNP	183
G/C	SNP	485
T/A	SNP	638
A/G	SNP	647
A/T	SNP	677
C/T	SNP	700
A/C	SNP	801
A/C	SNP	861
T/C	SNP	867
C/T	SNP	1245
A/AT/ATG(	INSERTION	1313
A/G	SNP	1315
GC/G	DELETION	1504
T/TGG	INSERTION	1509
C/A	SNP	1510
A/C	SNP	1587
T/G	SNP	1722
A/T	SNP	1761
T/G	SNP	1780
T/G	SNP	1789
A/T	SNP	1827
T/G	SNP	1846
C/T	SNP	1887
T/A	SNP	1888

		T/C	SNP	1893
		C/G	SNP	1894
		T/G	SNP	1895
		C/T	SNP	1897
		G/A	SNP	1924
		T/C	SNP	2019
		T/A	SNP	2053
		T/A	SNP	2077
XP_001955699.1 GF16109 [Drosophila ananassae]	GLOS_DANA_GF16109.1.1	A/G	SNP	97
		T/G	SNP	112
		G/A	SNP	115
		C/T	SNP	136
		G/A	SNP	225
		A/G/T	SNP	293
		A/G	SNP	489
		A/T	SNP	561
		C/T	SNP	677
		A/G	SNP	738
		T/G	SNP	786
		A/G	SNP	820
		T/C	SNP	900
		G/A	SNP	1256
		A/C	SNP	1353
		T/C	SNP	1404
		G/A	SNP	1665
		G/A	SNP	1713
		G/C	SNP	1741
		T/A	SNP	1755
		T/TA	INSERTION	1877
		T/C	SNP	1972
		A/G	SNP	1996
		T/G	SNP	2051
		C/G	SNP	2172
		A/C	SNP	2197
		G/T	SNP	2241
		T/C	SNP	2242

		A/T	SNP	2274
		T/A	SNP	2298
		A/G	SNP	2336
		T/C	SNP	2375
		T/C	SNP	2399
		T/C	SNP	2448
		A/C	SNP	2548
		C/T	SNP	2569
		A/T	SNP	2588
		A/T	SNP	2656
		T/G	SNP	2736
XP_001954716.1 GF16603 [Drosophila ananassae]	GLOS_DANA_GF16603.1.1	G/T	SNP	193
		A/G	SNP	194
		G/A	SNP	347
		A/G	SNP	483
		CAA/C	DELETION	501
		G/A	SNP	517
		G/A	SNP	522
		C/G	SNP	560
		G/A	SNP	889
		T/G	SNP	936
		G/A	SNP	967
		A/T	SNP	1030
		T/G	SNP	1383
		C/A	SNP	1447
		G/GA	INSERTION	2165
		C/T	SNP	2304
		G/A	SNP	2608
		C/T	SNP	2914
		C/T	SNP	2989
		C/T	SNP	3031
		G/A	SNP	3127
		C/T	SNP	3226
		T/G	SNP	3253
		T/C	SNP	3476
		A/T	SNP	3721

		C/T	SNP	3832
		G/A	SNP	4123
		C/T	SNP	4153
		T/C	SNP	4227
		A/G	SNP	4243
XP_001953259.1 GF17298 [Drosophila ananassae]	GLOS_DANA_GF17298.1.1	T/A	SNP	611
		A/T	SNP	632
		G/A	SNP	659
		A/G	SNP	683
		G/A	SNP	719
		A/G	SNP	734
		G/T	SNP	815
		G/A	SNP	830
		A/T	SNP	914
		ACATG/A	DELETION	1655
		A/G	SNP	1655
		C/T	SNP	1702
		A/AT	INSERTION	1786
		C/T	SNP	1981
		C/G	SNP	2266
		T/G	SNP	2338
		T/C	SNP	2365
		C/T	SNP	2581
		G/A	SNP	2599
		T/C	SNP	2620
		T/G	SNP	2681
		C/A	SNP	2709
		C/G	SNP	2742
		T/C	SNP	2767
		G/A	SNP	2806
		G/A	SNP	2812
		T/A	SNP	2829
		G/T	SNP	2831
		A/C	SNP	2832
		A/T	SNP	2890
		T/C	SNP	3409

		G/A	SNP	3512
		T/A	SNP	3531
	GLOS_DANA_GF17838.2.3	A/C	SNP	116
XP_001953827.1 GF17961 [Drosophila ananassae]	GLOS_DANA_GF17961.1.1	G/A	SNP	214
		G/A	SNP	337
		G/C	SNP	545
		G/C	SNP	2201
		A/C	SNP	2204
		A/G	SNP	2211
		A/C	SNP	2641
		A/C	SNP	2753
		A/T	SNP	2754
		A/T	SNP	2756
		A/T	SNP	2757
		G/GA	INSERTION	2760
		T/G	SNP	3449
		G/A	SNP	3470
		C/G	SNP	3961
		T/A	SNP	3963
		T/A	SNP	3964
		G/C	SNP	4100
		C/CG	INSERTION	4104
		A/ATC	INSERTION	4105
		C/T	SNP	4315
		C/T	SNP	4320
		T/C	SNP	4771
XP_001965577.1 GF22379 [Drosophila ananassae]	GLOS_DANA_GF22379.1.1	A/G	SNP	692
		A/G	SNP	1099
XP_001958284.1 GF23598 [Drosophila ananassae]	GLOS_DANA_GF23598.1.1	G/A	SNP	782
		A/G	SNP	1181
		G/C	SNP	1964
		A/C	SNP	2120
		A/T	SNP	2251
		C/T	SNP	2297
		G/A	SNP	2630
		C/A	SNP	2727

XP_001957802.1 GF23835 [Drosophila ananassae]	GLOS_DANA_GF23835.1.3	A/G	SNP	121
		T/C	SNP	208
		ATG/A	DELETION	1067
		T/A	SNP	1070
		T/C	SNP	1071
		A/G	SNP	1072
		A/G	SNP	1074
		T/A	SNP	1538
		T/A	SNP	1659
		G/T	SNP	1800
		A/G	SNP	1860
		A/G	SNP	2074
		T/C	SNP	3374
		T/C	SNP	3397
		G/GA	INSERTION	6226
		T/A	SNP	6668
		GA/G	DELETION	9383
		C/G	SNP	9552
		T/C	SNP	9553
		A/G	SNP	168
		A/C	SNP	232
		T/A	SNP	324
XP_001983080.1 GG10957 [Drosophila erecta]	GLOS_DERE_GG10957.1.2	T/C	SNP	271
		A/G	SNP	286
		A/G	SNP	727
		A/G	SNP	765
		C/G	SNP	799
		G/A	SNP	871
		T/C	SNP	874
XP_001971281.1 GG14514 [Drosophila erecta]	GLOS_DERE_GG14514.1.1	T/G	SNP	901
		G/T	SNP	1148
		A/C	SNP	1204
		CA/C	DELETION	1410
		A/AC	INSERTION	1499
		C/A	SNP	1526
XP_001978234.1 GG17809 [Drosophila erecta] ref|XP_002042722.1| GM17637	GLOS_DERE_GG17809.1.1	A/G	SNP	43

[Drosophila sechellia] ref|XP_002100509.1| GE17105 [Drosophila yakuba]					
			T/C	SNP	192
			T/C	SNP	291
			A/AT	INSERTION	379
			A/T	SNP	380
			C/A	SNP	387
			A/C	SNP	388
			G/A	SNP	747
			C/A	SNP	748
			A/G	SNP	749
			A/T	SNP	753
XP_001977467.1 GG19064 [Drosophila erecta]		GLOS_DERE_GG19064.1.3	C/T	SNP	261
			A/T	SNP	278
			C/A	SNP	291
			G/T	SNP	321
			A/G	SNP	325
			G/T	SNP	380
			T/C	SNP	419
			T/C	SNP	479
			G/A	SNP	551
			C/T	SNP	582
			T/C	SNP	692
			T/C	SNP	719
			G/A	SNP	723
			C/G	SNP	738
			T/C	SNP	789
			T/C	SNP	867
			C/A	SNP	878
			C/G	SNP	881
			C/T	SNP	980
			T/C	SNP	981
			C/A	SNP	994
			A/G	SNP	995
			A/G	SNP	1070
			T/C	SNP	1106
			T/C	SNP	1122

C/T	SNP	1190
T/C	SNP	1211
C/T	SNP	1286
T/C	SNP	1376
T/C	SNP	1394
A/G	SNP	1520
C/G	SNP	1535
C/T	SNP	1554
C/T	SNP	1590
C/A	SNP	1595
C/G	SNP	1635
T/C	SNP	1701
A/G	SNP	1726
G/A	SNP	22
T/G	SNP	40
A/G	SNP	114
G/A	SNP	149
T/A	SNP	162
G/A	SNP	165
T/C	SNP	198
A/G	SNP	250
C/G	SNP	255
C/T	SNP	264
C/T	SNP	469
G/A	SNP	471
G/A	SNP	483
C/G	SNP	526
T/C	SNP	534
C/T	SNP	615
A/G	SNP	639
G/A	SNP	708
T/C	SNP	828
A/C	SNP	60
T/A	SNP	72
C/T	SNP	78
T/C	SNP	81

		G/A	SNP	91
		A/G	SNP	114
		G/A	SNP	149
		A/G	SNP	192
		A/T	SNP	240
		A/G	SNP	387
		G/C	SNP	442
		A/G	SNP	573
		C/T	SNP	654
		T/C	SNP	792
		T/C	SNP	801
		A/T	SNP	884
		G/C	SNP	923
		A/G	SNP	939
		C/T	SNP	941
		A/C	SNP	1137
		G/A	SNP	1147
		T/C	SNP	1152
		T/A	SNP	1159
XP_001973896.1 GG21392 [Drosophila erecta]	GLOS_DERE_GG21392.1.1	C/CA	INSERTION	310
		C/T	SNP	1309
		G/A	SNP	1380
		G/A	SNP	3663
XP_001975829.1 GG22534 [Drosophila erecta]	GLOS_DERE_GG22534.1.1	C/T	SNP	748
		T/G	SNP	767
		G/A	SNP	1913
		CT/C	DELETION	1983
		T/A	SNP	2002
		G/A	SNP	2004
		T/C	SNP	2011
		C/G	SNP	2028
		A/G	SNP	2035
		A/G	SNP	2289
		T/C	SNP	2621
		A/G	SNP	2682
		T/G	SNP	2936

XP_001968601.1 GG24413 [Drosophila erecta]	GLOS_DERE_GG24413.1.1	C/T	SNP	46
		C/T	SNP	49
		T/C	SNP	133
		G/A	SNP	156
		A/G	SNP	234
		T/G	SNP	239
		G/A	SNP	241
		A/G	SNP	248
		C/T	SNP	252
		T/A	SNP	270
XP_001996119.1 GH13991 [Drosophila grimshawi]	GLOS_DGRI_GH13991.1.1	C/A	SNP	91
		A/G	SNP	124
XP_001985553.1 GH14440 [Drosophila grimshawi]	GLOS_DGRI_GH14440.1.1	G/C	SNP	236
XP_001994707.1 GH14582 [Drosophila grimshawi]	GLOS_DGRI_GH14582.1.1	C/G	SNP	797
		G/A	SNP	942
XP_001990298.1 GH18314 [Drosophila grimshawi]	GLOS_DGRI_GH18314.1.1	A/G	SNP	296
		T/C	SNP	357
		T/C	SNP	377
		T/C	SNP	378
		G/A	SNP	483
		T/C	SNP	642
		T/C	SNP	645
		A/G	SNP	652
		G/T	SNP	714
		T/C	SNP	744
		C/T	SNP	756
		T/C	SNP	777
		C/T	SNP	844
		G/A	SNP	862
		A/G	SNP	868
		C/T	SNP	900
		T/C	SNP	917
		G/T	SNP	927
		A/G	SNP	933
		T/C	SNP	969
		C/T	SNP	1015

		C/T	SNP	1053
		C/A	SNP	1087
		T/C	SNP	1154
		G/A	SNP	1158
		C/A	SNP	1161
		T/C	SNP	1169
		T/G	SNP	1200
		T/C	SNP	1209
		G/C	SNP	1312
		G/A	SNP	1313
		A/G	SNP	1374
		T/C	SNP	1542
		C/T	SNP	1555
		C/A	SNP	1566
		C/T	SNP	1615
		C/T	SNP	1618
		G/C	SNP	1697
		C/A	SNP	1743
		A/G	SNP	1744
		C/T	SNP	1758
		T/C	SNP	1779
		T/C	SNP	1785
		A/G	SNP	1818
		A/G	SNP	1860
		G/A	SNP	1887
		A/G	SNP	2072
		A/G	SNP	2113
XP_001993820.1 GH19078 [Drosophila grimshawi]	GLOS_DGRI_GH19078.1.1	G/C	SNP	104
		A/G	SNP	466
		T/C	SNP	519
		C/G	SNP	777
		C/G	SNP	847
		A/T	SNP	1119
XP_001985908.1 GH21074 [Drosophila grimshawi]	GLOS_DGRI_GH21074.1.2	T/A	SNP	850
		G/A	SNP	1184
		T/C	SNP	1465

XP_001986425.1 GH21360 [Drosophila grimshawi]	GLOS_DGRI_GH21360.1.1	C/T	SNP	87
		G/C	SNP	408
		G/A	SNP	570
		C/T	SNP	687
		A/G	SNP	789
		A/G	SNP	1083
		C/T	SNP	1109
		G/A	SNP	1113
		C/T	SNP	1125
		G/C	SNP	1199
		C/T	SNP	1707
		C/T	SNP	1764
		C/A	SNP	2187
		T/C	SNP	2325
		A/G	SNP	2721
		G/A	SNP	2862
		C/G	SNP	3202
XP_001986849.1 GH21600 [Drosophila grimshawi]	GLOS_DGRI_GH21600.3.3	C/T	SNP	9
	GLOS_DGRI_GH21600.3.3	A/T	SNP	10
	GLOS_DGRI_GH21600.3.3	A/T	SNP	861
	GLOS_DGRI_GH21600.3.3	C/G	SNP	862
	GLOS_DGRI_GH21600.3.3	G/C	SNP	932
	GLOS_DGRI_GH21600.3.3	A/G	SNP	957
	GLOS_DGRI_GH21600.3.3	G/A	SNP	1181
	GLOS_DGRI_GH21600.3.3	G/A	SNP	1239
		C/A	SNP	1288
XP_001995415.1 GH22644 [Drosophila grimshawi]	GLOS_DGRI_GH22644.1.1	G/A	SNP	348
XP_001996857.1 GH23671 [Drosophila grimshawi]	GLOS_DGRI_GH23671.1.1	G/T	SNP	472
XP_001992691.1 GH24075 [Drosophila grimshawi]	GLOS_DGRI_GH24075.1.1	C/T	SNP	500
		T/C	SNP	619
		G/A	SNP	818
		GA/G	DELETION	1274
XP_002011642.1 GI10981 [Drosophila mojavensis]	GLOS_DMOJ_GI10981.2.2	G/A	SNP	757
		C/T	SNP	1009
		A/T	SNP	1109
		G/A	SNP	1138

		A/T	SNP	1285
		A/T	SNP	1287
		A/G	SNP	1289
		A/G	SNP	1688
XP_002007161.1 GI12536 [Drosophila mojavensis]	GLOS_DMOJ_GI12536.1.1	G/T	SNP	98
		G/A	SNP	1350
		C/T	SNP	1433
		T/C	SNP	1499
		C/T	SNP	1522
		A/G	SNP	1527
		A/T	SNP	1548
		T/A	SNP	1550
XP_002002467.1 GI12593 [Drosophila mojavensis]	GLOS_DMOJ_GI12593.1.5	A/T	SNP	746
		T/C	SNP	748
		A/C	SNP	752
		C/T	SNP	753
		G/A	SNP	1104
		A/G	SNP	1106
XP_002006760.1 GI18413 [Drosophila mojavensis]	GLOS_DMOJ_GI18413.1.2	A/G	SNP	69
		A/T	SNP	138
		C/T	SNP	207
		G/T	SNP	508
		A/G	SNP	561
		G/A	SNP	562
		G/T	SNP	564
		C/G	SNP	566
		A/T	SNP	826
		T/A	SNP	827
		T/TCG	INSERTION	830
		AT/A	DELETION	833
		C/T	SNP	859
		T/C	SNP	864
		T/A	SNP	866
		T/G	SNP	870
		A/T	SNP	899
		A/C	SNP	992

		G/GAT	INSERTION	1185
		T/C	SNP	1189
		CCAA/C	DELETION	1191
		C/CG	INSERTION	1289
		G/GGC	INSERTION	1290
		G/C	SNP	1293
		T/A	SNP	1294
		T/G	SNP	1295
		T/C	SNP	1296
		C/T	SNP	1325
XP_002006760.1 GI18413 [Drosophila mojavensis]	GLOS_DMOJ_GI18413.2.2	G/C	SNP	55
		TC/T/TCAC	DELETION	107
		C/CA	INSERTION	108
		G/C	SNP	110
		C/A	SNP	111
		T/C	SNP	113
		C/A	SNP	116
		T/G	SNP	164
		T/A	SNP	165
		A/G	SNP	169
		G/C	SNP	322
		A/G	SNP	323
		AC/A	DELETION	327
		A/T	SNP	327
		G/C	SNP	376
		A/C	SNP	430
		A/G	SNP	433
		T/C	SNP	437
		C/A	SNP	1026
		G/T	SNP	1081
		A/T	SNP	1085
		A/C	SNP	1086
		A/G	SNP	1088
		A/C	SNP	1089
		C/G	SNP	1091
		A/AT	INSERTION	1092

		C/T	SNP	1094
		G/T	SNP	1156
		C/A	SNP	1210
		A/G	SNP	1271
		A/C	SNP	1288
		G/C	SNP	1317
		A/C	SNP	1318
		G/T	SNP	1321
		G/C	SNP	1378
		G/A	SNP	1663
		T/C	SNP	1718
		G/C	SNP	1719
		C/G	SNP	1721
		C/T	SNP	1884
		C/G	SNP	1887
		C/A	SNP	1940
		T/A	SNP	1941
		G/A	SNP	1945
		T/C	SNP	3146
		T/G	SNP	3147
		T/A	SNP	3150
		C/A	SNP	3945
		G/A	SNP	3946
		C/T	SNP	3967
		T/G	SNP	3968
XP_002004759.1 GI19420 [Drosophila mojavensis]	GLOS_DMOJ_GI19420.1.1	T/C	SNP	160
		T/C	SNP	238
		C/T	SNP	272
		A/G	SNP	341
		A/G	SNP	374
		T/A	SNP	397
		G/A	SNP	406
		C/G	SNP	422
		G/A	SNP	458
		G/T	SNP	467
		T/C	SNP	468

C/T	SNP	478
C/T	SNP	512
C/T	SNP	523
G/A	SNP	604
G/A	SNP	702
C/CA	INSERTION	783
A/G	SNP	913
A/G	SNP	920
G/A	SNP	936
G/A	SNP	1003
T/C	SNP	1052
C/T	SNP	1073
T/C	SNP	1111
C/G	SNP	1114
T/A	SNP	1169
G/A	SNP	1226
A/G	SNP	1294
G/A	SNP	1370
C/A	SNP	1463
C/T	SNP	1479
T/A	SNP	1483
C/T	SNP	1484
G/A	SNP	1540
C/T	SNP	1549
T/C	SNP	1658
G/C	SNP	1735
G/A	SNP	1745
T/C	SNP	1844
C/G	SNP	1899
A/C	SNP	2076
T/C	SNP	2172
A/G	SNP	2249
A/T	SNP	2352
T/G	SNP	2534
G/A	SNP	2574
T/G	SNP	2919

		T/C	SNP	2929
		A/G	SNP	2946
		T/C	SNP	3016
		T/C	SNP	3026
		G/T	SNP	3029
		C/T	SNP	3030
		A/T	SNP	3031
		A/ATCT	INSERTION	3045
XP_002004165.1 GI19764 [Drosophila mojavensis]	GLOS_DMOJ_GI19764.1.1	C/A	SNP	808
		A/G	SNP	1607
		A/T	SNP	1674
		C/A	SNP	1675
		CT/C	DELETION	1675
		C/G	SNP	1775
		T/C	SNP	1877
		A/C	SNP	1976
		T/A	SNP	2045
		C/A	SNP	2096
		A/G	SNP	2132
		C/T	SNP	2170
		G/A	SNP	2180
		G/T	SNP	2185
		A/G	SNP	2195
		A/G	SNP	2341
		A/T	SNP	2406
		G/GT	INSERTION	2432
		G/A	SNP	2553
		C/T	SNP	2586
		G/A	SNP	2659
		T/A	SNP	2660
		T/C	SNP	2683
		G/A	SNP	2778
		A/T	SNP	2800
		T/C	SNP	2833
		C/T	SNP	2841
		A/T	SNP	2872

		G/C	SNP	2909
		A/G	SNP	2951
		A/T	SNP	3053
		G/A	SNP	3064
		T/A	SNP	3069
		G/C	SNP	3147
		C/G	SNP	3270
		C/T	SNP	3372
		C/T	SNP	3419
		T/G	SNP	3527
		C/T	SNP	3548
		C/G	SNP	3553
		TA/T	DELETION	3645
		G/T	SNP	3772
		G/T	SNP	3806
	GLOS_DMOJ_GI19764.1.1	C/A	SNP	3818
		T/C	SNP	4137
		C/T	SNP	4171
		A/G	SNP	4227
XP_002004358.1 GI19892 [Drosophila mojavensis]	GLOS_DMOJ_GI19892.1.1	C/T	SNP	680
		C/T	SNP	988
		A/G	SNP	1016
XP_002004520.1 GI19977 [Drosophila mojavensis]	GLOS_DMOJ_GI19977.1.3	C/T	SNP	221
		T/C	SNP	245
		C/T	SNP	302
		G/T	SNP	519
		C/T	SNP	1747
		G/T	SNP	1876
		A/G	SNP	2065
		C/T	SNP	2104
		A/G	SNP	2106
		G/T	SNP	2115
		A/T	SNP	2127
XP_002003981.1 GI20119 [Drosophila mojavensis]	GLOS_DMOJ_GI20119.1.1	A/AG	INSERTION	313
		A/T	SNP	313
		A/T	SNP	315

		A/C	SNP	316
		A/G	SNP	317
		G/A	SNP	521
		G/C	SNP	522
		T/C	SNP	523
		G/A	SNP	525
		C/T	SNP	526
		TA/T	DELETION	528
		G/A	SNP	588
		T/C	SNP	768
		A/T	SNP	846
		T/C	SNP	874
XP_002005661.1 GI20590 [Drosophila mojavensis]	GLOS_DMOJ_GI20590.1.1	T/C	SNP	88
		G/GA	INSERTION	125
		T/C	SNP	159
		T/TA	INSERTION	267
		C/T	SNP	335
		C/CT	INSERTION	411
		T/A	SNP	827
		C/T	SNP	828
		T/C	SNP	3190
		G/A	SNP	3244
		GT/G	DELETION	3335
		T/TC	INSERTION	3337
		T/A	SNP	3361
XP_002005755.1 GI20641 [Drosophila mojavensis]	GLOS_DMOJ_GI20641.1.1	TA/T	DELETION	158
		A/C	SNP	262
		C/T	SNP	323
		A/G	SNP	587
		G/A	SNP	641
		G/A	SNP	800
		C/T	SNP	912
		A/C	SNP	1181
		C/T	SNP	1523
		A/G	SNP	1629
		A/G	SNP	1630

A/G	SNP	1638
C/G	SNP	1784
C/T	SNP	1811
C/T	SNP	1979
G/A	SNP	2247
A/G	SNP	2250
C/A	SNP	2284
C/G	SNP	2294
G/A	SNP	2319
TG/T	DELETION	2390
C/T	SNP	2398
T/G	SNP	2471
T/G	SNP	2476
A/G	SNP	2507
A/G	SNP	2519
A/C	SNP	2640
A/G	SNP	2645
T/C	SNP	2650
TA/T	DELETION	2704
A/G	SNP	2821
T/C	SNP	2833
T/G	SNP	2889
A/G	SNP	2910
C/G	SNP	2945
A/G	SNP	2949
A/T	SNP	2967
C/G	SNP	2970
G/C	SNP	2982
G/T	SNP	3000
G/A	SNP	3020
T/G	SNP	3040
C/T	SNP	3079
G/A	SNP	3160
T/C	SNP	3168
T/G	SNP	3176
C/T	SNP	3194

XP_002010850.1 GI21769 [Drosophila mojavensis]	GLOS_DMOJ_GI21769.1.1	A/T	SNP	13
		T/G	SNP	23
		A/G	SNP	294
		A/G	SNP	764
		G/A	SNP	789
		A/G	SNP	1149
		T/C	SNP	1648
		G/C	SNP	1821
		A/AT	INSERTION	2353
		T/G	SNP	2448
		A/T	SNP	2516
		T/C	SNP	2804
		A/T	SNP	2834
		G/A	SNP	2888
		A/T	SNP	2911
		G/T	SNP	3178
		T/C	SNP	3488
XP_002003524.1 GI22128 [Drosophila mojavensis]	GLOS_DMOJ_GI22128.1.1	C/T	SNP	74
		C/T	SNP	285
		T/G	SNP	410
		A/T	SNP	421
		C/T	SNP	430
		C/T	SNP	478
XP_002000268.1 GI22614 [Drosophila mojavensis]	GLOS_DMOJ_GI22614.1.1	G/C	SNP	608
		T/C	SNP	709
		A/G	SNP	892
		A/G	SNP	916
		T/A	SNP	975
		A/T	SNP	1426
		G/A	SNP	1515
		T/TCAA	INSERTION	1719
		A/T	SNP	1803
XP_001998941.1 GI24237 [Drosophila mojavensis]	GLOS_DMOJ_GI24237.1.1	G/A	SNP	30
		T/A	SNP	31
		C/G	SNP	56
		G/T	SNP	76

		A/C	SNP	113
		G/T	SNP	118
		G/C	SNP	130
		T/C	SNP	312
		C/T	SNP	389
		T/C	SNP	408
		T/C	SNP	669
		T/C	SNP	741
		A/T	SNP	798
		T/C	SNP	938
		AT/A	DELETION	1066
XP_001999095.1 GI24323 [Drosophila mojavensis]	GLOS_DMOJ_GI24323.1.1	T/A	SNP	599
		G/A	SNP	605
		C/A	SNP	672
XP_001999308.1 GI24442 [Drosophila mojavensis]	GLOS_DMOJ_GI24442.1.1	G/T	SNP	108
		A/G	SNP	596
		G/C	SNP	708
		C/A	SNP	729
		T/C	SNP	881
		G/A	SNP	907
		G/T	SNP	1238
		C/T	SNP	1286
		T/C	SNP	1423
		C/G	SNP	1447
		A/G	SNP	1463
		A/G	SNP	1515
		T/C	SNP	1552
		AAACAC/A	DELETION	1843
		T/G	SNP	1988
		A/C	SNP	2906
		C/T	SNP	3486
		A/G	SNP	3579
XP_002025973.1 GL10214 [Drosophila persimilis]	GLOS_DPER_GL10214.1.1	G/A	SNP	310
		G/A	SNP	316
		T/C	SNP	321
		G/C	SNP	336

		T/C	SNP	353
		G/A	SNP	494
		A/G	SNP	576
		C/T	SNP	588
		T/C	SNP	654
		A/C	SNP	688
		T/G	SNP	702
		G/C	SNP	721
		G/A	SNP	774
		G/A	SNP	839
		A/T	SNP	898
		T/C	SNP	914
		A/G	SNP	924
		T/G	SNP	947
		A/C	SNP	1287
		A/T	SNP	1509
		G/T	SNP	1899
		G/C	SNP	1901
		A/G	SNP	2095
		G/T	SNP	2134
		G/A	SNP	2826
XP_002015682.1 GL10892 [Drosophila persimilis]	GLOS_DPER_GL10892.1.1	C/T	SNP	31
		G/T	SNP	284
		T/A	SNP	319
		A/AT	INSERTION	364
		A/C	SNP	422
		A/T	SNP	423
		A/AAG	INSERTION	736
		G/T	SNP	987
		C/G	SNP	1060
		C/A	SNP	1484
		G/A	SNP	1552
		T/A	SNP	1696
		G/T	SNP	1714
		GA/G	DELETION	1823
		A/C	SNP	1856

		C/CA	INSERTION	1955
		A/G	SNP	1997
		G/GA	INSERTION	2070
		C/T	SNP	2152
		C/T	SNP	2275
		G/GA	INSERTION	2569
		GTCTC/G/(	DELETION	2611
		A/G	SNP	2741
		T/A	SNP	3030
XP_002019682.1 GL12526 [Drosophila persimilis]	GLOS_DPER_GL12526.1.1	A/G	SNP	764
		A/G	SNP	765
		A/G	SNP	766
		A/G	SNP	767
		C/A	SNP	780
		T/G	SNP	910
		A/G	SNP	964
		G/A	SNP	979
		G/A	SNP	1132
		G/A	SNP	1195
		G/A	SNP	1474
		C/A	SNP	1488
		G/A	SNP	1552
		G/T	SNP	1618
		G/A	SNP	1683
		T/C	SNP	1741
		A/C	SNP	1906
		A/G	SNP	1918
		T/C	SNP	1954
		G/A	SNP	1969
		G/T	SNP	2011
		C/A	SNP	2062
		T/C	SNP	2083
		A/G	SNP	2119
		C/T	SNP	2143
		G/A	SNP	2223
		G/A	SNP	2290

		G/A	SNP	2326
		G/A	SNP	2362
		A/G	SNP	2380
		C/T	SNP	2392
		A/G	SNP	2416
		G/C	SNP	2507
		A/C	SNP	2532
		C/T	SNP	2567
		C/T	SNP	2625
		A/G	SNP	2727
		C/A	SNP	2741
		C/T	SNP	2753
		A/G	SNP	2766
		C/A	SNP	2802
		G/A	SNP	2814
		T/C	SNP	2937
		C/A	SNP	3162
		C/T	SNP	3293
		A/C	SNP	3304
		A/T	SNP	3355
XP_002029322.1 GL15114 [Drosophila persimilis]	GLOS_DPER_GL15114.1.3	T/C	SNP	707
		C/T	SNP	1109
		G/A	SNP	1159
		T/C	SNP	1160
		C/A	SNP	1161
XP_002015053.1 GL19505 [Drosophila persimilis]	GLOS_DPER_GL19505.1.1	C/A	SNP	151
		CGGTAA/(	DELETION	204
		T/C	SNP	836
		T/G	SNP	839
		T/C	SNP	947
		C/G	SNP	1004
		C/G	SNP	1046
		C/G	SNP	1212
		A/T	SNP	1213
		G/A	SNP	1215
		A/T	SNP	1219

		T/C	SNP	1319
		G/A	SNP	1584
		A/G	SNP	1592
		T/C	SNP	1668
		G/A	SNP	1672
		A/G/T	SNP	1730
		G/C	SNP	1853
		A/G	SNP	1911
		A/C	SNP	1925
		T/G	SNP	2016
		T/G	SNP	2049
		G/GA	INSERTION	2057
		T/G	SNP	2072
		A/T	SNP	2086
		T/C	SNP	2092
		T/C	SNP	2155
XP_002023222.1 GL21241 [Drosophila persimilis]	GLOS_DPER_GL21241.1.4	A/T	SNP	756
		CA/C	DELETION	758
		T/A	SNP	913
		A/T	SNP	914
		C/A	SNP	1414
		C/G	SNP	1415
		T/G	SNP	1418
XP_002013379.1 GL24111 [Drosophila persimilis]	GLOS_DPER_GL24111.1.1	T/A	SNP	238
		C/A	SNP	243
		C/A	SNP	244
XP_001360538.1 GA11668 [Drosophila pseudoobscura pseudoobscura]	GLOS_DPSE_GA11668.1.3	T/C	SNP	58
		G/A	SNP	129
		A/C	SNP	2164
		A/C	SNP	2223
		G/GA	INSERTION	2226
		A/G	SNP	2230
		A/T	SNP	2319
		T/C	SNP	2320
		A/C	SNP	2321
		A/G	SNP	2322

		T/TC	INSERTION	2324
XP_002135879.1 GA22522 [Drosophila pseudoobscura pseudoobscura]	GLOS_DPSE_GA22522.1.1	G/A	SNP	82
		C/T	SNP	240
		T/C	SNP	246
		T/A	SNP	315
		C/T	SNP	329
		G/A	SNP	510
		C/A	SNP	528
		T/C	SNP	533
		G/T	SNP	549
		C/T	SNP	574
		C/T	SNP	617
		T/A	SNP	632
		T/C	SNP	676
		A/G	SNP	736
		A/T	SNP	741
		C/T	SNP	837
		G/T	SNP	861
		T/C	SNP	875
		T/G	SNP	911
		C/T	SNP	1027
		G/C	SNP	1089
		G/C	SNP	1122
		A/T	SNP	1131
		A/T	SNP	1139
		G/GT	INSERTION	1149
		A/G	SNP	1158
		T/C	SNP	1345
		G/C	SNP	1370
		C/T	SNP	1395
		G/C	SNP	1414
		C/A	SNP	1436
		C/T	SNP	1453
		G/C	SNP	1459
		G/A	SNP	1489
		G/T	SNP	1524

		C/A	SNP	1535
		T/C	SNP	1568
		A/G	SNP	1578
		A/G	SNP	1680
		G/A	SNP	1784
		T/A	SNP	1826
		G/A	SNP	1837
		A/T	SNP	1954
		C/T	SNP	1981
		G/A	SNP	2039
		A/C	SNP	2101
		G/A	SNP	2366
		A/C	SNP	2370
		C/CA	INSERTION	2372
		A/G	SNP	2395
		T/A	SNP	2414
		T/G	SNP	2579
		A/AAC	INSERTION	2596
		A/G	SNP	2610
		A/C	SNP	2639
		G/A	SNP	2643
		T/C	SNP	2947
		T/C	SNP	2974
		A/C	SNP	3008
		A/G	SNP	3046
XP_002138392.1 GA24742 [Drosophila pseudoobscura pseudoobscura]	GLOS_DPSE_GA24742.1.1	T/A	SNP	1202
XP_001358028.1 GA26760 [Drosophila pseudoobscura pseudoobscura]	GLOS_DPSE_GA26760.1.1	G/T	SNP	29
		T/A	SNP	31
		A/G	SNP	119
		T/C	SNP	132
		C/T	SNP	178
		T/A	SNP	180
		A/G	SNP	230
		G/A	SNP	242
		C/G	SNP	322
		G/A	SNP	390

T/C	SNP	418
G/A	SNP	427
T/C	SNP	466
G/T	SNP	508
T/C	SNP	529
A/G	SNP	568
C/T	SNP	616
A/T	SNP	623
T/C	SNP	625
G/C	SNP	643
C/T	SNP	1029
G/C	SNP	1124
C/T	SNP	1140
T/A	SNP	1171
G/T	SNP	1200
T/C	SNP	1204
C/G	SNP	1205
C/T	SNP	1206
G/A	SNP	1275
T/C	SNP	1285
G/A	SNP	1286
C/T	SNP	1287
A/G	SNP	1310
A/T	SNP	1332
T/C	SNP	1399
C/T	SNP	1504
A/G	SNP	1540
T/G	SNP	1541
T/A	SNP	1557
C/T	SNP	1567
C/T	SNP	1610
G/T	SNP	1656
G/T	SNP	1685
G/T	SNP	1708
C/G	SNP	1760
C/G	SNP	1764

		A/AC	INSERTION	1775
		T/C	SNP	1777
		A/T	SNP	1806
		A/G	SNP	1853
		T/A	SNP	1863
		A/G	SNP	1864
		T/C	SNP	1869
		G/A	SNP	1910
		T/G	SNP	1921
		T/G	SNP	1990
		T/C	SNP	2078
		C/A	SNP	2080
		A/C	SNP	2190
		G/C	SNP	2263
		C/CT	INSERTION	2285
		T/C	SNP	2320
		G/A	SNP	2496
		C/T	SNP	2624
		T/C	SNP	2627
		G/A	SNP	2656
		T/A	SNP	2725
XP_004444371.1 GA30464 [Drosophila pseudoobscura pseudoobscura]	GLOS_DPSE_GA30464.1.1	T/C	SNP	71
		C/T	SNP	74
		A/T	SNP	140
		G/C	SNP	141
		G/T	SNP	143
		A/C/G	SNP	145
		G/GT	INSERTION	146
		G/T	SNP	146
		C/T	SNP	414
		A/G	SNP	501
		G/C	SNP	536
		T/C	SNP	633
		C/T	SNP	639
		T/C	SNP	681
		A/C	SNP	888

		CA/C	DELETION	978
		A/C	SNP	1131
XP_002035771.1 GM15320 [Drosophila sechellia]	GLOS_DSEC_GM15320.1.1	GC/G	DELETION	483
		C/A	SNP	485
		C/G	SNP	486
		A/G/T	SNP	488
		A/ATC	INSERTION	489
		A/C	SNP	489
		T/A	SNP	519
		A/T	SNP	530
		C/T	SNP	585
		A/G	SNP	587
		G/T	SNP	589
		GT/G	DELETION	800
		A/C	SNP	804
		C/A	SNP	807
		A/G	SNP	809
		T/A	SNP	810
		T/A	SNP	1558
XP_002036375.1 GM17695 [Drosophila sechellia]	GLOS_DSEC_GM17695.1.1	CA/C	DELETION	605
		C/G	SNP	608
		C/G	SNP	611
		A/G	SNP	612
		T/G	SNP	613
		T/A	SNP	614
		T/G	SNP	626
		A/C	SNP	629
		G/T	SNP	630
		C/CA	INSERTION	772
		T/A	SNP	774
		A/G	SNP	777
		A/G	SNP	778
		T/A	SNP	779
		A/C	SNP	780
		G/T	SNP	830
		A/G	SNP	945

		A/T	SNP	969
		A/C	SNP	970
		C/T	SNP	972
		T/C	SNP	974
		T/TC	INSERTION	977
		C/G	SNP	1043
XP_002037661.1 GM18382 [Drosophila sechellia]	GLOS_DSEC_GM18382.1.1	T/C	SNP	28
		G/A	SNP	68
		G/C	SNP	169
		C/T	SNP	197
		T/G	SNP	237
		G/C	SNP	259
		A/G	SNP	285
		A/C	SNP	294
		T/C	SNP	420
		T/A	SNP	421
		T/C	SNP	434
		C/G	SNP	512
		G/A	SNP	531
		A/G	SNP	553
		TAA/T	DELETION	650
		TA/T	DELETION	653
		A/G	SNP	675
		A/G	SNP	905
		A/C	SNP	953
		G/A	SNP	1240
XP_002029827.1 GM25119 [Drosophila sechellia]	GLOS_DSEC_GM25119.1.2	T/TTATA/T	INSERTION	517
		G/A	SNP	597
		A/G	SNP	326
		T/C	SNP	434
XP_002030210.1 GM25313 [Drosophila sechellia]	GLOS_DSEC_GM25313.1.1	G/T	SNP	348
		G/C	SNP	350
		C/T	SNP	352
XP_002077642.1 crq [Drosophila simulans]	GLOS_DSIM_CRQ.1.1	A/C	SNP	72
		G/A	SNP	205
		T/A	SNP	249

		G/A	SNP	308
		G/A	SNP	625
		C/T	SNP	651
		C/G/T	SNP	666
		C/T	SNP	925
		A/G	SNP	1417
		C/T	SNP	1447
		T/C	SNP	1566
		G/A	SNP	1621
		G/T	SNP	1626
		A/T	SNP	1703
		G/A	SNP	1723
		A/G	SNP	1751
		C/T	SNP	1795
		C/G	SNP	1803
		T/A	SNP	1997
		C/CA	INSERTION	2083
XP_002085063.1 GD12510 [Drosophila simulans]	GLOS_DSIM_GD12510.1.1	A/G	SNP	315
		T/A	SNP	401
		G/A	SNP	427
		A/G	SNP	438
		C/T	SNP	441
		T/G	SNP	445
		A/G	SNP	944
		G/A	SNP	956
		C/T	SNP	962
		G/A	SNP	969
		G/T	SNP	998
		G/A	SNP	1010
		G/A	SNP	1030
		T/TA	INSERTION	1064
		A/AG	INSERTION	1067
		A/T	SNP	1069
		A/G	SNP	1104
		A/G	SNP	1149
		A/G	SNP	1162

		T/C	SNP	1171
		C/G	SNP	1662
		T/C	SNP	2348
		ACTAGTG	DELETION	2442
		A/C	SNP	3052
XP_002084740.1 GD14429 [Drosophila simulans]	GLOS_DSIM_GD14429.1.1	G/C	SNP	280
		C/T	SNP	286
		A/G	SNP	382
		T/C	SNP	693
		C/T	SNP	786
		A/T	SNP	1004
XP_002105969.1 Mct1 [Drosophila simulans]	GLOS_DSIM_MCT1.1.1	G/A	SNP	30
		G/A	SNP	52
		C/T	SNP	72
		T/C	SNP	73
		A/G	SNP	246
		C/T	SNP	345
		A/C	SNP	452
		A/G	SNP	550
		A/T	SNP	551
		T/G	SNP	554
		C/T	SNP	589
		G/A	SNP	626
		G/C	SNP	683
		A/T	SNP	764
		A/C	SNP	804
		G/T	SNP	812
		C/T	SNP	823
		C/CA	INSERTION	890
		AT/A	DELETION	901
		G/A	SNP	931
		C/T	SNP	1111
		C/CA	INSERTION	1134
		G/A	SNP	1299
		C/T	SNP	1422
		T/A	SNP	1556

		G/C	SNP	1663
		T/A	SNP	2829
		G/C	SNP	2845
XP_002056122.1 GJ10399 [Drosophila virilis]	GLOS_DVIR_GJ10399.1.1	C/T	SNP	36
		C/A	SNP	40
		A/G	SNP	154
		C/T	SNP	186
		A/T	SNP	347
		C/T	SNP	374
		C/T	SNP	387
		C/T	SNP	572
		C/T	SNP	638
		G/T	SNP	731
		G/A	SNP	804
		G/A	SNP	905
		C/T	SNP	932
		G/A	SNP	1160
		G/A	SNP	1304
		C/A	SNP	1320
		G/A	SNP	1340
		C/T	SNP	1452
		C/A	SNP	1978
		C/G	SNP	2001
		A/T	SNP	2075
XP_002055855.1 GJ10540 [Drosophila virilis]	GLOS_DVIR_GJ10540.1.1	T/C	SNP	139
		C/T	SNP	184
		A/G	SNP	193
		A/G	SNP	319
		C/G	SNP	532
		G/A	SNP	585
		G/A	SNP	605
		A/T	SNP	620
		C/A	SNP	622
XP_002046879.1 GJ13129 [Drosophila virilis]	GLOS_DVIR_GJ13129.1.1	G/A	SNP	1219
		A/AATAT	INSERTION	1222
		A/T	SNP	1222

		T/A	SNP	1247
		T/A	SNP	1255
		A/G	SNP	1438
		G/A	SNP	1543
		A/C	SNP	1555
		C/T	SNP	1561
		G/A	SNP	1639
		T/G	SNP	1648
		G/A	SNP	1787
		T/A	SNP	1830
		A/T	SNP	1831
XP_002047702.1 GJ13580 [Drosophila virilis]	GLOS_DVIR_GJ13580.1.1	C/T	SNP	73
		A/G	SNP	134
XP_002057045.1 GJ16564 [Drosophila virilis]	GLOS_DVIR_GJ16564.1.2	T/C	SNP	390
		C/G	SNP	513
		T/C	SNP	546
		C/T	SNP	549
		C/T	SNP	552
		C/T	SNP	595
		T/C	SNP	603
		G/T	SNP	671
		G/C	SNP	679
		C/T	SNP	756
		C/T	SNP	1327
XP_002057045.1 GJ16564 [Drosophila virilis]	GLOS_DVIR_GJ16564.2.2	T/G	SNP	78
		G/C	SNP	89
		C/T	SNP	92
		C/T	SNP	95
		G/A	SNP	115
		A/C	SNP	161
		A/T	SNP	504
		T/C	SNP	543
		T/C	SNP	557
		G/A	SNP	595
		T/G	SNP	635
		T/G	SNP	854

		A/C	SNP	860
		T/C	SNP	911
		C/T	SNP	971
		A/C	SNP	988
		C/T	SNP	1042
		T/C	SNP	1067
XP_002056942.1 GJ16799 [Drosophila virilis]	GLOS_DVIR_GJ16799.1.1	T/A	SNP	500
		T/C	SNP	815
		G/A	SNP	878
		A/G	SNP	935
		A/T	SNP	1166
		C/A	SNP	1580
		A/G	SNP	2133
XP_002052446.1 GJ17549 [Drosophila virilis]	GLOS_DVIR_GJ17549.1.1	C/A	SNP	29
		A/G	SNP	39
		C/T	SNP	56
		C/G	SNP	109
		C/T	SNP	410
		C/T	SNP	452
		C/A	SNP	495
		T/G	SNP	501
		T/A	SNP	593
		C/G	SNP	665
		A/G	SNP	686
		A/C	SNP	719
		T/G	SNP	881
		T/G	SNP	932
		A/T	SNP	1043
		T/A	SNP	1151
		G/A	SNP	1202
		A/G	SNP	1226
		G/A	SNP	1320
		G/GT	INSERTION	1356
		C/G	SNP	1369
		C/T	SNP	1415
XP_002059296.1 GJ18228 [Drosophila virilis]	GLOS_DVIR_GJ18228.1.1	G/A	SNP	215

		G/A	SNP	321
		G/C	SNP	354
		T/G	SNP	431
		T/G	SNP	434
		A/G	SNP	464
		T/C	SNP	472
		G/T	SNP	488
		C/T	SNP	489
		T/C	SNP	523
		G/A	SNP	527
		A/AC	INSERTION	1452
		C/A	SNP	1645
		G/C	SNP	1755
		T/G	SNP	1756
		C/A	SNP	1759
		A/C	SNP	1879
		T/G	SNP	2631
		C/A	SNP	2827
		A/C	SNP	2843
		T/C	SNP	2916
		T/A	SNP	2942
		CTT/C	DELETION	2959
		G/A	SNP	3062
		C/T	SNP	3068
		A/G	SNP	3080
		C/T	SNP	3128
		G/A	SNP	3254
		T/C	SNP	3266
		T/C	SNP	3335
XP_002055362.1 GJ19325 [Drosophila virilis]	GLOS_DVIR_GJ19325.1.1	C/T	SNP	311
		C/A	SNP	312
		T/C	SNP	472
		G/T	SNP	915
		A/T	SNP	934
		A/T	SNP	1022
		C/CA	INSERTION	1145

		G/A	SNP	1642
		C/T	SNP	1648
		C/CA	INSERTION	2701
		T/C	SNP	3392
		A/T	SNP	3517
XP_002049270.1 GJ21497 [Drosophila virilis]	GLOS_DVIR_GJ21497.1.3	A/T	SNP	2792
		C/A	SNP	2862
		T/C	SNP	2984
		C/T	SNP	3065
		A/AG	INSERTION	3088
		A/AT	INSERTION	3089
		G/A	SNP	3091
		A/G	SNP	3092
		A/G	SNP	3093
		A/G	SNP	3094
		T/A	SNP	3096
		CA/C	DELETION	3099
		T/C	SNP	3141
		A/T	SNP	3152
		T/TA	INSERTION	3193
		G/T	SNP	3194
		G/A/T	SNP	3195
		G/C	SNP	3197
		T/G	SNP	3198
		G/C	SNP	3585
		A/G	SNP	3586
		G/C	SNP	3589
		T/G	SNP	3592
		G/A	SNP	3628
		TC/T	DELETION	3639
		G/A	SNP	3653
XP_002049270.1 GJ21497 [Drosophila virilis]	GLOS_DVIR_GJ21497.2.3	G/C	SNP	794
		C/T	SNP	796
		C/A	SNP	861
		G/C	SNP	1004
		G/C	SNP	1005

		G/C	SNP	1090
		T/G	SNP	1106
		A/G	SNP	1183
		A/AT	INSERTION	1192
		A/ACT	INSERTION	1193
		A/T	SNP	1193
		A/G	SNP	1197
		T/C	SNP	1265
		TGG/T	DELETION	1326
		G/C	SNP	1369
		T/G	SNP	1370
		C/G	SNP	1371
		C/G	SNP	1481
		T/G	SNP	1484
		C/A	SNP	1486
		T/A	SNP	1487
XP_002049271.1 GJ21498 [Drosophila virilis]	GLOS_DVIR_GJ21498.1.1	G/C/T	SNP	69
		C/T	SNP	138
		A/T	SNP	200
		T/A	SNP	206
		G/T	SNP	327
		G/C	SNP	388
		T/C	SNP	468
		A/T	SNP	471
		A/T	SNP	474
		A/T	SNP	538
		G/A	SNP	624
		C/T	SNP	794
		A/G	SNP	863
		CTCCT/C	DELETION	928
		CTA/C	DELETION	996
		A/AT	INSERTION	998
		T/G	SNP	1155
		G/A	SNP	1157
		G/C	SNP	1476
		A/C/T	SNP	1545

		A/G	SNP	1613
		G/T	SNP	1614
		A/G	SNP	1681
		G/C	SNP	1685
		T/TGA	INSERTION	1751
		T/G	SNP	1763
XP_002049272.1 GJ21499 [D. virilis] ref|XP_002049273.1| Trypsin-1 [D. virilis]	GLOS_DVIR_GJ21499.1.1	T/A	SNP	65
		A/G	SNP	120
		A/G	SNP	121
		G/C	SNP	130
		G/A	SNP	190
		T/G	SNP	195
		A/G	SNP	196
		C/G	SNP	236
		C/T	SNP	245
		C/CA	INSERTION	313
		C/A	SNP	314
		G/A	SNP	315
		G/C	SNP	321
		G/C	SNP	322
		C/A	SNP	323
		T/G	SNP	324
		C/A	SNP	325
		C/CCGA	INSERTION	325
		CA/C	DELETION	327
		T/C	SNP	329
		A/C	SNP	398
		A/T	SNP	429
		T/G	SNP	494
		T/C	SNP	559
XP_002051013.1 GJ22461 [Drosophila virilis]	GLOS_DVIR_GJ22461.1.1	T/A	SNP	51
		C/T	SNP	53
		CA/C	DELETION	95
		G/C	SNP	269
		G/A	SNP	549
		C/T	SNP	801

		T/C	SNP	804
		A/G	SNP	960
		C/A	SNP	975
		G/A	SNP	1053
		C/T	SNP	1113
		T/G	SNP	1119
		C/T	SNP	1218
		G/A	SNP	1260
		C/T	SNP	1351
		C/T	SNP	1356
		C/T	SNP	1374
		G/A	SNP	1461
		A/G	SNP	1494
		T/C	SNP	1548
		T/C	SNP	1599
		T/A	SNP	1650
		C/G	SNP	1698
		C/G	SNP	1947
		T/C	SNP	1965
		A/T	SNP	2174
		C/T	SNP	2181
		A/G	SNP	2257
		A/G	SNP	3436
		T/G	SNP	3579
		GC/G	DELETION	3609
		C/A	SNP	3612
		A/T	SNP	3614
		A/T	SNP	3754
		G/A	SNP	3762
XM_002060180.1 Drosophila virilis GJ22515 (DvirGJ22515), mRNA	GLOS_DVIR_GJ22515.1.1	T/C	SNP	72
		T/C	SNP	81
		C/T	SNP	187
		A/G	SNP	202
		T/C	SNP	274
		T/C	SNP	283
		T/TC	INSERTION	296

		T/C	SNP	298
		T/A	SNP	1100
		A/C	SNP	1111
XP_002054581.1 GJ22731 [Drosophila virilis]	GLOS_DVIR_GJ22731.1.1	T/TA	INSERTION	84
		T/A	SNP	98
		T/C	SNP	239
		A/G	SNP	343
		A/G	SNP	347
		T/C	SNP	350
		C/A	SNP	456
		C/T	SNP	671
		T/C	SNP	716
		C/T	SNP	1523
		T/C	SNP	1826
		T/C	SNP	2039
		A/T	SNP	2049
XP_002068050.1 GK10672 [Drosophila willistoni]	GLOS_DWIL_GK10672.1.1	G/A	SNP	458
		G/A	SNP	549
		A/G	SNP	555
		G/A	SNP	723
		T/C	SNP	813
		T/A	SNP	990
		T/C	SNP	1146
		C/T	SNP	1359
		G/A	SNP	1371
		T/C	SNP	1584
		G/A	SNP	1823
		C/CA	INSERTION	2450
		T/TAC/TAC	INSERTION	2501
		A/C	SNP	2503
XP_002070627.1 GK10932 [Drosophila willistoni]	GLOS_DWIL_GK10932.1.1	A/G	SNP	66
		A/T	SNP	70
		T/G	SNP	124
		G/A	SNP	272
		G/A	SNP	749
		T/A	SNP	752

T/C	SNP	778
T/A	SNP	782
G/T	SNP	789
T/C	SNP	827
G/A	SNP	885
C/G	SNP	996
G/T	SNP	1043
T/C	SNP	1099
C/T	SNP	1128
C/T	SNP	1129
A/G	SNP	1175
C/T	SNP	1225
C/T	SNP	1238
A/T	SNP	1241
G/A	SNP	1272
ACTG/A	DELETION	1359
C/T	SNP	1363
G/C	SNP	1383
G/C	SNP	1389
C/G	SNP	1426
G/A	SNP	1430
A/G	SNP	1509
G/A/T	SNP	1559
C/T	SNP	1580
G/A	SNP	1587
T/C	SNP	1687
T/G	SNP	1715
T/C	SNP	1766
G/T	SNP	1859
G/A	SNP	1920
G/C	SNP	2029
C/G	SNP	2087
C/A	SNP	2147
C/G	SNP	2200
C/G	SNP	2218
A/G	SNP	2319

C/T	SNP	2456
C/T	SNP	2507
T/C	SNP	2587
T/C	SNP	2642
C/G	SNP	2755
G/A	SNP	2916
A/T	SNP	3046
A/G	SNP	3170
G/A	SNP	3428
C/T	SNP	3531
T/A	SNP	3546
C/T	SNP	3608
G/A	SNP	3965
G/A	SNP	4055
CT/CTT/C	INSERTION	4172
TG/T	DELETION	4290
T/C	SNP	4292
G/T	SNP	4305
T/A	SNP	4396
T/C	SNP	4460
C/CA	INSERTION	4462
C/G	SNP	4466
A/C	SNP	4511
A/G	SNP	4784
C/A	SNP	4862
C/G	SNP	4927
G/C	SNP	4928
A/C	SNP	4930
A/G	SNP	4931
G/A	SNP	4932
A/G	SNP	4933
A/T	SNP	5020
T/C	SNP	5117
G/T	SNP	5118
G/A	SNP	5185
T/TAG	INSERTION	5252

		G/C	SNP	5255
		C/G	SNP	5257
		G/A	SNP	5386
		GT/G	DELETION	5452
		T/G	SNP	5454
		C/T	SNP	5511
		T/G	SNP	5527
		A/C	SNP	5535
		G/T	SNP	5680
		GGC/G	DELETION	5746
		C/CG	INSERTION	5748
		C/A	SNP	5863
XP_002070501.1 GK10999 [Drosophila willistoni]	GLOS_DWIL_GK10999.1.1	C/T	SNP	6
		G/A	SNP	34
		G/A	SNP	38
		T/C	SNP	47
		T/C	SNP	57
		A/G	SNP	93
		C/T	SNP	98
		C/T	SNP	102
		C/T	SNP	108
		T/C	SNP	149
		C/T	SNP	159
		A/G	SNP	187
		A/G	SNP	191
XP_002070226.1 GK11147 [Drosophila willistoni]	GLOS_DWIL_GK11147.1.2	G/T	SNP	150
		A/T	SNP	152
		T/A	SNP	187
		C/T	SNP	189
		A/G	SNP	1120
		C/G	SNP	1121
		A/C	SNP	1160
		C/G	SNP	1163
		T/G	SNP	1166
		G/T	SNP	1167
		A/T	SNP	1836

		T/A	SNP	4
		A/G	SNP	97
XP_002069867.1 GK11338 [Drosophila willistoni]	GLOS_DWIL_GK11338.1.1	C/CA	INSERTION	687
		A/C	SNP	2273
		C/CT	INSERTION	2292
		G/T	SNP	2316
		A/T	SNP	4076
		A/G	SNP	4098
		C/CA	INSERTION	4112
		G/C	SNP	4476
		G/A	SNP	4521
		C/CT	INSERTION	5002
		T/G	SNP	5143
		C/G	SNP	5152
		G/T	SNP	6121
		C/A	SNP	6188
		C/T	SNP	6196
		T/C	SNP	6297
		T/A	SNP	6319
		T/C	SNP	6491
		G/A	SNP	7015
		T/A	SNP	7335
		C/A	SNP	7342
		G/T	SNP	7671
		C/G	SNP	7774
		G/A	SNP	7868
		T/A	SNP	7979
		C/T	SNP	8195
		A/T	SNP	8364
		T/C	SNP	8383
		T/TA	INSERTION	8557
		TA/T	DELETION	8987
		G/A	SNP	9001
		G/A	SNP	9306
		T/C	SNP	9509
		G/A	SNP	9664

		A/G	SNP	9695
		A/T	SNP	9791
		T/C	SNP	9936
		T/C	SNP	10153
		A/G	SNP	10209
		C/T	SNP	10226
XP_002069687.1 GK11657 [Drosophila willistoni]	GLOS_DWIL_GK11657.1.1	C/A	SNP	2
		C/T	SNP	95
		CTT/C	DELETION	113
		AC/A	DELETION	116
		C/G	SNP	118
		T/C	SNP	174
		C/T	SNP	177
		C/A	SNP	186
		C/T	SNP	222
		A/G	SNP	255
		T/C	SNP	285
		C/A	SNP	339
		A/G	SNP	345
		G/A	SNP	362
		C/T	SNP	435
		G/A	SNP	447
		A/G	SNP	450
		C/T	SNP	471
		A/T	SNP	477
		C/T	SNP	480
		T/C	SNP	520
		A/G	SNP	522
		T/C	SNP	523
		A/G	SNP	589
		T/G	SNP	593
XP_002067829.1 GK12510 [Drosophila willistoni]	GLOS_DWIL_GK12510.1.4	GA/G	DELETION	611
		AG/A	DELETION	612
		G/T	SNP	375
		G/T	SNP	395
		T/A	SNP	1030

		T/C	SNP	1032
		G/A	SNP	1070
XP_002072631.1 GK13707 [Drosophila willistoni]	GLOS_DWIL_GK13707.1.1	C/CA	INSERTION	1024
		C/T	SNP	1531
		C/CA	INSERTION	1844
		G/T	SNP	2236
		C/G	SNP	3451
		C/A	SNP	3810
		G/GA	INSERTION	4093
		C/G	SNP	6118
		G/GA	INSERTION	6401
		C/CA	INSERTION	6763
		A/G	SNP	9089
		C/CA	INSERTION	9616
XP_002072669.1 GK13726 [Drosophila willistoni]	GLOS_DWIL_GK13726.1.1	G/A	SNP	378
		T/C	SNP	720
		C/T	SNP	33
		G/A	SNP	38
		T/C	SNP	47
XP_002073839.1 GK14328 [Drosophila willistoni]	GLOS_DWIL_GK14328.1.2	G/A	SNP	77
		G/A	SNP	130
		A/G	SNP	330
		A/T	SNP	351
		C/T	SNP	668
		A/G	SNP	691
		C/T	SNP	696
		T/C	SNP	990
		C/T	SNP	999
		C/T	SNP	1058
		T/C	SNP	1140
		T/C	SNP	1155
		T/A	SNP	1247
		T/C	SNP	1313
		C/T	SNP	1535
		T/C	SNP	1608
		C/T	SNP	1676

G/T	SNP	1712
A/C	SNP	1748
A/G	SNP	1750
C/A	SNP	1802
G/T	SNP	1816
C/T	SNP	1830
C/G	SNP	1845
C/T	SNP	1936
C/T	SNP	2171
T/A	SNP	2222
T/A	SNP	2256
A/G	SNP	2294
G/T	SNP	2300
A/AT	INSERTION	2302
A/T	SNP	2303
A/G	SNP	2333
T/C	SNP	2342
G/GT	INSERTION	2379
T/A	SNP	2424
G/C	SNP	2472
C/A	SNP	2485
T/C	SNP	2510
C/T	SNP	2543
C/T	SNP	2562
C/T	SNP	2588
C/T	SNP	2590
C/T	SNP	2722
A/G	SNP	2724
C/T	SNP	2739
T/A	SNP	2750
G/A	SNP	2768
T/C	SNP	3021
A/G	SNP	3044
A/G	SNP	3174
G/A	SNP	3194
C/A	SNP	3270

		G/A	SNP	3271
XP_002073839.1 GK14328 [Drosophila willistoni]	GLOS_DWIL_GK14328.2.2	T/C	SNP	257
		T/C	SNP	317
		C/T	SNP	404
		G/A	SNP	407
		A/G	SNP	462
		T/C	SNP	467
		C/A	SNP	506
		G/A	SNP	510
		G/A	SNP	530
		G/A	SNP	578
		A/G	SNP	589
		T/C	SNP	617
		G/A	SNP	632
		T/C	SNP	634
		G/C	SNP	639
		A/C	SNP	653
		T/C	SNP	677
		A/G	SNP	691
		A/T	SNP	735
		G/A	SNP	793
		C/A	SNP	818
		A/G	SNP	834
		G/T	SNP	839
		C/T	SNP	857
		C/T	SNP	908
		C/T	SNP	920
		A/G	SNP	954
		A/T	SNP	1007
		T/C	SNP	1037
		G/T	SNP	1164
		T/A	SNP	1191
		C/CT	INSERTION	1210
		C/T	SNP	1246
		C/T	SNP	1326
		A/G	SNP	1335

		C/A	SNP	1351
		C/T	SNP	1377
		G/A	SNP	1392
XP_002074103.1 GK14466 [Drosophila willistoni]	GLOS_DWIL_GK14466.1.1	A/G	SNP	375
		G/A	SNP	519
		T/G	SNP	1162
		G/C	SNP	1452
		T/A	SNP	1789
XP_002064786.1 GK15016 [Drosophila willistoni]	GLOS_DWIL_GK15016.1.2	A/C	SNP	133
		G/C	SNP	205
		A/C	SNP	207
		A/G	SNP	212
		G/T	SNP	214
		C/CT	INSERTION	215
		C/T	SNP	273
		C/T	SNP	307
		C/A	SNP	587
XP_002075290.1 GK15974 [Drosophila willistoni]	GLOS_DWIL_GK15974.1.7	G/C	SNP	295
		T/C	SNP	299
		T/C	SNP	300
		T/C	SNP	301
		G/GAT	INSERTION	302
		T/C	SNP	304
		T/A	SNP	306
		G/T	SNP	409
		T/C	SNP	411
		C/T	SNP	413
		C/CGAT	INSERTION	414
		CGG/C	DELETION	415
		A/T	SNP	721
XP_002075290.1 GK15974 [Drosophila willistoni]	GLOS_DWIL_GK15974.3.7	G/T	SNP	333
		T/G	SNP	336
		G/A	SNP	337
		T/A	SNP	338
		G/C	SNP	339
XP_002066329.1 GK18237 [Drosophila willistoni]	GLOS_DWIL_GK18237.1.1	A/T	SNP	328

		G/C	SNP	356
		C/T	SNP	415
		A/T	SNP	442
		A/G	SNP	486
		T/G	SNP	569
		C/T	SNP	581
		G/A	SNP	587
		C/T	SNP	1268
		C/T	SNP	1351
		T/A	SNP	1380
		A/T	SNP	1591
XP_002070831.1 GK18649 [Drosophila willistoni]	GLOS_DWIL_GK18649.1.3	G/T	SNP	2680
		C/T	SNP	2710
		T/G	SNP	2779
		G/T	SNP	2864
		C/T	SNP	2897
		C/A	SNP	2900
XP_002062897.1 GK19454 [Drosophila willistoni]	GLOS_DWIL_GK19454.1.1	C/T	SNP	188
		C/T	SNP	198
		T/A	SNP	205
		C/T	SNP	210
		A/T	SNP	265
		A/T	SNP	266
		A/T	SNP	267
		C/A	SNP	387
		C/A	SNP	446
		T/C	SNP	447
		T/C	SNP	449
		A/G	SNP	450
		A/G	SNP	695
		C/G	SNP	755
		C/G	SNP	816
		T/G	SNP	877
		C/T	SNP	1024
		AT/A	DELETION	1095
		T/C	SNP	1097

XP_002069265.1 GK21058 [Drosophila willistoni]	GLOS_DWIL_GK21058.1.1	A/G	SNP	98
		G/GT	INSERTION	277
		C/G	SNP	292
		G/T	SNP	340
		T/C	SNP	386
		A/G	SNP	395
		G/A	SNP	463
		C/CT	INSERTION	596
		G/A	SNP	624
		C/T	SNP	644
		A/G	SNP	792
		G/A	SNP	879
		C/G	SNP	990
		G/T	SNP	1238
		G/A	SNP	1410
		T/A	SNP	1501
		G/T	SNP	1715
		A/T	SNP	1892
		T/C	SNP	1973
		T/A	SNP	2003
		T/A	SNP	2191
		C/A	SNP	2207
		T/C	SNP	2335
		A/C	SNP	2377
XP_002069253.1 GK21065 [Drosophila willistoni]	GLOS_DWIL_GK21065.1.1	T/C	SNP	121
		T/C	SNP	123
		A/T	SNP	155
		A/G	SNP	307
		A/C	SNP	380
		G/A	SNP	384
		T/C	SNP	476
		T/A	SNP	566
		G/A	SNP	596
		T/C	SNP	983
		A/T	SNP	986
		T/C	SNP	995

		T/C	SNP	1067
		C/T	SNP	1097
		C/T	SNP	1200
		G/A	SNP	1223
		G/A	SNP	1286
		C/T	SNP	1490
		T/C	SNP	1508
		A/G	SNP	1559
		T/G	SNP	1629
		A/G	SNP	1687
		T/C	SNP	1831
		A/C	SNP	1834
XP_002063538.1 GK21356 [Drosophila willistoni]	GLOS_DWIL_GK21356.4.5	A/C	SNP	1052
		C/A	SNP	1054
XP_002062967.1 GK21639 [Drosophila willistoni]	GLOS_DWIL_GK21639.1.5	G/A	SNP	1302
		G/A	SNP	1342
		CA/C	DELETION	1374
		T/A	SNP	1422
		A/G	SNP	1432
		T/A	SNP	1488
		C/T	SNP	1532
		A/G	SNP	1565
		A/G	SNP	1618
XP_002062967.1 GK21639 [Drosophila willistoni]	GLOS_DWIL_GK21639.2.5	T/C	SNP	101
		T/C	SNP	120
		G/A	SNP	148
		G/C	SNP	154
		C/T	SNP	159
		G/A	SNP	162
		T/A	SNP	170
XP_002062967.1 GK21639 [Drosophila willistoni]	GLOS_DWIL_GK21639.3.5	A/C	SNP	105
		C/T	SNP	115
		A/G	SNP	167
		C/G	SNP	173
		G/A	SNP	485
		G/T	SNP	573

		G/A	SNP	654
		G/A	SNP	690
		G/A	SNP	730
		T/G	SNP	752
		A/T	SNP	809
		G/A	SNP	819
		T/G	SNP	820
		C/T	SNP	857
		C/CT	INSERTION	877
		T/C	SNP	878
		T/C	SNP	881
		T/A	SNP	898
		T/A	SNP	899
		T/A	SNP	1917
		G/C	SNP	1998
		A/AG/AAG	INSERTION	2000
		T/G	SNP	2008
		A/G	SNP	3672
		T/A	SNP	3711
		T/G	SNP	3773
XP_002063649.1 GK22031 [Drosophila willistoni]	GLOS_DWIL_GK22031.1.1	A/G	SNP	107
		T/A	SNP	140
		T/C	SNP	571
		G/A	SNP	1459
		T/C	SNP	1733
		G/A	SNP	1885
		A/G	SNP	1961
		T/A	SNP	2279
XP_002074627.1 GK23174 [Drosophila willistoni]	GLOS_DWIL_GK23174.1.1	C/T	SNP	104
		A/G	SNP	127
		T/C	SNP	211
		GA/GAA/G	INSERTION	224
		G/A	SNP	292
		T/A	SNP	423
		G/T	SNP	475
		G/C	SNP	514

		T/C	SNP	555
		T/G	SNP	561
		T/C	SNP	573
		T/C	SNP	590
		A/T	SNP	621
		C/G	SNP	623
		A/C/T	SNP	624
		T/G	SNP	630
		C/T	SNP	660
		A/T	SNP	663
		A/G	SNP	693
		G/A	SNP	709
		G/T	SNP	711
		C/T	SNP	771
		T/C	SNP	894
		A/G	SNP	945
		C/T	SNP	967
		C/T	SNP	981
		C/T	SNP	1017
		A/C	SNP	1104
		G/T	SNP	1106
		G/GA	INSERTION	1121
		T/C	SNP	1147
		A/G	SNP	1151
		T/C	SNP	1206
		G/A	SNP	1211
		A/T	SNP	1232
		G/A	SNP	1242
		G/A	SNP	1995
		T/A	SNP	2017
		C/A	SNP	2045
		A/C	SNP	2281
		T/C	SNP	2324
		T/A	SNP	2343
		G/C/T	SNP	2349
XP_002067211.1 GK24139 [Drosophila willistoni]	GLOS_DWIL_GK24139.1.1	A/C	SNP	2267

A/G	SNP	2296
A/G	SNP	2350
G/T	SNP	2816
A/G	SNP	2817
C/T	SNP	2874
A/G	SNP	2939
C/T	SNP	2972
G/A	SNP	2996
T/A/G	SNP	2997
A/G	SNP	3259
A/AT	INSERTION	3261
T/G	SNP	3266
G/T	SNP	3298
A/G	SNP	3312
A/T	SNP	3352
T/G	SNP	3355
T/G	SNP	3374
G/A	SNP	3375
C/A	SNP	3376
G/T	SNP	3380
G/C	SNP	3382
A/C	SNP	3386
TC/T	DELETION	3387
G/A	SNP	3442
C/T	SNP	3446
T/A	SNP	3511
T/A	SNP	3513
T/G	SNP	3704
T/A	SNP	3708
C/A	SNP	3773
G/A	SNP	3781
A/C	SNP	3862
C/G/T	SNP	3863
C/A	SNP	3914
A/C	SNP	3979
T/G	SNP	3980

		C/A	SNP	3981
		C/T	SNP	4006
		T/G	SNP	4048
		T/A	SNP	4052
		A/AG	INSERTION	4246
		T/C	SNP	4298
		GC/G	DELETION	4315
		C/A	SNP	4317
		C/A	SNP	4319
		G/A	SNP	4327
		T/C	SNP	4557
		AG/A	DELETION	4599
		A/C	SNP	4601
		T/C	SNP	4602
		GA/G	DELETION	4607
		A/C	SNP	4610
		A/T	SNP	4611
		A/C	SNP	4616
		A/G	SNP	4654
		C/T	SNP	4693
		G/A	SNP	4712
		T/C	SNP	4738
		A/C	SNP	4753
		G/A	SNP	4757
		T/C	SNP	4790
		G/C	SNP	4805
		A/T	SNP	5073
		T/C	SNP	5113
		C/A	SNP	5135
		G/A	SNP	5143
		T/C	SNP	5189
		C/T	SNP	5197
		C/T	SNP	5356
		G/T	SNP	5359
		C/T	SNP	5371
XP_002067109.1 GK24194 [Drosophila willistoni]	GLOS_DWIL_GK24194.1.1	T/A	SNP	432

		C/G	SNP	480
		G/T	SNP	482
XP_002066964.1 GK24277 [Drosophila willistoni]	GLOS_DWIL_GK24277.1.1	C/G	SNP	422
		G/A	SNP	832
		C/T	SNP	1136
		G/A	SNP	1812
XP_002071201.1 GK25258 [Drosophila willistoni]	GLOS_DWIL_GK25258.2.2	GA/G	DELETION	81
		C/A	SNP	1396
		G/A	SNP	2291
		A/G	SNP	2294
		A/G	SNP	2305
		G/A	SNP	2306
		A/G	SNP	2308
XP_002071245.1 GK25686 [Drosophila willistoni]	GLOS_DWIL_GK25686.1.1	G/A	SNP	275
		G/C	SNP	354
		G/A	SNP	392
		A/G	SNP	461
		T/TA	INSERTION	593
		G/A	SNP	619
		T/C	SNP	662
		C/CA	INSERTION	697
		T/C	SNP	894
		A/G	SNP	913
		A/G	SNP	935
		T/A	SNP	954
		A/C	SNP	1110
		T/C	SNP	1177
		TA/T	DELETION	1215
		A/T	SNP	1223
		G/T	SNP	1254
		G/A	SNP	1264
		T/C	SNP	1479
		C/T	SNP	1739
		C/T	SNP	2998
		T/C	SNP	3020
		G/A	SNP	3272

XP_002091923.1 GE11964 [Drosophila yakuba]	GLOS_DYAK_GE11964.2.2	A/G	SNP	48
		G/A	SNP	50
		T/G	SNP	51
		A/G	SNP	121
		G/A	SNP	278
		A/G	SNP	310
		A/C	SNP	318
		T/C	SNP	424
		G/A	SNP	452
		T/G	SNP	468
		C/T	SNP	476
		G/A	SNP	509
		T/C	SNP	519
		G/C	SNP	628
		A/G	SNP	736
XP_002100904.1 GE15912 [Drosophila yakuba]	GLOS_DYAK_GE15912.2.4	A/C	SNP	167
		A/C	SNP	168
		A/AT	INSERTION	170
		A/T	SNP	170
		A/C	SNP	171
		G/T	SNP	172
		G/A	SNP	334
XP_002088313.1 GE18501 [Drosophila yakuba]	GLOS_DYAK_GE18501.1.1	A/C	SNP	203
		G/A	SNP	212
		A/G	SNP	235
		G/A	SNP	650
		A/G	SNP	1004
		A/G	SNP	1022
		G/A	SNP	1112
		C/T	SNP	1211
		G/T	SNP	1301
		A/G	SNP	1338
		T/C	SNP	1419
[BBH] EAST_DROME (sp|P13582) Serine protease easter OS=D. melanogaster	GLOS_EAST.2.2	A/G	SNP	17
		T/C	SNP	160
		G/T	SNP	518

		G/C	SNP	554
		G/A	SNP	587
		T/C	SNP	644
		A/G	SNP	887
		C/T	SNP	1082
		C/T	SNP	1142
[BBH] EF1A2_TRYB2 (sp|P86939) Elongation fact. 1-alpha 2; Tbb(927/4 GUTat10.1)	GLOS_EF1A2.1.2	G/T	SNP	139
		C/A	SNP	162
		C/G	SNP	165
		C/CT	INSERTION	168
		C/T	SNP	168
		C/T	SNP	169
		T/TG	INSERTION	1754
		A/G	SNP	1757
		A/AAG	INSERTION	1808
[BBH] ELP2_DROME (sp|Q7K4B3) Probable elongator complex protein 2 OS=D. m.	GLOS_ELP2.1.1	C/T	SNP	1536
		G/A	SNP	2050
		A/G	SNP	2461
		T/C	SNP	2775
		A/T	SNP	2776
		G/A	SNP	3219
		G/C	SNP	3246
		T/C	SNP	3310
		C/T	SNP	4863
		T/C	SNP	4934
		C/A	SNP	5293
[BBH] ENDOU_DROME (sp|Q9VZ49) Poly(U)-specific endoribonuclease homolog OS=D	GLOS_ENDOU.1.1	A/G	SNP	234
		C/A	SNP	1149
		A/G	SNP	1179
		G/A	SNP	1315
		A/G	SNP	1351
		C/T	SNP	1596
[BBH] ERF1_TRYBB (sp|Q9NAX8) Eukaryo. Pept. chain release fact. subunit 1 Tbb	GLOS_ERF1.1.2	G/GA	INSERTION	366
		G/T	SNP	2384
[BBH] EXPA_DROME (sp|Q07436) Protein expanded OS=D. m.GN=ex PE=1 SV=3	GLOS_EXPA.1.1	T/G	SNP	372
		G/A	SNP	609

		C/T	SNP	1115
		G/A	SNP	1196
		T/C	SNP	1310
		G/C	SNP	1388
		A/G	SNP	1676
		T/C	SNP	1823
		G/A	SNP	1877
		G/A	SNP	2032
		T/A	SNP	2504
		G/A	SNP	2564
		G/A	SNP	2741
		C/T	SNP	2774
		C/T	SNP	3290
		T/A	SNP	3959
		T/G	SNP	4409
		G/C	SNP	4679
		T/G	SNP	4959
		G/A	SNP	5170
		G/A	SNP	5881
FDL_DROME (sp|Q8WSF3) Probable beta-hexosaminidase fdl OS=D. m.	GLOS_FDL.1.2	TA/T	DELETION	174
		C/T	SNP	236
		A/G	SNP	583
		T/C	SNP	637
		G/A	SNP	690
		T/C	SNP	802
		T/C	SNP	817
		A/G	SNP	855
		A/C	SNP	1096
		G/A	SNP	1115
		A/G	SNP	1156
		C/T	SNP	1304
		G/A	SNP	1318
		C/T	SNP	1394
		C/T	SNP	1660
		G/A	SNP	1867
		A/G	SNP	2026

		G/A	SNP	2091
		T/G	SNP	2199
		T/A	SNP	2278
[BBH] GBLP_TRYBR (sp|P69104) Guanine nucleotide-binding protein subunit beta-like protein OS=Trypanosoma brucei rhodesiense PE=2 SV=1	GLOS_GBLP.3.4	G/A	SNP	16
		A/G	SNP	185
		G/A	SNP	332
[BBH] GCH1_DROME (sp|P48596) GTP cyclohydrolase 1 OS=D. melanogaster	GLOS_GCH1.1.1	G/A	SNP	361
		A/G	SNP	572
		C/G	SNP	1165
		A/AGAGG	INSERTION	1172
		G/C	SNP	1417
		G/A	SNP	1431
		C/T	SNP	1433
		G/T	SNP	1468
		T/A	SNP	1600
		G/C	SNP	1651
[BBH] GSK3B_TRYB2 (sp|Q388M1) Glycogen synthase kinase 3 OS=Tbb	GLOS_GSK3B.1.1	C/T	SNP	206
		AAAC/A	DELETION	3068
GSTT1_LUCCU (sp|P42860) Glutathione S-transferase 1-1 OS=L.uprina	GLOS_GSTT1.4.5	G/T	SNP	80
		C/T	SNP	81
		C/T	SNP	684
		G/A	SNP	802
		C/T	SNP	863
		T/A	SNP	1120
[BBH] H12_DROVI (sp|Q94555) Histone H1.2 OS=D. virilis GN=His1.2 PE=3 SV=1	GLOS_H12.1.1	G/A	SNP	68
		C/A	SNP	132
		A/G	SNP	136
		A/G	SNP	148
		A/G	SNP	165
		T/C	SNP	191
		C/T	SNP	224
		A/G	SNP	243
		C/T	SNP	288
		G/A	SNP	405
		G/A	SNP	429

		T/C	SNP	435
		A/AGCT	INSERTION	639
		G/C	SNP	663
		T/C	SNP	714
		G/A	SNP	717
		G/A	SNP	757
		T/A	SNP	837
		C/T	SNP	873
		A/T	SNP	895
		T/C	SNP	974
HBA_MOUSE (sp|P01942) Hemoglobin subunit alpha OS=M. musculus	GLOS_HBA.5.5	C/T	SNP	306
		G/A	SNP	310
		A/G	SNP	312
NM_008218.2 Mus musculus hemoglobin alpha, adult chain 1 (Hba-a1), mRNA	GLOS_HBA-A1.1.6	A/G	SNP	279
		C/T	SNP	522
NM_008218.2 Mus musculus hemoglobin alpha, adult chain 1 (Hba-a1), mRNA	GLOS_HBA-A1.2.6	C/A	SNP	51
NM_008218.2 Mus musculus hemoglobin alpha, adult chain 1 (Hba-a1), mRNA	GLOS_HBA-A1.4.6	T/C	SNP	840
NM_008218.2 Mus musculus hemoglobin alpha, adult chain 1 (Hba-a1), mRNA	GLOS_HBA-A1.4.6	T/G	SNP	930
		G/C	SNP	932
		C/T	SNP	1614
NM_008218.2 Mus musculus hemoglobin alpha, adult chain 1 (Hba-a1), mRNA	GLOS_HBA-A1.6.6	A/G	SNP	294
		C/G	SNP	329
HBB2_MOUSE (sp|P02089) Hemoglobin subunit beta-2 OS=Mus musculus	GLOS_HBB2.2.2	C/A	SNP	51
		A/G	SNP	114
NM_001278161.1 M. musculus hemoglobin, beta adult major chain (Hbb-b1), mRNA	GLOS_HBB-B1.1.2	C/A	SNP	59
		G/A	SNP	61
NM_001278161.1 M. musculus hemoglobin, beta adult major chain (Hbb-b1), mRNA	GLOS_HBB-B1.2.2	C/CT	INSERTION	373
		C/A	SNP	485
		C/T	SNP	487
		C/G	SNP	505
NM_008220.5 Mus musculus hemoglobin, beta adult t chain (Hbb-bt), mRNA	GLOS_HBB-BT.1.1	G/C	SNP	281
		T/G	SNP	328
		T/A	SNP	337
		C/A	SNP	338
		T/C	SNP	341
		G/A	SNP	348

		A/G	SNP	434
		T/C	SNP	435
		T/C	SNP	482
		A/T	SNP	508
[BBH] HH_DROWI (sp|B4NJP3) Prot hedgehog OS=D. willistoni GN=hh PE=3 SV=1	GLOS_HH.1.1	G/A	SNP	238
		G/A	SNP	391
		C/T	SNP	877
		G/A	SNP	1079
		C/T	SNP	1176
		G/A	SNP	1753
		T/G	SNP	1782
		A/G	SNP	1788
		C/T	SNP	1836
[BBH] HPRT_TRYBB (sp|Q07010) Hypoxanthine-guanine phosphoribosyltransferase OS=Trypanosoma brucei brucei GN=HGPRT PE=3 SV=1	GLOS_HPRT.1.1	T/C	SNP	24
		G/A	SNP	242
		A/G	SNP	243
		A/G	SNP	444
		A/T	SNP	787
		G/A	SNP	981
		A/G	SNP	1030
		T/C	SNP	1098
		C/T	SNP	1255
		C/T	SNP	1331
		C/A	SNP	1353
		T/G	SNP	1389
		T/C	SNP	1406
HYPA_HYPLI (sp|P35587) Hypodermin-A OS=Hypoderma lineatum PE=1 SV=2	GLOS_HYPA.1.1	A/T	SNP	396
		A/T	SNP	402
		C/T	SNP	607
		C/T	SNP	1743
		T/TTC	INSERTION	1749
[BBH] IF4A_TRYB2 (sp|Q38F76) Probable eukaryotic initiation factor 4A OS=Tbb	GLOS_IF4A.1.2	A/AT	INSERTION	1323
IMDH_DROME (sp|Q07152) Inosine-5'-monophosphate dehydrogenase OS=D. m.	GLOS_IMDH.2.2	C/T	SNP	269
		G/T	SNP	558
		T/C	SNP	678

		T/C	SNP	783
		A/T	SNP	882
		C/T	SNP	1407
		G/A	SNP	1623
		G/A	SNP	1731
		G/A	SNP	1732
		T/C	SNP	1903
		C/T	SNP	1928
		AG/A	DELETION	1947
		T/C	SNP	1955
		A/G	SNP	2152
		C/CT	INSERTION	2188
[BBH] KCC2A_DROME (sp|Q00168) Calcium/calmodulin-dependent protein kinase type II alpha chain OS=Drosophila melanogaster GN=CaMKII PE=1 SV=1	GLOS_KCC2A.2.2	G/T	SNP	29
		T/C	SNP	59
		C/G	SNP	60
		G/T	SNP	97
		T/C	SNP	118
		G/A	SNP	153
		A/T	SNP	173
		G/A	SNP	184
		G/T	SNP	195
		A/C	SNP	244
		A/G	SNP	256
		C/A	SNP	312
		T/C	SNP	321
		C/T	SNP	343
		C/T	SNP	415
		C/T	SNP	502
		G/A	SNP	514
		G/A	SNP	541
		A/G	SNP	643
		C/A	SNP	656
		G/A	SNP	712
		C/A	SNP	751
		A/G	SNP	815

C/T	SNP	856
C/T	SNP	857
G/C	SNP	875
C/G	SNP	1231
T/C	SNP	1387
C/T	SNP	1510
T/C	SNP	1558
G/A	SNP	1665
A/G	SNP	1726
A/G	SNP	1929
A/G	SNP	1954
A/G	SNP	1992
A/G	SNP	2016
T/A	SNP	2044
T/C	SNP	2091
C/G	SNP	2103
A/C	SNP	2114
T/G	SNP	2131
T/C	SNP	2141
T/A	SNP	2163
C/T	SNP	2235
C/T	SNP	2263
T/A	SNP	2268
A/C/T	SNP	2277
A/G	SNP	2305
T/G	SNP	2306
T/A	SNP	2310
A/C	SNP	2312
C/T	SNP	2316
G/A	SNP	2331
G/C	SNP	2359
C/G	SNP	2361
G/A	SNP	2373
G/C	SNP	2380
A/G	SNP	2407
G/C	SNP	2434

G/GT	INSERTION	2446
G/T	SNP	2460
C/T	SNP	2474
G/A	SNP	2477
A/T	SNP	2478
G/A	SNP	2488
G/A	SNP	2558
A/G	SNP	2564
A/G	SNP	2566
T/C	SNP	2586
C/A	SNP	2620
C/T	SNP	2647
G/A	SNP	2663
T/C	SNP	2778
A/G	SNP	2798
G/T	SNP	2805
T/A	SNP	2830
A/T	SNP	2869
G/C	SNP	2881
TA/T	DELETION	2885
G/C	SNP	2904
G/A	SNP	2956
C/T	SNP	2961
G/A	SNP	2972
TAA/TAAA	INSERTION	3024
C/T	SNP	3058
A/G	SNP	3081
T/C	SNP	3104
G/A	SNP	3113
A/T	SNP	3134
G/A	SNP	3142
C/G	SNP	3172
A/T	SNP	3174
A/C	SNP	3190
A/T	SNP	3198
A/G	SNP	3217

		T/A	SNP	3245
		G/A	SNP	3278
		T/G	SNP	3286
		G/A	SNP	3287
		G/A	SNP	3379
		CTGT/C	DELETION	3692
		G/C	SNP	3706
		A/G	SNP	3796
		G/A	SNP	3800
		A/T	SNP	4045
NP_001123818.2 uncharacterized protein LOC100170569 [X. (Silurana) tropicalis]	GLOS_LOC100170569.1.1	A/G	SNP	9
		C/G	SNP	10
		A/G	SNP	63
		C/G	SNP	64
		A/G	SNP	69
NM_001195093.1 Macaca mulatta MTRNR2-like (LOC100499562), mRNA	GLOS_LOC100499562.2.3	C/G	SNP	1804
		A/G	SNP	1826
		G/A	SNP	1830
		C/T	SNP	1886
		T/C	SNP	1925
		C/A	SNP	1967
		A/G	SNP	2078
		G/A	SNP	2083
		C/A	SNP	2135
		C/T	SNP	2167
		G/A	SNP	2181
		T/G	SNP	2285
		T/TA	INSERTION	3036
		C/T	SNP	3040
		G/A	SNP	3063
		G/C	SNP	3093
		G/A	SNP	3112
		T/C	SNP	3133
		G/A	SNP	3218
		C/T	SNP	3421
		TA/T	DELETION	3625

		C/CTT	INSERTION	3691
		G/C	SNP	3708
		C/T	SNP	3800
		G/C	SNP	4694
XP_003245892.1 PREDICTED: hypothetical protein LOC100575767 [A. pisum]	GLOS_LOC100575767.7.33	C/CT	INSERTION	2374
		A/AT	INSERTION	2980
		G/GT	INSERTION	3194
		T/C	SNP	4600
		A/G	SNP	4606
		C/T	SNP	5325
		G/A	SNP	5534
		C/T	SNP	5939
		T/C	SNP	7398
		T/C	SNP	7434
		T/C	SNP	7485
		A/AT	INSERTION	7800
		A/AT	INSERTION	8005
		G/T	SNP	8147
		C/CT	INSERTION	9072
XM_003581200.1 PREDICTED: B. distachyon uncharact. LOC100843429 mRNA	GLOS_LOC100843429.1.1	T/C	SNP	163
		A/G	SNP	1017
		T/TA	INSERTION	1163
		A/C	SNP	1169
		A/G	SNP	1296
		TA/T	DELETION	1507
		G/A	SNP	2151
		C/T	SNP	2683
		GT/G	DELETION	2688
		A/G	SNP	2999
		T/C	SNP	3017
		C/T	SNP	3102
		C/T	SNP	3166
		T/C	SNP	3441
		T/C	SNP	3466
		T/C	SNP	3503
		T/C	SNP	3564

XM_003689208.2 PRED: M. musc ferritin light chain 1-like, transcript variant1,mRNA		G/T	SNP	904
		A/C	SNP	906
XP_003693410.1 PREDICTED: TRAF-interacting protein-like, partial [Apis florea]	GLOS_LOC100869906.2.4	G/A	SNP	1424
		G/C	SNP	1425
		T/C	SNP	1778
		C/G	SNP	1858
		T/C	SNP	1923
		A/G	SNP	2308
XP_003708657.1 PREDICTED: chymotrypsin inhibitor-like [Megachile rotundata]	GLOS_LOC100876965.1.1	T/G	SNP	21
XM_003771971.1 PREDICTED: S. harrisii uncharacterized LOC100933241, mRNA	GLOS_LOC100933241.1.14	G/T	SNP	43
		T/G	SNP	70
XP_004085317.1 PRED.: uncharacterized protein LOC101169087, partial [O. latipes]	GLOS_LOC101169087.1.1	T/G	SNP	18
		A/G	SNP	177
		A/G	SNP	356
		C/T	SNP	476
		A/G	SNP	487
		G/A	SNP	492
		G/T	SNP	514
		T/C	SNP	912
		A/T	SNP	974
		T/C	SNP	987
		T/C	SNP	988
		G/A	SNP	1072
		A/C	SNP	1202
		T/C	SNP	1332
		A/G	SNP	1348
		G/T	SNP	1613
		G/C	SNP	1618
		G/A	SNP	1638
XP_004206943.1 PREDICTED: RNA-directed DNA polymerase from mobile element jockey-like [Hydra magnipapillata]	GLOS_LOC101236672.1.1	G/A	SNP	659
		C/T	SNP	918
		G/A	SNP	1337
		T/G	SNP	1728
		C/G	SNP	2285
XP_004531060.1 PREDICTED: protoporphyrinogen oxidase-like [Ceratitis capitata]	GLOS_LOC101448305.1.1	A/G	SNP	69

		C/T	SNP	284
		G/A	SNP	408
		C/T	SNP	518
		G/A	SNP	644
		G/A	SNP	666
		C/CT	INSERTION	677
		G/A	SNP	790
		C/G	SNP	857
		C/T	SNP	945
		C/T	SNP	983
XP_004526797.1 PREDICTED: arginine kinase-like isoform X1 [Ceratitis capitata]	GLOS_LOC101448504.1.1	G/GA	INSERTION	293
		A/G	SNP	315
		T/C	SNP	548
		T/C	SNP	680
		T/C	SNP	1032
		C/A	SNP	1039
		C/A	SNP	1040
		C/T	SNP	1343
		A/G	SNP	2361
		G/GT	INSERTION	2398
		C/A	SNP	2669
XP_004518567.1 PREDICTED: protein timeless-like isoform X1 [Ceratitis capitata] ref|XP_004518568.1| PREDICTED: protein timeless-like isoform X2 [C. capitata]	GLOS_LOC101448839.1.1	T/C	SNP	100
		TTTA/T	DELETION	202
		TTA/T	DELETION	203
		C/A	SNP	393
		G/A	SNP	522
		G/A	SNP	621
		T/C	SNP	629
		T/C	SNP	645
		T/C	SNP	741
		C/T	SNP	1012
		C/T	SNP	1202
		G/A	SNP	1245
		C/T	SNP	1272
		G/A	SNP	1403

		G/C	SNP	1471
		A/T	SNP	1479
		T/C	SNP	1872
		A/C/T	SNP	2151
		C/A	SNP	2244
		A/T	SNP	2259
		G/A	SNP	2937
		T/A	SNP	3153
		A/G	SNP	3210
		C/T	SNP	3342
		G/A	SNP	3429
		A/G	SNP	3438
		C/G	SNP	3519
		G/T	SNP	3539
		A/G	SNP	3659
		C/T	SNP	3702
		G/A	SNP	3709
		C/A	SNP	3825
		A/C	SNP	3966
		T/C	SNP	3999
		G/C	SNP	4167
		T/C	SNP	4257
		A/G	SNP	4557
		C/T	SNP	4719
		G/T	SNP	4881
		T/C	SNP	4889
		G/T	SNP	4975
		T/G	SNP	5204
		T/A	SNP	5209
		T/C	SNP	5211
		T/A	SNP	5455
XP_004523175.1 PREDICTED: lysM and putative peptidoglycan-binding domain-containing protein 1-like isoform X1 [Ceratitis capitata]	GLOS_LOC101449088.1.1	C/A	SNP	176
		A/C	SNP	200
		A/G	SNP	204
		GT/G	DELETION	295

		C/T	SNP	319
		A/C	SNP	437
		C/T	SNP	896
		T/C	SNP	901
		A/G	SNP	1195
		A/T	SNP	1694
		T/C	SNP	1865
		A/T	SNP	2133
		T/A	SNP	2135
		A/T	SNP	2245
		T/A	SNP	2368
		A/AT/ATT	INSERTION	2371
XP_004535877.1 PRED: uncharact. prot LOC101449597 isoform X2 [C. capitata]	GLOS_LOC101449597.1.1	G/GAA/GA	INSERTION	247
		G/C	SNP	853
XP_004524436.1 PRED: glyoxalase domain-containing prot 4-like isoform X1 [C. c.]	GLOS_LOC101449625.1.1	G/T	SNP	37
		G/A	SNP	88
		G/A	SNP	104
		C/T	SNP	490
		C/T	SNP	553
		C/T	SNP	568
		A/G	SNP	791
		T/C	SNP	827
		A/G	SNP	1030
		G/C	SNP	1052
		A/G	SNP	1071
		T/G	SNP	1155
XP_004524798.1 PRED: microsomal triglyceride transfer prot large subunit-like [C.c.]	GLOS_LOC101449626.1.1	G/A	SNP	37
		C/G	SNP	85
		T/C	SNP	279
		C/T	SNP	287
		G/A	SNP	289
		G/A	SNP	306
		C/T	SNP	321
		G/A	SNP	474
		A/G	SNP	837
		T/C	SNP	912

		G/T	SNP	914
		C/A	SNP	1002
		A/C	SNP	1071
		T/C	SNP	1086
		C/T	SNP	1161
		T/C	SNP	1203
		G/A	SNP	1214
		A/G	SNP	1245
		G/T	SNP	1288
		G/A	SNP	1353
		T/C	SNP	1366
		T/C	SNP	1497
		A/G	SNP	1515
		G/A	SNP	1545
		C/G	SNP	1610
		T/C	SNP	1674
		T/C	SNP	1914
		T/G	SNP	2241
		A/C	SNP	2352
		C/T	SNP	2637
		A/G	SNP	2688
		C/G	SNP	2707
		G/C	SNP	2720
XP_004529622.1 PREDICTED: protein xmas-2-like [Ceratitis capitata]	GLOS_LOC101449710.1.1	A/T	SNP	875
		G/T	SNP	932
		C/A	SNP	981
		C/T	SNP	1301
		C/T	SNP	1357
		G/A	SNP	1396
		C/T	SNP	1699
		T/A	SNP	1704
		T/C	SNP	1750
		C/A	SNP	2149
		T/C	SNP	2293
		T/C	SNP	2896
		T/C	SNP	3154

		G/A	SNP	3436
		G/A	SNP	3904
		G/A	SNP	4285
		A/G	SNP	4502
XP_004519690.1 PRED: uncharacterized protein LOC101449850 isoform X1 [C. c.] ref|XP_004519691.1| PRED: uncharact. Prot. LOC101449850 isoform X2 [C. c.]	GLOS_LOC101449850.1.3	G/A	SNP	81
XP_004519690.1 PRED.: uncharact. Prot. LOC101449850 isoform X1 [C. capitata] ref|XP_004519691.1| PRED: uncharact. Prot. LOC101449850 isoform X2 [C. c.]	GLOS_LOC101449850.3.3	A/T	SNP	42
		C/T	SNP	135
		T/A	SNP	154
		G/A	SNP	156
XP_004526049.1 PRED: bromodomain-containing prot. DDB_G0280777-like [C. c.]	GLOS_LOC101449871.1.1	G/C	SNP	232
		G/A	SNP	604
		A/G	SNP	645
		G/T	SNP	862
		A/G	SNP	865
		A/G	SNP	1051
		G/A	SNP	1078
		A/C	SNP	1116
		G/A	SNP	1126
		GTA/G	DELETION	1151
		G/A	SNP	1181
		G/C	SNP	1214
		G/A	SNP	1239
XP_004527004.1 PREDICTED: uncharacterized protein LOC101450058 [C. capitata]	GLOS_LOC101450058.1.3	C/T	SNP	369
		A/T	SNP	1019
		T/A	SNP	1020
		T/G	SNP	1106
		AAT/A	DELETION	1130
		A/T	SNP	1135
		T/A	SNP	1136
		T/TA	INSERTION	1136
		A/AG	INSERTION	1138
XP_004527004.1 PREDICTED: uncharacterized protein LOC101450058 [C. capitata]	GLOS_LOC101450058.3.3	A/T	SNP	337
		C/T	SNP	416
		A/G	SNP	600

		C/T	SNP	615
		C/T	SNP	665
		A/T	SNP	803
XP_004518575.1 PREDICTED: protein disulfide-isomerase A6-like [C. capitata]	GLOS_LOC101450103.1.1	C/T	SNP	106
		T/A	SNP	240
		A/G	SNP	708
		C/T	SNP	990
		T/C	SNP	1020
		C/T	SNP	1136
		T/A	SNP	1173
		T/C	SNP	1619
		C/T	SNP	1622
		C/G	SNP	1768
XP_004521926.1 PREDICTED: zinc carboxypeptidase A 1-like [Ceratitis capitata]	GLOS_LOC101450347.1.1	TTA/T	DELETION	1134
		TG/T	DELETION	1138
		A/C	SNP	1140
		T/C	SNP	1318
		T/A	SNP	1361
		T/G	SNP	1363
		C/T	SNP	1377
XP_004521991.1 PREDICTED: uncharacterized protein LOC101450402 [C. capitata]	GLOS_LOC101450402.10.11	T/C	SNP	606
		T/C	SNP	615
		T/C	SNP	619
		T/C	SNP	638
		T/C	SNP	679
		C/CA	INSERTION	690
		T/C	SNP	869
		C/G	SNP	1605
		A/G	SNP	1609
		A/G	SNP	1610
		T/A	SNP	1612
		A/C	SNP	1631
		G/A	SNP	1652
		G/A	SNP	2643
		G/A	SNP	2680
		AT/A	DELETION	2684

XP_004521991.1 PREDICTED: uncharacterized protein LOC101450402 [C. capitata]	GLOS_LOC101450402.2.11	G/T	SNP	98
		T/C	SNP	99
		A/G	SNP	167
		T/C	SNP	194
		A/C	SNP	228
		G/C	SNP	389
		T/G	SNP	390
		A/T	SNP	393
		G/A	SNP	581
		C/T	SNP	616
		C/G	SNP	843
		A/AT	INSERTION	844
		A/G	SNP	846
		C/G	SNP	847
		A/G	SNP	850
XP_004521991.1 PREDICTED: uncharacterized protein LOC101450402 [C. capitata]	GLOS_LOC101450402.4.11	C/A	SNP	762
		A/G	SNP	765
		T/A	SNP	768
XP_004521991.1 PREDICTED: uncharacterized protein LOC101450402 [C. capitata]	GLOS_LOC101450402.5.11	C/A	SNP	274
		C/G	SNP	275
		T/C	SNP	278
		C/T	SNP	501
		T/A	SNP	513
		T/A	SNP	514
		T/C	SNP	923
		T/C	SNP	1188
		T/C	SNP	1195
		T/C	SNP	1199
		T/C	SNP	1200
		C/T	SNP	1207
		T/C	SNP	1235
		C/T	SNP	1258
		T/C	SNP	1289
		G/C	SNP	1299
		T/C	SNP	1330
		T/C	SNP	1573

		A/G	SNP	1619
		A/C	SNP	1665
		G/A	SNP	1680
		A/T	SNP	1690
		T/A	SNP	1692
		A/T	SNP	2526
		G/C	SNP	2529
		G/A	SNP	2658
		A/G	SNP	2695
		T/C	SNP	2722
		G/GT	INSERTION	2771
		A/G	SNP	2788
		T/G	SNP	2823
		T/A	SNP	2824
XP_004521991.1 PREDICTED: uncharacterized protein LOC101450402 [C. capitata]	GLOS_LOC101450402.8.11	G/C	SNP	40
		G/T	SNP	42
		T/TA	INSERTION	96
		C/A	SNP	115
		G/A	SNP	121
		A/AT	INSERTION	123
XP_004521991.1 PREDICTED: uncharacterized protein LOC101450402 [C. capitata]	GLOS_LOC101450402.9.11	T/C	SNP	1282
		T/G	SNP	1487
XP_004520408.1 PRED: membrane-bound alkaline phosphatase-like [C. capitata]	GLOS_LOC101450467.1.1	C/G	SNP	133
		C/T	SNP	260
		T/A	SNP	581
		T/C	SNP	614
		A/G	SNP	615
		C/T	SNP	626
		G/A	SNP	656
		G/A	SNP	689
		C/G	SNP	690
		T/C	SNP	722
		C/G	SNP	999
		C/T	SNP	1367
		G/A	SNP	1463
		A/G	SNP	1466

		C/T	SNP	1517
		C/T	SNP	1568
		T/C	SNP	1598
		G/A	SNP	1742
		G/A	SNP	1827
XP_004535333.1 PRED: uncharact. prot LOC101450510 isoform X1 [C. capitata] ref|XP_004535334.1| PRED: uncharact. prot. LOC101450510 isoform X2 [C. c.] ref|XP_004535335.1| PRED.: uncharact. protein LOC101450510 isoform X3 [C. c.]	GLOS_LOC101450510.1.1	A/T	SNP	48
		C/T	SNP	260
		C/T	SNP	422
		G/A	SNP	444
		T/A	SNP	472
		G/C	SNP	848
		A/C	SNP	1196
		A/G	SNP	1297
		G/A	SNP	1330
		G/A	SNP	1454
		T/C	SNP	1562
		A/G	SNP	1584
		A/G	SNP	1937
		A/C	SNP	2435
		A/G	SNP	2456
		G/A	SNP	2508
		A/G	SNP	2537
		C/T	SNP	2582
		A/G	SNP	2741
		T/C	SNP	2810
		A/C	SNP	2837
XP_004521927.1 PREDICTED: zinc carboxypeptidase A 1-like [Ceratitis capitata]	GLOS_LOC101450530.2.4	G/T	SNP	958
		A/T	SNP	1256
		T/A	SNP	1257
		C/A	SNP	1383
		A/AT	INSERTION	1427
		C/T	SNP	1428
		T/A	SNP	2596
		A/T	SNP	2941

		A/C	SNP	2943
		A/G	SNP	2944
		T/A	SNP	2945
XP_004521927.1 PREDICTED: zinc carboxypeptidase A 1-like [Ceratitis capitata]	GLOS_LOC101450530.3.4	C/A	SNP	1049
		G/T	SNP	1063
XP_004521927.1 PREDICTED: zinc carboxypeptidase A 1-like [Ceratitis capitata]	GLOS_LOC101450530.4.4	T/C	SNP	138
		G/T	SNP	148
		G/A	SNP	179
		A/G	SNP	244
		A/T	SNP	248
		A/G	SNP	297
		A/C	SNP	301
		C/G	SNP	2994
		G/T	SNP	2996
		A/G	SNP	3001
		AC/A	DELETION	4486
XP_004521992.1 PREDICTED: uncharacterized protein LOC101450586 [C. capitata]	GLOS_LOC101450586.4.9	A/G	SNP	110
		C/T	SNP	137
		C/T	SNP	174
		T/G	SNP	274
XP_004521992.1 PREDICTED: uncharacterized protein LOC101450586 [C. capitata]	GLOS_LOC101450586.9.9	T/C	SNP	561
		T/A	SNP	578
		T/G	SNP	699
		A/G	SNP	702
		A/T	SNP	721
		G/A	SNP	722
		A/C	SNP	769
		G/A	SNP	784
		A/C	SNP	842
		T/G	SNP	1035
		G/T	SNP	1037
		T/C	SNP	1139
		C/A	SNP	1142
		G/C	SNP	1144
		T/G	SNP	1145
		G/A	SNP	1802

		G/A	SNP	1832
XP_004523930.1 PREDICTED: sialin-like [Ceratitis capitata]	GLOS_LOC101450592.2.2	T/C	SNP	161
		G/A	SNP	178
		C/A	SNP	227
XP_004536327.1 PREDICTED: uncharacterized protein LOC101451482 [C. capitata]	GLOS_LOC101451482.1.1	C/A	SNP	200
		A/G	SNP	309
		C/T	SNP	1302
		G/T	SNP	1330
		C/T	SNP	1682
		C/G	SNP	1688
		G/C	SNP	1740
		T/C	SNP	1752
		G/A	SNP	1899
		C/T	SNP	1953
		G/A	SNP	2109
		A/G	SNP	2388
		A/G	SNP	3213
		C/T	SNP	3680
		A/G	SNP	3814
		A/G	SNP	3942
		AG/A	DELETION	4060
		C/A	SNP	4466
		C/T	SNP	4485
		G/A	SNP	4602
		A/G	SNP	5072
		G/T	SNP	5204
		G/A	SNP	5208
		AG/A	DELETION	5641
		G/A	SNP	5643
		A/G	SNP	5894
XP_004523277.1 PREDICTED: WD repeat-containing prot. 81-like isoform X1 [C.c.]	GLOS_LOC101451574.1.1	G/A	SNP	1034
		C/T	SNP	1102
		T/C	SNP	1114
		G/A	SNP	1232
		G/T	SNP	1390
		C/T	SNP	1437

T/A	SNP	2129
G/C	SNP	2213
C/A	SNP	2218
C/T	SNP	2525
C/T	SNP	2840
C/T	SNP	3014
T/C	SNP	3040
T/C	SNP	3062
C/A	SNP	3143
A/G	SNP	3356
T/A	SNP	3586
G/A	SNP	3635
A/T	SNP	3641
T/A	SNP	3824
C/T	SNP	3839
T/C	SNP	3856
C/A	SNP	3954
C/T	SNP	4013
A/G	SNP	4052
T/C	SNP	4297
T/C	SNP	4313
T/C	SNP	4379
T/A	SNP	4400
C/T	SNP	4613
G/A	SNP	4634
C/G	SNP	4709
G/A	SNP	4712
G/A	SNP	4767
C/T	SNP	4838
T/C	SNP	5155
C/T	SNP	5197
A/G	SNP	5253
G/A	SNP	5270
T/C	SNP	5731
A/G	SNP	5831
T/TAACCG	INSERTION	6222

		A/T	SNP	6371
		G/A	SNP	6511
		G/T	SNP	6568
		G/GT	INSERTION	6638
		G/GT	INSERTION	6684
		C/T	SNP	6739
XP_004534769.1 PRED: solute carrier family 12 member 6-like isoform X5 [C. c.]	GLOS_LOC101451961.1.2	C/T	SNP	31
		T/C	SNP	99
		G/A	SNP	1061
		T/A	SNP	1286
		C/A	SNP	1349
		CT/C	DELETION	1415
		G/C	SNP	1452
		C/T	SNP	1623
		G/A	SNP	1678
		T/C	SNP	2197
		A/G	SNP	2318
		C/T	SNP	2400
		G/GT	INSERTION	2464
		G/C	SNP	2566
		A/G	SNP	2589
		T/C	SNP	2670
		C/T	SNP	2938
		T/C	SNP	3000
		T/C	SNP	3072
		T/C	SNP	3109
		C/T	SNP	3122
		C/T	SNP	3130
		G/A	SNP	3198
		C/T	SNP	3281
		G/C	SNP	3365
		A/G	SNP	3394
		C/A	SNP	3435
		A/G	SNP	3493
		A/T	SNP	3643
		A/T	SNP	3653

		C/A	SNP	3700
		G/A	SNP	3758
		C/G	SNP	3817
		T/C	SNP	4022
		G/T	SNP	4047
		G/A	SNP	4097
		G/T	SNP	4137
		G/A	SNP	4150
		T/A	SNP	4206
		G/A	SNP	4207
		C/T	SNP	4303
XP_004517444.1 PREDICTED: inositol-3-phosphate synthase-like [Ceratitis capitata]	GLOS_LOC101452090.1.1	A/G	SNP	828
		A/G	SNP	829
		G/A	SNP	849
		A/G	SNP	898
		T/C	SNP	1066
		C/T	SNP	1069
		C/T	SNP	1234
		T/C	SNP	1531
		G/A	SNP	1567
		A/C	SNP	1741
		A/G	SNP	1808
		C/T	SNP	1827
		C/A	SNP	1853
		G/A	SNP	1895
		G/A	SNP	1948
		T/A	SNP	2016
		G/A	SNP	2020
XP_004536688.1 PREDICTED: uncharacterized protein LOC101452450 [C. capitata]	GLOS_LOC101452450.1.1	G/A	SNP	82
		AATT/A	DELETION	233
		GATGATA	DELETION	260
		GATA/G	DELETION	263
XP_004533488.1 PREDICTED: alpha-N-acetylgalactosaminidase-like [C. capitata]	GLOS_LOC101452734.1.1	C/A	SNP	169
		C/T	SNP	442
		G/A	SNP	487
		A/G	SNP	982

XP_004537708.1 PREDICTED: adenosine kinase-like isoform X1 [Ceratitis capitata]	GLOS_LOC101452929.1.1	G/GA	INSERTION	148
		A/G	SNP	280
		TAA/TAAA	INSERTION	527
		G/A	SNP	1743
XP_004527472.1 PREDICTED: synaptic vesicle glycoprotein 2B-like [C. capitata]	GLOS_LOC101453078.1.2	A/AA	INSERTION	505
		G/A	SNP	508
		A/G	SNP	1932
		G/A	SNP	1933
		A/G	SNP	2107
XP_004535897.1 PREDICTED: protein ETHE1, mitochondrial-like [Ceratitis capitata]	GLOS_LOC101453109.1.1	A/T	SNP	109
		T/A	SNP	134
		T/TC	INSERTION	161
		G/C	SNP	202
		G/T	SNP	693
XP_004519981.1 PRED: histone-lysine N-methyltransferase Suv4-20-like isoform X1 [Ceratitis capitata] ref|XP_004519982.1| PREDICTED: histone-lysine N-methyltransferase Suv4-20-like isoform X2 [Ceratitis capitata]	GLOS_LOC101453125.1.2	T/G	SNP	100
		T/C	SNP	124
		C/T	SNP	845
		G/GT	INSERTION	1301
		A/G	SNP	1416
		C/T	SNP	2500
		C/CT	INSERTION	5214
XP_004527473.1 PREDICTED: synaptic vesicle glycoprotein 2A-like [C. capitata]	GLOS_LOC101453261.1.1	G/A	SNP	513
		T/C	SNP	948
		T/C	SNP	1449
		C/T	SNP	1463
		C/T	SNP	1580
XP_004524284.1 PREDICTED: pre-mRNA-processing factor 39-like [C. capitata]	GLOS_LOC101453313.1.1	A/G	SNP	260
		T/C	SNP	306
		T/C	SNP	308
		A/AAAAAG	INSERTION	356
		A/G	SNP	648
		A/T	SNP	990
		C/T	SNP	1083
		A/G	SNP	1221

C/T	SNP	1239
G/A	SNP	1250
G/A	SNP	1275
G/A	SNP	1694
A/C	SNP	1731
C/T	SNP	1752
G/T	SNP	2238
T/C	SNP	2451
C/T	SNP	2541
C/T	SNP	2562
T/C	SNP	2568
C/T	SNP	2715
A/T	SNP	2742
A/G	SNP	2808
C/T	SNP	2898
G/A	SNP	2985
G/T	SNP	3060
A/G	SNP	3213
G/T	SNP	3255
G/A	SNP	3604
T/C	SNP	3654
C/T	SNP	3660
C/A	SNP	3766
C/A	SNP	4437
T/A	SNP	4645
T/A	SNP	4843
T/TA	INSERTION	4843
G/A	SNP	4846
C/A	SNP	4890
G/A	SNP	4909
A/G	SNP	5100
A/G	SNP	5109
G/A	SNP	5124
C/A	SNP	5128
G/A	SNP	5129
A/G	SNP	5132

		T/C	SNP	5204
		G/A	SNP	5219
XP_004527026.1 PRED: glycine cleavage system H protein, mitochondrial-like [C. c.]	GLOS_LOC101453326.1.1	G/A	SNP	47
		T/C	SNP	467
		TA/T	DELETION	788
		G/A	SNP	861
		C/G	SNP	893
		G/T	SNP	901
		C/T	SNP	1042
		T/C	SNP	1061
		T/C	SNP	1088
		C/T	SNP	1155
		G/A	SNP	1338
XP_004537158.1 PREDICTED: transcription factor E2f-like [Ceratitis capitata]	GLOS_LOC101453541.1.1	T/C	SNP	143
		C/T	SNP	154
		G/C	SNP	258
		C/T	SNP	269
		G/A	SNP	303
		A/G	SNP	412
		C/G	SNP	473
		C/A	SNP	867
		A/C	SNP	873
		G/A	SNP	875
		C/T	SNP	876
		G/C	SNP	878
		G/A	SNP	881
		C/T	SNP	882
		C/A	SNP	883
		T/A	SNP	884
		C/A	SNP	887
		A/G	SNP	929
		T/C	SNP	1178
		G/A	SNP	1251
		T/C	SNP	1403
		G/T	SNP	1919
		C/G	SNP	2060

		T/C	SNP	2080
		C/G	SNP	2472
		C/G	SNP	2640
XP_004518597.1 PRED:putat. uncharact. prot DDB_G0279653-like isoform X3 [C.c.]	GLOS_LOC101453673.1.2	C/T	SNP	1568
		T/A	SNP	3078
		T/A	SNP	3079
XP_004531360.1 PREDICTED: protease inhibitor-like [Ceratitis capitata]	GLOS_LOC101453761.1.3	A/T	SNP	407
		G/A	SNP	428
		T/G	SNP	446
		T/TG	INSERTION	446
XP_004530529.1 PREDICTED: 60S ribosomal export protein NMD3-like [C. capitata]	GLOS_LOC101453821.1.1	G/A	SNP	119
		G/A	SNP	120
		T/C	SNP	243
		C/T	SNP	263
		C/T	SNP	469
		C/T	SNP	613
		C/G	SNP	973
		C/A	SNP	1090
		A/G	SNP	1157
		G/A	SNP	1282
		G/A	SNP	1351
		C/A	SNP	1358
		G/A	SNP	1405
		C/T	SNP	1636
		A/G	SNP	1643
		C/T	SNP	1774
		C/T	SNP	1775
		A/G	SNP	1872
		T/A	SNP	1875
XP_004536888.1 PREDICTED: uncharacterized protein LOC101453846 [C. capitata]	GLOS_LOC101453846.2.2	G/A	SNP	65
		A/ATAG	INSERTION	85
		T/C	SNP	245
		G/C	SNP	353
		A/T	SNP	377
		C/A	SNP	401
		A/T	SNP	841

A/T	SNP	926
C/G	SNP	928
T/C	SNP	956
A/G	SNP	1076
G/C	SNP	1265
C/T	SNP	1358
G/A	SNP	1477
A/G	SNP	1481
A/G	SNP	1673
G/A	SNP	1677
T/G	SNP	1766
T/C	SNP	1775
T/C	SNP	1885
G/T	SNP	2057
C/A	SNP	2129
C/T	SNP	2231
T/C	SNP	2326
C/T	SNP	2396
G/A	SNP	2426
C/G	SNP	2460
G/T	SNP	2480
G/A	SNP	2879
T/C	SNP	3026
C/T	SNP	3185
A/G	SNP	3471
A/C	SNP	3503
T/A	SNP	3529
C/T	SNP	3572
A/C	SNP	3608
C/T	SNP	3863
T/C	SNP	4055
G/A	SNP	4112
A/G	SNP	4210
C/A	SNP	4254
G/A	SNP	4308
C/A	SNP	4343

XP_004537801.1 PREDICTED: zinc metalloproteinase nas-4-like [Ceratitis capitata]	GLOS_LOC101454485.3.7	C/T	SNP	130
		C/CAG	INSERTION	190
		C/G	SNP	190
		C/A	SNP	191
		T/G	SNP	198
		AT/A	DELETION	515
		T/G	SNP	517
		G/C	SNP	520
		G/T	SNP	521
		C/G	SNP	525
		T/G	SNP	527
		C/T	SNP	551
		T/G	SNP	664
		T/G	SNP	1027
		ATT/A	DELETION	1057
		T/A	SNP	1073
		G/T	SNP	1074
		G/T	SNP	1119
		T/C	SNP	1124
XP_004537801.1 PREDICTED: zinc metalloproteinase nas-4-like [Ceratitis capitata]	GLOS_LOC101454485.5.7	T/C	SNP	643
		G/T	SNP	705
XP_004537801.1 PREDICTED: zinc metalloproteinase nas-4-like [Ceratitis capitata]	GLOS_LOC101454485.7.7	C/A	SNP	1353
		A/C	SNP	1900
		A/C	SNP	1914
		G/T	SNP	1937
XM_004517917.1 PREDICTED: Ceratitis capitata serine/threonine-protein kinase PAK 1-like (LOC101454487), transcript variant X4, mRNA	GLOS_LOC101454487.2.2	T/G	SNP	147
		T/A	SNP	396
		T/C	SNP	478
		G/A	SNP	506
		G/C	SNP	585
		A/T	SNP	605
		G/C	SNP	740
		C/T	SNP	990
		G/A	SNP	1005
		A/C	SNP	1044

		C/T	SNP	1062
		C/T	SNP	1079
		T/A	SNP	1103
		C/T	SNP	1120
		C/G	SNP	1168
		G/T	SNP	1528
		C/A	SNP	1759
		T/C	SNP	1784
		G/A	SNP	1815
		C/T	SNP	1901
		G/A	SNP	1947
		G/A	SNP	1965
		G/A	SNP	1966
		A/C	SNP	2141
		T/C	SNP	2294
		C/CACA	INSERTION	3272
		G/A	SNP	3588
		G/GA	INSERTION	3588
XP_004523031.1 PREDICTED: enkurin-like [Ceratitis capitata]	GLOS_LOC101454791.1.1	G/C	SNP	527
XP_004534042.1 PREDICTED: protein rolling stone-like [Ceratitis capitata]	GLOS_LOC101454883.1.1	C/G	SNP	141
		A/T	SNP	264
		C/T	SNP	318
		C/A	SNP	3537
		TGATTAA(	DELETION	3913
		A/G	SNP	4084
		A/G	SNP	4152
		A/G	SNP	4162
		A/G	SNP	4164
		A/G	SNP	4170
		A/G	SNP	4174
		A/G	SNP	4217
		A/G	SNP	4274
		G/A	SNP	4370
		G/C	SNP	4377
		C/T	SNP	4545
		C/T	SNP	4642

		A/G	SNP	4695
		G/A	SNP	4734
		C/G	SNP	4736
		T/G	SNP	4742
		C/T	SNP	5055
		T/C	SNP	5178
		A/G	SNP	5310
		A/G	SNP	5318
		G/A	SNP	5337
		A/G	SNP	5409
		A/G	SNP	5412
		A/G	SNP	5416
		A/G	SNP	5424
		A/G	SNP	5425
		A/G	SNP	6925
		A/G	SNP	6931
		A/G	SNP	6997
		A/G	SNP	7012
		A/G	SNP	7029
		A/G	SNP	7034
		T/C	SNP	9195
		A/G	SNP	9221
XP_004529560.1 PREDICTED: ras-related protein Ral-a-like isoform X3 [C. capitata]	GLOS_LOC101455051.1.1	A/C	SNP	262
		C/T	SNP	770
		TAG/T	DELETION	782
		G/A	SNP	799
		A/G	SNP	915
XP_004519344.1 PRED.: sphingolipid delta(4)-desaturase DES1-like [C. capitata]	GLOS_LOC101455374.1.1	C/T	SNP	378
		C/A	SNP	843
		G/A	SNP	918
		C/A	SNP	1157
		CT/C	DELETION	1164
		G/A	SNP	1203
		A/G	SNP	1315
		T/G	SNP	1330
XP_004526841.1 PRED: proton-coupled folate transporter-like isoform X1 [C. capit.]	GLOS_LOC101455387.1.1	A/G	SNP	91

ref|XP_004526842.1| PRED: proton-coupled folate transporter-like isoform X2[C.c.]				
ref|XP_004526843.1| PRED: proton-coupled folate transporter-like isoform X3[C.c.]				
		G/A	SNP	409
		G/A	SNP	410
		A/C	SNP	649
XP_004520095.1 PREDICTED: serine protease SP24D-like [Ceratitis capitata]	GLOS_LOC101455430.10.10	A/G	SNP	78
		T/TC	INSERTION	756
		C/G	SNP	761
		A/T	SNP	973
		A/T	SNP	976
		GGC/G	DELETION	979
		A/AT	INSERTION	982
		C/T	SNP	1158
		AG/A	DELETION	1174
		A/T	SNP	1185
		T/G	SNP	1250
		A/G	SNP	1251
		G/T	SNP	1397
		T/A	SNP	1627
		C/CA	INSERTION	1723
		T/TC	INSERTION	1726
		T/G	SNP	1732
		T/A	SNP	1753
		C/G	SNP	1756
		A/C	SNP	1957
		T/TG	INSERTION	2021
		C/G	SNP	2157
		A/G	SNP	2313
		A/G	SNP	2454
XP_004521067.1 PRED.: uncharact. protein LOC101455432 isoform X3 [C. capitata]	GLOS_LOC101455432.1.1	A/C	SNP	32
		A/G	SNP	396
		C/T	SNP	414
		G/A	SNP	510
		T/C	SNP	1528
		T/C	SNP	1541
		A/AT	INSERTION	2987

		A/AT	INSERTION	2998
		AT/A	DELETION	3023
		T/A	SNP	3027
		T/A	SNP	3091
		A/G	SNP	3112
XP_004530363.1 PRED: ATP-binding cassette sub-familyG member 1-like isoform X1 [C. capitata] ref|XP_004530364.1| PRED: ATP-binding cassette sub-family G member 1-like isoform X2 [C. capitata] ref|XP_004530365.1| PRED: ATP-binding cassette sub-family G member 1-like isoform X3 [C. capitata]	GLOS_LOC101455574.1.1	G/A	SNP	272
		A/T	SNP	274
		T/C	SNP	294
		A/G	SNP	723
		G/T	SNP	798
		C/T	SNP	851
		T/C	SNP	858
		A/T	SNP	943
		G/C	SNP	972
		TCCG/T	DELETION	991
		G/A	SNP	1033
		T/C	SNP	1143
		A/G	SNP	1146
		A/G	SNP	1260
		G/T	SNP	1323
		C/T	SNP	1527
		T/G	SNP	1731
		C/T	SNP	1740
		T/C	SNP	1764
		A/T	SNP	1806
		G/C	SNP	1815
		C/T	SNP	1824
		A/T	SNP	1994
		C/A	SNP	2024
XP_004520096.1 PREDICTED: serine protease SP24D-like [Ceratitis capitata]	GLOS_LOC101455604.4.10	T/A	SNP	690
		T/G	SNP	691
		T/C	SNP	787
		C/T	SNP	873

		T/TG	INSERTION	890
		T/G	SNP	891
		C/T	SNP	892
XP_004520096.1 PREDICTED: serine protease SP24D-like [Ceratitis capitata]	GLOS_LOC101455604.5.10	G/T	SNP	3387
		T/C	SNP	3452
		C/A	SNP	3555
		A/AT	INSERTION	3702
		T/C	SNP	3907
		T/C	SNP	3972
		C/CGAT	INSERTION	3976
		C/G	SNP	3977
		A/T	SNP	3978
		T/C	SNP	4114
		T/TA	INSERTION	4194
		G/GAT	INSERTION	4197
		G/T	SNP	4197
		C/G	SNP	4199
XP_004527398.1 PRED: synaptic vesicle glycoprotein 2A-like isoform X2 [C.capitata]	GLOS_LOC101455624.1.2	G/C	SNP	1520
		A/T	SNP	1543
		A/T	SNP	1420
		TA/T	DELETION	1427
XP_004521777.1 PREDICTED: CD109 antigen-like isoform X5 [Ceratitis capitata]	GLOS_LOC101455841.1.3	G/A	SNP	23
		T/C	SNP	113
		C/T	SNP	218
		C/T	SNP	236
		A/G	SNP	250
		C/T	SNP	263
		G/A/T	SNP	272
		A/G	SNP	286
		A/T	SNP	389
		C/T	SNP	420
		T/C	SNP	422
		A/G	SNP	452
		G/T	SNP	467
		A/G	SNP	489
		C/A	SNP	585

C/A	SNP	659
G/C	SNP	665
T/G	SNP	860
T/C	SNP	982
G/A	SNP	1025
C/T	SNP	1057
C/T	SNP	1094
G/A	SNP	1131
T/C	SNP	1170
A/T	SNP	1300
G/A	SNP	1372
C/T	SNP	1405
T/G	SNP	1494
T/C	SNP	1532
C/T	SNP	1562
A/G	SNP	1867
G/A	SNP	1886
G/A	SNP	2074
G/A	SNP	2097
G/A	SNP	2138
G/T	SNP	2143
A/G	SNP	2151
G/A	SNP	2240
A/T	SNP	2246
C/T	SNP	2262
C/T	SNP	2342
A/G	SNP	2349
T/A	SNP	2430
G/A	SNP	2462
T/C	SNP	2473
A/G	SNP	2517
C/T	SNP	2711
C/T	SNP	2744
C/T	SNP	2825
T/C	SNP	2861
A/G	SNP	2870

		T/C	SNP	2873
		T/C	SNP	2951
		G/A	SNP	2971
		G/A	SNP	2974
		C/T	SNP	2999
		T/C	SNP	3047
		C/T	SNP	3074
		C/T	SNP	3131
		T/C	SNP	3152
		A/G	SNP	3251
		A/G	SNP	3341
XP_004520628.1 PRED: uncharacter. Prot. LOC101456017 isoform X1 [C. capitata]	GLOS_LOC101456017.2.4	G/GT	INSERTION	115
		C/A	SNP	161
		G/C	SNP	169
		G/A	SNP	172
		T/A	SNP	269
		T/A	SNP	283
		C/T	SNP	302
XP_004520628.1 PRED: uncharact. protein LOC101456017 isoform X1 [C. capitata]	GLOS_LOC101456017.3.4	G/A	SNP	20
		C/CA	INSERTION	338
		T/C	SNP	393
		C/CAT	INSERTION	978
		A/G	SNP	1049
		C/T	SNP	1088
		A/G	SNP	1199
XP_004524646.1 PRED: retinoid-inducible serine carboxypeptidase-like [C. capitata]	GLOS_LOC101456159.4.4	A/G	SNP	383
		T/C	SNP	388
		G/T	SNP	550
		T/A	SNP	552
		C/A	SNP	553
		G/C	SNP	554
		A/T	SNP	1045
		T/G	SNP	1830
		A/G	SNP	1831
		C/G	SNP	1874
XP_004529473.1 PREDICTED: protein FAM188A homolog [Ceratitis capitata]	GLOS_LOC101456285.1.1	T/G	SNP	1212

		C/T	SNP	1230
		A/G	SNP	1252
		A/C	SNP	1366
		G/A	SNP	1371
		G/A	SNP	1444
		T/C	SNP	2099
		T/TG	INSERTION	2106
		G/A	SNP	2507
		A/C	SNP	4054
		T/A	SNP	4055
		T/A	SNP	4473
		C/T	SNP	4492
XP_004523400.1 PREDICTED: endoplasmin-like [Ceratitis capitata]	GLOS_LOC101456384.1.1	G/A	SNP	81
		T/G	SNP	196
		G/A	SNP	209
		T/A	SNP	210
		A/G	SNP	229
		A/C	SNP	237
		G/GAA/GA	INSERTION	248
		A/AAAG	INSERTION	251
		C/CAT	INSERTION	279
		A/G	SNP	559
		T/C	SNP	745
		T/C	SNP	748
		G/A	SNP	967
		C/T	SNP	994
		G/C	SNP	1087
		G/A	SNP	1204
		T/A	SNP	1360
		G/A	SNP	1414
		G/A	SNP	2046
		T/C	SNP	2094
		A/G	SNP	2134
		T/G	SNP	2158
		G/A	SNP	2220
		C/T	SNP	2347

		A/T	SNP	2377
		A/G	SNP	2389
		C/T	SNP	2644
		C/T	SNP	2689
		G/T	SNP	2946
		G/C	SNP	2951
		T/A	SNP	2954
		G/T	SNP	2956
		G/A	SNP	2957
XP_004525823.1 PREDICTED: fibroin heavy chain-like [Ceratitis capitata]	GLOS_LOC101456392.1.1	T/C	SNP	1530
		A/G	SNP	1806
		C/T	SNP	2137
		A/G	SNP	2358
		A/C	SNP	2554
		G/A	SNP	2637
		A/G	SNP	2880
		T/A	SNP	3189
		C/A	SNP	3191
		T/C	SNP	3236
		T/C	SNP	4009
		C/T	SNP	4125
		T/C	SNP	4232
		G/A	SNP	4509
		A/C	SNP	4533
		G/C	SNP	4834
XP_004535276.1 PREDICTED: inner centromere protein-like isoform X1 [C. capitata]	GLOS_LOC101456419.1.1	C/T	SNP	73
		A/T	SNP	76
		CA/C	DELETION	390
		A/G	SNP	590
		G/A	SNP	1175
		C/T	SNP	1695
		G/A	SNP	1727
		C/T	SNP	1746
		G/C	SNP	1825
		A/G	SNP	2078
		G/A	SNP	2097

		A/T	SNP	2165
		A/C	SNP	2201
		A/G	SNP	2329
		C/T	SNP	2387
		G/A	SNP	2684
		T/C	SNP	2956
		C/A	SNP	3075
		G/A	SNP	3076
		C/T	SNP	3079
		T/TA	INSERTION	3550
XP_004520728.1 PREDICTED: probable salivary secreted peptide-like [C. capitata]	GLOS_LOC101456499.1.1	T/C	SNP	88
		G/A	SNP	160
		C/T	SNP	201
		C/T	SNP	407
		A/T	SNP	419
XM_004521817.1 PRED: C. c. 60S ribosoml prot. L27a-like (LOC101456617),mRNA	GLOS_LOC101456617.4.5	C/T	SNP	34
		C/G	SNP	837
XP_004534236.1 PREDICTED: uncharacterized protein LOC101456777 [C. capitata]	GLOS_LOC101456777.3.6	C/T	SNP	306
		T/A	SNP	317
		T/G	SNP	345
		G/C	SNP	347
		T/C	SNP	348
		A/T	SNP	351
		T/G	SNP	435
		T/C	SNP	492
		C/A	SNP	493
		G/A	SNP	495
		T/G	SNP	496
		C/T	SNP	664
		C/A	SNP	709
		A/G	SNP	710
		TC/T	DELETION	805
		T/A	SNP	864
		A/T	SNP	869
		T/A	SNP	880
		T/A	SNP	881

XP_004536181.1 PREDICTED: UHRF1-binding protein 1-like isoform X2 [C. capitata]	GLOS_LOC101456970.1.1	A/C	SNP	541
		G/GT	INSERTION	937
		C/G	SNP	1940
		TA/T	DELETION	1944
		T/C	SNP	1984
		A/G	SNP	2017
		G/GT	INSERTION	2287
		G/C	SNP	2307
		T/C	SNP	2352
		T/C	SNP	2465
		T/C	SNP	2850
		A/G	SNP	3036
		G/A	SNP	3192
		C/T	SNP	3251
		A/G	SNP	3255
		A/G	SNP	3413
		T/C	SNP	3447
		T/C	SNP	3505
		G/A	SNP	3507
		T/A	SNP	3555
		G/A	SNP	3573
		T/C	SNP	3597
		T/C	SNP	3648
		T/C	SNP	3711
		G/C	SNP	4350
		C/T	SNP	4459
		G/T	SNP	4700
		T/C	SNP	5004
		T/C	SNP	5043
		A/G	SNP	5304
		G/T	SNP	5372
		G/A	SNP	5403
		C/A	SNP	5475
		C/G	SNP	5505
		A/G	SNP	5694
		A/G	SNP	5730

		A/C	SNP	5784
		C/T	SNP	5835
		G/A	SNP	6086
		G/A	SNP	6264
		G/A	SNP	6435
XP_004523041.1 PRED: phosphate-regulating neutral endopeptidase-like C.capitata]	GLOS_LOC101457066.1.1	T/G	SNP	77
		T/C	SNP	99
		C/T	SNP	127
		G/A	SNP	185
		G/A	SNP	367
		T/C	SNP	394
		C/A	SNP	449
		C/T	SNP	475
		G/A	SNP	634
		C/A	SNP	685
		G/A	SNP	703
		G/A	SNP	727
		A/G	SNP	739
		G/A	SNP	781
		A/T	SNP	1033
		C/T	SNP	1117
		T/C	SNP	1153
		G/A	SNP	1180
		C/T	SNP	1309
		A/G	SNP	1432
		C/T	SNP	1449
		C/T	SNP	1459
		C/G	SNP	1474
		C/T	SNP	1480
		A/C	SNP	1507
		T/C	SNP	1510
		C/T	SNP	1534
		A/G	SNP	1591
		T/C	SNP	1663
		T/C	SNP	1675
		A/G	SNP	1795

		G/A	SNP	1837
		A/G	SNP	2077
		G/A	SNP	2085
		G/A	SNP	2087
		C/A	SNP	2147
XP_004525658.1 PREDICTED: heat shock 70 kDa protein 14-like [Ceratitis capitata]	GLOS_LOC101457078.1.1	G/T	SNP	100
		A/T	SNP	532
		T/C	SNP	562
		G/A	SNP	563
		C/T	SNP	599
		A/G	SNP	974
		C/T	SNP	1098
		T/G	SNP	1110
		T/C	SNP	1157
		A/C	SNP	1199
		A/T	SNP	1208
		G/A	SNP	1229
		T/C	SNP	1265
		T/C	SNP	1292
		A/G	SNP	1409
		G/T	SNP	1431
		C/T	SNP	1433
		A/G	SNP	1436
		G/A	SNP	1561
		T/A	SNP	1607
		G/A	SNP	1608
		C/CA	INSERTION	1642
		C/T	SNP	1661
		G/A	SNP	1670
		A/G	SNP	1691
XP_004519837.1 PREDICTED: laccase-2-like [Ceratitis capitata]	GLOS_LOC101457181.1.5	T/C	SNP	559
		A/G	SNP	570
		T/C	SNP	574
		C/A	SNP	1393
		T/C	SNP	1522
		T/C	SNP	2275

		A/T	SNP	2329
		T/C	SNP	2744
		C/A	SNP	4243
		A/T	SNP	4244
		G/A	SNP	4246
		C/T	SNP	4247
XP_004519837.1 PREDICTED: laccase-2-like [Ceratitis capitata]	GLOS_LOC101457181.2.5	A/C	SNP	127
		C/T	SNP	1102
		G/C	SNP	1121
		T/C	SNP	1221
		C/A	SNP	1539
		T/C	SNP	4375
		G/A	SNP	4517
		G/A	SNP	4519
		T/A	SNP	4520
		G/A	SNP	4529
		T/A	SNP	4531
XP_004519837.1 PREDICTED: laccase-2-like [Ceratitis capitata]	GLOS_LOC101457181.3.5	G/A	SNP	273
		T/C	SNP	351
		G/A	SNP	410
		A/T	SNP	437
		G/A	SNP	441
		C/T	SNP	459
		T/A	SNP	518
		T/C	SNP	524
		G/A	SNP	2857
		G/T	SNP	2956
		T/A	SNP	3275
		T/A	SNP	3276
		T/G	SNP	3277
		A/C	SNP	3278
		A/AT	INSERTION	3281
XP_004519837.1 PREDICTED: laccase-2-like [Ceratitis capitata]	GLOS_LOC101457181.4.5	A/G	SNP	33
		T/C	SNP	688
		C/T	SNP	944
XP_004519837.1 PREDICTED: laccase-2-like [Ceratitis capitata]	GLOS_LOC101457181.5.5	A/G	SNP	685

		A/G	SNP	813
		A/G	SNP	849
		C/T	SNP	948
		T/C	SNP	1397
		C/T	SNP	1690
		A/G	SNP	1880
		A/C	SNP	2186
		A/T	SNP	2694
		A/G	SNP	2862
		T/G	SNP	3017
XP_004521782.1 PREDICTED: sodium- and chloride-dependent neutral and basic amino acid transporter B(0+)-like [Ceratitis capitata]	GLOS_LOC101457248.1.1	C/A	SNP	103
		A/T	SNP	180
		T/C	SNP	489
		G/C	SNP	702
		G/A	SNP	780
		T/C	SNP	1087
		G/A	SNP	1554
		A/G	SNP	1611
		C/T	SNP	1668
		A/T	SNP	1842
		A/G	SNP	1850
		G/C	SNP	1851
		A/C	SNP	1951
		A/G	SNP	1971
		A/G	SNP	2145
XP_004534337.1 PREDICTED: acyl-CoA Delta(11) desaturase-like [C. capitata]	GLOS_LOC101457705.1.2	G/GT/GTTT	INSERTION	122
		C/T	SNP	1045
		G/A	SNP	1521
		C/A	SNP	1523
		C/T	SNP	1689
		G/A	SNP	1998
		G/C	SNP	2048
		T/C	SNP	2074
		G/A	SNP	2118
		T/C	SNP	2155

		A/G	SNP	2220
		C/T	SNP	2450
		G/C	SNP	2571
		A/G	SNP	2592
		G/C	SNP	2610
		C/T	SNP	2618
		C/G	SNP	2625
		G/A	SNP	2810
		C/T	SNP	2868
XP_004534337.1 PREDICTED: acyl-CoA Delta(11) desaturase-like [C. capitata]	GLOS_LOC101457705.2.2	C/CA	INSERTION	1078
		G/A	SNP	1091
		A/G	SNP	1153
XP_004530636.1 PRED: bifunctional purine biosynthesis prot. PURH-like isoform X1 [C. capitata] ref|XP_004530637.1| PRED: bifunctional purine biosynthesis protein PURH-like isoform X2 [C. capitata] ref|XP_004530638.1| PRED: bifunctional purine biosynthesis protein PURH-like isoform X3 [C. capitata]	GLOS_LOC101457813.1.1	G/A	SNP	49
		AAC/A	DELETION	101
		TA/T	DELETION	111
		T/C	SNP	144
		C/T	SNP	168
		A/G	SNP	213
		T/C	SNP	234
		C/T	SNP	273
		A/C	SNP	288
		T/C	SNP	342
		G/C	SNP	435
		G/C	SNP	468
		A/G	SNP	513
		A/G	SNP	531
		T/A	SNP	549
		G/A	SNP	579
		T/G	SNP	583
		G/A	SNP	702
		C/T	SNP	741
		C/T	SNP	771
		A/G	SNP	786

		A/G	SNP	795
		C/T	SNP	800
		G/A/C	SNP	843
		T/C	SNP	930
		A/G	SNP	945
		A/G	SNP	1056
		G/A	SNP	1185
		T/C	SNP	1248
		G/T	SNP	1269
		T/C	SNP	1287
		C/CA	INSERTION	1328
		T/C	SNP	1530
		T/C	SNP	1629
		G/C	SNP	1903
		C/T	SNP	1922
XP_004518274.1 PREDICTED: serine protease easter-like [Ceratitis capitata]	GLOS_LOC101457953.1.5	C/A	SNP	29
		T/C	SNP	85
		C/G	SNP	95
		G/T	SNP	128
		C/T	SNP	190
		T/C	SNP	195
		C/A	SNP	205
		G/A	SNP	233
XP_004518274.1 PREDICTED: serine protease easter-like [Ceratitis capitata]	GLOS_LOC101457953.2.5	T/C	SNP	332
		T/C	SNP	778
		G/A	SNP	914
		C/T	SNP	932
		A/C	SNP	984
		C/A	SNP	1054
		G/A	SNP	1058
		G/A	SNP	1064
		CCTG/C	DELETION	1077
		A/G	SNP	1115
		A/G	SNP	1126
		A/G	SNP	1143
		T/A	SNP	1217

		C/A	SNP	1218
		G/C	SNP	1435
		T/C	SNP	1437
		C/A	SNP	1472
		G/A	SNP	1509
		T/G	SNP	1522
		A/G	SNP	1565
		A/T	SNP	1567
		C/A	SNP	1578
		A/G	SNP	1585
		A/G	SNP	1593
		A/T	SNP	1643
		C/G	SNP	1667
		G/A	SNP	1682
		A/G	SNP	1690
		C/G	SNP	1705
		A/C	SNP	1707
		C/G	SNP	1739
		C/G	SNP	1752
		A/G	SNP	1796
		C/T	SNP	1809
		C/T	SNP	1834
		T/G	SNP	1883
		T/C	SNP	2009
XP_004518274.1 PREDICTED: serine protease easter-like [Ceratitis capitata]	GLOS_LOC101457953.3.5	T/C	SNP	223
		T/C	SNP	301
		A/T	SNP	310
		T/G	SNP	377
		G/A	SNP	397
		T/C	SNP	453
		T/G	SNP	477
		A/G	SNP	524
		G/A	SNP	538
		A/G	SNP	634
		A/G	SNP	640
		C/T	SNP	768

		A/G	SNP	772
		C/G	SNP	842
		T/C	SNP	905
		G/A	SNP	916
		A/T	SNP	920
		A/G	SNP	953
		A/T	SNP	1077
		A/C	SNP	1190
		T/C	SNP	1202
		G/A	SNP	1242
XP_004518274.1 PREDICTED: serine protease easter-like [Ceratitis capitata]	GLOS_LOC101457953.5.5	T/C	SNP	493
		A/G	SNP	516
		T/C	SNP	525
XP_004521696.1 PREDICTED: uncharacterized protein LOC101458193 [C. capitata]	GLOS_LOC101458193.1.1	G/T	SNP	276
		G/A	SNP	277
		A/T	SNP	290
		G/A	SNP	292
		C/T	SNP	304
		T/C	SNP	328
		T/C	SNP	345
		A/G	SNP	364
		A/T	SNP	405
		A/G	SNP	515
		C/T	SNP	625
		G/A	SNP	649
		G/C	SNP	663
		A/G	SNP	744
		A/G	SNP	772
		A/G	SNP	832
XP_004519269.1 PRED: uncharact. prot. LOC101458246 isoform X2 [C. capitata]	GLOS_LOC101458246.1.1	A/AAAG	INSERTION	200
		GA/G	DELETION	218
		G/A	SNP	309
		T/C	SNP	453
		C/A	SNP	667
		A/G	SNP	675
		G/A	SNP	840

		C/T	SNP	1131
		G/A	SNP	1161
		G/A	SNP	1167
		G/T	SNP	1189
		C/G	SNP	1424
		A/G	SNP	1441
		G/A	SNP	1449
		A/G	SNP	1584
		G/A	SNP	1729
		G/A	SNP	1776
XP_004533341.1 PRED:ATP-binding cassette sub-familyG member 1-like isoform X1 [C. capitata] ref|XP_004533342.1| PRED: ATP-binding cassette sub-family G member 1-like isoform X2 [C. capitata]	GLOS_LOC101458352.1.1	C/A	SNP	18
		T/G	SNP	92
		T/A	SNP	102
		A/T	SNP	195
		G/A	SNP	205
		A/G	SNP	253
		A/G	SNP	342
		A/G	SNP	355
		G/A	SNP	679
		C/T	SNP	763
		A/G	SNP	955
		T/G	SNP	1384
		T/C	SNP	1753
		G/A	SNP	1813
		C/A	SNP	2326
		G/A	SNP	2770
		G/T	SNP	2917
XP_004535088.1 PREDICTED: inositol polyphosphate multikinase-like [C. capitata]	GLOS_LOC101458418.1.1	T/A	SNP	32
		G/A	SNP	74
		T/C	SNP	339
		C/A	SNP	431
		A/G	SNP	1197
XP_004520550.1 PRED: uncharact. protein LOC101458435 isoform X2 [C. capitata]	GLOS_LOC101458435.1.2	A/G	SNP	66
		T/A	SNP	249

		A/G	SNP	262
		G/A	SNP	281
		G/A	SNP	3235
		T/C	SNP	3354
		C/T	SNP	3810
		A/T	SNP	4011
		C/T	SNP	4085
		T/C	SNP	4143
		C/T	SNP	4383
		T/C	SNP	4425
		G/A	SNP	4454
		A/G	SNP	4689
		T/C	SNP	4764
		A/T	SNP	4771
		T/G	SNP	4790
		G/A	SNP	5628
		T/C	SNP	5658
		T/G	SNP	5782
		G/T	SNP	5970
		C/A	SNP	6147
		A/G	SNP	6279
		C/A	SNP	7164
		T/C	SNP	7254
		T/C	SNP	7278
		A/T	SNP	7579
		T/A	SNP	7611
		T/A	SNP	7616
		T/A	SNP	7881
		G/T	SNP	8372
		C/T	SNP	8412
		G/A	SNP	8416
		A/T	SNP	8431
		C/T	SNP	8438
		A/G	SNP	8611
XP_004520550.1 PRED: uncharact. protein LOC101458435 isoform X2 [C. capitata]	GLOS_LOC101458435.2.2	C/A	SNP	321
		G/GAT	INSERTION	322

		T/C	SNP	323
		G/C	SNP	324
		T/G	SNP	325
		A/G	SNP	326
		A/T	SNP	327
		A/T	SNP	399
		T/TG	INSERTION	1016
		T/A	SNP	1035
		C/G	SNP	3725
		T/A	SNP	3726
		C/A	SNP	10012
		A/T	SNP	10427
		G/A	SNP	10747
		G/C	SNP	10757
		G/A	SNP	10787
		A/T	SNP	10809
		T/G	SNP	10847
		T/A	SNP	10854
		T/A	SNP	10869
XP_004526962.1 PREDICTED: pyridoxal kinase-like [Ceratitis capitata]	GLOS_LOC101458811.1.1	C/T	SNP	53
		A/G	SNP	54
		C/A	SNP	56
		A/G	SNP	114
		G/A	SNP	210
		A/C	SNP	300
		A/G	SNP	539
		C/T	SNP	560
		T/C	SNP	710
		T/G	SNP	800
		C/T	SNP	836
XP_004527418.1 PREDICTED: type I inositol 3,4-bisphosphate 4-phosphatase-like isoform X4 [Ceratitis capitata]	GLOS_LOC101458989.1.1	A/T	SNP	218
		T/G	SNP	220
		TA/T	DELETION	641
		T/G	SNP	883
		C/T	SNP	924

		A/T	SNP	927
		G/A	SNP	1155
		TG/T	DELETION	1226
		G/T	SNP	1229
		C/T	SNP	1256
		T/G	SNP	1562
		A/G	SNP	1564
		A/G	SNP	1620
		T/TA	INSERTION	1678
		T/C	SNP	2218
		C/T	SNP	2371
		G/GGTGA	INSERTION	2420
		A/G	SNP	2421
		T/G	SNP	2424
		G/A	SNP	2623
		G/A	SNP	2866
		A/T	SNP	3376
		G/A	SNP	3879
		G/A	SNP	4285
		C/T	SNP	4510
		G/T	SNP	4657
		C/T	SNP	4798
		A/C	SNP	4838
		C/T	SNP	4903
		T/A	SNP	4990
		C/T	SNP	5087
		G/A/T	SNP	5128
		G/A	SNP	5444
		T/TA	INSERTION	5881
		A/G	SNP	5926
XP_004530196.1 PREDICTED: protein yellow-like [Ceratitis capitata]	GLOS_LOC101458997.1.1	G/T	SNP	29
		C/T	SNP	133
		A/C	SNP	163
		A/G	SNP	178
		G/A	SNP	274
		C/T	SNP	563

		T/C	SNP	588
		T/C	SNP	769
		T/C	SNP	1027
		A/C	SNP	1070
		T/C	SNP	1165
		T/G	SNP	1372
		A/G	SNP	1378
		C/T	SNP	1469
XP_004533346.1 PRED: ATP-binding cassette sub-familyG memb. 1-like isoform X1 [C. capitata] ref|XP_004533347.1| PRED: ATP-binding cassette sub-family G member 1-like isoform X2 [C. capitata]	GLOS_LOC101459058.1.1	G/T	SNP	48
		G/C	SNP	55
		G/T	SNP	56
		T/C	SNP	142
		A/T	SNP	150
		T/C	SNP	270
		T/G	SNP	345
		A/G	SNP	420
		T/C	SNP	521
		G/A	SNP	551
		G/A	SNP	593
		A/G	SNP	857
		C/T	SNP	920
		A/G	SNP	1085
		G/A	SNP	1289
		T/C	SNP	1388
		T/C	SNP	1409
		G/A	SNP	1455
		T/C	SNP	1460
		C/T	SNP	1763
		G/C	SNP	1949
		A/C	SNP	2144
		C/A	SNP	2201
		A/G	SNP	2237
		C/T	SNP	2321
		T/C	SNP	2546

		A/T	SNP	2557
		A/G	SNP	2558
		T/A	SNP	2568
		A/T	SNP	2610
		A/C	SNP	2611
		G/T	SNP	2624
		C/T	SNP	2740
		C/A	SNP	2757
		A/G	SNP	2797
		A/G	SNP	2821
		G/C	SNP	2822
		G/A	SNP	2863
		A/C	SNP	2888
		C/T	SNP	2953
		T/A	SNP	2973
XP_004526285.1 PRED: cytosolic non-specific dipeptidase-like isoform X1 [C. capitata] ref|XP_004526286.1| PRED: cytosolic non-specific dipeptidase-like isoform X2 [C. capitata] ref|XP_004526287.1| PRED: cytosolic non-specific dipeptidase-like isoform X3 [C. capitata]	GLOS_LOC101459287.4.5	C/T	SNP	79
		A/C	SNP	164
		A/G	SNP	165
		T/C	SNP	201
XP_004525673.1 PREDICTED: gamma-glutamyl hydrolase-like [Ceratitis capitata]	GLOS_LOC101459395.1.1	T/A	SNP	359
		A/ATG	INSERTION	439
		G/A	SNP	527
		C/T	SNP	601
		G/GTT	INSERTION	608
		G/T	SNP	608
		G/T	SNP	634
		GT/G	DELETION	634
		A/T	SNP	880
		A/G	SNP	902
		C/T	SNP	1228
		G/C	SNP	1262
		C/T	SNP	1286
		T/C	SNP	1364

		A/G	SNP	1406
		C/T	SNP	1454
		C/G	SNP	1632
		C/T	SNP	1639
XP_004533256.1 PREDICTED: zinc carboxypeptidase A 1-like [Ceratitis capitata]	GLOS_LOC101459419.1.2	G/GCCT	INSERTION	1357
		C/A	SNP	1364
		C/A	SNP	1366
		C/A	SNP	1389
		T/A	SNP	1480
		T/A	SNP	1576
		T/C	SNP	1580
		A/G	SNP	1581
		T/C	SNP	1703
		A/C	SNP	1704
		G/T	SNP	1707
		T/C	SNP	1710
		T/C	SNP	1913
		T/G	SNP	2284
XP_004537835.1 PREDICTED: zinc metalloproteinase nas-4-like [Ceratitis capitata]	GLOS_LOC101459622.10.22	G/C	SNP	175
		T/C	SNP	340
		C/G	SNP	342
		A/C	SNP	581
XP_004537835.1 PREDICTED: zinc metalloproteinase nas-4-like [Ceratitis capitata]	GLOS_LOC101459622.13.22	T/A	SNP	48
		T/C	SNP	69
		T/A/G	SNP	416
		T/A/G	SNP	572
		G/A	SNP	573
		G/C	SNP	610
		C/T	SNP	629
		C/T	SNP	638
		A/G	SNP	724
		A/G	SNP	725
		C/T	SNP	994
		G/A	SNP	1050
		G/T	SNP	1079
		C/T	SNP	2061

		T/G	SNP	3054
		T/A	SNP	3055
		T/C	SNP	3122
		G/T	SNP	3179
		C/T	SNP	3196
		C/G	SNP	3321
		T/A	SNP	3888
		G/A	SNP	3911
		T/A	SNP	3919
XP_004537835.1 PREDICTED: zinc metalloproteinase nas-4-like [Ceratitis capitata]	GLOS_LOC101459622.15.22	G/C	SNP	1879
		G/T	SNP	3644
		T/A	SNP	3645
		T/C	SNP	3646
		G/A	SNP	3656
		A/T	SNP	3926
		T/G	SNP	3929
		C/A	SNP	3930
		C/T	SNP	3933
		CA/C	DELETION	3936
		G/C	SNP	4045
		C/A	SNP	4048
		C/A	SNP	4049
		A/C	SNP	4165
XP_004537835.1 PREDICTED: zinc metalloproteinase nas-4-like [Ceratitis capitata]	GLOS_LOC101459622.16.22	G/A	SNP	65
		G/C	SNP	130
		A/G	SNP	197
		G/C	SNP	200
		C/G	SNP	201
		G/A	SNP	306
		A/AAAG/A(	INSERTION	307
		A/G	SNP	307
		G/C	SNP	310
		T/A	SNP	314
		A/G	SNP	315
		T/A	SNP	316
		A/G	SNP	357

		G/T	SNP	358
		G/C	SNP	384
		T/A	SNP	387
		A/G	SNP	490
		TG/T	DELETION	548
		G/GC	INSERTION	551
		T/TCA	INSERTION	552
		T/A	SNP	555
		T/G	SNP	620
		T/A	SNP	761
		C/T	SNP	762
XP_004537835.1 PREDICTED: zinc metalloproteinase nas-4-like [Ceratitis capitata]	GLOS_LOC101459622.18.22	C/T	SNP	77
		A/C	SNP	251
		C/G	SNP	252
XP_004537835.1 PREDICTED: zinc metalloproteinase nas-4-like [Ceratitis capitata]	GLOS_LOC101459622.19.22	A/G	SNP	898
		G/A	SNP	899
		G/A	SNP	1021
		G/A	SNP	1377
		G/A	SNP	1386
		G/C	SNP	1808
		A/C	SNP	1810
		G/A	SNP	1943
		C/G	SNP	1969
XP_004537835.1 PREDICTED: zinc metalloproteinase nas-4-like [Ceratitis capitata]	GLOS_LOC101459622.21.22	A/C	SNP	130
		T/G	SNP	192
		A/G	SNP	194
		A/G	SNP	261
		G/A	SNP	268
		G/T	SNP	316
		T/A	SNP	320
		A/C	SNP	322
		T/C	SNP	323
		A/T	SNP	324
		A/G	SNP	424
		A/G	SNP	426
		G/T	SNP	431

		C/G	SNP	432
		C/A	SNP	433
		G/GA	INSERTION	434
		G/C	SNP	436
		C/G	SNP	438
		T/A	SNP	440
		T/A	SNP	646
		G/T	SNP	648
		C/T	SNP	743
		C/T	SNP	752
		T/C	SNP	824
		A/G	SNP	835
		C/T	SNP	854
XP_004537835.1 PREDICTED: zinc metalloproteinase nas-4-like [Ceratitis capitata]	GLOS_LOC101459622.5.22	A/C	SNP	122
		A/C	SNP	185
		A/G	SNP	596
		G/A	SNP	603
		A/T	SNP	660
		T/TA	INSERTION	743
		A/T	SNP	746
		T/G	SNP	899
		C/T	SNP	908
		G/T	SNP	926
		A/G	SNP	1480
		T/G	SNP	1943
XP_004537835.1 PREDICTED: zinc metalloproteinase nas-4-like [Ceratitis capitata]	GLOS_LOC101459622.6.22	G/T	SNP	3789
		A/C	SNP	3791
		A/G	SNP	3793
		T/TC	INSERTION	3795
		G/T	SNP	3796
		TC/T	DELETION	3897
		C/G	SNP	3902
		C/T	SNP	3904
		A/G	SNP	3906
		A/G	SNP	3907
		G/T	SNP	3991

		C/A	SNP	3995
		CGA/C	DELETION	3997
		CA/C	DELETION	4077
		C/T	SNP	4080
		C/G	SNP	4081
		C/A	SNP	4084
		A/T	SNP	4085
		A/C	SNP	4086
		T/C	SNP	4210
		A/G	SNP	4212
		A/ATCG	INSERTION	4213
		A/ATC	INSERTION	4215
		A/G	SNP	4216
		C/A	SNP	4219
		A/C	SNP	4708
XP_004537835.1 PREDICTED: zinc metalloproteinase nas-4-like [Ceratitis capitata]	GLOS_LOC101459622.9.22	C/A	SNP	2258
		G/C	SNP	2577
		A/T	SNP	2578
		G/C	SNP	2581
		A/AT	INSERTION	2583
		A/T	SNP	2585
		C/A	SNP	2675
		G/A	SNP	2720
		C/A	SNP	2839
		C/T	SNP	3325
		T/C	SNP	3332
XP_004518098.1 PRED: exonuclease 3'-5' domain-containing prot. 2-like [C capitata]	GLOS_LOC101459623.1.5	G/A	SNP	132
		A/G	SNP	186
		C/T	SNP	212
		C/T	SNP	226
		G/C	SNP	283
		C/T	SNP	477
		G/A	SNP	698
		G/A	SNP	805
		C/A	SNP	979
		T/C	SNP	986

		C/T	SNP	1034
		T/C	SNP	1176
		G/A	SNP	1195
		G/T	SNP	1203
		C/T	SNP	1295
		C/T	SNP	1379
		T/C	SNP	1762
XP_004520747.1 PREDICTED: probable 3-hydroxyisobutyrate dehydrogenase, mitochondrial-like [Ceratitis capitata]	GLOS_LOC101459752.1.1	ACATGG/A	DELETION	311
		TG/T	DELETION	314
		G/T	SNP	320
		A/G	SNP	1008
		T/C	SNP	1242
		T/A	SNP	1269
XP_004526015.1 PREDICTED: alaserpin-like [Ceratitis capitata]	GLOS_LOC101459846.1.2	A/G	SNP	94
		A/G	SNP	140
		A/C	SNP	815
		G/A	SNP	1031
		G/A	SNP	1301
		A/G	SNP	1411
XP_004527242.1 PRED: uncharact. prot. LOC101459847 isoform X1 [C. capitata] ref|XP_004527243.1| PRED: uncharact. prot. LOC101459847 isoform X2 [C.c.] ref|XP_004527244.1| PRED: uncharact. prot. LOC101459847 isoform X3 [C.c] ref|XP_004527245.1| PRED: uncharact. prot. LOC101459847 isoform X4 [C.c.]	GLOS_LOC101459847.1.2	A/AT	INSERTION	222
		C/T	SNP	467
		T/A	SNP	1122
		G/A	SNP	1123
		T/A	SNP	1129
		C/T	SNP	1131
XP_004536554.1 PREDICTED: uncharacterized protein LOC101460221 [C. capitata]	GLOS_LOC101460221.1.1	C/T	SNP	293
		C/T	SNP	434
		C/T	SNP	500
		A/T	SNP	608
		A/G	SNP	673
		G/A	SNP	690
		A/G	SNP	741

	A/T	SNP	748
	C/G	SNP	871
	G/A	SNP	993
	C/G	SNP	1064
	C/CT	INSERTION	1159
	G/A	SNP	1190
	T/C	SNP	1192
	A/G	SNP	1465
	GA/G	DELETION	1782
XP_004531213.1 PRED: collagen alpha-1(IV) chain-like isoform X1 [C. capitata] ref|XP_ GLOS_LOC101460389.1.1	T/C	SNP	130
	A/G	SNP	204
	C/T	SNP	354
	T/C	SNP	399
	C/T	SNP	561
	A/G	SNP	750
	T/C	SNP	822
	C/T	SNP	1149
	T/C	SNP	1467
	C/T	SNP	1920
	T/C	SNP	2178
	G/A	SNP	2218
	A/T	SNP	2394
	T/A	SNP	2511
	A/G	SNP	3024
	A/G	SNP	3288
	T/C	SNP	3465
	T/C	SNP	3636
	T/C	SNP	3690
	C/T	SNP	3852
	T/C	SNP	3867
	T/C	SNP	3920
	T/C	SNP	3944
	A/G	SNP	4236
	C/T	SNP	4330
	T/C	SNP	4401
	G/T	SNP	4458

		A/G	SNP	4614
		G/GT	INSERTION	5515
		A/G	SNP	5582
XP_004537752.1 PRED.: methylenetetrahydrofolate reductase-like [C. capitata]	GLOS_LOC101460408.1.1	A/G	SNP	256
		C/T	SNP	1661
		G/A	SNP	1788
		C/T	SNP	1953
		CT/C	DELETION	2243
		A/G	SNP	2325
		C/T	SNP	2577
XP_004537383.1 PREDICTED: uncharacterized protein LOC101460522 [C. capitata]	GLOS_LOC101460522.2.3	C/T	SNP	1243
		A/G	SNP	1279
		A/T	SNP	1280
		T/C	SNP	1318
		C/T	SNP	1364
		C/A	SNP	1396
		C/T	SNP	1401
		CA/C	DELETION	1476
		A/G	SNP	1532
		G/A	SNP	1561
		G/C	SNP	1571
		G/A	SNP	1575
		A/G	SNP	1588
		C/T	SNP	1638
		G/T	SNP	1646
		C/T	SNP	1679
		A/G	SNP	1694
		G/C	SNP	1726
		T/G	SNP	1772
		T/A	SNP	1863
		G/T	SNP	2036
		G/A	SNP	2115
		G/A	SNP	2122
		G/A	SNP	2139
		G/T	SNP	2156
		CTTCACG	DELETION	2210

T/A	SNP	2302
T/C	SNP	2308
T/G	SNP	2370
C/T	SNP	2412
T/C	SNP	2449
T/C	SNP	2514
A/T	SNP	2687
T/C	SNP	2748
T/C	SNP	2771
A/G	SNP	2858
G/A	SNP	2872
C/A	SNP	2888
A/AT	INSERTION	2925
C/T	SNP	2976
C/T	SNP	2980
G/A	SNP	2991
C/T	SNP	3011
A/G	SNP	3038
A/G	SNP	3042
A/C	SNP	3167
A/T	SNP	3229
T/G	SNP	3255
C/T	SNP	3265
A/T	SNP	3281
C/T	SNP	3295
C/T	SNP	3325
C/T	SNP	3367
C/T	SNP	3453
A/G	SNP	3462
T/TA	INSERTION	3472
A/G	SNP	3566
C/A	SNP	3717
A/T	SNP	4065
A/C	SNP	4068
T/G	SNP	4245
T/C	SNP	4542

		G/A	SNP	4797
		A/G	SNP	4862
		C/T	SNP	4909
		T/A	SNP	4913
		C/T	SNP	4971
		T/A	SNP	138
		A/G	SNP	606
		C/T	SNP	624
		T/C	SNP	626
		C/G	SNP	966
		TA/T	DELETION	972
XM_004525528.1 PREDICTED: Ceratitis capitata phospholipid scramblase 1-like (LOC101460605), transcript variant X2, mRNA	GLOS_LOC101460605.2.3	C/T	SNP	4156
		C/T	SNP	4159
		C/T	SNP	4164
		T/C	SNP	4748
		A/T	SNP	4765
XP_004531215.1 PREDICTED: collagen alpha-1(IV) chain-like [Ceratitis capitata]	GLOS_LOC101460687.1.1	A/G	SNP	152
		GT/G	DELETION	298
		G/A	SNP	466
		A/C	SNP	541
		A/C	SNP	865
		G/A	SNP	910
		C/T	SNP	985
		A/G/T	SNP	1351
		C/T	SNP	1492
		A/G	SNP	1531
		C/T	SNP	1597
		A/G	SNP	1699
		G/A	SNP	1748
		T/C	SNP	1795
		G/T	SNP	1903
		G/A	SNP	2323
		G/C	SNP	2443
		G/A	SNP	2542
		T/A	SNP	2641

		A/C	SNP	2815
		G/A	SNP	2877
		C/T	SNP	2959
		G/A	SNP	3094
		G/T	SNP	3326
		T/A	SNP	3730
		G/C	SNP	3820
		T/C	SNP	3838
		G/T	SNP	3860
		C/T	SNP	3892
		A/G	SNP	3907
		A/G	SNP	3946
		C/T	SNP	4174
		T/C	SNP	4210
		G/T	SNP	4339
		A/G	SNP	4600
		G/A	SNP	4816
		C/G	SNP	5164
		A/G	SNP	5359
		A/G	SNP	5368
		C/G/T	SNP	5509
		A/T	SNP	5533
XP_004518643.1 PREDICTED: mucin-5AC-like [Ceratitis capitata]	GLOS_LOC101460827.2.2	A/T	SNP	544
		A/T	SNP	659
		G/A	SNP	692
XP_004522056.1 PRED: counting factor associated protein D-like [C. capitata]	GLOS_LOC101460835.1.1	G/A	SNP	745
		T/A	SNP	1021
		C/T	SNP	1239
		A/G	SNP	1627
		T/G	SNP	2032
		A/G	SNP	2077
XP_004521535.1 PREDICTED: serine proteinase stubble-like [Ceratitis capitata]	GLOS_LOC101461009.2.2	T/A	SNP	45
		G/A	SNP	148
		A/G	SNP	272
		T/G	SNP	315
		C/T	SNP	378

		G/A	SNP	406
		C/T	SNP	428
		A/G	SNP	429
		A/G	SNP	449
		C/T	SNP	480
		T/C	SNP	561
		G/A	SNP	714
		A/T	SNP	731
		G/A	SNP	762
		C/T	SNP	813
		G/A	SNP	834
		T/C	SNP	945
		A/G	SNP	951
		G/A	SNP	1049
		G/T	SNP	1095
		C/G	SNP	1129
		T/G	SNP	1151
		G/A	SNP	1158
		A/G	SNP	1166
		C/T	SNP	1169
		G/A	SNP	1196
XP_004530393.1 PREDICTED: acid trehalase-like protein 1-like [Ceratitis capitata]	GLOS_LOC101461034.1.1	C/T	SNP	115
		T/C	SNP	190
		G/C	SNP	202
		C/A	SNP	241
		T/C	SNP	262
		A/T	SNP	268
		A/C	SNP	284
		C/T	SNP	337
		C/T	SNP	347
		C/G	SNP	359
		A/G	SNP	377
		G/A	SNP	380
		G/A	SNP	430
		A/G	SNP	471
		T/C	SNP	505

C/A	SNP	507
G/T	SNP	566
A/G	SNP	580
C/T	SNP	621
C/G	SNP	631
C/T	SNP	697
C/T	SNP	730
C/G	SNP	734
T/C	SNP	772
A/T	SNP	805
A/G	SNP	816
G/A	SNP	874
T/C	SNP	877
C/T	SNP	953
T/C	SNP	962
A/G	SNP	988
C/T	SNP	991
C/T	SNP	1036
C/A	SNP	1079
G/C	SNP	1099
G/A	SNP	1119
A/T	SNP	1120
C/T	SNP	1132
C/G	SNP	1218
C/T	SNP	1282
G/A	SNP	1303
C/T	SNP	1312
A/G	SNP	1378
T/A	SNP	1390
A/T	SNP	1422
C/G	SNP	1463
C/T	SNP	1678
A/G	SNP	1720
T/C	SNP	1737
A/G	SNP	1873
G/A	SNP	2002

		A/C	SNP	2025
		T/C	SNP	2110
		A/G	SNP	2212
		G/T	SNP	2214
		T/A	SNP	2215
		T/C	SNP	2252
XP_004521971.1 PRED: epidermal growth factor-like protein 6-like [C. capitata]	GLOS_LOC101461076.2.2	C/T	SNP	47
XP_004523994.1 PREDICTED: stress response protein NST1-like [C. capitata]	GLOS_LOC101461145.1.1	C/T	SNP	243
		G/A	SNP	258
		G/A	SNP	275
		G/C	SNP	284
		G/T	SNP	289
		G/A	SNP	306
		C/A	SNP	317
		C/CT	INSERTION	333
		A/C	SNP	427
		A/G	SNP	448
		T/C	SNP	455
		G/C	SNP	484
		G/A	SNP	518
		A/G	SNP	530
		A/G	SNP	562
		G/A	SNP	791
		G/A	SNP	1474
		C/T	SNP	1549
		C/T	SNP	2263
		A/T	SNP	2409
		C/G	SNP	2457
		A/C	SNP	3190
		G/GT	INSERTION	3589
		C/CA	INSERTION	3696
		A/C	SNP	3774
		A/AT	INSERTION	3780
		C/CA	INSERTION	3833
		T/TA	INSERTION	4399
		A/G	SNP	4457

		C/CA	INSERTION	4705
XP_004519566.1 PREDICTED: myb-like protein Q-like [Ceratitis capitata]	GLOS_LOC101461187.1.1	G/T	SNP	28
		A/G	SNP	33
		T/TA	INSERTION	425
		C/T	SNP	752
		T/C	SNP	881
		A/T	SNP	1628
		A/G	SNP	1850
		C/A	SNP	1901
		T/G	SNP	2049
		A/G	SNP	2324
		A/G	SNP	2396
		T/C	SNP	2732
		G/A	SNP	2750
		C/T	SNP	2807
		A/G	SNP	2911
		G/A	SNP	2929
		T/C	SNP	2990
		C/T	SNP	2993
		T/C	SNP	3140
		TTA/T	DELETION	3189
		G/A	SNP	3509
		A/G	SNP	3567
		A/G	SNP	3604
		G/A	SNP	3765
		A/G	SNP	3785
		A/T	SNP	3790
		C/T	SNP	3836
		G/A	SNP	3841
		G/A	SNP	3900
		A/G	SNP	3908
		C/T	SNP	3917
		T/G	SNP	3925
		T/C	SNP	3933
		A/C	SNP	4042
		C/T	SNP	4100

		A/G	SNP	4138
		A/C	SNP	4143
		A/T	SNP	4191
		T/G	SNP	4206
		T/C	SNP	4207
		C/G	SNP	4287
		T/C	SNP	4298
		C/A	SNP	4303
		G/A	SNP	4321
		A/G	SNP	4325
		T/C	SNP	4327
XP_004535853.1 PRED: endoplasmic reticulum metallopeptidase 1-like isoform X1 [C. capitata] ref|XP_004535854.1| PRED: endoplasmic reticulum metallopeptidase1 -like isoform X2 [C. c.] ref|XP_004535855.1| PRED: endoplasmic reticulum metallopeptidase 1-like isoform X3 [C. c.] ref|XP_004535856.1| PRED: endoplasmic reticulum metallopeptidase 1-like isoform X4 [C. capitata]	GLOS_LOC101461359.1.1	AT/A	DELETION	230
		T/C	SNP	232
		T/G	SNP	233
		G/A	SNP	458
		G/A	SNP	466
		C/T	SNP	504
		A/G	SNP	620
		G/A	SNP	751
		T/C	SNP	822
		A/C	SNP	848
		T/A	SNP	990
		C/CA	INSERTION	1083
		A/G	SNP	1094
		T/A	SNP	1145
		T/A	SNP	1147
		G/T	SNP	1150
		T/C	SNP	1777
		C/T	SNP	2422
		T/C	SNP	2511
		C/T	SNP	2532
		G/A	SNP	2695

		T/C	SNP	2826
		G/T	SNP	2829
		A/G	SNP	2970
		C/A	SNP	3426
		A/G	SNP	3819
XP_004533269.1 PREDICTED: uncharacterized protein LOC101461462 [C. capitata]	GLOS_LOC101461462.1.1	G/T	SNP	123
		G/A	SNP	596
		T/A	SNP	662
		G/A	SNP	1373
		C/G	SNP	1696
		T/A	SNP	1761
		G/C	SNP	2249
		G/C	SNP	2256
		C/A	SNP	2352
		G/C	SNP	2587
		T/G	SNP	3303
		C/CG	INSERTION	3544
		A/G/T	SNP	3545
		T/C	SNP	3546
		A/T	SNP	3547
		T/A/G	SNP	3548
		G/T	SNP	3549
		A/T	SNP	3551
		G/A	SNP	4308
XP_004526501.1 PREDICTED: lysozyme-like [Ceratitis capitata]	GLOS_LOC101461912.1.1	G/A	SNP	135
		ACACACA	DELETION	484
		ACAAC/A	DELETION	488
		AAC/A	DELETION	490
XP_004534627.1 PREDICTED: uncharacterized protein LOC101462125 [C. capitata]	GLOS_LOC101462125.1.1	G/T	SNP	86
		G/A	SNP	573
		A/T	SNP	655
		T/C	SNP	738
		G/C	SNP	793
		C/T	SNP	863
		A/G	SNP	882
		A/G	SNP	996

XP_004518749.1 PREDICTED: probable chitinase 3-like [Ceratitis capitata]	GLOS_LOC101462140.1.1	T/C	SNP	365
		A/G	SNP	366
		C/G	SNP	400
XP_004534628.1 PREDICTED: uncharacterized protein LOC101462302 [C. capitata]	GLOS_LOC101462302.2.2	A/G	SNP	66
		A/G	SNP	131
		C/A	SNP	136
		T/C	SNP	147
		C/A	SNP	270
		T/C	SNP	338
		A/G	SNP	341
		T/C	SNP	343
		A/G	SNP	353
		A/G	SNP	470
		C/T	SNP	577
		T/G	SNP	625
		T/G	SNP	656
		T/G	SNP	699
		C/T	SNP	706
		T/C	SNP	721
		C/T	SNP	819
		A/G	SNP	864
		A/G	SNP	978
		A/G	SNP	1005
		G/C	SNP	1009
		T/C	SNP	1119
		G/C	SNP	1202
		G/A	SNP	1363
		G/T	SNP	1365
		C/T	SNP	1403
		G/A	SNP	1438
		A/G	SNP	1446
		G/A	SNP	1463
		C/T	SNP	1482
		C/A	SNP	1493
		G/A	SNP	1495
		G/A	SNP	1513

		A/G	SNP	1543
		G/A	SNP	1614
		T/C	SNP	1686
		G/A	SNP	1702
		C/T	SNP	1761
		T/C	SNP	1766
		A/G	SNP	1767
		G/A	SNP	1813
		A/C	SNP	1829
		A/G	SNP	1840
		C/T	SNP	1864
		A/G	SNP	1871
		A/G	SNP	1916
		C/T	SNP	1937
		C/A	SNP	1954
		G/T	SNP	2034
		G/T	SNP	2099
XP_004529335.1 PREDICTED: LOW QUALITY PROTEIN: activating signal cointegrator 1 complex subunit 3-like [Ceratitis capitata]	GLOS_LOC101462349.8.8	G/T	SNP	166
		T/C	SNP	168
		C/G	SNP	171
		T/C	SNP	196
		G/C	SNP	232
		G/A	SNP	283
		T/A	SNP	1005
		A/G	SNP	1490
		G/A	SNP	1511
		T/C	SNP	1512
		G/T	SNP	1523
		G/A	SNP	1572
		C/T	SNP	1614
		G/A	SNP	1945
		C/T	SNP	2059
		A/G	SNP	2100
		A/G	SNP	2102
		T/A	SNP	2109

XP_004527439.1 PREDICTED: sepiapterin reductase-like [Ceratitis capitata]	GLOS_LOC101462454.1.1	G/C	SNP	470
		G/A	SNP	652
		A/G	SNP	653
		C/G	SNP	669
		T/C	SNP	791
		C/G	SNP	828
		C/T	SNP	929
XP_004529143.1 PREDICTED: adenylosuccinate lyase-like [Ceratitis capitata]	GLOS_LOC101462532.1.1	T/C	SNP	7
		T/A	SNP	62
		TTA/T	DELETION	124
		T/C	SNP	167
		G/A	SNP	452
		G/A	SNP	545
		T/C	SNP	704
		G/A	SNP	707
		T/C	SNP	746
		G/A	SNP	821
		C/G	SNP	827
		T/A	SNP	980
		G/A	SNP	986
		C/G	SNP	998
		C/T	SNP	1010
		G/A	SNP	1037
		C/T	SNP	1109
		G/A	SNP	1118
		G/A	SNP	1136
		A/C	SNP	1220
		G/A	SNP	1370
		G/A	SNP	1401
		G/C	SNP	1566
		T/C	SNP	1568
XP_004521288.1 PRED: MD-2-related lipid-recognition protein-like [C. capitata]	GLOS_LOC101462556.1.1	C/T	SNP	82
		C/T	SNP	267
		T/C	SNP	429
		G/A	SNP	451
		C/A	SNP	454

		A/G	SNP	480
		G/A	SNP	504
		A/G	SNP	518
		T/C	SNP	548
		A/G	SNP	601
XP_004536656.1 PREDICTED: chymotrypsin-1-like [Ceratitis capitata]	GLOS_LOC101462601.1.5	G/A	SNP	34
		C/A	SNP	37
		C/G	SNP	38
		C/A	SNP	45
		T/G	SNP	130
		A/G	SNP	141
		C/T	SNP	202
		T/C	SNP	651
		A/T	SNP	672
		C/A	SNP	780
		T/A	SNP	922
		A/C	SNP	964
		C/A	SNP	1020
		A/T	SNP	1184
		T/C	SNP	1228
		A/G	SNP	1232
		T/C	SNP	1298
		C/T	SNP	1313
XP_004527440.1 PREDICTED: sepiapterin reductase-like [Ceratitis capitata]	GLOS_LOC101462636.1.1	A/G	SNP	87
		G/A	SNP	177
		G/GA	INSERTION	181
		T/TA	INSERTION	237
		T/C	SNP	540
		G/C	SNP	804
		G/A	SNP	882
		G/C	SNP	937
		A/G	SNP	969
		T/C	SNP	1002
		T/C	SNP	1005
XP_004529420.1 PRED: prob. isoaspartyl peptidase/L-asparaginase GA20639-like	GLOS_LOC101462766.1.14	G/A	SNP	1354
XP_004529420.1 PRED: prob. isoaspartyl peptidase/L-asparaginase GA20639-like	GLOS_LOC101462766.14.14	T/A	SNP	2345

		G/A	SNP	2348
		G/T	SNP	2349
		T/G	SNP	2351
		C/G	SNP	2680
		C/A	SNP	2681
XP_004529420.1 PRED: prob. isoaspartyl peptidase/L-asparaginase GA20639-like	GLOS_LOC101462766.5.14	CA/C	DELETION	2349
		A/T	SNP	2353
		G/A	SNP	2399
		T/C	SNP	2568
		C/A	SNP	2570
XP_004529420.1 PRED: prob. isoaspartyl peptidase/L-asparaginase GA20639-like	GLOS_LOC101462766.7.14	A/G	SNP	1429
XP_004529420.1 PRED: prob. isoaspartyl peptidase/L-asparaginase GA20639-like	GLOS_LOC101462766.9.14	T/G	SNP	683
		G/T	SNP	684
		C/A	SNP	910
		G/T	SNP	913
		C/G	SNP	915
		A/G	SNP	919
		C/G	SNP	926
		T/A	SNP	928
		T/A	SNP	929
XP_004536658.1 PREDICTED: chymotrypsin-2-like [Ceratitis capitata]	GLOS_LOC101462958.1.2	T/C	SNP	586
		C/T	SNP	735
		A/G	SNP	932
		G/A	SNP	986
XP_004524787.1 PREDICTED: serine protease easter-like [Ceratitis capitata]	GLOS_LOC101462986.1.1	G/A	SNP	72
		A/C	SNP	170
		G/A	SNP	223
		T/C	SNP	541
		T/C	SNP	667
		G/T	SNP	718
		C/A	SNP	743
		T/C	SNP	775
		C/G	SNP	1087
		G/T	SNP	1391
		G/A	SNP	1403
		G/C	SNP	1504

		C/G	SNP	1516
XP_004517889.1 PREDICTED: trypsin-like [Ceratitis capitata]	GLOS_LOC101463325.1.1	T/A	SNP	103
		G/T	SNP	169
		C/G	SNP	193
		A/T	SNP	197
		C/T	SNP	203
		G/C	SNP	272
		A/G	SNP	303
		G/A	SNP	434
		C/A	SNP	446
		T/G	SNP	527
		G/T	SNP	625
		G/T	SNP	630
		T/G	SNP	632
		G/A	SNP	794
		T/G	SNP	1103
		T/C	SNP	1104
		CA/C	DELETION	1106
		G/A	SNP	1112
		T/G	SNP	1113
		C/A	SNP	1273
		T/TA	INSERTION	1274
		C/A	SNP	1330
		C/G	SNP	1396
		G/T	SNP	1449
		T/A	SNP	1453
		A/G	SNP	1454
		C/A	SNP	1457
		T/C	SNP	1603
		ATTC/A	DELETION	1663
		A/G	SNP	2474
		A/C	SNP	2514
		C/T	SNP	2561
		T/G	SNP	2610
XP_004523823.1 PREDICTED: 2-aminoethanethiol dioxygenase-like [C. capitata]	GLOS_LOC101463338.1.1	T/C	SNP	155
		GTT/G	DELETION	250

		T/TA	INSERTION	448
		G/A	SNP	1184
XP_004534544.1 PREDICTED: uncharacterized protein LOC101463381 [C. capitata]	GLOS_LOC101463381.3.3	A/C	SNP	503
		C/G	SNP	2091
		A/G	SNP	2116
		C/A	SNP	2742
		G/T	SNP	3042
		T/A	SNP	3046
		A/T	SNP	3049
[BBH] LSD1_DROME (sp|Q9VCI3) Lipid storage droplets surface-binding protein 1 OS=Drosophila melanogaster GN=Lsd-1 PE=1 SV=2	GLOS_LSD1.1.1	A/G	SNP	232
		C/T	SNP	238
		G/A	SNP	348
		T/C	SNP	539
		C/CGCA	INSERTION	785
		C/T	SNP	884
		G/A	SNP	896
		C/A	SNP	1160
		T/A	SNP	1316
		C/T	SNP	1617
		G/GT	INSERTION	1635
[BBH] LTV1_DROME(sp|Q7KN79) Prot.LTV1 homolog D.m. GN=CG7686PE=1SV=1	GLOS_LTV1.1.1	A/C	SNP	265
		C/T	SNP	603
		C/T	SNP	772
		C/T	SNP	1063
		T/G	SNP	1147
		T/C	SNP	1210
		A/T	SNP	1226
YP_004400609.1 transmembrane protein [Mycoplasma mycoides subsp. Capri LC str. 95010] ref|WP_013729997.1| transmembrane protein [M. mycoides]	GLOS_MLC_9020.1.1	T/G	SNP	40
		C/T	SNP	89
		A/G	SNP	121
		A/T	SNP	143
		T/C	SNP	152
		A/G	SNP	194
		C/A	SNP	305

		C/A	SNP	320
		G/C	SNP	347
		A/C	SNP	381
		C/T	SNP	388
		G/T	SNP	477
		T/G	SNP	637
		C/T	SNP	674
		G/T	SNP	743
		C/CT	INSERTION	975
		G/GT	INSERTION	1161
		C/G	SNP	1381
		A/G	SNP	1410
		T/A	SNP	1545
		G/A	SNP	1602
		A/AT	INSERTION	1623
		T/A	SNP	1676
		C/A	SNP	1761
		T/C	SNP	1788
		A/G	SNP	1992
		A/G	SNP	2466
[BBH] MVL_DROME (sp|P49283) Protein Malvolio OS=D. m.r GN=Mvl PE=2 SV=2	GLOS_MVL.1.1	A/G	SNP	54
		CA/C	DELETION	156
		A/T	SNP	258
		T/C	SNP	387
		T/C	SNP	732
		T/A/C	SNP	747
		A/G	SNP	849
		C/A	SNP	855
		C/G	SNP	1473
		G/GA	INSERTION	2096
		T/A	SNP	2097
[BBH] MYSN_DROME (sp|Q99323) Myosin heavy chain, non-muscle OS=Drosophila me OS=Drosophila melanogaster GN=zip PE=1 SV=2	GLOS_MYSN.1.1	T/A	SNP	51
		G/GT	INSERTION	162
		G/GTT	INSERTION	282
		G/T	SNP	282

C/T	SNP	456
T/C	SNP	465
A/G	SNP	591
G/A	SNP	659
G/A	SNP	857
C/T	SNP	999
G/A	SNP	1026
G/T	SNP	1146
C/T	SNP	1161
G/A	SNP	1980
G/A	SNP	1986
C/T	SNP	2004
C/T	SNP	2232
A/G	SNP	2292
C/T	SNP	2293
G/T	SNP	2739
G/C	SNP	2823
C/G	SNP	2910
T/C	SNP	2979
G/C	SNP	2991
C/T	SNP	3006
C/T	SNP	3078
T/C	SNP	3132
T/A	SNP	3192
A/AT	INSERTION	3217
T/C	SNP	3222
C/T	SNP	3264
C/T	SNP	3333
C/T	SNP	3336
G/A	SNP	3372
T/C	SNP	3714
A/G	SNP	4116
T/C	SNP	4173
A/G	SNP	4281
T/A	SNP	4290
G/A	SNP	4494

		A/G	SNP	4506
		A/G	SNP	4593
		T/C	SNP	4608
		C/T	SNP	4626
		T/C	SNP	4935
		A/G	SNP	5148
		T/C	SNP	6063
		CT/C	DELETION	6298
NDUS2_TRYBB (sp|P21301) NADH-ubiquinone oxidoreductase 49 kDa subunit homolog OS=Trypanosoma brucei brucei GN=NAD7 PE=2 SV=2	GLOS_NDUS2.1.1	G/T	SNP	77
[BBH] NOG1_TRYBB (sp|Q9U6A9) Nucleolar GTP-binding protein 1 OS=Trypanosoma brucei brucei GN=NOG1 PE=1 SV=1	GLOS_NOG1.1.2	A/T	SNP	1553
[BBH] OB99B_DROME (sp|Q9VAI6) General odorant-binding protein 99b OS=Drosophil OS=Drosophila melanogaster GN=Obp99b PE=2 SV=1	GLOS_OB99B.1.1	C/A	SNP	73
		A/G	SNP	106
[BBH] P320_TRYBB (sp|P21787) Microtubule-associated protein P320 (Fragment) OS=Trypanosoma brucei brucei PE=4 SV=1	GLOS_P320.1.1	C/G	SNP	55
		T/C	SNP	69
		T/C	SNP	103
		G/A	SNP	111
		C/G	SNP	169
		T/C	SNP	183
		T/C	SNP	217
NP_730457.1 PAPS synthetase, isoform A [D. melanogaster] ref|NP_730458.1| PAPS synthetase, isoform B [D. melanogaster] ref|NP_730459.1| PAPS synthetase, isoform C [D. melanogaster] ref|NP_001262072.1| PAPS synthetase, isoform G [D. melanogaster]	GLOS_PAPSS.1.1	GTT/G/GT	DELETION	449
		T/C	SNP	454
		T/C	SNP	456
		G/C	SNP	477
		G/A	SNP	1322
		G/C	SNP	2222
		A/G	SNP	2344
		A/C	SNP	2378
		A/T	SNP	2522
[BBH] PCKG_DROME (sp|P20007) Phosphoenolpyruvate carboxykinase [GTP]	GLOS_PCKG.1.2	A/T	SNP	147

OS=Drosophila melanogaster GN=Pepck PE=2 SV=2				
		T/TTA	INSERTION	148
		G/T	SNP	188
		G/A	SNP	470
		G/A	SNP	485
		A/T	SNP	560
		G/A	SNP	596
		T/C	SNP	641
		C/T	SNP	707
		T/C	SNP	1304
		G/C	SNP	2138
		G/A	SNP	2180
		G/T	SNP	2191
		GT/GTT/G	INSERTION	2191
		C/T	SNP	2205
		G/GTA	INSERTION	2239
		A/T	SNP	2265
		G/A	SNP	2282
		T/A	SNP	2321
		A/C	SNP	2324
		C/T	SNP	2351
		T/TA	INSERTION	2362
		G/A	SNP	2363
		G/GA	INSERTION	2363
		G/A	SNP	2366
[BBH] PFR1_TRYBB (sp|P22225) 69 kDa paraflagellar rod protein OS=Tbb	GLOS_PFR1.1.1	C/T	SNP	1446
[BBH] PGKE_TRYBB (sp|P08893) Phosphoglycerate kinase, cytosolic OS=Tbb	GLOS_PGKE.1.1	T/A	SNP	902
		C/G	SNP	906
		T/A	SNP	908
		A/G	SNP	1065
NP_001161922.1 peritrophic matrix protein 3 precursor [Tribolium castaneum]	GLOS_PMP3.2.2	C/T	SNP	41
		C/T	SNP	47
		G/A	SNP	66
[BBH] PTER_DROGR (sp|B4J340)Phosphotriesterase-related prot.OS=D. grimshawi	GLOS_PTER.1.1	T/C	SNP	70
		A/G	SNP	245
		G/A	SNP	484

<table>
<tr><th></th><th></th><th></th><th></th><th></th></tr>
<tr><td></td><td></td><td>T/C</td><td>SNP</td><td>511</td></tr>
<tr><td></td><td></td><td>G/A</td><td>SNP</td><td>618</td></tr>
<tr><td></td><td></td><td>A/G</td><td>SNP</td><td>631</td></tr>
<tr><td></td><td></td><td>G/A</td><td>SNP</td><td>1007</td></tr>
<tr><td></td><td></td><td>CAT/C</td><td>DELETION</td><td>1192</td></tr>
<tr><td></td><td></td><td>G/T</td><td>SNP</td><td>1226</td></tr>
<tr><td></td><td></td><td>A/G</td><td>SNP</td><td>1303</td></tr>
<tr><td>NP_729109.1 glutaminyl cyclase [Drosophila melanogaster]</td><td>GLOS_QC.1.1</td><td>A/G</td><td>SNP</td><td>272</td></tr>
<tr><td></td><td></td><td>A/G</td><td>SNP</td><td>828</td></tr>
<tr><td></td><td></td><td>T/C</td><td>SNP</td><td>840</td></tr>
<tr><td></td><td></td><td>G/A</td><td>SNP</td><td>906</td></tr>
<tr><td></td><td></td><td>G/C</td><td>SNP</td><td>907</td></tr>
<tr><td></td><td></td><td>G/T</td><td>SNP</td><td>1086</td></tr>
<tr><td>[BBH] RIR2_TRYBB (sp|O15910) Ribonucleoside-diphosphate reductase small chain OS
small chain OS=Trypanosoma brucei brucei GN=RNR2 PE=2 SV=1</td><td>GLOS_RIR2.1.2</td><td>T/A</td><td>SNP</td><td>637</td></tr>
<tr><td></td><td></td><td>T/C</td><td>SNP</td><td>694</td></tr>
<tr><td></td><td></td><td>G/A</td><td>SNP</td><td>1555</td></tr>
<tr><td></td><td></td><td>G/A</td><td>SNP</td><td>1698</td></tr>
<tr><td></td><td></td><td>C/T</td><td>SNP</td><td>1699</td></tr>
<tr><td>[BBH] RIR2_DROME (sp|P48592) Ribonucleoside-diphosphate reductase
subunit M2 OS=Drosophila melanogaster GN=RnrS PE=1 SV=2</td><td>GLOS_RIR2.2.2</td><td>C/A</td><td>SNP</td><td>119</td></tr>
<tr><td></td><td></td><td>A/G</td><td>SNP</td><td>147</td></tr>
<tr><td></td><td></td><td>TTAC/T</td><td>DELETION</td><td>163</td></tr>
<tr><td></td><td></td><td>G/A</td><td>SNP</td><td>367</td></tr>
<tr><td></td><td></td><td>C/T</td><td>SNP</td><td>603</td></tr>
<tr><td></td><td></td><td>C/CT</td><td>INSERTION</td><td>632</td></tr>
<tr><td></td><td></td><td>T/A</td><td>SNP</td><td>714</td></tr>
<tr><td></td><td></td><td>T/C</td><td>SNP</td><td>738</td></tr>
<tr><td></td><td></td><td>C/T</td><td>SNP</td><td>868</td></tr>
<tr><td></td><td></td><td>G/A</td><td>SNP</td><td>951</td></tr>
<tr><td></td><td></td><td>G/A</td><td>SNP</td><td>1059</td></tr>
<tr><td></td><td></td><td>G/C</td><td>SNP</td><td>1096</td></tr>
<tr><td></td><td></td><td>C/A</td><td>SNP</td><td>1099</td></tr>
<tr><td></td><td></td><td>A/G</td><td>SNP</td><td>1101</td></tr>
<tr><td></td><td></td><td>T/C</td><td>SNP</td><td>1128</td></tr>
<tr><td></td><td></td><td>A/G</td><td>SNP</td><td>1233</td></tr>
</table>

		T/C	SNP	1389
RL23_DROME (sp|P48159) 60S ribosomal protein L23 OS=Drosophila melanogaster G[illegible]	GLOS_RL23.14.16	T/A	SNP	463
		A/C	SNP	657
		A/G	SNP	786
		C/T	SNP	910
		G/C	SNP	933
		G/T	SNP	2228
		A/C	SNP	2229
		C/T	SNP	2240
		C/T	SNP	2288
		T/C	SNP	2289
		G/A	SNP	2299
		A/G	SNP	2382
		G/A	SNP	2393
		G/A	SNP	2406
[BBH] RL27A_TRYBB (sp|O15883) 60S ribosomal protein L27a OS=Tbb	GLOS_RL27A.1.6	G/A	SNP	576
RL30_TRYBB (sp|P49153) 60S ribosomal prot.L30 OS=Tbb GN=RPL30 PE=3 SV=1	GLOS_RL30.1.1	C/A	SNP	405
		G/A	SNP	417
RL402_TRYCR (sp|P0CH27) Ubiquitin-60S ribosomal protein L40 OS=T. cruzi	GLOS_RL402.1.2	T/C	SNP	65
		G/A	SNP	461
RL40_TRYBB (sp|P21899) Ubiquitin-60S ribosomal protein L40 OS=Tbb	GLOS_RL40.3.8	T/A	SNP	118
		C/T	SNP	161
		A/G	SNP	302
[BBH] RN181_DROME (sp|Q9VE61) E3 ubiquitin-protein ligase RNF181 homolog OS=Drosophila melanogaster GN=CG7694 PE=2 SV=1	GLOS_RN181.1.1	A/T	SNP	393
		G/C	SNP	417
		T/TG	INSERTION	515
		T/G	SNP	516
		T/C	SNP	520
		T/C	SNP	521
		G/T	SNP	522
		C/T	SNP	523
NR_046235.1 Homo sapiens RNA, 45S pre-ribosomal 5 (RNA45S5), ribosomal RNA	GLOS_RNA45S5.1.2	A/C	SNP	306
		T/C	SNP	952
		T/A	SNP	1059
		C/T	SNP	1102

		A/G	SNP	1142
		C/T	SNP	1544
		G/A	SNP	2275
		C/A	SNP	2533
		G/A	SNP	2579
		T/C	SNP	2927
		C/CT	INSERTION	2969
		A/T	SNP	3188
		A/T	SNP	3524
		A/T	SNP	3557
		G/A	SNP	3627
		A/G	SNP	3733
		T/A	SNP	4198
[BBH] RPB1B_TRYBB (sp|P17545) DNA-directed RNA polymerase II subunit RPB1-B OS=Trypanosoma brucei brucei GN=TRP5.9 PE=1 SV=1	GLOS_RPB1B.1.1	T/C	SNP	1084
		G/T	SNP	1653
		C/T	SNP	2029
		C/T	SNP	3073
NP_649070.1 ribosomal prot. L26, isoform A [D. melanogaster] ref|NP_001262025.1| ribosomal protein L26, isoform B [D. melanogaster] ref|XP_001958190.1| GF23649 [D. ananassae] ref|XP_002042631.1| GM15002 [Drosophila sechellia] ref|XP_002095681.1| GE19578 [D. yakuba] ref|XP_002095689.1| GE19574 [D. yakuba] ref|XP_002085421.1| GD14780 [D. simulans]	GLOS_RPL26.1.3	A/C	SNP	92
		T/A	SNP	276
		A/T	SNP	277
		T/C	SNP	280
		G/C	SNP	282
		T/G	SNP	325
		T/C	SNP	333
		T/G	SNP	383
		T/A	SNP	501
		A/T	SNP	711
		T/G	SNP	712
		G/GT	INSERTION	713
		G/T	SNP	713
		T/C	SNP	715

		C/A	SNP	716
		C/A	SNP	717
[BBH] RPOA_LDVP (sp|Q83017) Replicase polyprotein 1ab OS=Lactate dehydrogenase elevating virus (strain Plagemann) GN=rep PE=1 SV=2	GLOS_RPOA.1.1	C/T	SNP	204
		T/C	SNP	1677
		T/C	SNP	1752
		T/C	SNP	2006
		G/A	SNP	2209
		C/T	SNP	2658
		A/C	SNP	2907
		T/C	SNP	3036
		C/T	SNP	4050
		T/G	SNP	4205
		C/A	SNP	4214
		C/T	SNP	4415
		G/A	SNP	5323
		G/A	SNP	5578
		T/C	SNP	5634
		T/C	SNP	6088
[BBH] RS12_TRYBB (sp|Q03253) 40S ribosomal protein S12 OS=Tbb	GLOS_RS12.1.4	A/G	SNP	400
[BBH] RUVB1_DROPS (sp|Q29AK9) RuvB-like helicase 1 OS=Drosophila pseudoobscura pseudoobscura GN=pont PE=3 SV=1	GLOS_RUVB1.1.1	G/A	SNP	99
		A/C	SNP	106
		G/GA	INSERTION	962
		A/G	SNP	1247
NP_729600.1 scramblase 1, isoform C [Drosophila melanogaster]	GLOS_SCRAMB1.1.1	T/A	SNP	1187
		T/G	SNP	1188
		A/G	SNP	1192
		A/G	SNP	1193
NP_610547.1 seele [D. melanogaster] ref|XP_002080855.1| GD26011 [D. simulans]	GLOS_SEL.1.1	T/A	SNP	1
		G/C	SNP	309
		C/T	SNP	596
		T/C	SNP	622
		C/T	SNP	702
		C/T	SNP	793
		T/A	SNP	859

XM_002581801.1 S. mansoni conserved hypoth. Prot. (Smp112590)mRNA,partial cds	GLOS_SMP_112590.1.1	C/G	SNP	92
		C/G	SNP	666
		C/T	SNP	729
		C/T	SNP	730
		C/A	SNP	866
		G/T	SNP	906
		G/A	SNP	908
		T/C	SNP	939
		T/C	SNP	2782
		C/T	SNP	2784
		A/T	SNP	2813
		T/G	SNP	2817
[BBH] SSRP1_DROPS (sp|Q293F6) FACT complex subunit Ssrp1 OS=Drosophila pseudoobscura pseudoobscura GN=Ssrp PE=3 SV=2	GLOS_SSRP1.1.1	C/T	SNP	902
		T/C	SNP	1145
		T/C	SNP	1490
		C/T	SNP	2087
XM_798344.1 Trypanosoma brucei brucei strain 927/4 GUTat10.1 hypothetical protein (Tb09.160.0430) partial mRNA	GLOS_TB09.160.0430.1.1	G/A	SNP	886
		T/C	SNP	2055
		G/A	SNP	2487
XM_798407.1 Trypanosoma brucei brucei strain 927/4 GUTat10.1 60S ribosomal protein L35 (Tb09.160.0710) partial mRNA	GLOS_TB09.160.0710.1.1	A/C	SNP	139
		C/G	SNP	146
		C/G	SNP	183
		T/A	SNP	573
		T/A	SNP	574
XM_798420.1 Tbb GUTat10.1 kynureninase (Tb09.160.0810) partial mRNA	GLOS_TB09.160.0810.1.1	GA/G	DELETION	632
		C/CA	INSERTION	816
		CT/C	DELETION	948
		T/A	SNP	3237
XM_798434.1 Tbb strain 927/4 GUTat10.1 prot. kinase (Tb09.160.0930) partial mRNA	GLOS_TB09.160.0930.1.1	C/CA	INSERTION	569
		CA/C	DELETION	712
XM_798466.1 Tbb strain 927/4 GUTat10.1 hypoth. Prot.(Tb09.160.1160)partial mRNA	GLOS_TB09.160.1160.1.1	A/G	SNP	556
		G/A	SNP	568
XP_803563.1 mitotubule-associated protein Gb4 [Tbb strain 927/4 GUTat10.1]	GLOS_TB09.160.1200.1.1	G/A	SNP	430

		T/C	SNP	647
		C/A	SNP	706
XM_798500.1 Tbb strain 927/4 GUTat10.1 hypoth prot (Tb09.160.1520) partial mRNA	GLOS_TB09.160.1520.1.1	A/C	SNP	21
		G/A	SNP	102
		C/T	SNP	146
		G/A	SNP	192
		C/A	SNP	198
		T/C	SNP	364
		A/C	SNP	535
		A/G	SNP	585
		T/C	SNP	633
		C/G	SNP	761
		C/T	SNP	817
		C/T	SNP	827
		C/T	SNP	992
		G/T	SNP	1035
		C/G	SNP	1288
		G/A	SNP	1505
		C/G	SNP	1868
		C/A	SNP	2031
		T/C	SNP	2047
		A/G	SNP	2135
XP_803698.1 ribosomal protein S7 [T. b. brucei strain 927/4 GUTat10.1]	GLOS_TB09.160.2550.1.1	C/T	SNP	10
		A/T	SNP	11
		C/T	SNP	15
		A/T	SNP	16
XP_803725.1 fatty acyl CoA synthetase 3 [T. brucei brucei strain 927/4 GUTat10.1]	GLOS_TB09.160.2810.1.2	G/C	SNP	1072
		C/A	SNP	1074
		C/G	SNP	1075
		C/T	SNP	1081
		G/A	SNP	958
		G/T	SNP	1515
		A/G	SNP	1517
		A/G	SNP	1695
		C/T	SNP	1696
XM_798715.1 Trypanosoma brucei brucei strain 927/4 GUTat10.1 cAMP-specific	GLOS_TB09.160.3590.1.1	GA/G	DELETION	1757

phosphodiesterase (Tb09.160.3590) partial mRNA				
		T/C	SNP	2146
		G/T	SNP	2149
		A/G	SNP	2150
		G/C	SNP	2647
		G/T	SNP	2648
		A/G	SNP	2817
		C/T	SNP	3184
		C/G	SNP	3259
		C/T	SNP	3853
		T/C	SNP	3858
		T/A	SNP	3859
XM_798741.1 Tbb GUTat10.1 hypothetical protein (Tb09.160.3780) partial mRNA	GLOS_TB09.160.3780.1.1	G/GT	INSERTION	1701
XM_821871.1 Trypanosoma brucei brucei strain 927/4 GUTat10.1 60S acidic ribosomalprotein (Tb09.160.4200) partial mRNA	GLOS_TB09.160.4200.1.1	G/GA	INSERTION	580
XP_826974.1 glutamate dehydrogenase [T. b. brucei strain 927/4 GUTat10.1]	GLOS_TB09.160.4310.1.1	C/CT	INSERTION	4508
XP_826981.1 succinate dehydrogenase [T. b. brucei strain 927/4 GUTat10.1]	GLOS_TB09.160.4380.1.1	C/A	SNP	16
		T/G	SNP	17
		C/CT	INSERTION	916
		GT/G	DELETION	1008
XM_821897.1 Tbb GUTat10.1 hypothetical protein (Tb09.160.4460) partial mRNA	GLOS_TB09.160.4460.1.1	T/C	SNP	798
		C/G	SNP	1384
		T/C	SNP	176
		C/T	SNP	225
		C/T	SNP	416
		T/C	SNP	446
		A/G	SNP	900
		T/C	SNP	914
XM_821907.1 Tbb GUTat10.1 hypothetical protein (Tb09.160.4580) partial mRNA	GLOS_TB09.160.4580.1.1	A/AT	INSERTION	1675
		A/G	SNP	1696
XM_821909.1 Tbb GUTat10.1 ABC transporter (Tb09.160.4600) partial mRNA	GLOS_TB09.160.4600.1.1	C/CA	INSERTION	126
		C/CT	INSERTION	3111
XM_821961.1 Tbb4 GUTat10.1 hypothetical protein (Tb09.160.5060) partial mRNA	GLOS_TB09.160.5060.1.1	T/TA	INSERTION	178
XM_822007.1 Tbb GUTat10.1 adenosine transporter (Tb09.160.5480) partial mRNA	GLOS_TB09.160.5480.1.1	A/AT	INSERTION	160
		T/TA	INSERTION	1387
XM_822018.1 Tbb GUTat10.1 60S ribosomal prot L11 (Tb09.160.5590) partial mRNA	GLOS_TB09.160.5590.1.1	T/C	SNP	698

		T/G	SNP	746
XM_822050.1 Tbb GUTat10.1 hypothetical protein (Tb09.211.0040) partial mRNA	GLOS_TB09.211.0040.1.1	T/TA	INSERTION	873
XP_827151.1 nascent polypeptide associated complex subunit [Trypanosoma brucei] ref ref|XP_827152.1| nascent polypeptide associated complex subunit [Tr. brucei]	GLOS_TB09.211.0120.1.2	G/C	SNP	428
XM_822080.1 Tbb GUTat10.1 hypothetical protein (Tb09.211.0320) partial mRNA	GLOS_TB09.211.0320.1.1	C/CT	INSERTION	2319
XM_822082.1 Tbb GUTat10.1 60S ribosomal prot L10 (Tb09.211.0340) partial mRNA	GLOS_TB09.211.0340.1.1	T/C	SNP	569
XM_822105.1 Tbb GUTat10.1 hypothetical protein (Tb09.211.0560) partial mRNA	GLOS_TB09.211.0560.1.1	G/A	SNP	1904
		G/GA	INSERTION	2277
		G/GA	INSERTION	2784
		T/TA	INSERTION	3170
		C/CT	INSERTION	3435
		G/GT	INSERTION	3662
		CAT/C	DELETION	3759
XM_822151.1 T. b. brucei strain 927/4 GUTat10.1 phosphatidylcholine:ceramide cholinephosphotransferase 2 (Tb09.211.1000) partial mRNA	GLOS_TB09.211.1000.1.1	T/C	SNP	301
		G/T	SNP	683
		A/G	SNP	687
		A/G	SNP	769
		G/T	SNP	901
		G/GT	INSERTION	917
		A/G	SNP	1695
		C/T	SNP	1909
		A/G	SNP	1910
		C/T	SNP	1940
		G/A	SNP	1956
		A/G	SNP	2154
		C/G/T	SNP	2180
		C/T	SNP	2182
		A/G	SNP	2192
		T/G	SNP	2296
		C/T	SNP	2306
XM_822158.1 Tbb GUTat10.1 hypothetical protein (Tb09.211.1070) partial mRNA	GLOS_TB09.211.1070.1.1	G/T	SNP	4102
		G/T	SNP	4112
		G/GT	INSERTION	4140
XM_822176.1 Tbb GUTat10.1 hypothetical protein (Tb09.211.1240) partial mRNA	GLOS_TB09.211.1240.1.1	C/CA	INSERTION	767
XM_822222.1 Tbb GUTat10.1 hypothetical protein (Tb09.211.1690) partial mRNA	GLOS_TB09.211.1690.1.1	C/A	SNP	324

XM_822228.1 Tbb strain 927/4 GUTat10.1 mitochondrial carrier protein (Tb09.211.1750) partial mRNA. nuclear gene for mitochondrial product	GLOS_TB09.211.1750.1.1	A/T	SNP	41
		A/T	SNP	42
		CA/C	DELETION	117
		G/GT	INSERTION	1441
		C/CT	INSERTION	1627
XM_822265.1 Tbb GUTat10.1 poly(A)-binding prot 1 (Tb09.211.2150) partial mRNA	GLOS_TB09.211.2150.1.1	T/A	SNP	40
		C/CT	INSERTION	2338
		GA/G	DELETION	3536
		GA/G	DELETION	4109
		C/CA	INSERTION	4255
		C/CA	INSERTION	4500
XM_822308.1 Tbb t-complex protein 1 subunit eta (Tb09.211.2570) partial mRNA	GLOS_TB09.211.2570.1.1	C/CA	INSERTION	417
XM_822323.1 Tbb hypothetical protein (Tb09.211.2700) partial mRNA	GLOS_TB09.211.2700.1.1	TA/T	DELETION	947
XP_827420.1 Gim5B protein [Trypanosoma brucei brucei strain 927/4 GUTat10.1]	GLOS_TB09.211.2740.1.1	G/A	SNP	289
		T/C	SNP	352
		C/CT	INSERTION	1340
		G/A	SNP	1434
		G/GA	INSERTION	1434
		CT/C	DELETION	1636
		A/AT	INSERTION	1678
		A/T	SNP	1678
XM_822340.1 Tbb GUTat10.1 hypothetical protein (Tb09.211.2880) partial mRNA	GLOS_TB09.211.2880.1.1	A/AT	INSERTION	3312
		C/CT	INSERTION	3955
XM_822342.1 Tbb GUTat10.1 hypothetical protein (Tb09.211.2900) partial mRNA	GLOS_TB09.211.2900.1.1	G/A	SNP	334
		G/A	SNP	654
		T/C	SNP	797
		A/G	SNP	1369
		T/C	SNP	1850
		T/C	SNP	1896
		T/C	SNP	1988
XM_822379.1 Tbb 60S ribosomal protein L31 (Tb09.211.3280) partial mRNA	GLOS_TB09.211.3280.1.1	G/T	SNP	759
		G/T	SNP	761
XM_822384.1 Tbb cystathione gamma lyase (Tb09.211.3330) partial mRNA	GLOS_TB09.211.3330.1.1	CT/C	DELETION	415
XM_822401.1 Tbb ATP-depend. DEAD/H RNA helicase (Tb09.211.3510) part. mRNA	GLOS_TB09.211.3510.1.1	GA/G	DELETION	2491
		C/CAT	INSERTION	3035

		C/G	SNP	3132
		TA/T	DELETION	3190
		G/GA	INSERTION	3443
		G/GT	INSERTION	3629
		A/G	SNP	3707
		C/CT	INSERTION	3743
		G/A	SNP	3883
		C/CT	INSERTION	3977
		G/GTT	INSERTION	4101
		G/T	SNP	4156
		G/GA	INSERTION	4574
XM_822405.1 Tbb glycerol kinase glycosomal (Tb09.211.3550) partial mRNA	GLOS_TB09.211.3550.1.1	C/A	SNP	131
		C/A	SNP	1908
		A/G	SNP	2107
		A/AT	INSERTION	2195
		G/T	SNP	2255
XM_822409.1 Tbb ubiquitin-activating enzyme E1 (Tb09.211.3610) partial mRNA	GLOS_TB09.211.3610.1.1	C/CT	INSERTION	378
XM_822456.1 Tbb hypothetical protein (Tb09.211.4070) partial mRNA	GLOS_TB09.211.4070.1.1	T/TA	INSERTION	235
XM_822482.1 Tbb GUTat10.1 hypothetical protein (Tb09.211.4360) partial mRNA	GLOS_TB09.211.4360.1.1	T/A	SNP	591
		T/C	SNP	592
		C/T	SNP	1132
		A/G	SNP	1743
		C/T	SNP	1982
		C/T	SNP	2144
		G/C	SNP	3018
XM_822500.1 T. b. brucei strain 927/4 GUTat10.1 kinetoplastid membrane protein KMP-11 (Tb09.211.4513) partial mRNA. nuclear gene for plastid product	GLOS_TB09.211.4513.1.1	C/T	SNP	218
		GA/G	DELETION	228
		C/T	SNP	240
		T/C	SNP	343
		A/G	SNP	351
		T/C	SNP	625
XM_822504.1 Tbb 60S ribosomal protein L12 (Tb09.211.4550) partial mRNA	GLOS_TB09.211.4550.1.1	A/G	SNP	257
		C/T	SNP	290
		A/C	SNP	356
		C/T	SNP	404

		A/G	SNP	413
		C/T	SNP	425
		C/T	SNP	446
		C/T	SNP	485
		G/T	SNP	539
		G/A	SNP	542
		C/G	SNP	548
		T/G	SNP	549
XP_827610.1 reiske iron-sulfur protein mitochondrial precursor [Trypanosoma brucei]	GLOS_TB09.211.4700.1.1	CATAT/CA	INSERTION	619
		T/TTCA	INSERTION	906
		TC/T	DELETION	965
		G/GA	INSERTION	1201
XM_822523.1 Tbb GUTat10.1 metacaspase 5 (Tb09.211.4760) partial mRNA	GLOS_TB09.211.4760.1.1	T/TC	INSERTION	283
		A/G	SNP	952
		G/A	SNP	976
XP_827634.1 hypothetical protein [T. b. brucei strain 927/4 GUTat10.1]	GLOS_TB09.211.4940.1.1	T/C	SNP	61
		G/A	SNP	199
		A/G	SNP	262
		T/G	SNP	440
		G/C	SNP	512
		A/T	SNP	690
		C/T	SNP	749
		C/T	SNP	773
		G/A	SNP	789
		T/C	SNP	821
		A/G	SNP	823
		T/G	SNP	953
		C/G	SNP	1007
		G/C	SNP	1054
		C/T	SNP	1109
		A/G	SNP	1146
		T/G	SNP	1182
		T/C	SNP	1296
		T/C	SNP	1299
		T/C	SNP	1402
		A/G	SNP	1463

		A/G	SNP	1541
		G/A	SNP	1564
		G/A	SNP	1641
		G/A	SNP	1709
		G/A	SNP	1757
		G/A	SNP	1918
		C/T	SNP	2128
		G/A	SNP	2263
		C/T	SNP	2403
		C/T	SNP	2429
XM_822625.1 Tbb GUTat10.1 hypothetical protein (Tb09.244.2170) partial mRNA	GLOS_TB09.244.2170.1.1	T/C	SNP	93
		C/CCA	INSERTION	207
		C/A	SNP	273
		T/G	SNP	477
		T/TAA	INSERTION	593
		C/A	SNP	600
		T/C	SNP	680
		T/C	SNP	698
		C/T	SNP	734
		A/G	SNP	735
		T/C	SNP	760
		A/G	SNP	836
		C/T	SNP	859
		C/T	SNP	895
		C/T	SNP	913
		C/T	SNP	1161
		C/T	SNP	1233
		A/G	SNP	1271
		A/T	SNP	1283
		C/T	SNP	1288
		C/T	SNP	1352
		C/T	SNP	1860
		T/C	SNP	1889
		A/G	SNP	1916
		A/G	SNP	2043
		T/A	SNP	2062

		T/G	SNP	2170
		G/A	SNP	2221
		T/C	SNP	2224
		T/C	SNP	2273
		C/T	SNP	2330
		C/A	SNP	2368
		A/T	SNP	2369
		C/T	SNP	2371
		A/G	SNP	2393
		A/C	SNP	2437
		A/C	SNP	2452
		C/A	SNP	2455
		C/T	SNP	2470
		G/A	SNP	2475
		G/A	SNP	2484
		C/G/T	SNP	2508
		C/A	SNP	2510
		TC/T	DELETION	2538
		C/T	SNP	2545
		T/C	SNP	2586
		T/C	SNP	2623
		AT/A	DELETION	2783
		T/C	SNP	2890
		G/A	SNP	2891
		T/C	SNP	2909
		A/C	SNP	2940
		A/C	SNP	2945
		G/A	SNP	3008
		G/A	SNP	3011
		C/T	SNP	3073
		C/G	SNP	3120
		C/T	SNP	3121
		G/A	SNP	3126
XM_822590.1 Tbb calcium motive p-type ATPase (Tb09.244.2570) partial mRNA	GLOS_TB09.244.2570.1.1	G/T	SNP	1232
XP_827681.1 60S ribosomal protein L32 [T. b. brucei strain 927/4 GUTat10.1]	GLOS_TB09.244.2590.1.1	C/T	SNP	74
		AAC/A	DELETION	447

		AAT/A	DELETION	511
XM_822584.1 Tbb 40S ribosomal protein S6 (Tb09.244.2630) partial mRNA	GLOS_TB09.244.2630.1.1	C/CT	INSERTION	304
		GAA/G	DELETION	419
		T/A	SNP	1280
		G/A	SNP	1281
XM_822578.1 Tbb GUTat10.1 hypothetical protein (Tb09.244.2660) partial mRNA	GLOS_TB09.244.2660.1.1	G/A	SNP	251
		C/A	SNP	4293
XM_822570.1 Tbb GUTat10.1 ribosomal protein L15 (Tb09.244.2720) partial mRNA	GLOS_TB09.244.2720.1.1	A/C	SNP	407
		G/A	SNP	413
		T/C	SNP	451
		A/C	SNP	503
		C/T	SNP	533
		C/G	SNP	569
		C/T	SNP	571
		A/G	SNP	592
		T/C	SNP	593
		T/C	SNP	632
		C/G	SNP	680
		T/C	SNP	710
		T/C	SNP	734
		A/G	SNP	740
		C/T	SNP	785
		C/G	SNP	812
		G/A	SNP	821
		C/T	SNP	830
		G/A	SNP	848
		G/A	SNP	899
XM_822571.1 Tbb ribosomal protein L36 (Tb09.244.2725) partial mRNA	GLOS_TB09.244.2725.1.1	G/T	SNP	493
		C/A	SNP	496
XM_822569.1 Tbb 60S ribosomal protein L5 (Tb09.244.2730) partial mRNA	GLOS_TB09.244.2730.1.1	C/T	SNP	35
		A/G	SNP	36
		A/G	SNP	65
		T/C	SNP	996
XM_798607.1 Tbb GUTat10.1 hypothetical protein (Tb09.v1.0150) partial mRNA	GLOS_TB09.V1.0150.1.1	T/C	SNP	3107
		A/G	SNP	3289
		T/G	SNP	3483

XM_821914.1 Tbb GUTat10.1 hypothetical protein (Tb09.v2.0030) partial mRNA	GLOS_TB09.V2.0030.1.1	C/T	SNP	121
		C/A	SNP	126
		A/G	SNP	129
		T/C	SNP	143
XP_827840.1 diphosphomevalonate decarboxylase [Tbb strain 927/4 GUTat10.1]	GLOS_TB10.05.0010.1.1	T/G	SNP	257
		G/T	SNP	328
		A/G	SNP	623
		G/A	SNP	633
		G/A	SNP	803
		G/A	SNP	1130
		T/C	SNP	1440
XM_822754.1 Tbb GUTat10.1 glucosidase (Tb10.05.0080) partial mRNA	GLOS_TB10.05.0080.1.1	T/C	SNP	840
		C/T	SNP	1433
		C/T	SNP	1817
		T/C	SNP	2730
XM_822757.1 T. b. brucei strain 927/4 GUTat10.1 serine/threonine protein phosphatase type 5 (Tb10.05.0110) partial mRNA	GLOS_TB10.05.0110.1.1	T/C	SNP	443
		A/G	SNP	2200
		CA/C	DELETION	2513
XM_822741.1 Tbb 60S ribosomal protein L10a (Tb10.05.0220) partial mRNA	GLOS_TB10.05.0220.1.1	GA/G	DELETION	176
		T/G	SNP	183
		C/A	SNP	447
		T/C	SNP	546
		G/A	SNP	831
		A/G	SNP	849
		C/A	SNP	1059
XP_822287.1 60S ribosomal protein L32 [T. b. brucei strain 927/4 GUTat10.1]	GLOS_TB10.100.0155.1.1	G/T	SNP	503
		G/T	SNP	504
		A/C	SNP	505
XP_823384.1 hypothetical protein [T. b. brucei strain 927/4 GUTat10.1]	GLOS_TB10.26.0100.1.1	T/TA	INSERTION	94
		C/CT	INSERTION	831
		C/CA	INSERTION	934
XM_818290.1 Tbb pumilio RNA-binding protein (Tb10.26.0140) partial mRNA	GLOS_TB10.26.0140.1.1	GAA/G	DELETION	131
		AAAT/A	DELETION	230
		A/T	SNP	565
		T/TTCA	INSERTION	719

		GA/G	DELETION	1660
		T/A	SNP	1758
		C/CT	INSERTION	4806
XP_823361.1 40S ribosomal protein S3 [T. brucei brucei strain 927/4 GUTat10.1]	GLOS_TB10.26.0370.1.1	G/T	SNP	165
		T/C	SNP	219
		T/A	SNP	252
XM_818259.1 Tbb GUTat10.1 CYC2-like cyclin (Tb10.26.0510) partial mRNA	GLOS_TB10.26.0510.1.1	C/A	SNP	230
		G/GT	INSERTION	686
		T/TA	INSERTION	1256
		G/GCA	INSERTION	1410
		C/G	SNP	1912
		A/G	SNP	1940
		A/T	SNP	1941
		A/T	SNP	1959
		A/G	SNP	2915
		C/T	SNP	5205
XM_818254.1 Tbb GUTat10.1 60S ribosomal protein L6 (Tb10.26.0560) partial mRNA	GLOS_TB10.26.0560.1.1	T/A	SNP	733
XP_823339.1 hypothetical protein [T. brucei brucei strain 927/4 GUTat10.1]	GLOS_TB10.26.0680.1.1	GA/G	DELETION	112
XM_818238.1 Tbb procyclic form surface glycoprotein (Tb10.26.0790) partial mRNA	GLOS_TB10.26.0790.1.1	C/T	SNP	127
		A/G	SNP	177
		A/T	SNP	218
		C/G	SNP	537
		T/C	SNP	594
		G/T	SNP	831
		T/A	SNP	1616
		G/A	SNP	1617
		G/A	SNP	2010
		G/A	SNP	2139
XM_818231.1 Tbb GUTat10.1 hypothetical protein (Tb10.26.0880) partial mRNA	GLOS_TB10.26.0880.1.1	C/T	SNP	271
		C/T	SNP	782
		TA/T	DELETION	949
		A/C	SNP	964
		C/T	SNP	1005
		G/GT	INSERTION	2054
		A/G	SNP	2069
		G/A	SNP	2116

XM_818214.1 Tbb GUTat10.1 heat shock protein 83 (Tb10.26.1080) partial mRNA	GLOS_TB10.26.1080.1.1	A/T	SNP	140
		G/A	SNP	178
		GAAA/GA/(	DELETION	212
		A/G	SNP	1930
		G/A	SNP	1957
		G/A	SNP	2086
		T/A	SNP	2475
		C/T	SNP	2476
		G/T	SNP	2477
		C/T	SNP	2479
		A/C	SNP	2482
XM_822728.1 Tbb GUTat10.1 elongation factor TU (Tb10.389.0070) partial mRNA	GLOS_TB10.389.0070.1.1	A/C	SNP	575
XM_822697.1 Trypanosoma brucei brucei strain 927/4 GUTat10.1 receptor-type adenylate cyclase GRESAG 4 (Tb10.389.0430) partial mRNA	GLOS_TB10.389.0430.1.1	A/G	SNP	244
		G/A	SNP	311
		A/G	SNP	812
		T/TG	INSERTION	3816
		A/C	SNP	3893
XP_827786.1 3' 5'-cyclic nucleotide phosphodiesterase [T. brucei TREU927]	GLOS_TB10.389.0510.1.1	G/GT	INSERTION	1191
XM_822687.1 Tbb GUTat10.1 hypothetical protein (Tb10.389.0570) partial mRNA	GLOS_TB10.389.0570.1.1	G/A	SNP	116
		G/A	SNP	369
		A/T	SNP	1226
		T/C	SNP	2546
XM_822682.1 Tbb prolyl-tRNA synthetase (Tb10.389.0630) partial mRNA	GLOS_TB10.389.0630.1.1	G/C	SNP	1287
		T/G	SNP	2696
XP_823487.1 hypothetical protein [T. brucei brucei strain 927/4 GUTat10.1]	GLOS_TB10.389.0680.1.1	G/A	SNP	116
		G/A	SNP	193
		GT/G	DELETION	253
		G/A	SNP	1587
		T/C	SNP	1765
XP_823486.1 mitochondrial carrier protein [T. brucei brucei strain 927/4 GUTat10.1]	GLOS_TB10.389.0690.1.1	T/G	SNP	90
		C/T	SNP	573
		C/T	SNP	969
		A/T	SNP	1107
XP_823483.1 cholinephosphate cytidylyltransferase A [T. brucei TREU927]	GLOS_TB10.389.0730.1.1	C/T	SNP	1116
		TA/T	DELETION	1634

		C/A	SNP	2018
		C/CA	INSERTION	2018
XM_818383.1 Tbb GUTat10.1 heat shock protein (Tb10.389.0880) partial mRNA	GLOS_TB10.389.0880.1.1	A/G	SNP	1705
		C/T	SNP	2040
		G/T	SNP	2833
XM_818380.1 Tbb 60S ribosomal protein L34 (Tb10.389.0910) partial mRNA	GLOS_TB10.389.0910.1.2	C/T	SNP	255
		T/C	SNP	271
		T/C	SNP	280
		C/T	SNP	289
		G/A	SNP	292
		G/A	SNP	794
		T/C	SNP	796
XM_818380.1 Tbb 60S ribosomal protein L34 (Tb10.389.0910) partial mRNA	GLOS_TB10.389.0910.2.2	A/C	SNP	37
		T/G	SNP	282
		T/C	SNP	290
		G/A	SNP	294
		G/T	SNP	510
		A/G	SNP	513
		G/A	SNP	516
		T/G	SNP	519
XM_818362.1 T. GUTat10.1 P-type H+-ATPase (Tb10.389.1180) partial mRNA	GLOS_TB10.389.1180.1.1	A/G	SNP	873
		T/C	SNP	874
		C/T	SNP	1681
		C/T	SNP	1682
		C/T	SNP	1709
		A/G	SNP	2112
		G/A	SNP	2118
		C/G	SNP	2495
		AT/A	DELETION	2843
		G/GT	INSERTION	2998
		T/G	SNP	3034
		CT/C	DELETION	3533
		C/CT	INSERTION	4130
		G/GA	INSERTION	4348
		G/A	SNP	4725
		C/CT	INSERTION	4888

XM_818338.1 Tbb cytosolic nonspecific dipeptidase (Tb10.389.1480) partial mRNA	GLOS_TB10.389.1480.1.1	A/G	SNP	1288
		AT/A	DELETION	1742
XP_823403.1 kynurenine aminotransferase [T. b. brucei strain 927/4 GUTat10.1]	GLOS_TB10.389.1810.1.1	A/G	SNP	227
		G/T	SNP	1058
		C/G	SNP	2075
		G/T	SNP	2231
		T/C	SNP	2399
XM_818174.1 T. b. brucei strain 927/4 GUTat10.1 histone H2B (Tb10.406.0450) partial mRNA ref|XM_818176.1| Tbb histone H2B (Tb10.406.0430) partial mRNA ref|XM_818177.1| Tbb histone H2B (Tb10.40.0420) partial mRNA ref|XM_818178.1| Tbb histone H2B (Tb10.406.0410) partial mRNA ref|XM_818179.1| Tbb histone H2B (Tb10.406.0400) partial mRNA ref|XM_818181.1| Tbb histone H2B (Tb10.406.0380) partial mRNA ref|XM_818182.1| Tbb histone H2B (Tb10.406.0370) partial mRNA ref|XM_818184.1| Tbb histone H2B (Tb10.406.0350) partial mRNA	GLOS_TB10.406.0450.1.1	T/A	SNP	28
		T/C	SNP	412
		C/T	SNP	513
		C/CGT/CG	INSERTION	578
		T/C	SNP	615
		C/T	SNP	621
		C/T	SNP	753
XM_818156.1 Tbb microtubule-associated protein (Tb10.406.0650) partial mRNA	GLOS_TB10.406.0650.1.1	G/A	SNP	43
		C/T	SNP	61
		G/A	SNP	100
		G/A	SNP	145
		C/T	SNP	153
		G/A	SNP	214
		T/C	SNP	246
		A/G	SNP	484
		A/AT	INSERTION	1335
		A/G	SNP	1361
		G/A	SNP	1629
XM_822953.1 Tbb GUTat10.1 hypothetical protein (Tb10.61.0540) partial mRNA	GLOS_TB10.61.0540.1.1	C/T	SNP	927
		GAT/G	DELETION	1491
XM_822919.1 Tbb glycosomal malate dehydrogenase (Tb10.61.0980) partial mRNA	GLOS_TB10.61.0980.1.1	G/GA	INSERTION	257
		G/GAAA	INSERTION	272
		G/A	SNP	279

		G/A	SNP	286
		T/TA	INSERTION	347
		C/CAT	INSERTION	433
		C/T	SNP	479
		A/T	SNP	495
		A/G	SNP	616
		T/C	SNP	727
		G/T	SNP	800
		T/C	SNP	884
		T/C	SNP	924
		C/A	SNP	945
		T/C	SNP	947
		T/C	SNP	949
		C/T	SNP	977
		C/T	SNP	987
		G/A	SNP	1058
		C/T	SNP	1136
		C/T	SNP	1228
		C/T	SNP	1329
		A/G	SNP	1718
		T/G	SNP	1775
		G/A	SNP	1889
		G/A	SNP	2092
XM_822905.1 Tbb GUTat10.1 hypothetical protein (Tb10.61.1260) partial mRNA	GLOS_TB10.61.1260.1.1	CT/C	DELETION	945
		TAC/T	DELETION	1183
XM_822898.1 Tbb nucleosome assembly protein (Tb10.61.1330) partial mRNA	GLOS_TB10.61.1330.1.1	G/T	SNP	227
		T/G	SNP	252
XP_827985.1 40S ribosomal protein S13 [T. b. brucei strain 927/4 GUTat10.1] ref|XP_951734.1| 40S ribosomal protein S13 [Tbb strain 927/4 GUTat10.1]	GLOS_TB10.61.1390.2.2	A/C	SNP	3
		G/GA	INSERTION	82
		C/T	SNP	143
		C/T	SNP	251
		C/A	SNP	350
XP_827964.1 hypothetical protein [T. brucei brucei strain 927/4 GUTat10.1]	GLOS_TB10.61.1790.1.1	G/T	SNP	108
		T/TA	INSERTION	109
		G/GAA	INSERTION	197

		G/T	SNP	474
		A/G	SNP	754
		A/G	SNP	796
		C/G	SNP	875
XM_822868.1 Tbb strain 927/4 GUTat10.1 mitochondrial carrier prot (Tb10.61.1820) partial mRNA. nuclear gene for mitoch. product ref|XM_822869.1| Tbb mitoch. carrier prot (Tb10.61.1810) partial mRNA. nuclear gene for mitochondrial product	GLOS_TB10.61.1820.1.1	ATT/A	DELETION	336
		C/T	SNP	482
		C/T	SNP	1299
XP_827959.1 hypothetical protein [T. brucei brucei strain 927/4 GUTat10.1]	GLOS_TB10.61.1840.1.1	T/A	SNP	212
		T/C	SNP	248
		G/A	SNP	351
XM_822864.1 Tbb GUTat10.1 aminopeptidase (Tb10.61.1870) partial mRNA	GLOS_TB10.61.1870.1.1	C/CT	INSERTION	1310
		T/C	SNP	1509
XM_822860.1 Tbb GUTat10.1 fibrillarin (Tb10.61.1920) partial mRNA	GLOS_TB10.61.1920.1.1	C/T	SNP	441
XM_822858.1 Tbb GUTat10.1 chaperone protein DnaJ (Tb10.61.1940) partial mRNA	GLOS_TB10.61.1940.1.1	C/A	SNP	101
		G/A	SNP	407
		A/G	SNP	527
		A/C	SNP	554
		G/GT	INSERTION	1587
XM_822856.1 Tbb 40S ribosomal protein S2 (Tb10.61.1960) partial mRNA	GLOS_TB10.61.1960.1.2	G/T	SNP	77
XM_822856.1 Tbb 40S ribosomal protein S2 (Tb10.61.1960) partial mRNA	GLOS_TB10.61.1960.2.2	C/T	SNP	81
XM_822855.1 Tbb GUTat10.1 hypothetical protein (Tb10.61.1970) partial mRNA	GLOS_TB10.61.1970.1.1	C/G	SNP	507
		C/A	SNP	970
		A/G	SNP	1185
XM_822844.1 Tbb 60S ribosomal protein L17 (Tb10.61.2090) partial mRNA	GLOS_TB10.61.2090.1.2	G/T	SNP	609
XM_822840.1 T. brucei brucei strain 927/4 GUTat10.1 proteasome regulatory non-ATPase subunit 8 (Tb10.61.2180) partial mRNA	GLOS_TB10.61.2180.1.1	C/G	SNP	453
		C/T	SNP	1343
XM_822837.1 T. GUTat10.1 hypothetical protein (Tb10.61.2210) partial mRNA	GLOS_TB10.61.2210.1.1	A/T	SNP	46
		C/T	SNP	77
		T/C	SNP	1102
		T/TC	INSERTION	1281
XM_822836.1 Tbb GUTat10.1 hypothetical protein (Tb10.61.2220) partial mRNA	GLOS_TB10.61.2220.1.1	G/A	SNP	871
		T/C	SNP	1207
		A/G	SNP	1393

		CAAA/C	DELETION	1625
		T/G	SNP	1741
		C/CT	INSERTION	2388
		C/CT	INSERTION	2693
XM_822833.1 Tbb GUTat10.1 hypothetical protein (Tb10.61.2270) partial mRNA	GLOS_TB10.61.2270.1.1	G/A	SNP	278
		A/G	SNP	783
		G/C	SNP	1106
		T/A	SNP	1982
		C/CT	INSERTION	3251
XM_822830.1 Tbb GUTat10.1 hypothetical protein (Tb10.61.2300) partial mRNA	GLOS_TB10.61.2300.1.1	CA/C	DELETION	298
		G/GA	INSERTION	630
		G/A	SNP	821
		G/A	SNP	2149
		C/G	SNP	2245
		G/A	SNP	3352
XM_822793.1 Tbb GUTat10.1 hypothetical protein (Tb10.61.2850) partial mRNA	GLOS_TB10.61.2850.1.1	A/T	SNP	53
XP_827883.1 aconitase [Trypanosoma brucei brucei strain 927/4 GUTat10.1]	GLOS_TB10.61.2880.1.1	T/A	SNP	475
		GA/G	DELETION	707
		GT/G	DELETION	767
		TAATA/T	DELETION	945
		C/CT	INSERTION	1092
		C/CTT	INSERTION	1354
XM_822770.1 Tbb GUTat10.1 hypothetical protein (Tb10.61.3120) partial mRNA	GLOS_TB10.61.3120.1.1	G/GA	INSERTION	803
		T/TA	INSERTION	1980
		A/G	SNP	2496
		C/CA	INSERTION	2504
		T/C	SNP	2566
		T/C	SNP	3185
		C/G	SNP	3545
XM_818154.1 Tbb EP1 procyclin precursor (Tb10.6k15.0020) partial mRNA	GLOS_TB10.6K15.0020.2.3	A/T	SNP	774
XM_818133.1 Tbb GUTat10.1 hypothetical protein (Tb10.6k15.0240) partial mRNA	GLOS_TB10.6K15.0240.1.1	G/T	SNP	83
		C/A	SNP	479
		C/A	SNP	481
		T/G	SNP	707
		CA/C	DELETION	758
		G/A	SNP	1508

		G/C	SNP	2457
		C/CGT	INSERTION	5165
		GA/G	DELETION	6320
XM_818129.1 Tbb GUTat10.1 hypothetical protein (Tb10.6k15.0280) partial mRNA	GLOS_TB10.6K15.0280.1.1	CA/C	DELETION	188
		CT/C	DELETION	401
XM_818120.1 Tbb GUTat10.1 hypothetical protein (Tb10.6k15.0380) partial mRNA	GLOS_TB10.6K15.0380.1.1	C/CA	INSERTION	315
		A/G	SNP	1656
		ATG/A	DELETION	3198
		A/AT	INSERTION	3490
XM_818117.1 Tbb 60S ribosomal protein L18 (Tb10.6k15.0410) partial mRNA	GLOS_TB10.6K15.0410.1.1	A/G	SNP	99
		T/C	SNP	138
		A/G	SNP	168
		G/A	SNP	192
		C/T	SNP	195
		T/C	SNP	198
		C/G	SNP	213
		C/T	SNP	240
		A/G	SNP	450
		C/T	SNP	534
XM_818108.1 Tbb GUTat10.1 hypothetical protein (Tb10.6k15.0520) partial mRNA	GLOS_TB10.6K15.0520.1.1	C/T	SNP	809
		A/T	SNP	810
		T/C	SNP	901
		C/T	SNP	919
		T/C	SNP	1018
		G/C	SNP	1021
		T/C	SNP	1042
		A/G	SNP	1075
		C/T	SNP	1116
		G/T	SNP	1117
		A/G	SNP	1120
		A/G	SNP	1189
		T/C	SNP	1210
		A/C	SNP	1222
		G/A	SNP	1231
		A/G	SNP	1237
		A/G	SNP	1300

		GT/G	DELETION	1636
XP_823188.1 hypothetical protein [T. brucei brucei strain 927/4 GUTat10.1]	GLOS_TB10.6K15.0690.1.1	C/G	SNP	1064
		C/CA	INSERTION	2650
XM_818050.1 Tbb isoleucyl-tRNA synthetase (Tb10.6k15.1220) partial mRNA	GLOS_TB10.6K15.1220.1.1	A/G	SNP	275
		G/GT	INSERTION	2320
XM_818039.1 Tbb GUTat10.1 pteridine transporter (Tb10.6k15.1350) partial mRNA	GLOS_TB10.6K15.1350.1.1	CA/C	DELETION	387
		GA/G	DELETION	573
		T/C	SNP	1415
XP_823117.1 hypothetical protein [T. brucei brucei strain 927/4 GUTat10.1]	GLOS_TB10.6K15.1510.1.1	G/A	SNP	304
		G/GA	INSERTION	316
		G/A	SNP	1001
		G/A	SNP	1060
		A/G	SNP	1097
		A/G	SNP	2111
XM_818023.1 Tbb GUTat10.1 small GTPase (Tb10.6k15.1520) partial mRNA	GLOS_TB10.6K15.1520.1.1	C/CT	INSERTION	1108
		T/C	SNP	1131
XM_818004.1 Tbb GUTat10.1 hypothetical protein (Tb10.6k15.1820) partial mRNA	GLOS_TB10.6K15.1820.1.1	GA/G	DELETION	583
XP_823066.1 cytochrome c oxidase subunit IX [T. b. brucei strain 927/4 GUTat10.1]	GLOS_TB10.6K15.2180.1.1	A/G	SNP	121
		ATAAT/A	DELETION	248
		C/CT	INSERTION	302
		A/T	SNP	728
XM_817968.1 Trypanosoma brucei brucei strain 927/4 GUTat10.1 eukaryotic translation initiation factor 3 subunit 8 (Tb10.6k15.2250) partial mRNA	GLOS_TB10.6K15.2250.1.1	T/C	SNP	520
		A/T	SNP	641
		G/GT	INSERTION	740
XP_823053.1 t-complex protein 1 subunit theta [Tbb strain 927/4 GUTat10.1]	GLOS_TB10.6K15.2330.1.1	A/G	SNP	1067
		C/CT	INSERTION	1832
XM_817944.1 Tbb GUTat10.1 hypothetical protein (Tb10.6k15.2510) partial mRNA	GLOS_TB10.6K15.2510.1.1	T/C	SNP	449
		G/A	SNP	1350
XP_823027.1 2,3-bisphosphoglycerate-independent phosphoglycerate mutase [Tbb]	GLOS_TB10.6K15.2620.1.1	A/G	SNP	81
		G/C	SNP	1930
		GA/G	DELETION	2277
XM_817930.1 Tbb GUTat10.1 hypothetical protein (Tb10.6k15.2660) partial mRNA	GLOS_TB10.6K15.2660.1.1	C/T	SNP	221
		CT/C	DELETION	422
		G/GA	INSERTION	614
		CA/C	DELETION	740

		G/GA	INSERTION	1036
		CT/C	DELETION	1287
		C/T	SNP	1338
		C/CT	INSERTION	1410
		G/GT	INSERTION	1581
		C/T	SNP	2776
		T/C	SNP	2807
XM_817927.1 Tbb GUTat10.1 hypothetical protein (Tb10.6k15.2690) partial mRNA	GLOS_TB10.6K15.2690.1.1	CT/C	DELETION	261
		C/CT	INSERTION	377
XM_817898.1 Trypanosoma brucei brucei strain 927/4 GUTat10.1 dihydrolipoamide acetyltransferase precursor (Tb10.6k15.3080) partial mRNA	GLOS_TB10.6K15.3080.1.1	C/G	SNP	529
XM_817883.1 Trypanosoma brucei brucei strain 927/4 GUTat10.1 succinyl-CoA ligase [GDP-forming] beta-chain (Tb10.6k15.3250) partial mRNA	GLOS_TB10.6K15.3250.1.1	A/T	SNP	175
		T/TC	INSERTION	483
XM_817875.1 Tbb 40S ribosomal protein S24e (Tb10.6k15.3350) partial mRNA	GLOS_TB10.6K15.3350.1.1	A/G	SNP	522
		C/T	SNP	523
		A/G	SNP	525
		A/C	SNP	530
		T/C	SNP	531
XP_822958.1 hypothetical protein [T. brucei brucei strain 927/4 GUTat10.1]	GLOS_TB10.6K15.3460.1.1	A/C	SNP	45
		G/A	SNP	2857
XM_817860.1 Trypanosoma brucei brucei strain 927/4 GUTat10.1 cysteine-rich acidic integral membrane protein precursor (Tb10.6k15.3510) partial mRNA	GLOS_TB10.6K15.3510.1.1	C/T	SNP	292
		A/G	SNP	293
		C/T	SNP	328
		AT/A	DELETION	751
XM_817839.1 Tbb dipeptidyl-peptidase 8-like serine peptidase, partial mRNA	GLOS_TB10.6K15.3800.1.1	C/A	SNP	654
		C/G	SNP	1039
		G/A	SNP	1080
		G/A	SNP	1085
		C/T	SNP	2147
		T/C	SNP	2589
		C/T	SNP	3540
XM_817837.1 Tbb sterol 24-c-methyltransferase (Tb10.6k15.3820) partial mRNA	GLOS_TB10.6K15.3820.1.1	G/A	SNP	86
		C/T	SNP	342
		C/T	SNP	1382

		G/A	SNP	2113
XM_817822.1 Tbb GUTat10.1 hypothetical protein (Tb10.70.0010) partial mRNA	GLOS_TB10.70.0010.1.1	G/C	SNP	429
		G/A	SNP	889
		C/A	SNP	890
		A/G	SNP	947
		C/T	SNP	948
XM_817788.1 Tbb GUTat10.1 hypothetical protein (Tb10.70.0440) partial mRNA	GLOS_TB10.70.0440.1.1	G/A	SNP	137
		C/T	SNP	196
		T/C	SNP	626
		T/G	SNP	701
		T/C	SNP	798
		G/A	SNP	801
		A/AT	INSERTION	866
		CAA/C	DELETION	1945
		T/TA	INSERTION	2167
XM_817786.1 Tbb 60S ribosomal proteins L37 (Tb10.70.0465) partial mRNA	GLOS_TB10.70.0465.1.1	C/A	SNP	342
		T/G	SNP	343
		G/A	SNP	375
		A/G	SNP	381
		C/T	SNP	537
XP_822855.1 hypothetical protein [T. brucei brucei strain 927/4 GUTat10.1]	GLOS_TB10.70.0730.1.1	TA/T	DELETION	502
		G/GA	INSERTION	1000
		A/G	SNP	1440
XM_817756.1 T. brucei brucei strain 927/4 GUTat10.1 universal minicircle sequence binding protein (UMSBP) (Tb10.70.0800) partial mRNA	GLOS_TB10.70.0800.1.2	CT/C	DELETION	1688
XP_822847.1 clathrin heavy chain [T. brucei brucei strain 927/4 GUTat10.1]	GLOS_TB10.70.0830.1.1	AT/A	DELETION	311
		GT/G	DELETION	651
		T/C	SNP	2441
XP_822838.1 protein kinase [Trypanosoma brucei brucei strain 927/4 GUTat10.1]	GLOS_TB10.70.0960.1.1	T/C	SNP	138
		G/GA	INSERTION	534
		C/G	SNP	815
		C/T	SNP	819
		T/C	SNP	893
		G/A	SNP	894
		G/A	SNP	1223
		T/C	SNP	1259

		G/C	SNP	1299
		G/A	SNP	1638
		T/C	SNP	2007
		C/T	SNP	2336
XM_817733.1 Tbb GUTat10.1 hypothetical protein (Tb10.70.1120) partial mRNA	GLOS_TB10.70.1120.1.1	C/T	SNP	2473
XM_817732.1 Tbb GUTat10.1 hypothetical protein (Tb10.70.1130) partial mRNA	GLOS_TB10.70.1130.1.1	GA/G	DELETION	82
		C/CT	INSERTION	386
	GLOS_TB10.70.1130.1.1	C/CT	INSERTION	858
XP_822821.1 valosin-containing protein homolog [Trypanosoma brucei TREU927]	GLOS_TB10.70.1190.1.1	G/GT	INSERTION	2656
XM_817699.1 Tbb 60S ribosomal protein L24 (Tb10.70.1540) partial mRNA	GLOS_TB10.70.1540.1.2	C/T	SNP	561
XM_817699.1 Tbb 60S ribosomal protein L24 (Tb10.70.1540) partial mRNA	GLOS_TB10.70.1540.2.2	A/G	SNP	174
XP_822783.1 hypothetical protein [T. brucei brucei strain 927/4 GUTat10.1]	GLOS_TB10.70.1660.1.1	G/A	SNP	1945
		CA/C	DELETION	4107
XM_817685.1 Tbb 40S ribosomal protein S18 (Tb10.70.1740) partial mRNA	GLOS_TB10.70.1740.1.1	A/T	SNP	258
		A/T	SNP	259
		A/G	SNP	270
		A/G	SNP	290
		T/A	SNP	751
XM_817671.1 Tbb GUTat10.1 hypothetical protein (Tb10.70.1930) partial mRNA	GLOS_TB10.70.1930.1.1	G/A	SNP	1633
		CT/C	DELETION	2184
XM_817657.1 Tbb ubiquitin/ribosomal protein S27a (Tb10.70.2170) partial mRNA	GLOS_TB10.70.2170.1.1	G/T	SNP	267
XP_822722.1 hypothetical protein [T. brucei brucei strain 927/4 GUTat10.1]	GLOS_TB10.70.2460.1.1	G/T	SNP	2270
		C/CT	INSERTION	3079
XP_822703.1 elongation factor 2 [T. brucei brucei strain 927/4 GUTat10.1] ref|XP_8227(	GLOS_TB10.70.2660.1.1	C/T	SNP	2342
XP_822676.1 hypothetical protein [T. brucei brucei strain 927/4 GUTat10.1]	GLOS_TB10.70.2970.1.1	G/A	SNP	40
		C/A	SNP	41
XP_822663.1 hypothetical protein [T. brucei brucei strain 927/4 GUTat10.1]	GLOS_TB10.70.3120.1.1	G/T	SNP	979
		A/T	SNP	1198
		T/TA	INSERTION	1218
XP_822661.1 hypothetical protein [T. brucei brucei strain 927/4 GUTat10.1]	GLOS_TB10.70.3150.1.1	GA/G	DELETION	169
		C/G	SNP	345
		A/G	SNP	1515
		A/ATGTG	INSERTION	1650
		G/A	SNP	1755
		C/T	SNP	1853
XM_817567.1 Tbb 60S ribosomal protein L30 (Tb10.70.3160) partial mRNA	GLOS_TB10.70.3160.1.2	A/T	SNP	625

		T/A	SNP	630
XM_817563.1 Tbb GUTat10.1 hypothetical protein (Tb10.70.3190) partial mRNA	GLOS_TB10.70.3190.1.1	T/C	SNP	112
		A/G	SNP	1025
		C/A	SNP	1378
		C/A	SNP	2437
XP_822647.1 ATP-dependent DEAD-box RNA helicase [T. brucei TREU927]	GLOS_TB10.70.3290.1.1	T/A	SNP	1047
		T/C	SNP	1134
		T/G	SNP	1681
		G/T	SNP	1682
		T/C	SNP	1953
		G/A	SNP	2000
XM_817548.1 Tbb 40S ribosomal protein S3a (Tb10.70.3370) partial mRNA	GLOS_TB10.70.3370.1.1	C/T	SNP	104
		T/C	SNP	888
XP_822632.1 60S ribosomal protein L18a [Tbb strain 927/4 GUTat10.1] ref|XP_829469.1| ribosomal protein L18 [Tbb strain 927/4 GUTat10.1]	GLOS_TB10.70.3510.1.1	G/T	SNP	11
		G/T	SNP	12
		A/T	SNP	15
		A/G	SNP	401
		A/G	SNP	404
		C/T	SNP	500
		A/G	SNP	554
		G/A	SNP	569
XM_817496.1 Tbb 60S acidic ribosomal protein P2 (Tb10.70.4060) partial mRNA	GLOS_TB10.70.4060.1.1	C/CA	INSERTION	93
		G/T	SNP	123
		T/A	SNP	468
		T/G	SNP	474
		A/G	SNP	1021
XP_822579.1 60S ribosomal protein L38 [T. brucei brucei strain 927/4 GUTat10.1]	GLOS_TB10.70.4155.1.2	A/G	SNP	29
XP_822573.1 delta-1-pyrroline-5-carboxylate dehydrogenase [Trypanosoma brucei]	GLOS_TB10.70.4280.1.1	G/T	SNP	120
		AT/A	DELETION	179
		A/G	SNP	278
		A/G	SNP	517
		T/C	SNP	565
		A/G	SNP	682
		T/C	SNP	1597
		A/G	SNP	1627

		C/T	SNP	1829
		C/T	SNP	2167
XP_822550.1 hypothetical protein [T. brucei brucei strain 927/4 GUTat10.1]	GLOS_TB10.70.4590.1.1	C/T	SNP	212
		C/CT	INSERTION	298
		T/C	SNP	1112
		T/C	SNP	1114
XM_817450.1 Tbb importin subunit beta-1 (Tb10.70.4720) partial mRNA	GLOS_TB10.70.4720.1.1	CTT/C	DELETION	667
		CT/C	DELETION	1341
		G/GA	INSERTION	1572
		C/CT	INSERTION	1780
		C/CT	INSERTION	2019
		C/T	SNP	4474
XM_817444.1 Tbb ribosomal protein S25 (Tb10.70.4800) partial mRNA	GLOS_TB10.70.4800.1.2	T/TA	INSERTION	163
		T/A	SNP	706
		T/A	SNP	707
XM_817437.1 Tbb eukaryotic translat. initiation fact 5 (Tb10.70.4880) partial mRNA	GLOS_TB10.70.4880.1.1	G/A	SNP	767
		A/T	SNP	787
		A/T	SNP	1283
		C/CT	INSERTION	1384
XM_817433.1 Tbb GUTat10.1 hypothetical protein (Tb10.70.4930) partial mRNA	GLOS_TB10.70.4930.1.1	C/CA	INSERTION	113
		T/TA	INSERTION	1279
		C/T	SNP	1383
		A/G	SNP	1395
		G/T	SNP	1696
		A/G	SNP	2305
XM_817417.1 Tbb lysosomal alpha-mannosidase precursor partial mRNA	GLOS_TB10.70.5100.1.1	C/G	SNP	1218
		C/A	SNP	1462
		C/T	SNP	2174
		C/T	SNP	2295
XM_817416.1 Tbb strain 927/4 GUTat10.1 mitochondrial malate dehydrogenase (Tb10.70.5110) partial mRNA. nuclear gene for mitochondrial product	GLOS_TB10.70.5110.1.1	A/G	SNP	144
		C/T	SNP	309
		A/G	SNP	988
		C/T	SNP	990
		G/T	SNP	1563
XP_822506.1 adenylate kinase [Trypanosoma brucei brucei strain 927/4 GUTat10.1]	GLOS_TB10.70.5150.1.1	C/T	SNP	464

		C/T	SNP	799
		A/T	SNP	886
		G/GA	INSERTION	1244
		G/C	SNP	1311
		C/CT	INSERTION	1399
XM_817398.1 Tbb GUTat10.1 La protein (Tb10.70.5360) partial mRNA	GLOS_TB10.70.5360.1.1	G/GA	INSERTION	663
		T/C	SNP	883
		G/A	SNP	1232
XM_817385.1 Tbb GUTat10.1 hypothetical protein (Tb10.70.5500) partial mRNA	GLOS_TB10.70.5500.1.1	C/T	SNP	453
		A/G	SNP	2107
		G/T	SNP	2350
XM_817380.1 Tbb GUTat10.1 hypothetical protein (Tb10.70.5560) partial mRNA	GLOS_TB10.70.5560.1.1	C/CA	INSERTION	106
		G/T	SNP	154
		G/GT	INSERTION	1110
		CTG/C	DELETION	1151
		C/CT	INSERTION	1296
XP_822456.1 hexokinase [Trypanosoma brucei brucei strain 927/4 GUTat10.1]	GLOS_TB10.70.5820.1.1	T/C	SNP	456
		G/C	SNP	483
		C/G	SNP	811
		C/A	SNP	812
		G/A	SNP	815
		A/T	SNP	816
		G/A	SNP	818
		G/A	SNP	961
		C/CT	INSERTION	2197
		G/GA	INSERTION	2611
		GAA/G	DELETION	2743
XM_817361.1 Tbb GUTat10.1 major vault protein (Tb10.70.5840) partial mRNA	GLOS_TB10.70.5840.1.1	G/A	SNP	166
		A/G	SNP	3387
XM_817326.1 Tbb dual specificity protein phosphatase (Tb10.70.6300) partial mRNA	GLOS_TB10.70.6300.1.1	A/G	SNP	112
		A/G	SNP	2166
		C/T	SNP	2190
		T/C	SNP	2235
		C/T	SNP	2860
		A/AT	INSERTION	2906
		T/A	SNP	2975

		A/G	SNP	3037
XM_817321.1 Tbb GUTat10.1 ATPase subunit 9 (Tb10.70.6340) partial mRNA	GLOS_TB10.70.6340.1.1	TC/T	DELETION	163
		T/C	SNP	528
		T/C	SNP	783
		A/T	SNP	809
		A/T	SNP	867
		A/G	SNP	1049
		A/G	SNP	1261
		ATCT/A	DELETION	1454
		G/GA	INSERTION	1474
XM_817315.1 Tbb transcriptional regulatory protein NOT1 partial mRNA	GLOS_TB10.70.6450.1.1	T/C	SNP	2730
		A/G	SNP	4660
		A/G	SNP	4682
		A/G	SNP	5115
		GA/G	DELETION	6069
		G/GT	INSERTION	6125
XM_817314.1 Tbb methionyl-tRNA synthetase (Tb10.70.6470) partial mRNA	GLOS_TB10.70.6470.1.1	GT/G	DELETION	3218
		T/G	SNP	3229
		CTTT/C	DELETION	3332
XM_817313.1 Tbb GUTat10.1 hypothetical protein (Tb10.70.6480) partial mRNA	GLOS_TB10.70.6480.1.1	A/AAAAAG	INSERTION	363
		CT/C	DELETION	517
		T/A	SNP	529
		AT/A	DELETION	584
		T/C	SNP	604
		A/G	SNP	660
		A/G	SNP	933
		C/A	SNP	3672
		GA/G	DELETION	4885
XM_817286.1 Tbb strain 927/4 GUTat10.1 katanin (Tb10.70.6880) partial mRNA	GLOS_TB10.70.6880.1.1	CA/CAA/C	INSERTION	159
		CA/C	DELETION	1704
		A/C	SNP	2633
XP_822368.1 60S ribosomal protein L9 [T. brucei brucei strain 927/4 GUTat10.1]	GLOS_TB10.70.7010.1.1	G/A	SNP	284
		C/T	SNP	293
		C/T	SNP	317
		G/A	SNP	335
		G/A	SNP	416

		G/A	SNP	443
		T/C	SNP	449
		G/C	SNP	461
		A/C	SNP	503
		T/A	SNP	551
		G/A	SNP	554
		T/C	SNP	593
		C/T	SNP	677
		A/G	SNP	680
XP_822364.1 t-complex protein 1 subunit delta [Tbb strain 927/4 GUTat10.1]	GLOS_TB10.70.7050.1.1	G/GA	INSERTION	612
		A/AT	INSERTION	755
XM_817258.1 Tbb GUTat10.1 hypothetical protein (Tb10.70.7220) partial mRNA	GLOS_TB10.70.7220.1.1	A/G	SNP	187
		T/C	SNP	349
		T/C	SNP	636
		A/T	SNP	1146
		T/G	SNP	2562
XM_817219.1 Tbb ATP-dependent DEAD/H RNA helicase partial mRNA	GLOS_TB10.70.7730.1.1	A/G	SNP	390
		C/T	SNP	417
		A/G	SNP	759
		C/CT	INSERTION	1724
		G/A	SNP	2064
		A/G	SNP	2629
		T/TA	INSERTION	2771
		G/A	SNP	2858
		GA/G	DELETION	2860
		G/T	SNP	2884
		A/T	SNP	3058
		G/A	SNP	3180
		A/T	SNP	3283
		G/T	SNP	3388
		A/G	SNP	3781
XP_822310.1 hypothetical protein [T. brucei brucei strain 927/4 GUTat10.1]	GLOS_TB10.70.7760.1.1	C/CT	INSERTION	1864
XM_823801.1 Tbb ribosomal protein S26 (Tb11.01.0355) partial mRNA	GLOS_TB11.01.0355.1.1	G/T	SNP	12
XP_828962.1 ribose 5-phosphate isomerase [Tbb strain 927/4 GUTat10.1]	GLOS_TB11.01.0700.1.1	G/A	SNP	22
XM_823871.1 Tbb GUTat10.1 cation transporter (Tb11.01.0720) partial mRNA	GLOS_TB11.01.0720.1.1	A/G	SNP	164
		A/AT	INSERTION	1021

		G/A	SNP	1444
XM_823894.Tbb proteasome regulatory non-ATPase subunit 2 partial mRNA	GLOS_TB11.01.0960.1.1	A/G	SNP	1405
		G/C	SNP	3245
XP_829017.1 hypothetical protein [T. brucei brucei strain 927/4 GUTat10.1]	GLOS_TB11.01.1290.1.1	G/A	SNP	389
		A/G	SNP	1148
		T/TTA	INSERTION	1356
XM_823930.1 Tbb S-adenosylhomocysteine hydrolase (Tb11.01.1350) partial mRNA	GLOS_TB11.01.1350.1.1	G/GA	INSERTION	217
		C/T	SNP	2455
		G/A	SNP	3898
		C/T	SNP	3956
XM_823941.1 Trypanosoma brucei brucei strain 927/4 GUTat10.1 nascent polypeptide associated complex subunit alpha (Tb11.01.1465) partial mRNA	GLOS_TB11.01.1465.1.1	A/T	SNP	36
		C/G	SNP	40
		G/A	SNP	41
XP_829036.1 40S ribosomal protein S27 [T. brucei brucei strain 927/4 GUTat10.1]	GLOS_TB11.01.1475.3.3	C/CT	INSERTION	515
		C/CT	INSERTION	559
XP_829050.1 hypothetical protein [T. brucei brucei strain 927/4 GUTat10.1]	GLOS_TB11.01.1625.1.1	C/G	SNP	69
		G/GA	INSERTION	542
XM_823969.1 Tbb 2-oxoglutarate dehydrogenase E1 component partial mRNA	GLOS_TB11.01.1740.1.1	G/GT	INSERTION	4981
		CTT/C	DELETION	5136
XM_823974.1 Tbb 60S ribosomal protein L29 (Tb11.01.1790) partial mRNA	GLOS_TB11.01.1790.1.2	A/G	SNP	201
		A/T	SNP	207
XM_823983.1 Tbb GUTat10.1 hypothetical protein (Tb11.01.1880) partial mRNA	GLOS_TB11.01.1880.1.1	G/T	SNP	1356
XM_823986.1 Tbb GUTat10.1 hypothetical protein (Tb11.01.1910) partial mRNA	GLOS_TB11.01.1910.1.1	C/CT	INSERTION	157
		G/A	SNP	1981
		A/G	SNP	2594
XM_824025.1 Tbb GUTat10.1 hypothetical protein (Tb11.01.2310) partial mRNA	GLOS_TB11.01.2310.1.1	G/A	SNP	1814
XM_824027.1 Tbb GUTat10.1 hypothetical protein (Tb11.01.2330) partial mRNA	GLOS_TB11.01.2330.1.1	G/A	SNP	1810
		T/TC	INSERTION	2367
		CT/C	DELETION	2518
XM_824043.1 Tbb GUTat10.1 hypothetical protein (Tb11.01.2490) partial mRNA	GLOS_TB11.01.2490.1.1	C/T	SNP	858
		T/C	SNP	2287
XM_824062.1 Tbb 40S ribosomal protein SA (Tb11.01.2680) partial mRNA	GLOS_TB11.01.2680.1.1	C/A	SNP	127
		T/A	SNP	128
		C/T	SNP	134
		C/T	SNP	975

		A/T	SNP	1101
XM_824073.1 Tbb GUTat10.1 hypothetical protein (Tb11.01.2800) partial mRNA	GLOS_TB11.01.2800.1.1	G/T	SNP	1568
XP_829173.1 hypothetical protein [T. brucei brucei strain 927/4 GUTat10.1]	GLOS_TB11.01.2880.1.1	C/G	SNP	213
		A/G	SNP	438
		G/A	SNP	895
		G/A	SNP	955
		G/A	SNP	960
		A/G	SNP	1000
		G/A	SNP	1020
XM_824095.1 Tbb 40S ribosomal protein L14 (Tb11.01.3020) partial mRNA	GLOS_TB11.01.3020.1.1	C/T	SNP	160
		C/A	SNP	185
		T/C	SNP	366
		C/T	SNP	387
		G/C	SNP	408
		T/C	SNP	411
		A/G	SNP	423
		C/T	SNP	444
		C/T	SNP	488
		G/A	SNP	492
XM_824105.1 Tbb GUTat10.1 heat shock protein 70 (Tb11.01.3110) partial mRNA	GLOS_TB11.01.3110.1.1	G/A	SNP	307
		A/G	SNP	396
		T/G	SNP	1855
		CGGTATG	DELETION	1948
		T/A	SNP	1972
		TGGTATG	DELETION	1972
XM_824118.1 Tbb GUTat10.1 hypothetical protein (Tb11.01.3290) partial mRNA	GLOS_TB11.01.3290.1.1	GA/G	DELETION	2271
		A/T	SNP	2283
		C/T	SNP	2590
XM_824120.1 Tbb strain 927/4 GUTat10.1 trichohyalin (Tb11.01.3320) partial mRNA	GLOS_TB11.01.3320.1.1	A/AGG	INSERTION	607
		A/G	SNP	978
		G/A	SNP	2202
		A/C	SNP	2481
		C/T	SNP	2495
		T/G	SNP	2496
		T/C	SNP	2956
XM_824124.1 Tbb glycosomal membrane protein (Tb11.01.3370) partial mRNA	GLOS_TB11.01.3370.1.1	A/T	SNP	80

		A/T	SNP	83
		G/A	SNP	240
		C/T	SNP	364
		G/A	SNP	538
		G/A	SNP	838
XP_829237.1 membrane-bound acid phosphatase [Tbb strain 927/4 GUTat10.1]	GLOS_TB11.01.3610.1.1	G/A	SNP	37
XP_829244.1 40S ribosomal prot S17 [Tbb strain 927/4 GUTat10.1] ref|XP_829245.1| 40S ribosomal protein S17 [Trypanosoma brucei brucei strain 927/4 GUTat10.1]	GLOS_TB11.01.3675.1.2	A/G	SNP	19
		C/T	SNP	21
		A/G	SNP	398
XM_824159.1 Tbb coatomer subunit gamma (Tb11.01.3740) partial mRNA	GLOS_TB11.01.3740.1.1	A/AT	INSERTION	2624
		A/G	SNP	3081
XP_829266.1 hypothetical protein [T. brucei brucei strain 927/4 GUTat10.1]	GLOS_TB11.01.3860.1.1	C/T	SNP	409
		G/GT	INSERTION	448
XP_829272.1 hypothetical protein [T. brucei brucei strain 927/4 GUTat10.1]	GLOS_TB11.01.3915.1.1	G/A	SNP	643
		C/CT	INSERTION	692
		T/A	SNP	940
		A/G	SNP	1167
		T/G	SNP	1168
		A/G	SNP	1170
		A/G	SNP	1171
		A/G	SNP	1172
		GGA/G	DELETION	1257
		G/T	SNP	1360
		AG/A	DELETION	1374
		T/C	SNP	1388
XM_824190.1 Tbb GUTat10.1 hypothetical protein (Tb11.01.4030) partial mRNA	GLOS_TB11.01.4030.1.1	A/G	SNP	217
		A/C	SNP	270
		G/A	SNP	286
		G/C	SNP	1440
		C/T	SNP	1736
		TA/T	DELETION	2387
XM_824200.1 Tbb GUTat10.1 protein kinase (Tb11.01.4130) partial mRNA	GLOS_TB11.01.4130.1.1	T/C	SNP	3110
		G/GT	INSERTION	3197
		C/T	SNP	3229
		GGAA/G	DELETION	3515

XP_829294.1 hypothetical protein [T. brucei brucei strain 927/4 GUTat10.1]	GLOS_TB11.01.4140.1.1	C/A	SNP	617
		A/G	SNP	680
		A/G	SNP	791
		T/C	SNP	1144
		T/C	SNP	1176
		A/T	SNP	1196
		G/C	SNP	1344
		C/T	SNP	1687
		A/G	SNP	1908
		CA/C	DELETION	2867
		A/G	SNP	2921
XM_824233.1 Tbb GUTat10.1 hypothetical protein (Tb11.01.4480) partial mRNA	GLOS_TB11.01.4480.1.1	C/T	SNP	625
		C/A	SNP	640
		T/G	SNP	750
		T/C	SNP	1098
		G/A	SNP	1752
		C/T	SNP	2516
		C/T	SNP	2780
XP_829371.1 cytochrome c oxidase subunit 10 [T. b. brucei strain 927/4 GUTat10.1]	GLOS_TB11.01.4702.1.1	T/A	SNP	49
		A/G	SNP	363
		C/CAA/CA	INSERTION	579
XM_824282.1 Tbb GUTat10.1 hypothetical protein (Tb11.01.4740) partial mRNA	GLOS_TB11.01.4740.1.1	G/GA	INSERTION	274
		CTTT/C/CT	DELETION	878
		TC/T	DELETION	1094
		G/GT	INSERTION	1785
		T/C	SNP	2575
		C/G	SNP	2823
XM_824283.1 Tbb elongation factor 1 gamma (Tb11.01.4750) partial mRNA	GLOS_TB11.01.4750.1.1	C/A	SNP	364
		C/T	SNP	443
		C/T	SNP	449
		G/A	SNP	633
XM_824291.1 Tbb GUTat10.1 hypothetical protein (Tb11.01.4850) partial mRNA	GLOS_TB11.01.4850.1.1	GA/G	DELETION	1210
		A/G	SNP	1228
		T/A	SNP	1324
		G/T	SNP	1397
		A/G	SNP	1572

		T/G	SNP	1634
		AAG/A	DELETION	1761
		GA/G	DELETION	2015
		GA/G	DELETION	2235
		CTT/C	DELETION	2399
		G/GA	INSERTION	2577
		A/AT	INSERTION	3726
		A/ATG	INSERTION	3815
XM_824313.1 Tbb paraflagellar rod component (Tb11.01.5100) partial mRNA	GLOS_TB11.01.5100.1.1	C/T	SNP	410
		C/CT	INSERTION	494
		A/G	SNP	613
		G/GA	INSERTION	879
		T/C	SNP	983
XM_824315.1 Tbb GUTat10.1 hypothetical protein (Tb11.01.5120) partial mRNA	GLOS_TB11.01.5120.1.1	C/T	SNP	185
		T/A	SNP	255
		T/C	SNP	263
		A/G	SNP	2321
		A/C	SNP	2352
		G/A	SNP	3499
		GA/G	DELETION	3795
XM_824337.1 Tbb receptor-type adenylate cyclase GRESAG 4 partial mRNA	GLOS_TB11.01.5310.1.1	C/T	SNP	131
		G/A	SNP	3347
		A/C	SNP	3526
		CT/C	DELETION	4074
		A/AT	INSERTION	4382
XM_824365.1 Tbb GUTat10.1 hypothetical protein (Tb11.01.5590) partial mRNA	GLOS_TB11.01.5590.1.1	C/T	SNP	261
		T/G	SNP	292
XM_824372.1 Tbb GUTat10.1 hypothetical protein (Tb11.01.5680) partial mRNA	GLOS_TB11.01.5680.1.1	T/C	SNP	93
		A/T	SNP	598
		C/G	SNP	2150
XM_824373.1 Tbb GUTat10.1 hypothetical protein (Tb11.01.5690) partial mRNA	GLOS_TB11.01.5690.1.1	GA/G	DELETION	394
		C/CT	INSERTION	906
		A/G	SNP	1501
		A/G	SNP	1794
		G/A	SNP	1861
		A/G	SNP	2224

		A/G	SNP	2320
		C/T	SNP	2350
		C/T	SNP	2940
		C/CT	INSERTION	2970
XP_829468.1 phenylalanyl-tRNA synthetase alpha subunit [T. brucei TREU927]	GLOS_TB11.01.5710.1.1	A/C	SNP	78
		A/C	SNP	106
		C/T	SNP	448
		T/C	SNP	619
		A/G	SNP	697
		A/T	SNP	703
		C/A	SNP	736
		C/T	SNP	742
		C/T	SNP	880
		A/G	SNP	934
		G/A	SNP	949
		G/C	SNP	1003
		G/A	SNP	1030
		G/A	SNP	1203
		T/C	SNP	1283
		A/G	SNP	1285
		C/T	SNP	1315
		C/A	SNP	1324
		AG/A	DELETION	1349
		T/C	SNP	1364
		C/G	SNP	1371
		C/CA	INSERTION	1494
		A/C	SNP	1499
		A/G	SNP	1545
		A/C	SNP	1550
		C/T	SNP	1738
		C/T	SNP	1743
		A/C	SNP	1824
		T/C	SNP	1874
		G/A	SNP	2251
XM_824382.1 Tbb GUTat10.1 hypothetical protein (Tb11.01.5780) partial mRNA	GLOS_TB11.01.5780.1.1	T/C	SNP	2032
		C/CT	INSERTION	3061

XP_829481.1 t-complex protein 1 subunit epsilon [Trypanosoma brucei TREU927]	GLOS_TB11.01.5860.1.1	T/TA	INSERTION	1880
XM_824396.1 Tbb GUTat10.1 hypothetical protein (Tb11.01.5930) partial mRNA	GLOS_TB11.01.5930.1.1	G/A	SNP	182
		A/G	SNP	267
		G/T	SNP	999
		T/C	SNP	2662
		G/A	SNP	2815
		C/T	SNP	2858
		A/G	SNP	3295
		G/A	SNP	3936
		A/G	SNP	3972
		G/A	SNP	4174
		T/C	SNP	4749
		C/T	SNP	5371
		G/A	SNP	5410
XM_824438.1 Tbb GUTat10.1 metalloprotease (Tb11.01.6360) partial mRNA	GLOS_TB11.01.6360.1.1	A/T	SNP	125
		A/C	SNP	267
		A/G	SNP	545
		C/A	SNP	625
		G/A	SNP	638
		G/A	SNP	1027
		T/C	SNP	1521
		T/G	SNP	2110
		G/A	SNP	2224
XM_824462.1 Tbb GUTat10.1 hypothetical protein (Tb11.01.6610) partial mRNA	GLOS_TB11.01.6610.1.1	C/T	SNP	108
		C/T	SNP	465
		C/T	SNP	519
		T/C	SNP	585
		G/A	SNP	684
		G/A	SNP	1005
		G/A	SNP	1155
		G/T	SNP	1198
		G/T	SNP	2190
XM_824467.1 Tbb iron superoxide dismutase (Tb11.01.6660) partial mRNA	GLOS_TB11.01.6660.1.1	C/T	SNP	226
		T/C	SNP	299
		G/A	SNP	386
		A/G	SNP	439

		T/C	SNP	647
		T/C	SNP	657
		G/A	SNP	1065
XM_824502.1 Tbb GUTat10.1 hypothetical protein (Tb11.01.7010) partial mRNA	GLOS_TB11.01.7010.1.1	A/G	SNP	37
		C/T	SNP	472
		A/G	SNP	493
		A/G	SNP	782
		A/C	SNP	2628
		T/A	SNP	3559
		C/T	SNP	3894
		G/T	SNP	4324
		T/C	SNP	4418
		G/A	SNP	4462
XP_829638.1 hypothetical protein [T. brucei brucei strain 927/4 GUTat10.1]	GLOS_TB11.01.7460.1.1	G/A	SNP	952
XM_824548.1 Tbb amino acid transporter (Tb11.01.7500) partial mRNA	GLOS_TB11.01.7500.1.1	G/C	SNP	139
		G/A	SNP	174
		C/T	SNP	703
		C/T	SNP	841
		A/T	SNP	879
		G/A	SNP	893
		T/C	SNP	1354
		T/C	SNP	1357
		A/C	SNP	1358
		G/A	SNP	1360
		T/A	SNP	1363
		T/C	SNP	1451
		T/G	SNP	2201
		T/C	SNP	2203
		T/C	SNP	2211
		G/A	SNP	2333
XP_829645.1 60S ribosomal protein L27 [Tbb GUTat10.1] ref|XP_829647.1| 60S ribosomal protein L27 [Trypanosoma brucei brucei strain 927/4 GUTat10.1]	GLOS_TB11.01.7535.1.1	G/A	SNP	136
		A/G	SNP	319
XM_824562.1 Tbb GUTat10.1 hypothetical protein (Tb11.01.7620) partial mRNA	GLOS_TB11.01.7620.1.1	G/GA	INSERTION	1221
		T/C	SNP	2237
		C/T	SNP	2552

XM_824563.1 Tbb GUTat10.1 hypothetical protein (Tb11.01.7630) partial mRNA	GLOS_TB11.01.7630.1.1	A/G	SNP	369
		G/C	SNP	500
		A/G	SNP	612
		T/C	SNP	713
		A/G	SNP	788
		C/G	SNP	1068
		C/T	SNP	1137
		G/A	SNP	1758
		C/T	SNP	1760
		T/C	SNP	2000
		G/A	SNP	2304
		C/T	SNP	2513
		T/C	SNP	2625
		T/G	SNP	2832
		GA/G	DELETION	3100
		G/C	SNP	3124
		G/A	SNP	3146
		T/TA	INSERTION	3259
		A/C	SNP	3354
		C/T	SNP	3374
		C/T	SNP	3414
		C/T	SNP	3447
		G/C	SNP	3481
		T/C	SNP	3508
		C/T	SNP	3509
		G/A	SNP	3527
XM_824577.1 Tbb nucleoside diphosphate kinase (Tb11.01.7800) partial mRNA	GLOS_TB11.01.7800.1.1	A/T	SNP	127
		G/A	SNP	143
		G/T	SNP	705
		CA/CAA/C	INSERTION	561
		C/CT	INSERTION	1188
XP_829708.1 hypothetical protein [T. brucei brucei strain 927/4 GUTat10.1]	GLOS_TB11.01.8225.1.1	GT/G	DELETION	738
		C/T	SNP	752
		C/G	SNP	970
XM_824637.1 Tbb dihydrolipoyl dehydrogenase (Tb11.01.8470) partial mRNA	GLOS_TB11.01.8470.1.1	T/C	SNP	433
		C/T	SNP	761

		C/T	SNP	1132
		C/T	SNP	1370
		C/A	SNP	1451
		G/T	SNP	1484
XM_824640.1 Tbb t-complex protein 1 subunit alpha (Tb11.01.8510) partial mRNA	GLOS_TB11.01.8510.1.1	G/A	SNP	139
		T/TA	INSERTION	250
		C/CT	INSERTION	397
		G/A	SNP	479
		G/GT	INSERTION	491
		G/T	SNP	494
		A/T	SNP	501
XM_824641.1 Tbb glucosamine-6-phosphate isomerase (Tb11.01.8520)partial mRNA	GLOS_TB11.01.8520.1.1	T/A	SNP	237
		T/A	SNP	238
		C/CT	INSERTION	413
		AC/A	DELETION	545
		C/A	SNP	585
		CT/C	DELETION	879
		T/C	SNP	1036
		T/C	SNP	1393
		G/C	SNP	1668
		C/T	SNP	1726
		C/T	SNP	1786
		T/C	SNP	2021
XP_829756.1 hypothetical protein [T. brucei brucei strain 927/4 GUTat10.1]	GLOS_TB11.01.8770.1.1	CTT/C/CT	DELETION	113
		A/C	SNP	868
		G/A	SNP	1112
		A/G	SNP	1268
		C/T	SNP	1269
		A/G	SNP	2258
		C/T	SNP	2876
XP_828324.1 hypothetical protein [T. brucei brucei strain 927/4 GUTat10.1]	GLOS_TB11.02.0010.1.1	AAAAT/A	DELETION	278
		A/T	SNP	638
XM_823251.1 Tbb GUTat10.1 hypothetical protein (Tb11.02.0210) partial mRNA	GLOS_TB11.02.0210.1.1	A/G	SNP	67
		A/G	SNP	97
		A/G	SNP	864
		A/G	SNP	1494

		A/G	SNP	1527
		AT/A	DELETION	2194
		A/G	SNP	2485
		T/A	SNP	2523
XP_828348.1 heat shock protein mitochondrial precursor [T. brucei TREU927]	GLOS_TB11.02.0250.1.1	C/A	SNP	56
		A/G	SNP	62
		G/A	SNP	77
		A/G	SNP	223
		A/G	SNP	244
		A/G	SNP	550
		C/T	SNP	1200
		A/G	SNP	1664
		A/G	SNP	2201
		C/T	SNP	2284
		T/C	SNP	2829
		G/GT	INSERTION	3279
XM_823282.1 Tbb GUTat10.1 hypothetical protein (Tb11.02.0445) partial mRNA	GLOS_TB11.02.0445.1.1	C/T	SNP	76
		A/T	SNP	77
		G/GA	INSERTION	116
		C/CA	INSERTION	217
		C/CT	INSERTION	916
XP_828407.1 dynein heavy chain [T. brucei brucei strain 927/4 GUTat10.1]	GLOS_TB11.02.0760.1.1	A/G	SNP	3203
XM_823316.1 Tbb squalene monooxygenase (Tb11.02.0780) partial mRNA	GLOS_TB11.02.0780.1.1	C/CA	INSERTION	662
		A/G	SNP	695
		C/A	SNP	696
XM_823317.1 Tbb strain 927/4 GUTat10.1 kinesin (Tb11.02.0790) partial mRNA	GLOS_TB11.02.0790.1.1	GGT/G	DELETION	424
		GA/G	DELETION	1159
		C/CCCT	INSERTION	1548
XM_823338.1 Tbb GUTat10.1 hypothetical protein (Tb11.02.0980) partial mRNA	GLOS_TB11.02.0980.1.1	C/T	SNP	2443
XP_828438.1 aminopeptidase [Trypanosoma brucei brucei strain 927/4 GUTat10.1]	GLOS_TB11.02.1070.1.1	C/T	SNP	238
XP_828440.1 40s ribosomal protein S4 [Tbb] ref|XP_828441.1| 40S ribosomal protein S4 [Trypanosoma brucei brucei strain 927/4 GUTat10.1]	GLOS_TB11.02.1085.1.1	C/T	SNP	31
		C/G	SNP	33
		C/G	SNP	732
		C/A	SNP	848
		T/C	SNP	849

XM_823349.1 Tbb nucleobase/nucleoside transporter 8.1 partial mRNA	GLOS_TB11.02.1100.1.1	T/C	SNP	190
		C/T	SNP	194
		A/C	SNP	204
		A/G	SNP	299
		G/A	SNP	377
		T/C	SNP	714
		A/G	SNP	737
		A/G	SNP	885
		T/G	SNP	1472
		A/G	SNP	1738
		AT/A	DELETION	1803
		T/C	SNP	1877
		A/G	SNP	1890
		C/CT	INSERTION	1913
		C/T	SNP	1917
XM_823359.1 Tbb GUTat10.1 hypothetical protein (Tb11.02.1190) partial mRNA	GLOS_TB11.02.1190.1.1	T/C	SNP	89
		G/A	SNP	1498
		T/C	SNP	2154
XM_823383.1 Tbb hypothetical protein (Tb11.02.1470) partial mRNA	GLOS_TB11.02.1470.1.1	A/G	SNP	257
		T/G	SNP	712
		A/G	SNP	1230
		G/A	SNP	1358
		C/A	SNP	2412
XM_823384.1 Tbb mitochondrial processing peptidase subunit alpha (Tb11.02.1480) partial mRNA. nuclear gene for mitochondrial product	GLOS_TB11.02.1480.1.1	CA/C	DELETION	120
		C/A	SNP	240
		A/G	SNP	871
		G/A	SNP	1805
		T/G	SNP	1864
		C/T	SNP	1929
		GT/G	DELETION	2073
		G/GGT	INSERTION	2310
XM_823403.1 Tbb strain 927/4 GUTat10.1 lectin (Tb11.02.1680) partial mRNA	GLOS_TB11.02.1680.1.1	A/G	SNP	1891
XM_823464.1 Tbb 60S ribosomal protein L17 (Tb11.02.2430) partial mRNA	GLOS_TB11.02.2430.1.1	C/T	SNP	295
XP_828580.1 fumarate hydratase class I [T. brucei brucei strain 927/4 GUTat10.1]	GLOS_TB11.02.2700.1.1	A/G	SNP	370
		A/T	SNP	802

		G/GAA	INSERTION	2176
XP_828602.1 ubiquitin carboxyl-terminal hydrolase [Trypanosoma brucei TREU927]	GLOS_TB11.02.2940.1.1	T/C	SNP	375
		T/C	SNP	2288
		G/A	SNP	2491
		C/CT	INSERTION	2961
XM_823517.1 Tbb GUTat10.1 sugar transporter (Tb11.02.3020) partial mRNA	GLOS_TB11.02.3020.1.1	GA/G	DELETION	2619
		A/G	SNP	3124
XM_823526.1 Tbb GUTat10.1 malic enzyme (Tb11.02.3120) partial mRNA	GLOS_TB11.02.3120.1.1	C/T	SNP	1477
		A/C	SNP	1480
		A/T	SNP	1481
		T/A	SNP	1483
		C/A	SNP	1486
		A/T	SNP	1764
		CAT/C	DELETION	2011
		T/C	SNP	2030
		AT/A	DELETION	2533
XM_823534.1 Tbb triosephosphate isomerase (Tb11.02.3210) partial mRNA	GLOS_TB11.02.3210.1.1	GTATA/G	DELETION	943
		G/GA	INSERTION	1084
		A/G	SNP	1157
XP_828635.1 hypothetical protein [T. brucei brucei strain 927/4 GUTat10.1]	GLOS_TB11.02.3310.1.1	TA/T	DELETION	93
		C/T	SNP	260
		T/C	SNP	537
		T/C	SNP	896
		T/A	SNP	1068
		G/GT	INSERTION	1108
		A/T	SNP	1116
		T/A	SNP	1117
		G/T	SNP	1118
		T/G	SNP	1307
		A/G	SNP	1337
		CT/C	DELETION	1388
		A/G	SNP	1427
		T/A	SNP	1442
XP_828657.1 hypothetical protein [T. brucei brucei strain 927/4 GUTat10.1]	GLOS_TB11.02.3570.1.1	CT/C	DELETION	1806
XM_823567.1 Tbb GUTat10.1 hypothetical protein (Tb11.02.3610) partial mRNA	GLOS_TB11.02.3610.1.1	A/AT	INSERTION	2257
XM_823578.1 Tbb GUTat10.1 hypothetical protein (Tb11.02.3770) partial mRNA	GLOS_TB11.02.3770.2.2	G/A	SNP	153

XP_828679.1 hypothetical protein [T. brucei brucei strain 927/4 GUTat10.1]	GLOS_TB11.02.3860.1.1	C/CT	INSERTION	2287
XM_823587.1 Tbb GUTat10.1 hypothetical protein (Tb11.02.3880) partial mRNA	GLOS_TB11.02.3880.1.1	GCTC/G	DELETION	3263
		G/T	SNP	4238
XM_823594.1 Tbb S-phase kinase-associated protein (Tb11.02.3990) partial mRNA	GLOS_TB11.02.3990.1.1	G/GA	INSERTION	737
		A/G	SNP	1280
XP_828688.1 40S ribosomal protein S15a [Tbb] ref|XP_844028.1| 40S ribosomal protein S15a [Trypanosoma brucei brucei strain 927/4 GUTat10.1]	GLOS_TB11.02.4000.1.1	T/A	SNP	625
XP_828691.1 protein transport protein Sec31 [Tbb strain 927/4 GUTat10.1]	GLOS_TB11.02.4040.1.1	G/C	SNP	1122
		A/G	SNP	2554
		A/G	SNP	4501
XM_823599.1 Tbb 60S ribosomal protein L28 (Tb11.02.4050) partial mRNA	GLOS_TB11.02.4050.1.1	T/C	SNP	405
		A/T	SNP	594
		C/T	SNP	595
XM_823604.1 Tbb pretranslocation prot subunit alpha (Tb11.02.4100) partial mRNA	GLOS_TB11.02.4100.1.1	C/CT	INSERTION	2511
XP_828702.1 pyruvate phosphate dikinase [Tbb strain 927/4 GUTat10.1]	GLOS_TB11.02.4150.1.1	C/CT	INSERTION	3126
		G/GT	INSERTION	3165
		G/GT	INSERTION	3332
		GT/G	DELETION	3569
XM_823611.1 Tbb 40S ribosomal protein S5 (Tb11.02.4170) partial mRNA	GLOS_TB11.02.4170.1.2	T/A	SNP	14
		T/A	SNP	16
		G/T	SNP	272
		A/G	SNP	669
XM_823611.1 Tbb 40S ribosomal protein S5 (Tb11.02.4170) partial mRNA	GLOS_TB11.02.4170.2.2	T/A	SNP	436
XM_823625.1 Tbb GUTat10.1 hypothetical protein (Tb11.02.4300) partial mRNA	GLOS_TB11.02.4300.1.1	CT/C	DELETION	352
		CA/C	DELETION	1547
		G/GA	INSERTION	1672
		CA/C	DELETION	2413
		G/GA	INSERTION	2692
		C/CT	INSERTION	4806
XM_823631.1 Tbb 40S ribosomal protein S21 (Tb11.02.4350) partial mRNA	GLOS_TB11.02.4350.1.1	T/A	SNP	405
		T/A	SNP	853
		T/A	SNP	854
XP_828733.1 aminopeptidase [Trypanosoma brucei brucei strain 927/4 GUTat10.1]	GLOS_TB11.02.4440.1.1	A/T	SNP	75
		A/AT	INSERTION	95
		A/G	SNP	164
		G/T	SNP	288

		G/C	SNP	816
		C/G	SNP	1158
		C/A	SNP	1191
		A/G	SNP	1521
		T/C	SNP	1997
		T/A	SNP	2001
		G/GA	INSERTION	4095
XM_823677.1 Tbb GUTat10.1 hypothetical protein (Tb11.02.4810) partial mRNA	GLOS_TB11.02.4810.1.1	C/T	SNP	802
XP_828778.1 acidocalcisomal pyrophosphatase [Tbb strain 927/4 GUTat10.1]	GLOS_TB11.02.4910.1.1	A/G	SNP	1258
		C/T	SNP	1315
XM_823706.1 Tbb GUTat10.1 hypothetical protein (Tb11.02.5120) partial mRNA	GLOS_TB11.02.5120.1.1	A/G	SNP	1945
		G/A	SNP	2875
		C/T	SNP	2908
XP_828807.1 pantothenate kinase subunit [T. brucei brucei strain 927/4 GUTat10.1]	GLOS_TB11.02.5190.1.1	T/C	SNP	1594
		T/C	SNP	3884
		A/G	SNP	4545
		C/T	SNP	4590
		A/G	SNP	5265
XM_823735.1 Tbb cystathionine beta-synthase (Tb11.02.5400) partial mRNA	GLOS_TB11.02.5400.1.1	T/A	SNP	2083
		C/G	SNP	2090
XM_823744.1 Tbb hypothetical protein (Tb11.02.5490) partial mRNA	GLOS_TB11.02.5490.1.1	A/AT	INSERTION	606
XM_823745.1 Tbb glucose-regulated protein 78 (Tb11.02.5500) partial mRNA	GLOS_TB11.02.5500.1.1	A/G	SNP	160
		C/G	SNP	640
		C/T	SNP	804
XM_823752.1 Tbb GUTat10.1 hypothetical protein (Tb11.02.5570) partial mRNA	GLOS_TB11.02.5570.1.1	A/T	SNP	172
		AT/A	DELETION	204
		CTT/C	DELETION	313
XM_823754.1 Tbb GUTat10.1 hypothetical protein (Tb11.02.5590) partial mRNA	GLOS_TB11.02.5590.1.1	C/T	SNP	44
		C/T	SNP	164
		CA/C	DELETION	167
		C/T	SNP	222
		A/G	SNP	338
		A/G	SNP	407
		A/G	SNP	505
		AT/A	DELETION	513
		C/T	SNP	745

		A/G	SNP	782
		A/C	SNP	786
		C/T	SNP	832
		A/G	SNP	869
		T/A	SNP	921
		T/C	SNP	1080
		G/A	SNP	1084
		C/T	SNP	1107
		G/A	SNP	1148
		G/A	SNP	1342
		C/T	SNP	1364
		G/A	SNP	1846
		A/G	SNP	1894
		T/C	SNP	1961
		C/T	SNP	1962
		G/A	SNP	2106
		C/T	SNP	2121
XM_823107.1 Tbb GUTat10.1 ABC transporter (Tb11.03.0030) partial mRNA	GLOS_TB11.03.0030.1.1	T/C	SNP	1176
		CA/C	DELETION	2810
XM_823102.1 Tbb GUTat10.1 ribokinase (Tb11.03.0090) partial mRNA	GLOS_TB11.03.0090.1.1	G/A	SNP	1233
		G/GT	INSERTION	2238
XM_823098.1 Tbb strain 927/4 GUTat10.1 nucleoporin (Tb11.03.0140) partial mRNA	GLOS_TB11.03.0140.1.1	G/A	SNP	118
		C/T	SNP	202
		T/C	SNP	238
		A/G	SNP	269
		T/C	SNP	274
XP_828183.1 isocitrate dehydrogenase [T. brucei brucei strain 927/4 GUTat10.1]	GLOS_TB11.03.0230.1.1	G/GA	INSERTION	749
		CAAAG/C	DELETION	940
		C/T	SNP	1017
XM_823089.1 Tbb GUTat10.1 hypothetical protein (Tb11.03.0240) partial mRNA	GLOS_TB11.03.0240.1.1	A/G	SNP	3704
		A/G	SNP	3720
		A/T	SNP	4186
		A/T	SNP	4187
XM_823088.1 Tbb GUTat10.1 cyclophilin A (Tb11.03.0250) partial mRNA	GLOS_TB11.03.0250.1.1	AG/A	DELETION	432
XM_823084.1 Tbb GUTat10.1 hypothetical protein (Tb11.03.0300) partial mRNA	GLOS_TB11.03.0300.1.1	T/A	SNP	474
		T/C	SNP	658

		A/G	SNP	1235
		T/C	SNP	2188
		A/C	SNP	2426
		T/G	SNP	2488
		T/C	SNP	2527
		C/T	SNP	2879
XP_828169.1 protein phosphatase 2C [T. brucei brucei strain 927/4 GUTat10.1]	GLOS_TB11.03.0390.1.1	C/T	SNP	221
		C/A	SNP	304
		CA/C	DELETION	1439
		G/GT	INSERTION	1554
		G/GTA	INSERTION	2312
XM_823075.1 Tbb GUTat10.1 DNA repair protein (Tb11.03.0400) partial mRNA	GLOS_TB11.03.0400.1.1	A/T	SNP	2559
XP_828152.1 hypothetical protein [T. brucei brucei strain 927/4 GUTat10.1]	GLOS_TB11.03.0475.1.1	T/A	SNP	588
		T/A	SNP	589
XM_823054.1 Tbb GUTat10.1 hypothetical protein (Tb11.03.0530) partial mRNA	GLOS_TB11.03.0530.1.1	T/A	SNP	438
		GA/G	DELETION	662
		T/TA	INSERTION	857
		A/AT	INSERTION	866
		A/T	SNP	1132
		C/T	SNP	1180
		C/T	SNP	1182
		G/GA	INSERTION	1263
		G/GA	INSERTION	1427
		CT/C	DELETION	2004
		CT/C	DELETION	2236
XM_823023.1 Tbb GUTat10.1 hypothetical protein (Tb11.03.0900) partial mRNA	GLOS_TB11.03.0900.1.1	T/G	SNP	732
		C/G	SNP	2083
		T/C	SNP	2158
		T/C	SNP	2546
		G/A	SNP	2681
		T/C	SNP	3061
		A/G	SNP	3168
		A/G	SNP	3257
		C/T	SNP	3279
		T/C	SNP	3384
XP_828111.1 elongation factor [Trypanosoma brucei brucei strain 927/4 GUTat10.1]	GLOS_TB11.03.0940.1.1	T/C	SNP	753

		G/A	SNP	1491
		C/T	SNP	1749
		C/G	SNP	1794
		A/C	SNP	2442
		A/AT	INSERTION	2488
		C/T	SNP	2550
		G/A	SNP	2610
		G/A	SNP	2616
XM_823219.1 Tbb GUTat10.1 hypothetical protein (Tb11.18.0002) partial mRNA	GLOS_TB11.18.0002.1.1	T/C	SNP	1164
XM_823216.1 Tbb GUTat10.1 hypothetical protein (Tb11.18.0005) partial mRNA	GLOS_TB11.18.0005.1.1	A/C	SNP	469
XM_823228.1 Tbb GUTat10.1 hypothetical protein (Tb11.22.0004) partial mRNA	GLOS_TB11.22.0004.1.1	G/A	SNP	696
XM_823228.1 Tbb GUTat10.1 hypothetical protein (Tb11.22.0004) partial mRNA		CT/C	DELETION	926
XM_823146.1 Tbb receptor-type adenylate cyclase GRESAG 4 partial mRNA	GLOS_TB11.27.0001.1.1	A/G	SNP	643
		A/G	SNP	1003
		A/G	SNP	1109
		G/A	SNP	1190
		G/A	SNP	1297
		G/A	SNP	1352
		T/C	SNP	1411
		C/T	SNP	1624
		A/G	SNP	1636
		T/C	SNP	1705
		A/T	SNP	1918
		T/C	SNP	1960
		A/G	SNP	2088
		G/A	SNP	2166
		A/G	SNP	2284
		A/G	SNP	2309
		C/T	SNP	2337
		C/T	SNP	2462
		A/G	SNP	2498
		C/T	SNP	2520
		T/C	SNP	2524
		G/A	SNP	2635
		C/T	SNP	2830
		T/C	SNP	3068

		T/C	SNP	3070
		G/A	SNP	3073
		A/G	SNP	3088
		C/T	SNP	3305
		T/C	SNP	3376
		C/T	SNP	3469
		A/G	SNP	3672
		T/C	SNP	3685
		G/A	SNP	3812
		T/C	SNP	3836
		G/A	SNP	3959
		C/CT	INSERTION	4111
		T/C	SNP	4216
		G/A	SNP	4376
		ATG/A	DELETION	4381
		GT/G	DELETION	4429
XM_823178.1 Tbb GUTat10.1 hypothetical protein (Tb11.39.0004) partial mRNA	GLOS_TB11.39.0004.1.1	C/CA	INSERTION	89
		G/GA	INSERTION	96
		C/CT	INSERTION	1497
XM_823177.1 Tbb GUTat10.1 hypothetical protein (Tb11.39.0005) partial mRNA	GLOS_TB11.39.0005.1.1	C/CT	INSERTION	368
		G/GA	INSERTION	749
		G/GA	INSERTION	3135
XP_828289.1 60S acidic ribosomal subunit protein [Trypanosoma brucei TREU927] ref|XP_828290.1| 60S acidic ribosomal subunit protein [T. brucei TREU927]	GLOS_TB11.46.0002.1.1	G/T	SNP	182
		G/A	SNP	462
		C/T	SNP	678
		G/T	SNP	974
XM_823189.1 Tbb GUTat10.1 hypothetical protein (Tb11.46.0009) partial mRNA	GLOS_TB11.46.0009.1.1	C/CT	INSERTION	3538
XP_828236.1 2-oxoglutarate dehydrogenase subunit [Trypanosoma brucei TREU927]	GLOS_TB11.47.0004.1.1	G/T	SNP	2538
		C/T	SNP	2543
		C/T	SNP	3126
		T/G	SNP	4586
		T/C	SNP	5197
XP_828234.1 hypothetical protein [T. brucei brucei strain 927/4 GUTat10.1]	GLOS_TB11.47.0006.1.1	T/TG	INSERTION	88
		T/C	SNP	150
		GA/G	DELETION	219

		C/T	SNP	999
		A/G	SNP	2874
		C/A	SNP	2957
		C/T	SNP	3045
		G/GAA	INSERTION	3051
XP_828218.1 hypothetical protein [T. brucei brucei strain 927/4 GUTat10.1]	GLOS_TB11.47.0022.1.1	A/G	SNP	107
		T/C	SNP	132
		C/T	SNP	312
		CACACAC	DELETION	367
		CA/C	DELETION	377
		C/CT	INSERTION	973
XP_828203.1 calpain, partial [Trypanosoma brucei brucei strain 927/4 GUTat10.1]	GLOS_TB11.47.0036.1.1	A/G	SNP	142
		C/A	SNP	145
		G/T	SNP	146
		A/G	SNP	187
		T/A	SNP	199
		A/G	SNP	201
		A/G	SNP	235
		G/C	SNP	260
		A/T	SNP	280
		A/G	SNP	281
		T/C	SNP	289
		C/T	SNP	296
		T/C	SNP	303
		A/G	SNP	391
		T/A	SNP	403
		A/G	SNP	405
		A/T	SNP	484
		A/G	SNP	485
		T/C	SNP	493
		C/T	SNP	500
		T/C	SNP	507
		T/A	SNP	508
		A/G	SNP	589
		T/A	SNP	607
		A/G	SNP	609

XM_823067.1 Tbb 60S ribosomal protein L21E (Tb11.50.0005) partial mRNA	GLOS_TB11.50.0005.1.1	A/G	SNP	33
		A/C	SNP	35
		C/T	SNP	135
		T/C	SNP	162
		T/A	SNP	165
		G/A	SNP	189
		C/T	SNP	195
		C/T	SNP	306
		T/C	SNP	387
		C/A	SNP	431
		G/A	SNP	465
XP_828164.1 dynein light chain [Trypanosoma brucei brucei strain 927/4 GUTat10.1]	GLOS_TB11.50.0007.1.1	T/TA	INSERTION	440
		GA/G	DELETION	610
		T/A	SNP	1072
		C/T	SNP	1076
		A/G	SNP	1096
XM_824256.1 Tbb GUTat10.1 hypothetical protein (Tb11.52.0008) partial mRNA	GLOS_TB11.52.0008.1.1	T/C	SNP	62
		A/G	SNP	69
		A/G	SNP	73
		C/T	SNP	89
		A/G	SNP	98
		A/G	SNP	132
		C/T	SNP	147
		C/G	SNP	152
		A/G	SNP	161
		G/A	SNP	164
		A/G	SNP	167
		A/G	SNP	186
		A/G	SNP	190
		C/T	SNP	206
		A/G	SNP	224
		A/G	SNP	249
		C/T	SNP	250
		A/C	SNP	267
		G/A	SNP	286
		A/G	SNP	484

		C/T	SNP	598
		A/C	SNP	768
XM_001218825.1 Tbb cytidine triphosphate synthase, putative partial mRNA	GLOS_TB927.1.1240.1.1	G/GA	INSERTION	246
XM_001218868.1 Tbb hypothetical protein, conserved (Tb927.1.1670) partial mRNA	GLOS_TB927.1.1670.1.1	C/T	SNP	249
XM_001218909.1 Tbb strain 927/4 GUTat10.1 calpain-like cysteine peptidase. cysteine peptidase, Clan CA, family C2 partial mRNA	GLOS_TB927.1.2100.1.1	C/CA	INSERTION	520
		G/A	SNP	1876
		C/CT	INSERTION	2074
		A/G	SNP	3259
		C/T	SNP	3286
		T/C	SNP	3301
		C/A	SNP	3304
		C/T	SNP	3305
		A/G	SNP	3310
		A/G	SNP	3358
		C/T	SNP	3368
		T/C	SNP	3380
XM_001218922.1 Tbb calpain-like protein fragment, putative partial mRNA	GLOS_TB927.1.2230.1.1	G/A	SNP	529
		GA/G	DELETION	587
		A/G	SNP	593
XM_841300.1 Tbb hypothetical protein, unlikely (Tb927.1.230) partial mRNA	GLOS_TB927.1.230.1.1	T/C	SNP	61
		T/A	SNP	94
		T/C	SNP	195
		C/T	SNP	215
		GA/G	DELETION	242
		T/C	SNP	249
		C/T	SNP	288
		A/G	SNP	299
		G/A	SNP	317
		T/TAA/TA	INSERTION	323
		A/T	SNP	361
		T/C	SNP	369
		C/T	SNP	371
		G/C	SNP	505
		G/T	SNP	507
		C/T	SNP	580

T/C	SNP	638
A/G	SNP	657
A/G	SNP	659
T/A	SNP	660
G/T	SNP	668
A/G	SNP	813
C/T	SNP	862
G/A	SNP	931
G/C	SNP	960
T/C	SNP	982
A/C	SNP	1022
T/A	SNP	1040
C/A	SNP	1057
CA/C	DELETION	1084
C/T	SNP	1129
T/C	SNP	1132
C/T	SNP	1136
T/A	SNP	1166
C/T	SNP	1183
A/T	SNP	1196
G/A	SNP	1238
G/C	SNP	1274
T/G	SNP	1280
C/T	SNP	1298
G/A	SNP	1312
A/G	SNP	1313
T/TTA	INSERTION	1327
A/T	SNP	1342
G/T	SNP	1382
T/C	SNP	1390
C/T	SNP	1421
G/T	SNP	1423
C/T	SNP	1424
G/A	SNP	1434
C/G	SNP	1443
A/G	SNP	1502

C/A	SNP	1509
G/T	SNP	1535
G/A	SNP	1551
G/A	SNP	1572
G/T	SNP	1581
C/A	SNP	1604
T/C	SNP	1618
C/T	SNP	1637
C/T	SNP	1675
T/C	SNP	1683
G/A	SNP	1709
G/T	SNP	1711
A/C	SNP	1732
C/T	SNP	1752
C/T	SNP	1759
C/T	SNP	1771
A/G	SNP	1786
C/T	SNP	1797
G/T	SNP	1832
A/T	SNP	1864
A/T	SNP	1865
C/T	SNP	1866
A/G	SNP	1887
C/T	SNP	1901
C/T	SNP	1907
A/G	SNP	1921
A/G	SNP	1928
G/T	SNP	1929
A/C	SNP	1931
CT/C	DELETION	1933
TC/T	DELETION	1937
A/C	SNP	1968
C/A	SNP	1993
C/T	SNP	2002
T/C	SNP	2015
C/T	SNP	2017

		C/T	SNP	2024
		A/T	SNP	2043
		G/T	SNP	2067
		T/C	SNP	2108
		TAC/T	DELETION	2121
		T/G	SNP	2150
		A/C	SNP	2151
		T/C	SNP	2188
		A/T	SNP	2189
		T/C	SNP	2198
XM_001218936.1 Tbb strain 927/4 GUTat10.1 beta tubulin partial mRNA	GLOS_TB927.1.2370.1.2	A/C	SNP	1303
XM_001218936.1 Tbb strain 927/4 GUTat10.1 beta tubulin partial mRNA	GLOS_TB927.1.2370.2.2	G/A	SNP	1423
		C/T	SNP	1487
		A/AT	INSERTION	1541
		A/T	SNP	1541
		A/G	SNP	1631
XM_001218941.1 Tbb strain 927/4 GUTat10.1 histone H3 partial mRNA	GLOS_TB927.1.2430.1.1	G/A	SNP	107
		G/T	SNP	612
		G/A	SNP	652
		C/T	SNP	803
		C/T	SNP	825
		A/T	SNP	1462
		T/A	SNP	1463
XP_001218979.1 pteridine transporter [T. brucei brucei strain 927/4 GUTat10.1]	GLOS_TB927.1.2820.1.1	C/T	SNP	34
		T/TA	INSERTION	383
		C/T	SNP	970
		C/T	SNP	1616
		T/C	SNP	2294
		T/C	SNP	2303
XP_001219016.1 40S ribosomal protein S11 [T. brucei brucei strain 927/4 GUTat10.1]	GLOS_TB927.1.3180.1.1	G/A	SNP	128
		C/T	SNP	137
		G/A	SNP	161
		T/C	SNP	200
		T/C	SNP	290
		A/G	SNP	296
		C/T	SNP	357

		T/C	SNP	461
		A/G	SNP	494
		T/C	SNP	524
		C/T	SNP	527
XP_001219089.1 alanine aminotransferase [T. brucei brucei strain 927/4 GUTat10.1]	GLOS_TB927.1.3950.1.1	C/CTT	INSERTION	264
		T/C	SNP	949
		A/G	SNP	1473
XM_001219129.1 Tbb hypothetical protein, conserved (Tb927.1.4370) partial mRNA	GLOS_TB927.1.4370.1.1	C/T	SNP	1453
		AAC/A	DELETION	2435
		C/G	SNP	2463
		C/G	SNP	2465
		CTT/C	DELETION	2634
XM_001219166.1 Tbb hypothetical protein, conserved (Tb927.1.4740) partial mRNA	GLOS_TB927.1.4740.1.1	G/GA	INSERTION	388
		G/GA	INSERTION	480
		T/C	SNP	2196
XM_001218756.1 Tbb DNA-directed RNA polymerase III, putative partial mRNA	GLOS_TB927.1.540.1.2	T/C	SNP	62
		T/C	SNP	64
		G/A	SNP	83
		C/T	SNP	162
		A/G	SNP	165
		C/A	SNP	174
		A/G	SNP	192
		G/A	SNP	231
		C/A	SNP	243
		A/T	SNP	343
		G/A	SNP	351
		T/G	SNP	357
		T/C	SNP	368
		G/T	SNP	386
		G/A	SNP	441
		A/C	SNP	508
		A/G	SNP	513
		A/C	SNP	521
		G/T	SNP	542
		C/T	SNP	582
		T/C	SNP	585

A/G	SNP	640
T/C	SNP	646
C/T	SNP	669
T/C	SNP	686
T/C	SNP	738
C/T	SNP	764
C/T	SNP	778
T/C	SNP	847
T/C	SNP	914
A/C	SNP	933
C/T	SNP	935
C/T	SNP	948
C/A	SNP	958
T/G	SNP	971
C/A	SNP	1011
A/G	SNP	1041
A/G	SNP	1052
T/C	SNP	1060
C/T	SNP	1068
T/A	SNP	1073
A/G	SNP	1076
G/A	SNP	1085
A/G	SNP	1103
C/G	SNP	1104
C/A	SNP	1122
A/G	SNP	1141
T/C	SNP	1149
G/A	SNP	1178
C/T	SNP	1213
G/T	SNP	1238
A/G	SNP	1251
C/T	SNP	1308
T/C	SNP	1324
T/C	SNP	1347
G/A	SNP	1528
T/C	SNP	1548

		C/T	SNP	1702
		A/C	SNP	1950
		G/A	SNP	1968
		A/G	SNP	1972
		T/C	SNP	1981
		C/G	SNP	2043
		C/CAT	INSERTION	2119
		T/C	SNP	2149
		A/C	SNP	2173
		A/G	SNP	2221
		G/C	SNP	2231
		A/G	SNP	2251
		G/A	SNP	2288
		T/G	SNP	2296
		A/G	SNP	2413
		C/A	SNP	2425
		CT/C	DELETION	2441
		A/G	SNP	2477
		G/C	SNP	2479
		C/T	SNP	2482
		G/T	SNP	2542
		C/T	SNP	2576
XM_001218756.1 Tbb DNA-directed RNA polymerase III, putative partial mRNA	GLOS_TB927.1.540.2.2	A/G	SNP	65
		C/G	SNP	79
		C/T	SNP	82
		G/T	SNP	90
		G/A	SNP	95
		A/G	SNP	101
		A/C	SNP	122
		A/C	SNP	124
		T/A	SNP	136
		C/T	SNP	141
		C/G	SNP	142
		G/T	SNP	144
		G/A	SNP	179
		T/C	SNP	183

G/A	SNP	214
C/T	SNP	226
G/C	SNP	235
G/A	SNP	271
C/G	SNP	272
G/A	SNP	310
A/T	SNP	317
A/G	SNP	372
G/T	SNP	406
C/A	SNP	408
C/G	SNP	470
T/G	SNP	488
C/T	SNP	489
T/C	SNP	515
T/C	SNP	535
A/C	SNP	566
G/C	SNP	591
A/G	SNP	649
G/A	SNP	661
A/G	SNP	676
G/C	SNP	732
T/G	SNP	749
T/G	SNP	752
T/G	SNP	781
T/A	SNP	803
T/C	SNP	804
C/G	SNP	827
T/C	SNP	861
G/A	SNP	864
G/T	SNP	872
G/T	SNP	881
C/T	SNP	919
A/T	SNP	927
C/T	SNP	928
A/T	SNP	936
A/G	SNP	985

T/C	SNP	1011
G/T	SNP	1023
T/C	SNP	1032
C/T	SNP	1042
G/T	SNP	1050
A/G	SNP	1066
C/G	SNP	1068
A/C	SNP	1074
G/T	SNP	1084
C/T	SNP	1094
T/G	SNP	1122
C/T	SNP	1130
T/A	SNP	1141
G/A	SNP	1144
A/T	SNP	1149
T/C	SNP	1202
A/G	SNP	1210
C/T	SNP	1216
A/C	SNP	1223
C/T	SNP	1256
T/A	SNP	1299
G/T	SNP	1311
G/A	SNP	1334
T/G	SNP	1351
T/C	SNP	1362
C/T	SNP	1366
G/A	SNP	1392
A/G	SNP	1406
G/A	SNP	1426
G/T	SNP	1461
C/G	SNP	1462
T/C	SNP	1508
T/C	SNP	1519
T/C	SNP	1522
A/G	SNP	1535
A/G	SNP	1592

A/G	SNP	1606
T/C	SNP	1613
G/T	SNP	1621
T/C	SNP	1651
A/G	SNP	1653
C/T	SNP	1656
G/T	SNP	1673
T/C	SNP	1679
C/T	SNP	1699
T/A	SNP	1702
A/G	SNP	1704
A/T	SNP	1707
A/G	SNP	1758
G/C	SNP	1912
T/C	SNP	1971
G/T	SNP	2075
T/C	SNP	2087
A/G	SNP	2106
G/C	SNP	2127
A/C	SNP	2144
A/G	SNP	2182
A/C	SNP	2205
A/G	SNP	2251
C/G	SNP	2270
T/C	SNP	2280
T/A	SNP	2305
T/G	SNP	2328
C/T	SNP	2337
A/C	SNP	2382
G/C	SNP	2457
A/G	SNP	2503
A/G	SNP	2507
A/G	SNP	2519
C/T	SNP	2532
G/T	SNP	2550
G/A	SNP	2620

		C/T	SNP	2624
XM_001218762.1 Tbb phosphate-repressible phosph. permease, putative part. mRNA	GLOS_TB927.1.600.1.1	A/G	SNP	239
		T/C	SNP	1353
		A/G	SNP	1362
		T/C	SNP	1706
		T/TC	INSERTION	1925
		C/T	SNP	2139
		AT/A	DELETION	2274
		T/TAA	INSERTION	2676
		T/C	SNP	2849
		G/GT	INSERTION	2892
		GA/G	DELETION	3090
		GA/G	DELETION	3410
XM_001218788.1 Tbb hypothetical protein, conserved (Tb927.1.860) partial mRNA	GLOS_TB927.1.860.1.1	G/A	SNP	917
XP_951477.1 retrotransposon hot spot (RHS) protein [Tbb TREU927]	GLOS_TB927.2.240.1.1	A/G	SNP	424
		A/C	SNP	427
		G/T	SNP	434
		A/G	SNP	454
		A/G	SNP	455
		A/G	SNP	458
		T/G	SNP	713
		A/G	SNP	729
		A/G	SNP	817
		C/T	SNP	824
		G/A	SNP	825
		A/G	SNP	880
		C/T	SNP	899
		G/A	SNP	1971
		A/G	SNP	2398
		A/G	SNP	2648
		G/A	SNP	3091
		G/GA	INSERTION	3185
XP_951552.1 hypothetical protein [T. brucei brucei strain 927/4 GUTat10.1] ref|XP_9515	GLOS_TB927.2.2510.1.1	A/G	SNP	147
		T/C	SNP	186
		C/T	SNP	858
XM_946502.1 Tbb D-alanyl-glycyl endopeptidase (Tb927.2.3460) partial mRNA	GLOS_TB927.2.3460.1.1	A/G	SNP	926

		C/T	SNP	947
		A/G	SNP	955
XM_946512.1 Tbb GUTat10.1 hypothetical protein (Tb927.2.3800) partial mRNA	GLOS_TB927.2.3800.1.1	T/TA	INSERTION	2494
XP_951481.1 retrotransposon hot spot (RHS) protein [T. brucei TREU927]	GLOS_TB927.2.380.1.1	T/G	SNP	35
		TTAA/T	DELETION	166
		C/T	SNP	179
		C/T	SNP	428
		A/G	SNP	437
		A/T	SNP	556
		G/A	SNP	756
		T/C	SNP	857
		T/G	SNP	892
		G/C	SNP	896
		T/A	SNP	901
		C/T	SNP	977
		A/T	SNP	1025
		C/G	SNP	1151
		G/A	SNP	1171
		T/C	SNP	1176
		T/G	SNP	1177
		C/T	SNP	1179
		C/G	SNP	1250
		T/C	SNP	1306
		C/G	SNP	1338
		A/G	SNP	1716
		C/T	SNP	1856
		C/T	SNP	1865
		T/C	SNP	1931
		G/C	SNP	1936
		G/C	SNP	1960
		G/A	SNP	2023
		T/G	SNP	2077
		G/A	SNP	2096
		G/T	SNP	2132
		A/T	SNP	2134
		G/A	SNP	2194

		A/G	SNP	2200
		T/C	SNP	2306
		A/T	SNP	2356
		A/G	SNP	2357
		T/C	SNP	2402
		G/T	SNP	2412
		G/A	SNP	2582
XP_951482.1 retrotransposon hot spot (RHS) protein [Trypanosoma brucei TREU927]	GLOS_TB927.2.400.1.1	T/G	SNP	55
		A/C	SNP	68
		T/C	SNP	71
		C/T	SNP	73
		A/T	SNP	217
		G/T	SNP	297
		T/C	SNP	303
		T/C	SNP	326
		A/G	SNP	368
		T/C	SNP	372
		C/T	SNP	519
		G/A	SNP	559
		T/C	SNP	602
		C/T	SNP	625
		A/C	SNP	652
		T/C	SNP	664
		T/A	SNP	670
		C/A	SNP	687
		CA/C	DELETION	714
		C/T	SNP	714
		A/G	SNP	717
		C/T	SNP	724
		C/T	SNP	759
		T/C	SNP	762
		A/C	SNP	781
		G/A	SNP	828
		T/C	SNP	834
		G/A	SNP	868
		T/A	SNP	900

G/C	SNP	904
C/T	SNP	928
A/T	SNP	966
T/A	SNP	972
T/C	SNP	1020
C/T	SNP	1049
C/A	SNP	1051
G/T	SNP	1053
C/T	SNP	1054
A/G	SNP	1063
G/A	SNP	1202
G/T	SNP	1228
T/C	SNP	1248
C/T	SNP	1267
A/T	SNP	1297
C/T	SNP	1298
G/A	SNP	1339
C/A	SNP	1360
T/G	SNP	1398
C/T	SNP	1427
G/T	SNP	1545
G/A	SNP	1567
A/C	SNP	1597
C/T	SNP	1779
A/G	SNP	1784
A/C	SNP	1805
A/T	SNP	1811
GA/G	DELETION	1814
A/G	SNP	1883
A/G	SNP	1901
AAT/A	DELETION	1957
A/T	SNP	1966
A/G	SNP	1979
C/T	SNP	2000
A/G	SNP	2098
C/T	SNP	2119

A/T	SNP	2226
C/A	SNP	2252
A/G	SNP	2300
C/A	SNP	2505
T/C	SNP	2515
G/T	SNP	2536
T/G	SNP	2568
C/T	SNP	2569
C/G	SNP	2622
A/G	SNP	2627
A/G	SNP	2628
C/G	SNP	2646
G/A	SNP	2756
A/G	SNP	2781
C/T	SNP	2791
G/A	SNP	2861
C/G	SNP	3244
A/C	SNP	3405
A/G	SNP	3406
C/G	SNP	3433
T/C	SNP	3657
C/T	SNP	3658
G/A	SNP	3659
A/T	SNP	3681
C/T	SNP	3785
T/G	SNP	3787
C/G	SNP	3827
A/G	SNP	3890
A/T	SNP	3894
C/T	SNP	3896
C/T	SNP	3902
C/T	SNP	3923
A/G	SNP	3947
C/T	SNP	3964
G/A	SNP	4160
T/C	SNP	4169

		C/A	SNP	4171
		G/T	SNP	4197
		T/G	SNP	4203
		G/C	SNP	4204
		T/C	SNP	4213
		T/C	SNP	4231
XM_946532.1 Tbb GUTat10.1 NUP-1 protein (Tb927.2.4230) partial mRNA	GLOS_TB927.2.4230.1.1	A/G	SNP	563
XP_951628.1 paraflagellar rod protein [T. brucei brucei strain 927/4 GUTat10.1]	GLOS_TB927.2.4330.1.1	C/T	SNP	2689
XP_951630.1 trypanothione synthetase [T. brucei brucei strain 927/4 GUTat10.1]	GLOS_TB927.2.4370.1.1	C/CT	INSERTION	225
		G/GT	INSERTION	2542
		C/CT	INSERTION	2601
		G/A	SNP	2956
XM_946554.1 Tbb GUTat10.1 hypothetical protein (Tb927.2.4580) partial mRNA	GLOS_TB927.2.4580.1.1	G/GA	INSERTION	281
		C/CA	INSERTION	2288
XM_946561.1 Tbb GUTat10.1 hypothetical protein (Tb927.2.4700) partial mRNA	GLOS_TB927.2.4700.1.1	GTA/G	DELETION	398
		T/TA	INSERTION	1190
		G/GT	INSERTION	1297
		C/CTT	INSERTION	1334
		G/GT	INSERTION	1528
		C/CT	INSERTION	1924
		C/CT	INSERTION	2008
		G/GA	INSERTION	2330
		C/CT	INSERTION	2424
XM_946391.1 Tbb retrotransposon hot spot (RHS) protein partial mRNA	GLOS_TB927.2.470.1.2	A/G	SNP	38
		G/C	SNP	95
		A/G	SNP	103
		G/A	SNP	154
		C/T	SNP	232
		A/C	SNP	270
		A/G	SNP	304
		A/C	SNP	317
		A/G	SNP	318
		G/A	SNP	320
		G/A	SNP	321
		A/G	SNP	354
		G/A	SNP	363

		A/C	SNP	393
		A/C	SNP	400
		C/T	SNP	454
		C/T	SNP	587
		G/A	SNP	589
		A/G	SNP	593
		T/G	SNP	616
		G/A	SNP	618
		A/G	SNP	1099
		G/A	SNP	1673
		A/G	SNP	1674
		C/A	SNP	1675
		C/A	SNP	1679
		C/CT	INSERTION	1750
		A/C	SNP	2404
		C/T	SNP	2409
		G/T	SNP	2490
		C/T	SNP	2534
XM_946391.1 Tbb retrotransposon hot spot protein (Tb927.2.470) partial mRNA	GLOS_TB927.2.470.2.2	C/A	SNP	69
		C/T	SNP	121
		A/G	SNP	132
		A/G	SNP	137
		T/A	SNP	139
		G/A	SNP	152
		G/A	SNP	414
		C/G	SNP	415
		T/A	SNP	435
		T/A	SNP	471
		G/A	SNP	497
		G/A	SNP	527
		G/A	SNP	622
		A/T	SNP	979
		A/T	SNP	991
		G/A	SNP	1256
		C/T	SNP	1264
		A/C	SNP	1530

		C/G	SNP	1572
		C/G	SNP	1574
		A/T	SNP	1576
		A/G	SNP	1593
		G/A	SNP	1926
		G/C	SNP	1991
		A/G	SNP	2111
		A/C	SNP	2121
		G/A	SNP	2659
		A/G	SNP	2702
		A/C	SNP	2722
XM_946562.1 Tbb GUTat10.1 RNA-binding protein (Tb927.2.4710) partial mRNA	GLOS_TB927.2.4710.1.1	C/CT	INSERTION	1084
		A/ATT	INSERTION	3801
		A/T	SNP	3801
		G/GA	INSERTION	3943
		C/CT	INSERTION	4068
XM_946615.1 Tbb GUTat10.1 hypothetical protein (Tb927.2.5360) partial mRNA	GLOS_TB927.2.5360.1.1	G/A	SNP	69
		C/A	SNP	104
		A/G	SNP	138
		A/T	SNP	143
		C/T	SNP	169
		A/C	SNP	200
		T/C	SNP	236
		G/T	SNP	270
		A/T	SNP	334
		G/A	SNP	337
		A/G	SNP	405
		T/C	SNP	408
		A/C	SNP	642
		A/G	SNP	645
		T/A	SNP	646
		T/G	SNP	647
		G/A	SNP	685
		T/A	SNP	837
		A/G	SNP	841
		A/G	SNP	880

		T/C	SNP	888
		C/T	SNP	925
		G/T	SNP	947
		T/C	SNP	952
		A/G	SNP	964
		G/A	SNP	1024
		C/T	SNP	1064
		T/C	SNP	1077
		C/A	SNP	1095
		A/G	SNP	1124
		C/T	SNP	1127
		A/G	SNP	1165
		T/C	SNP	1432
		A/G	SNP	1455
		T/C	SNP	1502
		C/T	SNP	1534
XM_946650.1 Tbb 60S ribosomal protein L44 (Tb927.2.6090) partial mRNA	GLOS_TB927.2.6090.1.1	TTAA/T	DELETION	513
XM_946397.1 Tbb GUTat10.1 hypothetical protein (Tb927.2.900) partial mRNA	GLOS_TB927.2.900.1.1	C/T	SNP	31
		A/T	SNP	105
		G/T	SNP	107
		C/T	SNP	122
		G/T	SNP	124
		G/A	SNP	130
		A/G	SNP	134
		T/C	SNP	168
		T/G	SNP	181
		C/G	SNP	190
		G/A	SNP	216
		T/C	SNP	254
		G/T	SNP	274
		T/C	SNP	305
		G/A	SNP	306
		C/G	SNP	313
		A/C	SNP	346
		C/A	SNP	356
		T/C	SNP	367

A/G	SNP	391
C/T	SNP	404
T/C	SNP	413
C/T	SNP	434
T/C	SNP	456
C/T	SNP	503
C/T	SNP	533
A/G	SNP	536
T/A	SNP	564
C/T	SNP	589
G/A	SNP	599
A/G	SNP	616
T/C	SNP	617
T/C	SNP	632
A/G	SNP	669
A/T	SNP	682
G/C	SNP	690
G/T	SNP	716
C/A	SNP	732
C/T	SNP	766
TTG/T	DELETION	771
C/T	SNP	787
C/G	SNP	793
C/T	SNP	794
C/T	SNP	796
A/G	SNP	818
A/C	SNP	828
A/T	SNP	832
C/T	SNP	865
A/G	SNP	871
C/T	SNP	884
G/T	SNP	921
A/C	SNP	956
A/G	SNP	970
G/A	SNP	1039
G/A	SNP	1045

A/G	SNP	1047
C/T	SNP	1096
A/G	SNP	1100
T/C	SNP	1101
A/G	SNP	1107
C/T	SNP	1219
C/T	SNP	1229
G/A	SNP	1235
G/A	SNP	1256
A/T	SNP	1296
C/T	SNP	1304
G/C	SNP	1326
A/G	SNP	1345
C/T	SNP	1351
T/G	SNP	1352
A/G	SNP	1368
C/T	SNP	1387
C/A	SNP	1396
C/T	SNP	1431
T/C	SNP	1438
C/T	SNP	1459
T/C	SNP	1489
G/T	SNP	1504
C/T	SNP	1523
A/G	SNP	1538
A/G	SNP	1540
A/G	SNP	1542
C/A	SNP	1557
C/T	SNP	1598
C/T	SNP	1613
A/C	SNP	1617
T/C	SNP	1634
C/A	SNP	1648
C/T	SNP	1652
A/T	SNP	1653
C/T	SNP	1656

		C/T	SNP	1666
		T/C	SNP	1668
		A/G	SNP	1734
		G/A	SNP	1749
		A/G	SNP	1769
		C/A	SNP	1771
		A/T	SNP	1781
		A/G	SNP	1803
		G/A	SNP	1829
		T/C	SNP	1846
		C/G	SNP	1858
		T/G	SNP	1872
		G/A	SNP	1927
		T/C	SNP	1939
		A/G	SNP	1947
		A/G	SNP	1966
		C/T	SNP	2008
		G/C	SNP	2016
XM_838604.1 Tbb hypothetical protein, conserved (Tb927.3.1010) partial mRNA	GLOS_TB927.3.1010.1.1	G/A	SNP	68
		TTTA/T	DELETION	2231
		C/CA	INSERTION	2685
		A/AT	INSERTION	3497
XM_838612.1 Tbb hypothetical protein, conserved (Tb927.3.1110) partial mRNA	GLOS_TB927.3.1110.1.1	C/CA	INSERTION	314
		G/GA	INSERTION	771
XP_843706.1 GTP-binding nuclear protein rtb2 [Tbb strain 927/4 GUTat10.1]	GLOS_TB927.3.1120.1.1	A/C	SNP	42
		G/A	SNP	44
		G/A	SNP	45
		C/CA	INSERTION	162
		T/TA	INSERTION	260
		G/GA	INSERTION	1266
XM_838622.1 Tbb protein transport protein Sec24C, putative partial mRNA	GLOS_TB927.3.1210.1.1	G/GT	INSERTION	806
		CT/C	DELETION	3212
XM_838639.1 Tbb ATP synthase beta chain, mitochondrial precursor partial mRNA	GLOS_TB927.3.1380.1.1	T/TA	INSERTION	124
XP_843763.1 hypothetical protein [T. brucei brucei strain 927/4 GUTat10.1]	GLOS_TB927.3.1690.1.1	G/C	SNP	465
		G/GA	INSERTION	652
		A/G	SNP	797

XM_838680.1 Tbb pyruvate dehydrogenase E1 beta subunit, putative partial mRNA	GLOS_TB927.3.1790.1.1	C/CA	INSERTION	86
		A/G	SNP	526
		G/GT	INSERTION	953
		T/C	SNP	1233
		G/A	SNP	2028
		TTTC/T	DELETION	2357
		TTC/T	DELETION	2364
XP_843778.1 3-oxo-5-alpha-steroid 4-dehydrogenase [Trypanosoma brucei TREU927]	GLOS_TB927.3.1840.1.1	C/CA	INSERTION	294
		AC/A	DELETION	432
		A/AT	INSERTION	646
		G/GA	INSERTION	1165
		C/CT	INSERTION	1316
		T/A	SNP	1453
		CA/C	DELETION	1541
		C/CT	INSERTION	1639
		TGTGTGT	DELETION	1740
		AAT/A	DELETION	2069
		A/G	SNP	2263
XP_843788.1 hypothetical protein [T. brucei brucei strain 927/4 GUTat10.1]	GLOS_TB927.3.1940.1.1	C/T	SNP	1799
XM_838711.1 Tbb hypothetical protein, conserved (Tb927.3.2100) partial mRNA	GLOS_TB927.3.2100.1.1	T/A	SNP	182
		A/G	SNP	209
		A/G	SNP	1135
		C/CT	INSERTION	1375
		GA/G	DELETION	1961
XP_843812.1 hypothetical protein [T. brucei brucei strain 927/4 GUTat10.1]	GLOS_TB927.3.2180.1.1	A/C	SNP	165
		GA/G	DELETION	185
XM_838724.1 Tbb succinyl-CoA synthetase alpha subunit, putative partial mRNA	GLOS_TB927.3.2230.1.1	C/T	SNP	809
		T/C	SNP	1006
		G/C	SNP	1078
		G/C	SNP	1124
XM_838750.1 Tbb hypothetical protein, conserved (Tb927.3.2490) partial mRNA	GLOS_TB927.3.2490.1.1	C/T	SNP	3054
XM_838760.1 Tbb ATP-dependent DEAD/H RNA helicase, putative partial mRNA	GLOS_TB927.3.2600.1.1	C/T	SNP	3278
		G/A	SNP	5702
XP_843881.1 hypothetical protein [T. brucei brucei strain 927/4 GUTat10.1]	GLOS_TB927.3.2880.1.1	A/T	SNP	606
XM_838790.1 Tbb elongation initiation factor 2 alpha subunit, putative partial mRNA	GLOS_TB927.3.2900.1.1	A/T	SNP	19
		A/G	SNP	1130

		CT/C	DELETION	1521
XM_838813.1 Tbb hypothetical protein, conserved (Tb927.3.3130) partial mRNA	GLOS_TB927.3.3130.1.1	G/T	SNP	275
		T/C	SNP	1204
		T/C	SNP	3248
		C/A	SNP	4755
XM_838827.1 Tbb ATP-dependent phosphofructokinase (Tb927.3.3270) partial mRNA	GLOS_TB927.3.3270.1.1	T/C	SNP	818
		A/C	SNP	3384
XM_838831.1 Tbb 60S ribosomal protein L13, putative (Tb927.3.3310) partial mRNA	GLOS_TB927.3.3310.1.1	T/C	SNP	133
		C/T	SNP	380
		C/T	SNP	794
		T/A	SNP	805
		T/A	SNP	806
XP_843934.1 aspartyl aminopeptidase [T. brucei brucei strain 927/4 GUTat10.1]	GLOS_TB927.3.3410.1.1	A/G	SNP	1946
XM_838857.1 Tbb lipophosphoglycan biosynthetic protein, putative partial mRNA	GLOS_TB927.3.3580.1.1	T/C	SNP	87
		T/C	SNP	2171
XM_838868.1 Tbb flagellar radial spoke protein-like, putative partial mRNA	GLOS_TB927.3.3690.1.1	GT/G	DELETION	1891
		GA/G	DELETION	1997
		G/GA	INSERTION	2160
XM_838874.1 Tbb hypothetical protein, conserved (Tb927.3.3750) partial mRNA	GLOS_TB927.3.3750.1.1	G/A	SNP	161
		AT/A	DELETION	1141
		G/GA	INSERTION	1758
XP_843969.1 hypothetical protein [Tbb] ref|XP_843971.1| hypothetical protein	GLOS_TB927.3.3770.1.1	G/A	SNP	142
		C/T	SNP	402
		C/A	SNP	425
		T/C	SNP	475
		G/A	SNP	685
		C/G	SNP	1146
XM_838889.1 Tbb carnitine O-palmitoyltransferase II, putative partial mRNA	GLOS_TB927.3.3900.1.1	A/G	SNP	292
XM_838903.1 Tbb hypothetical protein, conserved (Tb927.3.4040) partial mRNA	GLOS_TB927.3.4040.1.1	GTA/G	DELETION	2294
XM_838907.1 Tbbhypothetical protein, conserved (Tb927.3.4080) partial mRNA	GLOS_TB927.3.4080.1.1	T/C	SNP	40
		A/G	SNP	380
		T/C	SNP	502
		G/A	SNP	503
		C/T	SNP	526
		A/G	SNP	527
		T/C	SNP	631

		G/A	SNP	632
		A/G	SNP	655
		C/T	SNP	694
		C/T	SNP	985
		G/T	SNP	1029
		T/G	SNP	1030
		C/T	SNP	1111
		G/T	SNP	1375
		A/G	SNP	1430
		G/T	SNP	1451
		T/G	SNP	1453
		T/C	SNP	1454
		T/C	SNP	1459
		G/T	SNP	1482
		C/T	SNP	1531
		C/A	SNP	1532
		T/C	SNP	1579
		G/A	SNP	1617
		G/A	SNP	1714
		C/G	SNP	1735
XM_838915.1 Tbb hypothetical protein, conserved (Tb927.3.4160) partial mRNA	GLOS_TB927.3.4160.1.1	T/TA	INSERTION	216
		G/GA	INSERTION	971
XM_838928.1 Tbb 73 kDa paraflagellar rod protein (Tb927.3.4290) partial mRNA	GLOS_TB927.3.4290.1.1	G/A	SNP	574
		G/A	SNP	1943
XP_844042.1 fumarate hydratase [T. brucei brucei strain 927/4 GUTat10.1]	GLOS_TB927.3.4500.1.1	C/T	SNP	38
		C/T	SNP	39
		C/T	SNP	40
		G/GT	INSERTION	140
		G/GA	INSERTION	2510
		C/CT	INSERTION	2818
		A/AT	INSERTION	3019
		G/A	SNP	3129
		G/GA	INSERTION	3129
		A/AT	INSERTION	3276
XP_844067.1 aminopeptidase [Tbb] ref|XP_844071.1| aminopeptidase [Tbb]	GLOS_TB927.3.4750.1.1	G/A	SNP	3618
XM_838975.1 Tbb strain 927/4 GUTat10.1 dynamin partial mRNA	GLOS_TB927.3.4760.1.1	A/AT	INSERTION	239

		G/GA	INSERTION	2049
XP_844076.1 ubiquitin hydrolase [T. brucei brucei strain 927/4 GUTat10.1]	GLOS_TB927.3.4840.1.1	C/CA	INSERTION	886
XP_844108.1 cofilin/actin depolymerizing factor [Trypanosoma brucei TREU927]	GLOS_TB927.3.5180.1.1	G/GA	INSERTION	1363
XM_839049.1 Tbb 26S proteasome regulatory non-ATPase subunit partial mRNA	GLOS_TB927.3.5520.1.1	C/CA	INSERTION	3116
XM_838575.1 Tbb zinc finger protein 2, putative (Tb927.3.720) partial mRNA	GLOS_TB927.3.720.1.1	C/CT	INSERTION	204
XM_838577.1 Tbb hypothetical protein, conserved (Tb927.3.740) partial mRNA	GLOS_TB927.3.740.1.1	C/CTT	INSERTION	514
		CT/C	DELETION	970
		A/AT	INSERTION	1016
		C/CA	INSERTION	2475
		GT/G	DELETION	2559
XP_844280.1 hypothetical protein [T. brucei brucei strain 927/4 GUTat10.1]	GLOS_TB927.4.1300.1.1	G/A	SNP	23
		C/A	SNP	26
		T/G	SNP	27
		C/A	SNP	1561
		C/CA	INSERTION	1708
		AAT/A	DELETION	1873
		G/GA	INSERTION	1983
XP_844285.1 hydroxyacylglutathione hydrolase [Tbb strain 927/4 GUTat10.1]	GLOS_TB927.4.1350.1.1	TA/T	DELETION	1048
		G/GT	INSERTION	1295
XM_839236.1 Tbb GUTat10.1 ribosomal protein L3, putative partial mRNA	GLOS_TB927.4.1790.1.1	A/C	SNP	561
		G/T	SNP	578
		C/T	SNP	1450
XM_839242.1 Tbb hypothetical protein, conserved (Tb927.4.1850) partial mRNA	GLOS_TB927.4.1850.1.1	C/CT	INSERTION	2107
XM_839243.1 Tbb ribosomal protein S19, putative (Tb927.4.1860) partial mRNA	GLOS_TB927.4.1860.1.2	T/C	SNP	287
		C/CGT	INSERTION	546
		G/A	SNP	578
		C/CT	INSERTION	607
XP_844336.1 ribosomal protein S19 [T. brucei brucei strain 927/4 GUTat10.1]	GLOS_TB927.4.1860.2.2	G/A	SNP	85
		G/GA	INSERTION	150
		AAC/A	DELETION	202
		A/G	SNP	477
		ATTTTT/A/	DELETION	847
XM_839260.1 Tbb hypothetical protein, conserved (Tb927.4.2030) partial mRNA	GLOS_TB927.4.2030.1.1	G/C	SNP	142
		G/A	SNP	143
		C/T	SNP	149
		G/GT	INSERTION	933

		G/A	SNP	1297
XM_839265.1 Tbb hypothetical protein, conserved (Tb927.4.2080) partial mRNA	GLOS_TB927.4.2080.1.1	C/T	SNP	2364
XM_839298.1 Tbb hypothetical protein, conserved (Tb927.4.2410) partial mRNA	GLOS_TB927.4.2410.1.1	GA/G	DELETION	295
		T/A	SNP	647
		C/T	SNP	1515
XM_839310.1 Tbb hypothetical protein, conserved (Tb927.4.2530) partial mRNA	GLOS_TB927.4.2530.1.1	T/G	SNP	542
		A/G	SNP	594
		GA/G	DELETION	612
		TAG/T	DELETION	670
		G/GA	INSERTION	1345
		C/CA	INSERTION	1472
		TA/T	DELETION	1558
XM_839331.1 Tbb hypothetical protein, conserved (Tb927.4.2740) partial mRNA	GLOS_TB927.4.2740.1.1	A/T	SNP	475
		C/T	SNP	482
		CAA/C	DELETION	484
		T/C	SNP	1631
		C/CT	INSERTION	2325
		A/C	SNP	2456
XM_839346.1 Tbb hypothetical protein, conserved (Tb927.4.2890) partial mRNA	GLOS_TB927.4.2890.1.1	G/A	SNP	2709
XM_839363.1 Tbb hypothetical protein, conserved (Tb927.4.3060) partial mRNA	GLOS_TB927.4.3060.1.1	G/A	SNP	115
		TGACAAA	DELETION	248
		T/A	SNP	322
		G/T	SNP	323
		A/T	SNP	324
		T/C	SNP	394
		CT/C	DELETION	468
		T/A	SNP	470
		C/T	SNP	478
		C/T	SNP	537
		T/TCC	INSERTION	751
		G/A	SNP	766
		G/GT	INSERTION	1409
		C/T	SNP	1417
		C/A	SNP	1489
		G/A	SNP	2315
XM_839407.1 Tbb hypothetical protein, conserved (Tb927.4.3500) partial mRNA	GLOS_TB927.4.3500.1.1	G/A	SNP	177

		A/AT	INSERTION	1745
XM_839412.1 Tbb 60S ribosomal protein L13a, putative (Tb927.4.3550) partial mRNA	GLOS_TB927.4.3550.1.1	A/C	SNP	381
		C/G	SNP	417
		C/T	SNP	555
		C/G	SNP	744
XM_839416.1 Tbb translation elongation factor 1-beta, putative partial mRNA	GLOS_TB927.4.3590.1.1	T/G	SNP	961
XM_839419.1 Tbb serine/threonine-protein phosphatase PP1, putative partial mRNA	GLOS_TB927.4.3620.1.1	GA/G	DELETION	199
		C/CT	INSERTION	509
		TAGA/T	DELETION	867
		T/C	SNP	2883
XM_839431.1 Tbb hypothetical protein, conserved (Tb927.4.3740) partial mRNA	GLOS_TB927.4.3740.1.1	G/A	SNP	4
		C/T	SNP	30
		G/A	SNP	46
		C/T	SNP	72
		G/A	SNP	88
		C/T	SNP	114
		T/C	SNP	123
XM_839449.1 Tbb hypothetical protein, conserved (Tb927.4.3920) partial mRNA	GLOS_TB927.4.3920.1.1	C/A	SNP	354
		AT/A	DELETION	374
		C/A	SNP	420
		C/T	SNP	528
		A/G	SNP	3387
XM_839452.1 Tbb cytoskeleton-associated protein CAP5.5, putative partial mRNA	GLOS_TB927.4.3950.1.1	CA/C	DELETION	111
		C/CA	INSERTION	290
		C/A	SNP	2901
XM_839473.1 Tbb hypothetical protein, conserved (Tb927.4.4160) partial mRNA	GLOS_TB927.4.4160.1.1	C/A	SNP	214
		T/C	SNP	225
		T/C	SNP	238
		T/C	SNP	266
		C/T	SNP	281
		G/T	SNP	409
		T/C	SNP	670
		T/C	SNP	749
		G/A	SNP	750
		G/A	SNP	751
		T/C	SNP	761

		T/C	SNP	824
		C/T	SNP	957
		C/T	SNP	1047
		A/G	SNP	1067
		G/A	SNP	1230
		A/T	SNP	1246
		T/G	SNP	1304
		A/G	SNP	1321
		G/A	SNP	1326
		C/G	SNP	1354
		T/C	SNP	1355
		G/A	SNP	1398
		T/G	SNP	1399
		G/A	SNP	1403
		C/T	SNP	1898
		G/A	SNP	1913
		A/G	SNP	1938
		C/T	SNP	1966
		A/T	SNP	1998
		A/G	SNP	2040
		G/T	SNP	2055
		C/T	SNP	2056
XM_839501.1 Tbb receptor-type adenylate cyclase GRESAG 4, putat. partial mRNA	GLOS_TB927.4.4450.1.1	T/C	SNP	350
		A/T	SNP	395
		A/G	SNP	396
		G/T	SNP	397
		G/A	SNP	586
		C/G	SNP	590
		G/A	SNP	595
		T/C	SNP	621
		C/T	SNP	679
		T/C	SNP	684
		A/G	SNP	709
		A/G	SNP	812
		A/C	SNP	813
		G/T	SNP	930

T/G	SNP	1197
A/G	SNP	1225
G/A	SNP	1453
G/T	SNP	1513
A/T	SNP	1535
T/C	SNP	1634
A/G	SNP	1693
T/G	SNP	1771
G/A	SNP	1984
C/T	SNP	1989
C/T	SNP	1990
T/C	SNP	2055
C/T	SNP	2089
A/G	SNP	2110
A/C	SNP	2262
G/A	SNP	2269
A/C	SNP	2293
C/T	SNP	2296
C/T	SNP	2303
A/T	SNP	2344
C/T	SNP	2346
A/G	SNP	2369
C/T	SNP	2386
G/C	SNP	2394
A/G	SNP	2410
C/T	SNP	2430
C/T	SNP	2437
A/G	SNP	2450
C/G	SNP	2482
C/G	SNP	2483
A/G	SNP	2484
G/A	SNP	2563
T/C	SNP	2618
G/A	SNP	2746
C/T	SNP	2766
A/G	SNP	2788

		A/G	SNP	2790
		C/T	SNP	2791
		C/T	SNP	2805
		C/T	SNP	2807
		C/T	SNP	2974
		A/G	SNP	3028
		C/T	SNP	3029
		C/T	SNP	3030
		A/C	SNP	3031
		G/C	SNP	3044
		G/T	SNP	3070
		G/A	SNP	3087
		T/G	SNP	3257
		C/T	SNP	3270
		A/G	SNP	3271
		A/C	SNP	3340
		A/G	SNP	3346
		T/C	SNP	3417
		C/T	SNP	3424
		T/A	SNP	3454
		T/C	SNP	3455
		A/G	SNP	3550
		A/G	SNP	3561
		C/T	SNP	3577
		C/T	SNP	3640
		C/T	SNP	3643
		G/C	SNP	3662
		A/G	SNP	3710
		T/C	SNP	3792
		T/G	SNP	3801
		C/T	SNP	3818
		G/T	SNP	3852
		T/C	SNP	3895
		G/A	SNP	3926
XM_839511.1 Tbb hypothetical protein, conserved (Tb927.4.4550) partial mRNA	GLOS_TB927.4.4550.1.1	T/A	SNP	656
XM_839513.1 Tbb hypothetical protein, conserved (Tb927.4.4570) partial mRNA	GLOS_TB927.4.4570.1.1	G/GT	INSERTION	3492

XM_839529.1 Tbb amino acid transporter, putative (Tb927.4.4730) partial mRNA	GLOS_TB927.4.4730.1.1	C/CT	INSERTION	2142
XP_844648.1 ubiquinol-cytochrome C reductase [Tbb strain 927/4 GUTat10.1]	GLOS_TB927.4.4990.1.1	C/T	SNP	74
		C/A	SNP	76
		C/T	SNP	78
		G/C	SNP	86
		A/G	SNP	245
		G/A	SNP	322
		G/GA	INSERTION	330
		G/C	SNP	395
		A/G	SNP	397
		T/C	SNP	412
		A/G	SNP	455
		C/T	SNP	460
		C/T	SNP	562
		G/T	SNP	565
		C/T	SNP	580
XM_839133.1 Tbb gamma-adaptin 1, putative (Tb927.4.760) partial mRNA	GLOS_TB927.4.760.1.1	GT/G	DELETION	3023
XP_844774.1 ubiquitin-conjugating enzyme E2 [Tbb strain 927/4 GUTat10.1]	GLOS_TB927.5.1000.1.1	G/GT	INSERTION	857
XM_839687.1 Tbb mitochondrial processing peptidase, beta subunit partial mRNA	GLOS_TB927.5.1060.1.1	C/A	SNP	6
		G/GA	INSERTION	419
		CA/C	DELETION	788
		TA/T	DELETION	1010
		G/GT	INSERTION	2720
XP_844783.1 threonyl-tRNA synthetase [T. brucei brucei strain 927/4 GUTat10.1]	GLOS_TB927.5.1090.1.1	G/A	SNP	1466
XP_844785.1 60S ribosomal protein L2 [T. brucei brucei strain 927/4 GUTat10.1] ref|XP_ ref|XP_829685.1| 60S ribosomal protein L2 [Tbb strain 927/4 GUTat10.1]	GLOS_TB927.5.1110.1.1	A/G	SNP	586
		G/A	SNP	589
		G/A	SNP	631
		G/A	SNP	652
		G/C	SNP	769
		G/A	SNP	787
		A/G	SNP	790
		A/G	SNP	832
		T/C	SNP	859
		G/T	SNP	870
		G/A	SNP	874

		G/A	SNP	1105
		C/A	SNP	1123
		G/A	SNP	1147
		G/A	SNP	1150
		G/T	SNP	1231
		G/A	SNP	1249
XM_839706.1 Tbb strain 927/4 GUTat10.1 hypothetical protein partial mRNA	GLOS_TB927.5.1250.1.1	C/T	SNP	188
		CA/C	DELETION	204
		G/A	SNP	379
		G/A	SNP	559
		C/T	SNP	1303
		C/G	SNP	1957
		G/T	SNP	2346
		TC/T	DELETION	2450
		C/T	SNP	2459
XP_844821.1 NADH-cytochrome b5 reductase [Tbb strain 927/4 GUTat10.1]	GLOS_TB927.5.1470.1.1	GA/G	DELETION	1068
		A/T	SNP	1575
		C/CT	INSERTION	1984
		AG/A	DELETION	2041
XM_839738.1 Tbb strain 927/4 GUTat10.1 hypothetical protein partial mRNA	GLOS_TB927.5.1570.1.1	G/A	SNP	163
		G/GCA	INSERTION	392
		C/T	SNP	549
		C/CT	INSERTION	831
		C/T	SNP	1948
		C/A	SNP	3071
XM_839747.1 Tbb strain 927/4 GUTat10.1 protein phosphatase 2C partial mRNA	GLOS_TB927.5.1660.1.1	G/GA	INSERTION	312
		CA/C	DELETION	804
		A/G	SNP	2785
XM_839752.1 Tbb ribonucleoprotein p18, mitochondrial precursor partial mRNA	GLOS_TB927.5.1710.1.1	C/CT	INSERTION	960
		CAGAG/C	DELETION	1047
		G/T	SNP	1984
		GT/G	DELETION	1984
XM_839759.1 Tbb strain 927/4 GUTat10.1 hypothetical protein partial mRNA	GLOS_TB927.5.1780.1.1	G/A	SNP	1285
		C/T	SNP	1632
		T/C	SNP	1983
		A/G	SNP	2159

		C/G	SNP	2195
		C/T	SNP	2219
XP_844853.1 hypothetical protein [T. brucei brucei strain 927/4 GUTat10.1]	GLOS_TB927.5.1790.1.1	A/G	SNP	699
		A/G	SNP	1639
XM_839762.1 Tbb lysosomal/endosomal membrane protein p67 partial mRNA	GLOS_TB927.5.1810.1.1	C/CA	INSERTION	499
		T/A	SNP	500
		C/A	SNP	502
		G/A	SNP	699
		G/A	SNP	882
		G/C	SNP	988
		T/C	SNP	3039
		G/A	SNP	3301
XM_839791.1 Tbb strain 927/4 GUTat10.1 hypothetical protein partial mRNA	GLOS_TB927.5.2100.1.1	C/T	SNP	515
		AG/A	DELETION	1729
XM_839807.1 Tbb strain 927/4 GUTat10.1 hypothetical protein partial mRNA	GLOS_TB927.5.2260.1.2	G/A	SNP	311
		A/T	SNP	95
		A/ATT	INSERTION	96
		A/T	SNP	96
		G/A	SNP	299
		C/T	SNP	809
XM_839824.1 Tbb GUTat10.1 membrane transporter protein partial mRNA	GLOS_TB927.5.2430.1.1	A/G	SNP	193
		C/T	SNP	733
		T/C	SNP	1162
		C/T	SNP	1285
		T/A	SNP	1317
		C/T	SNP	1321
		C/T	SNP	1492
		A/T	SNP	2086
XM_839836.1 Tbb GUTat10.1 translation initiation factor partial mRNA	GLOS_TB927.5.2570.1.1	A/T	SNP	651
		C/G	SNP	754
		T/C	SNP	2617
XM_839864.1 Tbb strain 927/4 GUTat10.1 hypothetical protein partial mRNA	GLOS_TB927.5.2850.1.1	T/C	SNP	35
		G/A	SNP	39
		C/T	SNP	1953
XM_839872.1 Tbb strain 927/4 GUTat10.1 hypothetical protein partial mRNA	GLOS_TB927.5.2930.1.1	A/G	SNP	233
		T/C	SNP	277

XP_844968.1 phosphoribosylpyrophosphate synthetase [T. brucei TREU927]	GLOS_TB927.5.2960.1.1	A/T	SNP	96
		G/GT	INSERTION	157
		A/C	SNP	565
		T/A	SNP	764
XM_839891.1 Tbb GUTat10.1 translation initiation factor partial mRNA	GLOS_TB927.5.3120.1.1	T/A	SNP	1751
XM_839895.1 T. brucei brucei strain 927/4 GUTat10.1 protein kinase partial mRNA	GLOS_TB927.5.3160.1.1	T/C	SNP	470
		A/T	SNP	728
		A/T	SNP	1554
		A/G	SNP	1582
		G/T	SNP	3145
XM_839613.1 Tbb receptor-type adenylate cyclase GRESAG 4 partial mRNA	GLOS_TB927.5.320.1.1	G/GA	INSERTION	384
		C/T	SNP	461
		GA/G	DELETION	1150
		A/G	SNP	1335
		C/T	SNP	1480
		A/G	SNP	1991
		A/G	SNP	2052
		T/C	SNP	2134
		T/C	SNP	2652
		T/TA	INSERTION	2865
		C/T	SNP	2892
		A/G	SNP	2950
		C/T	SNP	3133
		T/G	SNP	3415
		A/T	SNP	3434
		A/T	SNP	3593
		A/G	SNP	3613
		G/C	SNP	3614
		T/C	SNP	3959
		A/G	SNP	4267
		G/A	SNP	4394
XM_839903.1 Tbb strain 927/4 GUTat10.1 hypothetical protein partial mRNA	GLOS_TB927.5.3240.1.1	A/C	SNP	100
		T/C	SNP	170
		C/T	SNP	688
		T/C	SNP	1251
XM_839936.1 Tbb strain 927/4 GUTat10.1 hypothetical protein partial mRNA	GLOS_TB927.5.3590.1.1	G/T	SNP	1032

		T/C	SNP	1237
		A/G	SNP	1248
		A/G	SNP	1287
		T/G	SNP	1887
		A/G	SNP	2788
		G/A	SNP	2893
XM_839617.1 Tbb GUTat10.1 75 kDa invariant surface glycoprotein partial mRNA	GLOS_TB927.5.360.1.1	A/G	SNP	155
		T/C	SNP	295
		C/T	SNP	571
		C/T	SNP	693
		T/C	SNP	734
		C/T	SNP	815
		C/A	SNP	1076
		C/T	SNP	1094
		C/A	SNP	1145
		C/T	SNP	1149
		C/T	SNP	1833
		G/C	SNP	1908
XP_845050.1 glutamine hydrolysing (not ammonia-dependent) carbomoyl phosphate synthase [Trypanosoma brucei	GLOS_TB927.5.3800.1.1	T/TTA	INSERTION	94
		G/GA	INSERTION	351
		G/GA	INSERTION	462
		C/CA	INSERTION	689
		A/AGTT	INSERTION	764
		T/G	SNP	868
		A/G	SNP	4136
		T/C	SNP	4715
		T/C	SNP	6592
		G/GT	INSERTION	7325
		T/C	SNP	7750
XM_839973.1 Tbb GUTat10.1 arginine N-methyltransferase partial mRNA	GLOS_TB927.5.3960.1.1	G/GT	INSERTION	273
XM_839979.1 Tbb GUTat10.1 hypothetical protein Tb927.5.4020 partial mRNA	GLOS_TB927.5.4020.1.1	G/GA	INSERTION	242
		C/CT	INSERTION	1381
XM_839996.1 T; brucei brucei strain 927/4 GUTat10.1 histone H4 partial mRNA	GLOS_TB927.5.4190.2.3	T/C	SNP	534
XM_839625.1 T. b. brucei strain 927/4 GUTat10.1 hypothetical protein partial mRNA	GLOS_TB927.5.440.1.1	C/CT	INSERTION	2822
XM_840023.1 T. b. brucei strain 927/4 GUTat10.1 major vault protein partial mRNA	GLOS_TB927.5.4460.1.1	C/CA	INSERTION	278

XP_844725.1 hypothetical protein [T. brucei brucei strain 927/4 GUTat10.1]	GLOS_TB927.5.510.1.1	G/A	SNP	484
		C/T	SNP	622
		A/G	SNP	628
		C/T	SNP	738
		GAA/G/GA	DELETION	934
		T/TA	INSERTION	1990
		C/T	SNP	2112
		G/C	SNP	2132
XM_839651.1 T. b. brucei strain 927/4 GUTat10.1 hypothetical protein partial mRNA	GLOS_TB927.5.700.1.1	T/G	SNP	149
		AT/A	DELETION	2601
		G/T	SNP	2735
XM_839667.1 T. b. brucei strain 927/4 GUTat10.1 hypothetical protein partial mRNA	GLOS_TB927.5.860.1.1	AT/A	DELETION	716
		C/A	SNP	2313
XP_844764.1 oligosaccharyl transferase subunit [Trypanosoma brucei TREU927]	GLOS_TB927.5.900.1.1	A/G	SNP	1048
		G/A	SNP	1378
		T/C	SNP	1403
		T/C	SNP	1577
		A/G	SNP	1592
		C/T	SNP	1968
		T/C	SNP	1969
		T/C	SNP	1973
		G/A	SNP	2374
		C/T	SNP	2645
		C/T	SNP	2747
XM_839674.1 Tbb GUTat10.1 NADH-dependent fumarate reductase partial mRNA	GLOS_TB927.5.930.1.1	T/C	SNP	1905
		A/G	SNP	1936
		T/C	SNP	2662
		C/CA	INSERTION	3590
XM_840131.1 Tbb GUTat10.1 cysteine peptidase precursor partial mRNA	GLOS_TB927.6.1020.1.1	C/A	SNP	46
		A/G	SNP	47
		G/T	SNP	352
		C/T	SNP	1327
		T/C	SNP	1329
		G/GT	INSERTION	1510
		G/A	SNP	1636
XP_845231.1 proteasome regulatory ATPase subunit 3 [T. brucei TREU927]	GLOS_TB927.6.1090.1.1	C/CA	INSERTION	196

		G/GAAGA	INSERTION	320
		GA/G	DELETION	1757
XM_840181.1 T. brucei brucei strain 927/4 GUTat10.1 aquaporin 3 partial mRNA	GLOS_TB927.6.1520.1.1	C/CA	INSERTION	1242
		C/CA	INSERTION	1549
		GA/G	DELETION	1828
		T/TA	INSERTION	2101
		AT/A	DELETION	2271
		C/CT	INSERTION	2492
		C/CT	INSERTION	2898
XM_840229.1 T. b. brucei strain 927/4 GUTat10.1 hypothetical protein partial mRNA	GLOS_TB927.6.2010.1.1	C/T	SNP	1455
XM_840251.1 T. b. brucei strain 927/4 GUTat10.1 hypothetical protein partial mRNA	GLOS_TB927.6.2230.1.1	A/G	SNP	376
		G/A	SNP	416
		G/A	SNP	503
		G/A	SNP	1433
		T/C	SNP	1601
		A/C	SNP	2237
		G/T	SNP	2253
XM_840257.1 T. b. brucei strain 927/4 GUTat10.1 hypothetical protein partial mRNA	GLOS_TB927.6.2290.1.1	C/A	SNP	323
		A/G	SNP	401
		C/A	SNP	765
		T/C	SNP	1106
		G/A	SNP	1282
		T/C	SNP	1479
		C/T	SNP	2271
		T/C	SNP	2392
		G/A	SNP	2501
XP_845395.1 pyridoxal kinase [Trypanosoma brucei brucei strain 927/4 GUTat10.1]	GLOS_TB927.6.2740.1.1	A/AGG	INSERTION	105
XP_845400.1 L-threonine 3-dehydrogenase [T. brucei brucei strain 927/4 GUTat10.1]	GLOS_TB927.6.2790.1.1	G/GT	INSERTION	116
		G/GA	INSERTION	1151
		C/CT	INSERTION	1619
		CA/C	DELETION	1819
		G/GT	INSERTION	1957
XM_840337.1 T. b. brucei strain 927/4 GUTat10.1 hypothetical protein partial mRNA	GLOS_TB927.6.3090.1.1	T/G	SNP	610
		GT/G	DELETION	4321
XP_845468.1 endosomal trafficking protein RME-8 [Trypanosoma brucei TREU927]	GLOS_TB927.6.3500.1.1	A/G	SNP	217
		C/T	SNP	336

		T/C	SNP	5283
XP_845483.1 ADP-ribosylation factor [T. brucei brucei strain 927/4 GUTat10.1]	GLOS_TB927.6.3650.1.1	A/C	SNP	209
		CA/C	DELETION	531
XM_840405.1 Tbb heat shock 70 kDa protein, mitochondrial precursor partial mRNA	GLOS_TB927.6.3800.1.1	T/C	SNP	84
		G/A	SNP	555
		TA/T	DELETION	2083
XM_840409.1 Tbb strain 927/4 GUTat10.1 reticulon domain protein partial mRNA	GLOS_TB927.6.3840.1.1	T/C	SNP	135
		G/A	SNP	163
		G/GA	INSERTION	163
		T/G	SNP	834
		C/CTT	INSERTION	1515
XP_845513.1 hypothetical protein [T. brucei brucei strain 927/4 GUTat10.1]	GLOS_TB927.6.3950.1.1	G/GT	INSERTION	233
		C/G	SNP	1874
		C/T	SNP	2021
		GT/G	DELETION	2060
		G/T	SNP	2070
XP_845532.1 hypothetical protein [T. brucei brucei strain 927/4 GUTat10.1]	GLOS_TB927.6.4140.1.1	ATT/A	DELETION	107
		G/GA	INSERTION	256
		G/GA	INSERTION	642
		CACA/C	DELETION	909
		CA/C	DELETION	911
		AT/A	DELETION	976
		G/A	SNP	1238
		GA/G	DELETION	1306
XP_845547.1 glyceraldehyde 3-phosphate dehydrogenase, glycosomal [Tbb]	GLOS_TB927.6.4300.1.1	T/C	SNP	923
		C/T	SNP	1061
		C/T	SNP	1224
		A/G	SNP	1410
		C/T	SNP	2097
XM_840468.1 Tbb strain 927/4 GUTat10.1 hypothetical protein partial mRNA	GLOS_TB927.6.4440.1.1	TAAA/T	DELETION	740
		GA/G	DELETION	802
		C/T	SNP	1485
		C/T	SNP	1541
		A/G	SNP	2002
		G/A	SNP	2149
		T/C	SNP	2151

XM_840472.1 Tbb strain 927/4 GUTat10.1 valyl-tRNA synthetase partial mRNA	GLOS_TB927.6.4480.1.1	A/T	SNP	54
		A/T	SNP	396
		C/T	SNP	1374
		G/A	SNP	1792
		C/T	SNP	2424
		C/G	SNP	2477
		A/G	SNP	2522
		T/C	SNP	2706
		G/A	SNP	2953
		G/A	SNP	3021
XP_845566.1 hypothetical protein [T. brucei brucei strain 927/4 GUTat10.1]	GLOS_TB927.6.4490.1.1	A/G	SNP	271
		CT/C	DELETION	823
		G/A	SNP	985
		A/AGT	INSERTION	1143
		T/G	SNP	1144
		C/CT	INSERTION	1275
		C/T	SNP	1633
		C/G	SNP	1720
XM_840478.1 Tbb 3-hydroxy-3-methylglutaryl-CoA reductase partial mRNA	GLOS_TB927.6.4540.1.1	GA/G	DELETION	278
		GA/G	DELETION	370
		C/A	SNP	396
		G/C	SNP	397
		T/TA	INSERTION	483
		T/C	SNP	1734
XM_840499.1 Tbb strain 927/4 GUTat10.1 hypothetical protein partial mRNA	GLOS_TB927.6.4750.1.1	G/GA	INSERTION	2186
		G/A	SNP	2419
		T/TA	INSERTION	2546
		T/C	SNP	2630
XM_840508.1 Tbb GUTat10.1 S-adenosylmethionine synthetase partial mRNA	GLOS_TB927.6.4840.1.1	T/C	SNP	214
		T/C	SNP	609
		C/T	SNP	741
		C/T	SNP	1227
		A/AAT	INSERTION	1580
		T/C	SNP	1808
XM_840522.1 Tbb 927/4 GUTat10.1 40S ribosomal protein S14 partial mRNA	GLOS_TB927.6.4980.1.2	A/G	SNP	305
		G/A	SNP	308

XM_840522.1 Tbb GUTat10.1 40S ribosomal protein S14 partial mRNA	GLOS_TB927.6.4980.2.2	A/C	SNP	47
		A/G	SNP	48
		C/T	SNP	407
XM_840531.1 Tbb strain 927/4 GUTat10.1 hypothetical protein partial mRNA	GLOS_TB927.6.5070.1.1	G/A	SNP	342
		G/GA	INSERTION	1529
		T/G	SNP	1667
		AT/A	DELETION	1725
		TA/T	DELETION	1836
		C/G	SNP	1932
		G/GAA	INSERTION	1952
		G/A	SNP	2008
XM_840532.1 Tbb strain 927/4 GUTat10.1 hypothetical protein partial mRNA	GLOS_TB927.6.5080.1.1	C/G	SNP	94
		TA/T	DELETION	112
		GA/G	DELETION	425
		T/C	SNP	1144
		C/T	SNP	1477
		C/CT	INSERTION	2293
		GA/G	DELETION	2740
XM_840533.1 Tbb strain 927/4 GUTat10.1 hypothetical protein partial mRNA	GLOS_TB927.6.5090.1.1	G/A	SNP	1150
		A/C	SNP	1549
		T/C	SNP	1950
		A/T	SNP	3041
		TTG/T	DELETION	3239
		C/T	SNP	3301
		C/T	SNP	3407
XM_840536.1 Tbb GUTat10.1 60S acidic ribosomal protein P2 partial mRNA	GLOS_TB927.6.5120.1.2	A/G	SNP	249
		G/A	SNP	258
XM_840082.1 Tbb strain 927/4 GUTat10.1 EP3-2 procyclin partial mRNA	GLOS_TB927.6.520.1.3	A/T	SNP	25
		G/A	SNP	488
		A/T	SNP	654
XM_840096.1 Tbb strain 927/4 GUTat10.1 hypothetical protein partial mRNA	GLOS_TB927.6.660.1.1	C/G	SNP	816
		A/G	SNP	1549
XM_840102.1 Tbb strain 927/4 GUTat10.1 40S ribosomal protein L14 partial mRNA	GLOS_TB927.6.720.1.1	G/T	SNP	20
		C/T	SNP	127
		C/T	SNP	286
		G/A	SNP	290

		G/A	SNP	334
		A/G	SNP	367
		T/A	SNP	747
XM_840109.1 Tbb receptor-type adenylate cyclase GRESAG 4 partial mRNA	GLOS_TB927.6.790.1.2	C/G	SNP	1394
		T/A	SNP	1428
		C/T	SNP	1429
		A/G	SNP	1430
		A/G	SNP	1492
		T/C	SNP	1705
		C/T	SNP	1785
		C/T	SNP	1884
		C/T	SNP	1887
		G/A	SNP	2001
		G/T	SNP	2012
		T/C	SNP	2163
		A/G	SNP	2631
		T/C	SNP	2969
XP_845202.1 receptor-type adenylate cyclase GRESAG 4 [T; brucei TREU927]	GLOS_TB927.6.790.2.2	C/A	SNP	55
		T/A	SNP	67
		C/T	SNP	69
		A/C	SNP	157
		T/A	SNP	161
		G/A	SNP	190
		A/G	SNP	192
		G/T	SNP	2017
		G/A	SNP	2038
		C/T	SNP	2066
XM_840124.1 Tbb GUTat10.1 cysteinyl-tRNA synthetase partial mRNA	GLOS_TB927.6.950.1.1	T/C	SNP	28
		T/A	SNP	2644
		A/C	SNP	2965
XP_845735.1 asparagine synthetase a [T. brucei brucei strain 927/4 GUTat10.1]	GLOS_TB927.7.1110.1.1	T/TTA	INSERTION	1939
XM_840660.1 Tbb hypothetical protein, conserved (Tb927.7.1290) partial mRNA	GLOS_TB927.7.1290.1.1	A/AT	INSERTION	131
		C/A	SNP	900
XP_845756.1 10 kDa heat shock protein [T. brucei brucei strain 927/4 GUTat10.1] ref|XP_845758.1| 10 kDa heat shock protein [T. b. brucei strain 927/4 GUTat10.1]	GLOS_TB927.7.1320.1.1	C/T	SNP	256
XM_840678.1 Tbbase subunit 9, putative (Tb927.7.1470) partial mRNA	GLOS_TB927.7.1470.1.1	A/AT	INSERTION	923

XM_840705.1 Tbb 60S ribosomal protein L7, putative (Tb927.7.1740) partial mRNA	GLOS_TB927.7.1740.1.1	A/G	SNP	261
		C/T	SNP	355
XP_845803.1 adenine phosphoribosyltransferase [T.b.brucei strain 927/4 GUTat10.1]	GLOS_TB927.7.1790.1.1	CA/C	DELETION	113
		G/GA	INSERTION	574
		T/A	SNP	1004
		CAAA/C	DELETION	1020
		C/T	SNP	1116
XP_845643.1 thimet oligopeptidase A [T. brucei brucei strain 927/4 GUTat10.1]	GLOS_TB927.7.190.1.1	C/CT	INSERTION	713
XM_840747.1 Tbb hypothetical protein, conserved (Tb927.7.2170) partial mRNA	GLOS_TB927.7.2170.1.1	T/C	SNP	493
		G/C	SNP	4025
		T/C	SNP	4573
XM_840749.1 T. hypothetical protein, conserved (Tb927.7.2190) partial mRNA	GLOS_TB927.7.2190.1.1	C/CA	INSERTION	123
		T/C	SNP	387
		G/C	SNP	802
		A/G	SNP	861
		A/G	SNP	960
		G/A	SNP	1175
		C/CT	INSERTION	1535
		A/G	SNP	1624
XM_840760.1 Tbb hypothetical protein, conserved (Tb927.7.2300) partial mRNA	GLOS_TB927.7.2300.1.1	A/G	SNP	539
		C/T	SNP	1528
		T/C	SNP	2635
		G/T	SNP	3040
		C/T	SNP	3142
		A/G	SNP	3240
XM_840554.1 Tbb 40S ribosomal protein S33, putative (Tb927.7.230) partial mRNA	GLOS_TB927.7.230.1.1	T/G	SNP	176
		A/T	SNP	520
		C/G	SNP	521
XM_840764.1 Tbb 40S ribosomal protein S15, putative (Tb927.7.2340) partial mRNA	GLOS_TB927.7.2340.1.1	A/T	SNP	16
		G/A	SNP	442
XM_840766.1 Tbb N-acetyltransferase, putative (Tb927.7.2360) partial mRNA	GLOS_TB927.7.2360.1.1	C/T	SNP	254
		G/A	SNP	587
		T/C	SNP	1060
		C/T	SNP	1111
		CT/C	DELETION	1325
		T/A	SNP	1512

		T/C	SNP	1583
		A/T	SNP	1780
		C/T	SNP	1998
		C/G	SNP	2062
		C/T	SNP	2068
XM_840769.1 Tbb hypothetical protein, conserved (Tb927.7.2390) partial mRNA	GLOS_TB927.7.2390.1.1	A/C	SNP	252
		A/C	SNP	253
		G/A	SNP	348
		GA/G	DELETION	464
		C/CT	INSERTION	1247
		C/T	SNP	1821
		A/G	SNP	1929
		G/A	SNP	3733
XP_845878.1 proteasome regulatory ATPase subunit 5 [T. brucei TREU927]	GLOS_TB927.7.2550.1.1	A/C	SNP	943
		G/GA	INSERTION	1166
		A/G	SNP	1587
		T/TA	INSERTION	2616
XP_845888.1 hypothetical protein [T. brucei brucei strain 927/4 GUTat10.1]	GLOS_TB927.7.2650.1.1	C/CA	INSERTION	615
		T/C	SNP	2191
		A/C	SNP	2192
		T/C	SNP	2225
		T/C	SNP	2227
XP_845905.1 histone H2A [Trypanosoma brucei brucei strain 927/4 GUTat10.1] ref|XP_845906.1| histone H2A [Tbb] ref|XP_845907.1| histone H2A [Tbb] ref|XP_845908.1| histone H2A [Tbb] ref|XP_845909.1| histone H2A [Tbb] ref|XP_845910.1| histone H2A [Tbb] ref|XP_845911.1| histone H2A [Tbb] ref|XP_845912.1| hist. H2A [Tbb] ref|XP_845913.1| histone H2A [Tbb] ref|XP_845914.1| histone H2A [Tbb] ref|XP_845915.1| histone H2A [Tbb] ref|XP_845916.1| histone H2A [Tbb] ref|XP_845917.1| histone H2A [Tbb]	GLOS_TB927.7.2820.2.2	G/A	SNP	100
		G/GA	INSERTION	210
		A/C	SNP	541
		A/C	SNP	837
		G/T	SNP	838
XP_845921.1 hypothetical protein [T. brucei brucei strain 927/4 GUTat10.1]	GLOS_TB927.7.2980.1.1	G/A	SNP	274
		G/A	SNP	384
		C/T	SNP	1055

XP_845978.1 hypothetical protein [T. brucei brucei strain 927/4 GUTat10.1]	GLOS_TB927.7.3550.1.2	G/A	SNP	17
		C/A	SNP	535
		C/T	SNP	2513
		A/G	SNP	2515
		A/C	SNP	2516
		C/CTTTGT	INSERTION	2288
XM_840892.1 Tbb tyrosyl-tRNA synthetase, putative (Tb927.7.3620) partial mRNA	GLOS_TB927.7.3620.1.1	T/C	SNP	134
		T/C	SNP	365
		A/G	SNP	510
		C/T	SNP	673
		A/G	SNP	914
		G/A	SNP	995
		A/C	SNP	1220
		A/G	SNP	2831
XM_840893.1 TbbTPR-repeat-containing chaperone prot. DNAJ, putat. partial mRNA	GLOS_TB927.7.3630.1.1	G/A	SNP	251
		C/G	SNP	561
		T/TGC	INSERTION	2418
		TTG/T	DELETION	2517
		T/C	SNP	2544
		A/G	SNP	2597
		T/C	SNP	2802
		C/T	SNP	2843
XM_840898.1 Tbb ubiquitin/ribosomal protein S27a, putative partial mRNA	GLOS_TB927.7.3680.1.2	CAA/C	DELETION	181
		G/GA	INSERTION	235
XM_840898.1 Tbb ubiquitin/ribosomal protein S27a, putative partial mRNA	GLOS_TB927.7.3680.2.2	G/T	SNP	740
XM_840904.1 Tbb hypothetical protein, conserved (Tb927.7.3740) partial mRNA	GLOS_TB927.7.3740.1.1	G/A	SNP	725
		G/A	SNP	1015
		GA/G	DELETION	1870
XM_840924.1 Tbb mitochondrial carrier prot., putative (Tb927.7.3940) partial mRNA	GLOS_TB927.7.3940.1.1	A/C	SNP	222
		G/A	SNP	336
		T/A	SNP	544
		T/C	SNP	717
		A/G	SNP	920
		G/A	SNP	1106
XM_840928.1 Tbb immunodominant antigen, putative (Tb927.7.3980) partial mRNA	GLOS_TB927.7.3980.1.1	A/G	SNP	3323
XP_846030.1 calpain-like cysteine peptidase [T. b. brucei strain 927/4 GUTat10.1]	GLOS_TB927.7.4070.1.1	C/CTTT	INSERTION	577

		G/GA	INSERTION	910
XM_840942.1 Tbb hypothetical protein, conserved (Tb927.7.4120) partial mRNA	GLOS_TB927.7.4120.1.1	A/T	SNP	1501
XM_840948.1 Tbb fatty acid elongase, putative (Tb927.7.4180) partial mRNA	GLOS_TB927.7.4180.1.1	C/T	SNP	533
		T/C	SNP	588
		C/T	SNP	1180
		C/CT	INSERTION	1478
		GA/G	DELETION	1575
		T/TTTTTG	INSERTION	1774
		G/GA	INSERTION	2143
XM_840957.1 T;; hypothetical protein, conserved (Tb927.7.4270) partial mRNA	GLOS_TB927.7.4270.1.1	G/A	SNP	79
		T/G	SNP	82
		T/C	SNP	765
		CT/C	DELETION	1016
		C/CTAT	INSERTION	1079
		GA/G	DELETION	1185
XP_846062.1 threonine synthase [T. brucei brucei strain 927/4 GUTat10.1]	GLOS_TB927.7.4390.1.1	A/G	SNP	374
XM_840975.1 Tbb hypothetical protein, conserved (Tb927.7.4450) partial mRNA	GLOS_TB927.7.4450.1.1	TA/T	DELETION	87
		G/T	SNP	338
		G/T	SNP	2061
XM_840980.1 Tbb hypothetical protein, conserved (Tb927.7.4500) partial mRNA	GLOS_TB927.7.4500.1.1	G/GA	INSERTION	2334
		T/C	SNP	2631
XM_840987.1 Tbb nucleoside hydrolase, putative (Tb927.7.4570) partial mRNA	GLOS_TB927.7.4570.1.1	G/C	SNP	1060
XM_841020.1 Tbb 5'-3' exonuclease XRNA, putative (Tb927.7.4900) partial mRNA	GLOS_TB927.7.4900.1.1	G/A	SNP	111
		CT/C	DELETION	116
		C/A	SNP	147
		A/G	SNP	318
		C/T	SNP	1055
XM_841030.1 Tbb 60S ribosomal protein L19, putative (Tb927.7.5000) partial mRNA	GLOS_TB927.7.5000.1.1	A/G	SNP	258
		T/C	SNP	327
		C/T	SNP	762
XM_841048.1 Tbb 60S ribosomal protein L23a, putative (Tb927.7.5180) partial mRNA	GLOS_TB927.7.5180.1.1	C/CT	INSERTION	906
XP_846144.1 hypothetical protein [T. brucei brucei strain 927/4 GUTat10.1]	GLOS_TB927.7.5210.1.1	T/A	SNP	380
		C/T	SNP	1354
		T/C	SNP	1886
		A/G	SNP	2324
		A/G	SNP	2577

		G/A	SNP	3256
		G/T	SNP	3783
XM_841053.1 Tbb lanosterol synthase (Tb927.7.5230) partial mRNA	GLOS_TB927.7.5230.1.1	G/A	SNP	2880
		G/GA	INSERTION	3524
		C/T	SNP	3590
XM_841058.1 Tbb hypothetical protein, conserved (Tb927.7.5280) partial mRNA	GLOS_TB927.7.5280.1.1	G/GA	INSERTION	120
		A/G	SNP	644
		G/A	SNP	3639
		A/G	SNP	4353
		G/A	SNP	4902
		G/A	SNP	5553
		G/A	SNP	5691
XM_841124.1 Tbbypothetical protein, conserved (Tb927.7.5940) partial mRNA	GLOS_TB927.7.5940.1.1	C/A	SNP	30
		G/T	SNP	31
		C/T	SNP	117
		G/A	SNP	169
		G/T	SNP	666
		C/T	SNP	903
		A/C	SNP	1070
		A/G	SNP	1117
		A/G	SNP	1123
		G/A	SNP	1131
		C/T	SNP	1267
		C/G	SNP	1268
		C/T	SNP	1277
		C/T	SNP	1292
		A/T	SNP	1295
		A/C	SNP	1301
		C/A	SNP	1361
		C/T	SNP	1364
		G/A	SNP	1398
		C/T	SNP	1400
		C/T	SNP	1469
		A/G	SNP	1538
		A/C	SNP	1559
		A/C	SNP	1586

		A/C	SNP	1592
		GT/G	DELETION	2396
		C/CT	INSERTION	2669
XM_841135.1 Tbb receptor-type adenylate cyclase GRESAG 4, putat. partial mRNA	GLOS_TB927.7.6050.1.1	T/C	SNP	140
		A/T	SNP	145
		C/T	SNP	292
		A/T	SNP	293
		A/G	SNP	406
		C/T	SNP	683
		T/C	SNP	902
		G/T	SNP	1370
		A/G	SNP	1549
		T/C	SNP	1979
		G/A	SNP	2282
		C/G	SNP	2919
		G/A	SNP	2921
		A/G	SNP	3021
		G/A	SNP	3022
		G/T	SNP	3131
XM_841139.1 Tbb hypothetical protein, conserved (Tb927.7.6090) partial mRNA	GLOS_TB927.7.6090.1.1	A/C	SNP	1802
		C/T	SNP	2513
		C/CT	INSERTION	2551
XM_841156.1 Tbb hypothetical protein, conserved (Tb927.7.6260) partial mRNA	GLOS_TB927.7.6260.1.1	CA/C	DELETION	228
		A/G	SNP	1271
		T/C	SNP	2261
		CT/C	DELETION	2315
XM_841172.1 Tbb hypothetical protein, conserved (Tb927.7.6420) partial mRNA	GLOS_TB927.7.6420.1.1	T/C	SNP	595
XP_846297.1 hypothetical protein [T. brucei brucei strain 927/4 GUTat10.1]	GLOS_TB927.7.6770.1.1	AG/A	DELETION	737
XM_841212.1 T. b. brucei strain 927/4 GUTat10.1 trans-sialidase partial mRNA	GLOS_TB927.7.6850.1.1	T/TA	INSERTION	237
		G/A	SNP	254
		A/C	SNP	431
		G/GA	INSERTION	684
		G/A	SNP	1009
		G/A	SNP	1335
		C/G	SNP	1461
		G/C	SNP	1941

		G/A	SNP	2491
		A/G	SNP	2700
		T/C	SNP	2822
		A/C	SNP	2888
		A/T	SNP	3090
XM_841217.1 Tbb double-strand-break repair prot rad21 homolog, putat parti mRNA	GLOS_TB927.7.6900.1.1	G/GAA	INSERTION	84
		G/T	SNP	113
		G/T	SNP	1008
XM_841224.1 Tbb paraflagellar rod protein, putative (Tb927.7.6970) partial mRNA	GLOS_TB927.7.6970.1.1	T/C	SNP	642
		G/GT	INSERTION	2625
XM_841236.1 Tbb hypothetical protein, conserved (Tb927.7.7090) partial mRNA	GLOS_TB927.7.7090.1.1	G/T	SNP	31
		A/T	SNP	32
		G/T	SNP	33
		C/CA	INSERTION	220
		G/A	SNP	257
		C/T	SNP	304
		GT/G	DELETION	833
		CT/C	DELETION	1056
		G/T	SNP	1116
		T/C	SNP	1309
		C/G	SNP	1361
XM_840602.1 Tbb heat shock 70 kDa protein, putative (Tb927.7.710) partial mRNA	GLOS_TB927.7.710.1.1	C/CA	INSERTION	815
XM_841238.1 Tbb leucine-rich repeat protein (LRRP), putative partial mRNA	GLOS_TB927.7.7110.1.1	A/G	SNP	670
		A/G	SNP	1474
		A/G	SNP	2327
		G/A	SNP	2383
		T/C	SNP	2975
XM_841269.1 Tbb ATP synthase alpha chain, mitochondri precursor partial mRNA	GLOS_TB927.7.7420.1.1	T/C	SNP	1569
XM_841274.1 Tbb receptor-type adenylate cyclase GRESAG 4, putati partial mRNA	GLOS_TB927.7.7470.1.1	TC/T	DELETION	421
		G/A	SNP	2252
		C/T	SNP	2641
		A/G	SNP	4378
XM_841855.1 T. 40S ribosomal protein S9, putative (Tb927.8.1110) partial mRNA	GLOS_TB927.8.1110.1.1	A/G	SNP	609
		A/C	SNP	681
		C/T	SNP	783
		A/C	SNP	807

		A/G	SNP	810
		G/C	SNP	867
		A/G	SNP	870
		G/A	SNP	897
		G/T	SNP	924
		T/C	SNP	927
		A/C	SNP	942
		A/G	SNP	945
XM_841876.1 Tbb 60S ribosomal protein L7a, putative (Tb927.8.1330) partial mRNA	GLOS_TB927.8.1330.1.1	G/A	SNP	42
		C/A	SNP	67
		C/G	SNP	77
XM_841891.1 Tbb hypothetical protein, conserved (Tb927.8.1500) partial mRNA	GLOS_TB927.8.1500.1.1	G/T	SNP	372
XM_841892.1 Tbb ATP-dependent DEAD/H RNA helicase, putative partial mRNA	GLOS_TB927.8.1510.1.1	A/G	SNP	305
		A/G	SNP	2306
		AT/A	DELETION	2320
		A/G	SNP	2505
		G/A	SNP	2591
		T/C	SNP	2827
XM_841896.1 Tbb hypothetical protein, conserved (Tb927.8.1550) partial mRNA	GLOS_TB927.8.1550.1.1	C/G	SNP	187
		C/T	SNP	719
		T/C	SNP	726
		CTT/C/CT	DELETION	737
		G/GA	INSERTION	838
		GA/G	DELETION	958
		GT/G	DELETION	1216
		TA/T	DELETION	1270
XP_846996.1 major surface protease gp63 [Tbb strain 927/4 GUTat10.1] ref|XP_84699 ref|XP_846997.1| major surface protease gp63 [Tbb 927/4 GUTat10.1]	GLOS_TB927.8.1620.1.1	A/G	SNP	34
		T/C	SNP	408
		C/T	SNP	493
		T/A	SNP	720
		C/T	SNP	825
		C/A	SNP	1330
		G/A	SNP	1380
		T/C	SNP	1738
		C/T	SNP	1746

		C/T	SNP	1771
		G/GT	INSERTION	1856
		C/T	SNP	2570
XM_841919.1 Tbb hypothetical protein, conserved (Tb927.8.1790) partial mRNA	GLOS_TB927.8.1790.1.1	CTT/C	DELETION	235
		T/C	SNP	1565
		A/T	SNP	1659
		G/A	SNP	1953
		G/T	SNP	1982
		T/C	SNP	2222
XM_841923.1 Tbb tRNA-methyl transferase, putative (Tb927.8.1830) partial mRNA	GLOS_TB927.8.1830.1.1	C/A	SNP	133
		T/A	SNP	149
		C/T	SNP	1141
		G/A	SNP	1951
		T/C	SNP	3141
		G/A	SNP	1631
		G/GA	INSERTION	2138
		TA/T	DELETION	2284
		A/T	SNP	2287
XP_847022.1 cytochrome c1, heme protein, mitochondrial precursor [Tbb]	GLOS_TB927.8.1890.1.1	T/C	SNP	439
		C/T	SNP	1412
XM_841943.1 Tbb hypothetical protein, conserved (Tb927.8.2030) partial mRNA	GLOS_TB927.8.2030.1.1	A/T	SNP	111
XM_841956.1 Tbb multidrug resistance protein A (Tb927.8.2160) partial mRNA	GLOS_TB927.8.2160.1.1	G/T	SNP	712
		T/C	SNP	1232
		G/A	SNP	2454
		T/TA	INSERTION	3233
		G/A	SNP	3796
XP_847080.1 hypothetical protein [T. brucei brucei strain 927/4 GUTat10.1]	GLOS_TB927.8.2470.1.1	C/G	SNP	39
		A/T	SNP	84
		G/T	SNP	124
		T/C	SNP	153
		T/C	SNP	215
		G/A	SNP	405
XM_841992.1 Tbb acetyl-CoA synthetase, putative (Tb927.8.2520) partial mRNA	GLOS_TB927.8.2520.1.1	C/T	SNP	967
		C/T	SNP	1693
		T/C	SNP	1969
		GA/G	DELETION	2531

		T/TA	INSERTION	2895
		A/G	SNP	2969
		G/T	SNP	3758
XM_841994.1 Tbb 3-ketoacyl-CoA thiolase, putative (Tb927.8.2540) partial mRNA	GLOS_TB927.8.2540.1.1	A/G	SNP	162
		T/A	SNP	336
		T/C	SNP	597
		T/C	SNP	781
		C/T	SNP	1675
XP_847096.1 kinesin [Trypanosoma brucei brucei strain 927/4 GUTat10.1]	GLOS_TB927.8.2630.1.1	C/CA	INSERTION	224
		T/TAA	INSERTION	373
		CTT/C	DELETION	520
		T/C	SNP	751
		T/TC	INSERTION	952
XP_847097.1 ubiquitin-activating enzyme E1 [T. b. brucei strain 927/4 GUTat10.1]	GLOS_TB927.8.2640.1.1	G/GA	INSERTION	332
		C/A	SNP	1905
		G/A	SNP	3510
		AG/A	DELETION	3744
		A/G	SNP	3755
		G/A	SNP	3782
		G/A	SNP	3879
		A/G	SNP	3880
		A/G	SNP	3907
		T/TC	INSERTION	4011
		C/CA	INSERTION	4131
		C/G	SNP	4245
XM_842029.1 Tbb mannosyl-oligosacchari 1,2-a-mannosidaseIB, putat parti mRNA	GLOS_TB927.8.2910.1.1	C/T	SNP	133
		G/A	SNP	424
		C/A	SNP	714
		G/A	SNP	924
		T/C	SNP	1179
		G/A	SNP	1240
		T/G	SNP	1602
XM_842044.1 Tbb cytosolic leucyl aminopeptidase, putative partial mRNA	GLOS_TB927.8.3060.1.1	A/G	SNP	370
		C/G	SNP	1921
		C/T	SNP	2884
		AT/A	DELETION	2885

XM_842048.1 Tbb GUTat10.1 coronin, putative (Tb927.8.3100) partial mRNA	GLOS_TB927.8.3100.1.1	T/TA	INSERTION	639
		C/T	SNP	906
		G/C	SNP	1093
		A/G	SNP	1727
		T/G	SNP	1736
		G/A	SNP	2231
		CAT/C	DELETION	2300
		GA/G	DELETION	2631
		G/A	SNP	2636
XM_842053.1 Tbb t-complex protein 1 gamma subunit, putative partial mRNA	GLOS_TB927.8.3150.1.1	G/A	SNP	62
		G/A	SNP	457
		CA/C	DELETION	504
		C/T	SNP	781
		CT/C	DELETION	808
		TAGAG/T	DELETION	1144
		C/T	SNP	1166
		G/A	SNP	2767
XP_847169.1 electron transfer protein [T. brucei brucei strain 927/4 GUTat10.1]	GLOS_TB927.8.3380.1.1	TC/T	DELETION	287
		A/C	SNP	442
		C/CT	INSERTION	467
		CT/CTT/C	INSERTION	1821
		C/T	SNP	1834
XM_842120.1 Tbb hypothetical protein, conserved (Tb927.8.3820) partial mRNA	GLOS_TB927.8.3820.1.1	C/CT	INSERTION	556
XM_842122.1 Tbb hypothetical protein, conserved (Tb927.8.3840) partial mRNA	GLOS_TB927.8.3840.1.1	C/CA	INSERTION	1804
		G/A	SNP	1945
		CA/C	DELETION	2873
XM_842139.1 Tbb flagellum-adhesion glycoprotein (Tb927.8.4010) partial mRNA	GLOS_TB927.8.4010.1.1	G/T	SNP	2226
XM_842143.1 Tbb hypothetical protein, conserved (Tb927.8.4050) partial mRNA	GLOS_TB927.8.4050.1.1	G/A	SNP	2311
		G/A	SNP	2533
XP_847264.1 small GTP-binding protein Rab11 [T. b. brucei strain 927/4 GUTat10.1]	GLOS_TB927.8.4330.1.1	C/T	SNP	227
		AAT/A	DELETION	304
		GAAA/G	DELETION	515
		CTTT/C	DELETION	929
		G/GT	INSERTION	1525
		C/T	SNP	2251
		C/T	SNP	2387

		G/T	SNP	2951
		C/T	SNP	2955
		C/T	SNP	2956
XM_842208.1 Tbb amino acid transporter, putative (Tb927.8.4700) partial mRNA	GLOS_TB927.8.4700.1.1	C/T	SNP	224
		T/G	SNP	282
		T/C	SNP	1533
		T/C	SNP	1602
		A/G	SNP	1625
		A/T	SNP	2118
		A/T	SNP	2120
		A/G	SNP	2536
		G/T	SNP	2632
		C/A	SNP	2660
		T/G	SNP	2742
		C/A	SNP	2743
		A/C	SNP	2749
XP_847308.1 hypothetical protein [T. brucei brucei strain 927/4 GUTat10.1]	GLOS_TB927.8.4780.1.1	G/A	SNP	1692
		C/T	SNP	1694
		G/T	SNP	1695
		A/C	SNP	1697
		G/A	SNP	1701
		G/T	SNP	2309
		A/G	SNP	2321
		C/CT	INSERTION	4228
XM_842244.1 Tbb hypothetical protein, conserved (Tb927.8.5070) partial mRNA	GLOS_TB927.8.5070.1.1	G/GA	INSERTION	494
		C/CT	INSERTION	557
		T/A	SNP	1092
		T/A	SNP	1093
XP_847342.1 cytochrome c [Trypanosoma brucei brucei strain 927/4 GUTat10.1]	GLOS_TB927.8.5120.1.1	G/GATA	INSERTION	223
		G/A	SNP	543
		C/CT	INSERTION	841
		A/C	SNP	903
		A/AG	INSERTION	964
		T/C	SNP	1335
XM_842263.1 Tbb 60S ribosomal protein L39, putative (Tb927.8.5260) partial mRNA	GLOS_TB927.8.5260.2.3	A/T	SNP	427
		A/T	SNP	429

XM_842284.1 Tbb flagellar calcium-binding protein (Tb927.8.5470) partial mRNA	GLOS_TB927.8.5470.1.1	A/C	SNP	490
		CA/C	DELETION	582
		T/C	SNP	738
		G/A	SNP	845
		G/A	SNP	854
XP_847390.1 transaldolase [Trypanosoma brucei brucei strain 927/4 GUTat10.1]	GLOS_TB927.8.5600.1.1	G/C	SNP	400
XP_847394.1 hypothetical protein [T. brucei brucei strain 927/4 GUTat10.1]	GLOS_TB927.8.5640.1.1	G/A	SNP	1114
		G/A	SNP	1889
		A/C	SNP	1890
XM_842315.1 Tbb protein tyrosine phosphatase, putative partial mRNA	GLOS_TB927.8.5780.1.1	G/A	SNP	900
		TC/T	DELETION	1548
		C/G	SNP	1823
XM_842337.1 Tbb fatty acid desaturase, putative (Tb927.8.6000) partial mRNA	GLOS_TB927.8.6000.1.1	C/A	SNP	1453
		C/T	SNP	1593
		G/A	SNP	1758
		T/A	SNP	1794
		TACATC/T	DELETION	2035
		GAAA/G	DELETION	2179
		G/C	SNP	2696
XM_842343.1 Tbb 2-amino-3-ketobutyrate coenzyme A ligase, putati partial mRNA	GLOS_TB927.8.6060.1.1	GA/G	DELETION	229
		A/G	SNP	239
		C/T	SNP	325
		T/TA	INSERTION	389
		G/A	SNP	694
		G/A	SNP	1478
		C/T	SNP	1570
XP_847441.1 hypothetical protein [T. brucei brucei strain 927/4 GUTat10.1]	GLOS_TB927.8.6110.1.1	A/G	SNP	47
		G/GA	INSERTION	1979
		T/A	SNP	2146
XM_842353.1 Tbb 40S ribosomal protein S8, putative (Tb927.8.6160) partial mRNA	GLOS_TB927.8.6160.1.1	T/G	SNP	262
XM_842354.1 Tbb GUTat10.1 transketolase, putative (Tb927.8.6170) partial mRNA	GLOS_TB927.8.6170.1.1	T/C	SNP	547
		G/A	SNP	1038
XM_842355.1 Tbb 60S ribosomal protein L26, putative (Tb927.8.6180) partial mRNA	GLOS_TB927.8.6180.1.2	A/G	SNP	44
		C/T	SNP	45
XP_847448.1 60S ribosomal protein L26 [T. brucei brucei strain 927/4 GUTat10.1]	GLOS_TB927.8.6180.2.2	A/T	SNP	10
		C/T	SNP	11

		C/T	SNP	327
		C/T	SNP	349
		A/G	SNP	382
		A/G	SNP	391
		T/C	SNP	433
		T/C	SNP	478
		T/C	SNP	508
		A/G	SNP	511
		A/G	SNP	637
XM_842358.1 Tbb phosphatidylinositol 3-kinase, putative (Tb927.8.6210) partial mRNA	GLOS_TB927.8.6210.1.1	A/G	SNP	1846
XP_847454.1 hypothetical protein [T. brucei brucei strain 927/4 GUTat10.1]	GLOS_TB927.8.6240.1.1	T/TA	INSERTION	96
		C/CA	INSERTION	155
		G/T	SNP	323
		GT/G	DELETION	1425
		C/CT	INSERTION	1760
		TA/T	DELETION	1904
XM_842381.1 Tbb RNA-binding protein, putative (Tb927.8.6440) partial mRNA	GLOS_TB927.8.6440.1.1	C/G	SNP	465
		TAAA/T	DELETION	953
		A/ATT	INSERTION	1155
XP_847475.1 inhibitor of cysteine peptidase [T. brucei brucei strain 927/4 GUTat10.1]	GLOS_TB927.8.6450.1.1	C/CG	INSERTION	522
		G/GT	INSERTION	893
XP_846902.1 cation-transporting ATPase [T. brucei brucei strain 927/4 GUTat10.1]	GLOS_TB927.8.650.1.1	T/C	SNP	1596
		G/T	SNP	3083
XM_842395.1 Tbb succinate dehydrogenase flavoprotein, putative partial mRNA	GLOS_TB927.8.6580.1.1	CAA/C	DELETION	143
		A/AT	INSERTION	256
		A/T	SNP	268
		A/AC	INSERTION	562
		C/A	SNP	977
		C/T	SNP	3162
XM_842401.1 Tbb hypothetical protein, conserved (Tb927.8.6640) partial mRNA	GLOS_TB927.8.6640.1.1	C/T	SNP	76
		GA/G	DELETION	274
		A/C	SNP	513
		CTA/C	DELETION	538
		G/A	SNP	1031
		G/A	SNP	1361
		G/C	SNP	1700

XM_842403.1 Tbb hypothetical protein, conserved (Tb927.8.6660) partial mRNA	GLOS_TB927.8.6660.1.1	G/A	SNP	45
		G/A	SNP	104
		G/A	SNP	120
		G/A	SNP	298
		G/C	SNP	856
		A/C	SNP	1088
		G/GGT	INSERTION	2251
		C/T	SNP	2890
XM_842412.1 Tbb translationally controlled tumor prot. (TCTP), putati partial mRNA	GLOS_TB927.8.6750.1.1	C/CCT	INSERTION	185
		T/C	SNP	196
		TA/T	DELETION	426
		G/A	SNP	864
		C/G	SNP	865
		C/A	SNP	959
XM_842434.1 Tbb 3-methylcrotonyl-CoA carboxylase, putative partial mRNA	GLOS_TB927.8.6970.1.1	G/A	SNP	231
		G/C	SNP	305
		T/C	SNP	340
		C/A	SNP	369
		G/C	SNP	376
		G/A	SNP	428
		C/T	SNP	439
		A/G	SNP	736
		T/C	SNP	847
		C/G	SNP	884
		C/T	SNP	1041
		T/C	SNP	1132
		G/A	SNP	1188
		A/T	SNP	1274
		G/A	SNP	1341
		C/G	SNP	1357
		C/G	SNP	1411
		C/T	SNP	1581
		A/G	SNP	1697
		A/C	SNP	1777
		C/T	SNP	1796
		G/A	SNP	1831

		G/A	SNP	1890
		C/T	SNP	1891
		C/T	SNP	2029
		G/A	SNP	2070
		T/C	SNP	2124
		T/C	SNP	2182
		A/G	SNP	2260
		C/T	SNP	2449
		C/T	SNP	2524
		G/A	SNP	2590
		C/T	SNP	2613
		T/G	SNP	2667
		G/C	SNP	2943
		A/C	SNP	3016
		A/T	SNP	3242
		T/G	SNP	3468
		T/A	SNP	3499
XP_847532.1 peptidase [Trypanosoma brucei brucei strain 927/4 GUTat10.1]	GLOS_TB927.8.7020.1.1	C/T	SNP	1599
		A/G	SNP	2347
XM_842447.1 Tbb acetyl-CoA carboxylase, putative (Tb927.8.7100) partial mRNA	GLOS_TB927.8.7100.1.1	T/TA	INSERTION	108
XM_842449.1 Tbb farnesyltransferase, putative (Tb927.8.7120) partial mRNA	GLOS_TB927.8.7120.1.1	G/C	SNP	179
XM_842452.1 Trypanosoma brucei brucei strain 927/4 GUTat10.1 UDP-Gal or UDP-Glc UDP-GlcNAc-dependent glycosyltransferase, putative partial mRNA	GLOS_TB927.8.7150.1.1	G/C	SNP	143
		A/G	SNP	160
		G/C	SNP	190
		C/T	SNP	768
		T/C	SNP	834
XM_842477.1 Tbb GUTat10.1 calreticulin, putative (Tb927.8.7410) partial mRNA	GLOS_TB927.8.7410.1.1	C/T	SNP	119
		G/T	SNP	120
		G/A	SNP	246
		G/A	SNP	255
		G/T	SNP	312
		C/T	SNP	486
		A/G	SNP	495
		A/G	SNP	501
		T/C	SNP	624

		G/A	SNP	629
		T/C	SNP	669
		C/T	SNP	866
		C/T	SNP	867
		C/A	SNP	882
		C/A	SNP	885
		C/T	SNP	984
		C/T	SNP	1025
		A/C	SNP	1341
		G/T	SNP	1438
		G/A	SNP	1453
		A/G	SNP	1477
		G/A	SNP	1509
		TAA/T	DELETION	1657
		A/G	SNP	1766
XM_842485.1 Tbb hypothetical protein, conserved (Tb927.8.7490) partial mRNA	GLOS_TB927.8.7490.1.1	A/G	SNP	126
		C/A	SNP	143
		A/G	SNP	150
		T/C	SNP	198
		A/G	SNP	258
		T/C	SNP	275
		T/C	SNP	940
		T/C	SNP	1127
		A/T	SNP	1135
		T/C	SNP	1285
		G/A	SNP	1311
		G/A	SNP	1497
		C/T	SNP	1608
		G/A	SNP	1677
		C/T	SNP	2325
		T/C	SNP	2424
		C/T	SNP	2874
		C/T	SNP	2916
		A/G	SNP	3105
		T/G	SNP	3308
		C/G	SNP	3558

		A/G	SNP	3573
		G/A	SNP	3712
		C/G	SNP	3730
		T/C	SNP	3765
		C/T	SNP	4001
		A/G	SNP	4086
		A/G	SNP	4157
		A/G	SNP	4172
		A/T	SNP	4211
		A/G	SNP	4234
		G/A	SNP	4245
XM_841820.1 Tbb nucleolar RNA-binding protein (Tb927.8.760) partial mRNA	GLOS_TB927.8.760.1.1	G/A	SNP	751
		A/G	SNP	1148
XM_842526.1 Tbb receptor-type adenylate cyclase GRESAG 4, putati partial mRNA	GLOS_TB927.8.7940.1.1	T/C	SNP	132
		A/G	SNP	558
		A/G	SNP	563
		C/G	SNP	564
		A/G	SNP	599
		C/T	SNP	695
		C/T	SNP	767
		C/T	SNP	988
		G/C	SNP	991
		G/A	SNP	1022
		A/G	SNP	1027
		A/T	SNP	1042
		T/C	SNP	1043
		G/A	SNP	1048
		G/A	SNP	1066
		C/G	SNP	1137
		G/A	SNP	1209
		A/G	SNP	1506
		A/G	SNP	1556
		C/T	SNP	1571
		T/C	SNP	1655
		G/C	SNP	1726
		A/C	SNP	2020

		C/T	SNP	2024
		G/A	SNP	2049
		G/A	SNP	2050
		A/G	SNP	2229
		A/G	SNP	2232
		T/A	SNP	2233
		T/C	SNP	2269
		G/A	SNP	2337
		G/C	SNP	2338
		G/T	SNP	3043
		T/C	SNP	3073
		G/A	SNP	3228
		A/G	SNP	3287
		C/G	SNP	3290
		A/G	SNP	3291
		T/G	SNP	3297
		G/A	SNP	3354
		C/T	SNP	3412
XM_842527.1 Tbb hypothetical protein, conserved (Tb927.8.7950) partial mRNA	GLOS_TB927.8.7950.1.1	C/CT	INSERTION	490
		C/T	SNP	2645
XM_842530.1 Tbb vacuolar-type proton translocating pyrophosphatase 1 parti mRNA	GLOS_TB927.8.7980.1.1	G/C	SNP	26
		T/C	SNP	382
		G/T	SNP	397
		GA/G	DELETION	407
		T/C	SNP	569
		ATTT/A	DELETION	621
		A/AT	INSERTION	815
		C/T	SNP	1992
		A/G	SNP	2232
		G/A	SNP	2247
		G/A	SNP	2253
		G/A	SNP	2400
		C/G	SNP	2466
		C/T	SNP	2535
		T/C	SNP	2541
		A/G	SNP	2682

		A/G	SNP	2853
		G/A	SNP	2916
		A/G	SNP	3235
		C/T	SNP	3384
XP_847643.1 hypothetical protein [T. brucei brucei strain 927/4 GUTat10.1]	GLOS_TB927.8.8200.1.1	C/T	SNP	2006
		G/C	SNP	2008
		A/G	SNP	2037
		A/C	SNP	2261
		G/A	SNP	2303
		G/A	SNP	2360
		T/C	SNP	2374
		C/T	SNP	2414
		A/G	SNP	2429
		T/C	SNP	2448
		A/G	SNP	2449
		C/G	SNP	2489
XM_842554.1 Tbb amino acid transporter, putative (Tb927.8.8240) partial mRNA	GLOS_TB927.8.8240.1.1	T/C	SNP	205
		G/A	SNP	209
		T/C	SNP	851
		C/T	SNP	1039
		T/C	SNP	1159
XM_842555.1 Tbb amino acid transporter, putative (Tb927.8.8250) partial mRNA	GLOS_TB927.8.8250.1.1	T/A	SNP	915
		G/C	SNP	916
		A/G	SNP	918
		T/C	SNP	1830
XP_847653.1 amino acid transporter [T. brucei brucei strain 927/4 GUTat10.1]	GLOS_TB927.8.8300.1.1	T/C	SNP	1847
		T/A	SNP	1854
		T/A	SNP	1855
		C/A	SNP	1893
		A/G	SNP	1927
		GA/G	DELETION	1975
		GA/G	DELETION	2456
		TA/T	DELETION	2661
		C/CAAA	INSERTION	2895
		G/GT	INSERTION	2978
		T/C	SNP	2997

[BBH] TBA_TRYBR (sp|P04106) Tubulin alpha chain T. b.rhodesiense PE=3 SV=1	GLOS_TBA.1.1	A/T	SNP	38
		C/G	SNP	40
		C/A	SNP	42
		A/C	SNP	43
		G/T	SNP	1490
		G/T	SNP	1491
XP_813091.1 ubiquitin hydrolase [Trypanosoma cruzi strain CL Brener]	GLOS_TC00.1047053507017.120.1.1	G/A	SNP	1232
		G/A	SNP	1557
XP_809984.1 hypothetical protein [Trypanosoma cruzi strain CL Brener]	GLOS_TC00.1047053508823.70.1.1	T/C	SNP	1398
		A/ATT	INSERTION	1488
XP_804510.1 ribosomal protein S29 [T. cruzi strain CL Brener] ref|XP_806920.1| ribosomal protein S29 [T. cruzi strain CL Brener] ref|XP_808328.1| ribosomal protein S29 [T. cruzi strain CL Brener]	GLOS_TC00.1047053511805.15.1.1	G/GA	INSERTION	172
		A/G	SNP	186
		G/A	SNP	241
		T/C	SNP	269
		G/A	SNP	374
		T/A	SNP	544
		G/A	SNP	545
[BBH] TDX_TRYBR (sp|Q26695) Thioredoxin peroxidase Tb rhodesiense	GLOS_TDX.1.1	A/G	SNP	195
		T/C	SNP	540
		C/CT	INSERTION	989
[BBH] TH2A_TRYBB (sp|Q06222) Glucose transporter 2A OS=Tbb	GLOS_TH2A.1.1	T/C	SNP	385
		G/A	SNP	2220
		A/C	SNP	2222
		A/T	SNP	2224
XM_001219103.1 Tbb cytochrome C oxidase subunit IV (trCOIV) partial mRNA	GLOS_TRCOIV.1.1	A/G	SNP	16
		G/A	SNP	555
		T/C	SNP	587
		T/G	SNP	591
		G/A	SNP	624
		G/GA	INSERTION	979
		AT/A	DELETION	2893
		G/A	SNP	3089
[BBH] TRF_SARPE (sp|Q26643) Transferrin OS=Sarcophaga peregrina PE=1 SV=1	GLOS_TRF.1.1	T/A	SNP	358
		GGT/G	DELETION	604

		C/T	SNP	755
		T/C	SNP	1355
		TC/T	DELETION	1355
		G/A	SNP	1438
		A/G	SNP	1720
		G/T	SNP	1915
TRYA_DROER (sp|P54624) Trypsin alpha OS=D. erecta GN=alphaTry PE=3 SV=1	GLOS_TRYA.1.5	A/T	SNP	1687
		A/AATCG	INSERTION	1739
		G/T	SNP	1802
		A/T	SNP	1910
		A/G	SNP	1930
		G/T	SNP	1994
		C/T	SNP	1995
		TAC/T	DELETION	2051
		G/C	SNP	2118
		T/G	SNP	2413
		CCG/C	DELETION	2433
		A/C	SNP	2472
		T/C	SNP	2475
		A/T	SNP	2555
		T/G	SNP	2619
		T/A	SNP	2684
		T/G	SNP	2687
TRYA4_LUCCU (sp|P35044) Trypsin alpha-4 OS=Lucilia cuprina PE=3 SV=1	GLOS_TRYA4.3.6	A/C	SNP	76
		T/A	SNP	81
		G/A	SNP	84
		C/A	SNP	86
		T/TG	INSERTION	99
		G/C	SNP	100
		A/C	SNP	180
		A/C	SNP	184
		G/A	SNP	191
		C/T	SNP	317
		T/C	SNP	318
		C/T	SNP	320
		G/GAT	INSERTION	321

		T/C	SNP	606
		A/T	SNP	677
		G/T	SNP	977
		C/T	SNP	1046
		C/A	SNP	1510
		T/G	SNP	1514
		T/C	SNP	1901
		G/T	SNP	1971
		A/G	SNP	2034
		T/G	SNP	2186
		T/G	SNP	2257
		TA/T	DELETION	2367
TRYDG_DROER (sp|P54626) Trypsin delta/gamma OS=D. erecta GN=deltaTry	GLOS_TRYDG.5.5	C/G	SNP	459
		G/T	SNP	784
		A/G	SNP	903
		T/G	SNP	1922
TRYT_DROME (sp|P42278) Trypsin theta OS=D. melanogaster GN=thetaTry	GLOS_TRYT.2.4	G/A	SNP	33
		G/C	SNP	41
		T/C	SNP	42
		G/A	SNP	43
		G/T	SNP	45
		C/T	SNP	182
		C/T	SNP	251
		C/T	SNP	256
		A/G	SNP	320
		G/A	SNP	370
		C/A	SNP	375
		A/G	SNP	376
		T/TC	INSERTION	378
		A/G	SNP	382
		C/G	SNP	383
		C/A	SNP	435
		T/A	SNP	516
		G/GA	INSERTION	517
		A/C	SNP	519
		G/T	SNP	521

		T/A	SNP	522
		G/A	SNP	523
		A/T	SNP	530
		G/A	SNP	539
		C/CG	INSERTION	588
		T/G	SNP	590
		A/G	SNP	594
		G/T	SNP	595
		C/A	SNP	599
		A/G	SNP	607
		A/C	SNP	656
		T/C	SNP	670
		G/T	SNP	673
		T/G	SNP	776
		T/C	SNP	777
		A/C/G	SNP	778
		A/T	SNP	847
		AC/A	DELETION	879
		A/C	SNP	884
		C/G	SNP	885
		T/A	SNP	887
		C/G	SNP	956
		C/T	SNP	1026
		G/A	SNP	1096
		T/A	SNP	1097
		A/G	SNP	1166
		T/A	SNP	1168
		C/A	SNP	1939
TRYT_DROER (sp|P54628) Trypsin theta OS=D. erecta GN=thetaTry PE=3 SV=1	GLOS_TRYT.4.4	C/T	SNP	905
		G/T	SNP	906
		T/C	SNP	1144
		T/C	SNP	1145
		A/C	SNP	1191
		T/C	SNP	1402
		C/G	SNP	1404
		T/A	SNP	1466

		G/A	SNP	1855
		T/C	SNP	1957
		C/G	SNP	1970
		C/A/G	SNP	2834
		C/T	SNP	2941
		A/T	SNP	2956
		C/CA	INSERTION	2958
		G/A	SNP	2959
		C/T	SNP	2962
		C/A	SNP	2963
		C/CA	INSERTION	2965
		G/A	SNP	2966
		G/GAA	INSERTION	2966
		C/T	SNP	2973
		C/T	SNP	3000
		G/T	SNP	3026
		C/A	SNP	3030
		T/G	SNP	3111
		T/G	SNP	3115
		C/T	SNP	3240
		G/T	SNP	3241
		A/T	SNP	3242
TTI_GLOMM (sp|O97373) Tsetse thrombin inhibitor OS=G.m. morsitans GN=TTI	GLOS_TTI.3.16	A/G	SNP	223
		C/T	SNP	265
		T/G	SNP	267
		C/T	SNP	305
		G/A	SNP	306
		A/G	SNP	355
XP_001288661.1 hypothetical protein [Trichomonas vaginalis G3]	GLOS_TVAG_157670.1.1	C/G	SNP	822
		AGG/A/AG	DELETION	1326
[BBH] TYPX_TRYBB (sp|O77404) Tryparedoxin OS=T. brucei brucei PE=1 SV=1	GLOS_TYPX.1.1	G/A	SNP	702
		G/C	SNP	799
		A/G	SNP	1005
[BBH] TYTR_TRYBB (sp|P39051) Trypanothione reductase OS=Tbb GN=TPR	GLOS_TYTR.1.1	A/G	SNP	1758
[BBH] U183_DROME (sp|Q9VSH9) UPF0183 protein CG7083 OS=D. melanogaster	GLOS_U183.1.1	A/C	SNP	239
		T/TA	INSERTION	327

		C/A	SNP	341
		C/CA	INSERTION	341
		T/C	SNP	1203
		C/T	SNP	1428
		T/C	SNP	1638
		T/C	SNP	2017
		T/C	SNP	2021
		A/C	SNP	2056
[BBH] WDR1_DROME (sp|Q9VU68) Actin-interacting protein 1 OS=D. melanogaster	GLOS_WDR1.1.1	T/G	SNP	32
		T/A	SNP	33
		A/G	SNP	34
		G/A	SNP	37
		G/A	SNP	93
		A/C	SNP	210
		T/C	SNP	797
		T/C	SNP	804
		C/T	SNP	928
		T/C	SNP	942
		C/T	SNP	1005
		T/C	SNP	1422
		T/A	SNP	1423
		C/A	SNP	1437
		G/A	SNP	1618
		C/T	SNP	1665
		G/C	SNP	1840
		A/G	SNP	1971
[BBH] Y816_DROME (sp|Q9VAF0) Uncharact. Prot. CG7816 OS=D. melanogaster	GLOS_Y816.1.1	T/C	SNP	326
		A/T	SNP	392
		A/ATTT	INSERTION	394
		A/T	SNP	394
		T/C	SNP	638
		G/A	SNP	685
		A/T	SNP	706
		A/G	SNP	721
		T/C	SNP	766
		C/T	SNP	868

A/G	SNP	1118
T/A	SNP	1562
A/C	SNP	1583
G/A	SNP	1593
C/A	SNP	1621
C/A	SNP	1641
T/A	SNP	1680
G/T	SNP	1684
T/G	SNP	1686
