# Supplementary material for: RNA-seq de novo Assembly Reveals Differential Gene Expression in Glossina palpalis gambiensis Infected with Trypanosoma brucei gambiense vs. Non-Infected and Self-Cured Flies
Source: Front Microbiol. 2015 Nov 13;6:1259. doi: 10.3389/fmicb.2015.01259 (PMC4643127; doi:10.3389/fmicb.2015.01259)
Supplement: Supplementary file 6 [file Table6.PDF]

Supplementary Table S6: SNP found in the differentially expressed genes from 10 days tsetse flies samples

Best hit description	Name	Alleles	Type	Position
NP_001036351.2 CG34109 [Drosophila melanogaster]	GLOS_CG34109.1.1	G/A	SNP	25
		C/T	SNP	32
		G/A	SNP	46
		C/T	SNP	65
		G/A	SNP	86
		C/G	SNP	91
		T/G	SNP	101
		C/T	SNP	200
		T/C	SNP	221
		T/C	SNP	279
		A/T	SNP	299
		G/C	SNP	443
		G/A	SNP	470
		A/G	SNP	524
		G/A	SNP	616
		C/T	SNP	711
		T/C	SNP	728
		T/C	SNP	854
		T/A	SNP	1079
		C/T	SNP	1238
		T/C	SNP	1358
		C/T	SNP	1373
		C/T	SNP	1952
		G/A	SNP	1982
		T/C	SNP	1997
		T/C	SNP	2065
		T/C	SNP	2192
		T/A	SNP	2270
		G/A	SNP	2305
		T/G	SNP	2399
		A/G	SNP	2405
		G/A	SNP	2428
		A/C	SNP	2458
		G/T	SNP	2459

		A/G	SNP	2467
		A/T	SNP	2576
XP_004529509.1 PREDICTED: uncharact. protein LOC101461922 isoform X1 [C. capitata]	GLOS_contig_000114	A/G	SNP	1441
		A/G	SNP	1481
		A/T	SNP	1506
		G/A	SNP	3134
		C/A	SNP	3165
		T/TA	INSERTION	3510
		G/A	SNP	3603
		C/A	SNP	3636
		T/A	SNP	3851
		T/G	SNP	3852
		C/T	SNP	3924
		C/CT	INSERTION	3983
		CT/C	DELETION	4008
		C/T	SNP	4102
		A/C	SNP	4162
		A/AT	INSERTION	4194
		G/A	SNP	4249
		C/T	SNP	4279
		GCAAA/G	DELETION	4333
		A/AT	INSERTION	4592
		CT/C	DELETION	4856
		C/T	SNP	4885
		AT/A	DELETION	5133
WP_021127132.1 hypothetical protein, partial [[Clostridium] sordellii]	GLOS_contig_000837	A/C	SNP	656
	GLOS_contig_002594	C/T	SNP	672
		A/G	SNP	889
	GLOS_contig_005481	T/A	SNP	528
		C/T	SNP	534
		T/G	SNP	538
		C/G	SNP	559
		A/G	SNP	591
	GLOS_contig_008288	C/T	SNP	44
		T/C	SNP	45
		T/C	SNP	46

	C/T	SNP	256
	A/G	SNP	269
	C/T	SNP	270
	C/T	SNP	334
	C/A	SNP	381
GLOS_contig_009185	C/T	SNP	298
	T/G	SNP	361
	T/C	SNP	424
	A/G	SNP	488
	C/A	SNP	571
	C/T	SNP	587
	T/C	SNP	598
	T/C	SNP	603
GLOS_contig_009515	T/A	SNP	75
	A/T	SNP	94
	A/T	SNP	146
	A/T	SNP	151
	T/A	SNP	243
	T/A	SNP	246
	T/A	SNP	247
	T/A	SNP	249
	T/A	SNP	250
GLOS_contig_010890	T/G	SNP	1697
	G/A	SNP	1765
	G/T	SNP	1796
	T/C	SNP	2065
	T/A	SNP	2077
GLOS_contig_011291	G/A	SNP	383
	C/G	SNP	585
GLOS_contig_011841	T/A	SNP	1
	T/C	SNP	4
	G/T	SNP	13
	A/G/T	SNP	25
	T/C	SNP	78
	G/T	SNP	143
	C/T	SNP	144

		T/G	SNP	147
		C/T	SNP	156
		T/C	SNP	174
	GLOS_contig_012866	G/A	SNP	12
		A/T	SNP	15
		G/A	SNP	36
		A/T	SNP	93
		C/A	SNP	101
		G/A	SNP	102
		A/G	SNP	105
		A/T	SNP	195
		G/A	SNP	204
		G/A	SNP	207
		C/A	SNP	223
		C/G	SNP	224
XP_001970283.1 GG10538 [Drosophila erecta]	GLOS_DERE_GG10538.1.1	A/G	SNP	70
		C/T	SNP	317
		C/T	SNP	393
		A/G	SNP	405
		A/G	SNP	438
		A/G	SNP	531
		C/A	SNP	618
		G/A	SNP	619
		T/A	SNP	705
		G/A	SNP	714
		G/A	SNP	850
		G/T	SNP	906
		G/A	SNP	988
		A/G	SNP	1024
		G/A	SNP	1027
		A/C	SNP	1037
		T/G	SNP	1068
		G/C	SNP	1161
		G/A	SNP	1185
		G/A	SNP	1218
XP_002004165.1 GI19764 [Drosophila mojavensis]	GLOS_DMOJ_GI19764.1.1	C/A	SNP	808

A/G	SNP	1607
A/T	SNP	1674
C/A	SNP	1675
CT/C	DELETION	1675
C/G	SNP	1775
T/C	SNP	1877
A/C	SNP	1976
T/A	SNP	2045
C/A	SNP	2096
A/G	SNP	2132
C/T	SNP	2170
G/A	SNP	2180
G/T	SNP	2185
A/G	SNP	2195
A/G	SNP	2341
A/T	SNP	2406
G/GT	INSERTION	2432
G/A	SNP	2553
C/T	SNP	2586
G/A	SNP	2659
T/A	SNP	2660
T/C	SNP	2683
G/A	SNP	2778
A/T	SNP	2800
T/C	SNP	2833
C/T	SNP	2841
A/T	SNP	2872
G/C	SNP	2909
A/G	SNP	2951
A/T	SNP	3053
G/A	SNP	3064
T/A	SNP	3069
G/C	SNP	3147
C/G	SNP	3270
C/T	SNP	3372
C/T	SNP	3419

		T/G	SNP	3527
		C/T	SNP	3548
		C/G	SNP	3553
		TA/T	DELETION	3645
		G/T	SNP	3772
		G/T	SNP	3806
		C/A	SNP	3818
		T/C	SNP	4137
		C/T	SNP	4171
		A/G	SNP	4227
XP_001999054.1 GI24301 [Drosophila mojavensis]	GLOS_DMOJ_GI24301.1.1	T/A	SNP	182
		C/G	SNP	374
		T/C	SNP	1210
		C/T	SNP	1363
		A/T	SNP	1390
		A/G	SNP	1465
		A/G	SNP	1519
		T/C	SNP	1685
		T/G	SNP	1804
		T/C	SNP	1952
		A/G	SNP	1992
		T/G	SNP	2027
		A/G	SNP	2194
		G/T	SNP	2255
		C/T	SNP	2273
XP_002022319.1 GL24331 [Drosophila persimilis]	GLOS_DPER_GL24331.1.3	A/G	SNP	142
		C/T	SNP	318
		C/G	SNP	320
		T/A	SNP	435
		T/A	SNP	439
		A/G	SNP	565
		G/A	SNP	593
		C/T	SNP	613
		T/C	SNP	936
		G/T	SNP	1134
		C/T	SNP	1478

		G/A	SNP	1647
		C/T	SNP	1822
		T/C	SNP	1841
		C/T	SNP	1902
		A/C	SNP	1952
		C/A	SNP	1955
		C/T	SNP	2018
		A/T	SNP	2057
		C/T	SNP	2338
		C/G	SNP	3012
		C/CA	INSERTION	3020
		CT/C	DELETION	3094
		C/T	SNP	3166
		C/A	SNP	3222
XP_002103263.1 GD20325 [Drosophila simulans]	GLOS_DSIM_GD20325.1.4	GTTT/G	DELETION	830
		T/C	SNP	834
		C/G	SNP	835
		CT/C	DELETION	835
		A/T	SNP	842
		G/T	SNP	885
		G/GA	INSERTION	963
		T/A/C	SNP	968
		G/C	SNP	969
		A/ATCG	INSERTION	970
		A/G	SNP	970
		G/A	SNP	975
		G/GA	INSERTION	980
		C/G	SNP	984
		C/A	SNP	985
		T/A	SNP	986
		A/G	SNP	1830
		AC/A	DELETION	1894
		C/A	SNP	1923
		G/A	SNP	1939
		G/T	SNP	1975
XP_002059296.1 GJ18228 [Drosophila virilis]	GLOS_DVIR_GJ18228.1.1	G/A	SNP	215

		G/A	SNP	321
		G/C	SNP	354
		T/G	SNP	431
		T/G	SNP	434
		A/G	SNP	464
		T/C	SNP	472
		G/T	SNP	488
		C/T	SNP	489
		T/C	SNP	523
		G/A	SNP	527
		A/AC	INSERTION	1452
		C/A	SNP	1645
		G/C	SNP	1755
		T/G	SNP	1756
		C/A	SNP	1759
		A/C	SNP	1879
		T/G	SNP	2631
		C/A	SNP	2827
		A/C	SNP	2843
		T/C	SNP	2916
		T/A	SNP	2942
		CTT/C	DELETION	2959
		G/A	SNP	3062
		C/T	SNP	3068
		A/G	SNP	3080
		C/T	SNP	3128
		G/A	SNP	3254
		T/C	SNP	3266
		T/C	SNP	3335
XP_002067848.1 GK12502 [Drosophila willistoni]	GLOS_DWIL_GK12502.1.1	T/C	SNP	92
		A/C	SNP	100
		T/C	SNP	148
		C/T	SNP	150
		G/A	SNP	153
		A/G	SNP	162
		T/C	SNP	230

		C/G	SNP	341
		AAGC/A	DELETION	355
		G/A	SNP	671
		A/T	SNP	913
		G/A	SNP	919
		T/A	SNP	973
XP_002072711.1 GK13541 [Drosophila willistoni]	GLOS_DWIL_GK13541.1.5	A/T	SNP	51
		G/T	SNP	52
		G/A	SNP	466
		C/T	SNP	1057
		T/A	SNP	1077
		A/C	SNP	1079
XP_002075290.1 GK15974 [Drosophila willistoni]	GLOS_DWIL_GK15974.5.7	A/AT	INSERTION	166
		A/T	SNP	167
		A/C	SNP	168
		G/C	SNP	169
		T/C	SNP	170
		G/C	SNP	270
		A/C	SNP	272
FDL_DROME (sp|Q8WSF3) Probable beta-hexosaminidase fdl OS=D. melanogaster	GLOS_FDL.1.2	TA/T	DELETION	174
		C/T	SNP	236
		A/G	SNP	583
		T/C	SNP	637
		G/A	SNP	690
		T/C	SNP	802
		T/C	SNP	817
		A/G	SNP	855
		A/C	SNP	1096
		G/A	SNP	1115
		A/G	SNP	1156
		C/T	SNP	1304
		G/A	SNP	1318
		C/T	SNP	1394
		C/T	SNP	1660
		G/A	SNP	1867
		A/G	SNP	2026

		G/A	SNP	2091
		T/G	SNP	2199
		T/A	SNP	2278
GPL_GLOAU (sp|Q4TTV7) Lectizyme OS=Glossina austeni GN=Gpl PE=2 SV=1	GLOS_GPL.11.22	C/T	SNP	59
		C/T	SNP	67
		G/A	SNP	269
		A/G	SNP	299
		T/C	SNP	397
		C/T	SNP	569
		G/A	SNP	597
		C/T	SNP	613
		T/C	SNP	1705
		A/G	SNP	1707
		AT/A	DELETION	1708
		T/TC	INSERTION	1709
		T/C	SNP	1711
		C/A	SNP	1771
		CT/C	DELETION	1831
		A/C	SNP	2401
		T/C	SNP	2402
		G/T	SNP	2403
		C/A	SNP	2404
		T/C	SNP	2405
		CA/C	DELETION	2406
		C/G	SNP	2406
		T/TC	INSERTION	2408
		G/T	SNP	2431
		T/C	SNP	2583
		T/G	SNP	3485
		T/G	SNP	3489
		A/G	SNP	3556
		T/A	SNP	3757
		C/T	SNP	3789
		C/A	SNP	3790
		T/A	SNP	3791
		T/C/G	SNP	3851

		T/A	SNP	3908
		C/G	SNP	3909
		T/C	SNP	3912
		A/T	SNP	4093
		A/G	SNP	4107
		T/A	SNP	4143
		C/T	SNP	4168
		CAA/C	DELETION	4169
		A/G	SNP	4181
		C/T	SNP	4228
		A/C	SNP	4293
		TC/T	DELETION	4400
		A/C	SNP	4590
GPL_GLOFF (sp|Q8MUG0) Lectizyme OS=Glossina fuscipes fuscipes GN=Gpl PE=2 SV=1	GLOS_GPL.16.22	C/G	SNP	69
		A/G	SNP	86
		A/T	SNP	87
		T/C	SNP	111
		C/T	SNP	138
		C/A	SNP	150
		A/T	SNP	189
		G/A	SNP	205
		T/C	SNP	206
		C/T	SNP	498
		G/A	SNP	710
		A/C	SNP	1010
		G/C	SNP	1013
		A/C	SNP	1014
		G/GC	INSERTION	1016
		A/T	SNP	1017
		A/G	SNP	1018
		C/CA	INSERTION	1121
		C/G	SNP	1122
		T/C	SNP	1125
		G/A	SNP	1126
		T/A	SNP	1128
		G/A	SNP	1129

		A/G	SNP	1243
		G/A	SNP	1249
[BBH] GST_MUSDO (sp|P46437) Glutathione S-transferase OS=M. domestica PE=2 SV=1	GLOS_GST.1.1	C/T	SNP	62
		A/T	SNP	592
		G/A	SNP	669
		G/A	SNP	689
		AT/A	DELETION	765
		T/C	SNP	1038
		G/C	SNP	1086
		A/T	SNP	1323
		G/A	SNP	1752
		T/A	SNP	1754
		G/C	SNP	1755
HYPB_HYPLI (sp|P35588) Hypodermin-B OS=Hypoderma lineatum PE=1 SV=1	GLOS_HYPB.1.2	T/A	SNP	56
		T/A	SNP	57
		C/A	SNP	58
		A/G	SNP	383
		C/T	SNP	421
		G/T	SNP	529
LECA_SARPE (sp|P05047) Lectin subunit alpha OS=Sarcophaga peregrina PE=1 SV=1	GLOS_LECA.3.13	A/AT	INSERTION	1313
		T/C	SNP	1322
		G/C	SNP	1349
		A/C	SNP	1350
		A/G	SNP	659
		G/A	SNP	660
		A/G	SNP	720
		G/A	SNP	744
		A/G	SNP	745
		A/G	SNP	761
		G/C	SNP	825
		T/G	SNP	843
		A/G	SNP	734
		G/T	SNP	119
		C/T	SNP	1016
		G/T	SNP	1135
		A/C	SNP	1136

		A/C	SNP	1137
		G/T	SNP	1141
		A/G	SNP	1375
		T/TA	INSERTION	1466
		C/A	SNP	1512
		C/CT	INSERTION	1800
		C/G	SNP	1815
		G/A	SNP	1838
		T/A	SNP	1874
XP_004521992.1 PREDICTED: uncharacterized protein LOC101450586 [Ceratitis capitata]	GLOS_LOC101450586.2.9	T/C	SNP	502
		T/C	SNP	512
		C/G	SNP	548
		T/C	SNP	552
		T/C	SNP	619
		T/A	SNP	958
		A/G	SNP	959
		G/A	SNP	960
		A/G	SNP	972
		T/C	SNP	1296
		G/A	SNP	1299
		A/G/T	SNP	1302
		G/T	SNP	1305
		A/G	SNP	1306
XP_004523338.1 PREDICTED: acyl-CoA Delta(11) desaturase-like isoform X3 [C. capitata] ref|XP_004523339.1| PRED: acyl-CoA Delta(11) desaturase-like isoform X4 [C. capitata]	GLOS_LOC101461142.7.9	T/C	SNP	192
		T/A	SNP	231
		C/T	SNP	232
		G/T	SNP	233
XP_004526299.1 PREDICTED: uncharacterized protein LOC101461327 [Ceratitis capitata]	GLOS_LOC101461327.1.1	A/G	SNP	157
		G/GA	INSERTION	808
		A/T	SNP	1479
		A/C	SNP	1776
		A/G	SNP	1814
		T/C	SNP	2505
		C/CA	INSERTION	3891
		C/A	SNP	4751

		A/C	SNP	5301
XP_004531314.1 PREDICTED: lysosomal aspartic protease-like [Ceratitis capitata]	GLOS_LOC101461571.2.2	C/A	SNP	145
		A/G	SNP	283
		A/C	SNP	347
		T/C	SNP	391
		A/G	SNP	445
		T/A	SNP	601
		C/A	SNP	610
		T/C	SNP	631
		C/A	SNP	702
		T/C	SNP	705
		T/C	SNP	747
		A/G	SNP	788
		A/G	SNP	808
		T/C	SNP	1056
		T/G	SNP	1297
		A/T	SNP	1312
		T/C	SNP	1318
		G/T	SNP	1332
		G/C	SNP	1365
		A/G	SNP	1369
		T/C	SNP	1374
		G/A	SNP	1396
		T/A	SNP	1836
		G/T	SNP	1842
XP_004522257.1 PREDICTED: uncharacterized protein LOC101462034 [Ceratitis capitata]	GLOS_LOC101462034.1.2	C/CA	INSERTION	362
		A/C	SNP	366
		A/G	SNP	421
		G/C	SNP	448
		C/T	SNP	501
YP_004400609.1 transmembrane protein [M. mycoides subsp. capri LC str. 95010] ref|WP_013729997.1| transmembrane protein [Mycoplasma mycoides]	GLOS_MLC_9020.1.1	T/G	SNP	40
		C/T	SNP	89
		A/G	SNP	121
		A/T	SNP	143
		T/C	SNP	152

		A/G	SNP	194
		C/A	SNP	305
		C/A	SNP	320
		G/C	SNP	347
		A/C	SNP	381
		C/T	SNP	388
		G/T	SNP	477
		T/G	SNP	637
		C/T	SNP	674
		G/T	SNP	743
		C/CT	INSERTION	975
		G/GT	INSERTION	1161
		C/G	SNP	1381
		A/G	SNP	1410
		T/A	SNP	1545
		G/A	SNP	1602
		A/AT	INSERTION	1623
		T/A	SNP	1676
		C/A	SNP	1761
		T/C	SNP	1788
		A/G	SNP	1992
		A/G	SNP	2466
NM_001193606.1 Pan troglodytes MTRNR2-like 10 (MTRNR2L10), mRNA	GLOS_MTRNR2L10.1.3	A/C	SNP	271
YP_007026263.1 NADH dehydrogenase subunit 1 (mitochondrion) [Chrysomya megacephala]	GLOS_ND1.2.4	A/G	SNP	79
		G/T	SNP	102
		A/T	SNP	113
		G/T	SNP	114
		G/A	SNP	120
		A/AG	INSERTION	279
		G/C	SNP	282
		C/G	SNP	284
		G/A	SNP	285
YP_007517102.1 NADH dehydrogenase subunit 3 (mitochondrion) [Procecidochares utilis]	GLOS_ND3.1.1	T/A	SNP	476
		T/A	SNP	479
		C/A	SNP	480
YP_007026246.1 NADH dehydrogenase subunit 4 (mitochondrion) [Chrysomya bezziana]	GLOS_ND4.5.6	A/T	SNP	397

		T/A	SNP	398
		A/C	SNP	523
		G/A	SNP	645
		C/T	SNP	694
		A/C	SNP	840
		A/G	SNP	1009
		C/T	SNP	1018
		T/A	SNP	1509
		T/A	SNP	1510
		T/G	SNP	1682
XP_002782358.1 conserved hypothetical protein [Perkinsus marinus ATCC 50983]	GLOS_PMAR_PMAR029216	T/C	SNP	740
		T/A	SNP	784
		A/G	SNP	811
		A/G	SNP	857
		A/G	SNP	973
		T/C	SNP	989
		G/A	SNP	1018
		G/C	SNP	1189
		G/T	SNP	1426
[BBH] RL31_DROME (sp|Q9V597) 60S ribosomal protein L31 OS=D. melanogaster	GLOS_RL31.2.3	T/C	SNP	21
		C/T	SNP	205
		C/T	SNP	244
		A/C	SNP	285
		C/T	SNP	294
		G/A	SNP	302
		A/G	SNP	324
		C/CG	INSERTION	333
		A/G	SNP	363
		A/G	SNP	383
		AT/A	DELETION	471
		C/T	SNP	489
		A/T	SNP	512
		G/A	SNP	529
		C/A	SNP	596
		C/T	SNP	863
		C/T	SNP	917

RS25_DROME (sp|P48588) 40S ribosomal prot S25 D.melanogaster GN=RpS25 PE=1 SV=3	GLOS_RS25.5.10	A/G	SNP	154
TRYDG_DROER (sp|P54626) Trypsin delta/gamma OS=D. erecta GN=deltaTry PE=3 SV=1	GLOS_TRYDG.5.5	C/G	SNP	459
		G/T	SNP	784
		A/G	SNP	903
		T/G	SNP	1922
TTI_GLOMM (sp|O97373) Tsetse thrombin inhibitor OS=G. m. morsitans GN=TTI PE=1 SV=1	GLOS_TTI.10.16	G/T	SNP	72
		G/A	SNP	118
