# Supplementary material for: RNA-seq de novo Assembly Reveals Differential Gene Expression in Glossina palpalis gambiensis Infected with Trypanosoma brucei gambiense vs. Non-Infected and Self-Cured Flies
Source: Front Microbiol. 2015 Nov 13;6:1259. doi: 10.3389/fmicb.2015.01259 (PMC4643127; doi:10.3389/fmicb.2015.01259)
Supplement: Supplementary file 7 [file Table7.PDF]

Supplementary Table S7: SNP evidenced in the differentially expressed genes from 20 days tsetse flies samples

Best hit description	Name	Alleles	Type	Position
XP_001653478.1 deoxyribonuclease I, putative [Aedes aegypti]	GLOS_AAEL_AAEL008876.1.1	T/C	SNP	5
		T/C	SNP	32
		T/A	SNP	173
		C/T	SNP	200
		T/G	SNP	203
		G/A	SNP	208
		C/G	SNP	217
		G/A	SNP	225
		T/C	SNP	261
XP_318675.2 AGAP009641-PA [Anopheles gambiae str. PEST]	GLOS_AGAP_AGAP009641.2.2	C/T	SNP	836
		A/C	SNP	920
		A/C	SNP	1149
		T/C	SNP	1161
		C/T	SNP	1173
		G/C	SNP	1669
		T/C	SNP	2514
		C/T	SNP	2630
		A/C	SNP	2693
[BBH] ALF_TRYBB (sp|P07752) Fructose-bisphosphate aldolase, glycosomal Tbb	GLOS_ALF.1.1	AGT/A	DELETION	1667
NP_610955.1 Activity-regulated cytoskeleton associated protein 1 [Drosophila melanogaster]	GLOS_ARC1.1.1	A/C	SNP	1713
		G/A	SNP	1728
		A/AG	INSERTION	2254
		T/A	SNP	2255
		A/G	SNP	2258
		T/A	SNP	2260
YP_001285409.1 polyprotein [Brevicoryne brassicae picorna-like virus]	GLOS_BBPLV_GP1.2.6	A/C	SNP	56
		T/A	SNP	57
		T/A	SNP	58
		T/C	SNP	59
		A/G	SNP	75
		A/C	SNP	294
		C/T	SNP	309
		G/A	SNP	324
		A/G	SNP	348
		C/T	SNP	740
		G/A	SNP	863
		A/G	SNP	936

G/A	SNP	1552
C/T	SNP	1572
T/C	SNP	1590
C/T	SNP	1593
A/G	SNP	1704
C/T	SNP	1806
C/T	SNP	1941
G/A	SNP	1984
A/G	SNP	1992
A/G	SNP	2182
G/A	SNP	2286
G/A	SNP	2406
G/A	SNP	2916
C/T	SNP	3044
G/A	SNP	3108
T/C	SNP	3360
T/C	SNP	3555
T/C	SNP	3570
T/C	SNP	3768
A/T	SNP	3788
A/G	SNP	4020
G/A	SNP	4281
G/A	SNP	4290
A/G	SNP	4409
T/A	SNP	4473
G/A	SNP	4608
A/G	SNP	4938
C/G	SNP	5381
A/G	SNP	5802
G/A	SNP	5865
A/G	SNP	5946
G/A	SNP	5972
C/T	SNP	5973
G/A	SNP	6176
A/G	SNP	6387
A/G/T	SNP	6438
A/G	SNP	6464
G/A	SNP	6550
C/T	SNP	6771

		G/A	SNP	7107
		A/G	SNP	7161
		C/T	SNP	7237
		A/G	SNP	7292
		C/T	SNP	7323
		T/C	SNP	7332
		T/C	SNP	7380
		T/C	SNP	7803
		T/C	SNP	7941
		A/G	SNP	7962
		G/A	SNP	8236
		C/T	SNP	8373
		A/G	SNP	8490
		C/T	SNP	8625
		A/G	SNP	8649
		C/A	SNP	8676
		C/T	SNP	8686
		A/G	SNP	8700
		C/T	SNP	8904
		G/A	SNP	8985
		G/A	SNP	9044
		G/T	SNP	9081
		G/C	SNP	9370
		C/T	SNP	9487
		G/A	SNP	9488
		C/T	SNP	9495
		C/T	SNP	9563
C12A2_MUSDO (sp|O18635) Cyt. P450 CYP12A2 OS=M. domestica GN=CYP12A2 PE=2 SV=1	GLOS_C12A2.3.4	A/C	SNP	120
		C/G	SNP	137
		CT/C	DELETION	143
		G/T	SNP	214
		T/A	SNP	376
		C/A	SNP	611
		C/T	SNP	675
		C/T	SNP	1124
		T/A	SNP	1879
		T/A	SNP	2514
		T/C	SNP	2649
		G/A	SNP	2743

		T/C	SNP	2870
		G/A	SNP	2966
		G/A	SNP	2973
		T/C	SNP	3421
		G/A	SNP	3455
		A/G	SNP	3481
		G/A	SNP	3482
[BBH] CALM_TRYBG (sp|P69098) Calmodulin OS=Trypanosoma brucei gambiense PE=3 SV=2	GLOS_CALM.1.1	A/G	SNP	448
		G/A	SNP	495
		A/G	SNP	840
		T/A	SNP	966
		C/T	SNP	1094
		C/T	SNP	1127
		A/C	SNP	1128
[BBH] CC2H2_TRYBB (sp|P54665) Cell division control protein 2 homolog 2 Tbb	GLOS_CC2H2.1.1	C/CT	INSERTION	302
		A/T	SNP	2298
		G/GT	INSERTION	2763
		C/T	SNP	2771
NP_001188973.1 CG15097, isoform C [Drosophila melanogaster]	GLOS_CG15097.2.5	A/G	SNP	1652
		G/T	SNP	1777
		T/A	SNP	1819
		T/C	SNP	2161
		G/A	SNP	2187
		T/A	SNP	2248
		T/A	SNP	2709
		A/T	SNP	2748
		C/T	SNP	3672
		A/G	SNP	3679
		T/C	SNP	3713
		CT/C	DELETION	3771
		G/T	SNP	3809
		C/T	SNP	3836
		G/A	SNP	3852
		G/A	SNP	3869
		T/C	SNP	3877
		T/C	SNP	4000
NP_001188973.1 CG15097, isoform C [Drosophila melanogaster]	GLOS_CG15097.4.5	A/G	SNP	669
		G/T	SNP	726
		T/A	SNP	3518

		C/G	SNP	3767
		C/CT	INSERTION	4386
[BBH] CH60_TRYBB (sp|Q37683) Chaperonin HSP60, mitochondrial Tbb GN=HSP60 PE=2 SV=2	GLOS_CH60.1.3	C/T	SNP	1684
[BBH] CLP_TRYBB (sp|P31543) Heat shock protein 100 OS=Tbb GN=HSP100 PE=3 SV=1	GLOS_CLP.1.1	T/C	SNP	1674
		T/C	SNP	2424
	GLOS_contig_000050	G/GA	INSERTION	145
		C/T	SNP	825
	GLOS_contig_000059	C/T	SNP	464
		A/T	SNP	522
		A/T	SNP	523
		G/GT	INSERTION	607
		CT/C	DELETION	818
		C/CT	INSERTION	1213
		T/TA	INSERTION	1266
		CT/C	DELETION	1480
		A/T	SNP	1549
		T/C	SNP	1598
		C/T	SNP	1600
	GLOS_contig_000389	T/C	SNP	928
		T/TTG/TTGTG	INSERTION	1191
		AT/A	DELETION	1266
	GLOS_contig_000427	A/G	SNP	146
		A/G	SNP	221
		A/G	SNP	790
		GC/G	DELETION	1031
	GLOS_contig_000441	A/T	SNP	92
		A/G	SNP	304
		C/T	SNP	331
		G/T	SNP	359
		A/T	SNP	364
		A/G	SNP	366
		A/G	SNP	473
		G/T	SNP	483
		T/TC	INSERTION	909
		C/T	SNP	1343
		GA/G	DELETION	1360
		C/T	SNP	1368
		A/G	SNP	1470
		A/G	SNP	1480

	A/T	SNP	1506
	A/G	SNP	1511
	C/T	SNP	1918
	C/T	SNP	1920
	A/T	SNP	1922
	C/T	SNP	1951
	A/G	SNP	1952
	C/T	SNP	1955
	A/G	SNP	1970
	C/T	SNP	2012
	G/T	SNP	2022
	A/T	SNP	2082
	G/A	SNP	2097
	G/A	SNP	2146
	T/A	SNP	2161
	A/G	SNP	2173
	G/A	SNP	2253
	G/A	SNP	2302
	T/C	SNP	2441
	C/A	SNP	2500
	C/T	SNP	2549
	C/G	SNP	3128
	C/T	SNP	3146
	C/T	SNP	3374
	A/C	SNP	3750
GLOS_contig_000530	G/A	SNP	325
	C/CT	INSERTION	564
	C/CT	INSERTION	1086
	TAA/T	DELETION	1239
GLOS_contig_000567	T/G	SNP	47
	T/G	SNP	48
	T/TG	INSERTION	117
	T/TG	INSERTION	118
	G/A	SNP	162
	T/C	SNP	171
	A/G	SNP	172
	A/G	SNP	379
	A/AT	INSERTION	385
	C/G	SNP	444

	G/A	SNP	643
	C/T	SNP	734
	G/T	SNP	758
	T/C	SNP	792
	C/T	SNP	839
	G/T	SNP	909
	A/G	SNP	1171
	A/T	SNP	1518
	A/G	SNP	1648
	A/G	SNP	1659
	G/A	SNP	1694
	C/A	SNP	1746
GLOS_contig_000712	A/C	SNP	601
GLOS_contig_000809	A/ATT	INSERTION	371
	A/T	SNP	379
	C/CT	INSERTION	519
	C/CA	INSERTION	662
	A/T	SNP	858
GLOS_contig_000926	A/G	SNP	268
	G/A	SNP	925
	T/C	SNP	1248
GLOS_contig_001123	A/G	SNP	61
	A/G	SNP	77
	T/G	SNP	170
	G/A	SNP	194
	A/T	SNP	255
	G/A	SNP	326
	A/G	SNP	339
	G/A	SNP	374
	A/C	SNP	403
	C/T	SNP	405
	G/T	SNP	464
	A/C	SNP	469
	G/A	SNP	470
	A/G	SNP	478
	A/G	SNP	494
	G/C	SNP	502
	C/A	SNP	594
	T/G	SNP	627

	A/T	SNP	629
	C/CCA	INSERTION	630
	G/A	SNP	639
	G/A	SNP	689
	G/A	SNP	707
	A/G	SNP	768
	A/C	SNP	770
	G/GT	INSERTION	777
	C/T	SNP	779
	C/T	SNP	837
	C/T	SNP	857
	A/AT	INSERTION	899
	T/C	SNP	1006
	A/G	SNP	1111
	G/A	SNP	1112
	T/A	SNP	1137
	A/C	SNP	1163
	C/T	SNP	1173
	C/G	SNP	1189
	T/C	SNP	1192
	G/A	SNP	1224
	A/G	SNP	1228
	C/T	SNP	1256
	T/G	SNP	1257
	G/A	SNP	1370
	G/A	SNP	1426
	G/T	SNP	1442
	A/T	SNP	1449
	A/G	SNP	1557
	G/A	SNP	1558
	A/G	SNP	1571
GLOS_contig_001171	TA/T	DELETION	214
	A/AT	INSERTION	372
	G/A	SNP	509
	AT/A	DELETION	529
	A/G	SNP	559
	A/G	SNP	672
	A/G	SNP	962
	G/A	SNP	965

	CT/C	DELETION	2060
	G/T	SNP	2595
	G/C	SNP	2752
GLOS_contig_001176	G/T	SNP	209
	CA/C	DELETION	374
	G/C	SNP	380
	A/T	SNP	383
	T/C	SNP	450
	C/T	SNP	504
	A/G	SNP	542
	C/T	SNP	598
	G/T	SNP	603
	A/T	SNP	612
	T/A	SNP	642
	A/C	SNP	717
	A/T	SNP	718
	T/G	SNP	763
	G/A	SNP	802
	C/T	SNP	803
	G/T	SNP	837
	A/G	SNP	849
	G/A	SNP	897
	G/C	SNP	951
	G/A	SNP	961
	A/C	SNP	1024
	C/G	SNP	1056
	C/T	SNP	1063
	C/T	SNP	1113
	C/T	SNP	1120
	T/C	SNP	1126
	G/A	SNP	1195
	T/A	SNP	1211
	T/G	SNP	1238
	C/A	SNP	1242
	CA/C	DELETION	1260
	T/A	SNP	1550
	T/C	SNP	1640
	C/T	SNP	1710
	T/A	SNP	1779

	A/T	SNP	1792
GLOS_contig_001185	C/G	SNP	191
	T/G	SNP	224
	G/A	SNP	278
	T/C	SNP	284
	G/C	SNP	738
	G/C	SNP	846
	A/T	SNP	853
GLOS_contig_001756	C/CT	INSERTION	114
	G/A	SNP	228
	A/G	SNP	271
	TTTCTC/T	DELETION	347
	GA/G	DELETION	1354
	T/C	SNP	2372
GLOS_contig_001921	GAA/G/GA	DELETION	620
	A/G	SNP	852
	A/G	SNP	908
	G/A	SNP	1289
	G/A	SNP	1377
	C/T	SNP	1622
GLOS_contig_002129	G/A	SNP	691
	G/GT	INSERTION	1062
	C/T	SNP	2232
	A/G	SNP	4262
	A/AT	INSERTION	5304
	A/G	SNP	5516
	T/TA	INSERTION	5986
	G/A	SNP	5993
	C/T	SNP	6248
GLOS_contig_002220	T/C	SNP	100
	C/T	SNP	105
	A/T	SNP	143
	C/G	SNP	151
	T/C	SNP	209
	T/A	SNP	230
	T/C	SNP	243
	A/C	SNP	331
	T/C	SNP	451
	C/T	SNP	453

G/C	SNP	469
C/T	SNP	485
C/G	SNP	546
T/C	SNP	638
T/A	SNP	666
T/TTC	INSERTION	672
T/A	SNP	678
G/GT	INSERTION	756
T/G	SNP	758
C/G	SNP	905
A/AT	INSERTION	986
G/A	SNP	987
T/G	SNP	1070
T/G	SNP	1115
A/G	SNP	1188
T/C	SNP	1199
A/G	SNP	1246
A/G	SNP	1407
T/C	SNP	1447
T/G	SNP	1449
T/A	SNP	1456
A/G	SNP	1487
A/C	SNP	1506
A/C	SNP	1522
C/A	SNP	1550
G/C	SNP	1552
G/A	SNP	1566
C/A	SNP	1572
G/A	SNP	1668
G/A	SNP	1735
G/A	SNP	1757
A/G	SNP	1761
G/A	SNP	1764
G/T	SNP	1778
C/G	SNP	1786
A/G	SNP	1847
C/T	SNP	1952
T/C	SNP	1954
A/G	SNP	2072

	T/C	SNP	2104
GLOS_contig_004073	T/C	SNP	363
	CTT/C	DELETION	481
	G/T	SNP	635
	T/TA	INSERTION	878
	C/CA	INSERTION	1005
	T/TA	INSERTION	1045
	GT/G	DELETION	1124
	C/CT	INSERTION	1276
	A/AT	INSERTION	1473
	C/CA	INSERTION	1602
GLOS_contig_004527	A/G	SNP	150
	G/A	SNP	470
	A/G	SNP	591
	G/C	SNP	623
	G/A	SNP	686
	G/T	SNP	836
	A/G	SNP	1446
GLOS_contig_005746	G/C	SNP	282
	C/T	SNP	395
	T/C	SNP	407
	C/G	SNP	466
	C/T	SNP	473
	A/AT	INSERTION	524
	A/T	SNP	619
	T/C	SNP	652
	C/T	SNP	926
	A/C	SNP	937
	C/G	SNP	962
	T/TA	INSERTION	1043
	C/T	SNP	1269
	T/G	SNP	1350
	G/A	SNP	1392
	A/G	SNP	1401
	T/C	SNP	1471
	C/A	SNP	1529
	A/G	SNP	1535
	A/T	SNP	1560
	C/CAA	INSERTION	1679

	G/A	SNP	1786
	C/T	SNP	1787
	A/G	SNP	1881
	T/G	SNP	1916
	G/A	SNP	2004
	C/T	SNP	2389
	G/A	SNP	2474
	C/T	SNP	2532
	A/C	SNP	2658
	A/G	SNP	2799
	G/A	SNP	2831
	A/AT	INSERTION	2962
	C/A	SNP	3038
	C/T	SNP	3180
	C/T	SNP	3209
	C/T	SNP	3310
	G/A	SNP	3329
	C/T	SNP	3518
	G/A	SNP	3520
	G/A	SNP	3521
GLOS_contig_005917	A/G	SNP	64
	C/A	SNP	125
	A/AT	INSERTION	126
	T/A	SNP	214
	G/T	SNP	238
	T/A	SNP	360
	A/G	SNP	432
	T/C	SNP	511
	G/T	SNP	530
	T/C	SNP	565
	T/C	SNP	728
	C/T	SNP	744
	A/T	SNP	792
	C/T	SNP	890
	G/C	SNP	975
	A/T	SNP	979
	G/C	SNP	1016
	C/T	SNP	1034
	A/G	SNP	1041

	T/C	SNP	1046
	G/A	SNP	1068
	C/G	SNP	1105
	A/T	SNP	1125
	C/G	SNP	1146
	A/G	SNP	1184
	C/A	SNP	1311
	G/A	SNP	1327
	G/A	SNP	1345
	A/T	SNP	1395
	A/G	SNP	1447
	T/G	SNP	1458
	G/C	SNP	1461
	C/T	SNP	1549
	G/A	SNP	1669
	C/CA	INSERTION	2020
	C/T	SNP	2061
	C/T	SNP	2080
	T/C	SNP	2106
	A/T	SNP	2180
	C/T	SNP	2207
	G/T	SNP	2209
	T/C	SNP	2224
	C/T	SNP	2233
	C/T	SNP	2258
	G/A	SNP	2358
	C/T	SNP	2363
GLOS_contig_006678	G/T	SNP	247
	A/G	SNP	262
	G/C	SNP	335
GLOS_contig_006723	G/T	SNP	239
	C/T	SNP	411
	T/C	SNP	427
	A/AT	INSERTION	636
GLOS_contig_006840	T/C	SNP	10
	A/G	SNP	11
	T/C	SNP	12
	C/T	SNP	13
	C/T	SNP	16

	A/G	SNP	23
	G/C	SNP	25
	A/T	SNP	31
	T/A	SNP	34
	T/A	SNP	37
	C/T	SNP	43
	T/G	SNP	44
	A/G	SNP	47
	T/C	SNP	199
GLOS_contig_006893	T/A	SNP	120
	A/G	SNP	205
	A/T	SNP	248
GLOS_contig_007349	G/A	SNP	161
	TA/T	DELETION	221
	A/AT	INSERTION	479
	G/GGT	INSERTION	548
	T/A	SNP	574
	C/T	SNP	612
	A/T	SNP	614
	A/G	SNP	619
	A/T	SNP	622
	A/C	SNP	1024
	G/T	SNP	1806
	CA/C	DELETION	1840
	T/C	SNP	1905
GLOS_contig_007356	A/T	SNP	100
	C/T	SNP	116
	C/T	SNP	284
	C/A	SNP	304
	C/T	SNP	322
	G/C	SNP	411
	C/A	SNP	435
	C/T	SNP	453
	T/G	SNP	730
	A/G	SNP	794
	G/A	SNP	844
	C/T	SNP	900
	C/A	SNP	915
	C/T	SNP	1062

	T/C	SNP	1065
	A/G	SNP	1066
	C/A	SNP	1136
	G/C	SNP	1176
	T/C	SNP	1225
	T/C	SNP	1271
	G/GT	INSERTION	1272
	C/A	SNP	1494
	A/C	SNP	1499
	C/G	SNP	1555
	C/T	SNP	1710
	T/A	SNP	1719
	T/G	SNP	1744
	G/A	SNP	1865
	C/T	SNP	1892
	T/G	SNP	1906
	C/T	SNP	2130
	A/T	SNP	2160
	G/C	SNP	2179
	T/C	SNP	2203
	A/C	SNP	2254
GLOS_contig_007423	A/G	SNP	433
	G/T	SNP	451
	C/T	SNP	529
GLOS_contig_007915	A/G	SNP	77
	T/A	SNP	84
	C/T	SNP	102
	TC/T	DELETION	109
	A/G	SNP	564
	A/G	SNP	743
	G/T	SNP	757
	C/T	SNP	783
GLOS_contig_007993	C/T	SNP	252
	G/A	SNP	375
	A/T	SNP	398
	A/C	SNP	746
	T/C	SNP	814
	G/C	SNP	882
	G/A	SNP	891

		G/A	SNP	939
		G/A	SNP	1016
		G/C	SNP	1055
		G/A	SNP	1119
		A/T	SNP	1305
		T/A	SNP	1827
		C/G	SNP	1949
		T/G	SNP	1983
		T/C	SNP	2035
		G/GT	INSERTION	2088
		C/G	SNP	2155
		A/T	SNP	2162
		A/T	SNP	2309
	GLOS_contig_008231	G/A	SNP	73
		T/C	SNP	168
		G/A	SNP	177
		T/C	SNP	197
		A/C	SNP	243
		C/T	SNP	963
		A/T	SNP	1011
		C/G	SNP	1015
	GLOS_contig_009274	A/G	SNP	837
		G/A	SNP	921
		G/T	SNP	1153
	GLOS_contig_009491	A/T	SNP	128
		T/G	SNP	143
		T/C	SNP	159
		T/G	SNP	160
		T/G	SNP	161
		T/G	SNP	164
	GLOS_contig_009631	T/C	SNP	55
		T/C	SNP	59
		A/T	SNP	295
		A/T	SNP	300
		G/C	SNP	302
NR_076090.1 Proteus mirabilis HI4320 strain HI4320 23S ribosomal RNA, complete sequence	GLOS_contig_009795	C/T	SNP	476
		C/T	SNP	495
		A/T	SNP	524
		C/T	SNP	537

C/T	SNP	617
C/T	SNP	633
GT/G	DELETION	634
A/G	SNP	640
T/A	SNP	649
A/T	SNP	675
A/G	SNP	685
G/T	SNP	768
A/C/T	SNP	795
A/G	SNP	797
C/G	SNP	869
T/A	SNP	876
A/G	SNP	898
G/T	SNP	906
C/T	SNP	924
A/G	SNP	937
A/G	SNP	995
C/T	SNP	1017
A/G	SNP	1019
A/G	SNP	1039
A/G	SNP	1040
A/T	SNP	1056
A/G	SNP	1063
G/T	SNP	1065
T/C	SNP	1067
C/T	SNP	1068
C/T	SNP	1142
C/G	SNP	1193
A/T	SNP	1197
T/C	SNP	1200
A/G	SNP	1242
T/C	SNP	1251
T/A	SNP	1298
A/G	SNP	1310
A/T	SNP	1316
T/C	SNP	1556
C/T	SNP	1751
G/A	SNP	1767
C/T	SNP	1768

		T/C	SNP	1865
		C/T	SNP	1891
		C/T	SNP	1903
		A/G	SNP	1917
		A/G	SNP	1936
		A/G	SNP	2183
		G/T	SNP	2320
		A/C	SNP	2322
		T/G	SNP	2336
		C/A	SNP	2338
		A/T	SNP	2350
	GLOS_contig_011049	A/G	SNP	319
		G/T	SNP	389
		G/A	SNP	423
		A/G	SNP	450
		A/T	SNP	475
		G/T	SNP	510
		T/G	SNP	647
	GLOS_contig_011116	A/G	SNP	123
		A/G	SNP	130
		AT/A	DELETION	212
		T/C	SNP	243
		G/T	SNP	422
		G/A	SNP	427
	GLOS_contig_011571	C/G	SNP	516
		A/G	SNP	547
		G/A	SNP	562
		A/C	SNP	576
		T/A	SNP	1234
		A/T	SNP	1294
		G/A	SNP	1296
	GLOS_contig_011873	G/A	SNP	194
		A/T	SNP	739
		A/G	SNP	740
	GLOS_contig_012940	A/G	SNP	301
		A/G	SNP	304
		A/T	SNP	305
		T/A	SNP	306
NR_074525.1 Sodalis glossinidius strain morsitans 16S ribosomal RNA, complete sequence	GLOS_contig_013399	A/C/T	SNP	161

		C/T	SNP	617
		C/T	SNP	619
		C/T	SNP	624
		C/T	SNP	651
		G/A	SNP	654
		G/A	SNP	661
		G/A	SNP	662
		C/T	SNP	684
	GLOS_contig_013569	G/C	SNP	133
		T/C	SNP	1040
		G/A	SNP	1297
		C/A	SNP	1298
NR_076264.1 Sodalis glossinidius strain morsitans 23S ribosomal RNA, complete sequence	GLOS_contig_014009	A/G	SNP	742
		T/A	SNP	990
		C/T	SNP	1720
		C/A	SNP	1721
	GLOS_contig_014337	G/T	SNP	55
		C/G	SNP	58
		A/T	SNP	59
	GLOS_contig_014405	C/T	SNP	287
		G/T	SNP	332
		C/G	SNP	352
		A/T	SNP	379
		C/A	SNP	380
		T/G	SNP	394
		A/C	SNP	427
		G/A	SNP	493
		A/G	SNP	508
		T/G	SNP	553
		A/G	SNP	582
		C/T	SNP	599
		G/A	SNP	715
		A/C	SNP	744
		CG/C	DELETION	754
		G/A	SNP	768
		A/G	SNP	769
		A/T	SNP	908
[BBH] COPB_TRYBB (sp|Q9NFU6) Coatomer subunit beta OS=T. brucei brucei PE=3 SV=1	GLOS_COPB.1.2	C/CT	INSERTION	418
		GT/G	DELETION	517

		A/T	SNP	1026
		G/A	SNP	1406
		G/A	SNP	2252
		A/C	SNP	2825
XP_001842487.1 multicopper oxidase [Culex quinquefasciatus]	GLOS_CPIPJ_CPIJ000864.1.1	A/T	SNP	56
		T/G	SNP	125
		T/C	SNP	126
		T/A	SNP	742
		T/C	SNP	917
		G/C	SNP	921
		A/G	SNP	922
		T/A	SNP	1092
		A/G	SNP	1095
		C/T	SNP	1141
		T/G	SNP	2002
CRAM_TRYBB (sp|Q03650) Cysteine-rich, acidic integral memb. prot. Tbb GN=CRAM PE=2 SV=1	GLOS_CRAM.1.1	G/A	SNP	24
		A/G	SNP	33
		T/C	SNP	59
		A/G	SNP	60
		A/G	SNP	69
		T/C	SNP	95
		G/A	SNP	141
		G/A	SNP	168
XP_001965963.1 GF11880 [Drosophila ananassae]	GLOS_DANA_GF11880.1.1	CT/C	DELETION	162
		CT/C	DELETION	239
		A/AATAT	INSERTION	609
		C/CT	INSERTION	1021
		A/AT	INSERTION	1154
		T/TTA	INSERTION	1421
		G/T	SNP	1527
		C/T	SNP	1530
		T/TA	INSERTION	1750
		C/G	SNP	1845
		G/A	SNP	1868
		CTTT/C	DELETION	2081
		CCCCA/C	DELETION	2116
		TAAA/T	DELETION	2297
		CA/C	DELETION	2792
		TCAG/T	DELETION	3351

T/A	SNP	3408
C/CT	INSERTION	3445
CAT/C	DELETION	3637
C/CT	INSERTION	3857
A/T	SNP	3864
A/T	SNP	3865
G/GA	INSERTION	4125
CTT/C	DELETION	4405
T/A	SNP	4585
T/A	SNP	4587
TA/T	DELETION	4588
A/ATAT	INSERTION	4811
A/AT	INSERTION	4900
C/T	SNP	5245
C/T	SNP	5332
G/C	SNP	5357
A/AAG	INSERTION	5687
A/G	SNP	5771
A/C	SNP	5890
G/A	SNP	6326
T/A	SNP	6555
G/A	SNP	6567
G/GAA	INSERTION	6567
C/A	SNP	6574
G/A	SNP	6577
G/A	SNP	6580
A/G	SNP	6581
A/C	SNP	6588
G/A	SNP	6590
C/A	SNP	6600
A/C	SNP	6652
A/C	SNP	6916
G/A	SNP	6985
T/C	SNP	7046
A/G	SNP	7129
G/A	SNP	7135
C/A	SNP	7210
T/C	SNP	7267
A/C	SNP	7315

		C/T	SNP	7330
		T/C	SNP	7486
		G/A	SNP	7507
		T/C	SNP	7525
		C/T	SNP	7549
		G/A	SNP	7576
		A/G	SNP	7579
		G/A	SNP	7606
		T/C	SNP	7639
		T/G	SNP	7705
		G/A	SNP	7867
		C/T	SNP	7894
		C/T	SNP	7921
		A/G	SNP	8035
		G/A	SNP	8180
XP_001955025.1 GF16453 [Drosophila ananassae]	GLOS_DANA_GF16453.1.1	A/G	SNP	427
		T/G	SNP	461
XP_001956624.1 GF24494 [Drosophila ananassae]	GLOS_DANA_GF24494.3.12	A/G	SNP	218
		A/G	SNP	262
XP_002001579.1 GI16517 [Drosophila mojavensis]	GLOS_DMOJ_GI16517.2.2	C/A	SNP	1166
XM_002016687.1 Drosophila persimilis GL10349 (DperGL10349), mRNA	GLOS_DPER_GL10349.1.1	G/A	SNP	4
		T/C	SNP	5
		C/G	SNP	17
		T/A	SNP	18
		C/T	SNP	76
		T/A	SNP	79
		G/C	SNP	140
		A/G	SNP	198
		A/G	SNP	199
XP_002023790.1 GL27219 [Drosophila persimilis]	GLOS_DPER_GL27219.1.2	C/A	SNP	75
		C/T	SNP	919
		C/G	SNP	1183
		A/T	SNP	1263
		C/T	SNP	1267
		C/T	SNP	1286
		A/G	SNP	1313
		A/G	SNP	1355
		A/G	SNP	1364
		T/G	SNP	1517

		G/T	SNP	1591
		A/G	SNP	1598
		C/T	SNP	1624
		G/C	SNP	1659
		A/G	SNP	1753
		C/T	SNP	1784
		A/AT	INSERTION	1810
		A/G	SNP	1841
		A/G	SNP	1907
		C/G	SNP	1923
		A/G	SNP	1927
		C/G	SNP	1969
		C/CT	INSERTION	2047
		G/C	SNP	2088
		T/G	SNP	2098
		C/CT	INSERTION	2202
XP_002023790.1 GL27219 [Drosophila persimilis]	GLOS_DPER_GL27219.2.2	G/C	SNP	1679
		C/A	SNP	1722
		A/G	SNP	1723
		G/A	SNP	1724
		C/G	SNP	1725
		T/C	SNP	1726
		T/C	SNP	1785
XP_001360538.1 GA11668 [Drosophila pseudoobscura pseudoobscura]	GLOS_DPSE_GA11668.1.3	T/C	SNP	58
		G/A	SNP	129
		A/C	SNP	2164
		A/C	SNP	2223
		G/GA	INSERTION	2226
		A/G	SNP	2230
		A/T	SNP	2319
		T/C	SNP	2320
		A/C	SNP	2321
		A/G	SNP	2322
		T/TC	INSERTION	2324
XP_001360538.1 GA11668 [Drosophila pseudoobscura pseudoobscura]	GLOS_DPSE_GA11668.2.3	G/C	SNP	40
		G/T	SNP	42
		AC/A	DELETION	111
		CT/C	DELETION	112
		T/A	SNP	122

		C/A	SNP	131
		G/T	SNP	1067
		T/G	SNP	1204
		C/A	SNP	1271
		A/T	SNP	1272
		C/T	SNP	1286
		C/A	SNP	1289
		A/C	SNP	1748
		C/CA	INSERTION	2065
		A/G	SNP	2096
		A/G	SNP	2119
		G/C	SNP	2123
		A/G	SNP	2124
		A/G	SNP	2140
		A/G	SNP	2144
		T/G	SNP	2178
		G/T	SNP	2201
		T/C	SNP	2262
		AT/A	DELETION	2333
		AT/A	DELETION	2591
		T/A	SNP	2593
		T/C	SNP	2646
		T/C	SNP	2647
		A/C	SNP	2649
		T/A	SNP	2651
		A/C	SNP	2652
		A/C	SNP	2653
		A/G	SNP	2654
		T/TC	INSERTION	2656
XP_001357586.2 GA13007 [Drosophila pseudoobscura pseudoobscura]	GLOS_DPSE_GA13007.1.1	A/G	SNP	192
		A/G	SNP	294
		C/G	SNP	299
		G/C	SNP	693
		T/A	SNP	762
		C/A	SNP	890
		C/CAA	INSERTION	890
		G/GA	INSERTION	950
		C/CA	INSERTION	1126
		T/A	SNP	1356

		T/A	SNP	1357
		T/A	SNP	1358
		A/C	SNP	1374
		A/G	SNP	1422
		T/C	SNP	1442
		T/A	SNP	1675
		G/T	SNP	1793
		G/T	SNP	1958
		T/C	SNP	2162
		A/G	SNP	2690
		T/C	SNP	2906
		C/T	SNP	3557
		G/A	SNP	3648
		A/C	SNP	3653
		AAC/A	DELETION	3656
		T/C	SNP	3772
		A/G	SNP	3950
XP_002041441.1 GM10148 [Drosophila sechellia]	GLOS_DSEC_GM10148.1.1	C/T	SNP	242
		G/T	SNP	461
XP_002041100.1 GM15369 [Drosophila sechellia]	GLOS_DSEC_GM15369.1.1	A/T	SNP	3
		A/T	SNP	4
		A/C	SNP	610
		A/C	SNP	612
		A/C	SNP	859
		C/T	SNP	862
XP_002047025.1 GJ12156 [Drosophila virilis]	GLOS_DVIR_GJ12156.1.1	G/T	SNP	242
		G/T	SNP	304
		G/A	SNP	558
		C/T	SNP	957
		C/T	SNP	1430
		C/G	SNP	1818
		G/A	SNP	1922
		G/A	SNP	2475
		G/A	SNP	2841
		G/C	SNP	2913
		C/G	SNP	2953
		G/T	SNP	3126
		G/A	SNP	3156
		T/C	SNP	3864

		G/A	SNP	4146
		G/A	SNP	4535
		T/C	SNP	4560
		T/C	SNP	4728
		C/T	SNP	5324
		G/A	SNP	5727
		G/C	SNP	6044
XP_002046266.1 GJ12604 [Drosophila virilis]	GLOS_DVIR_GJ12604.1.15	T/A	SNP	416
		A/T	SNP	432
		T/A	SNP	433
		T/A	SNP	455
		A/G	SNP	463
		C/T	SNP	608
XP_002046266.1 GJ12604 [Drosophila virilis]	GLOS_DVIR_GJ12604.2.15	C/T	SNP	30
		A/T	SNP	50
		AT/A	DELETION	314
		T/TA/TATA	INSERTION	315
		A/G	SNP	337
		G/A	SNP	339
		C/T	SNP	360
		T/G	SNP	361
		T/A	SNP	797
		A/AT	INSERTION	799
		A/C	SNP	844
XP_002046266.1 GJ12604 [Drosophila virilis]	GLOS_DVIR_GJ12604.6.15	A/C	SNP	286
		T/C	SNP	501
XP_002052322.1 GJ17492 [Drosophila virilis]	GLOS_DVIR_GJ17492.1.1	A/G	SNP	441
		G/A	SNP	688
		G/T	SNP	1008
		C/T	SNP	1342
		G/A	SNP	1464
		T/A	SNP	1466
		T/C	SNP	1468
		A/G	SNP	2429
		G/A	SNP	2462
		C/T	SNP	2529
		T/C	SNP	2530
		T/A	SNP	2549
		A/G	SNP	2559

		G/C	SNP	4200
		C/T	SNP	4220
		C/T	SNP	4298
		T/C	SNP	4738
		A/AC	INSERTION	4748
		G/A	SNP	4803
		C/T	SNP	4907
		A/C	SNP	4992
		G/A	SNP	5096
XP_002050776.1 GJ20013 [Drosophila virilis]	GLOS_DVIR_GJ20013.2.2	A/G	SNP	734
		A/G	SNP	2207
		T/A	SNP	2382
		T/A	SNP	2391
		C/T	SNP	2547
		A/C	SNP	2703
		T/G	SNP	2709
XP_002050345.1 GJ20262 [Drosophila virilis]	GLOS_DVIR_GJ20262.2.2	T/C	SNP	52
		G/GA	INSERTION	108
		T/C	SNP	385
		T/A	SNP	387
XP_002054598.1 GJ22718 [Drosophila virilis]	GLOS_DVIR_GJ22718.8.10	G/C	SNP	298
		A/T	SNP	1376
		G/T	SNP	1440
		A/G	SNP	1450
		G/C	SNP	1495
		G/C	SNP	1577
		A/G	SNP	1648
		A/G	SNP	1649
XP_002070501.1 GK10999 [Drosophila willistoni]	GLOS_DWIL_GK10999.1.1	C/T	SNP	6
		G/A	SNP	34
		G/A	SNP	38
		T/C	SNP	47
		T/C	SNP	57
		A/G	SNP	93
		C/T	SNP	98
		C/T	SNP	102
		C/T	SNP	108
		T/C	SNP	149
		C/T	SNP	159

		A/G	SNP	187
		A/G	SNP	191
XP_002070141.1 GK11188 [Drosophila willistoni]	GLOS_DWIL_GK11188.1.1	G/C	SNP	3126
		C/T	SNP	3128
		C/T	SNP	3174
		C/T	SNP	3416
		A/T	SNP	3419
		G/T	SNP	3527
		A/T	SNP	3541
		T/C	SNP	5196
		C/A	SNP	5234
		A/C	SNP	5947
		C/A	SNP	6004
		A/C	SNP	6005
		T/C	SNP	6100
		T/G	SNP	6104
		A/C	SNP	6156
		C/T	SNP	6331
		G/A	SNP	6509
		G/A	SNP	6563
		G/T	SNP	6579
		C/T	SNP	6632
XP_002067829.1 GK12510 [Drosophila willistoni]	GLOS_DWIL_GK12510.2.4	C/G	SNP	157
		G/A	SNP	213
		GA/G	DELETION	224
		AT/A	DELETION	829
		T/G	SNP	892
XP_002072711.1 GK13541 [Drosophila willistoni]	GLOS_DWIL_GK13541.2.5	T/C	SNP	90
		A/C	SNP	204
		T/A	SNP	256
		A/T	SNP	458
		C/G	SNP	467
		A/C	SNP	473
		C/T	SNP	537
		C/T	SNP	658
		C/T	SNP	730
		C/G	SNP	775
XP_002072711.1 GK13541 [Drosophila willistoni]	GLOS_DWIL_GK13541.3.5	A/AC	INSERTION	1466
		G/C	SNP	1468

		C/T	SNP	1913
XP_002072711.1 GK13541 [Drosophila willistoni]	GLOS_DWIL_GK13541.5.5	G/A	SNP	206
		C/G	SNP	277
		C/G	SNP	334
		C/T	SNP	381
		G/A	SNP	582
		A/T	SNP	583
		T/A	SNP	613
		AAT/A	DELETION	658
		T/A	SNP	660
		T/A	SNP	662
		T/A	SNP	675
		T/C	SNP	740
		G/T	SNP	751
		T/G	SNP	788
		C/T	SNP	1167
		A/G	SNP	1243
		A/G	SNP	1246
		A/G	SNP	1248
		T/A	SNP	1253
		A/C/G	SNP	1269
		A/C	SNP	1326
		C/T	SNP	1328
		G/A	SNP	1330
		A/G	SNP	1349
		A/T	SNP	1398
		C/T	SNP	1402
		C/T	SNP	1421
		C/A	SNP	1460
		A/G	SNP	1490
		A/G	SNP	1499
		G/T	SNP	1605
		G/T	SNP	1652
		C/T	SNP	1660
		C/T	SNP	1661
		C/A	SNP	1672
		C/G	SNP	1728
XP_002063538.1 GK21356 [Drosophila willistoni]	GLOS_DWIL_GK21356.5.5	TA/T	DELETION	436
		T/A	SNP	455

		A/T	SNP	467
		T/A	SNP	509
		T/A	SNP	512
		C/CGG	INSERTION	514
		A/G	SNP	515
		T/C	SNP	611
		G/A	SNP	1123
		C/T	SNP	1129
		A/C	SNP	1130
		T/G	SNP	1131
XP_002071201.1 GK25258 [Drosophila willistoni]	GLOS_DWIL_GK25258.2.2	GA/G	DELETION	81
		C/A	SNP	1396
		G/A	SNP	2291
		A/G	SNP	2294
		A/G	SNP	2305
		G/A	SNP	2306
		A/G	SNP	2308
[BBH] EF1A2_TRYB2 (sp|P86939) Elongation fact 1-alpha 2 OS=Tbb GN=Tb10.70.5670 PE=1 SV=1	GLOS_EF1A2.1.2	G/T	SNP	139
		C/A	SNP	162
		C/G	SNP	165
		C/CT	INSERTION	168
		C/T	SNP	168
		C/T	SNP	169
		T/TG	INSERTION	1754
		A/G	SNP	1757
		A/AAG	INSERTION	1808
[BBH] ERF1_TRYBB (sp|Q9NAX8) Eukaryotic peptide chain release factor subunit 1 OS=Tbb	GLOS_ERF1.1.2	G/GA	INSERTION	366
		G/T	SNP	2384
[BBH] GBLP_TRYBR (sp|P69104) Guanine nucleotide-binding protein subunit beta-like protein Tbb	GLOS_GBLP.3.4	G/A	SNP	16
		A/G	SNP	185
		G/A	SNP	332
[BBH] GSK3B_TRYB2 (sp|Q388M1) Glycogen synthase kinase 3 OS=Tbb GN=GSK3 PE=1 SV=1	GLOS_GSK3B.1.1	C/T	SNP	206
		AAAC/A	DELETION	3068
[BBH] HPRT_TRYBB (sp|Q07010) Hypoxanthine-guanine phosphoribosyltransferase OS=Tbb	GLOS_HPRT.1.1	T/C	SNP	24
		G/A	SNP	242
		A/G	SNP	243
		A/G	SNP	444
		A/T	SNP	787
		G/A	SNP	981

		A/G	SNP	1030
		T/C	SNP	1098
		C/T	SNP	1255
		C/T	SNP	1331
		C/A	SNP	1353
		T/G	SNP	1389
		T/C	SNP	1406
[BBH] HSP83_DROAV (sp|O02192) Heat shock protein 83 OS=D. auraria GN=Hsp83 PE=3 SV=1	GLOS_HSP83.1.1	A/G	SNP	476
		G/A	SNP	695
		G/A	SNP	935
		T/C	SNP	1217
		A/G	SNP	1382
		T/C	SNP	1862
		G/C	SNP	1991
		G/A	SNP	2387
		CT/C	DELETION	2510
		A/T	SNP	2644
		G/T	SNP	2645
		C/A	SNP	2808
		G/A	SNP	3074
		T/C	SNP	3593
		ACAG/A	DELETION	4218
		G/A	SNP	4330
		C/T	SNP	4427
		A/G	SNP	4656
		A/T	SNP	4727
[BBH] IF4A_TRYB2 (sp|Q38F76) Probable eukaryotic initiation factor 4A OS=Tbb	GLOS_IF4A.1.2	A/AT	INSERTION	1323
XP_001601177.1 PREDICTED: hypothetical protein LOC100116763 [Nasonia vitripennis]	GLOS_LOC100116763.1.1	C/T	SNP	20
		T/C	SNP	49
		A/G	SNP	113
		A/G	SNP	156
		C/T	SNP	159
		T/C	SNP	164
		T/C	SNP	191
		A/G	SNP	219
		C/G	SNP	263
		G/A	SNP	289
		A/G	SNP	321
		C/T	SNP	335

		C/T	SNP	398
		A/G	SNP	414
XM_003581200.1 PREDICTED: B. distachyon uncharacterized LOC100843429, mRNA	GLOS_LOC100843429.1.1	T/C	SNP	163
		A/G	SNP	1017
		T/TA	INSERTION	1163
		A/C	SNP	1169
		A/G	SNP	1296
		TA/T	DELETION	1507
		G/A	SNP	2151
		C/T	SNP	2683
		GT/G	DELETION	2688
		A/G	SNP	2999
		T/C	SNP	3017
		C/T	SNP	3102
		C/T	SNP	3166
		T/C	SNP	3441
		T/C	SNP	3466
		T/C	SNP	3503
		T/C	SNP	3564
XP_004521989.1 PREDICTED: uncharacterized protein LOC101450039 [Ceratitis capitata]	GLOS_LOC101450039.1.1	T/C	SNP	49
		A/G	SNP	58
		G/T	SNP	60
		G/A/C	SNP	61
		G/T	SNP	66
		G/A	SNP	101
		G/T	SNP	596
		T/G	SNP	623
		A/T	SNP	714
		A/C	SNP	754
		A/T	SNP	756
		T/A	SNP	796
		C/T	SNP	1151
		T/A	SNP	1492
		A/T	SNP	1494
		A/T	SNP	1500
		A/T	SNP	1530
		A/C	SNP	1531
		G/C	SNP	1640
		C/G	SNP	1642

		C/T	SNP	2277
		TC/T	DELETION	2349
		C/G	SNP	2352
		T/A	SNP	2354
		T/A	SNP	2355
		C/T	SNP	2944
		T/C	SNP	2998
		G/A	SNP	3024
		C/T	SNP	3045
		TA/T	DELETION	3088
		A/G	SNP	3118
		GC/G	DELETION	3393
		G/A	SNP	3401
		T/C	SNP	3431
		A/T	SNP	3660
XP_004521991.1 PREDICTED: uncharacterized protein LOC101450402 [Ceratitis capitata]	GLOS_LOC101450402.10.11	T/C	SNP	606
		T/C	SNP	615
		T/C	SNP	619
		T/C	SNP	638
		T/C	SNP	679
		C/CA	INSERTION	690
		T/C	SNP	869
		C/G	SNP	1605
		A/G	SNP	1609
		A/G	SNP	1610
		T/A	SNP	1612
		A/C	SNP	1631
		G/A	SNP	1652
		G/A	SNP	2643
		G/A	SNP	2680
		AT/A	DELETION	2684
XP_004521991.1 PREDICTED: uncharacterized protein LOC101450402 [Ceratitis capitata]	GLOS_LOC101450402.1.11	A/T	SNP	38
		G/T	SNP	39
		A/C	SNP	41
		T/C	SNP	45
		G/C	SNP	82
		G/T	SNP	84
		T/TA	INSERTION	138
		ATC/A	DELETION	162

		C/G	SNP	166
		T/C	SNP	167
		C/T	SNP	176
		T/C	SNP	347
		A/T	SNP	372
		A/G	SNP	695
XP_004521991.1 PREDICTED: uncharacterized protein LOC101450402 [Ceratitis capitata]	GLOS_LOC101450402.11.11	A/T	SNP	79
		A/G	SNP	1943
		A/G	SNP	1944
		A/G	SNP	1947
XP_004521991.1 PREDICTED: uncharacterized protein LOC101450402 [Ceratitis capitata]	GLOS_LOC101450402.2.11	G/T	SNP	98
		T/C	SNP	99
		A/G	SNP	167
		T/C	SNP	194
		A/C	SNP	228
		G/C	SNP	389
		T/G	SNP	390
		A/T	SNP	393
		G/A	SNP	581
		C/T	SNP	616
		C/G	SNP	843
		A/AT	INSERTION	844
		A/G	SNP	846
		C/G	SNP	847
		A/G	SNP	850
XP_004521991.1 PREDICTED: uncharacterized protein LOC101450402 [Ceratitis capitata]	GLOS_LOC101450402.3.11	A/G	SNP	375
		TA/T	DELETION	597
		A/G	SNP	630
		G/A	SNP	634
		T/C	SNP	637
		A/T	SNP	670
		T/A	SNP	671
		T/A	SNP	672
		C/CA	INSERTION	691
		C/A	SNP	703
		T/A	SNP	713
		T/C	SNP	714
		T/C	SNP	1173
		C/T	SNP	1174

		T/C	SNP	1183
		T/C	SNP	1223
		T/C	SNP	1248
		G/C	SNP	1287
		T/C	SNP	1290
		T/A	SNP	1316
		T/C	SNP	1333
		T/C	SNP	1338
		T/G	SNP	1642
		T/C	SNP	1899
		T/G	SNP	2267
		G/C	SNP	2268
		A/G	SNP	2632
		C/T	SNP	2748
		T/A	SNP	2764
		G/A	SNP	2841
XP_004521991.1 PREDICTED: uncharacterized protein LOC101450402 [Ceratitis capitata]	GLOS_LOC101450402.4.11	C/A	SNP	762
		A/G	SNP	765
		T/A	SNP	768
XP_004521991.1 PREDICTED: uncharacterized protein LOC101450402 [Ceratitis capitata]	GLOS_LOC101450402.5.11	C/A	SNP	274
		C/G	SNP	275
		T/C	SNP	278
		C/T	SNP	501
		T/A	SNP	513
		T/A	SNP	514
		T/C	SNP	923
		T/C	SNP	1188
		T/C	SNP	1195
		T/C	SNP	1199
		T/C	SNP	1200
		C/T	SNP	1207
		T/C	SNP	1235
		C/T	SNP	1258
		T/C	SNP	1289
		G/C	SNP	1299
		T/C	SNP	1330
		T/C	SNP	1573
		A/G	SNP	1619
		A/C	SNP	1665

		G/A	SNP	1680
		A/T	SNP	1690
		T/A	SNP	1692
		A/T	SNP	2526
		G/C	SNP	2529
		G/A	SNP	2658
		A/G	SNP	2695
		T/C	SNP	2722
		G/GT	INSERTION	2771
		A/G	SNP	2788
		T/G	SNP	2823
		T/A	SNP	2824
XP_004521991.1 PREDICTED: uncharacterized protein LOC101450402 [Ceratitis capitata]	GLOS_LOC101450402.6.11	T/TA	INSERTION	159
		T/G	SNP	1427
		G/T	SNP	1436
		AT/A	DELETION	1437
		T/C	SNP	1448
		C/A	SNP	1457
		A/G	SNP	1466
		A/T	SNP	2199
		C/A	SNP	2398
XP_004521991.1 PREDICTED: uncharacterized protein LOC101450402 [Ceratitis capitata]	GLOS_LOC101450402.7.11	A/T	SNP	721
		T/A	SNP	762
		T/A	SNP	1481
		T/C	SNP	1877
		AT/A	DELETION	1934
		T/G	SNP	1937
		C/A	SNP	1938
		T/G	SNP	1941
		T/G	SNP	1942
		T/C	SNP	2194
		G/A	SNP	2218
		A/C	SNP	2704
		T/G	SNP	5969
		T/C	SNP	6073
		T/C	SNP	6122
		C/A	SNP	6134
		T/C	SNP	6152
		T/A	SNP	6161

		G/A	SNP	6203
		G/T	SNP	6272
		C/T	SNP	6312
XP_004521991.1 PREDICTED: uncharacterized protein LOC101450402 [Ceratitis capitata]	GLOS_LOC101450402.8.11	G/C	SNP	40
		G/T	SNP	42
		T/TA	INSERTION	96
		C/A	SNP	115
		G/A	SNP	121
		A/AT	INSERTION	123
XP_004521992.1 PREDICTED: uncharacterized protein LOC101450586 [Ceratitis capitata]	GLOS_LOC101450586.3.9	G/C	SNP	155
		A/T	SNP	156
		C/CAA	INSERTION	690
		G/A	SNP	733
		G/T	SNP	742
		T/G	SNP	769
		G/A	SNP	1371
		T/A	SNP	1373
		A/G	SNP	1377
		T/G	SNP	1380
		C/A	SNP	1853
		G/A/T	SNP	2226
		T/C	SNP	2228
XP_004521992.1 PREDICTED: uncharacterized protein LOC101450586 [Ceratitis capitata]	GLOS_LOC101450586.4.9	A/G	SNP	110
		C/T	SNP	137
		C/T	SNP	174
		T/G	SNP	274
XP_004521992.1 PREDICTED: uncharacterized protein LOC101450586 [Ceratitis capitata]	GLOS_LOC101450586.9.9	T/C	SNP	561
		T/A	SNP	578
		T/G	SNP	699
		A/G	SNP	702
		A/T	SNP	721
		G/A	SNP	722
		A/C	SNP	769
		G/A	SNP	784
		A/C	SNP	842
		T/G	SNP	1035
		G/T	SNP	1037
		T/C	SNP	1139
		C/A	SNP	1142

		G/C	SNP	1144
		T/G	SNP	1145
		G/A	SNP	1802
		G/A	SNP	1832
XP_004520156.1 PREDICTED: transmembrane protease serine 9-like [Ceratitis capitata]	GLOS_LOC101450759.15.17	A/C	SNP	138
		A/G	SNP	1689
		T/G	SNP	1841
		T/A	SNP	1842
		A/G	SNP	1845
		T/A	SNP	1847
XP_004529547.1 PREDICTED: period circadian protein-like [Ceratitis capitata]	GLOS_LOC101453268.1.1	GTA/G/GTATA	DELETION	130
		C/G	SNP	291
		G/A	SNP	342
		C/T	SNP	546
		G/A	SNP	579
		A/G	SNP	1015
		C/T	SNP	1052
		A/AT	INSERTION	1191
		T/C	SNP	1773
		A/G	SNP	2016
		C/T	SNP	2505
		A/G	SNP	2640
		GCTA/G	DELETION	2776
		G/A	SNP	2832
		G/A	SNP	3108
		GT/G	DELETION	3337
XP_004518517.1 PRED: thiomorpholine-carboxylate dehydrogenase-like isoform X1 [C capitata]	GLOS_LOC101453488.1.1	C/CT	INSERTION	102
		C/T	SNP	126
		A/T	SNP	177
		T/TC	INSERTION	183
		A/G	SNP	305
		C/T	SNP	424
		G/A	SNP	476
		G/T	SNP	1111
		T/C	SNP	1177
		T/C	SNP	1346
XP_004525356.1 PREDICTED: acyl-CoA-binding protein homolog isoform X1 [Ceratitis capitata] ref|XP_004525357.1| PREDICTED: acyl-CoA-binding protein homolog isoform X2 [C. capitata]	GLOS_LOC101454212.1.1	A/G	SNP	425
		G/C	SNP	426

		A/C	SNP	427
XP_004522484.1 PREDICTED: uncharacterized protein LOC101454382 [Ceratitis capitata]	GLOS_LOC101454382.1.4	T/C	SNP	168
		G/C	SNP	295
		A/G	SNP	440
		G/A	SNP	442
		G/T	SNP	443
XP_004537801.1 PREDICTED: zinc metalloproteinase nas-4-like [Ceratitis capitata]	GLOS_LOC101454485.4.7	T/C	SNP	5163
		G/A	SNP	5255
		T/G	SNP	5301
		A/G	SNP	5302
		T/A	SNP	5307
		C/A	SNP	5469
XP_004527119.1 PREDICTED: uncharacterized protein LOC101454918 [Ceratitis capitata]	GLOS_LOC101454918.1.1	A/G	SNP	128
		C/CG	INSERTION	212
		T/TAA	INSERTION	213
		G/T	SNP	555
		A/G	SNP	589
		GTCGTCA/G	DELETION	627
		G/A	SNP	648
		C/T	SNP	999
		A/G	SNP	1071
		C/G	SNP	1440
		G/A	SNP	1689
		T/C	SNP	1790
XP_004536445.1 PREDICTED: protein halfway-like [Ceratitis capitata]	GLOS_LOC101455536.1.2	G/C	SNP	2134
		T/A	SNP	2136
XP_004520096.1 PREDICTED: serine protease SP24D-like [Ceratitis capitata]	GLOS_LOC101455604.7.10	T/C	SNP	83
		A/T	SNP	166
		A/G	SNP	170
		C/CT	INSERTION	173
		T/C	SNP	772
		G/GA	INSERTION	777
		TCA/T	DELETION	778
		T/G	SNP	782
		TA/T	DELETION	786
		C/CA	INSERTION	904
		C/G	SNP	908
		T/G	SNP	983
		T/G	SNP	988

		C/T	SNP	1054
		C/G	SNP	1154
		C/A	SNP	1155
		T/C	SNP	1157
		G/GA	INSERTION	1159
		A/G	SNP	1163
XP_004525558.1 PREDICTED: platelet binding protein GspB-like [Ceratitis capitata]	GLOS_LOC101456033.1.1	TA/T	DELETION	223
		C/T	SNP	360
		C/T	SNP	366
		G/C	SNP	387
		A/G	SNP	407
		A/G	SNP	469
		AT/A	DELETION	469
		A/T	SNP	892
		C/A	SNP	911
		A/T	SNP	918
		T/A	SNP	922
		T/G	SNP	928
		T/TCTCG	INSERTION	928
		A/G	SNP	984
		T/C	SNP	1118
		G/C	SNP	1157
		T/C	SNP	1549
		T/C	SNP	1921
		A/G	SNP	2006
		G/A	SNP	2021
		A/G	SNP	2214
		G/T	SNP	2288
		T/C	SNP	2360
		C/T	SNP	2377
		A/AT	INSERTION	2633
		C/T	SNP	2890
		A/T	SNP	2900
		T/A	SNP	2965
		G/A	SNP	3145
		A/G	SNP	3344
		T/C	SNP	3563
		T/A	SNP	3652
		CTGCTGT/C	DELETION	3722

		A/C	SNP	3770
		T/C	SNP	3773
		T/C	SNP	3776
		C/G	SNP	3785
		T/G	SNP	3953
		A/G	SNP	4259
		G/C	SNP	4296
		T/C	SNP	4601
		G/A	SNP	5153
		A/G	SNP	5711
		C/T	SNP	6169
		C/T	SNP	6457
		A/G	SNP	6851
		A/C	SNP	6986
XP_004519837.1 PREDICTED: laccase-2-like [Ceratitis capitata]	GLOS_LOC101457181.3.5	G/A	SNP	273
		T/C	SNP	351
		G/A	SNP	410
		A/T	SNP	437
		G/A	SNP	441
		C/T	SNP	459
		T/A	SNP	518
		T/C	SNP	524
		G/A	SNP	2857
		G/T	SNP	2956
		T/A	SNP	3275
		T/A	SNP	3276
		T/G	SNP	3277
		A/C	SNP	3278
		A/AT	INSERTION	3281
XP_004519837.1 PREDICTED: laccase-2-like [Ceratitis capitata]	GLOS_LOC101457181.5.5	A/G	SNP	685
		A/G	SNP	813
		A/G	SNP	849
		C/T	SNP	948
		T/C	SNP	1397
		C/T	SNP	1690
		A/G	SNP	1880
		A/C	SNP	2186
		A/T	SNP	2694
		A/G	SNP	2862

		T/G	SNP	3017
XP_004525673.1 PREDICTED: gamma-glutamyl hydrolase-like [Ceratitis capitata]	GLOS_LOC101459395.1.1	T/A	SNP	359
		A/ATG	INSERTION	439
		G/A	SNP	527
		C/T	SNP	601
		G/GTT	INSERTION	608
		G/T	SNP	608
		G/T	SNP	634
		GT/G	DELETION	634
		A/T	SNP	880
		A/G	SNP	902
		C/T	SNP	1228
		G/C	SNP	1262
		C/T	SNP	1286
		T/C	SNP	1364
		A/G	SNP	1406
		C/T	SNP	1454
		C/G	SNP	1632
		C/T	SNP	1639
XP_004534903.1 PRED: solute carrier family 2, facilit. glucose transporter memb. 1-like [C capitata]	GLOS_LOC101459427.1.1	T/C	SNP	24
		A/C	SNP	43
		G/C	SNP	50
		G/A	SNP	94
		A/G	SNP	109
		G/A	SNP	112
		C/A	SNP	208
		C/T	SNP	219
		A/C	SNP	223
		A/G	SNP	238
		T/C	SNP	277
		A/G	SNP	310
		G/A	SNP	319
		A/G	SNP	340
		A/T	SNP	345
		C/T	SNP	366
		G/T	SNP	367
		A/G	SNP	373
		C/T	SNP	397
		A/T	SNP	447

		A/G	SNP	633
		T/A	SNP	679
		C/A	SNP	690
		C/T	SNP	918
		G/A	SNP	973
		A/G	SNP	979
		T/C	SNP	984
		A/G	SNP	1107
		G/A	SNP	1174
		C/A	SNP	1188
		C/T	SNP	1192
		A/C	SNP	1296
		G/A	SNP	1324
		CTTG/C	DELETION	1418
		C/T	SNP	1440
XM_004521213.1 PRED: C. capitata eukaryotic translation initiation factor 3 subunit D-1-like, mRNA	GLOS_LOC101459449.2.2	G/A	SNP	1697
		A/G	SNP	1715
		C/G	SNP	1747
		G/A	SNP	1775
		C/G	SNP	1787
		G/A	SNP	1817
		A/G	SNP	1822
		G/A	SNP	1880
		C/G	SNP	1881
		G/A	SNP	1917
		G/A	SNP	1922
		G/T	SNP	1930
		A/G	SNP	1947
		A/G	SNP	1950
		C/T	SNP	1972
		A/G	SNP	1973
		T/C	SNP	2026
		T/C	SNP	2099
		G/A	SNP	2185
		G/A	SNP	2191
		A/C	SNP	2199
		C/T	SNP	2328
		C/T	SNP	2329
		C/A	SNP	2399

XP_004537835.1 PREDICTED: zinc metalloproteinase nas-4-like [Ceratitis capitata]	GLOS_LOC101459622.11.22	AT/A	DELETION	1367
		G/A	SNP	1371
		C/T	SNP	1372
		G/T	SNP	1375
		T/A	SNP	1376
		T/A	SNP	1377
		G/T	SNP	1381
		A/C	SNP	1391
		CG/C	DELETION	1392
		A/T	SNP	1482
		T/A/G	SNP	1557
		G/T	SNP	1562
		A/T	SNP	1579
		C/A	SNP	1934
		A/G	SNP	1935
		A/ATC	INSERTION	1936
		T/A	SNP	1942
		G/C	SNP	1944
		C/G	SNP	1945
		T/C	SNP	2047
		A/G	SNP	2275
		A/G	SNP	2317
		C/T	SNP	2324
		G/T	SNP	2364
		T/C	SNP	3726
		T/A	SNP	3766
XP_004537835.1 PREDICTED: zinc metalloproteinase nas-4-like [Ceratitis capitata]	GLOS_LOC101459622.1.22	A/G	SNP	254
		TG/T	DELETION	731
		G/A	SNP	840
		C/T	SNP	3399
		T/C	SNP	3407
		A/G	SNP	3408
		T/A	SNP	3416
		G/C	SNP	3456
		C/G	SNP	6468
		T/TAG	INSERTION	6549
		T/C	SNP	6552
		GC/G	DELETION	6554
		G/T	SNP	6678

		A/T	SNP	7982
XP_004537835.1 PREDICTED: zinc metalloproteinase nas-4-like [Ceratitis capitata]	GLOS_LOC101459622.12.22	T/C	SNP	1743
		C/T	SNP	1751
XP_004537835.1 PREDICTED: zinc metalloproteinase nas-4-like [Ceratitis capitata]	GLOS_LOC101459622.17.22	A/G	SNP	2226
		A/C	SNP	2276
		G/C	SNP	2321
		A/T	SNP	2383
		G/A	SNP	2412
		C/G	SNP	3544
		C/A	SNP	3585
XP_004537835.1 PREDICTED: zinc metalloproteinase nas-4-like [Ceratitis capitata]	GLOS_LOC101459622.7.22	T/G	SNP	887
		C/T	SNP	891
		C/A	SNP	5432
XP_004524083.1 PREDICTED: putative serine protease K12H4.7-like [Ceratitis capitata]	GLOS_LOC101459895.9.9	T/A	SNP	387
		A/AT	INSERTION	414
		A/C	SNP	1216
		T/A	SNP	1218
		G/T	SNP	1219
		T/A	SNP	1562
		A/G	SNP	1565
		A/G	SNP	1566
		T/A	SNP	1682
		C/T	SNP	1684
XP_004537921.1 PREDICTED: probable RNA helicase armi-like isoform X2 [Ceratitis capitata]	GLOS_LOC101460048.2.4	T/TA	INSERTION	4039
XP_004523332.1 PREDICTED: venom carboxylesterase-6-like [Ceratitis capitata]	GLOS_LOC101460475.2.2	C/T	SNP	62
		A/G	SNP	160
		C/T	SNP	187
		G/A	SNP	206
		C/T	SNP	207
		T/C	SNP	218
		G/A	SNP	281
		C/T	SNP	357
		C/T	SNP	440
		T/C	SNP	445
		T/A	SNP	453
		A/G	SNP	545
		A/G	SNP	548
		C/G	SNP	549
		T/C	SNP	557

		G/C	SNP	615
		T/C	SNP	663
		C/T	SNP	679
		A/G	SNP	775
		T/C	SNP	824
		G/A	SNP	907
		T/C	SNP	1062
		C/T	SNP	1135
		G/A	SNP	1196
		T/C	SNP	1225
		A/G	SNP	1339
		C/A	SNP	1378
		C/T	SNP	1522
		C/T	SNP	1538
		A/G	SNP	1543
		T/G	SNP	1565
		G/A	SNP	1789
		T/C	SNP	1850
		A/C	SNP	1936
		T/C	SNP	2100
		T/A	SNP	2113
		C/G	SNP	2121
		T/C	SNP	2139
XP_004530393.1 PREDICTED: acid trehalase-like protein 1-like [Ceratitis capitata]	GLOS_LOC101461034.1.1	C/T	SNP	115
		T/C	SNP	190
		G/C	SNP	202
		C/A	SNP	241
		T/C	SNP	262
		A/T	SNP	268
		A/C	SNP	284
		C/T	SNP	337
		C/T	SNP	347
		C/G	SNP	359
		A/G	SNP	377
		G/A	SNP	380
		G/A	SNP	430
		A/G	SNP	471
		T/C	SNP	505
		C/A	SNP	507

G/T	SNP	566
A/G	SNP	580
C/T	SNP	621
C/G	SNP	631
C/T	SNP	697
C/T	SNP	730
C/G	SNP	734
T/C	SNP	772
A/T	SNP	805
A/G	SNP	816
G/A	SNP	874
T/C	SNP	877
C/T	SNP	953
T/C	SNP	962
A/G	SNP	988
C/T	SNP	991
C/T	SNP	1036
C/A	SNP	1079
G/C	SNP	1099
G/A	SNP	1119
A/T	SNP	1120
C/T	SNP	1132
C/G	SNP	1218
C/T	SNP	1282
G/A	SNP	1303
C/T	SNP	1312
A/G	SNP	1378
T/A	SNP	1390
A/T	SNP	1422
C/G	SNP	1463
C/T	SNP	1678
A/G	SNP	1720
T/C	SNP	1737
A/G	SNP	1873
G/A	SNP	2002
A/C	SNP	2025
T/C	SNP	2110
A/G	SNP	2212
G/T	SNP	2214

		T/A	SNP	2215
		T/C	SNP	2252
XP_004518012.1 PREDICTED: probable fatty acid-binding protein-like [Ceratitis capitata]	GLOS_LOC101461063.7.16	T/C	SNP	113
		G/T	SNP	118
		T/G	SNP	131
XP_004535853.1 PRED: endoplasmic reticulum metallopeptidase 1-like isoform X1 [C. capitata] ref|XP_004535854.1| PRED: endoplasmic reticulum metallopeptidase 1-like isof. X2 [C. capitata] ref|XP_004535855.1| PRED: endoplasmic reticulum metallopeptidase 1-like isof. X3 [C. capitata] ref|XP_004535856.1| PRED: endoplasmic reticulum metallopeptidase 1-like isoform X4 [C. capitata]	GLOS_LOC101461359.1.1	AT/A	DELETION	230
		T/C	SNP	232
		T/G	SNP	233
		G/A	SNP	458
		G/A	SNP	466
		C/T	SNP	504
		A/G	SNP	620
		G/A	SNP	751
		T/C	SNP	822
		A/C	SNP	848
		T/A	SNP	990
		C/CA	INSERTION	1083
		A/G	SNP	1094
		T/A	SNP	1145
		T/A	SNP	1147
		G/T	SNP	1150
		T/C	SNP	1777
		C/T	SNP	2422
		T/C	SNP	2511
		C/T	SNP	2532
		G/A	SNP	2695
		T/C	SNP	2826
		G/T	SNP	2829
		A/G	SNP	2970
		C/A	SNP	3426
		A/G	SNP	3819
XP_004531052.1 PREDICTED: eukaryotic translation initiation factor 2D-like [Ceratitis capitata]	GLOS_LOC101462178.1.1	C/A	SNP	693
		T/G	SNP	694
		G/A	SNP	695
		G/T	SNP	702
		T/C	SNP	937

		T/C	SNP	1096
		C/T	SNP	1129
		C/T	SNP	1201
		C/T	SNP	1255
		G/C	SNP	1430
		G/A	SNP	1435
		C/G	SNP	1540
		C/T	SNP	1570
		C/T	SNP	1589
		A/G	SNP	1602
		A/G	SNP	1613
		A/G	SNP	1665
		T/A	SNP	1706
		C/A	SNP	1708
		T/C	SNP	1790
		C/T	SNP	1895
		A/G	SNP	1976
		C/T	SNP	2126
		C/T	SNP	2207
		C/T	SNP	2486
		C/A	SNP	2533
		A/T	SNP	2624
		A/G	SNP	2663
		T/C	SNP	2713
		A/C	SNP	2956
		T/A	SNP	2978
		C/T	SNP	2999
		C/A	SNP	3030
		G/A	SNP	3245
		C/A	SNP	3358
		T/A	SNP	3525
XP_004517510.1 PREDICTED: uncharacterized protein LOC101462313 [Ceratitis capitata]	GLOS_LOC101462313.1.1	A/G	SNP	55
		G/A	SNP	71
		G/T	SNP	76
		T/C	SNP	95
		G/A	SNP	106
		A/C	SNP	129
		G/A	SNP	159
		A/G	SNP	177

		C/T	SNP	191
		C/G	SNP	209
		G/A	SNP	237
		G/A	SNP	291
		C/A	SNP	348
		T/C	SNP	359
		G/C	SNP	410
		T/C	SNP	434
		A/G	SNP	435
		T/C	SNP	488
		G/A	SNP	501
		G/A	SNP	507
		T/C	SNP	561
		C/G	SNP	606
		T/C	SNP	614
		G/A	SNP	647
		G/A	SNP	653
		C/A	SNP	671
		T/G	SNP	686
		G/A	SNP	692
		G/C	SNP	764
		C/T	SNP	790
		C/T	SNP	791
		A/C	SNP	861
XM_004533853.1 PREDICTED: C. capitata 60S ribosomal protein L24-like (LOC101463546), mRNA	GLOS_LOC101463546.1.1	T/C	SNP	1136
		A/T	SNP	1138
		A/C	SNP	1139
		T/TC	INSERTION	1143
		T/C	SNP	1144
		A/G	SNP	1307
		A/G	SNP	1312
		A/G	SNP	1355
		A/G	SNP	1422
		A/G	SNP	1462
		C/T	SNP	1805
LRRX1_DICDI (sp|Q54G05) Putative leucine-rich repeat-containing protein DDB_G0290503 OS=Dictyostelium discoideum GN=DDB_G0290503 PE=4 SV=1	GLOS_LRRX1.1.4	G/C	SNP	275
		C/G	SNP	348
		G/GA	INSERTION	375

G/C	SNP	607
C/T	SNP	638
A/G	SNP	645
GAAAACTTA/G	DELETION	650
C/T	SNP	667
G/GAT	INSERTION	729
G/A	SNP	761
C/A	SNP	763
C/A	SNP	764
G/A	SNP	777
G/C	SNP	800
G/A	SNP	806
G/C	SNP	826
C/T	SNP	848
C/A	SNP	869
G/C	SNP	875
G/A	SNP	893
GA/GAA/G	INSERTION	908
T/A	SNP	946
G/A	SNP	956
G/T	SNP	959
A/G	SNP	964
T/G	SNP	994
C/G	SNP	995
A/G	SNP	1009
A/G	SNP	1028
GAGAA/G	DELETION	1031
C/T	SNP	1119
G/A	SNP	1128
A/T	SNP	1149
G/A	SNP	1180
A/C	SNP	1194
T/A	SNP	1204
T/G	SNP	1209
A/C	SNP	1232
T/A	SNP	1245
G/A	SNP	1264
C/T	SNP	1302
T/A	SNP	1303

G/GA	INSERTION	1317
C/T	SNP	1387
C/T	SNP	1390
G/T	SNP	1397
T/G	SNP	1419
T/A	SNP	1429
A/T	SNP	1455
A/T	SNP	1471
T/C	SNP	1497
A/C	SNP	1564
C/G	SNP	1572
G/C	SNP	1599
T/A	SNP	1626
A/G	SNP	1672
G/GA	INSERTION	1681
C/T	SNP	1710
G/A	SNP	1729
C/T	SNP	1732
T/G	SNP	1733
T/C	SNP	1753
A/C	SNP	1762
T/C	SNP	1767
A/C	SNP	1775
C/A	SNP	1786
G/C	SNP	1794
C/T	SNP	1802
A/G	SNP	1818
T/C	SNP	1871
C/T	SNP	1880
G/A	SNP	1883
C/T	SNP	1901
T/C	SNP	1925
G/T	SNP	1927
C/G	SNP	1932
C/G	SNP	1946
C/T	SNP	1966
AT/ATT/A	INSERTION	1974
G/A	SNP	2025
G/A	SNP	2080

		A/G	SNP	2083
		T/C	SNP	2099
		C/A	SNP	2122
		G/T	SNP	2123
		T/C	SNP	2129
		G/A	SNP	2130
		A/G	SNP	2151
		C/T	SNP	2161
		G/A	SNP	2326
NDUS2_TRYBB (sp|P21301) NADH-ubiquinone oxidoreductase 49 kDa subunit homolog OS=Tbb	GLOS_NDUS2.1.1	G/T	SNP	77
[BBH] NOG1_TRYBB (sp|Q9U6A9) Nucleolar GTP-binding protein 1 OS=Tbb	GLOS_NOG1.1.2	A/T	SNP	1553
[BBH] P320_TRYBB (sp|P21787) Microtubule-associated protein P320 (Fragment) OS=Tbb	GLOS_P320.1.1	C/G	SNP	55
		T/C	SNP	69
		T/C	SNP	103
		G/A	SNP	111
		C/G	SNP	169
		T/C	SNP	183
		T/C	SNP	217
YP_007639959.1 type IV pilus assembly FimV-related transmemb. prot. [Psychromonas sp. CNPT3]	GLOS_PCNPT3_05895.1.1	C/T	SNP	290
ref|WP_015465000.1| type IV pilus assembly FimV-related transmembrane prot. [Psychromon.sp]				
		T/G	SNP	471
		T/C	SNP	476
		A/G	SNP	483
		C/T	SNP	490
		T/C	SNP	493
[BBH] PFR1_TRYBB (sp|P22225) 69 kDa paraflagellar rod protein OS=Tbb	GLOS_PFR1.1.1	C/T	SNP	1446
[BBH] PGKE_TRYBB (sp|P08893) Phosphoglycerate kinase, cytosolic OS=Tbb	GLOS_PGKE.1.1	T/A	SNP	902
		C/G	SNP	906
		T/A	SNP	908
		A/G	SNP	1065
XP_002782358.1 conserved hypothetical protein [Perkinsus marinus ATCC 50983]	GLOS_PMAR_PMAR029216.2.2	T/C	SNP	429
		A/G	SNP	485
XM_004997989.1 Salpingoeca sp. ATCC 50818 hypothetical protein mRNA, complete cds	GLOS_PTSG_02057.1.1	A/T	SNP	219
		CT/C	DELETION	840
		A/G	SNP	967
		T/TC	INSERTION	1019
		A/AC	INSERTION	1309
		C/T	SNP	1659
		CT/C	DELETION	1678

		T/C	SNP	1781
		C/CA	INSERTION	1792
[BBH] RIR2_TRYBB (sp|O15910) Ribonucleoside-diphosphate reductase small chain OS=Tbb	GLOS_RIR2.1.2	T/A	SNP	637
		T/C	SNP	694
		G/A	SNP	1555
		G/A	SNP	1698
		C/T	SNP	1699
[BBH] RL27A_TRYBB (sp|O15883) 60S ribosomal protein L27a OS=Tbb	GLOS_RL27A.1.6	G/A	SNP	576
RL30_TRYBB (sp|P49153) 60S ribosomal protein L30 OS=Tbb GN=RPL30 PE=3 SV=1	GLOS_RL30.1.1	C/A	SNP	405
		G/A	SNP	417
RL402_TRYCR (sp|P0CH27) Ubiquitin-60S ribosomal protein L40 OS=T. cruzi PE=2 SV=1	GLOS_RL402.1.2	T/C	SNP	65
		G/A	SNP	461
RL40_TRYBB (sp|P21899) Ubiquitin-60S ribosomal protein L40 OS=Tbb PE=1 SV=2	GLOS_RL40.3.8	T/A	SNP	118
		C/T	SNP	161
		A/G	SNP	302
RL7_DROME (sp|P32100) 60S ribosomal protein L7 OS=D. melanogaster GN=RpL7 PE=1 SV=2	GLOS_RL7.1.5	C/A	SNP	381
		G/C	SNP	383
		A/G	SNP	384
		C/A	SNP	387
		A/T	SNP	754
		AT/A	DELETION	754
		T/A	SNP	756
		G/C	SNP	834
NR_046235.1 Homo sapiens RNA, 45S pre-ribosomal 5 (RNA45S5), ribosomal RNA	GLOS_RNA45S5.1.2	A/C	SNP	306
		T/C	SNP	952
		T/A	SNP	1059
		C/T	SNP	1102
		A/G	SNP	1142
		C/T	SNP	1544
		G/A	SNP	2275
		C/A	SNP	2533
		G/A	SNP	2579
		T/C	SNP	2927
		C/CT	INSERTION	2969
		A/T	SNP	3188
		A/T	SNP	3524
		A/T	SNP	3557
		G/A	SNP	3627
		A/G	SNP	3733

		T/A	SNP	4198
[BBH] RPB1B_TRYBB (sp|P17545) DNA-directed RNA polymerase II subunit RPB1-B OS=Tbb	GLOS_RPB1B.1.1	T/C	SNP	1084
		G/T	SNP	1653
		C/T	SNP	2029
		C/T	SNP	3073
NP_649070.1 ribosomal protein L26, isoform A [D. melanogaster] ref|NP_001262025.1| ribosomal protein L26, isoform B [D. melanogaster] ref|XP_001958190.1| GF23649 [D. ananassae] ref|XP_002042631.1| GM15002 [D. sechellia] ref|XP_002095681.1| GE19578 [D. yakuba] ref|XP_002095689.1| GE19574 [D. yakuba] ref|XP_002085421.1| GD14780 [D. simulans]	GLOS_RPL26.1.3	A/C	SNP	92
		T/A	SNP	276
		A/T	SNP	277
		T/C	SNP	280
		G/C	SNP	282
		T/G	SNP	325
		T/C	SNP	333
		T/G	SNP	383
		T/A	SNP	501
		A/T	SNP	711
		T/G	SNP	712
		G/GT	INSERTION	713
		G/T	SNP	713
		T/C	SNP	715
		C/A	SNP	716
		C/A	SNP	717
[BBH] RS12_TRYBB (sp|Q03253) 40S ribosomal protein S12 OS=Tbb GN=RPS12 PE=2 SV=2	GLOS_RS12.1.4	A/G	SNP	400
XM_002581801.1 S. mansoni conserved hypothetical protein (Smp_112590) mRNA, partial cds	GLOS_SMP_112590.1.1	C/G	SNP	92
		C/G	SNP	666
		C/T	SNP	729
		C/T	SNP	730
		C/A	SNP	866
		G/T	SNP	906
		G/A	SNP	908
		T/C	SNP	939
		T/C	SNP	2782
		C/T	SNP	2784
		A/T	SNP	2813
		T/G	SNP	2817
SODC_CHYAM (sp|Q07182) Superoxide dismutase [Cu-Zn] OS=C. amoena GN=Sod PE=3 SV=2	GLOS_SODC.1.5	C/T	SNP	539
XM_798344.1 Tbb strain 927/4 GUTat10.1 hypothetical protein (Tb09.160.0430) partial mRNA	GLOS_TB09.160.0430.1.1	G/A	SNP	886

		T/C	SNP	2055
		G/A	SNP	2487
XM_798407.1 Tbb GUTat10.1 60S ribosomal protein L35 (Tb09.160.0710) partial mRNA	GLOS_TB09.160.0710.1.1	A/C	SNP	139
		C/G	SNP	146
		C/G	SNP	183
		T/A	SNP	573
		T/A	SNP	574
XM_798420.1 T. b. brucei strain 927/4 GUTat10.1 kynureninase (Tb09.160.0810) partial mRNA	GLOS_TB09.160.0810.1.1	GA/G	DELETION	632
		C/CA	INSERTION	816
		CT/C	DELETION	948
		T/A	SNP	3237
XM_798434.1 T. b. brucei strain 927/4 GUTat10.1 protein kinase (Tb09.160.0930) partial mRNA	GLOS_TB09.160.0930.1.1	C/CA	INSERTION	569
		CA/C	DELETION	712
XM_798466.1 T. b. strain 927/4 GUTat10.1 hypothetical protein (Tb09.160.1160) partial mRNA	GLOS_TB09.160.1160.1.1	A/G	SNP	556
		G/A	SNP	568
XP_803563.1 mitotubule-associated protein Gb4 [T. brucei brucei strain 927/4 GUTat10.1]	GLOS_TB09.160.1200.1.1	G/A	SNP	430
		T/C	SNP	647
		C/A	SNP	706
XM_798500.1 Tbb strain 927/4 GUTat10.1 hypothetical protein (Tb09.160.1520) partial mRNA	GLOS_TB09.160.1520.1.1	A/C	SNP	21
		G/A	SNP	102
		C/T	SNP	146
		G/A	SNP	192
		C/A	SNP	198
		T/C	SNP	364
		A/C	SNP	535
		A/G	SNP	585
		T/C	SNP	633
		C/G	SNP	761
		C/T	SNP	817
		C/T	SNP	827
		C/T	SNP	992
		G/T	SNP	1035
		C/G	SNP	1288
		G/A	SNP	1505
		C/G	SNP	1868
		C/A	SNP	2031
		T/C	SNP	2047
		A/G	SNP	2135
XP_803698.1 ribosomal protein S7 [Trypanosoma brucei brucei strain 927/4 GUTat10.1]	GLOS_TB09.160.2550.1.1	C/T	SNP	10

		A/T	SNP	11
		C/T	SNP	15
		A/T	SNP	16
XP_803725.1 fatty acyl CoA synthetase 3 [Trypanosoma brucei brucei strain 927/4 GUTat10.1]	GLOS_TB09.160.2810.1.2	G/C	SNP	1072
		C/A	SNP	1074
		C/G	SNP	1075
		C/T	SNP	1081
XP_803725.1 fatty acyl CoA synthetase 3 [Trypanosoma brucei brucei strain 927/4 GUTat10.1]	GLOS_TB09.160.2810.2.2	G/A	SNP	958
		G/T	SNP	1515
		A/G	SNP	1517
		A/G	SNP	1695
		C/T	SNP	1696
XM_798715.1 Tbb GUTat10.1 cAMP-specific phosphodiesterase (Tb09.160.3590) partial mRNA	GLOS_TB09.160.3590.1.1	GA/G	DELETION	1757
		T/C	SNP	2146
		G/T	SNP	2149
		A/G	SNP	2150
		G/C	SNP	2647
		G/T	SNP	2648
		A/G	SNP	2817
		C/T	SNP	3184
		C/G	SNP	3259
		C/T	SNP	3853
		T/C	SNP	3858
		T/A	SNP	3859
XM_798741.1 Tbb strain 927/4 GUTat10.1 hypothetical protein (Tb09.160.3780) partial mRNA	GLOS_TB09.160.3780.1.1	G/GT	INSERTION	1701
XM_798745.1 Tbb strain 927/4 GUTat10.1 hypothetical protein (Tb09.160.3820) partial mRNA	GLOS_TB09.160.3820.1.1	G/GA	INSERTION	2020
XP_826963.1 inositol/phosphatidylinositol phosphatase [Trypanosoma brucei TREU927]	GLOS_TB09.160.4180.1.1	GTT/G	DELETION	2766
XM_821871.1 Tbb strain 927/4 GUTat10.1 60S acidic ribosomalprotein (Tb09.160.4200) partial mRNA	GLOS_TB09.160.4200.1.1	G/GA	INSERTION	580
XP_826974.1 glutamate dehydrogenase [Trypanosoma brucei brucei strain 927/4 GUTat10.1]	GLOS_TB09.160.4310.1.1	C/CT	INSERTION	4508
XP_826981.1 succinate dehydrogenase [Trypanosoma brucei brucei strain 927/4 GUTat10.1]	GLOS_TB09.160.4380.1.1	C/A	SNP	16
		T/G	SNP	17
		C/CT	INSERTION	916
		GT/G	DELETION	1008
XM_821897.1 Tbb strain 927/4 GUTat10.1 hypothetical protein (Tb09.160.4460) partial mRNA	GLOS_TB09.160.4460.1.1	T/C	SNP	798
		C/G	SNP	1384
XM_821905.1 Tbbi strain 927/4 GUTat10.1 arginine kinase (Tb09.160.4560) partial mRNA	GLOS_TB09.160.4560.1.1	T/C	SNP	176
		C/T	SNP	225
		C/T	SNP	416
		T/C	SNP	446

		A/G	SNP	900
		T/C	SNP	914
XM_821907.1 Tbb strain 927/4 GUTat10.1 hypothetical protein (Tb09.160.4580) partial mRNA	GLOS_TB09.160.4580.1.1	A/AT	INSERTION	1675
		A/G	SNP	1696
XM_821909.1 Tbb strain 927/4 GUTat10.1 ABC transporter (Tb09.160.4600) partial mRNA	GLOS_TB09.160.4600.1.1	C/CA	INSERTION	126
		C/CT	INSERTION	3111
XM_821961.1 Tbb strain 927/4 GUTat10.1 hypothetical protein (Tb09.160.5060) partial mRNA	GLOS_TB09.160.5060.1.1	T/TA	INSERTION	178
XM_822007.1 Tbb strain 927/4 GUTat10.1 adenosine transporter (Tb09.160.5480) partial mRNA	GLOS_TB09.160.5480.1.1	A/AT	INSERTION	160
		T/TA	INSERTION	1387
XM_822018.1 Tbb strain 927/4 GUTat10.1 60S ribosomal protein L11 (Tb09.160.5590) partial mRNA	GLOS_TB09.160.5590.1.1	T/C	SNP	698
		T/G	SNP	746
XM_822050.1 Tbb strain 927/4 GUTat10.1 hypothetical protein (Tb09.211.0040) partial mRNA	GLOS_TB09.211.0040.1.1	T/TA	INSERTION	873
XP_827151.1 nascent polypeptide associated complex subunit [Trypanosoma brucei] ref|XP_827152.1| nascent polypeptide associated complex subunit [Trypanosoma brucei]	GLOS_TB09.211.0120.1.2	G/C	SNP	428
XM_822060.1 Tbbstrain 927/4 GUTat10.1 chaperone protein DnaJ (Tb09.211.0140) partial mRNA	GLOS_TB09.211.0140.1.1	C/CA	INSERTION	258
XM_822080.1 Tbb strain 927/4 GUTat10.1 hypothetical protein (Tb09.211.0320) partial mRNA	GLOS_TB09.211.0320.1.1	C/CT	INSERTION	2319
XM_822082.1 Tbb strain 927/4 GUTat10.1 60S ribosomal protein L10 (Tb09.211.0340) partial mRNA	GLOS_TB09.211.0340.1.1	T/C	SNP	569
XM_822105.1 Tbb strain 927/4 GUTat10.1 hypothetical protein (Tb09.211.0560) partial mRNA	GLOS_TB09.211.0560.1.1	G/A	SNP	1904
		G/GA	INSERTION	2277
		G/GA	INSERTION	2784
		T/TA	INSERTION	3170
		C/CT	INSERTION	3435
		G/GT	INSERTION	3662
		CAT/C	DELETION	3759
XM_822151.1 Tbb phosphatidylcholine:ceramide cholinephosphotransferase 2, partial mRNA	GLOS_TB09.211.1000.1.1	T/C	SNP	301
		G/T	SNP	683
		A/G	SNP	687
		A/G	SNP	769
		G/T	SNP	901
		G/GT	INSERTION	917
		A/G	SNP	1695
		C/T	SNP	1909
		A/G	SNP	1910
		C/T	SNP	1940
		G/A	SNP	1956
		A/G	SNP	2154
		C/G/T	SNP	2180
		C/T	SNP	2182
		A/G	SNP	2192

		T/G	SNP	2296
		C/T	SNP	2306
XM_822158.1 Tbb strain 927/4 GUTat10.1 hypothetical protein (Tb09.211.1070) partial mRNA	GLOS_TB09.211.1070.1.1	G/T	SNP	4102
		G/T	SNP	4112
		G/GT	INSERTION	4140
XM_822176.1 Tbb strain 927/4 GUTat10.1 hypothetical protein (Tb09.211.1240) partial mRNA	GLOS_TB09.211.1240.1.1	C/CA	INSERTION	767
XM_822222.1 Tbb strain 927/4 GUTat10.1 hypothetical protein (Tb09.211.1690) partial mRNA	GLOS_TB09.211.1690.1.1	C/A	SNP	324
XM_822228.1 Tbb mitochondrial carrier protein, partial mRNA. nuclear gene for mitochondrial product	GLOS_TB09.211.1750.1.1	A/T	SNP	41
		A/T	SNP	42
		CA/C	DELETION	117
		G/GT	INSERTION	1441
		C/CT	INSERTION	1627
XM_822265.1 Tbb strain 927/4 GUTat10.1 poly(A)-binding protein 1 (Tb09.211.2150) partial mRNA	GLOS_TB09.211.2150.1.1	T/A	SNP	40
		C/CT	INSERTION	2338
		GA/G	DELETION	3536
		GA/G	DELETION	4109
		C/CA	INSERTION	4255
		C/CA	INSERTION	4500
XM_822266.1 Tbb strain 927/4 GUTat10.1 hypothetical protein (Tb09.211.2160) partial mRNA	GLOS_TB09.211.2160.1.1	GT/G	DELETION	1684
XM_822308.1 Tbb GUTat10.1 t-complex protein 1 subunit eta (Tb09.211.2570) partial mRNA	GLOS_TB09.211.2570.1.1	C/CA	INSERTION	417
XM_822323.1 Tbb strain 927/4 GUTat10.1 hypothetical protein (Tb09.211.2700) partial mRNA	GLOS_TB09.211.2700.1.1	TA/T	DELETION	947
XP_827420.1 Gim5B protein [Trypanosoma brucei brucei strain 927/4 GUTat10.1]	GLOS_TB09.211.2740.1.1	G/A	SNP	289
		T/C	SNP	352
		C/CT	INSERTION	1340
		G/A	SNP	1434
		G/GA	INSERTION	1434
		CT/C	DELETION	1636
		A/AT	INSERTION	1678
		A/T	SNP	1678
XM_822340.1 Tbb strain 927/4 GUTat10.1 hypothetical protein (Tb09.211.2880) partial mRNA	GLOS_TB09.211.2880.1.1	A/AT	INSERTION	3312
		C/CT	INSERTION	3955
XM_822342.1 Tbb strain 927/4 GUTat10.1 hypothetical protein (Tb09.211.2900) partial mRNA	GLOS_TB09.211.2900.1.1	G/A	SNP	334
		G/A	SNP	654
		T/C	SNP	797
		A/G	SNP	1369
		T/C	SNP	1850
		T/C	SNP	1896
		T/C	SNP	1988
XM_822379.1 Tbb strain 927/4 GUTat10.1 60S ribosomal protein L31 (Tb09.211.3280) partial mRNA	GLOS_TB09.211.3280.1.1	G/T	SNP	759

		G/T	SNP	761
XM_822384.1 Tbb strain 927/4 GUTat10.1 cystathione gamma lyase (Tb09.211.3330) partial mRNA	GLOS_TB09.211.3330.1.1	CT/C	DELETION	415
XP_827493.1 hypothetical protein [Trypanosoma brucei brucei strain 927/4 GUTat10.1]	GLOS_TB09.211.3500.1.1	GA/G	DELETION	132
XM_822401.1 Tbb GUTat10.1 ATP-dependent DEAD/H RNA helicase (Tb09.211.3510) partial mRNA	GLOS_TB09.211.3510.1.1	GA/G	DELETION	2491
		C/CAT	INSERTION	3035
		C/G	SNP	3132
		TA/T	DELETION	3190
		G/GA	INSERTION	3443
		G/GT	INSERTION	3629
		A/G	SNP	3707
		C/CT	INSERTION	3743
		G/A	SNP	3883
		C/CT	INSERTION	3977
		G/GTT	INSERTION	4101
		G/T	SNP	4156
		G/GA	INSERTION	4574
XM_822405.1 Tbb strain 927/4 GUTat10.1 glycerol kinase glycosomal (Tb09.211.3550) partial mRNA	GLOS_TB09.211.3550.1.1	C/A	SNP	131
		C/A	SNP	1908
		A/G	SNP	2107
		A/AT	INSERTION	2195
		G/T	SNP	2255
XM_822409.1 Tbb GUTat10.1 ubiquitin-activating enzyme E1 (Tb09.211.3610) partial mRNA	GLOS_TB09.211.3610.1.1	C/CT	INSERTION	378
XM_822445.1 Tbb strain 927/4 GUTat10.1 hypothetical protein (Tb09.211.3960) partial mRNA	GLOS_TB09.211.3960.1.1	CA/C	DELETION	460
		ATTG/A	DELETION	881
		G/GA	INSERTION	1035
		GA/G	DELETION	1462
XM_822456.1 Tbb strain 927/4 GUTat10.1 hypothetical protein (Tb09.211.4070) partial mRNA	GLOS_TB09.211.4070.1.1	T/TA	INSERTION	235
XM_822482.1 Tbb strain 927/4 GUTat10.1 hypothetical protein (Tb09.211.4360) partial mRNA	GLOS_TB09.211.4360.1.1	T/A	SNP	591
		T/C	SNP	592
		C/T	SNP	1132
		A/G	SNP	1743
		C/T	SNP	1982
		C/T	SNP	2144
		G/C	SNP	3018
XP_827586.1 ADP-ribosylation factor [Trypanosoma brucei brucei strain 927/4 GUTat10.1] ref|XP_827587.1| ADP-ribosylation factor [Tbb] ref|XP_827588.1| ADP-ribosylation factor [Tbb]	GLOS_TB09.211.4470.1.1	C/CTG	INSERTION	117
		A/G	SNP	336
		G/A	SNP	666
		G/A	SNP	759

		A/G	SNP	810
		C/T	SNP	851
		A/G	SNP	852
		G/A	SNP	873
XM_822500.1 Tbb kinetoplastid membr. prot. KMP-11 partial mRNA. nuclear gene for plastid product	GLOS_TB09.211.4513.1.1	C/T	SNP	218
		GA/G	DELETION	228
		C/T	SNP	240
		T/C	SNP	343
		A/G	SNP	351
		T/C	SNP	625
XM_822504.1 Tbb strain 927/4 GUTat10.1 60S ribosomal protein L12 (Tb09.211.4550) partial mRNA	GLOS_TB09.211.4550.1.1	A/G	SNP	257
		C/T	SNP	290
		A/C	SNP	356
		C/T	SNP	404
		A/G	SNP	413
		C/T	SNP	425
		C/T	SNP	446
		C/T	SNP	485
		G/T	SNP	539
		G/A	SNP	542
		C/G	SNP	548
		T/G	SNP	549
XP_827610.1 reiske iron-sulfur protein mitochondrial precursor [Trypanosoma brucei]	GLOS_TB09.211.4700.1.1	CATAT/CATATAT/C	INSERTION	619
		T/TTCA	INSERTION	906
		TC/T	DELETION	965
		G/GA	INSERTION	1201
XM_822523.1 Tbb strain 927/4 GUTat10.1 metacaspase 5 (Tb09.211.4760) partial mRNA	GLOS_TB09.211.4760.1.1	T/TC	INSERTION	283
		A/G	SNP	952
		G/A	SNP	976
XP_827634.1 hypothetical protein [Trypanosoma brucei brucei strain 927/4 GUTat10.1]	GLOS_TB09.211.4940.1.1	T/C	SNP	61
		G/A	SNP	199
		A/G	SNP	262
		T/G	SNP	440
		G/C	SNP	512
		A/T	SNP	690
		C/T	SNP	749
		C/T	SNP	773
		G/A	SNP	789
		T/C	SNP	821

		A/G	SNP	823
		T/G	SNP	953
		C/G	SNP	1007
		G/C	SNP	1054
		C/T	SNP	1109
		A/G	SNP	1146
		T/G	SNP	1182
		T/C	SNP	1296
		T/C	SNP	1299
		T/C	SNP	1402
		A/G	SNP	1463
		A/G	SNP	1541
		G/A	SNP	1564
		G/A	SNP	1641
		G/A	SNP	1709
		G/A	SNP	1757
		G/A	SNP	1918
		C/T	SNP	2128
		G/A	SNP	2263
		C/T	SNP	2403
		C/T	SNP	2429
XM_822625.1 Tbb strain 927/4 GUTat10.1 hypothetical protein (Tb09.244.2170) partial mRNA	GLOS_TB09.244.2170.1.1	T/C	SNP	93
		C/CCA	INSERTION	207
		C/A	SNP	273
		T/G	SNP	477
		T/TAA	INSERTION	593
		C/A	SNP	600
		T/C	SNP	680
		T/C	SNP	698
		C/T	SNP	734
		A/G	SNP	735
		T/C	SNP	760
		A/G	SNP	836
		C/T	SNP	859
		C/T	SNP	895
		C/T	SNP	913
		C/T	SNP	1161
		C/T	SNP	1233
		A/G	SNP	1271

A/T	SNP	1283
C/T	SNP	1288
C/T	SNP	1352
C/T	SNP	1860
T/C	SNP	1889
A/G	SNP	1916
A/G	SNP	2043
T/A	SNP	2062
T/G	SNP	2170
G/A	SNP	2221
T/C	SNP	2224
T/C	SNP	2273
C/T	SNP	2330
C/A	SNP	2368
A/T	SNP	2369
C/T	SNP	2371
A/G	SNP	2393
A/C	SNP	2437
A/C	SNP	2452
C/A	SNP	2455
C/T	SNP	2470
G/A	SNP	2475
G/A	SNP	2484
C/G/T	SNP	2508
C/A	SNP	2510
TC/T	DELETION	2538
C/T	SNP	2545
T/C	SNP	2586
T/C	SNP	2623
AT/A	DELETION	2783
T/C	SNP	2890
G/A	SNP	2891
T/C	SNP	2909
A/C	SNP	2940
A/C	SNP	2945
G/A	SNP	3008
G/A	SNP	3011
C/T	SNP	3073
C/G	SNP	3120

		C/T	SNP	3121
		G/A	SNP	3126
XM_822590.1 Tbb GUTat10.1 calcium motive p-type ATPase (Tb09.244.2570) partial mRNA	GLOS_TB09.244.2570.1.1	G/T	SNP	1232
XP_827681.1 60S ribosomal protein L32 [Trypanosoma brucei brucei strain 927/4 GUTat10.1]	GLOS_TB09.244.2590.1.1	C/T	SNP	74
		AAC/A	DELETION	447
		AAT/A	DELETION	511
XM_822584.1 Tbb strain 927/4 GUTat10.1 40S ribosomal protein S6 (Tb09.244.2630) partial mRNA	GLOS_TB09.244.2630.1.1	C/CT	INSERTION	304
		GAA/G	DELETION	419
		T/A	SNP	1280
		G/A	SNP	1281
XM_822578.1 Tbb strain 927/4 GUTat10.1 hypothetical protein (Tb09.244.2660) partial mRNA	GLOS_TB09.244.2660.1.1	G/A	SNP	251
		C/A	SNP	4293
XM_822570.1 Tbb strain 927/4 GUTat10.1 ribosomal protein L15 (Tb09.244.2720) partial mRNA	GLOS_TB09.244.2720.1.1	A/C	SNP	407
		G/A	SNP	413
		T/C	SNP	451
		A/C	SNP	503
		C/T	SNP	533
		C/G	SNP	569
		C/T	SNP	571
		A/G	SNP	592
		T/C	SNP	593
		T/C	SNP	632
		C/G	SNP	680
		T/C	SNP	710
		T/C	SNP	734
		A/G	SNP	740
		C/T	SNP	785
		C/G	SNP	812
		G/A	SNP	821
		C/T	SNP	830
		G/A	SNP	848
		G/A	SNP	899
XM_822571.1 Tbb strain 927/4 GUTat10.1 ribosomal protein L36 (Tb09.244.2725) partial mRNA	GLOS_TB09.244.2725.1.1	G/T	SNP	493
		C/A	SNP	496
XM_822569.1 Tbb strain 927/4 GUTat10.1 60S ribosomal protein L5 (Tb09.244.2730) partial mRNA	GLOS_TB09.244.2730.1.1	C/T	SNP	35
		A/G	SNP	36
		A/G	SNP	65
		T/C	SNP	996
XM_798607.1 Tbb strain 927/4 GUTat10.1 hypothetical protein (Tb09.v1.0150) partial mRNA	GLOS_TB09.V1.0150.1.1	T/C	SNP	3107

		A/G	SNP	3289
		T/G	SNP	3483
XM_821914.1 Tbb strain 927/4 GUTat10.1 hypothetical protein (Tb09.v2.0030) partial mRNA	GLOS_TB09.V2.0030.1.1	C/T	SNP	121
		C/A	SNP	126
		A/G	SNP	129
		T/C	SNP	143
XP_827840.1 diphosphomevalonate decarboxylase [T. brucei brucei strain 927/4 GUTat10.1]	GLOS_TB10.05.0010.1.1	T/G	SNP	257
		G/T	SNP	328
		A/G	SNP	623
		G/A	SNP	633
		G/A	SNP	803
		G/A	SNP	1130
		T/C	SNP	1440
XM_822754.1 T. brucei brucei strain 927/4 GUTat10.1 glucosidase (Tb10.05.0080) partial mRNA	GLOS_TB10.05.0080.1.1	T/C	SNP	840
		C/T	SNP	1433
		C/T	SNP	1817
		T/C	SNP	2730
XM_822757.1 Tbb GUTat10.1 serine/threonine prot. phosphatase type 5 (Tb10.05.0110) partial mRNA	GLOS_TB10.05.0110.1.1	T/C	SNP	443
		A/G	SNP	2200
		CA/C	DELETION	2513
XM_822741.1 Tbb strain 927/4 GUTat10.1 60S ribosomal protein L10a (Tb10.05.0220) partial mRNA	GLOS_TB10.05.0220.1.1	GA/G	DELETION	176
		T/G	SNP	183
		C/A	SNP	447
		T/C	SNP	546
		G/A	SNP	831
		A/G	SNP	849
		C/A	SNP	1059
XM_817190.1 Tbb GUTat10.1 proteasome subunit alpha 5 (Tb10.100.0120) partial mRNA	GLOS_TB10.100.0120.1.1	A/G	SNP	318
XP_822287.1 60S ribosomal protein L32 [Trypanosoma brucei brucei strain 927/4 GUTat10.1]	GLOS_TB10.100.0155.1.1	G/T	SNP	503
		G/T	SNP	504
		A/C	SNP	505
XP_823384.1 hypothetical protein [Trypanosoma brucei brucei strain 927/4 GUTat10.1]	GLOS_TB10.26.0100.1.1	T/TA	INSERTION	94
		C/CT	INSERTION	831
		C/CA	INSERTION	934
XM_818290.1 Tbb strain 927/4 GUTat10.1 pumilio RNA-binding protein (Tb10.26.0140) partial mRNA	GLOS_TB10.26.0140.1.1	GAA/G	DELETION	131
		AAAT/A	DELETION	230
		A/T	SNP	565
		T/TTCA	INSERTION	719
		GA/G	DELETION	1660

		T/A	SNP	1758
		C/CT	INSERTION	4806
XP_823361.1 40S ribosomal protein S3 [Trypanosoma brucei brucei strain 927/4 GUTat10.1]	GLOS_TB10.26.0370.1.1	G/T	SNP	165
		T/C	SNP	219
		T/A	SNP	252
XM_818259.1 Tbb strain 927/4 GUTat10.1 CYC2-like cyclin (Tb10.26.0510) partial mRNA	GLOS_TB10.26.0510.1.1	C/A	SNP	230
		G/GT	INSERTION	686
		T/TA	INSERTION	1256
		G/GCA	INSERTION	1410
		C/G	SNP	1912
		A/G	SNP	1940
		A/T	SNP	1941
		A/T	SNP	1959
		A/G	SNP	2915
		C/T	SNP	5205
XM_818254.1 Tbb strain 927/4 GUTat10.1 60S ribosomal protein L6 (Tb10.26.0560) partial mRNA	GLOS_TB10.26.0560.1.1	T/A	SNP	733
XP_823339.1 hypothetical protein [Trypanosoma brucei brucei strain 927/4 GUTat10.1]	GLOS_TB10.26.0680.1.1	GA/G	DELETION	112
XM_818238.1 Tbb GUTat10.1 procyclic form surface glycoprotein (Tb10.26.0790) partial mRNA	GLOS_TB10.26.0790.1.1	C/T	SNP	127
		A/G	SNP	177
		A/T	SNP	218
		C/G	SNP	537
		T/C	SNP	594
		G/T	SNP	831
		T/A	SNP	1616
		G/A	SNP	1617
		G/A	SNP	2010
		G/A	SNP	2139
XM_818231.1 Tbb strain 927/4 GUTat10.1 hypothetical protein (Tb10.26.0880) partial mRNA	GLOS_TB10.26.0880.1.1	C/T	SNP	271
		C/T	SNP	782
		TA/T	DELETION	949
		A/C	SNP	964
		C/T	SNP	1005
		G/GT	INSERTION	2054
		A/G	SNP	2069
		G/A	SNP	2116
XM_818214.1 Tbb strain 927/4 GUTat10.1 heat shock protein 83 (Tb10.26.1080) partial mRNA	GLOS_TB10.26.1080.1.1	A/T	SNP	140
		G/A	SNP	178
		GAAA/GA/G	DELETION	212
		A/G	SNP	1930

		G/A	SNP	1957
		G/A	SNP	2086
		T/A	SNP	2475
		C/T	SNP	2476
		G/T	SNP	2477
		C/T	SNP	2479
		A/C	SNP	2482
XM_822728.1 Tbb strain 927/4 GUTat10.1 elongation factor TU (Tb10.389.0070) partial mRNA	GLOS_TB10.389.0070.1.1	A/C	SNP	575
XM_822697.1 Tbb receptor-type adenylate cyclase GRESAG 4 (Tb10.389.0430) partial mRNA	GLOS_TB10.389.0430.1.1	A/G	SNP	244
		G/A	SNP	311
		A/G	SNP	812
		T/TG	INSERTION	3816
		A/C	SNP	3893
XP_827786.1 3' 5'-cyclic nucleotide phosphodiesterase [Trypanosoma brucei TREU927]	GLOS_TB10.389.0510.1.1	G/GT	INSERTION	1191
XM_822687.1 Tbb strain 927/4 GUTat10.1 hypothetical protein (Tb10.389.0570) partial mRNA	GLOS_TB10.389.0570.1.1	G/A	SNP	116
		G/A	SNP	369
		A/T	SNP	1226
		T/C	SNP	2546
XM_822682.1 Tbb strain 927/4 GUTat10.1 prolyl-tRNA synthetase (Tb10.389.0630) partial mRNA	GLOS_TB10.389.0630.1.1	G/C	SNP	1287
		T/G	SNP	2696
XP_823487.1 hypothetical protein [Trypanosoma brucei brucei strain 927/4 GUTat10.1]	GLOS_TB10.389.0680.1.1	G/A	SNP	116
		G/A	SNP	193
		GT/G	DELETION	253
		G/A	SNP	1587
		T/C	SNP	1765
XP_823486.1 mitochondrial carrier protein [Trypanosoma brucei brucei strain 927/4 GUTat10.1]	GLOS_TB10.389.0690.1.1	T/G	SNP	90
		C/T	SNP	573
		C/T	SNP	969
		A/T	SNP	1107
XP_823483.1 cholinephosphate cytidylyltransferase A [Trypanosoma brucei TREU927]	GLOS_TB10.389.0730.1.1	C/T	SNP	1116
		TA/T	DELETION	1634
		C/A	SNP	2018
		C/CA	INSERTION	2018
XM_818383.1 Tbb strain 927/4 GUTat10.1 heat shock protein (Tb10.389.0880) partial mRNA	GLOS_TB10.389.0880.1.1	A/G	SNP	1705
		C/T	SNP	2040
		G/T	SNP	2833
XM_818380.1 Tbb strain 927/4 GUTat10.1 60S ribosomal protein L34 (Tb10.389.0910) partial mRNA	GLOS_TB10.389.0910.1.2	C/T	SNP	255
		T/C	SNP	271
		T/C	SNP	280

		C/T	SNP	289
		G/A	SNP	292
		G/A	SNP	794
		T/C	SNP	796
		A/C	SNP	37
		T/G	SNP	282
		T/C	SNP	290
		G/A	SNP	294
		G/T	SNP	510
		A/G	SNP	513
		G/A	SNP	516
		T/G	SNP	519
XM_818362.1 Tbb strain 927/4 GUTat10.1 P-type H+-ATPase (Tb10.389.1180) partial mRNA	GLOS_TB10.389.1180.1.1	A/G	SNP	873
		T/C	SNP	874
		C/T	SNP	1681
		C/T	SNP	1682
		C/T	SNP	1709
		A/G	SNP	2112
		G/A	SNP	2118
		C/G	SNP	2495
		AT/A	DELETION	2843
		G/GT	INSERTION	2998
		T/G	SNP	3034
		CT/C	DELETION	3533
		C/CT	INSERTION	4130
		G/GA	INSERTION	4348
		G/A	SNP	4725
		C/CT	INSERTION	4888
XM_818338.1 Tbb GUTat10.1 cytosolic nonspecific dipeptidase (Tb10.389.1480) partial mRNA	GLOS_TB10.389.1480.1.1	A/G	SNP	1288
		AT/A	DELETION	1742
XP_823403.1 kynurenine aminotransferase [Trypanosoma brucei brucei strain 927/4 GUTat10.1]	GLOS_TB10.389.1810.1.1	A/G	SNP	227
		G/T	SNP	1058
		C/G	SNP	2075
		G/T	SNP	2231
		T/C	SNP	2399
XM_818174.1 Tbb strain 927/4 GUTat10.1 histone H2B (Tb10.406.0450) partial mRNA ref|XM_818176.1| Tbb histone H2B (Tb10.406.0430) partial mRNA ref|XM_818177.1| Tbb histone H2B (Tb10.406.0420) partial mRNA ref|XM_818178.1| Tbb histone H2B (Tb10.406.0410) partial mRNA ref|XM_818179.1| Tbb histone H2B (Tb10.406.0400) partial mRNA ref|XM_818181.1| Tbb histone H2B	GLOS_TB10.406.0450.1.1	T/A	SNP	28

(Tb10.406.0380) partial mRNA ref|XM_818182.1| Tbb histone H2B (Tb10.406.0370) partial mRNA ref|XM_818184.1| Tbb histone H2B (Tb10.406.0350) partial mRNA				
		T/C	SNP	412
		C/T	SNP	513
		C/CGT/CGTGT	INSERTION	578
		T/C	SNP	615
		C/T	SNP	621
		C/T	SNP	753
XM_818156.1 Tbb microtubule-associated protein (Tb10.406.0650) partial mRNA	GLOS_TB10.406.0650.1.1	G/A	SNP	43
		C/T	SNP	61
		G/A	SNP	100
		G/A	SNP	145
		C/T	SNP	153
		G/A	SNP	214
		T/C	SNP	246
		A/G	SNP	484
		A/AT	INSERTION	1335
		A/G	SNP	1361
		G/A	SNP	1629
XM_822953.1 Tbb strain 927/4 GUTat10.1 hypothetical protein (Tb10.61.0540) partial mRNA	GLOS_TB10.61.0540.1.1	C/T	SNP	927
		GAT/G	DELETION	1491
XM_822919.1 Tbb glycosomal malate dehydrogenase (Tb10.61.0980) partial mRNA	GLOS_TB10.61.0980.1.1	G/GA	INSERTION	257
		G/GAAA	INSERTION	272
		G/A	SNP	279
		G/A	SNP	286
		T/TA	INSERTION	347
		C/CAT	INSERTION	433
		C/T	SNP	479
		A/T	SNP	495
		A/G	SNP	616
		T/C	SNP	727
		G/T	SNP	800
		T/C	SNP	884
		T/C	SNP	924
		C/A	SNP	945
		T/C	SNP	947
		T/C	SNP	949
		C/T	SNP	977
		C/T	SNP	987

		G/A	SNP	1058
		C/T	SNP	1136
		C/T	SNP	1228
		C/T	SNP	1329
		A/G	SNP	1718
		T/G	SNP	1775
		G/A	SNP	1889
		G/A	SNP	2092
XM_822905.1 Tbb strain 927/4 GUTat10.1 hypothetical protein (Tb10.61.1260) partial mRNA	GLOS_TB10.61.1260.1.1	CT/C	DELETION	945
		TAC/T	DELETION	1183
XM_822898.1 Tbb nucleosome assembly protein (Tb10.61.1330) partial mRNA	GLOS_TB10.61.1330.1.1	G/T	SNP	227
		T/G	SNP	252
XP_827985.1 40S ribosomal protein S13 [Trypanosoma brucei brucei strain 927/4 GUTat10.1] ref|XP_951734.1| 40S ribosomal protein S13 [Trypanosoma brucei brucei strain 927/4 GUTat10.1]	GLOS_TB10.61.1390.2.2	A/C	SNP	3
		G/GA	INSERTION	82
		C/T	SNP	143
		C/T	SNP	251
		C/A	SNP	350
XP_827964.1 hypothetical protein [Trypanosoma brucei brucei strain 927/4 GUTat10.1]	GLOS_TB10.61.1790.1.1	G/T	SNP	108
		T/TA	INSERTION	109
		G/GAA	INSERTION	197
		G/T	SNP	474
		A/G	SNP	754
		A/G	SNP	796
		C/G	SNP	875
XM_822868.1 Tbb strain 927/4 GUTat10.1 mitochondrial carrier protein (Tb10.61.1820) partial mRNA. nuclear gene for mitochondrial product ref|XM_822869.1| Tbb 927/4 GUTat10.1 mitochondrial carrier protein (Tb10.61.1810) partial mRNA. nuclear gene for mitochondrial product	GLOS_TB10.61.1820.1.1	ATT/A	DELETION	336
		C/T	SNP	482
		C/T	SNP	1299
XP_827959.1 hypothetical protein [Trypanosoma brucei brucei strain 927/4 GUTat10.1]	GLOS_TB10.61.1840.1.1	T/A	SNP	212
		T/C	SNP	248
		G/A	SNP	351
XM_822864.1 Tbb strain 927/4 GUTat10.1 aminopeptidase (Tb10.61.1870) partial mRNA	GLOS_TB10.61.1870.1.1	C/CT	INSERTION	1310
		T/C	SNP	1509
XM_822860.1 Tbb strain 927/4 GUTat10.1 fibrillarin (Tb10.61.1920) partial mRNA	GLOS_TB10.61.1920.1.1	C/T	SNP	441
XM_822858.1 Tbb strain 927/4 GUTat10.1 chaperone protein DnaJ (Tb10.61.1940) partial mRNA	GLOS_TB10.61.1940.1.1	C/A	SNP	101
		G/A	SNP	407
		A/G	SNP	527

		A/C	SNP	554
		G/GT	INSERTION	1587
XM_822856.1 Tbb strain 927/4 GUTat10.1 40S ribosomal protein S2 (Tb10.61.1960) partial mRNA	GLOS_TB10.61.1960.1.2	G/T	SNP	77
		C/T	SNP	81
XM_822855.1 Tbb strain 927/4 GUTat10.1 hypothetical protein (Tb10.61.1970) partial mRNA	GLOS_TB10.61.1970.1.1	C/G	SNP	507
		C/A	SNP	970
		A/G	SNP	1185
XM_822844.1 Tbb 927/4 GUTat10.1 60S ribosomal protein L17 (Tb10.61.2090) partial mRNA	GLOS_TB10.61.2090.1.2	G/T	SNP	609
XM_822840.1 Tbb proteasome regulatory non-ATPase subunit 8 (Tb10.61.2180) partial mRNA	GLOS_TB10.61.2180.1.1	C/G	SNP	453
		C/T	SNP	1343
XM_822837.1 Tbb strain 927/4 GUTat10.1 hypothetical protein (Tb10.61.2210) partial mRNA	GLOS_TB10.61.2210.1.1	A/T	SNP	46
		C/T	SNP	77
		T/C	SNP	1102
		T/TC	INSERTION	1281
XM_822836.1 Tbb strain 927/4 GUTat10.1 hypothetical protein (Tb10.61.2220) partial mRNA	GLOS_TB10.61.2220.1.1	G/A	SNP	871
		T/C	SNP	1207
		A/G	SNP	1393
		CAAA/C	DELETION	1625
		T/G	SNP	1741
		C/CT	INSERTION	2388
		C/CT	INSERTION	2693
XM_822833.1 Tbb strain 927/4 GUTat10.1 hypothetical protein (Tb10.61.2270) partial mRNA	GLOS_TB10.61.2270.1.1	G/A	SNP	278
		A/G	SNP	783
		G/C	SNP	1106
		T/A	SNP	1982
		C/CT	INSERTION	3251
XM_822830.1 Tbb strain 927/4 GUTat10.1 hypothetical protein (Tb10.61.2300) partial mRNA	GLOS_TB10.61.2300.1.1	CA/C	DELETION	298
		G/GA	INSERTION	630
		G/A	SNP	821
		G/A	SNP	2149
		C/G	SNP	2245
		G/A	SNP	3352
XM_822793.1 Tbb strain 927/4 GUTat10.1 hypothetical protein (Tb10.61.2850) partial mRNA	GLOS_TB10.61.2850.1.1	A/T	SNP	53
XP_827883.1 aconitase [Trypanosoma brucei brucei strain 927/4 GUTat10.1]	GLOS_TB10.61.2880.1.1	T/A	SNP	475
		GA/G	DELETION	707
		GT/G	DELETION	767
		TAATA/T	DELETION	945
		C/CT	INSERTION	1092
		C/CTT	INSERTION	1354

XM_822770.1 Tbb strain 927/4 GUTat10.1 hypothetical protein (Tb10.61.3120) partial mRNA	GLOS_TB10.61.3120.1.1	G/GA	INSERTION	803
		T/TA	INSERTION	1980
		A/G	SNP	2496
		C/CA	INSERTION	2504
		T/C	SNP	2566
		T/C	SNP	3185
		C/G	SNP	3545
XM_818154.1 Tbb strain 927/4 GUTat10.1 EP1 procyclin precursor (Tb10.6k15.0020) partial mRNA	GLOS_TB10.6K15.0020.2.3	A/T	SNP	774
XM_818133.1 Tbb strain 927/4 GUTat10.1 hypothetical protein (Tb10.6k15.0240) partial mRNA	GLOS_TB10.6K15.0240.1.1	G/T	SNP	83
		C/A	SNP	479
		C/A	SNP	481
		T/G	SNP	707
		CA/C	DELETION	758
		G/A	SNP	1508
		G/C	SNP	2457
		C/CGT	INSERTION	5165
		GA/G	DELETION	6320
XM_818129.1 Tbb strain 927/4 GUTat10.1 hypothetical protein (Tb10.6k15.0280) partial mRNA	GLOS_TB10.6K15.0280.1.1	CA/C	DELETION	188
		CT/C	DELETION	401
XM_818120.1 Tbb strain 927/4 GUTat10.1 hypothetical protein (Tb10.6k15.0380) partial mRNA	GLOS_TB10.6K15.0380.1.1	C/CA	INSERTION	315
		A/G	SNP	1656
		ATG/A	DELETION	3198
		A/AT	INSERTION	3490
XM_818117.1 Tbb strain 927/4 GUTat10.1 60S ribosomal protein L18 (Tb10.6k15.0410) partial mRNA	GLOS_TB10.6K15.0410.1.1	A/G	SNP	99
		T/C	SNP	138
		A/G	SNP	168
		G/A	SNP	192
		C/T	SNP	195
		T/C	SNP	198
		C/G	SNP	213
		C/T	SNP	240
		A/G	SNP	450
		C/T	SNP	534
XM_818108.1 Tbb strain 927/4 GUTat10.1 hypothetical protein (Tb10.6k15.0520) partial mRNA	GLOS_TB10.6K15.0520.1.1	C/T	SNP	809
		A/T	SNP	810
		T/C	SNP	901
		C/T	SNP	919
		T/C	SNP	1018
		G/C	SNP	1021

		T/C	SNP	1042
		A/G	SNP	1075
		C/T	SNP	1116
		G/T	SNP	1117
		A/G	SNP	1120
		A/G	SNP	1189
		T/C	SNP	1210
		A/C	SNP	1222
		G/A	SNP	1231
		A/G	SNP	1237
		A/G	SNP	1300
		GT/G	DELETION	1636
XP_823188.1 hypothetical protein [Trypanosoma brucei brucei strain 927/4 GUTat10.1]	GLOS_TB10.6K15.0690.1.1	C/G	SNP	1064
		C/CA	INSERTION	2650
XP_823180.1 hypothetical protein [Trypanosoma brucei brucei strain 927/4 GUTat10.1]	GLOS_TB10.6K15.0810.1.1	G/A	SNP	629
XM_818050.1 Tbb strain 927/4 GUTat10.1 isoleucyl-tRNA synthetase (Tb10.6k15.1220) partial mRNA	GLOS_TB10.6K15.1220.1.1	A/G	SNP	275
		G/GT	INSERTION	2320
XM_818039.1 Tbb strain 927/4 GUTat10.1 pteridine transporter (Tb10.6k15.1350) partial mRNA	GLOS_TB10.6K15.1350.1.1	CA/C	DELETION	387
		GA/G	DELETION	573
		T/C	SNP	1415
XM_818025.1 Tbb strain 927/4 GUTat10.1 hypothetical protein (Tb10.6k15.1500) partial mRNA	GLOS_TB10.6K15.1500.1.1	A/C	SNP	1020
		G/T	SNP	1023
		T/C	SNP	1064
		G/C	SNP	1123
		A/G	SNP	1155
		AG/A	DELETION	1156
		A/G	SNP	1164
		A/G	SNP	1501
		A/T	SNP	1502
		A/G	SNP	1507
		A/G	SNP	1508
		A/G	SNP	2081
		C/CT	INSERTION	2853
		G/A	SNP	3206
		C/CT	INSERTION	3297
		T/TG	INSERTION	3602
		G/A	SNP	3930
		G/GA	INSERTION	3930
		G/GA	INSERTION	4078

XP_823117.1 hypothetical protein [Trypanosoma brucei brucei strain 927/4 GUTat10.1]	GLOS_TB10.6K15.1510.1.1	G/A	SNP	304
		G/GA	INSERTION	316
		G/A	SNP	1001
		G/A	SNP	1060
		A/G	SNP	1097
		A/G	SNP	2111
XM_818023.1 Tbb strain 927/4 GUTat10.1 small GTPase (Tb10.6k15.1520) partial mRNA	GLOS_TB10.6K15.1520.1.1	C/CT	INSERTION	1108
		T/C	SNP	1131
XM_818004.1 Tbb strain 927/4 GUTat10.1 hypothetical protein (Tb10.6k15.1820) partial mRNA	GLOS_TB10.6K15.1820.1.1	GA/G	DELETION	583
XP_823066.1 cytochrome c oxidase subunit IX [Trypanosoma brucei brucei strain 927/4 GUTat10.1]	GLOS_TB10.6K15.2180.1.1	A/G	SNP	121
		ATAAT/A	DELETION	248
		C/CT	INSERTION	302
		A/T	SNP	728
XM_817968.1 Tbb eukaryotic translation initiation factor 3 subunit 8 (Tb10.6k15.2250) partial mRNA	GLOS_TB10.6K15.2250.1.1	T/C	SNP	520
		A/T	SNP	641
		G/GT	INSERTION	740
XM_817964.1 Tbb strain 927/4 GUTat10.1 protein disulfide isomerase (Tb10.6k15.2290) partial mRNA	GLOS_TB10.6K15.2290.1.1	C/T	SNP	1093
XP_823053.1 t-complex protein 1 subunit theta [Trypanosoma brucei brucei strain 927/4 GUTat10.1]	GLOS_TB10.6K15.2330.1.1	A/G	SNP	1067
		C/CT	INSERTION	1832
XM_817944.1 Tbb strain 927/4 GUTat10.1 hypothetical protein (Tb10.6k15.2510) partial mRNA	GLOS_TB10.6K15.2510.1.1	T/C	SNP	449
		G/A	SNP	1350
XP_823027.1 2,3-bisphosphoglycerate-independent phosphoglycerate mutase [Tbb]	GLOS_TB10.6K15.2620.1.1	A/G	SNP	81
		G/C	SNP	1930
		GA/G	DELETION	2277
XM_817930.1 Tbb strain 927/4 GUTat10.1 hypothetical protein (Tb10.6k15.2660) partial mRNA	GLOS_TB10.6K15.2660.1.1	C/T	SNP	221
		CT/C	DELETION	422
		G/GA	INSERTION	614
		CA/C	DELETION	740
		G/GA	INSERTION	1036
		CT/C	DELETION	1287
		C/T	SNP	1338
		C/CT	INSERTION	1410
		G/GT	INSERTION	1581
		C/T	SNP	2776
		T/C	SNP	2807
XM_817927.1 Tbb strain 927/4 GUTat10.1 hypothetical protein (Tb10.6k15.2690) partial mRNA	GLOS_TB10.6K15.2690.1.1	CT/C	DELETION	261
		C/CT	INSERTION	377
XM_817898.1 Tbb dihydrolipoamide acetyltransferase precursor (Tb10.6k15.3080) partial mRNA	GLOS_TB10.6K15.3080.1.1	C/G	SNP	529
XM_817883.1 Tbb succinyl-CoA ligase [GDP-forming] beta-chain (Tb10.6k15.3250) partial mRNA	GLOS_TB10.6K15.3250.1.1	A/T	SNP	175

		T/TC	INSERTION	483
XM_817875.1 Tbb 40S ribosomal protein S24e (Tb10.6k15.3350) partial mRNA	GLOS_TB10.6K15.3350.1.1	A/G	SNP	522
		C/T	SNP	523
		A/G	SNP	525
		A/C	SNP	530
		T/C	SNP	531
XP_822958.1 hypothetical protein [Trypanosoma brucei brucei strain 927/4 GUTat10.1]	GLOS_TB10.6K15.3460.1.1	A/C	SNP	45
		G/A	SNP	2857
XM_817860.1 Tbb cysteine-rich acidic integral memb. prot. precursor (Tb10.6k15.3510) partial mRNA	GLOS_TB10.6K15.3510.1.1	C/T	SNP	292
		A/G	SNP	293
		C/T	SNP	328
		AT/A	DELETION	751
XM_817839.1 Tbb dipeptidyl-peptidase 8-like serine peptidase (Tb10.6k15.3800) partial mRNA	GLOS_TB10.6K15.3800.1.1	C/A	SNP	654
		C/G	SNP	1039
		G/A	SNP	1080
		G/A	SNP	1085
		C/T	SNP	2147
		T/C	SNP	2589
		C/T	SNP	3540
		G/A	SNP	86
		C/T	SNP	342
		C/T	SNP	1382
		G/A	SNP	2113
XM_817822.1 Tbb strain 927/4 GUTat10.1 hypothetical protein (Tb10.70.0010) partial mRNA	GLOS_TB10.70.0010.1.1	G/C	SNP	429
		G/A	SNP	889
		C/A	SNP	890
		A/G	SNP	947
		C/T	SNP	948
XM_817788.1 Tbb strain 927/4 GUTat10.1 hypothetical protein (Tb10.70.0440) partial mRNA	GLOS_TB10.70.0440.1.1	G/A	SNP	137
		C/T	SNP	196
		T/C	SNP	626
		T/G	SNP	701
		T/C	SNP	798
		G/A	SNP	801
		A/AT	INSERTION	866
		CAA/C	DELETION	1945
		T/TA	INSERTION	2167
XM_817786.1 Tbb strain 927/4 GUTat10.1 60S ribosomal proteins L37 (Tb10.70.0465) partial mRNA	GLOS_TB10.70.0465.1.1	C/A	SNP	342
		T/G	SNP	343

		G/A	SNP	375
		A/G	SNP	381
		C/T	SNP	537
XP_822855.1 hypothetical protein [Trypanosoma brucei brucei strain 927/4 GUTat10.1]	GLOS_TB10.70.0730.1.1	TA/T	DELETION	502
		G/GA	INSERTION	1000
		A/G	SNP	1440
XM_817756.1 Tbb universal minicircle sequence binding prot. (UMSBP) (Tb10.70.0800) partial mRNA	GLOS_TB10.70.0800.1.2	CT/C	DELETION	1688
XP_822847.1 clathrin heavy chain [Trypanosoma brucei brucei strain 927/4 GUTat10.1]	GLOS_TB10.70.0830.1.1	AT/A	DELETION	311
		GT/G	DELETION	651
		T/C	SNP	2441
XP_822838.1 protein kinase [Trypanosoma brucei brucei strain 927/4 GUTat10.1]	GLOS_TB10.70.0960.1.1	T/C	SNP	138
		G/GA	INSERTION	534
		C/G	SNP	815
		C/T	SNP	819
		T/C	SNP	893
		G/A	SNP	894
		G/A	SNP	1223
		T/C	SNP	1259
		G/C	SNP	1299
		G/A	SNP	1638
		T/C	SNP	2007
		C/T	SNP	2336
XM_817733.1 Tbb strain 927/4 GUTat10.1 hypothetical protein (Tb10.70.1120) partial mRNA	GLOS_TB10.70.1120.1.1	C/T	SNP	2473
XM_817732.1 Tbb strain 927/4 GUTat10.1 hypothetical protein (Tb10.70.1130) partial mRNA	GLOS_TB10.70.1130.1.1	GA/G	DELETION	82
		C/CT	INSERTION	386
		C/CT	INSERTION	858
XP_822821.1 valosin-containing protein homolog [Trypanosoma brucei TREU927]	GLOS_TB10.70.1190.1.1	G/GT	INSERTION	2656
XM_817699.1 Tbb strain 927/4 GUTat10.1 60S ribosomal protein L24 (Tb10.70.1540) partial mRNA	GLOS_TB10.70.1540.1.2	C/T	SNP	561
		A/G	SNP	174
XP_822783.1 hypothetical protein [Trypanosoma brucei brucei strain 927/4 GUTat10.1]	GLOS_TB10.70.1660.1.1	G/A	SNP	1945
		CA/C	DELETION	4107
XM_817685.1 Tbb strain 927/4 GUTat10.1 40S ribosomal protein S18 (Tb10.70.1740) partial mRNA	GLOS_TB10.70.1740.1.1	A/T	SNP	258
		A/T	SNP	259
		A/G	SNP	270
		A/G	SNP	290
		T/A	SNP	751
XM_817671.1 Tbb strain 927/4 GUTat10.1 hypothetical protein (Tb10.70.1930) partial mRNA	GLOS_TB10.70.1930.1.1	G/A	SNP	1633
		CT/C	DELETION	2184
XM_817657.1 Tbb ubiquitin/ribosomal protein S27a (Tb10.70.2170) partial mRNA	GLOS_TB10.70.2170.1.1	G/T	SNP	267

XP_822722.1 hypothetical protein [Trypanosoma brucei brucei strain 927/4 GUTat10.1]	GLOS_TB10.70.2460.1.1	G/T	SNP	2270
		C/CT	INSERTION	3079
XP_822703.1 elongation factor 2 [Trypanosoma brucei brucei strain 927/4 GUTat10.1] ref|XP_822704.1| elongation factor 2 [Trypanosoma brucei brucei strain 927/4 GUTat10.1]	GLOS_TB10.70.2660.1.1	C/T	SNP	2342
XP_822676.1 hypothetical protein [Trypanosoma brucei brucei strain 927/4 GUTat10.1]	GLOS_TB10.70.2970.1.1	G/A	SNP	40
		C/A	SNP	41
XP_822663.1 hypothetical protein [Trypanosoma brucei brucei strain 927/4 GUTat10.1]	GLOS_TB10.70.3120.1.1	G/T	SNP	979
		A/T	SNP	1198
		T/TA	INSERTION	1218
XP_822661.1 hypothetical protein [Trypanosoma brucei brucei strain 927/4 GUTat10.1]	GLOS_TB10.70.3150.1.1	GA/G	DELETION	169
		C/G	SNP	345
		A/G	SNP	1515
		A/ATGTG	INSERTION	1650
		G/A	SNP	1755
		C/T	SNP	1853
XM_817567.1 Tbb strain 927/4 GUTat10.1 60S ribosomal protein L30 (Tb10.70.3160) partial mRNA	GLOS_TB10.70.3160.1.2	A/T	SNP	625
		T/A	SNP	630
XM_817563.1 Tbb strain 927/4 GUTat10.1 hypothetical protein (Tb10.70.3190) partial mRNA	GLOS_TB10.70.3190.1.1	T/C	SNP	112
		A/G	SNP	1025
		C/A	SNP	1378
		C/A	SNP	2437
XP_822647.1 ATP-dependent DEAD-box RNA helicase [Trypanosoma brucei TREU927]	GLOS_TB10.70.3290.1.1	T/A	SNP	1047
		T/C	SNP	1134
		T/G	SNP	1681
		G/T	SNP	1682
		T/C	SNP	1953
		G/A	SNP	2000
XM_817548.1 Tbb strain 927/4 GUTat10.1 40S ribosomal protein S3a (Tb10.70.3370) partial mRNA	GLOS_TB10.70.3370.1.1	C/T	SNP	104
		T/C	SNP	888
XP_822632.1 60S ribosomal protein L18a [Trypanosoma brucei brucei strain 927/4 GUTat10.1] ref|XP_829469.1| ribosomal protein L18 [Trypanosoma brucei brucei strain 927/4 GUTat10.1]	GLOS_TB10.70.3510.1.1	G/T	SNP	11
		G/T	SNP	12
		A/T	SNP	15
		A/G	SNP	401
		A/G	SNP	404
		C/T	SNP	500
		A/G	SNP	554
		G/A	SNP	569
XM_817496.1 Tbb strain 927/4 GUTat10.1 60S acid. ribosomal prot. P2 (Tb10.70.4060) partial mRNA	GLOS_TB10.70.4060.1.1	C/CA	INSERTION	93

		G/T	SNP	123
		T/A	SNP	468
		T/G	SNP	474
		A/G	SNP	1021
XP_822579.1 60S ribosomal protein L38 [Trypanosoma brucei brucei strain 927/4 GUTat10.1]	GLOS_TB10.70.4155.1.2	A/G	SNP	29
XP_822573.1 delta-1-pyrroline-5-carboxylate dehydrogenase [Trypanosoma brucei]	GLOS_TB10.70.4280.1.1	G/T	SNP	120
		AT/A	DELETION	179
		A/G	SNP	278
		A/G	SNP	517
		T/C	SNP	565
		A/G	SNP	682
		T/C	SNP	1597
		A/G	SNP	1627
		C/T	SNP	1829
		C/T	SNP	2167
XP_822550.1 hypothetical protein [Trypanosoma brucei brucei strain 927/4 GUTat10.1]	GLOS_TB10.70.4590.1.1	C/T	SNP	212
		C/CT	INSERTION	298
		T/C	SNP	1112
		T/C	SNP	1114
XM_817450.1 Tbb strain 927/4 GUTat10.1 importin subunit beta-1 (Tb10.70.4720) partial mRNA	GLOS_TB10.70.4720.1.1	CTT/C	DELETION	667
		CT/C	DELETION	1341
		G/GA	INSERTION	1572
		C/CT	INSERTION	1780
		C/CT	INSERTION	2019
		C/T	SNP	4474
XM_817444.1 Tbb strain 927/4 GUTat10.1 ribosomal protein S25 (Tb10.70.4800) partial mRNA	GLOS_TB10.70.4800.1.2	T/TA	INSERTION	163
		T/A	SNP	706
		T/A	SNP	707
XM_817437.1 Tbb eukaryotic translation initiation factor 5 (Tb10.70.4880) partial mRNA	GLOS_TB10.70.4880.1.1	G/A	SNP	767
		A/T	SNP	787
		A/T	SNP	1283
		C/CT	INSERTION	1384
XM_817433.1 Tbb strain 927/4 GUTat10.1 hypothetical protein (Tb10.70.4930) partial mRNA	GLOS_TB10.70.4930.1.1	C/CA	INSERTION	113
		T/TA	INSERTION	1279
		C/T	SNP	1383
		A/G	SNP	1395
		G/T	SNP	1696
		A/G	SNP	2305
XM_817417.1 Tbb lysosomal alpha-mannosidase precursor (Tb10.70.5100) partial mRNA	GLOS_TB10.70.5100.1.1	C/G	SNP	1218

		C/A	SNP	1462
		C/T	SNP	2174
		C/T	SNP	2295
XM_817416.1 Trypanosoma brucei brucei strain 927/4 GUTat10.1 mitochondrial malate dehydrogenase (Tb10.70.5110) partial mRNA. nuclear gene for mitochondrial product	GLOS_TB10.70.5110.1.1	A/G	SNP	144
		C/T	SNP	309
		A/G	SNP	988
		C/T	SNP	990
		G/T	SNP	1563
XP_822506.1 adenylate kinase [Trypanosoma brucei brucei strain 927/4 GUTat10.1]	GLOS_TB10.70.5150.1.1	C/T	SNP	464
		C/T	SNP	799
		A/T	SNP	886
		G/GA	INSERTION	1244
		G/C	SNP	1311
		C/CT	INSERTION	1399
XM_817398.1 Tbb strain 927/4 GUTat10.1 La protein (Tb10.70.5360) partial mRNA	GLOS_TB10.70.5360.1.1	G/GA	INSERTION	663
		T/C	SNP	883
		G/A	SNP	1232
XM_817385.1 Tbb strain 927/4 GUTat10.1 hypothetical protein (Tb10.70.5500) partial mRNA	GLOS_TB10.70.5500.1.1	C/T	SNP	453
		A/G	SNP	2107
		G/T	SNP	2350
		C/CA	INSERTION	106
		G/T	SNP	154
		G/GT	INSERTION	1110
		CTG/C	DELETION	1151
		C/CT	INSERTION	1296
XP_822456.1 hexokinase [Trypanosoma brucei brucei strain 927/4 GUTat10.1]	GLOS_TB10.70.5820.1.1	T/C	SNP	456
		G/C	SNP	483
		C/G	SNP	811
		C/A	SNP	812
		G/A	SNP	815
		A/T	SNP	816
		G/A	SNP	818
		G/A	SNP	961
		C/CT	INSERTION	2197
		G/GA	INSERTION	2611
		GAA/G	DELETION	2743
XM_817361.1 Tbb strain 927/4 GUTat10.1 major vault protein (Tb10.70.5840) partial mRNA	GLOS_TB10.70.5840.1.1	G/A	SNP	166
		A/G	SNP	3387

XM_817326.1 Tbb strain 927/4 GUTat10.1 dual specificity protein phosphatase partial mRNA	GLOS_TB10.70.6300.1.1	A/G	SNP	112
		A/G	SNP	2166
		C/T	SNP	2190
		T/C	SNP	2235
		C/T	SNP	2860
		A/AT	INSERTION	2906
		T/A	SNP	2975
		A/G	SNP	3037
XM_817321.1 Trypanosoma brucei brucei strain 927/4 GUTat10.1 ATPase subunit 9 partial mRNA	GLOS_TB10.70.6340.1.1	TC/T	DELETION	163
		T/C	SNP	528
		T/C	SNP	783
		A/T	SNP	809
		A/T	SNP	867
		A/G	SNP	1049
		A/G	SNP	1261
		ATCT/A	DELETION	1454
		G/GA	INSERTION	1474
XM_817315.1 Tbb strain 927/4 GUTat10.1 transcriptional regulatory protein NOT1 partial mRNA	GLOS_TB10.70.6450.1.1	T/C	SNP	2730
		A/G	SNP	4660
		A/G	SNP	4682
		A/G	SNP	5115
		GA/G	DELETION	6069
		G/GT	INSERTION	6125
XM_817314.1 Tbb strain 927/4 GUTat10.1 methionyl-tRNA synthetase (Tb10.70.6470) partial mRNA	GLOS_TB10.70.6470.1.1	GT/G	DELETION	3218
		T/G	SNP	3229
		CTTT/C	DELETION	3332
XM_817313.1 Tbb strain 927/4 GUTat10.1 hypothetical protein (Tb10.70.6480) partial mRNA	GLOS_TB10.70.6480.1.1	A/AAAAAG	INSERTION	363
		CT/C	DELETION	517
		T/A	SNP	529
		AT/A	DELETION	584
		T/C	SNP	604
		A/G	SNP	660
		A/G	SNP	933
		C/A	SNP	3672
		GA/G	DELETION	4885
XM_817286.1 Tbb strain 927/4 GUTat10.1 katanin (Tb10.70.6880) partial mRNA	GLOS_TB10.70.6880.1.1	CA/CAA/C	INSERTION	159
		CA/C	DELETION	1704
		A/C	SNP	2633
XP_822368.1 60S ribosomal protein L9 [Trypanosoma brucei brucei strain 927/4 GUTat10.1]	GLOS_TB10.70.7010.1.1	G/A	SNP	284

		C/T	SNP	293
		C/T	SNP	317
		G/A	SNP	335
		G/A	SNP	416
		G/A	SNP	443
		T/C	SNP	449
		G/C	SNP	461
		A/C	SNP	503
		T/A	SNP	551
		G/A	SNP	554
		T/C	SNP	593
		C/T	SNP	677
		A/G	SNP	680
XP_822364.1 t-complex protein 1 subunit delta [Trypanosoma brucei brucei strain 927/4 GUTat10.1]	GLOS_TB10.70.7050.1.1	G/GA	INSERTION	612
		A/AT	INSERTION	755
XM_817258.1 Tbb strain 927/4 GUTat10.1 hypothetical protein (Tb10.70.7220) partial mRNA	GLOS_TB10.70.7220.1.1	A/G	SNP	187
		T/C	SNP	349
		T/C	SNP	636
		A/T	SNP	1146
		T/G	SNP	2562
XM_817219.1 Tbb strain 927/4 GUTat10.1 ATP-dependent DEAD/H RNA helicase partial mRNA	GLOS_TB10.70.7730.1.1	A/G	SNP	390
		C/T	SNP	417
		A/G	SNP	759
		C/CT	INSERTION	1724
		G/A	SNP	2064
		A/G	SNP	2629
		T/TA	INSERTION	2771
		G/A	SNP	2858
		GA/G	DELETION	2860
		G/T	SNP	2884
		A/T	SNP	3058
		G/A	SNP	3180
		A/T	SNP	3283
		G/T	SNP	3388
		A/G	SNP	3781
XP_822310.1 hypothetical protein [Trypanosoma brucei brucei strain 927/4 GUTat10.1]	GLOS_TB10.70.7760.1.1	C/CT	INSERTION	1864
XM_823801.1 Tbb strain 927/4 GUTat10.1 ribosomal protein S26 (Tb11.01.0355) partial mRNA	GLOS_TB11.01.0355.1.1	G/T	SNP	12
XP_828962.1 ribose 5-phosphate isomerase [Trypanosoma brucei brucei strain 927/4 GUTat10.1]	GLOS_TB11.01.0700.1.1	G/A	SNP	22
XM_823871.1 Tbb strain 927/4 GUTat10.1 cation transporter (Tb11.01.0720) partial mRNA	GLOS_TB11.01.0720.1.1	A/G	SNP	164

		A/AT	INSERTION	1021
		G/A	SNP	1444
XM_823872.1 Tbb strain 927/4 GUTat10.1 cation transporter (Tb11.01.0725) partial mRNA	GLOS_TB11.01.0725.1.1	C/T	SNP	679
		C/T	SNP	918
		A/T	SNP	919
		A/G	SNP	1100
		G/A	SNP	1112
XM_823894.1 Tbb proteasome regulatory non-ATPase subunit 2 (Tb11.01.0960) partial mRNA	GLOS_TB11.01.0960.1.1	A/G	SNP	1405
		G/C	SNP	3245
XM_823896.1 Tbb strain 927/4 GUTat10.1 hypothetical protein (Tb11.01.1000) partial mRNA	GLOS_TB11.01.1000.1.1	G/T	SNP	262
		C/T	SNP	2489
XP_829017.1 hypothetical protein [Trypanosoma brucei brucei strain 927/4 GUTat10.1]	GLOS_TB11.01.1290.1.1	G/A	SNP	389
		A/G	SNP	1148
		T/TTA	INSERTION	1356
XM_823930.1 Tbb strain 927/4 GUTat10.1 S-adenosylhomocysteine hydrolase partial mRNA	GLOS_TB11.01.1350.1.1	G/GA	INSERTION	217
		C/T	SNP	2455
		G/A	SNP	3898
		C/T	SNP	3956
XM_823941.1 Tbb nascent polypept. associated complex subunit alpha (Tb11.01.1465) partial mRNA	GLOS_TB11.01.1465.1.1	A/T	SNP	36
		C/G	SNP	40
		G/A	SNP	41
XP_829036.1 40S ribosomal protein S27 [Trypanosoma brucei brucei strain 927/4 GUTat10.1]	GLOS_TB11.01.1475.3.3	C/CT	INSERTION	515
		C/CT	INSERTION	559
XP_829050.1 hypothetical protein [Trypanosoma brucei brucei strain 927/4 GUTat10.1]	GLOS_TB11.01.1625.1.1	C/G	SNP	69
		G/GA	INSERTION	542
XM_823969.1 Tbb 2-oxoglutarate dehydrogenase E1 component (Tb11.01.1740) partial mRNA	GLOS_TB11.01.1740.1.1	G/GT	INSERTION	4981
		CTT/C	DELETION	5136
XM_823974.1 Tbb strain 927/4 GUTat10.1 60S ribosomal protein L29 (Tb11.01.1790) partial mRNA	GLOS_TB11.01.1790.1.2	A/G	SNP	201
		A/T	SNP	207
XM_823983.1 Tbb strain 927/4 GUTat10.1 hypothetical protein (Tb11.01.1880) partial mRNA	GLOS_TB11.01.1880.1.1	G/T	SNP	1356
XM_823986.1 Tbb strain 927/4 GUTat10.1 hypothetical protein (Tb11.01.1910) partial mRNA	GLOS_TB11.01.1910.1.1	C/CT	INSERTION	157
		G/A	SNP	1981
		A/G	SNP	2594
XM_824025.1 Tbb strain 927/4 GUTat10.1 hypothetical protein (Tb11.01.2310) partial mRNA	GLOS_TB11.01.2310.1.1	G/A	SNP	1814
XM_824027.1 Tbb strain 927/4 GUTat10.1 hypothetical protein (Tb11.01.2330) partial mRNA	GLOS_TB11.01.2330.1.1	G/A	SNP	1810
		T/TC	INSERTION	2367
		CT/C	DELETION	2518
XM_824043.1 Tbb strain 927/4 GUTat10.1 hypothetical protein (Tb11.01.2490) partial mRNA	GLOS_TB11.01.2490.1.1	C/T	SNP	858
		T/C	SNP	2287

XM_824062.1 Tbb strain 927/4 GUTat10.1 40S ribosomal protein SA (Tb11.01.2680) partial mRNA	GLOS_TB11.01.2680.1.1	C/A	SNP	127
		T/A	SNP	128
		C/T	SNP	134
		C/T	SNP	975
		A/T	SNP	1101
XM_824073.1 Tbb strain 927/4 GUTat10.1 hypothetical protein (Tb11.01.2800) partial mRNA	GLOS_TB11.01.2800.1.1	G/T	SNP	1568
XP_829173.1 hypothetical protein [Trypanosoma brucei brucei strain 927/4 GUTat10.1]	GLOS_TB11.01.2880.1.1	C/G	SNP	213
		A/G	SNP	438
		G/A	SNP	895
		G/A	SNP	955
		G/A	SNP	960
		A/G	SNP	1000
		G/A	SNP	1020
XM_824095.1 Tbb strain 927/4 GUTat10.1 40S ribosomal protein L14 (Tb11.01.3020) partial mRNA	GLOS_TB11.01.3020.1.1	C/T	SNP	160
		C/A	SNP	185
		T/C	SNP	366
		C/T	SNP	387
		G/C	SNP	408
		T/C	SNP	411
		A/G	SNP	423
		C/T	SNP	444
		C/T	SNP	488
		G/A	SNP	492
XM_824105.1 Tbb strain 927/4 GUTat10.1 heat shock protein 70 (Tb11.01.3110) partial mRNA	GLOS_TB11.01.3110.1.1	G/A	SNP	307
		A/G	SNP	396
		T/G	SNP	1855
		CGGTATGCCCGGA/C	DELETION	1948
		T/A	SNP	1972
		TGGTATGCCCGGA[illegible]	DELETION	1972
XM_824118.1 Tbb strain 927/4 GUTat10.1 hypothetical protein (Tb11.01.3290) partial mRNA	GLOS_TB11.01.3290.1.1	GA/G	DELETION	2271
		A/T	SNP	2283
		C/T	SNP	2590
XM_824120.1 Tbb strain 927/4 GUTat10.1 trichohyalin (Tb11.01.3320) partial mRNA	GLOS_TB11.01.3320.1.1	A/AGG	INSERTION	607
		A/G	SNP	978
		G/A	SNP	2202
		A/C	SNP	2481
		C/T	SNP	2495
		T/G	SNP	2496
		T/C	SNP	2956

XM_824122.1 Tbb cyclin dependent kinase-binding protein (Tb11.01.3350) partial mRNA	GLOS_TB11.01.3350.1.1	A/G	SNP	473
		G/A	SNP	807
		T/C	SNP	1060
		C/T	SNP	3037
XM_824124.1 Tbb 927/4 GUTat10.1 glycosomal membrane protein (Tb11.01.3370) partial mRNA	GLOS_TB11.01.3370.1.1	A/T	SNP	80
		A/T	SNP	83
		G/A	SNP	240
		C/T	SNP	364
		G/A	SNP	538
		G/A	SNP	838
XM_824139.1 Tbb strain 927/4 GUTat10.1 vacuolar ATP synthase subunit B partial mRNA	GLOS_TB11.01.3560.1.1	A/C	SNP	1416
		GT/G	DELETION	2294
XP_829237.1 membrane-bound acid phosphatase [T. brucei brucei strain 927/4 GUTat10.1]	GLOS_TB11.01.3610.1.1	G/A	SNP	37
XP_829244.1 40S ribosomal protein S17 [Trypanosoma brucei brucei strain 927/4 GUTat10.1]	GLOS_TB11.01.3675.1.2	A/G	SNP	19
ref|XP_829245.1| 40S ribosomal protein S17 [T. brucei brucei strain 927/4 GUTat10.1]				
		C/T	SNP	21
XP_829244.1 40S ribosomal protein S17 [Trypanosoma brucei brucei strain 927/4 GUTat10.1]	GLOS_TB11.01.3675.2.2	A/G	SNP	398
ref|XP_829245.1| 40S ribosomal protein S17 [T. brucei brucei strain 927/4 GUTat10.1]				
XM_824159.1 Tbb strain 927/4 GUTat10.1 coatomer subunit gamma (Tb11.01.3740) partial mRNA	GLOS_TB11.01.3740.1.1	A/AT	INSERTION	2624
		A/G	SNP	3081
XP_829266.1 hypothetical protein [Trypanosoma brucei brucei strain 927/4 GUTat10.1]	GLOS_TB11.01.3860.1.1	C/T	SNP	409
		G/GT	INSERTION	448
XP_829272.1 hypothetical protein [Trypanosoma brucei brucei strain 927/4 GUTat10.1]	GLOS_TB11.01.3915.1.1	G/A	SNP	643
		C/CT	INSERTION	692
		T/A	SNP	940
		A/G	SNP	1167
		T/G	SNP	1168
		A/G	SNP	1170
		A/G	SNP	1171
		A/G	SNP	1172
		GGA/G	DELETION	1257
		G/T	SNP	1360
		AG/A	DELETION	1374
		T/C	SNP	1388
XM_824190.1 Tbb strain 927/4 GUTat10.1 hypothetical protein (Tb11.01.4030) partial mRNA	GLOS_TB11.01.4030.1.1	A/G	SNP	217
		A/C	SNP	270
		G/A	SNP	286
		G/C	SNP	1440
		C/T	SNP	1736

		TA/T	DELETION	2387
XM_824200.1 Tbb strain 927/4 GUTat10.1 protein kinase (Tb11.01.4130) partial mRNA	GLOS_TB11.01.4130.1.1	T/C	SNP	3110
		G/GT	INSERTION	3197
		C/T	SNP	3229
		GGAA/G	DELETION	3515
XP_829294.1 hypothetical protein [Trypanosoma brucei brucei strain 927/4 GUTat10.1]	GLOS_TB11.01.4140.1.1	C/A	SNP	617
		A/G	SNP	680
		A/G	SNP	791
		T/C	SNP	1144
		T/C	SNP	1176
		A/T	SNP	1196
		G/C	SNP	1344
		C/T	SNP	1687
		A/G	SNP	1908
		CA/C	DELETION	2867
		A/G	SNP	2921
XM_824233.1 Tbb strain 927/4 GUTat10.1 hypothetical protein (Tb11.01.4480) partial mRNA	GLOS_TB11.01.4480.1.1	C/T	SNP	625
		C/A	SNP	640
		T/G	SNP	750
		T/C	SNP	1098
		G/A	SNP	1752
		C/T	SNP	2516
		C/T	SNP	2780
XP_829371.1 cytochrome c oxidase subunit 10 [Trypanosoma brucei brucei strain 927/4 GUTat10.1]	GLOS_TB11.01.4702.1.1	T/A	SNP	49
		A/G	SNP	363
		C/CAA/CA	INSERTION	579
XM_824282.1 Tbb strain 927/4 GUTat10.1 hypothetical protein (Tb11.01.4740) partial mRNA	GLOS_TB11.01.4740.1.1	G/GA	INSERTION	274
		CTTT/C/CTT	DELETION	878
		TC/T	DELETION	1094
		G/GT	INSERTION	1785
		T/C	SNP	2575
		C/G	SNP	2823
XM_824283.1 Tbb strain 927/4 GUTat10.1 elongation factor 1 gamma (Tb11.01.4750) partial mRNA	GLOS_TB11.01.4750.1.1	C/A	SNP	364
		C/T	SNP	443
		C/T	SNP	449
		G/A	SNP	633
XM_824291.1 Tbb strain 927/4 GUTat10.1 hypothetical protein (Tb11.01.4850) partial mRNA	GLOS_TB11.01.4850.1.1	GA/G	DELETION	1210
		A/G	SNP	1228
		T/A	SNP	1324

		G/T	SNP	1397
		A/G	SNP	1572
		T/G	SNP	1634
		AAG/A	DELETION	1761
		GA/G	DELETION	2015
		GA/G	DELETION	2235
		CTT/C	DELETION	2399
		G/GA	INSERTION	2577
		A/AT	INSERTION	3726
		A/ATG	INSERTION	3815
XM_824313.1 Tbb strain 927/4 GUTat10.1 paraflagellar rod component (Tb11.01.5100) partial mRNA	GLOS_TB11.01.5100.1.1	C/T	SNP	410
		C/CT	INSERTION	494
		A/G	SNP	613
		G/GA	INSERTION	879
		T/C	SNP	983
XM_824315.1 Tbb strain 927/4 GUTat10.1 hypothetical protein (Tb11.01.5120) partial mRNA	GLOS_TB11.01.5120.1.1	C/T	SNP	185
		T/A	SNP	255
		T/C	SNP	263
		A/G	SNP	2321
		A/C	SNP	2352
		G/A	SNP	3499
		GA/G	DELETION	3795
XM_824337.1 Tbb receptor-type adenylate cyclase GRESAG 4 (Tb11.01.5310) partial mRNA	GLOS_TB11.01.5310.1.1	C/T	SNP	131
		G/A	SNP	3347
		A/C	SNP	3526
		CT/C	DELETION	4074
		A/AT	INSERTION	4382
XM_824365.1 Tbb strain 927/4 GUTat10.1 hypothetical protein (Tb11.01.5590) partial mRNA	GLOS_TB11.01.5590.1.1	C/T	SNP	261
		T/G	SNP	292
XM_824372.1 Tbb strain 927/4 GUTat10.1 hypothetical protein (Tb11.01.5680) partial mRNA	GLOS_TB11.01.5680.1.1	T/C	SNP	93
		A/T	SNP	598
		C/G	SNP	2150
XM_824373.1 Tbb strain 927/4 GUTat10.1 hypothetical protein (Tb11.01.5690) partial mRNA	GLOS_TB11.01.5690.1.1	GA/G	DELETION	394
		C/CT	INSERTION	906
		A/G	SNP	1501
		A/G	SNP	1794
		G/A	SNP	1861
		A/G	SNP	2224
		A/G	SNP	2320

		C/T	SNP	2350
		C/T	SNP	2940
		C/CT	INSERTION	2970
XP_829468.1 phenylalanyl-tRNA synthetase alpha subunit [Trypanosoma brucei TREU927]	GLOS_TB11.01.5710.1.1	A/C	SNP	78
		A/C	SNP	106
		C/T	SNP	448
		T/C	SNP	619
		A/G	SNP	697
		A/T	SNP	703
		C/A	SNP	736
		C/T	SNP	742
		C/T	SNP	880
		A/G	SNP	934
		G/A	SNP	949
		G/C	SNP	1003
		G/A	SNP	1030
		G/A	SNP	1203
		T/C	SNP	1283
		A/G	SNP	1285
		C/T	SNP	1315
		C/A	SNP	1324
		AG/A	DELETION	1349
		T/C	SNP	1364
		C/G	SNP	1371
		C/CA	INSERTION	1494
		A/C	SNP	1499
		A/G	SNP	1545
		A/C	SNP	1550
		C/T	SNP	1738
		C/T	SNP	1743
		A/C	SNP	1824
		T/C	SNP	1874
		G/A	SNP	2251
XM_824382.1 Tbb strain 927/4 GUTat10.1 hypothetical protein (Tb11.01.5780) partial mRNA	GLOS_TB11.01.5780.1.1	T/C	SNP	2032
		C/CT	INSERTION	3061
XP_829481.1 t-complex protein 1 subunit epsilon [Trypanosoma brucei TREU927]	GLOS_TB11.01.5860.1.1	T/TA	INSERTION	1880
XM_824396.1 Tbb strain 927/4 GUTat10.1 hypothetical protein (Tb11.01.5930) partial mRNA	GLOS_TB11.01.5930.1.1	G/A	SNP	182
		A/G	SNP	267
		G/T	SNP	999

		T/C	SNP	2662
		G/A	SNP	2815
		C/T	SNP	2858
		A/G	SNP	3295
		G/A	SNP	3936
		A/G	SNP	3972
		G/A	SNP	4174
		T/C	SNP	4749
		C/T	SNP	5371
		G/A	SNP	5410
XM_824438.1 Tbb strain 927/4 GUTat10.1 metalloprotease (Tb11.01.6360) partial mRNA	GLOS_TB11.01.6360.1.1	A/T	SNP	125
		A/C	SNP	267
		A/G	SNP	545
		C/A	SNP	625
		G/A	SNP	638
		G/A	SNP	1027
		T/C	SNP	1521
		T/G	SNP	2110
		G/A	SNP	2224
XM_824460.1 Tbb strain 927/4 GUTat10.1 hypothetical protein (Tb11.01.6590) partial mRNA	GLOS_TB11.01.6590.1.1	CT/C	DELETION	171
		C/CA	INSERTION	573
		T/C	SNP	1211
		C/CA	INSERTION	1767
		A/C	SNP	1959
		A/C	SNP	2173
		AT/A	DELETION	2174
		A/G	SNP	2338
		T/A	SNP	2339
		C/A	SNP	2343
		T/A	SNP	2344
		T/A	SNP	2348
		G/A	SNP	2349
XM_824462.1 Tbb strain 927/4 GUTat10.1 hypothetical protein (Tb11.01.6610) partial mRNA	GLOS_TB11.01.6610.1.1	C/T	SNP	108
		C/T	SNP	465
		C/T	SNP	519
		T/C	SNP	585
		G/A	SNP	684
		G/A	SNP	1005
		G/A	SNP	1155

		G/T	SNP	1198
		G/T	SNP	2190
XM_824467.1 Tbb strain 927/4 GUTat10.1 iron superoxide dismutase (Tb11.01.6660) partial mRNA	GLOS_TB11.01.6660.1.1	C/T	SNP	226
		T/C	SNP	299
		G/A	SNP	386
		A/G	SNP	439
		T/C	SNP	647
		T/C	SNP	657
		G/A	SNP	1065
XM_824502.1 Tbb strain 927/4 GUTat10.1 hypothetical protein (Tb11.01.7010) partial mRNA	GLOS_TB11.01.7010.1.1	A/G	SNP	37
		C/T	SNP	472
		A/G	SNP	493
		A/G	SNP	782
		A/C	SNP	2628
		T/A	SNP	3559
		C/T	SNP	3894
		G/T	SNP	4324
		T/C	SNP	4418
		G/A	SNP	4462
XP_829613.1 NADH-cytochrome b5 reductase [Trypanosoma brucei brucei strain 927/4 GUTat10.1]	GLOS_TB11.01.7190.1.1	C/T	SNP	293
		C/T	SNP	306
		T/A	SNP	308
		T/C	SNP	424
		T/C	SNP	456
		G/A	SNP	494
		T/C	SNP	565
		A/T	SNP	588
		G/C	SNP	641
		A/T	SNP	648
		G/T	SNP	927
		GA/G	DELETION	1199
		T/G	SNP	1624
		A/G	SNP	1640
		T/C	SNP	1831
		C/T	SNP	1877
		T/A	SNP	1879
		G/T	SNP	1919
		G/A	SNP	1925
		G/C	SNP	1933

		A/G	SNP	1964
		T/TATA	INSERTION	2028
		T/A	SNP	2030
		G/GT	INSERTION	2370
		G/A	SNP	2602
		G/A	SNP	2936
XP_829638.1 hypothetical protein [Trypanosoma brucei brucei strain 927/4 GUTat10.1]	GLOS_TB11.01.7460.1.1	G/A	SNP	952
XM_824548.1 Tbb strain 927/4 GUTat10.1 amino acid transporter (Tb11.01.7500) partial mRNA	GLOS_TB11.01.7500.1.1	G/C	SNP	139
		G/A	SNP	174
		C/T	SNP	703
		C/T	SNP	841
		A/T	SNP	879
		G/A	SNP	893
		T/C	SNP	1354
		T/C	SNP	1357
		A/C	SNP	1358
		G/A	SNP	1360
		T/A	SNP	1363
		T/C	SNP	1451
		T/G	SNP	2201
		T/C	SNP	2203
		T/C	SNP	2211
		G/A	SNP	2333
XP_829645.1 60S ribosomal protein L27 [Trypanosoma brucei brucei strain 927/4 GUTat10.1] ref|XP_82 ref|XP_829647.1| 60S ribosomal protein L27 [Trypanosoma brucei brucei strain 927/4 GUTat10.1]	GLOS_TB11.01.7535.1.1	G/A	SNP	136
		A/G	SNP	319
XM_824562.1 Tbb strain 927/4 GUTat10.1 hypothetical protein (Tb11.01.7620) partial mRNA	GLOS_TB11.01.7620.1.1	G/GA	INSERTION	1221
		T/C	SNP	2237
		C/T	SNP	2552
XM_824563.1 Tbb strain 927/4 GUTat10.1 hypothetical protein (Tb11.01.7630) partial mRNA	GLOS_TB11.01.7630.1.1	A/G	SNP	369
		G/C	SNP	500
		A/G	SNP	612
		T/C	SNP	713
		A/G	SNP	788
		C/G	SNP	1068
		C/T	SNP	1137
		G/A	SNP	1758
		C/T	SNP	1760
		T/C	SNP	2000

		G/A	SNP	2304
		C/T	SNP	2513
		T/C	SNP	2625
		T/G	SNP	2832
		GA/G	DELETION	3100
		G/C	SNP	3124
		G/A	SNP	3146
		T/TA	INSERTION	3259
		A/C	SNP	3354
		C/T	SNP	3374
		C/T	SNP	3414
		C/T	SNP	3447
		G/C	SNP	3481
		T/C	SNP	3508
		C/T	SNP	3509
		G/A	SNP	3527
XM_824577.1 Tbbstrain 927/4 GUTat10.1 nucleoside diphosphate kinase partial mRNA	GLOS_TB11.01.7800.1.1	A/T	SNP	127
		G/A	SNP	143
		G/T	SNP	705
XM_824584.1 Tbb strain 927/4 GUTat10.1 microtubule-associated protein partial mRNA	GLOS_TB11.01.7880.1.1	CA/CAA/C	INSERTION	561
		C/CT	INSERTION	1188
XP_829708.1 hypothetical protein [Trypanosoma brucei brucei strain 927/4 GUTat10.1]	GLOS_TB11.01.8225.1.1	GT/G	DELETION	738
		C/T	SNP	752
		C/G	SNP	970
XM_824637.1 Tbb strain 927/4 GUTat10.1 dihydrolipoyl dehydrogenase (Tb11.01.8470) partial mRNA	GLOS_TB11.01.8470.1.1	T/C	SNP	433
		C/T	SNP	761
		C/T	SNP	1132
		C/T	SNP	1370
		C/A	SNP	1451
		G/T	SNP	1484
XM_824640.1 Tbb strain 927/4 GUTat10.1 t-complex protein 1 subunit alpha partial mRNA	GLOS_TB11.01.8510.1.1	G/A	SNP	139
		T/TA	INSERTION	250
		C/CT	INSERTION	397
		G/A	SNP	479
		G/GT	INSERTION	491
		G/T	SNP	494
		A/T	SNP	501
XM_824641.1 Tbb strain 927/4 GUTat10.1 glucosamine-6-phosphate isomerase partial mRNA	GLOS_TB11.01.8520.1.1	T/A	SNP	237
		T/A	SNP	238

		C/CT	INSERTION	413
		AC/A	DELETION	545
		C/A	SNP	585
		CT/C	DELETION	879
		T/C	SNP	1036
		T/C	SNP	1393
		G/C	SNP	1668
		C/T	SNP	1726
		C/T	SNP	1786
		T/C	SNP	2021
XP_829756.1 hypothetical protein [Trypanosoma brucei brucei strain 927/4 GUTat10.1]	GLOS_TB11.01.8770.1.1	CTT/C/CT	DELETION	113
		A/C	SNP	868
		G/A	SNP	1112
		A/G	SNP	1268
		C/T	SNP	1269
		A/G	SNP	2258
		C/T	SNP	2876
XM_824667.1 Tbb expression site-associated gene (ESAG) protein (Tb11.01.8820) partial mRNA	GLOS_TB11.01.8820.1.1	A/G	SNP	108
		G/A	SNP	156
		T/C	SNP	157
		C/T	SNP	163
		A/G	SNP	182
		T/C	SNP	513
		A/G	SNP	805
		T/C	SNP	830
		G/A	SNP	1096
		A/G	SNP	1119
		G/A	SNP	1149
		A/G	SNP	1217
		C/G	SNP	1239
		G/A	SNP	1265
		G/A	SNP	1285
		T/C	SNP	1325
		A/G	SNP	1681
		A/G	SNP	1685
		G/C	SNP	1727
		G/A	SNP	1729
		T/C	SNP	1769
		T/C	SNP	1778

		C/A	SNP	1806
		C/T	SNP	1850
		C/G	SNP	1870
		T/C	SNP	1883
		C/G	SNP	1898
		A/C	SNP	1910
		C/T	SNP	1915
		G/A	SNP	2024
		C/T	SNP	2032
		A/G	SNP	2078
		A/C	SNP	2120
		T/C	SNP	2189
		A/G	SNP	2247
		C/T	SNP	2335
		G/A	SNP	2347
		A/G	SNP	2573
		G/C	SNP	2747
		T/C	SNP	2750
		G/A	SNP	2760
		A/T	SNP	2768
		A/G	SNP	2786
		C/T	SNP	2825
		C/T	SNP	3012
		G/T	SNP	3204
		T/C	SNP	3275
		T/C	SNP	3332
		C/T	SNP	3555
		T/TTC	INSERTION	3883
		C/T	SNP	3887
		GTTA/G	DELETION	4204
		A/G	SNP	4541
XP_828324.1 hypothetical protein [Trypanosoma brucei brucei strain 927/4 GUTat10.1]	GLOS_TB11.02.0010.1.1	AAAAT/A	DELETION	278
		A/T	SNP	638
XM_823237.1 Tbb strain 927/4 GUTat10.1 aminopeptidase (Tb11.02.0070) partial mRNA	GLOS_TB11.02.0070.1.1	AT/A	DELETION	233
		CA/C	DELETION	358
		G/A	SNP	985
		G/A	SNP	1197
		A/C	SNP	2934
XM_823251.1 Tbb strain 927/4 GUTat10.1 hypothetical protein (Tb11.02.0210) partial mRNA	GLOS_TB11.02.0210.1.1	A/G	SNP	67

		A/G	SNP	97
		A/G	SNP	864
		A/G	SNP	1494
		A/G	SNP	1527
		AT/A	DELETION	2194
		A/G	SNP	2485
		T/A	SNP	2523
XP_828348.1 heat shock protein mitochondrial precursor [Trypanosoma brucei TREU927]	GLOS_TB11.02.0250.1.1	C/A	SNP	56
		A/G	SNP	62
		G/A	SNP	77
		A/G	SNP	223
		A/G	SNP	244
		A/G	SNP	550
		C/T	SNP	1200
		A/G	SNP	1664
		A/G	SNP	2201
		C/T	SNP	2284
		T/C	SNP	2829
		G/GT	INSERTION	3279
XM_823282.1 Tbb strain 927/4 GUTat10.1 hypothetical protein (Tb11.02.0445) partial mRNA	GLOS_TB11.02.0445.1.1	C/T	SNP	76
		A/T	SNP	77
		G/GA	INSERTION	116
		C/CA	INSERTION	217
		C/CT	INSERTION	916
XP_828407.1 dynein heavy chain [Trypanosoma brucei brucei strain 927/4 GUTat10.1]	GLOS_TB11.02.0760.1.1	A/G	SNP	3203
XM_823316.1 Tbb strain 927/4 GUTat10.1 squalene monooxygenase (Tb11.02.0780) partial mRNA	GLOS_TB11.02.0780.1.1	C/CA	INSERTION	662
		A/G	SNP	695
		C/A	SNP	696
XM_823317.1 Tbb strain 927/4 GUTat10.1 kinesin (Tb11.02.0790) partial mRNA	GLOS_TB11.02.0790.1.1	GGT/G	DELETION	424
		GA/G	DELETION	1159
		C/CCCT	INSERTION	1548
XP_828413.1 ubiquitin-conjugating enzyme [Trypanosoma brucei brucei strain 927/4 GUTat10.1]	GLOS_TB11.02.0815.1.1	CT/C	DELETION	540
		G/A	SNP	724
		G/T	SNP	834
		A/AC	INSERTION	884
		A/T	SNP	983
		G/GT	INSERTION	1127
		G/GA	INSERTION	1138
		C/CT	INSERTION	1384

		A/G	SNP	1408
		AT/A	DELETION	1537
		T/C	SNP	1548
		T/A	SNP	1550
		G/A	SNP	1558
		T/A	SNP	1631
		A/G	SNP	1658
		G/GA	INSERTION	1852
		A/AT	INSERTION	1905
		C/CAT	INSERTION	1956
		GTTTTTTTTGTGT(	DELETION	2056
		A/T	SNP	2195
		A/T	SNP	2196
		C/A	SNP	2294
XM_823338.1 Tbb strain 927/4 GUTat10.1 hypothetical protein (Tb11.02.0980) partial mRNA	GLOS_TB11.02.0980.1.1	C/T	SNP	2443
XP_828438.1 aminopeptidase [Trypanosoma brucei brucei strain 927/4 GUTat10.1]	GLOS_TB11.02.1070.1.1	C/T	SNP	238
XP_828440.1 40s ribosomal protein S4 [Trypanosoma brucei brucei strain 927/4 GUTat10.1] ref|XP_828441.1| 40S ribosomal protein S4 [Trypanosoma brucei brucei strain 927/4 GUTat10.1]	GLOS_TB11.02.1085.1.1	C/T	SNP	31
		C/G	SNP	33
		C/G	SNP	732
		C/A	SNP	848
		T/C	SNP	849
XM_823349.1 Tbb nucleobase/nucleoside transporter 8.1 (Tb11.02.1100) partial mRNA	GLOS_TB11.02.1100.1.1	T/C	SNP	190
		C/T	SNP	194
		A/C	SNP	204
		A/G	SNP	299
		G/A	SNP	377
		T/C	SNP	714
		A/G	SNP	737
		A/G	SNP	885
		T/G	SNP	1472
		A/G	SNP	1738
		AT/A	DELETION	1803
		T/C	SNP	1877
		A/G	SNP	1890
		C/CT	INSERTION	1913
		C/T	SNP	1917
XM_823359.1 Tbb strain 927/4 GUTat10.1 hypothetical protein (Tb11.02.1190) partial mRNA	GLOS_TB11.02.1190.1.1	T/C	SNP	89
		G/A	SNP	1498

		T/C	SNP	2154
XM_823383.1 Tbb strain 927/4 GUTat10.1 hypothetical protein (Tb11.02.1470) partial mRNA	GLOS_TB11.02.1470.1.1	A/G	SNP	257
		T/G	SNP	712
		A/G	SNP	1230
		G/A	SNP	1358
		C/A	SNP	2412
XM_823384.1 Trypanosoma brucei brucei strain 927/4 GUTat10.1 mitochondrial processing peptidase subunit alpha (Tb11.02.1480) partial mRNA. nuclear gene for mitochondrial product	GLOS_TB11.02.1480.1.1	CA/C	DELETION	120
		C/A	SNP	240
		A/G	SNP	871
		G/A	SNP	1805
		T/G	SNP	1864
		C/T	SNP	1929
		GT/G	DELETION	2073
		G/GGT	INSERTION	2310
XM_823403.1 Tbb strain 927/4 GUTat10.1 lectin (Tb11.02.1680) partial mRNA	GLOS_TB11.02.1680.1.1	A/G	SNP	1891
XP_828519.1 ferric reductase [Trypanosoma brucei brucei strain 927/4 GUTat10.1]	GLOS_TB11.02.1990.1.1	C/T	SNP	130
		A/C	SNP	389
		C/CA	INSERTION	777
		A/C	SNP	2570
		T/C	SNP	2585
		A/G	SNP	2690
XM_823464.1 Tbb strain 927/4 GUTat10.1 60S ribosomal protein L17 (Tb11.02.2430) partial mRNA	GLOS_TB11.02.2430.1.1	C/T	SNP	295
XP_828580.1 fumarate hydratase class I [Trypanosoma brucei brucei strain 927/4 GUTat10.1]	GLOS_TB11.02.2700.1.1	A/G	SNP	370
		A/T	SNP	802
		G/GAA	INSERTION	2176
XP_828602.1 ubiquitin carboxyl-terminal hydrolase [Trypanosoma brucei TREU927]	GLOS_TB11.02.2940.1.1	T/C	SNP	375
		T/C	SNP	2288
		G/A	SNP	2491
		C/CT	INSERTION	2961
XM_823517.1 Tbb strain 927/4 GUTat10.1 sugar transporter (Tb11.02.3020) partial mRNA	GLOS_TB11.02.3020.1.1	GA/G	DELETION	2619
		A/G	SNP	3124
XM_823526.1 Tbb strain 927/4 GUTat10.1 malic enzyme (Tb11.02.3120) partial mRNA	GLOS_TB11.02.3120.1.1	C/T	SNP	1477
		A/C	SNP	1480
		A/T	SNP	1481
		T/A	SNP	1483
		C/A	SNP	1486
		A/T	SNP	1764
		CAT/C	DELETION	2011

		T/C	SNP	2030
		AT/A	DELETION	2533
XM_823534.1 Tbb strain 927/4 GUTat10.1 triosephosphate isomerase (Tb11.02.3210) partial mRNA	GLOS_TB11.02.3210.1.1	GTATA/G	DELETION	943
		G/GA	INSERTION	1084
		A/G	SNP	1157
XP_828635.1 hypothetical protein [Trypanosoma brucei brucei strain 927/4 GUTat10.1]	GLOS_TB11.02.3310.1.1	TA/T	DELETION	93
		C/T	SNP	260
		T/C	SNP	537
		T/C	SNP	896
		T/A	SNP	1068
		G/GT	INSERTION	1108
		A/T	SNP	1116
		T/A	SNP	1117
		G/T	SNP	1118
		T/G	SNP	1307
		A/G	SNP	1337
		CT/C	DELETION	1388
		A/G	SNP	1427
		T/A	SNP	1442
XP_828657.1 hypothetical protein [Trypanosoma brucei brucei strain 927/4 GUTat10.1]	GLOS_TB11.02.3570.1.1	CT/C	DELETION	1806
XM_823567.1 Tbb strain 927/4 GUTat10.1 hypothetical protein (Tb11.02.3610) partial mRNA	GLOS_TB11.02.3610.1.1	A/AT	INSERTION	2257
XM_823578.1 Tbb strain 927/4 GUTat10.1 hypothetical protein (Tb11.02.3770) partial mRNA	GLOS_TB11.02.3770.2.2	G/A	SNP	153
XP_828679.1 hypothetical protein [Trypanosoma brucei brucei strain 927/4 GUTat10.1]	GLOS_TB11.02.3860.1.1	C/CT	INSERTION	2287
XM_823587.1 Tbb strain 927/4 GUTat10.1 hypothetical protein (Tb11.02.3880) partial mRNA	GLOS_TB11.02.3880.1.1	GCTC/G	DELETION	3263
		G/T	SNP	4238
XM_823594.1 Tbb S-phase kinase-associated protein (Tb11.02.3990) partial mRNA	GLOS_TB11.02.3990.1.1	G/GA	INSERTION	737
		A/G	SNP	1280
XP_828688.1 40S ribosomal protein S15a [Trypanosoma brucei brucei strain 927/4 GUTat10.1] ref|XP_844028.1| 40S ribosomal protein S15a [Trypanosoma brucei brucei strain 927/4 GUTat10.1]	GLOS_TB11.02.4000.1.1	T/A	SNP	625
XP_828691.1 protein transport protein Sec31 [Trypanosoma brucei brucei strain 927/4 GUTat10.1]	GLOS_TB11.02.4040.1.1	G/C	SNP	1122
		A/G	SNP	2554
		A/G	SNP	4501
XM_823599.1 Tbb strain 927/4 GUTat10.1 60S ribosomal protein L28 (Tb11.02.4050) partial mRNA	GLOS_TB11.02.4050.1.1	T/C	SNP	405
		A/T	SNP	594
		C/T	SNP	595
XM_823604.1 Tbb pretranslocation protein subunit alpha (Tb11.02.4100) partial mRNA	GLOS_TB11.02.4100.1.1	C/CT	INSERTION	2511
XP_828702.1 pyruvate phosphate dikinase [Trypanosoma brucei brucei strain 927/4 GUTat10.1]	GLOS_TB11.02.4150.1.1	C/CT	INSERTION	3126
		G/GT	INSERTION	3165
		G/GT	INSERTION	3332

		GT/G	DELETION	3569
XM_823611.1 Tbb strain 927/4 GUTat10.1 40S ribosomal protein S5 (Tb11.02.4170) partial mRNA	GLOS_TB11.02.4170.1.2	T/A	SNP	14
		T/A	SNP	16
		G/T	SNP	272
		A/G	SNP	669
		T/A	SNP	436
XM_823625.1 Tbb strain 927/4 GUTat10.1 hypothetical protein (Tb11.02.4300) partial mRNA	GLOS_TB11.02.4300.1.1	CT/C	DELETION	352
		CA/C	DELETION	1547
		G/GA	INSERTION	1672
		CA/C	DELETION	2413
		G/GA	INSERTION	2692
		C/CT	INSERTION	4806
XM_823631.1 Tbb strain 927/4 GUTat10.1 40S ribosomal protein S21 (Tb11.02.4350) partial mRNA	GLOS_TB11.02.4350.1.1	T/A	SNP	405
		T/A	SNP	853
		T/A	SNP	854
XM_823677.1 Tbb strain 927/4 GUTat10.1 hypothetical protein (Tb11.02.4810) partial mRNA	GLOS_TB11.02.4810.1.1	C/T	SNP	802
XP_828778.1 acidocalcisomal pyrophosphatase [T. brucei brucei strain 927/4 GUTat10.1]	GLOS_TB11.02.4910.1.1	A/G	SNP	1258
		C/T	SNP	1315
XM_823706.1 Tbb strain 927/4 GUTat10.1 hypothetical protein (Tb11.02.5120) partial mRNA	GLOS_TB11.02.5120.1.1	A/G	SNP	1945
		G/A	SNP	2875
		C/T	SNP	2908
XP_828807.1 pantothenate kinase subunit [Trypanosoma brucei brucei strain 927/4 GUTat10.1]	GLOS_TB11.02.5190.1.1	T/C	SNP	1594
		T/C	SNP	3884
		A/G	SNP	4545
		C/T	SNP	4590
		A/G	SNP	5265
XM_823735.1 Tbb strain 927/4 GUTat10.1 cystathionine beta-synthase partial mRNA	GLOS_TB11.02.5400.1.1	T/A	SNP	2083
		C/G	SNP	2090
XM_823744.1 Tbb strain 927/4 GUTat10.1 hypothetical protein (Tb11.02.5490) partial mRNA	GLOS_TB11.02.5490.1.1	A/AT	INSERTION	606
XM_823745.1 Tbb strain 927/4 GUTat10.1 glucose-regulated protein 78 (Tb11.02.5500) partial mRNA	GLOS_TB11.02.5500.1.1	A/G	SNP	160
		C/G	SNP	640
		C/T	SNP	804
XM_823752.1 Tbb strain 927/4 GUTat10.1 hypothetical protein (Tb11.02.5570) partial mRNA	GLOS_TB11.02.5570.1.1	A/T	SNP	172
		AT/A	DELETION	204
		CTT/C	DELETION	313
XM_823754.1 Tbb strain 927/4 GUTat10.1 hypothetical protein (Tb11.02.5590) partial mRNA	GLOS_TB11.02.5590.1.1	C/T	SNP	44
		C/T	SNP	164
		CA/C	DELETION	167
		C/T	SNP	222

		A/G	SNP	338
		A/G	SNP	407
		A/G	SNP	505
		AT/A	DELETION	513
		C/T	SNP	745
		A/G	SNP	782
		A/C	SNP	786
		C/T	SNP	832
		A/G	SNP	869
		T/A	SNP	921
		T/C	SNP	1080
		G/A	SNP	1084
		C/T	SNP	1107
		G/A	SNP	1148
		G/A	SNP	1342
		C/T	SNP	1364
		G/A	SNP	1846
		A/G	SNP	1894
		T/C	SNP	1961
		C/T	SNP	1962
		G/A	SNP	2106
		C/T	SNP	2121
XM_823107.1 Tbb strain 927/4 GUTat10.1 ABC transporter (Tb11.03.0030) partial mRNA	GLOS_TB11.03.0030.1.1	T/C	SNP	1176
		CA/C	DELETION	2810
XM_823102.1 Tbb strain 927/4 GUTat10.1 ribokinase (Tb11.03.0090) partial mRNA	GLOS_TB11.03.0090.1.1	G/A	SNP	1233
		G/GT	INSERTION	2238
XM_823098.1 Tbb strain 927/4 GUTat10.1 nucleoporin (Tb11.03.0140) partial mRNA	GLOS_TB11.03.0140.1.1	G/A	SNP	118
		C/T	SNP	202
		T/C	SNP	238
		A/G	SNP	269
		T/C	SNP	274
XP_828183.1 isocitrate dehydrogenase [Trypanosoma brucei brucei strain 927/4 GUTat10.1]	GLOS_TB11.03.0230.1.1	G/GA	INSERTION	749
		CAAAG/C	DELETION	940
		C/T	SNP	1017
XM_823089.1 Tbb strain 927/4 GUTat10.1 hypothetical protein (Tb11.03.0240) partial mRNA	GLOS_TB11.03.0240.1.1	A/G	SNP	3704
		A/G	SNP	3720
		A/T	SNP	4186
		A/T	SNP	4187
XM_823088.1 Tbb strain 927/4 GUTat10.1 cyclophilin A (Tb11.03.0250) partial mRNA	GLOS_TB11.03.0250.1.1	AG/A	DELETION	432

XM_823084.1 Tbb strain 927/4 GUTat10.1 hypothetical protein (Tb11.03.0300) partial mRNA	GLOS_TB11.03.0300.1.1	T/A	SNP	474
		T/C	SNP	658
		A/G	SNP	1235
		T/C	SNP	2188
		A/C	SNP	2426
		T/G	SNP	2488
		T/C	SNP	2527
		C/T	SNP	2879
XP_828169.1 protein phosphatase 2C [Trypanosoma brucei brucei strain 927/4 GUTat10.1]	GLOS_TB11.03.0390.1.1	C/T	SNP	221
		C/A	SNP	304
		CA/C	DELETION	1439
		G/GT	INSERTION	1554
		G/GTA	INSERTION	2312
XM_823075.1 Tbb strain 927/4 GUTat10.1 DNA repair protein (Tb11.03.0400) partial mRNA	GLOS_TB11.03.0400.1.1	A/T	SNP	2559
XP_828152.1 hypothetical protein [Trypanosoma brucei brucei strain 927/4 GUTat10.1]	GLOS_TB11.03.0475.1.1	T/A	SNP	588
		T/A	SNP	589
XM_823054.1 Tbb strain 927/4 GUTat10.1 hypothetical protein (Tb11.03.0530) partial mRNA	GLOS_TB11.03.0530.1.1	T/A	SNP	438
		GA/G	DELETION	662
		T/TA	INSERTION	857
		A/AT	INSERTION	866
		A/T	SNP	1132
		C/T	SNP	1180
		C/T	SNP	1182
		G/GA	INSERTION	1263
		G/GA	INSERTION	1427
		CT/C	DELETION	2004
		CT/C	DELETION	2236
XM_823023.1 Tbb strain 927/4 GUTat10.1 hypothetical protein (Tb11.03.0900) partial mRNA	GLOS_TB11.03.0900.1.1	T/G	SNP	732
		C/G	SNP	2083
		T/C	SNP	2158
		T/C	SNP	2546
		G/A	SNP	2681
		T/C	SNP	3061
		A/G	SNP	3168
		A/G	SNP	3257
		C/T	SNP	3279
		T/C	SNP	3384
XP_828111.1 elongation factor [Trypanosoma brucei brucei strain 927/4 GUTat10.1]	GLOS_TB11.03.0940.1.1	T/C	SNP	753
		G/A	SNP	1491

		C/T	SNP	1749
		C/G	SNP	1794
		A/C	SNP	2442
		A/AT	INSERTION	2488
		C/T	SNP	2550
		G/A	SNP	2610
		G/A	SNP	2616
XM_823219.1 Tbb strain 927/4 GUTat10.1 hypothetical protein (Tb11.18.0002) partial mRNA	GLOS_TB11.18.0002.1.1	T/C	SNP	1164
XM_823216.1 Tbb strain 927/4 GUTat10.1 hypothetical protein (Tb11.18.0005) partial mRNA	GLOS_TB11.18.0005.1.1	A/C	SNP	469
XM_823228.1 Tbb strain 927/4 GUTat10.1 hypothetical protein (Tb11.22.0004) partial mRNA	GLOS_TB11.22.0004.1.1	G/A	SNP	696
		CT/C	DELETION	926
XM_823146.1 Tbb receptor-type adenylate cyclase GRESAG 4 (Tb11.27.0001) partial mRNA	GLOS_TB11.27.0001.1.1	A/G	SNP	643
		A/G	SNP	1003
		A/G	SNP	1109
		G/A	SNP	1190
		G/A	SNP	1297
		G/A	SNP	1352
		T/C	SNP	1411
		C/T	SNP	1624
		A/G	SNP	1636
		T/C	SNP	1705
		A/T	SNP	1918
		T/C	SNP	1960
		A/G	SNP	2088
		G/A	SNP	2166
		A/G	SNP	2284
		A/G	SNP	2309
		C/T	SNP	2337
		C/T	SNP	2462
		A/G	SNP	2498
		C/T	SNP	2520
		T/C	SNP	2524
		G/A	SNP	2635
		C/T	SNP	2830
		T/C	SNP	3068
		T/C	SNP	3070
		G/A	SNP	3073
		A/G	SNP	3088
		C/T	SNP	3305

		T/C	SNP	3376
		C/T	SNP	3469
		A/G	SNP	3672
		T/C	SNP	3685
		G/A	SNP	3812
		T/C	SNP	3836
		G/A	SNP	3959
		C/CT	INSERTION	4111
		T/C	SNP	4216
		G/A	SNP	4376
		ATG/A	DELETION	4381
		GT/G	DELETION	4429
XM_823178.1 Tbb strain 927/4 GUTat10.1 hypothetical protein (Tb11.39.0004) partial mRNA	GLOS_TB11.39.0004.1.1	C/CA	INSERTION	89
		G/GA	INSERTION	96
		C/CT	INSERTION	1497
XM_823177.1 Tbb strain 927/4 GUTat10.1 hypothetical protein (Tb11.39.0005) partial mRNA	GLOS_TB11.39.0005.1.1	C/CT	INSERTION	368
		G/GA	INSERTION	749
		G/GA	INSERTION	3135
XP_828289.1 60S acidic ribosomal subunit protein [Trypanosoma brucei TREU927] ref|XP_828290.1| 60S acidic ribosomal subunit protein [Trypanosoma brucei TREU927]	GLOS_TB11.46.0002.1.1	G/T	SNP	182
		G/A	SNP	462
		C/T	SNP	678
		G/T	SNP	974
XM_823189.1 Tbb strain 927/4 GUTat10.1 hypothetical protein (Tb11.46.0009) partial mRNA	GLOS_TB11.46.0009.1.1	C/CT	INSERTION	3538
XP_828236.1 2-oxoglutarate dehydrogenase subunit [Trypanosoma brucei TREU927]	GLOS_TB11.47.0004.1.1	G/T	SNP	2538
		C/T	SNP	2543
		C/T	SNP	3126
		T/G	SNP	4586
		T/C	SNP	5197
XP_828234.1 hypothetical protein [Trypanosoma brucei brucei strain 927/4 GUTat10.1]	GLOS_TB11.47.0006.1.1	T/TG	INSERTION	88
		T/C	SNP	150
		GA/G	DELETION	219
		C/T	SNP	999
		A/G	SNP	2874
		C/A	SNP	2957
		C/T	SNP	3045
		G/GAA	INSERTION	3051
XP_828218.1 hypothetical protein [Trypanosoma brucei brucei strain 927/4 GUTat10.1]	GLOS_TB11.47.0022.1.1	A/G	SNP	107
		T/C	SNP	132

		C/T	SNP	312
		CACACACATACA/C	DELETION	367
		CA/C	DELETION	377
		C/CT	INSERTION	973
XP_828203.1 calpain, partial [Trypanosoma brucei brucei strain 927/4 GUTat10.1]	GLOS_TB11.47.0036.1.1	A/G	SNP	142
		C/A	SNP	145
		G/T	SNP	146
		A/G	SNP	187
		T/A	SNP	199
		A/G	SNP	201
		A/G	SNP	235
		G/C	SNP	260
		A/T	SNP	280
		A/G	SNP	281
		T/C	SNP	289
		C/T	SNP	296
		T/C	SNP	303
		A/G	SNP	391
		T/A	SNP	403
		A/G	SNP	405
		A/T	SNP	484
		A/G	SNP	485
		T/C	SNP	493
		C/T	SNP	500
		T/C	SNP	507
		T/A	SNP	508
		A/G	SNP	589
		T/A	SNP	607
		A/G	SNP	609
XM_823067.1 Tbb strain 927/4 GUTat10.1 60S ribosomal protein L21E (Tb11.50.0005) partial mRNA	GLOS_TB11.50.0005.1.1	A/G	SNP	33
		A/C	SNP	35
		C/T	SNP	135
		T/C	SNP	162
		T/A	SNP	165
		G/A	SNP	189
		C/T	SNP	195
		C/T	SNP	306
		T/C	SNP	387
		C/A	SNP	431

		G/A	SNP	465
XP_828164.1 dynein light chain [Trypanosoma brucei brucei strain 927/4 GUTat10.1]	GLOS_TB11.50.0007.1.1	T/TA	INSERTION	440
		GA/G	DELETION	610
		T/A	SNP	1072
		C/T	SNP	1076
		A/G	SNP	1096
XM_824256.1 Tbb strain 927/4 GUTat10.1 hypothetical protein (Tb11.52.0008) partial mRNA	GLOS_TB11.52.0008.1.1	T/C	SNP	62
		A/G	SNP	69
		A/G	SNP	73
		C/T	SNP	89
		A/G	SNP	98
		A/G	SNP	132
		C/T	SNP	147
		C/G	SNP	152
		A/G	SNP	161
		G/A	SNP	164
		A/G	SNP	167
		A/G	SNP	186
		A/G	SNP	190
		C/T	SNP	206
		A/G	SNP	224
		A/G	SNP	249
		C/T	SNP	250
		A/C	SNP	267
		G/A	SNP	286
		A/G	SNP	484
		C/T	SNP	598
		A/C	SNP	768
XM_001218825.1 T. brucei cytidine triphosphate synthase, putative (Tb927.1.1240) partial mRNA	GLOS_TB927.1.1240.1.1	G/GA	INSERTION	246
XM_001218868.1 Trypanosoma brucei hypothetical protein, conserved (Tb927.1.1670) partial mRNA	GLOS_TB927.1.1670.1.1	C/T	SNP	249
XM_001218909.1 Trypanosoma brucei brucei strain 927/4 GUTat10.1 calpain-like cysteine peptidase. cysteine peptidase, Clan CA, family C2 partial mRNA	GLOS_TB927.1.2100.1.1	C/CA	INSERTION	520
		G/A	SNP	1876
		C/CT	INSERTION	2074
		A/G	SNP	3259
		C/T	SNP	3286
		T/C	SNP	3301
		C/A	SNP	3304
		C/T	SNP	3305

		A/G	SNP	3310
		A/G	SNP	3358
		C/T	SNP	3368
		T/C	SNP	3380
XM_001218922.1 T. brucei calpain-like protein fragment, putative (Tb927.1.2230) partial mRNA	GLOS_TB927.1.2230.1.1	G/A	SNP	529
		GA/G	DELETION	587
		A/G	SNP	593
XM_001218924.1 Trypanosoma brucei hypothetical protein, unlikely (Tb927.1.2250) partial mRNA	GLOS_TB927.1.2250.1.1	C/CT	INSERTION	110
		C/CT	INSERTION	166
XM_841300.1 Trypanosoma brucei hypothetical protein, unlikely (Tb927.1.230) partial mRNA	GLOS_TB927.1.230.1.1	T/C	SNP	61
		T/A	SNP	94
		T/C	SNP	195
		C/T	SNP	215
		GA/G	DELETION	242
		T/C	SNP	249
		C/T	SNP	288
		A/G	SNP	299
		G/A	SNP	317
		T/TAA/TA	INSERTION	323
		A/T	SNP	361
		T/C	SNP	369
		C/T	SNP	371
		G/C	SNP	505
		G/T	SNP	507
		C/T	SNP	580
		T/C	SNP	638
		A/G	SNP	657
		A/G	SNP	659
		T/A	SNP	660
		G/T	SNP	668
		A/G	SNP	813
		C/T	SNP	862
		G/A	SNP	931
		G/C	SNP	960
		T/C	SNP	982
		A/C	SNP	1022
		T/A	SNP	1040
		C/A	SNP	1057
		CA/C	DELETION	1084

C/T	SNP	1129
T/C	SNP	1132
C/T	SNP	1136
T/A	SNP	1166
C/T	SNP	1183
A/T	SNP	1196
G/A	SNP	1238
G/C	SNP	1274
T/G	SNP	1280
C/T	SNP	1298
G/A	SNP	1312
A/G	SNP	1313
T/TTA	INSERTION	1327
A/T	SNP	1342
G/T	SNP	1382
T/C	SNP	1390
C/T	SNP	1421
G/T	SNP	1423
C/T	SNP	1424
G/A	SNP	1434
C/G	SNP	1443
A/G	SNP	1502
C/A	SNP	1509
G/T	SNP	1535
G/A	SNP	1551
G/A	SNP	1572
G/T	SNP	1581
C/A	SNP	1604
T/C	SNP	1618
C/T	SNP	1637
C/T	SNP	1675
T/C	SNP	1683
G/A	SNP	1709
G/T	SNP	1711
A/C	SNP	1732
C/T	SNP	1752
C/T	SNP	1759
C/T	SNP	1771
A/G	SNP	1786

		C/T	SNP	1797
		G/T	SNP	1832
		A/T	SNP	1864
		A/T	SNP	1865
		C/T	SNP	1866
		A/G	SNP	1887
		C/T	SNP	1901
		C/T	SNP	1907
		A/G	SNP	1921
		A/G	SNP	1928
		G/T	SNP	1929
		A/C	SNP	1931
		CT/C	DELETION	1933
		TC/T	DELETION	1937
		A/C	SNP	1968
		C/A	SNP	1993
		C/T	SNP	2002
		T/C	SNP	2015
		C/T	SNP	2017
		C/T	SNP	2024
		A/T	SNP	2043
		G/T	SNP	2067
		T/C	SNP	2108
		TAC/T	DELETION	2121
		T/G	SNP	2150
		A/C	SNP	2151
		T/C	SNP	2188
		A/T	SNP	2189
		T/C	SNP	2198
XM_001218936.1 Trypanosoma brucei brucei strain 927/4 GUTat10.1 beta tubulin partial mRNA	GLOS_TB927.1.2370.1.2	A/C	SNP	1303
XM_001218936.1 Trypanosoma brucei brucei strain 927/4 GUTat10.1 beta tubulin partial mRNA	GLOS_TB927.1.2370.2.2	G/A	SNP	1423
		C/T	SNP	1487
		A/AT	INSERTION	1541
		A/T	SNP	1541
		A/G	SNP	1631
XM_001218941.1 Trypanosoma brucei brucei strain 927/4 GUTat10.1 histone H3 partial mRNA	GLOS_TB927.1.2430.1.1	G/A	SNP	107
		G/T	SNP	612
		G/A	SNP	652
		C/T	SNP	803

		C/T	SNP	825
		A/T	SNP	1462
		T/A	SNP	1463
XP_001218979.1 pteridine transporter [Trypanosoma brucei brucei strain 927/4 GUTat10.1]	GLOS_TB927.1.2820.1.1	C/T	SNP	34
		T/TA	INSERTION	383
		C/T	SNP	970
		C/T	SNP	1616
		T/C	SNP	2294
		T/C	SNP	2303
XP_001219016.1 40S ribosomal protein S11 [Trypanosoma brucei brucei strain 927/4 GUTat10.1]	GLOS_TB927.1.3180.1.1	G/A	SNP	128
		C/T	SNP	137
		G/A	SNP	161
		T/C	SNP	200
		T/C	SNP	290
		A/G	SNP	296
		C/T	SNP	357
		T/C	SNP	461
		A/G	SNP	494
		T/C	SNP	524
		C/T	SNP	527
XM_001219028.1 Trypanosoma brucei hypothetical protein, conserved (Tb927.1.3310) partial mRNA	GLOS_TB927.1.3310.1.1	G/GA	INSERTION	1452
		G/GA	INSERTION	1647
XP_001219089.1 alanine aminotransferase [Trypanosoma brucei brucei strain 927/4 GUTat10.1]	GLOS_TB927.1.3950.1.1	C/CTT	INSERTION	264
		T/C	SNP	949
		A/G	SNP	1473
XM_001219129.1 Trypanosoma brucei hypothetical protein, conserved (Tb927.1.4370) partial mRNA	GLOS_TB927.1.4370.1.1	C/T	SNP	1453
		AAC/A	DELETION	2435
		C/G	SNP	2463
		C/G	SNP	2465
		CTT/C	DELETION	2634
XM_001219166.1 Trypanosoma brucei hypothetical protein, conserved (Tb927.1.4740) partial mRNA	GLOS_TB927.1.4740.1.1	G/GA	INSERTION	388
		G/GA	INSERTION	480
		T/C	SNP	2196
XM_001218756.1 T. brucei DNA-directed RNA polymerase III, putative (Tb927.1.540) partial mRNA	GLOS_TB927.1.540.1.2	T/C	SNP	62
		T/C	SNP	64
		G/A	SNP	83
		C/T	SNP	162
		A/G	SNP	165
		C/A	SNP	174

A/G	SNP	192
G/A	SNP	231
C/A	SNP	243
A/T	SNP	343
G/A	SNP	351
T/G	SNP	357
T/C	SNP	368
G/T	SNP	386
G/A	SNP	441
A/C	SNP	508
A/G	SNP	513
A/C	SNP	521
G/T	SNP	542
C/T	SNP	582
T/C	SNP	585
A/G	SNP	640
T/C	SNP	646
C/T	SNP	669
T/C	SNP	686
T/C	SNP	738
C/T	SNP	764
C/T	SNP	778
T/C	SNP	847
T/C	SNP	914
A/C	SNP	933
C/T	SNP	935
C/T	SNP	948
C/A	SNP	958
T/G	SNP	971
C/A	SNP	1011
A/G	SNP	1041
A/G	SNP	1052
T/C	SNP	1060
C/T	SNP	1068
T/A	SNP	1073
A/G	SNP	1076
G/A	SNP	1085
A/G	SNP	1103
C/G	SNP	1104

		C/A	SNP	1122
		A/G	SNP	1141
		T/C	SNP	1149
		G/A	SNP	1178
		C/T	SNP	1213
		G/T	SNP	1238
		A/G	SNP	1251
		C/T	SNP	1308
		T/C	SNP	1324
		T/C	SNP	1347
		G/A	SNP	1528
		T/C	SNP	1548
		C/T	SNP	1702
		A/C	SNP	1950
		G/A	SNP	1968
		A/G	SNP	1972
		T/C	SNP	1981
		C/G	SNP	2043
		C/CAT	INSERTION	2119
		T/C	SNP	2149
		A/C	SNP	2173
		A/G	SNP	2221
		G/C	SNP	2231
		A/G	SNP	2251
		G/A	SNP	2288
		T/G	SNP	2296
		A/G	SNP	2413
		C/A	SNP	2425
		CT/C	DELETION	2441
		A/G	SNP	2477
		G/C	SNP	2479
		C/T	SNP	2482
		G/T	SNP	2542
		C/T	SNP	2576
XM_001218756.1 T. brucei DNA-directed RNA polymerase III, putative (Tb927.1.540) partial mRNA	GLOS_TB927.1.540.2.2	A/G	SNP	65
		C/G	SNP	79
		C/T	SNP	82
		G/T	SNP	90
		G/A	SNP	95

A/G	SNP	101
A/C	SNP	122
A/C	SNP	124
T/A	SNP	136
C/T	SNP	141
C/G	SNP	142
G/T	SNP	144
G/A	SNP	179
T/C	SNP	183
G/A	SNP	214
C/T	SNP	226
G/C	SNP	235
G/A	SNP	271
C/G	SNP	272
G/A	SNP	310
A/T	SNP	317
A/G	SNP	372
G/T	SNP	406
C/A	SNP	408
C/G	SNP	470
T/G	SNP	488
C/T	SNP	489
T/C	SNP	515
T/C	SNP	535
A/C	SNP	566
G/C	SNP	591
A/G	SNP	649
G/A	SNP	661
A/G	SNP	676
G/C	SNP	732
T/G	SNP	749
T/G	SNP	752
T/G	SNP	781
T/A	SNP	803
T/C	SNP	804
C/G	SNP	827
T/C	SNP	861
G/A	SNP	864
G/T	SNP	872

G/T	SNP	881
C/T	SNP	919
A/T	SNP	927
C/T	SNP	928
A/T	SNP	936
A/G	SNP	985
T/C	SNP	1011
G/T	SNP	1023
T/C	SNP	1032
C/T	SNP	1042
G/T	SNP	1050
A/G	SNP	1066
C/G	SNP	1068
A/C	SNP	1074
G/T	SNP	1084
C/T	SNP	1094
T/G	SNP	1122
C/T	SNP	1130
T/A	SNP	1141
G/A	SNP	1144
A/T	SNP	1149
T/C	SNP	1202
A/G	SNP	1210
C/T	SNP	1216
A/C	SNP	1223
C/T	SNP	1256
T/A	SNP	1299
G/T	SNP	1311
G/A	SNP	1334
T/G	SNP	1351
T/C	SNP	1362
C/T	SNP	1366
G/A	SNP	1392
A/G	SNP	1406
G/A	SNP	1426
G/T	SNP	1461
C/G	SNP	1462
T/C	SNP	1508
T/C	SNP	1519

T/C	SNP	1522
A/G	SNP	1535
A/G	SNP	1592
A/G	SNP	1606
T/C	SNP	1613
G/T	SNP	1621
T/C	SNP	1651
A/G	SNP	1653
C/T	SNP	1656
G/T	SNP	1673
T/C	SNP	1679
C/T	SNP	1699
T/A	SNP	1702
A/G	SNP	1704
A/T	SNP	1707
A/G	SNP	1758
G/C	SNP	1912
T/C	SNP	1971
G/T	SNP	2075
T/C	SNP	2087
A/G	SNP	2106
G/C	SNP	2127
A/C	SNP	2144
A/G	SNP	2182
A/C	SNP	2205
A/G	SNP	2251
C/G	SNP	2270
T/C	SNP	2280
T/A	SNP	2305
T/G	SNP	2328
C/T	SNP	2337
A/C	SNP	2382
G/C	SNP	2457
A/G	SNP	2503
A/G	SNP	2507
A/G	SNP	2519
C/T	SNP	2532
G/T	SNP	2550
G/A	SNP	2620

		C/T	SNP	2624
XM_001218762.1 T. brucei phosphate-repressible phosphate permease, putative partial mRNA	GLOS_TB927.1.600.1.1	A/G	SNP	239
		T/C	SNP	1353
		A/G	SNP	1362
		T/C	SNP	1706
		T/TC	INSERTION	1925
		C/T	SNP	2139
		AT/A	DELETION	2274
		T/TAA	INSERTION	2676
		T/C	SNP	2849
		G/GT	INSERTION	2892
		GA/G	DELETION	3090
		GA/G	DELETION	3410
XM_001218788.1 Trypanosoma brucei hypothetical protein, conserved (Tb927.1.860) partial mRNA	GLOS_TB927.1.860.1.1	G/A	SNP	917
XP_951477.1 retrotransposon hot spot (RHS) protein [Trypanosoma brucei TREU927]	GLOS_TB927.2.240.1.1	A/G	SNP	424
		A/C	SNP	427
		G/T	SNP	434
		A/G	SNP	454
		A/G	SNP	455
		A/G	SNP	458
		T/G	SNP	713
		A/G	SNP	729
		A/G	SNP	817
		C/T	SNP	824
		G/A	SNP	825
		A/G	SNP	880
		C/T	SNP	899
		G/A	SNP	1971
		A/G	SNP	2398
		A/G	SNP	2648
		G/A	SNP	3091
		G/GA	INSERTION	3185
XP_951552.1 hypothetical protein [Trypanosoma brucei brucei strain 927/4 GUTat10.1] ref|XP_951553.1| hypothetical protein [Trypanosoma brucei brucei strain 927/4 GUTat10.1]	GLOS_TB927.2.2510.1.1	A/G	SNP	147
		T/C	SNP	186
		C/T	SNP	858
XM_946502.1 Tbb strain 927/4 GUTat10.1 D-alanyl-glycyl endopeptidase partial mRNA	GLOS_TB927.2.3460.1.1	A/G	SNP	926
		C/T	SNP	947
		A/G	SNP	955

XM_946511.1 T. strain 927/4 GUTat10.1 translation initiation factor IF-2 (Tb927.2.3780) partial mRNA	GLOS_TB927.2.3780.1.1	A/G	SNP	3357
XM_946512.1 Tbb strain 927/4 GUTat10.1 hypothetical protein (Tb927.2.3800) partial mRNA	GLOS_TB927.2.3800.1.1	T/TA	INSERTION	2494
XP_951481.1 retrotransposon hot spot (RHS) protein [Trypanosoma brucei TREU927]	GLOS_TB927.2.380.1.1	T/G	SNP	35
		TTAA/T	DELETION	166
		C/T	SNP	179
		C/T	SNP	428
		A/G	SNP	437
		A/T	SNP	556
		G/A	SNP	756
		T/C	SNP	857
		T/G	SNP	892
		G/C	SNP	896
		T/A	SNP	901
		C/T	SNP	977
		A/T	SNP	1025
		C/G	SNP	1151
		G/A	SNP	1171
		T/C	SNP	1176
		T/G	SNP	1177
		C/T	SNP	1179
		C/G	SNP	1250
		T/C	SNP	1306
		C/G	SNP	1338
		A/G	SNP	1716
		C/T	SNP	1856
		C/T	SNP	1865
		T/C	SNP	1931
		G/C	SNP	1936
		G/C	SNP	1960
		G/A	SNP	2023
		T/G	SNP	2077
		G/A	SNP	2096
		G/T	SNP	2132
		A/T	SNP	2134
		G/A	SNP	2194
		A/G	SNP	2200
		T/C	SNP	2306
		A/T	SNP	2356
		A/G	SNP	2357

		T/C	SNP	2402
		G/T	SNP	2412
		G/A	SNP	2582
XP_951482.1 retrotransposon hot spot (RHS) protein [Trypanosoma brucei TREU927]	GLOS_TB927.2.400.1.1	T/G	SNP	55
		A/C	SNP	68
		T/C	SNP	71
		C/T	SNP	73
		A/T	SNP	217
		G/T	SNP	297
		T/C	SNP	303
		T/C	SNP	326
		A/G	SNP	368
		T/C	SNP	372
		C/T	SNP	519
		G/A	SNP	559
		T/C	SNP	602
		C/T	SNP	625
		A/C	SNP	652
		T/C	SNP	664
		T/A	SNP	670
		C/A	SNP	687
		CA/C	DELETION	714
		C/T	SNP	714
		A/G	SNP	717
		C/T	SNP	724
		C/T	SNP	759
		T/C	SNP	762
		A/C	SNP	781
		G/A	SNP	828
		T/C	SNP	834
		G/A	SNP	868
		T/A	SNP	900
		G/C	SNP	904
		C/T	SNP	928
		A/T	SNP	966
		T/A	SNP	972
		T/C	SNP	1020
		C/T	SNP	1049
		C/A	SNP	1051

G/T	SNP	1053
C/T	SNP	1054
A/G	SNP	1063
G/A	SNP	1202
G/T	SNP	1228
T/C	SNP	1248
C/T	SNP	1267
A/T	SNP	1297
C/T	SNP	1298
G/A	SNP	1339
C/A	SNP	1360
T/G	SNP	1398
C/T	SNP	1427
G/T	SNP	1545
G/A	SNP	1567
A/C	SNP	1597
C/T	SNP	1779
A/G	SNP	1784
A/C	SNP	1805
A/T	SNP	1811
GA/G	DELETION	1814
A/G	SNP	1883
A/G	SNP	1901
AAT/A	DELETION	1957
A/T	SNP	1966
A/G	SNP	1979
C/T	SNP	2000
A/G	SNP	2098
C/T	SNP	2119
A/T	SNP	2226
C/A	SNP	2252
A/G	SNP	2300
C/A	SNP	2505
T/C	SNP	2515
G/T	SNP	2536
T/G	SNP	2568
C/T	SNP	2569
C/G	SNP	2622
A/G	SNP	2627

		A/G	SNP	2628
		C/G	SNP	2646
		G/A	SNP	2756
		A/G	SNP	2781
		C/T	SNP	2791
		G/A	SNP	2861
		C/G	SNP	3244
		A/C	SNP	3405
		A/G	SNP	3406
		C/G	SNP	3433
		T/C	SNP	3657
		C/T	SNP	3658
		G/A	SNP	3659
		A/T	SNP	3681
		C/T	SNP	3785
		T/G	SNP	3787
		C/G	SNP	3827
		A/G	SNP	3890
		A/T	SNP	3894
		C/T	SNP	3896
		C/T	SNP	3902
		C/T	SNP	3923
		A/G	SNP	3947
		C/T	SNP	3964
		G/A	SNP	4160
		T/C	SNP	4169
		C/A	SNP	4171
		G/T	SNP	4197
		T/G	SNP	4203
		G/C	SNP	4204
		T/C	SNP	4213
		T/C	SNP	4231
XM_946532.1 Tbb strain 927/4 GUTat10.1 NUP-1 protein (Tb927.2.4230) partial mRNA	GLOS_TB927.2.4230.1.1	A/G	SNP	563
XP_951628.1 paraflagellar rod protein [Trypanosoma brucei brucei strain 927/4 GUTat10.1]	GLOS_TB927.2.4330.1.1	C/T	SNP	2689
XP_951630.1 trypanothione synthetase [Trypanosoma brucei brucei strain 927/4 GUTat10.1]	GLOS_TB927.2.4370.1.1	C/CT	INSERTION	225
		G/GT	INSERTION	2542
		C/CT	INSERTION	2601
		G/A	SNP	2956
XM_946554.1 Tbb strain 927/4 GUTat10.1 hypothetical protein (Tb927.2.4580) partial mRNA	GLOS_TB927.2.4580.1.1	G/GA	INSERTION	281

		C/CA	INSERTION	2288
XP_951649.1 branched-chain amino acid aminotransferase [Trypanosoma brucei TREU927]	GLOS_TB927.2.4610.1.1	C/T	SNP	562
		A/G	SNP	921
		C/T	SNP	1035
		T/C	SNP	1230
XM_946561.1 Tbb strain 927/4 GUTat10.1 hypothetical protein (Tb927.2.4700) partial mRNA	GLOS_TB927.2.4700.1.1	GTA/G	DELETION	398
		T/TA	INSERTION	1190
		G/GT	INSERTION	1297
		C/CTT	INSERTION	1334
		G/GT	INSERTION	1528
		C/CT	INSERTION	1924
		C/CT	INSERTION	2008
		G/GA	INSERTION	2330
		C/CT	INSERTION	2424
XM_946391.1 Tbb retrotransposon hot spot (RHS) protein (Tb927.2.470) partial mRNA	GLOS_TB927.2.470.1.2	A/G	SNP	38
		G/C	SNP	95
		A/G	SNP	103
		G/A	SNP	154
		C/T	SNP	232
		A/C	SNP	270
		A/G	SNP	304
		A/C	SNP	317
		A/G	SNP	318
		G/A	SNP	320
		G/A	SNP	321
		A/G	SNP	354
		G/A	SNP	363
		A/C	SNP	393
		A/C	SNP	400
		C/T	SNP	454
		C/T	SNP	587
		G/A	SNP	589
		A/G	SNP	593
		T/G	SNP	616
		G/A	SNP	618
		A/G	SNP	1099
		G/A	SNP	1673
		A/G	SNP	1674
		C/A	SNP	1675

		C/A	SNP	1679
		C/CT	INSERTION	1750
		A/C	SNP	2404
		C/T	SNP	2409
		G/T	SNP	2490
		C/T	SNP	2534
XM_946391.1 Tbb retrotransposon hot spot (RHS) protein (Tb927.2.470) partial mRNA	GLOS_TB927.2.470.2.2	C/A	SNP	69
		C/T	SNP	121
		A/G	SNP	132
		A/G	SNP	137
		T/A	SNP	139
		G/A	SNP	152
		G/A	SNP	414
		C/G	SNP	415
		T/A	SNP	435
		T/A	SNP	471
		G/A	SNP	497
		G/A	SNP	527
		G/A	SNP	622
		A/T	SNP	979
		A/T	SNP	991
		G/A	SNP	1256
		C/T	SNP	1264
		A/C	SNP	1530
		C/G	SNP	1572
		C/G	SNP	1574
		A/T	SNP	1576
		A/G	SNP	1593
		G/A	SNP	1926
		G/C	SNP	1991
		A/G	SNP	2111
		A/C	SNP	2121
		G/A	SNP	2659
		A/G	SNP	2702
		A/C	SNP	2722
XM_946562.1 Tbb strain 927/4 GUTat10.1 RNA-binding protein (Tb927.2.4710) partial mRNA	GLOS_TB927.2.4710.1.1	C/CT	INSERTION	1084
		A/ATT	INSERTION	3801
		A/T	SNP	3801
		G/GA	INSERTION	3943

		C/CT	INSERTION	4068
XM_946615.1 Tbb strain 927/4 GUTat10.1 hypothetical protein (Tb927.2.5360) partial mRNA	GLOS_TB927.2.5360.1.1	G/A	SNP	69
		C/A	SNP	104
		A/G	SNP	138
		A/T	SNP	143
		C/T	SNP	169
		A/C	SNP	200
		T/C	SNP	236
		G/T	SNP	270
		A/T	SNP	334
		G/A	SNP	337
		A/G	SNP	405
		T/C	SNP	408
		A/C	SNP	642
		A/G	SNP	645
		T/A	SNP	646
		T/G	SNP	647
		G/A	SNP	685
		T/A	SNP	837
		A/G	SNP	841
		A/G	SNP	880
		T/C	SNP	888
		C/T	SNP	925
		G/T	SNP	947
		T/C	SNP	952
		A/G	SNP	964
		G/A	SNP	1024
		C/T	SNP	1064
		T/C	SNP	1077
		C/A	SNP	1095
		A/G	SNP	1124
		C/T	SNP	1127
		A/G	SNP	1165
		T/C	SNP	1432
		A/G	SNP	1455
		T/C	SNP	1502
		C/T	SNP	1534
XM_946650.1 Tbb strain 927/4 GUTat10.1 60S ribosomal protein L44 (Tb927.2.6090) partial mRNA	GLOS_TB927.2.6090.1.1	TTAA/T	DELETION	513
XM_946397.1 Tbb strain 927/4 GUTat10.1 hypothetical protein (Tb927.2.900) partial mRNA	GLOS_TB927.2.900.1.1	C/T	SNP	31

A/T	SNP	105
G/T	SNP	107
C/T	SNP	122
G/T	SNP	124
G/A	SNP	130
A/G	SNP	134
T/C	SNP	168
T/G	SNP	181
C/G	SNP	190
G/A	SNP	216
T/C	SNP	254
G/T	SNP	274
T/C	SNP	305
G/A	SNP	306
C/G	SNP	313
A/C	SNP	346
C/A	SNP	356
T/C	SNP	367
A/G	SNP	391
C/T	SNP	404
T/C	SNP	413
C/T	SNP	434
T/C	SNP	456
C/T	SNP	503
C/T	SNP	533
A/G	SNP	536
T/A	SNP	564
C/T	SNP	589
G/A	SNP	599
A/G	SNP	616
T/C	SNP	617
T/C	SNP	632
A/G	SNP	669
A/T	SNP	682
G/C	SNP	690
G/T	SNP	716
C/A	SNP	732
C/T	SNP	766
TTG/T	DELETION	771

C/T	SNP	787
C/G	SNP	793
C/T	SNP	794
C/T	SNP	796
A/G	SNP	818
A/C	SNP	828
A/T	SNP	832
C/T	SNP	865
A/G	SNP	871
C/T	SNP	884
G/T	SNP	921
A/C	SNP	956
A/G	SNP	970
G/A	SNP	1039
G/A	SNP	1045
A/G	SNP	1047
C/T	SNP	1096
A/G	SNP	1100
T/C	SNP	1101
A/G	SNP	1107
C/T	SNP	1219
C/T	SNP	1229
G/A	SNP	1235
G/A	SNP	1256
A/T	SNP	1296
C/T	SNP	1304
G/C	SNP	1326
A/G	SNP	1345
C/T	SNP	1351
T/G	SNP	1352
A/G	SNP	1368
C/T	SNP	1387
C/A	SNP	1396
C/T	SNP	1431
T/C	SNP	1438
C/T	SNP	1459
T/C	SNP	1489
G/T	SNP	1504
C/T	SNP	1523

		A/G	SNP	1538
		A/G	SNP	1540
		A/G	SNP	1542
		C/A	SNP	1557
		C/T	SNP	1598
		C/T	SNP	1613
		A/C	SNP	1617
		T/C	SNP	1634
		C/A	SNP	1648
		C/T	SNP	1652
		A/T	SNP	1653
		C/T	SNP	1656
		C/T	SNP	1666
		T/C	SNP	1668
		A/G	SNP	1734
		G/A	SNP	1749
		A/G	SNP	1769
		C/A	SNP	1771
		A/T	SNP	1781
		A/G	SNP	1803
		G/A	SNP	1829
		T/C	SNP	1846
		C/G	SNP	1858
		T/G	SNP	1872
		G/A	SNP	1927
		T/C	SNP	1939
		A/G	SNP	1947
		A/G	SNP	1966
		C/T	SNP	2008
		G/C	SNP	2016
XM_838604.1 Trypanosoma brucei hypothetical protein, conserved (Tb927.3.1010) partial mRNA	GLOS_TB927.3.1010.1.1	G/A	SNP	68
		TTTA/T	DELETION	2231
		C/CA	INSERTION	2685
		A/AT	INSERTION	3497
XM_838612.1 Trypanosoma brucei hypothetical protein, conserved (Tb927.3.1110) partial mRNA	GLOS_TB927.3.1110.1.1	C/CA	INSERTION	314
		G/GA	INSERTION	771
XP_843706.1 GTP-binding nuclear protein rtb2 [Trypanosoma brucei brucei strain 927/4 GUTat10.1]	GLOS_TB927.3.1120.1.1	A/C	SNP	42
		G/A	SNP	44
		G/A	SNP	45

		C/CA	INSERTION	162
		T/TA	INSERTION	260
		G/GA	INSERTION	1266
XM_838622.1 T. brucei protein transport protein Sec24C, putative (Tb927.3.1210) partial mRNA	GLOS_TB927.3.1210.1.1	G/GT	INSERTION	806
		CT/C	DELETION	3212
XM_838639.1 T. brucei ATP synthase beta chain, mitochondrial precursor partial mRNA	GLOS_TB927.3.1380.1.1	T/TA	INSERTION	124
XP_843763.1 hypothetical protein [Trypanosoma brucei brucei strain 927/4 GUTat10.1]	GLOS_TB927.3.1690.1.1	G/C	SNP	465
		G/GA	INSERTION	652
		A/G	SNP	797
XM_838680.1 T. brucei pyruvate dehydrogenase E1 beta subunit, putative partial mRNA	GLOS_TB927.3.1790.1.1	C/CA	INSERTION	86
		A/G	SNP	526
		G/GT	INSERTION	953
		T/C	SNP	1233
		G/A	SNP	2028
		TTTC/T	DELETION	2357
		TTC/T	DELETION	2364
XP_843778.1 3-oxo-5-alpha-steroid 4-dehydrogenase [Trypanosoma brucei TREU927]	GLOS_TB927.3.1840.1.1	C/CA	INSERTION	294
		AC/A	DELETION	432
		A/AT	INSERTION	646
		G/GA	INSERTION	1165
		C/CT	INSERTION	1316
		T/A	SNP	1453
		CA/C	DELETION	1541
		C/CT	INSERTION	1639
		TGTGTGTGTGTGT	DELETION	1740
		AAT/A	DELETION	2069
		A/G	SNP	2263
XP_843788.1 hypothetical protein [Trypanosoma brucei brucei strain 927/4 GUTat10.1]	GLOS_TB927.3.1940.1.1	C/T	SNP	1799
XM_838711.1 Trypanosoma brucei hypothetical protein, conserved (Tb927.3.2100) partial mRNA	GLOS_TB927.3.2100.1.1	T/A	SNP	182
		A/G	SNP	209
		A/G	SNP	1135
		C/CT	INSERTION	1375
		GA/G	DELETION	1961
XP_843812.1 hypothetical protein [Trypanosoma brucei brucei strain 927/4 GUTat10.1]	GLOS_TB927.3.2180.1.1	A/C	SNP	165
		GA/G	DELETION	185
XM_838724.1 T. brucei succinyl-CoA synthetase alpha subunit, putative partial mRNA	GLOS_TB927.3.2230.1.1	C/T	SNP	809
		T/C	SNP	1006
		G/C	SNP	1078
		G/C	SNP	1124

XM_838750.1 Trypanosoma brucei hypothetical protein, conserved (Tb927.3.2490) partial mRNA	GLOS_TB927.3.2490.1.1	C/T	SNP	3054
XM_838760.1 T. brucei ATP-dependent DEAD/H RNA helicase, putative partial mRNA	GLOS_TB927.3.2600.1.1	C/T	SNP	3278
		G/A	SNP	5702
XP_843881.1 hypothetical protein [Trypanosoma brucei brucei strain 927/4 GUTat10.1]	GLOS_TB927.3.2880.1.1	A/T	SNP	606
XM_838790.1 T. brucei elongation initiation factor 2 alpha subunit, putative partial mRNA	GLOS_TB927.3.2900.1.1	A/T	SNP	19
		A/G	SNP	1130
		CT/C	DELETION	1521
XM_838813.1 Trypanosoma brucei hypothetical protein, conserved (Tb927.3.3130) partial mRNA	GLOS_TB927.3.3130.1.1	G/T	SNP	275
		T/C	SNP	1204
		T/C	SNP	3248
		C/A	SNP	4755
XM_838827.1 T. brucei ATP-dependent phosphofructokinase (Tb927.3.3270) partial mRNA	GLOS_TB927.3.3270.1.1	T/C	SNP	818
		A/C	SNP	3384
XM_838831.1 Trypanosoma brucei 60S ribosomal protein L13, putative (Tb927.3.3310) partial mRNA	GLOS_TB927.3.3310.1.1	T/C	SNP	133
		C/T	SNP	380
		C/T	SNP	794
		T/A	SNP	805
		T/A	SNP	806
XP_843934.1 aspartyl aminopeptidase [Trypanosoma brucei brucei strain 927/4 GUTat10.1]	GLOS_TB927.3.3410.1.1	A/G	SNP	1946
XM_838857.1 T. brucei lipophosphoglycan biosynthetic protein, putative partial mRNA	GLOS_TB927.3.3580.1.1	T/C	SNP	87
		T/C	SNP	2171
XM_838868.1 T. brucei flagellar radial spoke protein-like, putative (Tb927.3.3690) partial mRNA	GLOS_TB927.3.3690.1.1	GT/G	DELETION	1891
		GA/G	DELETION	1997
		G/GA	INSERTION	2160
XM_838874.1 Trypanosoma brucei hypothetical protein, conserved (Tb927.3.3750) partial mRNA	GLOS_TB927.3.3750.1.1	G/A	SNP	161
		AT/A	DELETION	1141
		G/GA	INSERTION	1758
XP_843969.1 hypothetical protein [Trypanosoma brucei brucei strain 927/4 GUTat10.1] ref|XP_843971.1| hypothetical protein [Trypanosoma brucei brucei strain 927/4 GUTat10.1]	GLOS_TB927.3.3770.1.1	G/A	SNP	142
		C/T	SNP	402
		C/A	SNP	425
		T/C	SNP	475
		G/A	SNP	685
		C/G	SNP	1146
XM_838889.1 T. brucei carnitine O-palmitoyltransferase II, putative (Tb927.3.3900) partial mRNA	GLOS_TB927.3.3900.1.1	A/G	SNP	292
XM_838903.1 Trypanosoma brucei hypothetical protein, conserved (Tb927.3.4040) partial mRNA	GLOS_TB927.3.4040.1.1	GTA/G	DELETION	2294
XM_838907.1 Trypanosoma brucei hypothetical protein, conserved (Tb927.3.4080) partial mRNA	GLOS_TB927.3.4080.1.1	T/C	SNP	40
		A/G	SNP	380
		T/C	SNP	502

		G/A	SNP	503
		C/T	SNP	526
		A/G	SNP	527
		T/C	SNP	631
		G/A	SNP	632
		A/G	SNP	655
		C/T	SNP	694
		C/T	SNP	985
		G/T	SNP	1029
		T/G	SNP	1030
		C/T	SNP	1111
		G/T	SNP	1375
		A/G	SNP	1430
		G/T	SNP	1451
		T/G	SNP	1453
		T/C	SNP	1454
		T/C	SNP	1459
		G/T	SNP	1482
		C/T	SNP	1531
		C/A	SNP	1532
		T/C	SNP	1579
		G/A	SNP	1617
		G/A	SNP	1714
		C/G	SNP	1735
XM_838915.1 Trypanosoma brucei hypothetical protein, conserved (Tb927.3.4160) partial mRNA	GLOS_TB927.3.4160.1.1	T/TA	INSERTION	216
		G/GA	INSERTION	971
XM_838928.1 Trypanosoma brucei 73 kDa paraflagellar rod protein (Tb927.3.4290) partial mRNA	GLOS_TB927.3.4290.1.1	G/A	SNP	574
		G/A	SNP	1943
XP_844042.1 fumarate hydratase [Trypanosoma brucei brucei strain 927/4 GUTat10.1]	GLOS_TB927.3.4500.1.1	C/T	SNP	38
		C/T	SNP	39
		C/T	SNP	40
		G/GT	INSERTION	140
		G/GA	INSERTION	2510
		C/CT	INSERTION	2818
		A/AT	INSERTION	3019
		G/A	SNP	3129
		G/GA	INSERTION	3129
		A/AT	INSERTION	3276
XP_844067.1 aminopeptidase [Trypanosoma brucei brucei strain 927/4 GUTat10.1]	GLOS_TB927.3.4750.1.1	G/A	SNP	3618

ref|XP_843971.1| hypothetical protein [Trypanosoma brucei brucei strain 927/4 GUTat10.1]				
XM_838975.1 Trypanosoma brucei brucei strain 927/4 GUTat10.1 dynamin partial mRNA	GLOS_TB927.3.4760.1.1	A/AT	INSERTION	239
		G/GA	INSERTION	2049
XP_844076.1 ubiquitin hydrolase [Trypanosoma brucei brucei strain 927/4 GUTat10.1]	GLOS_TB927.3.4840.1.1	C/CA	INSERTION	886
XP_844108.1 cofilin/actin depolymerizing factor [Trypanosoma brucei TREU927]	GLOS_TB927.3.5180.1.1	G/GA	INSERTION	1363
XM_839049.1 T. brucei 26S proteasome regulatory non-ATPase subunit partial mRNA	GLOS_TB927.3.5520.1.1	C/CA	INSERTION	3116
XM_838575.1 Trypanosoma brucei zinc finger protein 2, putative (Tb927.3.720) partial mRNA	GLOS_TB927.3.720.1.1	C/CT	INSERTION	204
XM_838577.1 Trypanosoma brucei hypothetical protein, conserved (Tb927.3.740) partial mRNA	GLOS_TB927.3.740.1.1	C/CTT	INSERTION	514
		CT/C	DELETION	970
		A/AT	INSERTION	1016
		C/CA	INSERTION	2475
		GT/G	DELETION	2559
XP_844280.1 hypothetical protein [Trypanosoma brucei brucei strain 927/4 GUTat10.1]	GLOS_TB927.4.1300.1.1	G/A	SNP	23
		C/A	SNP	26
		T/G	SNP	27
		C/A	SNP	1561
		C/CA	INSERTION	1708
		AAT/A	DELETION	1873
		G/GA	INSERTION	1983
XP_844285.1 hydroxyacylglutathione hydrolase [Trypanosoma brucei brucei strain 927/4 GUTat10.1]	GLOS_TB927.4.1350.1.1	TA/T	DELETION	1048
		G/GT	INSERTION	1295
XM_839236.1 Tbb strain 927/4 GUTat10.1 ribosomal prot. L3, putative (Tb927.4.1790) partial mRNA	GLOS_TB927.4.1790.1.1	A/C	SNP	561
		G/T	SNP	578
		C/T	SNP	1450
XM_839242.1 Tbb strain 927/4 GUTat10.1 hypothetical prot., conserved (Tb927.4.1850) partial mRNA	GLOS_TB927.4.1850.1.1	C/CT	INSERTION	2107
XM_839243.1 T.strain 927/4 GUTat10.1 ribosomal protein S19, putative (Tb927.4.1860) partial mRNA	GLOS_TB927.4.1860.1.2	T/C	SNP	287
		C/CGT	INSERTION	546
		G/A	SNP	578
		C/CT	INSERTION	607
XP_844336.1 ribosomal protein S19 [Trypanosoma brucei brucei strain 927/4 GUTat10.1]	GLOS_TB927.4.1860.2.2	G/A	SNP	85
		G/GA	INSERTION	150
		AAC/A	DELETION	202
		A/G	SNP	477
		ATTTTT/A/ATTTT	DELETION	847
XM_839260.1 Tbb strain 927/4 GUTat10.1 hypothetical prot., conserved (Tb927.4.2030) partial mRNA	GLOS_TB927.4.2030.1.1	G/C	SNP	142
		G/A	SNP	143
		C/T	SNP	149
		G/GT	INSERTION	933
		G/A	SNP	1297

XM_839265.1 Tbbi strain 927/4 GUTat10.1 hypothetical protein, conserved partial mRNA	GLOS_TB927.4.2080.1.1	C/T	SNP	2364
XM_839298.1 Tbb strain 927/4 GUTat10.1 hypothetical prot., conserved (Tb927.4.2410) partial mRNA	GLOS_TB927.4.2410.1.1	GA/G	DELETION	295
		T/A	SNP	647
		C/T	SNP	1515
XM_839310.1 Tbb strain 927/4 GUTat10.1 hypothetical protein, conserved partial mRNA	GLOS_TB927.4.2530.1.1	T/G	SNP	542
		A/G	SNP	594
		GA/G	DELETION	612
		TAG/T	DELETION	670
		G/GA	INSERTION	1345
		C/CA	INSERTION	1472
		TA/T	DELETION	1558
XM_839331.1 Tbb strain 927/4 GUTat10.1 hypothetical prot., conserved (Tb927.4.2740) partial mRNA	GLOS_TB927.4.2740.1.1	A/T	SNP	475
		C/T	SNP	482
		CAA/C	DELETION	484
		T/C	SNP	1631
		C/CT	INSERTION	2325
		A/C	SNP	2456
XM_839346.1 Tbb 927/4 GUTat10.1 hypothetical protein, conserved (Tb927.4.2890) partial mRNA	GLOS_TB927.4.2890.1.1	G/A	SNP	2709
XM_839363.1 T. strain 927/4 GUTat10.1 hypothetical protein, conserved partial mRNA	GLOS_TB927.4.3060.1.1	G/A	SNP	115
		TGACAAAAGGA/T	DELETION	248
		T/A	SNP	322
		G/T	SNP	323
		A/T	SNP	324
		T/C	SNP	394
		CT/C	DELETION	468
		T/A	SNP	470
		C/T	SNP	478
		C/T	SNP	537
		T/TCC	INSERTION	751
		G/A	SNP	766
		G/GT	INSERTION	1409
		C/T	SNP	1417
		C/A	SNP	1489
		G/A	SNP	2315
XM_839407.1 Tbb strain 927/4 GUTat10.1 hypothetical prot., conserved (Tb927.4.3500) partial mRNA	GLOS_TB927.4.3500.1.1	G/A	SNP	177
		A/AT	INSERTION	1745
		A/C	SNP	381
		C/G	SNP	417
		C/T	SNP	555

		C/G	SNP	744
XM_839416.1 Tbb translation elongation factor 1-beta, putative (Tb927.4.3590) partial mRNA	GLOS_TB927.4.3590.1.1	T/G	SNP	961
XM_839419.1 Tbb serine/threonine-protein phosphatase PP1, putative (Tb927.4.3620) partial mRNA	GLOS_TB927.4.3620.1.1	GA/G	DELETION	199
		C/CT	INSERTION	509
		TAGA/T	DELETION	867
		T/C	SNP	2883
XM_839431.1 Tbb strain 927/4 GUTat10.1 hypothetical protein, conserved partial mRNA	GLOS_TB927.4.3740.1.1	G/A	SNP	4
		C/T	SNP	30
		G/A	SNP	46
		C/T	SNP	72
		G/A	SNP	88
		C/T	SNP	114
		T/C	SNP	123
XM_839449.1 Tbb strain 927/4 GUTat10.1 hypothetical prot., conserved (Tb927.4.3920) partial mRNA	GLOS_TB927.4.3920.1.1	C/A	SNP	354
		AT/A	DELETION	374
		C/A	SNP	420
		C/T	SNP	528
		A/G	SNP	3387
XM_839452.1 Tbb cytoskeleton-associated protein CAP5.5, putative (Tb927.4.3950) partial mRNA	GLOS_TB927.4.3950.1.1	CA/C	DELETION	111
		C/CA	INSERTION	290
		C/A	SNP	2901
XM_839473.1 Tbb strain 927/4 GUTat10.1 hypothetical protein, conserved partial mRNA	GLOS_TB927.4.4160.1.1	C/A	SNP	214
		T/C	SNP	225
		T/C	SNP	238
		T/C	SNP	266
		C/T	SNP	281
		G/T	SNP	409
		T/C	SNP	670
		T/C	SNP	749
		G/A	SNP	750
		G/A	SNP	751
		T/C	SNP	761
		T/C	SNP	824
		C/T	SNP	957
		C/T	SNP	1047
		A/G	SNP	1067
		G/A	SNP	1230
		A/T	SNP	1246
		T/G	SNP	1304

		A/G	SNP	1321
		G/A	SNP	1326
		C/G	SNP	1354
		T/C	SNP	1355
		G/A	SNP	1398
		T/G	SNP	1399
		G/A	SNP	1403
		C/T	SNP	1898
		G/A	SNP	1913
		A/G	SNP	1938
		C/T	SNP	1966
		A/T	SNP	1998
		A/G	SNP	2040
		G/T	SNP	2055
		C/T	SNP	2056
XM_839501.1 Tbbreceptor-type adenylate cyclase GRESAG 4, putative (Tb927.4.4450) partial mRNA	GLOS_TB927.4.4450.1.1	T/C	SNP	350
		A/T	SNP	395
		A/G	SNP	396
		G/T	SNP	397
		G/A	SNP	586
		C/G	SNP	590
		G/A	SNP	595
		T/C	SNP	621
		C/T	SNP	679
		T/C	SNP	684
		A/G	SNP	709
		A/G	SNP	812
		A/C	SNP	813
		G/T	SNP	930
		T/G	SNP	1197
		A/G	SNP	1225
		G/A	SNP	1453
		G/T	SNP	1513
		A/T	SNP	1535
		T/C	SNP	1634
		A/G	SNP	1693
		T/G	SNP	1771
		G/A	SNP	1984
		C/T	SNP	1989

C/T	SNP	1990
T/C	SNP	2055
C/T	SNP	2089
A/G	SNP	2110
A/C	SNP	2262
G/A	SNP	2269
A/C	SNP	2293
C/T	SNP	2296
C/T	SNP	2303
A/T	SNP	2344
C/T	SNP	2346
A/G	SNP	2369
C/T	SNP	2386
G/C	SNP	2394
A/G	SNP	2410
C/T	SNP	2430
C/T	SNP	2437
A/G	SNP	2450
C/G	SNP	2482
C/G	SNP	2483
A/G	SNP	2484
G/A	SNP	2563
T/C	SNP	2618
G/A	SNP	2746
C/T	SNP	2766
A/G	SNP	2788
A/G	SNP	2790
C/T	SNP	2791
C/T	SNP	2805
C/T	SNP	2807
C/T	SNP	2974
A/G	SNP	3028
C/T	SNP	3029
C/T	SNP	3030
A/C	SNP	3031
G/C	SNP	3044
G/T	SNP	3070
G/A	SNP	3087
T/G	SNP	3257

		C/T	SNP	3270
		A/G	SNP	3271
		A/C	SNP	3340
		A/G	SNP	3346
		T/C	SNP	3417
		C/T	SNP	3424
		T/A	SNP	3454
		T/C	SNP	3455
		A/G	SNP	3550
		A/G	SNP	3561
		C/T	SNP	3577
		C/T	SNP	3640
		C/T	SNP	3643
		G/C	SNP	3662
		A/G	SNP	3710
		T/C	SNP	3792
		T/G	SNP	3801
		C/T	SNP	3818
		G/T	SNP	3852
		T/C	SNP	3895
		G/A	SNP	3926
		T/A	SNP	656
XM_839513.1 Tbb hypothetical protein, conserved (Tb927.4.4570) partial mRNA	GLOS_TB927.4.4570.1.1	G/GT	INSERTION	3492
XM_839529.1 Tbb strain 927/4 GUTat10.1 amino acid transporter, putative partial mRNA	GLOS_TB927.4.4730.1.1	C/CT	INSERTION	2142
XP_844648.1 ubiquinol-cytochrome C reductase [T. brucei brucei strain 927/4 GUTat10.1]	GLOS_TB927.4.4990.1.1	C/T	SNP	74
		C/A	SNP	76
		C/T	SNP	78
		G/C	SNP	86
		A/G	SNP	245
		G/A	SNP	322
		G/GA	INSERTION	330
		G/C	SNP	395
		A/G	SNP	397
		T/C	SNP	412
		A/G	SNP	455
		C/T	SNP	460
		C/T	SNP	562
		G/T	SNP	565
		C/T	SNP	580

XM_839116.1 T. strain 927/4 GUTat10.1 hypothetical protein, conserved (Tb927.4.590) partial mRNA	GLOS_TB927.4.590.1.1	G/C	SNP	252
		A/G	SNP	1391
		A/G	SNP	2147
		G/A	SNP	2156
XM_839133.1 Tbb strain 927/4 GUTat10.1 gamma-adaptin 1, putative (Tb927.4.760) partial mRNA	GLOS_TB927.4.760.1.1	GT/G	DELETION	3023
XP_844774.1 ubiquitin-conjugating enzyme E2 [Trypanosoma brucei brucei strain 927/4 GUTat10.1]	GLOS_TB927.5.1000.1.1	G/GT	INSERTION	857
XM_839687.1 Tbb mitochondrial processing peptidase, beta subunit partial mRNA	GLOS_TB927.5.1060.1.1	C/A	SNP	6
		G/GA	INSERTION	419
		CA/C	DELETION	788
		TA/T	DELETION	1010
		G/GT	INSERTION	2720
XP_844783.1 threonyl-tRNA synthetase [Trypanosoma brucei brucei strain 927/4 GUTat10.1]	GLOS_TB927.5.1090.1.1	G/A	SNP	1466
XP_844785.1 60S ribosomal protein L2 [Trypanosoma brucei brucei strain 927/4 GUTat10.1]	GLOS_TB927.5.1110.1.1	A/G	SNP	586
ref|XP_829685.1| 60S ribosomal protein L2 [Trypanosoma brucei brucei strain 927/4 GUTat10.1]				
		G/A	SNP	589
		G/A	SNP	631
		G/A	SNP	652
		G/C	SNP	769
		G/A	SNP	787
		A/G	SNP	790
		A/G	SNP	832
		T/C	SNP	859
		G/T	SNP	870
		G/A	SNP	874
		G/A	SNP	1105
		C/A	SNP	1123
		G/A	SNP	1147
		G/A	SNP	1150
		G/T	SNP	1231
		G/A	SNP	1249
XM_839702.1 T. brucei brucei strain 927/4 GUTat10.1 short-chain dehydrogenase partial mRNA	GLOS_TB927.5.1210.1.1	A/C	SNP	98
		G/A	SNP	604
		G/T	SNP	686
		T/G	SNP	1028
XM_839706.1 Trypanosoma brucei brucei strain 927/4 GUTat10.1 hypothetical protein partial mRNA	GLOS_TB927.5.1250.1.1	C/T	SNP	188
		CA/C	DELETION	204
		G/A	SNP	379
		G/A	SNP	559
		C/T	SNP	1303

		C/G	SNP	1957
		G/T	SNP	2346
		TC/T	DELETION	2450
		C/T	SNP	2459
XP_844821.1 NADH-cytochrome b5 reductase [Trypanosoma brucei brucei strain 927/4 GUTat10.1]	GLOS_TB927.5.1470.1.1	GA/G	DELETION	1068
		A/T	SNP	1575
		C/CT	INSERTION	1984
		AG/A	DELETION	2041
XM_839738.1 Trypanosoma brucei brucei strain 927/4 GUTat10.1 hypothetical protein partial mRNA	GLOS_TB927.5.1570.1.1	G/A	SNP	163
		G/GCA	INSERTION	392
		C/T	SNP	549
		C/CT	INSERTION	831
		C/T	SNP	1948
		C/A	SNP	3071
XM_839747.1 T. brucei brucei strain 927/4 GUTat10.1 protein phosphatase 2C partial mRNA	GLOS_TB927.5.1660.1.1	G/GA	INSERTION	312
		CA/C	DELETION	804
		A/G	SNP	2785
XM_839752.1 T. strain 927/4 GUTat10.1 ribonucleoprotein p18, mitochondrial precursor partial mRNA	GLOS_TB927.5.1710.1.1	C/CT	INSERTION	960
		CAGAG/C	DELETION	1047
		G/T	SNP	1984
		GT/G	DELETION	1984
XM_839753.1 Trypanosoma brucei brucei strain 927/4 GUTat10.1 hypothetical protein partial mRNA	GLOS_TB927.5.1720.1.1	T/C	SNP	1215
		A/G	SNP	1992
		A/G	SNP	2008
		T/C	SNP	2062
XM_839759.1 Trypanosoma brucei brucei strain 927/4 GUTat10.1 hypothetical protein partial mRNA	GLOS_TB927.5.1780.1.1	G/A	SNP	1285
		C/T	SNP	1632
		T/C	SNP	1983
		A/G	SNP	2159
		C/G	SNP	2195
		C/T	SNP	2219
XP_844853.1 hypothetical protein [Trypanosoma brucei brucei strain 927/4 GUTat10.1]	GLOS_TB927.5.1790.1.1	A/G	SNP	699
		A/G	SNP	1639
XM_839762.1 Tbb lysosomal/endosomal membrane protein p67 partial mRNA	GLOS_TB927.5.1810.1.1	C/CA	INSERTION	499
		T/A	SNP	500
		C/A	SNP	502
		G/A	SNP	699
		G/A	SNP	882
		G/C	SNP	988

		T/C	SNP	3039
		G/A	SNP	3301
XM_839791.1 Trypanosoma brucei brucei strain 927/4 GUTat10.1 hypothetical protein partial mRNA	GLOS_TB927.5.2100.1.1	C/T	SNP	515
		AG/A	DELETION	1729
XM_839807.1 Trypanosoma brucei brucei strain 927/4 GUTat10.1 hypothetical protein partial mRNA	GLOS_TB927.5.2260.1.2	G/A	SNP	311
		A/T	SNP	95
		A/ATT	INSERTION	96
		A/T	SNP	96
		G/A	SNP	299
		C/T	SNP	809
XM_839824.1 Tbb strain 927/4 GUTat10.1 membrane transporter protein partial mRNA	GLOS_TB927.5.2430.1.1	A/G	SNP	193
		C/T	SNP	733
		T/C	SNP	1162
		C/T	SNP	1285
		T/A	SNP	1317
		C/T	SNP	1321
		C/T	SNP	1492
		A/T	SNP	2086
XM_839836.1 T. brucei brucei strain 927/4 GUTat10.1 translation initiation factor partial mRNA	GLOS_TB927.5.2570.1.1	A/T	SNP	651
		C/G	SNP	754
		T/C	SNP	2617
XM_839864.1 Trypanosoma brucei brucei strain 927/4 GUTat10.1 hypothetical protein partial mRNA	GLOS_TB927.5.2850.1.1	T/C	SNP	35
		G/A	SNP	39
		C/T	SNP	1953
XM_839872.1 Trypanosoma brucei brucei strain 927/4 GUTat10.1 hypothetical protein partial mRNA	GLOS_TB927.5.2930.1.1	A/G	SNP	233
		T/C	SNP	277
XM_839873.1 T. brucei brucei strain 927/4 GUTat10.1 stress-induced protein sti1 partial mRNA	GLOS_TB927.5.2940.1.1	G/GT	INSERTION	304
XP_844968.1 phosphoribosylpyrophosphate synthetase [Trypanosoma brucei TREU927]	GLOS_TB927.5.2960.1.1	A/T	SNP	96
		G/GT	INSERTION	157
		A/C	SNP	565
		T/A	SNP	764
XM_839891.1 T. brucei brucei strain 927/4 GUTat10.1 translation initiation factor partial mRNA	GLOS_TB927.5.3120.1.1	T/A	SNP	1751
XM_839895.1 Trypanosoma brucei brucei strain 927/4 GUTat10.1 protein kinase partial mRNA	GLOS_TB927.5.3160.1.1	T/C	SNP	470
		A/T	SNP	728
		A/T	SNP	1554
		A/G	SNP	1582
		G/T	SNP	3145
XM_839613.1 Tbb strain 927/4 GUTat10.1 receptor-type adenylate cyclase GRESAG 4 partial mRNA	GLOS_TB927.5.320.1.1	G/GA	INSERTION	384
		C/T	SNP	461

		GA/G	DELETION	1150
		A/G	SNP	1335
		C/T	SNP	1480
		A/G	SNP	1991
		A/G	SNP	2052
		T/C	SNP	2134
		T/C	SNP	2652
		T/TA	INSERTION	2865
		C/T	SNP	2892
		A/G	SNP	2950
		C/T	SNP	3133
		T/G	SNP	3415
		A/T	SNP	3434
		A/T	SNP	3593
		A/G	SNP	3613
		G/C	SNP	3614
		T/C	SNP	3959
		A/G	SNP	4267
		G/A	SNP	4394
XM_839903.1 Trypanosoma brucei brucei strain 927/4 GUTat10.1 hypothetical protein partial mRNA	GLOS_TB927.5.3240.1.1	A/C	SNP	100
		T/C	SNP	170
		C/T	SNP	688
		T/C	SNP	1251
XM_839928.1 Tbb strain 927/4 GUTat10.1 structural maintenance of chromosome 3 partial mRNA	GLOS_TB927.5.3510.1.1	T/C	SNP	1094
		G/A	SNP	1671
XM_839936.1 Trypanosoma brucei brucei strain 927/4 GUTat10.1 hypothetical protein partial mRNA	GLOS_TB927.5.3590.1.1	G/T	SNP	1032
		T/C	SNP	1237
		A/G	SNP	1248
		A/G	SNP	1287
		T/G	SNP	1887
		A/G	SNP	2788
		G/A	SNP	2893
XM_839617.1 Tbb strain 927/4 GUTat10.1 75 kDa invariant surface glycoprotein partial mRNA	GLOS_TB927.5.360.1.1	A/G	SNP	155
		T/C	SNP	295
		C/T	SNP	571
		C/T	SNP	693
		T/C	SNP	734
		C/T	SNP	815
		C/A	SNP	1076

		C/T	SNP	1094
		C/A	SNP	1145
		C/T	SNP	1149
		C/T	SNP	1833
		G/C	SNP	1908
XP_845050.1 glutamine hydrolysing (not ammonia-dependent) carbomoyl phosphate synthase [Tbb]	GLOS_TB927.5.3800.1.1	T/TTA	INSERTION	94
		G/GA	INSERTION	351
		G/GA	INSERTION	462
		C/CA	INSERTION	689
		A/AGTT	INSERTION	764
		T/G	SNP	868
		A/G	SNP	4136
		T/C	SNP	4715
		T/C	SNP	6592
		G/GT	INSERTION	7325
		T/C	SNP	7750
XM_839973.1 Tbb strain 927/4 GUTat10.1 arginine N-methyltransferase partial mRNA	GLOS_TB927.5.3960.1.1	G/GT	INSERTION	273
XM_839979.1 Tbb strain 927/4 GUTat10.1 hypothetical protein Tb927.5.4020 partial mRNA	GLOS_TB927.5.4020.1.1	G/GA	INSERTION	242
		C/CT	INSERTION	1381
XM_839996.1 Trypanosoma brucei brucei strain 927/4 GUTat10.1 histone H4 partial mRNA	GLOS_TB927.5.4190.2.3	T/C	SNP	534
XM_839625.1 Trypanosoma brucei brucei strain 927/4 GUTat10.1 hypothetical protein partial mRNA	GLOS_TB927.5.440.1.1	C/CT	INSERTION	2822
XM_840023.1 Trypanosoma brucei brucei strain 927/4 GUTat10.1 major vault protein partial mRNA	GLOS_TB927.5.4460.1.1	C/CA	INSERTION	278
XP_844725.1 hypothetical protein [Trypanosoma brucei brucei strain 927/4 GUTat10.1]	GLOS_TB927.5.510.1.1	G/A	SNP	484
		C/T	SNP	622
		A/G	SNP	628
		C/T	SNP	738
		GAA/G/GA	DELETION	934
		T/TA	INSERTION	1990
		C/T	SNP	2112
		G/C	SNP	2132
XM_839651.1 Trypanosoma brucei brucei strain 927/4 GUTat10.1 hypothetical protein partial mRNA	GLOS_TB927.5.700.1.1	T/G	SNP	149
		AT/A	DELETION	2601
		G/T	SNP	2735
XM_839667.1 Trypanosoma brucei brucei strain 927/4 GUTat10.1 hypothetical protein partial mRNA	GLOS_TB927.5.860.1.1	AT/A	DELETION	716
		C/A	SNP	2313
XP_844764.1 oligosaccharyl transferase subunit [Trypanosoma brucei TREU927]	GLOS_TB927.5.900.1.1	A/G	SNP	1048
		G/A	SNP	1378
		T/C	SNP	1403
		T/C	SNP	1577

		A/G	SNP	1592
		C/T	SNP	1968
		T/C	SNP	1969
		T/C	SNP	1973
		G/A	SNP	2374
		C/T	SNP	2645
		C/T	SNP	2747
XM_839674.1 Tbb strain 927/4 GUTat10.1 NADH-dependent fumarate reductase partial mRNA	GLOS_TB927.5.930.1.1	T/C	SNP	1905
		A/G	SNP	1936
		T/C	SNP	2662
		C/CA	INSERTION	3590
XM_840131.1 Tbb strain 927/4 GUTat10.1 cysteine peptidase precursor partial mRNA	GLOS_TB927.6.1020.1.1	C/A	SNP	46
		A/G	SNP	47
		G/T	SNP	352
		C/T	SNP	1327
		T/C	SNP	1329
		G/GT	INSERTION	1510
		G/A	SNP	1636
XP_845231.1 proteasome regulatory ATPase subunit 3 [Trypanosoma brucei TREU927]	GLOS_TB927.6.1090.1.1	C/CA	INSERTION	196
		G/GAAGA	INSERTION	320
		GA/G	DELETION	1757
XP_845270.1 hypothetical protein [Trypanosoma brucei brucei strain 927/4 GUTat10.1]	GLOS_TB927.6.1480.1.1	T/C	SNP	4593
		GA/G	DELETION	4951
		G/GA	INSERTION	5098
XM_840181.1 Trypanosoma brucei brucei strain 927/4 GUTat10.1 aquaporin 3 partial mRNA	GLOS_TB927.6.1520.1.1	C/CA	INSERTION	1242
		C/CA	INSERTION	1549
		GA/G	DELETION	1828
		T/TA	INSERTION	2101
		AT/A	DELETION	2271
		C/CT	INSERTION	2492
		C/CT	INSERTION	2898
XM_840229.1 Trypanosoma brucei brucei strain 927/4 GUTat10.1 hypothetical protein partial mRNA	GLOS_TB927.6.2010.1.1	C/T	SNP	1455
XM_840251.1 Trypanosoma brucei brucei strain 927/4 GUTat10.1 hypothetical protein partial mRNA	GLOS_TB927.6.2230.1.1	A/G	SNP	376
		G/A	SNP	416
		G/A	SNP	503
		G/A	SNP	1433
		T/C	SNP	1601
		A/C	SNP	2237
		G/T	SNP	2253

XM_840257.1 Trypanosoma brucei brucei strain 927/4 GUTat10.1 hypothetical protein partial mRNA	GLOS_TB927.6.2290.1.1	C/A	SNP	323
		A/G	SNP	401
		C/A	SNP	765
		T/C	SNP	1106
		G/A	SNP	1282
		T/C	SNP	1479
		C/T	SNP	2271
		T/C	SNP	2392
		G/A	SNP	2501
XP_845395.1 pyridoxal kinase [Trypanosoma brucei brucei strain 927/4 GUTat10.1]	GLOS_TB927.6.2740.1.1	A/AGG	INSERTION	105
XP_845400.1 L-threonine 3-dehydrogenase [Trypanosoma brucei brucei strain 927/4 GUTat10.1]	GLOS_TB927.6.2790.1.1	G/GT	INSERTION	116
		G/GA	INSERTION	1151
		C/CT	INSERTION	1619
		CA/C	DELETION	1819
		G/GT	INSERTION	1957
XM_840337.1 Trypanosoma brucei brucei strain 927/4 GUTat10.1 hypothetical protein partial mRNA	GLOS_TB927.6.3090.1.1	T/G	SNP	610
		GT/G	DELETION	4321
XP_845468.1 endosomal trafficking protein RME-8 [Trypanosoma brucei TREU927]	GLOS_TB927.6.3500.1.1	A/G	SNP	217
		C/T	SNP	336
		T/C	SNP	5283
XP_845483.1 ADP-ribosylation factor [Trypanosoma brucei brucei strain 927/4 GUTat10.1]	GLOS_TB927.6.3650.1.1	A/C	SNP	209
		CA/C	DELETION	531
XM_840405.1 Tbb heat shock 70 kDa protein, mitochondrial precursor partial mRNA	GLOS_TB927.6.3800.1.1	T/C	SNP	84
		G/A	SNP	555
		TA/T	DELETION	2083
XM_840409.1 T. brucei brucei strain 927/4 GUTat10.1 reticulon domain protein partial mRNA	GLOS_TB927.6.3840.1.1	T/C	SNP	135
		G/A	SNP	163
		G/GA	INSERTION	163
		T/G	SNP	834
		C/CTT	INSERTION	1515
XP_845513.1 hypothetical protein [Trypanosoma brucei brucei strain 927/4 GUTat10.1]	GLOS_TB927.6.3950.1.1	G/GT	INSERTION	233
		C/G	SNP	1874
		C/T	SNP	2021
		GT/G	DELETION	2060
		G/T	SNP	2070
XM_840425.1 Trypanosoma brucei brucei strain 927/4 GUTat10.1 hypothetical protein partial mRNA	GLOS_TB927.6.4000.1.1	A/T	SNP	22
		G/A	SNP	423
		C/T	SNP	546
		G/C	SNP	676

		T/TA	INSERTION	853
		C/T	SNP	2144
XP_845532.1 hypothetical protein [Trypanosoma brucei brucei strain 927/4 GUTat10.1]	GLOS_TB927.6.4140.1.1	ATT/A	DELETION	107
		G/GA	INSERTION	256
		G/GA	INSERTION	642
		CACA/C	DELETION	909
		CA/C	DELETION	911
		AT/A	DELETION	976
		G/A	SNP	1238
		GA/G	DELETION	1306
XP_845547.1 glyceraldehyde 3-phosphate dehydrogenase, glycosomal [Tbb strain 927/4 GUTat10.1]	GLOS_TB927.6.4300.1.1	T/C	SNP	923
		C/T	SNP	1061
		C/T	SNP	1224
		A/G	SNP	1410
		C/T	SNP	2097
XM_840468.1 Trypanosoma brucei brucei strain 927/4 GUTat10.1 hypothetical protein partial mRNA	GLOS_TB927.6.4440.1.1	TAAA/T	DELETION	740
		GA/G	DELETION	802
		C/T	SNP	1485
		C/T	SNP	1541
		A/G	SNP	2002
		G/A	SNP	2149
		T/C	SNP	2151
XM_840472.1 T. brucei brucei strain 927/4 GUTat10.1 valyl-tRNA synthetase partial mRNA	GLOS_TB927.6.4480.1.1	A/T	SNP	54
		A/T	SNP	396
		C/T	SNP	1374
		G/A	SNP	1792
		C/T	SNP	2424
		C/G	SNP	2477
		A/G	SNP	2522
		T/C	SNP	2706
		G/A	SNP	2953
		G/A	SNP	3021
XP_845566.1 hypothetical protein [Trypanosoma brucei brucei strain 927/4 GUTat10.1]	GLOS_TB927.6.4490.1.1	A/G	SNP	271
		CT/C	DELETION	823
		G/A	SNP	985
		A/AGT	INSERTION	1143
		T/G	SNP	1144
		C/CT	INSERTION	1275
		C/T	SNP	1633

		C/G	SNP	1720
XM_840478.1 Tbb strain 927/4 GUTat10.1 3-hydroxy-3-methylglutaryl-CoA reductase partial mRNA	GLOS_TB927.6.4540.1.1	GA/G	DELETION	278
		GA/G	DELETION	370
		C/A	SNP	396
		G/C	SNP	397
		T/TA	INSERTION	483
		T/C	SNP	1734
XM_840499.1 Trypanosoma brucei brucei strain 927/4 GUTat10.1 hypothetical protein partial mRNA	GLOS_TB927.6.4750.1.1	G/GA	INSERTION	2186
		G/A	SNP	2419
		T/TA	INSERTION	2546
		T/C	SNP	2630
XM_840508.1 Tbb strain 927/4 GUTat10.1 S-adenosylmethionine synthetase partial mRNA	GLOS_TB927.6.4840.1.1	T/C	SNP	214
		T/C	SNP	609
		C/T	SNP	741
		C/T	SNP	1227
		A/AAT	INSERTION	1580
		T/C	SNP	1808
XM_840522.1 T. brucei brucei strain 927/4 GUTat10.1 40S ribosomal protein S14 partial mRNA	GLOS_TB927.6.4980.1.2	A/G	SNP	305
		G/A	SNP	308
		A/C	SNP	47
		A/G	SNP	48
		C/T	SNP	407
XM_840531.1 Trypanosoma brucei brucei strain 927/4 GUTat10.1 hypothetical protein partial mRNA	GLOS_TB927.6.5070.1.1	G/A	SNP	342
		G/GA	INSERTION	1529
		T/G	SNP	1667
		AT/A	DELETION	1725
		TA/T	DELETION	1836
		C/G	SNP	1932
		G/GAA	INSERTION	1952
		G/A	SNP	2008
XM_840532.1 Trypanosoma brucei brucei strain 927/4 GUTat10.1 hypothetical protein partial mRNA	GLOS_TB927.6.5080.1.1	C/G	SNP	94
		TA/T	DELETION	112
		GA/G	DELETION	425
		T/C	SNP	1144
		C/T	SNP	1477
		C/CT	INSERTION	2293
		GA/G	DELETION	2740
XM_840533.1 Trypanosoma brucei brucei strain 927/4 GUTat10.1 hypothetical protein partial mRNA	GLOS_TB927.6.5090.1.1	G/A	SNP	1150
		A/C	SNP	1549

		T/C	SNP	1950
		A/T	SNP	3041
		TTG/T	DELETION	3239
		C/T	SNP	3301
		C/T	SNP	3407
XM_840536.1 Tbb strain 927/4 GUTat10.1 60S acidic ribosomal protein P2 partial mRNA	GLOS_TB927.6.5120.1.2	A/G	SNP	249
		G/A	SNP	258
XM_840082.1 Trypanosoma brucei brucei strain 927/4 GUTat10.1 EP3-2 procyclin partial mRNA	GLOS_TB927.6.520.1.3	A/T	SNP	25
		G/A	SNP	488
		A/T	SNP	654
XM_840096.1 Trypanosoma brucei brucei strain 927/4 GUTat10.1 hypothetical protein partial mRNA	GLOS_TB927.6.660.1.1	C/G	SNP	816
		A/G	SNP	1549
XM_840102.1 T. brucei brucei strain 927/4 GUTat10.1 40S ribosomal protein L14 partial mRNA	GLOS_TB927.6.720.1.1	G/T	SNP	20
		C/T	SNP	127
		C/T	SNP	286
		G/A	SNP	290
		G/A	SNP	334
		A/G	SNP	367
		T/A	SNP	747
XM_840109.1 Tbb strain 927/4 GUTat10.1 receptor-type adenylate cyclase GRESAG 4 partial mRNA	GLOS_TB927.6.790.1.2	C/G	SNP	1394
		T/A	SNP	1428
		C/T	SNP	1429
		A/G	SNP	1430
		A/G	SNP	1492
		T/C	SNP	1705
		C/T	SNP	1785
		C/T	SNP	1884
		C/T	SNP	1887
		G/A	SNP	2001
		G/T	SNP	2012
		T/C	SNP	2163
		A/G	SNP	2631
		T/C	SNP	2969
XP_845202.1 receptor-type adenylate cyclase GRESAG 4 [Trypanosoma brucei TREU927]	GLOS_TB927.6.790.2.2	C/A	SNP	55
		T/A	SNP	67
		C/T	SNP	69
		A/C	SNP	157
		T/A	SNP	161
		G/A	SNP	190

		A/G	SNP	192
		G/T	SNP	2017
		G/A	SNP	2038
		C/T	SNP	2066
XM_840124.1 T. brucei brucei strain 927/4 GUTat10.1 cysteinyl-tRNA synthetase partial mRNA	GLOS_TB927.6.950.1.1	T/C	SNP	28
		T/A	SNP	2644
		A/C	SNP	2965
XP_845735.1 asparagine synthetase a [Trypanosoma brucei brucei strain 927/4 GUTat10.1]	GLOS_TB927.7.1110.1.1	T/TTA	INSERTION	1939
XM_840660.1 Tbb strain 927/4 GUTat10.1 hypothetical prot., conserved (Tb927.7.1290) partial mRNA	GLOS_TB927.7.1290.1.1	A/AT	INSERTION	131
		C/A	SNP	900
XP_845756.1 10 kDa heat shock protein [Trypanosoma brucei brucei strain 927/4 GUTat10.1] ref|XP_845758.1| 10 kDa heat shock protein [Trypanosoma brucei brucei strain 927/4 GUTat10.1]	GLOS_TB927.7.1320.1.1	C/T	SNP	256
XM_840678.1 Tbb strain 927/4 GUTat10.1 ATPase subunit 9, putative (Tb927.7.1470) partial mRNA	GLOS_TB927.7.1470.1.1	A/AT	INSERTION	923
		A/G	SNP	261
		C/T	SNP	355
XP_845803.1 adenine phosphoribosyltransferase [T. brucei brucei strain 927/4 GUTat10.1]	GLOS_TB927.7.1790.1.1	CA/C	DELETION	113
		G/GA	INSERTION	574
		T/A	SNP	1004
		CAAA/C	DELETION	1020
		C/T	SNP	1116
XP_845643.1 thimet oligopeptidase A [Trypanosoma brucei brucei strain 927/4 GUTat10.1]	GLOS_TB927.7.190.1.1	C/CT	INSERTION	713
XM_840746.1 Tbb strain 927/4 GUTat10.1 hypothetical prot., conserved (Tb927.7.2160) partial mRNA	GLOS_TB927.7.2160.1.1	G/GA	INSERTION	373
		G/A	SNP	375
		G/A	SNP	379
		A/G	SNP	1100
		GA/G	DELETION	1636
XM_840747.1 Tbb strain 927/4 GUTat10.1 hypothetical prot., conserved (Tb927.7.2170) partial mRNA	GLOS_TB927.7.2170.1.1	T/C	SNP	493
		G/C	SNP	4025
		T/C	SNP	4573
XM_840749.1 Tbb strain 927/4 GUTat10.1 hypothetical prot, conserved (Tb927.7.2190) partial mRNA	GLOS_TB927.7.2190.1.1	C/CA	INSERTION	123
		T/C	SNP	387
		G/C	SNP	802
		A/G	SNP	861
		A/G	SNP	960
		G/A	SNP	1175
		C/CT	INSERTION	1535
		A/G	SNP	1624
XM_840754.1 Tbb strain 927/4 GUTat10.1 hypothetical prot., conserved (Tb927.7.2240) partial mRNA	GLOS_TB927.7.2240.1.1	G/A	SNP	30
		A/G	SNP	280

		T/C	SNP	990
		T/C	SNP	1232
XM_840760.1 Tbb strain 927/4 GUTat10.1 hypothetical protein, conserved partial mRNA	GLOS_TB927.7.2300.1.1	A/G	SNP	539
		C/T	SNP	1528
		T/C	SNP	2635
		G/T	SNP	3040
		C/T	SNP	3142
		A/G	SNP	3240
XM_840554.1 Tbb strain 927/4 GUTat10.1 40S ribosomal protein S33, putative partial mRNA	GLOS_TB927.7.230.1.1	T/G	SNP	176
		A/T	SNP	520
		C/G	SNP	521
XM_840764.1 Tbb strain 927/4 GUTat10.1 40S ribosomal protein S15, putative partial mRNA	GLOS_TB927.7.2340.1.1	A/T	SNP	16
		G/A	SNP	442
XM_840766.1 Tbb strain 927/4 GUTat10.1 N-acetyltransferase, putative (Tb927.7.2360) partial mRNA	GLOS_TB927.7.2360.1.1	C/T	SNP	254
		G/A	SNP	587
		T/C	SNP	1060
		C/T	SNP	1111
		CT/C	DELETION	1325
		T/A	SNP	1512
		T/C	SNP	1583
		A/T	SNP	1780
		C/T	SNP	1998
		C/G	SNP	2062
		C/T	SNP	2068
XM_840769.1 Tbb strain 927/4 GUTat10.1 hypothetical protein, conserved partial mRNA	GLOS_TB927.7.2390.1.1	A/C	SNP	252
		A/C	SNP	253
		G/A	SNP	348
		GA/G	DELETION	464
		C/CT	INSERTION	1247
		C/T	SNP	1821
		A/G	SNP	1929
		G/A	SNP	3733
XP_845878.1 proteasome regulatory ATPase subunit 5 [Trypanosoma brucei TREU927]	GLOS_TB927.7.2550.1.1	A/C	SNP	943
		G/GA	INSERTION	1166
		A/G	SNP	1587
		T/TA	INSERTION	2616
XM_840787.1 Tbb strain 927/4 GUTat10.1 hypothetical prot., conserved (Tb927.7.2570) partial mRNA	GLOS_TB927.7.2570.1.1	C/CA	INSERTION	452
		T/C	SNP	615
XP_845888.1 hypothetical protein [Trypanosoma brucei brucei strain 927/4 GUTat10.1]	GLOS_TB927.7.2650.1.1	C/CA	INSERTION	615

		T/C	SNP	2191
		A/C	SNP	2192
		T/C	SNP	2225
		T/C	SNP	2227
XP_845905.1 histone H2A [Trypanosoma brucei brucei strain 927/4 GUTat10.1] ref|XP_845906.1| histone H2A [Tbb] ref|XP_845907.1| histone H2A [Tbb] ref|XP_845908.1| histone H2A [Tbb] ref|XP_845909.1| histone H2A [Tbb] ref|XP_845910.1| histone H2A [Tbb] ref|XP_845911.1| histone H2A [Tbb] ref|XP_845912.1| histone H2A [Tbb] ref|XP_845913.1| histone H2A [Tbb] ref|XP_845914.1| histone H2A [Tbb| histone H2A [Tbb] ref|XP_845916.1| histone H2A [Tbb] ref|XP_845917.1| histone H2A [Tbb]	GLOS_TB927.7.2820.2.2	G/A	SNP	100
		G/GA	INSERTION	210
		A/C	SNP	541
		A/C	SNP	837
		G/T	SNP	838
XP_845921.1 hypothetical protein [Trypanosoma brucei brucei strain 927/4 GUTat10.1]	GLOS_TB927.7.2980.1.1	G/A	SNP	274
		G/A	SNP	384
		C/T	SNP	1055
XP_845978.1 hypothetical protein [Trypanosoma brucei brucei strain 927/4 GUTat10.1]	GLOS_TB927.7.3550.1.2	G/A	SNP	17
		C/A	SNP	535
		C/T	SNP	2513
		A/G	SNP	2515
		A/C	SNP	2516
		C/CTTTGT	INSERTION	2288
XM_840892.1 Tbb strain 927/4 GUTat10.1 tyrosyl-tRNA synthetase, putative partial mRNA	GLOS_TB927.7.3620.1.1	T/C	SNP	134
		T/C	SNP	365
		A/G	SNP	510
		C/T	SNP	673
		A/G	SNP	914
		G/A	SNP	995
		A/C	SNP	1220
		A/G	SNP	2831
XM_840893.1 Tbb TPR-repeat-containing chaperone protein DNAJ, putative partial mRNA	GLOS_TB927.7.3630.1.1	G/A	SNP	251
		C/G	SNP	561
		T/TGC	INSERTION	2418
		TTG/T	DELETION	2517
		T/C	SNP	2544
		A/G	SNP	2597
		T/C	SNP	2802
		C/T	SNP	2843

XM_840898.1 Tbb ubiquitin/ribosomal protein S27a, putative (Tb927.7.3680) partial mRNA	GLOS_TB927.7.3680.1.2	CAA/C	DELETION	181
		G/GA	INSERTION	235
XM_840898.1 Tbb ubiquitin/ribosomal protein S27a, putative (Tb927.7.3680) partial mRNA	GLOS_TB927.7.3680.2.2	G/T	SNP	740
XM_840904.1 Tbb strain 927/4 GUTat10.1 hypothetical prot., conserved (Tb927.7.3740) partial mRNA	GLOS_TB927.7.3740.1.1	G/A	SNP	725
		G/A	SNP	1015
		GA/G	DELETION	1870
XM_840924.1 Tbb strain 927/4 GUTat10.1 mitochondrial carrier protein, putative partial mRNA	GLOS_TB927.7.3940.1.1	A/C	SNP	222
		G/A	SNP	336
		T/A	SNP	544
		T/C	SNP	717
		A/G	SNP	920
		G/A	SNP	1106
XM_840928.1 Tbb immunodominant antigen, putative (Tb927.7.3980) partial mRNA	GLOS_TB927.7.3980.1.1	A/G	SNP	3323
XP_846030.1 calpain-like cysteine peptidase [Trypanosoma brucei brucei strain 927/4 GUTat10.1]	GLOS_TB927.7.4070.1.1	C/CTTT	INSERTION	577
		G/GA	INSERTION	910
XM_840942.1 Tbb strain 927/4 GUTat10.1 hypothetical prot, conserved (Tb927.7.4120) partial mRNA	GLOS_TB927.7.4120.1.1	A/T	SNP	1501
XM_840948.1 Tbb strain 927/4 GUTat10.1 fatty acid elongase, putative (Tb927.7.4180) partial mRNA	GLOS_TB927.7.4180.1.1	C/T	SNP	533
		T/C	SNP	588
		C/T	SNP	1180
		C/CT	INSERTION	1478
		GA/G	DELETION	1575
		T/TTTTTG	INSERTION	1774
		G/GA	INSERTION	2143
XM_840957.1 Tbb strain 927/4 GUTat10.1 hypothetical prot, conserved (Tb927.7.4270) partial mRNA	GLOS_TB927.7.4270.1.1	G/A	SNP	79
		T/G	SNP	82
		T/C	SNP	765
		CT/C	DELETION	1016
		C/CTAT	INSERTION	1079
		GA/G	DELETION	1185
XP_846062.1 threonine synthase [Trypanosoma brucei brucei strain 927/4 GUTat10.1]	GLOS_TB927.7.4390.1.1	A/G	SNP	374
XM_840975.1 Tbb strain 927/4 GUTat10.1 hypothetical prot, conserved (Tb927.7.4450) partial mRNA	GLOS_TB927.7.4450.1.1	TA/T	DELETION	87
		G/T	SNP	338
		G/T	SNP	2061
XM_840980.1 Tbb strain 927/4 GUTat10.1 hypothetical prot, conserved (Tb927.7.4500) partial mRNA	GLOS_TB927.7.4500.1.1	G/GA	INSERTION	2334
		T/C	SNP	2631
XM_840987.1 Tbb strain 927/4 GUTat10.1 nucleoside hydrolase, putative partial mRNA	GLOS_TB927.7.4570.1.1	G/C	SNP	1060
XM_841020.1 Tbb 5'-3' exonuclease XRNA, putative (Tb927.7.4900) partial mRNA	GLOS_TB927.7.4900.1.1	G/A	SNP	111
		CT/C	DELETION	116
		C/A	SNP	147

		A/G	SNP	318
		C/T	SNP	1055
XP_846120.1 glutamine synthetase [Trypanosoma brucei brucei strain 927/4 GUTat10.1]	GLOS_TB927.7.4970.1.1	AT/A	DELETION	150
		T/C	SNP	952
		A/C	SNP	1540
XM_841030.1 Tbb 60S ribosomal protein L19, putative (Tb927.7.5000) partial mRNA	GLOS_TB927.7.5000.1.1	A/G	SNP	258
		T/C	SNP	327
		C/T	SNP	762
XM_841048.1 Tbb 60S ribosomal protein L23a, putative (Tb927.7.5180) partial mRNA	GLOS_TB927.7.5180.1.1	C/CT	INSERTION	906
XP_846144.1 hypothetical protein [Trypanosoma brucei brucei strain 927/4 GUTat10.1]	GLOS_TB927.7.5210.1.1	T/A	SNP	380
		C/T	SNP	1354
		T/C	SNP	1886
		A/G	SNP	2324
		A/G	SNP	2577
		G/A	SNP	3256
		G/T	SNP	3783
XM_841053.1 Tbb strain 927/4 GUTat10.1 lanosterol synthase (Tb927.7.5230) partial mRNA	GLOS_TB927.7.5230.1.1	G/A	SNP	2880
		G/GA	INSERTION	3524
		C/T	SNP	3590
XM_841058.1 Tbb strain 927/4 GUTat10.1 hypothetical protein, conserved partial mRNA	GLOS_TB927.7.5280.1.1	G/GA	INSERTION	120
		A/G	SNP	644
		G/A	SNP	3639
		A/G	SNP	4353
		G/A	SNP	4902
		G/A	SNP	5553
		G/A	SNP	5691
XM_841077.1 Tbb strain 927/4 GUTat10.1 hypothetical prot, conserved (Tb927.7.5470) partial mRNA	GLOS_TB927.7.5470.1.1	C/T	SNP	255
		T/C	SNP	1611
		C/T	SNP	1706
		T/C	SNP	1935
		A/C	SNP	2267
XM_841124.1 Tbb strain 927/4 GUTat10.1 hypothetical prot, conserved (Tb927.7.5940) partial mRNA	GLOS_TB927.7.5940.1.1	C/A	SNP	30
		G/T	SNP	31
		C/T	SNP	117
		G/A	SNP	169
		G/T	SNP	666
		C/T	SNP	903
		A/C	SNP	1070
		A/G	SNP	1117

		A/G	SNP	1123
		G/A	SNP	1131
		C/T	SNP	1267
		C/G	SNP	1268
		C/T	SNP	1277
		C/T	SNP	1292
		A/T	SNP	1295
		A/C	SNP	1301
		C/A	SNP	1361
		C/T	SNP	1364
		G/A	SNP	1398
		C/T	SNP	1400
		C/T	SNP	1469
		A/G	SNP	1538
		A/C	SNP	1559
		A/C	SNP	1586
		A/C	SNP	1592
		GT/G	DELETION	2396
		C/CT	INSERTION	2669
XM_841135.1 Tbb receptor-type adenylate cyclase GRESAG 4, putative partial mRNA	GLOS_TB927.7.6050.1.1	T/C	SNP	140
		A/T	SNP	145
		C/T	SNP	292
		A/T	SNP	293
		A/G	SNP	406
		C/T	SNP	683
		T/C	SNP	902
		G/T	SNP	1370
		A/G	SNP	1549
		T/C	SNP	1979
		G/A	SNP	2282
		C/G	SNP	2919
		G/A	SNP	2921
		A/G	SNP	3021
		G/A	SNP	3022
		G/T	SNP	3131
XM_841139.1 Tbb strain 927/4 GUTat10.1 hypothetical protein, conserved partial mRNA	GLOS_TB927.7.6090.1.1	A/C	SNP	1802
		C/T	SNP	2513
		C/CT	INSERTION	2551
XM_841156.1 Tbb strain 927/4 GUTat10.1 hypothetical prot., conserved (Tb927.7.6260) partial mRNA	GLOS_TB927.7.6260.1.1	CA/C	DELETION	228

		A/G	SNP	1271
		T/C	SNP	2261
		CT/C	DELETION	2315
XM_841172.1 Tbb strain 927/4 GUTat10.1 hypothetical prot, conserved (Tb927.7.6420) partial mRNA	GLOS_TB927.7.6420.1.1	T/C	SNP	595
XP_846297.1 hypothetical protein [Trypanosoma brucei brucei strain 927/4 GUTat10.1]	GLOS_TB927.7.6770.1.1	AG/A	DELETION	737
XM_841206.1 Tbb strain 927/4 GUTat10.1 hypothetical prot, conserved (Tb927.7.6790) partial mRNA	GLOS_TB927.7.6790.1.1	A/T	SNP	575
		CAA/C	DELETION	1640
		A/G	SNP	3546
XM_841212.1 Trypanosoma brucei brucei strain 927/4 GUTat10.1 trans-sialidase partial mRNA	GLOS_TB927.7.6850.1.1	T/TA	INSERTION	237
		G/A	SNP	254
		A/C	SNP	431
		G/GA	INSERTION	684
		G/A	SNP	1009
		G/A	SNP	1335
		C/G	SNP	1461
		G/C	SNP	1941
		G/A	SNP	2491
		A/G	SNP	2700
		T/C	SNP	2822
		A/C	SNP	2888
		A/T	SNP	3090
XM_841217.1 Tbb double-strand-break repair protein rad21 homolog, putative partial mRNA	GLOS_TB927.7.6900.1.1	G/GAA	INSERTION	84
		G/T	SNP	113
		G/T	SNP	1008
XM_841224.1 Tbb strain 927/4 GUTat10.1 paraflagellar rod protein, putative partial mRNA	GLOS_TB927.7.6970.1.1	T/C	SNP	642
		G/GT	INSERTION	2625
XM_841232.1 Tbb strain 927/4 GUTat10.1 hypothetical protein, conserved partial mRNA	GLOS_TB927.7.7050.1.1	G/A	SNP	358
		T/TGAA	INSERTION	1388
XM_841236.1 Tbb strain 927/4 GUTat10.1 hypothetical protein, conserved partial mRNA	GLOS_TB927.7.7090.1.1	G/T	SNP	31
		A/T	SNP	32
		G/T	SNP	33
		C/CA	INSERTION	220
		G/A	SNP	257
		C/T	SNP	304
		GT/G	DELETION	833
		CT/C	DELETION	1056
		G/T	SNP	1116
		T/C	SNP	1309
		C/G	SNP	1361

XM_840602.1 Tbb heat shock 70 kDa protein, putative (Tb927.7.710) partial mRNA	GLOS_TB927.7.710.1.1	C/CA	INSERTION	815
XM_841238.1 Tbb leucine-rich repeat protein (LRRP), putative (Tb927.7.7110) partial mRNA	GLOS_TB927.7.7110.1.1	A/G	SNP	670
		A/G	SNP	1474
		A/G	SNP	2327
		G/A	SNP	2383
		T/C	SNP	2975
XM_841269.1 Tbb ATP synthase alpha chain, mitochondrial precursor (Tb927.7.7420) partial mRNA	GLOS_TB927.7.7420.1.1	T/C	SNP	1569
XM_841274.1 Tbb receptor-type adenylate cyclase GRESAG 4, putative partial mRNA	GLOS_TB927.7.7470.1.1	TC/T	DELETION	421
		G/A	SNP	2252
		C/T	SNP	2641
		A/G	SNP	4378
XM_841855.1 Tbb strain 927/4 GUTat10.1 40S ribosomal protein S9, putative partial mRNA	GLOS_TB927.8.1110.1.1	A/G	SNP	609
		A/C	SNP	681
		C/T	SNP	783
		A/C	SNP	807
		A/G	SNP	810
		G/C	SNP	867
		A/G	SNP	870
		G/A	SNP	897
		G/T	SNP	924
		T/C	SNP	927
		A/C	SNP	942
		A/G	SNP	945
XM_841876.1 Tbb 60S ribosomal protein L7a, putative (Tb927.8.1330) partial mRNA	GLOS_TB927.8.1330.1.1	G/A	SNP	42
		C/A	SNP	67
		C/G	SNP	77
XM_841891.1 Tbb strain 927/4 GUTat10.1 hypothetical protein, conserved partial mRNA	GLOS_TB927.8.1500.1.1	G/T	SNP	372
XM_841892.1 Tbb ATP-dependent DEAD/H RNA helicase, putative (Tb927.8.1510) partial mRNA	GLOS_TB927.8.1510.1.1	A/G	SNP	305
		A/G	SNP	2306
		AT/A	DELETION	2320
		A/G	SNP	2505
		G/A	SNP	2591
		T/C	SNP	2827
XM_841896.1 Tbb strain 927/4 GUTat10.1 hypothetical prot, conserved (Tb927.8.1550) partial mRNA	GLOS_TB927.8.1550.1.1	C/G	SNP	187
		C/T	SNP	719
		T/C	SNP	726
		CTT/C/CT	DELETION	737
		G/GA	INSERTION	838
		GA/G	DELETION	958

		GT/G	DELETION	1216
		TA/T	DELETION	1270
XP_846996.1 major surface protease gp63 [Trypanosoma brucei brucei strain 927/4 GUTat10.1] ref|XP_846997.1| major surface protease gp63 [T. brucei brucei strain 927/4 GUTat10.1]	GLOS_TB927.8.1620.1.1	A/G	SNP	34
		T/C	SNP	408
		C/T	SNP	493
		T/A	SNP	720
		C/T	SNP	825
		C/A	SNP	1330
		G/A	SNP	1380
		T/C	SNP	1738
		C/T	SNP	1746
		C/T	SNP	1771
		G/GT	INSERTION	1856
		C/T	SNP	2570
XM_841919.1 Tbb strain 927/4 GUTat10.1 hypothetical prot, conserved (Tb927.8.1790) partial mRNA	GLOS_TB927.8.1790.1.1	CTT/C	DELETION	235
		T/C	SNP	1565
		A/T	SNP	1659
		G/A	SNP	1953
		G/T	SNP	1982
		T/C	SNP	2222
XM_841923.1 Tbb strain 927/4 GUTat10.1 tRNA-methyl transferase, putative partial mRNA	GLOS_TB927.8.1830.1.1	C/A	SNP	133
		T/A	SNP	149
		C/T	SNP	1141
		G/A	SNP	1951
		T/C	SNP	3141
XM_841927.1 Tbb strain 927/4 GUTat10.1 Golgi/lysosome glycoprotein 1 partial mRNA	GLOS_TB927.8.1870.1.1	G/A	SNP	1631
		G/GA	INSERTION	2138
		TA/T	DELETION	2284
		A/T	SNP	2287
XP_847022.1 cytochrome c1, heme protein, mitochondrial precursor [Tbb strain 927/4 GUTat10.1]	GLOS_TB927.8.1890.1.1	T/C	SNP	439
		C/T	SNP	1412
XM_841943.1 Tbb strain 927/4 GUTat10.1 hypothetical prot, conserved (Tb927.8.2030) partial mRNA	GLOS_TB927.8.2030.1.1	A/T	SNP	111
XM_841956.1 Tbb strain 927/4 GUTat10.1 multidrug resistance prot A (Tb927.8.2160) partial mRNA	GLOS_TB927.8.2160.1.1	G/T	SNP	712
		T/C	SNP	1232
		G/A	SNP	2454
		T/TA	INSERTION	3233
		G/A	SNP	3796
XM_841972.1 Tbb strain 927/4 GUTat10.1 hypothetical protein (Tb927.8.2320) partial mRNA	GLOS_TB927.8.2320.1.1	C/T	SNP	504

		C/G	SNP	965
		A/C	SNP	980
		G/A	SNP	1011
		GT/G	DELETION	1076
		CAA/C	DELETION	1281
		G/A	SNP	1365
XP_847080.1 hypothetical protein [Trypanosoma brucei brucei strain 927/4 GUTat10.1]	GLOS_TB927.8.2470.1.1	C/G	SNP	39
		A/T	SNP	84
		G/T	SNP	124
		T/C	SNP	153
		T/C	SNP	215
		G/A	SNP	405
XM_841992.1 Tbb acetyl-CoA synthetase, putative (Tb927.8.2520) partial mRNA	GLOS_TB927.8.2520.1.1	C/T	SNP	967
		C/T	SNP	1693
		T/C	SNP	1969
		GA/G	DELETION	2531
		T/TA	INSERTION	2895
		A/G	SNP	2969
		G/T	SNP	3758
XP_847086.1 hypothetical protein [Trypanosoma brucei brucei strain 927/4 GUTat10.1]	GLOS_TB927.8.2530.1.1	T/C	SNP	44
		CT/C	DELETION	441
		G/C	SNP	1122
		T/G	SNP	1275
		A/T	SNP	1308
		C/T	SNP	1650
		A/C	SNP	1712
XM_841994.1 Tbb 3-ketoacyl-CoA thiolase, putative (Tb927.8.2540) partial mRNA	GLOS_TB927.8.2540.1.1	A/G	SNP	162
		T/A	SNP	336
		T/C	SNP	597
		T/C	SNP	781
		C/T	SNP	1675
XP_847096.1 kinesin [Trypanosoma brucei brucei strain 927/4 GUTat10.1]	GLOS_TB927.8.2630.1.1	C/CA	INSERTION	224
		T/TAA	INSERTION	373
		CTT/C	DELETION	520
		T/C	SNP	751
		T/TC	INSERTION	952
XP_847097.1 ubiquitin-activating enzyme E1 [Trypanosoma brucei brucei strain 927/4 GUTat10.1]	GLOS_TB927.8.2640.1.1	G/GA	INSERTION	332
		C/A	SNP	1905
		G/A	SNP	3510

		AG/A	DELETION	3744
		A/G	SNP	3755
		G/A	SNP	3782
		G/A	SNP	3879
		A/G	SNP	3880
		A/G	SNP	3907
		T/TC	INSERTION	4011
		C/CA	INSERTION	4131
		C/G	SNP	4245
XM_842029.1 Tbb mannosyl-oligosaccharide 1,2-alpha-mannosidase IB, putative partial mRNA	GLOS_TB927.8.2910.1.1	C/T	SNP	133
		G/A	SNP	424
		C/A	SNP	714
		G/A	SNP	924
		T/C	SNP	1179
		G/A	SNP	1240
		T/G	SNP	1602
XM_842044.1 Tbb cytosolic leucyl aminopeptidase, putative (Tb927.8.3060) partial mRNA	GLOS_TB927.8.3060.1.1	A/G	SNP	370
		C/G	SNP	1921
		C/T	SNP	2884
		AT/A	DELETION	2885
XM_842048.1 Tbb strain 927/4 GUTat10.1 coronin, putative (Tb927.8.3100) partial mRNA	GLOS_TB927.8.3100.1.1	T/TA	INSERTION	639
		C/T	SNP	906
		G/C	SNP	1093
		A/G	SNP	1727
		T/G	SNP	1736
		G/A	SNP	2231
		CAT/C	DELETION	2300
		GA/G	DELETION	2631
		G/A	SNP	2636
XM_842053.1 Tbb t-complex protein 1 gamma subunit, putative (Tb927.8.3150) partial mRNA	GLOS_TB927.8.3150.1.1	G/A	SNP	62
		G/A	SNP	457
		CA/C	DELETION	504
		C/T	SNP	781
		CT/C	DELETION	808
		TAGAG/T	DELETION	1144
		C/T	SNP	1166
		G/A	SNP	2767
XP_847169.1 electron transfer protein [Trypanosoma brucei brucei strain 927/4 GUTat10.1]	GLOS_TB927.8.3380.1.1	TC/T	DELETION	287
		A/C	SNP	442

		C/CT	INSERTION	467
		CT/CTT/C	INSERTION	1821
		C/T	SNP	1834
XM_842084.1 Tbb strain 927/4 GUTat10.1 hypothetical prot, conserved (Tb927.8.3460) partial mRNA	GLOS_TB927.8.3460.1.1	A/C	SNP	355
		G/A	SNP	849
		G/A	SNP	1459
		GA/G	DELETION	1526
		T/TA	INSERTION	1665
		A/T	SNP	1847
		T/A	SNP	1848
		A/G	SNP	1849
		G/A	SNP	1851
XM_842113.1 Tbb strain 927/4 GUTat10.1 nucleolar protein, putative (Tb927.8.3750) partial mRNA	GLOS_TB927.8.3750.1.1	G/T	SNP	205
		T/C	SNP	2373
		A/G	SNP	2379
XM_842120.1 Tbb strain 927/4 GUTat10.1 hypothetical prot, conserved (Tb927.8.3820) partial mRNA	GLOS_TB927.8.3820.1.1	C/CT	INSERTION	556
XM_842122.1 Tbb strain 927/4 GUTat10.1 hypothetical prot, conserved (Tb927.8.3840) partial mRNA	GLOS_TB927.8.3840.1.1	C/CA	INSERTION	1804
		G/A	SNP	1945
		CA/C	DELETION	2873
XM_842126.1 Tbb strain 927/4 GUTat10.1 hypothetical prot, conserved (Tb927.8.3880) partial mRNA	GLOS_TB927.8.3880.1.1	G/T	SNP	500
		T/TCA	INSERTION	677
		C/G	SNP	1806
		T/C	SNP	1807
		G/A	SNP	1814
		C/G	SNP	1818
		C/T	SNP	2019
		C/T	SNP	2190
XM_842139.1 Tbb strain 927/4 GUTat10.1 flagellum-adhesion glycoprotein partial mRNA	GLOS_TB927.8.4010.1.1	G/T	SNP	2226
XM_842143.1 Tbb strain 927/4 GUTat10.1 hypothetical protein, conserved partial mRNA	GLOS_TB927.8.4050.1.1	G/A	SNP	2311
		G/A	SNP	2533
XP_847264.1 small GTP-binding protein Rab11 [Trypanosoma brucei brucei strain 927/4 GUTat10.1]	GLOS_TB927.8.4330.1.1	C/T	SNP	227
		AAT/A	DELETION	304
		GAAA/G	DELETION	515
		CTTT/C	DELETION	929
		G/GT	INSERTION	1525
		C/T	SNP	2251
		C/T	SNP	2387
		G/T	SNP	2951
		C/T	SNP	2955

		C/T	SNP	2956
XM_842208.1 Tb strain 927/4 GUTat10.1 amino acid transporter, putative partial mRNA	GLOS_TB927.8.4700.1.1	C/T	SNP	224
		T/G	SNP	282
		T/C	SNP	1533
		T/C	SNP	1602
		A/G	SNP	1625
		A/T	SNP	2118
		A/T	SNP	2120
		A/G	SNP	2536
		G/T	SNP	2632
		C/A	SNP	2660
		T/G	SNP	2742
		C/A	SNP	2743
		A/C	SNP	2749
XP_847308.1 hypothetical protein [Trypanosoma brucei brucei strain 927/4 GUTat10.1]	GLOS_TB927.8.4780.1.1	G/A	SNP	1692
		C/T	SNP	1694
		G/T	SNP	1695
		A/C	SNP	1697
		G/A	SNP	1701
		G/T	SNP	2309
		A/G	SNP	2321
		C/CT	INSERTION	4228
XM_842244.1 Tbb strain 927/4 GUTat10.1 hypothetical protein, conserved partial mRNA	GLOS_TB927.8.5070.1.1	G/GA	INSERTION	494
		C/CT	INSERTION	557
		T/A	SNP	1092
		T/A	SNP	1093
XP_847342.1 cytochrome c [Trypanosoma brucei brucei strain 927/4 GUTat10.1]	GLOS_TB927.8.5120.1.1	G/GATA	INSERTION	223
		G/A	SNP	543
		C/CT	INSERTION	841
		A/C	SNP	903
		A/AG	INSERTION	964
		T/C	SNP	1335
XM_842263.1 Tbb strain 927/4 GUTat10.1 60S ribosomal protein L39, putative partial mRNA	GLOS_TB927.8.5260.2.3	A/T	SNP	427
		A/T	SNP	429
XM_842284.1 Tbb strain 927/4 GUTat10.1 flagellar calcium-binding protein partial mRNA	GLOS_TB927.8.5470.1.1	A/C	SNP	490
		CA/C	DELETION	582
		T/C	SNP	738
		G/A	SNP	845
		G/A	SNP	854

XP_847390.1 transaldolase [Trypanosoma brucei brucei strain 927/4 GUTat10.1]	GLOS_TB927.8.5600.1.1	G/C	SNP	400
XP_847394.1 hypothetical protein [Trypanosoma brucei brucei strain 927/4 GUTat10.1]	GLOS_TB927.8.5640.1.1	G/A	SNP	1114
		G/A	SNP	1889
		A/C	SNP	1890
XM_842315.1 Tbb strain 927/4 GUTat10.1 protein tyrosine phosphatase, putative partial mRNA	GLOS_TB927.8.5780.1.1	G/A	SNP	900
		TC/T	DELETION	1548
		C/G	SNP	1823
XM_842337.1 Tbb strain 927/4 GUTat10.1 fatty acid desaturase, putative partial mRNA	GLOS_TB927.8.6000.1.1	C/A	SNP	1453
		C/T	SNP	1593
		G/A	SNP	1758
		T/A	SNP	1794
		TACATC/T	DELETION	2035
		GAAA/G	DELETION	2179
		G/C	SNP	2696
XM_842343.1 Tbb 2-amino-3-ketobutyrate coenzyme A ligase, putative (Tb927.8.6060) partial mRNA	GLOS_TB927.8.6060.1.1	GA/G	DELETION	229
		A/G	SNP	239
		C/T	SNP	325
		T/TA	INSERTION	389
		G/A	SNP	694
		G/A	SNP	1478
		C/T	SNP	1570
XP_847441.1 hypothetical protein [Trypanosoma brucei brucei strain 927/4 GUTat10.1]	GLOS_TB927.8.6110.1.1	A/G	SNP	47
		G/GA	INSERTION	1979
		T/A	SNP	2146
XM_842353.1 Tbb 40S ribosomal protein S8, putative (Tb927.8.6160) partial mRNA	GLOS_TB927.8.6160.1.1	T/G	SNP	262
XM_842354.1 Tbb strain 927/4 GUTat10.1 transketolase, putative (Tb927.8.6170) partial mRNA	GLOS_TB927.8.6170.1.1	T/C	SNP	547
		G/A	SNP	1038
XM_842355.1 Tbb strain 927/4 GUTat10.1 60S ribosomal protein L26, putative partial mRNA	GLOS_TB927.8.6180.1.2	A/G	SNP	44
		C/T	SNP	45
XP_847448.1 60S ribosomal protein L26 [Trypanosoma brucei brucei strain 927/4 GUTat10.1]	GLOS_TB927.8.6180.2.2	A/T	SNP	10
		C/T	SNP	11
		C/T	SNP	327
		C/T	SNP	349
		A/G	SNP	382
		A/G	SNP	391
		T/C	SNP	433
		T/C	SNP	478
		T/C	SNP	508
		A/G	SNP	511

		A/G	SNP	637
XM_842358.1 Tbb strain 927/4 GUTat10.1 phosphatidylinositol 3-kinase, putative partial mRNA	GLOS_TB927.8.6210.1.1	A/G	SNP	1846
XP_847454.1 hypothetical protein [Trypanosoma brucei brucei strain 927/4 GUTat10.1]	GLOS_TB927.8.6240.1.1	T/TA	INSERTION	96
		C/CA	INSERTION	155
		G/T	SNP	323
		GT/G	DELETION	1425
		C/CT	INSERTION	1760
		TA/T	DELETION	1904
XM_842381.1 Tbb strain 927/4 GUTat10.1 RNA-binding prot, putative (Tb927.8.6440) partial mRNA	GLOS_TB927.8.6440.1.1	C/G	SNP	465
		TAAA/T	DELETION	953
		A/ATT	INSERTION	1155
XP_847475.1 inhibitor of cysteine peptidase [Trypanosoma brucei brucei strain 927/4 GUTat10.1]	GLOS_TB927.8.6450.1.1	C/CG	INSERTION	522
		G/GT	INSERTION	893
XP_846902.1 cation-transporting ATPase [Trypanosoma brucei brucei strain 927/4 GUTat10.1]	GLOS_TB927.8.650.1.1	T/C	SNP	1596
		G/T	SNP	3083
XM_842395.1 Tbb succinate dehydrogenase flavoprotein, putative (Tb927.8.6580) partial mRNA	GLOS_TB927.8.6580.1.1	CAA/C	DELETION	143
		A/AT	INSERTION	256
		A/T	SNP	268
		A/AC	INSERTION	562
		C/A	SNP	977
		C/T	SNP	3162
XM_842401.1 Tbb strain 927/4 GUTat10.1 hypothetical prot, conserved (Tb927.8.6640) partial mRNA	GLOS_TB927.8.6640.1.1	C/T	SNP	76
		GA/G	DELETION	274
		A/C	SNP	513
		CTA/C	DELETION	538
		G/A	SNP	1031
		G/A	SNP	1361
		G/C	SNP	1700
XM_842403.1 Tbb strain 927/4 GUTat10.1 hypothetical protein, conserved partial mRNA	GLOS_TB927.8.6660.1.1	G/A	SNP	45
		G/A	SNP	104
		G/A	SNP	120
		G/A	SNP	298
		G/C	SNP	856
		A/C	SNP	1088
		G/GGT	INSERTION	2251
		C/T	SNP	2890
XM_842412.1 Tbb translationally controlled tumor prot (TCTP), putative (Tb927.8.6750) partial mRNA	GLOS_TB927.8.6750.1.1	C/CCT	INSERTION	185
		T/C	SNP	196
		TA/T	DELETION	426

		G/A	SNP	864
		C/G	SNP	865
		C/A	SNP	959
XP_847519.1 hypothetical protein [Trypanosoma brucei brucei strain 927/4 GUTat10.1]	GLOS_TB927.8.6890.1.1	A/T	SNP	75
		T/TA	INSERTION	83
		C/G	SNP	327
XM_842434.1 Tbb 3-methylcrotonyl-CoA carboxylase, putative (Tb927.8.6970) partial mRNA	GLOS_TB927.8.6970.1.1	G/A	SNP	231
		G/C	SNP	305
		T/C	SNP	340
		C/A	SNP	369
		G/C	SNP	376
		G/A	SNP	428
		C/T	SNP	439
		A/G	SNP	736
		T/C	SNP	847
		C/G	SNP	884
		C/T	SNP	1041
		T/C	SNP	1132
		G/A	SNP	1188
		A/T	SNP	1274
		G/A	SNP	1341
		C/G	SNP	1357
		C/G	SNP	1411
		C/T	SNP	1581
		A/G	SNP	1697
		A/C	SNP	1777
		C/T	SNP	1796
		G/A	SNP	1831
		G/A	SNP	1890
		C/T	SNP	1891
		C/T	SNP	2029
		G/A	SNP	2070
		T/C	SNP	2124
		T/C	SNP	2182
		A/G	SNP	2260
		C/T	SNP	2449
		C/T	SNP	2524
		G/A	SNP	2590
		C/T	SNP	2613

		T/G	SNP	2667
		G/C	SNP	2943
		A/C	SNP	3016
		A/T	SNP	3242
		T/G	SNP	3468
		T/A	SNP	3499
XP_847532.1 peptidase [Trypanosoma brucei brucei strain 927/4 GUTat10.1]	GLOS_TB927.8.7020.1.1	C/T	SNP	1599
		A/G	SNP	2347
XM_842447.1 Tbb acetyl-CoA carboxylase, putative (Tb927.8.7100) partial mRNA	GLOS_TB927.8.7100.1.1	T/TA	INSERTION	108
XM_841815.1 Tbb RNA-binding protein, putative (Tb927.8.710) partial mRNA	GLOS_TB927.8.710.1.1	T/C	SNP	1418
		C/T	SNP	1508
		G/A	SNP	1830
		G/A	SNP	3929
		C/T	SNP	3974
XM_842449.1 Tbb strain 927/4 GUTat10.1 farnesyltransferase, putative (Tb927.8.7120) partial mRNA	GLOS_TB927.8.7120.1.1	G/C	SNP	179
XM_842452.1 Tbb UDP-Gal or UDP-GlcNAc-dependent glycosyltransferase, putative partial mRNA	GLOS_TB927.8.7150.1.1	G/C	SNP	143
		A/G	SNP	160
		G/C	SNP	190
		C/T	SNP	768
		T/C	SNP	834
XM_842477.1 Tbb strain 927/4 GUTat10.1 calreticulin, putative (Tb927.8.7410) partial mRNA	GLOS_TB927.8.7410.1.1	C/T	SNP	119
		G/T	SNP	120
		G/A	SNP	246
		G/A	SNP	255
		G/T	SNP	312
		C/T	SNP	486
		A/G	SNP	495
		A/G	SNP	501
		T/C	SNP	624
		G/A	SNP	629
		T/C	SNP	669
		C/T	SNP	866
		C/T	SNP	867
		C/A	SNP	882
		C/A	SNP	885
		C/T	SNP	984
		C/T	SNP	1025
		A/C	SNP	1341
		G/T	SNP	1438

		G/A	SNP	1453
		A/G	SNP	1477
		G/A	SNP	1509
		TAA/T	DELETION	1657
		A/G	SNP	1766
XM_842485.1 Tbb strain 927/4 GUTat10.1 hypothetical prot, conserved (Tb927.8.7490) partial mRNA	GLOS_TB927.8.7490.1.1	A/G	SNP	126
		C/A	SNP	143
		A/G	SNP	150
		T/C	SNP	198
		A/G	SNP	258
		T/C	SNP	275
		T/C	SNP	940
		T/C	SNP	1127
		A/T	SNP	1135
		T/C	SNP	1285
		G/A	SNP	1311
		G/A	SNP	1497
		C/T	SNP	1608
		G/A	SNP	1677
		C/T	SNP	2325
		T/C	SNP	2424
		C/T	SNP	2874
		C/T	SNP	2916
		A/G	SNP	3105
		T/G	SNP	3308
		C/G	SNP	3558
		A/G	SNP	3573
		G/A	SNP	3712
		C/G	SNP	3730
		T/C	SNP	3765
		C/T	SNP	4001
		A/G	SNP	4086
		A/G	SNP	4157
		A/G	SNP	4172
		A/T	SNP	4211
		A/G	SNP	4234
		G/A	SNP	4245
XM_841820.1 Tbb strain 927/4 GUTat10.1 nucleolar RNA-binding protein (Tb927.8.760) partial mRNA	GLOS_TB927.8.760.1.1	G/A	SNP	751
		A/G	SNP	1148

XM_842526.1 Tbb receptor-type adenylate cyclase GRESAG 4, putative partial mRNA	GLOS_TB927.8.7940.1.1	T/C	SNP	132
		A/G	SNP	558
		A/G	SNP	563
		C/G	SNP	564
		A/G	SNP	599
		C/T	SNP	695
		C/T	SNP	767
		C/T	SNP	988
		G/C	SNP	991
		G/A	SNP	1022
		A/G	SNP	1027
		A/T	SNP	1042
		T/C	SNP	1043
		G/A	SNP	1048
		G/A	SNP	1066
		C/G	SNP	1137
		G/A	SNP	1209
		A/G	SNP	1506
		A/G	SNP	1556
		C/T	SNP	1571
		T/C	SNP	1655
		G/C	SNP	1726
		A/C	SNP	2020
		C/T	SNP	2024
		G/A	SNP	2049
		G/A	SNP	2050
		A/G	SNP	2229
		A/G	SNP	2232
		T/A	SNP	2233
		T/C	SNP	2269
		G/A	SNP	2337
		G/C	SNP	2338
		G/T	SNP	3043
		T/C	SNP	3073
		G/A	SNP	3228
		A/G	SNP	3287
		C/G	SNP	3290
		A/G	SNP	3291
		T/G	SNP	3297

		G/A	SNP	3354
		C/T	SNP	3412
XM_842527.1 Tbb strain 927/4 GUTat10.1 hypothetical prot, conserved (Tb927.8.7950) partial mRNA	GLOS_TB927.8.7950.1.1	C/CT	INSERTION	490
		C/T	SNP	2645
XM_842530.1 Tbb vacuolar-type proton translocating pyrophosphatase 1 partial mRNA	GLOS_TB927.8.7980.1.1	G/C	SNP	26
		T/C	SNP	382
		G/T	SNP	397
		GA/G	DELETION	407
		T/C	SNP	569
		ATTT/A	DELETION	621
		A/AT	INSERTION	815
		C/T	SNP	1992
		A/G	SNP	2232
		G/A	SNP	2247
		G/A	SNP	2253
		G/A	SNP	2400
		C/G	SNP	2466
		C/T	SNP	2535
		T/C	SNP	2541
		A/G	SNP	2682
		A/G	SNP	2853
		G/A	SNP	2916
		A/G	SNP	3235
		C/T	SNP	3384
XP_847643.1 hypothetical protein [Trypanosoma brucei brucei strain 927/4 GUTat10.1]	GLOS_TB927.8.8200.1.1	C/T	SNP	2006
		G/C	SNP	2008
		A/G	SNP	2037
		A/C	SNP	2261
		G/A	SNP	2303
		G/A	SNP	2360
		T/C	SNP	2374
		C/T	SNP	2414
		A/G	SNP	2429
		T/C	SNP	2448
		A/G	SNP	2449
		C/G	SNP	2489
XM_842554.1 Tbb amino acid transporter, putative (Tb927.8.8240) partial mRNA	GLOS_TB927.8.8240.1.1	T/C	SNP	205
		G/A	SNP	209
		T/C	SNP	851

		C/T	SNP	1039
		T/C	SNP	1159
XM_842555.1 Tbb amino acid transporter, putative (Tb927.8.8250) partial mRNA	GLOS_TB927.8.8250.1.1	T/A	SNP	915
		G/C	SNP	916
		A/G	SNP	918
		T/C	SNP	1830
XP_847653.1 amino acid transporter [Trypanosoma brucei brucei strain 927/4 GUTat10.1]	GLOS_TB927.8.8300.1.1	T/C	SNP	1847
		T/A	SNP	1854
		T/A	SNP	1855
		C/A	SNP	1893
		A/G	SNP	1927
		GA/G	DELETION	1975
		GA/G	DELETION	2456
		TA/T	DELETION	2661
		C/CAAA	INSERTION	2895
		G/GT	INSERTION	2978
		T/C	SNP	2997
XP_846926.1 small GTP-binding protein Rab1 [Trypanosoma brucei brucei strain 927/4 GUTat10.1]	GLOS_TB927.8.890.1.1	C/CT	INSERTION	139
		TA/T	DELETION	940
		A/G	SNP	1025
		A/G	SNP	1026
[BBH] TBA_TRYBR (sp|P04106) Tubulin alpha chain OS=T. brucei rhodesiense PE=3 SV=1	GLOS_TBA.1.1	A/T	SNP	38
		C/G	SNP	40
		C/A	SNP	42
		A/C	SNP	43
		G/T	SNP	1490
		G/T	SNP	1491
XP_813091.1 ubiquitin hydrolase [Trypanosoma cruzi strain CL Brener]	GLOS_TC00.1047053507017.1	G/A	SNP	1232
		G/A	SNP	1557
XP_809984.1 hypothetical protein [Trypanosoma cruzi strain CL Brener]	GLOS_TC00.1047053508823.7	T/C	SNP	1398
		A/ATT	INSERTION	1488
XP_804510.1 ribosomal protein S29 [T. cruzi strain CL Brener] ref|XP_806920.1| ribosomal prot S29 [T. cruzi strain CL Brener] ref|XP_808328.1| ribosomal protein S29 [T. cruzi strain CL Brener]	GLOS_TC00.1047053511805.1	G/GA	INSERTION	172
		A/G	SNP	186
		G/A	SNP	241
		T/C	SNP	269
		G/A	SNP	374
		T/A	SNP	544
		G/A	SNP	545

[BBH] TDX_TRYBR (sp|Q26695) Thioredoxin peroxidase OS=T. brucei rhodesiense PE=2 SV=1	GLOS_TDX.1.1	A/G	SNP	195
		T/C	SNP	540
		C/CT	INSERTION	989
[BBH] TH2A_TRYBB (sp|Q06222) Glucose transporter 2A OS=T. b. brucei GN=THT2A PE=2 SV=1	GLOS_TH2A.1.1	T/C	SNP	385
		G/A	SNP	2220
		A/C	SNP	2222
		A/T	SNP	2224
[BBH] TM120_DROME (sp|Q9U1M2) Transmembrane protein 120 homolog OS=D. melanogaster	GLOS_TM120.1.1	TC/T	DELETION	137
		C/CA	INSERTION	302
		CT/C	DELETION	540
		GT/G	DELETION	751
		A/G	SNP	1472
		T/C	SNP	1473
		C/A	SNP	1524
		G/A	SNP	1832
		G/GA	INSERTION	2687
		T/A	SNP	2820
		G/GA	INSERTION	3069
XM_001219103.1 T. brucei cyt. C oxidase subunit IV (PMID:12467979) (trCOIV) partial mRNA	GLOS_TRCOIV.1.1	A/G	SNP	16
		G/A	SNP	555
		T/C	SNP	587
		T/G	SNP	591
		G/A	SNP	624
		G/GA	INSERTION	979
		AT/A	DELETION	2893
		G/A	SNP	3089
TRYDG_DROME (sp|P42276) Trypsin delta/gamma OS=D. melanogaster GN=deltaTry PE=2 SV=2	GLOS_TRYDG.4.5	C/T	SNP	40
		C/G	SNP	91
		T/C	SNP	119
		G/A	SNP	124
		T/C	SNP	125
		A/C	SNP	136
		G/A	SNP	184
		C/G	SNP	253
		T/A	SNP	273
		C/T	SNP	287
		T/C	SNP	341
		T/G	SNP	361
		G/T	SNP	414

		C/T	SNP	484
		T/A	SNP	509
		T/C	SNP	524
		C/A	SNP	588
		T/C	SNP	594
		T/C	SNP	626
		C/T	SNP	641
		A/G	SNP	686
		T/A	SNP	700
		T/C	SNP	734
		T/C	SNP	981
		T/C	SNP	988
		G/A	SNP	1120
		G/A	SNP	1194
		C/T	SNP	1225
		A/G	SNP	1241
		A/G	SNP	1264
		A/G	SNP	1294
		A/T	SNP	1309
		A/T	SNP	1323
		A/T	SNP	1348
		A/G	SNP	1389
		A/G	SNP	1403
		T/C	SNP	1455
		C/G	SNP	1525
		A/C	SNP	1528
TTI_GLOMM (sp|O97373) Tsetse thrombin inhibitor OS=G. morsitans morsitans GN=TTI PE=1 SV=1	GLOS_TTI.4.16	C/T	SNP	237
		G/C	SNP	536
		A/T	SNP	572
		A/T	SNP	577
XP_001288661.1 hypothetical protein [Trichomonas vaginalis G3]	GLOS_TVAG_157670.1.1	C/G	SNP	822
		AGG/A/AG	DELETION	1326
XP_001582404.1 viral A-type inclusion protein [Trichomonas vaginalis G3]	GLOS_TVAG_198570.1.2	T/G	SNP	133
		G/A	SNP	289
		A/G	SNP	308
		G/A	SNP	352
		T/G	SNP	369
		C/T	SNP	372
		A/G	SNP	422

		T/A	SNP	515
		G/A	SNP	596
		C/T	SNP	613
		T/A	SNP	677
		C/G	SNP	693
		G/T	SNP	695
		G/C	SNP	699
		T/C	SNP	704
		T/A	SNP	851
		C/T	SNP	892
		G/A	SNP	924
		C/A	SNP	954
		T/G	SNP	1022
		A/T	SNP	1044
		C/T	SNP	1105
		G/A	SNP	1188
		T/C	SNP	1422
		T/A	SNP	1522
		C/G	SNP	1547
		G/A	SNP	1699
		A/G	SNP	1753
		G/T	SNP	1774
		G/A	SNP	1793
		G/T	SNP	1796
		C/T	SNP	1808
		T/G	SNP	1817
		A/G	SNP	1875
		A/T	SNP	1888
		C/T	SNP	1934
		T/C	SNP	2030
		C/G	SNP	2076
		G/C	SNP	2115
		T/C	SNP	2226
		T/C	SNP	2253
		C/T	SNP	2412
		G/A	SNP	2604
		C/T	SNP	2749
[BBH] TYPX_TRYBB (sp|O77404) Tryparedoxin OS=Trypanosoma brucei brucei PE=1 SV=1	GLOS_TYPX.1.1	G/A	SNP	702
		G/C	SNP	799

		A/G	SNP	1005
[BBH] TYTR_TRYBB (sp|P39051) Trypanothione reductase OS=Tbb GN=TPR PE=1 SV=1	GLOS_TYTR.1.1	A/G	SNP	1758
USO1_YEAST (sp|P25386) Intracellular protein transport protein USO1 OS=S. cerevisiae	GLOS_USO1.1.1	A/G	SNP	25
		G/T	SNP	161
		A/G	SNP	164
		G/T	SNP	209
		G/T	SNP	221
		A/C	SNP	238
		G/GA	INSERTION	241
		C/A	SNP	269
		C/A	SNP	343
		C/CA	INSERTION	404
		A/T	SNP	427
		C/G	SNP	428
		G/A	SNP	429
		A/G	SNP	439
		A/C	SNP	476
		C/A	SNP	481
		A/T	SNP	484
		G/A	SNP	485
		T/C	SNP	492
		T/C	SNP	495
		A/G	SNP	496
		AGAATAAACT/A	DELETION	567
		A/C	SNP	605
		A/G	SNP	631
		C/A	SNP	635
		G/C	SNP	710
		A/T	SNP	724
		T/A	SNP	745
		G/T	SNP	787
		T/C	SNP	788
		G/T	SNP	824
		G/C	SNP	841
		GAAAATTTAAAGCAAGAAC	DELETION	842
		G/A	SNP	911
		G/A	SNP	1128
		A/T	SNP	1225
		T/G	SNP	1321

T/C	SNP	1381
T/C	SNP	1474
A/C	SNP	1558
A/T	SNP	1662
C/G	SNP	1858
G/A	SNP	1928
T/C	SNP	1936
C/T	SNP	2035
T/C	SNP	2175
A/C	SNP	2260
T/C	SNP	2325
A/T	SNP	2392
A/G	SNP	2482
A/T	SNP	2523
A/C	SNP	2679
C/G	SNP	2717
A/G	SNP	2947
G/T	SNP	3042
